# High-resolution spectroscopy of [H,C,N]^+^: III. Infrared Ã^2^Σ^+^ ← X̃^2^Π electronic transition of HCN^+^

**DOI:** 10.1039/d5cp04255k

**Published:** 2025-12-22

**Authors:** Samuel J. P. Marlton, Philipp C. Schmid, Weslley G. D. P. Silva, Oskar Asvany, Stephan Schlemmer

**Affiliations:** a I. Physikalisches Institut, Universität zu Köln Zülpicher Str. 77 50937 Cologne Germany marlton@ph1.uni-koeln.de schlemmer@ph1.uni-koeln.de

## Abstract

The Ã^2^Σ^+^ ← X̃^2^Π electronic transition of the fundamental HCN^+^ ion is reported. The spectrum exhibits a rotational, fine, and hyperfine structure that provides insights into the geometries and wavefunctions of the Ã^2^Σ^+^ and X̃^2^Π states. The extracted spectroscopic constants for the Ã state include (in cm^−1^) *T*_e_ = 3238.8224(2), *B* = 1.39568(3), *D* = 2.27(9) × 10^−5^, *γ* = −0.07332(7), *γ*_D_ = 1.87(3) × 10^−4^, and the Fermi contact hyperfine constant for the HCN^+^ nitrogen atom *b*_F_ = 0.0059(1). Based on this value, the Ã^2^Σ_1/2_^+^ state electronic wavefunction is estimated to have 11.5 ± 0.2% s orbital character. The spin–orbit coupling constant for the X̃ state was also determined with high precision *A*_SO_ = −49.3122(4) cm^−1^. This work illustrates that leak-out spectroscopy can be applied to measure high-resolution spectra of low energy electronic transitions.

## Abstract 2

Electronic spectra of HCN^+^ are measured using leak-out spectroscopy (LOS), providing insight into the nature of the excited state electronic wavefunction and demonstrating the effectiveness of LOS for high-resolution electronic spectroscopy.

## Graphical abstract

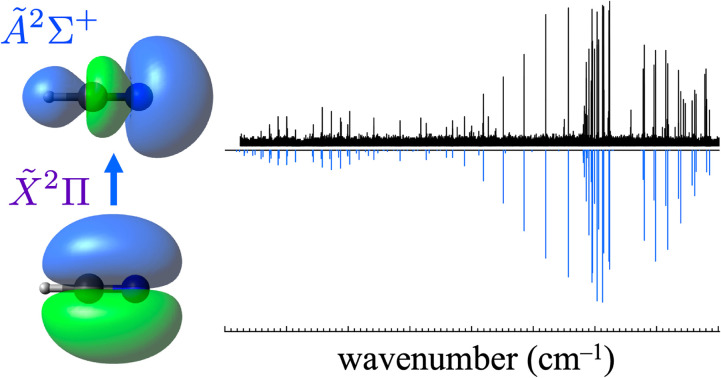

## Introduction

The HCN^+^ cation is a fundamental molecule with an interesting electronic structure; however, limited details have been reported about its electronic excited states. HCN^+^ is highly reactive,^1,2^ making it difficult to isolate for spectroscopic interrogation, so the only reported infrared spectra of HCN^+^ are vibrational spectra in inert Ne matrices^3^ and rovibrational and rotational spectra that have recently been measured in cryogenic ion traps as part of this series of publications.^4,5^

**Fig. 1:**
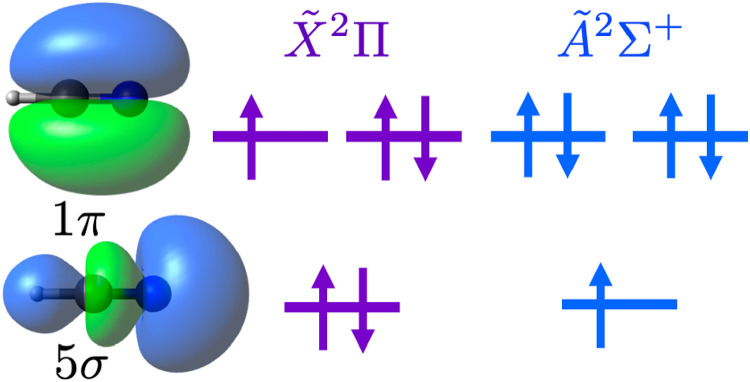
Relevant molecular orbitals of HCN^+^.

No electronic transitions of HCN^+^ have been directly measured; however, individual states of HCN^+^ have been observed by photoionisation and photoelectron spectroscopy of the less reactive neutral HCN.^6–10^ In these investigations, several bands from the HCN^+^ X̃^2^Π ground state and a low lying Ã^2^Σ^+^ state were observed. These states are separated by only ≈3250 cm^−1^ because of the similar energies of the 1π and 5σ orbitals, which are illustrated in Fig. 1.^6–10^

In most of these photoelectron and photoionisation spectra, only vibronic transitions are resolved.^7–10^ The highest resolution was achieved using zero electron kinetic energy (ZEKE) photoionisation spectroscopy (Δ*v̄* = 1 cm^−1^), which exhibited a partially resolved rotational structure.^6^ From the ZEKE spectra, it was possible to extract first experimental values of spin–orbit coupling of the ground state (*A*_SO_ = −49.8(1) cm^−1^), the rotational constant of the X̃^2^Π ground state (*B*″ = 1.36(1) cm^−1^), and the rotational constant of the Ã^2^Σ^+^ excited state (*B*′ = 1.37(1) cm^−1^). Despite being high-quality spectra, it is not clear from these results whether the rotational constant significantly changes in the Ã^2^Σ^+^ excited state. This is in contrast to high-level multireference configuration interaction calculations that include vibronic coupling, which predict a significant excited state CN bond contraction, which significantly changes the rotational constant in the excited state.^11^ Furthermore, higher resolution experimental studies are required to characterise the more subtle spectroscopic properties of HCN^+^. For example, the couplings between electronic orbital angular momentum (Λ), electron spin (*S*), nuclear rotation, and nuclear spin of the nitrogen (*I*_N_) and hydrogen (*I*_H_) are of fundamental interest.

In particular, the hyperfine coupling between electronic angular momenta and nuclear spin is valuable because it facilitates quantitative experimental characterisation of the electronic wavefunction.^12^ Some of these couplings have now been described for the X̃^2^Π state vibrational levels,^4,5^ but not for excited electronic states. The ability to characterise these subtle couplings in the excited state is also a necessary step towards finding promising candidate polyatomic molecular ions for which laser-cooling might be possible.^13^

It is often challenging to characterise the more subtle couplings between angular momenta for gas-phase ions in excited electronic states. For example, hyperfine splitting in excited electronic states is typically on the order of less than 0.1 cm^−1^ and has been observed for only a few gas-phase molecular ions, almost all of which are diatomics.^14–24^ To our knowledge, the only polyatomic ions for which excited state hyperfine splitting has been observed in the gas-phase are isotopologues of HCP^+^ and N_2_O^+^.^22,23^ One reason for this is that excited electronic states often have picosecond or femtosecond lifetimes, and so lifetime broadening makes small splittings between rovibronic lines unresolvable.^25^ Lifetime broadening becomes an increasingly common feature of larger molecules because they have more degrees of freedom and higher densities of states, which can facilitate fast excited state deactivation by dissociation, internal conversion, or intersystem crossing.

Well established methods for measuring electronic spectra of gas-phase ions are generally not suitable for resolving splittings smaller than 0.01 cm^−1^. For example, single-photon resonance enhanced photodissociation spectroscopy is often lifetime broadened,^25^ one-colour multiphoton dissociation can produce broad peaks that are slightly shifted,^26,27^ messenger tagging does not provide a spectrum of the bare ion,^28–30^ and one-colour photoionisation spectroscopy of neutral precursors with high energy photons is more Doppler broadened because this broadening increases proportionally to the photon energy. High-resolution infrared spectra of the HCN^+^ Ã^2^Σ^+^ ← X̃^2^Π transition are probably achievable using techniques such as laser induced reactions (LIR),^31^ laser induced inhibition of cluster growth (LIICG),^32^ and multiple-colour spectroscopy.^29,30^ However, these methods typically require sophisticated understanding of some reaction schemes, spectroscopic levels, or subtle experimental conditions to implement. Finally, ions like HCN^+^ are too reactive to be straightforwardly investigated in jets, discharges, and absorption cells.

In this study, we show that the recently developed leak-out spectroscopy (LOS) method^33^—which was recently applied to electronic spectroscopy for the first time^34^—overcomes some of these disadvantages and yields a high resolution single photon electronic spectrum of bare HCN^+^. This is the first spectrum of the Ã^2^Σ^+^ ← X̃^2^Π transition for HCN^+^, and the spectrum has a sufficiently high resolution to resolve a rotational, fine, and hyperfine structure. This is encouraging for the potential to measure single-photon electronic spectra of bare gas-phase ions far below the dissociation threshold using leak-out spectroscopy.

This article is a part of a series of publications reporting high-resolution infrared rovibrational^4^ and pure rotational^5^ spectra of HCN^+^. The previous parts of this series described the couplings between electronic and nuclear angular momenta of HCN^+^ in the X̃ electronic state. The electronic spectrum of this work was shown in Publication I of this series without analysis.^4^ In this part, we describe the couplings between electronic and nuclear angular momenta of HCN^+^ in the X̃ and Ã states. We then discuss the rovibronic structure of the experimental Ã^2^Σ^+^ ← X̃^2^Π LOS spectrum. The experimental spectrum is fitted with a rovibronic Hamiltonian to extract several spectroscopic constants. These results are contextualised by comparison with a calculated potential energy surface. We then discuss the hyperfine splitting observed in the Ã^2^Σ^+^ ← X̃^2^Π spectrum, which provides further insights into the electronic wavefunction of the HCN^+^ Ã state. Finally, we report a further rovibrational transition of HCN^+^ that overlaps with the rovibronic Ã^2^Σ^+^ ← X̃^2^Π spectrum. We argue that this rovibrational transition excites an upper (*κ*) Renner–Teller component of an X̃ state combination band composed of two quanta of *ν*_2_ and one quantum of *ν*_1_. Together with the previous studies and the earlier parts of this series,^4–6,11^ these results provide a detailed experimental foundation for understanding the Renner–Teller vibronic coupling in HCN^+^, as well as the vibronic, rovibronic, fine, and hyperfine structure of the HCN^+^ Ã electronic state.

## Experimental details

Spectra were measured using leak-out spectroscopy (LOS) in the cryogenically cooled 22-pole ion trap instrument referred to as LIRtrap.^31,35^ This method has been described in detail previously.^33^ Briefly, HCN^+^ ions were generated from acetonitrile vapour, which were bombarded with 70 eV electrons in a storage ion source. The ions in the source were pulsed out every 500 ms into a quadrupole mass filter (QMF1), which was set to select *m*/*z* 27. The mass selected ions exiting the QMF1 were then trapped and stored in a 22-pole ion trap mounted on a 10 K cold-head.^36^ After 10 ms of cooling with the neutral Ne (with a continuous number density of approximately 10^14^ cm^−3^) in the trap region, the ions were irradiated by light from a continuous optical parametric oscillator system (Toptica TOPO, ≈100 W cm^−2^, accuracy ≈10^−4^ cm^−1^) for 300 ms, the timing of which was controlled with a mechanical shutter. The light frequency was measured using a wavemeter (Bristol 621 A-IR, resolution ≈10^−3^ cm^−1^). Photoexcited ions could collide with neutral Ne buffer gas to convert some of their internal energy into kinetic energy. The ions with enhanced kinetic energy could overcome the low potential barrier at the trap exit and leak out of the trap. After exiting the trap, ions pass through a second quadrupole mass filter (QMF2) set to select *m*/*z* 27, before being detected using a Daly type detector.^37,38^ This process was repeated while scanning the OPO frequency to record a rovibrational LOS spectrum.

Several experimental configurations that differed slightly from one another were employed in this study. The spectrum was measured at a trap temperature of 27 K with continuous Ne as the buffer gas (*n* ≈ 2 × 10^14^ cm^−3^). This configuration was optimised to observe transitions involving the Ω = 1/2 component of the ground state, which requires elevated temperatures because the Ω = 3/2 component is lower in energy by ≈50 cm^−1^. This configuration was also optimised to suppress background peaks from isobaric C_2_H_3_^+^ by lowering the trap potential, which increased the trap strength and reduced the leak-out signal of C_2_H_3_^+^ more than that of HCN^+^. This difference between the leak-out signal of C_2_H_3_^+^ and HCN^+^ probably reflects a difference in their vibrational to kinetic energy transfer characteristics. A small range of the spectrum was measured at 9 K, with a 1 : 3 mixture of Ne : He that was pulsed into the ion trap. This configuration was optimised to provide a contour fit of the hyperfine splitting, which required low temperature to reduce Doppler broadening and care to avoid saturating the transitions as a result of near-total leak-out of the trapped ion population. Finally, the spectrum was remeasured at 4 K, with a 1 : 3 mixture of Ne : He that was pulsed into the ion trap, using the COLtrap machine.^38^ This configuration was optimised to provide the line positions of hyperfine components.

### Computational details

The geometries of the X̃^2^Π and Ã^2^Σ^+^ states were optimised using the CASPT2(9,8)/cc-pVQZ method^39,40^ in the OpenMolcas program.^41^ The PES in Fig. 4 is constructed by linear interpolation of the *xyz* coordinates from the optimised X̃^2^Π geometry (interpolated coordinate = 0) to the optimised Ã^2^Σ^+^ geometry (interpolated coordinate = 1). The electronic energies in Fig. 4 are calculated using the SOC+MRCI+Q(9,8)/cc-pVQZ method^40,42^ in the ORCA/5.0.2 program.^43^

## Results and discussion

### Angular momenta and splitting

**Fig. 2:**
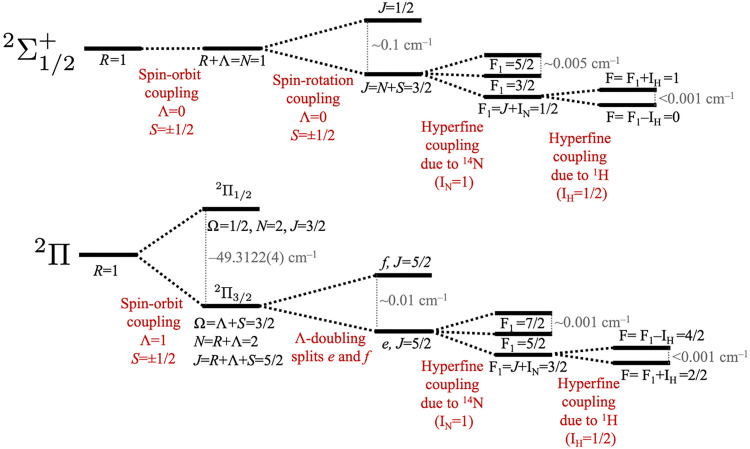
Summary of relevant splittings for HCN^+^.

HCN^+^ has a relatively complex electronic structure because it has several angular momenta that couple differently in the X̃ and Ã states. These include the rotation of the nuclear framework *R*, electronic orbital angular momentum Λ, electron spin *S*, nuclear spin of the nitrogen *I*_N_ and nuclear spin of the hydrogen *I*_H_. We also refer to the total angular momentum exclusive of electron and nuclear spin *N*, the total angular momentum exclusive of nuclear spin *J*, the total angular momentum exclusive of the hydrogen nuclear spin *F*_1_ and the total angular momentum *F*. The splittings in the ground X̃ and excited Ã states are illustrated in Fig. 2 for a level with one quantum of nuclear rotation (*R* = 1) and will be discussed with reference to the experimental spectrum.

The couplings between these angular momenta were described in detail in parts 1 and 2 of this series for the X̃ state,^4,5^ and the couplings of a Σ symmetry vibrationally excited state were also described in part 1 of this series.^4^ Nevertheless, it will be necessary to describe how these couplings split rovibrational levels in order to understand the experimental spectra reported here.

### Leak-out spectroscopy

**Fig. 3:**
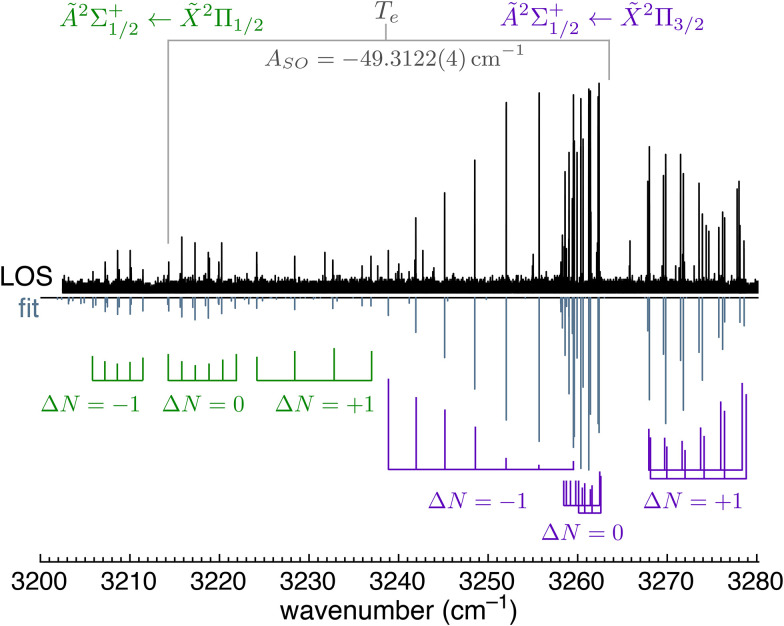
Electronic spectrum of the HCN^+^ Ã^2^Σ^+^ ← X̃^2^Π origin transition (black trace) measured using LOS at 27 K compared with a simulation (grey sticks) based on a fit using the PGOPHER program.^44^

The experimental spectrum of the Ã^2^Σ^+^ ← X̃^2^Π vibronic origin transition measured using leak-out spectroscopy (LOS) is shown in Fig. 3 (black trace). This spectrum was measured at 27 K. Spin–orbit coupling (SOC) splits the X̃^2^Π state into two components—X̃^2^Π_3/2_ and X̃^2^Π_1/2_—which are distinguished by the quantum number of the total electron angular momentum Ω = |Λ + Σ| = |1 ± 1/2|, where Λ and Σ are the quantum numbers for the orbital and spin angular momenta associated with *L̂*_*z*_ and *Ŝ*_*z*_, respectively. The Ω = 3/2 component is lower in energy because the …1π^3^ configuration is more than half-filled (Fig. 1). The experimental spectrum exhibits both the Ã^2^Σ_1/2_^+^ ← X̃^2^Π_3/2_ and Ã^2^Σ_1/2_^+^ ← X̃^2^Π_1/2_ components, with the Ã^2^Σ_1/2_^+^ ← X̃^2^Π_3/2_ transition being more intense. The Ã^2^Σ_1/2_^+^ ← X̃^2^Π_3/2_ transition is recognisable as a Ω = 1/2 ← 3/2 type transition because there are no transitions involving *J*″ = 1/2, but there are transitions involving *J*′ = 1/2.

Coupling of the angular momenta of HCN^+^ split the rovibronic levels of the ground and excited state, as summarised in Fig. 2. For the X̃^2^Π state, the largest splitting arises from spin orbit coupling, giving rise to X̃^2^Π_1/2_ and X̃^2^Π_3/2_ components with a spin–orbit constant of *A*_SO_ = −49.3122(4) cm^−1^ (indicated in Fig. 3). This value is reasonably close to previous MRCI calculations (−49.9 cm^−1^)^11^ and ZEKE measurements (−49.8 cm^−1^)^6^ and −49.3113(3) cm^−1^ calculated from the rovibrational spectra reported in part I of this series.^4^ Each *J* rotational level in the X̃^2^Π state is split by Λ-doubling into e and f components. The Ã^2^Σ^+^ state does not split by spin orbit coupling because it has Λ = 0. However, the spin (*S* = 1/2) couples with nuclear rotation to split each *N* rotational level into two components with *J* = |*N* ± *S*|. Additional splitting from hyperfine coupling will be discussed later.

These couplings between nuclear rotation and electronic angular momenta give rise to a level structure that is shown in the SI, which also illustrates the various possible branches that are observed in the rovibronic spectrum. The selection rules are Δ*J* = 0, ±1, with *e* ↔ *f* for Δ*J* = 0 or *e* ↔ *e* and *f* ↔ *f* for Δ*J* = ±1. This means that there are six branches expected for the Ã^2^Σ_1/2_^+^ ← X̃^2^Π_3/2_ transition and six branches for the Ã^2^Σ_1/2_^+^ ← X̃^2^Π_1/2_ transition, which we label by Δ*N* (in lower case) and Δ*J* (in upper case). Eleven of these twelve branches were observed, with the exception of the weak oP branch (Δ*N* = −2 and Δ*J* = −1) of the Ã^2^Σ_1/2_^+^ ← X̃^2^Π_1/2_ transition. The observed branches were included in a global fit with a nearby HCN^+^ combination band of Π symmetry that has not been reported before, with the rovibrational *ν*_1_ spectrum reported in ref. 4, the rovibrational spectrum of a combination band with Σ symmetry reported in ref. 4, and the rotational spectra reported in ref. 5. We refer to this fit as “fit 1”, which does not include hyperfine coupling. The observed rovibronic and rovibrational lines of HCN^+^ are captured well by fit 1, with average residuals lower than the wavemeter resolution (0.001 cm^−1^). Additional lines are observed that arise from isobaric C_2_H_3_^+^.

The experimental spectrum in Fig. 3 is compared to a fit produced using the PGOPHER program^44^ using the Hamiltonian:1
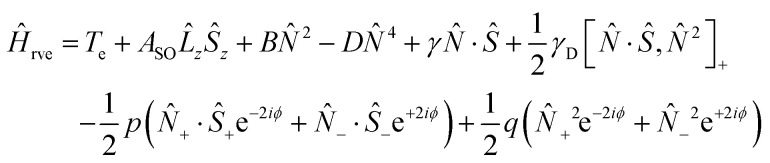
where *N* is the total angular momentum without nuclear spin or electron spin, *S* is the electron spin, *L* is the electron angular momentum, *T*_e_ is the term energy, *A*_SO_ is the spin–orbit coupling constant, *B* is the rotational constant, *D* is the centrifugal distortion constant, *γ* is the spin–rotation coupling constant, *γ*_D_ is the spin–rotation distortion constant, and *p* and *q* are the Λ doubling constants.

The spectroscopic constants from fit 1 are shown in Table 1. The *B* rotational constant is 3% larger in the Ã state (*B*′ = 1.39581(3) cm^−1^) than in the X̃ state (*B*′ = 1.35276(2) cm^−1^). This result is in good agreement with the *B* constants from sophisticated MRCI calculations that take into account vibronic coupling (*B*′ = 1.3974 cm^−1^ and *B*″ = 1.3533 cm^−1^).^11^ This agreement also indicates that the Ã state is mixed with the excited (*ν*_1_ + *ν*_2_)*μ* vibrational level of the X̃^2^Π ground state and that vibronic mixing beyond the Born–Oppenheimer approximation is particularly important for HCN^+^, as calculated in ref. 11. Because the rotational constant *B* is determined by the moment of inertia, which is determined by the molecular structure, these values validate the calculated result that the CN bond distance contracts significantly in the Ã state. Based on previous ZEKE experiments, it was ambiguous whether the CN bond length significantly contracts in the excited state (*B*′ = 1.37(1) cm^−1^ and *B*″ = 1.36(1) cm^−1^).^6^ This ambiguity between experiment and theory is now resolved by the higher resolution spectra presented here.

Spectroscopic constants of HCN^+^ states determined by a simultaneous fit of the Ã^2^Σ^+^ ← X̃^2^Π electronic spectrum, pure rotational transitions, rovibrational spectrum of the fundamental C–H stretch *ν*_1_, and the rovibrational spectra of two combination bands—*ν*_3_(σ) + 2*ν*_2_(π) and *ν*_1_(σ) + *ν*_2_(π). Fit 1 refers to this global fit. Fit 2 of the Ã state refers to a line fit of the 4 K spectrum, which includes hyperfine splitting. The symmetry of single quanta of the *ν*_1_(σ) and *ν*_2_(π) vibrations is indicated. All values are reported in cm^−1^. The hyperfine constants from fit 2 are shown in Table 2

**Table:** 

	X̃^2^Π					
	Fit 1^a^	Fit 2^a^	Rovib^b^	MRCI^c^	ZEKE^d^	Rot^e^
*B*	1.35276(1)	1.35278(2)	1.35275(1)	1.3533	1.36(1)	1.3527672(2)
*A*	−49.3120(3)	−49.3122(4)	−49.3113(3)	−49.9	−49.8(2)	
*A*_D_	−0.00145(4)	−0.00146(3)	−0.00144(1)			
*p*	0.0245(1)	0.0250(1)	0.02440(6)			
*q*	−0.00201(1)	−0.001995(6)	−0.002001(6)			−0.0020026(3)
*D* (× 10^−6^)	3.3(2)	3.1(4)	3.3(1)			3.28(3)

a Values obtained in this work.

b Values taken from Ref. 4.

c Values taken from Ref. 11.

d Values taken from Ref. 6.

e Values taken from Ref. 5.

**Table:** 

	Ã^2^Σ^+^				*ν*_3_(σ) + 2*ν*_2_(π)	
	Fit 1^a^	Fit 2^a^	MRCI^c^	ZEKE^d^	Fit 1^a^	MRCI^c^
*T*_e_	3238.8216(2)	3238.8224(2)	3233.1	3231	3272.955(3)	3269.0
*B*	1.39581(3)	1.39568(3)	1.3974	1.37(1)	1.3610(1)	1.3562
*A*					−4.587(8)	−4.6
*γ*	−0.07202(2)	−0.07332(7)				
*p*					−0.89(2)	
*q*					0.0034(1)	
*D* (× 10^−6^)	27(1)	22.7(9)			1.0(1)	
*H* (× 10^−7^)		70(8)				
*γ*_D_	0.00012(1)	0.000187(3)

**Table:** 

	*ν*_1_(σ)			*ν*_1_(σ) + *ν*_2_(π)		
	Fit 1^a^	Rovib^b^	MRCI^c^	Fit 1^a^	Rovib^b^	MRCI^c^
*T*_e_	3056.3413(2)	3056.3412(1)	3071.9	3340.8480(2)	3340.8480(2)	3352.0
*B*	1.34340(1)	1.343387(9)	1.3445	1.36993(2)	1.36993(1)	1.3710
*A*	−48.5992(3)	−48.5987(3)	−49.1			
*A*_D_	−0.00138(1)	−0.001364(6)				
*γ*			−0.07294(9)	−0.00463(5)	−0.00457(3)	
*p*	0.0287(1)	0.02862(6)				
*q*	−0.00236(1)	−0.002355(7)				
*D* (× 10^−6^)	3.0(1)	2.94(7)		2.8(4)	2.8(2)	

**Fig. 4:**
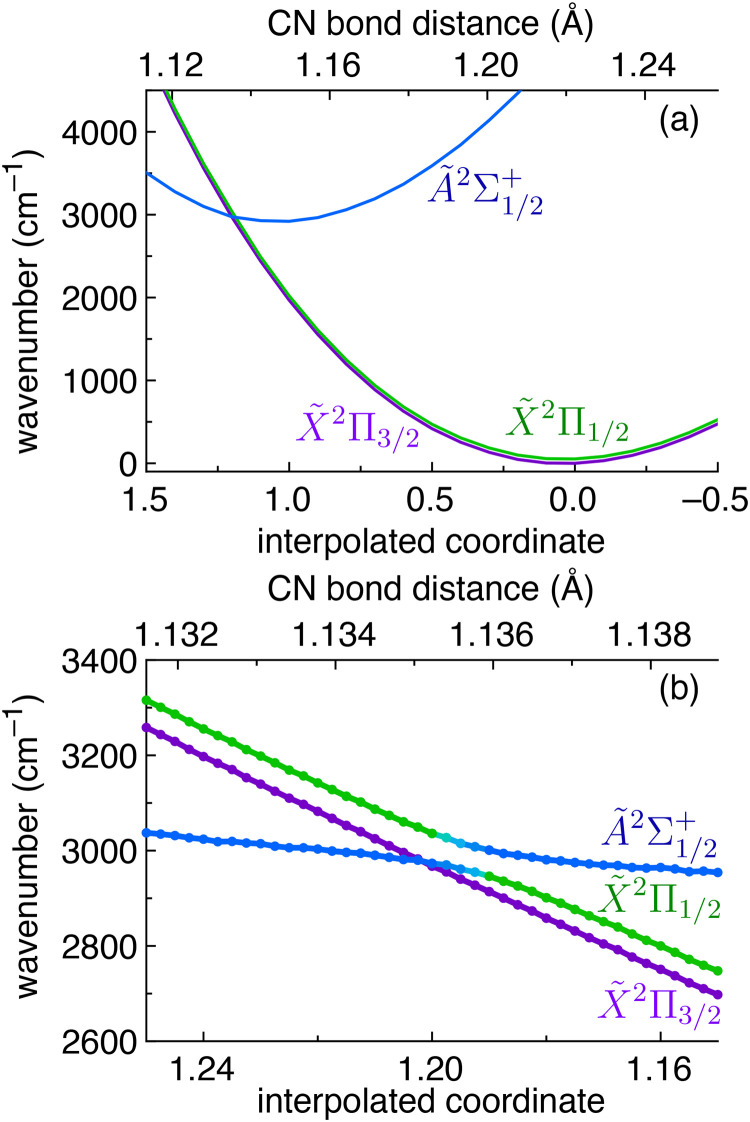
(a) Potential energy surface linearly interpolating between the X̃^2^Π and Ã^2^Σ^+^ optimised geometries. An enhanced view of the avoided crossing between the Ã^2^Σ_1/2_^+^ state and the X̃^2^Π_1/2_ component is shown in (b).

To illustrate the electronic states and their geometry changes investigated in this work, a potential energy surface (PES) for HCN^+^ is shown in Fig. 4. The PES follows the X̃^2^Π_3/2_ (purple curve) and X̃^2^Π_1/2_ (green curve) spin–orbit components of the ground electronic state and the Ã^2^Σ^+^ excited state (blue curve). The *x* axis in Fig. 4 shows the optimised X̃ state geometry at *x* = 0 and the optimised Ã state geometry at *x* = 1. Other *x* axis values represent interpolation (or extrapolation) between the *xyz* coordinates of the X̃ and Ã optimised geometries. Both states have linear *C*_∞v_ optimised geometries, and the CH bond length is similar in the X̃ ground state (CH_X̃_ = 1.087 Å) and the Ã excited state (CH_Ã_ = 1.086 Å). The calculated CN bond length is much larger in the X̃ ground state (CN_X̃_ = 1.222 Å) than in the Ã excited state (CN_Ã_ = 1.150 Å). Based on our calculations, the Ã^2^Σ^+^ ← X̃^2^Π origin transition can be expected to be weak because of a low oscillator strength (*f* = 0.0004 as calculated using MRCI+Q) and unfavorable Franck–Condon factors due to the change in the CN bond distance.

There is an avoided crossing between the Ã^2^Σ_1/2_^+^ state and the X̃^2^Π_1/2_ component close to the Ã^2^Σ_1/2_^+^ state minimum (see Fig. 4b), which involves the two states repelling each other and exchanging electronic character due to SOC (note that the selection rule for spin–orbit interactions between electronic states is ΔΩ = 0). Although the states do not cross, the lower energy Ω = 1/2 state has Ã^2^Σ_1/2_^+^ character at CN = 1.132 Å, the higher energy Ω = 1/2 state has Ã^2^Σ_1/2_^+^ character at CN = 1.138 Å, and the electronic character of both states is mixed at CN = 1.1355 Å as visualised by the changing colour in Fig. 4b. Although this avoided crossing is close to the minimum of the Ã^2^Σ_1/2_^+^ state, there are no apparent effects from this avoided crossing causing distortions of the experimental spectrum. It is possible that this is because the Ã^2^Σ^+^ ← X̃^2^Π origin transition we report here is actually below the energy of the avoided crossing. We also note that this crossing is avoided while the molecule is linear (as assumed in Fig. 4), but these states may cross if the bending motion is considered. Future studies could target transitions to vibrationally excited levels of the Ã state, which are probably more dramatically perturbed by this avoided crossing. Indeed, recent work by Jusko and coworkers investigates a transition to an excited vibrational level of the Ã electronic state.^45^

### Hyperfine splitting

**Fig. 5:**
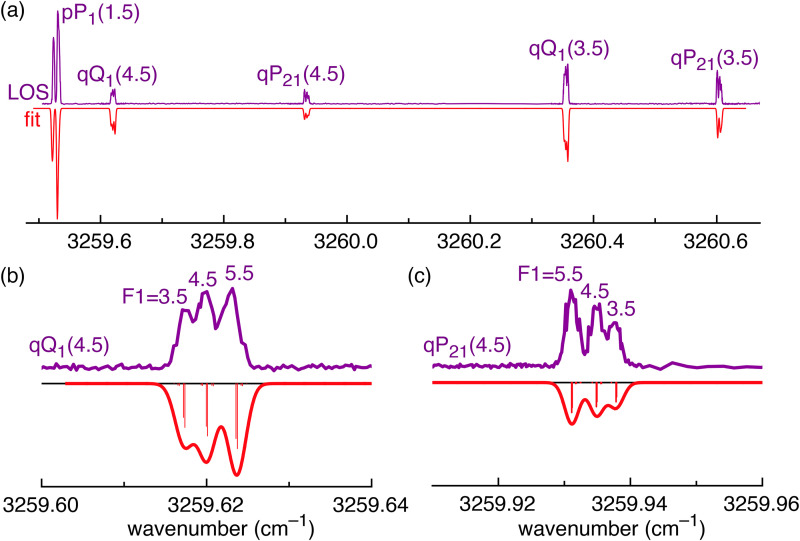
(a) Portion of the HCN^+^ Ã^2^Σ^+^ ← X̃^2^Π electronic spectrum (purple trace) measured using LOS at ≈9 K compared with a simulation (red trace) based on a contour fit using the PGOPHER program. Simulated rovibronic hyperfine transitions are shown as red sticks. The *P*, *Q*, and *R* branches are labelled with respect to Δ*N* (lower case) and Δ*J* (upper case). (b) and (c) A closer view of some of the lines shown in (a).

At low temperature (4 K and 9 K), the Doppler width of the lines is small enough that hyperfine splitting is observed (see Fig. 5). This splitting is on the order of 0.004 cm^−1^ and splits most lines into three observable components. To simulate the spectrum including hyperfine splitting, *Ĥ*_hfs_ was added to the Hamiltonian of eqn (1) as implemented in PGOPHER:^44^2
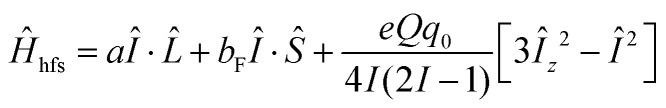
where *I* is the nuclear spin, *L* is the electronic orbital angular momentum, and *S* is the electron spin. The hyperfine constants quantify the splitting arising from coupling between the electron orbital angular momentum and the nuclear spin (*a*), the Fermi-contact parameter describing coupling between the electron spin and the nuclear spin (*b*_F_), and the nuclear quadrupole coupling constant (*eQq*_0_).^46^ The first and second terms in eqn (2) will appear once for the splitting arising from the nitrogen nuclear spin and once for the hydrogen nuclear spin. As illustrated in Fig. 2, the nitrogen (with nuclear spin *I*_N_ = 1) splits each *J* level into *F*_1_ = *J* + 1, *J*, and *J* − 1. Within the coupling scheme employed here, each of these *F*_1_ levels are split by the hydrogen (with nuclear spin *I*_H_ = 1/2) into *F* = *F*_1_ + 1/2 and *F* = *F*_1_ − 1/2. However, the hyperfine splitting from the hydrogen is too small to resolve transitions to different *F* levels in our spectrum. The hyperfine structure shown in Fig. 5 is dominated by transitions with Δ*F*_1_ = 0 and Δ*F* = 0.

The experimental electronic spectrum was then refit including hyperfine coupling in PGOPHER.^44^ This fit included lines from the pure rotational spectrum in ref. 5. This was done because fewer hyperfine constants were required to understand the hyperfine splitting for the electronic spectrum in this work than for the pure rotational spectrum in ref. 5, and this provided a good constraint of the ground state constants. This resulted in a fit with an average residual of less than 0.001 cm^−1^ and is referred to here as “fit 2” in Table 2. The magnitude of the hyperfine splitting arises mostly because of the isotropic interaction between the nuclear spin of the N atom and the electron spin in the Ã state, which is quantified by the Fermi-contact parameter *b*_F_. This large change in *b*_F_—five times smaller in the X̃ state than the Ã state—reflects the dramatic change in the electronic structure from X̃ to Ã. Therefore, the surprising fact that hyperfine splitting is resolved in these infrared spectra is definitive evidence that this is an electronic transition.

**Table 2:** Hyperfine constants included in the line fit of a 4 K Ã^2^Σ^+^ ← X̃^2^Π_3/2_ spectrum (cm^−1^). When sufficiently resolved, lines from a 35 K spectrum of the Ã^2^Σ^+^ ← X̃^2^Π_1/2_ transition and high *J* lines of the Ã^2^Σ^+^ ← X̃^2^Π_3/2_ transition were also included. Non-hyperfine constants from fit 2 are given in Table 1

	X̃^2^Π	X̃^2^Π	Ã^2^Σ^+^
	Fit 2	Ref. 5	Fit 2
*a*(N)	0.0013(2)	0.0013(1)	
*b*_F_(N)	0.0012(3)	0.0012(2)	0.0059(1)
*d*(N)		0.0019(1)	
*a*(H)	0.0013(4)	0.00013(3)	
*b*_F_(H)	−0.00192(7)	−0.0017(1)	0.0002(3)
*eQq*_0_	−0.00018(8)	−0.00019(4)	−0.0005(40)
*eQq*_2_		−0.0005(2)	

The Fermi contact parameter (*b*_F_ = 0.0059(1) cm^−1^) of the N atom in the Ã state provides a direct way of quantifying the electronic wavefunctions in the X̃ and Ã states. Comparing the *b*_F_ parameter in the Ã state of HCN^+^ with the *b*_orb_ of an isolated nitrogen (0.0514 cm^−1^)^12^ atom gives 
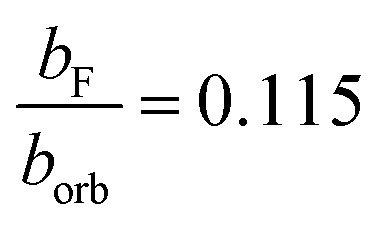
, which means that the Ã state has only 11.5 ± 0.1% s orbital character on the nitrogen. This is small compared to other similar nitrogen containing neutral molecules in ^2^Σ^+^ states.^47,48^ The relatively low s orbital character is because the singly occupied molecular orbital resembles mostly a p_*z*_ atomic orbital on the nitrogen atom (see Fig. 1).

Another possible effect that could be lowering the percentage of s orbital character in the ÃΣ_1/2_^+^ state could be mixing with the X̃Π_1/2_ state as a result of the avoided crossing in Fig. 4. This would result in the upper state having a linear combination of the Σ_1/2_^+^ and Π_1/2_ state configurations. Because the *b*_F_ is five times lower in the Π state (see Table 2), a greater mixing between the Π and Σ configurations will reduce the *b*_F_ value in the Ã state. However, this is quite speculative, and the subtle properties of avoided crossings are challenging to calculate.

We have also calculated the HCN^+^ nitrogen *b*_F_ parameter of the Ã state using the equation of motion coupled cluster method EOM-CCSD/cc-pVTZ in the Gaussian/16 program.^40,49–51^ This yielded an Ã state value of *b*_F_ = 0.014 cm^−1^, which is significantly larger than the experimental value (*b*_F_ = 0.0059(1) cm^−1^). This could be a further indication of the importance of vibronic coupling for HCN^+^ and suggests that the Ã state is not well described within the Born–Oppenheimer approximation.

There are very few polyatomic molecular ions for which the hyperfine structure is observed in excited electronic states. Serendipitously, one of these states is the Ã^2^Σ^+^ state of HCP^+^. Because phosphorus is directly below nitrogen in the periodic table, the Ã^2^Σ^+^ state of HCP^+^ are analogous to the Ã^2^Σ^+^ state of HCN^+^ investigated here. The Ã^2^Σ^+^ state of HCP^+^ have a Fermi contact parameter of *b*_F_ = 0.106(5) cm^−1^, which is over an order of magnitude larger than the value we measure for HCN^+^ (*b*_F_ = 0.0059(1) cm^−1^). Additionally, the HCP^+^ Ã^2^Σ^+^ state wavefunction has an s orbital character of 24%, which is significantly larger than that of HCN^+^ (11.5 ± 0.1%). This might reflect a larger mixing of the phosphorous p_*z*_ and s orbitals in the highest occupied molecular σ orbital of HCP^+^ or be the result of the HCN^+^ avoided crossing discussed above.

A contour fit of the experimental lines in Fig. 5 was attempted to capture some of the unresolved hyperfine structure from the hydrogen nucleus; however, no significant information about the hydrogen hyperfine splitting could be observed. Nevertheless, the contour fit was used to estimate the rotational and translational ion temperature. The rotational temperature was estimated to be 18 ± 5 K based on the relative intensities of the bands in Fig. 5, which arise from the differences in the population of the ground state *J* = 1.5, *J* = 3.5, and *J* = 4.5 levels. The fitted Gaussian width was 0.0020(5) cm^−1^, which corresponds to a translational temperature of 20 ± 5 K. As expected, these values are higher than the temperature of the trap itself (≈9 K) because of RF heating.^52^ Additionally, no Lorentzian lineshape was convoluted to account for lifetime broadening, which would further decrease the fitted Gaussian linewidth. The reason we did not include a Lorentzian in the fit is because there is considerable uncertainty in the splitting due to *I*_H_, which can lead to dubious results if one attempts to interpret subtle changes in the widths of these lines. It is clear from the narrow linewidths and the fact that the individual hyperfine lines have a similar width to the rovibrational lines that the excited state has a fairly long lifetime and, therefore, the low lying avoided crossing (Fig. 4) does not lead to fast internal conversion to the ground state. Assuming a lifetime broadening of 0.0025 cm^−1^ yields an extremely conservative lower limit for the excited state lifetime of 2 ns.

### Π[*ν*_3_(σ) + 2*ν*_2_(π)] combination band

**Fig. 6:**
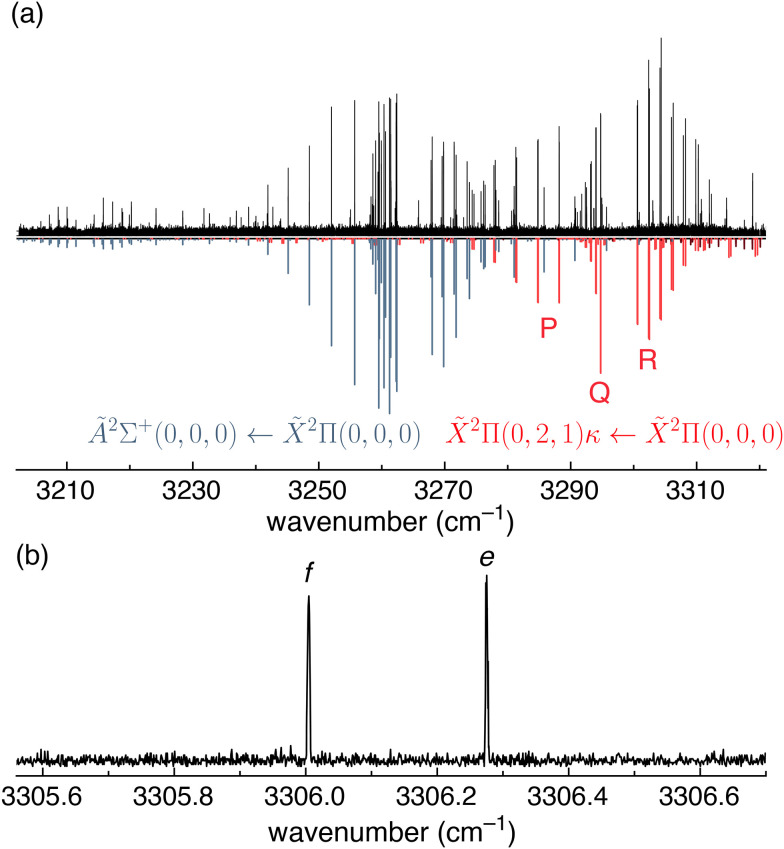
(a) Portion of the HCN^+^ Ã^2^Σ^+^ ← X̃^2^Π electronic spectrum and rovibrational spectrum exciting the *ν*_3_(σ) + 2*ν*_3_(π) combination band in the X̃ ground state (black trace) measured using LOS at ≈35 K. The experimental spectrum is compared with a simulation of the electronic transition (grey lines) and the vibronic transition (red lines) using the PGOPHER program. The vibrational levels are labelled by (*v*_1_,*v*_2_,*v*_3_), which are the vibrational quantum numbers of the CH stretch (*v*_1_), the HCN bend (*v*_2_), and the CN stretch (*v*_3_). The quantum number *κ* indicates that this band is the upper Renner–Teller component for this vibration (see the term diagram in Fig. 7). (b) Example of a Λ doublet in the *ν*_3_(σ) + 2*ν*_3_(π) combination band spectrum.

Although the focus of this article is the Ã ← X̃ transition of HCN^+^, we observed an additional vibrational transition that partially overlaps with the Ã ← X̃ band, which warrants further discussion. This vibrational transition is centered around 3295 cm^−1^ and was fit with the same Hamiltonian form as described in ref. 4 (red sticks in Fig. 6). The *P*, *Q*, and *R* branches are clearly apparent and feature Λ type doublets characteristic of a Π ← Π transition. We were unable to assign any lines associated with the Π_1/2_ ← Π_Ω_ components. Nevertheless, the *P*, *Q*, and *R* branches can only be reasonably fit to a Π ← Π transition—rather than a Δ ← Π or Σ ← Π transition—and so the upper state must have Π vibronic symmetry.

**Fig. 7:**
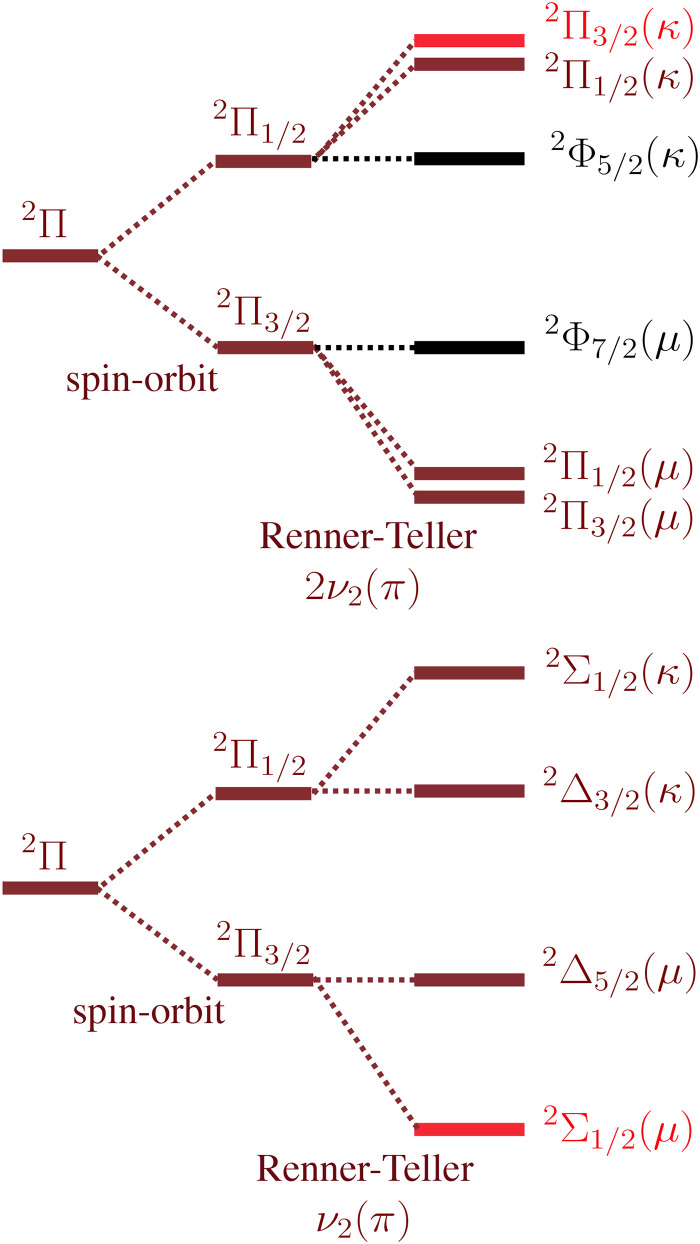
Schematic illustration of the Renner–Teller splitting for the X̃^2^Π state as a result of one (bottom) or two (top) quanta of the HCN^+^ bending mode *ν*_2_(π). The term symbols on the right hand side are labelled with quantum numbers by ^2*S*+1^*K*_P_. The levels observed in this study are highlighted in light red. Excitation from the ground state to levels with Φ symmetry is forbidden, and these states are shown in black.

The vibronic symmetry of HCN^+^ energy levels requires consideration of the Renner–Teller effect. Fig. 7 illustrates the states generated by Renner–Teller coupling in HCN^+^. The vibrational angular momentum from the *ν*_2_(π) bending mode (with quantum number *l* = *v*_2_, *v*_2_ − 2, …0 or 1) couples with the total electronic angular momentum (with quantum number Ω) to form the resulting vibronic angular momentum *P* = |±Ω ± *l*|. The quantum number *K* = |±Λ ± *l*| results from coupling between vibrational and orbital angular momenta. The vibronic term symbols are then ^2*S*+1^*K*_P_. One quantum of the *ν*_2_(π) mode (*l* = 1) couples with the orbital angular momentum (Λ = 1) to generate two Σ and two Δ vibronic states. Two quanta of the *ν*_2_(π) mode (*l* = 2 and 0) couples with the orbital angular momentum Λ = 1 to generate four Π and two Φ vibronic states. The upper components are labeled *κ* and the lower components are labeled *μ*.

The extracted spectroscopic constants from our fit (Table 1) agree well with the calculated values from ref. 11 for the upper (*κ*, Π_3/2_) Renner–Teller component of the *ν*_3_(σ) + 2*ν*_2_(π) combination band, including the surprisingly low spin–orbit coupling constant (*A*_SO_ = −4.91(1) cm^−1^). However, the *A*_SO_ value here only provides an effective fit, because the splitting of the Π_*p*=1/2_ and Π_*p*=3/2_ vibronic components will need to be treated different from the splitting of Π_Ω=1/2_ and Π_Ω=3/2_ spin–orbit components. This is consistent with the fact that transitions to Π_*p*=1/2_ levels were not observed. Considering the good agreement with calculations, we assign this band as excitation of the upper *κ*, Π Renner–Teller component of the *ν*_3_(σ) + 2*ν*_2_(π) combination band (Π(0, 2, 1)*κ*) as listed—but not analysed—in part I of this series of publications.^4^

The fact that this combination band is *ν*_3_(σ) + 2*ν*_2_(π) with Π symmetry validates the previous assignment in ref. 4 that a band with Σ vibronic symmetry corresponds to the lower (*μ*, Σ) Renner–Teller component of a *ν*_1_(σ) + *ν*_2_(π) combination band. If the Σ symmetry *ν*_3_(σ) + *ν*_2_(π) combination band from ref. 4 arose from the upper (*κ*) Renner–Teller component, then one would expect to observe Δ ← Π transitions slightly lower in energy. Because no such transitions are observed, the Σ symmetry *ν*_3_(σ) + *ν*_2_(π) combination band observed in ref. 4 can be assigned to the lower (*μ*) Renner–Teller component.

A thorough analysis of the Renner–Teller coupling of HCN^+^ is not within the scope of this work. Nevertheless, our results provide a promising indication that the calculations in ref. 11 are accurately capturing the strong Renner–Teller coupling of HCN^+^.

The Π(0, 2, 1)*κ* band reported here is the first experimental spectrum of a *κ* Renner–Teller component of HCN^+^. The Π(0, 2, 1)*μ* component has been measured at 2523 cm^−1^ using ZEKE spectroscopy in ref. 6. These experimental values enable a preliminary estimate of Renner–Teller parameters of HCN^+^ based on only experimental values. We employed the equations describing a Renner–Teller and spin–orbit active system given in ref. 53, the fundamental vibrational frequency of the CN stretching mode (0, 0, 1) measured at 1785 cm^−1^ by Wiedmann *et al.*, and the spin–orbit constant for HCN^+^ determined with our high-resolution spectra (*A*_SO_ = −49.3113 cm^−1^). From these results, we estimate an effective Renner–Teller vibrational constant of *ω* = 580 cm^−1^ and a splitting parameter of *ε* = 0.45. However, because of anharmonicity, these values differ from those required to fit the fundamental bending mode *μ* (292 cm^−1^) and *κ* components (821 cm^−1^).^6,11^

Future high-resolution experimental studies could provide a detailed characterisation of the Renner–Teller coupling of HCN^+^ by combining our results with measurements of the Δ(1, 1, 0) components and the Σ(1, 1, 0)*κ* component (calculated to occur at 3881 cm^−1^) as well as the Π(0, 2, 1)*μ* combination band component (measured at 2523 cm^−1^ using ZEKE).^6^ The Π(0, 2, 1)*κ* component in Fig. 6 is the first measured *κ* Renner–Teller component for HCN^+^ and serves as a valuable experimental reference point for analysing the vibronic coupling of HCN^+^.

## Conclusions and outlook

In summary, we have measured high resolution electronic spectra of the infrared Ã^2^Σ^+^ ← X̃^2^Π electronic transition origin band of HCN^+^, which resolved the rotational, fine, and hyperfine structure. Although both states have been observed before, the transition between these states has never been reported, and the spectra presented here have a significantly higher resolution. Furthermore, hyperfine splitting has not been observed before and provides a lens with which the electronic wavefunction can be interrogated. In this case, we find that the Ã state electronic wavefunction has 11.5(2)% s orbital character on the N atom. The fact that we can observe hyperfine splitting in the infrared is unusual and makes it clear that the observed spectrum corresponds to an electronic transition.

The quality of these spectroscopic constants easily affords accurate simulations of the rovibronic Ã → X̃ emission of HCN^+^ for comparison with astronomical measurements such as those possible with the James Webb Space Telescope.^54,55^ However, the fact that the electronic transition appears to have comparable intensity to vibrational excitation of a nearby *ν*_3_(σ) + 2*ν*_2_(π) combination band indicates that the electronic transition is fairly weak and will probably be challenging to detect astronomically.

We have recently reported electronic spectra measured using LOS with a low resolution white light fibre laser.^34^ The electronic spectrum of HCN^+^ presented here illustrate that electronic transitions can be measured using LOS with a very high resolution for a mass-selected, highly reactive bare ion that is excited well below the dissociation threshold.

## Conflicts of interest

There are no conflicts to declare.

## Supplementary Material

CP-028-D5CP04255K-s001

## Data Availability

The datasets generated and/or analysed during the current study are available from the corresponding authors upon reasonable request. All data supporting the findings of this study are available within the manuscript and its supplementary information (SI). Supplementary information: rovibrational scheme showing allowed rovibronic transitions and branches, the list of assigned rovibronic lines including hyperfine splitting, the list of assigned rovibronic lines from global fit without hyperfine splitting, and raw leak-out spectroscopy data for the electronic transition. See DOI: https://doi.org/10.1039/d5cp04255k.

## References

[cit1] Petrie S., Freeman C. G., Meot-Ner M., McEwan M. J., Ferguson E. E. (1990). Experimental study of HCN^+^ and HNC^+^ ion chemistry. J. Am. Chem. Soc..

[cit2] Dohnal P., Jusko P., Jiménez-Redondo M., Caselli P. (2023). Measurements of rate coefficients of CN^+^, HCN^+^, and HNC^+^ collisions with H_2_ at cryogenic temperatures. J. Chem. Phys..

[cit3] Forney D., Thompson W. E., Jacox M. E. (1992). The vibrational spectra of molecular ions isolated in solid neon. IX. HCN^+^, HNC^+^, and CN^−^. J. Chem. Phys..

[cit4] Schmid P. C., Marlton S. J. P., Silva W. G. D. P., Salomon T., Asvany O., Schlemmer S. (2026). High-resolution spectroscopy of [H,C,N]^+^: I. Rotationally resolved vibrational bands of HCN^+^ and HNC^+^. Phys. Chem. Chem. Phys..

[cit5] Silva W. G. D. P., Schmid P. C., Gupta D., Thorwirth S., Asvany O., Schlemmer S. (2026). High-resolution spectroscopy of [H,C,N]^+^: II. Ground state rotational spectrum of HCN^+^ (X̃^2^Π). Phys. Chem. Chem. Phys..

[cit6] Wiedmann R. T., White M. G. (1995). Vibronic coupling in the X̃^2^Π and Ã^2^Σ^+^ states of HCN^+^. J. Chem. Phys..

[cit7] Lorquet A., Lorquet J.-C., Delwiche J., Hubin-Franskin M.-J. (1982). Intramolecular dynamics by photoelectron spectroscopy. I. Application to N^+^_2_, HBr^+^, and HCN^+^. J. Chem. Phys..

[cit8] Fridh C., Åsbrink L. (1975). Photoelectron and electron impact spectrum of HCN. J. Electron Spectrosc. Relat. Phenom..

[cit9] Hollas J., Sutherley T. (1972). Geometry of C_2_N_2_^+^ and HCN^+^ from low-energy photoelectron spectroscopy. Mol. Phys..

[cit10] Eland J., Field T., Baltzer P., Hirst D. (1998). Photoelectron spectra, electronic structure, coincidence spectra and dissociation mechanisms of the hydrogen cyanide cation. Chem. Phys..

[cit11] Tarroni R., Mitrushenkov A., Palmieri P., Carter S. (2001). Energy levels of HCN^+^ and DCN^+^ in the vibronically coupled X^2^Π and A^2^Σ^+^ states. J. Chem. Phys..

[cit12] Fitzpatrick J. A., Manby F. R., Western C. M. (2005). The interpretation of molecular magnetic hyperfine interactions. J. Chem. Phys..

[cit13] Nötzold M., Wild R., Lochmann C., Wester R. (2022). Spectroscopy and ion thermometry of C 2- using laser-cooling transitions. Phys. Rev. A.

[cit14] Dabrowski I., Herzberg G. (1978). The spectrum of HeNe^+^. J. Mol. Spectrosc..

[cit15] Carrington A., Milverton D. R., Sarre P. J. (1978). Electronic absorption spectrum of CO^+^ in an ion beam: Analysis of the carbon nuclear hyperfine structure in ^13^CO^+^. Mol. Phys..

[cit16] Sarre P. J., Walmsley J. M., Whitham C. J. (1986). High-resolution laser photofragment spectroscopy of CH^+^. J. Chem. Soc., Faraday Trans. 2.

[cit17] Sarre P., Walmsley J., Whitham C. (1988). Laser photofragment spectroscopy of near-threshold resonances in SiH^+^. Philos. Trans. R. Soc., A.

[cit18] Edwards C., Maclean C., Sarre P. (1984). Laser predissociation spectrum of SH^+^ (A^3^Π–X^3^Σ^−^) Analysis of proton hyperfine structure. Mol. Phys..

[cit19] Edwards C., Jackson P., Sarre P., Milton D. (1986). Laser photofragment and emission spectroscopy of the A^2^Δ–X^2^Π system of PH^+^. Mol. Phys..

[cit20] Cosby P., Helm H., Larzilliere M. (1991). Photofragment spectroscopy of HF^+^. J. Chem. Phys..

[cit21] Boudjarane K., Lacoursière J., Larzillière M. (1994). Hyperfine structure of first negative system (B^2^Σ_u_^+^–X^2^Σ_g_^+^) of ^14^N_2_^+^ and ^15^N_2_^+^ from laser-induced fluorescence measurements. J. Chem. Phys..

[cit22] Larzillière M., Abed S., Carré M., Gaillard M., Lermé J., Broyer M. (1985). l-Type doubling hyperfine structure of N_2_O^+^. Chem. Phys. Lett..

[cit23] Sunahori F. X., Zhang X., Clouthier D. J. (2007). Electronic spectroscopy of jet-cooled HCP^+^: Molecular structure, phosphorus hyperfine structure, and Renner-Teller analysis. J. Chem. Phys..

[cit24] Hübers H.-W., Evenson K. M., Hill C., Brown J. M. (2009). The rotational spectrum of the NH^+^ radical in its X^2^Π and a^4^Σ^−^ states. J. Chem. Phys..

[cit25] Pino G. A., Feraud G., Broquier M., Grégoire G., Soorkia S., Dedonder C., Jouvet C. (2016). Non-radiative processes in protonated diazines, pyrimidine bases and an aromatic azine. Phys. Chem. Chem. Phys..

[cit26] Bomse D., Woodin R., Beauchamp J. (1979). Molecular activation with low-intensity CW infrared laser radiation. Multiphoton dissociation of ions derived from diethyl ether. J. Am. Chem. Soc..

[cit27] Zhao Y., de Beer E., Xu C., Taylor T., Neumark D. M. (1996). Spectroscopy and electron detachment dynamics of C_4_^−^, C_6_^−^, and C_8_^−^. J. Chem. Phys..

[cit28] Salomon T., Brackertz S., Asvany O., Savić I., Gerlich D., Harding M. E., Lipparini F., Gauss J., van der Avoird A., Schlemmer S. (2022). The He-H_3_^+^ complex. II. Infrared predissociation spectrum and energy term diagram. J. Chem. Phys..

[cit29] Marlton S. J., Buntine J. T., Watkins P., Liu C., Jacovella U., Carrascosa E., Bull J. N., Bieske E. J. (2023). Probing colossal carbon rings. J. Phys. Chem. A.

[cit30] Douglas-Walker T. E., Campbell E. K., Daly F. C., Douin S., Gans B., Jacovella U., Maurice C., Odant R., Palotas J. (2024). Ion Spectroscopy in the Context of the Diffuse Interstellar Bands: A Case Study with the Phenylacetylene Cation. ACS Earth Space Chem..

[cit31] Schlemmer S., Kuhn T., Lescop E., Gerlich D. (1999). Laser excited N_2_^+^ in a 22-pole ion trap:: Experimental studies of rotational relaxation processes. Int. J. Mass Spectrom..

[cit32] Chakrabarty S., Holz M., Campbell E. K., Banerjee A., Gerlich D., Maier J. P. (2013). A novel method to measure electronic spectra of cold molecular ions. J. Phys. Chem. Lett..

[cit33] Schmid P. C., Asvany O., Salomon T., Thorwirth S., Schlemmer S. (2022). Leak-out spectroscopy, a universal method of action spectroscopy in cold ion traps. J. Phys. Chem. A.

[cit34] Marlton S. J., Schmid P. C., Salomon T., Asvany O., Schlemmer S. (2025). Measuring Electronic Transitions Using Leak-Out Spectroscopy. J. Phys. Chem. Lett..

[cit35] Schlemmer S., Lescop E., von Richthofen J., Gerlich D., Smith M. A. (2002). Laser induced reactions in a 22-pole ion trap: C_2_H_2_^+^ + *hν* 3+ H_2_ → C_2_H_3_^+^ + H. J. Chem. Phys..

[cit36] Asvany O., Bielau F., Moratschke D., Krause J., Schlemmer S. (2010). Note: New design of a cryogenic linear radio frequency multipole trap. Rev. Sci. Instrum..

[cit37] Daly N. (1960). Scintillation type mass spectrometer ion detector. Rev. Sci. Instrum..

[cit38] Asvany O., Brünken S., Kluge L., Schlemmer S. (2014). COLTRAP: a 22-pole ion trapping machine for spectroscopy at 4 K. Appl. Phys. B:Lasers Opt..

[cit39] Andersson K., Malmqvist P. A., Roos B. O., Sadlej A. J., Wolinski K. (1990). Second-order perturbation theory with a CASSCF reference function. J. Phys. Chem..

[cit40] Dunning Jr T. H. (1989). Gaussian basis sets for use in correlated molecular calculations. I. The atoms boron through neon and hydrogen. J. Chem. Phys..

[cit41] Fdez Galvan I., Vacher M., Alavi A., Angeli C., Aquilante F., Autschbach J., Bao J. J., Bokarev S. I., Bogdanov N. A., Carlson R. K. (2019). *et al.*, OpenMolcas: From source code to insight. J. Chem. Theory Comput..

[cit42] Knowles P. J., Werner H.-J. (1988). An efficient method for the evaluation of coupling coefficients in configuration interaction calculations. Chem. Phys. Lett..

[cit43] Neese F. (2022). Software update: The ORCA program system–Version 5.0. Wiley Interdiscip. Rev.:Comput. Mol. Sci..

[cit44] Western C. M. (2017). PGOPHER: A program for simulating rotational, vibrational and electronic spectra. J. Quant. Spectrosc. Radiat. Transfer.

[cit45] Jiménez-Redondo M. ; Schleif C.; Palotás J.; Sarka J.; Bunn H.; Dohnal P.; Paola C. and Jusko P., High Resolution Overtone Spectroscopy of HCN^+^ and HNC^+^. Submitted, 2025 10.1016/j.saa.2025.127359 41412043

[cit46] Frosch R. A., Foley H. (1952). Magnetic hyperfine structure in diatomic molecules. Phys. Rev..

[cit47] Suzuki T., Saito S., Hirota E. (1986). Hyperfine coupling constants of NCO in Ã^2^Σ^+^ by sub-Doppler spectroscopy. J. Mol. Spectrosc..

[cit48] Radford H. (1964). Hyperfine Structure of the B^2^Σ^+^ State of CN. Phys. Rev..

[cit49] Frisch M. J. ; Trucks G. W.; Schlegel H. B.; Scuseria G. E.; Robb M. A.; Cheeseman J. R.; Scalmani G.; Barone V.; Petersson G. A.; Nakatsuji H.; et al. Gaussian 16 Revision C.01., 2016, Gaussian Inc., Wallingford CT, 2009

[cit50] Koch H., Jørgensen P. (1990). Coupled cluster response functions. J. Chem. Phys..

[cit51] Stanton J. F., Bartlett R. J. (1993). The equation of motion coupled-cluster method. A systematic biorthogonal approach to molecular excitation energies, transition probabilities, and excited state properties. J. Chem. Phys..

[cit52] Asvany O., Schlemmer S. (2009). Numerical simulations of kinetic ion temperature in a cryogenic linear multipole trap. Int. J. Mass Spectrom..

[cit53] Herzberg G. , Molecular Spectra and molecular structure-Vol III, Read Books Ltd, 2013, vol. 1

[cit54] Changala P. B., Chen N. L., Le H. L., Gans B., Steenbakkers K., Salomon T., Bonah L., Schroetter I., Canin A., Martin-Drumel M.-A. (2023). *et al.*, Astronomical CH_3_^+^ rovibrational assignments-A combined theoretical and experimental study validating observational findings in the d203-506 UV-irradiated protoplanetary disk. Astron. Astrophys..

[cit55] Berné O., Martin-Drumel M.-A., Schroetter I., Goicoechea J. R., Jacovella U., Gans B., Dartois E., Coudert L. H., Bergin E., Alarcon F. (2023). *et al.*, Formation of the methyl cation by photochemistry in a protoplanetary disk. Nature.

