# Supplementary material for: High-resolution spectroscopy of [H,C,N]+: III. Infrared Ã2Σ+ ← X̃2Π electronic transition of HCN+
Source: Phys Chem Chem Phys. 2025 Dec 22;28(7):4412–21. doi: 10.1039/d5cp04255k (PMC12885111; doi:10.1039/d5cp04255k)
Supplement: CP-028-D5CP04255K-s001 [file CP-028-D5CP04255K-s001.pdf]

Supplementary Information (SI) for Physical Chemistry Chemical Physics.
This journal is © the Owner Societies 2025

Supporting information for

# High-resolution spectroscopy of $[H,C,N]^+$: III. Infrared $A^2\Sigma^+ \leftarrow X\ ^2\Pi$ electronic transition of $HCN^+$

Samuel J. P. Marlton*, Philipp C. Schmid, Weslley G. D. P. Silva, Oskar Asvany, and Stephan Schlemmer*

I. Physikalisches Institut, Universität zu Köln, Z¨ülpicher Str. 77, 50937, Cologne, Germany.
E-mail: marlton@ph1.uni-koeln.de
E-mail: schlemmer@ph1.uni-koeln.de

1) Rovibrational Scheme ....... 2
2) Assigned rovibronic lines with hyperfine ....... 3
3) Assigned rovibronic lines without hyperfine from global fit ....... 4
4) Leak-out raw data ....... 6

# 1) Rovibrational Scheme

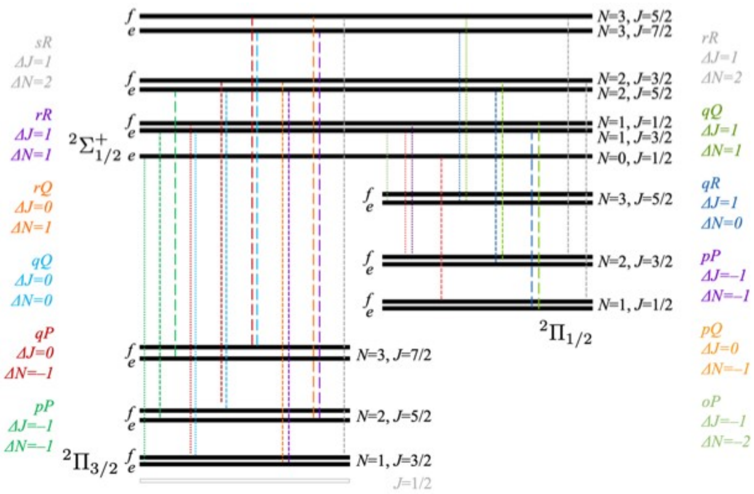


Figure S1: Rovibronic transition scheme illustrating the important transition selection rules, transition types, and branches for the A←X electronic transition of $HCN^+$.

# 2) Assigned rovibronic lines with hyperfine

```
molecule-name upper J' S' # lower J" S" # position stdev strength fit_width branch vibronic-level-upper(F1’,F’,F1”,F”) J' N' F1/F2-e/f F1’ F' J" N" F1/F2-e/f F1” F" measurement-type
LinearMolecule Excited 0 0 4 Ground 1 1 2 3259.523863 1 0.000895251615409448 0.0000 : pP1(1.5)0.5,0,1.5,1 : Excited Sigma 0.5 0 F1e 0.5 0 - Ground v=0 1.5 1 F1e 1.5 1 : LIRTRAP_35K_electronic
LinearMolecule Excited 0 1 5 Ground 1 0 2 3262.393906 1 0.00108428834705906 0.0000 : qP21(1.5)0.5,0,1.5,1 : Excited Sigma 0.5 1 F2f 0.5 0 - Ground v=0 1.5 1 F1f 1.5 1 : LIRTRAP_9K_electronic
LinearMolecule Excited 1 0 9 Ground 0 1 1 3259.523708 1 0.000822667064222177 0.0000 : pP1(1.5)0.5,1,0.5,0 : Excited Sigma 0.5 0 F1e 0.5 1 - Ground v=0 1.5 1 F1e 0.5 0 : LIRTRAP_9K_electronic
LinearMolecule Excited 1 0 9 Ground 1 1 1 3259.523708 1 0.0016154793325286 0.0000 : pP1(1.5)0.5,1,0.5,1 : Excited Sigma 0.5 0 F1e 0.5 1 - Ground v=0 1.5 1 F1e 0.5 1 : LIRTRAP_9K_electronic
LinearMolecule Excited 1 0 9 Ground 2 1 1 3259.523863 1 0.00248240841997698 0.0000 : pP1(1.5)0.5,1,1.5,2 : Excited Sigma 0.5 0 F1e 0.5 1 - Ground v=0 1.5 1 F1e 1.5 2 : LIRTRAP_35K_electronic
LinearMolecule Excited 1 0 11 Ground 2 1 5 3252.022522 1 0.0011518715663512 0.0000 : pP1(3.5)1.5,1,2.5,2 : Excited Sigma 2.5 2 F1e 1.5 1 - Ground v=0 3.5 3 F1e 2.5 2 : COLTRAP_4K_electronic
LinearMolecule Excited 1 0 12 Ground 2 1 4 3261.422739 1 0.00162519669223416 0.0000 : qP21(2.5)1.5,1,2.5,2 : Excited Sigma 1.5 2 F2f 1.5 1 - Ground v=0 2.5 2 F1f 2.5 2 : LIRTRAP_9K_electronic
LinearMolecule Excited 1 1 9 Ground 2 0 3 3255.69563 1 0.00183883738468658 0.0000 : pP1(2.5)0.5,1,1.5,2 : Excited Sigma 1.5 1 F1e 0.5 1 - Ground v=0 2.5 2 F1e 1.5 2 : COLTRAP_4K_electronic
LinearMolecule Excited 1 1 10 Ground 1 0 2 3262.281579 1 0.00119858614807528 0.0000 : qQ1(1.5)1.5,1,1.5,1 : Excited Sigma 1.5 1 F1e 1.5 1 - Ground v=0 1.5 1 F1f 1.5 1 : LIRTRAP_9K_electronic
LinearMolecule Excited 1 1 12 Ground 2 0 1 3262.393906 1 0.00234009161927613 0.0000 : qP21(1.5)0.5,1,1.5,2 : Excited Sigma 0.5 1 F2f 0.5 1 - Ground v=0 1.5 1 F1f 1.5 2 : LIRTRAP_9K_electronic
LinearMolecule Excited 1 1 13 Ground 1 0 1 3276.41887 1 0.000171895246566496 0.0000 : sR21(1.5)1.5,1,0.5,1 : Excited Sigma 2.5 3 F2f 1.5 1 - Ground v=0 1.5 1 F1f 0.5 1 : COLTRAP_4K_electronic
LinearMolecule Excited 1 1 13 Ground 0 0 1 3276.418882 1 0.000235384703844173 0.0000 : sR21(1.5)1.5,1,0.5,0 : Excited Sigma 2.5 3 F2f 1.5 1 - Ground v=0 1.5 1 F1f 0.5 0 : COLTRAP_4K_electronic
LinearMolecule Excited 1 1 13 Ground 2 0 5 3260.607347 1 0.000842750503387274 0.0000 : qP21(3.5)1.5,1,2.5,2 : Excited Sigma 2.5 3 F2f 1.5 1 - Ground v=0 3.5 3 F1f 2.5 2 : COLTRAP_4K_electronic
LinearMolecule Excited 2 0 12 Ground 2 1 1 3259.53144 1 0.00182870695978843 0.0000 : pP1(1.5)1.5,2,1.5,2 : Excited Sigma 0.5 0 F1e 1.5 2 - Ground v=0 1.5 1 F1e 1.5 2 : LIRTRAP_9K_electronic
LinearMolecule Excited 2 0 12 Ground 3 1 1 3259.530796 1 0.00640013013732401 0.0000 : pP1(1.5)1.5,2,2.5,3 : Excited Sigma 0.5 0 F1e 1.5 2 - Ground v=0 1.5 1 F1e 2.5 3 : LIRTRAP_35K_electronic
LinearMolecule Excited 2 0 13 Ground 3 1 4 3252.022522 1 0.00177121992904868 0.0000 : pP1(3.5)1.5,2,2.5,3 : Excited Sigma 2.5 2 F1e 1.5 2 - Ground v=0 3.5 3 F1e 2.5 3 : COLTRAP_4K_electronic
LinearMolecule Excited 2 0 14 Ground 3 1 5 3252.025137 1 0.00178200989052764 0.0000 : pP1(3.5)2.5,2,3.5,3 : Excited Sigma 2.5 2 F1e 2.5 2 - Ground v=0 3.5 3 F1e 3.5 3 : COLTRAP_4K_electronic
LinearMolecule Excited 2 0 15 Ground 3 1 2 3261.418708 1 0.00257900118310145 0.0000 : qP21(2.5)2.5,2,3.5,3 : Excited Sigma 1.5 2 F2f 2.5 2 - Ground v=0 2.5 2 F1f 3.5 3 : COLTRAP_4K_electronic
LinearMolecule Excited 2 0 15 Ground 2 1 8 3215.793041 1 0.000123250021798973 0.0000 : qQ2(1.5)2.5,2,1.5,2 : Excited Sigma 1.5 2 F2f 2.5 2 - Ground v=0 1.5 2 F2e 1.5 2 : LIRTRAP_35K_electronic
LinearMolecule Excited 2 0 16 Ground 3 1 2 3261.422739 1 0.00254067525188788 0.0000 : qP21(2.5)1.5,2,2.5,3 : Excited Sigma 1.5 2 F2f 1.5 2 - Ground v=0 2.5 2 F1f 2.5 3 : LIRTRAP_9K_electronic
LinearMolecule Excited 2 0 17 Ground 1 1 3 3281.023999 1 0.000505079117814123 0.0000 : sR21(2.5)2.5,2,1.5,1 : Excited Sigma 3.5 4 F2f 2.5 2 - Ground v=0 2.5 2 F1f 1.5 1 : COLTRAP_4K_electronic
LinearMolecule Excited 2 0 17 Ground 2 1 5 3271.807038 1 0.000988588674772621 0.0000 : rQ21(3.5)2.5,2,2.5,2 : Excited Sigma 3.5 4 F2f 2.5 2 - Ground v=0 3.5 3 F1e 2.5 2 : COLTRAP_4K_electronic
LinearMolecule Excited 2 0 17 Ground 3 1 6 3259.937672 1 0.000584090384491192 0.0000 : qP21(4.5)2.5,2,3.5,3 : Excited Sigma 3.5 4 F2f 2.5 2 - Ground v=0 4.5 4 F1f 3.5 3 : COLTRAP_4K_electronic
LinearMolecule Excited 2 1 12 Ground 3 0 2 3255.697547 1 0.00302903257446393 0.0000 : pP1(2.5)1.5,2,2.5,3 : Excited Sigma 1.5 1 F1e 1.5 2 - Ground v=0 2.5 2 F1e 2.5 3 : LIRTRAP_35K_electronic
LinearMolecule Excited 2 1 12 Ground 2 0 1 3262.281579 1 0.00196457736945485 0.0000 : qQ1(1.5)1.5,2,1.5,2 : Excited Sigma 1.5 1 F1e 1.5 2 - Ground v=0 1.5 1 F1f 1.5 2 : LIRTRAP_9K_electronic
LinearMolecule Excited 2 1 13 Ground 2 0 2 3262.28501 1 0.00305219860251525 0.0000 : qQ1(1.5)2.5,2,2.5,2 : Excited Sigma 1.5 1 F1e 2.5 2 - Ground v=0 1.5 1 F1f 2.5 2 : LIRTRAP_9K_electronic
LinearMolecule Excited 2 1 14 Ground 3 0 7 3210.134694 1 0.000108813257304208 0.0000 : pP2(1.5)1.5,2,2.5,3 : Excited Sigma 0.5 1 F2f 1.5 2 - Ground v=0 1.5 2 F2f 2.5 3 : LIRTRAP_35K_electronic
LinearMolecule Excited 2 1 14 Ground 3 0 1 3262.38921 1 0.00593638778396261 0.0000 : qP21(1.5)1.5,2,2.5,3 : Excited Sigma 0.5 1 F2f 1.5 2 - Ground v=0 1.5 1 F1f 2.5 3 : LIRTRAP_35K_electronic
LinearMolecule Excited 2 1 15 Ground 3 0 6 3248.503404 1 0.000884680966200552 0.0000 : pP1(4.5)2.5,2,3.5,3 : Excited Sigma 3.5 3 F1e 2.5 2 - Ground v=0 4.5 4 F1e 3.5 3 : COLTRAP_4K_electronic
LinearMolecule Excited 2 1 16 Ground 3 0 5 3260.604661 1 0.0012772130011611 0.0000 : qP21(3.5)2.5,2,3.5,3 : Excited Sigma 2.5 3 F2f 2.5 2 - Ground v=0 3.5 3 F1f 3.5 3 : COLTRAP_4K_electronic
LinearMolecule Excited 2 1 16 Ground 2 0 2 3276.413734 1 0.000098111372738689 0.0000 : sR21(1.5)2.5,2,2.5,2 : Excited Sigma 2.5 3 F2f 2.5 2 - Ground v=0 1.5 1 F1f 2.5 2 : COLTRAP_4K_electronic
LinearMolecule Excited 2 1 16 Ground 2 0 4 3269.830575 1 0.00210735620649376 0.0000 : rQ21(2.5)2.5,2,2.5,2 : Excited Sigma 2.5 3 F2f 2.5 2 - Ground v=0 2.5 2 F1e 2.5 2 : COLTRAP_4K_electronic
LinearMolecule Excited 2 1 17 Ground 1 0 1 3276.41887 1 0.000491641165658545 0.0000 : sR21(1.5)1.5,2,0.5,1 : Excited Sigma 2.5 3 F2f 1.5 2 - Ground v=0 1.5 1 F1f 0.5 1 : COLTRAP_4K_electronic
LinearMolecule Excited 2 1 17 Ground 3 0 4 3260.607347 1 0.0012863437878303 0.0000 : qP21(3.5)1.5,2,2.5,3 : Excited Sigma 2.5 3 F2f 1.5 2 - Ground v=0 3.5 3 F1f 2.5 3 : COLTRAP_4K_electronic
LinearMolecule Excited 2 1 17 Ground 2 0 3 3269.833816 1 0.00186442306816808 0.0000 : rQ21(2.5)1.5,2,1.5,2 : Excited Sigma 2.5 3 F2f 1.5 2 - Ground v=0 2.5 2 F1e 1.5 2 : COLTRAP_4K_electronic
LinearMolecule Excited 3 0 13 Ground 4 1 2 3252.025137 1 0.00237038566321636 0.0000 : pP1(3.5)2.5,3,3.5,4 : Excited Sigma 2.5 2 F1e 2.5 3 - Ground v=0 3.5 3 F1e 3.5 4 : COLTRAP_4K_electronic
LinearMolecule Excited 3 0 13 Ground 3 1 2 3261.241131 1 0.0033441160847484 0.0000 : qQ1(2.5)2.5,3,2.5,3 : Excited Sigma 2.5 2 F1e 2.5 3 - Ground v=0 2.5 2 F1f 2.5 3 : LIRTRAP_9K_electronic
LinearMolecule Excited 3 0 14 Ground 2 1 8 3215.618824 1 0.0000493059260558446 0.0000 : qR12(1.5)3.5,3,1.5,2 : Excited Sigma 2.5 2 F1e 3.5 3 - Ground v=0 1.5 2 F2e 1.5 2 : LIRTRAP_35K_electronic
LinearMolecule Excited 3 0 14 Ground 2 1 7 3215.618824 1 0.0000597371495295399 0.0000 : qR12(1.5)3.5,3,2.5,2 : Excited Sigma 2.5 2 F1e 3.5 3 - Ground v=0 1.5 2 F2e 2.5 2 : LIRTRAP_35K_electronic
LinearMolecule Excited 3 0 14 Ground 4 1 3 3252.028523 1 0.00253089788883461 0.0000 : pP1(3.5)3.5,3,4.5,4 : Excited Sigma 2.5 2 F1e 3.5 3 - Ground v=0 3.5 3 F1e 4.5 4 : COLTRAP_4K_electronic
LinearMolecule Excited 3 0 14 Ground 3 1 2 3261.244473 1 0.00336586280213082 0.0000 : qQ1(2.5)3.5,3,3.5,3 : Excited Sigma 2.5 2 F1e 3.5 3 - Ground v=0 2.5 2 F1f 3.5 3 : COLTRAP_4K_electronic
LinearMolecule Excited 3 0 14 Ground 3 1 9 3208.62394 1 0.000159321868508596 0.0000 : pQ12(2.5)3.5,3,3.5,3 : Excited Sigma 2.5 2 F1e 3.5 3 - Ground v=0 2.5 3 F2f 3.5 3 : LIRTRAP_35K_electronic
LinearMolecule Excited 3 0 15 Ground 3 1 7 3215.793041 1 0.000273139042039258 0.0000 : qQ2(1.5)2.5,3,2.5,3 : Excited Sigma 1.5 2 F2f 2.5 3 - Ground v=0 1.5 2 F2e 2.5 3 : LIRTRAP_35K_electronic
LinearMolecule Excited 3 0 15 Ground 4 1 7 3208.80084 1 0.000122679764336569 0.0000 : pP2(2.5)2.5,3,3.5,4 : Excited Sigma 1.5 2 F2f 2.5 3 - Ground v=0 2.5 3 F2f 3.5 4 : LIRTRAP_35K_electronic
LinearMolecule Excited 3 0 15 Ground 3 1 1 3268.00649 1 0.00352930738558567 0.0000 : rQ21(1.5)2.5,3,2.5,3 : Excited Sigma 1.5 2 F2f 2.5 3 - Ground v=0 1.5 1 F1e 2.5 3 : LIRTRAP_35K_electronic
LinearMolecule Excited 3 0 15 Ground 4 1 1 3261.418711 1 0.00389587499377196 0.0000 : qP21(2.5)2.5,3,3.5,4 : Excited Sigma 1.5 2 F2f 2.5 3 - Ground v=0 2.5 2 F1f 3.5 4 : LIRTRAP_9K_electronic
LinearMolecule Excited 3 0 16 Ground 2 1 5 3271.48715 1 0.000650463301761377 0.0000 : rR1(3.5)3.5,3,2.5,2 : Excited Sigma 4.5 4 F1e 3.5 3 - Ground v=0 3.5 3 F1e 2.5 2 : COLTRAP_4K_electronic
LinearMolecule Excited 3 0 16 Ground 4 1 6 3245.137433 1 0.000512011613895919 0.0000 : pP1(5.5)3.5,3,4.5,4 : Excited Sigma 4.5 4 F1e 3.5 3 - Ground v=0 5.5 5 F1e 4.5 4 : COLTRAP_4K_electronic
LinearMolecule Excited 3 0 16 Ground 3 1 6 3259.617194 1 0.00138751045109783 0.0000 : qQ1(4.5)3.5,3,3.5,3 : Excited Sigma 4.5 4 F1e 3.5 3 - Ground v=0 4.5 4 F1f 3.5 3 : COLTRAP_4K_electronic
LinearMolecule Excited 3 0 17 Ground 2 1 4 3281.02093 1 0.000759753985899709 0.0000 : sR21(2.5)3.5,3,2.5,2 : Excited Sigma 3.5 4 F2f 3.5 3 - Ground v=0 2.5 2 F1f 2.5 2 : COLTRAP_4K_electronic
LinearMolecule Excited 3 0 17 Ground 3 1 5 3271.804329 1 0.00137786383658319 0.0000 : rQ21(3.5)3.5,3,3.5,3 : Excited Sigma 3.5 4 F2f 3.5 3 - Ground v=0 3.5 3 F1e 3.5 3 : COLTRAP_4K_electronic
LinearMolecule Excited 3 0 17 Ground 4 1 5 3259.934899 1 0.00077317930307166 0.0000 : qP21(4.5)3.5,3,4.5,4 : Excited Sigma 3.5 4 F2f 3.5 3 - Ground v=0 4.5 4 F1f 4.5 4 : COLTRAP_4K_electronic
LinearMolecule Excited 3 0 18 Ground 4 1 4 3259.937672 1 0.000779730059771225 0.0000 : qP21(4.5)2.5,3,3.5,4 : Excited Sigma 3.5 4 F2f 2.5 3 - Ground v=0 4.5 4 F1f 3.5 4 : COLTRAP_4K_electronic
LinearMolecule Excited 3 0 18 Ground 2 1 3 3281.023999 1 0.000755252344018626 0.0000 : sR21(2.5)2.5,3,1.5,2 : Excited Sigma 3.5 4 F2f 2.5 3 - Ground v=0 2.5 2 F1f 1.5 2 : COLTRAP_4K_electronic
LinearMolecule Excited 3 0 18 Ground 3 1 4 3271.807114 1 0.00137975524171202 0.0000 : rQ21(3.5)2.5,3,2.5,3 : Excited Sigma 3.5 4 F2f 2.5 3 - Ground v=0 3.5 3 F1e 2.5 3 : COLTRAP_4K_electronic
LinearMolecule Excited 3 1 13 Ground 3 0 7 3210.029757 1 0.000236630786690282 0.0000 : pQ12(1.5)2.5,3,2.5,3 : Excited Sigma 1.5 1 F1e 2.5 3 - Ground v=0 1.5 2 F2f 2.5 3 : LIRTRAP_35K_electronic
LinearMolecule Excited 3 1 13 Ground 3 0 1 3262.28501 1 0.00430355297936899 0.0000 : qQ1(1.5)2.5,3,2.5,3 : Excited Sigma 1.5 1 F1e 2.5 3 - Ground v=0 1.5 1 F1f 2.5 3 : LIRTRAP_9K_electronic
LinearMolecule Excited 3 1 13 Ground 4 0 1 3255.701681 1 0.00472318885549998 0.0000 : pP1(2.5)2.5,3,3.5,4 : Excited Sigma 1.5 1 F1e 2.5 3 - Ground v=0 2.5 2 F1e 3.5 4 : COLTRAP_4K_electronic
LinearMolecule Excited 3 1 14 Ground 3 0 4 3260.352843 1 0.00247570967654976 0.0000 : qQ1(3.5)2.5,3,2.5,3 : Excited Sigma 3.5 3 F1e 2.5 3 - Ground v=0 3.5 3 F1f 2.5 3 : LIRTRAP_9K_electronic
LinearMolecule Excited 3 1 14 Ground 2 0 3 3269.579112 1 0.00110665042591335 0.0000 : rR1(2.5)2.5,3,1.5,2 : Excited Sigma 3.5 3 F1e 2.5 3 - Ground v=0 2.5 2 F1e 1.5 2 : COLTRAP_4K_electronic
LinearMolecule Excited 3 1 14 Ground 4 0 4 3248.503404 1 0.00119103149873246 0.0000 : pP1(4.5)2.5,3,3.5,4 : Excited Sigma 3.5 3 F1e 2.5 3 - Ground v=0 4.5 4 F1e 3.5 4 : COLTRAP_4K_electronic
LinearMolecule Excited 3 1 15 Ground 3 0 5 3260.354969 1 0.00132988524126086 0.0000 : qQ1(3.5)3.5,3,3.5,3 : Excited Sigma 3.5 3 F1e 3.5 3 - Ground v=0 3.5 3 F1f 3.5 3 : COLTRAP_4K_electronic
LinearMolecule Excited 3 1 15 Ground 4 0 5 3248.505857 1 0.00118145288052292 0.0000 : pP1(4.5)3.5,3,4.5,4 : Excited Sigma 3.5 3 F1e 3.5 3 - Ground v=0 4.5 4 F1e 4.5 4 : COLTRAP_4K_electronic
LinearMolecule Excited 3 1 15 Ground 3 0 4 3260.354969 1 0.00111690581499491 0.0000 : qQ1(3.5)3.5,3,2.5,3 : Excited Sigma 3.5 3 F1e 3.5 3 - Ground v=0 3.5 3 F1f 2.5 3 : COLTRAP_4K_electronic
LinearMolecule Excited 3 1 16 Ground 2 0 8 3224.159114 1 0.0000948138192496265 0.0000 : rR2(1.5)3.5,3,2.5,2 : Excited Sigma 2.5 3 F2f 3.5 3 - Ground v=0 1.5 2 F2f 2.5 2 : LIRTRAP_35K_electronic
LinearMolecule Excited 3 1 16 Ground 3 0 3 3269.826852 1 0.00357103037463171 0.0000 : rQ21(2.5)3.5,3,3.5,3 : Excited Sigma 2.5 3 F2f 3.5 3 - Ground v=0 2.5 2 F1e 3.5 3 : COLTRAP_4K_electronic
LinearMolecule Excited 3 1 16 Ground 4 0 3 3260.600563 1 0.00184986754644978 0.0000 : qP21(3.5)3.5,3,4.5,4 : Excited Sigma 2.5 3 F2f 3.5 3 - Ground v=0 3.5 3 F1f 4.5 4 : COLTRAP_4K_electronic
LinearMolecule Excited 3 1 16 Ground 2 0 2 3276.411217 1 0.00102718403855108 0.0000 : sR21(1.5)3.5,3,2.5,2 : Excited Sigma 2.5 3 F2f 3.5 3 - Ground v=0 1.5 1 F1f 2.5 2 : COLTRAP_4K_electronic
LinearMolecule Excited 3 1 17 Ground 4 0 2 3260.604661 1 0.00175096593563896 0.0000 : qP21(3.5)2.5,3,3.5,4 : Excited Sigma 2.5 3 F2f 2.5 3 - Ground v=0 3.5 3 F1f 3.5 4 : COLTRAP_4K_electronic
LinearMolecule Excited 3 1 17 Ground 2 0 1 3276.415567 1 0.000880623213283405 0.0000 : sR21(1.5)2.5,3,1.5,2 : Excited Sigma 2.5 3 F2f 2.5 3 - Ground v=0 1.5 1 F1f 1.5 2 : COLTRAP_4K_electronic
LinearMolecule Excited 3 1 17 Ground 2 0 2 3276.413734 1 0.0000358381110403391 0.0000 : sR21(1.5)2.5,3,2.5,2 : Excited Sigma 2.5 3 F2f 2.5 3 - Ground v=0 1.5 1 F1f 2.5 2 : COLTRAP_4K_electronic
LinearMolecule Excited 3 1 17 Ground 3 0 2 3269.830575 1 0.00289294833738558 0.0000 : rQ21(2.5)2.5,3,2.5,3 : Excited Sigma 2.5 3 F2f 2.5 3 - Ground v=0 2.5 2 F1e 2.5 3 : COLTRAP_4K_electronic
LinearMolecule Excited 3 1 17 Ground 3 0 1 3276.413666 1 0.000168179846112839 0.0000 : sR21(1.5)2.5,3,2.5,3 : Excited Sigma 2.5 3 F2f 2.5 3 - Ground v=0 1.5 1 F1f 2.5 3 : COLTRAP_4K_electronic
LinearMolecule Excited 3 1 21 Ground 3 0 6 3273.92599 1 0.000453592086379975 0.0000 : rQ21(4.5)3.5,3,3.5,3 : Excited Sigma 4.5 5 F2f 3.5 3 - Ground v=0 4.5 4 F1e 3.5 3 : LIRTRAP_35K_electronic
LinearMolecule Excited 3 1 21 Ground 2 0 5 3285.776819 1 0.000381074892516126 0.0000 : sR21(3.5)3.5,3,2.5,2 : Excited Sigma 4.5 5 F2f 3.5 3 - Ground v=0 3.5 3 F1f 2.5 2 : COLTRAP_4K_electronic
LinearMolecule Excited 3 1 21 Ground 4 0 6 3259.410878 1 0.00030546770737811 0.0000 : qP21(5.5)3.5,3,4.5,4 : Excited Sigma 4.5 5 F2f 3.5 3 - Ground v=0 5.5 5 F1f 4.5 4 : COLTRAP_4K_electronic
LinearMolecule Excited 4 0 13 Ground 4 1 7 3208.62394 1 0.000205161042973316 0.0000 : pQ12(2.5)3.5,4,3.5,4 : Excited Sigma 2.5 2 F1e 3.5 4 - Ground v=0 2.5 3 F2f 3.5 4 : LIRTRAP_35K_electronic
LinearMolecule Excited 4 0 13 Ground 4 1 1 3261.244512 1 0.00505850145151353 0.0000 : qQ1(2.5)3.5,4,3.5,4 : Excited Sigma 2.5 2 F1e 3.5 4 - Ground v=0 2.5 2 F1f 3.5 4 : COLTRAP_4K_electronic
LinearMolecule Excited 4 0 13 Ground 3 1 1 3267.832261 1 0.00121107039014324 0.0000 : rR1(1.5)3.5,4,2.5,3 : Excited Sigma 2.5 2 F1e 3.5 4 - Ground v=0 1.5 1 F1e 2.5 3 : LIRTRAP_35K_electronic
LinearMolecule Excited 4 0 13 Ground 5 1 1 3252.028523 1 0.00316836884946194 0.0000 : pP1(3.5)3.5,4,4.5,5 : Excited Sigma 2.5 2 F1e 3.5 4 - Ground v=0 3.5 3 F1e 4.5 5 : COLTRAP_4K_electronic
LinearMolecule Excited 4 0 13 Ground 3 1 7 3215.618824 1 0.000146878843543885 0.0000 : qR12(1.5)3.5,4,2.5,3 : Excited Sigma 2.5 2 F1e 3.5 4 - Ground v=0 1.5 2 F2e 2.5 3 : LIRTRAP_35K_electronic
LinearMolecule Excited 4 0 14 Ground 4 1 4 3259.617194 1 0.001789134519609 0.0000 : qQ1(4.5)3.5,4,3.5,4 : Excited Sigma 4.5 4 F1e 3.5 4 - Ground v=0 4.5 4 F1f 3.5 4 : COLTRAP_4K_electronic
LinearMolecule Excited 4 0 14 Ground 3 1 4 3271.48715 1 0.000862112794784524 0.0000 : rR1(3.5)3.5,4,2.5,3 : Excited Sigma 4.5 4 F1e 3.5 4 - Ground v=0 3.5 3 F1e 2.5 3 : COLTRAP_4K_electronic
LinearMolecule Excited 4 0 14 Ground 5 1 4 3245.137433 1 0.000643044718303359 0.0000 : pP1(5.5)3.5,4,4.5,5 : Excited Sigma 4.5 4 F1e 3.5 4 - Ground v=0 5.5 5 F1e 4.5 5 : COLTRAP_4K_electronic
LinearMolecule Excited 4 0 15 Ground 4 1 5 3259.61972 1 0.00173555400575911 0.0000 : qQ1(4.5)4.5,4,4.5,4 : Excited Sigma 4.5 4 F1e 4.5 4 - Ground v=0 4.5 4 F1f 4.5 4 : COLTRAP_4K_electronic
LinearMolecule Excited 4 0 15 Ground 3 1 5 3271.489798 1 0.000858062692685923 0.0000 : rR1(3.5)4.5,4,3.5,3 : Excited Sigma 4.5 4 F1e 4.5 4 - Ground v=0 3.5 3 F1e 3.5 3 : COLTRAP_4K_electronic
LinearMolecule Excited 4 0 15 Ground 5 1 5 3245.139867 1 0.000637077817031415 0.0000 : pP1(5.5)4.5,4,5.5,5 : Excited Sigma 4.5 4 F1e 4.5 4 - Ground v=0 5.5 5 F1e 5.5 5 : COLTRAP_4K_electronic
LinearMolecule Excited 4 0 16 Ground 4 1 3 3271.800525 1 0.00194282252903099 0.0000 : rQ21(3.5)4.5,4,4.5,4 : Excited Sigma 3.5 4 F2f 4.5 4 - Ground v=0 3.5 3 F1e 4.5 4 : COLTRAP_4K_electronic
LinearMolecule Excited 4 0 16 Ground 3 1 3 3281.016827 1 0.00109949628964149 0.0000 : sR21(2.5)4.5,4,3.5,3 : Excited Sigma 3.5 4 F2f 4.5 4 - Ground v=0 2.5 2 F1f 3.5 3 : COLTRAP_4K_electronic
LinearMolecule Excited 4 0 16 Ground 5 1 3 3259.930948 1 0.00101151432343621 0.0000 : qP21(4.5)4.5,4,5.5,5 : Excited Sigma 3.5 4 F2f 4.5 4 - Ground v=0 4.5 4 F1f 5.5 5 : COLTRAP_4K_electronic
LinearMolecule Excited 4 0 17 Ground 4 1 2 3271.804222 1 0.00176530592089571 0.0000 : rQ21(3.5)3.5,4,3.5,4 : Excited Sigma 3.5 4 F2f 3.5 4 - Ground v=0 3.5 3 F1e 3.5 4 : COLTRAP_4K_electronic
LinearMolecule Excited 4 0 17 Ground 5 1 2 3259.934899 1 0.000980972903775918 0.0000 : qP21(4.5)3.5,4,4.5,5 : Excited Sigma 3.5 4 F2f 3.5 4 - Ground v=0 4.5 4 F1f 4.5 5 : COLTRAP_4K_electronic
LinearMolecule Excited 4 0 22 Ground 5 1 6 3259.01841 1 0.000129723324880854 0.0000 : qP21(6.5)4.5,4,5.5,5 : Excited Sigma 5.5 6 F2f 4.5 4 - Ground v=0 6.5 6 F1f 5.5 5 : LIRTRAP_35K_electronic
LinearMolecule Excited 4 0 22 Ground 3 1 6 3290.669528 1 0.000175030469078293 0.0000 : sR21(4.5)4.5,4,3.5,3 : Excited Sigma 5.5 6 F2f 4.5 4 - Ground v=0 4.5 4 F1f 3.5 3 : COLTRAP_4K_electronic
LinearMolecule Excited 4 1 13 Ground 3 0 2 3269.581046 1 0.00158219430420904 0.0000 : rR1(2.5)3.5,4,2.5,3 : Excited Sigma 3.5 3 F1e 3.5 4 - Ground v=0 2.5 2 F1e 2.5 3 : LIRTRAP_35K_electronic
LinearMolecule Excited 4 1 13 Ground 5 0 2 3248.505857 1 0.00147812334976605 0.0000 : pP1(4.5)3.5,4,4.5,5 : Excited Sigma 3.5 3 F1e 3.5 4 - Ground v=0 4.5 4 F1e 4.5 5 : COLTRAP_4K_electronic
LinearMolecule Excited 4 1 13 Ground 4 0 2 3260.355134 1 0.00258181666559324 0.0000 : qQ1(3.5)3.5,4,3.5,4 : Excited Sigma 3.5 3 F1e 3.5 4 - Ground v=0 3.5 3 F1f 3.5 4 : COLTRAP_4K_electronic
LinearMolecule Excited 4 1 14 Ground 4 0 3 3260.358213 1 0.00264548936284845 0.0000 : qQ1(3.5)4.5,4,4.5,4 : Excited Sigma 3.5 3 F1e 4.5 4 - Ground v=0 3.5 3 F1f 4.5 4 : COLTRAP_4K_electronic
LinearMolecule Excited 4 1 14 Ground 4 0 2 3260.358213 1 0.000749199572686958 0.0000 : qQ1(3.5)4.5,4,3.5,4 : Excited Sigma 3.5 3 F1e 4.5 4 - Ground v=0 3.5 3 F1f 3.5 4 : COLTRAP_4K_electronic
LinearMolecule Excited 4 1 14 Ground 3 0 3 3269.584128 1 0.00166032690471846 0.0000 : rR1(2.5)4.5,4,3.5,3 : Excited Sigma 3.5 3 F1e 4.5 4 - Ground v=0 2.5 2 F1e 3.5 3 : COLTRAP_4K_electronic
LinearMolecule Excited 4 1 14 Ground 5 0 3 3248.509314 1 0.00153133626804682 0.0000 : pP1(4.5)4.5,4,5.5,5 : Excited Sigma 3.5 3 F1e 4.5 4 - Ground v=0 4.5 4 F1e 5.5 5 : COLTRAP_4K_electronic
LinearMolecule Excited 4 1 15 Ground 4 0 7 3217.26695 1 0.000269986602113186 0.0000 : qQ2(2.5)3.5,4,3.5,4 : Excited Sigma 2.5 3 F2f 3.5 4 - Ground v=0 2.5 3 F2e 3.5 4 : LIRTRAP_35K_electronic
LinearMolecule Excited 4 1 15 Ground 3 0 1 3276.410965 1 0.00141174674718508 0.0000 : sR21(1.5)3.5,4,2.5,3 : Excited Sigma 2.5 3 F2f 3.5 4 - Ground v=0 1.5 1 F1f 2.5 3 : COLTRAP_4K_electronic
LinearMolecule Excited 4 1 15 Ground 4 0 1 3269.826842 1 0.00461385275635197 0.0000 : rQ21(2.5)3.5,4,3.5,4 : Excited Sigma 2.5 3 F2f 3.5 4 - Ground v=0 2.5 2 F1e 3.5 4 : COLTRAP_4K_electronic
LinearMolecule Excited 4 1 15 Ground 5 0 1 3260.600563 1 0.00234746992535148 0.0000 : qP21(3.5)3.5,4,4.5,5 : Excited Sigma 2.5 3 F2f 3.5 4 - Ground v=0 3.5 3 F1f 4.5 5 : COLTRAP_4K_electronic
LinearMolecule Excited 4 1 15 Ground 3 0 7 3224.158946 1 0.000162579342458226 0.0000 : rR2(1.5)3.5,4,2.5,3 : Excited Sigma 2.5 3 F2f 3.5 4 - Ground v=0 1.5 2 F2f 2.5 3 : LIRTRAP_35K_electronic
LinearMolecule Excited 4 1 17 Ground 3 0 6 3273.547505 1 0.000329619458716049 0.0000 : rR1(4.5)4.5,4,3.5,3 : Excited Sigma 5.5 5 F1e 4.5 4 - Ground v=0 4.5 4 F1e 3.5 3 : COLTRAP_4K_electronic
LinearMolecule Excited 4 1 18 Ground 5 0 5 3259.40812 1 0.000379706806582114 0.0000 : qP21(5.5)4.5,4,5.5,5 : Excited Sigma 4.5 5 F2f 4.5 4 - Ground v=0 5.5 5 F1f 5.5 5 : COLTRAP_4K_electronic
LinearMolecule Excited 4 1 18 Ground 3 0 5 3285.773758 1 0.000505377579937986 0.0000 : sR21(3.5)4.5,4,3.5,3 : Excited Sigma 4.5 5 F2f 4.5 4 - Ground v=0 3.5 3 F1f 3.5 3 : COLTRAP_4K_electronic
LinearMolecule Excited 4 1 19 Ground 4 0 4 3273.92599 1 0.000585912806915978 0.0000 : rQ21(4.5)3.5,4,3.5,4 : Excited Sigma 4.5 5 F2f 3.5 4 - Ground v=0 4.5 4 F1e 3.5 4 : LIRTRAP_35K_electronic
LinearMolecule Excited 4 1 19 Ground 3 0 4 3285.776819 1 0.000509635448839458 0.0000 : sR21(3.5)3.5,4,2.5,3 : Excited Sigma 4.5 5 F2f 3.5 4 - Ground v=0 3.5 3 F1f 2.5 3 : COLTRAP_4K_electronic
LinearMolecule Excited 4 1 19 Ground 5 0 4 3259.410878 1 0.000382063875096269 0.0000 : qP21(5.5)3.5,4,4.5,5 : Excited Sigma 4.5 5 F2f 3.5 4 - Ground v=0 5.5 5 F1f 4.5 5 : COLTRAP_4K_electronic
LinearMolecule Excited 5 0 13 Ground 5 1 2 3259.619723 1 0.00214452164273589 0.0000 : qQ1(4.5)4.5,5,4.5,5 : Excited Sigma 4.5 4 F1e 4.5 5 - Ground v=0 4.5 4 F1f 4.5 5 : COLTRAP_4K_electronic
LinearMolecule Excited 5 0 13 Ground 6 1 2 3245.139867 1 0.000765688636324397 0.0000 : pP1(5.5)4.5,5,5.5,6 : Excited Sigma 4.5 4 F1e 4.5 5 - Ground v=0 5.5 5 F1e 5.5 6 : COLTRAP_4K_electronic
LinearMolecule Excited 5 0 13 Ground 4 1 2 3271.489798 1 0.00108197981005419 0.0000 : rR1(3.5)4.5,5,3.5,4 : Excited Sigma 4.5 4 F1e 4.5 5 - Ground v=0 3.5 3 F1e 3.5 4 : COLTRAP_4K_electronic
LinearMolecule Excited 5 0 14 Ground 4 1 3 3271.49256 1 0.0011092058876347 0.0000 : rR1(3.5)5.5,5,4.5,4 : Excited Sigma 4.5 4 F1e 5.5 5 - Ground v=0 3.5 3 F1e 4.5 4 : COLTRAP_4K_electronic
LinearMolecule Excited 5 0 14 Ground 5 1 3 3259.622694 1 0.00224403624733863 0.0000 : qQ1(4.5)5.5,5,5.5,5 : Excited Sigma 4.5 4 F1e 5.5 5 - Ground v=0 4.5 4 F1f 5.5 5 : COLTRAP_4K_electronic
```

LinearMolecule Excited 5 0 14 Ground 4 1 8 3218.44256 1 0.000052522919907094 0.0000 : qR12(3.5)5.5,5,4.5,4 : Excited Sigma 4.5 4 F1e 5.5 5 - Ground v=0 3.5 4 F2e 4.5 4 : LIRTRAP_35K_electronic
LinearMolecule Excited 5 0 14 Ground 6 1 3 3245.143315 1 0.000782631699287449 0.0000 : pP1(5.5)5.5,5,6.5,6 : Excited Sigma 4.5 4 F1e 5.5 5 - Ground v=0 5.5 5 F1e 6.5 6 : COLTRAP_4K_electronic
LinearMolecule Excited 5 0 15 Ground 4 1 1 3281.016827 1 0.00138850579094987 0.0000 : sR21(2.5)4.5,5,3.5,4 : Excited Sigma 3.5 4 F2f 4.5 5 - Ground v=0 2.5 2 F1f 3.5 4 : COLTRAP_4K_electronic
LinearMolecule Excited 5 0 15 Ground 4 1 7 3228.397871 1 0.000149445439532531 0.0000 : rR2(2.5)4.5,5,3.5,4 : Excited Sigma 3.5 4 F2f 4.5 5 - Ground v=0 2.5 3 F2f 3.5 4 : LIRTRAP_35K_electronic
LinearMolecule Excited 5 0 15 Ground 5 1 1 3271.800525 1 0.00237949305582866 0.0000 : rQ21(3.5)4.5,5,4.5,5 : Excited Sigma 3.5 4 F2f 4.5 5 - Ground v=0 3.5 3 F1e 4.5 5 : COLTRAP_4K_electronic
LinearMolecule Excited 5 0 15 Ground 6 1 1 3259.930948 1 0.00122600931152755 0.0000 : qP21(4.5)4.5,5,5.5,6 : Excited Sigma 3.5 4 F2f 4.5 5 - Ground v=0 4.5 4 F1f 5.5 6 : COLTRAP_4K_electronic
LinearMolecule Excited 5 0 18 Ground 4 1 5 3290.666474 1 0.00021756760242163 0.0000 : sR21(4.5)5.5,5,4.5,4 : Excited Sigma 5.5 6 F2f 5.5 5 - Ground v=0 4.5 4 F1f 4.5 4 : COLTRAP_4K_electronic
LinearMolecule Excited 5 0 19 Ground 4 1 4 3290.669528 1 0.000219810869665202 0.0000 : sR21(4.5)4.5,5,3.5,4 : Excited Sigma 5.5 6 F2f 4.5 5 - Ground v=0 4.5 4 F1f 3.5 4 : COLTRAP_4K_electronic
LinearMolecule Excited 5 0 19 Ground 6 1 4 3259.01841 1 0.000155807466691157 0.0000 : qP21(6.5)4.5,5,5.5,6 : Excited Sigma 5.5 6 F2f 4.5 5 - Ground v=0 6.5 6 F1f 5.5 6 : LIRTRAP_35K_electronic
LinearMolecule Excited 5 1 13 Ground 6 0 1 3248.509314 1 0.00184038258532726 0.0000 : pP1(4.5)4.5,5,5.5,6 : Excited Sigma 3.5 3 F1e 4.5 5 - Ground v=0 4.5 4 F1e 5.5 6 : COLTRAP_4K_electronic
LinearMolecule Excited 5 1 13 Ground 5 0 1 3260.358152 1 0.00412966606356648 0.0000 : qQ1(3.5)4.5,5,4.5,5 : Excited Sigma 3.5 3 F1e 4.5 5 - Ground v=0 3.5 3 F1f 4.5 5 : COLTRAP_4K_electronic
LinearMolecule Excited 5 1 13 Ground 4 0 1 3269.584128 1 0.00211344724868303 0.0000 : rR1(2.5)4.5,5,3.5,4 : Excited Sigma 3.5 3 F1e 4.5 5 - Ground v=0 2.5 2 F1e 3.5 4 : COLTRAP_4K_electronic
LinearMolecule Excited 5 1 14 Ground 4 0 4 3273.547505 1 0.000413215956270072 0.0000 : rR1(4.5)4.5,5,3.5,4 : Excited Sigma 5.5 5 F1e 4.5 5 - Ground v=0 4.5 4 F1e 3.5 4 : COLTRAP_4K_electronic
LinearMolecule Excited 5 1 14 Ground 5 0 4 3259.030405 1 0.00102134122430533 0.0000 : qQ1(5.5)4.5,5,4.5,5 : Excited Sigma 5.5 5 F1e 4.5 5 - Ground v=0 5.5 5 F1f 4.5 5 : COLTRAP_4K_electronic
LinearMolecule Excited 5 1 15 Ground 4 0 5 3273.550066 1 0.000409600005420979 0.0000 : rR1(4.5)5.5,5,4.5,4 : Excited Sigma 5.5 5 F1e 5.5 5 - Ground v=0 4.5 4 F1e 4.5 4 : COLTRAP_4K_electronic
LinearMolecule Excited 5 1 16 Ground 4 0 8 3232.650608 1 0.0000705770956350921 0.0000 : rR2(3.5)5.5,5,4.5,4 : Excited Sigma 4.5 5 F2f 5.5 5 - Ground v=0 3.5 4 F2f 4.5 4 : LIRTRAP_35K_electronic
LinearMolecule Excited 5 1 16 Ground 5 0 3 3273.921927 1 0.000743833816489124 0.0000 : rQ21(4.5)5.5,5,5.5,5 : Excited Sigma 4.5 5 F2f 5.5 5 - Ground v=0 4.5 4 F1e 5.5 5 : COLTRAP_4K_electronic
LinearMolecule Excited 5 1 16 Ground 6 0 3 3259.40442 1 0.000468162326701429 0.0000 : qP21(5.5)5.5,5,6.5,6 : Excited Sigma 4.5 5 F2f 5.5 5 - Ground v=0 5.5 5 F1f 6.5 6 : LIRTRAP_35K_electronic
LinearMolecule Excited 5 1 16 Ground 4 0 3 3285.769959 1 0.000657706065642796 0.0000 : sR21(3.5)5.5,5,4.5,4 : Excited Sigma 4.5 5 F2f 5.5 5 - Ground v=0 3.5 3 F1f 4.5 4 : COLTRAP_4K_electronic
LinearMolecule Excited 5 1 17 Ground 4 0 2 3285.773758 1 0.000635278643811832 0.0000 : sR21(3.5)4.5,5,3.5,4 : Excited Sigma 4.5 5 F2f 4.5 5 - Ground v=0 3.5 3 F1f 3.5 4 : COLTRAP_4K_electronic
LinearMolecule Excited 5 1 17 Ground 6 0 2 3259.40812 1 0.000459088882500895 0.0000 : qP21(5.5)4.5,5,5.5,6 : Excited Sigma 4.5 5 F2f 4.5 5 - Ground v=0 5.5 5 F1f 5.5 6 : COLTRAP_4K_electronic
LinearMolecule Excited 6 0 13 Ground 7 1 1 3245.143315 1 0.000914391396178645 0.0000 : pP1(5.5)5.5,6,6.5,7 : Excited Sigma 4.5 4 F1e 5.5 6 - Ground v=0 5.5 5 F1e 6.5 7 : COLTRAP_4K_electronic
LinearMolecule Excited 6 0 13 Ground 6 1 1 3259.622683 1 0.00267020440024448 0.0000 : qQ1(4.5)5.5,6,5.5,6 : Excited Sigma 4.5 4 F1e 5.5 6 - Ground v=0 4.5 4 F1f 5.5 6 : COLTRAP_4K_electronic
LinearMolecule Excited 6 0 13 Ground 5 1 1 3271.492501 1 0.00133776439467113 0.0000 : rR1(3.5)5.5,6,4.5,5 : Excited Sigma 4.5 4 F1e 5.5 6 - Ground v=0 3.5 3 F1e 4.5 5 : COLTRAP_4K_electronic
LinearMolecule Excited 6 0 13 Ground 5 1 7 3218.44256 1 0.0000805408044085134 0.0000 : qR12(3.5)5.5,6,4.5,5 : Excited Sigma 4.5 4 F1e 5.5 6 - Ground v=0 3.5 4 F2e 4.5 5 : LIRTRAP_35K_electronic
LinearMolecule Excited 6 0 16 Ground 7 1 3 3259.01246 1 0.000184012208421322 0.0000 : qP21(6.5)6.5,6,7.5,7 : Excited Sigma 5.5 6 F2f 6.5 6 - Ground v=0 6.5 6 F1f 7.5 7 : LIRTRAP_35K_electronic
LinearMolecule Excited 6 0 16 Ground 5 1 3 3290.662944 1 0.000267362795335395 0.0000 : sR21(4.5)6.5,6,5.5,5 : Excited Sigma 5.5 6 F2f 6.5 6 - Ground v=0 4.5 4 F1f 5.5 5 : COLTRAP_4K_electronic
LinearMolecule Excited 6 0 16 Ground 5 1 8 3236.911324 1 0.0000521028609434504 0.0000 : rR2(4.5)6.5,6,5.5,5 : Excited Sigma 5.5 6 F2f 6.5 6 - Ground v=0 4.5 5 F2f 5.5 5 : LIRTRAP_35K_electronic
LinearMolecule Excited 6 0 17 Ground 5 1 9 3236.915181 1 0.0000515957255095371 0.0000 : rR2(4.5)5.5,6,4.5,5 : Excited Sigma 5.5 6 F2f 5.5 6 - Ground v=0 4.5 5 F2f 4.5 5 : LIRTRAP_35K_electronic
LinearMolecule Excited 6 0 17 Ground 5 1 2 3290.666474 1 0.000261770665383579 0.0000 : sR21(4.5)5.5,6,4.5,5 : Excited Sigma 5.5 6 F2f 5.5 6 - Ground v=0 4.5 4 F1f 4.5 5 : COLTRAP_4K_electronic
LinearMolecule Excited 6 1 13 Ground 5 0 2 3273.550066 1 0.000492728470210019 0.0000 : rR1(4.5)5.5,6,4.5,5 : Excited Sigma 5.5 5 F1e 5.5 6 - Ground v=0 4.5 4 F1e 4.5 5 : COLTRAP_4K_electronic
LinearMolecule Excited 6 1 13 Ground 6 0 2 3259.03302 1 0.00118729220167106 0.0000 : qQ1(5.5)5.5,6,5.5,6 : Excited Sigma 5.5 5 F1e 5.5 6 - Ground v=0 5.5 5 F1f 5.5 6 : COLTRAP_4K_electronic
LinearMolecule Excited 6 1 14 Ground 5 0 3 3273.553045 1 0.000500177379643392 0.0000 : rR1(4.5)6.5,6,5.5,5 : Excited Sigma 5.5 5 F1e 6.5 6 - Ground v=0 4.5 4 F1e 5.5 5 : COLTRAP_4K_electronic
LinearMolecule Excited 6 1 15 Ground 7 0 1 3259.40409 1 0.000549342066478661 0.0000 : qP21(5.5)5.5,6,6.5,7 : Excited Sigma 4.5 5 F2f 5.5 6 - Ground v=0 5.5 5 F1f 6.5 7 : LIRTRAP_35K_electronic
LinearMolecule Excited 6 1 15 Ground 6 0 1 3273.92127 1 0.000880328537911369 0.0000 : rQ21(4.5)5.5,6,5.5,6 : Excited Sigma 4.5 5 F2f 5.5 6 - Ground v=0 4.5 4 F1e 5.5 6 : LIRTRAP_35K_electronic
LinearMolecule Excited 6 1 15 Ground 5 0 1 3285.769959 1 0.000792419857523321 0.0000 : sR21(3.5)5.5,6,4.5,5 : Excited Sigma 4.5 5 F2f 5.5 6 - Ground v=0 3.5 3 F1f 4.5 5 : COLTRAP_4K_electronic
LinearMolecule Excited 6 1 15 Ground 5 0 7 3232.650694 1 0.000109272206494345 0.0000 : rR2(3.5)5.5,6,4.5,5 : Excited Sigma 4.5 5 F2f 5.5 6 - Ground v=0 3.5 4 F2f 4.5 5 : LIRTRAP_35K_electronic
LinearMolecule Excited 7 0 15 Ground 6 1 1 3290.662944 1 0.000312483653535554 0.0000 : sR21(4.5)6.5,7,5.5,6 : Excited Sigma 5.5 6 F2f 6.5 7 - Ground v=0 4.5 4 F1f 5.5 6 : COLTRAP_4K_electronic
LinearMolecule Excited 7 0 15 Ground 8 1 1 3259.01246 1 0.000210988723315428 0.0000 : qP21(6.5)6.5,7,7.5,8 : Excited Sigma 5.5 6 F2f 6.5 7 - Ground v=0 6.5 6 F1f 7.5 8 : LIRTRAP_35K_electronic
LinearMolecule Excited 7 0 15 Ground 6 1 7 3236.910735 1 0.0000653801706468151 0.0000 : rR2(4.5)6.5,7,5.5,6 : Excited Sigma 5.5 6 F2f 6.5 7 - Ground v=0 4.5 5 F2f 5.5 6 : LIRTRAP_35K_electronic
LinearMolecule Excited 7 1 13 Ground 6 0 1 3273.553045 1 0.000584564658088722 0.0000 : rR1(4.5)6.5,7,5.5,6 : Excited Sigma 5.5 5 F1e 6.5 7 - Ground v=0 4.5 4 F1e 5.5 6 : COLTRAP_4K_electronic
LinearMolecule Excited 7 1 13 Ground 7 0 1 3259.036103 1 0.00141806425606701 0.0000 : qQ1(5.5)6.5,7,6.5,7 : Excited Sigma 5.5 5 F1e 6.5 7 - Ground v=0 5.5 5 F1f 6.5 7 : COLTRAP_4K_electronic
LinearMolecule Excited 7 1 13 Ground 8 0 1 3241.92632 1 0.000386369121830279 0.0000 : pP1(6.5)6.5,7,7.5,8 : Excited Sigma 5.5 5 F1e 6.5 7 - Ground v=0 6.5 6 F1e 7.5 8 : LIRTRAP_35K_electronic
LinearMolecule Excited 8 0 13 Ground 8 1 1 3258.592781 1 0.000626335942487888 0.0000 : qQ1(6.5)7.5,8,7.5,8 : Excited Sigma 6.5 6 F1e 7.5 8 - Ground v=0 6.5 6 F1f 7.5 8 : LIRTRAP_35K_electronic
LinearMolecule Excited 9 1 13 Ground 9 0 1 3258.29253 1 0.00023312550418959 0.0000 : qQ1(7.5)8.5,9,8.5,9 : Excited Sigma 7.5 7 F1e 8.5 9 - Ground v=0 7.5 7 F1f 8.5 9 : LIRTRAP_35K_electronic
LinearMolecule Excited 10 0 13 Ground 10 1 1 3258.12975 1 0.0000732868667558952 0.0000 : qQ1(8.5)9.5,10,9.5,10 : Excited Sigma 8.5 8 F1e 9.5 10 - Ground v=0 8.5 8 F1f 9.5 10 : LIRTRAP_35K_electronic
LinearMolecule Excited 11 1 13 Ground 11 0 1 3258.09921 1 0.0000195653128691757 0.0000 : qQ1(9.5)10.5,11,10.5,11 : Excited Sigma 9.5 9 F1e 10.5 11 - Ground v=0 9.5 9 F1f 10.5 11 : LIRTRAP_35K_electronic
LinearMolecule Ground 2 1 5 Ground 2 0 4 9.21890929 0.01 0.0000815214269746548 0.0000 : rR1e(2.5)2.5,2,2.5,2 : Ground v=0 3.5 3 F1e 2.5 2 - Ground v=0 2.5 2 F1e 2.5 2 : COLTRAP_4K_rotation
LinearMolecule Ground 3 0 6 Ground 3 1 5 11.85522249 0.01 0.000058776819805889 0.0000 : rR1e(3.5)3.5,3,3.5,3 : Ground v=0 4.5 4 F1e 3.5 3 - Ground v=0 3.5 3 F1e 3.5 3 : COLTRAP_4K_rotation
LinearMolecule Ground 3 0 6 Ground 2 1 5 11.85591433 0.01 0.000867018773865019 0.0000 : rR1e(3.5)3.5,3,2.5,2 : Ground v=0 4.5 4 F1e 3.5 3 - Ground v=0 3.5 3 F1e 2.5 2 : COLTRAP_4K_rotation
LinearMolecule Ground 3 1 4 Ground 2 0 3 9.21968108 0.01 0.00108241974886373 0.0000 : rR1e(2.5)2.5,3,1.5,2 : Ground v=0 3.5 3 F1e 2.5 3 - Ground v=0 2.5 2 F1e 1.5 2 : COLTRAP_4K_rotation
LinearMolecule Ground 3 1 4 Ground 3 0 2 9.21880489 0.01 0.000130028451275843 0.0000 : rR1e(2.5)2.5,3,2.5,3 : Ground v=0 3.5 3 F1e 2.5 3 - Ground v=0 2.5 2 F1e 2.5 3 : COLTRAP_4K_rotation
LinearMolecule Ground 3 1 5 Ground 2 0 4 9.21959468 0.01 0.00107545119709708 0.0000 : rR1e(2.5)3.5,3,2.5,2 : Ground v=0 3.5 3 F1e 3.5 3 - Ground v=0 2.5 2 F1e 2.5 2 : COLTRAP_4K_rotation
LinearMolecule Ground 3 1 5 Ground 3 0 3 9.21837244 0.01 0.0000930951037737865 0.0000 : rR1e(2.5)3.5,3,3.5,3 : Ground v=0 3.5 3 F1e 3.5 3 - Ground v=0 2.5 2 F1e 3.5 3 : COLTRAP_4K_rotation
LinearMolecule Ground 3 1 6 Ground 2 0 5 11.86266735 0.01 0.000867009580308674 0.0000 : rR1f(3.5)3.5,3,2.5,2 : Ground v=0 4.5 4 F1f 3.5 3 - Ground v=0 3.5 3 F1f 2.5 2 : COLTRAP_4K_rotation
LinearMolecule Ground 4 0 4 Ground 4 1 2 11.85514055 0.01 0.0000749996676786959 0.0000 : rR1e(3.5)3.5,4,3.5,4 : Ground v=0 4.5 4 F1e 3.5 4 - Ground v=0 3.5 3 F1e 3.5 4 : COLTRAP_4K_rotation
LinearMolecule Ground 4 0 5 Ground 4 1 3 11.85487301 0.01 0.0000607636604818788 0.0000 : rR1e(3.5)4.5,4,4.5,4 : Ground v=0 4.5 4 F1e 4.5 4 - Ground v=0 3.5 3 F1e 4.5 4 : COLTRAP_4K_rotation
LinearMolecule Ground 4 0 5 Ground 3 1 5 11.8558171 0.01 0.00115362229397057 0.0000 : rR1e(3.5)4.5,4,3.5,3 : Ground v=0 4.5 4 F1e 4.5 4 - Ground v=0 3.5 3 F1e 3.5 3 : COLTRAP_4K_rotation
LinearMolecule Ground 4 0 6 Ground 4 1 5 14.5032257 0.01 0.0000296228686019532 0.0000 : rR1f(4.5)4.5,4,4.5,4 : Ground v=0 5.5 5 F1f 4.5 4 - Ground v=0 4.5 4 F1f 4.5 4 : COLTRAP_4K_rotation
LinearMolecule Ground 4 1 2 Ground 4 0 1 9.21826342 0.01 0.000126813033925293 0.0000 : rR1e(2.5)3.5,4,3.5,4 : Ground v=0 3.5 3 F1e 3.5 4 - Ground v=0 2.5 2 F1e 3.5 4 : COLTRAP_4K_rotation
LinearMolecule Ground 4 1 2 Ground 3 0 2 9.21950189 0.01 0.00144488489655599 0.0000 : rR1e(2.5)3.5,4,2.5,3 : Ground v=0 3.5 3 F1e 3.5 4 - Ground v=0 2.5 2 F1e 2.5 3 : COLTRAP_4K_rotation
LinearMolecule Ground 4 1 3 Ground 3 0 3 9.21931601 0.01 0.00153499765307144 0.0000 : rR1e(2.5)4.5,4,3.5,3 : Ground v=0 3.5 3 F1e 4.5 4 - Ground v=0 2.5 2 F1e 3.5 3 : COLTRAP_4K_rotation
LinearMolecule Ground 4 1 4 Ground 3 0 4 11.86258477 0.01 0.00117130572615905 0.0000 : rR1f(3.5)3.5,4,2.5,3 : Ground v=0 4.5 4 F1f 3.5 4 - Ground v=0 3.5 3 F1f 2.5 3 : COLTRAP_4K_rotation
LinearMolecule Ground 4 1 4 Ground 4 0 2 11.86192287 0.01 0.0000750279946400194 0.0000 : rR1f(3.5)3.5,4,3.5,4 : Ground v=0 4.5 4 F1f 3.5 4 - Ground v=0 3.5 3 F1f 3.5 4 : COLTRAP_4K_rotation
LinearMolecule Ground 4 1 5 Ground 3 0 5 11.86257272 0.01 0.0011501370063689 0.0000 : rR1f(3.5)4.5,4,3.5,3 : Ground v=0 4.5 4 F1f 4.5 4 - Ground v=0 3.5 3 F1f 3.5 3 : COLTRAP_4K_rotation
LinearMolecule Ground 4 1 5 Ground 4 0 3 11.86156676 0.01 0.0000619108968332322 0.0000 : rR1f(3.5)4.5,4,4.5,4 : Ground v=0 4.5 4 F1f 4.5 4 - Ground v=0 3.5 3 F1f 4.5 4 : COLTRAP_4K_rotation
LinearMolecule Ground 4 1 6 Ground 4 0 5 14.49330269 0.01 0.0000296476765292963 0.0000 : rR1e(4.5)4.5,4,4.5,4 : Ground v=0 5.5 5 F1e 4.5 4 - Ground v=0 4.5 4 F1e 4.5 4 : COLTRAP_4K_rotation
LinearMolecule Ground 4 1 6 Ground 3 0 6 14.49389661 0.01 0.000714595376591275 0.0000 : rR1e(4.5)4.5,4,3.5,3 : Ground v=0 5.5 5 F1e 4.5 4 - Ground v=0 4.5 4 F1e 3.5 3 : COLTRAP_4K_rotation
LinearMolecule Ground 5 0 2 Ground 4 1 2 11.85573711 0.01 0.0014522498088946 0.0000 : rR1e(3.5)4.5,5,3.5,4 : Ground v=0 4.5 4 F1e 4.5 5 - Ground v=0 3.5 3 F1e 3.5 4 : COLTRAP_4K_rotation
LinearMolecule Ground 5 0 2 Ground 5 1 1 11.8547923 0.01 0.0000740450433879043 0.0000 : rR1e(3.5)4.5,5,4.5,5 : Ground v=0 4.5 4 F1e 4.5 5 - Ground v=0 3.5 3 F1e 4.5 5 : COLTRAP_4K_rotation
LinearMolecule Ground 5 0 3 Ground 4 1 3 11.85566338 0.01 0.00149802515196566 0.0000 : rR1e(3.5)5.5,5,4.5,4 : Ground v=0 4.5 4 F1e 5.5 5 - Ground v=0 3.5 3 F1e 4.5 4 : COLTRAP_4K_rotation
LinearMolecule Ground 5 0 4 Ground 5 1 2 14.503162 0.01 0.0000359421837520935 0.0000 : rR1f(4.5)4.5,5,4.5,5 : Ground v=0 5.5 5 F1f 4.5 5 - Ground v=0 4.5 4 F1f 4.5 5 : COLTRAP_4K_rotation
LinearMolecule Ground 5 0 5 Ground 5 1 3 14.5028696 0.01 0.000030408638765195 0.0000 : rR1f(4.5)5.5,5,5.5,5 : Ground v=0 5.5 5 F1f 5.5 5 - Ground v=0 4.5 4 F1f 5.5 5 : COLTRAP_4K_rotation
LinearMolecule Ground 5 0 6 Ground 4 1 6 17.13396535 0.01 0.000445392696208125 0.0000 : rR1e(5.5)5.5,5,4.5,4 : Ground v=0 6.5 6 F1e 5.5 5 - Ground v=0 5.5 5 F1e 4.5 4 : COLTRAP_4K_rotation
LinearMolecule Ground 5 1 1 Ground 4 0 1 9.21921234 0.01 0.00192812966230356 0.0000 : rR1e(2.5)4.5,5,3.5,4 : Ground v=0 3.5 3 F1e 4.5 5 - Ground v=0 2.5 2 F1e 3.5 4 : COLTRAP_4K_rotation
LinearMolecule Ground 5 1 2 Ground 4 0 2 11.86249722 0.01 0.00145222984480183 0.0000 : rR1f(3.5)4.5,5,3.5,4 : Ground v=0 4.5 4 F1f 4.5 5 - Ground v=0 3.5 3 F1f 3.5 4 : COLTRAP_4K_rotation
LinearMolecule Ground 5 1 2 Ground 5 0 1 11.86148654 0.01 0.0000740607280254557 0.0000 : rR1f(3.5)4.5,5,4.5,5 : Ground v=0 4.5 4 F1f 4.5 5 - Ground v=0 3.5 3 F1f 4.5 5 : COLTRAP_4K_rotation
LinearMolecule Ground 5 1 3 Ground 4 0 3 11.86244405 0.01 0.00149803793633426 0.0000 : rR1f(3.5)5.5,5,4.5,4 : Ground v=0 4.5 4 F1f 5.5 5 - Ground v=0 3.5 3 F1f 4.5 4 : COLTRAP_4K_rotation
LinearMolecule Ground 5 1 4 Ground 4 0 4 14.49383625 0.01 0.000899795638604681 0.0000 : rR1e(4.5)4.5,5,3.5,4 : Ground v=0 5.5 5 F1e 4.5 5 - Ground v=0 4.5 4 F1e 3.5 4 : COLTRAP_4K_rotation
LinearMolecule Ground 5 1 4 Ground 5 0 2 14.49323631 0.01 0.0000359387177564275 0.0000 : rR1e(4.5)4.5,5,4.5,5 : Ground v=0 5.5 5 F1e 4.5 5 - Ground v=0 4.5 4 F1e 4.5 5 : COLTRAP_4K_rotation
LinearMolecule Ground 5 1 5 Ground 5 0 3 14.49304755 0.01 0.0000304270435922997 0.0000 : rR1e(4.5)5.5,5,5.5,5 : Ground v=0 5.5 5 F1e 5.5 5 - Ground v=0 4.5 4 F1e 5.5 5 : COLTRAP_4K_rotation
LinearMolecule Ground 5 1 5 Ground 4 0 5 14.49383313 0.01 0.000886678117572048 0.0000 : rR1e(4.5)5.5,5,4.5,4 : Ground v=0 5.5 5 F1e 5.5 5 - Ground v=0 4.5 4 F1e 4.5 4 : COLTRAP_4K_rotation
LinearMolecule Ground 5 1 6 Ground 4 0 6 17.14745196 0.01 0.000444991875865616 0.0000 : rR1f(5.5)5.5,5,4.5,4 : Ground v=0 6.5 6 F1f 5.5 5 - Ground v=0 5.5 5 F1f 4.5 4 : COLTRAP_4K_rotation
LinearMolecule Ground 6 0 1 Ground 5 1 1 11.85558265 0.01 0.00180329475738451 0.0000 : rR1e(3.5)5.5,6,4.5,5 : Ground v=0 4.5 4 F1e 5.5 6 - Ground v=0 3.5 3 F1e 4.5 5 : COLTRAP_4K_rotation
LinearMolecule Ground 6 0 2 Ground 6 1 1 14.5028059 0.01 0.0000357943987484972 0.0000 : rR1f(4.5)5.5,6,5.5,6 : Ground v=0 5.5 5 F1f 5.5 6 - Ground v=0 4.5 4 F1f 5.5 6 : COLTRAP_4K_rotation
LinearMolecule Ground 6 0 3 Ground 5 1 3 14.50367598 1 0.00109015912486976 0.0000 : rR1f(4.5)6.5,6,5.5,5 : Ground v=0 5.5 5 F1f 6.5 6 - Ground v=0 4.5 4 F1f 5.5 5 : COLTRAP_4K_rotation
LinearMolecule Ground 6 0 5 Ground 5 1 5 17.13392692 0.01 0.00053066337934388 0.0000 : rR1e(5.5)6.5,6,5.5,5 : Ground v=0 6.5 6 F1e 6.5 6 - Ground v=0 5.5 5 F1e 5.5 5 : COLTRAP_4K_rotation
LinearMolecule Ground 6 1 1 Ground 5 0 1 11.86236623 0.01 0.00180328910423308 0.0000 : rR1f(3.5)5.5,6,4.5,5 : Ground v=0 4.5 4 F1f 5.5 6 - Ground v=0 3.5 3 F1f 4.5 5 : COLTRAP_4K_rotation
LinearMolecule Ground 6 1 2 Ground 6 0 1 14.49298308 0.01 0.0000357973246696225 0.0000 : rR1e(4.5)5.5,6,5.5,6 : Ground v=0 5.5 5 F1e 5.5 6 - Ground v=0 4.5 4 F1e 5.5 6 : COLTRAP_4K_rotation
LinearMolecule Ground 6 1 2 Ground 5 0 2 14.49376958 0.01 0.00107025635369371 0.0000 : rR1e(4.5)5.5,6,4.5,5 : Ground v=0 5.5 5 F1e 5.5 6 - Ground v=0 4.5 4 F1e 4.5 5 : COLTRAP_4K_rotation
LinearMolecule Ground 6 1 3 Ground 5 0 3 14.49374021 0.01 0.00109053287184468 0.0000 : rR1e(4.5)6.5,6,5.5,5 : Ground v=0 5.5 5 F1e 6.5 6 - Ground v=0 4.5 4 F1e 5.5 5 : COLTRAP_4K_rotation
LinearMolecule Ground 7 0 1 Ground 6 1 1 14.50361024 0.01 0.00127558967088461 0.0000 : rR1f(4.5)6.5,7,5.5,6 : Ground v=0 5.5 5 F1f 6.5 7 - Ground v=0 4.5 4 F1f 5.5 6 : COLTRAP_4K_rotation
LinearMolecule Ground 7 0 2 Ground 6 1 2 17.13386726 0.01 0.00062176447082173 0.0000 : rR1e(5.5)6.5,7,5.5,6 : Ground v=0 6.5 6 F1e 6.5 7 - Ground v=0 5.5 5 F1e 5.5 6 : COLTRAP_4K_rotation
LinearMolecule Ground 7 0 3 Ground 6 1 3 17.13386733 0.01 0.000629592543748738 0.0000 : rR1e(5.5)7.5,7,6.5,6 : Ground v=0 6.5 6 F1e 7.5 7 - Ground v=0 5.5 5 F1e 6.5 6 : COLTRAP_4K_rotation
LinearMolecule Ground 7 1 1 Ground 6 0 1 14.49367582 0.01 0.00127604012797931 0.0000 : rR1e(4.5)6.5,7,5.5,6 : Ground v=0 5.5 5 F1e 6.5 7 - Ground v=0 4.5 4 F1e 5.5 6 : COLTRAP_4K_rotation
LinearMolecule Ground 8 0 1 Ground 7 1 1 17.133806 0.01 0.000721079050061452 0.0000 : rR1e(5.5)7.5,8,6.5,7 : Ground v=0 6.5 6 F1e 7.5 8 - Ground v=0 5.5 5 F1e 6.5 7 : COLTRAP_4K_rotation
LinearMolecule Ground 8 1 1 Ground 7 0 1 17.14732922 0.01 0.000720430140392155 0.0000 : rR1f(5.5)7.5,8,6.5,7 : Ground v=0 6.5 6 F1f 7.5 8 - Ground v=0 5.5 5 F1f 6.5 7 : COLTRAP_4K_rotation
LinearMolecule Ground 5 0 5 Ground 4 1 5 14.50373727 0.01 0.000886709580041733 0.0000 : rR1f(4.5)5.5,5,4.5,4 : Ground v=0 5.5 5 F1f 5.5 5 - Ground v=0 4.5 4 F1f 4.5 4 : COLTRAP_4K_rotation
LinearMolecule Ground 5 0 4 Ground 4 1 4 14.50373728 0.01 0.000898742369210828 0.0000 : rR1f(4.5)4.5,5,3.5,4 : Ground v=0 5.5 5 F1f 4.5 5 - Ground v=0 4.5 4 F1f 3.5 4 : COLTRAP_4K_rotation
LinearMolecule Ground 4 0 6 Ground 3 1 6 14.50379415 0.01 0.000714359149324414 0.0000 : rR1f(4.5)4.5,4,3.5,3 : Ground v=0 5.5 5 F1f 4.5 4 - Ground v=0 4.5 4 F1f 3.5 3 : COLTRAP_4K_rotation
LinearMolecule Ground 6 0 2 Ground 5 1 2 14.50367589 0.01 0.00106937931798972 0.0000 : rR1f(4.5)5.5,6,4.5,5 : Ground v=0 5.5 5 F1f 5.5 6 - Ground v=0 4.5 4 F1f 4.5 5 : COLTRAP_4K_rotation

# 3) Assigned rovibronic lines without hyperfine from global fit

```
molecule-name upper J' S' # lower J" S" # position stdev strength width branch vibronic-level-upper J' N' F1/F2-e/f J" N" F1/F2-e/f data type
LinearMolecule Excited 1.5  0  1 Ground 1.5  1  1  3056.66979  1  0.0174089507105965  0.0000 : qQ1e(1.5) : Excited Linear 1.5  1 F1f - Ground v=0 1.5  1 F1e : LIRTRAP_35K
LinearMolecule Excited 1.5  1  1 Ground 1.5  0  1  3056.66979  1  0.0174085164907357  0.0000 : qQ1f(1.5) : Excited Linear 1.5  1 F1e - Ground v=0 1.5  1 F1f : LIRTRAP_35K
LinearMolecule Excited 2.5  1  1 Ground 2.5  0  1  3056.62553  1  0.00849945001518491 0.0000 : qQ1e(2.5) : Excited Linear 2.5  2 F1f - Ground v=0 2.5  2 F1e : LIRTRAP_35K
LinearMolecule Excited 2.5  0  1 Ground 2.5  1  1  3056.61992  1  0.00849860920665753 0.0000 : qQ1f(2.5) : Excited Linear 2.5  2 F1e - Ground v=0 2.5  2 F1f : LIRTRAP_35K
LinearMolecule Excited 3.5  0  1 Ground 3.5  1  1  3056.56479  1  0.004283515139588   0.0000 : qQ1e(3.5) : Excited Linear 3.5  3 F1f - Ground v=0 3.5  3 F1e : LIRTRAP_35K
LinearMolecule Excited 3.5  1  1 Ground 3.5  0  1  3056.55023  1  0.0042824680875317  0.0000 : qQ1f(3.5) : Excited Linear 3.5  3 F1e - Ground v=0 3.5  3 F1f : LIRTRAP_35K
LinearMolecule Excited 4.5  1  1 Ground 4.5  0  1  3056.48856  1  0.00207696544212161 0.0000 : qQ1e(4.5) : Excited Linear 4.5  4 F1f - Ground v=0 4.5  4 F1e : LIRTRAP_35K
LinearMolecule Excited 4.5  0  1 Ground 4.5  1  1  3056.4587  1  0.00207596487084713 0.0000 : qQ1f(4.5) : Excited Linear 4.5  4 F1e - Ground v=0 4.5  4 F1f : LIRTRAP_35K
LinearMolecule Excited 5.5  0  1 Ground 5.5  1  1  3056.39726  1  0.000942384922553608 0.0000 : qQ1e(5.5) : Excited Linear 5.5  5 F1f - Ground v=0 5.5  5 F1e : LIRTRAP_35K
LinearMolecule Excited 5.5  1  1 Ground 5.5  0  1  3056.34555  1  0.000941604177227193 0.0000 : qQ1f(5.5) : Excited Linear 5.5  5 F1e - Ground v=0 5.5  5 F1f : LIRTRAP_35K
LinearMolecule Excited 6.5  1  1 Ground 6.5  0  1  3056.29084  1  0.000394581759803592 0.0000 : qQ1e(6.5) : Excited Linear 6.5  6 F1f - Ground v=0 6.5  6 F1e : LIRTRAP_35K
LinearMolecule Excited 6.5  0  1 Ground 6.5  1  1  3056.20994  1  0.000394069037120828 0.0000 : qQ1f(6.5) : Excited Linear 6.5  6 F1e - Ground v=0 6.5  6 F1f : LIRTRAP_35K
LinearMolecule Excited 7.5  0  1 Ground 7.5  1  1  3056.17091  1  0.000232809715872659 0.0000 : qQ1e(7.5) : Excited Linear 7.5  7 F1f - Ground v=0 7.5  7 F1e : LIRTRAP_35K
LinearMolecule Excited 7.5  1  1 Ground 7.5  0  1  3056.0513  1  0.000232446270173121 0.0000 : qQ1f(7.5) : Excited Linear 7.5  7 F1e - Ground v=0 7.5  7 F1f : LIRTRAP_35K
LinearMolecule Excited 1.5  0  1 Ground 2.5  1  1  3050.08429  1  0.00705221168104699 0.0000 : pP1f(2.5) : Excited Linear 1.5  1 F1f - Ground v=0 2.5  2 F1f : LIRTRAP_35K
LinearMolecule Excited 1.5  1  1 Ground 2.5  0  1  3050.08429  1  0.00705269747287075 0.0000 : pP1e(2.5) : Excited Linear 1.5  1 F1e - Ground v=0 2.5  2 F1e : LIRTRAP_35K
LinearMolecule Excited 2.5  1  1 Ground 3.5  0  1  3047.40006  1  0.00937705352981706 0.0000 : pP1f(3.5) : Excited Linear 2.5  2 F1f - Ground v=0 3.5  3 F1f : LIRTRAP_35K
LinearMolecule Excited 2.5  0  1 Ground 3.5  1  1  3047.403219  1  0.00937861693416844 0.0000 : pP1e(3.5) : Excited Linear 2.5  2 F1e - Ground v=0 3.5  3 F1e : LIRTRAP_35K
LinearMolecule Excited 3.5  1  1 Ground 4.5  0  1  3044.70168  1  0.00898726485538887 0.0000 : pP1e(4.5) : Excited Linear 3.5  3 F1e - Ground v=0 4.5  4 F1e : LIRTRAP_35K
LinearMolecule Excited 3.5  0  1 Ground 4.5  1  1  3044.69598  1  0.00898434966089005 0.0000 : pP1f(4.5) : Excited Linear 3.5  3 F1f - Ground v=0 4.5  4 F1f : LIRTRAP_35K
LinearMolecule Excited 4.5  1  1 Ground 5.5  0  1  3041.97171  1  0.00719199617451911 0.0000 : pP1f(5.5) : Excited Linear 4.5  4 F1f - Ground v=0 5.5  5 F1f : LIRTRAP_35K
LinearMolecule Excited 4.5  0  1 Ground 5.5  1  1  3041.97894  1  0.00719597930963772 0.0000 : pP1e(5.5) : Excited Linear 4.5  4 F1e - Ground v=0 5.5  5 F1e : LIRTRAP_35K
LinearMolecule Excited 5.5  1  1 Ground 6.5  0  1  3039.23585  1  0.00502930785536518 0.0000 : pP1e(6.5) : Excited Linear 5.5  5 F1e - Ground v=0 6.5  6 F1e : LIRTRAP_35K
LinearMolecule Excited 5.5  0  1 Ground 6.5  1  1  3039.22662  1  0.00502496049332825 0.0000 : pP1f(6.5) : Excited Linear 5.5  5 F1f - Ground v=0 6.5  6 F1f : LIRTRAP_35K
LinearMolecule Excited 6.5  0  1 Ground 7.5  1  1  3036.47137  1  0.00312660107705034 0.0000 : pP1e(7.5) : Excited Linear 6.5  6 F1e - Ground v=0 7.5  7 F1e : LIRTRAP_35K
LinearMolecule Excited 6.5  1  1 Ground 7.5  0  1  3036.46076  1  0.00312264321119015 0.0000 : pP1f(7.5) : Excited Linear 6.5  6 F1f - Ground v=0 7.5  7 F1f : LIRTRAP_35K
LinearMolecule Excited 7.5  1  1 Ground 8.5  0  1  3033.685856  1  0.00174613974656014 0.0000 : pP1e(8.5) : Excited Linear 7.5  7 F1e - Ground v=0 8.5  8 F1e : LIRTRAP_35K
LinearMolecule Excited 7.5  0  1 Ground 8.5  1  1  3033.67391  1  0.00174305710702794 0.0000 : pP1f(8.5) : Excited Linear 7.5  7 F1f - Ground v=0 8.5  8 F1f : LIRTRAP_35K
LinearMolecule Excited 2.5  0  2 Ground 1.5  1  2  3062.818066  1  0.00244872567012587 0.0000 : rR2e(1.5) : Excited Linear 2.5  3 F2e - Ground v=0 1.5  2 F2e : LIRTRAP_35K
LinearMolecule Excited 2.5  1  2 Ground 1.5  0  2  3062.84646  1  0.00245308355967555 0.0000 : rR2f(1.5) : Excited Linear 2.5  3 F2f - Ground v=0 1.5  2 F2f : LIRTRAP_35K
LinearMolecule Excited 2.5  0  1 Ground 1.5  1  1  3063.20877  1  0.00870510167796312 0.0000 : rR1e(1.5) : Excited Linear 2.5  2 F1e - Ground v=0 1.5  1 F1e : LIRTRAP_35K
LinearMolecule Excited 2.5  1  1 Ground 1.5  0  1  3063.20878  1  0.00870503260474565 0.0000 : rR1f(1.5) : Excited Linear 2.5  2 F1f - Ground v=0 1.5  1 F1f : LIRTRAP_35K
LinearMolecule Excited 3.5  1  2 Ground 2.5  0  2  3065.52709  1  0.00280129584269736 0.0000 : rR2e(2.5) : Excited Linear 3.5  4 F2e - Ground v=0 2.5  3 F2e : LIRTRAP_35K
LinearMolecule Excited 3.5  0  2 Ground 2.5  1  2  3065.55635  1  0.00280888475083989 0.0000 : rR2f(2.5) : Excited Linear 3.5  4 F2f - Ground v=0 2.5  3 F2f : LIRTRAP_35K
LinearMolecule Excited 3.5  1  1 Ground 2.5  0  1  3065.77709  1  0.0125933198023518  0.0000 : rR1e(2.5) : Excited Linear 3.5  3 F1e - Ground v=0 2.5  2 F1e : LIRTRAP_35K
LinearMolecule Excited 3.5  0  1 Ground 2.5  1  1  3065.78269  1  0.0125927298518788  0.0000 : rR1f(2.5) : Excited Linear 3.5  3 F1f - Ground v=0 2.5  2 F1f : LIRTRAP_35K
LinearMolecule Excited 4.5  0  1 Ground 3.5  1  1  3068.32861  1  0.0131289429359598  0.0000 : rR1e(3.5) : Excited Linear 4.5  4 F1e - Ground v=0 3.5  3 F1e : LIRTRAP_35K
LinearMolecule Excited 4.5  1  1 Ground 3.5  0  1  3068.33757  1  0.0131272499916404  0.0000 : rR1f(3.5) : Excited Linear 4.5  4 F1f - Ground v=0 3.5  3 F1f : LIRTRAP_35K
LinearMolecule Excited 5.5  1  1 Ground 4.5  0  1  3070.86319  1  0.0114370946769143  0.0000 : rR1e(4.5) : Excited Linear 5.5  5 F1e - Ground v=0 4.5  4 F1e : LIRTRAP_35K
LinearMolecule Excited 5.5  0  1 Ground 4.5  1  1  3070.87761  1  0.0114340287667025  0.0000 : rR1f(4.5) : Excited Linear 5.5  5 F1f - Ground v=0 4.5  4 F1f : LIRTRAP_35K
LinearMolecule Excited 5.5  1  2 Ground 4.5  0  2  3070.88201  1  0.00219076014027181 0.0000 : rR2e(4.5) : Excited Linear 5.5  6 F2e - Ground v=0 4.5  5 F2e : LIRTRAP_35K
LinearMolecule Excited 5.5  0  2 Ground 4.5  1  2  3070.90883  1  0.00220110355042001 0.0000 : rR2f(4.5) : Excited Linear 5.5  6 F2f - Ground v=0 4.5  5 F2f : LIRTRAP_35K
LinearMolecule Excited 6.5  0  1 Ground 5.5  1  1  3073.38119  1  0.00869729512640995 0.0000 : rR1e(5.5) : Excited Linear 6.5  6 F1e - Ground v=0 5.5  5 F1e : LIRTRAP_35K
LinearMolecule Excited 6.5  1  1 Ground 5.5  0  1  3073.40142  1  0.00869314799093475 0.0000 : rR1f(5.5) : Excited Linear 6.5  6 F1f - Ground v=0 5.5  5 F1f : LIRTRAP_35K
LinearMolecule Excited 0.5  1  1 Ground 1.5  0  2  3051.791431  1  0.00136096097968262 0.0000 : pP2f(1.5) : Excited Linear 0.5  1 F2f - Ground v=0 1.5  2 F2f : LIRTRAP_35K
LinearMolecule Excited 0.5  0  1 Ground 1.5  1  2  3051.807704  1  0.00136230024822209 0.0000 : pP2e(1.5) : Excited Linear 0.5  1 F2e - Ground v=0 1.5  2 F2e : LIRTRAP_35K
LinearMolecule Excited 1.5  0  2 Ground 2.5  1  2  3048.990386  1  0.00196178171429948 0.0000 : pP2f(2.5) : Excited Linear 1.5  2 F2f - Ground v=0 2.5  3 F2f : LIRTRAP_35K
LinearMolecule Excited 1.5  1  2 Ground 2.5  0  2  3049.001271  1  0.00196541704960625 0.0000 : pP2e(2.5) : Excited Linear 1.5  2 F2e - Ground v=0 2.5  3 F2e : LIRTRAP_35K
LinearMolecule Excited 6.5  0  2 Ground 5.5  1  2  3073.5274  1  0.00160047112414677 0.0000 : rR2e(5.5) : Excited Linear 6.5  7 F2e - Ground v=0 5.5  6 F2e : LIRTRAP_35K
LinearMolecule Excited 6.5  1  2 Ground 5.5  0  2  3073.5524  1  0.00159536682740234 0.0000 : rR2f(5.5) : Excited Linear 6.5  7 F2f - Ground v=0 5.5  6 F2f : LIRTRAP_35K
LinearMolecule Excited 7.5  1  1 Ground 6.5  0  1  3075.88207  1  0.005883705064278   0.0000 : rR1e(6.5) : Excited Linear 7.5  7 F1e - Ground v=0 6.5  6 F1e : LIRTRAP_35K
LinearMolecule Excited 7.5  0  1 Ground 6.5  1  1  3075.9095  1  0.0058782945547335  0.0000 : rR1f(6.5) : Excited Linear 7.5  7 F1f - Ground v=0 6.5  6 F1f : LIRTRAP_35K
LinearMolecule Excited 8.5  0  1 Ground 7.5  1  1  3078.36841  1  0.00357587284713197 0.0000 : rR1e(7.5) : Excited Linear 8.5  8 F1e - Ground v=0 7.5  7 F1e : LIRTRAP_35K
LinearMolecule Excited 8.5  1  1 Ground 7.5  0  1  3078.40349  1  0.00357098162428989 0.0000 : rR1f(7.5) : Excited Linear 8.5  8 F1f - Ground v=0 7.5  7 F1f : LIRTRAP_35K
LinearMolecule Excited 9.5  1  1 Ground 8.5  0  1  3080.83692  1  0.00196405843494233 0.0000 : rR1e(8.5) : Excited Linear 9.5  9 F1e - Ground v=0 8.5  8 F1e : LIRTRAP_35K
LinearMolecule Excited 9.5  0  1 Ground 8.5  1  1  3080.88196  1  0.00196006772546706 0.0000 : rR1f(8.5) : Excited Linear 9.5  9 F1f - Ground v=0 8.5  8 F1f : LIRTRAP_35K
LinearMolecule Excited 1.5  1  3 Ground 2.5  0  1  3255.69958  1  0.0115901527559186  0.0000 : pP1(2.5) : Excited Sigma 1.5  1 F1e - Ground v=0 2.5  2 F1e : LIRTRAP_35K
LinearMolecule Excited 2.5  0  3 Ground 3.5  1  1  3252.02603  1  0.0107402644149782  0.0000 : pP1(3.5) : Excited Sigma 2.5  2 F1e - Ground v=0 3.5  3 F1e : LIRTRAP_35K
LinearMolecule Excited 3.5  1  3 Ground 4.5  0  1  3248.50809  1  0.00893808566114933 0.0000 : pP1(4.5) : Excited Sigma 3.5  3 F1e - Ground v=0 4.5  4 F1e : LIRTRAP_35K
LinearMolecule Excited 0.5  1  2 Ground 1.5  0  1  3262.3904  1  0.0102273208182034  0.0000 : qP21(1.5) : Excited Sigma 0.5  1 F2f - Ground v=0 1.5  1 F1f : LIRTRAP_35K
LinearMolecule Excited 1.5  1  3 Ground 1.5  0  1  3262.28189  1  0.00957004275578169 0.0000 : qQ1(1.5) : Excited Sigma 1.5  1 F1e - Ground v=0 1.5  1 F1f : LIRTRAP_35K
LinearMolecule Excited 1.5  0  3 Ground 2.5  1  1  3261.4194  1  0.00938156645040853 0.0000 : qP21(2.5) : Excited Sigma 1.5  2 F2f - Ground v=0 2.5  2 F1f : LIRTRAP_35K
LinearMolecule Excited 2.5  0  3 Ground 2.5  1  1  3261.24244  1  0.0139099050297562  0.0000 : qQ1(2.5) : Excited Sigma 2.5  2 F1e - Ground v=0 2.5  2 F1f : LIRTRAP_35K
LinearMolecule Excited 1.5  0  3 Ground 1.5  1  2  3215.79362  1  0.00242545518163722 0.0000 : qQ2(1.5) : Excited Sigma 1.5  2 F2f - Ground v=0 1.5  2 F2e : LIRTRAP_35K
LinearMolecule Excited 1.5  1  3 Ground 1.5  0  2  3210.02868  1  0.00206620877033326 0.0000 : pQ12(1.5) : Excited Sigma 1.5  1 F1e - Ground v=0 1.5  2 F2f : LIRTRAP_35K
LinearMolecule Excited 0.5  1  2 Ground 1.5  0  2  3210.13608  1  0.000815677902072579 0.0000 : pP2(1.5) : Excited Sigma 0.5  1 F2f - Ground v=0 1.5  2 F2f : LIRTRAP_35K
LinearMolecule Excited 2.5  0  3 Ground 2.5  1  2  3208.62374  1  0.00223548181117628 0.0000 : pQ12(2.5) : Excited Sigma 2.5  2 F1e - Ground v=0 2.5  3 F2f : LIRTRAP_35K
LinearMolecule Excited 2.5  1  3 Ground 2.5  0  2  3217.26698  1  0.00287929333971952 0.0000 : qQ2(2.5) : Excited Sigma 2.5  3 F2f - Ground v=0 2.5  3 F2e : LIRTRAP_35K
LinearMolecule Excited 4.5  1  3 Ground 4.5  0  2  3220.2471  1  0.00253462583440848 0.0000 : qQ2(4.5) : Excited Sigma 4.5  5 F2f - Ground v=0 4.5  5 F2e : LIRTRAP_35K
LinearMolecule Excited 3.5  0  3 Ground 3.5  1  2  3218.75244  1  0.00291306083543604 0.0000 : qQ2(3.5) : Excited Sigma 3.5  4 F2f - Ground v=0 3.5  4 F2e : LIRTRAP_35K
LinearMolecule Excited 4.5  0  3 Ground 5.5  1  1  3245.14049  1  0.00610205643426293 0.0000 : pP1(5.5) : Excited Sigma 4.5  4 F1e - Ground v=0 5.5  5 F1e : LIRTRAP_35K
LinearMolecule Excited 2.5  1  3 Ground 1.5  0  2  3224.16029  1  0.00106979217141546 0.0000 : rR2(1.5) : Excited Sigma 2.5  3 F2f - Ground v=0 1.5  2 F2f : LIRTRAP_35K
LinearMolecule Excited 3.5  0  3 Ground 2.5  1  2  3228.40116  1  0.00120301195278591 0.0000 : rR2(2.5) : Excited Sigma 3.5  4 F2f - Ground v=0 2.5  3 F2f : LIRTRAP_35K
LinearMolecule Excited 2.5  0  3 Ground 1.5  1  1  3267.82975  1  0.00337390760361129 0.0000 : rR1(1.5) : Excited Sigma 2.5  2 F1e - Ground v=0 1.5  1 F1e : LIRTRAP_35K
LinearMolecule Excited 1.5  0  3 Ground 1.5  1  1  3268.00796  1  0.0104389273965887  0.0000 : rQ21(1.5) : Excited Sigma 1.5  2 F2f - Ground v=0 1.5  1 F1e : LIRTRAP_35K
LinearMolecule Excited 3.5  1  3 Ground 2.5  0  1  3269.5826  1  0.005762105977923   0.0000 : rR1(2.5) : Excited Sigma 3.5  3 F1e - Ground v=0 2.5  2 F1e : LIRTRAP_35K
LinearMolecule Excited 2.5  1  3 Ground 2.5  0  1  3269.82989  1  0.0128756669878098  0.0000 : rQ21(2.5) : Excited Sigma 2.5  3 F2f - Ground v=0 2.5  2 F1e : LIRTRAP_35K
LinearMolecule Excited 4.5  0  3 Ground 3.5  1  1  3271.49014  1  0.00639184382852007 0.0000 : rR1(3.5) : Excited Sigma 4.5  4 F1e - Ground v=0 3.5  3 F1e : LIRTRAP_35K
LinearMolecule Excited 3.5  0  3 Ground 3.5  1  1  3271.80439  1  0.0115201180170258  0.0000 : rQ21(3.5) : Excited Sigma 3.5  4 F2f - Ground v=0 3.5  3 F1e : LIRTRAP_35K
LinearMolecule Excited 4.5  1  3 Ground 4.5  0  1  3273.92468  1  0.00845844730777474 0.0000 : rQ21(4.5) : Excited Sigma 4.5  5 F2f - Ground v=0 4.5  4 F1e : LIRTRAP_35K
LinearMolecule Excited 5.5  1  4 Ground 6.5  0  1  3274.64865  1  0.00333426814796752 0.0000 : pP1e(6.5) : Excited combopi 5.5  5 F1e - Ground v=0 6.5  6 F1e : LIRTRAP_35K
LinearMolecule Excited 5.5  0  4 Ground 6.5  1  1  3274.35256  1  0.00334077573955712 0.0000 : pP1f(6.5) : Excited combopi 5.5  5 F1f - Ground v=0 6.5  6 F1f : LIRTRAP_35K
LinearMolecule Excited 2.5  1  3 Ground 3.5  0  1  3260.60326  1  0.00915361791137903 0.0000 : qP21(3.5) : Excited Sigma 2.5  3 F2f - Ground v=0 3.5  3 F1f : LIRTRAP_35K
LinearMolecule Excited 3.5  1  3 Ground 3.5  0  1  3260.3558  1  0.0174137769003928  0.0000 : qQ1(3.5) : Excited Sigma 3.5  3 F1e - Ground v=0 3.5  3 F1f : LIRTRAP_35K
LinearMolecule Excited 3.5  0  3 Ground 4.5  1  1  3259.9337  1  0.00617265941480426 0.0000 : qP21(4.5) : Excited Sigma 3.5  4 F2f - Ground v=0 4.5  4 F1f : LIRTRAP_35K
LinearMolecule Excited 4.5  0  3 Ground 4.5  1  1  3259.62056  1  0.0141477624583206  0.0000 : qQ1(4.5) : Excited Sigma 4.5  4 F1e - Ground v=0 4.5  4 F1f : LIRTRAP_35K
LinearMolecule Excited 4.5  1  3 Ground 5.5  0  1  3259.40716  1  0.00366101008211047 0.0000 : qP21(5.5) : Excited Sigma 4.5  5 F2f - Ground v=0 5.5  5 F1f : LIRTRAP_35K
LinearMolecule Excited 5.5  0  3 Ground 5.5  1  1  3276.18577  1  0.00529576010349682 0.0000 : rQ21(5.5) : Excited Sigma 5.5  6 F2f - Ground v=0 5.5  5 F1e : LIRTRAP_35K
LinearMolecule Excited 2.5  1  3 Ground 1.5  0  1  3276.41199  1  0.00247004870440753 0.0000 : sR21(1.5) : Excited Sigma 2.5  3 F2f - Ground v=0 1.5  1 F1f : LIRTRAP_35K
LinearMolecule Excited 1.5  0  4 Ground 1.5  1  1  3294.74616  1  0.0161984675958642  0.0000 : qQ1e(1.5) : Excited combopi 1.5  1 F1f - Ground v=0 1.5  1 F1e : LIRTRAP_35K
LinearMolecule Excited 1.5  1  4 Ground 1.5  0  1  3294.76422  1  0.0161766191271604  0.0000 : qQ1f(1.5) : Excited combopi 1.5  1 F1e - Ground v=0 1.5  1 F1f : LIRTRAP_35K
LinearMolecule Excited 2.5  1  4 Ground 2.5  0  1  3293.98749  1  0.00736270172998529 0.0000 : qQ1e(2.5) : Excited combopi 2.5  2 F1f - Ground v=0 2.5  2 F1e : LIRTRAP_35K
LinearMolecule Excited 2.5  0  4 Ground 2.5  1  1  3294.0435  1  0.00733870430156065 0.0000 : qQ1f(2.5) : Excited combopi 2.5  2 F1e - Ground v=0 2.5  2 F1f : LIRTRAP_35K
LinearMolecule Excited 3.5  0  4 Ground 3.5  1  1  3293.15963  1  0.00350600647142919 0.0000 : qQ1e(3.5) : Excited combopi 3.5  3 F1f - Ground v=0 3.5  3 F1e : LIRTRAP_35K
LinearMolecule Excited 3.5  1  4 Ground 3.5  0  1  3293.26802  1  0.00348837961824852 0.0000 : qQ1f(3.5) : Excited combopi 3.5  3 F1e - Ground v=0 3.5  3 F1f : LIRTRAP_35K
LinearMolecule Excited 1.5  0  4 Ground 2.5  1  1  3288.1591  1  0.00856793613144778 0.0000 : pP1f(2.5) : Excited combopi 1.5  1 F1f - Ground v=0 2.5  2 F1f : LIRTRAP_35K
LinearMolecule Excited 1.5  1  4 Ground 2.5  0  1  3288.18093  1  0.00856249171797746 0.0000 : pP1e(2.5) : Excited combopi 1.5  1 F1e - Ground v=0 2.5  2 F1e : LIRTRAP_35K
LinearMolecule Excited 2.5  1  4 Ground 3.5  0  1  3284.76139  1  0.00996030764047719 0.0000 : pP1f(3.5) : Excited combopi 2.5  2 F1f - Ground v=0 3.5  3 F1f : LIRTRAP_35K
LinearMolecule Excited 2.5  0  4 Ground 3.5  1  1  3284.82727  1  0.00994370260595497 0.0000 : pP1e(3.5) : Excited combopi 2.5  2 F1e - Ground v=0 3.5  3 F1e : LIRTRAP_35K
LinearMolecule Excited 3.5  0  4 Ground 4.5  1  1  3281.29072  1  0.00826023691751508 0.0000 : pP1f(4.5) : Excited combopi 3.5  3 F1f - Ground v=0 4.5  4 F1f : LIRTRAP_35K
LinearMolecule Excited 3.5  1  4 Ground 4.5  0  1  3281.41968  1  0.00824146013266771 0.0000 : pP1e(4.5) : Excited combopi 3.5  3 F1e - Ground v=0 4.5  4 F1e : LIRTRAP_35K
LinearMolecule Excited 4.5  1  4 Ground 5.5  0  1  3277.80909  1  0.00566269454656987 0.0000 : pP1f(5.5) : Excited combopi 4.5  4 F1f - Ground v=0 5.5  5 F1f : LIRTRAP_35K
LinearMolecule Excited 4.5  0  4 Ground 5.5  1  1  3278.016291  1  0.00564942131074797 0.0000 : pP1e(5.5) : Excited combopi 4.5  4 F1e - Ground v=0 5.5  5 F1e : LIRTRAP_35K
LinearMolecule Excited 2.5  0  4 Ground 1.5  1  1  3300.63094  1  0.0102688959503254  0.0000 : rR1e(1.5) : Excited combopi 2.5  2 F1e - Ground v=0 1.5  1 F1e : LIRTRAP_35K
LinearMolecule Excited 2.5  1  4 Ground 1.5  0  1  3300.57147  1  0.0103013985891921  0.0000 : rR1f(1.5) : Excited combopi 2.5  2 F1f - Ground v=0 1.5  1 F1f : LIRTRAP_35K
LinearMolecule Excited 3.5  1  4 Ground 2.5  0  1  3302.49458  1  0.013421561776322   0.0000 : rR1e(2.5) : Excited combopi 3.5  3 F1e - Ground v=0 2.5  2 F1e : LIRTRAP_35K
LinearMolecule Excited 3.5  0  4 Ground 2.5  1  1  3302.3765  1  0.0134808713585932  0.0000 : rR1f(2.5) : Excited combopi 3.5  3 F1f - Ground v=0 2.5  2 F1f : LIRTRAP_35K
```

LinearMolecule Excited 4.5 0 4 Ground 3.5 1 1 3304.36604 1 0.012546445758444 0.0000 : rR1e(3.5) : Excited combopi 4.5 4 F1e - Ground v=0 3.5 3 F1e : LIRTRAP_35K
LinearMolecule Excited 4.5 1 4 Ground 3.5 0 1 3304.1758 1 0.012609623688488 0.0000 : rR1f(3.5) : Excited combopi 4.5 4 F1f - Ground v=0 3.5 3 F1f : LIRTRAP_35K
LinearMolecule Excited 5.5 1 4 Ground 4.5 0 1 3306.27502 1 0.0096843452106289 0.0000 : rR1e(4.5) : Excited combopi 5.5 5 F1e - Ground v=0 4.5 4 F1e : LIRTRAP_35K
LinearMolecule Excited 5.5 0 4 Ground 4.5 1 1 3306.00461 1 0.00973377259972093 0.0000 : rR1f(4.5) : Excited combopi 5.5 5 F1f - Ground v=0 4.5 4 F1f : LIRTRAP_35K
LinearMolecule Excited 0.5 1 4 Ground 1.5 0 1 3364.29279 1 0.0137757911876797 0.0000 : qP21(1.5) : Excited combosigma 0.5 1 F2f - Ground v=0 1.5 1 F1f : LIRTRAP_35K
LinearMolecule Excited 1.5 1 6 Ground 1.5 0 1 3364.29279 1 0.0128485370800942 0.0000 : qQ1(1.5) : Excited combosigma 1.5 1 F1e - Ground v=0 1.5 1 F1f : LIRTRAP_35K
LinearMolecule Excited 2.5 0 6 Ground 2.5 1 1 3363.18473 1 0.0175700083044382 0.0000 : qQ1(2.5) : Excited combosigma 2.5 2 F1e - Ground v=0 2.5 2 F1f : LIRTRAP_35K
LinearMolecule Excited 1.5 1 6 Ground 2.5 0 1 3357.70659 1 0.0146538084490458 0.0000 : pP1(2.5) : Excited combosigma 1.5 1 F1e - Ground v=0 2.5 2 F1e : LIRTRAP_35K
LinearMolecule Excited 2.5 0 6 Ground 3.5 1 1 3353.96439 1 0.0124724128016866 0.0000 : pP1(3.5) : Excited combosigma 2.5 2 F1e - Ground v=0 3.5 3 F1e : LIRTRAP_35K
LinearMolecule Excited 3.5 1 6 Ground 4.5 0 1 3350.32589 1 0.00930779283996231 0.0000 : pP1(4.5) : Excited combosigma 3.5 3 F1e - Ground v=0 4.5 4 F1e : LIRTRAP_35K
LinearMolecule Excited 4.5 0 6 Ground 5.5 1 1 3346.78863 1 0.00610144599434686 0.0000 : pP1(5.5) : Excited combosigma 4.5 4 F1e - Ground v=0 5.5 5 F1e : LIRTRAP_35K
LinearMolecule Excited 2.5 0 6 Ground 1.5 1 1 3369.76842 1 0.00337378592531594 0.0000 : rR1(1.5) : Excited combosigma 2.5 2 F1e - Ground v=0 1.5 1 F1e : LIRTRAP_35K
LinearMolecule Excited 1.5 0 6 Ground 1.5 1 1 3369.77924 1 0.0104391278546317 0.0000 : rQ21(1.5) : Excited combosigma 1.5 2 F2f - Ground v=0 1.5 1 F1e : LIRTRAP_35K
LinearMolecule Excited 3.5 0 6 Ground 2.5 1 1 3382.37487 1 0.00380478336056691 0.0000 : sR21(2.5) : Excited combosigma 3.5 4 F2f - Ground v=0 2.5 2 F1f : LIRTRAP_35K
LinearMolecule Excited 2.5 1 6 Ground 1.5 0 1 3378.00029 1 0.00246997575761762 0.0000 : sR21(1.5) : Excited combosigma 2.5 3 F2f - Ground v=0 1.5 1 F1f : LIRTRAP_35K
LinearMolecule Excited 4.5 1 6 Ground 3.5 0 1 3386.85178 1 0.00381000430982494 0.0000 : sR21(3.5) : Excited combosigma 4.5 5 F2f - Ground v=0 3.5 3 F1f : LIRTRAP_35K
LinearMolecule Excited 2.5 1 6 Ground 2.5 0 1 3371.41662 1 0.0128762926363564 0.0000 : rQ21(2.5) : Excited combosigma 2.5 3 F2f - Ground v=0 2.5 2 F1e : LIRTRAP_35K
LinearMolecule Excited 3.5 1 6 Ground 2.5 0 1 3371.40096 1 0.00576181043098108 0.0000 : rR1(2.5) : Excited combosigma 3.5 3 F1e - Ground v=0 2.5 2 F1e : LIRTRAP_35K
LinearMolecule Excited 3.5 0 6 Ground 3.5 1 1 3373.15834 1 0.0115211399867476 0.0000 : rQ21(3.5) : Excited combosigma 3.5 4 F2f - Ground v=0 3.5 3 F1e : LIRTRAP_35K
LinearMolecule Excited 4.5 0 6 Ground 3.5 1 1 3373.13806 1 0.00639143057390498 0.0000 : rR1(3.5) : Excited combosigma 4.5 4 F1e - Ground v=0 3.5 3 F1e : LIRTRAP_35K
LinearMolecule Excited 5.5 1 6 Ground 4.5 0 1 3374.9778 1 0.00556076791976567 0.0000 : rR1(4.5) : Excited combosigma 5.5 5 F1e - Ground v=0 4.5 4 F1e : LIRTRAP_35K
LinearMolecule Excited 4.5 1 6 Ground 4.5 0 1 3375.00321 1 0.00845958793542942 0.0000 : rQ21(4.5) : Excited combosigma 4.5 5 F2f - Ground v=0 4.5 4 F1e : LIRTRAP_35K
LinearMolecule Excited 6.5 0 6 Ground 5.5 1 1 3376.91881 1 0.00402893636122935 0.0000 : rR1(5.5) : Excited combosigma 6.5 6 F1e - Ground v=0 5.5 5 F1e : LIRTRAP_35K
LinearMolecule Excited 5.5 0 6 Ground 5.5 1 1 3376.94873 1 0.0052963556125739 0.0000 : rQ21(5.5) : Excited combosigma 5.5 6 F2f - Ground v=0 5.5 5 F1e : LIRTRAP_35K
LinearMolecule Excited 7.5 1 6 Ground 6.5 0 1 3378.9574 1 0.00249669342819545 0.0000 : rR1(6.5) : Excited combosigma 7.5 7 F1e - Ground v=0 6.5 6 F1e : LIRTRAP_35K
LinearMolecule Excited 6.5 1 6 Ground 6.5 0 1 3378.99304 1 0.00287708201136452 0.0000 : rQ21(6.5) : Excited combosigma 6.5 7 F2f - Ground v=0 6.5 6 F1e : LIRTRAP_35K
LinearMolecule Excited 5.5 1 6 Ground 6.5 0 1 3343.35101 1 0.00227011470900336 0.0000 : pP1(6.5) : Excited combosigma 5.5 5 F1e - Ground v=0 6.5 6 F1e : LIRTRAP_35K
LinearMolecule Excited 6.5 1 4 Ground 5.5 0 1 3307.88732 1 0.00638261652255751 0.0000 : rR1f(5.5) : Excited combopi 6.5 6 F1f - Ground v=0 5.5 5 F1f : LIRTRAP_35K
LinearMolecule Excited 6.5 0 4 Ground 5.5 1 1 3308.23703 1 0.00649285777129495 0.0000 : rR1e(5.5) : Excited combopi 6.5 6 F1e - Ground v=0 5.5 5 F1e : LIRTRAP_35K
LinearMolecule Excited 7.5 1 4 Ground 6.5 0 1 3310.26005 1 0.00376554643944893 0.0000 : rR1e(6.5) : Excited combopi 7.5 7 F1e - Ground v=0 6.5 6 F1e : LIRTRAP_35K
LinearMolecule Excited 4.5 1 4 Ground 4.5 0 1 3292.32706 1 0.00160289116830464 0.0000 : qQ1e(4.5) : Excited combopi 4.5 4 F1f - Ground v=0 4.5 4 F1e : LIRTRAP_35K
LinearMolecule Excited 5.5 0 3 Ground 4.5 1 1 3290.66513 1 0.00299571792444195 0.0000 : sR21(4.5) : Excited Sigma 5.5 6 F2f - Ground v=0 4.5 4 F1f : LIRTRAP_35K
LinearMolecule Excited 5.5 1 4 Ground 5.5 0 1 3291.75793 1 0.000722250535814234 0.0000 : qQ1f(5.5) : Excited combopi 5.5 5 F1e - Ground v=0 5.5 5 F1f : LIRTRAP_35K
LinearMolecule Excited 5.5 0 4 Ground 5.5 1 1 3291.52383 1 0.000705057367528508 0.0000 : qQ1e(5.5) : Excited combopi 5.5 5 F1f - Ground v=0 5.5 5 F1e : LIRTRAP_35K
LinearMolecule Excited 1.5 1 2 Ground 0.5 0 1 3060.09089 1 0.00134141430508774 0.0000 : rR2e(0.5) : Excited Linear 1.5 2 F2e - Ground v=0 0.5 1 F2e : LIRTRAP_35K
LinearMolecule Excited 1.5 0 2 Ground 0.5 1 1 3060.11674 1 0.0013405165419095 0.0000 : rR2f(0.5) : Excited Linear 1.5 2 F2f - Ground v=0 0.5 1 F2f : LIRTRAP_35K
LinearMolecule Excited 7.5 1 2 Ground 6.5 0 2 3076.15104 1 0.000488319888027541 0.0000 : rR2e(6.5) : Excited Linear 7.5 8 F2e - Ground v=0 6.5 7 F2e : LIRTRAP_35K
LinearMolecule Excited 7.5 0 2 Ground 6.5 1 2 3076.17146 1 0.000486691695602152 0.0000 : rR2f(6.5) : Excited Linear 7.5 8 F2f - Ground v=0 6.5 7 F2f : LIRTRAP_35K
LinearMolecule Excited 8.5 0 2 Ground 7.5 1 2 3078.75271 1 0.000235908370502129 0.0000 : rR2e(7.5) : Excited Linear 8.5 9 F2e - Ground v=0 7.5 8 F2e : LIRTRAP_35K
LinearMolecule Excited 8.5 1 2 Ground 7.5 0 2 3078.76938 1 0.000235117269387829 0.0000 : rR2f(7.5) : Excited Linear 8.5 9 F2f - Ground v=0 7.5 8 F2f : LIRTRAP_35K
LinearMolecule Excited 9.5 1 2 Ground 8.5 0 2 3081.33214 1 0.00010030650645092 0.0000 : rR2e(8.5) : Excited Linear 9.5 10 F2e - Ground v=0 8.5 9 F2e : LIRTRAP_35K
LinearMolecule Excited 9.5 0 2 Ground 8.5 1 2 3081.34304 1 9.99847940581238E-5 0.0000 : rR2f(8.5) : Excited Linear 9.5 10 F2f - Ground v=0 8.5 9 F2f : LIRTRAP_35K
LinearMolecule Excited 10.5 0 1 Ground 9.5 1 1 3083.29074 1 0.000411810374180728 0.0000 : rR1e(9.5) : Excited Linear 10.5 10 F1e - Ground v=0 9.5 9 F1e : LIRTRAP_35K
LinearMolecule Excited 10.5 1 1 Ground 9.5 0 1 3083.34583 1 0.000410245191084786 0.0000 : rR1f(9.5) : Excited Linear 10.5 10 F1f - Ground v=0 9.5 9 F1f : LIRTRAP_35K
LinearMolecule Excited 8.5 1 1 Ground 9.5 0 1 3030.866448 1 0.000369472683662383 0.0000 : pP1f(9.5) : Excited Linear 8.5 8 F1f - Ground v=0 9.5 9 F1f : LIRTRAP_35K
LinearMolecule Excited 8.5 0 1 Ground 9.5 1 1 3030.878652 1 0.000370930464931437 0.0000 : pP1e(9.5) : Excited Linear 8.5 8 F1e - Ground v=0 9.5 9 F1e : LIRTRAP_35K
LinearMolecule Excited 2.5 0 2 Ground 3.5 1 2 3046.17753 1 0.00146826121696098 0.0000 : pP2e(3.5) : Excited Linear 2.5 3 F2e - Ground v=0 3.5 4 F2e : LIRTRAP_35K
LinearMolecule Excited 2.5 1 2 Ground 3.5 0 2 3046.17146 1 0.00146539696155868 0.0000 : pP2f(3.5) : Excited Linear 2.5 3 F2f - Ground v=0 3.5 4 F2f : LIRTRAP_35K
LinearMolecule Excited 4.5 0 2 Ground 5.5 1 2 3040.481076 1 0.000748474004728975 0.0000 : pP2e(5.5) : Excited Linear 4.5 5 F2e - Ground v=0 5.5 6 F2e : LIRTRAP_35K
LinearMolecule Excited 4.5 1 2 Ground 5.5 0 2 3040.485473 1 0.000746660128816878 0.0000 : pP2f(5.5) : Excited Linear 4.5 5 F2f - Ground v=0 5.5 6 F2f : LIRTRAP_35K
LinearMolecule Excited 3.5 1 2 Ground 4.5 0 2 3043.337237 1 0.00113925562423935 0.0000 : pP2e(4.5) : Excited Linear 3.5 4 F2e - Ground v=0 4.5 5 F2e : LIRTRAP_35K
LinearMolecule Excited 3.5 0 2 Ground 4.5 1 2 3043.337237 1 0.0011351661931398 0.0000 : pP2f(4.5) : Excited Linear 3.5 4 F2f - Ground v=0 4.5 5 F2f : LIRTRAP_35K
LinearMolecule Excited 5.5 1 2 Ground 6.5 0 2 3037.609237 1 0.000421862682140921 0.0000 : pP2e(6.5) : Excited Linear 5.5 6 F2e - Ground v=0 6.5 7 F2e : LIRTRAP_35K
LinearMolecule Excited 5.5 0 2 Ground 6.5 1 2 3037.619038 1 0.000420026766783151 0.0000 : pP2f(6.5) : Excited Linear 5.5 6 F2f - Ground v=0 6.5 7 F2f : LIRTRAP_35K
LinearMolecule Excited 0.5 1 2 Ground 0.5 0 1 3214.33358 1 0.00134494401576964 0.0000 : qQ2(0.5) : Excited Sigma 0.5 1 F2f - Ground v=0 0.5 1 F2e : LIRTRAP_35K
LinearMolecule Excited 4.5 1 3 Ground 3.5 0 2 3232.653962 1 0.00109303018037399 0.0000 : rR2(3.5) : Excited Sigma 4.5 5 F2f - Ground v=0 3.5 4 F2f : LIRTRAP_35K
LinearMolecule Excited 5.5 0 3 Ground 6.5 1 1 3259.01576 1 0.0019085992161595 0.0000 : qP21(6.5) : Excited Sigma 5.5 6 F2f - Ground v=0 6.5 6 F1f : LIRTRAP_35K
LinearMolecule Excited 5.5 0 3 Ground 4.5 1 2 3236.913554 1 0.00083576575648432 0.0000 : rR2(4.5) : Excited Sigma 5.5 6 F2f - Ground v=0 4.5 5 F2f : LIRTRAP_35K
LinearMolecule Ground 6.5 1 1 Ground 5.5 0 1 17.1473748 1 0.00355992118796574 0.0000 : rR1f(5.5) : Ground v=0 6.5 6 F1f - Ground v=0 5.5 5 F1f : COLTRAP_rotations_Silva_et_al
LinearMolecule Ground 6.5 0 1 Ground 5.5 1 1 17.1338563 1 0.00356320955204285 0.0000 : rR1e(5.5) : Ground v=0 6.5 6 F1e - Ground v=0 5.5 5 F1e : COLTRAP_rotations_Silva_et_al
LinearMolecule Ground 5.5 1 1 Ground 4.5 0 1 14.4937791 1 0.00612512415067226 0.0000 : rR1e(4.5) : Ground v=0 5.5 5 F1e - Ground v=0 4.5 4 F1e : COLTRAP_rotations_Silva_et_al
LinearMolecule Ground 5.5 0 1 Ground 4.5 1 1 14.503691 1 0.00612290598279535 0.0000 : rR1f(4.5) : Ground v=0 5.5 5 F1f - Ground v=0 4.5 4 F1f : COLTRAP_rotations_Silva_et_al
LinearMolecule Ground 4.5 0 1 Ground 3.5 1 1 11.8557323 1 0.00832297385976414 0.0000 : rR1e(3.5) : Ground v=0 4.5 4 F1e - Ground v=0 3.5 3 F1e : COLTRAP_rotations_Silva_et_al
LinearMolecule Ground 4.5 1 1 Ground 3.5 0 1 11.86248329 1 0.00832294671662294 0.0000 : rR1f(3.5) : Ground v=0 4.5 4 F1f - Ground v=0 3.5 3 F1f : COLTRAP_rotations_Silva_et_al
LinearMolecule Ground 3.5 1 1 Ground 2.5 0 1 9.21946803 1 0.00841366312297274 0.0000 : rR1e(2.5) : Ground v=0 3.5 3 F1e - Ground v=0 2.5 2 F1e : COLTRAP_rotations_Silva_et_al
LinearMolecule Ground 3.5 0 1 Ground 2.5 1 1 9.2236722 1 0.00841519639136012 0.0000 : rR1f(2.5) : Ground v=0 3.5 3 F1f - Ground v=0 2.5 2 F1f : COLTRAP_rotations_Silva_et_al
LinearMolecule Excited 7.5 0 4 Ground 6.5 1 2 3254.41979588186 -0.0024410100263406 9.61795417587977E-5 0.0000 : qR12f(6.5) : Excited combopi 7.5 7 F1f - Ground v=0 6.5 7 F2f
LinearMolecule Excited 5.5 0 5 Ground 5.5 1 2 3254.71944976222 -0.0085110644625057 1.330000565509E-5 0.0000 : qQ2e(5.5) : Excited combopi 5.5 6 F2f - Ground v=0 5.5 6 F2e
LinearMolecule Excited 11.5 0 3 Ground 12.5 1 1 3254.63388880001 -0.0241071496174492 9.91711697322909E-6 0.0000 : pP1f(12.5) : Excited combopi 11.5 11 F1f - Ground v=0 12.5 12 F1f

# 4) Leak-out raw data

| | |
|---|---|
| 3202.7802583 | 2 |
| 3202.7818612 | 1 |
| 3202.7829431 | 3 |
| 3202.7850393 | 1 |
| 3202.7857597 | 2 |
| 3202.7864924 | 0 |
| 3202.7890298 | 1 |
| 3202.7897004 | 1 |
| 3202.7910106 | 0 |
| 3202.7913789 | 4 |
| 3202.7939768 | 2 |
| 3202.7960186 | 2 |
| 3202.7962313 | 0 |
| 3202.7980449 | 5 |
| 3202.799917 | 3 |
| 3202.800343 | 2 |
| 3202.802942 | 1 |
| 3202.8042104 | 0 |
| 3202.8060106 | 1 |
| 3202.8060366 | 2 |
| 3202.808249 | 2 |
| 3202.8099035 | 3 |
| 3202.8101677 | 1 |
| 3202.8121752 | 2 |
| 3202.8143647 | 1 |
| 3202.8146433 | 2 |
| 3202.8170518 | 3 |
| 3202.8172094 | 2 |
| 3202.8185994 | 1 |
| 3202.8203058 | 3 |
| 3202.8219203 | 0 |
| 3202.8239885 | 2 |
| 3202.8247218 | 1 |
| 3202.8265824 | 1 |
| 3202.8269628 | 0 |
| 3202.8285181 | 2 |
| 3202.830599 | 4 |
| 3202.8318156 | 2 |
| 3202.8323655 | 1 |
| 3202.8342554 | 2 |
| 3202.8356783 | 0 |
| 3202.8385348 | 3 |
| 3202.8390489 | 1 |
| 3202.8406617 | 0 |
| 3202.8408734 | 0 |
| 3202.8429625 | 3 |
| 3202.8435888 | 6 |
| 3202.8460474 | 0 |
| 3202.8471672 | 4 |
| 3202.8472029 | 2 |
| 3202.8497104 | 1 |
| 3202.8515238 | 3 |
| 3202.8516056 | 3 |
| 3202.8533062 | 0 |
| 3202.8548725 | 0 |
| 3202.8569781 | 2 |
| 3202.8586808 | 0 |
| 3202.8594806 | 0 |
| 3202.8604447 | 1 |
| 3202.8623063 | 1 |
| 3202.8636839 | 3 |
| 3202.8638393 | 1 |
| 3202.8647187 | 2 |
| 3202.8663147 | 0 |
| 3202.8681612 | 2 |
| 3202.8699345 | 2 |
| 3202.8725712 | 3 |
| 3202.8751205 | 0 |
| 3202.8753288 | 0 |
| 3202.8770895 | 1 |
| 3202.8802204 | 1 |
| 3202.8811519 | 1 |
| 3202.8830314 | 2 |
| 3202.8850218 | 3 |
| 3202.885246 | 2 |
| 3202.8862463 | 2 |
| 3202.886342 | 4 |
| 3202.8884794 | 2 |
| 3202.8887954 | 0 |
| 3202.8914505 | 1 |
| 3202.8928206 | 0 |
| 3202.8942954 | 0 |
| 3202.8961612 | 2 |
| 3202.8970814 | 0 |
| 3202.8983937 | 4 |
| 3202.8992055 | 1 |
| 3202.9007363 | 0 |
| 3202.9020273 | 1 |
| 3202.9036166 | 0 |
| 3202.9057267 | 0 |
| 3202.9062653 | 1 |
| 3202.9076153 | 1 |
| 3202.9077716 | 2 |
| 3202.9099055 | 4 |
| 3202.9101696 | 0 |
| 3202.9130081 | 1 |
| 3202.9141641 | 3 |
| 3202.915307 | 1 |
| 3202.9164851 | 4 |
| 3202.9171419 | 2 |
| 3202.9177372 | 0 |
| 3202.9215947 | 1 |
| 3202.9230537 | 3 |
| 3202.9238111 | 1 |
| 3202.9251506 | 2 |
| 3202.9273497 | 1 |
| 3202.9286894 | 1 |
| 3202.9296699 | 2 |
| 3202.9309991 | 4 |
| 3202.9334287 | 1 |
| 3202.9334669 | 3 |
| 3202.9346544 | 2 |
| 3202.9368307 | 5 |
| 3202.938497 | 0 |
| 3202.9393295 | 4 |
| 3202.9406042 | 3 |
| 3202.9409896 | 1 |
| 3202.9427148 | 1 |
| 3202.9440744 | 5 |
| 3202.9443537 | 0 |
| 3202.946418 | 2 |
| 3202.9480586 | 4 |
| 3202.949103 | 1 |
| 3202.9500614 | 0 |
| 3202.9515487 | 3 |
| 3202.9539672 | 0 |
| 3202.9552071 | 1 |
| 3202.9556343 | 2 |
| 3202.9574238 | 1 |
| 3202.9583737 | 1 |
| 3202.960981 | 1 |
| 3202.9621947 | 2 |
| 3202.9640485 | 2 |
| 3202.9650124 | 2 |
| 3202.9669163 | 2 |
| 3202.967708 | 4 |
| 3202.9692239 | 2 |
| 3202.9708227 | 0 |
| 3202.9722673 | 3 |
| 3202.9729378 | 0 |
| 3202.9750343 | 1 |
| 3202.9750694 | 5 |
| 3202.9765918 | 0 |
| 3202.9783088 | 0 |
| 3202.9799991 | 2 |
| 3202.9816137 | 3 |
| 3202.9830131 | 3 |
| 3202.9840128 | 0 |
| 3202.9846522 | 1 |
| 3202.9864008 | 1 |
| 3202.9870935 | 2 |
| 3202.9890018 | 3 |
| 3202.9909725 | 3 |
| 3202.9916325 | 0 |
| 3202.9934702 | 1 |
| 3202.9948012 | 0 |
| 3202.9970797 | 0 |
| 3202.9988782 | 1 |
| 3202.99925 | 4 |
| 3203.0011976 | 0 |
| 3203.0019244 | 1 |
| 3203.0029446 | 0 |
| 3203.0051327 | 1 |
| 3203.0062111 | 3 |
| 3203.0078008 | 1 |
| 3203.0083323 | 4 |
| 3203.0107282 | 0 |
| 3203.0119952 | 0 |
| 3203.013235 | 2 |
| 3203.0147991 | 3 |
| 3203.015955 | 5 |
| 3203.0172961 | 1 |
| 3203.0181961 | 2 |
| 3203.0199014 | 3 |
| 3203.0215232 | 0 |
| 3203.0222922 | 0 |
| 3203.0237255 | 1 |
| 3203.025219 | 1 |
| 3203.0261622 | 0 |
| 3203.0287746 | 0 |
| 3203.0298665 | 2 |
| 3203.0308332 | 2 |
| 3203.0315326 | 2 |
| 3203.0342167 | 0 |
| 3203.0353305 | 3 |
| 3203.0364348 | 1 |
| 3203.038044 | 0 |
| 3203.0388462 | 2 |
| 3203.0414635 | 1 |
| 3203.0421577 | 0 |
| 3203.0439366 | 1 |
| 3203.0457542 | 3 |
| 3203.046829 | 2 |
| 3203.048363 | 0 |
| 3203.0487193 | 4 |
| 3203.0508404 | 2 |
| 3203.0525399 | 2 |
| 3203.0532784 | 2 |
| 3203.0551461 | 1 |
| 3203.0559447 | 2 |
| 3203.0581554 | 4 |
| 3203.0592997 | 0 |
| 3203.0598186 | 0 |
| 3203.0615174 | 0 |
| 3203.0628443 | 3 |
| 3203.0642668 | 0 |
| 3203.0659756 | 3 |
| 3203.0666296 | 0 |
| 3203.0689396 | 1 |
| 3203.0691786 | 2 |
| 3203.0717842 | 1 |
| 3203.0725193 | 2 |
| 3203.0735174 | 3 |
| 3203.0752511 | 4 |
| 3203.076471 | 2 |
| 3203.0784437 | 3 |
| 3203.0793029 | 1 |
| 3203.0801768 | 0 |
| 3203.0816718 | 1 |
| 3203.0828778 | 1 |
| 3203.0833897 | 6 |
| 3203.0848642 | 3 |
| 3203.0873279 | 0 |
| 3203.0895539 | 6 |
| 3203.0920135 | 1 |
| 3203.0924202 | 0 |
| 3203.0936928 | 2 |
| 3203.0975845 | 0 |
| 3203.0987366 | 2 |
| 3203.1001179 | 2 |
| 3203.1023752 | 2 |
| 3203.1026646 | 0 |
| 3203.1046653 | 3 |
| 3203.1069808 | 3 |
| 3203.1071413 | 0 |
| 3203.1081882 | 4 |
| 3203.1099867 | 3 |
| 3203.1122619 | 3 |
| 3203.1131644 | 0 |
| 3203.1150862 | 1 |
| 3203.1152635 | 1 |
| 3203.1169964 | 2 |
| 3203.1177433 | 0 |
| 3203.1187864 | 3 |
| 3203.1213402 | 2 |
| 3203.1220401 | 1 |
| 3203.122062 | 1 |
| 3203.1230905 | 4 |
| 3203.1237582 | 1 |
| 3203.124949 | 4 |
| 3203.1273764 | 5 |
| 3203.1303788 | 3 |
| 3203.1327974 | 0 |
| 3203.1334677 | 5 |
| 3203.1339331 | 4 |
| 3203.1359881 | 4 |
| 3203.1364623 | 0 |
| 3203.1379232 | 3 |
| 3203.1404698 | 7 |
| 3203.1407134 | 8 |
| 3203.1424629 | 2 |
| 3203.1437991 | 3 |
| 3203.145819 | 1 |
| 3203.1470052 | 0 |
| 3203.1479211 | 0 |
| 3203.1499594 | 0 |
| 3203.1501587 | 3 |
| 3203.1528089 | 2 |
| 3203.154178 | 3 |
| 3203.1550511 | 1 |
| 3203.1562921 | 1 |
| 3203.1582921 | 0 |
| 3203.1584873 | 2 |
| 3203.160562 | 5 |
| 3203.1618988 | 4 |
| 3203.1627237 | 3 |
| 3203.1650125 | 1 |
| 3203.1658085 | 0 |
| 3203.1662178 | 3 |
| 3203.1675983 | 1 |
| 3203.16857 | 5 |
| 3203.1707281 | 0 |
| 3203.1719318 | 2 |
| 3203.1738469 | 1 |
| 3203.1741724 | 2 |
| 3203.175656 | 1 |
| 3203.1773192 | 0 |
| 3203.1792763 | 3 |
| 3203.1800686 | 1 |
| 3203.1812687 | 1 |
| 3203.182458 | 1 |
| 3203.1849222 | 2 |
| 3203.185684 | 1 |
| 3203.187787 | 1 |
| 3203.1886113 | 2 |
| 3203.1904357 | 0 |
| 3203.1912076 | 1 |
| 3203.1924496 | 1 |
| 3203.193036 | 2 |
| 3203.1972607 | 1 |
| 3203.1982019 | 5 |
| 3203.1996635 | 4 |
| 3203.2015082 | 3 |
| 3203.2021676 | 2 |
| 3203.2037932 | 3 |
| 3203.20495 | 4 |
| 3203.2065311 | 1 |
| 3203.2079173 | 4 |
| 3203.2087192 | 4 |
| 3203.2103516 | 0 |
| 3203.2118514 | 1 |
| 3203.2127841 | 2 |
| 3203.2154188 | 1 |
| 3203.2165829 | 0 |
| 3203.2180293 | 0 |
| 3203.21821 | 2 |
| 3203.2184947 | 2 |
| 3203.2205146 | 0 |
| 3203.2223842 | 3 |
| 3203.2233016 | 3 |
| 3203.2253346 | 1 |
| 3203.2271465 | 3 |
| 3203.2272592 | 1 |
| 3203.2294479 | 2 |
| 3203.2303735 | 5 |
| 3203.2318554 | 3 |
| 3203.2322711 | 3 |
| 3203.2323528 | 4 |
| 3203.2352073 | 2 |
| 3203.2362733 | 0 |
| 3203.2381914 | 0 |
| 3203.241871 | 0 |
| 3203.2436624 | 3 |

| | |
|---|---|
| 3203.2441768 | 1 |
| 3203.2455767 | 2 |
| 3203.2482807 | 0 |
| 3203.249234 | 1 |
| 3203.2498532 | 0 |
| 3203.2509488 | 2 |
| 3203.2539045 | 0 |
| 3203.2542294 | 3 |
| 3203.2558125 | 0 |
| 3203.2572878 | 2 |
| 3203.2583105 | 0 |
| 3203.2596599 | 3 |
| 3203.2609107 | 1 |
| 3203.2626324 | 2 |
| 3203.2637736 | 4 |
| 3203.2647039 | 3 |
| 3203.2667588 | 1 |
| 3203.2686776 | 2 |
| 3203.269472 | 3 |
| 3203.2713987 | 2 |
| 3203.2724393 | 1 |
| 3203.2739193 | 2 |
| 3203.2752934 | 2 |
| 3203.2766052 | 4 |
| 3203.2769562 | 3 |
| 3203.2790155 | 1 |
| 3203.2797742 | 1 |
| 3203.282013 | 3 |
| 3203.2823978 | 1 |
| 3203.2839527 | 1 |
| 3203.2854566 | 2 |
| 3203.28631 | 0 |
| 3203.2880186 | 1 |
| 3203.2895622 | 4 |
| 3203.2904359 | 1 |
| 3203.292156 | 1 |
| 3203.2921719 | 1 |
| 3203.2946479 | 0 |
| 3203.2963281 | 2 |
| 3203.2979415 | 1 |
| 3203.2991291 | 1 |
| 3203.2996065 | 5 |
| 3203.3017842 | 1 |
| 3203.3032856 | 1 |
| 3203.3042653 | 6 |
| 3203.3068318 | 5 |
| 3203.3068946 | 4 |
| 3203.3089295 | 1 |
| 3203.3095257 | 1 |
| 3203.3124727 | 0 |
| 3203.3135588 | 2 |
| 3203.3154369 | 1 |
| 3203.3165499 | 1 |
| 3203.3172445 | 1 |
| 3203.3187154 | 0 |
| 3203.3210652 | 4 |
| 3203.3219875 | 5 |
| 3203.3230961 | 4 |
| 3203.3256054 | 4 |
| 3203.3266029 | 2 |
| 3203.3282919 | 2 |
| 3203.3290396 | 1 |
| 3203.3302407 | 2 |
| 3203.3308967 | 3 |
| 3203.3339302 | 0 |
| 3203.334852 | 3 |
| 3203.3360331 | 3 |
| 3203.3379318 | 4 |
| 3203.3382188 | 4 |
| 3203.340732 | 1 |
| 3203.3415805 | 3 |
| 3203.3422701 | 1 |
| 3203.3447591 | 3 |
| 3203.3448502 | 2 |
| 3203.34759 | 2 |
| 3203.3482999 | 4 |
| 3203.3489456 | 0 |
| 3203.3518574 | 2 |
| 3203.3519526 | 1 |
| 3203.3544131 | 1 |
| 3203.3546892 | 2 |
| 3203.3563633 | 1 |
| 3203.3586245 | 0 |
| 3203.3596631 | 3 |
| 3203.3614614 | 2 |
| 3203.363106 | 2 |
| 3203.3642288 | 1 |
| 3203.3663651 | 5 |
| 3203.3666082 | 0 |
| 3203.368812 | 0 |
| 3203.3695745 | 3 |
| 3203.3711496 | 0 |
| 3203.3723786 | 2 |
| 3203.3736004 | 0 |
| 3203.3757583 | 0 |
| 3203.3760594 | 1 |
| 3203.3779169 | 3 |
| 3203.3800203 | 3 |
| 3203.3804466 | 1 |
| 3203.3819099 | 1 |
| 3203.3841163 | 1 |
| 3203.3847933 | 0 |
| 3203.3868697 | 1 |
| 3203.3875633 | 4 |
| 3203.3888383 | 0 |
| 3203.3896634 | 2 |
| 3203.3916091 | 0 |
| 3203.394614 | 1 |
| 3203.3948581 | 0 |
| 3203.3960855 | 2 |
| 3203.3969894 | 2 |
| 3203.3992081 | 2 |
| 3203.4008728 | 0 |
| 3203.4015978 | 2 |
| 3203.403535 | 2 |
| 3203.4041385 | 1 |
| 3203.4074721 | 1 |
| 3203.4075223 | 0 |
| 3203.4088489 | 1 |
| 3203.4111558 | 0 |
| 3203.4123708 | 5 |
| 3203.4133273 | 0 |
| 3203.4144049 | 2 |
| 3203.4160444 | 1 |
| 3203.4178983 | 2 |
| 3203.4184734 | 4 |
| 3203.41976 | 3 |
| 3203.42195 | 1 |
| 3203.4224924 | 2 |
| 3203.4251914 | 3 |
| 3203.4253686 | 3 |
| 3203.427292 | 2 |
| 3203.427583 | 2 |
| 3203.4298452 | 0 |
| 3203.4312082 | 2 |
| 3203.4320353 | 0 |
| 3203.4340431 | 1 |
| 3203.4355178 | 2 |
| 3203.4374621 | 0 |
| 3203.439484 | 5 |
| 3203.4395832 | 1 |
| 3203.4403578 | 4 |
| 3203.4408618 | 3 |
| 3203.4433694 | 2 |
| 3203.4452161 | 3 |
| 3203.4459259 | 2 |
| 3203.4482361 | 2 |
| 3203.4494844 | 2 |
| 3203.4512253 | 0 |
| 3203.4527684 | 0 |
| 3203.4537471 | 1 |
| 3203.4544358 | 0 |
| 3203.4557501 | 1 |
| 3203.4558885 | 0 |
| 3203.4573369 | 3 |
| 3203.4576843 | 2 |
| 3203.4577575 | 1 |
| 3203.4589337 | 0 |
| 3203.4593841 | 3 |
| 3203.4606826 | 2 |
| 3203.460944 | 1 |
| 3203.4619083 | 4 |
| 3203.4619682 | 2 |
| 3203.4639365 | 2 |
| 3203.4641393 | 2 |
| 3203.4645883 | 1 |
| 3203.4655193 | 1 |
| 3203.4655235 | 1 |
| 3203.4669608 | 1 |
| 3203.4673868 | 1 |
| 3203.4685563 | 1 |
| 3203.4689434 | 4 |
| 3203.4694794 | 0 |
| 3203.470296 | 0 |
| 3203.4711043 | 2 |
| 3203.4715196 | 3 |
| 3203.4716918 | 2 |
| 3203.4728916 | 0 |
| 3203.473566 | 0 |
| 3203.4739399 | 1 |
| 3203.4743855 | 1 |
| 3203.47612 | 2 |
| 3203.4761724 | 1 |
| 3203.4771657 | 2 |
| 3203.47772 | 2 |
| 3203.4784363 | 2 |
| 3203.4797012 | 1 |
| 3203.4799983 | 2 |
| 3203.4802962 | 2 |
| 3203.4810256 | 0 |
| 3203.4818018 | 2 |
| 3203.4818743 | 1 |
| 3203.482456 | 3 |
| 3203.483836 | 2 |
| 3203.4844211 | 1 |
| 3203.4851082 | 2 |
| 3203.4863994 | 2 |
| 3203.4864349 | 2 |
| 3203.4867049 | 2 |
| 3203.4881084 | 4 |
| 3203.4886865 | 1 |
| 3203.489928 | 2 |
| 3203.4900046 | 1 |
| 3203.4908179 | 2 |
| 3203.4909567 | 0 |
| 3203.4925968 | 3 |
| 3203.4930107 | 1 |
| 3203.4940772 | 1 |
| 3203.4944206 | 2 |
| 3203.4948968 | 1 |
| 3203.4955944 | 1 |
| 3203.4975488 | 3 |
| 3203.4981197 | 3 |
| 3203.4988116 | 2 |
| 3203.4990565 | 2 |
| 3203.4994164 | 4 |
| 3203.4995397 | 4 |
| 3203.5009058 | 3 |
| 3203.5014935 | 0 |
| 3203.5022749 | 0 |
| 3203.5029146 | 1 |
| 3203.504002 | 1 |
| 3203.5053551 | 1 |
| 3203.5053902 | 3 |
| 3203.5065613 | 3 |
| 3203.5067634 | 1 |
| 3203.5084214 | 1 |
| 3203.5091896 | 2 |
| 3203.5095671 | 1 |
| 3203.5095896 | 1 |
| 3203.5114995 | 0 |
| 3203.5115378 | 0 |
| 3203.5121102 | 2 |
| 3203.5121604 | 2 |
| 3203.5131102 | 1 |
| 3203.5137182 | 1 |
| 3203.5143905 | 1 |
| 3203.5153173 | 1 |
| 3203.5153918 | 2 |
| 3203.5158647 | 1 |
| 3203.5167569 | 2 |
| 3203.5183227 | 0 |
| 3203.518478 | 2 |
| 3203.5191592 | 1 |
| 3203.5192995 | 3 |
| 3203.5202953 | 0 |
| 3203.5211705 | 3 |
| 3203.5212104 | 1 |
| 3203.5221961 | 3 |
| 3203.5226355 | 4 |
| 3203.522691 | 1 |
| 3203.5248703 | 2 |
| 3203.5254332 | 1 |
| 3203.5254529 | 3 |
| 3203.5256303 | 6 |
| 3203.5273099 | 3 |
| 3203.527385 | 0 |
| 3203.5283201 | 5 |
| 3203.5287145 | 2 |
| 3203.5302395 | 0 |
| 3203.5315157 | 2 |
| 3203.5317035 | 2 |
| 3203.5317097 | 1 |
| 3203.5324597 | 1 |
| 3203.5335592 | 1 |
| 3203.5340628 | 0 |
| 3203.5350336 | 4 |
| 3203.5352024 | 1 |
| 3203.5354048 | 2 |
| 3203.5366432 | 0 |
| 3203.5374528 | 1 |
| 3203.5386735 | 1 |
| 3203.5390473 | 4 |
| 3203.5404376 | 3 |
| 3203.5407886 | 0 |
| 3203.541653 | 2 |
| 3203.5424675 | 1 |
| 3203.5426616 | 2 |
| 3203.5432495 | 3 |
| 3203.5444092 | 0 |
| 3203.5445638 | 1 |
| 3203.5456502 | 3 |
| 3203.5459324 | 0 |
| 3203.5472984 | 1 |
| 3203.5476228 | 0 |
| 3203.5495211 | 2 |
| 3203.5495647 | 0 |
| 3203.5499691 | 1 |
| 3203.5500408 | 2 |
| 3203.5513762 | 4 |
| 3203.5522283 | 0 |
| 3203.552309 | 1 |
| 3203.5528535 | 1 |
| 3203.5537293 | 0 |
| 3203.5555898 | 5 |
| 3203.5556582 | 1 |
| 3203.5566178 | 5 |
| 3203.5566867 | 1 |
| 3203.5579682 | 1 |
| 3203.5580188 | 0 |
| 3203.5593819 | 1 |
| 3203.5595027 | 3 |
| 3203.5598828 | 1 |
| 3203.5608152 | 1 |
| 3203.5620915 | 2 |
| 3203.5627427 | 2 |
| 3203.5636696 | 0 |
| 3203.5636972 | 2 |
| 3203.5644024 | 2 |
| 3203.5651063 | 0 |
| 3203.565505 | 4 |
| 3203.5664282 | 0 |
| 3203.5668387 | 2 |
| 3203.5669844 | 1 |
| 3203.5673683 | 1 |
| 3203.5690156 | 2 |
| 3203.5699533 | 2 |
| 3203.5701438 | 3 |
| 3203.5707566 | 0 |
| 3203.5707805 | 1 |
| 3203.5715179 | 1 |
| 3203.5724561 | 4 |
| 3203.5729243 | 2 |
| 3203.5750658 | 3 |
| 3203.5754231 | 2 |
| 3203.5766616 | 2 |
| 3203.5792899 | 1 |
| 3203.5796348 | 4 |
| 3203.5815173 | 1 |
| 3203.5823173 | 3 |
| 3203.5843755 | 2 |
| 3203.58689 | 1 |
| 3203.5871822 | 1 |
| 3203.5893773 | 1 |
| 3203.5898672 | 0 |
| 3203.5919766 | 1 |
| 3203.5930515 | 3 |
| 3203.5938707 | 2 |
| 3203.5954591 | 0 |
| 3203.5971538 | 0 |
| 3203.5988353 | 2 |
| 3203.6001281 | 3 |
| 3203.6015586 | 1 |
| 3203.6029604 | 1 |
| 3203.6036017 | 0 |
| 3203.6050465 | 5 |
| 3203.6069666 | 1 |
| 3203.608207 | 1 |
| 3203.6100875 | 1 |
| 3203.6103177 | 2 |
| 3203.6120106 | 3 |
| 3203.6121878 | 1 |
| 3203.6150794 | 3 |
| 3203.6165797 | 1 |
| 3203.6172731 | 3 |
| 3203.6196204 | 0 |
| 3203.6204256 | 5 |
| 3203.6220037 | 2 |
| 3203.6235067 | 0 |
| 3203.6247331 | 6 |
| 3203.6264271 | 1 |
| 3203.6273074 | 0 |
| 3203.629384 | 0 |

| | |
|---|---|
| 3203.63127 | 1 |
| 3203.6314806 | 3 |
| 3203.6335559 | 0 |
| 3203.6351891 | 2 |
| 3203.6361382 | 4 |
| 3203.637612 | 1 |
| 3203.6390088 | 1 |
| 3203.64015 | 1 |
| 3203.6413114 | 1 |
| 3203.6434792 | 1 |
| 3203.6454414 | 1 |
| 3203.6460215 | 5 |
| 3203.6480024 | 0 |
| 3203.6488331 | 2 |
| 3203.6503218 | 4 |
| 3203.6520793 | 2 |
| 3203.6528358 | 2 |
| 3203.6547412 | 1 |
| 3203.6558393 | 2 |
| 3203.6570726 | 1 |
| 3203.6577968 | 1 |
| 3203.6593235 | 2 |
| 3203.6615083 | 3 |
| 3203.6615856 | 2 |
| 3203.6641764 | 3 |
| 3203.6654482 | 1 |
| 3203.6661017 | 3 |
| 3203.6682223 | 2 |
| 3203.6692513 | 1 |
| 3203.6707898 | 0 |
| 3203.6725438 | 1 |
| 3203.6737697 | 3 |
| 3203.675421 | 4 |
| 3203.6766631 | 0 |
| 3203.6782312 | 1 |
| 3203.6794984 | 0 |
| 3203.6809462 | 1 |
| 3203.6819913 | 3 |
| 3203.6843996 | 3 |
| 3203.6853389 | 2 |
| 3203.6861289 | 5 |
| 3203.6886703 | 3 |
| 3203.6896589 | 2 |
| 3203.6904547 | 3 |
| 3203.6927154 | 3 |
| 3203.6932072 | 1 |
| 3203.6949658 | 2 |
| 3203.6959874 | 0 |
| 3203.6978034 | 0 |
| 3203.6992547 | 1 |
| 3203.7000917 | 2 |
| 3203.7018361 | 1 |
| 3203.7025453 | 3 |
| 3203.7047706 | 0 |
| 3203.7058826 | 2 |
| 3203.7076733 | 0 |
| 3203.7086128 | 0 |
| 3203.7095584 | 5 |
| 3203.7119913 | 1 |
| 3203.7130439 | 1 |
| 3203.7139701 | 2 |
| 3203.7158511 | 0 |
| 3203.7173484 | 2 |
| 3203.7185649 | 2 |
| 3203.7197999 | 1 |
| 3203.7215665 | 3 |
| 3203.7232776 | 2 |
| 3203.723973 | 0 |
| 3203.725398 | 1 |
| 3203.7273758 | 0 |
| 3203.7293611 | 3 |
| 3203.7304186 | 1 |
| 3203.7315874 | 1 |
| 3203.7327855 | 2 |
| 3203.7349518 | 2 |
| 3203.7360584 | 1 |
| 3203.737452 | 2 |
| 3203.7378734 | 0 |
| 3203.7404028 | 2 |
| 3203.7407295 | 0 |
| 3203.7425621 | 1 |
| 3203.7440266 | 5 |
| 3203.7446001 | 0 |
| 3203.746912 | 0 |
| 3203.747249 | 1 |
| 3203.7491464 | 1 |
| 3203.7511553 | 2 |
| 3203.7517664 | 2 |
| 3203.7536856 | 0 |
| 3203.75427 | 3 |
| 3203.7560148 | 1 |
| 3203.7568883 | 1 |
| 3203.7592509 | 2 |
| 3203.7616185 | 2 |
| 3203.7623431 | 0 |
| 3203.7637156 | 2 |
| 3203.7650612 | 1 |
| 3203.7661731 | 3 |
| 3203.7682827 | 0 |
| 3203.768623 | 3 |
| 3203.7705139 | 1 |
| 3203.7725271 | 1 |
| 3203.7733283 | 3 |
| 3203.7749968 | 0 |
| 3203.7763587 | 3 |
| 3203.7765358 | 0 |
| 3203.779936 | 2 |
| 3203.78092 | 1 |
| 3203.7825345 | 1 |
| 3203.7830408 | 2 |
| 3203.7848778 | 3 |
| 3203.7855288 | 3 |
| 3203.7873179 | 5 |
| 3203.7884619 | 0 |
| 3203.790084 | 2 |
| 3203.7914944 | 3 |
| 3203.7925229 | 2 |
| 3203.7938122 | 1 |
| 3203.7963058 | 3 |
| 3203.7969875 | 2 |
| 3203.7985396 | 0 |
| 3203.7991256 | 2 |
| 3203.8006809 | 0 |
| 3203.8034739 | 1 |
| 3203.8041627 | 1 |
| 3203.8059617 | 2 |
| 3203.8067705 | 1 |
| 3203.8081125 | 1 |
| 3203.8109824 | 2 |
| 3203.8112354 | 2 |
| 3203.8122685 | 1 |
| 3203.8140699 | 1 |
| 3203.815511 | 3 |
| 3203.8173951 | 2 |
| 3203.8187809 | 1 |
| 3203.8202656 | 2 |
| 3203.8206563 | 2 |
| 3203.822831 | 0 |
| 3203.8239901 | 3 |
| 3203.8249343 | 3 |
| 3203.8270211 | 0 |
| 3203.8281517 | 4 |
| 3203.8303398 | 1 |
| 3203.8303995 | 2 |
| 3203.8324806 | 0 |
| 3203.8342661 | 2 |
| 3203.8348739 | 5 |
| 3203.8362635 | 0 |
| 3203.8375669 | 5 |
| 3203.8381639 | 4 |
| 3203.840838 | 0 |
| 3203.8413368 | 2 |
| 3203.8431722 | 1 |
| 3203.8435288 | 0 |
| 3203.8453247 | 1 |
| 3203.8476704 | 3 |
| 3203.8484813 | 4 |
| 3203.849901 | 4 |
| 3203.8512674 | 2 |
| 3203.8528917 | 3 |
| 3203.8547681 | 3 |
| 3203.8556531 | 2 |
| 3203.8571184 | 2 |
| 3203.8595231 | 1 |
| 3203.8602166 | 4 |
| 3203.861186 | 3 |
| 3203.8627044 | 2 |
| 3203.8642597 | 2 |
| 3203.8657691 | 1 |
| 3203.867242 | 1 |
| 3203.8689757 | 1 |
| 3203.8700812 | 1 |
| 3203.8722262 | 0 |
| 3203.8725877 | 3 |
| 3203.8743182 | 3 |
| 3203.8764581 | 1 |
| 3203.8768148 | 1 |
| 3203.8782541 | 2 |
| 3203.8799326 | 1 |
| 3203.8804727 | 1 |
| 3203.8822321 | 2 |
| 3203.8837784 | 0 |
| 3203.8853006 | 1 |
| 3203.8869385 | 1 |
| 3203.8881715 | 3 |
| 3203.8894907 | 3 |
| 3203.8906459 | 4 |
| 3203.8925673 | 2 |
| 3203.8932129 | 3 |
| 3203.8944496 | 2 |
| 3203.8965739 | 4 |
| 3203.8974201 | 1 |
| 3203.8988025 | 2 |
| 3203.9003079 | 1 |
| 3203.9024222 | 2 |
| 3203.9028479 | 1 |
| 3203.9051073 | 2 |
| 3203.9072814 | 0 |
| 3203.9078756 | 1 |
| 3203.9099528 | 1 |
| 3203.9108537 | 0 |
| 3203.9128477 | 0 |
| 3203.9138872 | 0 |
| 3203.9147615 | 2 |
| 3203.9163641 | 1 |
| 3203.9171803 | 1 |
| 3203.9181925 | 2 |
| 3203.9206753 | 0 |
| 3203.921605 | 3 |
| 3203.9231271 | 0 |
| 3203.9246826 | 3 |
| 3203.9258463 | 0 |
| 3203.9270721 | 2 |
| 3203.9286565 | 4 |
| 3203.9303616 | 1 |
| 3203.931469 | 1 |
| 3203.9334056 | 3 |
| 3203.9346329 | 0 |
| 3203.9357131 | 0 |
| 3203.9368219 | 3 |
| 3203.9377307 | 2 |
| 3203.9401538 | 3 |
| 3203.9407173 | 0 |
| 3203.9427625 | 0 |
| 3203.944602 | 1 |
| 3203.9451809 | 4 |
| 3203.9470202 | 1 |
| 3203.9484991 | 2 |
| 3203.948726 | 1 |
| 3203.9510686 | 1 |
| 3203.951664 | 1 |
| 3203.9538127 | 0 |
| 3203.9555461 | 2 |
| 3203.9564502 | 2 |
| 3203.9580675 | 3 |
| 3203.9589512 | 1 |
| 3203.9612729 | 1 |
| 3203.961392 | 1 |
| 3203.9640823 | 2 |
| 3203.9652071 | 1 |
| 3203.9657259 | 3 |
| 3203.9679958 | 1 |
| 3203.9693376 | 1 |
| 3203.9702705 | 1 |
| 3203.9721251 | 2 |
| 3203.9731118 | 0 |
| 3203.9745862 | 2 |
| 3203.9751337 | 3 |
| 3203.9772077 | 2 |
| 3203.9788568 | 0 |
| 3203.9801186 | 1 |
| 3203.9814577 | 2 |
| 3203.9826075 | 2 |
| 3203.9839776 | 0 |
| 3203.9854664 | 1 |
| 3203.987109 | 0 |
| 3203.987321 | 3 |
| 3203.9897524 | 2 |
| 3203.9908198 | 1 |
| 3203.9931097 | 2 |
| 3203.9937235 | 1 |
| 3203.9953193 | 1 |
| 3203.9976447 | 2 |
| 3203.9984469 | 3 |
| 3203.9997993 | 2 |
| 3204.0009655 | 3 |
| 3204.0025886 | 2 |
| 3204.0044133 | 2 |
| 3204.0049827 | 4 |
| 3204.0068464 | 1 |
| 3204.0080165 | 1 |
| 3204.0093218 | 0 |
| 3204.0109762 | 1 |
| 3204.0117656 | 2 |
| 3204.0137724 | 0 |
| 3204.0147542 | 2 |
| 3204.0169963 | 1 |
| 3204.0172277 | 1 |
| 3204.0183435 | 0 |
| 3204.0210725 | 1 |
| 3204.0215938 | 2 |
| 3204.0226089 | 0 |
| 3204.0242049 | 3 |
| 3204.0251646 | 0 |
| 3204.027013 | 2 |
| 3204.0281606 | 3 |
| 3204.0295085 | 1 |
| 3204.0308512 | 1 |
| 3204.0330937 | 3 |
| 3204.0348756 | 1 |
| 3204.0353893 | 1 |
| 3204.0370385 | 2 |
| 3204.0374489 | 1 |
| 3204.0396948 | 0 |
| 3204.0417049 | 1 |
| 3204.042224 | 0 |
| 3204.0445748 | 1 |
| 3204.045322 | 1 |
| 3204.0468455 | 0 |
| 3204.0486393 | 2 |
| 3204.0493323 | 4 |
| 3204.0514705 | 1 |
| 3204.0516002 | 1 |
| 3204.0542417 | 3 |
| 3204.0543292 | 2 |
| 3204.0560195 | 0 |
| 3204.058109 | 0 |
| 3204.0586322 | 2 |
| 3204.0604779 | 2 |
| 3204.0627352 | 1 |
| 3204.0632611 | 0 |
| 3204.0644427 | 1 |
| 3204.0657838 | 3 |
| 3204.067537 | 2 |
| 3204.0691931 | 2 |
| 3204.0700285 | 3 |
| 3204.0718221 | 1 |
| 3204.0733819 | 1 |
| 3204.0737589 | 2 |
| 3204.0751343 | 0 |
| 3204.0771499 | 2 |
| 3204.0786311 | 0 |
| 3204.0796977 | 1 |
| 3204.0809103 | 3 |
| 3204.0820211 | 3 |
| 3204.0843856 | 2 |
| 3204.0860536 | 1 |
| 3204.0868511 | 1 |
| 3204.088895 | 1 |
| 3204.0895091 | 1 |
| 3204.0914089 | 3 |
| 3204.0922006 | 0 |
| 3204.0940533 | 0 |
| 3204.0961898 | 3 |
| 3204.0969499 | 3 |
| 3204.0981737 | 2 |
| 3204.0991312 | 1 |
| 3204.1009938 | 2 |
| 3204.1027065 | 5 |
| 3204.1039288 | 1 |
| 3204.1050836 | 1 |
| 3204.1062834 | 1 |
| 3204.1076523 | 0 |
| 3204.108522 | 2 |
| 3204.1100095 | 3 |
| 3204.111847 | 1 |
| 3204.1135719 | 3 |
| 3204.1140714 | 1 |
| 3204.1161477 | 2 |
| 3204.116384 | 3 |
| 3204.1185402 | 2 |
| 3204.118781 | 3 |
| 3204.1209286 | 1 |
| 3204.122955 | 1 |
| 3204.1241183 | 1 |
| 3204.1254819 | 1 |
| 3204.1263289 | 2 |
| 3204.127949 | 3 |
| 3204.1301393 | 3 |
| 3204.1309911 | 1 |
| 3204.132867 | 5 |
| 3204.1334579 | 2 |
| 3204.1356025 | 0 |

| | |
|---|---|
| 3204.1365972 | 0 |
| 3204.1377605 | 2 |
| 3204.1399721 | 1 |
| 3204.1413432 | 0 |
| 3204.1421701 | 1 |
| 3204.1436365 | 0 |
| 3204.1452423 | 0 |
| 3204.1464471 | 2 |
| 3204.1478431 | 4 |
| 3204.1492724 | 4 |
| 3204.1510953 | 3 |
| 3204.1514835 | 3 |
| 3204.153045 | 4 |
| 3204.1540616 | 2 |
| 3204.1559531 | 2 |
| 3204.1568057 | 2 |
| 3204.1586897 | 1 |
| 3204.1600112 | 0 |
| 3204.1607414 | 2 |
| 3204.1627633 | 2 |
| 3204.1641218 | 2 |
| 3204.1650365 | 0 |
| 3204.1669142 | 1 |
| 3204.1685087 | 2 |
| 3204.1695952 | 1 |
| 3204.1705393 | 2 |
| 3204.1729139 | 6 |
| 3204.1743371 | 1 |
| 3204.1750255 | 4 |
| 3204.1773608 | 0 |
| 3204.1781103 | 4 |
| 3204.1798614 | 1 |
| 3204.1820149 | 1 |
| 3204.1826907 | 2 |
| 3204.1843024 | 4 |
| 3204.1852452 | 1 |
| 3204.186977 | 1 |
| 3204.187786 | 1 |
| 3204.1900299 | 2 |
| 3204.1910719 | 0 |
| 3204.1928202 | 0 |
| 3204.1939693 | 1 |
| 3204.1953271 | 4 |
| 3204.1961272 | 3 |
| 3204.1984721 | 0 |
| 3204.1988679 | 2 |
| 3204.2006905 | 0 |
| 3204.2023079 | 1 |
| 3204.2030824 | 2 |
| 3204.2045633 | 1 |
| 3204.2051903 | 2 |
| 3204.2069697 | 2 |
| 3204.2074934 | 1 |
| 3204.2097918 | 2 |
| 3204.211453 | 1 |
| 3204.2124482 | 2 |
| 3204.2142432 | 2 |
| 3204.2155422 | 1 |
| 3204.2164828 | 3 |
| 3204.2191114 | 0 |
| 3204.2199468 | 1 |
| 3204.2220278 | 1 |
| 3204.2221348 | 1 |
| 3204.2244478 | 1 |
| 3204.2253834 | 0 |
| 3204.2272014 | 2 |
| 3204.2289868 | 1 |
| 3204.2298924 | 1 |
| 3204.2319738 | 1 |
| 3204.2324744 | 3 |
| 3204.2340212 | 0 |
| 3204.2361302 | 4 |
| 3204.2366877 | 1 |
| 3204.2381664 | 2 |
| 3204.2397967 | 2 |
| 3204.2405669 | 2 |
| 3204.2420839 | 1 |
| 3204.2430496 | 2 |
| 3204.2450189 | 1 |
| 3204.2467998 | 1 |
| 3204.2472545 | 3 |
| 3204.2492801 | 1 |
| 3204.2505652 | 3 |
| 3204.2518648 | 1 |
| 3204.2532268 | 2 |
| 3204.2551548 | 2 |
| 3204.2560858 | 0 |
| 3204.2568825 | 0 |
| 3204.2584079 | 1 |
| 3204.2591295 | 1 |
| 3204.2613941 | 2 |
| 3204.262867 | 0 |
| 3204.2644226 | 0 |
| 3204.2662131 | 1 |
| 3204.2674291 | 2 |
| 3204.2686619 | 1 |
| 3204.2696979 | 0 |
| 3204.2713125 | 1 |
| 3204.2734855 | 1 |
| 3204.2740135 | 1 |
| 3204.2762001 | 3 |
| 3204.2776467 | 1 |
| 3204.2786149 | 4 |
| 3204.279959 | 0 |
| 3204.2808474 | 0 |
| 3204.28335 | 0 |
| 3204.2838695 | 2 |
| 3204.2854794 | 0 |
| 3204.2875927 | 0 |
| 3204.2876359 | 0 |
| 3204.2896246 | 1 |
| 3204.291615 | 3 |
| 3204.2919395 | 0 |
| 3204.2933678 | 1 |
| 3204.2951218 | 2 |
| 3204.2960852 | 3 |
| 3204.2972006 | 2 |
| 3204.2990559 | 2 |
| 3204.3014191 | 1 |
| 3204.3015237 | 1 |
| 3204.303285 | 1 |
| 3204.3044995 | 4 |
| 3204.3062359 | 0 |
| 3204.3076721 | 4 |
| 3204.3086213 | 1 |
| 3204.3099647 | 1 |
| 3204.311243 | 4 |
| 3204.3113986 | 3 |
| 3204.3114315 | 3 |
| 3204.3122773 | 0 |
| 3204.3138492 | 2 |
| 3204.3138625 | 0 |
| 3204.3156124 | 2 |
| 3204.3163246 | 3 |
| 3204.3165579 | 2 |
| 3204.3167459 | 4 |
| 3204.3183507 | 2 |
| 3204.318471 | 2 |
| 3204.3188668 | 0 |
| 3204.3193005 | 3 |
| 3204.3205031 | 1 |
| 3204.3212774 | 1 |
| 3204.3216752 | 2 |
| 3204.323203 | 2 |
| 3204.32369 | 4 |
| 3204.3238362 | 3 |
| 3204.3251819 | 1 |
| 3204.3259358 | 3 |
| 3204.3260476 | 3 |
| 3204.3264008 | 2 |
| 3204.3272862 | 3 |
| 3204.3287766 | 2 |
| 3204.3289936 | 1 |
| 3204.329353 | 2 |
| 3204.3302313 | 3 |
| 3204.3314953 | 2 |
| 3204.3316821 | 2 |
| 3204.3329446 | 1 |
| 3204.333545 | 1 |
| 3204.3338394 | 3 |
| 3204.334909 | 6 |
| 3204.3351953 | 5 |
| 3204.3361018 | 1 |
| 3204.3363762 | 0 |
| 3204.3379355 | 4 |
| 3204.3382108 | 3 |
| 3204.3391467 | 1 |
| 3204.3394405 | 0 |
| 3204.3399405 | 2 |
| 3204.3402788 | 1 |
| 3204.3417991 | 0 |
| 3204.3423386 | 2 |
| 3204.3426318 | 0 |
| 3204.3435647 | 1 |
| 3204.3436542 | 0 |
| 3204.3448812 | 1 |
| 3204.3462504 | 0 |
| 3204.3466382 | 2 |
| 3204.3469984 | 3 |
| 3204.3472392 | 3 |
| 3204.3483407 | 2 |
| 3204.3490479 | 0 |
| 3204.3495303 | 1 |
| 3204.3511294 | 1 |
| 3204.3514673 | 4 |
| 3204.3518079 | 0 |
| 3204.3530502 | 5 |
| 3204.3539716 | 1 |
| 3204.3540309 | 0 |
| 3204.3542959 | 0 |
| 3204.3558763 | 3 |
| 3204.3566286 | 4 |
| 3204.356884 | 2 |
| 3204.3582434 | 4 |
| 3204.3585717 | 1 |
| 3204.3590467 | 1 |
| 3204.3590777 | 2 |
| 3204.3603183 | 3 |
| 3204.3611376 | 2 |
| 3204.361895 | 2 |
| 3204.3632184 | 3 |
| 3204.3633061 | 1 |
| 3204.3638687 | 1 |
| 3204.3639596 | 2 |
| 3204.3658478 | 1 |
| 3204.3661548 | 3 |
| 3204.3670873 | 3 |
| 3204.3682143 | 1 |
| 3204.3683181 | 4 |
| 3204.3692467 | 1 |
| 3204.369909 | 3 |
| 3204.3713132 | 1 |
| 3204.3713755 | 0 |
| 3204.3718752 | 1 |
| 3204.3735123 | 1 |
| 3204.3735781 | 2 |
| 3204.3737693 | 1 |
| 3204.3750476 | 1 |
| 3204.3753689 | 3 |
| 3204.3756267 | 3 |
| 3204.3772578 | 0 |
| 3204.3775654 | 4 |
| 3204.3778968 | 2 |
| 3204.3785761 | 2 |
| 3204.3798315 | 4 |
| 3204.3805732 | 0 |
| 3204.3807588 | 2 |
| 3204.3821726 | 1 |
| 3204.3822912 | 3 |
| 3204.3830588 | 2 |
| 3204.3842742 | 1 |
| 3204.3844304 | 2 |
| 3204.3848372 | 2 |
| 3204.3850761 | 2 |
| 3204.386569 | 3 |
| 3204.3873426 | 3 |
| 3204.3878156 | 1 |
| 3204.3889569 | 5 |
| 3204.3895388 | 0 |
| 3204.3898399 | 3 |
| 3204.3902419 | 1 |
| 3204.3917673 | 1 |
| 3204.391787 | 3 |
| 3204.391874 | 1 |
| 3204.3939632 | 4 |
| 3204.3941822 | 1 |
| 3204.3949958 | 2 |
| 3204.3957167 | 0 |
| 3204.3958117 | 1 |
| 3204.3971567 | 2 |
| 3204.3972453 | 2 |
| 3204.3984221 | 2 |
| 3204.3988797 | 4 |
| 3204.3997633 | 3 |
| 3204.4009858 | 1 |
| 3204.4016234 | 2 |
| 3204.40176 | 3 |
| 3204.4022892 | 2 |
| 3204.4031098 | 3 |
| 3204.4043035 | 1 |
| 3204.4046135 | 3 |
| 3204.4062413 | 0 |
| 3204.4064054 | 3 |
| 3204.4068868 | 2 |
| 3204.4076349 | 3 |
| 3204.4090393 | 1 |
| 3204.4094961 | 3 |
| 3204.4098191 | 3 |
| 3204.4113799 | 3 |
| 3204.4115062 | 5 |
| 3204.4119996 | 2 |
| 3204.4138684 | 3 |
| 3204.4140196 | 1 |
| 3204.4140889 | 1 |
| 3204.414745 | 1 |
| 3204.4158454 | 2 |
| 3204.4168205 | 0 |
| 3204.4173206 | 1 |
| 3204.4179713 | 1 |
| 3204.4188446 | 0 |
| 3204.4189766 | 1 |
| 3204.4195379 | 3 |
| 3204.4207464 | 2 |
| 3204.4211124 | 0 |
| 3204.4226049 | 2 |
| 3204.4228709 | 3 |
| 3204.4231311 | 1 |
| 3204.4238067 | 2 |
| 3204.4247226 | 1 |
| 3204.4259721 | 1 |
| 3204.4261246 | 3 |
| 3204.4270562 | 1 |
| 3204.4273471 | 2 |
| 3204.4281697 | 2 |
| 3204.4282254 | 1 |
| 3204.4296979 | 2 |
| 3204.4298025 | 1 |
| 3204.4299242 | 0 |
| 3204.4321231 | 4 |
| 3204.4322663 | 2 |
| 3204.4336377 | 1 |
| 3204.4338257 | 0 |
| 3204.4347758 | 0 |
| 3204.4353356 | 1 |
| 3204.4357112 | 1 |
| 3204.4360575 | 5 |
| 3204.4372362 | 2 |
| 3204.437627 | 0 |
| 3204.4385046 | 1 |
| 3204.4390625 | 3 |
| 3204.4397506 | 5 |
| 3204.4413237 | 2 |
| 3204.4416186 | 2 |
| 3204.4419612 | 3 |
| 3204.4429687 | 1 |
| 3204.4440349 | 0 |
| 3204.4441502 | 0 |
| 3204.4450054 | 1 |
| 3204.4457959 | 2 |
| 3204.4467967 | 2 |
| 3204.4470439 | 4 |
| 3204.4481485 | 0 |
| 3204.4486747 | 3 |
| 3204.4494429 | 0 |
| 3204.4495658 | 4 |
| 3204.4511057 | 1 |
| 3204.4519356 | 0 |
| 3204.4521449 | 2 |
| 3204.4522677 | 0 |
| 3204.4539519 | 2 |
| 3204.4546095 | 0 |
| 3204.4547042 | 3 |
| 3204.4558209 | 1 |
| 3204.4567282 | 4 |
| 3204.457151 | 2 |
| 3204.4583866 | 0 |
| 3204.4587931 | 1 |
| 3204.459167 | 2 |
| 3204.460393 | 2 |
| 3204.461026 | 2 |
| 3204.4617193 | 2 |
| 3204.4619377 | 0 |
| 3204.4631617 | 2 |
| 3204.4634209 | 1 |
| 3204.4641613 | 0 |
| 3204.4651665 | 2 |
| 3204.4658902 | 0 |
| 3204.4663411 | 2 |
| 3204.4663521 | 1 |
| 3204.4678146 | 2 |
| 3204.4684734 | 1 |
| 3204.4697572 | 1 |
| 3204.4699989 | 0 |
| 3204.4704933 | 5 |
| 3204.4711773 | 3 |
| 3204.4716462 | 1 |
| 3204.4722579 | 1 |
| 3204.4732562 | 1 |
| 3204.4740031 | 1 |
| 3204.4748999 | 4 |
| 3204.475283 | 4 |
| 3204.4759643 | 2 |
| 3204.4769909 | 1 |

| | |
|---|---|
| 3204.4771659 | 2 |
| 3204.4780414 | 4 |
| 3204.479686 | 1 |
| 3204.4800413 | 2 |
| 3204.4803588 | 0 |
| 3204.4808981 | 3 |
| 3204.4821195 | 0 |
| 3204.482333 | 3 |
| 3204.4824952 | 0 |
| 3204.4834037 | 0 |
| 3204.484643 | 0 |
| 3204.4847685 | 1 |
| 3204.4864151 | 2 |
| 3204.4869706 | 1 |
| 3204.4874428 | 1 |
| 3204.4877408 | 0 |
| 3204.4887719 | 2 |
| 3204.4894432 | 2 |
| 3204.489782 | 1 |
| 3204.4909544 | 3 |
| 3204.4915347 | 0 |
| 3204.4920887 | 0 |
| 3204.4934948 | 3 |
| 3204.4938366 | 1 |
| 3204.4948136 | 0 |
| 3204.4951081 | 0 |
| 3204.4964281 | 1 |
| 3204.4966326 | 7 |
| 3204.4977638 | 1 |
| 3204.4979917 | 1 |
| 3204.4990406 | 1 |
| 3204.4991968 | 2 |
| 3204.5005982 | 3 |
| 3204.5010247 | 2 |
| 3204.5015872 | 2 |
| 3204.5019698 | 0 |
| 3204.5034104 | 1 |
| 3204.5037485 | 1 |
| 3204.5041416 | 2 |
| 3204.5057199 | 0 |
| 3204.5060292 | 1 |
| 3204.5062376 | 1 |
| 3204.5081688 | 1 |
| 3204.5085012 | 1 |
| 3204.5085329 | 2 |
| 3204.5087314 | 2 |
| 3204.5104696 | 3 |
| 3204.510725 | 4 |
| 3204.5116308 | 0 |
| 3204.5124646 | 5 |
| 3204.5127126 | 1 |
| 3204.5130711 | 1 |
| 3204.5141469 | 1 |
| 3204.5143859 | 1 |
| 3204.5160728 | 3 |
| 3204.5161854 | 0 |
| 3204.5177583 | 4 |
| 3204.5177683 | 2 |
| 3204.518155 | 1 |
| 3204.519088 | 2 |
| 3204.5199032 | 2 |
| 3204.5204688 | 4 |
| 3204.5214406 | 5 |
| 3204.5215544 | 1 |
| 3204.5222079 | 4 |
| 3204.5235457 | 6 |
| 3204.5242884 | 6 |
| 3204.5243565 | 6 |
| 3204.5257498 | 3 |
| 3204.5258797 | 5 |
| 3204.5260797 | 1 |
| 3204.5265615 | 1 |
| 3204.5285045 | 0 |
| 3204.5288171 | 0 |
| 3204.5301299 | 2 |
| 3204.5306229 | 3 |
| 3204.530692 | 3 |
| 3204.531711 | 0 |
| 3204.5322789 | 1 |
| 3204.5323195 | 3 |
| 3204.5338817 | 2 |
| 3204.5345277 | 3 |
| 3204.5352966 | 2 |
| 3204.5365468 | 0 |
| 3204.5369453 | 4 |
| 3204.5371171 | 2 |
| 3204.5388856 | 1 |
| 3204.5391168 | 0 |
| 3204.5395351 | 1 |
| 3204.5398528 | 0 |
| 3204.5412651 | 3 |
| 3204.5413504 | 1 |
| 3204.5429376 | 1 |
| 3204.5430522 | 2 |
| 3204.5438007 | 0 |
| 3204.5447677 | 0 |
| 3204.5450469 | 1 |
| 3204.545659 | 3 |
| 3204.5464124 | 1 |
| 3204.5471364 | 6 |
| 3204.5483716 | 1 |
| 3204.549609 | 2 |
| 3204.5497088 | 2 |
| 3204.5500822 | 1 |
| 3204.5511061 | 0 |
| 3204.55208 | 4 |
| 3204.5527483 | 1 |
| 3204.5531085 | 0 |
| 3204.5532062 | 3 |
| 3204.5548746 | 2 |
| 3204.5554798 | 0 |
| 3204.5555808 | 2 |
| 3204.5563989 | 5 |
| 3204.5575175 | 0 |
| 3204.5581201 | 3 |
| 3204.5591137 | 3 |
| 3204.5595272 | 0 |
| 3204.5596607 | 1 |
| 3204.5607006 | 3 |
| 3204.5608089 | 3 |
| 3204.5630086 | 0 |
| 3204.5630406 | 3 |
| 3204.5640441 | 1 |
| 3204.5644745 | 1 |
| 3204.565334 | 1 |
| 3204.5653559 | 1 |
| 3204.5668501 | 0 |
| 3204.5670729 | 0 |
| 3204.5673837 | 1 |
| 3204.5688436 | 1 |
| 3204.5692682 | 0 |
| 3204.5701135 | 1 |
| 3204.5710681 | 1 |
| 3204.5711938 | 0 |
| 3204.5714948 | 0 |
| 3204.5721833 | 2 |
| 3204.5731994 | 0 |
| 3204.5737873 | 1 |
| 3204.5745417 | 1 |
| 3204.5758803 | 2 |
| 3204.5760611 | 1 |
| 3204.5763992 | 1 |
| 3204.5776106 | 1 |
| 3204.578074 | 1 |
| 3204.5787166 | 1 |
| 3204.579332 | 2 |
| 3204.5804455 | 3 |
| 3204.5811815 | 1 |
| 3204.5816823 | 1 |
| 3204.5827895 | 0 |
| 3204.5832789 | 4 |
| 3204.5844526 | 1 |
| 3204.5844849 | 2 |
| 3204.5853066 | 0 |
| 3204.5861527 | 2 |
| 3204.5868354 | 2 |
| 3204.5877439 | 1 |
| 3204.5883743 | 1 |
| 3204.5890326 | 3 |
| 3204.5905148 | 2 |
| 3204.590853 | 2 |
| 3204.5908842 | 2 |
| 3204.5921344 | 2 |
| 3204.5922446 | 1 |
| 3204.593715 | 2 |
| 3204.5938371 | 3 |
| 3204.5951922 | 2 |
| 3204.5952511 | 2 |
| 3204.5958081 | 2 |
| 3204.5968591 | 1 |
| 3204.5979768 | 2 |
| 3204.5982875 | 1 |
| 3204.5983638 | 3 |
| 3204.5995444 | 0 |
| 3204.600746 | 6 |
| 3204.6011877 | 1 |
| 3204.602281 | 1 |
| 3204.6023394 | 1 |
| 3204.6028853 | 1 |
| 3204.6038226 | 2 |
| 3204.6042078 | 2 |
| 3204.6050834 | 1 |
| 3204.6053118 | 5 |
| 3204.6061677 | 2 |
| 3204.6071059 | 1 |
| 3204.6071988 | 2 |
| 3204.6081591 | 1 |
| 3204.6088755 | 1 |
| 3204.610096 | 1 |
| 3204.6113677 | 3 |
| 3204.6116229 | 3 |
| 3204.6121372 | 0 |
| 3204.6128851 | 0 |
| 3204.6134967 | 4 |
| 3204.6141117 | 3 |
| 3204.6149875 | 3 |
| 3204.6152763 | 0 |
| 3204.6158169 | 1 |
| 3204.6171017 | 1 |
| 3204.6172486 | 0 |
| 3204.6185912 | 1 |
| 3204.6191605 | 2 |
| 3204.619533 | 2 |
| 3204.6197845 | 0 |
| 3204.6212913 | 0 |
| 3204.6217886 | 1 |
| 3204.6229937 | 1 |
| 3204.6233809 | 0 |
| 3204.6236347 | 1 |
| 3204.6257577 | 2 |
| 3204.6259296 | 3 |
| 3204.626001 | 1 |
| 3204.627364 | 2 |
| 3204.6275853 | 1 |
| 3204.6278875 | 4 |
| 3204.6288623 | 2 |
| 3204.62974 | 1 |
| 3204.6300519 | 3 |
| 3204.6310309 | 2 |
| 3204.631258 | 0 |
| 3204.6330984 | 1 |
| 3204.6331837 | 3 |
| 3204.633732 | 3 |
| 3204.6352134 | 1 |
| 3204.6354057 | 1 |
| 3204.6363266 | 4 |
| 3204.6364089 | 2 |
| 3204.6370732 | 0 |
| 3204.6381706 | 4 |
| 3204.6383881 | 0 |
| 3204.6404224 | 2 |
| 3204.6404247 | 3 |
| 3204.6412797 | 1 |
| 3204.6417125 | 2 |
| 3204.6420444 | 1 |
| 3204.6428148 | 1 |
| 3204.6437103 | 3 |
| 3204.6449625 | 0 |
| 3204.6452946 | 0 |
| 3204.6460391 | 1 |
| 3204.6467508 | 1 |
| 3204.6476178 | 2 |
| 3204.6478846 | 2 |
| 3204.6490387 | 7 |
| 3204.6492034 | 1 |
| 3204.6497765 | 2 |
| 3204.650598 | 2 |
| 3204.6517402 | 0 |
| 3204.6522326 | 2 |
| 3204.6522843 | 3 |
| 3204.6535273 | 2 |
| 3204.6539236 | 2 |
| 3204.6546484 | 2 |
| 3204.6563801 | 3 |
| 3204.6567333 | 4 |
| 3204.6570909 | 1 |
| 3204.6571849 | 2 |
| 3204.6582931 | 1 |
| 3204.658442 | 1 |
| 3204.6596593 | 1 |
| 3204.6605191 | 4 |
| 3204.6608408 | 3 |
| 3204.6614342 | 3 |
| 3204.6626484 | 4 |
| 3204.6629927 | 1 |
| 3204.6639086 | 3 |
| 3204.6642011 | 0 |
| 3204.6647789 | 0 |
| 3204.6661812 | 2 |
| 3204.666701 | 3 |
| 3204.6689595 | 1 |
| 3204.6701203 | 0 |
| 3204.6707096 | 0 |
| 3204.6731524 | 1 |
| 3204.6735449 | 1 |
| 3204.6754751 | 1 |
| 3204.6772404 | 1 |
| 3204.6785581 | 1 |
| 3204.6800397 | 0 |
| 3204.6808964 | 1 |
| 3204.6831287 | 0 |
| 3204.6834196 | 1 |
| 3204.6851797 | 1 |
| 3204.6873049 | 3 |
| 3204.6879613 | 1 |
| 3204.6897108 | 3 |
| 3204.6916243 | 2 |
| 3204.6923684 | 5 |
| 3204.6941387 | 2 |
| 3204.6946145 | 0 |
| 3204.6969983 | 0 |
| 3204.6985949 | 1 |
| 3204.6996797 | 2 |
| 3204.7001707 | 2 |
| 3204.7019502 | 0 |
| 3204.7037534 | 2 |
| 3204.704939 | 1 |
| 3204.7056242 | 1 |
| 3204.7076426 | 0 |
| 3204.7092933 | 2 |
| 3204.7104152 | 3 |
| 3204.712572 | 3 |
| 3204.712744 | 0 |
| 3204.7149843 | 1 |
| 3204.7157096 | 3 |
| 3204.7169194 | 2 |
| 3204.7188144 | 1 |
| 3204.7197888 | 0 |
| 3204.7217195 | 1 |
| 3204.7221779 | 1 |
| 3204.7247598 | 2 |
| 3204.7266425 | 1 |
| 3204.7273755 | 0 |
| 3204.7292935 | 0 |
| 3204.7299612 | 2 |
| 3204.7314792 | 1 |
| 3204.7322015 | 2 |
| 3204.7340259 | 0 |
| 3204.7356049 | 1 |
| 3204.7370349 | 1 |
| 3204.7382621 | 0 |
| 3204.739039 | 1 |
| 3204.7409281 | 0 |
| 3204.7424759 | 3 |
| 3204.7437176 | 1 |
| 3204.7450698 | 3 |
| 3204.7466452 | 2 |
| 3204.7481764 | 0 |
| 3204.7496769 | 3 |
| 3204.7504698 | 3 |
| 3204.7520238 | 1 |
| 3204.7531281 | 4 |
| 3204.7546773 | 3 |
| 3204.7559999 | 2 |
| 3204.7575251 | 2 |
| 3204.7592239 | 2 |
| 3204.7593068 | 4 |
| 3204.7617931 | 3 |
| 3204.7638021 | 3 |
| 3204.7649965 | 2 |
| 3204.7664992 | 2 |
| 3204.7671674 | 1 |
| 3204.7690666 | 2 |
| 3204.770724 | 2 |
| 3204.7714982 | 0 |
| 3204.7733411 | 1 |
| 3204.7741455 | 1 |
| 3204.77621 | 2 |
| 3204.7781427 | 3 |
| 3204.7782074 | 2 |
| 3204.7794821 | 3 |
| 3204.7805044 | 0 |
| 3204.7828958 | 2 |
| 3204.7844665 | 1 |
| 3204.7853638 | 2 |
| 3204.7870168 | 1 |
| 3204.7876201 | 2 |
| 3204.789618 | 0 |
| 3204.7912565 | 2 |
| 3204.7926272 | 5 |
| 3204.7938651 | 0 |
| 3204.7945118 | 0 |
| 3204.7961577 | 1 |

| | |
|---|---|
| 3204.7980846 | 2 |
| 3204.798943 | 1 |
| 3204.8012164 | 5 |
| 3204.8015958 | 2 |
| 3204.8028964 | 1 |
| 3204.8052807 | 3 |
| 3204.8061175 | 4 |
| 3204.8076037 | 0 |
| 3204.809181 | 2 |
| 3204.8103853 | 0 |
| 3204.8125838 | 2 |
| 3204.8130289 | 0 |
| 3204.8149447 | 2 |
| 3204.8156524 | 5 |
| 3204.8178701 | 0 |
| 3204.8192654 | 0 |
| 3204.8203685 | 1 |
| 3204.8221366 | 2 |
| 3204.8228305 | 1 |
| 3204.8249365 | 0 |
| 3204.82522 | 3 |
| 3204.8274578 | 1 |
| 3204.828626 | 1 |
| 3204.8300721 | 1 |
| 3204.8312046 | 1 |
| 3204.8333689 | 1 |
| 3204.8334719 | 2 |
| 3204.8356772 | 1 |
| 3204.835684 | 2 |
| 3204.837849 | 5 |
| 3204.8402355 | 1 |
| 3204.8406838 | 1 |
| 3204.8421651 | 1 |
| 3204.8437527 | 0 |
| 3204.84527 | 2 |
| 3204.8454952 | 0 |
| 3204.8473418 | 1 |
| 3204.8492016 | 0 |
| 3204.850405 | 0 |
| 3204.8514187 | 0 |
| 3204.8533086 | 1 |
| 3204.8548549 | 1 |
| 3204.8563729 | 0 |
| 3204.8576268 | 1 |
| 3204.8597147 | 1 |
| 3204.8601361 | 1 |
| 3204.8617973 | 0 |
| 3204.8626961 | 1 |
| 3204.8645186 | 2 |
| 3204.866448 | 0 |
| 3204.8671205 | 2 |
| 3204.86823 | 2 |
| 3204.8704631 | 3 |
| 3204.8711076 | 3 |
| 3204.873124 | 1 |
| 3204.8748716 | 1 |
| 3204.8761967 | 4 |
| 3204.8772616 | 0 |
| 3204.8778736 | 1 |
| 3204.8801566 | 2 |
| 3204.8810073 | 2 |
| 3204.8824306 | 3 |
| 3204.8842854 | 1 |
| 3204.8855982 | 0 |
| 3204.8864812 | 1 |
| 3204.8878017 | 0 |
| 3204.8893568 | 4 |
| 3204.8915526 | 1 |
| 3204.8918426 | 5 |
| 3204.8937006 | 3 |
| 3204.8952034 | 3 |
| 3204.8973804 | 2 |
| 3204.8977938 | 4 |
| 3204.8994525 | 2 |
| 3204.9007649 | 4 |
| 3204.901295 | 2 |
| 3204.9035884 | 0 |
| 3204.9055558 | 4 |
| 3204.9065029 | 4 |
| 3204.9077064 | 3 |
| 3204.9095206 | 2 |
| 3204.9104343 | 1 |
| 3204.9118531 | 2 |
| 3204.913034 | 1 |
| 3204.9140374 | 1 |
| 3204.9155602 | 0 |
| 3204.9174862 | 1 |
| 3204.9190747 | 2 |
| 3204.9199093 | 1 |
| 3204.9216014 | 4 |
| 3204.9229729 | 2 |
| 3204.9240903 | 1 |
| 3204.9259065 | 3 |
| 3204.9268666 | 3 |
| 3204.9288425 | 1 |
| 3204.930329 | 2 |
| 3204.9311724 | 1 |
| 3204.9327312 | 2 |
| 3204.9331299 | 0 |
| 3204.9358355 | 2 |
| 3204.9361019 | 0 |
| 3204.9379809 | 1 |
| 3204.940515 | 1 |
| 3204.9411391 | 1 |
| 3204.942378 | 2 |
| 3204.9435595 | 3 |
| 3204.9452405 | 5 |
| 3204.9471112 | 0 |
| 3204.9476912 | 1 |
| 3204.9491808 | 1 |
| 3204.9509955 | 2 |
| 3204.9520137 | 2 |
| 3204.9538245 | 1 |
| 3204.9541973 | 2 |
| 3204.9566132 | 1 |
| 3204.9580504 | 2 |
| 3204.9585454 | 2 |
| 3204.9607243 | 2 |
| 3204.9621712 | 1 |
| 3204.963575 | 1 |
| 3204.9653529 | 0 |
| 3204.9659631 | 1 |
| 3204.967736 | 1 |
| 3204.9685068 | 1 |
| 3204.9706149 | 4 |
| 3204.9723118 | 0 |
| 3204.9727198 | 2 |
| 3204.9748043 | 2 |
| 3204.9751163 | 2 |
| 3204.9773689 | 5 |
| 3204.9781675 | 2 |
| 3204.9801973 | 1 |
| 3204.9822455 | 0 |
| 3204.9827314 | 1 |
| 3204.9848964 | 1 |
| 3204.985121 | 0 |
| 3204.9872812 | 1 |
| 3204.988596 | 0 |
| 3204.9899509 | 0 |
| 3204.9915364 | 5 |
| 3204.9923101 | 1 |
| 3204.9947543 | 0 |
| 3204.9961125 | 2 |
| 3204.9962753 | 2 |
| 3204.9987686 | 2 |
| 3204.9992226 | 4 |
| 3205.0013247 | 2 |
| 3205.0028868 | 3 |
| 3205.0034973 | 1 |
| 3205.0056082 | 2 |
| 3205.0065398 | 2 |
| 3205.0083817 | 2 |
| 3205.0085678 | 1 |
| 3205.011039 | 1 |
| 3205.0128864 | 2 |
| 3205.0132938 | 1 |
| 3205.0149753 | 0 |
| 3205.0161341 | 2 |
| 3205.0174854 | 1 |
| 3205.0199691 | 3 |
| 3205.0201001 | 0 |
| 3205.0219317 | 2 |
| 3205.0229217 | 2 |
| 3205.0242435 | 1 |
| 3205.026457 | 1 |
| 3205.0277432 | 3 |
| 3205.0286638 | 3 |
| 3205.0295356 | 5 |
| 3205.0318989 | 4 |
| 3205.0339367 | 3 |
| 3205.0346793 | 0 |
| 3205.0365599 | 2 |
| 3205.0370974 | 4 |
| 3205.0388596 | 0 |
| 3205.0393621 | 1 |
| 3205.0420535 | 2 |
| 3205.0432896 | 0 |
| 3205.0448688 | 1 |
| 3205.0461065 | 1 |
| 3205.0461636 | 4 |
| 3205.0482025 | 1 |
| 3205.0500272 | 1 |
| 3205.0512233 | 2 |
| 3205.0518658 | 2 |
| 3205.0546229 | 0 |
| 3205.0556338 | 2 |
| 3205.0572532 | 1 |
| 3205.0579152 | 2 |
| 3205.0597586 | 3 |
| 3205.0608842 | 1 |
| 3205.0627331 | 2 |
| 3205.0643503 | 1 |
| 3205.0650668 | 0 |
| 3205.0670482 | 3 |
| 3205.0676797 | 0 |
| 3205.069362 | 0 |
| 3205.0715967 | 3 |
| 3205.0721376 | 0 |
| 3205.0736105 | 1 |
| 3205.0750812 | 2 |
| 3205.0763001 | 2 |
| 3205.0776501 | 1 |
| 3205.0793443 | 4 |
| 3205.081353 | 1 |
| 3205.0818546 | 1 |
| 3205.0838646 | 2 |
| 3205.0853293 | 3 |
| 3205.0860686 | 1 |
| 3205.0876063 | 2 |
| 3205.089306 | 3 |
| 3205.0905183 | 3 |
| 3205.0925586 | 3 |
| 3205.0928216 | 2 |
| 3205.0941564 | 4 |
| 3205.095887 | 1 |
| 3205.0981943 | 4 |
| 3205.0989145 | 0 |
| 3205.10083 | 3 |
| 3205.1027439 | 1 |
| 3205.1031756 | 2 |
| 3205.1046761 | 2 |
| 3205.1053543 | 0 |
| 3205.1069205 | 0 |
| 3205.109015 | 2 |
| 3205.1104388 | 0 |
| 3205.1121518 | 2 |
| 3205.1124482 | 1 |
| 3205.1149188 | 2 |
| 3205.1167677 | 1 |
| 3205.1169441 | 1 |
| 3205.118677 | 4 |
| 3205.1194169 | 3 |
| 3205.1210582 | 2 |
| 3205.1230735 | 1 |
| 3205.1237546 | 0 |
| 3205.1259526 | 3 |
| 3205.1267398 | 2 |
| 3205.1281349 | 1 |
| 3205.129696 | 3 |
| 3205.1306288 | 4 |
| 3205.1328167 | 2 |
| 3205.1340118 | 1 |
| 3205.135514 | 2 |
| 3205.1360759 | 2 |
| 3205.138239 | 0 |
| 3205.1397543 | 1 |
| 3205.1403793 | 1 |
| 3205.1427448 | 1 |
| 3205.1428038 | 0 |
| 3205.145896 | 3 |
| 3205.1470984 | 0 |
| 3205.1476339 | 0 |
| 3205.1502 | 2 |
| 3205.1502519 | 2 |
| 3205.1518809 | 3 |
| 3205.1528264 | 3 |
| 3205.1546516 | 1 |
| 3205.1566666 | 1 |
| 3205.1575931 | 4 |
| 3205.1590845 | 3 |
| 3205.1611615 | 1 |
| 3205.1614655 | 3 |
| 3205.1638839 | 3 |
| 3205.1646861 | 1 |
| 3205.1668062 | 0 |
| 3205.1677101 | 2 |
| 3205.169303 | 0 |
| 3205.1701375 | 2 |
| 3205.1716263 | 4 |
| 3205.173416 | 1 |
| 3205.1748753 | 1 |
| 3205.1763911 | 5 |
| 3205.178073 | 0 |
| 3205.1785284 | 1 |
| 3205.1801133 | 2 |
| 3205.18261 | 3 |
| 3205.1830137 | 1 |
| 3205.1840603 | 2 |
| 3205.1852564 | 0 |
| 3205.1876838 | 0 |
| 3205.1893388 | 2 |
| 3205.1897817 | 0 |
| 3205.1923856 | 3 |
| 3205.1927779 | 5 |
| 3205.1945229 | 0 |
| 3205.1948893 | 2 |
| 3205.1970473 | 2 |
| 3205.1987485 | 1 |
| 3205.1999851 | 1 |
| 3205.2010331 | 1 |
| 3205.2022453 | 4 |
| 3205.2041696 | 0 |
| 3205.2046416 | 1 |
| 3205.2063554 | 0 |
| 3205.2082744 | 1 |
| 3205.2089221 | 2 |
| 3205.2102584 | 0 |
| 3205.2124826 | 2 |
| 3205.2131761 | 1 |
| 3205.2153965 | 2 |
| 3205.2162078 | 0 |
| 3205.2181366 | 1 |
| 3205.219485 | 1 |
| 3205.2209485 | 0 |
| 3205.2218735 | 3 |
| 3205.2231735 | 3 |
| 3205.2255315 | 4 |
| 3205.2265549 | 1 |
| 3205.2281776 | 0 |
| 3205.229624 | 2 |
| 3205.2306002 | 2 |
| 3205.2321319 | 0 |
| 3205.2335805 | 1 |
| 3205.2350228 | 0 |
| 3205.2365327 | 0 |
| 3205.2373296 | 0 |
| 3205.2390872 | 3 |
| 3205.2398462 | 2 |
| 3205.2425571 | 2 |
| 3205.2439909 | 1 |
| 3205.2445945 | 4 |
| 3205.2464768 | 5 |
| 3205.2476975 | 3 |
| 3205.2483965 | 1 |
| 3205.2506119 | 3 |
| 3205.2516801 | 1 |
| 3205.2532606 | 0 |
| 3205.2541848 | 1 |
| 3205.2553233 | 2 |
| 3205.2571684 | 1 |
| 3205.2583983 | 2 |
| 3205.259706 | 4 |
| 3205.2614791 | 0 |
| 3205.2622256 | 3 |
| 3205.2637508 | 3 |
| 3205.265373 | 1 |
| 3205.2668116 | 0 |
| 3205.2687036 | 0 |
| 3205.2700474 | 0 |
| 3205.2717337 | 3 |
| 3205.2723282 | 2 |
| 3205.2744222 | 2 |
| 3205.2744847 | 1 |
| 3205.2772232 | 2 |
| 3205.2786116 | 2 |
| 3205.2797623 | 1 |
| 3205.280741 | 3 |
| 3205.2818906 | 1 |
| 3205.2838749 | 2 |
| 3205.2855512 | 0 |
| 3205.2859821 | 1 |
| 3205.2883912 | 1 |
| 3205.2891628 | 1 |
| 3205.2912166 | 1 |
| 3205.2922601 | 2 |
| 3205.293199 | 0 |
| 3205.2955709 | 2 |
| 3205.2960239 | 1 |
| 3205.2976892 | 4 |
| 3205.2980737 | 3 |
| 3205.3005459 | 1 |
| 3205.3021605 | 2 |
| 3205.3029877 | 5 |

| | |
|---|---|
| 3205.3041118 | 1 |
| 3205.3062512 | 1 |
| 3205.3071453 | 0 |
| 3205.3091101 | 1 |
| 3205.3097564 | 0 |
| 3205.3112015 | 1 |
| 3205.3131368 | 4 |
| 3205.3143194 | 3 |
| 3205.3163352 | 3 |
| 3205.3164504 | 3 |
| 3205.3192235 | 1 |
| 3205.3207563 | 1 |
| 3205.3207723 | 1 |
| 3205.3226318 | 1 |
| 3205.3236685 | 2 |
| 3205.3256804 | 2 |
| 3205.3273035 | 2 |
| 3205.3281606 | 3 |
| 3205.3297207 | 0 |
| 3205.3308022 | 0 |
| 3205.3320493 | 3 |
| 3205.3332805 | 1 |
| 3205.3350296 | 0 |
| 3205.3368553 | 2 |
| 3205.3379145 | 0 |
| 3205.3393435 | 1 |
| 3205.3412492 | 2 |
| 3205.3423753 | 0 |
| 3205.3435303 | 2 |
| 3205.3446735 | 0 |
| 3205.3462937 | 4 |
| 3205.3482004 | 0 |
| 3205.3487207 | 4 |
| 3205.3512308 | 1 |
| 3205.351258 | 0 |
| 3205.3527764 | 1 |
| 3205.353923 | 2 |
| 3205.3558984 | 2 |
| 3205.3572052 | 1 |
| 3205.3584167 | 1 |
| 3205.3608314 | 0 |
| 3205.3620425 | 0 |
| 3205.3622889 | 0 |
| 3205.3648351 | 3 |
| 3205.3652997 | 2 |
| 3205.3671922 | 3 |
| 3205.3682528 | 2 |
| 3205.3699785 | 1 |
| 3205.3715438 | 3 |
| 3205.3720691 | 7 |
| 3205.3739649 | 3 |
| 3205.3756093 | 1 |
| 3205.3762929 | 0 |
| 3205.3785263 | 2 |
| 3205.3790451 | 1 |
| 3205.3811043 | 1 |
| 3205.3830783 | 2 |
| 3205.3833836 | 3 |
| 3205.3852563 | 5 |
| 3205.3865286 | 2 |
| 3205.3878303 | 1 |
| 3205.3885016 | 0 |
| 3205.3910751 | 1 |
| 3205.392507 | 1 |
| 3205.3925234 | 1 |
| 3205.3946424 | 0 |
| 3205.3950126 | 2 |
| 3205.3975582 | 2 |
| 3205.3994475 | 1 |
| 3205.3999316 | 1 |
| 3205.4015251 | 0 |
| 3205.4031824 | 6 |
| 3205.4047787 | 3 |
| 3205.405861 | 2 |
| 3205.4069574 | 2 |
| 3205.408984 | 1 |
| 3205.4093683 | 2 |
| 3205.4109457 | 1 |
| 3205.4133896 | 3 |
| 3205.414105 | 1 |
| 3205.4158761 | 2 |
| 3205.4168391 | 0 |
| 3205.4183611 | 3 |
| 3205.4198204 | 5 |
| 3205.4212031 | 0 |
| 3205.422785 | 1 |
| 3205.4234234 | 1 |
| 3205.4253845 | 1 |
| 3205.4257196 | 0 |
| 3205.4278483 | 2 |
| 3205.4299929 | 3 |
| 3205.4303977 | 2 |
| 3205.4321914 | 2 |
| 3205.4334086 | 1 |
| 3205.4347557 | 4 |
| 3205.4362998 | 2 |
| 3205.4372857 | 2 |
| 3205.4380184 | 3 |
| 3205.4410718 | 0 |
| 3205.441653 | 2 |
| 3205.4433837 | 1 |
| 3205.4441858 | 0 |
| 3205.4458833 | 2 |
| 3205.4462031 | 3 |
| 3205.4486158 | 1 |
| 3205.450355 | 0 |
| 3205.4514375 | 1 |
| 3205.4529754 | 1 |
| 3205.4541402 | 3 |
| 3205.4563841 | 1 |
| 3205.4567738 | 1 |
| 3205.4584565 | 2 |
| 3205.4600431 | 1 |
| 3205.461313 | 0 |
| 3205.4630425 | 1 |
| 3205.4637212 | 1 |
| 3205.4651641 | 2 |
| 3205.4679329 | 3 |
| 3205.4680454 | 1 |
| 3205.4704207 | 3 |
| 3205.4713575 | 4 |
| 3205.4714708 | 3 |
| 3205.4733604 | 2 |
| 3205.4757331 | 2 |
| 3205.4766415 | 1 |
| 3205.477452 | 3 |
| 3205.479444 | 2 |
| 3205.4809847 | 1 |
| 3205.4814431 | 2 |
| 3205.4826991 | 6 |
| 3205.4834721 | 0 |
| 3205.4859114 | 0 |
| 3205.4877759 | 0 |
| 3205.4882541 | 2 |
| 3205.4903748 | 3 |
| 3205.4912599 | 1 |
| 3205.4926757 | 4 |
| 3205.4955205 | 1 |
| 3205.4958114 | 1 |
| 3205.4973202 | 3 |
| 3205.4984252 | 1 |
| 3205.4993756 | 3 |
| 3205.5015038 | 2 |
| 3205.5025216 | 2 |
| 3205.5037559 | 1 |
| 3205.5052348 | 0 |
| 3205.5070748 | 3 |
| 3205.5073563 | 2 |
| 3205.5093758 | 0 |
| 3205.5114657 | 2 |
| 3205.5121687 | 6 |
| 3205.5135446 | 2 |
| 3205.5145027 | 1 |
| 3205.5156757 | 1 |
| 3205.5169904 | 3 |
| 3205.5173882 | 3 |
| 3205.5330921 | 3 |
| 3205.5355168 | 1 |
| 3205.5366527 | 4 |
| 3205.5378349 | 3 |
| 3205.5386814 | 1 |
| 3205.5407731 | 1 |
| 3205.5417904 | 1 |
| 3205.5444905 | 0 |
| 3205.5446611 | 1 |
| 3205.5467673 | 3 |
| 3205.5477009 | 2 |
| 3205.5493759 | 2 |
| 3205.5498648 | 2 |
| 3205.5523533 | 3 |
| 3205.5536255 | 0 |
| 3205.5544378 | 6 |
| 3205.5564753 | 2 |
| 3205.5582872 | 0 |
| 3205.5595444 | 1 |
| 3205.5609222 | 2 |
| 3205.5617312 | 3 |
| 3205.5637265 | 2 |
| 3205.5641393 | 0 |
| 3205.5662457 | 0 |
| 3205.5677373 | 2 |
| 3205.5695252 | 1 |
| 3205.5702438 | 3 |
| 3205.5713474 | 2 |
| 3205.5723675 | 0 |
| 3205.5741681 | 2 |
| 3205.5744108 | 2 |
| 3205.5768693 | 0 |
| 3205.5786518 | 3 |
| 3205.5795723 | 1 |
| 3205.5811294 | 1 |
| 3205.5825539 | 0 |
| 3205.58423 | 4 |
| 3205.5850465 | 3 |
| 3205.5867835 | 0 |
| 3205.5890939 | 2 |
| 3205.5897042 | 5 |
| 3205.5908418 | 1 |
| 3205.5924952 | 1 |
| 3205.5938376 | 0 |
| 3205.5956884 | 3 |
| 3205.5969445 | 1 |
| 3205.5981074 | 4 |
| 3205.5984801 | 3 |
| 3205.6016393 | 0 |
| 3205.6022221 | 0 |
| 3205.6035086 | 0 |
| 3205.6053099 | 1 |
| 3205.6061323 | 3 |
| 3205.6077495 | 0 |
| 3205.6080785 | 2 |
| 3205.6100322 | 1 |
| 3205.6123684 | 1 |
| 3205.6128213 | 2 |
| 3205.6144536 | 2 |
| 3205.6157687 | 4 |
| 3205.6172501 | 1 |
| 3205.6192565 | 1 |
| 3205.6193235 | 3 |
| 3205.6213977 | 0 |
| 3205.623874 | 2 |
| 3205.6241808 | 6 |
| 3205.6259517 | 2 |
| 3205.6267654 | 3 |
| 3205.6282259 | 2 |
| 3205.6290963 | 3 |
| 3205.6315802 | 3 |
| 3205.6331381 | 1 |
| 3205.6333943 | 4 |
| 3205.6355142 | 3 |
| 3205.6371881 | 2 |
| 3205.6382355 | 3 |
| 3205.6398176 | 1 |
| 3205.6413545 | 5 |
| 3205.6427593 | 1 |
| 3205.6433584 | 1 |
| 3205.6449515 | 3 |
| 3205.6457299 | 1 |
| 3205.6484905 | 1 |
| 3205.6485389 | 2 |
| 3205.6504559 | 0 |
| 3205.652526 | 3 |
| 3205.6536215 | 5 |
| 3205.6545599 | 3 |
| 3205.6564737 | 2 |
| 3205.6578935 | 3 |
| 3205.6592001 | 1 |
| 3205.65996 | 5 |
| 3205.6613254 | 1 |
| 3205.6624249 | 2 |
| 3205.6637493 | 0 |
| 3205.6662559 | 3 |
| 3205.6662851 | 1 |
| 3205.6687703 | 3 |
| 3205.6694939 | 1 |
| 3205.6710073 | 4 |
| 3205.6737428 | 3 |
| 3205.6742555 | 3 |
| 3205.6761477 | 0 |
| 3205.6768417 | 1 |
| 3205.678695 | 1 |
| 3205.6803244 | 3 |
| 3205.6812345 | 1 |
| 3205.6830821 | 1 |
| 3205.6839931 | 0 |
| 3205.685967 | 4 |
| 3205.6869155 | 3 |
| 3205.6881876 | 1 |
| 3205.6902874 | 2 |
| 3205.6912144 | 1 |
| 3205.6931985 | 2 |
| 3205.6933734 | 0 |
| 3205.6948857 | 0 |
| 3205.6967191 | 2 |
| 3205.6978849 | 2 |
| 3205.6997668 | 0 |
| 3205.7004405 | 0 |
| 3205.7017501 | 3 |
| 3205.70396 | 2 |
| 3205.7041298 | 1 |
| 3205.7055217 | 0 |
| 3205.7083269 | 0 |
| 3205.7086542 | 4 |
| 3205.7102077 | 0 |
| 3205.7107913 | 1 |
| 3205.7131304 | 0 |
| 3205.7141441 | 6 |
| 3205.7154081 | 4 |
| 3205.7173335 | 4 |
| 3205.7188213 | 1 |
| 3205.71979 | 0 |
| 3205.7209532 | 1 |
| 3205.722483 | 1 |
| 3205.7247403 | 1 |
| 3205.7257376 | 0 |
| 3205.7276226 | 0 |
| 3205.7288517 | 3 |
| 3205.7289514 | 1 |
| 3205.7309527 | 3 |
| 3205.7328703 | 2 |
| 3205.7342396 | 3 |
| 3205.735955 | 0 |
| 3205.7371576 | 1 |
| 3205.7380759 | 1 |
| 3205.7393682 | 2 |
| 3205.7411798 | 0 |
| 3205.7425322 | 0 |
| 3205.7447026 | 0 |
| 3205.7457953 | 1 |
| 3205.7464926 | 1 |
| 3205.7483923 | 2 |
| 3205.7484429 | 0 |
| 3205.7506708 | 1 |
| 3205.7516058 | 1 |
| 3205.752966 | 2 |
| 3205.7552955 | 1 |
| 3205.7555081 | 4 |
| 3205.7572522 | 4 |
| 3205.7587882 | 0 |
| 3205.7595229 | 1 |
| 3205.7614448 | 0 |
| 3205.7624312 | 0 |
| 3205.7646224 | 2 |
| 3205.7656469 | 3 |
| 3205.7680458 | 2 |
| 3205.7683687 | 1 |
| 3205.7699361 | 0 |
| 3205.7719795 | 2 |
| 3205.7725119 | 3 |
| 3205.7742975 | 2 |
| 3205.7756474 | 3 |
| 3205.777835 | 2 |
| 3205.7792549 | 5 |
| 3205.7802171 | 1 |
| 3205.7816937 | 1 |
| 3205.7830825 | 1 |
| 3205.7845965 | 4 |
| 3205.7860841 | 2 |
| 3205.7869142 | 2 |
| 3205.788598 | 3 |
| 3205.7900025 | 1 |
| 3205.7909371 | 3 |
| 3205.792196 | 2 |
| 3205.7937497 | 2 |
| 3205.7953215 | 1 |
| 3205.7960401 | 3 |
| 3205.7978432 | 0 |
| 3205.798969 | 1 |
| 3205.8003138 | 1 |
| 3205.8018736 | 0 |
| 3205.8038496 | 0 |
| 3205.8050242 | 1 |
| 3205.8057248 | 3 |
| 3205.8078569 | 1 |
| 3205.8096954 | 4 |
| 3205.8101993 | 3 |
| 3205.8118524 | 4 |
| 3205.8123309 | 1 |
| 3205.8143567 | 1 |
| 3205.8160846 | 0 |
| 3205.8172877 | 1 |
| 3205.8190553 | 1 |
| 3205.8206095 | 1 |

| | |
|---|---|
| 3205.82242 | 0 |
| 3205.822453 | 3 |
| 3205.8245331 | 1 |
| 3205.8265619 | 3 |
| 3205.8271516 | 0 |
| 3205.8294991 | 4 |
| 3205.8300345 | 2 |
| 3205.8312963 | 2 |
| 3205.8335156 | 1 |
| 3205.8337069 | 2 |
| 3205.8351123 | 1 |
| 3205.8375372 | 1 |
| 3205.8381085 | 0 |
| 3205.8402345 | 2 |
| 3205.8408498 | 1 |
| 3205.8425714 | 1 |
| 3205.8431612 | 1 |
| 3205.8455599 | 2 |
| 3205.8461295 | 2 |
| 3205.846697 | 3 |
| 3205.8468796 | 3 |
| 3205.8469238 | 1 |
| 3205.8469912 | 1 |
| 3205.8470882 | 3 |
| 3205.8478756 | 3 |
| 3205.8479545 | 2 |
| 3205.8480703 | 0 |
| 3205.8497455 | 0 |
| 3205.8500783 | 1 |
| 3205.8501176 | 1 |
| 3205.8503677 | 2 |
| 3205.8503895 | 1 |
| 3205.8505987 | 3 |
| 3205.8508866 | 3 |
| 3205.8510503 | 3 |
| 3205.8513316 | 1 |
| 3205.8513554 | 3 |
| 3205.8515459 | 0 |
| 3205.8518676 | 3 |
| 3205.8519086 | 1 |
| 3205.8523851 | 2 |
| 3205.8528602 | 1 |
| 3205.8529169 | 0 |
| 3205.8530147 | 3 |
| 3205.8530582 | 2 |
| 3205.8531699 | 4 |
| 3205.8532944 | 2 |
| 3205.8535275 | 0 |
| 3205.8535718 | 3 |
| 3205.8539989 | 3 |
| 3205.8542038 | 0 |
| 3205.8544342 | 0 |
| 3205.8544635 | 1 |
| 3205.8545304 | 1 |
| 3205.8546242 | 2 |
| 3205.8546599 | 1 |
| 3205.8547168 | 2 |
| 3205.8549951 | 3 |
| 3205.8550559 | 3 |
| 3205.8554797 | 1 |
| 3205.8557387 | 0 |
| 3205.8560184 | 0 |
| 3205.8562797 | 3 |
| 3205.8565249 | 0 |
| 3205.8566517 | 2 |
| 3205.8566867 | 3 |
| 3205.8568239 | 1 |
| 3205.8568707 | 2 |
| 3205.8569407 | 2 |
| 3205.8573844 | 3 |
| 3205.8576514 | 3 |
| 3205.857727 | 3 |
| 3205.857746 | 3 |
| 3205.8579926 | 2 |
| 3205.858005 | 0 |
| 3205.8580112 | 0 |
| 3205.8582656 | 1 |
| 3205.8584329 | 0 |
| 3205.8590258 | 3 |
| 3205.8593023 | 2 |
| 3205.8593279 | 1 |
| 3205.8594154 | 2 |
| 3205.8598625 | 1 |
| 3205.8599974 | 0 |
| 3205.8600716 | 2 |
| 3205.8601277 | 1 |
| 3205.8602239 | 2 |
| 3205.8609847 | 1 |
| 3205.8612669 | 1 |
| 3205.8614805 | 2 |
| 3205.8616221 | 1 |
| 3205.861687 | 2 |
| 3205.8618079 | 1 |
| 3205.8618642 | 2 |
| 3205.861866 | 2 |
| 3205.8623951 | 1 |
| 3205.8624909 | 3 |
| 3205.8626059 | 0 |
| 3205.8627876 | 4 |
| 3205.8631449 | 1 |
| 3205.8631755 | 1 |
| 3205.8639005 | 3 |
| 3205.8639495 | 2 |
| 3205.8640301 | 2 |
| 3205.8641226 | 1 |
| 3205.8642891 | 4 |
| 3205.8644912 | 0 |
| 3205.8646158 | 0 |
| 3205.8650023 | 3 |
| 3205.8650528 | 1 |
| 3205.8651989 | 4 |
| 3205.865247 | 2 |
| 3205.8655624 | 1 |
| 3205.8659555 | 6 |
| 3205.8659661 | 3 |
| 3205.8660484 | 0 |
| 3205.8660803 | 2 |
| 3205.8662593 | 6 |
| 3205.8669936 | 4 |
| 3205.8669969 | 5 |
| 3205.8670507 | 8 |
| 3205.8671006 | 7 |
| 3205.8674057 | 8 |
| 3205.8676201 | 2 |
| 3205.8676313 | 7 |
| 3205.8681136 | 7 |
| 3205.8681498 | 7 |
| 3205.8685285 | 6 |
| 3205.8685563 | 3 |
| 3205.8686562 | 5 |
| 3205.8687309 | 4 |
| 3205.8688655 | 7 |
| 3205.8691893 | 7 |
| 3205.8692336 | 8 |
| 3205.869533 | 4 |
| 3205.8695578 | 6 |
| 3205.8699936 | 6 |
| 3205.8703639 | 3 |
| 3205.8704629 | 3 |
| 3205.8705685 | 8 |
| 3205.8706697 | 8 |
| 3205.8713686 | 9 |
| 3205.871419 | 6 |
| 3205.8714978 | 8 |
| 3205.8715524 | 2 |
| 3205.8716871 | 5 |
| 3205.8717369 | 2 |
| 3205.8718228 | 11 |
| 3205.8720498 | 4 |
| 3205.8725758 | 6 |
| 3205.8727117 | 1 |
| 3205.8728835 | 2 |
| 3205.8730203 | 5 |
| 3205.873203 | 6 |
| 3205.8735844 | 2 |
| 3205.873753 | 2 |
| 3205.874035 | 3 |
| 3205.8743004 | 1 |
| 3205.8743878 | 3 |
| 3205.8745145 | 1 |
| 3205.8746027 | 2 |
| 3205.8746634 | 2 |
| 3205.8746668 | 1 |
| 3205.875046 | 4 |
| 3205.8750983 | 0 |
| 3205.875472 | 2 |
| 3205.8756381 | 0 |
| 3205.875767 | 0 |
| 3205.8759447 | 0 |
| 3205.8760148 | 2 |
| 3205.8761073 | 3 |
| 3205.8762901 | 2 |
| 3205.8770083 | 2 |
| 3205.877149 | 1 |
| 3205.8772423 | 4 |
| 3205.8772615 | 1 |
| 3205.8775284 | 0 |
| 3205.8776813 | 1 |
| 3205.8777697 | 2 |
| 3205.8778445 | 2 |
| 3205.8779017 | 1 |
| 3205.8781999 | 1 |
| 3205.8787262 | 0 |
| 3205.8791981 | 4 |
| 3205.8792733 | 3 |
| 3205.8793293 | 3 |
| 3205.8796728 | 0 |
| 3205.8796848 | 1 |
| 3205.8797405 | 1 |
| 3205.8797935 | 2 |
| 3205.8798519 | 2 |
| 3205.8805245 | 1 |
| 3205.8805867 | 3 |
| 3205.8807344 | 1 |
| 3205.880814 | 1 |
| 3205.8811238 | 1 |
| 3205.8812046 | 4 |
| 3205.8813099 | 4 |
| 3205.8814789 | 0 |
| 3205.8815243 | 1 |
| 3205.8821187 | 2 |
| 3205.8822701 | 3 |
| 3205.8823903 | 0 |
| 3205.8825001 | 2 |
| 3205.8830225 | 1 |
| 3205.8830315 | 2 |
| 3205.883064 | 1 |
| 3205.8831972 | 1 |
| 3205.8835378 | 0 |
| 3205.8836881 | 0 |
| 3205.8841307 | 2 |
| 3205.8843723 | 0 |
| 3205.8844236 | 2 |
| 3205.8846765 | 1 |
| 3205.8851334 | 1 |
| 3205.8852384 | 1 |
| 3205.8852647 | 4 |
| 3205.8855725 | 1 |
| 3205.8857586 | 1 |
| 3205.8863065 | 3 |
| 3205.8864674 | 1 |
| 3205.8867575 | 5 |
| 3205.8868222 | 2 |
| 3205.8872054 | 0 |
| 3205.8872097 | 2 |
| 3205.8876882 | 4 |
| 3205.8879457 | 2 |
| 3205.8881299 | 1 |
| 3205.8883036 | 1 |
| 3205.8886786 | 1 |
| 3205.8891188 | 2 |
| 3205.889149 | 4 |
| 3205.8897126 | 1 |
| 3205.8897308 | 0 |
| 3205.8898319 | 2 |
| 3205.8898389 | 4 |
| 3205.890318 | 1 |
| 3205.8907726 | 2 |
| 3205.8910995 | 1 |
| 3205.8912086 | 0 |
| 3205.8912394 | 1 |
| 3205.8913324 | 1 |
| 3205.891845 | 1 |
| 3205.8925004 | 1 |
| 3205.8927628 | 2 |
| 3205.8928419 | 1 |
| 3205.8929547 | 2 |
| 3205.8931281 | 1 |
| 3205.8933254 | 1 |
| 3205.8934518 | 4 |
| 3205.8943001 | 3 |
| 3205.8945927 | 1 |
| 3205.8947378 | 2 |
| 3205.8950493 | 2 |
| 3205.8952184 | 2 |
| 3205.8956237 | 0 |
| 3205.8956364 | 2 |
| 3205.8960969 | 3 |
| 3205.8977415 | 2 |
| 3205.8989563 | 1 |
| 3205.8995824 | 2 |
| 3205.9006492 | 1 |
| 3205.9011196 | 2 |
| 3205.9024633 | 2 |
| 3205.9037517 | 3 |
| 3205.903815 | 4 |
| 3205.9046828 | 2 |
| 3205.9061524 | 2 |
| 3205.907068 | 1 |
| 3205.9086256 | 1 |
| 3205.9091578 | 1 |
| 3205.9095156 | 2 |
| 3205.9103396 | 0 |
| 3205.9121106 | 3 |
| 3205.9125029 | 1 |
| 3205.9139848 | 5 |
| 3205.9144739 | 1 |
| 3205.9152755 | 2 |
| 3205.9162836 | 4 |
| 3205.9173222 | 3 |
| 3205.9182491 | 1 |
| 3205.9195338 | 3 |
| 3205.91982 | 0 |
| 3205.9212416 | 1 |
| 3205.9222927 | 0 |
| 3205.9232631 | 3 |
| 3205.9239263 | 0 |
| 3205.9246712 | 5 |
| 3205.926117 | 1 |
| 3205.9263184 | 1 |
| 3205.9277487 | 3 |
| 3205.9291045 | 1 |
| 3205.929863 | 0 |
| 3205.930146 | 1 |
| 3205.9317105 | 3 |
| 3205.9322346 | 2 |
| 3205.9336105 | 0 |
| 3205.9343965 | 2 |
| 3205.9348247 | 2 |
| 3205.9353518 | 2 |
| 3205.9371131 | 3 |
| 3205.9380278 | 2 |
| 3205.9383742 | 0 |
| 3205.9391367 | 1 |
| 3205.9394644 | 3 |
| 3205.9416035 | 1 |
| 3205.9417095 | 1 |
| 3205.9431856 | 1 |
| 3205.9441363 | 1 |
| 3205.9449381 | 2 |
| 3205.9458284 | 2 |
| 3205.9462195 | 5 |
| 3205.9484176 | 1 |
| 3205.949212 | 0 |
| 3205.9492541 | 2 |
| 3205.9511126 | 2 |
| 3205.9513463 | 0 |
| 3205.9528702 | 3 |
| 3205.9535018 | 3 |
| 3205.9547754 | 0 |
| 3205.9556779 | 0 |
| 3205.9563864 | 2 |
| 3205.9577375 | 2 |
| 3205.9585868 | 2 |
| 3205.9593721 | 0 |
| 3205.9601744 | 2 |
| 3205.9611565 | 1 |
| 3205.9624615 | 5 |
| 3205.9637142 | 4 |
| 3205.9644445 | 0 |
| 3205.9651701 | 2 |
| 3205.9658886 | 3 |
| 3205.966045 | 2 |
| 3205.9675183 | 3 |
| 3205.968842 | 0 |
| 3205.9700028 | 2 |
| 3205.9704004 | 2 |
| 3205.9717794 | 3 |
| 3205.9719355 | 2 |
| 3205.9730019 | 1 |
| 3205.9736784 | 3 |
| 3205.9744164 | 0 |
| 3205.9748633 | 2 |
| 3205.9755894 | 1 |
| 3205.9757424 | 3 |
| 3205.9758507 | 0 |
| 3205.9770294 | 2 |
| 3205.977385 | 3 |
| 3205.9780683 | 3 |
| 3205.9786393 | 0 |
| 3205.9804157 | 1 |
| 3205.9807752 | 4 |
| 3205.9809641 | 2 |
| 3205.9813663 | 2 |
| 3205.9824044 | 1 |
| 3205.9829294 | 1 |
| 3205.9835138 | 1 |
| 3205.9836136 | 0 |
| 3205.9842269 | 4 |
| 3205.9854072 | 2 |
| 3205.9857291 | 2 |
| 3205.9862052 | 1 |
| 3205.9870233 | 2 |

| | |
|---|---|
| 3205.9876887 | 1 |
| 3205.9878102 | 0 |
| 3205.988804 | 1 |
| 3205.9888265 | 1 |
| 3205.9902242 | 3 |
| 3205.9903241 | 2 |
| 3205.9914693 | 4 |
| 3205.991553 | 1 |
| 3205.9921209 | 2 |
| 3205.9930162 | 1 |
| 3205.9936548 | 0 |
| 3205.9946713 | 1 |
| 3205.994978 | 4 |
| 3205.9951552 | 2 |
| 3205.9961106 | 2 |
| 3205.9968086 | 1 |
| 3205.9978821 | 1 |
| 3205.998128 | 1 |
| 3205.9987262 | 2 |
| 3205.9989126 | 1 |
| 3206.0001648 | 2 |
| 3206.0009639 | 3 |
| 3206.0009747 | 0 |
| 3206.0024076 | 3 |
| 3206.0031262 | 1 |
| 3206.0038229 | 1 |
| 3206.0039398 | 4 |
| 3206.0048642 | 0 |
| 3206.0052124 | 3 |
| 3206.0062595 | 2 |
| 3206.0068189 | 2 |
| 3206.0069268 | 3 |
| 3206.0077076 | 2 |
| 3206.0086056 | 2 |
| 3206.008678 | 0 |
| 3206.0089812 | 3 |
| 3206.0108139 | 0 |
| 3206.0109006 | 3 |
| 3206.0113104 | 3 |
| 3206.0121105 | 2 |
| 3206.0129224 | 3 |
| 3206.0130328 | 2 |
| 3206.0144469 | 2 |
| 3206.0148259 | 3 |
| 3206.014893 | 1 |
| 3206.015875 | 0 |
| 3206.0162926 | 1 |
| 3206.0166774 | 2 |
| 3206.0176856 | 1 |
| 3206.0178417 | 3 |
| 3206.0181227 | 0 |
| 3206.020065 | 4 |
| 3206.0205485 | 0 |
| 3206.0210485 | 3 |
| 3206.0211203 | 3 |
| 3206.0221934 | 2 |
| 3206.0226484 | 1 |
| 3206.0236756 | 2 |
| 3206.0241267 | 1 |
| 3206.0246559 | 1 |
| 3206.0252538 | 5 |
| 3206.0253032 | 2 |
| 3206.0263056 | 1 |
| 3206.0270298 | 1 |
| 3206.0277503 | 2 |
| 3206.0280729 | 3 |
| 3206.0289735 | 2 |
| 3206.0293144 | 3 |
| 3206.0301449 | 1 |
| 3206.0302694 | 1 |
| 3206.0315656 | 1 |
| 3206.0321899 | 1 |
| 3206.0327495 | 2 |
| 3206.032805 | 4 |
| 3206.0340383 | 2 |
| 3206.0346134 | 3 |
| 3206.0348192 | 2 |
| 3206.0349593 | 0 |
| 3206.0357007 | 1 |
| 3206.0364129 | 0 |
| 3206.0375697 | 1 |
| 3206.0377633 | 1 |
| 3206.0386748 | 1 |
| 3206.0391431 | 1 |
| 3206.0401795 | 1 |
| 3206.0404655 | 2 |
| 3206.0410245 | 0 |
| 3206.0417258 | 2 |
| 3206.0422346 | 4 |
| 3206.0430271 | 1 |
| 3206.0439854 | 2 |
| 3206.0446009 | 1 |
| 3206.0454034 | 0 |
| 3206.045594 | 3 |
| 3206.0463603 | 4 |
| 3206.0465098 | 3 |
| 3206.0472953 | 4 |
| 3206.0480853 | 2 |
| 3206.0484942 | 3 |
| 3206.0489626 | 1 |
| 3206.0497377 | 0 |
| 3206.0508655 | 1 |
| 3206.0513369 | 1 |
| 3206.051343 | 3 |
| 3206.0528045 | 2 |
| 3206.053066 | 1 |
| 3206.053672 | 2 |
| 3206.0541258 | 2 |
| 3206.0547885 | 0 |
| 3206.0561545 | 1 |
| 3206.0561881 | 1 |
| 3206.056492 | 2 |
| 3206.0581597 | 0 |
| 3206.0583143 | 2 |
| 3206.0590812 | 1 |
| 3206.0597812 | 1 |
| 3206.0608887 | 1 |
| 3206.0608919 | 2 |
| 3206.0614375 | 4 |
| 3206.0621027 | 5 |
| 3206.0627418 | 2 |
| 3206.063123 | 5 |
| 3206.0638516 | 2 |
| 3206.0640492 | 1 |
| 3206.0652632 | 3 |
| 3206.065429 | 1 |
| 3206.0662846 | 2 |
| 3206.0667801 | 0 |
| 3206.0675789 | 3 |
| 3206.067926 | 2 |
| 3206.0684877 | 4 |
| 3206.069268 | 1 |
| 3206.0697806 | 1 |
| 3206.0707837 | 4 |
| 3206.0708544 | 0 |
| 3206.0720634 | 0 |
| 3206.0721457 | 2 |
| 3206.0724783 | 2 |
| 3206.0735017 | 1 |
| 3206.0743275 | 2 |
| 3206.0748967 | 4 |
| 3206.0756103 | 1 |
| 3206.076089 | 0 |
| 3206.076445 | 0 |
| 3206.0777267 | 0 |
| 3206.0784023 | 1 |
| 3206.0790017 | 2 |
| 3206.0790803 | 1 |
| 3206.0800648 | 0 |
| 3206.0804138 | 3 |
| 3206.0809191 | 3 |
| 3206.081623 | 3 |
| 3206.0821527 | 4 |
| 3206.0829919 | 6 |
| 3206.0834393 | 2 |
| 3206.0839706 | 2 |
| 3206.0849977 | 2 |
| 3206.0850559 | 3 |
| 3206.0860905 | 1 |
| 3206.0863617 | 0 |
| 3206.0866174 | 0 |
| 3206.0879824 | 2 |
| 3206.0887216 | 0 |
| 3206.0890018 | 2 |
| 3206.0895848 | 2 |
| 3206.0905785 | 0 |
| 3206.0907351 | 2 |
| 3206.0918076 | 1 |
| 3206.0918276 | 3 |
| 3206.0928385 | 1 |
| 3206.0932753 | 0 |
| 3206.0942359 | 0 |
| 3206.0943054 | 2 |
| 3206.0956271 | 1 |
| 3206.095672 | 2 |
| 3206.0967781 | 1 |
| 3206.097373 | 1 |
| 3206.0980006 | 2 |
| 3206.0989121 | 2 |
| 3206.0990476 | 2 |
| 3206.1002684 | 3 |
| 3206.1007869 | 2 |
| 3206.1013038 | 1 |
| 3206.1021723 | 0 |
| 3206.1022172 | 2 |
| 3206.1034811 | 1 |
| 3206.1034828 | 2 |
| 3206.1041968 | 1 |
| 3206.1048157 | 1 |
| 3206.1056128 | 1 |
| 3206.1059199 | 1 |
| 3206.1061752 | 0 |
| 3206.1075301 | 1 |
| 3206.108344 | 0 |
| 3206.1085195 | 1 |
| 3206.1090871 | 0 |
| 3206.1092241 | 2 |
| 3206.1101341 | 1 |
| 3206.1108402 | 2 |
| 3206.1119993 | 2 |
| 3206.112343 | 0 |
| 3206.1127184 | 0 |
| 3206.1130143 | 1 |
| 3206.1140937 | 1 |
| 3206.1143558 | 1 |
| 3206.1158986 | 3 |
| 3206.1162628 | 1 |
| 3206.1162799 | 1 |
| 3206.1174927 | 0 |
| 3206.1176415 | 2 |
| 3206.118432 | 0 |
| 3206.1187472 | 3 |
| 3206.119112 | 2 |
| 3206.1197894 | 0 |
| 3206.1206098 | 2 |
| 3206.1212868 | 2 |
| 3206.1216385 | 1 |
| 3206.1222731 | 4 |
| 3206.1234803 | 2 |
| 3206.1235918 | 2 |
| 3206.1244358 | 3 |
| 3206.1248552 | 1 |
| 3206.1254888 | 2 |
| 3206.1264121 | 4 |
| 3206.1266974 | 3 |
| 3206.1274698 | 2 |
| 3206.1275686 | 3 |
| 3206.1289355 | 1 |
| 3206.1292578 | 3 |
| 3206.1298231 | 1 |
| 3206.1307504 | 4 |
| 3206.1311933 | 3 |
| 3206.1319379 | 3 |
| 3206.1323429 | 5 |
| 3206.1328367 | 1 |
| 3206.1335776 | 1 |
| 3206.1343445 | 1 |
| 3206.1350767 | 1 |
| 3206.1361814 | 2 |
| 3206.1364123 | 0 |
| 3206.1368119 | 2 |
| 3206.1369476 | 4 |
| 3206.1382639 | 1 |
| 3206.1390419 | 1 |
| 3206.1394225 | 2 |
| 3206.1395661 | 1 |
| 3206.1404378 | 1 |
| 3206.1411065 | 0 |
| 3206.1419807 | 1 |
| 3206.1423202 | 5 |
| 3206.1429781 | 1 |
| 3206.1434223 | 2 |
| 3206.1446828 | 1 |
| 3206.1449918 | 0 |
| 3206.1460004 | 2 |
| 3206.146209 | 1 |
| 3206.1464399 | 4 |
| 3206.1474773 | 0 |
| 3206.1485568 | 4 |
| 3206.1491526 | 4 |
| 3206.1501754 | 2 |
| 3206.1503586 | 6 |
| 3206.1505831 | 4 |
| 3206.1515918 | 1 |
| 3206.151957 | 1 |
| 3206.1527678 | 2 |
| 3206.1527874 | 2 |
| 3206.1539605 | 2 |
| 3206.1547653 | 3 |
| 3206.1551614 | 2 |
| 3206.1552773 | 1 |
| 3206.156355 | 5 |
| 3206.1566332 | 2 |
| 3206.1566922 | 3 |
| 3206.1580859 | 0 |
| 3206.1582548 | 0 |
| 3206.1598037 | 4 |
| 3206.1599284 | 3 |
| 3206.1604913 | 0 |
| 3206.1613431 | 2 |
| 3206.1615191 | 1 |
| 3206.1620937 | 0 |
| 3206.1629732 | 3 |
| 3206.1637005 | 1 |
| 3206.164272 | 0 |
| 3206.1651814 | 1 |
| 3206.1656477 | 2 |
| 3206.1663711 | 4 |
| 3206.1666534 | 1 |
| 3206.1670167 | 3 |
| 3206.1680797 | 2 |
| 3206.1681789 | 1 |
| 3206.1690783 | 1 |
| 3206.169751 | 0 |
| 3206.1698511 | 3 |
| 3206.1702976 | 3 |
| 3206.1715212 | 4 |
| 3206.1723976 | 1 |
| 3206.1728649 | 2 |
| 3206.172986 | 1 |
| 3206.1739578 | 3 |
| 3206.1751141 | 0 |
| 3206.1751203 | 3 |
| 3206.1754523 | 2 |
| 3206.176506 | 3 |
| 3206.1767293 | 1 |
| 3206.1775959 | 0 |
| 3206.1776268 | 1 |
| 3206.1787279 | 4 |
| 3206.1794287 | 1 |
| 3206.1808107 | 3 |
| 3206.1811161 | 4 |
| 3206.1813548 | 2 |
| 3206.182296 | 4 |
| 3206.1826428 | 3 |
| 3206.1832969 | 3 |
| 3206.1837261 | 5 |
| 3206.1838959 | 4 |
| 3206.1854585 | 3 |
| 3206.185475 | 2 |
| 3206.1874091 | 5 |
| 3206.1879753 | 2 |
| 3206.188006 | 4 |
| 3206.1883094 | 2 |
| 3206.1888852 | 5 |
| 3206.1897194 | 4 |
| 3206.1903945 | 1 |
| 3206.1912223 | 3 |
| 3206.19142 | 2 |
| 3206.1920165 | 4 |
| 3206.1926592 | 0 |
| 3206.1937646 | 2 |
| 3206.1940443 | 1 |
| 3206.1944925 | 0 |
| 3206.1945398 | 0 |
| 3206.1960618 | 2 |
| 3206.1967778 | 1 |
| 3206.1969215 | 1 |
| 3206.1974842 | 3 |
| 3206.1977226 | 4 |
| 3206.19847 | 5 |
| 3206.199538 | 1 |
| 3206.2004122 | 1 |
| 3206.2004564 | 3 |
| 3206.2012404 | 2 |
| 3206.2013267 | 1 |
| 3206.2022619 | 2 |
| 3206.2027583 | 0 |
| 3206.2032761 | 1 |
| 3206.2043284 | 2 |
| 3206.2046839 | 0 |
| 3206.2055988 | 2 |
| 3206.2066256 | 4 |
| 3206.2069926 | 3 |
| 3206.2072576 | 1 |
| 3206.2077688 | 5 |
| 3206.208273 | 0 |
| 3206.2091331 | 0 |
| 3206.2098794 | 1 |
| 3206.2102623 | 2 |
| 3206.2115848 | 0 |
| 3206.2116114 | 3 |
| 3206.2120406 | 1 |

| | |
|---|---|
| 3206.2126607 | 3 |
| 3206.2135084 | 1 |
| 3206.2141095 | 3 |
| 3206.2145642 | 1 |
| 3206.2150615 | 0 |
| 3206.2155994 | 4 |
| 3206.2165217 | 1 |
| 3206.2170074 | 2 |
| 3206.2170926 | 1 |
| 3206.2184942 | 2 |
| 3206.2186845 | 0 |
| 3206.2199265 | 5 |
| 3206.2202758 | 0 |
| 3206.2206874 | 2 |
| 3206.2212551 | 5 |
| 3206.2218143 | 0 |
| 3206.2222661 | 1 |
| 3206.2236263 | 3 |
| 3206.2237168 | 0 |
| 3206.2246808 | 2 |
| 3206.2249767 | 1 |
| 3206.2256654 | 1 |
| 3206.2265965 | 2 |
| 3206.2271881 | 4 |
| 3206.2274336 | 0 |
| 3206.2282307 | 4 |
| 3206.229001 | 1 |
| 3206.2292165 | 1 |
| 3206.229462 | 0 |
| 3206.2302648 | 3 |
| 3206.2306781 | 1 |
| 3206.2324709 | 5 |
| 3206.2325118 | 3 |
| 3206.2332301 | 0 |
| 3206.2339871 | 4 |
| 3206.2343111 | 0 |
| 3206.234547 | 1 |
| 3206.2357707 | 3 |
| 3206.2364748 | 1 |
| 3206.2369748 | 2 |
| 3206.2375526 | 0 |
| 3206.2383309 | 1 |
| 3206.2387401 | 1 |
| 3206.2392189 | 1 |
| 3206.2402745 | 7 |
| 3206.2409357 | 1 |
| 3206.2410331 | 1 |
| 3206.2411072 | 1 |
| 3206.2428587 | 3 |
| 3206.2432004 | 2 |
| 3206.2435524 | 0 |
| 3206.2443088 | 1 |
| 3206.2446079 | 1 |
| 3206.2454279 | 1 |
| 3206.2461842 | 5 |
| 3206.246809 | 1 |
| 3206.2478283 | 0 |
| 3206.2478342 | 0 |
| 3206.2488166 | 2 |
| 3206.2493673 | 2 |
| 3206.2496303 | 2 |
| 3206.2506078 | 0 |
| 3206.251174 | 1 |
| 3206.2518798 | 1 |
| 3206.2523874 | 2 |
| 3206.2526028 | 0 |
| 3206.2535303 | 1 |
| 3206.2537323 | 0 |
| 3206.2544988 | 0 |
| 3206.255041 | 2 |
| 3206.2556038 | 2 |
| 3206.2564798 | 1 |
| 3206.2572481 | 0 |
| 3206.2577385 | 3 |
| 3206.2582217 | 2 |
| 3206.2587753 | 2 |
| 3206.2589398 | 0 |
| 3206.2598265 | 2 |
| 3206.2607049 | 2 |
| 3206.2608687 | 2 |
| 3206.2623582 | 2 |
| 3206.2624335 | 1 |
| 3206.2631078 | 1 |
| 3206.2631104 | 2 |
| 3206.2645375 | 2 |
| 3206.2650349 | 2 |
| 3206.2653797 | 2 |
| 3206.2664239 | 1 |
| 3206.2664277 | 1 |
| 3206.2665281 | 4 |
| 3206.267875 | 2 |
| 3206.2696574 | 1 |
| 3206.2698186 | 0 |
| 3206.2698444 | 1 |
| 3206.2700407 | 2 |
| 3206.2713411 | 1 |
| 3206.2716556 | 2 |
| 3206.2729213 | 2 |
| 3206.2731824 | 3 |
| 3206.2738557 | 2 |
| 3206.2743694 | 1 |
| 3206.2744501 | 1 |
| 3206.2758241 | 1 |
| 3206.276214 | 3 |
| 3206.2764117 | 5 |
| 3206.2781467 | 3 |
| 3206.2784802 | 1 |
| 3206.2788214 | 1 |
| 3206.2792657 | 1 |
| 3206.280139 | 1 |
| 3206.280208 | 2 |
| 3206.2812328 | 0 |
| 3206.2818982 | 0 |
| 3206.2826533 | 3 |
| 3206.2829333 | 0 |
| 3206.2838568 | 4 |
| 3206.2845171 | 2 |
| 3206.2852338 | 1 |
| 3206.2860447 | 0 |
| 3206.2864671 | 4 |
| 3206.2866857 | 2 |
| 3206.2873323 | 1 |
| 3206.287931 | 3 |
| 3206.2886889 | 1 |
| 3206.289388 | 3 |
| 3206.2897423 | 2 |
| 3206.2898386 | 2 |
| 3206.2910765 | 1 |
| 3206.2911804 | 4 |
| 3206.2918219 | 4 |
| 3206.2927172 | 1 |
| 3206.2930772 | 0 |
| 3206.2937601 | 0 |
| 3206.2947901 | 2 |
| 3206.2955096 | 0 |
| 3206.2956343 | 0 |
| 3206.296815 | 2 |
| 3206.2972249 | 3 |
| 3206.2976861 | 1 |
| 3206.2984851 | 4 |
| 3206.2987398 | 0 |
| 3206.299358 | 2 |
| 3206.3002624 | 1 |
| 3206.3005871 | 0 |
| 3206.3015167 | 0 |
| 3206.3015836 | 2 |
| 3206.3030091 | 1 |
| 3206.3031097 | 2 |
| 3206.3040276 | 2 |
| 3206.304501 | 2 |
| 3206.3048515 | 0 |
| 3206.3060989 | 0 |
| 3206.3063499 | 1 |
| 3206.3067916 | 2 |
| 3206.3069348 | 1 |
| 3206.3073324 | 1 |
| 3206.3080533 | 0 |
| 3206.3094566 | 1 |
| 3206.3101344 | 3 |
| 3206.3102323 | 2 |
| 3206.3113106 | 1 |
| 3206.3121814 | 1 |
| 3206.3123234 | 1 |
| 3206.3125177 | 0 |
| 3206.3135302 | 1 |
| 3206.3137105 | 1 |
| 3206.3147132 | 1 |
| 3206.31541 | 1 |
| 3206.3158113 | 1 |
| 3206.3165338 | 2 |
| 3206.316955 | 4 |
| 3206.3182095 | 3 |
| 3206.3186085 | 1 |
| 3206.3195167 | 0 |
| 3206.3201538 | 2 |
| 3206.3208705 | 1 |
| 3206.321005 | 3 |
| 3206.3221988 | 3 |
| 3206.3223312 | 1 |
| 3206.3228594 | 2 |
| 3206.3241495 | 1 |
| 3206.3242973 | 2 |
| 3206.325003 | 3 |
| 3206.326034 | 4 |
| 3206.3263248 | 3 |
| 3206.3268703 | 0 |
| 3206.3276519 | 1 |
| 3206.3278405 | 3 |
| 3206.3285223 | 3 |
| 3206.3291759 | 0 |
| 3206.3302866 | 5 |
| 3206.3306663 | 2 |
| 3206.3318206 | 3 |
| 3206.3319986 | 1 |
| 3206.3325055 | 0 |
| 3206.3325316 | 2 |
| 3206.3336878 | 1 |
| 3206.3337668 | 2 |
| 3206.3344493 | 2 |
| 3206.3352873 | 2 |
| 3206.3360389 | 1 |
| 3206.3363963 | 3 |
| 3206.337598 | 1 |
| 3206.338391 | 1 |
| 3206.3387712 | 4 |
| 3206.3388149 | 2 |
| 3206.3397112 | 0 |
| 3206.3397471 | 0 |
| 3206.3413177 | 1 |
| 3206.3414869 | 1 |
| 3206.3417344 | 0 |
| 3206.3418017 | 1 |
| 3206.3435369 | 1 |
| 3206.3437637 | 1 |
| 3206.3441718 | 0 |
| 3206.3452432 | 2 |
| 3206.3453999 | 1 |
| 3206.3466445 | 0 |
| 3206.3468294 | 3 |
| 3206.347437 | 4 |
| 3206.3476848 | 4 |
| 3206.3489933 | 1 |
| 3206.3492983 | 0 |
| 3206.3493798 | 1 |
| 3206.3502415 | 2 |
| 3206.3507084 | 1 |
| 3206.3515774 | 3 |
| 3206.3522668 | 2 |
| 3206.3526113 | 3 |
| 3206.3529538 | 1 |
| 3206.3547172 | 2 |
| 3206.3551341 | 1 |
| 3206.3553618 | 3 |
| 3206.3559635 | 1 |
| 3206.3561165 | 2 |
| 3206.3567809 | 2 |
| 3206.3575259 | 3 |
| 3206.3582606 | 4 |
| 3206.3591201 | 2 |
| 3206.3593198 | 2 |
| 3206.360909 | 2 |
| 3206.3609489 | 1 |
| 3206.3612137 | 4 |
| 3206.362739 | 2 |
| 3206.3628279 | 1 |
| 3206.3632545 | 1 |
| 3206.3641424 | 3 |
| 3206.364949 | 1 |
| 3206.3650585 | 0 |
| 3206.3658227 | 1 |
| 3206.3661406 | 1 |
| 3206.3677473 | 3 |
| 3206.3679398 | 1 |
| 3206.3687045 | 2 |
| 3206.3690409 | 1 |
| 3206.3702051 | 1 |
| 3206.3711098 | 7 |
| 3206.3713496 | 4 |
| 3206.3715341 | 2 |
| 3206.3721638 | 1 |
| 3206.3725339 | 1 |
| 3206.3738863 | 0 |
| 3206.3742352 | 5 |
| 3206.3742815 | 4 |
| 3206.3747825 | 3 |
| 3206.3762327 | 0 |
| 3206.3765619 | 1 |
| 3206.3779935 | 4 |
| 3206.3782118 | 3 |
| 3206.3783136 | 2 |
| 3206.3787341 | 1 |
| 3206.3800578 | 1 |
| 3206.380517 | 3 |
| 3206.3809898 | 1 |
| 3206.3819587 | 0 |
| 3206.382158 | 2 |
| 3206.3829027 | 1 |
| 3206.3840724 | 1 |
| 3206.3845989 | 1 |
| 3206.3847724 | 4 |
| 3206.385436 | 1 |
| 3206.3856085 | 1 |
| 3206.3869028 | 1 |
| 3206.3871435 | 1 |
| 3206.3879781 | 1 |
| 3206.3881732 | 3 |
| 3206.3884831 | 2 |
| 3206.3898314 | 0 |
| 3206.3899991 | 3 |
| 3206.3902454 | 4 |
| 3206.3902981 | 4 |
| 3206.3918248 | 0 |
| 3206.392192 | 3 |
| 3206.3926339 | 0 |
| 3206.394105 | 0 |
| 3206.3945683 | 1 |
| 3206.3952787 | 2 |
| 3206.3955389 | 1 |
| 3206.3959201 | 2 |
| 3206.3973628 | 3 |
| 3206.3974202 | 2 |
| 3206.3977773 | 1 |
| 3206.3981073 | 3 |
| 3206.3987984 | 1 |
| 3206.3998235 | 4 |
| 3206.3999705 | 1 |
| 3206.4015329 | 6 |
| 3206.4023638 | 0 |
| 3206.402493 | 1 |
| 3206.4033858 | 2 |
| 3206.403938 | 1 |
| 3206.4043625 | 2 |
| 3206.4044557 | 1 |
| 3206.4054132 | 0 |
| 3206.4056022 | 3 |
| 3206.4064208 | 1 |
| 3206.4075367 | 2 |
| 3206.4079707 | 2 |
| 3206.4086444 | 2 |
| 3206.4096265 | 3 |
| 3206.4100974 | 3 |
| 3206.4101388 | 1 |
| 3206.4109348 | 3 |
| 3206.4121434 | 1 |
| 3206.4125319 | 2 |
| 3206.4132527 | 0 |
| 3206.4140444 | 1 |
| 3206.41454 | 3 |
| 3206.4146475 | 2 |
| 3206.4155249 | 0 |
| 3206.4157322 | 1 |
| 3206.4169578 | 3 |
| 3206.4179058 | 1 |
| 3206.4181883 | 2 |
| 3206.4182718 | 3 |
| 3206.4190318 | 3 |
| 3206.4197923 | 2 |
| 3206.4205698 | 0 |
| 3206.4212885 | 4 |
| 3206.4213017 | 2 |
| 3206.421716 | 2 |
| 3206.4228218 | 1 |
| 3206.4233862 | 1 |
| 3206.4243367 | 1 |
| 3206.4247416 | 0 |
| 3206.4251652 | 2 |
| 3206.4261122 | 3 |
| 3206.4263884 | 0 |
| 3206.4269987 | 1 |
| 3206.4279185 | 1 |
| 3206.4279688 | 3 |
| 3206.4294865 | 3 |
| 3206.4300397 | 4 |
| 3206.4303833 | 0 |
| 3206.4311803 | 3 |
| 3206.4313251 | 1 |
| 3206.431627 | 1 |
| 3206.4327211 | 2 |
| 3206.4327586 | 1 |
| 3206.4335431 | 4 |
| 3206.434589 | 2 |
| 3206.4350989 | 2 |
| 3206.4357848 | 1 |

| | |
|---|---|
| 3206.4363682 | 3 |
| 3206.4368521 | 4 |
| 3206.4369766 | 4 |
| 3206.4381216 | 0 |
| 3206.4388404 | 0 |
| 3206.4400687 | 2 |
| 3206.4402324 | 4 |
| 3206.441296 | 0 |
| 3206.4413482 | 0 |
| 3206.441601 | 5 |
| 3206.4418073 | 1 |
| 3206.4436282 | 1 |
| 3206.4436726 | 2 |
| 3206.4445813 | 1 |
| 3206.4450771 | 2 |
| 3206.4455485 | 3 |
| 3206.4456805 | 2 |
| 3206.4470397 | 4 |
| 3206.447364 | 3 |
| 3206.448484 | 3 |
| 3206.4487969 | 0 |
| 3206.4488433 | 2 |
| 3206.449336 | 4 |
| 3206.4500125 | 1 |
| 3206.4506749 | 2 |
| 3206.451923 | 0 |
| 3206.4521587 | 0 |
| 3206.4530227 | 0 |
| 3206.4535887 | 2 |
| 3206.4536984 | 4 |
| 3206.4552058 | 2 |
| 3206.4557792 | 1 |
| 3206.4563226 | 0 |
| 3206.4565205 | 3 |
| 3206.457009 | 0 |
| 3206.4581159 | 1 |
| 3206.4585245 | 1 |
| 3206.4595416 | 0 |
| 3206.4600223 | 5 |
| 3206.4606179 | 2 |
| 3206.4606351 | 2 |
| 3206.461434 | 1 |
| 3206.4629547 | 2 |
| 3206.4630818 | 3 |
| 3206.4630883 | 1 |
| 3206.4645201 | 3 |
| 3206.4654215 | 2 |
| 3206.4654786 | 2 |
| 3206.4657748 | 0 |
| 3206.4668071 | 3 |
| 3206.467455 | 3 |
| 3206.4679843 | 0 |
| 3206.4687749 | 0 |
| 3206.4692962 | 3 |
| 3206.4695096 | 1 |
| 3206.4712051 | 1 |
| 3206.4715209 | 1 |
| 3206.4716158 | 4 |
| 3206.4729938 | 2 |
| 3206.473032 | 0 |
| 3206.4730329 | 3 |
| 3206.4741033 | 1 |
| 3206.4749419 | 2 |
| 3206.4755885 | 4 |
| 3206.4761017 | 4 |
| 3206.4765497 | 1 |
| 3206.478051 | 1 |
| 3206.4781207 | 2 |
| 3206.4782715 | 1 |
| 3206.4792781 | 4 |
| 3206.4800122 | 1 |
| 3206.4803185 | 3 |
| 3206.480819 | 3 |
| 3206.4821724 | 1 |
| 3206.4823441 | 2 |
| 3206.4827733 | 0 |
| 3206.4832775 | 0 |
| 3206.4840885 | 1 |
| 3206.4846195 | 0 |
| 3206.4849567 | 0 |
| 3206.4860215 | 1 |
| 3206.4864641 | 3 |
| 3206.4868773 | 1 |
| 3206.4880183 | 3 |
| 3206.4881222 | 1 |
| 3206.4890317 | 0 |
| 3206.4897093 | 3 |
| 3206.4900116 | 1 |
| 3206.4906748 | 0 |
| 3206.4910479 | 5 |
| 3206.4925313 | 3 |
| 3206.4925939 | 1 |
| 3206.4934704 | 1 |
| 3206.494618 | 3 |
| 3206.4951491 | 1 |
| 3206.4955144 | 2 |
| 3206.4959574 | 0 |
| 3206.4961192 | 2 |
| 3206.4973986 | 0 |
| 3206.4976056 | 0 |
| 3206.4985291 | 1 |
| 3206.4985323 | 2 |
| 3206.4997098 | 1 |
| 3206.5004261 | 1 |
| 3206.5010619 | 0 |
| 3206.501325 | 1 |
| 3206.5021363 | 3 |
| 3206.5033254 | 2 |
| 3206.5036726 | 1 |
| 3206.5040286 | 0 |
| 3206.5046571 | 0 |
| 3206.5047425 | 2 |
| 3206.5060054 | 2 |
| 3206.5067717 | 1 |
| 3206.5071217 | 1 |
| 3206.5078445 | 5 |
| 3206.5087155 | 1 |
| 3206.509487 | 2 |
| 3206.5097118 | 1 |
| 3206.5106429 | 6 |
| 3206.5111612 | 2 |
| 3206.5118895 | 0 |
| 3206.5120751 | 0 |
| 3206.5126355 | 1 |
| 3206.5134843 | 3 |
| 3206.5144717 | 2 |
| 3206.5153295 | 1 |
| 3206.5156737 | 2 |
| 3206.5161076 | 2 |
| 3206.516292 | 0 |
| 3206.5173135 | 2 |
| 3206.5185165 | 2 |
| 3206.5188148 | 1 |
| 3206.5190854 | 3 |
| 3206.520155 | 1 |
| 3206.5202429 | 0 |
| 3206.52063 | 2 |
| 3206.5213696 | 3 |
| 3206.5218691 | 2 |
| 3206.522144 | 2 |
| 3206.5235268 | 1 |
| 3206.5242902 | 2 |
| 3206.5245832 | 3 |
| 3206.5257428 | 2 |
| 3206.5258464 | 1 |
| 3206.5264588 | 1 |
| 3206.5273801 | 2 |
| 3206.5279636 | 2 |
| 3206.5280144 | 3 |
| 3206.5289716 | 4 |
| 3206.5295116 | 3 |
| 3206.5303112 | 0 |
| 3206.5308793 | 4 |
| 3206.531249 | 0 |
| 3206.5319156 | 0 |
| 3206.5324575 | 1 |
| 3206.5328651 | 1 |
| 3206.5343576 | 1 |
| 3206.5347656 | 1 |
| 3206.5354774 | 2 |
| 3206.5356491 | 2 |
| 3206.5357098 | 1 |
| 3206.5371918 | 7 |
| 3206.537617 | 3 |
| 3206.5378526 | 3 |
| 3206.5388666 | 0 |
| 3206.539515 | 4 |
| 3206.5404486 | 3 |
| 3206.5405384 | 0 |
| 3206.5421664 | 2 |
| 3206.5421965 | 6 |
| 3206.5421992 | 0 |
| 3206.5434259 | 5 |
| 3206.5442944 | 1 |
| 3206.5443051 | 2 |
| 3206.5450789 | 0 |
| 3206.545737 | 2 |
| 3206.5464306 | 0 |
| 3206.5472281 | 2 |
| 3206.5481346 | 0 |
| 3206.5483906 | 4 |
| 3206.5497968 | 1 |
| 3206.5500674 | 3 |
| 3206.5505562 | 2 |
| 3206.5511953 | 1 |
| 3206.5518405 | 0 |
| 3206.5519551 | 2 |
| 3206.5527861 | 1 |
| 3206.5533756 | 2 |
| 3206.5543166 | 0 |
| 3206.5547695 | 0 |
| 3206.5559189 | 1 |
| 3206.5567378 | 2 |
| 3206.5567976 | 2 |
| 3206.5571914 | 2 |
| 3206.5585661 | 6 |
| 3206.5590617 | 3 |
| 3206.5594433 | 2 |
| 3206.5600565 | 3 |
| 3206.5605389 | 7 |
| 3206.5610891 | 0 |
| 3206.5619829 | 1 |
| 3206.5626974 | 1 |
| 3206.5631663 | 2 |
| 3206.5637388 | 0 |
| 3206.5639158 | 3 |
| 3206.5645082 | 2 |
| 3206.5651669 | 0 |
| 3206.5654218 | 1 |
| 3206.5663372 | 1 |
| 3206.5672962 | 2 |
| 3206.5681732 | 1 |
| 3206.5687624 | 1 |
| 3206.5693773 | 3 |
| 3206.5694167 | 2 |
| 3206.5700369 | 2 |
| 3206.5702663 | 0 |
| 3206.5719339 | 3 |
| 3206.5719978 | 1 |
| 3206.5732909 | 2 |
| 3206.5735736 | 1 |
| 3206.5740398 | 0 |
| 3206.5744097 | 2 |
| 3206.5749408 | 3 |
| 3206.5755362 | 3 |
| 3206.5757873 | 0 |
| 3206.5766021 | 2 |
| 3206.5768092 | 0 |
| 3206.5788194 | 3 |
| 3206.5791764 | 0 |
| 3206.579413 | 3 |
| 3206.5806781 | 2 |
| 3206.5807589 | 3 |
| 3206.5816708 | 4 |
| 3206.5817013 | 2 |
| 3206.5825711 | 0 |
| 3206.5831436 | 2 |
| 3206.5834985 | 0 |
| 3206.5855765 | 0 |
| 3206.5861214 | 2 |
| 3206.5893926 | 0 |
| 3206.591592 | 0 |
| 3206.593387 | 3 |
| 3206.5955807 | 2 |
| 3206.5958435 | 3 |
| 3206.5968561 | 1 |
| 3206.59728 | 2 |
| 3206.5976279 | 5 |
| 3206.5987095 | 3 |
| 3206.6006708 | 3 |
| 3206.6012063 | 4 |
| 3206.6023424 | 2 |
| 3206.6032152 | 2 |
| 3206.6032677 | 2 |
| 3206.6051156 | 3 |
| 3206.6062472 | 2 |
| 3206.6067794 | 1 |
| 3206.6070839 | 0 |
| 3206.6087243 | 2 |
| 3206.609134 | 0 |
| 3206.6110317 | 3 |
| 3206.6114764 | 3 |
| 3206.6123437 | 0 |
| 3206.6130249 | 1 |
| 3206.6141811 | 2 |
| 3206.6144624 | 0 |
| 3206.6152374 | 3 |
| 3206.6155293 | 2 |
| 3206.6170351 | 1 |
| 3206.6187152 | 5 |
| 3206.6197702 | 1 |
| 3206.6199641 | 2 |
| 3206.6204432 | 1 |
| 3206.6224428 | 1 |
| 3206.6232219 | 1 |
| 3206.6243419 | 0 |
| 3206.6247266 | 3 |
| 3206.6258097 | 0 |
| 3206.6270008 | 3 |
| 3206.6276976 | 3 |
| 3206.6292587 | 1 |
| 3206.6293313 | 2 |
| 3206.6302539 | 2 |
| 3206.630921 | 2 |
| 3206.6325011 | 2 |
| 3206.6332009 | 3 |
| 3206.6332815 | 1 |
| 3206.6353536 | 4 |
| 3206.6360971 | 1 |
| 3206.6378797 | 1 |
| 3206.638515 | 4 |
| 3206.6391513 | 3 |
| 3206.639703 | 0 |
| 3206.6412722 | 2 |
| 3206.6414361 | 3 |
| 3206.6431334 | 4 |
| 3206.643723 | 0 |
| 3206.6442723 | 1 |
| 3206.6456412 | 3 |
| 3206.6464691 | 2 |
| 3206.648047 | 0 |
| 3206.6485617 | 2 |
| 3206.6487153 | 2 |
| 3206.6495074 | 4 |
| 3206.6519978 | 1 |
| 3206.6525723 | 2 |
| 3206.6532279 | 2 |
| 3206.6540579 | 1 |
| 3206.6548676 | 1 |
| 3206.6554053 | 0 |
| 3206.6569787 | 1 |
| 3206.6570531 | 2 |
| 3206.658725 | 1 |
| 3206.6587825 | 0 |
| 3206.660934 | 1 |
| 3206.6617307 | 3 |
| 3206.6620132 | 0 |
| 3206.66267 | 2 |
| 3206.6644977 | 1 |
| 3206.6649963 | 0 |
| 3206.6661069 | 1 |
| 3206.6672807 | 1 |
| 3206.6673251 | 2 |
| 3206.6687789 | 1 |
| 3206.6702702 | 3 |
| 3206.6703614 | 1 |
| 3206.6719169 | 2 |
| 3206.6719736 | 4 |
| 3206.6738725 | 1 |
| 3206.6741112 | 3 |
| 3206.6752346 | 2 |
| 3206.6770299 | 2 |
| 3206.6776285 | 3 |
| 3206.6779832 | 5 |
| 3206.6785191 | 5 |
| 3206.6794475 | 2 |
| 3206.6807154 | 2 |
| 3206.6812186 | 1 |
| 3206.6834354 | 0 |
| 3206.6837149 | 1 |
| 3206.6849607 | 0 |
| 3206.6857438 | 1 |
| 3206.686374 | 0 |
| 3206.6866059 | 1 |
| 3206.6891394 | 1 |
| 3206.6892583 | 2 |
| 3206.690496 | 3 |
| 3206.6908613 | 0 |
| 3206.6932855 | 2 |
| 3206.693921 | 2 |
| 3206.6939793 | 1 |
| 3206.695657 | 0 |
| 3206.6963885 | 1 |
| 3206.6968408 | 1 |
| 3206.6987741 | 0 |
| 3206.6988952 | 2 |
| 3206.6999165 | 0 |
| 3206.7001925 | 5 |
| 3206.7019004 | 0 |
| 3206.7025249 | 3 |
| 3206.7037454 | 2 |
| 3206.7050209 | 3 |
| 3206.7055738 | 1 |

| | |
|---|---|
| 3206.7058059 | 2 |
| 3206.7079943 | 3 |
| 3206.7083594 | 1 |
| 3206.7087674 | 3 |
| 3206.7090931 | 3 |
| 3206.7107138 | 1 |
| 3206.7116235 | 0 |
| 3206.7130607 | 0 |
| 3206.7132247 | 4 |
| 3206.7150755 | 2 |
| 3206.7153403 | 1 |
| 3206.7164733 | 3 |
| 3206.7179332 | 4 |
| 3206.7182352 | 0 |
| 3206.7186337 | 4 |
| 3206.7206143 | 3 |
| 3206.7213168 | 1 |
| 3206.7213697 | 2 |
| 3206.7219041 | 1 |
| 3206.7241598 | 2 |
| 3206.7244008 | 2 |
| 3206.7258917 | 1 |
| 3206.7273834 | 1 |
| 3206.7278547 | 2 |
| 3206.728184 | 1 |
| 3206.7302834 | 1 |
| 3206.7314062 | 1 |
| 3206.7315647 | 3 |
| 3206.7322382 | 1 |
| 3206.7334616 | 2 |
| 3206.7338839 | 1 |
| 3206.7349241 | 1 |
| 3206.7364774 | 1 |
| 3206.7367922 | 1 |
| 3206.7369869 | 3 |
| 3206.7380318 | 2 |
| 3206.7384004 | 3 |
| 3206.7385399 | 1 |
| 3206.739508 | 4 |
| 3206.7412235 | 0 |
| 3206.7433534 | 2 |
| 3206.743886 | 0 |
| 3206.7469526 | 3 |
| 3206.7480195 | 2 |
| 3206.7495806 | 1 |
| 3206.7522413 | 2 |
| 3206.7532879 | 1 |
| 3206.7557123 | 1 |
| 3206.7582575 | 2 |
| 3206.7591774 | 3 |
| 3206.761525 | 0 |
| 3206.7617155 | 1 |
| 3206.7645336 | 0 |
| 3206.765741 | 0 |
| 3206.768475 | 2 |
| 3206.7713807 | 2 |
| 3206.7723215 | 2 |
| 3206.7747453 | 0 |
| 3206.7748257 | 2 |
| 3206.778261 | 1 |
| 3206.7803378 | 0 |
| 3206.7814318 | 4 |
| 3206.7839328 | 1 |
| 3206.7849193 | 1 |
| 3206.7872585 | 2 |
| 3206.7883304 | 1 |
| 3206.7903754 | 1 |
| 3206.7935165 | 2 |
| 3206.7948217 | 1 |
| 3206.7958849 | 3 |
| 3206.7977085 | 1 |
| 3206.7996738 | 0 |
| 3206.8019572 | 1 |
| 3206.802574 | 1 |
| 3206.8050343 | 0 |
| 3206.8075973 | 4 |
| 3206.8093145 | 1 |
| 3206.8112785 | 1 |
| 3206.8126765 | 2 |
| 3206.8153306 | 1 |
| 3206.8174179 | 1 |
| 3206.8181238 | 5 |
| 3206.8213046 | 0 |
| 3206.8221322 | 1 |
| 3206.8244384 | 1 |
| 3206.8267625 | 0 |
| 3206.8278297 | 3 |
| 3206.8301408 | 4 |
| 3206.8313967 | 2 |
| 3206.8339594 | 3 |
| 3206.8345177 | 0 |
| 3206.8375866 | 1 |
| 3206.8396248 | 1 |
| 3206.8406776 | 0 |
| 3206.8436453 | 2 |
| 3206.8447947 | 2 |
| 3206.8455806 | 2 |
| 3206.8487673 | 2 |
| 3206.8495867 | 0 |
| 3206.8521419 | 3 |
| 3206.8530387 | 2 |
| 3206.8560678 | 0 |
| 3206.8573723 | 3 |
| 3206.8590172 | 0 |
| 3206.8617679 | 3 |
| 3206.8619256 | 1 |
| 3206.8646324 | 1 |
| 3206.8672613 | 5 |
| 3206.8682628 | 1 |
| 3206.8707501 | 1 |
| 3206.8719248 | 1 |
| 3206.8747286 | 0 |
| 3206.8767111 | 1 |
| 3206.8781754 | 2 |
| 3206.8799928 | 0 |
| 3206.8812773 | 1 |
| 3206.8834183 | 1 |
| 3206.8860583 | 0 |
| 3206.8873601 | 1 |
| 3206.8890081 | 2 |
| 3206.8915505 | 1 |
| 3206.8927929 | 2 |
| 3206.8953502 | 2 |
| 3206.8959762 | 2 |
| 3206.8988437 | 2 |
| 3206.8998681 | 5 |
| 3206.9017967 | 1 |
| 3206.9033261 | 3 |
| 3206.9057604 | 1 |
| 3206.908957 | 4 |
| 3206.9098032 | 1 |
| 3206.9121795 | 1 |
| 3206.9134725 | 1 |
| 3206.9154629 | 1 |
| 3206.9180481 | 2 |
| 3206.919502 | 0 |
| 3206.9209833 | 1 |
| 3206.9225637 | 0 |
| 3206.9242939 | 1 |
| 3206.9268712 | 1 |
| 3206.9271001 | 1 |
| 3206.9301033 | 2 |
| 3206.931267 | 2 |
| 3206.9333694 | 2 |
| 3206.9356871 | 3 |
| 3206.9380109 | 1 |
| 3206.9391318 | 3 |
| 3206.9408569 | 1 |
| 3206.9429617 | 1 |
| 3206.9447891 | 2 |
| 3206.947952 | 1 |
| 3206.9491871 | 2 |
| 3206.9505325 | 1 |
| 3206.9521042 | 1 |
| 3206.9542402 | 0 |
| 3206.955933 | 4 |
| 3206.9582393 | 5 |
| 3206.9605301 | 1 |
| 3206.9618824 | 4 |
| 3206.964763 | 2 |
| 3206.9654209 | 0 |
| 3206.9678033 | 1 |
| 3206.9685377 | 1 |
| 3206.9698448 | 2 |
| 3206.9716789 | 1 |
| 3206.9726706 | 2 |
| 3206.9744778 | 1 |
| 3206.9761052 | 1 |
| 3206.9764062 | 1 |
| 3206.9789296 | 2 |
| 3206.9796915 | 3 |
| 3206.9809871 | 2 |
| 3206.9828207 | 1 |
| 3206.9839737 | 2 |
| 3206.984755 | 1 |
| 3206.9858225 | 1 |
| 3206.9883187 | 2 |
| 3206.9892927 | 2 |
| 3206.9902428 | 1 |
| 3206.992266 | 1 |
| 3206.9945696 | 1 |
| 3206.9949729 | 3 |
| 3206.9965434 | 4 |
| 3206.997352 | 1 |
| 3206.998979 | 3 |
| 3207.0005937 | 2 |
| 3207.0021369 | 2 |
| 3207.0036164 | 1 |
| 3207.0056341 | 2 |
| 3207.007161 | 2 |
| 3207.007454 | 1 |
| 3207.0096467 | 3 |
| 3207.0115161 | 2 |
| 3207.0125163 | 1 |
| 3207.013623 | 5 |
| 3207.0146816 | 2 |
| 3207.0163521 | 1 |
| 3207.0163572 | 2 |
| 3207.0192831 | 0 |
| 3207.0206295 | 2 |
| 3207.0211895 | 1 |
| 3207.0232174 | 2 |
| 3207.0247919 | 0 |
| 3207.0256239 | 2 |
| 3207.0277063 | 1 |
| 3207.0284187 | 2 |
| 3207.030267 | 3 |
| 3207.031256 | 4 |
| 3207.0329018 | 0 |
| 3207.0343891 | 1 |
| 3207.0355916 | 1 |
| 3207.0375191 | 2 |
| 3207.0380621 | 4 |
| 3207.0398955 | 1 |
| 3207.041792 | 1 |
| 3207.0426968 | 1 |
| 3207.044772 | 4 |
| 3207.0458144 | 3 |
| 3207.0480481 | 0 |
| 3207.0483202 | 0 |
| 3207.0494506 | 3 |
| 3207.051992 | 1 |
| 3207.0523795 | 0 |
| 3207.0542682 | 1 |
| 3207.055104 | 0 |
| 3207.056333 | 1 |
| 3207.0585865 | 0 |
| 3207.0598752 | 3 |
| 3207.0612238 | 1 |
| 3207.0628938 | 2 |
| 3207.0648903 | 1 |
| 3207.0649424 | 1 |
| 3207.0663708 | 2 |
| 3207.0684519 | 1 |
| 3207.0693461 | 0 |
| 3207.0704165 | 2 |
| 3207.0730065 | 2 |
| 3207.0737985 | 3 |
| 3207.0748983 | 2 |
| 3207.0758105 | 1 |
| 3207.0773005 | 0 |
| 3207.0785186 | 2 |
| 3207.0803176 | 1 |
| 3207.0823273 | 2 |
| 3207.0825307 | 2 |
| 3207.0853127 | 4 |
| 3207.0869367 | 2 |
| 3207.0869601 | 4 |
| 3207.0896199 | 0 |
| 3207.0899695 | 2 |
| 3207.0918546 | 2 |
| 3207.0932879 | 2 |
| 3207.0947344 | 5 |
| 3207.0968633 | 3 |
| 3207.0970991 | 0 |
| 3207.0991679 | 5 |
| 3207.0998604 | 2 |
| 3207.1015524 | 0 |
| 3207.103272 | 2 |
| 3207.1046687 | 1 |
| 3207.105851 | 4 |
| 3207.1074734 | 0 |
| 3207.108811 | 1 |
| 3207.110482 | 1 |
| 3207.1118315 | 2 |
| 3207.1131308 | 1 |
| 3207.1140155 | 0 |
| 3207.1158047 | 2 |
| 3207.1163884 | 2 |
| 3207.1180274 | 0 |
| 3207.1197884 | 0 |
| 3207.1207871 | 0 |
| 3207.1222607 | 3 |
| 3207.1243801 | 1 |
| 3207.1255873 | 4 |
| 3207.1266016 | 1 |
| 3207.1276245 | 1 |
| 3207.1282233 | 1 |
| 3207.1294113 | 4 |
| 3207.1296643 | 2 |
| 3207.1301169 | 1 |
| 3207.1310002 | 3 |
| 3207.1316036 | 3 |
| 3207.1325 | 2 |
| 3207.1328551 | 1 |
| 3207.1337067 | 0 |
| 3207.1340376 | 4 |
| 3207.1344876 | 0 |
| 3207.1354901 | 3 |
| 3207.1356236 | 0 |
| 3207.1365403 | 3 |
| 3207.1374822 | 2 |
| 3207.1375857 | 2 |
| 3207.1385186 | 2 |
| 3207.1393399 | 4 |
| 3207.1398481 | 2 |
| 3207.1404403 | 0 |
| 3207.1413444 | 0 |
| 3207.1416369 | 2 |
| 3207.141911 | 0 |
| 3207.1429358 | 4 |
| 3207.1431602 | 1 |
| 3207.1440019 | 3 |
| 3207.1442102 | 2 |
| 3207.1447836 | 4 |
| 3207.1456339 | 0 |
| 3207.1461207 | 2 |
| 3207.1476768 | 2 |
| 3207.1481401 | 2 |
| 3207.1484155 | 0 |
| 3207.1487789 | 1 |
| 3207.1495854 | 0 |
| 3207.1503761 | 2 |
| 3207.1510839 | 2 |
| 3207.1517862 | 2 |
| 3207.1518261 | 1 |
| 3207.1528137 | 2 |
| 3207.1539848 | 3 |
| 3207.1541648 | 1 |
| 3207.154538 | 1 |
| 3207.1553549 | 0 |
| 3207.1563446 | 4 |
| 3207.1568941 | 2 |
| 3207.1587179 | 4 |
| 3207.1600747 | 1 |
| 3207.1619166 | 2 |
| 3207.162188 | 2 |
| 3207.1641713 | 0 |
| 3207.1644448 | 0 |
| 3207.1664276 | 5 |
| 3207.1686309 | 1 |
| 3207.1688914 | 1 |
| 3207.1715286 | 1 |
| 3207.1716769 | 3 |
| 3207.173982 | 2 |
| 3207.1756331 | 2 |
| 3207.176368 | 1 |
| 3207.1776811 | 1 |
| 3207.1789019 | 0 |
| 3207.1804466 | 1 |
| 3207.1831258 | 3 |
| 3207.1832201 | 6 |
| 3207.1846938 | 1 |
| 3207.1857276 | 3 |
| 3207.1880311 | 5 |
| 3207.1896343 | 0 |
| 3207.1906385 | 0 |
| 3207.1923928 | 6 |
| 3207.1930973 | 0 |
| 3207.1952969 | 2 |
| 3207.1967219 | 1 |
| 3207.1969583 | 1 |
| 3207.198918 | 0 |
| 3207.1999407 | 2 |
| 3207.202047 | 2 |
| 3207.202655 | 1 |
| 3207.2036648 | 2 |
| 3207.204108 | 0 |
| 3207.2045306 | 1 |
| 3207.2047796 | 1 |
| 3207.2049376 | 0 |
| 3207.2061375 | 0 |
| 3207.2070655 | 1 |

| | |
|---|---|
| 3207.2078744 | 1 |
| 3207.2083922 | 3 |
| 3207.2087687 | 3 |
| 3207.2090714 | 1 |
| 3207.209518 | 2 |
| 3207.2102328 | 2 |
| 3207.2104844 | 2 |
| 3207.2119635 | 0 |
| 3207.2126071 | 1 |
| 3207.2138001 | 2 |
| 3207.2139202 | 2 |
| 3207.2143541 | 0 |
| 3207.2144502 | 4 |
| 3207.2158022 | 0 |
| 3207.216254 | 4 |
| 3207.2162958 | 4 |
| 3207.2163047 | 1 |
| 3207.2165063 | 4 |
| 3207.2168197 | 1 |
| 3207.2174209 | 0 |
| 3207.217553 | 1 |
| 3207.2176753 | 3 |
| 3207.2181171 | 2 |
| 3207.2182658 | 1 |
| 3207.2183254 | 1 |
| 3207.218772 | 4 |
| 3207.2193835 | 3 |
| 3207.2196395 | 0 |
| 3207.2198561 | 1 |
| 3207.2201186 | 1 |
| 3207.2203751 | 2 |
| 3207.2204458 | 1 |
| 3207.220746 | 3 |
| 3207.2214991 | 1 |
| 3207.2216746 | 0 |
| 3207.2218523 | 0 |
| 3207.2219145 | 0 |
| 3207.2219592 | 4 |
| 3207.2222664 | 1 |
| 3207.2229221 | 2 |
| 3207.2232964 | 2 |
| 3207.2233385 | 0 |
| 3207.2238621 | 1 |
| 3207.2239234 | 2 |
| 3207.2241463 | 0 |
| 3207.2244389 | 2 |
| 3207.2244978 | 2 |
| 3207.2245128 | 2 |
| 3207.2249612 | 2 |
| 3207.2253605 | 1 |
| 3207.2253761 | 2 |
| 3207.2254202 | 4 |
| 3207.2257475 | 0 |
| 3207.2263902 | 2 |
| 3207.2268619 | 0 |
| 3207.2270069 | 2 |
| 3207.2270248 | 1 |
| 3207.2272391 | 1 |
| 3207.2280886 | 2 |
| 3207.2282951 | 1 |
| 3207.2283259 | 5 |
| 3207.2284261 | 2 |
| 3207.2285218 | 2 |
| 3207.2293839 | 2 |
| 3207.2294258 | 4 |
| 3207.2296699 | 2 |
| 3207.2299975 | 1 |
| 3207.2303333 | 2 |
| 3207.2306299 | 1 |
| 3207.2309528 | 0 |
| 3207.2309833 | 3 |
| 3207.2310112 | 1 |
| 3207.2315586 | 1 |
| 3207.2316266 | 1 |
| 3207.2320409 | 3 |
| 3207.2321676 | 4 |
| 3207.2322774 | 5 |
| 3207.2323551 | 6 |
| 3207.2327994 | 11 |
| 3207.2335623 | 8 |
| 3207.2337895 | 5 |
| 3207.234244 | 11 |
| 3207.2343384 | 6 |
| 3207.2348258 | 15 |
| 3207.234846 | 6 |
| 3207.23517 | 5 |
| 3207.2354497 | 16 |
| 3207.2354526 | 7 |
| 3207.2361334 | 7 |
| 3207.2363667 | 5 |
| 3207.2365532 | 15 |
| 3207.2367517 | 9 |
| 3207.2367615 | 12 |
| 3207.2370956 | 13 |
| 3207.2375287 | 14 |
| 3207.2377486 | 10 |
| 3207.2379495 | 8 |
| 3207.2382759 | 9 |
| 3207.2384409 | 7 |
| 3207.2385058 | 11 |
| 3207.2385819 | 11 |
| 3207.2390989 | 6 |
| 3207.2394686 | 9 |
| 3207.2395607 | 10 |
| 3207.2401177 | 10 |
| 3207.2401389 | 13 |
| 3207.2405187 | 7 |
| 3207.2405403 | 6 |
| 3207.2408991 | 6 |
| 3207.2409598 | 3 |
| 3207.2411639 | 4 |
| 3207.2412799 | 7 |
| 3207.2415311 | 1 |
| 3207.241796 | 1 |
| 3207.2418658 | 0 |
| 3207.2419383 | 3 |
| 3207.2420975 | 4 |
| 3207.242979 | 6 |
| 3207.2429822 | 1 |
| 3207.2430772 | 1 |
| 3207.2435011 | 0 |
| 3207.2436927 | 2 |
| 3207.2438182 | 1 |
| 3207.2439789 | 4 |
| 3207.2442022 | 3 |
| 3207.2447367 | 0 |
| 3207.2449055 | 0 |
| 3207.2452769 | 2 |
| 3207.2455752 | 3 |
| 3207.2456864 | 1 |
| 3207.2457999 | 1 |
| 3207.2459269 | 3 |
| 3207.2461263 | 1 |
| 3207.2466018 | 5 |
| 3207.2466662 | 0 |
| 3207.2472354 | 2 |
| 3207.2482153 | 0 |
| 3207.2483799 | 0 |
| 3207.2497489 | 1 |
| 3207.2501438 | 2 |
| 3207.2516274 | 1 |
| 3207.2517149 | 1 |
| 3207.2530067 | 4 |
| 3207.2546666 | 3 |
| 3207.2548468 | 2 |
| 3207.2558908 | 0 |
| 3207.2568198 | 1 |
| 3207.2575889 | 0 |
| 3207.2585445 | 2 |
| 3207.2592903 | 1 |
| 3207.260033 | 4 |
| 3207.261704 | 3 |
| 3207.2623162 | 0 |
| 3207.2630899 | 5 |
| 3207.2637082 | 2 |
| 3207.2652195 | 5 |
| 3207.2659311 | 1 |
| 3207.2666975 | 2 |
| 3207.2676754 | 0 |
| 3207.2688837 | 2 |
| 3207.26935 | 2 |
| 3207.271691 | 1 |
| 3207.2717613 | 4 |
| 3207.2725401 | 2 |
| 3207.272546 | 2 |
| 3207.2747877 | 1 |
| 3207.275555 | 3 |
| 3207.2765194 | 2 |
| 3207.2776932 | 0 |
| 3207.2783552 | 6 |
| 3207.2794895 | 2 |
| 3207.280256 | 2 |
| 3207.2807196 | 0 |
| 3207.282302 | 1 |
| 3207.282863 | 5 |
| 3207.2842892 | 2 |
| 3207.2849973 | 3 |
| 3207.285703 | 0 |
| 3207.2871503 | 1 |
| 3207.2872628 | 2 |
| 3207.2884002 | 1 |
| 3207.2895197 | 2 |
| 3207.2904351 | 2 |
| 3207.2909654 | 3 |
| 3207.2926413 | 3 |
| 3207.2933123 | 3 |
| 3207.2933328 | 0 |
| 3207.2945221 | 1 |
| 3207.2959347 | 3 |
| 3207.2967362 | 1 |
| 3207.2974805 | 4 |
| 3207.2983939 | 1 |
| 3207.2995565 | 1 |
| 3207.3010057 | 1 |
| 3207.3013945 | 1 |
| 3207.3024295 | 1 |
| 3207.3029361 | 2 |
| 3207.3035312 | 2 |
| 3207.3050437 | 4 |
| 3207.3054385 | 4 |
| 3207.3070113 | 6 |
| 3207.307509 | 7 |
| 3207.30855 | 4 |
| 3207.3091158 | 7 |
| 3207.3109454 | 2 |
| 3207.3115473 | 1 |
| 3207.3122072 | 3 |
| 3207.3138267 | 1 |
| 3207.3139072 | 2 |
| 3207.3153166 | 2 |
| 3207.315592 | 7 |
| 3207.3172655 | 7 |
| 3207.3177012 | 5 |
| 3207.3186319 | 7 |
| 3207.3200407 | 7 |
| 3207.3214385 | 4 |
| 3207.3215068 | 1 |
| 3207.3227397 | 2 |
| 3207.3230148 | 3 |
| 3207.3242969 | 1 |
| 3207.3255961 | 3 |
| 3207.3263144 | 3 |
| 3207.3274029 | 0 |
| 3207.3279787 | 2 |
| 3207.3291457 | 1 |
| 3207.3303579 | 3 |
| 3207.3304817 | 4 |
| 3207.3315134 | 2 |
| 3207.3327634 | 2 |
| 3207.3335586 | 0 |
| 3207.3352215 | 3 |
| 3207.3360254 | 3 |
| 3207.336966 | 1 |
| 3207.3371717 | 3 |
| 3207.3381155 | 0 |
| 3207.3390339 | 4 |
| 3207.34067 | 3 |
| 3207.3413925 | 2 |
| 3207.3416646 | 0 |
| 3207.3429277 | 4 |
| 3207.3439406 | 0 |
| 3207.3444214 | 1 |
| 3207.3453729 | 0 |
| 3207.3462853 | 4 |
| 3207.3475073 | 5 |
| 3207.3485734 | 2 |
| 3207.3487226 | 1 |
| 3207.3505569 | 1 |
| 3207.3509856 | 3 |
| 3207.3523231 | 0 |
| 3207.3529471 | 2 |
| 3207.3536347 | 4 |
| 3207.3544016 | 1 |
| 3207.3549327 | 0 |
| 3207.3569873 | 1 |
| 3207.3575001 | 2 |
| 3207.3585125 | 1 |
| 3207.3596762 | 3 |
| 3207.3601335 | 1 |
| 3207.3616069 | 3 |
| 3207.3624446 | 2 |
| 3207.3630494 | 1 |
| 3207.3642408 | 3 |
| 3207.3645809 | 4 |
| 3207.3661351 | 3 |
| 3207.3672199 | 2 |
| 3207.3681384 | 0 |
| 3207.3688691 | 1 |
| 3207.369492 | 4 |
| 3207.3708266 | 1 |
| 3207.3710083 | 3 |
| 3207.372897 | 0 |
| 3207.3729266 | 2 |
| 3207.3737089 | 3 |
| 3207.3759115 | 0 |
| 3207.3762556 | 3 |
| 3207.3778972 | 3 |
| 3207.3786723 | 0 |
| 3207.3788908 | 0 |
| 3207.3808464 | 2 |
| 3207.3814875 | 2 |
| 3207.3817077 | 0 |
| 3207.3826662 | 2 |
| 3207.3836149 | 1 |
| 3207.3838949 | 0 |
| 3207.3850327 | 2 |
| 3207.3869701 | 2 |
| 3207.387601 | 3 |
| 3207.3878851 | 3 |
| 3207.3888967 | 1 |
| 3207.3895578 | 4 |
| 3207.3907828 | 2 |
| 3207.3917138 | 1 |
| 3207.3928067 | 4 |
| 3207.3939011 | 3 |
| 3207.3946169 | 0 |
| 3207.3956638 | 5 |
| 3207.3961399 | 3 |
| 3207.3967875 | 4 |
| 3207.3984762 | 2 |
| 3207.3986561 | 1 |
| 3207.3993989 | 1 |
| 3207.4010438 | 1 |
| 3207.4020619 | 1 |
| 3207.4021091 | 1 |
| 3207.4031575 | 2 |
| 3207.4043163 | 2 |
| 3207.4053993 | 2 |
| 3207.4061334 | 0 |
| 3207.4069331 | 4 |
| 3207.4086104 | 3 |
| 3207.4096195 | 3 |
| 3207.4100925 | 1 |
| 3207.4116911 | 1 |
| 3207.4122532 | 2 |
| 3207.4135276 | 0 |
| 3207.413842 | 2 |
| 3207.4148591 | 2 |
| 3207.4163782 | 1 |
| 3207.4172127 | 2 |
| 3207.417773 | 1 |
| 3207.4184134 | 2 |
| 3207.4204142 | 1 |
| 3207.4206304 | 1 |
| 3207.4217582 | 1 |
| 3207.4225565 | 4 |
| 3207.4231562 | 4 |
| 3207.4235654 | 0 |
| 3207.4252018 | 1 |
| 3207.4259911 | 1 |
| 3207.4267775 | 4 |
| 3207.4286673 | 1 |
| 3207.4290293 | 1 |
| 3207.4292251 | 0 |
| 3207.4307439 | 2 |
| 3207.4314298 | 1 |
| 3207.4326418 | 1 |
| 3207.4332613 | 3 |
| 3207.4346513 | 3 |
| 3207.4350971 | 2 |
| 3207.4366213 | 2 |
| 3207.4376588 | 0 |
| 3207.437968 | 0 |
| 3207.4384942 | 1 |
| 3207.440306 | 1 |
| 3207.4411234 | 3 |
| 3207.4421645 | 2 |
| 3207.4427585 | 4 |
| 3207.4433177 | 1 |
| 3207.4443284 | 0 |
| 3207.4450011 | 2 |
| 3207.4460348 | 0 |
| 3207.4465237 | 1 |
| 3207.4486231 | 6 |
| 3207.448707 | 2 |
| 3207.4497689 | 0 |
| 3207.4513875 | 1 |
| 3207.4523333 | 1 |
| 3207.4531189 | 2 |
| 3207.4539546 | 2 |
| 3207.4554031 | 2 |
| 3207.4557191 | 2 |
| 3207.4568845 | 2 |

| | |
|---|---|
| 3207.4579186 | 2 |
| 3207.4580056 | 3 |
| 3207.4590719 | 2 |
| 3207.4601358 | 2 |
| 3207.4617636 | 2 |
| 3207.4624154 | 2 |
| 3207.4636675 | 2 |
| 3207.4640105 | 2 |
| 3207.4647869 | 3 |
| 3207.4661935 | 4 |
| 3207.4666257 | 1 |
| 3207.4676315 | 0 |
| 3207.4685587 | 2 |
| 3207.4697822 | 4 |
| 3207.4700595 | 2 |
| 3207.4718919 | 0 |
| 3207.4722429 | 1 |
| 3207.4730302 | 1 |
| 3207.4740017 | 3 |
| 3207.47523 | 2 |
| 3207.4759494 | 1 |
| 3207.4768164 | 2 |
| 3207.4768471 | 2 |
| 3207.4769762 | 0 |
| 3207.4776294 | 1 |
| 3207.479167 | 1 |
| 3207.4793083 | 2 |
| 3207.4794093 | 5 |
| 3207.4799628 | 1 |
| 3207.480802 | 6 |
| 3207.4809667 | 3 |
| 3207.4810097 | 5 |
| 3207.4815763 | 7 |
| 3207.482071 | 4 |
| 3207.4831801 | 4 |
| 3207.483316 | 2 |
| 3207.483373 | 9 |
| 3207.4834832 | 4 |
| 3207.48418 | 4 |
| 3207.4846612 | 3 |
| 3207.4849294 | 3 |
| 3207.485466 | 1 |
| 3207.485815 | 3 |
| 3207.4867128 | 5 |
| 3207.4868 | 4 |
| 3207.4874737 | 5 |
| 3207.4879867 | 2 |
| 3207.4883344 | 2 |
| 3207.4890528 | 3 |
| 3207.4891098 | 3 |
| 3207.4903899 | 1 |
| 3207.4904228 | 5 |
| 3207.4908818 | 3 |
| 3207.4909432 | 4 |
| 3207.4914327 | 2 |
| 3207.4920947 | 2 |
| 3207.4921157 | 1 |
| 3207.4927457 | 1 |
| 3207.4932617 | 3 |
| 3207.4945369 | 0 |
| 3207.4947404 | 2 |
| 3207.4947554 | 3 |
| 3207.4957545 | 2 |
| 3207.4960757 | 1 |
| 3207.4962883 | 0 |
| 3207.4966399 | 0 |
| 3207.4969201 | 2 |
| 3207.4979057 | 2 |
| 3207.49821 | 2 |
| 3207.498466 | 1 |
| 3207.4990528 | 1 |
| 3207.4995938 | 2 |
| 3207.49997 | 1 |
| 3207.5007468 | 1 |
| 3207.5008414 | 3 |
| 3207.5019469 | 3 |
| 3207.5021445 | 1 |
| 3207.5026885 | 1 |
| 3207.503264 | 3 |
| 3207.5040301 | 2 |
| 3207.5043558 | 3 |
| 3207.5047941 | 1 |
| 3207.5048548 | 2 |
| 3207.5051056 | 1 |
| 3207.506211 | 1 |
| 3207.5069765 | 3 |
| 3207.507319 | 1 |
| 3207.5074982 | 3 |
| 3207.5075781 | 1 |
| 3207.5080095 | 0 |
| 3207.5091115 | 6 |
| 3207.5097766 | 1 |
| 3207.5100697 | 0 |
| 3207.5107424 | 0 |
| 3207.5109394 | 2 |
| 3207.5111084 | 0 |
| 3207.5114317 | 0 |
| 3207.5122619 | 4 |
| 3207.5131275 | 2 |
| 3207.5132147 | 1 |
| 3207.5138446 | 2 |
| 3207.5139342 | 1 |
| 3207.514222 | 2 |
| 3207.5143934 | 3 |
| 3207.5154235 | 2 |
| 3207.5159955 | 3 |
| 3207.5165949 | 0 |
| 3207.517584 | 4 |
| 3207.5176015 | 0 |
| 3207.5177324 | 3 |
| 3207.5177539 | 1 |
| 3207.5180551 | 1 |
| 3207.5186972 | 4 |
| 3207.5195883 | 0 |
| 3207.520415 | 1 |
| 3207.5204977 | 1 |
| 3207.5206537 | 2 |
| 3207.521486 | 0 |
| 3207.5220339 | 1 |
| 3207.5231818 | 1 |
| 3207.5231969 | 0 |
| 3207.5232681 | 2 |
| 3207.5232892 | 2 |
| 3207.5240347 | 1 |
| 3207.5241486 | 2 |
| 3207.5249308 | 0 |
| 3207.5255442 | 0 |
| 3207.5261361 | 0 |
| 3207.5262152 | 0 |
| 3207.5268632 | 4 |
| 3207.5270756 | 2 |
| 3207.5272045 | 2 |
| 3207.527278 | 2 |
| 3207.5288942 | 2 |
| 3207.5292748 | 1 |
| 3207.5293997 | 4 |
| 3207.5299561 | 1 |
| 3207.5305219 | 1 |
| 3207.5308803 | 1 |
| 3207.5318571 | 2 |
| 3207.531874 | 1 |
| 3207.5319742 | 5 |
| 3207.5331458 | 0 |
| 3207.5332708 | 3 |
| 3207.5332797 | 1 |
| 3207.533957 | 2 |
| 3207.5346934 | 3 |
| 3207.5351427 | 1 |
| 3207.5355163 | 3 |
| 3207.5357501 | 1 |
| 3207.5363141 | 2 |
| 3207.5364033 | 0 |
| 3207.5377457 | 5 |
| 3207.5387939 | 1 |
| 3207.5388269 | 4 |
| 3207.5388973 | 4 |
| 3207.5395196 | 1 |
| 3207.5398352 | 1 |
| 3207.5400085 | 1 |
| 3207.5407517 | 1 |
| 3207.5408768 | 1 |
| 3207.5413766 | 1 |
| 3207.5424246 | 4 |
| 3207.5428025 | 1 |
| 3207.5430695 | 0 |
| 3207.5436413 | 2 |
| 3207.544281 | 1 |
| 3207.5444103 | 0 |
| 3207.5450877 | 1 |
| 3207.5452535 | 4 |
| 3207.5458713 | 2 |
| 3207.5461551 | 3 |
| 3207.5463394 | 2 |
| 3207.5475558 | 0 |
| 3207.5481118 | 1 |
| 3207.5481922 | 2 |
| 3207.5484135 | 3 |
| 3207.54942 | 2 |
| 3207.5500814 | 1 |
| 3207.5501809 | 2 |
| 3207.5502595 | 1 |
| 3207.5508036 | 3 |
| 3207.5516354 | 4 |
| 3207.551987 | 2 |
| 3207.5525955 | 2 |
| 3207.5527424 | 3 |
| 3207.5535025 | 3 |
| 3207.5544871 | 3 |
| 3207.5547219 | 4 |
| 3207.555153 | 5 |
| 3207.5552537 | 0 |
| 3207.5559526 | 1 |
| 3207.5560527 | 1 |
| 3207.5570622 | 1 |
| 3207.5575627 | 4 |
| 3207.5582139 | 3 |
| 3207.5583633 | 2 |
| 3207.5586182 | 0 |
| 3207.5587264 | 1 |
| 3207.5588951 | 1 |
| 3207.5604686 | 4 |
| 3207.5605207 | 1 |
| 3207.5615302 | 4 |
| 3207.5616034 | 3 |
| 3207.5625691 | 0 |
| 3207.5627208 | 1 |
| 3207.5633026 | 1 |
| 3207.5637946 | 1 |
| 3207.5640226 | 0 |
| 3207.5643664 | 1 |
| 3207.5646649 | 2 |
| 3207.5648664 | 0 |
| 3207.5655138 | 2 |
| 3207.5661663 | 0 |
| 3207.5666498 | 3 |
| 3207.5672505 | 2 |
| 3207.5678047 | 1 |
| 3207.5680686 | 3 |
| 3207.5682411 | 0 |
| 3207.5684883 | 2 |
| 3207.5697577 | 1 |
| 3207.5700685 | 5 |
| 3207.5703145 | 0 |
| 3207.5707434 | 3 |
| 3207.5710664 | 0 |
| 3207.5713195 | 5 |
| 3207.5718888 | 0 |
| 3207.5726275 | 1 |
| 3207.572698 | 0 |
| 3207.5739319 | 1 |
| 3207.5739997 | 3 |
| 3207.5748025 | 3 |
| 3207.5749592 | 1 |
| 3207.575516 | 3 |
| 3207.5758588 | 1 |
| 3207.5762681 | 1 |
| 3207.5765946 | 1 |
| 3207.5770906 | 2 |
| 3207.5778121 | 1 |
| 3207.5778143 | 0 |
| 3207.5780715 | 0 |
| 3207.5789431 | 2 |
| 3207.5792938 | 1 |
| 3207.5802615 | 2 |
| 3207.5805894 | 3 |
| 3207.5811428 | 1 |
| 3207.5812721 | 1 |
| 3207.5825169 | 1 |
| 3207.5825181 | 1 |
| 3207.5829429 | 1 |
| 3207.5836067 | 2 |
| 3207.5843015 | 0 |
| 3207.5846516 | 4 |
| 3207.5848156 | 1 |
| 3207.585644 | 1 |
| 3207.5856968 | 2 |
| 3207.5862501 | 1 |
| 3207.5863259 | 3 |
| 3207.5865486 | 0 |
| 3207.5868209 | 2 |
| 3207.588085 | 2 |
| 3207.5887402 | 4 |
| 3207.5890055 | 2 |
| 3207.5895606 | 2 |
| 3207.5899617 | 3 |
| 3207.5901947 | 2 |
| 3207.5903156 | 2 |
| 3207.5921204 | 1 |
| 3207.5922555 | 2 |
| 3207.5923172 | 3 |
| 3207.592824 | 1 |
| 3207.5933575 | 1 |
| 3207.5937684 | 2 |
| 3207.5948255 | 3 |
| 3207.5948828 | 2 |
| 3207.5954927 | 4 |
| 3207.59626 | 2 |
| 3207.5964499 | 1 |
| 3207.5965889 | 0 |
| 3207.5970821 | 0 |
| 3207.5971727 | 2 |
| 3207.5982985 | 0 |
| 3207.5986279 | 5 |
| 3207.5997618 | 1 |
| 3207.5998091 | 2 |
| 3207.5998776 | 0 |
| 3207.6004604 | 2 |
| 3207.6005672 | 6 |
| 3207.6011977 | 4 |
| 3207.601588 | 1 |
| 3207.6024128 | 2 |
| 3207.6024771 | 6 |
| 3207.6032393 | 1 |
| 3207.604422 | 2 |
| 3207.6046117 | 2 |
| 3207.6048874 | 2 |
| 3207.6054122 | 0 |
| 3207.6056403 | 4 |
| 3207.6064268 | 3 |
| 3207.6065168 | 2 |
| 3207.6068341 | 2 |
| 3207.6079954 | 2 |
| 3207.6085358 | 4 |
| 3207.6090199 | 0 |
| 3207.6091168 | 4 |
| 3207.6093663 | 3 |
| 3207.6104079 | 2 |
| 3207.6105678 | 6 |
| 3207.611053 | 1 |
| 3207.612373 | 0 |
| 3207.6137858 | 1 |
| 3207.6141532 | 1 |
| 3207.6144732 | 2 |
| 3207.6144866 | 3 |
| 3207.6148048 | 2 |
| 3207.6159316 | 0 |
| 3207.6163013 | 1 |
| 3207.6169843 | 3 |
| 3207.6170839 | 4 |
| 3207.6183541 | 5 |
| 3207.6192313 | 2 |
| 3207.6215714 | 0 |
| 3207.6234433 | 0 |
| 3207.6251914 | 4 |
| 3207.6271807 | 1 |
| 3207.6296703 | 0 |
| 3207.6303113 | 1 |
| 3207.6333978 | 0 |
| 3207.6341877 | 1 |
| 3207.6365484 | 2 |
| 3207.6377756 | 2 |
| 3207.6408683 | 5 |
| 3207.6427936 | 0 |
| 3207.6442564 | 2 |
| 3207.6464236 | 0 |
| 3207.6478142 | 2 |
| 3207.6501266 | 1 |
| 3207.6524133 | 4 |
| 3207.6535778 | 2 |
| 3207.6558909 | 3 |
| 3207.6569354 | 0 |
| 3207.6597937 | 0 |
| 3207.661869 | 3 |
| 3207.6624879 | 2 |
| 3207.6647464 | 2 |
| 3207.6666239 | 1 |
| 3207.6682287 | 4 |
| 3207.6697779 | 1 |
| 3207.6720209 | 0 |
| 3207.674573 | 1 |
| 3207.67551 | 2 |
| 3207.6781364 | 3 |
| 3207.6794827 | 0 |
| 3207.6821748 | 3 |
| 3207.684168 | 0 |
| 3207.6852653 | 2 |
| 3207.6874024 | 1 |
| 3207.6887858 | 0 |
| 3207.6915808 | 2 |
| 3207.6927463 | 2 |
| 3207.6951363 | 0 |
| 3207.6971361 | 2 |
| 3207.6989314 | 1 |

| | |
|---|---|
| 3207.7006697 | 3 |
| 3207.702977 | 1 |
| 3207.7042007 | 2 |
| 3207.7068863 | 2 |
| 3207.707752 | 1 |
| 3207.7101864 | 1 |
| 3207.7125438 | 4 |
| 3207.7138523 | 1 |
| 3207.7159086 | 3 |
| 3207.7168057 | 1 |
| 3207.7193488 | 1 |
| 3207.7207959 | 3 |
| 3207.723307 | 2 |
| 3207.7258002 | 1 |
| 3207.7269522 | 1 |
| 3207.7297651 | 1 |
| 3207.7297774 | 4 |
| 3207.7327082 | 2 |
| 3207.7348306 | 2 |
| 3207.7361859 | 2 |
| 3207.7388433 | 3 |
| 3207.7401582 | 1 |
| 3207.742351 | 2 |
| 3207.744705 | 2 |
| 3207.7454357 | 1 |
| 3207.7480786 | 0 |
| 3207.7491682 | 0 |
| 3207.7508504 | 0 |
| 3207.7534994 | 2 |
| 3207.7554092 | 0 |
| 3207.7577036 | 1 |
| 3207.7586031 | 1 |
| 3207.7607099 | 1 |
| 3207.7612702 | 1 |
| 3207.7651251 | 1 |
| 3207.766805 | 1 |
| 3207.7683758 | 0 |
| 3207.7699813 | 1 |
| 3207.7729955 | 2 |
| 3207.7734049 | 2 |
| 3207.7759106 | 2 |
| 3207.7782054 | 4 |
| 3207.7805045 | 1 |
| 3207.7813854 | 1 |
| 3207.7834597 | 0 |
| 3207.7866175 | 2 |
| 3207.7875665 | 3 |
| 3207.7904902 | 1 |
| 3207.7916518 | 0 |
| 3207.7936117 | 2 |
| 3207.7956314 | 0 |
| 3207.7964736 | 0 |
| 3207.7992515 | 2 |
| 3207.8004844 | 0 |
| 3207.8029467 | 0 |
| 3207.8032654 | 4 |
| 3207.8052003 | 2 |
| 3207.8067096 | 0 |
| 3207.8083773 | 1 |
| 3207.8117996 | 0 |
| 3207.8123898 | 3 |
| 3207.8150082 | 1 |
| 3207.8155716 | 2 |
| 3207.8186423 | 1 |
| 3207.8208377 | 1 |
| 3207.8224234 | 1 |
| 3207.8250955 | 2 |
| 3207.8255793 | 2 |
| 3207.8283211 | 2 |
| 3207.8302605 | 1 |
| 3207.8312675 | 3 |
| 3207.8337502 | 1 |
| 3207.8350316 | 1 |
| 3207.8372447 | 1 |
| 3207.8399279 | 3 |
| 3207.8402909 | 2 |
| 3207.8439009 | 2 |
| 3207.8442726 | 0 |
| 3207.8470669 | 1 |
| 3207.8493955 | 2 |
| 3207.8500274 | 2 |
| 3207.8525367 | 0 |
| 3207.85373 | 3 |
| 3207.8563623 | 0 |
| 3207.8574999 | 2 |
| 3207.8602941 | 1 |
| 3207.8625618 | 4 |
| 3207.8633242 | 2 |
| 3207.8666779 | 2 |
| 3207.8673066 | 2 |
| 3207.8697709 | 1 |
| 3207.8720317 | 2 |
| 3207.8729104 | 1 |
| 3207.875805 | 2 |
| 3207.8770246 | 1 |
| 3207.8789047 | 0 |
| 3207.8813552 | 2 |
| 3207.8823564 | 3 |
| 3207.8846539 | 1 |
| 3207.8857791 | 1 |
| 3207.8878275 | 0 |
| 3207.8896195 | 3 |
| 3207.8920159 | 4 |
| 3207.8945358 | 1 |
| 3207.8945361 | 2 |
| 3207.8984557 | 2 |
| 3207.8987768 | 0 |
| 3207.9010529 | 0 |
| 3207.9034671 | 1 |
| 3207.9043388 | 4 |
| 3207.9076789 | 2 |
| 3207.9083846 | 3 |
| 3207.9109559 | 2 |
| 3207.913412 | 4 |
| 3207.914639 | 2 |
| 3207.9164422 | 3 |
| 3207.9175923 | 3 |
| 3207.9203451 | 0 |
| 3207.9228251 | 4 |
| 3207.9240521 | 1 |
| 3207.9264875 | 1 |
| 3207.9275328 | 2 |
| 3207.930179 | 1 |
| 3207.9327045 | 2 |
| 3207.9331523 | 2 |
| 3207.9353239 | 2 |
| 3207.9368264 | 2 |
| 3207.9391981 | 4 |
| 3207.9400349 | 3 |
| 3207.9432607 | 2 |
| 3207.9453466 | 1 |
| 3207.9460041 | 0 |
| 3207.9491563 | 1 |
| 3207.9508526 | 0 |
| 3207.9522405 | 2 |
| 3207.9544177 | 1 |
| 3207.955282 | 5 |
| 3207.958097 | 2 |
| 3207.960674 | 1 |
| 3207.9613297 | 1 |
| 3207.9640426 | 2 |
| 3207.9654672 | 0 |
| 3207.9674105 | 1 |
| 3207.970163 | 2 |
| 3207.9713189 | 0 |
| 3207.9731202 | 4 |
| 3207.9744601 | 3 |
| 3207.9764784 | 1 |
| 3207.9778807 | 1 |
| 3207.9807082 | 0 |
| 3207.9828789 | 0 |
| 3207.9841642 | 0 |
| 3207.9864532 | 2 |
| 3207.9872715 | 0 |
| 3207.9903451 | 1 |
| 3207.9920756 | 4 |
| 3207.993685 | 0 |
| 3207.9958066 | 1 |
| 3207.9974558 | 1 |
| 3207.9989503 | 1 |
| 3208.001728 | 2 |
| 3208.0027468 | 1 |
| 3208.0058422 | 2 |
| 3208.0065982 | 2 |
| 3208.0095244 | 1 |
| 3208.0101123 | 3 |
| 3208.0126418 | 2 |
| 3208.0150489 | 1 |
| 3208.0164267 | 2 |
| 3208.0192982 | 2 |
| 3208.0197954 | 2 |
| 3208.0219092 | 3 |
| 3208.0240752 | 1 |
| 3208.0248908 | 3 |
| 3208.027477 | 2 |
| 3208.0285385 | 4 |
| 3208.0310919 | 4 |
| 3208.0334405 | 0 |
| 3208.0349641 | 2 |
| 3208.0372907 | 4 |
| 3208.0384748 | 1 |
| 3208.0406623 | 4 |
| 3208.0434557 | 0 |
| 3208.0443187 | 4 |
| 3208.0468725 | 2 |
| 3208.0484473 | 5 |
| 3208.0502193 | 2 |
| 3208.0512598 | 0 |
| 3208.0539311 | 2 |
| 3208.0566223 | 1 |
| 3208.057197 | 1 |
| 3208.059936 | 1 |
| 3208.060903 | 2 |
| 3208.0633941 | 4 |
| 3208.065542 | 2 |
| 3208.066855 | 0 |
| 3208.068921 | 3 |
| 3208.069958 | 2 |
| 3208.0731062 | 1 |
| 3208.0742286 | 3 |
| 3208.0757379 | 0 |
| 3208.0777246 | 0 |
| 3208.0784208 | 2 |
| 3208.0816912 | 1 |
| 3208.0830045 | 0 |
| 3208.0854838 | 3 |
| 3208.0876783 | 1 |
| 3208.0885193 | 5 |
| 3208.0910133 | 3 |
| 3208.0923067 | 2 |
| 3208.0947886 | 3 |
| 3208.0964159 | 1 |
| 3208.098369 | 3 |
| 3208.1008043 | 3 |
| 3208.1018253 | 2 |
| 3208.1043094 | 3 |
| 3208.1056826 | 2 |
| 3208.1080805 | 0 |
| 3208.1101755 | 1 |
| 3208.1110915 | 0 |
| 3208.1136824 | 3 |
| 3208.1152433 | 3 |
| 3208.116845 | 1 |
| 3208.1185184 | 1 |
| 3208.1203233 | 1 |
| 3208.1226905 | 2 |
| 3208.1236659 | 1 |
| 3208.1263219 | 5 |
| 3208.1273428 | 2 |
| 3208.1303211 | 6 |
| 3208.1327853 | 1 |
| 3208.1335714 | 2 |
| 3208.1362049 | 2 |
| 3208.1367569 | 0 |
| 3208.1389686 | 2 |
| 3208.1420915 | 4 |
| 3208.1431023 | 1 |
| 3208.1455167 | 2 |
| 3208.1460519 | 2 |
| 3208.1489999 | 0 |
| 3208.150948 | 2 |
| 3208.1521043 | 1 |
| 3208.1541654 | 1 |
| 3208.1550704 | 1 |
| 3208.1577859 | 0 |
| 3208.1600813 | 1 |
| 3208.1615061 | 0 |
| 3208.163926 | 1 |
| 3208.1650927 | 2 |
| 3208.1673457 | 1 |
| 3208.169069 | 1 |
| 3208.170292 | 3 |
| 3208.1722727 | 3 |
| 3208.1744963 | 2 |
| 3208.1761118 | 2 |
| 3208.1776389 | 3 |
| 3208.1798037 | 1 |
| 3208.1821274 | 4 |
| 3208.1830573 | 2 |
| 3208.1859699 | 2 |
| 3208.1873086 | 1 |
| 3208.1896801 | 3 |
| 3208.1917513 | 2 |
| 3208.1932154 | 1 |
| 3208.1953557 | 0 |
| 3208.1969396 | 1 |
| 3208.1985074 | 1 |
| 3208.2004686 | 6 |
| 3208.2017617 | 3 |
| 3208.2037332 | 1 |
| 3208.2060215 | 3 |
| 3208.2071023 | 4 |
| 3208.2100668 | 1 |
| 3208.211038 | 1 |
| 3208.2134644 | 1 |
| 3208.2147495 | 1 |
| 3208.2172874 | 1 |
| 3208.2192513 | 3 |
| 3208.2200113 | 3 |
| 3208.2223181 | 2 |
| 3208.2239858 | 2 |
| 3208.2269416 | 1 |
| 3208.2276534 | 3 |
| 3208.2304533 | 4 |
| 3208.2324902 | 0 |
| 3208.2339591 | 2 |
| 3208.2356007 | 1 |
| 3208.2376083 | 1 |
| 3208.2395611 | 2 |
| 3208.24174 | 1 |
| 3208.2429997 | 1 |
| 3208.2451505 | 3 |
| 3208.2474011 | 1 |
| 3208.2482678 | 4 |
| 3208.2510715 | 3 |
| 3208.2516765 | 1 |
| 3208.2543134 | 3 |
| 3208.2562678 | 1 |
| 3208.2583724 | 0 |
| 3208.2599823 | 3 |
| 3208.2612435 | 2 |
| 3208.2640479 | 3 |
| 3208.2647492 | 2 |
| 3208.2671783 | 2 |
| 3208.2693454 | 0 |
| 3208.2702505 | 1 |
| 3208.2733325 | 0 |
| 3208.273675 | 1 |
| 3208.2764287 | 1 |
| 3208.2779831 | 1 |
| 3208.280056 | 4 |
| 3208.2823631 | 3 |
| 3208.2838805 | 3 |
| 3208.2865317 | 2 |
| 3208.2868214 | 0 |
| 3208.2893391 | 1 |
| 3208.2916899 | 1 |
| 3208.2925573 | 1 |
| 3208.29531 | 2 |
| 3208.296799 | 2 |
| 3208.2986818 | 4 |
| 3208.3004365 | 1 |
| 3208.3015447 | 3 |
| 3208.3041073 | 4 |
| 3208.3054457 | 0 |
| 3208.3070353 | 2 |
| 3208.3097721 | 5 |
| 3208.3113173 | 3 |
| 3208.3127597 | 3 |
| 3208.3145773 | 0 |
| 3208.3173619 | 4 |
| 3208.3196553 | 0 |
| 3208.3198918 | 1 |
| 3208.3232623 | 5 |
| 3208.3235577 | 1 |
| 3208.3271053 | 4 |
| 3208.3286648 | 2 |
| 3208.3306153 | 3 |
| 3208.3324554 | 1 |
| 3208.3332119 | 5 |
| 3208.3363714 | 4 |
| 3208.3381764 | 1 |
| 3208.339286 | 1 |
| 3208.3413127 | 2 |
| 3208.3422237 | 2 |
| 3208.3447568 | 0 |
| 3208.3460371 | 1 |
| 3208.3480169 | 6 |
| 3208.3507366 | 2 |
| 3208.3516678 | 6 |
| 3208.3541775 | 1 |
| 3208.3552763 | 3 |
| 3208.3573667 | 3 |
| 3208.3607934 | 3 |
| 3208.3616983 | 2 |
| 3208.3636862 | 3 |
| 3208.3659162 | 1 |
| 3208.3672971 | 3 |
| 3208.3695011 | 1 |
| 3208.3704979 | 3 |
| 3208.3731082 | 0 |
| 3208.3743268 | 1 |
| 3208.3756977 | 3 |

| | |
|---|---|
| 3208.3788228 | 1 |
| 3208.3796242 | 2 |
| 3208.3825101 | 2 |
| 3208.3834906 | 3 |
| 3208.3853301 | 2 |
| 3208.3878267 | 1 |
| 3208.3890882 | 4 |
| 3208.3908811 | 1 |
| 3208.3923398 | 0 |
| 3208.3949337 | 3 |
| 3208.3960582 | 2 |
| 3208.3981537 | 1 |
| 3208.4005363 | 2 |
| 3208.4019957 | 1 |
| 3208.4039572 | 2 |
| 3208.4057583 | 4 |
| 3208.4079932 | 2 |
| 3208.4099316 | 7 |
| 3208.411536 | 3 |
| 3208.4137687 | 5 |
| 3208.4161074 | 2 |
| 3208.4175111 | 1 |
| 3208.41899 | 0 |
| 3208.4207133 | 2 |
| 3208.4230191 | 4 |
| 3208.424003 | 1 |
| 3208.4267316 | 2 |
| 3208.4293677 | 0 |
| 3208.429877 | 1 |
| 3208.4323316 | 3 |
| 3208.4343879 | 0 |
| 3208.4356222 | 1 |
| 3208.4375824 | 3 |
| 3208.4382525 | 3 |
| 3208.4417872 | 1 |
| 3208.4425071 | 3 |
| 3208.4448294 | 3 |
| 3208.4467304 | 4 |
| 3208.4483584 | 3 |
| 3208.4514611 | 1 |
| 3208.4524529 | 1 |
| 3208.4544653 | 3 |
| 3208.4570886 | 0 |
| 3208.4579632 | 2 |
| 3208.460102 | 1 |
| 3208.462162 | 1 |
| 3208.4634393 | 1 |
| 3208.4662198 | 2 |
| 3208.4676913 | 1 |
| 3208.4698739 | 0 |
| 3208.4710643 | 0 |
| 3208.4739506 | 3 |
| 3208.4743324 | 0 |
| 3208.4766579 | 0 |
| 3208.4785669 | 3 |
| 3208.4799495 | 2 |
| 3208.482372 | 1 |
| 3208.484821 | 2 |
| 3208.4863446 | 0 |
| 3208.4887804 | 1 |
| 3208.4896601 | 1 |
| 3208.4920243 | 0 |
| 3208.493172 | 1 |
| 3208.4949433 | 4 |
| 3208.4971062 | 5 |
| 3208.4985747 | 2 |
| 3208.5015922 | 2 |
| 3208.5024943 | 4 |
| 3208.5047753 | 3 |
| 3208.5070591 | 3 |
| 3208.5084622 | 1 |
| 3208.5102023 | 0 |
| 3208.5120519 | 1 |
| 3208.5143449 | 1 |
| 3208.5155372 | 0 |
| 3208.518192 | 2 |
| 3208.5202171 | 3 |
| 3208.5215186 | 1 |
| 3208.5239069 | 3 |
| 3208.525431 | 7 |
| 3208.5273602 | 2 |
| 3208.5295083 | 0 |
| 3208.530772 | 2 |
| 3208.5325608 | 2 |
| 3208.5353866 | 3 |
| 3208.5366228 | 2 |
| 3208.5384349 | 3 |
| 3208.539434 | 0 |
| 3208.5426083 | 4 |
| 3208.5433384 | 2 |
| 3208.5463844 | 5 |
| 3208.5486726 | 5 |
| 3208.5490158 | 4 |
| 3208.5522817 | 1 |
| 3208.5529016 | 3 |
| 3208.555147 | 5 |
| 3208.5573804 | 2 |
| 3208.5589772 | 2 |
| 3208.5616432 | 0 |
| 3208.5627234 | 4 |
| 3208.5646375 | 0 |
| 3208.5669505 | 5 |
| 3208.5680576 | 5 |
| 3208.5701376 | 1 |
| 3208.5724938 | 2 |
| 3208.5743036 | 1 |
| 3208.5768279 | 4 |
| 3208.5782329 | 1 |
| 3208.5801069 | 1 |
| 3208.5805855 | 1 |
| 3208.5836255 | 1 |
| 3208.585701 | 5 |
| 3208.5869729 | 2 |
| 3208.589404 | 4 |
| 3208.5894871 | 3 |
| 3208.5897018 | 2 |
| 3208.5902085 | 2 |
| 3208.5910258 | 2 |
| 3208.5911588 | 2 |
| 3208.5914839 | 2 |
| 3208.5915846 | 6 |
| 3208.5919563 | 0 |
| 3208.592514 | 3 |
| 3208.5928091 | 2 |
| 3208.5928165 | 0 |
| 3208.5934737 | 1 |
| 3208.593514 | 1 |
| 3208.5937303 | 0 |
| 3208.5940762 | 1 |
| 3208.594553 | 4 |
| 3208.5948276 | 0 |
| 3208.595282 | 3 |
| 3208.5956313 | 3 |
| 3208.5957045 | 1 |
| 3208.5965421 | 1 |
| 3208.5970318 | 1 |
| 3208.5970937 | 4 |
| 3208.5971659 | 0 |
| 3208.5972644 | 2 |
| 3208.5978505 | 2 |
| 3208.5981265 | 2 |
| 3208.5984668 | 1 |
| 3208.5993888 | 0 |
| 3208.5995074 | 3 |
| 3208.5996558 | 1 |
| 3208.5998165 | 2 |
| 3208.5998647 | 4 |
| 3208.6006061 | 1 |
| 3208.6007098 | 1 |
| 3208.6009928 | 2 |
| 3208.6014155 | 1 |
| 3208.6015732 | 2 |
| 3208.601804 | 2 |
| 3208.6021786 | 3 |
| 3208.6026541 | 2 |
| 3208.6029045 | 2 |
| 3208.6029316 | 2 |
| 3208.603254 | 2 |
| 3208.6034769 | 0 |
| 3208.6037936 | 3 |
| 3208.6039481 | 4 |
| 3208.6044555 | 3 |
| 3208.6047034 | 3 |
| 3208.6049297 | 3 |
| 3208.6057508 | 1 |
| 3208.6060081 | 2 |
| 3208.6061484 | 3 |
| 3208.6062383 | 3 |
| 3208.6065622 | 2 |
| 3208.6074221 | 2 |
| 3208.6074923 | 2 |
| 3208.6077832 | 2 |
| 3208.607842 | 4 |
| 3208.6080103 | 2 |
| 3208.6083855 | 3 |
| 3208.6086845 | 1 |
| 3208.609083 | 3 |
| 3208.6097947 | 2 |
| 3208.6098916 | 2 |
| 3208.6104773 | 3 |
| 3208.6107355 | 4 |
| 3208.6108132 | 2 |
| 3208.6108221 | 2 |
| 3208.6114127 | 2 |
| 3208.6118016 | 1 |
| 3208.6123993 | 3 |
| 3208.6129909 | 1 |
| 3208.6131072 | 1 |
| 3208.6131287 | 1 |
| 3208.6131959 | 0 |
| 3208.6136466 | 2 |
| 3208.6137848 | 4 |
| 3208.6142096 | 1 |
| 3208.6147275 | 1 |
| 3208.6152279 | 2 |
| 3208.6156394 | 1 |
| 3208.6156559 | 3 |
| 3208.6160308 | 1 |
| 3208.6160785 | 0 |
| 3208.6162436 | 3 |
| 3208.6164992 | 1 |
| 3208.6167286 | 3 |
| 3208.6169576 | 0 |
| 3208.6170918 | 1 |
| 3208.6179429 | 1 |
| 3208.617964 | 1 |
| 3208.618342 | 1 |
| 3208.6185203 | 3 |
| 3208.6191369 | 1 |
| 3208.619464 | 4 |
| 3208.6195941 | 3 |
| 3208.6199694 | 6 |
| 3208.6203681 | 16 |
| 3208.6204899 | 7 |
| 3208.6208063 | 8 |
| 3208.6211337 | 11 |
| 3208.6211358 | 14 |
| 3208.6220102 | 22 |
| 3208.6220623 | 14 |
| 3208.6224516 | 14 |
| 3208.622965 | 17 |
| 3208.6232045 | 17 |
| 3208.6234608 | 17 |
| 3208.623555 | 22 |
| 3208.6241447 | 7 |
| 3208.6243233 | 15 |
| 3208.6250139 | 18 |
| 3208.625221 | 15 |
| 3208.6252312 | 8 |
| 3208.6257277 | 17 |
| 3208.6261409 | 14 |
| 3208.6262252 | 12 |
| 3208.6265465 | 15 |
| 3208.6270781 | 13 |
| 3208.6272942 | 12 |
| 3208.627391 | 9 |
| 3208.6274525 | 12 |
| 3208.6276412 | 7 |
| 3208.6278491 | 6 |
| 3208.6288723 | 7 |
| 3208.6293667 | 2 |
| 3208.6295252 | 2 |
| 3208.6303099 | 4 |
| 3208.6303893 | 7 |
| 3208.6307694 | 0 |
| 3208.6307816 | 1 |
| 3208.6309832 | 1 |
| 3208.6309838 | 2 |
| 3208.6317528 | 2 |
| 3208.6319016 | 2 |
| 3208.6324372 | 4 |
| 3208.6328266 | 0 |
| 3208.6329755 | 0 |
| 3208.633496 | 3 |
| 3208.6336503 | 2 |
| 3208.6340874 | 2 |
| 3208.6342128 | 2 |
| 3208.6346769 | 2 |
| 3208.6348593 | 4 |
| 3208.6348713 | 1 |
| 3208.6350181 | 3 |
| 3208.6357499 | 1 |
| 3208.6360858 | 1 |
| 3208.6363183 | 3 |
| 3208.6370194 | 1 |
| 3208.6371301 | 1 |
| 3208.6371949 | 3 |
| 3208.6376326 | 3 |
| 3208.6381598 | 1 |
| 3208.638779 | 2 |
| 3208.6388474 | 1 |
| 3208.6390136 | 3 |
| 3208.6390802 | 3 |
| 3208.6398113 | 6 |
| 3208.6399126 | 1 |
| 3208.6404201 | 3 |
| 3208.640424 | 1 |
| 3208.6404487 | 2 |
| 3208.6410654 | 2 |
| 3208.6412746 | 2 |
| 3208.6414014 | 3 |
| 3208.6418516 | 2 |
| 3208.6422459 | 1 |
| 3208.6423614 | 2 |
| 3208.6425903 | 3 |
| 3208.6431557 | 1 |
| 3208.6436992 | 2 |
| 3208.6440626 | 2 |
| 3208.6445555 | 1 |
| 3208.6445654 | 2 |
| 3208.6451438 | 4 |
| 3208.6453216 | 1 |
| 3208.6456036 | 1 |
| 3208.6459517 | 0 |
| 3208.6463762 | 7 |
| 3208.6464325 | 1 |
| 3208.6472465 | 3 |
| 3208.6473063 | 1 |
| 3208.6477872 | 3 |
| 3208.6478118 | 2 |
| 3208.6478502 | 4 |
| 3208.6482644 | 1 |
| 3208.6485871 | 2 |
| 3208.649346 | 3 |
| 3208.6496995 | 5 |
| 3208.6498738 | 3 |
| 3208.6501829 | 1 |
| 3208.6504532 | 2 |
| 3208.6504689 | 1 |
| 3208.6511672 | 3 |
| 3208.6513732 | 0 |
| 3208.6514938 | 4 |
| 3208.6516043 | 3 |
| 3208.6518165 | 2 |
| 3208.6525901 | 4 |
| 3208.6529254 | 2 |
| 3208.6529875 | 1 |
| 3208.6529915 | 1 |
| 3208.6537394 | 0 |
| 3208.6542412 | 3 |
| 3208.6544275 | 2 |
| 3208.6546675 | 4 |
| 3208.6548225 | 3 |
| 3208.6551637 | 3 |
| 3208.6558096 | 3 |
| 3208.6558626 | 0 |
| 3208.6559494 | 4 |
| 3208.656067 | 1 |
| 3208.6565952 | 2 |
| 3208.657129 | 1 |
| 3208.6574187 | 3 |
| 3208.6576329 | 5 |
| 3208.6579945 | 0 |
| 3208.6583091 | 7 |
| 3208.658522 | 0 |
| 3208.6587355 | 1 |
| 3208.6593711 | 1 |
| 3208.6593898 | 0 |
| 3208.6593922 | 3 |
| 3208.6602717 | 3 |
| 3208.6605917 | 2 |
| 3208.6608703 | 1 |
| 3208.6609633 | 3 |
| 3208.6610504 | 2 |
| 3208.6613977 | 1 |
| 3208.6617055 | 4 |
| 3208.6622973 | 3 |
| 3208.6629117 | 2 |
| 3208.6631672 | 3 |
| 3208.6631688 | 1 |
| 3208.6634937 | 0 |
| 3208.6637288 | 1 |
| 3208.6638432 | 1 |
| 3208.6641122 | 4 |
| 3208.6649288 | 1 |
| 3208.665066 | 1 |
| 3208.6652235 | 0 |
| 3208.6656691 | 2 |
| 3208.6660627 | 0 |
| 3208.6662948 | 2 |
| 3208.6665055 | 4 |
| 3208.66696 | 1 |
| 3208.6673925 | 1 |

| | |
|---|---|
| 3208.6676811 | 1 |
| 3208.6677392 | 2 |
| 3208.6678535 | 1 |
| 3208.66801 | 1 |
| 3208.6681361 | 2 |
| 3208.6691538 | 4 |
| 3208.6693724 | 1 |
| 3208.6696582 | 3 |
| 3208.6697762 | 4 |
| 3208.670156 | 2 |
| 3208.6703131 | 6 |
| 3208.6705098 | 0 |
| 3208.6709006 | 3 |
| 3208.6711521 | 4 |
| 3208.6714157 | 1 |
| 3208.6717482 | 3 |
| 3208.6723709 | 2 |
| 3208.6727583 | 4 |
| 3208.6728067 | 3 |
| 3208.6731117 | 8 |
| 3208.6737774 | 2 |
| 3208.6737912 | 3 |
| 3208.6740001 | 2 |
| 3208.6740721 | 1 |
| 3208.6748253 | 3 |
| 3208.675117 | 2 |
| 3208.6756052 | 2 |
| 3208.675654 | 0 |
| 3208.675817 | 2 |
| 3208.6763075 | 3 |
| 3208.6763553 | 2 |
| 3208.6769792 | 1 |
| 3208.677628 | 0 |
| 3208.6779128 | 1 |
| 3208.6781171 | 0 |
| 3208.6781686 | 4 |
| 3208.6787563 | 2 |
| 3208.6787952 | 0 |
| 3208.6788038 | 1 |
| 3208.6795015 | 1 |
| 3208.6797232 | 1 |
| 3208.6800095 | 2 |
| 3208.6801024 | 2 |
| 3208.6801506 | 4 |
| 3208.6810727 | 3 |
| 3208.6817002 | 4 |
| 3208.6819786 | 2 |
| 3208.6822918 | 2 |
| 3208.6824991 | 3 |
| 3208.6828905 | 2 |
| 3208.6829 | 0 |
| 3208.6831996 | 1 |
| 3208.683746 | 1 |
| 3208.6838357 | 3 |
| 3208.684586 | 4 |
| 3208.6845892 | 3 |
| 3208.6846565 | 1 |
| 3208.685353 | 0 |
| 3208.6859444 | 2 |
| 3208.6860846 | 3 |
| 3208.6861535 | 3 |
| 3208.6865066 | 3 |
| 3208.686743 | 1 |
| 3208.6869787 | 3 |
| 3208.6876656 | 1 |
| 3208.6878144 | 1 |
| 3208.6880822 | 3 |
| 3208.688687 | 0 |
| 3208.688815 | 2 |
| 3208.6891903 | 2 |
| 3208.6892168 | 0 |
| 3208.6899367 | 1 |
| 3208.6902837 | 2 |
| 3208.6904266 | 2 |
| 3208.6904359 | 4 |
| 3208.6904668 | 3 |
| 3208.691264 | 3 |
| 3208.6915349 | 2 |
| 3208.6921477 | 2 |
| 3208.6923462 | 5 |
| 3208.6927213 | 2 |
| 3208.6927222 | 0 |
| 3208.6933589 | 2 |
| 3208.6935259 | 3 |
| 3208.694043 | 0 |
| 3208.6942966 | 2 |
| 3208.6944046 | 3 |
| 3208.6946574 | 3 |
| 3208.6947305 | 0 |
| 3208.6949458 | 2 |
| 3208.6954555 | 3 |
| 3208.6955268 | 3 |
| 3208.6959723 | 3 |
| 3208.6964246 | 1 |
| 3208.6967396 | 3 |
| 3208.6972614 | 0 |
| 3208.6973378 | 1 |
| 3208.6978188 | 0 |
| 3208.6980938 | 0 |
| 3208.6984514 | 0 |
| 3208.6990334 | 0 |
| 3208.6990882 | 1 |
| 3208.699363 | 3 |
| 3208.6994672 | 1 |
| 3208.7002276 | 2 |
| 3208.7003232 | 3 |
| 3208.7005358 | 4 |
| 3208.7005837 | 2 |
| 3208.7008186 | 5 |
| 3208.7011269 | 0 |
| 3208.7017014 | 2 |
| 3208.7017794 | 3 |
| 3208.7027523 | 4 |
| 3208.7028846 | 4 |
| 3208.7031718 | 2 |
| 3208.7035001 | 3 |
| 3208.7039077 | 1 |
| 3208.7042474 | 0 |
| 3208.7042582 | 0 |
| 3208.7044005 | 1 |
| 3208.7049046 | 3 |
| 3208.7053083 | 1 |
| 3208.7053799 | 2 |
| 3208.7053857 | 1 |
| 3208.7060212 | 0 |
| 3208.7060701 | 4 |
| 3208.7063733 | 1 |
| 3208.7069195 | 2 |
| 3208.7069631 | 4 |
| 3208.7073501 | 1 |
| 3208.7079586 | 2 |
| 3208.7081702 | 1 |
| 3208.7085667 | 1 |
| 3208.7091567 | 3 |
| 3208.7093515 | 2 |
| 3208.7095449 | 1 |
| 3208.7096366 | 4 |
| 3208.7098125 | 1 |
| 3208.7101513 | 2 |
| 3208.7104622 | 3 |
| 3208.711315 | 3 |
| 3208.7113564 | 0 |
| 3208.7114884 | 3 |
| 3208.711907 | 2 |
| 3208.7119924 | 0 |
| 3208.7122786 | 3 |
| 3208.7124359 | 2 |
| 3208.7126347 | 0 |
| 3208.7135199 | 1 |
| 3208.7142364 | 2 |
| 3208.7145026 | 3 |
| 3208.7145962 | 3 |
| 3208.7146656 | 1 |
| 3208.714868 | 4 |
| 3208.7151612 | 4 |
| 3208.7155442 | 0 |
| 3208.7159044 | 0 |
| 3208.7160867 | 1 |
| 3208.716173 | 5 |
| 3208.7161905 | 5 |
| 3208.7165048 | 0 |
| 3208.7175238 | 0 |
| 3208.717752 | 4 |
| 3208.7178395 | 3 |
| 3208.7179123 | 3 |
| 3208.7184969 | 4 |
| 3208.7186313 | 2 |
| 3208.718794 | 5 |
| 3208.7192339 | 1 |
| 3208.7199495 | 4 |
| 3208.7202603 | 5 |
| 3208.7202711 | 2 |
| 3208.7205775 | 4 |
| 3208.7209283 | 6 |
| 3208.7214073 | 1 |
| 3208.721703 | 1 |
| 3208.721805 | 4 |
| 3208.7223481 | 2 |
| 3208.7226634 | 6 |
| 3208.722794 | 5 |
| 3208.7230643 | 1 |
| 3208.7235767 | 5 |
| 3208.7236144 | 5 |
| 3208.7241003 | 1 |
| 3208.7245207 | 5 |
| 3208.7249566 | 3 |
| 3208.7253361 | 1 |
| 3208.725502 | 2 |
| 3208.7258723 | 4 |
| 3208.7259317 | 2 |
| 3208.7266425 | 2 |
| 3208.7266473 | 2 |
| 3208.7269012 | 5 |
| 3208.7270105 | 0 |
| 3208.7276258 | 2 |
| 3208.7277048 | 2 |
| 3208.7278177 | 1 |
| 3208.7281903 | 0 |
| 3208.7288962 | 4 |
| 3208.7290678 | 1 |
| 3208.7294944 | 6 |
| 3208.7296547 | 0 |
| 3208.7301776 | 2 |
| 3208.7302289 | 1 |
| 3208.730541 | 2 |
| 3208.7310777 | 1 |
| 3208.731177 | 5 |
| 3208.7316385 | 1 |
| 3208.7320686 | 2 |
| 3208.7324797 | 1 |
| 3208.7331414 | 2 |
| 3208.7331549 | 0 |
| 3208.7333661 | 0 |
| 3208.7337422 | 2 |
| 3208.7343358 | 4 |
| 3208.7343553 | 3 |
| 3208.7344543 | 1 |
| 3208.7347145 | 6 |
| 3208.7351698 | 3 |
| 3208.7357324 | 2 |
| 3208.7360807 | 2 |
| 3208.7363938 | 4 |
| 3208.7363992 | 1 |
| 3208.7369141 | 2 |
| 3208.7370186 | 3 |
| 3208.7379886 | 3 |
| 3208.7380607 | 1 |
| 3208.738288 | 0 |
| 3208.7387713 | 2 |
| 3208.7387784 | 5 |
| 3208.7390381 | 4 |
| 3208.739609 | 2 |
| 3208.73976 | 1 |
| 3208.7398352 | 4 |
| 3208.7404531 | 2 |
| 3208.7405256 | 1 |
| 3208.7406448 | 1 |
| 3208.7412512 | 6 |
| 3208.7415457 | 2 |
| 3208.7416423 | 3 |
| 3208.7417371 | 2 |
| 3208.7421943 | 2 |
| 3208.7424356 | 2 |
| 3208.7429276 | 0 |
| 3208.7431318 | 5 |
| 3208.744073 | 2 |
| 3208.7442923 | 0 |
| 3208.7443274 | 3 |
| 3208.7447334 | 2 |
| 3208.7447693 | 0 |
| 3208.7448213 | 3 |
| 3208.7449703 | 1 |
| 3208.7458357 | 2 |
| 3208.745918 | 2 |
| 3208.7461494 | 1 |
| 3208.7461789 | 7 |
| 3208.7471023 | 4 |
| 3208.7471481 | 1 |
| 3208.7471971 | 0 |
| 3208.7478463 | 0 |
| 3208.7479153 | 2 |
| 3208.7485127 | 1 |
| 3208.7487504 | 1 |
| 3208.749013 | 2 |
| 3208.749034 | 3 |
| 3208.7497546 | 2 |
| 3208.7497956 | 1 |
| 3208.7501022 | 2 |
| 3208.7507011 | 2 |
| 3208.7508297 | 1 |
| 3208.7508332 | 1 |
| 3208.7514319 | 3 |
| 3208.7518346 | 4 |
| 3208.7520236 | 2 |
| 3208.7525835 | 4 |
| 3208.7527206 | 0 |
| 3208.7528519 | 2 |
| 3208.7528694 | 1 |
| 3208.7533394 | 2 |
| 3208.7537376 | 3 |
| 3208.753988 | 3 |
| 3208.7542113 | 0 |
| 3208.7546094 | 3 |
| 3208.754908 | 2 |
| 3208.7555261 | 1 |
| 3208.7558426 | 2 |
| 3208.7560168 | 1 |
| 3208.7562822 | 3 |
| 3208.7566443 | 2 |
| 3208.7567832 | 2 |
| 3208.7571574 | 0 |
| 3208.757289 | 2 |
| 3208.7583217 | 3 |
| 3208.7586126 | 3 |
| 3208.7586133 | 3 |
| 3208.7588582 | 1 |
| 3208.7594158 | 6 |
| 3208.7595208 | 1 |
| 3208.7597851 | 1 |
| 3208.7602879 | 2 |
| 3208.7605584 | 5 |
| 3208.7605765 | 2 |
| 3208.7613975 | 1 |
| 3208.7615076 | 2 |
| 3208.761669 | 3 |
| 3208.7617306 | 1 |
| 3208.7618138 | 0 |
| 3208.7623269 | 3 |
| 3208.7626488 | 2 |
| 3208.7628541 | 1 |
| 3208.7631706 | 1 |
| 3208.7639102 | 1 |
| 3208.7639528 | 1 |
| 3208.7644363 | 3 |
| 3208.7647639 | 1 |
| 3208.7647767 | 2 |
| 3208.7652177 | 1 |
| 3208.7652515 | 1 |
| 3208.7660762 | 1 |
| 3208.7663473 | 3 |
| 3208.7665284 | 1 |
| 3208.7666833 | 5 |
| 3208.7672918 | 4 |
| 3208.7674016 | 0 |
| 3208.7676172 | 2 |
| 3208.7676206 | 2 |
| 3208.7682435 | 2 |
| 3208.7687661 | 4 |
| 3208.7689689 | 2 |
| 3208.7691368 | 4 |
| 3208.7697605 | 1 |
| 3208.7702106 | 0 |
| 3208.7704136 | 3 |
| 3208.7705154 | 4 |
| 3208.7710057 | 2 |
| 3208.7713896 | 2 |
| 3208.7714118 | 3 |
| 3208.771626 | 4 |
| 3208.7723948 | 2 |
| 3208.7725256 | 5 |
| 3208.7725574 | 2 |
| 3208.7731394 | 1 |
| 3208.7732234 | 3 |
| 3208.7733833 | 1 |
| 3208.7740674 | 2 |
| 3208.7744692 | 1 |
| 3208.7748508 | 3 |
| 3208.7751407 | 4 |
| 3208.7752468 | 2 |
| 3208.7755414 | 2 |
| 3208.7759477 | 3 |
| 3208.7760984 | 2 |
| 3208.7768641 | 1 |
| 3208.7773367 | 2 |
| 3208.7774782 | 0 |
| 3208.7776911 | 2 |
| 3208.7776973 | 2 |
| 3208.7777231 | 2 |
| 3208.7790672 | 2 |
| 3208.7792752 | 1 |
| 3208.7796576 | 2 |
| 3208.7797053 | 2 |
| 3208.7800244 | 2 |

| | |
|---|---|
| 3208.7800328 | 2 |
| 3208.7802952 | 1 |
| 3208.7803203 | 3 |
| 3208.780918 | 2 |
| 3208.7816286 | 5 |
| 3208.7816671 | 0 |
| 3208.7819572 | 3 |
| 3208.7820983 | 4 |
| 3208.7826421 | 3 |
| 3208.7826575 | 1 |
| 3208.7831366 | 0 |
| 3208.7831993 | 0 |
| 3208.7843668 | 0 |
| 3208.7844959 | 2 |
| 3208.7845578 | 0 |
| 3208.7849038 | 4 |
| 3208.7849955 | 4 |
| 3208.7851528 | 2 |
| 3208.7857376 | 1 |
| 3208.7857638 | 0 |
| 3208.7862433 | 1 |
| 3208.7863922 | 1 |
| 3208.7864251 | 3 |
| 3208.7873703 | 0 |
| 3208.7874145 | 3 |
| 3208.7876228 | 2 |
| 3208.7882549 | 2 |
| 3208.7885076 | 3 |
| 3208.7891452 | 5 |
| 3208.7893745 | 3 |
| 3208.7896192 | 1 |
| 3208.7898379 | 1 |
| 3208.7900532 | 2 |
| 3208.7907368 | 2 |
| 3208.7907814 | 1 |
| 3208.7910904 | 2 |
| 3208.7911242 | 1 |
| 3208.7912105 | 2 |
| 3208.792008 | 2 |
| 3208.7924406 | 0 |
| 3208.7926536 | 3 |
| 3208.7927004 | 2 |
| 3208.7927729 | 1 |
| 3208.7934667 | 0 |
| 3208.7936389 | 4 |
| 3208.7937666 | 4 |
| 3208.7944274 | 1 |
| 3208.7947405 | 0 |
| 3208.7947625 | 1 |
| 3208.7949957 | 2 |
| 3208.7954988 | 0 |
| 3208.7955183 | 3 |
| 3208.7961209 | 0 |
| 3208.7964038 | 5 |
| 3208.7966768 | 0 |
| 3208.7968033 | 4 |
| 3208.7973915 | 5 |
| 3208.7974873 | 4 |
| 3208.7978448 | 3 |
| 3208.7979301 | 3 |
| 3208.7983639 | 4 |
| 3208.7983935 | 3 |
| 3208.799074 | 10 |
| 3208.7992072 | 5 |
| 3208.7995513 | 8 |
| 3208.7996067 | 7 |
| 3208.8001234 | 12 |
| 3208.8001495 | 6 |
| 3208.800518 | 7 |
| 3208.8013903 | 6 |
| 3208.8015941 | 6 |
| 3208.8019062 | 11 |
| 3208.8021791 | 5 |
| 3208.802667 | 7 |
| 3208.80276 | 7 |
| 3208.8031669 | 6 |
| 3208.8034987 | 5 |
| 3208.8035663 | 2 |
| 3208.8039769 | 7 |
| 3208.8040826 | 7 |
| 3208.804748 | 4 |
| 3208.8047655 | 3 |
| 3208.8053448 | 4 |
| 3208.8055486 | 5 |
| 3208.8055862 | 5 |
| 3208.8062208 | 5 |
| 3208.8066683 | 7 |
| 3208.8067206 | 8 |
| 3208.8070166 | 8 |
| 3208.8072498 | 7 |
| 3208.8080456 | 6 |
| 3208.8083155 | 3 |
| 3208.8086011 | 5 |
| 3208.8086803 | 3 |
| 3208.808937 | 4 |
| 3208.8090624 | 0 |
| 3208.8097915 | 1 |
| 3208.8098366 | 3 |
| 3208.8098567 | 4 |
| 3208.8098784 | 2 |
| 3208.8102858 | 4 |
| 3208.8114026 | 1 |
| 3208.811527 | 3 |
| 3208.8117093 | 1 |
| 3208.8118495 | 4 |
| 3208.8124428 | 1 |
| 3208.812462 | 5 |
| 3208.8128772 | 0 |
| 3208.8134344 | 3 |
| 3208.8138218 | 1 |
| 3208.8146946 | 1 |
| 3208.8156078 | 1 |
| 3208.8157252 | 2 |
| 3208.8159958 | 0 |
| 3208.8174545 | 1 |
| 3208.817464 | 0 |
| 3208.8184613 | 0 |
| 3208.8192155 | 5 |
| 3208.8195666 | 2 |
| 3208.8205503 | 3 |
| 3208.8206092 | 0 |
| 3208.8207745 | 0 |
| 3208.8220546 | 1 |
| 3208.8223658 | 1 |
| 3208.8234636 | 2 |
| 3208.8238927 | 2 |
| 3208.8250909 | 5 |
| 3208.8254624 | 2 |
| 3208.8259437 | 0 |
| 3208.8259994 | 3 |
| 3208.826992 | 3 |
| 3208.8273811 | 1 |
| 3208.8288337 | 1 |
| 3208.8292059 | 0 |
| 3208.8297807 | 1 |
| 3208.8310696 | 0 |
| 3208.8310823 | 3 |
| 3208.8316817 | 1 |
| 3208.8320838 | 1 |
| 3208.8328306 | 2 |
| 3208.8331326 | 4 |
| 3208.8340672 | 1 |
| 3208.8348912 | 1 |
| 3208.8353465 | 3 |
| 3208.8357649 | 4 |
| 3208.8359371 | 1 |
| 3208.8371439 | 1 |
| 3208.8378138 | 3 |
| 3208.8384014 | 0 |
| 3208.8390583 | 1 |
| 3208.8392457 | 0 |
| 3208.8402695 | 1 |
| 3208.8407311 | 2 |
| 3208.8413985 | 1 |
| 3208.8422128 | 2 |
| 3208.8424872 | 1 |
| 3208.8433985 | 3 |
| 3208.8439151 | 1 |
| 3208.8444049 | 3 |
| 3208.8451949 | 1 |
| 3208.8454832 | 1 |
| 3208.8465414 | 1 |
| 3208.8468432 | 1 |
| 3208.8473739 | 1 |
| 3208.8484635 | 0 |
| 3208.8486619 | 4 |
| 3208.8490506 | 2 |
| 3208.8492714 | 2 |
| 3208.8506768 | 1 |
| 3208.8513549 | 1 |
| 3208.851488 | 1 |
| 3208.85217 | 1 |
| 3208.853101 | 3 |
| 3208.8539827 | 0 |
| 3208.8543059 | 0 |
| 3208.8546286 | 1 |
| 3208.8555026 | 0 |
| 3208.8559675 | 0 |
| 3208.8568512 | 3 |
| 3208.8569672 | 0 |
| 3208.8576419 | 2 |
| 3208.8586331 | 0 |
| 3208.858779 | 4 |
| 3208.8595856 | 1 |
| 3208.8604146 | 1 |
| 3208.8606586 | 1 |
| 3208.8615263 | 2 |
| 3208.8618845 | 1 |
| 3208.8629008 | 1 |
| 3208.8635252 | 2 |
| 3208.8640948 | 5 |
| 3208.864365 | 1 |
| 3208.8650634 | 2 |
| 3208.8664029 | 1 |
| 3208.8665291 | 0 |
| 3208.8668834 | 3 |
| 3208.8684465 | 1 |
| 3208.8685755 | 2 |
| 3208.8693013 | 3 |
| 3208.8695194 | 1 |
| 3208.8703893 | 1 |
| 3208.8709478 | 2 |
| 3208.8713938 | 2 |
| 3208.8718858 | 3 |
| 3208.8724071 | 4 |
| 3208.8732324 | 4 |
| 3208.8737454 | 1 |
| 3208.8745971 | 2 |
| 3208.8755774 | 1 |
| 3208.8760438 | 2 |
| 3208.8764672 | 0 |
| 3208.877063 | 3 |
| 3208.8779344 | 2 |
| 3208.8784366 | 1 |
| 3208.8786727 | 1 |
| 3208.879541 | 4 |
| 3208.8804235 | 1 |
| 3208.8805511 | 1 |
| 3208.8818276 | 0 |
| 3208.8819574 | 0 |
| 3208.8822672 | 4 |
| 3208.8834348 | 2 |
| 3208.8838904 | 2 |
| 3208.884632 | 0 |
| 3208.8851058 | 1 |
| 3208.8858386 | 4 |
| 3208.8864634 | 1 |
| 3208.8869788 | 1 |
| 3208.8876597 | 2 |
| 3208.8881458 | 1 |
| 3208.8886368 | 3 |
| 3208.8892805 | 2 |
| 3208.8894531 | 0 |
| 3208.8904985 | 2 |
| 3208.8913076 | 2 |
| 3208.8920862 | 4 |
| 3208.8922289 | 2 |
| 3208.892861 | 2 |
| 3208.8933439 | 2 |
| 3208.8938247 | 0 |
| 3208.8947461 | 2 |
| 3208.8956244 | 4 |
| 3208.8962039 | 0 |
| 3208.8967519 | 2 |
| 3208.8969333 | 4 |
| 3208.8980128 | 0 |
| 3208.8983025 | 2 |
| 3208.8990736 | 3 |
| 3208.8994169 | 1 |
| 3208.8998962 | 5 |
| 3208.9005465 | 2 |
| 3208.901229 | 4 |
| 3208.9019704 | 3 |
| 3208.9025401 | 1 |
| 3208.9034843 | 2 |
| 3208.9040395 | 6 |
| 3208.9043362 | 1 |
| 3208.9046727 | 3 |
| 3208.9050964 | 1 |
| 3208.9064841 | 2 |
| 3208.9071858 | 3 |
| 3208.9083347 | 3 |
| 3208.908531 | 3 |
| 3208.9085397 | 1 |
| 3208.9095731 | 3 |
| 3208.9101131 | 0 |
| 3208.9107123 | 0 |
| 3208.9114026 | 1 |
| 3208.9121948 | 3 |
| 3208.9125167 | 1 |
| 3208.9136396 | 1 |
| 3208.9141915 | 2 |
| 3208.9151186 | 0 |
| 3208.9153944 | 2 |
| 3208.9159831 | 0 |
| 3208.9163366 | 2 |
| 3208.916961 | 1 |
| 3208.9176164 | 4 |
| 3208.9178958 | 1 |
| 3208.9191145 | 3 |
| 3208.9197686 | 1 |
| 3208.920324 | 2 |
| 3208.9208112 | 1 |
| 3208.9213565 | 1 |
| 3208.9217879 | 1 |
| 3208.9231979 | 1 |
| 3208.9235397 | 1 |
| 3208.9236968 | 0 |
| 3208.9243925 | 1 |
| 3208.9250561 | 2 |
| 3208.9254906 | 0 |
| 3208.9258419 | 2 |
| 3208.927499 | 0 |
| 3208.9276221 | 5 |
| 3208.9281264 | 1 |
| 3208.9288121 | 2 |
| 3208.9295423 | 3 |
| 3208.9300709 | 0 |
| 3208.9306102 | 2 |
| 3208.9317268 | 3 |
| 3208.9319416 | 0 |
| 3208.9324968 | 3 |
| 3208.9331741 | 2 |
| 3208.9338919 | 2 |
| 3208.9344056 | 1 |
| 3208.935483 | 1 |
| 3208.935673 | 1 |
| 3208.9358831 | 3 |
| 3208.9371121 | 2 |
| 3208.9373247 | 0 |
| 3208.9373592 | 1 |
| 3208.9383765 | 2 |
| 3208.9395194 | 5 |
| 3208.939689 | 3 |
| 3208.9406161 | 2 |
| 3208.9413438 | 1 |
| 3208.9420835 | 0 |
| 3208.9422289 | 2 |
| 3208.943018 | 1 |
| 3208.9436172 | 3 |
| 3208.9438801 | 1 |
| 3208.9452627 | 1 |
| 3208.9453514 | 1 |
| 3208.9455517 | 3 |
| 3208.9458042 | 3 |
| 3208.9462688 | 2 |
| 3208.9468745 | 1 |
| 3208.9473982 | 3 |
| 3208.9479312 | 0 |
| 3208.9484237 | 3 |
| 3208.9492338 | 2 |
| 3208.94999 | 4 |
| 3208.950158 | 1 |
| 3208.9507222 | 2 |
| 3208.9509254 | 2 |
| 3208.9515347 | 0 |
| 3208.9518385 | 1 |
| 3208.951947 | 1 |
| 3208.952463 | 3 |
| 3208.9530308 | 1 |
| 3208.9539208 | 2 |
| 3208.9542345 | 0 |
| 3208.9547408 | 0 |
| 3208.9551251 | 2 |
| 3208.9552355 | 2 |
| 3208.9560066 | 2 |
| 3208.9563208 | 1 |
| 3208.9567226 | 1 |
| 3208.957589 | 5 |
| 3208.9583154 | 5 |
| 3208.9588444 | 1 |
| 3208.958909 | 2 |
| 3208.958921 | 1 |
| 3208.9589945 | 1 |
| 3208.9606518 | 1 |
| 3208.960749 | 3 |
| 3208.9609358 | 1 |
| 3208.9619592 | 2 |
| 3208.9622773 | 2 |
| 3208.9622946 | 2 |
| 3208.9633792 | 1 |
| 3208.9637322 | 1 |
| 3208.9639377 | 2 |

| | |
|---|---|
| 3208.9646458 | 3 |
| 3208.9648616 | 2 |
| 3208.9655134 | 1 |
| 3208.9662835 | 4 |
| 3208.9669658 | 3 |
| 3208.9670265 | 3 |
| 3208.9674091 | 0 |
| 3208.9678842 | 0 |
| 3208.9681794 | 2 |
| 3208.9687508 | 5 |
| 3208.9692131 | 4 |
| 3208.9694998 | 3 |
| 3208.9702951 | 4 |
| 3208.970428 | 0 |
| 3208.9710667 | 0 |
| 3208.9713556 | 0 |
| 3208.971466 | 0 |
| 3208.9722212 | 2 |
| 3208.9727399 | 1 |
| 3208.9734647 | 0 |
| 3208.9742553 | 2 |
| 3208.9744684 | 2 |
| 3208.9745206 | 1 |
| 3208.9749422 | 2 |
| 3208.9752999 | 2 |
| 3208.9755997 | 2 |
| 3208.9763185 | 2 |
| 3208.9767971 | 1 |
| 3208.9774997 | 2 |
| 3208.9779839 | 3 |
| 3208.9782682 | 1 |
| 3208.9789056 | 2 |
| 3208.9794403 | 3 |
| 3208.9794736 | 3 |
| 3208.9801951 | 0 |
| 3208.9805945 | 2 |
| 3208.9808328 | 2 |
| 3208.9811089 | 2 |
| 3208.9817309 | 1 |
| 3208.9827347 | 1 |
| 3208.9829824 | 1 |
| 3208.9830892 | 4 |
| 3208.9836276 | 4 |
| 3208.9843748 | 4 |
| 3208.9846481 | 2 |
| 3208.9850861 | 2 |
| 3208.9859724 | 1 |
| 3208.9861667 | 1 |
| 3208.9867067 | 4 |
| 3208.9871429 | 3 |
| 3208.9873825 | 1 |
| 3208.9876768 | 3 |
| 3208.9884609 | 5 |
| 3208.9891415 | 2 |
| 3208.9894957 | 4 |
| 3208.9895464 | 0 |
| 3208.9898965 | 3 |
| 3208.9905047 | 3 |
| 3208.9908387 | 0 |
| 3208.9916399 | 0 |
| 3208.9923276 | 1 |
| 3208.9926419 | 0 |
| 3208.9933214 | 2 |
| 3208.9938745 | 3 |
| 3208.9943402 | 3 |
| 3208.9946588 | 1 |
| 3208.995012 | 0 |
| 3208.9954114 | 1 |
| 3208.9959439 | 2 |
| 3208.9967195 | 4 |
| 3208.9972324 | 4 |
| 3208.9973264 | 1 |
| 3208.9977922 | 0 |
| 3208.9979127 | 1 |
| 3208.9986345 | 2 |
| 3208.9989151 | 2 |
| 3208.9992894 | 2 |
| 3208.999735 | 3 |
| 3209.0000012 | 2 |
| 3209.0004929 | 0 |
| 3209.0010454 | 1 |
| 3209.0014277 | 1 |
| 3209.0020636 | 1 |
| 3209.0020866 | 2 |
| 3209.0028362 | 0 |
| 3209.0034123 | 3 |
| 3209.00351 | 1 |
| 3209.0044047 | 1 |
| 3209.004892 | 4 |
| 3209.0050374 | 1 |
| 3209.0057132 | 0 |
| 3209.0062937 | 3 |
| 3209.0069957 | 2 |
| 3209.0071354 | 3 |
| 3209.0075147 | 4 |
| 3209.0086865 | 1 |
| 3209.0088505 | 2 |
| 3209.008886 | 1 |
| 3209.0090231 | 1 |
| 3209.0099901 | 2 |
| 3209.0101792 | 1 |
| 3209.0106819 | 3 |
| 3209.0108464 | 2 |
| 3209.0108803 | 1 |
| 3209.011022 | 1 |
| 3209.0122143 | 0 |
| 3209.0125762 | 4 |
| 3209.0126246 | 1 |
| 3209.0128618 | 1 |
| 3209.0130595 | 4 |
| 3209.0141913 | 3 |
| 3209.0168625 | 1 |
| 3209.0175373 | 1 |
| 3209.0204053 | 2 |
| 3209.0214885 | 2 |
| 3209.0237317 | 7 |
| 3209.0263272 | 3 |
| 3209.0271531 | 2 |
| 3209.0294334 | 2 |
| 3209.0309963 | 2 |
| 3209.0330603 | 1 |
| 3209.0353976 | 4 |
| 3209.037113 | 1 |
| 3209.0395567 | 2 |
| 3209.0404785 | 1 |
| 3209.0420175 | 4 |
| 3209.0451341 | 0 |
| 3209.0464473 | 4 |
| 3209.0484853 | 2 |
| 3209.0495409 | 1 |
| 3209.0522122 | 2 |
| 3209.054746 | 0 |
| 3209.0549728 | 3 |
| 3209.0572289 | 1 |
| 3209.0599277 | 0 |
| 3209.0606157 | 1 |
| 3209.0633914 | 1 |
| 3209.064853 | 2 |
| 3209.0666408 | 1 |
| 3209.0689494 | 2 |
| 3209.0701409 | 1 |
| 3209.0729184 | 0 |
| 3209.0736197 | 2 |
| 3209.0754965 | 1 |
| 3209.0767199 | 1 |
| 3209.0801561 | 3 |
| 3209.0818717 | 0 |
| 3209.0832118 | 1 |
| 3209.0857943 | 3 |
| 3209.0865955 | 2 |
| 3209.0890512 | 2 |
| 3209.0917719 | 3 |
| 3209.0925854 | 4 |
| 3209.0945899 | 2 |
| 3209.0961618 | 1 |
| 3209.0988168 | 2 |
| 3209.099822 | 4 |
| 3209.1021676 | 0 |
| 3209.1044841 | 3 |
| 3209.1059395 | 2 |
| 3209.1077888 | 0 |
| 3209.1106824 | 0 |
| 3209.1116368 | 1 |
| 3209.1135623 | 2 |
| 3209.1152397 | 1 |
| 3209.1175699 | 0 |
| 3209.1192877 | 1 |
| 3209.1205859 | 3 |
| 3209.1235375 | 2 |
| 3209.1240501 | 5 |
| 3209.1262653 | 6 |
| 3209.1289658 | 1 |
| 3209.1296805 | 1 |
| 3209.1324385 | 2 |
| 3209.1336414 | 1 |
| 3209.1362827 | 3 |
| 3209.1365453 | 1 |
| 3209.1392218 | 2 |
| 3209.1414316 | 3 |
| 3209.143048 | 2 |
| 3209.1459666 | 1 |
| 3209.1476567 | 1 |
| 3209.1493814 | 4 |
| 3209.1516992 | 1 |
| 3209.1525437 | 4 |
| 3209.1553822 | 1 |
| 3209.1564327 | 1 |
| 3209.1584694 | 3 |
| 3209.1611482 | 0 |
| 3209.162407 | 3 |
| 3209.1639064 | 2 |
| 3209.165783 | 0 |
| 3209.1679649 | 2 |
| 3209.1702374 | 1 |
| 3209.1714636 | 1 |
| 3209.1740627 | 2 |
| 3209.1750313 | 3 |
| 3209.1773963 | 1 |
| 3209.1793379 | 1 |
| 3209.1806956 | 1 |
| 3209.1839477 | 0 |
| 3209.1842733 | 5 |
| 3209.1868677 | 5 |
| 3209.1889887 | 0 |
| 3209.1904234 | 6 |
| 3209.1931763 | 2 |
| 3209.1941772 | 2 |
| 3209.1964257 | 2 |
| 3209.1988843 | 5 |
| 3209.1997909 | 1 |
| 3209.2025779 | 3 |
| 3209.2033049 | 2 |
| 3209.2054276 | 1 |
| 3209.2068127 | 2 |
| 3209.2091712 | 3 |
| 3209.2114525 | 0 |
| 3209.2127945 | 2 |
| 3209.2156753 | 4 |
| 3209.2168746 | 2 |
| 3209.2191378 | 4 |
| 3209.2207605 | 1 |
| 3209.2217844 | 1 |
| 3209.2232904 | 3 |
| 3209.2267415 | 0 |
| 3209.2278593 | 1 |
| 3209.2303547 | 3 |
| 3209.2311737 | 1 |
| 3209.2332061 | 1 |
| 3209.2363099 | 2 |
| 3209.2372916 | 1 |
| 3209.2394362 | 3 |
| 3209.2406859 | 4 |
| 3209.243248 | 4 |
| 3209.2457241 | 4 |
| 3209.2469894 | 1 |
| 3209.2491357 | 3 |
| 3209.2503829 | 5 |
| 3209.2532184 | 1 |
| 3209.2533114 | 2 |
| 3209.2557078 | 1 |
| 3209.2587069 | 5 |
| 3209.2600818 | 3 |
| 3209.2619546 | 3 |
| 3209.263927 | 2 |
| 3209.26581 | 1 |
| 3209.268282 | 3 |
| 3209.2693033 | 5 |
| 3209.2719987 | 6 |
| 3209.2722331 | 5 |
| 3209.2749035 | 3 |
| 3209.277722 | 2 |
| 3209.2788453 | 2 |
| 3209.281481 | 2 |
| 3209.2823425 | 1 |
| 3209.2848242 | 0 |
| 3209.2872759 | 1 |
| 3209.2883153 | 3 |
| 3209.2902711 | 3 |
| 3209.2916621 | 1 |
| 3209.2943576 | 2 |
| 3209.2954813 | 3 |
| 3209.2974342 | 1 |
| 3209.3001791 | 2 |
| 3209.3006992 | 6 |
| 3209.3032005 | 2 |
| 3209.3045499 | 2 |
| 3209.3074361 | 5 |
| 3209.3096036 | 2 |
| 3209.309915 | 5 |
| 3209.3128551 | 1 |
| 3209.313776 | 4 |
| 3209.3154675 | 0 |
| 3209.3184636 | 0 |
| 3209.319723 | 6 |
| 3209.3221816 | 2 |
| 3209.3234332 | 3 |
| 3209.3261271 | 2 |
| 3209.3271317 | 0 |
| 3209.3294276 | 0 |
| 3209.3315744 | 1 |
| 3209.3331353 | 4 |
| 3209.3357282 | 1 |
| 3209.3374679 | 0 |
| 3209.3390362 | 2 |
| 3209.3411883 | 1 |
| 3209.3427491 | 2 |
| 3209.3439619 | 4 |
| 3209.3457505 | 3 |
| 3209.3477519 | 3 |
| 3209.3504421 | 3 |
| 3209.3514964 | 3 |
| 3209.353647 | 2 |
| 3209.3545573 | 3 |
| 3209.3572566 | 1 |
| 3209.3599908 | 1 |
| 3209.3605847 | 2 |
| 3209.3630523 | 3 |
| 3209.3646593 | 1 |
| 3209.3670718 | 3 |
| 3209.3693529 | 2 |
| 3209.370045 | 2 |
| 3209.3734006 | 4 |
| 3209.3740266 | 2 |
| 3209.3758228 | 1 |
| 3209.37776 | 2 |
| 3209.3804259 | 3 |
| 3209.3822042 | 1 |
| 3209.3843663 | 2 |
| 3209.385653 | 1 |
| 3209.3881345 | 3 |
| 3209.3889448 | 5 |
| 3209.3914958 | 1 |
| 3209.3928379 | 2 |
| 3209.3947913 | 3 |
| 3209.3965044 | 2 |
| 3209.3981049 | 2 |
| 3209.4013043 | 4 |
| 3209.4020887 | 4 |
| 3209.4046763 | 3 |
| 3209.4054541 | 1 |
| 3209.4080026 | 0 |
| 3209.4102199 | 3 |
| 3209.4116198 | 3 |
| 3209.4137888 | 0 |
| 3209.4164595 | 3 |
| 3209.4172657 | 2 |
| 3209.4200487 | 0 |
| 3209.4205808 | 4 |
| 3209.423673 | 3 |
| 3209.4254406 | 1 |
| 3209.4265675 | 1 |
| 3209.4297047 | 6 |
| 3209.4300753 | 3 |
| 3209.4323677 | 1 |
| 3209.435122 | 2 |
| 3209.4358377 | 2 |
| 3209.4376107 | 1 |
| 3209.4397976 | 0 |
| 3209.4409083 | 0 |
| 3209.443144 | 3 |
| 3209.4452869 | 0 |
| 3209.4476486 | 0 |
| 3209.4492572 | 2 |
| 3209.4509078 | 3 |
| 3209.4520674 | 1 |
| 3209.4551406 | 0 |
| 3209.4566065 | 1 |
| 3209.4580032 | 0 |
| 3209.4601062 | 2 |
| 3209.4622653 | 3 |
| 3209.4640194 | 2 |
| 3209.466836 | 3 |
| 3209.4668851 | 2 |
| 3209.4699588 | 2 |
| 3209.4704484 | 3 |
| 3209.4729991 | 3 |
| 3209.4760784 | 5 |
| 3209.4768462 | 2 |
| 3209.4795002 | 2 |
| 3209.480435 | 1 |
| 3209.4826706 | 1 |
| 3209.4843746 | 1 |
| 3209.4857351 | 0 |

| | |
|---|---|
| 3209.488538 | 3 |
| 3209.4894206 | 1 |
| 3209.4919604 | 2 |
| 3209.4935417 | 1 |
| 3209.4949263 | 1 |
| 3209.4978008 | 0 |
| 3209.4987521 | 3 |
| 3209.5009648 | 2 |
| 3209.5018167 | 0 |
| 3209.5042765 | 2 |
| 3209.507382 | 0 |
| 3209.5080225 | 3 |
| 3209.5103807 | 1 |
| 3209.5132339 | 0 |
| 3209.5139982 | 2 |
| 3209.5162995 | 0 |
| 3209.5177145 | 2 |
| 3209.5199931 | 2 |
| 3209.5211642 | 1 |
| 3209.5235417 | 4 |
| 3209.525767 | 2 |
| 3209.5274516 | 4 |
| 3209.5294755 | 2 |
| 3209.5303232 | 1 |
| 3209.5322158 | 2 |
| 3209.5344087 | 2 |
| 3209.5356989 | 2 |
| 3209.5379074 | 1 |
| 3209.5399279 | 1 |
| 3209.5414445 | 0 |
| 3209.5440735 | 7 |
| 3209.5451721 | 2 |
| 3209.5479058 | 5 |
| 3209.5493208 | 1 |
| 3209.5523306 | 3 |
| 3209.5525423 | 2 |
| 3209.5552984 | 1 |
| 3209.5576407 | 3 |
| 3209.5583516 | 0 |
| 3209.5610391 | 0 |
| 3209.5616909 | 2 |
| 3209.5637946 | 1 |
| 3209.5663683 | 2 |
| 3209.5678489 | 2 |
| 3209.5701951 | 2 |
| 3209.5713372 | 2 |
| 3209.5732327 | 3 |
| 3209.574214 | 2 |
| 3209.5769287 | 0 |
| 3209.5793224 | 3 |
| 3209.5805725 | 3 |
| 3209.582981 | 3 |
| 3209.5846005 | 2 |
| 3209.5862879 | 3 |
| 3209.588428 | 2 |
| 3209.589069 | 1 |
| 3209.5922286 | 2 |
| 3209.5945885 | 5 |
| 3209.5952486 | 2 |
| 3209.5981587 | 2 |
| 3209.5987771 | 0 |
| 3209.6011417 | 3 |
| 3209.6038772 | 4 |
| 3209.6057024 | 1 |
| 3209.6071413 | 1 |
| 3209.60859 | 4 |
| 3209.6107047 | 1 |
| 3209.6120211 | 4 |
| 3209.6146916 | 3 |
| 3209.6166936 | 2 |
| 3209.6184193 | 0 |
| 3209.6200547 | 0 |
| 3209.621363 | 4 |
| 3209.6234169 | 1 |
| 3209.6259181 | 3 |
| 3209.6268539 | 0 |
| 3209.6283341 | 2 |
| 3209.6303251 | 2 |
| 3209.6332128 | 0 |
| 3209.6345687 | 3 |
| 3209.6367555 | 0 |
| 3209.6387512 | 3 |
| 3209.6400744 | 1 |
| 3209.6418103 | 1 |
| 3209.6437144 | 2 |
| 3209.646049 | 2 |
| 3209.6481181 | 2 |
| 3209.6492014 | 1 |
| 3209.6513117 | 2 |
| 3209.6536509 | 1 |
| 3209.6547548 | 3 |
| 3209.656775 | 0 |
| 3209.6582416 | 1 |
| 3209.6612411 | 2 |
| 3209.6633529 | 2 |
| 3209.6635369 | 3 |
| 3209.6663025 | 1 |
| 3209.6675068 | 3 |
| 3209.6701275 | 3 |
| 3209.6716615 | 1 |
| 3209.6735103 | 1 |
| 3209.6750265 | 4 |
| 3209.6768158 | 1 |
| 3209.6796382 | 1 |
| 3209.6806485 | 4 |
| 3209.6830537 | 2 |
| 3209.6852093 | 2 |
| 3209.6865348 | 3 |
| 3209.6890094 | 1 |
| 3209.6898042 | 0 |
| 3209.692095 | 4 |
| 3209.6944404 | 3 |
| 3209.6959633 | 0 |
| 3209.6982989 | 7 |
| 3209.6983555 | 3 |
| 3209.7016599 | 2 |
| 3209.7041065 | 0 |
| 3209.704342 | 4 |
| 3209.7073278 | 8 |
| 3209.7084273 | 7 |
| 3209.7104858 | 3 |
| 3209.7127583 | 1 |
| 3209.7138081 | 2 |
| 3209.7163627 | 2 |
| 3209.7173089 | 2 |
| 3209.7201335 | 2 |
| 3209.7222877 | 3 |
| 3209.7235323 | 2 |
| 3209.7258803 | 1 |
| 3209.7278443 | 4 |
| 3209.7293487 | 1 |
| 3209.7303004 | 2 |
| 3209.7328394 | 5 |
| 3209.7352332 | 1 |
| 3209.7363087 | 2 |
| 3209.738613 | 4 |
| 3209.7404312 | 2 |
| 3209.7426032 | 1 |
| 3209.7448786 | 2 |
| 3209.7458654 | 1 |
| 3209.7482388 | 2 |
| 3209.7493125 | 2 |
| 3209.7515318 | 1 |
| 3209.754151 | 4 |
| 3209.7548396 | 2 |
| 3209.7577687 | 2 |
| 3209.7596771 | 1 |
| 3209.7607095 | 2 |
| 3209.7631413 | 1 |
| 3209.7642828 | 5 |
| 3209.7666122 | 1 |
| 3209.7683586 | 3 |
| 3209.7703005 | 1 |
| 3209.7724198 | 3 |
| 3209.7733403 | 0 |
| 3209.7761139 | 3 |
| 3209.7773002 | 4 |
| 3209.7796729 | 3 |
| 3209.7821595 | 1 |
| 3209.7832912 | 3 |
| 3209.7860009 | 0 |
| 3209.7875642 | 3 |
| 3209.789452 | 1 |
| 3209.7906694 | 1 |
| 3209.7933889 | 2 |
| 3209.7957919 | 0 |
| 3209.7962816 | 3 |
| 3209.7984285 | 1 |
| 3209.8004398 | 0 |
| 3209.8018693 | 4 |
| 3209.8043499 | 3 |
| 3209.8058227 | 5 |
| 3209.80808 | 0 |
| 3209.8091014 | 1 |
| 3209.8121593 | 2 |
| 3209.8139179 | 2 |
| 3209.8152755 | 0 |
| 3209.8180359 | 1 |
| 3209.8185584 | 2 |
| 3209.8206518 | 4 |
| 3209.8243624 | 3 |
| 3209.824736 | 3 |
| 3209.8274631 | 3 |
| 3209.8286897 | 3 |
| 3209.8314275 | 2 |
| 3209.8317549 | 1 |
| 3209.8354968 | 2 |
| 3209.8369257 | 1 |
| 3209.8383462 | 1 |
| 3209.841152 | 1 |
| 3209.8419113 | 0 |
| 3209.8444993 | 4 |
| 3209.8464647 | 2 |
| 3209.8476828 | 1 |
| 3209.849975 | 0 |
| 3209.8514006 | 3 |
| 3209.8539036 | 6 |
| 3209.8545227 | 1 |
| 3209.8566974 | 2 |
| 3209.859285 | 2 |
| 3209.8602692 | 0 |
| 3209.8630024 | 0 |
| 3209.8663924 | 2 |
| 3209.8665239 | 3 |
| 3209.8687809 | 2 |
| 3209.8700772 | 7 |
| 3209.8723168 | 1 |
| 3209.8738978 | 4 |
| 3209.875941 | 2 |
| 3209.878878 | 3 |
| 3209.8798784 | 1 |
| 3209.8815901 | 3 |
| 3209.883559 | 1 |
| 3209.8856038 | 2 |
| 3209.8874883 | 4 |
| 3209.8891042 | 0 |
| 3209.8910154 | 3 |
| 3209.892723 | 1 |
| 3209.8950595 | 1 |
| 3209.8973986 | 1 |
| 3209.8986539 | 1 |
| 3209.9002371 | 6 |
| 3209.9010225 | 3 |
| 3209.9021681 | 1 |
| 3209.9022936 | 1 |
| 3209.9027585 | 2 |
| 3209.9044457 | 3 |
| 3209.9049511 | 2 |
| 3209.9054716 | 2 |
| 3209.9065207 | 2 |
| 3209.9076147 | 1 |
| 3209.9080665 | 2 |
| 3209.9091975 | 2 |
| 3209.910633 | 1 |
| 3209.9109961 | 2 |
| 3209.9114823 | 0 |
| 3209.9117873 | 5 |
| 3209.9132363 | 4 |
| 3209.914095 | 2 |
| 3209.9143123 | 1 |
| 3209.9147544 | 2 |
| 3209.9162726 | 4 |
| 3209.9169823 | 1 |
| 3209.9179839 | 3 |
| 3209.9183412 | 1 |
| 3209.9188633 | 2 |
| 3209.9190089 | 3 |
| 3209.9206958 | 1 |
| 3209.9209551 | 2 |
| 3209.9230552 | 2 |
| 3209.924402 | 5 |
| 3209.9261998 | 1 |
| 3209.9281304 | 3 |
| 3209.9283889 | 5 |
| 3209.9302047 | 3 |
| 3209.9309782 | 3 |
| 3209.9331258 | 3 |
| 3209.9347477 | 1 |
| 3209.9358689 | 2 |
| 3209.9375536 | 3 |
| 3209.9387914 | 1 |
| 3209.9401877 | 2 |
| 3209.9405431 | 2 |
| 3209.9423311 | 4 |
| 3209.9445679 | 2 |
| 3209.9450889 | 0 |
| 3209.9464317 | 2 |
| 3209.9478177 | 2 |
| 3209.9495186 | 2 |
| 3209.9509904 | 2 |
| 3209.9522057 | 0 |
| 3209.953716 | 1 |
| 3209.9554002 | 0 |
| 3209.9566185 | 1 |
| 3209.9587655 | 2 |
| 3209.9591133 | 0 |
| 3209.9616495 | 3 |
| 3209.9618411 | 1 |
| 3209.9637414 | 2 |
| 3209.9652596 | 1 |
| 3209.9658699 | 2 |
| 3209.9684031 | 1 |
| 3209.9691605 | 2 |
| 3209.9706775 | 2 |
| 3209.9721414 | 1 |
| 3209.9731226 | 1 |
| 3209.9750252 | 1 |
| 3209.9755752 | 0 |
| 3209.9779383 | 2 |
| 3209.9784185 | 3 |
| 3209.9802095 | 5 |
| 3209.9821274 | 0 |
| 3209.983028 | 0 |
| 3209.984437 | 1 |
| 3209.9856501 | 6 |
| 3209.986118 | 2 |
| 3209.9865806 | 4 |
| 3209.9866901 | 3 |
| 3209.9867037 | 2 |
| 3209.9869598 | 0 |
| 3209.9873336 | 2 |
| 3209.98755 | 2 |
| 3209.9881152 | 1 |
| 3209.9882036 | 1 |
| 3209.9885774 | 2 |
| 3209.9890071 | 2 |
| 3209.9890314 | 1 |
| 3209.9896048 | 1 |
| 3209.9896312 | 3 |
| 3209.9897148 | 1 |
| 3209.9898543 | 3 |
| 3209.990324 | 0 |
| 3209.9904017 | 0 |
| 3209.9905098 | 0 |
| 3209.9907211 | 7 |
| 3209.9907858 | 1 |
| 3209.991402 | 1 |
| 3209.991921 | 1 |
| 3209.9921939 | 1 |
| 3209.9922164 | 1 |
| 3209.9922426 | 3 |
| 3209.9926368 | 0 |
| 3209.9926555 | 3 |
| 3209.9930018 | 4 |
| 3209.9936074 | 2 |
| 3209.993747 | 2 |
| 3209.9938238 | 1 |
| 3209.9943421 | 0 |
| 3209.9946262 | 3 |
| 3209.9947066 | 4 |
| 3209.9948611 | 2 |
| 3209.9948889 | 1 |
| 3209.9956997 | 3 |
| 3209.9959972 | 1 |
| 3209.9963253 | 1 |
| 3209.9964727 | 3 |
| 3209.9966938 | 0 |
| 3209.9967536 | 3 |
| 3209.9967868 | 2 |
| 3209.9976883 | 1 |
| 3209.9977427 | 1 |
| 3209.9979857 | 3 |
| 3209.9982203 | 2 |
| 3209.9982221 | 2 |
| 3209.99832 | 4 |
| 3209.9989263 | 3 |
| 3209.9993412 | 1 |
| 3209.9994116 | 1 |
| 3210.0001841 | 0 |
| 3210.000274 | 2 |
| 3210.0005035 | 3 |
| 3210.0006405 | 2 |
| 3210.000837 | 0 |
| 3210.0012742 | 3 |
| 3210.0015446 | 1 |
| 3210.0017036 | 4 |
| 3210.002042 | 2 |
| 3210.0020791 | 2 |
| 3210.0024189 | 2 |
| 3210.0026326 | 1 |
| 3210.0027075 | 3 |
| 3210.0031145 | 2 |
| 3210.0031202 | 0 |

| | |
|---|---|
| 3210.003206 | 1 |
| 3210.0037 | 3 |
| 3210.0041923 | 2 |
| 3210.004247 | 3 |
| 3210.0042589 | 1 |
| 3210.0046497 | 3 |
| 3210.0052609 | 1 |
| 3210.0055655 | 3 |
| 3210.0062564 | 5 |
| 3210.0063295 | 4 |
| 3210.0063411 | 1 |
| 3210.0064339 | 1 |
| 3210.0065732 | 2 |
| 3210.0069885 | 4 |
| 3210.0074073 | 1 |
| 3210.0075655 | 1 |
| 3210.0081759 | 1 |
| 3210.0082598 | 2 |
| 3210.0083289 | 4 |
| 3210.0083432 | 1 |
| 3210.008493 | 2 |
| 3210.0089324 | 3 |
| 3210.0090022 | 2 |
| 3210.0096961 | 1 |
| 3210.0098516 | 2 |
| 3210.0099103 | 2 |
| 3210.0104191 | 3 |
| 3210.0105591 | 0 |
| 3210.0110412 | 3 |
| 3210.0112313 | 1 |
| 3210.011455 | 0 |
| 3210.0116218 | 3 |
| 3210.0118481 | 2 |
| 3210.0120775 | 4 |
| 3210.0121221 | 1 |
| 3210.0124016 | 3 |
| 3210.0130758 | 2 |
| 3210.0130781 | 5 |
| 3210.0135779 | 4 |
| 3210.0139236 | 2 |
| 3210.0141152 | 3 |
| 3210.0141383 | 2 |
| 3210.0143474 | 0 |
| 3210.0145513 | 1 |
| 3210.0149159 | 5 |
| 3210.0154638 | 2 |
| 3210.0155413 | 1 |
| 3210.0158792 | 3 |
| 3210.0158855 | 5 |
| 3210.0159369 | 6 |
| 3210.0163997 | 1 |
| 3210.0171655 | 2 |
| 3210.0173497 | 7 |
| 3210.0173662 | 0 |
| 3210.017433 | 2 |
| 3210.0179246 | 4 |
| 3210.018193 | 2 |
| 3210.01843 | 1 |
| 3210.0185087 | 0 |
| 3210.0185239 | 2 |
| 3210.0185255 | 1 |
| 3210.0187472 | 3 |
| 3210.0193506 | 5 |
| 3210.0197507 | 0 |
| 3210.0201839 | 0 |
| 3210.020343 | 4 |
| 3210.0204654 | 3 |
| 3210.0207413 | 0 |
| 3210.0207452 | 1 |
| 3210.0209809 | 0 |
| 3210.0215747 | 3 |
| 3210.0218895 | 2 |
| 3210.0220316 | 1 |
| 3210.0223855 | 4 |
| 3210.0223914 | 4 |
| 3210.0226914 | 4 |
| 3210.0232762 | 4 |
| 3210.0233461 | 2 |
| 3210.0235436 | 8 |
| 3210.0240196 | 7 |
| 3210.0244586 | 9 |
| 3210.0244744 | 3 |
| 3210.0246767 | 4 |
| 3210.025082 | 7 |
| 3210.0251766 | 13 |
| 3210.0252387 | 4 |
| 3210.0256787 | 15 |
| 3210.0256898 | 10 |
| 3210.0261172 | 15 |
| 3210.0262954 | 13 |
| 3210.026713 | 18 |
| 3210.0271106 | 9 |
| 3210.0273201 | 8 |
| 3210.0276698 | 10 |
| 3210.0278405 | 11 |
| 3210.0278736 | 12 |
| 3210.0279972 | 15 |
| 3210.028148 | 10 |
| 3210.0288052 | 8 |
| 3210.0288508 | 12 |
| 3210.0290971 | 17 |
| 3210.0295442 | 13 |
| 3210.0295631 | 22 |
| 3210.0298111 | 14 |
| 3210.0299597 | 18 |
| 3210.0300612 | 9 |
| 3210.0301042 | 12 |
| 3210.0304478 | 18 |
| 3210.0306071 | 14 |
| 3210.0314662 | 8 |
| 3210.0314777 | 6 |
| 3210.0317065 | 2 |
| 3210.032108 | 7 |
| 3210.032546 | 8 |
| 3210.032603 | 5 |
| 3210.0327434 | 10 |
| 3210.032767 | 5 |
| 3210.033505 | 6 |
| 3210.0341066 | 5 |
| 3210.0341071 | 5 |
| 3210.0342062 | 12 |
| 3210.0343439 | 8 |
| 3210.0343832 | 5 |
| 3210.0347228 | 0 |
| 3210.0352632 | 5 |
| 3210.035493 | 2 |
| 3210.0356978 | 0 |
| 3210.0362853 | 0 |
| 3210.0363821 | 3 |
| 3210.0365152 | 2 |
| 3210.0365755 | 2 |
| 3210.0368722 | 3 |
| 3210.0374027 | 2 |
| 3210.0374296 | 4 |
| 3210.0374831 | 4 |
| 3210.0381296 | 1 |
| 3210.0382234 | 6 |
| 3210.0383382 | 3 |
| 3210.0388681 | 1 |
| 3210.038992 | 1 |
| 3210.0392099 | 1 |
| 3210.0392875 | 1 |
| 3210.0393449 | 3 |
| 3210.0396265 | 1 |
| 3210.0409068 | 2 |
| 3210.0409606 | 1 |
| 3210.0409666 | 1 |
| 3210.0411522 | 0 |
| 3210.0413827 | 1 |
| 3210.0414234 | 3 |
| 3210.041853 | 0 |
| 3210.0419404 | 2 |
| 3210.0422634 | 1 |
| 3210.042388 | 1 |
| 3210.0425411 | 1 |
| 3210.0426385 | 2 |
| 3210.0434016 | 0 |
| 3210.0435956 | 1 |
| 3210.0437233 | 0 |
| 3210.0444355 | 4 |
| 3210.0444707 | 0 |
| 3210.0445285 | 1 |
| 3210.0454064 | 4 |
| 3210.045427 | 0 |
| 3210.045641 | 2 |
| 3210.0457884 | 1 |
| 3210.045816 | 2 |
| 3210.0462264 | 1 |
| 3210.046647 | 2 |
| 3210.0468185 | 2 |
| 3210.047006 | 2 |
| 3210.0476457 | 0 |
| 3210.0476837 | 1 |
| 3210.0482239 | 1 |
| 3210.0482497 | 3 |
| 3210.0483787 | 0 |
| 3210.0484821 | 0 |
| 3210.048551 | 2 |
| 3210.0485516 | 3 |
| 3210.0488492 | 1 |
| 3210.0490406 | 0 |
| 3210.0493171 | 1 |
| 3210.0499065 | 2 |
| 3210.0501031 | 1 |
| 3210.0502607 | 0 |
| 3210.0502969 | 2 |
| 3210.0508142 | 1 |
| 3210.0515613 | 3 |
| 3210.0518132 | 3 |
| 3210.0518801 | 0 |
| 3210.0520232 | 1 |
| 3210.0523162 | 4 |
| 3210.0523419 | 2 |
| 3210.0527866 | 2 |
| 3210.0533292 | 2 |
| 3210.0535049 | 2 |
| 3210.0538546 | 2 |
| 3210.0541529 | 6 |
| 3210.0541598 | 1 |
| 3210.0545782 | 2 |
| 3210.0549873 | 0 |
| 3210.0551597 | 6 |
| 3210.0552236 | 3 |
| 3210.0553971 | 2 |
| 3210.0560483 | 0 |
| 3210.0561605 | 2 |
| 3210.0563994 | 2 |
| 3210.0566545 | 2 |
| 3210.0567444 | 1 |
| 3210.0569867 | 4 |
| 3210.0576962 | 1 |
| 3210.0579833 | 3 |
| 3210.0580296 | 3 |
| 3210.0583062 | 3 |
| 3210.0583939 | 5 |
| 3210.0584974 | 4 |
| 3210.0585257 | 4 |
| 3210.0587866 | 0 |
| 3210.0592712 | 2 |
| 3210.0597642 | 1 |
| 3210.0598009 | 3 |
| 3210.0604566 | 1 |
| 3210.0607054 | 4 |
| 3210.06079 | 0 |
| 3210.0611607 | 0 |
| 3210.0612008 | 3 |
| 3210.0613699 | 1 |
| 3210.0620374 | 1 |
| 3210.0621692 | 2 |
| 3210.0621802 | 4 |
| 3210.0627245 | 0 |
| 3210.0627944 | 1 |
| 3210.0630483 | 0 |
| 3210.0633923 | 1 |
| 3210.0635021 | 5 |
| 3210.063531 | 2 |
| 3210.0645601 | 5 |
| 3210.0645616 | 1 |
| 3210.0646705 | 3 |
| 3210.06474 | 0 |
| 3210.0650582 | 3 |
| 3210.065168 | 2 |
| 3210.0655656 | 2 |
| 3210.0660777 | 2 |
| 3210.0660994 | 3 |
| 3210.0665788 | 2 |
| 3210.0667935 | 1 |
| 3210.0669573 | 0 |
| 3210.0673867 | 4 |
| 3210.0676027 | 3 |
| 3210.0678357 | 3 |
| 3210.0678926 | 1 |
| 3210.068039 | 1 |
| 3210.0684714 | 2 |
| 3210.0684893 | 0 |
| 3210.0686542 | 4 |
| 3210.068815 | 2 |
| 3210.0690588 | 3 |
| 3210.0691197 | 1 |
| 3210.0700932 | 2 |
| 3210.070446 | 0 |
| 3210.0706853 | 0 |
| 3210.0707888 | 1 |
| 3210.0711619 | 0 |
| 3210.0712632 | 4 |
| 3210.0715749 | 1 |
| 3210.0715988 | 0 |
| 3210.0719182 | 3 |
| 3210.0724994 | 0 |
| 3210.0727876 | 1 |
| 3210.0728795 | 3 |
| 3210.0731012 | 2 |
| 3210.0734325 | 0 |
| 3210.0734915 | 2 |
| 3210.0735872 | 0 |
| 3210.0738624 | 2 |
| 3210.0742331 | 4 |
| 3210.0743683 | 2 |
| 3210.074708 | 2 |
| 3210.0749434 | 2 |
| 3210.0750926 | 5 |
| 3210.0754012 | 2 |
| 3210.0759945 | 0 |
| 3210.0763062 | 4 |
| 3210.0763498 | 3 |
| 3210.0763549 | 2 |
| 3210.0765455 | 2 |
| 3210.0766352 | 0 |
| 3210.0769389 | 2 |
| 3210.0772471 | 1 |
| 3210.077537 | 0 |
| 3210.0777176 | 3 |
| 3210.0777499 | 3 |
| 3210.0782914 | 2 |
| 3210.0788657 | 2 |
| 3210.0790855 | 3 |
| 3210.079383 | 1 |
| 3210.0795233 | 3 |
| 3210.0797191 | 2 |
| 3210.0798334 | 3 |
| 3210.0801428 | 3 |
| 3210.0801478 | 1 |
| 3210.0808804 | 6 |
| 3210.0809549 | 5 |
| 3210.0811477 | 3 |
| 3210.0812986 | 1 |
| 3210.0813562 | 4 |
| 3210.0815536 | 0 |
| 3210.0821192 | 4 |
| 3210.0821345 | 4 |
| 3210.0823945 | 0 |
| 3210.0831561 | 3 |
| 3210.0833463 | 2 |
| 3210.0836946 | 3 |
| 3210.0838643 | 4 |
| 3210.084266 | 4 |
| 3210.0845332 | 1 |
| 3210.0845545 | 4 |
| 3210.0849756 | 1 |
| 3210.0850436 | 1 |
| 3210.0855282 | 3 |
| 3210.0855921 | 0 |
| 3210.0856369 | 1 |
| 3210.0863991 | 4 |
| 3210.0869188 | 3 |
| 3210.0869903 | 1 |
| 3210.0871174 | 2 |
| 3210.0871385 | 3 |
| 3210.0874005 | 3 |
| 3210.0877057 | 0 |
| 3210.0878168 | 5 |
| 3210.0878288 | 0 |
| 3210.088012 | 3 |
| 3210.0882798 | 4 |
| 3210.0883867 | 2 |
| 3210.0894375 | 0 |
| 3210.0898342 | 1 |
| 3210.089906 | 3 |
| 3210.0899397 | 1 |
| 3210.0900836 | 3 |
| 3210.0902623 | 2 |
| 3210.0907091 | 2 |
| 3210.0915611 | 2 |
| 3210.0916984 | 1 |
| 3210.0917639 | 1 |
| 3210.0918957 | 4 |
| 3210.0921791 | 1 |
| 3210.0925637 | 1 |
| 3210.0928065 | 2 |
| 3210.0930219 | 0 |
| 3210.0933147 | 1 |
| 3210.0936088 | 3 |
| 3210.0936614 | 1 |
| 3210.0943884 | 4 |
| 3210.0946568 | 4 |
| 3210.0947089 | 1 |
| 3210.0950213 | 1 |
| 3210.095059 | 4 |
| 3210.095552 | 3 |
| 3210.0957086 | 0 |
| 3210.095869 | 5 |
| 3210.0961274 | 2 |
| 3210.0963725 | 2 |

| | |
|---|---|
| 3210.0965057 | 2 |
| 3210.096916 | 2 |
| 3210.09737 | 4 |
| 3210.0975245 | 4 |
| 3210.097684 | 4 |
| 3210.0981028 | 2 |
| 3210.0983687 | 2 |
| 3210.0985314 | 5 |
| 3210.0986372 | 0 |
| 3210.0992666 | 3 |
| 3210.0993446 | 4 |
| 3210.0993527 | 2 |
| 3210.0993718 | 4 |
| 3210.0999086 | 0 |
| 3210.100085 | 3 |
| 3210.1000967 | 4 |
| 3210.1007757 | 0 |
| 3210.1008206 | 6 |
| 3210.1010164 | 2 |
| 3210.1017433 | 1 |
| 3210.1020238 | 2 |
| 3210.1023236 | 2 |
| 3210.1024203 | 0 |
| 3210.1024966 | 3 |
| 3210.1024985 | 2 |
| 3210.1025379 | 2 |
| 3210.1031535 | 2 |
| 3210.1035799 | 2 |
| 3210.1037702 | 5 |
| 3210.1045122 | 2 |
| 3210.1045191 | 2 |
| 3210.1045984 | 2 |
| 3210.1047686 | 2 |
| 3210.105405 | 0 |
| 3210.1054732 | 3 |
| 3210.1058108 | 2 |
| 3210.1058157 | 1 |
| 3210.1062436 | 6 |
| 3210.1065408 | 3 |
| 3210.106994 | 1 |
| 3210.1075564 | 1 |
| 3210.1078039 | 0 |
| 3210.1078647 | 2 |
| 3210.1079339 | 0 |
| 3210.1084317 | 4 |
| 3210.1086855 | 3 |
| 3210.1087071 | 3 |
| 3210.1088852 | 3 |
| 3210.108936 | 2 |
| 3210.1090557 | 1 |
| 3210.1093162 | 1 |
| 3210.1099757 | 1 |
| 3210.1100926 | 0 |
| 3210.1101387 | 2 |
| 3210.1104376 | 3 |
| 3210.1106216 | 0 |
| 3210.111278 | 4 |
| 3210.1112951 | 4 |
| 3210.1117135 | 6 |
| 3210.1117865 | 4 |
| 3210.1120217 | 5 |
| 3210.1120614 | 1 |
| 3210.1125244 | 2 |
| 3210.1126158 | 2 |
| 3210.1130447 | 4 |
| 3210.1130668 | 3 |
| 3210.1132505 | 2 |
| 3210.1133395 | 1 |
| 3210.1140236 | 3 |
| 3210.1145858 | 3 |
| 3210.1147523 | 0 |
| 3210.1147642 | 2 |
| 3210.1148297 | 0 |
| 3210.1148946 | 5 |
| 3210.1150893 | 1 |
| 3210.1152121 | 2 |
| 3210.1154717 | 0 |
| 3210.1166644 | 4 |
| 3210.1169379 | 4 |
| 3210.1170779 | 2 |
| 3210.1171265 | 1 |
| 3210.1174755 | 1 |
| 3210.1176444 | 1 |
| 3210.1177509 | 3 |
| 3210.118227 | 2 |
| 3210.1183875 | 2 |
| 3210.1187455 | 2 |
| 3210.1188315 | 4 |
| 3210.118906 | 0 |
| 3210.1194105 | 1 |
| 3210.119468 | 1 |
| 3210.1198244 | 2 |
| 3210.1198913 | 0 |
| 3210.1206419 | 0 |
| 3210.1207959 | 4 |
| 3210.1210269 | 1 |
| 3210.1211393 | 1 |
| 3210.1215397 | 3 |
| 3210.1219301 | 3 |
| 3210.1222408 | 1 |
| 3210.1227234 | 2 |
| 3210.1227648 | 1 |
| 3210.122775 | 2 |
| 3210.1228631 | 0 |
| 3210.1229958 | 1 |
| 3210.1231911 | 2 |
| 3210.1235966 | 1 |
| 3210.1239817 | 1 |
| 3210.1239834 | 3 |
| 3210.1246012 | 1 |
| 3210.1246297 | 5 |
| 3210.1249141 | 3 |
| 3210.1255773 | 3 |
| 3210.1257728 | 1 |
| 3210.1258084 | 2 |
| 3210.1260495 | 3 |
| 3210.1262442 | 2 |
| 3210.1266853 | 1 |
| 3210.126821 | 5 |
| 3210.1268857 | 1 |
| 3210.126965 | 1 |
| 3210.1269869 | 0 |
| 3210.1277917 | 3 |
| 3210.1279742 | 3 |
| 3210.1282706 | 2 |
| 3210.1284164 | 4 |
| 3210.1289525 | 2 |
| 3210.1290537 | 2 |
| 3210.1290891 | 2 |
| 3210.1298186 | 1 |
| 3210.1298778 | 2 |
| 3210.1299749 | 1 |
| 3210.1301135 | 5 |
| 3210.1303019 | 1 |
| 3210.130303 | 2 |
| 3210.1303473 | 3 |
| 3210.1304411 | 2 |
| 3210.1305275 | 0 |
| 3210.1306286 | 2 |
| 3210.1307001 | 2 |
| 3210.1308661 | 1 |
| 3210.1311341 | 2 |
| 3210.131207 | 2 |
| 3210.1312906 | 2 |
| 3210.1313197 | 3 |
| 3210.1313928 | 1 |
| 3210.1313949 | 4 |
| 3210.1316879 | 4 |
| 3210.1317541 | 2 |
| 3210.1318962 | 1 |
| 3210.1319883 | 2 |
| 3210.132284 | 3 |
| 3210.132382 | 4 |
| 3210.1325 | 3 |
| 3210.1325347 | 2 |
| 3210.132673 | 4 |
| 3210.1327004 | 4 |
| 3210.1327007 | 2 |
| 3210.1331567 | 3 |
| 3210.1332317 | 2 |
| 3210.1332935 | 7 |
| 3210.1333182 | 3 |
| 3210.1333371 | 3 |
| 3210.1335078 | 6 |
| 3210.1335306 | 3 |
| 3210.1336958 | 6 |
| 3210.1338351 | 6 |
| 3210.1340045 | 11 |
| 3210.1341252 | 4 |
| 3210.134244 | 9 |
| 3210.1342966 | 12 |
| 3210.1343245 | 10 |
| 3210.1343719 | 9 |
| 3210.1344837 | 4 |
| 3210.1345569 | 9 |
| 3210.1345759 | 7 |
| 3210.134622 | 10 |
| 3210.1347266 | 7 |
| 3210.1348986 | 12 |
| 3210.1349959 | 7 |
| 3210.1351601 | 6 |
| 3210.1353186 | 8 |
| 3210.1354728 | 5 |
| 3210.135498 | 5 |
| 3210.1355401 | 8 |
| 3210.1355597 | 5 |
| 3210.1357791 | 5 |
| 3210.1358788 | 3 |
| 3210.1360703 | 6 |
| 3210.1361267 | 11 |
| 3210.1361761 | 6 |
| 3210.1362753 | 11 |
| 3210.136286 | 9 |
| 3210.1363039 | 8 |
| 3210.1364077 | 7 |
| 3210.1365404 | 3 |
| 3210.1368066 | 7 |
| 3210.1368255 | 5 |
| 3210.1368606 | 13 |
| 3210.1370389 | 4 |
| 3210.1370821 | 4 |
| 3210.1373408 | 6 |
| 3210.1375158 | 6 |
| 3210.1377061 | 8 |
| 3210.1378761 | 1 |
| 3210.1379367 | 2 |
| 3210.1379562 | 9 |
| 3210.1380091 | 7 |
| 3210.1380799 | 3 |
| 3210.1382887 | 4 |
| 3210.1383383 | 6 |
| 3210.138417 | 4 |
| 3210.1384518 | 2 |
| 3210.1384887 | 7 |
| 3210.1386713 | 7 |
| 3210.1388283 | 6 |
| 3210.1390485 | 9 |
| 3210.1391014 | 1 |
| 3210.1391592 | 6 |
| 3210.139394 | 5 |
| 3210.1394203 | 5 |
| 3210.1395112 | 6 |
| 3210.1395237 | 10 |
| 3210.1395378 | 3 |
| 3210.1397826 | 5 |
| 3210.1397885 | 4 |
| 3210.1399508 | 6 |
| 3210.1401488 | 5 |
| 3210.1405827 | 7 |
| 3210.140621 | 4 |
| 3210.1408933 | 6 |
| 3210.140939 | 7 |
| 3210.1410473 | 8 |
| 3210.1410656 | 5 |
| 3210.1411395 | 3 |
| 3210.1412126 | 5 |
| 3210.1412709 | 7 |
| 3210.1412783 | 5 |
| 3210.1417513 | 7 |
| 3210.1418292 | 0 |
| 3210.1420157 | 4 |
| 3210.142073 | 3 |
| 3210.1420795 | 3 |
| 3210.142088 | 2 |
| 3210.142096 | 4 |
| 3210.1421818 | 2 |
| 3210.1423685 | 4 |
| 3210.1423872 | 3 |
| 3210.1425819 | 2 |
| 3210.1427692 | 4 |
| 3210.1428225 | 5 |
| 3210.1429728 | 1 |
| 3210.1432023 | 1 |
| 3210.1432286 | 1 |
| 3210.1432999 | 5 |
| 3210.1433527 | 1 |
| 3210.1433908 | 3 |
| 3210.1434179 | 4 |
| 3210.1436508 | 1 |
| 3210.1437981 | 1 |
| 3210.1438173 | 0 |
| 3210.1439447 | 3 |
| 3210.1439766 | 2 |
| 3210.1441959 | 0 |
| 3210.1443808 | 3 |
| 3210.1444301 | 1 |
| 3210.1446737 | 0 |
| 3210.1447033 | 3 |
| 3210.1447184 | 1 |
| 3210.1447652 | 3 |
| 3210.1450083 | 0 |
| 3210.145161 | 2 |
| 3210.1458271 | 0 |
| 3210.1465676 | 5 |
| 3210.1466021 | 2 |
| 3210.1467725 | 4 |
| 3210.1468766 | 3 |
| 3210.1470038 | 2 |
| 3210.1471033 | 1 |
| 3210.147697 | 4 |
| 3210.1480312 | 1 |
| 3210.1490923 | 1 |
| 3210.1493266 | 0 |
| 3210.1500066 | 3 |
| 3210.1502577 | 3 |
| 3210.150997 | 2 |
| 3210.1517962 | 4 |
| 3210.1533601 | 1 |
| 3210.1534359 | 3 |
| 3210.1538517 | 3 |
| 3210.1547228 | 2 |
| 3210.1553579 | 1 |
| 3210.1553945 | 2 |
| 3210.156387 | 4 |
| 3210.156773 | 0 |
| 3210.1578149 | 3 |
| 3210.1582451 | 2 |
| 3210.1587738 | 4 |
| 3210.1598373 | 6 |
| 3210.1599655 | 3 |
| 3210.1611361 | 0 |
| 3210.1611486 | 5 |
| 3210.1613811 | 1 |
| 3210.1625233 | 1 |
| 3210.1627789 | 0 |
| 3210.1636671 | 3 |
| 3210.1646537 | 4 |
| 3210.1651763 | 2 |
| 3210.1653692 | 2 |
| 3210.1662356 | 1 |
| 3210.1666638 | 3 |
| 3210.1672491 | 0 |
| 3210.168292 | 1 |
| 3210.168716 | 3 |
| 3210.169227 | 1 |
| 3210.1701368 | 1 |
| 3210.1706945 | 3 |
| 3210.1715537 | 1 |
| 3210.1718551 | 0 |
| 3210.1723236 | 0 |
| 3210.1727499 | 2 |
| 3210.1740091 | 1 |
| 3210.1747116 | 3 |
| 3210.1747646 | 1 |
| 3210.175444 | 0 |
| 3210.1757433 | 4 |
| 3210.1763441 | 2 |
| 3210.1773906 | 3 |
| 3210.1777087 | 2 |
| 3210.1791354 | 5 |
| 3210.1794582 | 3 |
| 3210.1800143 | 3 |
| 3210.1805265 | 2 |
| 3210.1812939 | 2 |
| 3210.1816938 | 2 |
| 3210.182363 | 1 |
| 3210.1835571 | 4 |
| 3210.1850669 | 3 |
| 3210.1852522 | 2 |
| 3210.1875733 | 1 |
| 3210.1884481 | 3 |
| 3210.1902687 | 1 |
| 3210.1915047 | 1 |
| 3210.1933231 | 2 |
| 3210.1951767 | 3 |
| 3210.1959233 | 1 |
| 3210.1976702 | 2 |
| 3210.1982603 | 0 |
| 3210.2000844 | 3 |
| 3210.2017856 | 1 |
| 3210.2027197 | 1 |
| 3210.2044337 | 2 |
| 3210.2056796 | 2 |
| 3210.2068337 | 1 |
| 3210.2081112 | 1 |
| 3210.2093267 | 1 |
| 3210.2109978 | 2 |
| 3210.2122621 | 1 |
| 3210.213964 | 2 |
| 3210.2155931 | 4 |
| 3210.216789 | 4 |
| 3210.2188829 | 6 |
| 3210.2194279 | 2 |

| | |
|---|---|
| 3210.22069 | 5 |
| 3210.2228394 | 2 |
| 3210.2233633 | 1 |
| 3210.2249011 | 0 |
| 3210.2275764 | 2 |
| 3210.2276948 | 3 |
| 3210.2297271 | 1 |
| 3210.2302275 | 3 |
| 3210.2321868 | 0 |
| 3210.2337756 | 1 |
| 3210.236107 | 1 |
| 3210.2370367 | 3 |
| 3210.2380338 | 2 |
| 3210.2398706 | 3 |
| 3210.24127 | 2 |
| 3210.2416203 | 0 |
| 3210.2435382 | 3 |
| 3210.2450711 | 1 |
| 3210.2462877 | 3 |
| 3210.2471976 | 2 |
| 3210.2488867 | 2 |
| 3210.2511927 | 1 |
| 3210.2519217 | 1 |
| 3210.2540352 | 1 |
| 3210.2546569 | 1 |
| 3210.2559922 | 1 |
| 3210.2579257 | 1 |
| 3210.2587303 | 2 |
| 3210.2606518 | 1 |
| 3210.2622154 | 3 |
| 3210.2622694 | 1 |
| 3210.2652934 | 4 |
| 3210.2658046 | 2 |
| 3210.2669371 | 4 |
| 3210.2689551 | 5 |
| 3210.2704566 | 2 |
| 3210.27184 | 2 |
| 3210.2729507 | 2 |
| 3210.2748371 | 1 |
| 3210.2762774 | 1 |
| 3210.2771266 | 6 |
| 3210.2791064 | 0 |
| 3210.2804572 | 1 |
| 3210.2814478 | 5 |
| 3210.2825401 | 2 |
| 3210.2849143 | 1 |
| 3210.286482 | 2 |
| 3210.2867584 | 1 |
| 3210.2885358 | 1 |
| 3210.288875 | 3 |
| 3210.2914093 | 4 |
| 3210.2932593 | 4 |
| 3210.2941859 | 1 |
| 3210.2962464 | 3 |
| 3210.2962986 | 5 |
| 3210.2984067 | 0 |
| 3210.2999268 | 0 |
| 3210.3010658 | 0 |
| 3210.3025468 | 2 |
| 3210.3040868 | 3 |
| 3210.3054693 | 1 |
| 3210.3073342 | 1 |
| 3210.3077843 | 3 |
| 3210.3097211 | 0 |
| 3210.3113557 | 2 |
| 3210.3125759 | 3 |
| 3210.3136106 | 2 |
| 3210.3149749 | 1 |
| 3210.3166804 | 3 |
| 3210.3172232 | 2 |
| 3210.3197947 | 1 |
| 3210.3199961 | 4 |
| 3210.3227541 | 1 |
| 3210.3243359 | 1 |
| 3210.3245165 | 2 |
| 3210.3268069 | 1 |
| 3210.3277636 | 1 |
| 3210.329176 | 0 |
| 3210.3309666 | 2 |
| 3210.33133 | 4 |
| 3210.333734 | 3 |
| 3210.3344382 | 3 |
| 3210.3360947 | 5 |
| 3210.3378847 | 2 |
| 3210.3389804 | 2 |
| 3210.340567 | 4 |
| 3210.3413101 | 2 |
| 3210.3430503 | 1 |
| 3210.3441127 | 6 |
| 3210.3462991 | 1 |
| 3210.3477696 | 1 |
| 3210.3489293 | 5 |
| 3210.3505637 | 0 |
| 3210.3509728 | 2 |
| 3210.3525296 | 4 |
| 3210.3546931 | 5 |
| 3210.3551442 | 3 |
| 3210.3567738 | 1 |
| 3210.3582832 | 4 |
| 3210.3597221 | 2 |
| 3210.3615624 | 1 |
| 3210.362489 | 3 |
| 3210.3650137 | 0 |
| 3210.3652358 | 1 |
| 3210.3674196 | 1 |
| 3210.3685073 | 0 |
| 3210.3699008 | 3 |
| 3210.3712604 | 2 |
| 3210.3721283 | 2 |
| 3210.373687 | 4 |
| 3210.3744179 | 2 |
| 3210.376831 | 0 |
| 3210.3784471 | 2 |
| 3210.3796683 | 6 |
| 3210.3812308 | 3 |
| 3210.3822916 | 2 |
| 3210.3833462 | 0 |
| 3210.3855276 | 2 |
| 3210.3859773 | 3 |
| 3210.3881473 | 1 |
| 3210.3886344 | 2 |
| 3210.3908451 | 1 |
| 3210.3923989 | 5 |
| 3210.3934681 | 1 |
| 3210.3955009 | 2 |
| 3210.3962707 | 3 |
| 3210.3973613 | 1 |
| 3210.3997942 | 1 |
| 3210.3997962 | 2 |
| 3210.4022685 | 3 |
| 3210.4029352 | 0 |
| 3210.404268 | 2 |
| 3210.4064112 | 1 |
| 3210.4070466 | 3 |
| 3210.408893 | 3 |
| 3210.4100237 | 3 |
| 3210.4111787 | 1 |
| 3210.4131215 | 2 |
| 3210.4140387 | 0 |
| 3210.4165518 | 2 |
| 3210.4169857 | 5 |
| 3210.4191402 | 3 |
| 3210.419275 | 3 |
| 3210.4219422 | 4 |
| 3210.4231396 | 1 |
| 3210.4241569 | 3 |
| 3210.4258824 | 2 |
| 3210.427042 | 6 |
| 3210.4282671 | 2 |
| 3210.4302763 | 1 |
| 3210.4312175 | 5 |
| 3210.4329618 | 2 |
| 3210.4337877 | 0 |
| 3210.435965 | 2 |
| 3210.4368551 | 0 |
| 3210.4381807 | 1 |
| 3210.4399786 | 1 |
| 3210.4407416 | 2 |
| 3210.441452 | 0 |
| 3210.4441038 | 0 |
| 3210.4449443 | 2 |
| 3210.4464837 | 3 |
| 3210.4471013 | 0 |
| 3210.4492827 | 2 |
| 3210.4510046 | 3 |
| 3210.4514276 | 1 |
| 3210.4539987 | 3 |
| 3210.4549028 | 0 |
| 3210.4560836 | 2 |
| 3210.4576097 | 2 |
| 3210.4590307 | 0 |
| 3210.4607245 | 3 |
| 3210.4621164 | 2 |
| 3210.4637066 | 0 |
| 3210.4650983 | 2 |
| 3210.4660773 | 3 |
| 3210.4676772 | 4 |
| 3210.4693298 | 3 |
| 3210.4707377 | 3 |
| 3210.4719652 | 1 |
| 3210.4732412 | 2 |
| 3210.4744878 | 1 |
| 3210.4751648 | 2 |
| 3210.4766479 | 0 |
| 3210.4788025 | 2 |
| 3210.4803286 | 1 |
| 3210.4816483 | 2 |
| 3210.4826992 | 5 |
| 3210.4841976 | 0 |
| 3210.4862262 | 2 |
| 3210.4862778 | 0 |
| 3210.4885678 | 1 |
| 3210.4889817 | 4 |
| 3210.4914668 | 1 |
| 3210.4929112 | 0 |
| 3210.4939599 | 4 |
| 3210.4958648 | 3 |
| 3210.4966139 | 1 |
| 3210.4976075 | 1 |
| 3210.4990775 | 0 |
| 3210.5007439 | 0 |
| 3210.5025976 | 0 |
| 3210.5031284 | 0 |
| 3210.505677 | 0 |
| 3210.5062851 | 0 |
| 3210.5072824 | 1 |
| 3210.5087673 | 2 |
| 3210.5105366 | 2 |
| 3210.5124294 | 1 |
| 3210.5136451 | 1 |
| 3210.5151783 | 3 |
| 3210.5165453 | 3 |
| 3210.5172434 | 3 |
| 3210.5194363 | 1 |
| 3210.5204333 | 2 |
| 3210.5218154 | 1 |
| 3210.522832 | 1 |
| 3210.5246793 | 0 |
| 3210.5265352 | 2 |
| 3210.5275521 | 2 |
| 3210.529254 | 3 |
| 3210.5299409 | 4 |
| 3210.5323387 | 2 |
| 3210.5331835 | 1 |
| 3210.5336334 | 4 |
| 3210.5360932 | 4 |
| 3210.536858 | 1 |
| 3210.5382678 | 4 |
| 3210.540369 | 3 |
| 3210.5412074 | 1 |
| 3210.5423022 | 0 |
| 3210.5436531 | 2 |
| 3210.5453478 | 1 |
| 3210.5479767 | 1 |
| 3210.5485822 | 2 |
| 3210.5500684 | 2 |
| 3210.5502312 | 2 |
| 3210.5524438 | 1 |
| 3210.5536551 | 2 |
| 3210.5555913 | 0 |
| 3210.5570032 | 2 |
| 3210.5581565 | 3 |
| 3210.5597352 | 5 |
| 3210.5605403 | 0 |
| 3210.5623975 | 3 |
| 3210.5635234 | 2 |
| 3210.5646335 | 0 |
| 3210.5667017 | 3 |
| 3210.5674542 | 1 |
| 3210.5694157 | 2 |
| 3210.5711741 | 2 |
| 3210.5717526 | 1 |
| 3210.5728757 | 2 |
| 3210.5746734 | 2 |
| 3210.5771375 | 3 |
| 3210.5779563 | 2 |
| 3210.5789065 | 2 |
| 3210.5806424 | 0 |
| 3210.5806768 | 1 |
| 3210.5828593 | 3 |
| 3210.584565 | 2 |
| 3210.585184 | 3 |
| 3210.5874395 | 2 |
| 3210.5883183 | 3 |
| 3210.5897566 | 1 |
| 3210.5918896 | 1 |
| 3210.5926292 | 4 |
| 3210.5944357 | 1 |
| 3210.5954493 | 0 |
| 3210.5971559 | 1 |
| 3210.5979132 | 3 |
| 3210.6002474 | 1 |
| 3210.6016439 | 4 |
| 3210.6029828 | 0 |
| 3210.604704 | 1 |
| 3210.6051844 | 3 |
| 3210.6065737 | 3 |
| 3210.6084808 | 4 |
| 3210.6101833 | 2 |
| 3210.6113689 | 2 |
| 3210.612432 | 1 |
| 3210.6140386 | 2 |
| 3210.6156963 | 2 |
| 3210.6162732 | 0 |
| 3210.6185418 | 1 |
| 3210.619079 | 5 |
| 3210.6214809 | 0 |
| 3210.6215548 | 0 |
| 3210.6235378 | 0 |
| 3210.6249151 | 0 |
| 3210.6263638 | 3 |
| 3210.6276642 | 0 |
| 3210.629385 | 1 |
| 3210.6308106 | 2 |
| 3210.63252 | 1 |
| 3210.633453 | 1 |
| 3210.6349069 | 3 |
| 3210.6358716 | 4 |
| 3210.6373723 | 3 |
| 3210.6396551 | 1 |
| 3210.64025 | 3 |
| 3210.6426757 | 2 |
| 3210.6428866 | 0 |
| 3210.6455223 | 1 |
| 3210.6471138 | 3 |
| 3210.6471563 | 6 |
| 3210.649131 | 3 |
| 3210.6505269 | 2 |
| 3210.6519789 | 3 |
| 3210.6531825 | 2 |
| 3210.6546889 | 1 |
| 3210.6563289 | 1 |
| 3210.657605 | 2 |
| 3210.6586619 | 1 |
| 3210.6603812 | 0 |
| 3210.6613734 | 1 |
| 3210.6632917 | 3 |
| 3210.6637218 | 0 |
| 3210.6652177 | 5 |
| 3210.667917 | 1 |
| 3210.6686097 | 2 |
| 3210.6703349 | 5 |
| 3210.6711778 | 4 |
| 3210.672501 | 0 |
| 3210.6748594 | 3 |
| 3210.6751034 | 2 |
| 3210.6769367 | 4 |
| 3210.6782341 | 2 |
| 3210.6802959 | 0 |
| 3210.6809438 | 1 |
| 3210.6829528 | 5 |
| 3210.6845583 | 4 |
| 3210.6856746 | 0 |
| 3210.686626 | 1 |
| 3210.6885193 | 3 |
| 3210.6898401 | 0 |
| 3210.6908141 | 4 |
| 3210.6928257 | 1 |
| 3210.6951609 | 3 |
| 3210.6953195 | 4 |
| 3210.6964698 | 4 |
| 3210.6988814 | 3 |
| 3210.6991086 | 0 |
| 3210.7011776 | 1 |
| 3210.7020103 | 1 |
| 3210.7041234 | 2 |
| 3210.7047185 | 1 |
| 3210.7061067 | 3 |
| 3210.708462 | 2 |
| 3210.7091107 | 1 |
| 3210.7102931 | 1 |
| 3210.7119499 | 2 |
| 3210.714272 | 0 |
| 3210.7155269 | 2 |
| 3210.7160849 | 5 |
| 3210.7179889 | 1 |
| 3210.7182612 | 3 |
| 3210.7205421 | 1 |
| 3210.7220115 | 3 |
| 3210.723317 | 0 |
| 3210.7247536 | 2 |
| 3210.7260597 | 2 |
| 3210.7276416 | 2 |

| | |
|---|---|
| 3210.7294841 | 3 |
| 3210.7303091 | 0 |
| 3210.7318426 | 3 |
| 3210.7338678 | 3 |
| 3210.7348575 | 6 |
| 3210.7366891 | 1 |
| 3210.7379057 | 0 |
| 3210.7397967 | 3 |
| 3210.7401275 | 2 |
| 3210.7417459 | 2 |
| 3210.7423172 | 3 |
| 3210.7443182 | 0 |
| 3210.7462849 | 5 |
| 3210.7473501 | 3 |
| 3210.7491807 | 3 |
| 3210.7505089 | 1 |
| 3210.7516102 | 3 |
| 3210.7532089 | 2 |
| 3210.7539007 | 0 |
| 3210.7557998 | 1 |
| 3210.7576661 | 0 |
| 3210.7584214 | 1 |
| 3210.7605249 | 0 |
| 3210.7616604 | 2 |
| 3210.7631336 | 3 |
| 3210.7634869 | 3 |
| 3210.7658572 | 4 |
| 3210.7671633 | 1 |
| 3210.7681671 | 3 |
| 3210.7698307 | 1 |
| 3210.7707342 | 1 |
| 3210.7724993 | 1 |
| 3210.7746818 | 1 |
| 3210.7750361 | 1 |
| 3210.7767215 | 0 |
| 3210.77817 | 2 |
| 3210.779935 | 0 |
| 3210.780791 | 1 |
| 3210.7827911 | 2 |
| 3210.784766 | 4 |
| 3210.7855893 | 3 |
| 3210.7876253 | 1 |
| 3210.7885821 | 2 |
| 3210.7898016 | 1 |
| 3210.7914954 | 1 |
| 3210.7927835 | 2 |
| 3210.7941911 | 2 |
| 3210.7948841 | 2 |
| 3210.7963826 | 1 |
| 3210.7984714 | 1 |
| 3210.7996003 | 4 |
| 3210.8015953 | 3 |
| 3210.8018981 | 3 |
| 3210.8038588 | 1 |
| 3210.804482 | 2 |
| 3210.8062448 | 2 |
| 3210.8077853 | 1 |
| 3210.8094596 | 1 |
| 3210.8106324 | 2 |
| 3210.8116597 | 2 |
| 3210.8140422 | 5 |
| 3210.8150559 | 0 |
| 3210.8155986 | 2 |
| 3210.8183359 | 2 |
| 3210.8185937 | 3 |
| 3210.8203162 | 1 |
| 3210.8220033 | 3 |
| 3210.8236218 | 1 |
| 3210.825471 | 1 |
| 3210.8257424 | 1 |
| 3210.8278107 | 0 |
| 3210.828556 | 1 |
| 3210.8307669 | 4 |
| 3210.8318985 | 2 |
| 3210.8331469 | 0 |
| 3210.834601 | 3 |
| 3210.8357156 | 4 |
| 3210.8371578 | 2 |
| 3210.8392102 | 3 |
| 3210.8400556 | 1 |
| 3210.8418669 | 4 |
| 3210.8425065 | 0 |
| 3210.844325 | 4 |
| 3210.8462477 | 2 |
| 3210.8471508 | 3 |
| 3210.8486242 | 0 |
| 3210.8497714 | 2 |
| 3210.8515368 | 1 |
| 3210.8532955 | 2 |
| 3210.8545256 | 2 |
| 3210.8553241 | 3 |
| 3210.8568928 | 2 |
| 3210.858629 | 1 |
| 3210.8587506 | 2 |
| 3210.8613671 | 5 |
| 3210.8634294 | 0 |
| 3210.8635153 | 1 |
| 3210.8657557 | 2 |
| 3210.8668286 | 4 |
| 3210.8684846 | 1 |
| 3210.8699402 | 2 |
| 3210.871032 | 0 |
| 3210.8729294 | 5 |
| 3210.873549 | 0 |
| 3210.8757972 | 3 |
| 3210.8766969 | 2 |
| 3210.8782384 | 1 |
| 3210.8797882 | 0 |
| 3210.8802304 | 1 |
| 3210.8822954 | 0 |
| 3210.8843769 | 2 |
| 3210.8851097 | 2 |
| 3210.8871973 | 0 |
| 3210.8875523 | 0 |
| 3210.890195 | 2 |
| 3210.891533 | 0 |
| 3210.8918853 | 1 |
| 3210.8942979 | 0 |
| 3210.8950026 | 3 |
| 3210.8962506 | 2 |
| 3210.8966244 | 1 |
| 3210.8992336 | 7 |
| 3210.9004818 | 1 |
| 3210.9012907 | 4 |
| 3210.9034241 | 2 |
| 3210.9049028 | 6 |
| 3210.9066269 | 2 |
| 3210.9087663 | 2 |
| 3210.9089814 | 2 |
| 3210.911004 | 2 |
| 3210.9111203 | 2 |
| 3210.9129896 | 3 |
| 3210.9139972 | 1 |
| 3210.9158815 | 5 |
| 3210.9175953 | 1 |
| 3210.9187547 | 1 |
| 3210.9202728 | 1 |
| 3210.9218587 | 2 |
| 3210.92282 | 0 |
| 3210.9248015 | 1 |
| 3210.9258364 | 0 |
| 3210.9271062 | 1 |
| 3210.9286181 | 1 |
| 3210.9299568 | 1 |
| 3210.9322046 | 1 |
| 3210.9326417 | 0 |
| 3210.9332913 | 0 |
| 3210.9334113 | 1 |
| 3210.9338554 | 2 |
| 3210.9341412 | 4 |
| 3210.9343526 | 1 |
| 3210.9347369 | 0 |
| 3210.934794 | 0 |
| 3210.9350226 | 2 |
| 3210.9352754 | 0 |
| 3210.9358597 | 0 |
| 3210.9359813 | 1 |
| 3210.9360658 | 1 |
| 3210.9361377 | 2 |
| 3210.9366804 | 0 |
| 3210.936712 | 0 |
| 3210.9370432 | 1 |
| 3210.9370442 | 3 |
| 3210.93707 | 3 |
| 3210.9379186 | 1 |
| 3210.9380471 | 2 |
| 3210.9380765 | 3 |
| 3210.9385834 | 0 |
| 3210.9387014 | 2 |
| 3210.9387882 | 5 |
| 3210.9396795 | 0 |
| 3210.9397189 | 1 |
| 3210.9397196 | 1 |
| 3210.9397612 | 0 |
| 3210.9399589 | 1 |
| 3210.9411362 | 0 |
| 3210.9411964 | 2 |
| 3210.9412424 | 0 |
| 3210.9415273 | 2 |
| 3210.9416318 | 1 |
| 3210.941991 | 2 |
| 3210.9420908 | 2 |
| 3210.9423582 | 3 |
| 3210.9424327 | 0 |
| 3210.9425008 | 4 |
| 3210.9429773 | 0 |
| 3210.9431404 | 3 |
| 3210.9434762 | 0 |
| 3210.9437834 | 3 |
| 3210.943946 | 1 |
| 3210.9440295 | 0 |
| 3210.944552 | 1 |
| 3210.9446278 | 1 |
| 3210.9452265 | 2 |
| 3210.9452353 | 1 |
| 3210.9453286 | 1 |
| 3210.9455595 | 2 |
| 3210.9459317 | 2 |
| 3210.9462812 | 0 |
| 3210.9463183 | 1 |
| 3210.9467683 | 0 |
| 3210.9468463 | 0 |
| 3210.9470889 | 0 |
| 3210.9472569 | 3 |
| 3210.9474883 | 2 |
| 3210.9480288 | 2 |
| 3210.9486607 | 2 |
| 3210.9487982 | 1 |
| 3210.9488515 | 2 |
| 3210.9491319 | 0 |
| 3210.949347 | 1 |
| 3210.9494478 | 0 |
| 3210.9497045 | 1 |
| 3210.9497642 | 1 |
| 3210.9499817 | 1 |
| 3210.9505946 | 2 |
| 3210.9506368 | 2 |
| 3210.9511124 | 0 |
| 3210.9512374 | 1 |
| 3210.951723 | 7 |
| 3210.9519944 | 0 |
| 3210.9520464 | 2 |
| 3210.9527618 | 1 |
| 3210.9528495 | 0 |
| 3210.9529021 | 1 |
| 3210.9532066 | 3 |
| 3210.9533316 | 0 |
| 3210.9535999 | 2 |
| 3210.9541366 | 2 |
| 3210.95417 | 0 |
| 3210.954777 | 1 |
| 3210.9549317 | 4 |
| 3210.9551218 | 0 |
| 3210.9552657 | 3 |
| 3210.9553157 | 2 |
| 3210.955845 | 2 |
| 3210.9560372 | 4 |
| 3210.9560564 | 1 |
| 3210.9561262 | 1 |
| 3210.9564087 | 2 |
| 3210.9570143 | 2 |
| 3210.9571832 | 0 |
| 3210.9577351 | 2 |
| 3210.9580096 | 1 |
| 3210.9583245 | 0 |
| 3210.9585323 | 3 |
| 3210.9586752 | 0 |
| 3210.9588843 | 1 |
| 3210.9591768 | 1 |
| 3210.9592547 | 0 |
| 3210.9592651 | 1 |
| 3210.9593517 | 1 |
| 3210.9602963 | 1 |
| 3210.9606293 | 1 |
| 3210.9606998 | 1 |
| 3210.9608305 | 0 |
| 3210.9614621 | 0 |
| 3210.9614893 | 2 |
| 3210.9615411 | 0 |
| 3210.9616534 | 1 |
| 3210.9622188 | 1 |
| 3210.9626632 | 1 |
| 3210.9628141 | 0 |
| 3210.9629231 | 5 |
| 3210.9632092 | 1 |
| 3210.9633252 | 4 |
| 3210.9635661 | 5 |
| 3210.9638526 | 0 |
| 3210.9640651 | 1 |
| 3210.9641034 | 3 |
| 3210.9647933 | 0 |
| 3210.9648013 | 1 |
| 3210.9650708 | 0 |
| 3210.9661525 | 1 |
| 3210.9662139 | 3 |
| 3210.9664408 | 4 |
| 3210.9665745 | 3 |
| 3210.9666145 | 1 |
| 3210.966672 | 0 |
| 3210.9671373 | 3 |
| 3210.9671911 | 1 |
| 3210.9675814 | 1 |
| 3210.9678449 | 0 |
| 3210.9680723 | 2 |
| 3210.9680803 | 0 |
| 3210.9684772 | 2 |
| 3210.9685232 | 4 |
| 3210.9692083 | 1 |
| 3210.9692992 | 0 |
| 3210.9695533 | 1 |
| 3210.9696975 | 1 |
| 3210.9698029 | 6 |
| 3210.9702502 | 2 |
| 3210.9706403 | 2 |
| 3210.9710768 | 0 |
| 3210.9712641 | 0 |
| 3210.9714711 | 0 |
| 3210.9717287 | 2 |
| 3210.9718356 | 2 |
| 3210.97184 | 0 |
| 3210.9724462 | 2 |
| 3210.9730248 | 2 |
| 3210.9731797 | 0 |
| 3210.9732062 | 0 |
| 3210.9733201 | 1 |
| 3210.9739768 | 6 |
| 3210.9739978 | 4 |
| 3210.9742322 | 1 |
| 3210.9743305 | 1 |
| 3210.9743865 | 1 |
| 3210.9744158 | 3 |
| 3210.9748654 | 0 |
| 3210.9751608 | 0 |
| 3210.9757148 | 6 |
| 3210.9757741 | 2 |
| 3210.9764325 | 1 |
| 3210.9765018 | 1 |
| 3210.9767035 | 0 |
| 3210.9768864 | 1 |
| 3210.9769163 | 1 |
| 3210.9771336 | 1 |
| 3210.977533 | 1 |
| 3210.9778242 | 3 |
| 3210.9780236 | 2 |
| 3210.9782072 | 1 |
| 3210.9783933 | 1 |
| 3210.978847 | 1 |
| 3210.9793324 | 1 |
| 3210.9793753 | 1 |
| 3210.9797193 | 0 |
| 3210.9797635 | 2 |
| 3210.9797867 | 0 |
| 3210.9799276 | 1 |
| 3210.9804389 | 2 |
| 3210.9806198 | 1 |
| 3210.9806402 | 1 |
| 3210.9807811 | 0 |
| 3210.9814831 | 2 |
| 3210.9816304 | 2 |
| 3210.9817235 | 0 |
| 3210.9822663 | 2 |
| 3210.9826932 | 0 |
| 3210.9827431 | 0 |
| 3210.9829049 | 0 |
| 3210.9832826 | 3 |
| 3210.9836713 | 2 |
| 3210.9837817 | 1 |
| 3210.9838197 | 1 |
| 3210.9842433 | 2 |
| 3210.9844674 | 2 |
| 3210.9847055 | 1 |
| 3210.9847233 | 1 |
| 3210.9856489 | 1 |
| 3210.9856641 | 0 |
| 3210.9858301 | 2 |
| 3210.9860296 | 0 |
| 3210.9860624 | 3 |
| 3210.9863015 | 2 |
| 3210.986669 | 1 |
| 3210.9870194 | 2 |
| 3210.9875054 | 1 |
| 3210.9875098 | 3 |
| 3210.9877896 | 2 |

| | |
|---|---|
| 3210.9880982 | 3 |
| 3210.9882384 | 2 |
| 3210.988436 | 1 |
| 3210.9889188 | 0 |
| 3210.9892356 | 4 |
| 3210.9895491 | 0 |
| 3210.9896366 | 1 |
| 3210.989969 | 1 |
| 3210.9900799 | 1 |
| 3210.9902531 | 0 |
| 3210.9908367 | 3 |
| 3210.9908377 | 0 |
| 3210.9914278 | 3 |
| 3210.9917923 | 2 |
| 3210.9917938 | 2 |
| 3210.9919799 | 1 |
| 3210.9923965 | 2 |
| 3210.9924306 | 2 |
| 3210.9925618 | 0 |
| 3210.9931018 | 1 |
| 3210.9932219 | 1 |
| 3210.9933488 | 0 |
| 3210.9937118 | 3 |
| 3210.9937389 | 0 |
| 3210.9940266 | 2 |
| 3210.9943326 | 0 |
| 3210.9946629 | 0 |
| 3210.9947289 | 0 |
| 3210.9954989 | 1 |
| 3210.9955597 | 1 |
| 3210.9956402 | 2 |
| 3210.9962972 | 1 |
| 3210.9964124 | 0 |
| 3210.9965104 | 2 |
| 3210.9965885 | 1 |
| 3210.9968199 | 0 |
| 3210.9968298 | 1 |
| 3210.9974103 | 0 |
| 3210.9974882 | 1 |
| 3210.9982878 | 1 |
| 3210.998352 | 3 |
| 3210.9984313 | 3 |
| 3210.998712 | 0 |
| 3210.9987818 | 0 |
| 3210.9992996 | 0 |
| 3210.9993447 | 0 |
| 3210.9996025 | 2 |
| 3211.0001154 | 3 |
| 3211.0001413 | 1 |
| 3211.000488 | 4 |
| 3211.0006271 | 3 |
| 3211.0012212 | 0 |
| 3211.00141 | 4 |
| 3211.0015661 | 0 |
| 3211.0021035 | 2 |
| 3211.0021574 | 0 |
| 3211.0021995 | 0 |
| 3211.0022138 | 4 |
| 3211.0023933 | 1 |
| 3211.002884 | 1 |
| 3211.0030713 | 2 |
| 3211.0031437 | 2 |
| 3211.0033703 | 1 |
| 3211.0039362 | 1 |
| 3211.004044 | 3 |
| 3211.0047349 | 2 |
| 3211.0051683 | 0 |
| 3211.0051738 | 3 |
| 3211.005367 | 3 |
| 3211.0053749 | 0 |
| 3211.0055544 | 2 |
| 3211.0060591 | 2 |
| 3211.0062764 | 0 |
| 3211.0064765 | 0 |
| 3211.0068726 | 2 |
| 3211.0070091 | 1 |
| 3211.0073868 | 0 |
| 3211.0077595 | 0 |
| 3211.0078255 | 3 |
| 3211.0079948 | 0 |
| 3211.0083221 | 2 |
| 3211.008665 | 2 |
| 3211.0087199 | 1 |
| 3211.0089108 | 1 |
| 3211.009831 | 1 |
| 3211.0098755 | 1 |
| 3211.0099114 | 1 |
| 3211.0100166 | 0 |
| 3211.0100807 | 2 |
| 3211.010152 | 1 |
| 3211.0104003 | 1 |
| 3211.0112048 | 3 |
| 3211.0112184 | 0 |
| 3211.0115023 | 2 |
| 3211.0119128 | 0 |
| 3211.0119372 | 1 |
| 3211.0120227 | 0 |
| 3211.0123367 | 0 |
| 3211.0125015 | 0 |
| 3211.0127751 | 0 |
| 3211.0132742 | 3 |
| 3211.01335 | 3 |
| 3211.0138233 | 1 |
| 3211.0141561 | 2 |
| 3211.0143965 | 3 |
| 3211.0145921 | 1 |
| 3211.014849 | 1 |
| 3211.0149804 | 3 |
| 3211.0149891 | 0 |
| 3211.0151644 | 1 |
| 3211.0159564 | 0 |
| 3211.0161259 | 3 |
| 3211.0164319 | 3 |
| 3211.0168969 | 1 |
| 3211.0171022 | 1 |
| 3211.017154 | 1 |
| 3211.0172126 | 1 |
| 3211.0179671 | 3 |
| 3211.0180421 | 0 |
| 3211.0180457 | 2 |
| 3211.0183765 | 2 |
| 3211.0188981 | 4 |
| 3211.0191339 | 1 |
| 3211.0192989 | 1 |
| 3211.0193328 | 0 |
| 3211.019422 | 2 |
| 3211.0199026 | 4 |
| 3211.0201435 | 3 |
| 3211.0202263 | 2 |
| 3211.0213435 | 4 |
| 3211.0214167 | 2 |
| 3211.0214632 | 0 |
| 3211.0216459 | 0 |
| 3211.021668 | 2 |
| 3211.0218281 | 2 |
| 3211.0218515 | 2 |
| 3211.0225222 | 5 |
| 3211.0227167 | 1 |
| 3211.0231364 | 0 |
| 3211.0232397 | 2 |
| 3211.0235159 | 2 |
| 3211.0238805 | 1 |
| 3211.023942 | 1 |
| 3211.0241132 | 0 |
| 3211.0242447 | 2 |
| 3211.0244658 | 1 |
| 3211.0250164 | 1 |
| 3211.0251279 | 1 |
| 3211.025323 | 3 |
| 3211.025541 | 0 |
| 3211.0262606 | 0 |
| 3211.0263087 | 0 |
| 3211.0264347 | 2 |
| 3211.0269933 | 3 |
| 3211.027012 | 1 |
| 3211.0276011 | 0 |
| 3211.027682 | 3 |
| 3211.0277723 | 1 |
| 3211.0279327 | 0 |
| 3211.0282343 | 1 |
| 3211.0282929 | 2 |
| 3211.0285672 | 2 |
| 3211.0285866 | 0 |
| 3211.0291156 | 1 |
| 3211.0296321 | 1 |
| 3211.0296951 | 1 |
| 3211.0299625 | 3 |
| 3211.0300495 | 2 |
| 3211.030303 | 2 |
| 3211.0306055 | 0 |
| 3211.0308746 | 2 |
| 3211.031025 | 2 |
| 3211.0315184 | 0 |
| 3211.0315623 | 2 |
| 3211.0320824 | 3 |
| 3211.0321462 | 2 |
| 3211.0321638 | 0 |
| 3211.032958 | 2 |
| 3211.0331939 | 2 |
| 3211.0334035 | 1 |
| 3211.0334286 | 2 |
| 3211.0338101 | 1 |
| 3211.0339993 | 2 |
| 3211.0341885 | 0 |
| 3211.034464 | 1 |
| 3211.0346837 | 2 |
| 3211.034684 | 1 |
| 3211.0354087 | 0 |
| 3211.0355555 | 1 |
| 3211.0356171 | 2 |
| 3211.0361298 | 1 |
| 3211.0363704 | 1 |
| 3211.036646 | 3 |
| 3211.036664 | 2 |
| 3211.0371473 | 4 |
| 3211.0375215 | 1 |
| 3211.0376791 | 3 |
| 3211.0379171 | 2 |
| 3211.0381008 | 4 |
| 3211.0381626 | 2 |
| 3211.0381941 | 2 |
| 3211.0385558 | 4 |
| 3211.0389803 | 1 |
| 3211.0391429 | 1 |
| 3211.0391787 | 1 |
| 3211.0394726 | 2 |
| 3211.0396595 | 2 |
| 3211.0400761 | 0 |
| 3211.0404039 | 0 |
| 3211.0408471 | 3 |
| 3211.0408579 | 2 |
| 3211.041044 | 2 |
| 3211.0412374 | 1 |
| 3211.041806 | 3 |
| 3211.042086 | 1 |
| 3211.0421419 | 3 |
| 3211.0424201 | 4 |
| 3211.0425203 | 1 |
| 3211.0427844 | 2 |
| 3211.0431674 | 0 |
| 3211.0434159 | 3 |
| 3211.0437309 | 1 |
| 3211.0438316 | 1 |
| 3211.0440342 | 0 |
| 3211.0443783 | 0 |
| 3211.0452086 | 3 |
| 3211.0452154 | 0 |
| 3211.0454978 | 2 |
| 3211.0457405 | 1 |
| 3211.0458263 | 3 |
| 3211.0462802 | 2 |
| 3211.0462806 | 1 |
| 3211.0465806 | 2 |
| 3211.0470514 | 3 |
| 3211.0472657 | 1 |
| 3211.0475082 | 3 |
| 3211.0477721 | 1 |
| 3211.0478021 | 4 |
| 3211.0478284 | 0 |
| 3211.0482798 | 2 |
| 3211.0484278 | 1 |
| 3211.0489049 | 1 |
| 3211.0494106 | 3 |
| 3211.0494717 | 0 |
| 3211.0495289 | 1 |
| 3211.049997 | 1 |
| 3211.0501319 | 0 |
| 3211.0503411 | 0 |
| 3211.0505887 | 1 |
| 3211.0507509 | 2 |
| 3211.0513681 | 0 |
| 3211.0515385 | 4 |
| 3211.0516557 | 0 |
| 3211.0518768 | 1 |
| 3211.052038 | 1 |
| 3211.0522637 | 1 |
| 3211.0524705 | 3 |
| 3211.05258 | 1 |
| 3211.0529623 | 0 |
| 3211.053081 | 1 |
| 3211.0537802 | 0 |
| 3211.0541231 | 2 |
| 3211.0541451 | 4 |
| 3211.0542307 | 5 |
| 3211.0548287 | 0 |
| 3211.0550533 | 2 |
| 3211.0553677 | 0 |
| 3211.0554279 | 1 |
| 3211.0555172 | 0 |
| 3211.0559277 | 0 |
| 3211.0560528 | 2 |
| 3211.0563793 | 1 |
| 3211.0563953 | 1 |
| 3211.056988 | 2 |
| 3211.0571371 | 0 |
| 3211.057202 | 0 |
| 3211.0579773 | 0 |
| 3211.0581085 | 3 |
| 3211.0581502 | 0 |
| 3211.058479 | 0 |
| 3211.0585865 | 3 |
| 3211.0590459 | 1 |
| 3211.0592895 | 1 |
| 3211.0595211 | 4 |
| 3211.0596945 | 1 |
| 3211.0602393 | 0 |
| 3211.060432 | 0 |
| 3211.0605323 | 0 |
| 3211.0612139 | 3 |
| 3211.0614009 | 0 |
| 3211.0615377 | 0 |
| 3211.0616741 | 0 |
| 3211.0616903 | 1 |
| 3211.0617672 | 0 |
| 3211.0620889 | 2 |
| 3211.0625416 | 2 |
| 3211.0631422 | 1 |
| 3211.0631747 | 0 |
| 3211.0632531 | 1 |
| 3211.0637967 | 1 |
| 3211.0638955 | 2 |
| 3211.0641502 | 0 |
| 3211.0642394 | 3 |
| 3211.0646339 | 2 |
| 3211.0646818 | 3 |
| 3211.0650112 | 0 |
| 3211.0652077 | 1 |
| 3211.065767 | 2 |
| 3211.0658358 | 0 |
| 3211.0658663 | 1 |
| 3211.0664121 | 1 |
| 3211.0665069 | 0 |
| 3211.0666986 | 4 |
| 3211.0669389 | 0 |
| 3211.0672953 | 5 |
| 3211.0673443 | 0 |
| 3211.0675732 | 0 |
| 3211.0680264 | 0 |
| 3211.0682386 | 2 |
| 3211.0683256 | 1 |
| 3211.0689126 | 1 |
| 3211.0692055 | 1 |
| 3211.0693766 | 0 |
| 3211.0697816 | 6 |
| 3211.0698557 | 1 |
| 3211.0701745 | 4 |
| 3211.0703002 | 2 |
| 3211.0705696 | 1 |
| 3211.0706657 | 0 |
| 3211.0708749 | 2 |
| 3211.0713955 | 0 |
| 3211.071678 | 1 |
| 3211.0716944 | 0 |
| 3211.0718548 | 0 |
| 3211.0723832 | 1 |
| 3211.0724117 | 0 |
| 3211.0726476 | 2 |
| 3211.0730501 | 1 |
| 3211.0733916 | 1 |
| 3211.0734565 | 4 |
| 3211.073517 | 3 |
| 3211.0741388 | 2 |
| 3211.0743914 | 1 |
| 3211.0746872 | 0 |
| 3211.0748706 | 2 |
| 3211.0749178 | 1 |
| 3211.0751075 | 5 |
| 3211.0759344 | 1 |
| 3211.0760273 | 1 |
| 3211.0762131 | 1 |
| 3211.0762686 | 1 |
| 3211.0766433 | 2 |
| 3211.0767394 | 0 |
| 3211.0767558 | 0 |
| 3211.077542 | 0 |
| 3211.0776173 | 0 |
| 3211.077994 | 1 |
| 3211.0780011 | 1 |
| 3211.0786571 | 2 |
| 3211.0786821 | 1 |
| 3211.0790476 | 2 |
| 3211.0796998 | 2 |
| 3211.0797842 | 0 |

| | |
|---|---|
| 3211.0798477 | 3 |
| 3211.0799881 | 0 |
| 3211.0802968 | 1 |
| 3211.0805659 | 3 |
| 3211.0805839 | 0 |
| 3211.0806247 | 1 |
| 3211.0811362 | 5 |
| 3211.081531 | 1 |
| 3211.0815898 | 0 |
| 3211.0817665 | 0 |
| 3211.0817739 | 2 |
| 3211.0823676 | 4 |
| 3211.0824867 | 1 |
| 3211.0827582 | 0 |
| 3211.0828859 | 0 |
| 3211.0830048 | 2 |
| 3211.0838858 | 3 |
| 3211.0841508 | 0 |
| 3211.0843162 | 1 |
| 3211.0844994 | 0 |
| 3211.0845864 | 2 |
| 3211.08514 | 1 |
| 3211.0851856 | 2 |
| 3211.0852817 | 1 |
| 3211.0860864 | 1 |
| 3211.0861587 | 3 |
| 3211.0863474 | 1 |
| 3211.0866567 | 1 |
| 3211.0867992 | 0 |
| 3211.0871122 | 0 |
| 3211.0871986 | 0 |
| 3211.087518 | 1 |
| 3211.0876122 | 1 |
| 3211.0878757 | 1 |
| 3211.0884366 | 0 |
| 3211.0884805 | 2 |
| 3211.0889964 | 1 |
| 3211.089239 | 0 |
| 3211.0892586 | 0 |
| 3211.0893198 | 2 |
| 3211.0897611 | 2 |
| 3211.090073 | 2 |
| 3211.0904017 | 1 |
| 3211.0904347 | 2 |
| 3211.0907339 | 0 |
| 3211.0909795 | 2 |
| 3211.0914864 | 2 |
| 3211.0915912 | 0 |
| 3211.0917064 | 0 |
| 3211.0922669 | 3 |
| 3211.092616 | 0 |
| 3211.0928292 | 0 |
| 3211.0930513 | 1 |
| 3211.09325 | 0 |
| 3211.0934276 | 1 |
| 3211.0935927 | 2 |
| 3211.0937662 | 0 |
| 3211.0938964 | 3 |
| 3211.0944971 | 2 |
| 3211.0945176 | 0 |
| 3211.0946545 | 1 |
| 3211.0947832 | 1 |
| 3211.0954279 | 1 |
| 3211.0957492 | 3 |
| 3211.0959763 | 1 |
| 3211.0962333 | 0 |
| 3211.0963783 | 0 |
| 3211.0963819 | 2 |
| 3211.0967377 | 0 |
| 3211.0967689 | 1 |
| 3211.0972913 | 2 |
| 3211.0974824 | 1 |
| 3211.0978667 | 0 |
| 3211.0980479 | 1 |
| 3211.0986201 | 2 |
| 3211.0987339 | 0 |
| 3211.0990017 | 0 |
| 3211.099197 | 3 |
| 3211.0994318 | 4 |
| 3211.0994922 | 1 |
| 3211.0999395 | 0 |
| 3211.1002729 | 0 |
| 3211.1005961 | 0 |
| 3211.1009346 | 1 |
| 3211.1012269 | 1 |
| 3211.101398 | 0 |
| 3211.1016602 | 1 |
| 3211.1019878 | 0 |
| 3211.1021143 | 2 |
| 3211.1024778 | 2 |
| 3211.1026482 | 2 |
| 3211.1030841 | 4 |
| 3211.1033407 | 3 |
| 3211.1033892 | 0 |
| 3211.1035 | 3 |
| 3211.1039765 | 0 |
| 3211.1041726 | 1 |
| 3211.1043002 | 1 |
| 3211.1043359 | 1 |
| 3211.1044632 | 3 |
| 3211.1050516 | 2 |
| 3211.1052964 | 1 |
| 3211.105466 | 2 |
| 3211.1059623 | 0 |
| 3211.1061436 | 5 |
| 3211.106258 | 0 |
| 3211.106819 | 3 |
| 3211.1069758 | 0 |
| 3211.1069809 | 1 |
| 3211.1070777 | 1 |
| 3211.1072443 | 2 |
| 3211.1079758 | 1 |
| 3211.1081585 | 1 |
| 3211.1084529 | 1 |
| 3211.108927 | 2 |
| 3211.1089827 | 0 |
| 3211.1092374 | 1 |
| 3211.1092937 | 1 |
| 3211.1097282 | 1 |
| 3211.1099562 | 0 |
| 3211.1101029 | 0 |
| 3211.1105544 | 0 |
| 3211.1106523 | 1 |
| 3211.1108166 | 2 |
| 3211.1108736 | 1 |
| 3211.1111723 | 1 |
| 3211.1113826 | 1 |
| 3211.1117076 | 1 |
| 3211.111855 | 1 |
| 3211.112302 | 1 |
| 3211.1126998 | 0 |
| 3211.1130027 | 0 |
| 3211.1130388 | 0 |
| 3211.1134457 | 1 |
| 3211.1135057 | 4 |
| 3211.1136192 | 0 |
| 3211.1138097 | 3 |
| 3211.1142103 | 0 |
| 3211.1144508 | 0 |
| 3211.11456 | 0 |
| 3211.1147485 | 1 |
| 3211.1148183 | 2 |
| 3211.1152672 | 1 |
| 3211.1153447 | 3 |
| 3211.1159594 | 1 |
| 3211.116033 | 2 |
| 3211.116733 | 3 |
| 3211.1169503 | 1 |
| 3211.1172215 | 3 |
| 3211.1173673 | 2 |
| 3211.117432 | 4 |
| 3211.1179608 | 0 |
| 3211.1181453 | 1 |
| 3211.1185816 | 1 |
| 3211.1190551 | 2 |
| 3211.119354 | 0 |
| 3211.119485 | 2 |
| 3211.1200072 | 1 |
| 3211.1201573 | 2 |
| 3211.1204248 | 1 |
| 3211.1211243 | 0 |
| 3211.1213724 | 0 |
| 3211.121398 | 1 |
| 3211.1217427 | 2 |
| 3211.1218295 | 0 |
| 3211.1221289 | 2 |
| 3211.1226333 | 1 |
| 3211.1229119 | 0 |
| 3211.1231063 | 0 |
| 3211.1233751 | 0 |
| 3211.1241489 | 2 |
| 3211.1247386 | 1 |
| 3211.1249622 | 1 |
| 3211.1249815 | 3 |
| 3211.1250377 | 1 |
| 3211.1253877 | 0 |
| 3211.1259539 | 1 |
| 3211.1264363 | 2 |
| 3211.126626 | 0 |
| 3211.1266968 | 0 |
| 3211.1271385 | 0 |
| 3211.1276178 | 2 |
| 3211.1278028 | 2 |
| 3211.128059 | 1 |
| 3211.1281153 | 0 |
| 3211.128347 | 2 |
| 3211.1289549 | 1 |
| 3211.1294745 | 3 |
| 3211.1298148 | 1 |
| 3211.1300132 | 4 |
| 3211.1301518 | 5 |
| 3211.1304925 | 2 |
| 3211.1305409 | 2 |
| 3211.13116 | 1 |
| 3211.1312141 | 1 |
| 3211.131704 | 0 |
| 3211.1318726 | 0 |
| 3211.1324432 | 1 |
| 3211.1329567 | 3 |
| 3211.1332515 | 2 |
| 3211.1336415 | 0 |
| 3211.1337189 | 2 |
| 3211.1342453 | 1 |
| 3211.1343741 | 1 |
| 3211.1344141 | 2 |
| 3211.1349608 | 1 |
| 3211.1351513 | 1 |
| 3211.1353489 | 5 |
| 3211.1358901 | 1 |
| 3211.1359649 | 0 |
| 3211.1367694 | 2 |
| 3211.1367765 | 3 |
| 3211.1372098 | 0 |
| 3211.1374832 | 1 |
| 3211.1375439 | 1 |
| 3211.1381128 | 1 |
| 3211.1381587 | 0 |
| 3211.1383497 | 2 |
| 3211.1385153 | 3 |
| 3211.1388837 | 1 |
| 3211.1396116 | 0 |
| 3211.1398838 | 0 |
| 3211.1401047 | 1 |
| 3211.1403296 | 1 |
| 3211.1404251 | 3 |
| 3211.1404979 | 2 |
| 3211.1416858 | 0 |
| 3211.1417594 | 1 |
| 3211.1421166 | 1 |
| 3211.1430467 | 0 |
| 3211.1431602 | 0 |
| 3211.1432171 | 0 |
| 3211.1432431 | 1 |
| 3211.1434853 | 3 |
| 3211.1442509 | 1 |
| 3211.1444204 | 2 |
| 3211.1444349 | 3 |
| 3211.1446876 | 0 |
| 3211.1452402 | 1 |
| 3211.1453999 | 0 |
| 3211.1455506 | 2 |
| 3211.1460355 | 1 |
| 3211.1465852 | 3 |
| 3211.1469193 | 2 |
| 3211.1473884 | 3 |
| 3211.1475877 | 1 |
| 3211.1476129 | 7 |
| 3211.1476539 | 2 |
| 3211.1481951 | 2 |
| 3211.1484424 | 0 |
| 3211.1489775 | 1 |
| 3211.1495667 | 1 |
| 3211.1496189 | 0 |
| 3211.150093 | 1 |
| 3211.1503573 | 1 |
| 3211.1504649 | 3 |
| 3211.1506913 | 3 |
| 3211.1508719 | 0 |
| 3211.1512344 | 3 |
| 3211.1517048 | 3 |
| 3211.1521583 | 0 |
| 3211.1521675 | 3 |
| 3211.1525283 | 3 |
| 3211.1528187 | 2 |
| 3211.1531506 | 0 |
| 3211.1536108 | 2 |
| 3211.153906 | 1 |
| 3211.1540999 | 0 |
| 3211.1548213 | 5 |
| 3211.1549217 | 3 |
| 3211.154937 | 1 |
| 3211.1551438 | 3 |
| 3211.156041 | 0 |
| 3211.1560789 | 2 |
| 3211.1560873 | 1 |
| 3211.1564331 | 2 |
| 3211.1566087 | 1 |
| 3211.1572426 | 3 |
| 3211.1572617 | 2 |
| 3211.1580592 | 1 |
| 3211.1581066 | 1 |
| 3211.1585455 | 0 |
| 3211.1588699 | 4 |
| 3211.1589067 | 2 |
| 3211.1594417 | 0 |
| 3211.1598642 | 1 |
| 3211.1598707 | 1 |
| 3211.1601267 | 3 |
| 3211.1603836 | 1 |
| 3211.1611516 | 2 |
| 3211.1613721 | 1 |
| 3211.1617639 | 1 |
| 3211.1617986 | 0 |
| 3211.1625465 | 1 |
| 3211.1627656 | 0 |
| 3211.1628446 | 1 |
| 3211.1629647 | 0 |
| 3211.1633141 | 0 |
| 3211.1634787 | 0 |
| 3211.1639149 | 2 |
| 3211.1640834 | 0 |
| 3211.1645079 | 0 |
| 3211.1651151 | 2 |
| 3211.1652211 | 0 |
| 3211.1652227 | 2 |
| 3211.1657146 | 1 |
| 3211.1661515 | 2 |
| 3211.1665115 | 2 |
| 3211.1665256 | 2 |
| 3211.1665442 | 1 |
| 3211.1668926 | 4 |
| 3211.1678012 | 1 |
| 3211.1678378 | 0 |
| 3211.1679373 | 3 |
| 3211.1681177 | 0 |
| 3211.1683791 | 3 |
| 3211.1687988 | 1 |
| 3211.1691758 | 2 |
| 3211.1691945 | 1 |
| 3211.1701097 | 2 |
| 3211.1704996 | 0 |
| 3211.1707904 | 1 |
| 3211.1712287 | 2 |
| 3211.1713138 | 1 |
| 3211.1714002 | 1 |
| 3211.1719319 | 1 |
| 3211.1719696 | 2 |
| 3211.1725704 | 2 |
| 3211.1727331 | 1 |
| 3211.1727344 | 0 |
| 3211.1730396 | 0 |
| 3211.1737819 | 2 |
| 3211.173929 | 1 |
| 3211.174192 | 0 |
| 3211.1744269 | 1 |
| 3211.1745679 | 2 |
| 3211.1753136 | 3 |
| 3211.1758503 | 1 |
| 3211.1758657 | 1 |
| 3211.1761486 | 2 |
| 3211.1764131 | 2 |
| 3211.1766982 | 1 |
| 3211.177322 | 0 |
| 3211.177372 | 1 |
| 3211.1780712 | 0 |
| 3211.1783101 | 3 |
| 3211.1784169 | 1 |
| 3211.1791789 | 2 |
| 3211.1794041 | 2 |
| 3211.1794383 | 3 |
| 3211.179809 | 0 |
| 3211.1799299 | 0 |
| 3211.1802935 | 1 |
| 3211.1807295 | 2 |
| 3211.181029 | 0 |
| 3211.1811239 | 0 |
| 3211.1814248 | 2 |
| 3211.1820264 | 0 |
| 3211.1822206 | 0 |
| 3211.1824457 | 1 |
| 3211.1825863 | 1 |
| 3211.1831334 | 0 |
| 3211.1831815 | 1 |

| | |
|---|---|
| 3211.1833638 | 1 |
| 3211.1837166 | 4 |
| 3211.1844302 | 1 |
| 3211.1847477 | 1 |
| 3211.1852577 | 1 |
| 3211.1855284 | 3 |
| 3211.1856729 | 1 |
| 3211.1858343 | 1 |
| 3211.1858905 | 1 |
| 3211.1860742 | 1 |
| 3211.1869943 | 3 |
| 3211.1870867 | 0 |
| 3211.1872651 | 1 |
| 3211.1872784 | 0 |
| 3211.1878477 | 2 |
| 3211.1878973 | 1 |
| 3211.1884145 | 1 |
| 3211.1886746 | 0 |
| 3211.189561 | 3 |
| 3211.1899398 | 0 |
| 3211.1900516 | 1 |
| 3211.1902584 | 2 |
| 3211.1902956 | 0 |
| 3211.1907915 | 2 |
| 3211.1909492 | 2 |
| 3211.1916114 | 1 |
| 3211.191724 | 3 |
| 3211.1921086 | 2 |
| 3211.1923754 | 1 |
| 3211.1924098 | 0 |
| 3211.1928731 | 4 |
| 3211.1930734 | 3 |
| 3211.1933042 | 0 |
| 3211.194002 | 2 |
| 3211.1940122 | 1 |
| 3211.1945103 | 1 |
| 3211.1947375 | 1 |
| 3211.1950103 | 0 |
| 3211.195074 | 0 |
| 3211.1956551 | 2 |
| 3211.19572 | 1 |
| 3211.1966417 | 0 |
| 3211.1968664 | 3 |
| 3211.1971731 | 0 |
| 3211.1972201 | 0 |
| 3211.19737 | 3 |
| 3211.1979922 | 1 |
| 3211.1980117 | 0 |
| 3211.1984562 | 1 |
| 3211.1987555 | 2 |
| 3211.1992656 | 1 |
| 3211.1994315 | 2 |
| 3211.1996828 | 2 |
| 3211.2001805 | 0 |
| 3211.2004339 | 1 |
| 3211.2006018 | 2 |
| 3211.2010682 | 1 |
| 3211.2010961 | 1 |
| 3211.2011894 | 2 |
| 3211.2019511 | 1 |
| 3211.2024372 | 2 |
| 3211.2024864 | 1 |
| 3211.2031032 | 1 |
| 3211.2032524 | 0 |
| 3211.2035336 | 1 |
| 3211.2035789 | 1 |
| 3211.2036056 | 1 |
| 3211.2039898 | 0 |
| 3211.2044695 | 0 |
| 3211.2047844 | 0 |
| 3211.2051931 | 0 |
| 3211.2055887 | 1 |
| 3211.2057339 | 1 |
| 3211.2058899 | 1 |
| 3211.206539 | 0 |
| 3211.206578 | 1 |
| 3211.2070817 | 2 |
| 3211.207464 | 1 |
| 3211.2075564 | 0 |
| 3211.2082666 | 0 |
| 3211.2083658 | 1 |
| 3211.2085672 | 2 |
| 3211.2091604 | 1 |
| 3211.209302 | 1 |
| 3211.2094664 | 2 |
| 3211.2096995 | 1 |
| 3211.2104739 | 2 |
| 3211.2105027 | 0 |
| 3211.2106768 | 0 |
| 3211.2112115 | 1 |
| 3211.211286 | 2 |
| 3211.2115741 | 1 |
| 3211.2116931 | 1 |
| 3211.2120379 | 1 |
| 3211.2125975 | 1 |
| 3211.2130537 | 0 |
| 3211.2133064 | 1 |
| 3211.2137574 | 0 |
| 3211.2139725 | 1 |
| 3211.2140585 | 0 |
| 3211.2144622 | 0 |
| 3211.2150993 | 1 |
| 3211.2151286 | 1 |
| 3211.2154299 | 0 |
| 3211.215834 | 1 |
| 3211.2161289 | 1 |
| 3211.2164998 | 2 |
| 3211.2166225 | 4 |
| 3211.2169974 | 1 |
| 3211.2170075 | 5 |
| 3211.2179178 | 0 |
| 3211.2182493 | 0 |
| 3211.2182752 | 5 |
| 3211.2187316 | 1 |
| 3211.2189514 | 1 |
| 3211.2190852 | 2 |
| 3211.2193099 | 2 |
| 3211.2198835 | 0 |
| 3211.2203248 | 2 |
| 3211.2204068 | 2 |
| 3211.2205597 | 1 |
| 3211.2208582 | 3 |
| 3211.221453 | 1 |
| 3211.2216132 | 1 |
| 3211.2219288 | 1 |
| 3211.2222421 | 0 |
| 3211.2227336 | 0 |
| 3211.2227658 | 3 |
| 3211.2231079 | 1 |
| 3211.2232822 | 1 |
| 3211.2235353 | 1 |
| 3211.2242221 | 2 |
| 3211.2243198 | 0 |
| 3211.2247284 | 0 |
| 3211.2249643 | 2 |
| 3211.2250914 | 1 |
| 3211.2255724 | 3 |
| 3211.2256266 | 2 |
| 3211.2266148 | 3 |
| 3211.2271315 | 0 |
| 3211.2271392 | 1 |
| 3211.2272744 | 1 |
| 3211.2277657 | 0 |
| 3211.2277909 | 0 |
| 3211.2285949 | 1 |
| 3211.2286224 | 1 |
| 3211.2286883 | 1 |
| 3211.2287705 | 2 |
| 3211.2293654 | 0 |
| 3211.2293943 | 4 |
| 3211.2303509 | 1 |
| 3211.2305961 | 1 |
| 3211.2308473 | 0 |
| 3211.2309913 | 1 |
| 3211.2310143 | 2 |
| 3211.2317474 | 3 |
| 3211.2317672 | 2 |
| 3211.2322714 | 3 |
| 3211.2323006 | 1 |
| 3211.2327878 | 1 |
| 3211.2328009 | 1 |
| 3211.2332726 | 2 |
| 3211.2336289 | 0 |
| 3211.2342989 | 3 |
| 3211.2344491 | 0 |
| 3211.2345096 | 2 |
| 3211.2350299 | 0 |
| 3211.2355739 | 0 |
| 3211.2355872 | 2 |
| 3211.2358713 | 0 |
| 3211.236165 | 2 |
| 3211.2365247 | 0 |
| 3211.2366235 | 1 |
| 3211.2369151 | 3 |
| 3211.2373178 | 1 |
| 3211.2379448 | 1 |
| 3211.2381086 | 3 |
| 3211.2383733 | 0 |
| 3211.2385219 | 3 |
| 3211.2389234 | 1 |
| 3211.2389562 | 1 |
| 3211.239153 | 2 |
| 3211.2398485 | 1 |
| 3211.2400621 | 1 |
| 3211.2400722 | 0 |
| 3211.2403249 | 2 |
| 3211.2407374 | 1 |
| 3211.2411436 | 0 |
| 3211.2413077 | 0 |
| 3211.2418765 | 3 |
| 3211.2423831 | 0 |
| 3211.2425459 | 2 |
| 3211.2426461 | 1 |
| 3211.2434348 | 0 |
| 3211.2434405 | 0 |
| 3211.2435207 | 2 |
| 3211.2439136 | 3 |
| 3211.2439526 | 2 |
| 3211.2447855 | 0 |
| 3211.2448231 | 2 |
| 3211.2452404 | 1 |
| 3211.2458906 | 2 |
| 3211.2461119 | 0 |
| 3211.2464147 | 2 |
| 3211.2465535 | 2 |
| 3211.2468394 | 1 |
| 3211.2472879 | 0 |
| 3211.2475698 | 0 |
| 3211.2479814 | 3 |
| 3211.2481374 | 3 |
| 3211.2481658 | 1 |
| 3211.248545 | 0 |
| 3211.248681 | 1 |
| 3211.2491759 | 1 |
| 3211.2491771 | 1 |
| 3211.2495522 | 0 |
| 3211.2506589 | 1 |
| 3211.2507579 | 0 |
| 3211.2511407 | 4 |
| 3211.2511475 | 2 |
| 3211.2512442 | 0 |
| 3211.2516123 | 0 |
| 3211.2517227 | 2 |
| 3211.2524085 | 1 |
| 3211.2529245 | 2 |
| 3211.2531873 | 1 |
| 3211.2533869 | 1 |
| 3211.2536208 | 3 |
| 3211.2536652 | 0 |
| 3211.2543721 | 1 |
| 3211.2545472 | 3 |
| 3211.254698 | 0 |
| 3211.2549048 | 0 |
| 3211.2551031 | 0 |
| 3211.2551423 | 2 |
| 3211.2554796 | 3 |
| 3211.2555493 | 0 |
| 3211.2558632 | 4 |
| 3211.2561814 | 0 |
| 3211.2566672 | 2 |
| 3211.2568043 | 3 |
| 3211.256834 | 3 |
| 3211.2573067 | 1 |
| 3211.2574826 | 1 |
| 3211.2580868 | 3 |
| 3211.2583078 | 2 |
| 3211.2586557 | 1 |
| 3211.2587829 | 1 |
| 3211.2591527 | 1 |
| 3211.2592218 | 1 |
| 3211.2598659 | 1 |
| 3211.2601721 | 0 |
| 3211.2605043 | 0 |
| 3211.260612 | 0 |
| 3211.2608529 | 1 |
| 3211.260863 | 1 |
| 3211.2613697 | 0 |
| 3211.2616548 | 0 |
| 3211.2619461 | 1 |
| 3211.2620327 | 1 |
| 3211.2626372 | 0 |
| 3211.262744 | 3 |
| 3211.2632009 | 1 |
| 3211.2640994 | 1 |
| 3211.2641157 | 1 |
| 3211.2644701 | 2 |
| 3211.2649803 | 0 |
| 3211.2650891 | 0 |
| 3211.2654196 | 0 |
| 3211.2657916 | 0 |
| 3211.2661988 | 1 |
| 3211.2662732 | 0 |
| 3211.2662884 | 1 |
| 3211.266459 | 1 |
| 3211.2668482 | 1 |
| 3211.2670388 | 1 |
| 3211.2672271 | 0 |
| 3211.2673157 | 2 |
| 3211.2674535 | 1 |
| 3211.2677866 | 0 |
| 3211.2681504 | 0 |
| 3211.2687624 | 1 |
| 3211.2693059 | 4 |
| 3211.269325 | 1 |
| 3211.269515 | 2 |
| 3211.2695309 | 1 |
| 3211.2699966 | 1 |
| 3211.2701286 | 3 |
| 3211.2709253 | 3 |
| 3211.2709544 | 1 |
| 3211.2712393 | 1 |
| 3211.2713837 | 1 |
| 3211.2716896 | 2 |
| 3211.271788 | 3 |
| 3211.2721319 | 2 |
| 3211.2721576 | 1 |
| 3211.2730611 | 1 |
| 3211.2732898 | 3 |
| 3211.2733518 | 2 |
| 3211.273353 | 2 |
| 3211.2734549 | 2 |
| 3211.2736358 | 1 |
| 3211.2741655 | 2 |
| 3211.2742512 | 3 |
| 3211.2748193 | 2 |
| 3211.2750079 | 0 |
| 3211.2784341 | 1 |
| 3211.2814863 | 1 |
| 3211.2837837 | 0 |
| 3211.2839925 | 1 |
| 3211.2852076 | 2 |
| 3211.2853828 | 3 |
| 3211.2855046 | 1 |
| 3211.2855306 | 6 |
| 3211.2857426 | 2 |
| 3211.2871336 | 1 |
| 3211.2881438 | 1 |
| 3211.2881615 | 2 |
| 3211.2894558 | 2 |
| 3211.2897268 | 1 |
| 3211.2910127 | 5 |
| 3211.2914681 | 2 |
| 3211.2917938 | 5 |
| 3211.2918038 | 2 |
| 3211.2922941 | 1 |
| 3211.2926417 | 0 |
| 3211.2926467 | 1 |
| 3211.2930747 | 2 |
| 3211.2944531 | 5 |
| 3211.2954902 | 3 |
| 3211.2985729 | 3 |
| 3211.3005029 | 1 |
| 3211.3068824 | 0 |
| 3211.3084069 | 0 |
| 3211.3270616 | 1 |
| 3211.32857 | 2 |
| 3211.3383522 | 3 |
| 3211.3448794 | 1 |
| 3211.3451035 | 0 |
| 3211.3459165 | 3 |
| 3211.3459512 | 2 |
| 3211.3466686 | 0 |
| 3211.3468213 | 1 |
| 3211.3468278 | 1 |
| 3211.3475855 | 0 |
| 3211.347658 | 2 |
| 3211.3478947 | 1 |
| 3211.3481545 | 2 |
| 3211.3489181 | 1 |
| 3211.3491821 | 2 |
| 3211.3491854 | 1 |
| 3211.3492236 | 1 |
| 3211.3499982 | 2 |
| 3211.3502404 | 0 |
| 3211.3505648 | 1 |
| 3211.3506528 | 1 |
| 3211.3514283 | 0 |
| 3211.3516386 | 2 |
| 3211.3517689 | 2 |
| 3211.3520242 | 1 |
| 3211.3521838 | 1 |
| 3211.352858 | 1 |
| 3211.353243 | 1 |

| | |
|---|---|
| 3211.3534115 | 3 |
| 3211.3541252 | 0 |
| 3211.3544929 | 1 |
| 3211.3550463 | 2 |
| 3211.3552692 | 0 |
| 3211.3552899 | 1 |
| 3211.3554778 | 2 |
| 3211.3555907 | 1 |
| 3211.3560291 | 0 |
| 3211.3561453 | 2 |
| 3211.3564346 | 2 |
| 3211.356928 | 2 |
| 3211.3571841 | 1 |
| 3211.3575127 | 1 |
| 3211.3579315 | 0 |
| 3211.3583381 | 1 |
| 3211.3584716 | 1 |
| 3211.3589254 | 0 |
| 3211.3590915 | 0 |
| 3211.3591267 | 1 |
| 3211.3599551 | 0 |
| 3211.3601878 | 0 |
| 3211.3603947 | 0 |
| 3211.3606348 | 2 |
| 3211.3610225 | 1 |
| 3211.3612809 | 0 |
| 3211.3614386 | 1 |
| 3211.3621635 | 2 |
| 3211.3621915 | 4 |
| 3211.3628725 | 0 |
| 3211.363194 | 0 |
| 3211.3633908 | 2 |
| 3211.3636094 | 2 |
| 3211.3637295 | 1 |
| 3211.3637664 | 0 |
| 3211.3643094 | 2 |
| 3211.3647085 | 3 |
| 3211.3650936 | 0 |
| 3211.3658252 | 3 |
| 3211.3658471 | 0 |
| 3211.366039 | 0 |
| 3211.3663749 | 3 |
| 3211.3664561 | 1 |
| 3211.3673964 | 2 |
| 3211.3674016 | 2 |
| 3211.3676424 | 1 |
| 3211.368172 | 1 |
| 3211.3683744 | 1 |
| 3211.3685153 | 1 |
| 3211.3689535 | 1 |
| 3211.3691762 | 1 |
| 3211.3694756 | 2 |
| 3211.3696141 | 0 |
| 3211.3701387 | 5 |
| 3211.3702868 | 3 |
| 3211.3704533 | 2 |
| 3211.370888 | 1 |
| 3211.3710681 | 0 |
| 3211.371527 | 1 |
| 3211.3715528 | 0 |
| 3211.37199 | 5 |
| 3211.3722289 | 0 |
| 3211.3725433 | 2 |
| 3211.3728095 | 0 |
| 3211.3729545 | 1 |
| 3211.3734093 | 1 |
| 3211.3737192 | 1 |
| 3211.3739762 | 1 |
| 3211.3742526 | 2 |
| 3211.374785 | 1 |
| 3211.3748675 | 1 |
| 3211.3751127 | 1 |
| 3211.3758323 | 1 |
| 3211.3758651 | 1 |
| 3211.3764519 | 0 |
| 3211.3766402 | 2 |
| 3211.3767088 | 0 |
| 3211.3768969 | 1 |
| 3211.3772934 | 1 |
| 3211.3773583 | 2 |
| 3211.3776215 | 0 |
| 3211.378196 | 0 |
| 3211.3786478 | 0 |
| 3211.3787623 | 1 |
| 3211.3791458 | 0 |
| 3211.3793935 | 3 |
| 3211.379508 | 1 |
| 3211.3795501 | 1 |
| 3211.3799881 | 0 |
| 3211.3802227 | 2 |
| 3211.3804776 | 3 |
| 3211.3810625 | 1 |
| 3211.3812237 | 0 |
| 3211.3812654 | 1 |
| 3211.382057 | 0 |
| 3211.3825344 | 1 |
| 3211.3825852 | 0 |
| 3211.3829329 | 1 |
| 3211.3833662 | 0 |
| 3211.3836761 | 2 |
| 3211.3837413 | 1 |
| 3211.3839254 | 0 |
| 3211.3841646 | 0 |
| 3211.3844155 | 0 |
| 3211.3849756 | 0 |
| 3211.3851225 | 3 |
| 3211.3855367 | 2 |
| 3211.3862393 | 2 |
| 3211.3863075 | 0 |
| 3211.3864515 | 2 |
| 3211.3872996 | 1 |
| 3211.3873838 | 2 |
| 3211.3877593 | 0 |
| 3211.3878193 | 1 |
| 3211.3880417 | 1 |
| 3211.3880839 | 1 |
| 3211.3886507 | 3 |
| 3211.389055 | 0 |
| 3211.3898327 | 1 |
| 3211.3899315 | 1 |
| 3211.3900167 | 1 |
| 3211.3903934 | 0 |
| 3211.3907576 | 2 |
| 3211.3909945 | 0 |
| 3211.391549 | 0 |
| 3211.3916458 | 1 |
| 3211.3917976 | 1 |
| 3211.3921135 | 1 |
| 3211.3922384 | 1 |
| 3211.3927663 | 1 |
| 3211.3928154 | 0 |
| 3211.3928342 | 3 |
| 3211.3931322 | 1 |
| 3211.3933717 | 0 |
| 3211.3937839 | 1 |
| 3211.3940495 | 1 |
| 3211.3947411 | 0 |
| 3211.395105 | 0 |
| 3211.3952222 | 2 |
| 3211.3957787 | 2 |
| 3211.3959004 | 1 |
| 3211.3960913 | 2 |
| 3211.3965565 | 2 |
| 3211.3965947 | 2 |
| 3211.396858 | 0 |
| 3211.3969497 | 0 |
| 3211.3979065 | 1 |
| 3211.3979874 | 1 |
| 3211.3982042 | 0 |
| 3211.398538 | 0 |
| 3211.3990205 | 1 |
| 3211.3992528 | 1 |
| 3211.3998289 | 0 |
| 3211.4000843 | 1 |
| 3211.4004706 | 0 |
| 3211.4005862 | 3 |
| 3211.4010043 | 2 |
| 3211.4010443 | 1 |
| 3211.4016423 | 1 |
| 3211.4019783 | 0 |
| 3211.4022029 | 4 |
| 3211.4023781 | 1 |
| 3211.4026667 | 1 |
| 3211.4034225 | 2 |
| 3211.4035356 | 2 |
| 3211.4040225 | 2 |
| 3211.4043634 | 5 |
| 3211.4044346 | 1 |
| 3211.405111 | 1 |
| 3211.4052357 | 1 |
| 3211.4054583 | 1 |
| 3211.4059182 | 3 |
| 3211.4064864 | 2 |
| 3211.4067263 | 1 |
| 3211.4069923 | 0 |
| 3211.4073702 | 4 |
| 3211.4079535 | 0 |
| 3211.4080183 | 0 |
| 3211.408381 | 0 |
| 3211.4085584 | 1 |
| 3211.4087328 | 1 |
| 3211.40893 | 1 |
| 3211.4090916 | 1 |
| 3211.4097575 | 0 |
| 3211.4097959 | 3 |
| 3211.4098841 | 0 |
| 3211.4102236 | 0 |
| 3211.410645 | 2 |
| 3211.4109501 | 0 |
| 3211.4114139 | 7 |
| 3211.4117787 | 0 |
| 3211.4122072 | 1 |
| 3211.4124037 | 1 |
| 3211.4125108 | 3 |
| 3211.4127488 | 1 |
| 3211.4128206 | 2 |
| 3211.4130756 | 1 |
| 3211.4136773 | 1 |
| 3211.4137402 | 0 |
| 3211.4141397 | 2 |
| 3211.414197 | 0 |
| 3211.4143763 | 1 |
| 3211.4146189 | 0 |
| 3211.4156918 | 1 |
| 3211.4157158 | 4 |
| 3211.4161689 | 0 |
| 3211.4164962 | 2 |
| 3211.4165513 | 5 |
| 3211.4171434 | 1 |
| 3211.4171825 | 1 |
| 3211.417982 | 1 |
| 3211.4180876 | 2 |
| 3211.4182661 | 2 |
| 3211.4185438 | 2 |
| 3211.4187261 | 0 |
| 3211.4193774 | 0 |
| 3211.4194157 | 1 |
| 3211.4201684 | 1 |
| 3211.420218 | 1 |
| 3211.4202813 | 1 |
| 3211.4210188 | 1 |
| 3211.4212436 | 3 |
| 3211.4215248 | 2 |
| 3211.4219153 | 2 |
| 3211.4219913 | 1 |
| 3211.4224026 | 1 |
| 3211.4227671 | 3 |
| 3211.4231623 | 0 |
| 3211.4232173 | 2 |
| 3211.4235217 | 0 |
| 3211.4238496 | 1 |
| 3211.4241195 | 1 |
| 3211.4242637 | 1 |
| 3211.4245595 | 3 |
| 3211.4247045 | 3 |
| 3211.4251643 | 3 |
| 3211.4253379 | 2 |
| 3211.4253795 | 0 |
| 3211.425781 | 0 |
| 3211.4264187 | 1 |
| 3211.4265314 | 1 |
| 3211.4265503 | 0 |
| 3211.4270387 | 1 |
| 3211.4273807 | 1 |
| 3211.4276392 | 2 |
| 3211.4279437 | 1 |
| 3211.4280546 | 0 |
| 3211.4282746 | 1 |
| 3211.4286034 | 0 |
| 3211.4288951 | 0 |
| 3211.4290786 | 0 |
| 3211.4293863 | 1 |
| 3211.4298845 | 3 |
| 3211.4300359 | 2 |
| 3211.4307397 | 1 |
| 3211.4310547 | 2 |
| 3211.431087 | 2 |
| 3211.4316976 | 3 |
| 3211.4318634 | 1 |
| 3211.4319905 | 1 |
| 3211.4321955 | 2 |
| 3211.4325311 | 0 |
| 3211.4328181 | 2 |
| 3211.4335238 | 1 |
| 3211.4335332 | 1 |
| 3211.4338943 | 0 |
| 3211.4339352 | 0 |
| 3211.4340539 | 3 |
| 3211.4348564 | 1 |
| 3211.4351154 | 0 |
| 3211.4361049 | 1 |
| 3211.4361372 | 0 |
| 3211.4362539 | 1 |
| 3211.4365758 | 2 |
| 3211.4366315 | 3 |
| 3211.4373425 | 3 |
| 3211.4376986 | 0 |
| 3211.4378348 | 2 |
| 3211.4382501 | 0 |
| 3211.4383561 | 2 |
| 3211.4387403 | 1 |
| 3211.4390123 | 0 |
| 3211.4390253 | 1 |
| 3211.4397552 | 3 |
| 3211.4401991 | 4 |
| 3211.4402124 | 4 |
| 3211.4408247 | 4 |
| 3211.4411202 | 6 |
| 3211.4416799 | 9 |
| 3211.441743 | 5 |
| 3211.4423391 | 2 |
| 3211.4424559 | 0 |
| 3211.442569 | 6 |
| 3211.4426101 | 5 |
| 3211.44296 | 1 |
| 3211.4431615 | 3 |
| 3211.4435075 | 3 |
| 3211.4435299 | 3 |
| 3211.444012 | 1 |
| 3211.4443921 | 1 |
| 3211.4444556 | 1 |
| 3211.4446983 | 2 |
| 3211.4450099 | 1 |
| 3211.4453987 | 2 |
| 3211.4460381 | 2 |
| 3211.4460474 | 2 |
| 3211.4464622 | 2 |
| 3211.4467908 | 1 |
| 3211.4468569 | 3 |
| 3211.4468599 | 6 |
| 3211.4478085 | 0 |
| 3211.4480182 | 3 |
| 3211.4481952 | 2 |
| 3211.4482787 | 2 |
| 3211.4484531 | 2 |
| 3211.4498196 | 4 |
| 3211.4498596 | 10 |
| 3211.4500017 | 4 |
| 3211.4500181 | 6 |
| 3211.4506106 | 5 |
| 3211.4507544 | 9 |
| 3211.4513245 | 9 |
| 3211.4513929 | 8 |
| 3211.4519016 | 12 |
| 3211.4523165 | 5 |
| 3211.4523598 | 8 |
| 3211.4526713 | 3 |
| 3211.4527201 | 9 |
| 3211.4531441 | 5 |
| 3211.4532963 | 0 |
| 3211.4539091 | 5 |
| 3211.4540581 | 4 |
| 3211.4545583 | 6 |
| 3211.454854 | 6 |
| 3211.4549073 | 7 |
| 3211.455486 | 5 |
| 3211.4559805 | 4 |
| 3211.4561368 | 3 |
| 3211.4562909 | 8 |
| 3211.4569296 | 2 |
| 3211.4571013 | 3 |
| 3211.4574825 | 0 |
| 3211.4577407 | 3 |
| 3211.4581391 | 1 |
| 3211.4585803 | 1 |
| 3211.45886 | 2 |
| 3211.4590065 | 0 |
| 3211.4593053 | 1 |
| 3211.4593656 | 0 |
| 3211.4599765 | 3 |
| 3211.4601196 | 2 |
| 3211.4602375 | 0 |
| 3211.4613261 | 3 |
| 3211.4614623 | 1 |
| 3211.4615292 | 0 |
| 3211.4618936 | 0 |
| 3211.4620231 | 1 |
| 3211.4622835 | 1 |
| 3211.4625673 | 1 |
| 3211.4631347 | 1 |
| 3211.4631859 | 1 |
| 3211.4633575 | 1 |
| 3211.4636075 | 1 |

| | |
|---|---|
| 3211.464025 | 2 |
| 3211.4643489 | 1 |
| 3211.465034 | 2 |
| 3211.4650581 | 1 |
| 3211.465222 | 1 |
| 3211.4659807 | 1 |
| 3211.4661982 | 2 |
| 3211.4663582 | 0 |
| 3211.4666179 | 1 |
| 3211.4668662 | 1 |
| 3211.466927 | 2 |
| 3211.4672851 | 1 |
| 3211.4679359 | 0 |
| 3211.4680591 | 1 |
| 3211.4682779 | 2 |
| 3211.4689103 | 0 |
| 3211.4690309 | 0 |
| 3211.4693432 | 2 |
| 3211.4694044 | 0 |
| 3211.4702086 | 0 |
| 3211.4704575 | 0 |
| 3211.4707348 | 2 |
| 3211.4711631 | 1 |
| 3211.4713109 | 1 |
| 3211.4714981 | 1 |
| 3211.4718138 | 1 |
| 3211.4726187 | 1 |
| 3211.4726737 | 1 |
| 3211.4728188 | 1 |
| 3211.4729881 | 1 |
| 3211.4734262 | 1 |
| 3211.4735045 | 1 |
| 3211.4736728 | 1 |
| 3211.4745974 | 1 |
| 3211.4746244 | 0 |
| 3211.4747326 | 1 |
| 3211.4752315 | 2 |
| 3211.4752404 | 1 |
| 3211.4761546 | 2 |
| 3211.4762288 | 1 |
| 3211.4763024 | 3 |
| 3211.476381 | 2 |
| 3211.4769605 | 1 |
| 3211.4774234 | 1 |
| 3211.4776566 | 3 |
| 3211.4777107 | 2 |
| 3211.4782715 | 0 |
| 3211.4783979 | 1 |
| 3211.4789662 | 2 |
| 3211.4792417 | 0 |
| 3211.4792738 | 1 |
| 3211.4798223 | 0 |
| 3211.480354 | 1 |
| 3211.48043 | 0 |
| 3211.481021 | 0 |
| 3211.4810904 | 1 |
| 3211.481238 | 0 |
| 3211.4815029 | 1 |
| 3211.4817481 | 1 |
| 3211.4819182 | 0 |
| 3211.4825217 | 1 |
| 3211.4826425 | 0 |
| 3211.4832538 | 0 |
| 3211.4835837 | 0 |
| 3211.4836596 | 1 |
| 3211.4839014 | 1 |
| 3211.4843263 | 1 |
| 3211.4846643 | 4 |
| 3211.4854359 | 2 |
| 3211.4854472 | 1 |
| 3211.486036 | 1 |
| 3211.4860422 | 4 |
| 3211.4862684 | 1 |
| 3211.4868468 | 1 |
| 3211.4870761 | 1 |
| 3211.4873697 | 0 |
| 3211.4878627 | 0 |
| 3211.4881527 | 1 |
| 3211.4887462 | 0 |
| 3211.4887737 | 1 |
| 3211.4888459 | 2 |
| 3211.4892271 | 1 |
| 3211.489594 | 1 |
| 3211.4900987 | 4 |
| 3211.4902577 | 1 |
| 3211.4905102 | 2 |
| 3211.4910832 | 1 |
| 3211.4912035 | 2 |
| 3211.4918779 | 0 |
| 3211.4919292 | 0 |
| 3211.4920027 | 2 |
| 3211.4928165 | 0 |
| 3211.4932463 | 2 |
| 3211.4935477 | 0 |
| 3211.4935564 | 1 |
| 3211.4936573 | 1 |
| 3211.4943328 | 0 |
| 3211.4945774 | 1 |
| 3211.4946676 | 0 |
| 3211.4947837 | 3 |
| 3211.4953884 | 0 |
| 3211.4958488 | 1 |
| 3211.4960916 | 2 |
| 3211.496422 | 1 |
| 3211.496754 | 0 |
| 3211.4972104 | 0 |
| 3211.4973942 | 2 |
| 3211.4977045 | 1 |
| 3211.497759 | 2 |
| 3211.4982574 | 1 |
| 3211.4983021 | 1 |
| 3211.4990578 | 1 |
| 3211.4994138 | 2 |
| 3211.4996536 | 1 |
| 3211.4996625 | 0 |
| 3211.4998613 | 2 |
| 3211.5002627 | 1 |
| 3211.5008214 | 0 |
| 3211.5013023 | 4 |
| 3211.5013288 | 2 |
| 3211.5014505 | 0 |
| 3211.5016163 | 1 |
| 3211.5020746 | 1 |
| 3211.5026079 | 0 |
| 3211.5029085 | 1 |
| 3211.5030387 | 0 |
| 3211.5030552 | 0 |
| 3211.5035167 | 1 |
| 3211.5040836 | 2 |
| 3211.5045512 | 0 |
| 3211.5047832 | 2 |
| 3211.5048197 | 1 |
| 3211.5051319 | 2 |
| 3211.5054509 | 2 |
| 3211.5057252 | 0 |
| 3211.5060466 | 1 |
| 3211.5064529 | 1 |
| 3211.5065016 | 1 |
| 3211.5077007 | 0 |
| 3211.5077177 | 0 |
| 3211.5081291 | 0 |
| 3211.5084849 | 1 |
| 3211.5085656 | 0 |
| 3211.5089076 | 0 |
| 3211.5091485 | 2 |
| 3211.509564 | 1 |
| 3211.5095804 | 0 |
| 3211.5099395 | 0 |
| 3211.5099964 | 2 |
| 3211.5106428 | 2 |
| 3211.5110416 | 1 |
| 3211.5117319 | 0 |
| 3211.5117334 | 0 |
| 3211.5119391 | 1 |
| 3211.5120209 | 0 |
| 3211.5121999 | 0 |
| 3211.5124612 | 0 |
| 3211.5130633 | 1 |
| 3211.5133054 | 1 |
| 3211.5135706 | 3 |
| 3211.5139352 | 0 |
| 3211.514123 | 1 |
| 3211.5142053 | 1 |
| 3211.5151445 | 4 |
| 3211.5153358 | 3 |
| 3211.5154175 | 1 |
| 3211.5157738 | 1 |
| 3211.5158491 | 1 |
| 3211.5164086 | 1 |
| 3211.5171113 | 1 |
| 3211.5172124 | 0 |
| 3211.5175982 | 0 |
| 3211.5176612 | 2 |
| 3211.5180151 | 1 |
| 3211.5185174 | 1 |
| 3211.5185506 | 1 |
| 3211.5189504 | 1 |
| 3211.5190239 | 0 |
| 3211.5196634 | 0 |
| 3211.5197835 | 4 |
| 3211.5201411 | 3 |
| 3211.5204988 | 1 |
| 3211.5205463 | 0 |
| 3211.5207319 | 1 |
| 3211.5212202 | 1 |
| 3211.5214331 | 4 |
| 3211.5217332 | 2 |
| 3211.522331 | 0 |
| 3211.522543 | 1 |
| 3211.5225564 | 0 |
| 3211.5229694 | 3 |
| 3211.5233091 | 1 |
| 3211.5235516 | 1 |
| 3211.5242389 | 0 |
| 3211.5244058 | 0 |
| 3211.5244634 | 1 |
| 3211.5248134 | 1 |
| 3211.5252424 | 0 |
| 3211.5257103 | 0 |
| 3211.5258131 | 1 |
| 3211.5258557 | 3 |
| 3211.5263399 | 1 |
| 3211.5266625 | 1 |
| 3211.5273284 | 0 |
| 3211.5274863 | 5 |
| 3211.5278421 | 2 |
| 3211.5280521 | 1 |
| 3211.5281294 | 0 |
| 3211.5282514 | 2 |
| 3211.5287896 | 0 |
| 3211.5294159 | 1 |
| 3211.5295096 | 1 |
| 3211.5299728 | 1 |
| 3211.530115 | 0 |
| 3211.5302575 | 0 |
| 3211.5308904 | 1 |
| 3211.5310199 | 2 |
| 3211.5313855 | 0 |
| 3211.531473 | 1 |
| 3211.5317358 | 1 |
| 3211.5323428 | 0 |
| 3211.532559 | 0 |
| 3211.533296 | 1 |
| 3211.5335797 | 0 |
| 3211.533814 | 1 |
| 3211.5338319 | 1 |
| 3211.5344046 | 0 |
| 3211.5347456 | 1 |
| 3211.534995 | 0 |
| 3211.5352991 | 0 |
| 3211.5355287 | 2 |
| 3211.5358445 | 0 |
| 3211.5361274 | 0 |
| 3211.5362549 | 0 |
| 3211.5370445 | 2 |
| 3211.5370692 | 3 |
| 3211.5374725 | 0 |
| 3211.5382018 | 0 |
| 3211.5382206 | 1 |
| 3211.5384835 | 1 |
| 3211.5388676 | 0 |
| 3211.5390357 | 0 |
| 3211.5391622 | 3 |
| 3211.5393877 | 0 |
| 3211.5399253 | 1 |
| 3211.5400541 | 3 |
| 3211.5406059 | 1 |
| 3211.5406309 | 2 |
| 3211.541063 | 1 |
| 3211.5414964 | 2 |
| 3211.541519 | 2 |
| 3211.5422236 | 0 |
| 3211.5424308 | 0 |
| 3211.542459 | 1 |
| 3211.54278 | 0 |
| 3211.5428957 | 2 |
| 3211.5429522 | 1 |
| 3211.5434599 | 0 |
| 3211.5442729 | 0 |
| 3211.5444972 | 1 |
| 3211.5449247 | 0 |
| 3211.5449324 | 3 |
| 3211.5449702 | 2 |
| 3211.5452613 | 2 |
| 3211.5459677 | 2 |
| 3211.5460813 | 3 |
| 3211.5461504 | 1 |
| 3211.5466043 | 0 |
| 3211.5471248 | 1 |
| 3211.547515 | 1 |
| 3211.5481607 | 0 |
| 3211.5483952 | 1 |
| 3211.5484759 | 0 |
| 3211.5485001 | 0 |
| 3211.5489014 | 1 |
| 3211.549165 | 0 |
| 3211.5496302 | 0 |
| 3211.5498946 | 1 |
| 3211.5504822 | 1 |
| 3211.5506471 | 0 |
| 3211.5510699 | 0 |
| 3211.5511367 | 1 |
| 3211.5513094 | 1 |
| 3211.5514811 | 1 |
| 3211.5520041 | 1 |
| 3211.552131 | 1 |
| 3211.5524081 | 3 |
| 3211.5525662 | 1 |
| 3211.5532405 | 1 |
| 3211.5536546 | 1 |
| 3211.554265 | 3 |
| 3211.5547589 | 0 |
| 3211.5548145 | 2 |
| 3211.5548168 | 1 |
| 3211.5549208 | 3 |
| 3211.5550748 | 2 |
| 3211.5551078 | 2 |
| 3211.55584 | 0 |
| 3211.5561047 | 3 |
| 3211.5561674 | 2 |
| 3211.5570132 | 1 |
| 3211.5571485 | 0 |
| 3211.5574963 | 2 |
| 3211.5578514 | 1 |
| 3211.5579978 | 1 |
| 3211.5584544 | 2 |
| 3211.5586711 | 0 |
| 3211.5588546 | 1 |
| 3211.559422 | 0 |
| 3211.5598573 | 2 |
| 3211.5598921 | 2 |
| 3211.5603014 | 1 |
| 3211.5607536 | 2 |
| 3211.5610049 | 0 |
| 3211.5610533 | 1 |
| 3211.5613233 | 1 |
| 3211.5619711 | 1 |
| 3211.5621459 | 0 |
| 3211.5622308 | 2 |
| 3211.5625901 | 0 |
| 3211.5631411 | 1 |
| 3211.5634311 | 0 |
| 3211.5635333 | 0 |
| 3211.5639985 | 0 |
| 3211.5641265 | 1 |
| 3211.5644617 | 0 |
| 3211.5645539 | 1 |
| 3211.5649611 | 1 |
| 3211.5657466 | 1 |
| 3211.5658913 | 1 |
| 3211.56622 | 1 |
| 3211.5667335 | 0 |
| 3211.5667939 | 1 |
| 3211.5671359 | 2 |
| 3211.567217 | 0 |
| 3211.5676163 | 3 |
| 3211.5679326 | 1 |
| 3211.5680311 | 2 |
| 3211.5687162 | 1 |
| 3211.5688788 | 3 |
| 3211.5694335 | 0 |
| 3211.5695958 | 0 |
| 3211.569816 | 1 |
| 3211.56991 | 2 |
| 3211.5699157 | 1 |
| 3211.5709376 | 4 |
| 3211.5710903 | 1 |
| 3211.5716327 | 1 |
| 3211.5719916 | 0 |
| 3211.5720965 | 0 |
| 3211.5725574 | 1 |
| 3211.5726191 | 0 |
| 3211.5727077 | 2 |
| 3211.5728919 | 1 |
| 3211.573097 | 1 |
| 3211.5734147 | 1 |
| 3211.5737395 | 0 |
| 3211.574126 | 3 |
| 3211.574384 | 1 |
| 3211.5753232 | 0 |
| 3211.5753446 | 0 |
| 3211.5755962 | 0 |
| 3211.5759414 | 1 |

| | |
|---|---|
| 3211.5764741 | 1 |
| 3211.5765709 | 2 |
| 3211.5768959 | 3 |
| 3211.5773518 | 1 |
| 3211.577477 | 1 |
| 3211.5777264 | 0 |
| 3211.5781265 | 0 |
| 3211.5785996 | 1 |
| 3211.5788393 | 3 |
| 3211.5792455 | 2 |
| 3211.5793289 | 2 |
| 3211.5793774 | 0 |
| 3211.5797858 | 2 |
| 3211.580508 | 0 |
| 3211.5808252 | 2 |
| 3211.5808619 | 0 |
| 3211.5813494 | 1 |
| 3211.5815805 | 1 |
| 3211.5816953 | 0 |
| 3211.5824236 | 1 |
| 3211.5825493 | 0 |
| 3211.5830783 | 1 |
| 3211.5835369 | 3 |
| 3211.5838572 | 2 |
| 3211.5844029 | 4 |
| 3211.5845485 | 1 |
| 3211.5848119 | 2 |
| 3211.5850767 | 3 |
| 3211.5851724 | 0 |
| 3211.5853733 | 1 |
| 3211.5854285 | 0 |
| 3211.5864916 | 2 |
| 3211.5867164 | 2 |
| 3211.5870511 | 2 |
| 3211.5874048 | 1 |
| 3211.5874336 | 0 |
| 3211.5875859 | 1 |
| 3211.5883154 | 0 |
| 3211.5884509 | 4 |
| 3211.5884553 | 0 |
| 3211.588832 | 1 |
| 3211.5895323 | 1 |
| 3211.589567 | 0 |
| 3211.5895736 | 2 |
| 3211.5900439 | 1 |
| 3211.590362 | 0 |
| 3211.5907164 | 2 |
| 3211.5910297 | 2 |
| 3211.5918001 | 0 |
| 3211.5918524 | 0 |
| 3211.5919369 | 1 |
| 3211.5924026 | 0 |
| 3211.5927281 | 3 |
| 3211.5930608 | 2 |
| 3211.5933434 | 1 |
| 3211.5936628 | 2 |
| 3211.5938526 | 1 |
| 3211.5940762 | 1 |
| 3211.5940776 | 0 |
| 3211.5942425 | 0 |
| 3211.5952897 | 1 |
| 3211.5956251 | 1 |
| 3211.5959071 | 2 |
| 3211.5962932 | 1 |
| 3211.5964791 | 0 |
| 3211.5971112 | 3 |
| 3211.5972919 | 0 |
| 3211.5973401 | 0 |
| 3211.5974487 | 0 |
| 3211.5977541 | 1 |
| 3211.5980471 | 0 |
| 3211.5987552 | 0 |
| 3211.59884 | 0 |
| 3211.5988442 | 1 |
| 3211.5991145 | 0 |
| 3211.5997412 | 2 |
| 3211.6002388 | 1 |
| 3211.6006232 | 1 |
| 3211.6006286 | 1 |
| 3211.6011479 | 1 |
| 3211.6012645 | 2 |
| 3211.6014551 | 2 |
| 3211.6020397 | 2 |
| 3211.6023913 | 2 |
| 3211.6025904 | 0 |
| 3211.6030572 | 0 |
| 3211.6033926 | 0 |
| 3211.603395 | 1 |
| 3211.6039296 | 1 |
| 3211.6040248 | 0 |
| 3211.6047548 | 0 |
| 3211.6052049 | 0 |
| 3211.6053769 | 1 |
| 3211.6056457 | 1 |
| 3211.6056635 | 3 |
| 3211.6056664 | 0 |
| 3211.6060995 | 3 |
| 3211.6061777 | 3 |
| 3211.607246 | 1 |
| 3211.6073195 | 1 |
| 3211.6077387 | 0 |
| 3211.6078474 | 1 |
| 3211.607859 | 1 |
| 3211.6080245 | 2 |
| 3211.6082803 | 1 |
| 3211.6084943 | 0 |
| 3211.6087756 | 0 |
| 3211.6090459 | 1 |
| 3211.6096822 | 1 |
| 3211.6099168 | 0 |
| 3211.610582 | 0 |
| 3211.6109063 | 1 |
| 3211.6111777 | 0 |
| 3211.6114474 | 2 |
| 3211.6115886 | 1 |
| 3211.6116065 | 2 |
| 3211.6120211 | 0 |
| 3211.6121068 | 1 |
| 3211.6125616 | 1 |
| 3211.6130578 | 1 |
| 3211.6131626 | 1 |
| 3211.6135304 | 1 |
| 3211.6140388 | 1 |
| 3211.614341 | 0 |
| 3211.6146215 | 0 |
| 3211.6151813 | 1 |
| 3211.6152333 | 1 |
| 3211.615497 | 1 |
| 3211.615914 | 1 |
| 3211.6162423 | 0 |
| 3211.6166471 | 4 |
| 3211.6171249 | 0 |
| 3211.617186 | 3 |
| 3211.6173551 | 0 |
| 3211.6174758 | 3 |
| 3211.6176389 | 2 |
| 3211.6181126 | 0 |
| 3211.6184564 | 2 |
| 3211.6189616 | 0 |
| 3211.6191173 | 1 |
| 3211.619638 | 0 |
| 3211.6197839 | 0 |
| 3211.6203423 | 1 |
| 3211.6205632 | 0 |
| 3211.6207724 | 1 |
| 3211.6209655 | 2 |
| 3211.6210569 | 2 |
| 3211.6216654 | 2 |
| 3211.6224167 | 0 |
| 3211.6228543 | 2 |
| 3211.6228993 | 1 |
| 3211.6230746 | 2 |
| 3211.6232687 | 0 |
| 3211.6238279 | 1 |
| 3211.6243052 | 0 |
| 3211.6244438 | 1 |
| 3211.6247676 | 1 |
| 3211.6250578 | 1 |
| 3211.6251404 | 3 |
| 3211.6254378 | 1 |
| 3211.6256735 | 0 |
| 3211.6264053 | 1 |
| 3211.6264731 | 1 |
| 3211.6268952 | 2 |
| 3211.6272027 | 1 |
| 3211.6274936 | 3 |
| 3211.6281217 | 2 |
| 3211.6282499 | 0 |
| 3211.628374 | 0 |
| 3211.6285465 | 1 |
| 3211.6290744 | 2 |
| 3211.629266 | 1 |
| 3211.6294125 | 4 |
| 3211.6296226 | 2 |
| 3211.6300122 | 0 |
| 3211.6305299 | 0 |
| 3211.6306909 | 1 |
| 3211.6311947 | 0 |
| 3211.6312311 | 1 |
| 3211.6317714 | 0 |
| 3211.6319941 | 1 |
| 3211.6320513 | 3 |
| 3211.6323671 | 1 |
| 3211.6330272 | 3 |
| 3211.6330697 | 0 |
| 3211.6334864 | 2 |
| 3211.633656 | 1 |
| 3211.6344088 | 0 |
| 3211.6346391 | 0 |
| 3211.6350021 | 1 |
| 3211.6351783 | 0 |
| 3211.6354832 | 1 |
| 3211.6356135 | 2 |
| 3211.6359048 | 1 |
| 3211.6362537 | 0 |
| 3211.6365071 | 0 |
| 3211.636592 | 1 |
| 3211.6374799 | 2 |
| 3211.6376928 | 1 |
| 3211.6377284 | 0 |
| 3211.6380172 | 0 |
| 3211.6388696 | 1 |
| 3211.638913 | 3 |
| 3211.6392933 | 2 |
| 3211.6395819 | 0 |
| 3211.6396229 | 1 |
| 3211.6399049 | 3 |
| 3211.6403906 | 0 |
| 3211.6405447 | 1 |
| 3211.6408341 | 1 |
| 3211.6413798 | 0 |
| 3211.6418531 | 0 |
| 3211.6419157 | 1 |
| 3211.6421768 | 2 |
| 3211.6424406 | 0 |
| 3211.6429899 | 0 |
| 3211.6431408 | 3 |
| 3211.6432116 | 3 |
| 3211.6434276 | 1 |
| 3211.6439352 | 1 |
| 3211.6442633 | 0 |
| 3211.6443395 | 1 |
| 3211.6448935 | 0 |
| 3211.6453774 | 1 |
| 3211.6455019 | 0 |
| 3211.6455646 | 0 |
| 3211.6463332 | 0 |
| 3211.6466559 | 0 |
| 3211.6473383 | 1 |
| 3211.6474402 | 0 |
| 3211.6475346 | 1 |
| 3211.6476453 | 0 |
| 3211.6481052 | 1 |
| 3211.6483072 | 0 |
| 3211.6489773 | 0 |
| 3211.6489865 | 0 |
| 3211.6493408 | 1 |
| 3211.6497901 | 0 |
| 3211.6500121 | 0 |
| 3211.6501039 | 2 |
| 3211.6505247 | 1 |
| 3211.6508115 | 2 |
| 3211.6509333 | 1 |
| 3211.6511979 | 0 |
| 3211.6516652 | 0 |
| 3211.6522629 | 1 |
| 3211.6527792 | 0 |
| 3211.6528007 | 3 |
| 3211.6528329 | 1 |
| 3211.6533372 | 0 |
| 3211.653363 | 1 |
| 3211.6537489 | 1 |
| 3211.6538474 | 1 |
| 3211.6541879 | 1 |
| 3211.6548774 | 0 |
| 3211.6551377 | 0 |
| 3211.6551963 | 1 |
| 3211.6553103 | 2 |
| 3211.6562225 | 1 |
| 3211.6562428 | 0 |
| 3211.6571051 | 2 |
| 3211.6571809 | 0 |
| 3211.6572375 | 0 |
| 3211.6573948 | 0 |
| 3211.6576245 | 0 |
| 3211.6584055 | 1 |
| 3211.6584584 | 0 |
| 3211.6590206 | 0 |
| 3211.6592276 | 1 |
| 3211.6593393 | 1 |
| 3211.6597134 | 1 |
| 3211.6597234 | 0 |
| 3211.6604471 | 2 |
| 3211.6605029 | 0 |
| 3211.6610424 | 1 |
| 3211.6610642 | 1 |
| 3211.6614957 | 0 |
| 3211.6618736 | 0 |
| 3211.6622237 | 1 |
| 3211.6622368 | 1 |
| 3211.6631116 | 4 |
| 3211.6632136 | 0 |
| 3211.6634282 | 2 |
| 3211.6636252 | 1 |
| 3211.6637107 | 0 |
| 3211.6643286 | 0 |
| 3211.6646616 | 1 |
| 3211.6647232 | 2 |
| 3211.6651645 | 1 |
| 3211.6657996 | 1 |
| 3211.6658969 | 1 |
| 3211.6662374 | 1 |
| 3211.6665749 | 0 |
| 3211.6666175 | 1 |
| 3211.6667631 | 1 |
| 3211.667151 | 1 |
| 3211.667378 | 1 |
| 3211.6681441 | 2 |
| 3211.6683818 | 1 |
| 3211.6684226 | 2 |
| 3211.6690362 | 0 |
| 3211.6692144 | 0 |
| 3211.6699331 | 0 |
| 3211.6700963 | 0 |
| 3211.6702004 | 1 |
| 3211.6705786 | 1 |
| 3211.6706416 | 0 |
| 3211.6710784 | 0 |
| 3211.67133 | 1 |
| 3211.6717892 | 0 |
| 3211.6725905 | 1 |
| 3211.6727727 | 0 |
| 3211.6728885 | 2 |
| 3211.6730508 | 1 |
| 3211.6731205 | 0 |
| 3211.6739148 | 3 |
| 3211.6741938 | 0 |
| 3211.6748925 | 4 |
| 3211.6749627 | 1 |
| 3211.6752659 | 1 |
| 3211.6755606 | 1 |
| 3211.6757563 | 1 |
| 3211.6758106 | 1 |
| 3211.6764655 | 1 |
| 3211.6768291 | 1 |
| 3211.6768889 | 2 |
| 3211.6774742 | 3 |
| 3211.6775253 | 0 |
| 3211.6779505 | 0 |
| 3211.6783064 | 1 |
| 3211.6783358 | 0 |
| 3211.678445 | 2 |
| 3211.6787762 | 0 |
| 3211.6797939 | 0 |
| 3211.6798166 | 1 |
| 3211.6798857 | 1 |
| 3211.6805287 | 0 |
| 3211.6807598 | 0 |
| 3211.6809377 | 1 |
| 3211.6809432 | 1 |
| 3211.6810391 | 1 |
| 3211.6816894 | 0 |
| 3211.6819082 | 1 |
| 3211.682309 | 1 |
| 3211.6824209 | 0 |
| 3211.6826632 | 1 |
| 3211.6830631 | 0 |
| 3211.6834152 | 2 |
| 3211.683423 | 3 |
| 3211.6840193 | 1 |
| 3211.6843086 | 2 |
| 3211.6848643 | 1 |
| 3211.6850343 | 0 |
| 3211.6851412 | 1 |
| 3211.6855336 | 1 |
| 3211.6855653 | 1 |
| 3211.6861365 | 1 |
| 3211.6867052 | 0 |
| 3211.6868132 | 1 |
| 3211.6871483 | 0 |
| 3211.6875461 | 1 |
| 3211.6878099 | 0 |
| 3211.6880281 | 0 |

| | |
|---|---|
| 3211.6881503 | 1 |
| 3211.688653 | 1 |
| 3211.6892014 | 1 |
| 3211.6898076 | 2 |
| 3211.6898289 | 0 |
| 3211.6902293 | 1 |
| 3211.6902526 | 0 |
| 3211.6907059 | 3 |
| 3211.6909763 | 4 |
| 3211.6912326 | 1 |
| 3211.6918936 | 2 |
| 3211.6922861 | 0 |
| 3211.6924246 | 2 |
| 3211.6927438 | 0 |
| 3211.6933214 | 1 |
| 3211.6933764 | 1 |
| 3211.693504 | 0 |
| 3211.6938276 | 3 |
| 3211.6942576 | 2 |
| 3211.6943836 | 1 |
| 3211.6945165 | 2 |
| 3211.6949172 | 3 |
| 3211.6949675 | 2 |
| 3211.6958188 | 0 |
| 3211.6960533 | 1 |
| 3211.6961243 | 1 |
| 3211.6962461 | 1 |
| 3211.6962545 | 0 |
| 3211.6970514 | 0 |
| 3211.6973755 | 0 |
| 3211.6975757 | 0 |
| 3211.6979648 | 1 |
| 3211.6982465 | 0 |
| 3211.6988528 | 2 |
| 3211.6989619 | 1 |
| 3211.6991521 | 0 |
| 3211.6994163 | 0 |
| 3211.6994444 | 0 |
| 3211.6999317 | 3 |
| 3211.7004351 | 0 |
| 3211.7007614 | 2 |
| 3211.7007874 | 1 |
| 3211.7013724 | 1 |
| 3211.701576 | 4 |
| 3211.7016086 | 2 |
| 3211.7019448 | 1 |
| 3211.7022369 | 2 |
| 3211.702395 | 2 |
| 3211.7031154 | 0 |
| 3211.7032847 | 0 |
| 3211.7034321 | 0 |
| 3211.7038268 | 0 |
| 3211.7041442 | 0 |
| 3211.7043254 | 2 |
| 3211.7044573 | 2 |
| 3211.7051121 | 1 |
| 3211.705296 | 1 |
| 3211.7059754 | 0 |
| 3211.7061549 | 2 |
| 3211.70621 | 0 |
| 3211.7063549 | 0 |
| 3211.7068971 | 0 |
| 3211.7071383 | 0 |
| 3211.7078611 | 1 |
| 3211.708057 | 2 |
| 3211.7085759 | 0 |
| 3211.7086641 | 2 |
| 3211.7092476 | 1 |
| 3211.709288 | 0 |
| 3211.7098848 | 2 |
| 3211.7099844 | 2 |
| 3211.7100172 | 3 |
| 3211.7103665 | 1 |
| 3211.710597 | 1 |
| 3211.7110801 | 4 |
| 3211.7110987 | 1 |
| 3211.7120537 | 2 |
| 3211.712282 | 1 |
| 3211.7127011 | 0 |
| 3211.7129094 | 1 |
| 3211.7129388 | 1 |
| 3211.7136727 | 0 |
| 3211.713754 | 1 |
| 3211.7137685 | 1 |
| 3211.7145115 | 3 |
| 3211.7147122 | 2 |
| 3211.7150008 | 2 |
| 3211.7152222 | 1 |
| 3211.7154344 | 2 |
| 3211.7157308 | 0 |
| 3211.7157978 | 0 |
| 3211.7162486 | 0 |
| 3211.7163065 | 2 |
| 3211.7167361 | 2 |
| 3211.7175084 | 0 |
| 3211.7176182 | 0 |
| 3211.7180462 | 0 |
| 3211.7182767 | 0 |
| 3211.7185725 | 3 |
| 3211.7186084 | 0 |
| 3211.7187742 | 0 |
| 3211.7194346 | 1 |
| 3211.7195294 | 1 |
| 3211.7198382 | 1 |
| 3211.7205105 | 0 |
| 3211.7206475 | 1 |
| 3211.7208712 | 1 |
| 3211.7211903 | 2 |
| 3211.7217506 | 1 |
| 3211.7217515 | 1 |
| 3211.722403 | 2 |
| 3211.7227635 | 2 |
| 3211.7227841 | 1 |
| 3211.7231927 | 1 |
| 3211.7234688 | 0 |
| 3211.7237212 | 2 |
| 3211.7241687 | 2 |
| 3211.7246838 | 0 |
| 3211.7250638 | 0 |
| 3211.7251515 | 1 |
| 3211.7252409 | 3 |
| 3211.7260358 | 0 |
| 3211.7261166 | 1 |
| 3211.7264872 | 1 |
| 3211.7267939 | 0 |
| 3211.7273563 | 1 |
| 3211.7274609 | 2 |
| 3211.7278747 | 1 |
| 3211.728262 | 0 |
| 3211.7283621 | 1 |
| 3211.7287227 | 0 |
| 3211.7288441 | 1 |
| 3211.7290348 | 2 |
| 3211.7297418 | 0 |
| 3211.729911 | 3 |
| 3211.730209 | 1 |
| 3211.7307304 | 1 |
| 3211.7307712 | 1 |
| 3211.7311779 | 2 |
| 3211.7314163 | 1 |
| 3211.7319135 | 1 |
| 3211.7319544 | 1 |
| 3211.7322818 | 1 |
| 3211.7326247 | 1 |
| 3211.7327331 | 1 |
| 3211.7327409 | 0 |
| 3211.7330374 | 0 |
| 3211.7334758 | 0 |
| 3211.733723 | 1 |
| 3211.7344557 | 0 |
| 3211.734802 | 2 |
| 3211.7354862 | 0 |
| 3211.7356191 | 0 |
| 3211.7357317 | 2 |
| 3211.735746 | 2 |
| 3211.7362805 | 0 |
| 3211.7362963 | 2 |
| 3211.7363544 | 2 |
| 3211.7368686 | 1 |
| 3211.7371235 | 1 |
| 3211.7376552 | 0 |
| 3211.7379815 | 4 |
| 3211.7385058 | 1 |
| 3211.7386084 | 0 |
| 3211.7391349 | 1 |
| 3211.7394586 | 0 |
| 3211.7395007 | 0 |
| 3211.7397331 | 0 |
| 3211.73978 | 0 |
| 3211.7405873 | 1 |
| 3211.7405893 | 1 |
| 3211.7411322 | 0 |
| 3211.7415528 | 0 |
| 3211.7415624 | 2 |
| 3211.742152 | 0 |
| 3211.7422397 | 1 |
| 3211.742823 | 1 |
| 3211.7428456 | 2 |
| 3211.7430536 | 2 |
| 3211.7433002 | 2 |
| 3211.7437015 | 1 |
| 3211.7437451 | 2 |
| 3211.7443935 | 0 |
| 3211.7446377 | 1 |
| 3211.7453458 | 2 |
| 3211.7457541 | 0 |
| 3211.7461749 | 0 |
| 3211.7466679 | 0 |
| 3211.7467062 | 1 |
| 3211.7467366 | 1 |
| 3211.7467871 | 0 |
| 3211.7469753 | 0 |
| 3211.7470886 | 1 |
| 3211.7477992 | 2 |
| 3211.7481845 | 0 |
| 3211.7484362 | 0 |
| 3211.7485461 | 2 |
| 3211.7488601 | 1 |
| 3211.7489865 | 1 |
| 3211.7494578 | 1 |
| 3211.749814 | 0 |
| 3211.7502592 | 1 |
| 3211.7504785 | 0 |
| 3211.7506771 | 0 |
| 3211.7511117 | 1 |
| 3211.7512719 | 1 |
| 3211.7515318 | 0 |
| 3211.752142 | 2 |
| 3211.7521992 | 2 |
| 3211.7522155 | 1 |
| 3211.7531404 | 1 |
| 3211.7531416 | 1 |
| 3211.7537238 | 0 |
| 3211.7537654 | 0 |
| 3211.7541753 | 1 |
| 3211.7545605 | 2 |
| 3211.7546453 | 2 |
| 3211.755256 | 1 |
| 3211.755448 | 2 |
| 3211.7555194 | 0 |
| 3211.7562979 | 5 |
| 3211.7563556 | 1 |
| 3211.7564744 | 3 |
| 3211.7567444 | 1 |
| 3211.7573795 | 2 |
| 3211.7573994 | 1 |
| 3211.7581038 | 1 |
| 3211.758196 | 1 |
| 3211.7585711 | 2 |
| 3211.7588202 | 3 |
| 3211.7591858 | 0 |
| 3211.7595008 | 1 |
| 3211.7602314 | 2 |
| 3211.7602687 | 0 |
| 3211.7606313 | 1 |
| 3211.7609807 | 1 |
| 3211.7610747 | 1 |
| 3211.7611806 | 1 |
| 3211.7612878 | 3 |
| 3211.7622017 | 0 |
| 3211.7625584 | 0 |
| 3211.762618 | 0 |
| 3211.7635395 | 1 |
| 3211.7636293 | 0 |
| 3211.7638784 | 1 |
| 3211.7639844 | 0 |
| 3211.7644303 | 2 |
| 3211.7646889 | 2 |
| 3211.7648008 | 1 |
| 3211.7654143 | 0 |
| 3211.765753 | 1 |
| 3211.7658442 | 0 |
| 3211.7661718 | 0 |
| 3211.7663663 | 2 |
| 3211.7665648 | 2 |
| 3211.7670206 | 0 |
| 3211.7674362 | 1 |
| 3211.7677196 | 0 |
| 3211.768318 | 3 |
| 3211.7686865 | 1 |
| 3211.768819 | 1 |
| 3211.7689 | 0 |
| 3211.7691128 | 1 |
| 3211.7696736 | 1 |
| 3211.7698847 | 1 |
| 3211.7700441 | 1 |
| 3211.7701977 | 1 |
| 3211.7708348 | 2 |
| 3211.7709411 | 2 |
| 3211.7712695 | 2 |
| 3211.7717806 | 1 |
| 3211.7721957 | 0 |
| 3211.7726695 | 0 |
| 3211.7726827 | 3 |
| 3211.7729635 | 2 |
| 3211.7730328 | 0 |
| 3211.7734347 | 2 |
| 3211.7739833 | 0 |
| 3211.7739849 | 1 |
| 3211.7743945 | 1 |
| 3211.7746801 | 1 |
| 3211.7751035 | 4 |
| 3211.7751294 | 0 |
| 3211.7762148 | 0 |
| 3211.7762554 | 1 |
| 3211.7765773 | 1 |
| 3211.7767123 | 1 |
| 3211.7771833 | 0 |
| 3211.7771957 | 0 |
| 3211.777744 | 0 |
| 3211.7779 | 3 |
| 3211.7781549 | 2 |
| 3211.7787007 | 1 |
| 3211.7790984 | 2 |
| 3211.779453 | 1 |
| 3211.7797284 | 0 |
| 3211.7797714 | 0 |
| 3211.7804371 | 0 |
| 3211.7807443 | 0 |
| 3211.7808269 | 0 |
| 3211.7809854 | 1 |
| 3211.7812228 | 0 |
| 3211.7817673 | 0 |
| 3211.782006 | 1 |
| 3211.782561 | 3 |
| 3211.7827983 | 0 |
| 3211.7833329 | 0 |
| 3211.7835514 | 0 |
| 3211.7839081 | 0 |
| 3211.7839381 | 1 |
| 3211.7841016 | 0 |
| 3211.784332 | 1 |
| 3211.7849268 | 0 |
| 3211.785137 | 4 |
| 3211.7851745 | 4 |
| 3211.7854308 | 0 |
| 3211.7859023 | 1 |
| 3211.7859611 | 1 |
| 3211.7866395 | 2 |
| 3211.7869093 | 2 |
| 3211.7869853 | 0 |
| 3211.7874137 | 2 |
| 3211.7879896 | 2 |
| 3211.7881994 | 3 |
| 3211.7884186 | 0 |
| 3211.7889944 | 0 |
| 3211.7891193 | 0 |
| 3211.7892756 | 1 |
| 3211.7899735 | 2 |
| 3211.7900243 | 1 |
| 3211.7900414 | 1 |
| 3211.7906614 | 0 |
| 3211.7908669 | 2 |
| 3211.7913384 | 0 |
| 3211.7913448 | 1 |
| 3211.7913512 | 1 |
| 3211.7917166 | 2 |
| 3211.7920252 | 2 |
| 3211.7925854 | 2 |
| 3211.7931413 | 1 |
| 3211.7931585 | 0 |
| 3211.7934276 | 0 |
| 3211.7935864 | 0 |
| 3211.7939548 | 4 |
| 3211.7940741 | 1 |
| 3211.7948062 | 1 |
| 3211.7953292 | 1 |
| 3211.795365 | 3 |
| 3211.7955465 | 0 |
| 3211.7959193 | 0 |
| 3211.7960966 | 1 |
| 3211.7967692 | 1 |
| 3211.7968972 | 1 |
| 3211.7972749 | 2 |
| 3211.7974627 | 0 |
| 3211.7981382 | 1 |
| 3211.798154 | 0 |
| 3211.7987453 | 1 |
| 3211.7987566 | 0 |
| 3211.7988953 | 1 |
| 3211.7993836 | 0 |
| 3211.7994097 | 0 |
| 3211.8000129 | 2 |

| | |
|---|---|
| 3211.8002637 | 1 |
| 3211.8007863 | 2 |
| 3211.8009673 | 3 |
| 3211.8011871 | 0 |
| 3211.8015656 | 1 |
| 3211.8019532 | 1 |
| 3211.8019707 | 0 |
| 3211.8019717 | 0 |
| 3211.8028229 | 0 |
| 3211.8029526 | 3 |
| 3211.8034099 | 1 |
| 3211.8038769 | 1 |
| 3211.803968 | 0 |
| 3211.8042369 | 0 |
| 3211.8042558 | 1 |
| 3211.8043493 | 0 |
| 3211.8048448 | 2 |
| 3211.8049783 | 0 |
| 3211.8056082 | 0 |
| 3211.8057174 | 0 |
| 3211.8065152 | 1 |
| 3211.8069405 | 2 |
| 3211.8072577 | 1 |
| 3211.8072833 | 0 |
| 3211.8073218 | 0 |
| 3211.8077181 | 2 |
| 3211.807876 | 2 |
| 3211.8080035 | 0 |
| 3211.8086255 | 1 |
| 3211.808733 | 1 |
| 3211.8095285 | 2 |
| 3211.8099067 | 2 |
| 3211.8099877 | 0 |
| 3211.8102907 | 2 |
| 3211.8109134 | 2 |
| 3211.8110796 | 3 |
| 3211.8114491 | 1 |
| 3211.8118222 | 0 |
| 3211.8122467 | 1 |
| 3211.8127175 | 1 |
| 3211.8129634 | 3 |
| 3211.81311 | 0 |
| 3211.8134342 | 0 |
| 3211.8138076 | 0 |
| 3211.8143248 | 3 |
| 3211.8144597 | 1 |
| 3211.814907 | 1 |
| 3211.8149676 | 2 |
| 3211.8149688 | 2 |
| 3211.8157184 | 0 |
| 3211.816269 | 1 |
| 3211.8166966 | 0 |
| 3211.8167452 | 2 |
| 3211.8168402 | 2 |
| 3211.8169758 | 2 |
| 3211.8175286 | 3 |
| 3211.8177311 | 1 |
| 3211.8179808 | 2 |
| 3211.8181615 | 3 |
| 3211.8188951 | 0 |
| 3211.8192008 | 1 |
| 3211.8195735 | 1 |
| 3211.8197312 | 2 |
| 3211.8197932 | 2 |
| 3211.8198316 | 1 |
| 3211.8203082 | 1 |
| 3211.8204713 | 0 |
| 3211.8210074 | 0 |
| 3211.8214471 | 2 |
| 3211.8223732 | 0 |
| 3211.8228661 | 1 |
| 3211.8228712 | 1 |
| 3211.8228772 | 0 |
| 3211.8229399 | 2 |
| 3211.8233965 | 0 |
| 3211.8235526 | 1 |
| 3211.8237729 | 2 |
| 3211.8243475 | 3 |
| 3211.8246192 | 0 |
| 3211.8246304 | 4 |
| 3211.8250082 | 2 |
| 3211.8257256 | 2 |
| 3211.8261295 | 0 |
| 3211.8265433 | 0 |
| 3211.8265842 | 1 |
| 3211.8266047 | 2 |
| 3211.826761 | 2 |
| 3211.8268731 | 1 |
| 3211.8273985 | 0 |
| 3211.8280515 | 1 |
| 3211.8280577 | 0 |
| 3211.8285649 | 2 |
| 3211.828585 | 0 |
| 3211.8287441 | 2 |
| 3211.8293626 | 2 |
| 3211.8296158 | 0 |
| 3211.8300604 | 0 |
| 3211.8300839 | 1 |
| 3211.8303524 | 0 |
| 3211.8309171 | 4 |
| 3211.8312146 | 1 |
| 3211.8316649 | 0 |
| 3211.8319214 | 0 |
| 3211.8319521 | 1 |
| 3211.8325616 | 1 |
| 3211.8327934 | 1 |
| 3211.8328071 | 1 |
| 3211.833093 | 1 |
| 3211.8335372 | 1 |
| 3211.8337414 | 0 |
| 3211.8343366 | 1 |
| 3211.8348762 | 0 |
| 3211.8351823 | 1 |
| 3211.8354109 | 1 |
| 3211.8357595 | 0 |
| 3211.8360819 | 0 |
| 3211.8361636 | 2 |
| 3211.836375 | 2 |
| 3211.8367703 | 0 |
| 3211.8369781 | 1 |
| 3211.8373403 | 0 |
| 3211.8374416 | 1 |
| 3211.8382276 | 3 |
| 3211.8385213 | 1 |
| 3211.838647 | 1 |
| 3211.8389995 | 1 |
| 3211.8391304 | 0 |
| 3211.8397652 | 0 |
| 3211.8399172 | 0 |
| 3211.8402186 | 0 |
| 3211.8405095 | 0 |
| 3211.840773 | 4 |
| 3211.8408231 | 1 |
| 3211.8411047 | 0 |
| 3211.841332 | 2 |
| 3211.841528 | 2 |
| 3211.8417426 | 0 |
| 3211.8421987 | 0 |
| 3211.8425428 | 1 |
| 3211.8432567 | 1 |
| 3211.8433068 | 0 |
| 3211.8435014 | 1 |
| 3211.8440997 | 0 |
| 3211.844492 | 2 |
| 3211.8448497 | 1 |
| 3211.8448799 | 1 |
| 3211.8452678 | 2 |
| 3211.8455873 | 0 |
| 3211.8459698 | 1 |
| 3211.8460084 | 0 |
| 3211.8466165 | 2 |
| 3211.846709 | 0 |
| 3211.8468877 | 0 |
| 3211.847334 | 0 |
| 3211.847567 | 4 |
| 3211.8482017 | 1 |
| 3211.8482947 | 2 |
| 3211.8490279 | 1 |
| 3211.849112 | 1 |
| 3211.8495248 | 2 |
| 3211.8495932 | 0 |
| 3211.8497399 | 1 |
| 3211.8503566 | 1 |
| 3211.8504896 | 0 |
| 3211.8506785 | 1 |
| 3211.8514866 | 4 |
| 3211.8515334 | 1 |
| 3211.8515961 | 1 |
| 3211.8519672 | 2 |
| 3211.8525992 | 0 |
| 3211.8526136 | 1 |
| 3211.8526646 | 1 |
| 3211.8534724 | 1 |
| 3211.8535396 | 0 |
| 3211.8541322 | 2 |
| 3211.8543281 | 1 |
| 3211.8547516 | 0 |
| 3211.8548434 | 0 |
| 3211.8551493 | 3 |
| 3211.8555263 | 2 |
| 3211.8555632 | 2 |
| 3211.8563508 | 0 |
| 3211.8566564 | 1 |
| 3211.8569662 | 1 |
| 3211.8574125 | 4 |
| 3211.8574431 | 0 |
| 3211.8581263 | 0 |
| 3211.858378 | 2 |
| 3211.858396 | 0 |
| 3211.8586894 | 1 |
| 3211.8589281 | 0 |
| 3211.859435 | 0 |
| 3211.8597023 | 2 |
| 3211.8601907 | 1 |
| 3211.8604302 | 0 |
| 3211.8605739 | 1 |
| 3211.8609265 | 0 |
| 3211.8613967 | 0 |
| 3211.8617232 | 1 |
| 3211.861857 | 1 |
| 3211.8622359 | 2 |
| 3211.8627861 | 1 |
| 3211.8628642 | 1 |
| 3211.863618 | 3 |
| 3211.8636505 | 0 |
| 3211.86388 | 1 |
| 3211.8639525 | 1 |
| 3211.8641635 | 0 |
| 3211.8645694 | 0 |
| 3211.8648973 | 1 |
| 3211.8651258 | 1 |
| 3211.8656631 | 1 |
| 3211.8658731 | 0 |
| 3211.8664983 | 0 |
| 3211.8668664 | 1 |
| 3211.8671768 | 1 |
| 3211.8672724 | 0 |
| 3211.8676001 | 0 |
| 3211.8677599 | 2 |
| 3211.8679611 | 0 |
| 3211.8682431 | 1 |
| 3211.8682797 | 2 |
| 3211.8691428 | 1 |
| 3211.8694782 | 3 |
| 3211.8696592 | 2 |
| 3211.8702815 | 1 |
| 3211.8704813 | 2 |
| 3211.8708066 | 1 |
| 3211.8709407 | 0 |
| 3211.8709645 | 1 |
| 3211.8713541 | 2 |
| 3211.8715252 | 1 |
| 3211.872076 | 1 |
| 3211.8723727 | 1 |
| 3211.87289 | 1 |
| 3211.8728958 | 2 |
| 3211.8735783 | 1 |
| 3211.8736135 | 1 |
| 3211.8738986 | 1 |
| 3211.8743617 | 1 |
| 3211.8747661 | 1 |
| 3211.8749978 | 0 |
| 3211.8754962 | 1 |
| 3211.8755252 | 1 |
| 3211.8758266 | 5 |
| 3211.8759061 | 3 |
| 3211.8761318 | 1 |
| 3211.8764971 | 4 |
| 3211.8766135 | 1 |
| 3211.8772463 | 1 |
| 3211.8777593 | 1 |
| 3211.8781212 | 0 |
| 3211.8784658 | 1 |
| 3211.8785361 | 1 |
| 3211.8786082 | 1 |
| 3211.8787741 | 0 |
| 3211.8791358 | 2 |
| 3211.8793077 | 0 |
| 3211.8799602 | 2 |
| 3211.8800652 | 2 |
| 3211.8801026 | 3 |
| 3211.8804219 | 2 |
| 3211.8809839 | 2 |
| 3211.8812164 | 4 |
| 3211.8818311 | 0 |
| 3211.8819361 | 2 |
| 3211.8821934 | 1 |
| 3211.8828911 | 1 |
| 3211.8829707 | 1 |
| 3211.8832511 | 1 |
| 3211.8836239 | 0 |
| 3211.8837732 | 0 |
| 3211.8842911 | 0 |
| 3211.8843401 | 2 |
| 3211.8846283 | 2 |
| 3211.8855691 | 3 |
| 3211.8855757 | 1 |
| 3211.8859075 | 0 |
| 3211.8861712 | 0 |
| 3211.8866325 | 1 |
| 3211.8870134 | 1 |
| 3211.8871743 | 0 |
| 3211.887269 | 1 |
| 3211.8874361 | 2 |
| 3211.8878143 | 1 |
| 3211.8882925 | 1 |
| 3211.8883626 | 1 |
| 3211.8889514 | 3 |
| 3211.8892504 | 1 |
| 3211.8893906 | 4 |
| 3211.889724 | 0 |
| 3211.8901295 | 0 |
| 3211.8902305 | 0 |
| 3211.8908532 | 0 |
| 3211.8910527 | 1 |
| 3211.8911942 | 0 |
| 3211.8912365 | 1 |
| 3211.8915202 | 0 |
| 3211.8922803 | 2 |
| 3211.8928389 | 2 |
| 3211.8930266 | 1 |
| 3211.8936183 | 1 |
| 3211.8938714 | 1 |
| 3211.8940435 | 1 |
| 3211.8941195 | 1 |
| 3211.8943815 | 2 |
| 3211.8950834 | 0 |
| 3211.8952642 | 0 |
| 3211.8957135 | 2 |
| 3211.8958773 | 4 |
| 3211.8959228 | 1 |
| 3211.8960694 | 0 |
| 3211.8967376 | 2 |
| 3211.8967579 | 3 |
| 3211.8976687 | 3 |
| 3211.8977844 | 0 |
| 3211.8978147 | 1 |
| 3211.8980442 | 2 |
| 3211.898275 | 1 |
| 3211.8989254 | 0 |
| 3211.899009 | 1 |
| 3211.8995642 | 0 |
| 3211.8997186 | 0 |
| 3211.9002362 | 1 |
| 3211.9006509 | 1 |
| 3211.9010118 | 1 |
| 3211.9011758 | 1 |
| 3211.9014738 | 0 |
| 3211.901693 | 2 |
| 3211.9019717 | 0 |
| 3211.9022527 | 4 |
| 3211.9027589 | 2 |
| 3211.9032502 | 0 |
| 3211.9032823 | 3 |
| 3211.9037336 | 1 |
| 3211.9040239 | 1 |
| 3211.904085 | 0 |
| 3211.9044567 | 3 |
| 3211.9053843 | 0 |
| 3211.9055398 | 1 |
| 3211.9056026 | 3 |
| 3211.9056418 | 1 |
| 3211.9056553 | 1 |
| 3211.906651 | 2 |
| 3211.9066804 | 2 |
| 3211.9072202 | 0 |
| 3211.9072891 | 0 |
| 3211.9074352 | 0 |
| 3211.9075993 | 2 |
| 3211.908463 | 2 |
| 3211.9086098 | 1 |
| 3211.9088493 | 1 |
| 3211.9093297 | 1 |
| 3211.9095927 | 0 |
| 3211.9102045 | 3 |
| 3211.9105191 | 3 |
| 3211.9106362 | 2 |
| 3211.9106776 | 0 |
| 3211.9108682 | 1 |
| 3211.9111283 | 1 |
| 3211.9118277 | 0 |
| 3211.9118814 | 1 |
| 3211.9123039 | 1 |

| | |
|---|---|
| 3211.9130149 | 2 |
| 3211.9132972 | 1 |
| 3211.9134973 | 0 |
| 3211.9138379 | 2 |
| 3211.9141385 | 3 |
| 3211.914393 | 0 |
| 3211.9146104 | 1 |
| 3211.9147056 | 0 |
| 3211.9148927 | 1 |
| 3211.9155371 | 0 |
| 3211.9155574 | 2 |
| 3211.9161409 | 2 |
| 3211.9167016 | 1 |
| 3211.9168334 | 3 |
| 3211.9168709 | 1 |
| 3211.9171849 | 0 |
| 3211.9179656 | 0 |
| 3211.9182228 | 0 |
| 3211.9185293 | 0 |
| 3211.9188099 | 0 |
| 3211.9192033 | 1 |
| 3211.9194251 | 2 |
| 3211.9199021 | 2 |
| 3211.9200474 | 2 |
| 3211.9200757 | 2 |
| 3211.9206597 | 1 |
| 3211.9209791 | 1 |
| 3211.9213081 | 2 |
| 3211.9215686 | 0 |
| 3211.9222607 | 2 |
| 3211.9223466 | 2 |
| 3211.9227649 | 0 |
| 3211.9229001 | 2 |
| 3211.9233964 | 4 |
| 3211.9235479 | 1 |
| 3211.9235823 | 1 |
| 3211.9236832 | 0 |
| 3211.9243428 | 1 |
| 3211.9243887 | 1 |
| 3211.9243995 | 0 |
| 3211.9249089 | 0 |
| 3211.9255383 | 2 |
| 3211.9258643 | 0 |
| 3211.9262979 | 2 |
| 3211.9263009 | 1 |
| 3211.9266357 | 0 |
| 3211.9269214 | 2 |
| 3211.9269816 | 0 |
| 3211.9276991 | 1 |
| 3211.9280983 | 0 |
| 3211.9281022 | 1 |
| 3211.9283215 | 0 |
| 3211.9283389 | 1 |
| 3211.9288085 | 1 |
| 3211.9288314 | 2 |
| 3211.9293858 | 1 |
| 3211.9294793 | 3 |
| 3211.9297028 | 0 |
| 3211.9304959 | 2 |
| 3211.9309454 | 0 |
| 3211.9311312 | 1 |
| 3211.9312049 | 2 |
| 3211.9318145 | 1 |
| 3211.9321095 | 0 |
| 3211.9321621 | 2 |
| 3211.9325424 | 0 |
| 3211.9326842 | 0 |
| 3211.9332694 | 1 |
| 3211.9332764 | 1 |
| 3211.9333381 | 0 |
| 3211.9341924 | 0 |
| 3211.9344076 | 3 |
| 3211.9345255 | 5 |
| 3211.9348858 | 2 |
| 3211.9353382 | 0 |
| 3211.9357289 | 1 |
| 3211.9360924 | 0 |
| 3211.9362882 | 0 |
| 3211.9366677 | 0 |
| 3211.9369522 | 0 |
| 3211.9370087 | 2 |
| 3211.9374903 | 2 |
| 3211.9379714 | 0 |
| 3211.9382311 | 2 |
| 3211.9388387 | 1 |
| 3211.9391988 | 4 |
| 3211.9392495 | 0 |
| 3211.9393549 | 2 |
| 3211.9397305 | 1 |
| 3211.9403476 | 1 |
| 3211.9406576 | 2 |
| 3211.9414788 | 1 |
| 3211.9416041 | 1 |
| 3211.9419029 | 0 |
| 3211.942064 | 0 |
| 3211.9421252 | 0 |
| 3211.9421395 | 1 |
| 3211.9430885 | 0 |
| 3211.9433801 | 0 |
| 3211.9434234 | 2 |
| 3211.9436736 | 2 |
| 3211.9442383 | 1 |
| 3211.9442908 | 0 |
| 3211.944453 | 0 |
| 3211.9450596 | 1 |
| 3211.9456918 | 0 |
| 3211.9458221 | 4 |
| 3211.945889 | 3 |
| 3211.9459155 | 1 |
| 3211.9466409 | 0 |
| 3211.9470343 | 0 |
| 3211.9476966 | 2 |
| 3211.9478257 | 0 |
| 3211.9484179 | 3 |
| 3211.9484557 | 0 |
| 3211.9485441 | 2 |
| 3211.9486896 | 0 |
| 3211.9492691 | 0 |
| 3211.949592 | 1 |
| 3211.9497497 | 3 |
| 3211.9500327 | 2 |
| 3211.9501886 | 0 |
| 3211.950517 | 2 |
| 3211.9506944 | 1 |
| 3211.9512362 | 1 |
| 3211.9516099 | 0 |
| 3211.9519064 | 2 |
| 3211.9520063 | 0 |
| 3211.9523652 | 2 |
| 3211.9524356 | 1 |
| 3211.9528327 | 0 |
| 3211.9536437 | 1 |
| 3211.9536685 | 1 |
| 3211.9542911 | 0 |
| 3211.9543872 | 3 |
| 3211.955088 | 2 |
| 3211.9551907 | 2 |
| 3211.9553237 | 0 |
| 3211.95541 | 0 |
| 3211.9556149 | 1 |
| 3211.9562733 | 0 |
| 3211.9564334 | 2 |
| 3211.9566519 | 0 |
| 3211.9570302 | 2 |
| 3211.9572336 | 3 |
| 3211.9574109 | 0 |
| 3211.9583243 | 2 |
| 3211.9583374 | 0 |
| 3211.9588721 | 0 |
| 3211.9593973 | 2 |
| 3211.959683 | 1 |
| 3211.9599792 | 1 |
| 3211.9602821 | 0 |
| 3211.9604938 | 0 |
| 3211.9607236 | 1 |
| 3211.9611656 | 0 |
| 3211.9612181 | 1 |
| 3211.9612267 | 1 |
| 3211.9618354 | 1 |
| 3211.9619008 | 3 |
| 3211.9625829 | 0 |
| 3211.9630893 | 3 |
| 3211.9631268 | 2 |
| 3211.9634171 | 0 |
| 3211.9639279 | 0 |
| 3211.9640956 | 0 |
| 3211.9641303 | 2 |
| 3211.964228 | 0 |
| 3211.9651046 | 2 |
| 3211.9653479 | 2 |
| 3211.9655126 | 0 |
| 3211.9656539 | 3 |
| 3211.9659767 | 1 |
| 3211.9660887 | 1 |
| 3211.9662723 | 0 |
| 3211.9675847 | 1 |
| 3211.9676397 | 2 |
| 3211.9676553 | 1 |
| 3211.967868 | 2 |
| 3211.9687652 | 0 |
| 3211.9688489 | 3 |
| 3211.9690671 | 3 |
| 3211.9695138 | 3 |
| 3211.9698413 | 0 |
| 3211.969843 | 1 |
| 3211.9701312 | 1 |
| 3211.9705981 | 1 |
| 3211.9707979 | 2 |
| 3211.9709998 | 0 |
| 3211.9715259 | 0 |
| 3211.9716179 | 1 |
| 3211.972328 | 2 |
| 3211.9725243 | 4 |
| 3211.9726319 | 1 |
| 3211.9730071 | 1 |
| 3211.9731562 | 2 |
| 3211.9733209 | 0 |
| 3211.9739278 | 0 |
| 3211.9741693 | 0 |
| 3211.9742355 | 1 |
| 3211.9744031 | 0 |
| 3211.974759 | 1 |
| 3211.9754548 | 1 |
| 3211.9755536 | 3 |
| 3211.9760897 | 1 |
| 3211.976449 | 1 |
| 3211.9764665 | 3 |
| 3211.9767678 | 0 |
| 3211.9767726 | 0 |
| 3211.9774366 | 2 |
| 3211.9782343 | 0 |
| 3211.9783617 | 0 |
| 3211.9786768 | 1 |
| 3211.9790649 | 1 |
| 3211.9792788 | 2 |
| 3211.9793715 | 1 |
| 3211.9798825 | 2 |
| 3211.979981 | 1 |
| 3211.9801613 | 1 |
| 3211.9804359 | 0 |
| 3211.9805374 | 0 |
| 3211.9813053 | 1 |
| 3211.9813327 | 0 |
| 3211.9820213 | 1 |
| 3211.9824545 | 0 |
| 3211.982558 | 1 |
| 3211.9827237 | 0 |
| 3211.9831003 | 0 |
| 3211.9833224 | 1 |
| 3211.9839954 | 0 |
| 3211.9841409 | 1 |
| 3211.9845368 | 1 |
| 3211.98493 | 1 |
| 3211.9850053 | 1 |
| 3211.9852595 | 1 |
| 3211.9855141 | 3 |
| 3211.9862103 | 2 |
| 3211.9865207 | 2 |
| 3211.986597 | 1 |
| 3211.9866148 | 1 |
| 3211.9870594 | 1 |
| 3211.9876887 | 0 |
| 3211.9879372 | 0 |
| 3211.9883486 | 0 |
| 3211.9885175 | 2 |
| 3211.9889707 | 0 |
| 3211.9891041 | 1 |
| 3211.9893369 | 1 |
| 3211.9898793 | 1 |
| 3211.9902426 | 2 |
| 3211.9905046 | 1 |
| 3211.9906309 | 1 |
| 3211.9908907 | 1 |
| 3211.9912629 | 0 |
| 3211.9920007 | 0 |
| 3211.9922931 | 0 |
| 3211.9926171 | 2 |
| 3211.9928779 | 2 |
| 3211.9934795 | 0 |
| 3211.9938316 | 1 |
| 3211.993846 | 0 |
| 3211.994513 | 0 |
| 3211.9945347 | 0 |
| 3211.9947938 | 2 |
| 3211.9953399 | 1 |
| 3211.9953697 | 0 |
| 3211.9958489 | 0 |
| 3211.996224 | 2 |
| 3211.9963281 | 0 |
| 3211.9965154 | 2 |
| 3211.9970373 | 0 |
| 3211.9970779 | 1 |
| 3211.9977964 | 0 |
| 3211.9980915 | 0 |
| 3211.9986865 | 0 |
| 3211.9988044 | 0 |
| 3211.9992407 | 1 |
| 3211.9992743 | 0 |
| 3211.9995055 | 0 |
| 3211.9995258 | 2 |
| 3211.999896 | 2 |
| 3212.0003073 | 0 |
| 3212.000929 | 1 |
| 3212.0009481 | 0 |
| 3212.0017556 | 2 |
| 3212.0019644 | 1 |
| 3212.0022385 | 1 |
| 3212.0026772 | 1 |
| 3212.0029244 | 1 |
| 3212.0031299 | 1 |
| 3212.0032792 | 1 |
| 3212.0039849 | 1 |
| 3212.0041335 | 1 |
| 3212.0047645 | 1 |
| 3212.0048145 | 1 |
| 3212.0050884 | 1 |
| 3212.0054504 | 0 |
| 3212.0054883 | 0 |
| 3212.0059301 | 1 |
| 3212.0059377 | 2 |
| 3212.0064044 | 0 |
| 3212.006825 | 2 |
| 3212.006993 | 0 |
| 3212.0072094 | 2 |
| 3212.0074083 | 3 |
| 3212.007905 | 0 |
| 3212.0080864 | 2 |
| 3212.0082153 | 2 |
| 3212.008413 | 0 |
| 3212.0092971 | 0 |
| 3212.0095581 | 1 |
| 3212.0096963 | 2 |
| 3212.0099735 | 0 |
| 3212.0101336 | 0 |
| 3212.0104035 | 1 |
| 3212.0109604 | 2 |
| 3212.0110123 | 3 |
| 3212.0115693 | 0 |
| 3212.0117444 | 2 |
| 3212.0118072 | 1 |
| 3212.0122962 | 2 |
| 3212.0126665 | 1 |
| 3212.0130156 | 1 |
| 3212.0135552 | 1 |
| 3212.0137562 | 2 |
| 3212.0138893 | 0 |
| 3212.0143193 | 2 |
| 3212.0150394 | 0 |
| 3212.0150908 | 0 |
| 3212.0151303 | 0 |
| 3212.0151355 | 1 |
| 3212.0160293 | 1 |
| 3212.016107 | 1 |
| 3212.0168869 | 3 |
| 3212.0170878 | 2 |
| 3212.017318 | 0 |
| 3212.0175122 | 1 |
| 3212.0176368 | 1 |
| 3212.017736 | 3 |
| 3212.017755 | 0 |
| 3212.018904 | 3 |
| 3212.018948 | 2 |
| 3212.0192671 | 1 |
| 3212.0193357 | 1 |
| 3212.0194755 | 0 |
| 3212.0205408 | 1 |
| 3212.0206764 | 1 |
| 3212.0206872 | 1 |
| 3212.0209989 | 1 |
| 3212.0212513 | 0 |
| 3212.0217393 | 2 |
| 3212.0217686 | 1 |
| 3212.0227508 | 1 |
| 3212.0231062 | 1 |
| 3212.0231464 | 0 |
| 3212.0231558 | 1 |
| 3212.0234209 | 0 |
| 3212.0239626 | 2 |
| 3212.0242994 | 0 |
| 3212.0247935 | 0 |
| 3212.0248698 | 1 |
| 3212.0252002 | 0 |
| 3212.0255981 | 1 |

| | |
|---|---|
| 3212.0257082 | 0 |
| 3212.0259647 | 1 |
| 3212.0262676 | 2 |
| 3212.0266904 | 0 |
| 3212.0268531 | 5 |
| 3212.0268935 | 0 |
| 3212.0269616 | 1 |
| 3212.0277241 | 3 |
| 3212.0282738 | 0 |
| 3212.0284435 | 0 |
| 3212.0289988 | 2 |
| 3212.0291446 | 0 |
| 3212.0294855 | 0 |
| 3212.0300291 | 0 |
| 3212.0300376 | 0 |
| 3212.0306324 | 1 |
| 3212.030919 | 1 |
| 3212.0309694 | 1 |
| 3212.0316371 | 0 |
| 3212.0318165 | 2 |
| 3212.0322587 | 1 |
| 3212.0328219 | 2 |
| 3212.0329604 | 0 |
| 3212.0330164 | 2 |
| 3212.0333537 | 1 |
| 3212.0337256 | 0 |
| 3212.0337554 | 0 |
| 3212.0339688 | 0 |
| 3212.0343594 | 0 |
| 3212.0349988 | 2 |
| 3212.0350237 | 0 |
| 3212.035373 | 0 |
| 3212.0358851 | 1 |
| 3212.0365438 | 3 |
| 3212.036856 | 0 |
| 3212.0371166 | 1 |
| 3212.0373921 | 1 |
| 3212.0374716 | 2 |
| 3212.0376807 | 1 |
| 3212.0380909 | 1 |
| 3212.0382216 | 0 |
| 3212.0388193 | 1 |
| 3212.038946 | 1 |
| 3212.0392579 | 1 |
| 3212.0394146 | 1 |
| 3212.0400116 | 2 |
| 3212.0403539 | 4 |
| 3212.0404434 | 0 |
| 3212.0409333 | 1 |
| 3212.0414897 | 1 |
| 3212.0415272 | 1 |
| 3212.0422357 | 0 |
| 3212.0424345 | 2 |
| 3212.042585 | 0 |
| 3212.042735 | 4 |
| 3212.0430989 | 2 |
| 3212.0431363 | 0 |
| 3212.0435995 | 1 |
| 3212.0436956 | 0 |
| 3212.0447681 | 2 |
| 3212.0447992 | 0 |
| 3212.0447993 | 0 |
| 3212.0455448 | 2 |
| 3212.0455498 | 2 |
| 3212.0456535 | 1 |
| 3212.0465637 | 2 |
| 3212.046764 | 1 |
| 3212.0468774 | 0 |
| 3212.0471742 | 1 |
| 3212.0476775 | 1 |
| 3212.0480527 | 1 |
| 3212.0485326 | 1 |
| 3212.0490471 | 0 |
| 3212.0492314 | 2 |
| 3212.0495993 | 0 |
| 3212.0499003 | 0 |
| 3212.0499878 | 0 |
| 3212.0503406 | 2 |
| 3212.0507 | 0 |
| 3212.051097 | 0 |
| 3212.0511022 | 0 |
| 3212.0512169 | 0 |
| 3212.0518029 | 3 |
| 3212.0521251 | 1 |
| 3212.0522196 | 0 |
| 3212.0523782 | 1 |
| 3212.0530819 | 2 |
| 3212.0532348 | 0 |
| 3212.0533936 | 0 |
| 3212.0538946 | 1 |
| 3212.0542532 | 0 |
| 3212.0545162 | 0 |
| 3212.0545753 | 2 |
| 3212.0550086 | 0 |
| 3212.0554718 | 1 |
| 3212.0556986 | 1 |
| 3212.055842 | 0 |
| 3212.0562368 | 0 |
| 3212.0564472 | 2 |
| 3212.0566326 | 0 |
| 3212.0574418 | 5 |
| 3212.0580117 | 2 |
| 3212.0581152 | 1 |
| 3212.0582581 | 0 |
| 3212.0583697 | 2 |
| 3212.0585036 | 0 |
| 3212.0587275 | 1 |
| 3212.059583 | 4 |
| 3212.0599098 | 2 |
| 3212.0599723 | 1 |
| 3212.0609021 | 1 |
| 3212.0610093 | 3 |
| 3212.0610744 | 0 |
| 3212.0610837 | 1 |
| 3212.0616064 | 2 |
| 3212.0621751 | 0 |
| 3212.0625018 | 0 |
| 3212.0625463 | 1 |
| 3212.0628304 | 0 |
| 3212.062888 | 0 |
| 3212.0635364 | 0 |
| 3212.0638474 | 1 |
| 3212.0638664 | 3 |
| 3212.0644885 | 0 |
| 3212.0645235 | 1 |
| 3212.0648409 | 1 |
| 3212.0650497 | 0 |
| 3212.0655484 | 1 |
| 3212.0661592 | 2 |
| 3212.0665897 | 2 |
| 3212.066793 | 1 |
| 3212.066983 | 1 |
| 3212.0674507 | 1 |
| 3212.0675789 | 2 |
| 3212.0679086 | 1 |
| 3212.068196 | 2 |
| 3212.0682046 | 1 |
| 3212.0687911 | 2 |
| 3212.0688423 | 4 |
| 3212.0695164 | 1 |
| 3212.0698178 | 0 |
| 3212.0698464 | 1 |
| 3212.0703588 | 1 |
| 3212.0704514 | 1 |
| 3212.0708029 | 2 |
| 3212.0712317 | 0 |
| 3212.0713051 | 0 |
| 3212.0719405 | 1 |
| 3212.0720351 | 1 |
| 3212.0727962 | 2 |
| 3212.0728234 | 1 |
| 3212.0732855 | 0 |
| 3212.0735633 | 1 |
| 3212.0739304 | 2 |
| 3212.074026 | 0 |
| 3212.0742519 | 2 |
| 3212.0745211 | 4 |
| 3212.0745806 | 1 |
| 3212.0755623 | 2 |
| 3212.0759513 | 1 |
| 3212.0760081 | 1 |
| 3212.0763911 | 3 |
| 3212.0766275 | 1 |
| 3212.0766889 | 3 |
| 3212.0770389 | 0 |
| 3212.0775932 | 0 |
| 3212.0776316 | 0 |
| 3212.0782037 | 1 |
| 3212.0783686 | 0 |
| 3212.078491 | 0 |
| 3212.0792016 | 0 |
| 3212.0795192 | 1 |
| 3212.0802728 | 0 |
| 3212.0803672 | 0 |
| 3212.0803827 | 1 |
| 3212.0804987 | 3 |
| 3212.0807004 | 0 |
| 3212.0811169 | 2 |
| 3212.0816391 | 2 |
| 3212.0819614 | 0 |
| 3212.0825761 | 0 |
| 3212.0825879 | 0 |
| 3212.0830775 | 0 |
| 3212.0832441 | 1 |
| 3212.0834519 | 1 |
| 3212.0836499 | 2 |
| 3212.0842296 | 0 |
| 3212.0846374 | 1 |
| 3212.0846418 | 0 |
| 3212.0852102 | 2 |
| 3212.0856183 | 1 |
| 3212.0858875 | 1 |
| 3212.0862051 | 2 |
| 3212.0865089 | 2 |
| 3212.0865165 | 0 |
| 3212.086816 | 1 |
| 3212.0874903 | 0 |
| 3212.0877768 | 2 |
| 3212.088016 | 2 |
| 3212.0884464 | 0 |
| 3212.0888192 | 2 |
| 3212.0890336 | 2 |
| 3212.0894218 | 1 |
| 3212.0898278 | 0 |
| 3212.0900482 | 0 |
| 3212.0906856 | 1 |
| 3212.0907474 | 1 |
| 3212.0910065 | 0 |
| 3212.0913237 | 1 |
| 3212.0913517 | 0 |
| 3212.0919047 | 0 |
| 3212.0924798 | 0 |
| 3212.0925014 | 1 |
| 3212.0927241 | 1 |
| 3212.0931883 | 2 |
| 3212.0935933 | 0 |
| 3212.0937285 | 2 |
| 3212.0939516 | 0 |
| 3212.0944472 | 0 |
| 3212.0945952 | 0 |
| 3212.0954854 | 2 |
| 3212.0955325 | 0 |
| 3212.0956774 | 0 |
| 3212.0957519 | 0 |
| 3212.0962779 | 1 |
| 3212.096825 | 0 |
| 3212.0968338 | 1 |
| 3212.0974486 | 0 |
| 3212.0976896 | 0 |
| 3212.0979386 | 1 |
| 3212.0985351 | 2 |
| 3212.0987745 | 2 |
| 3212.0993647 | 1 |
| 3212.0997155 | 0 |
| 3212.0998105 | 0 |
| 3212.1000411 | 1 |
| 3212.1004996 | 1 |
| 3212.1005864 | 1 |
| 3212.1007565 | 1 |
| 3212.1010918 | 0 |
| 3212.1011486 | 0 |
| 3212.1016789 | 1 |
| 3212.1021485 | 2 |
| 3212.1023396 | 0 |
| 3212.1026641 | 0 |
| 3212.1028603 | 2 |
| 3212.1030053 | 2 |
| 3212.1036852 | 0 |
| 3212.1037166 | 1 |
| 3212.1043874 | 0 |
| 3212.1044013 | 1 |
| 3212.1050427 | 0 |
| 3212.1053065 | 3 |
| 3212.1054615 | 1 |
| 3212.1061771 | 1 |
| 3212.106197 | 1 |
| 3212.1064006 | 0 |
| 3212.1065083 | 1 |
| 3212.1069469 | 0 |
| 3212.1076102 | 1 |
| 3212.107716 | 0 |
| 3212.1077189 | 0 |
| 3212.1082894 | 3 |
| 3212.1084972 | 0 |
| 3212.1085788 | 2 |
| 3212.1090201 | 1 |
| 3212.1093121 | 0 |
| 3212.1095441 | 2 |
| 3212.11007 | 1 |
| 3212.1102652 | 0 |
| 3212.110387 | 2 |
| 3212.1105738 | 0 |
| 3212.1112823 | 0 |
| 3212.1113254 | 0 |
| 3212.1118271 | 0 |
| 3212.1120171 | 1 |
| 3212.1124248 | 1 |
| 3212.1125167 | 2 |
| 3212.1129142 | 0 |
| 3212.1134898 | 0 |
| 3212.1136427 | 1 |
| 3212.1137621 | 0 |
| 3212.1140945 | 1 |
| 3212.1146711 | 1 |
| 3212.1147112 | 3 |
| 3212.1152639 | 1 |
| 3212.115283 | 2 |
| 3212.1158917 | 0 |
| 3212.1159771 | 2 |
| 3212.1161006 | 1 |
| 3212.1167153 | 1 |
| 3212.1167398 | 2 |
| 3212.11724 | 1 |
| 3212.1175717 | 1 |
| 3212.1178394 | 2 |
| 3212.1182168 | 0 |
| 3212.118431 | 1 |
| 3212.1191194 | 3 |
| 3212.1191212 | 4 |
| 3212.1197208 | 2 |
| 3212.1197838 | 0 |
| 3212.1204535 | 1 |
| 3212.1207557 | 1 |
| 3212.12124 | 0 |
| 3212.1212401 | 0 |
| 3212.1213237 | 1 |
| 3212.1214994 | 1 |
| 3212.1224407 | 2 |
| 3212.1227783 | 0 |
| 3212.1228674 | 1 |
| 3212.1237141 | 1 |
| 3212.1237349 | 1 |
| 3212.1238463 | 1 |
| 3212.1238635 | 1 |
| 3212.1242695 | 1 |
| 3212.1245126 | 3 |
| 3212.1245907 | 1 |
| 3212.1253203 | 0 |
| 3212.1258529 | 0 |
| 3212.1260877 | 2 |
| 3212.126338 | 1 |
| 3212.1266393 | 0 |
| 3212.1268262 | 2 |
| 3212.1272933 | 0 |
| 3212.1273725 | 0 |
| 3212.1275534 | 1 |
| 3212.1280639 | 4 |
| 3212.1284138 | 1 |
| 3212.1285428 | 1 |
| 3212.1289054 | 2 |
| 3212.1291208 | 0 |
| 3212.1295354 | 1 |
| 3212.1304005 | 1 |
| 3212.1304313 | 0 |
| 3212.1305522 | 0 |
| 3212.1309639 | 0 |
| 3212.1311491 | 1 |
| 3212.1316357 | 0 |
| 3212.1321362 | 2 |
| 3212.1322161 | 2 |
| 3212.1322844 | 3 |
| 3212.1331339 | 1 |
| 3212.133266 | 1 |
| 3212.1337259 | 4 |
| 3212.1339313 | 2 |
| 3212.1342326 | 0 |
| 3212.1345464 | 1 |
| 3212.134754 | 0 |
| 3212.1351953 | 0 |
| 3212.1357984 | 0 |
| 3212.1358519 | 1 |
| 3212.1360025 | 0 |
| 3212.1368416 | 1 |
| 3212.1372631 | 1 |
| 3212.1372819 | 0 |
| 3212.1377364 | 1 |
| 3212.1379032 | 0 |
| 3212.1381296 | 2 |
| 3212.1382051 | 1 |
| 3212.1386756 | 0 |
| 3212.139179 | 2 |
| 3212.1395742 | 0 |
| 3212.1395994 | 0 |

| | |
|---|---|
| 3212.1396194 | 0 |
| 3212.1401988 | 2 |
| 3212.1402209 | 4 |
| 3212.1409102 | 1 |
| 3212.1410179 | 1 |
| 3212.1412041 | 1 |
| 3212.1413138 | 2 |
| 3212.1415144 | 1 |
| 3212.1422361 | 0 |
| 3212.1426666 | 0 |
| 3212.143058 | 0 |
| 3212.1433472 | 1 |
| 3212.143424 | 1 |
| 3212.1442875 | 1 |
| 3212.1444768 | 2 |
| 3212.1445131 | 1 |
| 3212.1447622 | 1 |
| 3212.1454597 | 1 |
| 3212.1457046 | 1 |
| 3212.1457731 | 1 |
| 3212.146155 | 2 |
| 3212.1466815 | 1 |
| 3212.146756 | 2 |
| 3212.1468352 | 1 |
| 3212.1476243 | 1 |
| 3212.1479203 | 0 |
| 3212.148231 | 2 |
| 3212.1482818 | 0 |
| 3212.1485265 | 0 |
| 3212.1490645 | 1 |
| 3212.14908 | 0 |
| 3212.1494179 | 0 |
| 3212.1503728 | 0 |
| 3212.150942 | 0 |
| 3212.1510065 | 1 |
| 3212.151079 | 0 |
| 3212.1512474 | 0 |
| 3212.1515446 | 1 |
| 3212.1516228 | 3 |
| 3212.1518989 | 0 |
| 3212.1524062 | 0 |
| 3212.1524301 | 1 |
| 3212.1529582 | 1 |
| 3212.1537332 | 1 |
| 3212.1537563 | 1 |
| 3212.153839 | 1 |
| 3212.1539655 | 1 |
| 3212.1541541 | 1 |
| 3212.154283 | 5 |
| 3212.1547624 | 1 |
| 3212.1552836 | 0 |
| 3212.1555213 | 1 |
| 3212.1563907 | 0 |
| 3212.1567361 | 1 |
| 3212.1568614 | 1 |
| 3212.1571051 | 2 |
| 3212.1572826 | 0 |
| 3212.1577767 | 0 |
| 3212.157868 | 1 |
| 3212.1582456 | 0 |
| 3212.1590807 | 1 |
| 3212.1590842 | 1 |
| 3212.1591928 | 3 |
| 3212.1594502 | 1 |
| 3212.1600854 | 1 |
| 3212.1603399 | 0 |
| 3212.1605268 | 0 |
| 3212.1608105 | 1 |
| 3212.1611346 | 0 |
| 3212.1612205 | 1 |
| 3212.161686 | 0 |
| 3212.1618497 | 3 |
| 3212.1621965 | 1 |
| 3212.162599 | 3 |
| 3212.162814 | 1 |
| 3212.1633767 | 0 |
| 3212.1640763 | 0 |
| 3212.164123 | 2 |
| 3212.1641699 | 1 |
| 3212.164484 | 1 |
| 3212.1646926 | 2 |
| 3212.1651873 | 0 |
| 3212.1654956 | 3 |
| 3212.1656212 | 3 |
| 3212.1661447 | 1 |
| 3212.1665259 | 2 |
| 3212.1666175 | 2 |
| 3212.1671165 | 0 |
| 3212.1671366 | 1 |
| 3212.1677091 | 0 |
| 3212.1679751 | 1 |
| 3212.1680058 | 1 |
| 3212.1684302 | 0 |
| 3212.1688841 | 0 |
| 3212.1690497 | 1 |
| 3212.1692549 | 1 |
| 3212.1694342 | 0 |
| 3212.1700803 | 2 |
| 3212.1702584 | 0 |
| 3212.1708175 | 1 |
| 3212.170871 | 0 |
| 3212.1713023 | 1 |
| 3212.1714023 | 1 |
| 3212.1721015 | 0 |
| 3212.1724732 | 0 |
| 3212.1727588 | 1 |
| 3212.173276 | 1 |
| 3212.1732909 | 1 |
| 3212.1735109 | 2 |
| 3212.1737244 | 1 |
| 3212.1739753 | 2 |
| 3212.1741247 | 1 |
| 3212.1746726 | 1 |
| 3212.1748269 | 0 |
| 3212.1756051 | 0 |
| 3212.1760219 | 2 |
| 3212.1761967 | 0 |
| 3212.1763186 | 2 |
| 3212.1773338 | 0 |
| 3212.1773814 | 2 |
| 3212.1774394 | 1 |
| 3212.1777063 | 2 |
| 3212.1785043 | 2 |
| 3212.1786616 | 1 |
| 3212.1787205 | 1 |
| 3212.1792806 | 3 |
| 3212.1793745 | 0 |
| 3212.1796188 | 0 |
| 3212.1799525 | 2 |
| 3212.1802235 | 1 |
| 3212.1807127 | 2 |
| 3212.1808566 | 1 |
| 3212.1810897 | 0 |
| 3212.1815231 | 0 |
| 3212.1816133 | 1 |
| 3212.1824402 | 1 |
| 3212.1824735 | 2 |
| 3212.1828945 | 1 |
| 3212.1829181 | 0 |
| 3212.1836001 | 0 |
| 3212.1840079 | 1 |
| 3212.1841652 | 0 |
| 3212.1845686 | 0 |
| 3212.1847304 | 1 |
| 3212.1853041 | 2 |
| 3212.1857649 | 1 |
| 3212.186005 | 0 |
| 3212.1863203 | 1 |
| 3212.1865977 | 0 |
| 3212.1867197 | 2 |
| 3212.1870562 | 1 |
| 3212.187339 | 2 |
| 3212.1874149 | 1 |
| 3212.1876444 | 1 |
| 3212.1880818 | 3 |
| 3212.1882971 | 1 |
| 3212.1883127 | 1 |
| 3212.188692 | 0 |
| 3212.1890576 | 1 |
| 3212.1893084 | 2 |
| 3212.189409 | 0 |
| 3212.1895392 | 0 |
| 3212.1900292 | 2 |
| 3212.1901193 | 0 |
| 3212.1902614 | 3 |
| 3212.1904877 | 2 |
| 3212.1909713 | 1 |
| 3212.190986 | 0 |
| 3212.1910239 | 1 |
| 3212.1914641 | 1 |
| 3212.1917725 | 1 |
| 3212.191862 | 5 |
| 3212.1920399 | 0 |
| 3212.1923201 | 0 |
| 3212.1928347 | 0 |
| 3212.1930309 | 1 |
| 3212.1933323 | 1 |
| 3212.1933507 | 0 |
| 3212.1934047 | 3 |
| 3212.1936146 | 1 |
| 3212.1937111 | 0 |
| 3212.1942458 | 1 |
| 3212.1944617 | 4 |
| 3212.1945368 | 1 |
| 3212.1950268 | 0 |
| 3212.1954938 | 2 |
| 3212.1954959 | 1 |
| 3212.1955442 | 0 |
| 3212.1956496 | 0 |
| 3212.1957525 | 1 |
| 3212.1962159 | 1 |
| 3212.1964204 | 1 |
| 3212.1965486 | 2 |
| 3212.1973227 | 0 |
| 3212.1973421 | 2 |
| 3212.1974251 | 2 |
| 3212.1981111 | 2 |
| 3212.1984315 | 0 |
| 3212.1998179 | 1 |
| 3212.200712 | 1 |
| 3212.2015643 | 0 |
| 3212.2021888 | 3 |
| 3212.2030856 | 1 |
| 3212.20435 | 0 |
| 3212.2055326 | 1 |
| 3212.2059112 | 2 |
| 3212.2073801 | 3 |
| 3212.208074 | 0 |
| 3212.2086935 | 1 |
| 3212.209582 | 0 |
| 3212.210631 | 1 |
| 3212.2123503 | 0 |
| 3212.2127772 | 0 |
| 3212.2140649 | 0 |
| 3212.2141072 | 2 |
| 3212.215848 | 0 |
| 3212.2168794 | 0 |
| 3212.2173019 | 2 |
| 3212.2185886 | 2 |
| 3212.2192487 | 1 |
| 3212.2206644 | 0 |
| 3212.2212505 | 0 |
| 3212.2220587 | 0 |
| 3212.2236366 | 1 |
| 3212.2240915 | 1 |
| 3212.2253856 | 1 |
| 3212.2264517 | 1 |
| 3212.2273092 | 2 |
| 3212.2275211 | 0 |
| 3212.2287925 | 2 |
| 3212.2295131 | 0 |
| 3212.2303186 | 2 |
| 3212.2311053 | 0 |
| 3212.2334013 | 4 |
| 3212.2336739 | 0 |
| 3212.2351793 | 1 |
| 3212.2354395 | 0 |
| 3212.2369498 | 1 |
| 3212.2370346 | 5 |
| 3212.2379817 | 1 |
| 3212.239441 | 1 |
| 3212.2399009 | 1 |
| 3212.241078 | 1 |
| 3212.242279 | 2 |
| 3212.2429557 | 2 |
| 3212.2434893 | 0 |
| 3212.244502 | 1 |
| 3212.2456933 | 1 |
| 3212.2465248 | 0 |
| 3212.2470634 | 0 |
| 3212.2484752 | 3 |
| 3212.2489178 | 1 |
| 3212.2503209 | 0 |
| 3212.2504539 | 2 |
| 3212.2519861 | 0 |
| 3212.2530713 | 2 |
| 3212.2537324 | 0 |
| 3212.2551583 | 1 |
| 3212.2555014 | 2 |
| 3212.2566605 | 2 |
| 3212.2579678 | 1 |
| 3212.258265 | 0 |
| 3212.2600954 | 3 |
| 3212.2601576 | 1 |
| 3212.2620889 | 1 |
| 3212.2622443 | 3 |
| 3212.2637076 | 1 |
| 3212.2648124 | 1 |
| 3212.2651998 | 1 |
| 3212.2663154 | 1 |
| 3212.2668978 | 1 |
| 3212.2680222 | 0 |
| 3212.2692431 | 1 |
| 3212.2698595 | 1 |
| 3212.2709464 | 2 |
| 3212.27179 | 0 |
| 3212.2727471 | 1 |
| 3212.2742721 | 1 |
| 3212.2745869 | 2 |
| 3212.2758343 | 1 |
| 3212.2767739 | 0 |
| 3212.276777 | 1 |
| 3212.2792753 | 0 |
| 3212.279563 | 1 |
| 3212.2802761 | 2 |
| 3212.281538 | 2 |
| 3212.2826145 | 1 |
| 3212.2832877 | 2 |
| 3212.28489 | 0 |
| 3212.2851486 | 2 |
| 3212.2854151 | 3 |
| 3212.2871341 | 0 |
| 3212.2872373 | 1 |
| 3212.2882144 | 0 |
| 3212.2885699 | 1 |
| 3212.2903107 | 2 |
| 3212.2912652 | 1 |
| 3212.292264 | 0 |
| 3212.2932524 | 2 |
| 3212.2936654 | 0 |
| 3212.2950872 | 2 |
| 3212.2959529 | 1 |
| 3212.2970304 | 1 |
| 3212.297666 | 0 |
| 3212.2994467 | 1 |
| 3212.2997369 | 1 |
| 3212.300382 | 0 |
| 3212.301022 | 0 |
| 3212.3029388 | 0 |
| 3212.3033114 | 2 |
| 3212.3039998 | 0 |
| 3212.3057712 | 2 |
| 3212.3060663 | 0 |
| 3212.3071503 | 1 |
| 3212.3077264 | 1 |
| 3212.3093572 | 1 |
| 3212.310014 | 2 |
| 3212.3111991 | 0 |
| 3212.3123589 | 1 |
| 3212.3126209 | 0 |
| 3212.314133 | 1 |
| 3212.3145427 | 1 |
| 3212.3161925 | 1 |
| 3212.316318 | 0 |
| 3212.3178078 | 0 |
| 3212.3187075 | 1 |
| 3212.319505 | 2 |
| 3212.3208838 | 2 |
| 3212.3214205 | 2 |
| 3212.3223298 | 1 |
| 3212.3234494 | 1 |
| 3212.3239471 | 0 |
| 3212.3244498 | 0 |
| 3212.3257738 | 2 |
| 3212.32632 | 0 |
| 3212.3281864 | 2 |
| 3212.3285389 | 0 |
| 3212.3299011 | 0 |
| 3212.3302697 | 0 |
| 3212.3320247 | 0 |
| 3212.3323811 | 2 |
| 3212.3335218 | 0 |
| 3212.3345332 | 1 |
| 3212.3349615 | 0 |
| 3212.3355512 | 3 |
| 3212.3372604 | 0 |
| 3212.3375814 | 1 |
| 3212.3393884 | 3 |
| 3212.3396558 | 1 |
| 3212.3409982 | 0 |
| 3212.3411349 | 1 |
| 3212.3423057 | 1 |
| 3212.3440666 | 0 |
| 3212.3444083 | 0 |
| 3212.345387 | 0 |
| 3212.3461133 | 1 |
| 3212.3480692 | 1 |
| 3212.3481465 | 0 |
| 3212.3491487 | 1 |
| 3212.3502984 | 3 |
| 3212.3506838 | 0 |
| 3212.351957 | 0 |
| 3212.3521675 | 1 |

| | |
|---|---|
| 3212.3543769 | 1 |
| 3212.354513 | 1 |
| 3212.3558381 | 0 |
| 3212.3566497 | 1 |
| 3212.3575167 | 0 |
| 3212.3584204 | 0 |
| 3212.3597871 | 0 |
| 3212.3602318 | 1 |
| 3212.3617813 | 1 |
| 3212.3623301 | 0 |
| 3212.363681 | 0 |
| 3212.3640392 | 0 |
| 3212.3653583 | 1 |
| 3212.3659364 | 0 |
| 3212.3666094 | 3 |
| 3212.3681761 | 2 |
| 3212.3687007 | 1 |
| 3212.3698811 | 1 |
| 3212.3712692 | 1 |
| 3212.3716245 | 1 |
| 3212.3733994 | 0 |
| 3212.3735319 | 2 |
| 3212.3738984 | 0 |
| 3212.3750863 | 2 |
| 3212.3767066 | 1 |
| 3212.3775882 | 0 |
| 3212.3782352 | 1 |
| 3212.3796444 | 1 |
| 3212.3798046 | 0 |
| 3212.3809777 | 1 |
| 3212.3811147 | 3 |
| 3212.3824577 | 0 |
| 3212.3833218 | 0 |
| 3212.3846084 | 0 |
| 3212.3851633 | 0 |
| 3212.3856873 | 1 |
| 3212.387011 | 2 |
| 3212.3883827 | 3 |
| 3212.3888031 | 0 |
| 3212.3899784 | 0 |
| 3212.3916949 | 1 |
| 3212.3920053 | 2 |
| 3212.3932783 | 1 |
| 3212.3936732 | 2 |
| 3212.3947932 | 2 |
| 3212.3955135 | 0 |
| 3212.3963815 | 1 |
| 3212.3974505 | 1 |
| 3212.3980968 | 2 |
| 3212.3991631 | 3 |
| 3212.4000642 | 1 |
| 3212.4008409 | 0 |
| 3212.4030031 | 3 |
| 3212.4030486 | 1 |
| 3212.4043791 | 3 |
| 3212.4051081 | 0 |
| 3212.4061746 | 2 |
| 3212.4071448 | 1 |
| 3212.4079981 | 1 |
| 3212.4089645 | 1 |
| 3212.4090459 | 0 |
| 3212.4107953 | 1 |
| 3212.412117 | 0 |
| 3212.4124026 | 0 |
| 3212.4135195 | 0 |
| 3212.4147234 | 2 |
| 3212.4153174 | 0 |
| 3212.4169454 | 1 |
| 3212.4177474 | 3 |
| 3212.4180967 | 0 |
| 3212.4192252 | 1 |
| 3212.419886 | 0 |
| 3212.4212865 | 1 |
| 3212.4217538 | 0 |
| 3212.4238199 | 0 |
| 3212.4243712 | 1 |
| 3212.4246968 | 1 |
| 3212.4259678 | 0 |
| 3212.4263017 | 0 |
| 3212.427429 | 2 |
| 3212.4281239 | 2 |
| 3212.4295205 | 0 |
| 3212.4302428 | 1 |
| 3212.431005 | 1 |
| 3212.432484 | 1 |
| 3212.4325525 | 0 |
| 3212.4341332 | 2 |
| 3212.4345547 | 0 |
| 3212.4360202 | 1 |
| 3212.4362066 | 2 |
| 3212.4377892 | 0 |
| 3212.4387882 | 2 |
| 3212.4394568 | 1 |
| 3212.4410927 | 0 |
| 3212.4412987 | 3 |
| 3212.4424425 | 3 |
| 3212.4432805 | 1 |
| 3212.4441972 | 0 |
| 3212.4451692 | 1 |
| 3212.4465972 | 2 |
| 3212.4471754 | 4 |
| 3212.4483092 | 2 |
| 3212.449248 | 0 |
| 3212.450361 | 0 |
| 3212.4508765 | 2 |
| 3212.4517524 | 0 |
| 3212.452955 | 2 |
| 3212.4533422 | 1 |
| 3212.4544737 | 1 |
| 3212.4554843 | 3 |
| 3212.4566769 | 1 |
| 3212.4575711 | 0 |
| 3212.4582446 | 0 |
| 3212.4593942 | 1 |
| 3212.4606149 | 2 |
| 3212.4612067 | 0 |
| 3212.4630302 | 1 |
| 3212.4630735 | 0 |
| 3212.4644529 | 3 |
| 3212.4647619 | 1 |
| 3212.4659896 | 0 |
| 3212.4671041 | 3 |
| 3212.4680188 | 0 |
| 3212.4681087 | 0 |
| 3212.4694106 | 0 |
| 3212.4702908 | 1 |
| 3212.4713351 | 0 |
| 3212.4721604 | 0 |
| 3212.4735384 | 1 |
| 3212.4737631 | 1 |
| 3212.4756965 | 0 |
| 3212.475829 | 1 |
| 3212.4762597 | 0 |
| 3212.4779378 | 0 |
| 3212.4783807 | 1 |
| 3212.4799835 | 0 |
| 3212.4804531 | 1 |
| 3212.4813846 | 0 |
| 3212.4831251 | 2 |
| 3212.4835405 | 3 |
| 3212.484206 | 2 |
| 3212.4850043 | 1 |
| 3212.4860309 | 2 |
| 3212.4863831 | 1 |
| 3212.487861 | 1 |
| 3212.4889862 | 1 |
| 3212.4900701 | 0 |
| 3212.4906667 | 0 |
| 3212.4919435 | 3 |
| 3212.4925468 | 3 |
| 3212.4935634 | 1 |
| 3212.4943555 | 0 |
| 3212.4957997 | 1 |
| 3212.4965378 | 2 |
| 3212.4979973 | 0 |
| 3212.4991922 | 1 |
| 3212.4993697 | 1 |
| 3212.5010422 | 2 |
| 3212.5012718 | 0 |
| 3212.5022333 | 2 |
| 3212.5034304 | 0 |
| 3212.5044156 | 0 |
| 3212.5055189 | 1 |
| 3212.505919 | 0 |
| 3212.5072968 | 3 |
| 3212.5081808 | 0 |
| 3212.5086614 | 0 |
| 3212.5095884 | 1 |
| 3212.5106119 | 0 |
| 3212.5115733 | 0 |
| 3212.5124985 | 1 |
| 3212.5140271 | 1 |
| 3212.5147909 | 0 |
| 3212.5153112 | 0 |
| 3212.516277 | 0 |
| 3212.5166324 | 1 |
| 3212.5183148 | 2 |
| 3212.5186794 | 1 |
| 3212.5200376 | 2 |
| 3212.5207062 | 0 |
| 3212.5213804 | 0 |
| 3212.5224668 | 0 |
| 3212.5231108 | 2 |
| 3212.5248446 | 1 |
| 3212.5250919 | 0 |
| 3212.5270358 | 0 |
| 3212.5274701 | 2 |
| 3212.5281518 | 0 |
| 3212.5286625 | 1 |
| 3212.5300882 | 0 |
| 3212.5311335 | 2 |
| 3212.5323807 | 0 |
| 3212.5324613 | 1 |
| 3212.5338895 | 2 |
| 3212.5345171 | 2 |
| 3212.5360685 | 1 |
| 3212.537023 | 0 |
| 3212.5376602 | 3 |
| 3212.5392124 | 0 |
| 3212.5394954 | 0 |
| 3212.5404502 | 0 |
| 3212.5415155 | 2 |
| 3212.5427525 | 1 |
| 3212.5435262 | 3 |
| 3212.5443949 | 1 |
| 3212.5449715 | 0 |
| 3212.5465932 | 3 |
| 3212.5469231 | 2 |
| 3212.5486729 | 0 |
| 3212.5487248 | 1 |
| 3212.5504582 | 2 |
| 3212.5509789 | 1 |
| 3212.5516916 | 2 |
| 3212.553049 | 0 |
| 3212.5537378 | 0 |
| 3212.5550108 | 0 |
| 3212.555402 | 0 |
| 3212.5570827 | 0 |
| 3212.5578849 | 0 |
| 3212.5585354 | 2 |
| 3212.5595163 | 1 |
| 3212.5599228 | 1 |
| 3212.56119 | 2 |
| 3212.5625091 | 1 |
| 3212.562742 | 1 |
| 3212.564074 | 2 |
| 3212.564158 | 0 |
| 3212.5657063 | 1 |
| 3212.5670368 | 0 |
| 3212.5677776 | 1 |
| 3212.5683441 | 3 |
| 3212.5694591 | 0 |
| 3212.570545 | 1 |
| 3212.5719857 | 1 |
| 3212.5724781 | 1 |
| 3212.5739697 | 1 |
| 3212.5742434 | 1 |
| 3212.5752268 | 1 |
| 3212.5757074 | 0 |
| 3212.577227 | 0 |
| 3212.5779513 | 1 |
| 3212.5786027 | 1 |
| 3212.5795514 | 1 |
| 3212.5802097 | 1 |
| 3212.5817479 | 0 |
| 3212.5824181 | 1 |
| 3212.5835787 | 0 |
| 3212.5847608 | 1 |
| 3212.5855263 | 0 |
| 3212.5864502 | 1 |
| 3212.5874827 | 0 |
| 3212.5884366 | 2 |
| 3212.5894621 | 0 |
| 3212.5902442 | 1 |
| 3212.5914869 | 2 |
| 3212.5926632 | 0 |
| 3212.5935825 | 0 |
| 3212.5941621 | 0 |
| 3212.5949778 | 0 |
| 3212.5954102 | 0 |
| 3212.5964598 | 0 |
| 3212.5980464 | 2 |
| 3212.5989977 | 0 |
| 3212.5998545 | 3 |
| 3212.600467 | 1 |
| 3212.6018591 | 1 |
| 3212.6023755 | 1 |
| 3212.6039243 | 0 |
| 3212.6043233 | 1 |
| 3212.605384 | 0 |
| 3212.6062701 | 1 |
| 3212.6067688 | 0 |
| 3212.6087705 | 0 |
| 3212.6088819 | 0 |
| 3212.6101791 | 0 |
| 3212.6106053 | 0 |
| 3212.6121537 | 1 |
| 3212.6121588 | 0 |
| 3212.6147079 | 0 |
| 3212.6147158 | 1 |
| 3212.615991 | 1 |
| 3212.6162913 | 1 |
| 3212.6165291 | 0 |
| 3212.6178992 | 0 |
| 3212.6196976 | 1 |
| 3212.6200797 | 0 |
| 3212.6213612 | 1 |
| 3212.621925 | 0 |
| 3212.623416 | 1 |
| 3212.6242522 | 1 |
| 3212.6243409 | 1 |
| 3212.6259419 | 1 |
| 3212.6265307 | 0 |
| 3212.6275826 | 2 |
| 3212.6285523 | 0 |
| 3212.6296344 | 2 |
| 3212.6309263 | 1 |
| 3212.6312167 | 0 |
| 3212.6329331 | 1 |
| 3212.6331479 | 0 |
| 3212.6343552 | 4 |
| 3212.6358424 | 2 |
| 3212.6366065 | 0 |
| 3212.6377676 | 3 |
| 3212.6378836 | 1 |
| 3212.6401214 | 1 |
| 3212.6404971 | 0 |
| 3212.6410322 | 2 |
| 3212.6424258 | 0 |
| 3212.6428066 | 0 |
| 3212.6441717 | 0 |
| 3212.6443552 | 1 |
| 3212.6455611 | 0 |
| 3212.6472571 | 1 |
| 3212.6477898 | 1 |
| 3212.6481752 | 0 |
| 3212.649264 | 0 |
| 3212.6502033 | 1 |
| 3212.6515688 | 1 |
| 3212.6519564 | 0 |
| 3212.653728 | 1 |
| 3212.6544827 | 3 |
| 3212.6552912 | 2 |
| 3212.6558127 | 2 |
| 3212.6569951 | 1 |
| 3212.6582991 | 1 |
| 3212.6592754 | 2 |
| 3212.6597607 | 2 |
| 3212.6605626 | 0 |
| 3212.6617131 | 3 |
| 3212.6624704 | 1 |
| 3212.663278 | 0 |
| 3212.6641565 | 2 |
| 3212.6650883 | 2 |
| 3212.6663856 | 2 |
| 3212.6669663 | 1 |
| 3212.6681484 | 1 |
| 3212.6689158 | 1 |
| 3212.6698238 | 0 |
| 3212.6707296 | 1 |
| 3212.6719226 | 1 |
| 3212.672935 | 1 |
| 3212.6746741 | 1 |
| 3212.6747933 | 0 |
| 3212.6763033 | 0 |
| 3212.6764773 | 2 |
| 3212.6774799 | 3 |
| 3212.6789348 | 0 |
| 3212.6797651 | 4 |
| 3212.6809176 | 0 |
| 3212.6815753 | 1 |
| 3212.6825949 | 1 |
| 3212.6831452 | 0 |
| 3212.6842555 | 0 |
| 3212.6844791 | 2 |
| 3212.6855919 | 1 |
| 3212.6870146 | 1 |
| 3212.6883759 | 3 |
| 3212.6886385 | 1 |
| 3212.6903047 | 0 |
| 3212.6905589 | 2 |
| 3212.6919407 | 1 |
| 3212.6930099 | 3 |

| | |
|---|---|
| 3212.6940328 | 1 |
| 3212.6942477 | 1 |
| 3212.6957803 | 1 |
| 3212.6959869 | 1 |
| 3212.6970995 | 0 |
| 3212.6990276 | 1 |
| 3212.6990471 | 1 |
| 3212.70041 | 1 |
| 3212.700749 | 2 |
| 3212.7020473 | 0 |
| 3212.7025729 | 2 |
| 3212.7037125 | 3 |
| 3212.7046022 | 1 |
| 3212.7057185 | 1 |
| 3212.7068006 | 1 |
| 3212.7076034 | 1 |
| 3212.7082758 | 1 |
| 3212.709998 | 1 |
| 3212.7110213 | 0 |
| 3212.7113715 | 0 |
| 3212.7126257 | 1 |
| 3212.7128037 | 0 |
| 3212.7144762 | 0 |
| 3212.7147368 | 0 |
| 3212.7160111 | 2 |
| 3212.7173068 | 2 |
| 3212.7180532 | 1 |
| 3212.7188679 | 1 |
| 3212.7197829 | 2 |
| 3212.7204062 | 1 |
| 3212.7213178 | 1 |
| 3212.7220642 | 0 |
| 3212.7241177 | 2 |
| 3212.724507 | 2 |
| 3212.7257186 | 4 |
| 3212.7260307 | 1 |
| 3212.7280084 | 0 |
| 3212.7282862 | 0 |
| 3212.7296186 | 1 |
| 3212.7306258 | 2 |
| 3212.7313043 | 1 |
| 3212.732287 | 0 |
| 3212.7332312 | 0 |
| 3212.734295 | 0 |
| 3212.7351358 | 0 |
| 3212.7359641 | 1 |
| 3212.7377469 | 0 |
| 3212.7378743 | 1 |
| 3212.7395038 | 1 |
| 3212.7399023 | 2 |
| 3212.7400238 | 1 |
| 3212.7415633 | 0 |
| 3212.7420228 | 1 |
| 3212.7435045 | 0 |
| 3212.744527 | 0 |
| 3212.7445561 | 0 |
| 3212.746838 | 1 |
| 3212.7474202 | 1 |
| 3212.7482402 | 1 |
| 3212.749129 | 2 |
| 3212.7498241 | 2 |
| 3212.7511486 | 1 |
| 3212.7517198 | 1 |
| 3212.7525399 | 0 |
| 3212.7541168 | 0 |
| 3212.7548749 | 2 |
| 3212.755106 | 0 |
| 3212.7562066 | 0 |
| 3212.7569623 | 0 |
| 3212.7572444 | 0 |
| 3212.7580359 | 0 |
| 3212.75847 | 1 |
| 3212.7585986 | 2 |
| 3212.7593137 | 0 |
| 3212.7594483 | 2 |
| 3212.7599986 | 3 |
| 3212.7606159 | 3 |
| 3212.7616418 | 0 |
| 3212.7618394 | 2 |
| 3212.762242 | 1 |
| 3212.7628208 | 1 |
| 3212.7633462 | 0 |
| 3212.7637441 | 0 |
| 3212.7643685 | 0 |
| 3212.7648504 | 1 |
| 3212.7648927 | 1 |
| 3212.7656835 | 0 |
| 3212.7660325 | 1 |
| 3212.7669994 | 4 |
| 3212.7670114 | 1 |
| 3212.7679395 | 2 |
| 3212.7683865 | 0 |
| 3212.7685436 | 1 |
| 3212.7690998 | 2 |
| 3212.7698982 | 0 |
| 3212.7699145 | 2 |
| 3212.7707788 | 1 |
| 3212.7708175 | 1 |
| 3212.7716496 | 0 |
| 3212.7717167 | 0 |
| 3212.7727247 | 1 |
| 3212.7727959 | 2 |
| 3212.7734906 | 1 |
| 3212.7739946 | 1 |
| 3212.7742119 | 0 |
| 3212.7748847 | 0 |
| 3212.7751641 | 1 |
| 3212.7756912 | 0 |
| 3212.7767014 | 2 |
| 3212.7768654 | 1 |
| 3212.7772608 | 1 |
| 3212.7776244 | 1 |
| 3212.7778054 | 1 |
| 3212.7783209 | 0 |
| 3212.779718 | 0 |
| 3212.7798068 | 1 |
| 3212.7804677 | 1 |
| 3212.7806652 | 1 |
| 3212.7806654 | 0 |
| 3212.78099 | 0 |
| 3212.7816823 | 1 |
| 3212.7821775 | 1 |
| 3212.7830455 | 1 |
| 3212.7831401 | 0 |
| 3212.7838321 | 0 |
| 3212.7849505 | 0 |
| 3212.7850707 | 0 |
| 3212.7851934 | 0 |
| 3212.7856328 | 1 |
| 3212.7860952 | 0 |
| 3212.7864982 | 3 |
| 3212.7872416 | 1 |
| 3212.7877474 | 0 |
| 3212.7879359 | 0 |
| 3212.7882246 | 2 |
| 3212.788986 | 3 |
| 3212.7892439 | 0 |
| 3212.789592 | 2 |
| 3212.789775 | 1 |
| 3212.7914102 | 0 |
| 3212.7915418 | 0 |
| 3212.7916488 | 0 |
| 3212.7924188 | 1 |
| 3212.7927002 | 1 |
| 3212.79353 | 0 |
| 3212.7937472 | 0 |
| 3212.7943941 | 0 |
| 3212.7946639 | 0 |
| 3212.7948688 | 3 |
| 3212.7948839 | 0 |
| 3212.7949591 | 1 |
| 3212.7961973 | 2 |
| 3212.7963815 | 1 |
| 3212.7964517 | 3 |
| 3212.7964886 | 1 |
| 3212.7969577 | 3 |
| 3212.7974613 | 1 |
| 3212.7978884 | 1 |
| 3212.7979795 | 2 |
| 3212.7982241 | 1 |
| 3212.7985499 | 2 |
| 3212.7994593 | 3 |
| 3212.7996178 | 0 |
| 3212.7997273 | 1 |
| 3212.7997631 | 0 |
| 3212.8007441 | 1 |
| 3212.8010133 | 1 |
| 3212.801037 | 1 |
| 3212.801298 | 1 |
| 3212.8016877 | 2 |
| 3212.8021459 | 2 |
| 3212.8023456 | 1 |
| 3212.8024598 | 0 |
| 3212.8037344 | 1 |
| 3212.8037411 | 2 |
| 3212.8037499 | 1 |
| 3212.8045019 | 1 |
| 3212.8051812 | 1 |
| 3212.8052908 | 2 |
| 3212.8053292 | 0 |
| 3212.8054901 | 1 |
| 3212.8062284 | 0 |
| 3212.8065268 | 3 |
| 3212.8066932 | 1 |
| 3212.8067823 | 2 |
| 3212.8068482 | 1 |
| 3212.8076902 | 0 |
| 3212.8079571 | 1 |
| 3212.8083862 | 2 |
| 3212.8091261 | 0 |
| 3212.8091278 | 2 |
| 3212.810048 | 2 |
| 3212.810093 | 0 |
| 3212.8101801 | 0 |
| 3212.8102007 | 0 |
| 3212.810738 | 1 |
| 3212.8108682 | 1 |
| 3212.8112929 | 0 |
| 3212.8118109 | 1 |
| 3212.8119662 | 2 |
| 3212.8120508 | 0 |
| 3212.8126235 | 1 |
| 3212.8131823 | 0 |
| 3212.8135415 | 3 |
| 3212.8140417 | 2 |
| 3212.8148087 | 1 |
| 3212.8148171 | 2 |
| 3212.8149096 | 1 |
| 3212.8157494 | 0 |
| 3212.8158135 | 1 |
| 3212.8163027 | 1 |
| 3212.8164195 | 1 |
| 3212.8168911 | 0 |
| 3212.8171261 | 2 |
| 3212.8173777 | 2 |
| 3212.8175366 | 1 |
| 3212.8184408 | 0 |
| 3212.8184441 | 1 |
| 3212.8190875 | 1 |
| 3212.8191066 | 0 |
| 3212.8193242 | 3 |
| 3212.8198602 | 0 |
| 3212.8205013 | 2 |
| 3212.8205359 | 2 |
| 3212.820928 | 2 |
| 3212.8212963 | 0 |
| 3212.8216611 | 0 |
| 3212.8222497 | 2 |
| 3212.8223312 | 1 |
| 3212.8226607 | 1 |
| 3212.8232837 | 1 |
| 3212.8234361 | 3 |
| 3212.8238696 | 3 |
| 3212.8239854 | 0 |
| 3212.8245696 | 2 |
| 3212.8248423 | 2 |
| 3212.8251176 | 2 |
| 3212.8251236 | 1 |
| 3212.8254864 | 1 |
| 3212.8255136 | 2 |
| 3212.8260427 | 0 |
| 3212.8268403 | 2 |
| 3212.8271735 | 0 |
| 3212.8275971 | 4 |
| 3212.8276061 | 0 |
| 3212.8281347 | 0 |
| 3212.8285191 | 2 |
| 3212.8287218 | 0 |
| 3212.8290872 | 1 |
| 3212.8298582 | 2 |
| 3212.8298867 | 2 |
| 3212.8303971 | 0 |
| 3212.8305384 | 0 |
| 3212.831262 | 0 |
| 3212.8315488 | 1 |
| 3212.8315972 | 0 |
| 3212.8320244 | 2 |
| 3212.8320836 | 1 |
| 3212.8326817 | 2 |
| 3212.8330271 | 1 |
| 3212.8337726 | 0 |
| 3212.8342139 | 4 |
| 3212.834479 | 1 |
| 3212.8346033 | 0 |
| 3212.8346138 | 2 |
| 3212.8347944 | 0 |
| 3212.8351018 | 0 |
| 3212.8359298 | 0 |
| 3212.8362141 | 1 |
| 3212.8362915 | 1 |
| 3212.8370381 | 1 |
| 3212.8371464 | 1 |
| 3212.8373994 | 1 |
| 3212.8377626 | 2 |
| 3212.8382141 | 0 |
| 3212.8383972 | 1 |
| 3212.8389118 | 1 |
| 3212.8390506 | 1 |
| 3212.8394862 | 3 |
| 3212.8397722 | 1 |
| 3212.8398771 | 2 |
| 3212.8406813 | 1 |
| 3212.8408562 | 0 |
| 3212.8410091 | 1 |
| 3212.8416705 | 2 |
| 3212.8424881 | 2 |
| 3212.8425415 | 1 |
| 3212.8428344 | 2 |
| 3212.8431365 | 1 |
| 3212.8436751 | 1 |
| 3212.8437505 | 0 |
| 3212.8438625 | 1 |
| 3212.8453154 | 2 |
| 3212.8457612 | 0 |
| 3212.846685 | 0 |
| 3212.8476849 | 0 |
| 3212.8480167 | 1 |
| 3212.8480484 | 0 |
| 3212.848345 | 1 |
| 3212.8485645 | 2 |
| 3212.8487937 | 0 |
| 3212.8490842 | 2 |
| 3212.8501015 | 2 |
| 3212.8503791 | 2 |
| 3212.8505408 | 0 |
| 3212.8505459 | 0 |
| 3212.8507413 | 0 |
| 3212.8515436 | 0 |
| 3212.8518621 | 1 |
| 3212.8523164 | 0 |
| 3212.8523235 | 6 |
| 3212.8525267 | 0 |
| 3212.8530994 | 2 |
| 3212.8531792 | 4 |
| 3212.8538401 | 1 |
| 3212.8543521 | 1 |
| 3212.8545107 | 0 |
| 3212.8547974 | 0 |
| 3212.8554451 | 0 |
| 3212.8555224 | 2 |
| 3212.8559071 | 0 |
| 3212.8562958 | 0 |
| 3212.8564196 | 0 |
| 3212.8572253 | 1 |
| 3212.8575356 | 2 |
| 3212.8576168 | 0 |
| 3212.8578992 | 0 |
| 3212.8583563 | 2 |
| 3212.8585495 | 0 |
| 3212.8588229 | 2 |
| 3212.8592689 | 1 |
| 3212.859384 | 1 |
| 3212.8598135 | 4 |
| 3212.8614617 | 1 |
| 3212.862168 | 0 |
| 3212.8632676 | 1 |
| 3212.8652956 | 1 |
| 3212.865666 | 2 |
| 3212.8677585 | 0 |
| 3212.8692254 | 2 |
| 3212.8697649 | 2 |
| 3212.8714253 | 0 |
| 3212.8718599 | 3 |
| 3212.8735876 | 4 |
| 3212.8751827 | 2 |
| 3212.8752631 | 0 |
| 3212.8774219 | 3 |
| 3212.8781572 | 2 |
| 3212.8801245 | 0 |
| 3212.8815688 | 3 |
| 3212.8821577 | 2 |
| 3212.883744 | 1 |
| 3212.8849596 | 1 |
| 3212.8860623 | 1 |
| 3212.8875821 | 0 |
| 3212.8887093 | 1 |
| 3212.889927 | 3 |
| 3212.8906371 | 0 |
| 3212.8919764 | 2 |
| 3212.8935854 | 2 |
| 3212.8944256 | 1 |
| 3212.8957968 | 3 |
| 3212.8968396 | 0 |

| | |
|---|---|
| 3212.898769 | 4 |
| 3212.8995804 | 3 |
| 3212.900387 | 0 |
| 3212.9024723 | 4 |
| 3212.9034673 | 2 |
| 3212.9042999 | 0 |
| 3212.906306 | 1 |
| 3212.9072577 | 3 |
| 3212.908212 | 2 |
| 3212.9097499 | 1 |
| 3212.9110685 | 3 |
| 3212.9121006 | 3 |
| 3212.913027 | 2 |
| 3212.915189 | 0 |
| 3212.9158661 | 2 |
| 3212.9173991 | 1 |
| 3212.9178733 | 2 |
| 3212.9202498 | 1 |
| 3212.9216025 | 1 |
| 3212.9216967 | 2 |
| 3212.9233737 | 1 |
| 3212.9256602 | 2 |
| 3212.9258161 | 4 |
| 3212.9271876 | 2 |
| 3212.9283955 | 2 |
| 3212.9284334 | 2 |
| 3212.9311784 | 2 |
| 3212.9317895 | 5 |
| 3212.933586 | 1 |
| 3212.9344574 | 3 |
| 3212.9364772 | 2 |
| 3212.9375258 | 2 |
| 3212.939081 | 0 |
| 3212.9399874 | 2 |
| 3212.9403575 | 3 |
| 3212.9426473 | 3 |
| 3212.9427575 | 5 |
| 3212.9449059 | 2 |
| 3212.9462075 | 2 |
| 3212.9462402 | 2 |
| 3212.9482837 | 1 |
| 3212.948777 | 3 |
| 3212.9497846 | 1 |
| 3212.9514729 | 1 |
| 3212.9526848 | 3 |
| 3212.9539317 | 3 |
| 3212.955733 | 3 |
| 3212.9567779 | 3 |
| 3212.9582702 | 2 |
| 3212.9590775 | 0 |
| 3212.9608644 | 3 |
| 3212.9622233 | 0 |
| 3212.9628618 | 0 |
| 3212.9647561 | 3 |
| 3212.9655247 | 0 |
| 3212.966953 | 3 |
| 3212.9678439 | 3 |
| 3212.9687843 | 1 |
| 3212.9705466 | 0 |
| 3212.9713729 | 1 |
| 3212.9736066 | 3 |
| 3212.9746767 | 1 |
| 3212.9753892 | 0 |
| 3212.9763809 | 0 |
| 3212.9779202 | 2 |
| 3212.9793948 | 2 |
| 3212.9799145 | 0 |
| 3212.982245 | 3 |
| 3212.9829601 | 0 |
| 3212.9836678 | 1 |
| 3212.9852309 | 3 |
| 3212.9866684 | 0 |
| 3212.9870212 | 2 |
| 3212.9889046 | 1 |
| 3212.9895108 | 0 |
| 3212.9907507 | 3 |
| 3212.9927023 | 3 |
| 3212.9936486 | 0 |
| 3212.9954144 | 2 |
| 3212.9961023 | 1 |
| 3212.9979126 | 0 |
| 3212.9987782 | 1 |
| 3213.0000216 | 1 |
| 3213.0012735 | 2 |
| 3213.0020111 | 2 |
| 3213.0031146 | 1 |
| 3213.0053967 | 4 |
| 3213.0056274 | 1 |
| 3213.0072884 | 4 |
| 3213.0089121 | 3 |
| 3213.0097865 | 2 |
| 3213.0116647 | 1 |
| 3213.01254 | 2 |
| 3213.0142926 | 3 |
| 3213.0143455 | 0 |
| 3213.0157735 | 2 |
| 3213.0178801 | 2 |
| 3213.0179979 | 1 |
| 3213.0192908 | 2 |
| 3213.0215024 | 0 |
| 3213.0222582 | 0 |
| 3213.0236279 | 0 |
| 3213.0247373 | 2 |
| 3213.0263471 | 0 |
| 3213.0271058 | 1 |
| 3213.0286559 | 1 |
| 3213.0299851 | 1 |
| 3213.0303545 | 2 |
| 3213.0326089 | 0 |
| 3213.0331651 | 3 |
| 3213.0342422 | 3 |
| 3213.0358172 | 2 |
| 3213.0367946 | 1 |
| 3213.038425 | 0 |
| 3213.0393769 | 0 |
| 3213.0403486 | 1 |
| 3213.0423645 | 1 |
| 3213.0433362 | 0 |
| 3213.0441836 | 3 |
| 3213.0456091 | 0 |
| 3213.0467062 | 3 |
| 3213.0484429 | 3 |
| 3213.0484824 | 2 |
| 3213.0500691 | 2 |
| 3213.0526213 | 2 |
| 3213.0528396 | 1 |
| 3213.0541199 | 1 |
| 3213.0559542 | 1 |
| 3213.0561959 | 2 |
| 3213.0581633 | 2 |
| 3213.0591403 | 2 |
| 3213.0608572 | 1 |
| 3213.0612637 | 0 |
| 3213.0631826 | 2 |
| 3213.0649149 | 3 |
| 3213.0658841 | 4 |
| 3213.0669962 | 3 |
| 3213.0680759 | 1 |
| 3213.069009 | 1 |
| 3213.069919 | 1 |
| 3213.0714196 | 1 |
| 3213.0726983 | 4 |
| 3213.0740501 | 1 |
| 3213.0751025 | 2 |
| 3213.0760219 | 1 |
| 3213.0774196 | 2 |
| 3213.0788799 | 4 |
| 3213.0799447 | 4 |
| 3213.081338 | 2 |
| 3213.0828315 | 3 |
| 3213.0835096 | 2 |
| 3213.0851348 | 3 |
| 3213.0857217 | 4 |
| 3213.0873057 | 2 |
| 3213.0890444 | 3 |
| 3213.0895786 | 1 |
| 3213.0913917 | 5 |
| 3213.0917326 | 1 |
| 3213.0935911 | 1 |
| 3213.0945822 | 2 |
| 3213.0959929 | 2 |
| 3213.0975775 | 1 |
| 3213.0979437 | 1 |
| 3213.1003057 | 0 |
| 3213.1011899 | 4 |
| 3213.1023052 | 1 |
| 3213.104028 | 3 |
| 3213.1045879 | 3 |
| 3213.1061601 | 1 |
| 3213.1078796 | 5 |
| 3213.1081964 | 1 |
| 3213.109422 | 3 |
| 3213.1107668 | 3 |
| 3213.1117228 | 2 |
| 3213.1137166 | 1 |
| 3213.1141137 | 3 |
| 3213.1158678 | 1 |
| 3213.1169955 | 2 |
| 3213.1181537 | 2 |
| 3213.11991 | 1 |
| 3213.1205018 | 2 |
| 3213.1223023 | 2 |
| 3213.1226962 | 1 |
| 3213.1246082 | 5 |
| 3213.1259935 | 8 |
| 3213.1267669 | 1 |
| 3213.1285632 | 2 |
| 3213.129051 | 2 |
| 3213.1303203 | 1 |
| 3213.1322123 | 2 |
| 3213.1328765 | 2 |
| 3213.1347693 | 1 |
| 3213.1348669 | 2 |
| 3213.1363764 | 1 |
| 3213.1376203 | 1 |
| 3213.1385893 | 2 |
| 3213.1406414 | 2 |
| 3213.1413543 | 3 |
| 3213.1434327 | 1 |
| 3213.1439959 | 1 |
| 3213.1456591 | 1 |
| 3213.1465255 | 1 |
| 3213.147867 | 1 |
| 3213.1492123 | 3 |
| 3213.1507133 | 3 |
| 3213.1515387 | 2 |
| 3213.1535735 | 2 |
| 3213.1536291 | 1 |
| 3213.1553762 | 2 |
| 3213.1566925 | 1 |
| 3213.1578285 | 2 |
| 3213.1592493 | 0 |
| 3213.1600704 | 2 |
| 3213.1621122 | 5 |
| 3213.1627371 | 1 |
| 3213.1636734 | 0 |
| 3213.1657446 | 2 |
| 3213.1662366 | 1 |
| 3213.1673103 | 4 |
| 3213.168705 | 1 |
| 3213.1698278 | 2 |
| 3213.1712639 | 1 |
| 3213.1725158 | 1 |
| 3213.1739587 | 0 |
| 3213.1746322 | 0 |
| 3213.1767469 | 0 |
| 3213.1774227 | 1 |
| 3213.1791708 | 4 |
| 3213.1792386 | 3 |
| 3213.181553 | 0 |
| 3213.1822812 | 1 |
| 3213.1834488 | 3 |
| 3213.1843551 | 0 |
| 3213.1856991 | 0 |
| 3213.1867006 | 2 |
| 3213.1880214 | 0 |
| 3213.1903486 | 1 |
| 3213.1904013 | 1 |
| 3213.1925479 | 1 |
| 3213.1932175 | 3 |
| 3213.1945932 | 1 |
| 3213.1963197 | 1 |
| 3213.1970413 | 2 |
| 3213.198242 | 0 |
| 3213.1997779 | 1 |
| 3213.2016582 | 3 |
| 3213.2023849 | 4 |
| 3213.2033432 | 3 |
| 3213.2053 | 1 |
| 3213.2057145 | 1 |
| 3213.2068757 | 1 |
| 3213.2086243 | 2 |
| 3213.2096709 | 3 |
| 3213.21087 | 1 |
| 3213.2116162 | 2 |
| 3213.2133789 | 1 |
| 3213.2145189 | 1 |
| 3213.2156859 | 0 |
| 3213.2165068 | 1 |
| 3213.2174447 | 2 |
| 3213.2187449 | 1 |
| 3213.2205959 | 0 |
| 3213.2215157 | 1 |
| 3213.2233873 | 3 |
| 3213.223457 | 1 |
| 3213.2252661 | 0 |
| 3213.2271493 | 1 |
| 3213.2272178 | 1 |
| 3213.2294741 | 1 |
| 3213.2305056 | 1 |
| 3213.2314492 | 1 |
| 3213.2331828 | 1 |
| 3213.2342617 | 0 |
| 3213.235873 | 1 |
| 3213.2373981 | 1 |
| 3213.2381367 | 3 |
| 3213.2394059 | 1 |
| 3213.2398439 | 3 |
| 3213.2418225 | 2 |
| 3213.2427185 | 1 |
| 3213.2440684 | 0 |
| 3213.2463823 | 0 |
| 3213.246504 | 3 |
| 3213.2485084 | 4 |
| 3213.2495478 | 2 |
| 3213.2509667 | 1 |
| 3213.2518917 | 2 |
| 3213.252823 | 1 |
| 3213.2540455 | 0 |
| 3213.2550757 | 1 |
| 3213.2566661 | 1 |
| 3213.258138 | 1 |
| 3213.2589295 | 1 |
| 3213.2608238 | 0 |
| 3213.2617762 | 1 |
| 3213.2622023 | 1 |
| 3213.2646033 | 2 |
| 3213.2649752 | 1 |
| 3213.2668657 | 2 |
| 3213.2680406 | 1 |
| 3213.2691762 | 2 |
| 3213.2702907 | 1 |
| 3213.2708247 | 2 |
| 3213.2721456 | 1 |
| 3213.2745568 | 1 |
| 3213.2750496 | 2 |
| 3213.2766579 | 4 |
| 3213.2772235 | 5 |
| 3213.2789179 | 2 |
| 3213.2799546 | 0 |
| 3213.2811739 | 3 |
| 3213.2833977 | 2 |
| 3213.2839689 | 3 |
| 3213.2854636 | 0 |
| 3213.2863476 | 1 |
| 3213.287795 | 2 |
| 3213.2897216 | 0 |
| 3213.2904008 | 0 |
| 3213.2917431 | 2 |
| 3213.2927398 | 3 |
| 3213.294127 | 1 |
| 3213.2956937 | 1 |
| 3213.2963821 | 1 |
| 3213.2989879 | 5 |
| 3213.2995911 | 1 |
| 3213.3005382 | 3 |
| 3213.3014232 | 1 |
| 3213.3029749 | 2 |
| 3213.3042803 | 1 |
| 3213.3054576 | 2 |
| 3213.306409 | 0 |
| 3213.3078085 | 1 |
| 3213.3085007 | 0 |
| 3213.3100635 | 0 |
| 3213.3113462 | 0 |
| 3213.3124525 | 1 |
| 3213.3135881 | 2 |
| 3213.3155444 | 2 |
| 3213.3164114 | 0 |
| 3213.3171171 | 5 |
| 3213.3185635 | 1 |
| 3213.3206414 | 0 |
| 3213.3211664 | 3 |
| 3213.3225511 | 2 |
| 3213.3239383 | 2 |
| 3213.3251667 | 2 |
| 3213.3268467 | 4 |
| 3213.3272313 | 2 |
| 3213.3293385 | 2 |
| 3213.3295126 | 4 |
| 3213.3312603 | 1 |
| 3213.3331185 | 3 |
| 3213.3339146 | 3 |
| 3213.3353909 | 2 |
| 3213.3362915 | 1 |
| 3213.3377952 | 2 |
| 3213.3392302 | 2 |
| 3213.3404158 | 1 |
| 3213.3418445 | 0 |
| 3213.342772 | 1 |
| 3213.3438945 | 2 |
| 3213.3455451 | 2 |
| 3213.3462864 | 1 |

| | |
|---|---|
| 3213.3479836 | 3 |
| 3213.3485137 | 2 |
| 3213.3498189 | 2 |
| 3213.3515777 | 1 |
| 3213.3525619 | 3 |
| 3213.3539815 | 1 |
| 3213.3551681 | 3 |
| 3213.3568521 | 3 |
| 3213.3571148 | 0 |
| 3213.3590872 | 3 |
| 3213.3604352 | 3 |
| 3213.3609206 | 0 |
| 3213.3630449 | 0 |
| 3213.363053 | 1 |
| 3213.3646595 | 2 |
| 3213.3664428 | 4 |
| 3213.3671067 | 0 |
| 3213.368955 | 4 |
| 3213.3695802 | 2 |
| 3213.3707181 | 3 |
| 3213.372327 | 4 |
| 3213.3733465 | 4 |
| 3213.3748496 | 1 |
| 3213.376605 | 1 |
| 3213.3776806 | 2 |
| 3213.378836 | 3 |
| 3213.3797167 | 2 |
| 3213.3811888 | 1 |
| 3213.383315 | 1 |
| 3213.3841151 | 1 |
| 3213.3858249 | 4 |
| 3213.3858867 | 3 |
| 3213.3880207 | 3 |
| 3213.3888786 | 1 |
| 3213.3901547 | 4 |
| 3213.3911595 | 2 |
| 3213.3924397 | 4 |
| 3213.3944164 | 1 |
| 3213.3951208 | 3 |
| 3213.3978755 | 1 |
| 3213.3981527 | 0 |
| 3213.3986763 | 1 |
| 3213.4004493 | 2 |
| 3213.4009469 | 0 |
| 3213.4027841 | 4 |
| 3213.4033376 | 3 |
| 3213.4046696 | 3 |
| 3213.4059657 | 1 |
| 3213.4074629 | 0 |
| 3213.4089246 | 2 |
| 3213.4104944 | 2 |
| 3213.410616 | 0 |
| 3213.4121188 | 5 |
| 3213.413354 | 0 |
| 3213.4149256 | 4 |
| 3213.4162295 | 0 |
| 3213.4179424 | 0 |
| 3213.4187069 | 1 |
| 3213.4194649 | 1 |
| 3213.4209432 | 1 |
| 3213.4227954 | 2 |
| 3213.4232346 | 0 |
| 3213.4249268 | 2 |
| 3213.4267875 | 3 |
| 3213.4272891 | 3 |
| 3213.4293433 | 1 |
| 3213.4301304 | 1 |
| 3213.4311807 | 2 |
| 3213.4334824 | 1 |
| 3213.4338764 | 3 |
| 3213.435062 | 1 |
| 3213.4361752 | 0 |
| 3213.437231 | 2 |
| 3213.4379685 | 2 |
| 3213.4403918 | 1 |
| 3213.4415276 | 1 |
| 3213.4426329 | 2 |
| 3213.4438333 | 1 |
| 3213.4439238 | 1 |
| 3213.4466875 | 3 |
| 3213.4480932 | 0 |
| 3213.4485474 | 2 |
| 3213.4504281 | 0 |
| 3213.4507059 | 2 |
| 3213.4525212 | 2 |
| 3213.4536345 | 1 |
| 3213.4540155 | 6 |
| 3213.4555962 | 3 |
| 3213.4570103 | 2 |
| 3213.4585683 | 3 |
| 3213.4597311 | 1 |
| 3213.4608163 | 1 |
| 3213.4614492 | 4 |
| 3213.4623884 | 0 |
| 3213.4628904 | 1 |
| 3213.4747118 | 2 |
| 3213.4752343 | 1 |
| 3213.4771874 | 0 |
| 3213.4794 | 0 |
| 3213.5072806 | 5 |
| 3213.5081347 | 0 |
| 3213.5092403 | 0 |
| 3213.5112609 | 1 |
| 3213.5123427 | 2 |
| 3213.5132542 | 1 |
| 3213.5152116 | 0 |
| 3213.5160231 | 0 |
| 3213.5176904 | 3 |
| 3213.5182025 | 1 |
| 3213.5198016 | 2 |
| 3213.521034 | 1 |
| 3213.5225374 | 2 |
| 3213.5242396 | 1 |
| 3213.5250998 | 2 |
| 3213.5258733 | 3 |
| 3213.5273151 | 1 |
| 3213.5280011 | 2 |
| 3213.5294738 | 2 |
| 3213.5307469 | 1 |
| 3213.5320605 | 3 |
| 3213.5339643 | 1 |
| 3213.5342225 | 0 |
| 3213.5360462 | 2 |
| 3213.5365167 | 1 |
| 3213.5382212 | 2 |
| 3213.5397435 | 3 |
| 3213.5404436 | 1 |
| 3213.5419796 | 2 |
| 3213.5432176 | 1 |
| 3213.5447262 | 2 |
| 3213.5457817 | 3 |
| 3213.5467061 | 3 |
| 3213.5477502 | 3 |
| 3213.5504107 | 1 |
| 3213.5507061 | 3 |
| 3213.5518666 | 0 |
| 3213.552986 | 5 |
| 3213.5545712 | 2 |
| 3213.5555265 | 0 |
| 3213.5573276 | 0 |
| 3213.558942 | 2 |
| 3213.5594356 | 2 |
| 3213.5611132 | 1 |
| 3213.5611866 | 3 |
| 3213.5630855 | 1 |
| 3213.5647527 | 1 |
| 3213.5659211 | 0 |
| 3213.5675251 | 1 |
| 3213.568117 | 1 |
| 3213.5700001 | 0 |
| 3213.5709571 | 2 |
| 3213.571603 | 1 |
| 3213.573322 | 0 |
| 3213.5748478 | 2 |
| 3213.5756671 | 1 |
| 3213.577186 | 4 |
| 3213.5776602 | 4 |
| 3213.5788841 | 4 |
| 3213.5802522 | 1 |
| 3213.5815341 | 3 |
| 3213.583289 | 3 |
| 3213.5838281 | 2 |
| 3213.5855585 | 1 |
| 3213.5869032 | 1 |
| 3213.5879743 | 1 |
| 3213.5893495 | 3 |
| 3213.5903159 | 3 |
| 3213.5917539 | 4 |
| 3213.592234 | 0 |
| 3213.5937954 | 2 |
| 3213.5954678 | 3 |
| 3213.5966822 | 0 |
| 3213.598069 | 2 |
| 3213.5987696 | 1 |
| 3213.6004735 | 4 |
| 3213.6020234 | 0 |
| 3213.6026338 | 3 |
| 3213.6048741 | 2 |
| 3213.6050137 | 1 |
| 3213.6072289 | 0 |
| 3213.6089309 | 1 |
| 3213.6090156 | 0 |
| 3213.6111294 | 2 |
| 3213.6120281 | 1 |
| 3213.6129586 | 2 |
| 3213.6144364 | 3 |
| 3213.6155658 | 2 |
| 3213.6170715 | 3 |
| 3213.6172638 | 2 |
| 3213.6190076 | 1 |
| 3213.6202341 | 3 |
| 3213.6217268 | 0 |
| 3213.6227194 | 5 |
| 3213.6241258 | 1 |
| 3213.6251036 | 4 |
| 3213.6265718 | 0 |
| 3213.62768 | 0 |
| 3213.6289056 | 2 |
| 3213.6291385 | 1 |
| 3213.6314729 | 2 |
| 3213.6325826 | 2 |
| 3213.6338947 | 1 |
| 3213.6356979 | 1 |
| 3213.636233 | 4 |
| 3213.6383375 | 1 |
| 3213.6391821 | 0 |
| 3213.6397312 | 2 |
| 3213.6414424 | 2 |
| 3213.6422348 | 4 |
| 3213.6437533 | 1 |
| 3213.6457666 | 1 |
| 3213.6465008 | 3 |
| 3213.6481245 | 3 |
| 3213.6482977 | 2 |
| 3213.6501258 | 0 |
| 3213.6518483 | 2 |
| 3213.6526096 | 2 |
| 3213.6541259 | 2 |
| 3213.65535 | 1 |
| 3213.6568078 | 4 |
| 3213.6578318 | 0 |
| 3213.6586844 | 2 |
| 3213.6602478 | 4 |
| 3213.6620682 | 0 |
| 3213.6625216 | 2 |
| 3213.6644483 | 1 |
| 3213.664684 | 2 |
| 3213.6659634 | 3 |
| 3213.6679127 | 1 |
| 3213.6683642 | 2 |
| 3213.6702218 | 4 |
| 3213.6711503 | 1 |
| 3213.6720086 | 3 |
| 3213.6737012 | 1 |
| 3213.6742649 | 4 |
| 3213.6762018 | 0 |
| 3213.6772484 | 1 |
| 3213.6787444 | 1 |
| 3213.6790257 | 1 |
| 3213.6808453 | 0 |
| 3213.6824993 | 1 |
| 3213.6827837 | 2 |
| 3213.6846994 | 0 |
| 3213.6857003 | 2 |
| 3213.6870868 | 1 |
| 3213.689837 | 3 |
| 3213.6916744 | 4 |
| 3213.6946271 | 1 |
| 3213.6948311 | 0 |
| 3213.6957402 | 3 |
| 3213.6970052 | 2 |
| 3213.6970926 | 1 |
| 3213.6988625 | 3 |
| 3213.699511 | 2 |
| 3213.7006821 | 4 |
| 3213.7013702 | 3 |
| 3213.7016542 | 1 |
| 3213.7031068 | 2 |
| 3213.7043693 | 1 |
| 3213.7047714 | 1 |
| 3213.7059418 | 0 |
| 3213.7062478 | 2 |
| 3213.7071485 | 4 |
| 3213.708331 | 3 |
| 3213.7094926 | 1 |
| 3213.7096807 | 3 |
| 3213.7102688 | 1 |
| 3213.7112838 | 1 |
| 3213.7120433 | 4 |
| 3213.7133962 | 1 |
| 3213.7145986 | 0 |
| 3213.7147814 | 0 |
| 3213.7160725 | 2 |
| 3213.7163729 | 0 |
| 3213.7178055 | 2 |
| 3213.718191 | 3 |
| 3213.7195364 | 4 |
| 3213.7195396 | 2 |
| 3213.7200206 | 0 |
| 3213.7212772 | 3 |
| 3213.7232053 | 5 |
| 3213.7232765 | 0 |
| 3213.7235112 | 1 |
| 3213.7242294 | 1 |
| 3213.7252775 | 4 |
| 3213.7266966 | 0 |
| 3213.72699 | 2 |
| 3213.7278365 | 3 |
| 3213.7293313 | 0 |
| 3213.730077 | 3 |
| 3213.7315657 | 3 |
| 3213.7320363 | 0 |
| 3213.7324411 | 1 |
| 3213.7333077 | 1 |
| 3213.7338584 | 2 |
| 3213.7354846 | 5 |
| 3213.7362417 | 3 |
| 3213.7370736 | 2 |
| 3213.7373619 | 3 |
| 3213.7385533 | 0 |
| 3213.7392767 | 0 |
| 3213.7400791 | 0 |
| 3213.7416881 | 1 |
| 3213.7417795 | 2 |
| 3213.7426677 | 0 |
| 3213.7440317 | 0 |
| 3213.7446848 | 0 |
| 3213.7459493 | 0 |
| 3213.746511 | 3 |
| 3213.7477342 | 1 |
| 3213.7477846 | 1 |
| 3213.7485746 | 7 |
| 3213.7505889 | 1 |
| 3213.7513835 | 1 |
| 3213.7516201 | 1 |
| 3213.7526144 | 1 |
| 3213.7535548 | 2 |
| 3213.7538437 | 2 |
| 3213.7550078 | 0 |
| 3213.7568522 | 1 |
| 3213.757104 | 1 |
| 3213.7575356 | 1 |
| 3213.7580946 | 0 |
| 3213.7590157 | 1 |
| 3213.7606789 | 3 |
| 3213.7610725 | 5 |
| 3213.7612714 | 3 |
| 3213.7630246 | 2 |
| 3213.7635527 | 0 |
| 3213.7653599 | 3 |
| 3213.7656388 | 1 |
| 3213.7662261 | 3 |
| 3213.7662609 | 3 |
| 3213.7679008 | 0 |
| 3213.7686039 | 0 |
| 3213.7694801 | 2 |
| 3213.7701029 | 2 |
| 3213.7716787 | 2 |
| 3213.7717997 | 3 |
| 3213.7726928 | 1 |
| 3213.7728678 | 1 |
| 3213.7734053 | 2 |
| 3213.7739729 | 1 |
| 3213.7751961 | 1 |
| 3213.7758171 | 1 |
| 3213.776089 | 2 |
| 3213.7771765 | 1 |
| 3213.7773555 | 5 |
| 3213.7776149 | 0 |
| 3213.7791186 | 1 |
| 3213.7794977 | 2 |
| 3213.7797249 | 4 |
| 3213.7808596 | 1 |
| 3213.7815311 | 1 |
| 3213.7817671 | 1 |
| 3213.7818807 | 0 |
| 3213.7829785 | 3 |
| 3213.7840902 | 0 |
| 3213.7842827 | 4 |
| 3213.7854928 | 1 |
| 3213.7858006 | 2 |
| 3213.7861772 | 1 |
| 3213.7872529 | 2 |

| | |
|---|---|
| 3213.7878152 | 2 |
| 3213.7881381 | 1 |
| 3213.7881942 | 1 |
| 3213.7894241 | 1 |
| 3213.7902921 | 3 |
| 3213.7912487 | 1 |
| 3213.7916058 | 1 |
| 3213.7919613 | 2 |
| 3213.7921548 | 2 |
| 3213.7933634 | 2 |
| 3213.7935356 | 1 |
| 3213.7937816 | 1 |
| 3213.7952027 | 0 |
| 3213.7953992 | 2 |
| 3213.7956937 | 1 |
| 3213.7965464 | 1 |
| 3213.7973611 | 3 |
| 3213.7980267 | 2 |
| 3213.7989073 | 1 |
| 3213.7990622 | 2 |
| 3213.8002778 | 1 |
| 3213.8004443 | 1 |
| 3213.8011834 | 2 |
| 3213.8019349 | 2 |
| 3213.8024058 | 1 |
| 3213.8028935 | 3 |
| 3213.803991 | 3 |
| 3213.8043084 | 3 |
| 3213.8045085 | 2 |
| 3213.8056219 | 9 |
| 3213.8065071 | 2 |
| 3213.8066117 | 1 |
| 3213.8079957 | 4 |
| 3213.8080147 | 3 |
| 3213.8083957 | 1 |
| 3213.8085609 | 1 |
| 3213.8102355 | 3 |
| 3213.810247 | 1 |
| 3213.8112816 | 3 |
| 3213.811832 | 1 |
| 3213.8122888 | 3 |
| 3213.8127404 | 6 |
| 3213.8133352 | 0 |
| 3213.8139946 | 1 |
| 3213.8146515 | 3 |
| 3213.8151407 | 0 |
| 3213.8162853 | 2 |
| 3213.8168456 | 4 |
| 3213.8171013 | 3 |
| 3213.8176302 | 2 |
| 3213.8185476 | 4 |
| 3213.819113 | 3 |
| 3213.8191242 | 0 |
| 3213.8195055 | 1 |
| 3213.8201474 | 2 |
| 3213.8203634 | 2 |
| 3213.8210331 | 2 |
| 3213.8216629 | 3 |
| 3213.8225241 | 0 |
| 3213.8228196 | 0 |
| 3213.8229318 | 2 |
| 3213.8237096 | 1 |
| 3213.8242712 | 1 |
| 3213.8245597 | 3 |
| 3213.8253782 | 1 |
| 3213.8256754 | 2 |
| 3213.82595 | 2 |
| 3213.8269452 | 2 |
| 3213.8270584 | 2 |
| 3213.8287507 | 1 |
| 3213.829275 | 3 |
| 3213.8296681 | 1 |
| 3213.8300674 | 0 |
| 3213.8309984 | 2 |
| 3213.8315478 | 4 |
| 3213.8322923 | 0 |
| 3213.8334934 | 4 |
| 3213.8339322 | 1 |
| 3213.835195 | 2 |
| 3213.8361558 | 1 |
| 3213.8372536 | 0 |
| 3213.8377814 | 2 |
| 3213.8387635 | 2 |
| 3213.8406908 | 1 |
| 3213.8422142 | 2 |
| 3213.8425672 | 0 |
| 3213.8449822 | 1 |
| 3213.8450495 | 3 |
| 3213.8460584 | 0 |
| 3213.8479368 | 0 |
| 3213.8491884 | 0 |
| 3213.8504051 | 3 |
| 3213.8512771 | 1 |
| 3213.8528652 | 2 |
| 3213.8537857 | 2 |
| 3213.8548805 | 2 |
| 3213.8568576 | 2 |
| 3213.857266 | 1 |
| 3213.8588243 | 1 |
| 3213.8603371 | 1 |
| 3213.8609622 | 1 |
| 3213.8626816 | 1 |
| 3213.8631946 | 2 |
| 3213.8651866 | 0 |
| 3213.8664616 | 3 |
| 3213.8672708 | 2 |
| 3213.8690131 | 1 |
| 3213.8700105 | 1 |
| 3213.8707451 | 3 |
| 3213.8721703 | 3 |
| 3213.8728947 | 0 |
| 3213.8730126 | 1 |
| 3213.8739587 | 0 |
| 3213.8739907 | 4 |
| 3213.8748919 | 4 |
| 3213.8754997 | 0 |
| 3213.8763596 | 1 |
| 3213.8764214 | 3 |
| 3213.8771507 | 0 |
| 3213.8774597 | 3 |
| 3213.8779977 | 2 |
| 3213.8788945 | 1 |
| 3213.8796888 | 2 |
| 3213.880143 | 2 |
| 3213.88017 | 0 |
| 3213.8808542 | 2 |
| 3213.8812243 | 4 |
| 3213.8819544 | 2 |
| 3213.8821527 | 1 |
| 3213.8827615 | 0 |
| 3213.8832485 | 1 |
| 3213.88355 | 2 |
| 3213.8850166 | 2 |
| 3213.8850221 | 0 |
| 3213.8852194 | 3 |
| 3213.8852625 | 3 |
| 3213.8863294 | 2 |
| 3213.8872582 | 2 |
| 3213.8872689 | 2 |
| 3213.887697 | 0 |
| 3213.8881123 | 1 |
| 3213.8883086 | 1 |
| 3213.8896789 | 1 |
| 3213.889947 | 0 |
| 3213.890504 | 2 |
| 3213.8906456 | 2 |
| 3213.890717 | 1 |
| 3213.8909831 | 1 |
| 3213.8917992 | 1 |
| 3213.8918687 | 2 |
| 3213.8929275 | 0 |
| 3213.8934246 | 4 |
| 3213.8938965 | 1 |
| 3213.893963 | 2 |
| 3213.8943715 | 1 |
| 3213.8947947 | 2 |
| 3213.8957145 | 3 |
| 3213.8965082 | 2 |
| 3213.8975069 | 0 |
| 3213.8982434 | 2 |
| 3213.8991406 | 3 |
| 3213.9000848 | 3 |
| 3213.901173 | 3 |
| 3213.9016553 | 3 |
| 3213.9025711 | 1 |
| 3213.9038495 | 5 |
| 3213.9044619 | 1 |
| 3213.9057645 | 3 |
| 3213.9060634 | 2 |
| 3213.9069191 | 5 |
| 3213.9079218 | 0 |
| 3213.9095503 | 2 |
| 3213.9099546 | 1 |
| 3213.9111453 | 2 |
| 3213.9126936 | 0 |
| 3213.9128669 | 3 |
| 3213.9144462 | 2 |
| 3213.9145469 | 1 |
| 3213.9152664 | 1 |
| 3213.9170534 | 2 |
| 3213.9180098 | 0 |
| 3213.9186116 | 3 |
| 3213.919647 | 0 |
| 3213.9205325 | 1 |
| 3213.9219461 | 1 |
| 3213.9219853 | 2 |
| 3213.9235787 | 1 |
| 3213.9235966 | 2 |
| 3213.9250158 | 3 |
| 3213.9264433 | 0 |
| 3213.9270293 | 2 |
| 3213.9278803 | 4 |
| 3213.928426 | 3 |
| 3213.930588 | 1 |
| 3213.931226 | 3 |
| 3213.9315885 | 2 |
| 3213.9329255 | 2 |
| 3213.9334298 | 1 |
| 3213.9343196 | 1 |
| 3213.9353277 | 2 |
| 3213.9358635 | 1 |
| 3213.9373416 | 3 |
| 3213.9377404 | 5 |
| 3213.9392644 | 2 |
| 3213.9398938 | 2 |
| 3213.9408966 | 2 |
| 3213.941816 | 0 |
| 3213.9422091 | 3 |
| 3213.9426603 | 3 |
| 3213.9445918 | 2 |
| 3213.9445925 | 1 |
| 3213.9463068 | 3 |
| 3213.9469389 | 1 |
| 3213.9483803 | 3 |
| 3213.9484508 | 0 |
| 3213.9499899 | 3 |
| 3213.9510156 | 4 |
| 3213.9514926 | 1 |
| 3213.9528071 | 3 |
| 3213.9529913 | 2 |
| 3213.9544132 | 2 |
| 3213.9561435 | 3 |
| 3213.9561866 | 3 |
| 3213.9578329 | 5 |
| 3213.9582952 | 3 |
| 3213.9586783 | 0 |
| 3213.9603851 | 0 |
| 3213.9607557 | 1 |
| 3213.9612782 | 2 |
| 3213.9628541 | 1 |
| 3213.9635075 | 1 |
| 3213.9645311 | 2 |
| 3213.965637 | 3 |
| 3213.966311 | 0 |
| 3213.9684881 | 5 |
| 3213.9688974 | 2 |
| 3213.9696317 | 0 |
| 3213.9701072 | 2 |
| 3213.9717421 | 0 |
| 3213.9726283 | 0 |
| 3213.9735606 | 0 |
| 3213.9742969 | 2 |
| 3213.9746436 | 2 |
| 3213.9756027 | 3 |
| 3213.9772329 | 2 |
| 3213.9777852 | 1 |
| 3213.9785494 | 1 |
| 3213.9797981 | 3 |
| 3213.9807656 | 2 |
| 3213.9812033 | 5 |
| 3213.9836227 | 0 |
| 3213.983727 | 2 |
| 3213.9840895 | 2 |
| 3213.985961 | 1 |
| 3213.9861525 | 0 |
| 3213.986745 | 2 |
| 3213.9876047 | 2 |
| 3213.9885791 | 0 |
| 3213.9890203 | 2 |
| 3213.9903956 | 3 |
| 3213.9920789 | 3 |
| 3213.9927864 | 4 |
| 3213.993573 | 3 |
| 3213.9941994 | 1 |
| 3213.9943948 | 1 |
| 3213.9958755 | 1 |
| 3213.9970761 | 2 |
| 3213.9975691 | 1 |
| 3213.9988214 | 1 |
| 3213.9993957 | 0 |
| 3214.0008942 | 5 |
| 3214.0019365 | 2 |
| 3214.0024886 | 3 |
| 3214.0033291 | 0 |
| 3214.0035246 | 1 |
| 3214.0051358 | 1 |
| 3214.006301 | 1 |
| 3214.0070541 | 0 |
| 3214.0078145 | 2 |
| 3214.0092474 | 3 |
| 3214.0096791 | 2 |
| 3214.0109111 | 4 |
| 3214.0123665 | 2 |
| 3214.0130821 | 2 |
| 3214.0139129 | 0 |
| 3214.0147144 | 3 |
| 3214.0160426 | 1 |
| 3214.0165306 | 1 |
| 3214.0180567 | 1 |
| 3214.0187536 | 2 |
| 3214.0197135 | 1 |
| 3214.0209313 | 1 |
| 3214.0213189 | 0 |
| 3214.0223138 | 1 |
| 3214.0236013 | 1 |
| 3214.0236115 | 2 |
| 3214.0253632 | 3 |
| 3214.0255245 | 0 |
| 3214.0270867 | 0 |
| 3214.0282233 | 3 |
| 3214.0287516 | 3 |
| 3214.0296582 | 1 |
| 3214.0306181 | 0 |
| 3214.0310418 | 1 |
| 3214.0325258 | 3 |
| 3214.0330446 | 4 |
| 3214.0341798 | 2 |
| 3214.0351973 | 1 |
| 3214.0359487 | 7 |
| 3214.0373498 | 2 |
| 3214.0378503 | 2 |
| 3214.0391177 | 2 |
| 3214.0391955 | 2 |
| 3214.0404618 | 3 |
| 3214.0419147 | 3 |
| 3214.0420737 | 3 |
| 3214.0431599 | 2 |
| 3214.0439594 | 3 |
| 3214.0452088 | 1 |
| 3214.0458931 | 1 |
| 3214.0469873 | 2 |
| 3214.0480671 | 4 |
| 3214.0488616 | 3 |
| 3214.0497176 | 1 |
| 3214.0508356 | 2 |
| 3214.0515114 | 2 |
| 3214.052632 | 2 |
| 3214.053199 | 4 |
| 3214.0538871 | 1 |
| 3214.0549285 | 4 |
| 3214.0559353 | 1 |
| 3214.0564527 | 2 |
| 3214.0581497 | 3 |
| 3214.0591049 | 0 |
| 3214.0601344 | 2 |
| 3214.0613258 | 5 |
| 3214.0621013 | 1 |
| 3214.0625737 | 2 |
| 3214.0641731 | 1 |
| 3214.0650736 | 1 |
| 3214.0652473 | 1 |
| 3214.0664485 | 1 |
| 3214.067476 | 6 |
| 3214.0690659 | 3 |
| 3214.0695537 | 3 |
| 3214.0704865 | 3 |
| 3214.0717147 | 1 |
| 3214.0719843 | 2 |
| 3214.0733009 | 2 |
| 3214.0742075 | 3 |
| 3214.0743962 | 1 |
| 3214.0762469 | 1 |
| 3214.076833 | 1 |
| 3214.0777763 | 5 |
| 3214.0778863 | 1 |
| 3214.0796871 | 2 |
| 3214.0810223 | 3 |
| 3214.0811476 | 1 |
| 3214.0820588 | 3 |
| 3214.0835307 | 1 |
| 3214.0836998 | 0 |
| 3214.0846672 | 2 |
| 3214.0858271 | 2 |

| | |
|---|---|
| 3214.086563 | 1 |
| 3214.0880031 | 0 |
| 3214.0885564 | 2 |
| 3214.089543 | 0 |
| 3214.090369 | 1 |
| 3214.0911886 | 6 |
| 3214.0926053 | 2 |
| 3214.0938478 | 4 |
| 3214.0941476 | 1 |
| 3214.094874 | 3 |
| 3214.09664 | 0 |
| 3214.0967138 | 1 |
| 3214.0979953 | 2 |
| 3214.0994501 | 1 |
| 3214.0997361 | 1 |
| 3214.1009389 | 1 |
| 3214.1018547 | 2 |
| 3214.1032543 | 2 |
| 3214.1036923 | 1 |
| 3214.1043271 | 1 |
| 3214.1057617 | 0 |
| 3214.1070304 | 2 |
| 3214.1074768 | 4 |
| 3214.1078957 | 1 |
| 3214.1097136 | 3 |
| 3214.1105868 | 2 |
| 3214.1109722 | 3 |
| 3214.1118629 | 1 |
| 3214.1135719 | 4 |
| 3214.1143031 | 1 |
| 3214.114983 | 3 |
| 3214.1155277 | 0 |
| 3214.1169034 | 0 |
| 3214.1181591 | 1 |
| 3214.11832 | 1 |
| 3214.1204041 | 1 |
| 3214.1206229 | 1 |
| 3214.1221082 | 4 |
| 3214.1224529 | 3 |
| 3214.1230929 | 0 |
| 3214.1241549 | 6 |
| 3214.1254169 | 2 |
| 3214.1267669 | 1 |
| 3214.1269272 | 2 |
| 3214.1280105 | 0 |
| 3214.128982 | 3 |
| 3214.1291369 | 3 |
| 3214.1306468 | 2 |
| 3214.1316162 | 0 |
| 3214.1326002 | 2 |
| 3214.1333508 | 0 |
| 3214.1345074 | 2 |
| 3214.1358301 | 1 |
| 3214.1366113 | 1 |
| 3214.1371988 | 5 |
| 3214.1387686 | 4 |
| 3214.139218 | 1 |
| 3214.1404155 | 4 |
| 3214.1412071 | 2 |
| 3214.1415033 | 2 |
| 3214.1429821 | 0 |
| 3214.1437189 | 0 |
| 3214.1447736 | 0 |
| 3214.1454341 | 1 |
| 3214.1469427 | 2 |
| 3214.1479951 | 3 |
| 3214.1486271 | 4 |
| 3214.1500205 | 2 |
| 3214.1508822 | 1 |
| 3214.1512986 | 3 |
| 3214.1525737 | 0 |
| 3214.1534666 | 3 |
| 3214.1550505 | 3 |
| 3214.1554049 | 1 |
| 3214.1567012 | 3 |
| 3214.1571816 | 0 |
| 3214.1581907 | 1 |
| 3214.1596364 | 2 |
| 3214.1599737 | 3 |
| 3214.1610451 | 0 |
| 3214.1621046 | 0 |
| 3214.1630768 | 4 |
| 3214.1641791 | 2 |
| 3214.1645587 | 1 |
| 3214.1654972 | 1 |
| 3214.1665271 | 2 |
| 3214.1671309 | 1 |
| 3214.1679672 | 2 |
| 3214.17002 | 3 |
| 3214.1704353 | 2 |
| 3214.1715723 | 3 |
| 3214.1720515 | 2 |
| 3214.1739094 | 2 |
| 3214.1741073 | 2 |
| 3214.1747216 | 1 |
| 3214.1760126 | 4 |
| 3214.1767818 | 0 |
| 3214.1779461 | 5 |
| 3214.1789459 | 2 |
| 3214.1800448 | 2 |
| 3214.1805044 | 3 |
| 3214.1815958 | 0 |
| 3214.1826988 | 1 |
| 3214.1838328 | 0 |
| 3214.1845782 | 2 |
| 3214.1850199 | 1 |
| 3214.1862716 | 3 |
| 3214.1869009 | 0 |
| 3214.1886976 | 2 |
| 3214.1888812 | 4 |
| 3214.1904141 | 0 |
| 3214.1911207 | 4 |
| 3214.192245 | 1 |
| 3214.1934533 | 0 |
| 3214.1937978 | 1 |
| 3214.1952589 | 3 |
| 3214.1958215 | 3 |
| 3214.1971976 | 0 |
| 3214.1979302 | 1 |
| 3214.1991303 | 6 |
| 3214.2004102 | 1 |
| 3214.200586 | 4 |
| 3214.2024951 | 0 |
| 3214.2033237 | 1 |
| 3214.203333 | 1 |
| 3214.2044589 | 5 |
| 3214.2052348 | 3 |
| 3214.2063066 | 1 |
| 3214.2075114 | 1 |
| 3214.2081657 | 1 |
| 3214.2093536 | 2 |
| 3214.2097408 | 0 |
| 3214.2113642 | 0 |
| 3214.2118026 | 1 |
| 3214.2122622 | 3 |
| 3214.2125684 | 3 |
| 3214.2126908 | 5 |
| 3214.2128364 | 1 |
| 3214.2128914 | 3 |
| 3214.2135626 | 1 |
| 3214.2137713 | 3 |
| 3214.2140784 | 3 |
| 3214.2142033 | 0 |
| 3214.2146713 | 2 |
| 3214.2152673 | 2 |
| 3214.2154604 | 2 |
| 3214.2157541 | 1 |
| 3214.2160695 | 3 |
| 3214.2162291 | 1 |
| 3214.2163813 | 2 |
| 3214.217025 | 0 |
| 3214.2172654 | 1 |
| 3214.2176934 | 4 |
| 3214.2178925 | 1 |
| 3214.2180174 | 5 |
| 3214.2183488 | 2 |
| 3214.2188665 | 2 |
| 3214.219302 | 2 |
| 3214.2195076 | 3 |
| 3214.2199366 | 1 |
| 3214.2200182 | 2 |
| 3214.2202169 | 8 |
| 3214.2207741 | 0 |
| 3214.2210469 | 3 |
| 3214.2210979 | 4 |
| 3214.2219021 | 6 |
| 3214.2221208 | 3 |
| 3214.2226669 | 3 |
| 3214.2231242 | 6 |
| 3214.2231618 | 6 |
| 3214.223319 | 2 |
| 3214.2233244 | 8 |
| 3214.2233796 | 6 |
| 3214.2238569 | 7 |
| 3214.2240431 | 3 |
| 3214.2241771 | 2 |
| 3214.2248263 | 1 |
| 3214.2253122 | 2 |
| 3214.2257891 | 3 |
| 3214.2258294 | 1 |
| 3214.2259638 | 0 |
| 3214.2260369 | 2 |
| 3214.2267353 | 0 |
| 3214.2271348 | 3 |
| 3214.2279628 | 3 |
| 3214.2280563 | 9 |
| 3214.2282517 | 9 |
| 3214.2284542 | 8 |
| 3214.2286981 | 9 |
| 3214.2291723 | 5 |
| 3214.2294829 | 2 |
| 3214.2296341 | 3 |
| 3214.2299528 | 9 |
| 3214.2299705 | 6 |
| 3214.2312436 | 6 |
| 3214.2314881 | 3 |
| 3214.2317135 | 5 |
| 3214.2317786 | 1 |
| 3214.2320452 | 3 |
| 3214.2325745 | 1 |
| 3214.2327099 | 1 |
| 3214.2332446 | 3 |
| 3214.2332907 | 4 |
| 3214.2336263 | 4 |
| 3214.233701 | 1 |
| 3214.2343649 | 0 |
| 3214.2343837 | 8 |
| 3214.2351332 | 1 |
| 3214.2353358 | 3 |
| 3214.2356345 | 1 |
| 3214.2358455 | 1 |
| 3214.2358635 | 3 |
| 3214.2362248 | 1 |
| 3214.2369485 | 2 |
| 3214.2369759 | 2 |
| 3214.2371817 | 1 |
| 3214.2374976 | 0 |
| 3214.2381107 | 0 |
| 3214.2385762 | 2 |
| 3214.2390374 | 6 |
| 3214.2390487 | 5 |
| 3214.2390495 | 5 |
| 3214.2394669 | 1 |
| 3214.2395695 | 2 |
| 3214.2401227 | 0 |
| 3214.240244 | 1 |
| 3214.2411748 | 3 |
| 3214.2413197 | 2 |
| 3214.2414109 | 0 |
| 3214.2419698 | 1 |
| 3214.2423105 | 3 |
| 3214.2424414 | 3 |
| 3214.2433813 | 4 |
| 3214.2434226 | 1 |
| 3214.2434892 | 3 |
| 3214.2438568 | 1 |
| 3214.2440516 | 1 |
| 3214.2443415 | 3 |
| 3214.2444849 | 1 |
| 3214.2448562 | 3 |
| 3214.2452394 | 1 |
| 3214.2453162 | 1 |
| 3214.2463758 | 1 |
| 3214.2467631 | 1 |
| 3214.2471843 | 2 |
| 3214.2472188 | 4 |
| 3214.2473994 | 1 |
| 3214.2474512 | 2 |
| 3214.2475502 | 1 |
| 3214.2485954 | 0 |
| 3214.2493399 | 0 |
| 3214.2504695 | 4 |
| 3214.2511243 | 3 |
| 3214.2521384 | 0 |
| 3214.2533078 | 4 |
| 3214.2537024 | 2 |
| 3214.2553466 | 2 |
| 3214.2554632 | 1 |
| 3214.2566053 | 0 |
| 3214.2580272 | 0 |
| 3214.2584337 | 2 |
| 3214.2590598 | 0 |
| 3214.2598213 | 2 |
| 3214.26047 | 2 |
| 3214.2623263 | 4 |
| 3214.2630464 | 4 |
| 3214.2640005 | 1 |
| 3214.2644634 | 2 |
| 3214.2659149 | 4 |
| 3214.266425 | 0 |
| 3214.2678619 | 2 |
| 3214.268845 | 0 |
| 3214.2694829 | 3 |
| 3214.2710066 | 2 |
| 3214.2712289 | 2 |
| 3214.2729923 | 3 |
| 3214.2738812 | 3 |
| 3214.2746185 | 2 |
| 3214.2752391 | 0 |
| 3214.275362 | 4 |
| 3214.2768971 | 0 |
| 3214.2780223 | 2 |
| 3214.2788083 | 3 |
| 3214.2795309 | 1 |
| 3214.2813124 | 1 |
| 3214.2814861 | 2 |
| 3214.2828323 | 3 |
| 3214.2830126 | 4 |
| 3214.2847496 | 2 |
| 3214.2850581 | 0 |
| 3214.2862073 | 2 |
| 3214.2877238 | 1 |
| 3214.2881574 | 1 |
| 3214.2892924 | 0 |
| 3214.2901868 | 2 |
| 3214.2912346 | 0 |
| 3214.2927934 | 1 |
| 3214.2931703 | 2 |
| 3214.2945577 | 2 |
| 3214.2945593 | 2 |
| 3214.2965626 | 3 |
| 3214.2970308 | 1 |
| 3214.2982388 | 1 |
| 3214.2991476 | 2 |
| 3214.2993721 | 2 |
| 3214.3002225 | 1 |
| 3214.301559 | 3 |
| 3214.3019435 | 2 |
| 3214.3029995 | 1 |
| 3214.3046037 | 2 |
| 3214.3057395 | 1 |
| 3214.3065656 | 0 |
| 3214.3073173 | 4 |
| 3214.3082938 | 2 |
| 3214.3087339 | 3 |
| 3214.3100431 | 3 |
| 3214.3109685 | 3 |
| 3214.3118703 | 2 |
| 3214.3119817 | 2 |
| 3214.3135434 | 2 |
| 3214.3135548 | 2 |
| 3214.3136569 | 1 |
| 3214.3141791 | 2 |
| 3214.3141832 | 2 |
| 3214.3146302 | 0 |
| 3214.3146421 | 4 |
| 3214.3148077 | 1 |
| 3214.3154587 | 0 |
| 3214.3157905 | 0 |
| 3214.3163741 | 3 |
| 3214.3164044 | 1 |
| 3214.3170492 | 3 |
| 3214.3173143 | 2 |
| 3214.3175177 | 2 |
| 3214.3177596 | 4 |
| 3214.317785 | 2 |
| 3214.318076 | 2 |
| 3214.3182099 | 1 |
| 3214.3188356 | 1 |
| 3214.3195291 | 2 |
| 3214.3196007 | 2 |
| 3214.319631 | 2 |
| 3214.3198158 | 3 |
| 3214.3203613 | 2 |
| 3214.3208393 | 2 |
| 3214.3208772 | 2 |
| 3214.3215221 | 3 |
| 3214.321918 | 0 |
| 3214.3219832 | 0 |
| 3214.3226604 | 1 |
| 3214.3228495 | 2 |
| 3214.323037 | 3 |
| 3214.3233391 | 1 |
| 3214.3235614 | 3 |
| 3214.3237036 | 1 |
| 3214.3245166 | 3 |
| 3214.3246385 | 3 |
| 3214.3249111 | 3 |
| 3214.325127 | 1 |
| 3214.3254155 | 0 |
| 3214.3257608 | 3 |
| 3214.3265461 | 2 |
| 3214.326643 | 2 |

| | |
|---|---|
| 3214.3268021 | 1 |
| 3214.3273354 | 0 |
| 3214.3275611 | 3 |
| 3214.3275688 | 2 |
| 3214.327613 | 0 |
| 3214.3283454 | 1 |
| 3214.3284565 | 2 |
| 3214.3290085 | 2 |
| 3214.3296716 | 5 |
| 3214.3301063 | 5 |
| 3214.3301549 | 4 |
| 3214.3304021 | 7 |
| 3214.3308827 | 3 |
| 3214.3312295 | 6 |
| 3214.3314323 | 10 |
| 3214.3316222 | 9 |
| 3214.3319475 | 13 |
| 3214.3319523 | 9 |
| 3214.332456 | 12 |
| 3214.3327423 | 9 |
| 3214.3332879 | 9 |
| 3214.3336624 | 16 |
| 3214.3337843 | 11 |
| 3214.3343955 | 7 |
| 3214.3344444 | 11 |
| 3214.3350303 | 12 |
| 3214.3350971 | 9 |
| 3214.3356897 | 7 |
| 3214.3357111 | 8 |
| 3214.3362262 | 12 |
| 3214.3364642 | 5 |
| 3214.3365551 | 3 |
| 3214.3365719 | 2 |
| 3214.3373309 | 7 |
| 3214.3376353 | 1 |
| 3214.3377132 | 0 |
| 3214.3379318 | 2 |
| 3214.3389537 | 2 |
| 3214.3390295 | 3 |
| 3214.3394338 | 4 |
| 3214.3395667 | 3 |
| 3214.3395778 | 0 |
| 3214.3402017 | 3 |
| 3214.3403041 | 7 |
| 3214.3407433 | 1 |
| 3214.3408757 | 1 |
| 3214.341347 | 0 |
| 3214.3416452 | 4 |
| 3214.3417837 | 2 |
| 3214.342203 | 3 |
| 3214.3427835 | 2 |
| 3214.3429033 | 1 |
| 3214.3432603 | 3 |
| 3214.3434896 | 1 |
| 3214.343623 | 0 |
| 3214.3438777 | 2 |
| 3214.3442749 | 1 |
| 3214.3442767 | 3 |
| 3214.344543 | 2 |
| 3214.3459691 | 2 |
| 3214.3459761 | 1 |
| 3214.3463796 | 1 |
| 3214.3467018 | 2 |
| 3214.3467501 | 2 |
| 3214.3468206 | 3 |
| 3214.3471386 | 4 |
| 3214.3471786 | 3 |
| 3214.3477865 | 2 |
| 3214.3483493 | 1 |
| 3214.3484063 | 1 |
| 3214.3485268 | 3 |
| 3214.3487107 | 3 |
| 3214.3490673 | 0 |
| 3214.3496611 | 1 |
| 3214.3500535 | 3 |
| 3214.3504075 | 2 |
| 3214.3506426 | 2 |
| 3214.350829 | 0 |
| 3214.3513253 | 1 |
| 3214.3518275 | 2 |
| 3214.351852 | 1 |
| 3214.3520563 | 2 |
| 3214.352648 | 5 |
| 3214.3528756 | 0 |
| 3214.3530014 | 1 |
| 3214.3531935 | 2 |
| 3214.353521 | 1 |
| 3214.3537048 | 2 |
| 3214.3540825 | 2 |
| 3214.3542362 | 3 |
| 3214.3543708 | 2 |
| 3214.3555063 | 1 |
| 3214.3555356 | 3 |
| 3214.3555601 | 0 |
| 3214.3556061 | 1 |
| 3214.3559507 | 2 |
| 3214.3568533 | 3 |
| 3214.356994 | 4 |
| 3214.3573083 | 1 |
| 3214.3574064 | 5 |
| 3214.3579077 | 1 |
| 3214.358164 | 2 |
| 3214.358256 | 2 |
| 3214.3588185 | 2 |
| 3214.3589021 | 0 |
| 3214.3590278 | 0 |
| 3214.359822 | 4 |
| 3214.3602799 | 4 |
| 3214.3603032 | 1 |
| 3214.3604098 | 5 |
| 3214.3605237 | 3 |
| 3214.3612304 | 0 |
| 3214.3617877 | 2 |
| 3214.3618108 | 3 |
| 3214.3621078 | 0 |
| 3214.3624051 | 3 |
| 3214.362507 | 1 |
| 3214.3631361 | 4 |
| 3214.3633454 | 2 |
| 3214.3638579 | 2 |
| 3214.3641055 | 3 |
| 3214.3642205 | 2 |
| 3214.3649151 | 4 |
| 3214.3650407 | 4 |
| 3214.3650905 | 4 |
| 3214.3652904 | 4 |
| 3214.3657069 | 1 |
| 3214.3659529 | 3 |
| 3214.3662901 | 4 |
| 3214.3666171 | 2 |
| 3214.3669643 | 2 |
| 3214.3669689 | 2 |
| 3214.3673257 | 0 |
| 3214.3683118 | 3 |
| 3214.3684656 | 4 |
| 3214.3685562 | 2 |
| 3214.3688291 | 1 |
| 3214.368962 | 1 |
| 3214.369824 | 2 |
| 3214.3699079 | 3 |
| 3214.3701973 | 3 |
| 3214.3702115 | 3 |
| 3214.3707135 | 2 |
| 3214.3712084 | 3 |
| 3214.3715138 | 1 |
| 3214.3715792 | 2 |
| 3214.3720124 | 0 |
| 3214.3721645 | 0 |
| 3214.3724424 | 1 |
| 3214.3728234 | 1 |
| 3214.3728383 | 1 |
| 3214.3734543 | 0 |
| 3214.3739272 | 0 |
| 3214.3741949 | 3 |
| 3214.3745928 | 4 |
| 3214.3747107 | 0 |
| 3214.3748959 | 3 |
| 3214.3759197 | 1 |
| 3214.376141 | 3 |
| 3214.3766449 | 2 |
| 3214.3767911 | 1 |
| 3214.3769047 | 3 |
| 3214.3769521 | 0 |
| 3214.3773386 | 1 |
| 3214.3778104 | 2 |
| 3214.3778681 | 1 |
| 3214.378162 | 2 |
| 3214.3789016 | 4 |
| 3214.3790642 | 3 |
| 3214.379068 | 5 |
| 3214.3798365 | 3 |
| 3214.3799585 | 1 |
| 3214.3802699 | 2 |
| 3214.3804067 | 5 |
| 3214.3807419 | 3 |
| 3214.3807731 | 5 |
| 3214.3818486 | 4 |
| 3214.381865 | 0 |
| 3214.3823659 | 4 |
| 3214.3824027 | 0 |
| 3214.3826766 | 3 |
| 3214.3829814 | 1 |
| 3214.3832586 | 2 |
| 3214.3838455 | 3 |
| 3214.3839509 | 1 |
| 3214.3842489 | 2 |
| 3214.3847855 | 2 |
| 3214.3848211 | 3 |
| 3214.3851162 | 3 |
| 3214.3851327 | 2 |
| 3214.3857999 | 2 |
| 3214.3863173 | 2 |
| 3214.3864039 | 3 |
| 3214.3864197 | 1 |
| 3214.3871324 | 2 |
| 3214.3875915 | 1 |
| 3214.3879864 | 3 |
| 3214.3886037 | 5 |
| 3214.3888392 | 4 |
| 3214.3889202 | 1 |
| 3214.3889557 | 2 |
| 3214.389506 | 0 |
| 3214.3897714 | 1 |
| 3214.3901454 | 0 |
| 3214.3904417 | 3 |
| 3214.3905201 | 1 |
| 3214.3905917 | 3 |
| 3214.391314 | 3 |
| 3214.3919753 | 2 |
| 3214.3919885 | 2 |
| 3214.3920049 | 2 |
| 3214.3921277 | 2 |
| 3214.3930354 | 3 |
| 3214.393061 | 0 |
| 3214.3932399 | 1 |
| 3214.3938512 | 1 |
| 3214.3941589 | 2 |
| 3214.3944162 | 3 |
| 3214.3944947 | 2 |
| 3214.3949575 | 5 |
| 3214.3951394 | 1 |
| 3214.3955225 | 0 |
| 3214.395904 | 2 |
| 3214.3960001 | 0 |
| 3214.3963939 | 1 |
| 3214.3965641 | 0 |
| 3214.3967048 | 0 |
| 3214.397305 | 2 |
| 3214.3974214 | 1 |
| 3214.397725 | 3 |
| 3214.3980524 | 2 |
| 3214.3982988 | 0 |
| 3214.3988508 | 3 |
| 3214.3988737 | 0 |
| 3214.3990911 | 1 |
| 3214.400559 | 2 |
| 3214.4007793 | 5 |
| 3214.4022775 | 0 |
| 3214.402525 | 0 |
| 3214.4036894 | 4 |
| 3214.4047642 | 2 |
| 3214.4058992 | 1 |
| 3214.4062973 | 3 |
| 3214.4073371 | 1 |
| 3214.4088661 | 1 |
| 3214.4089954 | 3 |
| 3214.4103621 | 1 |
| 3214.4116432 | 4 |
| 3214.4120366 | 2 |
| 3214.4132152 | 4 |
| 3214.4136862 | 2 |
| 3214.4152728 | 1 |
| 3214.4154643 | 0 |
| 3214.4167116 | 2 |
| 3214.4179543 | 4 |
| 3214.4183834 | 3 |
| 3214.4199484 | 2 |
| 3214.4205989 | 1 |
| 3214.421306 | 2 |
| 3214.4227687 | 2 |
| 3214.4236212 | 4 |
| 3214.4243184 | 3 |
| 3214.4256906 | 3 |
| 3214.4258824 | 0 |
| 3214.4269562 | 1 |
| 3214.4280116 | 2 |
| 3214.4289619 | 5 |
| 3214.4300981 | 1 |
| 3214.4313535 | 2 |
| 3214.4321176 | 2 |
| 3214.4324117 | 3 |
| 3214.4341782 | 0 |
| 3214.4348063 | 3 |
| 3214.4351488 | 4 |
| 3214.4368353 | 4 |
| 3214.4370574 | 2 |
| 3214.4379639 | 2 |
| 3214.4395565 | 0 |
| 3214.4401505 | 2 |
| 3214.4411165 | 0 |
| 3214.4418999 | 5 |
| 3214.4425165 | 4 |
| 3214.4434629 | 0 |
| 3214.4441888 | 0 |
| 3214.4453467 | 0 |
| 3214.4463373 | 2 |
| 3214.4471784 | 1 |
| 3214.4487152 | 2 |
| 3214.4495054 | 2 |
| 3214.4506528 | 1 |
| 3214.4507931 | 1 |
| 3214.4520029 | 3 |
| 3214.4523563 | 4 |
| 3214.4537216 | 2 |
| 3214.4549564 | 2 |
| 3214.4557245 | 1 |
| 3214.4563523 | 1 |
| 3214.4579495 | 1 |
| 3214.4584032 | 2 |
| 3214.4594831 | 2 |
| 3214.4600238 | 1 |
| 3214.460768 | 2 |
| 3214.4617175 | 3 |
| 3214.4626295 | 4 |
| 3214.4644101 | 2 |
| 3214.4650645 | 2 |
| 3214.4659683 | 2 |
| 3214.4665809 | 0 |
| 3214.4675738 | 1 |
| 3214.4688047 | 5 |
| 3214.4692003 | 0 |
| 3214.4705897 | 3 |
| 3214.4714983 | 2 |
| 3214.4725843 | 2 |
| 3214.4732218 | 2 |
| 3214.4742668 | 1 |
| 3214.4756029 | 2 |
| 3214.4756078 | 3 |
| 3214.4769928 | 1 |
| 3214.4777469 | 2 |
| 3214.4783778 | 0 |
| 3214.4801808 | 1 |
| 3214.4803752 | 1 |
| 3214.4815572 | 1 |
| 3214.4826258 | 1 |
| 3214.4835256 | 4 |
| 3214.4845215 | 2 |
| 3214.4853055 | 2 |
| 3214.4862425 | 4 |
| 3214.4874293 | 3 |
| 3214.4884534 | 1 |
| 3214.4894492 | 0 |
| 3214.4898075 | 1 |
| 3214.4916219 | 3 |
| 3214.4917566 | 2 |
| 3214.4922532 | 3 |
| 3214.4936482 | 3 |
| 3214.4938527 | 3 |
| 3214.4947951 | 0 |
| 3214.4961839 | 2 |
| 3214.4969529 | 0 |
| 3214.4984905 | 2 |
| 3214.4985986 | 1 |
| 3214.5001203 | 1 |
| 3214.5008591 | 2 |
| 3214.5018604 | 2 |
| 3214.502762 | 3 |
| 3214.5036532 | 1 |
| 3214.5046486 | 0 |
| 3214.5054721 | 1 |
| 3214.5060951 | 0 |
| 3214.507544 | 2 |
| 3214.5080514 | 2 |
| 3214.5095977 | 4 |
| 3214.5106768 | 1 |
| 3214.511065 | 2 |
| 3214.5118892 | 2 |
| 3214.5125163 | 0 |
| 3214.5139885 | 0 |
| 3214.5146844 | 2 |
| 3214.5159846 | 2 |
| 3214.5169005 | 1 |
| 3214.5171289 | 3 |

| | |
|---|---|
| 3214.5181696 | 1 |
| 3214.5192597 | 0 |
| 3214.5204844 | 1 |
| 3214.5216791 | 2 |
| 3214.5221091 | 1 |
| 3214.5228053 | 2 |
| 3214.5242535 | 0 |
| 3214.5256146 | 3 |
| 3214.5265485 | 1 |
| 3214.5266032 | 3 |
| 3214.5276686 | 3 |
| 3214.5286902 | 0 |
| 3214.529382 | 1 |
| 3214.5307444 | 0 |
| 3214.5317973 | 6 |
| 3214.5319864 | 3 |
| 3214.5331141 | 3 |
| 3214.5346145 | 2 |
| 3214.5357884 | 2 |
| 3214.5359159 | 6 |
| 3214.5373309 | 2 |
| 3214.5373349 | 0 |
| 3214.538809 | 3 |
| 3214.5402376 | 1 |
| 3214.5403809 | 3 |
| 3214.5417667 | 1 |
| 3214.5428152 | 1 |
| 3214.5428578 | 1 |
| 3214.5442673 | 6 |
| 3214.5451793 | 1 |
| 3214.5463289 | 1 |
| 3214.5468174 | 4 |
| 3214.548073 | 4 |
| 3214.5486007 | 4 |
| 3214.5495787 | 3 |
| 3214.5507418 | 1 |
| 3214.551699 | 2 |
| 3214.5531037 | 1 |
| 3214.5538473 | 0 |
| 3214.5544523 | 3 |
| 3214.5558581 | 1 |
| 3214.5559172 | 3 |
| 3214.5575509 | 3 |
| 3214.559049 | 2 |
| 3214.5598461 | 4 |
| 3214.5604532 | 1 |
| 3214.5611182 | 4 |
| 3214.5621858 | 2 |
| 3214.5623912 | 1 |
| 3214.5640346 | 2 |
| 3214.5649464 | 2 |
| 3214.5652914 | 1 |
| 3214.5669966 | 0 |
| 3214.5678052 | 2 |
| 3214.5681346 | 2 |
| 3214.5697824 | 1 |
| 3214.5703452 | 1 |
| 3214.5707798 | 1 |
| 3214.572608 | 2 |
| 3214.5729765 | 1 |
| 3214.5743853 | 1 |
| 3214.5748568 | 2 |
| 3214.5761269 | 1 |
| 3214.5773762 | 6 |
| 3214.5773966 | 1 |
| 3214.5792462 | 4 |
| 3214.5794974 | 2 |
| 3214.5805221 | 1 |
| 3214.581683 | 3 |
| 3214.5820575 | 2 |
| 3214.5833137 | 4 |
| 3214.5841341 | 4 |
| 3214.5849178 | 1 |
| 3214.5864415 | 2 |
| 3214.5874926 | 2 |
| 3214.5882993 | 3 |
| 3214.5887861 | 2 |
| 3214.5893008 | 0 |
| 3214.591043 | 2 |
| 3214.5912572 | 2 |
| 3214.5925019 | 2 |
| 3214.5932268 | 2 |
| 3214.5933948 | 2 |
| 3214.5952084 | 3 |
| 3214.5961597 | 1 |
| 3214.5972974 | 2 |
| 3214.5978483 | 2 |
| 3214.5991367 | 0 |
| 3214.5995524 | 2 |
| 3214.6003119 | 3 |
| 3214.6020331 | 1 |
| 3214.6024466 | 2 |
| 3214.6033814 | 0 |
| 3214.6051453 | 2 |
| 3214.605396 | 1 |
| 3214.6066354 | 2 |
| 3214.6073693 | 2 |
| 3214.6076862 | 1 |
| 3214.609282 | 1 |
| 3214.610297 | 4 |
| 3214.6118135 | 2 |
| 3214.6121534 | 3 |
| 3214.6134241 | 1 |
| 3214.6148032 | 0 |
| 3214.6148095 | 0 |
| 3214.6160873 | 3 |
| 3214.6168656 | 3 |
| 3214.6175923 | 0 |
| 3214.6185906 | 3 |
| 3214.6201339 | 0 |
| 3214.6210429 | 2 |
| 3214.6212417 | 2 |
| 3214.6225468 | 0 |
| 3214.6235516 | 1 |
| 3214.6243871 | 2 |
| 3214.6253853 | 2 |
| 3214.6260423 | 2 |
| 3214.6273445 | 3 |
| 3214.6281873 | 1 |
| 3214.6289017 | 1 |
| 3214.6294868 | 0 |
| 3214.6309614 | 3 |
| 3214.6314825 | 1 |
| 3214.6316698 | 1 |
| 3214.6331871 | 2 |
| 3214.6347423 | 0 |
| 3214.6349935 | 2 |
| 3214.6358968 | 1 |
| 3214.637422 | 1 |
| 3214.6381779 | 1 |
| 3214.6385421 | 2 |
| 3214.6399857 | 2 |
| 3214.6414599 | 1 |
| 3214.6415162 | 1 |
| 3214.6426342 | 1 |
| 3214.6437564 | 1 |
| 3214.6441191 | 2 |
| 3214.6455395 | 1 |
| 3214.6462068 | 1 |
| 3214.6471394 | 2 |
| 3214.6481055 | 0 |
| 3214.649661 | 0 |
| 3214.6508818 | 1 |
| 3214.6512346 | 2 |
| 3214.652313 | 2 |
| 3214.6535875 | 1 |
| 3214.6540043 | 2 |
| 3214.6552346 | 1 |
| 3214.6559135 | 0 |
| 3214.65602 | 2 |
| 3214.6577272 | 2 |
| 3214.6588419 | 1 |
| 3214.6599489 | 3 |
| 3214.6602205 | 1 |
| 3214.6614474 | 2 |
| 3214.6628853 | 2 |
| 3214.6635698 | 2 |
| 3214.6647275 | 3 |
| 3214.6649572 | 2 |
| 3214.666563 | 2 |
| 3214.6666304 | 3 |
| 3214.6678607 | 6 |
| 3214.6690132 | 3 |
| 3214.6701656 | 1 |
| 3214.6710851 | 5 |
| 3214.6714947 | 1 |
| 3214.6735206 | 1 |
| 3214.673889 | 3 |
| 3214.6740591 | 1 |
| 3214.675585 | 0 |
| 3214.6759221 | 1 |
| 3214.6767338 | 1 |
| 3214.6781062 | 1 |
| 3214.6790042 | 1 |
| 3214.6799815 | 1 |
| 3214.6807508 | 1 |
| 3214.6820084 | 0 |
| 3214.6824619 | 1 |
| 3214.6834163 | 0 |
| 3214.6846092 | 6 |
| 3214.6849459 | 2 |
| 3214.6864471 | 2 |
| 3214.6873936 | 2 |
| 3214.6877925 | 2 |
| 3214.6886155 | 0 |
| 3214.6900828 | 2 |
| 3214.6907505 | 3 |
| 3214.6918018 | 4 |
| 3214.6931388 | 3 |
| 3214.6943894 | 4 |
| 3214.6945821 | 4 |
| 3214.6963029 | 1 |
| 3214.697267 | 2 |
| 3214.6974879 | 0 |
| 3214.6991265 | 1 |
| 3214.6997026 | 0 |
| 3214.7006127 | 0 |
| 3214.7020334 | 2 |
| 3214.702809 | 2 |
| 3214.7037729 | 1 |
| 3214.7044089 | 2 |
| 3214.7054406 | 2 |
| 3214.7065565 | 0 |
| 3214.7074294 | 2 |
| 3214.7083794 | 3 |
| 3214.7091066 | 0 |
| 3214.7104559 | 2 |
| 3214.7106601 | 1 |
| 3214.7115439 | 3 |
| 3214.7130449 | 1 |
| 3214.7133472 | 3 |
| 3214.7145813 | 1 |
| 3214.7152446 | 1 |
| 3214.7164216 | 6 |
| 3214.717273 | 0 |
| 3214.7182459 | 1 |
| 3214.7197041 | 1 |
| 3214.7204504 | 0 |
| 3214.7206381 | 1 |
| 3214.7221679 | 1 |
| 3214.7228466 | 4 |
| 3214.7242291 | 1 |
| 3214.7251318 | 3 |
| 3214.725289 | 2 |
| 3214.7270013 | 1 |
| 3214.7276026 | 1 |
| 3214.7281442 | 5 |
| 3214.7298762 | 3 |
| 3214.7305817 | 2 |
| 3214.7316122 | 0 |
| 3214.7320351 | 2 |
| 3214.7324062 | 1 |
| 3214.7334591 | 2 |
| 3214.7345993 | 4 |
| 3214.7361544 | 4 |
| 3214.7368172 | 1 |
| 3214.7375389 | 3 |
| 3214.7388578 | 0 |
| 3214.7402696 | 3 |
| 3214.7410448 | 3 |
| 3214.7412137 | 2 |
| 3214.7429929 | 1 |
| 3214.7439341 | 0 |
| 3214.7443939 | 1 |
| 3214.7453336 | 0 |
| 3214.7463846 | 2 |
| 3214.7475671 | 4 |
| 3214.7482973 | 1 |
| 3214.7494538 | 1 |
| 3214.750568 | 3 |
| 3214.7510755 | 1 |
| 3214.752299 | 0 |
| 3214.7533877 | 2 |
| 3214.7543055 | 3 |
| 3214.754854 | 1 |
| 3214.755664 | 2 |
| 3214.7569775 | 0 |
| 3214.7579172 | 1 |
| 3214.7586138 | 2 |
| 3214.7600539 | 2 |
| 3214.7601664 | 2 |
| 3214.7613195 | 3 |
| 3214.7620949 | 0 |
| 3214.7633405 | 2 |
| 3214.7647851 | 1 |
| 3214.764861 | 4 |
| 3214.7662487 | 2 |
| 3214.7665629 | 3 |
| 3214.7680416 | 2 |
| 3214.7690874 | 4 |
| 3214.7697998 | 2 |
| 3214.7711095 | 2 |
| 3214.7713531 | 0 |
| 3214.7720979 | 1 |
| 3214.7735655 | 1 |
| 3214.7737455 | 3 |
| 3214.7752204 | 0 |
| 3214.7759682 | 2 |
| 3214.7773456 | 3 |
| 3214.7786245 | 2 |
| 3214.7791164 | 1 |
| 3214.7799187 | 3 |
| 3214.7806824 | 2 |
| 3214.7825849 | 1 |
| 3214.783432 | 2 |
| 3214.7840186 | 3 |
| 3214.7849067 | 0 |
| 3214.7856999 | 2 |
| 3214.7871817 | 1 |
| 3214.7879916 | 3 |
| 3214.7886069 | 1 |
| 3214.7900897 | 1 |
| 3214.7905453 | 2 |
| 3214.7923389 | 2 |
| 3214.792489 | 1 |
| 3214.7940453 | 0 |
| 3214.7950766 | 0 |
| 3214.7953317 | 1 |
| 3214.7957704 | 2 |
| 3214.797258 | 1 |
| 3214.7978501 | 3 |
| 3214.799484 | 0 |
| 3214.7997483 | 3 |
| 3214.8018546 | 1 |
| 3214.8021048 | 2 |
| 3214.802329 | 3 |
| 3214.8030225 | 2 |
| 3214.8031976 | 1 |
| 3214.8040086 | 1 |
| 3214.8046178 | 0 |
| 3214.8052202 | 1 |
| 3214.8059714 | 0 |
| 3214.806302 | 1 |
| 3214.8071469 | 2 |
| 3214.8075269 | 0 |
| 3214.8075666 | 1 |
| 3214.8094597 | 1 |
| 3214.8094635 | 2 |
| 3214.8103312 | 0 |
| 3214.8104601 | 0 |
| 3214.8107855 | 1 |
| 3214.8116368 | 2 |
| 3214.8123064 | 2 |
| 3214.8129071 | 0 |
| 3214.8133509 | 3 |
| 3214.8136209 | 4 |
| 3214.8147147 | 2 |
| 3214.8147415 | 2 |
| 3214.8156119 | 4 |
| 3214.8156436 | 2 |
| 3214.8167503 | 2 |
| 3214.8175427 | 1 |
| 3214.8181065 | 1 |
| 3214.8183863 | 2 |
| 3214.8184553 | 0 |
| 3214.8196448 | 0 |
| 3214.8205596 | 3 |
| 3214.8206818 | 1 |
| 3214.8207278 | 0 |
| 3214.822257 | 0 |
| 3214.8223992 | 5 |
| 3214.8234254 | 1 |
| 3214.824043 | 2 |
| 3214.8246281 | 3 |
| 3214.8251407 | 1 |
| 3214.8251578 | 1 |
| 3214.8261915 | 2 |
| 3214.8269372 | 0 |
| 3214.827663 | 2 |
| 3214.8277552 | 4 |
| 3214.8291961 | 0 |
| 3214.8292509 | 6 |
| 3214.8295041 | 3 |
| 3214.8304188 | 2 |
| 3214.8309002 | 3 |
| 3214.831738 | 1 |
| 3214.8328457 | 4 |
| 3214.8329345 | 0 |
| 3214.8337124 | 3 |
| 3214.8338198 | 2 |
| 3214.8346463 | 0 |
| 3214.8350335 | 2 |
| 3214.8357146 | 1 |

| | |
|---|---|
| 3214.8369241 | 0 |
| 3214.836984 | 2 |
| 3214.8375943 | 3 |
| 3214.8382049 | 4 |
| 3214.8386587 | 1 |
| 3214.8392206 | 3 |
| 3214.8392953 | 1 |
| 3214.8410806 | 2 |
| 3214.8413229 | 1 |
| 3214.8420547 | 2 |
| 3214.842326 | 0 |
| 3214.8424228 | 0 |
| 3214.8440088 | 1 |
| 3214.8440739 | 1 |
| 3214.844251 | 1 |
| 3214.8447711 | 0 |
| 3214.8458812 | 1 |
| 3214.8467929 | 3 |
| 3214.8468887 | 5 |
| 3214.847148 | 1 |
| 3214.8484194 | 4 |
| 3214.8489849 | 1 |
| 3214.8489929 | 2 |
| 3214.8500634 | 1 |
| 3214.8501715 | 2 |
| 3214.8513192 | 1 |
| 3214.8520531 | 0 |
| 3214.8521665 | 5 |
| 3214.8526347 | 1 |
| 3214.8532274 | 3 |
| 3214.8535139 | 1 |
| 3214.8544619 | 1 |
| 3214.8556506 | 1 |
| 3214.8557904 | 2 |
| 3214.8559542 | 2 |
| 3214.8568386 | 2 |
| 3214.8575117 | 1 |
| 3214.8576966 | 2 |
| 3214.8583959 | 2 |
| 3214.8589261 | 1 |
| 3214.8598025 | 0 |
| 3214.8604813 | 3 |
| 3214.8605408 | 2 |
| 3214.8606827 | 3 |
| 3214.8631867 | 1 |
| 3214.8633067 | 1 |
| 3214.8634442 | 1 |
| 3214.8640757 | 5 |
| 3214.8642658 | 0 |
| 3214.8649615 | 2 |
| 3214.8653567 | 0 |
| 3214.8656739 | 1 |
| 3214.8670568 | 1 |
| 3214.8672715 | 4 |
| 3214.868056 | 1 |
| 3214.8682808 | 0 |
| 3214.8691202 | 0 |
| 3214.8697361 | 2 |
| 3214.870035 | 2 |
| 3214.8717542 | 0 |
| 3214.8717867 | 1 |
| 3214.8719783 | 1 |
| 3214.8722591 | 0 |
| 3214.8734249 | 3 |
| 3214.874627 | 1 |
| 3214.8749215 | 3 |
| 3214.8750062 | 2 |
| 3214.8762449 | 2 |
| 3214.8765566 | 2 |
| 3214.8769485 | 0 |
| 3214.8776987 | 4 |
| 3214.8778461 | 2 |
| 3214.8789682 | 1 |
| 3214.879069 | 1 |
| 3214.8796591 | 1 |
| 3214.8801778 | 1 |
| 3214.8804829 | 1 |
| 3214.8817861 | 0 |
| 3214.8820922 | 1 |
| 3214.8826426 | 2 |
| 3214.8829439 | 1 |
| 3214.8840339 | 1 |
| 3214.884327 | 0 |
| 3214.8849961 | 4 |
| 3214.8854866 | 3 |
| 3214.8866439 | 3 |
| 3214.8870577 | 2 |
| 3214.8876098 | 3 |
| 3214.8886447 | 2 |
| 3214.8890742 | 5 |
| 3214.8898724 | 0 |
| 3214.8901664 | 1 |
| 3214.890836 | 0 |
| 3214.8910383 | 4 |
| 3214.8917022 | 1 |
| 3214.8923573 | 1 |
| 3214.8935438 | 2 |
| 3214.8938318 | 3 |
| 3214.8939104 | 1 |
| 3214.8942275 | 3 |
| 3214.8954103 | 4 |
| 3214.8957084 | 1 |
| 3214.8958658 | 2 |
| 3214.8971643 | 1 |
| 3214.8978099 | 0 |
| 3214.898004 | 3 |
| 3214.8983282 | 1 |
| 3214.8989551 | 0 |
| 3214.8996951 | 3 |
| 3214.9009557 | 2 |
| 3214.9010226 | 1 |
| 3214.901161 | 0 |
| 3214.9015932 | 1 |
| 3214.902749 | 3 |
| 3214.9031046 | 3 |
| 3214.9039613 | 3 |
| 3214.9041552 | 0 |
| 3214.9046623 | 4 |
| 3214.9051 | 1 |
| 3214.9057175 | 4 |
| 3214.9058531 | 0 |
| 3214.90618 | 3 |
| 3214.9066413 | 0 |
| 3214.9073136 | 2 |
| 3214.9073467 | 0 |
| 3214.9078143 | 3 |
| 3214.9089206 | 0 |
| 3214.9096142 | 0 |
| 3214.9097338 | 2 |
| 3214.9104002 | 0 |
| 3214.9111222 | 3 |
| 3214.9111345 | 4 |
| 3214.9114886 | 0 |
| 3214.9117402 | 0 |
| 3214.9124716 | 1 |
| 3214.913029 | 0 |
| 3214.9137966 | 2 |
| 3214.9140909 | 1 |
| 3214.9152101 | 5 |
| 3214.915384 | 0 |
| 3214.9156077 | 1 |
| 3214.9161014 | 1 |
| 3214.9167875 | 2 |
| 3214.9170908 | 1 |
| 3214.9170969 | 2 |
| 3214.9181434 | 0 |
| 3214.9187786 | 2 |
| 3214.9189068 | 1 |
| 3214.920031 | 1 |
| 3214.9201493 | 3 |
| 3214.9204318 | 1 |
| 3214.920648 | 1 |
| 3214.9212545 | 0 |
| 3214.9216869 | 2 |
| 3214.921746 | 2 |
| 3214.9229971 | 0 |
| 3214.9232551 | 3 |
| 3214.9235329 | 2 |
| 3214.9245055 | 2 |
| 3214.9245455 | 1 |
| 3214.9246765 | 2 |
| 3214.9249366 | 1 |
| 3214.9259217 | 1 |
| 3214.9261419 | 2 |
| 3214.9269572 | 2 |
| 3214.9273317 | 0 |
| 3214.9274991 | 0 |
| 3214.9280737 | 1 |
| 3214.9290913 | 0 |
| 3214.9291018 | 2 |
| 3214.9291161 | 1 |
| 3214.9298309 | 2 |
| 3214.9305719 | 0 |
| 3214.9311769 | 3 |
| 3214.9312009 | 0 |
| 3214.9314084 | 1 |
| 3214.9324612 | 0 |
| 3214.9329005 | 0 |
| 3214.9332255 | 1 |
| 3214.9342509 | 1 |
| 3214.9345146 | 0 |
| 3214.9346445 | 3 |
| 3214.9353247 | 1 |
| 3214.9354971 | 3 |
| 3214.9355784 | 2 |
| 3214.9361608 | 2 |
| 3214.9366206 | 0 |
| 3214.9374414 | 2 |
| 3214.938322 | 3 |
| 3214.9384729 | 1 |
| 3214.939015 | 0 |
| 3214.9392863 | 1 |
| 3214.9395725 | 0 |
| 3214.9406298 | 4 |
| 3214.9411364 | 1 |
| 3214.9415539 | 1 |
| 3214.9417152 | 2 |
| 3214.9423363 | 2 |
| 3214.9424922 | 1 |
| 3214.9425701 | 0 |
| 3214.943177 | 1 |
| 3214.9438912 | 1 |
| 3214.9441768 | 0 |
| 3214.9451123 | 0 |
| 3214.9452837 | 2 |
| 3214.9456262 | 2 |
| 3214.9459205 | 1 |
| 3214.94642 | 4 |
| 3214.9472403 | 2 |
| 3214.9476758 | 2 |
| 3214.9479666 | 0 |
| 3214.9483369 | 2 |
| 3214.9491766 | 2 |
| 3214.9492931 | 1 |
| 3214.9499081 | 0 |
| 3214.9499922 | 1 |
| 3214.9510423 | 0 |
| 3214.9514312 | 1 |
| 3214.9519056 | 1 |
| 3214.9524971 | 0 |
| 3214.9527668 | 3 |
| 3214.953141 | 3 |
| 3214.9540125 | 1 |
| 3214.9544068 | 1 |
| 3214.9546654 | 3 |
| 3214.9547744 | 2 |
| 3214.956143 | 1 |
| 3214.956328 | 2 |
| 3214.9569066 | 1 |
| 3214.9573999 | 1 |
| 3214.9578796 | 0 |
| 3214.9581538 | 5 |
| 3214.9586425 | 4 |
| 3214.9591461 | 0 |
| 3214.9594924 | 1 |
| 3214.9595598 | 0 |
| 3214.9604562 | 2 |
| 3214.9612064 | 0 |
| 3214.961889 | 1 |
| 3214.962271 | 0 |
| 3214.9623346 | 1 |
| 3214.9626651 | 0 |
| 3214.9633966 | 0 |
| 3214.963502 | 2 |
| 3214.964408 | 0 |
| 3214.9645036 | 0 |
| 3214.9656069 | 0 |
| 3214.9659482 | 1 |
| 3214.9661536 | 2 |
| 3214.9668852 | 1 |
| 3214.9669401 | 1 |
| 3214.9675758 | 1 |
| 3214.9683637 | 0 |
| 3214.9685142 | 2 |
| 3214.9689088 | 1 |
| 3214.9696654 | 0 |
| 3214.9700016 | 2 |
| 3214.9704853 | 0 |
| 3214.9708241 | 0 |
| 3214.9713256 | 0 |
| 3214.9718339 | 1 |
| 3214.9722604 | 3 |
| 3214.9725891 | 0 |
| 3214.9736917 | 1 |
| 3214.9738697 | 2 |
| 3214.9739855 | 5 |
| 3214.9746397 | 1 |
| 3214.9751389 | 1 |
| 3214.9756452 | 3 |
| 3214.9760469 | 0 |
| 3214.9765091 | 2 |
| 3214.9767089 | 0 |
| 3214.9777899 | 2 |
| 3214.9779026 | 5 |
| 3214.9780255 | 0 |
| 3214.9783677 | 1 |
| 3214.9795067 | 3 |
| 3214.9796851 | 2 |
| 3214.980064 | 1 |
| 3214.9804881 | 4 |
| 3214.9812967 | 2 |
| 3214.9814997 | 1 |
| 3214.9821093 | 0 |
| 3214.9822412 | 1 |
| 3214.9824535 | 2 |
| 3214.9829904 | 1 |
| 3214.9840272 | 1 |
| 3214.9842306 | 0 |
| 3214.9843932 | 6 |
| 3214.9854884 | 2 |
| 3214.9857155 | 1 |
| 3214.9864978 | 3 |
| 3214.9865883 | 4 |
| 3214.9872023 | 2 |
| 3214.9873993 | 4 |
| 3214.9878709 | 1 |
| 3214.9880163 | 2 |
| 3214.988206 | 1 |
| 3214.9892291 | 1 |
| 3214.989651 | 1 |
| 3214.9898828 | 1 |
| 3214.9905547 | 0 |
| 3214.9914875 | 2 |
| 3214.9915156 | 1 |
| 3214.992513 | 1 |
| 3214.9926644 | 2 |
| 3214.9934263 | 3 |
| 3214.9936887 | 0 |
| 3214.9943506 | 2 |
| 3214.9943716 | 0 |
| 3214.9952405 | 2 |
| 3214.9954626 | 2 |
| 3214.9959665 | 1 |
| 3214.9959767 | 0 |
| 3214.9971645 | 3 |
| 3214.9973385 | 3 |
| 3214.9978954 | 5 |
| 3214.9982397 | 0 |
| 3214.9985682 | 0 |
| 3214.9993244 | 1 |
| 3214.9998206 | 2 |
| 3215.0002901 | 1 |
| 3215.0010027 | 2 |
| 3215.0014433 | 1 |
| 3215.001449 | 0 |
| 3215.0021501 | 0 |
| 3215.0025937 | 1 |
| 3215.0028645 | 2 |
| 3215.0029573 | 4 |
| 3215.0037278 | 0 |
| 3215.004136 | 3 |
| 3215.0050506 | 2 |
| 3215.005388 | 3 |
| 3215.0058187 | 0 |
| 3215.0061651 | 2 |
| 3215.0070691 | 1 |
| 3215.0075434 | 4 |
| 3215.0080289 | 0 |
| 3215.0082405 | 1 |
| 3215.0088228 | 1 |
| 3215.0093996 | 1 |
| 3215.0102069 | 0 |
| 3215.0103858 | 3 |
| 3215.0104616 | 0 |
| 3215.0107891 | 1 |
| 3215.0115884 | 4 |
| 3215.012005 | 0 |
| 3215.012393 | 0 |
| 3215.0127614 | 2 |
| 3215.012957 | 3 |
| 3215.0137385 | 0 |
| 3215.0147794 | 1 |
| 3215.0148678 | 2 |
| 3215.0154019 | 1 |
| 3215.0156471 | 4 |
| 3215.0161887 | 0 |
| 3215.0166081 | 4 |
| 3215.0170514 | 3 |
| 3215.0170598 | 1 |
| 3215.0179069 | 1 |
| 3215.0183784 | 1 |
| 3215.0196105 | 1 |
| 3215.019659 | 0 |

| | |
|---|---|
| 3215.019885 | 1 |
| 3215.0203031 | 1 |
| 3215.0210817 | 1 |
| 3215.0216049 | 3 |
| 3215.0217336 | 2 |
| 3215.0220532 | 0 |
| 3215.0226347 | 1 |
| 3215.0228003 | 2 |
| 3215.0233934 | 2 |
| 3215.0238164 | 2 |
| 3215.0243784 | 0 |
| 3215.0255814 | 1 |
| 3215.0259334 | 0 |
| 3215.0259996 | 1 |
| 3215.0261096 | 3 |
| 3215.0262149 | 3 |
| 3215.0276065 | 1 |
| 3215.0281201 | 1 |
| 3215.028273 | 2 |
| 3215.0283064 | 3 |
| 3215.0290596 | 2 |
| 3215.029265 | 1 |
| 3215.0298788 | 1 |
| 3215.0305141 | 1 |
| 3215.030996 | 1 |
| 3215.0312552 | 2 |
| 3215.0318691 | 2 |
| 3215.0319124 | 0 |
| 3215.0322527 | 1 |
| 3215.0334251 | 4 |
| 3215.0336977 | 1 |
| 3215.0340498 | 3 |
| 3215.0348532 | 1 |
| 3215.0349323 | 3 |
| 3215.0353076 | 0 |
| 3215.0358641 | 1 |
| 3215.0362134 | 3 |
| 3215.036982 | 0 |
| 3215.0371983 | 2 |
| 3215.0376952 | 1 |
| 3215.0379288 | 3 |
| 3215.0384177 | 1 |
| 3215.0390492 | 0 |
| 3215.0400172 | 1 |
| 3215.040098 | 0 |
| 3215.040668 | 1 |
| 3215.0415471 | 1 |
| 3215.041918 | 1 |
| 3215.0421739 | 1 |
| 3215.0423598 | 2 |
| 3215.0434472 | 1 |
| 3215.0434896 | 2 |
| 3215.0441604 | 1 |
| 3215.0446709 | 1 |
| 3215.0446911 | 3 |
| 3215.0448928 | 0 |
| 3215.046414 | 1 |
| 3215.046456 | 1 |
| 3215.0471119 | 0 |
| 3215.0474676 | 2 |
| 3215.047799 | 1 |
| 3215.0485324 | 2 |
| 3215.0488918 | 2 |
| 3215.0489136 | 0 |
| 3215.0491847 | 2 |
| 3215.0502797 | 3 |
| 3215.0506868 | 1 |
| 3215.0511055 | 3 |
| 3215.0511832 | 0 |
| 3215.0521138 | 0 |
| 3215.0524415 | 1 |
| 3215.0533103 | 0 |
| 3215.0535326 | 1 |
| 3215.0538222 | 1 |
| 3215.0539966 | 2 |
| 3215.0546735 | 2 |
| 3215.05495 | 3 |
| 3215.0556278 | 0 |
| 3215.0562375 | 2 |
| 3215.0564507 | 2 |
| 3215.0570776 | 0 |
| 3215.0573998 | 2 |
| 3215.0582585 | 0 |
| 3215.0583339 | 1 |
| 3215.0585275 | 1 |
| 3215.0594471 | 1 |
| 3215.0595363 | 3 |
| 3215.059895 | 1 |
| 3215.0608589 | 1 |
| 3215.0610175 | 1 |
| 3215.0613923 | 1 |
| 3215.0623293 | 0 |
| 3215.0626351 | 1 |
| 3215.0628762 | 1 |
| 3215.0635684 | 0 |
| 3215.0640853 | 0 |
| 3215.064331 | 0 |
| 3215.0647596 | 2 |
| 3215.0648815 | 1 |
| 3215.0656121 | 0 |
| 3215.0661034 | 0 |
| 3215.0663413 | 0 |
| 3215.0674341 | 2 |
| 3215.067516 | 2 |
| 3215.0678259 | 0 |
| 3215.0689167 | 0 |
| 3215.0689413 | 1 |
| 3215.0691533 | 1 |
| 3215.0704497 | 1 |
| 3215.0705698 | 0 |
| 3215.0706233 | 1 |
| 3215.0709101 | 2 |
| 3215.0719464 | 1 |
| 3215.0722932 | 0 |
| 3215.0726776 | 2 |
| 3215.0729946 | 3 |
| 3215.0730515 | 0 |
| 3215.0733613 | 5 |
| 3215.0735972 | 3 |
| 3215.0739902 | 4 |
| 3215.0744725 | 2 |
| 3215.0749269 | 2 |
| 3215.0750734 | 0 |
| 3215.0754031 | 0 |
| 3215.0755658 | 0 |
| 3215.0760947 | 4 |
| 3215.0762416 | 3 |
| 3215.0766785 | 0 |
| 3215.0770224 | 1 |
| 3215.0770598 | 3 |
| 3215.0772406 | 2 |
| 3215.0774263 | 0 |
| 3215.0780112 | 0 |
| 3215.0780913 | 4 |
| 3215.0782446 | 2 |
| 3215.0791613 | 2 |
| 3215.0791662 | 1 |
| 3215.0792611 | 5 |
| 3215.0793983 | 1 |
| 3215.0797929 | 6 |
| 3215.0805164 | 1 |
| 3215.0810354 | 1 |
| 3215.0813172 | 2 |
| 3215.0813464 | 0 |
| 3215.0816059 | 0 |
| 3215.081716 | 4 |
| 3215.0818675 | 1 |
| 3215.0818848 | 0 |
| 3215.0820742 | 1 |
| 3215.0828906 | 0 |
| 3215.0832765 | 0 |
| 3215.0838176 | 2 |
| 3215.0840479 | 4 |
| 3215.0841866 | 1 |
| 3215.0851447 | 0 |
| 3215.0851471 | 3 |
| 3215.0858868 | 2 |
| 3215.0862832 | 3 |
| 3215.0868505 | 1 |
| 3215.0873192 | 2 |
| 3215.0880047 | 0 |
| 3215.0882366 | 0 |
| 3215.0892827 | 1 |
| 3215.0893594 | 0 |
| 3215.0894525 | 2 |
| 3215.0895393 | 2 |
| 3215.0908942 | 1 |
| 3215.0915005 | 1 |
| 3215.091558 | 0 |
| 3215.0920862 | 0 |
| 3215.0930927 | 4 |
| 3215.0933928 | 2 |
| 3215.0935029 | 1 |
| 3215.0942398 | 1 |
| 3215.0942676 | 1 |
| 3215.0948243 | 3 |
| 3215.0955307 | 0 |
| 3215.095691 | 0 |
| 3215.096014 | 1 |
| 3215.0964992 | 1 |
| 3215.0978063 | 0 |
| 3215.0979273 | 1 |
| 3215.0982895 | 1 |
| 3215.0985534 | 2 |
| 3215.0995294 | 0 |
| 3215.1001413 | 3 |
| 3215.1005037 | 1 |
| 3215.1009721 | 2 |
| 3215.1017793 | 0 |
| 3215.1020117 | 1 |
| 3215.1024288 | 0 |
| 3215.1024388 | 1 |
| 3215.1034516 | 1 |
| 3215.1035622 | 0 |
| 3215.1039124 | 2 |
| 3215.1044604 | 0 |
| 3215.1046377 | 2 |
| 3215.10505 | 2 |
| 3215.105667 | 2 |
| 3215.1065035 | 3 |
| 3215.1071386 | 0 |
| 3215.1076242 | 0 |
| 3215.1081807 | 2 |
| 3215.1082452 | 1 |
| 3215.1083739 | 0 |
| 3215.1097268 | 0 |
| 3215.1099453 | 0 |
| 3215.1102248 | 0 |
| 3215.1105281 | 1 |
| 3215.1113377 | 0 |
| 3215.1118094 | 0 |
| 3215.1119713 | 1 |
| 3215.1128345 | 3 |
| 3215.1130739 | 2 |
| 3215.1132573 | 0 |
| 3215.1134372 | 1 |
| 3215.1144971 | 3 |
| 3215.1145161 | 0 |
| 3215.1145738 | 1 |
| 3215.1155818 | 2 |
| 3215.1161544 | 3 |
| 3215.1165945 | 0 |
| 3215.117108 | 1 |
| 3215.1174432 | 4 |
| 3215.1179976 | 0 |
| 3215.1183914 | 1 |
| 3215.119154 | 2 |
| 3215.1193301 | 1 |
| 3215.119522 | 0 |
| 3215.1205747 | 2 |
| 3215.1208664 | 1 |
| 3215.1209928 | 2 |
| 3215.1218827 | 2 |
| 3215.1219408 | 2 |
| 3215.122919 | 1 |
| 3215.1229511 | 4 |
| 3215.1231196 | 2 |
| 3215.1233382 | 2 |
| 3215.1244549 | 1 |
| 3215.1249058 | 2 |
| 3215.1253902 | 0 |
| 3215.1256906 | 4 |
| 3215.1260334 | 1 |
| 3215.1264354 | 4 |
| 3215.1268634 | 1 |
| 3215.127589 | 0 |
| 3215.1279058 | 2 |
| 3215.1281461 | 1 |
| 3215.1290116 | 1 |
| 3215.1291807 | 0 |
| 3215.1299709 | 2 |
| 3215.1300108 | 0 |
| 3215.1301547 | 0 |
| 3215.1310526 | 2 |
| 3215.1312974 | 1 |
| 3215.1319382 | 0 |
| 3215.1322493 | 2 |
| 3215.1328783 | 2 |
| 3215.1333403 | 1 |
| 3215.1337731 | 1 |
| 3215.1346038 | 2 |
| 3215.1354396 | 2 |
| 3215.1355478 | 1 |
| 3215.1355702 | 3 |
| 3215.1367215 | 1 |
| 3215.1368888 | 0 |
| 3215.1369479 | 2 |
| 3215.1375322 | 1 |
| 3215.1383025 | 1 |
| 3215.138644 | 4 |
| 3215.1388788 | 0 |
| 3215.1399145 | 0 |
| 3215.1400183 | 2 |
| 3215.1407317 | 1 |
| 3215.1414953 | 1 |
| 3215.1415284 | 2 |
| 3215.1418931 | 0 |
| 3215.1424444 | 2 |
| 3215.1434387 | 2 |
| 3215.1434622 | 1 |
| 3215.1434945 | 2 |
| 3215.1445438 | 2 |
| 3215.1448505 | 1 |
| 3215.1458535 | 4 |
| 3215.145938 | 0 |
| 3215.1466888 | 3 |
| 3215.1468416 | 1 |
| 3215.1476041 | 0 |
| 3215.1480242 | 0 |
| 3215.1485515 | 0 |
| 3215.149116 | 0 |
| 3215.1493327 | 4 |
| 3215.1494678 | 1 |
| 3215.1505026 | 1 |
| 3215.1509915 | 1 |
| 3215.1514867 | 0 |
| 3215.1517804 | 2 |
| 3215.1520132 | 4 |
| 3215.1527144 | 1 |
| 3215.1531714 | 3 |
| 3215.1531971 | 2 |
| 3215.1541851 | 0 |
| 3215.1545897 | 0 |
| 3215.1550451 | 4 |
| 3215.1556714 | 1 |
| 3215.1559862 | 0 |
| 3215.15655 | 2 |
| 3215.1572344 | 2 |
| 3215.157596 | 0 |
| 3215.1576552 | 1 |
| 3215.1579991 | 1 |
| 3215.1586033 | 1 |
| 3215.1590193 | 1 |
| 3215.1598052 | 6 |
| 3215.1600379 | 0 |
| 3215.1603084 | 0 |
| 3215.1604825 | 5 |
| 3215.1614585 | 0 |
| 3215.1619559 | 3 |
| 3215.1623822 | 2 |
| 3215.1625852 | 2 |
| 3215.1632294 | 2 |
| 3215.1634628 | 2 |
| 3215.1645223 | 0 |
| 3215.1647096 | 2 |
| 3215.1652152 | 1 |
| 3215.1659617 | 1 |
| 3215.1659882 | 4 |
| 3215.1660678 | 0 |
| 3215.1670115 | 0 |
| 3215.1677389 | 1 |
| 3215.1677744 | 3 |
| 3215.1678517 | 0 |
| 3215.1685474 | 2 |
| 3215.1687118 | 2 |
| 3215.1692584 | 1 |
| 3215.1700016 | 3 |
| 3215.1704868 | 2 |
| 3215.1710977 | 3 |
| 3215.17166 | 1 |
| 3215.1722104 | 3 |
| 3215.1722433 | 3 |
| 3215.1724618 | 1 |
| 3215.1736735 | 1 |
| 3215.1737626 | 1 |
| 3215.1746625 | 2 |
| 3215.174873 | 2 |
| 3215.1754639 | 1 |
| 3215.1755272 | 1 |
| 3215.1764202 | 1 |
| 3215.1771016 | 2 |
| 3215.1771046 | 4 |
| 3215.1771862 | 1 |
| 3215.1777309 | 1 |
| 3215.1786472 | 1 |
| 3215.1790926 | 0 |
| 3215.1792793 | 1 |
| 3215.1796302 | 2 |
| 3215.1804926 | 0 |
| 3215.1812013 | 0 |
| 3215.1812874 | 1 |
| 3215.1817597 | 1 |
| 3215.1823408 | 0 |

| | |
|---|---|
| 3215.1824696 | 2 |
| 3215.1832962 | 2 |
| 3215.1839788 | 3 |
| 3215.1840553 | 1 |
| 3215.1846392 | 1 |
| 3215.1851337 | 2 |
| 3215.1858208 | 1 |
| 3215.1860293 | 2 |
| 3215.1866419 | 2 |
| 3215.1871745 | 0 |
| 3215.1877408 | 1 |
| 3215.1881892 | 1 |
| 3215.1891075 | 2 |
| 3215.18925 | 4 |
| 3215.1892965 | 1 |
| 3215.1897297 | 5 |
| 3215.1907174 | 4 |
| 3215.1911142 | 2 |
| 3215.1912196 | 1 |
| 3215.1913959 | 3 |
| 3215.1925459 | 2 |
| 3215.1925712 | 3 |
| 3215.193304 | 4 |
| 3215.1938709 | 2 |
| 3215.1944845 | 0 |
| 3215.1945107 | 2 |
| 3215.1952643 | 0 |
| 3215.1958938 | 0 |
| 3215.1959808 | 1 |
| 3215.1961826 | 0 |
| 3215.1969755 | 0 |
| 3215.1974878 | 1 |
| 3215.1982185 | 1 |
| 3215.1982651 | 1 |
| 3215.1987156 | 0 |
| 3215.1989233 | 4 |
| 3215.1998115 | 1 |
| 3215.1998997 | 5 |
| 3215.2001342 | 2 |
| 3215.2009398 | 1 |
| 3215.2018965 | 1 |
| 3215.2025 | 4 |
| 3215.2027014 | 2 |
| 3215.2032764 | 0 |
| 3215.2033996 | 0 |
| 3215.203838 | 1 |
| 3215.2046559 | 0 |
| 3215.2050779 | 2 |
| 3215.2054106 | 1 |
| 3215.2059111 | 4 |
| 3215.206279 | 0 |
| 3215.2064865 | 0 |
| 3215.2076813 | 0 |
| 3215.2077402 | 0 |
| 3215.2080556 | 1 |
| 3215.2088124 | 2 |
| 3215.209404 | 1 |
| 3215.2096623 | 2 |
| 3215.2097444 | 2 |
| 3215.2099075 | 1 |
| 3215.2106525 | 0 |
| 3215.2113816 | 0 |
| 3215.2118961 | 0 |
| 3215.2123763 | 1 |
| 3215.2126127 | 2 |
| 3215.2128036 | 0 |
| 3215.2140554 | 3 |
| 3215.2141837 | 1 |
| 3215.2143037 | 0 |
| 3215.2150373 | 1 |
| 3215.2154832 | 1 |
| 3215.2156013 | 0 |
| 3215.2159459 | 0 |
| 3215.2170209 | 2 |
| 3215.2171513 | 0 |
| 3215.2181605 | 3 |
| 3215.2183123 | 2 |
| 3215.2187272 | 2 |
| 3215.2187321 | 0 |
| 3215.2198615 | 1 |
| 3215.2199466 | 4 |
| 3215.2202804 | 1 |
| 3215.2207102 | 1 |
| 3215.2217487 | 2 |
| 3215.2218232 | 0 |
| 3215.2222786 | 2 |
| 3215.2222992 | 3 |
| 3215.2234488 | 2 |
| 3215.2238263 | 0 |
| 3215.2240301 | 1 |
| 3215.2247111 | 1 |
| 3215.2248697 | 2 |
| 3215.2251908 | 0 |
| 3215.225952 | 2 |
| 3215.2267044 | 0 |
| 3215.2268167 | 2 |
| 3215.2272282 | 1 |
| 3215.2277162 | 2 |
| 3215.2287797 | 1 |
| 3215.2289842 | 1 |
| 3215.2290841 | 2 |
| 3215.2296583 | 1 |
| 3215.2304778 | 0 |
| 3215.2305277 | 0 |
| 3215.2316643 | 0 |
| 3215.2319072 | 0 |
| 3215.2325458 | 2 |
| 3215.2330073 | 2 |
| 3215.2331121 | 0 |
| 3215.2332677 | 3 |
| 3215.2344561 | 1 |
| 3215.2347986 | 2 |
| 3215.2352322 | 0 |
| 3215.2353184 | 2 |
| 3215.2362912 | 1 |
| 3215.2363858 | 1 |
| 3215.2368141 | 1 |
| 3215.2371801 | 1 |
| 3215.2380698 | 1 |
| 3215.2386256 | 1 |
| 3215.2395167 | 5 |
| 3215.2401662 | 0 |
| 3215.2403289 | 0 |
| 3215.2405893 | 2 |
| 3215.2412553 | 1 |
| 3215.2420674 | 2 |
| 3215.2425444 | 2 |
| 3215.2428674 | 1 |
| 3215.2431694 | 6 |
| 3215.2432301 | 4 |
| 3215.2435098 | 1 |
| 3215.2441443 | 1 |
| 3215.2445141 | 2 |
| 3215.2445722 | 2 |
| 3215.2456269 | 0 |
| 3215.2461413 | 1 |
| 3215.2462695 | 2 |
| 3215.2471021 | 2 |
| 3215.2478795 | 1 |
| 3215.2481723 | 2 |
| 3215.2482527 | 3 |
| 3215.2492246 | 2 |
| 3215.2493054 | 5 |
| 3215.2496679 | 1 |
| 3215.2498207 | 2 |
| 3215.2507122 | 0 |
| 3215.2510309 | 1 |
| 3215.2521002 | 0 |
| 3215.2521943 | 1 |
| 3215.2525315 | 1 |
| 3215.252604 | 1 |
| 3215.2535938 | 1 |
| 3215.2540062 | 1 |
| 3215.2541094 | 4 |
| 3215.254283 | 0 |
| 3215.2554541 | 2 |
| 3215.2559549 | 0 |
| 3215.2560052 | 0 |
| 3215.2569011 | 1 |
| 3215.2571056 | 2 |
| 3215.2574417 | 1 |
| 3215.2583527 | 1 |
| 3215.258414 | 3 |
| 3215.2585846 | 0 |
| 3215.2590894 | 2 |
| 3215.259675 | 2 |
| 3215.2602641 | 1 |
| 3215.2609473 | 3 |
| 3215.2615203 | 1 |
| 3215.2616117 | 1 |
| 3215.2617079 | 0 |
| 3215.2624487 | 2 |
| 3215.263032 | 0 |
| 3215.2631444 | 1 |
| 3215.2640239 | 1 |
| 3215.2647935 | 4 |
| 3215.26488 | 2 |
| 3215.26587 | 2 |
| 3215.265941 | 0 |
| 3215.2661722 | 0 |
| 3215.2664224 | 1 |
| 3215.2665477 | 0 |
| 3215.2678103 | 2 |
| 3215.2679558 | 1 |
| 3215.2684778 | 2 |
| 3215.2685517 | 2 |
| 3215.2695582 | 1 |
| 3215.269745 | 1 |
| 3215.269924 | 1 |
| 3215.2709174 | 1 |
| 3215.2715691 | 1 |
| 3215.271711 | 1 |
| 3215.2720369 | 1 |
| 3215.2726145 | 1 |
| 3215.2733258 | 1 |
| 3215.2738451 | 2 |
| 3215.2743444 | 1 |
| 3215.2749491 | 1 |
| 3215.2751283 | 1 |
| 3215.2756337 | 2 |
| 3215.2761134 | 0 |
| 3215.2769477 | 0 |
| 3215.2770061 | 1 |
| 3215.2777169 | 0 |
| 3215.278541 | 1 |
| 3215.2788226 | 2 |
| 3215.2789523 | 0 |
| 3215.2799337 | 0 |
| 3215.2799636 | 3 |
| 3215.2802019 | 2 |
| 3215.2810761 | 0 |
| 3215.2817728 | 2 |
| 3215.2820833 | 1 |
| 3215.2829499 | 1 |
| 3215.2831152 | 4 |
| 3215.2831192 | 1 |
| 3215.2840006 | 0 |
| 3215.2845506 | 0 |
| 3215.2846493 | 1 |
| 3215.2851112 | 1 |
| 3215.2860166 | 5 |
| 3215.2860256 | 0 |
| 3215.2864005 | 1 |
| 3215.2875003 | 2 |
| 3215.2875978 | 2 |
| 3215.2883364 | 2 |
| 3215.2885065 | 1 |
| 3215.2894258 | 0 |
| 3215.2898435 | 2 |
| 3215.2898939 | 0 |
| 3215.2904358 | 1 |
| 3215.2912438 | 0 |
| 3215.2916907 | 2 |
| 3215.2921751 | 0 |
| 3215.2926518 | 1 |
| 3215.2927175 | 2 |
| 3215.2930339 | 0 |
| 3215.2938073 | 3 |
| 3215.2943796 | 2 |
| 3215.2944504 | 0 |
| 3215.295002 | 1 |
| 3215.2952356 | 4 |
| 3215.2954929 | 1 |
| 3215.2966757 | 2 |
| 3215.2966816 | 0 |
| 3215.2971366 | 1 |
| 3215.2976361 | 0 |
| 3215.2984175 | 0 |
| 3215.298769 | 3 |
| 3215.2992342 | 1 |
| 3215.2996155 | 1 |
| 3215.2999073 | 2 |
| 3215.3003248 | 1 |
| 3215.3009614 | 3 |
| 3215.3011655 | 0 |
| 3215.3015448 | 2 |
| 3215.3030413 | 1 |
| 3215.3032117 | 2 |
| 3215.3032171 | 0 |
| 3215.3037093 | 0 |
| 3215.3039472 | 1 |
| 3215.3043759 | 2 |
| 3215.3047033 | 0 |
| 3215.3057035 | 1 |
| 3215.3058778 | 1 |
| 3215.3061187 | 0 |
| 3215.3064731 | 0 |
| 3215.3075842 | 1 |
| 3215.307595 | 0 |
| 3215.3085327 | 3 |
| 3215.3090437 | 1 |
| 3215.3091097 | 1 |
| 3215.3096032 | 1 |
| 3215.3102157 | 1 |
| 3215.3104937 | 1 |
| 3215.3109355 | 1 |
| 3215.3122672 | 3 |
| 3215.3123595 | 2 |
| 3215.3127055 | 0 |
| 3215.3130663 | 2 |
| 3215.3131165 | 0 |
| 3215.3142662 | 3 |
| 3215.3142817 | 0 |
| 3215.3146929 | 2 |
| 3215.3149214 | 1 |
| 3215.3155538 | 0 |
| 3215.3165361 | 4 |
| 3215.3172642 | 1 |
| 3215.3173843 | 1 |
| 3215.3176577 | 1 |
| 3215.3178608 | 1 |
| 3215.3188727 | 1 |
| 3215.3190974 | 0 |
| 3215.3194756 | 1 |
| 3215.3200839 | 0 |
| 3215.3207338 | 2 |
| 3215.3208608 | 1 |
| 3215.3215452 | 2 |
| 3215.3218842 | 0 |
| 3215.3221932 | 3 |
| 3215.3227349 | 1 |
| 3215.3234919 | 3 |
| 3215.3241406 | 2 |
| 3215.3246423 | 1 |
| 3215.3249475 | 0 |
| 3215.325242 | 1 |
| 3215.3256111 | 1 |
| 3215.3266254 | 0 |
| 3215.3268968 | 1 |
| 3215.3275142 | 0 |
| 3215.327547 | 2 |
| 3215.3288051 | 0 |
| 3215.3290941 | 4 |
| 3215.3291156 | 3 |
| 3215.3292758 | 2 |
| 3215.3302749 | 2 |
| 3215.3304063 | 0 |
| 3215.3314025 | 1 |
| 3215.3316446 | 0 |
| 3215.3320538 | 0 |
| 3215.3325666 | 2 |
| 3215.3332728 | 0 |
| 3215.3333018 | 0 |
| 3215.3337031 | 0 |
| 3215.3342596 | 1 |
| 3215.3349065 | 0 |
| 3215.3355654 | 0 |
| 3215.3355869 | 2 |
| 3215.335606 | 3 |
| 3215.3365863 | 0 |
| 3215.3366219 | 4 |
| 3215.3380831 | 0 |
| 3215.3380847 | 5 |
| 3215.338403 | 4 |
| 3215.3387816 | 3 |
| 3215.3393935 | 2 |
| 3215.3396396 | 0 |
| 3215.3403419 | 2 |
| 3215.3405917 | 0 |
| 3215.341238 | 1 |
| 3215.3413049 | 1 |
| 3215.342448 | 0 |
| 3215.3428687 | 1 |
| 3215.3429887 | 1 |
| 3215.3438561 | 0 |
| 3215.3440436 | 0 |
| 3215.3440861 | 1 |
| 3215.3444751 | 0 |
| 3215.3454394 | 2 |
| 3215.3460865 | 1 |
| 3215.346331 | 1 |
| 3215.3471921 | 3 |
| 3215.3475353 | 1 |
| 3215.3476924 | 0 |
| 3215.347847 | 2 |
| 3215.3485676 | 0 |
| 3215.3490759 | 1 |
| 3215.3492016 | 3 |
| 3215.3494259 | 1 |
| 3215.3504142 | 0 |
| 3215.3506927 | 0 |
| 3215.3513006 | 2 |
| 3215.3517791 | 3 |

| | |
|---|---|
| 3215.3519767 | 1 |
| 3215.3525271 | 1 |
| 3215.3538979 | 1 |
| 3215.3539781 | 1 |
| 3215.3542336 | 1 |
| 3215.354532 | 2 |
| 3215.3554476 | 0 |
| 3215.3554849 | 2 |
| 3215.3557758 | 0 |
| 3215.3558681 | 1 |
| 3215.3569296 | 2 |
| 3215.357399 | 1 |
| 3215.3581527 | 1 |
| 3215.3582506 | 2 |
| 3215.3586726 | 4 |
| 3215.3587827 | 1 |
| 3215.3596631 | 1 |
| 3215.3598322 | 3 |
| 3215.3608033 | 1 |
| 3215.3612027 | 1 |
| 3215.3612037 | 1 |
| 3215.3618603 | 0 |
| 3215.3623227 | 1 |
| 3215.3625495 | 0 |
| 3215.3629803 | 1 |
| 3215.3636063 | 1 |
| 3215.3641376 | 1 |
| 3215.3651831 | 0 |
| 3215.365417 | 0 |
| 3215.3657636 | 0 |
| 3215.3657996 | 4 |
| 3215.3672355 | 1 |
| 3215.3672994 | 0 |
| 3215.3673927 | 1 |
| 3215.3684314 | 1 |
| 3215.3685722 | 1 |
| 3215.3695474 | 2 |
| 3215.3697614 | 1 |
| 3215.3705379 | 4 |
| 3215.3706449 | 3 |
| 3215.3713307 | 2 |
| 3215.3714336 | 0 |
| 3215.3718122 | 0 |
| 3215.3722869 | 0 |
| 3215.3727831 | 5 |
| 3215.3737594 | 1 |
| 3215.3737931 | 1 |
| 3215.3738952 | 1 |
| 3215.3752142 | 1 |
| 3215.3752157 | 0 |
| 3215.3755995 | 1 |
| 3215.3761192 | 1 |
| 3215.3767889 | 0 |
| 3215.3770184 | 0 |
| 3215.3776992 | 3 |
| 3215.378126 | 1 |
| 3215.3788228 | 1 |
| 3215.378967 | 0 |
| 3215.3794875 | 2 |
| 3215.3797888 | 3 |
| 3215.3806425 | 1 |
| 3215.3807035 | 1 |
| 3215.3813806 | 0 |
| 3215.3822454 | 0 |
| 3215.3823996 | 3 |
| 3215.3832625 | 1 |
| 3215.3835495 | 0 |
| 3215.383665 | 0 |
| 3215.3846205 | 1 |
| 3215.3846791 | 4 |
| 3215.3854331 | 1 |
| 3215.38553 | 2 |
| 3215.3860673 | 3 |
| 3215.3864133 | 1 |
| 3215.3872438 | 4 |
| 3215.387271 | 2 |
| 3215.3879838 | 1 |
| 3215.3883485 | 2 |
| 3215.3890462 | 0 |
| 3215.3892689 | 4 |
| 3215.3893005 | 0 |
| 3215.3897026 | 2 |
| 3215.3909653 | 0 |
| 3215.3910476 | 1 |
| 3215.3915572 | 2 |
| 3215.3919085 | 2 |
| 3215.3923285 | 0 |
| 3215.392607 | 3 |
| 3215.3936815 | 1 |
| 3215.3937585 | 5 |
| 3215.3943939 | 2 |
| 3215.3944144 | 1 |
| 3215.3946941 | 3 |
| 3215.3955517 | 0 |
| 3215.3960768 | 0 |
| 3215.3961587 | 1 |
| 3215.3966959 | 1 |
| 3215.3971193 | 0 |
| 3215.3982315 | 1 |
| 3215.3982589 | 0 |
| 3215.398914 | 1 |
| 3215.3991471 | 2 |
| 3215.3996242 | 3 |
| 3215.4005027 | 1 |
| 3215.4006264 | 2 |
| 3215.4011168 | 2 |
| 3215.4015341 | 3 |
| 3215.4018256 | 0 |
| 3215.4024993 | 5 |
| 3215.402905 | 0 |
| 3215.4032411 | 1 |
| 3215.4036343 | 1 |
| 3215.4043365 | 1 |
| 3215.404827 | 3 |
| 3215.4048492 | 0 |
| 3215.4055523 | 0 |
| 3215.4058168 | 1 |
| 3215.4065902 | 4 |
| 3215.406999 | 0 |
| 3215.4074405 | 0 |
| 3215.4080766 | 0 |
| 3215.4087819 | 1 |
| 3215.4093718 | 0 |
| 3215.4098714 | 1 |
| 3215.4105295 | 0 |
| 3215.4107128 | 2 |
| 3215.4109327 | 0 |
| 3215.4118219 | 0 |
| 3215.4121087 | 1 |
| 3215.4125124 | 3 |
| 3215.4126674 | 0 |
| 3215.4134963 | 2 |
| 3215.4136385 | 3 |
| 3215.4147906 | 1 |
| 3215.4147961 | 0 |
| 3215.415191 | 1 |
| 3215.4153761 | 1 |
| 3215.4161374 | 0 |
| 3215.4165012 | 0 |
| 3215.4171187 | 1 |
| 3215.4171519 | 3 |
| 3215.4183155 | 0 |
| 3215.4183361 | 0 |
| 3215.4196029 | 0 |
| 3215.4198589 | 0 |
| 3215.4199166 | 1 |
| 3215.4203792 | 2 |
| 3215.4211877 | 1 |
| 3215.4215264 | 2 |
| 3215.4217 | 0 |
| 3215.4225055 | 1 |
| 3215.4226592 | 3 |
| 3215.4233938 | 2 |
| 3215.4237896 | 2 |
| 3215.4240565 | 1 |
| 3215.4247752 | 1 |
| 3215.4249344 | 0 |
| 3215.4258239 | 1 |
| 3215.4266337 | 5 |
| 3215.4266937 | 2 |
| 3215.4268323 | 1 |
| 3215.427388 | 1 |
| 3215.4274689 | 2 |
| 3215.4283319 | 1 |
| 3215.4290241 | 1 |
| 3215.4292714 | 2 |
| 3215.429655 | 1 |
| 3215.4304328 | 2 |
| 3215.4307756 | 0 |
| 3215.4308402 | 0 |
| 3215.432011 | 1 |
| 3215.4320424 | 3 |
| 3215.4325682 | 3 |
| 3215.4332504 | 0 |
| 3215.4334254 | 1 |
| 3215.4335829 | 0 |
| 3215.4347209 | 1 |
| 3215.4347791 | 0 |
| 3215.4361932 | 0 |
| 3215.43621 | 0 |
| 3215.4363896 | 5 |
| 3215.4364968 | 2 |
| 3215.4367123 | 3 |
| 3215.4375819 | 0 |
| 3215.4381597 | 0 |
| 3215.4382711 | 0 |
| 3215.4388938 | 1 |
| 3215.4392411 | 2 |
| 3215.4401753 | 1 |
| 3215.4403486 | 0 |
| 3215.4412442 | 0 |
| 3215.4415439 | 0 |
| 3215.4418335 | 1 |
| 3215.4427936 | 1 |
| 3215.4428414 | 1 |
| 3215.4430933 | 1 |
| 3215.4432174 | 1 |
| 3215.4438692 | 1 |
| 3215.4441148 | 1 |
| 3215.4446821 | 0 |
| 3215.4449946 | 1 |
| 3215.4456127 | 0 |
| 3215.4462861 | 1 |
| 3215.4469828 | 1 |
| 3215.4474573 | 1 |
| 3215.4476043 | 1 |
| 3215.4477124 | 1 |
| 3215.4488689 | 0 |
| 3215.4493721 | 1 |
| 3215.4497371 | 1 |
| 3215.4501653 | 6 |
| 3215.4502728 | 0 |
| 3215.4512627 | 1 |
| 3215.4514287 | 2 |
| 3215.4521558 | 3 |
| 3215.4524846 | 2 |
| 3215.4532856 | 1 |
| 3215.453796 | 0 |
| 3215.4538058 | 1 |
| 3215.4543816 | 2 |
| 3215.4547907 | 0 |
| 3215.4549836 | 3 |
| 3215.4555912 | 2 |
| 3215.4561596 | 1 |
| 3215.4569454 | 3 |
| 3215.4574438 | 1 |
| 3215.4579271 | 1 |
| 3215.4579812 | 0 |
| 3215.4588882 | 1 |
| 3215.4593731 | 0 |
| 3215.4595653 | 2 |
| 3215.45981 | 3 |
| 3215.460593 | 0 |
| 3215.4606756 | 1 |
| 3215.461239 | 2 |
| 3215.4613694 | 6 |
| 3215.4623232 | 0 |
| 3215.4628254 | 1 |
| 3215.4637496 | 4 |
| 3215.4637721 | 2 |
| 3215.4641298 | 1 |
| 3215.4648688 | 0 |
| 3215.4652471 | 0 |
| 3215.4655751 | 2 |
| 3215.4659116 | 0 |
| 3215.4666967 | 1 |
| 3215.467475 | 2 |
| 3215.4675692 | 0 |
| 3215.4679593 | 1 |
| 3215.4690863 | 1 |
| 3215.4691248 | 2 |
| 3215.4694881 | 3 |
| 3215.4701932 | 0 |
| 3215.4706996 | 0 |
| 3215.4711463 | 0 |
| 3215.4712552 | 2 |
| 3215.4720416 | 0 |
| 3215.4720844 | 2 |
| 3215.4726641 | 6 |
| 3215.4734157 | 2 |
| 3215.4737855 | 0 |
| 3215.4742455 | 2 |
| 3215.4743759 | 2 |
| 3215.4752306 | 3 |
| 3215.4758139 | 0 |
| 3215.4761428 | 3 |
| 3215.4764929 | 0 |
| 3215.4766159 | 2 |
| 3215.4772071 | 3 |
| 3215.4775749 | 0 |
| 3215.4788499 | 0 |
| 3215.4788967 | 1 |
| 3215.4791185 | 2 |
| 3215.4798852 | 2 |
| 3215.480356 | 3 |
| 3215.4807803 | 1 |
| 3215.4816218 | 1 |
| 3215.4816418 | 0 |
| 3215.4818324 | 1 |
| 3215.4822674 | 1 |
| 3215.4828413 | 1 |
| 3215.4838232 | 0 |
| 3215.4843019 | 3 |
| 3215.484411 | 0 |
| 3215.4846384 | 0 |
| 3215.4848175 | 1 |
| 3215.4850085 | 0 |
| 3215.4856355 | 0 |
| 3215.4863845 | 3 |
| 3215.487388 | 0 |
| 3215.4877136 | 1 |
| 3215.4877441 | 1 |
| 3215.4885849 | 0 |
| 3215.4888731 | 4 |
| 3215.4893288 | 3 |
| 3215.4897591 | 1 |
| 3215.4898362 | 0 |
| 3215.4907454 | 3 |
| 3215.4910166 | 2 |
| 3215.4916741 | 2 |
| 3215.4919171 | 3 |
| 3215.4926857 | 4 |
| 3215.4927257 | 0 |
| 3215.4932595 | 2 |
| 3215.4941981 | 1 |
| 3215.4943143 | 2 |
| 3215.4953716 | 1 |
| 3215.4954161 | 0 |
| 3215.4958878 | 1 |
| 3215.4962778 | 2 |
| 3215.4966803 | 2 |
| 3215.4968997 | 1 |
| 3215.4981489 | 2 |
| 3215.4983674 | 2 |
| 3215.4984645 | 5 |
| 3215.4992104 | 0 |
| 3215.4995989 | 2 |
| 3215.4997561 | 2 |
| 3215.5001129 | 0 |
| 3215.5010826 | 1 |
| 3215.5013009 | 2 |
| 3215.5019119 | 0 |
| 3215.5024945 | 1 |
| 3215.5026368 | 1 |
| 3215.5033086 | 2 |
| 3215.5040377 | 0 |
| 3215.5045302 | 2 |
| 3215.5047067 | 1 |
| 3215.5055599 | 1 |
| 3215.5057101 | 1 |
| 3215.5064005 | 0 |
| 3215.5066501 | 1 |
| 3215.5068892 | 4 |
| 3215.5075206 | 1 |
| 3215.5082415 | 1 |
| 3215.509105 | 0 |
| 3215.5095662 | 2 |
| 3215.5097182 | 1 |
| 3215.5097747 | 1 |
| 3215.5106736 | 0 |
| 3215.5112426 | 3 |
| 3215.5116034 | 2 |
| 3215.5121121 | 3 |
| 3215.5124202 | 2 |
| 3215.5127844 | 3 |
| 3215.5129678 | 3 |
| 3215.513681 | 1 |
| 3215.5141868 | 1 |
| 3215.514408 | 4 |
| 3215.5153721 | 1 |
| 3215.5159579 | 0 |
| 3215.516313 | 2 |
| 3215.5169485 | 1 |
| 3215.5171035 | 2 |
| 3215.5180083 | 0 |
| 3215.518023 | 2 |
| 3215.5182136 | 0 |
| 3215.51839 | 2 |
| 3215.5193988 | 1 |
| 3215.5201273 | 2 |
| 3215.5203273 | 1 |
| 3215.5209673 | 0 |
| 3215.5212014 | 1 |

| | |
|---|---|
| 3215.5217634 | 1 |
| 3215.5221403 | 1 |
| 3215.5222719 | 1 |
| 3215.5236169 | 1 |
| 3215.5236428 | 0 |
| 3215.5238641 | 1 |
| 3215.5245219 | 1 |
| 3215.525092 | 1 |
| 3215.5254642 | 2 |
| 3215.5256886 | 5 |
| 3215.526323 | 0 |
| 3215.5266053 | 3 |
| 3215.526968 | 2 |
| 3215.5281064 | 0 |
| 3215.5281586 | 1 |
| 3215.5283502 | 0 |
| 3215.5286427 | 0 |
| 3215.5297133 | 0 |
| 3215.5297777 | 0 |
| 3215.5301751 | 3 |
| 3215.5304937 | 1 |
| 3215.5306131 | 1 |
| 3215.5316561 | 0 |
| 3215.5325847 | 2 |
| 3215.5326915 | 0 |
| 3215.5328325 | 2 |
| 3215.5334717 | 2 |
| 3215.5342943 | 0 |
| 3215.5344584 | 3 |
| 3215.5345896 | 1 |
| 3215.5347758 | 3 |
| 3215.5349283 | 0 |
| 3215.5366361 | 1 |
| 3215.5367823 | 4 |
| 3215.5372717 | 0 |
| 3215.5376701 | 2 |
| 3215.5377474 | 0 |
| 3215.5383812 | 3 |
| 3215.5391566 | 2 |
| 3215.5391779 | 1 |
| 3215.539389 | 1 |
| 3215.5408337 | 1 |
| 3215.5409249 | 2 |
| 3215.5412585 | 1 |
| 3215.5421976 | 0 |
| 3215.5424633 | 2 |
| 3215.5425869 | 1 |
| 3215.5436824 | 1 |
| 3215.543946 | 2 |
| 3215.5441742 | 3 |
| 3215.5450373 | 2 |
| 3215.5452067 | 2 |
| 3215.5454982 | 0 |
| 3215.5460143 | 1 |
| 3215.5466305 | 1 |
| 3215.5468518 | 2 |
| 3215.5475017 | 3 |
| 3215.5476134 | 2 |
| 3215.5479275 | 0 |
| 3215.5488218 | 0 |
| 3215.5495079 | 0 |
| 3215.5498189 | 2 |
| 3215.5505398 | 0 |
| 3215.551017 | 2 |
| 3215.5511987 | 4 |
| 3215.5521994 | 0 |
| 3215.5523197 | 1 |
| 3215.5523404 | 2 |
| 3215.5531145 | 2 |
| 3215.5536181 | 3 |
| 3215.5540087 | 2 |
| 3215.5545183 | 1 |
| 3215.5549686 | 1 |
| 3215.5551842 | 3 |
| 3215.5558787 | 7 |
| 3215.5565759 | 1 |
| 3215.5571522 | 2 |
| 3215.557265 | 2 |
| 3215.5582009 | 2 |
| 3215.5585901 | 1 |
| 3215.559032 | 0 |
| 3215.5597402 | 1 |
| 3215.5604698 | 1 |
| 3215.5605854 | 0 |
| 3215.5606829 | 1 |
| 3215.5614807 | 1 |
| 3215.5624067 | 0 |
| 3215.562859 | 2 |
| 3215.5630826 | 3 |
| 3215.5633905 | 4 |
| 3215.563432 | 2 |
| 3215.5638862 | 0 |
| 3215.5647706 | 0 |
| 3215.5648729 | 0 |
| 3215.5652775 | 2 |
| 3215.5656429 | 2 |
| 3215.5660791 | 0 |
| 3215.5671354 | 0 |
| 3215.567216 | 3 |
| 3215.5677478 | 1 |
| 3215.5677612 | 2 |
| 3215.5690261 | 1 |
| 3215.5690983 | 2 |
| 3215.5696336 | 0 |
| 3215.5698244 | 0 |
| 3215.5702489 | 0 |
| 3215.5709339 | 1 |
| 3215.5710139 | 2 |
| 3215.571905 | 2 |
| 3215.5724391 | 1 |
| 3215.5728244 | 2 |
| 3215.573009 | 1 |
| 3215.5738647 | 1 |
| 3215.5745503 | 2 |
| 3215.5745906 | 0 |
| 3215.5748669 | 0 |
| 3215.5758709 | 1 |
| 3215.5760326 | 2 |
| 3215.5766944 | 3 |
| 3215.5771612 | 0 |
| 3215.5776064 | 2 |
| 3215.5785068 | 0 |
| 3215.5786187 | 0 |
| 3215.5789881 | 2 |
| 3215.5790284 | 4 |
| 3215.5795794 | 1 |
| 3215.5800815 | 0 |
| 3215.5805233 | 3 |
| 3215.5807002 | 4 |
| 3215.5814078 | 1 |
| 3215.5817005 | 0 |
| 3215.5819431 | 0 |
| 3215.5830042 | 1 |
| 3215.5836626 | 1 |
| 3215.5839199 | 2 |
| 3215.5842791 | 1 |
| 3215.5847367 | 0 |
| 3215.5851775 | 3 |
| 3215.5858657 | 3 |
| 3215.5863922 | 2 |
| 3215.5865634 | 0 |
| 3215.5869833 | 1 |
| 3215.5878107 | 0 |
| 3215.5880192 | 4 |
| 3215.5883848 | 0 |
| 3215.5887588 | 0 |
| 3215.5895437 | 2 |
| 3215.5899699 | 0 |
| 3215.5901608 | 0 |
| 3215.5907814 | 1 |
| 3215.5912944 | 3 |
| 3215.5918507 | 0 |
| 3215.5921675 | 2 |
| 3215.5930636 | 1 |
| 3215.5935195 | 1 |
| 3215.5941599 | 2 |
| 3215.5945708 | 2 |
| 3215.5947103 | 0 |
| 3215.5949048 | 3 |
| 3215.5956524 | 0 |
| 3215.5965013 | 3 |
| 3215.5966449 | 2 |
| 3215.5967261 | 2 |
| 3215.5977228 | 1 |
| 3215.5980496 | 2 |
| 3215.598367 | 0 |
| 3215.5992404 | 0 |
| 3215.5995932 | 2 |
| 3215.6001593 | 0 |
| 3215.6006095 | 2 |
| 3215.60086 | 1 |
| 3215.6014441 | 0 |
| 3215.6017136 | 1 |
| 3215.6019404 | 2 |
| 3215.602324 | 1 |
| 3215.6024198 | 0 |
| 3215.6026289 | 2 |
| 3215.6027819 | 1 |
| 3215.6028376 | 3 |
| 3215.603103 | 1 |
| 3215.603243 | 1 |
| 3215.6034756 | 2 |
| 3215.6035686 | 3 |
| 3215.6036224 | 0 |
| 3215.6037493 | 1 |
| 3215.6037775 | 2 |
| 3215.6039945 | 1 |
| 3215.6041227 | 0 |
| 3215.6042718 | 0 |
| 3215.6043353 | 2 |
| 3215.6044635 | 1 |
| 3215.6045166 | 0 |
| 3215.6045904 | 0 |
| 3215.6046134 | 2 |
| 3215.6046486 | 4 |
| 3215.6046854 | 1 |
| 3215.6046955 | 0 |
| 3215.6049905 | 3 |
| 3215.6052406 | 1 |
| 3215.6052535 | 1 |
| 3215.6055073 | 1 |
| 3215.6056103 | 0 |
| 3215.6057052 | 3 |
| 3215.6057235 | 1 |
| 3215.6057958 | 3 |
| 3215.6058551 | 1 |
| 3215.6060644 | 2 |
| 3215.6061393 | 0 |
| 3215.6062031 | 1 |
| 3215.6062424 | 1 |
| 3215.6063567 | 0 |
| 3215.6065166 | 0 |
| 3215.6067785 | 3 |
| 3215.607034 | 0 |
| 3215.6070658 | 1 |
| 3215.6070829 | 1 |
| 3215.6071255 | 1 |
| 3215.6071797 | 1 |
| 3215.6072484 | 2 |
| 3215.607255 | 2 |
| 3215.6074508 | 1 |
| 3215.60746 | 0 |
| 3215.6077627 | 1 |
| 3215.6077732 | 0 |
| 3215.6080069 | 3 |
| 3215.6080376 | 1 |
| 3215.6080998 | 0 |
| 3215.6081763 | 2 |
| 3215.6082874 | 1 |
| 3215.6086595 | 3 |
| 3215.6086825 | 3 |
| 3215.6087773 | 0 |
| 3215.60879 | 1 |
| 3215.6087929 | 4 |
| 3215.6089696 | 0 |
| 3215.6091838 | 6 |
| 3215.6092854 | 6 |
| 3215.6092958 | 4 |
| 3215.6095496 | 2 |
| 3215.6095776 | 3 |
| 3215.6099235 | 3 |
| 3215.6100758 | 3 |
| 3215.6100805 | 4 |
| 3215.6100939 | 4 |
| 3215.6101241 | 2 |
| 3215.6103579 | 3 |
| 3215.6104322 | 3 |
| 3215.6105213 | 2 |
| 3215.6107505 | 5 |
| 3215.6108635 | 2 |
| 3215.6108774 | 5 |
| 3215.6109483 | 3 |
| 3215.6110949 | 3 |
| 3215.6112067 | 1 |
| 3215.6112736 | 4 |
| 3215.6112969 | 6 |
| 3215.6116697 | 3 |
| 3215.611692 | 6 |
| 3215.6117172 | 5 |
| 3215.611749 | 0 |
| 3215.6119784 | 3 |
| 3215.612066 | 4 |
| 3215.6121898 | 5 |
| 3215.6123006 | 3 |
| 3215.6123208 | 2 |
| 3215.6123563 | 4 |
| 3215.6125421 | 5 |
| 3215.6127228 | 3 |
| 3215.6127242 | 2 |
| 3215.6128324 | 7 |
| 3215.61296 | 6 |
| 3215.6129736 | 9 |
| 3215.6133168 | 4 |
| 3215.6134405 | 5 |
| 3215.613478 | 5 |
| 3215.6135378 | 6 |
| 3215.6137318 | 5 |
| 3215.6137779 | 7 |
| 3215.6139368 | 6 |
| 3215.6140502 | 3 |
| 3215.6140629 | 7 |
| 3215.6142057 | 6 |
| 3215.6142676 | 2 |
| 3215.6143264 | 5 |
| 3215.6143503 | 3 |
| 3215.6145912 | 3 |
| 3215.6147175 | 3 |
| 3215.6148279 | 6 |
| 3215.6149492 | 4 |
| 3215.6149788 | 5 |
| 3215.6150639 | 3 |
| 3215.6156908 | 3 |
| 3215.6157338 | 2 |
| 3215.6158125 | 1 |
| 3215.6158555 | 0 |
| 3215.6159872 | 5 |
| 3215.6161064 | 2 |
| 3215.6161874 | 1 |
| 3215.6163068 | 1 |
| 3215.6163716 | 0 |
| 3215.6164093 | 1 |
| 3215.6164237 | 2 |
| 3215.6165895 | 2 |
| 3215.6166426 | 0 |
| 3215.6167116 | 4 |
| 3215.6168382 | 3 |
| 3215.6169277 | 1 |
| 3215.6169674 | 4 |
| 3215.6170017 | 1 |
| 3215.6172315 | 1 |
| 3215.6172483 | 3 |
| 3215.6172724 | 7 |
| 3215.6175935 | 4 |
| 3215.6177375 | 5 |
| 3215.6178006 | 10 |
| 3215.6179106 | 5 |
| 3215.6179514 | 5 |
| 3215.6180247 | 8 |
| 3215.6184346 | 9 |
| 3215.6185233 | 7 |
| 3215.6185264 | 8 |
| 3215.6187227 | 6 |
| 3215.6187566 | 9 |
| 3215.6188781 | 6 |
| 3215.6190339 | 7 |
| 3215.6190943 | 4 |
| 3215.6191475 | 5 |
| 3215.6191616 | 6 |
| 3215.6193974 | 7 |
| 3215.6194226 | 6 |
| 3215.6194502 | 10 |
| 3215.6198757 | 10 |
| 3215.6199174 | 5 |
| 3215.6200778 | 5 |
| 3215.6201439 | 0 |
| 3215.6202277 | 4 |
| 3215.6202673 | 5 |
| 3215.6202712 | 1 |
| 3215.6203694 | 7 |
| 3215.6204901 | 1 |
| 3215.620594 | 4 |
| 3215.6207511 | 2 |
| 3215.6210009 | 4 |
| 3215.621059 | 0 |
| 3215.6210666 | 1 |
| 3215.6210747 | 1 |
| 3215.6210933 | 2 |
| 3215.6213884 | 3 |
| 3215.6216204 | 1 |
| 3215.6217828 | 1 |
| 3215.6218 | 1 |
| 3215.6219239 | 2 |
| 3215.6220988 | 0 |
| 3215.6221396 | 1 |
| 3215.6222142 | 1 |
| 3215.6222616 | 0 |
| 3215.622308 | 1 |
| 3215.6224969 | 0 |
| 3215.6226552 | 0 |
| 3215.6226754 | 1 |
| 3215.6227143 | 0 |
| 3215.6227844 | 1 |
| 3215.6228901 | 3 |

| | |
|---|---|
| 3215.622999 | 0 |
| 3215.6230271 | 0 |
| 3215.6231995 | 0 |
| 3215.623284 | 0 |
| 3215.6233659 | 0 |
| 3215.6234646 | 0 |
| 3215.6235561 | 1 |
| 3215.6238079 | 0 |
| 3215.623968 | 0 |
| 3215.6240451 | 2 |
| 3215.6240781 | 0 |
| 3215.6241687 | 1 |
| 3215.6246124 | 2 |
| 3215.6247569 | 1 |
| 3215.6247962 | 2 |
| 3215.6248388 | 2 |
| 3215.6249727 | 1 |
| 3215.6250022 | 0 |
| 3215.6250591 | 1 |
| 3215.6251143 | 1 |
| 3215.6251682 | 1 |
| 3215.6253385 | 0 |
| 3215.6254136 | 1 |
| 3215.6255306 | 1 |
| 3215.6256825 | 0 |
| 3215.6258088 | 2 |
| 3215.6258216 | 0 |
| 3215.6259822 | 2 |
| 3215.6260157 | 2 |
| 3215.6261177 | 1 |
| 3215.6261237 | 0 |
| 3215.6262996 | 1 |
| 3215.6263385 | 2 |
| 3215.6265238 | 1 |
| 3215.6266598 | 0 |
| 3215.6266612 | 2 |
| 3215.6267743 | 1 |
| 3215.6269441 | 0 |
| 3215.6269774 | 1 |
| 3215.6270419 | 1 |
| 3215.6274839 | 2 |
| 3215.6276189 | 2 |
| 3215.6276808 | 0 |
| 3215.6277481 | 0 |
| 3215.6279251 | 0 |
| 3215.6279856 | 1 |
| 3215.6280687 | 2 |
| 3215.6281128 | 0 |
| 3215.6281986 | 0 |
| 3215.6284402 | 2 |
| 3215.6285313 | 1 |
| 3215.6286227 | 1 |
| 3215.6287969 | 0 |
| 3215.6292884 | 6 |
| 3215.6294202 | 0 |
| 3215.6296266 | 1 |
| 3215.6297412 | 0 |
| 3215.6304145 | 2 |
| 3215.6304819 | 1 |
| 3215.6306999 | 0 |
| 3215.6307254 | 0 |
| 3215.631446 | 1 |
| 3215.6315274 | 2 |
| 3215.6316099 | 0 |
| 3215.6317431 | 0 |
| 3215.6320928 | 2 |
| 3215.632401 | 0 |
| 3215.6326626 | 0 |
| 3215.6327178 | 1 |
| 3215.6331351 | 1 |
| 3215.633139 | 1 |
| 3215.6332155 | 0 |
| 3215.6332534 | 4 |
| 3215.6338058 | 1 |
| 3215.6340819 | 1 |
| 3215.634465 | 0 |
| 3215.6345918 | 0 |
| 3215.6346299 | 2 |
| 3215.6348203 | 0 |
| 3215.6351319 | 0 |
| 3215.6356627 | 1 |
| 3215.6358082 | 1 |
| 3215.6363578 | 1 |
| 3215.6364804 | 1 |
| 3215.6367203 | 0 |
| 3215.6367591 | 2 |
| 3215.6369376 | 1 |
| 3215.6371689 | 0 |
| 3215.637638 | 0 |
| 3215.6377803 | 0 |
| 3215.6381908 | 2 |
| 3215.6382186 | 3 |
| 3215.6382664 | 0 |
| 3215.6385459 | 1 |
| 3215.638718 | 1 |
| 3215.6391461 | 1 |
| 3215.639441 | 0 |
| 3215.6396836 | 2 |
| 3215.6400229 | 3 |
| 3215.6400861 | 4 |
| 3215.6402512 | 2 |
| 3215.6408083 | 0 |
| 3215.6408341 | 1 |
| 3215.6409409 | 2 |
| 3215.64138 | 3 |
| 3215.6414796 | 1 |
| 3215.6415313 | 2 |
| 3215.6420265 | 1 |
| 3215.6422461 | 0 |
| 3215.6423602 | 0 |
| 3215.6425251 | 0 |
| 3215.6430593 | 0 |
| 3215.6430662 | 1 |
| 3215.6430948 | 0 |
| 3215.643582 | 3 |
| 3215.6436827 | 1 |
| 3215.6441055 | 0 |
| 3215.6444805 | 1 |
| 3215.6444965 | 0 |
| 3215.644698 | 1 |
| 3215.6448906 | 1 |
| 3215.6450538 | 2 |
| 3215.6456603 | 3 |
| 3215.645665 | 2 |
| 3215.6458116 | 1 |
| 3215.6460187 | 1 |
| 3215.6464566 | 0 |
| 3215.6465572 | 0 |
| 3215.6470909 | 2 |
| 3215.6474981 | 2 |
| 3215.647695 | 0 |
| 3215.6478576 | 4 |
| 3215.6479985 | 1 |
| 3215.647999 | 0 |
| 3215.6481531 | 0 |
| 3215.6484547 | 1 |
| 3215.648989 | 1 |
| 3215.6490098 | 2 |
| 3215.6494505 | 0 |
| 3215.6495571 | 6 |
| 3215.6500906 | 0 |
| 3215.6501981 | 3 |
| 3215.6502734 | 1 |
| 3215.650573 | 3 |
| 3215.6507363 | 1 |
| 3215.6508477 | 1 |
| 3215.6509548 | 0 |
| 3215.6511713 | 1 |
| 3215.651623 | 1 |
| 3215.6516679 | 1 |
| 3215.6524019 | 0 |
| 3215.6524535 | 1 |
| 3215.652509 | 0 |
| 3215.6526719 | 2 |
| 3215.6527632 | 1 |
| 3215.6536481 | 1 |
| 3215.6537413 | 0 |
| 3215.6537562 | 0 |
| 3215.6538945 | 3 |
| 3215.6545336 | 0 |
| 3215.6546367 | 1 |
| 3215.6546555 | 2 |
| 3215.6550523 | 1 |
| 3215.6553496 | 4 |
| 3215.6554852 | 2 |
| 3215.6554969 | 1 |
| 3215.6556678 | 2 |
| 3215.6558171 | 1 |
| 3215.6561805 | 0 |
| 3215.6564434 | 2 |
| 3215.6565387 | 2 |
| 3215.6568473 | 0 |
| 3215.6572544 | 8 |
| 3215.6573301 | 2 |
| 3215.6576868 | 0 |
| 3215.658095 | 1 |
| 3215.6581867 | 0 |
| 3215.6585113 | 1 |
| 3215.658592 | 2 |
| 3215.6587574 | 1 |
| 3215.6587674 | 0 |
| 3215.6593441 | 0 |
| 3215.6595791 | 0 |
| 3215.6597666 | 2 |
| 3215.6598412 | 0 |
| 3215.6603265 | 2 |
| 3215.6603326 | 2 |
| 3215.6605255 | 0 |
| 3215.6611838 | 0 |
| 3215.6612369 | 0 |
| 3215.6614292 | 2 |
| 3215.6616901 | 0 |
| 3215.6618259 | 1 |
| 3215.6622878 | 0 |
| 3215.6624181 | 3 |
| 3215.6625556 | 1 |
| 3215.6626112 | 3 |
| 3215.6627568 | 2 |
| 3215.6631134 | 6 |
| 3215.6631834 | 1 |
| 3215.663692 | 1 |
| 3215.6640985 | 0 |
| 3215.6641191 | 2 |
| 3215.6643802 | 1 |
| 3215.6643968 | 0 |
| 3215.664788 | 2 |
| 3215.6650164 | 0 |
| 3215.6652718 | 0 |
| 3215.6653441 | 1 |
| 3215.6654553 | 0 |
| 3215.6658765 | 4 |
| 3215.6660982 | 1 |
| 3215.6662227 | 0 |
| 3215.6666654 | 2 |
| 3215.6670587 | 3 |
| 3215.6671686 | 1 |
| 3215.6672745 | 1 |
| 3215.6674533 | 0 |
| 3215.6676806 | 2 |
| 3215.6677227 | 2 |
| 3215.6680263 | 0 |
| 3215.6683792 | 1 |
| 3215.6686521 | 0 |
| 3215.669054 | 0 |
| 3215.6692194 | 1 |
| 3215.6692625 | 3 |
| 3215.669289 | 2 |
| 3215.6698427 | 1 |
| 3215.6699682 | 0 |
| 3215.6700589 | 1 |
| 3215.6701816 | 0 |
| 3215.6709928 | 1 |
| 3215.6711753 | 1 |
| 3215.6713998 | 1 |
| 3215.671521 | 1 |
| 3215.6718436 | 0 |
| 3215.6720878 | 0 |
| 3215.6723086 | 5 |
| 3215.6724793 | 0 |
| 3215.6725339 | 0 |
| 3215.6726478 | 4 |
| 3215.6728998 | 1 |
| 3215.6729639 | 1 |
| 3215.6733823 | 0 |
| 3215.6736276 | 0 |
| 3215.6739435 | 2 |
| 3215.6743391 | 0 |
| 3215.6743506 | 0 |
| 3215.6743661 | 1 |
| 3215.6749545 | 1 |
| 3215.6752169 | 4 |
| 3215.6753479 | 1 |
| 3215.6756122 | 1 |
| 3215.675701 | 0 |
| 3215.6761545 | 3 |
| 3215.6762398 | 0 |
| 3215.6764585 | 2 |
| 3215.6769802 | 0 |
| 3215.6769837 | 1 |
| 3215.6772854 | 0 |
| 3215.6776034 | 1 |
| 3215.6776934 | 3 |
| 3215.678038 | 1 |
| 3215.678039 | 0 |
| 3215.6781029 | 2 |
| 3215.6783783 | 2 |
| 3215.6785647 | 1 |
| 3215.678917 | 0 |
| 3215.6793026 | 2 |
| 3215.6796493 | 1 |
| 3215.6800009 | 0 |
| 3215.6803145 | 2 |
| 3215.6804896 | 0 |
| 3215.6806607 | 2 |
| 3215.6807433 | 2 |
| 3215.6807904 | 0 |
| 3215.6812955 | 0 |
| 3215.6813182 | 2 |
| 3215.6815399 | 2 |
| 3215.6816214 | 2 |
| 3215.6816531 | 1 |
| 3215.682243 | 1 |
| 3215.6826159 | 1 |
| 3215.6828778 | 1 |
| 3215.6830599 | 2 |
| 3215.683149 | 1 |
| 3215.6831937 | 0 |
| 3215.6837843 | 1 |
| 3215.6840892 | 0 |
| 3215.6842699 | 1 |
| 3215.6844304 | 1 |
| 3215.6848103 | 0 |
| 3215.684925 | 1 |
| 3215.6849824 | 0 |
| 3215.6849861 | 2 |
| 3215.6856284 | 2 |
| 3215.6862311 | 1 |
| 3215.6863666 | 0 |
| 3215.6869398 | 1 |
| 3215.6869863 | 0 |
| 3215.6870971 | 2 |
| 3215.6872389 | 0 |
| 3215.6874492 | 2 |
| 3215.6875604 | 0 |
| 3215.6879821 | 0 |
| 3215.6883561 | 1 |
| 3215.6884879 | 1 |
| 3215.6887309 | 0 |
| 3215.6893215 | 1 |
| 3215.6893994 | 1 |
| 3215.6893995 | 0 |
| 3215.6895635 | 3 |
| 3215.6901921 | 1 |
| 3215.6902958 | 1 |
| 3215.690333 | 2 |
| 3215.6905582 | 1 |
| 3215.6906164 | 2 |
| 3215.6907046 | 1 |
| 3215.6909622 | 0 |
| 3215.6914666 | 0 |
| 3215.691798 | 1 |
| 3215.6918045 | 2 |
| 3215.6920623 | 1 |
| 3215.6920726 | 1 |
| 3215.6925248 | 1 |
| 3215.6925672 | 1 |
| 3215.693053 | 0 |
| 3215.6932898 | 0 |
| 3215.6933395 | 0 |
| 3215.6936783 | 0 |
| 3215.693881 | 3 |
| 3215.6940886 | 3 |
| 3215.6941296 | 0 |
| 3215.6945694 | 0 |
| 3215.6949426 | 5 |
| 3215.6952207 | 1 |
| 3215.6953617 | 1 |
| 3215.6953783 | 2 |
| 3215.695623 | 0 |
| 3215.6957548 | 2 |
| 3215.6962904 | 2 |
| 3215.6966388 | 0 |
| 3215.6967126 | 2 |
| 3215.6970226 | 1 |
| 3215.6972312 | 0 |
| 3215.6972327 | 0 |
| 3215.6978206 | 1 |
| 3215.6980203 | 0 |
| 3215.6980338 | 0 |
| 3215.6983655 | 3 |
| 3215.6984409 | 0 |
| 3215.698517 | 1 |
| 3215.6989957 | 0 |
| 3215.6991684 | 1 |
| 3215.6993004 | 1 |
| 3215.7000718 | 1 |
| 3215.7003905 | 4 |
| 3215.700834 | 1 |
| 3215.700997 | 2 |
| 3215.7010861 | 1 |
| 3215.7021656 | 0 |
| 3215.7024355 | 5 |
| 3215.7033844 | 0 |

| | |
|---|---|
| 3215.703682 | 2 |
| 3215.7038141 | 0 |
| 3215.7047785 | 1 |
| 3215.7052366 | 2 |
| 3215.7053249 | 0 |
| 3215.7056858 | 4 |
| 3215.7061704 | 1 |
| 3215.706539 | 0 |
| 3215.7067334 | 2 |
| 3215.7074049 | 1 |
| 3215.7077314 | 2 |
| 3215.7085167 | 1 |
| 3215.7090071 | 1 |
| 3215.7096816 | 0 |
| 3215.7097898 | 0 |
| 3215.7099822 | 0 |
| 3215.7113067 | 2 |
| 3215.7113556 | 2 |
| 3215.7113837 | 4 |
| 3215.7124734 | 0 |
| 3215.7124993 | 1 |
| 3215.7130861 | 1 |
| 3215.7137518 | 0 |
| 3215.7138663 | 2 |
| 3215.7141683 | 3 |
| 3215.7143365 | 1 |
| 3215.7150639 | 0 |
| 3215.7160228 | 0 |
| 3215.716478 | 1 |
| 3215.7168389 | 0 |
| 3215.7171526 | 0 |
| 3215.7175891 | 0 |
| 3215.7181886 | 3 |
| 3215.7184613 | 0 |
| 3215.7188037 | 4 |
| 3215.7192263 | 0 |
| 3215.720362 | 0 |
| 3215.7210015 | 1 |
| 3215.7215277 | 2 |
| 3215.7216214 | 3 |
| 3215.7216791 | 0 |
| 3215.7222631 | 2 |
| 3215.7232855 | 0 |
| 3215.7233252 | 2 |
| 3215.7235282 | 2 |
| 3215.7240533 | 1 |
| 3215.7247565 | 2 |
| 3215.725004 | 1 |
| 3215.725129 | 0 |
| 3215.7260409 | 0 |
| 3215.7262291 | 1 |
| 3215.727051 | 2 |
| 3215.7272163 | 1 |
| 3215.728078 | 0 |
| 3215.7283201 | 4 |
| 3215.7288284 | 1 |
| 3215.7292397 | 3 |
| 3215.7297927 | 0 |
| 3215.7300197 | 5 |
| 3215.7305449 | 0 |
| 3215.730579 | 2 |
| 3215.7317335 | 2 |
| 3215.7318369 | 1 |
| 3215.7327668 | 0 |
| 3215.733872 | 0 |
| 3215.7339289 | 5 |
| 3215.7339318 | 1 |
| 3215.7351708 | 1 |
| 3215.7353598 | 0 |
| 3215.7355335 | 1 |
| 3215.736263 | 0 |
| 3215.7365966 | 0 |
| 3215.7373898 | 2 |
| 3215.7377691 | 1 |
| 3215.7385281 | 1 |
| 3215.738597 | 2 |
| 3215.73875 | 3 |
| 3215.7390869 | 1 |
| 3215.7400973 | 0 |
| 3215.7402027 | 0 |
| 3215.7406435 | 3 |
| 3215.7416302 | 1 |
| 3215.7416835 | 0 |
| 3215.7425218 | 2 |
| 3215.7427814 | 3 |
| 3215.743011 | 2 |
| 3215.7432668 | 3 |
| 3215.7436933 | 2 |
| 3215.7437603 | 1 |
| 3215.744329 | 2 |
| 3215.7445962 | 1 |
| 3215.7452078 | 1 |
| 3215.7454434 | 0 |
| 3215.7456979 | 0 |
| 3215.7461391 | 0 |
| 3215.746934 | 1 |
| 3215.7470072 | 3 |
| 3215.7473057 | 5 |
| 3215.7477243 | 1 |
| 3215.7479381 | 1 |
| 3215.7486526 | 1 |
| 3215.7491473 | 2 |
| 3215.7492084 | 0 |
| 3215.7492643 | 1 |
| 3215.7505102 | 0 |
| 3215.750589 | 2 |
| 3215.7507966 | 3 |
| 3215.7510348 | 0 |
| 3215.7519626 | 1 |
| 3215.7519998 | 1 |
| 3215.752274 | 2 |
| 3215.7525586 | 1 |
| 3215.752885 | 2 |
| 3215.7533988 | 2 |
| 3215.7539168 | 0 |
| 3215.754082 | 0 |
| 3215.7546621 | 0 |
| 3215.7548036 | 1 |
| 3215.7553794 | 2 |
| 3215.7557119 | 1 |
| 3215.7560893 | 2 |
| 3215.7561356 | 1 |
| 3215.7564633 | 0 |
| 3215.7570735 | 4 |
| 3215.7572926 | 4 |
| 3215.7573787 | 4 |
| 3215.7578789 | 4 |
| 3215.7587947 | 2 |
| 3215.7588322 | 2 |
| 3215.7597158 | 1 |
| 3215.7598168 | 2 |
| 3215.7599781 | 1 |
| 3215.7601513 | 2 |
| 3215.7607803 | 2 |
| 3215.7612497 | 0 |
| 3215.761341 | 1 |
| 3215.7618667 | 0 |
| 3215.7621892 | 0 |
| 3215.7625096 | 2 |
| 3215.7628169 | 2 |
| 3215.7629957 | 2 |
| 3215.763325 | 3 |
| 3215.7639041 | 1 |
| 3215.7639995 | 2 |
| 3215.7649282 | 1 |
| 3215.7651178 | 2 |
| 3215.7657244 | 4 |
| 3215.7658674 | 0 |
| 3215.7659817 | 1 |
| 3215.766211 | 1 |
| 3215.7664076 | 3 |
| 3215.7665897 | 0 |
| 3215.7668571 | 3 |
| 3215.7669879 | 5 |
| 3215.7671753 | 1 |
| 3215.7673906 | 5 |
| 3215.767471 | 2 |
| 3215.7675797 | 0 |
| 3215.7679742 | 1 |
| 3215.7683343 | 0 |
| 3215.7686594 | 2 |
| 3215.7688972 | 1 |
| 3215.7689996 | 3 |
| 3215.7691224 | 0 |
| 3215.7691853 | 2 |
| 3215.7694802 | 2 |
| 3215.769893 | 0 |
| 3215.7699738 | 4 |
| 3215.7701665 | 3 |
| 3215.7702088 | 0 |
| 3215.7705497 | 2 |
| 3215.7707698 | 2 |
| 3215.7708954 | 1 |
| 3215.771094 | 2 |
| 3215.7714329 | 2 |
| 3215.7715548 | 4 |
| 3215.7715645 | 0 |
| 3215.7718659 | 0 |
| 3215.772093 | 2 |
| 3215.7721244 | 4 |
| 3215.7724064 | 4 |
| 3215.7727829 | 1 |
| 3215.7730011 | 1 |
| 3215.7731361 | 1 |
| 3215.7731858 | 0 |
| 3215.7732169 | 4 |
| 3215.7733472 | 0 |
| 3215.7734312 | 2 |
| 3215.7734333 | 0 |
| 3215.7737699 | 4 |
| 3215.7739343 | 2 |
| 3215.7740148 | 1 |
| 3215.7743003 | 1 |
| 3215.7746449 | 4 |
| 3215.7746581 | 2 |
| 3215.7746747 | 1 |
| 3215.7747564 | 0 |
| 3215.7749602 | 1 |
| 3215.7751336 | 2 |
| 3215.7751405 | 2 |
| 3215.7752837 | 2 |
| 3215.7753198 | 2 |
| 3215.7753283 | 0 |
| 3215.7753484 | 1 |
| 3215.7755857 | 0 |
| 3215.7757665 | 2 |
| 3215.7758678 | 0 |
| 3215.7761717 | 1 |
| 3215.7762068 | 1 |
| 3215.7765213 | 0 |
| 3215.7769819 | 2 |
| 3215.7770052 | 1 |
| 3215.7770267 | 4 |
| 3215.7771099 | 3 |
| 3215.7771517 | 0 |
| 3215.7771519 | 0 |
| 3215.7771538 | 0 |
| 3215.7772152 | 5 |
| 3215.7772467 | 2 |
| 3215.7772728 | 0 |
| 3215.7772916 | 1 |
| 3215.7778868 | 1 |
| 3215.7780593 | 0 |
| 3215.7781534 | 0 |
| 3215.7784456 | 1 |
| 3215.7784674 | 1 |
| 3215.7784773 | 0 |
| 3215.7788067 | 0 |
| 3215.778825 | 2 |
| 3215.7792393 | 1 |
| 3215.7793351 | 0 |
| 3215.7794046 | 0 |
| 3215.7795383 | 0 |
| 3215.7795504 | 1 |
| 3215.7795857 | 3 |
| 3215.7799131 | 2 |
| 3215.7799699 | 1 |
| 3215.7800046 | 0 |
| 3215.7800217 | 0 |
| 3215.7801723 | 4 |
| 3215.7802018 | 1 |
| 3215.7803737 | 4 |
| 3215.7803831 | 4 |
| 3215.7807241 | 1 |
| 3215.7808609 | 1 |
| 3215.7810136 | 0 |
| 3215.7810211 | 0 |
| 3215.7810392 | 1 |
| 3215.7815114 | 3 |
| 3215.7815864 | 0 |
| 3215.7818426 | 1 |
| 3215.7818534 | 0 |
| 3215.7819402 | 1 |
| 3215.7819623 | 1 |
| 3215.7820922 | 2 |
| 3215.782236 | 4 |
| 3215.7822453 | 1 |
| 3215.7824262 | 2 |
| 3215.7824629 | 0 |
| 3215.7825499 | 0 |
| 3215.7826769 | 0 |
| 3215.782752 | 3 |
| 3215.7827552 | 2 |
| 3215.783013 | 3 |
| 3215.7830847 | 1 |
| 3215.7831939 | 2 |
| 3215.7833245 | 1 |
| 3215.783391 | 0 |
| 3215.783626 | 0 |
| 3215.7836495 | 2 |
| 3215.7836617 | 1 |
| 3215.7840288 | 0 |
| 3215.7841152 | 2 |
| 3215.7842407 | 0 |
| 3215.7843074 | 0 |
| 3215.7843307 | 1 |
| 3215.7845247 | 1 |
| 3215.7845956 | 2 |
| 3215.7846472 | 1 |
| 3215.78472 | 1 |
| 3215.7848081 | 5 |
| 3215.7852007 | 2 |
| 3215.7853504 | 1 |
| 3215.7853713 | 0 |
| 3215.7856038 | 1 |
| 3215.7857882 | 0 |
| 3215.7859737 | 0 |
| 3215.7860576 | 1 |
| 3215.786111 | 0 |
| 3215.7861292 | 0 |
| 3215.7861482 | 3 |
| 3215.7861505 | 0 |
| 3215.7863694 | 0 |
| 3215.7864836 | 0 |
| 3215.7868345 | 1 |
| 3215.7868441 | 1 |
| 3215.7869673 | 2 |
| 3215.7869862 | 2 |
| 3215.7870765 | 0 |
| 3215.7871993 | 5 |
| 3215.7872075 | 2 |
| 3215.7872964 | 1 |
| 3215.7875479 | 1 |
| 3215.787569 | 0 |
| 3215.787656 | 2 |
| 3215.7878019 | 0 |
| 3215.7882128 | 0 |
| 3215.7882412 | 3 |
| 3215.7883009 | 4 |
| 3215.7883705 | 0 |
| 3215.7886631 | 3 |
| 3215.7887783 | 1 |
| 3215.7889106 | 0 |
| 3215.7889301 | 2 |
| 3215.7891606 | 3 |
| 3215.7892385 | 0 |
| 3215.7893152 | 3 |
| 3215.789427 | 4 |
| 3215.7894345 | 5 |
| 3215.7895642 | 2 |
| 3215.789912 | 1 |
| 3215.7899122 | 2 |
| 3215.790105 | 5 |
| 3215.7901469 | 8 |
| 3215.7903831 | 5 |
| 3215.7904129 | 2 |
| 3215.7904196 | 2 |
| 3215.7906508 | 3 |
| 3215.7908327 | 10 |
| 3215.7908385 | 5 |
| 3215.790897 | 13 |
| 3215.7909452 | 8 |
| 3215.79122 | 4 |
| 3215.791225 | 4 |
| 3215.7913346 | 16 |
| 3215.7915942 | 8 |
| 3215.7917631 | 10 |
| 3215.7919047 | 4 |
| 3215.7920518 | 18 |
| 3215.7920704 | 17 |
| 3215.7921777 | 8 |
| 3215.7924198 | 18 |
| 3215.7924747 | 5 |
| 3215.7924771 | 11 |
| 3215.7925055 | 17 |
| 3215.7925411 | 20 |
| 3215.792711 | 20 |
| 3215.7927134 | 10 |
| 3215.7927163 | 12 |
| 3215.793012 | 22 |
| 3215.7930796 | 10 |
| 3215.7933252 | 13 |
| 3215.7933365 | 29 |
| 3215.79341 | 16 |
| 3215.7938883 | 17 |
| 3215.7939312 | 9 |
| 3215.7939613 | 16 |
| 3215.7940325 | 12 |
| 3215.7940691 | 15 |
| 3215.7941735 | 11 |
| 3215.794448 | 9 |
| 3215.7945091 | 19 |
| 3215.7945974 | 14 |

| | |
|---|---|
| 3215.7947819 | 8 |
| 3215.7949027 | 11 |
| 3215.7949553 | 5 |
| 3215.7950895 | 22 |
| 3215.7951271 | 6 |
| 3215.795433 | 18 |
| 3215.7955759 | 19 |
| 3215.7956344 | 13 |
| 3215.7956376 | 14 |
| 3215.795818 | 14 |
| 3215.7958776 | 5 |
| 3215.7960849 | 16 |
| 3215.7961254 | 6 |
| 3215.7961651 | 10 |
| 3215.7965323 | 9 |
| 3215.7965396 | 9 |
| 3215.7965444 | 9 |
| 3215.7965697 | 16 |
| 3215.7967403 | 6 |
| 3215.7967897 | 11 |
| 3215.7971976 | 7 |
| 3215.7972554 | 1 |
| 3215.7973138 | 7 |
| 3215.7976036 | 14 |
| 3215.7976842 | 7 |
| 3215.797704 | 14 |
| 3215.7977311 | 6 |
| 3215.7978852 | 4 |
| 3215.7982293 | 1 |
| 3215.7983217 | 4 |
| 3215.7985492 | 2 |
| 3215.7985973 | 1 |
| 3215.7986039 | 0 |
| 3215.7986233 | 3 |
| 3215.7986296 | 2 |
| 3215.7986876 | 0 |
| 3215.7987126 | 3 |
| 3215.79894 | 4 |
| 3215.7991228 | 5 |
| 3215.7991672 | 3 |
| 3215.7993344 | 2 |
| 3215.7993419 | 4 |
| 3215.7996212 | 1 |
| 3215.7997914 | 1 |
| 3215.799792 | 0 |
| 3215.7999068 | 2 |
| 3215.7999455 | 1 |
| 3215.8001122 | 0 |
| 3215.8001693 | 0 |
| 3215.8002015 | 1 |
| 3215.8005316 | 2 |
| 3215.8006458 | 1 |
| 3215.8006818 | 1 |
| 3215.800939 | 1 |
| 3215.8009474 | 1 |
| 3215.8010744 | 0 |
| 3215.8011938 | 2 |
| 3215.8014789 | 1 |
| 3215.8016089 | 1 |
| 3215.8016849 | 0 |
| 3215.8020259 | 0 |
| 3215.8020678 | 2 |
| 3215.8021197 | 0 |
| 3215.8021239 | 1 |
| 3215.8021312 | 0 |
| 3215.8021706 | 2 |
| 3215.8021856 | 2 |
| 3215.8022541 | 2 |
| 3215.8022663 | 2 |
| 3215.8028464 | 2 |
| 3215.8028524 | 0 |
| 3215.8029466 | 1 |
| 3215.8029564 | 3 |
| 3215.8029868 | 2 |
| 3215.8029999 | 2 |
| 3215.803002 | 0 |
| 3215.8036031 | 3 |
| 3215.8037563 | 0 |
| 3215.803801 | 2 |
| 3215.8040165 | 1 |
| 3215.8040497 | 0 |
| 3215.8040709 | 0 |
| 3215.8042796 | 1 |
| 3215.8043313 | 0 |
| 3215.8044569 | 4 |
| 3215.8046939 | 0 |
| 3215.8047975 | 2 |
| 3215.8048864 | 0 |
| 3215.8048991 | 3 |
| 3215.8051453 | 0 |
| 3215.8051877 | 2 |
| 3215.8052213 | 0 |
| 3215.8052672 | 0 |
| 3215.8055483 | 1 |
| 3215.8056629 | 2 |
| 3215.80567 | 2 |
| 3215.8057869 | 1 |
| 3215.8058804 | 0 |
| 3215.8059187 | 2 |
| 3215.8060753 | 0 |
| 3215.806205 | 0 |
| 3215.8063495 | 3 |
| 3215.8064254 | 0 |
| 3215.8065547 | 1 |
| 3215.806569 | 2 |
| 3215.8066081 | 3 |
| 3215.8068703 | 1 |
| 3215.8071878 | 2 |
| 3215.807241 | 1 |
| 3215.8073921 | 0 |
| 3215.8075734 | 1 |
| 3215.8076462 | 1 |
| 3215.8077454 | 2 |
| 3215.8079012 | 3 |
| 3215.807934 | 2 |
| 3215.8079747 | 0 |
| 3215.8080893 | 1 |
| 3215.8081374 | 2 |
| 3215.808314 | 0 |
| 3215.8084566 | 0 |
| 3215.8084627 | 4 |
| 3215.808506 | 1 |
| 3215.8085784 | 1 |
| 3215.8088922 | 0 |
| 3215.8089837 | 1 |
| 3215.8090569 | 0 |
| 3215.8092429 | 6 |
| 3215.8092525 | 2 |
| 3215.8095562 | 1 |
| 3215.8096743 | 0 |
| 3215.809691 | 4 |
| 3215.8097811 | 3 |
| 3215.8099934 | 0 |
| 3215.8100702 | 1 |
| 3215.8100709 | 1 |
| 3215.8101392 | 1 |
| 3215.8104468 | 3 |
| 3215.8104532 | 1 |
| 3215.8105771 | 1 |
| 3215.8105792 | 1 |
| 3215.8106982 | 0 |
| 3215.8107759 | 1 |
| 3215.8110526 | 1 |
| 3215.8112446 | 0 |
| 3215.8113414 | 1 |
| 3215.811347 | 1 |
| 3215.8113606 | 0 |
| 3215.8115652 | 1 |
| 3215.8117578 | 0 |
| 3215.8118126 | 1 |
| 3215.8119136 | 2 |
| 3215.8120373 | 2 |
| 3215.8122336 | 4 |
| 3215.8122993 | 0 |
| 3215.8123518 | 3 |
| 3215.8123599 | 0 |
| 3215.8125288 | 1 |
| 3215.8125851 | 1 |
| 3215.812751 | 3 |
| 3215.8128662 | 0 |
| 3215.8129709 | 1 |
| 3215.8132353 | 1 |
| 3215.813278 | 3 |
| 3215.8134657 | 2 |
| 3215.8138081 | 1 |
| 3215.8138373 | 0 |
| 3215.8140888 | 0 |
| 3215.8141569 | 2 |
| 3215.8142867 | 3 |
| 3215.8143723 | 2 |
| 3215.8144187 | 3 |
| 3215.8144603 | 0 |
| 3215.8146131 | 0 |
| 3215.8146245 | 1 |
| 3215.8147984 | 3 |
| 3215.8149323 | 3 |
| 3215.8151793 | 0 |
| 3215.8153122 | 0 |
| 3215.8153276 | 2 |
| 3215.8155137 | 0 |
| 3215.815721 | 1 |
| 3215.8157853 | 1 |
| 3215.8158178 | 1 |
| 3215.8158863 | 0 |
| 3215.816002 | 2 |
| 3215.8160099 | 1 |
| 3215.8160119 | 0 |
| 3215.8162273 | 2 |
| 3215.8162538 | 3 |
| 3215.8163238 | 1 |
| 3215.8164174 | 1 |
| 3215.8168122 | 1 |
| 3215.8169204 | 2 |
| 3215.8170107 | 0 |
| 3215.8171775 | 1 |
| 3215.8173287 | 4 |
| 3215.817393 | 1 |
| 3215.817562 | 4 |
| 3215.8176015 | 1 |
| 3215.8178794 | 2 |
| 3215.8178823 | 1 |
| 3215.8179098 | 0 |
| 3215.8180365 | 2 |
| 3215.8182106 | 0 |
| 3215.8182924 | 1 |
| 3215.8187629 | 3 |
| 3215.8187694 | 2 |
| 3215.8189775 | 2 |
| 3215.8189913 | 1 |
| 3215.8190958 | 1 |
| 3215.8191062 | 3 |
| 3215.8191073 | 0 |
| 3215.8192932 | 1 |
| 3215.8194199 | 2 |
| 3215.8195367 | 1 |
| 3215.8197291 | 2 |
| 3215.8200241 | 1 |
| 3215.8200496 | 2 |
| 3215.8202745 | 2 |
| 3215.8202861 | 2 |
| 3215.8204447 | 0 |
| 3215.8204846 | 0 |
| 3215.820546 | 0 |
| 3215.820845 | 1 |
| 3215.820918 | 2 |
| 3215.8211041 | 1 |
| 3215.821145 | 1 |
| 3215.8211802 | 1 |
| 3215.8213873 | 1 |
| 3215.8214035 | 0 |
| 3215.8214565 | 3 |
| 3215.8215813 | 2 |
| 3215.821623 | 0 |
| 3215.8222133 | 0 |
| 3215.8222431 | 1 |
| 3215.8223494 | 3 |
| 3215.8230235 | 2 |
| 3215.82371 | 1 |
| 3215.8240658 | 0 |
| 3215.8244573 | 3 |
| 3215.825382 | 0 |
| 3215.8256553 | 2 |
| 3215.8260211 | 1 |
| 3215.8262151 | 4 |
| 3215.8270414 | 1 |
| 3215.8275152 | 0 |
| 3215.8276705 | 0 |
| 3215.8278008 | 2 |
| 3215.8285434 | 1 |
| 3215.8289757 | 1 |
| 3215.8294533 | 0 |
| 3215.8303409 | 0 |
| 3215.8306037 | 1 |
| 3215.8310195 | 1 |
| 3215.8320004 | 4 |
| 3215.8321672 | 0 |
| 3215.8330453 | 0 |
| 3215.8330764 | 0 |
| 3215.8331624 | 0 |
| 3215.8338824 | 0 |
| 3215.8344695 | 1 |
| 3215.8347353 | 1 |
| 3215.8350171 | 1 |
| 3215.835797 | 3 |
| 3215.8366018 | 0 |
| 3215.8366688 | 0 |
| 3215.8368513 | 1 |
| 3215.8369768 | 1 |
| 3215.8381003 | 0 |
| 3215.8387676 | 1 |
| 3215.8393935 | 0 |
| 3215.8397289 | 1 |
| 3215.840097 | 1 |
| 3215.8402648 | 2 |
| 3215.8404288 | 0 |
| 3215.8410825 | 3 |
| 3215.8414731 | 0 |
| 3215.8422379 | 0 |
| 3215.8424801 | 0 |
| 3215.843293 | 0 |
| 3215.8434857 | 1 |
| 3215.8445085 | 0 |
| 3215.8448167 | 1 |
| 3215.8454544 | 1 |
| 3215.8456091 | 1 |
| 3215.845862 | 1 |
| 3215.8463234 | 1 |
| 3215.8469161 | 1 |
| 3215.8470801 | 0 |
| 3215.84752 | 2 |
| 3215.8476729 | 1 |
| 3215.8487316 | 0 |
| 3215.8491264 | 1 |
| 3215.8493049 | 0 |
| 3215.8494844 | 2 |
| 3215.8503361 | 0 |
| 3215.851106 | 2 |
| 3215.8511155 | 2 |
| 3215.8514708 | 0 |
| 3215.8520846 | 1 |
| 3215.8528248 | 3 |
| 3215.8529881 | 0 |
| 3215.8538809 | 3 |
| 3215.8539876 | 2 |
| 3215.854528 | 1 |
| 3215.8549136 | 2 |
| 3215.8553391 | 7 |
| 3215.856349 | 2 |
| 3215.8564506 | 2 |
| 3215.8569028 | 0 |
| 3215.8570701 | 2 |
| 3215.8576807 | 1 |
| 3215.8582055 | 0 |
| 3215.8583533 | 2 |
| 3215.8587627 | 1 |
| 3215.8599609 | 1 |
| 3215.8603678 | 0 |
| 3215.860762 | 5 |
| 3215.8612959 | 0 |
| 3215.8615221 | 0 |
| 3215.8615429 | 5 |
| 3215.8627382 | 1 |
| 3215.8629862 | 0 |
| 3215.8634906 | 1 |
| 3215.8638878 | 0 |
| 3215.8642761 | 0 |
| 3215.8650866 | 4 |
| 3215.865132 | 7 |
| 3215.8659134 | 0 |
| 3215.8660031 | 2 |
| 3215.8664427 | 0 |
| 3215.8676714 | 0 |
| 3215.8678083 | 0 |
| 3215.867914 | 1 |
| 3215.8687166 | 2 |
| 3215.8693728 | 2 |
| 3215.8695196 | 1 |
| 3215.8703854 | 2 |
| 3215.8704586 | 1 |
| 3215.8713024 | 0 |
| 3215.8716402 | 1 |
| 3215.8724329 | 1 |
| 3215.8728258 | 2 |
| 3215.8730429 | 1 |
| 3215.8734736 | 1 |
| 3215.8741182 | 1 |
| 3215.8742173 | 3 |
| 3215.8752185 | 1 |
| 3215.875389 | 1 |
| 3215.8759582 | 0 |
| 3215.8767559 | 1 |
| 3215.8768334 | 0 |
| 3215.8773014 | 4 |
| 3215.8776071 | 0 |
| 3215.8777716 | 2 |
| 3215.878871 | 0 |
| 3215.8790466 | 1 |
| 3215.8790547 | 0 |
| 3215.8796522 | 2 |
| 3215.8803691 | 0 |
| 3215.8810076 | 3 |
| 3215.8817372 | 1 |
| 3215.881892 | 3 |

| | |
|---|---|
| 3215.8821158 | 3 |
| 3215.8831173 | 0 |
| 3215.8835691 | 1 |
| 3215.8839587 | 0 |
| 3215.8840428 | 3 |
| 3215.8846289 | 3 |
| 3215.8847453 | 0 |
| 3215.8856457 | 0 |
| 3215.8860418 | 3 |
| 3215.8867989 | 0 |
| 3215.8871532 | 2 |
| 3215.8872164 | 6 |
| 3215.8874941 | 0 |
| 3215.8883947 | 0 |
| 3215.889188 | 1 |
| 3215.8893114 | 1 |
| 3215.8896113 | 0 |
| 3215.8897044 | 2 |
| 3215.8909499 | 0 |
| 3215.8911615 | 0 |
| 3215.8912689 | 0 |
| 3215.8921183 | 1 |
| 3215.8925071 | 1 |
| 3215.89286 | 0 |
| 3215.8929109 | 0 |
| 3215.8939766 | 0 |
| 3215.8941852 | 1 |
| 3215.8943655 | 0 |
| 3215.8946123 | 1 |
| 3215.8956827 | 0 |
| 3215.8960656 | 0 |
| 3215.8964965 | 1 |
| 3215.8971875 | 5 |
| 3215.8976765 | 5 |
| 3215.8978871 | 1 |
| 3215.8983101 | 0 |
| 3215.8989383 | 2 |
| 3215.899252 | 4 |
| 3215.8993629 | 1 |
| 3215.899941 | 0 |
| 3215.9003551 | 2 |
| 3215.901465 | 1 |
| 3215.9019541 | 0 |
| 3215.9020686 | 4 |
| 3215.9021491 | 1 |
| 3215.9028995 | 1 |
| 3215.9035066 | 0 |
| 3215.9038434 | 0 |
| 3215.904675 | 0 |
| 3215.9052607 | 0 |
| 3215.9056433 | 2 |
| 3215.9056816 | 0 |
| 3215.9058843 | 1 |
| 3215.9069109 | 0 |
| 3215.907139 | 0 |
| 3215.9076834 | 2 |
| 3215.9081686 | 0 |
| 3215.908984 | 1 |
| 3215.9091358 | 0 |
| 3215.9101457 | 3 |
| 3215.9102413 | 0 |
| 3215.9103056 | 0 |
| 3215.9103155 | 5 |
| 3215.9118104 | 1 |
| 3215.9119334 | 1 |
| 3215.9124157 | 3 |
| 3215.9130383 | 1 |
| 3215.9134438 | 2 |
| 3215.9135619 | 2 |
| 3215.914257 | 2 |
| 3215.9145492 | 1 |
| 3215.9154102 | 1 |
| 3215.9156303 | 0 |
| 3215.9161602 | 0 |
| 3215.9167631 | 2 |
| 3215.9175197 | 2 |
| 3215.9177618 | 0 |
| 3215.9182189 | 0 |
| 3215.9185143 | 1 |
| 3215.9194439 | 0 |
| 3215.919526 | 2 |
| 3215.9200651 | 0 |
| 3215.9204752 | 0 |
| 3215.9205811 | 0 |
| 3215.9217835 | 0 |
| 3215.9218302 | 0 |
| 3215.9218512 | 1 |
| 3215.9228011 | 1 |
| 3215.9235693 | 1 |
| 3215.923922 | 1 |
| 3215.9245175 | 0 |
| 3215.9250116 | 2 |
| 3215.9252845 | 1 |
| 3215.9259782 | 0 |
| 3215.9260874 | 3 |
| 3215.9264622 | 0 |
| 3215.926692 | 2 |
| 3215.9275056 | 1 |
| 3215.9277071 | 4 |
| 3215.9287218 | 1 |
| 3215.9291335 | 2 |
| 3215.9292231 | 0 |
| 3215.9299653 | 0 |
| 3215.9308659 | 2 |
| 3215.9309913 | 3 |
| 3215.9310136 | 1 |
| 3215.931921 | 6 |
| 3215.9320507 | 0 |
| 3215.9328556 | 3 |
| 3215.9330018 | 1 |
| 3215.9335048 | 2 |
| 3215.9335844 | 1 |
| 3215.9347765 | 1 |
| 3215.9348813 | 1 |
| 3215.9354183 | 2 |
| 3215.9359419 | 0 |
| 3215.936258 | 1 |
| 3215.9365996 | 1 |
| 3215.9376727 | 0 |
| 3215.9377272 | 2 |
| 3215.9381478 | 0 |
| 3215.9384513 | 2 |
| 3215.9393824 | 1 |
| 3215.9394414 | 0 |
| 3215.9397771 | 1 |
| 3215.9402903 | 0 |
| 3215.9408208 | 4 |
| 3215.9415319 | 1 |
| 3215.9418227 | 1 |
| 3215.9419381 | 1 |
| 3215.9431256 | 1 |
| 3215.9434377 | 0 |
| 3215.9437182 | 2 |
| 3215.9440803 | 1 |
| 3215.9444208 | 0 |
| 3215.9451432 | 2 |
| 3215.9451443 | 0 |
| 3215.9459405 | 0 |
| 3215.9468439 | 0 |
| 3215.9468522 | 3 |
| 3215.9474153 | 1 |
| 3215.9477133 | 4 |
| 3215.9482236 | 2 |
| 3215.9485975 | 1 |
| 3215.9490074 | 3 |
| 3215.9494381 | 2 |
| 3215.9496816 | 3 |
| 3215.950606 | 1 |
| 3215.9513519 | 0 |
| 3215.9517612 | 1 |
| 3215.9523296 | 2 |
| 3215.952389 | 0 |
| 3215.9526533 | 3 |
| 3215.9532213 | 2 |
| 3215.9539623 | 1 |
| 3215.9541074 | 3 |
| 3215.954864 | 2 |
| 3215.9554492 | 0 |
| 3215.9559611 | 1 |
| 3215.9562648 | 1 |
| 3215.9565802 | 3 |
| 3215.9572229 | 0 |
| 3215.9573742 | 0 |
| 3215.9583306 | 0 |
| 3215.9587965 | 2 |
| 3215.9590324 | 1 |
| 3215.9601383 | 1 |
| 3215.9601985 | 1 |
| 3215.9604703 | 2 |
| 3215.9608065 | 0 |
| 3215.9616126 | 2 |
| 3215.961776 | 0 |
| 3215.9622433 | 1 |
| 3215.9632095 | 2 |
| 3215.963369 | 0 |
| 3215.9638281 | 1 |
| 3215.9644326 | 0 |
| 3215.9648967 | 2 |
| 3215.9653135 | 1 |
| 3215.9656882 | 1 |
| 3215.9663229 | 2 |
| 3215.9668135 | 0 |
| 3215.9674436 | 1 |
| 3215.9676319 | 1 |
| 3215.9684945 | 2 |
| 3215.9685657 | 0 |
| 3215.9691478 | 1 |
| 3215.9693457 | 0 |
| 3215.9701632 | 0 |
| 3215.9702535 | 2 |
| 3215.9708877 | 2 |
| 3215.9717182 | 3 |
| 3215.9719705 | 0 |
| 3215.9720933 | 0 |
| 3215.9723289 | 0 |
| 3215.9730894 | 3 |
| 3215.9733622 | 3 |
| 3215.973721 | 2 |
| 3215.9741574 | 0 |
| 3215.97487 | 1 |
| 3215.9755968 | 0 |
| 3215.9768105 | 1 |
| 3215.9768885 | 1 |
| 3215.97689 | 1 |
| 3215.9771789 | 0 |
| 3215.9781502 | 2 |
| 3215.9784368 | 0 |
| 3215.9785997 | 2 |
| 3215.9787209 | 0 |
| 3215.9797915 | 2 |
| 3215.9800958 | 1 |
| 3215.9809614 | 0 |
| 3215.9809856 | 0 |
| 3215.9815105 | 0 |
| 3215.9818591 | 0 |
| 3215.9826181 | 2 |
| 3215.9831225 | 1 |
| 3215.9833207 | 1 |
| 3215.9834829 | 0 |
| 3215.9840347 | 0 |
| 3215.9847616 | 1 |
| 3215.9852474 | 0 |
| 3215.9855411 | 1 |
| 3215.9860069 | 1 |
| 3215.9864393 | 0 |
| 3215.9871002 | 2 |
| 3215.9873264 | 4 |
| 3215.9875501 | 0 |
| 3215.9880937 | 0 |
| 3215.9888668 | 2 |
| 3215.9890657 | 0 |
| 3215.9892443 | 0 |
| 3215.9895683 | 1 |
| 3215.9909065 | 4 |
| 3215.9909894 | 0 |
| 3215.9913264 | 3 |
| 3215.99176 | 0 |
| 3215.9923306 | 0 |
| 3215.9928141 | 0 |
| 3215.9936038 | 6 |
| 3215.9936724 | 0 |
| 3215.994178 | 2 |
| 3215.9943305 | 1 |
| 3215.9946725 | 2 |
| 3215.9954965 | 1 |
| 3215.9961556 | 2 |
| 3215.9970705 | 0 |
| 3215.9972643 | 0 |
| 3215.9973563 | 2 |
| 3215.9979569 | 1 |
| 3215.9985809 | 3 |
| 3215.998877 | 1 |
| 3215.9994313 | 1 |
| 3215.9998939 | 2 |
| 3216.0005608 | 0 |
| 3216.0010687 | 0 |
| 3216.0011796 | 0 |
| 3216.0013032 | 2 |
| 3216.0022231 | 1 |
| 3216.0029154 | 4 |
| 3216.0032882 | 0 |
| 3216.0038507 | 3 |
| 3216.0041894 | 0 |
| 3216.0047293 | 0 |
| 3216.0052553 | 0 |
| 3216.0057776 | 1 |
| 3216.0061087 | 0 |
| 3216.0065817 | 3 |
| 3216.0071059 | 0 |
| 3216.0076953 | 0 |
| 3216.0080232 | 0 |
| 3216.0081526 | 1 |
| 3216.008915 | 0 |
| 3216.0093108 | 2 |
| 3216.0099399 | 0 |
| 3216.0103034 | 1 |
| 3216.0106768 | 6 |
| 3216.0110107 | 1 |
| 3216.0116907 | 0 |
| 3216.0118136 | 1 |
| 3216.0129272 | 2 |
| 3216.0130174 | 3 |
| 3216.0143528 | 7 |
| 3216.0157289 | 2 |
| 3216.0160605 | 1 |
| 3216.0169496 | 3 |
| 3216.0178696 | 1 |
| 3216.0183108 | 2 |
| 3216.0197097 | 3 |
| 3216.0209069 | 0 |
| 3216.0217559 | 1 |
| 3216.0231336 | 4 |
| 3216.023658 | 2 |
| 3216.025203 | 4 |
| 3216.0254892 | 0 |
| 3216.0260318 | 4 |
| 3216.026822 | 2 |
| 3216.028612 | 1 |
| 3216.029268 | 2 |
| 3216.0304478 | 2 |
| 3216.0308275 | 1 |
| 3216.0320557 | 1 |
| 3216.0334115 | 0 |
| 3216.0344715 | 1 |
| 3216.034679 | 0 |
| 3216.0353186 | 2 |
| 3216.0362798 | 1 |
| 3216.0376173 | 3 |
| 3216.0381339 | 3 |
| 3216.0397535 | 0 |
| 3216.0400839 | 0 |
| 3216.0411118 | 1 |
| 3216.0423525 | 3 |
| 3216.0428319 | 2 |
| 3216.0440909 | 6 |
| 3216.0450676 | 1 |
| 3216.0451677 | 3 |
| 3216.0458953 | 0 |
| 3216.0479079 | 1 |
| 3216.0483221 | 1 |
| 3216.0496331 | 3 |
| 3216.0507459 | 2 |
| 3216.0513779 | 1 |
| 3216.0522223 | 1 |
| 3216.0533788 | 2 |
| 3216.0541892 | 1 |
| 3216.0552228 | 2 |
| 3216.0570273 | 1 |
| 3216.0574339 | 2 |
| 3216.0576111 | 2 |
| 3216.0579952 | 1 |
| 3216.058682 | 2 |
| 3216.0595648 | 3 |
| 3216.0597805 | 1 |
| 3216.0606545 | 5 |
| 3216.0610462 | 1 |
| 3216.0614215 | 1 |
| 3216.061851 | 3 |
| 3216.0630787 | 3 |
| 3216.0632036 | 1 |
| 3216.0636886 | 3 |
| 3216.0636981 | 2 |
| 3216.0646133 | 0 |
| 3216.0656703 | 1 |
| 3216.0658572 | 3 |
| 3216.0668154 | 1 |
| 3216.0670525 | 1 |
| 3216.0671713 | 2 |
| 3216.0687697 | 1 |
| 3216.0688382 | 0 |
| 3216.0695289 | 1 |
| 3216.0702188 | 6 |
| 3216.0708001 | 3 |
| 3216.0708511 | 1 |
| 3216.0713981 | 4 |
| 3216.0723645 | 2 |
| 3216.0723751 | 1 |
| 3216.072889 | 4 |
| 3216.0742381 | 3 |
| 3216.0747458 | 6 |
| 3216.0751337 | 1 |
| 3216.0754013 | 1 |
| 3216.0758181 | 7 |

| | |
|---|---|
| 3216.0766905 | 2 |
| 3216.0775468 | 3 |
| 3216.0776295 | 3 |
| 3216.0780654 | 1 |
| 3216.0788554 | 1 |
| 3216.0792746 | 4 |
| 3216.0799006 | 2 |
| 3216.0802833 | 1 |
| 3216.0811034 | 2 |
| 3216.081566 | 2 |
| 3216.0824931 | 0 |
| 3216.0825192 | 0 |
| 3216.0832974 | 2 |
| 3216.0840078 | 3 |
| 3216.0845072 | 0 |
| 3216.0846338 | 2 |
| 3216.0853597 | 4 |
| 3216.0864953 | 3 |
| 3216.0871017 | 0 |
| 3216.0875343 | 2 |
| 3216.0878041 | 1 |
| 3216.0883056 | 3 |
| 3216.0892575 | 2 |
| 3216.0895103 | 1 |
| 3216.0902145 | 3 |
| 3216.0913268 | 2 |
| 3216.0915675 | 0 |
| 3216.092089 | 2 |
| 3216.09209 | 2 |
| 3216.0923727 | 3 |
| 3216.093479 | 1 |
| 3216.0939684 | 2 |
| 3216.0942026 | 1 |
| 3216.0955936 | 6 |
| 3216.0956922 | 0 |
| 3216.0958028 | 1 |
| 3216.0960615 | 2 |
| 3216.0976373 | 0 |
| 3216.0980266 | 2 |
| 3216.0985283 | 2 |
| 3216.098963 | 3 |
| 3216.0993962 | 1 |
| 3216.1004354 | 1 |
| 3216.1004357 | 1 |
| 3216.1007986 | 2 |
| 3216.1015682 | 1 |
| 3216.1019127 | 0 |
| 3216.103417 | 1 |
| 3216.1039044 | 0 |
| 3216.1039868 | 1 |
| 3216.1041179 | 0 |
| 3216.1051987 | 0 |
| 3216.1058525 | 2 |
| 3216.1058686 | 3 |
| 3216.1068457 | 6 |
| 3216.1076466 | 4 |
| 3216.1078871 | 1 |
| 3216.1088376 | 3 |
| 3216.109058 | 2 |
| 3216.1096405 | 1 |
| 3216.1105391 | 4 |
| 3216.1107002 | 3 |
| 3216.1118159 | 0 |
| 3216.1120695 | 1 |
| 3216.1130478 | 0 |
| 3216.1132066 | 4 |
| 3216.1133119 | 3 |
| 3216.1141605 | 7 |
| 3216.1150288 | 2 |
| 3216.1151985 | 4 |
| 3216.1157869 | 2 |
| 3216.1162857 | 0 |
| 3216.1170695 | 3 |
| 3216.1172415 | 1 |
| 3216.1179619 | 3 |
| 3216.1190729 | 2 |
| 3216.1191321 | 1 |
| 3216.1198061 | 2 |
| 3216.1204864 | 1 |
| 3216.1211106 | 0 |
| 3216.1219632 | 2 |
| 3216.1223205 | 2 |
| 3216.1224643 | 3 |
| 3216.1229756 | 3 |
| 3216.1233439 | 5 |
| 3216.1241794 | 4 |
| 3216.124875 | 5 |
| 3216.1256191 | 1 |
| 3216.1257454 | 0 |
| 3216.1265949 | 1 |
| 3216.1268589 | 1 |
| 3216.1273987 | 0 |
| 3216.1283292 | 1 |
| 3216.1283508 | 4 |
| 3216.1288058 | 1 |
| 3216.1293515 | 2 |
| 3216.1302641 | 3 |
| 3216.1312163 | 2 |
| 3216.131221 | 1 |
| 3216.1315619 | 2 |
| 3216.1326958 | 1 |
| 3216.1331781 | 3 |
| 3216.1337087 | 2 |
| 3216.1338381 | 1 |
| 3216.1341864 | 1 |
| 3216.1354177 | 0 |
| 3216.1357216 | 1 |
| 3216.1357385 | 4 |
| 3216.1364999 | 0 |
| 3216.1367228 | 1 |
| 3216.13795 | 2 |
| 3216.1383113 | 1 |
| 3216.1391148 | 2 |
| 3216.1396022 | 2 |
| 3216.1401813 | 1 |
| 3216.1405022 | 2 |
| 3216.1412819 | 1 |
| 3216.1417746 | 0 |
| 3216.1425899 | 2 |
| 3216.1429801 | 3 |
| 3216.1432223 | 5 |
| 3216.1440779 | 1 |
| 3216.1447154 | 0 |
| 3216.1451376 | 2 |
| 3216.1453557 | 2 |
| 3216.1468554 | 3 |
| 3216.1468739 | 2 |
| 3216.1476346 | 0 |
| 3216.147657 | 1 |
| 3216.1483017 | 1 |
| 3216.1493528 | 2 |
| 3216.1496546 | 1 |
| 3216.1500398 | 1 |
| 3216.1503755 | 1 |
| 3216.1509426 | 2 |
| 3216.1517228 | 1 |
| 3216.1520678 | 2 |
| 3216.1534015 | 3 |
| 3216.154312 | 5 |
| 3216.154378 | 3 |
| 3216.1548946 | 0 |
| 3216.1552661 | 1 |
| 3216.156288 | 1 |
| 3216.1567269 | 1 |
| 3216.1574515 | 2 |
| 3216.1579017 | 2 |
| 3216.1581531 | 1 |
| 3216.159054 | 4 |
| 3216.1597958 | 2 |
| 3216.1600074 | 5 |
| 3216.1612259 | 2 |
| 3216.1612409 | 1 |
| 3216.1615356 | 1 |
| 3216.1626268 | 1 |
| 3216.1627455 | 5 |
| 3216.162992 | 1 |
| 3216.1637264 | 2 |
| 3216.1639929 | 3 |
| 3216.1647319 | 0 |
| 3216.1654146 | 4 |
| 3216.1661704 | 3 |
| 3216.1667689 | 1 |
| 3216.166849 | 0 |
| 3216.1682704 | 8 |
| 3216.1686102 | 3 |
| 3216.1686982 | 3 |
| 3216.1694518 | 1 |
| 3216.1698095 | 0 |
| 3216.1707691 | 2 |
| 3216.1711944 | 4 |
| 3216.1716089 | 1 |
| 3216.1720477 | 0 |
| 3216.1726921 | 3 |
| 3216.1733509 | 2 |
| 3216.1740427 | 2 |
| 3216.1743959 | 2 |
| 3216.174718 | 0 |
| 3216.1750498 | 3 |
| 3216.1755205 | 4 |
| 3216.1763514 | 3 |
| 3216.1775563 | 2 |
| 3216.1779773 | 1 |
| 3216.178194 | 2 |
| 3216.1785934 | 2 |
| 3216.1787679 | 2 |
| 3216.1792693 | 1 |
| 3216.1802523 | 3 |
| 3216.1809044 | 1 |
| 3216.1816043 | 0 |
| 3216.1817444 | 1 |
| 3216.1827845 | 2 |
| 3216.1832925 | 1 |
| 3216.1835706 | 2 |
| 3216.1841678 | 2 |
| 3216.1845505 | 5 |
| 3216.1855817 | 2 |
| 3216.1859243 | 0 |
| 3216.1867864 | 1 |
| 3216.1871236 | 2 |
| 3216.1872361 | 2 |
| 3216.1879347 | 2 |
| 3216.1890553 | 1 |
| 3216.18911 | 1 |
| 3216.1897503 | 3 |
| 3216.1901683 | 4 |
| 3216.1910675 | 0 |
| 3216.1923849 | 3 |
| 3216.192459 | 2 |
| 3216.1927604 | 2 |
| 3216.1932841 | 0 |
| 3216.1939617 | 4 |
| 3216.1948374 | 0 |
| 3216.1950781 | 3 |
| 3216.1960568 | 2 |
| 3216.1963391 | 2 |
| 3216.1969153 | 4 |
| 3216.1969337 | 2 |
| 3216.1977406 | 3 |
| 3216.1981793 | 1 |
| 3216.1982437 | 3 |
| 3216.1991534 | 1 |
| 3216.1999737 | 1 |
| 3216.2007374 | 2 |
| 3216.2012936 | 0 |
| 3216.2013665 | 1 |
| 3216.2026268 | 2 |
| 3216.2028874 | 4 |
| 3216.2033777 | 3 |
| 3216.2044098 | 1 |
| 3216.2044459 | 0 |
| 3216.2049392 | 1 |
| 3216.2061098 | 1 |
| 3216.206144 | 3 |
| 3216.2072236 | 2 |
| 3216.2073428 | 5 |
| 3216.207685 | 1 |
| 3216.2086442 | 1 |
| 3216.2092569 | 2 |
| 3216.2094654 | 2 |
| 3216.2104088 | 0 |
| 3216.2108757 | 4 |
| 3216.2110493 | 2 |
| 3216.2114957 | 4 |
| 3216.2126933 | 4 |
| 3216.2131201 | 3 |
| 3216.213831 | 0 |
| 3216.2141296 | 2 |
| 3216.2141507 | 0 |
| 3216.214854 | 2 |
| 3216.2162237 | 1 |
| 3216.2165358 | 3 |
| 3216.2171448 | 1 |
| 3216.2174554 | 1 |
| 3216.2184375 | 1 |
| 3216.2186678 | 1 |
| 3216.2186862 | 2 |
| 3216.2195478 | 1 |
| 3216.2203632 | 1 |
| 3216.220882 | 2 |
| 3216.2210285 | 4 |
| 3216.2212217 | 1 |
| 3216.2226327 | 3 |
| 3216.2227587 | 3 |
| 3216.2230844 | 1 |
| 3216.2235216 | 1 |
| 3216.223766 | 1 |
| 3216.2253125 | 3 |
| 3216.2253315 | 0 |
| 3216.2264008 | 3 |
| 3216.2266411 | 0 |
| 3216.2269712 | 3 |
| 3216.2281759 | 2 |
| 3216.2282523 | 3 |
| 3216.2290576 | 2 |
| 3216.2295929 | 3 |
| 3216.2302362 | 1 |
| 3216.2305201 | 3 |
| 3216.2307454 | 2 |
| 3216.2315291 | 0 |
| 3216.2324732 | 3 |
| 3216.2333016 | 0 |
| 3216.2335599 | 2 |
| 3216.2343267 | 2 |
| 3216.2348863 | 1 |
| 3216.2351404 | 2 |
| 3216.2354596 | 3 |
| 3216.2363217 | 0 |
| 3216.2367 | 0 |
| 3216.23727 | 2 |
| 3216.237331 | 2 |
| 3216.2385531 | 4 |
| 3216.2393196 | 2 |
| 3216.239972 | 3 |
| 3216.2404466 | 1 |
| 3216.2409112 | 3 |
| 3216.2415428 | 2 |
| 3216.241592 | 2 |
| 3216.2418429 | 3 |
| 3216.2433242 | 0 |
| 3216.243776 | 2 |
| 3216.2443703 | 2 |
| 3216.2445608 | 0 |
| 3216.2449956 | 3 |
| 3216.2461757 | 2 |
| 3216.2462841 | 4 |
| 3216.2464528 | 3 |
| 3216.2478668 | 1 |
| 3216.2483503 | 1 |
| 3216.2485347 | 0 |
| 3216.2493184 | 3 |
| 3216.2496011 | 2 |
| 3216.2507992 | 1 |
| 3216.2511551 | 4 |
| 3216.2515121 | 2 |
| 3216.2523595 | 1 |
| 3216.2528354 | 4 |
| 3216.2529707 | 2 |
| 3216.2536425 | 3 |
| 3216.2546456 | 2 |
| 3216.2554494 | 1 |
| 3216.2555463 | 1 |
| 3216.2559804 | 2 |
| 3216.256855 | 1 |
| 3216.2573954 | 1 |
| 3216.2574994 | 0 |
| 3216.2586575 | 4 |
| 3216.2588204 | 3 |
| 3216.2590207 | 4 |
| 3216.2598916 | 0 |
| 3216.2607166 | 1 |
| 3216.2608974 | 1 |
| 3216.2618351 | 1 |
| 3216.2622362 | 1 |
| 3216.2632199 | 5 |
| 3216.2633715 | 2 |
| 3216.2636609 | 3 |
| 3216.2650181 | 3 |
| 3216.2652595 | 0 |
| 3216.2654701 | 0 |
| 3216.2663402 | 4 |
| 3216.2667594 | 2 |
| 3216.2673043 | 0 |
| 3216.2681482 | 0 |
| 3216.2682326 | 5 |
| 3216.2686207 | 5 |
| 3216.2688136 | 1 |
| 3216.2693399 | 1 |
| 3216.2698968 | 1 |
| 3216.2709716 | 3 |
| 3216.2721143 | 0 |
| 3216.2724612 | 3 |
| 3216.2725713 | 0 |
| 3216.2732798 | 1 |
| 3216.2736789 | 0 |
| 3216.2738387 | 0 |
| 3216.2745958 | 4 |
| 3216.2748991 | 4 |
| 3216.275732 | 2 |
| 3216.2764872 | 5 |
| 3216.276847 | 2 |
| 3216.2772347 | 0 |
| 3216.2784006 | 1 |
| 3216.2789794 | 4 |

| | |
|---|---|
| 3216.2791869 | 0 |
| 3216.2795026 | 1 |
| 3216.2799074 | 1 |
| 3216.2808967 | 2 |
| 3216.2819008 | 1 |
| 3216.2819699 | 4 |
| 3216.282923 | 3 |
| 3216.283459 | 2 |
| 3216.2835904 | 2 |
| 3216.2842639 | 0 |
| 3216.2846642 | 3 |
| 3216.2847348 | 1 |
| 3216.2858468 | 1 |
| 3216.2868199 | 0 |
| 3216.2888012 | 2 |
| 3216.2905895 | 1 |
| 3216.29102 | 3 |
| 3216.293414 | 0 |
| 3216.2940264 | 4 |
| 3216.2959106 | 3 |
| 3216.2963267 | 4 |
| 3216.2991706 | 3 |
| 3216.3008607 | 3 |
| 3216.301385 | 1 |
| 3216.3027881 | 2 |
| 3216.304529 | 2 |
| 3216.3054812 | 0 |
| 3216.3082051 | 5 |
| 3216.3085356 | 2 |
| 3216.3100177 | 1 |
| 3216.3109834 | 2 |
| 3216.3126476 | 1 |
| 3216.3143063 | 4 |
| 3216.3152277 | 0 |
| 3216.3166208 | 2 |
| 3216.3180986 | 1 |
| 3216.3194545 | 1 |
| 3216.3218694 | 2 |
| 3216.3226101 | 1 |
| 3216.3239992 | 1 |
| 3216.3246936 | 1 |
| 3216.3261029 | 3 |
| 3216.3279692 | 3 |
| 3216.3294117 | 1 |
| 3216.331149 | 2 |
| 3216.3320395 | 2 |
| 3216.3341923 | 3 |
| 3216.3350014 | 1 |
| 3216.3366215 | 4 |
| 3216.3384228 | 5 |
| 3216.3391125 | 0 |
| 3216.3413941 | 1 |
| 3216.3416798 | 0 |
| 3216.3433946 | 2 |
| 3216.3455973 | 3 |
| 3216.3462987 | 1 |
| 3216.3485093 | 3 |
| 3216.349538 | 2 |
| 3216.3505316 | 2 |
| 3216.3527128 | 5 |
| 3216.3529609 | 2 |
| 3216.3550421 | 0 |
| 3216.3567666 | 2 |
| 3216.3577529 | 1 |
| 3216.3592116 | 1 |
| 3216.3609241 | 2 |
| 3216.3621814 | 2 |
| 3216.3627259 | 5 |
| 3216.3652049 | 1 |
| 3216.3668208 | 1 |
| 3216.3674088 | 1 |
| 3216.3688287 | 0 |
| 3216.3703268 | 5 |
| 3216.3715172 | 2 |
| 3216.3738778 | 0 |
| 3216.3744917 | 4 |
| 3216.3764589 | 7 |
| 3216.3768559 | 4 |
| 3216.3785371 | 3 |
| 3216.3803233 | 3 |
| 3216.3816223 | 2 |
| 3216.3829237 | 4 |
| 3216.3845364 | 4 |
| 3216.3859749 | 5 |
| 3216.3878017 | 4 |
| 3216.3886378 | 0 |
| 3216.3907686 | 1 |
| 3216.3915191 | 2 |
| 3216.3927513 | 4 |
| 3216.3944289 | 3 |
| 3216.3956957 | 1 |
| 3216.3972523 | 4 |
| 3216.398299 | 2 |
| 3216.3997919 | 1 |
| 3216.4010697 | 3 |
| 3216.4027677 | 4 |
| 3216.4044757 | 2 |
| 3216.4050825 | 2 |
| 3216.4064167 | 1 |
| 3216.4086289 | 1 |
| 3216.4087214 | 2 |
| 3216.4105016 | 3 |
| 3216.4121947 | 5 |
| 3216.413881 | 0 |
| 3216.4157419 | 1 |
| 3216.4170196 | 4 |
| 3216.4177704 | 1 |
| 3216.4185676 | 3 |
| 3216.420743 | 3 |
| 3216.4226885 | 1 |
| 3216.4229439 | 2 |
| 3216.4252079 | 2 |
| 3216.4259689 | 0 |
| 3216.4272256 | 7 |
| 3216.4283994 | 0 |
| 3216.4303228 | 0 |
| 3216.4321385 | 3 |
| 3216.4332021 | 1 |
| 3216.4349777 | 1 |
| 3216.4363153 | 2 |
| 3216.438874 | 2 |
| 3216.4396734 | 1 |
| 3216.4405551 | 3 |
| 3216.4421169 | 2 |
| 3216.4437401 | 4 |
| 3216.4443042 | 1 |
| 3216.4462359 | 3 |
| 3216.4471719 | 0 |
| 3216.4489743 | 1 |
| 3216.4498011 | 0 |
| 3216.4511336 | 0 |
| 3216.4527724 | 1 |
| 3216.4537649 | 1 |
| 3216.4558183 | 5 |
| 3216.4570756 | 3 |
| 3216.4579373 | 1 |
| 3216.4602531 | 0 |
| 3216.4609471 | 1 |
| 3216.4620659 | 1 |
| 3216.4632611 | 4 |
| 3216.4653488 | 1 |
| 3216.4663348 | 1 |
| 3216.4675268 | 2 |
| 3216.4700612 | 2 |
| 3216.4704983 | 1 |
| 3216.4725477 | 2 |
| 3216.4728033 | 3 |
| 3216.4742581 | 5 |
| 3216.4769878 | 2 |
| 3216.4777069 | 1 |
| 3216.4791414 | 2 |
| 3216.4809838 | 2 |
| 3216.4823202 | 1 |
| 3216.4838687 | 2 |
| 3216.4847222 | 1 |
| 3216.4867457 | 0 |
| 3216.4874181 | 4 |
| 3216.4887093 | 0 |
| 3216.490809 | 0 |
| 3216.4913523 | 4 |
| 3216.493795 | 2 |
| 3216.4944648 | 2 |
| 3216.4957253 | 3 |
| 3216.498111 | 2 |
| 3216.4981381 | 2 |
| 3216.4999452 | 2 |
| 3216.5013553 | 3 |
| 3216.5030643 | 4 |
| 3216.5043842 | 1 |
| 3216.5051106 | 1 |
| 3216.5072434 | 0 |
| 3216.5079318 | 0 |
| 3216.5099201 | 2 |
| 3216.5107413 | 1 |
| 3216.5129077 | 2 |
| 3216.5139466 | 4 |
| 3216.5149537 | 0 |
| 3216.5162222 | 2 |
| 3216.5178471 | 2 |
| 3216.5190445 | 2 |
| 3216.5208587 | 2 |
| 3216.5223236 | 1 |
| 3216.5239918 | 1 |
| 3216.5256199 | 3 |
| 3216.5263524 | 1 |
| 3216.528107 | 0 |
| 3216.529057 | 5 |
| 3216.5314766 | 8 |
| 3216.5328168 | 5 |
| 3216.5330967 | 1 |
| 3216.5353295 | 3 |
| 3216.5357176 | 2 |
| 3216.5377918 | 0 |
| 3216.538918 | 1 |
| 3216.5407442 | 1 |
| 3216.5421985 | 3 |
| 3216.5430379 | 3 |
| 3216.5445106 | 1 |
| 3216.5453229 | 1 |
| 3216.5472429 | 2 |
| 3216.5484966 | 1 |
| 3216.5498744 | 0 |
| 3216.5515272 | 4 |
| 3216.5520881 | 3 |
| 3216.5536849 | 2 |
| 3216.5560145 | 2 |
| 3216.5564358 | 4 |
| 3216.5583374 | 1 |
| 3216.5599415 | 2 |
| 3216.5612539 | 3 |
| 3216.5627793 | 0 |
| 3216.5635324 | 2 |
| 3216.5658708 | 0 |
| 3216.5661377 | 2 |
| 3216.5679663 | 5 |
| 3216.5700889 | 1 |
| 3216.5709953 | 2 |
| 3216.5713778 | 2 |
| 3216.5730169 | 2 |
| 3216.5756102 | 0 |
| 3216.5771724 | 0 |
| 3216.5778326 | 2 |
| 3216.5800098 | 3 |
| 3216.5805221 | 2 |
| 3216.5824058 | 0 |
| 3216.583468 | 2 |
| 3216.5850776 | 4 |
| 3216.5862417 | 4 |
| 3216.5870465 | 4 |
| 3216.5892408 | 2 |
| 3216.5894055 | 3 |
| 3216.5913584 | 1 |
| 3216.5930205 | 2 |
| 3216.594151 | 3 |
| 3216.5953122 | 2 |
| 3216.5961886 | 0 |
| 3216.5978539 | 2 |
| 3216.6001451 | 0 |
| 3216.6011793 | 2 |
| 3216.6026939 | 6 |
| 3216.6035336 | 4 |
| 3216.6055906 | 1 |
| 3216.6077229 | 3 |
| 3216.6077894 | 2 |
| 3216.6096393 | 5 |
| 3216.6115134 | 3 |
| 3216.6121751 | 2 |
| 3216.6139009 | 3 |
| 3216.6148878 | 2 |
| 3216.6171751 | 0 |
| 3216.6178896 | 0 |
| 3216.6193907 | 1 |
| 3216.6207004 | 0 |
| 3216.6220513 | 2 |
| 3216.6237916 | 1 |
| 3216.6246922 | 4 |
| 3216.6268553 | 1 |
| 3216.6281937 | 3 |
| 3216.628665 | 3 |
| 3216.6310812 | 4 |
| 3216.6313526 | 1 |
| 3216.6331461 | 0 |
| 3216.6351726 | 2 |
| 3216.635548 | 3 |
| 3216.637472 | 4 |
| 3216.6384499 | 2 |
| 3216.6396858 | 4 |
| 3216.641825 | 1 |
| 3216.6424633 | 1 |
| 3216.6440545 | 1 |
| 3216.6460272 | 0 |
| 3216.6469342 | 1 |
| 3216.6487253 | 1 |
| 3216.6495994 | 2 |
| 3216.6506259 | 1 |
| 3216.6515364 | 0 |
| 3216.6537341 | 5 |
| 3216.6556788 | 2 |
| 3216.6565244 | 2 |
| 3216.658689 | 4 |
| 3216.6590774 | 0 |
| 3216.6608601 | 1 |
| 3216.6621287 | 2 |
| 3216.6634598 | 1 |
| 3216.6648671 | 2 |
| 3216.6661788 | 1 |
| 3216.6677063 | 3 |
| 3216.6695352 | 4 |
| 3216.6701012 | 2 |
| 3216.6717551 | 2 |
| 3216.6745573 | 1 |
| 3216.6750316 | 1 |
| 3216.6766672 | 1 |
| 3216.6768254 | 0 |
| 3216.6788008 | 4 |
| 3216.680752 | 2 |
| 3216.6812001 | 1 |
| 3216.6830743 | 2 |
| 3216.6838609 | 3 |
| 3216.6861607 | 0 |
| 3216.6863564 | 4 |
| 3216.688499 | 0 |
| 3216.6900233 | 2 |
| 3216.6910622 | 3 |
| 3216.6925321 | 3 |
| 3216.6931282 | 2 |
| 3216.6952501 | 2 |
| 3216.6970482 | 0 |
| 3216.6975902 | 5 |
| 3216.6992517 | 0 |
| 3216.7010068 | 4 |
| 3216.7024572 | 6 |
| 3216.7037526 | 1 |
| 3216.7046992 | 4 |
| 3216.7069181 | 3 |
| 3216.7089359 | 2 |
| 3216.7094805 | 1 |
| 3216.7110847 | 2 |
| 3216.7120536 | 1 |
| 3216.7142259 | 1 |
| 3216.7156611 | 5 |
| 3216.7164778 | 2 |
| 3216.7180134 | 1 |
| 3216.718573 | 1 |
| 3216.7187227 | 2 |
| 3216.7195754 | 3 |
| 3216.7200904 | 2 |
| 3216.7203549 | 2 |
| 3216.7207139 | 1 |
| 3216.7212869 | 0 |
| 3216.7216495 | 2 |
| 3216.7220518 | 5 |
| 3216.7226567 | 1 |
| 3216.7234387 | 1 |
| 3216.7235211 | 5 |
| 3216.7235569 | 2 |
| 3216.7245163 | 2 |
| 3216.7246246 | 2 |
| 3216.7249227 | 3 |
| 3216.7253343 | 1 |
| 3216.7257471 | 4 |
| 3216.7259244 | 4 |
| 3216.7265231 | 0 |
| 3216.7271343 | 0 |
| 3216.7272235 | 1 |
| 3216.7279431 | 1 |
| 3216.7283932 | 2 |
| 3216.728679 | 1 |
| 3216.7293266 | 1 |
| 3216.7296176 | 3 |
| 3216.7297412 | 0 |
| 3216.7304647 | 0 |
| 3216.7309392 | 3 |
| 3216.7316537 | 1 |
| 3216.7317036 | 6 |
| 3216.7320035 | 0 |
| 3216.7328714 | 1 |
| 3216.7330012 | 3 |
| 3216.7335188 | 1 |
| 3216.7335553 | 0 |
| 3216.7345313 | 4 |
| 3216.7348388 | 3 |
| 3216.7348875 | 2 |

| | |
|---|---|
| 3216.735135 | 1 |
| 3216.7358014 | 2 |
| 3216.7364972 | 1 |
| 3216.7366829 | 1 |
| 3216.7369518 | 2 |
| 3216.7379015 | 0 |
| 3216.7380145 | 4 |
| 3216.7383452 | 2 |
| 3216.7385236 | 2 |
| 3216.738953 | 2 |
| 3216.7392471 | 4 |
| 3216.7398734 | 2 |
| 3216.7406719 | 3 |
| 3216.7413448 | 1 |
| 3216.741796 | 1 |
| 3216.7419094 | 4 |
| 3216.741987 | 4 |
| 3216.7427519 | 2 |
| 3216.7436104 | 2 |
| 3216.7438943 | 2 |
| 3216.7438951 | 4 |
| 3216.7440361 | 0 |
| 3216.7450594 | 2 |
| 3216.7451816 | 2 |
| 3216.7458501 | 4 |
| 3216.746363 | 1 |
| 3216.746622 | 1 |
| 3216.7468324 | 1 |
| 3216.7475186 | 2 |
| 3216.7479822 | 0 |
| 3216.7485419 | 3 |
| 3216.7489754 | 2 |
| 3216.7491797 | 3 |
| 3216.7496686 | 2 |
| 3216.7499184 | 8 |
| 3216.7507505 | 0 |
| 3216.75107 | 6 |
| 3216.7514274 | 2 |
| 3216.7521936 | 4 |
| 3216.7527978 | 1 |
| 3216.752847 | 2 |
| 3216.7532192 | 4 |
| 3216.7533433 | 1 |
| 3216.7537361 | 5 |
| 3216.7547209 | 1 |
| 3216.755276 | 1 |
| 3216.7556272 | 0 |
| 3216.7558032 | 2 |
| 3216.7563542 | 2 |
| 3216.7567251 | 3 |
| 3216.7573195 | 0 |
| 3216.7577984 | 1 |
| 3216.7586216 | 1 |
| 3216.7586791 | 4 |
| 3216.7589112 | 0 |
| 3216.759185 | 1 |
| 3216.7597724 | 1 |
| 3216.760188 | 3 |
| 3216.7607821 | 2 |
| 3216.7608168 | 1 |
| 3216.7612433 | 3 |
| 3216.762501 | 1 |
| 3216.7625031 | 3 |
| 3216.7631724 | 3 |
| 3216.7632606 | 2 |
| 3216.763481 | 3 |
| 3216.7635073 | 2 |
| 3216.7647211 | 2 |
| 3216.7655838 | 4 |
| 3216.7658311 | 1 |
| 3216.7658367 | 3 |
| 3216.7661427 | 4 |
| 3216.7662099 | 1 |
| 3216.767229 | 1 |
| 3216.7673011 | 1 |
| 3216.7678748 | 1 |
| 3216.7682869 | 1 |
| 3216.7689284 | 3 |
| 3216.7695341 | 6 |
| 3216.7699417 | 2 |
| 3216.7700143 | 2 |
| 3216.7700651 | 3 |
| 3216.7702803 | 3 |
| 3216.7712805 | 0 |
| 3216.7717646 | 3 |
| 3216.7719154 | 5 |
| 3216.7721369 | 2 |
| 3216.772791 | 2 |
| 3216.773326 | 1 |
| 3216.7735318 | 3 |
| 3216.7736482 | 2 |
| 3216.7743222 | 0 |
| 3216.7747047 | 1 |
| 3216.7752079 | 1 |
| 3216.7755691 | 1 |
| 3216.7759096 | 2 |
| 3216.7761058 | 1 |
| 3216.77636 | 4 |
| 3216.7765887 | 3 |
| 3216.7768685 | 2 |
| 3216.7772221 | 3 |
| 3216.7778879 | 2 |
| 3216.7780554 | 1 |
| 3216.7783243 | 3 |
| 3216.7783615 | 2 |
| 3216.7785578 | 3 |
| 3216.7791221 | 2 |
| 3216.7791274 | 3 |
| 3216.7791916 | 5 |
| 3216.779287 | 1 |
| 3216.7800473 | 5 |
| 3216.7805902 | 1 |
| 3216.7813923 | 1 |
| 3216.7816542 | 1 |
| 3216.7821362 | 4 |
| 3216.7828187 | 1 |
| 3216.7841749 | 1 |
| 3216.7845369 | 0 |
| 3216.7852886 | 2 |
| 3216.7854845 | 0 |
| 3216.7858416 | 0 |
| 3216.7872913 | 2 |
| 3216.7876054 | 2 |
| 3216.7879079 | 1 |
| 3216.7888929 | 0 |
| 3216.7894616 | 1 |
| 3216.7902698 | 5 |
| 3216.7905241 | 2 |
| 3216.790785 | 2 |
| 3216.7920378 | 1 |
| 3216.7922836 | 3 |
| 3216.7936156 | 2 |
| 3216.7937698 | 1 |
| 3216.794348 | 1 |
| 3216.7955371 | 1 |
| 3216.7957831 | 2 |
| 3216.7964801 | 4 |
| 3216.796978 | 2 |
| 3216.7976271 | 1 |
| 3216.7986951 | 1 |
| 3216.7991804 | 1 |
| 3216.7993096 | 2 |
| 3216.8006107 | 1 |
| 3216.8013884 | 2 |
| 3216.8018764 | 1 |
| 3216.802346 | 4 |
| 3216.8032057 | 3 |
| 3216.8033979 | 3 |
| 3216.8045586 | 2 |
| 3216.8048858 | 5 |
| 3216.8055335 | 2 |
| 3216.8063761 | 1 |
| 3216.8066207 | 0 |
| 3216.8066505 | 2 |
| 3216.8082973 | 2 |
| 3216.8083728 | 2 |
| 3216.8093927 | 1 |
| 3216.8094858 | 2 |
| 3216.8108088 | 1 |
| 3216.8112457 | 2 |
| 3216.8118096 | 2 |
| 3216.8125082 | 3 |
| 3216.8128206 | 1 |
| 3216.8132358 | 2 |
| 3216.8147171 | 1 |
| 3216.814763 | 2 |
| 3216.8155235 | 0 |
| 3216.8164595 | 1 |
| 3216.8165362 | 1 |
| 3216.8169564 | 4 |
| 3216.8180587 | 0 |
| 3216.8180755 | 2 |
| 3216.8195011 | 3 |
| 3216.8199749 | 0 |
| 3216.8201292 | 1 |
| 3216.821024 | 4 |
| 3216.8215744 | 2 |
| 3216.8223025 | 1 |
| 3216.8228207 | 1 |
| 3216.8232497 | 0 |
| 3216.8232872 | 4 |
| 3216.8242535 | 0 |
| 3216.8247351 | 4 |
| 3216.8262674 | 1 |
| 3216.8264132 | 2 |
| 3216.826961 | 2 |
| 3216.8280222 | 3 |
| 3216.8285463 | 4 |
| 3216.8293381 | 1 |
| 3216.8297136 | 2 |
| 3216.8306273 | 3 |
| 3216.8310115 | 1 |
| 3216.8315599 | 0 |
| 3216.8316631 | 1 |
| 3216.8328555 | 1 |
| 3216.8335305 | 2 |
| 3216.8342714 | 2 |
| 3216.8346375 | 4 |
| 3216.8353026 | 3 |
| 3216.8358906 | 2 |
| 3216.836389 | 4 |
| 3216.8371439 | 0 |
| 3216.8377908 | 3 |
| 3216.838699 | 2 |
| 3216.8389759 | 1 |
| 3216.8399207 | 5 |
| 3216.8402842 | 1 |
| 3216.8415508 | 3 |
| 3216.8417495 | 1 |
| 3216.8425995 | 3 |
| 3216.8425997 | 4 |
| 3216.8437021 | 0 |
| 3216.8446039 | 0 |
| 3216.8449543 | 5 |
| 3216.8449553 | 2 |
| 3216.8453986 | 4 |
| 3216.8464065 | 2 |
| 3216.8465394 | 5 |
| 3216.8466434 | 0 |
| 3216.8476092 | 3 |
| 3216.8480499 | 3 |
| 3216.8482018 | 1 |
| 3216.8486642 | 1 |
| 3216.8527788 | 3 |
| 3216.8539521 | 2 |
| 3216.8542939 | 2 |
| 3216.8543139 | 3 |
| 3216.8548907 | 2 |
| 3216.8555498 | 2 |
| 3216.8561056 | 3 |
| 3216.8562612 | 4 |
| 3216.8567489 | 6 |
| 3216.8575335 | 1 |
| 3216.8579079 | 3 |
| 3216.8592363 | 4 |
| 3216.8593418 | 2 |
| 3216.8597963 | 0 |
| 3216.8607085 | 1 |
| 3216.8616743 | 2 |
| 3216.8618882 | 0 |
| 3216.8620871 | 5 |
| 3216.8632085 | 0 |
| 3216.8636944 | 2 |
| 3216.8640692 | 1 |
| 3216.8650405 | 1 |
| 3216.8655223 | 0 |
| 3216.8661547 | 2 |
| 3216.8667385 | 1 |
| 3216.8672294 | 1 |
| 3216.8679788 | 2 |
| 3216.8684331 | 6 |
| 3216.8690152 | 1 |
| 3216.8695379 | 3 |
| 3216.8705577 | 2 |
| 3216.8711498 | 2 |
| 3216.8718506 | 0 |
| 3216.8725606 | 1 |
| 3216.8727781 | 4 |
| 3216.8738646 | 0 |
| 3216.8740887 | 4 |
| 3216.874739 | 1 |
| 3216.8756302 | 0 |
| 3216.8758613 | 0 |
| 3216.8760903 | 1 |
| 3216.8770897 | 0 |
| 3216.8777788 | 3 |
| 3216.8783409 | 3 |
| 3216.8792926 | 0 |
| 3216.8802821 | 1 |
| 3216.8806786 | 0 |
| 3216.8809774 | 3 |
| 3216.8819239 | 2 |
| 3216.8827939 | 5 |
| 3216.883126 | 1 |
| 3216.8836133 | 3 |
| 3216.8838199 | 3 |
| 3216.8851025 | 2 |
| 3216.8852452 | 2 |
| 3216.8858598 | 2 |
| 3216.8869899 | 3 |
| 3216.8874734 | 2 |
| 3216.8881572 | 2 |
| 3216.8886612 | 6 |
| 3216.889429 | 3 |
| 3216.8899823 | 1 |
| 3216.8904727 | 1 |
| 3216.8913178 | 0 |
| 3216.8916238 | 0 |
| 3216.8923956 | 4 |
| 3216.8929551 | 1 |
| 3216.8929977 | 1 |
| 3216.8935696 | 1 |
| 3216.8947776 | 3 |
| 3216.8949225 | 2 |
| 3216.8960666 | 2 |
| 3216.8968245 | 2 |
| 3216.8970636 | 3 |
| 3216.8979545 | 2 |
| 3216.8980503 | 3 |
| 3216.8981582 | 3 |
| 3216.8997642 | 3 |
| 3216.8998179 | 2 |
| 3216.9003526 | 3 |
| 3216.9008495 | 2 |
| 3216.9020272 | 2 |
| 3216.9027481 | 0 |
| 3216.9031064 | 6 |
| 3216.9040771 | 3 |
| 3216.9042571 | 1 |
| 3216.9050619 | 1 |
| 3216.9052391 | 1 |
| 3216.9055728 | 2 |
| 3216.907047 | 1 |
| 3216.9073475 | 5 |
| 3216.9078794 | 2 |
| 3216.9089576 | 5 |
| 3216.9097397 | 1 |
| 3216.9097654 | 2 |
| 3216.9105123 | 0 |
| 3216.910948 | 3 |
| 3216.9116701 | 2 |
| 3216.9122462 | 0 |
| 3216.9128348 | 0 |
| 3216.9138119 | 1 |
| 3216.9143851 | 4 |
| 3216.9146492 | 1 |
| 3216.9155742 | 4 |
| 3216.9159666 | 1 |
| 3216.916755 | 3 |
| 3216.9175982 | 2 |
| 3216.9179467 | 4 |
| 3216.9184937 | 1 |
| 3216.9194885 | 0 |
| 3216.9196934 | 3 |
| 3216.9197502 | 2 |
| 3216.9211814 | 4 |
| 3216.9212377 | 3 |
| 3216.9221015 | 2 |
| 3216.9226287 | 1 |
| 3216.9236594 | 5 |
| 3216.9237862 | 3 |
| 3216.9250508 | 3 |
| 3216.9254571 | 2 |
| 3216.9258159 | 4 |
| 3216.9266424 | 3 |
| 3216.9270508 | 1 |
| 3216.9280685 | 3 |
| 3216.9284755 | 0 |
| 3216.9290118 | 2 |
| 3216.9300905 | 0 |
| 3216.9306154 | 0 |
| 3216.9313968 | 0 |
| 3216.9315421 | 1 |
| 3216.9319626 | 1 |
| 3216.93368 | 1 |
| 3216.9340394 | 3 |
| 3216.9346774 | 4 |
| 3216.9350554 | 0 |
| 3216.9361211 | 2 |
| 3216.9368294 | 2 |
| 3216.9368988 | 0 |
| 3216.9382526 | 3 |
| 3216.9384925 | 3 |

| | |
|---|---|
| 3216.9385875 | 3 |
| 3216.9393323 | 1 |
| 3216.9400657 | 2 |
| 3216.9410262 | 0 |
| 3216.9415421 | 2 |
| 3216.9419155 | 2 |
| 3216.9426008 | 1 |
| 3216.9435288 | 3 |
| 3216.9438382 | 0 |
| 3216.9441126 | 0 |
| 3216.9453191 | 4 |
| 3216.9459632 | 2 |
| 3216.9461234 | 1 |
| 3216.9474754 | 3 |
| 3216.9475362 | 4 |
| 3216.9481385 | 0 |
| 3216.9489338 | 1 |
| 3216.949768 | 1 |
| 3216.9500651 | 5 |
| 3216.9507391 | 1 |
| 3216.9514462 | 4 |
| 3216.9522997 | 2 |
| 3216.95236 | 3 |
| 3216.9529941 | 1 |
| 3216.953909 | 4 |
| 3216.9543787 | 3 |
| 3216.9544138 | 0 |
| 3216.9560622 | 1 |
| 3216.9564071 | 0 |
| 3216.9568759 | 3 |
| 3216.9572341 | 1 |
| 3216.957579 | 3 |
| 3216.9585982 | 2 |
| 3216.9589451 | 2 |
| 3216.9590538 | 2 |
| 3216.9600537 | 1 |
| 3216.9611876 | 1 |
| 3216.961648 | 1 |
| 3216.9622579 | 1 |
| 3216.9629738 | 1 |
| 3216.9636579 | 0 |
| 3216.9646679 | 1 |
| 3216.9653127 | 4 |
| 3216.9654543 | 2 |
| 3216.9659478 | 1 |
| 3216.9669101 | 3 |
| 3216.9671523 | 4 |
| 3216.9679642 | 3 |
| 3216.9682215 | 1 |
| 3216.9691519 | 0 |
| 3216.9699592 | 5 |
| 3216.970884 | 5 |
| 3216.9714759 | 0 |
| 3216.9720694 | 2 |
| 3216.9721116 | 1 |
| 3216.9729264 | 8 |
| 3216.9736316 | 3 |
| 3216.9748371 | 2 |
| 3216.9749642 | 4 |
| 3216.9757952 | 6 |
| 3216.9760614 | 3 |
| 3216.9769512 | 3 |
| 3216.9770936 | 4 |
| 3216.9783205 | 3 |
| 3216.9789156 | 2 |
| 3216.9796692 | 3 |
| 3216.980209 | 7 |
| 3216.9811066 | 2 |
| 3216.981709 | 2 |
| 3216.9817927 | 1 |
| 3216.9827166 | 1 |
| 3216.9828466 | 2 |
| 3216.9835933 | 1 |
| 3216.9839776 | 1 |
| 3216.9853862 | 3 |
| 3216.9857005 | 0 |
| 3216.9861136 | 1 |
| 3216.9870245 | 2 |
| 3216.9873934 | 1 |
| 3216.9878181 | 2 |
| 3216.9890393 | 3 |
| 3216.9892129 | 2 |
| 3216.9903449 | 1 |
| 3216.9904307 | 3 |
| 3216.9914806 | 2 |
| 3216.992467 | 0 |
| 3216.9927112 | 6 |
| 3216.9934668 | 1 |
| 3216.99373 | 2 |
| 3216.9941155 | 1 |
| 3216.9953258 | 0 |
| 3216.9954202 | 0 |
| 3216.9962973 | 4 |
| 3216.9966405 | 3 |
| 3216.9968765 | 2 |
| 3216.998248 | 2 |
| 3216.9982685 | 5 |
| 3216.9994884 | 3 |
| 3216.9995171 | 2 |
| 3217.00016 | 1 |
| 3217.001525 | 3 |
| 3217.0018799 | 3 |
| 3217.0024718 | 1 |
| 3217.0028938 | 3 |
| 3217.0038239 | 2 |
| 3217.0045028 | 2 |
| 3217.0047106 | 1 |
| 3217.0058828 | 2 |
| 3217.0059225 | 3 |
| 3217.0068819 | 1 |
| 3217.0073724 | 1 |
| 3217.0077599 | 2 |
| 3217.0088541 | 2 |
| 3217.0092146 | 0 |
| 3217.0097514 | 3 |
| 3217.0104356 | 1 |
| 3217.0113882 | 0 |
| 3217.011542 | 3 |
| 3217.0122396 | 3 |
| 3217.012955 | 0 |
| 3217.013471 | 2 |
| 3217.0137338 | 5 |
| 3217.0146271 | 7 |
| 3217.0148273 | 5 |
| 3217.016149 | 5 |
| 3217.0167964 | 5 |
| 3217.0169108 | 5 |
| 3217.0176807 | 9 |
| 3217.0186394 | 5 |
| 3217.0189221 | 5 |
| 3217.0197256 | 5 |
| 3217.020602 | 5 |
| 3217.0213077 | 4 |
| 3217.0213358 | 8 |
| 3217.0227141 | 3 |
| 3217.0228595 | 5 |
| 3217.0231049 | 6 |
| 3217.0244871 | 6 |
| 3217.0253778 | 6 |
| 3217.0262476 | 1 |
| 3217.0265159 | 2 |
| 3217.026919 | 1 |
| 3217.0275633 | 1 |
| 3217.0278548 | 1 |
| 3217.0285863 | 1 |
| 3217.0289365 | 0 |
| 3217.0298615 | 1 |
| 3217.0305731 | 0 |
| 3217.0310641 | 2 |
| 3217.0319062 | 2 |
| 3217.0320287 | 1 |
| 3217.0335559 | 2 |
| 3217.0336377 | 3 |
| 3217.0338236 | 2 |
| 3217.0345957 | 0 |
| 3217.0359115 | 0 |
| 3217.0360535 | 0 |
| 3217.0364597 | 6 |
| 3217.037565 | 6 |
| 3217.0382583 | 3 |
| 3217.0386229 | 3 |
| 3217.039789 | 3 |
| 3217.0400747 | 2 |
| 3217.0402147 | 1 |
| 3217.0409824 | 1 |
| 3217.0412214 | 1 |
| 3217.0421747 | 2 |
| 3217.0431054 | 3 |
| 3217.0434284 | 1 |
| 3217.0443863 | 4 |
| 3217.0447152 | 2 |
| 3217.0458519 | 4 |
| 3217.0459936 | 3 |
| 3217.0463767 | 7 |
| 3217.0470476 | 0 |
| 3217.0476205 | 1 |
| 3217.0482031 | 3 |
| 3217.0490384 | 2 |
| 3217.0498187 | 2 |
| 3217.0501451 | 1 |
| 3217.0504425 | 0 |
| 3217.0514912 | 0 |
| 3217.0519435 | 2 |
| 3217.0524798 | 6 |
| 3217.0534243 | 3 |
| 3217.0539768 | 3 |
| 3217.0543249 | 2 |
| 3217.0554662 | 3 |
| 3217.0558282 | 2 |
| 3217.0566291 | 1 |
| 3217.0567784 | 2 |
| 3217.0579719 | 2 |
| 3217.058418 | 2 |
| 3217.0588217 | 1 |
| 3217.059182 | 2 |
| 3217.0601726 | 6 |
| 3217.0610605 | 0 |
| 3217.0613167 | 1 |
| 3217.0620692 | 3 |
| 3217.0628495 | 3 |
| 3217.0629974 | 3 |
| 3217.0633111 | 1 |
| 3217.064291 | 1 |
| 3217.0649114 | 7 |
| 3217.0652313 | 1 |
| 3217.0665656 | 0 |
| 3217.0669797 | 2 |
| 3217.0675979 | 1 |
| 3217.0679201 | 2 |
| 3217.0687482 | 3 |
| 3217.069252 | 1 |
| 3217.0700947 | 3 |
| 3217.0708298 | 2 |
| 3217.0715189 | 2 |
| 3217.07178 | 1 |
| 3217.0730921 | 1 |
| 3217.0735516 | 1 |
| 3217.074085 | 4 |
| 3217.0743469 | 2 |
| 3217.0752592 | 1 |
| 3217.0756041 | 4 |
| 3217.0762112 | 2 |
| 3217.0769134 | 0 |
| 3217.0779996 | 3 |
| 3217.078579 | 1 |
| 3217.0791122 | 3 |
| 3217.0801614 | 3 |
| 3217.0801858 | 3 |
| 3217.0809456 | 1 |
| 3217.0812421 | 6 |
| 3217.0826828 | 4 |
| 3217.0827893 | 1 |
| 3217.0835603 | 5 |
| 3217.0840544 | 1 |
| 3217.0844303 | 1 |
| 3217.0846497 | 1 |
| 3217.085797 | 2 |
| 3217.0861259 | 1 |
| 3217.0869992 | 1 |
| 3217.087681 | 2 |
| 3217.0883874 | 3 |
| 3217.0885799 | 4 |
| 3217.0890646 | 0 |
| 3217.0902665 | 0 |
| 3217.0906398 | 2 |
| 3217.0911287 | 3 |
| 3217.0918385 | 1 |
| 3217.092228 | 2 |
| 3217.0927089 | 2 |
| 3217.0937166 | 1 |
| 3217.0940043 | 2 |
| 3217.0950215 | 1 |
| 3217.0951327 | 0 |
| 3217.0962509 | 0 |
| 3217.09651 | 1 |
| 3217.096775 | 2 |
| 3217.0980584 | 3 |
| 3217.0981193 | 1 |
| 3217.0992205 | 3 |
| 3217.0996093 | 1 |
| 3217.1005806 | 1 |
| 3217.1011532 | 2 |
| 3217.1013008 | 2 |
| 3217.1021426 | 2 |
| 3217.102525 | 2 |
| 3217.1028128 | 3 |
| 3217.1041867 | 3 |
| 3217.1050786 | 2 |
| 3217.1055685 | 2 |
| 3217.1056232 | 1 |
| 3217.1062834 | 1 |
| 3217.1071873 | 3 |
| 3217.1076214 | 3 |
| 3217.1083244 | 2 |
| 3217.108987 | 0 |
| 3217.1095115 | 0 |
| 3217.1105482 | 1 |
| 3217.1115242 | 2 |
| 3217.112258 | 0 |
| 3217.1122773 | 2 |
| 3217.1131123 | 5 |
| 3217.1133459 | 2 |
| 3217.1138079 | 0 |
| 3217.1149783 | 3 |
| 3217.1152442 | 2 |
| 3217.1164925 | 3 |
| 3217.1167025 | 4 |
| 3217.117259 | 0 |
| 3217.1182466 | 0 |
| 3217.118574 | 2 |
| 3217.1190561 | 3 |
| 3217.120114 | 4 |
| 3217.120462 | 1 |
| 3217.1212275 | 2 |
| 3217.1214866 | 2 |
| 3217.1227213 | 3 |
| 3217.1227945 | 3 |
| 3217.1240554 | 3 |
| 3217.1245733 | 2 |
| 3217.1246902 | 4 |
| 3217.1252216 | 1 |
| 3217.1260568 | 5 |
| 3217.1263852 | 2 |
| 3217.1274247 | 0 |
| 3217.1275669 | 3 |
| 3217.1288053 | 1 |
| 3217.1289891 | 1 |
| 3217.1298073 | 2 |
| 3217.1302927 | 5 |
| 3217.13114 | 2 |
| 3217.1314873 | 4 |
| 3217.1319865 | 1 |
| 3217.1332793 | 0 |
| 3217.1336564 | 3 |
| 3217.1339796 | 0 |
| 3217.1349718 | 5 |
| 3217.1350947 | 1 |
| 3217.1352299 | 4 |
| 3217.1360461 | 1 |
| 3217.1363808 | 1 |
| 3217.1380392 | 6 |
| 3217.1385718 | 2 |
| 3217.1390944 | 4 |
| 3217.1393358 | 2 |
| 3217.1404219 | 0 |
| 3217.1406327 | 1 |
| 3217.1410616 | 4 |
| 3217.1421658 | 0 |
| 3217.1425316 | 3 |
| 3217.1432343 | 2 |
| 3217.1437682 | 2 |
| 3217.144748 | 4 |
| 3217.1458434 | 1 |
| 3217.146079 | 1 |
| 3217.1461338 | 3 |
| 3217.1465143 | 1 |
| 3217.147405 | 1 |
| 3217.148066 | 1 |
| 3217.1485738 | 3 |
| 3217.1495609 | 1 |
| 3217.1499592 | 3 |
| 3217.1506267 | 1 |
| 3217.1516113 | 2 |
| 3217.1517754 | 2 |
| 3217.1530353 | 2 |
| 3217.1531733 | 2 |
| 3217.1541648 | 1 |
| 3217.1542102 | 2 |
| 3217.1550242 | 4 |
| 3217.1553165 | 1 |
| 3217.1560939 | 1 |
| 3217.1563639 | 1 |
| 3217.1578172 | 2 |
| 3217.1579075 | 4 |
| 3217.1584357 | 1 |
| 3217.1590738 | 2 |
| 3217.1601639 | 1 |
| 3217.1604244 | 1 |
| 3217.1614064 | 2 |
| 3217.1620966 | 3 |
| 3217.1627281 | 1 |
| 3217.1632366 | 5 |
| 3217.1637857 | 1 |

| | |
|---|---|
| 3217.1647552 | 3 |
| 3217.1652673 | 0 |
| 3217.1657842 | 0 |
| 3217.1666327 | 3 |
| 3217.1670167 | 0 |
| 3217.167588 | 1 |
| 3217.1678422 | 1 |
| 3217.1686709 | 1 |
| 3217.169656 | 3 |
| 3217.1698689 | 4 |
| 3217.1706361 | 1 |
| 3217.1713671 | 0 |
| 3217.1720213 | 1 |
| 3217.172469 | 2 |
| 3217.1732633 | 4 |
| 3217.1741631 | 2 |
| 3217.1744137 | 2 |
| 3217.1749748 | 1 |
| 3217.1754753 | 0 |
| 3217.1761825 | 2 |
| 3217.1770005 | 1 |
| 3217.1774223 | 2 |
| 3217.1777351 | 6 |
| 3217.1790486 | 1 |
| 3217.1792669 | 6 |
| 3217.1795699 | 0 |
| 3217.1806558 | 2 |
| 3217.1813032 | 1 |
| 3217.1820002 | 2 |
| 3217.1827242 | 1 |
| 3217.1831396 | 0 |
| 3217.1832405 | 0 |
| 3217.1833916 | 4 |
| 3217.1845368 | 1 |
| 3217.1851161 | 2 |
| 3217.186291 | 1 |
| 3217.1865382 | 1 |
| 3217.1872018 | 2 |
| 3217.1873525 | 3 |
| 3217.1882544 | 1 |
| 3217.1893998 | 5 |
| 3217.1894136 | 1 |
| 3217.1900165 | 3 |
| 3217.1907345 | 2 |
| 3217.1909435 | 2 |
| 3217.1916771 | 2 |
| 3217.1926346 | 1 |
| 3217.1933284 | 4 |
| 3217.1939869 | 3 |
| 3217.1943255 | 1 |
| 3217.195349 | 2 |
| 3217.1960386 | 1 |
| 3217.1965453 | 4 |
| 3217.1968731 | 3 |
| 3217.1978549 | 0 |
| 3217.198376 | 3 |
| 3217.1985135 | 1 |
| 3217.1997366 | 3 |
| 3217.2006597 | 1 |
| 3217.2007879 | 2 |
| 3217.2014628 | 3 |
| 3217.2015953 | 1 |
| 3217.2029192 | 1 |
| 3217.2032682 | 3 |
| 3217.2039689 | 4 |
| 3217.2051868 | 1 |
| 3217.2052805 | 4 |
| 3217.2052991 | 2 |
| 3217.2069635 | 2 |
| 3217.2071466 | 2 |
| 3217.2081164 | 5 |
| 3217.20817 | 2 |
| 3217.2088343 | 4 |
| 3217.2097132 | 2 |
| 3217.2102108 | 0 |
| 3217.2111424 | 2 |
| 3217.211322 | 2 |
| 3217.212737 | 4 |
| 3217.213292 | 5 |
| 3217.2141515 | 2 |
| 3217.21418 | 1 |
| 3217.2145342 | 2 |
| 3217.2158294 | 1 |
| 3217.2163152 | 0 |
| 3217.2165613 | 1 |
| 3217.2179654 | 4 |
| 3217.2184527 | 4 |
| 3217.2186251 | 1 |
| 3217.218763 | 3 |
| 3217.2201856 | 0 |
| 3217.2209238 | 5 |
| 3217.221016 | 1 |
| 3217.2219218 | 2 |
| 3217.2225382 | 3 |
| 3217.2230389 | 5 |
| 3217.2235075 | 1 |
| 3217.2248199 | 4 |
| 3217.2254514 | 1 |
| 3217.2254928 | 0 |
| 3217.2261561 | 2 |
| 3217.2261878 | 2 |
| 3217.2267415 | 2 |
| 3217.2279284 | 2 |
| 3217.2283972 | 3 |
| 3217.2291281 | 3 |
| 3217.2292354 | 1 |
| 3217.2299364 | 4 |
| 3217.2305393 | 3 |
| 3217.2311264 | 4 |
| 3217.232174 | 1 |
| 3217.2325578 | 2 |
| 3217.2327385 | 1 |
| 3217.2340555 | 1 |
| 3217.2344393 | 2 |
| 3217.2350738 | 4 |
| 3217.2350951 | 0 |
| 3217.2366524 | 3 |
| 3217.2370796 | 0 |
| 3217.2376276 | 1 |
| 3217.2378995 | 1 |
| 3217.2386652 | 3 |
| 3217.2387709 | 3 |
| 3217.2394736 | 2 |
| 3217.2407236 | 3 |
| 3217.2413778 | 2 |
| 3217.2416206 | 1 |
| 3217.2424155 | 3 |
| 3217.2424455 | 2 |
| 3217.2434665 | 2 |
| 3217.2442994 | 5 |
| 3217.2444901 | 2 |
| 3217.2457899 | 2 |
| 3217.2459365 | 0 |
| 3217.2466965 | 1 |
| 3217.2475275 | 0 |
| 3217.2477498 | 0 |
| 3217.2480813 | 1 |
| 3217.2489208 | 1 |
| 3217.2496988 | 1 |
| 3217.2507297 | 1 |
| 3217.2507437 | 0 |
| 3217.2516635 | 3 |
| 3217.2525599 | 3 |
| 3217.2532612 | 1 |
| 3217.2539192 | 2 |
| 3217.2543631 | 1 |
| 3217.2547225 | 0 |
| 3217.2554662 | 1 |
| 3217.2562653 | 1 |
| 3217.2569888 | 1 |
| 3217.2575399 | 3 |
| 3217.2580518 | 1 |
| 3217.2587912 | 1 |
| 3217.2591798 | 1 |
| 3217.260043 | 1 |
| 3217.2605167 | 2 |
| 3217.261683 | 4 |
| 3217.2619674 | 5 |
| 3217.262066 | 5 |
| 3217.2634418 | 6 |
| 3217.263951 | 3 |
| 3217.2644 | 12 |
| 3217.2655004 | 12 |
| 3217.2659311 | 19 |
| 3217.2667484 | 26 |
| 3217.2671009 | 22 |
| 3217.2677335 | 18 |
| 3217.2678353 | 16 |
| 3217.2689859 | 14 |
| 3217.2692051 | 12 |
| 3217.2704023 | 7 |
| 3217.2709851 | 2 |
| 3217.2710308 | 10 |
| 3217.2713102 | 4 |
| 3217.272199 | 2 |
| 3217.2731165 | 4 |
| 3217.2741348 | 0 |
| 3217.2742478 | 2 |
| 3217.2754218 | 3 |
| 3217.2759429 | 2 |
| 3217.2759629 | 2 |
| 3217.2770657 | 0 |
| 3217.2771785 | 2 |
| 3217.2778259 | 5 |
| 3217.278582 | 2 |
| 3217.2792466 | 2 |
| 3217.27976 | 4 |
| 3217.280049 | 1 |
| 3217.2807341 | 0 |
| 3217.2811721 | 3 |
| 3217.2817141 | 4 |
| 3217.2828574 | 2 |
| 3217.2835825 | 1 |
| 3217.2841293 | 0 |
| 3217.2845665 | 1 |
| 3217.2855794 | 2 |
| 3217.2858815 | 4 |
| 3217.2862166 | 5 |
| 3217.2870046 | 1 |
| 3217.287958 | 2 |
| 3217.2883821 | 1 |
| 3217.2891656 | 3 |
| 3217.28957 | 2 |
| 3217.2903529 | 5 |
| 3217.2904228 | 2 |
| 3217.291929 | 0 |
| 3217.2925037 | 1 |
| 3217.292631 | 1 |
| 3217.2940308 | 3 |
| 3217.2942976 | 1 |
| 3217.294321 | 1 |
| 3217.2949731 | 1 |
| 3217.2959206 | 3 |
| 3217.2966375 | 2 |
| 3217.2973101 | 2 |
| 3217.2980981 | 1 |
| 3217.2984002 | 1 |
| 3217.2993971 | 3 |
| 3217.2995811 | 0 |
| 3217.3003611 | 1 |
| 3217.3008823 | 0 |
| 3217.3015836 | 2 |
| 3217.3022207 | 2 |
| 3217.3023706 | 5 |
| 3217.3035995 | 0 |
| 3217.3044706 | 2 |
| 3217.3049081 | 2 |
| 3217.3049883 | 1 |
| 3217.3058193 | 2 |
| 3217.3071323 | 0 |
| 3217.3078748 | 2 |
| 3217.3080375 | 1 |
| 3217.3093362 | 0 |
| 3217.3093916 | 2 |
| 3217.3102603 | 1 |
| 3217.3108159 | 0 |
| 3217.3108833 | 1 |
| 3217.3121393 | 1 |
| 3217.3126465 | 1 |
| 3217.3126839 | 2 |
| 3217.3133333 | 7 |
| 3217.3138718 | 1 |
| 3217.3149753 | 1 |
| 3217.3152055 | 3 |
| 3217.3163637 | 2 |
| 3217.3169688 | 2 |
| 3217.3170456 | 1 |
| 3217.3174743 | 2 |
| 3217.3183113 | 1 |
| 3217.3185337 | 0 |
| 3217.319947 | 6 |
| 3217.3201966 | 2 |
| 3217.3209257 | 0 |
| 3217.3216726 | 1 |
| 3217.3224955 | 2 |
| 3217.3225002 | 4 |
| 3217.3234386 | 1 |
| 3217.3241873 | 6 |
| 3217.324245 | 0 |
| 3217.3250086 | 1 |
| 3217.325902 | 1 |
| 3217.3266945 | 2 |
| 3217.326744 | 4 |
| 3217.3275574 | 2 |
| 3217.328094 | 2 |
| 3217.3287274 | 1 |
| 3217.3289856 | 3 |
| 3217.3303613 | 2 |
| 3217.3306825 | 2 |
| 3217.3314015 | 2 |
| 3217.3314851 | 1 |
| 3217.3327047 | 5 |
| 3217.3330411 | 3 |
| 3217.3338987 | 1 |
| 3217.3344341 | 2 |
| 3217.3350801 | 1 |
| 3217.3360896 | 2 |
| 3217.3362935 | 1 |
| 3217.3377389 | 1 |
| 3217.3377696 | 2 |
| 3217.3383212 | 3 |
| 3217.3394395 | 0 |
| 3217.3396478 | 1 |
| 3217.3406503 | 1 |
| 3217.3408841 | 2 |
| 3217.340996 | 2 |
| 3217.3422981 | 2 |
| 3217.3430242 | 1 |
| 3217.3432613 | 5 |
| 3217.3440382 | 0 |
| 3217.3449362 | 2 |
| 3217.3453455 | 2 |
| 3217.3462655 | 2 |
| 3217.3464501 | 4 |
| 3217.3469931 | 3 |
| 3217.3475421 | 1 |
| 3217.3488057 | 4 |
| 3217.3491351 | 2 |
| 3217.3496968 | 0 |
| 3217.3502888 | 2 |
| 3217.3514414 | 0 |
| 3217.3516167 | 2 |
| 3217.3520721 | 0 |
| 3217.3528478 | 1 |
| 3217.3529318 | 0 |
| 3217.3540243 | 3 |
| 3217.3545508 | 0 |
| 3217.355788 | 4 |
| 3217.3562895 | 1 |
| 3217.3567304 | 1 |
| 3217.357443 | 2 |
| 3217.3580118 | 2 |
| 3217.3585778 | 1 |
| 3217.3590807 | 1 |
| 3217.3597984 | 2 |
| 3217.3604294 | 0 |
| 3217.3606324 | 1 |
| 3217.3613857 | 1 |
| 3217.362881 | 5 |
| 3217.3635839 | 5 |
| 3217.3637135 | 2 |
| 3217.3639275 | 1 |
| 3217.3642147 | 5 |
| 3217.3650912 | 2 |
| 3217.3662645 | 1 |
| 3217.3664252 | 2 |
| 3217.3675487 | 1 |
| 3217.3683896 | 2 |
| 3217.3685051 | 3 |
| 3217.3694013 | 1 |
| 3217.3696293 | 1 |
| 3217.3704079 | 3 |
| 3217.3706819 | 2 |
| 3217.3716091 | 1 |
| 3217.3719052 | 2 |
| 3217.3727962 | 0 |
| 3217.3731246 | 1 |
| 3217.3739716 | 3 |
| 3217.3744546 | 0 |
| 3217.3749119 | 2 |
| 3217.3759616 | 2 |
| 3217.3765896 | 2 |
| 3217.377495 | 2 |
| 3217.3776542 | 2 |
| 3217.3782654 | 1 |
| 3217.3786822 | 1 |
| 3217.379346 | 0 |
| 3217.3802412 | 1 |
| 3217.3805724 | 1 |
| 3217.3814787 | 0 |
| 3217.3818491 | 0 |
| 3217.3825913 | 2 |
| 3217.3835845 | 2 |
| 3217.3839828 | 2 |
| 3217.3846859 | 2 |
| 3217.3858356 | 3 |
| 3217.3859726 | 2 |
| 3217.3868718 | 5 |
| 3217.3874414 | 4 |
| 3217.3875441 | 3 |
| 3217.3886238 | 3 |
| 3217.3892601 | 1 |
| 3217.3893242 | 3 |

| | |
|---|---|
| 3217.3896484 | 3 |
| 3217.3906467 | 3 |
| 3217.3916987 | 2 |
| 3217.3927747 | 0 |
| 3217.3930371 | 1 |
| 3217.3938885 | 1 |
| 3217.3946286 | 0 |
| 3217.3952361 | 1 |
| 3217.3954186 | 2 |
| 3217.3960412 | 1 |
| 3217.3964356 | 0 |
| 3217.3970692 | 0 |
| 3217.3983938 | 3 |
| 3217.3988285 | 2 |
| 3217.3993703 | 2 |
| 3217.3996148 | 3 |
| 3217.4005772 | 1 |
| 3217.4019116 | 1 |
| 3217.4023296 | 1 |
| 3217.4034517 | 4 |
| 3217.403622 | 4 |
| 3217.4050166 | 4 |
| 3217.4062645 | 3 |
| 3217.406694 | 2 |
| 3217.4080527 | 0 |
| 3217.4086897 | 1 |
| 3217.4097038 | 1 |
| 3217.4103967 | 0 |
| 3217.4113868 | 1 |
| 3217.4128794 | 2 |
| 3217.4131633 | 4 |
| 3217.4143941 | 1 |
| 3217.4149571 | 3 |
| 3217.4161596 | 0 |
| 3217.4173155 | 1 |
| 3217.4180639 | 4 |
| 3217.4192615 | 0 |
| 3217.419397 | 1 |
| 3217.4206705 | 5 |
| 3217.4214739 | 1 |
| 3217.4228712 | 3 |
| 3217.4239775 | 1 |
| 3217.4245978 | 3 |
| 3217.4261763 | 1 |
| 3217.4269152 | 1 |
| 3217.4272778 | 1 |
| 3217.42849 | 1 |
| 3217.4292884 | 0 |
| 3217.4298875 | 2 |
| 3217.431063 | 2 |
| 3217.4321609 | 0 |
| 3217.4333969 | 4 |
| 3217.4340348 | 3 |
| 3217.4354207 | 1 |
| 3217.4358841 | 2 |
| 3217.437203 | 1 |
| 3217.4385908 | 1 |
| 3217.4385993 | 2 |
| 3217.4397861 | 2 |
| 3217.4405161 | 2 |
| 3217.442031 | 0 |
| 3217.4427752 | 0 |
| 3217.443267 | 4 |
| 3217.4445722 | 4 |
| 3217.4454175 | 0 |
| 3217.446411 | 0 |
| 3217.4477638 | 3 |
| 3217.4483963 | 2 |
| 3217.4491871 | 2 |
| 3217.4501368 | 2 |
| 3217.4513984 | 2 |
| 3217.4526432 | 1 |
| 3217.4529407 | 3 |
| 3217.4540158 | 1 |
| 3217.4542336 | 2 |
| 3217.4555379 | 3 |
| 3217.4563613 | 4 |
| 3217.4577475 | 5 |
| 3217.4584974 | 2 |
| 3217.4591365 | 1 |
| 3217.4598944 | 5 |
| 3217.4613407 | 0 |
| 3217.462287 | 2 |
| 3217.4633354 | 1 |
| 3217.4637827 | 2 |
| 3217.4651458 | 2 |
| 3217.4652013 | 1 |
| 3217.4666884 | 1 |
| 3217.4678774 | 4 |
| 3217.4684534 | 3 |
| 3217.4699604 | 2 |
| 3217.4702299 | 2 |
| 3217.4720041 | 2 |
| 3217.4727696 | 1 |
| 3217.4734693 | 0 |
| 3217.4744664 | 1 |
| 3217.4751856 | 0 |
| 3217.4761662 | 2 |
| 3217.4770059 | 2 |
| 3217.4782046 | 4 |
| 3217.4787188 | 4 |
| 3217.4793953 | 2 |
| 3217.4805174 | 4 |
| 3217.4816377 | 1 |
| 3217.4834228 | 3 |
| 3217.4847429 | 4 |
| 3217.4848579 | 3 |
| 3217.4861877 | 1 |
| 3217.4864458 | 0 |
| 3217.4877354 | 3 |
| 3217.4886673 | 1 |
| 3217.4897996 | 3 |
| 3217.4907564 | 2 |
| 3217.4910763 | 5 |
| 3217.4931754 | 1 |
| 3217.4937497 | 1 |
| 3217.494182 | 2 |
| 3217.4951582 | 5 |
| 3217.4962837 | 3 |
| 3217.4974199 | 2 |
| 3217.4984012 | 2 |
| 3217.4991241 | 1 |
| 3217.5000611 | 0 |
| 3217.5005184 | 2 |
| 3217.5014196 | 1 |
| 3217.5031713 | 2 |
| 3217.5032226 | 0 |
| 3217.5042578 | 0 |
| 3217.5053574 | 1 |
| 3217.5064345 | 2 |
| 3217.5075713 | 1 |
| 3217.5075958 | 2 |
| 3217.509338 | 3 |
| 3217.5102573 | 1 |
| 3217.5108728 | 0 |
| 3217.5122205 | 1 |
| 3217.5125037 | 5 |
| 3217.5135217 | 1 |
| 3217.5142747 | 5 |
| 3217.5149139 | 1 |
| 3217.5167965 | 3 |
| 3217.517382 | 0 |
| 3217.5184365 | 3 |
| 3217.5194574 | 1 |
| 3217.521166 | 2 |
| 3217.5215633 | 4 |
| 3217.5221932 | 3 |
| 3217.5233627 | 3 |
| 3217.5241981 | 1 |
| 3217.525311 | 1 |
| 3217.5253517 | 2 |
| 3217.526609 | 1 |
| 3217.5274363 | 0 |
| 3217.528349 | 1 |
| 3217.5297645 | 5 |
| 3217.5308672 | 2 |
| 3217.5316371 | 2 |
| 3217.5329871 | 2 |
| 3217.5333711 | 3 |
| 3217.5351503 | 1 |
| 3217.5352793 | 2 |
| 3217.5370767 | 2 |
| 3217.5378462 | 3 |
| 3217.5384437 | 0 |
| 3217.5396415 | 3 |
| 3217.5398595 | 2 |
| 3217.540938 | 0 |
| 3217.5426443 | 1 |
| 3217.5429205 | 3 |
| 3217.544033 | 1 |
| 3217.5446512 | 0 |
| 3217.545353 | 0 |
| 3217.5465483 | 1 |
| 3217.5471919 | 1 |
| 3217.5482259 | 3 |
| 3217.5491674 | 1 |
| 3217.5497132 | 0 |
| 3217.5513231 | 0 |
| 3217.551829 | 0 |
| 3217.5532047 | 1 |
| 3217.5541074 | 6 |
| 3217.5553449 | 4 |
| 3217.555394 | 1 |
| 3217.5565097 | 0 |
| 3217.5579544 | 2 |
| 3217.558367 | 2 |
| 3217.5598335 | 3 |
| 3217.5601816 | 0 |
| 3217.5618754 | 2 |
| 3217.5625371 | 2 |
| 3217.5625567 | 3 |
| 3217.5640853 | 1 |
| 3217.56492 | 0 |
| 3217.5660386 | 0 |
| 3217.5673681 | 4 |
| 3217.5680466 | 2 |
| 3217.5690293 | 4 |
| 3217.5695222 | 0 |
| 3217.5705982 | 3 |
| 3217.5716695 | 2 |
| 3217.5729383 | 2 |
| 3217.5741167 | 2 |
| 3217.5742146 | 0 |
| 3217.575715 | 0 |
| 3217.5762759 | 2 |
| 3217.5774593 | 3 |
| 3217.5788666 | 0 |
| 3217.5797465 | 2 |
| 3217.5807489 | 2 |
| 3217.5810532 | 2 |
| 3217.5821621 | 0 |
| 3217.5832253 | 2 |
| 3217.5841953 | 3 |
| 3217.5853695 | 0 |
| 3217.5856321 | 0 |
| 3217.5870966 | 3 |
| 3217.5873647 | 1 |
| 3217.5888301 | 1 |
| 3217.5892531 | 2 |
| 3217.5900931 | 2 |
| 3217.5915904 | 3 |
| 3217.5927299 | 2 |
| 3217.5930155 | 0 |
| 3217.5943748 | 1 |
| 3217.594736 | 1 |
| 3217.5959446 | 1 |
| 3217.596985 | 1 |
| 3217.597429 | 1 |
| 3217.5982743 | 3 |
| 3217.5989699 | 5 |
| 3217.6004228 | 1 |
| 3217.6019606 | 2 |
| 3217.6023175 | 2 |
| 3217.6035231 | 2 |
| 3217.6040696 | 1 |
| 3217.6055098 | 1 |
| 3217.6061172 | 1 |
| 3217.6066201 | 1 |
| 3217.6081784 | 2 |
| 3217.6087894 | 1 |
| 3217.6100879 | 1 |
| 3217.6109114 | 0 |
| 3217.6117325 | 0 |
| 3217.6128799 | 5 |
| 3217.6131828 | 2 |
| 3217.6145936 | 0 |
| 3217.6154919 | 2 |
| 3217.61638 | 0 |
| 3217.6175418 | 2 |
| 3217.6179618 | 1 |
| 3217.6193657 | 6 |
| 3217.6201684 | 1 |
| 3217.6205239 | 5 |
| 3217.6230392 | 1 |
| 3217.6234456 | 2 |
| 3217.6238897 | 3 |
| 3217.624111 | 2 |
| 3217.6252988 | 1 |
| 3217.6270336 | 0 |
| 3217.6275386 | 0 |
| 3217.628708 | 2 |
| 3217.6293419 | 1 |
| 3217.6301228 | 0 |
| 3217.6315824 | 0 |
| 3217.6319808 | 0 |
| 3217.6334443 | 4 |
| 3217.6341618 | 2 |
| 3217.6346065 | 2 |
| 3217.6362129 | 2 |
| 3217.6366789 | 2 |
| 3217.6376611 | 3 |
| 3217.638316 | 6 |
| 3217.6399155 | 5 |
| 3217.6411641 | 3 |
| 3217.6413501 | 3 |
| 3217.6424981 | 3 |
| 3217.6427092 | 2 |
| 3217.6438069 | 1 |
| 3217.6446941 | 2 |
| 3217.6463486 | 1 |
| 3217.6471147 | 3 |
| 3217.6479731 | 4 |
| 3217.6484254 | 4 |
| 3217.6500289 | 0 |
| 3217.6504865 | 3 |
| 3217.6518933 | 3 |
| 3217.6519251 | 3 |
| 3217.652811 | 2 |
| 3217.6544514 | 0 |
| 3217.6554669 | 2 |
| 3217.657068 | 1 |
| 3217.6574791 | 3 |
| 3217.6583552 | 3 |
| 3217.6586811 | 0 |
| 3217.6597235 | 3 |
| 3217.661249 | 2 |
| 3217.6613389 | 2 |
| 3217.6630039 | 2 |
| 3217.6633672 | 1 |
| 3217.6651289 | 3 |
| 3217.6659408 | 3 |
| 3217.667303 | 1 |
| 3217.6678627 | 5 |
| 3217.6682548 | 2 |
| 3217.6696591 | 2 |
| 3217.669681 | 0 |
| 3217.67109 | 2 |
| 3217.6721088 | 2 |
| 3217.6726245 | 1 |
| 3217.6736465 | 1 |
| 3217.6752105 | 1 |
| 3217.6755721 | 0 |
| 3217.6771154 | 2 |
| 3217.6775187 | 1 |
| 3217.6794221 | 0 |
| 3217.6799884 | 3 |
| 3217.6803414 | 2 |
| 3217.6812471 | 4 |
| 3217.682264 | 5 |
| 3217.6824149 | 1 |
| 3217.6842843 | 5 |
| 3217.6849586 | 2 |
| 3217.6864699 | 2 |
| 3217.6869503 | 0 |
| 3217.6877315 | 2 |
| 3217.6880348 | 1 |
| 3217.6890685 | 2 |
| 3217.6902782 | 1 |
| 3217.691279 | 0 |
| 3217.6923661 | 2 |
| 3217.6929096 | 2 |
| 3217.6938874 | 0 |
| 3217.6950959 | 1 |
| 3217.6955822 | 1 |
| 3217.6966928 | 1 |
| 3217.6978788 | 1 |
| 3217.6984857 | 3 |
| 3217.6995974 | 0 |
| 3217.7000872 | 1 |
| 3217.7009191 | 1 |
| 3217.7027373 | 2 |
| 3217.7033473 | 3 |
| 3217.7047418 | 2 |
| 3217.705125 | 2 |
| 3217.7066217 | 4 |
| 3217.7072746 | 2 |
| 3217.7082756 | 3 |
| 3217.7090654 | 4 |
| 3217.7102423 | 2 |
| 3217.7105996 | 4 |
| 3217.712074 | 1 |
| 3217.7128559 | 3 |
| 3217.7139792 | 1 |
| 3217.714334 | 0 |
| 3217.7158017 | 1 |
| 3217.7158877 | 3 |
| 3217.7170233 | 3 |
| 3217.7184925 | 0 |
| 3217.7195065 | 4 |
| 3217.7205001 | 3 |
| 3217.7209081 | 3 |
| 3217.7220608 | 1 |
| 3217.723558 | 2 |

| | |
|---|---|
| 3217.7237381 | 3 |
| 3217.7248513 | 5 |
| 3217.7262217 | 1 |
| 3217.7263713 | 1 |
| 3217.7276101 | 0 |
| 3217.728312 | 0 |
| 3217.7295122 | 1 |
| 3217.730236 | 1 |
| 3217.7313937 | 1 |
| 3217.7325314 | 4 |
| 3217.7328457 | 0 |
| 3217.7337084 | 2 |
| 3217.7342111 | 3 |
| 3217.7353822 | 1 |
| 3217.7365871 | 2 |
| 3217.7371193 | 8 |
| 3217.7381437 | 3 |
| 3217.7386849 | 4 |
| 3217.7402195 | 1 |
| 3217.7411251 | 4 |
| 3217.7417474 | 1 |
| 3217.7428647 | 2 |
| 3217.7431761 | 2 |
| 3217.7458845 | 2 |
| 3217.7458975 | 4 |
| 3217.7459037 | 1 |
| 3217.747285 | 2 |
| 3217.7485365 | 3 |
| 3217.748884 | 2 |
| 3217.7509536 | 2 |
| 3217.7511589 | 0 |
| 3217.7528477 | 3 |
| 3217.7532626 | 2 |
| 3217.7540319 | 3 |
| 3217.7556621 | 2 |
| 3217.7564711 | 0 |
| 3217.7572779 | 4 |
| 3217.7577181 | 1 |
| 3217.7591024 | 5 |
| 3217.760103 | 1 |
| 3217.7606791 | 0 |
| 3217.7621648 | 1 |
| 3217.7633911 | 2 |
| 3217.7634029 | 1 |
| 3217.7645381 | 0 |
| 3217.7653645 | 1 |
| 3217.7661746 | 3 |
| 3217.7678324 | 0 |
| 3217.768034 | 0 |
| 3217.7693022 | 2 |
| 3217.7695276 | 1 |
| 3217.7711974 | 0 |
| 3217.7714766 | 4 |
| 3217.7730134 | 5 |
| 3217.7738482 | 1 |
| 3217.7746136 | 2 |
| 3217.7759068 | 3 |
| 3217.7762741 | 3 |
| 3217.7769816 | 1 |
| 3217.7783276 | 3 |
| 3217.7791025 | 4 |
| 3217.7802589 | 1 |
| 3217.7812791 | 0 |
| 3217.7821701 | 0 |
| 3217.7823462 | 1 |
| 3217.7830253 | 1 |
| 3217.7846783 | 5 |
| 3217.7847917 | 0 |
| 3217.78683 | 1 |
| 3217.7869531 | 3 |
| 3217.7877714 | 1 |
| 3217.7893607 | 0 |
| 3217.7898361 | 1 |
| 3217.7911573 | 3 |
| 3217.7916405 | 2 |
| 3217.7929585 | 2 |
| 3217.793982 | 0 |
| 3217.7942463 | 5 |
| 3217.794923 | 1 |
| 3217.7966905 | 1 |
| 3217.7974767 | 2 |
| 3217.7990031 | 3 |
| 3217.7991737 | 4 |
| 3217.8008328 | 2 |
| 3217.8018065 | 1 |
| 3217.8021479 | 2 |
| 3217.803196 | 0 |
| 3217.8035138 | 0 |
| 3217.8050525 | 4 |
| 3217.8058684 | 2 |
| 3217.8071054 | 3 |
| 3217.8079988 | 0 |
| 3217.8087108 | 1 |
| 3217.8097229 | 0 |
| 3217.8106156 | 4 |
| 3217.8109045 | 1 |
| 3217.8128071 | 2 |
| 3217.8133075 | 0 |
| 3217.8142947 | 0 |
| 3217.8146661 | 4 |
| 3217.8165855 | 2 |
| 3217.8170925 | 2 |
| 3217.8182967 | 2 |
| 3217.8191653 | 1 |
| 3217.8193018 | 4 |
| 3217.8202254 | 1 |
| 3217.8214865 | 0 |
| 3217.8222869 | 1 |
| 3217.8228921 | 0 |
| 3217.8246635 | 3 |
| 3217.8252726 | 2 |
| 3217.825415 | 0 |
| 3217.8267711 | 1 |
| 3217.828441 | 1 |
| 3217.8284744 | 0 |
| 3217.8298921 | 1 |
| 3217.8306233 | 1 |
| 3217.8317213 | 1 |
| 3217.8318572 | 2 |
| 3217.8332017 | 0 |
| 3217.8346859 | 0 |
| 3217.8347886 | 0 |
| 3217.8362079 | 3 |
| 3217.8367817 | 0 |
| 3217.8374927 | 4 |
| 3217.8391308 | 0 |
| 3217.8404026 | 1 |
| 3217.8409113 | 1 |
| 3217.8416128 | 0 |
| 3217.8429853 | 4 |
| 3217.8431153 | 1 |
| 3217.8450272 | 2 |
| 3217.8456286 | 6 |
| 3217.8466114 | 2 |
| 3217.8481009 | 2 |
| 3217.8485169 | 5 |
| 3217.8487805 | 2 |
| 3217.8505935 | 1 |
| 3217.850676 | 0 |
| 3217.851606 | 3 |
| 3217.8528233 | 1 |
| 3217.8529965 | 5 |
| 3217.8551664 | 5 |
| 3217.8557283 | 1 |
| 3217.8565032 | 5 |
| 3217.8575361 | 2 |
| 3217.8586196 | 1 |
| 3217.8599903 | 1 |
| 3217.8603677 | 4 |
| 3217.8612869 | 0 |
| 3217.8620072 | 1 |
| 3217.8633625 | 2 |
| 3217.8640512 | 2 |
| 3217.8649455 | 4 |
| 3217.866623 | 0 |
| 3217.8667202 | 0 |
| 3217.8684632 | 0 |
| 3217.8685803 | 1 |
| 3217.8693306 | 2 |
| 3217.8706729 | 2 |
| 3217.8713191 | 1 |
| 3217.8724556 | 2 |
| 3217.8727998 | 2 |
| 3217.8735812 | 1 |
| 3217.8751962 | 2 |
| 3217.8753629 | 0 |
| 3217.8764345 | 2 |
| 3217.8775865 | 3 |
| 3217.8785366 | 2 |
| 3217.879785 | 1 |
| 3217.8803975 | 2 |
| 3217.8821355 | 1 |
| 3217.8822644 | 2 |
| 3217.8831342 | 1 |
| 3217.8844124 | 3 |
| 3217.8849061 | 0 |
| 3217.8861944 | 2 |
| 3217.8868345 | 0 |
| 3217.8879028 | 4 |
| 3217.8885064 | 3 |
| 3217.8899331 | 1 |
| 3217.8910535 | 2 |
| 3217.8918242 | 4 |
| 3217.8931107 | 1 |
| 3217.8937976 | 1 |
| 3217.8946238 | 4 |
| 3217.8954402 | 2 |
| 3217.8957724 | 3 |
| 3217.8966769 | 1 |
| 3217.8972979 | 1 |
| 3217.8974999 | 5 |
| 3217.8980022 | 4 |
| 3217.8981094 | 0 |
| 3217.8989078 | 2 |
| 3217.9002812 | 2 |
| 3217.9002858 | 2 |
| 3217.9003443 | 3 |
| 3217.900426 | 2 |
| 3217.9013347 | 3 |
| 3217.9017162 | 3 |
| 3217.9022267 | 1 |
| 3217.9027386 | 6 |
| 3217.9029271 | 4 |
| 3217.9037815 | 1 |
| 3217.9040302 | 1 |
| 3217.9044203 | 2 |
| 3217.9051948 | 1 |
| 3217.9054092 | 2 |
| 3217.9062029 | 2 |
| 3217.9066425 | 3 |
| 3217.9069996 | 3 |
| 3217.9072735 | 1 |
| 3217.9078347 | 2 |
| 3217.9081025 | 2 |
| 3217.908492 | 1 |
| 3217.9093587 | 2 |
| 3217.9095882 | 1 |
| 3217.9097647 | 2 |
| 3217.9101104 | 4 |
| 3217.9108031 | 2 |
| 3217.911439 | 0 |
| 3217.911638 | 2 |
| 3217.9118769 | 2 |
| 3217.9131872 | 4 |
| 3217.9136674 | 1 |
| 3217.913771 | 1 |
| 3217.9139678 | 0 |
| 3217.9149003 | 3 |
| 3217.915033 | 2 |
| 3217.9152762 | 2 |
| 3217.915296 | 2 |
| 3217.9163517 | 3 |
| 3217.9167184 | 0 |
| 3217.9169705 | 0 |
| 3217.9179921 | 1 |
| 3217.918582 | 4 |
| 3217.9188811 | 1 |
| 3217.9191271 | 1 |
| 3217.919508 | 4 |
| 3217.9202764 | 2 |
| 3217.9206407 | 1 |
| 3217.9206537 | 1 |
| 3217.9208477 | 2 |
| 3217.9219398 | 0 |
| 3217.9220165 | 3 |
| 3217.9223574 | 2 |
| 3217.922406 | 0 |
| 3217.923824 | 4 |
| 3217.9240983 | 2 |
| 3217.9249164 | 4 |
| 3217.9249461 | 0 |
| 3217.9251874 | 5 |
| 3217.9259594 | 2 |
| 3217.9262565 | 1 |
| 3217.9265416 | 1 |
| 3217.9274936 | 2 |
| 3217.9275992 | 2 |
| 3217.928652 | 0 |
| 3217.929041 | 2 |
| 3217.9290918 | 0 |
| 3217.9292485 | 0 |
| 3217.9296331 | 1 |
| 3217.9304739 | 2 |
| 3217.9312169 | 3 |
| 3217.9328062 | 1 |
| 3217.9339164 | 2 |
| 3217.9341529 | 2 |
| 3217.9355198 | 2 |
| 3217.9359251 | 2 |
| 3217.9372296 | 2 |
| 3217.9382251 | 3 |
| 3217.9389179 | 1 |
| 3217.9402443 | 1 |
| 3217.9409671 | 2 |
| 3217.9417654 | 0 |
| 3217.9425552 | 2 |
| 3217.9440367 | 0 |
| 3217.9452709 | 5 |
| 3217.9457044 | 1 |
| 3217.9466192 | 4 |
| 3217.9478933 | 1 |
| 3217.9488793 | 1 |
| 3217.9498392 | 1 |
| 3217.9503731 | 2 |
| 3217.9516124 | 0 |
| 3217.9520246 | 1 |
| 3217.9536083 | 0 |
| 3217.9537074 | 2 |
| 3217.9549568 | 2 |
| 3217.9559116 | 0 |
| 3217.9568496 | 2 |
| 3217.9576955 | 2 |
| 3217.9589339 | 6 |
| 3217.9594759 | 2 |
| 3217.9610929 | 0 |
| 3217.961482 | 1 |
| 3217.9622848 | 2 |
| 3217.9629291 | 2 |
| 3217.9644207 | 2 |
| 3217.965483 | 2 |
| 3217.9658887 | 2 |
| 3217.9671371 | 0 |
| 3217.9675171 | 1 |
| 3217.9682108 | 3 |
| 3217.9698768 | 4 |
| 3217.9705375 | 2 |
| 3217.9712015 | 3 |
| 3217.9719595 | 1 |
| 3217.9730125 | 2 |
| 3217.9737931 | 2 |
| 3217.9753896 | 0 |
| 3217.9766443 | 1 |
| 3217.9772295 | 0 |
| 3217.9787256 | 3 |
| 3217.978749 | 1 |
| 3217.9795459 | 1 |
| 3217.9815067 | 1 |
| 3217.9823733 | 2 |
| 3217.9831112 | 3 |
| 3217.9838714 | 2 |
| 3217.9856392 | 0 |
| 3217.9860213 | 1 |
| 3217.9866367 | 2 |
| 3217.9877681 | 0 |
| 3217.9887541 | 1 |
| 3217.9895449 | 2 |
| 3217.991238 | 2 |
| 3217.9914625 | 2 |
| 3217.9923978 | 3 |
| 3217.993959 | 0 |
| 3217.9949347 | 4 |
| 3217.9956898 | 1 |
| 3217.9968887 | 2 |
| 3217.997435 | 4 |
| 3217.9979425 | 0 |
| 3217.9989241 | 2 |
| 3218.0003029 | 1 |
| 3218.001336 | 2 |
| 3218.0019461 | 2 |
| 3218.002578 | 1 |
| 3218.0037056 | 3 |
| 3218.0039553 | 1 |
| 3218.0053299 | 3 |
| 3218.0065128 | 1 |
| 3218.0074176 | 0 |
| 3218.0080149 | 1 |
| 3218.009457 | 1 |
| 3218.0100772 | 4 |
| 3218.0101975 | 4 |
| 3218.0114585 | 4 |
| 3218.0123141 | 4 |
| 3218.0143334 | 4 |
| 3218.0145997 | 1 |
| 3218.0159956 | 0 |
| 3218.0164809 | 3 |
| 3218.018092 | 2 |
| 3218.0183279 | 0 |
| 3218.0198995 | 2 |
| 3218.0206016 | 2 |
| 3218.0216101 | 0 |
| 3218.0221539 | 0 |
| 3218.0227125 | 0 |
| 3218.02423 | 2 |

| | |
|---|---|
| 3218.0252252 | 3 |
| 3218.0254281 | 3 |
| 3218.0271754 | 2 |
| 3218.0277305 | 0 |
| 3218.0290134 | 3 |
| 3218.0302956 | 1 |
| 3218.0305106 | 4 |
| 3218.0321719 | 3 |
| 3218.0323542 | 2 |
| 3218.0337558 | 0 |
| 3218.0343725 | 4 |
| 3218.0361355 | 4 |
| 3218.0368006 | 2 |
| 3218.037791 | 3 |
| 3218.0389015 | 2 |
| 3218.0392576 | 2 |
| 3218.0405532 | 2 |
| 3218.0419213 | 2 |
| 3218.0419365 | 1 |
| 3218.0429338 | 2 |
| 3218.0437802 | 2 |
| 3218.0448199 | 2 |
| 3218.0458144 | 3 |
| 3218.0463986 | 3 |
| 3218.0475148 | 0 |
| 3218.0478288 | 1 |
| 3218.049132 | 3 |
| 3218.0503625 | 2 |
| 3218.0507722 | 2 |
| 3218.0522074 | 1 |
| 3218.0530263 | 2 |
| 3218.0538822 | 4 |
| 3218.0546165 | 0 |
| 3218.0561231 | 3 |
| 3218.0566197 | 1 |
| 3218.0576723 | 3 |
| 3218.0584892 | 0 |
| 3218.0590281 | 3 |
| 3218.0598965 | 4 |
| 3218.0615767 | 1 |
| 3218.0618491 | 3 |
| 3218.0636109 | 0 |
| 3218.0638521 | 1 |
| 3218.065418 | 2 |
| 3218.0663638 | 1 |
| 3218.066795 | 2 |
| 3218.0683129 | 2 |
| 3218.0686525 | 1 |
| 3218.0695517 | 0 |
| 3218.0708846 | 2 |
| 3218.0713762 | 1 |
| 3218.0730859 | 1 |
| 3218.073228 | 5 |
| 3218.0747566 | 1 |
| 3218.0756866 | 3 |
| 3218.076592 | 1 |
| 3218.077506 | 2 |
| 3218.0783423 | 6 |
| 3218.0792723 | 2 |
| 3218.0804766 | 3 |
| 3218.0811453 | 2 |
| 3218.082376 | 2 |
| 3218.0831252 | 3 |
| 3218.084707 | 2 |
| 3218.0852585 | 3 |
| 3218.085934 | 1 |
| 3218.087164 | 4 |
| 3218.0874566 | 2 |
| 3218.0889656 | 0 |
| 3218.0893809 | 2 |
| 3218.0906125 | 3 |
| 3218.0916236 | 4 |
| 3218.092157 | 0 |
| 3218.0934428 | 1 |
| 3218.0936884 | 0 |
| 3218.0946866 | 2 |
| 3218.096047 | 3 |
| 3218.096603 | 2 |
| 3218.0974147 | 2 |
| 3218.0991399 | 4 |
| 3218.099542 | 4 |
| 3218.100426 | 0 |
| 3218.101572 | 2 |
| 3218.1023342 | 0 |
| 3218.1031232 | 3 |
| 3218.1048213 | 1 |
| 3218.1049084 | 3 |
| 3218.1061123 | 1 |
| 3218.1075642 | 1 |
| 3218.1075945 | 3 |
| 3218.1085207 | 2 |
| 3218.1098705 | 3 |
| 3218.1107009 | 2 |
| 3218.111873 | 3 |
| 3218.1125526 | 1 |
| 3218.1131103 | 1 |
| 3218.1146772 | 1 |
| 3218.1153977 | 2 |
| 3218.1164257 | 2 |
| 3218.1170581 | 0 |
| 3218.1176114 | 3 |
| 3218.1191962 | 0 |
| 3218.1199266 | 2 |
| 3218.1212717 | 2 |
| 3218.1221852 | 1 |
| 3218.1233963 | 2 |
| 3218.1237515 | 2 |
| 3218.1250984 | 2 |
| 3218.1260647 | 1 |
| 3218.1265093 | 2 |
| 3218.1279001 | 0 |
| 3218.128599 | 1 |
| 3218.1296724 | 1 |
| 3218.1299083 | 1 |
| 3218.1307845 | 3 |
| 3218.132769 | 1 |
| 3218.133451 | 2 |
| 3218.1335788 | 5 |
| 3218.1351486 | 2 |
| 3218.1358687 | 3 |
| 3218.1364184 | 2 |
| 3218.1379145 | 2 |
| 3218.1387213 | 2 |
| 3218.1398439 | 1 |
| 3218.1399839 | 3 |
| 3218.1412797 | 1 |
| 3218.1417472 | 2 |
| 3218.1435283 | 2 |
| 3218.144152 | 2 |
| 3218.1451195 | 3 |
| 3218.1465593 | 1 |
| 3218.1467844 | 2 |
| 3218.1479054 | 3 |
| 3218.1491245 | 0 |
| 3218.1497871 | 2 |
| 3218.1509229 | 6 |
| 3218.1513975 | 1 |
| 3218.1527851 | 0 |
| 3218.1535214 | 2 |
| 3218.1543686 | 4 |
| 3218.1559558 | 0 |
| 3218.1561284 | 2 |
| 3218.1571271 | 4 |
| 3218.1586364 | 3 |
| 3218.1587148 | 1 |
| 3218.159854 | 3 |
| 3218.1607261 | 3 |
| 3218.1616977 | 4 |
| 3218.1634183 | 5 |
| 3218.1637123 | 4 |
| 3218.1649887 | 1 |
| 3218.165733 | 1 |
| 3218.1661873 | 5 |
| 3218.1676217 | 1 |
| 3218.168853 | 2 |
| 3218.1698644 | 3 |
| 3218.1705426 | 2 |
| 3218.1716056 | 0 |
| 3218.1728038 | 1 |
| 3218.173319 | 2 |
| 3218.1742928 | 0 |
| 3218.175452 | 2 |
| 3218.1758356 | 1 |
| 3218.1769251 | 1 |
| 3218.1780315 | 3 |
| 3218.1792473 | 0 |
| 3218.1795719 | 4 |
| 3218.1807172 | 1 |
| 3218.18162 | 0 |
| 3218.1820132 | 1 |
| 3218.1836203 | 3 |
| 3218.1845018 | 2 |
| 3218.1855073 | 0 |
| 3218.1855084 | 1 |
| 3218.1871704 | 2 |
| 3218.1875786 | 4 |
| 3218.1887377 | 1 |
| 3218.1903559 | 2 |
| 3218.1906253 | 1 |
| 3218.1917972 | 2 |
| 3218.1927307 | 0 |
| 3218.1929702 | 1 |
| 3218.1942205 | 0 |
| 3218.1947448 | 2 |
| 3218.19632 | 1 |
| 3218.1974226 | 1 |
| 3218.1982354 | 3 |
| 3218.1991507 | 1 |
| 3218.1995601 | 0 |
| 3218.2008286 | 1 |
| 3218.2015151 | 3 |
| 3218.2022282 | 1 |
| 3218.2040737 | 3 |
| 3218.2047167 | 2 |
| 3218.2058246 | 2 |
| 3218.2062241 | 4 |
| 3218.2077524 | 1 |
| 3218.2090322 | 2 |
| 3218.2091611 | 0 |
| 3218.2106505 | 0 |
| 3218.2107131 | 1 |
| 3218.2122574 | 2 |
| 3218.2137912 | 0 |
| 3218.2141369 | 4 |
| 3218.215322 | 2 |
| 3218.2155448 | 2 |
| 3218.2172569 | 5 |
| 3218.2183689 | 4 |
| 3218.2184354 | 2 |
| 3218.2202755 | 3 |
| 3218.2207551 | 2 |
| 3218.2217647 | 3 |
| 3218.2222932 | 2 |
| 3218.2229703 | 2 |
| 3218.2250008 | 3 |
| 3218.2255413 | 0 |
| 3218.2261434 | 1 |
| 3218.2271418 | 3 |
| 3218.2282637 | 1 |
| 3218.2292157 | 4 |
| 3218.2293104 | 2 |
| 3218.2306439 | 1 |
| 3218.2321642 | 0 |
| 3218.2327022 | 5 |
| 3218.2338063 | 1 |
| 3218.2345689 | 1 |
| 3218.2352743 | 2 |
| 3218.2359413 | 0 |
| 3218.2376098 | 3 |
| 3218.2385564 | 3 |
| 3218.2385734 | 2 |
| 3218.2395684 | 2 |
| 3218.2404103 | 2 |
| 3218.2407966 | 1 |
| 3218.2428404 | 1 |
| 3218.2438557 | 2 |
| 3218.2451782 | 1 |
| 3218.2453244 | 1 |
| 3218.2467466 | 4 |
| 3218.247261 | 2 |
| 3218.2483995 | 2 |
| 3218.2494065 | 1 |
| 3218.2505228 | 4 |
| 3218.2513886 | 2 |
| 3218.2528488 | 4 |
| 3218.2528683 | 1 |
| 3218.2541092 | 2 |
| 3218.2545549 | 2 |
| 3218.2557209 | 2 |
| 3218.2572665 | 1 |
| 3218.2575715 | 1 |
| 3218.2584782 | 0 |
| 3218.2593733 | 3 |
| 3218.2605305 | 1 |
| 3218.2618622 | 3 |
| 3218.2630978 | 0 |
| 3218.2642887 | 1 |
| 3218.2647259 | 1 |
| 3218.265658 | 0 |
| 3218.2659361 | 9 |
| 3218.2674267 | 1 |
| 3218.2683856 | 4 |
| 3218.2695094 | 4 |
| 3218.2697109 | 0 |
| 3218.2698578 | 2 |
| 3218.2711513 | 0 |
| 3218.2712461 | 2 |
| 3218.2718954 | 1 |
| 3218.2730416 | 2 |
| 3218.2769289 | 2 |
| 3218.2781142 | 1 |
| 3218.2782338 | 2 |
| 3218.2795706 | 1 |
| 3218.2806917 | 4 |
| 3218.280888 | 2 |
| 3218.2821898 | 1 |
| 3218.2823399 | 3 |
| 3218.2836304 | 1 |
| 3218.2848942 | 2 |
| 3218.2857499 | 1 |
| 3218.2863845 | 0 |
| 3218.2871545 | 0 |
| 3218.2885109 | 1 |
| 3218.2894363 | 2 |
| 3218.2905454 | 1 |
| 3218.2912266 | 5 |
| 3218.2914767 | 2 |
| 3218.2933148 | 1 |
| 3218.2940498 | 2 |
| 3218.2949418 | 3 |
| 3218.2962569 | 0 |
| 3218.296636 | 1 |
| 3218.2981454 | 2 |
| 3218.2981504 | 3 |
| 3218.2994383 | 1 |
| 3218.3011325 | 0 |
| 3218.3017704 | 1 |
| 3218.3028044 | 3 |
| 3218.3031325 | 1 |
| 3218.3045891 | 0 |
| 3218.3047097 | 1 |
| 3218.3064522 | 2 |
| 3218.3075108 | 2 |
| 3218.3077806 | 3 |
| 3218.3079743 | 2 |
| 3218.3091307 | 1 |
| 3218.3094823 | 1 |
| 3218.3107462 | 2 |
| 3218.3122399 | 1 |
| 3218.3131213 | 4 |
| 3218.3144693 | 0 |
| 3218.3149552 | 3 |
| 3218.3156342 | 2 |
| 3218.3178103 | 3 |
| 3218.3178269 | 2 |
| 3218.3187039 | 2 |
| 3218.3190271 | 1 |
| 3218.3199878 | 1 |
| 3218.3215339 | 0 |
| 3218.3223112 | 0 |
| 3218.3230557 | 1 |
| 3218.3242634 | 2 |
| 3218.3251762 | 4 |
| 3218.3253148 | 2 |
| 3218.3262868 | 1 |
| 3218.3275525 | 2 |
| 3218.3284287 | 3 |
| 3218.3297446 | 4 |
| 3218.3304143 | 2 |
| 3218.3314188 | 3 |
| 3218.3315679 | 1 |
| 3218.333722 | 0 |
| 3218.3342941 | 1 |
| 3218.3351586 | 1 |
| 3218.3364692 | 0 |
| 3218.3375324 | 1 |
| 3218.3379863 | 4 |
| 3218.338488 | 2 |
| 3218.3397687 | 0 |
| 3218.3411594 | 1 |
| 3218.3420738 | 4 |
| 3218.3423397 | 1 |
| 3218.343933 | 2 |
| 3218.344433 | 3 |
| 3218.3452826 | 2 |
| 3218.346242 | 2 |
| 3218.3470133 | 0 |
| 3218.3490282 | 0 |
| 3218.3491402 | 2 |
| 3218.3501203 | 3 |
| 3218.3510031 | 1 |
| 3218.3526487 | 3 |
| 3218.3538723 | 3 |
| 3218.3544143 | 0 |
| 3218.3555142 | 2 |
| 3218.356521 | 1 |
| 3218.3575638 | 3 |
| 3218.3577083 | 0 |
| 3218.3591282 | 1 |
| 3218.3601296 | 1 |
| 3218.3603182 | 3 |
| 3218.3617389 | 0 |
| 3218.3623749 | 1 |

| | |
|---|---|
| 3218.3638295 | 2 |
| 3218.3647592 | 3 |
| 3218.3658843 | 2 |
| 3218.367112 | 2 |
| 3218.3671448 | 3 |
| 3218.3685317 | 1 |
| 3218.3696762 | 3 |
| 3218.3700741 | 4 |
| 3218.3710558 | 1 |
| 3218.371675 | 1 |
| 3218.3727466 | 4 |
| 3218.3731627 | 0 |
| 3218.37399 | 2 |
| 3218.3757651 | 3 |
| 3218.3764056 | 2 |
| 3218.3773013 | 2 |
| 3218.3781675 | 1 |
| 3218.3791542 | 1 |
| 3218.3804421 | 2 |
| 3218.3813373 | 3 |
| 3218.3823293 | 3 |
| 3218.3830637 | 3 |
| 3218.3838679 | 0 |
| 3218.3854349 | 0 |
| 3218.3854722 | 3 |
| 3218.3868515 | 3 |
| 3218.3871071 | 3 |
| 3218.3887812 | 0 |
| 3218.3895698 | 3 |
| 3218.3905518 | 1 |
| 3218.3915408 | 1 |
| 3218.3927405 | 5 |
| 3218.3935673 | 1 |
| 3218.3943597 | 2 |
| 3218.3946733 | 4 |
| 3218.3956805 | 1 |
| 3218.3975462 | 4 |
| 3218.3982467 | 1 |
| 3218.3996947 | 1 |
| 3218.4000926 | 2 |
| 3218.4012163 | 1 |
| 3218.4022599 | 1 |
| 3218.4031121 | 2 |
| 3218.4040162 | 3 |
| 3218.404987 | 0 |
| 3218.4062122 | 2 |
| 3218.4074926 | 1 |
| 3218.4078775 | 3 |
| 3218.4087632 | 1 |
| 3218.4097899 | 0 |
| 3218.4107592 | 2 |
| 3218.4117085 | 1 |
| 3218.4118182 | 2 |
| 3218.4124229 | 0 |
| 3218.412905 | 2 |
| 3218.4130121 | 0 |
| 3218.4133564 | 3 |
| 3218.4134239 | 0 |
| 3218.4140237 | 0 |
| 3218.4142774 | 2 |
| 3218.4144296 | 6 |
| 3218.4145403 | 0 |
| 3218.4147291 | 2 |
| 3218.4156558 | 3 |
| 3218.4159752 | 2 |
| 3218.4163807 | 1 |
| 3218.4164623 | 5 |
| 3218.416767 | 1 |
| 3218.4171561 | 0 |
| 3218.4184936 | 1 |
| 3218.4185002 | 3 |
| 3218.4185355 | 1 |
| 3218.4187131 | 3 |
| 3218.4191388 | 2 |
| 3218.4193538 | 3 |
| 3218.4202036 | 0 |
| 3218.4204718 | 1 |
| 3218.4206228 | 3 |
| 3218.4207432 | 1 |
| 3218.4208441 | 2 |
| 3218.4214874 | 3 |
| 3218.4217454 | 2 |
| 3218.4222408 | 3 |
| 3218.4229172 | 4 |
| 3218.4229219 | 1 |
| 3218.4235772 | 1 |
| 3218.4236648 | 4 |
| 3218.4241567 | 2 |
| 3218.4242104 | 0 |
| 3218.4249093 | 1 |
| 3218.425409 | 1 |
| 3218.4256273 | 1 |
| 3218.4260329 | 1 |
| 3218.4261969 | 1 |
| 3218.4264462 | 1 |
| 3218.4267011 | 1 |
| 3218.4269414 | 0 |
| 3218.4276239 | 2 |
| 3218.4282122 | 0 |
| 3218.4283222 | 3 |
| 3218.428642 | 2 |
| 3218.4293471 | 1 |
| 3218.4295179 | 3 |
| 3218.4299338 | 3 |
| 3218.4303971 | 2 |
| 3218.4304128 | 1 |
| 3218.4304673 | 4 |
| 3218.4307187 | 3 |
| 3218.4317717 | 5 |
| 3218.4318419 | 5 |
| 3218.4324775 | 3 |
| 3218.4325376 | 4 |
| 3218.4327277 | 9 |
| 3218.4329567 | 7 |
| 3218.4336915 | 8 |
| 3218.4338346 | 7 |
| 3218.4345721 | 5 |
| 3218.434782 | 5 |
| 3218.4352088 | 3 |
| 3218.4353451 | 2 |
| 3218.4358249 | 3 |
| 3218.4360719 | 2 |
| 3218.436147 | 3 |
| 3218.4369025 | 8 |
| 3218.4369953 | 6 |
| 3218.4371781 | 5 |
| 3218.4381351 | 5 |
| 3218.4385047 | 5 |
| 3218.4388077 | 4 |
| 3218.4393032 | 5 |
| 3218.439448 | 4 |
| 3218.4394913 | 4 |
| 3218.4400383 | 3 |
| 3218.4405443 | 2 |
| 3218.4408791 | 8 |
| 3218.4414419 | 3 |
| 3218.4415101 | 9 |
| 3218.4419028 | 8 |
| 3218.4424617 | 8 |
| 3218.4425553 | 6 |
| 3218.4426225 | 5 |
| 3218.4433906 | 7 |
| 3218.4437356 | 7 |
| 3218.4437921 | 4 |
| 3218.4440784 | 10 |
| 3218.4441854 | 0 |
| 3218.4454805 | 0 |
| 3218.4454989 | 2 |
| 3218.44629 | 0 |
| 3218.4463822 | 2 |
| 3218.4464174 | 1 |
| 3218.4467583 | 1 |
| 3218.4474852 | 2 |
| 3218.4477547 | 2 |
| 3218.4477612 | 0 |
| 3218.4484942 | 2 |
| 3218.4485339 | 2 |
| 3218.4490228 | 2 |
| 3218.4496422 | 1 |
| 3218.4497132 | 0 |
| 3218.4503441 | 2 |
| 3218.4506176 | 3 |
| 3218.4508435 | 2 |
| 3218.4511288 | 0 |
| 3218.4514796 | 2 |
| 3218.451989 | 3 |
| 3218.4520948 | 3 |
| 3218.4521652 | 1 |
| 3218.4532716 | 2 |
| 3218.4532717 | 5 |
| 3218.4537026 | 2 |
| 3218.4539093 | 3 |
| 3218.4543886 | 1 |
| 3218.4545561 | 2 |
| 3218.4547749 | 1 |
| 3218.4551435 | 0 |
| 3218.4554104 | 4 |
| 3218.4564549 | 3 |
| 3218.4565527 | 2 |
| 3218.4567142 | 3 |
| 3218.4572086 | 0 |
| 3218.4573603 | 1 |
| 3218.4575425 | 1 |
| 3218.4598651 | 1 |
| 3218.4613831 | 0 |
| 3218.4625937 | 0 |
| 3218.4637021 | 3 |
| 3218.4643017 | 1 |
| 3218.4652873 | 3 |
| 3218.4668142 | 3 |
| 3218.4679228 | 2 |
| 3218.470644 | 1 |
| 3218.471252 | 1 |
| 3218.473176 | 4 |
| 3218.474127 | 1 |
| 3218.476038 | 4 |
| 3218.4777316 | 3 |
| 3218.4793554 | 4 |
| 3218.480518 | 1 |
| 3218.4807973 | 0 |
| 3218.4832654 | 5 |
| 3218.484915 | 1 |
| 3218.4859563 | 0 |
| 3218.4868062 | 1 |
| 3218.4884306 | 0 |
| 3218.4898088 | 3 |
| 3218.4911006 | 1 |
| 3218.491821 | 2 |
| 3218.4931029 | 4 |
| 3218.493619 | 2 |
| 3218.4950457 | 1 |
| 3218.4961328 | 1 |
| 3218.4968116 | 3 |
| 3218.4975554 | 1 |
| 3218.4982743 | 2 |
| 3218.4994078 | 4 |
| 3218.4995229 | 3 |
| 3218.5015048 | 2 |
| 3218.5024656 | 1 |
| 3218.5033699 | 3 |
| 3218.5044103 | 2 |
| 3218.5044246 | 0 |
| 3218.5061018 | 3 |
| 3218.5071949 | 2 |
| 3218.507325 | 3 |
| 3218.508973 | 1 |
| 3218.5093158 | 1 |
| 3218.5103835 | 0 |
| 3218.5114196 | 3 |
| 3218.5120815 | 3 |
| 3218.5133477 | 1 |
| 3218.5136959 | 2 |
| 3218.515194 | 2 |
| 3218.5167685 | 0 |
| 3218.5170663 | 0 |
| 3218.5179301 | 0 |
| 3218.5182297 | 2 |
| 3218.5193571 | 1 |
| 3218.5208095 | 1 |
| 3218.5213312 | 3 |
| 3218.5222673 | 2 |
| 3218.5232187 | 0 |
| 3218.5241825 | 7 |
| 3218.524794 | 3 |
| 3218.5266585 | 1 |
| 3218.5277127 | 3 |
| 3218.5287625 | 1 |
| 3218.5297606 | 4 |
| 3218.5299834 | 2 |
| 3218.5311603 | 1 |
| 3218.5321122 | 1 |
| 3218.533097 | 1 |
| 3218.534217 | 2 |
| 3218.5348364 | 0 |
| 3218.5367777 | 3 |
| 3218.5370242 | 1 |
| 3218.538012 | 3 |
| 3218.5392278 | 1 |
| 3218.539484 | 3 |
| 3218.5401457 | 2 |
| 3218.5413412 | 4 |
| 3218.5415787 | 1 |
| 3218.5439468 | 2 |
| 3218.5445904 | 1 |
| 3218.5450107 | 2 |
| 3218.5458908 | 1 |
| 3218.5474615 | 5 |
| 3218.5485282 | 3 |
| 3218.5486648 | 1 |
| 3218.5507185 | 3 |
| 3218.5508244 | 2 |
| 3218.5516935 | 3 |
| 3218.5530367 | 0 |
| 3218.5534844 | 2 |
| 3218.5542918 | 3 |
| 3218.5553974 | 2 |
| 3218.5565884 | 1 |
| 3218.557227 | 2 |
| 3218.5580858 | 1 |
| 3218.5591193 | 1 |
| 3218.5594907 | 4 |
| 3218.5607437 | 4 |
| 3218.5615665 | 0 |
| 3218.5627616 | 0 |
| 3218.5637644 | 1 |
| 3218.564012 | 1 |
| 3218.5658703 | 2 |
| 3218.5663668 | 3 |
| 3218.5669944 | 3 |
| 3218.5687357 | 1 |
| 3218.5694213 | 1 |
| 3218.5705266 | 1 |
| 3218.5709604 | 1 |
| 3218.5718685 | 1 |
| 3218.573218 | 2 |
| 3218.5737999 | 0 |
| 3218.5752746 | 2 |
| 3218.5763269 | 2 |
| 3218.5767344 | 2 |
| 3218.578004 | 0 |
| 3218.5785558 | 4 |
| 3218.5799886 | 1 |
| 3218.5803923 | 3 |
| 3218.5819386 | 2 |
| 3218.5830395 | 3 |
| 3218.5838883 | 2 |
| 3218.5844129 | 1 |
| 3218.5852224 | 0 |
| 3218.5871645 | 1 |
| 3218.5876708 | 5 |
| 3218.5883092 | 2 |
| 3218.5896212 | 4 |
| 3218.5897241 | 1 |
| 3218.5910147 | 2 |
| 3218.5913765 | 2 |
| 3218.5924935 | 1 |
| 3218.5943153 | 1 |
| 3218.5944154 | 0 |
| 3218.5956619 | 4 |
| 3218.5969608 | 2 |
| 3218.5975522 | 1 |
| 3218.5977806 | 0 |
| 3218.5988831 | 1 |
| 3218.5999616 | 2 |
| 3218.6015154 | 2 |
| 3218.6019671 | 0 |
| 3218.6029238 | 0 |
| 3218.6038647 | 1 |
| 3218.6048284 | 2 |
| 3218.6054925 | 3 |
| 3218.6062965 | 4 |
| 3218.6075759 | 0 |
| 3218.6082348 | 2 |
| 3218.6090699 | 1 |
| 3218.6098424 | 1 |
| 3218.6110626 | 1 |
| 3218.6119277 | 0 |
| 3218.6127915 | 0 |
| 3218.6141051 | 3 |
| 3218.6144777 | 1 |
| 3218.6157394 | 3 |
| 3218.6170378 | 1 |
| 3218.6178582 | 1 |
| 3218.61843 | 0 |
| 3218.6199041 | 3 |
| 3218.621298 | 2 |
| 3218.6218619 | 2 |
| 3218.6225257 | 4 |
| 3218.6240552 | 1 |
| 3218.6247503 | 7 |
| 3218.6254364 | 1 |
| 3218.6269351 | 3 |
| 3218.6272678 | 1 |
| 3218.6282783 | 3 |
| 3218.6292438 | 2 |
| 3218.630571 | 4 |
| 3218.6310713 | 2 |
| 3218.631681 | 0 |
| 3218.6335834 | 1 |
| 3218.6338388 | 3 |
| 3218.6348454 | 0 |
| 3218.6361537 | 2 |
| 3218.6366135 | 2 |

| | |
|---|---|
| 3218.6378511 | 2 |
| 3218.6387792 | 3 |
| 3218.6399207 | 3 |
| 3218.6407827 | 2 |
| 3218.640884 | 0 |
| 3218.6421383 | 4 |
| 3218.6430703 | 4 |
| 3218.6436604 | 1 |
| 3218.6447181 | 2 |
| 3218.6459437 | 3 |
| 3218.6468465 | 3 |
| 3218.6471634 | 5 |
| 3218.6480874 | 4 |
| 3218.6489148 | 1 |
| 3218.6498339 | 3 |
| 3218.6518913 | 3 |
| 3218.651937 | 3 |
| 3218.6531019 | 0 |
| 3218.6535362 | 2 |
| 3218.6551566 | 2 |
| 3218.6562594 | 4 |
| 3218.6571526 | 4 |
| 3218.6576599 | 1 |
| 3218.658598 | 1 |
| 3218.6594905 | 3 |
| 3218.6607899 | 3 |
| 3218.6610291 | 4 |
| 3218.6622282 | 1 |
| 3218.6629764 | 5 |
| 3218.6639188 | 2 |
| 3218.665693 | 2 |
| 3218.6658198 | 1 |
| 3218.667114 | 1 |
| 3218.6675753 | 1 |
| 3218.6692219 | 1 |
| 3218.6701445 | 0 |
| 3218.6714082 | 2 |
| 3218.6723423 | 4 |
| 3218.672782 | 2 |
| 3218.6739838 | 1 |
| 3218.6745307 | 1 |
| 3218.6756034 | 1 |
| 3218.6769871 | 0 |
| 3218.6774776 | 2 |
| 3218.6789658 | 1 |
| 3218.6791702 | 4 |
| 3218.6802394 | 0 |
| 3218.6817773 | 2 |
| 3218.6821892 | 2 |
| 3218.6833892 | 1 |
| 3218.6835125 | 2 |
| 3218.6848436 | 4 |
| 3218.6859194 | 1 |
| 3218.6864764 | 3 |
| 3218.6866575 | 4 |
| 3218.68824 | 4 |
| 3218.6897336 | 3 |
| 3218.6909877 | 3 |
| 3218.6910001 | 2 |
| 3218.6920463 | 1 |
| 3218.6932339 | 1 |
| 3218.6933099 | 2 |
| 3218.6950006 | 1 |
| 3218.6959069 | 3 |
| 3218.6970118 | 2 |
| 3218.6972087 | 3 |
| 3218.6978089 | 1 |
| 3218.6990834 | 5 |
| 3218.7000852 | 3 |
| 3218.7016223 | 2 |
| 3218.7019295 | 4 |
| 3218.7029505 | 0 |
| 3218.7040937 | 0 |
| 3218.7056269 | 0 |
| 3218.7059903 | 0 |
| 3218.7067458 | 0 |
| 3218.7071739 | 2 |
| 3218.708237 | 1 |
| 3218.7093786 | 1 |
| 3218.7107328 | 6 |
| 3218.7119061 | 2 |
| 3218.7129747 | 0 |
| 3218.7132064 | 4 |
| 3218.7145436 | 3 |
| 3218.7149733 | 2 |
| 3218.7161024 | 4 |
| 3218.7163257 | 3 |
| 3218.716993 | 1 |
| 3218.7171814 | 7 |
| 3218.71738 | 4 |
| 3218.7183161 | 7 |
| 3218.7187541 | 1 |
| 3218.7188094 | 3 |
| 3218.7190643 | 1 |
| 3218.7197177 | 3 |
| 3218.7199222 | 2 |
| 3218.7201097 | 2 |
| 3218.7207265 | 4 |
| 3218.7209161 | 1 |
| 3218.7209581 | 0 |
| 3218.7219014 | 3 |
| 3218.7220186 | 1 |
| 3218.7220788 | 1 |
| 3218.7223307 | 2 |
| 3218.7229302 | 0 |
| 3218.7231164 | 3 |
| 3218.7239551 | 1 |
| 3218.7243041 | 1 |
| 3218.7245939 | 0 |
| 3218.7246192 | 4 |
| 3218.7248705 | 2 |
| 3218.7249332 | 0 |
| 3218.7256055 | 1 |
| 3218.7258033 | 1 |
| 3218.7260473 | 2 |
| 3218.7262546 | 1 |
| 3218.7268173 | 1 |
| 3218.7273298 | 5 |
| 3218.7278546 | 2 |
| 3218.7283877 | 1 |
| 3218.7284269 | 0 |
| 3218.7293009 | 1 |
| 3218.7293443 | 1 |
| 3218.7294453 | 1 |
| 3218.729775 | 5 |
| 3218.7298593 | 2 |
| 3218.7309719 | 6 |
| 3218.7311704 | 0 |
| 3218.7312987 | 1 |
| 3218.7316175 | 2 |
| 3218.7316242 | 2 |
| 3218.7322529 | 1 |
| 3218.7327597 | 0 |
| 3218.7330238 | 5 |
| 3218.7331343 | 2 |
| 3218.7337815 | 3 |
| 3218.7340183 | 3 |
| 3218.7345263 | 6 |
| 3218.734604 | 0 |
| 3218.7347703 | 3 |
| 3218.7355013 | 1 |
| 3218.7357445 | 1 |
| 3218.7359934 | 1 |
| 3218.7362298 | 0 |
| 3218.736842 | 3 |
| 3218.7372068 | 5 |
| 3218.7376035 | 1 |
| 3218.7378681 | 1 |
| 3218.7385244 | 2 |
| 3218.7386257 | 3 |
| 3218.739028 | 2 |
| 3218.7391293 | 1 |
| 3218.7394058 | 1 |
| 3218.7395495 | 0 |
| 3218.7397685 | 3 |
| 3218.7409729 | 5 |
| 3218.7412801 | 2 |
| 3218.7413221 | 2 |
| 3218.7418325 | 0 |
| 3218.7420034 | 2 |
| 3218.7424325 | 4 |
| 3218.7426978 | 2 |
| 3218.7426982 | 2 |
| 3218.7434382 | 2 |
| 3218.7436349 | 0 |
| 3218.7441103 | 1 |
| 3218.7448361 | 4 |
| 3218.7449652 | 2 |
| 3218.7455088 | 2 |
| 3218.7456281 | 1 |
| 3218.7457633 | 2 |
| 3218.7466153 | 2 |
| 3218.7466418 | 2 |
| 3218.747388 | 2 |
| 3218.7474135 | 4 |
| 3218.7474261 | 2 |
| 3218.747545 | 0 |
| 3218.7483172 | 6 |
| 3218.7488734 | 5 |
| 3218.7489477 | 3 |
| 3218.7492371 | 3 |
| 3218.7499186 | 15 |
| 3218.7501157 | 11 |
| 3218.7509809 | 21 |
| 3218.7510404 | 7 |
| 3218.7513009 | 14 |
| 3218.7521634 | 20 |
| 3218.752379 | 18 |
| 3218.7527185 | 17 |
| 3218.7530962 | 18 |
| 3218.7534697 | 20 |
| 3218.753512 | 16 |
| 3218.75406 | 13 |
| 3218.7543741 | 15 |
| 3218.7546216 | 9 |
| 3218.754985 | 8 |
| 3218.7553779 | 8 |
| 3218.7561741 | 5 |
| 3218.7562573 | 3 |
| 3218.7562744 | 9 |
| 3218.7564782 | 0 |
| 3218.756974 | 0 |
| 3218.7575173 | 1 |
| 3218.7579974 | 1 |
| 3218.7585892 | 3 |
| 3218.7587553 | 0 |
| 3218.7589464 | 5 |
| 3218.7591002 | 2 |
| 3218.7600091 | 2 |
| 3218.7601299 | 3 |
| 3218.7605728 | 4 |
| 3218.7610025 | 4 |
| 3218.7614376 | 5 |
| 3218.7615067 | 3 |
| 3218.7618962 | 0 |
| 3218.7622823 | 1 |
| 3218.7626713 | 1 |
| 3218.763311 | 1 |
| 3218.7634585 | 1 |
| 3218.7636063 | 2 |
| 3218.7642733 | 3 |
| 3218.7646948 | 2 |
| 3218.7650386 | 2 |
| 3218.7651429 | 1 |
| 3218.7655521 | 5 |
| 3218.7656209 | 3 |
| 3218.765794 | 2 |
| 3218.7663378 | 1 |
| 3218.7669045 | 1 |
| 3218.7673771 | 2 |
| 3218.7677842 | 2 |
| 3218.7680164 | 0 |
| 3218.7680732 | 1 |
| 3218.7681377 | 2 |
| 3218.7683697 | 2 |
| 3218.7688087 | 3 |
| 3218.7711612 | 0 |
| 3218.7720523 | 3 |
| 3218.7739237 | 0 |
| 3218.7757734 | 4 |
| 3218.7766039 | 5 |
| 3218.7788428 | 1 |
| 3218.7797308 | 3 |
| 3218.780494 | 3 |
| 3218.783408 | 1 |
| 3218.7843467 | 2 |
| 3218.785761 | 1 |
| 3218.7876605 | 0 |
| 3218.7889398 | 1 |
| 3218.7905284 | 1 |
| 3218.7915911 | 0 |
| 3218.793542 | 0 |
| 3218.794655 | 1 |
| 3218.7967708 | 2 |
| 3218.7991158 | 2 |
| 3218.7998733 | 2 |
| 3218.8021113 | 5 |
| 3218.8029721 | 3 |
| 3218.8045354 | 1 |
| 3218.8064087 | 3 |
| 3218.8072994 | 0 |
| 3218.8098828 | 4 |
| 3218.8105873 | 3 |
| 3218.8126542 | 0 |
| 3218.814564 | 2 |
| 3218.8159358 | 3 |
| 3218.8172404 | 0 |
| 3218.8182251 | 3 |
| 3218.8202458 | 3 |
| 3218.8224295 | 3 |
| 3218.8230401 | 2 |
| 3218.8247701 | 0 |
| 3218.8257634 | 0 |
| 3218.8275888 | 4 |
| 3218.8298404 | 3 |
| 3218.8303633 | 0 |
| 3218.831825 | 0 |
| 3218.8318951 | 2 |
| 3218.8319717 | 4 |
| 3218.832294 | 2 |
| 3218.8327554 | 1 |
| 3218.8336344 | 1 |
| 3218.8342133 | 5 |
| 3218.8352648 | 1 |
| 3218.8354422 | 3 |
| 3218.8360569 | 3 |
| 3218.8361336 | 0 |
| 3218.836218 | 1 |
| 3218.8371137 | 2 |
| 3218.8381114 | 2 |
| 3218.8382169 | 1 |
| 3218.8382881 | 2 |
| 3218.839941 | 4 |
| 3218.8402371 | 1 |
| 3218.8402854 | 1 |
| 3218.8408688 | 2 |
| 3218.8409391 | 2 |
| 3218.8417227 | 4 |
| 3218.8426914 | 3 |
| 3218.8429914 | 2 |
| 3218.8432219 | 0 |
| 3218.8435322 | 1 |
| 3218.8436466 | 3 |
| 3218.8441439 | 1 |
| 3218.8446929 | 5 |
| 3218.8457707 | 3 |
| 3218.8458778 | 0 |
| 3218.8465209 | 2 |
| 3218.8483901 | 3 |
| 3218.8486029 | 6 |
| 3218.8487123 | 0 |
| 3218.8488487 | 1 |
| 3218.849543 | 2 |
| 3218.8507434 | 4 |
| 3218.8509156 | 2 |
| 3218.8513225 | 2 |
| 3218.851594 | 0 |
| 3218.8520102 | 1 |
| 3218.8528054 | 3 |
| 3218.8528528 | 1 |
| 3218.8538909 | 3 |
| 3218.8541004 | 1 |
| 3218.8545574 | 3 |
| 3218.854993 | 0 |
| 3218.8557564 | 1 |
| 3218.856799 | 3 |
| 3218.8568218 | 2 |
| 3218.8570963 | 2 |
| 3218.8578446 | 3 |
| 3218.8581383 | 6 |
| 3218.8585305 | 3 |
| 3218.8586053 | 3 |
| 3218.8595137 | 1 |
| 3218.8596523 | 3 |
| 3218.8598819 | 2 |
| 3218.860369 | 3 |
| 3218.8604167 | 2 |
| 3218.8607172 | 1 |
| 3218.8610412 | 1 |
| 3218.8623789 | 1 |
| 3218.8624188 | 0 |
| 3218.8626407 | 1 |
| 3218.8631492 | 2 |
| 3218.8636901 | 1 |
| 3218.8643445 | 4 |
| 3218.8648339 | 1 |
| 3218.8652682 | 2 |
| 3218.8653359 | 2 |
| 3218.8657703 | 2 |
| 3218.8658935 | 0 |
| 3218.8668754 | 2 |
| 3218.8671027 | 2 |
| 3218.8672552 | 2 |
| 3218.867978 | 0 |
| 3218.8680171 | 1 |
| 3218.8684907 | 2 |
| 3218.8690407 | 1 |
| 3218.869461 | 6 |
| 3218.8696572 | 4 |
| 3218.8703979 | 4 |
| 3218.8711113 | 7 |
| 3218.8711286 | 4 |
| 3218.8716384 | 3 |

| | |
|---|---|
| 3218.8719783 | 7 |
| 3218.8722385 | 18 |
| 3218.872704 | 15 |
| 3218.8734575 | 12 |
| 3218.8741555 | 5 |
| 3218.8742798 | 10 |
| 3218.8743919 | 4 |
| 3218.8745122 | 4 |
| 3218.8752932 | 2 |
| 3218.8760604 | 4 |
| 3218.8761883 | 5 |
| 3218.877042 | 2 |
| 3218.8772835 | 1 |
| 3218.8775297 | 2 |
| 3218.8775519 | 1 |
| 3218.8785783 | 5 |
| 3218.8788364 | 0 |
| 3218.8794208 | 4 |
| 3218.8795638 | 2 |
| 3218.8797475 | 5 |
| 3218.8805467 | 1 |
| 3218.8810538 | 4 |
| 3218.881132 | 1 |
| 3218.8813638 | 4 |
| 3218.8822127 | 1 |
| 3218.8824443 | 5 |
| 3218.8825192 | 4 |
| 3218.8830867 | 2 |
| 3218.883613 | 5 |
| 3218.8839748 | 4 |
| 3218.8841757 | 2 |
| 3218.8848898 | 1 |
| 3218.8853119 | 2 |
| 3218.8856748 | 2 |
| 3218.8856977 | 0 |
| 3218.885887 | 2 |
| 3218.8871134 | 3 |
| 3218.8879006 | 0 |
| 3218.8880044 | 0 |
| 3218.8884647 | 3 |
| 3218.8886931 | 4 |
| 3218.8897633 | 2 |
| 3218.8905125 | 3 |
| 3218.8905421 | 2 |
| 3218.8906983 | 2 |
| 3218.890744 | 2 |
| 3218.891693 | 1 |
| 3218.8917455 | 4 |
| 3218.8923674 | 5 |
| 3218.8930741 | 2 |
| 3218.8932646 | 2 |
| 3218.8933517 | 1 |
| 3218.8934578 | 0 |
| 3218.8943904 | 5 |
| 3218.8948726 | 3 |
| 3218.8951547 | 1 |
| 3218.8953834 | 1 |
| 3218.8961282 | 4 |
| 3218.8972878 | 0 |
| 3218.8974709 | 5 |
| 3218.897779 | 4 |
| 3218.8984157 | 3 |
| 3218.8985325 | 0 |
| 3218.8987785 | 1 |
| 3218.8989166 | 0 |
| 3218.8992314 | 3 |
| 3218.8999044 | 1 |
| 3218.9007505 | 3 |
| 3218.901408 | 1 |
| 3218.9015473 | 3 |
| 3218.901825 | 1 |
| 3218.9022028 | 0 |
| 3218.9030985 | 4 |
| 3218.9035434 | 2 |
| 3218.9036764 | 1 |
| 3218.9038442 | 2 |
| 3218.9048418 | 2 |
| 3218.9050766 | 5 |
| 3218.9056731 | 0 |
| 3218.9060368 | 2 |
| 3218.9070311 | 2 |
| 3218.9077733 | 0 |
| 3218.9080206 | 2 |
| 3218.9083487 | 2 |
| 3218.9085035 | 1 |
| 3218.9098491 | 3 |
| 3218.9099656 | 1 |
| 3218.9103647 | 1 |
| 3218.9108521 | 1 |
| 3218.911117 | 1 |
| 3218.9115246 | 1 |
| 3218.9122472 | 0 |
| 3218.9133298 | 1 |
| 3218.9137417 | 5 |
| 3218.9138972 | 2 |
| 3218.9143696 | 1 |
| 3218.9145851 | 7 |
| 3218.9150299 | 3 |
| 3218.915853 | 3 |
| 3218.9164251 | 1 |
| 3218.918206 | 1 |
| 3218.9208324 | 2 |
| 3218.9211259 | 0 |
| 3218.923841 | 2 |
| 3218.9245511 | 3 |
| 3218.9264392 | 4 |
| 3218.9284288 | 1 |
| 3218.9294147 | 1 |
| 3218.9309637 | 1 |
| 3218.9317959 | 1 |
| 3218.9336503 | 1 |
| 3218.9358742 | 1 |
| 3218.9369306 | 5 |
| 3218.9387137 | 2 |
| 3218.9400703 | 2 |
| 3218.9422905 | 2 |
| 3218.9434009 | 4 |
| 3218.9454353 | 3 |
| 3218.9471851 | 2 |
| 3218.9483239 | 0 |
| 3218.9501019 | 2 |
| 3218.9514538 | 0 |
| 3218.9531039 | 4 |
| 3218.9549564 | 0 |
| 3218.955865 | 0 |
| 3218.9583059 | 4 |
| 3218.9586708 | 3 |
| 3218.9609833 | 6 |
| 3218.9621496 | 4 |
| 3218.9633193 | 4 |
| 3218.9650364 | 2 |
| 3218.9661261 | 2 |
| 3218.9678532 | 3 |
| 3218.9704223 | 1 |
| 3218.9710115 | 3 |
| 3218.973093 | 1 |
| 3218.973765 | 5 |
| 3218.9756153 | 1 |
| 3218.9777615 | 5 |
| 3218.9787599 | 2 |
| 3218.9814769 | 3 |
| 3218.982494 | 4 |
| 3218.9842424 | 2 |
| 3218.9849419 | 1 |
| 3218.9872938 | 3 |
| 3218.9888826 | 3 |
| 3218.9903582 | 2 |
| 3218.9911553 | 1 |
| 3218.9931324 | 1 |
| 3218.9950361 | 3 |
| 3218.9972271 | 2 |
| 3218.9984392 | 4 |
| 3219.0006492 | 2 |
| 3219.0007217 | 2 |
| 3219.0033879 | 1 |
| 3219.0035737 | 2 |
| 3219.00582 | 1 |
| 3219.0069929 | 1 |
| 3219.0096562 | 1 |
| 3219.0104058 | 2 |
| 3219.0128276 | 4 |
| 3219.0132904 | 4 |
| 3219.0150171 | 2 |
| 3219.0159823 | 1 |
| 3219.0185955 | 3 |
| 3219.020208 | 1 |
| 3219.0215816 | 1 |
| 3219.0231734 | 3 |
| 3219.0243011 | 3 |
| 3219.0256308 | 7 |
| 3219.0282808 | 0 |
| 3219.0291818 | 2 |
| 3219.0314268 | 1 |
| 3219.0316675 | 2 |
| 3219.033956 | 0 |
| 3219.0363019 | 2 |
| 3219.0373804 | 6 |
| 3219.0394095 | 2 |
| 3219.0404351 | 1 |
| 3219.0430645 | 3 |
| 3219.0431587 | 4 |
| 3219.0450786 | 1 |
| 3219.0467957 | 1 |
| 3219.0480961 | 3 |
| 3219.0493206 | 2 |
| 3219.0518378 | 3 |
| 3219.0526154 | 2 |
| 3219.0544473 | 5 |
| 3219.0556679 | 3 |
| 3219.0579254 | 3 |
| 3219.0584615 | 5 |
| 3219.0609113 | 0 |
| 3219.0623664 | 3 |
| 3219.0636722 | 1 |
| 3219.0657941 | 3 |
| 3219.0658653 | 0 |
| 3219.0681671 | 2 |
| 3219.0704767 | 2 |
| 3219.0710753 | 5 |
| 3219.0729072 | 2 |
| 3219.0743176 | 0 |
| 3219.0749934 | 2 |
| 3219.0763704 | 2 |
| 3219.0782626 | 0 |
| 3219.0797264 | 6 |
| 3219.0834155 | 2 |
| 3219.085872 | 0 |
| 3219.0876198 | 2 |
| 3219.0894741 | 4 |
| 3219.0903248 | 1 |
| 3219.090458 | 2 |
| 3219.0921409 | 1 |
| 3219.0924088 | 1 |
| 3219.0924227 | 1 |
| 3219.0938896 | 4 |
| 3219.093966 | 7 |
| 3219.0948413 | 1 |
| 3219.0949829 | 1 |
| 3219.0952485 | 2 |
| 3219.0965637 | 2 |
| 3219.0966725 | 2 |
| 3219.0970108 | 2 |
| 3219.0982909 | 0 |
| 3219.0983263 | 2 |
| 3219.0996161 | 4 |
| 3219.0996408 | 0 |
| 3219.1000692 | 2 |
| 3219.1006295 | 3 |
| 3219.1010015 | 0 |
| 3219.1022175 | 4 |
| 3219.102387 | 1 |
| 3219.1023871 | 3 |
| 3219.1041436 | 2 |
| 3219.1044954 | 2 |
| 3219.1046521 | 1 |
| 3219.1056929 | 3 |
| 3219.1057084 | 3 |
| 3219.1063739 | 3 |
| 3219.1066224 | 1 |
| 3219.1067797 | 3 |
| 3219.1078204 | 1 |
| 3219.1086208 | 1 |
| 3219.1092425 | 1 |
| 3219.1105724 | 3 |
| 3219.1112681 | 4 |
| 3219.1119584 | 0 |
| 3219.1128632 | 2 |
| 3219.113183 | 3 |
| 3219.1145985 | 0 |
| 3219.1159197 | 1 |
| 3219.1163634 | 2 |
| 3219.1176812 | 2 |
| 3219.1182585 | 3 |
| 3219.1189496 | 3 |
| 3219.1200891 | 2 |
| 3219.1209106 | 1 |
| 3219.1227211 | 2 |
| 3219.1227558 | 2 |
| 3219.1240623 | 6 |
| 3219.1244635 | 1 |
| 3219.1246011 | 3 |
| 3219.1253424 | 3 |
| 3219.1267876 | 2 |
| 3219.1277032 | 2 |
| 3219.1286736 | 3 |
| 3219.12906 | 2 |
| 3219.130924 | 0 |
| 3219.1313127 | 2 |
| 3219.1324498 | 2 |
| 3219.1332693 | 1 |
| 3219.1338248 | 4 |
| 3219.1354358 | 2 |
| 3219.1357387 | 3 |
| 3219.1365052 | 0 |
| 3219.1379439 | 4 |
| 3219.1390214 | 3 |
| 3219.1396167 | 0 |
| 3219.1406701 | 2 |
| 3219.1411169 | 4 |
| 3219.1424622 | 3 |
| 3219.143048 | 3 |
| 3219.1442877 | 1 |
| 3219.1450491 | 0 |
| 3219.1459892 | 1 |
| 3219.147421 | 0 |
| 3219.1481487 | 1 |
| 3219.1482603 | 2 |
| 3219.1498986 | 1 |
| 3219.1506439 | 2 |
| 3219.1513364 | 3 |
| 3219.1522818 | 3 |
| 3219.1539255 | 3 |
| 3219.1547192 | 1 |
| 3219.1553296 | 1 |
| 3219.1563938 | 1 |
| 3219.1568282 | 1 |
| 3219.1581253 | 4 |
| 3219.1595963 | 0 |
| 3219.1596069 | 3 |
| 3219.1610362 | 2 |
| 3219.1616686 | 1 |
| 3219.1624757 | 2 |
| 3219.1639551 | 4 |
| 3219.1642677 | 3 |
| 3219.1657251 | 1 |
| 3219.1667065 | 0 |
| 3219.167579 | 3 |
| 3219.1684488 | 2 |
| 3219.1696134 | 1 |
| 3219.1710095 | 1 |
| 3219.1712427 | 0 |
| 3219.1723134 | 1 |
| 3219.1730984 | 1 |
| 3219.1734196 | 4 |
| 3219.1751922 | 1 |
| 3219.1763588 | 4 |
| 3219.1764759 | 1 |
| 3219.1783298 | 3 |
| 3219.1784852 | 3 |
| 3219.1801805 | 1 |
| 3219.1812694 | 2 |
| 3219.1817168 | 1 |
| 3219.182271 | 2 |
| 3219.183393 | 0 |
| 3219.184363 | 0 |
| 3219.1857758 | 1 |
| 3219.1866937 | 1 |
| 3219.1876343 | 1 |
| 3219.1877862 | 2 |
| 3219.1884597 | 1 |
| 3219.1894624 | 2 |
| 3219.19107 | 4 |
| 3219.1917466 | 3 |
| 3219.1929407 | 5 |
| 3219.1936906 | 3 |
| 3219.1944072 | 2 |
| 3219.1957159 | 1 |
| 3219.1965182 | 0 |
| 3219.1966211 | 2 |
| 3219.198569 | 0 |
| 3219.199048 | 1 |
| 3219.1997427 | 1 |
| 3219.2015456 | 1 |
| 3219.2022681 | 0 |
| 3219.2028043 | 4 |
| 3219.2034575 | 1 |
| 3219.2056106 | 2 |
| 3219.2060252 | 1 |
| 3219.2072626 | 1 |
| 3219.2080314 | 2 |
| 3219.20862 | 0 |
| 3219.209141 | 1 |
| 3219.2099095 | 4 |
| 3219.2115806 | 2 |
| 3219.2120824 | 2 |
| 3219.2129066 | 1 |
| 3219.2144088 | 1 |
| 3219.2153665 | 1 |
| 3219.2154813 | 3 |
| 3219.2168291 | 2 |
| 3219.2178123 | 3 |
| 3219.2190197 | 0 |
| 3219.2191633 | 0 |

| | |
|---|---|
| 3219.2205454 | 2 |
| 3219.2217009 | 2 |
| 3219.2229401 | 3 |
| 3219.2234084 | 5 |
| 3219.2250272 | 4 |
| 3219.2251117 | 0 |
| 3219.2257597 | 4 |
| 3219.2266829 | 1 |
| 3219.2277154 | 0 |
| 3219.2296088 | 4 |
| 3219.2298955 | 1 |
| 3219.230943 | 1 |
| 3219.2313239 | 2 |
| 3219.2329367 | 0 |
| 3219.2340668 | 2 |
| 3219.2341305 | 1 |
| 3219.2360221 | 2 |
| 3219.2363837 | 0 |
| 3219.2369242 | 2 |
| 3219.2383387 | 4 |
| 3219.2392294 | 1 |
| 3219.2404601 | 1 |
| 3219.2413032 | 4 |
| 3219.2425554 | 5 |
| 3219.2428662 | 1 |
| 3219.2437219 | 4 |
| 3219.2450214 | 2 |
| 3219.2456262 | 1 |
| 3219.2469325 | 6 |
| 3219.2481976 | 3 |
| 3219.2488707 | 4 |
| 3219.2496969 | 2 |
| 3219.2499815 | 3 |
| 3219.2512841 | 2 |
| 3219.2530688 | 2 |
| 3219.253519 | 0 |
| 3219.2541695 | 2 |
| 3219.2547257 | 2 |
| 3219.2554299 | 2 |
| 3219.25652 | 0 |
| 3219.2577193 | 4 |
| 3219.258923 | 4 |
| 3219.259729 | 3 |
| 3219.2608125 | 1 |
| 3219.2616357 | 0 |
| 3219.262718 | 1 |
| 3219.2632579 | 1 |
| 3219.2642679 | 4 |
| 3219.2655452 | 0 |
| 3219.266552 | 4 |
| 3219.2670329 | 2 |
| 3219.2682536 | 2 |
| 3219.2692804 | 2 |
| 3219.2696494 | 1 |
| 3219.270655 | 0 |
| 3219.2717957 | 2 |
| 3219.2726838 | 3 |
| 3219.2728217 | 3 |
| 3219.2739996 | 6 |
| 3219.2759149 | 4 |
| 3219.2761315 | 0 |
| 3219.2773023 | 1 |
| 3219.2780335 | 1 |
| 3219.2788709 | 3 |
| 3219.2803955 | 1 |
| 3219.2808695 | 2 |
| 3219.2815096 | 5 |
| 3219.2824365 | 1 |
| 3219.2830157 | 1 |
| 3219.2846405 | 4 |
| 3219.2858592 | 6 |
| 3219.2870708 | 1 |
| 3219.2880402 | 1 |
| 3219.2889317 | 0 |
| 3219.289894 | 5 |
| 3219.2907747 | 1 |
| 3219.2915585 | 1 |
| 3219.2926929 | 2 |
| 3219.2935939 | 0 |
| 3219.2943067 | 3 |
| 3219.2949521 | 2 |
| 3219.2967407 | 0 |
| 3219.2971755 | 0 |
| 3219.2979235 | 4 |
| 3219.2985541 | 1 |
| 3219.3003527 | 3 |
| 3219.3004731 | 0 |
| 3219.3014785 | 1 |
| 3219.3023252 | 1 |
| 3219.3036631 | 1 |
| 3219.304727 | 2 |
| 3219.3056992 | 1 |
| 3219.3057368 | 1 |
| 3219.3070399 | 0 |
| 3219.3081102 | 1 |
| 3219.3096116 | 2 |
| 3219.3098331 | 0 |
| 3219.3108338 | 5 |
| 3219.3122196 | 0 |
| 3219.3123241 | 0 |
| 3219.3141379 | 0 |
| 3219.3145568 | 4 |
| 3219.3152515 | 2 |
| 3219.3161141 | 2 |
| 3219.3169186 | 3 |
| 3219.3180113 | 0 |
| 3219.3198949 | 4 |
| 3219.3201346 | 3 |
| 3219.3208308 | 4 |
| 3219.3220624 | 1 |
| 3219.3233734 | 3 |
| 3219.3238855 | 1 |
| 3219.3248522 | 0 |
| 3219.3264398 | 0 |
| 3219.3265532 | 2 |
| 3219.3275106 | 1 |
| 3219.3279741 | 0 |
| 3219.3290266 | 2 |
| 3219.3304938 | 2 |
| 3219.3310503 | 0 |
| 3219.3330322 | 3 |
| 3219.3334001 | 1 |
| 3219.3344953 | 1 |
| 3219.3358485 | 1 |
| 3219.3360798 | 0 |
| 3219.3371125 | 0 |
| 3219.3382753 | 2 |
| 3219.3388168 | 5 |
| 3219.3400225 | 0 |
| 3219.3404745 | 1 |
| 3219.3412051 | 1 |
| 3219.3423389 | 1 |
| 3219.343805 | 3 |
| 3219.3440842 | 0 |
| 3219.3455056 | 4 |
| 3219.3464255 | 2 |
| 3219.3475001 | 2 |
| 3219.3483999 | 2 |
| 3219.3494099 | 1 |
| 3219.3497062 | 2 |
| 3219.3510463 | 0 |
| 3219.3516624 | 1 |
| 3219.3527246 | 2 |
| 3219.3538694 | 3 |
| 3219.3539915 | 1 |
| 3219.3554996 | 0 |
| 3219.3562291 | 2 |
| 3219.3570603 | 3 |
| 3219.3584682 | 1 |
| 3219.3589169 | 3 |
| 3219.3601624 | 3 |
| 3219.3617304 | 1 |
| 3219.361844 | 3 |
| 3219.3628717 | 1 |
| 3219.3634392 | 1 |
| 3219.3650496 | 3 |
| 3219.3652552 | 1 |
| 3219.366551 | 0 |
| 3219.3677899 | 5 |
| 3219.3678762 | 3 |
| 3219.3688019 | 2 |
| 3219.3707346 | 6 |
| 3219.3709783 | 2 |
| 3219.3721916 | 0 |
| 3219.3728308 | 0 |
| 3219.3740131 | 2 |
| 3219.3753033 | 0 |
| 3219.3754305 | 1 |
| 3219.3769921 | 3 |
| 3219.3777892 | 0 |
| 3219.3784131 | 1 |
| 3219.3799203 | 0 |
| 3219.3811139 | 1 |
| 3219.3819244 | 2 |
| 3219.3829948 | 0 |
| 3219.3840687 | 3 |
| 3219.3845893 | 0 |
| 3219.3854268 | 2 |
| 3219.3866885 | 2 |
| 3219.3876567 | 0 |
| 3219.3885343 | 2 |
| 3219.3889534 | 0 |
| 3219.3899942 | 1 |
| 3219.3911507 | 1 |
| 3219.3915083 | 1 |
| 3219.39263 | 3 |
| 3219.3943626 | 4 |
| 3219.3944028 | 2 |
| 3219.3958416 | 1 |
| 3219.3969001 | 0 |
| 3219.3975697 | 3 |
| 3219.3984915 | 0 |
| 3219.3991671 | 1 |
| 3219.4005697 | 2 |
| 3219.4018031 | 2 |
| 3219.4020809 | 2 |
| 3219.4026446 | 2 |
| 3219.4033653 | 2 |
| 3219.4048654 | 1 |
| 3219.4057707 | 1 |
| 3219.4065925 | 1 |
| 3219.4081773 | 1 |
| 3219.4087162 | 2 |
| 3219.410112 | 2 |
| 3219.4111844 | 3 |
| 3219.4116138 | 1 |
| 3219.4126598 | 4 |
| 3219.4134833 | 0 |
| 3219.4143247 | 1 |
| 3219.415107 | 0 |
| 3219.4163473 | 2 |
| 3219.4174862 | 1 |
| 3219.4184929 | 3 |
| 3219.4195917 | 1 |
| 3219.4207037 | 0 |
| 3219.4210791 | 2 |
| 3219.422113 | 3 |
| 3219.4225856 | 1 |
| 3219.424045 | 2 |
| 3219.4243878 | 0 |
| 3219.426141 | 2 |
| 3219.4269327 | 2 |
| 3219.4276478 | 1 |
| 3219.4288503 | 2 |
| 3219.4299856 | 2 |
| 3219.4303112 | 3 |
| 3219.4320339 | 3 |
| 3219.432516 | 4 |
| 3219.4343223 | 1 |
| 3219.4347603 | 2 |
| 3219.4351636 | 3 |
| 3219.4364588 | 1 |
| 3219.4368689 | 1 |
| 3219.4380931 | 2 |
| 3219.439313 | 2 |
| 3219.4398409 | 3 |
| 3219.4410101 | 4 |
| 3219.4411739 | 2 |
| 3219.4428888 | 0 |
| 3219.4435743 | 1 |
| 3219.4443802 | 1 |
| 3219.4460103 | 3 |
| 3219.4464033 | 0 |
| 3219.4476829 | 3 |
| 3219.4494506 | 2 |
| 3219.4496216 | 1 |
| 3219.4506455 | 2 |
| 3219.4515928 | 0 |
| 3219.4518098 | 1 |
| 3219.4535392 | 3 |
| 3219.4536342 | 0 |
| 3219.4550013 | 1 |
| 3219.4561788 | 2 |
| 3219.4568293 | 1 |
| 3219.4580267 | 2 |
| 3219.4588153 | 0 |
| 3219.4600603 | 1 |
| 3219.4600981 | 3 |
| 3219.4618169 | 2 |
| 3219.4628762 | 0 |
| 3219.463107 | 0 |
| 3219.4642529 | 2 |
| 3219.4659388 | 1 |
| 3219.466083 | 2 |
| 3219.467209 | 3 |
| 3219.4682447 | 2 |
| 3219.4698522 | 3 |
| 3219.4699303 | 1 |
| 3219.4717117 | 3 |
| 3219.4729764 | 1 |
| 3219.4729964 | 1 |
| 3219.4738721 | 3 |
| 3219.4751305 | 2 |
| 3219.4757717 | 3 |
| 3219.476992 | 1 |
| 3219.477174 | 2 |
| 3219.4779454 | 0 |
| 3219.4801497 | 3 |
| 3219.4808544 | 1 |
| 3219.4813976 | 2 |
| 3219.4825434 | 1 |
| 3219.4836316 | 1 |
| 3219.4850162 | 2 |
| 3219.4859381 | 0 |
| 3219.4861431 | 3 |
| 3219.4874031 | 0 |
| 3219.4879732 | 3 |
| 3219.4887778 | 3 |
| 3219.48953 | 3 |
| 3219.4914184 | 1 |
| 3219.4914568 | 4 |
| 3219.4929082 | 0 |
| 3219.4940938 | 0 |
| 3219.4941472 | 0 |
| 3219.4952863 | 1 |
| 3219.4963021 | 1 |
| 3219.4975471 | 0 |
| 3219.498448 | 2 |
| 3219.499329 | 0 |
| 3219.5005967 | 5 |
| 3219.501026 | 5 |
| 3219.5020064 | 2 |
| 3219.5029858 | 2 |
| 3219.504201 | 1 |
| 3219.5050869 | 1 |
| 3219.505548 | 0 |
| 3219.5065775 | 1 |
| 3219.5079383 | 2 |
| 3219.5082975 | 2 |
| 3219.5093733 | 1 |
| 3219.5104403 | 1 |
| 3219.511663 | 1 |
| 3219.5129261 | 2 |
| 3219.5134507 | 2 |
| 3219.5145033 | 4 |
| 3219.5159611 | 1 |
| 3219.5162529 | 2 |
| 3219.5171341 | 1 |
| 3219.5180485 | 2 |
| 3219.5192306 | 3 |
| 3219.5200626 | 2 |
| 3219.5207852 | 0 |
| 3219.5216805 | 2 |
| 3219.5228974 | 1 |
| 3219.5237184 | 2 |
| 3219.5241313 | 2 |
| 3219.5256154 | 1 |
| 3219.5268589 | 4 |
| 3219.5273456 | 2 |
| 3219.528939 | 3 |
| 3219.5291901 | 1 |
| 3219.5301025 | 0 |
| 3219.5309802 | 1 |
| 3219.5317395 | 1 |
| 3219.5325618 | 3 |
| 3219.5341699 | 4 |
| 3219.5354854 | 3 |
| 3219.5363406 | 4 |
| 3219.536783 | 0 |
| 3219.5379324 | 2 |
| 3219.5384114 | 3 |
| 3219.5387408 | 2 |
| 3219.5407888 | 3 |
| 3219.5413065 | 3 |
| 3219.5419673 | 4 |
| 3219.5428451 | 4 |
| 3219.5443181 | 1 |
| 3219.5451913 | 1 |
| 3219.5461062 | 2 |
| 3219.5469762 | 1 |
| 3219.5484056 | 5 |
| 3219.5485696 | 3 |
| 3219.5502996 | 0 |
| 3219.5508201 | 1 |
| 3219.5514281 | 0 |
| 3219.5523816 | 2 |
| 3219.5533957 | 5 |
| 3219.5548793 | 3 |
| 3219.5549565 | 0 |
| 3219.5563371 | 0 |
| 3219.5568187 | 3 |
| 3219.5582991 | 0 |
| 3219.5596829 | 1 |

| | |
|---|---|
| 3219.5602951 | 3 |
| 3219.5613918 | 1 |
| 3219.5619704 | 0 |
| 3219.5631056 | 1 |
| 3219.5638403 | 0 |
| 3219.564685 | 0 |
| 3219.56621 | 0 |
| 3219.5673445 | 2 |
| 3219.5680165 | 1 |
| 3219.5688424 | 3 |
| 3219.5695713 | 2 |
| 3219.570751 | 0 |
| 3219.5711987 | 4 |
| 3219.5724722 | 2 |
| 3219.5733247 | 3 |
| 3219.5742174 | 1 |
| 3219.5753662 | 2 |
| 3219.5763633 | 3 |
| 3219.5768478 | 2 |
| 3219.5782185 | 2 |
| 3219.5794834 | 2 |
| 3219.5798339 | 1 |
| 3219.5807696 | 1 |
| 3219.5818349 | 1 |
| 3219.5827613 | 2 |
| 3219.5837756 | 1 |
| 3219.5845717 | 3 |
| 3219.5856431 | 3 |
| 3219.5864833 | 1 |
| 3219.5873689 | 1 |
| 3219.5885572 | 2 |
| 3219.5891947 | 0 |
| 3219.5897703 | 0 |
| 3219.5910875 | 2 |
| 3219.5921958 | 3 |
| 3219.5929994 | 2 |
| 3219.5937387 | 1 |
| 3219.5941392 | 5 |
| 3219.5954107 | 1 |
| 3219.5969228 | 0 |
| 3219.597093 | 1 |
| 3219.5985296 | 3 |
| 3219.5986722 | 2 |
| 3219.5995507 | 2 |
| 3219.6016666 | 3 |
| 3219.6027077 | 1 |
| 3219.6030398 | 0 |
| 3219.6043098 | 0 |
| 3219.6049462 | 3 |
| 3219.6063978 | 4 |
| 3219.6064107 | 2 |
| 3219.6073726 | 0 |
| 3219.609125 | 2 |
| 3219.60923 | 1 |
| 3219.6107646 | 3 |
| 3219.611759 | 2 |
| 3219.6128184 | 0 |
| 3219.6140276 | 5 |
| 3219.6145524 | 1 |
| 3219.6154186 | 2 |
| 3219.615908 | 0 |
| 3219.6168976 | 0 |
| 3219.6176263 | 0 |
| 3219.6186967 | 1 |
| 3219.6200767 | 0 |
| 3219.6209389 | 1 |
| 3219.6214363 | 4 |
| 3219.6226564 | 3 |
| 3219.6236927 | 1 |
| 3219.6245281 | 0 |
| 3219.6252845 | 2 |
| 3219.6259272 | 1 |
| 3219.6272447 | 2 |
| 3219.627719 | 2 |
| 3219.6294097 | 1 |
| 3219.6304731 | 3 |
| 3219.6306967 | 1 |
| 3219.6318622 | 2 |
| 3219.6328641 | 1 |
| 3219.6334383 | 0 |
| 3219.6345925 | 2 |
| 3219.6355177 | 3 |
| 3219.6363549 | 2 |
| 3219.6371122 | 2 |
| 3219.6390235 | 4 |
| 3219.639085 | 0 |
| 3219.6398581 | 5 |
| 3219.6414226 | 4 |
| 3219.6419386 | 1 |
| 3219.6428273 | 0 |
| 3219.643984 | 1 |
| 3219.6453856 | 0 |
| 3219.6463713 | 1 |
| 3219.6464832 | 2 |
| 3219.6478445 | 5 |
| 3219.6479468 | 1 |
| 3219.6495988 | 1 |
| 3219.6502945 | 0 |
| 3219.6514636 | 2 |
| 3219.6525148 | 3 |
| 3219.6530542 | 0 |
| 3219.6542098 | 5 |
| 3219.6554949 | 0 |
| 3219.6557992 | 3 |
| 3219.6568242 | 1 |
| 3219.6580972 | 0 |
| 3219.6591263 | 5 |
| 3219.66023 | 1 |
| 3219.6602666 | 4 |
| 3219.6615351 | 0 |
| 3219.6622093 | 1 |
| 3219.6634281 | 3 |
| 3219.664813 | 1 |
| 3219.6650858 | 1 |
| 3219.666448 | 1 |
| 3219.6672451 | 0 |
| 3219.6680175 | 1 |
| 3219.6691949 | 2 |
| 3219.6701824 | 3 |
| 3219.671361 | 2 |
| 3219.6719851 | 1 |
| 3219.6724159 | 3 |
| 3219.6735492 | 2 |
| 3219.6745024 | 3 |
| 3219.67571 | 1 |
| 3219.6764098 | 2 |
| 3219.6775401 | 1 |
| 3219.6788704 | 2 |
| 3219.6792756 | 1 |
| 3219.6804107 | 1 |
| 3219.6810968 | 1 |
| 3219.6820269 | 1 |
| 3219.6823645 | 4 |
| 3219.6836482 | 2 |
| 3219.6849414 | 1 |
| 3219.686171 | 3 |
| 3219.6862194 | 0 |
| 3219.6881493 | 1 |
| 3219.6883503 | 2 |
| 3219.6897709 | 3 |
| 3219.6906733 | 1 |
| 3219.6909744 | 0 |
| 3219.6923868 | 1 |
| 3219.6934274 | 3 |
| 3219.6942676 | 1 |
| 3219.6944182 | 0 |
| 3219.695748 | 1 |
| 3219.6970403 | 3 |
| 3219.6978982 | 0 |
| 3219.6984074 | 2 |
| 3219.6992834 | 2 |
| 3219.7010224 | 0 |
| 3219.701864 | 2 |
| 3219.7028048 | 0 |
| 3219.7036334 | 0 |
| 3219.7044599 | 2 |
| 3219.7054245 | 2 |
| 3219.7061704 | 2 |
| 3219.7069441 | 2 |
| 3219.7081902 | 4 |
| 3219.7088525 | 1 |
| 3219.709988 | 3 |
| 3219.7114213 | 1 |
| 3219.7114951 | 3 |
| 3219.7133988 | 1 |
| 3219.7139957 | 0 |
| 3219.7150453 | 5 |
| 3219.7164252 | 1 |
| 3219.7165889 | 3 |
| 3219.7174252 | 2 |
| 3219.7177577 | 2 |
| 3219.7195402 | 2 |
| 3219.7204626 | 2 |
| 3219.7205389 | 2 |
| 3219.7224588 | 1 |
| 3219.7233582 | 2 |
| 3219.7239196 | 1 |
| 3219.7255105 | 2 |
| 3219.725533 | 6 |
| 3219.7268457 | 1 |
| 3219.7270203 | 0 |
| 3219.7276998 | 1 |
| 3219.7299312 | 1 |
| 3219.7301866 | 2 |
| 3219.7318186 | 2 |
| 3219.7318839 | 1 |
| 3219.7333638 | 0 |
| 3219.7343177 | 2 |
| 3219.7348321 | 0 |
| 3219.7359619 | 0 |
| 3219.7370721 | 1 |
| 3219.7384141 | 2 |
| 3219.7394515 | 2 |
| 3219.7400676 | 2 |
| 3219.7407277 | 0 |
| 3219.7417289 | 2 |
| 3219.7420303 | 3 |
| 3219.7441891 | 7 |
| 3219.7441943 | 2 |
| 3219.7456204 | 2 |
| 3219.7467774 | 3 |
| 3219.7468445 | 0 |
| 3219.7479388 | 1 |
| 3219.7491101 | 2 |
| 3219.749855 | 2 |
| 3219.7510911 | 0 |
| 3219.7523129 | 1 |
| 3219.7535416 | 4 |
| 3219.7537366 | 1 |
| 3219.7553249 | 0 |
| 3219.7555263 | 2 |
| 3219.7568 | 3 |
| 3219.7578143 | 2 |
| 3219.7587156 | 4 |
| 3219.7598737 | 3 |
| 3219.7605797 | 4 |
| 3219.7612121 | 1 |
| 3219.7618522 | 1 |
| 3219.7625868 | 4 |
| 3219.7645871 | 1 |
| 3219.7648478 | 2 |
| 3219.7662831 | 2 |
| 3219.7664851 | 4 |
| 3219.7676464 | 4 |
| 3219.7694453 | 0 |
| 3219.7694613 | 2 |
| 3219.7703441 | 3 |
| 3219.7717342 | 2 |
| 3219.7725046 | 1 |
| 3219.7737305 | 0 |
| 3219.7740677 | 2 |
| 3219.7749457 | 1 |
| 3219.7768497 | 2 |
| 3219.7771374 | 3 |
| 3219.7779942 | 4 |
| 3219.7789448 | 6 |
| 3219.7796705 | 1 |
| 3219.7807959 | 1 |
| 3219.7816336 | 7 |
| 3219.7830865 | 2 |
| 3219.7835013 | 2 |
| 3219.7850653 | 0 |
| 3219.7852075 | 5 |
| 3219.7861601 | 2 |
| 3219.7878603 | 2 |
| 3219.7887314 | 0 |
| 3219.7893565 | 0 |
| 3219.7904416 | 1 |
| 3219.7913622 | 0 |
| 3219.7926164 | 1 |
| 3219.7931814 | 1 |
| 3219.7938871 | 1 |
| 3219.7945643 | 2 |
| 3219.7957205 | 1 |
| 3219.796455 | 2 |
| 3219.7976674 | 1 |
| 3219.7990843 | 2 |
| 3219.7998651 | 0 |
| 3219.800615 | 1 |
| 3219.8018136 | 1 |
| 3219.8018267 | 2 |
| 3219.8035282 | 0 |
| 3219.8036836 | 0 |
| 3219.804939 | 1 |
| 3219.8062134 | 2 |
| 3219.8067212 | 4 |
| 3219.8077269 | 0 |
| 3219.808806 | 3 |
| 3219.8097849 | 1 |
| 3219.8108522 | 1 |
| 3219.81139 | 1 |
| 3219.8130084 | 6 |
| 3219.8135851 | 2 |
| 3219.8141986 | 1 |
| 3219.8154989 | 2 |
| 3219.816368 | 2 |
| 3219.8172136 | 2 |
| 3219.8182507 | 0 |
| 3219.8193496 | 3 |
| 3219.820551 | 1 |
| 3219.8209066 | 3 |
| 3219.8217844 | 0 |
| 3219.8224598 | 3 |
| 3219.8240953 | 0 |
| 3219.8250062 | 3 |
| 3219.8263444 | 2 |
| 3219.8271119 | 1 |
| 3219.8273784 | 2 |
| 3219.8288354 | 2 |
| 3219.8297849 | 4 |
| 3219.8300016 | 0 |
| 3219.8315319 | 1 |
| 3219.8323646 | 1 |
| 3219.8329974 | 1 |
| 3219.8343913 | 4 |
| 3219.8350984 | 0 |
| 3219.836819 | 3 |
| 3219.8373622 | 3 |
| 3219.8379043 | 2 |
| 3219.8392325 | 1 |
| 3219.8397816 | 1 |
| 3219.8406374 | 1 |
| 3219.8419986 | 0 |
| 3219.8430341 | 2 |
| 3219.8433657 | 1 |
| 3219.8446556 | 3 |
| 3219.8455988 | 3 |
| 3219.8470871 | 1 |
| 3219.8473545 | 2 |
| 3219.8488075 | 2 |
| 3219.8492553 | 2 |
| 3219.8501559 | 3 |
| 3219.8514417 | 1 |
| 3219.8517218 | 1 |
| 3219.8531696 | 2 |
| 3219.8544875 | 0 |
| 3219.8547951 | 0 |
| 3219.8560441 | 0 |
| 3219.8564234 | 2 |
| 3219.8575864 | 1 |
| 3219.8583978 | 4 |
| 3219.8597353 | 4 |
| 3219.8601852 | 0 |
| 3219.8607794 | 1 |
| 3219.862326 | 1 |
| 3219.8625561 | 2 |
| 3219.8636585 | 3 |
| 3219.8652979 | 0 |
| 3219.865907 | 1 |
| 3219.8669624 | 5 |
| 3219.867795 | 5 |
| 3219.8683836 | 4 |
| 3219.8693963 | 5 |
| 3219.8705698 | 1 |
| 3219.8719543 | 3 |
| 3219.8725604 | 3 |
| 3219.8735945 | 6 |
| 3219.873969 | 3 |
| 3219.8760851 | 5 |
| 3219.8769612 | 4 |
| 3219.8776934 | 9 |
| 3219.8782135 | 4 |
| 3219.879516 | 0 |
| 3219.879548 | 6 |
| 3219.8799635 | 1 |
| 3219.8800881 | 3 |
| 3219.8804542 | 2 |
| 3219.8806813 | 2 |
| 3219.8810887 | 1 |
| 3219.8812585 | 3 |
| 3219.8815572 | 3 |
| 3219.8821795 | 2 |
| 3219.8822217 | 4 |
| 3219.8829727 | 5 |
| 3219.8830878 | 2 |
| 3219.883335 | 1 |
| 3219.88359 | 2 |
| 3219.8837107 | 3 |
| 3219.8840805 | 1 |
| 3219.8847687 | 1 |
| 3219.884977 | 0 |
| 3219.8850245 | 3 |
| 3219.8852435 | 0 |

| | |
|---|---|
| 3219.8854006 | 1 |
| 3219.8859315 | 1 |
| 3219.8859815 | 2 |
| 3219.8863643 | 4 |
| 3219.8867428 | 0 |
| 3219.8868944 | 1 |
| 3219.887282 | 1 |
| 3219.8877316 | 1 |
| 3219.8877322 | 1 |
| 3219.8879778 | 1 |
| 3219.8881586 | 2 |
| 3219.8882688 | 3 |
| 3219.8888253 | 3 |
| 3219.8889832 | 1 |
| 3219.8892915 | 0 |
| 3219.8895316 | 5 |
| 3219.8897268 | 2 |
| 3219.8899737 | 2 |
| 3219.8901169 | 1 |
| 3219.8907734 | 1 |
| 3219.8908187 | 6 |
| 3219.8917623 | 2 |
| 3219.8917972 | 0 |
| 3219.8919675 | 4 |
| 3219.8920443 | 1 |
| 3219.8921697 | 2 |
| 3219.8925749 | 4 |
| 3219.8927241 | 0 |
| 3219.8929985 | 1 |
| 3219.8930606 | 0 |
| 3219.8935509 | 2 |
| 3219.8939555 | 1 |
| 3219.8941367 | 1 |
| 3219.8942701 | 5 |
| 3219.8950592 | 3 |
| 3219.8952579 | 2 |
| 3219.8953979 | 2 |
| 3219.8957531 | 2 |
| 3219.8962366 | 1 |
| 3219.8963006 | 1 |
| 3219.8963409 | 1 |
| 3219.8964049 | 2 |
| 3219.8965382 | 3 |
| 3219.8968892 | 0 |
| 3219.8974111 | 1 |
| 3219.897645 | 1 |
| 3219.897823 | 0 |
| 3219.8981873 | 2 |
| 3219.8984635 | 3 |
| 3219.8985848 | 2 |
| 3219.8989533 | 0 |
| 3219.8990084 | 1 |
| 3219.899646 | 1 |
| 3219.8998339 | 3 |
| 3219.9000048 | 1 |
| 3219.9002012 | 1 |
| 3219.9002466 | 6 |
| 3219.9006814 | 3 |
| 3219.9009038 | 1 |
| 3219.9010776 | 0 |
| 3219.9016152 | 0 |
| 3219.9019254 | 1 |
| 3219.9021332 | 0 |
| 3219.9027257 | 2 |
| 3219.9030292 | 2 |
| 3219.9031234 | 3 |
| 3219.9034373 | 1 |
| 3219.9034556 | 1 |
| 3219.9035769 | 4 |
| 3219.9037627 | 3 |
| 3219.9038647 | 0 |
| 3219.9046574 | 1 |
| 3219.9046897 | 4 |
| 3219.9051791 | 1 |
| 3219.9053065 | 2 |
| 3219.9055681 | 2 |
| 3219.9059633 | 1 |
| 3219.9060428 | 1 |
| 3219.9064792 | 1 |
| 3219.9069471 | 2 |
| 3219.9070421 | 0 |
| 3219.9071428 | 1 |
| 3219.9077651 | 0 |
| 3219.9078404 | 2 |
| 3219.908116 | 3 |
| 3219.9081594 | 2 |
| 3219.9086101 | 2 |
| 3219.9088267 | 1 |
| 3219.9089948 | 3 |
| 3219.909524 | 2 |
| 3219.9096168 | 2 |
| 3219.9097587 | 3 |
| 3219.9103009 | 3 |
| 3219.9104738 | 2 |
| 3219.9107168 | 1 |
| 3219.9110713 | 1 |
| 3219.9111402 | 3 |
| 3219.9112595 | 2 |
| 3219.9119811 | 1 |
| 3219.9120261 | 0 |
| 3219.9121936 | 1 |
| 3219.9124165 | 2 |
| 3219.9129164 | 1 |
| 3219.9129567 | 2 |
| 3219.9135902 | 4 |
| 3219.9135909 | 1 |
| 3219.9142492 | 1 |
| 3219.9143909 | 4 |
| 3219.9146435 | 1 |
| 3219.914775 | 1 |
| 3219.9150344 | 4 |
| 3219.9152442 | 3 |
| 3219.9157003 | 2 |
| 3219.9157296 | 5 |
| 3219.9160339 | 5 |
| 3219.9160918 | 3 |
| 3219.9164138 | 2 |
| 3219.9173186 | 5 |
| 3219.9175618 | 4 |
| 3219.9176105 | 0 |
| 3219.9178852 | 3 |
| 3219.9179693 | 4 |
| 3219.9182279 | 3 |
| 3219.9184402 | 0 |
| 3219.9184928 | 2 |
| 3219.9190074 | 1 |
| 3219.919246 | 2 |
| 3219.9198884 | 1 |
| 3219.9199086 | 1 |
| 3219.9199291 | 1 |
| 3219.9205349 | 2 |
| 3219.9207265 | 3 |
| 3219.9210032 | 1 |
| 3219.9210771 | 2 |
| 3219.9219143 | 2 |
| 3219.9220649 | 2 |
| 3219.9220734 | 1 |
| 3219.9220871 | 1 |
| 3219.9225902 | 4 |
| 3219.9228327 | 2 |
| 3219.9229071 | 0 |
| 3219.9231803 | 7 |
| 3219.9236554 | 3 |
| 3219.9236639 | 7 |
| 3219.9238892 | 8 |
| 3219.9240987 | 8 |
| 3219.9249393 | 14 |
| 3219.9255117 | 9 |
| 3219.9256711 | 10 |
| 3219.9259113 | 12 |
| 3219.9259305 | 16 |
| 3219.9261973 | 8 |
| 3219.9263855 | 11 |
| 3219.9264759 | 12 |
| 3219.9268135 | 13 |
| 3219.9272395 | 10 |
| 3219.9272726 | 8 |
| 3219.9276517 | 10 |
| 3219.9278488 | 8 |
| 3219.9285654 | 10 |
| 3219.9286654 | 10 |
| 3219.9288575 | 11 |
| 3219.9291817 | 6 |
| 3219.9292212 | 10 |
| 3219.929885 | 5 |
| 3219.9300627 | 7 |
| 3219.9303089 | 4 |
| 3219.9306018 | 9 |
| 3219.9308403 | 3 |
| 3219.9311291 | 4 |
| 3219.9312211 | 1 |
| 3219.9314658 | 3 |
| 3219.9314726 | 4 |
| 3219.9315073 | 6 |
| 3219.9319713 | 5 |
| 3219.9320335 | 7 |
| 3219.9325339 | 9 |
| 3219.9325866 | 9 |
| 3219.9328582 | 5 |
| 3219.9333183 | 9 |
| 3219.9334585 | 11 |
| 3219.9336475 | 6 |
| 3219.9342039 | 7 |
| 3219.9346548 | 15 |
| 3219.9346815 | 6 |
| 3219.9350164 | 12 |
| 3219.9350712 | 9 |
| 3219.9354019 | 3 |
| 3219.9354329 | 4 |
| 3219.935712 | 6 |
| 3219.9358203 | 4 |
| 3219.9358485 | 7 |
| 3219.9360359 | 3 |
| 3219.9364277 | 5 |
| 3219.9365299 | 2 |
| 3219.9366986 | 4 |
| 3219.9370404 | 3 |
| 3219.9373269 | 2 |
| 3219.9375813 | 1 |
| 3219.9376605 | 2 |
| 3219.9376722 | 4 |
| 3219.9380548 | 3 |
| 3219.9384194 | 4 |
| 3219.9387402 | 3 |
| 3219.938987 | 1 |
| 3219.9390783 | 2 |
| 3219.9394282 | 1 |
| 3219.9394633 | 1 |
| 3219.9395229 | 1 |
| 3219.9400408 | 2 |
| 3219.9402277 | 0 |
| 3219.9407315 | 1 |
| 3219.9408394 | 3 |
| 3219.9408699 | 2 |
| 3219.9411391 | 2 |
| 3219.9414508 | 1 |
| 3219.9414741 | 1 |
| 3219.9416553 | 0 |
| 3219.942201 | 0 |
| 3219.9422329 | 4 |
| 3219.9425576 | 2 |
| 3219.942797 | 2 |
| 3219.9428363 | 4 |
| 3219.9428647 | 3 |
| 3219.9430496 | 0 |
| 3219.9436972 | 2 |
| 3219.9438717 | 1 |
| 3219.9439734 | 2 |
| 3219.9440288 | 2 |
| 3219.9442963 | 1 |
| 3219.9443676 | 1 |
| 3219.9450228 | 2 |
| 3219.9452008 | 3 |
| 3219.945319 | 1 |
| 3219.9453289 | 2 |
| 3219.9453598 | 1 |
| 3219.9458686 | 1 |
| 3219.946112 | 0 |
| 3219.9461268 | 3 |
| 3219.9466444 | 1 |
| 3219.9471125 | 1 |
| 3219.9472515 | 3 |
| 3219.9476682 | 0 |
| 3219.947689 | 1 |
| 3219.9478068 | 4 |
| 3219.9478318 | 1 |
| 3219.9479127 | 3 |
| 3219.9482162 | 5 |
| 3219.9482714 | 3 |
| 3219.9487696 | 1 |
| 3219.949524 | 0 |
| 3219.9496765 | 2 |
| 3219.9498713 | 2 |
| 3219.9501109 | 0 |
| 3219.9504651 | 2 |
| 3219.9506702 | 2 |
| 3219.9506984 | 2 |
| 3219.9507683 | 0 |
| 3219.9509287 | 1 |
| 3219.951133 | 2 |
| 3219.9514261 | 7 |
| 3219.9515029 | 1 |
| 3219.9517706 | 0 |
| 3219.9520296 | 4 |
| 3219.9524753 | 6 |
| 3219.9525225 | 2 |
| 3219.9527222 | 1 |
| 3219.9528308 | 1 |
| 3219.9528429 | 4 |
| 3219.9532659 | 1 |
| 3219.9536709 | 1 |
| 3219.9537666 | 4 |
| 3219.953918 | 1 |
| 3219.9542592 | 0 |
| 3219.9543781 | 4 |
| 3219.9549379 | 0 |
| 3219.9550154 | 0 |
| 3219.9553662 | 3 |
| 3219.9553817 | 1 |
| 3219.9554124 | 3 |
| 3219.9559941 | 5 |
| 3219.9561603 | 1 |
| 3219.9562014 | 3 |
| 3219.9562876 | 1 |
| 3219.9566475 | 3 |
| 3219.9570275 | 3 |
| 3219.9570475 | 1 |
| 3219.9572331 | 1 |
| 3219.9575117 | 3 |
| 3219.9578293 | 6 |
| 3219.9578304 | 2 |
| 3219.9582825 | 4 |
| 3219.958489 | 0 |
| 3219.9586498 | 1 |
| 3219.9588094 | 1 |
| 3219.9590242 | 1 |
| 3219.9597515 | 1 |
| 3219.9598552 | 3 |
| 3219.9598983 | 1 |
| 3219.9600381 | 0 |
| 3219.9603298 | 1 |
| 3219.9607023 | 3 |
| 3219.9607131 | 2 |
| 3219.9607962 | 1 |
| 3219.9610155 | 1 |
| 3219.9610786 | 1 |
| 3219.961706 | 0 |
| 3219.9617156 | 0 |
| 3219.9617249 | 3 |
| 3219.9622744 | 1 |
| 3219.9623766 | 3 |
| 3219.9627684 | 1 |
| 3219.9631029 | 6 |
| 3219.9635631 | 4 |
| 3219.9636658 | 0 |
| 3219.9638718 | 0 |
| 3219.9638865 | 3 |
| 3219.9639022 | 2 |
| 3219.9643847 | 4 |
| 3219.9645672 | 1 |
| 3219.9649641 | 2 |
| 3219.9651744 | 3 |
| 3219.965358 | 3 |
| 3219.9653699 | 0 |
| 3219.9659338 | 2 |
| 3219.9659907 | 3 |
| 3219.9664257 | 1 |
| 3219.9664815 | 1 |
| 3219.9666566 | 1 |
| 3219.9671632 | 2 |
| 3219.967408 | 0 |
| 3219.9675016 | 2 |
| 3219.9676611 | 4 |
| 3219.9677682 | 3 |
| 3219.9680248 | 1 |
| 3219.968025 | 3 |
| 3219.9683721 | 0 |
| 3219.9684733 | 0 |
| 3219.9689465 | 3 |
| 3219.969175 | 1 |
| 3219.9693324 | 2 |
| 3219.9697943 | 2 |
| 3219.9698164 | 6 |
| 3219.969948 | 2 |
| 3219.9704624 | 4 |
| 3219.9705117 | 1 |
| 3219.9707248 | 0 |
| 3219.9709172 | 1 |
| 3219.9711914 | 2 |
| 3219.9712145 | 1 |
| 3219.9712329 | 2 |
| 3219.9718928 | 5 |
| 3219.9721638 | 3 |
| 3219.9722945 | 2 |
| 3219.9724006 | 2 |
| 3219.9727094 | 3 |
| 3219.9730151 | 1 |
| 3219.9731091 | 3 |
| 3219.9735475 | 2 |
| 3219.9737135 | 1 |
| 3219.9739135 | 1 |
| 3219.9742711 | 3 |
| 3219.9743801 | 2 |

| | |
|---|---|
| 3219.9746324 | 2 |
| 3219.9746967 | 5 |
| 3219.9748859 | 1 |
| 3219.9753146 | 1 |
| 3219.9753783 | 1 |
| 3219.9757712 | 2 |
| 3219.9757874 | 4 |
| 3219.9761644 | 3 |
| 3219.9770361 | 3 |
| 3219.9780327 | 1 |
| 3219.9790224 | 1 |
| 3219.97981 | 2 |
| 3219.9802976 | 2 |
| 3219.9808587 | 1 |
| 3219.9820815 | 2 |
| 3219.9824113 | 1 |
| 3219.9835667 | 2 |
| 3219.9845474 | 0 |
| 3219.9847135 | 2 |
| 3219.9856794 | 0 |
| 3219.9858452 | 2 |
| 3219.9874001 | 0 |
| 3219.9881796 | 2 |
| 3219.988324 | 1 |
| 3219.9895137 | 2 |
| 3219.9899118 | 2 |
| 3219.9912904 | 2 |
| 3219.9920427 | 5 |
| 3219.9924418 | 3 |
| 3219.9934657 | 3 |
| 3219.9940649 | 3 |
| 3219.9953194 | 3 |
| 3219.9955379 | 1 |
| 3219.9969218 | 3 |
| 3219.9974687 | 0 |
| 3219.9979709 | 1 |
| 3219.9985952 | 0 |
| 3219.9995145 | 3 |
| 3219.999746 | 1 |
| 3220.0013612 | 2 |
| 3220.001744 | 4 |
| 3220.0031735 | 2 |
| 3220.0038485 | 3 |
| 3220.004245 | 1 |
| 3220.0053948 | 1 |
| 3220.0057691 | 0 |
| 3220.0072258 | 3 |
| 3220.007853 | 1 |
| 3220.0082855 | 2 |
| 3220.0096851 | 1 |
| 3220.0098811 | 2 |
| 3220.0106686 | 2 |
| 3220.0110084 | 0 |
| 3220.0119697 | 2 |
| 3220.0133271 | 1 |
| 3220.013661 | 5 |
| 3220.0147897 | 2 |
| 3220.0156163 | 1 |
| 3220.0166477 | 0 |
| 3220.0169823 | 5 |
| 3220.0180776 | 0 |
| 3220.018924 | 1 |
| 3220.0189639 | 0 |
| 3220.0208197 | 1 |
| 3220.021657 | 1 |
| 3220.0220687 | 7 |
| 3220.0226727 | 2 |
| 3220.0241052 | 1 |
| 3220.0242644 | 3 |
| 3220.025198 | 2 |
| 3220.0256802 | 2 |
| 3220.0262752 | 1 |
| 3220.0274431 | 1 |
| 3220.0282289 | 0 |
| 3220.0289089 | 1 |
| 3220.0294619 | 1 |
| 3220.0305637 | 1 |
| 3220.031085 | 0 |
| 3220.0322905 | 0 |
| 3220.0332816 | 2 |
| 3220.0340835 | 1 |
| 3220.0350064 | 2 |
| 3220.0355224 | 0 |
| 3220.0357879 | 0 |
| 3220.0370158 | 1 |
| 3220.0371186 | 1 |
| 3220.0387512 | 1 |
| 3220.0392511 | 0 |
| 3220.0395899 | 2 |
| 3220.0407972 | 2 |
| 3220.0414722 | 1 |
| 3220.0423268 | 4 |
| 3220.0430101 | 2 |
| 3220.0440808 | 2 |
| 3220.0444972 | 2 |
| 3220.0452595 | 6 |
| 3220.0463931 | 1 |
| 3220.0469544 | 1 |
| 3220.0476854 | 2 |
| 3220.0485617 | 0 |
| 3220.0487636 | 1 |
| 3220.0504743 | 1 |
| 3220.050804 | 1 |
| 3220.0510205 | 2 |
| 3220.0527134 | 3 |
| 3220.0531382 | 2 |
| 3220.0541061 | 1 |
| 3220.0545572 | 0 |
| 3220.056 | 4 |
| 3220.0565582 | 1 |
| 3220.0571772 | 3 |
| 3220.0579651 | 0 |
| 3220.0590351 | 2 |
| 3220.0595408 | 1 |
| 3220.0604641 | 0 |
| 3220.0615977 | 0 |
| 3220.061686 | 1 |
| 3220.0625979 | 0 |
| 3220.0641611 | 2 |
| 3220.0648236 | 2 |
| 3220.0651329 | 3 |
| 3220.0657695 | 3 |
| 3220.0663547 | 4 |
| 3220.067543 | 3 |
| 3220.0680589 | 0 |
| 3220.0687842 | 2 |
| 3220.0695795 | 2 |
| 3220.0707965 | 1 |
| 3220.0716229 | 2 |
| 3220.0721143 | 1 |
| 3220.0737702 | 2 |
| 3220.0738666 | 1 |
| 3220.0754034 | 3 |
| 3220.075634 | 2 |
| 3220.0763884 | 2 |
| 3220.0770469 | 2 |
| 3220.0784244 | 1 |
| 3220.0785816 | 0 |
| 3220.0797268 | 3 |
| 3220.0803967 | 3 |
| 3220.0807765 | 2 |
| 3220.0818645 | 0 |
| 3220.0827112 | 1 |
| 3220.0830601 | 0 |
| 3220.0841705 | 3 |
| 3220.0850123 | 2 |
| 3220.0857569 | 1 |
| 3220.0859261 | 1 |
| 3220.0868448 | 3 |
| 3220.0874833 | 3 |
| 3220.0883387 | 3 |
| 3220.0895064 | 3 |
| 3220.0905182 | 0 |
| 3220.0913847 | 0 |
| 3220.0920125 | 0 |
| 3220.0925629 | 2 |
| 3220.093978 | 1 |
| 3220.0941901 | 4 |
| 3220.0953366 | 1 |
| 3220.0955821 | 2 |
| 3220.0960883 | 4 |
| 3220.0972231 | 1 |
| 3220.0975695 | 1 |
| 3220.0984955 | 1 |
| 3220.0992983 | 2 |
| 3220.1001869 | 1 |
| 3220.1015572 | 3 |
| 3220.1015573 | 2 |
| 3220.1026972 | 1 |
| 3220.1041046 | 1 |
| 3220.104531 | 0 |
| 3220.1052247 | 1 |
| 3220.1058167 | 1 |
| 3220.1072633 | 0 |
| 3220.1082879 | 1 |
| 3220.1084397 | 1 |
| 3220.1088816 | 2 |
| 3220.1102308 | 1 |
| 3220.1107954 | 2 |
| 3220.1120955 | 3 |
| 3220.112656 | 1 |
| 3220.1135475 | 5 |
| 3220.1137638 | 1 |
| 3220.1150104 | 1 |
| 3220.1151265 | 2 |
| 3220.1162661 | 1 |
| 3220.1170256 | 1 |
| 3220.1179457 | 0 |
| 3220.1187565 | 3 |
| 3220.1193271 | 3 |
| 3220.1199947 | 0 |
| 3220.1207501 | 1 |
| 3220.1216364 | 1 |
| 3220.1229755 | 1 |
| 3220.1237566 | 1 |
| 3220.12434 | 1 |
| 3220.1253982 | 2 |
| 3220.1254933 | 1 |
| 3220.1270553 | 0 |
| 3220.1271207 | 4 |
| 3220.1278282 | 1 |
| 3220.1292977 | 0 |
| 3220.1293666 | 1 |
| 3220.1300943 | 0 |
| 3220.1307218 | 3 |
| 3220.1311716 | 2 |
| 3220.132313 | 1 |
| 3220.133172 | 0 |
| 3220.1340087 | 1 |
| 3220.1345886 | 2 |
| 3220.1354453 | 1 |
| 3220.1368869 | 4 |
| 3220.1371123 | 2 |
| 3220.138001 | 2 |
| 3220.1383852 | 0 |
| 3220.1389904 | 1 |
| 3220.140428 | 4 |
| 3220.1411834 | 0 |
| 3220.1423293 | 2 |
| 3220.1426853 | 2 |
| 3220.1435231 | 1 |
| 3220.1446047 | 0 |
| 3220.1448933 | 1 |
| 3220.1461133 | 1 |
| 3220.1463458 | 2 |
| 3220.1477149 | 2 |
| 3220.147995 | 2 |
| 3220.1487639 | 2 |
| 3220.1501823 | 2 |
| 3220.1503304 | 2 |
| 3220.1513641 | 3 |
| 3220.1519515 | 2 |
| 3220.1526548 | 1 |
| 3220.1533448 | 2 |
| 3220.1544657 | 2 |
| 3220.1553155 | 2 |
| 3220.1564124 | 5 |
| 3220.1564367 | 3 |
| 3220.157768 | 1 |
| 3220.1582054 | 1 |
| 3220.1594331 | 1 |
| 3220.1602738 | 3 |
| 3220.1607638 | 1 |
| 3220.1614119 | 1 |
| 3220.1615669 | 3 |
| 3220.1624181 | 1 |
| 3220.1625106 | 3 |
| 3220.1627028 | 1 |
| 3220.163318 | 2 |
| 3220.1633305 | 0 |
| 3220.1634378 | 3 |
| 3220.1642104 | 4 |
| 3220.1647909 | 4 |
| 3220.1648995 | 1 |
| 3220.1656294 | 3 |
| 3220.1657381 | 2 |
| 3220.1660165 | 3 |
| 3220.1664255 | 4 |
| 3220.1665858 | 4 |
| 3220.1670153 | 4 |
| 3220.1674177 | 2 |
| 3220.1675354 | 1 |
| 3220.1678501 | 1 |
| 3220.1681604 | 2 |
| 3220.1687638 | 0 |
| 3220.1693413 | 1 |
| 3220.1699471 | 4 |
| 3220.1700737 | 3 |
| 3220.1702307 | 2 |
| 3220.1704876 | 2 |
| 3220.1706758 | 1 |
| 3220.1708019 | 2 |
| 3220.1712705 | 3 |
| 3220.1719862 | 3 |
| 3220.1721236 | 1 |
| 3220.1725212 | 1 |
| 3220.1729777 | 1 |
| 3220.1732204 | 2 |
| 3220.1735686 | 1 |
| 3220.1736891 | 2 |
| 3220.1744874 | 2 |
| 3220.1747738 | 2 |
| 3220.1753849 | 2 |
| 3220.1754624 | 2 |
| 3220.1755683 | 3 |
| 3220.1765986 | 6 |
| 3220.1766247 | 2 |
| 3220.1767318 | 2 |
| 3220.1769128 | 3 |
| 3220.1772743 | 0 |
| 3220.1776642 | 1 |
| 3220.177921 | 0 |
| 3220.1785164 | 2 |
| 3220.1786877 | 2 |
| 3220.1795163 | 2 |
| 3220.1795882 | 2 |
| 3220.1799549 | 1 |
| 3220.1803659 | 4 |
| 3220.1807228 | 0 |
| 3220.1811364 | 0 |
| 3220.1817714 | 0 |
| 3220.1823143 | 1 |
| 3220.1826395 | 0 |
| 3220.1827102 | 1 |
| 3220.1835253 | 2 |
| 3220.1835358 | 3 |
| 3220.1836263 | 1 |
| 3220.183856 | 3 |
| 3220.1849393 | 4 |
| 3220.1850766 | 1 |
| 3220.1854281 | 2 |
| 3220.1861054 | 3 |
| 3220.1864559 | 1 |
| 3220.1865675 | 4 |
| 3220.1868449 | 1 |
| 3220.1871476 | 1 |
| 3220.1878881 | 2 |
| 3220.1880393 | 0 |
| 3220.188763 | 2 |
| 3220.1891288 | 3 |
| 3220.1891977 | 5 |
| 3220.1900404 | 1 |
| 3220.1901306 | 1 |
| 3220.1901457 | 2 |
| 3220.1911933 | 4 |
| 3220.1912769 | 0 |
| 3220.1913114 | 1 |
| 3220.1918711 | 7 |
| 3220.1927691 | 2 |
| 3220.1928467 | 2 |
| 3220.1931156 | 5 |
| 3220.1934697 | 1 |
| 3220.1940878 | 5 |
| 3220.1946513 | 1 |
| 3220.1946876 | 0 |
| 3220.1949855 | 1 |
| 3220.1955472 | 1 |
| 3220.1958082 | 1 |
| 3220.1960956 | 0 |
| 3220.1966386 | 8 |
| 3220.1968207 | 1 |
| 3220.197493 | 3 |
| 3220.1978863 | 2 |
| 3220.1986817 | 2 |
| 3220.1988332 | 2 |
| 3220.198899 | 1 |
| 3220.1998327 | 1 |
| 3220.2001018 | 3 |
| 3220.200156 | 1 |
| 3220.2010825 | 3 |
| 3220.2014489 | 1 |
| 3220.2014762 | 1 |
| 3220.2020227 | 1 |
| 3220.2021609 | 4 |
| 3220.2030313 | 2 |
| 3220.2033729 | 1 |
| 3220.2035116 | 2 |
| 3220.2040533 | 1 |
| 3220.2041912 | 0 |
| 3220.2043552 | 0 |
| 3220.2048185 | 0 |
| 3220.2049955 | 1 |
| 3220.2059495 | 1 |

| | |
|---|---|
| 3220.206308 | 1 |
| 3220.2066885 | 4 |
| 3220.2067112 | 0 |
| 3220.207348 | 3 |
| 3220.2074327 | 2 |
| 3220.2080697 | 0 |
| 3220.2086333 | 3 |
| 3220.2091058 | 4 |
| 3220.2091435 | 1 |
| 3220.2095385 | 0 |
| 3220.209928 | 4 |
| 3220.2099638 | 1 |
| 3220.21042 | 1 |
| 3220.2106387 | 0 |
| 3220.2111996 | 2 |
| 3220.211862 | 3 |
| 3220.2121075 | 0 |
| 3220.2126151 | 1 |
| 3220.212766 | 2 |
| 3220.2129721 | 2 |
| 3220.2136081 | 1 |
| 3220.2144669 | 6 |
| 3220.2145308 | 3 |
| 3220.2147459 | 1 |
| 3220.2150632 | 1 |
| 3220.2162869 | 0 |
| 3220.2165115 | 2 |
| 3220.216585 | 1 |
| 3220.2167097 | 0 |
| 3220.2167599 | 3 |
| 3220.2167918 | 2 |
| 3220.217041 | 1 |
| 3220.2170712 | 1 |
| 3220.2172932 | 0 |
| 3220.2176687 | 3 |
| 3220.2179035 | 1 |
| 3220.2182803 | 1 |
| 3220.2184102 | 0 |
| 3220.2185541 | 2 |
| 3220.218697 | 1 |
| 3220.2190811 | 2 |
| 3220.2196736 | 2 |
| 3220.2197292 | 0 |
| 3220.2197509 | 0 |
| 3220.2197772 | 2 |
| 3220.219851 | 3 |
| 3220.2201153 | 4 |
| 3220.2203019 | 0 |
| 3220.2206168 | 2 |
| 3220.2208785 | 2 |
| 3220.2211899 | 2 |
| 3220.2216263 | 1 |
| 3220.2217355 | 2 |
| 3220.2220846 | 2 |
| 3220.2220987 | 1 |
| 3220.2221111 | 1 |
| 3220.2221288 | 3 |
| 3220.2221801 | 6 |
| 3220.222676 | 0 |
| 3220.2228479 | 4 |
| 3220.2229276 | 0 |
| 3220.2231502 | 0 |
| 3220.2233094 | 0 |
| 3220.2235154 | 3 |
| 3220.2237606 | 2 |
| 3220.2239934 | 0 |
| 3220.2244559 | 2 |
| 3220.2247247 | 3 |
| 3220.2249042 | 1 |
| 3220.2250175 | 5 |
| 3220.225488 | 1 |
| 3220.2256004 | 2 |
| 3220.225762 | 3 |
| 3220.2258576 | 1 |
| 3220.2259173 | 1 |
| 3220.2259233 | 3 |
| 3220.2261091 | 2 |
| 3220.2267708 | 1 |
| 3220.2270252 | 2 |
| 3220.2270382 | 1 |
| 3220.2274081 | 2 |
| 3220.2279037 | 2 |
| 3220.2280098 | 1 |
| 3220.2282301 | 2 |
| 3220.2284773 | 2 |
| 3220.2285477 | 1 |
| 3220.2286351 | 1 |
| 3220.2288077 | 3 |
| 3220.2291136 | 0 |
| 3220.2292276 | 2 |
| 3220.2293003 | 0 |
| 3220.2297156 | 6 |
| 3220.2297897 | 3 |
| 3220.2300899 | 2 |
| 3220.230732 | 2 |
| 3220.2307531 | 1 |
| 3220.2308071 | 4 |
| 3220.2308525 | 0 |
| 3220.230916 | 2 |
| 3220.2314962 | 1 |
| 3220.2318189 | 1 |
| 3220.2319135 | 2 |
| 3220.2319572 | 0 |
| 3220.2320896 | 2 |
| 3220.2322048 | 1 |
| 3220.2322281 | 1 |
| 3220.2326809 | 3 |
| 3220.2326831 | 1 |
| 3220.2328865 | 5 |
| 3220.2330783 | 1 |
| 3220.2331446 | 3 |
| 3220.2333341 | 2 |
| 3220.2333586 | 3 |
| 3220.2336111 | 3 |
| 3220.2336826 | 2 |
| 3220.2340432 | 1 |
| 3220.23405 | 2 |
| 3220.2341261 | 2 |
| 3220.2343219 | 1 |
| 3220.2345869 | 2 |
| 3220.2347495 | 1 |
| 3220.2347568 | 0 |
| 3220.2349501 | 2 |
| 3220.2351031 | 3 |
| 3220.2353187 | 4 |
| 3220.2354335 | 2 |
| 3220.2355593 | 2 |
| 3220.2355788 | 0 |
| 3220.2359269 | 1 |
| 3220.2360023 | 1 |
| 3220.2361614 | 4 |
| 3220.2361793 | 2 |
| 3220.2362546 | 1 |
| 3220.2363606 | 1 |
| 3220.2365226 | 1 |
| 3220.2365797 | 3 |
| 3220.2370276 | 1 |
| 3220.2372228 | 2 |
| 3220.237225 | 0 |
| 3220.2374106 | 1 |
| 3220.2375087 | 3 |
| 3220.2376234 | 4 |
| 3220.237796 | 1 |
| 3220.2378649 | 2 |
| 3220.2379032 | 2 |
| 3220.2380241 | 0 |
| 3220.2383282 | 0 |
| 3220.2384239 | 2 |
| 3220.2384348 | 1 |
| 3220.2388197 | 1 |
| 3220.2392618 | 7 |
| 3220.2393288 | 1 |
| 3220.239384 | 1 |
| 3220.2393984 | 2 |
| 3220.2396158 | 2 |
| 3220.2397531 | 0 |
| 3220.2397801 | 2 |
| 3220.2399659 | 2 |
| 3220.2400215 | 1 |
| 3220.2402261 | 2 |
| 3220.2402307 | 2 |
| 3220.240238 | 0 |
| 3220.2404328 | 0 |
| 3220.24067 | 0 |
| 3220.2409042 | 2 |
| 3220.2410175 | 0 |
| 3220.2411157 | 1 |
| 3220.2412149 | 1 |
| 3220.2412154 | 1 |
| 3220.2412174 | 1 |
| 3220.2414444 | 3 |
| 3220.2416911 | 5 |
| 3220.2417159 | 2 |
| 3220.2417227 | 1 |
| 3220.2418928 | 5 |
| 3220.2420594 | 2 |
| 3220.2421448 | 2 |
| 3220.2421458 | 0 |
| 3220.2422417 | 2 |
| 3220.2423858 | 3 |
| 3220.2425411 | 5 |
| 3220.2425458 | 1 |
| 3220.2427492 | 0 |
| 3220.243016 | 1 |
| 3220.2430729 | 3 |
| 3220.2431545 | 3 |
| 3220.2431854 | 4 |
| 3220.2432018 | 4 |
| 3220.2434521 | 2 |
| 3220.2436283 | 5 |
| 3220.2438595 | 8 |
| 3220.2439455 | 9 |
| 3220.2439806 | 6 |
| 3220.2439936 | 2 |
| 3220.2440048 | 2 |
| 3220.2443577 | 5 |
| 3220.2443691 | 6 |
| 3220.2443771 | 10 |
| 3220.2445468 | 10 |
| 3220.2448441 | 11 |
| 3220.2448661 | 8 |
| 3220.2449579 | 9 |
| 3220.2450221 | 11 |
| 3220.2452726 | 10 |
| 3220.2453258 | 11 |
| 3220.2455356 | 12 |
| 3220.2458363 | 18 |
| 3220.2458733 | 13 |
| 3220.2459034 | 14 |
| 3220.2459657 | 14 |
| 3220.2459663 | 21 |
| 3220.2461256 | 16 |
| 3220.2462534 | 9 |
| 3220.2465418 | 17 |
| 3220.246557 | 11 |
| 3220.2466394 | 16 |
| 3220.2467319 | 26 |
| 3220.2467485 | 9 |
| 3220.2468677 | 19 |
| 3220.246911 | 19 |
| 3220.2471524 | 15 |
| 3220.2471742 | 14 |
| 3220.2472597 | 14 |
| 3220.2474443 | 9 |
| 3220.2476877 | 16 |
| 3220.2477245 | 8 |
| 3220.2477439 | 11 |
| 3220.2477732 | 9 |
| 3220.2480078 | 11 |
| 3220.2481909 | 15 |
| 3220.2482619 | 13 |
| 3220.248472 | 10 |
| 3220.2486619 | 9 |
| 3220.248671 | 10 |
| 3220.2487612 | 17 |
| 3220.2489243 | 22 |
| 3220.2490481 | 14 |
| 3220.2491159 | 14 |
| 3220.2491785 | 12 |
| 3220.2492023 | 8 |
| 3220.2493165 | 8 |
| 3220.2494244 | 9 |
| 3220.2496265 | 9 |
| 3220.2496479 | 9 |
| 3220.2498785 | 6 |
| 3220.2502308 | 9 |
| 3220.2502607 | 9 |
| 3220.2503596 | 9 |
| 3220.2503597 | 9 |
| 3220.2505151 | 6 |
| 3220.250704 | 4 |
| 3220.2507191 | 6 |
| 3220.2508111 | 9 |
| 3220.2509255 | 5 |
| 3220.2510273 | 1 |
| 3220.2511978 | 4 |
| 3220.2513241 | 2 |
| 3220.2513505 | 2 |
| 3220.2513956 | 6 |
| 3220.2516254 | 2 |
| 3220.2517976 | 2 |
| 3220.2518094 | 1 |
| 3220.2521689 | 2 |
| 3220.2523038 | 3 |
| 3220.252306 | 1 |
| 3220.2524924 | 0 |
| 3220.2526274 | 2 |
| 3220.2526563 | 1 |
| 3220.2527182 | 2 |
| 3220.2527778 | 1 |
| 3220.2527874 | 1 |
| 3220.2528884 | 2 |
| 3220.2528919 | 2 |
| 3220.2529085 | 2 |
| 3220.2533954 | 1 |
| 3220.2535333 | 3 |
| 3220.2536586 | 1 |
| 3220.2536665 | 1 |
| 3220.2536708 | 1 |
| 3220.2539387 | 0 |
| 3220.2539773 | 0 |
| 3220.2541502 | 2 |
| 3220.2541872 | 1 |
| 3220.2541952 | 2 |
| 3220.2543656 | 2 |
| 3220.254382 | 3 |
| 3220.2546789 | 1 |
| 3220.2547444 | 4 |
| 3220.2547574 | 2 |
| 3220.2550026 | 3 |
| 3220.2551854 | 2 |
| 3220.2553597 | 5 |
| 3220.2553993 | 2 |
| 3220.255499 | 2 |
| 3220.2555903 | 0 |
| 3220.2558581 | 2 |
| 3220.2558725 | 1 |
| 3220.256034 | 1 |
| 3220.2560531 | 2 |
| 3220.2564607 | 0 |
| 3220.2571875 | 2 |
| 3220.2572764 | 0 |
| 3220.2575828 | 1 |
| 3220.2577378 | 2 |
| 3220.258867 | 3 |
| 3220.258884 | 2 |
| 3220.258936 | 1 |
| 3220.2597209 | 1 |
| 3220.2601103 | 2 |
| 3220.2606155 | 4 |
| 3220.2612567 | 3 |
| 3220.2613924 | 2 |
| 3220.2617559 | 1 |
| 3220.2621444 | 0 |
| 3220.2627996 | 3 |
| 3220.2631322 | 2 |
| 3220.2636093 | 1 |
| 3220.2637669 | 1 |
| 3220.2644076 | 1 |
| 3220.2647647 | 3 |
| 3220.2652709 | 3 |
| 3220.2653786 | 1 |
| 3220.2662397 | 0 |
| 3220.2665436 | 1 |
| 3220.266971 | 3 |
| 3220.2670536 | 0 |
| 3220.2682595 | 3 |
| 3220.2684308 | 0 |
| 3220.2690585 | 1 |
| 3220.2695548 | 3 |
| 3220.269566 | 3 |
| 3220.2695847 | 2 |
| 3220.2702527 | 1 |
| 3220.2706653 | 2 |
| 3220.2713338 | 0 |
| 3220.2715221 | 1 |
| 3220.2715569 | 2 |
| 3220.2724372 | 0 |
| 3220.2724726 | 1 |
| 3220.2730096 | 0 |
| 3220.2734775 | 0 |
| 3220.2737067 | 1 |
| 3220.2743204 | 1 |
| 3220.2743861 | 4 |
| 3220.275429 | 1 |
| 3220.2755558 | 3 |
| 3220.2759915 | 0 |
| 3220.2763294 | 2 |
| 3220.2768327 | 1 |
| 3220.277193 | 3 |
| 3220.2777223 | 1 |
| 3220.2781858 | 1 |
| 3220.2782691 | 4 |
| 3220.2786121 | 4 |
| 3220.279797 | 2 |
| 3220.2800337 | 5 |
| 3220.2801649 | 2 |
| 3220.2806338 | 3 |
| 3220.2811725 | 1 |
| 3220.2812505 | 1 |
| 3220.281905 | 0 |
| 3220.2823801 | 2 |
| 3220.2831805 | 1 |

| | |
|---|---|
| 3220.2832115 | 1 |
| 3220.2835624 | 3 |
| 3220.2839072 | 2 |
| 3220.2846513 | 0 |
| 3220.2848877 | 1 |
| 3220.2857554 | 1 |
| 3220.2863256 | 1 |
| 3220.2864558 | 0 |
| 3220.2865506 | 3 |
| 3220.2869861 | 2 |
| 3220.2877418 | 0 |
| 3220.2881983 | 2 |
| 3220.2884455 | 1 |
| 3220.288476 | 2 |
| 3220.2891254 | 3 |
| 3220.2895411 | 4 |
| 3220.2904944 | 3 |
| 3220.2907543 | 1 |
| 3220.2915641 | 1 |
| 3220.2917212 | 1 |
| 3220.2923277 | 2 |
| 3220.2925306 | 2 |
| 3220.2927498 | 4 |
| 3220.2927689 | 1 |
| 3220.2934143 | 2 |
| 3220.2939889 | 4 |
| 3220.2944204 | 4 |
| 3220.2947587 | 3 |
| 3220.2947716 | 0 |
| 3220.2958478 | 1 |
| 3220.2959095 | 2 |
| 3220.2963742 | 3 |
| 3220.2971923 | 1 |
| 3220.2974613 | 1 |
| 3220.2981777 | 3 |
| 3220.2983003 | 3 |
| 3220.2988331 | 1 |
| 3220.2988667 | 2 |
| 3220.3000156 | 3 |
| 3220.3000996 | 4 |
| 3220.300698 | 0 |
| 3220.3007759 | 2 |
| 3220.3011161 | 1 |
| 3220.3018654 | 1 |
| 3220.3021723 | 2 |
| 3220.3026381 | 2 |
| 3220.3027369 | 0 |
| 3220.3036747 | 3 |
| 3220.3038362 | 2 |
| 3220.3041359 | 1 |
| 3220.3046747 | 3 |
| 3220.3048069 | 2 |
| 3220.305289 | 1 |
| 3220.3054301 | 1 |
| 3220.3070718 | 4 |
| 3220.3072912 | 2 |
| 3220.3083079 | 0 |
| 3220.3090655 | 1 |
| 3220.3100482 | 2 |
| 3220.3116067 | 4 |
| 3220.3122154 | 3 |
| 3220.3129212 | 0 |
| 3220.3144431 | 2 |
| 3220.3150332 | 1 |
| 3220.3159853 | 3 |
| 3220.316436 | 2 |
| 3220.3178138 | 1 |
| 3220.3179648 | 1 |
| 3220.3195521 | 1 |
| 3220.3208119 | 3 |
| 3220.320848 | 3 |
| 3220.3225266 | 0 |
| 3220.3228166 | 1 |
| 3220.3243554 | 4 |
| 3220.325534 | 3 |
| 3220.3257547 | 3 |
| 3220.3271132 | 3 |
| 3220.3279743 | 0 |
| 3220.3286115 | 2 |
| 3220.330217 | 1 |
| 3220.3302419 | 0 |
| 3220.3319911 | 0 |
| 3220.3328526 | 1 |
| 3220.33318 | 5 |
| 3220.3342615 | 4 |
| 3220.3354508 | 1 |
| 3220.3365938 | 1 |
| 3220.3366686 | 3 |
| 3220.3384229 | 4 |
| 3220.3391641 | 3 |
| 3220.3393741 | 3 |
| 3220.3408623 | 3 |
| 3220.3425113 | 2 |
| 3220.3427342 | 3 |
| 3220.3440655 | 1 |
| 3220.344826 | 3 |
| 3220.3455119 | 0 |
| 3220.3466266 | 2 |
| 3220.347976 | 1 |
| 3220.3488937 | 2 |
| 3220.3490613 | 4 |
| 3220.3507565 | 4 |
| 3220.3508653 | 3 |
| 3220.3523292 | 0 |
| 3220.3531039 | 1 |
| 3220.3541848 | 1 |
| 3220.3546425 | 1 |
| 3220.3555988 | 0 |
| 3220.356734 | 2 |
| 3220.3580276 | 3 |
| 3220.3582233 | 1 |
| 3220.3600215 | 4 |
| 3220.3603397 | 2 |
| 3220.3618789 | 0 |
| 3220.3627325 | 4 |
| 3220.3627773 | 0 |
| 3220.3642388 | 2 |
| 3220.3658358 | 5 |
| 3220.3660223 | 2 |
| 3220.3675362 | 1 |
| 3220.3675709 | 0 |
| 3220.3694863 | 1 |
| 3220.3703683 | 1 |
| 3220.3704912 | 1 |
| 3220.3717436 | 1 |
| 3220.3726858 | 2 |
| 3220.373633 | 2 |
| 3220.3750572 | 2 |
| 3220.3760097 | 1 |
| 3220.3768963 | 0 |
| 3220.3770178 | 3 |
| 3220.3785361 | 0 |
| 3220.3791332 | 3 |
| 3220.3802163 | 1 |
| 3220.381525 | 1 |
| 3220.3825331 | 0 |
| 3220.383759 | 1 |
| 3220.3841671 | 1 |
| 3220.385068 | 1 |
| 3220.386229 | 1 |
| 3220.3867122 | 1 |
| 3220.3879745 | 3 |
| 3220.3889806 | 2 |
| 3220.3897031 | 2 |
| 3220.3907683 | 3 |
| 3220.3915194 | 2 |
| 3220.3932623 | 2 |
| 3220.3943009 | 2 |
| 3220.3943888 | 1 |
| 3220.3955718 | 3 |
| 3220.3955894 | 2 |
| 3220.3972496 | 2 |
| 3220.3976372 | 1 |
| 3220.3990033 | 1 |
| 3220.4000568 | 3 |
| 3220.4008794 | 2 |
| 3220.4018055 | 2 |
| 3220.402668 | 1 |
| 3220.404505 | 0 |
| 3220.4046423 | 0 |
| 3220.4053698 | 1 |
| 3220.4063478 | 1 |
| 3220.4068587 | 2 |
| 3220.4078754 | 3 |
| 3220.4092639 | 0 |
| 3220.4105019 | 2 |
| 3220.4111878 | 1 |
| 3220.4113981 | 3 |
| 3220.4131253 | 4 |
| 3220.414438 | 1 |
| 3220.4147877 | 2 |
| 3220.4160187 | 3 |
| 3220.4165703 | 1 |
| 3220.4179431 | 3 |
| 3220.4187605 | 0 |
| 3220.4202377 | 1 |
| 3220.4209496 | 1 |
| 3220.421117 | 1 |
| 3220.422776 | 1 |
| 3220.4231041 | 1 |
| 3220.4240551 | 1 |
| 3220.4251169 | 0 |
| 3220.4258661 | 5 |
| 3220.4270418 | 3 |
| 3220.428088 | 1 |
| 3220.4287032 | 5 |
| 3220.4294864 | 1 |
| 3220.4314459 | 1 |
| 3220.4320095 | 1 |
| 3220.4327448 | 2 |
| 3220.4334431 | 2 |
| 3220.4348755 | 2 |
| 3220.435641 | 0 |
| 3220.436743 | 3 |
| 3220.4377228 | 0 |
| 3220.4382775 | 0 |
| 3220.4396336 | 2 |
| 3220.439755 | 1 |
| 3220.4409729 | 1 |
| 3220.4416027 | 1 |
| 3220.443121 | 4 |
| 3220.4443262 | 1 |
| 3220.4446987 | 2 |
| 3220.4457001 | 1 |
| 3220.4471835 | 1 |
| 3220.4472029 | 1 |
| 3220.4487735 | 3 |
| 3220.4496605 | 2 |
| 3220.4504744 | 2 |
| 3220.4514457 | 2 |
| 3220.4522876 | 1 |
| 3220.4527507 | 1 |
| 3220.4540803 | 2 |
| 3220.4549858 | 1 |
| 3220.4563393 | 0 |
| 3220.4570694 | 2 |
| 3220.4586638 | 2 |
| 3220.4590226 | 1 |
| 3220.4596801 | 2 |
| 3220.4611122 | 2 |
| 3220.4616557 | 2 |
| 3220.4627432 | 0 |
| 3220.4631326 | 1 |
| 3220.4644002 | 0 |
| 3220.4657391 | 2 |
| 3220.4660693 | 2 |
| 3220.4668827 | 0 |
| 3220.4679521 | 3 |
| 3220.4685792 | 1 |
| 3220.4699388 | 1 |
| 3220.4713516 | 2 |
| 3220.4720261 | 4 |
| 3220.4729171 | 1 |
| 3220.4737844 | 1 |
| 3220.4749897 | 1 |
| 3220.4759004 | 1 |
| 3220.4762999 | 3 |
| 3220.4782592 | 0 |
| 3220.4783995 | 0 |
| 3220.4797572 | 5 |
| 3220.4801562 | 1 |
| 3220.4811865 | 2 |
| 3220.4820627 | 6 |
| 3220.4830381 | 1 |
| 3220.4840268 | 1 |
| 3220.4847828 | 3 |
| 3220.4867243 | 4 |
| 3220.4868347 | 0 |
| 3220.4881999 | 3 |
| 3220.4888653 | 2 |
| 3220.4895983 | 2 |
| 3220.4905075 | 1 |
| 3220.491704 | 3 |
| 3220.4922325 | 2 |
| 3220.4936034 | 1 |
| 3220.494611 | 3 |
| 3220.4954455 | 1 |
| 3220.4958852 | 2 |
| 3220.4971894 | 3 |
| 3220.4986402 | 2 |
| 3220.4992415 | 2 |
| 3220.4997196 | 1 |
| 3220.5012833 | 1 |
| 3220.5017928 | 3 |
| 3220.5028739 | 2 |
| 3220.5036539 | 0 |
| 3220.50514 | 1 |
| 3220.5053465 | 1 |
| 3220.5067278 | 2 |
| 3220.5080013 | 5 |
| 3220.508098 | 0 |
| 3220.5096752 | 2 |
| 3220.5100999 | 1 |
| 3220.5111447 | 4 |
| 3220.512159 | 1 |
| 3220.5132187 | 1 |
| 3220.5137159 | 2 |
| 3220.5151778 | 3 |
| 3220.515814 | 2 |
| 3220.517324 | 1 |
| 3220.5173917 | 1 |
| 3220.5187808 | 5 |
| 3220.5200402 | 2 |
| 3220.5203339 | 0 |
| 3220.5215623 | 1 |
| 3220.5222372 | 1 |
| 3220.5233832 | 4 |
| 3220.5240414 | 2 |
| 3220.5254677 | 2 |
| 3220.5263136 | 2 |
| 3220.5273486 | 2 |
| 3220.5286872 | 1 |
| 3220.5287795 | 1 |
| 3220.5301277 | 1 |
| 3220.5314777 | 3 |
| 3220.5316859 | 0 |
| 3220.5325558 | 2 |
| 3220.5332612 | 1 |
| 3220.5348491 | 2 |
| 3220.5360664 | 0 |
| 3220.536542 | 2 |
| 3220.5379243 | 2 |
| 3220.5380505 | 2 |
| 3220.53915 | 2 |
| 3220.5405485 | 1 |
| 3220.5406542 | 4 |
| 3220.5418082 | 0 |
| 3220.5426229 | 5 |
| 3220.5437717 | 2 |
| 3220.5451137 | 4 |
| 3220.5460891 | 0 |
| 3220.5470419 | 1 |
| 3220.5479335 | 3 |
| 3220.5483906 | 4 |
| 3220.5498825 | 3 |
| 3220.5501772 | 1 |
| 3220.551591 | 0 |
| 3220.5520421 | 0 |
| 3220.553417 | 1 |
| 3220.5545964 | 1 |
| 3220.5549894 | 2 |
| 3220.5564704 | 1 |
| 3220.5568341 | 0 |
| 3220.5580219 | 2 |
| 3220.5588192 | 3 |
| 3220.559715 | 0 |
| 3220.5608092 | 3 |
| 3220.5619467 | 2 |
| 3220.5624971 | 1 |
| 3220.5631626 | 0 |
| 3220.5646684 | 1 |
| 3220.5657706 | 2 |
| 3220.5666036 | 3 |
| 3220.5676048 | 0 |
| 3220.5679452 | 3 |
| 3220.5695024 | 3 |
| 3220.5705398 | 1 |
| 3220.5707935 | 1 |
| 3220.5723146 | 2 |
| 3220.5725972 | 2 |
| 3220.5730998 | 1 |
| 3220.5748452 | 1 |
| 3220.5750771 | 3 |
| 3220.5762469 | 3 |
| 3220.577992 | 1 |
| 3220.5780941 | 5 |
| 3220.5792222 | 2 |
| 3220.5801008 | 0 |
| 3220.5809314 | 1 |
| 3220.5822699 | 3 |
| 3220.5829836 | 1 |
| 3220.5834517 | 1 |
| 3220.5847068 | 1 |
| 3220.5859042 | 0 |
| 3220.5868123 | 4 |
| 3220.5877063 | 1 |
| 3220.5888166 | 0 |
| 3220.5893505 | 1 |
| 3220.5903448 | 3 |
| 3220.5914418 | 2 |
| 3220.5923364 | 4 |
| 3220.592888 | 2 |
| 3220.5946615 | 3 |

| | |
|---|---|
| 3220.5948807 | 2 |
| 3220.5963659 | 0 |
| 3220.5971664 | 3 |
| 3220.5985154 | 1 |
| 3220.5986271 | 2 |
| 3220.5995417 | 1 |
| 3220.6006994 | 1 |
| 3220.6013881 | 3 |
| 3220.6026098 | 2 |
| 3220.6033241 | 2 |
| 3220.604538 | 4 |
| 3220.6054297 | 3 |
| 3220.6060723 | 5 |
| 3220.6073121 | 1 |
| 3220.6081573 | 0 |
| 3220.6088786 | 2 |
| 3220.6103889 | 0 |
| 3220.6110285 | 3 |
| 3220.6127427 | 3 |
| 3220.6130159 | 4 |
| 3220.6136836 | 2 |
| 3220.6150503 | 2 |
| 3220.6155533 | 3 |
| 3220.6171533 | 4 |
| 3220.6176777 | 2 |
| 3220.6185792 | 2 |
| 3220.6195686 | 1 |
| 3220.6209337 | 3 |
| 3220.6211303 | 0 |
| 3220.6224258 | 2 |
| 3220.6234379 | 6 |
| 3220.6240017 | 5 |
| 3220.6253251 | 2 |
| 3220.6261913 | 1 |
| 3220.6267969 | 1 |
| 3220.6282331 | 2 |
| 3220.6294453 | 4 |
| 3220.629844 | 1 |
| 3220.6310905 | 3 |
| 3220.6314124 | 2 |
| 3220.6323165 | 0 |
| 3220.633004 | 2 |
| 3220.6341685 | 3 |
| 3220.6355411 | 0 |
| 3220.6359072 | 1 |
| 3220.637187 | 2 |
| 3220.6385103 | 1 |
| 3220.6388132 | 4 |
| 3220.6400177 | 0 |
| 3220.6402004 | 0 |
| 3220.6416149 | 2 |
| 3220.6421101 | 3 |
| 3220.6433242 | 1 |
| 3220.6446093 | 0 |
| 3220.6449494 | 2 |
| 3220.6462452 | 1 |
| 3220.6473731 | 1 |
| 3220.648025 | 1 |
| 3220.64903 | 2 |
| 3220.6494629 | 3 |
| 3220.651199 | 6 |
| 3220.6512943 | 1 |
| 3220.652718 | 1 |
| 3220.6542945 | 1 |
| 3220.6545958 | 3 |
| 3220.6557297 | 3 |
| 3220.6565874 | 3 |
| 3220.6574696 | 1 |
| 3220.6582812 | 0 |
| 3220.6593785 | 2 |
| 3220.6598972 | 1 |
| 3220.6611223 | 1 |
| 3220.6620968 | 1 |
| 3220.6633521 | 3 |
| 3220.6639055 | 2 |
| 3220.6651686 | 3 |
| 3220.6664528 | 2 |
| 3220.6667524 | 6 |
| 3220.6674765 | 1 |
| 3220.6683405 | 1 |
| 3220.6695533 | 3 |
| 3220.6699437 | 3 |
| 3220.6712443 | 3 |
| 3220.6728667 | 1 |
| 3220.6731134 | 4 |
| 3220.6738688 | 2 |
| 3220.6751226 | 2 |
| 3220.6756018 | 0 |
| 3220.6769126 | 0 |
| 3220.6779361 | 4 |
| 3220.6786552 | 3 |
| 3220.6791721 | 3 |
| 3220.6805672 | 0 |
| 3220.6815953 | 0 |
| 3220.6824963 | 1 |
| 3220.6833935 | 5 |
| 3220.6837374 | 0 |
| 3220.6850651 | 0 |
| 3220.6861602 | 3 |
| 3220.6870466 | 3 |
| 3220.6877109 | 2 |
| 3220.6889423 | 0 |
| 3220.6899352 | 1 |
| 3220.6911103 | 2 |
| 3220.692085 | 3 |
| 3220.6924997 | 1 |
| 3220.6933198 | 2 |
| 3220.6946391 | 3 |
| 3220.6955212 | 1 |
| 3220.6963758 | 2 |
| 3220.6971107 | 5 |
| 3220.6977379 | 0 |
| 3220.6991154 | 2 |
| 3220.6999384 | 2 |
| 3220.7009136 | 1 |
| 3220.7020409 | 2 |
| 3220.7031582 | 3 |
| 3220.703224 | 1 |
| 3220.7045802 | 3 |
| 3220.7055964 | 2 |
| 3220.706864 | 3 |
| 3220.7074645 | 0 |
| 3220.7083111 | 1 |
| 3220.709378 | 2 |
| 3220.7101712 | 2 |
| 3220.7117619 | 2 |
| 3220.7122285 | 0 |
| 3220.7131172 | 1 |
| 3220.7139626 | 1 |
| 3220.7151742 | 0 |
| 3220.7162303 | 2 |
| 3220.7171142 | 0 |
| 3220.7179339 | 4 |
| 3220.7180122 | 1 |
| 3220.7191466 | 2 |
| 3220.720846 | 1 |
| 3220.7216 | 1 |
| 3220.7223635 | 0 |
| 3220.7230582 | 1 |
| 3220.7245705 | 3 |
| 3220.7255469 | 0 |
| 3220.7257144 | 1 |
| 3220.7272808 | 1 |
| 3220.7273487 | 1 |
| 3220.7287274 | 1 |
| 3220.7302449 | 1 |
| 3220.7303829 | 1 |
| 3220.7316919 | 1 |
| 3220.7324328 | 3 |
| 3220.7333558 | 1 |
| 3220.7338738 | 0 |
| 3220.7351346 | 0 |
| 3220.7365809 | 2 |
| 3220.7370098 | 1 |
| 3220.7383315 | 0 |
| 3220.7393641 | 2 |
| 3220.7403319 | 1 |
| 3220.7407935 | 4 |
| 3220.7417796 | 1 |
| 3220.7430601 | 2 |
| 3220.7431844 | 3 |
| 3220.7447924 | 1 |
| 3220.7460181 | 1 |
| 3220.7461493 | 2 |
| 3220.7473012 | 0 |
| 3220.7488224 | 2 |
| 3220.7494469 | 0 |
| 3220.7507941 | 2 |
| 3220.7508885 | 1 |
| 3220.7526849 | 1 |
| 3220.7533018 | 2 |
| 3220.754132 | 1 |
| 3220.7545731 | 2 |
| 3220.7557453 | 3 |
| 3220.7563177 | 1 |
| 3220.7574864 | 0 |
| 3220.7588055 | 1 |
| 3220.7595796 | 3 |
| 3220.7608826 | 3 |
| 3220.7618718 | 2 |
| 3220.7622716 | 1 |
| 3220.7636695 | 0 |
| 3220.7645877 | 0 |
| 3220.7647004 | 0 |
| 3220.7665622 | 1 |
| 3220.7669519 | 0 |
| 3220.7675918 | 1 |
| 3220.7690845 | 0 |
| 3220.769286 | 0 |
| 3220.770854 | 1 |
| 3220.77133 | 1 |
| 3220.7722134 | 2 |
| 3220.7736826 | 1 |
| 3220.7742168 | 0 |
| 3220.7758262 | 2 |
| 3220.7762955 | 1 |
| 3220.7770525 | 1 |
| 3220.7774498 | 2 |
| 3220.7788844 | 2 |
| 3220.7796502 | 5 |
| 3220.7809613 | 5 |
| 3220.7823052 | 2 |
| 3220.7828967 | 3 |
| 3220.7833123 | 2 |
| 3220.7844284 | 3 |
| 3220.7853597 | 1 |
| 3220.7859703 | 2 |
| 3220.7871427 | 1 |
| 3220.7881083 | 1 |
| 3220.7895421 | 3 |
| 3220.7902957 | 1 |
| 3220.7910355 | 2 |
| 3220.7920607 | 1 |
| 3220.7934556 | 1 |
| 3220.7941869 | 1 |
| 3220.7948665 | 1 |
| 3220.7961323 | 1 |
| 3220.7966899 | 1 |
| 3220.7972943 | 2 |
| 3220.7989191 | 0 |
| 3220.7994938 | 0 |
| 3220.8005445 | 1 |
| 3220.8016634 | 5 |
| 3220.8030937 | 0 |
| 3220.8037018 | 1 |
| 3220.8041666 | 2 |
| 3220.8051475 | 2 |
| 3220.8067259 | 0 |
| 3220.8067511 | 1 |
| 3220.8080336 | 1 |
| 3220.8082007 | 0 |
| 3220.809665 | 0 |
| 3220.8101962 | 1 |
| 3220.811299 | 2 |
| 3220.8125316 | 1 |
| 3220.8127356 | 2 |
| 3220.8139335 | 3 |
| 3220.8157686 | 0 |
| 3220.8159654 | 2 |
| 3220.8172333 | 4 |
| 3220.8183844 | 3 |
| 3220.8190451 | 1 |
| 3220.8196057 | 1 |
| 3220.8208594 | 3 |
| 3220.8221122 | 3 |
| 3220.8224655 | 1 |
| 3220.8240114 | 1 |
| 3220.8240905 | 3 |
| 3220.8253103 | 1 |
| 3220.8268241 | 2 |
| 3220.8274748 | 0 |
| 3220.8283339 | 5 |
| 3220.8292182 | 0 |
| 3220.8306226 | 0 |
| 3220.8316388 | 0 |
| 3220.8319399 | 4 |
| 3220.8336942 | 3 |
| 3220.8339549 | 1 |
| 3220.8352206 | 2 |
| 3220.8361814 | 1 |
| 3220.8369603 | 2 |
| 3220.8377921 | 1 |
| 3220.8387494 | 0 |
| 3220.8394282 | 1 |
| 3220.8400961 | 1 |
| 3220.8417386 | 2 |
| 3220.8431355 | 4 |
| 3220.8437366 | 6 |
| 3220.8442632 | 3 |
| 3220.845522 | 4 |
| 3220.8461643 | 4 |
| 3220.8472463 | 2 |
| 3220.8478842 | 4 |
| 3220.8487427 | 4 |
| 3220.8502121 | 2 |
| 3220.8510028 | 1 |
| 3220.8517995 | 1 |
| 3220.8528166 | 3 |
| 3220.8538901 | 1 |
| 3220.8543788 | 2 |
| 3220.8558357 | 0 |
| 3220.8563865 | 0 |
| 3220.8570869 | 0 |
| 3220.8579541 | 2 |
| 3220.8593265 | 2 |
| 3220.8596741 | 2 |
| 3220.8612016 | 1 |
| 3220.8612645 | 3 |
| 3220.8629332 | 1 |
| 3220.8637379 | 3 |
| 3220.8643027 | 4 |
| 3220.8651039 | 2 |
| 3220.8672557 | 0 |
| 3220.8674431 | 0 |
| 3220.868276 | 2 |
| 3220.8700573 | 2 |
| 3220.870116 | 3 |
| 3220.8718172 | 2 |
| 3220.871987 | 2 |
| 3220.8735968 | 1 |
| 3220.8740971 | 2 |
| 3220.875146 | 3 |
| 3220.8760647 | 0 |
| 3220.8768415 | 5 |
| 3220.8776954 | 2 |
| 3220.8787828 | 0 |
| 3220.8801461 | 2 |
| 3220.8812153 | 1 |
| 3220.8815208 | 2 |
| 3220.8829075 | 2 |
| 3220.8832364 | 0 |
| 3220.8840488 | 2 |
| 3220.8855296 | 1 |
| 3220.887061 | 3 |
| 3220.8877236 | 1 |
| 3220.887822 | 3 |
| 3220.8895731 | 1 |
| 3220.8908182 | 2 |
| 3220.8908781 | 1 |
| 3220.8918859 | 1 |
| 3220.8931458 | 2 |
| 3220.894033 | 1 |
| 3220.8951272 | 0 |
| 3220.8959939 | 0 |
| 3220.8970628 | 2 |
| 3220.8977463 | 0 |
| 3220.8985958 | 3 |
| 3220.8996657 | 1 |
| 3220.9004306 | 0 |
| 3220.9013595 | 1 |
| 3220.9021738 | 2 |
| 3220.903333 | 3 |
| 3220.9048899 | 3 |
| 3220.9049223 | 5 |
| 3220.9063773 | 0 |
| 3220.9067892 | 1 |
| 3220.9080444 | 4 |
| 3220.9092678 | 0 |
| 3220.9097931 | 2 |
| 3220.9108019 | 2 |
| 3220.9116396 | 3 |
| 3220.9123298 | 2 |
| 3220.9138068 | 2 |
| 3220.9143311 | 2 |
| 3220.9153299 | 0 |
| 3220.9160715 | 2 |
| 3220.9173701 | 2 |
| 3220.918405 | 2 |
| 3220.9191052 | 2 |
| 3220.9199878 | 3 |
| 3220.9206555 | 0 |
| 3220.9216241 | 2 |
| 3220.9230131 | 2 |
| 3220.923649 | 0 |
| 3220.9246002 | 3 |
| 3220.9258395 | 1 |
| 3220.92629 | 4 |
| 3220.927978 | 2 |
| 3220.9282751 | 2 |
| 3220.9294727 | 1 |
| 3220.9308536 | 2 |
| 3220.9310587 | 0 |
| 3220.9323456 | 1 |

| | |
|---|---|
| 3220.9328697 | 2 |
| 3220.9342597 | 0 |
| 3220.9349706 | 1 |
| 3220.9363781 | 0 |
| 3220.9377796 | 2 |
| 3220.9377858 | 1 |
| 3220.9394973 | 2 |
| 3220.9398372 | 2 |
| 3220.941056 | 1 |
| 3220.9422171 | 0 |
| 3220.9429468 | 1 |
| 3220.9435571 | 1 |
| 3220.9445184 | 0 |
| 3220.9455414 | 0 |
| 3220.9470768 | 0 |
| 3220.9472289 | 1 |
| 3220.9486076 | 4 |
| 3220.9489713 | 1 |
| 3220.9502095 | 2 |
| 3220.9510653 | 4 |
| 3220.9519581 | 1 |
| 3220.9533736 | 2 |
| 3220.9538475 | 0 |
| 3220.9555699 | 1 |
| 3220.9560622 | 0 |
| 3220.9562956 | 0 |
| 3220.9578909 | 3 |
| 3220.9583571 | 2 |
| 3220.9591329 | 2 |
| 3220.96086 | 0 |
| 3220.9612405 | 2 |
| 3220.9622625 | 1 |
| 3220.9632962 | 1 |
| 3220.9643269 | 4 |
| 3220.9653543 | 0 |
| 3220.9659795 | 1 |
| 3220.9673743 | 1 |
| 3220.9684394 | 3 |
| 3220.9687474 | 2 |
| 3220.9697677 | 1 |
| 3220.9712953 | 2 |
| 3220.9722823 | 0 |
| 3220.972361 | 2 |
| 3220.9739411 | 5 |
| 3220.9753009 | 0 |
| 3220.9756981 | 1 |
| 3220.9768755 | 1 |
| 3220.9775016 | 0 |
| 3220.9783986 | 2 |
| 3220.979546 | 1 |
| 3220.9802839 | 5 |
| 3220.9818379 | 1 |
| 3220.9819673 | 0 |
| 3220.9832221 | 1 |
| 3220.9844279 | 2 |
| 3220.9850614 | 0 |
| 3220.9860726 | 5 |
| 3220.9869446 | 1 |
| 3220.9878286 | 3 |
| 3220.9887979 | 2 |
| 3220.9895663 | 0 |
| 3220.9907266 | 2 |
| 3220.99083 | 1 |
| 3220.9930015 | 5 |
| 3220.9940868 | 1 |
| 3220.994513 | 2 |
| 3220.9954518 | 1 |
| 3220.9958187 | 6 |
| 3220.9968827 | 1 |
| 3220.9974127 | 3 |
| 3220.9988593 | 3 |
| 3221.0000535 | 7 |
| 3221.0004632 | 0 |
| 3221.0019761 | 6 |
| 3221.0030454 | 2 |
| 3221.0034918 | 2 |
| 3221.004725 | 2 |
| 3221.0049285 | 1 |
| 3221.0062926 | 0 |
| 3221.0069996 | 1 |
| 3221.0086342 | 0 |
| 3221.0094206 | 3 |
| 3221.0105088 | 2 |
| 3221.0115119 | 1 |
| 3221.0123703 | 4 |
| 3221.0128939 | 1 |
| 3221.0144105 | 1 |
| 3221.0150208 | 2 |
| 3221.0162619 | 2 |
| 3221.0166793 | 1 |
| 3221.0180665 | 3 |
| 3221.0188972 | 1 |
| 3221.0200913 | 0 |
| 3221.0209311 | 6 |
| 3221.0219896 | 1 |
| 3221.0224184 | 1 |
| 3221.0230949 | 3 |
| 3221.0240841 | 2 |
| 3221.025097 | 2 |
| 3221.0265585 | 3 |
| 3221.0273379 | 5 |
| 3221.0282417 | 2 |
| 3221.0290579 | 2 |
| 3221.0306447 | 0 |
| 3221.0318564 | 2 |
| 3221.0321636 | 1 |
| 3221.0332378 | 1 |
| 3221.0335253 | 2 |
| 3221.0351145 | 1 |
| 3221.036139 | 1 |
| 3221.0368119 | 1 |
| 3221.0382172 | 1 |
| 3221.0383729 | 0 |
| 3221.0396783 | 4 |
| 3221.0408387 | 3 |
| 3221.0415955 | 2 |
| 3221.0421612 | 1 |
| 3221.0436631 | 1 |
| 3221.0446731 | 1 |
| 3221.0449128 | 5 |
| 3221.0459534 | 1 |
| 3221.0475179 | 0 |
| 3221.047907 | 0 |
| 3221.0493677 | 1 |
| 3221.0499603 | 2 |
| 3221.0503995 | 3 |
| 3221.0512063 | 1 |
| 3221.0525567 | 0 |
| 3221.0540928 | 0 |
| 3221.0550985 | 2 |
| 3221.0558007 | 1 |
| 3221.0569088 | 3 |
| 3221.0572744 | 2 |
| 3221.0587074 | 1 |
| 3221.0599431 | 1 |
| 3221.0603673 | 4 |
| 3221.0612045 | 5 |
| 3221.0621913 | 2 |
| 3221.0630943 | 1 |
| 3221.0641699 | 1 |
| 3221.0646758 | 0 |
| 3221.0662516 | 2 |
| 3221.066882 | 0 |
| 3221.0678753 | 2 |
| 3221.0690253 | 2 |
| 3221.0695151 | 3 |
| 3221.0713895 | 1 |
| 3221.071406 | 3 |
| 3221.072622 | 1 |
| 3221.074176 | 0 |
| 3221.0743353 | 3 |
| 3221.0756091 | 3 |
| 3221.0766667 | 2 |
| 3221.0774181 | 1 |
| 3221.0785063 | 2 |
| 3221.0796364 | 0 |
| 3221.0799607 | 3 |
| 3221.0801746 | 0 |
| 3221.0820649 | 0 |
| 3221.0832005 | 3 |
| 3221.0833759 | 3 |
| 3221.0849452 | 4 |
| 3221.0851262 | 4 |
| 3221.0863671 | 3 |
| 3221.0876109 | 0 |
| 3221.0885529 | 2 |
| 3221.0897469 | 4 |
| 3221.0904729 | 1 |
| 3221.0916919 | 5 |
| 3221.092916 | 0 |
| 3221.092995 | 0 |
| 3221.0946631 | 3 |
| 3221.0949385 | 0 |
| 3221.0960572 | 2 |
| 3221.0967311 | 1 |
| 3221.09847 | 0 |
| 3221.0990274 | 0 |
| 3221.0993217 | 1 |
| 3221.1013318 | 3 |
| 3221.1018899 | 2 |
| 3221.10219 | 2 |
| 3221.1033156 | 1 |
| 3221.1042455 | 3 |
| 3221.105835 | 2 |
| 3221.1070202 | 3 |
| 3221.107595 | 2 |
| 3221.1086651 | 1 |
| 3221.1087139 | 0 |
| 3221.1104527 | 1 |
| 3221.111689 | 1 |
| 3221.1121039 | 0 |
| 3221.1129144 | 2 |
| 3221.1139947 | 2 |
| 3221.1159987 | 1 |
| 3221.1161148 | 0 |
| 3221.1170111 | 0 |
| 3221.1184239 | 1 |
| 3221.1184793 | 1 |
| 3221.1199511 | 1 |
| 3221.1206171 | 1 |
| 3221.1224255 | 2 |
| 3221.1229982 | 2 |
| 3221.123329 | 1 |
| 3221.1245069 | 2 |
| 3221.1249972 | 5 |
| 3221.1258869 | 2 |
| 3221.1270692 | 2 |
| 3221.1281375 | 3 |
| 3221.12927 | 1 |
| 3221.1303523 | 3 |
| 3221.130892 | 0 |
| 3221.1319404 | 1 |
| 3221.1322727 | 2 |
| 3221.1336973 | 2 |
| 3221.1343724 | 0 |
| 3221.1355775 | 0 |
| 3221.1367376 | 1 |
| 3221.1373498 | 1 |
| 3221.1379754 | 1 |
| 3221.1393195 | 2 |
| 3221.1402432 | 0 |
| 3221.1413529 | 0 |
| 3221.1423389 | 2 |
| 3221.1432038 | 1 |
| 3221.1437783 | 1 |
| 3221.1445005 | 1 |
| 3221.1462481 | 1 |
| 3221.1469056 | 2 |
| 3221.1481995 | 0 |
| 3221.1483336 | 5 |
| 3221.1495812 | 4 |
| 3221.151117 | 5 |
| 3221.1513486 | 3 |
| 3221.1523052 | 7 |
| 3221.1530484 | 2 |
| 3221.1539833 | 3 |
| 3221.1542061 | 1 |
| 3221.1551127 | 3 |
| 3221.1555129 | 2 |
| 3221.1565311 | 2 |
| 3221.1578911 | 1 |
| 3221.15818 | 0 |
| 3221.1594987 | 1 |
| 3221.1606815 | 1 |
| 3221.160998 | 2 |
| 3221.1620634 | 0 |
| 3221.1622489 | 2 |
| 3221.1636911 | 4 |
| 3221.1638839 | 0 |
| 3221.165155 | 3 |
| 3221.1660831 | 2 |
| 3221.1661887 | 1 |
| 3221.1682256 | 0 |
| 3221.1683283 | 3 |
| 3221.1699825 | 1 |
| 3221.1714111 | 2 |
| 3221.1714607 | 1 |
| 3221.1726056 | 2 |
| 3221.173483 | 0 |
| 3221.1742767 | 1 |
| 3221.178116 | 1 |
| 3221.1782767 | 1 |
| 3221.1798297 | 2 |
| 3221.1814729 | 0 |
| 3221.1820447 | 1 |
| 3221.1841638 | 3 |
| 3221.1883317 | 0 |
| 3221.1890529 | 1 |
| 3221.1907521 | 2 |
| 3221.1910427 | 2 |
| 3221.1930612 | 2 |
| 3221.1937601 | 1 |
| 3221.1937705 | 1 |
| 3221.1948457 | 2 |
| 3221.1961681 | 0 |
| 3221.1966567 | 0 |
| 3221.1973187 | 4 |
| 3221.1997339 | 3 |
| 3221.2003039 | 1 |
| 3221.2005154 | 1 |
| 3221.2015035 | 3 |
| 3221.20367 | 1 |
| 3221.2065094 | 0 |
| 3221.2084416 | 4 |
| 3221.2101105 | 2 |
| 3221.2103314 | 1 |
| 3221.2109377 | 0 |
| 3221.2127978 | 3 |
| 3221.2129267 | 2 |
| 3221.2163913 | 3 |
| 3221.2172237 | 0 |
| 3221.2172407 | 0 |
| 3221.2186243 | 0 |
| 3221.219274 | 3 |
| 3221.2197359 | 1 |
| 3221.2213773 | 1 |
| 3221.2237999 | 3 |
| 3221.2241127 | 1 |
| 3221.2256355 | 1 |
| 3221.2259812 | 0 |
| 3221.2263889 | 1 |
| 3221.2271224 | 3 |
| 3221.2275164 | 2 |
| 3221.2278464 | 2 |
| 3221.2287943 | 2 |
| 3221.2289886 | 2 |
| 3221.230453 | 1 |
| 3221.2307256 | 1 |
| 3221.231277 | 0 |
| 3221.2321216 | 2 |
| 3221.2321338 | 0 |
| 3221.233308 | 6 |
| 3221.2333864 | 1 |
| 3221.2337236 | 2 |
| 3221.2350667 | 1 |
| 3221.2352468 | 0 |
| 3221.2358349 | 2 |
| 3221.2369065 | 2 |
| 3221.2393349 | 0 |
| 3221.2393401 | 2 |
| 3221.2394595 | 0 |
| 3221.240103 | 1 |
| 3221.2408193 | 3 |
| 3221.2413031 | 1 |
| 3221.2414278 | 0 |
| 3221.2422565 | 2 |
| 3221.2427902 | 3 |
| 3221.242956 | 0 |
| 3221.2446561 | 1 |
| 3221.2458709 | 1 |
| 3221.2465098 | 1 |
| 3221.2466491 | 5 |
| 3221.247361 | 1 |
| 3221.2476705 | 5 |
| 3221.2505568 | 1 |
| 3221.2514525 | 0 |
| 3221.2557598 | 1 |
| 3221.2563417 | 3 |
| 3221.2581183 | 2 |
| 3221.2593524 | 5 |
| 3221.2633152 | 4 |
| 3221.2686052 | 2 |
| 3221.2687036 | 1 |
| 3221.2693769 | 4 |
| 3221.2701003 | 3 |
| 3221.2701147 | 1 |
| 3221.2702247 | 2 |
| 3221.2706993 | 1 |
| 3221.2707138 | 0 |
| 3221.2713078 | 3 |
| 3221.2718122 | 1 |
| 3221.2730584 | 1 |
| 3221.2732771 | 1 |
| 3221.2747168 | 3 |
| 3221.2749688 | 1 |
| 3221.2752939 | 1 |
| 3221.2760369 | 2 |
| 3221.2777881 | 2 |
| 3221.2778643 | 2 |
| 3221.2779655 | 4 |
| 3221.2796401 | 0 |
| 3221.2798585 | 1 |
| 3221.2816249 | 3 |

| | |
|---|---|
| 3221.2824983 | 2 |
| 3221.2832578 | 2 |
| 3221.2837176 | 1 |
| 3221.283899 | 0 |
| 3221.2842428 | 1 |
| 3221.2849365 | 1 |
| 3221.2862522 | 2 |
| 3221.2863435 | 2 |
| 3221.2865589 | 4 |
| 3221.2877191 | 1 |
| 3221.2878344 | 2 |
| 3221.2885475 | 1 |
| 3221.2888586 | 4 |
| 3221.2908782 | 4 |
| 3221.2916445 | 0 |
| 3221.29167 | 3 |
| 3221.2927033 | 0 |
| 3221.2933658 | 0 |
| 3221.2942778 | 2 |
| 3221.2948038 | 2 |
| 3221.2955336 | 1 |
| 3221.2968271 | 1 |
| 3221.2970766 | 2 |
| 3221.2982594 | 3 |
| 3221.299765 | 1 |
| 3221.303119 | 0 |
| 3221.3048835 | 1 |
| 3221.3066948 | 1 |
| 3221.3067701 | 0 |
| 3221.309592 | 1 |
| 3221.3103286 | 1 |
| 3221.3112233 | 1 |
| 3221.3117568 | 4 |
| 3221.3135856 | 2 |
| 3221.3146522 | 1 |
| 3221.3151189 | 0 |
| 3221.3151506 | 2 |
| 3221.3182033 | 2 |
| 3221.3187966 | 7 |
| 3221.3192968 | 1 |
| 3221.3193147 | 1 |
| 3221.3205352 | 3 |
| 3221.3206434 | 3 |
| 3221.3212086 | 5 |
| 3221.3217454 | 1 |
| 3221.322001 | 4 |
| 3221.3233262 | 5 |
| 3221.3239351 | 1 |
| 3221.3257267 | 5 |
| 3221.3258936 | 2 |
| 3221.3265471 | 5 |
| 3221.3280249 | 6 |
| 3221.3280968 | 1 |
| 3221.3285904 | 1 |
| 3221.3298046 | 1 |
| 3221.3310893 | 4 |
| 3221.3311861 | 1 |
| 3221.3319956 | 5 |
| 3221.3331091 | 1 |
| 3221.3332566 | 4 |
| 3221.3337053 | 4 |
| 3221.3337682 | 5 |
| 3221.3342523 | 1 |
| 3221.3347723 | 0 |
| 3221.3351137 | 6 |
| 3221.3355237 | 2 |
| 3221.3364919 | 5 |
| 3221.3375111 | 0 |
| 3221.3380437 | 1 |
| 3221.3385752 | 1 |
| 3221.3406012 | 2 |
| 3221.3412137 | 3 |
| 3221.3422036 | 2 |
| 3221.3426312 | 3 |
| 3221.3459084 | 0 |
| 3221.3509398 | 3 |
| 3221.3518665 | 3 |
| 3221.3529916 | 1 |
| 3221.3538283 | 2 |
| 3221.3566331 | 4 |
| 3221.3566802 | 1 |
| 3221.3568535 | 1 |
| 3221.3571116 | 2 |
| 3221.3577671 | 2 |
| 3221.3588038 | 1 |
| 3221.3588089 | 4 |
| 3221.3596629 | 2 |
| 3221.360172 | 2 |
| 3221.3606664 | 0 |
| 3221.3620442 | 1 |
| 3221.3621827 | 2 |
| 3221.3639469 | 3 |
| 3221.3640481 | 0 |
| 3221.364513 | 0 |
| 3221.3649155 | 1 |
| 3221.3649483 | 0 |
| 3221.3658053 | 0 |
| 3221.366074 | 0 |
| 3221.3661171 | 1 |
| 3221.3666192 | 1 |
| 3221.3671484 | 1 |
| 3221.3677805 | 0 |
| 3221.3681753 | 3 |
| 3221.3684387 | 0 |
| 3221.3685379 | 2 |
| 3221.3694313 | 1 |
| 3221.3695685 | 1 |
| 3221.3701337 | 2 |
| 3221.3701685 | 2 |
| 3221.3712059 | 0 |
| 3221.3722957 | 2 |
| 3221.3723456 | 2 |
| 3221.3726315 | 0 |
| 3221.374139 | 0 |
| 3221.3753007 | 2 |
| 3221.375865 | 1 |
| 3221.3768223 | 3 |
| 3221.3771303 | 1 |
| 3221.3787004 | 1 |
| 3221.3794619 | 2 |
| 3221.3798608 | 3 |
| 3221.3801102 | 3 |
| 3221.3812507 | 2 |
| 3221.382011 | 0 |
| 3221.3827717 | 1 |
| 3221.3830443 | 1 |
| 3221.3842065 | 1 |
| 3221.3849019 | 1 |
| 3221.3849543 | 1 |
| 3221.3859291 | 1 |
| 3221.386787 | 1 |
| 3221.3877873 | 1 |
| 3221.3888527 | 1 |
| 3221.3900781 | 1 |
| 3221.3910371 | 0 |
| 3221.3916222 | 0 |
| 3221.3926959 | 0 |
| 3221.3931585 | 2 |
| 3221.3942953 | 1 |
| 3221.395869 | 3 |
| 3221.3960761 | 0 |
| 3221.3972047 | 0 |
| 3221.3982357 | 1 |
| 3221.3988939 | 4 |
| 3221.4001302 | 2 |
| 3221.4005831 | 2 |
| 3221.4017674 | 0 |
| 3221.4018148 | 0 |
| 3221.4034699 | 1 |
| 3221.4050636 | 1 |
| 3221.4051873 | 4 |
| 3221.406339 | 2 |
| 3221.4076304 | 2 |
| 3221.407756 | 3 |
| 3221.4095596 | 2 |
| 3221.4096249 | 2 |
| 3221.4105351 | 1 |
| 3221.4113635 | 2 |
| 3221.4123145 | 0 |
| 3221.4128812 | 1 |
| 3221.4136734 | 1 |
| 3221.4145536 | 3 |
| 3221.4156474 | 1 |
| 3221.4164838 | 2 |
| 3221.4169141 | 1 |
| 3221.4184826 | 2 |
| 3221.4192172 | 3 |
| 3221.4202285 | 0 |
| 3221.4218467 | 2 |
| 3221.4225134 | 1 |
| 3221.4236372 | 2 |
| 3221.424003 | 2 |
| 3221.4251975 | 0 |
| 3221.4256685 | 0 |
| 3221.4272449 | 0 |
| 3221.4282905 | 2 |
| 3221.4284348 | 3 |
| 3221.4298059 | 1 |
| 3221.4311088 | 2 |
| 3221.4312846 | 1 |
| 3221.4327236 | 1 |
| 3221.4333392 | 0 |
| 3221.4343893 | 1 |
| 3221.4355907 | 0 |
| 3221.4360616 | 1 |
| 3221.4369467 | 3 |
| 3221.4380987 | 3 |
| 3221.4391536 | 2 |
| 3221.4400834 | 1 |
| 3221.441128 | 3 |
| 3221.4420678 | 1 |
| 3221.4427684 | 1 |
| 3221.4437563 | 3 |
| 3221.4449431 | 1 |
| 3221.4458017 | 0 |
| 3221.4469177 | 0 |
| 3221.4474376 | 2 |
| 3221.4486771 | 3 |
| 3221.4494757 | 1 |
| 3221.4501773 | 0 |
| 3221.4515292 | 1 |
| 3221.4519136 | 2 |
| 3221.4532637 | 3 |
| 3221.4543745 | 2 |
| 3221.4548985 | 3 |
| 3221.456152 | 2 |
| 3221.4565105 | 2 |
| 3221.4580148 | 1 |
| 3221.4591167 | 2 |
| 3221.4593138 | 0 |
| 3221.4607015 | 1 |
| 3221.4612096 | 0 |
| 3221.4625044 | 0 |
| 3221.4637633 | 2 |
| 3221.4642734 | 1 |
| 3221.4650984 | 1 |
| 3221.4658725 | 2 |
| 3221.4667329 | 4 |
| 3221.467941 | 3 |
| 3221.4688015 | 0 |
| 3221.4696633 | 0 |
| 3221.4714315 | 2 |
| 3221.4716836 | 0 |
| 3221.4731157 | 1 |
| 3221.4732004 | 3 |
| 3221.4745749 | 0 |
| 3221.4758528 | 1 |
| 3221.4767079 | 2 |
| 3221.4775227 | 1 |
| 3221.4784898 | 3 |
| 3221.4793076 | 2 |
| 3221.4801909 | 1 |
| 3221.4812262 | 1 |
| 3221.4826567 | 0 |
| 3221.482924 | 1 |
| 3221.484411 | 1 |
| 3221.485382 | 0 |
| 3221.4860472 | 3 |
| 3221.4870902 | 4 |
| 3221.4875951 | 2 |
| 3221.4888169 | 1 |
| 3221.4894341 | 1 |
| 3221.4907614 | 1 |
| 3221.4917285 | 3 |
| 3221.4924776 | 1 |
| 3221.493432 | 0 |
| 3221.4947214 | 3 |
| 3221.4949133 | 1 |
| 3221.4965139 | 1 |
| 3221.4972418 | 1 |
| 3221.4982086 | 1 |
| 3221.4994034 | 1 |
| 3221.4998497 | 0 |
| 3221.5012594 | 4 |
| 3221.5014278 | 3 |
| 3221.50277 | 3 |
| 3221.5039361 | 2 |
| 3221.5043907 | 4 |
| 3221.505641 | 0 |
| 3221.5066563 | 1 |
| 3221.5077788 | 1 |
| 3221.5083107 | 4 |
| 3221.5094248 | 1 |
| 3221.510412 | 1 |
| 3221.5116026 | 3 |
| 3221.5119609 | 1 |
| 3221.5130208 | 2 |
| 3221.5138995 | 1 |
| 3221.5152493 | 0 |
| 3221.5156792 | 0 |
| 3221.5167494 | 2 |
| 3221.5179967 | 5 |
| 3221.5185131 | 3 |
| 3221.5199188 | 0 |
| 3221.5205152 | 2 |
| 3221.5215932 | 2 |
| 3221.5225726 | 0 |
| 3221.5234252 | 1 |
| 3221.5243422 | 2 |
| 3221.5249571 | 1 |
| 3221.5265092 | 0 |
| 3221.5276563 | 0 |
| 3221.5277884 | 1 |
| 3221.5293691 | 0 |
| 3221.5297458 | 3 |
| 3221.5310381 | 2 |
| 3221.5321283 | 0 |
| 3221.5330825 | 1 |
| 3221.5338958 | 0 |
| 3221.5344326 | 2 |
| 3221.5357584 | 3 |
| 3221.5371291 | 3 |
| 3221.5372006 | 0 |
| 3221.5384714 | 1 |
| 3221.5391986 | 1 |
| 3221.540178 | 0 |
| 3221.5412679 | 4 |
| 3221.5423922 | 0 |
| 3221.5434955 | 2 |
| 3221.5438218 | 5 |
| 3221.5447611 | 2 |
| 3221.5454571 | 0 |
| 3221.5467489 | 1 |
| 3221.5476793 | 0 |
| 3221.5490916 | 1 |
| 3221.5497678 | 2 |
| 3221.5504268 | 1 |
| 3221.5515736 | 2 |
| 3221.5527109 | 0 |
| 3221.5532207 | 2 |
| 3221.5542814 | 2 |
| 3221.5553153 | 0 |
| 3221.5561724 | 1 |
| 3221.5572895 | 1 |
| 3221.5576906 | 0 |
| 3221.5591078 | 1 |
| 3221.5594855 | 0 |
| 3221.5610146 | 3 |
| 3221.561968 | 2 |
| 3221.5628091 | 1 |
| 3221.5636443 | 1 |
| 3221.564277 | 3 |
| 3221.5658747 | 3 |
| 3221.5670002 | 2 |
| 3221.5674848 | 3 |
| 3221.5686758 | 0 |
| 3221.5690589 | 1 |
| 3221.5705212 | 2 |
| 3221.5719523 | 3 |
| 3221.5722723 | 0 |
| 3221.5732099 | 0 |
| 3221.5740292 | 3 |
| 3221.5750096 | 2 |
| 3221.5760648 | 1 |
| 3221.5766729 | 3 |
| 3221.5781403 | 2 |
| 3221.5785828 | 0 |
| 3221.5794496 | 1 |
| 3221.5810883 | 1 |
| 3221.5814591 | 2 |
| 3221.5823774 | 1 |
| 3221.5834497 | 4 |
| 3221.5846403 | 2 |
| 3221.585055 | 3 |
| 3221.5867424 | 1 |
| 3221.5875392 | 0 |
| 3221.5877247 | 1 |
| 3221.5889574 | 1 |
| 3221.5899373 | 2 |
| 3221.5909004 | 1 |
| 3221.5917513 | 0 |
| 3221.5928572 | 1 |
| 3221.5939499 | 1 |
| 3221.5940709 | 3 |
| 3221.5951995 | 2 |
| 3221.5965176 | 0 |
| 3221.5973708 | 0 |
| 3221.5983632 | 4 |
| 3221.598779 | 0 |
| 3221.5999063 | 3 |
| 3221.6009548 | 0 |
| 3221.6017196 | 1 |
| 3221.6030301 | 4 |

| | |
|---|---|
| 3221.6035538 | 1 |
| 3221.604785 | 3 |
| 3221.6051294 | 5 |
| 3221.6063742 | 1 |
| 3221.6077201 | 2 |
| 3221.6088989 | 1 |
| 3221.6097503 | 2 |
| 3221.6101925 | 2 |
| 3221.611231 | 1 |
| 3221.612845 | 0 |
| 3221.6128954 | 0 |
| 3221.6139925 | 3 |
| 3221.6154483 | 0 |
| 3221.6161697 | 1 |
| 3221.6166714 | 0 |
| 3221.6178053 | 3 |
| 3221.6191802 | 1 |
| 3221.6200795 | 2 |
| 3221.6208251 | 5 |
| 3221.621867 | 2 |
| 3221.6224077 | 1 |
| 3221.6231899 | 3 |
| 3221.6241237 | 4 |
| 3221.6248402 | 3 |
| 3221.6264267 | 0 |
| 3221.6272902 | 1 |
| 3221.628048 | 1 |
| 3221.6290963 | 0 |
| 3221.6301522 | 3 |
| 3221.6313477 | 0 |
| 3221.6315436 | 2 |
| 3221.6325927 | 0 |
| 3221.6335155 | 1 |
| 3221.6345256 | 0 |
| 3221.6354963 | 3 |
| 3221.636765 | 1 |
| 3221.6378442 | 0 |
| 3221.6386554 | 1 |
| 3221.6392593 | 3 |
| 3221.6407088 | 5 |
| 3221.6408482 | 0 |
| 3221.6421151 | 2 |
| 3221.6429237 | 0 |
| 3221.6441858 | 1 |
| 3221.6454185 | 4 |
| 3221.6455915 | 0 |
| 3221.6467201 | 1 |
| 3221.6478856 | 3 |
| 3221.6479935 | 2 |
| 3221.6494892 | 1 |
| 3221.6502286 | 1 |
| 3221.6516856 | 1 |
| 3221.6526549 | 1 |
| 3221.6530459 | 1 |
| 3221.654374 | 5 |
| 3221.6547056 | 0 |
| 3221.6559175 | 0 |
| 3221.6570576 | 3 |
| 3221.657871 | 3 |
| 3221.6590135 | 4 |
| 3221.659398 | 1 |
| 3221.6605667 | 0 |
| 3221.6612547 | 1 |
| 3221.6624507 | 1 |
| 3221.6634224 | 0 |
| 3221.664285 | 0 |
| 3221.6650837 | 3 |
| 3221.6666988 | 1 |
| 3221.6671158 | 4 |
| 3221.6683885 | 0 |
| 3221.6689761 | 3 |
| 3221.6700485 | 1 |
| 3221.6707485 | 2 |
| 3221.6717718 | 2 |
| 3221.67313 | 5 |
| 3221.673994 | 2 |
| 3221.6749046 | 0 |
| 3221.6753997 | 4 |
| 3221.6763806 | 4 |
| 3221.6776618 | 0 |
| 3221.6781801 | 1 |
| 3221.6797372 | 3 |
| 3221.6800186 | 2 |
| 3221.6810329 | 0 |
| 3221.6824844 | 2 |
| 3221.6829948 | 0 |
| 3221.6838001 | 1 |
| 3221.6848053 | 2 |
| 3221.6859399 | 1 |
| 3221.6868731 | 0 |
| 3221.6880936 | 1 |
| 3221.6889447 | 4 |
| 3221.6895469 | 2 |
| 3221.690696 | 0 |
| 3221.6916579 | 0 |
| 3221.6918572 | 4 |
| 3221.693209 | 2 |
| 3221.6939871 | 3 |
| 3221.6951985 | 0 |
| 3221.6966691 | 3 |
| 3221.6969082 | 2 |
| 3221.6984027 | 1 |
| 3221.6984217 | 0 |
| 3221.6999951 | 0 |
| 3221.7008657 | 2 |
| 3221.7015339 | 1 |
| 3221.7024621 | 2 |
| 3221.7035094 | 1 |
| 3221.7047776 | 2 |
| 3221.7055351 | 2 |
| 3221.7059655 | 3 |
| 3221.7073867 | 3 |
| 3221.7078542 | 1 |
| 3221.7090899 | 3 |
| 3221.7102649 | 1 |
| 3221.7103952 | 3 |
| 3221.712118 | 3 |
| 3221.7126341 | 0 |
| 3221.7137342 | 0 |
| 3221.7148606 | 2 |
| 3221.715464 | 1 |
| 3221.7168294 | 0 |
| 3221.7169198 | 2 |
| 3221.7178473 | 0 |
| 3221.7188879 | 2 |
| 3221.7202583 | 2 |
| 3221.7210664 | 3 |
| 3221.7219624 | 4 |
| 3221.7227885 | 1 |
| 3221.7234777 | 3 |
| 3221.7243692 | 0 |
| 3221.7257558 | 3 |
| 3221.7258726 | 0 |
| 3221.7274816 | 0 |
| 3221.7285341 | 3 |
| 3221.7290171 | 0 |
| 3221.7301787 | 1 |
| 3221.7311001 | 2 |
| 3221.7318423 | 1 |
| 3221.7333531 | 1 |
| 3221.7335357 | 2 |
| 3221.734907 | 1 |
| 3221.7356233 | 3 |
| 3221.736459 | 4 |
| 3221.7379083 | 3 |
| 3221.7380413 | 1 |
| 3221.7390123 | 0 |
| 3221.739997 | 0 |
| 3221.7411689 | 1 |
| 3221.7419153 | 0 |
| 3221.7433116 | 3 |
| 3221.7438516 | 3 |
| 3221.7448705 | 2 |
| 3221.7459188 | 4 |
| 3221.7465064 | 4 |
| 3221.7481385 | 4 |
| 3221.7489103 | 5 |
| 3221.7494141 | 9 |
| 3221.7504385 | 6 |
| 3221.7508305 | 7 |
| 3221.7521943 | 2 |
| 3221.7532365 | 2 |
| 3221.7540268 | 2 |
| 3221.7553738 | 0 |
| 3221.7563707 | 4 |
| 3221.7571577 | 0 |
| 3221.7579326 | 3 |
| 3221.7584223 | 0 |
| 3221.7596586 | 1 |
| 3221.7599812 | 1 |
| 3221.7614837 | 3 |
| 3221.7626841 | 0 |
| 3221.7634429 | 0 |
| 3221.7641953 | 2 |
| 3221.7656909 | 4 |
| 3221.7659701 | 1 |
| 3221.7673895 | 1 |
| 3221.7674198 | 1 |
| 3221.7683939 | 2 |
| 3221.7695442 | 2 |
| 3221.7709538 | 4 |
| 3221.7718603 | 1 |
| 3221.7724276 | 0 |
| 3221.7739317 | 2 |
| 3221.7742559 | 1 |
| 3221.7754036 | 4 |
| 3221.7761207 | 2 |
| 3221.7770263 | 2 |
| 3221.7782231 | 1 |
| 3221.7786226 | 0 |
| 3221.779507 | 2 |
| 3221.7809078 | 1 |
| 3221.7811511 | 5 |
| 3221.7825464 | 2 |
| 3221.7829339 | 0 |
| 3221.784661 | 0 |
| 3221.7854125 | 0 |
| 3221.7863995 | 1 |
| 3221.7871902 | 2 |
| 3221.7881631 | 1 |
| 3221.7895247 | 1 |
| 3221.7901478 | 1 |
| 3221.7907331 | 3 |
| 3221.7918862 | 1 |
| 3221.7937206 | 2 |
| 3221.7938049 | 1 |
| 3221.7946729 | 1 |
| 3221.795411 | 1 |
| 3221.7964453 | 1 |
| 3221.7973214 | 3 |
| 3221.7985239 | 3 |
| 3221.7994126 | 2 |
| 3221.8001644 | 4 |
| 3221.8011172 | 4 |
| 3221.8025991 | 2 |
| 3221.8027534 | 3 |
| 3221.8042605 | 0 |
| 3221.8047788 | 2 |
| 3221.8060664 | 1 |
| 3221.8069097 | 0 |
| 3221.8077271 | 1 |
| 3221.8092087 | 2 |
| 3221.8093337 | 0 |
| 3221.8106662 | 1 |
| 3221.8119587 | 4 |
| 3221.8121348 | 2 |
| 3221.8131625 | 3 |
| 3221.8142349 | 2 |
| 3221.8153291 | 1 |
| 3221.8162013 | 2 |
| 3221.8169173 | 2 |
| 3221.8179525 | 0 |
| 3221.8187216 | 2 |
| 3221.8196043 | 1 |
| 3221.8205925 | 4 |
| 3221.8212965 | 2 |
| 3221.8222774 | 2 |
| 3221.823634 | 2 |
| 3221.8242819 | 2 |
| 3221.8251967 | 3 |
| 3221.8255154 | 1 |
| 3221.826922 | 3 |
| 3221.8276989 | 0 |
| 3221.828521 | 0 |
| 3221.8295966 | 2 |
| 3221.830528 | 1 |
| 3221.8316204 | 4 |
| 3221.8321161 | 2 |
| 3221.8334539 | 1 |
| 3221.8346976 | 1 |
| 3221.8349411 | 1 |
| 3221.8364696 | 2 |
| 3221.8366174 | 0 |
| 3221.8380125 | 3 |
| 3221.839023 | 0 |
| 3221.8394654 | 0 |
| 3221.8407026 | 0 |
| 3221.8412749 | 0 |
| 3221.8427663 | 3 |
| 3221.8435924 | 0 |
| 3221.8440679 | 0 |
| 3221.8451375 | 1 |
| 3221.8460929 | 2 |
| 3221.8471501 | 2 |
| 3221.8481317 | 1 |
| 3221.8490852 | 2 |
| 3221.8502733 | 4 |
| 3221.8508969 | 1 |
| 3221.8522756 | 2 |
| 3221.8530289 | 3 |
| 3221.8532857 | 2 |
| 3221.8548128 | 3 |
| 3221.8551336 | 0 |
| 3221.8562926 | 1 |
| 3221.8579957 | 3 |
| 3221.8580461 | 1 |
| 3221.8593189 | 0 |
| 3221.8600024 | 2 |
| 3221.8609563 | 0 |
| 3221.8617796 | 2 |
| 3221.8632813 | 1 |
| 3221.8639967 | 4 |
| 3221.8646815 | 1 |
| 3221.8657188 | 4 |
| 3221.8657462 | 0 |
| 3221.8673075 | 1 |
| 3221.8687361 | 4 |
| 3221.8691478 | 4 |
| 3221.8701291 | 5 |
| 3221.8711654 | 2 |
| 3221.8715809 | 1 |
| 3221.8732026 | 1 |
| 3221.8736423 | 1 |
| 3221.8750456 | 1 |
| 3221.8761466 | 2 |
| 3221.8764123 | 2 |
| 3221.8778529 | 1 |
| 3221.8785437 | 2 |
| 3221.8789671 | 1 |
| 3221.8803765 | 1 |
| 3221.880973 | 1 |
| 3221.8819527 | 2 |
| 3221.8830442 | 2 |
| 3221.8839013 | 1 |
| 3221.884817 | 1 |
| 3221.8859948 | 0 |
| 3221.8871913 | 2 |
| 3221.8873017 | 0 |
| 3221.8890438 | 1 |
| 3221.8900516 | 1 |
| 3221.8905656 | 3 |
| 3221.8916799 | 1 |
| 3221.8918662 | 2 |
| 3221.8934315 | 1 |
| 3221.8945479 | 0 |
| 3221.8949783 | 1 |
| 3221.896548 | 1 |
| 3221.896985 | 1 |
| 3221.8980552 | 1 |
| 3221.8994766 | 1 |
| 3221.8997149 | 2 |
| 3221.9011871 | 1 |
| 3221.9012689 | 4 |
| 3221.9024982 | 1 |
| 3221.9031484 | 2 |
| 3221.9040177 | 0 |
| 3221.9055485 | 1 |
| 3221.9065656 | 1 |
| 3221.9070986 | 1 |
| 3221.9082085 | 4 |
| 3221.9084744 | 1 |
| 3221.9099546 | 1 |
| 3221.9105293 | 4 |
| 3221.9115566 | 2 |
| 3221.9128377 | 2 |
| 3221.9132033 | 3 |
| 3221.9148528 | 1 |
| 3221.9149446 | 1 |
| 3221.9160998 | 0 |
| 3221.9169623 | 0 |
| 3221.9177091 | 2 |
| 3221.9190235 | 1 |
| 3221.9196939 | 1 |
| 3221.9207781 | 0 |
| 3221.9216078 | 1 |
| 3221.922439 | 0 |
| 3221.9239654 | 2 |
| 3221.9243461 | 1 |
| 3221.9258688 | 2 |
| 3221.9259849 | 3 |
| 3221.9272224 | 3 |
| 3221.9286585 | 3 |
| 3221.9291302 | 0 |
| 3221.9298173 | 3 |
| 3221.9311313 | 2 |
| 3221.9320224 | 4 |
| 3221.9332406 | 1 |
| 3221.933855 | 0 |
| 3221.9348951 | 2 |
| 3221.9363431 | 1 |
| 3221.9363571 | 0 |
| 3221.9377968 | 2 |
| 3221.9384133 | 1 |

| | |
|---|---|
| 3221.9392972 | 0 |
| 3221.9411181 | 3 |
| 3221.9412767 | 4 |
| 3221.9421762 | 0 |
| 3221.9430179 | 0 |
| 3221.9443399 | 1 |
| 3221.9443758 | 1 |
| 3221.9458449 | 0 |
| 3221.9473486 | 3 |
| 3221.9475811 | 2 |
| 3221.9483607 | 2 |
| 3221.9496916 | 2 |
| 3221.9506121 | 2 |
| 3221.9515301 | 1 |
| 3221.9517798 | 1 |
| 3221.9533246 | 3 |
| 3221.9538858 | 1 |
| 3221.9550151 | 3 |
| 3221.9560277 | 1 |
| 3221.9566529 | 0 |
| 3221.9579938 | 0 |
| 3221.9588367 | 2 |
| 3221.9597312 | 2 |
| 3221.9608536 | 0 |
| 3221.961012 | 2 |
| 3221.9625574 | 2 |
| 3221.9630539 | 2 |
| 3221.9643681 | 4 |
| 3221.965324 | 0 |
| 3221.9660813 | 2 |
| 3221.9674232 | 3 |
| 3221.9684809 | 2 |
| 3221.9687697 | 3 |
| 3221.9699588 | 2 |
| 3221.9704068 | 1 |
| 3221.9720995 | 1 |
| 3221.9726168 | 2 |
| 3221.9739124 | 3 |
| 3221.9749066 | 2 |
| 3221.9754876 | 3 |
| 3221.9769028 | 0 |
| 3221.9770045 | 0 |
| 3221.9780671 | 1 |
| 3221.9794015 | 1 |
| 3221.9799399 | 3 |
| 3221.9812668 | 3 |
| 3221.9817627 | 4 |
| 3221.9834197 | 3 |
| 3221.9840231 | 1 |
| 3221.9844402 | 0 |
| 3221.9862164 | 4 |
| 3221.9863393 | 0 |
| 3221.9874693 | 1 |
| 3221.9887192 | 4 |
| 3221.9890362 | 2 |
| 3221.9898912 | 1 |
| 3221.9913907 | 1 |
| 3221.9925032 | 1 |
| 3221.9929123 | 1 |
| 3221.9945736 | 1 |
| 3221.9953349 | 8 |
| 3221.9958321 | 2 |
| 3221.9967064 | 1 |
| 3221.9979449 | 0 |
| 3221.9981681 | 1 |
| 3221.9991505 | 2 |
| 3222.0004501 | 1 |
| 3222.0013579 | 0 |
| 3222.0025077 | 1 |
| 3222.0031577 | 1 |
| 3222.004258 | 1 |
| 3222.004745 | 1 |
| 3222.0060237 | 2 |
| 3222.0069141 | 2 |
| 3222.0082116 | 3 |
| 3222.0085554 | 2 |
| 3222.0094501 | 2 |
| 3222.0107859 | 1 |
| 3222.0119585 | 3 |
| 3222.012005 | 3 |
| 3222.0135134 | 1 |
| 3222.013737 | 6 |
| 3222.0147521 | 0 |
| 3222.0163686 | 3 |
| 3222.0167162 | 1 |
| 3222.0180288 | 1 |
| 3222.0188201 | 1 |
| 3222.0201198 | 2 |
| 3222.0211769 | 3 |
| 3222.0217749 | 1 |
| 3222.0232286 | 4 |
| 3222.0236571 | 2 |
| 3222.0248202 | 2 |
| 3222.0254665 | 2 |
| 3222.026015 | 0 |
| 3222.027666 | 3 |
| 3222.0282713 | 1 |
| 3222.0293581 | 2 |
| 3222.0299833 | 0 |
| 3222.031436 | 0 |
| 3222.0322919 | 4 |
| 3222.0324905 | 3 |
| 3222.0342604 | 2 |
| 3222.0347439 | 2 |
| 3222.035674 | 3 |
| 3222.0368254 | 0 |
| 3222.0376853 | 4 |
| 3222.0388161 | 1 |
| 3222.0393166 | 2 |
| 3222.040492 | 2 |
| 3222.0416698 | 3 |
| 3222.042603 | 0 |
| 3222.0436977 | 4 |
| 3222.043819 | 2 |
| 3222.0450852 | 3 |
| 3222.0462136 | 4 |
| 3222.0467715 | 3 |
| 3222.0475617 | 6 |
| 3222.0487184 | 5 |
| 3222.0494706 | 0 |
| 3222.0508466 | 1 |
| 3222.0514183 | 1 |
| 3222.0523321 | 1 |
| 3222.0532655 | 2 |
| 3222.0542378 | 1 |
| 3222.0552603 | 0 |
| 3222.0564945 | 3 |
| 3222.0571896 | 1 |
| 3222.0576035 | 1 |
| 3222.0591403 | 0 |
| 3222.0600703 | 2 |
| 3222.0603669 | 0 |
| 3222.0616333 | 2 |
| 3222.0623412 | 2 |
| 3222.0638997 | 2 |
| 3222.0648806 | 1 |
| 3222.065883 | 0 |
| 3222.0668735 | 2 |
| 3222.0670972 | 0 |
| 3222.0686512 | 2 |
| 3222.0698014 | 1 |
| 3222.0699121 | 2 |
| 3222.0717747 | 1 |
| 3222.0717968 | 1 |
| 3222.0727301 | 1 |
| 3222.0745035 | 4 |
| 3222.0747351 | 1 |
| 3222.075893 | 2 |
| 3222.076721 | 0 |
| 3222.0772874 | 1 |
| 3222.0788069 | 1 |
| 3222.0793361 | 2 |
| 3222.0804277 | 1 |
| 3222.0814893 | 0 |
| 3222.0821364 | 0 |
| 3222.0835626 | 1 |
| 3222.0841318 | 3 |
| 3222.085391 | 4 |
| 3222.0859074 | 1 |
| 3222.0872181 | 3 |
| 3222.0884563 | 0 |
| 3222.0885364 | 2 |
| 3222.0901319 | 1 |
| 3222.0903562 | 2 |
| 3222.0916081 | 1 |
| 3222.09312 | 0 |
| 3222.0937063 | 0 |
| 3222.094555 | 2 |
| 3222.0952988 | 3 |
| 3222.0965499 | 0 |
| 3222.0973631 | 1 |
| 3222.0986221 | 2 |
| 3222.0993953 | 0 |
| 3222.0999468 | 2 |
| 3222.1013218 | 1 |
| 3222.1015981 | 5 |
| 3222.1030433 | 3 |
| 3222.1036338 | 1 |
| 3222.1045152 | 0 |
| 3222.1058323 | 1 |
| 3222.107278 | 2 |
| 3222.107314 | 2 |
| 3222.1083627 | 0 |
| 3222.1096135 | 1 |
| 3222.110537 | 2 |
| 3222.1113167 | 2 |
| 3222.1123976 | 3 |
| 3222.1138117 | 2 |
| 3222.1138723 | 1 |
| 3222.1151498 | 1 |
| 3222.1161731 | 2 |
| 3222.1171254 | 1 |
| 3222.117893 | 0 |
| 3222.1191473 | 1 |
| 3222.120132 | 0 |
| 3222.1211526 | 0 |
| 3222.1216683 | 2 |
| 3222.1224966 | 1 |
| 3222.1233054 | 1 |
| 3222.1252337 | 1 |
| 3222.1255371 | 1 |
| 3222.1263768 | 0 |
| 3222.1277926 | 0 |
| 3222.1278584 | 2 |
| 3222.1289161 | 2 |
| 3222.1306505 | 5 |
| 3222.1308525 | 1 |
| 3222.1320851 | 1 |
| 3222.1330268 | 4 |
| 3222.133862 | 2 |
| 3222.1347448 | 4 |
| 3222.1360369 | 3 |
| 3222.1365234 | 2 |
| 3222.1377485 | 0 |
| 3222.1386316 | 1 |
| 3222.139698 | 2 |
| 3222.1404346 | 2 |
| 3222.1416855 | 1 |
| 3222.1425127 | 0 |
| 3222.1433557 | 1 |
| 3222.144666 | 0 |
| 3222.1449426 | 4 |
| 3222.1462146 | 0 |
| 3222.1477874 | 4 |
| 3222.1479455 | 1 |
| 3222.1494682 | 2 |
| 3222.1496998 | 4 |
| 3222.1512113 | 1 |
| 3222.1514232 | 3 |
| 3222.152256 | 1 |
| 3222.1533721 | 0 |
| 3222.1537825 | 0 |
| 3222.1553476 | 1 |
| 3222.1564398 | 2 |
| 3222.1571175 | 1 |
| 3222.1585354 | 0 |
| 3222.1594722 | 3 |
| 3222.1601932 | 3 |
| 3222.1605513 | 3 |
| 3222.1620943 | 1 |
| 3222.1630232 | 0 |
| 3222.1635737 | 2 |
| 3222.1649593 | 0 |
| 3222.1659584 | 2 |
| 3222.1666774 | 2 |
| 3222.1674649 | 1 |
| 3222.1684785 | 2 |
| 3222.16931 | 3 |
| 3222.1709459 | 7 |
| 3222.1715193 | 2 |
| 3222.1721311 | 3 |
| 3222.1728723 | 2 |
| 3222.1739108 | 2 |
| 3222.1752554 | 1 |
| 3222.1761162 | 4 |
| 3222.1772193 | 2 |
| 3222.1777946 | 1 |
| 3222.1790568 | 1 |
| 3222.1798209 | 1 |
| 3222.1806077 | 0 |
| 3222.181354 | 3 |
| 3222.1824106 | 3 |
| 3222.1834285 | 0 |
| 3222.1844029 | 0 |
| 3222.1850144 | 1 |
| 3222.1864001 | 2 |
| 3222.1877417 | 3 |
| 3222.1882772 | 0 |
| 3222.1893107 | 1 |
| 3222.1898291 | 3 |
| 3222.1909122 | 3 |
| 3222.1921827 | 0 |
| 3222.1929752 | 0 |
| 3222.1941661 | 0 |
| 3222.1945865 | 0 |
| 3222.1959404 | 3 |
| 3222.1963139 | 1 |
| 3222.197898 | 1 |
| 3222.1989179 | 0 |
| 3222.1993629 | 3 |
| 3222.2008044 | 0 |
| 3222.2008805 | 2 |
| 3222.2021138 | 2 |
| 3222.2033911 | 3 |
| 3222.203737 | 4 |
| 3222.2050461 | 2 |
| 3222.2065082 | 2 |
| 3222.2069374 | 1 |
| 3222.2086548 | 1 |
| 3222.2088079 | 1 |
| 3222.2097306 | 1 |
| 3222.2100732 | 4 |
| 3222.2114735 | 2 |
| 3222.2130083 | 1 |
| 3222.2134929 | 1 |
| 3222.2145894 | 2 |
| 3222.215747 | 0 |
| 3222.2163042 | 3 |
| 3222.2177758 | 2 |
| 3222.218502 | 4 |
| 3222.2191179 | 6 |
| 3222.2198205 | 8 |
| 3222.2213973 | 4 |
| 3222.2220444 | 1 |
| 3222.222872 | 0 |
| 3222.2238987 | 2 |
| 3222.2244108 | 1 |
| 3222.2257997 | 2 |
| 3222.2266766 | 0 |
| 3222.2269566 | 2 |
| 3222.228748 | 4 |
| 3222.2296077 | 1 |
| 3222.2298714 | 0 |
| 3222.2311985 | 3 |
| 3222.2320108 | 0 |
| 3222.2329549 | 0 |
| 3222.2342624 | 1 |
| 3222.2351086 | 0 |
| 3222.2360679 | 2 |
| 3222.236509 | 1 |
| 3222.2378029 | 1 |
| 3222.2386995 | 0 |
| 3222.2389031 | 1 |
| 3222.2403841 | 1 |
| 3222.2409092 | 3 |
| 3222.2420965 | 2 |
| 3222.2432715 | 0 |
| 3222.2443657 | 3 |
| 3222.2455902 | 3 |
| 3222.2459239 | 1 |
| 3222.2472472 | 1 |
| 3222.2480476 | 2 |
| 3222.2488169 | 1 |
| 3222.2497849 | 0 |
| 3222.2511699 | 2 |
| 3222.2516418 | 1 |
| 3222.2527396 | 2 |
| 3222.2537938 | 2 |
| 3222.2545922 | 1 |
| 3222.2549866 | 1 |
| 3222.2564767 | 0 |
| 3222.257464 | 3 |
| 3222.2580048 | 2 |
| 3222.2595579 | 0 |
| 3222.2595735 | 0 |
| 3222.2607194 | 0 |
| 3222.2621845 | 2 |
| 3222.2625268 | 1 |
| 3222.2637021 | 3 |
| 3222.2647054 | 1 |
| 3222.2659915 | 0 |
| 3222.2660636 | 2 |
| 3222.2676532 | 3 |
| 3222.2686107 | 3 |
| 3222.2689369 | 3 |
| 3222.2701381 | 1 |
| 3222.2713213 | 1 |
| 3222.2726164 | 2 |
| 3222.2736877 | 2 |
| 3222.2741196 | 2 |
| 3222.2750962 | 2 |
| 3222.2753456 | 2 |
| 3222.2764767 | 4 |

| | |
|---|---|
| 3222.2780628 | 2 |
| 3222.2781129 | 0 |
| 3222.2796687 | 3 |
| 3222.280269 | 4 |
| 3222.2811227 | 2 |
| 3222.2825679 | 2 |
| 3222.2830739 | 1 |
| 3222.284073 | 1 |
| 3222.2851268 | 3 |
| 3222.2861557 | 1 |
| 3222.2875093 | 2 |
| 3222.2878777 | 2 |
| 3222.2891785 | 2 |
| 3222.2901574 | 2 |
| 3222.2907744 | 1 |
| 3222.2924442 | 3 |
| 3222.2924546 | 2 |
| 3222.2938074 | 1 |
| 3222.2950629 | 0 |
| 3222.2955838 | 2 |
| 3222.2964135 | 2 |
| 3222.2970005 | 3 |
| 3222.2982198 | 1 |
| 3222.2994956 | 2 |
| 3222.2997043 | 2 |
| 3222.3012427 | 0 |
| 3222.3018477 | 3 |
| 3222.3027251 | 1 |
| 3222.3040921 | 2 |
| 3222.3051027 | 3 |
| 3222.3059317 | 2 |
| 3222.3065597 | 2 |
| 3222.3078614 | 2 |
| 3222.3084233 | 2 |
| 3222.3093216 | 0 |
| 3222.3107439 | 1 |
| 3222.3109614 | 2 |
| 3222.3119492 | 1 |
| 3222.3133302 | 3 |
| 3222.3139856 | 2 |
| 3222.3149019 | 0 |
| 3222.31567 | 0 |
| 3222.3168259 | 2 |
| 3222.3182662 | 0 |
| 3222.3185161 | 2 |
| 3222.3200597 | 0 |
| 3222.3201966 | 0 |
| 3222.3214096 | 3 |
| 3222.3226614 | 4 |
| 3222.3231021 | 1 |
| 3222.3239765 | 1 |
| 3222.3254848 | 2 |
| 3222.3261023 | 2 |
| 3222.3270297 | 2 |
| 3222.3275311 | 0 |
| 3222.3290864 | 3 |
| 3222.3302394 | 5 |
| 3222.3307927 | 1 |
| 3222.3321151 | 2 |
| 3222.3322063 | 0 |
| 3222.3335498 | 2 |
| 3222.3346044 | 2 |
| 3222.3351405 | 3 |
| 3222.3367643 | 2 |
| 3222.3370926 | 0 |
| 3222.3387831 | 2 |
| 3222.3391317 | 3 |
| 3222.3400156 | 2 |
| 3222.3415384 | 0 |
| 3222.3424396 | 0 |
| 3222.3430611 | 0 |
| 3222.344347 | 0 |
| 3222.344527 | 1 |
| 3222.34592 | 3 |
| 3222.3464629 | 3 |
| 3222.3477413 | 1 |
| 3222.3487018 | 0 |
| 3222.3493529 | 4 |
| 3222.3502531 | 4 |
| 3222.3509974 | 1 |
| 3222.3519456 | 3 |
| 3222.3534412 | 0 |
| 3222.3541567 | 2 |
| 3222.3551264 | 2 |
| 3222.3558586 | 0 |
| 3222.3566937 | 1 |
| 3222.3574892 | 0 |
| 3222.3585539 | 1 |
| 3222.3594877 | 1 |
| 3222.3603023 | 0 |
| 3222.3614673 | 2 |
| 3222.3624559 | 1 |
| 3222.3634303 | 1 |
| 3222.3643344 | 5 |
| 3222.3652357 | 1 |
| 3222.3663787 | 6 |
| 3222.3664246 | 0 |
| 3222.3674932 | 2 |
| 3222.3685974 | 2 |
| 3222.3693757 | 0 |
| 3222.3703734 | 2 |
| 3222.3711814 | 0 |
| 3222.3726901 | 1 |
| 3222.3739375 | 0 |
| 3222.3742454 | 2 |
| 3222.3754443 | 2 |
| 3222.375599 | 1 |
| 3222.3770083 | 0 |
| 3222.3782916 | 0 |
| 3222.3786153 | 1 |
| 3222.3799332 | 2 |
| 3222.3806772 | 3 |
| 3222.3819489 | 0 |
| 3222.3831291 | 3 |
| 3222.3838279 | 0 |
| 3222.3844036 | 2 |
| 3222.3855739 | 3 |
| 3222.3859851 | 1 |
| 3222.3873736 | 1 |
| 3222.3879952 | 3 |
| 3222.3894104 | 3 |
| 3222.389627 | 1 |
| 3222.3909143 | 4 |
| 3222.392291 | 3 |
| 3222.3928482 | 0 |
| 3222.3936665 | 2 |
| 3222.3948148 | 2 |
| 3222.3959235 | 2 |
| 3222.3968398 | 2 |
| 3222.3973036 | 2 |
| 3222.3987931 | 2 |
| 3222.3991392 | 2 |
| 3222.4003813 | 1 |
| 3222.401486 | 2 |
| 3222.401709 | 0 |
| 3222.4030986 | 2 |
| 3222.4034317 | 2 |
| 3222.4049271 | 1 |
| 3222.4061363 | 1 |
| 3222.4065893 | 2 |
| 3222.4079233 | 1 |
| 3222.4086539 | 2 |
| 3222.4092866 | 0 |
| 3222.4104379 | 1 |
| 3222.4116139 | 2 |
| 3222.4122351 | 1 |
| 3222.4131665 | 2 |
| 3222.4143337 | 2 |
| 3222.4154511 | 2 |
| 3222.4157013 | 0 |
| 3222.4172108 | 5 |
| 3222.4174384 | 1 |
| 3222.4186508 | 2 |
| 3222.419266 | 5 |
| 3222.4205225 | 1 |
| 3222.4216383 | 2 |
| 3222.4223467 | 1 |
| 3222.4232804 | 1 |
| 3222.4248413 | 2 |
| 3222.4250158 | 4 |
| 3222.4264347 | 1 |
| 3222.4265543 | 1 |
| 3222.4277004 | 0 |
| 3222.4293887 | 0 |
| 3222.4298251 | 0 |
| 3222.4307217 | 1 |
| 3222.4316588 | 0 |
| 3222.4326872 | 1 |
| 3222.4338429 | 1 |
| 3222.4342676 | 1 |
| 3222.4353782 | 2 |
| 3222.4362802 | 2 |
| 3222.4377436 | 0 |
| 3222.438574 | 2 |
| 3222.4392641 | 2 |
| 3222.4402488 | 1 |
| 3222.4407037 | 1 |
| 3222.4418604 | 1 |
| 3222.4432518 | 3 |
| 3222.4437833 | 1 |
| 3222.4449392 | 1 |
| 3222.4457062 | 3 |
| 3222.4465378 | 1 |
| 3222.447362 | 2 |
| 3222.4485406 | 0 |
| 3222.4496292 | 3 |
| 3222.4502065 | 1 |
| 3222.4515179 | 2 |
| 3222.4527388 | 2 |
| 3222.4527467 | 3 |
| 3222.4541674 | 1 |
| 3222.454625 | 3 |
| 3222.455345 | 2 |
| 3222.4565139 | 1 |
| 3222.4581576 | 4 |
| 3222.4582538 | 2 |
| 3222.4593336 | 1 |
| 3222.4603783 | 2 |
| 3222.4611652 | 0 |
| 3222.4622706 | 2 |
| 3222.4636307 | 3 |
| 3222.4638849 | 1 |
| 3222.4651686 | 1 |
| 3222.466238 | 2 |
| 3222.4662516 | 2 |
| 3222.4678336 | 1 |
| 3222.4684631 | 7 |
| 3222.4692967 | 3 |
| 3222.4705483 | 1 |
| 3222.4713834 | 2 |
| 3222.4722873 | 2 |
| 3222.4729943 | 2 |
| 3222.474462 | 3 |
| 3222.4748146 | 3 |
| 3222.4757653 | 2 |
| 3222.4773308 | 2 |
| 3222.4774877 | 2 |
| 3222.4790358 | 4 |
| 3222.4796084 | 2 |
| 3222.4803579 | 1 |
| 3222.4818463 | 1 |
| 3222.4823078 | 0 |
| 3222.4832032 | 3 |
| 3222.4844602 | 3 |
| 3222.485312 | 0 |
| 3222.4859583 | 1 |
| 3222.4869016 | 1 |
| 3222.4883533 | 3 |
| 3222.4893354 | 3 |
| 3222.4896649 | 2 |
| 3222.4912621 | 1 |
| 3222.4917 | 2 |
| 3222.4927448 | 2 |
| 3222.4937729 | 0 |
| 3222.4944516 | 1 |
| 3222.4955509 | 2 |
| 3222.4969136 | 0 |
| 3222.4970128 | 1 |
| 3222.4987002 | 1 |
| 3222.499149 | 2 |
| 3222.5002882 | 0 |
| 3222.5012112 | 4 |
| 3222.5018927 | 0 |
| 3222.5030023 | 2 |
| 3222.5038796 | 2 |
| 3222.5052905 | 0 |
| 3222.5054325 | 3 |
| 3222.5067313 | 1 |
| 3222.5074583 | 3 |
| 3222.5081251 | 3 |
| 3222.509499 | 0 |
| 3222.5106953 | 2 |
| 3222.511347 | 0 |
| 3222.5124544 | 1 |
| 3222.5127734 | 2 |
| 3222.5142833 | 2 |
| 3222.5147347 | 2 |
| 3222.5161049 | 1 |
| 3222.5170516 | 1 |
| 3222.517169 | 3 |
| 3222.5181182 | 0 |
| 3222.51975 | 1 |
| 3222.5200557 | 3 |
| 3222.521022 | 5 |
| 3222.5221544 | 3 |
| 3222.5232144 | 0 |
| 3222.5240283 | 1 |
| 3222.5248887 | 6 |
| 3222.5260852 | 0 |
| 3222.526575 | 2 |
| 3222.5284137 | 1 |
| 3222.5288616 | 1 |
| 3222.5295251 | 3 |
| 3222.5306725 | 1 |
| 3222.5308916 | 2 |
| 3222.5323065 | 1 |
| 3222.5338119 | 0 |
| 3222.5338432 | 0 |
| 3222.5347956 | 3 |
| 3222.535441 | 2 |
| 3222.5374546 | 1 |
| 3222.5379764 | 0 |
| 3222.5385276 | 2 |
| 3222.5390413 | 1 |
| 3222.5395099 | 2 |
| 3222.5396968 | 1 |
| 3222.5403609 | 1 |
| 3222.5406488 | 1 |
| 3222.541547 | 1 |
| 3222.5417723 | 2 |
| 3222.5426117 | 3 |
| 3222.5427359 | 2 |
| 3222.543568 | 1 |
| 3222.5436499 | 2 |
| 3222.5440308 | 2 |
| 3222.5443981 | 2 |
| 3222.5449939 | 0 |
| 3222.5456777 | 1 |
| 3222.5459711 | 1 |
| 3222.5465394 | 3 |
| 3222.5467502 | 2 |
| 3222.547147 | 0 |
| 3222.5475954 | 0 |
| 3222.5481838 | 1 |
| 3222.5488806 | 2 |
| 3222.5489433 | 1 |
| 3222.5494513 | 0 |
| 3222.5496215 | 1 |
| 3222.5506411 | 1 |
| 3222.5510195 | 1 |
| 3222.5516546 | 1 |
| 3222.5521295 | 2 |
| 3222.5523044 | 1 |
| 3222.5527019 | 0 |
| 3222.5533133 | 3 |
| 3222.5539771 | 0 |
| 3222.5543574 | 2 |
| 3222.5544319 | 0 |
| 3222.5551029 | 1 |
| 3222.5555323 | 3 |
| 3222.5562388 | 0 |
| 3222.5571142 | 2 |
| 3222.557205 | 3 |
| 3222.5576616 | 2 |
| 3222.5585405 | 3 |
| 3222.5585517 | 1 |
| 3222.5585727 | 0 |
| 3222.5590256 | 2 |
| 3222.5600415 | 2 |
| 3222.5602238 | 0 |
| 3222.5604059 | 2 |
| 3222.5609175 | 2 |
| 3222.5617781 | 1 |
| 3222.5623264 | 2 |
| 3222.5628591 | 3 |
| 3222.5633281 | 1 |
| 3222.5633931 | 3 |
| 3222.5639148 | 1 |
| 3222.5646895 | 3 |
| 3222.5647713 | 2 |
| 3222.5653998 | 2 |
| 3222.5658208 | 1 |
| 3222.5664145 | 1 |
| 3222.566675 | 4 |
| 3222.5673006 | 4 |
| 3222.5675318 | 1 |
| 3222.5681255 | 4 |
| 3222.5682802 | 1 |
| 3222.5694062 | 1 |
| 3222.5697027 | 0 |
| 3222.5698755 | 4 |
| 3222.5702533 | 0 |
| 3222.5705348 | 2 |
| 3222.5709522 | 3 |
| 3222.5722402 | 1 |
| 3222.5729162 | 4 |
| 3222.5740862 | 1 |
| 3222.5747023 | 3 |
| 3222.5760001 | 3 |
| 3222.5771898 | 0 |
| 3222.5775148 | 1 |
| 3222.5787327 | 0 |
| 3222.5793192 | 0 |

| | |
|---|---|
| 3222.5801699 | 1 |
| 3222.5810657 | 0 |
| 3222.5822089 | 1 |
| 3222.5830762 | 1 |
| 3222.5839096 | 0 |
| 3222.5853179 | 1 |
| 3222.5865456 | 1 |
| 3222.5866314 | 0 |
| 3222.5879255 | 2 |
| 3222.5886932 | 5 |
| 3222.589616 | 1 |
| 3222.5907642 | 1 |
| 3222.5916325 | 1 |
| 3222.5930815 | 2 |
| 3222.5931494 | 0 |
| 3222.5945283 | 1 |
| 3222.5954887 | 1 |
| 3222.5964199 | 3 |
| 3222.5967565 | 6 |
| 3222.5978698 | 1 |
| 3222.5988481 | 2 |
| 3222.6000816 | 3 |
| 3222.6007683 | 0 |
| 3222.6022125 | 1 |
| 3222.6022858 | 0 |
| 3222.6032629 | 1 |
| 3222.6049425 | 1 |
| 3222.6052009 | 1 |
| 3222.6064 | 2 |
| 3222.60742 | 2 |
| 3222.6084995 | 0 |
| 3222.6097858 | 0 |
| 3222.6103359 | 3 |
| 3222.6115889 | 2 |
| 3222.6118448 | 0 |
| 3222.6130096 | 1 |
| 3222.6133948 | 1 |
| 3222.6146332 | 2 |
| 3222.615829 | 4 |
| 3222.6164042 | 2 |
| 3222.6173145 | 4 |
| 3222.6182056 | 0 |
| 3222.6198374 | 2 |
| 3222.6203057 | 1 |
| 3222.6215707 | 0 |
| 3222.6223618 | 2 |
| 3222.6232249 | 2 |
| 3222.6249696 | 1 |
| 3222.6251831 | 5 |
| 3222.6259665 | 3 |
| 3222.6272145 | 1 |
| 3222.6280939 | 2 |
| 3222.6287098 | 0 |
| 3222.6301917 | 0 |
| 3222.6303908 | 2 |
| 3222.6317797 | 3 |
| 3222.6330692 | 2 |
| 3222.6333843 | 0 |
| 3222.6344142 | 4 |
| 3222.6350081 | 1 |
| 3222.6363212 | 2 |
| 3222.6367805 | 1 |
| 3222.6377493 | 1 |
| 3222.6392154 | 2 |
| 3222.6392506 | 2 |
| 3222.640997 | 6 |
| 3222.6418312 | 2 |
| 3222.6430711 | 0 |
| 3222.6439899 | 0 |
| 3222.6444323 | 2 |
| 3222.6452383 | 1 |
| 3222.6463364 | 1 |
| 3222.6474275 | 2 |
| 3222.6483877 | 0 |
| 3222.6494277 | 0 |
| 3222.6505282 | 4 |
| 3222.6511937 | 0 |
| 3222.6522298 | 1 |
| 3222.6530678 | 0 |
| 3222.6535841 | 4 |
| 3222.6550462 | 0 |
| 3222.6550546 | 1 |
| 3222.6567247 | 0 |
| 3222.6578209 | 2 |
| 3222.658354 | 1 |
| 3222.6595286 | 1 |
| 3222.6602904 | 0 |
| 3222.6609898 | 1 |
| 3222.6621706 | 0 |
| 3222.6632916 | 2 |
| 3222.6639328 | 0 |
| 3222.6650162 | 0 |
| 3222.6658362 | 1 |
| 3222.6673582 | 2 |
| 3222.6676994 | 5 |
| 3222.669023 | 3 |
| 3222.6692102 | 4 |
| 3222.670604 | 0 |
| 3222.6716944 | 0 |
| 3222.6718718 | 2 |
| 3222.6735271 | 2 |
| 3222.6742169 | 3 |
| 3222.6751032 | 3 |
| 3222.6759036 | 2 |
| 3222.6769732 | 0 |
| 3222.6783429 | 1 |
| 3222.6783966 | 0 |
| 3222.6797912 | 1 |
| 3222.6809225 | 3 |
| 3222.6816037 | 1 |
| 3222.6825375 | 5 |
| 3222.6833269 | 0 |
| 3222.6848335 | 3 |
| 3222.6856331 | 1 |
| 3222.6860288 | 4 |
| 3222.6867721 | 0 |
| 3222.6880421 | 0 |
| 3222.6888863 | 1 |
| 3222.6901889 | 1 |
| 3222.6902852 | 2 |
| 3222.6917856 | 2 |
| 3222.6923107 | 5 |
| 3222.6935313 | 3 |
| 3222.6946343 | 0 |
| 3222.6954548 | 1 |
| 3222.6964574 | 2 |
| 3222.697147 | 2 |
| 3222.6982432 | 3 |
| 3222.6995641 | 2 |
| 3222.7001457 | 1 |
| 3222.7014071 | 0 |
| 3222.7018662 | 1 |
| 3222.7029355 | 2 |
| 3222.7043324 | 1 |
| 3222.7044375 | 4 |
| 3222.7063702 | 1 |
| 3222.7065414 | 1 |
| 3222.7078581 | 2 |
| 3222.7085099 | 2 |
| 3222.7094778 | 3 |
| 3222.7106385 | 1 |
| 3222.7107701 | 2 |
| 3222.71216 | 1 |
| 3222.7136879 | 2 |
| 3222.7139582 | 1 |
| 3222.715149 | 0 |
| 3222.7156728 | 0 |
| 3222.7170282 | 1 |
| 3222.7179582 | 0 |
| 3222.7186465 | 1 |
| 3222.7198821 | 2 |
| 3222.7199651 | 2 |
| 3222.7209926 | 3 |
| 3222.7224524 | 2 |
| 3222.722987 | 2 |
| 3222.7237633 | 0 |
| 3222.7246819 | 0 |
| 3222.7261036 | 0 |
| 3222.7270555 | 0 |
| 3222.7275117 | 4 |
| 3222.7290341 | 1 |
| 3222.7291928 | 1 |
| 3222.7302092 | 5 |
| 3222.731703 | 1 |
| 3222.7322598 | 1 |
| 3222.7335297 | 1 |
| 3222.734331 | 2 |
| 3222.735249 | 2 |
| 3222.7366904 | 1 |
| 3222.7367628 | 1 |
| 3222.7377678 | 0 |
| 3222.7386515 | 3 |
| 3222.739765 | 3 |
| 3222.7408512 | 2 |
| 3222.7416355 | 0 |
| 3222.7427377 | 3 |
| 3222.7428992 | 5 |
| 3222.7441508 | 3 |
| 3222.745523 | 0 |
| 3222.7459471 | 3 |
| 3222.7474072 | 1 |
| 3222.7481071 | 2 |
| 3222.7490253 | 3 |
| 3222.7503343 | 1 |
| 3222.7508971 | 1 |
| 3222.7520797 | 2 |
| 3222.7527227 | 4 |
| 3222.754023 | 2 |
| 3222.7552231 | 3 |
| 3222.7558615 | 1 |
| 3222.7565967 | 2 |
| 3222.7571722 | 2 |
| 3222.7584731 | 0 |
| 3222.759447 | 3 |
| 3222.7600427 | 4 |
| 3222.7611863 | 0 |
| 3222.761728 | 1 |
| 3222.7632403 | 1 |
| 3222.7643576 | 4 |
| 3222.7646698 | 0 |
| 3222.7659884 | 2 |
| 3222.7667724 | 3 |
| 3222.7672652 | 3 |
| 3222.7682247 | 3 |
| 3222.7693142 | 2 |
| 3222.7703477 | 1 |
| 3222.771527 | 1 |
| 3222.7725003 | 3 |
| 3222.7731061 | 2 |
| 3222.773573 | 3 |
| 3222.7752356 | 2 |
| 3222.7758577 | 1 |
| 3222.7767931 | 0 |
| 3222.7779285 | 1 |
| 3222.7785692 | 1 |
| 3222.7798975 | 2 |
| 3222.7800808 | 3 |
| 3222.7812351 | 1 |
| 3222.7827881 | 5 |
| 3222.7829732 | 1 |
| 3222.7846158 | 2 |
| 3222.784774 | 1 |
| 3222.7858818 | 1 |
| 3222.7869151 | 1 |
| 3222.7877212 | 4 |
| 3222.7887425 | 1 |
| 3222.7897488 | 1 |
| 3222.7907169 | 0 |
| 3222.7919126 | 3 |
| 3222.792567 | 0 |
| 3222.7939048 | 1 |
| 3222.7943418 | 1 |
| 3222.7953005 | 1 |
| 3222.7964961 | 3 |
| 3222.7968488 | 1 |
| 3222.7983281 | 0 |
| 3222.799378 | 3 |
| 3222.7997414 | 2 |
| 3222.8008491 | 3 |
| 3222.8012054 | 6 |
| 3222.8028397 | 2 |
| 3222.8033228 | 1 |
| 3222.804313 | 1 |
| 3222.8052422 | 5 |
| 3222.8060343 | 1 |
| 3222.8070888 | 2 |
| 3222.8080893 | 3 |
| 3222.80907 | 4 |
| 3222.8100744 | 3 |
| 3222.8111048 | 7 |
| 3222.8123211 | 2 |
| 3222.8124848 | 2 |
| 3222.8137265 | 3 |
| 3222.8147478 | 1 |
| 3222.8152719 | 0 |
| 3222.8168213 | 1 |
| 3222.8179056 | 2 |
| 3222.8181315 | 2 |
| 3222.8194714 | 2 |
| 3222.8196357 | 3 |
| 3222.820904 | 2 |
| 3222.821908 | 1 |
| 3222.8228078 | 0 |
| 3222.8234032 | 1 |
| 3222.8246958 | 3 |
| 3222.8263371 | 1 |
| 3222.8263703 | 0 |
| 3222.8276909 | 0 |
| 3222.8286484 | 1 |
| 3222.8286706 | 2 |
| 3222.8301668 | 3 |
| 3222.831488 | 0 |
| 3222.8317541 | 2 |
| 3222.8332018 | 3 |
| 3222.8338271 | 1 |
| 3222.8350194 | 2 |
| 3222.83607 | 1 |
| 3222.8366282 | 1 |
| 3222.8372121 | 1 |
| 3222.8388208 | 2 |
| 3222.8396351 | 4 |
| 3222.8401379 | 1 |
| 3222.8414016 | 1 |
| 3222.8428892 | 4 |
| 3222.8430176 | 1 |
| 3222.8444016 | 2 |
| 3222.845508 | 1 |
| 3222.8457739 | 0 |
| 3222.8469203 | 2 |
| 3222.8477572 | 2 |
| 3222.8488437 | 0 |
| 3222.8499837 | 4 |
| 3222.8506668 | 2 |
| 3222.8520141 | 0 |
| 3222.8526476 | 4 |
| 3222.8538804 | 1 |
| 3222.8547096 | 4 |
| 3222.8551863 | 4 |
| 3222.8562884 | 0 |
| 3222.8576359 | 2 |
| 3222.8577034 | 0 |
| 3222.8590793 | 0 |
| 3222.8597412 | 1 |
| 3222.8609458 | 1 |
| 3222.861242 | 1 |
| 3222.8625659 | 4 |
| 3222.8636185 | 1 |
| 3222.8641912 | 0 |
| 3222.8653381 | 0 |
| 3222.8659104 | 3 |
| 3222.8670393 | 1 |
| 3222.8682405 | 1 |
| 3222.8686484 | 3 |
| 3222.8699588 | 0 |
| 3222.8706793 | 1 |
| 3222.8717006 | 1 |
| 3222.8723385 | 2 |
| 3222.873092 | 3 |
| 3222.8750255 | 3 |
| 3222.8757955 | 1 |
| 3222.8767991 | 1 |
| 3222.8772362 | 1 |
| 3222.8781975 | 1 |
| 3222.8793654 | 1 |
| 3222.8794479 | 1 |
| 3222.8807336 | 1 |
| 3222.8819794 | 3 |
| 3222.8828757 | 2 |
| 3222.884364 | 1 |
| 3222.884436 | 4 |
| 3222.8858722 | 5 |
| 3222.8871546 | 0 |
| 3222.8876027 | 0 |
| 3222.8883747 | 2 |
| 3222.8891095 | 1 |
| 3222.8909361 | 1 |
| 3222.89161 | 2 |
| 3222.8921684 | 0 |
| 3222.8931423 | 4 |
| 3222.8937061 | 1 |
| 3222.8946764 | 3 |
| 3222.8961826 | 5 |
| 3222.8971034 | 1 |
| 3222.8981882 | 1 |
| 3222.8986432 | 0 |
| 3222.8999414 | 2 |
| 3222.9000098 | 1 |
| 3222.901223 | 2 |
| 3222.9022569 | 1 |
| 3222.9029068 | 0 |
| 3222.9047107 | 1 |
| 3222.9056608 | 0 |
| 3222.9059393 | 3 |
| 3222.9071193 | 1 |
| 3222.9077 | 1 |
| 3222.9085161 | 2 |
| 3222.9094444 | 0 |
| 3222.9108472 | 1 |
| 3222.9118037 | 2 |
| 3222.9123547 | 2 |
| 3222.913501 | 2 |
| 3222.9143414 | 3 |
| 3222.9148209 | 2 |

| | |
|---|---|
| 3222.9165283 | 2 |
| 3222.9171999 | 2 |
| 3222.9181712 | 1 |
| 3222.9193684 | 1 |
| 3222.9196429 | 0 |
| 3222.92072 | 1 |
| 3222.921542 | 3 |
| 3222.922387 | 1 |
| 3222.9232012 | 3 |
| 3222.9248431 | 1 |
| 3222.9256976 | 2 |
| 3222.9258262 | 2 |
| 3222.9273863 | 3 |
| 3222.9281858 | 1 |
| 3222.929399 | 1 |
| 3222.9303986 | 2 |
| 3222.9313824 | 2 |
| 3222.9324982 | 0 |
| 3222.9328011 | 2 |
| 3222.934007 | 6 |
| 3222.9352817 | 0 |
| 3222.9360781 | 0 |
| 3222.9368948 | 2 |
| 3222.9376549 | 2 |
| 3222.9387146 | 0 |
| 3222.9398011 | 2 |
| 3222.9403313 | 1 |
| 3222.9413686 | 2 |
| 3222.9421917 | 1 |
| 3222.9429746 | 2 |
| 3222.9440166 | 2 |
| 3222.945093 | 1 |
| 3222.9462257 | 0 |
| 3222.9467046 | 1 |
| 3222.9479149 | 2 |
| 3222.9490693 | 2 |
| 3222.9494802 | 1 |
| 3222.9511678 | 2 |
| 3222.9512999 | 2 |
| 3222.9525503 | 2 |
| 3222.9533756 | 3 |
| 3222.9539006 | 1 |
| 3222.9555855 | 1 |
| 3222.9566123 | 1 |
| 3222.9568333 | 0 |
| 3222.9583457 | 2 |
| 3222.9584083 | 4 |
| 3222.9594826 | 0 |
| 3222.9608306 | 1 |
| 3222.9615757 | 1 |
| 3222.9624684 | 1 |
| 3222.9634105 | 2 |
| 3222.9644832 | 2 |
| 3222.9659866 | 3 |
| 3222.966039 | 2 |
| 3222.967382 | 0 |
| 3222.9679243 | 0 |
| 3222.9695714 | 0 |
| 3222.9701777 | 1 |
| 3222.9711526 | 0 |
| 3222.9723213 | 0 |
| 3222.9732912 | 1 |
| 3222.9736977 | 2 |
| 3222.9752744 | 1 |
| 3222.9753046 | 0 |
| 3222.976419 | 5 |
| 3222.9772318 | 0 |
| 3222.9784472 | 1 |
| 3222.9793932 | 2 |
| 3222.9802683 | 2 |
| 3222.9814896 | 0 |
| 3222.9822553 | 3 |
| 3222.9835666 | 2 |
| 3222.9845769 | 2 |
| 3222.9852039 | 2 |
| 3222.9863274 | 1 |
| 3222.9867761 | 1 |
| 3222.9873408 | 1 |
| 3222.9891078 | 2 |
| 3222.989647 | 1 |
| 3222.9905386 | 3 |
| 3222.9916071 | 2 |
| 3222.9926343 | 0 |
| 3222.9932287 | 1 |
| 3222.9944543 | 0 |
| 3222.9953914 | 2 |
| 3222.9965616 | 0 |
| 3222.9971874 | 0 |
| 3222.9985589 | 0 |
| 3222.9987844 | 0 |
| 3222.9998758 | 3 |
| 3223.001117 | 1 |
| 3223.0016297 | 2 |
| 3223.0029676 | 0 |
| 3223.0037893 | 1 |
| 3223.0048996 | 0 |
| 3223.0057313 | 1 |
| 3223.006279 | 0 |
| 3223.0074417 | 4 |
| 3223.0080608 | 1 |
| 3223.0090403 | 2 |
| 3223.0103556 | 0 |
| 3223.010609 | 0 |
| 3223.0122064 | 0 |
| 3223.0127569 | 1 |
| 3223.0138676 | 0 |
| 3223.0149159 | 0 |
| 3223.0155448 | 0 |
| 3223.016683 | 1 |
| 3223.0172731 | 2 |
| 3223.0183905 | 0 |
| 3223.0196891 | 2 |
| 3223.0208103 | 1 |
| 3223.0215437 | 0 |
| 3223.0221238 | 1 |
| 3223.0233443 | 3 |
| 3223.024772 | 1 |
| 3223.0253669 | 3 |
| 3223.026297 | 1 |
| 3223.0272129 | 2 |
| 3223.0281941 | 1 |
| 3223.0286434 | 1 |
| 3223.0300178 | 2 |
| 3223.0309396 | 2 |
| 3223.0312946 | 0 |
| 3223.0329198 | 2 |
| 3223.0338983 | 1 |
| 3223.0342187 | 1 |
| 3223.0355674 | 2 |
| 3223.0364226 | 2 |
| 3223.037185 | 2 |
| 3223.0384969 | 3 |
| 3223.0397287 | 0 |
| 3223.0408488 | 3 |
| 3223.0410145 | 2 |
| 3223.0422396 | 3 |
| 3223.0423756 | 2 |
| 3223.0435688 | 0 |
| 3223.0449829 | 0 |
| 3223.045815 | 2 |
| 3223.0466678 | 1 |
| 3223.0474695 | 2 |
| 3223.0484111 | 0 |
| 3223.0498362 | 1 |
| 3223.0501155 | 1 |
| 3223.0515239 | 0 |
| 3223.0516084 | 1 |
| 3223.053078 | 1 |
| 3223.0544891 | 1 |
| 3223.0547987 | 3 |
| 3223.0558328 | 0 |
| 3223.0572187 | 2 |
| 3223.0582199 | 3 |
| 3223.0586096 | 2 |
| 3223.0595189 | 3 |
| 3223.0604319 | 1 |
| 3223.0615529 | 0 |
| 3223.0626619 | 2 |
| 3223.0638992 | 1 |
| 3223.0648461 | 1 |
| 3223.0655555 | 0 |
| 3223.065932 | 3 |
| 3223.0674321 | 5 |
| 3223.0683587 | 10 |
| 3223.0686328 | 7 |
| 3223.0701454 | 2 |
| 3223.0710197 | 1 |
| 3223.0720656 | 0 |
| 3223.0732096 | 1 |
| 3223.0740349 | 0 |
| 3223.0747872 | 1 |
| 3223.0755064 | 1 |
| 3223.0767317 | 5 |
| 3223.0779818 | 2 |
| 3223.0784203 | 2 |
| 3223.079151 | 2 |
| 3223.0799187 | 1 |
| 3223.0812425 | 3 |
| 3223.0822685 | 0 |
| 3223.0831902 | 2 |
| 3223.0842297 | 2 |
| 3223.0846213 | 3 |
| 3223.0859755 | 0 |
| 3223.0872719 | 1 |
| 3223.0874548 | 0 |
| 3223.0888965 | 1 |
| 3223.0898754 | 3 |
| 3223.0907078 | 0 |
| 3223.0910658 | 3 |
| 3223.0923444 | 1 |
| 3223.093414 | 3 |
| 3223.0944068 | 2 |
| 3223.0953923 | 0 |
| 3223.0955862 | 2 |
| 3223.0971946 | 2 |
| 3223.0982224 | 0 |
| 3223.0987105 | 0 |
| 3223.0998226 | 4 |
| 3223.100568 | 2 |
| 3223.1019033 | 3 |
| 3223.1028312 | 4 |
| 3223.103293 | 2 |
| 3223.1041046 | 2 |
| 3223.1053843 | 1 |
| 3223.1065735 | 1 |
| 3223.1079194 | 1 |
| 3223.1082786 | 0 |
| 3223.1094232 | 1 |
| 3223.1100046 | 1 |
| 3223.1113764 | 3 |
| 3223.1122249 | 2 |
| 3223.1133661 | 2 |
| 3223.1144384 | 3 |
| 3223.1148031 | 2 |
| 3223.1158499 | 4 |
| 3223.1169952 | 7 |
| 3223.1172474 | 3 |
| 3223.1187534 | 2 |
| 3223.119379 | 1 |
| 3223.1208044 | 0 |
| 3223.1211416 | 2 |
| 3223.1224095 | 2 |
| 3223.1235098 | 0 |
| 3223.1242707 | 4 |
| 3223.1254412 | 0 |
| 3223.1259497 | 3 |
| 3223.1274307 | 2 |
| 3223.1285407 | 2 |
| 3223.1290153 | 1 |
| 3223.1301078 | 1 |
| 3223.1307824 | 5 |
| 3223.1317878 | 2 |
| 3223.1331881 | 2 |
| 3223.1337268 | 3 |
| 3223.134997 | 3 |
| 3223.1352565 | 0 |
| 3223.1366581 | 1 |
| 3223.1373779 | 2 |
| 3223.1383343 | 2 |
| 3223.1394669 | 0 |
| 3223.1401114 | 1 |
| 3223.1413183 | 4 |
| 3223.1426311 | 3 |
| 3223.1426674 | 0 |
| 3223.1440996 | 3 |
| 3223.1446922 | 3 |
| 3223.1456307 | 0 |
| 3223.1466278 | 2 |
| 3223.1480989 | 1 |
| 3223.1493726 | 2 |
| 3223.1496196 | 1 |
| 3223.1508574 | 1 |
| 3223.1519768 | 3 |
| 3223.1524405 | 1 |
| 3223.15381 | 5 |
| 3223.1540246 | 3 |
| 3223.1554612 | 4 |
| 3223.1559561 | 0 |
| 3223.157169 | 0 |
| 3223.1583322 | 1 |
| 3223.1588182 | 3 |
| 3223.1602682 | 0 |
| 3223.1608218 | 2 |
| 3223.1618783 | 1 |
| 3223.1631227 | 2 |
| 3223.1641179 | 1 |
| 3223.1651742 | 0 |
| 3223.1659268 | 3 |
| 3223.1668758 | 0 |
| 3223.1669958 | 2 |
| 3223.1687161 | 4 |
| 3223.1694448 | 6 |
| 3223.1703005 | 2 |
| 3223.170911 | 3 |
| 3223.1721966 | 1 |
| 3223.1731084 | 0 |
| 3223.174222 | 2 |
| 3223.1744069 | 3 |
| 3223.1759966 | 3 |
| 3223.1770935 | 1 |
| 3223.1778672 | 3 |
| 3223.178816 | 2 |
| 3223.1801265 | 3 |
| 3223.180818 | 2 |
| 3223.1818005 | 2 |
| 3223.1820911 | 3 |
| 3223.1835978 | 4 |
| 3223.1838387 | 1 |
| 3223.1851718 | 2 |
| 3223.1864319 | 1 |
| 3223.1870938 | 2 |
| 3223.188027 | 2 |
| 3223.1888967 | 2 |
| 3223.1901133 | 1 |
| 3223.191056 | 2 |
| 3223.1919951 | 6 |
| 3223.193179 | 4 |
| 3223.1934456 | 1 |
| 3223.1949672 | 2 |
| 3223.195857 | 1 |
| 3223.1964806 | 0 |
| 3223.1974664 | 1 |
| 3223.1983704 | 2 |
| 3223.1996033 | 0 |
| 3223.2003652 | 1 |
| 3223.2015438 | 0 |
| 3223.2020739 | 1 |
| 3223.2030862 | 0 |
| 3223.2040698 | 2 |
| 3223.2050157 | 2 |
| 3223.2062554 | 1 |
| 3223.207255 | 2 |
| 3223.2076087 | 1 |
| 3223.2087026 | 1 |
| 3223.2099992 | 1 |
| 3223.2104643 | 1 |
| 3223.2113953 | 2 |
| 3223.2124152 | 1 |
| 3223.2137743 | 1 |
| 3223.2148394 | 1 |
| 3223.2154228 | 2 |
| 3223.2166914 | 4 |
| 3223.2172182 | 2 |
| 3223.2184438 | 1 |
| 3223.2190605 | 0 |
| 3223.2198485 | 0 |
| 3223.2208697 | 2 |
| 3223.2220861 | 4 |
| 3223.2221963 | 1 |
| 3223.2236309 | 5 |
| 3223.2252064 | 1 |
| 3223.2255476 | 3 |
| 3223.2267311 | 2 |
| 3223.2273943 | 1 |
| 3223.2284687 | 1 |
| 3223.2287303 | 0 |
| 3223.2302175 | 1 |
| 3223.2308351 | 1 |
| 3223.2317329 | 1 |
| 3223.2333591 | 1 |
| 3223.2338067 | 0 |
| 3223.2347202 | 1 |
| 3223.2363294 | 0 |
| 3223.2363984 | 1 |
| 3223.2375155 | 1 |
| 3223.2383726 | 0 |
| 3223.2396131 | 1 |
| 3223.2404113 | 2 |
| 3223.2410166 | 1 |
| 3223.2426209 | 3 |
| 3223.2430918 | 1 |
| 3223.2444894 | 0 |
| 3223.2454939 | 1 |
| 3223.2461177 | 1 |
| 3223.2472809 | 2 |
| 3223.2476857 | 3 |
| 3223.2487049 | 0 |
| 3223.2495131 | 6 |
| 3223.2510539 | 7 |
| 3223.2516869 | 7 |
| 3223.2527725 | 7 |
| 3223.2540635 | 1 |
| 3223.2544303 | 2 |

| | |
|---|---|
| 3223.2558735 | 1 |
| 3223.2568844 | 1 |
| 3223.2574384 | 1 |
| 3223.2583244 | 1 |
| 3223.2593959 | 1 |
| 3223.2600972 | 0 |
| 3223.2611239 | 3 |
| 3223.2620119 | 0 |
| 3223.2628142 | 1 |
| 3223.2640261 | 1 |
| 3223.2647462 | 1 |
| 3223.2658721 | 2 |
| 3223.2662937 | 3 |
| 3223.2675058 | 2 |
| 3223.2682514 | 2 |
| 3223.2694373 | 2 |
| 3223.2705373 | 1 |
| 3223.2714647 | 1 |
| 3223.2726942 | 3 |
| 3223.2732294 | 0 |
| 3223.2741586 | 2 |
| 3223.2752635 | 1 |
| 3223.2757167 | 1 |
| 3223.2770918 | 1 |
| 3223.2775959 | 0 |
| 3223.2790023 | 1 |
| 3223.2798528 | 3 |
| 3223.2804949 | 0 |
| 3223.2817729 | 0 |
| 3223.2819662 | 2 |
| 3223.2833046 | 3 |
| 3223.284045 | 3 |
| 3223.2851343 | 1 |
| 3223.2863534 | 0 |
| 3223.2873128 | 2 |
| 3223.2882346 | 0 |
| 3223.2893888 | 2 |
| 3223.2898953 | 2 |
| 3223.2909313 | 1 |
| 3223.2914848 | 3 |
| 3223.2928517 | 3 |
| 3223.2938238 | 0 |
| 3223.2947761 | 1 |
| 3223.2959157 | 0 |
| 3223.2966275 | 0 |
| 3223.2976345 | 5 |
| 3223.2987411 | 6 |
| 3223.2988286 | 1 |
| 3223.3008181 | 1 |
| 3223.3009601 | 5 |
| 3223.3021133 | 1 |
| 3223.3027439 | 3 |
| 3223.3042872 | 1 |
| 3223.3053141 | 4 |
| 3223.3057251 | 2 |
| 3223.3072607 | 1 |
| 3223.3074467 | 2 |
| 3223.3090853 | 1 |
| 3223.3103267 | 1 |
| 3223.3107217 | 0 |
| 3223.3117847 | 1 |
| 3223.3120046 | 1 |
| 3223.3134793 | 1 |
| 3223.3147494 | 3 |
| 3223.315114 | 0 |
| 3223.3162818 | 1 |
| 3223.3172967 | 1 |
| 3223.3182739 | 1 |
| 3223.3193179 | 0 |
| 3223.3201575 | 1 |
| 3223.3209433 | 0 |
| 3223.3212517 | 3 |
| 3223.3227167 | 2 |
| 3223.3238102 | 1 |
| 3223.3249558 | 0 |
| 3223.325967 | 1 |
| 3223.3263781 | 2 |
| 3223.3275309 | 6 |
| 3223.328651 | 3 |
| 3223.3291073 | 1 |
| 3223.3303607 | 0 |
| 3223.3307444 | 3 |
| 3223.3319365 | 2 |
| 3223.3331349 | 0 |
| 3223.3337827 | 3 |
| 3223.3340638 | 1 |
| 3223.3345724 | 1 |
| 3223.3350744 | 1 |
| 3223.3353532 | 2 |
| 3223.3356901 | 4 |
| 3223.3366076 | 2 |
| 3223.3366838 | 0 |
| 3223.3372002 | 0 |
| 3223.3379463 | 2 |
| 3223.3380435 | 1 |
| 3223.338631 | 0 |
| 3223.3392134 | 0 |
| 3223.339532 | 0 |
| 3223.3399514 | 1 |
| 3223.3406018 | 1 |
| 3223.3412664 | 0 |
| 3223.3414722 | 1 |
| 3223.3419877 | 0 |
| 3223.3422213 | 1 |
| 3223.3424307 | 0 |
| 3223.3430559 | 1 |
| 3223.3438738 | 0 |
| 3223.3443787 | 1 |
| 3223.3447369 | 0 |
| 3223.3448962 | 2 |
| 3223.345949 | 1 |
| 3223.3461878 | 1 |
| 3223.3465644 | 0 |
| 3223.3467798 | 4 |
| 3223.347729 | 1 |
| 3223.3479914 | 1 |
| 3223.3485894 | 3 |
| 3223.3487668 | 0 |
| 3223.3494269 | 2 |
| 3223.3495068 | 0 |
| 3223.3504671 | 1 |
| 3223.350711 | 1 |
| 3223.3509652 | 0 |
| 3223.3518136 | 1 |
| 3223.3518932 | 3 |
| 3223.3529663 | 1 |
| 3223.3533305 | 0 |
| 3223.3538878 | 1 |
| 3223.3540415 | 1 |
| 3223.3541581 | 0 |
| 3223.3555412 | 0 |
| 3223.3556605 | 2 |
| 3223.3563587 | 0 |
| 3223.3567423 | 1 |
| 3223.3568823 | 2 |
| 3223.3580714 | 2 |
| 3223.3582556 | 2 |
| 3223.3585795 | 2 |
| 3223.3586423 | 2 |
| 3223.3590014 | 1 |
| 3223.359627 | 0 |
| 3223.3602948 | 2 |
| 3223.3603652 | 0 |
| 3223.3603744 | 2 |
| 3223.3606047 | 2 |
| 3223.3612912 | 0 |
| 3223.3617557 | 1 |
| 3223.3620272 | 2 |
| 3223.3624982 | 3 |
| 3223.3627446 | 0 |
| 3223.3628333 | 1 |
| 3223.3633776 | 2 |
| 3223.3634079 | 1 |
| 3223.3635271 | 4 |
| 3223.3645842 | 2 |
| 3223.3646166 | 1 |
| 3223.3646867 | 0 |
| 3223.3651284 | 0 |
| 3223.3651327 | 1 |
| 3223.3654337 | 2 |
| 3223.3662055 | 1 |
| 3223.3663162 | 3 |
| 3223.3665443 | 1 |
| 3223.3666337 | 1 |
| 3223.3670896 | 0 |
| 3223.3674288 | 0 |
| 3223.3676488 | 0 |
| 3223.3677999 | 1 |
| 3223.3681816 | 2 |
| 3223.3686219 | 1 |
| 3223.3689541 | 1 |
| 3223.369201 | 1 |
| 3223.3693713 | 3 |
| 3223.3695472 | 2 |
| 3223.3698876 | 0 |
| 3223.3705316 | 1 |
| 3223.3707311 | 0 |
| 3223.3708312 | 0 |
| 3223.3711856 | 0 |
| 3223.3720629 | 0 |
| 3223.3723323 | 0 |
| 3223.372483 | 0 |
| 3223.3725507 | 2 |
| 3223.3728294 | 0 |
| 3223.373393 | 1 |
| 3223.3734869 | 1 |
| 3223.373556 | 1 |
| 3223.3740031 | 3 |
| 3223.3742237 | 3 |
| 3223.3749661 | 2 |
| 3223.3753674 | 2 |
| 3223.3753815 | 3 |
| 3223.3755709 | 2 |
| 3223.376229 | 1 |
| 3223.376645 | 1 |
| 3223.3768724 | 2 |
| 3223.3770918 | 1 |
| 3223.3773473 | 1 |
| 3223.3774118 | 4 |
| 3223.3779817 | 1 |
| 3223.3781211 | 2 |
| 3223.3783677 | 1 |
| 3223.3787617 | 1 |
| 3223.3790517 | 1 |
| 3223.3796604 | 4 |
| 3223.3799194 | 3 |
| 3223.380305 | 1 |
| 3223.3803093 | 2 |
| 3223.380454 | 0 |
| 3223.3810689 | 2 |
| 3223.3812275 | 2 |
| 3223.3814766 | 2 |
| 3223.38165 | 1 |
| 3223.3817206 | 1 |
| 3223.3818068 | 1 |
| 3223.381855 | 0 |
| 3223.3820118 | 1 |
| 3223.3827879 | 1 |
| 3223.3835126 | 1 |
| 3223.3836797 | 1 |
| 3223.384585 | 0 |
| 3223.3846842 | 4 |
| 3223.3846941 | 4 |
| 3223.3847267 | 0 |
| 3223.3847622 | 2 |
| 3223.3857313 | 3 |
| 3223.3857806 | 1 |
| 3223.3865919 | 1 |
| 3223.3867073 | 1 |
| 3223.3873981 | 4 |
| 3223.38803 | 2 |
| 3223.3880402 | 3 |
| 3223.3882714 | 1 |
| 3223.3893292 | 0 |
| 3223.3894563 | 0 |
| 3223.3905569 | 0 |
| 3223.3908819 | 2 |
| 3223.3912503 | 2 |
| 3223.3914076 | 0 |
| 3223.3918654 | 4 |
| 3223.3919858 | 2 |
| 3223.39261 | 2 |
| 3223.3930906 | 2 |
| 3223.3937069 | 1 |
| 3223.3940867 | 1 |
| 3223.3949586 | 2 |
| 3223.395566 | 2 |
| 3223.3959191 | 5 |
| 3223.396226 | 4 |
| 3223.3969751 | 1 |
| 3223.3971359 | 2 |
| 3223.3972227 | 1 |
| 3223.3975126 | 1 |
| 3223.3975906 | 2 |
| 3223.398889 | 2 |
| 3223.3994549 | 2 |
| 3223.3998202 | 1 |
| 3223.4002023 | 2 |
| 3223.4002311 | 4 |
| 3223.4005395 | 0 |
| 3223.4013312 | 0 |
| 3223.401664 | 1 |
| 3223.4016984 | 3 |
| 3223.4020555 | 4 |
| 3223.4026695 | 2 |
| 3223.4035627 | 3 |
| 3223.404234 | 1 |
| 3223.4046318 | 0 |
| 3223.4048651 | 2 |
| 3223.4049468 | 1 |
| 3223.4061777 | 3 |
| 3223.4063325 | 2 |
| 3223.4064843 | 3 |
| 3223.4069723 | 0 |
| 3223.4079074 | 3 |
| 3223.4080379 | 3 |
| 3223.409069 | 0 |
| 3223.409245 | 3 |
| 3223.4096018 | 2 |
| 3223.4097863 | 0 |
| 3223.4107957 | 3 |
| 3223.4110327 | 0 |
| 3223.4115934 | 0 |
| 3223.4116876 | 0 |
| 3223.4125342 | 0 |
| 3223.4128343 | 2 |
| 3223.4132066 | 2 |
| 3223.4141201 | 3 |
| 3223.4146284 | 1 |
| 3223.414641 | 1 |
| 3223.4155184 | 1 |
| 3223.4160755 | 0 |
| 3223.4161258 | 0 |
| 3223.4164166 | 0 |
| 3223.4173406 | 2 |
| 3223.4173659 | 1 |
| 3223.418704 | 2 |
| 3223.4187148 | 4 |
| 3223.4195637 | 3 |
| 3223.4197079 | 1 |
| 3223.4203298 | 4 |
| 3223.4205811 | 1 |
| 3223.4212903 | 2 |
| 3223.4214744 | 1 |
| 3223.4222876 | 0 |
| 3223.4226091 | 1 |
| 3223.4227764 | 3 |
| 3223.4235292 | 1 |
| 3223.4236548 | 1 |
| 3223.4238096 | 1 |
| 3223.4248697 | 2 |
| 3223.4253008 | 3 |
| 3223.4254753 | 1 |
| 3223.4255342 | 0 |
| 3223.4271419 | 2 |
| 3223.4271454 | 2 |
| 3223.4277457 | 0 |
| 3223.4277886 | 0 |
| 3223.4287874 | 1 |
| 3223.4290953 | 0 |
| 3223.4297418 | 0 |
| 3223.430079 | 2 |
| 3223.4303386 | 2 |
| 3223.4308656 | 1 |
| 3223.4318593 | 0 |
| 3223.4319215 | 4 |
| 3223.432419 | 2 |
| 3223.4326413 | 1 |
| 3223.4330438 | 0 |
| 3223.4342872 | 1 |
| 3223.4343284 | 2 |
| 3223.4354629 | 3 |
| 3223.4354802 | 5 |
| 3223.4358614 | 1 |
| 3223.4365322 | 3 |
| 3223.4366358 | 2 |
| 3223.4370385 | 2 |
| 3223.437561 | 2 |
| 3223.4380319 | 1 |
| 3223.4382204 | 1 |
| 3223.4396189 | 2 |
| 3223.4396473 | 1 |
| 3223.4397467 | 1 |
| 3223.4401539 | 4 |
| 3223.4412327 | 3 |
| 3223.4414086 | 4 |
| 3223.4414088 | 2 |
| 3223.4418692 | 3 |
| 3223.441903 | 2 |
| 3223.4422007 | 5 |
| 3223.4426502 | 1 |
| 3223.4426517 | 2 |
| 3223.4428561 | 2 |
| 3223.4436132 | 3 |
| 3223.443663 | 3 |
| 3223.4440981 | 1 |
| 3223.4442641 | 3 |
| 3223.4447745 | 1 |
| 3223.4451874 | 6 |
| 3223.4456424 | 3 |
| 3223.4468672 | 1 |
| 3223.4473499 | 3 |
| 3223.4486715 | 5 |

| | |
|---|---|
| 3223.4492034 | 3 |
| 3223.4503394 | 2 |
| 3223.4513995 | 4 |
| 3223.4519322 | 0 |
| 3223.4536035 | 0 |
| 3223.4541113 | 5 |
| 3223.4552186 | 3 |
| 3223.4557495 | 0 |
| 3223.457023 | 0 |
| 3223.4584567 | 1 |
| 3223.4591506 | 1 |
| 3223.4600375 | 2 |
| 3223.4610004 | 1 |
| 3223.4612829 | 0 |
| 3223.462654 | 2 |
| 3223.4633913 | 0 |
| 3223.4646441 | 2 |
| 3223.4653529 | 0 |
| 3223.4661231 | 1 |
| 3223.4673969 | 2 |
| 3223.4680953 | 0 |
| 3223.4695205 | 1 |
| 3223.470228 | 2 |
| 3223.4710475 | 0 |
| 3223.472535 | 1 |
| 3223.4727246 | 2 |
| 3223.4740896 | 1 |
| 3223.4750549 | 3 |
| 3223.4754834 | 1 |
| 3223.4766427 | 0 |
| 3223.4773274 | 2 |
| 3223.4787484 | 4 |
| 3223.4797153 | 0 |
| 3223.480528 | 2 |
| 3223.4814643 | 3 |
| 3223.4821922 | 1 |
| 3223.4832978 | 0 |
| 3223.4842776 | 1 |
| 3223.4853132 | 1 |
| 3223.4864477 | 4 |
| 3223.4871171 | 3 |
| 3223.4883169 | 3 |
| 3223.4894217 | 1 |
| 3223.4904229 | 1 |
| 3223.4912399 | 3 |
| 3223.4920485 | 1 |
| 3223.4930146 | 2 |
| 3223.4941989 | 1 |
| 3223.4945067 | 1 |
| 3223.4957322 | 1 |
| 3223.4966887 | 1 |
| 3223.4969207 | 2 |
| 3223.498113 | 1 |
| 3223.4992234 | 0 |
| 3223.5004454 | 3 |
| 3223.5011653 | 3 |
| 3223.5024161 | 2 |
| 3223.50387 | 2 |
| 3223.5043128 | 3 |
| 3223.5053809 | 1 |
| 3223.5056505 | 1 |
| 3223.5070311 | 0 |
| 3223.5078175 | 1 |
| 3223.508881 | 0 |
| 3223.5100156 | 2 |
| 3223.5106104 | 2 |
| 3223.5113435 | 2 |
| 3223.5130166 | 2 |
| 3223.5130994 | 4 |
| 3223.5147398 | 2 |
| 3223.5155678 | 0 |
| 3223.5168209 | 1 |
| 3223.5173406 | 0 |
| 3223.5180516 | 1 |
| 3223.5193991 | 1 |
| 3223.5207035 | 2 |
| 3223.5209425 | 0 |
| 3223.5221976 | 1 |
| 3223.5228382 | 1 |
| 3223.5243945 | 1 |
| 3223.5258605 | 2 |
| 3223.5259703 | 0 |
| 3223.527239 | 0 |
| 3223.5277589 | 3 |
| 3223.5294271 | 3 |
| 3223.5295151 | 0 |
| 3223.5308996 | 1 |
| 3223.531839 | 2 |
| 3223.5321972 | 0 |
| 3223.5332517 | 0 |
| 3223.5340353 | 0 |
| 3223.5357578 | 2 |
| 3223.5367468 | 0 |
| 3223.5371978 | 1 |
| 3223.538521 | 3 |
| 3223.5393421 | 5 |
| 3223.5400774 | 1 |
| 3223.5412012 | 0 |
| 3223.5420686 | 0 |
| 3223.5434893 | 3 |
| 3223.5436674 | 3 |
| 3223.5454569 | 3 |
| 3223.5461887 | 1 |
| 3223.5471828 | 2 |
| 3223.5479841 | 0 |
| 3223.5486258 | 0 |
| 3223.5495549 | 0 |
| 3223.5506043 | 1 |
| 3223.5515731 | 1 |
| 3223.5524875 | 3 |
| 3223.5535334 | 2 |
| 3223.5543114 | 0 |
| 3223.5556818 | 4 |
| 3223.5559395 | 1 |
| 3223.55725 | 1 |
| 3223.5586455 | 1 |
| 3223.5589648 | 2 |
| 3223.560105 | 2 |
| 3223.5611367 | 2 |
| 3223.5617011 | 1 |
| 3223.5631059 | 1 |
| 3223.5641446 | 5 |
| 3223.5649155 | 2 |
| 3223.565185 | 1 |
| 3223.5664945 | 1 |
| 3223.5681098 | 1 |
| 3223.5683682 | 1 |
| 3223.5696279 | 1 |
| 3223.5701439 | 1 |
| 3223.5711734 | 1 |
| 3223.5724968 | 3 |
| 3223.5732478 | 1 |
| 3223.5741632 | 3 |
| 3223.5749933 | 0 |
| 3223.5766737 | 1 |
| 3223.5767171 | 1 |
| 3223.5781461 | 0 |
| 3223.579037 | 1 |
| 3223.579551 | 2 |
| 3223.5806508 | 6 |
| 3223.5820614 | 4 |
| 3223.5827732 | 0 |
| 3223.5838164 | 2 |
| 3223.5844653 | 1 |
| 3223.5860885 | 3 |
| 3223.5865831 | 1 |
| 3223.5875236 | 2 |
| 3223.5887744 | 1 |
| 3223.589807 | 3 |
| 3223.5902049 | 0 |
| 3223.5915364 | 1 |
| 3223.5917127 | 2 |
| 3223.5933032 | 1 |
| 3223.5940611 | 1 |
| 3223.5952126 | 1 |
| 3223.596181 | 2 |
| 3223.5969237 | 0 |
| 3223.5978943 | 1 |
| 3223.5983152 | 2 |
| 3223.5996127 | 1 |
| 3223.6008789 | 0 |
| 3223.6013705 | 0 |
| 3223.602672 | 0 |
| 3223.6036453 | 0 |
| 3223.6051058 | 0 |
| 3223.6053713 | 1 |
| 3223.6056422 | 1 |
| 3223.6649445 | 0 |
| 3223.6659144 | 5 |
| 3223.666782 | 1 |
| 3223.6677378 | 2 |
| 3223.6682976 | 3 |
| 3223.6694313 | 0 |
| 3223.6704397 | 3 |
| 3223.6709243 | 2 |
| 3223.6724557 | 2 |
| 3223.6731429 | 3 |
| 3223.6738702 | 2 |
| 3223.6746284 | 1 |
| 3223.6757438 | 3 |
| 3223.6770097 | 2 |
| 3223.6775393 | 0 |
| 3223.6792167 | 3 |
| 3223.6801777 | 2 |
| 3223.680223 | 3 |
| 3223.6828284 | 3 |
| 3223.6839974 | 3 |
| 3223.6842251 | 3 |
| 3223.6848851 | 2 |
| 3223.6854583 | 1 |
| 3223.6863703 | 1 |
| 3223.6871947 | 2 |
| 3223.6882026 | 1 |
| 3223.6896262 | 1 |
| 3223.689897 | 1 |
| 3223.6910197 | 2 |
| 3223.6921645 | 3 |
| 3223.6927971 | 3 |
| 3223.6947772 | 3 |
| 3223.6952545 | 1 |
| 3223.6964835 | 2 |
| 3223.6965922 | 2 |
| 3223.6988818 | 0 |
| 3223.6990537 | 3 |
| 3223.7004105 | 1 |
| 3223.7021913 | 3 |
| 3223.7027324 | 0 |
| 3223.7033831 | 4 |
| 3223.7047687 | 2 |
| 3223.7052208 | 1 |
| 3223.7063486 | 5 |
| 3223.7071438 | 2 |
| 3223.7072806 | 2 |
| 3223.7076418 | 0 |
| 3223.7088563 | 3 |
| 3223.7102518 | 2 |
| 3223.7102552 | 3 |
| 3223.7113567 | 1 |
| 3223.7120197 | 0 |
| 3223.7134931 | 2 |
| 3223.7143415 | 2 |
| 3223.7151042 | 2 |
| 3223.7164872 | 1 |
| 3223.7165949 | 2 |
| 3223.718157 | 5 |
| 3223.7189851 | 2 |
| 3223.7202651 | 1 |
| 3223.7211148 | 3 |
| 3223.7217015 | 1 |
| 3223.7224982 | 1 |
| 3223.7242477 | 4 |
| 3223.7246122 | 2 |
| 3223.7255297 | 0 |
| 3223.7271116 | 1 |
| 3223.7278685 | 3 |
| 3223.7289459 | 1 |
| 3223.7299385 | 3 |
| 3223.730908 | 1 |
| 3223.7322348 | 3 |
| 3223.7335509 | 2 |
| 3223.7343196 | 1 |
| 3223.7351099 | 1 |
| 3223.7363571 | 4 |
| 3223.7377944 | 0 |
| 3223.7379173 | 2 |
| 3223.7397275 | 1 |
| 3223.7402734 | 3 |
| 3223.7411147 | 1 |
| 3223.7424063 | 3 |
| 3223.7424991 | 2 |
| 3223.7429995 | 2 |
| 3223.7441727 | 1 |
| 3223.7450549 | 4 |
| 3223.7467322 | 1 |
| 3223.7470006 | 3 |
| 3223.7487221 | 3 |
| 3223.7490693 | 4 |
| 3223.7502855 | 2 |
| 3223.7503891 | 0 |
| 3223.7518218 | 4 |
| 3223.7533171 | 4 |
| 3223.7539765 | 1 |
| 3223.7545587 | 1 |
| 3223.7562199 | 0 |
| 3223.7567069 | 3 |
| 3223.7572536 | 4 |
| 3223.7581134 | 2 |
| 3223.7588851 | 2 |
| 3223.7598872 | 1 |
| 3223.760959 | 2 |
| 3223.761221 | 2 |
| 3223.7623645 | 0 |
| 3223.7633162 | 5 |
| 3223.7643373 | 4 |
| 3223.7650785 | 1 |
| 3223.7666479 | 1 |
| 3223.7677521 | 4 |
| 3223.7678484 | 2 |
| 3223.7693271 | 5 |
| 3223.7699132 | 3 |
| 3223.7717899 | 2 |
| 3223.772009 | 1 |
| 3223.7731433 | 3 |
| 3223.7747737 | 3 |
| 3223.7754916 | 2 |
| 3223.776102 | 1 |
| 3223.777552 | 2 |
| 3223.7782676 | 1 |
| 3223.7793483 | 2 |
| 3223.7807797 | 3 |
| 3223.7820515 | 3 |
| 3223.782273 | 2 |
| 3223.7833628 | 5 |
| 3223.7851912 | 3 |
| 3223.7854257 | 3 |
| 3223.786773 | 4 |
| 3223.7881301 | 4 |
| 3223.7891476 | 4 |
| 3223.7895864 | 3 |
| 3223.7904156 | 2 |
| 3223.791453 | 3 |
| 3223.7929448 | 0 |
| 3223.7936485 | 2 |
| 3223.7942528 | 1 |
| 3223.7953123 | 3 |
| 3223.7958535 | 0 |
| 3223.797076 | 1 |
| 3223.797349 | 0 |
| 3223.7983861 | 5 |
| 3223.7984141 | 2 |
| 3223.8002657 | 5 |
| 3223.800954 | 3 |
| 3223.8024458 | 2 |
| 3223.8031505 | 5 |
| 3223.8031737 | 1 |
| 3223.8046192 | 1 |
| 3223.8057195 | 2 |
| 3223.8059123 | 2 |
| 3223.8069344 | 2 |
| 3223.8082286 | 4 |
| 3223.8085422 | 1 |
| 3223.8102793 | 2 |
| 3223.8111292 | 1 |
| 3223.8122238 | 3 |
| 3223.8127407 | 2 |
| 3223.8139553 | 1 |
| 3223.8150332 | 1 |
| 3223.8158187 | 1 |
| 3223.8165748 | 2 |
| 3223.8169324 | 1 |
| 3223.8180477 | 3 |
| 3223.8205175 | 1 |
| 3223.8205635 | 1 |
| 3223.8222731 | 1 |
| 3223.8224035 | 0 |
| 3223.8238323 | 2 |
| 3223.824629 | 2 |
| 3223.8263222 | 1 |
| 3223.8273946 | 1 |
| 3223.8285301 | 2 |
| 3223.8298316 | 1 |
| 3223.8305108 | 2 |
| 3223.8314968 | 2 |
| 3223.8328229 | 1 |
| 3223.8329479 | 1 |
| 3223.8344039 | 2 |
| 3223.8352905 | 1 |
| 3223.8364327 | 2 |
| 3223.8372957 | 3 |
| 3223.8382239 | 5 |
| 3223.8389811 | 1 |
| 3223.8394452 | 4 |
| 3223.841278 | 1 |
| 3223.8415973 | 1 |
| 3223.8424982 | 3 |
| 3223.8440854 | 1 |
| 3223.8443105 | 1 |
| 3223.8458665 | 2 |
| 3223.846034 | 2 |
| 3223.8468751 | 2 |
| 3223.8481324 | 0 |
| 3223.8481614 | 0 |
| 3223.8492172 | 1 |

| | |
|---|---|
| 3223.84982 | 2 |
| 3223.8506931 | 1 |
| 3223.8511831 | 4 |
| 3223.8523583 | 0 |
| 3223.8535892 | 0 |
| 3223.8543798 | 0 |
| 3223.8558464 | 3 |
| 3223.8563133 | 3 |
| 3223.8569798 | 3 |
| 3223.8583334 | 1 |
| 3223.8596198 | 3 |
| 3223.860077 | 5 |
| 3223.8619057 | 1 |
| 3223.8625492 | 1 |
| 3223.8640275 | 2 |
| 3223.8653123 | 1 |
| 3223.8655671 | 2 |
| 3223.8674275 | 4 |
| 3223.8683874 | 2 |
| 3223.8685508 | 1 |
| 3223.8703616 | 0 |
| 3223.8715382 | 2 |
| 3223.8724061 | 2 |
| 3223.8726241 | 1 |
| 3223.8738003 | 2 |
| 3223.8747185 | 3 |
| 3223.8758793 | 3 |
| 3223.8762366 | 3 |
| 3223.8780647 | 2 |
| 3223.8783252 | 2 |
| 3223.879651 | 1 |
| 3223.8802953 | 4 |
| 3223.8818022 | 2 |
| 3223.8829363 | 2 |
| 3223.8829617 | 0 |
| 3223.8845224 | 3 |
| 3223.8852225 | 6 |
| 3223.8859894 | 3 |
| 3223.8877706 | 3 |
| 3223.8885717 | 0 |
| 3223.8889139 | 1 |
| 3223.8896212 | 2 |
| 3223.8903568 | 1 |
| 3223.8916694 | 1 |
| 3223.8919588 | 3 |
| 3223.893432 | 1 |
| 3223.8938848 | 0 |
| 3223.8952793 | 2 |
| 3223.895457 | 0 |
| 3223.8962565 | 3 |
| 3223.8978236 | 4 |
| 3223.8985745 | 2 |
| 3223.8994045 | 0 |
| 3223.8997331 | 0 |
| 3223.9004302 | 2 |
| 3223.9021239 | 1 |
| 3223.9027183 | 3 |
| 3223.904473 | 3 |
| 3223.9051258 | 4 |
| 3223.9061226 | 0 |
| 3223.9068683 | 2 |
| 3223.9069336 | 2 |
| 3223.9092723 | 1 |
| 3223.9106517 | 1 |
| 3223.9106561 | 2 |
| 3223.9122576 | 0 |
| 3223.912681 | 0 |
| 3223.914033 | 1 |
| 3223.9156858 | 0 |
| 3223.9170001 | 1 |
| 3223.9179748 | 3 |
| 3223.918958 | 2 |
| 3223.9198384 | 1 |
| 3223.9203597 | 5 |
| 3223.9215581 | 2 |
| 3223.9218593 | 3 |
| 3223.9232753 | 3 |
| 3223.9242022 | 1 |
| 3223.9255741 | 1 |
| 3223.9259014 | 2 |
| 3223.9273557 | 3 |
| 3223.9281011 | 1 |
| 3223.9288135 | 0 |
| 3223.9291994 | 0 |
| 3223.9302416 | 5 |
| 3223.9313898 | 2 |
| 3223.9318698 | 1 |
| 3223.9329887 | 2 |
| 3223.9339162 | 2 |
| 3223.9350819 | 3 |
| 3223.9361632 | 2 |
| 3223.9366554 | 0 |
| 3223.9384813 | 2 |
| 3223.9396048 | 1 |
| 3223.9396063 | 0 |
| 3223.9406975 | 1 |
| 3223.9412006 | 1 |
| 3223.9425823 | 2 |
| 3223.9427182 | 6 |
| 3223.9434151 | 1 |
| 3223.944584 | 1 |
| 3223.9454383 | 2 |
| 3223.9463637 | 2 |
| 3223.9466513 | 4 |
| 3223.9486371 | 2 |
| 3223.949746 | 2 |
| 3223.9501498 | 4 |
| 3223.9517225 | 2 |
| 3223.9517671 | 0 |
| 3223.9527761 | 1 |
| 3223.9541143 | 1 |
| 3223.954767 | 1 |
| 3223.9564423 | 1 |
| 3223.9570888 | 4 |
| 3223.9584787 | 1 |
| 3223.9589067 | 3 |
| 3223.9603149 | 3 |
| 3223.9615501 | 1 |
| 3223.9629585 | 2 |
| 3223.9637968 | 1 |
| 3223.9644416 | 5 |
| 3223.9644992 | 2 |
| 3223.9668554 | 3 |
| 3223.9682527 | 3 |
| 3223.9685225 | 2 |
| 3223.9693745 | 1 |
| 3223.9701354 | 1 |
| 3223.9716825 | 3 |
| 3223.9726027 | 0 |
| 3223.9729157 | 1 |
| 3223.9738309 | 2 |
| 3223.9756508 | 1 |
| 3223.9759549 | 2 |
| 3223.9770234 | 2 |
| 3223.977787 | 0 |
| 3223.978713 | 2 |
| 3223.9788761 | 4 |
| 3223.9794145 | 3 |
| 3223.9802845 | 1 |
| 3223.9813704 | 1 |
| 3223.9822594 | 3 |
| 3223.9827755 | 5 |
| 3223.984793 | 3 |
| 3223.9851914 | 2 |
| 3223.9863339 | 4 |
| 3223.9867658 | 3 |
| 3223.9884601 | 1 |
| 3223.9893629 | 2 |
| 3223.9905743 | 0 |
| 3223.9907593 | 1 |
| 3223.9924787 | 1 |
| 3223.9931074 | 8 |
| 3223.9933671 | 2 |
| 3223.9950901 | 1 |
| 3223.9953992 | 1 |
| 3223.9957422 | 0 |
| 3223.9961381 | 5 |
| 3223.9980882 | 1 |
| 3223.9993511 | 0 |
| 3224.000245 | 2 |
| 3224.0008771 | 0 |
| 3224.0009912 | 1 |
| 3224.0035523 | 1 |
| 3224.0045483 | 2 |
| 3224.0049993 | 1 |
| 3224.005953 | 1 |
| 3224.0072588 | 0 |
| 3224.009139 | 1 |
| 3224.0096149 | 2 |
| 3224.0101984 | 0 |
| 3224.0117059 | 0 |
| 3224.0119127 | 0 |
| 3224.0134803 | 6 |
| 3224.0136646 | 1 |
| 3224.015097 | 3 |
| 3224.0163561 | 2 |
| 3224.0164181 | 7 |
| 3224.0178466 | 1 |
| 3224.0185673 | 1 |
| 3224.0195415 | 4 |
| 3224.0211246 | 2 |
| 3224.0220769 | 2 |
| 3224.0223572 | 2 |
| 3224.0230782 | 1 |
| 3224.0240555 | 1 |
| 3224.0251231 | 1 |
| 3224.0262319 | 1 |
| 3224.0270023 | 3 |
| 3224.0277995 | 1 |
| 3224.0283187 | 2 |
| 3224.0295442 | 4 |
| 3224.0296746 | 3 |
| 3224.0310623 | 4 |
| 3224.0320655 | 1 |
| 3224.032298 | 1 |
| 3224.0330246 | 3 |
| 3224.034411 | 1 |
| 3224.0344785 | 1 |
| 3224.0362852 | 4 |
| 3224.0365292 | 3 |
| 3224.0375083 | 0 |
| 3224.0383892 | 2 |
| 3224.0400739 | 3 |
| 3224.0406747 | 0 |
| 3224.0418346 | 3 |
| 3224.0430363 | 0 |
| 3224.0446478 | 0 |
| 3224.0452607 | 2 |
| 3224.0459251 | 2 |
| 3224.0472653 | 3 |
| 3224.0485008 | 2 |
| 3224.0487221 | 1 |
| 3224.0495437 | 2 |
| 3224.0512487 | 2 |
| 3224.0517373 | 2 |
| 3224.0533114 | 3 |
| 3224.0538171 | 0 |
| 3224.0547806 | 3 |
| 3224.0558335 | 5 |
| 3224.0568571 | 3 |
| 3224.0579791 | 2 |
| 3224.0585673 | 2 |
| 3224.0592304 | 1 |
| 3224.0607385 | 2 |
| 3224.0607727 | 4 |
| 3224.0618687 | 0 |
| 3224.0631981 | 3 |
| 3224.0639217 | 0 |
| 3224.0655688 | 0 |
| 3224.0666415 | 2 |
| 3224.0670572 | 2 |
| 3224.0684168 | 2 |
| 3224.0684937 | 5 |
| 3224.0690677 | 6 |
| 3224.0698256 | 6 |
| 3224.0709645 | 8 |
| 3224.0716037 | 4 |
| 3224.0728145 | 2 |
| 3224.0741006 | 6 |
| 3224.0750412 | 5 |
| 3224.0755684 | 2 |
| 3224.0764214 | 2 |
| 3224.0774879 | 2 |
| 3224.0790585 | 3 |
| 3224.0791741 | 3 |
| 3224.0805814 | 2 |
| 3224.0812149 | 1 |
| 3224.0819605 | 1 |
| 3224.0827305 | 3 |
| 3224.0840935 | 1 |
| 3224.084294 | 4 |
| 3224.0855649 | 3 |
| 3224.0856355 | 3 |
| 3224.0865394 | 2 |
| 3224.0875754 | 0 |
| 3224.0882709 | 5 |
| 3224.08945 | 0 |
| 3224.0909536 | 2 |
| 3224.0913601 | 0 |
| 3224.0928093 | 4 |
| 3224.0936564 | 1 |
| 3224.0951472 | 2 |
| 3224.0964948 | 3 |
| 3224.0973356 | 4 |
| 3224.0982159 | 4 |
| 3224.1004571 | 2 |
| 3224.1010368 | 1 |
| 3224.1014629 | 1 |
| 3224.1026885 | 2 |
| 3224.1028576 | 2 |
| 3224.1044782 | 2 |
| 3224.1050412 | 1 |
| 3224.1059877 | 3 |
| 3224.1064709 | 1 |
| 3224.1075249 | 0 |
| 3224.1086185 | 2 |
| 3224.1096943 | 0 |
| 3224.1105905 | 0 |
| 3224.111598 | 3 |
| 3224.1117635 | 2 |
| 3224.1131296 | 2 |
| 3224.1145335 | 2 |
| 3224.11511 | 0 |
| 3224.1158818 | 1 |
| 3224.1169654 | 3 |
| 3224.1180299 | 1 |
| 3224.1190322 | 2 |
| 3224.1196173 | 3 |
| 3224.12031 | 3 |
| 3224.121121 | 5 |
| 3224.1226026 | 0 |
| 3224.1236129 | 1 |
| 3224.1237077 | 1 |
| 3224.12461 | 2 |
| 3224.1250967 | 1 |
| 3224.1252793 | 3 |
| 3224.1270673 | 2 |
| 3224.1281341 | 3 |
| 3224.1287298 | 2 |
| 3224.1295639 | 3 |
| 3224.1305981 | 1 |
| 3224.1313543 | 1 |
| 3224.1317573 | 2 |
| 3224.1326732 | 4 |
| 3224.1332567 | 3 |
| 3224.1335656 | 1 |
| 3224.1337295 | 2 |
| 3224.1345503 | 4 |
| 3224.1354522 | 3 |
| 3224.1361587 | 1 |
| 3224.1366499 | 1 |
| 3224.1366981 | 1 |
| 3224.1367175 | 3 |
| 3224.136951 | 1 |
| 3224.1370717 | 2 |
| 3224.137083 | 1 |
| 3224.1370955 | 2 |
| 3224.1371716 | 1 |
| 3224.1372113 | 2 |
| 3224.137338 | 2 |
| 3224.1373439 | 0 |
| 3224.1373684 | 0 |
| 3224.1374577 | 0 |
| 3224.1375525 | 1 |
| 3224.137689 | 0 |
| 3224.1376913 | 3 |
| 3224.1377598 | 0 |
| 3224.1379401 | 2 |
| 3224.1379934 | 1 |
| 3224.1381649 | 1 |
| 3224.138358 | 1 |
| 3224.1384598 | 0 |
| 3224.1385034 | 1 |
| 3224.138566 | 0 |
| 3224.1386838 | 1 |
| 3224.1387052 | 0 |
| 3224.1387421 | 1 |
| 3224.1388139 | 5 |
| 3224.1388268 | 2 |
| 3224.1393457 | 0 |
| 3224.1393482 | 2 |
| 3224.1394289 | 1 |
| 3224.1394344 | 3 |
| 3224.1396288 | 3 |
| 3224.1399029 | 0 |
| 3224.1400584 | 1 |
| 3224.1401454 | 1 |
| 3224.1402499 | 5 |
| 3224.1402668 | 1 |
| 3224.1403509 | 4 |
| 3224.1404877 | 1 |
| 3224.1406278 | 0 |
| 3224.1407178 | 2 |
| 3224.1408848 | 1 |
| 3224.1409122 | 2 |
| 3224.1409536 | 1 |
| 3224.1409536 | 4 |
| 3224.1410145 | 5 |
| 3224.1411097 | 0 |
| 3224.1412859 | 1 |
| 3224.1413199 | 2 |
| 3224.1415055 | 2 |
| 3224.1415752 | 2 |

| | |
|---|---|
| 3224.1417907 | 2 |
| 3224.1420188 | 2 |
| 3224.1422409 | 2 |
| 3224.1423443 | 1 |
| 3224.1424732 | 2 |
| 3224.1426906 | 1 |
| 3224.1428491 | 2 |
| 3224.1430035 | 3 |
| 3224.143008 | 2 |
| 3224.1430878 | 1 |
| 3224.1431611 | 5 |
| 3224.1434017 | 2 |
| 3224.1434732 | 4 |
| 3224.1434807 | 2 |
| 3224.1434924 | 1 |
| 3224.1441743 | 2 |
| 3224.144175 | 3 |
| 3224.1443048 | 2 |
| 3224.1443243 | 2 |
| 3224.1444723 | 2 |
| 3224.1446848 | 2 |
| 3224.1448195 | 2 |
| 3224.1449978 | 4 |
| 3224.1450631 | 6 |
| 3224.1450663 | 2 |
| 3224.1451796 | 3 |
| 3224.1453094 | 1 |
| 3224.1460473 | 1 |
| 3224.1461112 | 2 |
| 3224.1463269 | 4 |
| 3224.1463856 | 1 |
| 3224.1464164 | 2 |
| 3224.1464239 | 0 |
| 3224.146449 | 3 |
| 3224.14651 | 3 |
| 3224.1465529 | 1 |
| 3224.1466135 | 3 |
| 3224.1470833 | 2 |
| 3224.1471522 | 1 |
| 3224.1472039 | 2 |
| 3224.147352 | 4 |
| 3224.1474257 | 0 |
| 3224.1474512 | 3 |
| 3224.1476955 | 0 |
| 3224.1480346 | 2 |
| 3224.1481404 | 0 |
| 3224.1481886 | 1 |
| 3224.148392 | 1 |
| 3224.148578 | 1 |
| 3224.1485962 | 4 |
| 3224.148663 | 1 |
| 3224.1486713 | 2 |
| 3224.1488379 | 5 |
| 3224.1490132 | 3 |
| 3224.1490141 | 2 |
| 3224.1490154 | 3 |
| 3224.1491407 | 1 |
| 3224.1492546 | 2 |
| 3224.1494471 | 0 |
| 3224.1494824 | 5 |
| 3224.1497099 | 0 |
| 3224.1498419 | 2 |
| 3224.1499369 | 7 |
| 3224.1500555 | 3 |
| 3224.1501944 | 1 |
| 3224.1502049 | 2 |
| 3224.1502662 | 5 |
| 3224.1505697 | 5 |
| 3224.1506226 | 3 |
| 3224.1508828 | 3 |
| 3224.1509437 | 1 |
| 3224.1510049 | 4 |
| 3224.1510972 | 3 |
| 3224.1514393 | 4 |
| 3224.1514403 | 1 |
| 3224.1514856 | 6 |
| 3224.1517236 | 2 |
| 3224.151734 | 0 |
| 3224.1518475 | 4 |
| 3224.1518568 | 4 |
| 3224.1519979 | 2 |
| 3224.152116 | 0 |
| 3224.1521519 | 2 |
| 3224.1521553 | 1 |
| 3224.1521633 | 2 |
| 3224.152411 | 1 |
| 3224.1524713 | 3 |
| 3224.152543 | 0 |
| 3224.152863 | 4 |
| 3224.152869 | 2 |
| 3224.1529111 | 1 |
| 3224.1529461 | 3 |
| 3224.1531613 | 3 |
| 3224.1532409 | 3 |
| 3224.1533949 | 3 |
| 3224.1534663 | 3 |
| 3224.1536508 | 3 |
| 3224.1536703 | 0 |
| 3224.1537041 | 1 |
| 3224.1537799 | 2 |
| 3224.1538009 | 4 |
| 3224.153813 | 4 |
| 3224.1538199 | 4 |
| 3224.1541921 | 3 |
| 3224.1543278 | 0 |
| 3224.1545624 | 5 |
| 3224.1546546 | 0 |
| 3224.1547755 | 2 |
| 3224.1548511 | 1 |
| 3224.1549777 | 3 |
| 3224.1550963 | 2 |
| 3224.155201 | 3 |
| 3224.1553087 | 4 |
| 3224.1553107 | 5 |
| 3224.1554536 | 5 |
| 3224.1555035 | 3 |
| 3224.155524 | 4 |
| 3224.1555785 | 0 |
| 3224.155662 | 4 |
| 3224.1556833 | 2 |
| 3224.1557524 | 5 |
| 3224.155808 | 3 |
| 3224.1559731 | 11 |
| 3224.1560288 | 3 |
| 3224.1560393 | 1 |
| 3224.1563197 | 4 |
| 3224.156399 | 3 |
| 3224.1564329 | 4 |
| 3224.1564764 | 17 |
| 3224.1565618 | 2 |
| 3224.1566638 | 5 |
| 3224.1567849 | 2 |
| 3224.1568581 | 2 |
| 3224.1571929 | 3 |
| 3224.1572059 | 2 |
| 3224.1572334 | 4 |
| 3224.1573829 | 6 |
| 3224.1574766 | 14 |
| 3224.157515 | 3 |
| 3224.1575488 | 11 |
| 3224.1575855 | 4 |
| 3224.157717 | 19 |
| 3224.1577205 | 5 |
| 3224.1577655 | 6 |
| 3224.1578701 | 10 |
| 3224.1579387 | 10 |
| 3224.1579784 | 8 |
| 3224.1580547 | 3 |
| 3224.1580603 | 12 |
| 3224.1583637 | 16 |
| 3224.1583694 | 13 |
| 3224.1585523 | 3 |
| 3224.1585952 | 13 |
| 3224.1586276 | 17 |
| 3224.1586514 | 10 |
| 3224.1586617 | 21 |
| 3224.1587916 | 18 |
| 3224.1588652 | 8 |
| 3224.1590489 | 11 |
| 3224.1590746 | 10 |
| 3224.1591196 | 10 |
| 3224.1591673 | 7 |
| 3224.1592166 | 15 |
| 3224.1593207 | 16 |
| 3224.1594109 | 14 |
| 3224.1596842 | 18 |
| 3224.1597136 | 15 |
| 3224.1598117 | 8 |
| 3224.1599135 | 11 |
| 3224.1599388 | 19 |
| 3224.1600119 | 9 |
| 3224.1602259 | 11 |
| 3224.1603148 | 9 |
| 3224.1604168 | 10 |
| 3224.1606253 | 12 |
| 3224.1606328 | 5 |
| 3224.1607563 | 4 |
| 3224.1607678 | 6 |
| 3224.1608227 | 9 |
| 3224.1608279 | 6 |
| 3224.161009 | 7 |
| 3224.1610111 | 8 |
| 3224.161123 | 11 |
| 3224.1611553 | 7 |
| 3224.1612396 | 2 |
| 3224.1612695 | 7 |
| 3224.1613924 | 3 |
| 3224.1614396 | 6 |
| 3224.1615493 | 11 |
| 3224.1615859 | 9 |
| 3224.1617565 | 8 |
| 3224.1619791 | 4 |
| 3224.1619939 | 10 |
| 3224.1620342 | 12 |
| 3224.1621294 | 9 |
| 3224.1621397 | 13 |
| 3224.1622332 | 11 |
| 3224.1623209 | 10 |
| 3224.1623655 | 12 |
| 3224.1624219 | 9 |
| 3224.1624653 | 14 |
| 3224.1625561 | 7 |
| 3224.1626289 | 10 |
| 3224.1626451 | 11 |
| 3224.1628052 | 12 |
| 3224.1631241 | 9 |
| 3224.1634338 | 11 |
| 3224.1636584 | 9 |
| 3224.1637684 | 12 |
| 3224.1639393 | 10 |
| 3224.1639498 | 11 |
| 3224.1642545 | 12 |
| 3224.1643291 | 6 |
| 3224.1644497 | 10 |
| 3224.1644872 | 10 |
| 3224.1645165 | 13 |
| 3224.1645318 | 11 |
| 3224.1646 | 15 |
| 3224.1646232 | 8 |
| 3224.1647079 | 11 |
| 3224.16471 | 9 |
| 3224.1647347 | 10 |
| 3224.1648712 | 10 |
| 3224.1650077 | 10 |
| 3224.1650184 | 7 |
| 3224.1651689 | 6 |
| 3224.1653851 | 11 |
| 3224.1655583 | 10 |
| 3224.1656049 | 9 |
| 3224.1657015 | 8 |
| 3224.16571 | 13 |
| 3224.1657892 | 11 |
| 3224.1659585 | 4 |
| 3224.1660804 | 10 |
| 3224.1661724 | 8 |
| 3224.1662448 | 12 |
| 3224.1664391 | 13 |
| 3224.1665304 | 12 |
| 3224.1666226 | 13 |
| 3224.1666865 | 10 |
| 3224.1667024 | 8 |
| 3224.1667936 | 11 |
| 3224.1668617 | 10 |
| 3224.1669517 | 10 |
| 3224.1671707 | 5 |
| 3224.1675713 | 8 |
| 3224.167618 | 7 |
| 3224.1676441 | 4 |
| 3224.167739 | 10 |
| 3224.1679085 | 7 |
| 3224.1679168 | 9 |
| 3224.1679414 | 6 |
| 3224.1679957 | 6 |
| 3224.1680246 | 6 |
| 3224.168285 | 3 |
| 3224.1683177 | 1 |
| 3224.1683461 | 8 |
| 3224.1684188 | 5 |
| 3224.1684638 | 9 |
| 3224.1685137 | 0 |
| 3224.1685922 | 3 |
| 3224.1686009 | 3 |
| 3224.1686469 | 4 |
| 3224.1688219 | 6 |
| 3224.1688479 | 6 |
| 3224.1689069 | 5 |
| 3224.1690534 | 1 |
| 3224.1690751 | 4 |
| 3224.1692598 | 4 |
| 3224.1694141 | 4 |
| 3224.1694527 | 2 |
| 3224.1695442 | 3 |
| 3224.169639 | 2 |
| 3224.169823 | 0 |
| 3224.170161 | 1 |
| 3224.1703624 | 3 |
| 3224.1703687 | 2 |
| 3224.1703963 | 1 |
| 3224.1704056 | 4 |
| 3224.1704213 | 3 |
| 3224.1706125 | 2 |
| 3224.170842 | 1 |
| 3224.1710591 | 3 |
| 3224.1710637 | 1 |
| 3224.1713263 | 0 |
| 3224.1713343 | 1 |
| 3224.171454 | 1 |
| 3224.1714915 | 3 |
| 3224.171557 | 2 |
| 3224.1715717 | 2 |
| 3224.1716898 | 1 |
| 3224.1717165 | 1 |
| 3224.1717179 | 1 |
| 3224.1717385 | 2 |
| 3224.1717974 | 3 |
| 3224.1718088 | 0 |
| 3224.1719252 | 3 |
| 3224.171937 | 1 |
| 3224.1720519 | 1 |
| 3224.1723149 | 2 |
| 3224.172354 | 0 |
| 3224.1723782 | 1 |
| 3224.1724014 | 1 |
| 3224.1725145 | 1 |
| 3224.172785 | 4 |
| 3224.1733982 | 3 |
| 3224.1737063 | 1 |
| 3224.174518 | 2 |
| 3224.1746079 | 1 |
| 3224.1746224 | 1 |
| 3224.1757732 | 0 |
| 3224.1766435 | 2 |
| 3224.1766509 | 0 |
| 3224.176683 | 2 |
| 3224.1770986 | 1 |
| 3224.1775037 | 2 |
| 3224.1783765 | 1 |
| 3224.1787155 | 2 |
| 3224.1794911 | 3 |
| 3224.1796928 | 0 |
| 3224.1801298 | 2 |
| 3224.180268 | 2 |
| 3224.1810305 | 1 |
| 3224.1814872 | 0 |
| 3224.1820228 | 0 |
| 3224.182322 | 1 |
| 3224.1830322 | 1 |
| 3224.1835878 | 1 |
| 3224.1840767 | 0 |
| 3224.1846793 | 0 |
| 3224.1855948 | 2 |
| 3224.1859586 | 2 |
| 3224.1859622 | 0 |
| 3224.1866782 | 3 |
| 3224.1867089 | 3 |
| 3224.1874678 | 2 |
| 3224.1882172 | 2 |
| 3224.1888275 | 2 |
| 3224.1888306 | 1 |
| 3224.1900479 | 5 |
| 3224.1901118 | 4 |
| 3224.1904256 | 2 |
| 3224.1908704 | 1 |
| 3224.1912387 | 2 |
| 3224.1915522 | 0 |
| 3224.1919127 | 2 |
| 3224.1926687 | 0 |
| 3224.1930609 | 2 |
| 3224.193592 | 0 |
| 3224.1943057 | 0 |
| 3224.1946815 | 1 |
| 3224.194754 | 0 |
| 3224.1955258 | 3 |
| 3224.1958841 | 0 |
| 3224.1961743 | 2 |
| 3224.1961761 | 2 |
| 3224.197304 | 0 |
| 3224.1973123 | 2 |
| 3224.1980804 | 0 |
| 3224.1981151 | 2 |
| 3224.1995027 | 3 |
| 3224.2000168 | 2 |
| 3224.2002146 | 0 |

| | |
|---|---|
| 3224.2005602 | 2 |
| 3224.2012028 | 0 |
| 3224.2014489 | 5 |
| 3224.2025627 | 1 |
| 3224.2027521 | 5 |
| 3224.2029759 | 0 |
| 3224.2035825 | 2 |
| 3224.2035988 | 3 |
| 3224.2039368 | 4 |
| 3224.2050031 | 3 |
| 3224.2050204 | 1 |
| 3224.2059182 | 0 |
| 3224.2064207 | 1 |
| 3224.2065633 | 4 |
| 3224.206646 | 3 |
| 3224.2072047 | 4 |
| 3224.2083513 | 0 |
| 3224.2084019 | 4 |
| 3224.2085427 | 2 |
| 3224.2089282 | 2 |
| 3224.2090255 | 3 |
| 3224.2094585 | 2 |
| 3224.2096967 | 1 |
| 3224.2105621 | 4 |
| 3224.2107499 | 2 |
| 3224.2108451 | 4 |
| 3224.2118914 | 2 |
| 3224.2120479 | 3 |
| 3224.212635 | 2 |
| 3224.2126723 | 3 |
| 3224.2140959 | 2 |
| 3224.2142514 | 2 |
| 3224.2144482 | 0 |
| 3224.2150514 | 3 |
| 3224.2153968 | 2 |
| 3224.215648 | 1 |
| 3224.2165532 | 1 |
| 3224.2165668 | 0 |
| 3224.2171386 | 4 |
| 3224.2180031 | 1 |
| 3224.2183927 | 4 |
| 3224.2187311 | 2 |
| 3224.2190139 | 0 |
| 3224.2196893 | 3 |
| 3224.2205499 | 1 |
| 3224.2211418 | 0 |
| 3224.2215475 | 3 |
| 3224.2217122 | 1 |
| 3224.2222826 | 3 |
| 3224.2228555 | 3 |
| 3224.2228771 | 2 |
| 3224.2235107 | 1 |
| 3224.2237016 | 0 |
| 3224.2247313 | 2 |
| 3224.2248248 | 1 |
| 3224.2254522 | 1 |
| 3224.2256014 | 1 |
| 3224.2260452 | 3 |
| 3224.2263806 | 4 |
| 3224.227509 | 4 |
| 3224.2277264 | 3 |
| 3224.2278079 | 0 |
| 3224.2284113 | 2 |
| 3224.2285588 | 2 |
| 3224.2294893 | 3 |
| 3224.2296819 | 1 |
| 3224.2298002 | 2 |
| 3224.230843 | 4 |
| 3224.2315365 | 0 |
| 3224.231647 | 2 |
| 3224.2319122 | 2 |
| 3224.232482 | 8 |
| 3224.232483 | 2 |
| 3224.2325593 | 1 |
| 3224.2337641 | 3 |
| 3224.2338091 | 2 |
| 3224.2345733 | 2 |
| 3224.2354766 | 3 |
| 3224.2359216 | 4 |
| 3224.2360751 | 2 |
| 3224.2364093 | 0 |
| 3224.2365892 | 2 |
| 3224.2366326 | 1 |
| 3224.2370047 | 5 |
| 3224.2373344 | 1 |
| 3224.2378134 | 4 |
| 3224.2378464 | 1 |
| 3224.2386415 | 0 |
| 3224.2386774 | 1 |
| 3224.2389469 | 2 |
| 3224.2399186 | 2 |
| 3224.2410806 | 1 |
| 3224.2423124 | 0 |
| 3224.2424508 | 1 |
| 3224.2443242 | 5 |
| 3224.2460061 | 2 |
| 3224.2460564 | 3 |
| 3224.2474926 | 4 |
| 3224.2480543 | 4 |
| 3224.248901 | 2 |
| 3224.2495884 | 2 |
| 3224.2508173 | 1 |
| 3224.2524371 | 3 |
| 3224.2525246 | 0 |
| 3224.2537329 | 6 |
| 3224.2538895 | 3 |
| 3224.2544587 | 6 |
| 3224.2556507 | 3 |
| 3224.2564237 | 3 |
| 3224.257171 | 4 |
| 3224.2580495 | 5 |
| 3224.2594574 | 1 |
| 3224.260647 | 1 |
| 3224.2608417 | 2 |
| 3224.262071 | 2 |
| 3224.2628354 | 1 |
| 3224.2639209 | 2 |
| 3224.2646509 | 0 |
| 3224.2655246 | 1 |
| 3224.2670654 | 3 |
| 3224.2675096 | 1 |
| 3224.2691207 | 1 |
| 3224.2701495 | 0 |
| 3224.2707809 | 0 |
| 3224.2723674 | 3 |
| 3224.2733326 | 0 |
| 3224.2743136 | 4 |
| 3224.2752402 | 3 |
| 3224.2761061 | 3 |
| 3224.2766607 | 2 |
| 3224.2779281 | 2 |
| 3224.2786232 | 2 |
| 3224.2795147 | 3 |
| 3224.2800437 | 2 |
| 3224.2810131 | 2 |
| 3224.2810352 | 3 |
| 3224.2826571 | 1 |
| 3224.2838875 | 0 |
| 3224.2839238 | 3 |
| 3224.2857351 | 2 |
| 3224.2858326 | 0 |
| 3224.2874879 | 1 |
| 3224.2889781 | 6 |
| 3224.2891541 | 2 |
| 3224.2898583 | 1 |
| 3224.2916647 | 3 |
| 3224.2918582 | 3 |
| 3224.2928145 | 1 |
| 3224.2944871 | 0 |
| 3224.2955935 | 1 |
| 3224.2960289 | 2 |
| 3224.2973218 | 3 |
| 3224.2984179 | 0 |
| 3224.2985737 | 1 |
| 3224.2992516 | 2 |
| 3224.3010135 | 3 |
| 3224.3010414 | 4 |
| 3224.3031212 | 3 |
| 3224.3035841 | 2 |
| 3224.3038244 | 0 |
| 3224.3053143 | 3 |
| 3224.3056275 | 2 |
| 3224.306992 | 2 |
| 3224.3078577 | 1 |
| 3224.3089445 | 1 |
| 3224.310031 | 0 |
| 3224.3108304 | 0 |
| 3224.3111118 | 3 |
| 3224.3128798 | 3 |
| 3224.3133931 | 2 |
| 3224.3144754 | 1 |
| 3224.3151881 | 0 |
| 3224.3170538 | 2 |
| 3224.3171009 | 2 |
| 3224.3180073 | 1 |
| 3224.3197757 | 2 |
| 3224.3202044 | 0 |
| 3224.3220588 | 0 |
| 3224.3224181 | 3 |
| 3224.3233999 | 1 |
| 3224.3243449 | 0 |
| 3224.3248473 | 5 |
| 3224.3260297 | 0 |
| 3224.3272122 | 0 |
| 3224.3278517 | 1 |
| 3224.3291887 | 2 |
| 3224.3299639 | 2 |
| 3224.3313883 | 1 |
| 3224.3316977 | 0 |
| 3224.3328173 | 0 |
| 3224.3338806 | 2 |
| 3224.3339364 | 1 |
| 3224.3353978 | 0 |
| 3224.335695 | 1 |
| 3224.3369614 | 0 |
| 3224.3380914 | 0 |
| 3224.3387642 | 1 |
| 3224.3398015 | 2 |
| 3224.3399416 | 0 |
| 3224.3415135 | 3 |
| 3224.3426841 | 2 |
| 3224.34318 | 5 |
| 3224.3440183 | 2 |
| 3224.3457419 | 1 |
| 3224.346915 | 2 |
| 3224.3471397 | 1 |
| 3224.3480203 | 1 |
| 3224.349538 | 2 |
| 3224.350212 | 5 |
| 3224.3516814 | 2 |
| 3224.3524989 | 2 |
| 3224.3533529 | 4 |
| 3224.3536431 | 6 |
| 3224.3552257 | 2 |
| 3224.3562554 | 1 |
| 3224.3575107 | 3 |
| 3224.3576495 | 1 |
| 3224.3589813 | 2 |
| 3224.3592931 | 0 |
| 3224.3602788 | 4 |
| 3224.3618115 | 0 |
| 3224.3627944 | 2 |
| 3224.3628396 | 2 |
| 3224.3638444 | 3 |
| 3224.3655473 | 2 |
| 3224.3657246 | 1 |
| 3224.3663858 | 0 |
| 3224.3683596 | 2 |
| 3224.3683653 | 0 |
| 3224.3703846 | 0 |
| 3224.370734 | 2 |
| 3224.3721438 | 3 |
| 3224.3731632 | 2 |
| 3224.3733267 | 3 |
| 3224.3749681 | 1 |
| 3224.375661 | 1 |
| 3224.3766909 | 4 |
| 3224.3776324 | 2 |
| 3224.3780811 | 1 |
| 3224.3791664 | 0 |
| 3224.3805572 | 1 |
| 3224.3817578 | 0 |
| 3224.3825979 | 0 |
| 3224.3831218 | 2 |
| 3224.3842442 | 1 |
| 3224.3849208 | 1 |
| 3224.3857983 | 1 |
| 3224.387087 | 0 |
| 3224.387423 | 0 |
| 3224.3888123 | 1 |
| 3224.3891492 | 2 |
| 3224.3910028 | 2 |
| 3224.391152 | 2 |
| 3224.3922429 | 1 |
| 3224.3931372 | 0 |
| 3224.3932741 | 3 |
| 3224.3956555 | 1 |
| 3224.3964834 | 1 |
| 3224.3966211 | 3 |
| 3224.3978367 | 2 |
| 3224.3987278 | 1 |
| 3224.4000865 | 0 |
| 3224.4004426 | 2 |
| 3224.4023679 | 3 |
| 3224.4033111 | 2 |
| 3224.4038826 | 2 |
| 3224.4049704 | 3 |
| 3224.4057156 | 3 |
| 3224.4069273 | 1 |
| 3224.4077485 | 2 |
| 3224.4091907 | 3 |
| 3224.4093802 | 2 |
| 3224.4106128 | 2 |
| 3224.4108883 | 1 |
| 3224.4118305 | 2 |
| 3224.4125111 | 2 |
| 3224.4136551 | 1 |
| 3224.4152422 | 2 |
| 3224.4153235 | 2 |
| 3224.4163936 | 1 |
| 3224.4173503 | 1 |
| 3224.4185373 | 3 |
| 3224.4190864 | 2 |
| 3224.4201854 | 2 |
| 3224.421423 | 1 |
| 3224.4216913 | 1 |
| 3224.4231863 | 4 |
| 3224.42375 | 1 |
| 3224.4251482 | 0 |
| 3224.4267335 | 2 |
| 3224.4273686 | 2 |
| 3224.4281175 | 4 |
| 3224.4289042 | 4 |
| 3224.4301769 | 5 |
| 3224.4318446 | 2 |
| 3224.4320925 | 4 |
| 3224.4329504 | 0 |
| 3224.4336359 | 2 |
| 3224.4351742 | 1 |
| 3224.4364566 | 3 |
| 3224.4369644 | 0 |
| 3224.4378999 | 3 |
| 3224.4386278 | 2 |
| 3224.4396009 | 3 |
| 3224.4403306 | 1 |
| 3224.4412259 | 3 |
| 3224.4423748 | 1 |
| 3224.443187 | 2 |
| 3224.443819 | 5 |
| 3224.4453686 | 3 |
| 3224.4455327 | 2 |
| 3224.446808 | 1 |
| 3224.4482799 | 1 |
| 3224.4487257 | 0 |
| 3224.4499091 | 2 |
| 3224.4508583 | 2 |
| 3224.451965 | 0 |
| 3224.4524721 | 3 |
| 3224.4534601 | 0 |
| 3224.4551663 | 2 |
| 3224.4552767 | 4 |
| 3224.456516 | 2 |
| 3224.4574169 | 2 |
| 3224.458468 | 1 |
| 3224.459614 | 2 |
| 3224.4607273 | 1 |
| 3224.4613002 | 3 |
| 3224.462118 | 0 |
| 3224.4623951 | 1 |
| 3224.4640454 | 1 |
| 3224.4645312 | 2 |
| 3224.4652759 | 4 |
| 3224.4665182 | 1 |
| 3224.4670257 | 2 |
| 3224.4679943 | 2 |
| 3224.4689926 | 3 |
| 3224.4700245 | 3 |
| 3224.4715071 | 5 |
| 3224.4717652 | 0 |
| 3224.4733294 | 5 |
| 3224.4739525 | 3 |
| 3224.4750031 | 3 |
| 3224.4762956 | 0 |
| 3224.4767283 | 1 |
| 3224.4782889 | 2 |
| 3224.4788531 | 2 |
| 3224.4800349 | 0 |
| 3224.4808635 | 2 |
| 3224.4816808 | 2 |
| 3224.4827187 | 0 |
| 3224.4836046 | 1 |
| 3224.4841337 | 1 |
| 3224.4855021 | 2 |
| 3224.4856553 | 1 |
| 3224.4873808 | 3 |
| 3224.4883352 | 3 |
| 3224.4894052 | 1 |
| 3224.4901817 | 2 |
| 3224.4908852 | 2 |
| 3224.4918147 | 1 |
| 3224.4925459 | 2 |
| 3224.4936579 | 2 |
| 3224.4943989 | 0 |

| | |
|---|---|
| 3224.4952133 | 4 |
| 3224.4954774 | 3 |
| 3224.4957294 | 2 |
| 3224.4973603 | 2 |
| 3224.4976432 | 2 |
| 3224.4983598 | 3 |
| 3224.4983922 | 1 |
| 3224.4984041 | 1 |
| 3224.499524 | 3 |
| 3224.5002073 | 0 |
| 3224.500296 | 2 |
| 3224.5008684 | 1 |
| 3224.5012025 | 2 |
| 3224.501558 | 1 |
| 3224.502279 | 2 |
| 3224.5027332 | 5 |
| 3224.5030386 | 2 |
| 3224.5048215 | 2 |
| 3224.5050022 | 2 |
| 3224.5051274 | 3 |
| 3224.5066798 | 3 |
| 3224.5070285 | 5 |
| 3224.507054 | 4 |
| 3224.5083949 | 2 |
| 3224.5098692 | 5 |
| 3224.5101045 | 2 |
| 3224.5112696 | 0 |
| 3224.5112718 | 1 |
| 3224.5113479 | 1 |
| 3224.512837 | 1 |
| 3224.5130127 | 2 |
| 3224.513782 | 2 |
| 3224.513819 | 2 |
| 3224.5148311 | 0 |
| 3224.5152981 | 1 |
| 3224.5161936 | 0 |
| 3224.5166015 | 2 |
| 3224.5168351 | 2 |
| 3224.5175493 | 3 |
| 3224.5180068 | 5 |
| 3224.51821 | 3 |
| 3224.5188799 | 4 |
| 3224.5198587 | 2 |
| 3224.5201467 | 3 |
| 3224.5207287 | 4 |
| 3224.5207734 | 4 |
| 3224.5217034 | 1 |
| 3224.5217568 | 0 |
| 3224.5227307 | 3 |
| 3224.523891 | 5 |
| 3224.5239306 | 2 |
| 3224.5246989 | 7 |
| 3224.5252844 | 0 |
| 3224.5261248 | 0 |
| 3224.5269183 | 3 |
| 3224.5270082 | 1 |
| 3224.5281682 | 1 |
| 3224.5286002 | 4 |
| 3224.5289728 | 2 |
| 3224.5292554 | 4 |
| 3224.5297904 | 2 |
| 3224.5308885 | 2 |
| 3224.5313421 | 3 |
| 3224.5314402 | 1 |
| 3224.5324165 | 5 |
| 3224.5327707 | 1 |
| 3224.5336299 | 0 |
| 3224.5341536 | 4 |
| 3224.5347368 | 2 |
| 3224.535086 | 1 |
| 3224.5361655 | 0 |
| 3224.5362946 | 3 |
| 3224.5373163 | 3 |
| 3224.5378355 | 2 |
| 3224.5387276 | 0 |
| 3224.5388086 | 1 |
| 3224.5395654 | 3 |
| 3224.5399011 | 0 |
| 3224.5403053 | 2 |
| 3224.5409686 | 5 |
| 3224.541232 | 2 |
| 3224.5425226 | 2 |
| 3224.542765 | 2 |
| 3224.5428485 | 1 |
| 3224.5439423 | 3 |
| 3224.5442252 | 1 |
| 3224.5446101 | 4 |
| 3224.5462369 | 0 |
| 3224.5465371 | 3 |
| 3224.5473518 | 0 |
| 3224.5473923 | 2 |
| 3224.5481208 | 2 |
| 3224.549054 | 1 |
| 3224.5494893 | 1 |
| 3224.550501 | 1 |
| 3224.5505873 | 4 |
| 3224.5506844 | 2 |
| 3224.5521568 | 2 |
| 3224.5522813 | 2 |
| 3224.5531028 | 3 |
| 3224.5532235 | 1 |
| 3224.5539939 | 0 |
| 3224.5552112 | 2 |
| 3224.5552749 | 3 |
| 3224.5559385 | 1 |
| 3224.5560649 | 0 |
| 3224.5574952 | 1 |
| 3224.5579378 | 1 |
| 3224.5583582 | 1 |
| 3224.5583938 | 1 |
| 3224.5595049 | 3 |
| 3224.5601298 | 0 |
| 3224.5603378 | 2 |
| 3224.5607817 | 0 |
| 3224.5614239 | 0 |
| 3224.5628737 | 4 |
| 3224.5631265 | 1 |
| 3224.5636936 | 2 |
| 3224.5642482 | 0 |
| 3224.5645165 | 1 |
| 3224.564683 | 2 |
| 3224.5656711 | 3 |
| 3224.5661989 | 0 |
| 3224.5667862 | 3 |
| 3224.5676981 | 2 |
| 3224.5681769 | 4 |
| 3224.5683207 | 0 |
| 3224.5698945 | 2 |
| 3224.5701521 | 1 |
| 3224.570458 | 2 |
| 3224.5708051 | 0 |
| 3224.5715041 | 1 |
| 3224.5718294 | 3 |
| 3224.5724621 | 1 |
| 3224.5725411 | 1 |
| 3224.5741848 | 4 |
| 3224.5750501 | 3 |
| 3224.5751396 | 3 |
| 3224.5753773 | 2 |
| 3224.5755758 | 1 |
| 3224.5759511 | 1 |
| 3224.577128 | 4 |
| 3224.5780838 | 2 |
| 3224.5789211 | 2 |
| 3224.5790153 | 3 |
| 3224.5799056 | 1 |
| 3224.5801762 | 0 |
| 3224.581232 | 2 |
| 3224.5813198 | 3 |
| 3224.5819162 | 0 |
| 3224.5823994 | 0 |
| 3224.5829571 | 2 |
| 3224.5839708 | 0 |
| 3224.5842102 | 2 |
| 3224.5847656 | 2 |
| 3224.5858785 | 5 |
| 3224.5861123 | 2 |
| 3224.586745 | 2 |
| 3224.5873726 | 2 |
| 3224.5886401 | 0 |
| 3224.5888795 | 1 |
| 3224.5889059 | 2 |
| 3224.589697 | 1 |
| 3224.5897254 | 3 |
| 3224.5901778 | 2 |
| 3224.5918505 | 0 |
| 3224.59238 | 0 |
| 3224.5928367 | 2 |
| 3224.5933788 | 3 |
| 3224.5936276 | 0 |
| 3224.5941963 | 1 |
| 3224.5942295 | 1 |
| 3224.5957529 | 3 |
| 3224.5958921 | 1 |
| 3224.5961712 | 2 |
| 3224.5973514 | 3 |
| 3224.597788 | 1 |
| 3224.5988128 | 3 |
| 3224.5990764 | 1 |
| 3224.5997491 | 1 |
| 3224.6004479 | 1 |
| 3224.6004765 | 1 |
| 3224.6018712 | 4 |
| 3224.6021963 | 1 |
| 3224.6030802 | 0 |
| 3224.6033794 | 1 |
| 3224.6034235 | 3 |
| 3224.6034858 | 1 |
| 3224.6035838 | 1 |
| 3224.6039327 | 1 |
| 3224.604176 | 1 |
| 3224.6043834 | 4 |
| 3224.6047441 | 2 |
| 3224.6048231 | 3 |
| 3224.6051198 | 3 |
| 3224.6055942 | 3 |
| 3224.6058342 | 0 |
| 3224.6060742 | 1 |
| 3224.6063017 | 1 |
| 3224.6067823 | 1 |
| 3224.6068058 | 1 |
| 3224.6069956 | 2 |
| 3224.6072302 | 3 |
| 3224.6074235 | 3 |
| 3224.6081395 | 3 |
| 3224.6082281 | 1 |
| 3224.6083272 | 0 |
| 3224.6086111 | 5 |
| 3224.6090729 | 3 |
| 3224.6093603 | 4 |
| 3224.6097698 | 2 |
| 3224.6098918 | 3 |
| 3224.6099251 | 1 |
| 3224.6101419 | 3 |
| 3224.6102148 | 0 |
| 3224.6106061 | 2 |
| 3224.6106967 | 5 |
| 3224.611359 | 2 |
| 3224.6116873 | 1 |
| 3224.6117753 | 2 |
| 3224.6119824 | 1 |
| 3224.6121827 | 1 |
| 3224.6123071 | 1 |
| 3224.6129106 | 2 |
| 3224.6130134 | 1 |
| 3224.6131853 | 1 |
| 3224.6132751 | 1 |
| 3224.6137754 | 2 |
| 3224.6139451 | 0 |
| 3224.6146474 | 2 |
| 3224.6146756 | 1 |
| 3224.6147519 | 3 |
| 3224.6148146 | 1 |
| 3224.6153801 | 1 |
| 3224.6158463 | 1 |
| 3224.6160141 | 2 |
| 3224.6162487 | 0 |
| 3224.6165597 | 0 |
| 3224.6166766 | 3 |
| 3224.616745 | 1 |
| 3224.6169626 | 0 |
| 3224.6171922 | 4 |
| 3224.6175901 | 2 |
| 3224.6178774 | 1 |
| 3224.6178903 | 0 |
| 3224.6180746 | 1 |
| 3224.6185386 | 1 |
| 3224.6190056 | 1 |
| 3224.6192476 | 4 |
| 3224.6194241 | 0 |
| 3224.6195845 | 2 |
| 3224.619778 | 2 |
| 3224.6197804 | 1 |
| 3224.6202313 | 8 |
| 3224.6204827 | 1 |
| 3224.6206203 | 2 |
| 3224.6213389 | 3 |
| 3224.6218908 | 2 |
| 3224.6220221 | 4 |
| 3224.6220615 | 4 |
| 3224.6221118 | 2 |
| 3224.6224917 | 1 |
| 3224.6227185 | 4 |
| 3224.6227309 | 3 |
| 3224.6238196 | 0 |
| 3224.623926 | 0 |
| 3224.6240061 | 3 |
| 3224.6243563 | 2 |
| 3224.6244045 | 1 |
| 3224.6245611 | 2 |
| 3224.6245982 | 0 |
| 3224.6250129 | 1 |
| 3224.6253811 | 1 |
| 3224.6253971 | 1 |
| 3224.6258456 | 3 |
| 3224.6258557 | 3 |
| 3224.6265447 | 1 |
| 3224.6265454 | 2 |
| 3224.6269426 | 0 |
| 3224.6270614 | 0 |
| 3224.6271165 | 2 |
| 3224.6273538 | 0 |
| 3224.6283315 | 3 |
| 3224.6283457 | 2 |
| 3224.6288332 | 3 |
| 3224.6289129 | 3 |
| 3224.6289416 | 3 |
| 3224.628968 | 1 |
| 3224.6290673 | 3 |
| 3224.6291247 | 2 |
| 3224.6295343 | 1 |
| 3224.6299958 | 0 |
| 3224.6303326 | 0 |
| 3224.6307057 | 0 |
| 3224.6312333 | 1 |
| 3224.6313463 | 1 |
| 3224.6314242 | 2 |
| 3224.6314468 | 2 |
| 3224.6315707 | 1 |
| 3224.6320425 | 2 |
| 3224.6322522 | 1 |
| 3224.6326578 | 1 |
| 3224.6327713 | 3 |
| 3224.6334025 | 3 |
| 3224.6334851 | 3 |
| 3224.6334932 | 1 |
| 3224.6335596 | 1 |
| 3224.6338239 | 4 |
| 3224.6343769 | 1 |
| 3224.6344798 | 1 |
| 3224.6349674 | 3 |
| 3224.6353839 | 1 |
| 3224.6358489 | 3 |
| 3224.6359667 | 2 |
| 3224.6362257 | 1 |
| 3224.6364426 | 3 |
| 3224.6376477 | 4 |
| 3224.6384577 | 2 |
| 3224.6386483 | 0 |
| 3224.6390074 | 2 |
| 3224.6390924 | 1 |
| 3224.6403134 | 1 |
| 3224.6417309 | 5 |
| 3224.6417602 | 4 |
| 3224.6428662 | 2 |
| 3224.6435022 | 2 |
| 3224.6439141 | 5 |
| 3224.6445777 | 3 |
| 3224.6445918 | 2 |
| 3224.6446121 | 0 |
| 3224.6458904 | 2 |
| 3224.6462447 | 6 |
| 3224.6467097 | 0 |
| 3224.6473089 | 1 |
| 3224.6485676 | 0 |
| 3224.6490064 | 4 |
| 3224.6494606 | 2 |
| 3224.6502143 | 1 |
| 3224.6503194 | 1 |
| 3224.6503202 | 1 |
| 3224.6519277 | 1 |
| 3224.6519446 | 0 |
| 3224.6535235 | 4 |
| 3224.653714 | 2 |
| 3224.6545307 | 1 |
| 3224.6547305 | 4 |
| 3224.6551119 | 2 |
| 3224.6552587 | 1 |
| 3224.6560277 | 1 |
| 3224.6570129 | 0 |
| 3224.6575165 | 3 |
| 3224.6583672 | 2 |
| 3224.6590154 | 1 |
| 3224.6590204 | 3 |
| 3224.6596056 | 2 |
| 3224.6604824 | 1 |
| 3224.6608056 | 1 |
| 3224.6613906 | 1 |
| 3224.6623261 | 0 |
| 3224.6630562 | 4 |
| 3224.6630592 | 6 |
| 3224.6637921 | 1 |
| 3224.6640194 | 2 |

| | |
|---|---|
| 3224.6647126 | 2 |
| 3224.6661211 | 0 |
| 3224.6662246 | 2 |
| 3224.6668436 | 3 |
| 3224.6670468 | 2 |
| 3224.6673057 | 1 |
| 3224.668625 | 0 |
| 3224.6686347 | 0 |
| 3224.6697491 | 1 |
| 3224.6700859 | 3 |
| 3224.6701595 | 1 |
| 3224.6713799 | 2 |
| 3224.671672 | 4 |
| 3224.6718159 | 5 |
| 3224.672702 | 5 |
| 3224.6728191 | 0 |
| 3224.6738601 | 5 |
| 3224.6746463 | 1 |
| 3224.6750133 | 0 |
| 3224.6752385 | 1 |
| 3224.6766154 | 0 |
| 3224.6767956 | 4 |
| 3224.6774426 | 2 |
| 3224.6780945 | 4 |
| 3224.6783624 | 5 |
| 3224.6792421 | 1 |
| 3224.6792629 | 2 |
| 3224.6802141 | 1 |
| 3224.6807201 | 2 |
| 3224.6811227 | 4 |
| 3224.6826109 | 1 |
| 3224.6828107 | 2 |
| 3224.6829692 | 0 |
| 3224.6832982 | 2 |
| 3224.6850181 | 0 |
| 3224.6852785 | 4 |
| 3224.6858564 | 2 |
| 3224.6861768 | 0 |
| 3224.6872794 | 2 |
| 3224.6873471 | 1 |
| 3224.6880149 | 3 |
| 3224.6888599 | 0 |
| 3224.6890986 | 1 |
| 3224.6891381 | 2 |
| 3224.6900461 | 1 |
| 3224.6910889 | 4 |
| 3224.6913311 | 0 |
| 3224.6922356 | 0 |
| 3224.6931369 | 3 |
| 3224.6931555 | 1 |
| 3224.6937969 | 6 |
| 3224.694255 | 1 |
| 3224.6948523 | 2 |
| 3224.6954691 | 2 |
| 3224.6962417 | 3 |
| 3224.6962553 | 1 |
| 3224.6970458 | 3 |
| 3224.6978787 | 2 |
| 3224.6981309 | 2 |
| 3224.6988442 | 4 |
| 3224.6997419 | 0 |
| 3224.7003202 | 1 |
| 3224.7009718 | 1 |
| 3224.7012809 | 1 |
| 3224.7013516 | 1 |
| 3224.7027094 | 3 |
| 3224.702896 | 1 |
| 3224.7037159 | 1 |
| 3224.7041234 | 0 |
| 3224.7043603 | 0 |
| 3224.7052857 | 4 |
| 3224.7059975 | 3 |
| 3224.7066935 | 3 |
| 3224.7074831 | 3 |
| 3224.7078676 | 1 |
| 3224.7086389 | 0 |
| 3224.7089432 | 1 |
| 3224.709429 | 3 |
| 3224.7104529 | 2 |
| 3224.7111266 | 1 |
| 3224.7113216 | 1 |
| 3224.7120982 | 1 |
| 3224.7124235 | 1 |
| 3224.7125525 | 1 |
| 3224.7136184 | 2 |
| 3224.7137118 | 1 |
| 3224.7139514 | 1 |
| 3224.7153441 | 3 |
| 3224.7157491 | 3 |
| 3224.7163933 | 0 |
| 3224.7166304 | 3 |
| 3224.7170455 | 1 |
| 3224.7184545 | 1 |
| 3224.718855 | 4 |
| 3224.7196616 | 1 |
| 3224.7198624 | 2 |
| 3224.7205556 | 2 |
| 3224.7209275 | 3 |
| 3224.7219876 | 3 |
| 3224.722038 | 4 |
| 3224.7221352 | 3 |
| 3224.7230469 | 4 |
| 3224.7239614 | 4 |
| 3224.7242515 | 5 |
| 3224.7248966 | 5 |
| 3224.7259969 | 5 |
| 3224.7260258 | 2 |
| 3224.7266922 | 0 |
| 3224.7273471 | 3 |
| 3224.7275329 | 2 |
| 3224.7280757 | 3 |
| 3224.7292937 | 4 |
| 3224.7295439 | 3 |
| 3224.7303045 | 2 |
| 3224.7305876 | 5 |
| 3224.7305979 | 3 |
| 3224.7314115 | 3 |
| 3224.7324175 | 1 |
| 3224.7331131 | 1 |
| 3224.7334527 | 3 |
| 3224.7345108 | 1 |
| 3224.7347488 | 2 |
| 3224.735519 | 1 |
| 3224.7355964 | 1 |
| 3224.7370366 | 2 |
| 3224.7376549 | 0 |
| 3224.7379333 | 1 |
| 3224.7383227 | 3 |
| 3224.7390832 | 2 |
| 3224.7396204 | 2 |
| 3224.7400813 | 1 |
| 3224.7401663 | 3 |
| 3224.7413557 | 4 |
| 3224.741497 | 3 |
| 3224.7415566 | 0 |
| 3224.7430085 | 0 |
| 3224.7432835 | 1 |
| 3224.7443543 | 0 |
| 3224.7446852 | 1 |
| 3224.7454574 | 5 |
| 3224.7457744 | 3 |
| 3224.7460211 | 1 |
| 3224.7473345 | 1 |
| 3224.7481819 | 2 |
| 3224.7482323 | 2 |
| 3224.7486399 | 2 |
| 3224.7491938 | 3 |
| 3224.7493424 | 5 |
| 3224.7511205 | 2 |
| 3224.7511626 | 0 |
| 3224.7514018 | 4 |
| 3224.752469 | 0 |
| 3224.7530708 | 1 |
| 3224.7537544 | 2 |
| 3224.7538052 | 2 |
| 3224.7543156 | 0 |
| 3224.7555596 | 3 |
| 3224.7559502 | 2 |
| 3224.7565012 | 1 |
| 3224.7568917 | 1 |
| 3224.757673 | 3 |
| 3224.7581355 | 2 |
| 3224.7583283 | 1 |
| 3224.7593675 | 1 |
| 3224.76031 | 3 |
| 3224.761189 | 0 |
| 3224.7612776 | 4 |
| 3224.7616411 | 3 |
| 3224.7617185 | 1 |
| 3224.7630313 | 0 |
| 3224.7632121 | 2 |
| 3224.7637149 | 2 |
| 3224.7640405 | 2 |
| 3224.7654826 | 0 |
| 3224.7660535 | 2 |
| 3224.766603 | 1 |
| 3224.7667626 | 8 |
| 3224.7680615 | 6 |
| 3224.7686016 | 0 |
| 3224.7688178 | 0 |
| 3224.7692046 | 3 |
| 3224.7698023 | 0 |
| 3224.7705333 | 4 |
| 3224.7712638 | 9 |
| 3224.772545 | 1 |
| 3224.7726785 | 4 |
| 3224.7728119 | 1 |
| 3224.7737008 | 0 |
| 3224.7748546 | 2 |
| 3224.775431 | 2 |
| 3224.7757791 | 0 |
| 3224.7765576 | 2 |
| 3224.7765826 | 1 |
| 3224.77706 | 5 |
| 3224.7782516 | 0 |
| 3224.7788831 | 3 |
| 3224.7791241 | 2 |
| 3224.7804583 | 2 |
| 3224.7804615 | 3 |
| 3224.7808926 | 1 |
| 3224.7825792 | 2 |
| 3224.7829378 | 5 |
| 3224.7831652 | 4 |
| 3224.7837508 | 2 |
| 3224.7842488 | 3 |
| 3224.7847483 | 1 |
| 3224.7856688 | 1 |
| 3224.7861607 | 1 |
| 3224.7877218 | 0 |
| 3224.7879151 | 3 |
| 3224.7887894 | 3 |
| 3224.7889724 | 1 |
| 3224.7904855 | 2 |
| 3224.7912389 | 0 |
| 3224.7916039 | 1 |
| 3224.7929201 | 3 |
| 3224.7934362 | 0 |
| 3224.7936189 | 1 |
| 3224.7956279 | 3 |
| 3224.7975607 | 2 |
| 3224.7991155 | 4 |
| 3224.8005312 | 4 |
| 3224.802127 | 2 |
| 3224.8032178 | 1 |
| 3224.8051845 | 1 |
| 3224.8059326 | 2 |
| 3224.8076162 | 2 |
| 3224.8101741 | 4 |
| 3224.8113854 | 2 |
| 3224.8130043 | 1 |
| 3224.8139933 | 3 |
| 3224.815884 | 5 |
| 3224.817934 | 0 |
| 3224.8186886 | 2 |
| 3224.8203856 | 2 |
| 3224.8210325 | 2 |
| 3224.8238482 | 1 |
| 3224.8258934 | 1 |
| 3224.8259078 | 3 |
| 3224.8266152 | 3 |
| 3224.8287217 | 0 |
| 3224.8297299 | 2 |
| 3224.8316553 | 3 |
| 3224.8334479 | 4 |
| 3224.8348975 | 2 |
| 3224.8369078 | 2 |
| 3224.8371848 | 2 |
| 3224.8394462 | 1 |
| 3224.8416194 | 1 |
| 3224.8422256 | 0 |
| 3224.8441639 | 1 |
| 3224.8451278 | 2 |
| 3224.8473683 | 2 |
| 3224.8481374 | 4 |
| 3224.8500998 | 2 |
| 3224.851817 | 0 |
| 3224.8528099 | 3 |
| 3224.8541459 | 2 |
| 3224.8566188 | 1 |
| 3224.8577448 | 2 |
| 3224.8594409 | 0 |
| 3224.8603198 | 2 |
| 3224.8624833 | 7 |
| 3224.864567 | 5 |
| 3224.8654501 | 2 |
| 3224.8677443 | 3 |
| 3224.868449 | 3 |
| 3224.870337 | 1 |
| 3224.8724815 | 1 |
| 3224.8737425 | 1 |
| 3224.8751253 | 4 |
| 3224.8766713 | 2 |
| 3224.8782388 | 1 |
| 3224.8790641 | 0 |
| 3224.8817154 | 5 |
| 3224.8832585 | 1 |
| 3224.8843616 | 2 |
| 3224.8859906 | 1 |
| 3224.8873489 | 3 |
| 3224.8895802 | 3 |
| 3224.8901107 | 5 |
| 3224.8909549 | 2 |
| 3224.892155 | 2 |
| 3224.8922056 | 1 |
| 3224.8935258 | 2 |
| 3224.8937458 | 1 |
| 3224.8947496 | 2 |
| 3224.8953074 | 2 |
| 3224.8962028 | 2 |
| 3224.8968584 | 2 |
| 3224.8986531 | 4 |
| 3224.8987773 | 3 |
| 3224.8991126 | 2 |
| 3224.900304 | 3 |
| 3224.9015382 | 2 |
| 3224.9016533 | 2 |
| 3224.9020303 | 1 |
| 3224.9028624 | 2 |
| 3224.9045154 | 0 |
| 3224.9048967 | 1 |
| 3224.9057553 | 3 |
| 3224.9066916 | 2 |
| 3224.9067663 | 1 |
| 3224.9077976 | 0 |
| 3224.9091781 | 2 |
| 3224.9098167 | 2 |
| 3224.9105605 | 2 |
| 3224.9107536 | 3 |
| 3224.9130646 | 0 |
| 3224.9134314 | 2 |
| 3224.9145336 | 1 |
| 3224.9148528 | 2 |
| 3224.9155947 | 1 |
| 3224.9160606 | 1 |
| 3224.917029 | 2 |
| 3224.9181117 | 0 |
| 3224.9183806 | 3 |
| 3224.918959 | 1 |
| 3224.9198783 | 3 |
| 3224.920601 | 3 |
| 3224.9226272 | 3 |
| 3224.9227268 | 2 |
| 3224.9233386 | 3 |
| 3224.9235188 | 1 |
| 3224.9258235 | 1 |
| 3224.9258323 | 1 |
| 3224.9259099 | 1 |
| 3224.9272711 | 3 |
| 3224.9281604 | 1 |
| 3224.9287376 | 1 |
| 3224.9292644 | 0 |
| 3224.9294686 | 2 |
| 3224.9312225 | 1 |
| 3224.9317889 | 2 |
| 3224.9330959 | 2 |
| 3224.9336959 | 4 |
| 3224.9346106 | 3 |
| 3224.9349804 | 1 |
| 3224.9358565 | 4 |
| 3224.936348 | 4 |
| 3224.9371457 | 3 |
| 3224.9381318 | 4 |
| 3224.9386564 | 0 |
| 3224.9397381 | 2 |
| 3224.9405669 | 3 |
| 3224.9415709 | 2 |
| 3224.9418235 | 4 |
| 3224.942427 | 2 |
| 3224.9438856 | 1 |
| 3224.9447172 | 2 |
| 3224.9448408 | 2 |
| 3224.9452306 | 0 |
| 3224.9464871 | 3 |
| 3224.9473434 | 2 |
| 3224.9492124 | 3 |
| 3224.949586 | 1 |
| 3224.9499504 | 2 |
| 3224.9507443 | 4 |
| 3224.9521312 | 1 |
| 3224.9523071 | 0 |
| 3224.9529517 | 1 |
| 3224.9531985 | 1 |
| 3224.9547002 | 4 |

| | |
|---|---|
| 3224.9555005 | 4 |
| 3224.9569387 | 6 |
| 3224.9574201 | 3 |
| 3224.9578692 | 0 |
| 3224.9580714 | 0 |
| 3224.9599347 | 1 |
| 3224.9605041 | 2 |
| 3224.9605675 | 1 |
| 3224.962242 | 2 |
| 3224.9628826 | 2 |
| 3224.9633646 | 2 |
| 3224.9649835 | 7 |
| 3224.9654738 | 1 |
| 3224.9657773 | 2 |
| 3224.9659895 | 3 |
| 3224.9673331 | 2 |
| 3224.9684028 | 1 |
| 3224.9688973 | 2 |
| 3224.9693815 | 0 |
| 3224.9703909 | 3 |
| 3224.9708466 | 4 |
| 3224.9713862 | 3 |
| 3224.9726964 | 0 |
| 3224.9731242 | 1 |
| 3224.9742638 | 3 |
| 3224.9754602 | 4 |
| 3224.9760517 | 6 |
| 3224.9768983 | 3 |
| 3224.9770007 | 1 |
| 3224.9785971 | 3 |
| 3224.979067 | 3 |
| 3224.9791352 | 1 |
| 3224.9810865 | 3 |
| 3224.9815042 | 2 |
| 3224.9821543 | 1 |
| 3224.9824268 | 2 |
| 3224.9837633 | 3 |
| 3224.9844703 | 4 |
| 3224.9853059 | 0 |
| 3224.9863302 | 2 |
| 3224.987096 | 3 |
| 3224.9871453 | 4 |
| 3224.9874008 | 1 |
| 3224.9889768 | 4 |
| 3224.9910616 | 1 |
| 3224.9917197 | 3 |
| 3224.9944184 | 4 |
| 3224.9948698 | 0 |
| 3224.9971985 | 1 |
| 3224.9981175 | 1 |
| 3225.0002119 | 2 |
| 3225.0022159 | 2 |
| 3225.003357 | 0 |
| 3225.0058529 | 1 |
| 3225.0063515 | 0 |
| 3225.0078433 | 2 |
| 3225.0106253 | 1 |
| 3225.010769 | 0 |
| 3225.0129517 | 1 |
| 3225.0139427 | 4 |
| 3225.0155586 | 1 |
| 3225.0181205 | 2 |
| 3225.0189746 | 2 |
| 3225.0207092 | 3 |
| 3225.0216964 | 2 |
| 3225.0240609 | 4 |
| 3225.024302 | 0 |
| 3225.02745 | 0 |
| 3225.0290718 | 1 |
| 3225.0297403 | 1 |
| 3225.0314347 | 4 |
| 3225.0330561 | 0 |
| 3225.0350372 | 3 |
| 3225.0366967 | 3 |
| 3225.0377176 | 2 |
| 3225.0400919 | 0 |
| 3225.040282 | 3 |
| 3225.0425581 | 3 |
| 3225.043466 | 1 |
| 3225.0461138 | 0 |
| 3225.0480543 | 2 |
| 3225.0490796 | 0 |
| 3225.0508274 | 0 |
| 3225.0516414 | 2 |
| 3225.0532018 | 3 |
| 3225.0551499 | 1 |
| 3225.0558366 | 3 |
| 3225.0583556 | 0 |
| 3225.060322 | 1 |
| 3225.0612315 | 3 |
| 3225.0632161 | 2 |
| 3225.0635731 | 3 |
| 3225.0659244 | 1 |
| 3225.0670334 | 0 |
| 3225.0690033 | 0 |
| 3225.070816 | 0 |
| 3225.0722429 | 3 |
| 3225.0736843 | 4 |
| 3225.0748615 | 3 |
| 3225.076853 | 0 |
| 3225.0782661 | 4 |
| 3225.0792213 | 3 |
| 3225.0814337 | 0 |
| 3225.0822678 | 3 |
| 3225.0838084 | 2 |
| 3225.0852296 | 1 |
| 3225.0862672 | 0 |
| 3225.0895266 | 1 |
| 3225.0902208 | 3 |
| 3225.0916744 | 2 |
| 3225.0944881 | 3 |
| 3225.0956515 | 3 |
| 3225.0975952 | 2 |
| 3225.0981475 | 2 |
| 3225.0993256 | 2 |
| 3225.1019921 | 2 |
| 3225.1034064 | 1 |
| 3225.1050129 | 1 |
| 3225.1051404 | 3 |
| 3225.1073154 | 4 |
| 3225.1093361 | 4 |
| 3225.1111286 | 2 |
| 3225.1119502 | 0 |
| 3225.1147472 | 0 |
| 3225.1157768 | 1 |
| 3225.1176429 | 2 |
| 3225.1185608 | 3 |
| 3225.1203976 | 7 |
| 3225.1206428 | 2 |
| 3225.1237173 | 1 |
| 3225.1244483 | 3 |
| 3225.1259742 | 3 |
| 3225.1271789 | 0 |
| 3225.1290105 | 3 |
| 3225.1307457 | 4 |
| 3225.133009 | 2 |
| 3225.1340277 | 2 |
| 3225.1356823 | 3 |
| 3225.1371273 | 0 |
| 3225.1382744 | 6 |
| 3225.1391275 | 2 |
| 3225.1427522 | 1 |
| 3225.1444652 | 1 |
| 3225.1454008 | 0 |
| 3225.1463817 | 1 |
| 3225.1477994 | 3 |
| 3225.1491631 | 1 |
| 3225.150969 | 2 |
| 3225.1531771 | 3 |
| 3225.1540432 | 1 |
| 3225.1558618 | 3 |
| 3225.1573096 | 4 |
| 3225.1591702 | 3 |
| 3225.159716 | 1 |
| 3225.1620767 | 1 |
| 3225.1643943 | 1 |
| 3225.1644995 | 3 |
| 3225.1667934 | 3 |
| 3225.1680508 | 4 |
| 3225.1693515 | 1 |
| 3225.1701635 | 3 |
| 3225.1718731 | 2 |
| 3225.1749767 | 4 |
| 3225.1751446 | 2 |
| 3225.1769463 | 4 |
| 3225.1782214 | 1 |
| 3225.1809598 | 5 |
| 3225.1818576 | 1 |
| 3225.1831029 | 2 |
| 3225.1854079 | 1 |
| 3225.1861295 | 1 |
| 3225.1878956 | 2 |
| 3225.189573 | 1 |
| 3225.1910691 | 1 |
| 3225.1934772 | 1 |
| 3225.1947288 | 3 |
| 3225.1961939 | 2 |
| 3225.1976827 | 0 |
| 3225.1988735 | 1 |
| 3225.2007743 | 3 |
| 3225.2014055 | 1 |
| 3225.2037215 | 1 |
| 3225.2057223 | 2 |
| 3225.2060991 | 3 |
| 3225.2076652 | 2 |
| 3225.2090705 | 1 |
| 3225.2107001 | 5 |
| 3225.21292 | 4 |
| 3225.2144561 | 1 |
| 3225.215193 | 2 |
| 3225.217429 | 3 |
| 3225.218769 | 3 |
| 3225.2198357 | 1 |
| 3225.22248 | 3 |
| 3225.2241765 | 1 |
| 3225.2245468 | 2 |
| 3225.2268889 | 3 |
| 3225.2277293 | 3 |
| 3225.2293419 | 2 |
| 3225.231629 | 1 |
| 3225.2318177 | 2 |
| 3225.2350853 | 0 |
| 3225.2361707 | 0 |
| 3225.2375379 | 3 |
| 3225.2388503 | 5 |
| 3225.2402669 | 2 |
| 3225.2424043 | 1 |
| 3225.2431737 | 5 |
| 3225.2446104 | 2 |
| 3225.2460406 | 5 |
| 3225.2478798 | 1 |
| 3225.2502123 | 1 |
| 3225.2507484 | 2 |
| 3225.2521592 | 0 |
| 3225.2543444 | 2 |
| 3225.2550893 | 2 |
| 3225.2570447 | 4 |
| 3225.2587877 | 2 |
| 3225.2607817 | 3 |
| 3225.2614615 | 0 |
| 3225.2632147 | 0 |
| 3225.2648417 | 2 |
| 3225.2656927 | 1 |
| 3225.2676455 | 1 |
| 3225.2690345 | 2 |
| 3225.2704625 | 3 |
| 3225.2730681 | 3 |
| 3225.2735678 | 3 |
| 3225.2756383 | 1 |
| 3225.2776759 | 3 |
| 3225.2783777 | 3 |
| 3225.281099 | 0 |
| 3225.2812412 | 5 |
| 3225.2841637 | 4 |
| 3225.2853896 | 0 |
| 3225.2867768 | 1 |
| 3225.2882559 | 1 |
| 3225.2896002 | 4 |
| 3225.2917846 | 2 |
| 3225.2920014 | 1 |
| 3225.2948367 | 0 |
| 3225.2954847 | 1 |
| 3225.2973174 | 5 |
| 3225.2990418 | 2 |
| 3225.2998806 | 2 |
| 3225.3021271 | 0 |
| 3225.3035953 | 3 |
| 3225.3045561 | 4 |
| 3225.3059915 | 0 |
| 3225.3079578 | 3 |
| 3225.3089828 | 6 |
| 3225.3116057 | 3 |
| 3225.3123706 | 3 |
| 3225.3148812 | 1 |
| 3225.3152614 | 3 |
| 3225.3173775 | 4 |
| 3225.3193963 | 2 |
| 3225.3196741 | 2 |
| 3225.3228514 | 3 |
| 3225.3231247 | 0 |
| 3225.3249792 | 1 |
| 3225.3269307 | 3 |
| 3225.3281229 | 1 |
| 3225.3305053 | 1 |
| 3225.331023 | 2 |
| 3225.3328801 | 4 |
| 3225.3354589 | 2 |
| 3225.3365611 | 4 |
| 3225.337745 | 3 |
| 3225.3388554 | 2 |
| 3225.3407574 | 3 |
| 3225.3411006 | 2 |
| 3225.344224 | 1 |
| 3225.3454651 | 1 |
| 3225.3465269 | 3 |
| 3225.3474959 | 2 |
| 3225.3496089 | 2 |
| 3225.3509012 | 3 |
| 3225.353121 | 2 |
| 3225.3535378 | 3 |
| 3225.3563705 | 0 |
| 3225.3583202 | 2 |
| 3225.358417 | 2 |
| 3225.3609144 | 3 |
| 3225.3613343 | 0 |
| 3225.3638998 | 4 |
| 3225.3653193 | 2 |
| 3225.3662072 | 3 |
| 3225.3690753 | 0 |
| 3225.3699653 | 3 |
| 3225.3716722 | 0 |
| 3225.3731183 | 1 |
| 3225.3746043 | 6 |
| 3225.3763586 | 4 |
| 3225.3772051 | 1 |
| 3225.3793698 | 3 |
| 3225.3804864 | 2 |
| 3225.3825041 | 4 |
| 3225.3838111 | 1 |
| 3225.385254 | 3 |
| 3225.3870891 | 4 |
| 3225.3879986 | 1 |
| 3225.3894363 | 2 |
| 3225.3913168 | 3 |
| 3225.3929922 | 3 |
| 3225.3947944 | 1 |
| 3225.3957245 | 2 |
| 3225.3986552 | 4 |
| 3225.3995392 | 2 |
| 3225.4002988 | 5 |
| 3225.4016758 | 1 |
| 3225.4047028 | 2 |
| 3225.4048339 | 0 |
| 3225.4071098 | 2 |
| 3225.4085652 | 1 |
| 3225.4099675 | 5 |
| 3225.4117299 | 3 |
| 3225.4132252 | 1 |
| 3225.4154132 | 2 |
| 3225.4161605 | 2 |
| 3225.4181371 | 3 |
| 3225.4183065 | 0 |
| 3225.4208678 | 4 |
| 3225.4226926 | 2 |
| 3225.4235065 | 2 |
| 3225.4261213 | 3 |
| 3225.4266206 | 3 |
| 3225.4281246 | 1 |
| 3225.4306919 | 3 |
| 3225.431354 | 5 |
| 3225.4340388 | 3 |
| 3225.4353001 | 4 |
| 3225.4363215 | 1 |
| 3225.4383402 | 4 |
| 3225.439057 | 3 |
| 3225.4415994 | 1 |
| 3225.4420729 | 4 |
| 3225.4442467 | 4 |
| 3225.4465775 | 3 |
| 3225.4468682 | 3 |
| 3225.4492039 | 3 |
| 3225.4502529 | 1 |
| 3225.4523208 | 3 |
| 3225.4535223 | 0 |
| 3225.4555257 | 1 |
| 3225.4570722 | 2 |
| 3225.4596391 | 2 |
| 3225.4598443 | 1 |
| 3225.461678 | 2 |
| 3225.4623975 | 1 |
| 3225.4648073 | 2 |
| 3225.467059 | 3 |
| 3225.4674793 | 3 |
| 3225.4700431 | 3 |
| 3225.4705497 | 2 |
| 3225.4725463 | 1 |
| 3225.4736997 | 1 |
| 3225.4762848 | 4 |
| 3225.4781657 | 1 |
| 3225.4784569 | 0 |
| 3225.4801813 | 3 |
| 3225.4817726 | 0 |
| 3225.4823684 | 2 |

| | |
|---|---|
| 3225.4850705 | 2 |
| 3225.4862042 | 0 |
| 3225.4882682 | 3 |
| 3225.4899489 | 1 |
| 3225.4905365 | 1 |
| 3225.4928515 | 1 |
| 3225.4933125 | 1 |
| 3225.4962028 | 2 |
| 3225.4970973 | 1 |
| 3225.4985586 | 1 |
| 3225.5003224 | 0 |
| 3225.5019786 | 2 |
| 3225.5045192 | 1 |
| 3225.5060505 | 4 |
| 3225.5068782 | 2 |
| 3225.5088742 | 1 |
| 3225.5098523 | 3 |
| 3225.5115242 | 1 |
| 3225.5124257 | 1 |
| 3225.5154875 | 3 |
| 3225.5163185 | 2 |
| 3225.5176296 | 1 |
| 3225.5200646 | 2 |
| 3225.5203871 | 3 |
| 3225.5232408 | 2 |
| 3225.5243632 | 4 |
| 3225.5248472 | 1 |
| 3225.5268281 | 1 |
| 3225.5282165 | 2 |
| 3225.5299853 | 3 |
| 3225.5313017 | 3 |
| 3225.5326356 | 4 |
| 3225.5347085 | 3 |
| 3225.5358163 | 2 |
| 3225.5377954 | 2 |
| 3225.5400397 | 1 |
| 3225.5413034 | 1 |
| 3225.5425962 | 1 |
| 3225.5431709 | 1 |
| 3225.5454996 | 1 |
| 3225.5473334 | 3 |
| 3225.5483193 | 1 |
| 3225.5502878 | 1 |
| 3225.551725 | 2 |
| 3225.5542042 | 1 |
| 3225.5558548 | 2 |
| 3225.556655 | 2 |
| 3225.5588043 | 2 |
| 3225.5600228 | 5 |
| 3225.5615347 | 1 |
| 3225.5645223 | 3 |
| 3225.5646781 | 3 |
| 3225.5665899 | 3 |
| 3225.5667907 | 1 |
| 3225.5689443 | 1 |
| 3225.5707745 | 5 |
| 3225.5723488 | 2 |
| 3225.5741593 | 0 |
| 3225.5755435 | 3 |
| 3225.5773992 | 2 |
| 3225.5780732 | 0 |
| 3225.5803823 | 2 |
| 3225.582318 | 1 |
| 3225.5827508 | 0 |
| 3225.5850195 | 3 |
| 3225.5854449 | 1 |
| 3225.5878334 | 4 |
| 3225.589826 | 3 |
| 3225.5905357 | 3 |
| 3225.5930559 | 4 |
| 3225.5935912 | 2 |
| 3225.5956716 | 0 |
| 3225.5982641 | 2 |
| 3225.5989735 | 3 |
| 3225.6007134 | 3 |
| 3225.6022856 | 3 |
| 3225.6039077 | 5 |
| 3225.6062134 | 1 |
| 3225.6069126 | 2 |
| 3225.6089915 | 3 |
| 3225.6093744 | 1 |
| 3225.6117148 | 1 |
| 3225.6132287 | 1 |
| 3225.6150617 | 3 |
| 3225.6166903 | 3 |
| 3225.6176511 | 3 |
| 3225.6200905 | 1 |
| 3225.6204637 | 3 |
| 3225.6222151 | 2 |
| 3225.6242327 | 3 |
| 3225.6248036 | 4 |
| 3225.6271771 | 0 |
| 3225.6286811 | 1 |
| 3225.6302639 | 2 |
| 3225.6320791 | 5 |
| 3225.6325236 | 2 |
| 3225.6352459 | 4 |
| 3225.6356693 | 2 |
| 3225.6371479 | 2 |
| 3225.6389181 | 0 |
| 3225.6414598 | 3 |
| 3225.6432922 | 6 |
| 3225.6444394 | 4 |
| 3225.6458083 | 2 |
| 3225.6480032 | 0 |
| 3225.6480795 | 3 |
| 3225.6504393 | 4 |
| 3225.65187 | 3 |
| 3225.6537543 | 2 |
| 3225.656413 | 4 |
| 3225.6565286 | 5 |
| 3225.6584478 | 0 |
| 3225.6596144 | 1 |
| 3225.6613147 | 1 |
| 3225.6634713 | 0 |
| 3225.6645417 | 0 |
| 3225.6669036 | 3 |
| 3225.6683134 | 2 |
| 3225.6699415 | 2 |
| 3225.6713975 | 3 |
| 3225.6719888 | 6 |
| 3225.6742502 | 3 |
| 3225.6748332 | 0 |
| 3225.6778468 | 3 |
| 3225.6783114 | 1 |
| 3225.6809416 | 2 |
| 3225.6825143 | 2 |
| 3225.6829034 | 0 |
| 3225.6854379 | 1 |
| 3225.6865858 | 1 |
| 3225.6883592 | 1 |
| 3225.6900118 | 0 |
| 3225.6904126 | 4 |
| 3225.6929704 | 1 |
| 3225.6947183 | 2 |
| 3225.6950163 | 3 |
| 3225.6970899 | 2 |
| 3225.6976828 | 4 |
| 3225.7005824 | 0 |
| 3225.7015846 | 2 |
| 3225.7031764 | 2 |
| 3225.7049595 | 1 |
| 3225.70631 | 5 |
| 3225.7094109 | 2 |
| 3225.7094459 | 3 |
| 3225.7111224 | 2 |
| 3225.7130581 | 1 |
| 3225.7138176 | 1 |
| 3225.7160555 | 1 |
| 3225.7172646 | 2 |
| 3225.7193834 | 1 |
| 3225.7201101 | 2 |
| 3225.7204448 | 2 |
| 3225.7241268 | 4 |
| 3225.7247673 | 1 |
| 3225.7270142 | 0 |
| 3225.7286504 | 3 |
| 3225.730217 | 3 |
| 3225.7316531 | 5 |
| 3225.7326476 | 1 |
| 3225.7346656 | 3 |
| 3225.7361929 | 2 |
| 3225.7376989 | 3 |
| 3225.7394038 | 3 |
| 3225.7409433 | 1 |
| 3225.7425872 | 5 |
| 3225.7435146 | 4 |
| 3225.745307 | 3 |
| 3225.7469283 | 2 |
| 3225.7483375 | 2 |
| 3225.7500273 | 2 |
| 3225.7517693 | 4 |
| 3225.752822 | 3 |
| 3225.7546473 | 0 |
| 3225.7553691 | 4 |
| 3225.7567902 | 0 |
| 3225.7591941 | 3 |
| 3225.76123 | 3 |
| 3225.7627447 | 1 |
| 3225.7632561 | 1 |
| 3225.7655505 | 0 |
| 3225.7674839 | 1 |
| 3225.7675978 | 6 |
| 3225.7701652 | 2 |
| 3225.7713786 | 2 |
| 3225.7735343 | 4 |
| 3225.7754253 | 1 |
| 3225.7757096 | 1 |
| 3225.7787187 | 3 |
| 3225.7788846 | 0 |
| 3225.7816004 | 4 |
| 3225.7823724 | 1 |
| 3225.7842979 | 3 |
| 3225.7866644 | 1 |
| 3225.7879746 | 3 |
| 3225.7886979 | 3 |
| 3225.7896008 | 2 |
| 3225.7925901 | 0 |
| 3225.7937269 | 2 |
| 3225.7945157 | 0 |
| 3225.7968755 | 2 |
| 3225.7981232 | 1 |
| 3225.8000404 | 3 |
| 3225.801279 | 5 |
| 3225.8020584 | 2 |
| 3225.8043289 | 2 |
| 3225.8059431 | 5 |
| 3225.8066677 | 7 |
| 3225.8092393 | 1 |
| 3225.8098125 | 3 |
| 3225.8117432 | 1 |
| 3225.8126937 | 3 |
| 3225.8152019 | 2 |
| 3225.8162267 | 3 |
| 3225.8183136 | 3 |
| 3225.8194998 | 2 |
| 3225.8211795 | 3 |
| 3225.8227109 | 3 |
| 3225.8247842 | 2 |
| 3225.8252244 | 2 |
| 3225.8271276 | 1 |
| 3225.829165 | 3 |
| 3225.8309638 | 4 |
| 3225.8320871 | 3 |
| 3225.833475 | 2 |
| 3225.8355545 | 4 |
| 3225.8357646 | 2 |
| 3225.8380743 | 4 |
| 3225.8395553 | 2 |
| 3225.8405934 | 3 |
| 3225.8432871 | 3 |
| 3225.8445433 | 0 |
| 3225.846005 | 2 |
| 3225.8475511 | 1 |
| 3225.8487963 | 1 |
| 3225.8504306 | 3 |
| 3225.8518485 | 3 |
| 3225.8537035 | 2 |
| 3225.8556394 | 1 |
| 3225.8563318 | 1 |
| 3225.8590747 | 1 |
| 3225.8595911 | 0 |
| 3225.8609146 | 1 |
| 3225.8635399 | 2 |
| 3225.8638101 | 3 |
| 3225.865748 | 2 |
| 3225.8670149 | 3 |
| 3225.8690716 | 4 |
| 3225.8710352 | 2 |
| 3225.8722709 | 0 |
| 3225.8739096 | 4 |
| 3225.8752095 | 2 |
| 3225.8769224 | 5 |
| 3225.8782053 | 3 |
| 3225.8800565 | 4 |
| 3225.8822787 | 2 |
| 3225.8824728 | 0 |
| 3225.8838505 | 6 |
| 3225.8869498 | 3 |
| 3225.8870013 | 1 |
| 3225.8896585 | 2 |
| 3225.8901518 | 4 |
| 3225.8922667 | 2 |
| 3225.8931639 | 0 |
| 3225.8948116 | 2 |
| 3225.8972556 | 6 |
| 3225.8982981 | 3 |
| 3225.8996216 | 4 |
| 3225.9008199 | 2 |
| 3225.9022388 | 0 |
| 3225.9056806 | 2 |
| 3225.906334 | 1 |
| 3225.9079547 | 4 |
| 3225.9086082 | 2 |
| 3225.9112914 | 1 |
| 3225.9126913 | 4 |
| 3225.9136839 | 4 |
| 3225.9160706 | 1 |
| 3225.9171634 | 2 |
| 3225.9184287 | 3 |
| 3225.9198185 | 2 |
| 3225.9213342 | 1 |
| 3225.9229469 | 3 |
| 3225.9255173 | 1 |
| 3225.9269418 | 5 |
| 3225.9282062 | 1 |
| 3225.9292187 | 3 |
| 3225.9311591 | 1 |
| 3225.9321139 | 2 |
| 3225.934001 | 3 |
| 3225.9353864 | 2 |
| 3225.9367141 | 2 |
| 3225.9389642 | 1 |
| 3225.9391506 | 0 |
| 3225.9414348 | 2 |
| 3225.9433295 | 3 |
| 3225.945109 | 4 |
| 3225.9460308 | 0 |
| 3225.9474546 | 1 |
| 3225.950043 | 2 |
| 3225.9500777 | 3 |
| 3225.9516016 | 3 |
| 3225.9547747 | 5 |
| 3225.9550042 | 3 |
| 3225.9568427 | 0 |
| 3225.956864 | 2 |
| 3225.957491 | 1 |
| 3225.9582361 | 3 |
| 3225.959177 | 1 |
| 3225.9605278 | 2 |
| 3225.9610145 | 1 |
| 3225.962279 | 1 |
| 3225.9626033 | 1 |
| 3225.96288 | 2 |
| 3225.964057 | 3 |
| 3225.9644493 | 2 |
| 3225.9665099 | 1 |
| 3225.967067 | 0 |
| 3225.9674982 | 0 |
| 3225.9676567 | 5 |
| 3225.9691421 | 6 |
| 3225.969673 | 2 |
| 3225.9700632 | 2 |
| 3225.9718008 | 5 |
| 3225.9719453 | 1 |
| 3225.9730045 | 0 |
| 3225.9738272 | 3 |
| 3225.9740012 | 0 |
| 3225.9751927 | 2 |
| 3225.9759547 | 1 |
| 3225.9765272 | 1 |
| 3225.9770976 | 1 |
| 3225.9778205 | 3 |
| 3225.9784882 | 4 |
| 3225.9793207 | 2 |
| 3225.979336 | 1 |
| 3225.9802421 | 2 |
| 3225.9805791 | 2 |
| 3225.9811913 | 1 |
| 3225.9820913 | 0 |
| 3225.9835202 | 0 |
| 3225.9845463 | 2 |
| 3225.984606 | 3 |
| 3225.9855781 | 1 |
| 3225.986417 | 2 |
| 3225.9871122 | 0 |
| 3225.9879974 | 0 |
| 3225.9884894 | 2 |
| 3225.9901701 | 1 |
| 3225.9903461 | 1 |
| 3225.9911483 | 2 |
| 3225.9924064 | 3 |
| 3225.9930554 | 1 |
| 3225.9934029 | 1 |
| 3225.9940501 | 1 |
| 3225.9953249 | 0 |
| 3225.996074 | 3 |
| 3225.9962612 | 4 |
| 3225.9974425 | 1 |
| 3225.9979958 | 1 |
| 3225.999391 | 2 |
| 3225.9996298 | 1 |
| 3226.0006363 | 3 |

| | |
|---|---|
| 3226.0007936 | 1 |
| 3226.002179 | 2 |
| 3226.0026668 | 1 |
| 3226.0036824 | 1 |
| 3226.0041335 | 0 |
| 3226.0042724 | 1 |
| 3226.0057046 | 2 |
| 3226.0065496 | 2 |
| 3226.0077678 | 4 |
| 3226.0085611 | 4 |
| 3226.0088307 | 3 |
| 3226.0095794 | 4 |
| 3226.0105504 | 3 |
| 3226.0117091 | 4 |
| 3226.0123933 | 4 |
| 3226.0130537 | 3 |
| 3226.0136668 | 4 |
| 3226.014288 | 4 |
| 3226.0155582 | 2 |
| 3226.0162031 | 2 |
| 3226.0173761 | 3 |
| 3226.0173947 | 2 |
| 3226.0185167 | 2 |
| 3226.0188982 | 1 |
| 3226.0194857 | 3 |
| 3226.0210968 | 1 |
| 3226.0212057 | 4 |
| 3226.0219472 | 1 |
| 3226.0224202 | 0 |
| 3226.0240427 | 3 |
| 3226.0241273 | 1 |
| 3226.0257615 | 1 |
| 3226.025898 | 1 |
| 3226.0271409 | 2 |
| 3226.027674 | 1 |
| 3226.0279933 | 1 |
| 3226.0293747 | 2 |
| 3226.0293926 | 4 |
| 3226.0302701 | 0 |
| 3226.0321162 | 1 |
| 3226.0321678 | 4 |
| 3226.032901 | 2 |
| 3226.0337879 | 4 |
| 3226.0346873 | 2 |
| 3226.0353863 | 1 |
| 3226.0361567 | 1 |
| 3226.0363409 | 3 |
| 3226.0370831 | 2 |
| 3226.0380601 | 0 |
| 3226.0399123 | 1 |
| 3226.0400408 | 1 |
| 3226.0410514 | 0 |
| 3226.0412605 | 5 |
| 3226.0420623 | 3 |
| 3226.04284 | 4 |
| 3226.0431097 | 1 |
| 3226.0445348 | 2 |
| 3226.0448493 | 1 |
| 3226.0454434 | 0 |
| 3226.0473015 | 3 |
| 3226.0488036 | 3 |
| 3226.0490235 | 3 |
| 3226.0497075 | 3 |
| 3226.0507419 | 3 |
| 3226.0511168 | 3 |
| 3226.0515729 | 3 |
| 3226.052667 | 1 |
| 3226.0534711 | 2 |
| 3226.0542906 | 6 |
| 3226.0546586 | 3 |
| 3226.0549981 | 1 |
| 3226.0568915 | 1 |
| 3226.056956 | 4 |
| 3226.0583277 | 0 |
| 3226.0589719 | 2 |
| 3226.0596881 | 1 |
| 3226.0600292 | 2 |
| 3226.0613812 | 0 |
| 3226.0619159 | 2 |
| 3226.0629839 | 0 |
| 3226.0637766 | 1 |
| 3226.0644027 | 4 |
| 3226.0644972 | 1 |
| 3226.0655693 | 1 |
| 3226.0663043 | 2 |
| 3226.067377 | 3 |
| 3226.0674211 | 4 |
| 3226.0688349 | 5 |
| 3226.0693031 | 4 |
| 3226.0700095 | 1 |
| 3226.0710156 | 5 |
| 3226.0716453 | 3 |
| 3226.0724499 | 3 |
| 3226.0728134 | 3 |
| 3226.0728686 | 3 |
| 3226.0734998 | 3 |
| 3226.0738879 | 4 |
| 3226.0746909 | 3 |
| 3226.0754819 | 3 |
| 3226.0755023 | 1 |
| 3226.076291 | 2 |
| 3226.0771185 | 1 |
| 3226.0783981 | 1 |
| 3226.0784568 | 0 |
| 3226.0789781 | 4 |
| 3226.0803139 | 2 |
| 3226.0807966 | 2 |
| 3226.0833322 | 3 |
| 3226.0842966 | 3 |
| 3226.0858979 | 3 |
| 3226.0870127 | 3 |
| 3226.0889701 | 3 |
| 3226.0906357 | 0 |
| 3226.0913389 | 0 |
| 3226.0924074 | 1 |
| 3226.0933695 | 2 |
| 3226.0939 | 4 |
| 3226.0942709 | 1 |
| 3226.0947428 | 0 |
| 3226.0953934 | 4 |
| 3226.0964988 | 1 |
| 3226.0971189 | 4 |
| 3226.0981051 | 5 |
| 3226.0983818 | 3 |
| 3226.0999085 | 1 |
| 3226.1002682 | 2 |
| 3226.1002898 | 1 |
| 3226.1005526 | 2 |
| 3226.1013394 | 3 |
| 3226.101718 | 2 |
| 3226.1026481 | 2 |
| 3226.1044029 | 3 |
| 3226.1058827 | 2 |
| 3226.1075359 | 3 |
| 3226.1095702 | 4 |
| 3226.1107704 | 2 |
| 3226.1130335 | 1 |
| 3226.1134376 | 2 |
| 3226.1157436 | 3 |
| 3226.1171988 | 3 |
| 3226.1184099 | 1 |
| 3226.1202247 | 5 |
| 3226.120883 | 3 |
| 3226.1230652 | 2 |
| 3226.1246523 | 5 |
| 3226.1253815 | 1 |
| 3226.1279969 | 0 |
| 3226.1292634 | 1 |
| 3226.1310925 | 1 |
| 3226.1332988 | 2 |
| 3226.1340929 | 5 |
| 3226.1363196 | 3 |
| 3226.1367165 | 4 |
| 3226.1388274 | 1 |
| 3226.1398538 | 3 |
| 3226.1420332 | 4 |
| 3226.1434728 | 2 |
| 3226.1448649 | 0 |
| 3226.1461365 | 3 |
| 3226.1479322 | 3 |
| 3226.1497277 | 1 |
| 3226.1519228 | 1 |
| 3226.1525718 | 4 |
| 3226.1549171 | 1 |
| 3226.1553898 | 0 |
| 3226.1576909 | 2 |
| 3226.1591725 | 0 |
| 3226.1602064 | 4 |
| 3226.1619664 | 0 |
| 3226.1640398 | 4 |
| 3226.1655865 | 2 |
| 3226.1670404 | 2 |
| 3226.1679782 | 0 |
| 3226.1703304 | 4 |
| 3226.1717717 | 3 |
| 3226.1725063 | 5 |
| 3226.1747607 | 4 |
| 3226.1758016 | 4 |
| 3226.1782197 | 4 |
| 3226.1793826 | 1 |
| 3226.1813593 | 3 |
| 3226.1828754 | 2 |
| 3226.183636 | 2 |
| 3226.18615 | 1 |
| 3226.1866309 | 0 |
| 3226.188817 | 2 |
| 3226.1913076 | 2 |
| 3226.1916817 | 4 |
| 3226.1935634 | 3 |
| 3226.1945927 | 4 |
| 3226.1968749 | 2 |
| 3226.1977508 | 2 |
| 3226.1996656 | 1 |
| 3226.2017375 | 2 |
| 3226.203012 | 1 |
| 3226.2037725 | 3 |
| 3226.2053808 | 2 |
| 3226.2072044 | 0 |
| 3226.2091579 | 2 |
| 3226.2101965 | 2 |
| 3226.2123357 | 2 |
| 3226.2130347 | 1 |
| 3226.215581 | 0 |
| 3226.2168924 | 1 |
| 3226.2184624 | 3 |
| 3226.2198637 | 3 |
| 3226.221724 | 3 |
| 3226.223576 | 9 |
| 3226.2240197 | 3 |
| 3226.2261892 | 4 |
| 3226.2284195 | 2 |
| 3226.228914 | 3 |
| 3226.2314534 | 5 |
| 3226.2321738 | 0 |
| 3226.2348734 | 0 |
| 3226.2363671 | 2 |
| 3226.2377735 | 1 |
| 3226.2390006 | 5 |
| 3226.240177 | 3 |
| 3226.2417638 | 3 |
| 3226.2437407 | 1 |
| 3226.244645 | 2 |
| 3226.2473039 | 4 |
| 3226.2475074 | 3 |
| 3226.2492467 | 2 |
| 3226.2521082 | 3 |
| 3226.2530551 | 4 |
| 3226.2550667 | 1 |
| 3226.2558106 | 3 |
| 3226.2575644 | 0 |
| 3226.2587066 | 2 |
| 3226.26062 | 1 |
| 3226.2617233 | 4 |
| 3226.263571 | 2 |
| 3226.2655065 | 1 |
| 3226.2657336 | 1 |
| 3226.2684107 | 1 |
| 3226.2707125 | 5 |
| 3226.271346 | 2 |
| 3226.2733791 | 1 |
| 3226.2738218 | 1 |
| 3226.2768084 | 1 |
| 3226.278261 | 3 |
| 3226.2792446 | 1 |
| 3226.2808131 | 0 |
| 3226.2822728 | 0 |
| 3226.2846409 | 2 |
| 3226.2853813 | 1 |
| 3226.2873043 | 0 |
| 3226.2890345 | 1 |
| 3226.2895908 | 1 |
| 3226.2917303 | 0 |
| 3226.2927348 | 5 |
| 3226.2952407 | 3 |
| 3226.296782 | 3 |
| 3226.2980586 | 3 |
| 3226.2993378 | 2 |
| 3226.3007656 | 2 |
| 3226.3022305 | 3 |
| 3226.3040794 | 1 |
| 3226.3053964 | 3 |
| 3226.3077858 | 0 |
| 3226.3090219 | 0 |
| 3226.3103467 | 1 |
| 3226.3124077 | 3 |
| 3226.3131022 | 4 |
| 3226.3148658 | 1 |
| 3226.3170566 | 5 |
| 3226.3178356 | 3 |
| 3226.3201074 | 5 |
| 3226.3211346 | 1 |
| 3226.3228752 | 1 |
| 3226.324437 | 1 |
| 3226.3265922 | 2 |
| 3226.3282029 | 2 |
| 3226.3286619 | 2 |
| 3226.3311563 | 3 |
| 3226.3317865 | 0 |
| 3226.3336075 | 4 |
| 3226.33545 | 2 |
| 3226.3370804 | 5 |
| 3226.3386091 | 2 |
| 3226.3395606 | 0 |
| 3226.341893 | 2 |
| 3226.3435086 | 3 |
| 3226.3444937 | 2 |
| 3226.3461378 | 3 |
| 3226.3470971 | 1 |
| 3226.3489515 | 2 |
| 3226.3508509 | 4 |
| 3226.3525615 | 2 |
| 3226.3544123 | 3 |
| 3226.3560314 | 2 |
| 3226.3575413 | 3 |
| 3226.359159 | 0 |
| 3226.3601342 | 1 |
| 3226.3625498 | 1 |
| 3226.3628632 | 4 |
| 3226.365475 | 5 |
| 3226.3673347 | 2 |
| 3226.3681213 | 3 |
| 3226.369292 | 3 |
| 3226.3710815 | 1 |
| 3226.3723308 | 4 |
| 3226.3753757 | 4 |
| 3226.3762919 | 2 |
| 3226.3777893 | 5 |
| 3226.378673 | 1 |
| 3226.3805751 | 4 |
| 3226.3818796 | 2 |
| 3226.3837489 | 3 |
| 3226.3854665 | 1 |
| 3226.386518 | 1 |
| 3226.3886356 | 0 |
| 3226.3907025 | 2 |
| 3226.3917252 | 3 |
| 3226.393546 | 4 |
| 3226.3942747 | 1 |
| 3226.3965944 | 1 |
| 3226.3974182 | 5 |
| 3226.3988162 | 3 |
| 3226.4010244 | 2 |
| 3226.4023994 | 4 |
| 3226.4039255 | 4 |
| 3226.4061413 | 1 |
| 3226.4068305 | 0 |
| 3226.4093058 | 1 |
| 3226.4094087 | 1 |
| 3226.4118168 | 0 |
| 3226.4140945 | 3 |
| 3226.4154182 | 1 |
| 3226.4171825 | 0 |
| 3226.4182942 | 2 |
| 3226.4200613 | 1 |
| 3226.4211365 | 0 |
| 3226.4226439 | 1 |
| 3226.4243998 | 3 |
| 3226.4256517 | 4 |
| 3226.4275511 | 2 |
| 3226.4285096 | 1 |
| 3226.430407 | 2 |
| 3226.4322538 | 1 |
| 3226.4334491 | 2 |
| 3226.4355487 | 2 |
| 3226.4363492 | 0 |
| 3226.4381855 | 4 |
| 3226.4400824 | 2 |
| 3226.4413463 | 3 |
| 3226.4426425 | 3 |
| 3226.4434861 | 1 |
| 3226.4450807 | 1 |
| 3226.4476251 | 1 |
| 3226.4487421 | 4 |
| 3226.4514108 | 2 |
| 3226.4528194 | 3 |
| 3226.453738 | 2 |
| 3226.4554367 | 3 |
| 3226.4564082 | 2 |
| 3226.4582862 | 1 |
| 3226.4598638 | 1 |
| 3226.4619379 | 4 |
| 3226.4636023 | 4 |
| 3226.4644551 | 4 |

| | |
|---|---|
| 3226.4670783 | 0 |
| 3226.4672205 | 2 |
| 3226.4696144 | 1 |
| 3226.4712488 | 3 |
| 3226.4725173 | 3 |
| 3226.4741589 | 1 |
| 3226.4754443 | 1 |
| 3226.4772587 | 1 |
| 3226.479093 | 3 |
| 3226.4797421 | 1 |
| 3226.4819026 | 1 |
| 3226.4827874 | 2 |
| 3226.4845811 | 3 |
| 3226.4859751 | 0 |
| 3226.4875738 | 1 |
| 3226.4893656 | 0 |
| 3226.4905707 | 2 |
| 3226.4925536 | 1 |
| 3226.4942426 | 1 |
| 3226.4952128 | 5 |
| 3226.4971809 | 6 |
| 3226.4978726 | 0 |
| 3226.5000969 | 2 |
| 3226.5013609 | 5 |
| 3226.502996 | 1 |
| 3226.5054583 | 0 |
| 3226.5057954 | 3 |
| 3226.508238 | 1 |
| 3226.5092204 | 1 |
| 3226.5109735 | 2 |
| 3226.5127281 | 1 |
| 3226.513416 | 6 |
| 3226.5155708 | 3 |
| 3226.5166769 | 1 |
| 3226.5183612 | 1 |
| 3226.5202746 | 2 |
| 3226.5219643 | 2 |
| 3226.523395 | 2 |
| 3226.5247509 | 1 |
| 3226.5260937 | 0 |
| 3226.5279574 | 1 |
| 3226.5293456 | 0 |
| 3226.5315678 | 3 |
| 3226.5320588 | 2 |
| 3226.5341275 | 2 |
| 3226.5348822 | 4 |
| 3226.5371708 | 2 |
| 3226.5388357 | 2 |
| 3226.5403147 | 2 |
| 3226.5419348 | 1 |
| 3226.5437538 | 4 |
| 3226.5445342 | 1 |
| 3226.5465206 | 1 |
| 3226.5473121 | 0 |
| 3226.5491496 | 2 |
| 3226.5519655 | 1 |
| 3226.5522769 | 0 |
| 3226.5545438 | 4 |
| 3226.5552949 | 3 |
| 3226.5571959 | 0 |
| 3226.5590265 | 0 |
| 3226.5602916 | 1 |
| 3226.5622055 | 5 |
| 3226.5627457 | 0 |
| 3226.5648637 | 4 |
| 3226.5671166 | 4 |
| 3226.5679486 | 5 |
| 3226.5695016 | 3 |
| 3226.5705693 | 2 |
| 3226.572981 | 0 |
| 3226.5743992 | 1 |
| 3226.5755326 | 1 |
| 3226.5776783 | 4 |
| 3226.5786069 | 2 |
| 3226.5809874 | 0 |
| 3226.5814469 | 5 |
| 3226.5832087 | 2 |
| 3226.5850336 | 8 |
| 3226.5859644 | 4 |
| 3226.5883065 | 1 |
| 3226.5889618 | 0 |
| 3226.5918593 | 2 |
| 3226.593198 | 4 |
| 3226.5944658 | 1 |
| 3226.5963272 | 3 |
| 3226.5973318 | 2 |
| 3226.5995575 | 4 |
| 3226.6010986 | 2 |
| 3226.6023178 | 2 |
| 3226.6040674 | 2 |
| 3226.6051108 | 1 |
| 3226.6061669 | 3 |
| 3226.6085094 | 2 |
| 3226.6096936 | 5 |
| 3226.6112975 | 2 |
| 3226.6126154 | 1 |
| 3226.614428 | 2 |
| 3226.6160648 | 3 |
| 3226.6171182 | 3 |
| 3226.6187832 | 0 |
| 3226.6197451 | 1 |
| 3226.6221439 | 4 |
| 3226.6238132 | 4 |
| 3226.6242018 | 2 |
| 3226.6268773 | 3 |
| 3226.6274072 | 0 |
| 3226.6295748 | 2 |
| 3226.6316897 | 4 |
| 3226.6327661 | 4 |
| 3226.6344222 | 2 |
| 3226.6356114 | 1 |
| 3226.6370643 | 2 |
| 3226.6399412 | 3 |
| 3226.6404097 | 1 |
| 3226.6422196 | 1 |
| 3226.6435426 | 3 |
| 3226.6454247 | 3 |
| 3226.6470516 | 4 |
| 3226.6482206 | 4 |
| 3226.6503297 | 1 |
| 3226.6509478 | 1 |
| 3226.6536412 | 3 |
| 3226.6549093 | 6 |
| 3226.6559631 | 1 |
| 3226.6580347 | 1 |
| 3226.6588354 | 2 |
| 3226.6605486 | 6 |
| 3226.6623426 | 5 |
| 3226.6630415 | 1 |
| 3226.665177 | 3 |
| 3226.666705 | 5 |
| 3226.6684048 | 1 |
| 3226.6691956 | 2 |
| 3226.6714555 | 0 |
| 3226.6737597 | 2 |
| 3226.674345 | 2 |
| 3226.6760223 | 2 |
| 3226.6775584 | 3 |
| 3226.6794088 | 0 |
| 3226.6805032 | 2 |
| 3226.6824056 | 0 |
| 3226.6841041 | 1 |
| 3226.6850211 | 2 |
| 3226.6871087 | 3 |
| 3226.6883317 | 1 |
| 3226.6898931 | 2 |
| 3226.6921916 | 1 |
| 3226.693269 | 2 |
| 3226.6944488 | 2 |
| 3226.6966472 | 1 |
| 3226.697818 | 2 |
| 3226.6997466 | 2 |
| 3226.7011031 | 1 |
| 3226.7028124 | 2 |
| 3226.7036062 | 0 |
| 3226.7059606 | 1 |
| 3226.7075109 | 3 |
| 3226.7082109 | 5 |
| 3226.71075 | 3 |
| 3226.7123704 | 3 |
| 3226.7128631 | 4 |
| 3226.7151669 | 0 |
| 3226.7162357 | 3 |
| 3226.7174647 | 5 |
| 3226.7193242 | 4 |
| 3226.7210109 | 1 |
| 3226.7228399 | 2 |
| 3226.7238188 | 1 |
| 3226.7260204 | 3 |
| 3226.7272126 | 2 |
| 3226.7291601 | 3 |
| 3226.729696 | 5 |
| 3226.7318701 | 1 |
| 3226.7339503 | 0 |
| 3226.7345754 | 1 |
| 3226.7369712 | 2 |
| 3226.7385372 | 3 |
| 3226.7396436 | 3 |
| 3226.7417353 | 1 |
| 3226.7426928 | 1 |
| 3226.744644 | 0 |
| 3226.7456856 | 3 |
| 3226.7473158 | 0 |
| 3226.7492411 | 2 |
| 3226.7506776 | 4 |
| 3226.7524997 | 2 |
| 3226.7542815 | 2 |
| 3226.7549655 | 3 |
| 3226.7568289 | 1 |
| 3226.7578115 | 5 |
| 3226.7599743 | 2 |
| 3226.7616984 | 5 |
| 3226.7632732 | 1 |
| 3226.7647927 | 0 |
| 3226.7658243 | 3 |
| 3226.7678055 | 1 |
| 3226.7689676 | 5 |
| 3226.7705005 | 2 |
| 3226.7729167 | 1 |
| 3226.7730028 | 3 |
| 3226.7756907 | 3 |
| 3226.776543 | 2 |
| 3226.7792183 | 5 |
| 3226.7807034 | 3 |
| 3226.7816171 | 2 |
| 3226.7835963 | 3 |
| 3226.7844742 | 3 |
| 3226.7864107 | 5 |
| 3226.7879466 | 2 |
| 3226.7897929 | 1 |
| 3226.791706 | 2 |
| 3226.7919565 | 2 |
| 3226.7938816 | 0 |
| 3226.7961252 | 1 |
| 3226.7969452 | 0 |
| 3226.7990779 | 2 |
| 3226.8013443 | 4 |
| 3226.8019639 | 4 |
| 3226.8034857 | 1 |
| 3226.8047944 | 6 |
| 3226.8066628 | 2 |
| 3226.807705 | 3 |
| 3226.8103716 | 4 |
| 3226.8114749 | 4 |
| 3226.8127852 | 1 |
| 3226.8148595 | 3 |
| 3226.8157679 | 2 |
| 3226.8179814 | 4 |
| 3226.8198276 | 3 |
| 3226.8203898 | 3 |
| 3226.8222121 | 2 |
| 3226.8238327 | 0 |
| 3226.825329 | 1 |
| 3226.8272278 | 2 |
| 3226.8282352 | 0 |
| 3226.8307015 | 3 |
| 3226.8314569 | 3 |
| 3226.833765 | 2 |
| 3226.8351451 | 2 |
| 3226.8364648 | 5 |
| 3226.8385823 | 4 |
| 3226.8393958 | 1 |
| 3226.840476 | 1 |
| 3226.8429026 | 5 |
| 3226.8441132 | 3 |
| 3226.8462632 | 1 |
| 3226.8472969 | 2 |
| 3226.8495202 | 5 |
| 3226.8503679 | 4 |
| 3226.8522567 | 3 |
| 3226.8536558 | 2 |
| 3226.8546144 | 5 |
| 3226.8568097 | 2 |
| 3226.8590008 | 2 |
| 3226.8600901 | 1 |
| 3226.8615337 | 0 |
| 3226.8627125 | 2 |
| 3226.8646472 | 1 |
| 3226.8656061 | 2 |
| 3226.8683011 | 2 |
| 3226.8697418 | 3 |
| 3226.8707654 | 3 |
| 3226.872801 | 2 |
| 3226.8734909 | 4 |
| 3226.8755796 | 2 |
| 3226.8774311 | 5 |
| 3226.878316 | 1 |
| 3226.8807643 | 1 |
| 3226.8815099 | 3 |
| 3226.8835559 | 2 |
| 3226.8853791 | 1 |
| 3226.8866729 | 3 |
| 3226.8887278 | 4 |
| 3226.8893161 | 2 |
| 3226.8916437 | 3 |
| 3226.8937085 | 1 |
| 3226.8937826 | 2 |
| 3226.8964751 | 4 |
| 3226.8977509 | 3 |
| 3226.8990443 | 2 |
| 3226.9010225 | 2 |
| 3226.9024078 | 4 |
| 3226.9039025 | 1 |
| 3226.9049559 | 2 |
| 3226.9070423 | 3 |
| 3226.9090792 | 4 |
| 3226.9100016 | 2 |
| 3226.9123439 | 0 |
| 3226.9134279 | 3 |
| 3226.9152039 | 4 |
| 3226.917373 | 1 |
| 3226.9180135 | 2 |
| 3226.9199043 | 2 |
| 3226.9208726 | 1 |
| 3226.9223063 | 3 |
| 3226.9239925 | 1 |
| 3226.9264925 | 0 |
| 3226.927894 | 1 |
| 3226.9288108 | 2 |
| 3226.9309593 | 3 |
| 3226.9318681 | 5 |
| 3226.9337897 | 3 |
| 3226.9357676 | 2 |
| 3226.9361757 | 5 |
| 3226.9385056 | 2 |
| 3226.9406633 | 0 |
| 3226.9411208 | 0 |
| 3226.9434308 | 1 |
| 3226.9443771 | 2 |
| 3226.9457792 | 2 |
| 3226.9472825 | 3 |
| 3226.9494082 | 2 |
| 3226.95126 | 0 |
| 3226.9514569 | 1 |
| 3226.9520133 | 2 |
| 3226.9534675 | 4 |
| 3226.9543065 | 2 |
| 3226.9546968 | 1 |
| 3226.9553167 | 4 |
| 3226.956365 | 1 |
| 3226.95731 | 1 |
| 3226.9584134 | 2 |
| 3226.9591476 | 3 |
| 3226.9594398 | 0 |
| 3226.9608378 | 2 |
| 3226.9615862 | 2 |
| 3226.9627042 | 0 |
| 3226.9630221 | 4 |
| 3226.9634704 | 1 |
| 3226.9646386 | 3 |
| 3226.9654435 | 4 |
| 3226.9663614 | 1 |
| 3226.9664905 | 0 |
| 3226.9668184 | 4 |
| 3226.9682316 | 3 |
| 3226.9685758 | 2 |
| 3226.9705385 | 4 |
| 3226.9706878 | 6 |
| 3226.9712515 | 0 |
| 3226.9716281 | 2 |
| 3226.9733898 | 2 |
| 3226.9736107 | 0 |
| 3226.9747866 | 2 |
| 3226.9756369 | 4 |
| 3226.9759884 | 2 |
| 3226.9764178 | 4 |
| 3226.9781311 | 2 |
| 3226.9791892 | 3 |
| 3226.9791914 | 1 |
| 3226.979281 | 1 |
| 3226.9815779 | 0 |
| 3226.9820683 | 2 |
| 3226.9827154 | 2 |
| 3226.9831158 | 2 |
| 3226.9835755 | 2 |
| 3226.98414 | 3 |
| 3226.9855511 | 1 |
| 3226.9862766 | 4 |
| 3226.986861 | 1 |
| 3226.98719 | 1 |
| 3226.9885656 | 3 |
| 3226.9889253 | 2 |
| 3226.990246 | 3 |

| | |
|---|---|
| 3226.9913748 | 2 |
| 3226.9915721 | 2 |
| 3226.9918868 | 5 |
| 3226.993529 | 2 |
| 3226.9942547 | 7 |
| 3226.9951351 | 1 |
| 3226.9952994 | 1 |
| 3226.9968141 | 4 |
| 3226.9970645 | 2 |
| 3226.9974164 | 3 |
| 3226.9986775 | 2 |
| 3226.9999221 | 5 |
| 3226.9999284 | 3 |
| 3227.0011989 | 1 |
| 3227.0018799 | 2 |
| 3227.0024535 | 1 |
| 3227.0030152 | 2 |
| 3227.0046424 | 2 |
| 3227.0050971 | 4 |
| 3227.0055025 | 1 |
| 3227.005648 | 1 |
| 3227.0074393 | 0 |
| 3227.0075055 | 1 |
| 3227.0085357 | 1 |
| 3227.0097713 | 3 |
| 3227.0103073 | 3 |
| 3227.010746 | 3 |
| 3227.01289 | 1 |
| 3227.0142007 | 6 |
| 3227.0158656 | 4 |
| 3227.0162989 | 3 |
| 3227.0166142 | 2 |
| 3227.0184023 | 3 |
| 3227.0185955 | 0 |
| 3227.0204975 | 1 |
| 3227.021845 | 2 |
| 3227.0238648 | 3 |
| 3227.0249347 | 4 |
| 3227.0249615 | 1 |
| 3227.025214 | 4 |
| 3227.0268863 | 1 |
| 3227.0272502 | 4 |
| 3227.0281578 | 2 |
| 3227.0291732 | 1 |
| 3227.0300572 | 2 |
| 3227.0314567 | 3 |
| 3227.0329717 | 2 |
| 3227.0347277 | 1 |
| 3227.0354961 | 4 |
| 3227.0370765 | 6 |
| 3227.03903 | 2 |
| 3227.0396302 | 2 |
| 3227.0413569 | 1 |
| 3227.0439809 | 3 |
| 3227.0448589 | 1 |
| 3227.0470422 | 2 |
| 3227.0479082 | 1 |
| 3227.0504039 | 1 |
| 3227.0510028 | 4 |
| 3227.0530837 | 3 |
| 3227.0554219 | 0 |
| 3227.0558951 | 5 |
| 3227.0584473 | 2 |
| 3227.0590217 | 0 |
| 3227.0604305 | 3 |
| 3227.0629798 | 3 |
| 3227.0639432 | 2 |
| 3227.0656782 | 1 |
| 3227.067308 | 1 |
| 3227.0685715 | 0 |
| 3227.0706478 | 1 |
| 3227.0714866 | 1 |
| 3227.0736985 | 3 |
| 3227.0740232 | 3 |
| 3227.0767867 | 2 |
| 3227.0771341 | 2 |
| 3227.0795641 | 2 |
| 3227.0814382 | 3 |
| 3227.0819549 | 1 |
| 3227.0844361 | 3 |
| 3227.08521 | 3 |
| 3227.0868961 | 4 |
| 3227.0893938 | 1 |
| 3227.0902374 | 3 |
| 3227.0924419 | 2 |
| 3227.0933162 | 1 |
| 3227.0954722 | 1 |
| 3227.0980335 | 1 |
| 3227.0984399 | 1 |
| 3227.1001135 | 4 |
| 3227.1012272 | 0 |
| 3227.1030796 | 1 |
| 3227.1048021 | 0 |
| 3227.1065717 | 3 |
| 3227.107757 | 1 |
| 3227.1085169 | 1 |
| 3227.1109968 | 3 |
| 3227.1127134 | 2 |
| 3227.1134551 | 3 |
| 3227.1157616 | 5 |
| 3227.1165582 | 2 |
| 3227.1180864 | 2 |
| 3227.1194654 | 4 |
| 3227.1217796 | 4 |
| 3227.1234233 | 1 |
| 3227.1245604 | 4 |
| 3227.1262738 | 1 |
| 3227.1275176 | 2 |
| 3227.1292565 | 2 |
| 3227.1302939 | 0 |
| 3227.1308971 | 1 |
| 3227.1326589 | 2 |
| 3227.1332948 | 1 |
| 3227.1341505 | 3 |
| 3227.1344162 | 2 |
| 3227.1349343 | 0 |
| 3227.1365739 | 0 |
| 3227.1378689 | 2 |
| 3227.1379583 | 0 |
| 3227.1387332 | 0 |
| 3227.1402494 | 3 |
| 3227.1404069 | 1 |
| 3227.1406424 | 3 |
| 3227.1417487 | 4 |
| 3227.1421494 | 1 |
| 3227.1425141 | 3 |
| 3227.1433472 | 4 |
| 3227.144405 | 1 |
| 3227.1452347 | 2 |
| 3227.1454252 | 2 |
| 3227.1471951 | 4 |
| 3227.147344 | 3 |
| 3227.1483166 | 3 |
| 3227.1484103 | 1 |
| 3227.1496929 | 1 |
| 3227.1504798 | 5 |
| 3227.1512168 | 3 |
| 3227.1527175 | 1 |
| 3227.1528945 | 2 |
| 3227.1535539 | 1 |
| 3227.1552372 | 2 |
| 3227.155785 | 5 |
| 3227.1560078 | 3 |
| 3227.1566429 | 2 |
| 3227.1581985 | 2 |
| 3227.1583239 | 2 |
| 3227.1595946 | 2 |
| 3227.1599331 | 5 |
| 3227.160778 | 4 |
| 3227.1622067 | 2 |
| 3227.1624825 | 1 |
| 3227.1625316 | 5 |
| 3227.162909 | 3 |
| 3227.1652622 | 4 |
| 3227.1653121 | 3 |
| 3227.1669695 | 6 |
| 3227.1670967 | 2 |
| 3227.1675517 | 0 |
| 3227.1681937 | 3 |
| 3227.1699005 | 2 |
| 3227.170231 | 2 |
| 3227.1705143 | 0 |
| 3227.1715486 | 1 |
| 3227.1737055 | 1 |
| 3227.1748465 | 2 |
| 3227.1768083 | 3 |
| 3227.1787672 | 0 |
| 3227.1799439 | 0 |
| 3227.18158 | 1 |
| 3227.1827008 | 3 |
| 3227.1846327 | 4 |
| 3227.1867836 | 2 |
| 3227.1873446 | 1 |
| 3227.1897157 | 4 |
| 3227.1901089 | 5 |
| 3227.1925407 | 0 |
| 3227.1942292 | 2 |
| 3227.1956485 | 3 |
| 3227.1978988 | 3 |
| 3227.1981651 | 1 |
| 3227.1993423 | 2 |
| 3227.2022442 | 1 |
| 3227.203013 | 2 |
| 3227.204756 | 0 |
| 3227.2060642 | 1 |
| 3227.2076512 | 1 |
| 3227.2098865 | 1 |
| 3227.2107057 | 1 |
| 3227.212663 | 4 |
| 3227.2128922 | 3 |
| 3227.2144586 | 3 |
| 3227.2160794 | 4 |
| 3227.2184909 | 0 |
| 3227.2201475 | 4 |
| 3227.2215601 | 1 |
| 3227.2229834 | 2 |
| 3227.2242802 | 3 |
| 3227.2263642 | 2 |
| 3227.2283667 | 2 |
| 3227.2290948 | 2 |
| 3227.2312512 | 2 |
| 3227.2334793 | 2 |
| 3227.2344819 | 2 |
| 3227.2360183 | 2 |
| 3227.2376162 | 0 |
| 3227.2393097 | 1 |
| 3227.2401547 | 3 |
| 3227.2420088 | 1 |
| 3227.243644 | 4 |
| 3227.2451758 | 6 |
| 3227.2472452 | 4 |
| 3227.2479951 | 2 |
| 3227.2505684 | 3 |
| 3227.2516607 | 2 |
| 3227.2526157 | 1 |
| 3227.254199 | 1 |
| 3227.2552779 | 5 |
| 3227.2573383 | 3 |
| 3227.2582197 | 1 |
| 3227.2598298 | 3 |
| 3227.2623339 | 1 |
| 3227.2635861 | 0 |
| 3227.2656041 | 2 |
| 3227.2674908 | 1 |
| 3227.2680144 | 3 |
| 3227.2702802 | 3 |
| 3227.2713718 | 2 |
| 3227.2731182 | 1 |
| 3227.2752059 | 0 |
| 3227.2763883 | 1 |
| 3227.2783341 | 1 |
| 3227.2797698 | 1 |
| 3227.2814425 | 1 |
| 3227.2822499 | 4 |
| 3227.2847384 | 4 |
| 3227.2864252 | 4 |
| 3227.2872639 | 3 |
| 3227.2891174 | 1 |
| 3227.290292 | 0 |
| 3227.2920444 | 2 |
| 3227.293953 | 2 |
| 3227.2946785 | 0 |
| 3227.2975585 | 2 |
| 3227.297816 | 4 |
| 3227.3003504 | 1 |
| 3227.3008056 | 8 |
| 3227.3020706 | 1 |
| 3227.3034389 | 1 |
| 3227.3046092 | 3 |
| 3227.3063353 | 2 |
| 3227.3072732 | 2 |
| 3227.3077002 | 2 |
| 3227.3098336 | 2 |
| 3227.3105342 | 4 |
| 3227.3124386 | 3 |
| 3227.3133051 | 1 |
| 3227.3153873 | 0 |
| 3227.3169367 | 3 |
| 3227.3172107 | 1 |
| 3227.3186053 | 0 |
| 3227.3198766 | 2 |
| 3227.3212603 | 4 |
| 3227.3230939 | 2 |
| 3227.3235804 | 2 |
| 3227.3248232 | 2 |
| 3227.3267678 | 1 |
| 3227.3280188 | 2 |
| 3227.3291763 | 3 |
| 3227.3293501 | 1 |
| 3227.3318023 | 1 |
| 3227.3320494 | 1 |
| 3227.3335161 | 1 |
| 3227.3351714 | 3 |
| 3227.3364163 | 3 |
| 3227.3382662 | 1 |
| 3227.3387003 | 3 |
| 3227.340117 | 3 |
| 3227.341845 | 3 |
| 3227.3419998 | 0 |
| 3227.3436823 | 3 |
| 3227.3448941 | 3 |
| 3227.3464208 | 2 |
| 3227.346855 | 3 |
| 3227.3484192 | 1 |
| 3227.3506215 | 1 |
| 3227.3512981 | 1 |
| 3227.3523037 | 5 |
| 3227.3541224 | 1 |
| 3227.3547873 | 1 |
| 3227.3560807 | 2 |
| 3227.3577255 | 4 |
| 3227.3587875 | 1 |
| 3227.3600122 | 6 |
| 3227.3606876 | 1 |
| 3227.3620119 | 1 |
| 3227.3632035 | 2 |
| 3227.3648527 | 3 |
| 3227.366129 | 0 |
| 3227.3676531 | 4 |
| 3227.3687976 | 2 |
| 3227.3707905 | 2 |
| 3227.3714611 | 4 |
| 3227.3732349 | 3 |
| 3227.3733393 | 1 |
| 3227.3753817 | 3 |
| 3227.3761815 | 4 |
| 3227.3776714 | 3 |
| 3227.3789429 | 2 |
| 3227.3796106 | 4 |
| 3227.3818925 | 3 |
| 3227.3820656 | 3 |
| 3227.383909 | 1 |
| 3227.3852866 | 3 |
| 3227.3861784 | 3 |
| 3227.3874573 | 6 |
| 3227.3891687 | 5 |
| 3227.3898372 | 2 |
| 3227.3916969 | 0 |
| 3227.3920924 | 6 |
| 3227.3941937 | 0 |
| 3227.3944522 | 1 |
| 3227.3959962 | 0 |
| 3227.3975471 | 3 |
| 3227.3984303 | 3 |
| 3227.4002166 | 6 |
| 3227.4007969 | 0 |
| 3227.4022177 | 2 |
| 3227.4040657 | 1 |
| 3227.4046932 | 2 |
| 3227.4062943 | 6 |
| 3227.4073961 | 1 |
| 3227.4092593 | 0 |
| 3227.4105251 | 1 |
| 3227.4111146 | 2 |
| 3227.4129808 | 4 |
| 3227.413487 | 2 |
| 3227.4152323 | 1 |
| 3227.4161079 | 2 |
| 3227.4172317 | 2 |
| 3227.4194282 | 3 |
| 3227.4202589 | 4 |
| 3227.4218115 | 1 |
| 3227.4223086 | 2 |
| 3227.4240376 | 5 |
| 3227.4253002 | 3 |
| 3227.4264714 | 3 |
| 3227.4274803 | 5 |
| 3227.4284327 | 1 |
| 3227.4302865 | 3 |
| 3227.4308154 | 2 |
| 3227.4326568 | 3 |
| 3227.4334849 | 3 |
| 3227.4341671 | 2 |
| 3227.4359656 | 1 |
| 3227.4380013 | 3 |
| 3227.4380324 | 2 |
| 3227.4394747 | 3 |
| 3227.4408567 | 1 |
| 3227.4419972 | 0 |
| 3227.4420438 | 2 |
| 3227.442901 | 2 |
| 3227.4435819 | 4 |
| 3227.4435961 | 3 |

| | |
|---|---|
| 3227.4447138 | 2 |
| 3227.4449593 | 4 |
| 3227.4452178 | 2 |
| 3227.4457421 | 6 |
| 3227.4467438 | 2 |
| 3227.4467453 | 1 |
| 3227.4481728 | 1 |
| 3227.4483422 | 1 |
| 3227.4485226 | 1 |
| 3227.4494584 | 1 |
| 3227.44966 | 1 |
| 3227.4502847 | 1 |
| 3227.4504634 | 0 |
| 3227.4515355 | 1 |
| 3227.4524178 | 1 |
| 3227.4525069 | 1 |
| 3227.4529319 | 1 |
| 3227.4534215 | 3 |
| 3227.4544057 | 1 |
| 3227.4549047 | 0 |
| 3227.455391 | 2 |
| 3227.4554714 | 3 |
| 3227.4556191 | 3 |
| 3227.4570406 | 3 |
| 3227.4570555 | 0 |
| 3227.4571942 | 0 |
| 3227.4587601 | 5 |
| 3227.4590949 | 1 |
| 3227.4594011 | 2 |
| 3227.459997 | 1 |
| 3227.4601876 | 4 |
| 3227.4609839 | 2 |
| 3227.4612549 | 2 |
| 3227.4622112 | 0 |
| 3227.4622472 | 2 |
| 3227.4634867 | 3 |
| 3227.4635645 | 1 |
| 3227.464196 | 1 |
| 3227.4650472 | 0 |
| 3227.4652459 | 2 |
| 3227.4654675 | 0 |
| 3227.4661736 | 4 |
| 3227.4663637 | 4 |
| 3227.4673169 | 2 |
| 3227.4678813 | 3 |
| 3227.468374 | 1 |
| 3227.4687477 | 4 |
| 3227.4694134 | 1 |
| 3227.4696094 | 2 |
| 3227.4707224 | 0 |
| 3227.4707625 | 4 |
| 3227.4710688 | 1 |
| 3227.4719727 | 1 |
| 3227.4720999 | 0 |
| 3227.4729597 | 1 |
| 3227.4736129 | 3 |
| 3227.4742513 | 3 |
| 3227.4743947 | 1 |
| 3227.4754287 | 2 |
| 3227.4757381 | 3 |
| 3227.4762196 | 5 |
| 3227.4770984 | 0 |
| 3227.4777792 | 2 |
| 3227.4778726 | 4 |
| 3227.4784325 | 6 |
| 3227.4787443 | 1 |
| 3227.4799476 | 1 |
| 3227.4802522 | 5 |
| 3227.4804794 | 0 |
| 3227.4807685 | 1 |
| 3227.4819215 | 2 |
| 3227.4821396 | 3 |
| 3227.4821713 | 3 |
| 3227.4831567 | 3 |
| 3227.4839393 | 3 |
| 3227.4839651 | 4 |
| 3227.484362 | 2 |
| 3227.4848945 | 5 |
| 3227.4857674 | 4 |
| 3227.4861538 | 3 |
| 3227.4866409 | 3 |
| 3227.4869621 | 2 |
| 3227.4874901 | 5 |
| 3227.4880913 | 2 |
| 3227.4889577 | 2 |
| 3227.4891123 | 2 |
| 3227.4898194 | 0 |
| 3227.4904623 | 1 |
| 3227.4909059 | 2 |
| 3227.491788 | 1 |
| 3227.4924987 | 5 |
| 3227.4930979 | 0 |
| 3227.4933167 | 0 |
| 3227.4939558 | 3 |
| 3227.4942547 | 1 |
| 3227.4948421 | 3 |
| 3227.4955426 | 5 |
| 3227.4957282 | 5 |
| 3227.4957572 | 3 |
| 3227.4970607 | 3 |
| 3227.4971547 | 2 |
| 3227.4980761 | 0 |
| 3227.4988713 | 4 |
| 3227.499124 | 1 |
| 3227.4991516 | 2 |
| 3227.5002156 | 0 |
| 3227.5009313 | 2 |
| 3227.5014041 | 0 |
| 3227.5018616 | 3 |
| 3227.5021394 | 2 |
| 3227.5031043 | 1 |
| 3227.5034248 | 1 |
| 3227.5034389 | 0 |
| 3227.5047776 | 2 |
| 3227.5048871 | 2 |
| 3227.5053432 | 0 |
| 3227.506294 | 1 |
| 3227.5067501 | 2 |
| 3227.5076047 | 3 |
| 3227.5076411 | 4 |
| 3227.5083352 | 0 |
| 3227.5086076 | 1 |
| 3227.5098312 | 0 |
| 3227.510273 | 2 |
| 3227.51083 | 2 |
| 3227.5115054 | 4 |
| 3227.5121283 | 1 |
| 3227.512351 | 5 |
| 3227.5123786 | 1 |
| 3227.5129708 | 3 |
| 3227.5140695 | 3 |
| 3227.5141159 | 4 |
| 3227.5147394 | 3 |
| 3227.5148365 | 4 |
| 3227.5156546 | 1 |
| 3227.516013 | 3 |
| 3227.5164297 | 3 |
| 3227.5173662 | 2 |
| 3227.5173786 | 2 |
| 3227.5177565 | 6 |
| 3227.5184034 | 2 |
| 3227.5194251 | 3 |
| 3227.5198276 | 1 |
| 3227.5202713 | 4 |
| 3227.5205344 | 4 |
| 3227.521508 | 4 |
| 3227.5215737 | 1 |
| 3227.5219294 | 2 |
| 3227.5227905 | 2 |
| 3227.5237764 | 2 |
| 3227.524384 | 4 |
| 3227.524529 | 3 |
| 3227.5252925 | 3 |
| 3227.5260647 | 1 |
| 3227.5264486 | 4 |
| 3227.5266156 | 1 |
| 3227.5267326 | 4 |
| 3227.5275783 | 3 |
| 3227.5280506 | 3 |
| 3227.5287792 | 2 |
| 3227.529634 | 3 |
| 3227.5296615 | 2 |
| 3227.530605 | 1 |
| 3227.5307396 | 1 |
| 3227.5318085 | 2 |
| 3227.5326311 | 5 |
| 3227.5329466 | 4 |
| 3227.5336996 | 5 |
| 3227.5338353 | 4 |
| 3227.5345165 | 5 |
| 3227.5348417 | 4 |
| 3227.5354106 | 4 |
| 3227.5356071 | 5 |
| 3227.5364103 | 0 |
| 3227.5367804 | 4 |
| 3227.5373843 | 1 |
| 3227.5377767 | 5 |
| 3227.5385184 | 2 |
| 3227.5393819 | 5 |
| 3227.5396357 | 7 |
| 3227.5396594 | 5 |
| 3227.5408842 | 7 |
| 3227.541078 | 5 |
| 3227.5416473 | 3 |
| 3227.542118 | 1 |
| 3227.5424949 | 3 |
| 3227.5428353 | 0 |
| 3227.5445857 | 1 |
| 3227.5448169 | 1 |
| 3227.544942 | 1 |
| 3227.5458106 | 1 |
| 3227.5460024 | 5 |
| 3227.5465656 | 1 |
| 3227.5465826 | 1 |
| 3227.5478806 | 5 |
| 3227.5481908 | 4 |
| 3227.5484906 | 1 |
| 3227.5489417 | 1 |
| 3227.5495912 | 0 |
| 3227.5499661 | 1 |
| 3227.5504375 | 2 |
| 3227.5514348 | 0 |
| 3227.5517454 | 1 |
| 3227.5521736 | 2 |
| 3227.5529597 | 3 |
| 3227.5534611 | 3 |
| 3227.5541167 | 2 |
| 3227.5542449 | 0 |
| 3227.5550589 | 2 |
| 3227.5558642 | 4 |
| 3227.5559283 | 3 |
| 3227.5565085 | 2 |
| 3227.5571857 | 2 |
| 3227.5577548 | 0 |
| 3227.5584665 | 1 |
| 3227.5586703 | 4 |
| 3227.5597645 | 1 |
| 3227.5598874 | 0 |
| 3227.5605282 | 2 |
| 3227.5606011 | 2 |
| 3227.5613217 | 2 |
| 3227.562119 | 1 |
| 3227.5621763 | 2 |
| 3227.5628972 | 1 |
| 3227.5639159 | 2 |
| 3227.5640394 | 2 |
| 3227.564795 | 3 |
| 3227.5653678 | 2 |
| 3227.5656764 | 2 |
| 3227.5661093 | 1 |
| 3227.5673572 | 2 |
| 3227.5673805 | 2 |
| 3227.5674491 | 1 |
| 3227.568687 | 0 |
| 3227.5687297 | 4 |
| 3227.569476 | 2 |
| 3227.5701638 | 2 |
| 3227.5705501 | 3 |
| 3227.5707235 | 0 |
| 3227.5718373 | 1 |
| 3227.5722815 | 1 |
| 3227.5725782 | 3 |
| 3227.5726307 | 1 |
| 3227.5733759 | 1 |
| 3227.574339 | 2 |
| 3227.5747137 | 1 |
| 3227.5753448 | 3 |
| 3227.5756582 | 1 |
| 3227.5764877 | 2 |
| 3227.5765948 | 3 |
| 3227.5769712 | 1 |
| 3227.5778719 | 0 |
| 3227.5783848 | 2 |
| 3227.5785844 | 4 |
| 3227.579456 | 2 |
| 3227.5797924 | 1 |
| 3227.5809903 | 2 |
| 3227.5815568 | 2 |
| 3227.5815689 | 2 |
| 3227.581663 | 1 |
| 3227.582836 | 2 |
| 3227.5832745 | 0 |
| 3227.5836089 | 1 |
| 3227.58438 | 1 |
| 3227.5851521 | 2 |
| 3227.58566 | 1 |
| 3227.5857923 | 3 |
| 3227.5864692 | 0 |
| 3227.5866948 | 2 |
| 3227.5877885 | 0 |
| 3227.5879798 | 2 |
| 3227.588632 | 2 |
| 3227.5889852 | 4 |
| 3227.589163 | 4 |
| 3227.5906974 | 0 |
| 3227.5909141 | 4 |
| 3227.5911396 | 2 |
| 3227.591593 | 2 |
| 3227.5925765 | 2 |
| 3227.5930704 | 1 |
| 3227.5936678 | 7 |
| 3227.5940622 | 2 |
| 3227.5940742 | 0 |
| 3227.5947768 | 2 |
| 3227.5954403 | 4 |
| 3227.5957565 | 2 |
| 3227.5967602 | 3 |
| 3227.5969997 | 3 |
| 3227.5978352 | 4 |
| 3227.5980553 | 2 |
| 3227.5985546 | 4 |
| 3227.5998285 | 1 |
| 3227.6000604 | 1 |
| 3227.6004485 | 0 |
| 3227.6005132 | 2 |
| 3227.6016624 | 3 |
| 3227.6017779 | 4 |
| 3227.6023009 | 3 |
| 3227.6034785 | 0 |
| 3227.6037376 | 0 |
| 3227.6037956 | 2 |
| 3227.604514 | 3 |
| 3227.605139 | 3 |
| 3227.6060063 | 3 |
| 3227.6062556 | 3 |
| 3227.6063267 | 1 |
| 3227.606501 | 2 |
| 3227.6084534 | 0 |
| 3227.6085997 | 0 |
| 3227.6092817 | 2 |
| 3227.6094369 | 2 |
| 3227.6100509 | 0 |
| 3227.6103401 | 2 |
| 3227.6103814 | 6 |
| 3227.6118354 | 1 |
| 3227.6122425 | 1 |
| 3227.6124404 | 1 |
| 3227.6126876 | 2 |
| 3227.6131851 | 1 |
| 3227.6139593 | 2 |
| 3227.6143231 | 0 |
| 3227.6149019 | 2 |
| 3227.6154079 | 0 |
| 3227.6166868 | 2 |
| 3227.6167027 | 1 |
| 3227.6173744 | 5 |
| 3227.617714 | 3 |
| 3227.6183239 | 4 |
| 3227.6185997 | 1 |
| 3227.6191424 | 1 |
| 3227.6202299 | 4 |
| 3227.6204426 | 4 |
| 3227.6212806 | 4 |
| 3227.6214164 | 1 |
| 3227.6216434 | 1 |
| 3227.6225037 | 0 |
| 3227.6228442 | 0 |
| 3227.6235326 | 2 |
| 3227.6240924 | 3 |
| 3227.624479 | 4 |
| 3227.6252103 | 3 |
| 3227.6257573 | 1 |
| 3227.6262112 | 3 |
| 3227.6264502 | 0 |
| 3227.6277013 | 3 |
| 3227.6277949 | 1 |
| 3227.6282041 | 1 |
| 3227.6291902 | 2 |
| 3227.6292948 | 2 |
| 3227.6295815 | 4 |
| 3227.6305953 | 1 |
| 3227.6312941 | 0 |
| 3227.6313048 | 2 |
| 3227.6328498 | 2 |
| 3227.6329415 | 2 |
| 3227.6330305 | 2 |
| 3227.6333407 | 1 |
| 3227.6342873 | 5 |
| 3227.6346557 | 1 |
| 3227.6349331 | 2 |
| 3227.6356959 | 2 |
| 3227.6366167 | 2 |
| 3227.6368689 | 3 |

| | |
|---|---|
| 3227.6375614 | 1 |
| 3227.6380714 | 4 |
| 3227.6386163 | 2 |
| 3227.6390316 | 1 |
| 3227.6400525 | 1 |
| 3227.6401087 | 0 |
| 3227.6411971 | 1 |
| 3227.6417652 | 3 |
| 3227.6418799 | 4 |
| 3227.6419217 | 8 |
| 3227.6424972 | 2 |
| 3227.643863 | 3 |
| 3227.644007 | 1 |
| 3227.6443831 | 3 |
| 3227.6451001 | 2 |
| 3227.6457168 | 1 |
| 3227.6459387 | 0 |
| 3227.6463363 | 0 |
| 3227.6464509 | 3 |
| 3227.6474792 | 6 |
| 3227.6482056 | 1 |
| 3227.6486788 | 0 |
| 3227.6492796 | 4 |
| 3227.6497396 | 1 |
| 3227.6501627 | 0 |
| 3227.6506644 | 0 |
| 3227.6517058 | 1 |
| 3227.651936 | 1 |
| 3227.6523644 | 4 |
| 3227.6527871 | 2 |
| 3227.6539736 | 1 |
| 3227.6539921 | 1 |
| 3227.6543815 | 1 |
| 3227.6544546 | 3 |
| 3227.6556172 | 0 |
| 3227.6557036 | 2 |
| 3227.6560767 | 3 |
| 3227.6564693 | 2 |
| 3227.6575254 | 2 |
| 3227.6583141 | 1 |
| 3227.6587188 | 2 |
| 3227.6591675 | 1 |
| 3227.660299 | 0 |
| 3227.6605783 | 5 |
| 3227.6608376 | 1 |
| 3227.6611852 | 1 |
| 3227.6622138 | 1 |
| 3227.6622853 | 0 |
| 3227.6628571 | 1 |
| 3227.6631052 | 2 |
| 3227.6639211 | 1 |
| 3227.6647017 | 1 |
| 3227.6647212 | 1 |
| 3227.6657484 | 3 |
| 3227.6662931 | 2 |
| 3227.6669206 | 2 |
| 3227.6670175 | 4 |
| 3227.6672842 | 2 |
| 3227.6684785 | 3 |
| 3227.6689164 | 3 |
| 3227.6694031 | 4 |
| 3227.670305 | 0 |
| 3227.6703054 | 1 |
| 3227.6712994 | 3 |
| 3227.6717498 | 2 |
| 3227.6724794 | 2 |
| 3227.6727248 | 2 |
| 3227.673026 | 4 |
| 3227.6730636 | 0 |
| 3227.6744843 | 2 |
| 3227.6746728 | 0 |
| 3227.6747311 | 3 |
| 3227.6753793 | 1 |
| 3227.6760345 | 4 |
| 3227.6771064 | 0 |
| 3227.6772811 | 3 |
| 3227.6778564 | 1 |
| 3227.6786243 | 4 |
| 3227.679116 | 1 |
| 3227.6796161 | 4 |
| 3227.680027 | 1 |
| 3227.6807649 | 3 |
| 3227.6809019 | 1 |
| 3227.68171 | 0 |
| 3227.6818651 | 5 |
| 3227.6828838 | 1 |
| 3227.6833477 | 2 |
| 3227.6843309 | 2 |
| 3227.6846804 | 5 |
| 3227.6848111 | 2 |
| 3227.6855667 | 2 |
| 3227.6856778 | 3 |
| 3227.6867537 | 2 |
| 3227.6868352 | 1 |
| 3227.6872182 | 1 |
| 3227.6878237 | 2 |
| 3227.6884922 | 0 |
| 3227.6888917 | 1 |
| 3227.6896638 | 1 |
| 3227.6896799 | 1 |
| 3227.6900269 | 1 |
| 3227.6911738 | 1 |
| 3227.6923265 | 3 |
| 3227.6923383 | 4 |
| 3227.6930196 | 1 |
| 3227.6934345 | 4 |
| 3227.6934995 | 2 |
| 3227.6940644 | 2 |
| 3227.6947719 | 0 |
| 3227.6955853 | 3 |
| 3227.6960251 | 2 |
| 3227.6961835 | 5 |
| 3227.6964323 | 3 |
| 3227.6971902 | 3 |
| 3227.6982747 | 1 |
| 3227.6983948 | 3 |
| 3227.6988496 | 1 |
| 3227.6995541 | 0 |
| 3227.700463 | 4 |
| 3227.7006319 | 3 |
| 3227.7014796 | 5 |

| | |
|---|---|
| 3227.7018058 | 3 |
| 3227.7024114 | 4 |
| 3227.7027355 | 3 |
| 3227.7028583 | 3 |
| 3227.7043331 | 3 |
| 3227.7043876 | 1 |
| 3227.7051195 | 1 |
| 3227.7059743 | 3 |
| 3227.7059816 | 1 |
| 3227.7065333 | 2 |
| 3227.7066619 | 2 |
| 3227.7080901 | 5 |
| 3227.7082499 | 2 |
| 3227.7083184 | 3 |
| 3227.7084928 | 0 |
| 3227.7100704 | 2 |
| 3227.7102763 | 3 |
| 3227.7103898 | 1 |
| 3227.7114424 | 2 |
| 3227.7121108 | 4 |
| 3227.7121343 | 2 |
| 3227.7126557 | 1 |
| 3227.713307 | 2 |
| 3227.7140552 | 3 |
| 3227.7148732 | 3 |
| 3227.7153687 | 1 |
| 3227.7158107 | 0 |
| 3227.7160987 | 3 |
| 3227.7166526 | 2 |
| 3227.7170528 | 1 |
| 3227.7178146 | 1 |
| 3227.7181507 | 1 |
| 3227.7181806 | 1 |
| 3227.7193137 | 2 |
| 3227.7195061 | 1 |
| 3227.7200567 | 3 |
| 3227.7210497 | 2 |
| 3227.7211185 | 5 |
| 3227.7220003 | 3 |
| 3227.7223148 | 1 |
| 3227.7230657 | 2 |
| 3227.7238601 | 3 |
| 3227.7243361 | 1 |
| 3227.7250273 | 3 |
| 3227.7252241 | 2 |
| 3227.7259923 | 1 |
| 3227.7261709 | 2 |
| 3227.7269114 | 1 |
| 3227.7273249 | 0 |
| 3227.7278169 | 2 |
| 3227.7287557 | 2 |
| 3227.7288041 | 4 |
| 3227.7296148 | 2 |
| 3227.730501 | 4 |
| 3227.7310822 | 0 |
| 3227.7312569 | 0 |
| 3227.7314383 | 2 |
| 3227.7324536 | 0 |
| 3227.7328303 | 2 |
| 3227.7331812 | 0 |
| 3227.7332136 | 4 |
| 3227.7343103 | 4 |
| 3227.7351865 | 2 |
| 3227.7354411 | 0 |
| 3227.7354657 | 3 |
| 3227.7357287 | 3 |
| 3227.7369907 | 3 |
| 3227.7376438 | 0 |
| 3227.7383578 | 1 |
| 3227.738402 | 4 |
| 3227.7393871 | 1 |
| 3227.7398862 | 4 |
| 3227.7400846 | 1 |
| 3227.7405733 | 1 |
| 3227.7412804 | 1 |
| 3227.7416846 | 3 |
| 3227.7422828 | 2 |
| 3227.7427645 | 2 |
| 3227.7430756 | 1 |
| 3227.7438506 | 5 |
| 3227.7444712 | 2 |
| 3227.7451156 | 1 |
| 3227.7455181 | 4 |
| 3227.7459145 | 1 |
| 3227.7462507 | 1 |
| 3227.7471947 | 0 |
| 3227.7476296 | 1 |
| 3227.7477123 | 1 |
| 3227.7491694 | 5 |
| 3227.749418 | 4 |
| 3227.7494487 | 1 |
| 3227.749698 | 2 |
| 3227.7509929 | 0 |
| 3227.7510674 | 0 |
| 3227.7516209 | 1 |
| 3227.7522608 | 3 |
| 3227.7530255 | 3 |
| 3227.7533251 | 1 |
| 3227.7541515 | 4 |
| 3227.7542758 | 2 |
| 3227.7546302 | 1 |
| 3227.7554178 | 1 |
| 3227.7554411 | 4 |
| 3227.7564354 | 2 |
| 3227.7569104 | 4 |
| 3227.7571793 | 0 |
| 3227.7577003 | 3 |
| 3227.758615 | 2 |
| 3227.7593363 | 1 |
| 3227.7593778 | 2 |
| 3227.7597608 | 1 |
| 3227.7603069 | 3 |
| 3227.7616083 | 1 |
| 3227.7617657 | 4 |
| 3227.7619359 | 1 |
| 3227.7631193 | 2 |
| 3227.7633826 | 1 |
| 3227.7637224 | 1 |
| 3227.7642237 | 4 |
| 3227.7651989 | 6 |
| 3227.765611 | 3 |

| | |
|---|---|
| 3227.7665619 | 1 |
| 3227.7665749 | 2 |
| 3227.7667847 | 1 |
| 3227.7681179 | 2 |
| 3227.7682049 | 1 |
| 3227.7687255 | 2 |
| 3227.7695469 | 4 |
| 3227.7700957 | 1 |
| 3227.7706112 | 0 |
| 3227.7708088 | 1 |
| 3227.7718389 | 2 |
| 3227.7721624 | 3 |
| 3227.7723554 | 2 |
| 3227.7726109 | 1 |
| 3227.7736255 | 1 |
| 3227.7740969 | 1 |
| 3227.7746067 | 3 |
| 3227.77499 | 1 |
| 3227.775872 | 1 |
| 3227.7766046 | 2 |
| 3227.7766148 | 1 |
| 3227.7769143 | 0 |
| 3227.7780328 | 2 |
| 3227.778145 | 2 |
| 3227.7790861 | 5 |
| 3227.7792355 | 3 |
| 3227.7800205 | 2 |
| 3227.7806076 | 6 |
| 3227.7806399 | 0 |
| 3227.7819501 | 1 |
| 3227.7820309 | 1 |
| 3227.7825443 | 1 |
| 3227.7827706 | 0 |
| 3227.7839064 | 1 |
| 3227.7843396 | 2 |
| 3227.7845303 | 2 |
| 3227.7853925 | 2 |
| 3227.785878 | 0 |
| 3227.7866923 | 1 |
| 3227.7868078 | 2 |
| 3227.7868365 | 1 |
| 3227.788591 | 2 |
| 3227.788814 | 0 |
| 3227.7889499 | 1 |
| 3227.7894399 | 5 |
| 3227.790495 | 2 |
| 3227.790739 | 0 |
| 3227.7910734 | 0 |
| 3227.7916654 | 2 |
| 3227.7923134 | 6 |
| 3227.792625 | 1 |
| 3227.7934105 | 1 |
| 3227.793555 | 2 |
| 3227.7940442 | 2 |
| 3227.7953705 | 3 |
| 3227.7957826 | 2 |
| 3227.7965627 | 6 |
| 3227.7965668 | 3 |
| 3227.7965963 | 1 |
| 3227.7976269 | 1 |
| 3227.7978051 | 3 |
| 3227.7982036 | 1 |
| 3227.7995684 | 2 |
| 3227.799623 | 0 |
| 3227.7998748 | 1 |
| 3227.8007842 | 6 |
| 3227.8013263 | 0 |
| 3227.8014074 | 1 |
| 3227.8025258 | 0 |
| 3227.8028107 | 0 |
| 3227.8030553 | 2 |
| 3227.8036522 | 0 |
| 3227.8044582 | 1 |
| 3227.8055153 | 0 |
| 3227.8057861 | 0 |
| 3227.8061452 | 3 |
| 3227.8062092 | 1 |
| 3227.8076533 | 2 |
| 3227.8076632 | 3 |
| 3227.8079542 | 3 |
| 3227.808749 | 1 |
| 3227.8095221 | 0 |
| 3227.8095498 | 1 |
| 3227.8105054 | 2 |
| 3227.8106547 | 3 |
| 3227.8114551 | 1 |
| 3227.8117304 | 0 |
| 3227.812257 | 2 |
| 3227.8127973 | 2 |
| 3227.8136277 | 3 |
| 3227.8138594 | 2 |
| 3227.8145885 | 0 |
| 3227.8146507 | 4 |
| 3227.8160513 | 1 |
| 3227.8165575 | 3 |
| 3227.816788 | 1 |
| 3227.8173523 | 0 |
| 3227.8177723 | 1 |
| 3227.8190125 | 0 |
| 3227.8193217 | 1 |
| 3227.8200638 | 3 |
| 3227.8202737 | 6 |
| 3227.8203166 | 1 |
| 3227.8210361 | 3 |
| 3227.8215197 | 1 |
| 3227.8218694 | 4 |
| 3227.8227389 | 0 |
| 3227.8232031 | 2 |
| 3227.8241435 | 1 |
| 3227.8247138 | 3 |
| 3227.8248214 | 4 |
| 3227.8253394 | 3 |
| 3227.8264355 | 0 |
| 3227.8266094 | 3 |
| 3227.8266372 | 4 |
| 3227.8273036 | 2 |
| 3227.8278146 | 0 |
| 3227.8283621 | 2 |
| 3227.8289515 | 1 |
| 3227.8292734 | 2 |
| 3227.8298964 | 4 |

| | |
|---|---|
| 3227.8308477 | 2 |
| 3227.8311959 | 1 |
| 3227.831229 | 3 |
| 3227.8319567 | 0 |
| 3227.8325304 | 2 |
| 3227.8336046 | 5 |
| 3227.8338629 | 3 |
| 3227.8346022 | 3 |
| 3227.8351383 | 3 |
| 3227.8352677 | 2 |
| 3227.8357796 | 4 |
| 3227.8364574 | 2 |
| 3227.8365306 | 1 |
| 3227.8377029 | 4 |
| 3227.8378902 | 3 |
| 3227.8385418 | 0 |
| 3227.8386051 | 0 |
| 3227.8395433 | 2 |
| 3227.8400262 | 6 |
| 3227.8410311 | 1 |
| 3227.8414949 | 4 |
| 3227.8421394 | 1 |
| 3227.8427668 | 1 |
| 3227.8431831 | 2 |
| 3227.843188 | 3 |
| 3227.8437114 | 1 |
| 3227.8447746 | 4 |
| 3227.8449848 | 1 |
| 3227.8452309 | 1 |
| 3227.8454749 | 1 |
| 3227.8468976 | 2 |
| 3227.8472917 | 0 |
| 3227.8473494 | 4 |
| 3227.8482212 | 5 |
| 3227.848422 | 1 |
| 3227.8498089 | 5 |
| 3227.8500501 | 1 |
| 3227.8507745 | 3 |
| 3227.8514276 | 1 |
| 3227.851619 | 3 |
| 3227.852012 | 2 |
| 3227.8524215 | 1 |
| 3227.8528743 | 4 |
| 3227.8532869 | 1 |
| 3227.8544851 | 2 |
| 3227.8546497 | 1 |
| 3227.8550697 | 2 |
| 3227.8561142 | 0 |
| 3227.856502 | 2 |
| 3227.8569667 | 1 |
| 3227.8576572 | 1 |
| 3227.8580651 | 2 |
| 3227.8582512 | 0 |
| 3227.8587338 | 0 |
| 3227.8597663 | 3 |
| 3227.859787 | 2 |
| 3227.8603781 | 1 |
| 3227.860591 | 1 |
| 3227.8617202 | 3 |
| 3227.8622089 | 4 |
| 3227.862499 | 5 |
| 3227.8635879 | 2 |
| 3227.8640429 | 2 |
| 3227.8643001 | 3 |
| 3227.8643954 | 1 |
| 3227.8653078 | 3 |
| 3227.8666071 | 2 |
| 3227.8667152 | 2 |
| 3227.8670906 | 2 |
| 3227.867204 | 1 |
| 3227.868623 | 1 |
| 3227.8687623 | 0 |
| 3227.8689163 | 1 |
| 3227.86944 | 0 |
| 3227.8703313 | 4 |
| 3227.8704107 | 1 |
| 3227.8714477 | 1 |
| 3227.8715654 | 3 |
| 3227.8727167 | 2 |
| 3227.8731476 | 0 |
| 3227.8732098 | 3 |
| 3227.8733669 | 5 |
| 3227.8741512 | 3 |
| 3227.8746625 | 3 |
| 3227.8761294 | 3 |
| 3227.8763575 | 2 |
| 3227.8764483 | 0 |
| 3227.8768994 | 3 |
| 3227.8775175 | 0 |
| 3227.8778647 | 3 |
| 3227.878501 | 3 |
| 3227.8789555 | 4 |
| 3227.8796991 | 1 |
| 3227.8798513 | 0 |
| 3227.8804227 | 2 |
| 3227.880999 | 4 |
| 3227.8820653 | 3 |
| 3227.8825069 | 2 |
| 3227.8825751 | 2 |
| 3227.8832653 | 1 |
| 3227.8841177 | 2 |
| 3227.8841664 | 0 |
| 3227.8845998 | 2 |
| 3227.8852326 | 5 |
| 3227.8858086 | 3 |
| 3227.8868457 | 1 |
| 3227.8871987 | 2 |
| 3227.8873446 | 3 |
| 3227.8881859 | 2 |
| 3227.8884095 | 3 |
| 3227.889068 | 1 |
| 3227.8896305 | 2 |
| 3227.8909038 | 1 |
| 3227.8912684 | 1 |
| 3227.8913029 | 2 |
| 3227.891649 | 2 |
| 3227.8922848 | 2 |
| 3227.892847 | 2 |
| 3227.8935342 | 4 |
| 3227.893625 | 0 |
| 3227.8945861 | 2 |
| 3227.8950528 | 1 |
| 3227.8951784 | 2 |
| 3227.8955979 | 0 |
| 3227.8972119 | 1 |
| 3227.8972853 | 4 |
| 3227.8973031 | 3 |
| 3227.8980876 | 3 |
| 3227.8986953 | 6 |
| 3227.8997871 | 1 |
| 3227.9000307 | 0 |
| 3227.9007543 | 2 |
| 3227.9012593 | 4 |
| 3227.9014039 | 0 |
| 3227.9024277 | 1 |
| 3227.9033275 | 1 |
| 3227.9035182 | 1 |
| 3227.9037784 | 1 |
| 3227.9044197 | 1 |
| 3227.9051829 | 2 |
| 3227.9051888 | 0 |
| 3227.905859 | 1 |
| 3227.906432 | 3 |
| 3227.9064524 | 2 |
| 3227.9072343 | 1 |
| 3227.9074161 | 2 |
| 3227.9085029 | 2 |
| 3227.9093822 | 1 |
| 3227.9098742 | 5 |
| 3227.9105902 | 1 |
| 3227.9107661 | 5 |
| 3227.911694 | 2 |
| 3227.9119163 | 0 |
| 3227.9122183 | 0 |
| 3227.912605 | 1 |
| 3227.9132155 | 5 |
| 3227.9143464 | 2 |
| 3227.9145118 | 2 |
| 3227.9146332 | 1 |
| 3227.9155297 | 7 |
| 3227.9162264 | 2 |
| 3227.9166597 | 3 |
| 3227.9170712 | 2 |
| 3227.91762 | 1 |
| 3227.9181916 | 1 |
| 3227.9188214 | 3 |
| 3227.919382 | 1 |
| 3227.9195211 | 2 |
| 3227.9206738 | 3 |
| 3227.9210555 | 2 |
| 3227.9212147 | 0 |
| 3227.9216395 | 2 |
| 3227.9219592 | 2 |
| 3227.9232881 | 1 |
| 3227.923908 | 2 |
| 3227.924324 | 1 |
| 3227.9249973 | 1 |
| 3227.9251809 | 1 |
| 3227.9257004 | 3 |
| 3227.9257566 | 2 |
| 3227.9266754 | 4 |
| 3227.9272409 | 2 |
| 3227.9279669 | 3 |
| 3227.9282361 | 2 |
| 3227.9285912 | 0 |
| 3227.9295036 | 4 |
| 3227.9298336 | 4 |
| 3227.9302067 | 1 |
| 3227.930776 | 0 |
| 3227.9315474 | 1 |
| 3227.931762 | 3 |
| 3227.932618 | 4 |
| 3227.9328595 | 3 |
| 3227.9330575 | 2 |
| 3227.9338958 | 0 |
| 3227.9350932 | 2 |
| 3227.9355072 | 2 |
| 3227.9355834 | 2 |
| 3227.935967 | 1 |
| 3227.9363093 | 1 |
| 3227.9374626 | 1 |
| 3227.9374803 | 1 |
| 3227.9375206 | 3 |
| 3227.9389599 | 3 |
| 3227.9393107 | 2 |
| 3227.9397613 | 4 |
| 3227.9400235 | 3 |
| 3227.9405974 | 4 |
| 3227.9414369 | 2 |
| 3227.9423264 | 4 |
| 3227.9428137 | 1 |
| 3227.9433198 | 5 |
| 3227.9435299 | 2 |
| 3227.9439841 | 5 |
| 3227.9443635 | 2 |
| 3227.9456328 | 3 |
| 3227.9457764 | 3 |
| 3227.9459019 | 3 |
| 3227.9469727 | 1 |
| 3227.9474518 | 1 |
| 3227.9475962 | 1 |
| 3227.9487074 | 0 |
| 3227.9493321 | 1 |
| 3227.9493824 | 5 |
| 3227.9502365 | 0 |
| 3227.9506202 | 1 |
| 3227.9515308 | 0 |
| 3227.952138 | 1 |
| 3227.9525122 | 2 |
| 3227.9527675 | 3 |
| 3227.9532516 | 1 |
| 3227.954115 | 2 |
| 3227.9543604 | 2 |
| 3227.9546597 | 5 |
| 3227.955277 | 3 |
| 3227.9562836 | 1 |
| 3227.9564811 | 2 |
| 3227.9570922 | 3 |
| 3227.9581052 | 2 |
| 3227.9582868 | 2 |
| 3227.9585145 | 4 |
| 3227.9587589 | 2 |
| 3227.9598825 | 0 |
| 3227.9603074 | 2 |
| 3227.9611166 | 5 |
| 3227.9612567 | 1 |
| 3227.9619578 | 3 |
| 3227.9627556 | 1 |
| 3227.9629087 | 0 |
| 3227.9635078 | 0 |
| 3227.9643821 | 0 |
| 3227.964474 | 2 |
| 3227.9650039 | 2 |
| 3227.9652758 | 4 |
| 3227.9661824 | 1 |
| 3227.9668051 | 4 |
| 3227.9671524 | 1 |
| 3227.9679799 | 0 |
| 3227.9680215 | 4 |
| 3227.9685291 | 2 |
| 3227.9690072 | 0 |
| 3227.9697242 | 1 |
| 3227.9700833 | 1 |
| 3227.9707655 | 1 |
| 3227.9712626 | 0 |
| 3227.9717749 | 4 |
| 3227.9723837 | 2 |
| 3227.973359 | 6 |
| 3227.9735917 | 1 |
| 3227.9738923 | 5 |
| 3227.9746248 | 1 |
| 3227.9752701 | 4 |
| 3227.9757502 | 3 |
| 3227.9761591 | 3 |
| 3227.9769382 | 1 |
| 3227.9771406 | 3 |
| 3227.9778168 | 2 |
| 3227.9782242 | 2 |
| 3227.9792296 | 4 |
| 3227.9794042 | 0 |
| 3227.9795432 | 1 |
| 3227.9796844 | 0 |
| 3227.9808525 | 2 |
| 3227.9814053 | 1 |
| 3227.9814624 | 0 |
| 3227.9824746 | 0 |
| 3227.9829109 | 2 |
| 3227.9837385 | 1 |
| 3227.9842939 | 3 |
| 3227.9851149 | 3 |
| 3227.9851379 | 3 |
| 3227.9860448 | 0 |
| 3227.9862336 | 5 |
| 3227.9867828 | 0 |
| 3227.9873244 | 0 |
| 3227.9880226 | 1 |
| 3227.9885794 | 0 |
| 3227.9892504 | 1 |
| 3227.9896187 | 3 |
| 3227.9896343 | 3 |
| 3227.990981 | 1 |
| 3227.9911124 | 1 |
| 3227.9914779 | 0 |
| 3227.992344 | 2 |
| 3227.992446 | 1 |
| 3227.9936414 | 3 |
| 3227.9939466 | 5 |
| 3227.9942936 | 5 |
| 3227.994854 | 1 |
| 3227.9952394 | 2 |
| 3227.9962211 | 1 |
| 3227.9969578 | 0 |
| 3227.9969969 | 0 |
| 3227.9982971 | 1 |
| 3227.9984962 | 3 |
| 3227.9985038 | 3 |
| 3227.9988775 | 1 |
| 3228.0000003 | 0 |
| 3228.0004926 | 1 |
| 3228.0009519 | 2 |
| 3228.0013726 | 2 |
| 3228.0021475 | 2 |
| 3228.0022896 | 1 |
| 3228.0032181 | 2 |
| 3228.0035914 | 2 |
| 3228.004364 | 0 |
| 3228.0045606 | 2 |
| 3228.0050617 | 2 |
| 3228.0051144 | 1 |
| 3228.0064881 | 1 |
| 3228.0067278 | 3 |
| 3228.0070647 | 2 |
| 3228.007571 | 2 |
| 3228.0081015 | 3 |
| 3228.0086138 | 1 |
| 3228.0092127 | 1 |
| 3228.0097198 | 3 |
| 3228.0105764 | 1 |
| 3228.0108648 | 1 |
| 3228.0116792 | 2 |
| 3228.0121212 | 1 |
| 3228.0126308 | 3 |
| 3228.0135063 | 3 |
| 3228.0140229 | 4 |
| 3228.0142575 | 1 |
| 3228.0143009 | 3 |
| 3228.0155899 | 2 |
| 3228.0156595 | 1 |
| 3228.0158918 | 3 |
| 3228.0167644 | 0 |
| 3228.0170192 | 2 |
| 3228.0175495 | 2 |
| 3228.0185836 | 1 |
| 3228.0186238 | 3 |
| 3228.0194243 | 1 |
| 3228.0199304 | 4 |
| 3228.020286 | 2 |
| 3228.020951 | 3 |
| 3228.0213116 | 1 |
| 3228.0225402 | 2 |
| 3228.0233329 | 0 |
| 3228.0235224 | 5 |
| 3228.0237059 | 0 |

| | |
|---|---|
| 3228.0243198 | 1 |
| 3228.025405 | 2 |
| 3228.0254098 | 2 |
| 3228.0256553 | 2 |
| 3228.0260985 | 2 |
| 3228.0262921 | 2 |
| 3228.0276491 | 2 |
| 3228.0282035 | 0 |
| 3228.0282793 | 3 |
| 3228.0292907 | 1 |
| 3228.0294401 | 0 |
| 3228.0301714 | 2 |
| 3228.0306006 | 1 |
| 3228.0307209 | 2 |
| 3228.0317054 | 3 |
| 3228.0322748 | 1 |
| 3228.0326507 | 4 |
| 3228.0328572 | 2 |
| 3228.0339613 | 1 |
| 3228.0344425 | 3 |
| 3228.034479 | 1 |
| 3228.0351362 | 3 |
| 3228.0360455 | 2 |
| 3228.0360932 | 3 |
| 3228.0371742 | 2 |
| 3228.037439 | 1 |
| 3228.0378483 | 1 |
| 3228.0386477 | 0 |
| 3228.0389966 | 3 |
| 3228.0393761 | 2 |
| 3228.0396154 | 6 |
| 3228.0406279 | 0 |
| 3228.0410692 | 6 |
| 3228.0413237 | 2 |
| 3228.0423484 | 2 |
| 3228.0428402 | 2 |
| 3228.0438407 | 1 |
| 3228.0441122 | 2 |
| 3228.0441217 | 2 |
| 3228.0452988 | 0 |
| 3228.0457693 | 1 |
| 3228.0463096 | 2 |
| 3228.046314 | 2 |
| 3228.0475104 | 3 |
| 3228.0483619 | 2 |
| 3228.048559 | 2 |
| 3228.0491322 | 4 |
| 3228.049375 | 1 |
| 3228.0498752 | 2 |
| 3228.0501102 | 4 |
| 3228.0502834 | 2 |
| 3228.0514334 | 0 |
| 3228.0516588 | 4 |
| 3228.0528747 | 0 |
| 3228.0530693 | 4 |
| 3228.0536117 | 2 |
| 3228.0537647 | 2 |
| 3228.0537862 | 1 |
| 3228.0554587 | 0 |
| 3228.0555738 | 3 |
| 3228.0558926 | 3 |
| 3228.057456 | 1 |
| 3228.057554 | 2 |
| 3228.0576789 | 3 |
| 3228.0588497 | 2 |
| 3228.0593585 | 5 |
| 3228.0593826 | 4 |
| 3228.0594743 | 0 |
| 3228.060954 | 2 |
| 3228.0610193 | 3 |
| 3228.0618188 | 2 |
| 3228.0619231 | 3 |
| 3228.062069 | 2 |
| 3228.0631085 | 4 |
| 3228.063559 | 1 |
| 3228.064161 | 1 |
| 3228.0651833 | 2 |
| 3228.0653315 | 2 |
| 3228.0661484 | 2 |
| 3228.0665472 | 3 |
| 3228.0666775 | 2 |
| 3228.0668534 | 2 |
| 3228.0683327 | 3 |
| 3228.0683976 | 3 |
| 3228.0685196 | 3 |
| 3228.0691048 | 1 |
| 3228.0698678 | 3 |
| 3228.070088 | 0 |
| 3228.0714298 | 0 |
| 3228.0716521 | 0 |
| 3228.0718108 | 1 |
| 3228.0725609 | 0 |
| 3228.0733739 | 0 |
| 3228.0735542 | 1 |
| 3228.0741811 | 7 |
| 3228.0742808 | 0 |
| 3228.0748769 | 1 |
| 3228.0757971 | 1 |
| 3228.0765651 | 1 |
| 3228.0767489 | 1 |
| 3228.0775605 | 5 |
| 3228.0781579 | 3 |
| 3228.0787658 | 1 |
| 3228.0788119 | 0 |
| 3228.0793024 | 0 |
| 3228.080746 | 1 |
| 3228.080783 | 1 |
| 3228.0808045 | 3 |
| 3228.0814109 | 1 |
| 3228.0825041 | 2 |
| 3228.0827402 | 2 |
| 3228.0831785 | 3 |
| 3228.08342 | 3 |
| 3228.0839859 | 0 |
| 3228.0854493 | 2 |
| 3228.0857329 | 3 |
| 3228.085872 | 1 |
| 3228.0869967 | 2 |
| 3228.0871641 | 1 |
| 3228.0880819 | 5 |
| 3228.0882865 | 0 |
| 3228.0889508 | 4 |
| 3228.089414 | 4 |
| 3228.0904955 | 3 |
| 3228.0905441 | 3 |
| 3228.0908156 | 2 |
| 3228.0915241 | 2 |
| 3228.0920333 | 0 |
| 3228.0928606 | 4 |
| 3228.0932918 | 0 |
| 3228.0938835 | 2 |
| 3228.0943652 | 2 |
| 3228.0949871 | 3 |
| 3228.0952242 | 2 |
| 3228.0958134 | 1 |
| 3228.0959251 | 1 |
| 3228.0963793 | 1 |
| 3228.0969125 | 1 |
| 3228.0980882 | 3 |
| 3228.0982537 | 1 |
| 3228.0986812 | 3 |
| 3228.0991071 | 2 |
| 3228.1001515 | 2 |
| 3228.1006677 | 2 |
| 3228.1012104 | 2 |
| 3228.10171 | 2 |
| 3228.102319 | 4 |
| 3228.1024226 | 1 |
| 3228.1030038 | 1 |
| 3228.1033294 | 1 |
| 3228.1041405 | 0 |
| 3228.1046741 | 1 |
| 3228.1049859 | 3 |
| 3228.1051701 | 1 |
| 3228.106197 | 0 |
| 3228.106994 | 3 |
| 3228.1070719 | 2 |
| 3228.1082603 | 2 |
| 3228.1088033 | 5 |
| 3228.1088282 | 3 |
| 3228.1094414 | 3 |
| 3228.1101076 | 1 |
| 3228.1107514 | 1 |
| 3228.1108737 | 2 |
| 3228.1116454 | 1 |
| 3228.1124846 | 1 |
| 3228.112903 | 1 |
| 3228.1130249 | 2 |
| 3228.1136756 | 0 |
| 3228.1143721 | 0 |
| 3228.1143953 | 1 |
| 3228.1152295 | 4 |
| 3228.1153938 | 1 |
| 3228.1162555 | 1 |
| 3228.1171433 | 5 |
| 3228.1172631 | 1 |
| 3228.117497 | 3 |
| 3228.1187886 | 3 |
| 3228.1195622 | 0 |
| 3228.1199701 | 2 |
| 3228.1200983 | 2 |
| 3228.1206043 | 1 |
| 3228.1211525 | 1 |
| 3228.1216001 | 1 |
| 3228.1224884 | 1 |
| 3228.1231945 | 2 |
| 3228.1234602 | 0 |
| 3228.1238016 | 0 |
| 3228.12429 | 0 |
| 3228.1250872 | 1 |
| 3228.1253697 | 3 |
| 3228.1260405 | 2 |
| 3228.126518 | 0 |
| 3228.1270548 | 1 |
| 3228.1277507 | 2 |
| 3228.1282822 | 1 |
| 3228.129278 | 3 |
| 3228.1294967 | 3 |
| 3228.1297889 | 0 |
| 3228.1304913 | 1 |
| 3228.1308723 | 4 |
| 3228.1319133 | 0 |
| 3228.1320014 | 1 |
| 3228.1325371 | 2 |
| 3228.132542 | 2 |
| 3228.1337476 | 0 |
| 3228.1337694 | 0 |
| 3228.1337902 | 4 |
| 3228.1348505 | 4 |
| 3228.1354467 | 2 |
| 3228.136024 | 1 |
| 3228.1366229 | 3 |
| 3228.136983 | 3 |
| 3228.137706 | 2 |
| 3228.1384097 | 0 |
| 3228.1384222 | 1 |
| 3228.1395904 | 1 |
| 3228.1396398 | 0 |
| 3228.1406719 | 3 |
| 3228.1410233 | 2 |
| 3228.1414681 | 0 |
| 3228.1417721 | 2 |
| 3228.1424364 | 1 |
| 3228.1431179 | 1 |
| 3228.143287 | 1 |
| 3228.1445734 | 2 |
| 3228.1450419 | 1 |
| 3228.1452585 | 2 |
| 3228.1452671 | 3 |
| 3228.14556 | 0 |
| 3228.1470315 | 0 |
| 3228.1475594 | 1 |
| 3228.1476052 | 2 |
| 3228.1487836 | 2 |
| 3228.1490067 | 5 |
| 3228.1490498 | 1 |
| 3228.1501093 | 0 |
| 3228.150173 | 0 |
| 3228.1505845 | 2 |
| 3228.1512781 | 1 |
| 3228.1515709 | 2 |
| 3228.1525012 | 2 |
| 3228.1529232 | 0 |
| 3228.1529774 | 2 |
| 3228.153987 | 2 |
| 3228.1540107 | 2 |
| 3228.1555484 | 1 |
| 3228.1557015 | 5 |
| 3228.1561019 | 0 |
| 3228.1571808 | 3 |
| 3228.1574106 | 3 |
| 3228.1579859 | 4 |
| 3228.1582888 | 1 |
| 3228.1590386 | 3 |
| 3228.1593555 | 0 |
| 3228.1604853 | 2 |
| 3228.1604865 | 4 |
| 3228.1610879 | 2 |
| 3228.1616338 | 2 |
| 3228.1620828 | 7 |
| 3228.1629805 | 2 |
| 3228.1629893 | 1 |
| 3228.1640616 | 1 |
| 3228.1641107 | 1 |
| 3228.1648244 | 1 |
| 3228.1653984 | 1 |
| 3228.1658841 | 1 |
| 3228.1661488 | 3 |
| 3228.1665505 | 1 |
| 3228.1671408 | 2 |
| 3228.1679386 | 2 |
| 3228.167968 | 2 |
| 3228.1687195 | 4 |
| 3228.1694464 | 0 |
| 3228.1702624 | 0 |
| 3228.1705763 | 2 |
| 3228.1708405 | 4 |
| 3228.1721023 | 3 |
| 3228.1722171 | 0 |
| 3228.1724605 | 1 |
| 3228.1734918 | 1 |
| 3228.1741745 | 0 |
| 3228.174191 | 0 |
| 3228.1750975 | 3 |
| 3228.1758718 | 2 |
| 3228.1762993 | 2 |
| 3228.1765993 | 6 |
| 3228.1768459 | 5 |
| 3228.177689 | 3 |
| 3228.1783251 | 1 |
| 3228.1783279 | 5 |
| 3228.178727 | 1 |
| 3228.1799829 | 1 |
| 3228.1804424 | 3 |
| 3228.1805693 | 2 |
| 3228.1811257 | 2 |
| 3228.1815264 | 3 |
| 3228.1825177 | 2 |
| 3228.1831267 | 1 |
| 3228.1834727 | 2 |
| 3228.1839958 | 2 |
| 3228.184589 | 0 |
| 3228.1849782 | 6 |
| 3228.1851402 | 2 |
| 3228.1864133 | 4 |
| 3228.1872687 | 1 |
| 3228.1873153 | 2 |
| 3228.1875658 | 1 |
| 3228.187839 | 3 |
| 3228.1886272 | 0 |
| 3228.1888371 | 1 |
| 3228.1894066 | 5 |
| 3228.1901678 | 3 |
| 3228.190318 | 1 |
| 3228.1912477 | 1 |
| 3228.1918842 | 4 |
| 3228.1918938 | 1 |
| 3228.1930267 | 0 |
| 3228.1933438 | 1 |
| 3228.1935655 | 2 |
| 3228.1942062 | 3 |
| 3228.1943503 | 1 |
| 3228.1955027 | 3 |
| 3228.1957938 | 3 |
| 3228.1960586 | 0 |
| 3228.1964443 | 1 |
| 3228.1967214 | 3 |
| 3228.1971108 | 2 |
| 3228.1973578 | 2 |
| 3228.1982633 | 3 |
| 3228.1983408 | 3 |
| 3228.1984279 | 1 |
| 3228.1997899 | 3 |
| 3228.2001128 | 4 |
| 3228.2001286 | 0 |
| 3228.2005455 | 3 |
| 3228.2014045 | 1 |
| 3228.2019279 | 6 |
| 3228.2025409 | 2 |
| 3228.2029367 | 3 |
| 3228.2034554 | 1 |
| 3228.2044925 | 0 |
| 3228.2048601 | 1 |
| 3228.2048637 | 2 |
| 3228.2056515 | 3 |
| 3228.2060286 | 2 |
| 3228.2066773 | 3 |
| 3228.2075759 | 1 |
| 3228.2079574 | 3 |
| 3228.2081162 | 2 |
| 3228.2089537 | 2 |
| 3228.2090777 | 3 |
| 3228.2099436 | 4 |
| 3228.210474 | 4 |
| 3228.2111159 | 3 |
| 3228.2118986 | 1 |
| 3228.2122563 | 1 |
| 3228.212519 | 3 |
| 3228.2134071 | 2 |
| 3228.2140615 | 0 |
| 3228.2143501 | 0 |
| 3228.2147855 | 4 |
| 3228.2151402 | 1 |

| | |
|---|---|
| 3228.2152152 | 2 |
| 3228.2162935 | 1 |
| 3228.2165003 | 2 |
| 3228.2178432 | 1 |
| 3228.2178811 | 2 |
| 3228.2186052 | 1 |
| 3228.2187738 | 3 |
| 3228.2196899 | 0 |
| 3228.2204342 | 5 |
| 3228.2209187 | 2 |
| 3228.2212478 | 3 |
| 3228.2217514 | 2 |
| 3228.2223744 | 2 |
| 3228.2228524 | 1 |
| 3228.2235862 | 1 |
| 3228.224051 | 2 |
| 3228.2245546 | 1 |
| 3228.2252912 | 2 |
| 3228.2253578 | 3 |
| 3228.2267692 | 1 |
| 3228.2267776 | 0 |
| 3228.2269187 | 3 |
| 3228.2278053 | 1 |
| 3228.228116 | 2 |
| 3228.2284515 | 3 |
| 3228.2284559 | 4 |
| 3228.2301239 | 0 |
| 3228.2310292 | 1 |
| 3228.2312068 | 3 |
| 3228.2315541 | 3 |
| 3228.2316661 | 0 |
| 3228.2325709 | 1 |
| 3228.2327873 | 1 |
| 3228.2330009 | 2 |
| 3228.2343848 | 1 |
| 3228.2344589 | 1 |
| 3228.234974 | 3 |
| 3228.2357164 | 1 |
| 3228.2362026 | 4 |
| 3228.236253 | 2 |
| 3228.2375238 | 1 |
| 3228.2379199 | 2 |
| 3228.2382571 | 1 |
| 3228.2383619 | 0 |
| 3228.2396066 | 3 |
| 3228.2400279 | 0 |
| 3228.2401542 | 3 |
| 3228.2403196 | 2 |
| 3228.2419438 | 5 |
| 3228.2419774 | 0 |
| 3228.2420016 | 1 |
| 3228.2425633 | 3 |
| 3228.2439733 | 1 |
| 3228.2441538 | 2 |
| 3228.2450653 | 3 |
| 3228.2459839 | 3 |
| 3228.2462358 | 2 |
| 3228.2466111 | 4 |
| 3228.2467784 | 1 |
| 3228.2471975 | 2 |
| 3228.247548 | 2 |
| 3228.2484665 | 1 |
| 3228.248951 | 1 |
| 3228.2496979 | 1 |
| 3228.2497857 | 2 |
| 3228.250976 | 2 |
| 3228.2510917 | 4 |
| 3228.2511499 | 3 |
| 3228.251784 | 2 |
| 3228.253137 | 2 |
| 3228.2532476 | 0 |
| 3228.2533757 | 2 |
| 3228.254513 | 0 |
| 3228.2550109 | 0 |
| 3228.2557299 | 0 |
| 3228.2559772 | 1 |
| 3228.2560207 | 4 |
| 3228.2567178 | 2 |
| 3228.2577688 | 1 |
| 3228.2580981 | 1 |
| 3228.2583208 | 1 |
| 3228.2598233 | 3 |
| 3228.2600752 | 1 |
| 3228.2604796 | 3 |
| 3228.2611089 | 1 |
| 3228.2613741 | 1 |
| 3228.2615851 | 3 |
| 3228.2626743 | 3 |
| 3228.263153 | 2 |
| 3228.2635295 | 4 |
| 3228.2646151 | 1 |
| 3228.2646758 | 0 |
| 3228.2648895 | 2 |
| 3228.2659546 | 1 |
| 3228.2660376 | 6 |
| 3228.2666152 | 0 |
| 3228.2671577 | 3 |
| 3228.2674018 | 5 |
| 3228.2677844 | 1 |
| 3228.2687697 | 3 |
| 3228.2693572 | 2 |
| 3228.2702496 | 2 |
| 3228.2704662 | 1 |
| 3228.2713194 | 1 |
| 3228.2721546 | 2 |
| 3228.2735338 | 4 |
| 3228.2738949 | 3 |
| 3228.2755569 | 1 |
| 3228.2764459 | 3 |
| 3228.2764924 | 1 |
| 3228.2784989 | 3 |
| 3228.278792 | 1 |
| 3228.2794417 | 1 |
| 3228.2809934 | 2 |
| 3228.2814631 | 1 |
| 3228.2827352 | 3 |
| 3228.2835103 | 5 |
| 3228.2840559 | 2 |
| 3228.2851478 | 1 |
| 3228.2861585 | 1 |
| 3228.2871392 | 2 |
| 3228.2880949 | 1 |
| 3228.2887587 | 0 |
| 3228.2895598 | 0 |
| 3228.2906121 | 0 |
| 3228.2914563 | 0 |
| 3228.2928752 | 6 |
| 3228.2930094 | 2 |
| 3228.2951267 | 5 |
| 3228.2953896 | 5 |
| 3228.2966456 | 1 |
| 3228.2971835 | 4 |
| 3228.2981701 | 0 |
| 3228.2996618 | 2 |
| 3228.300298 | 1 |
| 3228.3009702 | 1 |
| 3228.3024328 | 0 |
| 3228.3029467 | 0 |
| 3228.3046124 | 2 |
| 3228.3051545 | 1 |
| 3228.3059251 | 1 |
| 3228.3066418 | 1 |
| 3228.3082361 | 1 |
| 3228.3094448 | 1 |
| 3228.3094947 | 3 |
| 3228.3110469 | 2 |
| 3228.3122982 | 2 |
| 3228.3126363 | 4 |
| 3228.3140177 | 4 |
| 3228.3147008 | 2 |
| 3228.3152236 | 1 |
| 3228.3161858 | 2 |
| 3228.3174463 | 1 |
| 3228.3181295 | 1 |
| 3228.3195497 | 0 |
| 3228.3201445 | 1 |
| 3228.3211279 | 1 |
| 3228.3224488 | 0 |
| 3228.3234794 | 2 |
| 3228.3235049 | 2 |
| 3228.3251847 | 1 |
| 3228.3253736 | 0 |
| 3228.3261839 | 1 |
| 3228.3275783 | 0 |
| 3228.3286789 | 0 |
| 3228.3292796 | 3 |
| 3228.3305933 | 2 |
| 3228.3317178 | 4 |
| 3228.3319185 | 2 |
| 3228.3332325 | 2 |
| 3228.3338856 | 2 |
| 3228.3344084 | 4 |
| 3228.3354891 | 3 |
| 3228.3371669 | 1 |
| 3228.3372932 | 2 |
| 3228.3385594 | 0 |
| 3228.338717 | 3 |
| 3228.340664 | 2 |
| 3228.3412807 | 2 |
| 3228.342687 | 3 |
| 3228.3430855 | 2 |
| 3228.3438262 | 2 |
| 3228.3446282 | 0 |
| 3228.3467062 | 3 |
| 3228.3472373 | 2 |
| 3228.3484949 | 2 |
| 3228.3485298 | 0 |
| 3228.3499992 | 0 |
| 3228.350798 | 3 |
| 3228.3515243 | 2 |
| 3228.352837 | 5 |
| 3228.3531584 | 0 |
| 3228.3547484 | 1 |
| 3228.3554523 | 3 |
| 3228.3567564 | 1 |
| 3228.3578246 | 2 |
| 3228.3581392 | 1 |
| 3228.3591138 | 1 |
| 3228.3597303 | 1 |
| 3228.3612241 | 2 |
| 3228.3625252 | 3 |
| 3228.363129 | 7 |
| 3228.3645627 | 2 |
| 3228.3648644 | 1 |
| 3228.3660046 | 2 |
| 3228.3671671 | 0 |
| 3228.3676517 | 1 |
| 3228.3692768 | 1 |
| 3228.3693203 | 0 |
| 3228.3710396 | 0 |
| 3228.3713183 | 3 |
| 3228.3721083 | 3 |
| 3228.3729268 | 3 |
| 3228.3732862 | 2 |
| 3228.3735578 | 2 |
| 3228.3735931 | 5 |
| 3228.3735971 | 0 |
| 3228.3737937 | 4 |
| 3228.3738737 | 3 |
| 3228.3740097 | 1 |
| 3228.3743686 | 0 |
| 3228.3743694 | 3 |
| 3228.3745882 | 4 |
| 3228.3746433 | 1 |
| 3228.3747107 | 3 |
| 3228.3747437 | 3 |
| 3228.3748755 | 1 |
| 3228.3749093 | 2 |
| 3228.375435 | 2 |
| 3228.375463 | 2 |
| 3228.3756622 | 2 |
| 3228.375795 | 1 |
| 3228.3758945 | 0 |
| 3228.3762467 | 4 |
| 3228.3766378 | 3 |
| 3228.3766483 | 0 |
| 3228.3766742 | 2 |
| 3228.3769048 | 2 |
| 3228.3770329 | 2 |
| 3228.3773409 | 2 |
| 3228.3777155 | 4 |
| 3228.3777568 | 1 |
| 3228.3779678 | 1 |
| 3228.3780013 | 0 |
| 3228.3784329 | 2 |
| 3228.3784451 | 4 |
| 3228.3785233 | 4 |
| 3228.3792293 | 2 |
| 3228.3793258 | 1 |
| 3228.3795213 | 5 |
| 3228.3796994 | 0 |
| 3228.3797499 | 2 |
| 3228.3797677 | 2 |
| 3228.3799621 | 1 |
| 3228.3799898 | 0 |
| 3228.3803614 | 1 |
| 3228.3803781 | 2 |
| 3228.3808138 | 2 |
| 3228.3810052 | 2 |
| 3228.3810119 | 2 |
| 3228.3814499 | 4 |
| 3228.3814873 | 2 |
| 3228.3815516 | 1 |
| 3228.3818163 | 2 |
| 3228.3823939 | 3 |
| 3228.3825532 | 4 |
| 3228.3828062 | 2 |
| 3228.3829692 | 3 |
| 3228.3830592 | 0 |
| 3228.3830675 | 1 |
| 3228.3833005 | 0 |
| 3228.3833985 | 3 |
| 3228.3834022 | 2 |
| 3228.3836391 | 2 |
| 3228.3840981 | 4 |
| 3228.3842698 | 3 |
| 3228.3844099 | 3 |
| 3228.3845524 | 5 |
| 3228.3849571 | 1 |
| 3228.3850337 | 1 |
| 3228.3854124 | 0 |
| 3228.3856386 | 1 |
| 3228.3858038 | 0 |
| 3228.3858827 | 1 |
| 3228.3860482 | 2 |
| 3228.3863226 | 1 |
| 3228.3865397 | 4 |
| 3228.3865723 | 2 |
| 3228.386955 | 2 |
| 3228.3869557 | 0 |
| 3228.3872435 | 2 |
| 3228.3875997 | 1 |
| 3228.3877321 | 4 |
| 3228.3878008 | 2 |
| 3228.3879707 | 1 |
| 3228.3880068 | 3 |
| 3228.3881006 | 2 |
| 3228.3883583 | 0 |
| 3228.3884326 | 2 |
| 3228.3887549 | 0 |
| 3228.3888313 | 0 |
| 3228.3891596 | 2 |
| 3228.3893063 | 2 |
| 3228.3894448 | 0 |
| 3228.3895426 | 1 |
| 3228.3896347 | 1 |
| 3228.3900567 | 5 |
| 3228.3900752 | 4 |
| 3228.3900962 | 4 |
| 3228.3904433 | 2 |
| 3228.3906614 | 0 |
| 3228.3907221 | 1 |
| 3228.3907633 | 0 |
| 3228.3908707 | 0 |
| 3228.3910037 | 1 |
| 3228.3910518 | 1 |
| 3228.3910522 | 3 |
| 3228.391581 | 1 |
| 3228.3915866 | 2 |
| 3228.3915996 | 0 |
| 3228.3919053 | 3 |
| 3228.3919955 | 0 |
| 3228.3920805 | 5 |
| 3228.3923209 | 2 |
| 3228.3926556 | 2 |
| 3228.3926968 | 0 |
| 3228.3930288 | 1 |
| 3228.3930892 | 0 |
| 3228.3931119 | 0 |
| 3228.3932071 | 0 |
| 3228.3932112 | 1 |
| 3228.3935155 | 2 |
| 3228.3935885 | 2 |
| 3228.3938073 | 3 |
| 3228.3940018 | 1 |
| 3228.3940322 | 5 |
| 3228.3940513 | 4 |
| 3228.3941403 | 5 |
| 3228.394175 | 0 |
| 3228.3942274 | 1 |
| 3228.3945384 | 2 |
| 3228.3947922 | 5 |
| 3228.3949834 | 3 |
| 3228.3951128 | 3 |
| 3228.3952451 | 5 |
| 3228.395409 | 5 |
| 3228.3955454 | 3 |
| 3228.3956862 | 3 |
| 3228.3959129 | 8 |
| 3228.3960494 | 4 |
| 3228.3962271 | 2 |
| 3228.3962628 | 7 |
| 3228.3963177 | 8 |
| 3228.3964331 | 10 |
| 3228.3965896 | 9 |
| 3228.396595 | 15 |
| 3228.397022 | 12 |
| 3228.3972259 | 14 |
| 3228.397227 | 17 |
| 3228.3974801 | 13 |
| 3228.39753 | 15 |
| 3228.397606 | 12 |
| 3228.3976378 | 11 |

| | |
|---|---|
| 3228.3979026 | 17 |
| 3228.397953 | 16 |
| 3228.3979819 | 15 |
| 3228.3981082 | 17 |
| 3228.3981679 | 18 |
| 3228.398285 | 17 |
| 3228.3985235 | 6 |
| 3228.3986236 | 14 |
| 3228.3986563 | 10 |
| 3228.3988054 | 14 |
| 3228.3991577 | 13 |
| 3228.3993937 | 5 |
| 3228.3996504 | 6 |
| 3228.3996812 | 5 |
| 3228.3998624 | 7 |
| 3228.3999805 | 8 |
| 3228.4001847 | 7 |
| 3228.4002777 | 3 |
| 3228.4003804 | 10 |
| 3228.4006043 | 5 |
| 3228.4006179 | 8 |
| 3228.401111 | 8 |
| 3228.401228 | 8 |
| 3228.4012538 | 5 |
| 3228.4013865 | 7 |
| 3228.4017056 | 11 |
| 3228.4017092 | 8 |
| 3228.4017478 | 10 |
| 3228.4017737 | 13 |
| 3228.4020292 | 16 |
| 3228.4021418 | 7 |
| 3228.4023275 | 6 |
| 3228.4024192 | 4 |
| 3228.4024335 | 10 |
| 3228.4025991 | 10 |
| 3228.4026679 | 14 |
| 3228.4028192 | 19 |
| 3228.4029903 | 10 |
| 3228.4030505 | 14 |
| 3228.4031907 | 9 |
| 3228.4032258 | 17 |
| 3228.4036803 | 18 |
| 3228.4038447 | 8 |
| 3228.4039163 | 8 |
| 3228.4039254 | 7 |
| 3228.403938 | 8 |
| 3228.4041721 | 14 |
| 3228.4044028 | 13 |
| 3228.4044043 | 14 |
| 3228.4047304 | 11 |
| 3228.4047464 | 16 |
| 3228.405102 | 7 |
| 3228.4052471 | 12 |
| 3228.4055572 | 10 |
| 3228.4056922 | 10 |
| 3228.4057673 | 13 |
| 3228.4057763 | 13 |
| 3228.4062247 | 11 |
| 3228.4062334 | 11 |
| 3228.4063306 | 13 |
| 3228.4063592 | 6 |
| 3228.4064269 | 14 |
| 3228.4065064 | 8 |
| 3228.4066284 | 7 |
| 3228.4067559 | 5 |
| 3228.4071436 | 4 |
| 3228.4071887 | 12 |
| 3228.4073285 | 8 |
| 3228.4073397 | 10 |
| 3228.4074106 | 7 |
| 3228.4075011 | 10 |
| 3228.4076484 | 6 |
| 3228.4076766 | 5 |
| 3228.4079118 | 13 |
| 3228.4079642 | 0 |
| 3228.4081291 | 4 |
| 3228.4081367 | 3 |
| 3228.4084365 | 2 |
| 3228.4084375 | 4 |
| 3228.4086808 | 1 |
| 3228.4087095 | 2 |
| 3228.4087885 | 2 |
| 3228.4088191 | 2 |
| 3228.4090718 | 2 |
| 3228.4091808 | 0 |
| 3228.4093463 | 3 |
| 3228.409502 | 1 |
| 3228.4096439 | 2 |
| 3228.4098987 | 0 |
| 3228.4100231 | 1 |
| 3228.4102587 | 3 |
| 3228.4103034 | 2 |
| 3228.4104891 | 5 |
| 3228.4105684 | 2 |
| 3228.4105866 | 2 |
| 3228.4106201 | 2 |
| 3228.4108121 | 0 |
| 3228.4109278 | 2 |
| 3228.4111941 | 0 |
| 3228.4112738 | 0 |
| 3228.4115976 | 3 |
| 3228.4116446 | 1 |
| 3228.4118264 | 1 |
| 3228.411855 | 1 |
| 3228.4119903 | 3 |
| 3228.4120558 | 1 |
| 3228.4122295 | 3 |
| 3228.4122522 | 2 |
| 3228.4123411 | 5 |
| 3228.4123507 | 2 |
| 3228.4124395 | 1 |
| 3228.4124843 | 4 |
| 3228.4127978 | 2 |
| 3228.4132238 | 2 |
| 3228.4133449 | 3 |
| 3228.4133739 | 0 |
| 3228.4134312 | 3 |
| 3228.4135796 | 1 |
| 3228.4136949 | 1 |
| 3228.4142809 | 2 |
| 3228.4150768 | 2 |
| 3228.4160243 | 1 |
| 3228.4162656 | 4 |
| 3228.4171266 | 5 |
| 3228.4183339 | 1 |
| 3228.4186834 | 4 |
| 3228.4199531 | 1 |
| 3228.4208199 | 2 |
| 3228.4216263 | 0 |
| 3228.4226998 | 4 |
| 3228.4239398 | 3 |
| 3228.4247043 | 2 |
| 3228.4259151 | 5 |
| 3228.4262313 | 2 |
| 3228.427642 | 0 |
| 3228.4287387 | 3 |
| 3228.429053 | 1 |
| 3228.4308137 | 3 |
| 3228.4311192 | 2 |
| 3228.4322388 | 1 |
| 3228.4337375 | 3 |
| 3228.4339469 | 2 |
| 3228.4352344 | 1 |
| 3228.4357029 | 1 |
| 3228.4370115 | 0 |
| 3228.4382412 | 0 |
| 3228.4386574 | 1 |
| 3228.4405104 | 0 |
| 3228.4406221 | 0 |
| 3228.4418365 | 3 |
| 3228.4426572 | 0 |
| 3228.4438467 | 5 |
| 3228.4446766 | 2 |
| 3228.4454712 | 2 |
| 3228.4468075 | 2 |
| 3228.447599 | 2 |
| 3228.4488291 | 3 |
| 3228.4497835 | 3 |
| 3228.4506942 | 3 |
| 3228.4514715 | 0 |
| 3228.4515947 | 3 |
| 3228.4530413 | 1 |
| 3228.4542428 | 1 |
| 3228.4544542 | 2 |
| 3228.455681 | 1 |
| 3228.4567971 | 1 |
| 3228.4570352 | 2 |
| 3228.4583878 | 3 |
| 3228.4591275 | 3 |
| 3228.4603426 | 2 |
| 3228.4614813 | 4 |
| 3228.4619522 | 3 |
| 3228.4628001 | 0 |
| 3228.4636839 | 1 |
| 3228.4648753 | 4 |
| 3228.4660789 | 3 |
| 3228.4669933 | 4 |
| 3228.467828 | 0 |
| 3228.4683668 | 1 |
| 3228.4694985 | 3 |
| 3228.4701782 | 2 |
| 3228.4714352 | 1 |
| 3228.4725491 | 3 |
| 3228.4726375 | 1 |
| 3228.4738391 | 1 |
| 3228.4749481 | 2 |
| 3228.4758731 | 1 |
| 3228.4772138 | 2 |
| 3228.4777799 | 1 |
| 3228.4790457 | 2 |
| 3228.4794629 | 1 |
| 3228.4799162 | 5 |
| 3228.4820787 | 1 |
| 3228.4821728 | 3 |
| 3228.4833877 | 0 |
| 3228.4844046 | 2 |
| 3228.4853655 | 2 |
| 3228.4867583 | 2 |
| 3228.4873361 | 1 |
| 3228.4882968 | 1 |
| 3228.488879 | 2 |
| 3228.4896107 | 2 |
| 3228.4911672 | 2 |
| 3228.4916519 | 2 |
| 3228.4935484 | 7 |
| 3228.4937101 | 5 |
| 3228.4948272 | 3 |
| 3228.496131 | 0 |
| 3228.4964031 | 5 |
| 3228.4977163 | 3 |
| 3228.4982638 | 0 |
| 3228.4999185 | 1 |
| 3228.5007314 | 1 |
| 3228.5011146 | 0 |
| 3228.5025484 | 2 |
| 3228.503264 | 0 |
| 3228.504488 | 5 |
| 3228.5049564 | 1 |
| 3228.5054936 | 2 |
| 3228.5065856 | 2 |
| 3228.5079489 | 0 |
| 3228.5082531 | 4 |
| 3228.5092657 | 2 |
| 3228.5100796 | 1 |
| 3228.5114067 | 3 |
| 3228.5115016 | 2 |
| 3228.5130474 | 2 |
| 3228.5142333 | 2 |
| 3228.5151275 | 0 |
| 3228.515847 | 2 |
| 3228.5160566 | 1 |
| 3228.5185775 | 3 |
| 3228.5189877 | 4 |
| 3228.5193609 | 4 |
| 3228.5203726 | 4 |
| 3228.5208136 | 3 |
| 3228.5227166 | 4 |
| 3228.5236033 | 3 |
| 3228.5241371 | 3 |
| 3228.52585 | 0 |
| 3228.525871 | 2 |
| 3228.5269992 | 3 |
| 3228.5283705 | 3 |
| 3228.5287721 | 3 |
| 3228.5302617 | 1 |
| 3228.5304627 | 2 |
| 3228.5326098 | 2 |
| 3228.5327751 | 1 |
| 3228.5334091 | 2 |
| 3228.534905 | 2 |
| 3228.5350636 | 2 |
| 3228.5368911 | 2 |
| 3228.53761 | 0 |
| 3228.53824 | 1 |
| 3228.5397161 | 1 |
| 3228.5400184 | 0 |
| 3228.5414763 | 1 |
| 3228.5426261 | 2 |
| 3228.5428536 | 2 |
| 3228.5439783 | 1 |
| 3228.5442061 | 3 |
| 3228.5458054 | 2 |
| 3228.5472242 | 2 |
| 3228.5474142 | 2 |
| 3228.5481836 | 2 |
| 3228.5497691 | 2 |
| 3228.5500837 | 2 |
| 3228.5505675 | 3 |
| 3228.5514147 | 1 |
| 3228.5526419 | 2 |
| 3228.5540704 | 5 |
| 3228.5543622 | 3 |
| 3228.5552036 | 2 |
| 3228.5565225 | 4 |
| 3228.5575681 | 2 |
| 3228.5581504 | 3 |
| 3228.5595402 | 3 |
| 3228.5604914 | 0 |
| 3228.5608544 | 2 |
| 3228.5623788 | 3 |
| 3228.5625548 | 3 |
| 3228.5637094 | 2 |
| 3228.5656529 | 0 |
| 3228.5657051 | 2 |
| 3228.5669058 | 2 |
| 3228.5673481 | 0 |
| 3228.5686808 | 2 |
| 3228.5695427 | 0 |
| 3228.5700355 | 3 |
| 3228.5709267 | 2 |
| 3228.5724617 | 2 |
| 3228.5725486 | 1 |
| 3228.5744416 | 0 |
| 3228.5756037 | 4 |
| 3228.5756243 | 2 |
| 3228.5769348 | 2 |
| 3228.5776048 | 1 |
| 3228.57898 | 0 |
| 3228.5798678 | 3 |
| 3228.5806859 | 1 |
| 3228.5813952 | 4 |
| 3228.5829682 | 2 |
| 3228.5838796 | 2 |
| 3228.5838858 | 3 |
| 3228.5857673 | 1 |
| 3228.587136 | 2 |
| 3228.5878426 | 1 |
| 3228.5889706 | 0 |
| 3228.5890009 | 1 |
| 3228.5903953 | 0 |
| 3228.5911874 | 2 |
| 3228.5915337 | 2 |
| 3228.5916288 | 3 |
| 3228.5921741 | 2 |
| 3228.5927982 | 1 |
| 3228.5934213 | 4 |
| 3228.593461 | 0 |
| 3228.5943006 | 1 |
| 3228.595177 | 1 |
| 3228.5954785 | 2 |
| 3228.5961364 | 5 |
| 3228.5963499 | 0 |
| 3228.5969997 | 1 |
| 3228.5978106 | 0 |
| 3228.5979352 | 0 |
| 3228.5981883 | 0 |
| 3228.5994203 | 4 |
| 3228.6001873 | 2 |
| 3228.600607 | 1 |
| 3228.6010101 | 2 |
| 3228.6014716 | 1 |
| 3228.6019865 | 3 |
| 3228.6022208 | 3 |
| 3228.6026764 | 2 |
| 3228.6040447 | 1 |
| 3228.6043868 | 1 |
| 3228.6047366 | 1 |
| 3228.6050685 | 4 |
| 3228.6054141 | 1 |
| 3228.6061155 | 1 |
| 3228.6069014 | 4 |
| 3228.6069606 | 2 |
| 3228.6077627 | 1 |
| 3228.6079996 | 0 |
| 3228.6083278 | 2 |
| 3228.6094695 | 2 |
| 3228.6101097 | 3 |
| 3228.6106161 | 1 |
| 3228.6106518 | 2 |
| 3228.611639 | 3 |
| 3228.612052 | 4 |
| 3228.6122922 | 1 |
| 3228.6134479 | 1 |
| 3228.61367 | 2 |
| 3228.6139579 | 3 |
| 3228.61451 | 4 |
| 3228.615088 | 2 |
| 3228.6155361 | 2 |
| 3228.6162435 | 4 |
| 3228.6164126 | 3 |
| 3228.6177666 | 3 |
| 3228.618025 | 0 |
| 3228.618307 | 3 |

| | |
|---|---|
| 3228.6192407 | 3 |
| 3228.6192917 | 2 |
| 3228.6193379 | 0 |
| 3228.6204036 | 2 |
| 3228.6208595 | 1 |
| 3228.6211942 | 4 |
| 3228.6219593 | 5 |
| 3228.6227861 | 2 |
| 3228.6230716 | 1 |
| 3228.6233505 | 3 |
| 3228.6244582 | 4 |
| 3228.6247822 | 1 |
| 3228.625782 | 0 |
| 3228.626044 | 1 |
| 3228.6261576 | 3 |
| 3228.6265773 | 3 |
| 3228.6273239 | 4 |
| 3228.6282179 | 3 |
| 3228.6288213 | 2 |
| 3228.629248 | 0 |
| 3228.6294167 | 2 |
| 3228.6305194 | 1 |
| 3228.630725 | 1 |
| 3228.6313374 | 1 |
| 3228.6324852 | 2 |
| 3228.6325372 | 1 |
| 3228.6329339 | 2 |
| 3228.6335816 | 1 |
| 3228.6336176 | 1 |
| 3228.6348316 | 1 |
| 3228.6353888 | 5 |
| 3228.6359613 | 2 |
| 3228.6364396 | 2 |
| 3228.6372157 | 3 |
| 3228.6374128 | 4 |
| 3228.6386192 | 1 |
| 3228.6391608 | 1 |
| 3228.6395676 | 1 |
| 3228.6398037 | 2 |
| 3228.6405687 | 0 |
| 3228.6408548 | 1 |
| 3228.6411795 | 1 |
| 3228.6423705 | 1 |
| 3228.6426146 | 1 |
| 3228.6426275 | 4 |
| 3228.6435866 | 1 |
| 3228.6441972 | 2 |
| 3228.6452316 | 1 |
| 3228.645396 | 0 |
| 3228.646231 | 1 |
| 3228.6464464 | 4 |
| 3228.6476928 | 3 |
| 3228.647779 | 2 |
| 3228.6481295 | 2 |
| 3228.6497832 | 2 |
| 3228.6501946 | 1 |
| 3228.6504037 | 2 |
| 3228.6513283 | 1 |
| 3228.6518072 | 2 |
| 3228.6520745 | 0 |
| 3228.6521725 | 1 |
| 3228.6533198 | 5 |
| 3228.653831 | 0 |
| 3228.6544764 | 1 |
| 3228.656984 | 2 |
| 3228.6575592 | 1 |
| 3228.6588112 | 4 |
| 3228.6604921 | 0 |
| 3228.661104 | 3 |
| 3228.6628249 | 1 |
| 3228.6644755 | 3 |
| 3228.6646515 | 3 |
| 3228.6665595 | 1 |
| 3228.6677356 | 2 |
| 3228.6685997 | 3 |
| 3228.6696269 | 1 |
| 3228.6705272 | 4 |
| 3228.6717409 | 1 |
| 3228.6734009 | 5 |
| 3228.6742949 | 3 |
| 3228.6763599 | 0 |
| 3228.6775934 | 1 |
| 3228.6787746 | 0 |
| 3228.6799236 | 3 |
| 3228.6811956 | 1 |
| 3228.6830149 | 2 |
| 3228.6836035 | 1 |
| 3228.6849352 | 0 |
| 3228.6862203 | 1 |
| 3228.6882947 | 3 |
| 3228.689244 | 0 |
| 3228.6907734 | 4 |
| 3228.6917538 | 4 |
| 3228.6920861 | 2 |
| 3228.6938561 | 1 |
| 3228.6945885 | 3 |
| 3228.6964123 | 3 |
| 3228.6980478 | 1 |
| 3228.6990133 | 4 |
| 3228.7002494 | 3 |
| 3228.7012676 | 0 |
| 3228.702591 | 1 |
| 3228.7046479 | 2 |
| 3228.7051511 | 3 |
| 3228.7065923 | 2 |
| 3228.7069315 | 4 |
| 3228.7084768 | 2 |
| 3228.709574 | 1 |
| 3228.7112812 | 1 |
| 3228.7130147 | 0 |
| 3228.713824 | 2 |
| 3228.7152845 | 2 |
| 3228.716557 | 1 |
| 3228.7172858 | 3 |
| 3228.7192879 | 2 |
| 3228.7198496 | 1 |
| 3228.7203199 | 1 |
| 3228.7229883 | 2 |
| 3228.7232105 | 1 |
| 3228.7254104 | 3 |
| 3228.7264171 | 3 |
| 3228.7269178 | 2 |
| 3228.7293502 | 1 |
| 3228.7301302 | 1 |
| 3228.7314449 | 1 |
| 3228.7326764 | 1 |
| 3228.7344669 | 0 |
| 3228.7363736 | 5 |
| 3228.7366838 | 2 |
| 3228.7391514 | 2 |
| 3228.7392365 | 2 |
| 3228.7411958 | 1 |
| 3228.7415505 | 1 |
| 3228.7430062 | 1 |
| 3228.7450208 | 0 |
| 3228.7456159 | 0 |
| 3228.7467176 | 3 |
| 3228.7474197 | 2 |
| 3228.7489779 | 2 |
| 3228.750388 | 1 |
| 3228.7512749 | 2 |
| 3228.7529112 | 3 |
| 3228.753456 | 4 |
| 3228.7551291 | 0 |
| 3228.7564167 | 4 |
| 3228.7573103 | 3 |
| 3228.7587421 | 5 |
| 3228.7605394 | 2 |
| 3228.7614814 | 1 |
| 3228.7625651 | 0 |
| 3228.7636956 | 2 |
| 3228.7653238 | 2 |
| 3228.7659946 | 0 |
| 3228.7675253 | 1 |
| 3228.7692744 | 4 |
| 3228.7696702 | 0 |
| 3228.7716249 | 3 |
| 3228.7725109 | 1 |
| 3228.7739356 | 2 |
| 3228.7754569 | 1 |
| 3228.7763824 | 3 |
| 3228.7776095 | 4 |
| 3228.779003 | 1 |
| 3228.7809884 | 0 |
| 3228.7818776 | 1 |
| 3228.7831143 | 4 |
| 3228.7846552 | 0 |
| 3228.7851234 | 1 |
| 3228.7867451 | 0 |
| 3228.7881737 | 0 |
| 3228.7894101 | 4 |
| 3228.7907688 | 0 |
| 3228.7920172 | 5 |
| 3228.7936056 | 0 |
| 3228.7945912 | 1 |
| 3228.7957472 | 0 |
| 3228.7966825 | 2 |
| 3228.797926 | 1 |
| 3228.7992652 | 3 |
| 3228.8001314 | 2 |
| 3228.801211 | 2 |
| 3228.8026662 | 2 |
| 3228.8039434 | 1 |
| 3228.8056457 | 4 |
| 3228.8064711 | 0 |
| 3228.8073159 | 1 |
| 3228.8086296 | 5 |
| 3228.809627 | 5 |
| 3228.8118672 | 0 |
| 3228.8132415 | 1 |
| 3228.8133371 | 1 |
| 3228.8156054 | 3 |
| 3228.815996 | 2 |
| 3228.8177613 | 5 |
| 3228.8192389 | 2 |
| 3228.8205003 | 1 |
| 3228.8223185 | 4 |
| 3228.8228213 | 1 |
| 3228.8249111 | 1 |
| 3228.825559 | 1 |
| 3228.8266478 | 2 |
| 3228.828401 | 3 |
| 3228.8289446 | 1 |
| 3228.8308581 | 1 |
| 3228.8314809 | 2 |
| 3228.8328807 | 3 |
| 3228.8345508 | 2 |
| 3228.8350896 | 2 |
| 3228.8366282 | 0 |
| 3228.8377243 | 5 |
| 3228.8391871 | 3 |
| 3228.8407087 | 2 |
| 3228.8417975 | 2 |
| 3228.8428976 | 3 |
| 3228.8444781 | 2 |
| 3228.8452399 | 1 |
| 3228.8466047 | 3 |
| 3228.8473976 | 1 |
| 3228.8492279 | 0 |
| 3228.8501249 | 4 |
| 3228.851151 | 4 |
| 3228.8523969 | 0 |
| 3228.8531134 | 1 |
| 3228.8547101 | 5 |
| 3228.8561006 | 8 |
| 3228.8575867 | 2 |
| 3228.8590889 | 2 |
| 3228.8591992 | 1 |
| 3228.8601143 | 3 |
| 3228.8606936 | 2 |
| 3228.8612385 | 3 |
| 3228.8625102 | 6 |
| 3228.8627342 | 1 |
| 3228.8637399 | 1 |
| 3228.8645136 | 2 |
| 3228.8645817 | 2 |
| 3228.8651282 | 1 |
| 3228.8656646 | 0 |
| 3228.8665114 | 1 |
| 3228.8673107 | 1 |
| 3228.8683245 | 1 |
| 3228.8687292 | 1 |
| 3228.8687484 | 3 |
| 3228.8699989 | 2 |
| 3228.8703555 | 1 |
| 3228.8711735 | 1 |
| 3228.8714304 | 2 |
| 3228.8726507 | 4 |
| 3228.8731323 | 4 |
| 3228.8742048 | 1 |
| 3228.8743598 | 3 |
| 3228.8749002 | 0 |
| 3228.8754411 | 2 |
| 3228.8765393 | 2 |
| 3228.8766427 | 2 |
| 3228.8768684 | 3 |
| 3228.8769064 | 1 |
| 3228.8778751 | 1 |
| 3228.8779045 | 4 |
| 3228.8780279 | 4 |
| 3228.8782535 | 1 |
| 3228.8790777 | 5 |
| 3228.8795689 | 2 |
| 3228.880068 | 0 |
| 3228.8812806 | 2 |
| 3228.8812854 | 1 |
| 3228.8820926 | 2 |
| 3228.8824917 | 1 |
| 3228.8833209 | 5 |
| 3228.8835198 | 4 |
| 3228.8835439 | 3 |
| 3228.8836156 | 4 |
| 3228.8848794 | 8 |
| 3228.8849693 | 0 |
| 3228.8850279 | 4 |
| 3228.8853976 | 2 |
| 3228.8856196 | 5 |
| 3228.8857536 | 1 |
| 3228.8866582 | 4 |
| 3228.8870358 | 2 |
| 3228.8872441 | 2 |
| 3228.8873193 | 0 |
| 3228.8890639 | 4 |
| 3228.8892879 | 3 |
| 3228.8895906 | 3 |
| 3228.8900018 | 5 |
| 3228.8902809 | 3 |
| 3228.8911157 | 4 |
| 3228.8912608 | 1 |
| 3228.8922413 | 1 |
| 3228.8930769 | 1 |
| 3228.8933441 | 1 |
| 3228.8952215 | 0 |
| 3228.8955785 | 2 |
| 3228.8959864 | 0 |
| 3228.8974113 | 4 |
| 3228.8979708 | 4 |
| 3228.8998843 | 1 |
| 3228.9008801 | 2 |
| 3228.9025697 | 3 |
| 3228.9033215 | 1 |
| 3228.9050734 | 4 |
| 3228.9061128 | 1 |
| 3228.9074724 | 4 |
| 3228.9082197 | 4 |
| 3228.9082464 | 0 |
| 3228.9092129 | 6 |
| 3228.9109995 | 2 |
| 3228.9125359 | 1 |
| 3228.9131532 | 0 |
| 3228.9140305 | 3 |
| 3228.9149165 | 2 |
| 3228.9156571 | 1 |
| 3228.9161806 | 1 |
| 3228.9164796 | 0 |
| 3228.9165501 | 0 |
| 3228.9170807 | 2 |
| 3228.9185628 | 4 |
| 3228.9193619 | 2 |
| 3228.9201024 | 1 |
| 3228.9207426 | 1 |
| 3228.9209841 | 4 |
| 3228.9210887 | 3 |
| 3228.9219593 | 5 |
| 3228.9226016 | 4 |
| 3228.9228079 | 4 |
| 3228.9230548 | 1 |
| 3228.9233941 | 2 |
| 3228.9248682 | 3 |
| 3228.9248689 | 1 |
| 3228.9251397 | 2 |
| 3228.9258054 | 8 |
| 3228.9265474 | 1 |
| 3228.926549 | 2 |
| 3228.9266854 | 2 |
| 3228.9273399 | 5 |
| 3228.9273501 | 1 |
| 3228.9282571 | 2 |
| 3228.9285402 | 5 |
| 3228.9288053 | 2 |
| 3228.9295068 | 2 |
| 3228.929664 | 4 |
| 3228.9297859 | 4 |
| 3228.9303382 | 1 |
| 3228.9305608 | 2 |
| 3228.9308775 | 0 |
| 3228.9318263 | 2 |
| 3228.9325205 | 3 |
| 3228.9326995 | 1 |
| 3228.9327876 | 1 |
| 3228.932998 | 2 |
| 3228.9336211 | 1 |
| 3228.9348694 | 1 |
| 3228.9351164 | 2 |
| 3228.9354339 | 2 |
| 3228.9355026 | 1 |
| 3228.9357238 | 3 |
| 3228.9363775 | 1 |
| 3228.9364489 | 1 |
| 3228.9370342 | 0 |
| 3228.9370477 | 0 |
| 3228.9373043 | 0 |
| 3228.9381501 | 1 |

| | |
|---|---|
| 3228.9384456 | 1 |
| 3228.938739 | 0 |
| 3228.9390072 | 0 |
| 3228.9398605 | 1 |
| 3228.9400382 | 1 |
| 3228.9403166 | 2 |
| 3228.9405814 | 1 |
| 3228.9412989 | 2 |
| 3228.9418174 | 3 |
| 3228.9418187 | 2 |
| 3228.9418689 | 4 |
| 3228.9420629 | 1 |
| 3228.943148 | 5 |
| 3228.9436304 | 3 |
| 3228.9437073 | 3 |
| 3228.9442545 | 1 |
| 3228.9446106 | 1 |
| 3228.9446418 | 0 |
| 3228.94548 | 1 |
| 3228.945808 | 0 |
| 3228.9464135 | 2 |
| 3228.947352 | 2 |
| 3228.9473942 | 0 |
| 3228.9478488 | 1 |
| 3228.9486328 | 1 |
| 3228.9495322 | 3 |
| 3228.9500282 | 1 |
| 3228.9504874 | 2 |
| 3228.9514728 | 1 |
| 3228.9518923 | 4 |
| 3228.9527706 | 1 |
| 3228.9538505 | 0 |
| 3228.9546845 | 3 |
| 3228.9550913 | 3 |
| 3228.9551234 | 0 |
| 3228.9559825 | 0 |
| 3228.9567566 | 1 |
| 3228.9576039 | 1 |
| 3228.957605 | 3 |
| 3228.958662 | 2 |
| 3228.9595813 | 3 |
| 3228.9600445 | 0 |
| 3228.9605426 | 1 |
| 3228.9606192 | 0 |
| 3228.9617585 | 1 |
| 3228.9618972 | 0 |
| 3228.9628425 | 2 |
| 3228.963328 | 3 |
| 3228.9642741 | 1 |
| 3228.9645814 | 2 |
| 3228.9663886 | 3 |
| 3228.9665155 | 2 |
| 3228.966896 | 3 |
| 3228.9675265 | 2 |
| 3228.9682214 | 1 |
| 3228.9686839 | 0 |
| 3228.9695348 | 2 |
| 3228.9697449 | 0 |
| 3228.9704315 | 3 |
| 3228.9717012 | 4 |
| 3228.9720107 | 0 |
| 3228.9721974 | 3 |
| 3228.9734119 | 2 |
| 3228.9739099 | 0 |
| 3228.9744921 | 2 |
| 3228.9753598 | 2 |
| 3228.9758609 | 2 |
| 3228.9759438 | 1 |
| 3228.9768221 | 0 |
| 3228.9768331 | 1 |
| 3228.9773127 | 2 |
| 3228.9778275 | 3 |
| 3228.9779796 | 4 |
| 3228.9796411 | 1 |
| 3228.9800091 | 0 |
| 3228.980704 | 1 |
| 3228.9808917 | 0 |
| 3228.9810669 | 2 |
| 3228.9822272 | 3 |
| 3228.9830467 | 2 |
| 3228.9838971 | 3 |
| 3228.9843347 | 2 |
| 3228.9844962 | 0 |
| 3228.9851865 | 2 |
| 3228.985866 | 1 |
| 3228.9867467 | 1 |
| 3228.9868786 | 1 |
| 3228.9878773 | 0 |
| 3228.9882692 | 2 |
| 3228.9894584 | 1 |
| 3228.9894744 | 3 |
| 3228.9896129 | 0 |
| 3228.98994 | 1 |
| 3228.9918745 | 2 |
| 3228.992577 | 4 |
| 3228.9928763 | 0 |
| 3228.9929721 | 0 |
| 3228.9941562 | 0 |
| 3228.994262 | 2 |
| 3228.9956369 | 0 |
| 3228.9962291 | 3 |
| 3228.996882 | 3 |
| 3228.9974393 | 1 |
| 3228.9978014 | 1 |
| 3228.9980677 | 1 |
| 3228.9983396 | 3 |
| 3228.9995509 | 5 |
| 3229.0000649 | 0 |
| 3229.0000702 | 3 |
| 3229.0000724 | 1 |
| 3229.0010679 | 2 |
| 3229.0019639 | 5 |
| 3229.002278 | 2 |
| 3229.0028492 | 0 |
| 3229.0029133 | 6 |
| 3229.0036094 | 0 |
| 3229.0041977 | 3 |
| 3229.0047533 | 2 |
| 3229.0055258 | 3 |
| 3229.0056203 | 1 |
| 3229.006464 | 2 |
| 3229.0074194 | 2 |
| 3229.0080815 | 1 |
| 3229.0082328 | 5 |
| 3229.0091183 | 0 |
| 3229.010337 | 0 |
| 3229.0106118 | 3 |
| 3229.0110108 | 1 |
| 3229.0122172 | 3 |
| 3229.0123875 | 0 |
| 3229.0130016 | 2 |
| 3229.0143367 | 3 |
| 3229.0151115 | 2 |
| 3229.0160607 | 0 |
| 3229.0166257 | 2 |
| 3229.0171569 | 3 |
| 3229.0181703 | 4 |
| 3229.0195818 | 1 |
| 3229.020622 | 0 |
| 3229.0224802 | 5 |
| 3229.0228402 | 2 |
| 3229.025079 | 1 |
| 3229.0252222 | 1 |
| 3229.0264525 | 1 |
| 3229.028718 | 4 |
| 3229.0289091 | 2 |
| 3229.0300598 | 2 |
| 3229.0313794 | 3 |
| 3229.0325188 | 2 |
| 3229.0342116 | 3 |
| 3229.0348818 | 1 |
| 3229.0363489 | 0 |
| 3229.0368105 | 1 |
| 3229.038798 | 2 |
| 3229.0406102 | 3 |
| 3229.0413947 | 2 |
| 3229.0431869 | 1 |
| 3229.0440812 | 3 |
| 3229.0455927 | 3 |
| 3229.0466584 | 1 |
| 3229.0481225 | 3 |
| 3229.0490729 | 1 |
| 3229.0502881 | 3 |
| 3229.0512678 | 1 |
| 3229.0530712 | 1 |
| 3229.0545678 | 0 |
| 3229.0554298 | 1 |
| 3229.0567683 | 1 |
| 3229.0585187 | 3 |
| 3229.0598859 | 1 |
| 3229.060489 | 1 |
| 3229.0617292 | 2 |
| 3229.0640712 | 2 |
| 3229.0641607 | 1 |
| 3229.0662688 | 0 |
| 3229.0668944 | 2 |
| 3229.0680505 | 1 |
| 3229.069874 | 3 |
| 3229.0698824 | 1 |
| 3229.071331 | 2 |
| 3229.0731048 | 1 |
| 3229.0742941 | 3 |
| 3229.0754087 | 1 |
| 3229.0768779 | 1 |
| 3229.0782239 | 3 |
| 3229.0790662 | 2 |
| 3229.0806483 | 3 |
| 3229.0818133 | 3 |
| 3229.0830432 | 1 |
| 3229.0836423 | 4 |
| 3229.0847172 | 2 |
| 3229.0860276 | 0 |
| 3229.0875492 | 3 |
| 3229.0881583 | 3 |
| 3229.0902595 | 2 |
| 3229.0910424 | 1 |
| 3229.0931982 | 1 |
| 3229.0942486 | 1 |
| 3229.0962194 | 3 |
| 3229.0971752 | 0 |
| 3229.0981088 | 2 |
| 3229.1002556 | 3 |
| 3229.1006748 | 0 |
| 3229.1018547 | 3 |
| 3229.104176 | 0 |
| 3229.1044914 | 0 |
| 3229.1058789 | 0 |
| 3229.1066159 | 5 |
| 3229.1084512 | 1 |
| 3229.1107097 | 1 |
| 3229.111287 | 0 |
| 3229.1123365 | 1 |
| 3229.1130569 | 0 |
| 3229.1142432 | 3 |
| 3229.1156331 | 3 |
| 3229.1166765 | 2 |
| 3229.1177258 | 1 |
| 3229.1192277 | 2 |
| 3229.1198212 | 2 |
| 3229.1215049 | 0 |
| 3229.1223646 | 2 |
| 3229.124109 | 3 |
| 3229.1246029 | 1 |
| 3229.1264508 | 0 |
| 3229.1282148 | 3 |
| 3229.1297063 | 1 |
| 3229.1308477 | 1 |
| 3229.132098 | 2 |
| 3229.1333779 | 1 |
| 3229.1338975 | 2 |
| 3229.1351571 | 1 |
| 3229.1369501 | 1 |
| 3229.1375686 | 2 |
| 3229.1386797 | 0 |
| 3229.1407855 | 0 |
| 3229.1417103 | 1 |
| 3229.1437492 | 4 |
| 3229.1440607 | 1 |
| 3229.146559 | 1 |
| 3229.1466671 | 2 |
| 3229.1486199 | 0 |
| 3229.1500941 | 3 |
| 3229.1511105 | 2 |
| 3229.1523849 | 0 |
| 3229.1532205 | 2 |
| 3229.1550058 | 1 |
| 3229.1562833 | 2 |
| 3229.1571938 | 2 |
| 3229.1590564 | 1 |
| 3229.1592958 | 4 |
| 3229.1607796 | 3 |
| 3229.1623625 | 3 |
| 3229.1630149 | 2 |
| 3229.1653246 | 2 |
| 3229.1661837 | 1 |
| 3229.1676453 | 2 |
| 3229.1690132 | 1 |
| 3229.1698658 | 0 |
| 3229.170703 | 2 |
| 3229.1714115 | 1 |
| 3229.1731473 | 3 |
| 3229.1743169 | 3 |
| 3229.1748958 | 0 |
| 3229.1769555 | 2 |
| 3229.1776956 | 3 |
| 3229.17899 | 3 |
| 3229.1802864 | 1 |
| 3229.1818775 | 3 |
| 3229.1834144 | 2 |
| 3229.1841111 | 2 |
| 3229.1860699 | 3 |
| 3229.1873981 | 2 |
| 3229.1883711 | 4 |
| 3229.1895 | 1 |
| 3229.1905386 | 2 |
| 3229.1927259 | 1 |
| 3229.1941661 | 2 |
| 3229.1945805 | 5 |
| 3229.1964893 | 0 |
| 3229.1973571 | 3 |
| 3229.1987147 | 3 |
| 3229.2002822 | 3 |
| 3229.2013015 | 4 |
| 3229.2028435 | 3 |
| 3229.2031417 | 1 |
| 3229.2044971 | 2 |
| 3229.2062086 | 0 |
| 3229.2071881 | 1 |
| 3229.2082116 | 2 |
| 3229.209566 | 0 |
| 3229.2109669 | 1 |
| 3229.2119054 | 3 |
| 3229.2134671 | 0 |
| 3229.2145741 | 5 |
| 3229.2151418 | 3 |
| 3229.216693 | 2 |
| 3229.2186575 | 1 |
| 3229.2191057 | 2 |
| 3229.2210951 | 0 |
| 3229.2214402 | 6 |
| 3229.2237843 | 0 |
| 3229.2247635 | 1 |
| 3229.2256278 | 1 |
| 3229.2272277 | 1 |
| 3229.2274968 | 1 |
| 3229.2288841 | 0 |
| 3229.2297106 | 1 |
| 3229.2312452 | 0 |
| 3229.2332049 | 2 |
| 3229.234236 | 1 |
| 3229.236678 | 0 |
| 3229.2367416 | 0 |
| 3229.2377896 | 2 |
| 3229.2398396 | 1 |
| 3229.2410015 | 0 |
| 3229.2424763 | 4 |
| 3229.2434317 | 1 |
| 3229.2442933 | 0 |
| 3229.2463562 | 2 |
| 3229.2472983 | 4 |
| 3229.2488319 | 0 |
| 3229.2490269 | 1 |
| 3229.2510794 | 3 |
| 3229.2521371 | 1 |
| 3229.25271 | 1 |
| 3229.2543274 | 3 |
| 3229.2555658 | 1 |
| 3229.2569537 | 2 |
| 3229.2584513 | 0 |
| 3229.2587096 | 3 |
| 3229.260973 | 3 |
| 3229.2612703 | 2 |
| 3229.2627163 | 4 |
| 3229.2643737 | 0 |
| 3229.2654165 | 2 |
| 3229.2668674 | 0 |
| 3229.267114 | 1 |
| 3229.2691359 | 2 |
| 3229.2710972 | 3 |
| 3229.2717997 | 3 |
| 3229.2732754 | 1 |
| 3229.2738067 | 1 |
| 3229.2754131 | 4 |
| 3229.277182 | 1 |
| 3229.2773149 | 1 |
| 3229.2797631 | 0 |
| 3229.2801993 | 2 |
| 3229.281946 | 6 |
| 3229.2828259 | 2 |
| 3229.2839478 | 1 |
| 3229.2853906 | 3 |
| 3229.2861882 | 1 |
| 3229.2880982 | 4 |
| 3229.2895885 | 2 |
| 3229.2907268 | 1 |
| 3229.2921297 | 1 |
| 3229.2927712 | 1 |
| 3229.2946198 | 1 |
| 3229.2960652 | 5 |
| 3229.2968384 | 3 |
| 3229.2978077 | 0 |
| 3229.2985563 | 0 |
| 3229.3012165 | 3 |

| | |
|---|---|
| 3229.3017521 | 2 |
| 3229.3025726 | 2 |
| 3229.3045289 | 0 |
| 3229.3049947 | 3 |
| 3229.3062863 | 3 |
| 3229.3083663 | 2 |
| 3229.3087353 | 3 |
| 3229.3100835 | 3 |
| 3229.3108384 | 3 |
| 3229.312976 | 2 |
| 3229.3131741 | 0 |
| 3229.3147326 | 4 |
| 3229.3164463 | 3 |
| 3229.3170104 | 5 |
| 3229.3181809 | 0 |
| 3229.3198511 | 1 |
| 3229.3209108 | 2 |
| 3229.3226005 | 3 |
| 3229.3239617 | 3 |
| 3229.3250141 | 4 |
| 3229.3269469 | 1 |
| 3229.3277636 | 3 |
| 3229.3292087 | 2 |
| 3229.3298644 | 4 |
| 3229.3317598 | 2 |
| 3229.3328346 | 2 |
| 3229.3338511 | 3 |
| 3229.3357418 | 2 |
| 3229.336288 | 3 |
| 3229.3379166 | 4 |
| 3229.3386179 | 2 |
| 3229.3399604 | 1 |
| 3229.3414814 | 1 |
| 3229.3422802 | 1 |
| 3229.343762 | 2 |
| 3229.345225 | 3 |
| 3229.3459898 | 3 |
| 3229.3472135 | 0 |
| 3229.3488298 | 0 |
| 3229.3495683 | 2 |
| 3229.3512966 | 1 |
| 3229.3519328 | 4 |
| 3229.3532686 | 2 |
| 3229.3547893 | 3 |
| 3229.3557611 | 3 |
| 3229.3566585 | 1 |
| 3229.3582355 | 1 |
| 3229.3598272 | 1 |
| 3229.3608446 | 4 |
| 3229.3621694 | 1 |
| 3229.3638797 | 4 |
| 3229.3647348 | 5 |
| 3229.3649499 | 0 |
| 3229.3673189 | 1 |
| 3229.3688942 | 2 |
| 3229.3692433 | 2 |
| 3229.3709886 | 2 |
| 3229.3725309 | 1 |
| 3229.3730499 | 1 |
| 3229.3752493 | 2 |
| 3229.3756936 | 2 |
| 3229.3766456 | 3 |
| 3229.3776402 | 2 |
| 3229.3799361 | 3 |
| 3229.3811632 | 2 |
| 3229.3817826 | 1 |
| 3229.3834554 | 1 |
| 3229.3844108 | 1 |
| 3229.3854242 | 2 |
| 3229.3871202 | 0 |
| 3229.387428 | 1 |
| 3229.3895721 | 1 |
| 3229.3904386 | 3 |
| 3229.3919435 | 0 |
| 3229.3933319 | 4 |
| 3229.3937288 | 1 |
| 3229.3951447 | 4 |
| 3229.3964184 | 3 |
| 3229.3976581 | 0 |
| 3229.3998042 | 1 |
| 3229.3998498 | 2 |
| 3229.4018527 | 1 |
| 3229.4023364 | 1 |
| 3229.4036348 | 0 |
| 3229.4049276 | 0 |
| 3229.406568 | 1 |
| 3229.4070398 | 3 |
| 3229.408058 | 3 |
| 3229.4094383 | 3 |
| 3229.4118592 | 1 |
| 3229.4124342 | 2 |
| 3229.413329 | 5 |
| 3229.414603 | 3 |
| 3229.4167315 | 1 |
| 3229.4178838 | 4 |
| 3229.418627 | 2 |
| 3229.4206436 | 2 |
| 3229.4212971 | 2 |
| 3229.4228193 | 6 |
| 3229.423337 | 0 |
| 3229.4253143 | 1 |
| 3229.4265378 | 1 |
| 3229.4274333 | 2 |
| 3229.4293342 | 3 |
| 3229.4295895 | 5 |
| 3229.4314249 | 2 |
| 3229.4330929 | 4 |
| 3229.4334271 | 2 |
| 3229.4347476 | 0 |
| 3229.4366835 | 4 |
| 3229.4369566 | 1 |
| 3229.4384582 | 1 |
| 3229.4396713 | 2 |
| 3229.4414471 | 4 |
| 3229.4423238 | 2 |
| 3229.4432513 | 3 |
| 3229.4447262 | 0 |
| 3229.4451318 | 3 |
| 3229.4468365 | 2 |
| 3229.4484575 | 1 |
| 3229.449398 | 2 |
| 3229.4504921 | 1 |
| 3229.4512702 | 1 |
| 3229.4526457 | 2 |
| 3229.4547061 | 2 |
| 3229.4559193 | 1 |
| 3229.45662 | 2 |
| 3229.4577069 | 1 |
| 3229.4594498 | 0 |
| 3229.4608049 | 0 |
| 3229.4614322 | 3 |
| 3229.4628693 | 1 |
| 3229.4639713 | 2 |
| 3229.4660802 | 7 |
| 3229.4669082 | 1 |
| 3229.4684127 | 2 |
| 3229.4699143 | 0 |
| 3229.470305 | 4 |
| 3229.4726133 | 2 |
| 3229.472707 | 5 |
| 3229.4741171 | 1 |
| 3229.4758332 | 2 |
| 3229.4767845 | 3 |
| 3229.4779515 | 3 |
| 3229.4779656 | 3 |
| 3229.4781015 | 5 |
| 3229.4797885 | 2 |
| 3229.4798041 | 1 |
| 3229.4802856 | 3 |
| 3229.4806946 | 1 |
| 3229.4816866 | 5 |
| 3229.4819674 | 0 |
| 3229.4823961 | 2 |
| 3229.4829875 | 1 |
| 3229.4836484 | 3 |
| 3229.4841171 | 0 |
| 3229.4843653 | 1 |
| 3229.4849888 | 0 |
| 3229.4849935 | 3 |
| 3229.4859563 | 1 |
| 3229.4861768 | 2 |
| 3229.4867345 | 3 |
| 3229.4877087 | 1 |
| 3229.4882166 | 1 |
| 3229.4884645 | 2 |
| 3229.4885905 | 3 |
| 3229.4896089 | 1 |
| 3229.4896491 | 1 |
| 3229.4903059 | 2 |
| 3229.4904792 | 4 |
| 3229.4915579 | 2 |
| 3229.4927259 | 4 |
| 3229.4929596 | 0 |
| 3229.4931225 | 1 |
| 3229.4937036 | 3 |
| 3229.4944077 | 0 |
| 3229.4946499 | 0 |
| 3229.494988 | 4 |
| 3229.4961891 | 2 |
| 3229.4965131 | 3 |
| 3229.4969025 | 1 |
| 3229.4974705 | 2 |
| 3229.4976403 | 3 |
| 3229.4985083 | 3 |
| 3229.4991743 | 6 |
| 3229.4994362 | 3 |
| 3229.499442 | 0 |
| 3229.5008841 | 2 |
| 3229.5012278 | 2 |
| 3229.5016085 | 1 |
| 3229.5028543 | 3 |
| 3229.5029176 | 5 |
| 3229.5034218 | 1 |
| 3229.5034896 | 4 |
| 3229.5044118 | 2 |
| 3229.5049116 | 1 |
| 3229.5055635 | 3 |
| 3229.5064723 | 1 |
| 3229.5067756 | 2 |
| 3229.5076114 | 3 |
| 3229.5077785 | 2 |
| 3229.508867 | 1 |
| 3229.5091219 | 3 |
| 3229.509152 | 1 |
| 3229.5098451 | 2 |
| 3229.5099312 | 2 |
| 3229.5116164 | 1 |
| 3229.5117442 | 3 |
| 3229.5118574 | 1 |
| 3229.5122967 | 1 |
| 3229.5134903 | 5 |
| 3229.5137874 | 1 |
| 3229.5144976 | 2 |
| 3229.5153103 | 2 |
| 3229.5153832 | 2 |
| 3229.5160699 | 1 |
| 3229.516426 | 3 |
| 3229.5171438 | 0 |
| 3229.517884 | 1 |
| 3229.5179292 | 4 |
| 3229.5186446 | 2 |
| 3229.5189606 | 1 |
| 3229.5193127 | 2 |
| 3229.5202926 | 0 |
| 3229.5207331 | 3 |
| 3229.521595 | 1 |
| 3229.5216587 | 2 |
| 3229.5223796 | 4 |
| 3229.5224485 | 2 |
| 3229.5235509 | 1 |
| 3229.5242303 | 1 |
| 3229.5243166 | 1 |
| 3229.5248873 | 2 |
| 3229.5250213 | 2 |
| 3229.5262529 | 0 |
| 3229.5263886 | 1 |
| 3229.5275158 | 0 |
| 3229.527523 | 2 |
| 3229.5275623 | 3 |
| 3229.5282243 | 2 |
| 3229.528895 | 1 |
| 3229.5300712 | 3 |
| 3229.5303935 | 3 |
| 3229.530593 | 1 |
| 3229.5306318 | 1 |
| 3229.5319913 | 1 |
| 3229.5321389 | 1 |
| 3229.5323608 | 1 |
| 3229.5331693 | 3 |
| 3229.5333309 | 1 |
| 3229.534525 | 0 |
| 3229.5349514 | 1 |
| 3229.5354475 | 1 |
| 3229.5362766 | 4 |
| 3229.5365162 | 1 |
| 3229.5366729 | 2 |
| 3229.5369923 | 0 |
| 3229.5384847 | 1 |
| 3229.5387117 | 1 |
| 3229.5388936 | 3 |
| 3229.5389152 | 2 |
| 3229.5400379 | 1 |
| 3229.5407863 | 3 |
| 3229.5409437 | 1 |
| 3229.5415653 | 1 |
| 3229.5417318 | 3 |
| 3229.5427195 | 1 |
| 3229.5429511 | 1 |
| 3229.5435846 | 0 |
| 3229.5444958 | 1 |
| 3229.5447207 | 3 |
| 3229.5453518 | 3 |
| 3229.5454399 | 6 |
| 3229.5463512 | 1 |
| 3229.547102 | 3 |
| 3229.5472736 | 0 |
| 3229.5485785 | 2 |
| 3229.5487769 | 2 |
| 3229.5489056 | 4 |
| 3229.5493067 | 1 |
| 3229.5506224 | 4 |
| 3229.5506866 | 3 |
| 3229.5511603 | 2 |
| 3229.5516996 | 3 |
| 3229.5519744 | 0 |
| 3229.5525555 | 0 |
| 3229.5531242 | 2 |
| 3229.5538659 | 1 |
| 3229.5546088 | 2 |
| 3229.5551383 | 2 |
| 3229.5558046 | 1 |
| 3229.556229 | 4 |
| 3229.5569972 | 0 |
| 3229.557207 | 4 |
| 3229.5578563 | 2 |
| 3229.5583654 | 3 |
| 3229.5584359 | 2 |
| 3229.5599622 | 3 |
| 3229.5600739 | 1 |
| 3229.5607052 | 3 |
| 3229.5612706 | 2 |
| 3229.5613502 | 4 |
| 3229.5615869 | 2 |
| 3229.5626057 | 3 |
| 3229.5633058 | 2 |
| 3229.5635874 | 0 |
| 3229.5644128 | 4 |
| 3229.5644292 | 3 |
| 3229.5649828 | 1 |
| 3229.5661117 | 2 |
| 3229.5661625 | 3 |
| 3229.5674106 | 1 |
| 3229.567549 | 2 |
| 3229.5681453 | 1 |
| 3229.5686436 | 0 |
| 3229.5696433 | 2 |
| 3229.5698604 | 0 |
| 3229.5700152 | 1 |
| 3229.5700949 | 1 |
| 3229.5710636 | 3 |
| 3229.5719155 | 2 |
| 3229.5721061 | 2 |
| 3229.5726044 | 1 |
| 3229.572803 | 4 |
| 3229.5740762 | 5 |
| 3229.5742256 | 4 |
| 3229.5747208 | 2 |
| 3229.5749299 | 1 |
| 3229.575378 | 3 |
| 3229.5762984 | 2 |
| 3229.5770728 | 3 |
| 3229.5771619 | 3 |
| 3229.5779426 | 2 |
| 3229.5785465 | 2 |
| 3229.5787188 | 2 |
| 3229.5791487 | 1 |
| 3229.5802489 | 2 |
| 3229.5802525 | 2 |
| 3229.5804053 | 3 |
| 3229.5810443 | 1 |
| 3229.581528 | 1 |
| 3229.5826191 | 1 |
| 3229.5831584 | 1 |
| 3229.5835556 | 1 |
| 3229.5837061 | 2 |
| 3229.584569 | 2 |
| 3229.584927 | 1 |
| 3229.5855681 | 1 |
| 3229.5860863 | 2 |
| 3229.587413 | 0 |
| 3229.5874572 | 1 |
| 3229.5877542 | 0 |
| 3229.5885333 | 1 |
| 3229.5887614 | 1 |
| 3229.5898283 | 1 |
| 3229.5898811 | 2 |
| 3229.5905661 | 3 |
| 3229.591104 | 2 |
| 3229.5913615 | 2 |
| 3229.5925051 | 2 |
| 3229.5926936 | 1 |
| 3229.5928076 | 4 |
| 3229.5939769 | 0 |

| | |
|---|---|
| 3229.5941373 | 2 |
| 3229.5945541 | 4 |
| 3229.5956452 | 3 |
| 3229.5956907 | 4 |
| 3229.5963706 | 2 |
| 3229.5971022 | 0 |
| 3229.5974524 | 1 |
| 3229.5980836 | 1 |
| 3229.5981912 | 3 |
| 3229.5989823 | 2 |
| 3229.5996562 | 1 |
| 3229.6002512 | 2 |
| 3229.6008242 | 1 |
| 3229.6015397 | 5 |
| 3229.6018092 | 1 |
| 3229.6025117 | 3 |
| 3229.6029838 | 2 |
| 3229.6035722 | 0 |
| 3229.6039596 | 2 |
| 3229.6041267 | 0 |
| 3229.6051245 | 5 |
| 3229.6056329 | 3 |
| 3229.6068716 | 4 |
| 3229.6070005 | 1 |
| 3229.607277 | 0 |
| 3229.6079413 | 4 |
| 3229.6086449 | 4 |
| 3229.6087954 | 0 |
| 3229.6094622 | 3 |
| 3229.6099425 | 0 |
| 3229.6100344 | 6 |
| 3229.6113712 | 2 |
| 3229.6116566 | 5 |
| 3229.6119337 | 4 |
| 3229.6124666 | 3 |
| 3229.6131893 | 1 |
| 3229.6134597 | 0 |
| 3229.6138967 | 1 |
| 3229.6149297 | 5 |
| 3229.6158712 | 0 |
| 3229.6160433 | 2 |
| 3229.6163151 | 2 |
| 3229.6167585 | 2 |
| 3229.61792 | 3 |
| 3229.6179251 | 0 |
| 3229.6188814 | 1 |
| 3229.6190494 | 3 |
| 3229.619087 | 2 |
| 3229.6208421 | 3 |
| 3229.6208588 | 4 |
| 3229.621104 | 2 |
| 3229.6213705 | 2 |
| 3229.6220919 | 0 |
| 3229.6227946 | 1 |
| 3229.6238282 | 2 |
| 3229.6246841 | 2 |
| 3229.6259079 | 0 |
| 3229.6259576 | 2 |
| 3229.6281019 | 1 |
| 3229.6282056 | 2 |
| 3229.6294355 | 2 |
| 3229.6303718 | 3 |
| 3229.6314722 | 0 |
| 3229.6319843 | 2 |
| 3229.633167 | 0 |
| 3229.6340536 | 1 |
| 3229.6346576 | 3 |
| 3229.6357677 | 0 |
| 3229.6365007 | 3 |
| 3229.6375289 | 1 |
| 3229.6391959 | 0 |
| 3229.6396894 | 1 |
| 3229.6405542 | 3 |
| 3229.6411637 | 2 |
| 3229.6427483 | 2 |
| 3229.6430977 | 1 |
| 3229.6443621 | 1 |
| 3229.6451904 | 1 |
| 3229.6463625 | 1 |
| 3229.6469453 | 2 |
| 3229.6480012 | 2 |
| 3229.649364 | 2 |
| 3229.6506576 | 1 |
| 3229.6508281 | 4 |
| 3229.6518785 | 2 |
| 3229.652724 | 2 |
| 3229.6538251 | 2 |
| 3229.6548533 | 2 |
| 3229.6553913 | 0 |
| 3229.656375 | 1 |
| 3229.6573481 | 1 |
| 3229.658064 | 3 |
| 3229.6596594 | 4 |
| 3229.6602276 | 4 |
| 3229.6610191 | 1 |
| 3229.6626687 | 1 |
| 3229.6632249 | 4 |
| 3229.6642143 | 4 |
| 3229.6646062 | 2 |
| 3229.6652502 | 2 |
| 3229.6664066 | 4 |
| 3229.6675465 | 2 |
| 3229.6689298 | 1 |
| 3229.6690034 | 0 |
| 3229.6701152 | 3 |
| 3229.6710699 | 1 |
| 3229.6712972 | 2 |
| 3229.6735 | 2 |
| 3229.6741253 | 1 |
| 3229.6751421 | 3 |
| 3229.675637 | 5 |
| 3229.6756422 | 2 |
| 3229.6777681 | 1 |
| 3229.6784276 | 1 |
| 3229.6786552 | 3 |
| 3229.6803411 | 2 |
| 3229.6813396 | 3 |
| 3229.6823522 | 1 |
| 3229.6827164 | 3 |
| 3229.6843344 | 1 |
| 3229.6844972 | 3 |
| 3229.6857503 | 0 |
| 3229.6872799 | 3 |
| 3229.6878978 | 1 |
| 3229.6891375 | 2 |
| 3229.6899249 | 4 |
| 3229.69113 | 1 |
| 3229.6923133 | 5 |
| 3229.6925264 | 1 |
| 3229.6940689 | 2 |
| 3229.6941776 | 2 |
| 3229.6950949 | 1 |
| 3229.6968241 | 1 |
| 3229.6970455 | 2 |
| 3229.6986494 | 1 |
| 3229.6994374 | 1 |
| 3229.6999337 | 2 |
| 3229.7006823 | 3 |
| 3229.701995 | 5 |
| 3229.7033663 | 2 |
| 3229.7035087 | 1 |
| 3229.7048537 | 1 |
| 3229.7059817 | 1 |
| 3229.7064934 | 3 |
| 3229.7068356 | 3 |
| 3229.7081527 | 2 |
| 3229.7090102 | 0 |
| 3229.7104013 | 2 |
| 3229.7112147 | 0 |
| 3229.712551 | 3 |
| 3229.7129229 | 2 |
| 3229.7139227 | 4 |
| 3229.7153174 | 1 |
| 3229.7157867 | 3 |
| 3229.7161786 | 1 |
| 3229.7175301 | 4 |
| 3229.7185064 | 1 |
| 3229.7199719 | 1 |
| 3229.7201892 | 0 |
| 3229.7215408 | 2 |
| 3229.7222254 | 1 |
| 3229.7229964 | 2 |
| 3229.7244856 | 1 |
| 3229.7251221 | 0 |
| 3229.7259901 | 2 |
| 3229.7269934 | 1 |
| 3229.7278802 | 1 |
| 3229.7292896 | 4 |
| 3229.7298033 | 1 |
| 3229.7310538 | 2 |
| 3229.7311223 | 1 |
| 3229.732561 | 2 |
| 3229.7327334 | 4 |
| 3229.7345632 | 1 |
| 3229.7355512 | 2 |
| 3229.7363635 | 0 |
| 3229.7372475 | 2 |
| 3229.7380991 | 3 |
| 3229.7388353 | 1 |
| 3229.7401012 | 3 |
| 3229.7407544 | 1 |
| 3229.7420425 | 2 |
| 3229.7431453 | 4 |
| 3229.7437421 | 0 |
| 3229.7449085 | 2 |
| 3229.7454083 | 0 |
| 3229.7465125 | 2 |
| 3229.7480575 | 1 |
| 3229.7489535 | 2 |
| 3229.7495353 | 2 |
| 3229.7499733 | 3 |
| 3229.7517085 | 2 |
| 3229.7519076 | 3 |
| 3229.7535611 | 2 |
| 3229.7544132 | 2 |
| 3229.7544547 | 4 |
| 3229.7562973 | 3 |
| 3229.7567391 | 3 |
| 3229.757401 | 2 |
| 3229.7588326 | 0 |
| 3229.7597317 | 2 |
| 3229.7606275 | 2 |
| 3229.7609933 | 1 |
| 3229.7624076 | 2 |
| 3229.7633986 | 1 |
| 3229.7636981 | 4 |
| 3229.7652943 | 1 |
| 3229.7653373 | 2 |
| 3229.7666126 | 0 |
| 3229.7678046 | 3 |
| 3229.768636 | 1 |
| 3229.7701405 | 0 |
| 3229.7704049 | 3 |
| 3229.7725034 | 6 |
| 3229.7730451 | 1 |
| 3229.7735921 | 2 |
| 3229.77469 | 1 |
| 3229.7747439 | 0 |
| 3229.7761416 | 4 |
| 3229.7775775 | 4 |
| 3229.7782044 | 4 |
| 3229.7791754 | 1 |
| 3229.7802697 | 3 |
| 3229.781187 | 1 |
| 3229.7823284 | 3 |
| 3229.7827663 | 3 |
| 3229.7840275 | 0 |
| 3229.7848059 | 2 |
| 3229.7863705 | 2 |
| 3229.7864948 | 1 |
| 3229.787662 | 1 |
| 3229.7888337 | 1 |
| 3229.7889852 | 2 |
| 3229.7904148 | 4 |
| 3229.7909119 | 3 |
| 3229.7919432 | 5 |
| 3229.7932111 | 6 |
| 3229.7936987 | 3 |
| 3229.7951264 | 3 |
| 3229.7960752 | 1 |
| 3229.7970663 | 1 |
| 3229.7982277 | 2 |
| 3229.7985202 | 4 |
| 3229.7998285 | 2 |
| 3229.8008261 | 1 |
| 3229.8014612 | 3 |
| 3229.8026593 | 4 |
| 3229.803408 | 2 |
| 3229.8045094 | 5 |
| 3229.8050225 | 5 |
| 3229.8058654 | 3 |
| 3229.8075614 | 0 |
| 3229.8078015 | 4 |
| 3229.8091487 | 5 |
| 3229.8092605 | 2 |
| 3229.8104701 | 2 |
| 3229.8115641 | 7 |
| 3229.8125069 | 0 |
| 3229.813768 | 0 |
| 3229.8140917 | 0 |
| 3229.8152965 | 3 |
| 3229.8160269 | 5 |
| 3229.8161767 | 3 |
| 3229.8179612 | 3 |
| 3229.8189578 | 2 |
| 3229.8196543 | 1 |
| 3229.8207528 | 3 |
| 3229.8219694 | 3 |
| 3229.8232683 | 2 |
| 3229.823309 | 1 |
| 3229.8254387 | 3 |
| 3229.8256881 | 1 |
| 3229.82666 | 1 |
| 3229.8277013 | 3 |
| 3229.8278463 | 3 |
| 3229.8292947 | 2 |
| 3229.8301156 | 2 |
| 3229.8312925 | 2 |
| 3229.8324933 | 3 |
| 3229.832652 | 4 |
| 3229.8344753 | 3 |
| 3229.8344951 | 5 |
| 3229.8355587 | 3 |
| 3229.8370871 | 0 |
| 3229.8373371 | 5 |
| 3229.8385284 | 1 |
| 3229.8398167 | 2 |
| 3229.8408248 | 0 |
| 3229.8416414 | 0 |
| 3229.8423817 | 0 |
| 3229.843207 | 1 |
| 3229.843916 | 2 |
| 3229.8453304 | 0 |
| 3229.8462741 | 0 |
| 3229.8467265 | 0 |
| 3229.8479686 | 1 |
| 3229.8494445 | 1 |
| 3229.8500841 | 3 |
| 3229.851018 | 6 |
| 3229.8516689 | 6 |
| 3229.8521975 | 4 |
| 3229.85332 | 2 |
| 3229.854344 | 1 |
| 3229.8552632 | 1 |
| 3229.8563145 | 3 |
| 3229.8577235 | 1 |
| 3229.8579866 | 4 |
| 3229.8589367 | 0 |
| 3229.8602182 | 0 |
| 3229.8603515 | 0 |
| 3229.8616856 | 4 |
| 3229.8628347 | 2 |
| 3229.8635538 | 1 |
| 3229.8645031 | 1 |
| 3229.8653244 | 3 |
| 3229.8664766 | 0 |
| 3229.8675659 | 1 |
| 3229.8684948 | 3 |
| 3229.869488 | 1 |
| 3229.8700383 | 4 |
| 3229.8713735 | 1 |
| 3229.8715493 | 5 |
| 3229.8728009 | 1 |
| 3229.8742352 | 1 |
| 3229.8751167 | 0 |
| 3229.8763155 | 4 |
| 3229.8769668 | 1 |
| 3229.8783901 | 0 |
| 3229.8790707 | 1 |
| 3229.8794541 | 1 |
| 3229.8807905 | 0 |
| 3229.8816623 | 3 |
| 3229.8831989 | 3 |
| 3229.8834555 | 3 |
| 3229.884381 | 1 |
| 3229.8854429 | 1 |
| 3229.8859056 | 0 |
| 3229.8872349 | 1 |
| 3229.8882702 | 2 |
| 3229.8889708 | 2 |
| 3229.8902038 | 2 |
| 3229.891158 | 1 |
| 3229.8927486 | 1 |
| 3229.8936791 | 2 |
| 3229.8938406 | 3 |
| 3229.8948719 | 1 |
| 3229.8953762 | 1 |
| 3229.8963357 | 3 |
| 3229.8978907 | 1 |
| 3229.8982829 | 3 |
| 3229.8994571 | 1 |
| 3229.8997261 | 4 |
| 3229.9015519 | 3 |
| 3229.9022425 | 0 |
| 3229.9030693 | 1 |
| 3229.9042916 | 3 |
| 3229.9046777 | 0 |
| 3229.9061708 | 2 |
| 3229.9061741 | 2 |
| 3229.9080447 | 2 |
| 3229.9087966 | 3 |
| 3229.9097738 | 0 |
| 3229.9107474 | 1 |

| | |
|---|---|
| 3229.9111753 | 1 |
| 3229.9127746 | 3 |
| 3229.9139538 | 1 |
| 3229.9148673 | 3 |
| 3229.9155575 | 0 |
| 3229.9161488 | 1 |
| 3229.9180323 | 3 |
| 3229.9180653 | 0 |
| 3229.9195423 | 3 |
| 3229.9202662 | 2 |
| 3229.9207509 | 3 |
| 3229.9224418 | 2 |
| 3229.9231581 | 2 |
| 3229.9239945 | 0 |
| 3229.9251078 | 1 |
| 3229.9256759 | 0 |
| 3229.9273166 | 3 |
| 3229.9274525 | 3 |
| 3229.9289374 | 1 |
| 3229.9301325 | 4 |
| 3229.9307606 | 1 |
| 3229.9318041 | 2 |
| 3229.9324518 | 3 |
| 3229.933372 | 4 |
| 3229.9351777 | 1 |
| 3229.9355565 | 2 |
| 3229.9365956 | 0 |
| 3229.9372604 | 0 |
| 3229.9379508 | 1 |
| 3229.9389715 | 1 |
| 3229.9397849 | 5 |
| 3229.9412124 | 1 |
| 3229.9413032 | 2 |
| 3229.9435194 | 2 |
| 3229.9437027 | 2 |
| 3229.9448386 | 2 |
| 3229.9458441 | 2 |
| 3229.9459113 | 1 |
| 3229.9473059 | 1 |
| 3229.9490126 | 2 |
| 3229.9491535 | 2 |
| 3229.9501188 | 2 |
| 3229.9515477 | 4 |
| 3229.9527872 | 2 |
| 3229.9529865 | 2 |
| 3229.9539067 | 4 |
| 3229.9557192 | 1 |
| 3229.9558202 | 0 |
| 3229.9568268 | 2 |
| 3229.9582729 | 4 |
| 3229.9585903 | 2 |
| 3229.9598918 | 0 |
| 3229.9609192 | 1 |
| 3229.9621449 | 2 |
| 3229.962438 | 2 |
| 3229.9634718 | 0 |
| 3229.9650433 | 2 |
| 3229.9650565 | 3 |
| 3229.9671715 | 2 |
| 3229.9672919 | 2 |
| 3229.9683944 | 1 |
| 3229.9698069 | 0 |
| 3229.9706439 | 1 |
| 3229.9716116 | 3 |
| 3229.971641 | 2 |
| 3229.9732197 | 1 |
| 3229.9746098 | 1 |
| 3229.9750896 | 1 |
| 3229.9758502 | 0 |
| 3229.97669 | 1 |
| 3229.978005 | 3 |
| 3229.9788607 | 1 |
| 3229.9797549 | 1 |
| 3229.9808882 | 2 |
| 3229.981693 | 3 |
| 3229.9834707 | 1 |
| 3229.9839392 | 1 |
| 3229.9846895 | 4 |
| 3229.9854262 | 1 |
| 3229.9862557 | 2 |
| 3229.9874126 | 1 |
| 3229.9886423 | 1 |
| 3229.9890205 | 1 |
| 3229.990624 | 5 |
| 3229.9907239 | 2 |
| 3229.9918235 | 2 |
| 3229.992986 | 1 |
| 3229.9937876 | 1 |
| 3229.9944779 | 0 |
| 3229.995426 | 1 |
| 3229.9970519 | 1 |
| 3229.9974578 | 2 |
| 3229.9979502 | 3 |
| 3229.9997959 | 1 |
| 3229.9999616 | 1 |
| 3230.0012533 | 4 |
| 3230.0028455 | 1 |
| 3230.0028477 | 1 |
| 3230.0040328 | 0 |
| 3230.0051386 | 2 |
| 3230.0061856 | 1 |
| 3230.0070023 | 4 |
| 3230.0083778 | 2 |
| 3230.0087643 | 4 |
| 3230.0102888 | 1 |
| 3230.0108756 | 0 |
| 3230.0118095 | 0 |
| 3230.0128163 | 1 |
| 3230.0141013 | 1 |
| 3230.0145236 | 2 |
| 3230.0161731 | 4 |
| 3230.0172246 | 3 |
| 3230.0172951 | 3 |
| 3230.0187415 | 1 |
| 3230.0198874 | 1 |
| 3230.0202736 | 2 |
| 3230.0214881 | 2 |
| 3230.0223163 | 0 |
| 3230.0239451 | 3 |
| 3230.0239861 | 2 |
| 3230.0254584 | 1 |
| 3230.0262047 | 0 |
| 3230.0273565 | 0 |
| 3230.0280055 | 2 |
| 3230.0283814 | 4 |
| 3230.0296017 | 1 |
| 3230.0305095 | 2 |
| 3230.0315315 | 0 |
| 3230.0328518 | 1 |
| 3230.0333912 | 2 |
| 3230.0346792 | 3 |
| 3230.0348096 | 0 |
| 3230.0363112 | 2 |
| 3230.0375255 | 3 |
| 3230.0380706 | 0 |
| 3230.0396127 | 2 |
| 3230.0404104 | 3 |
| 3230.040532 | 1 |
| 3230.0419233 | 2 |
| 3230.0426226 | 1 |
| 3230.0436505 | 0 |
| 3230.04486 | 2 |
| 3230.0463201 | 1 |
| 3230.0463436 | 3 |
| 3230.0472278 | 2 |
| 3230.0480635 | 0 |
| 3230.0498191 | 3 |
| 3230.0500162 | 1 |
| 3230.051818 | 1 |
| 3230.0521713 | 0 |
| 3230.0534054 | 2 |
| 3230.0548181 | 1 |
| 3230.0553651 | 2 |
| 3230.0562592 | 5 |
| 3230.0568226 | 1 |
| 3230.0575402 | 1 |
| 3230.0590012 | 3 |
| 3230.0598421 | 1 |
| 3230.0608873 | 1 |
| 3230.0618577 | 5 |
| 3230.062769 | 1 |
| 3230.0639634 | 0 |
| 3230.0646814 | 0 |
| 3230.0660494 | 0 |
| 3230.066424 | 2 |
| 3230.0673016 | 2 |
| 3230.0685581 | 2 |
| 3230.0698103 | 1 |
| 3230.0699101 | 2 |
| 3230.0716318 | 3 |
| 3230.0721109 | 3 |
| 3230.073347 | 1 |
| 3230.0746403 | 2 |
| 3230.0747844 | 1 |
| 3230.0759898 | 2 |
| 3230.0767678 | 2 |
| 3230.077826 | 4 |
| 3230.0791959 | 1 |
| 3230.0792178 | 3 |
| 3230.0808642 | 2 |
| 3230.0812619 | 0 |
| 3230.0824664 | 0 |
| 3230.0833523 | 2 |
| 3230.084575 | 1 |
| 3230.085397 | 1 |
| 3230.0861081 | 2 |
| 3230.0871921 | 1 |
| 3230.0878971 | 1 |
| 3230.0896231 | 1 |
| 3230.0902529 | 1 |
| 3230.0910683 | 1 |
| 3230.0923571 | 0 |
| 3230.0929134 | 1 |
| 3230.0940405 | 1 |
| 3230.0947077 | 0 |
| 3230.0954315 | 2 |
| 3230.0962137 | 1 |
| 3230.0979655 | 3 |
| 3230.0980811 | 0 |
| 3230.0998717 | 2 |
| 3230.100214 | 1 |
| 3230.1011893 | 3 |
| 3230.1015827 | 1 |
| 3230.103315 | 2 |
| 3230.1040563 | 2 |
| 3230.10492 | 0 |
| 3230.1067692 | 2 |
| 3230.1068 | 3 |
| 3230.1087128 | 0 |
| 3230.109328 | 1 |
| 3230.1099493 | 1 |
| 3230.1109089 | 3 |
| 3230.1116725 | 6 |
| 3230.112815 | 4 |
| 3230.1135952 | 1 |
| 3230.1150532 | 2 |
| 3230.1158337 | 4 |
| 3230.1164393 | 4 |
| 3230.1177843 | 4 |
| 3230.1178409 | 0 |
| 3230.1192865 | 0 |
| 3230.120427 | 0 |
| 3230.1210236 | 3 |
| 3230.122194 | 1 |
| 3230.1228555 | 2 |
| 3230.1244995 | 2 |
| 3230.1248808 | 2 |
| 3230.1258281 | 1 |
| 3230.1268835 | 1 |
| 3230.1272278 | 0 |
| 3230.1281566 | 3 |
| 3230.1298377 | 2 |
| 3230.1302697 | 3 |
| 3230.1312792 | 2 |
| 3230.1321344 | 1 |
| 3230.1333387 | 1 |
| 3230.1345594 | 2 |
| 3230.1352777 | 1 |
| 3230.1360971 | 2 |
| 3230.1365049 | 2 |
| 3230.1373556 | 0 |
| 3230.1386248 | 4 |
| 3230.1395301 | 2 |
| 3230.1410774 | 3 |
| 3230.1419623 | 5 |
| 3230.1430603 | 1 |
| 3230.1434401 | 2 |
| 3230.144343 | 6 |
| 3230.1451568 | 4 |
| 3230.1463424 | 1 |
| 3230.1475221 | 0 |
| 3230.1482 | 0 |
| 3230.1492849 | 0 |
| 3230.1499309 | 1 |
| 3230.1506008 | 2 |
| 3230.15202 | 3 |
| 3230.1524402 | 2 |
| 3230.1539765 | 0 |
| 3230.1541076 | 2 |
| 3230.1557517 | 0 |
| 3230.1567175 | 2 |
| 3230.1575861 | 5 |
| 3230.1582794 | 2 |
| 3230.1593566 | 1 |
| 3230.1603934 | 1 |
| 3230.1616307 | 0 |
| 3230.1624639 | 2 |
| 3230.1628268 | 3 |
| 3230.1636366 | 3 |
| 3230.1650401 | 4 |
| 3230.1664165 | 2 |
| 3230.1665927 | 6 |
| 3230.1674061 | 3 |
| 3230.1683769 | 3 |
| 3230.1692557 | 4 |
| 3230.170588 | 1 |
| 3230.1711409 | 4 |
| 3230.1724892 | 2 |
| 3230.1727878 | 1 |
| 3230.1740535 | 4 |
| 3230.17583 | 3 |
| 3230.1762887 | 0 |
| 3230.1770925 | 1 |
| 3230.177863 | 1 |
| 3230.1790355 | 3 |
| 3230.1792932 | 4 |
| 3230.180244 | 2 |
| 3230.1823412 | 0 |
| 3230.1823821 | 3 |
| 3230.1833741 | 2 |
| 3230.1840653 | 0 |
| 3230.184819 | 0 |
| 3230.1862117 | 1 |
| 3230.1871778 | 3 |
| 3230.188664 | 2 |
| 3230.1889841 | 1 |
| 3230.1902414 | 2 |
| 3230.1911523 | 4 |
| 3230.1912758 | 0 |
| 3230.1929476 | 1 |
| 3230.1934713 | 2 |
| 3230.1949303 | 1 |
| 3230.1957097 | 1 |
| 3230.1967626 | 3 |
| 3230.1977075 | 1 |
| 3230.1984433 | 2 |
| 3230.2000881 | 4 |
| 3230.200966 | 0 |
| 3230.201218 | 4 |
| 3230.2020698 | 0 |
| 3230.2029767 | 2 |
| 3230.203759 | 0 |
| 3230.2048001 | 3 |
| 3230.2058466 | 1 |
| 3230.2069424 | 1 |
| 3230.207799 | 5 |
| 3230.2091449 | 3 |
| 3230.2093852 | 2 |
| 3230.2103943 | 1 |
| 3230.2115888 | 0 |
| 3230.2118954 | 0 |
| 3230.213585 | 1 |
| 3230.2138678 | 0 |
| 3230.2149436 | 2 |
| 3230.2162369 | 1 |
| 3230.2162977 | 3 |
| 3230.2184557 | 5 |
| 3230.218894 | 1 |
| 3230.2196084 | 1 |
| 3230.2205299 | 2 |
| 3230.2213104 | 1 |
| 3230.2219351 | 1 |
| 3230.2229073 | 2 |
| 3230.2240769 | 0 |
| 3230.2251542 | 1 |
| 3230.2257168 | 0 |
| 3230.2271476 | 3 |
| 3230.2271612 | 1 |
| 3230.2283479 | 1 |
| 3230.2301387 | 3 |
| 3230.2305415 | 3 |
| 3230.2323061 | 0 |
| 3230.2323415 | 1 |
| 3230.234316 | 3 |
| 3230.2344439 | 2 |
| 3230.2354234 | 2 |
| 3230.2368317 | 1 |
| 3230.2370935 | 1 |
| 3230.2382147 | 2 |
| 3230.239461 | 1 |
| 3230.2404312 | 3 |
| 3230.2407598 | 3 |
| 3230.2422202 | 0 |
| 3230.2429325 | 1 |
| 3230.2443603 | 1 |
| 3230.2449587 | 2 |
| 3230.2464745 | 0 |
| 3230.2464986 | 1 |
| 3230.24809 | 1 |
| 3230.2482177 | 1 |
| 3230.249415 | 1 |
| 3230.2501833 | 0 |
| 3230.2508728 | 2 |

| | |
|---|---|
| 3230.2522273 | 1 |
| 3230.2534709 | 0 |
| 3230.2538792 | 1 |
| 3230.2550939 | 2 |
| 3230.2557673 | 2 |
| 3230.2571717 | 2 |
| 3230.2573996 | 1 |
| 3230.2588529 | 0 |
| 3230.260093 | 3 |
| 3230.2602304 | 2 |
| 3230.2614644 | 1 |
| 3230.2618633 | 5 |
| 3230.2625614 | 3 |
| 3230.2641853 | 2 |
| 3230.2649152 | 2 |
| 3230.2661775 | 2 |
| 3230.2671685 | 1 |
| 3230.267961 | 2 |
| 3230.268326 | 3 |
| 3230.270009 | 2 |
| 3230.2704848 | 2 |
| 3230.2711239 | 4 |
| 3230.2725249 | 3 |
| 3230.2728674 | 0 |
| 3230.2741342 | 4 |
| 3230.2753543 | 1 |
| 3230.2760344 | 1 |
| 3230.2773497 | 1 |
| 3230.2773728 | 3 |
| 3230.2789221 | 1 |
| 3230.2802352 | 2 |
| 3230.2809784 | 1 |
| 3230.2818536 | 3 |
| 3230.2824138 | 3 |
| 3230.2838493 | 1 |
| 3230.2838727 | 2 |
| 3230.2853447 | 3 |
| 3230.286837 | 1 |
| 3230.287249 | 4 |
| 3230.2876316 | 1 |
| 3230.2897144 | 1 |
| 3230.2905185 | 2 |
| 3230.2912001 | 2 |
| 3230.2915354 | 0 |
| 3230.2928557 | 1 |
| 3230.2932851 | 0 |
| 3230.2947249 | 2 |
| 3230.2955595 | 2 |
| 3230.2966074 | 1 |
| 3230.2974044 | 1 |
| 3230.2986201 | 4 |
| 3230.2994041 | 0 |
| 3230.3006192 | 2 |
| 3230.3010573 | 0 |
| 3230.3024263 | 0 |
| 3230.3031189 | 2 |
| 3230.3040283 | 4 |
| 3230.3047428 | 1 |
| 3230.305965 | 2 |
| 3230.3067697 | 1 |
| 3230.3070792 | 2 |
| 3230.3084497 | 2 |
| 3230.3097901 | 1 |
| 3230.3107831 | 3 |
| 3230.3112025 | 2 |
| 3230.3125545 | 0 |
| 3230.3132132 | 1 |
| 3230.3136692 | 0 |
| 3230.3152629 | 2 |
| 3230.3153146 | 1 |
| 3230.3167818 | 1 |
| 3230.317681 | 3 |
| 3230.3184403 | 3 |
| 3230.3196452 | 3 |
| 3230.3204694 | 2 |
| 3230.321564 | 3 |
| 3230.3226907 | 4 |
| 3230.3231798 | 2 |
| 3230.3246014 | 3 |
| 3230.3249682 | 1 |
| 3230.3261211 | 3 |
| 3230.3271977 | 6 |
| 3230.3276339 | 0 |
| 3230.329437 | 2 |
| 3230.3302361 | 2 |
| 3230.3305328 | 1 |
| 3230.3320027 | 2 |
| 3230.3326259 | 4 |
| 3230.333866 | 3 |
| 3230.3339924 | 4 |
| 3230.3356105 | 2 |
| 3230.3364102 | 2 |
| 3230.3370205 | 4 |
| 3230.3379467 | 0 |
| 3230.339209 | 2 |
| 3230.3398472 | 1 |
| 3230.3403857 | 4 |
| 3230.3417569 | 0 |
| 3230.342863 | 0 |
| 3230.3438754 | 2 |
| 3230.3446961 | 0 |
| 3230.346016 | 2 |
| 3230.3460895 | 2 |
| 3230.3473944 | 1 |
| 3230.3482044 | 3 |
| 3230.348738 | 0 |
| 3230.3501058 | 3 |
| 3230.3510109 | 4 |
| 3230.3510776 | 3 |
| 3230.352807 | 3 |
| 3230.3533992 | 0 |
| 3230.3548506 | 0 |
| 3230.3553986 | 4 |
| 3230.3562447 | 3 |
| 3230.3567541 | 4 |
| 3230.3576727 | 0 |
| 3230.3588114 | 2 |
| 3230.3595241 | 2 |
| 3230.3604137 | 2 |
| 3230.3614744 | 1 |
| 3230.3623568 | 0 |
| 3230.3632487 | 1 |
| 3230.3640729 | 1 |
| 3230.3649334 | 1 |
| 3230.3668918 | 0 |
| 3230.3669197 | 1 |
| 3230.3681078 | 0 |
| 3230.3695284 | 1 |
| 3230.3698143 | 1 |
| 3230.3711455 | 5 |
| 3230.3715538 | 1 |
| 3230.3729986 | 1 |
| 3230.3736509 | 1 |
| 3230.3745872 | 1 |
| 3230.3760969 | 4 |
| 3230.3766095 | 3 |
| 3230.3777745 | 2 |
| 3230.3782369 | 1 |
| 3230.3790672 | 0 |
| 3230.3803266 | 0 |
| 3230.3812498 | 3 |
| 3230.3824461 | 6 |
| 3230.3835582 | 3 |
| 3230.3842823 | 1 |
| 3230.3849533 | 4 |
| 3230.3861111 | 0 |
| 3230.3872051 | 1 |
| 3230.3876588 | 0 |
| 3230.3884169 | 2 |
| 3230.3891146 | 3 |
| 3230.3901438 | 2 |
| 3230.3917271 | 0 |
| 3230.3922562 | 1 |
| 3230.3930747 | 3 |
| 3230.3935836 | 1 |
| 3230.3951063 | 1 |
| 3230.3963982 | 0 |
| 3230.3967317 | 2 |
| 3230.3973012 | 1 |
| 3230.398529 | 0 |
| 3230.3995178 | 2 |
| 3230.4010236 | 2 |
| 3230.4010597 | 2 |
| 3230.4028114 | 2 |
| 3230.4030194 | 1 |
| 3230.4044273 | 3 |
| 3230.4049698 | 2 |
| 3230.4053011 | 3 |
| 3230.4070606 | 1 |
| 3230.4079646 | 4 |
| 3230.4089399 | 0 |
| 3230.4093542 | 1 |
| 3230.4105804 | 4 |
| 3230.411565 | 2 |
| 3230.4117988 | 2 |
| 3230.4136776 | 0 |
| 3230.41407 | 1 |
| 3230.4150444 | 3 |
| 3230.4167416 | 0 |
| 3230.4172323 | 1 |
| 3230.4179483 | 1 |
| 3230.4185082 | 2 |
| 3230.4204641 | 0 |
| 3230.4213386 | 2 |
| 3230.4214033 | 2 |
| 3230.4232937 | 4 |
| 3230.4233747 | 0 |
| 3230.4242335 | 2 |
| 3230.4255354 | 3 |
| 3230.4264472 | 0 |
| 3230.427671 | 1 |
| 3230.4286393 | 2 |
| 3230.4294731 | 4 |
| 3230.4308384 | 2 |
| 3230.4313056 | 1 |
| 3230.4323172 | 2 |
| 3230.4326208 | 3 |
| 3230.4342332 | 2 |
| 3230.4346479 | 2 |
| 3230.4355915 | 1 |
| 3230.4366014 | 2 |
| 3230.4373536 | 0 |
| 3230.4388855 | 1 |
| 3230.4389135 | 3 |
| 3230.4403297 | 3 |
| 3230.441421 | 2 |
| 3230.4420278 | 0 |
| 3230.4432363 | 1 |
| 3230.4435432 | 1 |
| 3230.4446077 | 3 |
| 3230.445983 | 2 |
| 3230.4465703 | 2 |
| 3230.4475962 | 1 |
| 3230.4486195 | 0 |
| 3230.4496342 | 3 |
| 3230.4507911 | 1 |
| 3230.4509224 | 4 |
| 3230.4522638 | 1 |
| 3230.4527442 | 0 |
| 3230.4537814 | 3 |
| 3230.4553972 | 1 |
| 3230.4555803 | 2 |
| 3230.4565906 | 0 |
| 3230.4580328 | 2 |
| 3230.4584679 | 0 |
| 3230.4595578 | 2 |
| 3230.4607232 | 2 |
| 3230.4617805 | 1 |
| 3230.4623335 | 1 |
| 3230.463043 | 2 |
| 3230.464999 | 1 |
| 3230.4654873 | 5 |
| 3230.4660156 | 1 |
| 3230.4665414 | 2 |
| 3230.4679573 | 1 |
| 3230.4686894 | 3 |
| 3230.4697904 | 2 |
| 3230.470505 | 4 |
| 3230.472174 | 2 |
| 3230.4733583 | 2 |
| 3230.4741655 | 0 |
| 3230.4741847 | 2 |
| 3230.475892 | 2 |
| 3230.4759869 | 0 |
| 3230.4769473 | 0 |
| 3230.478318 | 1 |
| 3230.4787506 | 2 |
| 3230.4799854 | 4 |
| 3230.4807333 | 6 |
| 3230.4822059 | 2 |
| 3230.4832262 | 4 |
| 3230.4840905 | 2 |
| 3230.4852329 | 4 |
| 3230.4853055 | 1 |
| 3230.4872713 | 4 |
| 3230.4875475 | 3 |
| 3230.4884196 | 3 |
| 3230.4898563 | 1 |
| 3230.4899843 | 4 |
| 3230.4913059 | 2 |
| 3230.4927298 | 2 |
| 3230.4927398 | 1 |
| 3230.4938898 | 3 |
| 3230.4947116 | 3 |
| 3230.4948558 | 1 |
| 3230.4971799 | 1 |
| 3230.4979431 | 3 |
| 3230.4984042 | 1 |
| 3230.4998097 | 4 |
| 3230.5006901 | 0 |
| 3230.5015767 | 1 |
| 3230.5023993 | 2 |
| 3230.503874 | 2 |
| 3230.5038923 | 4 |
| 3230.5052104 | 2 |
| 3230.5068028 | 3 |
| 3230.5068054 | 0 |
| 3230.5080779 | 3 |
| 3230.5089796 | 3 |
| 3230.509956 | 2 |
| 3230.5109809 | 3 |
| 3230.5118074 | 0 |
| 3230.5130905 | 0 |
| 3230.5131255 | 0 |
| 3230.5146527 | 2 |
| 3230.5159551 | 1 |
| 3230.5160026 | 2 |
| 3230.5176011 | 1 |
| 3230.5180182 | 2 |
| 3230.5196728 | 0 |
| 3230.5207647 | 0 |
| 3230.5210025 | 1 |
| 3230.522279 | 2 |
| 3230.5230452 | 2 |
| 3230.5239076 | 3 |
| 3230.5250945 | 6 |
| 3230.5266185 | 3 |
| 3230.5269749 | 2 |
| 3230.5273441 | 1 |
| 3230.5288632 | 4 |
| 3230.5290166 | 5 |
| 3230.5305949 | 3 |
| 3230.5313681 | 3 |
| 3230.5320638 | 0 |
| 3230.5335959 | 1 |
| 3230.5343336 | 0 |
| 3230.5348513 | 4 |
| 3230.5362518 | 0 |
| 3230.5366672 | 5 |
| 3230.5380901 | 1 |
| 3230.5391971 | 3 |
| 3230.5396045 | 5 |
| 3230.5409557 | 1 |
| 3230.5410184 | 2 |
| 3230.5423162 | 2 |
| 3230.5438268 | 0 |
| 3230.5446465 | 1 |
| 3230.5455532 | 0 |
| 3230.5460276 | 1 |
| 3230.5465638 | 1 |
| 3230.5475883 | 0 |
| 3230.5486614 | 1 |
| 3230.5503661 | 1 |
| 3230.5506411 | 2 |
| 3230.5521286 | 2 |
| 3230.5524934 | 3 |
| 3230.5532581 | 1 |
| 3230.5550826 | 4 |
| 3230.5553544 | 3 |
| 3230.5566992 | 1 |
| 3230.5573052 | 4 |
| 3230.5578542 | 1 |
| 3230.5594714 | 2 |
| 3230.5599712 | 3 |
| 3230.5610211 | 0 |
| 3230.5626336 | 1 |
| 3230.5631886 | 3 |
| 3230.5646552 | 0 |
| 3230.5648489 | 2 |
| 3230.5666866 | 0 |
| 3230.5671704 | 1 |
| 3230.5674311 | 1 |
| 3230.5688679 | 0 |
| 3230.5694242 | 2 |
| 3230.5706725 | 4 |
| 3230.5715296 | 4 |
| 3230.5722099 | 0 |
| 3230.5738473 | 0 |
| 3230.5739628 | 1 |
| 3230.5751723 | 3 |
| 3230.5762955 | 2 |
| 3230.5770193 | 5 |
| 3230.5780684 | 5 |
| 3230.5789803 | 2 |
| 3230.5799746 | 3 |
| 3230.5799935 | 0 |
| 3230.581892 | 3 |
| 3230.5821814 | 2 |
| 3230.5829172 | 1 |
| 3230.5841438 | 1 |
| 3230.585451 | 3 |
| 3230.5861711 | 4 |
| 3230.5874434 | 3 |

| | |
|---|---|
| 3230.5884734 | 2 |
| 3230.5892301 | 2 |
| 3230.5901819 | 2 |
| 3230.5910936 | 0 |
| 3230.5919415 | 2 |
| 3230.5931264 | 1 |
| 3230.5936787 | 1 |
| 3230.5950155 | 1 |
| 3230.5955838 | 1 |
| 3230.5967095 | 2 |
| 3230.5967156 | 2 |
| 3230.5983991 | 1 |
| 3230.5995199 | 2 |
| 3230.5998937 | 1 |
| 3230.6016472 | 1 |
| 3230.6018164 | 2 |
| 3230.6027405 | 0 |
| 3230.6048273 | 0 |
| 3230.6050755 | 1 |
| 3230.6063414 | 5 |
| 3230.6064181 | 1 |
| 3230.6077653 | 0 |
| 3230.6092839 | 1 |
| 3230.6098839 | 1 |
| 3230.6108043 | 3 |
| 3230.6113647 | 4 |
| 3230.6130511 | 0 |
| 3230.6137705 | 1 |
| 3230.6144593 | 2 |
| 3230.6158318 | 4 |
| 3230.6163676 | 6 |
| 3230.6174145 | 0 |
| 3230.6179911 | 0 |
| 3230.6195269 | 2 |
| 3230.6196848 | 2 |
| 3230.6205273 | 3 |
| 3230.622165 | 2 |
| 3230.6230253 | 0 |
| 3230.623404 | 3 |
| 3230.6240322 | 1 |
| 3230.6248998 | 1 |
| 3230.6261335 | 3 |
| 3230.6274823 | 2 |
| 3230.6280989 | 2 |
| 3230.6291412 | 1 |
| 3230.6303634 | 0 |
| 3230.6311576 | 3 |
| 3230.6318423 | 1 |
| 3230.6326938 | 2 |
| 3230.6335786 | 2 |
| 3230.6341217 | 1 |
| 3230.6358553 | 3 |
| 3230.6371755 | 2 |
| 3230.6373187 | 1 |
| 3230.6381141 | 1 |
| 3230.6390298 | 1 |
| 3230.6401198 | 2 |
| 3230.6419131 | 0 |
| 3230.6421253 | 1 |
| 3230.6432688 | 1 |
| 3230.6445747 | 4 |
| 3230.6449187 | 1 |
| 3230.6459444 | 1 |
| 3230.6464577 | 0 |
| 3230.6474329 | 2 |
| 3230.6487238 | 2 |
| 3230.6497497 | 2 |
| 3230.6509005 | 2 |
| 3230.6518269 | 2 |
| 3230.6529823 | 4 |
| 3230.6538646 | 2 |
| 3230.6547137 | 2 |
| 3230.655458 | 1 |
| 3230.655868 | 1 |
| 3230.6569019 | 0 |
| 3230.6584203 | 3 |
| 3230.6590822 | 4 |
| 3230.6601421 | 2 |
| 3230.6613419 | 0 |
| 3230.6619836 | 3 |
| 3230.6634579 | 0 |
| 3230.6635997 | 3 |
| 3230.6651936 | 2 |
| 3230.6655637 | 2 |
| 3230.6665898 | 2 |
| 3230.6678669 | 1 |
| 3230.668309 | 1 |
| 3230.6693239 | 2 |
| 3230.6697785 | 4 |
| 3230.6716754 | 3 |
| 3230.6722627 | 4 |
| 3230.6724056 | 2 |
| 3230.6735814 | 3 |
| 3230.6741578 | 3 |
| 3230.6745179 | 4 |
| 3230.6750842 | 5 |
| 3230.675113 | 0 |
| 3230.6756625 | 2 |
| 3230.6760254 | 1 |
| 3230.6766856 | 2 |
| 3230.6768583 | 2 |
| 3230.6773933 | 3 |
| 3230.6779564 | 0 |
| 3230.6791554 | 2 |
| 3230.6792695 | 2 |
| 3230.6794303 | 0 |
| 3230.67963 | 3 |
| 3230.6810659 | 1 |
| 3230.6811163 | 6 |
| 3230.681472 | 3 |
| 3230.6820111 | 2 |
| 3230.6820928 | 2 |
| 3230.6825156 | 0 |
| 3230.6835114 | 3 |
| 3230.6835841 | 0 |
| 3230.6839413 | 3 |
| 3230.6843241 | 2 |
| 3230.685224 | 1 |
| 3230.685248 | 0 |
| 3230.6857071 | 2 |
| 3230.6857208 | 1 |
| 3230.6868785 | 2 |
| 3230.6874829 | 2 |
| 3230.6879008 | 2 |
| 3230.6880011 | 1 |
| 3230.6883627 | 2 |
| 3230.6887574 | 2 |
| 3230.6899954 | 0 |
| 3230.6906835 | 4 |
| 3230.6914547 | 1 |
| 3230.6927917 | 1 |
| 3230.6931792 | 2 |
| 3230.6946374 | 1 |
| 3230.6960184 | 1 |
| 3230.696977 | 2 |
| 3230.697359 | 2 |
| 3230.6982362 | 5 |
| 3230.6994226 | 2 |
| 3230.7007623 | 2 |
| 3230.7015638 | 1 |
| 3230.701692 | 3 |
| 3230.703375 | 0 |
| 3230.703463 | 2 |
| 3230.7055178 | 1 |
| 3230.7057767 | 2 |
| 3230.7071248 | 2 |
| 3230.7079245 | 2 |
| 3230.7082589 | 0 |
| 3230.709841 | 3 |
| 3230.7111726 | 1 |
| 3230.7118867 | 2 |
| 3230.7126485 | 0 |
| 3230.713601 | 2 |
| 3230.7147542 | 4 |
| 3230.7148351 | 1 |
| 3230.715813 | 2 |
| 3230.7165677 | 3 |
| 3230.7177607 | 1 |
| 3230.7196556 | 4 |
| 3230.7201791 | 2 |
| 3230.7213611 | 2 |
| 3230.7214495 | 1 |
| 3230.7227642 | 0 |
| 3230.7232386 | 0 |
| 3230.7242874 | 0 |
| 3230.7254665 | 1 |
| 3230.7259925 | 1 |
| 3230.7272742 | 4 |
| 3230.7281536 | 3 |
| 3230.7284891 | 2 |
| 3230.7295276 | 0 |
| 3230.7313415 | 1 |
| 3230.7317039 | 4 |
| 3230.7329192 | 4 |
| 3230.7335552 | 1 |
| 3230.7350343 | 1 |
| 3230.735303 | 0 |
| 3230.7367486 | 0 |
| 3230.7376805 | 1 |
| 3230.7385496 | 2 |
| 3230.7394074 | 2 |
| 3230.7404661 | 1 |
| 3230.7410498 | 2 |
| 3230.7423892 | 2 |
| 3230.7428682 | 1 |
| 3230.7444914 | 2 |
| 3230.74471 | 5 |
| 3230.7460464 | 4 |
| 3230.7465978 | 1 |
| 3230.7481179 | 2 |
| 3230.7484805 | 0 |
| 3230.7497194 | 2 |
| 3230.7504027 | 3 |
| 3230.7515446 | 4 |
| 3230.7522309 | 1 |
| 3230.7536882 | 5 |
| 3230.7540802 | 0 |
| 3230.75509 | 0 |
| 3230.7562237 | 2 |
| 3230.7562884 | 2 |
| 3230.7581197 | 4 |
| 3230.7587091 | 4 |
| 3230.7595723 | 4 |
| 3230.7605557 | 3 |
| 3230.7615635 | 3 |
| 3230.7628328 | 4 |
| 3230.763271 | 1 |
| 3230.7641629 | 2 |
| 3230.7649773 | 5 |
| 3230.7660041 | 3 |
| 3230.7671595 | 1 |
| 3230.7677674 | 0 |
| 3230.7688499 | 1 |
| 3230.7697292 | 1 |
| 3230.7706748 | 1 |
| 3230.771662 | 0 |
| 3230.7724942 | 2 |
| 3230.7734216 | 4 |
| 3230.7745681 | 2 |
| 3230.7751458 | 5 |
| 3230.7770382 | 1 |
| 3230.7770799 | 1 |
| 3230.778482 | 4 |
| 3230.7791905 | 5 |
| 3230.7796588 | 1 |
| 3230.7812372 | 2 |
| 3230.7819111 | 2 |
| 3230.7824655 | 2 |
| 3230.7835023 | 0 |
| 3230.7847534 | 2 |
| 3230.7857332 | 3 |
| 3230.7869349 | 2 |
| 3230.7876534 | 2 |
| 3230.7884058 | 3 |
| 3230.7897738 | 2 |
| 3230.7903864 | 4 |
| 3230.7916447 | 2 |
| 3230.7927244 | 2 |
| 3230.7927289 | 2 |
| 3230.7939011 | 0 |
| 3230.7956673 | 1 |
| 3230.795872 | 2 |
| 3230.7969609 | 3 |
| 3230.7981939 | 2 |
| 3230.7991281 | 3 |
| 3230.7996307 | 2 |
| 3230.8004032 | 1 |
| 3230.801908 | 1 |
| 3230.8021483 | 3 |
| 3230.8036587 | 1 |
| 3230.8049208 | 1 |
| 3230.8050306 | 2 |
| 3230.8061422 | 0 |
| 3230.8069625 | 3 |
| 3230.8081879 | 1 |
| 3230.8086638 | 1 |
| 3230.8097424 | 0 |
| 3230.8112621 | 6 |
| 3230.8114949 | 1 |
| 3230.8128163 | 3 |
| 3230.8135445 | 1 |
| 3230.8142005 | 3 |
| 3230.815486 | 2 |
| 3230.8170898 | 0 |
| 3230.8172676 | 1 |
| 3230.8183713 | 2 |
| 3230.8190397 | 0 |
| 3230.8200902 | 0 |
| 3230.8209204 | 1 |
| 3230.821814 | 0 |
| 3230.8232015 | 1 |
| 3230.8236863 | 1 |
| 3230.8249925 | 0 |
| 3230.8253641 | 4 |
| 3230.8269735 | 3 |
| 3230.8279508 | 2 |
| 3230.8286855 | 4 |
| 3230.8299346 | 2 |
| 3230.8300683 | 2 |
| 3230.8316676 | 2 |
| 3230.8332701 | 0 |
| 3230.8332729 | 4 |
| 3230.834355 | 1 |
| 3230.8347838 | 3 |
| 3230.8360146 | 1 |
| 3230.8365228 | 0 |
| 3230.838116 | 3 |
| 3230.8393606 | 1 |
| 3230.8398709 | 0 |
| 3230.8407514 | 3 |
| 3230.8416756 | 2 |
| 3230.8430933 | 3 |
| 3230.8440387 | 2 |
| 3230.8444352 | 1 |
| 3230.8455596 | 4 |
| 3230.8466894 | 1 |
| 3230.8468416 | 1 |
| 3230.8484033 | 4 |
| 3230.8490861 | 0 |
| 3230.8499066 | 1 |
| 3230.8507931 | 2 |
| 3230.8515913 | 3 |
| 3230.8523006 | 2 |
| 3230.8533996 | 3 |
| 3230.8540724 | 2 |
| 3230.8557599 | 1 |
| 3230.8563424 | 0 |
| 3230.8579136 | 2 |
| 3230.8580068 | 2 |
| 3230.8590304 | 0 |
| 3230.8606026 | 2 |
| 3230.8609093 | 2 |
| 3230.862004 | 4 |
| 3230.8624045 | 2 |
| 3230.8634932 | 4 |
| 3230.8650493 | 0 |
| 3230.8659806 | 5 |
| 3230.8667589 | 2 |
| 3230.8674942 | 2 |
| 3230.8685861 | 0 |
| 3230.8699048 | 3 |
| 3230.8705843 | 2 |
| 3230.8715665 | 1 |
| 3230.8727082 | 2 |
| 3230.8735179 | 3 |
| 3230.873839 | 2 |
| 3230.8749623 | 2 |
| 3230.8761432 | 1 |
| 3230.8769892 | 1 |
| 3230.8783376 | 1 |
| 3230.8786896 | 1 |
| 3230.8795905 | 4 |
| 3230.8811646 | 1 |
| 3230.8815783 | 2 |
| 3230.88294 | 3 |
| 3230.88345 | 5 |
| 3230.8846547 | 3 |
| 3230.8858489 | 2 |
| 3230.8859698 | 2 |
| 3230.8871245 | 2 |
| 3230.8881883 | 1 |
| 3230.8888883 | 3 |
| 3230.8906363 | 1 |
| 3230.8912021 | 4 |
| 3230.8917509 | 3 |
| 3230.89286 | 3 |
| 3230.8938141 | 0 |
| 3230.8945668 | 1 |
| 3230.8954981 | 2 |
| 3230.8967126 | 3 |
| 3230.897983 | 3 |
| 3230.8982361 | 1 |
| 3230.8999659 | 2 |
| 3230.9008299 | 4 |
| 3230.9015831 | 3 |
| 3230.9021851 | 5 |
| 3230.9031154 | 0 |
| 3230.9039899 | 1 |
| 3230.904983 | 3 |
| 3230.9062162 | 0 |
| 3230.9067409 | 2 |
| 3230.9076444 | 0 |
| 3230.9087419 | 1 |

| | |
|---|---|
| 3230.9095864 | 2 |
| 3230.910673 | 0 |
| 3230.9119802 | 2 |
| 3230.9124845 | 2 |
| 3230.9137474 | 4 |
| 3230.9137654 | 1 |
| 3230.9151536 | 0 |
| 3230.9164746 | 1 |
| 3230.9169225 | 3 |
| 3230.9182188 | 2 |
| 3230.9187994 | 3 |
| 3230.9198201 | 2 |
| 3230.9205387 | 4 |
| 3230.9217608 | 3 |
| 3230.9228477 | 2 |
| 3230.9238763 | 0 |
| 3230.9248972 | 3 |
| 3230.9258435 | 0 |
| 3230.9268049 | 2 |
| 3230.9273803 | 3 |
| 3230.9279888 | 4 |
| 3230.9296375 | 2 |
| 3230.9304354 | 2 |
| 3230.9312272 | 4 |
| 3230.932504 | 2 |
| 3230.9333287 | 4 |
| 3230.9344704 | 3 |
| 3230.9351738 | 3 |
| 3230.9360412 | 2 |
| 3230.9368106 | 1 |
| 3230.9381359 | 1 |
| 3230.9391545 | 1 |
| 3230.9402383 | 1 |
| 3230.9406806 | 2 |
| 3230.9416545 | 2 |
| 3230.9418455 | 1 |
| 3230.9431075 | 1 |
| 3230.9441667 | 2 |
| 3230.9444631 | 1 |
| 3230.9464869 | 2 |
| 3230.9468302 | 3 |
| 3230.9472227 | 2 |
| 3230.9490648 | 3 |
| 3230.9497773 | 1 |
| 3230.9507111 | 2 |
| 3230.9514627 | 1 |
| 3230.9526278 | 2 |
| 3230.9538549 | 3 |
| 3230.9541888 | 1 |
| 3230.9554581 | 3 |
| 3230.9560288 | 5 |
| 3230.9569902 | 2 |
| 3230.9586659 | 1 |
| 3230.9589062 | 1 |
| 3230.9604523 | 2 |
| 3230.9609933 | 2 |
| 3230.9622947 | 3 |
| 3230.9623666 | 3 |
| 3230.9638365 | 0 |
| 3230.9653895 | 1 |
| 3230.9654851 | 0 |
| 3230.9662377 | 2 |
| 3230.9672952 | 2 |
| 3230.9683052 | 1 |
| 3230.9698068 | 1 |
| 3230.9704858 | 3 |
| 3230.9720612 | 1 |
| 3230.9721037 | 3 |
| 3230.9733502 | 1 |
| 3230.9746969 | 2 |
| 3230.9756424 | 1 |
| 3230.9757436 | 1 |
| 3230.9772664 | 1 |
| 3230.9780107 | 5 |
| 3230.9791198 | 0 |
| 3230.9797757 | 3 |
| 3230.9805959 | 1 |
| 3230.9819469 | 4 |
| 3230.9819834 | 0 |
| 3230.9840519 | 3 |
| 3230.9846448 | 1 |
| 3230.9853941 | 2 |
| 3230.9864989 | 3 |
| 3230.9870742 | 0 |
| 3230.9882572 | 2 |
| 3230.9891098 | 4 |
| 3230.9906786 | 1 |
| 3230.9914949 | 3 |
| 3230.9921261 | 1 |
| 3230.993307 | 1 |
| 3230.9935103 | 3 |
| 3230.9952929 | 5 |
| 3230.9957332 | 0 |
| 3230.9967104 | 0 |
| 3230.9978938 | 5 |
| 3230.9983786 | 2 |
| 3230.9999748 | 2 |
| 3231.0001404 | 4 |
| 3231.0016104 | 1 |
| 3231.0028303 | 2 |
| 3231.0028884 | 1 |
| 3231.0045236 | 2 |
| 3231.0049351 | 2 |
| 3231.0054348 | 4 |
| 3231.0076649 | 1 |
| 3231.0081056 | 0 |
| 3231.0091985 | 4 |
| 3231.0101729 | 4 |
| 3231.010749 | 2 |
| 3231.0113874 | 0 |
| 3231.0129431 | 2 |
| 3231.0141289 | 4 |
| 3231.0148086 | 3 |
| 3231.0157342 | 4 |
| 3231.0167507 | 2 |
| 3231.0175568 | 2 |
| 3231.0188609 | 4 |
| 3231.0194898 | 3 |
| 3231.0215276 | 3 |
| 3231.0215906 | 1 |
| 3231.0225739 | 1 |
| 3231.0240467 | 2 |
| 3231.0241411 | 0 |
| 3231.0248659 | 2 |
| 3231.0261816 | 3 |
| 3231.0268959 | 1 |
| 3231.0278479 | 1 |
| 3231.0284322 | 3 |
| 3231.0302743 | 2 |
| 3231.0312125 | 2 |
| 3231.0313064 | 1 |
| 3231.0331127 | 0 |
| 3231.0332772 | 1 |
| 3231.0342914 | 1 |
| 3231.0359213 | 0 |
| 3231.0360475 | 3 |
| 3231.0372541 | 1 |
| 3231.0379092 | 1 |
| 3231.0397518 | 1 |
| 3231.0407589 | 4 |
| 3231.0410018 | 1 |
| 3231.0426514 | 0 |
| 3231.0427524 | 2 |
| 3231.0436174 | 1 |
| 3231.0443733 | 1 |
| 3231.0461871 | 2 |
| 3231.0471938 | 2 |
| 3231.0478988 | 3 |
| 3231.0487328 | 5 |
| 3231.0488953 | 1 |
| 3231.0501717 | 1 |
| 3231.0516747 | 1 |
| 3231.0523705 | 1 |
| 3231.0533552 | 6 |
| 3231.0542804 | 2 |
| 3231.0553774 | 1 |
| 3231.0566241 | 3 |
| 3231.0570891 | 4 |
| 3231.0578424 | 4 |
| 3231.0596257 | 2 |
| 3231.0601617 | 1 |
| 3231.0612507 | 2 |
| 3231.061986 | 3 |
| 3231.0628934 | 1 |
| 3231.0633977 | 0 |
| 3231.0651763 | 0 |
| 3231.0659857 | 2 |
| 3231.0666903 | 2 |
| 3231.0676754 | 1 |
| 3231.0683285 | 3 |
| 3231.0692701 | 3 |
| 3231.070996 | 2 |
| 3231.0714341 | 1 |
| 3231.0722382 | 0 |
| 3231.0731199 | 1 |
| 3231.0736603 | 3 |
| 3231.0751874 | 2 |
| 3231.0762868 | 1 |
| 3231.0772131 | 1 |
| 3231.0777462 | 0 |
| 3231.0789962 | 3 |
| 3231.0798275 | 1 |
| 3231.0804198 | 2 |
| 3231.0819709 | 4 |
| 3231.0827043 | 2 |
| 3231.083598 | 0 |
| 3231.0849469 | 4 |
| 3231.0851049 | 2 |
| 3231.0862784 | 2 |
| 3231.0869822 | 4 |
| 3231.0883823 | 1 |
| 3231.0889706 | 0 |
| 3231.0898718 | 2 |
| 3231.091167 | 2 |
| 3231.091321 | 5 |
| 3231.092631 | 0 |
| 3231.0942452 | 4 |
| 3231.0947268 | 0 |
| 3231.0960594 | 1 |
| 3231.0964498 | 2 |
| 3231.0979166 | 2 |
| 3231.0984244 | 1 |
| 3231.0995373 | 2 |
| 3231.1011507 | 5 |
| 3231.1012955 | 2 |
| 3231.1020921 | 3 |
| 3231.1030506 | 2 |
| 3231.1043886 | 1 |
| 3231.1054655 | 7 |
| 3231.10596 | 4 |
| 3231.1074022 | 0 |
| 3231.1076702 | 1 |
| 3231.109598 | 2 |
| 3231.1106123 | 2 |
| 3231.1112407 | 1 |
| 3231.1120807 | 1 |
| 3231.1133542 | 1 |
| 3231.114148 | 0 |
| 3231.1150041 | 5 |
| 3231.1155754 | 2 |
| 3231.1168891 | 2 |
| 3231.1181438 | 0 |
| 3231.1184374 | 4 |
| 3231.1199383 | 2 |
| 3231.1199848 | 0 |
| 3231.1215585 | 4 |
| 3231.1215855 | 3 |
| 3231.1232469 | 1 |
| 3231.1243083 | 5 |
| 3231.1248775 | 2 |
| 3231.1262376 | 0 |
| 3231.1266732 | 4 |
| 3231.1273519 | 4 |
| 3231.1291471 | 2 |
| 3231.1295543 | 3 |
| 3231.1303991 | 3 |
| 3231.1310897 | 5 |
| 3231.1323767 | 1 |
| 3231.1341466 | 2 |
| 3231.1342284 | 1 |
| 3231.1354909 | 2 |
| 3231.1357621 | 0 |
| 3231.1368927 | 0 |
| 3231.1380771 | 4 |
| 3231.1390057 | 2 |
| 3231.1396382 | 2 |
| 3231.1408476 | 3 |
| 3231.1414444 | 1 |
| 3231.1425327 | 2 |
| 3231.1438321 | 3 |
| 3231.1448271 | 1 |
| 3231.1454912 | 1 |
| 3231.1468145 | 2 |
| 3231.1480676 | 4 |
| 3231.1482238 | 2 |
| 3231.1491028 | 3 |
| 3231.1512196 | 1 |
| 3231.1514364 | 1 |
| 3231.1528229 | 1 |
| 3231.1531135 | 1 |
| 3231.1539519 | 1 |
| 3231.1550039 | 2 |
| 3231.156456 | 0 |
| 3231.1574422 | 2 |
| 3231.1578733 | 2 |
| 3231.1592552 | 5 |
| 3231.1592626 | 2 |
| 3231.1609584 | 1 |
| 3231.1617216 | 0 |
| 3231.1626641 | 2 |
| 3231.1640642 | 2 |
| 3231.1644581 | 0 |
| 3231.1658971 | 2 |
| 3231.1667441 | 2 |
| 3231.1676753 | 3 |
| 3231.1686297 | 2 |
| 3231.1692831 | 2 |
| 3231.1699809 | 2 |
| 3231.1708865 | 3 |
| 3231.1721679 | 0 |
| 3231.1726677 | 3 |
| 3231.1737384 | 2 |
| 3231.1747422 | 2 |
| 3231.176175 | 2 |
| 3231.1770197 | 1 |
| 3231.1780488 | 0 |
| 3231.1780629 | 0 |
| 3231.1796916 | 1 |
| 3231.180458 | 2 |
| 3231.1810469 | 0 |
| 3231.182169 | 2 |
| 3231.1832643 | 4 |
| 3231.1844052 | 3 |
| 3231.184782 | 0 |
| 3231.1863436 | 0 |
| 3231.1866761 | 1 |
| 3231.1881554 | 2 |
| 3231.1893754 | 1 |
| 3231.1902206 | 1 |
| 3231.1910759 | 3 |
| 3231.1912667 | 1 |
| 3231.192699 | 2 |
| 3231.1940805 | 2 |
| 3231.1942469 | 4 |
| 3231.1957904 | 3 |
| 3231.1963717 | 5 |
| 3231.1970802 | 5 |
| 3231.1988777 | 4 |
| 3231.1988945 | 2 |
| 3231.2006879 | 1 |
| 3231.2008059 | 3 |
| 3231.2020578 | 6 |
| 3231.2033756 | 3 |
| 3231.2036471 | 4 |
| 3231.2053382 | 4 |
| 3231.2057056 | 1 |
| 3231.207322 | 2 |
| 3231.2080021 | 0 |
| 3231.2083678 | 1 |
| 3231.2095666 | 1 |
| 3231.2103828 | 2 |
| 3231.2111657 | 6 |
| 3231.2127789 | 0 |
| 3231.213068 | 0 |
| 3231.2145184 | 2 |
| 3231.2148054 | 1 |
| 3231.2157982 | 0 |
| 3231.2162731 | 0 |
| 3231.2186333 | 2 |
| 3231.2192342 | 2 |
| 3231.2197391 | 0 |
| 3231.2207142 | 3 |
| 3231.2213154 | 0 |
| 3231.2222061 | 3 |
| 3231.2235135 | 3 |
| 3231.224755 | 0 |
| 3231.2261704 | 3 |
| 3231.2262599 | 1 |
| 3231.2279678 | 3 |
| 3231.2280876 | 1 |
| 3231.2286315 | 2 |
| 3231.2301851 | 2 |
| 3231.2310925 | 1 |
| 3231.2319256 | 2 |
| 3231.2324932 | 1 |
| 3231.2339623 | 1 |
| 3231.2348143 | 1 |
| 3231.2359922 | 2 |
| 3231.2372013 | 1 |
| 3231.2376677 | 1 |
| 3231.2384215 | 3 |
| 3231.2395849 | 1 |
| 3231.2403081 | 0 |
| 3231.2419653 | 2 |
| 3231.2422379 | 1 |
| 3231.2433453 | 3 |
| 3231.2444346 | 0 |
| 3231.2449334 | 1 |
| 3231.2465343 | 1 |
| 3231.2465511 | 3 |
| 3231.2479214 | 3 |
| 3231.2488641 | 1 |
| 3231.2498879 | 2 |

| | |
|---|---|
| 3231.2510944 | 1 |
| 3231.2514047 | 3 |
| 3231.2536073 | 0 |
| 3231.2539014 | 1 |
| 3231.2545798 | 3 |
| 3231.2555076 | 6 |
| 3231.2558622 | 2 |
| 3231.2575598 | 4 |
| 3231.2579987 | 2 |
| 3231.2592256 | 1 |
| 3231.2603547 | 2 |
| 3231.2611124 | 0 |
| 3231.2618143 | 2 |
| 3231.2628514 | 2 |
| 3231.2635679 | 1 |
| 3231.2647579 | 4 |
| 3231.2653606 | 4 |
| 3231.2664078 | 2 |
| 3231.2672563 | 2 |
| 3231.2682582 | 5 |
| 3231.2691278 | 1 |
| 3231.2695663 | 1 |
| 3231.2713342 | 0 |
| 3231.2723074 | 3 |
| 3231.2728511 | 4 |
| 3231.2744052 | 0 |
| 3231.2744324 | 4 |
| 3231.2761251 | 4 |
| 3231.2771254 | 1 |
| 3231.2777007 | 2 |
| 3231.2787064 | 0 |
| 3231.2794541 | 3 |
| 3231.2804923 | 3 |
| 3231.2807841 | 1 |
| 3231.2819242 | 1 |
| 3231.2836797 | 0 |
| 3231.2843155 | 6 |
| 3231.2853541 | 4 |
| 3231.2862389 | 0 |
| 3231.2869025 | 2 |
| 3231.2878957 | 3 |
| 3231.2887175 | 3 |
| 3231.2902827 | 3 |
| 3231.2908604 | 2 |
| 3231.2920604 | 0 |
| 3231.293228 | 2 |
| 3231.2937119 | 3 |
| 3231.2945349 | 4 |
| 3231.2950356 | 2 |
| 3231.2962275 | 3 |
| 3231.2976996 | 1 |
| 3231.2978237 | 2 |
| 3231.2990434 | 1 |
| 3231.3003581 | 5 |
| 3231.3007422 | 2 |
| 3231.3021896 | 3 |
| 3231.3026108 | 0 |
| 3231.3035255 | 0 |
| 3231.3046592 | 1 |
| 3231.3061426 | 6 |
| 3231.3068652 | 2 |
| 3231.3079427 | 1 |
| 3231.3084416 | 1 |
| 3231.3088449 | 0 |
| 3231.3101981 | 0 |
| 3231.3111801 | 1 |
| 3231.3119755 | 2 |
| 3231.3131275 | 2 |
| 3231.3141196 | 2 |
| 3231.3152504 | 0 |
| 3231.3164231 | 1 |
| 3231.3166866 | 0 |
| 3231.3168613 | 0 |
| 3231.3184259 | 3 |
| 3231.3193302 | 4 |
| 3231.320414 | 1 |
| 3231.321432 | 1 |
| 3231.3226702 | 1 |
| 3231.323208 | 0 |
| 3231.323989 | 4 |
| 3231.3247461 | 2 |
| 3231.3256409 | 1 |
| 3231.3271216 | 0 |
| 3231.3283663 | 2 |
| 3231.3289847 | 2 |
| 3231.3299459 | 1 |
| 3231.329996 | 2 |
| 3231.3316427 | 2 |
| 3231.3328858 | 0 |
| 3231.3332888 | 1 |
| 3231.3346486 | 0 |
| 3231.3352078 | 3 |
| 3231.3367361 | 3 |
| 3231.3376963 | 1 |
| 3231.3380159 | 2 |
| 3231.3396275 | 1 |
| 3231.3397237 | 2 |
| 3231.341012 | 3 |
| 3231.342044 | 1 |
| 3231.3427408 | 1 |
| 3231.3436678 | 0 |
| 3231.344645 | 1 |
| 3231.3453933 | 2 |
| 3231.3468143 | 2 |
| 3231.3477159 | 2 |
| 3231.3488579 | 2 |
| 3231.3491734 | 0 |
| 3231.3500146 | 0 |
| 3231.3512581 | 5 |
| 3231.3514758 | 0 |
| 3231.3524839 | 1 |
| 3231.353314 | 1 |
| 3231.3551177 | 2 |
| 3231.3556786 | 2 |
| 3231.356415 | 1 |
| 3231.3577673 | 1 |
| 3231.3581204 | 2 |
| 3231.3593625 | 3 |
| 3231.3597959 | 2 |
| 3231.361264 | 2 |
| 3231.3621037 | 2 |
| 3231.3628308 | 1 |
| 3231.3634325 | 1 |
| 3231.3646456 | 3 |
| 3231.3659928 | 1 |
| 3231.3668712 | 0 |
| 3231.3674679 | 1 |
| 3231.3686631 | 0 |
| 3231.3693328 | 2 |
| 3231.3707746 | 0 |
| 3231.371603 | 2 |
| 3231.3729053 | 1 |
| 3231.3732328 | 4 |
| 3231.3735315 | 5 |
| 3231.3752207 | 2 |
| 3231.3761254 | 2 |
| 3231.3765109 | 3 |
| 3231.3778393 | 2 |
| 3231.3790764 | 3 |
| 3231.3795686 | 2 |
| 3231.3811371 | 3 |
| 3231.3820541 | 3 |
| 3231.3831513 | 4 |
| 3231.3834697 | 2 |
| 3231.384392 | 2 |
| 3231.3849896 | 3 |
| 3231.3862108 | 3 |
| 3231.3871682 | 1 |
| 3231.3880291 | 1 |
| 3231.3890134 | 4 |
| 3231.3899174 | 2 |
| 3231.3908748 | 1 |
| 3231.3919843 | 0 |
| 3231.3919897 | 1 |
| 3231.3935519 | 1 |
| 3231.3942676 | 0 |
| 3231.3950499 | 2 |
| 3231.3967902 | 2 |
| 3231.3969841 | 2 |
| 3231.3985674 | 3 |
| 3231.3988449 | 0 |
| 3231.3998709 | 4 |
| 3231.4014881 | 3 |
| 3231.4015988 | 1 |
| 3231.4030755 | 1 |
| 3231.4036784 | 3 |
| 3231.403709 | 2 |
| 3231.406112 | 0 |
| 3231.4062057 | 2 |
| 3231.4075523 | 0 |
| 3231.4081576 | 1 |
| 3231.4088197 | 2 |
| 3231.4098033 | 3 |
| 3231.4107331 | 4 |
| 3231.4114622 | 3 |
| 3231.4125362 | 2 |
| 3231.4136534 | 2 |
| 3231.4147188 | 3 |
| 3231.415747 | 3 |
| 3231.4165963 | 1 |
| 3231.4175093 | 1 |
| 3231.4185991 | 2 |
| 3231.4198813 | 3 |
| 3231.4200811 | 2 |
| 3231.4218586 | 1 |
| 3231.4220386 | 0 |
| 3231.4225962 | 2 |
| 3231.4240259 | 3 |
| 3231.4248353 | 3 |
| 3231.4258786 | 1 |
| 3231.4271089 | 0 |
| 3231.4276797 | 0 |
| 3231.4286931 | 1 |
| 3231.4294589 | 4 |
| 3231.4305257 | 3 |
| 3231.4313948 | 3 |
| 3231.4328688 | 2 |
| 3231.4332678 | 3 |
| 3231.4346119 | 1 |
| 3231.4350709 | 3 |
| 3231.4356714 | 2 |
| 3231.4368396 | 1 |
| 3231.4385746 | 2 |
| 3231.4385966 | 1 |
| 3231.4395781 | 3 |
| 3231.4406906 | 2 |
| 3231.4411851 | 0 |
| 3231.44257 | 5 |
| 3231.443333 | 3 |
| 3231.4447679 | 1 |
| 3231.444801 | 0 |
| 3231.446242 | 4 |
| 3231.4471184 | 3 |
| 3231.4474178 | 5 |
| 3231.448679 | 1 |
| 3231.4499037 | 1 |
| 3231.4507734 | 1 |
| 3231.4514094 | 1 |
| 3231.4525781 | 0 |
| 3231.4534189 | 4 |
| 3231.4543197 | 3 |
| 3231.4553598 | 4 |
| 3231.4567718 | 1 |
| 3231.4570247 | 2 |
| 3231.4579753 | 1 |
| 3231.4599426 | 1 |
| 3231.4601526 | 2 |
| 3231.4607241 | 1 |
| 3231.4621823 | 0 |
| 3231.4631882 | 3 |
| 3231.4634615 | 2 |
| 3231.4648837 | 1 |
| 3231.4656911 | 3 |
| 3231.4663494 | 1 |
| 3231.4674377 | 6 |
| 3231.468854 | 2 |
| 3231.4692135 | 2 |
| 3231.4706294 | 0 |
| 3231.4709274 | 1 |
| 3231.4725581 | 0 |
| 3231.4727694 | 0 |
| 3231.4738212 | 1 |
| 3231.4751366 | 0 |
| 3231.4756145 | 0 |
| 3231.476966 | 2 |
| 3231.477939 | 1 |
| 3231.4787163 | 2 |
| 3231.4797546 | 0 |
| 3231.479839 | 3 |
| 3231.4818006 | 3 |
| 3231.4826078 | 2 |
| 3231.4830375 | 5 |
| 3231.4844445 | 1 |
| 3231.4845183 | 2 |
| 3231.4856699 | 0 |
| 3231.4864291 | 2 |
| 3231.4880353 | 1 |
| 3231.4889537 | 0 |
| 3231.4894426 | 0 |
| 3231.4904904 | 1 |
| 3231.4907797 | 1 |
| 3231.4918259 | 1 |
| 3231.4932702 | 3 |
| 3231.4940705 | 3 |
| 3231.4948678 | 2 |
| 3231.4960162 | 2 |
| 3231.4969429 | 2 |
| 3231.4975526 | 4 |
| 3231.4980392 | 0 |
| 3231.4994554 | 3 |
| 3231.5009086 | 3 |
| 3231.5011782 | 2 |
| 3231.5023577 | 1 |
| 3231.5034094 | 1 |
| 3231.5041607 | 0 |
| 3231.5051406 | 2 |
| 3231.5062614 | 0 |
| 3231.5072267 | 1 |
| 3231.5075816 | 4 |
| 3231.5091353 | 3 |
| 3231.5098791 | 1 |
| 3231.5106502 | 2 |
| 3231.5121018 | 3 |
| 3231.5121704 | 3 |
| 3231.5136262 | 4 |
| 3231.5142884 | 2 |
| 3231.5158913 | 3 |
| 3231.5166342 | 1 |
| 3231.517254 | 1 |
| 3231.5187713 | 2 |
| 3231.5188237 | 3 |
| 3231.5194526 | 2 |
| 3231.5200727 | 1 |
| 3231.5208374 | 0 |
| 3231.5210284 | 2 |
| 3231.5212519 | 0 |
| 3231.5214223 | 3 |
| 3231.5214837 | 3 |
| 3231.5214962 | 1 |
| 3231.5222363 | 3 |
| 3231.5223019 | 2 |
| 3231.522849 | 1 |
| 3231.5228833 | 3 |
| 3231.5232437 | 3 |
| 3231.5235299 | 6 |
| 3231.5243291 | 1 |
| 3231.524408 | 1 |
| 3231.5244126 | 5 |
| 3231.5246742 | 0 |
| 3231.5250409 | 0 |
| 3231.5258036 | 0 |
| 3231.5258068 | 2 |
| 3231.5261366 | 1 |
| 3231.5265196 | 2 |
| 3231.5267918 | 1 |
| 3231.5269888 | 1 |
| 3231.5272861 | 2 |
| 3231.5275913 | 1 |
| 3231.527869 | 1 |
| 3231.5281079 | 2 |
| 3231.528481 | 3 |
| 3231.5288403 | 3 |
| 3231.5290106 | 0 |
| 3231.5292584 | 2 |
| 3231.5300167 | 3 |
| 3231.5301652 | 4 |
| 3231.5303017 | 4 |
| 3231.5303619 | 2 |
| 3231.5304341 | 2 |
| 3231.5314163 | 2 |
| 3231.5316082 | 3 |
| 3231.5319154 | 2 |
| 3231.5324554 | 2 |
| 3231.5327443 | 1 |
| 3231.5327659 | 2 |
| 3231.5328942 | 3 |
| 3231.5330347 | 1 |
| 3231.5336905 | 2 |
| 3231.5338026 | 2 |
| 3231.5339706 | 3 |
| 3231.5342846 | 1 |
| 3231.5352708 | 2 |
| 3231.5355306 | 1 |
| 3231.5356383 | 3 |
| 3231.5356596 | 4 |
| 3231.5361347 | 3 |
| 3231.5364009 | 2 |
| 3231.5368004 | 6 |
| 3231.5369986 | 1 |
| 3231.5373447 | 3 |
| 3231.5374016 | 3 |
| 3231.5377679 | 0 |
| 3231.5384126 | 4 |
| 3231.5386809 | 1 |
| 3231.5393859 | 0 |
| 3231.5395304 | 1 |
| 3231.5395848 | 6 |
| 3231.5396618 | 1 |
| 3231.5396918 | 3 |
| 3231.5403321 | 0 |
| 3231.5404577 | 2 |
| 3231.5409118 | 0 |
| 3231.541439 | 4 |

| | |
|---|---|
| 3231.5415744 | 1 |
| 3231.54162 | 2 |
| 3231.5423355 | 9 |
| 3231.5427017 | 4 |
| 3231.5431407 | 2 |
| 3231.5433904 | 2 |
| 3231.5434049 | 2 |
| 3231.5435993 | 0 |
| 3231.544187 | 0 |
| 3231.5444137 | 0 |
| 3231.5449258 | 1 |
| 3231.54513 | 2 |
| 3231.5456585 | 1 |
| 3231.5458092 | 3 |
| 3231.5458163 | 0 |
| 3231.546222 | 1 |
| 3231.5467627 | 0 |
| 3231.5468068 | 1 |
| 3231.5473845 | 2 |
| 3231.5478032 | 2 |
| 3231.54795 | 2 |
| 3231.5482727 | 2 |
| 3231.5485102 | 6 |
| 3231.5486037 | 3 |
| 3231.5486888 | 2 |
| 3231.5493067 | 0 |
| 3231.5493881 | 0 |
| 3231.5494461 | 1 |
| 3231.5502383 | 3 |
| 3231.5502825 | 5 |
| 3231.5505081 | 1 |
| 3231.5513862 | 3 |
| 3231.5515896 | 0 |
| 3231.5518904 | 1 |
| 3231.5518914 | 1 |
| 3231.5523488 | 4 |
| 3231.5535453 | 2 |
| 3231.5544987 | 3 |
| 3231.5545533 | 1 |
| 3231.5558461 | 2 |
| 3231.5568695 | 3 |
| 3231.5577978 | 2 |
| 3231.5590208 | 1 |
| 3231.5594739 | 3 |
| 3231.5611043 | 1 |
| 3231.5617573 | 1 |
| 3231.5627088 | 3 |
| 3231.5637205 | 1 |
| 3231.5637636 | 2 |
| 3231.5652429 | 4 |
| 3231.5668583 | 0 |
| 3231.5668656 | 3 |
| 3231.5682384 | 0 |
| 3231.5689152 | 3 |
| 3231.5699123 | 1 |
| 3231.5701814 | 1 |
| 3231.5716371 | 1 |
| 3231.5728499 | 5 |
| 3231.5730723 | 2 |
| 3231.5741094 | 1 |
| 3231.5754727 | 1 |
| 3231.5763561 | 1 |
| 3231.5778494 | 3 |
| 3231.5778997 | 1 |
| 3231.5793279 | 1 |
| 3231.5805737 | 2 |
| 3231.5811043 | 1 |
| 3231.5825139 | 3 |
| 3231.5828463 | 5 |
| 3231.5835414 | 0 |
| 3231.5844215 | 2 |
| 3231.5855636 | 2 |
| 3231.5864122 | 2 |
| 3231.587504 | 1 |
| 3231.5884372 | 0 |
| 3231.5895023 | 2 |
| 3231.5899904 | 1 |
| 3231.5917918 | 0 |
| 3231.5920356 | 2 |
| 3231.5931138 | 2 |
| 3231.5932808 | 0 |
| 3231.5949151 | 1 |
| 3231.5956841 | 0 |
| 3231.5965349 | 0 |
| 3231.5977174 | 1 |
| 3231.5981779 | 1 |
| 3231.5988908 | 4 |
| 3231.6010933 | 2 |
| 3231.6013194 | 2 |
| 3231.6025675 | 0 |
| 3231.6029233 | 1 |
| 3231.6043064 | 2 |
| 3231.6056165 | 0 |
| 3231.6060338 | 3 |
| 3231.6072325 | 1 |
| 3231.6079338 | 3 |
| 3231.6092062 | 6 |
| 3231.6096887 | 1 |
| 3231.6100533 | 2 |
| 3231.6117807 | 2 |
| 3231.6128738 | 0 |
| 3231.613481 | 4 |
| 3231.6142035 | 1 |
| 3231.6158089 | 0 |
| 3231.6165195 | 0 |
| 3231.617258 | 3 |
| 3231.61848 | 2 |
| 3231.6188387 | 3 |
| 3231.6201847 | 2 |
| 3231.6212873 | 1 |
| 3231.6218629 | 6 |
| 3231.6227754 | 2 |
| 3231.623474 | 0 |
| 3231.6247455 | 2 |
| 3231.6253878 | 2 |
| 3231.6263705 | 1 |
| 3231.6283349 | 2 |
| 3231.6284888 | 1 |
| 3231.629342 | 3 |
| 3231.6305902 | 4 |
| 3231.6312768 | 4 |
| 3231.632588 | 2 |
| 3231.6328695 | 2 |
| 3231.634118 | 2 |
| 3231.6341343 | 1 |
| 3231.6349768 | 2 |
| 3231.6371045 | 3 |
| 3231.6378214 | 0 |
| 3231.6384669 | 2 |
| 3231.6403638 | 5 |
| 3231.6406806 | 2 |
| 3231.6417083 | 5 |
| 3231.6430206 | 5 |
| 3231.6431568 | 4 |
| 3231.6443468 | 2 |
| 3231.6448328 | 1 |
| 3231.6459707 | 1 |
| 3231.647263 | 2 |
| 3231.6476735 | 3 |
| 3231.6488249 | 1 |
| 3231.6501394 | 2 |
| 3231.6509626 | 3 |
| 3231.6516708 | 5 |
| 3231.6523936 | 0 |
| 3231.6535351 | 3 |
| 3231.6550075 | 2 |
| 3231.6558827 | 0 |
| 3231.6567938 | 2 |
| 3231.657072 | 1 |
| 3231.6583818 | 1 |
| 3231.6589855 | 3 |
| 3231.6601979 | 1 |
| 3231.6613344 | 0 |
| 3231.6618824 | 1 |
| 3231.6631769 | 0 |
| 3231.6641511 | 3 |
| 3231.6650212 | 2 |
| 3231.6657405 | 3 |
| 3231.6667802 | 2 |
| 3231.6673516 | 4 |
| 3231.6681617 | 1 |
| 3231.6695585 | 3 |
| 3231.6705371 | 3 |
| 3231.671118 | 3 |
| 3231.6723069 | 2 |
| 3231.6731082 | 1 |
| 3231.6742734 | 0 |
| 3231.6748654 | 2 |
| 3231.6760496 | 4 |
| 3231.6771232 | 1 |
| 3231.677177 | 0 |
| 3231.6787472 | 4 |
| 3231.6797424 | 6 |
| 3231.6806708 | 2 |
| 3231.6815105 | 0 |
| 3231.6829269 | 1 |
| 3231.6835003 | 0 |
| 3231.6835376 | 0 |
| 3231.6852812 | 0 |
| 3231.6865412 | 2 |
| 3231.6873101 | 1 |
| 3231.6878981 | 3 |
| 3231.6890837 | 3 |
| 3231.6902264 | 4 |
| 3231.6906265 | 0 |
| 3231.6916619 | 4 |
| 3231.6928383 | 0 |
| 3231.6936286 | 1 |
| 3231.6949284 | 2 |
| 3231.6959447 | 5 |
| 3231.6960857 | 2 |
| 3231.698372 | 3 |
| 3231.6990543 | 2 |
| 3231.6995529 | 2 |
| 3231.7008074 | 1 |
| 3231.700865 | 3 |
| 3231.7028039 | 1 |
| 3231.7029905 | 4 |
| 3231.70407 | 2 |
| 3231.705377 | 2 |
| 3231.7058546 | 0 |
| 3231.7063021 | 5 |
| 3231.7078038 | 1 |
| 3231.7086786 | 1 |
| 3231.7100172 | 2 |
| 3231.7109132 | 2 |
| 3231.7121401 | 1 |
| 3231.7126517 | 2 |
| 3231.7133282 | 2 |
| 3231.7144448 | 2 |
| 3231.7144978 | 5 |
| 3231.7164352 | 3 |
| 3231.7175787 | 1 |
| 3231.7176158 | 1 |
| 3231.7188723 | 3 |
| 3231.7195395 | 3 |
| 3231.7208034 | 2 |
| 3231.7220572 | 3 |
| 3231.7225726 | 3 |
| 3231.7239172 | 0 |
| 3231.7241297 | 4 |
| 3231.7257909 | 2 |
| 3231.7266183 | 3 |
| 3231.727135 | 1 |
| 3231.7283607 | 0 |
| 3231.7293675 | 3 |
| 3231.7303459 | 3 |
| 3231.7307519 | 0 |
| 3231.7319304 | 3 |
| 3231.7334061 | 1 |
| 3231.733434 | 3 |
| 3231.7349837 | 0 |
| 3231.7356704 | 1 |
| 3231.7370976 | 1 |
| 3231.7375646 | 5 |
| 3231.7386657 | 1 |
| 3231.7398105 | 1 |
| 3231.7402726 | 3 |
| 3231.7418877 | 3 |
| 3231.7428367 | 5 |
| 3231.7428578 | 2 |
| 3231.7447145 | 4 |
| 3231.7455556 | 2 |
| 3231.7465036 | 1 |
| 3231.7477687 | 1 |
| 3231.7477829 | 2 |
| 3231.7481651 | 3 |
| 3231.7487648 | 3 |
| 3231.748939 | 3 |
| 3231.7490187 | 4 |
| 3231.7491127 | 1 |
| 3231.7493755 | 2 |
| 3231.7498913 | 1 |
| 3231.7499161 | 2 |
| 3231.7500576 | 3 |
| 3231.7501422 | 2 |
| 3231.7504899 | 0 |
| 3231.7505305 | 1 |
| 3231.7506858 | 3 |
| 3231.7507274 | 3 |
| 3231.751314 | 1 |
| 3231.7514285 | 1 |
| 3231.751835 | 1 |
| 3231.7518466 | 3 |
| 3231.7520737 | 2 |
| 3231.7521397 | 1 |
| 3231.7523409 | 2 |
| 3231.7525283 | 2 |
| 3231.7527089 | 3 |
| 3231.7527201 | 2 |
| 3231.7528828 | 2 |
| 3231.7529591 | 4 |
| 3231.7529956 | 3 |
| 3231.7536015 | 1 |
| 3231.753632 | 2 |
| 3231.7536768 | 0 |
| 3231.7536883 | 2 |
| 3231.7539583 | 0 |
| 3231.753984 | 1 |
| 3231.7542808 | 1 |
| 3231.7544492 | 0 |
| 3231.7546541 | 3 |
| 3231.7546704 | 3 |
| 3231.7552627 | 3 |
| 3231.755284 | 2 |
| 3231.755484 | 4 |
| 3231.7556805 | 2 |
| 3231.7559304 | 2 |
| 3231.7560396 | 1 |
| 3231.756107 | 3 |
| 3231.7565629 | 3 |
| 3231.7566059 | 3 |
| 3231.7567279 | 4 |
| 3231.7567567 | 2 |
| 3231.7568698 | 2 |
| 3231.7572631 | 0 |
| 3231.7572825 | 1 |
| 3231.7574618 | 0 |
| 3231.7578682 | 2 |
| 3231.7579193 | 2 |
| 3231.7581956 | 4 |
| 3231.7582018 | 0 |
| 3231.7583043 | 1 |
| 3231.7584802 | 2 |
| 3231.7588433 | 2 |
| 3231.7590027 | 1 |
| 3231.7591621 | 2 |
| 3231.7591738 | 2 |
| 3231.7592448 | 2 |
| 3231.7596694 | 4 |
| 3231.759796 | 2 |
| 3231.759958 | 1 |
| 3231.7601898 | 0 |
| 3231.7602199 | 1 |
| 3231.7603745 | 4 |
| 3231.7604045 | 2 |
| 3231.7608389 | 5 |
| 3231.761127 | 4 |
| 3231.761151 | 2 |
| 3231.7615313 | 1 |
| 3231.7615342 | 3 |
| 3231.7616678 | 3 |
| 3231.761771 | 1 |
| 3231.7618363 | 2 |
| 3231.7621974 | 4 |
| 3231.7623142 | 2 |
| 3231.7624275 | 3 |
| 3231.7625444 | 0 |
| 3231.7627462 | 0 |
| 3231.7629867 | 2 |
| 3231.7630068 | 1 |
| 3231.7630761 | 1 |
| 3231.7631083 | 2 |
| 3231.7631838 | 3 |
| 3231.7633636 | 3 |
| 3231.7636392 | 1 |
| 3231.763834 | 0 |
| 3231.7640444 | 0 |
| 3231.7643242 | 3 |
| 3231.7645351 | 1 |
| 3231.7647203 | 3 |
| 3231.7648188 | 3 |
| 3231.76484 | 5 |
| 3231.7651362 | 3 |
| 3231.7652399 | 3 |
| 3231.7652873 | 3 |
| 3231.7655046 | 3 |
| 3231.7662659 | 2 |
| 3231.7663569 | 2 |
| 3231.7663657 | 2 |
| 3231.7663819 | 1 |
| 3231.7666509 | 3 |
| 3231.7667585 | 1 |
| 3231.7669343 | 4 |
| 3231.7673009 | 4 |
| 3231.7673147 | 4 |
| 3231.7674582 | 3 |
| 3231.7677826 | 3 |
| 3231.7678193 | 6 |
| 3231.767841 | 6 |
| 3231.7681299 | 5 |
| 3231.7683307 | 5 |
| 3231.7687934 | 10 |

| | |
|---|---|
| 3231.7689181 | 8 |
| 3231.7690225 | 11 |
| 3231.7692429 | 10 |
| 3231.7692788 | 16 |
| 3231.7694349 | 21 |
| 3231.7695191 | 16 |
| 3231.7696067 | 11 |
| 3231.7697526 | 13 |
| 3231.770111 | 13 |
| 3231.7701213 | 9 |
| 3231.7702191 | 7 |
| 3231.7702891 | 16 |
| 3231.770328 | 7 |
| 3231.7707038 | 10 |
| 3231.7707463 | 12 |
| 3231.7707793 | 16 |
| 3231.7711348 | 4 |
| 3231.771393 | 8 |
| 3231.7715337 | 7 |
| 3231.7719287 | 4 |
| 3231.771993 | 3 |
| 3231.7720749 | 5 |
| 3231.7722495 | 1 |
| 3231.7724024 | 2 |
| 3231.7728541 | 4 |
| 3231.7728658 | 5 |
| 3231.7733581 | 3 |
| 3231.7734285 | 2 |
| 3231.7734651 | 1 |
| 3231.7734756 | 5 |
| 3231.7739347 | 4 |
| 3231.7739761 | 2 |
| 3231.7741832 | 3 |
| 3231.7743475 | 5 |
| 3231.774434 | 3 |
| 3231.7744861 | 4 |
| 3231.7747085 | 2 |
| 3231.7748714 | 1 |
| 3231.7750432 | 1 |
| 3231.7750711 | 1 |
| 3231.7751887 | 3 |
| 3231.7752386 | 3 |
| 3231.7757014 | 5 |
| 3231.7757719 | 2 |
| 3231.7762522 | 1 |
| 3231.7764406 | 2 |
| 3231.776554 | 2 |
| 3231.7766348 | 3 |
| 3231.7768177 | 1 |
| 3231.7768665 | 1 |
| 3231.7771806 | 0 |
| 3231.7773476 | 4 |
| 3231.7776459 | 2 |
| 3231.7777233 | 5 |
| 3231.7780549 | 5 |
| 3231.7782652 | 1 |
| 3231.7782958 | 6 |
| 3231.7783407 | 3 |
| 3231.7785684 | 3 |
| 3231.7787911 | 2 |
| 3231.7790312 | 3 |
| 3231.7791914 | 2 |
| 3231.7791964 | 3 |
| 3231.7794254 | 1 |
| 3231.7794901 | 0 |
| 3231.7796651 | 1 |
| 3231.7796763 | 2 |
| 3231.7797526 | 0 |
| 3231.7804677 | 3 |
| 3231.7806465 | 2 |
| 3231.7807743 | 1 |
| 3231.7807929 | 2 |
| 3231.7809065 | 2 |
| 3231.7811139 | 4 |
| 3231.7812624 | 1 |
| 3231.7813926 | 2 |
| 3231.781656 | 1 |
| 3231.7816719 | 2 |
| 3231.7816821 | 3 |
| 3231.7820836 | 0 |
| 3231.7826017 | 2 |
| 3231.782651 | 1 |
| 3231.782963 | 1 |
| 3231.7830456 | 2 |
| 3231.783078 | 1 |
| 3231.7832259 | 0 |
| 3231.7834982 | 0 |
| 3231.7835954 | 1 |
| 3231.7837206 | 1 |
| 3231.7841632 | 3 |
| 3231.7841663 | 1 |
| 3231.7842617 | 1 |
| 3231.7842731 | 2 |
| 3231.7845589 | 3 |
| 3231.7847327 | 5 |
| 3231.7849288 | 1 |
| 3231.7850761 | 4 |
| 3231.7850824 | 2 |
| 3231.7852265 | 2 |
| 3231.7854453 | 2 |
| 3231.785492 | 1 |
| 3231.7861247 | 2 |
| 3231.7862324 | 2 |
| 3231.7862533 | 2 |
| 3231.7864678 | 3 |
| 3231.7865004 | 3 |
| 3231.7866215 | 0 |
| 3231.7870647 | 2 |
| 3231.7871563 | 6 |
| 3231.7874563 | 2 |
| 3231.7876361 | 2 |
| 3231.7877896 | 2 |
| 3231.7878044 | 1 |
| 3231.7881833 | 2 |
| 3231.7882492 | 1 |
| 3231.7883147 | 1 |
| 3231.7884776 | 4 |
| 3231.7884945 | 2 |
| 3231.7887621 | 2 |
| 3231.7892157 | 1 |
| 3231.7892701 | 4 |
| 3231.7893274 | 2 |
| 3231.7896468 | 2 |
| 3231.789686 | 2 |
| 3231.789892 | 2 |
| 3231.7904148 | 2 |
| 3231.7904791 | 1 |
| 3231.7905836 | 0 |
| 3231.7912084 | 0 |
| 3231.7924871 | 1 |
| 3231.7936402 | 1 |
| 3231.7936654 | 3 |
| 3231.7950398 | 1 |
| 3231.7959201 | 0 |
| 3231.7961122 | 3 |
| 3231.7978562 | 3 |
| 3231.7991419 | 1 |
| 3231.7993178 | 3 |
| 3231.8005421 | 1 |
| 3231.8011699 | 5 |
| 3231.8025631 | 0 |
| 3231.8030402 | 2 |
| 3231.8041238 | 2 |
| 3231.8044489 | 7 |
| 3231.8059232 | 4 |
| 3231.8064431 | 4 |
| 3231.8070814 | 3 |
| 3231.8071309 | 1 |
| 3231.8074014 | 4 |
| 3231.8075555 | 3 |
| 3231.8084502 | 3 |
| 3231.8089544 | 0 |
| 3231.8102092 | 3 |
| 3231.8103301 | 2 |
| 3231.8104873 | 1 |
| 3231.8111102 | 0 |
| 3231.8112212 | 0 |
| 3231.8114696 | 0 |
| 3231.8118179 | 1 |
| 3231.8128803 | 1 |
| 3231.8131209 | 1 |
| 3231.8137996 | 0 |
| 3231.8139155 | 0 |
| 3231.8141202 | 3 |
| 3231.8148306 | 2 |
| 3231.8153981 | 4 |
| 3231.8156352 | 1 |
| 3231.8161404 | 4 |
| 3231.8168222 | 2 |
| 3231.8171454 | 2 |
| 3231.8178315 | 4 |
| 3231.8181882 | 1 |
| 3231.8191071 | 0 |
| 3231.819306 | 3 |
| 3231.8196517 | 2 |
| 3231.8201676 | 0 |
| 3231.820533 | 1 |
| 3231.8208536 | 0 |
| 3231.8215299 | 1 |
| 3231.8218068 | 1 |
| 3231.8222582 | 4 |
| 3231.823188 | 0 |
| 3231.823529 | 3 |
| 3231.8235586 | 0 |
| 3231.8244779 | 2 |
| 3231.8251276 | 1 |
| 3231.8252179 | 2 |
| 3231.8254613 | 2 |
| 3231.8261901 | 1 |
| 3231.8263658 | 4 |
| 3231.8271303 | 2 |
| 3231.8273732 | 3 |
| 3231.8280955 | 3 |
| 3231.828259 | 2 |
| 3231.8291234 | 2 |
| 3231.829209 | 2 |
| 3231.8298231 | 0 |
| 3231.829998 | 1 |
| 3231.8312378 | 1 |
| 3231.8319033 | 2 |
| 3231.8319207 | 1 |
| 3231.8324208 | 1 |
| 3231.8330916 | 0 |
| 3231.8332861 | 4 |
| 3231.8335099 | 1 |
| 3231.8347946 | 2 |
| 3231.8349081 | 1 |
| 3231.8355958 | 2 |
| 3231.835687 | 2 |
| 3231.8361429 | 2 |
| 3231.8364734 | 0 |
| 3231.8368136 | 2 |
| 3231.8380172 | 3 |
| 3231.8387636 | 6 |
| 3231.8389445 | 1 |
| 3231.8390552 | 2 |
| 3231.8391775 | 1 |
| 3231.8397927 | 1 |
| 3231.8408167 | 1 |
| 3231.8408689 | 2 |
| 3231.841014 | 1 |
| 3231.8411517 | 2 |
| 3231.842504 | 2 |
| 3231.842683 | 0 |
| 3231.8428833 | 3 |
| 3231.8436932 | 2 |
| 3231.8438927 | 2 |
| 3231.8439565 | 1 |
| 3231.8444657 | 2 |
| 3231.8457358 | 3 |
| 3231.8460583 | 4 |
| 3231.8462782 | 4 |
| 3231.8471489 | 3 |
| 3231.8472472 | 2 |
| 3231.8478835 | 4 |
| 3231.8481739 | 1 |
| 3231.8485209 | 0 |
| 3231.8487216 | 3 |
| 3231.8495691 | 3 |
| 3231.8500701 | 3 |
| 3231.8503661 | 2 |
| 3231.8507197 | 1 |
| 3231.8514235 | 1 |
| 3231.8517657 | 1 |
| 3231.8521909 | 3 |
| 3231.8530925 | 4 |
| 3231.8533198 | 1 |
| 3231.8533556 | 1 |
| 3231.8543755 | 0 |
| 3231.8544681 | 0 |
| 3231.8548988 | 2 |
| 3231.8554305 | 2 |
| 3231.8558583 | 0 |
| 3231.8558746 | 4 |
| 3231.8567424 | 0 |
| 3231.8574579 | 2 |
| 3231.8576528 | 2 |
| 3231.8579725 | 1 |
| 3231.8591027 | 1 |
| 3231.8591063 | 2 |
| 3231.8601744 | 3 |
| 3231.8604278 | 2 |
| 3231.8606483 | 2 |
| 3231.8610984 | 2 |
| 3231.8612141 | 0 |
| 3231.8622613 | 2 |
| 3231.8625068 | 1 |
| 3231.8628191 | 1 |
| 3231.8633586 | 3 |
| 3231.8634426 | 2 |
| 3231.8642446 | 2 |
| 3231.8645288 | 8 |
| 3231.8652955 | 4 |
| 3231.865587 | 0 |
| 3231.8663315 | 4 |
| 3231.8665053 | 3 |
| 3231.8667131 | 1 |
| 3231.8674591 | 2 |
| 3231.8688989 | 4 |
| 3231.868958 | 0 |
| 3231.869153 | 0 |
| 3231.8695684 | 1 |
| 3231.8701465 | 3 |
| 3231.8702165 | 1 |
| 3231.871109 | 2 |
| 3231.8715403 | 0 |
| 3231.8717301 | 2 |
| 3231.8720046 | 4 |
| 3231.8725344 | 2 |
| 3231.8727412 | 1 |
| 3231.8739173 | 1 |
| 3231.8741951 | 2 |
| 3231.8747127 | 1 |
| 3231.8748308 | 4 |
| 3231.8755897 | 2 |
| 3231.8760143 | 3 |
| 3231.8762842 | 2 |
| 3231.8769469 | 1 |
| 3231.8773343 | 0 |
| 3231.8774713 | 4 |
| 3231.8784751 | 3 |
| 3231.8785252 | 2 |
| 3231.8789494 | 3 |
| 3231.8796371 | 3 |
| 3231.8804125 | 1 |
| 3231.880628 | 2 |
| 3231.8812144 | 1 |
| 3231.881507 | 3 |
| 3231.8823511 | 3 |
| 3231.8825231 | 1 |
| 3231.8830431 | 3 |
| 3231.883245 | 1 |
| 3231.8835734 | 2 |
| 3231.8838897 | 4 |
| 3231.8851965 | 6 |
| 3231.885459 | 2 |
| 3231.8874777 | 1 |
| 3231.887478 | 2 |
| 3231.8889267 | 2 |
| 3231.890452 | 0 |
| 3231.8905734 | 2 |
| 3231.8921691 | 2 |
| 3231.8923397 | 2 |
| 3231.893591 | 1 |
| 3231.8944743 | 2 |
| 3231.8951633 | 3 |
| 3231.8968094 | 0 |
| 3231.8972688 | 1 |
| 3231.8978854 | 4 |
| 3231.8989658 | 2 |
| 3231.8997294 | 0 |
| 3231.90053 | 3 |
| 3231.9020353 | 2 |
| 3231.9026689 | 2 |
| 3231.9037446 | 6 |
| 3231.9041612 | 5 |
| 3231.9058604 | 3 |
| 3231.9061778 | 4 |
| 3231.9070367 | 0 |
| 3231.9077822 | 5 |
| 3231.9087539 | 3 |
| 3231.9101364 | 3 |
| 3231.9104914 | 2 |
| 3231.9121892 | 1 |
| 3231.9130214 | 1 |
| 3231.9137858 | 5 |
| 3231.9150094 | 1 |
| 3231.9154303 | 1 |
| 3231.9167437 | 1 |
| 3231.9184332 | 1 |
| 3231.9189219 | 3 |
| 3231.9200875 | 1 |
| 3231.9204446 | 4 |
| 3231.9214515 | 2 |
| 3231.9227474 | 0 |
| 3231.9233923 | 4 |
| 3231.9246786 | 1 |
| 3231.9250896 | 2 |
| 3231.9264369 | 1 |
| 3231.9273313 | 1 |
| 3231.928228 | 0 |
| 3231.929498 | 2 |
| 3231.929833 | 6 |

| | |
|---|---|
| 3231.9305508 | 3 |
| 3231.9320966 | 1 |
| 3231.9330204 | 2 |
| 3231.9338485 | 3 |
| 3231.9348226 | 2 |
| 3231.9357242 | 1 |
| 3231.9362043 | 2 |
| 3231.9374206 | 5 |
| 3231.9381154 | 4 |
| 3231.938862 | 1 |
| 3231.9403083 | 0 |
| 3231.9409869 | 1 |
| 3231.9422843 | 0 |
| 3231.9428605 | 3 |
| 3231.944169 | 4 |
| 3231.9444757 | 2 |
| 3231.9458631 | 2 |
| 3231.9463636 | 4 |
| 3231.9474727 | 1 |
| 3231.9485679 | 1 |
| 3231.9491988 | 2 |
| 3231.9501021 | 6 |
| 3231.9512118 | 1 |
| 3231.9518374 | 5 |
| 3231.9531521 | 3 |
| 3231.9538774 | 2 |
| 3231.9548317 | 2 |
| 3231.9557969 | 0 |
| 3231.9565303 | 2 |
| 3231.95804 | 1 |
| 3231.9583951 | 3 |
| 3231.9598987 | 1 |
| 3231.9608306 | 3 |
| 3231.9617698 | 2 |
| 3231.9623687 | 3 |
| 3231.9638372 | 2 |
| 3231.9642428 | 2 |
| 3231.9659457 | 2 |
| 3231.9659826 | 0 |
| 3231.9679338 | 2 |
| 3231.9690294 | 1 |
| 3231.9690727 | 2 |
| 3231.9702088 | 0 |
| 3231.9709644 | 2 |
| 3231.9715952 | 1 |
| 3231.9730493 | 1 |
| 3231.9736523 | 4 |
| 3231.9746869 | 2 |
| 3231.9765659 | 2 |
| 3231.9765886 | 2 |
| 3231.977497 | 1 |
| 3231.9793668 | 4 |
| 3231.9795673 | 1 |
| 3231.9808101 | 3 |
| 3231.9812755 | 0 |
| 3231.9825728 | 2 |
| 3231.9837313 | 3 |
| 3231.9839517 | 0 |
| 3231.9853085 | 2 |
| 3231.9856653 | 2 |
| 3231.9869145 | 1 |
| 3231.9886738 | 1 |
| 3231.9887257 | 3 |
| 3231.9901898 | 2 |
| 3231.9904034 | 1 |
| 3231.9920956 | 1 |
| 3231.9925405 | 4 |
| 3231.9928198 | 0 |
| 3231.9942573 | 2 |
| 3231.9955787 | 4 |
| 3231.9959754 | 1 |
| 3231.9967009 | 1 |
| 3231.9980347 | 1 |
| 3231.9989993 | 2 |
| 3231.9995306 | 2 |
| 3232.000942 | 0 |
| 3232.0021525 | 1 |
| 3232.0022175 | 3 |
| 3232.0041005 | 4 |
| 3232.0041785 | 1 |
| 3232.0054471 | 1 |
| 3232.0063641 | 0 |
| 3232.0078034 | 3 |
| 3232.0083705 | 2 |
| 3232.0093158 | 4 |
| 3232.0104053 | 1 |
| 3232.0115775 | 2 |
| 3232.0125285 | 2 |
| 3232.0131377 | 2 |
| 3232.0144274 | 0 |
| 3232.0155664 | 2 |
| 3232.0156679 | 3 |
| 3232.0176033 | 0 |
| 3232.0185654 | 3 |
| 3232.0186599 | 1 |
| 3232.0196089 | 2 |
| 3232.0210567 | 1 |
| 3232.0216196 | 2 |
| 3232.0227303 | 2 |
| 3232.023783 | 1 |
| 3232.0246345 | 1 |
| 3232.0252487 | 5 |
| 3232.0267371 | 1 |
| 3232.0274023 | 4 |
| 3232.0283123 | 1 |
| 3232.0296738 | 4 |
| 3232.0299164 | 2 |
| 3232.0316116 | 1 |
| 3232.0327622 | 2 |
| 3232.0327809 | 4 |
| 3232.033942 | 3 |
| 3232.0347108 | 1 |
| 3232.0359792 | 1 |
| 3232.0364223 | 1 |
| 3232.037749 | 1 |
| 3232.0388357 | 1 |
| 3232.0391789 | 3 |
| 3232.0406512 | 2 |
| 3232.0410021 | 3 |
| 3232.0417666 | 5 |
| 3232.0427787 | 3 |
| 3232.0438734 | 2 |
| 3232.0451781 | 2 |
| 3232.0460669 | 4 |
| 3232.0470361 | 5 |
| 3232.0478386 | 3 |
| 3232.0491125 | 1 |
| 3232.049744 | 1 |
| 3232.0503135 | 1 |
| 3232.051983 | 3 |
| 3232.0526775 | 0 |
| 3232.0534405 | 3 |
| 3232.0548113 | 1 |
| 3232.0554874 | 2 |
| 3232.056187 | 0 |
| 3232.0579619 | 2 |
| 3232.0581609 | 1 |
| 3232.0594201 | 0 |
| 3232.0601515 | 0 |
| 3232.0611936 | 4 |
| 3232.0624862 | 3 |
| 3232.0627636 | 4 |
| 3232.0649217 | 3 |
| 3232.0649677 | 1 |
| 3232.0658072 | 2 |
| 3232.0672701 | 2 |
| 3232.0679452 | 1 |
| 3232.0686989 | 4 |
| 3232.0695195 | 1 |
| 3232.0711748 | 1 |
| 3232.0719773 | 1 |
| 3232.0728281 | 0 |
| 3232.0734933 | 0 |
| 3232.0742935 | 4 |
| 3232.075491 | 1 |
| 3232.0768129 | 2 |
| 3232.0773563 | 2 |
| 3232.0784956 | 3 |
| 3232.0786001 | 4 |
| 3232.0802283 | 4 |
| 3232.0813052 | 2 |
| 3232.0816765 | 3 |
| 3232.0829016 | 2 |
| 3232.0833784 | 2 |
| 3232.0845424 | 0 |
| 3232.0855752 | 1 |
| 3232.0860555 | 2 |
| 3232.0874016 | 0 |
| 3232.0889168 | 3 |
| 3232.089375 | 1 |
| 3232.090608 | 2 |
| 3232.091334 | 1 |
| 3232.092355 | 2 |
| 3232.0928839 | 5 |
| 3232.0939281 | 1 |
| 3232.0957654 | 4 |
| 3232.0958258 | 1 |
| 3232.09665 | 3 |
| 3232.0975266 | 0 |
| 3232.0988724 | 2 |
| 3232.1002097 | 2 |
| 3232.1006744 | 1 |
| 3232.1019107 | 1 |
| 3232.1027671 | 1 |
| 3232.1041286 | 1 |
| 3232.1045454 | 3 |
| 3232.1056012 | 1 |
| 3232.1067129 | 1 |
| 3232.1077063 | 3 |
| 3232.1087376 | 1 |
| 3232.1091515 | 4 |
| 3232.1101572 | 5 |
| 3232.1116357 | 0 |
| 3232.1123884 | 2 |
| 3232.1133317 | 1 |
| 3232.1143948 | 3 |
| 3232.1150564 | 5 |
| 3232.1160875 | 3 |
| 3232.1171534 | 1 |
| 3232.1180069 | 4 |
| 3232.1193817 | 1 |
| 3232.1199419 | 2 |
| 3232.1211868 | 1 |
| 3232.122182 | 0 |
| 3232.1221894 | 2 |
| 3232.1232086 | 2 |
| 3232.1241615 | 2 |
| 3232.1252149 | 1 |
| 3232.1262949 | 0 |
| 3232.1273012 | 1 |
| 3232.127835 | 2 |
| 3232.1289731 | 6 |
| 3232.1302251 | 4 |
| 3232.1304148 | 2 |
| 3232.1324238 | 2 |
| 3232.133163 | 2 |
| 3232.133772 | 3 |
| 3232.1346734 | 0 |
| 3232.1359633 | 0 |
| 3232.1363253 | 2 |
| 3232.1373833 | 1 |
| 3232.1383956 | 1 |
| 3232.139504 | 6 |
| 3232.1407582 | 1 |
| 3232.1413298 | 1 |
| 3232.1423655 | 0 |
| 3232.1426449 | 2 |
| 3232.1434707 | 2 |
| 3232.1447313 | 3 |
| 3232.1464455 | 1 |
| 3232.147362 | 0 |
| 3232.147588 | 2 |
| 3232.1485328 | 1 |
| 3232.1499804 | 1 |
| 3232.1506906 | 1 |
| 3232.1520301 | 2 |
| 3232.1530506 | 2 |
| 3232.1535115 | 0 |
| 3232.1542163 | 3 |
| 3232.1557013 | 4 |
| 3232.1568573 | 2 |
| 3232.1573498 | 3 |
| 3232.1586624 | 6 |
| 3232.1597181 | 2 |
| 3232.1603431 | 1 |
| 3232.1614299 | 3 |
| 3232.1619157 | 4 |
| 3232.162913 | 2 |
| 3232.1636647 | 4 |
| 3232.1655253 | 3 |
| 3232.1665801 | 3 |
| 3232.1666051 | 1 |
| 3232.1680866 | 1 |
| 3232.1684594 | 3 |
| 3232.1695301 | 4 |
| 3232.1705826 | 2 |
| 3232.171229 | 3 |
| 3232.1723731 | 0 |
| 3232.1731305 | 4 |
| 3232.1742942 | 5 |
| 3232.1754795 | 1 |
| 3232.1760841 | 2 |
| 3232.1765642 | 0 |
| 3232.1777438 | 1 |
| 3232.1791269 | 4 |
| 3232.1803317 | 2 |
| 3232.1811086 | 2 |
| 3232.1815358 | 3 |
| 3232.1821299 | 0 |
| 3232.1839607 | 2 |
| 3232.1840196 | 1 |
| 3232.1854102 | 4 |
| 3232.1866242 | 4 |
| 3232.1872146 | 3 |
| 3232.1881291 | 2 |
| 3232.1889439 | 2 |
| 3232.1902383 | 1 |
| 3232.1915015 | 2 |
| 3232.1925537 | 1 |
| 3232.1932433 | 2 |
| 3232.1941056 | 1 |
| 3232.194709 | 2 |
| 3232.1964006 | 0 |
| 3232.1967643 | 3 |
| 3232.197884 | 0 |
| 3232.1993742 | 1 |
| 3232.199531 | 3 |
| 3232.2006377 | 4 |
| 3232.201468 | 4 |
| 3232.20271 | 2 |
| 3232.2035416 | 1 |
| 3232.204665 | 0 |
| 3232.2057605 | 2 |
| 3232.2064788 | 2 |
| 3232.2077366 | 2 |
| 3232.2088695 | 3 |
| 3232.2088797 | 3 |
| 3232.2097848 | 3 |
| 3232.211136 | 4 |
| 3232.2121839 | 3 |
| 3232.2123247 | 0 |
| 3232.2138425 | 1 |
| 3232.2154533 | 1 |
| 3232.2157358 | 1 |
| 3232.216068 | 2 |
| 3232.2173142 | 0 |
| 3232.2187628 | 2 |
| 3232.2195811 | 2 |
| 3232.2200445 | 5 |
| 3232.2214637 | 3 |
| 3232.2217965 | 1 |
| 3232.2227633 | 4 |
| 3232.2240352 | 2 |
| 3232.2249431 | 2 |
| 3232.2252695 | 4 |
| 3232.2264352 | 0 |
| 3232.2285599 | 4 |
| 3232.2289737 | 0 |
| 3232.2299478 | 2 |
| 3232.2309659 | 4 |
| 3232.2311707 | 2 |
| 3232.2325911 | 3 |
| 3232.2335311 | 4 |
| 3232.2339075 | 1 |
| 3232.2358923 | 1 |
| 3232.2371151 | 1 |
| 3232.2371677 | 0 |
| 3232.2385967 | 1 |
| 3232.2387891 | 0 |
| 3232.2402723 | 1 |
| 3232.2415077 | 3 |
| 3232.2421638 | 0 |
| 3232.2432399 | 4 |
| 3232.243669 | 2 |
| 3232.2449731 | 1 |
| 3232.2451688 | 2 |
| 3232.2464058 | 2 |
| 3232.248186 | 1 |
| 3232.2482796 | 1 |
| 3232.2496201 | 2 |
| 3232.250786 | 2 |
| 3232.2517538 | 1 |
| 3232.2527093 | 1 |
| 3232.2535926 | 1 |
| 3232.2549044 | 3 |
| 3232.2554232 | 0 |
| 3232.2561419 | 2 |
| 3232.2572515 | 2 |
| 3232.2578246 | 4 |
| 3232.258596 | 0 |
| 3232.259829 | 0 |
| 3232.2611628 | 2 |
| 3232.2624582 | 1 |
| 3232.2624885 | 2 |
| 3232.2635495 | 4 |
| 3232.2645273 | 3 |
| 3232.2653982 | 1 |
| 3232.2667322 | 2 |
| 3232.2674444 | 1 |
| 3232.2683748 | 0 |
| 3232.2684841 | 2 |
| 3232.2699151 | 1 |
| 3232.2713326 | 0 |

| | |
|---|---|
| 3232.2716799 | 0 |
| 3232.2728529 | 2 |
| 3232.2736936 | 4 |
| 3232.2747893 | 5 |
| 3232.275985 | 1 |
| 3232.2766501 | 3 |
| 3232.277754 | 0 |
| 3232.2778444 | 1 |
| 3232.28002 | 0 |
| 3232.280607 | 3 |
| 3232.2814601 | 3 |
| 3232.281964 | 1 |
| 3232.2832377 | 2 |
| 3232.2837359 | 5 |
| 3232.2852231 | 0 |
| 3232.2861698 | 0 |
| 3232.2871647 | 2 |
| 3232.2879506 | 4 |
| 3232.2891699 | 4 |
| 3232.2905792 | 0 |
| 3232.2906952 | 4 |
| 3232.2925483 | 1 |
| 3232.2927815 | 3 |
| 3232.2937483 | 3 |
| 3232.2950777 | 1 |
| 3232.2955502 | 3 |
| 3232.2957146 | 2 |
| 3232.2973709 | 2 |
| 3232.2980277 | 0 |
| 3232.2999482 | 2 |
| 3232.3003836 | 0 |
| 3232.3011494 | 4 |
| 3232.3023773 | 2 |
| 3232.3033279 | 1 |
| 3232.3041855 | 4 |
| 3232.3054204 | 5 |
| 3232.3064796 | 1 |
| 3232.3064914 | 1 |
| 3232.307946 | 1 |
| 3232.3085471 | 0 |
| 3232.3089211 | 1 |
| 3232.3108233 | 1 |
| 3232.3109608 | 2 |
| 3232.3118359 | 2 |
| 3232.3127184 | 1 |
| 3232.3136353 | 1 |
| 3232.3149147 | 3 |
| 3232.3159466 | 3 |
| 3232.317001 | 3 |
| 3232.317603 | 1 |
| 3232.318649 | 0 |
| 3232.3198206 | 4 |
| 3232.3202753 | 3 |
| 3232.3219566 | 1 |
| 3232.3229701 | 3 |
| 3232.3236825 | 2 |
| 3232.3248994 | 1 |
| 3232.3251809 | 4 |
| 3232.3264868 | 0 |
| 3232.3267612 | 1 |
| 3232.3276142 | 3 |
| 3232.3288283 | 1 |
| 3232.3290656 | 1 |
| 3232.3307993 | 3 |
| 3232.332199 | 4 |
| 3232.3322888 | 0 |
| 3232.3340629 | 2 |
| 3232.334672 | 3 |
| 3232.3354361 | 0 |
| 3232.3364982 | 2 |
| 3232.3372725 | 3 |
| 3232.3384013 | 2 |
| 3232.3394888 | 2 |
| 3232.3403483 | 2 |
| 3232.341333 | 2 |
| 3232.3422913 | 4 |
| 3232.3434145 | 1 |
| 3232.3438992 | 6 |
| 3232.3454379 | 1 |
| 3232.3461736 | 3 |
| 3232.3471197 | 1 |
| 3232.3481625 | 1 |
| 3232.34824 | 0 |
| 3232.3498028 | 3 |
| 3232.3504316 | 2 |
| 3232.3514548 | 1 |
| 3232.3522605 | 2 |
| 3232.3526569 | 4 |
| 3232.3538439 | 1 |
| 3232.3554174 | 2 |
| 3232.3558453 | 4 |
| 3232.3568312 | 4 |
| 3232.3574469 | 0 |
| 3232.3586836 | 1 |
| 3232.3598419 | 4 |
| 3232.3605212 | 2 |
| 3232.3613793 | 2 |
| 3232.3625787 | 2 |
| 3232.3633861 | 2 |
| 3232.3637735 | 1 |
| 3232.3649802 | 4 |
| 3232.3664816 | 2 |
| 3232.3671034 | 5 |
| 3232.3680898 | 4 |
| 3232.3686691 | 0 |
| 3232.3698905 | 0 |
| 3232.3704225 | 1 |
| 3232.3716069 | 5 |
| 3232.3731107 | 4 |
| 3232.373287 | 2 |
| 3232.3748697 | 2 |
| 3232.3752522 | 1 |
| 3232.3767548 | 4 |
| 3232.3772482 | 4 |
| 3232.3776704 | 1 |
| 3232.3794388 | 3 |
| 3232.380215 | 3 |
| 3232.3807583 | 0 |
| 3232.382086 | 1 |
| 3232.3831836 | 3 |
| 3232.3837219 | 1 |
| 3232.3852213 | 1 |
| 3232.385637 | 3 |
| 3232.3873313 | 2 |
| 3232.3882955 | 3 |
| 3232.3889925 | 3 |
| 3232.3891912 | 0 |
| 3232.3907982 | 2 |
| 3232.3918217 | 1 |
| 3232.392011 | 2 |
| 3232.393355 | 4 |
| 3232.3942583 | 0 |
| 3232.3951692 | 3 |
| 3232.3958934 | 1 |
| 3232.3967085 | 2 |
| 3232.3979593 | 2 |
| 3232.3985008 | 3 |
| 3232.4001743 | 4 |
| 3232.4008203 | 2 |
| 3232.4015577 | 1 |
| 3232.4024561 | 2 |
| 3232.4030857 | 3 |
| 3232.4042263 | 0 |
| 3232.4052924 | 2 |
| 3232.4055105 | 2 |
| 3232.4072192 | 2 |
| 3232.4072967 | 3 |
| 3232.4091925 | 4 |
| 3232.4101344 | 0 |
| 3232.4103298 | 3 |
| 3232.4115806 | 2 |
| 3232.4124506 | 2 |
| 3232.4131235 | 6 |
| 3232.4145445 | 4 |
| 3232.4158089 | 3 |
| 3232.4164045 | 1 |
| 3232.4169566 | 3 |
| 3232.4178617 | 2 |
| 3232.4193304 | 4 |
| 3232.4196989 | 1 |
| 3232.4209756 | 6 |
| 3232.4215103 | 2 |
| 3232.4237473 | 1 |
| 3232.4239949 | 2 |
| 3232.4249951 | 3 |
| 3232.4262916 | 3 |
| 3232.4265517 | 0 |
| 3232.4279888 | 4 |
| 3232.4290056 | 3 |
| 3232.4298839 | 1 |
| 3232.4304479 | 3 |
| 3232.4312351 | 4 |
| 3232.4324988 | 4 |
| 3232.4326015 | 3 |
| 3232.4338309 | 0 |
| 3232.4349452 | 3 |
| 3232.4356702 | 2 |
| 3232.4373267 | 2 |
| 3232.4378629 | 0 |
| 3232.4388398 | 1 |
| 3232.4393434 | 0 |
| 3232.440189 | 2 |
| 3232.4417026 | 2 |
| 3232.4424763 | 3 |
| 3232.4433234 | 1 |
| 3232.444918 | 4 |
| 3232.4449578 | 2 |
| 3232.4455967 | 1 |
| 3232.4467336 | 1 |
| 3232.4473004 | 1 |
| 3232.4482225 | 4 |
| 3232.4495288 | 1 |
| 3232.4501046 | 1 |
| 3232.4509611 | 1 |
| 3232.4525581 | 1 |
| 3232.4533924 | 3 |
| 3232.4544069 | 0 |
| 3232.455283 | 1 |
| 3232.455941 | 1 |
| 3232.4573099 | 1 |
| 3232.4581676 | 1 |
| 3232.4587523 | 3 |
| 3232.4601205 | 3 |
| 3232.4602756 | 2 |
| 3232.4619214 | 1 |
| 3232.4633801 | 2 |
| 3232.4638085 | 2 |
| 3232.4646788 | 0 |
| 3232.4660989 | 2 |
| 3232.466574 | 2 |
| 3232.4673893 | 3 |
| 3232.4680919 | 2 |
| 3232.4691028 | 2 |
| 3232.4699467 | 2 |
| 3232.4708328 | 0 |
| 3232.4721582 | 0 |
| 3232.4724124 | 2 |
| 3232.4739172 | 2 |
| 3232.4748007 | 0 |
| 3232.4750879 | 1 |
| 3232.4766753 | 1 |
| 3232.4774749 | 4 |
| 3232.4789177 | 3 |
| 3232.4789686 | 0 |
| 3232.4806459 | 5 |
| 3232.48144 | 3 |
| 3232.4820788 | 1 |
| 3232.483227 | 4 |
| 3232.4840202 | 4 |
| 3232.4844278 | 1 |
| 3232.486116 | 1 |
| 3232.486917 | 6 |
| 3232.4870352 | 4 |
| 3232.4892423 | 4 |
| 3232.4894065 | 1 |
| 3232.4899899 | 3 |
| 3232.4906001 | 0 |
| 3232.4920485 | 5 |
| 3232.4926531 | 4 |
| 3232.4941109 | 3 |
| 3232.4949938 | 0 |
| 3232.4957159 | 3 |
| 3232.4969175 | 0 |
| 3232.4971123 | 3 |
| 3232.4979467 | 0 |
| 3232.4991388 | 4 |
| 3232.4996242 | 1 |
| 3232.5011589 | 1 |
| 3232.5021147 | 1 |
| 3232.5032496 | 1 |
| 3232.5042779 | 1 |
| 3232.5048601 | 3 |
| 3232.505813 | 4 |
| 3232.5073661 | 2 |
| 3232.5073984 | 4 |
| 3232.5088476 | 4 |
| 3232.5096545 | 1 |
| 3232.5104266 | 4 |
| 3232.5113223 | 1 |
| 3232.5130591 | 4 |
| 3232.5145304 | 2 |
| 3232.5145353 | 3 |
| 3232.5151307 | 1 |
| 3232.5160934 | 3 |
| 3232.5173329 | 1 |
| 3232.5181389 | 2 |
| 3232.5191205 | 1 |
| 3232.5200494 | 4 |
| 3232.5203176 | 3 |
| 3232.5218835 | 0 |
| 3232.5229352 | 1 |
| 3232.5237712 | 3 |
| 3232.5246927 | 0 |
| 3232.5259055 | 2 |
| 3232.5265977 | 2 |
| 3232.5271966 | 3 |
| 3232.5278374 | 1 |
| 3232.5291591 | 2 |
| 3232.5299138 | 2 |
| 3232.5310527 | 3 |
| 3232.5321462 | 3 |
| 3232.5328321 | 3 |
| 3232.5341072 | 1 |
| 3232.5342185 | 1 |
| 3232.5352382 | 2 |
| 3232.53713 | 0 |
| 3232.5374469 | 1 |
| 3232.5378833 | 1 |
| 3232.5388982 | 0 |
| 3232.5404078 | 4 |
| 3232.5415644 | 3 |
| 3232.5416725 | 1 |
| 3232.5431578 | 3 |
| 3232.5431871 | 2 |
| 3232.5448525 | 3 |
| 3232.5457087 | 3 |
| 3232.5460985 | 3 |
| 3232.5472973 | 4 |
| 3232.548073 | 0 |
| 3232.5488461 | 1 |
| 3232.5502161 | 0 |
| 3232.5507572 | 4 |
| 3232.5517601 | 2 |
| 3232.5527938 | 2 |
| 3232.553788 | 1 |
| 3232.5539916 | 2 |
| 3232.5548713 | 4 |
| 3232.5555812 | 1 |
| 3232.5560357 | 2 |
| 3232.5569589 | 1 |
| 3232.55748 | 2 |
| 3232.5585659 | 1 |
| 3232.5597281 | 1 |
| 3232.5602963 | 2 |
| 3232.5610863 | 7 |
| 3232.5630337 | 2 |
| 3232.5636453 | 3 |
| 3232.5646034 | 1 |
| 3232.5650212 | 1 |
| 3232.5662441 | 4 |
| 3232.5666512 | 2 |
| 3232.5684148 | 0 |
| 3232.5696078 | 1 |
| 3232.5698853 | 3 |
| 3232.5710477 | 1 |
| 3232.5721241 | 2 |
| 3232.572244 | 7 |
| 3232.573195 | 1 |
| 3232.5740408 | 1 |
| 3232.5752399 | 1 |
| 3232.5756451 | 2 |
| 3232.575897 | 3 |
| 3232.5762249 | 3 |
| 3232.5774562 | 2 |
| 3232.5775391 | 1 |
| 3232.5781037 | 3 |
| 3232.5781893 | 2 |
| 3232.5782159 | 2 |
| 3232.5786354 | 2 |
| 3232.5791832 | 2 |
| 3232.580604 | 1 |
| 3232.5807709 | 3 |
| 3232.5811261 | 1 |
| 3232.5821103 | 1 |
| 3232.582575 | 3 |
| 3232.5831803 | 4 |
| 3232.5837199 | 3 |
| 3232.5843707 | 2 |
| 3232.5852101 | 0 |
| 3232.5856519 | 2 |
| 3232.5859443 | 1 |
| 3232.5872497 | 2 |
| 3232.5876452 | 1 |
| 3232.5888186 | 1 |
| 3232.5897805 | 0 |
| 3232.5905956 | 1 |
| 3232.5908899 | 3 |
| 3232.5915459 | 2 |
| 3232.5917781 | 2 |
| 3232.5925575 | 2 |
| 3232.5926187 | 2 |
| 3232.5928768 | 2 |
| 3232.5933346 | 4 |

| | |
|---|---|
| 3232.5939228 | 5 |
| 3232.5949289 | 1 |
| 3232.5950898 | 6 |
| 3232.595531 | 0 |
| 3232.5956771 | 0 |
| 3232.5958293 | 2 |
| 3232.5970146 | 2 |
| 3232.5970266 | 1 |
| 3232.5971279 | 3 |
| 3232.5973181 | 2 |
| 3232.5979832 | 3 |
| 3232.5979993 | 3 |
| 3232.598224 | 4 |
| 3232.5993624 | 3 |
| 3232.5998503 | 2 |
| 3232.6003692 | 1 |
| 3232.6004892 | 2 |
| 3232.6015318 | 2 |
| 3232.6015716 | 1 |
| 3232.6023639 | 3 |
| 3232.6025618 | 2 |
| 3232.6031715 | 1 |
| 3232.6037911 | 1 |
| 3232.6038242 | 3 |
| 3232.603928 | 1 |
| 3232.6050656 | 1 |
| 3232.6053154 | 3 |
| 3232.6061786 | 3 |
| 3232.6063077 | 2 |
| 3232.6067114 | 2 |
| 3232.6067293 | 2 |
| 3232.6076957 | 2 |
| 3232.6080356 | 3 |
| 3232.6089156 | 2 |
| 3232.6089738 | 4 |
| 3232.6099374 | 3 |
| 3232.6100563 | 2 |
| 3232.6100646 | 3 |
| 3232.6112555 | 0 |
| 3232.6115432 | 2 |
| 3232.6117157 | 2 |
| 3232.6123761 | 2 |
| 3232.6131961 | 1 |
| 3232.613468 | 1 |
| 3232.6136251 | 2 |
| 3232.6137146 | 4 |
| 3232.6138007 | 1 |
| 3232.613835 | 2 |
| 3232.6143895 | 3 |
| 3232.6148986 | 4 |
| 3232.6152243 | 0 |
| 3232.6152337 | 1 |
| 3232.6159221 | 3 |
| 3232.6161044 | 0 |
| 3232.6162662 | 2 |
| 3232.6163459 | 5 |
| 3232.6165519 | 2 |
| 3232.6166045 | 1 |
| 3232.6166465 | 3 |
| 3232.6169336 | 1 |
| 3232.617026 | 2 |
| 3232.6170281 | 3 |
| 3232.6171228 | 3 |
| 3232.6176276 | 4 |
| 3232.6178199 | 2 |
| 3232.6178374 | 0 |
| 3232.6178956 | 3 |
| 3232.6186577 | 2 |
| 3232.6188258 | 0 |
| 3232.6189463 | 2 |
| 3232.6193142 | 5 |
| 3232.6193244 | 2 |
| 3232.6194398 | 4 |
| 3232.619565 | 2 |
| 3232.6196574 | 5 |
| 3232.6202486 | 5 |
| 3232.6203865 | 0 |
| 3232.6207528 | 1 |
| 3232.6208349 | 1 |
| 3232.6208922 | 2 |
| 3232.6209696 | 1 |
| 3232.6210381 | 4 |
| 3232.6211467 | 2 |
| 3232.6213852 | 1 |
| 3232.6215534 | 2 |
| 3232.6221525 | 3 |
| 3232.6221688 | 1 |
| 3232.6222931 | 0 |
| 3232.622415 | 0 |
| 3232.6225439 | 3 |
| 3232.6227668 | 0 |
| 3232.6233496 | 2 |
| 3232.6235512 | 1 |
| 3232.6237021 | 0 |
| 3232.6237413 | 2 |
| 3232.6247329 | 1 |
| 3232.6248184 | 1 |
| 3232.6249593 | 1 |
| 3232.6251758 | 0 |
| 3232.6252039 | 2 |
| 3232.6252068 | 1 |
| 3232.6253003 | 1 |
| 3232.6256145 | 3 |
| 3232.6256193 | 1 |
| 3232.6258531 | 2 |
| 3232.6258967 | 2 |
| 3232.6259113 | 0 |
| 3232.6262594 | 2 |
| 3232.6263183 | 3 |
| 3232.6265567 | 1 |
| 3232.6266389 | 1 |
| 3232.6267391 | 3 |
| 3232.6270468 | 0 |
| 3232.6270993 | 4 |
| 3232.6271142 | 0 |
| 3232.6271802 | 3 |
| 3232.6271894 | 1 |
| 3232.6275302 | 1 |
| 3232.6275837 | 1 |
| 3232.627856 | 4 |
| 3232.627935 | 1 |
| 3232.628051 | 1 |
| 3232.628116 | 4 |
| 3232.6284878 | 1 |
| 3232.6285923 | 2 |
| 3232.628613 | 1 |
| 3232.6289206 | 1 |
| 3232.6289363 | 1 |
| 3232.6292259 | 1 |
| 3232.6292341 | 2 |
| 3232.6295665 | 2 |
| 3232.6296071 | 2 |
| 3232.6296977 | 1 |
| 3232.6297589 | 2 |
| 3232.6299333 | 3 |
| 3232.6300619 | 1 |
| 3232.6301608 | 0 |
| 3232.6302401 | 2 |
| 3232.6302655 | 2 |
| 3232.6305829 | 2 |
| 3232.6306712 | 0 |
| 3232.6306929 | 2 |
| 3232.6310332 | 0 |
| 3232.6310768 | 2 |
| 3232.6312525 | 1 |
| 3232.6314399 | 1 |
| 3232.6316313 | 0 |
| 3232.63188 | 2 |
| 3232.6320339 | 2 |
| 3232.6320744 | 1 |
| 3232.632116 | 2 |
| 3232.6323614 | 1 |
| 3232.6324952 | 3 |
| 3232.6325294 | 3 |
| 3232.6325762 | 4 |
| 3232.6327496 | 3 |
| 3232.6327942 | 4 |
| 3232.6329756 | 2 |
| 3232.6332358 | 3 |
| 3232.6333139 | 2 |
| 3232.6333692 | 2 |
| 3232.6336841 | 1 |
| 3232.6339191 | 3 |
| 3232.6339855 | 2 |
| 3232.6340313 | 1 |
| 3232.6341294 | 1 |
| 3232.6342809 | 4 |
| 3232.6344283 | 0 |
| 3232.6345758 | 0 |
| 3232.6350485 | 1 |
| 3232.6350648 | 1 |
| 3232.6351858 | 1 |
| 3232.6351873 | 1 |
| 3232.6351973 | 1 |
| 3232.6352278 | 1 |
| 3232.6356239 | 4 |
| 3232.6358461 | 3 |
| 3232.6359602 | 2 |
| 3232.6361409 | 1 |
| 3232.6362187 | 2 |
| 3232.636364 | 2 |
| 3232.6365599 | 1 |
| 3232.6367117 | 2 |
| 3232.6367488 | 3 |
| 3232.6368922 | 1 |
| 3232.6368946 | 1 |
| 3232.6369628 | 4 |
| 3232.6370168 | 2 |
| 3232.6370589 | 0 |
| 3232.637567 | 1 |
| 3232.6380186 | 3 |
| 3232.6382422 | 3 |
| 3232.6382772 | 0 |
| 3232.6383369 | 1 |
| 3232.6386931 | 2 |
| 3232.6387132 | 0 |
| 3232.6387376 | 1 |
| 3232.6390203 | 1 |
| 3232.6392995 | 0 |
| 3232.6393735 | 1 |
| 3232.6394187 | 0 |
| 3232.6394475 | 2 |
| 3232.6396842 | 2 |
| 3232.6397788 | 2 |
| 3232.6399114 | 5 |
| 3232.6399713 | 3 |
| 3232.6399833 | 3 |
| 3232.6400487 | 2 |
| 3232.6401435 | 6 |
| 3232.6404221 | 2 |
| 3232.6404355 | 4 |
| 3232.6406304 | 0 |
| 3232.6406816 | 4 |
| 3232.6408042 | 2 |
| 3232.6411503 | 0 |
| 3232.6413171 | 1 |
| 3232.6413307 | 2 |
| 3232.6413599 | 2 |
| 3232.6417612 | 2 |
| 3232.64206 | 0 |
| 3232.6421884 | 5 |
| 3232.642236 | 1 |
| 3232.6423373 | 2 |
| 3232.6424991 | 2 |
| 3232.6425376 | 2 |
| 3232.6429706 | 1 |
| 3232.6430535 | 2 |
| 3232.6432118 | 0 |
| 3232.6433032 | 0 |
| 3232.6433377 | 1 |
| 3232.6433843 | 1 |
| 3232.6435807 | 5 |
| 3232.6436752 | 1 |
| 3232.6439514 | 2 |
| 3232.6439662 | 1 |
| 3232.6441146 | 1 |
| 3232.644205 | 1 |
| 3232.6445327 | 1 |
| 3232.644563 | 0 |
| 3232.6448222 | 1 |
| 3232.6448276 | 2 |
| 3232.6451699 | 4 |
| 3232.6453066 | 1 |
| 3232.6453769 | 0 |
| 3232.6454311 | 0 |
| 3232.6458944 | 0 |
| 3232.6460762 | 4 |
| 3232.6460842 | 3 |
| 3232.6460923 | 3 |
| 3232.6462867 | 2 |
| 3232.6464148 | 1 |
| 3232.6466314 | 0 |
| 3232.6472018 | 3 |
| 3232.6472091 | 1 |
| 3232.6472798 | 1 |
| 3232.6473067 | 3 |
| 3232.6474387 | 2 |
| 3232.6474976 | 1 |
| 3232.6475327 | 1 |
| 3232.6476477 | 2 |
| 3232.6477808 | 5 |
| 3232.6479315 | 0 |
| 3232.6482854 | 6 |
| 3232.6484097 | 4 |
| 3232.6484976 | 4 |
| 3232.6485347 | 0 |
| 3232.6487661 | 1 |
| 3232.6488132 | 4 |
| 3232.649013 | 6 |
| 3232.649082 | 4 |
| 3232.6490972 | 5 |
| 3232.6495192 | 3 |
| 3232.649594 | 14 |
| 3232.6499684 | 7 |
| 3232.6500933 | 8 |
| 3232.6500933 | 9 |
| 3232.6502363 | 15 |
| 3232.6502585 | 15 |
| 3232.6503554 | 14 |
| 3232.6506151 | 12 |
| 3232.6509063 | 15 |
| 3232.651093 | 10 |
| 3232.6510973 | 16 |
| 3232.6512052 | 10 |
| 3232.6513382 | 6 |
| 3232.6515429 | 4 |
| 3232.6517233 | 9 |
| 3232.6518399 | 8 |
| 3232.6519146 | 14 |
| 3232.6519187 | 11 |
| 3232.6519368 | 9 |
| 3232.6519553 | 8 |
| 3232.6520211 | 6 |
| 3232.6521679 | 9 |
| 3232.652482 | 7 |
| 3232.6529582 | 11 |
| 3232.6529861 | 6 |
| 3232.6530456 | 4 |
| 3232.6531274 | 2 |
| 3232.6533852 | 8 |
| 3232.6536693 | 2 |
| 3232.6537679 | 9 |
| 3232.6539682 | 10 |
| 3232.6539783 | 8 |
| 3232.6544552 | 7 |
| 3232.6544814 | 12 |
| 3232.6546074 | 11 |
| 3232.6547055 | 17 |
| 3232.6549121 | 11 |
| 3232.6549689 | 9 |
| 3232.6549858 | 8 |
| 3232.6550779 | 8 |
| 3232.6552356 | 9 |
| 3232.6555066 | 10 |
| 3232.6558199 | 11 |
| 3232.6558496 | 10 |
| 3232.6560601 | 15 |
| 3232.6560894 | 12 |
| 3232.656239 | 15 |
| 3232.6563308 | 13 |
| 3232.6567733 | 9 |
| 3232.6568109 | 8 |
| 3232.6570252 | 6 |
| 3232.6571367 | 7 |
| 3232.6572065 | 8 |
| 3232.6575718 | 12 |
| 3232.6576431 | 5 |
| 3232.6578365 | 8 |
| 3232.6578909 | 7 |
| 3232.658001 | 11 |
| 3232.6580707 | 11 |
| 3232.6582195 | 5 |
| 3232.6584663 | 13 |
| 3232.6585629 | 10 |
| 3232.6587913 | 8 |
| 3232.6588254 | 7 |
| 3232.6588775 | 5 |
| 3232.6590613 | 7 |
| 3232.6592695 | 8 |
| 3232.6594795 | 8 |
| 3232.6595298 | 4 |
| 3232.6596005 | 11 |
| 3232.6597715 | 10 |
| 3232.660065 | 10 |
| 3232.6602243 | 8 |
| 3232.6603068 | 9 |
| 3232.6603557 | 4 |
| 3232.6606152 | 4 |
| 3232.6607079 | 2 |
| 3232.6607227 | 2 |
| 3232.6607789 | 5 |
| 3232.6608509 | 5 |
| 3232.660872 | 8 |
| 3232.6608916 | 3 |
| 3232.6614429 | 10 |
| 3232.6615627 | 6 |
| 3232.661671 | 2 |
| 3232.662088 | 2 |
| 3232.6621609 | 0 |
| 3232.6623119 | 1 |
| 3232.6623668 | 1 |
| 3232.6624859 | 5 |
| 3232.6626295 | 2 |

| | |
|---|---|
| 3232.6626942 | 3 |
| 3232.6632641 | 1 |
| 3232.6633113 | 1 |
| 3232.6633321 | 2 |
| 3232.6635026 | 0 |
| 3232.6637377 | 2 |
| 3232.6639643 | 2 |
| 3232.6641655 | 1 |
| 3232.6642986 | 3 |
| 3232.6643759 | 0 |
| 3232.6643999 | 2 |
| 3232.6646254 | 0 |
| 3232.6647668 | 2 |
| 3232.6649705 | 2 |
| 3232.6650115 | 1 |
| 3232.6650859 | 2 |
| 3232.6650904 | 1 |
| 3232.6652368 | 4 |
| 3232.6652411 | 0 |
| 3232.6654696 | 0 |
| 3232.6655733 | 0 |
| 3232.6657281 | 3 |
| 3232.6658911 | 1 |
| 3232.6662508 | 1 |
| 3232.6664016 | 3 |
| 3232.6666594 | 3 |
| 3232.6667388 | 2 |
| 3232.6667791 | 4 |
| 3232.6667913 | 3 |
| 3232.6670342 | 2 |
| 3232.6671471 | 2 |
| 3232.6674206 | 2 |
| 3232.6674606 | 1 |
| 3232.6675763 | 2 |
| 3232.6677258 | 2 |
| 3232.6678295 | 3 |
| 3232.6678623 | 1 |
| 3232.6680347 | 0 |
| 3232.6681988 | 1 |
| 3232.6684935 | 5 |
| 3232.6685193 | 1 |
| 3232.6685675 | 4 |
| 3232.6689328 | 2 |
| 3232.6690057 | 2 |
| 3232.6691694 | 0 |
| 3232.6691786 | 0 |
| 3232.6695784 | 3 |
| 3232.669677 | 2 |
| 3232.6697715 | 3 |
| 3232.6698188 | 1 |
| 3232.6701259 | 3 |
| 3232.6701463 | 1 |
| 3232.6701871 | 0 |
| 3232.6703266 | 3 |
| 3232.6703442 | 1 |
| 3232.6706061 | 1 |
| 3232.6708937 | 0 |
| 3232.6712432 | 1 |
| 3232.6712786 | 4 |
| 3232.6713036 | 2 |
| 3232.6714968 | 2 |
| 3232.671525 | 2 |
| 3232.6717579 | 0 |
| 3232.6718565 | 2 |
| 3232.6718953 | 0 |
| 3232.6718995 | 2 |
| 3232.6720101 | 4 |
| 3232.6731461 | 0 |
| 3232.6732383 | 2 |
| 3232.6732631 | 1 |
| 3232.6737258 | 0 |
| 3232.6741191 | 5 |
| 3232.6744406 | 5 |
| 3232.6753198 | 2 |
| 3232.6753378 | 2 |
| 3232.6757876 | 0 |
| 3232.6760155 | 2 |
| 3232.6765778 | 0 |
| 3232.6774147 | 2 |
| 3232.6777702 | 1 |
| 3232.6786272 | 1 |
| 3232.6787332 | 1 |
| 3232.6790233 | 5 |
| 3232.6795828 | 2 |
| 3232.680004 | 2 |
| 3232.6806364 | 3 |
| 3232.6810024 | 2 |
| 3232.6821614 | 3 |
| 3232.6826299 | 1 |
| 3232.6827382 | 1 |
| 3232.682754 | 1 |
| 3232.6837402 | 3 |
| 3232.6842086 | 3 |
| 3232.6844489 | 2 |
| 3232.6847171 | 4 |
| 3232.6851651 | 2 |
| 3232.6856173 | 2 |
| 3232.6866969 | 3 |
| 3232.6868681 | 3 |
| 3232.6873055 | 1 |
| 3232.6876513 | 5 |
| 3232.6882274 | 2 |
| 3232.6886619 | 1 |
| 3232.688976 | 3 |
| 3232.6895708 | 1 |
| 3232.6902902 | 1 |
| 3232.6912224 | 1 |
| 3232.6912646 | 1 |
| 3232.6915693 | 2 |
| 3232.692187 | 5 |
| 3232.6923595 | 4 |
| 3232.6931052 | 0 |
| 3232.693292 | 4 |
| 3232.6942863 | 3 |
| 3232.6944296 | 0 |
| 3232.6947052 | 3 |
| 3232.6952075 | 1 |
| 3232.6959573 | 2 |
| 3232.6963438 | 2 |
| 3232.6969511 | 0 |
| 3232.6970841 | 3 |
| 3232.6971114 | 2 |
| 3232.698177 | 1 |
| 3232.6985105 | 0 |
| 3232.6989332 | 2 |
| 3232.6993576 | 2 |
| 3232.6997259 | 2 |
| 3232.6998388 | 2 |
| 3232.7006974 | 2 |
| 3232.7007712 | 2 |
| 3232.7016831 | 2 |
| 3232.7017317 | 1 |
| 3232.7029918 | 1 |
| 3232.7038084 | 3 |
| 3232.7038102 | 3 |
| 3232.704216 | 3 |
| 3232.7046528 | 2 |
| 3232.7052354 | 3 |
| 3232.7058144 | 2 |
| 3232.7061945 | 1 |
| 3232.7065247 | 1 |
| 3232.7067376 | 3 |
| 3232.7080288 | 2 |
| 3232.7080514 | 3 |
| 3232.7080627 | 3 |
| 3232.708737 | 1 |
| 3232.7092229 | 2 |
| 3232.7096465 | 1 |
| 3232.7106061 | 0 |
| 3232.7108113 | 0 |
| 3232.7109964 | 3 |
| 3232.7110887 | 2 |
| 3232.7117668 | 4 |
| 3232.7122307 | 4 |
| 3232.7126762 | 2 |
| 3232.7128971 | 2 |
| 3232.7140191 | 1 |
| 3232.7141697 | 2 |
| 3232.7148636 | 2 |
| 3232.715604 | 0 |
| 3232.7157288 | 1 |
| 3232.716182 | 6 |
| 3232.7168492 | 1 |
| 3232.7169589 | 0 |
| 3232.7171484 | 5 |
| 3232.7186509 | 2 |
| 3232.7187388 | 1 |
| 3232.7191527 | 2 |
| 3232.7195531 | 2 |
| 3232.7201599 | 1 |
| 3232.720671 | 2 |
| 3232.7207856 | 0 |
| 3232.7214473 | 2 |
| 3232.7216652 | 2 |
| 3232.7225207 | 2 |
| 3232.7230285 | 1 |
| 3232.7232557 | 1 |
| 3232.7236622 | 0 |
| 3232.7238663 | 0 |
| 3232.7245549 | 4 |
| 3232.7248656 | 0 |
| 3232.7253556 | 3 |
| 3232.7261062 | 4 |
| 3232.7261894 | 1 |
| 3232.7264796 | 2 |
| 3232.727585 | 4 |
| 3232.7276006 | 3 |
| 3232.7285824 | 0 |
| 3232.7286633 | 2 |
| 3232.7291797 | 1 |
| 3232.7295743 | 4 |
| 3232.7296018 | 2 |
| 3232.7310137 | 0 |
| 3232.7311286 | 3 |
| 3232.7312154 | 2 |
| 3232.7319909 | 0 |
| 3232.732357 | 4 |
| 3232.7324279 | 1 |
| 3232.7333961 | 1 |
| 3232.7339603 | 1 |
| 3232.7340742 | 2 |
| 3232.7349186 | 4 |
| 3232.7356694 | 0 |
| 3232.7360992 | 3 |
| 3232.7362086 | 0 |
| 3232.7363013 | 5 |
| 3232.7371427 | 3 |
| 3232.7375474 | 1 |
| 3232.7382728 | 2 |
| 3232.7391514 | 2 |
| 3232.7393968 | 1 |
| 3232.7394322 | 1 |
| 3232.7401104 | 3 |
| 3232.7404831 | 1 |
| 3232.7412486 | 1 |
| 3232.7412779 | 1 |
| 3232.7421834 | 3 |
| 3232.7428552 | 3 |
| 3232.7431234 | 0 |
| 3232.7434389 | 5 |
| 3232.7436664 | 4 |
| 3232.7442295 | 2 |
| 3232.7451773 | 5 |
| 3232.7457695 | 4 |
| 3232.7457979 | 1 |
| 3232.7465649 | 1 |
| 3232.746761 | 3 |
| 3232.7468412 | 0 |
| 3232.7473716 | 2 |
| 3232.7479625 | 2 |
| 3232.7483103 | 2 |
| 3232.748549 | 1 |
| 3232.7500844 | 7 |
| 3232.750129 | 1 |
| 3232.7501743 | 1 |
| 3232.7504801 | 2 |
| 3232.7514908 | 1 |
| 3232.7522237 | 6 |
| 3232.7522535 | 3 |
| 3232.7529191 | 4 |
| 3232.7530741 | 2 |
| 3232.7536485 | 3 |
| 3232.7544253 | 2 |
| 3232.7546823 | 2 |
| 3232.7550544 | 2 |
| 3232.755211 | 1 |
| 3232.7559312 | 3 |
| 3232.7562083 | 3 |
| 3232.757215 | 2 |
| 3232.7574635 | 3 |
| 3232.7576045 | 1 |
| 3232.7578275 | 2 |
| 3232.7591959 | 1 |
| 3232.7594454 | 5 |
| 3232.7596336 | 2 |
| 3232.7602849 | 9 |
| 3232.7608764 | 3 |
| 3232.7612 | 1 |
| 3232.7620795 | 3 |
| 3232.7622105 | 7 |
| 3232.7622265 | 1 |
| 3232.7629982 | 1 |
| 3232.7637257 | 6 |
| 3232.7640012 | 1 |
| 3232.7641012 | 1 |
| 3232.7644144 | 0 |
| 3232.7651328 | 5 |
| 3232.7660591 | 0 |
| 3232.7664549 | 1 |
| 3232.7666936 | 2 |
| 3232.7670124 | 1 |
| 3232.7682318 | 2 |
| 3232.7684239 | 3 |
| 3232.7687066 | 0 |
| 3232.7691938 | 1 |
| 3232.769947 | 2 |
| 3232.7699778 | 0 |
| 3232.7699851 | 2 |
| 3232.7712209 | 2 |
| 3232.7712542 | 5 |
| 3232.7712959 | 1 |
| 3232.772816 | 2 |
| 3232.7729804 | 0 |
| 3232.7733033 | 5 |
| 3232.7736347 | 2 |
| 3232.7738582 | 3 |
| 3232.7740977 | 0 |
| 3232.7745623 | 1 |
| 3232.7759386 | 1 |
| 3232.7760662 | 0 |
| 3232.7764051 | 0 |
| 3232.7764692 | 1 |
| 3232.7776609 | 6 |
| 3232.7776633 | 5 |
| 3232.7783156 | 1 |
| 3232.7788334 | 1 |
| 3232.77979 | 0 |
| 3232.7803116 | 1 |
| 3232.7803612 | 5 |
| 3232.7805564 | 4 |
| 3232.7812457 | 4 |
| 3232.781449 | 2 |
| 3232.7822526 | 2 |
| 3232.7827901 | 0 |
| 3232.7834586 | 1 |
| 3232.783813 | 2 |
| 3232.7840398 | 1 |
| 3232.7847852 | 0 |
| 3232.7850865 | 1 |
| 3232.7851142 | 2 |
| 3232.7861053 | 3 |
| 3232.7862061 | 6 |
| 3232.7871248 | 2 |
| 3232.7874617 | 2 |
| 3232.7878463 | 4 |
| 3232.7879233 | 2 |
| 3232.7886789 | 0 |
| 3232.7889404 | 0 |
| 3232.7898255 | 3 |
| 3232.7900982 | 0 |
| 3232.7909185 | 3 |
| 3232.7910622 | 1 |
| 3232.7917758 | 1 |
| 3232.7922321 | 1 |
| 3232.7923902 | 0 |
| 3232.7940247 | 2 |
| 3232.7941776 | 3 |
| 3232.7943323 | 2 |
| 3232.7947622 | 4 |
| 3232.7949357 | 2 |
| 3232.7954539 | 4 |
| 3232.7962735 | 3 |
| 3232.7973131 | 1 |
| 3232.7973389 | 4 |
| 3232.7974799 | 1 |
| 3232.7978641 | 1 |
| 3232.7985305 | 4 |
| 3232.7987918 | 3 |
| 3232.7995202 | 3 |
| 3232.7997514 | 4 |
| 3232.8004104 | 2 |
| 3232.8011441 | 3 |
| 3232.8015346 | 2 |
| 3232.8018883 | 3 |
| 3232.8020604 | 0 |
| 3232.8021006 | 2 |
| 3232.8031159 | 3 |
| 3232.8033904 | 1 |
| 3232.8037196 | 0 |
| 3232.803961 | 3 |
| 3232.8046977 | 0 |
| 3232.8048236 | 6 |
| 3232.8061414 | 3 |
| 3232.8062245 | 2 |
| 3232.8065658 | 0 |
| 3232.8069197 | 3 |
| 3232.807839 | 3 |
| 3232.8083295 | 2 |
| 3232.8083937 | 4 |
| 3232.8086575 | 2 |
| 3232.8092254 | 2 |
| 3232.8098107 | 2 |
| 3232.8103698 | 1 |

| | |
|---|---|
| 3232.8106596 | 4 |
| 3232.8109197 | 5 |
| 3232.8118808 | 2 |
| 3232.8122575 | 2 |
| 3232.8124561 | 2 |
| 3232.8134348 | 1 |
| 3232.8135586 | 5 |
| 3232.813572 | 0 |
| 3232.8143262 | 4 |
| 3232.8150835 | 4 |
| 3232.8157466 | 3 |
| 3232.8160655 | 3 |
| 3232.8175396 | 3 |
| 3232.8175659 | 3 |
| 3232.8176315 | 1 |
| 3232.8176567 | 0 |
| 3232.8188074 | 3 |
| 3232.8188393 | 1 |
| 3232.8193563 | 4 |
| 3232.8196951 | 2 |
| 3232.8203051 | 2 |
| 3232.8207814 | 1 |
| 3232.8217812 | 0 |
| 3232.8217868 | 1 |
| 3232.8224186 | 3 |
| 3232.8227328 | 2 |
| 3232.8233064 | 5 |
| 3232.8238548 | 2 |
| 3232.8240133 | 0 |
| 3232.8248221 | 1 |
| 3232.8252062 | 1 |
| 3232.8253346 | 4 |
| 3232.8254732 | 2 |
| 3232.826111 | 4 |
| 3232.8267138 | 3 |
| 3232.8268653 | 2 |
| 3232.8277072 | 4 |
| 3232.8282593 | 0 |
| 3232.8284225 | 3 |
| 3232.8292104 | 1 |
| 3232.8294115 | 1 |
| 3232.8297431 | 0 |
| 3232.8299896 | 1 |
| 3232.8313542 | 1 |
| 3232.8315584 | 6 |
| 3232.8318077 | 1 |
| 3232.8322645 | 1 |
| 3232.8330751 | 2 |
| 3232.8332182 | 1 |
| 3232.8341388 | 3 |
| 3232.8342146 | 2 |
| 3232.8346637 | 2 |
| 3232.8351522 | 6 |
| 3232.8358294 | 1 |
| 3232.8363009 | 1 |
| 3232.8365552 | 0 |
| 3232.8371122 | 1 |
| 3232.8377374 | 2 |
| 3232.8381199 | 1 |
| 3232.8387121 | 0 |
| 3232.8393934 | 1 |
| 3232.8394913 | 2 |
| 3232.8395931 | 2 |
| 3232.8405193 | 6 |
| 3232.8411622 | 2 |
| 3232.8417637 | 4 |
| 3232.8418494 | 0 |
| 3232.8426337 | 3 |
| 3232.8430343 | 2 |
| 3232.8430536 | 0 |
| 3232.8441014 | 2 |
| 3232.8446784 | 0 |
| 3232.8450057 | 2 |
| 3232.8457448 | 0 |
| 3232.8462805 | 0 |
| 3232.846429 | 1 |
| 3232.8465192 | 2 |
| 3232.8472717 | 0 |
| 3232.8478122 | 2 |
| 3232.8480485 | 2 |
| 3232.8485576 | 2 |
| 3232.8486023 | 0 |
| 3232.8490349 | 1 |
| 3232.8502489 | 4 |
| 3232.8506997 | 1 |
| 3232.8507722 | 1 |
| 3232.8517754 | 2 |
| 3232.8518595 | 1 |
| 3232.8529849 | 3 |
| 3232.8532396 | 4 |
| 3232.8533286 | 1 |
| 3232.8536199 | 2 |
| 3232.8540831 | 3 |
| 3232.8544139 | 1 |
| 3232.855087 | 2 |
| 3232.8551274 | 1 |
| 3232.8554021 | 1 |
| 3232.8563741 | 1 |
| 3232.8571228 | 1 |
| 3232.8573152 | 2 |
| 3232.8574538 | 1 |
| 3232.8579216 | 1 |
| 3232.8584764 | 5 |
| 3232.8592882 | 2 |
| 3232.8599061 | 1 |
| 3232.8599305 | 1 |
| 3232.8601953 | 0 |
| 3232.861137 | 5 |
| 3232.8611458 | 1 |
| 3232.8619447 | 2 |
| 3232.86216 | 2 |
| 3232.8627497 | 0 |
| 3232.863293 | 3 |
| 3232.8640268 | 1 |
| 3232.8641474 | 0 |
| 3232.8644562 | 2 |
| 3232.8653247 | 1 |
| 3232.8656663 | 1 |
| 3232.8660416 | 4 |
| 3232.8664447 | 2 |
| 3232.8671261 | 1 |
| 3232.8674109 | 4 |
| 3232.8675409 | 5 |
| 3232.8683452 | 1 |
| 3232.8688664 | 3 |
| 3232.8694527 | 2 |
| 3232.8695307 | 4 |
| 3232.8701296 | 0 |
| 3232.8706122 | 0 |
| 3232.871108 | 2 |
| 3232.8712617 | 1 |
| 3232.8724216 | 1 |
| 3232.8725378 | 2 |
| 3232.8736703 | 4 |
| 3232.873704 | 2 |
| 3232.8740787 | 2 |
| 3232.8750379 | 1 |
| 3232.8750849 | 3 |
| 3232.8755089 | 0 |
| 3232.8756712 | 1 |
| 3232.8767026 | 4 |
| 3232.8770982 | 2 |
| 3232.8777046 | 2 |
| 3232.8782203 | 1 |
| 3232.8782765 | 2 |
| 3232.8791363 | 1 |
| 3232.8797776 | 4 |
| 3232.8799987 | 1 |
| 3232.8804913 | 1 |
| 3232.880967 | 3 |
| 3232.8810704 | 2 |
| 3232.8819509 | 2 |
| 3232.8820582 | 0 |
| 3232.8828714 | 4 |
| 3232.8831163 | 2 |
| 3232.8837166 | 3 |
| 3232.8841421 | 4 |
| 3232.8845502 | 4 |
| 3232.884791 | 1 |
| 3232.8852705 | 1 |
| 3232.8861671 | 2 |
| 3232.8862441 | 1 |
| 3232.8864163 | 1 |
| 3232.887644 | 3 |
| 3232.8880609 | 2 |
| 3232.8880945 | 2 |
| 3232.8887724 | 3 |
| 3232.8893092 | 3 |
| 3232.8895803 | 3 |
| 3232.8897702 | 1 |
| 3232.8907274 | 3 |
| 3232.8910733 | 2 |
| 3232.8911789 | 0 |
| 3232.8912349 | 4 |
| 3232.8924078 | 0 |
| 3232.8931068 | 2 |
| 3232.8931718 | 3 |
| 3232.8939687 | 1 |
| 3232.8943171 | 1 |
| 3232.8945803 | 3 |
| 3232.8950974 | 3 |
| 3232.8958374 | 0 |
| 3232.8958854 | 3 |
| 3232.8967765 | 1 |
| 3232.8974942 | 2 |
| 3232.8976417 | 1 |
| 3232.8984953 | 3 |
| 3232.8986223 | 1 |
| 3232.8989306 | 1 |
| 3232.8993066 | 5 |
| 3232.899573 | 1 |
| 3232.9003136 | 1 |
| 3232.9009308 | 0 |
| 3232.9012875 | 0 |
| 3232.9015948 | 5 |
| 3232.9017965 | 0 |
| 3232.9022246 | 0 |
| 3232.902481 | 0 |
| 3232.9038241 | 3 |
| 3232.9038829 | 0 |
| 3232.9043883 | 2 |
| 3232.9047294 | 1 |
| 3232.9048698 | 0 |
| 3232.9061107 | 0 |
| 3232.9062201 | 0 |
| 3232.9064101 | 0 |
| 3232.9069803 | 3 |
| 3232.9074903 | 5 |
| 3232.9076023 | 2 |
| 3232.9082212 | 2 |
| 3232.9090598 | 4 |
| 3232.9095911 | 1 |
| 3232.9102766 | 4 |
| 3232.9102867 | 1 |
| 3232.9106971 | 4 |
| 3232.9116499 | 1 |
| 3232.9120317 | 0 |
| 3232.9127553 | 5 |
| 3232.9129453 | 3 |
| 3232.9129982 | 0 |
| 3232.9140138 | 0 |
| 3232.9140989 | 4 |
| 3232.9149196 | 1 |
| 3232.9149289 | 2 |
| 3232.9155832 | 1 |
| 3232.9164274 | 1 |
| 3232.9166228 | 2 |
| 3232.916887 | 1 |
| 3232.9170474 | 1 |
| 3232.9174033 | 2 |
| 3232.9185399 | 2 |
| 3232.9185886 | 2 |
| 3232.9193866 | 4 |
| 3232.9202422 | 0 |
| 3232.9209577 | 2 |
| 3232.9209626 | 1 |
| 3232.9215861 | 3 |
| 3232.9217643 | 2 |
| 3232.9223733 | 0 |
| 3232.9225129 | 2 |
| 3232.9234233 | 2 |
| 3232.9236919 | 2 |
| 3232.9237713 | 1 |
| 3232.9243312 | 5 |
| 3232.9250445 | 1 |
| 3232.9257546 | 0 |
| 3232.9258038 | 2 |
| 3232.9263896 | 0 |
| 3232.9268601 | 0 |
| 3232.92742 | 1 |
| 3232.9280249 | 3 |
| 3232.9285484 | 3 |
| 3232.9290897 | 0 |
| 3232.9291793 | 0 |
| 3232.9295894 | 4 |
| 3232.930138 | 2 |
| 3232.9303893 | 2 |
| 3232.9313319 | 1 |
| 3232.9317545 | 2 |
| 3232.9322212 | 3 |
| 3232.9327033 | 3 |
| 3232.9327872 | 0 |
| 3232.9335147 | 2 |
| 3232.9340451 | 2 |
| 3232.9348074 | 1 |
| 3232.9348927 | 2 |
| 3232.9352643 | 2 |
| 3232.9361076 | 2 |
| 3232.9362065 | 1 |
| 3232.9367651 | 0 |
| 3232.9371627 | 3 |
| 3232.9378687 | 2 |
| 3232.9382851 | 3 |
| 3232.9386201 | 0 |
| 3232.938929 | 1 |
| 3232.9397666 | 0 |
| 3232.9405416 | 3 |
| 3232.9406567 | 3 |
| 3232.9408369 | 1 |
| 3232.9418478 | 2 |
| 3232.9418485 | 2 |
| 3232.9423723 | 1 |
| 3232.9427542 | 1 |
| 3232.9429231 | 2 |
| 3232.9441134 | 0 |
| 3232.9441981 | 3 |
| 3232.9451125 | 1 |
| 3232.9452841 | 2 |
| 3232.9452874 | 6 |
| 3232.94546 | 3 |
| 3232.9467423 | 3 |
| 3232.9467495 | 4 |
| 3232.9467628 | 2 |
| 3232.9477587 | 2 |
| 3232.9480435 | 1 |
| 3232.9488782 | 4 |
| 3232.9490001 | 0 |
| 3232.9498842 | 1 |
| 3232.9500422 | 3 |
| 3232.9503473 | 3 |
| 3232.9512658 | 2 |
| 3232.9513421 | 0 |
| 3232.9517305 | 3 |
| 3232.9524765 | 2 |
| 3232.952845 | 0 |
| 3232.9536751 | 0 |
| 3232.9538033 | 4 |
| 3232.9538305 | 2 |
| 3232.954924 | 1 |
| 3232.9549835 | 4 |
| 3232.9560093 | 1 |
| 3232.956048 | 2 |
| 3232.9564265 | 2 |
| 3232.9564922 | 1 |
| 3232.9573194 | 2 |
| 3232.9580045 | 2 |
| 3232.9588216 | 2 |
| 3232.9591882 | 3 |
| 3232.9595306 | 1 |
| 3232.9596055 | 2 |
| 3232.9598741 | 3 |
| 3232.9608193 | 0 |
| 3232.9616046 | 0 |
| 3232.9616462 | 3 |
| 3232.9620194 | 3 |
| 3232.9626538 | 2 |
| 3232.9630588 | 1 |
| 3232.9638683 | 2 |
| 3232.9642133 | 1 |
| 3232.9646407 | 2 |
| 3232.9652826 | 2 |
| 3232.965678 | 2 |
| 3232.9659074 | 0 |
| 3232.9664903 | 3 |
| 3232.9668645 | 0 |
| 3232.9669632 | 2 |
| 3232.9681021 | 1 |
| 3232.9681039 | 4 |
| 3232.9684467 | 1 |
| 3232.9694726 | 4 |
| 3232.9700792 | 1 |
| 3232.9701986 | 4 |
| 3232.9703643 | 1 |
| 3232.9706205 | 0 |
| 3232.9717009 | 2 |
| 3232.9725743 | 1 |
| 3232.9725908 | 3 |
| 3232.9730738 | 1 |
| 3232.9734828 | 0 |
| 3232.9736368 | 3 |
| 3232.9745819 | 1 |
| 3232.9746283 | 4 |
| 3232.974869 | 5 |
| 3232.97543 | 1 |
| 3232.975597 | 2 |
| 3232.9761631 | 0 |
| 3232.9774292 | 6 |
| 3232.9774618 | 2 |
| 3232.9777687 | 3 |
| 3232.9783201 | 0 |
| 3232.9788106 | 1 |
| 3232.9794087 | 0 |
| 3232.9797547 | 1 |

| | |
|---|---|
| 3232.9801341 | 2 |
| 3232.9805164 | 0 |
| 3232.9814629 | 1 |
| 3232.9817258 | 1 |
| 3232.9824247 | 4 |
| 3232.9825149 | 1 |
| 3232.9831656 | 3 |
| 3232.9835469 | 3 |
| 3232.9843008 | 2 |
| 3232.9844414 | 1 |
| 3232.9850527 | 2 |
| 3232.985096 | 0 |
| 3232.9859766 | 3 |
| 3232.9862408 | 0 |
| 3232.9863834 | 1 |
| 3232.987116 | 2 |
| 3232.9872899 | 1 |
| 3232.9887889 | 2 |
| 3232.988838 | 3 |
| 3232.9888508 | 3 |
| 3232.9895323 | 5 |
| 3232.9902578 | 2 |
| 3232.9907155 | 3 |
| 3232.9908058 | 1 |
| 3232.9913896 | 0 |
| 3232.9915209 | 0 |
| 3232.991824 | 2 |
| 3232.9920429 | 2 |
| 3232.9921123 | 0 |
| 3232.9924566 | 2 |
| 3232.9926708 | 1 |
| 3232.9932587 | 1 |
| 3232.9935861 | 4 |
| 3232.9937844 | 2 |
| 3232.9938277 | 3 |
| 3232.9941236 | 1 |
| 3232.9948705 | 1 |
| 3232.9953285 | 1 |
| 3232.9956279 | 1 |
| 3232.9957299 | 3 |
| 3232.9958147 | 3 |
| 3232.9965327 | 1 |
| 3232.9966108 | 3 |
| 3232.9972185 | 3 |
| 3232.9973527 | 3 |
| 3232.9976676 | 5 |
| 3232.9977254 | 2 |
| 3232.9983275 | 2 |
| 3232.9985114 | 1 |
| 3232.9985173 | 3 |
| 3232.9986062 | 6 |
| 3232.9994522 | 1 |
| 3232.9997343 | 0 |
| 3233.0000921 | 1 |
| 3233.0002909 | 3 |
| 3233.0009321 | 3 |
| 3233.0009548 | 2 |
| 3233.0009679 | 1 |
| 3233.0010738 | 3 |
| 3233.0017188 | 2 |
| 3233.0024505 | 2 |
| 3233.0026482 | 0 |
| 3233.0030379 | 4 |
| 3233.0031423 | 3 |
| 3233.0032487 | 2 |
| 3233.0033358 | 3 |
| 3233.0034271 | 1 |
| 3233.0041834 | 3 |
| 3233.0044814 | 2 |
| 3233.0049359 | 2 |
| 3233.0050446 | 1 |
| 3233.0053119 | 1 |
| 3233.0056931 | 2 |
| 3233.0060834 | 2 |
| 3233.0063209 | 3 |
| 3233.0063242 | 2 |
| 3233.0071082 | 1 |
| 3233.0071453 | 0 |
| 3233.007358 | 1 |
| 3233.0076106 | 3 |
| 3233.0082456 | 1 |
| 3233.0089884 | 3 |
| 3233.0098846 | 0 |
| 3233.0100747 | 1 |
| 3233.0101469 | 3 |
| 3233.0108125 | 2 |
| 3233.01096 | 4 |
| 3233.0117503 | 2 |
| 3233.0121461 | 2 |
| 3233.0128711 | 2 |
| 3233.0131423 | 2 |
| 3233.0140954 | 2 |
| 3233.0141208 | 3 |
| 3233.0141291 | 2 |
| 3233.0148626 | 3 |
| 3233.0156338 | 1 |
| 3233.016277 | 3 |
| 3233.0163399 | 3 |
| 3233.017453 | 2 |
| 3233.0175313 | 0 |
| 3233.0180589 | 1 |
| 3233.0183053 | 2 |
| 3233.0184547 | 3 |
| 3233.0188121 | 2 |
| 3233.0193685 | 2 |
| 3233.0207714 | 3 |
| 3233.0211206 | 2 |
| 3233.0217866 | 1 |
| 3233.02383 | 2 |
| 3233.0247332 | 0 |
| 3233.0250572 | 3 |
| 3233.0264001 | 3 |
| 3233.0264333 | 2 |
| 3233.0270079 | 1 |
| 3233.0290788 | 3 |
| 3233.0292803 | 0 |
| 3233.0307079 | 7 |
| 3233.0318194 | 3 |
| 3233.0325026 | 1 |
| 3233.0329521 | 3 |
| 3233.0343855 | 3 |
| 3233.0349856 | 1 |
| 3233.0357702 | 4 |
| 3233.0364338 | 4 |
| 3233.0377457 | 4 |
| 3233.0381878 | 2 |
| 3233.0404311 | 2 |
| 3233.0404493 | 0 |
| 3233.0417162 | 4 |
| 3233.0423519 | 2 |
| 3233.0431892 | 0 |
| 3233.044501 | 5 |
| 3233.045297 | 2 |
| 3233.0460939 | 3 |
| 3233.0473434 | 2 |
| 3233.0475857 | 6 |
| 3233.0488081 | 3 |
| 3233.0494966 | 1 |
| 3233.0505431 | 3 |
| 3233.0516227 | 6 |
| 3233.0528675 | 1 |
| 3233.0541472 | 4 |
| 3233.0542888 | 1 |
| 3233.0554032 | 3 |
| 3233.0560367 | 4 |
| 3233.0574161 | 1 |
| 3233.0582345 | 1 |
| 3233.058712 | 1 |
| 3233.0605453 | 5 |
| 3233.0611216 | 1 |
| 3233.062082 | 3 |
| 3233.0631228 | 4 |
| 3233.0636754 | 2 |
| 3233.0652847 | 0 |
| 3233.0656083 | 5 |
| 3233.0664127 | 2 |
| 3233.0680079 | 2 |
| 3233.0684361 | 4 |
| 3233.0691423 | 1 |
| 3233.0697725 | 3 |
| 3233.0713066 | 2 |
| 3233.0723444 | 1 |
| 3233.0726612 | 1 |
| 3233.0740152 | 3 |
| 3233.0751842 | 1 |
| 3233.0760061 | 2 |
| 3233.0772239 | 2 |
| 3233.0779989 | 6 |
| 3233.0788737 | 3 |
| 3233.0794227 | 2 |
| 3233.0804681 | 5 |
| 3233.0818387 | 1 |
| 3233.0826242 | 1 |
| 3233.0833616 | 1 |
| 3233.0841244 | 3 |
| 3233.0853929 | 0 |
| 3233.0862161 | 4 |
| 3233.0868313 | 0 |
| 3233.0882641 | 2 |
| 3233.0885412 | 2 |
| 3233.0896609 | 1 |
| 3233.0906817 | 4 |
| 3233.0917038 | 3 |
| 3233.0929836 | 3 |
| 3233.0930891 | 2 |
| 3233.0942837 | 1 |
| 3233.0954142 | 5 |
| 3233.0962346 | 3 |
| 3233.0968889 | 1 |
| 3233.0980055 | 0 |
| 3233.099405 | 1 |
| 3233.1006692 | 5 |
| 3233.1010296 | 0 |
| 3233.1019675 | 1 |
| 3233.1019793 | 3 |
| 3233.1038834 | 1 |
| 3233.1050029 | 5 |
| 3233.1057837 | 2 |
| 3233.106322 | 1 |
| 3233.1072439 | 1 |
| 3233.1084562 | 3 |
| 3233.1094315 | 4 |
| 3233.1098692 | 3 |
| 3233.1112676 | 1 |
| 3233.1124236 | 1 |
| 3233.1128447 | 0 |
| 3233.1135694 | 1 |
| 3233.1148692 | 1 |
| 3233.1159537 | 3 |
| 3233.1163078 | 2 |
| 3233.117702 | 5 |
| 3233.1188477 | 2 |
| 3233.1191968 | 1 |
| 3233.1208283 | 0 |
| 3233.1217701 | 2 |
| 3233.1222519 | 1 |
| 3233.1235629 | 2 |
| 3233.1243137 | 2 |
| 3233.1254978 | 4 |
| 3233.1256004 | 4 |
| 3233.1268412 | 1 |
| 3233.1276246 | 3 |
| 3233.1286926 | 1 |
| 3233.1294607 | 4 |
| 3233.1311572 | 2 |
| 3233.1316495 | 1 |
| 3233.1324735 | 4 |
| 3233.132989 | 3 |
| 3233.1344546 | 4 |
| 3233.1349681 | 6 |
| 3233.1362569 | 9 |
| 3233.1377442 | 5 |
| 3233.1379385 | 4 |
| 3233.1389549 | 1 |
| 3233.1398628 | 4 |
| 3233.1409601 | 3 |
| 3233.1422908 | 3 |
| 3233.1424368 | 1 |
| 3233.1437994 | 0 |
| 3233.1446536 | 3 |
| 3233.1453012 | 1 |
| 3233.1469072 | 0 |
| 3233.1474709 | 0 |
| 3233.1480961 | 3 |
| 3233.1498491 | 3 |
| 3233.1500871 | 4 |
| 3233.1513334 | 3 |
| 3233.1517262 | 1 |
| 3233.1530195 | 2 |
| 3233.1542322 | 2 |
| 3233.1549304 | 2 |
| 3233.1556495 | 5 |
| 3233.1563061 | 2 |
| 3233.1581147 | 1 |
| 3233.1585942 | 0 |
| 3233.1598494 | 1 |
| 3233.1606687 | 2 |
| 3233.1608915 | 3 |
| 3233.1622571 | 1 |
| 3233.1625363 | 3 |
| 3233.16401 | 2 |
| 3233.1655418 | 4 |
| 3233.1659729 | 2 |
| 3233.1668789 | 2 |
| 3233.1680299 | 2 |
| 3233.1686988 | 2 |
| 3233.169611 | 1 |
| 3233.1705692 | 0 |
| 3233.1720414 | 4 |
| 3233.1726526 | 1 |
| 3233.173364 | 2 |
| 3233.1751231 | 0 |
| 3233.1752722 | 1 |
| 3233.1762916 | 1 |
| 3233.1769333 | 1 |
| 3233.1776002 | 4 |
| 3233.1790884 | 0 |
| 3233.1795186 | 0 |
| 3233.1811897 | 0 |
| 3233.1814925 | 2 |
| 3233.1824877 | 0 |
| 3233.1836124 | 0 |
| 3233.1847595 | 3 |
| 3233.1853019 | 1 |
| 3233.1864106 | 2 |
| 3233.1873649 | 3 |
| 3233.1888578 | 4 |
| 3233.1890903 | 0 |
| 3233.1905961 | 4 |
| 3233.1909987 | 2 |
| 3233.1923595 | 2 |
| 3233.1930856 | 3 |
| 3233.1942962 | 1 |
| 3233.1945419 | 3 |
| 3233.1951857 | 4 |
| 3233.1968027 | 2 |
| 3233.198092 | 4 |
| 3233.1984908 | 1 |
| 3233.1996738 | 1 |
| 3233.2003061 | 0 |
| 3233.2012628 | 1 |
| 3233.2028202 | 1 |
| 3233.2031787 | 3 |
| 3233.2038655 | 1 |
| 3233.2053751 | 1 |
| 3233.2057627 | 3 |
| 3233.2071749 | 2 |
| 3233.2079535 | 0 |
| 3233.2089632 | 2 |
| 3233.2099154 | 3 |
| 3233.2107922 | 0 |
| 3233.2121923 | 0 |
| 3233.212691 | 2 |
| 3233.2134843 | 5 |
| 3233.2146106 | 1 |
| 3233.2152627 | 0 |
| 3233.2162372 | 4 |
| 3233.2169348 | 1 |
| 3233.2182202 | 4 |
| 3233.2194586 | 4 |
| 3233.2200068 | 1 |
| 3233.2213338 | 3 |
| 3233.2217827 | 5 |
| 3233.2231146 | 1 |
| 3233.2234434 | 1 |
| 3233.2244987 | 1 |
| 3233.2262031 | 2 |
| 3233.2266639 | 3 |
| 3233.2275428 | 3 |
| 3233.2284097 | 2 |
| 3233.2296351 | 0 |
| 3233.2306228 | 0 |
| 3233.2310764 | 0 |
| 3233.2323627 | 1 |
| 3233.2333527 | 1 |
| 3233.2340683 | 0 |
| 3233.2351458 | 2 |
| 3233.2359325 | 4 |
| 3233.2368999 | 2 |
| 3233.2381749 | 2 |
| 3233.2386354 | 2 |
| 3233.2398429 | 1 |
| 3233.2404284 | 3 |
| 3233.241191 | 1 |
| 3233.242746 | 3 |
| 3233.2434784 | 4 |
| 3233.2445932 | 2 |
| 3233.2448825 | 2 |
| 3233.2461886 | 1 |
| 3233.247565 | 1 |
| 3233.2479434 | 1 |
| 3233.2491488 | 1 |
| 3233.249889 | 2 |
| 3233.2506717 | 2 |
| 3233.251842 | 3 |
| 3233.252281 | 0 |
| 3233.2543758 | 1 |
| 3233.2544073 | 2 |
| 3233.2557681 | 0 |
| 3233.2567004 | 1 |
| 3233.2571585 | 4 |
| 3233.2585767 | 1 |
| 3233.2592458 | 0 |

| | |
|---|---|
| 3233.2603629 | 2 |
| 3233.2611647 | 1 |
| 3233.262752 | 4 |
| 3233.2633944 | 3 |
| 3233.2637118 | 1 |
| 3233.2652617 | 0 |
| 3233.2657122 | 4 |
| 3233.2662634 | 1 |
| 3233.2676437 | 0 |
| 3233.267894 | 3 |
| 3233.2691283 | 2 |
| 3233.270108 | 2 |
| 3233.2712573 | 3 |
| 3233.2720331 | 2 |
| 3233.2730621 | 2 |
| 3233.2745671 | 2 |
| 3233.2756618 | 3 |
| 3233.2760031 | 0 |
| 3233.2775417 | 0 |
| 3233.2779858 | 1 |
| 3233.2788453 | 2 |
| 3233.2802693 | 3 |
| 3233.280811 | 2 |
| 3233.2819274 | 0 |
| 3233.2828236 | 0 |
| 3233.2833872 | 1 |
| 3233.2846331 | 1 |
| 3233.2857966 | 2 |
| 3233.2859334 | 1 |
| 3233.2873571 | 1 |
| 3233.2884084 | 1 |
| 3233.2896469 | 2 |
| 3233.29007 | 0 |
| 3233.2911602 | 0 |
| 3233.2919596 | 1 |
| 3233.292833 | 1 |
| 3233.2940398 | 2 |
| 3233.2950805 | 1 |
| 3233.2954873 | 3 |
| 3233.2969122 | 2 |
| 3233.2973733 | 2 |
| 3233.2984075 | 1 |
| 3233.2997468 | 2 |
| 3233.3003411 | 2 |
| 3233.3014739 | 2 |
| 3233.3024824 | 0 |
| 3233.3031071 | 3 |
| 3233.3042458 | 3 |
| 3233.3044228 | 1 |
| 3233.3065924 | 5 |
| 3233.306787 | 2 |
| 3233.3081529 | 2 |
| 3233.30941 | 2 |
| 3233.3097413 | 1 |
| 3233.3107234 | 0 |
| 3233.3116477 | 2 |
| 3233.3130413 | 1 |
| 3233.3131168 | 2 |
| 3233.3147365 | 3 |
| 3233.3154361 | 0 |
| 3233.315938 | 2 |
| 3233.3169308 | 3 |
| 3233.3184058 | 2 |
| 3233.3185454 | 6 |
| 3233.3200685 | 1 |
| 3233.3207698 | 3 |
| 3233.3223525 | 3 |
| 3233.323443 | 0 |
| 3233.3240828 | 0 |
| 3233.3247766 | 1 |
| 3233.3252942 | 2 |
| 3233.3262586 | 3 |
| 3233.3279027 | 2 |
| 3233.3280732 | 2 |
| 3233.3299429 | 2 |
| 3233.3302943 | 2 |
| 3233.3312773 | 1 |
| 3233.3326914 | 2 |
| 3233.3330786 | 1 |
| 3233.3339463 | 5 |
| 3233.3350803 | 4 |
| 3233.3363377 | 1 |
| 3233.3368375 | 1 |
| 3233.3374733 | 2 |
| 3233.3389565 | 9 |
| 3233.3395083 | 4 |
| 3233.3409586 | 2 |
| 3233.3417982 | 3 |
| 3233.3425665 | 1 |
| 3233.3435516 | 2 |
| 3233.3447267 | 3 |
| 3233.3455584 | 1 |
| 3233.3468222 | 3 |
| 3233.3472923 | 3 |
| 3233.3486951 | 4 |
| 3233.3490014 | 0 |
| 3233.3499701 | 2 |
| 3233.3510412 | 2 |
| 3233.3515091 | 2 |
| 3233.3527226 | 3 |
| 3233.3537486 | 3 |
| 3233.3545011 | 3 |
| 3233.3558094 | 2 |
| 3233.3560841 | 4 |
| 3233.3575926 | 1 |
| 3233.357971 | 0 |
| 3233.3597753 | 3 |
| 3233.3605766 | 1 |
| 3233.3614327 | 2 |
| 3233.36217 | 6 |
| 3233.362689 | 2 |
| 3233.3635715 | 2 |
| 3233.3648064 | 3 |
| 3233.3656702 | 2 |
| 3233.3671174 | 2 |
| 3233.3678214 | 1 |
| 3233.3691794 | 2 |
| 3233.3696862 | 1 |
| 3233.3710896 | 1 |
| 3233.372048 | 0 |
| 3233.3725838 | 3 |
| 3233.373248 | 0 |
| 3233.3741524 | 1 |
| 3233.3745611 | 3 |
| 3233.3762881 | 4 |
| 3233.3776132 | 1 |
| 3233.3784283 | 3 |
| 3233.379477 | 2 |
| 3233.3796207 | 0 |
| 3233.381182 | 1 |
| 3233.381482 | 3 |
| 3233.3828623 | 1 |
| 3233.384474 | 3 |
| 3233.3847403 | 0 |
| 3233.3859462 | 1 |
| 3233.3862824 | 0 |
| 3233.3877705 | 0 |
| 3233.3885413 | 1 |
| 3233.3893933 | 3 |
| 3233.3906332 | 3 |
| 3233.3912576 | 1 |
| 3233.3926355 | 4 |
| 3233.3927035 | 1 |
| 3233.3937505 | 4 |
| 3233.3950698 | 3 |
| 3233.3959442 | 0 |
| 3233.3971981 | 3 |
| 3233.3980507 | 3 |
| 3233.3988472 | 7 |
| 3233.3996021 | 2 |
| 3233.4000681 | 2 |
| 3233.4012762 | 1 |
| 3233.4026374 | 1 |
| 3233.4036761 | 5 |
| 3233.4047759 | 1 |
| 3233.4051449 | 1 |
| 3233.4064236 | 2 |
| 3233.4072587 | 7 |
| 3233.4076659 | 4 |
| 3233.409079 | 5 |
| 3233.4096988 | 2 |
| 3233.4111847 | 2 |
| 3233.411461 | 1 |
| 3233.4127926 | 4 |
| 3233.4138592 | 0 |
| 3233.4145223 | 1 |
| 3233.4158643 | 3 |
| 3233.4166801 | 1 |
| 3233.4174858 | 1 |
| 3233.4187752 | 1 |
| 3233.419193 | 2 |
| 3233.4206919 | 1 |
| 3233.4209056 | 1 |
| 3233.421898 | 1 |
| 3233.4232071 | 4 |
| 3233.4239309 | 3 |
| 3233.4247146 | 3 |
| 3233.4265719 | 1 |
| 3233.42663 | 6 |
| 3233.4283505 | 6 |
| 3233.4283593 | 2 |
| 3233.4299186 | 4 |
| 3233.430602 | 5 |
| 3233.4313543 | 2 |
| 3233.4326427 | 3 |
| 3233.433451 | 3 |
| 3233.4342285 | 1 |
| 3233.435587 | 1 |
| 3233.4365002 | 1 |
| 3233.4376337 | 0 |
| 3233.4380623 | 3 |
| 3233.4388038 | 0 |
| 3233.4405831 | 2 |
| 3233.4408727 | 0 |
| 3233.4420646 | 3 |
| 3233.4429619 | 6 |
| 3233.443614 | 1 |
| 3233.4448041 | 2 |
| 3233.445875 | 2 |
| 3233.4471995 | 2 |
| 3233.4473175 | 1 |
| 3233.4487877 | 2 |
| 3233.4495584 | 3 |
| 3233.4497417 | 1 |
| 3233.4509635 | 2 |
| 3233.4516107 | 2 |
| 3233.4531555 | 9 |
| 3233.4543896 | 0 |
| 3233.4555994 | 2 |
| 3233.4562381 | 5 |
| 3233.4567937 | 2 |
| 3233.4580275 | 3 |
| 3233.4584292 | 2 |
| 3233.4593235 | 1 |
| 3233.4606861 | 1 |
| 3233.46193 | 2 |
| 3233.4622014 | 3 |
| 3233.4631763 | 1 |
| 3233.464211 | 2 |
| 3233.4654146 | 4 |
| 3233.4660059 | 1 |
| 3233.4670104 | 1 |
| 3233.4682795 | 0 |
| 3233.4692557 | 1 |
| 3233.4704526 | 2 |
| 3233.4707926 | 0 |
| 3233.472115 | 3 |
| 3233.4733116 | 2 |
| 3233.4734047 | 3 |
| 3233.474904 | 2 |
| 3233.4758927 | 2 |
| 3233.4767969 | 1 |
| 3233.4776696 | 4 |
| 3233.4780568 | 2 |
| 3233.4793514 | 2 |
| 3233.4803854 | 4 |
| 3233.4813037 | 3 |
| 3233.4817094 | 3 |
| 3233.482863 | 1 |
| 3233.4843267 | 3 |
| 3233.4845979 | 3 |
| 3233.4858928 | 0 |
| 3233.4869557 | 1 |
| 3233.488056 | 5 |
| 3233.4889391 | 4 |
| 3233.4894737 | 4 |
| 3233.4907262 | 4 |
| 3233.491656 | 2 |
| 3233.4923738 | 2 |
| 3233.493889 | 4 |
| 3233.4940247 | 2 |
| 3233.4952575 | 1 |
| 3233.4963729 | 3 |
| 3233.496685 | 1 |
| 3233.4979317 | 1 |
| 3233.4988206 | 1 |
| 3233.5001302 | 3 |
| 3233.5005791 | 2 |
| 3233.5015713 | 4 |
| 3233.5025906 | 2 |
| 3233.5032194 | 2 |
| 3233.5042992 | 3 |
| 3233.5048757 | 0 |
| 3233.5067341 | 4 |
| 3233.5072885 | 1 |
| 3233.5080271 | 1 |
| 3233.5091827 | 0 |
| 3233.5096771 | 2 |
| 3233.5106686 | 2 |
| 3233.5126718 | 1 |
| 3233.5129753 | 3 |
| 3233.5138813 | 0 |
| 3233.514638 | 0 |
| 3233.5155733 | 2 |
| 3233.5165246 | 1 |
| 3233.5175865 | 4 |
| 3233.5184825 | 2 |
| 3233.519112 | 2 |
| 3233.5205039 | 0 |
| 3233.5220198 | 0 |
| 3233.5220214 | 1 |
| 3233.523631 | 2 |
| 3233.524158 | 5 |
| 3233.5252034 | 0 |
| 3233.5261026 | 1 |
| 3233.5266533 | 3 |
| 3233.5277218 | 0 |
| 3233.5292913 | 3 |
| 3233.5294 | 2 |
| 3233.5306459 | 3 |
| 3233.5318558 | 0 |
| 3233.5324711 | 4 |
| 3233.5335657 | 3 |
| 3233.5345561 | 3 |
| 3233.5360228 | 6 |
| 3233.5360654 | 1 |
| 3233.5373261 | 2 |
| 3233.5378547 | 2 |
| 3233.5390462 | 0 |
| 3233.5402263 | 2 |
| 3233.5407618 | 1 |
| 3233.5421026 | 1 |
| 3233.5425056 | 3 |
| 3233.5436128 | 3 |
| 3233.5451157 | 0 |
| 3233.5452207 | 0 |
| 3233.5465104 | 2 |
| 3233.5473729 | 1 |
| 3233.5485456 | 4 |
| 3233.5494352 | 3 |
| 3233.5501495 | 5 |
| 3233.5514302 | 2 |
| 3233.5522585 | 1 |
| 3233.5526144 | 2 |
| 3233.5539427 | 3 |
| 3233.5545584 | 3 |
| 3233.5562052 | 0 |
| 3233.557492 | 2 |
| 3233.5581116 | 0 |
| 3233.558816 | 2 |
| 3233.5592642 | 2 |
| 3233.5609089 | 2 |
| 3233.5611353 | 2 |
| 3233.5627678 | 4 |
| 3233.5636939 | 3 |
| 3233.5642698 | 1 |
| 3233.5652586 | 3 |
| 3233.5660059 | 2 |
| 3233.5672181 | 1 |
| 3233.5681941 | 1 |
| 3233.5690375 | 2 |
| 3233.5699386 | 2 |
| 3233.5704376 | 1 |
| 3233.5720133 | 3 |
| 3233.5732325 | 6 |
| 3233.5736875 | 1 |
| 3233.5747839 | 1 |
| 3233.575307 | 3 |
| 3233.5761142 | 2 |
| 3233.5775047 | 2 |
| 3233.5779056 | 3 |
| 3233.5790212 | 1 |
| 3233.5797429 | 2 |
| 3233.5805103 | 0 |
| 3233.5821635 | 4 |
| 3233.582973 | 2 |
| 3233.5845832 | 5 |
| 3233.5847751 | 0 |
| 3233.5859983 | 0 |
| 3233.586346 | 3 |
| 3233.5874976 | 1 |
| 3233.5885047 | 1 |
| 3233.5890668 | 1 |
| 3233.5901596 | 2 |
| 3233.5912941 | 1 |
| 3233.5922726 | 5 |
| 3233.5929978 | 2 |
| 3233.5941634 | 1 |
| 3233.5952392 | 1 |
| 3233.5952619 | 2 |
| 3233.596681 | 3 |
| 3233.5983107 | 2 |
| 3233.5987959 | 1 |

| | |
|---|---|
| 3233.6000342 | 2 |
| 3233.6000434 | 0 |
| 3233.6017416 | 4 |
| 3233.6027973 | 1 |
| 3233.6031079 | 2 |
| 3233.6044545 | 2 |
| 3233.6052001 | 1 |
| 3233.6062636 | 2 |
| 3233.6072561 | 1 |
| 3233.6077976 | 0 |
| 3233.6096611 | 2 |
| 3233.6096906 | 1 |
| 3233.6110768 | 4 |
| 3233.6122625 | 2 |
| 3233.6123382 | 4 |
| 3233.6136549 | 3 |
| 3233.6140778 | 2 |
| 3233.6154327 | 3 |
| 3233.6172588 | 1 |
| 3233.6176128 | 1 |
| 3233.6186696 | 1 |
| 3233.6189341 | 4 |
| 3233.6201042 | 2 |
| 3233.6214511 | 1 |
| 3233.6221087 | 2 |
| 3233.6235401 | 0 |
| 3233.6236509 | 2 |
| 3233.6248759 | 1 |
| 3233.6258576 | 5 |
| 3233.6265002 | 0 |
| 3233.6275318 | 0 |
| 3233.6284517 | 1 |
| 3233.6295974 | 1 |
| 3233.6299155 | 5 |
| 3233.6309648 | 3 |
| 3233.6324808 | 1 |
| 3233.6330592 | 1 |
| 3233.6344169 | 2 |
| 3233.6346929 | 1 |
| 3233.6361027 | 6 |
| 3233.6374616 | 4 |
| 3233.6379353 | 2 |
| 3233.6388718 | 2 |
| 3233.6397419 | 3 |
| 3233.6405368 | 1 |
| 3233.6417446 | 3 |
| 3233.6418373 | 0 |
| 3233.6427748 | 2 |
| 3233.644574 | 2 |
| 3233.6450742 | 1 |
| 3233.6459898 | 1 |
| 3233.6474942 | 2 |
| 3233.6479342 | 1 |
| 3233.6490205 | 1 |
| 3233.6496604 | 2 |
| 3233.6505068 | 0 |
| 3233.6521626 | 1 |
| 3233.652632 | 4 |
| 3233.6533143 | 1 |
| 3233.6542802 | 5 |
| 3233.6562304 | 1 |
| 3233.6564317 | 0 |
| 3233.6576752 | 4 |
| 3233.6580201 | 1 |
| 3233.6596265 | 1 |
| 3233.6603624 | 4 |
| 3233.6611 | 3 |
| 3233.6619938 | 4 |
| 3233.6630491 | 1 |
| 3233.6633374 | 1 |
| 3233.6647682 | 3 |
| 3233.6653854 | 1 |
| 3233.6666823 | 1 |
| 3233.6671902 | 2 |
| 3233.6680754 | 3 |
| 3233.6695428 | 2 |
| 3233.6704269 | 4 |
| 3233.6713669 | 4 |
| 3233.6721835 | 1 |
| 3233.6735122 | 0 |
| 3233.6744802 | 1 |
| 3233.6747684 | 3 |
| 3233.6755919 | 3 |
| 3233.676458 | 1 |
| 3233.678095 | 1 |
| 3233.678424 | 3 |
| 3233.6794466 | 0 |
| 3233.6807543 | 4 |
| 3233.6809459 | 1 |
| 3233.6823887 | 2 |
| 3233.6837895 | 0 |
| 3233.6838636 | 1 |
| 3233.6853155 | 2 |
| 3233.6858624 | 1 |
| 3233.6871043 | 3 |
| 3233.6877594 | 4 |
| 3233.6884317 | 3 |
| 3233.6899907 | 2 |
| 3233.6902151 | 2 |
| 3233.6911798 | 1 |
| 3233.6929268 | 3 |
| 3233.6933187 | 2 |
| 3233.6943671 | 1 |
| 3233.6954507 | 3 |
| 3233.6969632 | 2 |
| 3233.6974366 | 3 |
| 3233.6980316 | 2 |
| 3233.699307 | 3 |
| 3233.6999638 | 1 |
| 3233.7006955 | 0 |
| 3233.7021188 | 5 |
| 3233.7025033 | 4 |
| 3233.7039796 | 1 |
| 3233.7046174 | 3 |
| 3233.7053406 | 4 |
| 3233.7063479 | 3 |
| 3233.7071821 | 2 |
| 3233.708927 | 3 |
| 3233.7090787 | 1 |
| 3233.7102232 | 1 |
| 3233.7114318 | 2 |
| 3233.7124271 | 2 |
| 3233.7129048 | 1 |
| 3233.713649 | 2 |
| 3233.7150842 | 1 |
| 3233.7161937 | 0 |
| 3233.7163066 | 0 |
| 3233.7176865 | 6 |
| 3233.718036 | 2 |
| 3233.7191748 | 3 |
| 3233.7207523 | 2 |
| 3233.7209057 | 1 |
| 3233.7223978 | 1 |
| 3233.7227281 | 2 |
| 3233.7237123 | 1 |
| 3233.7253944 | 6 |
| 3233.7255134 | 1 |
| 3233.7266032 | 2 |
| 3233.728163 | 1 |
| 3233.7284621 | 4 |
| 3233.7294547 | 7 |
| 3233.7303594 | 0 |
| 3233.731692 | 4 |
| 3233.732388 | 2 |
| 3233.733253 | 2 |
| 3233.7346408 | 0 |
| 3233.7346647 | 1 |
| 3233.7359983 | 3 |
| 3233.7373949 | 4 |
| 3233.7374947 | 3 |
| 3233.7389072 | 1 |
| 3233.7394967 | 0 |
| 3233.7406274 | 1 |
| 3233.7412881 | 3 |
| 3233.7427401 | 0 |
| 3233.7440611 | 0 |
| 3233.7441974 | 2 |
| 3233.7454798 | 1 |
| 3233.7461254 | 2 |
| 3233.7474562 | 3 |
| 3233.7483936 | 3 |
| 3233.7489984 | 1 |
| 3233.7502489 | 2 |
| 3233.7506068 | 4 |
| 3233.7520741 | 3 |
| 3233.7531237 | 1 |
| 3233.7535603 | 1 |
| 3233.7550609 | 4 |
| 3233.7560884 | 3 |
| 3233.7565949 | 2 |
| 3233.7577931 | 2 |
| 3233.7581775 | 2 |
| 3233.7594105 | 4 |
| 3233.7603392 | 2 |
| 3233.7610088 | 2 |
| 3233.7618345 | 1 |
| 3233.7632292 | 1 |
| 3233.7644084 | 3 |
| 3233.7654544 | 2 |
| 3233.7657561 | 2 |
| 3233.7669907 | 3 |
| 3233.767199 | 0 |
| 3233.7689176 | 3 |
| 3233.7694897 | 1 |
| 3233.7703064 | 0 |
| 3233.7715652 | 0 |
| 3233.7727133 | 3 |
| 3233.7734603 | 2 |
| 3233.7743725 | 2 |
| 3233.7748933 | 2 |
| 3233.7765983 | 1 |
| 3233.7776038 | 2 |
| 3233.7779063 | 2 |
| 3233.7792766 | 0 |
| 3233.7797789 | 3 |
| 3233.7807464 | 1 |
| 3233.7817641 | 1 |
| 3233.7823793 | 3 |
| 3233.7831971 | 2 |
| 3233.7848537 | 0 |
| 3233.7855148 | 0 |
| 3233.7864402 | 2 |
| 3233.7869887 | 3 |
| 3233.7885616 | 3 |
| 3233.7891537 | 2 |
| 3233.7896782 | 2 |
| 3233.7915486 | 3 |
| 3233.7916348 | 1 |
| 3233.7935307 | 3 |
| 3233.7941623 | 1 |
| 3233.7949287 | 2 |
| 3233.7955989 | 3 |
| 3233.7965194 | 2 |
| 3233.7978539 | 3 |
| 3233.7983688 | 0 |
| 3233.8001327 | 1 |
| 3233.80044 | 2 |
| 3233.8018732 | 1 |
| 3233.8026161 | 3 |
| 3233.8028916 | 1 |
| 3233.8043719 | 2 |
| 3233.8049163 | 3 |
| 3233.8063481 | 0 |
| 3233.8073282 | 0 |
| 3233.8078437 | 1 |
| 3233.8090555 | 3 |
| 3233.8094224 | 0 |
| 3233.8107516 | 3 |
| 3233.8119004 | 3 |
| 3233.8125889 | 5 |
| 3233.8137484 | 5 |
| 3233.8144136 | 1 |
| 3233.815303 | 1 |
| 3233.8165863 | 1 |
| 3233.8168697 | 1 |
| 3233.8180239 | 2 |
| 3233.8186532 | 5 |
| 3233.8195818 | 2 |
| 3233.8207721 | 3 |
| 3233.8221215 | 1 |
| 3233.8228102 | 3 |
| 3233.8230625 | 2 |
| 3233.824408 | 3 |
| 3233.825672 | 1 |
| 3233.8259448 | 2 |
| 3233.827889 | 2 |
| 3233.8279984 | 1 |
| 3233.8296655 | 0 |
| 3233.8297309 | 2 |
| 3233.831285 | 1 |
| 3233.8323604 | 0 |
| 3233.8325233 | 2 |
| 3233.8337295 | 1 |
| 3233.8353639 | 6 |
| 3233.8355271 | 0 |
| 3233.8366898 | 0 |
| 3233.8370667 | 2 |
| 3233.8388248 | 2 |
| 3233.8393436 | 2 |
| 3233.840652 | 1 |
| 3233.8420402 | 4 |
| 3233.8422982 | 0 |
| 3233.8433849 | 4 |
| 3233.8436974 | 0 |
| 3233.8453682 | 1 |
| 3233.8458963 | 2 |
| 3233.8467575 | 1 |
| 3233.8484899 | 5 |
| 3233.8490345 | 5 |
| 3233.8501011 | 1 |
| 3233.8509146 | 3 |
| 3233.8515151 | 1 |
| 3233.8526617 | 1 |
| 3233.8533433 | 1 |
| 3233.8547849 | 1 |
| 3233.8555076 | 5 |
| 3233.8559817 | 3 |
| 3233.8575589 | 2 |
| 3233.8578152 | 3 |
| 3233.8588291 | 1 |
| 3233.8602696 | 2 |
| 3233.8610757 | 3 |
| 3233.8623813 | 2 |
| 3233.8624266 | 2 |
| 3233.8638714 | 3 |
| 3233.8650436 | 1 |
| 3233.8653273 | 0 |
| 3233.866547 | 0 |
| 3233.8665646 | 0 |
| 3233.8686758 | 2 |
| 3233.8692581 | 2 |
| 3233.8702687 | 0 |
| 3233.8709718 | 1 |
| 3233.872393 | 0 |
| 3233.8728982 | 1 |
| 3233.8738553 | 3 |
| 3233.8747998 | 1 |
| 3233.876122 | 0 |
| 3233.876774 | 2 |
| 3233.8777697 | 2 |
| 3233.8790585 | 1 |
| 3233.8799237 | 5 |
| 3233.8803636 | 0 |
| 3233.8813515 | 2 |
| 3233.8821434 | 3 |
| 3233.8836513 | 3 |
| 3233.8841101 | 2 |
| 3233.8853421 | 2 |
| 3233.8861848 | 3 |
| 3233.8872822 | 5 |
| 3233.8885368 | 2 |
| 3233.889166 | 1 |
| 3233.8901666 | 3 |
| 3233.8903915 | 3 |
| 3233.892267 | 4 |
| 3233.8932968 | 1 |
| 3233.8937214 | 4 |
| 3233.89467 | 4 |
| 3233.8954912 | 0 |
| 3233.8961353 | 1 |
| 3233.8979511 | 0 |
| 3233.8981758 | 3 |
| 3233.8995779 | 2 |
| 3233.9001402 | 2 |
| 3233.9007696 | 1 |
| 3233.9018823 | 1 |
| 3233.9031384 | 2 |
| 3233.9041694 | 1 |
| 3233.9046756 | 2 |
| 3233.905976 | 1 |
| 3233.9068902 | 1 |
| 3233.9074778 | 1 |
| 3233.9089173 | 1 |
| 3233.9100282 | 2 |
| 3233.9102716 | 1 |
| 3233.911655 | 0 |
| 3233.9127757 | 2 |
| 3233.9135627 | 1 |
| 3233.9139665 | 1 |
| 3233.9157016 | 1 |
| 3233.916475 | 1 |
| 3233.9171987 | 3 |
| 3233.9184792 | 2 |
| 3233.9192922 | 2 |
| 3233.9198333 | 1 |
| 3233.9212952 | 1 |
| 3233.9216677 | 2 |
| 3233.923466 | 1 |
| 3233.9235633 | 4 |
| 3233.9246629 | 4 |
| 3233.9258539 | 2 |
| 3233.9263392 | 3 |
| 3233.9274983 | 1 |
| 3233.9286473 | 2 |
| 3233.929878 | 2 |
| 3233.9300822 | 1 |
| 3233.9309927 | 2 |
| 3233.9319849 | 3 |
| 3233.9336364 | 4 |
| 3233.9342926 | 4 |
| 3233.9354681 | 1 |
| 3233.9355635 | 1 |
| 3233.9367592 | 2 |

| | |
|---|---|
| 3233.9384301 | 1 |
| 3233.9392991 | 2 |
| 3233.9395729 | 0 |
| 3233.940404 | 3 |
| 3233.9422958 | 2 |
| 3233.9426732 | 3 |
| 3233.9436687 | 1 |
| 3233.9448617 | 3 |
| 3233.9451762 | 4 |
| 3233.9462386 | 2 |
| 3233.9467604 | 2 |
| 3233.9483717 | 6 |
| 3233.9496952 | 3 |
| 3233.9497393 | 2 |
| 3233.951097 | 3 |
| 3233.9515465 | 2 |
| 3233.9531935 | 1 |
| 3233.9534205 | 3 |
| 3233.9547082 | 1 |
| 3233.9558431 | 2 |
| 3233.9570139 | 1 |
| 3233.9578905 | 1 |
| 3233.9589477 | 2 |
| 3233.9594372 | 1 |
| 3233.9605622 | 3 |
| 3233.9607847 | 2 |
| 3233.962022 | 2 |
| 3233.9628486 | 3 |
| 3233.9640579 | 3 |
| 3233.9653615 | 3 |
| 3233.9654864 | 1 |
| 3233.9667583 | 1 |
| 3233.9680849 | 3 |
| 3233.968102 | 1 |
| 3233.9694788 | 1 |
| 3233.9708181 | 3 |
| 3233.971646 | 3 |
| 3233.9727341 | 2 |
| 3233.9739964 | 1 |
| 3233.9749209 | 6 |
| 3233.9749855 | 1 |
| 3233.9764497 | 4 |
| 3233.9774684 | 3 |
| 3233.9778514 | 3 |
| 3233.9778775 | 1 |
| 3233.9791878 | 2 |
| 3233.9796511 | 3 |
| 3233.9798564 | 5 |
| 3233.9806435 | 6 |
| 3233.981755 | 5 |
| 3233.9820927 | 4 |
| 3233.9824214 | 4 |
| 3233.9830494 | 0 |
| 3233.9841454 | 1 |
| 3233.9843531 | 3 |
| 3233.9847961 | 2 |
| 3233.9850986 | 1 |
| 3233.9856096 | 1 |
| 3233.9872496 | 2 |
| 3233.9874813 | 1 |
| 3233.9875717 | 4 |
| 3233.9891235 | 1 |
| 3233.9893772 | 4 |
| 3233.9894149 | 0 |
| 3233.9901889 | 1 |
| 3233.9906528 | 4 |
| 3233.9913331 | 1 |
| 3233.9922739 | 1 |
| 3233.9925592 | 2 |
| 3233.9935553 | 0 |
| 3233.9937319 | 2 |
| 3233.9947404 | 4 |
| 3233.9950296 | 2 |
| 3233.9954392 | 0 |
| 3233.9960593 | 4 |
| 3233.9966961 | 1 |
| 3233.9967071 | 1 |
| 3233.9978295 | 3 |
| 3233.9981935 | 1 |
| 3233.999226 | 1 |
| 3233.9994956 | 0 |
| 3234.0000237 | 4 |
| 3234.0009373 | 1 |
| 3234.001465 | 5 |
| 3234.002276 | 2 |
| 3234.0023996 | 0 |
| 3234.0027645 | 4 |
| 3234.0034274 | 1 |
| 3234.0036819 | 1 |
| 3234.0044721 | 2 |
| 3234.0053021 | 3 |
| 3234.0056115 | 3 |
| 3234.0060359 | 3 |
| 3234.0073194 | 1 |
| 3234.0078576 | 3 |
| 3234.0080945 | 1 |
| 3234.008579 | 3 |
| 3234.0091759 | 0 |
| 3234.0103633 | 3 |
| 3234.0107086 | 3 |
| 3234.0111685 | 5 |
| 3234.012074 | 1 |
| 3234.0123272 | 2 |
| 3234.0125983 | 3 |
| 3234.0132854 | 2 |
| 3234.0140245 | 0 |
| 3234.0154251 | 4 |
| 3234.0156584 | 4 |
| 3234.0156756 | 1 |
| 3234.0158402 | 7 |
| 3234.0166154 | 2 |
| 3234.0184173 | 3 |
| 3234.0186279 | 2 |
| 3234.0187828 | 2 |
| 3234.0196165 | 3 |
| 3234.0203192 | 0 |
| 3234.0209395 | 4 |
| 3234.0212304 | 3 |
| 3234.0221742 | 2 |
| 3234.0226059 | 3 |
| 3234.0232457 | 1 |
| 3234.0235233 | 3 |
| 3234.024163 | 4 |
| 3234.024987 | 1 |
| 3234.025003 | 1 |
| 3234.0257022 | 3 |
| 3234.0263006 | 1 |
| 3234.0271214 | 0 |
| 3234.0278024 | 2 |
| 3234.027921 | 3 |
| 3234.0291862 | 7 |
| 3234.0296373 | 0 |
| 3234.0298354 | 1 |
| 3234.0310174 | 2 |
| 3234.0313554 | 3 |
| 3234.0313776 | 0 |
| 3234.0319033 | 3 |
| 3234.0329208 | 0 |
| 3234.0338475 | 4 |
| 3234.0341784 | 2 |
| 3234.0344552 | 0 |
| 3234.035043 | 2 |
| 3234.0360055 | 2 |
| 3234.0363859 | 1 |
| 3234.037311 | 2 |
| 3234.0374653 | 4 |
| 3234.038623 | 3 |
| 3234.0388581 | 4 |
| 3234.039679 | 0 |
| 3234.0400748 | 2 |
| 3234.0400796 | 3 |
| 3234.0409301 | 4 |
| 3234.041746 | 1 |
| 3234.042255 | 4 |
| 3234.0423934 | 2 |
| 3234.0434571 | 6 |
| 3234.044075 | 4 |
| 3234.0452708 | 2 |
| 3234.045689 | 1 |
| 3234.0458654 | 2 |
| 3234.0460083 | 1 |
| 3234.0468033 | 3 |
| 3234.0473608 | 1 |
| 3234.0475571 | 0 |
| 3234.0484225 | 5 |
| 3234.0499745 | 2 |
| 3234.050083 | 2 |
| 3234.0504118 | 2 |
| 3234.0504448 | 3 |
| 3234.0519659 | 3 |
| 3234.0523299 | 2 |
| 3234.0526069 | 2 |
| 3234.0527893 | 3 |
| 3234.0542046 | 3 |
| 3234.054701 | 3 |
| 3234.0554206 | 6 |
| 3234.055712 | 1 |
| 3234.0560656 | 2 |
| 3234.0568673 | 1 |
| 3234.0574272 | 2 |
| 3234.0580135 | 2 |
| 3234.0586207 | 2 |
| 3234.0589093 | 2 |
| 3234.0595646 | 2 |
| 3234.0605956 | 3 |
| 3234.0609533 | 2 |
| 3234.0619788 | 2 |
| 3234.0623304 | 3 |
| 3234.0628772 | 6 |
| 3234.0630671 | 3 |
| 3234.0640696 | 2 |
| 3234.0651047 | 2 |
| 3234.0653111 | 5 |
| 3234.0655433 | 4 |
| 3234.0657796 | 1 |
| 3234.0663899 | 2 |
| 3234.0675901 | 4 |
| 3234.0679488 | 1 |
| 3234.0689058 | 2 |
| 3234.069711 | 1 |
| 3234.0698892 | 2 |
| 3234.0706669 | 5 |
| 3234.0707482 | 2 |
| 3234.071506 | 2 |
| 3234.0725645 | 1 |
| 3234.0728143 | 0 |
| 3234.0733189 | 1 |
| 3234.0735515 | 5 |
| 3234.0740082 | 1 |
| 3234.0747324 | 2 |
| 3234.075639 | 1 |
| 3234.0763882 | 4 |
| 3234.0765958 | 4 |
| 3234.0776876 | 1 |
| 3234.0778009 | 4 |
| 3234.0785705 | 2 |
| 3234.0790584 | 0 |
| 3234.0800133 | 0 |
| 3234.0806108 | 1 |
| 3234.0808046 | 2 |
| 3234.0813296 | 3 |
| 3234.0814629 | 2 |
| 3234.0823666 | 2 |
| 3234.0831777 | 4 |
| 3234.083753 | 3 |
| 3234.0844274 | 3 |
| 3234.0854052 | 3 |
| 3234.0856258 | 3 |
| 3234.0857964 | 2 |
| 3234.0862829 | 1 |
| 3234.0876013 | 2 |
| 3234.0880705 | 1 |
| 3234.088498 | 1 |
| 3234.0889801 | 1 |
| 3234.0897233 | 0 |
| 3234.0898753 | 4 |
| 3234.090509 | 3 |
| 3234.0911571 | 0 |
| 3234.0916414 | 4 |
| 3234.0920864 | 1 |
| 3234.0930683 | 2 |
| 3234.0935215 | 7 |
| 3234.0946431 | 4 |
| 3234.0950506 | 1 |
| 3234.0955237 | 1 |
| 3234.0962213 | 1 |
| 3234.0967212 | 1 |
| 3234.0967445 | 0 |
| 3234.0969309 | 2 |
| 3234.0983703 | 3 |
| 3234.0984344 | 1 |
| 3234.1001053 | 5 |
| 3234.1003061 | 2 |
| 3234.1003379 | 2 |
| 3234.101404 | 1 |
| 3234.1016182 | 2 |
| 3234.1025723 | 2 |
| 3234.10275 | 2 |
| 3234.1035713 | 3 |
| 3234.1043206 | 3 |
| 3234.1043877 | 2 |
| 3234.1048218 | 2 |
| 3234.1054559 | 3 |
| 3234.1069547 | 1 |
| 3234.1070557 | 3 |
| 3234.10748 | 1 |
| 3234.1080376 | 3 |
| 3234.108962 | 4 |
| 3234.109576 | 1 |
| 3234.1097802 | 0 |
| 3234.1107319 | 2 |
| 3234.111595 | 1 |
| 3234.112398 | 3 |
| 3234.1126087 | 1 |
| 3234.112635 | 1 |
| 3234.1140788 | 1 |
| 3234.1141952 | 2 |
| 3234.1142745 | 0 |
| 3234.1156397 | 2 |
| 3234.116259 | 2 |
| 3234.116289 | 3 |
| 3234.1170106 | 2 |
| 3234.1172485 | 2 |
| 3234.1183814 | 2 |
| 3234.1189103 | 3 |
| 3234.1191726 | 2 |
| 3234.1199802 | 0 |
| 3234.1203424 | 0 |
| 3234.1212009 | 2 |
| 3234.1220515 | 1 |
| 3234.1222445 | 3 |
| 3234.1230362 | 0 |
| 3234.1233921 | 4 |
| 3234.1243555 | 1 |
| 3234.1248769 | 0 |
| 3234.1250081 | 1 |
| 3234.1256339 | 1 |
| 3234.1268021 | 3 |
| 3234.1271459 | 0 |
| 3234.1273218 | 3 |
| 3234.1281699 | 1 |
| 3234.1283822 | 1 |
| 3234.1295891 | 3 |
| 3234.129698 | 0 |
| 3234.1299551 | 0 |
| 3234.1308456 | 0 |
| 3234.1320431 | 0 |
| 3234.1328937 | 2 |
| 3234.1329231 | 1 |
| 3234.1329423 | 1 |
| 3234.1344007 | 0 |
| 3234.1345783 | 3 |
| 3234.1348235 | 1 |
| 3234.1355079 | 0 |
| 3234.1359965 | 1 |
| 3234.1373641 | 0 |
| 3234.1377253 | 2 |
| 3234.1379732 | 1 |
| 3234.1386516 | 4 |
| 3234.1390914 | 1 |
| 3234.13933 | 1 |
| 3234.1404051 | 0 |
| 3234.140531 | 0 |
| 3234.1411492 | 1 |
| 3234.1419943 | 3 |
| 3234.1420142 | 3 |
| 3234.1436134 | 3 |
| 3234.1439162 | 0 |
| 3234.1439475 | 0 |
| 3234.1451617 | 0 |
| 3234.1452454 | 3 |
| 3234.1462042 | 2 |
| 3234.146425 | 0 |
| 3234.1469715 | 3 |
| 3234.1479484 | 1 |
| 3234.1481825 | 6 |
| 3234.1488064 | 2 |
| 3234.1492041 | 1 |
| 3234.1497768 | 1 |
| 3234.1506775 | 0 |
| 3234.1510009 | 6 |
| 3234.15133 | 3 |
| 3234.1526928 | 1 |
| 3234.1531944 | 0 |
| 3234.1535344 | 2 |
| 3234.1535618 | 0 |
| 3234.1541759 | 1 |
| 3234.1554846 | 4 |
| 3234.1560045 | 3 |
| 3234.1565959 | 2 |
| 3234.1579192 | 6 |
| 3234.1581593 | 3 |
| 3234.1583724 | 4 |
| 3234.1584826 | 1 |
| 3234.1595378 | 1 |
| 3234.1601606 | 1 |
| 3234.1606403 | 2 |
| 3234.1609892 | 0 |
| 3234.1615902 | 0 |
| 3234.1622143 | 1 |
| 3234.163053 | 0 |
| 3234.163484 | 1 |
| 3234.1642197 | 1 |

| | |
|---|---|
| 3234.1646568 | 2 |
| 3234.1648479 | 0 |
| 3234.1655284 | 2 |
| 3234.1668901 | 3 |
| 3234.1672874 | 4 |
| 3234.1675268 | 4 |
| 3234.1685369 | 5 |
| 3234.1686635 | 0 |
| 3234.1693263 | 1 |
| 3234.1699789 | 3 |
| 3234.1707553 | 3 |
| 3234.1714836 | 5 |
| 3234.1714927 | 3 |
| 3234.1720916 | 2 |
| 3234.1727944 | 4 |
| 3234.1734043 | 7 |
| 3234.174095 | 5 |
| 3234.1746414 | 3 |
| 3234.1749529 | 4 |
| 3234.17597 | 3 |
| 3234.1761637 | 2 |
| 3234.1764531 | 2 |
| 3234.1770816 | 1 |
| 3234.1781467 | 1 |
| 3234.1790355 | 0 |
| 3234.1794423 | 0 |
| 3234.1796733 | 2 |
| 3234.1801693 | 4 |
| 3234.1807564 | 3 |
| 3234.1814888 | 3 |
| 3234.18197 | 3 |
| 3234.1824982 | 5 |
| 3234.1829609 | 1 |
| 3234.183077 | 3 |
| 3234.1839199 | 3 |
| 3234.1845301 | 2 |
| 3234.1850378 | 4 |
| 3234.1855906 | 3 |
| 3234.1864672 | 1 |
| 3234.186865 | 2 |
| 3234.1878068 | 5 |
| 3234.1879264 | 1 |
| 3234.1892066 | 1 |
| 3234.1898437 | 3 |
| 3234.1905502 | 4 |
| 3234.1905611 | 1 |
| 3234.1908794 | 1 |
| 3234.1917473 | 3 |
| 3234.1923403 | 3 |
| 3234.1932675 | 0 |
| 3234.1938905 | 3 |
| 3234.1943971 | 1 |
| 3234.1953915 | 7 |
| 3234.1954191 | 3 |
| 3234.1954286 | 1 |
| 3234.1967677 | 2 |
| 3234.1972879 | 2 |
| 3234.1976875 | 2 |
| 3234.1981684 | 0 |
| 3234.1988693 | 2 |
| 3234.1989053 | 1 |
| 3234.2001789 | 2 |
| 3234.200233 | 3 |
| 3234.201575 | 1 |
| 3234.2018745 | 2 |
| 3234.2023332 | 2 |
| 3234.2029989 | 0 |
| 3234.2033423 | 4 |
| 3234.2039791 | 4 |
| 3234.2046631 | 0 |
| 3234.206239 | 2 |
| 3234.2081699 | 2 |
| 3234.2094457 | 3 |
| 3234.2108376 | 4 |
| 3234.2123932 | 2 |
| 3234.2147327 | 0 |
| 3234.2154979 | 0 |
| 3234.2177023 | 0 |
| 3234.2185423 | 2 |
| 3234.2203552 | 4 |
| 3234.2221744 | 3 |
| 3234.2232678 | 1 |
| 3234.2251371 | 1 |
| 3234.2263326 | 1 |
| 3234.2282516 | 2 |
| 3234.2305515 | 2 |
| 3234.2310448 | 0 |
| 3234.2327105 | 2 |
| 3234.2336042 | 0 |
| 3234.2359538 | 2 |
| 3234.2367714 | 3 |
| 3234.2386592 | 5 |
| 3234.2407512 | 3 |
| 3234.2409486 | 2 |
| 3234.2434011 | 0 |
| 3234.2443579 | 2 |
| 3234.246481 | 3 |
| 3234.2480095 | 2 |
| 3234.2497914 | 2 |
| 3234.251346 | 2 |
| 3234.2522939 | 2 |
| 3234.2538562 | 1 |
| 3234.2565389 | 4 |
| 3234.256989 | 3 |
| 3234.2590762 | 3 |
| 3234.2606991 | 2 |
| 3234.2616511 | 3 |
| 3234.2642493 | 1 |
| 3234.2649334 | 3 |
| 3234.2666141 | 3 |
| 3234.2679307 | 1 |
| 3234.2692638 | 2 |
| 3234.2713499 | 2 |
| 3234.2720569 | 1 |
| 3234.2746897 | 0 |
| 3234.2751035 | 4 |
| 3234.2768829 | 5 |
| 3234.2788532 | 4 |
| 3234.2800459 | 3 |
| 3234.2819848 | 4 |
| 3234.2828244 | 2 |
| 3234.2849862 | 0 |
| 3234.2865641 | 2 |
| 3234.2880231 | 0 |
| 3234.2895589 | 2 |
| 3234.2904394 | 4 |
| 3234.2926895 | 0 |
| 3234.2943499 | 3 |
| 3234.2954228 | 3 |
| 3234.2975491 | 0 |
| 3234.298776 | 1 |
| 3234.2998604 | 1 |
| 3234.3020812 | 1 |
| 3234.303069 | 2 |
| 3234.3046887 | 1 |
| 3234.3070425 | 1 |
| 3234.3080873 | 1 |
| 3234.3100674 | 2 |
| 3234.3107893 | 4 |
| 3234.313096 | 1 |
| 3234.3145251 | 1 |
| 3234.3157692 | 1 |
| 3234.317181 | 1 |
| 3234.3185313 | 2 |
| 3234.3209975 | 1 |
| 3234.3212581 | 3 |
| 3234.3230845 | 1 |
| 3234.3254452 | 0 |
| 3234.3265087 | 6 |
| 3234.3277265 | 1 |
| 3234.3295283 | 1 |
| 3234.3310535 | 1 |
| 3234.3337762 | 4 |
| 3234.3342654 | 2 |
| 3234.3360538 | 2 |
| 3234.3370589 | 2 |
| 3234.3386614 | 1 |
| 3234.3406972 | 4 |
| 3234.3416287 | 3 |
| 3234.3434854 | 2 |
| 3234.3445901 | 3 |
| 3234.3466504 | 3 |
| 3234.3487994 | 4 |
| 3234.3497113 | 3 |
| 3234.3515053 | 2 |
| 3234.3527901 | 5 |
| 3234.3547627 | 2 |
| 3234.3562217 | 0 |
| 3234.3572621 | 2 |
| 3234.3588868 | 0 |
| 3234.360199 | 1 |
| 3234.3620986 | 4 |
| 3234.3641002 | 1 |
| 3234.36504 | 0 |
| 3234.3672024 | 0 |
| 3234.3684119 | 0 |
| 3234.3701743 | 2 |
| 3234.3717959 | 2 |
| 3234.3728184 | 0 |
| 3234.3747938 | 1 |
| 3234.3759761 | 0 |
| 3234.3775159 | 4 |
| 3234.3784386 | 1 |
| 3234.3800533 | 4 |
| 3234.3827343 | 2 |
| 3234.3835885 | 3 |
| 3234.3852704 | 2 |
| 3234.3875258 | 1 |
| 3234.3883644 | 4 |
| 3234.3906121 | 1 |
| 3234.3914309 | 4 |
| 3234.3933724 | 1 |
| 3234.3946269 | 0 |
| 3234.3963845 | 4 |
| 3234.3979869 | 1 |
| 3234.3990566 | 4 |
| 3234.4011487 | 4 |
| 3234.4022448 | 3 |
| 3234.4041548 | 2 |
| 3234.4059803 | 0 |
| 3234.4065415 | 2 |
| 3234.4087271 | 3 |
| 3234.4101112 | 3 |
| 3234.4113004 | 2 |
| 3234.4138388 | 4 |
| 3234.415114 | 3 |
| 3234.4170734 | 2 |
| 3234.4174517 | 2 |
| 3234.4193729 | 1 |
| 3234.4214534 | 2 |
| 3234.4222226 | 3 |
| 3234.4242152 | 1 |
| 3234.4256579 | 3 |
| 3234.4277416 | 3 |
| 3234.4291394 | 0 |
| 3234.4306502 | 2 |
| 3234.4324072 | 5 |
| 3234.4326513 | 1 |
| 3234.4353922 | 3 |
| 3234.4360411 | 3 |
| 3234.437539 | 1 |
| 3234.4399212 | 4 |
| 3234.4410703 | 2 |
| 3234.4428768 | 3 |
| 3234.444917 | 4 |
| 3234.445696 | 2 |
| 3234.4474337 | 2 |
| 3234.4484248 | 2 |
| 3234.4505263 | 2 |
| 3234.4528366 | 0 |
| 3234.4541941 | 1 |
| 3234.4553402 | 4 |
| 3234.456914 | 2 |
| 3234.4587324 | 3 |
| 3234.459172 | 3 |
| 3234.4615117 | 1 |
| 3234.4632913 | 3 |
| 3234.4639767 | 3 |
| 3234.4664241 | 2 |
| 3234.4675896 | 2 |
| 3234.4694366 | 2 |
| 3234.4709793 | 0 |
| 3234.4724338 | 4 |
| 3234.4739537 | 0 |
| 3234.4752599 | 3 |
| 3234.47732 | 2 |
| 3234.4785542 | 0 |
| 3234.4801836 | 3 |
| 3234.4819438 | 2 |
| 3234.4825184 | 2 |
| 3234.4852625 | 3 |
| 3234.4868694 | 3 |
| 3234.4878766 | 2 |
| 3234.4898998 | 1 |
| 3234.4910547 | 3 |
| 3234.493094 | 1 |
| 3234.4946885 | 1 |
| 3234.4957253 | 3 |
| 3234.4978843 | 4 |
| 3234.4983772 | 3 |
| 3234.4996534 | 0 |
| 3234.5022126 | 4 |
| 3234.5030892 | 1 |
| 3234.5044651 | 2 |
| 3234.5064244 | 1 |
| 3234.5084919 | 2 |
| 3234.5097328 | 4 |
| 3234.5116069 | 1 |
| 3234.5135036 | 1 |
| 3234.5140338 | 1 |
| 3234.517331 | 5 |
| 3234.5179868 | 1 |
| 3234.5191072 | 3 |
| 3234.5210782 | 2 |
| 3234.5222221 | 4 |
| 3234.523998 | 0 |
| 3234.5252876 | 3 |
| 3234.5270348 | 2 |
| 3234.52927 | 2 |
| 3234.5299261 | 5 |
| 3234.5323876 | 0 |
| 3234.5325749 | 3 |
| 3234.5347756 | 4 |
| 3234.53689 | 3 |
| 3234.5376812 | 2 |
| 3234.539309 | 2 |
| 3234.5409645 | 2 |
| 3234.542333 | 1 |
| 3234.5444427 | 3 |
| 3234.54542 | 3 |
| 3234.547219 | 1 |
| 3234.5495124 | 1 |
| 3234.5503434 | 1 |
| 3234.5520522 | 4 |
| 3234.5527518 | 1 |
| 3234.5548033 | 2 |
| 3234.55717 | 1 |
| 3234.5574501 | 2 |
| 3234.5602692 | 0 |
| 3234.5611464 | 3 |
| 3234.5631119 | 0 |
| 3234.5649008 | 3 |
| 3234.5660585 | 1 |
| 3234.5676038 | 1 |
| 3234.5684557 | 3 |
| 3234.5708952 | 3 |
| 3234.5730905 | 3 |
| 3234.5736232 | 0 |
| 3234.5758738 | 5 |
| 3234.5769173 | 5 |
| 3234.5784502 | 4 |
| 3234.5802135 | 7 |
| 3234.5820527 | 2 |
| 3234.5834347 | 3 |
| 3234.5845985 | 2 |
| 3234.5863454 | 3 |
| 3234.5885653 | 0 |
| 3234.5892033 | 6 |
| 3234.590989 | 4 |
| 3234.5925972 | 0 |
| 3234.5939412 | 1 |
| 3234.5962242 | 0 |
| 3234.5970223 | 1 |
| 3234.5994141 | 1 |
| 3234.6003876 | 4 |
| 3234.6023869 | 1 |
| 3234.6025619 | 3 |
| 3234.6050217 | 2 |
| 3234.6066699 | 2 |
| 3234.6076165 | 2 |
| 3234.6095771 | 4 |
| 3234.6110838 | 3 |
| 3234.6132664 | 2 |
| 3234.6154202 | 4 |
| 3234.6154603 | 2 |
| 3234.6181358 | 2 |
| 3234.618738 | 2 |
| 3234.6202945 | 3 |
| 3234.6222421 | 3 |
| 3234.6241076 | 3 |
| 3234.6260285 | 2 |
| 3234.6269155 | 0 |
| 3234.6288511 | 3 |
| 3234.6305765 | 3 |
| 3234.6314992 | 4 |
| 3234.6332643 | 3 |
| 3234.6349571 | 1 |
| 3234.6358613 | 3 |
| 3234.6375503 | 1 |
| 3234.6390287 | 5 |
| 3234.6413418 | 3 |
| 3234.6419208 | 4 |
| 3234.6446279 | 1 |
| 3234.6451052 | 2 |
| 3234.647436 | 2 |
| 3234.649173 | 2 |
| 3234.6498616 | 2 |
| 3234.6511927 | 3 |
| 3234.6539981 | 1 |
| 3234.6552883 | 5 |
| 3234.6568737 | 0 |
| 3234.658131 | 2 |
| 3234.6601566 | 4 |

| | |
|---|---|
| 3234.6607014 | 1 |
| 3234.6633323 | 1 |
| 3234.6651001 | 4 |
| 3234.6662358 | 1 |
| 3234.6679473 | 4 |
| 3234.6686862 | 4 |
| 3234.6705819 | 0 |
| 3234.6727113 | 2 |
| 3234.6736262 | 0 |
| 3234.6757634 | 1 |
| 3234.6764828 | 2 |
| 3234.6789898 | 3 |
| 3234.6795555 | 2 |
| 3234.6823291 | 1 |
| 3234.6832792 | 4 |
| 3234.6851685 | 0 |
| 3234.6861132 | 3 |
| 3234.6867594 | 2 |
| 3234.6894969 | 4 |
| 3234.6914446 | 1 |
| 3234.6915812 | 2 |
| 3234.6944769 | 1 |
| 3234.6950084 | 1 |
| 3234.6966403 | 2 |
| 3234.6984945 | 2 |
| 3234.7000675 | 2 |
| 3234.7021006 | 3 |
| 3234.7031306 | 2 |
| 3234.705473 | 4 |
| 3234.70548 | 2 |
| 3234.7077737 | 2 |
| 3234.7097516 | 2 |
| 3234.7117008 | 3 |
| 3234.7127521 | 4 |
| 3234.7151031 | 2 |
| 3234.7151082 | 2 |
| 3234.7178697 | 1 |
| 3234.7187947 | 2 |
| 3234.7211027 | 2 |
| 3234.7221822 | 3 |
| 3234.7237811 | 3 |
| 3234.7248035 | 1 |
| 3234.726151 | 1 |
| 3234.7282946 | 4 |
| 3234.730182 | 1 |
| 3234.7313315 | 1 |
| 3234.7326469 | 4 |
| 3234.7346741 | 2 |
| 3234.7355123 | 2 |
| 3234.7373627 | 0 |
| 3234.7384885 | 2 |
| 3234.7401847 | 2 |
| 3234.7421392 | 8 |
| 3234.7436242 | 3 |
| 3234.7451592 | 3 |
| 3234.7467986 | 2 |
| 3234.7479585 | 1 |
| 3234.7504119 | 2 |
| 3234.7512255 | 7 |
| 3234.7534329 | 3 |
| 3234.7553452 | 1 |
| 3234.7563976 | 2 |
| 3234.758089 | 2 |
| 3234.7591025 | 1 |
| 3234.7604284 | 2 |
| 3234.7628238 | 5 |
| 3234.7637426 | 2 |
| 3234.7657236 | 2 |
| 3234.766599 | 6 |
| 3234.7687694 | 2 |
| 3234.7692803 | 3 |
| 3234.771676 | 1 |
| 3234.7735694 | 2 |
| 3234.774612 | 1 |
| 3234.776247 | 2 |
| 3234.7772922 | 2 |
| 3234.7793378 | 6 |
| 3234.7811131 | 2 |
| 3234.7820228 | 3 |
| 3234.784234 | 3 |
| 3234.7850044 | 3 |
| 3234.7871943 | 0 |
| 3234.7888411 | 2 |
| 3234.7897075 | 6 |
| 3234.791934 | 4 |
| 3234.7929312 | 1 |
| 3234.7948059 | 2 |
| 3234.7964809 | 2 |
| 3234.7976454 | 1 |
| 3234.7979382 | 1 |
| 3234.7982821 | 5 |
| 3234.7995085 | 2 |
| 3234.8006079 | 5 |
| 3234.8006084 | 2 |
| 3234.8017065 | 4 |
| 3234.8023837 | 3 |
| 3234.8037609 | 3 |
| 3234.8044529 | 2 |
| 3234.8053008 | 0 |
| 3234.8055452 | 2 |
| 3234.8063766 | 4 |
| 3234.8076102 | 2 |
| 3234.8085972 | 1 |
| 3234.8089134 | 1 |
| 3234.8092639 | 0 |
| 3234.8099725 | 4 |
| 3234.8109037 | 2 |
| 3234.8126755 | 2 |
| 3234.8132326 | 4 |
| 3234.8134066 | 3 |
| 3234.8144512 | 4 |
| 3234.8154772 | 2 |
| 3234.8156844 | 1 |
| 3234.8165009 | 0 |
| 3234.8174986 | 2 |
| 3234.8184959 | 3 |
| 3234.8188242 | 3 |
| 3234.8195245 | 0 |
| 3234.8208315 | 3 |
| 3234.8211196 | 1 |
| 3234.8220389 | 6 |
| 3234.8229302 | 1 |
| 3234.8241837 | 4 |
| 3234.8243134 | 2 |
| 3234.82487 | 3 |
| 3234.8256625 | 1 |
| 3234.8265557 | 2 |
| 3234.8266955 | 1 |
| 3234.8284378 | 2 |
| 3234.8284749 | 3 |
| 3234.8296778 | 2 |
| 3234.8306894 | 4 |
| 3234.8315693 | 1 |
| 3234.8319806 | 0 |
| 3234.8327965 | 3 |
| 3234.8338713 | 1 |
| 3234.8346293 | 3 |
| 3234.8350051 | 3 |
| 3234.836201 | 4 |
| 3234.8367587 | 1 |
| 3234.836787 | 2 |
| 3234.8384219 | 3 |
| 3234.8391516 | 2 |
| 3234.8395424 | 2 |
| 3234.8402902 | 1 |
| 3234.8417022 | 4 |
| 3234.842279 | 2 |
| 3234.8432448 | 1 |
| 3234.8435278 | 3 |
| 3234.8443467 | 3 |
| 3234.8451706 | 2 |
| 3234.8459946 | 2 |
| 3234.8472036 | 1 |
| 3234.8475516 | 4 |
| 3234.8478676 | 4 |
| 3234.849309 | 2 |
| 3234.8501574 | 1 |
| 3234.850435 | 1 |
| 3234.8513831 | 2 |
| 3234.8521458 | 2 |
| 3234.853678 | 1 |
| 3234.8541393 | 3 |
| 3234.8550359 | 0 |
| 3234.8552435 | 1 |
| 3234.8560973 | 1 |
| 3234.8573492 | 3 |
| 3234.8578961 | 4 |
| 3234.8580533 | 0 |
| 3234.8590781 | 5 |
| 3234.8601224 | 3 |
| 3234.8610535 | 1 |
| 3234.8621272 | 1 |
| 3234.8623742 | 1 |
| 3234.862436 | 0 |
| 3234.8635275 | 0 |
| 3234.8646178 | 0 |
| 3234.8655369 | 0 |
| 3234.8660195 | 2 |
| 3234.8675695 | 0 |
| 3234.8678483 | 1 |
| 3234.8685123 | 4 |
| 3234.8694889 | 1 |
| 3234.8701153 | 2 |
| 3234.8705162 | 4 |
| 3234.8712934 | 4 |
| 3234.8725224 | 3 |
| 3234.8730932 | 0 |
| 3234.8736099 | 3 |
| 3234.8750976 | 2 |
| 3234.8754864 | 0 |
| 3234.8760241 | 3 |
| 3234.8775628 | 3 |
| 3234.8782027 | 4 |
| 3234.8782718 | 1 |
| 3234.8790827 | 5 |
| 3234.8803521 | 4 |
| 3234.8804988 | 7 |
| 3234.8812634 | 2 |
| 3234.8822874 | 2 |
| 3234.8838002 | 3 |
| 3234.883803 | 2 |
| 3234.8855613 | 1 |
| 3234.8858629 | 1 |
| 3234.8862661 | 1 |
| 3234.8869522 | 3 |
| 3234.8885803 | 1 |
| 3234.8891541 | 2 |
| 3234.889458 | 0 |
| 3234.8898522 | 1 |
| 3234.891691 | 1 |
| 3234.8917425 | 1 |
| 3234.8939188 | 2 |
| 3234.8942148 | 2 |
| 3234.8945881 | 0 |
| 3234.8948754 | 5 |
| 3234.8965064 | 1 |
| 3234.896592 | 3 |
| 3234.8976893 | 0 |
| 3234.8980286 | 2 |
| 3234.8994095 | 4 |
| 3234.9000377 | 0 |
| 3234.9006522 | 2 |
| 3234.9018486 | 7 |
| 3234.9023146 | 0 |
| 3234.9049501 | 2 |
| 3234.9065988 | 1 |
| 3234.9072666 | 0 |
| 3234.9089279 | 3 |
| 3234.9105738 | 2 |
| 3234.9127952 | 1 |
| 3234.9139805 | 1 |
| 3234.91566 | 0 |
| 3234.9173873 | 3 |
| 3234.9180021 | 3 |
| 3234.9199172 | 7 |
| 3234.9219195 | 2 |
| 3234.9226131 | 1 |
| 3234.9243994 | 1 |
| 3234.9254259 | 4 |
| 3234.9273274 | 3 |
| 3234.9282414 | 2 |
| 3234.9309076 | 1 |
| 3234.932407 | 5 |
| 3234.9331993 | 1 |
| 3234.9352109 | 2 |
| 3234.9363695 | 3 |
| 3234.9385639 | 1 |
| 3234.9396194 | 1 |
| 3234.9406886 | 3 |
| 3234.9427242 | 2 |
| 3234.9443649 | 2 |
| 3234.9458299 | 4 |
| 3234.9483179 | 0 |
| 3234.9491906 | 3 |
| 3234.9501802 | 0 |
| 3234.9514754 | 3 |
| 3234.9530137 | 4 |
| 3234.9553489 | 3 |
| 3234.9568176 | 2 |
| 3234.9588467 | 1 |
| 3234.9599139 | 2 |
| 3234.9616164 | 1 |
| 3234.9633962 | 3 |
| 3234.9641644 | 6 |
| 3234.9665301 | 5 |
| 3234.9677263 | 1 |
| 3234.9698009 | 4 |
| 3234.97131 | 1 |
| 3234.9720247 | 2 |
| 3234.9737224 | 1 |
| 3234.9741889 | 2 |
| 3234.9761575 | 0 |
| 3234.9791679 | 4 |
| 3234.9796633 | 2 |
| 3234.9820157 | 1 |
| 3234.9828671 | 1 |
| 3234.9842552 | 3 |
| 3234.9862732 | 2 |
| 3234.9875215 | 3 |
| 3234.9892715 | 2 |
| 3234.9901087 | 2 |
| 3234.9927262 | 4 |
| 3234.993034 | 4 |
| 3234.994969 | 3 |
| 3234.997532 | 5 |
| 3234.9985668 | 1 |
| 3235.0000602 | 3 |
| 3235.0015267 | 2 |
| 3235.0027705 | 1 |
| 3235.0050301 | 4 |
| 3235.0058654 | 1 |
| 3235.0079981 | 2 |
| 3235.0094211 | 1 |
| 3235.0107574 | 3 |
| 3235.0125617 | 4 |
| 3235.0132741 | 3 |
| 3235.0156844 | 1 |
| 3235.0166227 | 2 |
| 3235.0181375 | 2 |
| 3235.0204246 | 2 |
| 3235.0212696 | 4 |
| 3235.0234035 | 0 |
| 3235.0236686 | 0 |
| 3235.0263965 | 0 |
| 3235.0280137 | 4 |
| 3235.0285208 | 4 |
| 3235.0306259 | 5 |
| 3235.031804 | 1 |
| 3235.0338076 | 0 |
| 3235.0348737 | 2 |
| 3235.0371059 | 2 |
| 3235.0389457 | 2 |
| 3235.0399907 | 3 |
| 3235.0414751 | 2 |
| 3235.0431522 | 2 |
| 3235.0448703 | 0 |
| 3235.0466267 | 2 |
| 3235.0473771 | 4 |
| 3235.0499501 | 2 |
| 3235.050199 | 0 |
| 3235.0525869 | 4 |
| 3235.0543348 | 2 |
| 3235.0556833 | 3 |
| 3235.0571474 | 2 |
| 3235.0591 | 2 |
| 3235.0606122 | 2 |
| 3235.0606615 | 1 |
| 3235.0633515 | 2 |
| 3235.0648414 | 3 |
| 3235.0655098 | 3 |
| 3235.066962 | 1 |
| 3235.0690166 | 0 |
| 3235.0703995 | 0 |
| 3235.0725526 | 0 |
| 3235.0732129 | 2 |
| 3235.0760609 | 1 |
| 3235.0773061 | 2 |
| 3235.0781871 | 3 |
| 3235.0802733 | 2 |
| 3235.0808347 | 0 |
| 3235.0831344 | 2 |
| 3235.0856596 | 1 |
| 3235.086184 | 1 |
| 3235.0883495 | 5 |
| 3235.0889092 | 4 |
| 3235.0911908 | 2 |
| 3235.0921883 | 3 |
| 3235.0939864 | 4 |
| 3235.0961122 | 1 |
| 3235.0973706 | 1 |
| 3235.0985584 | 7 |
| 3235.1000164 | 3 |
| 3235.1010885 | 1 |
| 3235.1037033 | 2 |
| 3235.104691 | 4 |
| 3235.1063174 | 3 |
| 3235.1077802 | 0 |
| 3235.1084645 | 5 |
| 3235.1098166 | 3 |
| 3235.1116792 | 2 |
| 3235.1132301 | 4 |
| 3235.1153907 | 3 |
| 3235.1166409 | 0 |

| | |
|---|---|
| 3235.1192318 | 4 |
| 3235.1193933 | 2 |
| 3235.122158 | 8 |
| 3235.1229842 | 2 |
| 3235.1243832 | 2 |
| 3235.1266599 | 2 |
| 3235.1273215 | 1 |
| 3235.1297648 | 2 |
| 3235.1303844 | 4 |
| 3235.1329956 | 4 |
| 3235.1344167 | 0 |
| 3235.1356732 | 1 |
| 3235.1374714 | 3 |
| 3235.138197 | 2 |
| 3235.1401732 | 2 |
| 3235.1415198 | 1 |
| 3235.1429365 | 1 |
| 3235.1450955 | 5 |
| 3235.1459913 | 5 |
| 3235.1474095 | 6 |
| 3235.1491301 | 0 |
| 3235.1501626 | 5 |
| 3235.1528496 | 3 |
| 3235.1532647 | 1 |
| 3235.1554074 | 2 |
| 3235.1571483 | 4 |
| 3235.1580862 | 2 |
| 3235.1605269 | 1 |
| 3235.1613488 | 2 |
| 3235.1627811 | 4 |
| 3235.1650555 | 1 |
| 3235.1657935 | 1 |
| 3235.1681424 | 4 |
| 3235.1691511 | 3 |
| 3235.1711272 | 2 |
| 3235.1726166 | 2 |
| 3235.1740199 | 1 |
| 3235.1756706 | 1 |
| 3235.1773979 | 6 |
| 3235.1787175 | 2 |
| 3235.1798736 | 1 |
| 3235.1819692 | 2 |
| 3235.1837552 | 6 |
| 3235.1846408 | 4 |
| 3235.1865008 | 1 |
| 3235.1882706 | 2 |
| 3235.1900062 | 1 |
| 3235.1913537 | 2 |
| 3235.1928136 | 2 |
| 3235.1941661 | 4 |
| 3235.1951047 | 1 |
| 3235.1970268 | 4 |
| 3235.1987926 | 0 |
| 3235.2003079 | 0 |
| 3235.2020118 | 2 |
| 3235.2031899 | 2 |
| 3235.2047729 | 2 |
| 3235.2066224 | 5 |
| 3235.2072864 | 4 |
| 3235.2093705 | 1 |
| 3235.2117833 | 2 |
| 3235.2127722 | 2 |
| 3235.2145981 | 2 |
| 3235.2155422 | 1 |
| 3235.2180315 | 1 |
| 3235.218191 | 4 |
| 3235.2207479 | 1 |
| 3235.2227158 | 2 |
| 3235.2238113 | 0 |
| 3235.2251705 | 1 |
| 3235.2265598 | 1 |
| 3235.2279234 | 0 |
| 3235.2308328 | 3 |
| 3235.2319364 | 3 |
| 3235.2334559 | 2 |
| 3235.2342723 | 2 |
| 3235.236514 | 4 |
| 3235.2373993 | 2 |
| 3235.238589 | 2 |
| 3235.2413297 | 2 |
| 3235.2421468 | 3 |
| 3235.2442379 | 3 |
| 3235.2451304 | 1 |
| 3235.246513 | 3 |
| 3235.2486763 | 2 |
| 3235.2495317 | 3 |
| 3235.2515041 | 2 |
| 3235.2535014 | 2 |
| 3235.2545181 | 1 |
| 3235.2566537 | 2 |
| 3235.2576694 | 2 |
| 3235.259588 | 2 |
| 3235.2618958 | 1 |
| 3235.2624256 | 2 |
| 3235.2649893 | 1 |
| 3235.2656521 | 1 |
| 3235.2672879 | 2 |
| 3235.2691213 | 1 |
| 3235.2710498 | 0 |
| 3235.2724171 | 6 |
| 3235.2729266 | 2 |
| 3235.2753064 | 1 |
| 3235.2772645 | 2 |
| 3235.2777957 | 3 |
| 3235.2800239 | 1 |
| 3235.2810445 | 2 |
| 3235.2833991 | 1 |
| 3235.2851852 | 2 |
| 3235.2863661 | 1 |
| 3235.2877682 | 2 |
| 3235.2890095 | 2 |
| 3235.290863 | 3 |
| 3235.2918334 | 3 |
| 3235.2938192 | 2 |
| 3235.295615 | 2 |
| 3235.2975253 | 2 |
| 3235.2983919 | 2 |
| 3235.3007185 | 3 |
| 3235.3012234 | 0 |
| 3235.3029557 | 0 |
| 3235.3055091 | 0 |
| 3235.307099 | 1 |
| 3235.3078134 | 0 |
| 3235.3094724 | 3 |
| 3235.3120565 | 2 |
| 3235.3124848 | 1 |
| 3235.3141371 | 5 |
| 3235.3164416 | 1 |
| 3235.3174529 | 1 |
| 3235.3191503 | 1 |
| 3235.3203512 | 3 |
| 3235.3224721 | 0 |
| 3235.3244686 | 4 |
| 3235.3253515 | 1 |
| 3235.3268383 | 1 |
| 3235.3277839 | 2 |
| 3235.3301496 | 1 |
| 3235.3306812 | 2 |
| 3235.3329877 | 3 |
| 3235.3351526 | 3 |
| 3235.335637 | 1 |
| 3235.3366558 | 1 |
| 3235.3387429 | 2 |
| 3235.340876 | 1 |
| 3235.3427439 | 1 |
| 3235.3439476 | 3 |
| 3235.3459564 | 2 |
| 3235.3461644 | 3 |
| 3235.3480631 | 4 |
| 3235.3502807 | 4 |
| 3235.3513299 | 2 |
| 3235.3536209 | 1 |
| 3235.3550423 | 7 |
| 3235.3565975 | 2 |
| 3235.3588089 | 3 |
| 3235.3593696 | 1 |
| 3235.361233 | 5 |
| 3235.3627594 | 1 |
| 3235.3649332 | 2 |
| 3235.3660888 | 3 |
| 3235.367505 | 2 |
| 3235.3692327 | 0 |
| 3235.3707896 | 3 |
| 3235.372057 | 2 |
| 3235.373492 | 0 |
| 3235.3755034 | 5 |
| 3235.3771293 | 1 |
| 3235.3781184 | 0 |
| 3235.3799671 | 3 |
| 3235.380935 | 1 |
| 3235.3830259 | 1 |
| 3235.3846446 | 2 |
| 3235.3864229 | 0 |
| 3235.388202 | 2 |
| 3235.3889476 | 4 |
| 3235.3907059 | 2 |
| 3235.3921533 | 2 |
| 3235.3945107 | 3 |
| 3235.3960257 | 2 |
| 3235.3972728 | 1 |
| 3235.3986059 | 0 |
| 3235.4008267 | 1 |
| 3235.4016089 | 5 |
| 3235.4038064 | 4 |
| 3235.4044152 | 1 |
| 3235.4062778 | 4 |
| 3235.4088983 | 3 |
| 3235.4097943 | 0 |
| 3235.4117074 | 1 |
| 3235.4121673 | 3 |
| 3235.4152811 | 3 |
| 3235.4156566 | 2 |
| 3235.4170374 | 2 |
| 3235.4188693 | 0 |
| 3235.420371 | 1 |
| 3235.4220552 | 0 |
| 3235.4238391 | 0 |
| 3235.4254619 | 0 |
| 3235.4268861 | 1 |
| 3235.4284637 | 6 |
| 3235.4305598 | 2 |
| 3235.4309279 | 2 |
| 3235.4330657 | 2 |
| 3235.4345655 | 2 |
| 3235.4361037 | 0 |
| 3235.4377842 | 1 |
| 3235.4393439 | 3 |
| 3235.4405109 | 0 |
| 3235.4430705 | 1 |
| 3235.4440741 | 3 |
| 3235.4463341 | 2 |
| 3235.4472003 | 0 |
| 3235.4484171 | 2 |
| 3235.4509828 | 2 |
| 3235.4522411 | 3 |
| 3235.4538547 | 3 |
| 3235.4550754 | 1 |
| 3235.4570328 | 3 |
| 3235.4574869 | 4 |
| 3235.4604971 | 5 |
| 3235.462198 | 0 |
| 3235.4624553 | 5 |
| 3235.4643592 | 2 |
| 3235.4656162 | 3 |
| 3235.467513 | 4 |
| 3235.4697246 | 4 |
| 3235.4701644 | 1 |
| 3235.4719651 | 2 |
| 3235.4732996 | 3 |
| 3235.4753492 | 5 |
| 3235.4763293 | 2 |
| 3235.4783301 | 2 |
| 3235.4807682 | 2 |
| 3235.4814971 | 1 |
| 3235.4834461 | 1 |
| 3235.4852177 | 1 |
| 3235.486031 | 2 |
| 3235.4882771 | 1 |
| 3235.4892387 | 1 |
| 3235.4912369 | 3 |
| 3235.492313 | 1 |
| 3235.4941999 | 2 |
| 3235.4963691 | 4 |
| 3235.4972271 | 3 |
| 3235.498802 | 3 |
| 3235.5000124 | 2 |
| 3235.5019469 | 2 |
| 3235.5039991 | 2 |
| 3235.5045252 | 3 |
| 3235.5065655 | 1 |
| 3235.5087317 | 1 |
| 3235.509568 | 4 |
| 3235.5113728 | 3 |
| 3235.5127274 | 0 |
| 3235.5145204 | 0 |
| 3235.5162297 | 0 |
| 3235.5176909 | 0 |
| 3235.5190384 | 0 |
| 3235.5205735 | 1 |
| 3235.5219832 | 3 |
| 3235.5229455 | 1 |
| 3235.5259807 | 1 |
| 3235.5272478 | 3 |
| 3235.5281622 | 2 |
| 3235.5299969 | 0 |
| 3235.5310454 | 5 |
| 3235.5336681 | 4 |
| 3235.5344938 | 0 |
| 3235.5363577 | 4 |
| 3235.5384865 | 2 |
| 3235.53901 | 1 |
| 3235.5413068 | 4 |
| 3235.5421585 | 1 |
| 3235.5430513 | 1 |
| 3235.5460551 | 4 |
| 3235.5466371 | 2 |
| 3235.5483988 | 1 |
| 3235.5504777 | 0 |
| 3235.5521221 | 2 |
| 3235.5536053 | 2 |
| 3235.5545747 | 1 |
| 3235.5563669 | 2 |
| 3235.5579749 | 1 |
| 3235.5597085 | 0 |
| 3235.5611972 | 4 |
| 3235.5624112 | 4 |
| 3235.5642908 | 0 |
| 3235.5648345 | 0 |
| 3235.5673298 | 4 |
| 3235.5694702 | 4 |
| 3235.5699673 | 1 |
| 3235.5725062 | 2 |
| 3235.5734506 | 3 |
| 3235.5753899 | 3 |
| 3235.5770899 | 1 |
| 3235.5789485 | 0 |
| 3235.5803209 | 3 |
| 3235.581249 | 3 |
| 3235.5840049 | 4 |
| 3235.5843219 | 3 |
| 3235.5860272 | 3 |
| 3235.5885936 | 1 |
| 3235.589383 | 5 |
| 3235.5913571 | 1 |
| 3235.5918075 | 1 |
| 3235.5935943 | 3 |
| 3235.5960794 | 2 |
| 3235.5967689 | 3 |
| 3235.5986429 | 2 |
| 3235.6004779 | 3 |
| 3235.6016809 | 3 |
| 3235.6031007 | 4 |
| 3235.6042365 | 3 |
| 3235.6060344 | 3 |
| 3235.6072733 | 2 |
| 3235.6098187 | 1 |
| 3235.6109152 | 2 |
| 3235.6115949 | 1 |
| 3235.6142288 | 3 |
| 3235.615286 | 1 |
| 3235.6169504 | 1 |
| 3235.6187979 | 4 |
| 3235.6204153 | 3 |
| 3235.6221022 | 0 |
| 3235.622273 | 2 |
| 3235.6250973 | 2 |
| 3235.6266164 | 4 |
| 3235.6280655 | 3 |
| 3235.6304043 | 2 |
| 3235.6307164 | 0 |
| 3235.6328466 | 0 |
| 3235.6347909 | 2 |
| 3235.6351868 | 1 |
| 3235.6381936 | 1 |
| 3235.6385243 | 2 |
| 3235.6403372 | 6 |
| 3235.6426982 | 3 |
| 3235.6439332 | 3 |
| 3235.6455668 | 7 |
| 3235.6465061 | 1 |
| 3235.6478672 | 1 |
| 3235.6490602 | 1 |
| 3235.6506612 | 2 |
| 3235.6532209 | 2 |
| 3235.6541211 | 4 |
| 3235.6559901 | 3 |
| 3235.6564828 | 1 |
| 3235.6588271 | 2 |
| 3235.6605647 | 2 |
| 3235.6611557 | 3 |
| 3235.6629029 | 2 |
| 3235.6648164 | 2 |
| 3235.6666929 | 2 |
| 3235.6684182 | 2 |
| 3235.6697063 | 0 |
| 3235.6712057 | 0 |
| 3235.672761 | 1 |
| 3235.6739169 | 3 |
| 3235.6767552 | 2 |
| 3235.6777493 | 1 |
| 3235.6798845 | 2 |
| 3235.6799975 | 1 |
| 3235.6819829 | 2 |

| | |
|---|---|
| 3235.6837008 | 0 |
| 3235.6857845 | 2 |
| 3235.6871695 | 2 |
| 3235.6882625 | 1 |
| 3235.6898381 | 2 |
| 3235.6908974 | 2 |
| 3235.6932007 | 0 |
| 3235.694773 | 1 |
| 3235.6955377 | 2 |
| 3235.6971172 | 4 |
| 3235.6995749 | 2 |
| 3235.7002563 | 2 |
| 3235.7026379 | 1 |
| 3235.7037255 | 1 |
| 3235.7054086 | 1 |
| 3235.706221 | 0 |
| 3235.7086308 | 3 |
| 3235.7100536 | 3 |
| 3235.7111909 | 7 |
| 3235.7134139 | 1 |
| 3235.7141197 | 2 |
| 3235.7158322 | 1 |
| 3235.7180266 | 3 |
| 3235.7190987 | 3 |
| 3235.7212165 | 3 |
| 3235.722461 | 3 |
| 3235.72412 | 3 |
| 3235.7253159 | 5 |
| 3235.7273383 | 4 |
| 3235.7291546 | 3 |
| 3235.7297034 | 1 |
| 3235.7319694 | 2 |
| 3235.733658 | 2 |
| 3235.7342351 | 3 |
| 3235.7361901 | 1 |
| 3235.7378979 | 2 |
| 3235.7389161 | 0 |
| 3235.7403517 | 1 |
| 3235.7424125 | 1 |
| 3235.7446956 | 2 |
| 3235.745505 | 3 |
| 3235.7474727 | 5 |
| 3235.7484538 | 1 |
| 3235.7506607 | 1 |
| 3235.7517487 | 2 |
| 3235.7533468 | 4 |
| 3235.7551095 | 4 |
| 3235.7557552 | 1 |
| 3235.7575107 | 3 |
| 3235.7597966 | 2 |
| 3235.7606832 | 1 |
| 3235.7627011 | 5 |
| 3235.7640076 | 0 |
| 3235.7656932 | 1 |
| 3235.767033 | 2 |
| 3235.7691724 | 2 |
| 3235.770457 | 2 |
| 3235.7723048 | 3 |
| 3235.7735125 | 4 |
| 3235.7746421 | 2 |
| 3235.7771808 | 5 |
| 3235.7790529 | 4 |
| 3235.7796287 | 7 |
| 3235.7817203 | 3 |
| 3235.7819801 | 3 |
| 3235.7838255 | 7 |
| 3235.7858915 | 4 |
| 3235.787063 | 2 |
| 3235.7890138 | 4 |
| 3235.7901044 | 4 |
| 3235.7922612 | 1 |
| 3235.7935581 | 6 |
| 3235.7944065 | 2 |
| 3235.7968532 | 1 |
| 3235.7976102 | 1 |
| 3235.7997929 | 1 |
| 3235.8011924 | 1 |
| 3235.8025017 | 1 |
| 3235.8047584 | 1 |
| 3235.805797 | 3 |
| 3235.8079351 | 3 |
| 3235.8099912 | 4 |
| 3235.8105835 | 3 |
| 3235.8126879 | 3 |
| 3235.8135837 | 3 |
| 3235.815399 | 0 |
| 3235.8175316 | 0 |
| 3235.8185601 | 1 |
| 3235.8197263 | 2 |
| 3235.821186 | 1 |
| 3235.8230992 | 2 |
| 3235.8241668 | 0 |
| 3235.8264551 | 3 |
| 3235.8284309 | 4 |
| 3235.8288383 | 2 |
| 3235.831274 | 1 |
| 3235.8323559 | 3 |
| 3235.8337045 | 0 |
| 3235.8355417 | 3 |
| 3235.8367893 | 6 |
| 3235.8388513 | 3 |
| 3235.8392959 | 7 |
| 3235.8409743 | 1 |
| 3235.8430858 | 1 |
| 3235.8438921 | 1 |
| 3235.8461659 | 3 |
| 3235.8482859 | 0 |
| 3235.8488342 | 1 |
| 3235.8495218 | 1 |
| 3235.8496928 | 1 |
| 3235.8513026 | 1 |
| 3235.8516444 | 1 |
| 3235.8523621 | 2 |
| 3235.8526433 | 5 |
| 3235.8532537 | 3 |
| 3235.8541575 | 0 |
| 3235.8543124 | 0 |
| 3235.8549024 | 0 |
| 3235.8556566 | 4 |
| 3235.8559087 | 3 |
| 3235.856743 | 2 |
| 3235.8568896 | 3 |
| 3235.8573685 | 1 |
| 3235.8589787 | 2 |
| 3235.8590477 | 1 |
| 3235.8592983 | 1 |
| 3235.8602001 | 2 |
| 3235.8604649 | 1 |
| 3235.8616069 | 2 |
| 3235.8619249 | 1 |
| 3235.862017 | 2 |
| 3235.8632544 | 0 |
| 3235.8641616 | 2 |
| 3235.864533 | 3 |
| 3235.8646753 | 2 |
| 3235.865075 | 5 |
| 3235.8659191 | 1 |
| 3235.8662778 | 3 |
| 3235.8672392 | 1 |
| 3235.8677117 | 0 |
| 3235.8682227 | 2 |
| 3235.8688613 | 3 |
| 3235.8690636 | 1 |
| 3235.8690867 | 2 |
| 3235.8708235 | 0 |
| 3235.8713633 | 4 |
| 3235.8713828 | 8 |
| 3235.8725997 | 4 |
| 3235.8726877 | 1 |
| 3235.8727654 | 4 |
| 3235.8741818 | 3 |
| 3235.8744675 | 5 |
| 3235.8755127 | 0 |
| 3235.8759524 | 2 |
| 3235.876014 | 3 |
| 3235.8769044 | 1 |
| 3235.8774597 | 2 |
| 3235.8775342 | 0 |
| 3235.8785956 | 3 |
| 3235.8788299 | 1 |
| 3235.8804012 | 2 |
| 3235.8805035 | 2 |
| 3235.8806422 | 3 |
| 3235.8811752 | 4 |
| 3235.8824312 | 0 |
| 3235.8825367 | 2 |
| 3235.8828923 | 1 |
| 3235.8837759 | 1 |
| 3235.8845514 | 2 |
| 3235.8853897 | 2 |
| 3235.8856009 | 3 |
| 3235.8864064 | 1 |
| 3235.886483 | 3 |
| 3235.8866332 | 2 |
| 3235.8882583 | 2 |
| 3235.8883417 | 2 |
| 3235.8894989 | 2 |
| 3235.8900447 | 3 |
| 3235.8901647 | 1 |
| 3235.8908987 | 1 |
| 3235.8910249 | 0 |
| 3235.8924489 | 3 |
| 3235.8927221 | 3 |
| 3235.893452 | 2 |
| 3235.8941432 | 0 |
| 3235.8947627 | 1 |
| 3235.8953666 | 2 |
| 3235.8954069 | 4 |
| 3235.8957162 | 1 |
| 3235.8965985 | 3 |
| 3235.8966217 | 3 |
| 3235.8973157 | 1 |
| 3235.8975274 | 3 |
| 3235.898123 | 2 |
| 3235.8986009 | 3 |
| 3235.8990958 | 4 |
| 3235.8995383 | 2 |
| 3235.9006531 | 3 |
| 3235.9007094 | 2 |
| 3235.9010435 | 1 |
| 3235.9020391 | 2 |
| 3235.902094 | 6 |
| 3235.9022186 | 0 |
| 3235.9034484 | 1 |
| 3235.9034815 | 1 |
| 3235.903814 | 1 |
| 3235.9044471 | 1 |
| 3235.9048153 | 6 |
| 3235.9048906 | 3 |
| 3235.9053892 | 1 |
| 3235.9063183 | 4 |
| 3235.9068318 | 1 |
| 3235.9076613 | 3 |
| 3235.9081597 | 0 |
| 3235.9083291 | 3 |
| 3235.9085886 | 0 |
| 3235.9089737 | 2 |
| 3235.9096533 | 1 |
| 3235.9098097 | 2 |
| 3235.9102061 | 1 |
| 3235.9102935 | 1 |
| 3235.9112519 | 2 |
| 3235.9114732 | 3 |
| 3235.9115354 | 4 |
| 3235.9116021 | 0 |
| 3235.9116345 | 3 |
| 3235.9116946 | 0 |
| 3235.9117684 | 3 |
| 3235.9118546 | 2 |
| 3235.9119274 | 1 |
| 3235.9121652 | 6 |
| 3235.9127135 | 1 |
| 3235.912784 | 1 |
| 3235.9128835 | 2 |
| 3235.9129502 | 3 |
| 3235.9132212 | 1 |
| 3235.9133015 | 6 |
| 3235.9134109 | 2 |
| 3235.913541 | 1 |
| 3235.9135994 | 3 |
| 3235.9136404 | 1 |
| 3235.9137979 | 2 |
| 3235.9138361 | 1 |
| 3235.9139888 | 4 |
| 3235.9141302 | 0 |
| 3235.9141894 | 1 |
| 3235.9146566 | 2 |
| 3235.9147679 | 3 |
| 3235.9150891 | 2 |
| 3235.9155015 | 1 |
| 3235.9156072 | 3 |
| 3235.9157176 | 0 |
| 3235.9157274 | 3 |
| 3235.9158412 | 1 |
| 3235.9158637 | 4 |
| 3235.9159527 | 2 |
| 3235.9160299 | 2 |
| 3235.9160417 | 4 |
| 3235.9163813 | 4 |
| 3235.916579 | 2 |
| 3235.916792 | 2 |
| 3235.9167934 | 6 |
| 3235.9170533 | 3 |
| 3235.9172165 | 1 |
| 3235.9174013 | 2 |
| 3235.9176642 | 2 |
| 3235.9177155 | 2 |
| 3235.9177397 | 1 |
| 3235.9178184 | 2 |
| 3235.9178529 | 2 |
| 3235.9180133 | 4 |
| 3235.9180554 | 0 |
| 3235.9181097 | 2 |
| 3235.9181686 | 1 |
| 3235.9184847 | 3 |
| 3235.9187437 | 3 |
| 3235.918962 | 0 |
| 3235.9190779 | 2 |
| 3235.9191162 | 2 |
| 3235.9193842 | 1 |
| 3235.9194677 | 1 |
| 3235.9196352 | 4 |
| 3235.9197743 | 4 |
| 3235.9197802 | 1 |
| 3235.9198561 | 5 |
| 3235.9201587 | 1 |
| 3235.920192 | 6 |
| 3235.9203893 | 3 |
| 3235.9207565 | 5 |
| 3235.9208709 | 6 |
| 3235.9211813 | 7 |
| 3235.9213004 | 9 |
| 3235.9213329 | 6 |
| 3235.9215783 | 6 |
| 3235.921664 | 5 |
| 3235.9218851 | 9 |
| 3235.921984 | 7 |
| 3235.9220071 | 9 |
| 3235.9220675 | 7 |
| 3235.9221639 | 5 |
| 3235.9223905 | 7 |
| 3235.9226991 | 8 |
| 3235.9227177 | 10 |
| 3235.9228294 | 13 |
| 3235.9228324 | 5 |
| 3235.9229872 | 12 |
| 3235.923191 | 14 |
| 3235.9233098 | 9 |
| 3235.9235454 | 8 |
| 3235.9237556 | 13 |
| 3235.9238331 | 8 |
| 3235.9240973 | 12 |
| 3235.9241022 | 2 |
| 3235.9241566 | 7 |
| 3235.9244582 | 7 |
| 3235.9244825 | 8 |
| 3235.9244871 | 11 |
| 3235.9245604 | 7 |
| 3235.9249664 | 13 |
| 3235.9251128 | 7 |
| 3235.9253288 | 5 |
| 3235.9256256 | 11 |
| 3235.9256952 | 5 |
| 3235.9257452 | 10 |
| 3235.9259097 | 9 |
| 3235.9259975 | 6 |
| 3235.9260096 | 3 |
| 3235.9261158 | 5 |
| 3235.9262495 | 8 |
| 3235.9264559 | 6 |
| 3235.9264979 | 7 |
| 3235.9268668 | 5 |
| 3235.927017 | 13 |
| 3235.9270271 | 9 |
| 3235.927404 | 10 |
| 3235.927571 | 9 |
| 3235.927765 | 9 |
| 3235.9278379 | 8 |
| 3235.9278866 | 9 |
| 3235.9280247 | 9 |
| 3235.9280933 | 7 |
| 3235.9281015 | 7 |
| 3235.9284424 | 5 |
| 3235.9286035 | 5 |
| 3235.9286646 | 3 |
| 3235.9287137 | 7 |
| 3235.9287741 | 7 |
| 3235.9292246 | 5 |
| 3235.9294311 | 6 |
| 3235.929703 | 6 |
| 3235.9297342 | 9 |
| 3235.9298779 | 3 |
| 3235.9301363 | 3 |
| 3235.9303276 | 6 |
| 3235.9304338 | 0 |
| 3235.9307635 | 4 |
| 3235.9308198 | 8 |
| 3235.9309385 | 3 |
| 3235.9310763 | 2 |
| 3235.9310911 | 2 |
| 3235.9311018 | 3 |
| 3235.9312444 | 2 |
| 3235.9315178 | 3 |

| | |
|---|---|
| 3235.9317851 | 3 |
| 3235.9318369 | 2 |
| 3235.93188 | 0 |
| 3235.9319097 | 3 |
| 3235.9321951 | 3 |
| 3235.9322923 | 0 |
| 3235.9324125 | 4 |
| 3235.9325754 | 1 |
| 3235.9325773 | 3 |
| 3235.9327832 | 1 |
| 3235.9329044 | 0 |
| 3235.9329173 | 4 |
| 3235.9329876 | 1 |
| 3235.9330084 | 2 |
| 3235.9330515 | 4 |
| 3235.933206 | 2 |
| 3235.9334899 | 3 |
| 3235.9335414 | 2 |
| 3235.9337963 | 2 |
| 3235.9339191 | 0 |
| 3235.9341796 | 4 |
| 3235.9342075 | 1 |
| 3235.9342487 | 1 |
| 3235.9343555 | 1 |
| 3235.9343755 | 3 |
| 3235.9347974 | 5 |
| 3235.9354293 | 6 |
| 3235.9357949 | 0 |
| 3235.9358026 | 1 |
| 3235.9361608 | 4 |
| 3235.9362366 | 4 |
| 3235.9369717 | 1 |
| 3235.9379876 | 1 |
| 3235.9381394 | 2 |
| 3235.9390944 | 0 |
| 3235.9395172 | 0 |
| 3235.9397044 | 1 |
| 3235.939986 | 1 |
| 3235.9411141 | 2 |
| 3235.9416052 | 4 |
| 3235.9418481 | 1 |
| 3235.9428948 | 0 |
| 3235.9429014 | 2 |
| 3235.9437685 | 2 |
| 3235.9442067 | 2 |
| 3235.9445339 | 3 |
| 3235.9451445 | 1 |
| 3235.9454916 | 1 |
| 3235.946633 | 2 |
| 3235.9471131 | 0 |
| 3235.9473203 | 3 |
| 3235.9481425 | 2 |
| 3235.9490227 | 1 |
| 3235.9490727 | 0 |
| 3235.9495384 | 1 |
| 3235.9502749 | 0 |
| 3235.9510403 | 1 |
| 3235.9511629 | 0 |
| 3235.95158 | 0 |
| 3235.9523661 | 1 |
| 3235.9527408 | 4 |
| 3235.9529216 | 2 |
| 3235.9538732 | 1 |
| 3235.9544176 | 2 |
| 3235.9545482 | 2 |
| 3235.9554547 | 3 |
| 3235.9555292 | 6 |
| 3235.956617 | 1 |
| 3235.9571644 | 2 |
| 3235.9571798 | 1 |
| 3235.9583865 | 1 |
| 3235.9587854 | 2 |
| 3235.9589637 | 2 |
| 3235.9594655 | 2 |
| 3235.9598568 | 1 |
| 3235.9605184 | 0 |
| 3235.9611618 | 0 |
| 3235.9616087 | 2 |
| 3235.9620759 | 1 |
| 3235.9625972 | 0 |
| 3235.9628554 | 2 |
| 3235.9630703 | 2 |
| 3235.9639842 | 1 |
| 3235.9643974 | 0 |
| 3235.9649695 | 0 |
| 3235.9662179 | 1 |
| 3235.9664649 | 1 |
| 3235.9668867 | 1 |
| 3235.9677206 | 1 |
| 3235.9678121 | 2 |
| 3235.9680519 | 1 |
| 3235.9691617 | 2 |
| 3235.9696017 | 2 |
| 3235.9704775 | 2 |
| 3235.9706224 | 1 |
| 3235.9713086 | 2 |
| 3235.9722624 | 3 |
| 3235.9722798 | 2 |
| 3235.9724425 | 1 |
| 3235.9732156 | 3 |
| 3235.9738849 | 2 |
| 3235.9748017 | 2 |
| 3235.97519 | 1 |
| 3235.9754504 | 2 |
| 3235.9757504 | 6 |
| 3235.9764905 | 1 |
| 3235.9767488 | 4 |
| 3235.9774674 | 3 |
| 3235.9780995 | 1 |
| 3235.9788181 | 3 |
| 3235.9793997 | 5 |
| 3235.9797794 | 0 |
| 3235.9804835 | 5 |
| 3235.9806122 | 2 |
| 3235.981564 | 2 |
| 3235.982073 | 3 |
| 3235.9828175 | 3 |
| 3235.9828755 | 4 |
| 3235.9836164 | 2 |
| 3235.9844283 | 2 |
| 3235.9845119 | 3 |
| 3235.9853323 | 1 |
| 3235.9855842 | 4 |
| 3235.9862089 | 1 |
| 3235.9869979 | 3 |
| 3235.987388 | 4 |
| 3235.9880175 | 1 |
| 3235.9883223 | 2 |
| 3235.9886744 | 0 |
| 3235.989425 | 1 |
| 3235.9897439 | 2 |
| 3235.990859 | 0 |
| 3235.9913855 | 1 |
| 3235.9915156 | 2 |
| 3235.9921636 | 1 |
| 3235.9926977 | 2 |
| 3235.9930893 | 4 |
| 3235.993873 | 0 |
| 3235.9943158 | 2 |
| 3235.995134 | 1 |
| 3235.9951784 | 3 |
| 3235.9959641 | 3 |
| 3235.9961409 | 3 |
| 3235.9970349 | 4 |
| 3235.9971666 | 2 |
| 3235.9981938 | 1 |
| 3235.9988143 | 4 |
| 3235.9993951 | 3 |
| 3235.9998183 | 1 |
| 3235.9998727 | 3 |
| 3236.0008529 | 1 |
| 3236.0012838 | 2 |
| 3236.0016538 | 3 |
| 3236.0018929 | 2 |
| 3236.0026944 | 2 |
| 3236.0033641 | 1 |
| 3236.0041053 | 1 |
| 3236.0044498 | 0 |
| 3236.0049033 | 1 |
| 3236.0050828 | 2 |
| 3236.0058592 | 2 |
| 3236.0059422 | 3 |
| 3236.0072075 | 3 |
| 3236.0073786 | 2 |
| 3236.008043 | 0 |
| 3236.0093749 | 1 |
| 3236.0099734 | 3 |
| 3236.0107054 | 0 |
| 3236.0122803 | 2 |
| 3236.0124378 | 6 |
| 3236.0137482 | 1 |
| 3236.0144256 | 3 |
| 3236.0153476 | 2 |
| 3236.0162438 | 2 |
| 3236.0171751 | 2 |
| 3236.0187839 | 1 |
| 3236.0188761 | 1 |
| 3236.0198192 | 2 |
| 3236.0208062 | 2 |
| 3236.022237 | 1 |
| 3236.0228514 | 2 |
| 3236.0235509 | 3 |
| 3236.0249383 | 1 |
| 3236.0258081 | 0 |
| 3236.0266278 | 2 |
| 3236.0280417 | 0 |
| 3236.0290351 | 2 |
| 3236.0290887 | 1 |
| 3236.0303266 | 2 |
| 3236.031294 | 1 |
| 3236.032862 | 2 |
| 3236.0334099 | 2 |
| 3236.0341467 | 5 |
| 3236.0347477 | 0 |
| 3236.0361574 | 1 |
| 3236.037272 | 2 |
| 3236.0375903 | 1 |
| 3236.0391059 | 1 |
| 3236.0391504 | 3 |
| 3236.0409299 | 3 |
| 3236.0419412 | 2 |
| 3236.042439 | 4 |
| 3236.0436119 | 2 |
| 3236.0441075 | 0 |
| 3236.0451172 | 3 |
| 3236.0463706 | 3 |
| 3236.0467374 | 1 |
| 3236.0478529 | 0 |
| 3236.0486428 | 4 |
| 3236.0503457 | 5 |
| 3236.0509205 | 2 |
| 3236.0515629 | 0 |
| 3236.0526108 | 2 |
| 3236.0527497 | 1 |
| 3236.0543871 | 2 |
| 3236.0553939 | 0 |
| 3236.0556111 | 1 |
| 3236.0575183 | 0 |
| 3236.0585003 | 2 |
| 3236.0589195 | 2 |
| 3236.0600426 | 1 |
| 3236.0612873 | 1 |
| 3236.0623638 | 2 |
| 3236.0627497 | 6 |
| 3236.0638341 | 5 |
| 3236.0646881 | 2 |
| 3236.0654414 | 1 |
| 3236.066025 | 2 |
| 3236.0672346 | 0 |
| 3236.0686092 | 3 |
| 3236.0695354 | 4 |
| 3236.0700645 | 3 |
| 3236.0716629 | 1 |
| 3236.0721234 | 2 |
| 3236.0732134 | 4 |
| 3236.0745279 | 5 |
| 3236.0752614 | 1 |
| 3236.0760463 | 1 |
| 3236.0767156 | 2 |
| 3236.0776955 | 1 |
| 3236.0790165 | 1 |
| 3236.079453 | 0 |
| 3236.0804725 | 6 |
| 3236.0813599 | 2 |
| 3236.0820977 | 2 |
| 3236.0827548 | 1 |
| 3236.0843392 | 1 |
| 3236.085585 | 2 |
| 3236.0862557 | 1 |
| 3236.086801 | 1 |
| 3236.0883619 | 3 |
| 3236.0885398 | 1 |
| 3236.0895842 | 4 |
| 3236.091163 | 2 |
| 3236.0916832 | 0 |
| 3236.09245 | 0 |
| 3236.0938829 | 4 |
| 3236.0942191 | 2 |
| 3236.0951084 | 1 |
| 3236.0961184 | 6 |
| 3236.0976426 | 1 |
| 3236.0982462 | 2 |
| 3236.099253 | 3 |
| 3236.0993689 | 3 |
| 3236.1011395 | 3 |
| 3236.1015934 | 3 |
| 3236.1022162 | 1 |
| 3236.1039932 | 3 |
| 3236.1043385 | 1 |
| 3236.1053711 | 1 |
| 3236.1062879 | 4 |
| 3236.106795 | 0 |
| 3236.1084496 | 5 |
| 3236.1089391 | 2 |
| 3236.1106103 | 1 |
| 3236.1106834 | 0 |
| 3236.1118063 | 3 |
| 3236.1132557 | 3 |
| 3236.1142289 | 1 |
| 3236.1148797 | 0 |
| 3236.1162483 | 0 |
| 3236.1166499 | 1 |
| 3236.1174501 | 4 |
| 3236.1183797 | 2 |
| 3236.1201689 | 4 |
| 3236.1208644 | 3 |
| 3236.1214758 | 0 |
| 3236.1223177 | 2 |
| 3236.1226213 | 3 |
| 3236.124266 | 1 |
| 3236.1252552 | 2 |
| 3236.1258967 | 0 |
| 3236.1267308 | 2 |
| 3236.1279914 | 0 |
| 3236.1287278 | 1 |
| 3236.1300867 | 1 |
| 3236.1304144 | 0 |
| 3236.1322939 | 3 |
| 3236.1324643 | 2 |
| 3236.1327837 | 7 |
| 3236.1344437 | 6 |
| 3236.1354893 | 5 |
| 3236.136238 | 5 |
| 3236.1373204 | 2 |
| 3236.1379597 | 2 |
| 3236.1390489 | 1 |
| 3236.1397842 | 1 |
| 3236.1413097 | 2 |
| 3236.1415007 | 4 |
| 3236.1428738 | 2 |
| 3236.1438231 | 2 |
| 3236.1443699 | 2 |
| 3236.1459244 | 4 |
| 3236.1462663 | 2 |
| 3236.1474677 | 1 |
| 3236.1486937 | 3 |
| 3236.1489641 | 3 |
| 3236.1505447 | 0 |
| 3236.1515621 | 4 |
| 3236.1519655 | 3 |
| 3236.1528546 | 1 |
| 3236.1532447 | 0 |
| 3236.1545236 | 2 |
| 3236.1560961 | 1 |
| 3236.156485 | 2 |
| 3236.1580197 | 3 |
| 3236.15839 | 2 |
| 3236.1591323 | 0 |
| 3236.1597308 | 5 |
| 3236.1612184 | 1 |
| 3236.162383 | 1 |
| 3236.1636071 | 4 |
| 3236.1645821 | 1 |
| 3236.1650782 | 2 |
| 3236.1667051 | 2 |
| 3236.1677496 | 1 |
| 3236.1677725 | 0 |
| 3236.168721 | 3 |
| 3236.1692231 | 4 |
| 3236.1710997 | 5 |
| 3236.1724193 | 3 |
| 3236.1724607 | 3 |
| 3236.1739036 | 1 |
| 3236.1749077 | 3 |
| 3236.1755492 | 3 |
| 3236.1758233 | 4 |
| 3236.177081 | 0 |
| 3236.178484 | 0 |
| 3236.1789092 | 3 |
| 3236.1803834 | 2 |
| 3236.1810509 | 2 |
| 3236.1812982 | 2 |
| 3236.1827311 | 2 |
| 3236.1836245 | 1 |
| 3236.1845653 | 2 |
| 3236.1854866 | 1 |
| 3236.1864341 | 2 |
| 3236.18768 | 6 |
| 3236.1885031 | 1 |
| 3236.189337 | 2 |
| 3236.1903364 | 2 |
| 3236.1905267 | 4 |
| 3236.1921464 | 0 |

| | |
|---|---|
| 3236.1928195 | 1 |
| 3236.1942867 | 2 |
| 3236.1955117 | 1 |
| 3236.1955299 | 4 |
| 3236.196764 | 1 |
| 3236.197611 | 1 |
| 3236.1984427 | 2 |
| 3236.1996612 | 1 |
| 3236.2008274 | 2 |
| 3236.2019792 | 0 |
| 3236.2020574 | 3 |
| 3236.2042531 | 1 |
| 3236.2043533 | 4 |
| 3236.2053769 | 1 |
| 3236.2062161 | 3 |
| 3236.2069945 | 2 |
| 3236.2086192 | 0 |
| 3236.2094981 | 3 |
| 3236.2102549 | 2 |
| 3236.2110949 | 2 |
| 3236.2118356 | 2 |
| 3236.2126387 | 1 |
| 3236.2137998 | 1 |
| 3236.2145371 | 4 |
| 3236.2156703 | 0 |
| 3236.2159617 | 5 |
| 3236.217418 | 0 |
| 3236.2190512 | 1 |
| 3236.2191294 | 4 |
| 3236.2205991 | 3 |
| 3236.2212775 | 1 |
| 3236.2225596 | 3 |
| 3236.2228296 | 2 |
| 3236.2247149 | 3 |
| 3236.2255492 | 7 |
| 3236.225852 | 1 |
| 3236.2270914 | 1 |
| 3236.2273034 | 0 |
| 3236.2287684 | 3 |
| 3236.2297406 | 2 |
| 3236.230266 | 0 |
| 3236.2314917 | 1 |
| 3236.2325331 | 0 |
| 3236.2333497 | 1 |
| 3236.2342288 | 1 |
| 3236.2346344 | 2 |
| 3236.2364983 | 1 |
| 3236.2372738 | 1 |
| 3236.2377536 | 1 |
| 3236.2389145 | 1 |
| 3236.2396579 | 4 |
| 3236.2408618 | 1 |
| 3236.2422908 | 0 |
| 3236.243216 | 1 |
| 3236.2434665 | 0 |
| 3236.2446221 | 1 |
| 3236.245261 | 2 |
| 3236.2470076 | 0 |
| 3236.2471509 | 3 |
| 3236.2486822 | 1 |
| 3236.2489256 | 0 |
| 3236.2504459 | 1 |
| 3236.2515587 | 2 |
| 3236.2518269 | 2 |
| 3236.2533507 | 3 |
| 3236.2536942 | 2 |
| 3236.2548024 | 1 |
| 3236.255575 | 2 |
| 3236.2567319 | 2 |
| 3236.2580281 | 0 |
| 3236.2585554 | 1 |
| 3236.2596543 | 1 |
| 3236.2609595 | 4 |
| 3236.2613522 | 0 |
| 3236.2628553 | 2 |
| 3236.2633054 | 3 |
| 3236.2644066 | 1 |
| 3236.26561 | 1 |
| 3236.2662566 | 1 |
| 3236.2671896 | 2 |
| 3236.2679845 | 1 |
| 3236.268571 | 4 |
| 3236.2700209 | 2 |
| 3236.2704742 | 0 |
| 3236.27201 | 1 |
| 3236.2729316 | 3 |
| 3236.2735258 | 1 |
| 3236.274925 | 3 |
| 3236.2752042 | 3 |
| 3236.2762588 | 1 |
| 3236.2773069 | 2 |
| 3236.2783042 | 4 |
| 3236.2794621 | 1 |
| 3236.2800524 | 1 |
| 3236.2816771 | 3 |
| 3236.2824371 | 0 |
| 3236.2827478 | 1 |
| 3236.284094 | 2 |
| 3236.2841117 | 5 |
| 3236.2859664 | 1 |
| 3236.287094 | 2 |
| 3236.287646 | 0 |
| 3236.2889106 | 1 |
| 3236.2895071 | 5 |
| 3236.2908369 | 2 |
| 3236.2915179 | 1 |
| 3236.2919231 | 1 |
| 3236.29356 | 0 |
| 3236.2940294 | 0 |
| 3236.2950515 | 2 |
| 3236.2962438 | 5 |
| 3236.2966469 | 1 |
| 3236.2981585 | 1 |
| 3236.2988852 | 0 |
| 3236.3000575 | 1 |
| 3236.3011315 | 1 |
| 3236.3021745 | 1 |
| 3236.3032505 | 1 |
| 3236.303597 | 0 |
| 3236.3044337 | 3 |
| 3236.3062554 | 3 |
| 3236.3063199 | 3 |
| 3236.3075667 | 2 |
| 3236.3088272 | 3 |
| 3236.3094797 | 2 |
| 3236.3099625 | 1 |
| 3236.3112161 | 1 |
| 3236.3125569 | 1 |
| 3236.3128251 | 4 |
| 3236.3141222 | 1 |
| 3236.3145331 | 1 |
| 3236.3159091 | 0 |
| 3236.3169527 | 3 |
| 3236.3176588 | 0 |
| 3236.3192277 | 0 |
| 3236.3197342 | 3 |
| 3236.3205453 | 1 |
| 3236.3212811 | 1 |
| 3236.3225616 | 3 |
| 3236.3234612 | 1 |
| 3236.3243153 | 1 |
| 3236.3256566 | 6 |
| 3236.3266323 | 4 |
| 3236.3273432 | 3 |
| 3236.3282118 | 1 |
| 3236.3288611 | 1 |
| 3236.3302775 | 2 |
| 3236.3312026 | 3 |
| 3236.3317849 | 2 |
| 3236.3323561 | 4 |
| 3236.3332667 | 2 |
| 3236.3344687 | 2 |
| 3236.3356635 | 1 |
| 3236.3360626 | 1 |
| 3236.3381018 | 1 |
| 3236.3385889 | 3 |
| 3236.3392625 | 2 |
| 3236.340646 | 0 |
| 3236.3407721 | 0 |
| 3236.3421657 | 2 |
| 3236.342667 | 3 |
| 3236.3437539 | 1 |
| 3236.34528 | 0 |
| 3236.3462955 | 1 |
| 3236.3470957 | 1 |
| 3236.3478742 | 3 |
| 3236.3488151 | 2 |
| 3236.3502106 | 1 |
| 3236.3505069 | 1 |
| 3236.3517212 | 3 |
| 3236.3524775 | 2 |
| 3236.3533651 | 1 |
| 3236.3547644 | 0 |
| 3236.3549633 | 0 |
| 3236.356451 | 2 |
| 3236.3567537 | 2 |
| 3236.3584584 | 1 |
| 3236.3593035 | 1 |
| 3236.3599617 | 2 |
| 3236.3609428 | 0 |
| 3236.3617544 | 1 |
| 3236.3627661 | 1 |
| 3236.3638588 | 1 |
| 3236.3647308 | 3 |
| 3236.3661341 | 2 |
| 3236.3662967 | 1 |
| 3236.3670393 | 3 |
| 3236.3683493 | 3 |
| 3236.3683626 | 1 |
| 3236.370318 | 1 |
| 3236.3709633 | 0 |
| 3236.3717404 | 1 |
| 3236.3724629 | 5 |
| 3236.3744971 | 1 |
| 3236.3750339 | 1 |
| 3236.375953 | 2 |
| 3236.3770483 | 2 |
| 3236.3774621 | 0 |
| 3236.3787073 | 2 |
| 3236.3797097 | 1 |
| 3236.3803194 | 1 |
| 3236.3812517 | 2 |
| 3236.3829121 | 2 |
| 3236.3831257 | 3 |
| 3236.384401 | 8 |
| 3236.3849313 | 2 |
| 3236.3855428 | 2 |
| 3236.3874113 | 0 |
| 3236.3878814 | 0 |
| 3236.3885471 | 2 |
| 3236.390319 | 3 |
| 3236.3904341 | 3 |
| 3236.3918211 | 4 |
| 3236.3925211 | 2 |
| 3236.3941306 | 2 |
| 3236.3941824 | 2 |
| 3236.3959508 | 4 |
| 3236.3966268 | 2 |
| 3236.3970186 | 0 |
| 3236.3982902 | 2 |
| 3236.3988818 | 1 |
| 3236.4002745 | 4 |
| 3236.4008522 | 3 |
| 3236.402633 | 4 |
| 3236.4037612 | 3 |
| 3236.4038908 | 3 |
| 3236.4051268 | 4 |
| 3236.4061788 | 0 |
| 3236.4068783 | 2 |
| 3236.4080717 | 0 |
| 3236.4082069 | 5 |
| 3236.4096327 | 1 |
| 3236.4100169 | 3 |
| 3236.4111418 | 2 |
| 3236.4124291 | 3 |
| 3236.4130357 | 2 |
| 3236.4145617 | 5 |
| 3236.4148181 | 1 |
| 3236.4161192 | 1 |
| 3236.4172788 | 3 |
| 3236.4179653 | 2 |
| 3236.4186954 | 1 |
| 3236.4199711 | 5 |
| 3236.420188 | 1 |
| 3236.4216664 | 0 |
| 3236.4223083 | 2 |
| 3236.4238141 | 2 |
| 3236.4245692 | 3 |
| 3236.4250692 | 4 |
| 3236.4265314 | 3 |
| 3236.4270985 | 1 |
| 3236.4283561 | 0 |
| 3236.4288228 | 2 |
| 3236.4298092 | 2 |
| 3236.4316728 | 2 |
| 3236.4317265 | 3 |
| 3236.4323547 | 1 |
| 3236.4335149 | 1 |
| 3236.4346107 | 0 |
| 3236.435894 | 0 |
| 3236.4367486 | 0 |
| 3236.4381141 | 4 |
| 3236.438537 | 3 |
| 3236.4396541 | 1 |
| 3236.4406148 | 1 |
| 3236.4412007 | 0 |
| 3236.4419907 | 1 |
| 3236.4434731 | 2 |
| 3236.4447529 | 3 |
| 3236.44556 | 0 |
| 3236.4457848 | 2 |
| 3236.447081 | 2 |
| 3236.4475829 | 0 |
| 3236.4488248 | 2 |
| 3236.4500434 | 0 |
| 3236.4505103 | 2 |
| 3236.4520642 | 4 |
| 3236.4523173 | 5 |
| 3236.453508 | 1 |
| 3236.4544649 | 0 |
| 3236.455081 | 2 |
| 3236.4561097 | 0 |
| 3236.4563657 | 2 |
| 3236.4580607 | 1 |
| 3236.4589063 | 3 |
| 3236.4597051 | 0 |
| 3236.460858 | 3 |
| 3236.4617892 | 2 |
| 3236.4630432 | 4 |
| 3236.4634574 | 6 |
| 3236.4645212 | 0 |
| 3236.4654599 | 2 |
| 3236.4666416 | 2 |
| 3236.467521 | 1 |
| 3236.4681991 | 3 |
| 3236.4691446 | 4 |
| 3236.4704902 | 3 |
| 3236.4705743 | 1 |
| 3236.4722419 | 1 |
| 3236.4727895 | 1 |
| 3236.4742792 | 2 |
| 3236.4755964 | 2 |
| 3236.4758532 | 1 |
| 3236.4766457 | 0 |
| 3236.4770833 | 0 |
| 3236.4779262 | 1 |
| 3236.4795994 | 0 |
| 3236.4798246 | 3 |
| 3236.4813673 | 1 |
| 3236.4820858 | 4 |
| 3236.4835794 | 0 |
| 3236.484313 | 0 |
| 3236.4847318 | 2 |
| 3236.486666 | 3 |
| 3236.4868963 | 1 |
| 3236.4878897 | 2 |
| 3236.4894299 | 0 |
| 3236.4894455 | 2 |
| 3236.4905707 | 2 |
| 3236.4913709 | 1 |
| 3236.4924953 | 1 |
| 3236.4933341 | 4 |
| 3236.4947453 | 2 |
| 3236.495365 | 0 |
| 3236.4960577 | 2 |
| 3236.4975754 | 2 |
| 3236.4978273 | 0 |
| 3236.4991703 | 0 |
| 3236.500249 | 3 |
| 3236.5002668 | 0 |
| 3236.5013047 | 1 |
| 3236.5027441 | 0 |
| 3236.5028552 | 3 |
| 3236.5043141 | 2 |
| 3236.5054898 | 2 |
| 3236.5064367 | 1 |
| 3236.5076283 | 1 |
| 3236.5083408 | 3 |
| 3236.5090987 | 3 |
| 3236.5095277 | 2 |
| 3236.5115206 | 1 |
| 3236.5120739 | 4 |
| 3236.5125113 | 6 |
| 3236.5134416 | 2 |
| 3236.5142118 | 0 |
| 3236.5154207 | 0 |
| 3236.5169687 | 3 |
| 3236.5174816 | 0 |
| 3236.5182339 | 3 |
| 3236.5187784 | 2 |
| 3236.5204456 | 2 |
| 3236.5205968 | 0 |
| 3236.5223321 | 3 |
| 3236.5232358 | 1 |
| 3236.5238886 | 0 |
| 3236.5247864 | 1 |
| 3236.5259 | 2 |
| 3236.5271117 | 5 |
| 3236.5281443 | 2 |
| 3236.5286912 | 1 |
| 3236.5295169 | 0 |
| 3236.5299565 | 2 |
| 3236.5310913 | 1 |

| | |
|---|---|
| 3236.5325545 | 1 |
| 3236.5333649 | 3 |
| 3236.5343584 | 2 |
| 3236.5347473 | 0 |
| 3236.5359333 | 0 |
| 3236.537103 | 2 |
| 3236.5376397 | 5 |
| 3236.5385545 | 2 |
| 3236.5397212 | 0 |
| 3236.5404543 | 2 |
| 3236.5414923 | 3 |
| 3236.5422432 | 2 |
| 3236.5431579 | 5 |
| 3236.5438039 | 3 |
| 3236.5452107 | 0 |
| 3236.5462001 | 0 |
| 3236.5467645 | 2 |
| 3236.5479232 | 3 |
| 3236.5484886 | 2 |
| 3236.5496103 | 2 |
| 3236.5509926 | 1 |
| 3236.5514371 | 1 |
| 3236.5524581 | 1 |
| 3236.5528347 | 3 |
| 3236.5544596 | 0 |
| 3236.5554655 | 0 |
| 3236.5560746 | 0 |
| 3236.5567743 | 2 |
| 3236.5579726 | 0 |
| 3236.558509 | 1 |
| 3236.5597841 | 2 |
| 3236.5606309 | 0 |
| 3236.5616723 | 0 |
| 3236.5629133 | 0 |
| 3236.5630622 | 2 |
| 3236.564606 | 0 |
| 3236.5655573 | 5 |
| 3236.5659383 | 1 |
| 3236.5675785 | 3 |
| 3236.5680902 | 2 |
| 3236.5696082 | 1 |
| 3236.5696659 | 1 |
| 3236.5708174 | 3 |
| 3236.5721117 | 1 |
| 3236.5723133 | 1 |
| 3236.5737853 | 2 |
| 3236.5740713 | 0 |
| 3236.5756795 | 4 |
| 3236.5768054 | 1 |
| 3236.5770116 | 0 |
| 3236.5781817 | 2 |
| 3236.5782213 | 1 |
| 3236.5802598 | 0 |
| 3236.581652 | 0 |
| 3236.5818367 | 3 |
| 3236.5828784 | 2 |
| 3236.5836189 | 1 |
| 3236.5847168 | 3 |
| 3236.5859871 | 2 |
| 3236.5862226 | 1 |
| 3236.587857 | 4 |
| 3236.5879284 | 3 |
| 3236.5897176 | 1 |
| 3236.5903139 | 0 |
| 3236.5912358 | 1 |
| 3236.5924534 | 3 |
| 3236.5930073 | 2 |
| 3236.5939663 | 1 |
| 3236.5949892 | 0 |
| 3236.5954217 | 1 |
| 3236.5963801 | 5 |
| 3236.5976205 | 2 |
| 3236.5982625 | 1 |
| 3236.599533 | 2 |
| 3236.6000848 | 2 |
| 3236.6014368 | 1 |
| 3236.6019049 | 4 |
| 3236.6030024 | 2 |
| 3236.6039594 | 1 |
| 3236.6047625 | 1 |
| 3236.6062431 | 6 |
| 3236.6062992 | 3 |
| 3236.6077635 | 1 |
| 3236.6086518 | 2 |
| 3236.6095204 | 0 |
| 3236.6110042 | 3 |
| 3236.6116829 | 3 |
| 3236.6128737 | 1 |
| 3236.6136983 | 2 |
| 3236.6144775 | 3 |
| 3236.6152953 | 0 |
| 3236.6156217 | 3 |
| 3236.6169753 | 2 |
| 3236.6170883 | 2 |
| 3236.6183821 | 2 |
| 3236.619009 | 1 |
| 3236.6199899 | 2 |
| 3236.6212915 | 3 |
| 3236.6225153 | 2 |
| 3236.6233613 | 5 |
| 3236.6247627 | 1 |
| 3236.625499 | 1 |
| 3236.6258533 | 1 |
| 3236.6273649 | 1 |
| 3236.6283521 | 3 |
| 3236.6287353 | 3 |
| 3236.6290369 | 2 |
| 3236.630835 | 2 |
| 3236.6315393 | 3 |
| 3236.6316484 | 1 |
| 3236.6334014 | 4 |
| 3236.6347095 | 2 |
| 3236.6351865 | 4 |
| 3236.6362545 | 2 |
| 3236.6372992 | 1 |
| 3236.6382979 | 1 |
| 3236.6387218 | 1 |
| 3236.6397617 | 4 |
| 3236.6407777 | 4 |
| 3236.6415435 | 2 |
| 3236.6427139 | 2 |
| 3236.6435591 | 2 |
| 3236.6450734 | 0 |
| 3236.6451073 | 0 |
| 3236.6462766 | 2 |
| 3236.6466324 | 6 |
| 3236.6468428 | 2 |
| 3236.6476546 | 1 |
| 3236.6477219 | 0 |
| 3236.648226 | 2 |
| 3236.6483109 | 1 |
| 3236.6489212 | 1 |
| 3236.6491578 | 1 |
| 3236.6494886 | 3 |
| 3236.649573 | 3 |
| 3236.6505401 | 4 |
| 3236.6508905 | 2 |
| 3236.6513146 | 0 |
| 3236.6514416 | 1 |
| 3236.6520435 | 2 |
| 3236.6523079 | 3 |
| 3236.6525026 | 3 |
| 3236.6529572 | 1 |
| 3236.653207 | 1 |
| 3236.6536559 | 1 |
| 3236.6538584 | 1 |
| 3236.6544427 | 5 |
| 3236.6545793 | 3 |
| 3236.6549553 | 2 |
| 3236.6554172 | 2 |
| 3236.6556404 | 0 |
| 3236.6563508 | 1 |
| 3236.6569658 | 2 |
| 3236.6570777 | 1 |
| 3236.6572328 | 2 |
| 3236.6572587 | 4 |
| 3236.6582629 | 1 |
| 3236.6584136 | 1 |
| 3236.6588733 | 2 |
| 3236.6596721 | 1 |
| 3236.6599821 | 0 |
| 3236.6603998 | 1 |
| 3236.6605376 | 1 |
| 3236.6606522 | 1 |
| 3236.6609376 | 2 |
| 3236.6609638 | 0 |
| 3236.6617777 | 2 |
| 3236.6620082 | 0 |
| 3236.662524 | 2 |
| 3236.6631776 | 1 |
| 3236.6632736 | 3 |
| 3236.6632979 | 2 |
| 3236.6636944 | 2 |
| 3236.6644474 | 0 |
| 3236.6646997 | 1 |
| 3236.6651892 | 2 |
| 3236.6654979 | 2 |
| 3236.6655226 | 0 |
| 3236.665783 | 0 |
| 3236.666266 | 2 |
| 3236.6665596 | 1 |
| 3236.6667887 | 0 |
| 3236.667296 | 4 |
| 3236.6674239 | 2 |
| 3236.6678407 | 1 |
| 3236.6680672 | 1 |
| 3236.6687563 | 2 |
| 3236.6691944 | 1 |
| 3236.6697814 | 2 |
| 3236.6700225 | 2 |
| 3236.6701737 | 1 |
| 3236.6702675 | 5 |
| 3236.6714098 | 2 |
| 3236.6714423 | 0 |
| 3236.6722328 | 2 |
| 3236.6723295 | 2 |
| 3236.6730296 | 3 |
| 3236.6732702 | 1 |
| 3236.6737054 | 0 |
| 3236.6737876 | 3 |
| 3236.6740849 | 4 |
| 3236.6749462 | 1 |
| 3236.6755508 | 1 |
| 3236.6760178 | 2 |
| 3236.6760907 | 1 |
| 3236.6762138 | 1 |
| 3236.6764115 | 2 |
| 3236.677026 | 1 |
| 3236.6770292 | 2 |
| 3236.6771749 | 1 |
| 3236.6777721 | 3 |
| 3236.6785725 | 0 |
| 3236.6789184 | 1 |
| 3236.6794659 | 1 |
| 3236.6798036 | 2 |
| 3236.6800261 | 1 |
| 3236.6800596 | 0 |
| 3236.6801855 | 1 |
| 3236.6809685 | 0 |
| 3236.6811761 | 1 |
| 3236.6815186 | 0 |
| 3236.6818891 | 1 |
| 3236.6829769 | 3 |
| 3236.6830999 | 2 |
| 3236.6835503 | 3 |
| 3236.6838498 | 2 |
| 3236.6842218 | 2 |
| 3236.6842693 | 1 |
| 3236.6846687 | 2 |
| 3236.6851906 | 2 |
| 3236.6854085 | 1 |
| 3236.686325 | 1 |
| 3236.6864257 | 4 |
| 3236.6865249 | 4 |
| 3236.6868241 | 2 |
| 3236.6872535 | 0 |
| 3236.687629 | 2 |
| 3236.6876957 | 0 |
| 3236.6883028 | 2 |
| 3236.6890893 | 3 |
| 3236.6893666 | 2 |
| 3236.6893996 | 1 |
| 3236.6899961 | 0 |
| 3236.6904202 | 3 |
| 3236.6908731 | 0 |
| 3236.6910038 | 1 |
| 3236.6914278 | 0 |
| 3236.6917749 | 4 |
| 3236.69211 | 4 |
| 3236.6922845 | 3 |
| 3236.6926874 | 3 |
| 3236.6930416 | 1 |
| 3236.6935764 | 6 |
| 3236.6936384 | 1 |
| 3236.6946207 | 6 |
| 3236.6947102 | 8 |
| 3236.6953419 | 3 |
| 3236.6953818 | 2 |
| 3236.6957422 | 1 |
| 3236.6964686 | 3 |
| 3236.6966604 | 4 |
| 3236.6967709 | 1 |
| 3236.6972531 | 2 |
| 3236.6977576 | 2 |
| 3236.698214 | 0 |
| 3236.6984856 | 3 |
| 3236.6986742 | 2 |
| 3236.6990311 | 3 |
| 3236.6997976 | 1 |
| 3236.6999662 | 1 |
| 3236.7004849 | 2 |
| 3236.7008946 | 0 |
| 3236.7013116 | 3 |
| 3236.7016606 | 1 |
| 3236.7020089 | 1 |
| 3236.7022446 | 7 |
| 3236.7028211 | 0 |
| 3236.7028468 | 2 |
| 3236.7028682 | 3 |
| 3236.7040673 | 2 |
| 3236.7042123 | 0 |
| 3236.7045041 | 2 |
| 3236.7051933 | 1 |
| 3236.7052811 | 2 |
| 3236.7058956 | 1 |
| 3236.7059023 | 2 |
| 3236.7067142 | 0 |
| 3236.7071746 | 4 |
| 3236.7071791 | 3 |
| 3236.7074334 | 1 |
| 3236.7077257 | 1 |
| 3236.7083285 | 1 |
| 3236.7084491 | 1 |
| 3236.7092398 | 1 |
| 3236.7093243 | 3 |
| 3236.7099516 | 0 |
| 3236.7099541 | 4 |
| 3236.7102562 | 4 |
| 3236.7106732 | 1 |
| 3236.7113853 | 4 |
| 3236.7114664 | 1 |
| 3236.71159 | 2 |
| 3236.7123627 | 0 |
| 3236.7126436 | 1 |
| 3236.7132999 | 2 |
| 3236.713532 | 1 |
| 3236.7143009 | 1 |
| 3236.7146114 | 1 |
| 3236.7149556 | 0 |
| 3236.7152955 | 4 |
| 3236.7155456 | 7 |
| 3236.7155957 | 4 |
| 3236.7158689 | 0 |
| 3236.716011 | 1 |
| 3236.7160546 | 0 |
| 3236.7169857 | 0 |
| 3236.7177558 | 2 |
| 3236.7177923 | 1 |
| 3236.7181767 | 4 |
| 3236.7186864 | 3 |
| 3236.7187458 | 0 |
| 3236.7188027 | 1 |
| 3236.7193887 | 3 |
| 3236.7196397 | 1 |
| 3236.720527 | 5 |
| 3236.7207077 | 1 |
| 3236.7209704 | 0 |
| 3236.7211433 | 1 |
| 3236.7216134 | 1 |
| 3236.7221947 | 2 |
| 3236.7228502 | 2 |
| 3236.7231284 | 4 |
| 3236.7240247 | 0 |
| 3236.7240525 | 2 |
| 3236.7241806 | 2 |
| 3236.7242446 | 0 |
| 3236.7250997 | 1 |
| 3236.7254908 | 2 |
| 3236.7258562 | 2 |
| 3236.7259211 | 3 |
| 3236.7262543 | 1 |
| 3236.7270396 | 2 |
| 3236.7271269 | 0 |
| 3236.7271644 | 0 |
| 3236.7280613 | 1 |
| 3236.7282521 | 0 |
| 3236.7287053 | 3 |
| 3236.7293207 | 0 |
| 3236.7296776 | 1 |
| 3236.7299711 | 1 |
| 3236.7301402 | 2 |
| 3236.7303474 | 2 |
| 3236.7309402 | 1 |
| 3236.7314336 | 1 |
| 3236.7322518 | 0 |
| 3236.7322819 | 1 |
| 3236.732588 | 1 |
| 3236.7331343 | 4 |
| 3236.7332653 | 2 |
| 3236.7335453 | 3 |
| 3236.7335701 | 1 |
| 3236.7339204 | 1 |
| 3236.7348816 | 2 |

| | |
|---|---|
| 3236.7351402 | 0 |
| 3236.7353472 | 0 |
| 3236.7358653 | 0 |
| 3236.7364113 | 0 |
| 3236.736634 | 3 |
| 3236.736857 | 4 |
| 3236.7371233 | 2 |
| 3236.7374049 | 2 |
| 3236.7377626 | 0 |
| 3236.7378486 | 0 |
| 3236.7381999 | 3 |
| 3236.7389598 | 0 |
| 3236.7395513 | 4 |
| 3236.7397504 | 1 |
| 3236.739848 | 4 |
| 3236.7400918 | 1 |
| 3236.7408967 | 1 |
| 3236.7413916 | 3 |
| 3236.7414876 | 3 |
| 3236.7419959 | 0 |
| 3236.7423953 | 1 |
| 3236.7424175 | 0 |
| 3236.7430158 | 1 |
| 3236.7436792 | 1 |
| 3236.7439666 | 1 |
| 3236.744031 | 0 |
| 3236.744949 | 1 |
| 3236.7451135 | 1 |
| 3236.7456428 | 4 |
| 3236.7458433 | 0 |
| 3236.7461338 | 0 |
| 3236.7464188 | 5 |
| 3236.7469612 | 1 |
| 3236.746973 | 1 |
| 3236.7480589 | 2 |
| 3236.7482221 | 2 |
| 3236.7484861 | 1 |
| 3236.7489556 | 0 |
| 3236.7491146 | 1 |
| 3236.7492178 | 2 |
| 3236.7500093 | 5 |
| 3236.7502108 | 1 |
| 3236.7508865 | 2 |
| 3236.7512274 | 2 |
| 3236.7512421 | 1 |
| 3236.7512958 | 2 |
| 3236.7522534 | 1 |
| 3236.7523276 | 2 |
| 3236.7536383 | 0 |
| 3236.7537297 | 3 |
| 3236.7537977 | 3 |
| 3236.7540522 | 0 |
| 3236.7547187 | 3 |
| 3236.7550816 | 0 |
| 3236.755134 | 4 |
| 3236.7553444 | 1 |
| 3236.7561616 | 3 |
| 3236.7564503 | 1 |
| 3236.7566587 | 1 |
| 3236.7567077 | 7 |
| 3236.757423 | 1 |
| 3236.7577997 | 1 |
| 3236.758029 | 1 |
| 3236.7585672 | 2 |
| 3236.7592389 | 1 |
| 3236.7593385 | 1 |
| 3236.7597585 | 1 |
| 3236.7602699 | 1 |
| 3236.7605287 | 1 |
| 3236.7609928 | 4 |
| 3236.7612989 | 2 |
| 3236.761659 | 3 |
| 3236.7622676 | 2 |
| 3236.7626577 | 1 |
| 3236.7629871 | 2 |
| 3236.7631451 | 2 |
| 3236.763518 | 2 |
| 3236.7638329 | 2 |
| 3236.76415 | 1 |
| 3236.7652355 | 3 |
| 3236.7652897 | 2 |
| 3236.7653673 | 1 |
| 3236.7656351 | 1 |
| 3236.7660073 | 1 |
| 3236.7666293 | 1 |
| 3236.7667327 | 0 |
| 3236.7673655 | 3 |
| 3236.7678709 | 0 |
| 3236.767932 | 1 |
| 3236.768837 | 2 |
| 3236.7689789 | 2 |
| 3236.7693679 | 2 |
| 3236.7695042 | 1 |
| 3236.7697191 | 0 |
| 3236.7704542 | 0 |
| 3236.7704944 | 3 |
| 3236.7714026 | 1 |
| 3236.7717028 | 3 |
| 3236.7718851 | 0 |
| 3236.7724114 | 0 |
| 3236.7728808 | 1 |
| 3236.77302 | 1 |
| 3236.7732011 | 3 |
| 3236.7732643 | 3 |
| 3236.7743798 | 0 |
| 3236.7745544 | 0 |
| 3236.7748143 | 0 |
| 3236.7749592 | 4 |
| 3236.7753379 | 0 |
| 3236.7759622 | 1 |
| 3236.7761491 | 1 |
| 3236.7764159 | 1 |
| 3236.7768458 | 0 |
| 3236.777106 | 2 |
| 3236.7774434 | 2 |
| 3236.777523 | 1 |
| 3236.7776502 | 1 |
| 3236.7788664 | 4 |
| 3236.7790372 | 1 |
| 3236.7792521 | 0 |
| 3236.7800436 | 2 |
| 3236.7801841 | 1 |
| 3236.7802551 | 1 |
| 3236.7808756 | 1 |
| 3236.7812337 | 1 |
| 3236.7819405 | 1 |
| 3236.7821018 | 0 |
| 3236.7826607 | 3 |
| 3236.7828969 | 2 |
| 3236.7830166 | 0 |
| 3236.7836221 | 0 |
| 3236.7841364 | 1 |
| 3236.7841441 | 6 |
| 3236.7843921 | 0 |
| 3236.7853703 | 0 |
| 3236.7854581 | 2 |
| 3236.7857813 | 0 |
| 3236.7863727 | 2 |
| 3236.7865282 | 1 |
| 3236.7871507 | 1 |
| 3236.7872733 | 3 |
| 3236.7873543 | 3 |
| 3236.7878246 | 4 |
| 3236.7882384 | 1 |
| 3236.7889137 | 2 |
| 3236.7890999 | 3 |
| 3236.7894939 | 0 |
| 3236.7896211 | 3 |
| 3236.7901369 | 1 |
| 3236.7907977 | 2 |
| 3236.7909939 | 4 |
| 3236.7912569 | 0 |
| 3236.7917315 | 1 |
| 3236.7922382 | 0 |
| 3236.7927996 | 1 |
| 3236.7929528 | 3 |
| 3236.7936427 | 3 |
| 3236.7938515 | 2 |
| 3236.7939462 | 2 |
| 3236.7943103 | 0 |
| 3236.79521 | 1 |
| 3236.7952947 | 2 |
| 3236.7960012 | 2 |
| 3236.7961424 | 3 |
| 3236.7964088 | 2 |
| 3236.7964907 | 1 |
| 3236.7971478 | 2 |
| 3236.797468 | 2 |
| 3236.7977723 | 1 |
| 3236.7981009 | 0 |
| 3236.7984185 | 0 |
| 3236.7991745 | 2 |
| 3236.7993954 | 1 |
| 3236.7994352 | 0 |
| 3236.8002987 | 1 |
| 3236.8003313 | 2 |
| 3236.800908 | 2 |
| 3236.8010296 | 2 |
| 3236.8016998 | 1 |
| 3236.8022346 | 3 |
| 3236.8022947 | 0 |
| 3236.8031429 | 2 |
| 3236.8035357 | 1 |
| 3236.8035408 | 3 |
| 3236.8041064 | 2 |
| 3236.8041266 | 3 |
| 3236.8048794 | 1 |
| 3236.8051374 | 0 |
| 3236.805529 | 1 |
| 3236.8059361 | 3 |
| 3236.8060078 | 1 |
| 3236.8067695 | 3 |
| 3236.8068006 | 1 |
| 3236.8072747 | 5 |
| 3236.8077439 | 1 |
| 3236.8080155 | 1 |
| 3236.8082161 | 1 |
| 3236.8082976 | 2 |
| 3236.809252 | 1 |
| 3236.8100984 | 4 |
| 3236.8101896 | 1 |
| 3236.8104976 | 2 |
| 3236.8105893 | 2 |
| 3236.8109143 | 0 |
| 3236.8110563 | 2 |
| 3236.8113528 | 4 |
| 3236.8123583 | 2 |
| 3236.8124756 | 2 |
| 3236.8130102 | 0 |
| 3236.8130309 | 2 |
| 3236.8131541 | 2 |
| 3236.8143198 | 2 |
| 3236.8144787 | 0 |
| 3236.8146618 | 5 |
| 3236.814989 | 3 |
| 3236.8153651 | 3 |
| 3236.815979 | 4 |
| 3236.8160625 | 0 |
| 3236.8166292 | 4 |
| 3236.8171833 | 1 |
| 3236.8172181 | 0 |
| 3236.8172665 | 1 |
| 3236.8178254 | 1 |
| 3236.8182893 | 1 |
| 3236.8187499 | 2 |
| 3236.8190077 | 1 |
| 3236.8199227 | 3 |
| 3236.8200915 | 2 |
| 3236.8203524 | 0 |
| 3236.8209084 | 1 |
| 3236.8216186 | 4 |
| 3236.8217839 | 0 |
| 3236.8217872 | 1 |
| 3236.8220506 | 1 |
| 3236.8226319 | 0 |
| 3236.8230575 | 2 |
| 3236.8232385 | 0 |
| 3236.8236512 | 3 |
| 3236.8239389 | 1 |
| 3236.8246986 | 3 |
| 3236.8247051 | 1 |
| 3236.8247879 | 2 |
| 3236.8258806 | 2 |
| 3236.8262915 | 0 |
| 3236.8263119 | 3 |
| 3236.8264218 | 0 |
| 3236.8270835 | 2 |
| 3236.827755 | 0 |
| 3236.8278435 | 0 |
| 3236.8281151 | 2 |
| 3236.8287516 | 2 |
| 3236.8288611 | 3 |
| 3236.8293025 | 3 |
| 3236.8294585 | 0 |
| 3236.8297919 | 2 |
| 3236.8303866 | 1 |
| 3236.8309612 | 0 |
| 3236.8310309 | 1 |
| 3236.8310802 | 0 |
| 3236.8316421 | 2 |
| 3236.8325964 | 2 |
| 3236.8330722 | 0 |
| 3236.8332598 | 0 |
| 3236.8333726 | 2 |
| 3236.8338884 | 3 |
| 3236.8344486 | 2 |
| 3236.8345135 | 1 |
| 3236.835075 | 0 |
| 3236.8355986 | 3 |
| 3236.8356381 | 0 |
| 3236.8359925 | 0 |
| 3236.8365324 | 1 |
| 3236.8368117 | 3 |
| 3236.8372934 | 1 |
| 3236.8374058 | 2 |
| 3236.8374097 | 1 |
| 3236.8387389 | 0 |
| 3236.8389625 | 0 |
| 3236.838994 | 2 |
| 3236.8396714 | 3 |
| 3236.8399651 | 0 |
| 3236.8404923 | 1 |
| 3236.8407143 | 0 |
| 3236.8410541 | 4 |
| 3236.8411802 | 0 |
| 3236.8413441 | 2 |
| 3236.8417849 | 2 |
| 3236.8420263 | 2 |
| 3236.8426099 | 2 |
| 3236.8436208 | 2 |
| 3236.843701 | 2 |
| 3236.8440404 | 3 |
| 3236.8448074 | 0 |
| 3236.8449823 | 0 |
| 3236.8450462 | 2 |
| 3236.8451266 | 0 |
| 3236.8460117 | 1 |
| 3236.8462372 | 1 |
| 3236.8467588 | 1 |
| 3236.8469541 | 2 |
| 3236.8472792 | 3 |
| 3236.8478603 | 2 |
| 3236.8478996 | 1 |
| 3236.8480727 | 2 |
| 3236.8489579 | 2 |
| 3236.849916 | 0 |
| 3236.8500249 | 2 |
| 3236.8501469 | 2 |
| 3236.8505979 | 0 |
| 3236.8508501 | 2 |
| 3236.8511384 | 3 |
| 3236.8516737 | 1 |
| 3236.8520012 | 2 |
| 3236.8520587 | 1 |
| 3236.8529054 | 1 |
| 3236.8530392 | 1 |
| 3236.8535185 | 1 |
| 3236.8539642 | 2 |
| 3236.8546945 | 3 |
| 3236.8548438 | 0 |
| 3236.8549615 | 0 |
| 3236.8554609 | 1 |
| 3236.855886 | 2 |
| 3236.8559469 | 1 |
| 3236.8567721 | 1 |
| 3236.8570144 | 2 |
| 3236.8570552 | 0 |
| 3236.8572892 | 1 |
| 3236.8582405 | 0 |
| 3236.8583705 | 1 |
| 3236.8585938 | 2 |
| 3236.8593749 | 1 |
| 3236.8596519 | 1 |
| 3236.8600795 | 1 |
| 3236.8607098 | 0 |
| 3236.8608441 | 1 |
| 3236.8609011 | 0 |
| 3236.8611994 | 2 |
| 3236.8616719 | 4 |
| 3236.8618214 | 0 |
| 3236.8623916 | 1 |
| 3236.8626463 | 1 |
| 3236.8628621 | 2 |
| 3236.8636937 | 1 |
| 3236.8640253 | 0 |
| 3236.86403 | 3 |
| 3236.8648128 | 4 |
| 3236.8648618 | 2 |
| 3236.8652114 | 2 |
| 3236.8656248 | 2 |
| 3236.866093 | 1 |
| 3236.8662322 | 3 |
| 3236.8667948 | 2 |
| 3236.8669771 | 0 |
| 3236.8677444 | 2 |
| 3236.8684941 | 1 |
| 3236.8686435 | 1 |
| 3236.8688182 | 2 |
| 3236.8690044 | 3 |
| 3236.8694037 | 1 |
| 3236.8700527 | 1 |
| 3236.8702384 | 2 |
| 3236.8707321 | 3 |

| | |
|---|---|
| 3236.8711743 | 0 |
| 3236.8715957 | 1 |
| 3236.8724179 | 5 |
| 3236.8724673 | 1 |
| 3236.8724796 | 3 |
| 3236.8726208 | 1 |
| 3236.873616 | 3 |
| 3236.8736176 | 0 |
| 3236.8740489 | 2 |
| 3236.8748431 | 1 |
| 3236.874857 | 4 |
| 3236.8755481 | 0 |
| 3236.8756945 | 1 |
| 3236.8757849 | 1 |
| 3236.8765773 | 2 |
| 3236.8767366 | 2 |
| 3236.877149 | 2 |
| 3236.8775414 | 4 |
| 3236.8777053 | 1 |
| 3236.8783137 | 2 |
| 3236.8789265 | 1 |
| 3236.8792415 | 4 |
| 3236.87955 | 1 |
| 3236.8796248 | 6 |
| 3236.8799349 | 2 |
| 3236.8806976 | 1 |
| 3236.8814436 | 1 |
| 3236.8816438 | 2 |
| 3236.8816768 | 0 |
| 3236.8818295 | 1 |
| 3236.882442 | 0 |
| 3236.8825905 | 2 |
| 3236.8826972 | 0 |
| 3236.8827942 | 3 |
| 3236.8833762 | 0 |
| 3236.8833949 | 1 |
| 3236.8835155 | 1 |
| 3236.8835732 | 1 |
| 3236.8838012 | 2 |
| 3236.884528 | 1 |
| 3236.8846978 | 0 |
| 3236.8848368 | 3 |
| 3236.88487 | 2 |
| 3236.8852704 | 1 |
| 3236.8852868 | 2 |
| 3236.8854441 | 0 |
| 3236.8858902 | 1 |
| 3236.8860597 | 0 |
| 3236.8862552 | 1 |
| 3236.8864741 | 3 |
| 3236.886858 | 3 |
| 3236.8870376 | 1 |
| 3236.8873686 | 1 |
| 3236.887473 | 2 |
| 3236.8878232 | 3 |
| 3236.8879961 | 4 |
| 3236.8880568 | 0 |
| 3236.8880597 | 1 |
| 3236.8884797 | 1 |
| 3236.8887109 | 2 |
| 3236.8890398 | 2 |
| 3236.8893146 | 1 |
| 3236.8895844 | 1 |
| 3236.8896936 | 3 |
| 3236.8897095 | 0 |
| 3236.8899599 | 2 |
| 3236.8903366 | 3 |
| 3236.8903957 | 2 |
| 3236.8905944 | 1 |
| 3236.8906631 | 0 |
| 3236.890714 | 0 |
| 3236.8909894 | 2 |
| 3236.8910459 | 4 |
| 3236.891119 | 2 |
| 3236.8915266 | 2 |
| 3236.8916681 | 0 |
| 3236.8917667 | 3 |
| 3236.8920051 | 1 |
| 3236.8920701 | 1 |
| 3236.8923139 | 2 |
| 3236.8925022 | 5 |
| 3236.89259 | 3 |
| 3236.8926347 | 0 |
| 3236.8927063 | 1 |
| 3236.8927562 | 1 |
| 3236.8928301 | 1 |
| 3236.8928724 | 2 |
| 3236.8931011 | 2 |
| 3236.8932596 | 0 |
| 3236.8934642 | 2 |
| 3236.8936562 | 3 |
| 3236.8936882 | 1 |
| 3236.8940484 | 2 |
| 3236.8942887 | 2 |
| 3236.8943497 | 2 |
| 3236.8944112 | 0 |
| 3236.8944116 | 2 |
| 3236.8946957 | 3 |
| 3236.8947755 | 3 |
| 3236.8948766 | 1 |
| 3236.8950012 | 0 |
| 3236.8952388 | 3 |
| 3236.8955134 | 2 |
| 3236.8957624 | 3 |
| 3236.8960369 | 2 |
| 3236.8961208 | 2 |
| 3236.8961219 | 2 |
| 3236.8962931 | 1 |
| 3236.8963391 | 3 |
| 3236.8964698 | 4 |
| 3236.8965613 | 3 |
| 3236.8966129 | 2 |
| 3236.897064 | 1 |
| 3236.8971078 | 1 |
| 3236.8974291 | 0 |
| 3236.8975445 | 1 |
| 3236.8976586 | 2 |
| 3236.8978229 | 0 |
| 3236.8979259 | 2 |
| 3236.8980881 | 1 |
| 3236.8981683 | 0 |
| 3236.898191 | 3 |
| 3236.8984465 | 1 |
| 3236.8985776 | 2 |
| 3236.8986707 | 1 |
| 3236.8988171 | 0 |
| 3236.8988439 | 1 |
| 3236.898861 | 1 |
| 3236.8988745 | 2 |
| 3236.8992779 | 1 |
| 3236.8994977 | 1 |
| 3236.8995976 | 1 |
| 3236.8997378 | 4 |
| 3236.8998513 | 1 |
| 3236.8999596 | 1 |
| 3236.8999725 | 2 |
| 3236.9001007 | 0 |
| 3236.900114 | 1 |
| 3236.9003001 | 1 |
| 3236.9003132 | 2 |
| 3236.9004199 | 1 |
| 3236.9005297 | 4 |
| 3236.9006431 | 2 |
| 3236.9007245 | 2 |
| 3236.9007783 | 0 |
| 3236.9008055 | 1 |
| 3236.9009121 | 1 |
| 3236.9009143 | 3 |
| 3236.9009638 | 0 |
| 3236.9009847 | 4 |
| 3236.9010666 | 2 |
| 3236.9010977 | 2 |
| 3236.9011034 | 2 |
| 3236.9013173 | 1 |
| 3236.9013316 | 2 |
| 3236.9013817 | 5 |
| 3236.9016287 | 0 |
| 3236.9017078 | 0 |
| 3236.9017483 | 3 |
| 3236.9019432 | 2 |
| 3236.9020809 | 1 |
| 3236.9020939 | 0 |
| 3236.9021521 | 2 |
| 3236.9021766 | 3 |
| 3236.9022243 | 1 |
| 3236.9023311 | 2 |
| 3236.9024103 | 3 |
| 3236.9024573 | 2 |
| 3236.9025748 | 1 |
| 3236.9027033 | 2 |
| 3236.9027383 | 0 |
| 3236.9027397 | 1 |
| 3236.9027708 | 1 |
| 3236.9029212 | 0 |
| 3236.9030654 | 2 |
| 3236.9031431 | 3 |
| 3236.9032044 | 2 |
| 3236.9033601 | 5 |
| 3236.903556 | 3 |
| 3236.9036684 | 0 |
| 3236.9037398 | 1 |
| 3236.903782 | 1 |
| 3236.9038392 | 3 |
| 3236.9038587 | 4 |
| 3236.9039472 | 0 |
| 3236.9040619 | 2 |
| 3236.9041141 | 1 |
| 3236.9041496 | 2 |
| 3236.9043303 | 1 |
| 3236.9044086 | 0 |
| 3236.9044186 | 3 |
| 3236.9044705 | 2 |
| 3236.9045806 | 0 |
| 3236.90462 | 1 |
| 3236.9048642 | 0 |
| 3236.9050382 | 0 |
| 3236.905039 | 1 |
| 3236.9050856 | 5 |
| 3236.9050975 | 4 |
| 3236.9051786 | 0 |
| 3236.9051943 | 3 |
| 3236.9051974 | 1 |
| 3236.9053125 | 0 |
| 3236.9053334 | 4 |
| 3236.905755 | 1 |
| 3236.9059379 | 2 |
| 3236.9059637 | 1 |
| 3236.9060305 | 3 |
| 3236.9060517 | 3 |
| 3236.9061369 | 2 |
| 3236.9062657 | 2 |
| 3236.9063384 | 4 |
| 3236.9064547 | 1 |
| 3236.9065444 | 1 |
| 3236.9065528 | 0 |
| 3236.9065735 | 1 |
| 3236.9065999 | 0 |
| 3236.906617 | 2 |
| 3236.906634 | 2 |
| 3236.9066718 | 1 |
| 3236.9067711 | 1 |
| 3236.906928 | 1 |
| 3236.9070759 | 0 |
| 3236.9071274 | 2 |
| 3236.9072667 | 3 |
| 3236.907314 | 2 |
| 3236.9073422 | 4 |
| 3236.9075385 | 1 |
| 3236.9077049 | 4 |
| 3236.9077991 | 1 |
| 3236.9079276 | 2 |
| 3236.907938 | 1 |
| 3236.9079931 | 3 |
| 3236.9080009 | 2 |
| 3236.9081144 | 2 |
| 3236.9081863 | 2 |
| 3236.9082095 | 2 |
| 3236.9082619 | 1 |
| 3236.9082954 | 3 |
| 3236.9083686 | 2 |
| 3236.9084884 | 3 |
| 3236.9085107 | 1 |
| 3236.9086389 | 6 |
| 3236.9087445 | 2 |
| 3236.9089293 | 6 |
| 3236.909018 | 8 |
| 3236.9091435 | 7 |
| 3236.9091485 | 10 |
| 3236.9091616 | 4 |
| 3236.9092312 | 9 |
| 3236.9093432 | 5 |
| 3236.9094577 | 5 |
| 3236.9096051 | 3 |
| 3236.9097677 | 12 |
| 3236.9098358 | 7 |
| 3236.9099334 | 1 |
| 3236.9100438 | 12 |
| 3236.9102756 | 10 |
| 3236.9102795 | 6 |
| 3236.910364 | 15 |
| 3236.910525 | 5 |
| 3236.9105395 | 7 |
| 3236.9106091 | 12 |
| 3236.9106585 | 8 |
| 3236.9106771 | 11 |
| 3236.9107266 | 8 |
| 3236.910757 | 11 |
| 3236.9110452 | 7 |
| 3236.9110572 | 10 |
| 3236.9111198 | 11 |
| 3236.9111599 | 10 |
| 3236.9112499 | 7 |
| 3236.9112741 | 11 |
| 3236.9112883 | 11 |
| 3236.9113812 | 8 |
| 3236.9115436 | 14 |
| 3236.9116855 | 8 |
| 3236.9118366 | 8 |
| 3236.9118858 | 9 |
| 3236.9119034 | 10 |
| 3236.91195 | 8 |
| 3236.9119677 | 13 |
| 3236.9120385 | 13 |
| 3236.9120545 | 15 |
| 3236.9120745 | 9 |
| 3236.9122111 | 9 |
| 3236.91222 | 4 |
| 3236.9124793 | 4 |
| 3236.912662 | 7 |
| 3236.9128965 | 3 |
| 3236.9129518 | 7 |
| 3236.9130297 | 5 |
| 3236.9130439 | 4 |
| 3236.913219 | 4 |
| 3236.9132291 | 4 |
| 3236.9133032 | 1 |
| 3236.9133048 | 4 |
| 3236.9133167 | 4 |
| 3236.9133619 | 7 |
| 3236.9134358 | 9 |
| 3236.9134604 | 3 |
| 3236.9136043 | 7 |
| 3236.9136166 | 13 |
| 3236.9136699 | 4 |
| 3236.9137283 | 4 |
| 3236.914185 | 6 |
| 3236.9142719 | 7 |
| 3236.9142727 | 5 |
| 3236.9142834 | 6 |
| 3236.9144974 | 6 |
| 3236.9146089 | 7 |
| 3236.914635 | 9 |
| 3236.9146892 | 4 |
| 3236.9147058 | 10 |
| 3236.9147335 | 7 |
| 3236.914743 | 10 |
| 3236.9147601 | 10 |
| 3236.9149108 | 8 |
| 3236.9150644 | 6 |
| 3236.9151273 | 9 |
| 3236.9152333 | 11 |
| 3236.9152518 | 5 |
| 3236.9153006 | 19 |
| 3236.915528 | 10 |
| 3236.9155938 | 6 |
| 3236.9156706 | 11 |
| 3236.9156976 | 5 |
| 3236.9157119 | 9 |
| 3236.9157626 | 8 |
| 3236.9157746 | 6 |
| 3236.9158891 | 8 |
| 3236.9160441 | 10 |
| 3236.9161504 | 13 |
| 3236.9162008 | 8 |
| 3236.9163026 | 7 |
| 3236.9165581 | 6 |
| 3236.9166197 | 6 |
| 3236.9166417 | 5 |
| 3236.9167632 | 6 |
| 3236.9167922 | 5 |
| 3236.9168038 | 15 |
| 3236.916868 | 3 |
| 3236.9169593 | 9 |
| 3236.9169848 | 6 |
| 3236.9171494 | 7 |
| 3236.9173527 | 5 |
| 3236.9174666 | 3 |
| 3236.9174688 | 4 |
| 3236.9174719 | 6 |
| 3236.9174797 | 4 |
| 3236.9175299 | 1 |
| 3236.9175404 | 6 |
| 3236.9175427 | 9 |
| 3236.9175982 | 12 |
| 3236.9176595 | 3 |
| 3236.9176628 | 8 |
| 3236.9179116 | 13 |
| 3236.9179219 | 7 |
| 3236.9179386 | 13 |
| 3236.9180388 | 9 |
| 3236.9181784 | 8 |
| 3236.9184659 | 7 |
| 3236.9185416 | 6 |

| | |
|---|---|
| 3236.9185853 | 7 |
| 3236.918598 | 8 |
| 3236.9186405 | 6 |
| 3236.9186758 | 6 |
| 3236.9188806 | 6 |
| 3236.9190343 | 5 |
| 3236.9190749 | 6 |
| 3236.919097 | 7 |
| 3236.9191511 | 3 |
| 3236.9194639 | 9 |
| 3236.9195464 | 9 |
| 3236.9195702 | 8 |
| 3236.9196117 | 4 |
| 3236.9198201 | 3 |
| 3236.9198468 | 2 |
| 3236.9198581 | 3 |
| 3236.9199474 | 10 |
| 3236.9200276 | 7 |
| 3236.9200607 | 5 |
| 3236.9200927 | 8 |
| 3236.9200932 | 6 |
| 3236.9201109 | 9 |
| 3236.9204599 | 2 |
| 3236.9205656 | 0 |
| 3236.9205688 | 6 |
| 3236.9206108 | 4 |
| 3236.9206156 | 4 |
| 3236.9208231 | 1 |
| 3236.9208262 | 5 |
| 3236.9209159 | 4 |
| 3236.920958 | 0 |
| 3236.9209828 | 4 |
| 3236.9209831 | 2 |
| 3236.9213316 | 1 |
| 3236.9213348 | 2 |
| 3236.9214347 | 5 |
| 3236.9215117 | 6 |
| 3236.9216671 | 1 |
| 3236.9217116 | 0 |
| 3236.9218137 | 2 |
| 3236.9218296 | 3 |
| 3236.9218746 | 1 |
| 3236.9222423 | 3 |
| 3236.9222456 | 2 |
| 3236.9222502 | 2 |
| 3236.9222628 | 1 |
| 3236.9223289 | 1 |
| 3236.9223976 | 2 |
| 3236.9225288 | 3 |
| 3236.922586 | 1 |
| 3236.9226671 | 2 |
| 3236.9227812 | 2 |
| 3236.9227887 | 5 |
| 3236.9228662 | 0 |
| 3236.9228932 | 1 |
| 3236.9229382 | 2 |
| 3236.9230125 | 1 |
| 3236.9231316 | 0 |
| 3236.9233381 | 4 |
| 3236.9233617 | 2 |
| 3236.923392 | 2 |
| 3236.9234457 | 1 |
| 3236.9237543 | 1 |
| 3236.9239292 | 2 |
| 3236.9239618 | 1 |
| 3236.9239783 | 2 |
| 3236.9241101 | 3 |
| 3236.924159 | 1 |
| 3236.9242389 | 2 |
| 3236.9243481 | 0 |
| 3236.9243818 | 4 |
| 3236.9244278 | 2 |
| 3236.9245874 | 2 |
| 3236.9247767 | 1 |
| 3236.9247894 | 1 |
| 3236.9248116 | 2 |
| 3236.9248178 | 3 |
| 3236.924868 | 0 |
| 3236.9249518 | 3 |
| 3236.9250571 | 3 |
| 3236.9251253 | 2 |
| 3236.9252511 | 0 |
| 3236.925383 | 2 |
| 3236.9254968 | 1 |
| 3236.9256219 | 2 |
| 3236.9257685 | 2 |
| 3236.9258445 | 2 |
| 3236.9260589 | 2 |
| 3236.9260616 | 0 |
| 3236.9260619 | 3 |
| 3236.9261307 | 2 |
| 3236.9261697 | 0 |
| 3236.926192 | 1 |
| 3236.9262146 | 3 |
| 3236.9263029 | 0 |
| 3236.9263438 | 0 |
| 3236.9263884 | 3 |
| 3236.9264135 | 0 |
| 3236.926421 | 1 |
| 3236.9266603 | 1 |
| 3236.9268719 | 1 |
| 3236.9269341 | 2 |
| 3236.9270815 | 4 |
| 3236.9272311 | 2 |
| 3236.9273214 | 1 |
| 3236.9273799 | 3 |
| 3236.9273816 | 0 |
| 3236.927384 | 3 |
| 3236.9275698 | 0 |
| 3236.9276432 | 2 |
| 3236.9276566 | 1 |
| 3236.9277152 | 0 |
| 3236.9277489 | 0 |
| 3236.9279039 | 1 |
| 3236.9279714 | 0 |
| 3236.9280775 | 3 |
| 3236.9281058 | 5 |
| 3236.928113 | 0 |
| 3236.9282245 | 0 |
| 3236.9282758 | 0 |
| 3236.9284228 | 1 |
| 3236.928631 | 1 |
| 3236.9286316 | 2 |
| 3236.9286514 | 3 |
| 3236.9288466 | 1 |
| 3236.9289284 | 1 |
| 3236.9289324 | 1 |
| 3236.9289693 | 2 |
| 3236.929045 | 3 |
| 3236.9291797 | 3 |
| 3236.9292122 | 0 |
| 3236.929418 | 0 |
| 3236.9294731 | 3 |
| 3236.9295198 | 0 |
| 3236.9295209 | 1 |
| 3236.9295742 | 2 |
| 3236.9296314 | 0 |
| 3236.9297445 | 3 |
| 3236.9298942 | 1 |
| 3236.9303646 | 4 |
| 3236.9303667 | 2 |
| 3236.9303898 | 1 |
| 3236.9304234 | 3 |
| 3236.9305094 | 0 |
| 3236.9305434 | 2 |
| 3236.9305721 | 5 |
| 3236.9305965 | 1 |
| 3236.9306089 | 1 |
| 3236.9306812 | 3 |
| 3236.9307707 | 2 |
| 3236.9308683 | 5 |
| 3236.9309736 | 2 |
| 3236.9309805 | 1 |
| 3236.9311796 | 4 |
| 3236.9313293 | 3 |
| 3236.9314451 | 1 |
| 3236.9315223 | 1 |
| 3236.9315443 | 2 |
| 3236.9315658 | 2 |
| 3236.9315786 | 0 |
| 3236.9316023 | 0 |
| 3236.9316592 | 1 |
| 3236.931709 | 1 |
| 3236.9317282 | 0 |
| 3236.9318059 | 4 |
| 3236.9319854 | 2 |
| 3236.9320027 | 2 |
| 3236.9320402 | 1 |
| 3236.9322628 | 2 |
| 3236.9323484 | 0 |
| 3236.9327758 | 2 |
| 3236.9328001 | 0 |
| 3236.932864 | 2 |
| 3236.9330019 | 2 |
| 3236.9330383 | 2 |
| 3236.9330393 | 7 |
| 3236.9330565 | 1 |
| 3236.9330621 | 1 |
| 3236.9333502 | 2 |
| 3236.9335117 | 0 |
| 3236.9335285 | 1 |
| 3236.9335619 | 3 |
| 3236.933646 | 3 |
| 3236.9337554 | 0 |
| 3236.9338126 | 3 |
| 3236.9338659 | 0 |
| 3236.9339628 | 1 |
| 3236.9340252 | 0 |
| 3236.9340608 | 3 |
| 3236.9342563 | 0 |
| 3236.934391 | 3 |
| 3236.9346266 | 0 |
| 3236.934752 | 1 |
| 3236.9349153 | 2 |
| 3236.9350039 | 3 |
| 3236.9358069 | 1 |
| 3236.9358611 | 2 |
| 3236.9359687 | 2 |
| 3236.93598 | 1 |
| 3236.9360539 | 1 |
| 3236.9366415 | 3 |
| 3236.936933 | 2 |
| 3236.9372357 | 2 |
| 3236.9372775 | 1 |
| 3236.9373466 | 0 |
| 3236.9378766 | 1 |
| 3236.9383074 | 1 |
| 3236.9386574 | 2 |
| 3236.9386957 | 1 |
| 3236.9388129 | 3 |
| 3236.9392851 | 1 |
| 3236.9393605 | 3 |
| 3236.9393835 | 2 |
| 3236.9398738 | 2 |
| 3236.9402427 | 1 |
| 3236.9405484 | 4 |
| 3236.9406156 | 5 |
| 3236.9406953 | 0 |
| 3236.9407605 | 1 |
| 3236.9408409 | 0 |
| 3236.9410587 | 1 |
| 3236.9416089 | 2 |
| 3236.9418086 | 2 |
| 3236.9421139 | 1 |
| 3236.9424303 | 2 |
| 3236.9424676 | 0 |
| 3236.9429752 | 4 |
| 3236.9430084 | 0 |
| 3236.9430742 | 2 |
| 3236.9435844 | 0 |
| 3236.9436172 | 0 |
| 3236.9440399 | 1 |
| 3236.9444449 | 2 |
| 3236.9444757 | 3 |
| 3236.944682 | 1 |
| 3236.9447228 | 3 |
| 3236.9449321 | 0 |
| 3236.9451353 | 5 |
| 3236.9452548 | 0 |
| 3236.9455261 | 1 |
| 3236.9462837 | 2 |
| 3236.9464777 | 1 |
| 3236.9466066 | 3 |
| 3236.9467626 | 2 |
| 3236.947179 | 2 |
| 3236.9472115 | 3 |
| 3236.9473045 | 2 |
| 3236.9477337 | 1 |
| 3236.9477923 | 1 |
| 3236.9480123 | 0 |
| 3236.9485017 | 0 |
| 3236.9485039 | 2 |
| 3236.9488299 | 1 |
| 3236.9489925 | 0 |
| 3236.9492228 | 0 |
| 3236.9495396 | 0 |
| 3236.9495591 | 0 |
| 3236.9496173 | 0 |
| 3236.9501614 | 3 |
| 3236.9502165 | 1 |
| 3236.9504907 | 1 |
| 3236.9505399 | 0 |
| 3236.9510043 | 0 |
| 3236.9512159 | 3 |
| 3236.9513099 | 2 |
| 3236.9513643 | 1 |
| 3236.952326 | 1 |
| 3236.9524057 | 1 |
| 3236.9524608 | 0 |
| 3236.9529644 | 0 |
| 3236.9529709 | 4 |
| 3236.9533432 | 4 |
| 3236.9533655 | 1 |
| 3236.9540073 | 0 |
| 3236.9542629 | 1 |
| 3236.9543589 | 3 |
| 3236.9544745 | 0 |
| 3236.9546114 | 2 |
| 3236.9546513 | 2 |
| 3236.9549485 | 1 |
| 3236.9549706 | 1 |
| 3236.9551323 | 2 |
| 3236.9555343 | 2 |
| 3236.9560595 | 1 |
| 3236.9560969 | 2 |
| 3236.9562815 | 3 |
| 3236.956627 | 1 |
| 3236.9567264 | 0 |
| 3236.9573525 | 0 |
| 3236.9574042 | 1 |
| 3236.9575247 | 1 |
| 3236.9577444 | 2 |
| 3236.9578896 | 2 |
| 3236.9582352 | 1 |
| 3236.9584859 | 0 |
| 3236.958604 | 2 |
| 3236.959012 | 3 |
| 3236.959073 | 1 |
| 3236.9593206 | 2 |
| 3236.9594674 | 3 |
| 3236.9594846 | 3 |
| 3236.9600033 | 1 |
| 3236.9605127 | 2 |
| 3236.9606552 | 0 |
| 3236.9607698 | 1 |
| 3236.9608541 | 2 |
| 3236.9616009 | 1 |
| 3236.9618408 | 1 |
| 3236.9619356 | 2 |
| 3236.9620034 | 2 |
| 3236.962151 | 0 |
| 3236.9623883 | 1 |
| 3236.9625696 | 2 |
| 3236.9626862 | 4 |
| 3236.9630763 | 4 |
| 3236.9634272 | 1 |
| 3236.9636656 | 2 |
| 3236.9637483 | 1 |
| 3236.9637982 | 2 |
| 3236.9644526 | 2 |
| 3236.9647542 | 2 |
| 3236.9648131 | 1 |
| 3236.9652063 | 2 |
| 3236.9652978 | 4 |
| 3236.9656566 | 1 |
| 3236.9656632 | 0 |
| 3236.9657425 | 2 |
| 3236.9658534 | 0 |
| 3236.9661158 | 4 |
| 3236.966391 | 1 |
| 3236.9666546 | 1 |
| 3236.9669753 | 2 |
| 3236.9670726 | 3 |
| 3236.9672688 | 0 |
| 3236.9675341 | 0 |
| 3236.9676254 | 0 |
| 3236.9680082 | 0 |
| 3236.9681953 | 3 |
| 3236.968267 | 0 |
| 3236.9688471 | 1 |
| 3236.9690589 | 3 |
| 3236.9691478 | 1 |
| 3236.9693282 | 1 |
| 3236.9697094 | 2 |
| 3236.969761 | 2 |
| 3236.9698636 | 0 |
| 3236.9702891 | 1 |
| 3236.9704302 | 4 |
| 3236.9706243 | 2 |
| 3236.9713964 | 1 |
| 3236.9715943 | 4 |
| 3236.9717097 | 1 |
| 3236.9719846 | 4 |
| 3236.9722234 | 1 |
| 3236.9722894 | 0 |
| 3236.9727198 | 0 |
| 3236.9730472 | 2 |
| 3236.9730523 | 2 |
| 3236.9732611 | 2 |
| 3236.9732789 | 1 |
| 3236.9733854 | 2 |
| 3236.9737884 | 2 |
| 3236.9742415 | 4 |

| | |
|---|---|
| 3236.9744281 | 4 |
| 3236.9747595 | 1 |
| 3236.9749155 | 1 |
| 3236.9749412 | 2 |
| 3236.9749792 | 0 |
| 3236.9756289 | 1 |
| 3236.9758158 | 2 |
| 3236.9760057 | 1 |
| 3236.9762307 | 0 |
| 3236.9762585 | 1 |
| 3236.9764458 | 2 |
| 3236.9769148 | 2 |
| 3236.9770422 | 3 |
| 3236.9775251 | 1 |
| 3236.9777213 | 0 |
| 3236.9778204 | 0 |
| 3236.9778581 | 1 |
| 3236.9786446 | 2 |
| 3236.9786955 | 3 |
| 3236.9788024 | 3 |
| 3236.9793899 | 4 |
| 3236.9795139 | 0 |
| 3236.979527 | 1 |
| 3236.9795845 | 3 |
| 3236.9800271 | 4 |
| 3236.9801386 | 1 |
| 3236.9805715 | 2 |
| 3236.9807659 | 0 |
| 3236.9810025 | 0 |
| 3236.9814012 | 0 |
| 3236.9814502 | 1 |
| 3236.9814973 | 5 |
| 3236.9815217 | 0 |
| 3236.9820803 | 2 |
| 3236.9825477 | 2 |
| 3236.9826492 | 2 |
| 3236.9828578 | 1 |
| 3236.983035 | 2 |
| 3236.9830936 | 0 |
| 3236.9832092 | 1 |
| 3236.9834565 | 2 |
| 3236.9836231 | 0 |
| 3236.9842991 | 3 |
| 3236.9845094 | 0 |
| 3236.9848929 | 0 |
| 3236.9849247 | 1 |
| 3236.9852287 | 1 |
| 3236.9853299 | 1 |
| 3236.9855144 | 2 |
| 3236.9861438 | 1 |
| 3236.9862624 | 1 |
| 3236.9863285 | 1 |
| 3236.9865166 | 1 |
| 3236.9866469 | 1 |
| 3236.9870962 | 0 |
| 3236.9871313 | 1 |
| 3236.9876028 | 2 |
| 3236.9876521 | 3 |
| 3236.9878968 | 2 |
| 3236.9879051 | 1 |
| 3236.9882934 | 2 |
| 3236.988806 | 0 |
| 3236.9888973 | 1 |
| 3236.9890608 | 0 |
| 3236.9891943 | 2 |
| 3236.9895608 | 1 |
| 3236.9895704 | 1 |
| 3236.9900295 | 3 |
| 3236.9900381 | 0 |
| 3236.9904235 | 3 |
| 3236.9907544 | 1 |
| 3236.990778 | 1 |
| 3236.9908456 | 3 |
| 3236.9912571 | 2 |
| 3236.9914666 | 3 |
| 3236.9919944 | 3 |
| 3236.9921941 | 1 |
| 3236.9922127 | 1 |
| 3236.9923781 | 1 |
| 3236.9928956 | 1 |
| 3236.9929272 | 3 |
| 3236.9931829 | 3 |
| 3236.9933272 | 1 |
| 3236.9936429 | 1 |
| 3236.9939826 | 0 |
| 3236.9940961 | 2 |
| 3236.99427 | 4 |
| 3236.99458 | 4 |
| 3236.9948555 | 2 |
| 3236.9949202 | 3 |
| 3236.9951621 | 1 |
| 3236.9954877 | 1 |
| 3236.9958721 | 1 |
| 3236.9958914 | 2 |
| 3236.9965068 | 4 |
| 3236.9967408 | 3 |
| 3236.996755 | 3 |
| 3236.9969966 | 0 |
| 3236.9973246 | 0 |
| 3236.9974751 | 1 |
| 3236.9976406 | 2 |
| 3236.9977541 | 0 |
| 3236.9981112 | 1 |
| 3236.9985036 | 2 |
| 3236.9985896 | 1 |
| 3236.9987111 | 1 |
| 3236.9988071 | 1 |
| 3236.9992546 | 2 |
| 3236.9995631 | 1 |
| 3236.9996997 | 2 |
| 3236.9998126 | 3 |
| 3237.0001381 | 2 |
| 3237.0002443 | 1 |
| 3237.0004375 | 0 |
| 3237.0008744 | 4 |
| 3237.0009937 | 2 |
| 3237.0013823 | 1 |
| 3237.0015274 | 2 |
| 3237.0015605 | 0 |
| 3237.0023575 | 3 |
| 3237.0025012 | 1 |
| 3237.0025287 | 3 |
| 3237.0030691 | 1 |
| 3237.0034716 | 2 |
| 3237.00353 | 1 |
| 3237.0036705 | 0 |
| 3237.0037103 | 0 |
| 3237.0042159 | 1 |
| 3237.0044531 | 0 |
| 3237.0046455 | 1 |
| 3237.0047231 | 2 |
| 3237.0050587 | 2 |
| 3237.0051222 | 1 |
| 3237.005339 | 1 |
| 3237.0053537 | 0 |
| 3237.0058161 | 1 |
| 3237.0062358 | 0 |
| 3237.0064256 | 1 |
| 3237.0065522 | 2 |
| 3237.0066003 | 1 |
| 3237.0070222 | 1 |
| 3237.0074422 | 2 |
| 3237.0075459 | 0 |
| 3237.0077634 | 1 |
| 3237.0079012 | 3 |
| 3237.0081425 | 1 |
| 3237.0087051 | 2 |
| 3237.0088149 | 2 |
| 3237.0088513 | 2 |
| 3237.0088757 | 1 |
| 3237.009312 | 2 |
| 3237.0094367 | 1 |
| 3237.0099496 | 2 |
| 3237.0099627 | 2 |
| 3237.010196 | 0 |
| 3237.0105225 | 1 |
| 3237.0107914 | 1 |
| 3237.0109047 | 0 |
| 3237.0117415 | 1 |
| 3237.0118842 | 3 |
| 3237.012011 | 0 |
| 3237.0122729 | 2 |
| 3237.012582 | 1 |
| 3237.0128141 | 5 |
| 3237.0129372 | 1 |
| 3237.0130346 | 3 |
| 3237.0135095 | 2 |
| 3237.0137846 | 3 |
| 3237.0141417 | 2 |
| 3237.014174 | 1 |
| 3237.0142846 | 2 |
| 3237.0142854 | 0 |
| 3237.0150622 | 2 |
| 3237.0150885 | 0 |
| 3237.015318 | 1 |
| 3237.0153528 | 2 |
| 3237.0155507 | 1 |
| 3237.0157862 | 2 |
| 3237.0159193 | 2 |
| 3237.0162772 | 3 |
| 3237.0168283 | 3 |
| 3237.0169094 | 1 |
| 3237.0170389 | 2 |
| 3237.0170661 | 1 |
| 3237.0178323 | 2 |
| 3237.0178444 | 2 |
| 3237.0182036 | 1 |
| 3237.0183425 | 1 |
| 3237.0198255 | 0 |
| 3237.0206444 | 4 |
| 3237.0211454 | 5 |
| 3237.0222338 | 3 |
| 3237.0236497 | 3 |
| 3237.0239989 | 2 |
| 3237.0250973 | 1 |
| 3237.0252822 | 2 |
| 3237.0270512 | 3 |
| 3237.0279886 | 5 |
| 3237.028504 | 1 |
| 3237.0295231 | 1 |
| 3237.0307562 | 1 |
| 3237.031081 | 0 |
| 3237.0323214 | 1 |
| 3237.0335885 | 1 |
| 3237.0344015 | 2 |
| 3237.0348796 | 2 |
| 3237.0366826 | 0 |
| 3237.037591 | 3 |
| 3237.0384039 | 4 |
| 3237.0394891 | 3 |
| 3237.03983 | 1 |
| 3237.0416762 | 2 |
| 3237.0426146 | 1 |
| 3237.0426579 | 4 |
| 3237.0437009 | 6 |
| 3237.044483 | 2 |
| 3237.0450187 | 2 |
| 3237.0450469 | 3 |
| 3237.0456623 | 4 |
| 3237.0457583 | 0 |
| 3237.0470394 | 0 |
| 3237.047044 | 1 |
| 3237.0482247 | 2 |
| 3237.048618 | 2 |
| 3237.0491893 | 2 |
| 3237.049904 | 1 |
| 3237.0500892 | 1 |
| 3237.0507937 | 2 |
| 3237.0513192 | 1 |
| 3237.0518851 | 1 |
| 3237.0519917 | 1 |
| 3237.0524075 | 0 |
| 3237.0531034 | 1 |
| 3237.0538858 | 3 |
| 3237.0544218 | 2 |
| 3237.0544676 | 0 |
| 3237.0555358 | 1 |
| 3237.0556988 | 1 |
| 3237.0570235 | 1 |
| 3237.0571765 | 2 |
| 3237.0573114 | 2 |
| 3237.0582099 | 2 |
| 3237.0590206 | 4 |
| 3237.0597517 | 3 |
| 3237.0597973 | 0 |
| 3237.0605971 | 0 |
| 3237.0607756 | 0 |
| 3237.0618606 | 1 |
| 3237.0621406 | 0 |
| 3237.0621445 | 1 |
| 3237.0635222 | 3 |
| 3237.0643883 | 2 |
| 3237.06587 | 2 |
| 3237.0664668 | 4 |
| 3237.0683175 | 1 |
| 3237.0688096 | 1 |
| 3237.070121 | 1 |
| 3237.0714886 | 2 |
| 3237.072715 | 3 |
| 3237.0741973 | 3 |
| 3237.0743957 | 2 |
| 3237.0768472 | 2 |
| 3237.0779075 | 3 |
| 3237.0792556 | 3 |
| 3237.0803826 | 1 |
| 3237.0812757 | 1 |
| 3237.0826182 | 2 |
| 3237.0844985 | 0 |
| 3237.0851107 | 3 |
| 3237.0869465 | 1 |
| 3237.087982 | 0 |
| 3237.0896058 | 4 |
| 3237.0911417 | 1 |
| 3237.0912159 | 1 |
| 3237.0926416 | 1 |
| 3237.0947991 | 2 |
| 3237.0953348 | 3 |
| 3237.0966754 | 0 |
| 3237.0977272 | 4 |
| 3237.0991775 | 1 |
| 3237.0998319 | 2 |
| 3237.1017834 | 1 |
| 3237.1029965 | 3 |
| 3237.1041599 | 1 |
| 3237.1053774 | 2 |
| 3237.1056321 | 0 |
| 3237.1067267 | 0 |
| 3237.108727 | 0 |
| 3237.1096101 | 1 |
| 3237.1105812 | 5 |
| 3237.1122398 | 3 |
| 3237.1137202 | 4 |
| 3237.1151639 | 5 |
| 3237.1159911 | 2 |
| 3237.1176498 | 1 |
| 3237.1179179 | 1 |
| 3237.1198588 | 3 |
| 3237.1214243 | 4 |
| 3237.1221843 | 0 |
| 3237.1231584 | 0 |
| 3237.1248435 | 3 |
| 3237.1258553 | 0 |
| 3237.1274854 | 0 |
| 3237.1280216 | 3 |
| 3237.129567 | 2 |
| 3237.131456 | 3 |
| 3237.1319549 | 1 |
| 3237.1340002 | 4 |
| 3237.1344702 | 0 |
| 3237.1359713 | 4 |
| 3237.1369603 | 0 |
| 3237.1384498 | 3 |
| 3237.1396301 | 1 |
| 3237.141295 | 4 |
| 3237.1425789 | 2 |
| 3237.144113 | 3 |
| 3237.1448447 | 2 |
| 3237.1459606 | 0 |
| 3237.1471191 | 0 |
| 3237.1481571 | 1 |
| 3237.1490917 | 0 |
| 3237.1507665 | 3 |
| 3237.152135 | 2 |
| 3237.1526759 | 4 |
| 3237.1545276 | 4 |
| 3237.1554961 | 5 |
| 3237.1567183 | 0 |
| 3237.1589372 | 2 |
| 3237.1592395 | 1 |
| 3237.1608504 | 1 |
| 3237.1617306 | 0 |
| 3237.1630449 | 2 |
| 3237.1646496 | 2 |
| 3237.1651223 | 1 |
| 3237.1667536 | 4 |
| 3237.1679605 | 3 |
| 3237.1698406 | 2 |
| 3237.1703829 | 4 |
| 3237.1718339 | 0 |
| 3237.1735552 | 0 |
| 3237.1739368 | 0 |
| 3237.1755758 | 1 |
| 3237.1760651 | 1 |
| 3237.1782104 | 2 |
| 3237.1799988 | 1 |
| 3237.1805685 | 0 |
| 3237.1819974 | 2 |
| 3237.1831074 | 1 |
| 3237.1848807 | 0 |
| 3237.1853325 | 0 |
| 3237.1872498 | 1 |
| 3237.1882594 | 3 |
| 3237.1890081 | 2 |
| 3237.1909798 | 1 |
| 3237.1924115 | 1 |
| 3237.1929427 | 4 |
| 3237.1946447 | 3 |
| 3237.1949433 | 1 |
| 3237.1967611 | 3 |
| 3237.1983536 | 1 |
| 3237.1991715 | 2 |
| 3237.200616 | 3 |
| 3237.2013673 | 3 |

| | |
|---|---|
| 3237.2028326 | 3 |
| 3237.2042127 | 2 |
| 3237.2050493 | 5 |
| 3237.2067102 | 2 |
| 3237.2075479 | 1 |
| 3237.209851 | 1 |
| 3237.2100147 | 1 |
| 3237.2115622 | 0 |
| 3237.2129412 | 2 |
| 3237.213898 | 4 |
| 3237.2153366 | 2 |
| 3237.2158531 | 3 |
| 3237.2178844 | 0 |
| 3237.2197218 | 0 |
| 3237.2201521 | 3 |
| 3237.2215663 | 4 |
| 3237.2225057 | 2 |
| 3237.2236955 | 0 |
| 3237.2249526 | 1 |
| 3237.2269243 | 3 |
| 3237.228309 | 1 |
| 3237.2283335 | 1 |
| 3237.230545 | 1 |
| 3237.2310699 | 4 |
| 3237.2329533 | 2 |
| 3237.23407 | 2 |
| 3237.2350325 | 5 |
| 3237.2366429 | 2 |
| 3237.2379423 | 3 |
| 3237.238105 | 4 |
| 3237.2400024 | 5 |
| 3237.2410349 | 0 |
| 3237.2425527 | 2 |
| 3237.2443252 | 5 |
| 3237.2447136 | 2 |
| 3237.2462181 | 3 |
| 3237.2472579 | 0 |
| 3237.2484358 | 3 |
| 3237.25051 | 4 |
| 3237.25075 | 2 |
| 3237.2521119 | 2 |
| 3237.2532904 | 1 |
| 3237.2548845 | 0 |
| 3237.2558022 | 3 |
| 3237.2575773 | 1 |
| 3237.2593274 | 2 |
| 3237.2597152 | 5 |
| 3237.2618994 | 3 |
| 3237.2622037 | 2 |
| 3237.2632824 | 1 |
| 3237.2656593 | 2 |
| 3237.2662932 | 5 |
| 3237.2676123 | 3 |
| 3237.268587 | 4 |
| 3237.2702996 | 1 |
| 3237.2714294 | 3 |
| 3237.2724573 | 2 |
| 3237.2737189 | 3 |
| 3237.2743448 | 0 |
| 3237.2772001 | 4 |
| 3237.2779914 | 1 |
| 3237.278485 | 2 |
| 3237.2805037 | 1 |
| 3237.2813006 | 1 |
| 3237.2830224 | 1 |
| 3237.2835582 | 3 |
| 3237.2849298 | 3 |
| 3237.286622 | 4 |
| 3237.2872392 | 1 |
| 3237.2888085 | 4 |
| 3237.2897687 | 3 |
| 3237.2914411 | 1 |
| 3237.2931417 | 2 |
| 3237.2934595 | 3 |
| 3237.2945734 | 2 |
| 3237.2959539 | 3 |
| 3237.297238 | 3 |
| 3237.2985846 | 1 |
| 3237.299739 | 5 |
| 3237.301165 | 1 |
| 3237.3031373 | 2 |
| 3237.3034348 | 4 |
| 3237.3054159 | 3 |
| 3237.305606 | 3 |
| 3237.3070496 | 1 |
| 3237.3082449 | 3 |
| 3237.3099286 | 1 |
| 3237.3109971 | 2 |
| 3237.3125378 | 0 |
| 3237.3140951 | 3 |
| 3237.3149328 | 3 |
| 3237.316487 | 2 |
| 3237.3176409 | 3 |
| 3237.3183454 | 0 |
| 3237.320147 | 2 |
| 3237.3220729 | 3 |
| 3237.3229386 | 4 |
| 3237.3237155 | 3 |
| 3237.3249474 | 0 |
| 3237.3259424 | 1 |
| 3237.3277625 | 4 |
| 3237.328452 | 2 |
| 3237.3302954 | 2 |
| 3237.3308432 | 3 |
| 3237.3324039 | 0 |
| 3237.3336335 | 0 |
| 3237.3347742 | 1 |
| 3237.3363018 | 3 |
| 3237.3364779 | 1 |
| 3237.3389159 | 3 |
| 3237.3393056 | 3 |
| 3237.3405532 | 2 |
| 3237.3425459 | 2 |
| 3237.3431236 | 1 |
| 3237.3447304 | 1 |
| 3237.3458795 | 3 |
| 3237.3476158 | 2 |
| 3237.3485812 | 3 |
| 3237.3497113 | 2 |
| 3237.3508823 | 2 |
| 3237.3528834 | 1 |
| 3237.3538563 | 3 |
| 3237.3546348 | 3 |
| 3237.3563059 | 3 |
| 3237.3579083 | 3 |
| 3237.358318 | 0 |
| 3237.3595195 | 0 |
| 3237.3617536 | 2 |
| 3237.3621175 | 2 |
| 3237.3640237 | 1 |
| 3237.3647731 | 2 |
| 3237.3663495 | 5 |
| 3237.3675116 | 3 |
| 3237.3680529 | 2 |
| 3237.3700468 | 3 |
| 3237.3704473 | 0 |
| 3237.3727421 | 3 |
| 3237.3737602 | 2 |
| 3237.3744309 | 1 |
| 3237.3765982 | 4 |
| 3237.3770191 | 2 |
| 3237.3784594 | 2 |
| 3237.3792397 | 2 |
| 3237.3804231 | 2 |
| 3237.381981 | 2 |
| 3237.3826197 | 1 |
| 3237.3846703 | 2 |
| 3237.3861925 | 3 |
| 3237.3873118 | 1 |
| 3237.3883239 | 1 |
| 3237.3889672 | 5 |
| 3237.390581 | 1 |
| 3237.3916843 | 1 |
| 3237.393584 | 0 |
| 3237.3949017 | 3 |
| 3237.3962991 | 0 |
| 3237.3970253 | 2 |
| 3237.397588 | 0 |
| 3237.3999914 | 1 |
| 3237.4010445 | 1 |
| 3237.4019656 | 4 |
| 3237.4034801 | 1 |
| 3237.4041299 | 1 |
| 3237.4059385 | 6 |
| 3237.4071668 | 3 |
| 3237.408824 | 2 |
| 3237.4101349 | 1 |
| 3237.4110843 | 4 |
| 3237.4124191 | 2 |
| 3237.4135361 | 1 |
| 3237.4147441 | 2 |
| 3237.4152434 | 1 |
| 3237.4166355 | 5 |
| 3237.4187026 | 1 |
| 3237.4192462 | 2 |
| 3237.420028 | 1 |
| 3237.4215848 | 2 |
| 3237.4226373 | 3 |
| 3237.4246177 | 2 |
| 3237.425988 | 1 |
| 3237.4269378 | 4 |
| 3237.4277521 | 2 |
| 3237.4300371 | 3 |
| 3237.4300965 | 4 |
| 3237.43138 | 3 |
| 3237.4330884 | 1 |
| 3237.4338132 | 1 |
| 3237.4354457 | 3 |
| 3237.4370652 | 3 |
| 3237.437562 | 2 |
| 3237.4393306 | 2 |
| 3237.4406562 | 3 |
| 3237.4416239 | 2 |
| 3237.4426104 | 1 |
| 3237.4442155 | 2 |
| 3237.4461914 | 1 |
| 3237.4466862 | 1 |
| 3237.4484741 | 1 |
| 3237.4488235 | 2 |
| 3237.4503538 | 2 |
| 3237.4527096 | 3 |
| 3237.4533546 | 2 |
| 3237.4547413 | 0 |
| 3237.4553202 | 2 |
| 3237.4573924 | 3 |
| 3237.4578745 | 0 |
| 3237.4591571 | 3 |
| 3237.4615202 | 1 |
| 3237.4620608 | 1 |
| 3237.4632567 | 3 |
| 3237.4640161 | 2 |
| 3237.465154 | 3 |
| 3237.4669605 | 0 |
| 3237.46763 | 1 |
| 3237.4696637 | 2 |
| 3237.4710443 | 3 |
| 3237.4715069 | 1 |
| 3237.4728483 | 1 |
| 3237.4741545 | 1 |
| 3237.4751501 | 3 |
| 3237.4761361 | 4 |
| 3237.478338 | 2 |
| 3237.479134 | 2 |
| 3237.4800902 | 0 |
| 3237.4821106 | 1 |
| 3237.4826512 | 1 |
| 3237.4837946 | 1 |
| 3237.4859337 | 1 |
| 3237.48604 | 1 |
| 3237.4878538 | 1 |
| 3237.4889654 | 0 |
| 3237.4905417 | 3 |
| 3237.4914321 | 2 |
| 3237.4926149 | 1 |
| 3237.4941747 | 0 |
| 3237.4949372 | 2 |
| 3237.4966413 | 1 |
| 3237.4982336 | 3 |
| 3237.4986514 | 5 |
| 3237.5006946 | 3 |
| 3237.5020186 | 1 |
| 3237.502643 | 3 |
| 3237.5038944 | 1 |
| 3237.5059142 | 4 |
| 3237.5064748 | 0 |
| 3237.5078621 | 2 |
| 3237.5088374 | 6 |
| 3237.5103425 | 1 |
| 3237.5110398 | 4 |
| 3237.5129888 | 0 |
| 3237.5142919 | 1 |
| 3237.5153336 | 0 |
| 3237.5159493 | 4 |
| 3237.5173678 | 4 |
| 3237.5190151 | 2 |
| 3237.5203428 | 1 |
| 3237.5206417 | 2 |
| 3237.5224693 | 1 |
| 3237.5226367 | 0 |
| 3237.5247979 | 4 |
| 3237.5257653 | 3 |
| 3237.5272628 | 3 |
| 3237.5288084 | 2 |
| 3237.5295353 | 3 |
| 3237.5307092 | 3 |
| 3237.5323825 | 2 |
| 3237.5333293 | 1 |
| 3237.5347839 | 1 |
| 3237.5361281 | 1 |
| 3237.5380198 | 2 |
| 3237.5384448 | 4 |
| 3237.5397069 | 3 |
| 3237.5413564 | 2 |
| 3237.542245 | 2 |
| 3237.5435527 | 2 |
| 3237.5443007 | 5 |
| 3237.5459189 | 3 |
| 3237.5478265 | 5 |
| 3237.5488528 | 0 |
| 3237.5500819 | 2 |
| 3237.5512901 | 2 |
| 3237.5522561 | 3 |
| 3237.5536257 | 2 |
| 3237.5542731 | 1 |
| 3237.5552647 | 1 |
| 3237.5568709 | 1 |
| 3237.5582741 | 2 |
| 3237.5597873 | 2 |
| 3237.5605945 | 2 |
| 3237.5619408 | 4 |
| 3237.5632156 | 4 |
| 3237.5652088 | 1 |
| 3237.5658752 | 3 |
| 3237.5667802 | 3 |
| 3237.5688569 | 1 |
| 3237.5691596 | 1 |
| 3237.5706549 | 3 |
| 3237.5721616 | 0 |
| 3237.5731732 | 1 |
| 3237.5743597 | 3 |
| 3237.5749449 | 4 |
| 3237.5764045 | 4 |
| 3237.578726 | 2 |
| 3237.578925 | 6 |
| 3237.5800638 | 3 |
| 3237.5812261 | 2 |
| 3237.5825248 | 1 |
| 3237.5843428 | 3 |
| 3237.5851859 | 1 |
| 3237.5869511 | 3 |
| 3237.588353 | 0 |
| 3237.5895026 | 2 |
| 3237.590793 | 2 |
| 3237.591322 | 3 |
| 3237.5934465 | 0 |
| 3237.5946775 | 3 |
| 3237.5952265 | 1 |
| 3237.5969079 | 2 |
| 3237.597513 | 2 |
| 3237.5994024 | 1 |
| 3237.6002354 | 0 |
| 3237.6010375 | 3 |
| 3237.6030105 | 0 |
| 3237.6036859 | 2 |
| 3237.6052119 | 5 |
| 3237.606146 | 2 |
| 3237.6082143 | 3 |
| 3237.6095818 | 2 |
| 3237.6102933 | 1 |
| 3237.6113511 | 1 |
| 3237.6123947 | 4 |
| 3237.6138278 | 1 |
| 3237.6155837 | 3 |
| 3237.6161089 | 2 |
| 3237.6176288 | 1 |
| 3237.6179775 | 0 |
| 3237.6199667 | 5 |
| 3237.6212963 | 4 |
| 3237.6223384 | 4 |
| 3237.6241767 | 2 |
| 3237.6245833 | 3 |
| 3237.6261471 | 0 |
| 3237.6274423 | 2 |
| 3237.6286106 | 3 |
| 3237.629986 | 3 |
| 3237.6307479 | 4 |
| 3237.6317896 | 0 |
| 3237.6332172 | 2 |
| 3237.6349561 | 1 |
| 3237.636049 | 2 |
| 3237.636894 | 2 |
| 3237.6388692 | 1 |
| 3237.6392598 | 1 |
| 3237.6411384 | 1 |
| 3237.642457 | 2 |
| 3237.6434582 | 3 |
| 3237.6449307 | 1 |
| 3237.6457222 | 1 |
| 3237.6476279 | 4 |
| 3237.647908 | 1 |
| 3237.6491567 | 1 |
| 3237.6510737 | 3 |
| 3237.6518963 | 2 |

| | |
|---|---|
| 3237.652982 | 4 |
| 3237.653889 | 2 |
| 3237.6554847 | 3 |
| 3237.6563598 | 2 |
| 3237.6565373 | 5 |
| 3237.656936 | 0 |
| 3237.6569483 | 6 |
| 3237.6570332 | 4 |
| 3237.6578931 | 2 |
| 3237.658519 | 1 |
| 3237.6585772 | 3 |
| 3237.6588727 | 5 |
| 3237.6590331 | 1 |
| 3237.6596866 | 2 |
| 3237.6600001 | 3 |
| 3237.6604402 | 6 |
| 3237.6605779 | 2 |
| 3237.6608135 | 1 |
| 3237.6613516 | 4 |
| 3237.6614342 | 2 |
| 3237.6621935 | 3 |
| 3237.662783 | 0 |
| 3237.6629534 | 2 |
| 3237.6635556 | 1 |
| 3237.6635618 | 2 |
| 3237.6635873 | 2 |
| 3237.6637197 | 4 |
| 3237.664174 | 4 |
| 3237.6652595 | 1 |
| 3237.6654799 | 3 |
| 3237.665538 | 4 |
| 3237.6656927 | 4 |
| 3237.6657915 | 2 |
| 3237.6663826 | 1 |
| 3237.6667039 | 4 |
| 3237.6672783 | 3 |
| 3237.6676984 | 4 |
| 3237.6678582 | 0 |
| 3237.6680887 | 2 |
| 3237.6685756 | 1 |
| 3237.6688407 | 1 |
| 3237.6691213 | 1 |
| 3237.6694739 | 5 |
| 3237.6697259 | 6 |
| 3237.6700087 | 2 |
| 3237.6706586 | 3 |
| 3237.671104 | 14 |
| 3237.6713075 | 3 |
| 3237.6717154 | 2 |
| 3237.6720254 | 2 |
| 3237.6722336 | 0 |
| 3237.6727634 | 2 |
| 3237.6730398 | 0 |
| 3237.6730784 | 2 |
| 3237.6740415 | 4 |
| 3237.6742398 | 1 |
| 3237.6742801 | 1 |
| 3237.6744116 | 3 |
| 3237.6747472 | 3 |
| 3237.674916 | 3 |
| 3237.675459 | 4 |
| 3237.6755081 | 1 |
| 3237.6758118 | 2 |
| 3237.6758511 | 6 |
| 3237.6762852 | 2 |
| 3237.6766454 | 0 |
| 3237.6768632 | 4 |
| 3237.6771869 | 2 |
| 3237.6773414 | 2 |
| 3237.6775468 | 2 |
| 3237.6778764 | 0 |
| 3237.6784212 | 0 |
| 3237.6788195 | 3 |
| 3237.6789867 | 3 |
| 3237.679018 | 1 |
| 3237.6790558 | 0 |
| 3237.6791079 | 2 |
| 3237.6797329 | 1 |
| 3237.6797639 | 3 |
| 3237.6802654 | 1 |
| 3237.6804905 | 3 |
| 3237.6805852 | 3 |
| 3237.6813124 | 0 |
| 3237.6815001 | 1 |
| 3237.6818771 | 3 |
| 3237.6822413 | 1 |
| 3237.6824609 | 2 |
| 3237.6825283 | 1 |
| 3237.6827616 | 1 |
| 3237.6829291 | 0 |
| 3237.6830113 | 3 |
| 3237.6834498 | 3 |
| 3237.6841471 | 4 |
| 3237.684262 | 2 |
| 3237.6847858 | 2 |
| 3237.6847949 | 0 |
| 3237.6848467 | 2 |
| 3237.6851382 | 0 |
| 3237.6855738 | 4 |
| 3237.68601 | 2 |
| 3237.686268 | 2 |
| 3237.686679 | 4 |
| 3237.6866983 | 3 |
| 3237.6866989 | 1 |
| 3237.6867038 | 4 |
| 3237.6871045 | 0 |
| 3237.6872951 | 0 |
| 3237.6878743 | 4 |
| 3237.6879344 | 3 |
| 3237.6884484 | 1 |
| 3237.6886454 | 5 |
| 3237.6888097 | 2 |
| 3237.6888585 | 3 |
| 3237.689282 | 4 |
| 3237.6901818 | 2 |
| 3237.6904463 | 5 |
| 3237.6905528 | 2 |
| 3237.6907362 | 2 |
| 3237.6907954 | 3 |
| 3237.691113 | 5 |
| 3237.6912093 | 2 |
| 3237.6913054 | 0 |
| 3237.6913298 | 4 |
| 3237.6920753 | 1 |
| 3237.692562 | 7 |
| 3237.6926305 | 3 |
| 3237.6928443 | 5 |
| 3237.6931534 | 1 |
| 3237.6932564 | 1 |
| 3237.693301 | 4 |
| 3237.6935594 | 0 |
| 3237.6939681 | 1 |
| 3237.6947102 | 2 |
| 3237.6950043 | 4 |
| 3237.6955025 | 2 |
| 3237.6955628 | 5 |
| 3237.6964994 | 3 |
| 3237.6967004 | 5 |
| 3237.6972879 | 1 |
| 3237.6977572 | 3 |
| 3237.6984666 | 3 |
| 3237.6992885 | 1 |
| 3237.6995005 | 0 |
| 3237.6995318 | 0 |
| 3237.7015486 | 0 |
| 3237.7025787 | 1 |
| 3237.7030144 | 2 |
| 3237.7033093 | 3 |
| 3237.7040723 | 1 |
| 3237.7048873 | 4 |
| 3237.7050493 | 0 |
| 3237.7055689 | 2 |
| 3237.7060054 | 2 |
| 3237.7069434 | 1 |
| 3237.7069976 | 4 |
| 3237.7078444 | 2 |
| 3237.7083707 | 3 |
| 3237.7092603 | 3 |
| 3237.7098076 | 1 |
| 3237.710406 | 2 |
| 3237.7112487 | 1 |
| 3237.7118965 | 2 |
| 3237.7121064 | 1 |
| 3237.7133114 | 3 |
| 3237.7136372 | 2 |
| 3237.7143615 | 4 |
| 3237.7146845 | 2 |
| 3237.7156866 | 1 |
| 3237.7158891 | 0 |
| 3237.7162158 | 3 |
| 3237.7167362 | 0 |
| 3237.7171311 | 3 |
| 3237.7177329 | 3 |
| 3237.71829 | 2 |
| 3237.7192329 | 4 |
| 3237.7199113 | 2 |
| 3237.7201067 | 3 |
| 3237.7210063 | 0 |
| 3237.7216301 | 6 |
| 3237.7216518 | 2 |
| 3237.721849 | 0 |
| 3237.7230232 | 2 |
| 3237.7239113 | 2 |
| 3237.7241078 | 1 |
| 3237.7245414 | 3 |
| 3237.725735 | 2 |
| 3237.7268976 | 3 |
| 3237.7285637 | 4 |
| 3237.729845 | 0 |
| 3237.7313266 | 1 |
| 3237.7328105 | 3 |
| 3237.7329362 | 1 |
| 3237.7348654 | 6 |
| 3237.7365507 | 3 |
| 3237.7369777 | 2 |
| 3237.7382615 | 2 |
| 3237.739425 | 1 |
| 3237.7422798 | 3 |
| 3237.7429078 | 3 |
| 3237.7444831 | 5 |
| 3237.747085 | 3 |
| 3237.7477876 | 1 |
| 3237.7494681 | 4 |
| 3237.7502108 | 6 |
| 3237.7507514 | 3 |
| 3237.7508511 | 4 |
| 3237.7518639 | 3 |
| 3237.7533168 | 1 |
| 3237.754584 | 3 |
| 3237.7555079 | 3 |
| 3237.7570764 | 3 |
| 3237.7576782 | 1 |
| 3237.7589368 | 4 |
| 3237.7596642 | 4 |
| 3237.7620667 | 3 |
| 3237.7632594 | 1 |
| 3237.765532 | 4 |
| 3237.767611 | 5 |
| 3237.7679275 | 1 |
| 3237.7693867 | 3 |
| 3237.7698223 | 3 |
| 3237.7706831 | 1 |
| 3237.7721422 | 3 |
| 3237.7729755 | 3 |
| 3237.775074 | 3 |
| 3237.7758445 | 0 |
| 3237.7770058 | 3 |
| 3237.7778649 | 1 |
| 3237.7791657 | 3 |
| 3237.7807696 | 1 |
| 3237.7818409 | 2 |
| 3237.783459 | 1 |
| 3237.7845607 | 3 |
| 3237.7852616 | 1 |
| 3237.7871659 | 0 |
| 3237.7883664 | 1 |
| 3237.7907053 | 2 |
| 3237.7927902 | 2 |
| 3237.7930745 | 0 |
| 3237.7938571 | 0 |
| 3237.794109 | 4 |
| 3237.7946334 | 2 |
| 3237.7965495 | 2 |
| 3237.7973676 | 1 |
| 3237.7991934 | 0 |
| 3237.799766 | 1 |
| 3237.8014561 | 3 |
| 3237.8028529 | 2 |
| 3237.8037604 | 2 |
| 3237.8057777 | 2 |
| 3237.8063688 | 3 |
| 3237.8077386 | 1 |
| 3237.809305 | 5 |
| 3237.8097793 | 3 |
| 3237.8117372 | 2 |
| 3237.8128314 | 4 |
| 3237.8144724 | 1 |
| 3237.8169781 | 4 |
| 3237.8177959 | 4 |
| 3237.8188254 | 2 |
| 3237.8197386 | 0 |
| 3237.8205676 | 1 |
| 3237.8218026 | 1 |
| 3237.8230833 | 3 |
| 3237.8243803 | 0 |
| 3237.8248948 | 0 |
| 3237.8265696 | 6 |
| 3237.8291807 | 0 |
| 3237.8295566 | 5 |
| 3237.8323569 | 1 |
| 3237.8328239 | 1 |
| 3237.8355819 | 0 |
| 3237.8362236 | 1 |
| 3237.8367563 | 2 |
| 3237.8378854 | 2 |
| 3237.838691 | 2 |
| 3237.8392098 | 3 |
| 3237.8398709 | 1 |
| 3237.8416195 | 2 |
| 3237.8419088 | 3 |
| 3237.8427981 | 2 |
| 3237.8448025 | 1 |
| 3237.8465168 | 1 |
| 3237.8465888 | 4 |
| 3237.8479208 | 0 |
| 3237.8505863 | 2 |
| 3237.8507519 | 3 |
| 3237.8522547 | 1 |
| 3237.8534127 | 2 |
| 3237.8559282 | 3 |
| 3237.8573419 | 1 |
| 3237.8584499 | 3 |
| 3237.8598028 | 3 |
| 3237.8603058 | 3 |
| 3237.8616443 | 0 |
| 3237.8639713 | 6 |
| 3237.8648368 | 1 |
| 3237.8663136 | 2 |
| 3237.8678404 | 3 |
| 3237.8694064 | 2 |
| 3237.8697209 | 2 |
| 3237.8723333 | 2 |
| 3237.8728105 | 2 |
| 3237.8736816 | 1 |
| 3237.8740534 | 2 |
| 3237.8765105 | 2 |
| 3237.8787239 | 1 |
| 3237.8805574 | 0 |
| 3237.8809307 | 0 |
| 3237.8817366 | 2 |
| 3237.8817859 | 1 |
| 3237.8828828 | 2 |
| 3237.8833262 | 3 |
| 3237.8853052 | 0 |
| 3237.8868466 | 0 |
| 3237.8870355 | 5 |
| 3237.8889493 | 4 |
| 3237.8896997 | 2 |
| 3237.8906918 | 4 |
| 3237.8924117 | 0 |
| 3237.8936243 | 2 |
| 3237.8951924 | 3 |
| 3237.8959051 | 3 |
| 3237.8973475 | 2 |
| 3237.8989135 | 3 |
| 3237.8991789 | 3 |
| 3237.9015888 | 4 |
| 3237.9020379 | 0 |
| 3237.9040441 | 2 |
| 3237.9043092 | 2 |
| 3237.9065915 | 2 |
| 3237.9090277 | 1 |
| 3237.9103234 | 3 |
| 3237.9118874 | 6 |
| 3237.9142609 | 3 |
| 3237.9146648 | 1 |
| 3237.9166824 | 4 |
| 3237.9167407 | 4 |
| 3237.9190602 | 1 |
| 3237.9193563 | 1 |
| 3237.9200173 | 1 |
| 3237.9218843 | 7 |
| 3237.9230427 | 1 |
| 3237.9236909 | 7 |
| 3237.9248969 | 1 |
| 3237.9266479 | 4 |
| 3237.9273572 | 1 |
| 3237.9275247 | 1 |
| 3237.9292262 | 0 |
| 3237.9295081 | 3 |
| 3237.9318239 | 0 |
| 3237.9327353 | 2 |
| 3237.9338244 | 2 |
| 3237.9357745 | 3 |
| 3237.9359088 | 2 |
| 3237.9374129 | 0 |
| 3237.9384911 | 2 |
| 3237.9396796 | 0 |
| 3237.9412884 | 1 |
| 3237.9425178 | 0 |
| 3237.9443735 | 2 |
| 3237.9449084 | 1 |
| 3237.9465436 | 2 |

| | |
|---|---|
| 3237.9478023 | 1 |
| 3237.9485666 | 1 |
| 3237.9507085 | 0 |
| 3237.9514009 | 1 |
| 3237.9531527 | 1 |
| 3237.9553845 | 2 |
| 3237.9560409 | 1 |
| 3237.9574899 | 0 |
| 3237.958379 | 3 |
| 3237.960776 | 2 |
| 3237.963156 | 0 |
| 3237.9635076 | 2 |
| 3237.9651966 | 2 |
| 3237.9656305 | 1 |
| 3237.9666627 | 1 |
| 3237.9680588 | 8 |
| 3237.9680773 | 4 |
| 3237.9692498 | 2 |
| 3237.9700869 | 4 |
| 3237.9710572 | 2 |
| 3237.9728055 | 2 |
| 3237.9738222 | 0 |
| 3237.9757046 | 3 |
| 3237.9757912 | 0 |
| 3237.9780317 | 1 |
| 3237.9783654 | 3 |
| 3237.980023 | 3 |
| 3237.9816798 | 4 |
| 3237.9829533 | 2 |
| 3237.9844973 | 3 |
| 3237.9845514 | 1 |
| 3237.9864976 | 1 |
| 3237.9881217 | 6 |
| 3237.9886134 | 4 |
| 3237.9902192 | 5 |
| 3237.9912781 | 5 |
| 3237.9935222 | 3 |
| 3237.9937059 | 1 |
| 3237.9948349 | 7 |
| 3237.9970417 | 2 |
| 3237.9980624 | 3 |
| 3237.9998858 | 2 |
| 3238.0007517 | 1 |
| 3238.0019973 | 0 |
| 3238.0046259 | 2 |
| 3238.0053456 | 3 |
| 3238.0057502 | 5 |
| 3238.0079121 | 3 |
| 3238.0084019 | 2 |
| 3238.0097971 | 3 |
| 3238.0109245 | 3 |
| 3238.0123196 | 3 |
| 3238.0130579 | 1 |
| 3238.0141685 | 1 |
| 3238.0159389 | 1 |
| 3238.0165382 | 2 |
| 3238.0180985 | 3 |
| 3238.0186455 | 2 |
| 3238.0203915 | 3 |
| 3238.0213482 | 4 |
| 3238.0224923 | 5 |
| 3238.0240523 | 5 |
| 3238.0251779 | 5 |
| 3238.0264815 | 2 |
| 3238.0274621 | 2 |
| 3238.0286863 | 0 |
| 3238.0304275 | 5 |
| 3238.0313629 | 0 |
| 3238.0331129 | 4 |
| 3238.03359 | 2 |
| 3238.0350426 | 1 |
| 3238.0365503 | 1 |
| 3238.0374492 | 1 |
| 3238.0393548 | 2 |
| 3238.040435 | 1 |
| 3238.041559 | 3 |
| 3238.0434215 | 1 |
| 3238.0437075 | 3 |
| 3238.0458608 | 2 |
| 3238.046649 | 4 |
| 3238.0477414 | 1 |
| 3238.0492446 | 1 |
| 3238.0507889 | 3 |
| 3238.051783 | 3 |
| 3238.0532506 | 4 |
| 3238.0556996 | 1 |
| 3238.0560907 | 5 |
| 3238.0568302 | 1 |
| 3238.0582725 | 1 |
| 3238.0587165 | 2 |
| 3238.060476 | 2 |
| 3238.0617357 | 1 |
| 3238.0624376 | 0 |
| 3238.0645543 | 4 |
| 3238.0652612 | 1 |
| 3238.066935 | 1 |
| 3238.0673527 | 2 |
| 3238.0688747 | 4 |
| 3238.0703751 | 2 |
| 3238.0705126 | 1 |
| 3238.0730252 | 3 |
| 3238.0731934 | 3 |
| 3238.0755604 | 3 |
| 3238.0762708 | 1 |
| 3238.0775218 | 3 |
| 3238.0789507 | 2 |
| 3238.0796195 | 1 |
| 3238.0813526 | 2 |
| 3238.0831245 | 3 |
| 3238.0833925 | 3 |
| 3238.0851841 | 0 |
| 3238.0859123 | 3 |
| 3238.0874669 | 4 |
| 3238.0894536 | 2 |
| 3238.0896657 | 6 |
| 3238.0915193 | 1 |
| 3238.0923389 | 1 |
| 3238.0947381 | 0 |
| 3238.0955083 | 0 |
| 3238.0960527 | 1 |
| 3238.0961977 | 2 |
| 3238.0978331 | 3 |
| 3238.0988511 | 1 |
| 3238.0992458 | 3 |
| 3238.1003541 | 2 |
| 3238.1010523 | 8 |
| 3238.1017212 | 6 |
| 3238.1031732 | 2 |
| 3238.1042559 | 5 |
| 3238.1043899 | 4 |
| 3238.1049284 | 2 |
| 3238.1054925 | 4 |
| 3238.1067287 | 4 |
| 3238.1074459 | 8 |
| 3238.1082519 | 5 |
| 3238.1086128 | 3 |
| 3238.1111811 | 4 |
| 3238.1112862 | 2 |
| 3238.1116204 | 4 |
| 3238.1127349 | 3 |
| 3238.1130189 | 2 |
| 3238.1138169 | 1 |
| 3238.1147541 | 1 |
| 3238.115828 | 3 |
| 3238.1161231 | 2 |
| 3238.1174802 | 2 |
| 3238.1176978 | 2 |
| 3238.1193125 | 2 |
| 3238.1195358 | 3 |
| 3238.1205462 | 0 |
| 3238.1213085 | 6 |
| 3238.1232679 | 2 |
| 3238.123286 | 3 |
| 3238.1233702 | 7 |
| 3238.1247251 | 4 |
| 3238.1255195 | 2 |
| 3238.1262281 | 1 |
| 3238.1273226 | 3 |
| 3238.1280827 | 2 |
| 3238.1297107 | 2 |
| 3238.1297652 | 1 |
| 3238.13091 | 1 |
| 3238.131338 | 5 |
| 3238.1326268 | 4 |
| 3238.1329293 | 8 |
| 3238.1338355 | 0 |
| 3238.135144 | 4 |
| 3238.1358587 | 1 |
| 3238.1359307 | 0 |
| 3238.1379434 | 3 |
| 3238.1380624 | 7 |
| 3238.1380899 | 2 |
| 3238.1403207 | 0 |
| 3238.1403883 | 1 |
| 3238.141946 | 2 |
| 3238.142512 | 2 |
| 3238.143557 | 2 |
| 3238.1439321 | 1 |
| 3238.1448769 | 1 |
| 3238.145561 | 1 |
| 3238.1463528 | 3 |
| 3238.147967 | 4 |
| 3238.1482139 | 3 |
| 3238.1485437 | 3 |
| 3238.1502465 | 1 |
| 3238.1502868 | 2 |
| 3238.1508648 | 2 |
| 3238.1519449 | 0 |
| 3238.153361 | 1 |
| 3238.1539369 | 0 |
| 3238.1545749 | 5 |
| 3238.154744 | 4 |
| 3238.1554217 | 2 |
| 3238.1567068 | 2 |
| 3238.1577425 | 1 |
| 3238.1581124 | 1 |
| 3238.1599093 | 0 |
| 3238.1610099 | 1 |
| 3238.1614263 | 4 |
| 3238.1622247 | 1 |
| 3238.1627644 | 4 |
| 3238.1632156 | 4 |
| 3238.1643563 | 4 |
| 3238.1654479 | 5 |
| 3238.1660503 | 2 |
| 3238.1665721 | 3 |
| 3238.1669101 | 1 |
| 3238.1680078 | 0 |
| 3238.168854 | 2 |
| 3238.1693785 | 5 |
| 3238.1710168 | 3 |
| 3238.1724457 | 4 |
| 3238.1726133 | 2 |
| 3238.1726951 | 1 |
| 3238.1742852 | 4 |
| 3238.1748005 | 3 |
| 3238.1754185 | 0 |
| 3238.1770321 | 3 |
| 3238.1774122 | 3 |
| 3238.1794554 | 0 |
| 3238.179688 | 3 |
| 3238.1802437 | 5 |
| 3238.1823758 | 2 |
| 3238.182845 | 2 |
| 3238.182852 | 3 |
| 3238.185068 | 3 |
| 3238.1855394 | 5 |
| 3238.1859831 | 3 |
| 3238.1874085 | 2 |
| 3238.188297 | 1 |
| 3238.1893108 | 1 |
| 3238.1908028 | 2 |
| 3238.1910181 | 1 |
| 3238.1924663 | 0 |
| 3238.1932661 | 1 |
| 3238.1941801 | 3 |
| 3238.1950324 | 4 |
| 3238.1956001 | 1 |
| 3238.1964454 | 0 |
| 3238.197917 | 1 |
| 3238.1984401 | 3 |
| 3238.199473 | 2 |
| 3238.2030215 | 1 |
| 3238.20402 | 0 |
| 3238.2068673 | 2 |
| 3238.2103359 | 3 |
| 3238.2118508 | 1 |
| 3238.2147291 | 4 |
| 3238.2168351 | 3 |
| 3238.2193032 | 6 |
| 3238.2218286 | 1 |
| 3238.2241102 | 1 |
| 3238.227263 | 4 |
| 3238.2283466 | 1 |
| 3238.2321702 | 2 |
| 3238.2354465 | 4 |
| 3238.2362162 | 2 |
| 3238.2391437 | 2 |
| 3238.2413194 | 4 |
| 3238.2439306 | 2 |
| 3238.2472432 | 3 |
| 3238.2493944 | 2 |
| 3238.2523972 | 5 |
| 3238.2531072 | 3 |
| 3238.2566482 | 3 |
| 3238.2592293 | 2 |
| 3238.2604767 | 3 |
| 3238.2634301 | 3 |
| 3238.2650838 | 2 |
| 3238.2687118 | 2 |
| 3238.2719022 | 3 |
| 3238.2736713 | 4 |
| 3238.2770548 | 5 |
| 3238.2783789 | 3 |
| 3238.2816982 | 0 |
| 3238.2844265 | 4 |
| 3238.2861048 | 1 |
| 3238.2894069 | 3 |
| 3238.2920436 | 3 |
| 3238.2931933 | 1 |
| 3238.2957801 | 0 |
| 3238.2979126 | 2 |
| 3238.300709 | 1 |
| 3238.3025042 | 2 |
| 3238.3054885 | 3 |
| 3238.3081258 | 3 |
| 3238.3104174 | 1 |
| 3238.3136457 | 1 |
| 3238.314613 | 2 |
| 3238.3179115 | 3 |
| 3238.3216206 | 4 |
| 3238.3230858 | 3 |
| 3238.326536 | 1 |
| 3238.3273998 | 5 |
| 3238.3308683 | 2 |
| 3238.3341437 | 0 |
| 3238.3349412 | 1 |
| 3238.3380643 | 3 |
| 3238.3404321 | 0 |
| 3238.342977 | 3 |
| 3238.3442629 | 3 |
| 3238.3475617 | 1 |
| 3238.3503701 | 3 |
| 3238.3517822 | 2 |
| 3238.3543419 | 1 |
| 3238.3583108 | 1 |
| 3238.3593136 | 1 |
| 3238.362829 | 1 |
| 3238.3640259 | 6 |
| 3238.3674943 | 3 |
| 3238.3708516 | 5 |
| 3238.3731732 | 4 |
| 3238.375592 | 1 |
| 3238.3773979 | 3 |
| 3238.3802876 | 2 |
| 3238.3833746 | 4 |
| 3238.3849751 | 2 |
| 3238.3881603 | 2 |
| 3238.3893359 | 0 |
| 3238.3916165 | 2 |
| 3238.3921567 | 5 |
| 3238.3944805 | 3 |
| 3238.3965936 | 3 |
| 3238.397088 | 7 |
| 3238.3991799 | 2 |
| 3238.4003771 | 6 |
| 3238.4019312 | 0 |
| 3238.4036343 | 3 |
| 3238.4052758 | 2 |
| 3238.4078003 | 5 |
| 3238.4088294 | 2 |
| 3238.4100932 | 6 |
| 3238.4121399 | 3 |
| 3238.4128181 | 4 |
| 3238.4148896 | 3 |
| 3238.4158531 | 2 |
| 3238.4177639 | 1 |
| 3238.4195364 | 4 |
| 3238.4206352 | 1 |
| 3238.4228086 | 3 |
| 3238.4238284 | 3 |
| 3238.4255572 | 1 |
| 3238.4273296 | 2 |
| 3238.428708 | 2 |
| 3238.4307357 | 1 |
| 3238.4308662 | 3 |
| 3238.4331396 | 1 |
| 3238.4348827 | 4 |
| 3238.4358104 | 5 |
| 3238.4383961 | 3 |
| 3238.4385959 | 1 |
| 3238.4402677 | 2 |
| 3238.4426296 | 0 |
| 3238.4432768 | 3 |
| 3238.4452006 | 1 |
| 3238.4468839 | 5 |
| 3238.4486832 | 4 |
| 3238.450327 | 4 |
| 3238.4516749 | 3 |
| 3238.453753 | 1 |
| 3238.4554786 | 2 |
| 3238.4575877 | 1 |
| 3238.4577638 | 1 |

| | |
|---|---|
| 3238.4603656 | 4 |
| 3238.4616331 | 2 |
| 3238.4625863 | 3 |
| 3238.4645366 | 3 |
| 3238.4670382 | 3 |
| 3238.4672806 | 2 |
| 3238.4697068 | 1 |
| 3238.4701275 | 4 |
| 3238.4718232 | 1 |
| 3238.4740061 | 2 |
| 3238.4749159 | 3 |
| 3238.4767861 | 1 |
| 3238.4780721 | 4 |
| 3238.4799316 | 1 |
| 3238.4817858 | 4 |
| 3238.4827878 | 4 |
| 3238.4846031 | 0 |
| 3238.4857112 | 3 |
| 3238.4875455 | 4 |
| 3238.488055 | 2 |
| 3238.4904982 | 2 |
| 3238.4930628 | 3 |
| 3238.4935528 | 1 |
| 3238.4949688 | 1 |
| 3238.4967238 | 2 |
| 3238.4978375 | 6 |
| 3238.5005818 | 1 |
| 3238.5019747 | 1 |
| 3238.5039038 | 2 |
| 3238.5045607 | 2 |
| 3238.5065962 | 0 |
| 3238.5081163 | 5 |
| 3238.5090491 | 3 |
| 3238.5116556 | 4 |
| 3238.5131138 | 3 |
| 3238.5140261 | 3 |
| 3238.5158156 | 4 |
| 3238.5167445 | 3 |
| 3238.5184927 | 4 |
| 3238.5196098 | 0 |
| 3238.5215872 | 2 |
| 3238.5237262 | 0 |
| 3238.5242089 | 3 |
| 3238.525731 | 0 |
| 3238.528111 | 1 |
| 3238.5292922 | 4 |
| 3238.5313896 | 0 |
| 3238.5328344 | 3 |
| 3238.5349627 | 2 |
| 3238.5350481 | 2 |
| 3238.5378625 | 2 |
| 3238.5395928 | 4 |
| 3238.5401347 | 2 |
| 3238.5422794 | 3 |
| 3238.5433052 | 6 |
| 3238.5454212 | 4 |
| 3238.5474401 | 0 |
| 3238.5483057 | 3 |
| 3238.550429 | 4 |
| 3238.5514736 | 3 |
| 3238.5534777 | 3 |
| 3238.5551954 | 2 |
| 3238.5565962 | 3 |
| 3238.5583495 | 0 |
| 3238.5595491 | 3 |
| 3238.5615691 | 0 |
| 3238.5620802 | 1 |
| 3238.5636725 | 6 |
| 3238.5665044 | 3 |
| 3238.5665772 | 2 |
| 3238.5688108 | 3 |
| 3238.5706453 | 2 |
| 3238.5711712 | 2 |
| 3238.5731943 | 2 |
| 3238.574749 | 3 |
| 3238.576748 | 3 |
| 3238.5787787 | 1 |
| 3238.5800499 | 2 |
| 3238.5816883 | 4 |
| 3238.5822839 | 2 |
| 3238.5847396 | 7 |
| 3238.5866988 | 3 |
| 3238.5873528 | 4 |
| 3238.5898971 | 3 |
| 3238.5903633 | 2 |
| 3238.592533 | 3 |
| 3238.5936133 | 2 |
| 3238.595322 | 2 |
| 3238.5971971 | 1 |
| 3238.5988801 | 3 |
| 3238.6003994 | 1 |
| 3238.6017054 | 2 |
| 3238.6039653 | 4 |
| 3238.6053411 | 2 |
| 3238.6066055 | 2 |
| 3238.6085189 | 1 |
| 3238.6102226 | 0 |
| 3238.6106787 | 4 |
| 3238.6130276 | 2 |
| 3238.6136125 | 1 |
| 3238.6153257 | 4 |
| 3238.6174481 | 0 |
| 3238.6185314 | 1 |
| 3238.6199661 | 2 |
| 3238.6217328 | 2 |
| 3238.6237864 | 1 |
| 3238.6249615 | 0 |
| 3238.6259323 | 2 |
| 3238.6286119 | 3 |
| 3238.6288561 | 3 |
| 3238.6315842 | 2 |
| 3238.6335804 | 1 |
| 3238.6339918 | 2 |
| 3238.6364843 | 3 |
| 3238.6376795 | 4 |
| 3238.6395574 | 0 |
| 3238.6411187 | 4 |
| 3238.6428059 | 3 |
| 3238.6444213 | 3 |
| 3238.6453647 | 5 |
| 3238.6467995 | 2 |
| 3238.6491764 | 1 |
| 3238.6498442 | 5 |
| 3238.6519576 | 1 |
| 3238.65269 | 4 |
| 3238.6543811 | 1 |
| 3238.6555335 | 0 |
| 3238.657539 | 4 |
| 3238.6598764 | 0 |
| 3238.6608766 | 5 |
| 3238.6630002 | 0 |
| 3238.6638797 | 2 |
| 3238.6663151 | 4 |
| 3238.6675442 | 1 |
| 3238.6687868 | 3 |
| 3238.669882 | 1 |
| 3238.6722429 | 3 |
| 3238.672548 | 6 |
| 3238.6745124 | 0 |
| 3238.6773203 | 3 |
| 3238.6777525 | 0 |
| 3238.679525 | 2 |
| 3238.6812814 | 5 |
| 3238.6828588 | 2 |
| 3238.6849757 | 4 |
| 3238.6858784 | 1 |
| 3238.6882585 | 1 |
| 3238.6883292 | 3 |
| 3238.6912982 | 6 |
| 3238.6920009 | 1 |
| 3238.6940686 | 6 |
| 3238.6953481 | 0 |
| 3238.6967518 | 0 |
| 3238.6981446 | 3 |
| 3238.7004476 | 5 |
| 3238.7016746 | 1 |
| 3238.7036432 | 3 |
| 3238.7044017 | 4 |
| 3238.7065781 | 3 |
| 3238.7074324 | 3 |
| 3238.709669 | 4 |
| 3238.7112304 | 1 |
| 3238.7121236 | 1 |
| 3238.7139651 | 5 |
| 3238.7149772 | 2 |
| 3238.7166743 | 5 |
| 3238.7186805 | 2 |
| 3238.7200725 | 3 |
| 3238.7222863 | 1 |
| 3238.7226957 | 5 |
| 3238.7250832 | 2 |
| 3238.7264957 | 1 |
| 3238.7273183 | 2 |
| 3238.7298889 | 3 |
| 3238.7309232 | 0 |
| 3238.732839 | 1 |
| 3238.734803 | 5 |
| 3238.7358729 | 3 |
| 3238.7379191 | 2 |
| 3238.738656 | 3 |
| 3238.7406513 | 5 |
| 3238.7416776 | 3 |
| 3238.7440535 | 2 |
| 3238.7457243 | 1 |
| 3238.7470252 | 6 |
| 3238.74858 | 1 |
| 3238.7490794 | 2 |
| 3238.7516184 | 1 |
| 3238.752685 | 6 |
| 3238.7533648 | 1 |
| 3238.7536866 | 2 |
| 3238.7538026 | 0 |
| 3238.7540108 | 5 |
| 3238.7543179 | 4 |
| 3238.7543869 | 3 |
| 3238.755461 | 3 |
| 3238.7560597 | 2 |
| 3238.7564127 | 4 |
| 3238.7564311 | 1 |
| 3238.7565681 | 1 |
| 3238.7568998 | 3 |
| 3238.7572766 | 1 |
| 3238.7583865 | 4 |
| 3238.7585581 | 4 |
| 3238.7594065 | 2 |
| 3238.7595562 | 1 |
| 3238.7596991 | 0 |
| 3238.7607774 | 1 |
| 3238.7608625 | 3 |
| 3238.7614255 | 2 |
| 3238.7618366 | 0 |
| 3238.7620701 | 3 |
| 3238.7621171 | 6 |
| 3238.7627959 | 0 |
| 3238.763532 | 2 |
| 3238.76398 | 2 |
| 3238.7655675 | 1 |
| 3238.7656945 | 2 |
| 3238.7659152 | 1 |
| 3238.766563 | 1 |
| 3238.7665635 | 1 |
| 3238.7669721 | 3 |
| 3238.7672462 | 3 |
| 3238.7680935 | 4 |
| 3238.7681342 | 1 |
| 3238.768874 | 2 |
| 3238.7691342 | 2 |
| 3238.7693954 | 2 |
| 3238.7700164 | 4 |
| 3238.7708133 | 2 |
| 3238.7708601 | 2 |
| 3238.7716154 | 4 |
| 3238.7718821 | 3 |
| 3238.7720186 | 0 |
| 3238.7724801 | 1 |
| 3238.7730663 | 1 |
| 3238.7735405 | 4 |
| 3238.7743783 | 2 |
| 3238.7745198 | 0 |
| 3238.7748509 | 4 |
| 3238.7751335 | 1 |
| 3238.7761483 | 3 |
| 3238.7766546 | 2 |
| 3238.7770223 | 1 |
| 3238.777269 | 1 |
| 3238.777646 | 4 |
| 3238.7781985 | 0 |
| 3238.7790565 | 4 |
| 3238.7792632 | 0 |
| 3238.7796587 | 3 |
| 3238.7801796 | 3 |
| 3238.780792 | 2 |
| 3238.7815027 | 2 |
| 3238.7817627 | 3 |
| 3238.7818564 | 1 |
| 3238.7821548 | 2 |
| 3238.7822719 | 1 |
| 3238.7838259 | 0 |
| 3238.7841489 | 3 |
| 3238.7844633 | 3 |
| 3238.7847264 | 4 |
| 3238.7850117 | 5 |
| 3238.7850618 | 1 |
| 3238.7857915 | 1 |
| 3238.7860693 | 5 |
| 3238.7871812 | 2 |
| 3238.7873969 | 1 |
| 3238.7878323 | 2 |
| 3238.7881282 | 1 |
| 3238.7886684 | 2 |
| 3238.7887347 | 1 |
| 3238.7892782 | 4 |
| 3238.789842 | 1 |
| 3238.7900323 | 1 |
| 3238.7907126 | 0 |
| 3238.7914431 | 4 |
| 3238.7916703 | 2 |
| 3238.792246 | 2 |
| 3238.79238 | 4 |
| 3238.7927551 | 0 |
| 3238.7928852 | 3 |
| 3238.7937127 | 0 |
| 3238.7944243 | 2 |
| 3238.7947459 | 2 |
| 3238.7948956 | 1 |
| 3238.7958047 | 4 |
| 3238.7958465 | 3 |
| 3238.7958894 | 3 |
| 3238.7967292 | 0 |
| 3238.7969076 | 2 |
| 3238.7977341 | 3 |
| 3238.7979622 | 1 |
| 3238.7982185 | 2 |
| 3238.7993981 | 3 |
| 3238.7995281 | 3 |
| 3238.7997114 | 2 |
| 3238.8003455 | 0 |
| 3238.8004888 | 3 |
| 3238.8005792 | 3 |
| 3238.8013543 | 2 |
| 3238.8018089 | 3 |
| 3238.8023628 | 1 |
| 3238.8026832 | 1 |
| 3238.8028924 | 0 |
| 3238.8035177 | 3 |
| 3238.8042077 | 1 |
| 3238.8044674 | 2 |
| 3238.8051038 | 1 |
| 3238.8052663 | 2 |
| 3238.8055494 | 3 |
| 3238.806381 | 2 |
| 3238.8069329 | 1 |
| 3238.8073634 | 1 |
| 3238.8075921 | 1 |
| 3238.808629 | 3 |
| 3238.8087446 | 2 |
| 3238.8088821 | 1 |
| 3238.8092529 | 4 |
| 3238.8095697 | 1 |
| 3238.8098918 | 3 |
| 3238.8103522 | 3 |
| 3238.8108498 | 0 |
| 3238.8117661 | 4 |
| 3238.8117925 | 4 |
| 3238.8124447 | 2 |
| 3238.8125495 | 5 |
| 3238.8131114 | 1 |
| 3238.813343 | 5 |
| 3238.8136705 | 2 |
| 3238.8137915 | 3 |
| 3238.8145707 | 0 |
| 3238.8146828 | 1 |
| 3238.8148476 | 2 |
| 3238.8152848 | 4 |
| 3238.8156958 | 0 |
| 3238.8157566 | 3 |
| 3238.8160044 | 5 |
| 3238.8163473 | 2 |
| 3238.8166955 | 2 |
| 3238.8175505 | 1 |
| 3238.8176683 | 3 |
| 3238.8180302 | 5 |
| 3238.8183039 | 2 |
| 3238.8183157 | 1 |
| 3238.8183535 | 4 |
| 3238.8196203 | 2 |
| 3238.8197102 | 2 |
| 3238.8197276 | 2 |
| 3238.8199505 | 1 |
| 3238.820805 | 1 |
| 3238.8212211 | 0 |
| 3238.8214257 | 7 |
| 3238.8219543 | 2 |
| 3238.822086 | 2 |
| 3238.8228405 | 2 |
| 3238.8230444 | 3 |
| 3238.823179 | 1 |
| 3238.8235639 | 2 |
| 3238.8237169 | 2 |
| 3238.8239904 | 1 |
| 3238.8240512 | 6 |
| 3238.8249144 | 2 |
| 3238.8250304 | 0 |

| | |
|---|---|
| 3238.8254327 | 4 |
| 3238.8262144 | 0 |
| 3238.8262254 | 0 |
| 3238.8265724 | 3 |
| 3238.8266014 | 1 |
| 3238.8270308 | 0 |
| 3238.827303 | 2 |
| 3238.8275678 | 1 |
| 3238.8277239 | 3 |
| 3238.8277945 | 4 |
| 3238.8283326 | 1 |
| 3238.8285865 | 1 |
| 3238.8290799 | 2 |
| 3238.8296476 | 1 |
| 3238.8296605 | 2 |
| 3238.8296717 | 3 |
| 3238.830225 | 5 |
| 3238.8303994 | 3 |
| 3238.831287 | 1 |
| 3238.8314604 | 6 |
| 3238.8316253 | 4 |
| 3238.8316907 | 4 |
| 3238.8321309 | 2 |
| 3238.8322752 | 3 |
| 3238.8334219 | 1 |
| 3238.833532 | 0 |
| 3238.8338232 | 5 |
| 3238.833884 | 3 |
| 3238.8339896 | 5 |
| 3238.8339912 | 5 |
| 3238.8344225 | 3 |
| 3238.8349546 | 4 |
| 3238.8351196 | 1 |
| 3238.8354309 | 1 |
| 3238.8361355 | 3 |
| 3238.8362399 | 3 |
| 3238.8363224 | 1 |
| 3238.8363922 | 2 |
| 3238.8367972 | 5 |
| 3238.8374616 | 0 |
| 3238.8376458 | 1 |
| 3238.8378872 | 3 |
| 3238.837941 | 2 |
| 3238.8382198 | 2 |
| 3238.8382835 | 2 |
| 3238.8385509 | 2 |
| 3238.8390686 | 2 |
| 3238.8392915 | 2 |
| 3238.8393741 | 2 |
| 3238.8397511 | 3 |
| 3238.8401743 | 2 |
| 3238.8403334 | 3 |
| 3238.8404526 | 1 |
| 3238.841053 | 2 |
| 3238.8410661 | 2 |
| 3238.8412214 | 3 |
| 3238.8415102 | 1 |
| 3238.8416037 | 3 |
| 3238.841717 | 3 |
| 3238.8425625 | 0 |
| 3238.8425642 | 5 |
| 3238.8428895 | 2 |
| 3238.8429016 | 1 |
| 3238.8429671 | 0 |
| 3238.8434111 | 1 |
| 3238.8435529 | 7 |
| 3238.8439427 | 1 |
| 3238.844305 | 3 |
| 3238.844623 | 4 |
| 3238.844883 | 4 |
| 3238.8449486 | 3 |
| 3238.8455403 | 0 |
| 3238.8456489 | 3 |
| 3238.8458207 | 2 |
| 3238.8459318 | 4 |
| 3238.8460618 | 1 |
| 3238.8461246 | 2 |
| 3238.8466602 | 2 |
| 3238.8466917 | 2 |
| 3238.846875 | 0 |
| 3238.8470505 | 4 |
| 3238.8478853 | 4 |
| 3238.8479355 | 7 |
| 3238.848172 | 9 |
| 3238.8485092 | 9 |
| 3238.84863 | 12 |
| 3238.8489776 | 17 |
| 3238.8494137 | 11 |
| 3238.8494774 | 12 |
| 3238.8496004 | 18 |
| 3238.8500733 | 11 |
| 3238.8501563 | 5 |
| 3238.8502388 | 15 |
| 3238.8505613 | 17 |
| 3238.8510062 | 12 |
| 3238.8510333 | 15 |
| 3238.8515632 | 12 |
| 3238.8516339 | 20 |
| 3238.8520876 | 12 |
| 3238.8521122 | 11 |
| 3238.8521626 | 13 |
| 3238.8523931 | 22 |
| 3238.8528919 | 14 |
| 3238.8529652 | 20 |
| 3238.8532277 | 14 |
| 3238.8533968 | 10 |
| 3238.8534212 | 13 |
| 3238.8536692 | 7 |
| 3238.8540368 | 9 |
| 3238.8546382 | 16 |
| 3238.854683 | 21 |
| 3238.8546886 | 16 |
| 3238.8551546 | 12 |
| 3238.8555427 | 14 |
| 3238.8555841 | 11 |
| 3238.8556133 | 14 |
| 3238.8562348 | 16 |
| 3238.8564004 | 15 |
| 3238.8564354 | 13 |
| 3238.8571145 | 5 |
| 3238.8572699 | 15 |
| 3238.8573481 | 3 |
| 3238.8573792 | 6 |
| 3238.8579474 | 3 |
| 3238.8580594 | 5 |
| 3238.8581572 | 0 |
| 3238.8584638 | 3 |
| 3238.8592985 | 1 |
| 3238.8594071 | 4 |
| 3238.8595686 | 5 |
| 3238.8596581 | 4 |
| 3238.8600005 | 1 |
| 3238.8603369 | 3 |
| 3238.8605785 | 0 |
| 3238.8606995 | 2 |
| 3238.8610721 | 2 |
| 3238.8611755 | 1 |
| 3238.8613857 | 2 |
| 3238.8615437 | 2 |
| 3238.861783 | 3 |
| 3238.8622733 | 1 |
| 3238.8625554 | 2 |
| 3238.8628096 | 5 |
| 3238.8634176 | 4 |
| 3238.8635949 | 2 |
| 3238.8636333 | 3 |
| 3238.863787 | 4 |
| 3238.8640872 | 1 |
| 3238.8641232 | 6 |
| 3238.8642384 | 4 |
| 3238.8645875 | 2 |
| 3238.8653498 | 3 |
| 3238.8655048 | 2 |
| 3238.8656336 | 2 |
| 3238.8659147 | 2 |
| 3238.8661591 | 1 |
| 3238.8665213 | 2 |
| 3238.8666692 | 1 |
| 3238.8669792 | 2 |
| 3238.8672735 | 0 |
| 3238.8675449 | 4 |
| 3238.8677214 | 2 |
| 3238.8682604 | 1 |
| 3238.8683286 | 3 |
| 3238.8687531 | 1 |
| 3238.8697444 | 5 |
| 3238.870886 | 4 |
| 3238.8719176 | 6 |
| 3238.8739601 | 1 |
| 3238.8743303 | 4 |
| 3238.8761944 | 2 |
| 3238.8774942 | 1 |
| 3238.8786021 | 4 |
| 3238.8799157 | 2 |
| 3238.8810443 | 3 |
| 3238.8820708 | 5 |
| 3238.8834663 | 2 |
| 3238.8844586 | 0 |
| 3238.8864878 | 3 |
| 3238.8866861 | 2 |
| 3238.8884707 | 0 |
| 3238.8901494 | 1 |
| 3238.8903037 | 1 |
| 3238.8915109 | 5 |
| 3238.8929186 | 1 |
| 3238.8941805 | 3 |
| 3238.8953474 | 4 |
| 3238.897038 | 3 |
| 3238.8981868 | 3 |
| 3238.8988097 | 4 |
| 3238.9007028 | 2 |
| 3238.9007741 | 0 |
| 3238.9021681 | 3 |
| 3238.9050199 | 5 |
| 3238.9053377 | 2 |
| 3238.9068983 | 3 |
| 3238.9077385 | 2 |
| 3238.9090407 | 1 |
| 3238.9101559 | 1 |
| 3238.9119987 | 3 |
| 3238.9131671 | 2 |
| 3238.9140844 | 2 |
| 3238.9159345 | 3 |
| 3238.9173331 | 1 |
| 3238.9178884 | 4 |
| 3238.9198245 | 7 |
| 3238.9198349 | 0 |
| 3238.9212101 | 5 |
| 3238.9234839 | 5 |
| 3238.9247775 | 1 |
| 3238.9260359 | 4 |
| 3238.9261426 | 2 |
| 3238.9283274 | 3 |
| 3238.9293716 | 4 |
| 3238.9304958 | 2 |
| 3238.9318964 | 3 |
| 3238.9328135 | 3 |
| 3238.9343577 | 4 |
| 3238.935508 | 1 |
| 3238.9363283 | 2 |
| 3238.9379489 | 4 |
| 3238.9387325 | 4 |
| 3238.9405362 | 2 |
| 3238.9406126 | 2 |
| 3238.9429074 | 1 |
| 3238.9438008 | 3 |
| 3238.9441028 | 3 |
| 3238.9460804 | 6 |
| 3238.9480576 | 6 |
| 3238.9490042 | 0 |
| 3238.950057 | 3 |
| 3238.9508112 | 2 |
| 3238.9529258 | 0 |
| 3238.9539101 | 1 |
| 3238.9545241 | 5 |
| 3238.9567107 | 3 |
| 3238.9572731 | 4 |
| 3238.9594471 | 1 |
| 3238.9598643 | 1 |
| 3238.9614106 | 2 |
| 3238.9627325 | 1 |
| 3238.9631969 | 1 |
| 3238.9654904 | 3 |
| 3238.9672435 | 1 |
| 3238.9678247 | 1 |
| 3238.9692732 | 2 |
| 3238.9698568 | 4 |
| 3238.9711944 | 4 |
| 3238.9731479 | 2 |
| 3238.9736662 | 2 |
| 3238.9752605 | 1 |
| 3238.9757633 | 0 |
| 3238.9772897 | 2 |
| 3238.9783552 | 4 |
| 3238.9797205 | 4 |
| 3238.9810266 | 2 |
| 3238.9821689 | 2 |
| 3238.9839639 | 5 |
| 3238.9840815 | 3 |
| 3238.9857281 | 4 |
| 3238.987245 | 1 |
| 3238.9876742 | 3 |
| 3238.9890723 | 4 |
| 3238.9911213 | 4 |
| 3238.9912755 | 1 |
| 3238.9927407 | 2 |
| 3238.9941542 | 1 |
| 3238.9954457 | 0 |
| 3238.9971444 | 3 |
| 3238.9985956 | 4 |
| 3238.9998715 | 3 |
| 3239.0010283 | 1 |
| 3239.0030163 | 3 |
| 3239.0031571 | 2 |
| 3239.0041354 | 2 |
| 3239.0061282 | 4 |
| 3239.0062336 | 4 |
| 3239.0080858 | 4 |
| 3239.0091505 | 4 |
| 3239.0108919 | 0 |
| 3239.0122476 | 2 |
| 3239.0136494 | 2 |
| 3239.0144137 | 2 |
| 3239.0156403 | 2 |
| 3239.0173457 | 2 |
| 3239.0175715 | 3 |
| 3239.0191207 | 2 |
| 3239.0210663 | 1 |
| 3239.0218381 | 4 |
| 3239.0230234 | 4 |
| 3239.0237719 | 1 |
| 3239.0255182 | 0 |
| 3239.0269819 | 4 |
| 3239.0275263 | 0 |
| 3239.0296684 | 4 |
| 3239.0306784 | 1 |
| 3239.0313178 | 3 |
| 3239.0326787 | 2 |
| 3239.0338076 | 0 |
| 3239.0350054 | 1 |
| 3239.0367756 | 4 |
| 3239.0377387 | 3 |
| 3239.039256 | 2 |
| 3239.0397591 | 0 |
| 3239.0410473 | 2 |
| 3239.0432289 | 4 |
| 3239.0438557 | 3 |
| 3239.0449852 | 7 |
| 3239.0469021 | 0 |
| 3239.0484521 | 5 |
| 3239.0497922 | 2 |
| 3239.0505191 | 6 |
| 3239.0525518 | 1 |
| 3239.052562 | 4 |
| 3239.054659 | 1 |
| 3239.0550401 | 1 |
| 3239.0568451 | 2 |
| 3239.0577598 | 3 |
| 3239.0592683 | 2 |
| 3239.0603484 | 3 |
| 3239.0616964 | 0 |
| 3239.0630372 | 5 |
| 3239.0645512 | 5 |
| 3239.0650266 | 3 |
| 3239.0672834 | 3 |
| 3239.0675356 | 0 |
| 3239.0689131 | 3 |
| 3239.0706933 | 3 |
| 3239.070887 | 3 |
| 3239.0730158 | 3 |
| 3239.0737904 | 3 |
| 3239.0753664 | 1 |
| 3239.0765662 | 1 |
| 3239.0774969 | 2 |
| 3239.0788743 | 4 |
| 3239.0802751 | 5 |
| 3239.081286 | 2 |
| 3239.0828795 | 3 |
| 3239.0835335 | 3 |
| 3239.0856808 | 3 |
| 3239.086905 | 1 |
| 3239.0874147 | 0 |
| 3239.089608 | 3 |
| 3239.0900137 | 2 |
| 3239.0908789 | 5 |
| 3239.0925049 | 0 |
| 3239.0943205 | 1 |
| 3239.0960181 | 1 |
| 3239.0968289 | 1 |
| 3239.0983049 | 2 |
| 3239.0985911 | 5 |
| 3239.1000669 | 3 |
| 3239.1021491 | 2 |
| 3239.1023107 | 3 |
| 3239.1042158 | 2 |
| 3239.1052006 | 2 |
| 3239.1067134 | 3 |
| 3239.1079062 | 2 |
| 3239.1088097 | 2 |
| 3239.1106523 | 1 |
| 3239.1121291 | 2 |
| 3239.112202 | 0 |
| 3239.1147965 | 2 |

| | |
|---|---|
| 3239.1151564 | 4 |
| 3239.1162105 | 1 |
| 3239.1177286 | 1 |
| 3239.1197307 | 5 |
| 3239.1203794 | 4 |
| 3239.1217679 | 1 |
| 3239.1223869 | 4 |
| 3239.1243895 | 4 |
| 3239.1249161 | 2 |
| 3239.1260857 | 3 |
| 3239.1276334 | 3 |
| 3239.1286118 | 1 |
| 3239.1299997 | 3 |
| 3239.1318522 | 0 |
| 3239.1327336 | 0 |
| 3239.1337863 | 5 |
| 3239.1353502 | 4 |
| 3239.1360353 | 2 |
| 3239.1371974 | 0 |
| 3239.1387275 | 2 |
| 3239.1399407 | 4 |
| 3239.141615 | 2 |
| 3239.1429146 | 1 |
| 3239.1440842 | 5 |
| 3239.146306 | 5 |
| 3239.1470038 | 4 |
| 3239.1481287 | 5 |
| 3239.1498229 | 1 |
| 3239.1500403 | 4 |
| 3239.1519136 | 2 |
| 3239.1527644 | 0 |
| 3239.1535019 | 3 |
| 3239.1552116 | 3 |
| 3239.1565861 | 3 |
| 3239.1577215 | 0 |
| 3239.1587961 | 2 |
| 3239.1609117 | 3 |
| 3239.1621531 | 1 |
| 3239.1629987 | 3 |
| 3239.1648073 | 4 |
| 3239.1648237 | 2 |
| 3239.1667069 | 1 |
| 3239.1670712 | 2 |
| 3239.1683436 | 3 |
| 3239.1699471 | 1 |
| 3239.1710612 | 2 |
| 3239.17252 | 0 |
| 3239.1739849 | 1 |
| 3239.1746745 | 2 |
| 3239.1764054 | 3 |
| 3239.1775926 | 1 |
| 3239.1788663 | 1 |
| 3239.1799434 | 3 |
| 3239.1818364 | 1 |
| 3239.1834889 | 7 |
| 3239.1839827 | 2 |
| 3239.1856317 | 2 |
| 3239.1864648 | 3 |
| 3239.1875758 | 2 |
| 3239.1879974 | 1 |
| 3239.1890239 | 0 |
| 3239.1893142 | 1 |
| 3239.1905891 | 1 |
| 3239.1910415 | 2 |
| 3239.1919996 | 2 |
| 3239.1920383 | 0 |
| 3239.1938635 | 3 |
| 3239.1940817 | 2 |
| 3239.1945073 | 5 |
| 3239.1945595 | 3 |
| 3239.1960747 | 3 |
| 3239.1965377 | 1 |
| 3239.1968105 | 2 |
| 3239.1981817 | 3 |
| 3239.198532 | 4 |
| 3239.1991753 | 2 |
| 3239.2002518 | 0 |
| 3239.2007683 | 4 |
| 3239.2007967 | 2 |
| 3239.202537 | 2 |
| 3239.2026448 | 1 |
| 3239.2032792 | 3 |
| 3239.204471 | 7 |
| 3239.2045056 | 2 |
| 3239.2057061 | 3 |
| 3239.2064632 | 3 |
| 3239.2068152 | 2 |
| 3239.2072372 | 3 |
| 3239.208386 | 2 |
| 3239.2089338 | 1 |
| 3239.2094938 | 1 |
| 3239.2101918 | 3 |
| 3239.2107459 | 1 |
| 3239.2113608 | 1 |
| 3239.2123046 | 3 |
| 3239.2128962 | 2 |
| 3239.2135034 | 3 |
| 3239.2148428 | 3 |
| 3239.2150434 | 4 |
| 3239.2151105 | 2 |
| 3239.2162904 | 0 |
| 3239.2168628 | 5 |
| 3239.2170286 | 0 |
| 3239.2178524 | 3 |
| 3239.219377 | 1 |
| 3239.2199192 | 0 |
| 3239.2207281 | 1 |
| 3239.2216607 | 7 |
| 3239.2218309 | 4 |
| 3239.2231571 | 5 |
| 3239.2234636 | 4 |
| 3239.2239384 | 5 |
| 3239.2252434 | 3 |
| 3239.2255954 | 2 |
| 3239.2257675 | 2 |
| 3239.2268333 | 2 |
| 3239.2275403 | 1 |
| 3239.2280784 | 5 |
| 3239.2298138 | 1 |
| 3239.2299325 | 3 |
| 3239.2304751 | 3 |
| 3239.2310071 | 1 |
| 3239.2321894 | 1 |
| 3239.2324997 | 4 |
| 3239.2333644 | 0 |
| 3239.2337524 | 4 |
| 3239.2348106 | 1 |
| 3239.2351187 | 1 |
| 3239.2364721 | 1 |
| 3239.2368039 | 5 |
| 3239.2369756 | 1 |
| 3239.2382347 | 2 |
| 3239.2382801 | 3 |
| 3239.2399862 | 1 |
| 3239.2405016 | 4 |
| 3239.2406135 | 1 |
| 3239.2411888 | 2 |
| 3239.241907 | 6 |
| 3239.2426842 | 3 |
| 3239.2428442 | 1 |
| 3239.2441002 | 5 |
| 3239.2447615 | 1 |
| 3239.2460993 | 0 |
| 3239.2461004 | 2 |
| 3239.2469533 | 0 |
| 3239.248112 | 0 |
| 3239.2485456 | 2 |
| 3239.2491309 | 2 |
| 3239.2496125 | 1 |
| 3239.2514515 | 1 |
| 3239.2514528 | 3 |
| 3239.2521538 | 2 |
| 3239.2526702 | 2 |
| 3239.2533455 | 5 |
| 3239.2535464 | 3 |
| 3239.254731 | 1 |
| 3239.2556613 | 2 |
| 3239.2559424 | 1 |
| 3239.2565276 | 3 |
| 3239.2568976 | 4 |
| 3239.2585686 | 1 |
| 3239.2586491 | 0 |
| 3239.2589859 | 0 |
| 3239.2599444 | 3 |
| 3239.2604397 | 2 |
| 3239.26127 | 1 |
| 3239.26271 | 2 |
| 3239.2629919 | 4 |
| 3239.2637279 | 3 |
| 3239.263779 | 0 |
| 3239.2647724 | 3 |
| 3239.2658985 | 7 |
| 3239.2670075 | 5 |
| 3239.2671563 | 2 |
| 3239.2677728 | 3 |
| 3239.2679011 | 2 |
| 3239.2700763 | 1 |
| 3239.2708838 | 0 |
| 3239.270904 | 3 |
| 3239.2712832 | 3 |
| 3239.2715906 | 3 |
| 3239.2725956 | 2 |
| 3239.2733302 | 1 |
| 3239.2746383 | 1 |
| 3239.2747042 | 3 |
| 3239.2747768 | 3 |
| 3239.2754014 | 2 |
| 3239.2767114 | 1 |
| 3239.2772993 | 4 |
| 3239.2776632 | 2 |
| 3239.2797592 | 2 |
| 3239.2798103 | 1 |
| 3239.2803146 | 3 |
| 3239.2812213 | 1 |
| 3239.282144 | 4 |
| 3239.2825567 | 2 |
| 3239.2830036 | 3 |
| 3239.2839307 | 3 |
| 3239.2844643 | 5 |
| 3239.2853438 | 2 |
| 3239.2858531 | 3 |
| 3239.2865249 | 4 |
| 3239.2871782 | 2 |
| 3239.2878363 | 1 |
| 3239.2881665 | 4 |
| 3239.2896534 | 3 |
| 3239.2903061 | 2 |
| 3239.2903872 | 1 |
| 3239.290485 | 3 |
| 3239.2912489 | 1 |
| 3239.2923611 | 2 |
| 3239.293636 | 1 |
| 3239.2936649 | 5 |
| 3239.2939455 | 2 |
| 3239.2952834 | 2 |
| 3239.2957159 | 2 |
| 3239.2962095 | 2 |
| 3239.2973484 | 3 |
| 3239.297786 | 1 |
| 3239.2979985 | 0 |
| 3239.2987232 | 3 |
| 3239.2994295 | 6 |
| 3239.300402 | 1 |
| 3239.3008727 | 1 |
| 3239.3014483 | 6 |
| 3239.3017502 | 0 |
| 3239.3017917 | 1 |
| 3239.3040473 | 4 |
| 3239.3056018 | 3 |
| 3239.3064678 | 2 |
| 3239.3086298 | 1 |
| 3239.3095265 | 2 |
| 3239.311755 | 0 |
| 3239.3123982 | 2 |
| 3239.3142504 | 1 |
| 3239.3164041 | 1 |
| 3239.3173503 | 4 |
| 3239.3199016 | 1 |
| 3239.3210813 | 3 |
| 3239.3228427 | 3 |
| 3239.324179 | 1 |
| 3239.3249444 | 2 |
| 3239.32702 | 4 |
| 3239.3282725 | 3 |
| 3239.3293596 | 3 |
| 3239.332052 | 1 |
| 3239.3325829 | 4 |
| 3239.3343813 | 2 |
| 3239.3372817 | 0 |
| 3239.3373316 | 3 |
| 3239.339544 | 3 |
| 3239.3406133 | 3 |
| 3239.3427476 | 2 |
| 3239.3435609 | 2 |
| 3239.3460102 | 3 |
| 3239.3478871 | 0 |
| 3239.3484092 | 3 |
| 3239.3503904 | 3 |
| 3239.351661 | 2 |
| 3239.3537567 | 5 |
| 3239.3549905 | 3 |
| 3239.3562114 | 1 |
| 3239.35797 | 3 |
| 3239.3602377 | 2 |
| 3239.3608528 | 1 |
| 3239.3635273 | 2 |
| 3239.364323 | 1 |
| 3239.3662821 | 3 |
| 3239.3675123 | 4 |
| 3239.3688187 | 1 |
| 3239.3706622 | 3 |
| 3239.3719736 | 1 |
| 3239.3739947 | 3 |
| 3239.3746883 | 3 |
| 3239.3763702 | 2 |
| 3239.3784966 | 2 |
| 3239.3792316 | 0 |
| 3239.3816286 | 3 |
| 3239.3832295 | 4 |
| 3239.3843508 | 1 |
| 3239.3864737 | 3 |
| 3239.3871618 | 2 |
| 3239.3896424 | 1 |
| 3239.3904276 | 1 |
| 3239.3916614 | 1 |
| 3239.3942374 | 5 |
| 3239.3953903 | 2 |
| 3239.3972129 | 1 |
| 3239.3980809 | 3 |
| 3239.4001989 | 2 |
| 3239.4021477 | 3 |
| 3239.4029368 | 3 |
| 3239.4055601 | 4 |
| 3239.4060183 | 0 |
| 3239.4072682 | 0 |
| 3239.4101882 | 0 |
| 3239.4115139 | 3 |
| 3239.4131813 | 2 |
| 3239.4142352 | 1 |
| 3239.4158456 | 3 |
| 3239.4178988 | 1 |
| 3239.4184262 | 5 |
| 3239.4200311 | 1 |
| 3239.4218652 | 1 |
| 3239.4233568 | 5 |
| 3239.4249849 | 2 |
| 3239.4264475 | 4 |
| 3239.4286731 | 4 |
| 3239.4292656 | 0 |
| 3239.4313571 | 6 |
| 3239.4329921 | 1 |
| 3239.4339235 | 1 |
| 3239.4356056 | 1 |
| 3239.4357571 | 1 |
| 3239.4373749 | 2 |
| 3239.4377455 | 0 |
| 3239.4379517 | 2 |
| 3239.4389458 | 5 |
| 3239.4399692 | 1 |
| 3239.44046 | 1 |
| 3239.4411443 | 4 |
| 3239.4419736 | 1 |
| 3239.4435091 | 4 |
| 3239.4439526 | 3 |
| 3239.4444921 | 3 |
| 3239.4453854 | 1 |
| 3239.446305 | 3 |
| 3239.4467822 | 1 |
| 3239.4480688 | 2 |
| 3239.4483284 | 2 |
| 3239.4494052 | 3 |
| 3239.4495814 | 1 |
| 3239.4508727 | 1 |
| 3239.4512954 | 0 |
| 3239.4519245 | 4 |
| 3239.4530762 | 4 |
| 3239.4540236 | 3 |
| 3239.4550276 | 3 |
| 3239.4555301 | 6 |
| 3239.4561334 | 2 |
| 3239.4569273 | 2 |
| 3239.4574807 | 1 |
| 3239.459018 | 0 |
| 3239.4593376 | 3 |
| 3239.4599855 | 3 |
| 3239.4602583 | 2 |
| 3239.4609452 | 2 |
| 3239.4618727 | 2 |
| 3239.4643032 | 1 |
| 3239.4646493 | 2 |
| 3239.4667498 | 1 |
| 3239.467714 | 1 |
| 3239.4696169 | 3 |
| 3239.4713344 | 2 |
| 3239.4729571 | 5 |
| 3239.4740224 | 3 |
| 3239.4753225 | 2 |
| 3239.4776512 | 4 |
| 3239.479497 | 3 |
| 3239.479691 | 3 |
| 3239.482086 | 6 |
| 3239.4829826 | 2 |
| 3239.484637 | 3 |

| | |
|---|---|
| 3239.4868242 | 3 |
| 3239.4880427 | 3 |
| 3239.4900803 | 1 |
| 3239.49099 | 1 |
| 3239.4923984 | 1 |
| 3239.494841 | 3 |
| 3239.4954886 | 2 |
| 3239.498316 | 1 |
| 3239.4984734 | 1 |
| 3239.5003695 | 3 |
| 3239.5024564 | 3 |
| 3239.503721 | 3 |
| 3239.504942 | 2 |
| 3239.5065723 | 2 |
| 3239.5087031 | 5 |
| 3239.5105423 | 2 |
| 3239.5116374 | 5 |
| 3239.5132031 | 2 |
| 3239.5140563 | 2 |
| 3239.516251 | 3 |
| 3239.5174182 | 4 |
| 3239.5188391 | 0 |
| 3239.520774 | 2 |
| 3239.5216913 | 4 |
| 3239.5242157 | 1 |
| 3239.5248015 | 5 |
| 3239.5265562 | 1 |
| 3239.5287994 | 0 |
| 3239.5293884 | 3 |
| 3239.5315055 | 3 |
| 3239.5322641 | 4 |
| 3239.5344344 | 2 |
| 3239.5363893 | 1 |
| 3239.5373889 | 3 |
| 3239.5394835 | 2 |
| 3239.5405363 | 1 |
| 3239.5426866 | 2 |
| 3239.5442573 | 1 |
| 3239.5453356 | 2 |
| 3239.5474003 | 4 |
| 3239.547869 | 1 |
| 3239.5506397 | 2 |
| 3239.5521369 | 2 |
| 3239.5527891 | 0 |
| 3239.5551573 | 2 |
| 3239.5558323 | 0 |
| 3239.5577854 | 0 |
| 3239.5600508 | 1 |
| 3239.5606154 | 2 |
| 3239.5621318 | 4 |
| 3239.5632526 | 4 |
| 3239.5656695 | 2 |
| 3239.5677032 | 2 |
| 3239.5680698 | 1 |
| 3239.5702224 | 2 |
| 3239.571177 | 2 |
| 3239.5730327 | 6 |
| 3239.5738251 | 1 |
| 3239.5755921 | 3 |
| 3239.5781396 | 1 |
| 3239.5787466 | 4 |
| 3239.5803923 | 2 |
| 3239.5827215 | 4 |
| 3239.5840582 | 0 |
| 3239.5857363 | 3 |
| 3239.5867265 | 2 |
| 3239.5884527 | 2 |
| 3239.5902137 | 1 |
| 3239.5915859 | 1 |
| 3239.5935527 | 0 |
| 3239.5946381 | 1 |
| 3239.5963292 | 1 |
| 3239.5970994 | 2 |
| 3239.5994861 | 0 |
| 3239.6012802 | 1 |
| 3239.6022337 | 1 |
| 3239.6041922 | 0 |
| 3239.604862 | 2 |
| 3239.6068304 | 2 |
| 3239.6091765 | 0 |
| 3239.6099371 | 3 |
| 3239.611854 | 5 |
| 3239.612938 | 5 |
| 3239.6153503 | 1 |
| 3239.6165995 | 0 |
| 3239.6175687 | 3 |
| 3239.6192815 | 0 |
| 3239.6206128 | 4 |
| 3239.622368 | 1 |
| 3239.6243446 | 1 |
| 3239.6250396 | 1 |
| 3239.6271382 | 2 |
| 3239.6279431 | 3 |
| 3239.630445 | 2 |
| 3239.6314929 | 3 |
| 3239.6338119 | 3 |
| 3239.6353219 | 3 |
| 3239.6363381 | 6 |
| 3239.6377997 | 2 |
| 3239.6390375 | 3 |
| 3239.6404875 | 3 |
| 3239.6427364 | 3 |
| 3239.643968 | 1 |
| 3239.6459819 | 1 |
| 3239.6477156 | 2 |
| 3239.6488654 | 2 |
| 3239.6502383 | 6 |
| 3239.6515168 | 0 |
| 3239.6538299 | 3 |
| 3239.6548086 | 2 |
| 3239.6563833 | 1 |
| 3239.6583315 | 3 |
| 3239.6585118 | 5 |
| 3239.6593585 | 0 |
| 3239.6597232 | 3 |
| 3239.6607056 | 4 |
| 3239.6610177 | 5 |
| 3239.6612392 | 1 |
| 3239.6617296 | 2 |
| 3239.6620312 | 3 |
| 3239.6629219 | 1 |
| 3239.6636024 | 1 |
| 3239.6636625 | 0 |
| 3239.663681 | 4 |
| 3239.6650532 | 1 |
| 3239.6658184 | 3 |
| 3239.6659135 | 1 |
| 3239.6663251 | 2 |
| 3239.6668259 | 1 |
| 3239.6675554 | 2 |
| 3239.6677361 | 1 |
| 3239.6682988 | 1 |
| 3239.6684205 | 1 |
| 3239.6689989 | 0 |
| 3239.6694019 | 0 |
| 3239.6695565 | 1 |
| 3239.6697856 | 5 |
| 3239.6702013 | 2 |
| 3239.671114 | 2 |
| 3239.6712135 | 4 |
| 3239.6719696 | 2 |
| 3239.6727381 | 3 |
| 3239.6727607 | 2 |
| 3239.6728164 | 3 |
| 3239.6729849 | 3 |
| 3239.674413 | 2 |
| 3239.6752144 | 2 |
| 3239.6752369 | 0 |
| 3239.6753264 | 2 |
| 3239.6756852 | 1 |
| 3239.6767615 | 1 |
| 3239.6769721 | 3 |
| 3239.6772755 | 1 |
| 3239.6776161 | 4 |
| 3239.6779852 | 1 |
| 3239.6781887 | 0 |
| 3239.6784116 | 1 |
| 3239.6789501 | 2 |
| 3239.6792385 | 1 |
| 3239.6793312 | 0 |
| 3239.6794728 | 6 |
| 3239.6803629 | 0 |
| 3239.6806938 | 3 |
| 3239.6812869 | 10 |
| 3239.6817022 | 2 |
| 3239.6818496 | 1 |
| 3239.6820839 | 2 |
| 3239.6826003 | 2 |
| 3239.6829099 | 1 |
| 3239.6833894 | 1 |
| 3239.6838606 | 2 |
| 3239.684075 | 5 |
| 3239.6843023 | 1 |
| 3239.684586 | 2 |
| 3239.6854287 | 1 |
| 3239.6859037 | 4 |
| 3239.6861636 | 2 |
| 3239.6862843 | 3 |
| 3239.6867434 | 6 |
| 3239.6869133 | 2 |
| 3239.6869545 | 4 |
| 3239.6871407 | 1 |
| 3239.68787 | 2 |
| 3239.6887824 | 0 |
| 3239.6887856 | 1 |
| 3239.6888383 | 3 |
| 3239.688902 | 1 |
| 3239.6889921 | 3 |
| 3239.6897499 | 4 |
| 3239.6912149 | 1 |
| 3239.6934823 | 0 |
| 3239.6948321 | 3 |
| 3239.6966905 | 2 |
| 3239.6984095 | 1 |
| 3239.6990832 | 2 |
| 3239.7019293 | 1 |
| 3239.7025029 | 1 |
| 3239.7048975 | 2 |
| 3239.7059216 | 4 |
| 3239.7073868 | 2 |
| 3239.7089466 | 2 |
| 3239.7099135 | 5 |
| 3239.7119656 | 4 |
| 3239.7137191 | 4 |
| 3239.7142885 | 2 |
| 3239.7165144 | 2 |
| 3239.7173214 | 1 |
| 3239.7197017 | 4 |
| 3239.721758 | 2 |
| 3239.7226757 | 2 |
| 3239.7238866 | 2 |
| 3239.7259564 | 0 |
| 3239.7280409 | 0 |
| 3239.7296332 | 0 |
| 3239.7303358 | 0 |
| 3239.7326781 | 4 |
| 3239.7333222 | 5 |
| 3239.7353393 | 3 |
| 3239.7373444 | 2 |
| 3239.7380739 | 5 |
| 3239.7390739 | 2 |
| 3239.7393106 | 3 |
| 3239.7404019 | 2 |
| 3239.7415666 | 1 |
| 3239.7423939 | 2 |
| 3239.7436962 | 0 |
| 3239.7443228 | 3 |
| 3239.745385 | 6 |
| 3239.7461175 | 1 |
| 3239.7463388 | 2 |
| 3239.747835 | 2 |
| 3239.7484144 | 0 |
| 3239.7493912 | 0 |
| 3239.7508511 | 4 |
| 3239.7511815 | 1 |
| 3239.7520185 | 3 |
| 3239.7531023 | 2 |
| 3239.7540813 | 2 |
| 3239.7555953 | 5 |
| 3239.7563422 | 2 |
| 3239.7572318 | 0 |
| 3239.757488 | 1 |
| 3239.7590312 | 2 |
| 3239.7596273 | 1 |
| 3239.7605982 | 7 |
| 3239.7613322 | 2 |
| 3239.7625651 | 3 |
| 3239.7636855 | 1 |
| 3239.7641801 | 1 |
| 3239.7656021 | 0 |
| 3239.7662615 | 0 |
| 3239.7672911 | 2 |
| 3239.7679198 | 2 |
| 3239.7690877 | 1 |
| 3239.7701076 | 2 |
| 3239.7712975 | 1 |
| 3239.7718536 | 1 |
| 3239.7733222 | 2 |
| 3239.7734791 | 1 |
| 3239.7752953 | 2 |
| 3239.7759331 | 1 |
| 3239.7768979 | 3 |
| 3239.7777468 | 1 |
| 3239.778406 | 2 |
| 3239.7796265 | 5 |
| 3239.7808436 | 3 |
| 3239.781332 | 0 |
| 3239.7824533 | 3 |
| 3239.7826252 | 2 |
| 3239.7840059 | 2 |
| 3239.7854204 | 2 |
| 3239.7866043 | 2 |
| 3239.7871105 | 3 |
| 3239.7881904 | 1 |
| 3239.7888582 | 1 |
| 3239.7900124 | 1 |
| 3239.7905719 | 0 |
| 3239.7917743 | 2 |
| 3239.792672 | 6 |
| 3239.7932895 | 3 |
| 3239.7938628 | 4 |
| 3239.7947655 | 1 |
| 3239.7960312 | 1 |
| 3239.7968049 | 0 |
| 3239.7981587 | 1 |
| 3239.7987013 | 1 |
| 3239.7997063 | 3 |
| 3239.8005833 | 1 |
| 3239.8007856 | 5 |
| 3239.8019334 | 4 |
| 3239.8038582 | 2 |
| 3239.8040863 | 3 |
| 3239.8054037 | 4 |
| 3239.8057699 | 2 |
| 3239.8073171 | 1 |
| 3239.8080989 | 2 |
| 3239.8090665 | 3 |
| 3239.8099325 | 3 |
| 3239.810421 | 4 |
| 3239.8119369 | 2 |
| 3239.8130627 | 4 |
| 3239.8135599 | 0 |
| 3239.8153377 | 3 |
| 3239.8156684 | 3 |
| 3239.8169059 | 3 |
| 3239.8179167 | 3 |
| 3239.8186969 | 3 |
| 3239.8197231 | 2 |
| 3239.8199787 | 6 |
| 3239.8217761 | 1 |
| 3239.8221983 | 0 |
| 3239.8228903 | 2 |
| 3239.8242043 | 2 |
| 3239.8256765 | 3 |
| 3239.8261697 | 1 |
| 3239.8267352 | 3 |
| 3239.8277209 | 2 |
| 3239.8290504 | 1 |
| 3239.8296478 | 3 |
| 3239.8308064 | 2 |
| 3239.831703 | 4 |
| 3239.8326247 | 5 |
| 3239.8332342 | 2 |
| 3239.8343718 | 0 |
| 3239.8353536 | 3 |
| 3239.8365839 | 2 |
| 3239.8372228 | 3 |
| 3239.8381224 | 4 |
| 3239.8389382 | 0 |
| 3239.839519 | 0 |
| 3239.8410029 | 3 |
| 3239.8417953 | 4 |
| 3239.8426053 | 1 |
| 3239.8436377 | 1 |
| 3239.8447703 | 2 |
| 3239.8453359 | 0 |
| 3239.8465059 | 2 |
| 3239.8479927 | 1 |
| 3239.8481249 | 2 |
| 3239.8492789 | 2 |
| 3239.8503615 | 2 |
| 3239.8507575 | 1 |
| 3239.8518803 | 2 |
| 3239.8529919 | 0 |
| 3239.854389 | 2 |
| 3239.8544124 | 3 |
| 3239.8552655 | 0 |
| 3239.8566643 | 1 |
| 3239.8572373 | 3 |
| 3239.8585183 | 2 |
| 3239.8595703 | 3 |
| 3239.8611055 | 1 |
| 3239.8614116 | 3 |
| 3239.8627336 | 1 |
| 3239.8635747 | 0 |
| 3239.8643002 | 0 |
| 3239.8656587 | 1 |
| 3239.8657958 | 4 |
| 3239.8669058 | 4 |
| 3239.8683596 | 3 |
| 3239.8693188 | 3 |
| 3239.8701454 | 2 |
| 3239.8711738 | 1 |

| | |
|---|---|
| 3239.8722058 | 1 |
| 3239.8732151 | 1 |
| 3239.8738155 | 2 |
| 3239.8753732 | 2 |
| 3239.8755754 | 4 |
| 3239.8769908 | 2 |
| 3239.8772467 | 4 |
| 3239.8783489 | 2 |
| 3239.8794702 | 0 |
| 3239.8802018 | 6 |
| 3239.8814534 | 1 |
| 3239.8818799 | 0 |
| 3239.8834386 | 3 |
| 3239.8843698 | 3 |
| 3239.8848216 | 2 |
| 3239.8862399 | 3 |
| 3239.8873296 | 2 |
| 3239.8874283 | 3 |
| 3239.8886051 | 3 |
| 3239.8892708 | 1 |
| 3239.891044 | 0 |
| 3239.8914533 | 2 |
| 3239.8921924 | 0 |
| 3239.8937498 | 5 |
| 3239.8941128 | 2 |
| 3239.8954468 | 4 |
| 3239.8956542 | 0 |
| 3239.8967622 | 2 |
| 3239.8972604 | 6 |
| 3239.8978552 | 0 |
| 3239.8980141 | 0 |
| 3239.8987485 | 4 |
| 3239.8989292 | 3 |
| 3239.9002829 | 2 |
| 3239.9004096 | 3 |
| 3239.900626 | 3 |
| 3239.9009423 | 3 |
| 3239.9018871 | 3 |
| 3239.9021067 | 4 |
| 3239.9030272 | 6 |
| 3239.9032485 | 8 |
| 3239.9036156 | 9 |
| 3239.9037474 | 7 |
| 3239.9048583 | 6 |
| 3239.9052897 | 11 |
| 3239.9055103 | 10 |
| 3239.9057847 | 10 |
| 3239.9060587 | 7 |
| 3239.9068719 | 1 |
| 3239.9071288 | 4 |
| 3239.9082142 | 5 |
| 3239.9083273 | 3 |
| 3239.9088267 | 2 |
| 3239.9094084 | 3 |
| 3239.9097298 | 1 |
| 3239.9103155 | 3 |
| 3239.910442 | 5 |
| 3239.91142 | 1 |
| 3239.9115737 | 1 |
| 3239.9116348 | 5 |
| 3239.9122247 | 3 |
| 3239.9132802 | 1 |
| 3239.9134406 | 2 |
| 3239.9140927 | 2 |
| 3239.9144173 | 1 |
| 3239.9145744 | 3 |
| 3239.9146219 | 6 |
| 3239.9160687 | 1 |
| 3239.91639 | 2 |
| 3239.9166547 | 2 |
| 3239.9169701 | 2 |
| 3239.9176204 | 0 |
| 3239.9182361 | 1 |
| 3239.9189245 | 3 |
| 3239.9193953 | 2 |
| 3239.9197236 | 1 |
| 3239.9198857 | 2 |
| 3239.9208831 | 1 |
| 3239.9209157 | 1 |
| 3239.9213631 | 3 |
| 3239.9224418 | 5 |
| 3239.922484 | 0 |
| 3239.9225073 | 0 |
| 3239.923977 | 0 |
| 3239.9240872 | 3 |
| 3239.9243295 | 3 |
| 3239.9250314 | 3 |
| 3239.9251192 | 5 |
| 3239.9253073 | 3 |
| 3239.9258379 | 1 |
| 3239.9260503 | 5 |
| 3239.926269 | 1 |
| 3239.9269123 | 4 |
| 3239.9271585 | 5 |
| 3239.9271723 | 2 |
| 3239.9279304 | 3 |
| 3239.9286591 | 6 |
| 3239.9289301 | 2 |
| 3239.9296289 | 1 |
| 3239.9297553 | 3 |
| 3239.9301537 | 1 |
| 3239.9305525 | 1 |
| 3239.9322186 | 4 |
| 3239.932316 | 5 |
| 3239.9335801 | 2 |
| 3239.9348484 | 1 |
| 3239.9353179 | 6 |
| 3239.9357447 | 0 |
| 3239.9368976 | 2 |
| 3239.9382609 | 1 |
| 3239.9395385 | 1 |
| 3239.9399961 | 1 |
| 3239.9410887 | 4 |
| 3239.9412956 | 0 |
| 3239.9428104 | 4 |
| 3239.9442574 | 3 |
| 3239.9445303 | 1 |
| 3239.9457302 | 1 |
| 3239.9474112 | 6 |
| 3239.947885 | 3 |
| 3239.9491219 | 2 |
| 3239.9493025 | 4 |
| 3239.9508918 | 1 |
| 3239.9515993 | 1 |
| 3239.9522906 | 2 |
| 3239.9535896 | 2 |
| 3239.9545571 | 1 |
| 3239.9558765 | 2 |
| 3239.9560761 | 2 |
| 3239.9573089 | 4 |
| 3239.9584316 | 2 |
| 3239.9591778 | 2 |
| 3239.9604843 | 4 |
| 3239.9606978 | 2 |
| 3239.9621216 | 1 |
| 3239.9622153 | 1 |
| 3239.9642836 | 2 |
| 3239.9651871 | 2 |
| 3239.965554 | 0 |
| 3239.9671602 | 0 |
| 3239.9677269 | 2 |
| 3239.9683457 | 3 |
| 3239.9697778 | 1 |
| 3239.9705613 | 2 |
| 3239.9715449 | 4 |
| 3239.9721871 | 1 |
| 3239.9731723 | 4 |
| 3239.9743503 | 2 |
| 3239.9750111 | 3 |
| 3239.9763287 | 2 |
| 3239.9767662 | 3 |
| 3239.9779591 | 2 |
| 3239.9789835 | 4 |
| 3239.9794337 | 0 |
| 3239.9810268 | 0 |
| 3239.9813287 | 1 |
| 3239.9824706 | 3 |
| 3239.9837632 | 3 |
| 3239.9846769 | 1 |
| 3239.9853032 | 1 |
| 3239.9859098 | 1 |
| 3239.9870761 | 6 |
| 3239.9873585 | 3 |
| 3239.9887817 | 1 |
| 3239.9901516 | 1 |
| 3239.9905636 | 0 |
| 3239.9924803 | 2 |
| 3239.9928492 | 2 |
| 3239.9931146 | 5 |
| 3239.9934044 | 4 |
| 3239.9935832 | 2 |
| 3239.9940029 | 3 |
| 3239.9947187 | 0 |
| 3239.9948922 | 1 |
| 3239.9949018 | 2 |
| 3239.9949802 | 2 |
| 3239.9951457 | 2 |
| 3239.9956451 | 1 |
| 3239.9960368 | 1 |
| 3239.9971735 | 4 |
| 3239.9973704 | 4 |
| 3239.9975253 | 0 |
| 3239.9977219 | 0 |
| 3239.9977435 | 1 |
| 3239.9978418 | 2 |
| 3239.9982854 | 3 |
| 3239.9988315 | 2 |
| 3239.9995679 | 1 |
| 3239.999748 | 1 |
| 3240.0001013 | 1 |
| 3240.0002008 | 1 |
| 3240.0012814 | 2 |
| 3240.0016194 | 3 |
| 3240.0018924 | 1 |
| 3240.0019124 | 6 |
| 3240.0020109 | 4 |
| 3240.0022018 | 0 |
| 3240.0026676 | 2 |
| 3240.0033602 | 2 |
| 3240.0036833 | 2 |
| 3240.004282 | 5 |
| 3240.0043231 | 1 |
| 3240.0046346 | 0 |
| 3240.0048259 | 4 |
| 3240.0051011 | 2 |
| 3240.0060477 | 1 |
| 3240.006109 | 2 |
| 3240.0063447 | 1 |
| 3240.0065533 | 3 |
| 3240.0069218 | 3 |
| 3240.0073583 | 1 |
| 3240.0075223 | 0 |
| 3240.0076958 | 0 |
| 3240.008544 | 3 |
| 3240.0091424 | 1 |
| 3240.0093673 | 4 |
| 3240.0097027 | 5 |
| 3240.0098353 | 0 |
| 3240.009963 | 3 |
| 3240.0103479 | 2 |
| 3240.01036 | 3 |
| 3240.0108949 | 1 |
| 3240.0111368 | 3 |
| 3240.0116245 | 1 |
| 3240.0122836 | 4 |
| 3240.0124257 | 2 |
| 3240.012793 | 1 |
| 3240.0131021 | 4 |
| 3240.013276 | 3 |
| 3240.0140664 | 4 |
| 3240.0142239 | 2 |
| 3240.0144673 | 3 |
| 3240.0146274 | 4 |
| 3240.0155306 | 3 |
| 3240.0159868 | 1 |
| 3240.0160417 | 0 |
| 3240.016205 | 2 |
| 3240.0166463 | 2 |
| 3240.0166594 | 4 |
| 3240.016955 | 1 |
| 3240.0179917 | 5 |
| 3240.0182784 | 4 |
| 3240.0183376 | 3 |
| 3240.0184078 | 2 |
| 3240.018744 | 3 |
| 3240.0190592 | 0 |
| 3240.0191614 | 1 |
| 3240.0194829 | 5 |
| 3240.020564 | 1 |
| 3240.0206295 | 2 |
| 3240.0208888 | 3 |
| 3240.020964 | 4 |
| 3240.0214169 | 2 |
| 3240.0221818 | 7 |
| 3240.0224853 | 3 |
| 3240.0228046 | 5 |
| 3240.0228606 | 7 |
| 3240.0230404 | 5 |
| 3240.0237245 | 7 |
| 3240.0239146 | 10 |
| 3240.0246538 | 15 |
| 3240.0246661 | 13 |
| 3240.0251249 | 10 |
| 3240.025306 | 13 |
| 3240.0255213 | 6 |
| 3240.0255808 | 9 |
| 3240.0263679 | 9 |
| 3240.0265369 | 6 |
| 3240.0269468 | 2 |
| 3240.0270612 | 1 |
| 3240.0279447 | 3 |
| 3240.0282741 | 1 |
| 3240.0283946 | 4 |
| 3240.0284802 | 5 |
| 3240.0293844 | 2 |
| 3240.0295011 | 1 |
| 3240.0297168 | 3 |
| 3240.0302555 | 2 |
| 3240.0303037 | 1 |
| 3240.030637 | 2 |
| 3240.0312244 | 2 |
| 3240.0313119 | 4 |
| 3240.0313298 | 1 |
| 3240.0320219 | 1 |
| 3240.0325506 | 6 |
| 3240.0330834 | 6 |
| 3240.0331371 | 2 |
| 3240.0336667 | 2 |
| 3240.0338016 | 2 |
| 3240.0341154 | 2 |
| 3240.0342678 | 1 |
| 3240.0344528 | 4 |
| 3240.0352281 | 3 |
| 3240.0358454 | 3 |
| 3240.0360391 | 0 |
| 3240.0361404 | 4 |
| 3240.0365471 | 4 |
| 3240.0365766 | 1 |
| 3240.0368295 | 1 |
| 3240.0377395 | 1 |
| 3240.038123 | 3 |
| 3240.0385403 | 1 |
| 3240.0387442 | 5 |
| 3240.0389243 | 2 |
| 3240.0390578 | 1 |
| 3240.0395956 | 1 |
| 3240.0396606 | 3 |
| 3240.0403215 | 1 |
| 3240.0405374 | 2 |
| 3240.0409279 | 6 |
| 3240.0409363 | 2 |
| 3240.0418419 | 3 |
| 3240.0419883 | 2 |
| 3240.0426627 | 1 |
| 3240.0430376 | 0 |
| 3240.0432653 | 3 |
| 3240.0434545 | 3 |
| 3240.043745 | 4 |
| 3240.0441732 | 3 |
| 3240.0442608 | 2 |
| 3240.0447257 | 3 |
| 3240.0449666 | 3 |
| 3240.0459402 | 2 |
| 3240.0461101 | 3 |
| 3240.0462791 | 2 |
| 3240.0465046 | 3 |
| 3240.0467672 | 3 |
| 3240.0472157 | 2 |
| 3240.0477461 | 1 |
| 3240.0481655 | 2 |
| 3240.0488559 | 5 |
| 3240.0488802 | 2 |
| 3240.0495258 | 1 |
| 3240.0509402 | 2 |
| 3240.0523561 | 4 |
| 3240.0532513 | 3 |
| 3240.0545569 | 0 |
| 3240.0556137 | 1 |
| 3240.0570357 | 2 |
| 3240.0584763 | 3 |
| 3240.0588065 | 3 |
| 3240.0605469 | 2 |
| 3240.0620105 | 0 |
| 3240.063386 | 2 |
| 3240.0645638 | 3 |
| 3240.0658142 | 1 |
| 3240.0669679 | 2 |
| 3240.0674201 | 4 |
| 3240.0688712 | 1 |
| 3240.0708407 | 4 |
| 3240.0712075 | 3 |
| 3240.0733181 | 2 |
| 3240.0734886 | 1 |
| 3240.0757948 | 1 |
| 3240.076678 | 1 |
| 3240.0773834 | 5 |
| 3240.0797806 | 4 |
| 3240.0803833 | 1 |
| 3240.0816983 | 0 |
| 3240.0825842 | 1 |
| 3240.0837296 | 3 |
| 3240.0860236 | 1 |
| 3240.0866966 | 2 |

| | |
|---|---|
| 3240.0886351 | 2 |
| 3240.0888045 | 4 |
| 3240.0905623 | 1 |
| 3240.0918241 | 3 |
| 3240.093025 | 4 |
| 3240.094835 | 1 |
| 3240.0953068 | 1 |
| 3240.0964303 | 2 |
| 3240.0986355 | 3 |
| 3240.0991072 | 2 |
| 3240.1007362 | 2 |
| 3240.1025378 | 1 |
| 3240.103126 | 3 |
| 3240.104875 | 3 |
| 3240.1050072 | 1 |
| 3240.1062307 | 4 |
| 3240.1085257 | 0 |
| 3240.1092053 | 4 |
| 3240.1106528 | 1 |
| 3240.1116268 | 3 |
| 3240.1127652 | 1 |
| 3240.1133455 | 3 |
| 3240.1150154 | 3 |
| 3240.1168808 | 1 |
| 3240.1177328 | 0 |
| 3240.1194255 | 3 |
| 3240.1197688 | 0 |
| 3240.1221093 | 2 |
| 3240.1236972 | 2 |
| 3240.1244414 | 3 |
| 3240.1256603 | 0 |
| 3240.1262584 | 1 |
| 3240.1276448 | 3 |
| 3240.1289179 | 3 |
| 3240.1304077 | 1 |
| 3240.1321861 | 0 |
| 3240.1327579 | 4 |
| 3240.1343695 | 2 |
| 3240.1358337 | 1 |
| 3240.1366494 | 5 |
| 3240.1382184 | 2 |
| 3240.1392992 | 3 |
| 3240.1409632 | 2 |
| 3240.1421181 | 1 |
| 3240.1432208 | 3 |
| 3240.1451875 | 2 |
| 3240.145229 | 2 |
| 3240.1474514 | 3 |
| 3240.1482281 | 3 |
| 3240.1488629 | 7 |
| 3240.1504181 | 1 |
| 3240.1516981 | 3 |
| 3240.153195 | 2 |
| 3240.1542036 | 1 |
| 3240.1556791 | 1 |
| 3240.1563793 | 3 |
| 3240.1582769 | 5 |
| 3240.1595119 | 0 |
| 3240.1606052 | 2 |
| 3240.1615569 | 2 |
| 3240.1624271 | 3 |
| 3240.1637223 | 6 |
| 3240.1655018 | 1 |
| 3240.1665408 | 6 |
| 3240.1678227 | 2 |
| 3240.1685199 | 3 |
| 3240.1703905 | 0 |
| 3240.1712092 | 3 |
| 3240.1728572 | 3 |
| 3240.1744695 | 1 |
| 3240.1753752 | 3 |
| 3240.1769601 | 4 |
| 3240.1773675 | 2 |
| 3240.1791615 | 1 |
| 3240.1807349 | 3 |
| 3240.1815341 | 1 |
| 3240.1829768 | 2 |
| 3240.1847109 | 3 |
| 3240.1856644 | 2 |
| 3240.1873034 | 2 |
| 3240.1878524 | 2 |
| 3240.1892468 | 2 |
| 3240.1902109 | 5 |
| 3240.1920325 | 2 |
| 3240.1931348 | 2 |
| 3240.1941086 | 2 |
| 3240.1954068 | 0 |
| 3240.1964644 | 4 |
| 3240.1982505 | 1 |
| 3240.1988475 | 2 |
| 3240.2004976 | 2 |
| 3240.2017058 | 3 |
| 3240.2029334 | 3 |
| 3240.2040661 | 2 |
| 3240.2053536 | 1 |
| 3240.2066756 | 2 |
| 3240.2086153 | 0 |
| 3240.2089474 | 1 |
| 3240.2106948 | 3 |
| 3240.2113254 | 1 |
| 3240.2130901 | 1 |
| 3240.2141735 | 3 |
| 3240.2154143 | 1 |
| 3240.2165741 | 4 |
| 3240.2172893 | 2 |
| 3240.2188337 | 2 |
| 3240.2204184 | 4 |
| 3240.2207214 | 0 |
| 3240.2222487 | 3 |
| 3240.2235003 | 4 |
| 3240.2250412 | 3 |
| 3240.226892 | 3 |
| 3240.2276821 | 0 |
| 3240.2294414 | 1 |
| 3240.2297768 | 2 |
| 3240.2311386 | 3 |
| 3240.233238 | 3 |
| 3240.2339233 | 2 |
| 3240.2354682 | 1 |
| 3240.2366189 | 3 |
| 3240.2378626 | 2 |
| 3240.2393639 | 2 |
| 3240.2401502 | 1 |
| 3240.2414195 | 4 |
| 3240.2427823 | 2 |
| 3240.2440626 | 0 |
| 3240.2454257 | 1 |
| 3240.2459776 | 0 |
| 3240.248007 | 2 |
| 3240.2489037 | 1 |
| 3240.250088 | 1 |
| 3240.2514424 | 0 |
| 3240.2523251 | 4 |
| 3240.2544344 | 3 |
| 3240.2544444 | 2 |
| 3240.2560532 | 3 |
| 3240.2578193 | 2 |
| 3240.2591414 | 3 |
| 3240.260413 | 1 |
| 3240.2606744 | 3 |
| 3240.2626576 | 0 |
| 3240.2639681 | 2 |
| 3240.2641011 | 2 |
| 3240.2664571 | 4 |
| 3240.2671603 | 1 |
| 3240.268489 | 5 |
| 3240.2705121 | 2 |
| 3240.2710259 | 3 |
| 3240.2731091 | 3 |
| 3240.2736928 | 1 |
| 3240.2747552 | 1 |
| 3240.2758301 | 0 |
| 3240.277597 | 1 |
| 3240.2789774 | 3 |
| 3240.2796172 | 6 |
| 3240.281038 | 3 |
| 3240.2824649 | 0 |
| 3240.2840018 | 3 |
| 3240.2857517 | 2 |
| 3240.2864409 | 0 |
| 3240.2880666 | 4 |
| 3240.2882721 | 0 |
| 3240.2897512 | 2 |
| 3240.2905945 | 1 |
| 3240.2924118 | 1 |
| 3240.2934288 | 2 |
| 3240.2939053 | 2 |
| 3240.2956049 | 1 |
| 3240.2973192 | 0 |
| 3240.2981774 | 4 |
| 3240.2995907 | 1 |
| 3240.3011299 | 2 |
| 3240.3022759 | 3 |
| 3240.3036249 | 2 |
| 3240.3045297 | 1 |
| 3240.305685 | 0 |
| 3240.3066274 | 0 |
| 3240.3083358 | 1 |
| 3240.3094392 | 1 |
| 3240.3106196 | 2 |
| 3240.3124054 | 3 |
| 3240.3131791 | 2 |
| 3240.3150211 | 1 |
| 3240.3158881 | 1 |
| 3240.3176047 | 2 |
| 3240.3186803 | 2 |
| 3240.3191382 | 2 |
| 3240.321518 | 0 |
| 3240.3220422 | 1 |
| 3240.3225671 | 2 |
| 3240.32523 | 1 |
| 3240.3253855 | 2 |
| 3240.3265709 | 0 |
| 3240.326799 | 0 |
| 3240.3271932 | 4 |
| 3240.3273416 | 4 |
| 3240.3286048 | 3 |
| 3240.3287937 | 1 |
| 3240.3292255 | 4 |
| 3240.3296542 | 1 |
| 3240.3300087 | 3 |
| 3240.3301001 | 3 |
| 3240.3310517 | 4 |
| 3240.3318509 | 3 |
| 3240.331863 | 1 |
| 3240.3319026 | 1 |
| 3240.3325524 | 0 |
| 3240.3326965 | 1 |
| 3240.3334113 | 2 |
| 3240.3335008 | 4 |
| 3240.3341724 | 0 |
| 3240.3347373 | 3 |
| 3240.3348865 | 2 |
| 3240.3350242 | 3 |
| 3240.3350558 | 0 |
| 3240.3355489 | 4 |
| 3240.3360433 | 2 |
| 3240.3369603 | 5 |
| 3240.3371524 | 5 |
| 3240.3376108 | 2 |
| 3240.3382201 | 5 |
| 3240.3382331 | 2 |
| 3240.3385248 | 4 |
| 3240.3390394 | 1 |
| 3240.3395741 | 0 |
| 3240.3403965 | 1 |
| 3240.3404233 | 2 |
| 3240.3409394 | 2 |
| 3240.3413484 | 1 |
| 3240.3416349 | 3 |
| 3240.3417132 | 5 |
| 3240.3426533 | 3 |
| 3240.342942 | 10 |
| 3240.3431686 | 3 |
| 3240.3437606 | 4 |
| 3240.3442704 | 1 |
| 3240.3446601 | 4 |
| 3240.3452347 | 1 |
| 3240.3453906 | 5 |
| 3240.3458604 | 1 |
| 3240.345873 | 1 |
| 3240.346513 | 5 |
| 3240.3470918 | 4 |
| 3240.3475788 | 3 |
| 3240.3478595 | 4 |
| 3240.3481834 | 0 |
| 3240.3483846 | 5 |
| 3240.3491762 | 0 |
| 3240.3500695 | 4 |
| 3240.3504925 | 5 |
| 3240.3508394 | 0 |
| 3240.3510159 | 1 |
| 3240.3514232 | 1 |
| 3240.3515067 | 4 |
| 3240.3523669 | 2 |
| 3240.3524394 | 2 |
| 3240.3527392 | 3 |
| 3240.353058 | 1 |
| 3240.3533448 | 2 |
| 3240.3534689 | 3 |
| 3240.354258 | 2 |
| 3240.3545265 | 1 |
| 3240.3551545 | 1 |
| 3240.3552456 | 3 |
| 3240.3561934 | 5 |
| 3240.3566088 | 0 |
| 3240.3574028 | 3 |
| 3240.3575057 | 3 |
| 3240.358115 | 2 |
| 3240.3581364 | 1 |
| 3240.3581598 | 2 |
| 3240.3591756 | 0 |
| 3240.3591892 | 4 |
| 3240.3604881 | 3 |
| 3240.3613768 | 4 |
| 3240.3633311 | 1 |
| 3240.3646696 | 4 |
| 3240.3659922 | 3 |
| 3240.3663437 | 1 |
| 3240.3682995 | 1 |
| 3240.3694305 | 4 |
| 3240.3707506 | 2 |
| 3240.3725124 | 2 |
| 3240.3725888 | 5 |
| 3240.3747199 | 1 |
| 3240.3759792 | 3 |
| 3240.3768178 | 3 |
| 3240.3778278 | 2 |
| 3240.3791667 | 2 |
| 3240.3799819 | 5 |
| 3240.3821369 | 2 |
| 3240.3823161 | 1 |
| 3240.3847583 | 0 |
| 3240.3850977 | 4 |
| 3240.3868001 | 1 |
| 3240.3878956 | 3 |
| 3240.3889387 | 2 |
| 3240.3904374 | 5 |
| 3240.3911008 | 1 |
| 3240.3926572 | 0 |
| 3240.39431 | 0 |
| 3240.3951631 | 1 |
| 3240.3969245 | 0 |
| 3240.3969746 | 5 |
| 3240.3993761 | 2 |
| 3240.4005296 | 3 |
| 3240.4015949 | 5 |
| 3240.403434 | 1 |
| 3240.4036618 | 1 |
| 3240.4060088 | 3 |
| 3240.406206 | 1 |
| 3240.4076935 | 3 |
| 3240.4091573 | 2 |
| 3240.4099076 | 2 |
| 3240.4118172 | 0 |
| 3240.4129227 | 0 |
| 3240.413803 | 2 |
| 3240.4161722 | 0 |
| 3240.4169376 | 2 |
| 3240.4183887 | 2 |
| 3240.4183921 | 2 |
| 3240.4203014 | 1 |
| 3240.4217717 | 3 |
| 3240.4230246 | 2 |
| 3240.4240421 | 0 |
| 3240.4252409 | 2 |
| 3240.4266502 | 1 |
| 3240.4282106 | 2 |
| 3240.4284019 | 0 |
| 3240.4300783 | 0 |
| 3240.4309285 | 4 |
| 3240.4325579 | 0 |
| 3240.4344675 | 0 |
| 3240.4349227 | 2 |
| 3240.43646 | 2 |
| 3240.4368403 | 1 |
| 3240.439263 | 3 |
| 3240.4403196 | 2 |
| 3240.4416687 | 6 |
| 3240.4429545 | 2 |
| 3240.4433057 | 2 |
| 3240.4456828 | 0 |
| 3240.4458102 | 1 |
| 3240.4474316 | 1 |
| 3240.4491042 | 4 |
| 3240.4502316 | 3 |
| 3240.4522311 | 1 |
| 3240.4522681 | 3 |
| 3240.4541533 | 1 |
| 3240.4551149 | 4 |
| 3240.4567708 | 0 |
| 3240.4580255 | 2 |
| 3240.4587342 | 1 |
| 3240.4607975 | 2 |
| 3240.4618443 | 4 |
| 3240.4624687 | 1 |
| 3240.4645791 | 2 |
| 3240.4650173 | 3 |
| 3240.4663603 | 3 |
| 3240.4684065 | 2 |
| 3240.4688605 | 7 |
| 3240.4703072 | 3 |
| 3240.4722823 | 3 |

| | |
|---|---|
| 3240.4725634 | 4 |
| 3240.4744578 | 2 |
| 3240.4751485 | 0 |
| 3240.4762501 | 2 |
| 3240.4770137 | 2 |
| 3240.4791343 | 5 |
| 3240.4802573 | 2 |
| 3240.4811515 | 2 |
| 3240.4825161 | 2 |
| 3240.4837621 | 2 |
| 3240.4857892 | 1 |
| 3240.4860628 | 1 |
| 3240.4877158 | 2 |
| 3240.4888024 | 4 |
| 3240.489621 | 4 |
| 3240.4913747 | 3 |
| 3240.4929039 | 4 |
| 3240.4941055 | 5 |
| 3240.4951962 | 2 |
| 3240.4960479 | 3 |
| 3240.4979967 | 3 |
| 3240.4984537 | 1 |
| 3240.5000436 | 0 |
| 3240.5020327 | 0 |
| 3240.5026228 | 2 |
| 3240.5045401 | 1 |
| 3240.5047976 | 2 |
| 3240.5063697 | 3 |
| 3240.5079419 | 0 |
| 3240.5085166 | 1 |
| 3240.5099447 | 0 |
| 3240.5118442 | 1 |
| 3240.5130848 | 1 |
| 3240.5141898 | 1 |
| 3240.5154447 | 4 |
| 3240.5162453 | 4 |
| 3240.5174224 | 3 |
| 3240.5185911 | 2 |
| 3240.5203694 | 1 |
| 3240.5213209 | 0 |
| 3240.522737 | 4 |
| 3240.5234156 | 4 |
| 3240.5253586 | 7 |
| 3240.5271638 | 1 |
| 3240.5274066 | 2 |
| 3240.5292617 | 2 |
| 3240.5296329 | 1 |
| 3240.5315817 | 3 |
| 3240.5331153 | 0 |
| 3240.5341411 | 2 |
| 3240.5355513 | 3 |
| 3240.5358129 | 0 |
| 3240.5377341 | 1 |
| 3240.5387768 | 0 |
| 3240.5399286 | 2 |
| 3240.5410708 | 2 |
| 3240.5425115 | 6 |
| 3240.5438144 | 1 |
| 3240.545705 | 1 |
| 3240.5466634 | 0 |
| 3240.5477945 | 2 |
| 3240.5488207 | 1 |
| 3240.5509744 | 0 |
| 3240.5525555 | 3 |
| 3240.5530427 | 3 |
| 3240.5546211 | 2 |
| 3240.5548246 | 4 |
| 3240.5565859 | 0 |
| 3240.5581281 | 3 |
| 3240.5589343 | 2 |
| 3240.5605608 | 1 |
| 3240.5616715 | 4 |
| 3240.5632835 | 1 |
| 3240.5649313 | 2 |
| 3240.5655476 | 1 |
| 3240.567468 | 4 |
| 3240.5680655 | 3 |
| 3240.5687438 | 4 |
| 3240.5708003 | 7 |
| 3240.5715364 | 1 |
| 3240.5731354 | 2 |
| 3240.5737775 | 0 |
| 3240.5755421 | 1 |
| 3240.5769759 | 3 |
| 3240.578123 | 0 |
| 3240.5792741 | 3 |
| 3240.580316 | 0 |
| 3240.581914 | 0 |
| 3240.5825634 | 1 |
| 3240.58415 | 2 |
| 3240.586187 | 1 |
| 3240.586437 | 2 |
| 3240.5871054 | 2 |
| 3240.5877657 | 0 |
| 3240.587901 | 1 |
| 3240.5890702 | 2 |
| 3240.5898665 | 3 |
| 3240.5900067 | 2 |
| 3240.5910817 | 2 |
| 3240.5915323 | 1 |
| 3240.5917392 | 3 |
| 3240.593007 | 2 |
| 3240.5930242 | 0 |
| 3240.593269 | 2 |
| 3240.5936116 | 0 |
| 3240.5943753 | 2 |
| 3240.5951684 | 0 |
| 3240.5956858 | 1 |
| 3240.5959431 | 0 |
| 3240.5966609 | 1 |
| 3240.5967985 | 2 |
| 3240.597737 | 2 |
| 3240.5981825 | 3 |
| 3240.5986415 | 1 |
| 3240.5994483 | 1 |
| 3240.5996 | 1 |
| 3240.6009026 | 7 |
| 3240.6010465 | 0 |
| 3240.6013136 | 2 |
| 3240.6020331 | 0 |
| 3240.6022789 | 2 |
| 3240.6032623 | 1 |
| 3240.603728 | 1 |
| 3240.6039541 | 1 |
| 3240.6046181 | 3 |
| 3240.605034 | 3 |
| 3240.6061998 | 3 |
| 3240.6064223 | 3 |
| 3240.6074506 | 1 |
| 3240.607558 | 1 |
| 3240.6078346 | 1 |
| 3240.6082419 | 1 |
| 3240.6090252 | 0 |
| 3240.6095461 | 3 |
| 3240.6102284 | 2 |
| 3240.610418 | 1 |
| 3240.6114174 | 2 |
| 3240.6115111 | 3 |
| 3240.6116112 | 3 |
| 3240.6125858 | 0 |
| 3240.6133101 | 1 |
| 3240.6149542 | 3 |
| 3240.6150979 | 2 |
| 3240.6161829 | 0 |
| 3240.6175867 | 2 |
| 3240.6181714 | 2 |
| 3240.618849 | 4 |
| 3240.6201728 | 1 |
| 3240.621158 | 0 |
| 3240.6213082 | 2 |
| 3240.6227569 | 2 |
| 3240.6241947 | 3 |
| 3240.6250209 | 1 |
| 3240.6251876 | 3 |
| 3240.6272158 | 2 |
| 3240.6274785 | 2 |
| 3240.6287113 | 1 |
| 3240.630108 | 0 |
| 3240.6301754 | 3 |
| 3240.6319347 | 0 |
| 3240.6321233 | 1 |
| 3240.6334826 | 2 |
| 3240.6340643 | 1 |
| 3240.6349448 | 3 |
| 3240.6360455 | 3 |
| 3240.63678 | 1 |
| 3240.6380138 | 1 |
| 3240.6396583 | 2 |
| 3240.639827 | 3 |
| 3240.6405366 | 2 |
| 3240.6415771 | 3 |
| 3240.6426336 | 1 |
| 3240.6437267 | 1 |
| 3240.6448617 | 2 |
| 3240.6456453 | 3 |
| 3240.6458963 | 1 |
| 3240.6467778 | 2 |
| 3240.6481207 | 4 |
| 3240.6493393 | 2 |
| 3240.6498958 | 1 |
| 3240.6512141 | 1 |
| 3240.651998 | 2 |
| 3240.6527315 | 1 |
| 3240.6542181 | 1 |
| 3240.6548771 | 2 |
| 3240.6554368 | 1 |
| 3240.6563029 | 1 |
| 3240.6573149 | 4 |
| 3240.6577642 | 1 |
| 3240.6599282 | 3 |
| 3240.6604921 | 3 |
| 3240.6617465 | 0 |
| 3240.6617723 | 2 |
| 3240.6629688 | 3 |
| 3240.6639919 | 2 |
| 3240.6646175 | 1 |
| 3240.6660051 | 1 |
| 3240.6674346 | 3 |
| 3240.668062 | 5 |
| 3240.6690408 | 3 |
| 3240.6694183 | 1 |
| 3240.6709446 | 4 |
| 3240.6710502 | 1 |
| 3240.6730905 | 0 |
| 3240.6736753 | 2 |
| 3240.6748713 | 2 |
| 3240.67576 | 3 |
| 3240.6761658 | 0 |
| 3240.6774527 | 3 |
| 3240.6789268 | 1 |
| 3240.6794676 | 1 |
| 3240.6800012 | 3 |
| 3240.6812182 | 0 |
| 3240.6821372 | 2 |
| 3240.6833105 | 1 |
| 3240.6842941 | 2 |
| 3240.6858639 | 5 |
| 3240.6859601 | 1 |
| 3240.6871646 | 0 |
| 3240.6880931 | 0 |
| 3240.6881833 | 1 |
| 3240.6899215 | 1 |
| 3240.6899292 | 4 |
| 3240.6918708 | 0 |
| 3240.6931112 | 1 |
| 3240.6933263 | 3 |
| 3240.6942468 | 1 |
| 3240.6949239 | 4 |
| 3240.696387 | 0 |
| 3240.6974384 | 1 |
| 3240.6979942 | 1 |
| 3240.6985354 | 2 |
| 3240.6993861 | 3 |
| 3240.7007081 | 0 |
| 3240.7018173 | 3 |
| 3240.7025477 | 0 |
| 3240.7034989 | 3 |
| 3240.7041736 | 1 |
| 3240.7049587 | 1 |
| 3240.7061258 | 1 |
| 3240.7070747 | 2 |
| 3240.7089752 | 5 |
| 3240.7091598 | 4 |
| 3240.710066 | 1 |
| 3240.7110366 | 1 |
| 3240.7114431 | 0 |
| 3240.7127028 | 2 |
| 3240.7138593 | 4 |
| 3240.7152865 | 1 |
| 3240.7154101 | 1 |
| 3240.7169428 | 0 |
| 3240.7180264 | 1 |
| 3240.7184198 | 2 |
| 3240.7197959 | 1 |
| 3240.72004 | 1 |
| 3240.7214787 | 0 |
| 3240.7222184 | 2 |
| 3240.7231112 | 3 |
| 3240.7245853 | 1 |
| 3240.7255355 | 0 |
| 3240.7267011 | 1 |
| 3240.7268499 | 1 |
| 3240.7278455 | 0 |
| 3240.7290363 | 1 |
| 3240.7293036 | 2 |
| 3240.7310023 | 2 |
| 3240.73233 | 0 |
| 3240.7327177 | 1 |
| 3240.733547 | 1 |
| 3240.7342491 | 0 |
| 3240.7350055 | 2 |
| 3240.7362056 | 0 |
| 3240.7373437 | 3 |
| 3240.738726 | 2 |
| 3240.7396084 | 0 |
| 3240.7403518 | 1 |
| 3240.7406656 | 3 |
| 3240.7422518 | 1 |
| 3240.7429886 | 1 |
| 3240.743695 | 0 |
| 3240.7446569 | 2 |
| 3240.7454363 | 2 |
| 3240.7470806 | 2 |
| 3240.7476278 | 1 |
| 3240.7480025 | 2 |
| 3240.7491976 | 1 |
| 3240.7495095 | 3 |
| 3240.7509289 | 2 |
| 3240.7519894 | 4 |
| 3240.7535104 | 1 |
| 3240.7544094 | 1 |
| 3240.7544212 | 0 |
| 3240.7563312 | 1 |
| 3240.7570075 | 1 |
| 3240.7574281 | 0 |
| 3240.7587436 | 0 |
| 3240.759398 | 2 |
| 3240.7605888 | 0 |
| 3240.7612032 | 2 |
| 3240.762822 | 2 |
| 3240.7634316 | 1 |
| 3240.7637453 | 2 |
| 3240.76503 | 0 |
| 3240.7664626 | 3 |
| 3240.7673452 | 1 |
| 3240.7685367 | 1 |
| 3240.7690191 | 0 |
| 3240.7698977 | 3 |
| 3240.7701724 | 3 |
| 3240.7714903 | 1 |
| 3240.7726619 | 3 |
| 3240.773729 | 2 |
| 3240.7749854 | 1 |
| 3240.77606 | 0 |
| 3240.7768081 | 0 |
| 3240.7770534 | 3 |
| 3240.7777378 | 0 |
| 3240.779873 | 4 |
| 3240.7803999 | 1 |
| 3240.7809294 | 2 |
| 3240.7820079 | 3 |
| 3240.7828071 | 2 |
| 3240.7842822 | 1 |
| 3240.7850126 | 2 |
| 3240.7858488 | 1 |
| 3240.7866538 | 2 |
| 3240.7870258 | 1 |
| 3240.7883873 | 2 |
| 3240.789632 | 2 |
| 3240.7904425 | 5 |
| 3240.7914395 | 3 |
| 3240.7926361 | 2 |
| 3240.7931385 | 3 |
| 3240.7942302 | 3 |
| 3240.7944254 | 1 |
| 3240.7962607 | 0 |
| 3240.7968484 | 2 |
| 3240.7976779 | 0 |
| 3240.7991188 | 3 |
| 3240.7996162 | 1 |
| 3240.8005514 | 1 |
| 3240.8012442 | 0 |
| 3240.8028965 | 3 |
| 3240.8035987 | 2 |
| 3240.8044612 | 0 |
| 3240.8057054 | 1 |
| 3240.8065112 | 2 |
| 3240.8074398 | 0 |
| 3240.8076738 | 1 |
| 3240.8092665 | 4 |
| 3240.8105574 | 0 |
| 3240.810811 | 1 |
| 3240.8120676 | 1 |
| 3240.8126864 | 5 |
| 3240.8141788 | 1 |
| 3240.8155482 | 2 |
| 3240.8158101 | 1 |
| 3240.8166339 | 0 |
| 3240.8178842 | 2 |
| 3240.8183996 | 1 |
| 3240.8197213 | 1 |
| 3240.8208922 | 1 |
| 3240.8215722 | 2 |

| | |
|---|---|
| 3240.8224797 | 0 |
| 3240.823412 | 1 |
| 3240.8243552 | 0 |
| 3240.8251106 | 3 |
| 3240.8269162 | 0 |
| 3240.8269293 | 0 |
| 3240.8282823 | 4 |
| 3240.8292263 | 3 |
| 3240.8300012 | 3 |
| 3240.8308005 | 3 |
| 3240.8309739 | 2 |
| 3240.8325596 | 2 |
| 3240.8329663 | 2 |
| 3240.8343386 | 2 |
| 3240.836312 | 0 |
| 3240.8363198 | 4 |
| 3240.837193 | 1 |
| 3240.838045 | 0 |
| 3240.8391175 | 1 |
| 3240.8396883 | 0 |
| 3240.8410404 | 3 |
| 3240.8418392 | 0 |
| 3240.8421802 | 1 |
| 3240.8434507 | 3 |
| 3240.8448551 | 0 |
| 3240.8452282 | 1 |
| 3240.8470121 | 1 |
| 3240.8470997 | 1 |
| 3240.8480697 | 3 |
| 3240.849425 | 1 |
| 3240.8501163 | 0 |
| 3240.8509962 | 3 |
| 3240.8514234 | 0 |
| 3240.8533533 | 2 |
| 3240.8540769 | 0 |
| 3240.8549207 | 0 |
| 3240.8551771 | 0 |
| 3240.8566403 | 2 |
| 3240.8571093 | 3 |
| 3240.8589057 | 0 |
| 3240.8595128 | 2 |
| 3240.8604154 | 1 |
| 3240.8607334 | 2 |
| 3240.8619617 | 2 |
| 3240.8627108 | 3 |
| 3240.8639443 | 1 |
| 3240.8655632 | 0 |
| 3240.8658204 | 0 |
| 3240.867176 | 0 |
| 3240.8674032 | 1 |
| 3240.8689685 | 0 |
| 3240.8695991 | 1 |
| 3240.8708435 | 2 |
| 3240.8717844 | 1 |
| 3240.8723128 | 1 |
| 3240.873708 | 2 |
| 3240.8738235 | 2 |
| 3240.8751152 | 1 |
| 3240.8762771 | 1 |
| 3240.8768947 | 3 |
| 3240.8778115 | 4 |
| 3240.8783564 | 0 |
| 3240.8798245 | 1 |
| 3240.8813818 | 1 |
| 3240.88176 | 1 |
| 3240.8831652 | 3 |
| 3240.8831852 | 2 |
| 3240.8843622 | 5 |
| 3240.8860182 | 1 |
| 3240.8861517 | 0 |
| 3240.8873232 | 3 |
| 3240.8874936 | 3 |
| 3240.8888068 | 1 |
| 3240.8903126 | 0 |
| 3240.8908813 | 0 |
| 3240.8915066 | 4 |
| 3240.892809 | 0 |
| 3240.8937671 | 3 |
| 3240.8948635 | 1 |
| 3240.8960742 | 0 |
| 3240.8964406 | 2 |
| 3240.8970507 | 1 |
| 3240.8987708 | 2 |
| 3240.8988283 | 0 |
| 3240.9003192 | 0 |
| 3240.9014492 | 1 |
| 3240.902064 | 2 |
| 3240.902993 | 3 |
| 3240.9039114 | 1 |
| 3240.9053336 | 3 |
| 3240.906377 | 2 |
| 3240.9067562 | 3 |
| 3240.9075609 | 2 |
| 3240.9082205 | 0 |
| 3240.9091515 | 2 |
| 3240.9106241 | 3 |
| 3240.9117375 | 1 |
| 3240.9128259 | 3 |
| 3240.9136805 | 0 |
| 3240.9142017 | 2 |
| 3240.9153772 | 2 |
| 3240.915677 | 1 |
| 3240.9165836 | 2 |
| 3240.9181924 | 2 |
| 3240.919038 | 0 |
| 3240.9200555 | 1 |
| 3240.9207116 | 2 |
| 3240.9221575 | 1 |
| 3240.9223979 | 2 |
| 3240.923107 | 3 |
| 3240.9249102 | 4 |
| 3240.9251269 | 2 |
| 3240.9260112 | 1 |
| 3240.9277696 | 2 |
| 3240.92875 | 2 |
| 3240.9294639 | 6 |
| 3240.9299771 | 0 |
| 3240.9309007 | 4 |
| 3240.9315806 | 1 |
| 3240.93257 | 2 |
| 3240.9339917 | 0 |
| 3240.9347119 | 1 |
| 3240.9351615 | 0 |
| 3240.9358376 | 2 |
| 3240.9370205 | 0 |
| 3240.9386538 | 1 |
| 3240.9388373 | 3 |
| 3240.9400194 | 2 |
| 3240.9406822 | 3 |
| 3240.9414178 | 2 |
| 3240.9432757 | 2 |
| 3240.9438248 | 0 |
| 3240.9449838 | 1 |
| 3240.946297 | 2 |
| 3240.9465005 | 1 |
| 3240.9471102 | 2 |
| 3240.9479984 | 0 |
| 3240.9497063 | 1 |
| 3240.9501065 | 1 |
| 3240.9510577 | 2 |
| 3240.9518931 | 0 |
| 3240.953189 | 2 |
| 3240.954245 | 2 |
| 3240.9550819 | 1 |
| 3240.9564634 | 2 |
| 3240.9566518 | 2 |
| 3240.9577707 | 0 |
| 3240.9590619 | 0 |
| 3240.9600343 | 1 |
| 3240.960776 | 1 |
| 3240.962025 | 3 |
| 3240.962805 | 2 |
| 3240.9637985 | 3 |
| 3240.9643163 | 0 |
| 3240.9657975 | 0 |
| 3240.9664985 | 2 |
| 3240.9667356 | 0 |
| 3240.9685968 | 0 |
| 3240.9688141 | 0 |
| 3240.9696453 | 0 |
| 3240.9706475 | 1 |
| 3240.9721682 | 4 |
| 3240.9728097 | 0 |
| 3240.9735416 | 1 |
| 3240.9745151 | 2 |
| 3240.97584 | 1 |
| 3240.9765725 | 2 |
| 3240.9769273 | 0 |
| 3240.9788015 | 1 |
| 3240.9793802 | 2 |
| 3240.9799267 | 1 |
| 3240.9815114 | 0 |
| 3240.9815135 | 0 |
| 3240.9828288 | 1 |
| 3240.9844088 | 1 |
| 3240.9845732 | 0 |
| 3240.9858864 | 5 |
| 3240.9864891 | 1 |
| 3240.9874975 | 3 |
| 3240.9884266 | 3 |
| 3240.9897917 | 0 |
| 3240.9904507 | 3 |
| 3240.9916011 | 0 |
| 3240.9924319 | 2 |
| 3240.9938001 | 2 |
| 3240.9940375 | 0 |
| 3240.9956611 | 3 |
| 3240.9960124 | 0 |
| 3240.9970242 | 0 |
| 3240.9982251 | 1 |
| 3240.9990147 | 1 |
| 3240.9999633 | 0 |
| 3241.001027 | 0 |
| 3241.0011419 | 2 |
| 3241.0029459 | 1 |
| 3241.0037644 | 2 |
| 3241.0046417 | 3 |
| 3241.0051712 | 1 |
| 3241.0066284 | 0 |
| 3241.0071383 | 3 |
| 3241.0080915 | 2 |
| 3241.0092462 | 2 |
| 3241.0100737 | 3 |
| 3241.0109009 | 5 |
| 3241.0118429 | 3 |
| 3241.01232 | 0 |
| 3241.0140896 | 1 |
| 3241.0144098 | 3 |
| 3241.0152451 | 0 |
| 3241.0167686 | 2 |
| 3241.01764 | 0 |
| 3241.0187241 | 2 |
| 3241.0191332 | 3 |
| 3241.0198677 | 2 |
| 3241.0207827 | 1 |
| 3241.0223713 | 2 |
| 3241.0227419 | 1 |
| 3241.0241647 | 3 |
| 3241.0251826 | 2 |
| 3241.0255034 | 0 |
| 3241.0268058 | 2 |
| 3241.0280596 | 1 |
| 3241.0289362 | 1 |
| 3241.0300916 | 2 |
| 3241.0302829 | 1 |
| 3241.0315821 | 1 |
| 3241.0325532 | 1 |
| 3241.0327023 | 1 |
| 3241.0339615 | 0 |
| 3241.0352187 | 3 |
| 3241.0365121 | 2 |
| 3241.0375298 | 1 |
| 3241.0381376 | 1 |
| 3241.0389065 | 0 |
| 3241.0398946 | 0 |
| 3241.0409937 | 1 |
| 3241.0414911 | 2 |
| 3241.0429843 | 1 |
| 3241.0441332 | 2 |
| 3241.0446731 | 0 |
| 3241.0457007 | 3 |
| 3241.0466863 | 7 |
| 3241.0472435 | 1 |
| 3241.0489222 | 1 |
| 3241.0493554 | 0 |
| 3241.0500294 | 2 |
| 3241.0511664 | 0 |
| 3241.0527081 | 2 |
| 3241.0533741 | 0 |
| 3241.0535416 | 1 |
| 3241.0556289 | 0 |
| 3241.055727 | 0 |
| 3241.0561593 | 1 |
| 3241.0572288 | 1 |
| 3241.0580398 | 1 |
| 3241.05994 | 3 |
| 3241.0609364 | 0 |
| 3241.0615556 | 1 |
| 3241.0630886 | 2 |
| 3241.0633003 | 0 |
| 3241.0644495 | 1 |
| 3241.0647133 | 2 |
| 3241.066121 | 2 |
| 3241.0672845 | 0 |
| 3241.0674813 | 0 |
| 3241.0691851 | 1 |
| 3241.069499 | 2 |
| 3241.0704055 | 1 |
| 3241.0718905 | 1 |
| 3241.0727579 | 3 |
| 3241.0733652 | 1 |
| 3241.0749523 | 0 |
| 3241.075583 | 5 |
| 3241.0765945 | 2 |
| 3241.0774069 | 2 |
| 3241.0786962 | 2 |
| 3241.079026 | 1 |
| 3241.0803882 | 2 |
| 3241.081623 | 2 |
| 3241.0818379 | 3 |
| 3241.0830997 | 1 |
| 3241.0841545 | 2 |
| 3241.0849275 | 2 |
| 3241.0862202 | 1 |
| 3241.0868637 | 2 |
| 3241.0882358 | 2 |
| 3241.0885413 | 4 |
| 3241.0895129 | 0 |
| 3241.0904621 | 1 |
| 3241.0908096 | 1 |
| 3241.0925354 | 0 |
| 3241.0938799 | 0 |
| 3241.0949902 | 1 |
| 3241.0960296 | 1 |
| 3241.0961735 | 4 |
| 3241.097513 | 2 |
| 3241.0976539 | 2 |
| 3241.0986671 | 4 |
| 3241.1001179 | 1 |
| 3241.1009302 | 0 |
| 3241.1020373 | 1 |
| 3241.1025205 | 1 |
| 3241.1037943 | 1 |
| 3241.1055217 | 1 |
| 3241.1055884 | 1 |
| 3241.1068891 | 3 |
| 3241.1070819 | 1 |
| 3241.1083078 | 1 |
| 3241.1092277 | 1 |
| 3241.1103042 | 2 |
| 3241.1106404 | 2 |
| 3241.1117952 | 2 |
| 3241.1136144 | 2 |
| 3241.113733 | 1 |
| 3241.1151151 | 2 |
| 3241.1160683 | 0 |
| 3241.1171898 | 1 |
| 3241.1182641 | 0 |
| 3241.1187361 | 2 |
| 3241.1198896 | 2 |
| 3241.1201872 | 0 |
| 3241.121207 | 4 |
| 3241.1228303 | 2 |
| 3241.1231475 | 1 |
| 3241.1243003 | 4 |
| 3241.1245688 | 1 |
| 3241.124767 | 1 |
| 3241.1248828 | 1 |
| 3241.1253233 | 5 |
| 3241.1256436 | 1 |
| 3241.1264625 | 0 |
| 3241.1267216 | 2 |
| 3241.1268035 | 1 |
| 3241.1277364 | 1 |
| 3241.1278818 | 1 |
| 3241.1281642 | 2 |
| 3241.1281949 | 2 |
| 3241.1286987 | 1 |
| 3241.1288318 | 1 |
| 3241.1293688 | 3 |
| 3241.1296265 | 4 |
| 3241.1301791 | 0 |
| 3241.1304246 | 0 |
| 3241.1304884 | 2 |
| 3241.1307868 | 1 |
| 3241.131354 | 2 |
| 3241.1319136 | 2 |
| 3241.1320336 | 2 |
| 3241.132107 | 1 |
| 3241.1321841 | 0 |
| 3241.1323045 | 0 |
| 3241.1331723 | 1 |
| 3241.1331763 | 4 |
| 3241.1340216 | 1 |
| 3241.1341179 | 3 |
| 3241.1346694 | 0 |
| 3241.1347397 | 2 |
| 3241.1348122 | 2 |
| 3241.1353412 | 2 |
| 3241.1354566 | 2 |
| 3241.1357385 | 1 |
| 3241.1362904 | 3 |
| 3241.1367496 | 3 |

| | |
|---|---|
| 3241.1372145 | 4 |
| 3241.1372697 | 4 |
| 3241.1373575 | 1 |
| 3241.1378272 | 0 |
| 3241.1378963 | 3 |
| 3241.1380284 | 0 |
| 3241.1383769 | 1 |
| 3241.1384365 | 2 |
| 3241.1389002 | 1 |
| 3241.1390858 | 2 |
| 3241.1395143 | 0 |
| 3241.1397363 | 2 |
| 3241.1406962 | 2 |
| 3241.1408765 | 0 |
| 3241.1413638 | 1 |
| 3241.1414216 | 4 |
| 3241.1420738 | 2 |
| 3241.1422338 | 0 |
| 3241.1422858 | 1 |
| 3241.1427647 | 3 |
| 3241.1428798 | 0 |
| 3241.143118 | 1 |
| 3241.1435258 | 2 |
| 3241.1438444 | 0 |
| 3241.1440744 | 0 |
| 3241.1442224 | 4 |
| 3241.1452268 | 1 |
| 3241.145426 | 1 |
| 3241.1454611 | 3 |
| 3241.1456834 | 3 |
| 3241.1462283 | 6 |
| 3241.1465015 | 0 |
| 3241.1467068 | 1 |
| 3241.1467943 | 2 |
| 3241.1473028 | 3 |
| 3241.1478522 | 2 |
| 3241.1478536 | 1 |
| 3241.1480804 | 3 |
| 3241.1487158 | 1 |
| 3241.1487265 | 2 |
| 3241.1490325 | 0 |
| 3241.149316 | 2 |
| 3241.150367 | 2 |
| 3241.1508265 | 6 |
| 3241.1509582 | 2 |
| 3241.1509602 | 0 |
| 3241.1510118 | 2 |
| 3241.1511403 | 1 |
| 3241.152353 | 1 |
| 3241.1524257 | 1 |
| 3241.1527017 | 1 |
| 3241.1531412 | 0 |
| 3241.153308 | 1 |
| 3241.153519 | 1 |
| 3241.1539352 | 0 |
| 3241.1539837 | 1 |
| 3241.1542832 | 3 |
| 3241.1548085 | 2 |
| 3241.1550157 | 0 |
| 3241.1550246 | 3 |
| 3241.1554811 | 5 |
| 3241.1555224 | 2 |
| 3241.1563228 | 1 |
| 3241.1563229 | 2 |
| 3241.1569266 | 3 |
| 3241.1569823 | 1 |
| 3241.1574556 | 5 |
| 3241.1577866 | 1 |
| 3241.1578217 | 1 |
| 3241.1578595 | 0 |
| 3241.158503 | 2 |
| 3241.1591243 | 1 |
| 3241.1595206 | 1 |
| 3241.1595581 | 0 |
| 3241.1596468 | 1 |
| 3241.1597332 | 0 |
| 3241.160488 | 0 |
| 3241.160691 | 4 |
| 3241.1609056 | 3 |
| 3241.1612027 | 0 |
| 3241.1620679 | 1 |
| 3241.1620817 | 5 |
| 3241.1625677 | 1 |
| 3241.1630044 | 2 |
| 3241.1632541 | 1 |
| 3241.1634971 | 3 |
| 3241.1635363 | 4 |
| 3241.1636651 | 2 |
| 3241.1642857 | 1 |
| 3241.1644738 | 2 |
| 3241.1646548 | 3 |
| 3241.165116 | 0 |
| 3241.1654962 | 2 |
| 3241.1660379 | 0 |
| 3241.1660762 | 1 |
| 3241.1666315 | 2 |
| 3241.1667971 | 6 |
| 3241.1672423 | 3 |
| 3241.1672738 | 5 |
| 3241.1676867 | 8 |
| 3241.1685698 | 7 |
| 3241.1685858 | 3 |
| 3241.1685922 | 6 |
| 3241.1687203 | 3 |
| 3241.1688344 | 6 |
| 3241.1699619 | 6 |
| 3241.1699818 | 11 |
| 3241.1704207 | 4 |
| 3241.1706718 | 5 |
| 3241.1710244 | 4 |
| 3241.1711547 | 3 |
| 3241.1712544 | 4 |
| 3241.1714153 | 8 |
| 3241.1721084 | 1 |
| 3241.1723732 | 4 |
| 3241.1727859 | 11 |
| 3241.1729383 | 7 |
| 3241.1731163 | 6 |
| 3241.173668 | 4 |
| 3241.1737375 | 7 |
| 3241.1738047 | 5 |
| 3241.1743218 | 2 |
| 3241.1749976 | 6 |
| 3241.1750434 | 3 |
| 3241.1751819 | 5 |
| 3241.1757133 | 1 |
| 3241.1762467 | 4 |
| 3241.1764388 | 7 |
| 3241.1764465 | 5 |
| 3241.1768058 | 8 |
| 3241.1772565 | 5 |
| 3241.1776075 | 7 |
| 3241.1777159 | 3 |
| 3241.17799 | 1 |
| 3241.1784489 | 3 |
| 3241.1788705 | 5 |
| 3241.1790954 | 3 |
| 3241.1791178 | 1 |
| 3241.1795731 | 2 |
| 3241.1797125 | 1 |
| 3241.1810261 | 0 |
| 3241.1812379 | 3 |
| 3241.1812752 | 2 |
| 3241.1815982 | 3 |
| 3241.181629 | 0 |
| 3241.1820147 | 1 |
| 3241.1825773 | 2 |
| 3241.1826806 | 1 |
| 3241.1828903 | 3 |
| 3241.1830836 | 2 |
| 3241.1836464 | 2 |
| 3241.1839253 | 2 |
| 3241.1843926 | 3 |
| 3241.1847139 | 1 |
| 3241.1851221 | 1 |
| 3241.1853994 | 3 |
| 3241.1854814 | 1 |
| 3241.1858673 | 1 |
| 3241.1860487 | 0 |
| 3241.1865341 | 4 |
| 3241.1871588 | 1 |
| 3241.1872545 | 1 |
| 3241.1874421 | 4 |
| 3241.1879064 | 1 |
| 3241.1880648 | 1 |
| 3241.1888631 | 3 |
| 3241.1889458 | 1 |
| 3241.1891434 | 2 |
| 3241.1894585 | 1 |
| 3241.189672 | 3 |
| 3241.1900827 | 1 |
| 3241.1904063 | 1 |
| 3241.1907867 | 4 |
| 3241.1909333 | 2 |
| 3241.1910669 | 2 |
| 3241.1916953 | 2 |
| 3241.191852 | 2 |
| 3241.1918942 | 1 |
| 3241.1923931 | 1 |
| 3241.1926365 | 2 |
| 3241.1929184 | 0 |
| 3241.1934739 | 1 |
| 3241.1936828 | 3 |
| 3241.1940648 | 1 |
| 3241.1942159 | 0 |
| 3241.1948202 | 0 |
| 3241.1950361 | 2 |
| 3241.195673 | 0 |
| 3241.1956933 | 1 |
| 3241.1957767 | 0 |
| 3241.1957825 | 0 |
| 3241.1958297 | 6 |
| 3241.1965952 | 1 |
| 3241.1967572 | 1 |
| 3241.1973166 | 3 |
| 3241.1976264 | 3 |
| 3241.1979271 | 0 |
| 3241.198338 | 3 |
| 3241.198533 | 2 |
| 3241.1985445 | 1 |
| 3241.1995171 | 1 |
| 3241.1996859 | 1 |
| 3241.2000234 | 0 |
| 3241.2003186 | 3 |
| 3241.2004958 | 1 |
| 3241.2010522 | 2 |
| 3241.2011883 | 0 |
| 3241.2015644 | 2 |
| 3241.20182 | 0 |
| 3241.2019926 | 0 |
| 3241.2021572 | 2 |
| 3241.2027439 | 1 |
| 3241.2033399 | 2 |
| 3241.2038118 | 2 |
| 3241.2041 | 2 |
| 3241.2043049 | 1 |
| 3241.2045749 | 3 |
| 3241.2048299 | 1 |
| 3241.2051608 | 0 |
| 3241.2053941 | 1 |
| 3241.2054693 | 0 |
| 3241.2060288 | 3 |
| 3241.2060646 | 1 |
| 3241.2061735 | 2 |
| 3241.2067336 | 1 |
| 3241.207019 | 2 |
| 3241.2076236 | 3 |
| 3241.207742 | 0 |
| 3241.208575 | 3 |
| 3241.208675 | 2 |
| 3241.2087464 | 1 |
| 3241.2090358 | 0 |
| 3241.2092407 | 1 |
| 3241.2092637 | 1 |
| 3241.2099989 | 1 |
| 3241.2102783 | 1 |
| 3241.2105107 | 1 |
| 3241.2109301 | 0 |
| 3241.2109638 | 3 |
| 3241.2109782 | 0 |
| 3241.211186 | 0 |
| 3241.2119525 | 1 |
| 3241.2123962 | 0 |
| 3241.2126326 | 3 |
| 3241.2129962 | 1 |
| 3241.213137 | 1 |
| 3241.2136702 | 0 |
| 3241.2145209 | 0 |
| 3241.2146561 | 0 |
| 3241.214733 | 2 |
| 3241.2150298 | 3 |
| 3241.2151169 | 1 |
| 3241.2152296 | 0 |
| 3241.2160874 | 3 |
| 3241.2163515 | 2 |
| 3241.2165172 | 1 |
| 3241.21695 | 0 |
| 3241.2169911 | 0 |
| 3241.2173682 | 0 |
| 3241.2183349 | 1 |
| 3241.2184016 | 4 |
| 3241.2184557 | 2 |
| 3241.2189219 | 1 |
| 3241.2190586 | 1 |
| 3241.2197559 | 4 |
| 3241.2197644 | 1 |
| 3241.2200228 | 3 |
| 3241.220663 | 5 |
| 3241.2208894 | 3 |
| 3241.2209983 | 1 |
| 3241.2216214 | 3 |
| 3241.2217245 | 1 |
| 3241.2226282 | 1 |
| 3241.2226293 | 1 |
| 3241.2226666 | 0 |
| 3241.2230827 | 1 |
| 3241.2233937 | 1 |
| 3241.2237867 | 2 |
| 3241.2240622 | 4 |
| 3241.2246338 | 2 |
| 3241.2246485 | 1 |
| 3241.2247802 | 0 |
| 3241.224809 | 2 |
| 3241.225283 | 1 |
| 3241.2260162 | 2 |
| 3241.2261767 | 3 |
| 3241.2266306 | 3 |
| 3241.2269734 | 2 |
| 3241.2269999 | 4 |
| 3241.2271799 | 3 |
| 3241.227313 | 3 |
| 3241.2280651 | 3 |
| 3241.2284747 | 1 |
| 3241.2286022 | 2 |
| 3241.2289708 | 4 |
| 3241.2290424 | 4 |
| 3241.2292199 | 0 |
| 3241.2294112 | 1 |
| 3241.2300646 | 3 |
| 3241.2301857 | 1 |
| 3241.2308233 | 1 |
| 3241.2312283 | 0 |
| 3241.2314953 | 4 |
| 3241.2315559 | 1 |
| 3241.2319139 | 2 |
| 3241.2327585 | 2 |
| 3241.2327697 | 0 |
| 3241.2329198 | 3 |
| 3241.2333344 | 1 |
| 3241.2335179 | 5 |
| 3241.233715 | 0 |
| 3241.2338174 | 1 |
| 3241.2348153 | 0 |
| 3241.2349609 | 1 |
| 3241.2352876 | 3 |
| 3241.2355759 | 2 |
| 3241.2357021 | 2 |
| 3241.2362542 | 3 |
| 3241.2364654 | 0 |
| 3241.2365204 | 1 |
| 3241.237306 | 2 |
| 3241.2375549 | 0 |
| 3241.2377078 | 3 |
| 3241.2381124 | 1 |
| 3241.2383444 | 2 |
| 3241.2384294 | 2 |
| 3241.2386449 | 1 |
| 3241.239042 | 1 |
| 3241.2394407 | 2 |
| 3241.2398235 | 1 |
| 3241.2403466 | 1 |
| 3241.2406108 | 2 |
| 3241.240689 | 1 |
| 3241.2409667 | 2 |
| 3241.2412484 | 3 |
| 3241.2419031 | 1 |
| 3241.2419771 | 0 |
| 3241.242693 | 0 |
| 3241.2429124 | 0 |
| 3241.2435869 | 1 |
| 3241.2436115 | 1 |
| 3241.2436872 | 1 |
| 3241.2439865 | 2 |
| 3241.2443512 | 1 |
| 3241.2443727 | 0 |
| 3241.2445751 | 1 |
| 3241.2449556 | 2 |
| 3241.245512 | 0 |
| 3241.2459866 | 1 |
| 3241.2462161 | 4 |
| 3241.246776 | 1 |
| 3241.2468022 | 0 |
| 3241.2468154 | 2 |
| 3241.2474585 | 6 |
| 3241.2480297 | 2 |
| 3241.248099 | 2 |
| 3241.248652 | 0 |
| 3241.2487295 | 0 |
| 3241.2491647 | 1 |
| 3241.2493286 | 1 |
| 3241.249512 | 2 |
| 3241.2497248 | 2 |
| 3241.2499699 | 3 |

| | |
|---|---|
| 3241.2502326 | 1 |
| 3241.2505696 | 0 |
| 3241.2509182 | 2 |
| 3241.2513972 | 2 |
| 3241.2514919 | 2 |
| 3241.2523014 | 0 |
| 3241.2527005 | 4 |
| 3241.2528219 | 2 |
| 3241.2528915 | 1 |
| 3241.2532419 | 1 |
| 3241.2536591 | 1 |
| 3241.2536729 | 1 |
| 3241.2544975 | 3 |
| 3241.2549116 | 1 |
| 3241.2552032 | 1 |
| 3241.2553498 | 2 |
| 3241.2553659 | 1 |
| 3241.2554681 | 1 |
| 3241.2558161 | 0 |
| 3241.2566104 | 1 |
| 3241.2567948 | 3 |
| 3241.2570321 | 2 |
| 3241.2574225 | 2 |
| 3241.2576093 | 2 |
| 3241.2578883 | 0 |
| 3241.2579538 | 2 |
| 3241.2587348 | 0 |
| 3241.2589967 | 3 |
| 3241.2598555 | 1 |
| 3241.2598653 | 0 |
| 3241.2599295 | 3 |
| 3241.2604017 | 0 |
| 3241.2604035 | 0 |
| 3241.2609578 | 2 |
| 3241.2610879 | 3 |
| 3241.2617265 | 0 |
| 3241.2618989 | 1 |
| 3241.2620012 | 1 |
| 3241.2625089 | 3 |
| 3241.2627188 | 0 |
| 3241.2632859 | 0 |
| 3241.2633087 | 1 |
| 3241.2635478 | 1 |
| 3241.2641807 | 3 |
| 3241.2642723 | 3 |
| 3241.2650116 | 0 |
| 3241.2650148 | 2 |
| 3241.2651428 | 1 |
| 3241.2651613 | 1 |
| 3241.2658562 | 2 |
| 3241.2663148 | 0 |
| 3241.2664985 | 3 |
| 3241.2666362 | 4 |
| 3241.2669737 | 1 |
| 3241.2675854 | 2 |
| 3241.2676467 | 1 |
| 3241.2678027 | 1 |
| 3241.2686065 | 2 |
| 3241.2687445 | 4 |
| 3241.2688029 | 2 |
| 3241.2692788 | 0 |
| 3241.2693314 | 5 |
| 3241.2703084 | 3 |
| 3241.2703639 | 2 |
| 3241.2707542 | 3 |
| 3241.270817 | 1 |
| 3241.271603 | 2 |
| 3241.2716287 | 1 |
| 3241.2717499 | 2 |
| 3241.2722361 | 1 |
| 3241.2728128 | 1 |
| 3241.2731111 | 2 |
| 3241.2731155 | 0 |
| 3241.273349 | 1 |
| 3241.274287 | 2 |
| 3241.2743417 | 0 |
| 3241.2743427 | 0 |
| 3241.2745536 | 0 |
| 3241.2749867 | 4 |
| 3241.2756294 | 2 |
| 3241.2758471 | 0 |
| 3241.2761969 | 3 |
| 3241.276712 | 1 |
| 3241.2769555 | 0 |
| 3241.2770935 | 2 |
| 3241.2771264 | 0 |
| 3241.2774007 | 2 |
| 3241.2779776 | 1 |
| 3241.2785292 | 3 |
| 3241.2786178 | 3 |
| 3241.2788462 | 7 |
| 3241.2794518 | 1 |
| 3241.2796298 | 0 |
| 3241.279775 | 1 |
| 3241.279841 | 5 |
| 3241.2800409 | 2 |
| 3241.2806699 | 0 |
| 3241.2807311 | 0 |
| 3241.2808668 | 4 |
| 3241.2809438 | 2 |
| 3241.2822016 | 3 |
| 3241.2822643 | 1 |
| 3241.2829623 | 1 |
| 3241.2830717 | 1 |
| 3241.2833164 | 1 |
| 3241.2834634 | 2 |
| 3241.2840968 | 1 |
| 3241.2841693 | 4 |
| 3241.2842564 | 2 |
| 3241.2848293 | 1 |
| 3241.2851336 | 1 |
| 3241.285243 | 1 |
| 3241.2855227 | 0 |
| 3241.2856367 | 2 |
| 3241.2865155 | 3 |
| 3241.2868294 | 2 |
| 3241.2868296 | 0 |
| 3241.28694 | 1 |
| 3241.2878311 | 1 |
| 3241.2879658 | 0 |
| 3241.2882365 | 0 |
| 3241.2883533 | 0 |
| 3241.2889208 | 0 |
| 3241.2896288 | 3 |
| 3241.2896342 | 3 |
| 3241.2898082 | 1 |
| 3241.2900524 | 3 |
| 3241.2901039 | 0 |
| 3241.2903812 | 3 |
| 3241.2907559 | 1 |
| 3241.2912685 | 4 |
| 3241.2918937 | 1 |
| 3241.291972 | 1 |
| 3241.2921374 | 0 |
| 3241.2926321 | 2 |
| 3241.2932895 | 0 |
| 3241.2933508 | 1 |
| 3241.2934765 | 1 |
| 3241.2937268 | 3 |
| 3241.294209 | 3 |
| 3241.2942331 | 2 |
| 3241.2949425 | 1 |
| 3241.2952588 | 2 |
| 3241.2956509 | 1 |
| 3241.2958643 | 2 |
| 3241.2959078 | 0 |
| 3241.2963136 | 4 |
| 3241.2966728 | 4 |
| 3241.2967531 | 4 |
| 3241.2969802 | 3 |
| 3241.2974174 | 1 |
| 3241.2979619 | 4 |
| 3241.2984303 | 3 |
| 3241.2985895 | 2 |
| 3241.298922 | 2 |
| 3241.2991931 | 2 |
| 3241.2992576 | 0 |
| 3241.2993937 | 0 |
| 3241.2996928 | 2 |
| 3241.2999104 | 0 |
| 3241.3001352 | 4 |
| 3241.3007253 | 1 |
| 3241.3010943 | 1 |
| 3241.3012503 | 1 |
| 3241.3017875 | 2 |
| 3241.3020928 | 0 |
| 3241.3022692 | 2 |
| 3241.3025812 | 0 |
| 3241.30297 | 0 |
| 3241.3031665 | 3 |
| 3241.3037468 | 1 |
| 3241.303776 | 3 |
| 3241.3040483 | 2 |
| 3241.3049399 | 2 |
| 3241.3053171 | 0 |
| 3241.305411 | 1 |
| 3241.305462 | 0 |
| 3241.3056743 | 0 |
| 3241.3063383 | 0 |
| 3241.306591 | 1 |
| 3241.3066072 | 0 |
| 3241.3071401 | 2 |
| 3241.3076272 | 2 |
| 3241.3079671 | 3 |
| 3241.3079788 | 1 |
| 3241.3083218 | 0 |
| 3241.3087066 | 2 |
| 3241.3091568 | 1 |
| 3241.3091886 | 2 |
| 3241.3102239 | 2 |
| 3241.3103 | 2 |
| 3241.3103535 | 2 |
| 3241.31051 | 1 |
| 3241.3105463 | 2 |
| 3241.3113976 | 2 |
| 3241.3114213 | 2 |
| 3241.3115053 | 1 |
| 3241.3123668 | 1 |
| 3241.3124776 | 4 |
| 3241.3125495 | 0 |
| 3241.3133105 | 1 |
| 3241.3134608 | 3 |
| 3241.3137205 | 1 |
| 3241.3140114 | 2 |
| 3241.3144625 | 2 |
| 3241.3146607 | 3 |
| 3241.3149776 | 1 |
| 3241.3156346 | 0 |
| 3241.3156589 | 5 |
| 3241.3160455 | 2 |
| 3241.3162029 | 2 |
| 3241.3165732 | 1 |
| 3241.3169896 | 1 |
| 3241.3173064 | 3 |
| 3241.3179958 | 2 |
| 3241.3180392 | 1 |
| 3241.3180515 | 0 |
| 3241.3187481 | 0 |
| 3241.318964 | 3 |
| 3241.3190789 | 1 |
| 3241.3192489 | 1 |
| 3241.3199084 | 3 |
| 3241.320083 | 1 |
| 3241.3202942 | 1 |
| 3241.3205687 | 1 |
| 3241.3208331 | 1 |
| 3241.3211561 | 0 |
| 3241.3220047 | 3 |
| 3241.3220766 | 0 |
| 3241.3221826 | 1 |
| 3241.3227441 | 0 |
| 3241.3229282 | 3 |
| 3241.323215 | 1 |
| 3241.3234753 | 2 |
| 3241.3239659 | 1 |
| 3241.3241311 | 1 |
| 3241.3247527 | 0 |
| 3241.3250151 | 1 |
| 3241.3253932 | 1 |
| 3241.3253939 | 0 |
| 3241.3258123 | 1 |
| 3241.3259523 | 1 |
| 3241.3263139 | 5 |
| 3241.3266646 | 0 |
| 3241.3269704 | 2 |
| 3241.3275903 | 1 |
| 3241.32764 | 2 |
| 3241.3280173 | 2 |
| 3241.328466 | 4 |
| 3241.328496 | 1 |
| 3241.328543 | 3 |
| 3241.3293019 | 1 |
| 3241.329951 | 2 |
| 3241.3299678 | 1 |
| 3241.3303884 | 0 |
| 3241.3303906 | 1 |
| 3241.3311165 | 0 |
| 3241.3311526 | 0 |
| 3241.3312177 | 2 |
| 3241.3312438 | 1 |
| 3241.3314639 | 1 |
| 3241.3319428 | 2 |
| 3241.3321984 | 1 |
| 3241.3328927 | 0 |
| 3241.3330097 | 1 |
| 3241.333181 | 1 |
| 3241.3339375 | 1 |
| 3241.3344306 | 1 |
| 3241.334493 | 0 |
| 3241.3349737 | 2 |
| 3241.3350845 | 2 |
| 3241.3353152 | 3 |
| 3241.3353773 | 2 |
| 3241.3355749 | 1 |
| 3241.3361333 | 0 |
| 3241.3369763 | 1 |
| 3241.3373666 | 1 |
| 3241.337399 | 0 |
| 3241.3375183 | 2 |
| 3241.3379096 | 0 |
| 3241.3381658 | 1 |
| 3241.3382136 | 0 |
| 3241.3382187 | 0 |
| 3241.3382561 | 1 |
| 3241.3394781 | 2 |
| 3241.3396723 | 1 |
| 3241.340246 | 4 |
| 3241.3403049 | 2 |
| 3241.3405983 | 1 |
| 3241.340686 | 0 |
| 3241.3410934 | 1 |
| 3241.3416139 | 3 |
| 3241.3417514 | 1 |
| 3241.3417914 | 2 |
| 3241.3423202 | 0 |
| 3241.3427387 | 2 |
| 3241.3429288 | 4 |
| 3241.3433583 | 0 |
| 3241.3434324 | 1 |
| 3241.3435397 | 1 |
| 3241.3438461 | 4 |
| 3241.3449166 | 2 |
| 3241.3449822 | 2 |
| 3241.3450321 | 1 |
| 3241.3458066 | 3 |
| 3241.3464635 | 0 |
| 3241.3467835 | 1 |
| 3241.347064 | 0 |
| 3241.3471078 | 2 |
| 3241.3473388 | 1 |
| 3241.3476039 | 1 |
| 3241.3480861 | 1 |
| 3241.348117 | 1 |
| 3241.348336 | 1 |
| 3241.349193 | 1 |
| 3241.3494113 | 4 |
| 3241.3495271 | 2 |
| 3241.3502073 | 2 |
| 3241.3505498 | 1 |
| 3241.3505606 | 0 |
| 3241.3506483 | 2 |
| 3241.3508271 | 4 |
| 3241.3511621 | 2 |
| 3241.3512716 | 0 |
| 3241.3514822 | 1 |
| 3241.3520531 | 2 |
| 3241.3524948 | 1 |
| 3241.352798 | 1 |
| 3241.3527989 | 1 |
| 3241.3538202 | 3 |
| 3241.3538908 | 1 |
| 3241.3539031 | 1 |
| 3241.3545627 | 3 |
| 3241.3546964 | 1 |
| 3241.3550915 | 3 |
| 3241.3552642 | 3 |
| 3241.355323 | 1 |
| 3241.3555127 | 5 |
| 3241.356205 | 1 |
| 3241.3563624 | 2 |
| 3241.3570351 | 2 |
| 3241.3571209 | 2 |
| 3241.3574065 | 1 |
| 3241.3576249 | 1 |
| 3241.3578694 | 2 |
| 3241.358334 | 2 |
| 3241.3584747 | 1 |
| 3241.3592885 | 2 |
| 3241.3595694 | 1 |
| 3241.360029 | 1 |
| 3241.3602978 | 0 |
| 3241.360652 | 1 |
| 3241.3608294 | 1 |
| 3241.3610309 | 4 |
| 3241.3613575 | 2 |
| 3241.361664 | 3 |
| 3241.3618294 | 2 |
| 3241.362097 | 2 |
| 3241.362544 | 0 |
| 3241.3626234 | 3 |
| 3241.3632231 | 0 |
| 3241.3634074 | 1 |
| 3241.3637143 | 3 |

| | |
|---|---|
| 3241.3640481 | 3 |
| 3241.36467 | 5 |
| 3241.3647193 | 4 |
| 3241.3649962 | 1 |
| 3241.3651086 | 0 |
| 3241.3652698 | 1 |
| 3241.3659942 | 0 |
| 3241.3663958 | 1 |
| 3241.3667341 | 1 |
| 3241.3672923 | 0 |
| 3241.3677811 | 3 |
| 3241.3677873 | 2 |
| 3241.368161 | 3 |
| 3241.3683747 | 1 |
| 3241.3689755 | 1 |
| 3241.3691189 | 4 |
| 3241.3694811 | 1 |
| 3241.369503 | 1 |
| 3241.3697749 | 2 |
| 3241.3699544 | 4 |
| 3241.3705035 | 2 |
| 3241.3705483 | 0 |
| 3241.3709147 | 3 |
| 3241.3714011 | 0 |
| 3241.3715525 | 3 |
| 3241.3721093 | 1 |
| 3241.3724948 | 3 |
| 3241.3725023 | 1 |
| 3241.3728144 | 2 |
| 3241.3729516 | 2 |
| 3241.3732908 | 1 |
| 3241.3737897 | 1 |
| 3241.3738935 | 1 |
| 3241.374535 | 2 |
| 3241.3747856 | 3 |
| 3241.3751303 | 2 |
| 3241.3752579 | 1 |
| 3241.3759573 | 1 |
| 3241.3760397 | 4 |
| 3241.3761943 | 2 |
| 3241.3763711 | 2 |
| 3241.3765222 | 1 |
| 3241.3772186 | 3 |
| 3241.3779903 | 1 |
| 3241.378069 | 2 |
| 3241.3782247 | 2 |
| 3241.3783101 | 0 |
| 3241.3787384 | 2 |
| 3241.3788428 | 1 |
| 3241.3790898 | 6 |
| 3241.3792718 | 2 |
| 3241.380003 | 0 |
| 3241.3801419 | 0 |
| 3241.3801908 | 3 |
| 3241.3810994 | 1 |
| 3241.381489 | 1 |
| 3241.3820572 | 1 |
| 3241.3820716 | 3 |
| 3241.3821298 | 1 |
| 3241.3824272 | 3 |
| 3241.3825858 | 0 |
| 3241.3829512 | 2 |
| 3241.3830179 | 3 |
| 3241.3837152 | 1 |
| 3241.3842112 | 3 |
| 3241.3842476 | 1 |
| 3241.3849596 | 2 |
| 3241.3850533 | 1 |
| 3241.3851215 | 4 |
| 3241.385467 | 3 |
| 3241.3858333 | 2 |
| 3241.38623 | 4 |
| 3241.3864491 | 3 |
| 3241.3869382 | 2 |
| 3241.3870508 | 1 |
| 3241.3875887 | 2 |
| 3241.3879962 | 0 |
| 3241.3880003 | 0 |
| 3241.3880245 | 1 |
| 3241.3882942 | 3 |
| 3241.3886526 | 0 |
| 3241.3893135 | 1 |
| 3241.3894982 | 1 |
| 3241.3898437 | 1 |
| 3241.3898503 | 3 |
| 3241.389937 | 6 |
| 3241.390449 | 2 |
| 3241.3912699 | 3 |
| 3241.3917722 | 1 |
| 3241.391921 | 2 |
| 3241.3919694 | 5 |
| 3241.3921715 | 0 |
| 3241.3927382 | 6 |
| 3241.3927767 | 1 |
| 3241.3935844 | 3 |
| 3241.3937929 | 3 |
| 3241.3938348 | 2 |
| 3241.3942745 | 2 |
| 3241.3944031 | 2 |
| 3241.3945834 | 2 |
| 3241.3952448 | 1 |
| 3241.3953755 | 0 |
| 3241.3956567 | 1 |
| 3241.3962136 | 2 |
| 3241.3962829 | 3 |
| 3241.3969689 | 3 |
| 3241.3970095 | 1 |
| 3241.3975445 | 2 |
| 3241.397614 | 1 |
| 3241.3981232 | 1 |
| 3241.3983056 | 1 |
| 3241.3984642 | 1 |
| 3241.3987759 | 4 |
| 3241.3989242 | 1 |
| 3241.3989898 | 1 |
| 3241.4000586 | 2 |
| 3241.4004047 | 1 |
| 3241.4007071 | 1 |
| 3241.4009942 | 0 |
| 3241.4010479 | 0 |
| 3241.4011794 | 3 |
| 3241.4013249 | 2 |
| 3241.4022953 | 1 |
| 3241.4024508 | 1 |
| 3241.4026166 | 0 |
| 3241.4027505 | 1 |
| 3241.4027529 | 3 |
| 3241.4034404 | 1 |
| 3241.4038731 | 0 |
| 3241.4040925 | 1 |
| 3241.4045449 | 2 |
| 3241.4050335 | 3 |
| 3241.4051807 | 2 |
| 3241.4052763 | 3 |
| 3241.4054521 | 3 |
| 3241.4062569 | 1 |
| 3241.4062588 | 2 |
| 3241.4066362 | 2 |
| 3241.4073761 | 0 |
| 3241.4073795 | 1 |
| 3241.4076626 | 1 |
| 3241.4080148 | 2 |
| 3241.4081205 | 3 |
| 3241.4084916 | 3 |
| 3241.4086697 | 0 |
| 3241.4090606 | 0 |
| 3241.4095891 | 2 |
| 3241.409901 | 0 |
| 3241.4101812 | 3 |
| 3241.4102988 | 1 |
| 3241.4107773 | 3 |
| 3241.4113333 | 3 |
| 3241.4115724 | 0 |
| 3241.4115793 | 1 |
| 3241.4124275 | 1 |
| 3241.412545 | 1 |
| 3241.4125825 | 0 |
| 3241.4133827 | 5 |
| 3241.4134456 | 0 |
| 3241.4139329 | 1 |
| 3241.4141924 | 2 |
| 3241.4142616 | 1 |
| 3241.4142803 | 1 |
| 3241.4143617 | 2 |
| 3241.41468 | 2 |
| 3241.4154161 | 1 |
| 3241.4157127 | 1 |
| 3241.4163839 | 3 |
| 3241.4165648 | 1 |
| 3241.4168571 | 4 |
| 3241.4170607 | 1 |
| 3241.417144 | 4 |
| 3241.4175537 | 0 |
| 3241.4179028 | 1 |
| 3241.4184827 | 1 |
| 3241.4187516 | 1 |
| 3241.4188433 | 2 |
| 3241.4188975 | 0 |
| 3241.4196512 | 3 |
| 3241.4197966 | 0 |
| 3241.4199058 | 1 |
| 3241.4205113 | 3 |
| 3241.4208833 | 1 |
| 3241.4211127 | 0 |
| 3241.4214103 | 1 |
| 3241.4214425 | 1 |
| 3241.4216168 | 1 |
| 3241.4219557 | 3 |
| 3241.4222505 | 2 |
| 3241.422675 | 1 |
| 3241.4228563 | 1 |
| 3241.4231354 | 0 |
| 3241.4233124 | 5 |
| 3241.424219 | 2 |
| 3241.4242354 | 1 |
| 3241.4242738 | 5 |
| 3241.4249383 | 0 |
| 3241.4254228 | 4 |
| 3241.4256243 | 0 |
| 3241.4259087 | 3 |
| 3241.4260766 | 2 |
| 3241.426585 | 6 |
| 3241.4268064 | 3 |
| 3241.4268928 | 1 |
| 3241.4273411 | 4 |
| 3241.4274964 | 1 |
| 3241.4282788 | 1 |
| 3241.428378 | 1 |
| 3241.4287963 | 2 |
| 3241.4289136 | 3 |
| 3241.4289632 | 1 |
| 3241.4296288 | 1 |
| 3241.4296816 | 1 |
| 3241.4297695 | 0 |
| 3241.4305091 | 0 |
| 3241.4306332 | 1 |
| 3241.4309567 | 2 |
| 3241.4313346 | 1 |
| 3241.4317063 | 1 |
| 3241.4324004 | 1 |
| 3241.4324881 | 6 |
| 3241.43277 | 2 |
| 3241.4331314 | 1 |
| 3241.4334627 | 1 |
| 3241.4336338 | 2 |
| 3241.4339328 | 2 |
| 3241.4341177 | 0 |
| 3241.4343683 | 1 |
| 3241.4351131 | 1 |
| 3241.4351313 | 0 |
| 3241.435284 | 1 |
| 3241.4357146 | 3 |
| 3241.4359481 | 1 |
| 3241.4364069 | 4 |
| 3241.4369254 | 2 |
| 3241.436938 | 2 |
| 3241.4374717 | 1 |
| 3241.4377384 | 3 |
| 3241.4378281 | 4 |
| 3241.4386766 | 3 |
| 3241.4386981 | 2 |
| 3241.439141 | 1 |
| 3241.4392439 | 2 |
| 3241.4393716 | 1 |
| 3241.4398492 | 1 |
| 3241.440142 | 1 |
| 3241.4403811 | 1 |
| 3241.4409392 | 1 |
| 3241.4412655 | 1 |
| 3241.4416727 | 2 |
| 3241.4417742 | 2 |
| 3241.4418171 | 3 |
| 3241.4418822 | 4 |
| 3241.4426669 | 0 |
| 3241.4429426 | 0 |
| 3241.4430018 | 4 |
| 3241.4432845 | 0 |
| 3241.4439321 | 1 |
| 3241.4445564 | 3 |
| 3241.4448346 | 3 |
| 3241.4449012 | 1 |
| 3241.4453636 | 2 |
| 3241.4457934 | 2 |
| 3241.4459611 | 2 |
| 3241.4460218 | 1 |
| 3241.446423 | 1 |
| 3241.4468683 | 0 |
| 3241.447086 | 2 |
| 3241.4472143 | 0 |
| 3241.4474598 | 0 |
| 3241.4480096 | 1 |
| 3241.4483447 | 2 |
| 3241.448694 | 1 |
| 3241.448985 | 1 |
| 3241.4491788 | 0 |
| 3241.4492721 | 3 |
| 3241.4496732 | 1 |
| 3241.4499828 | 2 |
| 3241.4502979 | 2 |
| 3241.450337 | 3 |
| 3241.4506949 | 2 |
| 3241.4512358 | 2 |
| 3241.4512576 | 3 |
| 3241.4520129 | 2 |
| 3241.4523236 | 1 |
| 3241.4523716 | 1 |
| 3241.4534029 | 0 |
| 3241.4535658 | 1 |
| 3241.4536076 | 0 |
| 3241.4538971 | 0 |
| 3241.4540036 | 1 |
| 3241.4543555 | 1 |
| 3241.454665 | 2 |
| 3241.4550686 | 1 |
| 3241.4553489 | 4 |
| 3241.4553805 | 1 |
| 3241.4560844 | 5 |
| 3241.456499 | 1 |
| 3241.4567507 | 2 |
| 3241.4571021 | 4 |
| 3241.4573338 | 4 |
| 3241.4578382 | 1 |
| 3241.4579638 | 0 |
| 3241.458263 | 0 |
| 3241.4583749 | 2 |
| 3241.458519 | 0 |
| 3241.4586655 | 1 |
| 3241.4588407 | 0 |
| 3241.4593672 | 1 |
| 3241.4594373 | 1 |
| 3241.4598837 | 0 |
| 3241.4602762 | 2 |
| 3241.4609225 | 1 |
| 3241.4611409 | 1 |
| 3241.4612558 | 1 |
| 3241.4618799 | 0 |
| 3241.461906 | 2 |
| 3241.4621264 | 1 |
| 3241.4626843 | 1 |
| 3241.4628014 | 0 |
| 3241.4630503 | 2 |
| 3241.4637432 | 1 |
| 3241.4638994 | 3 |
| 3241.4641227 | 1 |
| 3241.4642264 | 1 |
| 3241.4645284 | 1 |
| 3241.464953 | 1 |
| 3241.4651346 | 1 |
| 3241.46537 | 3 |
| 3241.4660971 | 2 |
| 3241.4662018 | 0 |
| 3241.4664737 | 1 |
| 3241.466757 | 2 |
| 3241.4673337 | 4 |
| 3241.4675248 | 2 |
| 3241.4678847 | 0 |
| 3241.4683088 | 1 |
| 3241.468362 | 1 |
| 3241.4688715 | 1 |
| 3241.4691248 | 0 |
| 3241.4692984 | 0 |
| 3241.4697746 | 1 |
| 3241.4700327 | 2 |
| 3241.4704288 | 1 |
| 3241.4707589 | 1 |
| 3241.4711762 | 1 |
| 3241.4713983 | 1 |
| 3241.4716567 | 2 |
| 3241.4721026 | 3 |
| 3241.4724529 | 0 |
| 3241.4725681 | 1 |
| 3241.4728166 | 1 |
| 3241.4733178 | 1 |
| 3241.4733746 | 2 |
| 3241.4738909 | 3 |
| 3241.474047 | 1 |
| 3241.4741317 | 2 |
| 3241.4741447 | 0 |
| 3241.4745702 | 3 |
| 3241.4750427 | 4 |
| 3241.4750957 | 0 |
| 3241.4756241 | 0 |
| 3241.4758261 | 4 |

| | |
|---|---|
| 3241.4765244 | 2 |
| 3241.4769257 | 2 |
| 3241.4770266 | 1 |
| 3241.477601 | 1 |
| 3241.4776092 | 1 |
| 3241.4779955 | 1 |
| 3241.4782328 | 1 |
| 3241.4783365 | 3 |
| 3241.4790308 | 1 |
| 3241.4796287 | 3 |
| 3241.4797248 | 1 |
| 3241.4801289 | 2 |
| 3241.4804003 | 1 |
| 3241.480484 | 1 |
| 3241.4808241 | 0 |
| 3241.4808384 | 2 |
| 3241.4812296 | 0 |
| 3241.4813864 | 1 |
| 3241.4816527 | 1 |
| 3241.4821826 | 1 |
| 3241.4825306 | 1 |
| 3241.4828301 | 2 |
| 3241.4831664 | 3 |
| 3241.4834411 | 1 |
| 3241.4837955 | 1 |
| 3241.4843114 | 0 |
| 3241.4843429 | 0 |
| 3241.484581 | 1 |
| 3241.4850981 | 2 |
| 3241.4854296 | 0 |
| 3241.485918 | 4 |
| 3241.4862779 | 2 |
| 3241.486592 | 3 |
| 3241.4868109 | 3 |
| 3241.4870921 | 1 |
| 3241.4872072 | 1 |
| 3241.4875722 | 1 |
| 3241.487611 | 2 |
| 3241.4879584 | 1 |
| 3241.4881818 | 3 |
| 3241.4886043 | 3 |
| 3241.4889655 | 0 |
| 3241.4892678 | 2 |
| 3241.4895076 | 1 |
| 3241.4901982 | 0 |
| 3241.4906989 | 3 |
| 3241.4908366 | 1 |
| 3241.4910999 | 2 |
| 3241.4915258 | 1 |
| 3241.4915261 | 1 |
| 3241.4915714 | 3 |
| 3241.4924381 | 1 |
| 3241.492499 | 5 |
| 3241.4930652 | 1 |
| 3241.4932681 | 1 |
| 3241.493517 | 2 |
| 3241.4936802 | 1 |
| 3241.4940054 | 1 |
| 3241.4942512 | 4 |
| 3241.4947536 | 3 |
| 3241.4948178 | 2 |
| 3241.495595 | 0 |
| 3241.4956795 | 1 |
| 3241.4959387 | 5 |
| 3241.4963373 | 1 |
| 3241.4966726 | 1 |
| 3241.4970899 | 0 |
| 3241.4974429 | 3 |
| 3241.4977771 | 1 |
| 3241.4979152 | 2 |
| 3241.4982394 | 2 |
| 3241.4987532 | 1 |
| 3241.4988471 | 2 |
| 3241.4990135 | 2 |
| 3241.4998 | 3 |
| 3241.4998113 | 1 |
| 3241.500256 | 0 |
| 3241.5005085 | 0 |
| 3241.5006212 | 4 |
| 3241.5007828 | 3 |
| 3241.5012039 | 2 |
| 3241.5014643 | 3 |
| 3241.5017056 | 0 |
| 3241.5023659 | 2 |
| 3241.5025658 | 1 |
| 3241.5030573 | 0 |
| 3241.5036296 | 1 |
| 3241.5038072 | 2 |
| 3241.5040313 | 3 |
| 3241.5042574 | 0 |
| 3241.5043174 | 1 |
| 3241.5047685 | 2 |
| 3241.5050846 | 1 |
| 3241.5052521 | 1 |
| 3241.5054013 | 1 |
| 3241.5058 | 2 |
| 3241.5058312 | 2 |
| 3241.5062992 | 3 |
| 3241.5067086 | 4 |
| 3241.5069652 | 0 |
| 3241.5073764 | 2 |
| 3241.5075305 | 1 |
| 3241.5076319 | 3 |
| 3241.5078066 | 0 |
| 3241.5080409 | 4 |
| 3241.5081113 | 2 |
| 3241.508219 | 1 |
| 3241.5085279 | 0 |
| 3241.5088778 | 2 |
| 3241.5094287 | 0 |
| 3241.5098267 | 4 |
| 3241.5100572 | 2 |
| 3241.5103951 | 1 |
| 3241.5104135 | 0 |
| 3241.5104687 | 0 |
| 3241.5107447 | 1 |
| 3241.5107474 | 3 |
| 3241.5110364 | 2 |
| 3241.5113667 | 2 |
| 3241.5115053 | 2 |
| 3241.5116342 | 2 |
| 3241.5121952 | 3 |
| 3241.5123557 | 2 |
| 3241.5129711 | 5 |
| 3241.5129834 | 3 |
| 3241.5131103 | 2 |
| 3241.5134402 | 1 |
| 3241.5135463 | 1 |
| 3241.5136083 | 1 |
| 3241.5144555 | 1 |
| 3241.5145374 | 1 |
| 3241.5145425 | 0 |
| 3241.5148644 | 2 |
| 3241.515233 | 3 |
| 3241.5155892 | 2 |
| 3241.5156105 | 1 |
| 3241.5156949 | 2 |
| 3241.51606 | 1 |
| 3241.5162337 | 2 |
| 3241.5163542 | 3 |
| 3241.5165102 | 1 |
| 3241.5170514 | 0 |
| 3241.517376 | 1 |
| 3241.5174828 | 0 |
| 3241.5177643 | 3 |
| 3241.5181939 | 0 |
| 3241.5183362 | 2 |
| 3241.5184737 | 2 |
| 3241.5186728 | 2 |
| 3241.5188861 | 2 |
| 3241.5197119 | 0 |
| 3241.5197538 | 2 |
| 3241.5198219 | 2 |
| 3241.519899 | 0 |
| 3241.5203473 | 1 |
| 3241.5203947 | 1 |
| 3241.5206549 | 1 |
| 3241.5211286 | 2 |
| 3241.521184 | 2 |
| 3241.5214621 | 0 |
| 3241.5218893 | 0 |
| 3241.5220065 | 2 |
| 3241.5220515 | 2 |
| 3241.5223981 | 5 |
| 3241.5224155 | 1 |
| 3241.5230496 | 2 |
| 3241.5231461 | 0 |
| 3241.5233176 | 0 |
| 3241.5235952 | 4 |
| 3241.5241358 | 2 |
| 3241.5243662 | 0 |
| 3241.5244533 | 8 |
| 3241.524506 | 2 |
| 3241.5245395 | 5 |
| 3241.5249656 | 5 |
| 3241.5254193 | 3 |
| 3241.5257377 | 2 |
| 3241.5258876 | 1 |
| 3241.5259942 | 4 |
| 3241.5263218 | 4 |
| 3241.5266836 | 2 |
| 3241.5268273 | 1 |
| 3241.526945 | 3 |
| 3241.5274129 | 3 |
| 3241.5279104 | 2 |
| 3241.5279988 | 2 |
| 3241.5281074 | 3 |
| 3241.5282185 | 1 |
| 3241.5284618 | 2 |
| 3241.529075 | 3 |
| 3241.5291065 | 2 |
| 3241.5292083 | 3 |
| 3241.5292574 | 2 |
| 3241.5292644 | 2 |
| 3241.5297489 | 0 |
| 3241.5302535 | 0 |
| 3241.5304132 | 3 |
| 3241.5306848 | 3 |
| 3241.5313975 | 0 |
| 3241.531417 | 1 |
| 3241.5315196 | 0 |
| 3241.5317149 | 2 |
| 3241.5319514 | 3 |
| 3241.5321523 | 1 |
| 3241.5327248 | 1 |
| 3241.5328902 | 1 |
| 3241.5330945 | 0 |
| 3241.5332504 | 2 |
| 3241.5336796 | 0 |
| 3241.5339644 | 0 |
| 3241.5346177 | 3 |
| 3241.5346443 | 2 |
| 3241.5346572 | 5 |
| 3241.5348524 | 5 |
| 3241.5351345 | 2 |
| 3241.5351562 | 1 |
| 3241.5356563 | 0 |
| 3241.5361543 | 2 |
| 3241.5361996 | 3 |
| 3241.5363269 | 0 |
| 3241.5363433 | 2 |
| 3241.5364599 | 1 |
| 3241.5368066 | 0 |
| 3241.5369157 | 2 |
| 3241.5371632 | 1 |
| 3241.5376004 | 3 |
| 3241.5379904 | 1 |
| 3241.5380388 | 5 |
| 3241.5387542 | 1 |
| 3241.5389055 | 0 |
| 3241.5390165 | 2 |
| 3241.5394511 | 1 |
| 3241.5396315 | 1 |
| 3241.5399221 | 2 |
| 3241.5400835 | 3 |
| 3241.5401784 | 2 |
| 3241.5402491 | 2 |
| 3241.5407633 | 0 |
| 3241.5408076 | 2 |
| 3241.5412221 | 2 |
| 3241.5413732 | 0 |
| 3241.5415228 | 0 |
| 3241.5415871 | 4 |
| 3241.5424407 | 2 |
| 3241.5427203 | 2 |
| 3241.5427633 | 2 |
| 3241.5429229 | 3 |
| 3241.5430795 | 2 |
| 3241.5435861 | 2 |
| 3241.5438401 | 3 |
| 3241.5440845 | 3 |
| 3241.5443413 | 0 |
| 3241.5447149 | 4 |
| 3241.5447499 | 1 |
| 3241.5447993 | 3 |
| 3241.5448226 | 2 |
| 3241.5455364 | 2 |
| 3241.5458798 | 3 |
| 3241.5461097 | 1 |
| 3241.5462523 | 0 |
| 3241.5464642 | 4 |
| 3241.5467216 | 1 |
| 3241.5469818 | 2 |
| 3241.547001 | 1 |
| 3241.5471761 | 2 |
| 3241.5473633 | 2 |
| 3241.5478311 | 1 |
| 3241.5479773 | 1 |
| 3241.5487498 | 3 |
| 3241.5487686 | 1 |
| 3241.548825 | 1 |
| 3241.5491341 | 2 |
| 3241.5491779 | 0 |
| 3241.5497037 | 2 |
| 3241.549939 | 3 |
| 3241.5501576 | 2 |
| 3241.5503677 | 3 |
| 3241.5504035 | 0 |
| 3241.5506474 | 0 |
| 3241.5509321 | 0 |
| 3241.5510691 | 0 |
| 3241.5515238 | 1 |
| 3241.5519628 | 1 |
| 3241.5519943 | 1 |
| 3241.5519956 | 0 |
| 3241.5529279 | 1 |
| 3241.5529352 | 0 |
| 3241.5531403 | 1 |
| 3241.5534924 | 1 |
| 3241.553649 | 2 |
| 3241.5538288 | 1 |
| 3241.5541421 | 1 |
| 3241.554366 | 1 |
| 3241.5545394 | 4 |
| 3241.5545487 | 3 |
| 3241.5548199 | 1 |
| 3241.555406 | 2 |
| 3241.5556625 | 5 |
| 3241.5556814 | 1 |
| 3241.5558136 | 3 |
| 3241.5561371 | 2 |
| 3241.5563739 | 4 |
| 3241.5564531 | 2 |
| 3241.5567778 | 1 |
| 3241.5569828 | 5 |
| 3241.557263 | 1 |
| 3241.5577055 | 0 |
| 3241.5581009 | 1 |
| 3241.5581934 | 3 |
| 3241.5584969 | 0 |
| 3241.5586958 | 1 |
| 3241.5589234 | 1 |
| 3241.5590946 | 0 |
| 3241.5592648 | 3 |
| 3241.5597096 | 1 |
| 3241.5597163 | 0 |
| 3241.5601405 | 0 |
| 3241.5604226 | 4 |
| 3241.5607404 | 2 |
| 3241.560898 | 2 |
| 3241.5612957 | 1 |
| 3241.5614314 | 2 |
| 3241.5615591 | 2 |
| 3241.5618166 | 5 |
| 3241.5619177 | 1 |
| 3241.5621496 | 1 |
| 3241.5622542 | 0 |
| 3241.56239 | 1 |
| 3241.5635532 | 3 |
| 3241.5636835 | 2 |
| 3241.5638554 | 3 |
| 3241.5638893 | 3 |
| 3241.5639407 | 2 |
| 3241.5645244 | 2 |
| 3241.5646349 | 3 |
| 3241.5651529 | 1 |
| 3241.5652236 | 3 |
| 3241.5656194 | 0 |
| 3241.5656921 | 1 |
| 3241.5661037 | 1 |
| 3241.5661573 | 1 |
| 3241.5664241 | 4 |
| 3241.5666175 | 2 |
| 3241.56679 | 0 |
| 3241.5670584 | 0 |
| 3241.5671518 | 1 |
| 3241.5675888 | 2 |
| 3241.5680144 | 3 |
| 3241.5682086 | 1 |
| 3241.5682668 | 4 |
| 3241.5683307 | 2 |
| 3241.5690077 | 0 |
| 3241.5690569 | 0 |
| 3241.5690933 | 0 |
| 3241.5694163 | 2 |
| 3241.5695236 | 1 |
| 3241.5700514 | 1 |
| 3241.5706645 | 3 |
| 3241.5706743 | 3 |
| 3241.5706992 | 0 |
| 3241.5708632 | 1 |
| 3241.5714811 | 4 |
| 3241.5715171 | 2 |

| | |
|---|---|
| 3241.5718466 | 2 |
| 3241.5721216 | 1 |
| 3241.5723309 | 0 |
| 3241.5723343 | 2 |
| 3241.5726734 | 3 |
| 3241.5730959 | 1 |
| 3241.5732136 | 2 |
| 3241.5732547 | 0 |
| 3241.5734116 | 1 |
| 3241.5739781 | 0 |
| 3241.5740183 | 1 |
| 3241.5744753 | 0 |
| 3241.5745014 | 2 |
| 3241.5749385 | 1 |
| 3241.5749889 | 2 |
| 3241.5751065 | 5 |
| 3241.5752882 | 1 |
| 3241.5756958 | 1 |
| 3241.5762994 | 1 |
| 3241.5763133 | 2 |
| 3241.5764587 | 2 |
| 3241.5766405 | 4 |
| 3241.5771557 | 2 |
| 3241.5772033 | 2 |
| 3241.5775548 | 3 |
| 3241.5776548 | 1 |
| 3241.577938 | 1 |
| 3241.5779641 | 0 |
| 3241.5782504 | 3 |
| 3241.5785514 | 0 |
| 3241.5785809 | 3 |
| 3241.57881 | 2 |
| 3241.5795123 | 2 |
| 3241.5796505 | 3 |
| 3241.5799884 | 1 |
| 3241.5800416 | 3 |
| 3241.5805955 | 3 |
| 3241.5807342 | 2 |
| 3241.580954 | 2 |
| 3241.5810607 | 1 |
| 3241.5814654 | 3 |
| 3241.5817692 | 2 |
| 3241.5819639 | 2 |
| 3241.5822893 | 1 |
| 3241.5825163 | 0 |
| 3241.5826303 | 2 |
| 3241.58294 | 1 |
| 3241.5830148 | 0 |
| 3241.5833107 | 1 |
| 3241.5840395 | 2 |
| 3241.5840972 | 2 |
| 3241.5841138 | 0 |
| 3241.5842068 | 1 |
| 3241.5845717 | 0 |
| 3241.5847505 | 2 |
| 3241.5848139 | 2 |
| 3241.5850842 | 0 |
| 3241.5864629 | 2 |
| 3241.5866087 | 0 |
| 3241.5867801 | 2 |
| 3241.5867967 | 1 |
| 3241.586851 | 3 |
| 3241.5870008 | 1 |
| 3241.5870405 | 3 |
| 3241.5871523 | 1 |
| 3241.5873772 | 3 |
| 3241.587567 | 2 |
| 3241.5880893 | 1 |
| 3241.5883778 | 4 |
| 3241.5886843 | 1 |
| 3241.5890327 | 1 |
| 3241.5892651 | 1 |
| 3241.5896988 | 2 |
| 3241.5898593 | 1 |
| 3241.5903478 | 2 |
| 3241.590438 | 4 |
| 3241.5904512 | 0 |
| 3241.5908869 | 2 |
| 3241.5910249 | 0 |
| 3241.5910716 | 6 |
| 3241.5912541 | 2 |
| 3241.5920278 | 1 |
| 3241.5921409 | 3 |
| 3241.5921652 | 3 |
| 3241.5923963 | 2 |
| 3241.5926028 | 2 |
| 3241.5926552 | 1 |
| 3241.5933358 | 6 |
| 3241.5936176 | 1 |
| 3241.5937734 | 3 |
| 3241.5938818 | 2 |
| 3241.5941972 | 0 |
| 3241.5943677 | 1 |
| 3241.5946914 | 0 |
| 3241.5947755 | 2 |
| 3241.5951159 | 2 |
| 3241.595708 | 2 |
| 3241.5958615 | 3 |
| 3241.5960737 | 0 |
| 3241.5961764 | 2 |
| 3241.596397 | 7 |
| 3241.5968977 | 0 |
| 3241.5969684 | 0 |
| 3241.5974415 | 3 |
| 3241.5975687 | 2 |
| 3241.597771 | 3 |
| 3241.5981775 | 2 |
| 3241.5984081 | 0 |
| 3241.5986937 | 1 |
| 3241.5987639 | 1 |
| 3241.598863 | 3 |
| 3241.5992466 | 2 |
| 3241.5994418 | 1 |
| 3241.5995149 | 1 |
| 3241.5996248 | 2 |
| 3241.6000148 | 3 |
| 3241.6002193 | 2 |
| 3241.6004769 | 3 |
| 3241.6006785 | 1 |
| 3241.6007834 | 5 |
| 3241.600963 | 0 |
| 3241.601229 | 4 |
| 3241.6016065 | 4 |
| 3241.6018484 | 0 |
| 3241.6019007 | 0 |
| 3241.6021799 | 3 |
| 3241.6022435 | 0 |
| 3241.6026243 | 0 |
| 3241.6031586 | 1 |
| 3241.6035095 | 2 |
| 3241.6036427 | 3 |
| 3241.6039038 | 3 |
| 3241.6039276 | 0 |
| 3241.6042856 | 3 |
| 3241.6047282 | 0 |
| 3241.6050581 | 3 |
| 3241.6051664 | 0 |
| 3241.6055405 | 1 |
| 3241.6056528 | 1 |
| 3241.6064657 | 1 |
| 3241.606478 | 2 |
| 3241.6064967 | 1 |
| 3241.6066597 | 4 |
| 3241.6067566 | 3 |
| 3241.6067624 | 0 |
| 3241.6075732 | 3 |
| 3241.6076896 | 1 |
| 3241.6081157 | 1 |
| 3241.6082851 | 2 |
| 3241.6083839 | 3 |
| 3241.6083912 | 0 |
| 3241.6087736 | 4 |
| 3241.6091547 | 0 |
| 3241.6094229 | 2 |
| 3241.6095613 | 1 |
| 3241.6098686 | 5 |
| 3241.6104465 | 4 |
| 3241.6106786 | 0 |
| 3241.6110235 | 3 |
| 3241.6113473 | 3 |
| 3241.6115492 | 1 |
| 3241.6116565 | 1 |
| 3241.6116713 | 1 |
| 3241.611865 | 2 |
| 3241.6119931 | 1 |
| 3241.6122158 | 1 |
| 3241.6122499 | 1 |
| 3241.6127414 | 2 |
| 3241.6128818 | 1 |
| 3241.613353 | 3 |
| 3241.6139456 | 1 |
| 3241.6139776 | 2 |
| 3241.6140542 | 2 |
| 3241.6141377 | 2 |
| 3241.6147413 | 1 |
| 3241.6147981 | 3 |
| 3241.6147984 | 0 |
| 3241.6153453 | 2 |
| 3241.6154129 | 2 |
| 3241.6154316 | 0 |
| 3241.6163429 | 2 |
| 3241.6164882 | 2 |
| 3241.6167246 | 1 |
| 3241.616739 | 1 |
| 3241.6172688 | 2 |
| 3241.6172793 | 2 |
| 3241.6175486 | 2 |
| 3241.6179374 | 0 |
| 3241.6180363 | 2 |
| 3241.6182443 | 1 |
| 3241.6186373 | 1 |
| 3241.6188412 | 5 |
| 3241.6189339 | 1 |
| 3241.6191994 | 0 |
| 3241.6197522 | 0 |
| 3241.6197634 | 1 |
| 3241.6198795 | 3 |
| 3241.6202713 | 2 |
| 3241.6203067 | 3 |
| 3241.6203262 | 0 |
| 3241.6207587 | 2 |
| 3241.6211441 | 2 |
| 3241.6212838 | 3 |
| 3241.6213063 | 2 |
| 3241.6219795 | 5 |
| 3241.622498 | 5 |
| 3241.6226521 | 4 |
| 3241.6226657 | 1 |
| 3241.6227771 | 4 |
| 3241.6236055 | 1 |
| 3241.6237211 | 2 |
| 3241.6238002 | 1 |
| 3241.6238361 | 1 |
| 3241.6240184 | 0 |
| 3241.6242585 | 2 |
| 3241.6245884 | 3 |
| 3241.6247292 | 2 |
| 3241.6250661 | 2 |
| 3241.6255783 | 2 |
| 3241.6257581 | 5 |
| 3241.6259584 | 3 |
| 3241.6260731 | 3 |
| 3241.6265321 | 1 |
| 3241.6266838 | 1 |
| 3241.6267731 | 2 |
| 3241.6271181 | 2 |
| 3241.627436 | 1 |
| 3241.627701 | 1 |
| 3241.6277485 | 1 |
| 3241.6278125 | 4 |
| 3241.6279932 | 3 |
| 3241.6281227 | 1 |
| 3241.6288911 | 1 |
| 3241.629313 | 2 |
| 3241.6295716 | 1 |
| 3241.6296499 | 0 |
| 3241.6298402 | 0 |
| 3241.6298494 | 2 |
| 3241.6302874 | 0 |
| 3241.6302978 | 3 |
| 3241.6306037 | 1 |
| 3241.6308111 | 1 |
| 3241.6312076 | 1 |
| 3241.6316117 | 3 |
| 3241.6320461 | 2 |
| 3241.6320827 | 1 |
| 3241.6321734 | 1 |
| 3241.632286 | 1 |
| 3241.6329816 | 2 |
| 3241.6330253 | 0 |
| 3241.633497 | 5 |
| 3241.6335118 | 1 |
| 3241.6335686 | 7 |
| 3241.6341766 | 1 |
| 3241.6342131 | 1 |
| 3241.6346047 | 4 |
| 3241.6347046 | 1 |
| 3241.6348767 | 0 |
| 3241.6354316 | 2 |
| 3241.6358868 | 1 |
| 3241.6359066 | 1 |
| 3241.6360565 | 0 |
| 3241.6360754 | 2 |
| 3241.6360921 | 3 |
| 3241.6364597 | 3 |
| 3241.6371121 | 1 |
| 3241.63756 | 0 |
| 3241.6375957 | 3 |
| 3241.6378354 | 3 |
| 3241.6378387 | 4 |
| 3241.638448 | 1 |
| 3241.63849 | 1 |
| 3241.6388368 | 2 |
| 3241.6389394 | 1 |
| 3241.6391255 | 1 |
| 3241.6392292 | 2 |
| 3241.6395167 | 1 |
| 3241.6400387 | 2 |
| 3241.6400886 | 2 |
| 3241.6402207 | 3 |
| 3241.6403762 | 1 |
| 3241.6407688 | 2 |
| 3241.6409387 | 2 |
| 3241.6418159 | 0 |
| 3241.641859 | 2 |
| 3241.642014 | 2 |
| 3241.6421633 | 3 |
| 3241.6422679 | 3 |
| 3241.6425193 | 5 |
| 3241.6430652 | 0 |
| 3241.6436754 | 1 |
| 3241.6439425 | 1 |
| 3241.6440149 | 2 |
| 3241.6440944 | 4 |
| 3241.6441148 | 3 |
| 3241.6444758 | 4 |
| 3241.6445084 | 4 |
| 3241.6447691 | 1 |
| 3241.6453822 | 3 |
| 3241.6454673 | 1 |
| 3241.6457132 | 1 |
| 3241.6460013 | 0 |
| 3241.6464189 | 3 |
| 3241.6465384 | 2 |
| 3241.6469944 | 2 |
| 3241.6471724 | 1 |
| 3241.6472395 | 1 |
| 3241.6472583 | 1 |
| 3241.6475961 | 4 |
| 3241.6481872 | 3 |
| 3241.6483658 | 4 |
| 3241.6485153 | 1 |
| 3241.6487852 | 1 |
| 3241.6489258 | 4 |
| 3241.6492921 | 1 |
| 3241.6494158 | 5 |
| 3241.6496882 | 3 |
| 3241.6500226 | 1 |
| 3241.6504015 | 2 |
| 3241.6504172 | 1 |
| 3241.6508329 | 0 |
| 3241.6508468 | 3 |
| 3241.6509546 | 0 |
| 3241.6513496 | 1 |
| 3241.6518948 | 0 |
| 3241.6519024 | 0 |
| 3241.6525203 | 4 |
| 3241.652715 | 2 |
| 3241.6530244 | 0 |
| 3241.6531252 | 1 |
| 3241.6532526 | 0 |
| 3241.6535168 | 2 |
| 3241.6537313 | 4 |
| 3241.6538074 | 3 |
| 3241.6543812 | 2 |
| 3241.6545899 | 2 |
| 3241.6545998 | 0 |
| 3241.6546701 | 1 |
| 3241.6555613 | 0 |
| 3241.6556007 | 3 |
| 3241.655673 | 0 |
| 3241.6557674 | 4 |
| 3241.6559398 | 0 |
| 3241.6560453 | 0 |
| 3241.6563241 | 0 |
| 3241.6566636 | 2 |
| 3241.6569946 | 0 |
| 3241.6570105 | 1 |
| 3241.6573722 | 1 |
| 3241.6577988 | 0 |
| 3241.6578279 | 3 |
| 3241.6581628 | 0 |
| 3241.6583817 | 5 |
| 3241.659056 | 1 |
| 3241.6591661 | 3 |
| 3241.6591752 | 2 |
| 3241.6597734 | 1 |
| 3241.6599056 | 3 |
| 3241.6600028 | 2 |
| 3241.6601019 | 3 |
| 3241.6607248 | 3 |
| 3241.6608744 | 4 |
| 3241.6609636 | 2 |

| | |
|---|---|
| 3241.6609977 | 0 |
| 3241.661071 | 2 |
| 3241.6619615 | 0 |
| 3241.6620622 | 3 |
| 3241.6621591 | 3 |
| 3241.6622228 | 0 |
| 3241.6627545 | 0 |
| 3241.6630063 | 1 |
| 3241.6632999 | 2 |
| 3241.6633729 | 1 |
| 3241.6635815 | 3 |
| 3241.6636419 | 0 |
| 3241.6636574 | 1 |
| 3241.6644212 | 3 |
| 3241.6647542 | 2 |
| 3241.6647596 | 2 |
| 3241.665147 | 1 |
| 3241.6651876 | 0 |
| 3241.665562 | 2 |
| 3241.6660089 | 3 |
| 3241.6662024 | 3 |
| 3241.666425 | 1 |
| 3241.667039 | 1 |
| 3241.6672053 | 1 |
| 3241.667302 | 1 |
| 3241.6674364 | 2 |
| 3241.6675847 | 1 |
| 3241.6679879 | 2 |
| 3241.6681804 | 2 |
| 3241.6682796 | 5 |
| 3241.6684515 | 0 |
| 3241.6690601 | 3 |
| 3241.6691495 | 2 |
| 3241.6691785 | 2 |
| 3241.6694434 | 6 |
| 3241.6699559 | 1 |
| 3241.6699987 | 0 |
| 3241.6700212 | 2 |
| 3241.6709782 | 0 |
| 3241.6711875 | 1 |
| 3241.6712385 | 5 |
| 3241.6713131 | 1 |
| 3241.6716405 | 1 |
| 3241.6716598 | 0 |
| 3241.6721066 | 2 |
| 3241.6722026 | 1 |
| 3241.6724466 | 2 |
| 3241.6729912 | 2 |
| 3241.6730517 | 0 |
| 3241.6731901 | 3 |
| 3241.6736412 | 2 |
| 3241.6737643 | 3 |
| 3241.6738035 | 1 |
| 3241.6738518 | 0 |
| 3241.6745732 | 2 |
| 3241.6746228 | 2 |
| 3241.6748839 | 2 |
| 3241.6752362 | 0 |
| 3241.6754501 | 2 |
| 3241.6756966 | 2 |
| 3241.6758096 | 2 |
| 3241.6761541 | 2 |
| 3241.6762288 | 2 |
| 3241.6766751 | 2 |
| 3241.6768137 | 2 |
| 3241.6772189 | 1 |
| 3241.6773051 | 1 |
| 3241.6773175 | 2 |
| 3241.6780066 | 5 |
| 3241.6780592 | 2 |
| 3241.6782752 | 0 |
| 3241.6789347 | 3 |
| 3241.6791224 | 1 |
| 3241.6792983 | 0 |
| 3241.6796755 | 3 |
| 3241.6797373 | 1 |
| 3241.6798625 | 4 |
| 3241.6800632 | 2 |
| 3241.6803246 | 5 |
| 3241.6809415 | 1 |
| 3241.6814201 | 3 |
| 3241.681435 | 2 |
| 3241.6816058 | 1 |
| 3241.6817503 | 1 |
| 3241.6820286 | 2 |
| 3241.6824512 | 3 |
| 3241.68254 | 2 |
| 3241.6825696 | 3 |
| 3241.6829337 | 1 |
| 3241.6832503 | 0 |
| 3241.6836973 | 3 |
| 3241.6838117 | 0 |
| 3241.6838453 | 4 |
| 3241.6838517 | 1 |
| 3241.6846266 | 2 |
| 3241.6848233 | 3 |
| 3241.6848301 | 1 |
| 3241.684854 | 3 |
| 3241.6855124 | 1 |
| 3241.6855226 | 2 |
| 3241.6859221 | 1 |
| 3241.6862685 | 3 |
| 3241.6863518 | 1 |
| 3241.6865407 | 0 |
| 3241.6868434 | 4 |
| 3241.6871126 | 2 |
| 3241.6873548 | 2 |
| 3241.6877017 | 2 |
| 3241.6881462 | 0 |
| 3241.6883102 | 5 |
| 3241.6884029 | 3 |
| 3241.6885332 | 4 |
| 3241.6886118 | 2 |
| 3241.6888128 | 4 |
| 3241.6895454 | 1 |
| 3241.6895836 | 1 |
| 3241.6900426 | 1 |
| 3241.6901854 | 1 |
| 3241.6903874 | 2 |
| 3241.6904202 | 5 |
| 3241.6906983 | 2 |
| 3241.6911934 | 5 |
| 3241.6915157 | 0 |
| 3241.6915381 | 0 |
| 3241.6917792 | 1 |
| 3241.692195 | 1 |
| 3241.6923876 | 1 |
| 3241.6928214 | 4 |
| 3241.6933217 | 4 |
| 3241.6933773 | 2 |
| 3241.6936902 | 0 |
| 3241.6937664 | 1 |
| 3241.6938177 | 3 |
| 3241.6941008 | 3 |
| 3241.6943598 | 4 |
| 3241.6943767 | 3 |
| 3241.6946058 | 2 |
| 3241.695315 | 1 |
| 3241.6954192 | 1 |
| 3241.6954646 | 0 |
| 3241.6958184 | 1 |
| 3241.6960607 | 0 |
| 3241.6962302 | 0 |
| 3241.6965874 | 2 |
| 3241.6966828 | 2 |
| 3241.6969367 | 5 |
| 3241.6971508 | 3 |
| 3241.6975519 | 1 |
| 3241.6981491 | 3 |
| 3241.6982096 | 1 |
| 3241.6984367 | 2 |
| 3241.6986058 | 1 |
| 3241.6987135 | 2 |
| 3241.6988455 | 0 |
| 3241.6988688 | 3 |
| 3241.6990876 | 1 |
| 3241.6999977 | 3 |
| 3241.7001482 | 2 |
| 3241.7009161 | 0 |
| 3241.7009927 | 2 |
| 3241.7010266 | 2 |
| 3241.7013262 | 2 |
| 3241.701486 | 5 |
| 3241.7015454 | 1 |
| 3241.7018083 | 3 |
| 3241.7019676 | 5 |
| 3241.7019892 | 3 |
| 3241.7020549 | 1 |
| 3241.7020923 | 3 |
| 3241.702996 | 3 |
| 3241.7030974 | 0 |
| 3241.703118 | 0 |
| 3241.7032281 | 2 |
| 3241.7038513 | 2 |
| 3241.7039725 | 3 |
| 3241.7043689 | 2 |
| 3241.7045852 | 5 |
| 3241.704624 | 1 |
| 3241.7048855 | 0 |
| 3241.7054933 | 2 |
| 3241.7055802 | 1 |
| 3241.7058555 | 3 |
| 3241.7063906 | 2 |
| 3241.7066435 | 1 |
| 3241.7067452 | 4 |
| 3241.7067584 | 0 |
| 3241.7068996 | 3 |
| 3241.7071 | 1 |
| 3241.7075829 | 0 |
| 3241.7077311 | 2 |
| 3241.7078728 | 3 |
| 3241.7084739 | 0 |
| 3241.70861 | 2 |
| 3241.7091186 | 2 |
| 3241.7092334 | 3 |
| 3241.7095789 | 3 |
| 3241.7096018 | 2 |
| 3241.7096397 | 1 |
| 3241.7100024 | 0 |
| 3241.7104346 | 2 |
| 3241.7107671 | 3 |
| 3241.7108057 | 2 |
| 3241.7111913 | 2 |
| 3241.7117046 | 2 |
| 3241.7117064 | 1 |
| 3241.7117413 | 2 |
| 3241.7121145 | 4 |
| 3241.7121789 | 4 |
| 3241.7128909 | 0 |
| 3241.7129497 | 3 |
| 3241.7132448 | 3 |
| 3241.7133959 | 2 |
| 3241.7137474 | 5 |
| 3241.7139639 | 1 |
| 3241.713986 | 0 |
| 3241.7143016 | 0 |
| 3241.7150045 | 2 |
| 3241.7151256 | 0 |
| 3241.7151655 | 1 |
| 3241.7152868 | 2 |
| 3241.7155926 | 2 |
| 3241.7157183 | 1 |
| 3241.7159496 | 0 |
| 3241.7163007 | 2 |
| 3241.7163621 | 0 |
| 3241.7167787 | 1 |
| 3241.716919 | 0 |
| 3241.7171357 | 2 |
| 3241.7171645 | 0 |
| 3241.7173607 | 3 |
| 3241.7175799 | 2 |
| 3241.7179954 | 0 |
| 3241.7180891 | 1 |
| 3241.7187458 | 1 |
| 3241.7191195 | 1 |
| 3241.719261 | 3 |
| 3241.7193213 | 2 |
| 3241.719576 | 2 |
| 3241.7198688 | 2 |
| 3241.7202472 | 0 |
| 3241.7202801 | 2 |
| 3241.7208694 | 2 |
| 3241.7210018 | 1 |
| 3241.7211176 | 3 |
| 3241.7211772 | 0 |
| 3241.7215672 | 3 |
| 3241.7221707 | 1 |
| 3241.7223526 | 1 |
| 3241.7224531 | 4 |
| 3241.7225111 | 2 |
| 3241.7229472 | 4 |
| 3241.7230941 | 3 |
| 3241.7238137 | 4 |
| 3241.7239019 | 0 |
| 3241.724122 | 2 |
| 3241.7242372 | 3 |
| 3241.7244515 | 3 |
| 3241.7252665 | 1 |
| 3241.7253557 | 3 |
| 3241.7255397 | 1 |
| 3241.7255781 | 2 |
| 3241.7256377 | 1 |
| 3241.7262824 | 3 |
| 3241.7262979 | 0 |
| 3241.7264345 | 3 |
| 3241.7265509 | 5 |
| 3241.726937 | 2 |
| 3241.7269911 | 1 |
| 3241.7272632 | 2 |
| 3241.7275136 | 4 |
| 3241.7275923 | 1 |
| 3241.7283677 | 3 |
| 3241.7285203 | 2 |
| 3241.728803 | 8 |
| 3241.728892 | 2 |
| 3241.7291344 | 1 |
| 3241.7295277 | 3 |
| 3241.7301932 | 5 |
| 3241.7302404 | 2 |
| 3241.7304069 | 2 |
| 3241.730507 | 0 |
| 3241.7305739 | 1 |
| 3241.7310111 | 2 |
| 3241.7312081 | 4 |
| 3241.7312142 | 0 |
| 3241.7312713 | 2 |
| 3241.7319472 | 3 |
| 3241.7323095 | 2 |
| 3241.7325322 | 1 |
| 3241.7327848 | 1 |
| 3241.7330056 | 0 |
| 3241.7334095 | 0 |
| 3241.7337424 | 3 |
| 3241.7338116 | 1 |
| 3241.7338526 | 6 |
| 3241.7340576 | 0 |
| 3241.734618 | 1 |
| 3241.7346478 | 1 |
| 3241.735023 | 0 |
| 3241.7351289 | 1 |
| 3241.7356391 | 1 |
| 3241.7358071 | 0 |
| 3241.7359129 | 3 |
| 3241.7361111 | 2 |
| 3241.7363178 | 7 |
| 3241.736412 | 1 |
| 3241.7365266 | 2 |
| 3241.7372578 | 1 |
| 3241.7373063 | 0 |
| 3241.7379409 | 4 |
| 3241.7380645 | 3 |
| 3241.7381327 | 1 |
| 3241.7383567 | 2 |
| 3241.7384409 | 0 |
| 3241.7385106 | 3 |
| 3241.7391403 | 3 |
| 3241.7394525 | 2 |
| 3241.7396699 | 2 |
| 3241.7399826 | 3 |
| 3241.7400749 | 1 |
| 3241.740382 | 1 |
| 3241.7405859 | 1 |
| 3241.7408355 | 1 |
| 3241.7408719 | 0 |
| 3241.741022 | 2 |
| 3241.7415367 | 5 |
| 3241.7417862 | 3 |
| 3241.742197 | 2 |
| 3241.7423168 | 4 |
| 3241.7425237 | 3 |
| 3241.7428298 | 5 |
| 3241.7429924 | 2 |
| 3241.74337 | 0 |
| 3241.7438713 | 4 |
| 3241.7439393 | 4 |
| 3241.7441666 | 3 |
| 3241.7446665 | 2 |
| 3241.7449605 | 2 |
| 3241.7450352 | 1 |
| 3241.7450446 | 1 |
| 3241.7450722 | 0 |
| 3241.7454611 | 1 |
| 3241.7459271 | 3 |
| 3241.7460975 | 3 |
| 3241.7463692 | 1 |
| 3241.7464605 | 2 |
| 3241.7467134 | 0 |
| 3241.747133 | 1 |
| 3241.7477408 | 3 |
| 3241.7477884 | 1 |
| 3241.7478075 | 2 |
| 3241.7479455 | 3 |
| 3241.7481584 | 2 |
| 3241.7481811 | 0 |
| 3241.7490843 | 1 |
| 3241.7490979 | 1 |
| 3241.7491178 | 1 |
| 3241.7496369 | 1 |
| 3241.7498897 | 1 |
| 3241.7502103 | 3 |
| 3241.7502724 | 2 |
| 3241.7503854 | 2 |
| 3241.7504225 | 1 |

| | |
|---|---|
| 3241.7508216 | 1 |
| 3241.7510397 | 3 |
| 3241.7512428 | 3 |
| 3241.7513295 | 1 |
| 3241.7521905 | 0 |
| 3241.7521913 | 1 |
| 3241.7521983 | 1 |
| 3241.7524684 | 1 |
| 3241.7531017 | 3 |
| 3241.7532268 | 1 |
| 3241.7533892 | 2 |
| 3241.7536142 | 1 |
| 3241.7536696 | 2 |
| 3241.7543569 | 2 |
| 3241.7548261 | 2 |
| 3241.7550232 | 3 |
| 3241.7552659 | 1 |
| 3241.7553255 | 1 |
| 3241.7555986 | 0 |
| 3241.7561941 | 1 |
| 3241.756262 | 1 |
| 3241.756381 | 2 |
| 3241.7565471 | 2 |
| 3241.75656 | 2 |
| 3241.7568953 | 1 |
| 3241.7571939 | 3 |
| 3241.7574568 | 2 |
| 3241.7575907 | 1 |
| 3241.7579855 | 1 |
| 3241.7581897 | 1 |
| 3241.758319 | 1 |
| 3241.7583849 | 4 |
| 3241.7584389 | 0 |
| 3241.7589982 | 3 |
| 3241.7594521 | 2 |
| 3241.7595254 | 2 |
| 3241.7599115 | 2 |
| 3241.7600863 | 3 |
| 3241.760181 | 1 |
| 3241.7607713 | 4 |
| 3241.7608563 | 0 |
| 3241.7609295 | 3 |
| 3241.7612854 | 5 |
| 3241.7613367 | 2 |
| 3241.761976 | 1 |
| 3241.7623998 | 2 |
| 3241.7624408 | 2 |
| 3241.762662 | 2 |
| 3241.7626664 | 0 |
| 3241.7633473 | 0 |
| 3241.7634191 | 4 |
| 3241.7636555 | 1 |
| 3241.7638603 | 3 |
| 3241.764236 | 3 |
| 3241.7647785 | 2 |
| 3241.7649711 | 1 |
| 3241.7651592 | 5 |
| 3241.7654155 | 2 |
| 3241.7655368 | 4 |
| 3241.7656358 | 1 |
| 3241.765734 | 1 |
| 3241.7663535 | 2 |
| 3241.7664998 | 1 |
| 3241.7667493 | 0 |
| 3241.7669305 | 4 |
| 3241.7669657 | 1 |
| 3241.7673993 | 1 |
| 3241.7674733 | 2 |
| 3241.7676672 | 1 |
| 3241.7678286 | 0 |
| 3241.7684431 | 2 |
| 3241.7685847 | 1 |
| 3241.7686117 | 2 |
| 3241.7690539 | 0 |
| 3241.769148 | 4 |
| 3241.7694561 | 3 |
| 3241.7695522 | 3 |
| 3241.7699935 | 2 |
| 3241.7702536 | 2 |
| 3241.7703665 | 1 |
| 3241.7709317 | 2 |
| 3241.7710323 | 2 |
| 3241.7711465 | 2 |
| 3241.7713113 | 0 |
| 3241.7717057 | 1 |
| 3241.7719064 | 4 |
| 3241.7720846 | 5 |
| 3241.7725627 | 2 |
| 3241.772573 | 1 |
| 3241.77273 | 4 |
| 3241.7733852 | 3 |
| 3241.7734365 | 3 |
| 3241.773787 | 4 |
| 3241.7738764 | 0 |
| 3241.7743003 | 1 |
| 3241.7744096 | 1 |
| 3241.7744939 | 2 |
| 3241.7745045 | 0 |
| 3241.774981 | 2 |
| 3241.7750281 | 1 |
| 3241.7751077 | 0 |
| 3241.7751551 | 2 |
| 3241.7753971 | 3 |
| 3241.7754573 | 1 |
| 3241.7757245 | 4 |
| 3241.7758333 | 2 |
| 3241.7758458 | 2 |
| 3241.7761977 | 1 |
| 3241.7761992 | 0 |
| 3241.7767921 | 1 |
| 3241.7770209 | 1 |
| 3241.777423 | 1 |
| 3241.7777308 | 2 |
| 3241.7781217 | 0 |
| 3241.7781278 | 1 |
| 3241.7786486 | 1 |
| 3241.7789503 | 3 |
| 3241.7791514 | 1 |
| 3241.7791969 | 3 |
| 3241.7792113 | 1 |
| 3241.7792949 | 3 |
| 3241.779363 | 2 |
| 3241.7793812 | 1 |
| 3241.7794962 | 0 |
| 3241.7798535 | 2 |
| 3241.7801764 | 1 |
| 3241.7805575 | 2 |
| 3241.7809646 | 4 |
| 3241.7810584 | 1 |
| 3241.7810626 | 2 |
| 3241.7814413 | 2 |
| 3241.7815787 | 3 |
| 3241.7816016 | 0 |
| 3241.7817196 | 1 |
| 3241.7819133 | 1 |
| 3241.7821988 | 3 |
| 3241.7827871 | 4 |
| 3241.7830234 | 5 |
| 3241.783211 | 0 |
| 3241.7834869 | 1 |
| 3241.7835075 | 0 |
| 3241.7835181 | 3 |
| 3241.7835387 | 0 |
| 3241.7838622 | 3 |
| 3241.7841196 | 0 |
| 3241.7842129 | 2 |
| 3241.784296 | 0 |
| 3241.7847837 | 0 |
| 3241.7851427 | 2 |
| 3241.7851673 | 5 |
| 3241.78525 | 1 |
| 3241.7855772 | 4 |
| 3241.7856962 | 3 |
| 3241.786169 | 1 |
| 3241.7862177 | 1 |
| 3241.7862584 | 4 |
| 3241.786405 | 2 |
| 3241.7866569 | 1 |
| 3241.7868632 | 1 |
| 3241.7872671 | 2 |
| 3241.7873426 | 1 |
| 3241.7874821 | 2 |
| 3241.7876197 | 1 |
| 3241.7878482 | 1 |
| 3241.7878904 | 1 |
| 3241.7880233 | 0 |
| 3241.7882328 | 2 |
| 3241.7884408 | 1 |
| 3241.7884597 | 2 |
| 3241.7886011 | 1 |
| 3241.7889694 | 2 |
| 3241.7892623 | 1 |
| 3241.7896344 | 1 |
| 3241.7897313 | 1 |
| 3241.789925 | 1 |
| 3241.790133 | 1 |
| 3241.7903152 | 2 |
| 3241.7903803 | 0 |
| 3241.7906331 | 1 |
| 3241.7910314 | 0 |
| 3241.7912334 | 4 |
| 3241.7913469 | 1 |
| 3241.7913603 | 0 |
| 3241.7917786 | 2 |
| 3241.791979 | 1 |
| 3241.7923543 | 0 |
| 3241.7924187 | 2 |
| 3241.7924428 | 3 |
| 3241.7924824 | 6 |
| 3241.7925835 | 4 |
| 3241.7927433 | 2 |
| 3241.7929325 | 2 |
| 3241.7931215 | 2 |
| 3241.793143 | 1 |
| 3241.7935752 | 2 |
| 3241.7939611 | 1 |
| 3241.7940244 | 0 |
| 3241.7941569 | 4 |
| 3241.7943463 | 2 |
| 3241.7945306 | 2 |
| 3241.7948435 | 1 |
| 3241.795014 | 4 |
| 3241.7954338 | 1 |
| 3241.7955999 | 1 |
| 3241.7956727 | 2 |
| 3241.7957512 | 4 |
| 3241.7960854 | 3 |
| 3241.7961688 | 3 |
| 3241.796508 | 2 |
| 3241.7966939 | 3 |
| 3241.7967 | 1 |
| 3241.7967527 | 4 |
| 3241.7968648 | 2 |
| 3241.7972121 | 4 |
| 3241.7973836 | 1 |
| 3241.7975037 | 1 |
| 3241.7976727 | 2 |
| 3241.7977881 | 3 |
| 3241.7977979 | 0 |
| 3241.7979818 | 1 |
| 3241.7986745 | 1 |
| 3241.7986842 | 1 |
| 3241.7987738 | 4 |
| 3241.7988813 | 1 |
| 3241.7994866 | 4 |
| 3241.8003315 | 1 |
| 3241.8003337 | 3 |
| 3241.8006345 | 5 |
| 3241.8009575 | 3 |
| 3241.8014949 | 2 |
| 3241.8020833 | 2 |
| 3241.8030476 | 4 |
| 3241.8031044 | 4 |
| 3241.8035777 | 0 |
| 3241.8044569 | 1 |
| 3241.8045839 | 2 |
| 3241.8047909 | 2 |
| 3241.8050488 | 4 |
| 3241.8054225 | 2 |
| 3241.8054702 | 0 |
| 3241.8065719 | 2 |
| 3241.8066311 | 4 |
| 3241.8069702 | 2 |
| 3241.8082571 | 7 |
| 3241.8083347 | 5 |
| 3241.8086074 | 2 |
| 3241.8087216 | 2 |
| 3241.8096306 | 2 |
| 3241.8101421 | 4 |
| 3241.8107595 | 0 |
| 3241.8109082 | 2 |
| 3241.8111582 | 5 |
| 3241.8116921 | 1 |
| 3241.812453 | 3 |
| 3241.8125091 | 2 |
| 3241.8127484 | 1 |
| 3241.8133621 | 1 |
| 3241.8136571 | 1 |
| 3241.8140841 | 1 |
| 3241.81439 | 4 |
| 3241.815223 | 2 |
| 3241.815544 | 3 |
| 3241.8159444 | 2 |
| 3241.815972 | 2 |
| 3241.8164028 | 5 |
| 3241.8167098 | 4 |
| 3241.8171946 | 6 |
| 3241.8175273 | 6 |
| 3241.8184579 | 9 |
| 3241.8185431 | 4 |
| 3241.8198784 | 17 |
| 3241.8200498 | 14 |
| 3241.8204049 | 11 |
| 3241.8207955 | 14 |
| 3241.8210122 | 13 |
| 3241.8215722 | 9 |
| 3241.8219344 | 6 |
| 3241.8221141 | 6 |
| 3241.8225358 | 4 |
| 3241.8227325 | 3 |
| 3241.8234263 | 1 |
| 3241.8248021 | 2 |
| 3241.8249616 | 4 |
| 3241.82526 | 1 |
| 3241.8258747 | 1 |
| 3241.8265949 | 5 |
| 3241.8268482 | 1 |
| 3241.8268687 | 4 |
| 3241.8271013 | 1 |
| 3241.8287049 | 1 |
| 3241.8289066 | 1 |
| 3241.8289508 | 7 |
| 3241.8290411 | 1 |
| 3241.8298144 | 5 |
| 3241.8299146 | 2 |
| 3241.8304098 | 7 |
| 3241.8310502 | 1 |
| 3241.8310716 | 2 |
| 3241.8317954 | 1 |
| 3241.8330227 | 6 |
| 3241.8331857 | 4 |
| 3241.8339456 | 2 |
| 3241.8342481 | 6 |
| 3241.8346364 | 3 |
| 3241.8350469 | 2 |
| 3241.8353203 | 3 |
| 3241.835834 | 2 |
| 3241.8362083 | 1 |
| 3241.8366298 | 3 |
| 3241.8372912 | 1 |
| 3241.8375141 | 3 |
| 3241.8378228 | 0 |
| 3241.8379748 | 1 |
| 3241.8394231 | 2 |
| 3241.8395952 | 4 |
| 3241.8397411 | 1 |
| 3241.8400492 | 1 |
| 3241.8415663 | 5 |
| 3241.8430925 | 3 |
| 3241.8460395 | 4 |
| 3241.8476724 | 3 |
| 3241.851023 | 2 |
| 3241.8536154 | 1 |
| 3241.855312 | 4 |
| 3241.8587338 | 2 |
| 3241.859616 | 2 |
| 3241.8630805 | 2 |
| 3241.8666808 | 1 |
| 3241.8675258 | 2 |
| 3241.8701818 | 2 |
| 3241.8737849 | 6 |
| 3241.8754786 | 1 |
| 3241.8795166 | 0 |
| 3241.8808706 | 4 |
| 3241.8837813 | 1 |
| 3241.8870313 | 3 |
| 3241.8880591 | 3 |
| 3241.8912497 | 1 |
| 3241.8930075 | 1 |
| 3241.8954577 | 2 |
| 3241.8961148 | 5 |
| 3241.8963057 | 1 |
| 3241.896508 | 3 |
| 3241.8969745 | 2 |
| 3241.8970336 | 0 |
| 3241.897669 | 3 |
| 3241.8978669 | 2 |
| 3241.8979621 | 6 |
| 3241.8980008 | 2 |
| 3241.8988334 | 3 |
| 3241.8993473 | 3 |
| 3241.9003564 | 1 |
| 3241.9003804 | 1 |
| 3241.9004273 | 3 |
| 3241.9007677 | 2 |
| 3241.9010895 | 2 |
| 3241.901286 | 1 |
| 3241.9018305 | 0 |
| 3241.9023873 | 2 |
| 3241.9027449 | 7 |
| 3241.9027895 | 2 |
| 3241.9035367 | 2 |
| 3241.9035943 | 3 |

| | |
|---|---|
| 3241.9037125 | 2 |
| 3241.9043051 | 1 |
| 3241.9043923 | 2 |
| 3241.9045085 | 5 |
| 3241.9049042 | 5 |
| 3241.9056287 | 1 |
| 3241.9056945 | 2 |
| 3241.9060292 | 2 |
| 3241.9062195 | 2 |
| 3241.9072381 | 1 |
| 3241.9073576 | 0 |
| 3241.9075689 | 1 |
| 3241.9080945 | 0 |
| 3241.9081138 | 1 |
| 3241.9081655 | 2 |
| 3241.9088442 | 3 |
| 3241.9090834 | 3 |
| 3241.9094043 | 5 |
| 3241.910149 | 4 |
| 3241.9109862 | 1 |
| 3241.9111067 | 1 |
| 3241.9113823 | 1 |
| 3241.9115254 | 2 |
| 3241.9119401 | 3 |
| 3241.9121126 | 3 |
| 3241.9130619 | 2 |
| 3241.9131787 | 1 |
| 3241.9136883 | 2 |
| 3241.9137649 | 6 |
| 3241.91385 | 2 |
| 3241.9143001 | 2 |
| 3241.9143671 | 2 |
| 3241.9146685 | 1 |
| 3241.9150327 | 3 |
| 3241.9160105 | 4 |
| 3241.9160533 | 6 |
| 3241.916123 | 5 |
| 3241.9161424 | 1 |
| 3241.916288 | 2 |
| 3241.9164998 | 2 |
| 3241.9165993 | 4 |
| 3241.9170069 | 3 |
| 3241.9177111 | 5 |
| 3241.9178734 | 1 |
| 3241.9179511 | 2 |
| 3241.9179873 | 8 |
| 3241.9183371 | 5 |
| 3241.9187359 | 5 |
| 3241.9192165 | 5 |
| 3241.9193852 | 17 |
| 3241.9199063 | 10 |
| 3241.9200524 | 10 |
| 3241.9201261 | 22 |
| 3241.9202799 | 17 |
| 3241.9204022 | 12 |
| 3241.9206837 | 14 |
| 3241.9213077 | 24 |
| 3241.9213646 | 31 |
| 3241.9217537 | 21 |
| 3241.9221855 | 21 |
| 3241.9222993 | 26 |
| 3241.9223617 | 25 |
| 3241.9223985 | 17 |
| 3241.9224479 | 14 |
| 3241.9232679 | 24 |
| 3241.9233076 | 39 |
| 3241.9235201 | 16 |
| 3241.9237846 | 26 |
| 3241.9239342 | 18 |
| 3241.9239492 | 24 |
| 3241.9243623 | 23 |
| 3241.9245806 | 11 |
| 3241.9252555 | 23 |
| 3241.925298 | 25 |
| 3241.9256765 | 24 |
| 3241.9262573 | 22 |
| 3241.9262651 | 28 |
| 3241.9262703 | 26 |
| 3241.927025 | 29 |
| 3241.9270495 | 18 |
| 3241.9271781 | 16 |
| 3241.9277414 | 8 |
| 3241.9282405 | 5 |
| 3241.9284602 | 19 |
| 3241.928568 | 8 |
| 3241.9285707 | 13 |
| 3241.9290738 | 1 |
| 3241.9290939 | 4 |
| 3241.9291416 | 2 |
| 3241.9296456 | 3 |
| 3241.9301547 | 3 |
| 3241.9305297 | 5 |
| 3241.9307255 | 6 |
| 3241.9307793 | 2 |
| 3241.9310544 | 3 |
| 3241.9311017 | 3 |
| 3241.9312618 | 0 |
| 3241.9317303 | 2 |
| 3241.9317948 | 3 |
| 3241.932091 | 3 |
| 3241.9322953 | 2 |
| 3241.9323538 | 3 |
| 3241.9326918 | 3 |
| 3241.9331795 | 5 |
| 3241.9333866 | 1 |
| 3241.9337042 | 6 |
| 3241.933794 | 4 |
| 3241.9340238 | 2 |
| 3241.9343759 | 3 |
| 3241.9346117 | 1 |
| 3241.9350777 | 1 |
| 3241.9351871 | 2 |
| 3241.9356692 | 0 |
| 3241.9376158 | 6 |
| 3241.9390191 | 1 |
| 3241.9402918 | 3 |
| 3241.9414886 | 1 |
| 3241.9429472 | 2 |
| 3241.9430795 | 0 |
| 3241.945172 | 4 |
| 3241.9466598 | 2 |
| 3241.9470658 | 7 |
| 3241.9493412 | 6 |
| 3241.9500008 | 8 |
| 3241.9515727 | 10 |
| 3241.9527956 | 1 |
| 3241.9535511 | 4 |
| 3241.9552038 | 3 |
| 3241.9559181 | 3 |
| 3241.9576484 | 2 |
| 3241.9585781 | 2 |
| 3241.9603241 | 3 |
| 3241.961506 | 5 |
| 3241.9625812 | 3 |
| 3241.964414 | 2 |
| 3241.9645121 | 1 |
| 3241.9662552 | 0 |
| 3241.9683062 | 4 |
| 3241.9691218 | 1 |
| 3241.9709836 | 2 |
| 3241.9715952 | 2 |
| 3241.9732477 | 1 |
| 3241.974705 | 6 |
| 3241.9747127 | 4 |
| 3241.9770733 | 0 |
| 3241.9775837 | 1 |
| 3241.9792487 | 2 |
| 3241.9800992 | 2 |
| 3241.9817251 | 0 |
| 3241.9829201 | 0 |
| 3241.9838141 | 1 |
| 3241.9858814 | 0 |
| 3241.9872898 | 3 |
| 3241.9874456 | 0 |
| 3241.9891902 | 2 |
| 3241.9910275 | 1 |
| 3241.9917495 | 2 |
| 3241.9930086 | 5 |
| 3241.9937717 | 1 |
| 3241.9952314 | 0 |
| 3241.997242 | 2 |
| 3241.9972998 | 4 |
| 3241.9991845 | 2 |
| 3242.000519 | 0 |
| 3242.0010533 | 1 |
| 3242.0018757 | 3 |
| 3242.0047545 | 2 |
| 3242.0051776 | 2 |
| 3242.006257 | 4 |
| 3242.0080351 | 3 |
| 3242.0086122 | 3 |
| 3242.0102881 | 3 |
| 3242.012328 | 4 |
| 3242.0128986 | 0 |
| 3242.0147916 | 3 |
| 3242.0156803 | 3 |
| 3242.0168791 | 1 |
| 3242.0176304 | 1 |
| 3242.0197303 | 4 |
| 3242.0209447 | 5 |
| 3242.0214747 | 7 |
| 3242.0232615 | 2 |
| 3242.0248967 | 2 |
| 3242.0252349 | 3 |
| 3242.0272257 | 3 |
| 3242.027863 | 4 |
| 3242.0297147 | 3 |
| 3242.0312465 | 2 |
| 3242.0316892 | 0 |
| 3242.0337471 | 0 |
| 3242.0338218 | 4 |
| 3242.0349741 | 3 |
| 3242.0366019 | 1 |
| 3242.0376469 | 3 |
| 3242.0388115 | 2 |
| 3242.0405186 | 5 |
| 3242.0418582 | 3 |
| 3242.0439206 | 2 |
| 3242.0446354 | 2 |
| 3242.0457835 | 1 |
| 3242.0463537 | 1 |
| 3242.048344 | 2 |
| 3242.0492939 | 2 |
| 3242.0501334 | 4 |
| 3242.0517833 | 1 |
| 3242.0533363 | 1 |
| 3242.0538682 | 3 |
| 3242.0556549 | 3 |
| 3242.0572082 | 4 |
| 3242.0583533 | 0 |
| 3242.0592764 | 2 |
| 3242.0610837 | 3 |
| 3242.0627253 | 1 |
| 3242.0628867 | 2 |
| 3242.0647599 | 2 |
| 3242.065652 | 4 |
| 3242.067539 | 1 |
| 3242.0687622 | 1 |
| 3242.0694827 | 2 |
| 3242.0713482 | 1 |
| 3242.0718974 | 1 |
| 3242.0734038 | 3 |
| 3242.0750713 | 4 |
| 3242.0760333 | 1 |
| 3242.0776749 | 3 |
| 3242.0779867 | 1 |
| 3242.0796144 | 2 |
| 3242.0811103 | 4 |
| 3242.0817102 | 3 |
| 3242.083658 | 3 |
| 3242.0839738 | 1 |
| 3242.0859911 | 1 |
| 3242.0871945 | 1 |
| 3242.0881063 | 2 |
| 3242.0896342 | 0 |
| 3242.089975 | 3 |
| 3242.0918073 | 3 |
| 3242.0928725 | 0 |
| 3242.0942427 | 2 |
| 3242.0963556 | 3 |
| 3242.096731 | 1 |
| 3242.098702 | 5 |
| 3242.0992034 | 2 |
| 3242.1007466 | 1 |
| 3242.1024521 | 3 |
| 3242.1032809 | 3 |
| 3242.1049997 | 5 |
| 3242.1054886 | 2 |
| 3242.1064937 | 4 |
| 3242.1086177 | 1 |
| 3242.1094379 | 7 |
| 3242.1114067 | 4 |
| 3242.11263 | 1 |
| 3242.1140057 | 1 |
| 3242.1154626 | 2 |
| 3242.1160065 | 2 |
| 3242.1175895 | 3 |
| 3242.1181954 | 0 |
| 3242.1198809 | 3 |
| 3242.1214711 | 3 |
| 3242.1218386 | 1 |
| 3242.1235409 | 2 |
| 3242.1246713 | 2 |
| 3242.1261472 | 2 |
| 3242.1270156 | 1 |
| 3242.1282197 | 3 |
| 3242.1300065 | 2 |
| 3242.1301304 | 2 |
| 3242.1319029 | 2 |
| 3242.1332449 | 2 |
| 3242.1339597 | 0 |
| 3242.1350796 | 1 |
| 3242.1366235 | 2 |
| 3242.1381204 | 1 |
| 3242.1388241 | 0 |
| 3242.1403132 | 3 |
| 3242.1421668 | 3 |
| 3242.1423319 | 1 |
| 3242.1441364 | 2 |
| 3242.1448811 | 4 |
| 3242.1471361 | 3 |
| 3242.148699 | 1 |
| 3242.1495255 | 1 |
| 3242.1510032 | 3 |
| 3242.1525177 | 4 |
| 3242.1531542 | 5 |
| 3242.1546959 | 4 |
| 3242.1555574 | 5 |
| 3242.1571408 | 1 |
| 3242.1585302 | 2 |
| 3242.1596304 | 2 |
| 3242.1613609 | 4 |
| 3242.1625959 | 3 |
| 3242.1638397 | 1 |
| 3242.164218 | 1 |
| 3242.1658231 | 1 |
| 3242.1666907 | 3 |
| 3242.1686488 | 2 |
| 3242.1702446 | 1 |
| 3242.1702558 | 3 |
| 3242.1720668 | 3 |
| 3242.1733242 | 4 |
| 3242.1742291 | 3 |
| 3242.1754708 | 2 |
| 3242.1764499 | 2 |
| 3242.1782061 | 6 |
| 3242.1783844 | 6 |
| 3242.1804678 | 1 |
| 3242.1819702 | 2 |
| 3242.1823777 | 3 |
| 3242.1838984 | 1 |
| 3242.1847787 | 2 |
| 3242.1858866 | 1 |
| 3242.1879868 | 0 |
| 3242.1890798 | 3 |
| 3242.1901242 | 4 |
| 3242.1906856 | 1 |
| 3242.1921249 | 2 |
| 3242.1940595 | 2 |
| 3242.1949179 | 1 |
| 3242.1968754 | 5 |
| 3242.1977376 | 7 |
| 3242.1997313 | 3 |
| 3242.2003724 | 0 |
| 3242.2018432 | 1 |
| 3242.2035017 | 3 |
| 3242.2038468 | 2 |
| 3242.2059216 | 2 |
| 3242.2061747 | 1 |
| 3242.2079748 | 1 |
| 3242.2091095 | 3 |
| 3242.2098237 | 2 |
| 3242.2114736 | 2 |
| 3242.2119452 | 2 |
| 3242.2135288 | 1 |
| 3242.2154269 | 1 |
| 3242.2163828 | 1 |
| 3242.2177375 | 2 |
| 3242.2186178 | 0 |
| 3242.2197866 | 2 |
| 3242.2217121 | 3 |
| 3242.2219751 | 2 |
| 3242.2234718 | 3 |
| 3242.224019 | 5 |
| 3242.2260881 | 1 |
| 3242.2274932 | 2 |
| 3242.2282429 | 3 |
| 3242.2294351 | 3 |
| 3242.2303909 | 1 |
| 3242.2322313 | 3 |
| 3242.2336001 | 4 |
| 3242.234534 | 3 |
| 3242.2361423 | 3 |
| 3242.2369097 | 3 |
| 3242.2386016 | 2 |
| 3242.2400766 | 1 |
| 3242.2410383 | 4 |
| 3242.242711 | 1 |
| 3242.2428786 | 4 |
| 3242.244988 | 6 |
| 3242.2464959 | 5 |
| 3242.2466344 | 2 |
| 3242.2488775 | 2 |

| | |
|---|---|
| 3242.249773 | 3 |
| 3242.2510266 | 3 |
| 3242.2532298 | 3 |
| 3242.2537924 | 3 |
| 3242.2551952 | 2 |
| 3242.2559806 | 3 |
| 3242.257254 | 0 |
| 3242.2585023 | 2 |
| 3242.2596394 | 2 |
| 3242.2607665 | 2 |
| 3242.2615756 | 2 |
| 3242.2632203 | 4 |
| 3242.2648318 | 2 |
| 3242.2657098 | 1 |
| 3242.2668592 | 1 |
| 3242.2673984 | 2 |
| 3242.2695589 | 2 |
| 3242.270267 | 3 |
| 3242.271173 | 2 |
| 3242.2723977 | 3 |
| 3242.2738463 | 3 |
| 3242.2752568 | 0 |
| 3242.2755656 | 2 |
| 3242.2780878 | 4 |
| 3242.2793209 | 3 |
| 3242.2797727 | 0 |
| 3242.2809073 | 0 |
| 3242.2823085 | 4 |
| 3242.2841037 | 2 |
| 3242.2856208 | 0 |
| 3242.2866458 | 2 |
| 3242.2878426 | 5 |
| 3242.289143 | 2 |
| 3242.290692 | 1 |
| 3242.2911542 | 3 |
| 3242.2925555 | 1 |
| 3242.2943896 | 3 |
| 3242.2954992 | 4 |
| 3242.2967121 | 5 |
| 3242.297897 | 3 |
| 3242.2986623 | 2 |
| 3242.3008208 | 4 |
| 3242.3011634 | 6 |
| 3242.3026155 | 2 |
| 3242.3045322 | 3 |
| 3242.305187 | 1 |
| 3242.3060911 | 3 |
| 3242.3070039 | 2 |
| 3242.3088591 | 2 |
| 3242.3100679 | 6 |
| 3242.3104114 | 2 |
| 3242.3123016 | 0 |
| 3242.3130297 | 5 |
| 3242.314096 | 4 |
| 3242.3152297 | 0 |
| 3242.3169278 | 1 |
| 3242.3184221 | 3 |
| 3242.3186293 | 1 |
| 3242.3204299 | 2 |
| 3242.3222979 | 1 |
| 3242.3223602 | 1 |
| 3242.3240291 | 2 |
| 3242.3255377 | 0 |
| 3242.326514 | 2 |
| 3242.3282619 | 2 |
| 3242.3296836 | 1 |
| 3242.3311871 | 3 |
| 3242.3312723 | 1 |
| 3242.3335007 | 2 |
| 3242.335425 | 4 |
| 3242.335461 | 5 |
| 3242.3374071 | 5 |
| 3242.3378987 | 2 |
| 3242.3397116 | 1 |
| 3242.3404547 | 2 |
| 3242.3423082 | 2 |
| 3242.3427168 | 5 |
| 3242.3440069 | 2 |
| 3242.345267 | 4 |
| 3242.3473669 | 2 |
| 3242.3478006 | 0 |
| 3242.349504 | 1 |
| 3242.3504926 | 2 |
| 3242.3525575 | 2 |
| 3242.352878 | 3 |
| 3242.3541552 | 4 |
| 3242.3555085 | 0 |
| 3242.3562347 | 1 |
| 3242.3581896 | 3 |
| 3242.3592056 | 3 |
| 3242.3598833 | 3 |
| 3242.3617476 | 4 |
| 3242.362414 | 5 |
| 3242.3636831 | 2 |
| 3242.3648008 | 3 |
| 3242.3661405 | 1 |
| 3242.3671068 | 3 |
| 3242.3679402 | 1 |
| 3242.3696342 | 1 |
| 3242.3717044 | 3 |
| 3242.3721209 | 2 |
| 3242.374587 | 4 |
| 3242.3747353 | 1 |
| 3242.3761282 | 2 |
| 3242.3773004 | 1 |
| 3242.3788329 | 2 |
| 3242.3803853 | 5 |
| 3242.3809406 | 4 |
| 3242.3826101 | 3 |
| 3242.3840836 | 1 |
| 3242.3843291 | 1 |
| 3242.3865096 | 1 |
| 3242.3875446 | 2 |
| 3242.3888018 | 2 |
| 3242.3900102 | 1 |
| 3242.3912288 | 0 |
| 3242.3922496 | 1 |
| 3242.393044 | 4 |
| 3242.3946739 | 1 |
| 3242.3966438 | 5 |
| 3242.3972737 | 1 |
| 3242.398293 | 2 |
| 3242.3991018 | 1 |
| 3242.4004209 | 2 |
| 3242.4020851 | 3 |
| 3242.4029411 | 6 |
| 3242.4048219 | 2 |
| 3242.4051857 | 3 |
| 3242.4064579 | 2 |
| 3242.4078303 | 1 |
| 3242.4085128 | 4 |
| 3242.4098518 | 0 |
| 3242.4122032 | 3 |
| 3242.412467 | 2 |
| 3242.4144501 | 0 |
| 3242.414883 | 3 |
| 3242.4167486 | 1 |
| 3242.4177416 | 3 |
| 3242.4188539 | 1 |
| 3242.4203466 | 5 |
| 3242.4211893 | 2 |
| 3242.4229946 | 2 |
| 3242.4244784 | 4 |
| 3242.4250854 | 3 |
| 3242.4265147 | 3 |
| 3242.4274137 | 2 |
| 3242.4282494 | 2 |
| 3242.4308875 | 1 |
| 3242.4319715 | 3 |
| 3242.433404 | 0 |
| 3242.434574 | 3 |
| 3242.4356035 | 3 |
| 3242.4366014 | 4 |
| 3242.4378675 | 2 |
| 3242.4388319 | 1 |
| 3242.4404734 | 2 |
| 3242.4415811 | 2 |
| 3242.442389 | 5 |
| 3242.444633 | 4 |
| 3242.4460651 | 1 |
| 3242.4463347 | 3 |
| 3242.4477601 | 3 |
| 3242.4492811 | 3 |
| 3242.4496423 | 1 |
| 3242.4514216 | 2 |
| 3242.4524402 | 1 |
| 3242.4532447 | 3 |
| 3242.4550601 | 0 |
| 3242.4558736 | 2 |
| 3242.4575218 | 4 |
| 3242.4589597 | 1 |
| 3242.4598017 | 0 |
| 3242.4616126 | 4 |
| 3242.4616535 | 1 |
| 3242.462869 | 1 |
| 3242.4649051 | 3 |
| 3242.4664768 | 1 |
| 3242.4678134 | 2 |
| 3242.4683498 | 3 |
| 3242.4703305 | 5 |
| 3242.4709032 | 3 |
| 3242.4727264 | 1 |
| 3242.4741153 | 1 |
| 3242.4758038 | 5 |
| 3242.4769356 | 2 |
| 3242.4773852 | 4 |
| 3242.4794611 | 3 |
| 3242.4806276 | 2 |
| 3242.4813702 | 2 |
| 3242.4831709 | 1 |
| 3242.4843966 | 2 |
| 3242.484636 | 1 |
| 3242.4868915 | 2 |
| 3242.4869284 | 2 |
| 3242.4892624 | 0 |
| 3242.4904885 | 0 |
| 3242.4904964 | 1 |
| 3242.4922978 | 0 |
| 3242.4932326 | 4 |
| 3242.4937947 | 2 |
| 3242.496236 | 3 |
| 3242.4973081 | 3 |
| 3242.4986043 | 1 |
| 3242.499368 | 1 |
| 3242.5010236 | 3 |
| 3242.5024259 | 1 |
| 3242.5027126 | 0 |
| 3242.5038878 | 0 |
| 3242.5054934 | 2 |
| 3242.5064568 | 1 |
| 3242.5083088 | 6 |
| 3242.5092816 | 2 |
| 3242.5110373 | 3 |
| 3242.5115687 | 1 |
| 3242.5135961 | 2 |
| 3242.5141779 | 2 |
| 3242.5161001 | 5 |
| 3242.5173245 | 1 |
| 3242.5190245 | 2 |
| 3242.5199393 | 6 |
| 3242.5208236 | 2 |
| 3242.5227956 | 1 |
| 3242.5241766 | 3 |
| 3242.5242343 | 1 |
| 3242.526012 | 2 |
| 3242.5280383 | 0 |
| 3242.5284413 | 1 |
| 3242.5298945 | 3 |
| 3242.5307735 | 1 |
| 3242.5321392 | 3 |
| 3242.5336101 | 3 |
| 3242.5348781 | 3 |
| 3242.5362723 | 0 |
| 3242.5368095 | 3 |
| 3242.5384014 | 2 |
| 3242.5385563 | 4 |
| 3242.5407061 | 1 |
| 3242.5422399 | 2 |
| 3242.542266 | 6 |
| 3242.5446209 | 2 |
| 3242.5451375 | 4 |
| 3242.5472507 | 1 |
| 3242.5487403 | 1 |
| 3242.5488562 | 1 |
| 3242.5501294 | 6 |
| 3242.5514545 | 3 |
| 3242.5523917 | 1 |
| 3242.5537936 | 1 |
| 3242.5554124 | 3 |
| 3242.5572468 | 1 |
| 3242.5572984 | 1 |
| 3242.5597969 | 3 |
| 3242.5613698 | 3 |
| 3242.561735 | 0 |
| 3242.5632576 | 2 |
| 3242.5642146 | 0 |
| 3242.5659592 | 2 |
| 3242.5670938 | 3 |
| 3242.5687081 | 2 |
| 3242.5703394 | 2 |
| 3242.5707683 | 1 |
| 3242.5728594 | 2 |
| 3242.5739074 | 1 |
| 3242.5746449 | 3 |
| 3242.5759084 | 1 |
| 3242.5771252 | 1 |
| 3242.5785617 | 1 |
| 3242.5790237 | 2 |
| 3242.581103 | 2 |
| 3242.5827281 | 2 |
| 3242.5829762 | 3 |
| 3242.5841209 | 1 |
| 3242.5855247 | 3 |
| 3242.5867232 | 3 |
| 3242.5880633 | 3 |
| 3242.5894381 | 3 |
| 3242.5905744 | 1 |
| 3242.59112 | 2 |
| 3242.593105 | 1 |
| 3242.5945252 | 4 |
| 3242.5949623 | 3 |
| 3242.5969848 | 4 |
| 3242.5971604 | 5 |
| 3242.5972739 | 0 |
| 3242.5977165 | 3 |
| 3242.5985154 | 4 |
| 3242.5985858 | 2 |
| 3242.599038 | 1 |
| 3242.6000271 | 1 |
| 3242.6009629 | 1 |
| 3242.6014299 | 2 |
| 3242.6014808 | 1 |
| 3242.602415 | 2 |
| 3242.6027816 | 1 |
| 3242.6033814 | 3 |
| 3242.6041839 | 2 |
| 3242.6041926 | 3 |
| 3242.6055548 | 1 |
| 3242.6057738 | 3 |
| 3242.6061575 | 3 |
| 3242.6062687 | 1 |
| 3242.6072807 | 5 |
| 3242.6082581 | 3 |
| 3242.6087747 | 3 |
| 3242.6092404 | 0 |
| 3242.6100319 | 1 |
| 3242.6101125 | 4 |
| 3242.6103387 | 3 |
| 3242.6104327 | 3 |
| 3242.6114501 | 0 |
| 3242.6120708 | 3 |
| 3242.612548 | 4 |
| 3242.6132191 | 0 |
| 3242.6139482 | 1 |
| 3242.6146065 | 4 |
| 3242.6146578 | 3 |
| 3242.6156743 | 4 |
| 3242.6159755 | 2 |
| 3242.6168239 | 2 |
| 3242.6170156 | 3 |
| 3242.6171077 | 0 |
| 3242.6182942 | 2 |
| 3242.6188424 | 3 |
| 3242.6196729 | 0 |
| 3242.6200884 | 2 |
| 3242.6201659 | 1 |
| 3242.6209405 | 3 |
| 3242.6218423 | 4 |
| 3242.6219704 | 0 |
| 3242.6227225 | 4 |
| 3242.6230355 | 2 |
| 3242.6230849 | 3 |
| 3242.6242575 | 1 |
| 3242.624808 | 0 |
| 3242.6250878 | 1 |
| 3242.6259181 | 4 |
| 3242.6260813 | 3 |
| 3242.6263017 | 2 |
| 3242.626941 | 2 |
| 3242.6275442 | 1 |
| 3242.6283884 | 1 |
| 3242.6289262 | 5 |
| 3242.6292526 | 2 |
| 3242.6292557 | 3 |
| 3242.6305613 | 2 |
| 3242.6307729 | 4 |
| 3242.6311702 | 0 |
| 3242.631632 | 1 |
| 3242.6319746 | 4 |
| 3242.6332359 | 2 |
| 3242.6334059 | 3 |
| 3242.6339052 | 1 |
| 3242.6347206 | 3 |
| 3242.6351161 | 2 |
| 3242.6355586 | 3 |
| 3242.6356161 | 6 |
| 3242.6365869 | 3 |
| 3242.6367409 | 0 |
| 3242.6372549 | 0 |
| 3242.6382657 | 1 |
| 3242.6389189 | 2 |
| 3242.6391563 | 5 |
| 3242.639984 | 2 |

| | |
|---|---|
| 3242.6406096 | 4 |
| 3242.6406469 | 0 |
| 3242.6417627 | 2 |
| 3242.6418522 | 2 |
| 3242.6428107 | 1 |
| 3242.6431043 | 2 |
| 3242.6431595 | 1 |
| 3242.6446948 | 3 |
| 3242.6447367 | 2 |
| 3242.6452506 | 0 |
| 3242.6454399 | 4 |
| 3242.6463546 | 2 |
| 3242.6471047 | 0 |
| 3242.64799 | 0 |
| 3242.6481736 | 3 |
| 3242.6481773 | 4 |
| 3242.649579 | 2 |
| 3242.6496855 | 1 |
| 3242.6498914 | 4 |
| 3242.6507768 | 2 |
| 3242.65111 | 3 |
| 3242.6524452 | 1 |
| 3242.6531027 | 0 |
| 3242.6531266 | 1 |
| 3242.6541172 | 5 |
| 3242.6542711 | 3 |
| 3242.654273 | 0 |
| 3242.6543118 | 2 |
| 3242.6562041 | 6 |
| 3242.6562796 | 0 |
| 3242.6564437 | 0 |
| 3242.6577872 | 3 |
| 3242.6578938 | 2 |
| 3242.6581188 | 3 |
| 3242.6589677 | 0 |
| 3242.6597272 | 5 |
| 3242.6598503 | 2 |
| 3242.660883 | 1 |
| 3242.6610398 | 2 |
| 3242.6613039 | 2 |
| 3242.6622778 | 5 |
| 3242.6624427 | 3 |
| 3242.6634298 | 5 |
| 3242.6640057 | 1 |
| 3242.6642416 | 3 |
| 3242.6647218 | 1 |
| 3242.6653364 | 2 |
| 3242.6660316 | 5 |
| 3242.6664499 | 5 |
| 3242.6668334 | 1 |
| 3242.6669641 | 2 |
| 3242.6681172 | 2 |
| 3242.6685199 | 1 |
| 3242.6690052 | 3 |
| 3242.6698346 | 2 |
| 3242.6704083 | 1 |
| 3242.6711326 | 4 |
| 3242.6717059 | 4 |
| 3242.6719841 | 4 |
| 3242.6723778 | 0 |
| 3242.6725102 | 1 |
| 3242.6735696 | 0 |
| 3242.6737905 | 1 |
| 3242.6746084 | 7 |
| 3242.6749941 | 2 |
| 3242.6751494 | 1 |
| 3242.6761155 | 2 |
| 3242.6761729 | 4 |
| 3242.6769712 | 0 |
| 3242.6771952 | 5 |
| 3242.6780421 | 1 |
| 3242.6782415 | 2 |
| 3242.6790263 | 1 |
| 3242.6794261 | 1 |
| 3242.6800426 | 2 |
| 3242.6810795 | 1 |
| 3242.6817413 | 2 |
| 3242.6817542 | 4 |
| 3242.6827557 | 3 |
| 3242.6831156 | 1 |
| 3242.6831374 | 3 |
| 3242.6834585 | 3 |
| 3242.6846344 | 1 |
| 3242.6846508 | 2 |
| 3242.6850215 | 3 |
| 3242.6858194 | 2 |
| 3242.6865195 | 2 |
| 3242.6872451 | 2 |
| 3242.687972 | 1 |
| 3242.6882444 | 4 |
| 3242.6892872 | 5 |
| 3242.6893629 | 3 |
| 3242.6894246 | 5 |
| 3242.6903904 | 4 |
| 3242.6911199 | 1 |
| 3242.6912849 | 2 |
| 3242.6918626 | 1 |
| 3242.6923515 | 3 |
| 3242.6934969 | 0 |
| 3242.693813 | 2 |
| 3242.694625 | 3 |
| 3242.694946 | 1 |
| 3242.6951598 | 5 |
| 3242.6954658 | 0 |
| 3242.6955761 | 0 |
| 3242.695637 | 4 |
| 3242.6959388 | 2 |
| 3242.6964037 | 0 |
| 3242.6964972 | 2 |
| 3242.6965928 | 3 |
| 3242.6968199 | 2 |
| 3242.6970957 | 1 |
| 3242.6974043 | 3 |
| 3242.6976318 | 1 |
| 3242.6980424 | 4 |
| 3242.6981129 | 2 |
| 3242.6981546 | 0 |
| 3242.6988598 | 3 |
| 3242.6992755 | 3 |
| 3242.6993176 | 1 |
| 3242.6994642 | 1 |
| 3242.6996151 | 3 |
| 3242.6998528 | 2 |
| 3242.70012 | 0 |
| 3242.7005389 | 4 |
| 3242.7008824 | 2 |
| 3242.7008946 | 1 |
| 3242.7010976 | 3 |
| 3242.7015438 | 1 |
| 3242.7016327 | 0 |
| 3242.7019358 | 1 |
| 3242.7024366 | 1 |
| 3242.7026434 | 3 |
| 3242.7028997 | 1 |
| 3242.703127 | 1 |
| 3242.7034808 | 4 |
| 3242.7036265 | 3 |
| 3242.7038802 | 1 |
| 3242.7040593 | 2 |
| 3242.7042699 | 5 |
| 3242.7047245 | 2 |
| 3242.7049184 | 4 |
| 3242.7052454 | 3 |
| 3242.705318 | 4 |
| 3242.7054715 | 0 |
| 3242.7057483 | 3 |
| 3242.7060761 | 1 |
| 3242.7064819 | 1 |
| 3242.7067638 | 6 |
| 3242.7068153 | 4 |
| 3242.7069713 | 6 |
| 3242.7070708 | 9 |
| 3242.7071592 | 2 |
| 3242.7077009 | 6 |
| 3242.7082568 | 15 |
| 3242.7084161 | 10 |
| 3242.7084866 | 10 |
| 3242.7090206 | 22 |
| 3242.7091481 | 9 |
| 3242.7093026 | 11 |
| 3242.7100149 | 11 |
| 3242.7100682 | 15 |
| 3242.7101232 | 17 |
| 3242.7101893 | 11 |
| 3242.7104799 | 9 |
| 3242.710741 | 3 |
| 3242.7112577 | 4 |
| 3242.7114467 | 3 |
| 3242.7114541 | 11 |
| 3242.7116125 | 2 |
| 3242.7121215 | 4 |
| 3242.7121488 | 2 |
| 3242.7122412 | 8 |
| 3242.7128903 | 1 |
| 3242.7129373 | 1 |
| 3242.7130656 | 2 |
| 3242.7135544 | 3 |
| 3242.7137152 | 4 |
| 3242.713777 | 4 |
| 3242.7142296 | 3 |
| 3242.7143963 | 1 |
| 3242.7144094 | 1 |
| 3242.7150688 | 3 |
| 3242.7152733 | 1 |
| 3242.7158137 | 2 |
| 3242.7158652 | 2 |
| 3242.7164147 | 4 |
| 3242.7165797 | 6 |
| 3242.7172158 | 3 |
| 3242.7172925 | 1 |
| 3242.717683 | 2 |
| 3242.7177607 | 2 |
| 3242.7181578 | 5 |
| 3242.7184257 | 1 |
| 3242.7191518 | 3 |
| 3242.7194678 | 2 |
| 3242.7195676 | 1 |
| 3242.7200486 | 1 |
| 3242.7200574 | 4 |
| 3242.7204886 | 1 |
| 3242.7210004 | 4 |
| 3242.7212989 | 3 |
| 3242.7213021 | 0 |
| 3242.7215061 | 3 |
| 3242.7221995 | 0 |
| 3242.7233682 | 2 |
| 3242.7235815 | 1 |
| 3242.7254616 | 5 |
| 3242.7259044 | 3 |
| 3242.7271247 | 1 |
| 3242.7274299 | 0 |
| 3242.7293745 | 4 |
| 3242.7295737 | 4 |
| 3242.730778 | 1 |
| 3242.7323511 | 6 |
| 3242.7326381 | 2 |
| 3242.7335296 | 1 |
| 3242.735135 | 3 |
| 3242.7360447 | 0 |
| 3242.7367416 | 2 |
| 3242.7371651 | 2 |
| 3242.7385407 | 2 |
| 3242.7389039 | 2 |
| 3242.7409492 | 3 |
| 3242.7414672 | 3 |
| 3242.7419636 | 2 |
| 3242.7434302 | 3 |
| 3242.7439631 | 0 |
| 3242.7447654 | 2 |
| 3242.7460879 | 4 |
| 3242.7466481 | 1 |
| 3242.747896 | 4 |
| 3242.7493667 | 1 |
| 3242.7494056 | 3 |
| 3242.7509001 | 0 |
| 3242.7511527 | 2 |
| 3242.7528497 | 2 |
| 3242.7529268 | 1 |
| 3242.7547643 | 2 |
| 3242.7554983 | 5 |
| 3242.756218 | 2 |
| 3242.7575564 | 3 |
| 3242.7579313 | 0 |
| 3242.7594046 | 0 |
| 3242.7599585 | 4 |
| 3242.7604878 | 1 |
| 3242.761056 | 2 |
| 3242.76286 | 2 |
| 3242.7638437 | 3 |
| 3242.7650797 | 3 |
| 3242.7651759 | 2 |
| 3242.7666795 | 2 |
| 3242.7669572 | 0 |
| 3242.7683331 | 4 |
| 3242.7692774 | 3 |
| 3242.7701987 | 2 |
| 3242.7713571 | 4 |
| 3242.7721418 | 5 |
| 3242.7731671 | 2 |
| 3242.7734554 | 1 |
| 3242.7750427 | 2 |
| 3242.7764421 | 0 |
| 3242.7770075 | 2 |
| 3242.7771278 | 4 |
| 3242.7786801 | 1 |
| 3242.780263 | 6 |
| 3242.7805032 | 1 |
| 3242.78174 | 2 |
| 3242.7831514 | 2 |
| 3242.7836883 | 3 |
| 3242.7845139 | 2 |
| 3242.7852956 | 0 |
| 3242.7866539 | 2 |
| 3242.7867933 | 3 |
| 3242.7885575 | 2 |
| 3242.7898092 | 2 |
| 3242.7901875 | 8 |
| 3242.7912865 | 1 |
| 3242.7912912 | 4 |
| 3242.7928073 | 3 |
| 3242.7938451 | 3 |
| 3242.79473 | 3 |
| 3242.7959922 | 0 |
| 3242.7968566 | 4 |
| 3242.7978978 | 4 |
| 3242.798429 | 0 |
| 3242.7993408 | 3 |
| 3242.8006244 | 1 |
| 3242.800735 | 3 |
| 3242.8023136 | 1 |
| 3242.8031264 | 0 |
| 3242.803498 | 1 |
| 3242.8052358 | 0 |
| 3242.805826 | 1 |
| 3242.8069966 | 4 |
| 3242.8072643 | 1 |
| 3242.8088392 | 5 |
| 3242.8095621 | 1 |
| 3242.8105587 | 1 |
| 3242.8112664 | 2 |
| 3242.81217 | 2 |
| 3242.8131311 | 1 |
| 3242.8140251 | 4 |
| 3242.8148255 | 2 |
| 3242.8160914 | 2 |
| 3242.8170048 | 2 |
| 3242.8184989 | 5 |
| 3242.8185333 | 0 |
| 3242.8197291 | 1 |
| 3242.8204204 | 4 |
| 3242.8215809 | 4 |
| 3242.8228413 | 2 |
| 3242.8229657 | 3 |
| 3242.8249421 | 4 |
| 3242.825717 | 1 |
| 3242.8260107 | 0 |
| 3242.8277102 | 2 |
| 3242.828067 | 1 |
| 3242.8290872 | 4 |
| 3242.8308356 | 1 |
| 3242.8310252 | 2 |
| 3242.8322888 | 4 |
| 3242.8328316 | 1 |
| 3242.8341515 | 0 |
| 3242.8347675 | 2 |
| 3242.8361489 | 0 |
| 3242.8369033 | 2 |
| 3242.8376067 | 2 |
| 3242.8390921 | 2 |
| 3242.8398989 | 2 |
| 3242.8403735 | 0 |
| 3242.8422417 | 3 |
| 3242.8423667 | 1 |
| 3242.843918 | 1 |
| 3242.8444432 | 1 |
| 3242.8454286 | 3 |
| 3242.8463668 | 3 |
| 3242.846732 | 2 |
| 3242.8480488 | 3 |
| 3242.8492865 | 6 |
| 3242.8493384 | 1 |
| 3242.8509071 | 2 |
| 3242.8515439 | 4 |
| 3242.8526204 | 2 |
| 3242.8535332 | 4 |
| 3242.8544188 | 4 |
| 3242.8555276 | 2 |
| 3242.8558826 | 3 |
| 3242.8570103 | 0 |
| 3242.8577844 | 3 |
| 3242.8588457 | 3 |
| 3242.8596337 | 4 |
| 3242.860598 | 0 |
| 3242.8623091 | 3 |
| 3242.8629207 | 0 |
| 3242.8632774 | 2 |
| 3242.8647585 | 2 |
| 3242.8652243 | 3 |
| 3242.8666798 | 3 |
| 3242.8680092 | 3 |
| 3242.8681184 | 2 |
| 3242.869095 | 1 |
| 3242.8702524 | 1 |

| | |
|---|---|
| 3242.8709133 | 3 |
| 3242.8717781 | 2 |
| 3242.8727454 | 2 |
| 3242.8743925 | 1 |
| 3242.874608 | 0 |
| 3242.8758843 | 3 |
| 3242.8770649 | 1 |
| 3242.877739 | 0 |
| 3242.878719 | 2 |
| 3242.8793975 | 1 |
| 3242.8808736 | 2 |
| 3242.8816127 | 1 |
| 3242.8827579 | 3 |
| 3242.883717 | 1 |
| 3242.8846316 | 1 |
| 3242.885057 | 1 |
| 3242.8864376 | 1 |
| 3242.8872734 | 1 |
| 3242.8880094 | 0 |
| 3242.8889345 | 2 |
| 3242.8899326 | 1 |
| 3242.8913037 | 1 |
| 3242.8918131 | 3 |
| 3242.8927335 | 1 |
| 3242.8936769 | 4 |
| 3242.8947973 | 3 |
| 3242.8959778 | 0 |
| 3242.8963511 | 1 |
| 3242.8974461 | 2 |
| 3242.8979353 | 3 |
| 3242.8986176 | 3 |
| 3242.8996779 | 0 |
| 3242.900482 | 2 |
| 3242.9020729 | 0 |
| 3242.9021277 | 0 |
| 3242.9037026 | 4 |
| 3242.9044597 | 1 |
| 3242.9053231 | 0 |
| 3242.9067128 | 1 |
| 3242.9071962 | 5 |
| 3242.9083307 | 2 |
| 3242.9089838 | 6 |
| 3242.9102219 | 4 |
| 3242.9111866 | 0 |
| 3242.9122937 | 0 |
| 3242.9139402 | 2 |
| 3242.9144907 | 2 |
| 3242.9152088 | 1 |
| 3242.9161267 | 2 |
| 3242.916212 | 2 |
| 3242.9172902 | 1 |
| 3242.918776 | 0 |
| 3242.9196213 | 0 |
| 3242.9201521 | 2 |
| 3242.9218595 | 3 |
| 3242.9229095 | 1 |
| 3242.9230694 | 1 |
| 3242.9248152 | 2 |
| 3242.925185 | 1 |
| 3242.9265361 | 2 |
| 3242.9271586 | 4 |
| 3242.9277095 | 1 |
| 3242.9292313 | 1 |
| 3242.9303 | 3 |
| 3242.9303723 | 1 |
| 3242.9319045 | 3 |
| 3242.9323338 | 3 |
| 3242.933973 | 0 |
| 3242.9342508 | 3 |
| 3242.9351831 | 1 |
| 3242.9366637 | 0 |
| 3242.9370679 | 1 |
| 3242.9380751 | 2 |
| 3242.9388424 | 2 |
| 3242.9401583 | 3 |
| 3242.9411397 | 3 |
| 3242.9423809 | 5 |
| 3242.9431747 | 3 |
| 3242.9437289 | 0 |
| 3242.9446596 | 4 |
| 3242.9454573 | 1 |
| 3242.9466102 | 2 |
| 3242.9476001 | 2 |
| 3242.948408 | 1 |
| 3242.949066 | 2 |
| 3242.9504446 | 2 |
| 3242.9511107 | 3 |
| 3242.9520509 | 1 |
| 3242.9529713 | 1 |
| 3242.9543583 | 2 |
| 3242.9554931 | 2 |
| 3242.9557036 | 3 |
| 3242.9559153 | 2 |
| 3242.9574463 | 4 |
| 3242.9584178 | 2 |
| 3242.9598061 | 1 |
| 3242.960959 | 1 |
| 3242.9620644 | 2 |
| 3242.9622012 | 1 |
| 3242.9636198 | 2 |
| 3242.9645738 | 3 |
| 3242.9646992 | 4 |
| 3242.9660247 | 4 |
| 3242.9661713 | 3 |
| 3242.9677038 | 2 |
| 3242.9690037 | 1 |
| 3242.9699708 | 1 |
| 3242.9707312 | 1 |
| 3242.9716856 | 5 |
| 3242.9723743 | 2 |
| 3242.9737584 | 1 |
| 3242.9752009 | 4 |
| 3242.9753618 | 3 |
| 3242.9765735 | 6 |
| 3242.9772957 | 0 |
| 3242.977521 | 0 |
| 3242.9789621 | 1 |
| 3242.9805779 | 2 |
| 3242.9810982 | 2 |
| 3242.9818079 | 3 |
| 3242.9826114 | 1 |
| 3242.9840438 | 2 |
| 3242.9844731 | 0 |
| 3242.985781 | 2 |
| 3242.98672 | 3 |
| 3242.9879194 | 2 |
| 3242.9879385 | 3 |
| 3242.9894748 | 1 |
| 3242.990339 | 1 |
| 3242.991421 | 1 |
| 3242.9921835 | 3 |
| 3242.9921896 | 4 |
| 3242.9935687 | 0 |
| 3242.995371 | 2 |
| 3242.9953766 | 1 |
| 3242.9966667 | 2 |
| 3242.9974866 | 1 |
| 3242.998112 | 3 |
| 3242.9997502 | 2 |
| 3242.9998993 | 1 |
| 3243.0012215 | 3 |
| 3243.0021371 | 3 |
| 3243.0038857 | 1 |
| 3243.0040591 | 5 |
| 3243.0048326 | 5 |
| 3243.0058208 | 3 |
| 3243.0060456 | 2 |
| 3243.0078248 | 1 |
| 3243.008691 | 2 |
| 3243.0102998 | 3 |
| 3243.0108058 | 2 |
| 3243.0117952 | 2 |
| 3243.012513 | 2 |
| 3243.0136591 | 1 |
| 3243.0146894 | 1 |
| 3243.0156819 | 2 |
| 3243.0164484 | 2 |
| 3243.0173882 | 4 |
| 3243.0178798 | 2 |
| 3243.0194165 | 4 |
| 3243.0200942 | 2 |
| 3243.0212989 | 1 |
| 3243.0223184 | 2 |
| 3243.0228245 | 7 |
| 3243.0237758 | 3 |
| 3243.0245405 | 2 |
| 3243.0255718 | 1 |
| 3243.0267286 | 3 |
| 3243.0274159 | 0 |
| 3243.0285282 | 1 |
| 3243.0286027 | 1 |
| 3243.0302126 | 4 |
| 3243.0311871 | 0 |
| 3243.031812 | 4 |
| 3243.0330259 | 2 |
| 3243.0341972 | 3 |
| 3243.0342436 | 3 |
| 3243.0359485 | 2 |
| 3243.0363914 | 3 |
| 3243.0379626 | 2 |
| 3243.0387903 | 3 |
| 3243.0390656 | 2 |
| 3243.0406411 | 2 |
| 3243.0407673 | 1 |
| 3243.0422236 | 0 |
| 3243.0430289 | 1 |
| 3243.043878 | 0 |
| 3243.0449403 | 1 |
| 3243.0457154 | 1 |
| 3243.0462553 | 5 |
| 3243.0477336 | 4 |
| 3243.048662 | 1 |
| 3243.0493489 | 1 |
| 3243.0506218 | 0 |
| 3243.0519792 | 3 |
| 3243.0520005 | 2 |
| 3243.0536817 | 5 |
| 3243.0543233 | 5 |
| 3243.0550486 | 2 |
| 3243.0564028 | 4 |
| 3243.0570455 | 0 |
| 3243.058471 | 5 |
| 3243.0593862 | 2 |
| 3243.0596315 | 4 |
| 3243.0612495 | 1 |
| 3243.0615114 | 1 |
| 3243.063379 | 1 |
| 3243.063386 | 2 |
| 3243.0649569 | 5 |
| 3243.0656445 | 1 |
| 3243.0663697 | 1 |
| 3243.0676123 | 1 |
| 3243.0689587 | 1 |
| 3243.0690772 | 0 |
| 3243.0708368 | 1 |
| 3243.0709733 | 2 |
| 3243.0717906 | 0 |
| 3243.0733783 | 4 |
| 3243.0739853 | 3 |
| 3243.0750876 | 1 |
| 3243.0756864 | 5 |
| 3243.076852 | 3 |
| 3243.0771646 | 2 |
| 3243.0787724 | 0 |
| 3243.0797804 | 2 |
| 3243.0799266 | 0 |
| 3243.0811348 | 3 |
| 3243.0818464 | 3 |
| 3243.083486 | 1 |
| 3243.0845079 | 2 |
| 3243.0846532 | 7 |
| 3243.0863288 | 1 |
| 3243.0863603 | 1 |
| 3243.0877596 | 1 |
| 3243.0882142 | 3 |
| 3243.0885998 | 4 |
| 3243.0906327 | 1 |
| 3243.0909315 | 4 |
| 3243.0923266 | 1 |
| 3243.0929644 | 2 |
| 3243.0943022 | 5 |
| 3243.0948869 | 1 |
| 3243.096049 | 0 |
| 3243.0973029 | 3 |
| 3243.0977435 | 1 |
| 3243.0990641 | 3 |
| 3243.1003595 | 0 |
| 3243.1004308 | 1 |
| 3243.1021997 | 3 |
| 3243.1031624 | 2 |
| 3243.1036384 | 2 |
| 3243.1044482 | 1 |
| 3243.1057631 | 2 |
| 3243.1066173 | 1 |
| 3243.1077004 | 2 |
| 3243.1079365 | 2 |
| 3243.1090663 | 4 |
| 3243.1101446 | 2 |
| 3243.1111525 | 3 |
| 3243.1121003 | 2 |
| 3243.1136439 | 3 |
| 3243.1143395 | 3 |
| 3243.1143802 | 2 |
| 3243.1152129 | 2 |
| 3243.1160975 | 2 |
| 3243.1162428 | 3 |
| 3243.1167474 | 1 |
| 3243.1171237 | 0 |
| 3243.1178676 | 2 |
| 3243.1179269 | 1 |
| 3243.1187283 | 1 |
| 3243.1190658 | 2 |
| 3243.119244 | 0 |
| 3243.119877 | 5 |
| 3243.1200445 | 0 |
| 3243.120565 | 0 |
| 3243.1211498 | 2 |
| 3243.1213421 | 0 |
| 3243.1220189 | 0 |
| 3243.1222479 | 3 |
| 3243.1230182 | 2 |
| 3243.1231396 | 5 |
| 3243.1242501 | 5 |
| 3243.1244516 | 1 |
| 3243.1252042 | 3 |
| 3243.125309 | 3 |
| 3243.1256718 | 2 |
| 3243.1266749 | 2 |
| 3243.1268042 | 8 |
| 3243.127051 | 4 |
| 3243.1282837 | 3 |
| 3243.1284643 | 1 |
| 3243.1284784 | 0 |
| 3243.1285707 | 0 |
| 3243.1295352 | 1 |
| 3243.1304055 | 3 |
| 3243.130896 | 2 |
| 3243.1309656 | 1 |
| 3243.1310232 | 2 |
| 3243.131864 | 1 |
| 3243.1324848 | 0 |
| 3243.1332224 | 3 |
| 3243.1332662 | 0 |
| 3243.1334371 | 2 |
| 3243.1343153 | 1 |
| 3243.1348037 | 2 |
| 3243.1349669 | 5 |
| 3243.1350637 | 1 |
| 3243.1362193 | 2 |
| 3243.1365797 | 2 |
| 3243.137027 | 2 |
| 3243.1376533 | 4 |
| 3243.1377492 | 3 |
| 3243.1385047 | 3 |
| 3243.1387943 | 1 |
| 3243.1391802 | 1 |
| 3243.1394665 | 2 |
| 3243.1405392 | 5 |
| 3243.1408989 | 2 |
| 3243.1412172 | 3 |
| 3243.1416637 | 4 |
| 3243.1423442 | 2 |
| 3243.1424014 | 2 |
| 3243.1426611 | 1 |
| 3243.1435622 | 0 |
| 3243.1438547 | 1 |
| 3243.1442547 | 3 |
| 3243.1452073 | 3 |
| 3243.145232 | 4 |
| 3243.1459958 | 0 |
| 3243.1464352 | 1 |
| 3243.1466385 | 1 |
| 3243.1466774 | 0 |
| 3243.1477193 | 1 |
| 3243.1485243 | 5 |
| 3243.1486262 | 1 |
| 3243.148726 | 2 |
| 3243.14993 | 3 |
| 3243.1504584 | 0 |
| 3243.1506926 | 2 |
| 3243.1516777 | 2 |
| 3243.1517584 | 1 |
| 3243.1520359 | 3 |
| 3243.1521584 | 3 |
| 3243.1533094 | 0 |
| 3243.1534436 | 0 |
| 3243.1534896 | 0 |
| 3243.1546362 | 2 |
| 3243.1546487 | 1 |
| 3243.1552465 | 3 |
| 3243.1561723 | 2 |
| 3243.1565264 | 0 |
| 3243.1566188 | 2 |
| 3243.1573341 | 1 |
| 3243.1576029 | 3 |
| 3243.1577123 | 3 |
| 3243.1584683 | 2 |
| 3243.1586565 | 5 |
| 3243.1589468 | 4 |
| 3243.1602231 | 2 |
| 3243.1607113 | 2 |
| 3243.1609133 | 3 |
| 3243.1613148 | 3 |

| | |
|---|---|
| 3243.161399 | 2 |
| 3243.1623833 | 3 |
| 3243.1625329 | 1 |
| 3243.1631131 | 5 |
| 3243.1634976 | 2 |
| 3243.163822 | 3 |
| 3243.1645799 | 2 |
| 3243.1649631 | 3 |
| 3243.16548 | 3 |
| 3243.1659576 | 3 |
| 3243.166464 | 3 |
| 3243.1671698 | 0 |
| 3243.1680154 | 2 |
| 3243.1680231 | 2 |
| 3243.1686324 | 2 |
| 3243.1688542 | 3 |
| 3243.168933 | 4 |
| 3243.1693025 | 5 |
| 3243.1703165 | 1 |
| 3243.170478 | 2 |
| 3243.1708745 | 1 |
| 3243.1718573 | 5 |
| 3243.1721528 | 5 |
| 3243.172486 | 2 |
| 3243.1725738 | 1 |
| 3243.1735933 | 1 |
| 3243.1738044 | 3 |
| 3243.1739152 | 6 |
| 3243.1752714 | 1 |
| 3243.1754702 | 2 |
| 3243.1757168 | 6 |
| 3243.1761933 | 5 |
| 3243.1768196 | 3 |
| 3243.1768643 | 1 |
| 3243.1777932 | 5 |
| 3243.1779618 | 2 |
| 3243.179108 | 6 |
| 3243.179356 | 4 |
| 3243.1798971 | 1 |
| 3243.1800558 | 1 |
| 3243.1805759 | 5 |
| 3243.1813168 | 3 |
| 3243.1813975 | 3 |
| 3243.1822196 | 2 |
| 3243.1822425 | 0 |
| 3243.1833451 | 3 |
| 3243.1835213 | 4 |
| 3243.1835875 | 2 |
| 3243.1844244 | 2 |
| 3243.1848629 | 2 |
| 3243.1849623 | 4 |
| 3243.1856114 | 1 |
| 3243.1861361 | 3 |
| 3243.1863171 | 0 |
| 3243.1865863 | 0 |
| 3243.1873122 | 5 |
| 3243.1878619 | 2 |
| 3243.1881726 | 1 |
| 3243.1893397 | 2 |
| 3243.189631 | 3 |
| 3243.1903124 | 1 |
| 3243.1908328 | 6 |
| 3243.1911053 | 1 |
| 3243.1916347 | 0 |
| 3243.1920471 | 2 |
| 3243.1930157 | 0 |
| 3243.1932696 | 2 |
| 3243.1933007 | 2 |
| 3243.193541 | 0 |
| 3243.1942806 | 1 |
| 3243.1947529 | 3 |
| 3243.1956886 | 1 |
| 3243.1963279 | 1 |
| 3243.1964423 | 1 |
| 3243.1965097 | 8 |
| 3243.1975039 | 0 |
| 3243.1975233 | 2 |
| 3243.1983533 | 7 |
| 3243.199075 | 2 |
| 3243.1994835 | 1 |
| 3243.1995766 | 1 |
| 3243.2002123 | 1 |
| 3243.2002513 | 4 |
| 3243.2010833 | 3 |
| 3243.2020362 | 1 |
| 3243.2038165 | 4 |
| 3243.2039801 | 0 |
| 3243.2053117 | 4 |
| 3243.2065532 | 1 |
| 3243.207078 | 0 |
| 3243.2079542 | 1 |
| 3243.2097466 | 2 |
| 3243.2098472 | 1 |
| 3243.2106072 | 2 |
| 3243.2117402 | 0 |
| 3243.2124315 | 1 |
| 3243.2135595 | 6 |
| 3243.2151485 | 4 |
| 3243.2152624 | 0 |
| 3243.2165584 | 4 |
| 3243.2172237 | 1 |
| 3243.2178508 | 0 |
| 3243.2186986 | 2 |
| 3243.2204595 | 2 |
| 3243.2212151 | 0 |
| 3243.222026 | 0 |
| 3243.223125 | 1 |
| 3243.2233934 | 1 |
| 3243.2245802 | 0 |
| 3243.2263326 | 2 |
| 3243.2264991 | 3 |
| 3243.2277456 | 3 |
| 3243.2286848 | 4 |
| 3243.2296231 | 2 |
| 3243.2304716 | 1 |
| 3243.2312254 | 1 |
| 3243.2325787 | 4 |
| 3243.2338167 | 1 |
| 3243.2345634 | 2 |
| 3243.2359074 | 1 |
| 3243.2362991 | 1 |
| 3243.2371129 | 2 |
| 3243.2379786 | 3 |
| 3243.2386652 | 4 |
| 3243.2406904 | 1 |
| 3243.2411994 | 0 |
| 3243.2419445 | 0 |
| 3243.242791 | 4 |
| 3243.243586 | 0 |
| 3243.245149 | 4 |
| 3243.2460535 | 1 |
| 3243.2468302 | 6 |
| 3243.2473363 | 1 |
| 3243.2488087 | 3 |
| 3243.2494532 | 1 |
| 3243.2499429 | 2 |
| 3243.251506 | 4 |
| 3243.2524739 | 0 |
| 3243.2531516 | 2 |
| 3243.2549379 | 1 |
| 3243.2552819 | 3 |
| 3243.2562044 | 1 |
| 3243.2572341 | 2 |
| 3243.2578675 | 0 |
| 3243.259272 | 1 |
| 3243.2597275 | 3 |
| 3243.261122 | 6 |
| 3243.2618194 | 4 |
| 3243.2625043 | 5 |
| 3243.2636991 | 1 |
| 3243.2644346 | 0 |
| 3243.2656784 | 5 |
| 3243.2662316 | 0 |
| 3243.2675797 | 3 |
| 3243.2684796 | 2 |
| 3243.2692684 | 5 |
| 3243.2706118 | 1 |
| 3243.2708052 | 2 |
| 3243.2721192 | 1 |
| 3243.2725668 | 1 |
| 3243.2740963 | 0 |
| 3243.2751384 | 1 |
| 3243.2756131 | 1 |
| 3243.2767386 | 2 |
| 3243.2773959 | 4 |
| 3243.2786842 | 1 |
| 3243.2802265 | 2 |
| 3243.2805895 | 1 |
| 3243.2819927 | 4 |
| 3243.2829896 | 1 |
| 3243.2834733 | 2 |
| 3243.284662 | 3 |
| 3243.2855355 | 0 |
| 3243.2859444 | 1 |
| 3243.2876683 | 3 |
| 3243.2878648 | 4 |
| 3243.2890854 | 3 |
| 3243.2900068 | 2 |
| 3243.2912301 | 2 |
| 3243.2922572 | 3 |
| 3243.2926967 | 2 |
| 3243.2938305 | 2 |
| 3243.294549 | 2 |
| 3243.2957819 | 3 |
| 3243.2960876 | 4 |
| 3243.2976857 | 1 |
| 3243.2991068 | 4 |
| 3243.2991742 | 2 |
| 3243.3003889 | 2 |
| 3243.3009386 | 2 |
| 3243.3026972 | 1 |
| 3243.3036431 | 3 |
| 3243.3042398 | 2 |
| 3243.3052033 | 3 |
| 3243.3057142 | 2 |
| 3243.3072203 | 2 |
| 3243.307723 | 1 |
| 3243.3086439 | 1 |
| 3243.3099367 | 2 |
| 3243.3101693 | 2 |
| 3243.3116801 | 5 |
| 3243.3119952 | 4 |
| 3243.3134066 | 4 |
| 3243.3147009 | 1 |
| 3243.3149921 | 0 |
| 3243.316283 | 4 |
| 3243.3172858 | 7 |
| 3243.318166 | 3 |
| 3243.3191583 | 1 |
| 3243.3193468 | 4 |
| 3243.3212495 | 1 |
| 3243.3215608 | 2 |
| 3243.3223671 | 2 |
| 3243.3237794 | 4 |
| 3243.3243817 | 3 |
| 3243.3259727 | 3 |
| 3243.3260763 | 0 |
| 3243.3275476 | 2 |
| 3243.3285767 | 1 |
| 3243.3298707 | 4 |
| 3243.330577 | 3 |
| 3243.3310124 | 3 |
| 3243.3325512 | 1 |
| 3243.3336988 | 4 |
| 3243.334007 | 0 |
| 3243.3350178 | 3 |
| 3243.3363045 | 2 |
| 3243.3376072 | 5 |
| 3243.3379328 | 3 |
| 3243.3396388 | 1 |
| 3243.3400525 | 3 |
| 3243.3405813 | 1 |
| 3243.3418032 | 1 |
| 3243.3424392 | 2 |
| 3243.3437588 | 4 |
| 3243.3444323 | 1 |
| 3243.3451637 | 4 |
| 3243.3467999 | 3 |
| 3243.3476799 | 1 |
| 3243.3480953 | 4 |
| 3243.3493381 | 2 |
| 3243.3497164 | 4 |
| 3243.3510873 | 2 |
| 3243.3522949 | 3 |
| 3243.352363 | 1 |
| 3243.3535984 | 2 |
| 3243.3547029 | 1 |
| 3243.3560726 | 2 |
| 3243.3563602 | 6 |
| 3243.3571367 | 1 |
| 3243.3587891 | 1 |
| 3243.3595263 | 4 |
| 3243.3605686 | 4 |
| 3243.3611272 | 0 |
| 3243.3630481 | 1 |
| 3243.3636805 | 1 |
| 3243.363855 | 4 |
| 3243.3650819 | 1 |
| 3243.3657793 | 1 |
| 3243.3668548 | 0 |
| 3243.3675286 | 2 |
| 3243.369061 | 1 |
| 3243.3699768 | 1 |
| 3243.3702996 | 1 |
| 3243.3714383 | 3 |
| 3243.3726808 | 4 |
| 3243.3731418 | 2 |
| 3243.3747096 | 2 |
| 3243.3753483 | 3 |
| 3243.3760119 | 2 |
| 3243.3775672 | 1 |
| 3243.378184 | 3 |
| 3243.3792417 | 5 |
| 3243.3797773 | 1 |
| 3243.3808033 | 1 |
| 3243.3819458 | 2 |
| 3243.3828947 | 2 |
| 3243.3838685 | 1 |
| 3243.3848861 | 2 |
| 3243.3864358 | 4 |
| 3243.3870208 | 1 |
| 3243.3872801 | 5 |
| 3243.3887308 | 1 |
| 3243.389448 | 1 |
| 3243.390597 | 4 |
| 3243.3910679 | 3 |
| 3243.3921884 | 0 |
| 3243.3933735 | 1 |
| 3243.3940708 | 4 |
| 3243.3947599 | 3 |
| 3243.3958947 | 4 |
| 3243.3967387 | 1 |
| 3243.3980484 | 2 |
| 3243.3982644 | 2 |
| 3243.3997116 | 3 |
| 3243.4002288 | 2 |
| 3243.4015566 | 2 |
| 3243.4022803 | 2 |
| 3243.4027648 | 2 |
| 3243.4041502 | 2 |
| 3243.4049525 | 0 |
| 3243.4060776 | 2 |
| 3243.4073754 | 1 |
| 3243.4075118 | 2 |
| 3243.408763 | 4 |
| 3243.4092491 | 2 |
| 3243.410997 | 2 |
| 3243.412001 | 3 |
| 3243.4123395 | 1 |
| 3243.4135764 | 2 |
| 3243.4140842 | 1 |
| 3243.4150934 | 2 |
| 3243.4162951 | 6 |
| 3243.4173917 | 6 |
| 3243.4186306 | 3 |
| 3243.418861 | 2 |
| 3243.4201661 | 1 |
| 3243.420859 | 1 |
| 3243.4215554 | 1 |
| 3243.4229397 | 3 |
| 3243.4238696 | 2 |
| 3243.4251236 | 5 |
| 3243.4252313 | 2 |
| 3243.4270623 | 2 |
| 3243.4278146 | 2 |
| 3243.4281124 | 3 |
| 3243.4289514 | 2 |
| 3243.4304151 | 0 |
| 3243.4312663 | 3 |
| 3243.4319782 | 3 |
| 3243.4329897 | 2 |
| 3243.4345279 | 3 |
| 3243.4351886 | 1 |
| 3243.4361187 | 0 |
| 3243.4372136 | 3 |
| 3243.4377768 | 4 |
| 3243.4389784 | 2 |
| 3243.4397465 | 1 |
| 3243.4404259 | 3 |
| 3243.4421317 | 5 |
| 3243.442504 | 4 |
| 3243.4433745 | 1 |
| 3243.4439817 | 2 |
| 3243.4452516 | 2 |
| 3243.4464463 | 2 |
| 3243.446656 | 6 |
| 3243.448368 | 1 |
| 3243.4489039 | 6 |
| 3243.4496928 | 3 |
| 3243.4506998 | 0 |
| 3243.4515859 | 2 |
| 3243.4527581 | 3 |
| 3243.4529638 | 1 |
| 3243.4540277 | 0 |
| 3243.4552669 | 3 |
| 3243.4561968 | 0 |
| 3243.4565581 | 0 |
| 3243.4581909 | 3 |
| 3243.4589754 | 3 |
| 3243.4602381 | 2 |
| 3243.4606389 | 1 |
| 3243.4617867 | 3 |
| 3243.4625214 | 1 |

| | |
|---|---|
| 3243.4636023 | 2 |
| 3243.4646665 | 2 |
| 3243.4657117 | 1 |
| 3243.4670187 | 2 |
| 3243.4675217 | 3 |
| 3243.4688111 | 1 |
| 3243.4699062 | 3 |
| 3243.4701897 | 0 |
| 3243.471213 | 2 |
| 3243.4714169 | 2 |
| 3243.473446 | 2 |
| 3243.4742718 | 0 |
| 3243.4747271 | 3 |
| 3243.4760023 | 2 |
| 3243.4766664 | 2 |
| 3243.4777347 | 4 |
| 3243.4786552 | 2 |
| 3243.4794687 | 1 |
| 3243.4802572 | 4 |
| 3243.480786 | 2 |
| 3243.4824452 | 1 |
| 3243.4835752 | 3 |
| 3243.4838222 | 7 |
| 3243.4850279 | 3 |
| 3243.4854492 | 1 |
| 3243.4867639 | 2 |
| 3243.4880103 | 4 |
| 3243.488854 | 2 |
| 3243.4899299 | 3 |
| 3243.4901941 | 0 |
| 3243.4909988 | 0 |
| 3243.4923767 | 2 |
| 3243.4928476 | 3 |
| 3243.4941896 | 2 |
| 3243.4952354 | 2 |
| 3243.4956341 | 0 |
| 3243.4967218 | 2 |
| 3243.4978571 | 3 |
| 3243.4986769 | 4 |
| 3243.4993216 | 2 |
| 3243.5006467 | 5 |
| 3243.5018553 | 0 |
| 3243.5018588 | 0 |
| 3243.5035354 | 4 |
| 3243.5040078 | 5 |
| 3243.5047085 | 1 |
| 3243.5065179 | 1 |
| 3243.506774 | 1 |
| 3243.5083255 | 2 |
| 3243.5089512 | 2 |
| 3243.5107254 | 1 |
| 3243.5108837 | 4 |
| 3243.5119145 | 2 |
| 3243.5132296 | 1 |
| 3243.513402 | 4 |
| 3243.5145094 | 2 |
| 3243.5157087 | 1 |
| 3243.5164278 | 1 |
| 3243.5175685 | 1 |
| 3243.5184949 | 0 |
| 3243.519421 | 1 |
| 3243.520536 | 2 |
| 3243.5207779 | 3 |
| 3243.5225711 | 2 |
| 3243.5232467 | 1 |
| 3243.523373 | 1 |
| 3243.5246802 | 3 |
| 3243.5254189 | 4 |
| 3243.526533 | 1 |
| 3243.5272704 | 1 |
| 3243.5284746 | 3 |
| 3243.5292648 | 0 |
| 3243.5300862 | 0 |
| 3243.5311655 | 1 |
| 3243.5319715 | 1 |
| 3243.5335052 | 3 |
| 3243.5337541 | 1 |
| 3243.5349651 | 2 |
| 3243.5361061 | 3 |
| 3243.5361215 | 4 |
| 3243.5377151 | 3 |
| 3243.5387124 | 3 |
| 3243.5392725 | 1 |
| 3243.5403752 | 1 |
| 3243.5413801 | 1 |
| 3243.5423058 | 5 |
| 3243.5431426 | 2 |
| 3243.5445284 | 5 |
| 3243.5454367 | 7 |
| 3243.5458075 | 2 |
| 3243.5470077 | 5 |
| 3243.5474117 | 0 |
| 3243.549288 | 1 |
| 3243.5496262 | 4 |
| 3243.5507025 | 2 |
| 3243.5517966 | 2 |
| 3243.5525688 | 5 |
| 3243.5531372 | 1 |
| 3243.5544457 | 2 |
| 3243.5550608 | 2 |
| 3243.5561871 | 2 |
| 3243.5566711 | 2 |
| 3243.5584741 | 2 |
| 3243.5591817 | 2 |
| 3243.5605881 | 2 |
| 3243.5611817 | 0 |
| 3243.561593 | 3 |
| 3243.5628564 | 1 |
| 3243.5632359 | 0 |
| 3243.5646763 | 1 |
| 3243.5659758 | 1 |
| 3243.5663895 | 3 |
| 3243.5680548 | 1 |
| 3243.5684698 | 2 |
| 3243.5691493 | 2 |
| 3243.5703597 | 2 |
| 3243.5714449 | 4 |
| 3243.5728801 | 4 |
| 3243.5729459 | 2 |
| 3243.5741051 | 1 |
| 3243.5748309 | 0 |
| 3243.5757715 | 1 |
| 3243.5770425 | 3 |
| 3243.5776319 | 2 |
| 3243.5783033 | 4 |
| 3243.5797803 | 2 |
| 3243.5801131 | 2 |
| 3243.5814525 | 3 |
| 3243.5816104 | 2 |
| 3243.5827215 | 0 |
| 3243.5843261 | 3 |
| 3243.5846123 | 2 |
| 3243.585081 | 1 |
| 3243.5866355 | 1 |
| 3243.5876532 | 2 |
| 3243.5888975 | 1 |
| 3243.5893203 | 2 |
| 3243.5909119 | 2 |
| 3243.5910184 | 3 |
| 3243.5921937 | 2 |
| 3243.5933519 | 1 |
| 3243.594212 | 2 |
| 3243.5954345 | 3 |
| 3243.5959271 | 0 |
| 3243.5971631 | 1 |
| 3243.5977275 | 1 |
| 3243.5984123 | 3 |
| 3243.599527 | 3 |
| 3243.6014365 | 2 |
| 3243.601658 | 1 |
| 3243.6029347 | 2 |
| 3243.6032195 | 3 |
| 3243.6048375 | 8 |
| 3243.6051049 | 2 |
| 3243.6063569 | 5 |
| 3243.6075853 | 0 |
| 3243.6078293 | 2 |
| 3243.6093592 | 5 |
| 3243.609587 | 0 |
| 3243.610973 | 3 |
| 3243.611836 | 2 |
| 3243.6121139 | 1 |
| 3243.6140948 | 3 |
| 3243.614464 | 2 |
| 3243.6160069 | 1 |
| 3243.6167393 | 1 |
| 3243.617416 | 3 |
| 3243.6186714 | 2 |
| 3243.6190577 | 3 |
| 3243.6198615 | 0 |
| 3243.6206077 | 5 |
| 3243.6216758 | 0 |
| 3243.6222976 | 0 |
| 3243.6234948 | 4 |
| 3243.6247574 | 2 |
| 3243.6257673 | 1 |
| 3243.6261089 | 3 |
| 3243.6276723 | 2 |
| 3243.6280921 | 2 |
| 3243.6287173 | 3 |
| 3243.6302157 | 0 |
| 3243.6308665 | 3 |
| 3243.6321729 | 1 |
| 3243.6329092 | 1 |
| 3243.6338217 | 1 |
| 3243.6350908 | 4 |
| 3243.6352083 | 3 |
| 3243.6369967 | 1 |
| 3243.6373837 | 0 |
| 3243.6383024 | 2 |
| 3243.6393999 | 3 |
| 3243.6398214 | 2 |
| 3243.6415838 | 2 |
| 3243.6422192 | 5 |
| 3243.6436511 | 3 |
| 3243.6438433 | 6 |
| 3243.6452951 | 2 |
| 3243.6457559 | 1 |
| 3243.646846 | 2 |
| 3243.647729 | 4 |
| 3243.6483612 | 1 |
| 3243.6499621 | 2 |
| 3243.6510275 | 1 |
| 3243.6518825 | 0 |
| 3243.6529659 | 4 |
| 3243.6531923 | 1 |
| 3243.6544676 | 2 |
| 3243.6555912 | 2 |
| 3243.6557375 | 4 |
| 3243.6574304 | 1 |
| 3243.6579689 | 2 |
| 3243.6592257 | 5 |
| 3243.6602452 | 1 |
| 3243.6607817 | 3 |
| 3243.661601 | 1 |
| 3243.6622391 | 1 |
| 3243.6638394 | 0 |
| 3243.6648729 | 4 |
| 3243.6654427 | 0 |
| 3243.6666751 | 3 |
| 3243.6672697 | 4 |
| 3243.6687127 | 2 |
| 3243.6696383 | 2 |
| 3243.6699666 | 2 |
| 3243.6712968 | 0 |
| 3243.6713155 | 2 |
| 3243.6730366 | 3 |
| 3243.6737893 | 5 |
| 3243.6746818 | 7 |
| 3243.6755351 | 2 |
| 3243.6764497 | 1 |
| 3243.6776014 | 1 |
| 3243.6789749 | 1 |
| 3243.6796819 | 4 |
| 3243.6807946 | 0 |
| 3243.6809561 | 1 |
| 3243.6818057 | 2 |
| 3243.6832588 | 2 |
| 3243.6840566 | 5 |
| 3243.6850461 | 2 |
| 3243.6855651 | 1 |
| 3243.6870983 | 3 |
| 3243.6874284 | 3 |
| 3243.6887582 | 1 |
| 3243.6897408 | 5 |
| 3243.690204 | 1 |
| 3243.6919263 | 3 |
| 3243.6923535 | 1 |
| 3243.6934471 | 2 |
| 3243.6945757 | 3 |
| 3243.6950646 | 7 |
| 3243.6959603 | 1 |
| 3243.6970792 | 0 |
| 3243.6980962 | 2 |
| 3243.6992513 | 3 |
| 3243.6998473 | 4 |
| 3243.7009374 | 0 |
| 3243.7019552 | 2 |
| 3243.7023476 | 1 |
| 3243.7038515 | 1 |
| 3243.7041063 | 2 |
| 3243.7056309 | 1 |
| 3243.7060158 | 2 |
| 3243.7076887 | 0 |
| 3243.7086348 | 3 |
| 3243.7096155 | 0 |
| 3243.710466 | 2 |
| 3243.7109234 | 4 |
| 3243.7119616 | 5 |
| 3243.7130689 | 1 |
| 3243.7139149 | 1 |
| 3243.7152868 | 2 |
| 3243.716059 | 1 |
| 3243.716994 | 3 |
| 3243.7170326 | 0 |
| 3243.718209 | 2 |
| 3243.7194664 | 2 |
| 3243.7199593 | 1 |
| 3243.7214188 | 2 |
| 3243.7216028 | 1 |
| 3243.7230331 | 2 |
| 3243.7239306 | 0 |
| 3243.7249295 | 2 |
| 3243.7261343 | 2 |
| 3243.7265382 | 2 |
| 3243.7278301 | 5 |
| 3243.7288704 | 3 |
| 3243.7292225 | 1 |
| 3243.7303885 | 0 |
| 3243.7314617 | 4 |
| 3243.7325984 | 3 |
| 3243.7334007 | 3 |
| 3243.7344097 | 2 |
| 3243.7355384 | 4 |
| 3243.7359493 | 0 |
| 3243.7374826 | 1 |
| 3243.7379465 | 3 |
| 3243.7387784 | 3 |
| 3243.7404328 | 0 |
| 3243.7404418 | 1 |
| 3243.7419434 | 2 |
| 3243.742707 | 3 |
| 3243.7440891 | 5 |
| 3243.7447894 | 0 |
| 3243.7457737 | 1 |
| 3243.7463293 | 0 |
| 3243.7475762 | 2 |
| 3243.7481499 | 0 |
| 3243.7499935 | 1 |
| 3243.7502927 | 2 |
| 3243.7515034 | 4 |
| 3243.7518239 | 1 |
| 3243.7529329 | 2 |
| 3243.7537127 | 2 |
| 3243.7543878 | 2 |
| 3243.7555129 | 1 |
| 3243.7569293 | 0 |
| 3243.7575069 | 1 |
| 3243.7589887 | 1 |
| 3243.7591413 | 1 |
| 3243.7606938 | 3 |
| 3243.7610109 | 4 |
| 3243.7619672 | 4 |
| 3243.7633154 | 5 |
| 3243.7644739 | 0 |
| 3243.7655944 | 3 |
| 3243.7657695 | 4 |
| 3243.7667677 | 3 |
| 3243.7680324 | 1 |
| 3243.7687174 | 2 |
| 3243.7692035 | 1 |
| 3243.7705006 | 0 |
| 3243.7721378 | 1 |
| 3243.7724395 | 0 |
| 3243.7739747 | 0 |
| 3243.7749395 | 2 |
| 3243.7752607 | 1 |
| 3243.7760996 | 1 |
| 3243.776846 | 0 |
| 3243.7778241 | 1 |
| 3243.7795306 | 2 |
| 3243.7799131 | 3 |
| 3243.780677 | 1 |
| 3243.7818456 | 3 |
| 3243.7832901 | 1 |
| 3243.7840266 | 4 |
| 3243.7848392 | 3 |
| 3243.7860405 | 1 |
| 3243.7864283 | 2 |
| 3243.787473 | 4 |
| 3243.788153 | 2 |
| 3243.7896167 | 2 |
| 3243.7901758 | 2 |
| 3243.7911731 | 3 |
| 3243.7926267 | 4 |
| 3243.7937468 | 2 |
| 3243.7939406 | 2 |
| 3243.7953326 | 0 |
| 3243.7956322 | 3 |
| 3243.79716 | 1 |
| 3243.7976851 | 7 |
| 3243.7987873 | 1 |
| 3243.7996878 | 2 |

| | |
|---|---|
| 3243.8005236 | 3 |
| 3243.8014386 | 2 |
| 3243.8022307 | 1 |
| 3243.8033643 | 4 |
| 3243.804443 | 1 |
| 3243.8047798 | 2 |
| 3243.8060719 | 0 |
| 3243.8066608 | 3 |
| 3243.8076996 | 3 |
| 3243.8091973 | 6 |
| 3243.8094279 | 1 |
| 3243.8108792 | 2 |
| 3243.8118768 | 3 |
| 3243.8131984 | 4 |
| 3243.8136388 | 2 |
| 3243.8147733 | 2 |
| 3243.8152416 | 1 |
| 3243.816618 | 2 |
| 3243.8168755 | 4 |
| 3243.8185662 | 1 |
| 3243.8188323 | 5 |
| 3243.8199502 | 2 |
| 3243.8212182 | 1 |
| 3243.8219613 | 3 |
| 3243.8228093 | 10 |
| 3243.8235896 | 9 |
| 3243.8248753 | 8 |
| 3243.825566 | 9 |
| 3243.8269086 | 11 |
| 3243.8281534 | 9 |
| 3243.8282061 | 4 |
| 3243.8295713 | 5 |
| 3243.8297906 | 1 |
| 3243.8311458 | 3 |
| 3243.8328359 | 3 |
| 3243.8331597 | 1 |
| 3243.8345951 | 4 |
| 3243.834675 | 2 |
| 3243.835962 | 2 |
| 3243.8373078 | 5 |
| 3243.8380434 | 1 |
| 3243.838935 | 3 |
| 3243.8400479 | 6 |
| 3243.8410998 | 1 |
| 3243.8422908 | 3 |
| 3243.842895 | 2 |
| 3243.8441351 | 4 |
| 3243.8450625 | 2 |
| 3243.8454008 | 3 |
| 3243.8463675 | 3 |
| 3243.8475164 | 1 |
| 3243.8483704 | 1 |
| 3243.8488984 | 3 |
| 3243.8504011 | 1 |
| 3243.8515181 | 2 |
| 3243.8521026 | 4 |
| 3243.8527088 | 5 |
| 3243.853741 | 3 |
| 3243.8552602 | 3 |
| 3243.8554051 | 1 |
| 3243.8564357 | 2 |
| 3243.8578415 | 5 |
| 3243.8579134 | 1 |
| 3243.8592843 | 2 |
| 3243.8607602 | 4 |
| 3243.8610755 | 0 |
| 3243.8628208 | 1 |
| 3243.8629935 | 5 |
| 3243.8639229 | 1 |
| 3243.8653053 | 1 |
| 3243.8655351 | 1 |
| 3243.8670637 | 2 |
| 3243.8674926 | 2 |
| 3243.8682067 | 3 |
| 3243.8700013 | 0 |
| 3243.8701674 | 2 |
| 3243.8719397 | 1 |
| 3243.8722552 | 3 |
| 3243.8730129 | 2 |
| 3243.8740592 | 1 |
| 3243.875415 | 5 |
| 3243.8757257 | 1 |
| 3243.8768628 | 3 |
| 3243.8782261 | 1 |
| 3243.8785754 | 3 |
| 3243.8797068 | 0 |
| 3243.8798361 | 1 |
| 3243.8800679 | 3 |
| 3243.8812663 | 3 |
| 3243.8816855 | 2 |
| 3243.8818608 | 2 |
| 3243.8824075 | 1 |
| 3243.8826803 | 2 |
| 3243.8831897 | 1 |
| 3243.8835823 | 1 |
| 3243.8840814 | 3 |
| 3243.8843028 | 5 |
| 3243.8845701 | 2 |
| 3243.8848211 | 1 |
| 3243.8855289 | 2 |
| 3243.8865943 | 4 |
| 3243.8868164 | 2 |
| 3243.8868243 | 2 |
| 3243.8871305 | 4 |
| 3243.8880843 | 1 |
| 3243.8880952 | 1 |
| 3243.8885153 | 4 |
| 3243.8890799 | 1 |
| 3243.8894056 | 1 |
| 3243.8896512 | 2 |
| 3243.8897743 | 2 |
| 3243.8903699 | 3 |
| 3243.8905389 | 3 |
| 3243.8913237 | 7 |
| 3243.8919289 | 8 |
| 3243.892166 | 6 |
| 3243.8922847 | 8 |
| 3243.8926123 | 4 |
| 3243.8928283 | 4 |
| 3243.8935306 | 1 |
| 3243.8945126 | 1 |
| 3243.8945231 | 3 |
| 3243.8945562 | 2 |
| 3243.8948776 | 2 |
| 3243.8955642 | 1 |
| 3243.8955831 | 2 |
| 3243.895628 | 3 |
| 3243.8962182 | 2 |
| 3243.8966355 | 2 |
| 3243.8971458 | 3 |
| 3243.8975504 | 1 |
| 3243.8975695 | 2 |
| 3243.89774 | 1 |
| 3243.8987184 | 2 |
| 3243.8992054 | 3 |
| 3243.899286 | 5 |
| 3243.8994977 | 1 |
| 3243.8998689 | 2 |
| 3243.9003252 | 4 |
| 3243.9006907 | 2 |
| 3243.9009419 | 2 |
| 3243.9010107 | 2 |
| 3243.901933 | 2 |
| 3243.9022718 | 4 |
| 3243.902395 | 2 |
| 3243.9028256 | 1 |
| 3243.9028994 | 1 |
| 3243.9036281 | 1 |
| 3243.9038835 | 0 |
| 3243.9039579 | 1 |
| 3243.9043308 | 3 |
| 3243.9047644 | 1 |
| 3243.9052536 | 1 |
| 3243.9052579 | 6 |
| 3243.9061132 | 5 |
| 3243.9062609 | 2 |
| 3243.9064311 | 5 |
| 3243.906537 | 2 |
| 3243.9069717 | 1 |
| 3243.9078385 | 4 |
| 3243.9080856 | 1 |
| 3243.908315 | 0 |
| 3243.9086033 | 2 |
| 3243.908723 | 0 |
| 3243.9095011 | 2 |
| 3243.909809 | 2 |
| 3243.910173 | 2 |
| 3243.9103567 | 3 |
| 3243.9106849 | 3 |
| 3243.9111703 | 1 |
| 3243.9114351 | 2 |
| 3243.9114816 | 3 |
| 3243.9122528 | 1 |
| 3243.9130519 | 4 |
| 3243.9132457 | 3 |
| 3243.9133481 | 3 |
| 3243.9136696 | 1 |
| 3243.9144535 | 0 |
| 3243.9144883 | 1 |
| 3243.9153129 | 2 |
| 3243.9153834 | 2 |
| 3243.9156209 | 0 |
| 3243.9158913 | 1 |
| 3243.9162302 | 4 |
| 3243.9163933 | 1 |
| 3243.917302 | 2 |
| 3243.9174801 | 5 |
| 3243.917494 | 2 |
| 3243.9181729 | 0 |
| 3243.918328 | 2 |
| 3243.9192672 | 1 |
| 3243.9194097 | 2 |
| 3243.9196349 | 3 |
| 3243.9201219 | 1 |
| 3243.9205854 | 1 |
| 3243.9207726 | 0 |
| 3243.9210725 | 2 |
| 3243.9210899 | 1 |
| 3243.9221483 | 4 |
| 3243.92231 | 4 |
| 3243.9223316 | 3 |
| 3243.9224398 | 3 |
| 3243.9229165 | 1 |
| 3243.9236569 | 1 |
| 3243.9241703 | 5 |
| 3243.9246265 | 5 |
| 3243.924741 | 2 |
| 3243.9249573 | 0 |
| 3243.9257277 | 4 |
| 3243.9259526 | 1 |
| 3243.9264014 | 1 |
| 3243.9266283 | 3 |
| 3243.9270346 | 2 |
| 3243.9273023 | 3 |
| 3243.9275514 | 1 |
| 3243.928039 | 4 |
| 3243.9283708 | 1 |
| 3243.9285172 | 0 |
| 3243.9290716 | 0 |
| 3243.9294168 | 1 |
| 3243.9299163 | 5 |
| 3243.930021 | 0 |
| 3243.9301703 | 0 |
| 3243.9306407 | 3 |
| 3243.9313181 | 3 |
| 3243.9318237 | 0 |
| 3243.9319469 | 3 |
| 3243.9319481 | 1 |
| 3243.9320837 | 1 |
| 3243.9328003 | 1 |
| 3243.9335222 | 2 |
| 3243.9337231 | 0 |
| 3243.9344219 | 0 |
| 3243.934869 | 2 |
| 3243.9349891 | 2 |
| 3243.9351352 | 2 |
| 3243.9352436 | 2 |
| 3243.9359514 | 1 |
| 3243.9363119 | 2 |
| 3243.9363287 | 3 |
| 3243.9368067 | 3 |
| 3243.9373712 | 2 |
| 3243.9376101 | 0 |
| 3243.9378523 | 2 |
| 3243.9379943 | 1 |
| 3243.9384711 | 1 |
| 3243.9388389 | 1 |
| 3243.9393166 | 2 |
| 3243.9397666 | 0 |
| 3243.9399478 | 1 |
| 3243.9407202 | 6 |
| 3243.9411767 | 12 |
| 3243.9413052 | 7 |
| 3243.9415601 | 3 |
| 3243.9416293 | 10 |
| 3243.942616 | 13 |
| 3243.9426828 | 7 |
| 3243.9428286 | 12 |
| 3243.9433377 | 8 |
| 3243.9434933 | 9 |
| 3243.944046 | 10 |
| 3243.9444529 | 5 |
| 3243.9446554 | 9 |
| 3243.9452791 | 1 |
| 3243.9460058 | 1 |
| 3243.946019 | 6 |
| 3243.9461435 | 2 |
| 3243.9465127 | 2 |
| 3243.946654 | 1 |
| 3243.9473563 | 6 |
| 3243.9478361 | 2 |
| 3243.9480339 | 3 |
| 3243.9480794 | 3 |
| 3243.9484136 | 2 |
| 3243.9493153 | 1 |
| 3243.9493357 | 1 |
| 3243.949623 | 1 |
| 3243.949702 | 2 |
| 3243.9500408 | 3 |
| 3243.9501591 | 1 |
| 3243.9516411 | 5 |
| 3243.9516644 | 6 |
| 3243.9523218 | 0 |
| 3243.9524944 | 3 |
| 3243.9525037 | 1 |
| 3243.9525987 | 3 |
| 3243.9530963 | 3 |
| 3243.9532737 | 1 |
| 3243.9536232 | 8 |
| 3243.9540403 | 1 |
| 3243.9545891 | 0 |
| 3243.955342 | 2 |
| 3243.9557064 | 1 |
| 3243.9557735 | 1 |
| 3243.9560766 | 2 |
| 3243.9569526 | 1 |
| 3243.9572611 | 4 |
| 3243.9573141 | 4 |
| 3243.9575329 | 2 |
| 3243.9577579 | 2 |
| 3243.9579233 | 2 |
| 3243.958588 | 2 |
| 3243.9590394 | 2 |
| 3243.9596044 | 1 |
| 3243.9602426 | 1 |
| 3243.9610693 | 1 |
| 3243.9628929 | 5 |
| 3243.9657744 | 2 |
| 3243.9660433 | 0 |
| 3243.9678742 | 2 |
| 3243.9689003 | 2 |
| 3243.9706853 | 4 |
| 3243.9717241 | 1 |
| 3243.9739964 | 4 |
| 3243.9762956 | 2 |
| 3243.9763219 | 2 |
| 3243.978806 | 1 |
| 3243.9798479 | 2 |
| 3243.9818375 | 8 |
| 3243.9840339 | 1 |
| 3243.9849097 | 2 |
| 3243.9861302 | 2 |
| 3243.9873024 | 1 |
| 3243.9889373 | 1 |
| 3243.9910121 | 3 |
| 3243.9918982 | 4 |
| 3243.993695 | 1 |
| 3243.9957118 | 7 |
| 3243.9965093 | 3 |
| 3243.9990354 | 3 |
| 3244.0001369 | 4 |
| 3244.0017523 | 3 |
| 3244.0028956 | 3 |
| 3244.0055386 | 2 |
| 3244.0069197 | 4 |
| 3244.0070974 | 0 |
| 3244.0093943 | 3 |
| 3244.0106891 | 2 |
| 3244.0126594 | 2 |
| 3244.0145252 | 6 |
| 3244.0157924 | 3 |
| 3244.0175658 | 1 |
| 3244.0186263 | 1 |
| 3244.020316 | 2 |
| 3244.0225835 | 2 |
| 3244.0238485 | 4 |
| 3244.0256509 | 3 |
| 3244.0266008 | 2 |
| 3244.0280068 | 1 |
| 3244.0297838 | 1 |
| 3244.0310765 | 1 |
| 3244.0323407 | 1 |
| 3244.034352 | 2 |
| 3244.0358928 | 1 |
| 3244.0380594 | 2 |
| 3244.0388284 | 6 |
| 3244.0406725 | 0 |
| 3244.0418028 | 3 |
| 3244.0433856 | 4 |
| 3244.0460594 | 1 |
| 3244.0463906 | 3 |
| 3244.0487347 | 1 |
| 3244.0497965 | 2 |

| | |
|---|---|
| 3244.0509232 | 1 |
| 3244.0531834 | 1 |
| 3244.0542595 | 3 |
| 3244.0560717 | 3 |
| 3244.0582344 | 4 |
| 3244.0591911 | 6 |
| 3244.0612956 | 2 |
| 3244.0626789 | 2 |
| 3244.0642304 | 0 |
| 3244.0649027 | 2 |
| 3244.0672083 | 4 |
| 3244.0690267 | 2 |
| 3244.069829 | 2 |
| 3244.0715805 | 3 |
| 3244.0727304 | 3 |
| 3244.074678 | 1 |
| 3244.0767073 | 0 |
| 3244.0772508 | 4 |
| 3244.0795977 | 3 |
| 3244.0804436 | 2 |
| 3244.0821304 | 2 |
| 3244.0838805 | 1 |
| 3244.0852036 | 2 |
| 3244.0874228 | 3 |
| 3244.0881793 | 3 |
| 3244.0899062 | 4 |
| 3244.0916598 | 1 |
| 3244.0926295 | 1 |
| 3244.0947905 | 1 |
| 3244.0958605 | 1 |
| 3244.0981798 | 1 |
| 3244.0995552 | 0 |
| 3244.100406 | 3 |
| 3244.103047 | 2 |
| 3244.1037493 | 2 |
| 3244.1057682 | 5 |
| 3244.1072945 | 1 |
| 3244.1085444 | 6 |
| 3244.110287 | 2 |
| 3244.1109828 | 2 |
| 3244.1132449 | 0 |
| 3244.1153626 | 2 |
| 3244.116396 | 0 |
| 3244.1182711 | 2 |
| 3244.1194614 | 2 |
| 3244.1212416 | 3 |
| 3244.1232363 | 2 |
| 3244.123858 | 1 |
| 3244.1260211 | 1 |
| 3244.1266869 | 5 |
| 3244.1285214 | 3 |
| 3244.130425 | 6 |
| 3244.131678 | 3 |
| 3244.1335912 | 3 |
| 3244.134926 | 1 |
| 3244.135785 | 3 |
| 3244.1383423 | 1 |
| 3244.1387917 | 2 |
| 3244.1412727 | 3 |
| 3244.1424667 | 6 |
| 3244.1443499 | 6 |
| 3244.1462161 | 2 |
| 3244.1469527 | 3 |
| 3244.1487572 | 5 |
| 3244.1502282 | 2 |
| 3244.1521577 | 2 |
| 3244.1535237 | 2 |
| 3244.1549591 | 2 |
| 3244.1570686 | 4 |
| 3244.1573557 | 2 |
| 3244.1600649 | 3 |
| 3244.1606386 | 6 |
| 3244.162955 | 1 |
| 3244.1646486 | 2 |
| 3244.1655965 | 6 |
| 3244.1674376 | 1 |
| 3244.1686899 | 2 |
| 3244.1702442 | 1 |
| 3244.1716462 | 1 |
| 3244.1730226 | 2 |
| 3244.1748563 | 1 |
| 3244.176825 | 1 |
| 3244.1777079 | 1 |
| 3244.1794289 | 2 |
| 3244.180773 | 0 |
| 3244.1828233 | 2 |
| 3244.1843602 | 2 |
| 3244.1852802 | 1 |
| 3244.1875817 | 0 |
| 3244.1878411 | 2 |
| 3244.1898214 | 3 |
| 3244.1916086 | 0 |
| 3244.1934566 | 3 |
| 3244.1951131 | 1 |
| 3244.1967529 | 6 |
| 3244.198152 | 1 |
| 3244.2005995 | 1 |
| 3244.2009959 | 3 |
| 3244.2031866 | 4 |
| 3244.2037586 | 1 |
| 3244.2061794 | 2 |
| 3244.2079926 | 1 |
| 3244.2088745 | 3 |
| 3244.2108694 | 3 |
| 3244.2111834 | 1 |
| 3244.2130455 | 2 |
| 3244.2149684 | 0 |
| 3244.2162482 | 2 |
| 3244.21801 | 1 |
| 3244.2188012 | 1 |
| 3244.2209065 | 1 |
| 3244.2230593 | 0 |
| 3244.2238566 | 4 |
| 3244.2257618 | 1 |
| 3244.2273183 | 3 |
| 3244.2288305 | 2 |
| 3244.2298455 | 1 |
| 3244.2320085 | 3 |
| 3244.2336689 | 2 |
| 3244.2343902 | 2 |
| 3244.2368551 | 3 |
| 3244.2384413 | 2 |
| 3244.2392852 | 2 |
| 3244.2415982 | 3 |
| 3244.2425055 | 3 |
| 3244.2440135 | 3 |
| 3244.2463719 | 3 |
| 3244.247075 | 2 |
| 3244.249372 | 2 |
| 3244.2496844 | 3 |
| 3244.2527701 | 2 |
| 3244.253992 | 3 |
| 3244.2552412 | 4 |
| 3244.257384 | 3 |
| 3244.2582816 | 0 |
| 3244.2599056 | 5 |
| 3244.2604007 | 2 |
| 3244.2624918 | 3 |
| 3244.2646557 | 1 |
| 3244.2654799 | 3 |
| 3244.2671057 | 2 |
| 3244.2684308 | 1 |
| 3244.2711216 | 1 |
| 3244.2722073 | 4 |
| 3244.2739603 | 1 |
| 3244.2751305 | 1 |
| 3244.2756866 | 2 |
| 3244.2781791 | 4 |
| 3244.279143 | 3 |
| 3244.2810337 | 1 |
| 3244.2826328 | 3 |
| 3244.2842015 | 5 |
| 3244.2852605 | 0 |
| 3244.2878264 | 1 |
| 3244.2887263 | 1 |
| 3244.2901766 | 0 |
| 3244.2909624 | 1 |
| 3244.291604 | 5 |
| 3244.291832 | 1 |
| 3244.2933267 | 2 |
| 3244.2958826 | 1 |
| 3244.2962517 | 0 |
| 3244.2967054 | 2 |
| 3244.2979587 | 1 |
| 3244.2986035 | 0 |
| 3244.2991903 | 1 |
| 3244.2994359 | 1 |
| 3244.3010203 | 3 |
| 3244.3023667 | 3 |
| 3244.3030559 | 3 |
| 3244.303841 | 1 |
| 3244.304477 | 0 |
| 3244.3062294 | 2 |
| 3244.3073195 | 1 |
| 3244.3076779 | 2 |
| 3244.3089674 | 4 |
| 3244.3101033 | 1 |
| 3244.3108247 | 6 |
| 3244.3121958 | 2 |
| 3244.3124709 | 4 |
| 3244.3145353 | 0 |
| 3244.3151527 | 3 |
| 3244.3154286 | 1 |
| 3244.316849 | 2 |
| 3244.3169091 | 1 |
| 3244.3180613 | 2 |
| 3244.3198246 | 1 |
| 3244.3199425 | 3 |
| 3244.3210009 | 3 |
| 3244.3222879 | 4 |
| 3244.3226061 | 1 |
| 3244.3244589 | 1 |
| 3244.3248781 | 2 |
| 3244.3252691 | 1 |
| 3244.3281408 | 1 |
| 3244.3291617 | 5 |
| 3244.3323824 | 2 |
| 3244.3338812 | 3 |
| 3244.3368365 | 3 |
| 3244.3403842 | 3 |
| 3244.3419145 | 2 |
| 3244.345335 | 3 |
| 3244.3467493 | 2 |
| 3244.3502354 | 1 |
| 3244.3530259 | 3 |
| 3244.3545049 | 3 |
| 3244.3577987 | 2 |
| 3244.3591991 | 6 |
| 3244.3625089 | 2 |
| 3244.3650634 | 2 |
| 3244.3664213 | 3 |
| 3244.3702521 | 1 |
| 3244.3711008 | 2 |
| 3244.3749751 | 2 |
| 3244.3777287 | 3 |
| 3244.3801437 | 0 |
| 3244.3828448 | 2 |
| 3244.3848521 | 2 |
| 3244.3880896 | 3 |
| 3244.3915092 | 0 |
| 3244.3923306 | 2 |
| 3244.3956253 | 3 |
| 3244.396023 | 3 |
| 3244.3999623 | 3 |
| 3244.4029286 | 3 |
| 3244.4048444 | 3 |
| 3244.4078553 | 2 |
| 3244.4094606 | 4 |
| 3244.4122133 | 3 |
| 3244.4158264 | 1 |
| 3244.4171322 | 2 |
| 3244.4206266 | 3 |
| 3244.4219908 | 3 |
| 3244.4251383 | 2 |
| 3244.4266175 | 4 |
| 3244.4302546 | 1 |
| 3244.4333566 | 1 |
| 3244.4351137 | 2 |
| 3244.4371963 | 3 |
| 3244.4392658 | 0 |
| 3244.4430798 | 3 |
| 3244.445507 | 1 |
| 3244.4477638 | 3 |
| 3244.4507226 | 2 |
| 3244.4516561 | 3 |
| 3244.4554851 | 4 |
| 3244.4565221 | 5 |
| 3244.4591248 | 1 |
| 3244.462858 | 1 |
| 3244.4656579 | 2 |
| 3244.4672961 | 5 |
| 3244.4701222 | 3 |
| 3244.4716131 | 4 |
| 3244.475198 | 2 |
| 3244.4769178 | 5 |
| 3244.4802393 | 1 |
| 3244.4826874 | 1 |
| 3244.4846911 | 2 |
| 3244.4882808 | 2 |
| 3244.4885507 | 6 |
| 3244.4921893 | 1 |
| 3244.4956145 | 2 |
| 3244.4965109 | 0 |
| 3244.4997287 | 5 |
| 3244.5012824 | 4 |
| 3244.5050129 | 0 |
| 3244.5072132 | 3 |
| 3244.5100247 | 3 |
| 3244.5125601 | 2 |
| 3244.5145758 | 1 |
| 3244.5170685 | 0 |
| 3244.5188406 | 0 |
| 3244.522897 | 2 |
| 3244.5255823 | 1 |
| 3244.5264992 | 2 |
| 3244.5294746 | 3 |
| 3244.5328286 | 5 |
| 3244.5337966 | 1 |
| 3244.5367424 | 1 |
| 3244.5388235 | 4 |
| 3244.5418638 | 1 |
| 3244.5452594 | 4 |
| 3244.5468578 | 5 |
| 3244.549773 | 4 |
| 3244.5512837 | 4 |
| 3244.5554883 | 3 |
| 3244.5559793 | 3 |
| 3244.5586449 | 5 |
| 3244.5623361 | 2 |
| 3244.5636545 | 0 |
| 3244.5668532 | 1 |
| 3244.5697233 | 0 |
| 3244.5714282 | 4 |
| 3244.5746348 | 2 |
| 3244.5766658 | 2 |
| 3244.5800408 | 3 |
| 3244.5813275 | 4 |
| 3244.5842775 | 2 |
| 3244.5874755 | 2 |
| 3244.5885897 | 2 |
| 3244.5913487 | 5 |
| 3244.5943833 | 0 |
| 3244.5955854 | 3 |
| 3244.5987331 | 3 |
| 3244.6004453 | 2 |
| 3244.6032155 | 3 |
| 3244.6070708 | 1 |
| 3244.60861 | 2 |
| 3244.6125195 | 3 |
| 3244.6133713 | 4 |
| 3244.6169133 | 1 |
| 3244.6197 | 1 |
| 3244.6204773 | 5 |
| 3244.6241694 | 1 |
| 3244.6254036 | 1 |
| 3244.6283692 | 2 |
| 3244.6318409 | 5 |
| 3244.6335411 | 2 |
| 3244.6362019 | 0 |
| 3244.6382865 | 2 |
| 3244.6413857 | 2 |
| 3244.6440648 | 2 |
| 3244.6452994 | 3 |
| 3244.6490839 | 3 |
| 3244.6506699 | 1 |
| 3244.6534279 | 2 |
| 3244.6555578 | 1 |
| 3244.6588507 | 1 |
| 3244.6616687 | 0 |
| 3244.6626708 | 1 |
| 3244.6663595 | 3 |
| 3244.667666 | 1 |
| 3244.6707817 | 4 |
| 3244.6735378 | 2 |
| 3244.6757341 | 1 |
| 3244.6782496 | 1 |
| 3244.6799107 | 2 |
| 3244.6840746 | 3 |
| 3244.6844104 | 2 |
| 3244.6872949 | 1 |
| 3244.6907329 | 2 |
| 3244.6940233 | 5 |
| 3244.694742 | 3 |
| 3244.698723 | 2 |
| 3244.7001805 | 0 |
| 3244.702938 | 4 |
| 3244.7060467 | 0 |
| 3244.7080362 | 2 |
| 3244.7107396 | 1 |
| 3244.7119885 | 2 |
| 3244.7157658 | 2 |
| 3244.7190658 | 2 |
| 3244.7205994 | 2 |
| 3244.7233261 | 4 |
| 3244.7243871 | 1 |
| 3244.7273665 | 0 |
| 3244.729615 | 0 |
| 3244.7318943 | 4 |
| 3244.7356612 | 3 |
| 3244.737528 | 1 |
| 3244.7405388 | 4 |
| 3244.7421855 | 0 |
| 3244.7461613 | 3 |

| | |
|---|---|
| 3244.749414 | 1 |
| 3244.7504782 | 2 |
| 3244.7531919 | 3 |
| 3244.7546026 | 3 |
| 3244.7567946 | 1 |
| 3244.7605384 | 2 |
| 3244.7619848 | 1 |
| 3244.7649725 | 0 |
| 3244.7666289 | 0 |
| 3244.7699203 | 0 |
| 3244.7728314 | 0 |
| 3244.7744355 | 1 |
| 3244.7779952 | 1 |
| 3244.7792197 | 2 |
| 3244.782207 | 2 |
| 3244.7853178 | 2 |
| 3244.7871406 | 4 |
| 3244.7898254 | 2 |
| 3244.7917656 | 3 |
| 3244.7948022 | 3 |
| 3244.7980135 | 1 |
| 3244.7994163 | 2 |
| 3244.8029769 | 2 |
| 3244.8040541 | 4 |
| 3244.8076257 | 3 |
| 3244.8105474 | 1 |
| 3244.8123133 | 0 |
| 3244.814973 | 5 |
| 3244.8158789 | 2 |
| 3244.8192652 | 4 |
| 3244.8225053 | 2 |
| 3244.8237342 | 2 |
| 3244.8279795 | 3 |
| 3244.8291915 | 2 |
| 3244.8325756 | 2 |
| 3244.835361 | 3 |
| 3244.8372933 | 4 |
| 3244.8402814 | 2 |
| 3244.8420785 | 0 |
| 3244.845082 | 1 |
| 3244.8479256 | 2 |
| 3244.8490827 | 2 |
| 3244.8526643 | 2 |
| 3244.8536235 | 3 |
| 3244.8567818 | 1 |
| 3244.8599787 | 2 |
| 3244.8614502 | 3 |
| 3244.8645628 | 3 |
| 3244.8668073 | 6 |
| 3244.8694253 | 3 |
| 3244.8705389 | 2 |
| 3244.8739646 | 6 |
| 3244.8774859 | 3 |
| 3244.8791078 | 3 |
| 3244.8818662 | 4 |
| 3244.8852031 | 0 |
| 3244.8869892 | 2 |
| 3244.889605 | 1 |
| 3244.8909993 | 3 |
| 3244.8943825 | 5 |
| 3244.8963712 | 2 |
| 3244.8989285 | 2 |
| 3244.9025662 | 1 |
| 3244.9039022 | 3 |
| 3244.9071385 | 2 |
| 3244.9086143 | 3 |
| 3244.910786 | 1 |
| 3244.914307 | 2 |
| 3244.9159925 | 5 |
| 3244.9186965 | 1 |
| 3244.921973 | 4 |
| 3244.9233441 | 2 |
| 3244.9270836 | 2 |
| 3244.9280473 | 2 |
| 3244.931635 | 2 |
| 3244.9353908 | 1 |
| 3244.9370244 | 0 |
| 3244.939221 | 0 |
| 3244.9414101 | 5 |
| 3244.9443275 | 2 |
| 3244.9455782 | 0 |
| 3244.9483323 | 0 |
| 3244.9520611 | 6 |
| 3244.9536061 | 1 |
| 3244.9571284 | 4 |
| 3244.9584353 | 1 |
| 3244.9608554 | 5 |
| 3244.9651087 | 1 |
| 3244.9660931 | 3 |
| 3244.969008 | 6 |
| 3244.9714006 | 4 |
| 3244.9749137 | 3 |
| 3244.9757819 | 3 |
| 3244.9792536 | 0 |
| 3244.9820181 | 0 |
| 3244.9835707 | 1 |
| 3244.9870478 | 3 |
| 3244.9899149 | 1 |
| 3244.9917942 | 1 |
| 3244.9946183 | 2 |
| 3244.9957253 | 3 |
| 3244.9996818 | 2 |
| 3245.0011249 | 1 |
| 3245.0045406 | 3 |
| 3245.0077991 | 2 |
| 3245.009439 | 1 |
| 3245.0125326 | 3 |
| 3245.0153124 | 2 |
| 3245.016752 | 0 |
| 3245.0201327 | 2 |
| 3245.0219214 | 4 |
| 3245.0243312 | 0 |
| 3245.0280689 | 4 |
| 3245.029836 | 3 |
| 3245.0332709 | 2 |
| 3245.0347246 | 2 |
| 3245.0378011 | 2 |
| 3245.0407124 | 4 |
| 3245.0417341 | 1 |
| 3245.0448408 | 2 |
| 3245.0471423 | 1 |
| 3245.0501075 | 1 |
| 3245.0533335 | 1 |
| 3245.0544381 | 5 |
| 3245.0581209 | 4 |
| 3245.0596205 | 3 |
| 3245.0627302 | 1 |
| 3245.0659893 | 5 |
| 3245.0678474 | 1 |
| 3245.0710835 | 1 |
| 3245.0720917 | 4 |
| 3245.0733665 | 3 |
| 3245.0744485 | 3 |
| 3245.076153 | 2 |
| 3245.0765782 | 4 |
| 3245.0775243 | 0 |
| 3245.0791816 | 1 |
| 3245.0796918 | 1 |
| 3245.0798251 | 1 |
| 3245.0815054 | 2 |
| 3245.082708 | 3 |
| 3245.0827176 | 4 |
| 3245.0847016 | 2 |
| 3245.08509 | 1 |
| 3245.0855061 | 0 |
| 3245.0872833 | 1 |
| 3245.0875229 | 3 |
| 3245.0898212 | 3 |
| 3245.0899512 | 2 |
| 3245.0913979 | 4 |
| 3245.092233 | 4 |
| 3245.0923309 | 0 |
| 3245.0938156 | 2 |
| 3245.095094 | 0 |
| 3245.0952748 | 2 |
| 3245.0967482 | 3 |
| 3245.0977584 | 4 |
| 3245.0981755 | 3 |
| 3245.0998831 | 2 |
| 3245.1003693 | 3 |
| 3245.1008682 | 2 |
| 3245.1031025 | 2 |
| 3245.1036057 | 2 |
| 3245.1054925 | 0 |
| 3245.1055018 | 2 |
| 3245.1061173 | 2 |
| 3245.1081442 | 4 |
| 3245.1081615 | 2 |
| 3245.1085988 | 3 |
| 3245.1106501 | 1 |
| 3245.1112073 | 5 |
| 3245.1117421 | 0 |
| 3245.1119966 | 5 |
| 3245.1123142 | 5 |
| 3245.1124913 | 3 |
| 3245.1125608 | 3 |
| 3245.1126418 | 2 |
| 3245.1133906 | 3 |
| 3245.1134364 | 3 |
| 3245.1135245 | 3 |
| 3245.1137956 | 4 |
| 3245.1138321 | 1 |
| 3245.1140831 | 3 |
| 3245.1144439 | 5 |
| 3245.1150177 | 1 |
| 3245.1154818 | 4 |
| 3245.1155621 | 1 |
| 3245.1155888 | 1 |
| 3245.1156919 | 1 |
| 3245.116462 | 2 |
| 3245.1167332 | 2 |
| 3245.1169786 | 4 |
| 3245.1170935 | 3 |
| 3245.1171375 | 0 |
| 3245.1175094 | 2 |
| 3245.1181696 | 4 |
| 3245.1181828 | 2 |
| 3245.1183694 | 3 |
| 3245.1184564 | 0 |
| 3245.1184759 | 2 |
| 3245.1191422 | 0 |
| 3245.1195547 | 0 |
| 3245.1199852 | 1 |
| 3245.120314 | 3 |
| 3245.1206251 | 3 |
| 3245.1207652 | 3 |
| 3245.120789 | 3 |
| 3245.1214442 | 4 |
| 3245.121834 | 3 |
| 3245.1219692 | 3 |
| 3245.1223028 | 0 |
| 3245.1224584 | 5 |
| 3245.1226093 | 3 |
| 3245.1229397 | 3 |
| 3245.1232315 | 0 |
| 3245.1236138 | 1 |
| 3245.1236576 | 5 |
| 3245.1238178 | 3 |
| 3245.1239653 | 2 |
| 3245.1243695 | 3 |
| 3245.1246963 | 4 |
| 3245.1248532 | 4 |
| 3245.1255135 | 3 |
| 3245.1255973 | 4 |
| 3245.125675 | 2 |
| 3245.1260163 | 2 |
| 3245.1260196 | 3 |
| 3245.1262824 | 2 |
| 3245.1267594 | 4 |
| 3245.1268837 | 3 |
| 3245.1271423 | 3 |
| 3245.1272241 | 0 |
| 3245.1273601 | 2 |
| 3245.1278749 | 2 |
| 3245.1279497 | 2 |
| 3245.1279552 | 5 |
| 3245.128073 | 0 |
| 3245.1281859 | 1 |
| 3245.1284667 | 2 |
| 3245.1288618 | 2 |
| 3245.1289446 | 3 |
| 3245.1294614 | 2 |
| 3245.1296297 | 1 |
| 3245.1297185 | 2 |
| 3245.1297972 | 3 |
| 3245.1299609 | 2 |
| 3245.1301914 | 1 |
| 3245.1302501 | 5 |
| 3245.1308339 | 0 |
| 3245.1310704 | 2 |
| 3245.1313241 | 4 |
| 3245.1313768 | 6 |
| 3245.1315301 | 3 |
| 3245.131629 | 5 |
| 3245.1322611 | 2 |
| 3245.1322986 | 2 |
| 3245.1326382 | 4 |
| 3245.1328923 | 2 |
| 3245.1330637 | 2 |
| 3245.1331425 | 0 |
| 3245.1331431 | 0 |
| 3245.1333183 | 3 |
| 3245.1334668 | 2 |
| 3245.1335514 | 5 |
| 3245.1336341 | 5 |
| 3245.1337719 | 6 |
| 3245.1338027 | 4 |
| 3245.1339518 | 2 |
| 3245.1349598 | 7 |
| 3245.135071 | 11 |
| 3245.1351163 | 24 |
| 3245.1351829 | 4 |
| 3245.1352083 | 14 |
| 3245.1353284 | 10 |
| 3245.1356274 | 9 |
| 3245.1356892 | 23 |
| 3245.136092 | 30 |
| 3245.1369541 | 29 |
| 3245.1370276 | 37 |
| 3245.1370844 | 27 |
| 3245.137115 | 38 |
| 3245.1371254 | 37 |
| 3245.137502 | 35 |
| 3245.137615 | 40 |
| 3245.1377051 | 36 |
| 3245.1381807 | 37 |
| 3245.1382702 | 41 |
| 3245.138474 | 36 |
| 3245.1387206 | 33 |
| 3245.1389027 | 39 |
| 3245.1390917 | 43 |
| 3245.1391764 | 49 |
| 3245.13936 | 44 |
| 3245.1397766 | 33 |
| 3245.1399833 | 41 |
| 3245.1402413 | 51 |
| 3245.1402679 | 29 |
| 3245.1403738 | 50 |
| 3245.1405266 | 38 |
| 3245.1407877 | 47 |
| 3245.1409428 | 50 |
| 3245.1413183 | 48 |
| 3245.1414882 | 45 |
| 3245.141563 | 42 |
| 3245.1415755 | 44 |
| 3245.1419006 | 52 |
| 3245.1419451 | 44 |
| 3245.1423154 | 38 |
| 3245.1425691 | 49 |
| 3245.1429662 | 37 |
| 3245.1430804 | 42 |
| 3245.1431402 | 30 |
| 3245.1434021 | 44 |
| 3245.1434597 | 44 |
| 3245.1439439 | 25 |
| 3245.1447729 | 35 |
| 3245.1448505 | 15 |
| 3245.1448916 | 26 |
| 3245.1451406 | 27 |
| 3245.145214 | 6 |
| 3245.1453521 | 13 |
| 3245.1454643 | 9 |
| 3245.1455826 | 5 |
| 3245.1456073 | 15 |
| 3245.1459336 | 4 |
| 3245.1460245 | 6 |
| 3245.1463416 | 2 |
| 3245.146396 | 0 |
| 3245.1468074 | 1 |
| 3245.1469025 | 7 |
| 3245.1469973 | 2 |
| 3245.1470048 | 3 |
| 3245.1478384 | 5 |
| 3245.1480837 | 5 |
| 3245.1482588 | 1 |
| 3245.148552 | 5 |
| 3245.1486332 | 4 |
| 3245.1490774 | 2 |
| 3245.1492564 | 4 |
| 3245.1492835 | 1 |
| 3245.1493439 | 1 |
| 3245.1496394 | 2 |
| 3245.1498481 | 4 |
| 3245.1500135 | 1 |
| 3245.1501829 | 2 |
| 3245.1503207 | 3 |
| 3245.1505152 | 1 |
| 3245.1512123 | 6 |
| 3245.1512509 | 2 |
| 3245.151363 | 2 |
| 3245.1514088 | 4 |
| 3245.1514885 | 4 |
| 3245.1515887 | 4 |
| 3245.152013 | 1 |
| 3245.1520939 | 3 |
| 3245.1522644 | 5 |
| 3245.1523574 | 0 |
| 3245.152656 | 3 |
| 3245.1528506 | 1 |
| 3245.1544117 | 2 |
| 3245.1548689 | 4 |
| 3245.1551897 | 3 |
| 3245.1557257 | 4 |

| | |
|---|---|
| 3245.1560642 | 3 |
| 3245.1566918 | 3 |
| 3245.1575501 | 0 |
| 3245.1577034 | 1 |
| 3245.1586771 | 2 |
| 3245.1591233 | 1 |
| 3245.1594309 | 0 |
| 3245.1606508 | 1 |
| 3245.1609276 | 2 |
| 3245.1614345 | 0 |
| 3245.1620849 | 2 |
| 3245.162196 | 0 |
| 3245.1635928 | 4 |
| 3245.1639216 | 2 |
| 3245.1644047 | 1 |
| 3245.1649284 | 1 |
| 3245.165363 | 2 |
| 3245.1657761 | 0 |
| 3245.1670911 | 2 |
| 3245.1674842 | 3 |
| 3245.1677063 | 1 |
| 3245.167915 | 1 |
| 3245.1689245 | 4 |
| 3245.1700557 | 8 |
| 3245.1701624 | 2 |
| 3245.1705462 | 4 |
| 3245.1716306 | 6 |
| 3245.1718368 | 2 |
| 3245.1725094 | 2 |
| 3245.1730431 | 2 |
| 3245.1730935 | 2 |
| 3245.174349 | 0 |
| 3245.1749501 | 1 |
| 3245.1750669 | 2 |
| 3245.1757131 | 2 |
| 3245.1766659 | 3 |
| 3245.1766863 | 3 |
| 3245.1775774 | 2 |
| 3245.1778439 | 1 |
| 3245.178926 | 4 |
| 3245.1796964 | 2 |
| 3245.180205 | 2 |
| 3245.1802898 | 1 |
| 3245.1806043 | 0 |
| 3245.1821389 | 5 |
| 3245.1822607 | 2 |
| 3245.182288 | 2 |
| 3245.1832651 | 2 |
| 3245.1837581 | 2 |
| 3245.1837635 | 4 |
| 3245.184755 | 1 |
| 3245.185967 | 3 |
| 3245.1865227 | 2 |
| 3245.1871018 | 2 |
| 3245.1872887 | 5 |
| 3245.1882111 | 4 |
| 3245.1882275 | 3 |
| 3245.1890679 | 2 |
| 3245.1900235 | 5 |
| 3245.1906313 | 3 |
| 3245.1908887 | 1 |
| 3245.1919551 | 7 |
| 3245.191975 | 2 |
| 3245.1930724 | 1 |
| 3245.1931971 | 3 |
| 3245.1934125 | 3 |
| 3245.1945631 | 1 |
| 3245.1949773 | 1 |
| 3245.1962659 | 1 |
| 3245.1964767 | 4 |
| 3245.1966338 | 3 |
| 3245.1976236 | 4 |
| 3245.1977159 | 0 |
| 3245.1982829 | 1 |
| 3245.1988214 | 2 |
| 3245.1994038 | 0 |
| 3245.2001617 | 3 |
| 3245.2012147 | 2 |
| 3245.2015729 | 2 |
| 3245.202508 | 4 |
| 3245.2030954 | 2 |
| 3245.2031775 | 3 |
| 3245.2044445 | 4 |
| 3245.204597 | 3 |
| 3245.2048821 | 0 |
| 3245.2055764 | 1 |
| 3245.2058649 | 2 |
| 3245.2065056 | 1 |
| 3245.2066535 | 2 |
| 3245.2079863 | 1 |
| 3245.2086626 | 2 |
| 3245.2093052 | 5 |
| 3245.2098458 | 2 |
| 3245.2100284 | 1 |
| 3245.2107966 | 1 |
| 3245.2117921 | 2 |
| 3245.2122417 | 6 |
| 3245.2124478 | 2 |
| 3245.2134836 | 5 |
| 3245.2137137 | 0 |
| 3245.2140603 | 3 |
| 3245.2148405 | 1 |
| 3245.2151502 | 2 |
| 3245.2157179 | 1 |
| 3245.2168609 | 1 |
| 3245.217174 | 3 |
| 3245.2179204 | 6 |
| 3245.2182247 | 1 |
| 3245.2184467 | 2 |
| 3245.2195561 | 5 |
| 3245.2196848 | 4 |
| 3245.2203926 | 3 |
| 3245.2217955 | 2 |
| 3245.2218702 | 5 |
| 3245.2220426 | 2 |
| 3245.2223518 | 3 |
| 3245.2230913 | 1 |
| 3245.224146 | 2 |
| 3245.2244733 | 1 |
| 3245.2252719 | 2 |
| 3245.2254551 | 6 |
| 3245.2259574 | 1 |
| 3245.2267553 | 2 |
| 3245.226904 | 3 |
| 3245.2276931 | 3 |
| 3245.2288198 | 3 |
| 3245.229051 | 2 |
| 3245.2298942 | 5 |
| 3245.2302277 | 3 |
| 3245.2302639 | 3 |
| 3245.2313847 | 3 |
| 3245.232001 | 2 |
| 3245.2322764 | 3 |
| 3245.2326385 | 2 |
| 3245.2332837 | 1 |
| 3245.23392 | 2 |
| 3245.2344568 | 3 |
| 3245.2351286 | 2 |
| 3245.2365803 | 4 |
| 3245.2367703 | 2 |
| 3245.2368471 | 6 |
| 3245.2379827 | 6 |
| 3245.2385774 | 1 |
| 3245.2388839 | 0 |
| 3245.2393265 | 1 |
| 3245.2406698 | 4 |
| 3245.2412431 | 2 |
| 3245.2415474 | 6 |
| 3245.2416438 | 1 |
| 3245.2420845 | 4 |
| 3245.2426212 | 5 |
| 3245.2434414 | 5 |
| 3245.2444238 | 2 |
| 3245.2446766 | 3 |
| 3245.2456517 | 3 |
| 3245.2458274 | 1 |
| 3245.2465899 | 4 |
| 3245.2471972 | 0 |
| 3245.2477863 | 2 |
| 3245.2487609 | 2 |
| 3245.2490531 | 2 |
| 3245.2490574 | 4 |
| 3245.2502542 | 4 |
| 3245.2506272 | 3 |
| 3245.2513729 | 3 |
| 3245.2522749 | 2 |
| 3245.2527951 | 5 |
| 3245.2529947 | 4 |
| 3245.2532822 | 3 |
| 3245.2535606 | 2 |
| 3245.2552745 | 1 |
| 3245.2553538 | 1 |
| 3245.2559913 | 0 |
| 3245.2566346 | 2 |
| 3245.2568327 | 2 |
| 3245.2575175 | 3 |
| 3245.2581588 | 4 |
| 3245.2588033 | 4 |
| 3245.2596185 | 0 |
| 3245.260075 | 0 |
| 3245.2601384 | 4 |
| 3245.2609073 | 4 |
| 3245.2616492 | 3 |
| 3245.2621365 | 2 |
| 3245.2626026 | 1 |
| 3245.2637706 | 7 |
| 3245.2640536 | 3 |
| 3245.264783 | 1 |
| 3245.2654482 | 2 |
| 3245.2655547 | 5 |
| 3245.2664158 | 1 |
| 3245.2664875 | 2 |
| 3245.267254 | 2 |
| 3245.2678472 | 1 |
| 3245.2681952 | 1 |
| 3245.2683992 | 1 |
| 3245.2695056 | 1 |
| 3245.2704372 | 2 |
| 3245.2706467 | 1 |
| 3245.2712444 | 0 |
| 3245.2723661 | 5 |
| 3245.272939 | 2 |
| 3245.2733488 | 1 |
| 3245.2735831 | 3 |
| 3245.2740271 | 0 |
| 3245.2750512 | 2 |
| 3245.2750989 | 1 |
| 3245.2754078 | 4 |
| 3245.2762651 | 2 |
| 3245.2769496 | 1 |
| 3245.2772532 | 2 |
| 3245.2783153 | 3 |
| 3245.2790373 | 3 |
| 3245.2796169 | 2 |
| 3245.2804166 | 2 |
| 3245.280573 | 4 |
| 3245.2810705 | 7 |
| 3245.281612 | 0 |
| 3245.2825773 | 1 |
| 3245.2828103 | 3 |
| 3245.2829166 | 1 |
| 3245.2839265 | 1 |
| 3245.2840473 | 1 |
| 3245.2850251 | 3 |
| 3245.2858567 | 2 |
| 3245.2865702 | 0 |
| 3245.2872526 | 4 |
| 3245.287381 | 3 |
| 3245.288118 | 2 |
| 3245.2885272 | 2 |
| 3245.2891576 | 4 |
| 3245.2897342 | 3 |
| 3245.2908426 | 4 |
| 3245.2910537 | 0 |
| 3245.2914961 | 1 |
| 3245.2920607 | 1 |
| 3245.2924402 | 2 |
| 3245.2935104 | 5 |
| 3245.2940454 | 2 |
| 3245.2946723 | 1 |
| 3245.2956796 | 1 |
| 3245.2958986 | 2 |
| 3245.2965509 | 1 |
| 3245.296995 | 1 |
| 3245.2972011 | 2 |
| 3245.2986624 | 2 |
| 3245.2988812 | 2 |
| 3245.2990807 | 4 |
| 3245.2994907 | 2 |
| 3245.3003329 | 4 |
| 3245.3009816 | 1 |
| 3245.3012474 | 5 |
| 3245.3022087 | 3 |
| 3245.3029825 | 3 |
| 3245.3029925 | 3 |
| 3245.3039768 | 2 |
| 3245.3048009 | 2 |
| 3245.3048688 | 1 |
| 3245.3052774 | 0 |
| 3245.3069183 | 3 |
| 3245.3069282 | 3 |
| 3245.3069536 | 3 |
| 3245.3080143 | 1 |
| 3245.3081611 | 2 |
| 3245.3092919 | 3 |
| 3245.3095152 | 2 |
| 3245.3106756 | 1 |
| 3245.3109299 | 3 |
| 3245.3110608 | 1 |
| 3245.3117053 | 0 |
| 3245.3121225 | 1 |
| 3245.3126899 | 4 |
| 3245.3138704 | 1 |
| 3245.3141428 | 3 |
| 3245.3145081 | 2 |
| 3245.314612 | 3 |
| 3245.3159189 | 1 |
| 3245.3170257 | 5 |
| 3245.3172645 | 4 |
| 3245.3173388 | 1 |
| 3245.3176497 | 2 |
| 3245.3189221 | 1 |
| 3245.3190836 | 3 |
| 3245.3192536 | 2 |
| 3245.3199405 | 0 |
| 3245.3214555 | 3 |
| 3245.3215247 | 3 |
| 3245.3218164 | 3 |
| 3245.3230025 | 2 |
| 3245.3233415 | 1 |
| 3245.3240281 | 3 |
| 3245.3240536 | 2 |
| 3245.3246864 | 2 |
| 3245.3254917 | 4 |
| 3245.3257779 | 2 |
| 3245.3266663 | 2 |
| 3245.3271587 | 4 |
| 3245.3276902 | 2 |
| 3245.3286458 | 0 |
| 3245.3290978 | 2 |
| 3245.329244 | 4 |
| 3245.3299895 | 3 |
| 3245.3308109 | 4 |
| 3245.3315882 | 5 |
| 3245.3319144 | 4 |
| 3245.3326128 | 2 |
| 3245.3333271 | 0 |
| 3245.3336153 | 2 |
| 3245.3337853 | 3 |
| 3245.3348712 | 4 |
| 3245.3353736 | 2 |
| 3245.3358862 | 3 |
| 3245.3374179 | 1 |
| 3245.3374853 | 1 |
| 3245.3377221 | 0 |
| 3245.3387513 | 3 |
| 3245.3390736 | 1 |
| 3245.3401495 | 5 |
| 3245.3402036 | 5 |
| 3245.3406738 | 4 |
| 3245.3411085 | 3 |
| 3245.3418568 | 3 |
| 3245.3431098 | 4 |
| 3245.3433046 | 2 |
| 3245.3435408 | 3 |
| 3245.3437176 | 4 |
| 3245.3446302 | 0 |
| 3245.3450314 | 2 |
| 3245.345642 | 2 |
| 3245.3461802 | 3 |
| 3245.3471596 | 6 |
| 3245.3478752 | 3 |
| 3245.3482503 | 1 |
| 3245.3487871 | 3 |
| 3245.349241 | 1 |
| 3245.3498191 | 3 |
| 3245.3499052 | 1 |
| 3245.3512801 | 2 |
| 3245.3518969 | 3 |
| 3245.3522733 | 4 |
| 3245.3523776 | 5 |
| 3245.3538476 | 7 |
| 3245.3538651 | 0 |
| 3245.3544599 | 2 |
| 3245.3550442 | 1 |
| 3245.355632 | 0 |
| 3245.3557284 | 2 |
| 3245.3566268 | 3 |
| 3245.3568539 | 1 |
| 3245.3582482 | 0 |
| 3245.3586956 | 1 |
| 3245.3587838 | 7 |
| 3245.3593354 | 2 |
| 3245.3598097 | 4 |
| 3245.3604611 | 2 |
| 3245.3611301 | 2 |
| 3245.3620067 | 3 |
| 3245.362509 | 2 |
| 3245.3632997 | 3 |
| 3245.3637696 | 0 |
| 3245.3642893 | 1 |
| 3245.3649085 | 2 |
| 3245.3652347 | 1 |

| | |
|---|---|
| 3245.3659548 | 2 |
| 3245.3662269 | 1 |
| 3245.3666908 | 2 |
| 3245.367525 | 1 |
| 3245.3676122 | 3 |
| 3245.3687133 | 1 |
| 3245.3694689 | 3 |
| 3245.3700948 | 3 |
| 3245.3708394 | 2 |
| 3245.3712669 | 2 |
| 3245.3718267 | 3 |
| 3245.372302 | 0 |
| 3245.372793 | 2 |
| 3245.3738739 | 0 |
| 3245.3739044 | 5 |
| 3245.3743346 | 2 |
| 3245.3750647 | 1 |
| 3245.3750653 | 5 |
| 3245.3770235 | 0 |
| 3245.3770595 | 2 |
| 3245.3776361 | 2 |
| 3245.3782301 | 3 |
| 3245.3782622 | 2 |
| 3245.3790404 | 2 |
| 3245.379846 | 2 |
| 3245.3799394 | 3 |
| 3245.3813676 | 3 |
| 3245.3819774 | 2 |
| 3245.3820234 | 3 |
| 3245.3827636 | 2 |
| 3245.3832498 | 3 |
| 3245.3839375 | 1 |
| 3245.3850688 | 2 |
| 3245.3852635 | 2 |
| 3245.3853066 | 2 |
| 3245.3867117 | 2 |
| 3245.3867267 | 0 |
| 3245.3877611 | 1 |
| 3245.3879919 | 2 |
| 3245.388441 | 0 |
| 3245.3894578 | 2 |
| 3245.3896718 | 3 |
| 3245.3900861 | 2 |
| 3245.3904575 | 2 |
| 3245.3913834 | 1 |
| 3245.3919442 | 0 |
| 3245.3920314 | 0 |
| 3245.3928258 | 2 |
| 3245.3938393 | 2 |
| 3245.3941772 | 0 |
| 3245.3956377 | 0 |
| 3245.3956809 | 1 |
| 3245.3958338 | 2 |
| 3245.3968789 | 3 |
| 3245.3974936 | 1 |
| 3245.3977416 | 1 |
| 3245.3983061 | 0 |
| 3245.3994776 | 7 |
| 3245.399694 | 4 |
| 3245.4000115 | 3 |
| 3245.4002032 | 4 |
| 3245.4016434 | 5 |
| 3245.4018224 | 6 |
| 3245.4019291 | 2 |
| 3245.402734 | 3 |
| 3245.4030843 | 8 |
| 3245.4043603 | 6 |
| 3245.4049267 | 6 |
| 3245.4055547 | 8 |
| 3245.4058178 | 2 |
| 3245.4061992 | 1 |
| 3245.4065394 | 5 |
| 3245.4081719 | 5 |
| 3245.4083665 | 3 |
| 3245.4087053 | 6 |
| 3245.4088246 | 4 |
| 3245.4092636 | 3 |
| 3245.4106914 | 1 |
| 3245.4109602 | 2 |
| 3245.4114972 | 4 |
| 3245.4119357 | 1 |
| 3245.4126493 | 2 |
| 3245.4133057 | 2 |
| 3245.41374 | 2 |
| 3245.4146802 | 1 |
| 3245.4152469 | 2 |
| 3245.4156501 | 1 |
| 3245.4159725 | 1 |
| 3245.4167994 | 1 |
| 3245.4173646 | 1 |
| 3245.4176471 | 3 |
| 3245.4178728 | 5 |
| 3245.4182576 | 2 |
| 3245.4197785 | 1 |
| 3245.4199328 | 2 |
| 3245.4206913 | 2 |
| 3245.4216518 | 5 |
| 3245.422028 | 2 |
| 3245.4222209 | 1 |
| 3245.4233988 | 3 |
| 3245.4234258 | 4 |
| 3245.4244227 | 4 |
| 3245.4245477 | 3 |
| 3245.4252573 | 1 |
| 3245.4264792 | 1 |
| 3245.4265659 | 3 |
| 3245.4274084 | 0 |
| 3245.4282854 | 1 |
| 3245.4284239 | 1 |
| 3245.429002 | 0 |
| 3245.4291612 | 1 |
| 3245.4297607 | 5 |
| 3245.4310884 | 6 |
| 3245.431356 | 1 |
| 3245.4317729 | 2 |
| 3245.4324097 | 0 |
| 3245.4328451 | 3 |
| 3245.4340294 | 4 |
| 3245.4345415 | 5 |
| 3245.4347512 | 2 |
| 3245.4359204 | 2 |
| 3245.4363721 | 1 |
| 3245.4363775 | 2 |
| 3245.4368194 | 1 |
| 3245.4379388 | 1 |
| 3245.4385192 | 1 |
| 3245.4391385 | 2 |
| 3245.4397172 | 1 |
| 3245.4397355 | 2 |
| 3245.4408288 | 2 |
| 3245.4413798 | 4 |
| 3245.4422872 | 1 |
| 3245.4423625 | 3 |
| 3245.4428397 | 3 |
| 3245.4437829 | 3 |
| 3245.4440821 | 3 |
| 3245.4445839 | 3 |
| 3245.4449864 | 1 |
| 3245.4460843 | 7 |
| 3245.4466551 | 2 |
| 3245.4471603 | 0 |
| 3245.4473992 | 3 |
| 3245.4476332 | 1 |
| 3245.4493522 | 4 |
| 3245.4494722 | 3 |
| 3245.4497593 | 4 |
| 3245.4502158 | 2 |
| 3245.4512237 | 3 |
| 3245.4515591 | 6 |
| 3245.4518081 | 3 |
| 3245.4519265 | 2 |
| 3245.4531185 | 2 |
| 3245.4540589 | 1 |
| 3245.4543974 | 3 |
| 3245.4551115 | 4 |
| 3245.4555517 | 2 |
| 3245.4556996 | 0 |
| 3245.456603 | 0 |
| 3245.4576174 | 2 |
| 3245.458007 | 3 |
| 3245.4589863 | 3 |
| 3245.4591315 | 5 |
| 3245.4592638 | 0 |
| 3245.4601284 | 2 |
| 3245.4605923 | 4 |
| 3245.4613689 | 5 |
| 3245.4619756 | 3 |
| 3245.4624424 | 6 |
| 3245.4632224 | 2 |
| 3245.4635763 | 1 |
| 3245.4640088 | 2 |
| 3245.4644901 | 3 |
| 3245.4655644 | 0 |
| 3245.4658068 | 2 |
| 3245.4667909 | 7 |
| 3245.4672477 | 2 |
| 3245.4675011 | 2 |
| 3245.4681171 | 3 |
| 3245.4686922 | 7 |
| 3245.4697368 | 1 |
| 3245.4702639 | 3 |
| 3245.4704279 | 2 |
| 3245.4704521 | 2 |
| 3245.4717802 | 1 |
| 3245.4723461 | 2 |
| 3245.4727733 | 2 |
| 3245.4736707 | 3 |
| 3245.4747036 | 4 |
| 3245.4749848 | 1 |
| 3245.4750199 | 3 |
| 3245.4759045 | 1 |
| 3245.4762451 | 3 |
| 3245.4778539 | 0 |
| 3245.4780461 | 2 |
| 3245.4783875 | 2 |
| 3245.4788021 | 3 |
| 3245.4791436 | 3 |
| 3245.4804828 | 3 |
| 3245.4805485 | 3 |
| 3245.4815454 | 2 |
| 3245.4817086 | 2 |
| 3245.4826399 | 3 |
| 3245.4829045 | 0 |
| 3245.4835558 | 1 |
| 3245.4844824 | 2 |
| 3245.4845056 | 3 |
| 3245.4858055 | 2 |
| 3245.4864407 | 2 |
| 3245.4866826 | 1 |
| 3245.4870229 | 0 |
| 3245.4875003 | 1 |
| 3245.4886218 | 4 |
| 3245.4887654 | 3 |
| 3245.4899211 | 5 |
| 3245.4899617 | 1 |
| 3245.4906064 | 2 |
| 3245.4912248 | 2 |
| 3245.4918785 | 2 |
| 3245.4921722 | 1 |
| 3245.4933313 | 3 |
| 3245.4934919 | 2 |
| 3245.4942472 | 0 |
| 3245.4943012 | 2 |
| 3245.4948504 | 2 |
| 3245.496193 | 2 |
| 3245.4963216 | 1 |
| 3245.4967073 | 2 |
| 3245.4971457 | 4 |
| 3245.4981789 | 1 |
| 3245.4982095 | 2 |
| 3245.4983679 | 3 |
| 3245.4993531 | 1 |
| 3245.500875 | 3 |
| 3245.5019934 | 2 |
| 3245.5038442 | 4 |
| 3245.505164 | 4 |
| 3245.5072218 | 2 |
| 3245.5087146 | 1 |
| 3245.5103322 | 2 |
| 3245.5115322 | 3 |
| 3245.5124592 | 4 |
| 3245.514869 | 2 |
| 3245.5161803 | 3 |
| 3245.5181914 | 0 |
| 3245.5200194 | 3 |
| 3245.5213329 | 2 |
| 3245.5230268 | 3 |
| 3245.5245484 | 1 |
| 3245.5266226 | 3 |
| 3245.5282487 | 1 |
| 3245.5288993 | 2 |
| 3245.5312383 | 1 |
| 3245.5314144 | 3 |
| 3245.5335112 | 1 |
| 3245.5353861 | 5 |
| 3245.5363519 | 2 |
| 3245.538593 | 4 |
| 3245.5394488 | 3 |
| 3245.5411192 | 1 |
| 3245.5434627 | 1 |
| 3245.5439417 | 1 |
| 3245.5462461 | 2 |
| 3245.5469669 | 1 |
| 3245.5492307 | 3 |
| 3245.5507773 | 2 |
| 3245.5519039 | 1 |
| 3245.5540368 | 3 |
| 3245.5550549 | 1 |
| 3245.5563973 | 3 |
| 3245.5588535 | 4 |
| 3245.5597107 | 2 |
| 3245.5617644 | 3 |
| 3245.5629328 | 4 |
| 3245.5645549 | 3 |
| 3245.5669773 | 3 |
| 3245.5683089 | 3 |
| 3245.5696132 | 3 |
| 3245.5709003 | 1 |
| 3245.5730534 | 1 |
| 3245.5739153 | 0 |
| 3245.5756826 | 2 |
| 3245.5775918 | 3 |
| 3245.5785349 | 2 |
| 3245.5803751 | 4 |
| 3245.5821084 | 1 |
| 3245.582758 | 2 |
| 3245.5850469 | 3 |
| 3245.5861739 | 2 |
| 3245.588108 | 1 |
| 3245.5902779 | 3 |
| 3245.5913212 | 1 |
| 3245.5925807 | 2 |
| 3245.5938813 | 3 |
| 3245.5955558 | 4 |
| 3245.5972429 | 1 |
| 3245.5989086 | 2 |
| 3245.6009504 | 0 |
| 3245.6017492 | 1 |
| 3245.6042361 | 1 |
| 3245.6050854 | 1 |
| 3245.6064199 | 3 |
| 3245.6088333 | 2 |
| 3245.6095039 | 1 |
| 3245.6117351 | 3 |
| 3245.6124788 | 3 |
| 3245.6148286 | 6 |
| 3245.6159029 | 2 |
| 3245.6178197 | 2 |
| 3245.619482 | 1 |
| 3245.6210616 | 1 |
| 3245.6225776 | 4 |
| 3245.6244141 | 0 |
| 3245.625209 | 1 |
| 3245.6277496 | 1 |
| 3245.6280639 | 1 |
| 3245.6299612 | 3 |
| 3245.632273 | 4 |
| 3245.6337913 | 6 |
| 3245.6356575 | 2 |
| 3245.6365017 | 3 |
| 3245.6379443 | 1 |
| 3245.6389605 | 3 |
| 3245.6409312 | 3 |
| 3245.6434856 | 1 |
| 3245.6435209 | 2 |
| 3245.6462957 | 3 |
| 3245.6477824 | 1 |
| 3245.6483759 | 4 |
| 3245.6507449 | 0 |
| 3245.651509 | 3 |
| 3245.6537422 | 0 |
| 3245.654557 | 3 |
| 3245.6579946 | 2 |
| 3245.658646 | 3 |
| 3245.6596236 | 3 |
| 3245.6618755 | 4 |
| 3245.6625313 | 2 |
| 3245.6648842 | 2 |
| 3245.6666943 | 2 |
| 3245.6674584 | 1 |
| 3245.6697949 | 3 |
| 3245.6707915 | 2 |
| 3245.6728001 | 1 |
| 3245.6739839 | 0 |
| 3245.6757898 | 3 |
| 3245.6780947 | 0 |
| 3245.6784221 | 2 |
| 3245.6803815 | 2 |
| 3245.6812895 | 4 |
| 3245.6829134 | 4 |
| 3245.6851925 | 2 |
| 3245.6857141 | 2 |
| 3245.6877536 | 0 |
| 3245.6898283 | 4 |
| 3245.6910066 | 1 |
| 3245.6932584 | 4 |
| 3245.6935752 | 0 |
| 3245.6959994 | 2 |
| 3245.696834 | 1 |
| 3245.6990316 | 3 |
| 3245.7004583 | 1 |
| 3245.7020581 | 1 |
| 3245.7033866 | 2 |

| | |
|---|---|
| 3245.7042235 | 2 |
| 3245.7066291 | 3 |
| 3245.7085802 | 3 |
| 3245.70964 | 3 |
| 3245.7113783 | 1 |
| 3245.712494 | 1 |
| 3245.7142231 | 2 |
| 3245.7158231 | 4 |
| 3245.7172549 | 3 |
| 3245.719361 | 2 |
| 3245.7203817 | 5 |
| 3245.7222382 | 2 |
| 3245.7238705 | 1 |
| 3245.725296 | 4 |
| 3245.7270062 | 1 |
| 3245.7275922 | 2 |
| 3245.7295342 | 2 |
| 3245.7317731 | 2 |
| 3245.7327088 | 4 |
| 3245.7347596 | 3 |
| 3245.7357816 | 3 |
| 3245.7375542 | 1 |
| 3245.7388303 | 1 |
| 3245.7404965 | 4 |
| 3245.7423537 | 5 |
| 3245.7437194 | 1 |
| 3245.745829 | 0 |
| 3245.7465748 | 3 |
| 3245.7492271 | 0 |
| 3245.7508269 | 7 |
| 3245.7509023 | 3 |
| 3245.7535803 | 5 |
| 3245.7546308 | 3 |
| 3245.7561262 | 2 |
| 3245.7584944 | 1 |
| 3245.7597106 | 2 |
| 3245.761211 | 5 |
| 3245.7620903 | 2 |
| 3245.764251 | 1 |
| 3245.7660369 | 1 |
| 3245.7666214 | 1 |
| 3245.7686528 | 6 |
| 3245.7705139 | 2 |
| 3245.771415 | 1 |
| 3245.7735716 | 3 |
| 3245.7742492 | 0 |
| 3245.7758851 | 3 |
| 3245.7778364 | 0 |
| 3245.7791735 | 1 |
| 3245.7805736 | 1 |
| 3245.7825029 | 0 |
| 3245.7838033 | 2 |
| 3245.7858266 | 2 |
| 3245.7870882 | 1 |
| 3245.7886095 | 4 |
| 3245.7905867 | 2 |
| 3245.7915373 | 3 |
| 3245.7937508 | 2 |
| 3245.794042 | 3 |
| 3245.7964441 | 3 |
| 3245.7973395 | 3 |
| 3245.7996793 | 3 |
| 3245.8018876 | 0 |
| 3245.8020325 | 1 |
| 3245.8047939 | 3 |
| 3245.8055862 | 3 |
| 3245.8075776 | 5 |
| 3245.8090436 | 1 |
| 3245.8106306 | 3 |
| 3245.81188 | 3 |
| 3245.8128324 | 5 |
| 3245.8149317 | 2 |
| 3245.8157381 | 1 |
| 3245.8175113 | 2 |
| 3245.8194853 | 1 |
| 3245.8203131 | 1 |
| 3245.8227485 | 1 |
| 3245.8239242 | 2 |
| 3245.8255442 | 0 |
| 3245.8275341 | 3 |
| 3245.8280216 | 0 |
| 3245.8305807 | 1 |
| 3245.8309283 | 0 |
| 3245.8325915 | 1 |
| 3245.835183 | 0 |
| 3245.8361326 | 3 |
| 3245.838326 | 3 |
| 3245.840255 | 1 |
| 3245.8410479 | 5 |
| 3245.8429459 | 1 |
| 3245.8441248 | 1 |
| 3245.846185 | 4 |
| 3245.8468316 | 4 |
| 3245.84856 | 4 |
| 3245.8509902 | 1 |
| 3245.8517954 | 2 |
| 3245.8535454 | 2 |
| 3245.8548617 | 2 |
| 3245.856773 | 4 |
| 3245.8585554 | 0 |
| 3245.8594539 | 2 |
| 3245.8612461 | 1 |
| 3245.8624087 | 1 |
| 3245.863614 | 2 |
| 3245.8654787 | 3 |
| 3245.867041 | 0 |
| 3245.8688324 | 1 |
| 3245.8698839 | 2 |
| 3245.8718355 | 0 |
| 3245.8738118 | 1 |
| 3245.8746421 | 2 |
| 3245.876852 | 0 |
| 3245.8776371 | 1 |
| 3245.8797241 | 1 |
| 3245.8805196 | 2 |
| 3245.882464 | 1 |
| 3245.8843914 | 1 |
| 3245.8855491 | 3 |
| 3245.8871787 | 1 |
| 3245.8887192 | 1 |
| 3245.890516 | 2 |
| 3245.8924013 | 1 |
| 3245.8930246 | 3 |
| 3245.8947251 | 2 |
| 3245.8962283 | 4 |
| 3245.8982258 | 0 |
| 3245.9001153 | 1 |
| 3245.9013944 | 1 |
| 3245.9028118 | 1 |
| 3245.9035429 | 0 |
| 3245.9053522 | 3 |
| 3245.9075836 | 2 |
| 3245.9081603 | 0 |
| 3245.9107638 | 0 |
| 3245.9112455 | 1 |
| 3245.9129645 | 0 |
| 3245.9147968 | 2 |
| 3245.916292 | 4 |
| 3245.9184705 | 3 |
| 3245.9194399 | 1 |
| 3245.9211574 | 3 |
| 3245.9227429 | 1 |
| 3245.9237613 | 2 |
| 3245.9253781 | 3 |
| 3245.9277789 | 2 |
| 3245.9284978 | 1 |
| 3245.9307023 | 0 |
| 3245.931153 | 4 |
| 3245.9334389 | 1 |
| 3245.9354779 | 2 |
| 3245.9363062 | 1 |
| 3245.9386454 | 0 |
| 3245.9395871 | 7 |
| 3245.9414922 | 3 |
| 3245.9422391 | 3 |
| 3245.9445933 | 3 |
| 3245.9461518 | 4 |
| 3245.9468638 | 5 |
| 3245.9494781 | 3 |
| 3245.9496817 | 1 |
| 3245.9521519 | 1 |
| 3245.9537483 | 2 |
| 3245.9546892 | 2 |
| 3245.9562814 | 3 |
| 3245.9578628 | 3 |
| 3245.9595684 | 4 |
| 3245.961367 | 6 |
| 3245.961607 | 2 |
| 3245.9644318 | 0 |
| 3245.965268 | 2 |
| 3245.9670568 | 3 |
| 3245.9691198 | 2 |
| 3245.9698807 | 3 |
| 3245.9720402 | 3 |
| 3245.9730103 | 3 |
| 3245.9747886 | 1 |
| 3245.9756219 | 0 |
| 3245.9779768 | 2 |
| 3245.9798468 | 2 |
| 3245.979876 | 0 |
| 3245.982537 | 1 |
| 3245.9842129 | 2 |
| 3245.9854974 | 5 |
| 3245.9867854 | 3 |
| 3245.9884975 | 1 |
| 3245.9906441 | 1 |
| 3245.9924346 | 0 |
| 3245.9931093 | 2 |
| 3245.9953063 | 3 |
| 3245.9960375 | 0 |
| 3245.9979048 | 2 |
| 3246.0000467 | 1 |
| 3246.0000996 | 3 |
| 3246.0010777 | 0 |
| 3246.0017692 | 2 |
| 3246.0030845 | 2 |
| 3246.0031651 | 0 |
| 3246.0037273 | 1 |
| 3246.0050229 | 1 |
| 3246.0051378 | 1 |
| 3246.0056935 | 1 |
| 3246.0065957 | 1 |
| 3246.0077711 | 1 |
| 3246.0088157 | 1 |
| 3246.0097683 | 4 |
| 3246.0104784 | 2 |
| 3246.0107053 | 2 |
| 3246.0109601 | 2 |
| 3246.0121197 | 1 |
| 3246.0136516 | 0 |
| 3246.0143075 | 1 |
| 3246.0143497 | 4 |
| 3246.0150388 | 1 |
| 3246.0166238 | 2 |
| 3246.0172246 | 0 |
| 3246.0182339 | 1 |
| 3246.0183484 | 2 |
| 3246.0193937 | 2 |
| 3246.0198789 | 0 |
| 3246.0216762 | 4 |
| 3246.0217457 | 1 |
| 3246.0233852 | 4 |
| 3246.0237231 | 1 |
| 3246.0238537 | 2 |
| 3246.0253326 | 0 |
| 3246.0260259 | 1 |
| 3246.0264686 | 0 |
| 3246.0268673 | 0 |
| 3246.0282496 | 2 |
| 3246.0292819 | 1 |
| 3246.0294245 | 1 |
| 3246.0295127 | 3 |
| 3246.031399 | 1 |
| 3246.0316189 | 4 |
| 3246.0331709 | 2 |
| 3246.0336652 | 2 |
| 3246.0341216 | 6 |
| 3246.0346516 | 9 |
| 3246.0367812 | 7 |
| 3246.0369227 | 8 |
| 3246.0369513 | 3 |
| 3246.0377058 | 8 |
| 3246.0388697 | 8 |
| 3246.0391486 | 5 |
| 3246.0402479 | 2 |
| 3246.041329 | 2 |
| 3246.0421687 | 2 |
| 3246.0422443 | 1 |
| 3246.0439732 | 4 |
| 3246.044395 | 2 |
| 3246.0447987 | 4 |
| 3246.0450155 | 2 |
| 3246.0466851 | 2 |
| 3246.0470208 | 0 |
| 3246.0480866 | 2 |
| 3246.0484699 | 2 |
| 3246.0493111 | 2 |
| 3246.0498073 | 4 |
| 3246.0515938 | 1 |
| 3246.0520933 | 4 |
| 3246.0525987 | 2 |
| 3246.0533822 | 2 |
| 3246.0544844 | 2 |
| 3246.0555215 | 1 |
| 3246.0566247 | 4 |
| 3246.0566367 | 1 |
| 3246.0572867 | 0 |
| 3246.0580109 | 4 |
| 3246.0594775 | 2 |
| 3246.059907 | 1 |
| 3246.0604736 | 0 |
| 3246.0608288 | 5 |
| 3246.0617389 | 0 |
| 3246.0627108 | 3 |
| 3246.0636532 | 5 |
| 3246.0647325 | 6 |
| 3246.0654203 | 3 |
| 3246.0656079 | 5 |
| 3246.0675548 | 3 |
| 3246.0678721 | 3 |
| 3246.0684153 | 1 |
| 3246.068746 | 2 |
| 3246.0701639 | 2 |
| 3246.0705589 | 1 |
| 3246.0709243 | 2 |
| 3246.0724904 | 3 |
| 3246.073202 | 3 |
| 3246.0734084 | 3 |
| 3246.075688 | 0 |
| 3246.0757658 | 2 |
| 3246.0766124 | 1 |
| 3246.076628 | 3 |
| 3246.0778888 | 2 |
| 3246.0782404 | 1 |
| 3246.0799482 | 3 |
| 3246.0803707 | 1 |
| 3246.0809147 | 0 |
| 3246.0819626 | 3 |
| 3246.0821674 | 2 |
| 3246.0835841 | 0 |
| 3246.0845362 | 1 |
| 3246.0848371 | 2 |
| 3246.0855076 | 3 |
| 3246.0864525 | 1 |
| 3246.0869083 | 1 |
| 3246.0875359 | 5 |
| 3246.08851 | 2 |
| 3246.0896069 | 4 |
| 3246.0905575 | 3 |
| 3246.0905635 | 4 |
| 3246.092061 | 1 |
| 3246.0924168 | 1 |
| 3246.0933386 | 1 |
| 3246.0938255 | 5 |
| 3246.0952639 | 0 |
| 3246.0960379 | 0 |
| 3246.096663 | 1 |
| 3246.0976245 | 1 |
| 3246.0982674 | 3 |
| 3246.0984232 | 0 |
| 3246.0994012 | 4 |
| 3246.0996981 | 5 |
| 3246.1014988 | 2 |
| 3246.1020743 | 4 |
| 3246.10229 | 3 |
| 3246.1033617 | 4 |
| 3246.1044136 | 2 |
| 3246.1045512 | 4 |
| 3246.1046811 | 1 |
| 3246.1054427 | 5 |
| 3246.1055064 | 1 |
| 3246.1059175 | 4 |
| 3246.1061797 | 6 |
| 3246.106187 | 3 |
| 3246.1064821 | 3 |
| 3246.1070362 | 1 |
| 3246.107627 | 4 |
| 3246.1080285 | 0 |
| 3246.1084725 | 3 |
| 3246.1088112 | 3 |
| 3246.1094198 | 1 |
| 3246.1094308 | 5 |
| 3246.1096109 | 4 |
| 3246.109967 | 1 |
| 3246.1106292 | 1 |
| 3246.1106367 | 0 |
| 3246.1113106 | 3 |
| 3246.1113233 | 4 |
| 3246.11161 | 3 |
| 3246.1122172 | 1 |
| 3246.1127563 | 5 |
| 3246.1129522 | 4 |
| 3246.1129894 | 1 |
| 3246.1130756 | 3 |
| 3246.1145504 | 5 |
| 3246.1145582 | 4 |
| 3246.1147702 | 5 |
| 3246.1150421 | 2 |
| 3246.1152155 | 10 |
| 3246.1155033 | 7 |
| 3246.1164125 | 10 |
| 3246.1168692 | 10 |
| 3246.1169481 | 10 |

| | |
|---|---|
| 3246.1173194 | 7 |
| 3246.1179982 | 7 |
| 3246.1181802 | 6 |
| 3246.1182091 | 5 |
| 3246.1186332 | 3 |
| 3246.11868 | 6 |
| 3246.1192658 | 6 |
| 3246.1197568 | 3 |
| 3246.1200642 | 1 |
| 3246.1204617 | 2 |
| 3246.1206845 | 1 |
| 3246.120944 | 7 |
| 3246.1209731 | 0 |
| 3246.1217032 | 2 |
| 3246.1220128 | 3 |
| 3246.1221636 | 1 |
| 3246.1227323 | 8 |
| 3246.1229971 | 1 |
| 3246.1234315 | 1 |
| 3246.1234775 | 0 |
| 3246.1241638 | 1 |
| 3246.1250075 | 2 |
| 3246.1250079 | 3 |
| 3246.1250759 | 6 |
| 3246.125306 | 1 |
| 3246.1255048 | 2 |
| 3246.1258561 | 1 |
| 3246.1266968 | 1 |
| 3246.1270809 | 2 |
| 3246.1273037 | 3 |
| 3246.128024 | 1 |
| 3246.1280844 | 0 |
| 3246.1281532 | 2 |
| 3246.1290513 | 1 |
| 3246.129259 | 3 |
| 3246.1292912 | 1 |
| 3246.1296211 | 2 |
| 3246.1300059 | 0 |
| 3246.1301785 | 4 |
| 3246.1306473 | 3 |
| 3246.1313298 | 2 |
| 3246.1313349 | 2 |
| 3246.1317782 | 0 |
| 3246.1318721 | 2 |
| 3246.132605 | 3 |
| 3246.1326736 | 3 |
| 3246.1326903 | 1 |
| 3246.1328431 | 0 |
| 3246.1333045 | 5 |
| 3246.1342164 | 4 |
| 3246.1347323 | 0 |
| 3246.1347527 | 1 |
| 3246.1348107 | 4 |
| 3246.1354134 | 2 |
| 3246.1358079 | 2 |
| 3246.1358337 | 4 |
| 3246.1364578 | 4 |
| 3246.13689 | 0 |
| 3246.1374549 | 1 |
| 3246.1377952 | 2 |
| 3246.1384483 | 2 |
| 3246.1385754 | 0 |
| 3246.1386346 | 1 |
| 3246.1386839 | 2 |
| 3246.1397956 | 2 |
| 3246.1399304 | 10 |
| 3246.140055 | 2 |
| 3246.1401091 | 3 |
| 3246.140776 | 0 |
| 3246.1409545 | 2 |
| 3246.1409988 | 1 |
| 3246.1418545 | 3 |
| 3246.1423271 | 0 |
| 3246.1424438 | 3 |
| 3246.1425877 | 1 |
| 3246.1428727 | 6 |
| 3246.1433619 | 4 |
| 3246.143578 | 2 |
| 3246.1440774 | 1 |
| 3246.1441253 | 2 |
| 3246.1449046 | 1 |
| 3246.1450181 | 1 |
| 3246.1457001 | 0 |
| 3246.1457373 | 1 |
| 3246.1459489 | 1 |
| 3246.1467629 | 6 |
| 3246.1470097 | 2 |
| 3246.1472594 | 1 |
| 3246.1477596 | 3 |
| 3246.1478245 | 6 |
| 3246.1480972 | 1 |
| 3246.1485941 | 2 |
| 3246.1486744 | 4 |
| 3246.1487688 | 3 |
| 3246.1492145 | 3 |
| 3246.1495261 | 1 |
| 3246.1507564 | 1 |
| 3246.1507586 | 0 |
| 3246.150865 | 1 |
| 3246.1511859 | 3 |
| 3246.1512112 | 1 |
| 3246.1514346 | 3 |
| 3246.1528997 | 0 |
| 3246.1532524 | 4 |
| 3246.1536403 | 4 |
| 3246.1542659 | 0 |
| 3246.1547873 | 1 |
| 3246.1549088 | 1 |
| 3246.1563726 | 3 |
| 3246.1575466 | 2 |
| 3246.15861 | 2 |
| 3246.1598915 | 1 |
| 3246.1616254 | 5 |
| 3246.1622229 | 1 |
| 3246.1630184 | 0 |
| 3246.1643165 | 1 |
| 3246.1665898 | 4 |
| 3246.1676441 | 4 |
| 3246.168997 | 3 |
| 3246.170353 | 5 |
| 3246.1712926 | 0 |
| 3246.1729502 | 1 |
| 3246.1744782 | 1 |
| 3246.1747093 | 2 |
| 3246.1766602 | 2 |
| 3246.176737 | 3 |
| 3246.1792742 | 5 |
| 3246.1803243 | 3 |
| 3246.1811802 | 3 |
| 3246.1823883 | 3 |
| 3246.1830495 | 2 |
| 3246.184565 | 2 |
| 3246.1853807 | 3 |
| 3246.1867014 | 3 |
| 3246.188661 | 2 |
| 3246.1891178 | 2 |
| 3246.1906794 | 4 |
| 3246.1923824 | 2 |
| 3246.192761 | 0 |
| 3246.194619 | 1 |
| 3246.1962454 | 1 |
| 3246.1974687 | 2 |
| 3246.1983737 | 0 |
| 3246.1994387 | 4 |
| 3246.201415 | 1 |
| 3246.2020303 | 1 |
| 3246.2032597 | 2 |
| 3246.2039207 | 1 |
| 3246.205989 | 2 |
| 3246.2073398 | 1 |
| 3246.2076224 | 3 |
| 3246.2100065 | 1 |
| 3246.2112545 | 2 |
| 3246.211995 | 0 |
| 3246.2139154 | 2 |
| 3246.2139668 | 2 |
| 3246.2167319 | 4 |
| 3246.2167841 | 6 |
| 3246.2187882 | 3 |
| 3246.2206559 | 4 |
| 3246.2212233 | 2 |
| 3246.2224959 | 5 |
| 3246.2232984 | 3 |
| 3246.2242667 | 0 |
| 3246.2260905 | 0 |
| 3246.2267841 | 5 |
| 3246.2286356 | 1 |
| 3246.2292678 | 1 |
| 3246.2305291 | 1 |
| 3246.2319642 | 2 |
| 3246.2332007 | 2 |
| 3246.2340149 | 2 |
| 3246.235226 | 2 |
| 3246.2372329 | 2 |
| 3246.2385792 | 4 |
| 3246.2393488 | 4 |
| 3246.240966 | 3 |
| 3246.2424149 | 3 |
| 3246.2424286 | 1 |
| 3246.2444011 | 0 |
| 3246.245289 | 2 |
| 3246.2468101 | 0 |
| 3246.2476861 | 1 |
| 3246.2495001 | 2 |
| 3246.2513106 | 5 |
| 3246.251445 | 0 |
| 3246.2534392 | 2 |
| 3246.2544587 | 2 |
| 3246.2558544 | 4 |
| 3246.2570827 | 1 |
| 3246.2581643 | 3 |
| 3246.2596276 | 1 |
| 3246.2599545 | 1 |
| 3246.2619612 | 2 |
| 3246.2632465 | 4 |
| 3246.2639925 | 1 |
| 3246.2656553 | 4 |
| 3246.2664548 | 1 |
| 3246.2685984 | 1 |
| 3246.2696785 | 3 |
| 3246.2702766 | 3 |
| 3246.2716791 | 1 |
| 3246.2730405 | 3 |
| 3246.2740073 | 2 |
| 3246.2752015 | 5 |
| 3246.2760673 | 3 |
| 3246.2777229 | 0 |
| 3246.2786365 | 0 |
| 3246.2801617 | 3 |
| 3246.2813451 | 0 |
| 3246.2819403 | 2 |
| 3246.2841414 | 4 |
| 3246.2846951 | 5 |
| 3246.2862589 | 1 |
| 3246.2874158 | 1 |
| 3246.2883146 | 2 |
| 3246.2898106 | 1 |
| 3246.2914382 | 3 |
| 3246.2921874 | 1 |
| 3246.294205 | 3 |
| 3246.2946086 | 3 |
| 3246.2964696 | 0 |
| 3246.2970363 | 0 |
| 3246.2984601 | 2 |
| 3246.3006536 | 4 |
| 3246.301247 | 2 |
| 3246.3028573 | 2 |
| 3246.3043588 | 3 |
| 3246.3050835 | 8 |
| 3246.3062155 | 1 |
| 3246.3068161 | 3 |
| 3246.3092545 | 3 |
| 3246.309569 | 1 |
| 3246.3107592 | 3 |
| 3246.3127463 | 3 |
| 3246.3138168 | 2 |
| 3246.3147011 | 0 |
| 3246.3158197 | 1 |
| 3246.3172774 | 2 |
| 3246.3187589 | 1 |
| 3246.3193056 | 2 |
| 3246.3209474 | 2 |
| 3246.3223414 | 3 |
| 3246.3231201 | 1 |
| 3246.3245301 | 3 |
| 3246.3251686 | 1 |
| 3246.3271081 | 1 |
| 3246.3283389 | 4 |
| 3246.3291049 | 3 |
| 3246.3309451 | 2 |
| 3246.3314785 | 0 |
| 3246.3330035 | 1 |
| 3246.3344286 | 1 |
| 3246.3357954 | 2 |
| 3246.3368452 | 5 |
| 3246.3374294 | 0 |
| 3246.3389718 | 1 |
| 3246.3403819 | 1 |
| 3246.3416175 | 0 |
| 3246.3428975 | 6 |
| 3246.3443612 | 2 |
| 3246.3452492 | 3 |
| 3246.346252 | 5 |
| 3246.3484552 | 0 |
| 3246.3499155 | 3 |
| 3246.3505599 | 2 |
| 3246.3519325 | 0 |
| 3246.3522305 | 2 |
| 3246.3540143 | 3 |
| 3246.3556399 | 2 |
| 3246.3563707 | 4 |
| 3246.3579598 | 4 |
| 3246.3589707 | 4 |
| 3246.3601475 | 3 |
| 3246.3618555 | 2 |
| 3246.3625109 | 1 |
| 3246.3641106 | 4 |
| 3246.3650054 | 1 |
| 3246.3664003 | 2 |
| 3246.3683004 | 0 |
| 3246.3691581 | 3 |
| 3246.3700353 | 1 |
| 3246.3708008 | 3 |
| 3246.372434 | 2 |
| 3246.3736177 | 2 |
| 3246.3746496 | 2 |
| 3246.3758774 | 2 |
| 3246.3771115 | 1 |
| 3246.3788132 | 1 |
| 3246.3803114 | 1 |
| 3246.381055 | 2 |
| 3246.3824166 | 2 |
| 3246.3833608 | 2 |
| 3246.3841861 | 3 |
| 3246.3861684 | 2 |
| 3246.3870617 | 3 |
| 3246.3889965 | 1 |
| 3246.3893554 | 0 |
| 3246.391603 | 2 |
| 3246.392673 | 0 |
| 3246.393148 | 3 |
| 3246.3948372 | 2 |
| 3246.3965139 | 2 |
| 3246.3969464 | 1 |
| 3246.3986652 | 3 |
| 3246.4001865 | 0 |
| 3246.4012281 | 0 |
| 3246.4027568 | 1 |
| 3246.4035236 | 1 |
| 3246.4050317 | 3 |
| 3246.4053715 | 5 |
| 3246.4074818 | 1 |
| 3246.4085325 | 4 |
| 3246.4097602 | 1 |
| 3246.411011 | 2 |
| 3246.4113805 | 1 |
| 3246.4134622 | 1 |
| 3246.4146323 | 4 |
| 3246.4160282 | 3 |
| 3246.4169061 | 1 |
| 3246.4178501 | 2 |
| 3246.4194977 | 0 |
| 3246.4198796 | 1 |
| 3246.4214523 | 2 |
| 3246.4233337 | 1 |
| 3246.4242807 | 4 |
| 3246.4250165 | 0 |
| 3246.426329 | 2 |
| 3246.4278793 | 2 |
| 3246.429559 | 2 |
| 3246.4303749 | 4 |
| 3246.4320684 | 2 |
| 3246.4332772 | 1 |
| 3246.4343839 | 1 |
| 3246.4362071 | 3 |
| 3246.436793 | 3 |
| 3246.4385095 | 1 |
| 3246.4393199 | 1 |
| 3246.4408384 | 2 |
| 3246.4422667 | 3 |
| 3246.4429935 | 1 |
| 3246.4442951 | 1 |
| 3246.4460934 | 5 |
| 3246.4466744 | 6 |
| 3246.4482448 | 0 |
| 3246.44913 | 0 |
| 3246.4505694 | 1 |
| 3246.4512076 | 3 |
| 3246.4528767 | 1 |
| 3246.4544067 | 0 |
| 3246.4554029 | 1 |
| 3246.4572287 | 1 |
| 3246.4575134 | 4 |
| 3246.4585504 | 1 |
| 3246.4605763 | 3 |
| 3246.4610775 | 2 |
| 3246.4625499 | 2 |
| 3246.4634658 | 1 |
| 3246.4647976 | 2 |
| 3246.4663663 | 1 |
| 3246.4675689 | 3 |
| 3246.4686578 | 4 |
| 3246.4699104 | 0 |

| | |
|---|---|
| 3246.4715765 | 2 |
| 3246.4722164 | 1 |
| 3246.4739023 | 2 |
| 3246.4751842 | 1 |
| 3246.476476 | 0 |
| 3246.4771139 | 5 |
| 3246.4787163 | 3 |
| 3246.4793336 | 3 |
| 3246.4802121 | 1 |
| 3246.4807377 | 3 |
| 3246.4814111 | 4 |
| 3246.4817549 | 1 |
| 3246.4820151 | 1 |
| 3246.4830341 | 1 |
| 3246.4840364 | 0 |
| 3246.484126 | 3 |
| 3246.4844604 | 4 |
| 3246.4852771 | 1 |
| 3246.4855735 | 3 |
| 3246.4861893 | 4 |
| 3246.4863459 | 5 |
| 3246.4879079 | 2 |
| 3246.4879249 | 4 |
| 3246.4888706 | 2 |
| 3246.4890111 | 3 |
| 3246.4891096 | 4 |
| 3246.4899839 | 2 |
| 3246.4905187 | 0 |
| 3246.4912799 | 1 |
| 3246.4916872 | 1 |
| 3246.492147 | 3 |
| 3246.4923208 | 3 |
| 3246.4936352 | 3 |
| 3246.4938664 | 2 |
| 3246.4944861 | 4 |
| 3246.4946386 | 2 |
| 3246.4951237 | 1 |
| 3246.4959895 | 1 |
| 3246.4963981 | 1 |
| 3246.4969457 | 0 |
| 3246.4973318 | 3 |
| 3246.4980211 | 3 |
| 3246.498388 | 4 |
| 3246.4985711 | 1 |
| 3246.499273 | 3 |
| 3246.5002894 | 5 |
| 3246.5004912 | 2 |
| 3246.501139 | 4 |
| 3246.5016951 | 3 |
| 3246.5018914 | 3 |
| 3246.5027225 | 1 |
| 3246.5027322 | 1 |
| 3246.5033045 | 2 |
| 3246.5041082 | 0 |
| 3246.5046596 | 1 |
| 3246.5055635 | 3 |
| 3246.5058512 | 2 |
| 3246.5061505 | 1 |
| 3246.5066323 | 0 |
| 3246.5072368 | 2 |
| 3246.5074722 | 2 |
| 3246.5083873 | 2 |
| 3246.5088531 | 4 |
| 3246.5099733 | 2 |
| 3246.510402 | 1 |
| 3246.5106968 | 0 |
| 3246.5107652 | 2 |
| 3246.5111476 | 4 |
| 3246.5118984 | 4 |
| 3246.5124655 | 0 |
| 3246.5126882 | 1 |
| 3246.5132557 | 2 |
| 3246.514063 | 3 |
| 3246.5141859 | 1 |
| 3246.5154418 | 1 |
| 3246.5155324 | 0 |
| 3246.5157725 | 3 |
| 3246.5164903 | 4 |
| 3246.5170403 | 6 |
| 3246.5176668 | 7 |
| 3246.5187726 | 0 |
| 3246.5193249 | 4 |
| 3246.5194544 | 0 |
| 3246.5200896 | 1 |
| 3246.5204943 | 0 |
| 3246.5214482 | 3 |
| 3246.5215806 | 0 |
| 3246.5217323 | 2 |
| 3246.5230277 | 2 |
| 3246.5232939 | 1 |
| 3246.5239743 | 3 |
| 3246.5249725 | 1 |
| 3246.5251236 | 2 |
| 3246.5251266 | 2 |
| 3246.5260149 | 2 |
| 3246.5260747 | 4 |
| 3246.5271771 | 3 |
| 3246.5276969 | 2 |
| 3246.5280515 | 0 |
| 3246.5286249 | 1 |
| 3246.5294531 | 2 |
| 3246.5297437 | 3 |
| 3246.5300679 | 5 |
| 3246.5310059 | 6 |
| 3246.5314308 | 3 |
| 3246.532116 | 4 |
| 3246.5322254 | 2 |
| 3246.5329253 | 1 |
| 3246.5332807 | 0 |
| 3246.5341544 | 3 |
| 3246.5343296 | 2 |
| 3246.5344649 | 1 |
| 3246.5360817 | 0 |
| 3246.5362793 | 0 |
| 3246.5366227 | 2 |
| 3246.5376429 | 3 |
| 3246.5382846 | 3 |
| 3246.5385318 | 1 |
| 3246.5388124 | 3 |
| 3246.5393205 | 1 |
| 3246.5398416 | 5 |
| 3246.5403753 | 7 |
| 3246.5404036 | 1 |
| 3246.5418257 | 1 |
| 3246.542417 | 2 |
| 3246.5425581 | 5 |
| 3246.542993 | 1 |
| 3246.5437103 | 3 |
| 3246.5438897 | 0 |
| 3246.5445144 | 2 |
| 3246.5449122 | 3 |
| 3246.5457249 | 0 |
| 3246.5460618 | 2 |
| 3246.5470783 | 1 |
| 3246.5472082 | 0 |
| 3246.5478712 | 1 |
| 3246.5487164 | 6 |
| 3246.5487237 | 1 |
| 3246.5493708 | 2 |
| 3246.5494352 | 3 |
| 3246.5506956 | 1 |
| 3246.5511676 | 5 |
| 3246.551828 | 2 |
| 3246.5523144 | 2 |
| 3246.5525949 | 1 |
| 3246.5530289 | 1 |
| 3246.5532726 | 2 |
| 3246.5542354 | 1 |
| 3246.5546837 | 2 |
| 3246.5552229 | 0 |
| 3246.5553914 | 2 |
| 3246.556559 | 1 |
| 3246.556838 | 2 |
| 3246.5569584 | 1 |
| 3246.5577972 | 1 |
| 3246.5579916 | 3 |
| 3246.5585306 | 4 |
| 3246.5589106 | 4 |
| 3246.5601057 | 4 |
| 3246.5606075 | 3 |
| 3246.5612236 | 1 |
| 3246.5614641 | 2 |
| 3246.5619385 | 2 |
| 3246.5624155 | 2 |
| 3246.5630372 | 1 |
| 3246.5636056 | 1 |
| 3246.5642432 | 3 |
| 3246.5643905 | 1 |
| 3246.5650992 | 3 |
| 3246.5662821 | 1 |
| 3246.5668794 | 1 |
| 3246.5670516 | 3 |
| 3246.5673914 | 1 |
| 3246.5674065 | 1 |
| 3246.5678939 | 2 |
| 3246.5696692 | 2 |
| 3246.5698927 | 4 |
| 3246.5705454 | 2 |
| 3246.5711294 | 4 |
| 3246.5715326 | 1 |
| 3246.5716127 | 3 |
| 3246.5717487 | 2 |
| 3246.5728636 | 4 |
| 3246.5731764 | 3 |
| 3246.5738394 | 0 |
| 3246.5738538 | 0 |
| 3246.575447 | 5 |
| 3246.5755401 | 2 |
| 3246.576511 | 4 |
| 3246.5766603 | 2 |
| 3246.576856 | 3 |
| 3246.57756 | 1 |
| 3246.5783254 | 4 |
| 3246.5797292 | 2 |
| 3246.5797398 | 1 |
| 3246.5798157 | 2 |
| 3246.5803519 | 2 |
| 3246.5810788 | 0 |
| 3246.5818811 | 2 |
| 3246.5822823 | 2 |
| 3246.5825916 | 1 |
| 3246.5826838 | 2 |
| 3246.5838999 | 3 |
| 3246.5851245 | 1 |
| 3246.5852015 | 3 |
| 3246.5856345 | 2 |
| 3246.585738 | 0 |
| 3246.5860412 | 1 |
| 3246.5865062 | 4 |
| 3246.5866831 | 1 |
| 3246.5881818 | 1 |
| 3246.5883561 | 3 |
| 3246.5892819 | 1 |
| 3246.5895994 | 3 |
| 3246.5897667 | 2 |
| 3246.590049 | 3 |
| 3246.5905512 | 0 |
| 3246.592212 | 3 |
| 3246.5924101 | 3 |
| 3246.592416 | 2 |
| 3246.5927758 | 1 |
| 3246.594064 | 0 |
| 3246.5943994 | 2 |
| 3246.5945929 | 4 |
| 3246.5950578 | 1 |
| 3246.5954647 | 1 |
| 3246.5962963 | 0 |
| 3246.596343 | 2 |
| 3246.5975908 | 2 |
| 3246.5983087 | 0 |
| 3246.5984235 | 5 |
| 3246.5986221 | 1 |
| 3246.5991469 | 5 |
| 3246.599716 | 4 |
| 3246.600753 | 6 |
| 3246.600922 | 1 |
| 3246.6013283 | 0 |
| 3246.6017407 | 5 |
| 3246.6028326 | 1 |
| 3246.6033614 | 1 |
| 3246.6040281 | 4 |
| 3246.6043097 | 3 |
| 3246.6045327 | 1 |
| 3246.6047131 | 3 |
| 3246.6054443 | 1 |
| 3246.6068548 | 2 |
| 3246.6068643 | 3 |
| 3246.6072432 | 2 |
| 3246.6074599 | 3 |
| 3246.6084654 | 1 |
| 3246.6085604 | 3 |
| 3246.6088728 | 0 |
| 3246.6102193 | 3 |
| 3246.6109452 | 1 |
| 3246.6110608 | 3 |
| 3246.6122952 | 3 |
| 3246.6132084 | 1 |
| 3246.6132369 | 1 |
| 3246.6134662 | 5 |
| 3246.6142249 | 4 |
| 3246.6143 | 2 |
| 3246.6148257 | 2 |
| 3246.6150862 | 2 |
| 3246.6164888 | 3 |
| 3246.6165987 | 1 |
| 3246.6172913 | 3 |
| 3246.6177868 | 2 |
| 3246.6179709 | 3 |
| 3246.6180318 | 1 |
| 3246.6189368 | 2 |
| 3246.6194419 | 1 |
| 3246.6199125 | 4 |
| 3246.6199383 | 1 |
| 3246.6208351 | 0 |
| 3246.6209833 | 4 |
| 3246.6221043 | 7 |
| 3246.6221422 | 2 |
| 3246.6222762 | 4 |
| 3246.6225127 | 1 |
| 3246.6229054 | 1 |
| 3246.6238135 | 2 |
| 3246.6241333 | 0 |
| 3246.6241733 | 1 |
| 3246.6252586 | 2 |
| 3246.6256599 | 3 |
| 3246.6260797 | 2 |
| 3246.6262353 | 1 |
| 3246.6263737 | 3 |
| 3246.6268244 | 3 |
| 3246.6271411 | 1 |
| 3246.6277791 | 2 |
| 3246.6279742 | 0 |
| 3246.628417 | 4 |
| 3246.6288394 | 5 |
| 3246.6297759 | 1 |
| 3246.6300878 | 3 |
| 3246.6301048 | 2 |
| 3246.6303307 | 7 |
| 3246.6312861 | 2 |
| 3246.6313437 | 2 |
| 3246.6315415 | 0 |
| 3246.632328 | 0 |
| 3246.6330925 | 1 |
| 3246.63312 | 1 |
| 3246.6338532 | 1 |
| 3246.6340327 | 1 |
| 3246.6340633 | 2 |
| 3246.6348724 | 2 |
| 3246.6348821 | 1 |
| 3246.6357902 | 1 |
| 3246.6363061 | 1 |
| 3246.6363358 | 2 |
| 3246.6365988 | 2 |
| 3246.6366515 | 1 |
| 3246.6376368 | 1 |
| 3246.6378181 | 5 |
| 3246.638506 | 1 |
| 3246.6385495 | 0 |
| 3246.6390179 | 6 |
| 3246.6392305 | 1 |
| 3246.6394558 | 1 |
| 3246.6403861 | 0 |
| 3246.640476 | 0 |
| 3246.6412706 | 1 |
| 3246.6418224 | 5 |
| 3246.6420391 | 3 |
| 3246.6422612 | 3 |
| 3246.6426299 | 2 |
| 3246.6432744 | 3 |
| 3246.6434369 | 3 |
| 3246.6436761 | 6 |
| 3246.6448796 | 0 |
| 3246.644967 | 1 |
| 3246.6450565 | 1 |
| 3246.6458416 | 1 |
| 3246.6462189 | 1 |
| 3246.646589 | 3 |
| 3246.6471448 | 2 |
| 3246.6475375 | 0 |
| 3246.6478439 | 0 |
| 3246.6486046 | 0 |
| 3246.6486092 | 3 |
| 3246.6488414 | 4 |
| 3246.6496955 | 2 |
| 3246.6499364 | 1 |
| 3246.6500688 | 4 |
| 3246.6504333 | 3 |
| 3246.6505703 | 3 |
| 3246.6518568 | 1 |
| 3246.6522058 | 2 |
| 3246.6524835 | 2 |
| 3246.6527589 | 0 |
| 3246.6530605 | 3 |
| 3246.6543157 | 5 |
| 3246.6544041 | 2 |
| 3246.6546055 | 1 |
| 3246.654794 | 3 |
| 3246.6551713 | 1 |
| 3246.6552727 | 1 |
| 3246.6559824 | 1 |
| 3246.6562477 | 6 |
| 3246.6571434 | 2 |
| 3246.6572271 | 1 |

| | |
|---|---|
| 3246.6572301 | 1 |
| 3246.6582403 | 2 |
| 3246.6587979 | 2 |
| 3246.6589992 | 0 |
| 3246.6596486 | 4 |
| 3246.6597205 | 0 |
| 3246.6598405 | 3 |
| 3246.6613005 | 2 |
| 3246.6613846 | 2 |
| 3246.6614025 | 3 |
| 3246.6626228 | 3 |
| 3246.662944 | 1 |
| 3246.6633668 | 0 |
| 3246.6636536 | 3 |
| 3246.6637136 | 1 |
| 3246.6638989 | 1 |
| 3246.6649129 | 3 |
| 3246.6649195 | 0 |
| 3246.665349 | 2 |
| 3246.6654009 | 2 |
| 3246.6666566 | 3 |
| 3246.6673247 | 6 |
| 3246.6675805 | 1 |
| 3246.667694 | 4 |
| 3246.6678841 | 3 |
| 3246.6681241 | 2 |
| 3246.6690372 | 1 |
| 3246.669093 | 2 |
| 3246.6692772 | 3 |
| 3246.6702517 | 3 |
| 3246.6703835 | 3 |
| 3246.671657 | 0 |
| 3246.6719083 | 3 |
| 3246.6720612 | 1 |
| 3246.6724082 | 2 |
| 3246.6725153 | 2 |
| 3246.6729936 | 1 |
| 3246.6735698 | 2 |
| 3246.6738548 | 1 |
| 3246.6740854 | 2 |
| 3246.6747474 | 2 |
| 3246.6749743 | 3 |
| 3246.6750558 | 6 |
| 3246.6753282 | 2 |
| 3246.6763155 | 4 |
| 3246.6765464 | 2 |
| 3246.6771128 | 2 |
| 3246.6774475 | 1 |
| 3246.6776672 | 2 |
| 3246.6777581 | 0 |
| 3246.6782115 | 5 |
| 3246.678271 | 3 |
| 3246.6783706 | 2 |
| 3246.6796061 | 2 |
| 3246.6799112 | 1 |
| 3246.6799357 | 3 |
| 3246.6799654 | 0 |
| 3246.680087 | 1 |
| 3246.6812995 | 1 |
| 3246.6815229 | 1 |
| 3246.6815851 | 1 |
| 3246.6827315 | 1 |
| 3246.6829532 | 1 |
| 3246.6831433 | 5 |
| 3246.683272 | 2 |
| 3246.683497 | 1 |
| 3246.6840415 | 2 |
| 3246.684195 | 3 |
| 3246.6848951 | 5 |
| 3246.6852744 | 0 |
| 3246.68584 | 0 |
| 3246.6860463 | 2 |
| 3246.6860665 | 0 |
| 3246.686225 | 3 |
| 3246.6870413 | 1 |
| 3246.6871885 | 0 |
| 3246.6874607 | 1 |
| 3246.6876543 | 4 |
| 3246.6886564 | 3 |
| 3246.6887981 | 2 |
| 3246.6890998 | 1 |
| 3246.6895403 | 4 |
| 3246.6905037 | 2 |
| 3246.6905532 | 4 |
| 3246.6906314 | 1 |
| 3246.6906411 | 6 |
| 3246.691155 | 2 |
| 3246.6916817 | 3 |
| 3246.6925232 | 2 |
| 3246.6925344 | 2 |
| 3246.6930254 | 2 |
| 3246.6930935 | 2 |
| 3246.6932995 | 0 |
| 3246.6934441 | 3 |
| 3246.6938325 | 2 |
| 3246.6950272 | 2 |
| 3246.6951636 | 3 |
| 3246.6951672 | 2 |
| 3246.6956347 | 2 |
| 3246.6959659 | 2 |
| 3246.6967311 | 1 |
| 3246.6968888 | 1 |
| 3246.6972775 | 3 |
| 3246.6979809 | 1 |
| 3246.6982422 | 4 |
| 3246.698611 | 0 |
| 3246.6988815 | 3 |
| 3246.6988832 | 2 |
| 3246.6995364 | 1 |
| 3246.6997532 | 1 |
| 3246.7003945 | 2 |
| 3246.7004919 | 1 |
| 3246.7013829 | 1 |
| 3246.7015451 | 4 |
| 3246.7016117 | 1 |
| 3246.7024841 | 0 |
| 3246.7029349 | 3 |
| 3246.7030004 | 5 |
| 3246.7033417 | 0 |
| 3246.703345 | 0 |
| 3246.704278 | 1 |
| 3246.7047083 | 1 |
| 3246.7049598 | 2 |
| 3246.7051282 | 4 |
| 3246.7063675 | 7 |
| 3246.7064424 | 6 |
| 3246.7067355 | 1 |
| 3246.7068281 | 1 |
| 3246.7071841 | 4 |
| 3246.7081531 | 1 |
| 3246.7082451 | 2 |
| 3246.7085523 | 3 |
| 3246.7086078 | 3 |
| 3246.7096526 | 5 |
| 3246.7097417 | 2 |
| 3246.7098437 | 0 |
| 3246.7099304 | 2 |
| 3246.7104072 | 2 |
| 3246.7105628 | 2 |
| 3246.7118061 | 1 |
| 3246.7118074 | 3 |
| 3246.7121439 | 6 |
| 3246.7127252 | 1 |
| 3246.7129303 | 2 |
| 3246.7132522 | 1 |
| 3246.7134439 | 3 |
| 3246.7140575 | 0 |
| 3246.7144229 | 5 |
| 3246.7146509 | 3 |
| 3246.7154306 | 1 |
| 3246.7155761 | 0 |
| 3246.7158271 | 1 |
| 3246.7162287 | 5 |
| 3246.7165434 | 2 |
| 3246.7171133 | 2 |
| 3246.7173116 | 2 |
| 3246.7177466 | 4 |
| 3246.7181211 | 1 |
| 3246.7188625 | 1 |
| 3246.7188763 | 1 |
| 3246.7193104 | 6 |
| 3246.7193773 | 0 |
| 3246.7203156 | 3 |
| 3246.7206723 | 3 |
| 3246.7207455 | 1 |
| 3246.7211193 | 3 |
| 3246.7216048 | 3 |
| 3246.7217467 | 1 |
| 3246.7222316 | 1 |
| 3246.7228736 | 2 |
| 3246.7229506 | 4 |
| 3246.7231118 | 3 |
| 3246.724037 | 0 |
| 3246.7244507 | 1 |
| 3246.724926 | 2 |
| 3246.724989 | 2 |
| 3246.7250436 | 3 |
| 3246.7254242 | 1 |
| 3246.7257841 | 3 |
| 3246.7264938 | 4 |
| 3246.7268446 | 2 |
| 3246.7271426 | 3 |
| 3246.727179 | 0 |
| 3246.7282332 | 2 |
| 3246.7282371 | 1 |
| 3246.7283139 | 3 |
| 3246.7285566 | 1 |
| 3246.7295154 | 2 |
| 3246.7296512 | 4 |
| 3246.7298034 | 1 |
| 3246.7304086 | 2 |
| 3246.7308343 | 3 |
| 3246.7311457 | 2 |
| 3246.7312911 | 2 |
| 3246.7316374 | 6 |
| 3246.73223 | 0 |
| 3246.7325497 | 3 |
| 3246.7329703 | 4 |
| 3246.7330022 | 3 |
| 3246.733957 | 2 |
| 3246.7341217 | 2 |
| 3246.7344153 | 0 |
| 3246.7348354 | 2 |
| 3246.7353144 | 1 |
| 3246.735853 | 1 |
| 3246.7360977 | 3 |
| 3246.7367269 | 1 |
| 3246.7370619 | 3 |
| 3246.7371323 | 1 |
| 3246.7372911 | 3 |
| 3246.7373452 | 3 |
| 3246.7374863 | 1 |
| 3246.7383929 | 2 |
| 3246.739272 | 2 |
| 3246.739374 | 0 |
| 3246.7394679 | 1 |
| 3246.7400504 | 1 |
| 3246.7404652 | 1 |
| 3246.7404688 | 2 |
| 3246.7410582 | 3 |
| 3246.7416407 | 0 |
| 3246.7418644 | 2 |
| 3246.7422693 | 1 |
| 3246.7425568 | 1 |
| 3246.7429769 | 2 |
| 3246.7433076 | 0 |
| 3246.7437177 | 1 |
| 3246.7442736 | 2 |
| 3246.7443979 | 3 |
| 3246.744925 | 1 |
| 3246.7453174 | 1 |
| 3246.745635 | 1 |
| 3246.7462078 | 3 |
| 3246.747069 | 4 |
| 3246.7471049 | 1 |
| 3246.7471635 | 3 |
| 3246.7472573 | 5 |
| 3246.747742 | 3 |
| 3246.7483785 | 0 |
| 3246.7485132 | 4 |
| 3246.7490997 | 2 |
| 3246.7492727 | 2 |
| 3246.7498852 | 2 |
| 3246.750104 | 1 |
| 3246.7502036 | 4 |
| 3246.7503062 | 0 |
| 3246.7511658 | 0 |
| 3246.7516334 | 0 |
| 3246.7520538 | 1 |
| 3246.7524515 | 0 |
| 3246.7526784 | 0 |
| 3246.7532537 | 2 |
| 3246.753271 | 2 |
| 3246.7541338 | 1 |
| 3246.7549422 | 2 |
| 3246.7550434 | 1 |
| 3246.755361 | 2 |
| 3246.7566901 | 1 |
| 3246.7590452 | 2 |
| 3246.7599266 | 1 |
| 3246.7634832 | 6 |
| 3246.7640321 | 2 |
| 3246.7662153 | 2 |
| 3246.7682378 | 0 |
| 3246.7695549 | 0 |
| 3246.7721578 | 0 |
| 3246.7732189 | 2 |
| 3246.7751766 | 5 |
| 3246.7779234 | 1 |
| 3246.7786006 | 0 |
| 3246.7804103 | 2 |
| 3246.7832755 | 2 |
| 3246.7842911 | 1 |
| 3246.7865866 | 2 |
| 3246.7883021 | 4 |
| 3246.7909701 | 2 |
| 3246.7915107 | 1 |
| 3246.7939796 | 1 |
| 3246.7963346 | 2 |
| 3246.7969971 | 2 |
| 3246.799993 | 3 |
| 3246.8020975 | 2 |
| 3246.8034955 | 3 |
| 3246.805363 | 2 |
| 3246.806638 | 0 |
| 3246.8094392 | 3 |
| 3246.810842 | 1 |
| 3246.8126413 | 4 |
| 3246.8151989 | 2 |
| 3246.8159408 | 2 |
| 3246.8182765 | 5 |
| 3246.8199235 | 3 |
| 3246.8215104 | 4 |
| 3246.82334 | 2 |
| 3246.8249325 | 1 |
| 3246.8277633 | 1 |
| 3246.8285 | 4 |
| 3246.8314523 | 4 |
| 3246.8334429 | 3 |
| 3246.8344683 | 1 |
| 3246.8365158 | 0 |
| 3246.8379705 | 4 |
| 3246.8407617 | 6 |
| 3246.842707 | 4 |
| 3246.8445327 | 3 |
| 3246.8462729 | 3 |
| 3246.8472725 | 0 |
| 3246.84959 | 3 |
| 3246.8522178 | 3 |
| 3246.8531628 | 0 |
| 3246.8552667 | 3 |
| 3246.8570285 | 0 |
| 3246.859121 | 2 |
| 3246.8614203 | 5 |
| 3246.8623279 | 2 |
| 3246.8646263 | 1 |
| 3246.8654758 | 1 |
| 3246.8681103 | 1 |
| 3246.8702283 | 4 |
| 3246.8715192 | 5 |
| 3246.8738344 | 4 |
| 3246.8750822 | 2 |
| 3246.8777318 | 0 |
| 3246.8786763 | 4 |
| 3246.880847 | 0 |
| 3246.8832375 | 2 |
| 3246.8843772 | 2 |
| 3246.8867046 | 1 |
| 3246.8882848 | 1 |
| 3246.8905126 | 5 |
| 3246.8928334 | 2 |
| 3246.8940115 | 3 |
| 3246.8965022 | 4 |
| 3246.898724 | 1 |
| 3246.8995905 | 2 |
| 3246.9021038 | 3 |
| 3246.9032483 | 0 |
| 3246.9053114 | 1 |
| 3246.9064329 | 0 |
| 3246.9087075 | 3 |
| 3246.9110941 | 3 |
| 3246.9122822 | 2 |
| 3246.9150384 | 2 |
| 3246.9151766 | 1 |
| 3246.918176 | 0 |
| 3246.920913 | 2 |
| 3246.9217037 | 2 |
| 3246.923653 | 2 |
| 3246.9250097 | 4 |
| 3246.9275543 | 0 |
| 3246.9284696 | 3 |
| 3246.9310586 | 0 |
| 3246.9332678 | 2 |
| 3246.9343953 | 2 |
| 3246.9372288 | 3 |
| 3246.9377985 | 2 |
| 3246.9402251 | 2 |
| 3246.9427311 | 2 |
| 3246.9438914 | 4 |
| 3246.9458058 | 3 |
| 3246.9475504 | 1 |
| 3246.9492917 | 4 |
| 3246.9511433 | 2 |

| | |
|---|---|
| 3246.9522186 | 1 |
| 3246.9555093 | 2 |
| 3246.9562548 | 4 |
| 3246.9587409 | 1 |
| 3246.9611374 | 1 |
| 3246.961373 | 2 |
| 3246.9642463 | 1 |
| 3246.9652554 | 1 |
| 3246.967324 | 2 |
| 3246.9702343 | 2 |
| 3246.9715031 | 2 |
| 3246.9731176 | 6 |
| 3246.9748352 | 1 |
| 3246.9767197 | 3 |
| 3246.9794085 | 2 |
| 3246.9806573 | 1 |
| 3246.9826707 | 2 |
| 3246.9840749 | 1 |
| 3246.9864013 | 1 |
| 3246.9889414 | 0 |
| 3246.9906169 | 2 |
| 3246.9922661 | 1 |
| 3246.9933671 | 2 |
| 3246.9960859 | 3 |
| 3246.9969116 | 1 |
| 3246.9994136 | 0 |
| 3247.0016636 | 1 |
| 3247.0022241 | 5 |
| 3247.0047953 | 2 |
| 3247.0068256 | 2 |
| 3247.0084929 | 1 |
| 3247.0101007 | 3 |
| 3247.011464 | 2 |
| 3247.0140777 | 0 |
| 3247.0166431 | 7 |
| 3247.0169872 | 2 |
| 3247.0196422 | 3 |
| 3247.0210085 | 4 |
| 3247.022977 | 1 |
| 3247.0257355 | 2 |
| 3247.0271668 | 3 |
| 3247.0293883 | 3 |
| 3247.030442 | 0 |
| 3247.0329229 | 1 |
| 3247.0347744 | 1 |
| 3247.0355895 | 1 |
| 3247.039051 | 0 |
| 3247.0395337 | 4 |
| 3247.0427177 | 1 |
| 3247.0430758 | 2 |
| 3247.0454333 | 4 |
| 3247.0476305 | 1 |
| 3247.0486323 | 3 |
| 3247.0505711 | 1 |
| 3247.0521939 | 4 |
| 3247.0551148 | 2 |
| 3247.0567831 | 3 |
| 3247.0582586 | 3 |
| 3247.0608301 | 2 |
| 3247.0617793 | 2 |
| 3247.0636517 | 5 |
| 3247.0661689 | 2 |
| 3247.0676123 | 2 |
| 3247.0698362 | 1 |
| 3247.0712285 | 1 |
| 3247.0727844 | 0 |
| 3247.0755572 | 2 |
| 3247.0765084 | 3 |
| 3247.0789516 | 4 |
| 3247.0802979 | 5 |
| 3247.0825024 | 3 |
| 3247.0840711 | 2 |
| 3247.0863419 | 2 |
| 3247.0884539 | 3 |
| 3247.0887716 | 1 |
| 3247.0919528 | 2 |
| 3247.0943212 | 1 |
| 3247.0953035 | 1 |
| 3247.0976372 | 5 |
| 3247.0983954 | 2 |
| 3247.1005296 | 1 |
| 3247.1020473 | 1 |
| 3247.1041705 | 2 |
| 3247.1064483 | 1 |
| 3247.1079123 | 2 |
| 3247.1104581 | 3 |
| 3247.1116376 | 1 |
| 3247.1143141 | 4 |
| 3247.1163509 | 1 |
| 3247.1176353 | 1 |
| 3247.1195163 | 4 |
| 3247.1217252 | 3 |
| 3247.1229563 | 3 |
| 3247.1257441 | 3 |
| 3247.1268586 | 4 |
| 3247.1291429 | 1 |
| 3247.1301223 | 2 |
| 3247.132384 | 4 |
| 3247.1346179 | 2 |
| 3247.13609 | 3 |
| 3247.1384692 | 2 |
| 3247.139463 | 3 |
| 3247.141609 | 3 |
| 3247.143933 | 5 |
| 3247.14453 | 3 |
| 3247.1465651 | 0 |
| 3247.1489975 | 1 |
| 3247.150684 | 2 |
| 3247.153137 | 4 |
| 3247.1539713 | 3 |
| 3247.1563867 | 3 |
| 3247.1576763 | 1 |
| 3247.1596982 | 2 |
| 3247.1620049 | 3 |
| 3247.1633726 | 2 |
| 3247.1660127 | 8 |
| 3247.1668114 | 6 |
| 3247.1690046 | 2 |
| 3247.1699501 | 0 |
| 3247.1703058 | 1 |
| 3247.1718334 | 1 |
| 3247.1723361 | 2 |
| 3247.1731314 | 7 |
| 3247.1732302 | 4 |
| 3247.1751125 | 2 |
| 3247.1756349 | 1 |
| 3247.176809 | 2 |
| 3247.1773689 | 2 |
| 3247.1788541 | 1 |
| 3247.1792357 | 3 |
| 3247.1804953 | 1 |
| 3247.1816369 | 2 |
| 3247.1825804 | 0 |
| 3247.1829397 | 2 |
| 3247.1849108 | 4 |
| 3247.1849245 | 3 |
| 3247.1865181 | 3 |
| 3247.1870248 | 0 |
| 3247.1883749 | 1 |
| 3247.188524 | 2 |
| 3247.1897647 | 3 |
| 3247.1911368 | 2 |
| 3247.1918086 | 1 |
| 3247.1927812 | 2 |
| 3247.1942663 | 4 |
| 3247.1946152 | 3 |
| 3247.1957273 | 1 |
| 3247.196756 | 3 |
| 3247.197813 | 3 |
| 3247.1983646 | 2 |
| 3247.1990816 | 0 |
| 3247.2003584 | 0 |
| 3247.2015152 | 1 |
| 3247.2015864 | 1 |
| 3247.2040566 | 2 |
| 3247.2042525 | 2 |
| 3247.2051839 | 2 |
| 3247.2062092 | 1 |
| 3247.2063292 | 2 |
| 3247.2072312 | 3 |
| 3247.2091012 | 0 |
| 3247.2107737 | 3 |
| 3247.2133053 | 4 |
| 3247.2141892 | 4 |
| 3247.2166398 | 0 |
| 3247.2188452 | 3 |
| 3247.2194851 | 2 |
| 3247.222108 | 3 |
| 3247.2238986 | 0 |
| 3247.2253419 | 1 |
| 3247.2274397 | 0 |
| 3247.2288277 | 1 |
| 3247.2310121 | 2 |
| 3247.2334661 | 2 |
| 3247.2346965 | 1 |
| 3247.2366057 | 2 |
| 3247.2383644 | 3 |
| 3247.239553 | 2 |
| 3247.2420144 | 3 |
| 3247.2437419 | 1 |
| 3247.2465766 | 6 |
| 3247.247564 | 6 |
| 3247.2501119 | 5 |
| 3247.2512276 | 2 |
| 3247.2531243 | 3 |
| 3247.2558666 | 0 |
| 3247.2568357 | 1 |
| 3247.259834 | 3 |
| 3247.2605086 | 3 |
| 3247.2627908 | 0 |
| 3247.2637663 | 2 |
| 3247.2663451 | 3 |
| 3247.2686123 | 3 |
| 3247.2697367 | 1 |
| 3247.272184 | 2 |
| 3247.2735186 | 1 |
| 3247.2753871 | 4 |
| 3247.2778294 | 5 |
| 3247.278781 | 1 |
| 3247.2813393 | 3 |
| 3247.2825834 | 5 |
| 3247.2852501 | 3 |
| 3247.2870793 | 1 |
| 3247.2883642 | 1 |
| 3247.2909806 | 2 |
| 3247.2920379 | 5 |
| 3247.294204 | 1 |
| 3247.2969344 | 2 |
| 3247.297599 | 2 |
| 3247.2999482 | 2 |
| 3247.3015653 | 1 |
| 3247.3028756 | 2 |
| 3247.3047616 | 2 |
| 3247.3070366 | 1 |
| 3247.3098014 | 1 |
| 3247.3107731 | 2 |
| 3247.3130985 | 4 |
| 3247.3137885 | 2 |
| 3247.3169935 | 2 |
| 3247.3190543 | 2 |
| 3247.3204416 | 5 |
| 3247.3216717 | 1 |
| 3247.322994 | 3 |
| 3247.3255513 | 5 |
| 3247.3279635 | 2 |
| 3247.3292129 | 1 |
| 3247.3315257 | 1 |
| 3247.334066 | 1 |
| 3247.3349299 | 1 |
| 3247.3373442 | 0 |
| 3247.3385128 | 4 |
| 3247.3410529 | 1 |
| 3247.3415608 | 0 |
| 3247.3446231 | 1 |
| 3247.3469971 | 3 |
| 3247.3477684 | 4 |
| 3247.3491579 | 1 |
| 3247.351656 | 2 |
| 3247.3530389 | 3 |
| 3247.3559347 | 1 |
| 3247.3568132 | 3 |
| 3247.3596482 | 2 |
| 3247.3603855 | 6 |
| 3247.3621143 | 4 |
| 3247.3651007 | 2 |
| 3247.3662058 | 3 |
| 3247.3684657 | 2 |
| 3247.3697764 | 4 |
| 3247.3719347 | 4 |
| 3247.3744383 | 1 |
| 3247.3756716 | 2 |
| 3247.3784491 | 5 |
| 3247.3799961 | 0 |
| 3247.3807579 | 1 |
| 3247.3838514 | 2 |
| 3247.3854213 | 3 |
| 3247.3867992 | 0 |
| 3247.3887962 | 6 |
| 3247.3906066 | 5 |
| 3247.3932995 | 2 |
| 3247.3939077 | 4 |
| 3247.3962708 | 4 |
| 3247.3978653 | 2 |
| 3247.400412 | 0 |
| 3247.4019982 | 0 |
| 3247.4037957 | 3 |
| 3247.4062706 | 1 |
| 3247.406894 | 1 |
| 3247.4087103 | 0 |
| 3247.4119293 | 1 |
| 3247.4130707 | 0 |
| 3247.4152006 | 2 |
| 3247.4162754 | 2 |
| 3247.4188955 | 2 |
| 3247.421106 | 2 |
| 3247.422155 | 0 |
| 3247.4245733 | 1 |
| 3247.4262413 | 1 |
| 3247.4284256 | 3 |
| 3247.4305872 | 2 |
| 3247.4311706 | 1 |
| 3247.4342596 | 3 |
| 3247.4347753 | 1 |
| 3247.4368282 | 3 |
| 3247.4392759 | 2 |
| 3247.4419818 | 5 |
| 3247.443327 | 1 |
| 3247.4453049 | 1 |
| 3247.4464768 | 3 |
| 3247.4491016 | 1 |
| 3247.4499423 | 3 |
| 3247.4526089 | 3 |
| 3247.4547286 | 2 |
| 3247.4560263 | 4 |
| 3247.4581552 | 2 |
| 3247.4595625 | 2 |
| 3247.461633 | 1 |
| 3247.4639036 | 5 |
| 3247.464828 | 4 |
| 3247.4671862 | 1 |
| 3247.468653 | 3 |
| 3247.4707334 | 1 |
| 3247.4732804 | 3 |
| 3247.4747828 | 3 |
| 3247.4769308 | 3 |
| 3247.4782552 | 4 |
| 3247.4807751 | 5 |
| 3247.4822683 | 3 |
| 3247.4842612 | 1 |
| 3247.4865249 | 3 |
| 3247.4873887 | 2 |
| 3247.4901384 | 2 |
| 3247.4911725 | 3 |
| 3247.4935046 | 2 |
| 3247.4958981 | 3 |
| 3247.4972681 | 0 |
| 3247.499679 | 1 |
| 3247.5004429 | 3 |
| 3247.5035798 | 2 |
| 3247.5050929 | 1 |
| 3247.5066475 | 2 |
| 3247.5086317 | 3 |
| 3247.5101746 | 0 |
| 3247.512032 | 0 |
| 3247.514341 | 3 |
| 3247.51583 | 1 |
| 3247.5184344 | 5 |
| 3247.5191103 | 2 |
| 3247.5215683 | 4 |
| 3247.5243879 | 4 |
| 3247.5255197 | 1 |
| 3247.5279192 | 2 |
| 3247.5288573 | 1 |
| 3247.5310921 | 2 |
| 3247.5333958 | 2 |
| 3247.5346722 | 1 |
| 3247.5367396 | 2 |
| 3247.5385204 | 2 |
| 3247.5407086 | 3 |
| 3247.5416657 | 4 |
| 3247.5444934 | 2 |
| 3247.5467831 | 2 |
| 3247.5475324 | 3 |
| 3247.550112 | 3 |
| 3247.5510959 | 1 |
| 3247.5530866 | 2 |
| 3247.5556333 | 3 |
| 3247.5568148 | 5 |
| 3247.5595094 | 1 |
| 3247.5616924 | 2 |
| 3247.5628859 | 1 |
| 3247.5655409 | 1 |
| 3247.5660498 | 1 |
| 3247.5690221 | 2 |
| 3247.5717261 | 1 |
| 3247.5728256 | 1 |
| 3247.575265 | 1 |
| 3247.5759674 | 4 |
| 3247.5784478 | 1 |
| 3247.5792121 | 1 |
| 3247.5814232 | 1 |
| 3247.5838584 | 1 |
| 3247.5851832 | 1 |

| | |
|---|---|
| 3247.5876327 | 1 |
| 3247.5900274 | 4 |
| 3247.5911101 | 0 |
| 3247.5934081 | 2 |
| 3247.5947922 | 2 |
| 3247.5967797 | 4 |
| 3247.5994464 | 3 |
| 3247.6007591 | 3 |
| 3247.6028846 | 3 |
| 3247.6029931 | 2 |
| 3247.6043568 | 3 |
| 3247.6043997 | 0 |
| 3247.6062602 | 3 |
| 3247.6074298 | 4 |
| 3247.6086743 | 1 |
| 3247.6089784 | 1 |
| 3247.6096393 | 3 |
| 3247.6127486 | 1 |
| 3247.6128606 | 4 |
| 3247.6136918 | 3 |
| 3247.6160114 | 7 |
| 3247.6160791 | 1 |
| 3247.6171889 | 0 |
| 3247.6172424 | 5 |
| 3247.6202348 | 5 |
| 3247.6203959 | 2 |
| 3247.6217663 | 4 |
| 3247.6218576 | 2 |
| 3247.6234414 | 1 |
| 3247.6250864 | 4 |
| 3247.6254528 | 3 |
| 3247.6266962 | 2 |
| 3247.6275168 | 3 |
| 3247.6294507 | 2 |
| 3247.63014 | 3 |
| 3247.6312503 | 3 |
| 3247.6326637 | 2 |
| 3247.6327629 | 2 |
| 3247.6345812 | 3 |
| 3247.6347189 | 4 |
| 3247.6366675 | 2 |
| 3247.6372025 | 2 |
| 3247.6384518 | 1 |
| 3247.6406186 | 1 |
| 3247.6410706 | 2 |
| 3247.6418529 | 2 |
| 3247.6421778 | 4 |
| 3247.6449113 | 1 |
| 3247.6453315 | 3 |
| 3247.64643 | 2 |
| 3247.6471128 | 2 |
| 3247.6477267 | 3 |
| 3247.6498144 | 2 |
| 3247.6504268 | 5 |
| 3247.6513411 | 4 |
| 3247.6517444 | 1 |
| 3247.6539756 | 3 |
| 3247.6544921 | 1 |
| 3247.655305 | 1 |
| 3247.6578551 | 1 |
| 3247.6581054 | 2 |
| 3247.659463 | 5 |
| 3247.66039 | 1 |
| 3247.6609677 | 3 |
| 3247.6616939 | 3 |
| 3247.6631287 | 4 |
| 3247.664702 | 6 |
| 3247.6654842 | 3 |
| 3247.6666179 | 1 |
| 3247.6675619 | 4 |
| 3247.6695038 | 5 |
| 3247.6701351 | 5 |
| 3247.6707096 | 3 |
| 3247.6716818 | 1 |
| 3247.6731037 | 2 |
| 3247.6740057 | 2 |
| 3247.6749856 | 1 |
| 3247.6765464 | 2 |
| 3247.6765722 | 3 |
| 3247.6776617 | 6 |
| 3247.6802453 | 2 |
| 3247.6807843 | 2 |
| 3247.6826997 | 1 |
| 3247.6828003 | 3 |
| 3247.6837657 | 4 |
| 3247.6846516 | 2 |
| 3247.6863779 | 3 |
| 3247.6867733 | 0 |
| 3247.6884797 | 5 |
| 3247.6891101 | 2 |
| 3247.6906395 | 2 |
| 3247.6916247 | 2 |
| 3247.6925192 | 1 |
| 3247.6930312 | 3 |
| 3247.6950534 | 1 |
| 3247.6953816 | 3 |
| 3247.6963908 | 1 |
| 3247.6977854 | 1 |
| 3247.6988839 | 1 |
| 3247.6995688 | 0 |
| 3247.7010038 | 0 |
| 3247.70185 | 0 |
| 3247.7028333 | 3 |
| 3247.7046625 | 2 |
| 3247.7051061 | 2 |
| 3247.7060897 | 1 |
| 3247.7075107 | 4 |
| 3247.708626 | 2 |
| 3247.709946 | 1 |
| 3247.7105203 | 1 |
| 3247.7119672 | 1 |
| 3247.7123971 | 1 |
| 3247.7140222 | 0 |
| 3247.7146117 | 3 |
| 3247.7149991 | 1 |
| 3247.717346 | 2 |
| 3247.7174991 | 4 |
| 3247.7200213 | 2 |
| 3247.7201949 | 2 |
| 3247.7203874 | 0 |
| 3247.7220824 | 2 |
| 3247.7230229 | 3 |
| 3247.7248091 | 2 |
| 3247.7252267 | 2 |
| 3247.7269171 | 2 |
| 3247.7274372 | 4 |
| 3247.7295035 | 3 |
| 3247.7307988 | 3 |
| 3247.730904 | 1 |
| 3247.7326965 | 5 |
| 3247.7332809 | 1 |
| 3247.7339896 | 6 |
| 3247.7345697 | 3 |
| 3247.7361666 | 2 |
| 3247.7385908 | 2 |
| 3247.7388803 | 2 |
| 3247.7390218 | 2 |
| 3247.7404067 | 2 |
| 3247.7421879 | 3 |
| 3247.742488 | 2 |
| 3247.7438603 | 0 |
| 3247.7458988 | 1 |
| 3247.7460934 | 3 |
| 3247.7478371 | 1 |
| 3247.7480498 | 2 |
| 3247.749273 | 2 |
| 3247.75049 | 4 |
| 3247.7521089 | 2 |
| 3247.7521799 | 3 |
| 3247.7528301 | 2 |
| 3247.7551266 | 1 |
| 3247.7554 | 4 |
| 3247.7572209 | 3 |
| 3247.7580855 | 5 |
| 3247.7586758 | 2 |
| 3247.7597553 | 4 |
| 3247.7615491 | 2 |
| 3247.7623775 | 0 |
| 3247.7631184 | 4 |
| 3247.7646525 | 3 |
| 3247.7649055 | 3 |
| 3247.7659794 | 3 |
| 3247.767567 | 1 |
| 3247.7681399 | 3 |
| 3247.7700373 | 3 |
| 3247.7708829 | 2 |
| 3247.7713199 | 3 |
| 3247.7725985 | 2 |
| 3247.7741244 | 3 |
| 3247.7758368 | 2 |
| 3247.7766185 | 1 |
| 3247.7768732 | 2 |
| 3247.7778287 | 3 |
| 3247.7800806 | 1 |
| 3247.7806043 | 2 |
| 3247.7812112 | 1 |
| 3247.7827517 | 0 |
| 3247.7833588 | 5 |
| 3247.784953 | 2 |
| 3247.7853717 | 1 |
| 3247.7869002 | 0 |
| 3247.7886202 | 1 |
| 3247.7893075 | 2 |
| 3247.7893801 | 4 |
| 3247.7903882 | 1 |
| 3247.7923714 | 0 |
| 3247.7934907 | 7 |
| 3247.7952823 | 2 |
| 3247.7964376 | 3 |
| 3247.7966259 | 3 |
| 3247.7973869 | 2 |
| 3247.798317 | 1 |
| 3247.8001388 | 1 |
| 3247.8009545 | 2 |
| 3247.8017871 | 3 |
| 3247.802645 | 1 |
| 3247.8035851 | 1 |
| 3247.80566 | 2 |
| 3247.8059141 | 3 |
| 3247.8072804 | 2 |
| 3247.8073822 | 1 |
| 3247.8089924 | 3 |
| 3247.8105924 | 2 |
| 3247.8114812 | 2 |
| 3247.8125842 | 3 |
| 3247.813559 | 0 |
| 3247.8151731 | 1 |
| 3247.8152312 | 0 |
| 3247.8170997 | 2 |
| 3247.8181165 | 1 |
| 3247.8186514 | 2 |
| 3247.8196726 | 5 |
| 3247.8212216 | 0 |
| 3247.8226935 | 7 |
| 3247.8230163 | 0 |
| 3247.8239988 | 1 |
| 3247.8260924 | 3 |
| 3247.8264588 | 1 |
| 3247.8272231 | 3 |
| 3247.8275744 | 4 |
| 3247.8305867 | 1 |
| 3247.8306252 | 4 |
| 3247.8312119 | 6 |
| 3247.8330777 | 3 |
| 3247.8336648 | 3 |
| 3247.834853 | 2 |
| 3247.8359684 | 1 |
| 3247.8370661 | 2 |
| 3247.8385163 | 0 |
| 3247.8393623 | 2 |
| 3247.8396181 | 3 |
| 3247.8409715 | 7 |
| 3247.8426085 | 5 |
| 3247.8428618 | 2 |
| 3247.8452187 | 0 |
| 3247.8459333 | 2 |
| 3247.8464994 | 3 |
| 3247.8471361 | 3 |
| 3247.8492194 | 2 |
| 3247.8496032 | 1 |
| 3247.8505885 | 1 |
| 3247.8519287 | 2 |
| 3247.8527124 | 2 |
| 3247.8531259 | 1 |
| 3247.8558503 | 2 |
| 3247.8562494 | 0 |
| 3247.8578851 | 3 |
| 3247.8592329 | 1 |
| 3247.85934 | 3 |
| 3247.8602306 | 2 |
| 3247.8612499 | 2 |
| 3247.8622716 | 2 |
| 3247.8641428 | 2 |
| 3247.8646921 | 2 |
| 3247.8658152 | 2 |
| 3247.8673364 | 4 |
| 3247.8687256 | 1 |
| 3247.8690152 | 8 |
| 3247.8692702 | 3 |
| 3247.870643 | 2 |
| 3247.8730369 | 1 |
| 3247.8735267 | 4 |
| 3247.8735998 | 3 |
| 3247.8760663 | 1 |
| 3247.876547 | 1 |
| 3247.8772803 | 0 |
| 3247.8779338 | 1 |
| 3247.8793004 | 3 |
| 3247.8807693 | 1 |
| 3247.8827287 | 1 |
| 3247.8830473 | 1 |
| 3247.8834268 | 1 |
| 3247.88544 | 3 |
| 3247.885731 | 2 |
| 3247.8872072 | 2 |
| 3247.8874089 | 0 |
| 3247.8891775 | 0 |
| 3247.8897725 | 3 |
| 3247.8912906 | 5 |
| 3247.8931174 | 1 |
| 3247.8932801 | 2 |
| 3247.8942233 | 1 |
| 3247.8952627 | 3 |
| 3247.8960628 | 1 |
| 3247.8981309 | 3 |
| 3247.8983057 | 1 |
| 3247.900152 | 3 |
| 3247.9018982 | 5 |
| 3247.9019898 | 5 |
| 3247.9028767 | 3 |
| 3247.9041324 | 2 |
| 3247.9052268 | 0 |
| 3247.9058375 | 1 |
| 3247.9074598 | 1 |
| 3247.9079279 | 1 |
| 3247.9090429 | 2 |
| 3247.910691 | 1 |
| 3247.9110596 | 3 |
| 3247.913282 | 1 |
| 3247.9136047 | 2 |
| 3247.9148887 | 3 |
| 3247.9157729 | 4 |
| 3247.9172157 | 1 |
| 3247.9185443 | 4 |
| 3247.9185585 | 0 |
| 3247.9203003 | 1 |
| 3247.9204885 | 0 |
| 3247.9225054 | 6 |
| 3247.9225291 | 2 |
| 3247.9239902 | 5 |
| 3247.9258348 | 3 |
| 3247.9262262 | 3 |
| 3247.9271896 | 4 |
| 3247.9282691 | 2 |
| 3247.9300913 | 2 |
| 3247.9305322 | 4 |
| 3247.932245 | 1 |
| 3247.9323367 | 2 |
| 3247.933295 | 3 |
| 3247.9353641 | 3 |
| 3247.936071 | 2 |
| 3247.9368149 | 3 |
| 3247.9389637 | 4 |
| 3247.9392582 | 1 |
| 3247.9406832 | 2 |
| 3247.940786 | 3 |
| 3247.9428056 | 3 |
| 3247.9432741 | 3 |
| 3247.9443334 | 1 |
| 3247.9448735 | 1 |
| 3247.9458676 | 2 |
| 3247.9480246 | 1 |
| 3247.9486458 | 0 |
| 3247.9508224 | 4 |
| 3247.9514663 | 3 |
| 3247.9517664 | 0 |
| 3247.9542114 | 4 |
| 3247.9543623 | 1 |
| 3247.9557211 | 3 |
| 3247.9560996 | 1 |
| 3247.9573529 | 0 |
| 3247.9578487 | 1 |
| 3247.958718 | 1 |
| 3247.9607155 | 2 |
| 3247.9614868 | 2 |
| 3247.9620783 | 1 |
| 3247.9637967 | 2 |
| 3247.9646943 | 0 |
| 3247.964785 | 3 |
| 3247.9674061 | 2 |
| 3247.968025 | 2 |
| 3247.9680481 | 1 |
| 3247.9701237 | 3 |
| 3247.9701708 | 5 |
| 3247.9727307 | 2 |
| 3247.9728688 | 3 |
| 3247.9737319 | 4 |
| 3247.9746027 | 1 |
| 3247.9765235 | 0 |
| 3247.9782413 | 0 |
| 3247.9789377 | 2 |
| 3247.979945 | 3 |
| 3247.9805633 | 3 |

| | |
|---|---|
| 3247.9820887 | 2 |
| 3247.9824392 | 1 |
| 3247.9829708 | 2 |
| 3247.9857948 | 1 |
| 3247.9862955 | 4 |
| 3247.9863706 | 3 |
| 3247.9868217 | 3 |
| 3247.9889012 | 2 |
| 3247.9902598 | 1 |
| 3247.9910378 | 4 |
| 3247.9931073 | 1 |
| 3247.9934352 | 4 |
| 3247.9945996 | 1 |
| 3247.9947616 | 2 |
| 3247.9957603 | 2 |
| 3247.9981858 | 2 |
| 3247.9986801 | 2 |
| 3247.9996157 | 1 |
| 3248.0005155 | 2 |
| 3248.0013171 | 1 |
| 3248.0020823 | 0 |
| 3248.0045099 | 2 |
| 3248.0052051 | 0 |
| 3248.0060739 | 3 |
| 3248.007487 | 3 |
| 3248.0076425 | 0 |
| 3248.0098556 | 2 |
| 3248.0101923 | 3 |
| 3248.0110848 | 0 |
| 3248.0118893 | 5 |
| 3248.0135501 | 1 |
| 3248.0147559 | 2 |
| 3248.0154036 | 1 |
| 3248.0165679 | 0 |
| 3248.0184489 | 5 |
| 3248.0191583 | 2 |
| 3248.0192164 | 1 |
| 3248.0205084 | 0 |
| 3248.022268 | 4 |
| 3248.0233039 | 5 |
| 3248.0237569 | 3 |
| 3248.0240365 | 4 |
| 3248.0262606 | 4 |
| 3248.027487 | 2 |
| 3248.0286829 | 2 |
| 3248.0295731 | 2 |
| 3248.0299114 | 1 |
| 3248.0317961 | 3 |
| 3248.0318481 | 4 |
| 3248.0326533 | 5 |
| 3248.0350509 | 2 |
| 3248.0351654 | 1 |
| 3248.0371536 | 4 |
| 3248.0373328 | 2 |
| 3248.0390427 | 0 |
| 3248.0400457 | 2 |
| 3248.0410791 | 1 |
| 3248.0414594 | 2 |
| 3248.0425479 | 2 |
| 3248.0443494 | 3 |
| 3248.0450592 | 1 |
| 3248.0455687 | 3 |
| 3248.0475752 | 0 |
| 3248.0478804 | 1 |
| 3248.0492877 | 1 |
| 3248.0506611 | 2 |
| 3248.0512992 | 2 |
| 3248.052725 | 4 |
| 3248.053722 | 2 |
| 3248.0541561 | 6 |
| 3248.055665 | 1 |
| 3248.0569247 | 2 |
| 3248.0573179 | 1 |
| 3248.058831 | 2 |
| 3248.0595683 | 1 |
| 3248.0606091 | 2 |
| 3248.06148 | 2 |
| 3248.0637741 | 3 |
| 3248.0641193 | 2 |
| 3248.0660921 | 1 |
| 3248.0663305 | 0 |
| 3248.0668958 | 2 |
| 3248.0684119 | 1 |
| 3248.0689368 | 4 |
| 3248.0703364 | 2 |
| 3248.0716795 | 2 |
| 3248.0724215 | 5 |
| 3248.0731195 | 1 |
| 3248.0739003 | 6 |
| 3248.0763481 | 2 |
| 3248.0769771 | 2 |
| 3248.078068 | 0 |
| 3248.0784026 | 0 |
| 3248.0796649 | 1 |
| 3248.0810725 | 1 |
| 3248.0821297 | 3 |
| 3248.0830556 | 2 |
| 3248.0844101 | 2 |
| 3248.0854723 | 0 |
| 3248.0860786 | 0 |
| 3248.0877525 | 2 |
| 3248.0887098 | 1 |
| 3248.0888489 | 3 |
| 3248.0908088 | 2 |
| 3248.0915186 | 0 |
| 3248.0922853 | 5 |
| 3248.0940153 | 4 |
| 3248.0949183 | 0 |
| 3248.096535 | 4 |
| 3248.0967674 | 4 |
| 3248.0977638 | 2 |
| 3248.0979781 | 2 |
| 3248.1002084 | 3 |
| 3248.1015559 | 1 |
| 3248.1022613 | 2 |
| 3248.103346 | 4 |
| 3248.1048257 | 2 |
| 3248.1055857 | 1 |
| 3248.1064 | 0 |
| 3248.106922 | 4 |
| 3248.1084837 | 6 |
| 3248.109691 | 4 |
| 3248.1104832 | 1 |
| 3248.1115623 | 4 |
| 3248.1127318 | 2 |
| 3248.1135336 | 0 |
| 3248.1154572 | 2 |
| 3248.1166277 | 0 |
| 3248.1171077 | 3 |
| 3248.1184616 | 2 |
| 3248.1193832 | 2 |
| 3248.1202543 | 1 |
| 3248.1211 | 1 |
| 3248.1224352 | 3 |
| 3248.1226111 | 1 |
| 3248.125097 | 2 |
| 3248.1255382 | 1 |
| 3248.1263928 | 2 |
| 3248.1267543 | 1 |
| 3248.1282526 | 4 |
| 3248.1300403 | 3 |
| 3248.1300906 | 1 |
| 3248.1314529 | 2 |
| 3248.1334229 | 1 |
| 3248.134111 | 1 |
| 3248.135234 | 5 |
| 3248.1362849 | 2 |
| 3248.1375037 | 1 |
| 3248.1378498 | 2 |
| 3248.1384821 | 8 |
| 3248.1409164 | 2 |
| 3248.1409547 | 1 |
| 3248.1421854 | 4 |
| 3248.143285 | 1 |
| 3248.1446406 | 0 |
| 3248.1465227 | 0 |
| 3248.146551 | 4 |
| 3248.1474296 | 1 |
| 3248.1480275 | 1 |
| 3248.1496846 | 2 |
| 3248.1507501 | 6 |
| 3248.1525977 | 3 |
| 3248.1527462 | 3 |
| 3248.153608 | 1 |
| 3248.1553549 | 3 |
| 3248.1563187 | 1 |
| 3248.1580544 | 2 |
| 3248.1585785 | 4 |
| 3248.1590399 | 3 |
| 3248.1594989 | 4 |
| 3248.1625815 | 1 |
| 3248.1625943 | 7 |
| 3248.1629896 | 2 |
| 3248.1648808 | 0 |
| 3248.1655215 | 0 |
| 3248.1661696 | 2 |
| 3248.167921 | 3 |
| 3248.1692389 | 1 |
| 3248.1699144 | 1 |
| 3248.1710045 | 2 |
| 3248.1714997 | 2 |
| 3248.1725337 | 3 |
| 3248.1746126 | 5 |
| 3248.174795 | 1 |
| 3248.1761312 | 3 |
| 3248.1779651 | 4 |
| 3248.1781757 | 2 |
| 3248.1793069 | 1 |
| 3248.1803676 | 3 |
| 3248.1813623 | 4 |
| 3248.1825694 | 2 |
| 3248.1843299 | 1 |
| 3248.1846821 | 2 |
| 3248.1851704 | 3 |
| 3248.1867893 | 0 |
| 3248.1880354 | 0 |
| 3248.1901014 | 3 |
| 3248.1901575 | 2 |
| 3248.1912652 | 5 |
| 3248.1921568 | 2 |
| 3248.1933055 | 0 |
| 3248.1947127 | 3 |
| 3248.1951096 | 4 |
| 3248.1963116 | 1 |
| 3248.1964734 | 1 |
| 3248.1996278 | 3 |
| 3248.1998515 | 4 |
| 3248.2005303 | 0 |
| 3248.2030146 | 4 |
| 3248.2031674 | 2 |
| 3248.2035987 | 1 |
| 3248.2051355 | 2 |
| 3248.2063845 | 2 |
| 3248.206821 | 2 |
| 3248.2086397 | 3 |
| 3248.2088308 | 3 |
| 3248.2099999 | 1 |
| 3248.2118696 | 1 |
| 3248.2121965 | 2 |
| 3248.2143775 | 3 |
| 3248.2144894 | 0 |
| 3248.2154522 | 0 |
| 3248.2171671 | 4 |
| 3248.2172123 | 4 |
| 3248.2196589 | 1 |
| 3248.2203242 | 4 |
| 3248.2210787 | 2 |
| 3248.2212991 | 3 |
| 3248.2241937 | 1 |
| 3248.2242936 | 2 |
| 3248.224853 | 2 |
| 3248.2266886 | 3 |
| 3248.2272836 | 1 |
| 3248.2289398 | 1 |
| 3248.2293381 | 2 |
| 3248.230594 | 4 |
| 3248.2324542 | 3 |
| 3248.2330563 | 2 |
| 3248.2343808 | 3 |
| 3248.2347974 | 2 |
| 3248.2360762 | 2 |
| 3248.2364693 | 4 |
| 3248.2375964 | 1 |
| 3248.2389881 | 1 |
| 3248.2401751 | 4 |
| 3248.2407654 | 2 |
| 3248.2424685 | 3 |
| 3248.2434532 | 0 |
| 3248.2440002 | 2 |
| 3248.2453573 | 4 |
| 3248.2457559 | 4 |
| 3248.2474346 | 1 |
| 3248.2490937 | 0 |
| 3248.250104 | 2 |
| 3248.251853 | 4 |
| 3248.2521665 | 2 |
| 3248.2530438 | 5 |
| 3248.2534413 | 1 |
| 3248.255826 | 2 |
| 3248.2561674 | 3 |
| 3248.256417 | 2 |
| 3248.2591233 | 2 |
| 3248.2593916 | 4 |
| 3248.260735 | 2 |
| 3248.2610591 | 3 |
| 3248.2626256 | 3 |
| 3248.2645897 | 2 |
| 3248.2652463 | 0 |
| 3248.2656974 | 2 |
| 3248.2664959 | 0 |
| 3248.2683357 | 3 |
| 3248.268911 | 3 |
| 3248.2707722 | 1 |
| 3248.2718972 | 3 |
| 3248.2721001 | 1 |
| 3248.2739067 | 2 |
| 3248.2739858 | 2 |
| 3248.2756105 | 2 |
| 3248.2773315 | 2 |
| 3248.2778863 | 4 |
| 3248.2786461 | 4 |
| 3248.2791704 | 4 |
| 3248.2818494 | 2 |
| 3248.2822348 | 1 |
| 3248.282542 | 1 |
| 3248.2840578 | 2 |
| 3248.2849475 | 4 |
| 3248.286016 | 2 |
| 3248.2874863 | 3 |
| 3248.2886779 | 4 |
| 3248.288879 | 1 |
| 3248.2901099 | 0 |
| 3248.2912283 | 1 |
| 3248.293477 | 2 |
| 3248.2936022 | 1 |
| 3248.2945999 | 2 |
| 3248.2949917 | 0 |
| 3248.2966948 | 2 |
| 3248.2979986 | 3 |
| 3248.2980403 | 4 |
| 3248.3005023 | 7 |
| 3248.3007423 | 3 |
| 3248.3021271 | 3 |
| 3248.303 | 4 |
| 3248.304086 | 0 |
| 3248.3051793 | 3 |
| 3248.3065242 | 2 |
| 3248.3068804 | 2 |
| 3248.309381 | 1 |
| 3248.3100119 | 2 |
| 3248.3101633 | 0 |
| 3248.3122002 | 2 |
| 3248.3134339 | 5 |
| 3248.3135726 | 0 |
| 3248.3153676 | 0 |
| 3248.3165907 | 0 |
| 3248.3170168 | 3 |
| 3248.3178891 | 2 |
| 3248.3186758 | 5 |
| 3248.3191414 | 6 |
| 3248.3194815 | 3 |
| 3248.3214491 | 1 |
| 3248.3218778 | 3 |
| 3248.3226216 | 1 |
| 3248.322623 | 1 |
| 3248.3254006 | 1 |
| 3248.3254604 | 5 |
| 3248.326633 | 1 |
| 3248.327756 | 2 |
| 3248.3288387 | 1 |
| 3248.3305542 | 1 |
| 3248.3312387 | 3 |
| 3248.3332124 | 3 |
| 3248.3336062 | 2 |
| 3248.3346131 | 0 |
| 3248.3352742 | 2 |
| 3248.3359887 | 1 |
| 3248.3383842 | 4 |
| 3248.3387182 | 5 |
| 3248.33935 | 1 |
| 3248.3397175 | 3 |
| 3248.34174 | 4 |
| 3248.3430622 | 4 |
| 3248.3444882 | 1 |
| 3248.3451908 | 3 |
| 3248.3464024 | 2 |
| 3248.347432 | 1 |
| 3248.3480483 | 0 |
| 3248.3506438 | 1 |
| 3248.3508011 | 3 |
| 3248.3519173 | 3 |
| 3248.3529868 | 6 |
| 3248.3534295 | 1 |
| 3248.3549905 | 2 |
| 3248.3550664 | 2 |
| 3248.3568572 | 5 |
| 3248.3570894 | 2 |
| 3248.3602694 | 3 |
| 3248.3604107 | 2 |
| 3248.3608851 | 1 |
| 3248.3634544 | 5 |
| 3248.3636767 | 2 |
| 3248.364178 | 5 |

| | |
|---|---|
| 3248.3651951 | 2 |
| 3248.3655273 | 1 |
| 3248.3665432 | 3 |
| 3248.3679981 | 1 |
| 3248.3682498 | 1 |
| 3248.3702237 | 0 |
| 3248.3706756 | 0 |
| 3248.3727665 | 1 |
| 3248.3731142 | 3 |
| 3248.373747 | 3 |
| 3248.3764927 | 4 |
| 3248.3773652 | 1 |
| 3248.3802112 | 3 |
| 3248.3835278 | 3 |
| 3248.3845091 | 1 |
| 3248.3875945 | 3 |
| 3248.3893543 | 4 |
| 3248.3932126 | 2 |
| 3248.3964603 | 1 |
| 3248.3972969 | 2 |
| 3248.4012885 | 2 |
| 3248.4023881 | 4 |
| 3248.4060291 | 2 |
| 3248.4075536 | 0 |
| 3248.4103081 | 3 |
| 3248.4134953 | 5 |
| 3248.4149776 | 1 |
| 3248.4174285 | 2 |
| 3248.4207146 | 1 |
| 3248.4221399 | 0 |
| 3248.4256088 | 4 |
| 3248.426803 | 3 |
| 3248.430156 | 6 |
| 3248.4333984 | 0 |
| 3248.4339285 | 3 |
| 3248.4377233 | 3 |
| 3248.4397631 | 1 |
| 3248.442405 | 5 |
| 3248.4456439 | 0 |
| 3248.4478953 | 0 |
| 3248.4502831 | 5 |
| 3248.4519136 | 4 |
| 3248.4553052 | 1 |
| 3248.4587367 | 4 |
| 3248.4596468 | 5 |
| 3248.462908 | 3 |
| 3248.466015 | 3 |
| 3248.4675228 | 5 |
| 3248.4708334 | 3 |
| 3248.4720178 | 5 |
| 3248.4752933 | 2 |
| 3248.4781202 | 3 |
| 3248.4792222 | 3 |
| 3248.4803791 | 3 |
| 3248.4808983 | 4 |
| 3248.4809445 | 1 |
| 3248.4812419 | 2 |
| 3248.4820048 | 2 |
| 3248.4820078 | 2 |
| 3248.4822592 | 0 |
| 3248.4822849 | 3 |
| 3248.4824547 | 2 |
| 3248.4828888 | 2 |
| 3248.4830324 | 3 |
| 3248.483735 | 2 |
| 3248.4838187 | 4 |
| 3248.4838201 | 5 |
| 3248.4838677 | 2 |
| 3248.4844229 | 3 |
| 3248.4851256 | 3 |
| 3248.4851729 | 1 |
| 3248.4854137 | 1 |
| 3248.4858255 | 5 |
| 3248.4858567 | 1 |
| 3248.4862445 | 4 |
| 3248.4868863 | 2 |
| 3248.4869394 | 2 |
| 3248.4876213 | 3 |
| 3248.4877508 | 2 |
| 3248.4877653 | 1 |
| 3248.4879436 | 5 |
| 3248.4887269 | 2 |
| 3248.4888899 | 3 |
| 3248.4891961 | 0 |
| 3248.4897201 | 4 |
| 3248.4899107 | 0 |
| 3248.4903795 | 4 |
| 3248.4906093 | 3 |
| 3248.4906989 | 2 |
| 3248.4907163 | 2 |
| 3248.4914444 | 4 |
| 3248.4919421 | 1 |
| 3248.4919986 | 2 |
| 3248.4920702 | 0 |
| 3248.4920959 | 2 |
| 3248.4924278 | 1 |
| 3248.4937058 | 0 |
| 3248.4938136 | 2 |
| 3248.4938656 | 3 |
| 3248.4939999 | 0 |
| 3248.4942812 | 2 |
| 3248.4944836 | 2 |
| 3248.4945142 | 3 |
| 3248.4946616 | 2 |
| 3248.4953489 | 2 |
| 3248.4956339 | 2 |
| 3248.4960007 | 2 |
| 3248.4960615 | 1 |
| 3248.4965591 | 4 |
| 3248.4967085 | 3 |
| 3248.4967757 | 2 |
| 3248.4974545 | 1 |
| 3248.4978609 | 7 |
| 3248.4979016 | 1 |
| 3248.4980705 | 1 |
| 3248.498097 | 4 |
| 3248.4981296 | 3 |
| 3248.498308 | 2 |
| 3248.4987939 | 2 |
| 3248.498856 | 3 |
| 3248.4989535 | 2 |
| 3248.499492 | 1 |
| 3248.4995978 | 2 |
| 3248.4996193 | 1 |
| 3248.4996581 | 5 |
| 3248.4996664 | 4 |
| 3248.4998168 | 1 |
| 3248.5001769 | 5 |
| 3248.5002867 | 2 |
| 3248.5002987 | 0 |
| 3248.5010686 | 2 |
| 3248.5011379 | 2 |
| 3248.5011768 | 3 |
| 3248.5013474 | 0 |
| 3248.5015409 | 5 |
| 3248.5016135 | 10 |
| 3248.5018465 | 10 |
| 3248.5020506 | 4 |
| 3248.5020798 | 8 |
| 3248.5022633 | 24 |
| 3248.5024146 | 15 |
| 3248.5027933 | 26 |
| 3248.5029106 | 18 |
| 3248.5029192 | 16 |
| 3248.5030153 | 27 |
| 3248.5030747 | 28 |
| 3248.5032979 | 34 |
| 3248.5033444 | 27 |
| 3248.5037652 | 35 |
| 3248.5039314 | 38 |
| 3248.5041748 | 25 |
| 3248.504309 | 39 |
| 3248.5044661 | 40 |
| 3248.5047394 | 36 |
| 3248.5050052 | 35 |
| 3248.5050448 | 52 |
| 3248.5050562 | 42 |
| 3248.5054814 | 40 |
| 3248.505494 | 39 |
| 3248.5054979 | 46 |
| 3248.5055565 | 47 |
| 3248.5056764 | 46 |
| 3248.5056962 | 44 |
| 3248.5061728 | 40 |
| 3248.5062909 | 37 |
| 3248.5066132 | 53 |
| 3248.5071555 | 33 |
| 3248.5072624 | 38 |
| 3248.5073411 | 42 |
| 3248.5074173 | 46 |
| 3248.5074422 | 39 |
| 3248.507569 | 42 |
| 3248.5075719 | 30 |
| 3248.507983 | 35 |
| 3248.5081974 | 37 |
| 3248.5083614 | 35 |
| 3248.5085361 | 41 |
| 3248.5086779 | 30 |
| 3248.508722 | 48 |
| 3248.5087371 | 56 |
| 3248.5088819 | 42 |
| 3248.5089202 | 54 |
| 3248.5094139 | 52 |
| 3248.5096415 | 64 |
| 3248.509918 | 69 |
| 3248.5099725 | 52 |
| 3248.5100249 | 68 |
| 3248.5100479 | 38 |
| 3248.510669 | 40 |
| 3248.5107347 | 49 |
| 3248.5108023 | 27 |
| 3248.5111029 | 36 |
| 3248.5111346 | 21 |
| 3248.511719 | 17 |
| 3248.511733 | 18 |
| 3248.5119567 | 17 |
| 3248.5119782 | 29 |
| 3248.5122473 | 5 |
| 3248.5126058 | 8 |
| 3248.5126133 | 6 |
| 3248.5128916 | 8 |
| 3248.512973 | 2 |
| 3248.5130038 | 4 |
| 3248.5132887 | 1 |
| 3248.5135126 | 4 |
| 3248.5135145 | 3 |
| 3248.5139625 | 4 |
| 3248.5140811 | 1 |
| 3248.5141259 | 0 |
| 3248.5141459 | 0 |
| 3248.5141999 | 0 |
| 3248.5150557 | 2 |
| 3248.5153838 | 3 |
| 3248.5154819 | 2 |
| 3248.5157703 | 3 |
| 3248.5160733 | 0 |
| 3248.5161449 | 2 |
| 3248.5161764 | 3 |
| 3248.5163687 | 2 |
| 3248.5166126 | 1 |
| 3248.5167926 | 6 |
| 3248.5170177 | 3 |
| 3248.5170379 | 1 |
| 3248.5176302 | 2 |
| 3248.51769 | 2 |
| 3248.5178053 | 3 |
| 3248.5178319 | 2 |
| 3248.5185105 | 3 |
| 3248.5187198 | 2 |
| 3248.5191261 | 3 |
| 3248.5191845 | 5 |
| 3248.5192035 | 2 |
| 3248.5197685 | 4 |
| 3248.5198102 | 3 |
| 3248.5199912 | 1 |
| 3248.5202008 | 3 |
| 3248.5202128 | 1 |
| 3248.5210782 | 3 |
| 3248.5211269 | 4 |
| 3248.5216162 | 5 |
| 3248.5219398 | 3 |
| 3248.5232225 | 3 |
| 3248.5237536 | 3 |
| 3248.5249473 | 3 |
| 3248.5262945 | 5 |
| 3248.5271198 | 5 |
| 3248.5276283 | 1 |
| 3248.5281162 | 2 |
| 3248.5302044 | 2 |
| 3248.5306459 | 1 |
| 3248.5315949 | 1 |
| 3248.5330548 | 1 |
| 3248.5334251 | 2 |
| 3248.5346086 | 1 |
| 3248.5356553 | 3 |
| 3248.5365942 | 1 |
| 3248.5367881 | 1 |
| 3248.5381994 | 5 |
| 3248.5390384 | 1 |
| 3248.5404297 | 1 |
| 3248.5410337 | 2 |
| 3248.5420151 | 0 |
| 3248.5432203 | 4 |
| 3248.5441446 | 8 |
| 3248.5451378 | 4 |
| 3248.5459394 | 8 |
| 3248.5468114 | 0 |
| 3248.5483399 | 3 |
| 3248.548359 | 1 |
| 3248.5501293 | 4 |
| 3248.5505054 | 2 |
| 3248.5516743 | 3 |
| 3248.5523617 | 2 |
| 3248.5536487 | 1 |
| 3248.5549285 | 1 |
| 3248.5552212 | 2 |
| 3248.5566744 | 2 |
| 3248.557205 | 4 |
| 3248.558649 | 3 |
| 3248.5597103 | 1 |
| 3248.5597544 | 2 |
| 3248.5606581 | 2 |
| 3248.5618272 | 0 |
| 3248.5624751 | 2 |
| 3248.5638141 | 1 |
| 3248.5640793 | 0 |
| 3248.5656902 | 1 |
| 3248.5661373 | 2 |
| 3248.5669411 | 2 |
| 3248.5679853 | 2 |
| 3248.5689575 | 2 |
| 3248.5702508 | 0 |
| 3248.5704133 | 3 |
| 3248.571 | 1 |
| 3248.5721434 | 1 |
| 3248.5734863 | 2 |
| 3248.5743406 | 1 |
| 3248.5753782 | 4 |
| 3248.5762934 | 5 |
| 3248.5776027 | 1 |
| 3248.577992 | 3 |
| 3248.5790864 | 0 |
| 3248.5798717 | 2 |
| 3248.5811776 | 1 |
| 3248.5824298 | 3 |
| 3248.5832683 | 3 |
| 3248.5838762 | 0 |
| 3248.5848085 | 3 |
| 3248.5858863 | 2 |
| 3248.5874467 | 1 |
| 3248.5877405 | 3 |
| 3248.5893753 | 1 |
| 3248.5895232 | 2 |
| 3248.5907539 | 2 |
| 3248.5914598 | 1 |
| 3248.5925093 | 1 |
| 3248.5938906 | 3 |
| 3248.5942508 | 5 |
| 3248.5955133 | 2 |
| 3248.5959892 | 0 |
| 3248.5966784 | 2 |
| 3248.5985567 | 2 |
| 3248.5993253 | 3 |
| 3248.6006688 | 1 |
| 3248.60093 | 2 |
| 3248.6017514 | 4 |
| 3248.6032596 | 1 |
| 3248.6034225 | 3 |
| 3248.6047995 | 4 |
| 3248.6056045 | 2 |
| 3248.6065243 | 4 |
| 3248.6072641 | 2 |
| 3248.6081929 | 3 |
| 3248.6092911 | 2 |
| 3248.6093312 | 3 |
| 3248.6109669 | 1 |
| 3248.6115093 | 0 |
| 3248.6128502 | 0 |
| 3248.6139672 | 5 |
| 3248.6143645 | 1 |
| 3248.6155682 | 2 |
| 3248.6156095 | 1 |
| 3248.6182361 | 2 |
| 3248.618637 | 1 |
| 3248.6194627 | 2 |
| 3248.6205731 | 2 |
| 3248.621497 | 2 |
| 3248.6218788 | 3 |
| 3248.6235476 | 4 |
| 3248.6237303 | 2 |
| 3248.6248541 | 2 |
| 3248.6265095 | 1 |
| 3248.6271796 | 3 |
| 3248.6277574 | 1 |
| 3248.6287394 | 4 |
| 3248.6291472 | 3 |
| 3248.6300047 | 0 |
| 3248.63141 | 2 |
| 3248.6326783 | 2 |
| 3248.6331037 | 3 |
| 3248.6342916 | 5 |
| 3248.6356366 | 3 |
| 3248.6365649 | 1 |

| | |
|---|---|
| 3248.6377828 | 2 |
| 3248.6381241 | 0 |
| 3248.6387766 | 3 |
| 3248.6395254 | 0 |
| 3248.6413509 | 5 |
| 3248.6424909 | 4 |
| 3248.6431204 | 1 |
| 3248.6441344 | 1 |
| 3248.644957 | 1 |
| 3248.6457108 | 1 |
| 3248.6463595 | 4 |
| 3248.6472879 | 3 |
| 3248.648601 | 1 |
| 3248.649026 | 4 |
| 3248.6500962 | 3 |
| 3248.6510016 | 1 |
| 3248.6516328 | 1 |
| 3248.6527687 | 2 |
| 3248.6536113 | 2 |
| 3248.6551483 | 5 |
| 3248.655826 | 2 |
| 3248.6566658 | 4 |
| 3248.6574114 | 2 |
| 3248.6581156 | 1 |
| 3248.6591659 | 2 |
| 3248.6596119 | 1 |
| 3248.6609394 | 4 |
| 3248.6620421 | 1 |
| 3248.6627591 | 3 |
| 3248.6639426 | 6 |
| 3248.6648865 | 0 |
| 3248.6652776 | 2 |
| 3248.6665938 | 2 |
| 3248.6668093 | 2 |
| 3248.6686524 | 5 |
| 3248.6689192 | 1 |
| 3248.6704083 | 2 |
| 3248.6718934 | 6 |
| 3248.6722234 | 3 |
| 3248.6735652 | 2 |
| 3248.6748627 | 2 |
| 3248.6754325 | 2 |
| 3248.6766272 | 3 |
| 3248.6774216 | 3 |
| 3248.6781443 | 4 |
| 3248.6787207 | 3 |
| 3248.6799139 | 3 |
| 3248.6810229 | 2 |
| 3248.6814546 | 4 |
| 3248.6831205 | 2 |
| 3248.6833542 | 1 |
| 3248.6844269 | 1 |
| 3248.6858282 | 1 |
| 3248.6866379 | 3 |
| 3248.6874751 | 3 |
| 3248.6886702 | 3 |
| 3248.6893902 | 4 |
| 3248.6902511 | 2 |
| 3248.6910024 | 3 |
| 3248.691629 | 4 |
| 3248.6934632 | 1 |
| 3248.693821 | 1 |
| 3248.6946897 | 5 |
| 3248.6952526 | 4 |
| 3248.6961902 | 4 |
| 3248.6977668 | 2 |
| 3248.6981695 | 4 |
| 3248.6992642 | 3 |
| 3248.7000664 | 3 |
| 3248.7010288 | 1 |
| 3248.7018304 | 6 |
| 3248.7025727 | 2 |
| 3248.7042834 | 1 |
| 3248.7046182 | 4 |
| 3248.7060608 | 6 |
| 3248.7065902 | 2 |
| 3248.7075241 | 6 |
| 3248.708227 | 1 |
| 3248.7086947 | 1 |
| 3248.7099605 | 0 |
| 3248.7112846 | 2 |
| 3248.7123842 | 1 |
| 3248.7126425 | 2 |
| 3248.7134764 | 3 |
| 3248.7144616 | 1 |
| 3248.7153339 | 6 |
| 3248.7163767 | 1 |
| 3248.7177786 | 1 |
| 3248.7188458 | 7 |
| 3248.7204079 | 2 |
| 3248.7209539 | 4 |
| 3248.7210542 | 4 |
| 3248.7226789 | 1 |
| 3248.7234808 | 1 |
| 3248.7245585 | 3 |
| 3248.724904 | 2 |
| 3248.7266426 | 2 |
| 3248.7274147 | 1 |
| 3248.7285014 | 2 |
| 3248.7294603 | 2 |
| 3248.7299486 | 2 |
| 3248.7312953 | 1 |
| 3248.7321158 | 4 |
| 3248.7328764 | 1 |
| 3248.733314 | 3 |
| 3248.7346575 | 1 |
| 3248.7356716 | 4 |
| 3248.7358492 | 4 |
| 3248.7375069 | 1 |
| 3248.7380891 | 3 |
| 3248.7394706 | 5 |
| 3248.741011 | 2 |
| 3248.741344 | 4 |
| 3248.7420923 | 6 |
| 3248.7431014 | 1 |
| 3248.7434383 | 2 |
| 3248.7451498 | 1 |
| 3248.7452177 | 3 |
| 3248.7462534 | 0 |
| 3248.7479206 | 4 |
| 3248.7482855 | 1 |
| 3248.7491445 | 2 |
| 3248.7500857 | 2 |
| 3248.7501168 | 5 |
| 3248.7514778 | 0 |
| 3248.7516313 | 1 |
| 3248.7537077 | 2 |
| 3248.7547516 | 2 |
| 3248.7556453 | 1 |
| 3248.7566246 | 4 |
| 3248.7571255 | 2 |
| 3248.7582277 | 1 |
| 3248.7595316 | 1 |
| 3248.7597652 | 1 |
| 3248.7615589 | 2 |
| 3248.7619539 | 1 |
| 3248.7633742 | 1 |
| 3248.7635358 | 2 |
| 3248.7659641 | 2 |
| 3248.7660925 | 3 |
| 3248.766511 | 3 |
| 3248.7675562 | 2 |
| 3248.7693626 | 1 |
| 3248.7696024 | 3 |
| 3248.7708391 | 0 |
| 3248.771475 | 1 |
| 3248.773018 | 4 |
| 3248.7738755 | 2 |
| 3248.7742838 | 2 |
| 3248.7757622 | 1 |
| 3248.7764175 | 7 |
| 3248.7775456 | 0 |
| 3248.7786947 | 2 |
| 3248.7791711 | 2 |
| 3248.7804171 | 1 |
| 3248.7810965 | 4 |
| 3248.781582 | 1 |
| 3248.7824969 | 3 |
| 3248.7834966 | 0 |
| 3248.7850953 | 2 |
| 3248.7856567 | 2 |
| 3248.7861285 | 0 |
| 3248.7861542 | 3 |
| 3248.7878203 | 0 |
| 3248.7894089 | 1 |
| 3248.7894466 | 1 |
| 3248.7908828 | 2 |
| 3248.791927 | 1 |
| 3248.7929125 | 4 |
| 3248.7938264 | 0 |
| 3248.7940974 | 0 |
| 3248.795306 | 2 |
| 3248.7959219 | 2 |
| 3248.7961278 | 1 |
| 3248.796269 | 4 |
| 3248.7978313 | 2 |
| 3248.7982745 | 0 |
| 3248.798765 | 1 |
| 3248.7997897 | 2 |
| 3248.8000612 | 1 |
| 3248.8002765 | 3 |
| 3248.801241 | 1 |
| 3248.8012883 | 1 |
| 3248.803075 | 1 |
| 3248.8036234 | 1 |
| 3248.8038294 | 0 |
| 3248.8044656 | 3 |
| 3248.8054349 | 1 |
| 3248.8057915 | 2 |
| 3248.8072295 | 2 |
| 3248.8074951 | 2 |
| 3248.8081638 | 0 |
| 3248.8087945 | 3 |
| 3248.8094293 | 1 |
| 3248.8101223 | 4 |
| 3248.8109604 | 3 |
| 3248.8117947 | 6 |
| 3248.8136135 | 2 |
| 3248.8170652 | 2 |
| 3248.8196662 | 3 |
| 3248.8215743 | 3 |
| 3248.8244201 | 5 |
| 3248.8256304 | 1 |
| 3248.8286799 | 4 |
| 3248.8323709 | 2 |
| 3248.8336573 | 3 |
| 3248.8363287 | 2 |
| 3248.838048 | 1 |
| 3248.8406114 | 1 |
| 3248.8434499 | 3 |
| 3248.8454549 | 3 |
| 3248.8491896 | 4 |
| 3248.8498676 | 3 |
| 3248.8537999 | 4 |
| 3248.8569922 | 2 |
| 3248.8581288 | 1 |
| 3248.8616734 | 3 |
| 3248.862998 | 2 |
| 3248.8657622 | 3 |
| 3248.8677006 | 3 |
| 3248.8704494 | 2 |
| 3248.8733379 | 3 |
| 3248.8748645 | 3 |
| 3248.8782774 | 2 |
| 3248.8813896 | 2 |
| 3248.8820858 | 4 |
| 3248.8856283 | 1 |
| 3248.8875527 | 2 |
| 3248.8903742 | 0 |
| 3248.8924206 | 0 |
| 3248.8951355 | 4 |
| 3248.8987858 | 2 |
| 3248.8994666 | 2 |
| 3248.9027564 | 1 |
| 3248.9041096 | 4 |
| 3248.9077644 | 5 |
| 3248.910987 | 3 |
| 3248.9122044 | 2 |
| 3248.9154432 | 1 |
| 3248.9166422 | 4 |
| 3248.9200587 | 0 |
| 3248.9228012 | 1 |
| 3248.9245859 | 3 |
| 3248.9275506 | 2 |
| 3248.9289689 | 0 |
| 3248.9325985 | 2 |
| 3248.9350048 | 3 |
| 3248.9369573 | 3 |
| 3248.9400472 | 0 |
| 3248.9427714 | 3 |
| 3248.9445262 | 3 |
| 3248.9478689 | 3 |
| 3248.9491654 | 3 |
| 3248.9528091 | 4 |
| 3248.9552403 | 2 |
| 3248.9572381 | 4 |
| 3248.9606504 | 2 |
| 3248.9617717 | 2 |
| 3248.9647043 | 4 |
| 3248.9659198 | 3 |
| 3248.969041 | 1 |
| 3248.9722004 | 3 |
| 3248.9740563 | 3 |
| 3248.9768648 | 3 |
| 3248.9788292 | 3 |
| 3248.9819367 | 2 |
| 3248.9848622 | 3 |
| 3248.9865335 | 2 |
| 3248.9899434 | 4 |
| 3248.9916215 | 3 |
| 3248.994874 | 1 |
| 3248.9969064 | 3 |
| 3248.9995781 | 1 |
| 3249.00291 | 1 |
| 3249.0038015 | 1 |
| 3249.0059911 | 3 |
| 3249.0101077 | 3 |
| 3249.0106389 | 2 |
| 3249.0135162 | 2 |
| 3249.0162211 | 1 |
| 3249.0189142 | 2 |
| 3249.0218577 | 0 |
| 3249.0239879 | 1 |
| 3249.0264524 | 1 |
| 3249.0282612 | 3 |
| 3249.0316192 | 1 |
| 3249.034829 | 6 |
| 3249.0357278 | 1 |
| 3249.0397953 | 1 |
| 3249.0409958 | 2 |
| 3249.0450755 | 0 |
| 3249.0456291 | 2 |
| 3249.0493097 | 4 |
| 3249.0527289 | 4 |
| 3249.0536436 | 1 |
| 3249.0568844 | 3 |
| 3249.0583194 | 2 |
| 3249.0612758 | 4 |
| 3249.064904 | 4 |
| 3249.0657236 | 3 |
| 3249.0688794 | 1 |
| 3249.0717072 | 1 |
| 3249.0730239 | 2 |
| 3249.0764343 | 2 |
| 3249.0782373 | 2 |
| 3249.0822769 | 2 |
| 3249.0851426 | 1 |
| 3249.0865669 | 3 |
| 3249.089864 | 2 |
| 3249.0911526 | 3 |
| 3249.0935214 | 2 |
| 3249.0957328 | 2 |
| 3249.0983604 | 2 |
| 3249.1018505 | 0 |
| 3249.1028717 | 2 |
| 3249.1058691 | 7 |
| 3249.109328 | 3 |
| 3249.1104703 | 4 |
| 3249.1143096 | 2 |
| 3249.1163163 | 2 |
| 3249.1187436 | 1 |
| 3249.1221904 | 3 |
| 3249.1233939 | 1 |
| 3249.1265616 | 7 |
| 3249.1286497 | 2 |
| 3249.1315494 | 2 |
| 3249.133936 | 3 |
| 3249.1362722 | 0 |
| 3249.1391714 | 1 |
| 3249.1407234 | 2 |
| 3249.144594 | 3 |
| 3249.1457453 | 1 |
| 3249.148641 | 3 |
| 3249.1516342 | 0 |
| 3249.153803 | 2 |
| 3249.1563746 | 0 |
| 3249.1584023 | 3 |
| 3249.1610761 | 1 |
| 3249.1643385 | 1 |
| 3249.1653526 | 2 |
| 3249.1686467 | 2 |
| 3249.1722188 | 3 |
| 3249.1730386 | 2 |
| 3249.1764925 | 0 |
| 3249.1784487 | 1 |
| 3249.1814317 | 1 |
| 3249.1840114 | 0 |
| 3249.1859673 | 1 |
| 3249.1892514 | 2 |
| 3249.1907121 | 0 |
| 3249.1938571 | 3 |
| 3249.1956803 | 1 |
| 3249.1977485 | 3 |
| 3249.2012473 | 1 |
| 3249.2031996 | 6 |
| 3249.2064146 | 2 |
| 3249.207863 | 2 |
| 3249.211504 | 0 |
| 3249.2139219 | 2 |
| 3249.2156192 | 1 |
| 3249.2191389 | 3 |
| 3249.2204453 | 3 |
| 3249.2243952 | 2 |

| | |
|---|---|
| 3249.2255216 | 7 |
| 3249.228327 | 1 |
| 3249.2319384 | 2 |
| 3249.2332303 | 2 |
| 3249.2366144 | 1 |
| 3249.2389766 | 3 |
| 3249.2414311 | 4 |
| 3249.2436316 | 1 |
| 3249.2454622 | 4 |
| 3249.2491326 | 2 |
| 3249.2493902 | 2 |
| 3249.2539179 | 2 |
| 3249.2567442 | 1 |
| 3249.2587088 | 1 |
| 3249.2607667 | 1 |
| 3249.2633578 | 1 |
| 3249.2653683 | 0 |
| 3249.269014 | 0 |
| 3249.270698 | 1 |
| 3249.274223 | 2 |
| 3249.2754329 | 3 |
| 3249.2783995 | 4 |
| 3249.2812105 | 3 |
| 3249.2830405 | 2 |
| 3249.2858451 | 2 |
| 3249.2879797 | 1 |
| 3249.2904968 | 3 |
| 3249.2943946 | 1 |
| 3249.2952548 | 2 |
| 3249.2984864 | 0 |
| 3249.3002272 | 1 |
| 3249.3036507 | 1 |
| 3249.3066322 | 3 |
| 3249.3084409 | 2 |
| 3249.3117129 | 3 |
| 3249.3133037 | 1 |
| 3249.3160561 | 2 |
| 3249.3180355 | 2 |
| 3249.3210926 | 6 |
| 3249.324336 | 1 |
| 3249.3256116 | 1 |
| 3249.3282258 | 2 |
| 3249.3309764 | 1 |
| 3249.3325884 | 4 |
| 3249.3349638 | 2 |
| 3249.3373015 | 3 |
| 3249.3405666 | 1 |
| 3249.3434461 | 2 |
| 3249.3456735 | 2 |
| 3249.3484966 | 1 |
| 3249.3498338 | 0 |
| 3249.3532474 | 3 |
| 3249.3568062 | 4 |
| 3249.3576007 | 2 |
| 3249.361085 | 1 |
| 3249.3627682 | 5 |
| 3249.3660558 | 1 |
| 3249.3675325 | 2 |
| 3249.3708667 | 2 |
| 3249.3740671 | 3 |
| 3249.3750746 | 3 |
| 3249.3781313 | 2 |
| 3249.3793152 | 3 |
| 3249.3824652 | 2 |
| 3249.3859068 | 3 |
| 3249.3872555 | 4 |
| 3249.391179 | 5 |
| 3249.3928007 | 4 |
| 3249.3960051 | 2 |
| 3249.3976833 | 0 |
| 3249.4004838 | 1 |
| 3249.4029302 | 2 |
| 3249.4043886 | 1 |
| 3249.4077267 | 2 |
| 3249.4112102 | 4 |
| 3249.4124654 | 1 |
| 3249.4155509 | 2 |
| 3249.4165657 | 1 |
| 3249.4200072 | 2 |
| 3249.4232888 | 5 |
| 3249.4251283 | 4 |
| 3249.4280918 | 0 |
| 3249.4292298 | 1 |
| 3249.4327413 | 1 |
| 3249.4351822 | 0 |
| 3249.4372341 | 2 |
| 3249.4401027 | 3 |
| 3249.4414251 | 0 |
| 3249.4448479 | 1 |
| 3249.4462089 | 3 |
| 3249.4499983 | 1 |
| 3249.4530089 | 1 |
| 3249.4547831 | 3 |
| 3249.4576705 | 4 |
| 3249.4591302 | 1 |
| 3249.4626454 | 7 |
| 3249.4661139 | 3 |
| 3249.4666536 | 5 |
| 3249.4701579 | 3 |
| 3249.4733699 | 3 |
| 3249.474104 | 1 |
| 3249.4769338 | 1 |
| 3249.4788255 | 1 |
| 3249.4811983 | 0 |
| 3249.4850865 | 1 |
| 3249.4870372 | 1 |
| 3249.4896738 | 3 |
| 3249.4913122 | 2 |
| 3249.4949187 | 5 |
| 3249.4957693 | 2 |
| 3249.4988191 | 3 |
| 3249.5023035 | 1 |
| 3249.5036325 | 3 |
| 3249.507239 | 2 |
| 3249.5097669 | 1 |
| 3249.5120256 | 3 |
| 3249.514576 | 3 |
| 3249.5162986 | 5 |
| 3249.5189601 | 3 |
| 3249.5206498 | 4 |
| 3249.52409 | 3 |
| 3249.5268217 | 5 |
| 3249.5282638 | 6 |
| 3249.5317361 | 2 |
| 3249.5340807 | 3 |
| 3249.5361442 | 0 |
| 3249.5396947 | 0 |
| 3249.5409853 | 1 |
| 3249.5439228 | 1 |
| 3249.5463843 | 1 |
| 3249.5490166 | 1 |
| 3249.5519553 | 1 |
| 3249.5534704 | 3 |
| 3249.55633 | 3 |
| 3249.557776 | 4 |
| 3249.5610388 | 5 |
| 3249.5645765 | 1 |
| 3249.5648658 | 0 |
| 3249.5684227 | 1 |
| 3249.5711527 | 3 |
| 3249.5735853 | 1 |
| 3249.5765795 | 2 |
| 3249.5778687 | 2 |
| 3249.5815269 | 2 |
| 3249.5845858 | 2 |
| 3249.5860435 | 0 |
| 3249.5892193 | 5 |
| 3249.5909868 | 0 |
| 3249.5934488 | 1 |
| 3249.5947083 | 2 |
| 3249.5989119 | 5 |
| 3249.6018244 | 3 |
| 3249.6027485 | 1 |
| 3249.6062623 | 0 |
| 3249.6095903 | 4 |
| 3249.6105304 | 6 |
| 3249.6146169 | 1 |
| 3249.6152366 | 2 |
| 3249.6184032 | 4 |
| 3249.6197023 | 0 |
| 3249.6228611 | 0 |
| 3249.626677 | 1 |
| 3249.6275658 | 8 |
| 3249.6305823 | 1 |
| 3249.6334655 | 4 |
| 3249.6356257 | 4 |
| 3249.6375401 | 2 |
| 3249.6414828 | 0 |
| 3249.6431301 | 1 |
| 3249.6461043 | 2 |
| 3249.6475221 | 1 |
| 3249.6511643 | 4 |
| 3249.6535308 | 4 |
| 3249.6555841 | 2 |
| 3249.6586683 | 3 |
| 3249.6592823 | 1 |
| 3249.6634771 | 2 |
| 3249.6663099 | 2 |
| 3249.6674376 | 4 |
| 3249.6702449 | 0 |
| 3249.6726793 | 4 |
| 3249.6756617 | 4 |
| 3249.6777262 | 1 |
| 3249.6806905 | 3 |
| 3249.6842619 | 4 |
| 3249.6853892 | 2 |
| 3249.6883543 | 2 |
| 3249.6896356 | 2 |
| 3249.6932057 | 3 |
| 3249.696699 | 1 |
| 3249.6978951 | 2 |
| 3249.7012774 | 1 |
| 3249.7028166 | 2 |
| 3249.7056902 | 4 |
| 3249.7082714 | 1 |
| 3249.7093364 | 5 |
| 3249.7131181 | 2 |
| 3249.7167103 | 0 |
| 3249.7174521 | 3 |
| 3249.7210564 | 1 |
| 3249.7230049 | 2 |
| 3249.7254191 | 0 |
| 3249.727684 | 4 |
| 3249.7306713 | 2 |
| 3249.7340029 | 2 |
| 3249.7350241 | 2 |
| 3249.7381566 | 2 |
| 3249.7415099 | 2 |
| 3249.7426187 | 0 |
| 3249.7458522 | 3 |
| 3249.746972 | 3 |
| 3249.750309 | 1 |
| 3249.752463 | 1 |
| 3249.7556927 | 2 |
| 3249.758832 | 4 |
| 3249.7605405 | 4 |
| 3249.7635753 | 3 |
| 3249.764791 | 1 |
| 3249.7685175 | 2 |
| 3249.7711366 | 5 |
| 3249.7724694 | 2 |
| 3249.7759649 | 0 |
| 3249.7794166 | 2 |
| 3249.7803083 | 3 |
| 3249.7833607 | 0 |
| 3249.7854153 | 4 |
| 3249.7886512 | 5 |
| 3249.7917783 | 2 |
| 3249.7933789 | 2 |
| 3249.7964738 | 3 |
| 3249.7973321 | 1 |
| 3249.8005728 | 3 |
| 3249.8042444 | 2 |
| 3249.8049523 | 2 |
| 3249.8081755 | 2 |
| 3249.8125354 | 2 |
| 3249.813266 | 4 |
| 3249.8170489 | 4 |
| 3249.8187495 | 3 |
| 3249.8216587 | 2 |
| 3249.8231161 | 3 |
| 3249.8270338 | 2 |
| 3249.8298293 | 0 |
| 3249.8312258 | 1 |
| 3249.8335835 | 1 |
| 3249.8368074 | 3 |
| 3249.8381172 | 3 |
| 3249.841084 | 2 |
| 3249.843353 | 2 |
| 3249.8459675 | 4 |
| 3249.8480971 | 1 |
| 3249.8513868 | 2 |
| 3249.8545108 | 2 |
| 3249.8559285 | 1 |
| 3249.8591146 | 1 |
| 3249.8609558 | 2 |
| 3249.8642898 | 3 |
| 3249.86683 | 1 |
| 3249.8686161 | 1 |
| 3249.8725466 | 2 |
| 3249.8746412 | 1 |
| 3249.875314 | 0 |
| 3249.8788083 | 2 |
| 3249.8804706 | 0 |
| 3249.8833323 | 2 |
| 3249.8852475 | 2 |
| 3249.8881032 | 2 |
| 3249.8918944 | 1 |
| 3249.8929126 | 0 |
| 3249.8958837 | 1 |
| 3249.8978445 | 4 |
| 3249.9011903 | 1 |
| 3249.9040619 | 2 |
| 3249.9060761 | 1 |
| 3249.9090303 | 1 |
| 3249.9105687 | 2 |
| 3249.9139226 | 0 |
| 3249.9176076 | 2 |
| 3249.9185988 | 1 |
| 3249.922144 | 1 |
| 3249.9233614 | 3 |
| 3249.926562 | 5 |
| 3249.9280169 | 3 |
| 3249.9303729 | 3 |
| 3249.9339297 | 2 |
| 3249.9368637 | 4 |
| 3249.9378394 | 1 |
| 3249.9419553 | 2 |
| 3249.9428993 | 2 |
| 3249.9465391 | 1 |
| 3249.9482953 | 1 |
| 3249.951466 | 1 |
| 3249.9545846 | 4 |
| 3249.9564701 | 2 |
| 3249.959043 | 2 |
| 3249.9604997 | 1 |
| 3249.9641378 | 3 |
| 3249.9669822 | 1 |
| 3249.9681499 | 2 |
| 3249.9713064 | 0 |
| 3249.9721997 | 6 |
| 3249.9758206 | 4 |
| 3249.9776098 | 3 |
| 3249.9809508 | 0 |
| 3249.981056 | 6 |
| 3249.9838515 | 0 |
| 3249.9843885 | 3 |
| 3249.9860297 | 3 |
| 3249.9868282 | 1 |
| 3249.9882459 | 3 |
| 3249.9888835 | 2 |
| 3249.9904136 | 6 |
| 3249.9916814 | 3 |
| 3249.9927943 | 1 |
| 3249.993248 | 1 |
| 3249.9964614 | 3 |
| 3249.9967095 | 2 |
| 3249.9976256 | 2 |
| 3249.9980571 | 2 |
| 3250.0012314 | 1 |
| 3250.0013407 | 3 |
| 3250.0023698 | 3 |
| 3250.0037271 | 1 |
| 3250.0044662 | 1 |
| 3250.0060509 | 0 |
| 3250.0091888 | 2 |
| 3250.0106427 | 4 |
| 3250.01301 | 3 |
| 3250.016826 | 2 |
| 3250.0182467 | 1 |
| 3250.0217749 | 2 |
| 3250.0233262 | 0 |
| 3250.0263745 | 5 |
| 3250.0280388 | 3 |
| 3250.0314474 | 1 |
| 3250.0335411 | 3 |
| 3250.0356773 | 1 |
| 3250.0391114 | 2 |
| 3250.0416386 | 0 |
| 3250.0432246 | 2 |
| 3250.0460981 | 3 |
| 3250.0478049 | 2 |
| 3250.0504329 | 1 |
| 3250.0536026 | 5 |
| 3250.0555568 | 5 |
| 3250.0583954 | 4 |
| 3250.0604299 | 2 |
| 3250.0636699 | 1 |
| 3250.0663963 | 3 |
| 3250.0679121 | 0 |
| 3250.0715286 | 1 |
| 3250.0727592 | 3 |
| 3250.0758206 | 3 |
| 3250.0771601 | 3 |
| 3250.0809363 | 2 |
| 3250.0835678 | 0 |
| 3250.0853173 | 3 |
| 3250.0886259 | 2 |
| 3250.0898524 | 4 |
| 3250.0937218 | 3 |
| 3250.0963416 | 3 |
| 3250.0980087 | 1 |
| 3250.1008219 | 0 |

| | |
|---|---|
| 3250.1018411 | 3 |
| 3250.1049107 | 3 |
| 3250.1086213 | 2 |
| 3250.1099636 | 2 |
| 3250.1130691 | 2 |
| 3250.1148729 | 2 |
| 3250.1174099 | 4 |
| 3250.1202813 | 4 |
| 3250.121886 | 4 |
| 3250.1255406 | 3 |
| 3250.126667 | 2 |
| 3250.1302325 | 3 |
| 3250.1332246 | 2 |
| 3250.1345967 | 3 |
| 3250.1378062 | 1 |
| 3250.139652 | 4 |
| 3250.1422986 | 4 |
| 3250.1452688 | 1 |
| 3250.1471466 | 2 |
| 3250.1498567 | 1 |
| 3250.1512918 | 4 |
| 3250.1545383 | 2 |
| 3250.1578574 | 3 |
| 3250.1592197 | 5 |
| 3250.1623021 | 1 |
| 3250.1639634 | 3 |
| 3250.1668633 | 0 |
| 3250.1701545 | 4 |
| 3250.172619 | 1 |
| 3250.1757021 | 2 |
| 3250.176955 | 5 |
| 3250.1795432 | 3 |
| 3250.1829667 | 2 |
| 3250.1842713 | 1 |
| 3250.1870825 | 0 |
| 3250.1886886 | 5 |
| 3250.191868 | 4 |
| 3250.1936425 | 2 |
| 3250.1969691 | 3 |
| 3250.1996576 | 3 |
| 3250.2015165 | 2 |
| 3250.2044388 | 1 |
| 3250.206212 | 2 |
| 3250.2087736 | 6 |
| 3250.2124161 | 1 |
| 3250.2131332 | 1 |
| 3250.216764 | 1 |
| 3250.2200827 | 1 |
| 3250.2214959 | 6 |
| 3250.2247476 | 3 |
| 3250.2265106 | 0 |
| 3250.229851 | 1 |
| 3250.2322581 | 3 |
| 3250.2337664 | 7 |
| 3250.2374966 | 3 |
| 3250.2380211 | 3 |
| 3250.2409304 | 2 |
| 3250.243011 | 1 |
| 3250.2456441 | 4 |
| 3250.2484783 | 4 |
| 3250.2510622 | 5 |
| 3250.2535068 | 8 |
| 3250.2550506 | 1 |
| 3250.2583064 | 1 |
| 3250.261356 | 3 |
| 3250.2632573 | 4 |
| 3250.2662113 | 3 |
| 3250.2682141 | 3 |
| 3250.2713703 | 3 |
| 3250.2728297 | 5 |
| 3250.2756426 | 2 |
| 3250.2779221 | 0 |
| 3250.2811132 | 1 |
| 3250.2833659 | 2 |
| 3250.2865564 | 2 |
| 3250.287458 | 2 |
| 3250.2907182 | 3 |
| 3250.2921532 | 3 |
| 3250.2952406 | 2 |
| 3250.2979252 | 0 |
| 3250.3002537 | 1 |
| 3250.3031177 | 3 |
| 3250.3041303 | 2 |
| 3250.3075676 | 4 |
| 3250.3113387 | 2 |
| 3250.3117324 | 3 |
| 3250.3153179 | 1 |
| 3250.3186537 | 4 |
| 3250.3191594 | 6 |
| 3250.3223832 | 2 |
| 3250.3247638 | 1 |
| 3250.3272983 | 3 |
| 3250.3303925 | 4 |
| 3250.3326047 | 3 |
| 3250.3354412 | 1 |
| 3250.3365684 | 0 |
| 3250.3403238 | 1 |
| 3250.3412042 | 1 |
| 3250.3448859 | 2 |
| 3250.3475225 | 1 |
| 3250.3494471 | 1 |
| 3250.3518278 | 0 |
| 3250.3554167 | 1 |
| 3250.3571665 | 1 |
| 3250.3600876 | 5 |
| 3250.3616107 | 4 |
| 3250.3648126 | 2 |
| 3250.3661907 | 2 |
| 3250.3692681 | 1 |
| 3250.3718249 | 1 |
| 3250.3736902 | 1 |
| 3250.3767542 | 4 |
| 3250.3788372 | 3 |
| 3250.3818115 | 3 |
| 3250.3852542 | 3 |
| 3250.386002 | 2 |
| 3250.3893265 | 1 |
| 3250.3905725 | 1 |
| 3250.3942035 | 2 |
| 3250.3971369 | 3 |
| 3250.3991391 | 3 |
| 3250.4019756 | 1 |
| 3250.4041448 | 1 |
| 3250.4075715 | 4 |
| 3250.4100669 | 1 |
| 3250.4115188 | 2 |
| 3250.4153015 | 3 |
| 3250.4157502 | 2 |
| 3250.4195111 | 2 |
| 3250.4224681 | 3 |
| 3250.4240822 | 3 |
| 3250.4268839 | 1 |
| 3250.4285886 | 2 |
| 3250.4325508 | 2 |
| 3250.4343756 | 3 |
| 3250.4365311 | 5 |
| 3250.4400008 | 1 |
| 3250.4407931 | 5 |
| 3250.4446801 | 1 |
| 3250.4458825 | 1 |
| 3250.4483628 | 3 |
| 3250.4515277 | 0 |
| 3250.4548271 | 1 |
| 3250.4561749 | 5 |
| 3250.4600553 | 3 |
| 3250.4610511 | 2 |
| 3250.4633622 | 1 |
| 3250.4673823 | 0 |
| 3250.4679719 | 1 |
| 3250.4715958 | 5 |
| 3250.4738392 | 3 |
| 3250.4764417 | 3 |
| 3250.4795571 | 0 |
| 3250.4817412 | 9 |
| 3250.4845193 | 3 |
| 3250.4860086 | 0 |
| 3250.4888923 | 6 |
| 3250.492493 | 0 |
| 3250.493535 | 2 |
| 3250.4965162 | 5 |
| 3250.4984617 | 1 |
| 3250.5017487 | 5 |
| 3250.5034045 | 0 |
| 3250.5068124 | 2 |
| 3250.5101168 | 2 |
| 3250.5109186 | 2 |
| 3250.5144788 | 5 |
| 3250.5159313 | 1 |
| 3250.5189114 | 2 |
| 3250.5219226 | 4 |
| 3250.5235597 | 1 |
| 3250.527095 | 2 |
| 3250.5286179 | 2 |
| 3250.5307841 | 3 |
| 3250.5340105 | 5 |
| 3250.53602 | 2 |
| 3250.5388203 | 1 |
| 3250.5422605 | 1 |
| 3250.5439574 | 3 |
| 3250.5472457 | 1 |
| 3250.5483303 | 2 |
| 3250.5518014 | 2 |
| 3250.5533805 | 0 |
| 3250.5556972 | 2 |
| 3250.5596862 | 1 |
| 3250.5611309 | 1 |
| 3250.5641196 | 2 |
| 3250.565484 | 1 |
| 3250.5686794 | 3 |
| 3250.5722225 | 1 |
| 3250.5738558 | 2 |
| 3250.5768962 | 3 |
| 3250.5790971 | 3 |
| 3250.5819708 | 1 |
| 3250.5828212 | 3 |
| 3250.5867614 | 1 |
| 3250.5896755 | 3 |
| 3250.5911597 | 4 |
| 3250.5938341 | 5 |
| 3250.5962504 | 2 |
| 3250.5988611 | 0 |
| 3250.6019706 | 2 |
| 3250.603038 | 4 |
| 3250.6066111 | 4 |
| 3250.6098137 | 5 |
| 3250.6109715 | 2 |
| 3250.6145594 | 3 |
| 3250.6157366 | 6 |
| 3250.6197077 | 4 |
| 3250.6222178 | 1 |
| 3250.6239338 | 5 |
| 3250.6272235 | 4 |
| 3250.6285711 | 3 |
| 3250.6315232 | 2 |
| 3250.634848 | 2 |
| 3250.636505 | 5 |
| 3250.6392051 | 2 |
| 3250.6410661 | 1 |
| 3250.6441105 | 2 |
| 3250.6471098 | 1 |
| 3250.6487538 | 3 |
| 3250.6521798 | 3 |
| 3250.6537616 | 4 |
| 3250.6566822 | 1 |
| 3250.6583407 | 3 |
| 3250.6615158 | 2 |
| 3250.6647481 | 3 |
| 3250.6664949 | 2 |
| 3250.6698688 | 3 |
| 3250.6705913 | 0 |
| 3250.6736037 | 2 |
| 3250.6768801 | 3 |
| 3250.6782031 | 1 |
| 3250.6809532 | 2 |
| 3250.6847889 | 2 |
| 3250.686377 | 2 |
| 3250.6894098 | 3 |
| 3250.6904665 | 0 |
| 3250.6943965 | 3 |
| 3250.6969966 | 4 |
| 3250.6988611 | 1 |
| 3250.7019583 | 6 |
| 3250.7033126 | 0 |
| 3250.7066958 | 1 |
| 3250.7082629 | 4 |
| 3250.7112847 | 2 |
| 3250.714541 | 3 |
| 3250.715615 | 2 |
| 3250.7190487 | 3 |
| 3250.7204964 | 2 |
| 3250.7243616 | 1 |
| 3250.7274198 | 1 |
| 3250.7286656 | 1 |
| 3250.7321158 | 2 |
| 3250.7327125 | 1 |
| 3250.7360799 | 2 |
| 3250.7377905 | 4 |
| 3250.7407172 | 1 |
| 3250.7443567 | 1 |
| 3250.7455466 | 1 |
| 3250.7481526 | 3 |
| 3250.7512256 | 0 |
| 3250.7532871 | 4 |
| 3250.7569778 | 3 |
| 3250.7579134 | 1 |
| 3250.7618396 | 4 |
| 3250.7625365 | 5 |
| 3250.7659163 | 2 |
| 3250.7688693 | 3 |
| 3250.7710565 | 3 |
| 3250.7739663 | 3 |
| 3250.775866 | 0 |
| 3250.778838 | 3 |
| 3250.782238 | 4 |
| 3250.7833155 | 2 |
| 3250.7862315 | 6 |
| 3250.7879582 | 1 |
| 3250.790259 | 2 |
| 3250.7926433 | 3 |
| 3250.7958413 | 4 |
| 3250.7989664 | 2 |
| 3250.8007947 | 4 |
| 3250.8038715 | 2 |
| 3250.8047409 | 3 |
| 3250.8081563 | 5 |
| 3250.8115536 | 1 |
| 3250.812616 | 3 |
| 3250.8162153 | 4 |
| 3250.8179057 | 2 |
| 3250.8211178 | 1 |
| 3250.8234988 | 1 |
| 3250.8253222 | 2 |
| 3250.8278997 | 5 |
| 3250.829326 | 1 |
| 3250.8326207 | 2 |
| 3250.8361761 | 5 |
| 3250.8371865 | 6 |
| 3250.8400408 | 4 |
| 3250.8426576 | 6 |
| 3250.8447669 | 3 |
| 3250.8472775 | 6 |
| 3250.8487162 | 4 |
| 3250.852327 | 0 |
| 3250.8560182 | 0 |
| 3250.8572393 | 0 |
| 3250.8602606 | 3 |
| 3250.8624928 | 1 |
| 3250.8654816 | 2 |
| 3250.867041 | 2 |
| 3250.8702487 | 0 |
| 3250.8733787 | 1 |
| 3250.8741381 | 3 |
| 3250.8779256 | 2 |
| 3250.8790037 | 1 |
| 3250.8821613 | 2 |
| 3250.8847655 | 1 |
| 3250.8871207 | 0 |
| 3250.8899574 | 4 |
| 3250.8920962 | 1 |
| 3250.8948456 | 1 |
| 3250.8964613 | 6 |
| 3250.8998075 | 3 |
| 3250.9029749 | 3 |
| 3250.9044081 | 1 |
| 3250.9077662 | 0 |
| 3250.9087649 | 1 |
| 3250.9119738 | 3 |
| 3250.9149702 | 1 |
| 3250.9159199 | 1 |
| 3250.9195864 | 1 |
| 3250.9227135 | 2 |
| 3250.9229195 | 2 |
| 3250.9269922 | 2 |
| 3250.9283699 | 3 |
| 3250.9322449 | 1 |
| 3250.9345688 | 1 |
| 3250.9365852 | 7 |
| 3250.9399622 | 1 |
| 3250.9402291 | 2 |
| 3250.9440045 | 2 |
| 3250.9471847 | 3 |
| 3250.9485681 | 1 |
| 3250.9518791 | 0 |
| 3250.954068 | 3 |
| 3250.9571433 | 2 |
| 3250.9602875 | 6 |
| 3250.9617663 | 1 |
| 3250.9646846 | 4 |
| 3250.966023 | 1 |
| 3250.9692077 | 2 |
| 3250.971066 | 2 |
| 3250.9739851 | 1 |
| 3250.9769352 | 5 |
| 3250.9775739 | 1 |
| 3250.9815771 | 2 |
| 3250.9843853 | 0 |
| 3250.986385 | 3 |
| 3250.989613 | 0 |
| 3250.9915175 | 4 |
| 3250.9939447 | 2 |
| 3250.9959517 | 1 |
| 3250.9989309 | 4 |
| 3251.0017541 | 2 |

| | |
|---|---|
| 3251.0031415 | 3 |
| 3251.0061467 | 4 |
| 3251.0096871 | 4 |
| 3251.0111015 | 1 |
| 3251.0136671 | 3 |
| 3251.0155094 | 3 |
| 3251.0187871 | 0 |
| 3251.0200775 | 2 |
| 3251.0231489 | 3 |
| 3251.0267281 | 0 |
| 3251.0285668 | 1 |
| 3251.0312933 | 2 |
| 3251.0330686 | 3 |
| 3251.0364984 | 0 |
| 3251.0396048 | 2 |
| 3251.0408787 | 3 |
| 3251.0437376 | 4 |
| 3251.0458011 | 2 |
| 3251.0491666 | 1 |
| 3251.050252 | 1 |
| 3251.0534171 | 5 |
| 3251.057181 | 3 |
| 3251.0578217 | 3 |
| 3251.0613651 | 4 |
| 3251.0632951 | 3 |
| 3251.0665078 | 4 |
| 3251.0695095 | 1 |
| 3251.0707394 | 3 |
| 3251.0737392 | 2 |
| 3251.0771055 | 1 |
| 3251.0780287 | 1 |
| 3251.0817449 | 2 |
| 3251.0830346 | 2 |
| 3251.0858126 | 2 |
| 3251.0893809 | 2 |
| 3251.0908529 | 2 |
| 3251.0941646 | 2 |
| 3251.096059 | 1 |
| 3251.0996123 | 3 |
| 3251.1004182 | 0 |
| 3251.103216 | 4 |
| 3251.1064022 | 2 |
| 3251.1075754 | 5 |
| 3251.1107157 | 2 |
| 3251.1131052 | 6 |
| 3251.1163159 | 3 |
| 3251.1193975 | 1 |
| 3251.1208955 | 2 |
| 3251.1242666 | 0 |
| 3251.1254741 | 3 |
| 3251.1287481 | 3 |
| 3251.1324734 | 0 |
| 3251.1335589 | 3 |
| 3251.1367177 | 2 |
| 3251.1383575 | 2 |
| 3251.1412936 | 2 |
| 3251.1444376 | 4 |
| 3251.1459712 | 0 |
| 3251.1496176 | 2 |
| 3251.1500184 | 4 |
| 3251.1538225 | 4 |
| 3251.1547485 | 4 |
| 3251.156833 | 3 |
| 3251.1571512 | 1 |
| 3251.1583351 | 2 |
| 3251.1583626 | 1 |
| 3251.1606559 | 3 |
| 3251.1609234 | 2 |
| 3251.1628315 | 2 |
| 3251.1629738 | 3 |
| 3251.164854 | 1 |
| 3251.1663585 | 0 |
| 3251.1666357 | 2 |
| 3251.1673547 | 3 |
| 3251.1694984 | 2 |
| 3251.1700738 | 4 |
| 3251.1711184 | 4 |
| 3251.1716532 | 3 |
| 3251.1738204 | 3 |
| 3251.1749921 | 1 |
| 3251.1757179 | 3 |
| 3251.1759193 | 4 |
| 3251.1778417 | 4 |
| 3251.1785291 | 1 |
| 3251.1793225 | 5 |
| 3251.1809046 | 4 |
| 3251.1823396 | 0 |
| 3251.1828237 | 4 |
| 3251.1832947 | 2 |
| 3251.1854815 | 3 |
| 3251.1862442 | 4 |
| 3251.186734 | 2 |
| 3251.1883254 | 1 |
| 3251.1889455 | 3 |
| 3251.1897038 | 3 |
| 3251.1916202 | 6 |
| 3251.192808 | 1 |
| 3251.1948903 | 2 |
| 3251.1954816 | 2 |
| 3251.1976861 | 0 |
| 3251.200586 | 3 |
| 3251.2009548 | 0 |
| 3251.2033439 | 4 |
| 3251.2052223 | 5 |
| 3251.2067617 | 2 |
| 3251.2086662 | 4 |
| 3251.2109946 | 3 |
| 3251.2133443 | 2 |
| 3251.2138264 | 1 |
| 3251.2164506 | 3 |
| 3251.218908 | 2 |
| 3251.219557 | 2 |
| 3251.2218807 | 3 |
| 3251.2234958 | 1 |
| 3251.2255971 | 3 |
| 3251.2275974 | 5 |
| 3251.2298295 | 3 |
| 3251.2316898 | 4 |
| 3251.2321645 | 0 |
| 3251.2349713 | 2 |
| 3251.2374453 | 2 |
| 3251.2376383 | 0 |
| 3251.2403297 | 8 |
| 3251.2416169 | 2 |
| 3251.2437441 | 1 |
| 3251.2457923 | 2 |
| 3251.247711 | 1 |
| 3251.2499564 | 0 |
| 3251.2515055 | 3 |
| 3251.2533375 | 3 |
| 3251.2556145 | 0 |
| 3251.2564372 | 6 |
| 3251.2598828 | 2 |
| 3251.2609224 | 4 |
| 3251.2627234 | 2 |
| 3251.2637656 | 2 |
| 3251.2660324 | 4 |
| 3251.2689012 | 0 |
| 3251.2695765 | 2 |
| 3251.2722293 | 1 |
| 3251.2742859 | 3 |
| 3251.2755101 | 1 |
| 3251.2778101 | 3 |
| 3251.2792522 | 2 |
| 3251.2813625 | 2 |
| 3251.2821933 | 3 |
| 3251.2843393 | 3 |
| 3251.2870449 | 4 |
| 3251.2879914 | 3 |
| 3251.2900973 | 2 |
| 3251.2924623 | 2 |
| 3251.2938342 | 0 |
| 3251.2967378 | 4 |
| 3251.2968141 | 1 |
| 3251.2995459 | 2 |
| 3251.3016799 | 2 |
| 3251.302734 | 1 |
| 3251.3047864 | 2 |
| 3251.3059948 | 1 |
| 3251.3089066 | 6 |
| 3251.3115101 | 4 |
| 3251.3121421 | 6 |
| 3251.3147811 | 5 |
| 3251.3155385 | 4 |
| 3251.3179001 | 4 |
| 3251.3203144 | 0 |
| 3251.3215185 | 3 |
| 3251.3236971 | 1 |
| 3251.3244171 | 3 |
| 3251.3275825 | 1 |
| 3251.3298434 | 5 |
| 3251.3304143 | 0 |
| 3251.3330694 | 3 |
| 3251.3348506 | 4 |
| 3251.3369592 | 2 |
| 3251.3377905 | 3 |
| 3251.340843 | 2 |
| 3251.3425577 | 0 |
| 3251.3438348 | 1 |
| 3251.3458151 | 3 |
| 3251.3481426 | 1 |
| 3251.3493598 | 3 |
| 3251.3516103 | 0 |
| 3251.3532822 | 1 |
| 3251.3551455 | 1 |
| 3251.3575234 | 2 |
| 3251.3589745 | 2 |
| 3251.3608215 | 1 |
| 3251.3617729 | 2 |
| 3251.3646147 | 0 |
| 3251.3673588 | 3 |
| 3251.3677639 | 1 |
| 3251.3708291 | 5 |
| 3251.3714217 | 3 |
| 3251.3736813 | 2 |
| 3251.3750815 | 0 |
| 3251.3768572 | 6 |
| 3251.379142 | 1 |
| 3251.3802787 | 0 |
| 3251.3836483 | 1 |
| 3251.3841969 | 4 |
| 3251.3866269 | 3 |
| 3251.3892106 | 0 |
| 3251.3900164 | 1 |
| 3251.3922969 | 1 |
| 3251.3932985 | 1 |
| 3251.3951614 | 1 |
| 3251.3979289 | 4 |
| 3251.3990932 | 0 |
| 3251.4016163 | 1 |
| 3251.4028657 | 3 |
| 3251.4058443 | 0 |
| 3251.4077482 | 2 |
| 3251.4087468 | 5 |
| 3251.4108639 | 6 |
| 3251.4124693 | 2 |
| 3251.41432 | 4 |
| 3251.4173264 | 1 |
| 3251.4182735 | 2 |
| 3251.4205496 | 3 |
| 3251.4212692 | 2 |
| 3251.4236253 | 3 |
| 3251.4260603 | 3 |
| 3251.4278933 | 2 |
| 3251.4296347 | 0 |
| 3251.4307109 | 4 |
| 3251.4340791 | 2 |
| 3251.4360105 | 2 |
| 3251.4363888 | 3 |
| 3251.4391716 | 7 |
| 3251.4402246 | 0 |
| 3251.4420884 | 3 |
| 3251.444136 | 0 |
| 3251.4459592 | 2 |
| 3251.4483154 | 2 |
| 3251.4497432 | 1 |
| 3251.4526103 | 1 |
| 3251.4526355 | 2 |
| 3251.4557405 | 2 |
| 3251.4582545 | 3 |
| 3251.4587826 | 1 |
| 3251.461488 | 1 |
| 3251.4627778 | 0 |
| 3251.4644577 | 1 |
| 3251.4656359 | 3 |
| 3251.4684505 | 3 |
| 3251.4701265 | 1 |
| 3251.4728724 | 4 |
| 3251.4741307 | 4 |
| 3251.4762489 | 2 |
| 3251.4780316 | 0 |
| 3251.4799485 | 3 |
| 3251.4810122 | 2 |
| 3251.4834565 | 1 |
| 3251.4859609 | 5 |
| 3251.486615 | 5 |
| 3251.4887742 | 4 |
| 3251.4902524 | 1 |
| 3251.4929813 | 1 |
| 3251.4942189 | 3 |
| 3251.4964252 | 5 |
| 3251.499151 | 2 |
| 3251.500116 | 4 |
| 3251.5021396 | 6 |
| 3251.5047238 | 1 |
| 3251.5052103 | 5 |
| 3251.5084268 | 3 |
| 3251.5093026 | 2 |
| 3251.51187 | 1 |
| 3251.5131583 | 1 |
| 3251.5150207 | 3 |
| 3251.5169191 | 1 |
| 3251.5184189 | 2 |
| 3251.5209762 | 2 |
| 3251.5230588 | 3 |
| 3251.5236806 | 1 |
| 3251.5265496 | 4 |
| 3251.5278244 | 1 |
| 3251.5298314 | 2 |
| 3251.5311457 | 3 |
| 3251.5346586 | 3 |
| 3251.5361588 | 1 |
| 3251.5372036 | 2 |
| 3251.539638 | 4 |
| 3251.5422391 | 3 |
| 3251.5427141 | 8 |
| 3251.5451936 | 3 |
| 3251.546078 | 1 |
| 3251.5487073 | 5 |
| 3251.5503184 | 2 |
| 3251.5520709 | 5 |
| 3251.5543312 | 3 |
| 3251.5558821 | 1 |
| 3251.5583654 | 3 |
| 3251.5595043 | 2 |
| 3251.5619921 | 5 |
| 3251.5640018 | 1 |
| 3251.5649771 | 5 |
| 3251.5677307 | 4 |
| 3251.5687022 | 0 |
| 3251.5714033 | 5 |
| 3251.5734881 | 1 |
| 3251.5743448 | 3 |
| 3251.5770344 | 2 |
| 3251.5786297 | 3 |
| 3251.5807381 | 3 |
| 3251.5830086 | 1 |
| 3251.5847256 | 0 |
| 3251.5870114 | 0 |
| 3251.5876648 | 1 |
| 3251.5907598 | 4 |
| 3251.5927325 | 2 |
| 3251.593642 | 5 |
| 3251.5966982 | 2 |
| 3251.5978281 | 6 |
| 3251.5996441 | 1 |
| 3251.601926 | 9 |
| 3251.6028844 | 5 |
| 3251.6058287 | 1 |
| 3251.6060473 | 0 |
| 3251.6089344 | 2 |
| 3251.6112886 | 4 |
| 3251.6122049 | 3 |
| 3251.6145307 | 1 |
| 3251.6162703 | 1 |
| 3251.6190103 | 1 |
| 3251.6204914 | 2 |
| 3251.6218773 | 2 |
| 3251.6241763 | 1 |
| 3251.6250577 | 4 |
| 3251.6270794 | 0 |
| 3251.6304106 | 3 |
| 3251.6314357 | 1 |
| 3251.6338028 | 0 |
| 3251.6351825 | 2 |
| 3251.6372157 | 2 |
| 3251.6388949 | 1 |
| 3251.6407157 | 1 |
| 3251.6431514 | 8 |
| 3251.64449 | 2 |
| 3251.6463884 | 3 |
| 3251.6483109 | 3 |
| 3251.6490621 | 2 |
| 3251.6517268 | 2 |
| 3251.6531739 | 2 |
| 3251.6553776 | 1 |
| 3251.6579009 | 1 |
| 3251.6587791 | 3 |
| 3251.6617405 | 4 |
| 3251.6626722 | 1 |
| 3251.6654897 | 2 |
| 3251.6667148 | 0 |
| 3251.6687612 | 3 |
| 3251.6704711 | 4 |
| 3251.6721528 | 4 |
| 3251.6745721 | 3 |
| 3251.6760267 | 0 |
| 3251.6780868 | 5 |
| 3251.6805915 | 4 |
| 3251.6817168 | 2 |
| 3251.6839899 | 3 |
| 3251.6855318 | 3 |
| 3251.6876583 | 2 |

| | |
|---|---|
| 3251.6904257 | 0 |
| 3251.6915865 | 1 |
| 3251.6937414 | 0 |
| 3251.6943531 | 8 |
| 3251.6969406 | 4 |
| 3251.6994262 | 2 |
| 3251.7001629 | 3 |
| 3251.7025809 | 2 |
| 3251.7047816 | 3 |
| 3251.7057892 | 1 |
| 3251.7080196 | 2 |
| 3251.7104034 | 1 |
| 3251.711849 | 1 |
| 3251.713973 | 3 |
| 3251.7154735 | 2 |
| 3251.7174413 | 1 |
| 3251.7191674 | 2 |
| 3251.7215307 | 4 |
| 3251.7243726 | 1 |
| 3251.7246804 | 0 |
| 3251.727372 | 2 |
| 3251.7283863 | 3 |
| 3251.7308463 | 1 |
| 3251.7333622 | 1 |
| 3251.7344324 | 1 |
| 3251.7370481 | 3 |
| 3251.7378708 | 0 |
| 3251.7401697 | 3 |
| 3251.7414828 | 3 |
| 3251.7436172 | 5 |
| 3251.7459161 | 5 |
| 3251.7478361 | 7 |
| 3251.7497546 | 1 |
| 3251.7509597 | 3 |
| 3251.7530622 | 1 |
| 3251.7552861 | 2 |
| 3251.7564041 | 3 |
| 3251.7587912 | 1 |
| 3251.76136 | 2 |
| 3251.7623019 | 2 |
| 3251.7645386 | 4 |
| 3251.7659997 | 1 |
| 3251.7682061 | 4 |
| 3251.7703248 | 1 |
| 3251.7718438 | 0 |
| 3251.7743218 | 3 |
| 3251.7750623 | 2 |
| 3251.7780024 | 3 |
| 3251.7789879 | 3 |
| 3251.7809066 | 3 |
| 3251.7828228 | 3 |
| 3251.7833065 | 5 |
| 3251.7857877 | 1 |
| 3251.7866848 | 1 |
| 3251.7884679 | 5 |
| 3251.7901608 | 2 |
| 3251.7917053 | 7 |
| 3251.7930428 | 2 |
| 3251.7940202 | 3 |
| 3251.796469 | 1 |
| 3251.7980751 | 3 |
| 3251.7988822 | 1 |
| 3251.8005245 | 0 |
| 3251.8017432 | 2 |
| 3251.8041629 | 2 |
| 3251.806263 | 3 |
| 3251.8066732 | 2 |
| 3251.8083869 | 5 |
| 3251.8096626 | 2 |
| 3251.8118657 | 4 |
| 3251.8134811 | 2 |
| 3251.8149164 | 2 |
| 3251.8168927 | 4 |
| 3251.8177 | 0 |
| 3251.8192734 | 2 |
| 3251.8218082 | 1 |
| 3251.8227791 | 1 |
| 3251.8250285 | 4 |
| 3251.8255986 | 4 |
| 3251.8275357 | 4 |
| 3251.8292384 | 1 |
| 3251.8296968 | 4 |
| 3251.8319001 | 0 |
| 3251.8330747 | 1 |
| 3251.8347773 | 5 |
| 3251.8367913 | 2 |
| 3251.8377532 | 3 |
| 3251.8401376 | 6 |
| 3251.8404033 | 1 |
| 3251.8432709 | 3 |
| 3251.8445994 | 4 |
| 3251.8455334 | 5 |
| 3251.8480948 | 0 |
| 3251.8488796 | 3 |
| 3251.8508436 | 2 |
| 3251.8517825 | 4 |
| 3251.853712 | 1 |
| 3251.8552689 | 4 |
| 3251.8566289 | 1 |
| 3251.8582722 | 2 |
| 3251.8602007 | 3 |
| 3251.8616054 | 2 |
| 3251.8627843 | 1 |
| 3251.8642309 | 3 |
| 3251.8665045 | 2 |
| 3251.8682211 | 1 |
| 3251.8693484 | 3 |
| 3251.8716315 | 0 |
| 3251.8716465 | 1 |
| 3251.874394 | 0 |
| 3251.8759677 | 1 |
| 3251.8762738 | 3 |
| 3251.8783458 | 1 |
| 3251.8796118 | 3 |
| 3251.881278 | 2 |
| 3251.8829026 | 1 |
| 3251.8849982 | 6 |
| 3251.886028 | 2 |
| 3251.8871327 | 5 |
| 3251.8892488 | 1 |
| 3251.8899525 | 4 |
| 3251.8923433 | 1 |
| 3251.8943794 | 1 |
| 3251.8950764 | 2 |
| 3251.8969375 | 3 |
| 3251.8981977 | 2 |
| 3251.8999083 | 1 |
| 3251.9025311 | 3 |
| 3251.9030431 | 2 |
| 3251.9052959 | 5 |
| 3251.9070344 | 4 |
| 3251.9074661 | 3 |
| 3251.909516 | 2 |
| 3251.911421 | 4 |
| 3251.913114 | 3 |
| 3251.9143195 | 4 |
| 3251.9165343 | 2 |
| 3251.9179854 | 1 |
| 3251.9186029 | 0 |
| 3251.9206676 | 1 |
| 3251.9217693 | 3 |
| 3251.9232719 | 4 |
| 3251.92488 | 1 |
| 3251.9258555 | 2 |
| 3251.9276175 | 3 |
| 3251.9293105 | 3 |
| 3251.9312496 | 2 |
| 3251.933144 | 4 |
| 3251.9340633 | 3 |
| 3251.935953 | 0 |
| 3251.9365884 | 3 |
| 3251.9382802 | 1 |
| 3251.9407029 | 4 |
| 3251.9421267 | 3 |
| 3251.9434291 | 1 |
| 3251.9448524 | 4 |
| 3251.9466108 | 1 |
| 3251.9482574 | 5 |
| 3251.9499315 | 2 |
| 3251.9514777 | 2 |
| 3251.9529702 | 2 |
| 3251.9547105 | 0 |
| 3251.9565684 | 6 |
| 3251.9578418 | 3 |
| 3251.9588308 | 1 |
| 3251.9608446 | 2 |
| 3251.9617088 | 3 |
| 3251.963254 | 3 |
| 3251.9661832 | 0 |
| 3251.9662309 | 2 |
| 3251.9685381 | 4 |
| 3251.9695659 | 1 |
| 3251.9706545 | 3 |
| 3251.9729529 | 2 |
| 3251.9746742 | 2 |
| 3251.975627 | 3 |
| 3251.9763146 | 0 |
| 3251.9780112 | 2 |
| 3251.9781963 | 3 |
| 3251.9804741 | 2 |
| 3251.9820846 | 1 |
| 3251.982741 | 3 |
| 3251.9838406 | 4 |
| 3251.9847966 | 7 |
| 3251.9862606 | 4 |
| 3251.987704 | 2 |
| 3251.9890073 | 1 |
| 3251.990416 | 4 |
| 3251.9924045 | 1 |
| 3251.9928415 | 0 |
| 3251.9943384 | 7 |
| 3251.9955395 | 1 |
| 3251.9965387 | 0 |
| 3251.9982911 | 3 |
| 3251.9987809 | 5 |
| 3252.0008726 | 2 |
| 3252.0010764 | 4 |
| 3252.0034304 | 2 |
| 3252.0046006 | 1 |
| 3252.0048773 | 4 |
| 3252.0070801 | 0 |
| 3252.0081236 | 4 |
| 3252.0082155 | 1 |
| 3252.0082955 | 2 |
| 3252.0083272 | 1 |
| 3252.0086701 | 4 |
| 3252.0088508 | 3 |
| 3252.0091884 | 2 |
| 3252.0093142 | 1 |
| 3252.0095858 | 0 |
| 3252.0099452 | 2 |
| 3252.0102359 | 7 |
| 3252.0105668 | 5 |
| 3252.0107631 | 2 |
| 3252.011086 | 4 |
| 3252.0111546 | 0 |
| 3252.0111708 | 0 |
| 3252.0112504 | 0 |
| 3252.0121256 | 6 |
| 3252.0122216 | 2 |
| 3252.0123214 | 5 |
| 3252.0123946 | 2 |
| 3252.0125014 | 1 |
| 3252.0129824 | 2 |
| 3252.013358 | 4 |
| 3252.0134339 | 0 |
| 3252.0139044 | 3 |
| 3252.0140117 | 3 |
| 3252.0141104 | 0 |
| 3252.014404 | 3 |
| 3252.0150461 | 2 |
| 3252.0151074 | 5 |
| 3252.0152764 | 3 |
| 3252.0154256 | 3 |
| 3252.0155961 | 3 |
| 3252.0160464 | 4 |
| 3252.0161108 | 3 |
| 3252.0161692 | 1 |
| 3252.0166158 | 2 |
| 3252.0166189 | 1 |
| 3252.0167959 | 5 |
| 3252.0167995 | 2 |
| 3252.0176957 | 2 |
| 3252.0178238 | 1 |
| 3252.0181112 | 2 |
| 3252.0183176 | 2 |
| 3252.018505 | 4 |
| 3252.0187724 | 3 |
| 3252.0188077 | 5 |
| 3252.0192226 | 3 |
| 3252.0194462 | 1 |
| 3252.0196903 | 8 |
| 3252.0201276 | 20 |
| 3252.0201497 | 7 |
| 3252.0201927 | 7 |
| 3252.020331 | 25 |
| 3252.0205677 | 27 |
| 3252.0207589 | 28 |
| 3252.0208263 | 35 |
| 3252.0213398 | 53 |
| 3252.0214217 | 39 |
| 3252.0216174 | 69 |
| 3252.02178 | 49 |
| 3252.022003 | 68 |
| 3252.022238 | 57 |
| 3252.0226636 | 61 |
| 3252.0229051 | 63 |
| 3252.0232487 | 75 |
| 3252.0234406 | 96 |
| 3252.023575 | 81 |
| 3252.0235999 | 71 |
| 3252.0241836 | 75 |
| 3252.0243566 | 88 |
| 3252.0249554 | 73 |
| 3252.0249761 | 72 |
| 3252.0251747 | 73 |
| 3252.025214 | 87 |
| 3252.0253304 | 83 |
| 3252.0257769 | 77 |
| 3252.0259829 | 66 |
| 3252.0264829 | 76 |
| 3252.0266833 | 69 |
| 3252.026892 | 70 |
| 3252.0270064 | 83 |
| 3252.0270177 | 80 |
| 3252.0272225 | 74 |
| 3252.0277354 | 80 |
| 3252.0279312 | 86 |
| 3252.027955 | 83 |
| 3252.0286579 | 72 |
| 3252.0287378 | 99 |
| 3252.0289097 | 75 |
| 3252.0289099 | 78 |
| 3252.0289868 | 53 |
| 3252.0292522 | 71 |
| 3252.0293849 | 76 |
| 3252.029791 | 53 |
| 3252.0303031 | 35 |
| 3252.0306751 | 40 |
| 3252.0309481 | 23 |
| 3252.0309541 | 13 |
| 3252.0309585 | 12 |
| 3252.0317903 | 1 |
| 3252.0318056 | 6 |
| 3252.0319582 | 5 |
| 3252.0320322 | 4 |
| 3252.0321409 | 5 |
| 3252.032821 | 2 |
| 3252.0328239 | 1 |
| 3252.0329423 | 3 |
| 3252.0335077 | 1 |
| 3252.0337791 | 3 |
| 3252.0341113 | 0 |
| 3252.0341565 | 4 |
| 3252.0342626 | 5 |
| 3252.0345983 | 4 |
| 3252.035195 | 2 |
| 3252.0353303 | 2 |
| 3252.0354648 | 1 |
| 3252.0355642 | 2 |
| 3252.0361662 | 1 |
| 3252.0361773 | 1 |
| 3252.0365437 | 2 |
| 3252.0367342 | 2 |
| 3252.0368206 | 1 |
| 3252.0369137 | 1 |
| 3252.0370721 | 3 |
| 3252.0371697 | 1 |
| 3252.0375925 | 1 |
| 3252.0380088 | 2 |
| 3252.038016 | 1 |
| 3252.03822 | 1 |
| 3252.0385152 | 5 |
| 3252.0387493 | 0 |
| 3252.0388417 | 4 |
| 3252.0392063 | 2 |
| 3252.0393542 | 5 |
| 3252.0398124 | 3 |
| 3252.0398193 | 2 |
| 3252.0400396 | 5 |
| 3252.0400817 | 3 |
| 3252.040863 | 3 |
| 3252.041286 | 2 |
| 3252.041543 | 1 |
| 3252.04206 | 3 |
| 3252.0430704 | 1 |
| 3252.0433016 | 2 |
| 3252.0440203 | 2 |
| 3252.0446354 | 4 |
| 3252.045073 | 2 |
| 3252.0461047 | 1 |
| 3252.0469971 | 2 |
| 3252.0474726 | 3 |
| 3252.0475169 | 4 |
| 3252.0490754 | 1 |
| 3252.0495644 | 6 |
| 3252.0497641 | 2 |
| 3252.0504619 | 2 |
| 3252.0509011 | 2 |
| 3252.051826 | 1 |
| 3252.0527316 | 4 |
| 3252.0529928 | 4 |
| 3252.0532284 | 4 |

| | |
|---|---|
| 3252.0537402 | 3 |
| 3252.0546076 | 3 |
| 3252.0550544 | 2 |
| 3252.0559 | 1 |
| 3252.0566837 | 0 |
| 3252.0571409 | 4 |
| 3252.0581294 | 1 |
| 3252.0581463 | 0 |
| 3252.0591555 | 1 |
| 3252.059514 | 3 |
| 3252.0595556 | 1 |
| 3252.060984 | 3 |
| 3252.0617801 | 2 |
| 3252.0618096 | 2 |
| 3252.0627045 | 1 |
| 3252.0628236 | 1 |
| 3252.0629858 | 4 |
| 3252.0640136 | 2 |
| 3252.0647268 | 3 |
| 3252.0661455 | 1 |
| 3252.0665967 | 6 |
| 3252.0668374 | 1 |
| 3252.0671735 | 1 |
| 3252.0683847 | 4 |
| 3252.0686135 | 1 |
| 3252.0692017 | 1 |
| 3252.0699605 | 0 |
| 3252.0703856 | 1 |
| 3252.0716336 | 4 |
| 3252.0721514 | 2 |
| 3252.0723766 | 3 |
| 3252.0731536 | 1 |
| 3252.0738361 | 2 |
| 3252.0747654 | 3 |
| 3252.074884 | 3 |
| 3252.0755664 | 1 |
| 3252.0763698 | 4 |
| 3252.0769741 | 3 |
| 3252.0771437 | 4 |
| 3252.0780063 | 1 |
| 3252.0786145 | 1 |
| 3252.0792162 | 4 |
| 3252.0796598 | 3 |
| 3252.0809593 | 3 |
| 3252.0815237 | 1 |
| 3252.0819656 | 3 |
| 3252.0825747 | 3 |
| 3252.0827583 | 3 |
| 3252.0836522 | 0 |
| 3252.0850852 | 1 |
| 3252.0851335 | 5 |
| 3252.0854779 | 5 |
| 3252.0865313 | 1 |
| 3252.0869305 | 5 |
| 3252.0875402 | 8 |
| 3252.0889292 | 2 |
| 3252.0889381 | 1 |
| 3252.0900586 | 4 |
| 3252.0900883 | 1 |
| 3252.0910032 | 3 |
| 3252.0912837 | 2 |
| 3252.092085 | 3 |
| 3252.0922672 | 4 |
| 3252.0932658 | 1 |
| 3252.0940593 | 2 |
| 3252.0948922 | 2 |
| 3252.095259 | 3 |
| 3252.0962229 | 6 |
| 3252.0963809 | 5 |
| 3252.0969058 | 0 |
| 3252.0973474 | 4 |
| 3252.098102 | 3 |
| 3252.0989052 | 0 |
| 3252.0991131 | 2 |
| 3252.0993261 | 1 |
| 3252.1002506 | 2 |
| 3252.101423 | 2 |
| 3252.1019807 | 1 |
| 3252.103071 | 1 |
| 3252.1035761 | 5 |
| 3252.1036788 | 2 |
| 3252.1044272 | 2 |
| 3252.1052975 | 3 |
| 3252.10539 | 3 |
| 3252.1062767 | 0 |
| 3252.1069262 | 4 |
| 3252.1077083 | 2 |
| 3252.1081742 | 2 |
| 3252.1089532 | 1 |
| 3252.109005 | 1 |
| 3252.1099375 | 2 |
| 3252.1104731 | 1 |
| 3252.1111958 | 5 |
| 3252.111741 | 0 |
| 3252.1125636 | 0 |
| 3252.1135344 | 2 |
| 3252.1140968 | 3 |
| 3252.1145683 | 2 |
| 3252.1146589 | 3 |
| 3252.1153363 | 3 |
| 3252.115543 | 2 |
| 3252.1164228 | 1 |
| 3252.1175099 | 6 |
| 3252.1182653 | 3 |
| 3252.1184758 | 1 |
| 3252.1192886 | 6 |
| 3252.1199543 | 3 |
| 3252.1211589 | 3 |
| 3252.1212111 | 3 |
| 3252.1224608 | 1 |
| 3252.1230654 | 3 |
| 3252.1231141 | 3 |
| 3252.1241133 | 2 |
| 3252.1243106 | 5 |
| 3252.1248754 | 6 |
| 3252.125856 | 4 |
| 3252.1261193 | 2 |
| 3252.1270598 | 3 |
| 3252.1279472 | 2 |
| 3252.1279935 | 6 |
| 3252.1291415 | 0 |
| 3252.1296447 | 2 |
| 3252.1298559 | 3 |
| 3252.1309259 | 1 |
| 3252.1315839 | 4 |
| 3252.131695 | 2 |
| 3252.1324205 | 2 |
| 3252.1334248 | 0 |
| 3252.133484 | 4 |
| 3252.1344267 | 2 |
| 3252.1350261 | 5 |
| 3252.1357755 | 2 |
| 3252.1362802 | 2 |
| 3252.1373701 | 2 |
| 3252.1381874 | 1 |
| 3252.1383291 | 2 |
| 3252.138454 | 3 |
| 3252.13924 | 3 |
| 3252.1398059 | 4 |
| 3252.1406338 | 6 |
| 3252.1411637 | 4 |
| 3252.1420518 | 2 |
| 3252.1426451 | 1 |
| 3252.1429004 | 6 |
| 3252.1436651 | 3 |
| 3252.1444862 | 3 |
| 3252.14572 | 1 |
| 3252.1459446 | 5 |
| 3252.14623 | 2 |
| 3252.147092 | 3 |
| 3252.1473813 | 2 |
| 3252.148272 | 0 |
| 3252.1491158 | 2 |
| 3252.1493017 | 4 |
| 3252.149798 | 3 |
| 3252.1508005 | 5 |
| 3252.1513931 | 4 |
| 3252.1522041 | 3 |
| 3252.1527549 | 2 |
| 3252.1537277 | 1 |
| 3252.1538298 | 4 |
| 3252.1539752 | 0 |
| 3252.1552429 | 3 |
| 3252.1552856 | 3 |
| 3252.1562337 | 2 |
| 3252.1567562 | 3 |
| 3252.1567897 | 2 |
| 3252.1579115 | 1 |
| 3252.1591281 | 2 |
| 3252.1591431 | 4 |
| 3252.1598063 | 1 |
| 3252.1611526 | 4 |
| 3252.1617222 | 1 |
| 3252.162023 | 2 |
| 3252.1624067 | 0 |
| 3252.1634134 | 3 |
| 3252.1635727 | 3 |
| 3252.164387 | 3 |
| 3252.1646519 | 3 |
| 3252.1655816 | 1 |
| 3252.1657056 | 3 |
| 3252.166785 | 1 |
| 3252.1676518 | 2 |
| 3252.1678115 | 3 |
| 3252.1691373 | 2 |
| 3252.1694938 | 2 |
| 3252.1699837 | 6 |
| 3252.1708009 | 3 |
| 3252.1709622 | 2 |
| 3252.1723386 | 4 |
| 3252.172386 | 2 |
| 3252.1733255 | 2 |
| 3252.1742597 | 1 |
| 3252.1749295 | 2 |
| 3252.1753548 | 0 |
| 3252.1758477 | 3 |
| 3252.1760569 | 6 |
| 3252.1766349 | 1 |
| 3252.1777499 | 1 |
| 3252.1788235 | 1 |
| 3252.1788782 | 3 |
| 3252.1789583 | 4 |
| 3252.1806417 | 6 |
| 3252.1808063 | 1 |
| 3252.1812358 | 6 |
| 3252.1820229 | 1 |
| 3252.1826677 | 1 |
| 3252.1832133 | 1 |
| 3252.1841311 | 3 |
| 3252.1850458 | 2 |
| 3252.1850868 | 0 |
| 3252.1859017 | 0 |
| 3252.1859135 | 0 |
| 3252.1870257 | 1 |
| 3252.1877536 | 5 |
| 3252.1883757 | 4 |
| 3252.1887641 | 1 |
| 3252.1898772 | 0 |
| 3252.1904348 | 2 |
| 3252.1907818 | 0 |
| 3252.1914743 | 2 |
| 3252.1920253 | 3 |
| 3252.1920959 | 3 |
| 3252.1933451 | 3 |
| 3252.194457 | 2 |
| 3252.1945665 | 3 |
| 3252.195358 | 2 |
| 3252.1959851 | 2 |
| 3252.1961443 | 5 |
| 3252.1970426 | 9 |
| 3252.1976604 | 3 |
| 3252.1982508 | 0 |
| 3252.1986165 | 2 |
| 3252.1999038 | 5 |
| 3252.2001703 | 2 |
| 3252.2007241 | 3 |
| 3252.2010488 | 0 |
| 3252.2016915 | 5 |
| 3252.2017149 | 2 |
| 3252.2029567 | 2 |
| 3252.2038834 | 6 |
| 3252.2045111 | 3 |
| 3252.2049245 | 4 |
| 3252.2059471 | 5 |
| 3252.2064102 | 6 |
| 3252.2065646 | 2 |
| 3252.2073853 | 2 |
| 3252.2085326 | 1 |
| 3252.2087301 | 4 |
| 3252.2097013 | 2 |
| 3252.2098217 | 3 |
| 3252.2110716 | 3 |
| 3252.2115353 | 3 |
| 3252.211872 | 4 |
| 3252.2123954 | 1 |
| 3252.2134019 | 2 |
| 3252.2136295 | 1 |
| 3252.2144562 | 4 |
| 3252.2156762 | 2 |
| 3252.216294 | 3 |
| 3252.2165454 | 1 |
| 3252.2172458 | 0 |
| 3252.2183134 | 3 |
| 3252.2183429 | 2 |
| 3252.2188946 | 2 |
| 3252.2200226 | 5 |
| 3252.2202875 | 2 |
| 3252.2216807 | 3 |
| 3252.2217282 | 5 |
| 3252.2223601 | 0 |
| 3252.2225531 | 0 |
| 3252.223597 | 1 |
| 3252.2241097 | 1 |
| 3252.2241786 | 2 |
| 3252.2250957 | 3 |
| 3252.225901 | 2 |
| 3252.2262645 | 3 |
| 3252.2267667 | 0 |
| 3252.2280742 | 2 |
| 3252.228259 | 3 |
| 3252.2296445 | 1 |
| 3252.2297785 | 4 |
| 3252.2303775 | 0 |
| 3252.2304831 | 3 |
| 3252.2316624 | 1 |
| 3252.232812 | 3 |
| 3252.2328536 | 3 |
| 3252.2334248 | 0 |
| 3252.2340711 | 1 |
| 3252.2344648 | 4 |
| 3252.2351342 | 1 |
| 3252.2355164 | 2 |
| 3252.2364113 | 1 |
| 3252.2366899 | 6 |
| 3252.2378071 | 1 |
| 3252.2381889 | 2 |
| 3252.2386772 | 3 |
| 3252.239397 | 2 |
| 3252.2398735 | 4 |
| 3252.2408437 | 1 |
| 3252.2414096 | 1 |
| 3252.2422467 | 0 |
| 3252.2428505 | 5 |
| 3252.2430625 | 4 |
| 3252.2437407 | 3 |
| 3252.2449732 | 1 |
| 3252.2450141 | 3 |
| 3252.2460289 | 2 |
| 3252.2468104 | 1 |
| 3252.2469618 | 2 |
| 3252.2473771 | 3 |
| 3252.2482659 | 3 |
| 3252.2486365 | 2 |
| 3252.2493244 | 4 |
| 3252.249953 | 5 |
| 3252.2502245 | 0 |
| 3252.2515159 | 7 |
| 3252.252241 | 4 |
| 3252.2524973 | 1 |
| 3252.2532783 | 7 |
| 3252.2537069 | 5 |
| 3252.2539163 | 6 |
| 3252.2551874 | 4 |
| 3252.2558592 | 2 |
| 3252.2559762 | 3 |
| 3252.2571114 | 3 |
| 3252.2578989 | 1 |
| 3252.2582255 | 5 |
| 3252.2588709 | 6 |
| 3252.2591132 | 2 |
| 3252.2598695 | 3 |
| 3252.2607113 | 1 |
| 3252.261387 | 3 |
| 3252.262154 | 4 |
| 3252.2622713 | 1 |
| 3252.2635192 | 4 |
| 3252.2643716 | 1 |
| 3252.2645723 | 8 |
| 3252.2656096 | 1 |
| 3252.2657801 | 1 |
| 3252.266284 | 3 |
| 3252.266999 | 4 |
| 3252.2678382 | 1 |
| 3252.2685998 | 4 |
| 3252.2691005 | 3 |
| 3252.2693736 | 2 |
| 3252.2699582 | 4 |
| 3252.2713873 | 3 |
| 3252.2715862 | 4 |
| 3252.2721959 | 5 |
| 3252.272762 | 1 |
| 3252.2734156 | 0 |
| 3252.2734208 | 4 |
| 3252.2748021 | 2 |
| 3252.2753436 | 1 |
| 3252.276139 | 4 |
| 3252.2766349 | 4 |
| 3252.2766492 | 3 |
| 3252.2779149 | 5 |
| 3252.2782158 | 2 |
| 3252.2787412 | 0 |
| 3252.2789406 | 3 |
| 3252.2803681 | 2 |

| | |
|---|---|
| 3252.2815998 | 2 |
| 3252.2817509 | 1 |
| 3252.2817907 | 1 |
| 3252.282658 | 5 |
| 3252.2827924 | 3 |
| 3252.2834981 | 3 |
| 3252.2842763 | 2 |
| 3252.2854434 | 1 |
| 3252.2858412 | 2 |
| 3252.286057 | 3 |
| 3252.2869304 | 1 |
| 3252.2872774 | 2 |
| 3252.2873675 | 4 |
| 3252.2879359 | 4 |
| 3252.2888036 | 2 |
| 3252.2889883 | 1 |
| 3252.2898222 | 1 |
| 3252.2899232 | 1 |
| 3252.2900077 | 0 |
| 3252.2907899 | 3 |
| 3252.2918625 | 2 |
| 3252.2919282 | 5 |
| 3252.2926448 | 0 |
| 3252.2926677 | 1 |
| 3252.2935749 | 1 |
| 3252.293836 | 8 |
| 3252.2946919 | 5 |
| 3252.2949334 | 1 |
| 3252.295149 | 1 |
| 3252.2952061 | 2 |
| 3252.2961435 | 3 |
| 3252.2968504 | 4 |
| 3252.2970459 | 3 |
| 3252.2974724 | 3 |
| 3252.2982172 | 0 |
| 3252.2982879 | 5 |
| 3252.2982891 | 7 |
| 3252.2991806 | 2 |
| 3252.2992182 | 4 |
| 3252.2998519 | 0 |
| 3252.3001449 | 1 |
| 3252.3011526 | 5 |
| 3252.3017514 | 0 |
| 3252.3018708 | 3 |
| 3252.302394 | 2 |
| 3252.3026012 | 1 |
| 3252.3035873 | 2 |
| 3252.3039355 | 2 |
| 3252.304082 | 2 |
| 3252.304335 | 5 |
| 3252.305005 | 1 |
| 3252.3050487 | 4 |
| 3252.3053845 | 2 |
| 3252.3054947 | 2 |
| 3252.3057828 | 3 |
| 3252.3072783 | 4 |
| 3252.3072921 | 2 |
| 3252.3073112 | 2 |
| 3252.3079661 | 2 |
| 3252.3086268 | 1 |
| 3252.3086297 | 6 |
| 3252.3094367 | 3 |
| 3252.3097423 | 2 |
| 3252.3103489 | 1 |
| 3252.3104614 | 0 |
| 3252.311498 | 2 |
| 3252.3121992 | 3 |
| 3252.312606 | 2 |
| 3252.3136786 | 5 |
| 3252.3139618 | 1 |
| 3252.3148757 | 1 |
| 3252.3149749 | 1 |
| 3252.3160078 | 2 |
| 3252.3170362 | 1 |
| 3252.3170807 | 0 |
| 3252.3178114 | 1 |
| 3252.3184401 | 0 |
| 3252.3189471 | 5 |
| 3252.3191666 | 2 |
| 3252.3193203 | 3 |
| 3252.3202356 | 3 |
| 3252.3207852 | 1 |
| 3252.3213296 | 3 |
| 3252.3219523 | 1 |
| 3252.3225431 | 2 |
| 3252.323459 | 2 |
| 3252.3236326 | 6 |
| 3252.3238921 | 1 |
| 3252.3247522 | 6 |
| 3252.324851 | 1 |
| 3252.3253133 | 4 |
| 3252.325451 | 1 |
| 3252.3261655 | 5 |
| 3252.3266593 | 0 |
| 3252.3271431 | 1 |
| 3252.3278019 | 4 |
| 3252.3288077 | 1 |
| 3252.3288255 | 4 |
| 3252.3289241 | 2 |
| 3252.3290243 | 1 |
| 3252.3290964 | 2 |
| 3252.3294082 | 0 |
| 3252.3301542 | 1 |
| 3252.330305 | 2 |
| 3252.3306653 | 2 |
| 3252.3312213 | 1 |
| 3252.3312455 | 3 |
| 3252.3319721 | 3 |
| 3252.3319882 | 2 |
| 3252.3326087 | 4 |
| 3252.3328904 | 2 |
| 3252.333791 | 2 |
| 3252.3343773 | 3 |
| 3252.3346998 | 1 |
| 3252.3348995 | 2 |
| 3252.3350632 | 5 |
| 3252.3359396 | 2 |
| 3252.336285 | 4 |
| 3252.3365338 | 1 |
| 3252.3368312 | 1 |
| 3252.3374894 | 2 |
| 3252.3376474 | 0 |
| 3252.3378994 | 1 |
| 3252.3384401 | 3 |
| 3252.3385886 | 1 |
| 3252.3385974 | 1 |
| 3252.3391057 | 2 |
| 3252.339349 | 2 |
| 3252.340127 | 2 |
| 3252.3414822 | 2 |
| 3252.3415413 | 3 |
| 3252.3419177 | 2 |
| 3252.3428363 | 6 |
| 3252.3434014 | 1 |
| 3252.3445464 | 4 |
| 3252.3449036 | 6 |
| 3252.3456287 | 3 |
| 3252.3458076 | 1 |
| 3252.3458929 | 3 |
| 3252.3473259 | 2 |
| 3252.3477374 | 4 |
| 3252.3481765 | 1 |
| 3252.3487296 | 1 |
| 3252.3491468 | 1 |
| 3252.3492645 | 2 |
| 3252.3501494 | 1 |
| 3252.3505416 | 4 |
| 3252.3518442 | 2 |
| 3252.3519867 | 0 |
| 3252.3526128 | 2 |
| 3252.3529584 | 0 |
| 3252.3538136 | 1 |
| 3252.3543028 | 1 |
| 3252.3544326 | 0 |
| 3252.3552184 | 3 |
| 3252.3560001 | 3 |
| 3252.3563897 | 1 |
| 3252.3564713 | 6 |
| 3252.3571927 | 1 |
| 3252.3575747 | 2 |
| 3252.3577134 | 0 |
| 3252.358009 | 3 |
| 3252.3581947 | 3 |
| 3252.3589788 | 4 |
| 3252.358994 | 1 |
| 3252.3590417 | 3 |
| 3252.3591943 | 1 |
| 3252.3593376 | 3 |
| 3252.3594738 | 0 |
| 3252.3595056 | 2 |
| 3252.3603732 | 0 |
| 3252.3603973 | 0 |
| 3252.3618604 | 2 |
| 3252.3624894 | 3 |
| 3252.3625666 | 7 |
| 3252.3630939 | 2 |
| 3252.3638819 | 1 |
| 3252.3643812 | 0 |
| 3252.3652676 | 0 |
| 3252.3654325 | 1 |
| 3252.3662315 | 1 |
| 3252.3667147 | 2 |
| 3252.370437 | 4 |
| 3252.3748848 | 3 |
| 3252.3753357 | 5 |
| 3252.377266 | 2 |
| 3252.3782182 | 4 |
| 3252.3793721 | 2 |
| 3252.3806563 | 4 |
| 3252.3841491 | 4 |
| 3252.3899036 | 1 |
| 3252.391249 | 2 |
| 3252.3983504 | 1 |
| 3252.3986578 | 3 |
| 3252.4013371 | 1 |
| 3252.4045409 | 4 |
| 3252.40461 | 5 |
| 3252.4058477 | 5 |
| 3252.4063752 | 6 |
| 3252.4083463 | 2 |
| 3252.409272 | 4 |
| 3252.4108829 | 2 |
| 3252.4132535 | 2 |
| 3252.4157734 | 5 |
| 3252.41994 | 3 |
| 3252.4200932 | 3 |
| 3252.4223743 | 2 |
| 3252.4226393 | 0 |
| 3252.423501 | 1 |
| 3252.4266579 | 1 |
| 3252.4268281 | 4 |
| 3252.4300319 | 4 |
| 3252.4307111 | 4 |
| 3252.4339944 | 4 |
| 3252.4373185 | 2 |
| 3252.4391593 | 1 |
| 3252.4422865 | 0 |
| 3252.4432767 | 1 |
| 3252.4460956 | 4 |
| 3252.4488495 | 4 |
| 3252.4491614 | 1 |
| 3252.4515895 | 1 |
| 3252.4540684 | 1 |
| 3252.4549129 | 4 |
| 3252.4571854 | 2 |
| 3252.4589502 | 1 |
| 3252.4604888 | 2 |
| 3252.461518 | 3 |
| 3252.4617858 | 2 |
| 3252.4619757 | 4 |
| 3252.4638917 | 1 |
| 3252.4647181 | 3 |
| 3252.4661527 | 4 |
| 3252.46912 | 1 |
| 3252.4695332 | 4 |
| 3252.4719083 | 4 |
| 3252.4734402 | 3 |
| 3252.4757487 | 1 |
| 3252.4788892 | 1 |
| 3252.480425 | 2 |
| 3252.483984 | 1 |
| 3252.4846853 | 2 |
| 3252.4891885 | 3 |
| 3252.4898331 | 1 |
| 3252.4915689 | 2 |
| 3252.494909 | 2 |
| 3252.4970552 | 1 |
| 3252.4989082 | 4 |
| 3252.500575 | 2 |
| 3252.502296 | 1 |
| 3252.5032726 | 6 |
| 3252.5051234 | 2 |
| 3252.5066187 | 4 |
| 3252.5081242 | 2 |
| 3252.5082287 | 2 |
| 3252.510434 | 0 |
| 3252.5107032 | 0 |
| 3252.5133656 | 3 |
| 3252.5157051 | 3 |
| 3252.516553 | 0 |
| 3252.5177069 | 2 |
| 3252.5193062 | 3 |
| 3252.5193657 | 5 |
| 3252.5213973 | 3 |
| 3252.5216693 | 6 |
| 3252.524454 | 3 |
| 3252.5251265 | 2 |
| 3252.5278461 | 5 |
| 3252.5307067 | 5 |
| 3252.5313412 | 1 |
| 3252.5421327 | 6 |
| 3252.5428893 | 6 |
| 3252.5435913 | 3 |
| 3252.544728 | 2 |
| 3252.5477986 | 3 |
| 3252.5484626 | 2 |
| 3252.5495329 | 5 |
| 3252.5509271 | 3 |
| 3252.5529085 | 2 |
| 3252.5535285 | 2 |
| 3252.5547494 | 4 |
| 3252.5561941 | 5 |
| 3252.5583119 | 3 |
| 3252.5596504 | 3 |
| 3252.561372 | 6 |
| 3252.562563 | 4 |
| 3252.5629369 | 0 |
| 3252.5636909 | 4 |
| 3252.5641084 | 2 |
| 3252.5680891 | 4 |
| 3252.5705853 | 0 |
| 3252.5709179 | 2 |
| 3252.5726556 | 3 |
| 3252.5737617 | 1 |
| 3252.5774886 | 2 |
| 3252.5776896 | 4 |
| 3252.5857595 | 1 |
| 3252.5875856 | 3 |
| 3252.5905157 | 0 |
| 3252.5920943 | 2 |
| 3252.5936775 | 2 |
| 3252.5943097 | 2 |
| 3252.5960332 | 2 |
| 3252.5965318 | 1 |
| 3252.5982152 | 4 |
| 3252.6010355 | 2 |
| 3252.6014926 | 0 |
| 3252.6028164 | 3 |
| 3252.6049443 | 5 |
| 3252.6062118 | 4 |
| 3252.6081337 | 2 |
| 3252.6090989 | 1 |
| 3252.6115177 | 5 |
| 3252.6124816 | 4 |
| 3252.6135198 | 4 |
| 3252.7417822 | 2 |
| 3252.7421192 | 1 |
| 3252.7458186 | 1 |
| 3252.7475299 | 2 |
| 3252.7507124 | 1 |
| 3252.7539002 | 0 |
| 3252.7552801 | 2 |
| 3252.7579555 | 3 |
| 3252.7584301 | 1 |
| 3252.7602193 | 1 |
| 3252.7623383 | 4 |
| 3252.7638704 | 2 |
| 3252.7640569 | 0 |
| 3252.765934 | 2 |
| 3252.7676547 | 3 |
| 3252.7690033 | 4 |
| 3252.7714531 | 2 |
| 3252.7732866 | 2 |
| 3252.7746872 | 3 |
| 3252.776263 | 4 |
| 3252.7769344 | 7 |
| 3252.7795631 | 2 |
| 3252.7821643 | 0 |
| 3252.78357 | 3 |
| 3252.787334 | 3 |
| 3252.7907164 | 0 |
| 3252.7918524 | 3 |
| 3252.7930508 | 0 |
| 3252.7942178 | 3 |
| 3252.7964552 | 1 |
| 3252.7996196 | 3 |
| 3252.8016218 | 4 |
| 3252.8043101 | 3 |
| 3252.8051698 | 2 |
| 3252.8078722 | 4 |
| 3252.8102562 | 2 |
| 3252.8102869 | 0 |
| 3252.8116002 | 4 |
| 3252.812265 | 6 |
| 3252.812951 | 1 |
| 3252.8160875 | 2 |
| 3252.8174225 | 2 |
| 3252.8201086 | 2 |
| 3252.8212427 | 1 |
| 3252.8231137 | 1 |
| 3252.8250023 | 1 |
| 3252.825814 | 0 |
| 3252.8274371 | 0 |

| | |
|---|---|
| 3252.8295711 | 4 |
| 3252.83173 | 3 |
| 3252.8340746 | 1 |
| 3252.8363548 | 2 |
| 3252.8400938 | 1 |
| 3252.8431707 | 5 |
| 3252.8433643 | 3 |
| 3252.8457239 | 2 |
| 3252.8479635 | 4 |
| 3252.8504192 | 5 |
| 3252.8521467 | 2 |
| 3252.8545116 | 3 |
| 3252.8569699 | 5 |
| 3252.8572591 | 1 |
| 3252.8576111 | 3 |
| 3252.8585784 | 1 |
| 3252.8599592 | 2 |
| 3252.8622392 | 1 |
| 3252.8636195 | 3 |
| 3252.8667305 | 3 |
| 3252.867469 | 4 |
| 3252.869711 | 2 |
| 3252.8729074 | 0 |
| 3252.8734399 | 1 |
| 3252.8754887 | 0 |
| 3252.8767727 | 1 |
| 3252.8800333 | 1 |
| 3252.8820759 | 2 |
| 3252.8835883 | 1 |
| 3252.8861115 | 1 |
| 3252.8868633 | 1 |
| 3252.8885694 | 2 |
| 3252.8927534 | 0 |
| 3252.893406 | 0 |
| 3252.8959709 | 3 |
| 3252.8977392 | 3 |
| 3252.9008105 | 3 |
| 3252.9010521 | 5 |
| 3252.9034204 | 1 |
| 3252.9036639 | 1 |
| 3252.9046623 | 3 |
| 3252.9065693 | 2 |
| 3252.9084032 | 2 |
| 3252.9093297 | 2 |
| 3252.9117299 | 2 |
| 3252.912812 | 0 |
| 3252.9162662 | 1 |
| 3252.9184998 | 2 |
| 3252.9201493 | 1 |
| 3252.9223445 | 4 |
| 3252.9242674 | 2 |
| 3252.925635 | 1 |
| 3252.9279562 | 0 |
| 3252.9302532 | 2 |
| 3252.9326459 | 4 |
| 3252.9338955 | 2 |
| 3252.9360618 | 4 |
| 3252.9384049 | 3 |
| 3252.9405255 | 2 |
| 3252.940579 | 0 |
| 3252.9423617 | 2 |
| 3252.9457048 | 3 |
| 3252.947491 | 2 |
| 3252.9480975 | 2 |
| 3252.9492346 | 1 |
| 3252.9505418 | 2 |
| 3252.9529369 | 3 |
| 3252.9549546 | 6 |
| 3252.9555897 | 3 |
| 3252.9581572 | 2 |
| 3252.9595177 | 2 |
| 3252.9610481 | 2 |
| 3252.9637426 | 1 |
| 3252.9647907 | 2 |
| 3252.9675568 | 2 |
| 3252.9700254 | 3 |
| 3252.9731694 | 1 |
| 3252.9761967 | 2 |
| 3252.9776462 | 1 |
| 3252.9788497 | 4 |
| 3252.9813689 | 2 |
| 3252.9822327 | 2 |
| 3252.9857994 | 4 |
| 3252.9861973 | 3 |
| 3252.9890194 | 2 |
| 3252.9907666 | 2 |
| 3252.9908309 | 1 |
| 3252.9921816 | 4 |
| 3252.9946307 | 4 |
| 3252.9953955 | 4 |
| 3252.996398 | 3 |
| 3252.9980461 | 3 |
| 3253.0006333 | 4 |
| 3253.0030379 | 3 |
| 3253.0053194 | 1 |
| 3253.0054941 | 1 |
| 3253.0076317 | 2 |
| 3253.0100316 | 4 |
| 3253.0106294 | 1 |
| 3253.0130408 | 0 |
| 3253.0145284 | 5 |
| 3253.0174846 | 0 |
| 3253.019627 | 3 |
| 3253.0206159 | 4 |
| 3253.024812 | 1 |
| 3253.0266891 | 2 |
| 3253.0276618 | 0 |
| 3253.031374 | 6 |
| 3253.0324972 | 3 |
| 3253.0354915 | 4 |
| 3253.0379568 | 4 |
| 3253.0385755 | 0 |
| 3253.039478 | 2 |
| 3253.0405122 | 3 |
| 3253.0416968 | 2 |
| 3253.0445316 | 2 |
| 3253.0447988 | 4 |
| 3253.0470246 | 3 |
| 3253.0481594 | 3 |
| 3253.0509971 | 2 |
| 3253.0526816 | 2 |
| 3253.0542684 | 2 |
| 3253.0566174 | 4 |
| 3253.0575941 | 3 |
| 3253.0598042 | 4 |
| 3253.0617066 | 4 |
| 3253.06436 | 1 |
| 3253.0666595 | 3 |
| 3253.0683046 | 2 |
| 3253.0711361 | 1 |
| 3253.0712863 | 1 |
| 3253.0737744 | 0 |
| 3253.0770638 | 1 |
| 3253.0774736 | 5 |
| 3253.0790811 | 1 |
| 3253.0818353 | 2 |
| 3253.0835582 | 1 |
| 3253.0845945 | 1 |
| 3253.0870318 | 2 |
| 3253.0883511 | 1 |
| 3253.0888302 | 2 |
| 3253.0914216 | 2 |
| 3253.0934531 | 3 |
| 3253.0945953 | 3 |
| 3253.0962612 | 2 |
| 3253.098638 | 3 |
| 3253.1002714 | 2 |
| 3253.1029634 | 1 |
| 3253.1043443 | 2 |
| 3253.1068898 | 3 |
| 3253.1082256 | 2 |
| 3253.109783 | 2 |
| 3253.1133267 | 0 |
| 3253.1149178 | 1 |
| 3253.1169789 | 2 |
| 3253.1185484 | 7 |
| 3253.121371 | 2 |
| 3253.1225586 | 4 |
| 3253.1236884 | 0 |
| 3253.1244952 | 2 |
| 3253.1255226 | 3 |
| 3253.1301498 | 3 |
| 3253.1306033 | 0 |
| 3253.1324582 | 3 |
| 3253.1349391 | 3 |
| 3253.1362163 | 0 |
| 3253.138138 | 3 |
| 3253.1395319 | 2 |
| 3253.1405199 | 1 |
| 3253.1425026 | 1 |
| 3253.1435274 | 1 |
| 3253.1453602 | 3 |
| 3253.148635 | 3 |
| 3253.1497832 | 2 |
| 3253.153063 | 4 |
| 3253.1549279 | 0 |
| 3253.1575436 | 0 |
| 3253.1601132 | 2 |
| 3253.1604743 | 9 |
| 3253.1642597 | 1 |
| 3253.1652311 | 5 |
| 3253.1691914 | 4 |
| 3253.170401 | 1 |
| 3253.1728131 | 2 |
| 3253.1729642 | 0 |
| 3253.1739803 | 2 |
| 3253.1746424 | 3 |
| 3253.1759677 | 2 |
| 3253.1790408 | 0 |
| 3253.1810943 | 4 |
| 3253.1826246 | 1 |
| 3253.1847958 | 3 |
| 3253.1861631 | 1 |
| 3253.1888978 | 2 |
| 3253.1900845 | 3 |
| 3253.1908756 | 2 |
| 3253.1925279 | 0 |
| 3253.194904 | 2 |
| 3253.19633 | 1 |
| 3253.1993781 | 1 |
| 3253.1999384 | 2 |
| 3253.2032318 | 6 |
| 3253.2035378 | 7 |
| 3253.2062326 | 3 |
| 3253.2099495 | 2 |
| 3253.2112861 | 3 |
| 3253.213416 | 0 |
| 3253.2175991 | 2 |
| 3253.2193489 | 4 |
| 3253.2216125 | 3 |
| 3253.2217727 | 0 |
| 3253.2222585 | 2 |
| 3253.2228401 | 2 |
| 3253.2249863 | 1 |
| 3253.2276094 | 1 |
| 3253.2285504 | 2 |
| 3253.2309888 | 4 |
| 3253.2320404 | 2 |
| 3253.2350444 | 5 |
| 3253.2381282 | 0 |
| 3253.2389678 | 2 |
| 3253.2405025 | 2 |
| 3253.241516 | 2 |
| 3253.2429711 | 0 |
| 3253.2471041 | 3 |
| 3253.2479888 | 0 |
| 3253.250667 | 2 |
| 3253.2526123 | 5 |
| 3253.2561618 | 4 |
| 3253.2570333 | 2 |
| 3253.2598639 | 0 |
| 3253.2613556 | 0 |
| 3253.2615513 | 1 |
| 3253.2650589 | 1 |
| 3253.2669109 | 2 |
| 3253.267485 | 0 |
| 3253.2691035 | 4 |
| 3253.2703792 | 1 |
| 3253.2719575 | 3 |
| 3253.2737896 | 0 |
| 3253.2756792 | 1 |
| 3253.2774034 | 4 |
| 3253.2784933 | 2 |
| 3253.2814927 | 2 |
| 3253.2844239 | 2 |
| 3253.2854911 | 3 |
| 3253.2880447 | 3 |
| 3253.2896742 | 3 |
| 3253.2907048 | 2 |
| 3253.2918109 | 2 |
| 3253.2958139 | 1 |
| 3253.2979235 | 3 |
| 3253.2997677 | 0 |
| 3253.3023426 | 1 |
| 3253.3050038 | 1 |
| 3253.3075045 | 0 |
| 3253.3100171 | 4 |
| 3253.3103053 | 4 |
| 3253.3115032 | 2 |
| 3253.3125891 | 0 |
| 3253.3136059 | 2 |
| 3253.3166539 | 3 |
| 3253.3168563 | 2 |
| 3253.3192761 | 6 |
| 3253.3206011 | 1 |
| 3253.3234575 | 3 |
| 3253.3246271 | 3 |
| 3253.3260548 | 2 |
| 3253.3283564 | 6 |
| 3253.328969 | 3 |
| 3253.3319057 | 2 |
| 3253.3345621 | 3 |
| 3253.3352794 | 2 |
| 3253.3384713 | 3 |
| 3253.3405564 | 9 |
| 3253.3422355 | 0 |
| 3253.3466427 | 3 |
| 3253.3473077 | 5 |
| 3253.3511117 | 0 |
| 3253.3519156 | 3 |
| 3253.3548688 | 3 |
| 3253.3567409 | 3 |
| 3253.3568128 | 4 |
| 3253.3574195 | 1 |
| 3253.3578694 | 5 |
| 3253.3609522 | 4 |
| 3253.3626631 | 1 |
| 3253.3644538 | 1 |
| 3253.3669207 | 1 |
| 3253.3680687 | 1 |
| 3253.3713678 | 4 |
| 3253.3713901 | 2 |
| 3253.3738318 | 2 |
| 3253.3759901 | 2 |
| 3253.3767699 | 4 |
| 3253.3798387 | 3 |
| 3253.3808689 | 0 |
| 3253.3842249 | 0 |
| 3253.3857866 | 1 |
| 3253.3867867 | 5 |
| 3253.388632 | 3 |
| 3253.3908271 | 1 |
| 3253.3931299 | 1 |
| 3253.3969105 | 3 |
| 3253.4003578 | 3 |
| 3253.4013397 | 1 |
| 3253.4044703 | 2 |
| 3253.4045594 | 2 |
| 3253.4052069 | 2 |
| 3253.4054511 | 3 |
| 3253.4073147 | 1 |
| 3253.4093787 | 0 |
| 3253.410725 | 3 |
| 3253.4134632 | 2 |
| 3253.4145649 | 2 |
| 3253.4174903 | 0 |
| 3253.4200807 | 1 |
| 3253.4221556 | 3 |
| 3253.4245001 | 2 |
| 3253.4247893 | 5 |
| 3253.426383 | 1 |
| 3253.4303434 | 3 |
| 3253.4309338 | 1 |
| 3253.4344151 | 1 |
| 3253.4358237 | 0 |
| 3253.4382839 | 4 |
| 3253.4406134 | 5 |
| 3253.4412232 | 3 |
| 3253.4419776 | 1 |
| 3253.4468391 | 0 |
| 3253.4470094 | 2 |
| 3253.4474366 | 3 |
| 3253.4492247 | 1 |
| 3253.4498592 | 0 |
| 3253.4538476 | 2 |
| 3253.4547963 | 2 |
| 3253.4565455 | 2 |
| 3253.4576574 | 5 |
| 3253.4597704 | 1 |
| 3253.4618027 | 1 |
| 3253.4637363 | 2 |
| 3253.4661523 | 3 |
| 3253.4684473 | 2 |
| 3253.4703953 | 1 |
| 3253.472484 | 1 |
| 3253.4754761 | 2 |
| 3253.4775563 | 2 |
| 3253.4782589 | 2 |
| 3253.4812633 | 4 |
| 3253.4820605 | 1 |
| 3253.4856848 | 1 |
| 3253.4886586 | 2 |
| 3253.4903469 | 3 |
| 3253.4925816 | 2 |
| 3253.4925858 | 3 |
| 3253.4932379 | 3 |
| 3253.494474 | 1 |
| 3253.4946096 | 1 |
| 3253.4973139 | 1 |
| 3253.4980613 | 3 |
| 3253.5021187 | 0 |
| 3253.503767 | 1 |
| 3253.5053811 | 4 |

| | |
|---|---|
| 3253.507505 | 1 |
| 3253.5086193 | 3 |
| 3253.5104953 | 2 |
| 3253.512665 | 3 |
| 3253.5132782 | 2 |
| 3253.5164815 | 5 |
| 3253.5178498 | 2 |
| 3253.523006 | 3 |
| 3253.5261454 | 1 |
| 3253.5271257 | 4 |
| 3253.5292659 | 1 |
| 3253.5295524 | 3 |
| 3253.5320579 | 1 |
| 3253.5355664 | 4 |
| 3253.5366768 | 3 |
| 3253.539724 | 1 |
| 3253.5408079 | 2 |
| 3253.5418176 | 2 |
| 3253.5418611 | 1 |
| 3253.5425798 | 2 |
| 3253.544461 | 2 |
| 3253.5450277 | 1 |
| 3253.5481735 | 1 |
| 3253.5504777 | 1 |
| 3253.5520715 | 2 |
| 3253.5555702 | 2 |
| 3253.55578 | 2 |
| 3253.5588045 | 4 |
| 3253.558854 | 0 |
| 3253.5617394 | 1 |
| 3253.565033 | 3 |
| 3253.5670879 | 1 |
| 3253.5684978 | 2 |
| 3253.5717647 | 1 |
| 3253.5718838 | 2 |
| 3253.5763658 | 3 |
| 3253.5770521 | 2 |
| 3253.5787752 | 3 |
| 3253.5825702 | 4 |
| 3253.5830566 | 0 |
| 3253.5862314 | 4 |
| 3253.5868355 | 3 |
| 3253.5880461 | 6 |
| 3253.5882423 | 3 |
| 3253.5889826 | 0 |
| 3253.5907039 | 1 |
| 3253.5918768 | 3 |
| 3253.5943037 | 5 |
| 3253.5967086 | 6 |
| 3253.5990449 | 3 |
| 3253.6014877 | 2 |
| 3253.6027594 | 1 |
| 3253.6049319 | 1 |
| 3253.6064067 | 5 |
| 3253.6083215 | 1 |
| 3253.6106425 | 2 |
| 3253.6136422 | 1 |
| 3253.6149191 | 4 |
| 3253.6170089 | 1 |
| 3253.6193118 | 2 |
| 3253.6218087 | 1 |
| 3253.6248852 | 1 |
| 3253.6258923 | 3 |
| 3253.627367 | 6 |
| 3253.6288857 | 1 |
| 3253.6288939 | 1 |
| 3253.6302443 | 2 |
| 3253.6310552 | 4 |
| 3253.6337684 | 2 |
| 3253.6340793 | 4 |
| 3253.6378285 | 3 |
| 3253.6396595 | 5 |
| 3253.6410249 | 3 |
| 3253.6428179 | 1 |
| 3253.6444572 | 3 |
| 3253.6473014 | 3 |
| 3253.649388 | 3 |
| 3253.6504939 | 3 |
| 3253.6536115 | 4 |
| 3253.6547592 | 4 |
| 3253.6588494 | 3 |
| 3253.6602875 | 2 |
| 3253.661409 | 0 |
| 3253.6644803 | 1 |
| 3253.6654666 | 3 |
| 3253.668718 | 2 |
| 3253.6717303 | 0 |
| 3253.6728243 | 0 |
| 3253.674815 | 2 |
| 3253.6751461 | 3 |
| 3253.675556 | 3 |
| 3253.675686 | 2 |
| 3253.6776635 | 0 |
| 3253.6798141 | 0 |
| 3253.6811096 | 3 |
| 3253.6839089 | 1 |
| 3253.6857278 | 6 |
| 3253.6868485 | 2 |
| 3253.6898139 | 0 |
| 3253.6905885 | 6 |
| 3253.6930716 | 4 |
| 3253.6952077 | 9 |
| 3253.6964798 | 6 |
| 3253.6987768 | 5 |
| 3253.7005077 | 0 |
| 3253.7047346 | 2 |
| 3253.7069424 | 1 |
| 3253.7102769 | 2 |
| 3253.7122474 | 1 |
| 3253.712361 | 2 |
| 3253.715494 | 0 |
| 3253.7155514 | 5 |
| 3253.7158493 | 1 |
| 3253.7224295 | 2 |
| 3253.7225572 | 1 |
| 3253.7233442 | 1 |
| 3253.723543 | 2 |
| 3253.7236875 | 1 |
| 3253.7258916 | 2 |
| 3253.7270315 | 2 |
| 3253.7296032 | 2 |
| 3253.7304115 | 4 |
| 3253.7331117 | 1 |
| 3253.7363422 | 3 |
| 3253.7376267 | 1 |
| 3253.7397729 | 3 |
| 3253.7410539 | 3 |
| 3253.7430727 | 2 |
| 3253.7465945 | 0 |
| 3253.7480616 | 2 |
| 3253.7506181 | 0 |
| 3253.7520815 | 4 |
| 3253.7553923 | 2 |
| 3253.7579554 | 2 |
| 3253.7584603 | 2 |
| 3253.7608795 | 3 |
| 3253.7632966 | 3 |
| 3253.7637424 | 0 |
| 3253.768414 | 0 |
| 3253.7685341 | 4 |
| 3253.7695073 | 1 |
| 3253.7695086 | 2 |
| 3253.7711128 | 4 |
| 3253.7729888 | 1 |
| 3253.7739712 | 1 |
| 3253.7763887 | 1 |
| 3253.7773761 | 1 |
| 3253.7801716 | 2 |
| 3253.7821031 | 2 |
| 3253.7840337 | 3 |
| 3253.7860425 | 1 |
| 3253.7882367 | 2 |
| 3253.7901164 | 3 |
| 3253.793382 | 4 |
| 3253.7952537 | 4 |
| 3253.7979412 | 2 |
| 3253.799138 | 2 |
| 3253.8017142 | 7 |
| 3253.8049539 | 1 |
| 3253.8057385 | 3 |
| 3253.8090474 | 2 |
| 3253.8098173 | 2 |
| 3253.8100119 | 2 |
| 3253.8101185 | 6 |
| 3253.8103654 | 0 |
| 3253.8132754 | 0 |
| 3253.8154021 | 0 |
| 3253.817596 | 5 |
| 3253.8197814 | 1 |
| 3253.8214551 | 4 |
| 3253.8233992 | 6 |
| 3253.8244425 | 2 |
| 3253.8267558 | 0 |
| 3253.8289454 | 5 |
| 3253.830265 | 2 |
| 3253.8326013 | 1 |
| 3253.8342396 | 5 |
| 3253.8375492 | 1 |
| 3253.8408476 | 2 |
| 3253.8420649 | 1 |
| 3253.8444833 | 4 |
| 3253.8455048 | 2 |
| 3253.8490298 | 1 |
| 3253.8508418 | 3 |
| 3253.850885 | 3 |
| 3253.8532569 | 3 |
| 3253.8577605 | 2 |
| 3253.8579497 | 0 |
| 3253.8582663 | 0 |
| 3253.859286 | 2 |
| 3253.8603744 | 4 |
| 3253.8626907 | 1 |
| 3253.8633121 | 6 |
| 3253.8661037 | 3 |
| 3253.8671912 | 5 |
| 3253.8694581 | 2 |
| 3253.8723196 | 1 |
| 3253.872743 | 1 |
| 3253.8757765 | 3 |
| 3253.8760756 | 2 |
| 3253.8785381 | 5 |
| 3253.8810545 | 0 |
| 3253.8828054 | 3 |
| 3253.8846711 | 0 |
| 3253.8866819 | 4 |
| 3253.8908102 | 4 |
| 3253.8942573 | 2 |
| 3253.8947668 | 3 |
| 3253.897668 | 2 |
| 3253.8993368 | 1 |
| 3253.8996585 | 4 |
| 3253.9050612 | 4 |
| 3253.9051517 | 3 |
| 3253.9055546 | 1 |
| 3253.9057382 | 2 |
| 3253.9063951 | 1 |
| 3253.9084426 | 2 |
| 3253.9085083 | 3 |
| 3253.9091532 | 2 |
| 3253.9095477 | 1 |
| 3253.9122748 | 1 |
| 3253.9123871 | 0 |
| 3253.9136054 | 7 |
| 3253.9140475 | 1 |
| 3253.9153751 | 0 |
| 3253.9163751 | 2 |
| 3253.9180416 | 1 |
| 3253.9189328 | 2 |
| 3253.9197135 | 2 |
| 3253.9212845 | 1 |
| 3253.9216568 | 1 |
| 3253.9224646 | 3 |
| 3253.9233812 | 1 |
| 3253.9233868 | 4 |
| 3253.9245157 | 4 |
| 3253.9271322 | 3 |
| 3253.927401 | 3 |
| 3253.9281611 | 0 |
| 3253.9302191 | 1 |
| 3253.9303441 | 4 |
| 3253.9311219 | 3 |
| 3253.9321993 | 5 |
| 3253.9337639 | 4 |
| 3253.9338335 | 3 |
| 3253.9349009 | 1 |
| 3253.9352438 | 2 |
| 3253.9369165 | 5 |
| 3253.9381256 | 4 |
| 3253.9388515 | 2 |
| 3253.9403124 | 2 |
| 3253.94113 | 5 |
| 3253.9420882 | 2 |
| 3253.9424768 | 5 |
| 3253.9446612 | 1 |
| 3253.9452081 | 2 |
| 3253.9454648 | 6 |
| 3253.946388 | 4 |
| 3253.9477033 | 3 |
| 3253.9480031 | 0 |
| 3253.948641 | 1 |
| 3253.9486757 | 2 |
| 3253.9496917 | 2 |
| 3253.9511973 | 2 |
| 3253.9515916 | 6 |
| 3253.9543674 | 3 |
| 3253.9559079 | 0 |
| 3253.9586146 | 3 |
| 3253.9605001 | 1 |
| 3253.9616449 | 6 |
| 3253.9641697 | 2 |
| 3253.9650077 | 4 |
| 3253.9668598 | 2 |
| 3253.9701468 | 1 |
| 3253.9711018 | 0 |
| 3253.9735042 | 2 |
| 3253.9748208 | 2 |
| 3253.9771659 | 2 |
| 3253.9792271 | 1 |
| 3253.9804352 | 3 |
| 3253.9825323 | 1 |
| 3253.9835496 | 0 |
| 3253.9856512 | 2 |
| 3253.9878917 | 4 |
| 3253.9887233 | 2 |
| 3253.9918587 | 2 |
| 3253.9924833 | 2 |
| 3253.9945514 | 2 |
| 3253.9966083 | 4 |
| 3253.9985532 | 2 |
| 3254.0015766 | 3 |
| 3254.0021853 | 2 |
| 3254.0051946 | 2 |
| 3254.0070277 | 1 |
| 3254.0076848 | 6 |
| 3254.01062 | 1 |
| 3254.0116782 | 1 |
| 3254.0148937 | 4 |
| 3254.0164358 | 2 |
| 3254.0174694 | 5 |
| 3254.0198189 | 4 |
| 3254.0211501 | 3 |
| 3254.0234038 | 3 |
| 3254.0244387 | 2 |
| 3254.0273052 | 2 |
| 3254.0292258 | 5 |
| 3254.0301811 | 3 |
| 3254.0324739 | 1 |
| 3254.0345044 | 6 |
| 3254.0354888 | 4 |
| 3254.038345 | 2 |
| 3254.0398809 | 2 |
| 3254.04189 | 4 |
| 3254.0444784 | 4 |
| 3254.0450713 | 2 |
| 3254.0475666 | 0 |
| 3254.049087 | 1 |
| 3254.0510082 | 2 |
| 3254.0533874 | 1 |
| 3254.0552176 | 1 |
| 3254.0571784 | 3 |
| 3254.0582422 | 2 |
| 3254.0603742 | 2 |
| 3254.062893 | 0 |
| 3254.063987 | 3 |
| 3254.0661796 | 3 |
| 3254.0676099 | 3 |
| 3254.069787 | 3 |
| 3254.0717582 | 3 |
| 3254.0733706 | 2 |
| 3254.0755223 | 2 |
| 3254.0769191 | 2 |
| 3254.0789134 | 1 |
| 3254.0812894 | 4 |
| 3254.0823713 | 0 |
| 3254.0849455 | 3 |
| 3254.0856034 | 3 |
| 3254.0880455 | 3 |
| 3254.0902101 | 1 |
| 3254.0915449 | 2 |
| 3254.0942038 | 4 |
| 3254.095446 | 5 |
| 3254.0980593 | 3 |
| 3254.1002207 | 1 |
| 3254.1014882 | 1 |
| 3254.1037831 | 3 |
| 3254.1047752 | 3 |
| 3254.1067008 | 4 |
| 3254.1081566 | 3 |
| 3254.1101926 | 3 |
| 3254.1130698 | 1 |
| 3254.1150329 | 1 |
| 3254.1159681 | 4 |
| 3254.1184579 | 4 |
| 3254.1188036 | 0 |
| 3254.1221609 | 3 |
| 3254.1235222 | 6 |
| 3254.1257059 | 3 |
| 3254.1280786 | 0 |
| 3254.1288615 | 3 |
| 3254.1313819 | 0 |
| 3254.1320392 | 5 |
| 3254.1351079 | 1 |
| 3254.1369169 | 2 |

| | |
|---|---|
| 3254.1388028 | 3 |
| 3254.1409557 | 1 |
| 3254.1420109 | 3 |
| 3254.1453369 | 2 |
| 3254.1474709 | 5 |
| 3254.148024 | 4 |
| 3254.1506603 | 3 |
| 3254.1513725 | 5 |
| 3254.153414 | 6 |
| 3254.1561183 | 2 |
| 3254.157499 | 4 |
| 3254.1591335 | 6 |
| 3254.1608323 | 1 |
| 3254.1631215 | 2 |
| 3254.1652282 | 4 |
| 3254.166831 | 1 |
| 3254.168807 | 1 |
| 3254.1701937 | 6 |
| 3254.1736328 | 1 |
| 3254.1751798 | 1 |
| 3254.17621 | 3 |
| 3254.1787861 | 2 |
| 3254.1798057 | 2 |
| 3254.182157 | 2 |
| 3254.1845086 | 4 |
| 3254.1856478 | 1 |
| 3254.1885729 | 2 |
| 3254.1891115 | 1 |
| 3254.192232 | 4 |
| 3254.1936231 | 1 |
| 3254.1961515 | 3 |
| 3254.1981167 | 3 |
| 3254.1990442 | 2 |
| 3254.202256 | 8 |
| 3254.2028996 | 1 |
| 3254.2048561 | 2 |
| 3254.2072222 | 3 |
| 3254.2091703 | 0 |
| 3254.2108319 | 4 |
| 3254.2124354 | 3 |
| 3254.214156 | 4 |
| 3254.2172571 | 4 |
| 3254.2179112 | 5 |
| 3254.2207821 | 2 |
| 3254.2216026 | 1 |
| 3254.2242962 | 3 |
| 3254.2266543 | 3 |
| 3254.2274838 | 3 |
| 3254.23015 | 0 |
| 3254.230532 | 3 |
| 3254.2334577 | 1 |
| 3254.2353991 | 3 |
| 3254.2370753 | 3 |
| 3254.2393187 | 2 |
| 3254.2416993 | 2 |
| 3254.2433439 | 2 |
| 3254.2451104 | 4 |
| 3254.2462899 | 2 |
| 3254.2491409 | 1 |
| 3254.2504198 | 1 |
| 3254.2524292 | 4 |
| 3254.2551231 | 2 |
| 3254.2557516 | 2 |
| 3254.2585655 | 3 |
| 3254.2593282 | 3 |
| 3254.2619906 | 4 |
| 3254.2640533 | 2 |
| 3254.2647764 | 6 |
| 3254.2680127 | 5 |
| 3254.2687719 | 4 |
| 3254.2709679 | 2 |
| 3254.2737022 | 2 |
| 3254.2747016 | 4 |
| 3254.2769859 | 1 |
| 3254.2785677 | 1 |
| 3254.2806614 | 1 |
| 3254.2834291 | 1 |
| 3254.2842328 | 1 |
| 3254.2868684 | 2 |
| 3254.2879913 | 4 |
| 3254.2901232 | 0 |
| 3254.2923185 | 2 |
| 3254.2930878 | 1 |
| 3254.2956823 | 2 |
| 3254.2980845 | 0 |
| 3254.298979 | 4 |
| 3254.3015381 | 3 |
| 3254.3031474 | 1 |
| 3254.305104 | 2 |
| 3254.306325 | 1 |
| 3254.3081714 | 2 |
| 3254.3109574 | 3 |
| 3254.311344 | 2 |
| 3254.3144631 | 5 |
| 3254.3165168 | 5 |
| 3254.3177071 | 4 |
| 3254.3202967 | 2 |
| 3254.3212453 | 5 |
| 3254.3234567 | 3 |
| 3254.3263989 | 2 |
| 3254.3277359 | 6 |
| 3254.3302166 | 2 |
| 3254.3312713 | 3 |
| 3254.3335666 | 6 |
| 3254.3358717 | 5 |
| 3254.3368561 | 2 |
| 3254.3391562 | 2 |
| 3254.3405385 | 1 |
| 3254.343037 | 6 |
| 3254.344881 | 2 |
| 3254.3459394 | 1 |
| 3254.3484852 | 3 |
| 3254.3495555 | 1 |
| 3254.3521684 | 4 |
| 3254.3539656 | 1 |
| 3254.3552132 | 1 |
| 3254.3577574 | 1 |
| 3254.3589194 | 2 |
| 3254.3611309 | 1 |
| 3254.362349 | 6 |
| 3254.3647781 | 0 |
| 3254.3671565 | 2 |
| 3254.3680568 | 1 |
| 3254.37078 | 1 |
| 3254.3718976 | 3 |
| 3254.3743599 | 1 |
| 3254.3767062 | 1 |
| 3254.3783143 | 5 |
| 3254.3803335 | 2 |
| 3254.3816572 | 1 |
| 3254.3841284 | 3 |
| 3254.3860533 | 3 |
| 3254.3864765 | 1 |
| 3254.3894502 | 4 |
| 3254.3904608 | 1 |
| 3254.3924953 | 2 |
| 3254.3946332 | 2 |
| 3254.3964026 | 2 |
| 3254.398244 | 4 |
| 3254.3999973 | 3 |
| 3254.4020408 | 2 |
| 3254.4045173 | 3 |
| 3254.4057874 | 4 |
| 3254.4083538 | 1 |
| 3254.4091407 | 1 |
| 3254.4118411 | 5 |
| 3254.4133071 | 4 |
| 3254.4150708 | 5 |
| 3254.4171364 | 4 |
| 3254.418206 | 2 |
| 3254.4204486 | 2 |
| 3254.4231473 | 2 |
| 3254.4239402 | 6 |
| 3254.4266728 | 3 |
| 3254.4277989 | 1 |
| 3254.4302454 | 4 |
| 3254.4310536 | 3 |
| 3254.4339241 | 2 |
| 3254.4356795 | 2 |
| 3254.4372251 | 0 |
| 3254.4391523 | 1 |
| 3254.4415639 | 2 |
| 3254.4424782 | 5 |
| 3254.4448174 | 2 |
| 3254.4461771 | 2 |
| 3254.4486058 | 1 |
| 3254.4501184 | 4 |
| 3254.4518077 | 3 |
| 3254.4541455 | 5 |
| 3254.455696 | 3 |
| 3254.4583422 | 3 |
| 3254.4593712 | 2 |
| 3254.4614284 | 1 |
| 3254.4641435 | 2 |
| 3254.465148 | 3 |
| 3254.4675791 | 2 |
| 3254.4683289 | 3 |
| 3254.4707648 | 2 |
| 3254.473251 | 2 |
| 3254.4741299 | 3 |
| 3254.4761737 | 3 |
| 3254.4777632 | 1 |
| 3254.4801524 | 2 |
| 3254.4824483 | 7 |
| 3254.4836342 | 2 |
| 3254.4859734 | 1 |
| 3254.487155 | 2 |
| 3254.4891366 | 3 |
| 3254.4916742 | 1 |
| 3254.492577 | 0 |
| 3254.494946 | 0 |
| 3254.4962191 | 4 |
| 3254.4985467 | 2 |
| 3254.5010251 | 1 |
| 3254.5018339 | 1 |
| 3254.5045126 | 2 |
| 3254.5060001 | 3 |
| 3254.5075971 | 0 |
| 3254.5094708 | 2 |
| 3254.5118331 | 1 |
| 3254.5140928 | 0 |
| 3254.5148683 | 0 |
| 3254.517976 | 0 |
| 3254.5198424 | 2 |
| 3254.5207482 | 0 |
| 3254.5232695 | 3 |
| 3254.5245451 | 2 |
| 3254.5269224 | 1 |
| 3254.5275681 | 2 |
| 3254.5298497 | 5 |
| 3254.5326672 | 2 |
| 3254.5333967 | 5 |
| 3254.5358083 | 1 |
| 3254.5371811 | 2 |
| 3254.5388329 | 4 |
| 3254.5422705 | 1 |
| 3254.5427436 | 2 |
| 3254.5452878 | 0 |
| 3254.5465917 | 2 |
| 3254.5487298 | 2 |
| 3254.5498029 | 7 |
| 3254.5530703 | 3 |
| 3254.5549968 | 2 |
| 3254.5562913 | 1 |
| 3254.5584223 | 3 |
| 3254.559296 | 1 |
| 3254.5617302 | 5 |
| 3254.5644622 | 1 |
| 3254.5648743 | 2 |
| 3254.5675162 | 1 |
| 3254.5695565 | 2 |
| 3254.5709013 | 3 |
| 3254.5732027 | 1 |
| 3254.5746613 | 1 |
| 3254.5766026 | 2 |
| 3254.5787766 | 3 |
| 3254.5795371 | 4 |
| 3254.5829335 | 3 |
| 3254.5830106 | 2 |
| 3254.5855672 | 2 |
| 3254.5882654 | 4 |
| 3254.5894956 | 0 |
| 3254.5918363 | 2 |
| 3254.5927871 | 2 |
| 3254.5948708 | 5 |
| 3254.5974211 | 5 |
| 3254.5982981 | 2 |
| 3254.6012952 | 4 |
| 3254.6026027 | 4 |
| 3254.6047816 | 4 |
| 3254.6057319 | 3 |
| 3254.6085986 | 2 |
| 3254.6108665 | 1 |
| 3254.6118074 | 0 |
| 3254.6134958 | 3 |
| 3254.6152266 | 5 |
| 3254.6174675 | 6 |
| 3254.6194088 | 5 |
| 3254.6207473 | 4 |
| 3254.6230429 | 2 |
| 3254.6254438 | 0 |
| 3254.6260722 | 3 |
| 3254.6286526 | 3 |
| 3254.6298932 | 2 |
| 3254.6321929 | 0 |
| 3254.634704 | 2 |
| 3254.6360949 | 3 |
| 3254.6383882 | 0 |
| 3254.6390863 | 3 |
| 3254.6422412 | 2 |
| 3254.6431073 | 1 |
| 3254.6455397 | 2 |
| 3254.6478889 | 0 |
| 3254.6491536 | 1 |
| 3254.6509198 | 0 |
| 3254.6518364 | 0 |
| 3254.654716 | 4 |
| 3254.6574747 | 3 |
| 3254.6584958 | 0 |
| 3254.6609204 | 0 |
| 3254.6618445 | 4 |
| 3254.6639748 | 1 |
| 3254.666655 | 3 |
| 3254.667563 | 2 |
| 3254.6693736 | 4 |
| 3254.6717954 | 1 |
| 3254.6732139 | 3 |
| 3254.6754303 | 0 |
| 3254.6765172 | 2 |
| 3254.6790682 | 2 |
| 3254.6801804 | 0 |
| 3254.6825798 | 3 |
| 3254.6850026 | 2 |
| 3254.6866051 | 1 |
| 3254.6884538 | 2 |
| 3254.6900827 | 1 |
| 3254.6918978 | 2 |
| 3254.6946841 | 1 |
| 3254.6955949 | 2 |
| 3254.6984339 | 3 |
| 3254.699454 | 2 |
| 3254.7019196 | 2 |
| 3254.7032196 | 1 |
| 3254.7049408 | 2 |
| 3254.7075358 | 2 |
| 3254.7086574 | 3 |
| 3254.7109155 | 2 |
| 3254.7122564 | 1 |
| 3254.7148388 | 1 |
| 3254.7166888 | 1 |
| 3254.7180122 | 3 |
| 3254.720472 | 2 |
| 3254.7216497 | 1 |
| 3254.7238137 | 2 |
| 3254.7256214 | 3 |
| 3254.7273085 | 5 |
| 3254.7299621 | 3 |
| 3254.7312722 | 4 |
| 3254.7331274 | 1 |
| 3254.7349285 | 0 |
| 3254.7369938 | 2 |
| 3254.7390065 | 2 |
| 3254.7405631 | 2 |
| 3254.7424312 | 6 |
| 3254.7435517 | 2 |
| 3254.7460863 | 2 |
| 3254.748437 | 4 |
| 3254.7493703 | 3 |
| 3254.7519067 | 3 |
| 3254.753182 | 1 |
| 3254.7559911 | 1 |
| 3254.7578356 | 1 |
| 3254.7592068 | 5 |
| 3254.761453 | 2 |
| 3254.7624141 | 2 |
| 3254.7652851 | 0 |
| 3254.7657712 | 2 |
| 3254.7683916 | 3 |
| 3254.7705625 | 2 |
| 3254.7720454 | 0 |
| 3254.7747352 | 3 |
| 3254.7752956 | 3 |
| 3254.7780604 | 4 |
| 3254.7788416 | 3 |
| 3254.7814416 | 2 |
| 3254.7839304 | 2 |
| 3254.7850369 | 4 |
| 3254.7873779 | 4 |
| 3254.7899473 | 1 |
| 3254.7905902 | 2 |
| 3254.793192 | 2 |
| 3254.7942295 | 4 |
| 3254.7966119 | 1 |
| 3254.7985734 | 1 |
| 3254.8000831 | 2 |
| 3254.8025221 | 2 |
| 3254.8032017 | 1 |
| 3254.805821 | 0 |
| 3254.8082856 | 3 |
| 3254.8094279 | 1 |
| 3254.8117845 | 1 |
| 3254.8130659 | 1 |
| 3254.8140766 | 2 |

| | |
|---|---|
| 3254.8160992 | 0 |
| 3254.8187597 | 5 |
| 3254.821542 | 4 |
| 3254.8224697 | 3 |
| 3254.824665 | 3 |
| 3254.8259428 | 0 |
| 3254.8283405 | 1 |
| 3254.8305371 | 1 |
| 3254.8329143 | 0 |
| 3254.8342721 | 0 |
| 3254.835291 | 5 |
| 3254.8382485 | 2 |
| 3254.8388539 | 1 |
| 3254.8408334 | 4 |
| 3254.8441279 | 4 |
| 3254.8447793 | 3 |
| 3254.8469953 | 1 |
| 3254.8479453 | 1 |
| 3254.8504915 | 4 |
| 3254.8527451 | 2 |
| 3254.8544691 | 2 |
| 3254.8568513 | 0 |
| 3254.8578024 | 2 |
| 3254.8596502 | 7 |
| 3254.8618464 | 4 |
| 3254.8628515 | 2 |
| 3254.8659236 | 2 |
| 3254.8667772 | 5 |
| 3254.8691487 | 3 |
| 3254.8719433 | 1 |
| 3254.8728064 | 1 |
| 3254.8757031 | 2 |
| 3254.8759976 | 2 |
| 3254.8798597 | 3 |
| 3254.8801365 | 2 |
| 3254.8823922 | 0 |
| 3254.8850968 | 1 |
| 3254.8857512 | 3 |
| 3254.886941 | 0 |
| 3254.8893151 | 5 |
| 3254.8912328 | 1 |
| 3254.8939075 | 3 |
| 3254.894978 | 2 |
| 3254.8975012 | 5 |
| 3254.898458 | 0 |
| 3254.9004362 | 2 |
| 3254.9013215 | 1 |
| 3254.9045238 | 2 |
| 3254.9063088 | 1 |
| 3254.9074435 | 2 |
| 3254.9076843 | 6 |
| 3254.9079187 | 2 |
| 3254.9087484 | 5 |
| 3254.9100196 | 0 |
| 3254.91012 | 4 |
| 3254.9114555 | 3 |
| 3254.9115272 | 0 |
| 3254.9117971 | 3 |
| 3254.9130241 | 8 |
| 3254.9135197 | 3 |
| 3254.9138067 | 3 |
| 3254.9150388 | 3 |
| 3254.9152565 | 2 |
| 3254.9162211 | 2 |
| 3254.9162212 | 2 |
| 3254.9171431 | 1 |
| 3254.9174622 | 0 |
| 3254.9185093 | 3 |
| 3254.9196777 | 1 |
| 3254.9202682 | 4 |
| 3254.9207119 | 2 |
| 3254.920991 | 2 |
| 3254.9217239 | 3 |
| 3254.9217708 | 0 |
| 3254.9232209 | 5 |
| 3254.9233342 | 2 |
| 3254.9245206 | 3 |
| 3254.9257073 | 1 |
| 3254.9260926 | 5 |
| 3254.9267482 | 1 |
| 3254.9268038 | 0 |
| 3254.9269176 | 4 |
| 3254.9282094 | 3 |
| 3254.9288866 | 1 |
| 3254.9290342 | 2 |
| 3254.929865 | 1 |
| 3254.9301895 | 4 |
| 3254.9315173 | 2 |
| 3254.9317543 | 3 |
| 3254.932131 | 4 |
| 3254.9323432 | 3 |
| 3254.9340544 | 4 |
| 3254.9344335 | 1 |
| 3254.9348344 | 9 |
| 3254.9349837 | 2 |
| 3254.9359673 | 2 |
| 3254.9363404 | 2 |
| 3254.9369416 | 2 |
| 3254.9377564 | 4 |
| 3254.9386809 | 3 |
| 3254.9393998 | 2 |
| 3254.9397783 | 1 |
| 3254.9399632 | 5 |
| 3254.9400049 | 2 |
| 3254.9420164 | 4 |
| 3254.9420238 | 3 |
| 3254.9429809 | 3 |
| 3254.9436327 | 3 |
| 3254.9444679 | 2 |
| 3254.9445166 | 2 |
| 3254.9445502 | 4 |
| 3254.945687 | 1 |
| 3254.9462212 | 1 |
| 3254.9463675 | 1 |
| 3254.9472671 | 2 |
| 3254.9486494 | 4 |
| 3254.9487432 | 3 |
| 3254.9487814 | 4 |
| 3254.9504122 | 14 |
| 3254.9504998 | 9 |
| 3254.951151 | 10 |
| 3254.9525166 | 8 |
| 3254.9533879 | 9 |
| 3254.953398 | 7 |
| 3254.9535116 | 6 |
| 3254.9542645 | 9 |
| 3254.9543842 | 7 |
| 3254.9562112 | 5 |
| 3254.9565854 | 8 |
| 3254.9569588 | 1 |
| 3254.9569822 | 7 |
| 3254.9578421 | 4 |
| 3254.9582711 | 0 |
| 3254.9592968 | 1 |
| 3254.959993 | 5 |
| 3254.9605375 | 0 |
| 3254.9626381 | 3 |
| 3254.9627035 | 1 |
| 3254.9633887 | 5 |
| 3254.9635525 | 3 |
| 3254.9640617 | 2 |
| 3254.9641227 | 2 |
| 3254.9652254 | 2 |
| 3254.9664488 | 4 |
| 3254.9664491 | 3 |
| 3254.9668646 | 4 |
| 3254.9673027 | 1 |
| 3254.9677152 | 2 |
| 3254.9687572 | 0 |
| 3254.9696914 | 2 |
| 3254.9704195 | 4 |
| 3254.9710949 | 1 |
| 3254.9718945 | 0 |
| 3254.9720046 | 1 |
| 3254.9722545 | 2 |
| 3254.972958 | 1 |
| 3254.9735184 | 0 |
| 3254.9751862 | 5 |
| 3254.9764221 | 5 |
| 3254.9787878 | 2 |
| 3254.9812178 | 2 |
| 3254.982114 | 1 |
| 3254.9842798 | 2 |
| 3254.985491 | 6 |
| 3254.987877 | 2 |
| 3254.9896327 | 3 |
| 3254.9911693 | 3 |
| 3254.994194 | 3 |
| 3254.9944275 | 3 |
| 3254.9971613 | 2 |
| 3254.999909 | 3 |
| 3255.0004991 | 3 |
| 3255.0026935 | 3 |
| 3255.0043998 | 3 |
| 3255.0062097 | 1 |
| 3255.0064458 | 2 |
| 3255.0068224 | 2 |
| 3255.0068615 | 1 |
| 3255.0070177 | 3 |
| 3255.0071234 | 3 |
| 3255.0078258 | 3 |
| 3255.0079409 | 2 |
| 3255.0081436 | 5 |
| 3255.0083196 | 3 |
| 3255.0084999 | 3 |
| 3255.0092452 | 5 |
| 3255.0093539 | 3 |
| 3255.0095571 | 2 |
| 3255.009967 | 2 |
| 3255.0101445 | 1 |
| 3255.01035 | 0 |
| 3255.0105893 | 1 |
| 3255.010813 | 2 |
| 3255.0110432 | 3 |
| 3255.0111822 | 1 |
| 3255.0117505 | 3 |
| 3255.0117602 | 2 |
| 3255.0124305 | 0 |
| 3255.0125922 | 1 |
| 3255.0127518 | 3 |
| 3255.0130912 | 2 |
| 3255.0135886 | 3 |
| 3255.0141015 | 3 |
| 3255.0141651 | 2 |
| 3255.0142562 | 1 |
| 3255.0144958 | 3 |
| 3255.0152704 | 4 |
| 3255.0157807 | 2 |
| 3255.0158052 | 2 |
| 3255.0160106 | 0 |
| 3255.0162348 | 2 |
| 3255.0169879 | 1 |
| 3255.017313 | 3 |
| 3255.0175282 | 3 |
| 3255.0176863 | 4 |
| 3255.0178295 | 2 |
| 3255.0183194 | 3 |
| 3255.0186822 | 2 |
| 3255.0190596 | 1 |
| 3255.0192984 | 1 |
| 3255.0200494 | 2 |
| 3255.0200664 | 6 |
| 3255.0207675 | 3 |
| 3255.0211237 | 4 |
| 3255.0212634 | 4 |
| 3255.0218561 | 5 |
| 3255.0218613 | 2 |
| 3255.0225466 | 1 |
| 3255.0229663 | 2 |
| 3255.0230346 | 2 |
| 3255.023107 | 0 |
| 3255.0234601 | 3 |
| 3255.0237933 | 2 |
| 3255.0244336 | 1 |
| 3255.0246388 | 2 |
| 3255.0252113 | 3 |
| 3255.0255356 | 5 |
| 3255.0255887 | 7 |
| 3255.0256922 | 0 |
| 3255.0262088 | 3 |
| 3255.0266405 | 1 |
| 3255.0270182 | 2 |
| 3255.0270292 | 3 |
| 3255.027068 | 4 |
| 3255.0277756 | 0 |
| 3255.0284965 | 6 |
| 3255.0285579 | 0 |
| 3255.0289413 | 9 |
| 3255.0291124 | 6 |
| 3255.0291706 | 6 |
| 3255.0302468 | 8 |
| 3255.0303211 | 7 |
| 3255.0303987 | 10 |
| 3255.0308762 | 16 |
| 3255.0313206 | 18 |
| 3255.0317966 | 14 |
| 3255.0319569 | 12 |
| 3255.0324458 | 20 |
| 3255.0324943 | 14 |
| 3255.0325664 | 6 |
| 3255.0332742 | 8 |
| 3255.033515 | 8 |
| 3255.0337614 | 3 |
| 3255.0339893 | 3 |
| 3255.0346571 | 10 |
| 3255.0348335 | 5 |
| 3255.0350455 | 1 |
| 3255.0352944 | 0 |
| 3255.0358622 | 4 |
| 3255.0358973 | 4 |
| 3255.0365479 | 3 |
| 3255.0373753 | 0 |
| 3255.0374465 | 2 |
| 3255.0376009 | 2 |
| 3255.0379412 | 1 |
| 3255.0381844 | 1 |
| 3255.0385997 | 2 |
| 3255.0391132 | 3 |
| 3255.0392638 | 3 |
| 3255.039449 | 2 |
| 3255.0394542 | 10 |
| 3255.039661 | 3 |
| 3255.040073 | 2 |
| 3255.0404434 | 0 |
| 3255.0415927 | 0 |
| 3255.0416321 | 0 |
| 3255.0418744 | 3 |
| 3255.0423197 | 1 |
| 3255.0426304 | 0 |
| 3255.0434321 | 3 |
| 3255.0436606 | 1 |
| 3255.0437953 | 3 |
| 3255.0438097 | 4 |
| 3255.0440502 | 1 |
| 3255.0442513 | 1 |
| 3255.0449547 | 3 |
| 3255.0453958 | 6 |
| 3255.0457316 | 1 |
| 3255.0459081 | 4 |
| 3255.0466187 | 1 |
| 3255.046711 | 3 |
| 3255.0473121 | 2 |
| 3255.0488487 | 4 |
| 3255.050426 | 3 |
| 3255.0535731 | 1 |
| 3255.0539504 | 2 |
| 3255.0566977 | 4 |
| 3255.0589745 | 2 |
| 3255.0595322 | 2 |
| 3255.0618385 | 1 |
| 3255.0638747 | 3 |
| 3255.0659812 | 2 |
| 3255.0667426 | 3 |
| 3255.0692727 | 7 |
| 3255.0719151 | 7 |
| 3255.0725743 | 1 |
| 3255.0745453 | 2 |
| 3255.0760497 | 2 |
| 3255.0779916 | 4 |
| 3255.080492 | 4 |
| 3255.0813812 | 1 |
| 3255.0839006 | 3 |
| 3255.0849396 | 0 |
| 3255.0875599 | 1 |
| 3255.0895235 | 0 |
| 3255.0909666 | 2 |
| 3255.0935466 | 3 |
| 3255.0940101 | 2 |
| 3255.0966837 | 0 |
| 3255.099017 | 2 |
| 3255.0996313 | 1 |
| 3255.10212 | 1 |
| 3255.1034439 | 4 |
| 3255.1038748 | 1 |
| 3255.1050653 | 3 |
| 3255.1054828 | 1 |
| 3255.1066469 | 1 |
| 3255.1076221 | 5 |
| 3255.1082625 | 0 |
| 3255.1093561 | 1 |
| 3255.1095518 | 3 |
| 3255.109681 | 2 |
| 3255.1101328 | 0 |
| 3255.1114637 | 0 |
| 3255.1121959 | 2 |
| 3255.1125722 | 2 |
| 3255.1126463 | 3 |
| 3255.1130916 | 3 |
| 3255.1146126 | 1 |
| 3255.114808 | 3 |
| 3255.1154626 | 5 |
| 3255.1159824 | 7 |
| 3255.117792 | 4 |
| 3255.1189516 | 4 |
| 3255.119888 | 4 |
| 3255.1214239 | 4 |
| 3255.122345 | 2 |
| 3255.1233758 | 1 |
| 3255.1252147 | 2 |
| 3255.125708 | 0 |
| 3255.1281547 | 4 |
| 3255.1286685 | 2 |
| 3255.1300522 | 3 |

| | |
|---|---|
| 3255.131747 | 4 |
| 3255.1322039 | 4 |
| 3255.1340511 | 1 |
| 3255.1347599 | 2 |
| 3255.1368097 | 7 |
| 3255.1379745 | 3 |
| 3255.1391513 | 1 |
| 3255.1406528 | 1 |
| 3255.1426297 | 1 |
| 3255.1431783 | 5 |
| 3255.1443211 | 2 |
| 3255.1459017 | 5 |
| 3255.1466022 | 4 |
| 3255.1485073 | 5 |
| 3255.1490282 | 2 |
| 3255.1505833 | 4 |
| 3255.1510369 | 1 |
| 3255.1524667 | 0 |
| 3255.1533466 | 3 |
| 3255.1548975 | 2 |
| 3255.1565215 | 7 |
| 3255.1572029 | 4 |
| 3255.1592504 | 3 |
| 3255.1603643 | 2 |
| 3255.1609651 | 2 |
| 3255.1627005 | 2 |
| 3255.1636648 | 5 |
| 3255.1657544 | 3 |
| 3255.1659792 | 5 |
| 3255.1675791 | 2 |
| 3255.1689504 | 3 |
| 3255.1700021 | 1 |
| 3255.1716955 | 3 |
| 3255.1746676 | 2 |
| 3255.1755097 | 2 |
| 3255.1763212 | 3 |
| 3255.1770071 | 2 |
| 3255.1775618 | 3 |
| 3255.179172 | 3 |
| 3255.1801532 | 1 |
| 3255.1808765 | 3 |
| 3255.182462 | 5 |
| 3255.1828415 | 2 |
| 3255.1846325 | 3 |
| 3255.1851572 | 1 |
| 3255.1858419 | 3 |
| 3255.186764 | 2 |
| 3255.1872325 | 4 |
| 3255.1886648 | 3 |
| 3255.189492 | 0 |
| 3255.1902016 | 1 |
| 3255.1925853 | 2 |
| 3255.1932257 | 2 |
| 3255.1951307 | 4 |
| 3255.1953099 | 1 |
| 3255.196488 | 1 |
| 3255.1980979 | 3 |
| 3255.2001046 | 2 |
| 3255.2022481 | 1 |
| 3255.2060388 | 2 |
| 3255.2162549 | 5 |
| 3255.217544 | 0 |
| 3255.2184881 | 4 |
| 3255.2191512 | 4 |
| 3255.2199904 | 1 |
| 3255.2208713 | 2 |
| 3255.2220298 | 4 |
| 3255.2226474 | 4 |
| 3255.2231381 | 5 |
| 3255.2240528 | 4 |
| 3255.2254184 | 1 |
| 3255.2256059 | 4 |
| 3255.226221 | 3 |
| 3255.2272518 | 3 |
| 3255.2291531 | 3 |
| 3255.2293132 | 2 |
| 3255.2307626 | 4 |
| 3255.2316182 | 1 |
| 3255.2317549 | 5 |
| 3255.2328834 | 3 |
| 3255.23463 | 1 |
| 3255.2360397 | 1 |
| 3255.2372737 | 2 |
| 3255.2374598 | 6 |
| 3255.2398497 | 4 |
| 3255.2402174 | 4 |
| 3255.2413814 | 3 |
| 3255.2433364 | 3 |
| 3255.243761 | 2 |
| 3255.2446904 | 1 |
| 3255.245054 | 2 |
| 3255.2475084 | 4 |
| 3255.248055 | 2 |
| 3255.249465 | 4 |
| 3255.2502812 | 3 |
| 3255.2523661 | 2 |
| 3255.2545589 | 1 |
| 3255.2547007 | 4 |
| 3255.2571523 | 1 |
| 3255.2591112 | 4 |
| 3255.2602168 | 2 |
| 3255.2620029 | 3 |
| 3255.2631409 | 3 |
| 3255.2639946 | 1 |
| 3255.2641149 | 3 |
| 3255.2641922 | 1 |
| 3255.2660125 | 2 |
| 3255.2661557 | 3 |
| 3255.2678323 | 1 |
| 3255.2687623 | 1 |
| 3255.2711221 | 2 |
| 3255.2721476 | 2 |
| 3255.2730374 | 4 |
| 3255.2747649 | 6 |
| 3255.2758407 | 3 |
| 3255.2774544 | 4 |
| 3255.2788258 | 2 |
| 3255.2796313 | 0 |
| 3255.2813204 | 1 |
| 3255.2817311 | 2 |
| 3255.2833047 | 1 |
| 3255.2846776 | 0 |
| 3255.2861815 | 1 |
| 3255.2877141 | 3 |
| 3255.2881668 | 2 |
| 3255.2884964 | 2 |
| 3255.2908645 | 1 |
| 3255.2914732 | 3 |
| 3255.29295 | 1 |
| 3255.2945416 | 1 |
| 3255.2974369 | 1 |
| 3255.2991534 | 5 |
| 3255.2995397 | 2 |
| 3255.3010276 | 2 |
| 3255.3020259 | 1 |
| 3255.3034545 | 3 |
| 3255.3036504 | 0 |
| 3255.3053506 | 3 |
| 3255.3068487 | 1 |
| 3255.307514 | 6 |
| 3255.3079947 | 8 |
| 3255.3099593 | 5 |
| 3255.3104107 | 3 |
| 3255.3113267 | 4 |
| 3255.313933 | 2 |
| 3255.3141249 | 1 |
| 3255.3159105 | 2 |
| 3255.3164927 | 2 |
| 3255.3171615 | 4 |
| 3255.3181925 | 2 |
| 3255.3187792 | 2 |
| 3255.3208543 | 1 |
| 3255.3215409 | 2 |
| 3255.3231502 | 0 |
| 3255.3243337 | 5 |
| 3255.3254458 | 2 |
| 3255.3271044 | 2 |
| 3255.3278784 | 2 |
| 3255.3294364 | 2 |
| 3255.3306363 | 2 |
| 3255.3320038 | 2 |
| 3255.3323795 | 2 |
| 3255.3331508 | 2 |
| 3255.3343877 | 2 |
| 3255.336724 | 6 |
| 3255.336739 | 1 |
| 3255.3386658 | 4 |
| 3255.339826 | 1 |
| 3255.3405844 | 3 |
| 3255.3429876 | 2 |
| 3255.3443743 | 4 |
| 3255.346829 | 2 |
| 3255.3473773 | 1 |
| 3255.3499008 | 1 |
| 3255.3506741 | 3 |
| 3255.3513983 | 2 |
| 3255.3526895 | 1 |
| 3255.3535687 | 2 |
| 3255.3538588 | 2 |
| 3255.3545418 | 1 |
| 3255.3569851 | 2 |
| 3255.3579674 | 4 |
| 3255.358225 | 5 |
| 3255.3597881 | 3 |
| 3255.3613427 | 1 |
| 3255.3626185 | 1 |
| 3255.3632009 | 4 |
| 3255.3643981 | 4 |
| 3255.3663209 | 2 |
| 3255.3671345 | 2 |
| 3255.3685966 | 3 |
| 3255.3705291 | 2 |
| 3255.3713141 | 3 |
| 3255.3730801 | 4 |
| 3255.3732159 | 3 |
| 3255.375132 | 2 |
| 3255.3763092 | 1 |
| 3255.3777753 | 1 |
| 3255.3794059 | 2 |
| 3255.379499 | 4 |
| 3255.3812262 | 1 |
| 3255.3819287 | 8 |
| 3255.3833195 | 2 |
| 3255.3846075 | 2 |
| 3255.3856851 | 3 |
| 3255.3868472 | 2 |
| 3255.3881466 | 3 |
| 3255.3888796 | 3 |
| 3255.3914336 | 3 |
| 3255.3925929 | 4 |
| 3255.3948282 | 3 |
| 3255.3953975 | 6 |
| 3255.397637 | 2 |
| 3255.3987992 | 2 |
| 3255.4002598 | 1 |
| 3255.4010351 | 1 |
| 3255.4012631 | 2 |
| 3255.4028651 | 4 |
| 3255.4032923 | 3 |
| 3255.4049603 | 1 |
| 3255.4052399 | 1 |
| 3255.406083 | 0 |
| 3255.4079262 | 2 |
| 3255.4098809 | 6 |
| 3255.4102668 | 2 |
| 3255.4120907 | 1 |
| 3255.4133586 | 4 |
| 3255.4145224 | 4 |
| 3255.4158353 | 6 |
| 3255.4166772 | 4 |
| 3255.4185146 | 1 |
| 3255.4190789 | 5 |
| 3255.4214066 | 5 |
| 3255.4223786 | 5 |
| 3255.4230187 | 2 |
| 3255.4244406 | 3 |
| 3255.4260064 | 2 |
| 3255.4272226 | 3 |
| 3255.4281398 | 4 |
| 3255.4290114 | 5 |
| 3255.4307091 | 3 |
| 3255.4310741 | 3 |
| 3255.4334235 | 2 |
| 3255.4350974 | 1 |
| 3255.4368985 | 2 |
| 3255.4386181 | 2 |
| 3255.4388176 | 2 |
| 3255.440674 | 1 |
| 3255.4416651 | 2 |
| 3255.443118 | 3 |
| 3255.4445238 | 0 |
| 3255.4450456 | 2 |
| 3255.4463243 | 1 |
| 3255.4473649 | 4 |
| 3255.4477506 | 3 |
| 3255.4496549 | 1 |
| 3255.4509925 | 3 |
| 3255.4511281 | 4 |
| 3255.4539397 | 4 |
| 3255.4541773 | 2 |
| 3255.4560813 | 2 |
| 3255.4567043 | 3 |
| 3255.4574748 | 2 |
| 3255.4590081 | 4 |
| 3255.4605222 | 0 |
| 3255.4619301 | 1 |
| 3255.4627592 | 1 |
| 3255.4642837 | 3 |
| 3255.4652044 | 1 |
| 3255.4668371 | 2 |
| 3255.4681825 | 4 |
| 3255.4695766 | 2 |
| 3255.4709171 | 2 |
| 3255.4717613 | 3 |
| 3255.4723549 | 2 |
| 3255.4739298 | 2 |
| 3255.4750097 | 5 |
| 3255.4763982 | 1 |
| 3255.4774497 | 2 |
| 3255.4785045 | 1 |
| 3255.4801383 | 3 |
| 3255.4805913 | 3 |
| 3255.4831466 | 3 |
| 3255.4845126 | 2 |
| 3255.4868636 | 1 |
| 3255.4883062 | 7 |
| 3255.4893601 | 4 |
| 3255.4899608 | 2 |
| 3255.4908756 | 2 |
| 3255.4924075 | 2 |
| 3255.4934636 | 3 |
| 3255.4939141 | 3 |
| 3255.4957005 | 4 |
| 3255.4966819 | 5 |
| 3255.4972008 | 3 |
| 3255.4989785 | 5 |
| 3255.4998779 | 2 |
| 3255.5022821 | 3 |
| 3255.5023218 | 0 |
| 3255.5038277 | 0 |
| 3255.5054345 | 3 |
| 3255.5062103 | 1 |
| 3255.5078732 | 3 |
| 3255.508646 | 6 |
| 3255.5105676 | 3 |
| 3255.511342 | 4 |
| 3255.5125234 | 3 |
| 3255.5143393 | 3 |
| 3255.5146165 | 3 |
| 3255.5163073 | 1 |
| 3255.5174247 | 2 |
| 3255.5182448 | 6 |
| 3255.5202774 | 3 |
| 3255.5205277 | 5 |
| 3255.5218897 | 2 |
| 3255.5231105 | 5 |
| 3255.5245471 | 1 |
| 3255.526282 | 2 |
| 3255.5281225 | 2 |
| 3255.5302352 | 2 |
| 3255.5304885 | 4 |
| 3255.5324423 | 6 |
| 3255.5336451 | 1 |
| 3255.5350674 | 2 |
| 3255.5364143 | 2 |
| 3255.5368598 | 2 |
| 3255.5385314 | 3 |
| 3255.5392737 | 5 |
| 3255.5402102 | 1 |
| 3255.5415935 | 3 |
| 3255.5424721 | 1 |
| 3255.542901 | 2 |
| 3255.544108 | 3 |
| 3255.5455533 | 2 |
| 3255.5475667 | 1 |
| 3255.5484747 | 1 |
| 3255.5502474 | 3 |
| 3255.5505793 | 3 |
| 3255.5527058 | 1 |
| 3255.5543212 | 0 |
| 3255.5545566 | 2 |
| 3255.5563665 | 1 |
| 3255.5573455 | 0 |
| 3255.5585613 | 7 |
| 3255.5601781 | 2 |
| 3255.5609065 | 1 |
| 3255.5621081 | 3 |
| 3255.564258 | 1 |
| 3255.5649769 | 2 |
| 3255.5663513 | 6 |
| 3255.5665444 | 1 |
| 3255.568026 | 2 |
| 3255.5689366 | 2 |
| 3255.5702025 | 2 |
| 3255.5717132 | 2 |
| 3255.5725762 | 1 |
| 3255.5750764 | 3 |
| 3255.5769388 | 1 |
| 3255.5780212 | 3 |
| 3255.5795151 | 1 |
| 3255.5801683 | 1 |
| 3255.5823125 | 2 |
| 3255.5827836 | 2 |

| | |
|---|---|
| 3255.5829809 | 5 |
| 3255.5843217 | 2 |
| 3255.5854736 | 4 |
| 3255.5873145 | 1 |
| 3255.5875976 | 5 |
| 3255.5894304 | 1 |
| 3255.5911242 | 4 |
| 3255.591664 | 4 |
| 3255.5936918 | 7 |
| 3255.5944155 | 5 |
| 3255.595825 | 2 |
| 3255.5976737 | 1 |
| 3255.598421 | 1 |
| 3255.5995193 | 2 |
| 3255.6004262 | 2 |
| 3255.6023698 | 2 |
| 3255.6036874 | 1 |
| 3255.6045685 | 0 |
| 3255.6055276 | 1 |
| 3255.6066413 | 4 |
| 3255.607901 | 2 |
| 3255.6098563 | 2 |
| 3255.6108317 | 4 |
| 3255.6115425 | 0 |
| 3255.611876 | 7 |
| 3255.6135344 | 4 |
| 3255.6150164 | 5 |
| 3255.615923 | 2 |
| 3255.6189763 | 1 |
| 3255.6199054 | 4 |
| 3255.6225539 | 2 |
| 3255.6238772 | 2 |
| 3255.624682 | 1 |
| 3255.6265732 | 4 |
| 3255.6281084 | 4 |
| 3255.629322 | 0 |
| 3255.6293981 | 2 |
| 3255.6305101 | 0 |
| 3255.6317768 | 3 |
| 3255.6335244 | 0 |
| 3255.635188 | 5 |
| 3255.6355727 | 2 |
| 3255.6369215 | 2 |
| 3255.6373391 | 1 |
| 3255.638332 | 3 |
| 3255.639487 | 2 |
| 3255.6402247 | 1 |
| 3255.6413505 | 2 |
| 3255.6435838 | 1 |
| 3255.6436968 | 4 |
| 3255.6458349 | 2 |
| 3255.6473398 | 3 |
| 3255.6483726 | 4 |
| 3255.6493458 | 2 |
| 3255.651242 | 3 |
| 3255.6517909 | 2 |
| 3255.6531559 | 4 |
| 3255.6533342 | 1 |
| 3255.6557055 | 1 |
| 3255.655901 | 0 |
| 3255.6581897 | 4 |
| 3255.6589229 | 2 |
| 3255.6600163 | 1 |
| 3255.6613853 | 3 |
| 3255.6616881 | 3 |
| 3255.6632846 | 0 |
| 3255.6657213 | 3 |
| 3255.6660717 | 3 |
| 3255.6671919 | 2 |
| 3255.6697795 | 2 |
| 3255.6708348 | 2 |
| 3255.6709512 | 5 |
| 3255.6723999 | 2 |
| 3255.673691 | 5 |
| 3255.6738983 | 1 |
| 3255.6743167 | 4 |
| 3255.6748689 | 0 |
| 3255.6750583 | 4 |
| 3255.6752243 | 2 |
| 3255.6752616 | 3 |
| 3255.6763234 | 2 |
| 3255.6763402 | 1 |
| 3255.6764225 | 3 |
| 3255.677015 | 6 |
| 3255.6772416 | 7 |
| 3255.6780218 | 2 |
| 3255.6780581 | 0 |
| 3255.6780799 | 1 |
| 3255.6784152 | 4 |
| 3255.6784559 | 2 |
| 3255.6787933 | 3 |
| 3255.6793838 | 1 |
| 3255.679575 | 1 |
| 3255.6799474 | 2 |
| 3255.6802302 | 1 |
| 3255.6805962 | 3 |
| 3255.6807844 | 2 |
| 3255.6810459 | 3 |
| 3255.6810817 | 2 |
| 3255.681643 | 3 |
| 3255.6827767 | 4 |
| 3255.6829475 | 0 |
| 3255.6831374 | 6 |
| 3255.683652 | 2 |
| 3255.6838575 | 2 |
| 3255.6840998 | 3 |
| 3255.6844052 | 1 |
| 3255.6849715 | 1 |
| 3255.6850132 | 3 |
| 3255.6853633 | 1 |
| 3255.6856469 | 2 |
| 3255.6857542 | 4 |
| 3255.6858164 | 3 |
| 3255.6858688 | 5 |
| 3255.6867011 | 1 |
| 3255.6869264 | 3 |
| 3255.687066 | 2 |
| 3255.6872143 | 3 |
| 3255.6874664 | 5 |
| 3255.6875135 | 1 |
| 3255.6881703 | 3 |
| 3255.6883773 | 1 |
| 3255.6886206 | 5 |
| 3255.6889929 | 0 |
| 3255.6891768 | 2 |
| 3255.6893061 | 2 |
| 3255.6897048 | 4 |
| 3255.6897138 | 0 |
| 3255.6899091 | 2 |
| 3255.6901986 | 4 |
| 3255.6906251 | 2 |
| 3255.6906458 | 3 |
| 3255.6907989 | 6 |
| 3255.6909275 | 1 |
| 3255.6917196 | 3 |
| 3255.691823 | 7 |
| 3255.6922419 | 8 |
| 3255.6924966 | 9 |
| 3255.6925526 | 7 |
| 3255.6927293 | 12 |
| 3255.6927963 | 2 |
| 3255.6931183 | 18 |
| 3255.6932888 | 20 |
| 3255.6938551 | 30 |
| 3255.6938581 | 38 |
| 3255.6941431 | 49 |
| 3255.6949174 | 37 |
| 3255.6949697 | 57 |
| 3255.6949796 | 55 |
| 3255.6952601 | 85 |
| 3255.6955038 | 67 |
| 3255.6955182 | 62 |
| 3255.6961166 | 63 |
| 3255.696373 | 90 |
| 3255.6964344 | 71 |
| 3255.6965963 | 91 |
| 3255.6969895 | 87 |
| 3255.6975053 | 80 |
| 3255.6975466 | 101 |
| 3255.6978609 | 81 |
| 3255.6978669 | 88 |
| 3255.6983674 | 73 |
| 3255.6984303 | 103 |
| 3255.6992136 | 68 |
| 3255.6992454 | 78 |
| 3255.6993396 | 61 |
| 3255.6994425 | 64 |
| 3255.6996929 | 56 |
| 3255.700039 | 78 |
| 3255.7000626 | 74 |
| 3255.7001683 | 71 |
| 3255.7006036 | 88 |
| 3255.7007319 | 90 |
| 3255.7008985 | 83 |
| 3255.7012026 | 95 |
| 3255.7016152 | 90 |
| 3255.7019851 | 76 |
| 3255.702062 | 99 |
| 3255.7021031 | 101 |
| 3255.7022515 | 104 |
| 3255.7022582 | 82 |
| 3255.7024547 | 77 |
| 3255.7025068 | 86 |
| 3255.7032789 | 75 |
| 3255.7035399 | 29 |
| 3255.7038501 | 49 |
| 3255.7039783 | 43 |
| 3255.7044209 | 26 |
| 3255.7046714 | 18 |
| 3255.7048426 | 5 |
| 3255.705072 | 25 |
| 3255.7051372 | 14 |
| 3255.7056751 | 3 |
| 3255.7056895 | 4 |
| 3255.7061066 | 2 |
| 3255.7063105 | 3 |
| 3255.7065653 | 3 |
| 3255.7069406 | 5 |
| 3255.7073941 | 3 |
| 3255.7075691 | 5 |
| 3255.7078479 | 4 |
| 3255.7079614 | 4 |
| 3255.7080763 | 0 |
| 3255.7084768 | 5 |
| 3255.7085605 | 3 |
| 3255.7086741 | 3 |
| 3255.7088308 | 2 |
| 3255.7088494 | 0 |
| 3255.7099291 | 4 |
| 3255.7100489 | 2 |
| 3255.7100936 | 0 |
| 3255.7104956 | 4 |
| 3255.7113461 | 3 |
| 3255.7129092 | 2 |
| 3255.7142042 | 0 |
| 3255.7157507 | 4 |
| 3255.7160113 | 2 |
| 3255.7176635 | 5 |
| 3255.7177362 | 2 |
| 3255.7197063 | 4 |
| 3255.7207919 | 4 |
| 3255.7210823 | 1 |
| 3255.7223539 | 1 |
| 3255.7236001 | 4 |
| 3255.7250819 | 4 |
| 3255.7263169 | 2 |
| 3255.7265771 | 3 |
| 3255.7295254 | 0 |
| 3255.7296371 | 2 |
| 3255.7315223 | 3 |
| 3255.7328021 | 4 |
| 3255.7339641 | 2 |
| 3255.7357406 | 2 |
| 3255.7361206 | 3 |
| 3255.7379472 | 2 |
| 3255.7393226 | 2 |
| 3255.7403288 | 2 |
| 3255.7416605 | 1 |
| 3255.7425106 | 1 |
| 3255.7439621 | 1 |
| 3255.7449678 | 1 |
| 3255.7457764 | 3 |
| 3255.7477078 | 3 |
| 3255.748106 | 3 |
| 3255.7500511 | 6 |
| 3255.7511113 | 3 |
| 3255.7525553 | 0 |
| 3255.7537027 | 3 |
| 3255.7539429 | 1 |
| 3255.7566626 | 3 |
| 3255.7567519 | 1 |
| 3255.7592667 | 4 |
| 3255.7610776 | 3 |
| 3255.7623914 | 6 |
| 3255.7639401 | 1 |
| 3255.7642551 | 4 |
| 3255.7658783 | 2 |
| 3255.7659198 | 4 |
| 3255.7670552 | 1 |
| 3255.7681861 | 2 |
| 3255.7699284 | 1 |
| 3255.7708453 | 2 |
| 3255.7719262 | 6 |
| 3255.7740589 | 2 |
| 3255.7756074 | 5 |
| 3255.7757483 | 1 |
| 3255.778154 | 0 |
| 3255.7792535 | 3 |
| 3255.7794707 | 3 |
| 3255.7815374 | 3 |
| 3255.7818809 | 1 |
| 3255.7835683 | 1 |
| 3255.7856433 | 1 |
| 3255.785824 | 1 |
| 3255.7875361 | 1 |
| 3255.7888922 | 2 |
| 3255.7902034 | 3 |
| 3255.7919632 | 3 |
| 3255.7922067 | 3 |
| 3255.7936825 | 2 |
| 3255.7944158 | 1 |
| 3255.7960926 | 2 |
| 3255.7963793 | 2 |
| 3255.7990068 | 2 |
| 3255.801086 | 1 |
| 3255.8015995 | 3 |
| 3255.8035913 | 3 |
| 3255.8043675 | 5 |
| 3255.8059461 | 2 |
| 3255.8076364 | 2 |
| 3255.8079168 | 4 |
| 3255.8098232 | 4 |
| 3255.8107329 | 1 |
| 3255.8126636 | 1 |
| 3255.8138037 | 5 |
| 3255.8140388 | 1 |
| 3255.8148285 | 0 |
| 3255.8164872 | 7 |
| 3255.8165387 | 2 |
| 3255.81789 | 0 |
| 3255.8186101 | 5 |
| 3255.8207676 | 4 |
| 3255.8222897 | 3 |
| 3255.8232902 | 2 |
| 3255.8245568 | 1 |
| 3255.8259572 | 2 |
| 3255.8271047 | 1 |
| 3255.8278069 | 3 |
| 3255.8295605 | 4 |
| 3255.8311997 | 2 |
| 3255.8318233 | 2 |
| 3255.8339692 | 3 |
| 3255.8344364 | 4 |
| 3255.8348535 | 1 |
| 3255.8364236 | 5 |
| 3255.8374388 | 1 |
| 3255.8389499 | 3 |
| 3255.8399558 | 3 |
| 3255.8420515 | 1 |
| 3255.8430789 | 1 |
| 3255.8439452 | 3 |
| 3255.8451808 | 5 |
| 3255.8460712 | 1 |
| 3255.8494225 | 4 |
| 3255.8499939 | 2 |
| 3255.8511808 | 0 |
| 3255.8528991 | 3 |
| 3255.8538003 | 5 |
| 3255.853847 | 2 |
| 3255.8553248 | 1 |
| 3255.8565641 | 2 |
| 3255.8581971 | 0 |
| 3255.8588863 | 1 |
| 3255.8606913 | 4 |
| 3255.8607378 | 0 |
| 3255.8632198 | 1 |
| 3255.8642416 | 3 |
| 3255.8655055 | 4 |
| 3255.8663295 | 3 |
| 3255.8676874 | 4 |
| 3255.869388 | 1 |
| 3255.8709896 | 0 |
| 3255.8714031 | 2 |
| 3255.873394 | 3 |
| 3255.8744109 | 1 |
| 3255.8761893 | 3 |
| 3255.8773213 | 2 |
| 3255.878146 | 2 |
| 3255.8797065 | 0 |
| 3255.8804426 | 2 |
| 3255.8823219 | 1 |
| 3255.8829969 | 1 |
| 3255.8835343 | 5 |
| 3255.8854056 | 2 |
| 3255.8859619 | 2 |
| 3255.8864983 | 4 |
| 3255.8891733 | 3 |
| 3255.8913139 | 3 |
| 3255.8928008 | 5 |
| 3255.8936218 | 1 |
| 3255.8958247 | 3 |
| 3255.8959349 | 3 |
| 3255.8969933 | 3 |

| | |
|---|---|
| 3255.8991243 | 4 |
| 3255.8993396 | 3 |
| 3255.9009797 | 0 |
| 3255.902284 | 4 |
| 3255.9037942 | 3 |
| 3255.9045151 | 2 |
| 3255.9050385 | 3 |
| 3255.9070716 | 1 |
| 3255.907107 | 5 |
| 3255.9082366 | 2 |
| 3255.9103104 | 2 |
| 3255.91099 | 2 |
| 3255.9129382 | 1 |
| 3255.9133844 | 2 |
| 3255.9149246 | 0 |
| 3255.9167359 | 5 |
| 3255.9171545 | 1 |
| 3255.9190156 | 3 |
| 3255.9197278 | 2 |
| 3255.9216702 | 1 |
| 3255.9221959 | 1 |
| 3255.9238747 | 3 |
| 3255.9254737 | 1 |
| 3255.9258625 | 1 |
| 3255.9272617 | 1 |
| 3255.9276732 | 0 |
| 3255.9294974 | 1 |
| 3255.9309162 | 1 |
| 3255.9317537 | 5 |
| 3255.9329989 | 2 |
| 3255.9343064 | 4 |
| 3255.9357611 | 3 |
| 3255.9372538 | 0 |
| 3255.9379108 | 3 |
| 3255.9405027 | 2 |
| 3255.941331 | 3 |
| 3255.9434864 | 3 |
| 3255.9437019 | 6 |
| 3255.9453173 | 0 |
| 3255.9471463 | 4 |
| 3255.9471859 | 3 |
| 3255.9474746 | 2 |
| 3255.9492358 | 3 |
| 3255.9499691 | 2 |
| 3255.9508686 | 2 |
| 3255.9525254 | 2 |
| 3255.9542413 | 0 |
| 3255.9547631 | 9 |
| 3255.9569382 | 2 |
| 3255.9580699 | 0 |
| 3255.9589891 | 2 |
| 3255.9596551 | 1 |
| 3255.9611516 | 3 |
| 3255.9626613 | 3 |
| 3255.964531 | 4 |
| 3255.9655624 | 5 |
| 3255.9668303 | 2 |
| 3255.9675217 | 4 |
| 3255.9691224 | 2 |
| 3255.9698574 | 6 |
| 3255.9717696 | 3 |
| 3255.9735646 | 4 |
| 3255.9740238 | 2 |
| 3255.9751243 | 3 |
| 3255.9758771 | 4 |
| 3255.9766932 | 1 |
| 3255.9780448 | 0 |
| 3255.9791363 | 1 |
| 3255.9807074 | 4 |
| 3255.9821618 | 2 |
| 3255.9833269 | 2 |
| 3255.9849903 | 1 |
| 3255.9861439 | 2 |
| 3255.987367 | 1 |
| 3255.9888913 | 1 |
| 3255.9902074 | 3 |
| 3255.9911815 | 3 |
| 3255.9925265 | 2 |
| 3255.994244 | 2 |
| 3255.9942594 | 4 |
| 3255.9958651 | 4 |
| 3255.9977079 | 2 |
| 3255.9984035 | 3 |
| 3255.9988953 | 0 |
| 3256.0006095 | 1 |
| 3256.0020258 | 4 |
| 3256.003401 | 2 |
| 3256.0045973 | 4 |
| 3256.0060426 | 3 |
| 3256.0066927 | 4 |
| 3256.0085428 | 1 |
| 3256.0092945 | 3 |
| 3256.0106692 | 4 |
| 3256.0125513 | 3 |
| 3256.0127245 | 3 |
| 3256.0143927 | 1 |
| 3256.0161123 | 2 |
| 3256.0170247 | 4 |
| 3256.0174471 | 8 |
| 3256.018837 | 2 |
| 3256.0201663 | 2 |
| 3256.0213347 | 3 |
| 3256.0227381 | 3 |
| 3256.0234178 | 2 |
| 3256.0244847 | 5 |
| 3256.0269168 | 3 |
| 3256.0276853 | 3 |
| 3256.0288978 | 1 |
| 3256.0302511 | 2 |
| 3256.0306748 | 3 |
| 3256.0321766 | 2 |
| 3256.0338592 | 0 |
| 3256.0347044 | 1 |
| 3256.0367907 | 2 |
| 3256.0368156 | 2 |
| 3256.0384509 | 4 |
| 3256.0390882 | 1 |
| 3256.040941 | 3 |
| 3256.0425319 | 2 |
| 3256.0433312 | 0 |
| 3256.0447694 | 2 |
| 3256.0452035 | 6 |
| 3256.0473602 | 0 |
| 3256.0488345 | 2 |
| 3256.0493019 | 1 |
| 3256.0511872 | 2 |
| 3256.0515932 | 4 |
| 3256.0531189 | 1 |
| 3256.0551932 | 5 |
| 3256.0557298 | 2 |
| 3256.0578148 | 4 |
| 3256.0581678 | 3 |
| 3256.0601363 | 1 |
| 3256.0614752 | 4 |
| 3256.0622979 | 3 |
| 3256.0633396 | 1 |
| 3256.0639735 | 2 |
| 3256.0658518 | 5 |
| 3256.0667693 | 2 |
| 3256.0674634 | 2 |
| 3256.0693637 | 3 |
| 3256.0701847 | 2 |
| 3256.0712701 | 3 |
| 3256.0729991 | 1 |
| 3256.074405 | 1 |
| 3256.0757949 | 3 |
| 3256.07601 | 5 |
| 3256.0779644 | 5 |
| 3256.0794373 | 4 |
| 3256.079915 | 3 |
| 3256.0815034 | 3 |
| 3256.0830607 | 3 |
| 3256.0833197 | 3 |
| 3256.0845588 | 4 |
| 3256.0863656 | 2 |
| 3256.0880082 | 2 |
| 3256.0884735 | 1 |
| 3256.0902813 | 1 |
| 3256.0912828 | 2 |
| 3256.0926815 | 2 |
| 3256.0942127 | 1 |
| 3256.0951577 | 2 |
| 3256.0960483 | 2 |
| 3256.097908 | 2 |
| 3256.0988439 | 3 |
| 3256.0997394 | 0 |
| 3256.1009392 | 3 |
| 3256.1030871 | 5 |
| 3256.1041564 | 2 |
| 3256.1046045 | 7 |
| 3256.1055837 | 3 |
| 3256.1071081 | 4 |
| 3256.1079532 | 1 |
| 3256.1099838 | 5 |
| 3256.1110995 | 3 |
| 3256.1128515 | 7 |
| 3256.1133389 | 1 |
| 3256.1144875 | 3 |
| 3256.1165083 | 2 |
| 3256.1168511 | 5 |
| 3256.1183047 | 4 |
| 3256.1197619 | 3 |
| 3256.1223702 | 6 |
| 3256.1224343 | 4 |
| 3256.1234705 | 3 |
| 3256.1239701 | 3 |
| 3256.1264467 | 3 |
| 3256.127183 | 1 |
| 3256.1288337 | 4 |
| 3256.129288 | 3 |
| 3256.1310603 | 4 |
| 3256.1324172 | 4 |
| 3256.1335031 | 2 |
| 3256.1351632 | 3 |
| 3256.1354548 | 2 |
| 3256.1380426 | 2 |
| 3256.1387203 | 1 |
| 3256.140309 | 3 |
| 3256.140824 | 3 |
| 3256.1423542 | 4 |
| 3256.1438561 | 3 |
| 3256.1455076 | 1 |
| 3256.1460101 | 0 |
| 3256.148126 | 6 |
| 3256.1486002 | 2 |
| 3256.1502303 | 2 |
| 3256.1508458 | 3 |
| 3256.1520971 | 1 |
| 3256.1541767 | 0 |
| 3256.155133 | 0 |
| 3256.1559881 | 3 |
| 3256.1570483 | 1 |
| 3256.1587255 | 3 |
| 3256.1599636 | 3 |
| 3256.1607431 | 1 |
| 3256.1622283 | 2 |
| 3256.1624691 | 2 |
| 3256.16341 | 2 |
| 3256.165359 | 2 |
| 3256.16604 | 4 |
| 3256.169473 | 2 |
| 3256.1705558 | 4 |
| 3256.1727703 | 3 |
| 3256.1740736 | 1 |
| 3256.174207 | 1 |
| 3256.1762088 | 3 |
| 3256.177111 | 3 |
| 3256.1779998 | 1 |
| 3256.1789907 | 4 |
| 3256.1795082 | 2 |
| 3256.1817242 | 2 |
| 3256.1826391 | 4 |
| 3256.1829761 | 4 |
| 3256.1849861 | 2 |
| 3256.1856693 | 1 |
| 3256.1871361 | 3 |
| 3256.1883665 | 0 |
| 3256.1899167 | 0 |
| 3256.1915184 | 1 |
| 3256.1917547 | 3 |
| 3256.1938527 | 1 |
| 3256.1946031 | 4 |
| 3256.1961167 | 1 |
| 3256.1969675 | 0 |
| 3256.1984543 | 4 |
| 3256.1998266 | 2 |
| 3256.2006894 | 2 |
| 3256.2023737 | 3 |
| 3256.2024543 | 2 |
| 3256.2039029 | 3 |
| 3256.2059976 | 2 |
| 3256.2065799 | 3 |
| 3256.2089032 | 1 |
| 3256.2110603 | 1 |
| 3256.2126729 | 3 |
| 3256.2144245 | 2 |
| 3256.2166901 | 3 |
| 3256.2169811 | 3 |
| 3256.2183201 | 1 |
| 3256.2189604 | 2 |
| 3256.2205081 | 1 |
| 3256.2218302 | 4 |
| 3256.2222008 | 2 |
| 3256.2232312 | 6 |
| 3256.2236023 | 2 |
| 3256.2247163 | 5 |
| 3256.2265829 | 1 |
| 3256.2279865 | 0 |
| 3256.228998 | 0 |
| 3256.2296195 | 4 |
| 3256.2307021 | 1 |
| 3256.2319497 | 2 |
| 3256.2329904 | 7 |
| 3256.2353153 | 0 |
| 3256.2355507 | 0 |
| 3256.2375168 | 2 |
| 3256.2384738 | 3 |
| 3256.2400975 | 2 |
| 3256.2405757 | 6 |
| 3256.2421407 | 3 |
| 3256.2440262 | 5 |
| 3256.244126 | 2 |
| 3256.2454577 | 1 |
| 3256.2473116 | 0 |
| 3256.2479767 | 3 |
| 3256.2488287 | 4 |
| 3256.2516264 | 0 |
| 3256.252425 | 3 |
| 3256.2543493 | 0 |
| 3256.2562164 | 2 |
| 3256.2584492 | 1 |
| 3256.2585618 | 3 |
| 3256.262945 | 1 |
| 3256.2640711 | 2 |
| 3256.2654066 | 5 |
| 3256.2668518 | 2 |
| 3256.2677314 | 1 |
| 3256.2677987 | 2 |
| 3256.2678188 | 2 |
| 3256.268931 | 3 |
| 3256.2701066 | 2 |
| 3256.2701913 | 3 |
| 3256.2704359 | 2 |
| 3256.2708133 | 2 |
| 3256.2708709 | 4 |
| 3256.2721512 | 5 |
| 3256.2721692 | 2 |
| 3256.2730363 | 3 |
| 3256.273482 | 3 |
| 3256.2735166 | 2 |
| 3256.2744698 | 3 |
| 3256.2747751 | 3 |
| 3256.2757082 | 3 |
| 3256.2760816 | 1 |
| 3256.2762038 | 2 |
| 3256.2766866 | 2 |
| 3256.2774178 | 0 |
| 3256.2779027 | 1 |
| 3256.278806 | 3 |
| 3256.2788546 | 1 |
| 3256.279695 | 3 |
| 3256.2805025 | 3 |
| 3256.2809955 | 3 |
| 3256.2816513 | 4 |
| 3256.2822488 | 5 |
| 3256.2828868 | 0 |
| 3256.2829703 | 0 |
| 3256.2843432 | 1 |
| 3256.284358 | 2 |
| 3256.2856773 | 3 |
| 3256.2857695 | 2 |
| 3256.287306 | 5 |
| 3256.2879371 | 7 |
| 3256.2880051 | 1 |
| 3256.2881218 | 1 |
| 3256.2895001 | 1 |
| 3256.2895742 | 2 |
| 3256.2899878 | 2 |
| 3256.2911464 | 2 |
| 3256.2913911 | 1 |
| 3256.2927683 | 2 |
| 3256.2929175 | 3 |
| 3256.2939098 | 1 |
| 3256.2942655 | 2 |
| 3256.2955325 | 2 |
| 3256.2960477 | 4 |
| 3256.2963334 | 3 |
| 3256.2969191 | 3 |
| 3256.297225 | 2 |
| 3256.2975944 | 2 |
| 3256.2985685 | 4 |
| 3256.2987766 | 5 |
| 3256.3001371 | 6 |
| 3256.3018514 | 2 |
| 3256.302169 | 4 |
| 3256.3026965 | 4 |
| 3256.3031383 | 1 |
| 3256.3041334 | 4 |
| 3256.3047834 | 2 |
| 3256.3051716 | 1 |
| 3256.3055427 | 1 |
| 3256.3064501 | 3 |
| 3256.3066763 | 3 |

| | |
|---|---|
| 3256.3073669 | 3 |
| 3256.3088179 | 5 |
| 3256.3090984 | 4 |
| 3256.3091541 | 3 |
| 3256.3102856 | 3 |
| 3256.31073 | 4 |
| 3256.3112984 | 2 |
| 3256.3114029 | 6 |
| 3256.3120002 | 1 |
| 3256.3128205 | 3 |
| 3256.3137914 | 3 |
| 3256.3140236 | 3 |
| 3256.3145942 | 1 |
| 3256.3151969 | 3 |
| 3256.3172433 | 2 |
| 3256.3172775 | 1 |
| 3256.3191088 | 4 |
| 3256.320151 | 3 |
| 3256.3213628 | 2 |
| 3256.3231738 | 1 |
| 3256.3241444 | 0 |
| 3256.325679 | 0 |
| 3256.326522 | 0 |
| 3256.3279142 | 3 |
| 3256.329292 | 5 |
| 3256.3300769 | 1 |
| 3256.330903 | 2 |
| 3256.3327521 | 3 |
| 3256.3340963 | 2 |
| 3256.3355893 | 3 |
| 3256.3362259 | 4 |
| 3256.3377674 | 6 |
| 3256.3384141 | 5 |
| 3256.3399014 | 2 |
| 3256.3414968 | 2 |
| 3256.3425386 | 3 |
| 3256.3438131 | 2 |
| 3256.3450235 | 1 |
| 3256.3464179 | 8 |
| 3256.3478224 | 1 |
| 3256.3481666 | 3 |
| 3256.3502285 | 3 |
| 3256.3508258 | 3 |
| 3256.3523926 | 2 |
| 3256.3539265 | 1 |
| 3256.3551447 | 0 |
| 3256.3568392 | 4 |
| 3256.3574221 | 0 |
| 3256.358936 | 2 |
| 3256.3607936 | 6 |
| 3256.3610615 | 2 |
| 3256.3623938 | 4 |
| 3256.3645657 | 2 |
| 3256.3651408 | 1 |
| 3256.3660465 | 3 |
| 3256.3675778 | 2 |
| 3256.3676147 | 3 |
| 3256.3701895 | 4 |
| 3256.3711216 | 2 |
| 3256.3729165 | 0 |
| 3256.3735281 | 1 |
| 3256.3753599 | 4 |
| 3256.3758874 | 4 |
| 3256.3770374 | 2 |
| 3256.3788667 | 1 |
| 3256.3800187 | 3 |
| 3256.3810948 | 4 |
| 3256.381985 | 3 |
| 3256.3840214 | 3 |
| 3256.3850863 | 2 |
| 3256.38595 | 3 |
| 3256.3876713 | 3 |
| 3256.3879435 | 0 |
| 3256.3888541 | 1 |
| 3256.3902501 | 3 |
| 3256.391656 | 4 |
| 3256.3927256 | 1 |
| 3256.3939547 | 2 |
| 3256.3955522 | 2 |
| 3256.3976193 | 2 |
| 3256.3981934 | 1 |
| 3256.3994562 | 2 |
| 3256.400591 | 3 |
| 3256.4026929 | 2 |
| 3256.4035941 | 5 |
| 3256.404853 | 1 |
| 3256.4065745 | 5 |
| 3256.4067653 | 4 |
| 3256.4081102 | 1 |
| 3256.4104115 | 1 |
| 3256.4105168 | 4 |
| 3256.4123662 | 3 |
| 3256.4133507 | 5 |
| 3256.4149905 | 1 |
| 3256.4156571 | 4 |
| 3256.4177359 | 2 |
| 3256.418848 | 1 |
| 3256.4201758 | 4 |
| 3256.4213041 | 2 |
| 3256.4217989 | 2 |
| 3256.4232482 | 0 |
| 3256.4247529 | 1 |
| 3256.4257129 | 1 |
| 3256.4271723 | 3 |
| 3256.4284966 | 3 |
| 3256.4296793 | 1 |
| 3256.4312756 | 2 |
| 3256.4319358 | 1 |
| 3256.4334035 | 2 |
| 3256.4349312 | 3 |
| 3256.4351143 | 2 |
| 3256.4364791 | 3 |
| 3256.43793 | 3 |
| 3256.4389583 | 1 |
| 3256.4403905 | 2 |
| 3256.4423172 | 4 |
| 3256.4433625 | 4 |
| 3256.4439636 | 2 |
| 3256.4460739 | 6 |
| 3256.4469156 | 3 |
| 3256.447977 | 3 |
| 3256.4499833 | 2 |
| 3256.4502259 | 3 |
| 3256.4519851 | 4 |
| 3256.4528406 | 2 |
| 3256.4543991 | 2 |
| 3256.4560292 | 2 |
| 3256.4567352 | 1 |
| 3256.4580448 | 1 |
| 3256.4589587 | 3 |
| 3256.4606597 | 1 |
| 3256.462203 | 2 |
| 3256.4633572 | 3 |
| 3256.4645522 | 2 |
| 3256.465014 | 3 |
| 3256.4670587 | 0 |
| 3256.4687099 | 5 |
| 3256.4691647 | 2 |
| 3256.4705651 | 1 |
| 3256.4722782 | 4 |
| 3256.4724812 | 2 |
| 3256.4743155 | 1 |
| 3256.474966 | 1 |
| 3256.477036 | 0 |
| 3256.4777953 | 3 |
| 3256.4790613 | 2 |
| 3256.4804093 | 1 |
| 3256.4812729 | 1 |
| 3256.4825422 | 3 |
| 3256.4841832 | 3 |
| 3256.4856813 | 2 |
| 3256.4866294 | 3 |
| 3256.4868858 | 6 |
| 3256.4896949 | 1 |
| 3256.4897182 | 1 |
| 3256.4913871 | 4 |
| 3256.4923228 | 11 |
| 3256.4936651 | 1 |
| 3256.4957153 | 4 |
| 3256.4957527 | 1 |
| 3256.4984906 | 0 |
| 3256.4991275 | 4 |
| 3256.5003402 | 2 |
| 3256.5011212 | 0 |
| 3256.5030018 | 2 |
| 3256.5038897 | 2 |
| 3256.5052132 | 2 |
| 3256.5063899 | 4 |
| 3256.508057 | 3 |
| 3256.5088245 | 1 |
| 3256.5101054 | 3 |
| 3256.5115523 | 2 |
| 3256.5126652 | 1 |
| 3256.5144707 | 3 |
| 3256.5152131 | 1 |
| 3256.5162767 | 2 |
| 3256.5180608 | 1 |
| 3256.5189421 | 4 |
| 3256.5195425 | 3 |
| 3256.5205436 | 3 |
| 3256.5221019 | 4 |
| 3256.5241402 | 5 |
| 3256.5250672 | 0 |
| 3256.5261043 | 3 |
| 3256.5270925 | 2 |
| 3256.5288311 | 0 |
| 3256.5305401 | 5 |
| 3256.5306718 | 4 |
| 3256.5324599 | 4 |
| 3256.5340127 | 4 |
| 3256.5349069 | 2 |
| 3256.5355232 | 3 |
| 3256.5371471 | 2 |
| 3256.5390059 | 2 |
| 3256.5398997 | 2 |
| 3256.5412782 | 3 |
| 3256.5418784 | 3 |
| 3256.5437021 | 2 |
| 3256.5455593 | 3 |
| 3256.5455896 | 4 |
| 3256.5472604 | 1 |
| 3256.5483797 | 2 |
| 3256.5502045 | 2 |
| 3256.5512148 | 1 |
| 3256.551916 | 3 |
| 3256.5539028 | 0 |
| 3256.5555696 | 5 |
| 3256.5562965 | 4 |
| 3256.5580634 | 3 |
| 3256.558284 | 1 |
| 3256.5604183 | 7 |
| 3256.5610693 | 3 |
| 3256.5627878 | 6 |
| 3256.5640193 | 2 |
| 3256.5647163 | 1 |
| 3256.5659383 | 3 |
| 3256.5666554 | 1 |
| 3256.5685545 | 1 |
| 3256.5699155 | 2 |
| 3256.5711534 | 0 |
| 3256.5724739 | 4 |
| 3256.572991 | 3 |
| 3256.5747609 | 4 |
| 3256.5764723 | 2 |
| 3256.5770997 | 3 |
| 3256.5782809 | 3 |
| 3256.579382 | 1 |
| 3256.5812535 | 1 |
| 3256.5828185 | 3 |
| 3256.5840246 | 4 |
| 3256.5861537 | 5 |
| 3256.5862642 | 2 |
| 3256.5870509 | 4 |
| 3256.5881574 | 0 |
| 3256.590487 | 4 |
| 3256.5905282 | 2 |
| 3256.5923397 | 0 |
| 3256.5935761 | 7 |
| 3256.5952362 | 0 |
| 3256.5960848 | 3 |
| 3256.5976896 | 1 |
| 3256.5989412 | 4 |
| 3256.5999598 | 1 |
| 3256.6014131 | 2 |
| 3256.6031425 | 1 |
| 3256.6033849 | 3 |
| 3256.6049802 | 1 |
| 3256.6058887 | 3 |
| 3256.6078366 | 2 |
| 3256.6083321 | 3 |
| 3256.6106423 | 3 |
| 3256.6117547 | 1 |
| 3256.6124754 | 5 |
| 3256.6134306 | 0 |
| 3256.6143617 | 1 |
| 3256.6157058 | 3 |
| 3256.6176705 | 4 |
| 3256.6180459 | 1 |
| 3256.6202199 | 4 |
| 3256.6202983 | 1 |
| 3256.6218387 | 1 |
| 3256.6232086 | 1 |
| 3256.6246641 | 3 |
| 3256.6263296 | 2 |
| 3256.6270204 | 1 |
| 3256.629937 | 3 |
| 3256.6302032 | 5 |
| 3256.6308321 | 4 |
| 3256.6323905 | 1 |
| 3256.633366 | 2 |
| 3256.635216 | 0 |
| 3256.6367566 | 1 |
| 3256.6368886 | 3 |
| 3256.6384896 | 3 |
| 3256.6398151 | 2 |
| 3256.6416156 | 0 |
| 3256.642528 | 2 |
| 3256.6437662 | 2 |
| 3256.6453564 | 1 |
| 3256.6462276 | 4 |
| 3256.6474018 | 5 |
| 3256.6491701 | 0 |
| 3256.6499757 | 2 |
| 3256.6519938 | 1 |
| 3256.6520964 | 0 |
| 3256.6542122 | 3 |
| 3256.6545782 | 3 |
| 3256.6561978 | 2 |
| 3256.6576554 | 1 |
| 3256.6585526 | 2 |
| 3256.6599981 | 1 |
| 3256.6605921 | 0 |
| 3256.6622221 | 3 |
| 3256.663608 | 6 |
| 3256.6638776 | 3 |
| 3256.6659971 | 0 |
| 3256.6668252 | 4 |
| 3256.6688253 | 4 |
| 3256.6699559 | 3 |
| 3256.6709174 | 1 |
| 3256.6718831 | 2 |
| 3256.6740526 | 1 |
| 3256.6745846 | 7 |
| 3256.6763755 | 4 |
| 3256.677325 | 8 |
| 3256.6791156 | 3 |
| 3256.6793282 | 2 |
| 3256.6817459 | 2 |
| 3256.6826795 | 2 |
| 3256.6832448 | 1 |
| 3256.6843713 | 1 |
| 3256.6859316 | 3 |
| 3256.6875229 | 3 |
| 3256.6892004 | 1 |
| 3256.6903849 | 2 |
| 3256.6917447 | 3 |
| 3256.6920485 | 2 |
| 3256.6938274 | 1 |
| 3256.6944911 | 2 |
| 3256.6962424 | 3 |
| 3256.6974786 | 0 |
| 3256.6988014 | 2 |
| 3256.7000294 | 1 |
| 3256.7007598 | 1 |
| 3256.702411 | 5 |
| 3256.7042815 | 0 |
| 3256.7043735 | 5 |
| 3256.7061474 | 2 |
| 3256.7079663 | 4 |
| 3256.708017 | 1 |
| 3256.709584 | 3 |
| 3256.7106975 | 5 |
| 3256.7112102 | 5 |
| 3256.7135356 | 1 |
| 3256.7150976 | 4 |
| 3256.716143 | 2 |
| 3256.716687 | 2 |
| 3256.7181296 | 2 |
| 3256.7192757 | 4 |
| 3256.7206709 | 2 |
| 3256.7225765 | 0 |
| 3256.7233641 | 1 |
| 3256.7251956 | 1 |
| 3256.7254038 | 1 |
| 3256.7284403 | 0 |
| 3256.7286723 | 1 |
| 3256.7295268 | 4 |
| 3256.7315542 | 1 |
| 3256.7319185 | 3 |
| 3256.7333576 | 2 |
| 3256.735199 | 3 |
| 3256.7358416 | 2 |
| 3256.7379653 | 2 |
| 3256.7379981 | 3 |
| 3256.7399521 | 0 |
| 3256.7412115 | 1 |
| 3256.7426919 | 1 |
| 3256.7438287 | 0 |
| 3256.7445856 | 1 |
| 3256.746877 | 3 |
| 3256.7472514 | 3 |
| 3256.7490604 | 3 |
| 3256.7499502 | 3 |

| | |
|---|---|
| 3256.7508733 | 0 |
| 3256.7520736 | 2 |
| 3256.7538912 | 3 |
| 3256.7540168 | 2 |
| 3256.7560298 | 3 |
| 3256.757077 | 4 |
| 3256.7579894 | 1 |
| 3256.7590751 | 2 |
| 3256.7613432 | 6 |
| 3256.7624211 | 0 |
| 3256.7633032 | 1 |
| 3256.764367 | 2 |
| 3256.7656232 | 0 |
| 3256.7667212 | 5 |
| 3256.7683348 | 2 |
| 3256.7698159 | 2 |
| 3256.7708 | 2 |
| 3256.7719663 | 2 |
| 3256.7732375 | 1 |
| 3256.7738006 | 2 |
| 3256.77605 | 3 |
| 3256.7776126 | 2 |
| 3256.7786452 | 4 |
| 3256.7798594 | 4 |
| 3256.7809432 | 3 |
| 3256.7820297 | 0 |
| 3256.7832963 | 2 |
| 3256.7848814 | 4 |
| 3256.7857297 | 1 |
| 3256.7873833 | 4 |
| 3256.7885088 | 4 |
| 3256.7901346 | 3 |
| 3256.7906535 | 3 |
| 3256.7922611 | 2 |
| 3256.794058 | 1 |
| 3256.7942979 | 2 |
| 3256.7958315 | 2 |
| 3256.7969302 | 2 |
| 3256.7977506 | 2 |
| 3256.7996355 | 5 |
| 3256.8003138 | 2 |
| 3256.8016537 | 4 |
| 3256.8027571 | 4 |
| 3256.8046705 | 5 |
| 3256.8049382 | 2 |
| 3256.8069855 | 6 |
| 3256.8087559 | 3 |
| 3256.8089532 | 2 |
| 3256.8106817 | 5 |
| 3256.8110452 | 4 |
| 3256.8132446 | 3 |
| 3256.8143464 | 5 |
| 3256.8153388 | 5 |
| 3256.8173471 | 0 |
| 3256.8180872 | 1 |
| 3256.8203824 | 3 |
| 3256.8207647 | 1 |
| 3256.8217032 | 1 |
| 3256.8233372 | 1 |
| 3256.8233563 | 3 |
| 3256.8260794 | 6 |
| 3256.8275082 | 4 |
| 3256.8280983 | 2 |
| 3256.8297576 | 2 |
| 3256.8304585 | 0 |
| 3256.8322488 | 4 |
| 3256.8337384 | 1 |
| 3256.8339506 | 3 |
| 3256.8355601 | 3 |
| 3256.8365008 | 4 |
| 3256.837672 | 2 |
| 3256.8392828 | 2 |
| 3256.8405551 | 1 |
| 3256.842069 | 3 |
| 3256.8429838 | 2 |
| 3256.844052 | 2 |
| 3256.8456289 | 3 |
| 3256.8464395 | 4 |
| 3256.8473487 | 5 |
| 3256.8484419 | 2 |
| 3256.8510721 | 1 |
| 3256.8512932 | 0 |
| 3256.8521108 | 1 |
| 3256.8539488 | 3 |
| 3256.8547454 | 2 |
| 3256.8563142 | 1 |
| 3256.8574014 | 1 |
| 3256.8585949 | 5 |
| 3256.8602919 | 1 |
| 3256.8610238 | 1 |
| 3256.8625738 | 2 |
| 3256.8635605 | 5 |
| 3256.8655389 | 1 |
| 3256.8667366 | 2 |
| 3256.8678708 | 1 |
| 3256.8688531 | 3 |
| 3256.8706981 | 1 |
| 3256.8714931 | 3 |
| 3256.8729655 | 2 |
| 3256.8737486 | 3 |
| 3256.875805 | 2 |
| 3256.8762229 | 6 |
| 3256.8781733 | 1 |
| 3256.8793805 | 1 |
| 3256.8798137 | 5 |
| 3256.8822475 | 2 |
| 3256.88344 | 0 |
| 3256.8834562 | 1 |
| 3256.8849868 | 3 |
| 3256.8862415 | 2 |
| 3256.887897 | 3 |
| 3256.8886396 | 1 |
| 3256.8902961 | 2 |
| 3256.8917908 | 2 |
| 3256.89236 | 2 |
| 3256.894365 | 0 |
| 3256.8943774 | 3 |
| 3256.8957389 | 2 |
| 3256.8973324 | 3 |
| 3256.8984386 | 2 |
| 3256.8993973 | 2 |
| 3256.9010902 | 2 |
| 3256.9030125 | 3 |
| 3256.9039488 | 2 |
| 3256.9044363 | 3 |
| 3256.9062241 | 3 |
| 3256.906423 | 0 |
| 3256.9089435 | 1 |
| 3256.9094894 | 2 |
| 3256.910484 | 1 |
| 3256.9122826 | 6 |
| 3256.9133596 | 5 |
| 3256.9148626 | 0 |
| 3256.9159803 | 2 |
| 3256.9172535 | 1 |
| 3256.918975 | 1 |
| 3256.9190417 | 1 |
| 3256.9214938 | 0 |
| 3256.9227159 | 1 |
| 3256.9227442 | 2 |
| 3256.9249294 | 0 |
| 3256.9254017 | 3 |
| 3256.9270269 | 1 |
| 3256.9284675 | 4 |
| 3256.9294977 | 3 |
| 3256.9309605 | 2 |
| 3256.9317562 | 2 |
| 3256.9336548 | 1 |
| 3256.934654 | 2 |
| 3256.9351189 | 3 |
| 3256.9371864 | 1 |
| 3256.9378206 | 2 |
| 3256.9378596 | 0 |
| 3256.9405881 | 3 |
| 3256.942064 | 2 |
| 3256.9429499 | 1 |
| 3256.9434601 | 3 |
| 3256.945096 | 4 |
| 3256.9469195 | 1 |
| 3256.9470022 | 2 |
| 3256.9495616 | 2 |
| 3256.9500833 | 2 |
| 3256.9510096 | 6 |
| 3256.9532486 | 3 |
| 3256.9546884 | 5 |
| 3256.9556223 | 3 |
| 3256.9563855 | 5 |
| 3256.9577962 | 2 |
| 3256.959236 | 1 |
| 3256.9597796 | 2 |
| 3256.9617853 | 6 |
| 3256.9627929 | 5 |
| 3256.9641904 | 0 |
| 3256.9653563 | 3 |
| 3256.9663188 | 1 |
| 3256.9681618 | 4 |
| 3256.9682991 | 3 |
| 3256.9698601 | 1 |
| 3256.9716981 | 5 |
| 3256.9730487 | 2 |
| 3256.9740508 | 2 |
| 3256.9746263 | 3 |
| 3256.9765015 | 0 |
| 3256.9781444 | 1 |
| 3256.9783303 | 1 |
| 3256.9797212 | 3 |
| 3256.9806936 | 2 |
| 3256.9819304 | 0 |
| 3256.9834518 | 3 |
| 3256.9850735 | 3 |
| 3256.9859038 | 3 |
| 3256.9874184 | 2 |
| 3256.9881852 | 3 |
| 3256.9895018 | 3 |
| 3256.9905164 | 3 |
| 3256.991318 | 3 |
| 3256.9930686 | 1 |
| 3256.9949785 | 3 |
| 3256.9961794 | 3 |
| 3256.9970197 | 2 |
| 3256.9985828 | 1 |
| 3256.9990521 | 2 |
| 3257.0005779 | 1 |
| 3257.002591 | 1 |
| 3257.0029008 | 3 |
| 3257.0048924 | 2 |
| 3257.0055472 | 2 |
| 3257.0070472 | 1 |
| 3257.0078941 | 2 |
| 3257.0098917 | 4 |
| 3257.0108835 | 1 |
| 3257.0120903 | 2 |
| 3257.0135375 | 3 |
| 3257.0144915 | 4 |
| 3257.0151952 | 4 |
| 3257.0171252 | 2 |
| 3257.0177529 | 4 |
| 3257.0190404 | 1 |
| 3257.0205798 | 1 |
| 3257.0215253 | 4 |
| 3257.0225842 | 1 |
| 3257.0236414 | 2 |
| 3257.0258146 | 3 |
| 3257.027329 | 6 |
| 3257.0274673 | 3 |
| 3257.0294555 | 2 |
| 3257.0296181 | 0 |
| 3257.0313715 | 1 |
| 3257.0326817 | 1 |
| 3257.0340598 | 3 |
| 3257.0353198 | 1 |
| 3257.0357833 | 5 |
| 3257.0369013 | 1 |
| 3257.0382766 | 3 |
| 3257.0402183 | 4 |
| 3257.0413527 | 4 |
| 3257.0425389 | 1 |
| 3257.0450099 | 4 |
| 3257.0455162 | 2 |
| 3257.0463057 | 2 |
| 3257.0484833 | 1 |
| 3257.0485275 | 2 |
| 3257.0509237 | 4 |
| 3257.0515124 | 3 |
| 3257.0521488 | 2 |
| 3257.0541005 | 2 |
| 3257.0550335 | 2 |
| 3257.0565944 | 3 |
| 3257.0574188 | 1 |
| 3257.0595587 | 5 |
| 3257.060893 | 0 |
| 3257.0610048 | 1 |
| 3257.0624395 | 3 |
| 3257.0641882 | 4 |
| 3257.0642412 | 2 |
| 3257.0663216 | 2 |
| 3257.0671364 | 0 |
| 3257.0691664 | 2 |
| 3257.0699426 | 1 |
| 3257.071375 | 3 |
| 3257.0729919 | 2 |
| 3257.073228 | 1 |
| 3257.075136 | 3 |
| 3257.0753109 | 1 |
| 3257.0764001 | 1 |
| 3257.0784192 | 4 |
| 3257.079396 | 0 |
| 3257.0800948 | 0 |
| 3257.082164 | 2 |
| 3257.0830891 | 1 |
| 3257.0851645 | 3 |
| 3257.08595 | 1 |
| 3257.0874867 | 3 |
| 3257.0876128 | 2 |
| 3257.0896328 | 3 |
| 3257.0907436 | 1 |
| 3257.0918994 | 4 |
| 3257.0931969 | 1 |
| 3257.0946575 | 2 |
| 3257.0961347 | 1 |
| 3257.0970895 | 4 |
| 3257.0982479 | 7 |
| 3257.0997569 | 0 |
| 3257.1002326 | 2 |
| 3257.1016303 | 2 |
| 3257.1036794 | 2 |
| 3257.1040258 | 3 |
| 3257.1060588 | 1 |
| 3257.1064053 | 4 |
| 3257.1074922 | 3 |
| 3257.1092627 | 1 |
| 3257.1107524 | 3 |
| 3257.1112238 | 2 |
| 3257.1122411 | 2 |
| 3257.1147913 | 0 |
| 3257.1157367 | 5 |
| 3257.1165992 | 2 |
| 3257.1182046 | 0 |
| 3257.118739 | 0 |
| 3257.1202698 | 3 |
| 3257.1219626 | 5 |
| 3257.1227411 | 2 |
| 3257.1239156 | 1 |
| 3257.1247049 | 4 |
| 3257.126351 | 2 |
| 3257.1277859 | 3 |
| 3257.1281075 | 3 |
| 3257.1299772 | 2 |
| 3257.1307031 | 1 |
| 3257.1326806 | 2 |
| 3257.1344346 | 0 |
| 3257.135247 | 6 |
| 3257.1366971 | 1 |
| 3257.1373331 | 2 |
| 3257.1395539 | 1 |
| 3257.1396237 | 3 |
| 3257.141028 | 2 |
| 3257.1432599 | 2 |
| 3257.1433768 | 4 |
| 3257.1452968 | 3 |
| 3257.1466335 | 2 |
| 3257.1475003 | 2 |
| 3257.1490599 | 0 |
| 3257.149712 | 2 |
| 3257.1512794 | 3 |
| 3257.1525543 | 0 |
| 3257.1536144 | 0 |
| 3257.1556414 | 2 |
| 3257.1557213 | 3 |
| 3257.158381 | 3 |
| 3257.1584977 | 0 |
| 3257.1600683 | 1 |
| 3257.1613293 | 2 |
| 3257.1620125 | 7 |
| 3257.1635108 | 3 |
| 3257.1651751 | 5 |
| 3257.1658412 | 2 |
| 3257.1673254 | 1 |
| 3257.1681633 | 1 |
| 3257.1699539 | 3 |
| 3257.1705493 | 1 |
| 3257.1725349 | 1 |
| 3257.174415 | 1 |
| 3257.1747143 | 1 |
| 3257.1757317 | 4 |
| 3257.1767824 | 1 |
| 3257.1782591 | 2 |
| 3257.180096 | 1 |
| 3257.1806084 | 1 |
| 3257.1823759 | 1 |
| 3257.1824663 | 1 |
| 3257.184616 | 0 |
| 3257.1868277 | 3 |
| 3257.1869638 | 1 |
| 3257.1890576 | 3 |
| 3257.1892555 | 7 |
| 3257.1907781 | 4 |
| 3257.1924181 | 7 |
| 3257.1935422 | 1 |
| 3257.1946985 | 2 |
| 3257.1956428 | 1 |
| 3257.1967887 | 4 |
| 3257.1978492 | 0 |

| | |
|---|---|
| 3257.1997727 | 1 |
| 3257.2011555 | 0 |
| 3257.2015448 | 3 |
| 3257.2035916 | 3 |
| 3257.2038992 | 1 |
| 3257.2054309 | 3 |
| 3257.2070813 | 1 |
| 3257.2086492 | 3 |
| 3257.2094386 | 0 |
| 3257.2113843 | 3 |
| 3257.212181 | 3 |
| 3257.2121962 | 4 |
| 3257.2144463 | 3 |
| 3257.2158874 | 4 |
| 3257.2162198 | 5 |
| 3257.2176078 | 2 |
| 3257.2196623 | 2 |
| 3257.2201151 | 2 |
| 3257.2218399 | 5 |
| 3257.2227946 | 5 |
| 3257.2249518 | 0 |
| 3257.2257839 | 3 |
| 3257.2269948 | 1 |
| 3257.2283817 | 2 |
| 3257.2294074 | 0 |
| 3257.2310039 | 3 |
| 3257.2325851 | 3 |
| 3257.2331021 | 4 |
| 3257.2345085 | 3 |
| 3257.2359483 | 2 |
| 3257.2368358 | 1 |
| 3257.2382925 | 3 |
| 3257.239992 | 1 |
| 3257.2410007 | 3 |
| 3257.2414071 | 2 |
| 3257.2436048 | 2 |
| 3257.2443089 | 2 |
| 3257.2451172 | 2 |
| 3257.2473478 | 2 |
| 3257.2482832 | 2 |
| 3257.2487617 | 2 |
| 3257.2504106 | 1 |
| 3257.2522299 | 1 |
| 3257.2535783 | 4 |
| 3257.2542473 | 4 |
| 3257.256357 | 2 |
| 3257.2576428 | 4 |
| 3257.2578626 | 2 |
| 3257.2595958 | 6 |
| 3257.2606312 | 1 |
| 3257.2618419 | 0 |
| 3257.2627427 | 2 |
| 3257.2647351 | 2 |
| 3257.2661126 | 2 |
| 3257.2665228 | 1 |
| 3257.2685191 | 2 |
| 3257.2693273 | 2 |
| 3257.2708239 | 4 |
| 3257.2723158 | 1 |
| 3257.2734974 | 1 |
| 3257.2756884 | 4 |
| 3257.2759081 | 1 |
| 3257.2781595 | 4 |
| 3257.2781941 | 2 |
| 3257.2802732 | 1 |
| 3257.2814833 | 2 |
| 3257.2819395 | 3 |
| 3257.2835812 | 0 |
| 3257.2852079 | 2 |
| 3257.2858797 | 2 |
| 3257.2882256 | 6 |
| 3257.2882838 | 2 |
| 3257.2903575 | 2 |
| 3257.2916635 | 1 |
| 3257.2920705 | 5 |
| 3257.2936626 | 2 |
| 3257.2947659 | 1 |
| 3257.2962105 | 3 |
| 3257.2980882 | 3 |
| 3257.2985228 | 5 |
| 3257.3006336 | 0 |
| 3257.3009009 | 1 |
| 3257.3021407 | 1 |
| 3257.3034151 | 1 |
| 3257.3045363 | 2 |
| 3257.3060757 | 4 |
| 3257.3066648 | 4 |
| 3257.3086502 | 4 |
| 3257.3090329 | 2 |
| 3257.3109506 | 0 |
| 3257.3128498 | 1 |
| 3257.3133981 | 6 |
| 3257.3149588 | 3 |
| 3257.316762 | 3 |
| 3257.3177175 | 2 |
| 3257.3187014 | 2 |
| 3257.3190472 | 3 |
| 3257.3212175 | 2 |
| 3257.3220628 | 5 |
| 3257.3238628 | 3 |
| 3257.3256142 | 2 |
| 3257.3266212 | 2 |
| 3257.3281438 | 0 |
| 3257.3287934 | 3 |
| 3257.3304014 | 2 |
| 3257.3317224 | 3 |
| 3257.3328201 | 1 |
| 3257.3342302 | 4 |
| 3257.3354322 | 1 |
| 3257.3362746 | 1 |
| 3257.3381545 | 1 |
| 3257.339014 | 5 |
| 3257.3398313 | 0 |
| 3257.3416861 | 1 |
| 3257.3429252 | 2 |
| 3257.3443557 | 2 |
| 3257.3454653 | 3 |
| 3257.3469083 | 1 |
| 3257.3474214 | 0 |
| 3257.3489604 | 1 |
| 3257.349963 | 3 |
| 3257.3510212 | 3 |
| 3257.3522015 | 2 |
| 3257.3543051 | 4 |
| 3257.3553093 | 2 |
| 3257.3566332 | 4 |
| 3257.3576012 | 3 |
| 3257.3598438 | 3 |
| 3257.3602498 | 4 |
| 3257.3611048 | 3 |
| 3257.3636385 | 5 |
| 3257.3640712 | 5 |
| 3257.3657526 | 2 |
| 3257.3667638 | 4 |
| 3257.3679841 | 1 |
| 3257.369744 | 3 |
| 3257.3709267 | 1 |
| 3257.3726095 | 3 |
| 3257.3727161 | 4 |
| 3257.3751219 | 4 |
| 3257.3765638 | 2 |
| 3257.3766196 | 3 |
| 3257.3787259 | 2 |
| 3257.3796219 | 6 |
| 3257.3809681 | 3 |
| 3257.3823793 | 1 |
| 3257.3833478 | 3 |
| 3257.3848172 | 3 |
| 3257.3858528 | 3 |
| 3257.3871402 | 5 |
| 3257.3881115 | 3 |
| 3257.389393 | 4 |
| 3257.3915199 | 2 |
| 3257.3918689 | 1 |
| 3257.3935907 | 2 |
| 3257.3943263 | 2 |
| 3257.3959887 | 4 |
| 3257.3965514 | 2 |
| 3257.3985635 | 1 |
| 3257.4001908 | 6 |
| 3257.4003808 | 0 |
| 3257.4017677 | 3 |
| 3257.4027236 | 3 |
| 3257.4046821 | 3 |
| 3257.4057061 | 1 |
| 3257.4074968 | 3 |
| 3257.4076509 | 1 |
| 3257.4079768 | 2 |
| 3257.4089441 | 3 |
| 3257.409607 | 1 |
| 3257.4096071 | 4 |
| 3257.4101704 | 3 |
| 3257.4107488 | 1 |
| 3257.4114996 | 0 |
| 3257.4119206 | 5 |
| 3257.4126006 | 2 |
| 3257.4129313 | 0 |
| 3257.413355 | 4 |
| 3257.4145568 | 1 |
| 3257.415161 | 1 |
| 3257.4155581 | 0 |
| 3257.4155966 | 1 |
| 3257.4161559 | 2 |
| 3257.4171079 | 1 |
| 3257.417495 | 1 |
| 3257.4183531 | 2 |
| 3257.4188364 | 4 |
| 3257.4192998 | 2 |
| 3257.4196332 | 2 |
| 3257.4202491 | 1 |
| 3257.4203875 | 4 |
| 3257.421352 | 4 |
| 3257.4218228 | 3 |
| 3257.4223503 | 2 |
| 3257.4227391 | 1 |
| 3257.4233555 | 3 |
| 3257.4236113 | 2 |
| 3257.424794 | 5 |
| 3257.4248808 | 7 |
| 3257.4250834 | 9 |
| 3257.4263347 | 4 |
| 3257.4266938 | 9 |
| 3257.4277284 | 2 |
| 3257.427884 | 0 |
| 3257.4280158 | 3 |
| 3257.4285012 | 2 |
| 3257.429202 | 5 |
| 3257.4294763 | 0 |
| 3257.4305693 | 1 |
| 3257.4307783 | 3 |
| 3257.4320157 | 3 |
| 3257.4321261 | 2 |
| 3257.4325072 | 1 |
| 3257.4326872 | 2 |
| 3257.4336983 | 1 |
| 3257.4343668 | 3 |
| 3257.434556 | 2 |
| 3257.4348036 | 2 |
| 3257.4354436 | 5 |
| 3257.436165 | 6 |
| 3257.4366241 | 4 |
| 3257.4371142 | 0 |
| 3257.4375205 | 2 |
| 3257.4384302 | 2 |
| 3257.4390509 | 2 |
| 3257.4393402 | 1 |
| 3257.440238 | 3 |
| 3257.4404888 | 3 |
| 3257.4405235 | 4 |
| 3257.4406566 | 3 |
| 3257.4407455 | 2 |
| 3257.4418953 | 1 |
| 3257.4426478 | 5 |
| 3257.4436284 | 2 |
| 3257.443809 | 4 |
| 3257.4442149 | 2 |
| 3257.4444632 | 1 |
| 3257.4448366 | 1 |
| 3257.4454985 | 1 |
| 3257.446062 | 2 |
| 3257.4466325 | 1 |
| 3257.4469032 | 0 |
| 3257.4480802 | 2 |
| 3257.4481421 | 2 |
| 3257.448263 | 6 |
| 3257.4498646 | 3 |
| 3257.450287 | 2 |
| 3257.4506989 | 5 |
| 3257.4507853 | 2 |
| 3257.4516742 | 1 |
| 3257.4523614 | 2 |
| 3257.4524634 | 4 |
| 3257.4527028 | 2 |
| 3257.4534584 | 3 |
| 3257.4539138 | 5 |
| 3257.4545287 | 1 |
| 3257.4551117 | 1 |
| 3257.4559755 | 1 |
| 3257.4565153 | 1 |
| 3257.4567933 | 3 |
| 3257.4573984 | 3 |
| 3257.4576004 | 3 |
| 3257.4580011 | 2 |
| 3257.4588271 | 1 |
| 3257.4592034 | 6 |
| 3257.4596934 | 5 |
| 3257.4597702 | 0 |
| 3257.460559 | 5 |
| 3257.4615787 | 3 |
| 3257.462243 | 5 |
| 3257.4632169 | 2 |
| 3257.4632761 | 2 |
| 3257.463708 | 5 |
| 3257.4637621 | 2 |
| 3257.4640774 | 2 |
| 3257.4654558 | 1 |
| 3257.4656303 | 0 |
| 3257.4661389 | 1 |
| 3257.4664004 | 4 |
| 3257.4674045 | 0 |
| 3257.4678278 | 3 |
| 3257.4678481 | 1 |
| 3257.4687012 | 1 |
| 3257.4689624 | 2 |
| 3257.4701361 | 5 |
| 3257.4703841 | 2 |
| 3257.4706166 | 2 |
| 3257.4718513 | 1 |
| 3257.4720835 | 3 |
| 3257.4726128 | 5 |
| 3257.4732224 | 2 |
| 3257.4735146 | 4 |
| 3257.4735231 | 1 |
| 3257.4736255 | 1 |
| 3257.4737366 | 2 |
| 3257.4743907 | 6 |
| 3257.4749606 | 2 |
| 3257.4752294 | 1 |
| 3257.4753561 | 2 |
| 3257.476095 | 1 |
| 3257.4772138 | 3 |
| 3257.4773592 | 3 |
| 3257.4778566 | 0 |
| 3257.4779328 | 1 |
| 3257.479276 | 2 |
| 3257.4804063 | 0 |
| 3257.4811434 | 3 |
| 3257.4826174 | 1 |
| 3257.4826699 | 1 |
| 3257.484335 | 1 |
| 3257.4855398 | 2 |
| 3257.4858026 | 4 |
| 3257.487005 | 3 |
| 3257.4876769 | 2 |
| 3257.4892374 | 0 |
| 3257.4896779 | 1 |
| 3257.4904515 | 1 |
| 3257.4917745 | 5 |
| 3257.4923876 | 1 |
| 3257.493257 | 5 |
| 3257.494897 | 3 |
| 3257.4953189 | 4 |
| 3257.4967883 | 2 |
| 3257.4971148 | 3 |
| 3257.4983532 | 5 |
| 3257.4996176 | 1 |
| 3257.4999782 | 4 |
| 3257.500635 | 6 |
| 3257.5018786 | 0 |
| 3257.5032021 | 3 |
| 3257.5039073 | 1 |
| 3257.5048628 | 3 |
| 3257.5059631 | 0 |
| 3257.5064945 | 1 |
| 3257.5072979 | 6 |
| 3257.5087033 | 1 |
| 3257.5092382 | 0 |
| 3257.5103664 | 3 |
| 3257.5113382 | 2 |
| 3257.5124852 | 2 |
| 3257.5130391 | 2 |
| 3257.514919 | 2 |
| 3257.5151366 | 4 |
| 3257.5159164 | 2 |
| 3257.5167181 | 1 |
| 3257.5179461 | 5 |
| 3257.5187466 | 5 |
| 3257.5201052 | 6 |
| 3257.5205593 | 1 |
| 3257.5210375 | 3 |
| 3257.5228737 | 2 |
| 3257.5232936 | 1 |
| 3257.5246193 | 5 |
| 3257.5251072 | 4 |
| 3257.5265561 | 1 |
| 3257.5268929 | 0 |
| 3257.5280398 | 3 |
| 3257.5291231 | 1 |
| 3257.5297717 | 2 |
| 3257.5310434 | 1 |
| 3257.5319077 | 2 |
| 3257.5335636 | 3 |
| 3257.5343632 | 2 |

| | |
|---|---|
| 3257.5345694 | 2 |
| 3257.5360575 | 3 |
| 3257.5362979 | 4 |
| 3257.5372812 | 2 |
| 3257.5388407 | 1 |
| 3257.5394539 | 1 |
| 3257.5401562 | 2 |
| 3257.5412169 | 1 |
| 3257.5423678 | 5 |
| 3257.5433868 | 5 |
| 3257.5441069 | 3 |
| 3257.5454431 | 5 |
| 3257.5460225 | 3 |
| 3257.5472496 | 1 |
| 3257.5483211 | 2 |
| 3257.5485842 | 2 |
| 3257.5501731 | 2 |
| 3257.5517211 | 2 |
| 3257.5518868 | 5 |
| 3257.553253 | 0 |
| 3257.5538253 | 5 |
| 3257.5551629 | 4 |
| 3257.5552142 | 2 |
| 3257.5564396 | 1 |
| 3257.5574642 | 5 |
| 3257.558405 | 0 |
| 3257.5597692 | 3 |
| 3257.5599533 | 1 |
| 3257.5616412 | 3 |
| 3257.5623537 | 2 |
| 3257.5632324 | 1 |
| 3257.5640158 | 0 |
| 3257.5642776 | 2 |
| 3257.5656122 | 3 |
| 3257.5665938 | 0 |
| 3257.5671189 | 2 |
| 3257.5686337 | 1 |
| 3257.5693345 | 1 |
| 3257.5704046 | 3 |
| 3257.5714819 | 3 |
| 3257.5717108 | 1 |
| 3257.5733627 | 2 |
| 3257.5738453 | 2 |
| 3257.5753618 | 2 |
| 3257.5762203 | 3 |
| 3257.5766042 | 5 |
| 3257.578038 | 1 |
| 3257.5787108 | 0 |
| 3257.5799234 | 2 |
| 3257.5806733 | 1 |
| 3257.5815281 | 1 |
| 3257.5834389 | 4 |
| 3257.5835007 | 1 |
| 3257.5846776 | 3 |
| 3257.5861941 | 3 |
| 3257.5864022 | 2 |
| 3257.5875481 | 8 |
| 3257.5876281 | 3 |
| 3257.5893956 | 3 |
| 3257.589645 | 0 |
| 3257.5912529 | 3 |
| 3257.5921014 | 3 |
| 3257.5930103 | 0 |
| 3257.5936912 | 0 |
| 3257.594962 | 3 |
| 3257.5962435 | 0 |
| 3257.5969903 | 2 |
| 3257.5971653 | 0 |
| 3257.5988525 | 1 |
| 3257.5996792 | 2 |
| 3257.6005477 | 1 |
| 3257.6013929 | 2 |
| 3257.6024963 | 0 |
| 3257.6031051 | 1 |
| 3257.6039783 | 3 |
| 3257.6049705 | 4 |
| 3257.6064216 | 2 |
| 3257.6069338 | 4 |
| 3257.6077051 | 4 |
| 3257.6087588 | 1 |
| 3257.6096086 | 0 |
| 3257.6111537 | 3 |
| 3257.6114485 | 2 |
| 3257.612198 | 2 |
| 3257.6129199 | 1 |
| 3257.6144949 | 2 |
| 3257.6151103 | 1 |
| 3257.6161797 | 1 |
| 3257.6174218 | 1 |
| 3257.6176629 | 1 |
| 3257.6185891 | 1 |
| 3257.6200185 | 5 |
| 3257.6208625 | 1 |
| 3257.6217868 | 3 |
| 3257.6226828 | 1 |
| 3257.6244331 | 1 |
| 3257.6244645 | 1 |
| 3257.6256602 | 5 |
| 3257.6264482 | 2 |
| 3257.627707 | 3 |
| 3257.6288859 | 6 |
| 3257.6297705 | 3 |
| 3257.6298323 | 3 |
| 3257.6317161 | 3 |
| 3257.6326712 | 0 |
| 3257.6329989 | 4 |
| 3257.6341708 | 3 |
| 3257.6352007 | 4 |
| 3257.6364209 | 1 |
| 3257.6368363 | 6 |
| 3257.6379379 | 5 |
| 3257.6392801 | 2 |
| 3257.6396125 | 5 |
| 3257.6413552 | 0 |
| 3257.6425318 | 2 |
| 3257.6425836 | 2 |
| 3257.6438718 | 3 |
| 3257.6446121 | 4 |
| 3257.6449481 | 3 |
| 3257.6464182 | 3 |
| 3257.6474611 | 5 |
| 3257.6483239 | 1 |
| 3257.6492523 | 3 |
| 3257.6509475 | 1 |
| 3257.6511752 | 3 |
| 3257.6524758 | 4 |
| 3257.6530358 | 2 |
| 3257.6531519 | 2 |
| 3257.6552326 | 1 |
| 3257.6558259 | 2 |
| 3257.6561269 | 6 |
| 3257.6576812 | 2 |
| 3257.6589713 | 3 |
| 3257.6597215 | 3 |
| 3257.6605774 | 1 |
| 3257.661268 | 2 |
| 3257.6626049 | 3 |
| 3257.6633403 | 4 |
| 3257.6644335 | 5 |
| 3257.6648375 | 4 |
| 3257.6660509 | 5 |
| 3257.6670173 | 1 |
| 3257.6676271 | 1 |
| 3257.6686971 | 2 |
| 3257.6698578 | 2 |
| 3257.6707921 | 2 |
| 3257.6721696 | 8 |
| 3257.6721804 | 2 |
| 3257.6738757 | 1 |
| 3257.6739422 | 3 |
| 3257.6759987 | 1 |
| 3257.6768882 | 0 |
| 3257.6771312 | 2 |
| 3257.678302 | 1 |
| 3257.6795776 | 2 |
| 3257.6797988 | 2 |
| 3257.6811314 | 2 |
| 3257.681956 | 3 |
| 3257.6829392 | 1 |
| 3257.6843232 | 4 |
| 3257.6850053 | 2 |
| 3257.6859338 | 3 |
| 3257.687145 | 3 |
| 3257.6874951 | 3 |
| 3257.688618 | 2 |
| 3257.6898631 | 1 |
| 3257.6903528 | 2 |
| 3257.6914258 | 2 |
| 3257.6922847 | 3 |
| 3257.6934573 | 1 |
| 3257.6935855 | 3 |
| 3257.6952097 | 1 |
| 3257.6962646 | 5 |
| 3257.696286 | 3 |
| 3257.6981418 | 2 |
| 3257.6986623 | 0 |
| 3257.6988472 | 2 |
| 3257.7009849 | 3 |
| 3257.701354 | 2 |
| 3257.7023735 | 2 |
| 3257.7032114 | 2 |
| 3257.7041927 | 2 |
| 3257.7048356 | 1 |
| 3257.7056798 | 3 |
| 3257.7074289 | 1 |
| 3257.7077277 | 4 |
| 3257.7089716 | 3 |
| 3257.709915 | 5 |
| 3257.7111 | 1 |
| 3257.7117865 | 1 |
| 3257.7122494 | 1 |
| 3257.7135103 | 2 |
| 3257.7146363 | 1 |
| 3257.7155689 | 1 |
| 3257.7164716 | 1 |
| 3257.7170528 | 1 |
| 3257.7184394 | 0 |
| 3257.7192918 | 3 |
| 3257.7202392 | 3 |
| 3257.7210939 | 2 |
| 3257.7221069 | 1 |
| 3257.7227915 | 1 |
| 3257.723528 | 9 |
| 3257.7243946 | 1 |
| 3257.7260064 | 8 |
| 3257.7265869 | 2 |
| 3257.7278609 | 3 |
| 3257.728621 | 0 |
| 3257.7301018 | 2 |
| 3257.7305086 | 4 |
| 3257.7315079 | 1 |
| 3257.7325541 | 2 |
| 3257.7336598 | 1 |
| 3257.7348408 | 4 |
| 3257.7354054 | 1 |
| 3257.7359908 | 1 |
| 3257.737229 | 6 |
| 3257.7381391 | 1 |
| 3257.7388041 | 0 |
| 3257.7396138 | 2 |
| 3257.7404686 | 5 |
| 3257.7417739 | 2 |
| 3257.7423564 | 2 |
| 3257.7433415 | 6 |
| 3257.7438461 | 3 |
| 3257.7457572 | 0 |
| 3257.7468131 | 1 |
| 3257.7472709 | 4 |
| 3257.748049 | 3 |
| 3257.7491659 | 2 |
| 3257.7492222 | 2 |
| 3257.7504667 | 2 |
| 3257.7515212 | 1 |
| 3257.7525227 | 0 |
| 3257.7530698 | 3 |
| 3257.7548811 | 2 |
| 3257.7553695 | 2 |
| 3257.7560479 | 1 |
| 3257.7575299 | 2 |
| 3257.7586492 | 2 |
| 3257.7588572 | 5 |
| 3257.7598034 | 0 |
| 3257.7606612 | 2 |
| 3257.7612398 | 2 |
| 3257.7630268 | 3 |
| 3257.7637241 | 2 |
| 3257.7649868 | 1 |
| 3257.7651059 | 2 |
| 3257.7658785 | 5 |
| 3257.7675809 | 1 |
| 3257.7685302 | 0 |
| 3257.7695038 | 0 |
| 3257.7702648 | 2 |
| 3257.7716437 | 6 |
| 3257.7720724 | 6 |
| 3257.7728306 | 7 |
| 3257.7740948 | 3 |
| 3257.7752437 | 3 |
| 3257.7760821 | 2 |
| 3257.7772457 | 3 |
| 3257.7776111 | 4 |
| 3257.7783368 | 1 |
| 3257.7794116 | 3 |
| 3257.7803169 | 3 |
| 3257.7814951 | 1 |
| 3257.7822443 | 2 |
| 3257.7836107 | 4 |
| 3257.7844333 | 2 |
| 3257.7846292 | 1 |
| 3257.7861148 | 1 |
| 3257.7862717 | 4 |
| 3257.7877367 | 0 |
| 3257.7883441 | 3 |
| 3257.7892574 | 5 |
| 3257.7907851 | 4 |
| 3257.7912557 | 0 |
| 3257.7922955 | 4 |
| 3257.7929156 | 3 |
| 3257.7936363 | 2 |
| 3257.7950707 | 1 |
| 3257.7959557 | 1 |
| 3257.7971392 | 6 |
| 3257.7972213 | 2 |
| 3257.7994799 | 3 |
| 3257.8001425 | 3 |
| 3257.8003619 | 1 |
| 3257.8015623 | 1 |
| 3257.8024024 | 3 |
| 3257.8043221 | 2 |
| 3257.8047861 | 5 |
| 3257.8058851 | 1 |
| 3257.8062212 | 2 |
| 3257.8071672 | 3 |
| 3257.8081541 | 2 |
| 3257.8090417 | 0 |
| 3257.8102941 | 3 |
| 3257.8112961 | 6 |
| 3257.8118551 | 2 |
| 3257.8127488 | 2 |
| 3257.8140504 | 2 |
| 3257.8146013 | 1 |
| 3257.8157463 | 3 |
| 3257.8164292 | 4 |
| 3257.8173565 | 3 |
| 3257.8188163 | 2 |
| 3257.8193381 | 3 |
| 3257.8207127 | 4 |
| 3257.8212224 | 3 |
| 3257.8224348 | 2 |
| 3257.8227096 | 1 |
| 3257.8238192 | 3 |
| 3257.8253078 | 5 |
| 3257.8253965 | 2 |
| 3257.8270532 | 1 |
| 3257.8277777 | 2 |
| 3257.8291011 | 2 |
| 3257.829812 | 1 |
| 3257.8303805 | 2 |
| 3257.8320421 | 2 |
| 3257.832378 | 2 |
| 3257.8327797 | 1 |
| 3257.8342571 | 5 |
| 3257.8343986 | 2 |
| 3257.8359922 | 4 |
| 3257.8370528 | 1 |
| 3257.8372197 | 3 |
| 3257.8378737 | 3 |
| 3257.8386135 | 4 |
| 3257.8387387 | 0 |
| 3257.8388001 | 3 |
| 3257.8394353 | 0 |
| 3257.8394668 | 3 |
| 3257.8396102 | 2 |
| 3257.8405794 | 1 |
| 3257.8408645 | 2 |
| 3257.8412918 | 2 |
| 3257.8415377 | 0 |
| 3257.8416356 | 4 |
| 3257.8420228 | 3 |
| 3257.8423158 | 4 |
| 3257.8425254 | 3 |
| 3257.842709 | 1 |
| 3257.8432525 | 2 |
| 3257.8432779 | 1 |
| 3257.8436287 | 0 |
| 3257.8436859 | 4 |
| 3257.8440049 | 2 |
| 3257.8446375 | 5 |
| 3257.8447628 | 0 |
| 3257.8449245 | 3 |
| 3257.8453294 | 3 |
| 3257.8458424 | 3 |
| 3257.8458901 | 2 |
| 3257.8459712 | 6 |
| 3257.8469093 | 2 |
| 3257.8475246 | 0 |
| 3257.8475329 | 4 |
| 3257.8477446 | 1 |
| 3257.8483116 | 2 |
| 3257.8484925 | 1 |
| 3257.8487734 | 3 |
| 3257.848845 | 4 |
| 3257.8490219 | 2 |

| | |
|---|---|
| 3257.8492131 | 4 |
| 3257.8494824 | 0 |
| 3257.8498557 | 3 |
| 3257.8502597 | 1 |
| 3257.8507387 | 3 |
| 3257.8510326 | 2 |
| 3257.8515761 | 2 |
| 3257.8516301 | 0 |
| 3257.8519672 | 2 |
| 3257.8524402 | 5 |
| 3257.8525444 | 6 |
| 3257.852677 | 1 |
| 3257.8534775 | 1 |
| 3257.853787 | 2 |
| 3257.8542776 | 1 |
| 3257.8545216 | 2 |
| 3257.8546842 | 3 |
| 3257.8551016 | 1 |
| 3257.855356 | 3 |
| 3257.8557437 | 3 |
| 3257.856063 | 2 |
| 3257.856224 | 0 |
| 3257.8564135 | 3 |
| 3257.8569383 | 2 |
| 3257.857194 | 0 |
| 3257.8572858 | 2 |
| 3257.8579868 | 2 |
| 3257.8580421 | 3 |
| 3257.8582258 | 1 |
| 3257.8586758 | 2 |
| 3257.8587066 | 0 |
| 3257.8594845 | 2 |
| 3257.8595175 | 3 |
| 3257.8602711 | 1 |
| 3257.8603219 | 3 |
| 3257.8606397 | 1 |
| 3257.8610002 | 4 |
| 3257.8610857 | 1 |
| 3257.8612988 | 3 |
| 3257.8615341 | 1 |
| 3257.8621138 | 1 |
| 3257.8624066 | 4 |
| 3257.8625819 | 0 |
| 3257.8630022 | 8 |
| 3257.8634591 | 2 |
| 3257.8636689 | 2 |
| 3257.8638438 | 3 |
| 3257.8643723 | 3 |
| 3257.864545 | 3 |
| 3257.8650967 | 1 |
| 3257.8653263 | 2 |
| 3257.8654035 | 4 |
| 3257.8656247 | 2 |
| 3257.8660016 | 2 |
| 3257.8665858 | 3 |
| 3257.866994 | 5 |
| 3257.8671424 | 1 |
| 3257.8672977 | 1 |
| 3257.8673388 | 1 |
| 3257.8676754 | 3 |
| 3257.8678258 | 0 |
| 3257.8685464 | 2 |
| 3257.8691454 | 1 |
| 3257.8691649 | 2 |
| 3257.8694453 | 2 |
| 3257.8695512 | 4 |
| 3257.8698487 | 2 |
| 3257.8704554 | 5 |
| 3257.8704788 | 2 |
| 3257.8708511 | 3 |
| 3257.8712666 | 3 |
| 3257.8713165 | 0 |
| 3257.8720138 | 2 |
| 3257.8722257 | 2 |
| 3257.8729297 | 2 |
| 3257.8730601 | 1 |
| 3257.8732086 | 3 |
| 3257.8732863 | 1 |
| 3257.8735503 | 2 |
| 3257.8742579 | 4 |
| 3257.8742786 | 2 |
| 3257.8751571 | 1 |
| 3257.8752249 | 1 |
| 3257.875339 | 2 |
| 3257.8754999 | 1 |
| 3257.8757074 | 2 |
| 3257.8762374 | 2 |
| 3257.8764133 | 1 |
| 3257.8767993 | 4 |
| 3257.8770554 | 3 |
| 3257.8774489 | 2 |
| 3257.8776997 | 3 |
| 3257.8778087 | 1 |
| 3257.8780212 | 2 |
| 3257.8787744 | 3 |
| 3257.8788128 | 1 |
| 3257.8793101 | 2 |
| 3257.8796102 | 1 |
| 3257.8799088 | 1 |
| 3257.879965 | 3 |
| 3257.8800785 | 1 |
| 3257.8804615 | 0 |
| 3257.8806336 | 2 |
| 3257.8809294 | 0 |
| 3257.881564 | 1 |
| 3257.8819619 | 3 |
| 3257.8821038 | 5 |
| 3257.8827845 | 4 |
| 3257.8829813 | 3 |
| 3257.8830539 | 3 |
| 3257.8830653 | 2 |
| 3257.8839171 | 5 |
| 3257.8840821 | 4 |
| 3257.8842038 | 2 |
| 3257.8847989 | 0 |
| 3257.885048 | 2 |
| 3257.8851611 | 0 |
| 3257.8854163 | 1 |
| 3257.8857388 | 1 |
| 3257.886646 | 0 |
| 3257.8868242 | 0 |
| 3257.8869153 | 4 |
| 3257.8871039 | 6 |
| 3257.8873112 | 3 |
| 3257.8879672 | 1 |
| 3257.8881225 | 0 |
| 3257.8884721 | 0 |
| 3257.8885764 | 1 |
| 3257.8891982 | 1 |
| 3257.8893278 | 3 |
| 3257.8897648 | 1 |
| 3257.8898266 | 3 |
| 3257.8901301 | 1 |
| 3257.890715 | 2 |
| 3257.8908014 | 2 |
| 3257.8911436 | 3 |
| 3257.8914468 | 1 |
| 3257.8917447 | 0 |
| 3257.8922071 | 0 |
| 3257.8925157 | 2 |
| 3257.8927243 | 4 |
| 3257.8931782 | 4 |
| 3257.8932917 | 1 |
| 3257.8937393 | 2 |
| 3257.893862 | 0 |
| 3257.894197 | 2 |
| 3257.8944327 | 2 |
| 3257.8947498 | 1 |
| 3257.8954201 | 4 |
| 3257.8957687 | 2 |
| 3257.8960771 | 3 |
| 3257.8961322 | 4 |
| 3257.8962055 | 3 |
| 3257.8963681 | 0 |
| 3257.896892 | 4 |
| 3257.8976209 | 4 |
| 3257.8976413 | 0 |
| 3257.8978292 | 4 |
| 3257.8984295 | 2 |
| 3257.8985374 | 0 |
| 3257.8985655 | 2 |
| 3257.8992604 | 4 |
| 3257.8997725 | 3 |
| 3257.9002898 | 2 |
| 3257.9003306 | 1 |
| 3257.9009899 | 3 |
| 3257.9010043 | 7 |
| 3257.9010254 | 0 |
| 3257.9013973 | 0 |
| 3257.9020041 | 1 |
| 3257.9020985 | 3 |
| 3257.9022252 | 2 |
| 3257.9027017 | 4 |
| 3257.9032266 | 4 |
| 3257.9032653 | 0 |
| 3257.9035587 | 2 |
| 3257.9040612 | 2 |
| 3257.9043362 | 5 |
| 3257.9050873 | 0 |
| 3257.9051111 | 1 |
| 3257.9053483 | 2 |
| 3257.9056108 | 2 |
| 3257.9057622 | 3 |
| 3257.9064182 | 2 |
| 3257.9066309 | 3 |
| 3257.9067109 | 2 |
| 3257.9071552 | 1 |
| 3257.9072558 | 2 |
| 3257.908112 | 1 |
| 3257.9081896 | 2 |
| 3257.9084508 | 1 |
| 3257.9085748 | 0 |
| 3257.9087246 | 4 |
| 3257.9088853 | 3 |
| 3257.909069 | 1 |
| 3257.9098298 | 2 |
| 3257.9103586 | 1 |
| 3257.9105894 | 1 |
| 3257.9108888 | 5 |
| 3257.9111392 | 2 |
| 3257.9115726 | 3 |
| 3257.9116212 | 5 |
| 3257.9121089 | 1 |
| 3257.9123777 | 1 |
| 3257.9125072 | 0 |
| 3257.9126368 | 2 |
| 3257.9130762 | 5 |
| 3257.9132991 | 1 |
| 3257.9140461 | 4 |
| 3257.9143669 | 3 |
| 3257.9144191 | 2 |
| 3257.914609 | 2 |
| 3257.9147153 | 4 |
| 3257.9151749 | 2 |
| 3257.9158187 | 1 |
| 3257.9162415 | 3 |
| 3257.9163944 | 5 |
| 3257.9164102 | 2 |
| 3257.9171206 | 6 |
| 3257.9172966 | 1 |
| 3257.9175587 | 1 |
| 3257.9178777 | 1 |
| 3257.9180868 | 1 |
| 3257.918245 | 3 |
| 3257.9187828 | 3 |
| 3257.9188073 | 2 |
| 3257.9190866 | 2 |
| 3257.9197864 | 3 |
| 3257.9199574 | 1 |
| 3257.9200812 | 3 |
| 3257.9207543 | 1 |
| 3257.9208567 | 2 |
| 3257.9209326 | 4 |
| 3257.9216053 | 5 |
| 3257.9216643 | 3 |
| 3257.9222702 | 6 |
| 3257.922568 | 3 |
| 3257.9228935 | 2 |
| 3257.9229022 | 4 |
| 3257.9230507 | 1 |
| 3257.9234633 | 2 |
| 3257.9243313 | 1 |
| 3257.9243981 | 0 |
| 3257.9244797 | 1 |
| 3257.9246896 | 3 |
| 3257.9249137 | 3 |
| 3257.9254742 | 1 |
| 3257.925582 | 1 |
| 3257.9261123 | 0 |
| 3257.9263958 | 1 |
| 3257.9268333 | 3 |
| 3257.9268692 | 2 |
| 3257.9270603 | 1 |
| 3257.9276538 | 0 |
| 3257.9278486 | 4 |
| 3257.9282715 | 0 |
| 3257.92846 | 3 |
| 3257.9289186 | 1 |
| 3257.9291451 | 3 |
| 3257.9293834 | 2 |
| 3257.9294684 | 3 |
| 3257.9299219 | 5 |
| 3257.9301203 | 1 |
| 3257.9307615 | 2 |
| 3257.9307767 | 1 |
| 3257.930833 | 2 |
| 3257.9310228 | 3 |
| 3257.9318803 | 2 |
| 3257.932213 | 2 |
| 3257.9322313 | 2 |
| 3257.9323371 | 0 |
| 3257.9325588 | 3 |
| 3257.9336969 | 4 |
| 3257.9337885 | 2 |
| 3257.9338732 | 3 |
| 3257.93394 | 1 |
| 3257.9341213 | 3 |
| 3257.934317 | 2 |
| 3257.9344354 | 1 |
| 3257.9356926 | 1 |
| 3257.936177 | 4 |
| 3257.9361869 | 1 |
| 3257.9363757 | 3 |
| 3257.9366011 | 0 |
| 3257.9367695 | 2 |
| 3257.9370956 | 1 |
| 3257.937409 | 1 |
| 3257.9375696 | 3 |
| 3257.9376596 | 2 |
| 3257.937923 | 3 |
| 3257.9386713 | 2 |
| 3257.9391118 | 0 |
| 3257.9393917 | 2 |
| 3257.9395748 | 0 |
| 3257.9398626 | 5 |
| 3257.9406657 | 2 |
| 3257.9409017 | 2 |
| 3257.9411777 | 3 |
| 3257.9412701 | 3 |
| 3257.9413662 | 1 |
| 3257.9414628 | 2 |
| 3257.9416784 | 3 |
| 3257.9422217 | 1 |
| 3257.9426293 | 4 |
| 3257.9434557 | 2 |
| 3257.9434595 | 1 |
| 3257.9436032 | 1 |
| 3257.9441436 | 2 |
| 3257.9442539 | 3 |
| 3257.9443006 | 1 |
| 3257.9451398 | 1 |
| 3257.945177 | 1 |
| 3257.9457565 | 2 |
| 3257.945891 | 0 |
| 3257.9465995 | 5 |
| 3257.9466112 | 1 |
| 3257.9467723 | 1 |
| 3257.946926 | 3 |
| 3257.9472376 | 5 |
| 3257.9476612 | 4 |
| 3257.9479517 | 1 |
| 3257.948017 | 3 |
| 3257.9485504 | 1 |
| 3257.9487404 | 2 |
| 3257.9490237 | 1 |
| 3257.9496619 | 4 |
| 3257.9497635 | 3 |
| 3257.9503651 | 0 |
| 3257.9504975 | 1 |
| 3257.9507556 | 2 |
| 3257.9508363 | 2 |
| 3257.951386 | 1 |
| 3257.9518469 | 1 |
| 3257.9520403 | 1 |
| 3257.9526292 | 3 |
| 3257.952705 | 2 |
| 3257.9531249 | 4 |
| 3257.9538325 | 5 |
| 3257.9538451 | 3 |
| 3257.9543831 | 4 |
| 3257.9544087 | 4 |
| 3257.9547223 | 2 |
| 3257.9550195 | 2 |
| 3257.9554139 | 4 |
| 3257.9558473 | 2 |
| 3257.9560553 | 3 |
| 3257.956487 | 3 |
| 3257.9565308 | 1 |
| 3257.9568004 | 2 |
| 3257.9569125 | 2 |
| 3257.9573153 | 4 |
| 3257.9575259 | 1 |
| 3257.957679 | 3 |
| 3257.9588941 | 0 |
| 3257.9590188 | 3 |
| 3257.9590457 | 1 |
| 3257.9591645 | 3 |
| 3257.9595078 | 2 |
| 3257.9602236 | 1 |
| 3257.9605168 | 3 |
| 3257.9608434 | 6 |
| 3257.9609241 | 3 |
| 3257.9610931 | 2 |

| | |
|---|---|
| 3257.9616537 | 2 |
| 3257.9616839 | 1 |
| 3257.962043 | 2 |
| 3257.9625391 | 4 |
| 3257.9626105 | 1 |
| 3257.9628552 | 1 |
| 3257.9630702 | 4 |
| 3257.9636523 | 2 |
| 3257.9640164 | 4 |
| 3257.9645446 | 3 |
| 3257.9647536 | 2 |
| 3257.9650313 | 2 |
| 3257.9651847 | 3 |
| 3257.9653321 | 3 |
| 3257.9656777 | 5 |
| 3257.9656962 | 2 |
| 3257.9659855 | 3 |
| 3257.9660521 | 2 |
| 3257.9669616 | 3 |
| 3257.9673744 | 0 |
| 3257.9678545 | 3 |
| 3257.9682191 | 0 |
| 3257.968304 | 3 |
| 3257.9686967 | 3 |
| 3257.968762 | 3 |
| 3257.9690388 | 1 |
| 3257.9690723 | 1 |
| 3257.9696087 | 0 |
| 3257.9703837 | 1 |
| 3257.9703887 | 3 |
| 3257.9708218 | 7 |
| 3257.9708991 | 1 |
| 3257.9710178 | 1 |
| 3257.9713746 | 0 |
| 3257.9715548 | 2 |
| 3257.9717596 | 3 |
| 3257.9724565 | 1 |
| 3257.9725096 | 2 |
| 3257.9728883 | 9 |
| 3257.9730908 | 4 |
| 3257.9737177 | 2 |
| 3257.9738196 | 3 |
| 3257.9742077 | 1 |
| 3257.9745079 | 1 |
| 3257.9750343 | 5 |
| 3257.9752619 | 3 |
| 3257.9756355 | 1 |
| 3257.9762489 | 1 |
| 3257.9762983 | 4 |
| 3257.9764324 | 0 |
| 3257.9766858 | 1 |
| 3257.9768964 | 2 |
| 3257.9769182 | 1 |
| 3257.9780005 | 3 |
| 3257.9781705 | 0 |
| 3257.9783271 | 1 |
| 3257.978853 | 1 |
| 3257.9790274 | 3 |
| 3257.9791826 | 1 |
| 3257.979598 | 1 |
| 3257.9801428 | 2 |
| 3257.9802664 | 0 |
| 3257.9805143 | 0 |
| 3257.980532 | 0 |
| 3257.9808063 | 3 |
| 3257.9814399 | 5 |
| 3257.9815957 | 1 |
| 3257.9817569 | 3 |
| 3257.9824496 | 1 |
| 3257.9826469 | 3 |
| 3257.9827366 | 1 |
| 3257.9827924 | 1 |
| 3257.9829003 | 2 |
| 3257.9835859 | 2 |
| 3257.9839849 | 2 |
| 3257.9842695 | 1 |
| 3257.9850314 | 0 |
| 3257.985076 | 1 |
| 3257.985306 | 1 |
| 3257.98533 | 3 |
| 3257.9861123 | 2 |
| 3257.9861628 | 2 |
| 3257.9862236 | 4 |
| 3257.9868884 | 1 |
| 3257.9870801 | 3 |
| 3257.9871499 | 4 |
| 3257.9880009 | 2 |
| 3257.9885304 | 3 |
| 3257.9886389 | 3 |
| 3257.9887155 | 1 |
| 3257.9895269 | 2 |
| 3257.9895323 | 2 |
| 3257.9898189 | 0 |
| 3257.9899991 | 4 |
| 3257.9905908 | 2 |
| 3257.9906793 | 3 |
| 3257.9910052 | 2 |
| 3257.9912371 | 2 |
| 3257.9913977 | 2 |
| 3257.9920776 | 2 |
| 3257.9921415 | 3 |
| 3257.9927135 | 1 |
| 3257.9927482 | 2 |
| 3257.9933124 | 1 |
| 3257.993507 | 2 |
| 3257.9937544 | 3 |
| 3257.9942177 | 3 |
| 3257.9943202 | 2 |
| 3257.9947425 | 0 |
| 3257.9949132 | 0 |
| 3257.9955596 | 1 |
| 3257.9958867 | 1 |
| 3257.996517 | 6 |
| 3257.9965453 | 2 |
| 3257.9969689 | 2 |
| 3257.9970191 | 2 |
| 3257.9970354 | 3 |
| 3257.9978664 | 1 |
| 3257.9980309 | 4 |
| 3257.9982533 | 1 |
| 3257.9984651 | 3 |
| 3257.9990161 | 1 |
| 3257.9990471 | 2 |
| 3257.9994101 | 2 |
| 3257.9994365 | 3 |
| 3257.9998877 | 5 |
| 3257.9999597 | 1 |
| 3258.0003776 | 1 |
| 3258.001166 | 0 |
| 3258.0013333 | 2 |
| 3258.0016283 | 3 |
| 3258.0023061 | 1 |
| 3258.002359 | 3 |
| 3258.0023684 | 4 |
| 3258.0029808 | 7 |
| 3258.0031135 | 2 |
| 3258.0036759 | 0 |
| 3258.0041488 | 2 |
| 3258.0043707 | 3 |
| 3258.0043981 | 2 |
| 3258.0046952 | 3 |
| 3258.004708 | 2 |
| 3258.0052669 | 2 |
| 3258.0054583 | 1 |
| 3258.0061543 | 1 |
| 3258.0064085 | 3 |
| 3258.0065623 | 0 |
| 3258.0070466 | 1 |
| 3258.0073321 | 6 |
| 3258.0073696 | 1 |
| 3258.007593 | 3 |
| 3258.0076066 | 4 |
| 3258.008368 | 2 |
| 3258.0084211 | 0 |
| 3258.0089965 | 1 |
| 3258.0090256 | 1 |
| 3258.0094519 | 1 |
| 3258.0099741 | 1 |
| 3258.0105066 | 1 |
| 3258.0107015 | 3 |
| 3258.0107456 | 5 |
| 3258.0112624 | 2 |
| 3258.0114201 | 1 |
| 3258.0116296 | 1 |
| 3258.0118954 | 1 |
| 3258.0127082 | 1 |
| 3258.0127761 | 2 |
| 3258.0129746 | 3 |
| 3258.013058 | 2 |
| 3258.0133055 | 2 |
| 3258.0141034 | 0 |
| 3258.0142715 | 2 |
| 3258.0148396 | 1 |
| 3258.0149479 | 2 |
| 3258.0153566 | 3 |
| 3258.0156476 | 0 |
| 3258.0158576 | 0 |
| 3258.0159808 | 2 |
| 3258.0165598 | 2 |
| 3258.0167218 | 0 |
| 3258.0167507 | 1 |
| 3258.0168604 | 1 |
| 3258.0174446 | 2 |
| 3258.0177505 | 0 |
| 3258.0180847 | 0 |
| 3258.0187673 | 0 |
| 3258.0187841 | 5 |
| 3258.0192369 | 3 |
| 3258.0194037 | 2 |
| 3258.0199733 | 1 |
| 3258.0200872 | 3 |
| 3258.0202419 | 1 |
| 3258.020246 | 2 |
| 3258.020723 | 2 |
| 3258.0208854 | 1 |
| 3258.0215135 | 2 |
| 3258.0218283 | 2 |
| 3258.0223032 | 1 |
| 3258.0223464 | 5 |
| 3258.0223489 | 1 |
| 3258.0232681 | 1 |
| 3258.0237165 | 4 |
| 3258.0237209 | 3 |
| 3258.0238449 | 3 |
| 3258.0240654 | 1 |
| 3258.0241355 | 0 |
| 3258.0250293 | 2 |
| 3258.0252314 | 4 |
| 3258.026002 | 4 |
| 3258.0260102 | 2 |
| 3258.0261419 | 1 |
| 3258.0261437 | 4 |
| 3258.0266851 | 4 |
| 3258.027083 | 1 |
| 3258.0274294 | 3 |
| 3258.0279796 | 4 |
| 3258.0280913 | 4 |
| 3258.0288087 | 1 |
| 3258.0288755 | 2 |
| 3258.0290443 | 0 |
| 3258.0292082 | 3 |
| 3258.0294043 | 4 |
| 3258.030147 | 5 |
| 3258.0302617 | 3 |
| 3258.0305249 | 6 |
| 3258.0311571 | 4 |
| 3258.0312331 | 2 |
| 3258.0318566 | 4 |
| 3258.0319635 | 1 |
| 3258.0320343 | 1 |
| 3258.0321645 | 2 |
| 3258.0326314 | 2 |
| 3258.0331541 | 3 |
| 3258.0336516 | 2 |
| 3258.0339166 | 3 |
| 3258.034083 | 2 |
| 3258.0344085 | 5 |
| 3258.0347396 | 3 |
| 3258.035015 | 0 |
| 3258.0354662 | 3 |
| 3258.0354928 | 2 |
| 3258.0355953 | 1 |
| 3258.036279 | 1 |
| 3258.036449 | 3 |
| 3258.0364881 | 0 |
| 3258.0372946 | 1 |
| 3258.0374248 | 5 |
| 3258.0374811 | 3 |
| 3258.0377515 | 3 |
| 3258.0377982 | 3 |
| 3258.0385888 | 4 |
| 3258.0388806 | 2 |
| 3258.0390107 | 3 |
| 3258.0397094 | 3 |
| 3258.0398657 | 3 |
| 3258.0403463 | 4 |
| 3258.0404187 | 2 |
| 3258.0406937 | 0 |
| 3258.0407138 | 1 |
| 3258.0413466 | 2 |
| 3258.0416238 | 1 |
| 3258.0419073 | 2 |
| 3258.0425574 | 3 |
| 3258.0425997 | 3 |
| 3258.0426904 | 1 |
| 3258.0431603 | 2 |
| 3258.0438072 | 1 |
| 3258.0439094 | 0 |
| 3258.0443727 | 3 |
| 3258.0445817 | 1 |
| 3258.0446325 | 1 |
| 3258.0450902 | 0 |
| 3258.045388 | 1 |
| 3258.0453958 | 1 |
| 3258.0464418 | 0 |
| 3258.0465002 | 2 |
| 3258.0465513 | 2 |
| 3258.046984 | 1 |
| 3258.0472313 | 0 |
| 3258.04745 | 0 |
| 3258.0483611 | 2 |
| 3258.0484508 | 1 |
| 3258.0484514 | 3 |
| 3258.0489658 | 1 |
| 3258.0491731 | 0 |
| 3258.0496212 | 1 |
| 3258.0503128 | 0 |
| 3258.0505255 | 1 |
| 3258.0508748 | 2 |
| 3258.0511151 | 3 |
| 3258.0513252 | 2 |
| 3258.0513817 | 0 |
| 3258.052 | 1 |
| 3258.0521793 | 4 |
| 3258.0521943 | 4 |
| 3258.052736 | 2 |
| 3258.0531317 | 2 |
| 3258.0534712 | 1 |
| 3258.0538726 | 1 |
| 3258.0539282 | 3 |
| 3258.0545389 | 1 |
| 3258.0545445 | 1 |
| 3258.0545854 | 1 |
| 3258.0548341 | 0 |
| 3258.055555 | 1 |
| 3258.0560237 | 4 |
| 3258.056228 | 1 |
| 3258.0562866 | 2 |
| 3258.0564983 | 5 |
| 3258.0567423 | 3 |
| 3258.0572143 | 2 |
| 3258.0576878 | 1 |
| 3258.0577904 | 0 |
| 3258.0583392 | 3 |
| 3258.0585713 | 0 |
| 3258.058627 | 6 |
| 3258.0591227 | 0 |
| 3258.0594832 | 5 |
| 3258.0595741 | 4 |
| 3258.0602506 | 2 |
| 3258.0602909 | 3 |
| 3258.0604107 | 4 |
| 3258.060535 | 1 |
| 3258.0610535 | 1 |
| 3258.0615747 | 3 |
| 3258.0616741 | 0 |
| 3258.0617166 | 5 |
| 3258.0625125 | 3 |
| 3258.0626387 | 1 |
| 3258.0631487 | 1 |
| 3258.0632762 | 2 |
| 3258.0639684 | 1 |
| 3258.0640638 | 4 |
| 3258.0643152 | 2 |
| 3258.064406 | 5 |
| 3258.0647945 | 1 |
| 3258.0651745 | 5 |
| 3258.0652418 | 0 |
| 3258.0660943 | 1 |
| 3258.0664694 | 2 |
| 3258.066498 | 0 |
| 3258.0667 | 3 |
| 3258.0669361 | 1 |
| 3258.0673588 | 1 |
| 3258.0677304 | 1 |
| 3258.0682417 | 0 |
| 3258.0683831 | 3 |
| 3258.0687236 | 3 |
| 3258.0688386 | 3 |
| 3258.0694629 | 3 |
| 3258.0695871 | 1 |
| 3258.0700398 | 2 |
| 3258.0702695 | 5 |
| 3258.0706707 | 1 |
| 3258.0709176 | 2 |
| 3258.0710289 | 2 |
| 3258.0710544 | 2 |
| 3258.0717218 | 4 |
| 3258.0721768 | 5 |
| 3258.0722909 | 4 |
| 3258.0727939 | 3 |
| 3258.0729698 | 1 |
| 3258.073252 | 6 |

| | |
|---|---|
| 3258.0733758 | 1 |
| 3258.073739 | 1 |
| 3258.0738941 | 0 |
| 3258.0743741 | 2 |
| 3258.0746884 | 1 |
| 3258.0748006 | 1 |
| 3258.0750898 | 2 |
| 3258.0755871 | 1 |
| 3258.0757195 | 1 |
| 3258.0763296 | 4 |
| 3258.0767074 | 1 |
| 3258.0768844 | 3 |
| 3258.0772238 | 1 |
| 3258.0779181 | 2 |
| 3258.0780292 | 5 |
| 3258.0786582 | 1 |
| 3258.0787296 | 1 |
| 3258.0789828 | 3 |
| 3258.0790895 | 2 |
| 3258.0792002 | 1 |
| 3258.0796807 | 3 |
| 3258.0801236 | 3 |
| 3258.0802381 | 1 |
| 3258.0804379 | 0 |
| 3258.0808194 | 1 |
| 3258.0814938 | 0 |
| 3258.0816983 | 1 |
| 3258.0823599 | 3 |
| 3258.0824308 | 4 |
| 3258.0827129 | 2 |
| 3258.0831233 | 2 |
| 3258.0831633 | 5 |
| 3258.0834306 | 1 |
| 3258.0836771 | 1 |
| 3258.0841408 | 2 |
| 3258.0847697 | 1 |
| 3258.0851482 | 1 |
| 3258.0853124 | 5 |
| 3258.0854303 | 4 |
| 3258.085527 | 3 |
| 3258.0863054 | 2 |
| 3258.0865841 | 1 |
| 3258.0870153 | 4 |
| 3258.0873218 | 0 |
| 3258.0873695 | 1 |
| 3258.0880417 | 1 |
| 3258.0881032 | 2 |
| 3258.08816 | 4 |
| 3258.0883738 | 2 |
| 3258.0888177 | 3 |
| 3258.0891149 | 2 |
| 3258.0897414 | 0 |
| 3258.0897943 | 2 |
| 3258.0902745 | 2 |
| 3258.0906217 | 1 |
| 3258.0908737 | 2 |
| 3258.0909888 | 0 |
| 3258.0917084 | 3 |
| 3258.0919997 | 3 |
| 3258.0924999 | 4 |
| 3258.0925654 | 3 |
| 3258.092717 | 1 |
| 3258.092827 | 2 |
| 3258.0931036 | 4 |
| 3258.0937141 | 3 |
| 3258.0940713 | 7 |
| 3258.094331 | 4 |
| 3258.0945047 | 3 |
| 3258.0951677 | 4 |
| 3258.0953198 | 7 |
| 3258.0956646 | 10 |
| 3258.0956706 | 5 |
| 3258.0960352 | 6 |
| 3258.0966416 | 5 |
| 3258.0966664 | 2 |
| 3258.0968271 | 6 |
| 3258.0974063 | 6 |
| 3258.0977134 | 3 |
| 3258.0979289 | 9 |
| 3258.0980648 | 5 |
| 3258.098492 | 4 |
| 3258.0986139 | 7 |
| 3258.0990514 | 5 |
| 3258.0993251 | 9 |
| 3258.0999822 | 5 |
| 3258.100654 | 5 |
| 3258.1008154 | 5 |
| 3258.1016691 | 5 |
| 3258.102321 | 3 |
| 3258.1030058 | 3 |
| 3258.1032254 | 5 |
| 3258.10434 | 2 |
| 3258.1051261 | 5 |
| 3258.1052143 | 1 |
| 3258.1056425 | 3 |
| 3258.1057068 | 4 |
| 3258.1065254 | 1 |
| 3258.1071437 | 4 |
| 3258.1082238 | 1 |
| 3258.1092794 | 0 |
| 3258.1097229 | 0 |
| 3258.1103492 | 3 |
| 3258.1110655 | 2 |
| 3258.1113894 | 5 |
| 3258.1120863 | 3 |
| 3258.1126373 | 4 |
| 3258.1133095 | 4 |
| 3258.113531 | 3 |
| 3258.1142692 | 3 |
| 3258.1150095 | 4 |
| 3258.1158193 | 4 |
| 3258.1162143 | 1 |
| 3258.1172062 | 2 |
| 3258.1172071 | 4 |
| 3258.1183724 | 1 |
| 3258.1186316 | 3 |
| 3258.1187989 | 3 |
| 3258.1201173 | 2 |
| 3258.120283 | 0 |
| 3258.1209904 | 1 |
| 3258.1214703 | 5 |
| 3258.1226076 | 8 |
| 3258.1236186 | 2 |
| 3258.1238945 | 3 |
| 3258.1247164 | 7 |
| 3258.1251535 | 6 |
| 3258.1255231 | 12 |
| 3258.1260343 | 9 |
| 3258.127025 | 9 |
| 3258.1276933 | 7 |
| 3258.1280414 | 15 |
| 3258.1291387 | 9 |
| 3258.1298691 | 13 |
| 3258.1301798 | 13 |
| 3258.1308887 | 16 |
| 3258.1309915 | 6 |
| 3258.1317866 | 10 |
| 3258.1325057 | 4 |
| 3258.1330395 | 7 |
| 3258.1338201 | 4 |
| 3258.1342797 | 3 |
| 3258.1352624 | 2 |
| 3258.136284 | 4 |
| 3258.1362997 | 3 |
| 3258.1373072 | 2 |
| 3258.1375607 | 0 |
| 3258.1383682 | 1 |
| 3258.1389597 | 3 |
| 3258.1393595 | 1 |
| 3258.1400202 | 1 |
| 3258.1408446 | 3 |
| 3258.1412739 | 5 |
| 3258.1420384 | 5 |
| 3258.143026 | 3 |
| 3258.143502 | 2 |
| 3258.1439667 | 1 |
| 3258.1449236 | 1 |
| 3258.1457029 | 0 |
| 3258.1458789 | 0 |
| 3258.1465766 | 0 |
| 3258.1470761 | 2 |
| 3258.1478027 | 1 |
| 3258.1482343 | 3 |
| 3258.149737 | 0 |
| 3258.1505367 | 1 |
| 3258.1513456 | 3 |
| 3258.1522447 | 1 |
| 3258.1526201 | 5 |
| 3258.1544012 | 2 |
| 3258.1551554 | 2 |
| 3258.1556257 | 1 |
| 3258.1564138 | 3 |
| 3258.1576413 | 1 |
| 3258.1586142 | 5 |
| 3258.1593113 | 2 |
| 3258.1597806 | 2 |
| 3258.1612641 | 2 |
| 3258.1628503 | 2 |
| 3258.1632606 | 3 |
| 3258.164033 | 1 |
| 3258.1650565 | 2 |
| 3258.1665539 | 3 |
| 3258.1668094 | 3 |
| 3258.1680724 | 3 |
| 3258.1689952 | 1 |
| 3258.1692772 | 2 |
| 3258.1707017 | 3 |
| 3258.171829 | 2 |
| 3258.1728156 | 1 |
| 3258.1739315 | 1 |
| 3258.1747632 | 0 |
| 3258.1755843 | 1 |
| 3258.1758996 | 3 |
| 3258.1774231 | 6 |
| 3258.1782334 | 4 |
| 3258.1791694 | 3 |
| 3258.1805289 | 1 |
| 3258.180761 | 1 |
| 3258.1817895 | 1 |
| 3258.1830698 | 0 |
| 3258.1837794 | 1 |
| 3258.1846209 | 1 |
| 3258.1858626 | 1 |
| 3258.1864091 | 1 |
| 3258.1882589 | 3 |
| 3258.1890029 | 4 |
| 3258.1894891 | 1 |
| 3258.190772 | 0 |
| 3258.1913369 | 2 |
| 3258.1923939 | 2 |
| 3258.1934007 | 3 |
| 3258.1941593 | 1 |
| 3258.1951491 | 2 |
| 3258.1959372 | 7 |
| 3258.1974602 | 3 |
| 3258.1976899 | 2 |
| 3258.1982055 | 3 |
| 3258.200151 | 2 |
| 3258.20057 | 4 |
| 3258.2017916 | 0 |
| 3258.2020013 | 2 |
| 3258.204433 | 3 |
| 3258.2045175 | 0 |
| 3258.2053366 | 3 |
| 3258.2063409 | 0 |
| 3258.2072513 | 2 |
| 3258.2080832 | 0 |
| 3258.208836 | 3 |
| 3258.2101759 | 0 |
| 3258.2114631 | 0 |
| 3258.2115796 | 4 |
| 3258.2126807 | 1 |
| 3258.2138679 | 0 |
| 3258.214521 | 0 |
| 3258.2159202 | 0 |
| 3258.2165768 | 6 |
| 3258.2176489 | 2 |
| 3258.2183805 | 2 |
| 3258.2196427 | 0 |
| 3258.2206818 | 2 |
| 3258.2216081 | 1 |
| 3258.2223679 | 2 |
| 3258.2233518 | 1 |
| 3258.223631 | 3 |
| 3258.2252525 | 2 |
| 3258.225628 | 2 |
| 3258.2270639 | 2 |
| 3258.2281219 | 6 |
| 3258.2287356 | 4 |
| 3258.2302873 | 0 |
| 3258.2306505 | 1 |
| 3258.2321448 | 3 |
| 3258.2335856 | 2 |
| 3258.2336528 | 3 |
| 3258.2350045 | 1 |
| 3258.2350523 | 3 |
| 3258.2361918 | 0 |
| 3258.2373015 | 0 |
| 3258.2385076 | 1 |
| 3258.2392746 | 3 |
| 3258.2400789 | 2 |
| 3258.2403486 | 1 |
| 3258.2416151 | 4 |
| 3258.2427244 | 2 |
| 3258.2438204 | 2 |
| 3258.2447703 | 0 |
| 3258.2464197 | 5 |
| 3258.246636 | 3 |
| 3258.2481487 | 1 |
| 3258.2487893 | 2 |
| 3258.2491181 | 1 |
| 3258.2506474 | 1 |
| 3258.2507264 | 5 |
| 3258.2513487 | 5 |
| 3258.2529421 | 4 |
| 3258.2537594 | 2 |
| 3258.2549604 | 1 |
| 3258.2560147 | 1 |
| 3258.2576804 | 4 |
| 3258.2577192 | 2 |
| 3258.259128 | 2 |
| 3258.2593819 | 1 |
| 3258.2599708 | 2 |
| 3258.2609975 | 1 |
| 3258.2624999 | 2 |
| 3258.2635199 | 2 |
| 3258.264613 | 3 |
| 3258.2650041 | 1 |
| 3258.2660777 | 2 |
| 3258.2669437 | 2 |
| 3258.2681831 | 6 |
| 3258.26914 | 1 |
| 3258.2702583 | 4 |
| 3258.2710119 | 1 |
| 3258.2716809 | 3 |
| 3258.2724581 | 1 |
| 3258.2737725 | 4 |
| 3258.2743568 | 1 |
| 3258.2762166 | 4 |
| 3258.2767599 | 5 |
| 3258.2777317 | 5 |
| 3258.2782622 | 4 |
| 3258.2786305 | 0 |
| 3258.2786315 | 3 |
| 3258.2787252 | 5 |
| 3258.2793849 | 2 |
| 3258.2796603 | 0 |
| 3258.2800539 | 0 |
| 3258.2805519 | 5 |
| 3258.280574 | 1 |
| 3258.2807824 | 1 |
| 3258.2813292 | 4 |
| 3258.281602 | 2 |
| 3258.2816642 | 5 |
| 3258.2819625 | 2 |
| 3258.282034 | 4 |
| 3258.282669 | 1 |
| 3258.2828823 | 1 |
| 3258.2829988 | 4 |
| 3258.2834943 | 4 |
| 3258.2835201 | 2 |
| 3258.2844936 | 0 |
| 3258.2846894 | 4 |
| 3258.2846965 | 4 |
| 3258.2851855 | 4 |
| 3258.2854141 | 2 |
| 3258.2859371 | 1 |
| 3258.2860314 | 6 |
| 3258.2863406 | 6 |
| 3258.2866153 | 8 |
| 3258.2871997 | 16 |
| 3258.2872654 | 9 |
| 3258.2880261 | 14 |
| 3258.2880982 | 8 |
| 3258.2881428 | 23 |
| 3258.2884473 | 18 |
| 3258.2885607 | 19 |
| 3258.2891839 | 26 |
| 3258.2892442 | 22 |
| 3258.2896341 | 24 |
| 3258.2901783 | 22 |
| 3258.2902925 | 23 |
| 3258.2908274 | 25 |
| 3258.2909564 | 23 |
| 3258.2910954 | 23 |
| 3258.2911696 | 22 |
| 3258.2922894 | 20 |
| 3258.2923647 | 30 |
| 3258.2924909 | 10 |
| 3258.2925826 | 24 |
| 3258.2926121 | 16 |
| 3258.2932904 | 22 |
| 3258.2938106 | 17 |
| 3258.2940502 | 21 |
| 3258.2944118 | 15 |
| 3258.2946602 | 13 |
| 3258.2948446 | 14 |
| 3258.2955152 | 15 |
| 3258.2955471 | 6 |
| 3258.2958452 | 5 |
| 3258.2960164 | 8 |
| 3258.2966873 | 2 |
| 3258.2969548 | 2 |

| | |
|---|---|
| 3258.2971865 | 1 |
| 3258.2972269 | 3 |
| 3258.2975187 | 3 |
| 3258.2979188 | 2 |
| 3258.2982071 | 4 |
| 3258.2985465 | 2 |
| 3258.298701 | 2 |
| 3258.2992431 | 1 |
| 3258.2994606 | 4 |
| 3258.3000224 | 3 |
| 3258.3004294 | 5 |
| 3258.3006699 | 2 |
| 3258.3010871 | 0 |
| 3258.3014986 | 2 |
| 3258.3015492 | 2 |
| 3258.30188 | 4 |
| 3258.302186 | 3 |
| 3258.3028507 | 2 |
| 3258.3028723 | 0 |
| 3258.3029361 | 1 |
| 3258.303809 | 1 |
| 3258.3038821 | 4 |
| 3258.3045118 | 3 |
| 3258.3046661 | 0 |
| 3258.3049328 | 3 |
| 3258.3050381 | 1 |
| 3258.3050858 | 3 |
| 3258.3052494 | 3 |
| 3258.3056893 | 5 |
| 3258.3064761 | 4 |
| 3258.3067548 | 3 |
| 3258.3070758 | 5 |
| 3258.3071138 | 2 |
| 3258.3072923 | 0 |
| 3258.3076776 | 1 |
| 3258.3082163 | 3 |
| 3258.3087374 | 1 |
| 3258.3087802 | 2 |
| 3258.3092211 | 0 |
| 3258.3092824 | 3 |
| 3258.3096861 | 3 |
| 3258.3098484 | 3 |
| 3258.3102117 | 0 |
| 3258.3110052 | 0 |
| 3258.311122 | 1 |
| 3258.3113533 | 1 |
| 3258.3113856 | 3 |
| 3258.3116822 | 6 |
| 3258.3123107 | 2 |
| 3258.3125804 | 0 |
| 3258.3129838 | 1 |
| 3258.3133271 | 3 |
| 3258.3135384 | 4 |
| 3258.3139256 | 1 |
| 3258.3140862 | 4 |
| 3258.3142056 | 3 |
| 3258.3148451 | 2 |
| 3258.3153989 | 0 |
| 3258.3158893 | 0 |
| 3258.3162542 | 3 |
| 3258.3163616 | 0 |
| 3258.3167562 | 0 |
| 3258.3168989 | 1 |
| 3258.3172882 | 4 |
| 3258.3174984 | 0 |
| 3258.3175462 | 0 |
| 3258.3180003 | 1 |
| 3258.3181478 | 3 |
| 3258.318632 | 0 |
| 3258.3187918 | 2 |
| 3258.319125 | 3 |
| 3258.3192219 | 2 |
| 3258.3194938 | 0 |
| 3258.32024 | 2 |
| 3258.3204786 | 3 |
| 3258.3210457 | 2 |
| 3258.321422 | 1 |
| 3258.3216663 | 1 |
| 3258.3218026 | 2 |
| 3258.3220181 | 3 |
| 3258.3234632 | 2 |
| 3258.3239698 | 1 |
| 3258.3249273 | 3 |
| 3258.3262598 | 1 |
| 3258.3271211 | 1 |
| 3258.328263 | 3 |
| 3258.3285617 | 6 |
| 3258.3303486 | 1 |
| 3258.3305478 | 5 |
| 3258.3314233 | 3 |
| 3258.3325756 | 2 |
| 3258.3338243 | 0 |
| 3258.3345957 | 2 |
| 3258.335868 | 0 |
| 3258.3361023 | 0 |
| 3258.3376118 | 1 |
| 3258.3381165 | 0 |
| 3258.3392697 | 2 |
| 3258.3408747 | 0 |
| 3258.3409656 | 2 |
| 3258.342116 | 3 |
| 3258.3425784 | 5 |
| 3258.3437483 | 1 |
| 3258.3446687 | 1 |
| 3258.3456295 | 0 |
| 3258.3469597 | 1 |
| 3258.3469774 | 0 |
| 3258.3487436 | 1 |
| 3258.3496722 | 2 |
| 3258.3506296 | 1 |
| 3258.351898 | 2 |
| 3258.352036 | 1 |
| 3258.3532066 | 4 |
| 3258.3536923 | 2 |
| 3258.3548482 | 1 |
| 3258.3563226 | 2 |
| 3258.3563403 | 1 |
| 3258.3575975 | 3 |
| 3258.35872 | 3 |
| 3258.3597421 | 0 |
| 3258.3607836 | 1 |
| 3258.3618188 | 1 |
| 3258.3628989 | 2 |
| 3258.3632102 | 2 |
| 3258.3648773 | 3 |
| 3258.3657181 | 1 |
| 3258.3664787 | 3 |
| 3258.3673142 | 6 |
| 3258.3682493 | 6 |
| 3258.3698813 | 5 |
| 3258.3704782 | 5 |
| 3258.3707667 | 5 |
| 3258.3720567 | 4 |
| 3258.3723276 | 7 |
| 3258.373687 | 8 |
| 3258.3752016 | 2 |
| 3258.3759556 | 2 |
| 3258.3771552 | 4 |
| 3258.3773155 | 0 |
| 3258.3784025 | 4 |
| 3258.3794666 | 3 |
| 3258.380097 | 1 |
| 3258.3815714 | 2 |
| 3258.3817001 | 4 |
| 3258.3822126 | 1 |
| 3258.3843965 | 5 |
| 3258.3845707 | 3 |
| 3258.3860916 | 2 |
| 3258.3866266 | 1 |
| 3258.3880212 | 3 |
| 3258.3888638 | 2 |
| 3258.3899657 | 5 |
| 3258.3908036 | 2 |
| 3258.391132 | 1 |
| 3258.3927072 | 2 |
| 3258.3936225 | 1 |
| 3258.3941569 | 2 |
| 3258.3951813 | 3 |
| 3258.3960241 | 6 |
| 3258.3972694 | 3 |
| 3258.3984106 | 5 |
| 3258.3985386 | 4 |
| 3258.399663 | 2 |
| 3258.4011233 | 2 |
| 3258.4020874 | 3 |
| 3258.4027711 | 0 |
| 3258.4037299 | 0 |
| 3258.4050549 | 2 |
| 3258.40534 | 1 |
| 3258.4068863 | 2 |
| 3258.4072547 | 1 |
| 3258.4085472 | 0 |
| 3258.4093301 | 3 |
| 3258.4102035 | 4 |
| 3258.4112198 | 2 |
| 3258.4119435 | 0 |
| 3258.4136915 | 1 |
| 3258.4140058 | 3 |
| 3258.414766 | 4 |
| 3258.4158877 | 1 |
| 3258.4170002 | 6 |
| 3258.4175428 | 3 |
| 3258.4189072 | 2 |
| 3258.4190468 | 3 |
| 3258.4210672 | 3 |
| 3258.4216409 | 1 |
| 3258.4223216 | 3 |
| 3258.4235766 | 1 |
| 3258.4236506 | 1 |
| 3258.4252474 | 1 |
| 3258.4261575 | 1 |
| 3258.4267028 | 5 |
| 3258.4276989 | 2 |
| 3258.4288012 | 1 |
| 3258.4301025 | 4 |
| 3258.4311209 | 2 |
| 3258.4311489 | 3 |
| 3258.4327706 | 3 |
| 3258.4332201 | 1 |
| 3258.4347654 | 2 |
| 3258.4352243 | 2 |
| 3258.4359888 | 0 |
| 3258.4370663 | 1 |
| 3258.4373301 | 2 |
| 3258.4385745 | 1 |
| 3258.4397927 | 3 |
| 3258.4405201 | 2 |
| 3258.4416843 | 2 |
| 3258.4421717 | 1 |
| 3258.4440017 | 2 |
| 3258.4449513 | 0 |
| 3258.4454143 | 2 |
| 3258.4460698 | 5 |
| 3258.4475328 | 2 |
| 3258.448679 | 1 |
| 3258.4487266 | 3 |
| 3258.4501189 | 2 |
| 3258.4511701 | 5 |
| 3258.4518649 | 3 |
| 3258.4531139 | 4 |
| 3258.4535011 | 3 |
| 3258.4549328 | 1 |
| 3258.4562027 | 1 |
| 3258.4562629 | 3 |
| 3258.4571921 | 1 |
| 3258.4585858 | 1 |
| 3258.459128 | 3 |
| 3258.460338 | 2 |
| 3258.4614594 | 3 |
| 3258.4623015 | 1 |
| 3258.4632775 | 4 |
| 3258.4644577 | 2 |
| 3258.4655081 | 2 |
| 3258.4662719 | 0 |
| 3258.4669718 | 5 |
| 3258.4678655 | 2 |
| 3258.4691505 | 1 |
| 3258.4697891 | 1 |
| 3258.4708932 | 3 |
| 3258.4717031 | 1 |
| 3258.4717468 | 3 |
| 3258.471962 | 1 |
| 3258.47203 | 2 |
| 3258.4725857 | 2 |
| 3258.473532 | 0 |
| 3258.4735486 | 4 |
| 3258.473767 | 1 |
| 3258.4743054 | 4 |
| 3258.4743928 | 3 |
| 3258.4745975 | 1 |
| 3258.4746777 | 1 |
| 3258.475464 | 3 |
| 3258.4756193 | 3 |
| 3258.4756424 | 4 |
| 3258.4758672 | 3 |
| 3258.4763821 | 1 |
| 3258.4764555 | 0 |
| 3258.4767109 | 0 |
| 3258.4777219 | 1 |
| 3258.478032 | 3 |
| 3258.4780412 | 3 |
| 3258.4784199 | 0 |
| 3258.4784955 | 2 |
| 3258.4786702 | 3 |
| 3258.4793363 | 3 |
| 3258.4797751 | 2 |
| 3258.4798727 | 1 |
| 3258.4800395 | 4 |
| 3258.4804644 | 0 |
| 3258.4807186 | 4 |
| 3258.4808246 | 3 |
| 3258.4808859 | 0 |
| 3258.4812956 | 0 |
| 3258.4816377 | 1 |
| 3258.4818904 | 0 |
| 3258.4824242 | 2 |
| 3258.4832121 | 0 |
| 3258.4834082 | 2 |
| 3258.4836701 | 1 |
| 3258.4838377 | 2 |
| 3258.4841806 | 1 |
| 3258.4842736 | 1 |
| 3258.4849487 | 2 |
| 3258.485364 | 2 |
| 3258.4855949 | 1 |
| 3258.4857228 | 2 |
| 3258.4857811 | 5 |
| 3258.4859004 | 4 |
| 3258.4868143 | 1 |
| 3258.4868658 | 1 |
| 3258.4868953 | 3 |
| 3258.487313 | 0 |
| 3258.4875877 | 4 |
| 3258.4882963 | 2 |
| 3258.4890368 | 1 |
| 3258.4890619 | 2 |
| 3258.4892331 | 0 |
| 3258.4892723 | 3 |
| 3258.4896889 | 1 |
| 3258.4900717 | 3 |
| 3258.4904395 | 7 |
| 3258.490684 | 3 |
| 3258.4910081 | 1 |
| 3258.4916305 | 7 |
| 3258.4917181 | 6 |
| 3258.4919699 | 10 |
| 3258.4922062 | 11 |
| 3258.4927332 | 8 |
| 3258.4928739 | 18 |
| 3258.4933976 | 17 |
| 3258.4935489 | 19 |
| 3258.4935524 | 17 |
| 3258.4942843 | 14 |
| 3258.494376 | 23 |
| 3258.4946965 | 23 |
| 3258.4949666 | 24 |
| 3258.495064 | 13 |
| 3258.4958351 | 18 |
| 3258.4960726 | 15 |
| 3258.4964347 | 13 |
| 3258.4966332 | 17 |
| 3258.4969201 | 9 |
| 3258.4969851 | 17 |
| 3258.4973267 | 11 |
| 3258.498252 | 6 |
| 3258.4984905 | 2 |
| 3258.498779 | 6 |
| 3258.4987857 | 4 |
| 3258.4989516 | 1 |
| 3258.4993879 | 3 |
| 3258.4998854 | 5 |
| 3258.5003389 | 0 |
| 3258.5005207 | 2 |
| 3258.5014771 | 1 |
| 3258.501479 | 3 |
| 3258.5015431 | 3 |
| 3258.5015738 | 3 |
| 3258.5016911 | 2 |
| 3258.5016916 | 3 |
| 3258.50237 | 2 |
| 3258.5028432 | 6 |
| 3258.5036011 | 0 |
| 3258.5037914 | 4 |
| 3258.5038376 | 3 |
| 3258.5045796 | 1 |
| 3258.5048443 | 4 |
| 3258.5060992 | 3 |
| 3258.5064708 | 1 |
| 3258.5073591 | 1 |
| 3258.508386 | 2 |
| 3258.5093328 | 1 |
| 3258.5101695 | 1 |
| 3258.5117561 | 0 |
| 3258.5118754 | 2 |
| 3258.5130252 | 1 |
| 3258.5139492 | 3 |
| 3258.5152695 | 0 |
| 3258.5155647 | 1 |
| 3258.5168842 | 1 |
| 3258.5168922 | 3 |
| 3258.5185484 | 0 |
| 3258.5198901 | 2 |
| 3258.520593 | 1 |

| | |
|---|---|
| 3258.5213663 | 1 |
| 3258.5220111 | 4 |
| 3258.5236502 | 3 |
| 3258.5238908 | 0 |
| 3258.5250236 | 2 |
| 3258.5258358 | 0 |
| 3258.5266811 | 2 |
| 3258.5277564 | 2 |
| 3258.5279277 | 0 |
| 3258.5296154 | 2 |
| 3258.5306625 | 2 |
| 3258.5311131 | 3 |
| 3258.5320202 | 1 |
| 3258.5331996 | 2 |
| 3258.5337621 | 0 |
| 3258.5337872 | 1 |
| 3258.5337922 | 3 |
| 3258.5342027 | 3 |
| 3258.5347119 | 0 |
| 3258.5350039 | 1 |
| 3258.5356273 | 0 |
| 3258.5359482 | 5 |
| 3258.5362351 | 2 |
| 3258.5363236 | 3 |
| 3258.537303 | 0 |
| 3258.5373433 | 4 |
| 3258.5374396 | 4 |
| 3258.5378125 | 5 |
| 3258.5382891 | 1 |
| 3258.538548 | 0 |
| 3258.5386935 | 0 |
| 3258.539022 | 2 |
| 3258.5390816 | 3 |
| 3258.5395603 | 1 |
| 3258.5401607 | 2 |
| 3258.540393 | 1 |
| 3258.5406294 | 2 |
| 3258.5406608 | 2 |
| 3258.5409767 | 3 |
| 3258.5415497 | 3 |
| 3258.5416693 | 2 |
| 3258.5418943 | 1 |
| 3258.5421315 | 2 |
| 3258.542429 | 4 |
| 3258.542965 | 2 |
| 3258.5434642 | 7 |
| 3258.5436489 | 3 |
| 3258.5436554 | 0 |
| 3258.5440803 | 3 |
| 3258.5446424 | 5 |
| 3258.544985 | 6 |
| 3258.5452834 | 1 |
| 3258.545703 | 1 |
| 3258.5460235 | 4 |
| 3258.5463949 | 1 |
| 3258.5466307 | 3 |
| 3258.5468129 | 1 |
| 3258.5473199 | 0 |
| 3258.5473285 | 3 |
| 3258.5481186 | 1 |
| 3258.5484116 | 1 |
| 3258.5484217 | 3 |
| 3258.5485196 | 3 |
| 3258.5486943 | 1 |
| 3258.5493437 | 3 |
| 3258.5495981 | 1 |
| 3258.5497081 | 1 |
| 3258.550137 | 0 |
| 3258.5506186 | 2 |
| 3258.5507587 | 7 |
| 3258.5512847 | 2 |
| 3258.5515845 | 1 |
| 3258.5516915 | 3 |
| 3258.5518195 | 1 |
| 3258.5525359 | 3 |
| 3258.5530544 | 5 |
| 3258.5530759 | 8 |
| 3258.5534121 | 8 |
| 3258.5534989 | 3 |
| 3258.5537845 | 3 |
| 3258.5542709 | 11 |
| 3258.5543695 | 13 |
| 3258.554781 | 5 |
| 3258.5552345 | 12 |
| 3258.5556406 | 8 |
| 3258.5561703 | 6 |
| 3258.5562171 | 3 |
| 3258.5564584 | 4 |
| 3258.5565328 | 4 |
| 3258.5573694 | 7 |
| 3258.5573813 | 6 |
| 3258.5577758 | 8 |
| 3258.5580333 | 4 |
| 3258.5582923 | 9 |
| 3258.5588454 | 5 |
| 3258.5588462 | 9 |
| 3258.5594447 | 6 |
| 3258.559495 | 8 |
| 3258.559715 | 2 |
| 3258.559876 | 14 |
| 3258.5608655 | 11 |
| 3258.5610036 | 5 |
| 3258.5612989 | 9 |
| 3258.5613929 | 4 |
| 3258.5615369 | 5 |
| 3258.5621514 | 3 |
| 3258.5621776 | 5 |
| 3258.5622984 | 8 |
| 3258.5628152 | 5 |
| 3258.5634482 | 1 |
| 3258.5637351 | 4 |
| 3258.5638928 | 1 |
| 3258.5639865 | 2 |
| 3258.5640047 | 1 |
| 3258.5640203 | 5 |
| 3258.5644871 | 1 |
| 3258.5645172 | 4 |
| 3258.5645206 | 1 |
| 3258.5646849 | 3 |
| 3258.564794 | 1 |
| 3258.5649014 | 0 |
| 3258.5649578 | 3 |
| 3258.5649744 | 2 |
| 3258.565629 | 3 |
| 3258.5658554 | 3 |
| 3258.5663027 | 3 |
| 3258.5663856 | 0 |
| 3258.5665751 | 6 |
| 3258.5669509 | 3 |
| 3258.5670047 | 1 |
| 3258.5671668 | 0 |
| 3258.5671905 | 2 |
| 3258.5673552 | 3 |
| 3258.5681291 | 1 |
| 3258.5684276 | 0 |
| 3258.5685872 | 0 |
| 3258.5688237 | 0 |
| 3258.5689474 | 2 |
| 3258.5690017 | 2 |
| 3258.5690051 | 3 |
| 3258.5697717 | 1 |
| 3258.5699448 | 3 |
| 3258.5703601 | 6 |
| 3258.5707869 | 2 |
| 3258.5710464 | 2 |
| 3258.571121 | 0 |
| 3258.5714467 | 4 |
| 3258.5714612 | 1 |
| 3258.5715271 | 2 |
| 3258.5719236 | 2 |
| 3258.5722935 | 2 |
| 3258.5725615 | 5 |
| 3258.5730605 | 2 |
| 3258.5730616 | 2 |
| 3258.5733088 | 2 |
| 3258.5737533 | 3 |
| 3258.5738132 | 0 |
| 3258.5740182 | 1 |
| 3258.5741146 | 2 |
| 3258.5743596 | 4 |
| 3258.5744332 | 2 |
| 3258.5745575 | 1 |
| 3258.5747106 | 2 |
| 3258.575116 | 1 |
| 3258.5753017 | 3 |
| 3258.5757115 | 3 |
| 3258.5760286 | 1 |
| 3258.5760831 | 0 |
| 3258.5761871 | 1 |
| 3258.5765794 | 2 |
| 3258.5772018 | 4 |
| 3258.5775826 | 1 |
| 3258.5775966 | 2 |
| 3258.5776904 | 0 |
| 3258.5778164 | 1 |
| 3258.5778241 | 1 |
| 3258.5781087 | 2 |
| 3258.5786391 | 2 |
| 3258.5788522 | 0 |
| 3258.5790438 | 4 |
| 3258.5792697 | 0 |
| 3258.5794393 | 3 |
| 3258.5798047 | 3 |
| 3258.5800114 | 1 |
| 3258.5800406 | 2 |
| 3258.5801705 | 1 |
| 3258.5805669 | 3 |
| 3258.5811931 | 0 |
| 3258.581279 | 5 |
| 3258.5814489 | 3 |
| 3258.5814783 | 1 |
| 3258.5818315 | 2 |
| 3258.5821475 | 1 |
| 3258.5824829 | 2 |
| 3258.5826001 | 2 |
| 3258.5831544 | 3 |
| 3258.5833379 | 1 |
| 3258.5836288 | 1 |
| 3258.5837782 | 1 |
| 3258.583826 | 3 |
| 3258.583929 | 0 |
| 3258.5839375 | 1 |
| 3258.5839917 | 1 |
| 3258.5844512 | 3 |
| 3258.5850093 | 5 |
| 3258.5852396 | 2 |
| 3258.5852628 | 2 |
| 3258.5854201 | 5 |
| 3258.5858788 | 3 |
| 3258.5859859 | 4 |
| 3258.5863126 | 7 |
| 3258.5864435 | 7 |
| 3258.5870498 | 16 |
| 3258.5871719 | 19 |
| 3258.5873347 | 13 |
| 3258.587389 | 30 |
| 3258.5876418 | 19 |
| 3258.5880771 | 42 |
| 3258.5881538 | 37 |
| 3258.5883914 | 29 |
| 3258.5888519 | 40 |
| 3258.5895856 | 30 |
| 3258.5896 | 55 |
| 3258.5896299 | 57 |
| 3258.5898081 | 54 |
| 3258.5901358 | 54 |
| 3258.5903491 | 63 |
| 3258.5904261 | 56 |
| 3258.5904912 | 51 |
| 3258.5912206 | 47 |
| 3258.5912637 | 45 |
| 3258.5913344 | 52 |
| 3258.5913715 | 51 |
| 3258.5916298 | 42 |
| 3258.5923415 | 37 |
| 3258.5923823 | 56 |
| 3258.5927293 | 59 |
| 3258.5928524 | 32 |
| 3258.5931924 | 42 |
| 3258.5933682 | 51 |
| 3258.5933816 | 43 |
| 3258.5935672 | 38 |
| 3258.5938746 | 40 |
| 3258.5940692 | 33 |
| 3258.5942403 | 32 |
| 3258.5944906 | 24 |
| 3258.5946994 | 30 |
| 3258.5953428 | 26 |
| 3258.595412 | 29 |
| 3258.5957482 | 26 |
| 3258.595823 | 23 |
| 3258.5958816 | 12 |
| 3258.5966446 | 13 |
| 3258.5967109 | 11 |
| 3258.596766 | 5 |
| 3258.5970327 | 6 |
| 3258.5971485 | 5 |
| 3258.5971664 | 2 |
| 3258.5971706 | 4 |
| 3258.597548 | 6 |
| 3258.5976306 | 3 |
| 3258.5981967 | 1 |
| 3258.5982208 | 2 |
| 3258.5982359 | 0 |
| 3258.5985433 | 2 |
| 3258.5986594 | 3 |
| 3258.5986925 | 4 |
| 3258.5987351 | 3 |
| 3258.5988221 | 3 |
| 3258.5990901 | 2 |
| 3258.5993227 | 0 |
| 3258.599393 | 6 |
| 3258.5996514 | 1 |
| 3258.6000798 | 1 |
| 3258.6003514 | 2 |
| 3258.6005931 | 4 |
| 3258.6005967 | 2 |
| 3258.6008156 | 1 |
| 3258.6009041 | 0 |
| 3258.6009891 | 3 |
| 3258.6010924 | 3 |
| 3258.6011382 | 0 |
| 3258.6011385 | 2 |
| 3258.6013454 | 2 |
| 3258.601845 | 4 |
| 3258.6022079 | 2 |
| 3258.6023006 | 0 |
| 3258.6023715 | 1 |
| 3258.6024101 | 0 |
| 3258.6025097 | 1 |
| 3258.602707 | 2 |
| 3258.6030456 | 3 |
| 3258.6031291 | 1 |
| 3258.6032414 | 4 |
| 3258.6035955 | 3 |
| 3258.6038685 | 1 |
| 3258.6047928 | 4 |
| 3258.6050378 | 2 |
| 3258.605325 | 2 |
| 3258.6055405 | 4 |
| 3258.6055754 | 1 |
| 3258.6064052 | 2 |
| 3258.6070208 | 0 |
| 3258.6073369 | 1 |
| 3258.6075373 | 3 |
| 3258.607686 | 4 |
| 3258.6078975 | 3 |
| 3258.6081851 | 1 |
| 3258.60872 | 2 |
| 3258.6092101 | 3 |
| 3258.6103997 | 2 |
| 3258.6104092 | 3 |
| 3258.6104472 | 1 |
| 3258.6106432 | 3 |
| 3258.6114436 | 4 |
| 3258.6114457 | 1 |
| 3258.6121453 | 2 |
| 3258.6123389 | 1 |
| 3258.6124974 | 4 |
| 3258.6125379 | 6 |
| 3258.6127789 | 3 |
| 3258.6133781 | 0 |
| 3258.6139476 | 0 |
| 3258.6144516 | 3 |
| 3258.6144556 | 1 |
| 3258.6146381 | 2 |
| 3258.6150934 | 5 |
| 3258.615321 | 1 |
| 3258.6159825 | 4 |
| 3258.6167832 | 2 |
| 3258.6176308 | 1 |
| 3258.6177241 | 5 |
| 3258.6188029 | 2 |
| 3258.6191043 | 0 |
| 3258.6193128 | 2 |
| 3258.6205035 | 1 |
| 3258.6211607 | 3 |
| 3258.6221667 | 3 |
| 3258.6222817 | 3 |
| 3258.622724 | 1 |
| 3258.6233239 | 0 |
| 3258.6245644 | 2 |
| 3258.6246342 | 3 |
| 3258.6258292 | 1 |
| 3258.6260858 | 3 |
| 3258.626516 | 0 |
| 3258.6276107 | 5 |
| 3258.6286547 | 2 |
| 3258.6286979 | 3 |
| 3258.6292895 | 1 |
| 3258.6304004 | 2 |
| 3258.6305206 | 1 |
| 3258.6311535 | 2 |
| 3258.6320126 | 4 |
| 3258.6328833 | 4 |
| 3258.6328948 | 3 |
| 3258.6333438 | 6 |
| 3258.6344259 | 1 |
| 3258.634527 | 4 |
| 3258.6357795 | 1 |
| 3258.6359865 | 4 |
| 3258.6368627 | 2 |
| 3258.6378181 | 2 |

| | |
|---|---|
| 3258.6380036 | 2 |
| 3258.6390265 | 3 |
| 3258.6392996 | 3 |
| 3258.6396163 | 1 |
| 3258.6411587 | 2 |
| 3258.6413732 | 6 |
| 3258.6422043 | 2 |
| 3258.6423577 | 0 |
| 3258.6433457 | 1 |
| 3258.6436272 | 1 |
| 3258.6440031 | 4 |
| 3258.6447509 | 1 |
| 3258.6450854 | 2 |
| 3258.6460467 | 4 |
| 3258.6462781 | 0 |
| 3258.6472714 | 2 |
| 3258.6480223 | 2 |
| 3258.6486178 | 1 |
| 3258.6494392 | 0 |
| 3258.6498327 | 3 |
| 3258.6500571 | 2 |
| 3258.6512706 | 1 |
| 3258.6516469 | 3 |
| 3258.6524763 | 3 |
| 3258.6528601 | 2 |
| 3258.6537757 | 2 |
| 3258.6543346 | 4 |
| 3258.654405 | 1 |
| 3258.6554856 | 2 |
| 3258.6563649 | 3 |
| 3258.6572016 | 3 |
| 3258.6573007 | 0 |
| 3258.6577451 | 3 |
| 3258.6586091 | 1 |
| 3258.6587132 | 2 |
| 3258.6594004 | 2 |
| 3258.6603909 | 4 |
| 3258.6611145 | 0 |
| 3258.6614903 | 1 |
| 3258.6621716 | 2 |
| 3258.6627561 | 2 |
| 3258.663152 | 1 |
| 3258.6632295 | 1 |
| 3258.6640271 | 1 |
| 3258.6646613 | 2 |
| 3258.665474 | 2 |
| 3258.6665759 | 3 |
| 3258.6675801 | 2 |
| 3258.6679674 | 4 |
| 3258.6682921 | 0 |
| 3258.6689474 | 2 |
| 3258.6690483 | 1 |
| 3258.670152 | 1 |
| 3258.6705501 | 1 |
| 3258.6712279 | 4 |
| 3258.6722848 | 6 |
| 3258.672443 | 4 |
| 3258.6735965 | 1 |
| 3258.6737835 | 1 |
| 3258.6738031 | 1 |
| 3258.6750241 | 1 |
| 3258.6760464 | 2 |
| 3258.6763816 | 1 |
| 3258.6768136 | 6 |
| 3258.6774954 | 1 |
| 3258.6786664 | 2 |
| 3258.6788316 | 2 |
| 3258.6801159 | 1 |
| 3258.6804258 | 6 |
| 3258.680482 | 1 |
| 3258.6812578 | 3 |
| 3258.6818486 | 3 |
| 3258.6831645 | 1 |
| 3258.6835106 | 1 |
| 3258.6837779 | 1 |
| 3258.6849009 | 3 |
| 3258.6849797 | 1 |
| 3258.6854539 | 1 |
| 3258.6859931 | 2 |
| 3258.6866904 | 4 |
| 3258.687728 | 5 |
| 3258.6880623 | 5 |
| 3258.6888522 | 3 |
| 3258.6894838 | 0 |
| 3258.689729 | 1 |
| 3258.6909033 | 5 |
| 3258.6910565 | 0 |
| 3258.6921787 | 3 |
| 3258.6926888 | 2 |
| 3258.6931284 | 1 |
| 3258.693811 | 3 |
| 3258.6944904 | 0 |
| 3258.6954694 | 1 |
| 3258.6955329 | 6 |
| 3258.6965005 | 1 |
| 3258.6965322 | 4 |
| 3258.6973065 | 3 |
| 3258.6982381 | 2 |
| 3258.6988192 | 6 |
| 3258.6998196 | 2 |
| 3258.6999771 | 3 |
| 3258.7009273 | 1 |
| 3258.701085 | 4 |
| 3258.7014153 | 4 |
| 3258.7022272 | 3 |
| 3258.703225 | 3 |
| 3258.7033164 | 2 |
| 3258.7039509 | 3 |
| 3258.7049858 | 1 |
| 3258.7050846 | 3 |
| 3258.7061202 | 2 |
| 3258.7066793 | 3 |
| 3258.7075659 | 6 |
| 3258.7078399 | 5 |
| 3258.7083163 | 4 |
| 3258.709306 | 7 |
| 3258.70962 | 0 |
| 3258.7106277 | 4 |
| 3258.7108602 | 1 |
| 3258.7114522 | 0 |
| 3258.7125391 | 2 |
| 3258.7126791 | 3 |
| 3258.7131669 | 1 |
| 3258.7134336 | 3 |
| 3258.7136525 | 6 |
| 3258.7136682 | 1 |
| 3258.7140232 | 2 |
| 3258.7140396 | 0 |
| 3258.7146467 | 4 |
| 3258.7146525 | 4 |
| 3258.7151293 | 2 |
| 3258.7152163 | 2 |
| 3258.715241 | 2 |
| 3258.7152759 | 2 |
| 3258.7154222 | 2 |
| 3258.7157118 | 2 |
| 3258.715949 | 0 |
| 3258.71633 | 1 |
| 3258.7164538 | 1 |
| 3258.7165379 | 2 |
| 3258.7168655 | 1 |
| 3258.7170877 | 0 |
| 3258.7171544 | 1 |
| 3258.7173701 | 4 |
| 3258.7176508 | 3 |
| 3258.7176935 | 3 |
| 3258.7179813 | 2 |
| 3258.7183678 | 3 |
| 3258.7185306 | 4 |
| 3258.7188936 | 1 |
| 3258.7190822 | 1 |
| 3258.7191531 | 0 |
| 3258.7192527 | 2 |
| 3258.7196116 | 4 |
| 3258.7198248 | 4 |
| 3258.7200359 | 1 |
| 3258.7200559 | 4 |
| 3258.720065 | 5 |
| 3258.7206225 | 1 |
| 3258.7209082 | 1 |
| 3258.7210826 | 2 |
| 3258.7214479 | 4 |
| 3258.7217146 | 2 |
| 3258.7217721 | 1 |
| 3258.7221821 | 1 |
| 3258.722291 | 2 |
| 3258.722397 | 2 |
| 3258.7226396 | 4 |
| 3258.7227192 | 0 |
| 3258.7228453 | 2 |
| 3258.7229165 | 1 |
| 3258.7233651 | 4 |
| 3258.7234487 | 5 |
| 3258.7236599 | 3 |
| 3258.7241879 | 1 |
| 3258.7244141 | 3 |
| 3258.7245726 | 1 |
| 3258.7247696 | 4 |
| 3258.7248634 | 2 |
| 3258.7251921 | 1 |
| 3258.7252166 | 4 |
| 3258.7254903 | 3 |
| 3258.7255347 | 2 |
| 3258.72578 | 5 |
| 3258.7264251 | 2 |
| 3258.7264847 | 3 |
| 3258.7265743 | 2 |
| 3258.7266504 | 4 |
| 3258.7269903 | 3 |
| 3258.7272026 | 8 |
| 3258.7274433 | 3 |
| 3258.7275709 | 4 |
| 3258.7280589 | 0 |
| 3258.7282351 | 3 |
| 3258.7282663 | 2 |
| 3258.7288094 | 3 |
| 3258.72888 | 3 |
| 3258.7288983 | 4 |
| 3258.7293205 | 4 |
| 3258.7295 | 1 |
| 3258.729613 | 3 |
| 3258.7298732 | 3 |
| 3258.7301774 | 1 |
| 3258.7302333 | 1 |
| 3258.7304242 | 2 |
| 3258.7304595 | 4 |
| 3258.7305721 | 2 |
| 3258.7311233 | 3 |
| 3258.7312252 | 2 |
| 3258.7315144 | 6 |
| 3258.7316858 | 2 |
| 3258.7318418 | 2 |
| 3258.731985 | 0 |
| 3258.7322737 | 0 |
| 3258.7324656 | 1 |
| 3258.7324945 | 4 |
| 3258.7326613 | 2 |
| 3258.733053 | 2 |
| 3258.7335592 | 4 |
| 3258.7337307 | 3 |
| 3258.7338243 | 2 |
| 3258.7338735 | 1 |
| 3258.7339983 | 1 |
| 3258.7347397 | 1 |
| 3258.7348202 | 5 |
| 3258.7348804 | 5 |
| 3258.7348931 | 1 |
| 3258.7349831 | 1 |
| 3258.7352936 | 2 |
| 3258.7353453 | 1 |
| 3258.7354654 | 2 |
| 3258.7359321 | 5 |
| 3258.7362903 | 3 |
| 3258.7364828 | 3 |
| 3258.7364894 | 3 |
| 3258.7365875 | 4 |
| 3258.7370737 | 6 |
| 3258.7375838 | 14 |
| 3258.7378723 | 9 |
| 3258.7379362 | 8 |
| 3258.7379461 | 4 |
| 3258.7381223 | 10 |
| 3258.7381483 | 12 |
| 3258.7382414 | 13 |
| 3258.7388306 | 14 |
| 3258.7391207 | 13 |
| 3258.7392413 | 16 |
| 3258.739302 | 13 |
| 3258.7393363 | 13 |
| 3258.7394113 | 22 |
| 3258.7394938 | 16 |
| 3258.7396946 | 8 |
| 3258.7400467 | 11 |
| 3258.740635 | 14 |
| 3258.7409723 | 11 |
| 3258.7410801 | 12 |
| 3258.7413387 | 14 |
| 3258.7413483 | 15 |
| 3258.7414502 | 12 |
| 3258.7416254 | 13 |
| 3258.7423542 | 18 |
| 3258.742362 | 19 |
| 3258.7424987 | 14 |
| 3258.7425277 | 19 |
| 3258.7426204 | 20 |
| 3258.7431562 | 15 |
| 3258.7433607 | 14 |
| 3258.7435471 | 12 |
| 3258.7435874 | 18 |
| 3258.7438171 | 16 |
| 3258.7442152 | 14 |
| 3258.7446655 | 12 |
| 3258.7447668 | 15 |
| 3258.7448403 | 23 |
| 3258.7453105 | 14 |
| 3258.7453127 | 13 |
| 3258.7453957 | 16 |
| 3258.7455484 | 16 |
| 3258.745633 | 18 |
| 3258.7459202 | 5 |
| 3258.7464008 | 11 |
| 3258.7464784 | 7 |
| 3258.7465064 | 14 |
| 3258.746693 | 15 |
| 3258.7467462 | 7 |
| 3258.7470636 | 8 |
| 3258.7474318 | 9 |
| 3258.747614 | 5 |
| 3258.7481028 | 2 |
| 3258.7482778 | 7 |
| 3258.748417 | 0 |
| 3258.7487239 | 4 |
| 3258.7488606 | 1 |
| 3258.7488676 | 5 |
| 3258.7490969 | 1 |
| 3258.749145 | 2 |
| 3258.7493339 | 1 |
| 3258.7497475 | 0 |
| 3258.7498476 | 2 |
| 3258.7499078 | 4 |
| 3258.7502265 | 1 |
| 3258.7503784 | 2 |
| 3258.7505066 | 2 |
| 3258.7509624 | 4 |
| 3258.7511046 | 5 |
| 3258.7512045 | 1 |
| 3258.7513248 | 2 |
| 3258.7516242 | 3 |
| 3258.7516527 | 3 |
| 3258.7520312 | 3 |
| 3258.7522 | 1 |
| 3258.7525062 | 3 |
| 3258.7526636 | 1 |
| 3258.7528404 | 2 |
| 3258.7532701 | 1 |
| 3258.7534395 | 1 |
| 3258.7537368 | 2 |
| 3258.7538849 | 5 |
| 3258.7540632 | 3 |
| 3258.7542357 | 5 |
| 3258.7547748 | 2 |
| 3258.7547781 | 1 |
| 3258.7548645 | 3 |
| 3258.7549716 | 0 |
| 3258.7550326 | 0 |
| 3258.7551245 | 5 |
| 3258.7551828 | 5 |
| 3258.7557067 | 2 |
| 3258.7557754 | 3 |
| 3258.7560852 | 2 |
| 3258.7565415 | 1 |
| 3258.7566274 | 2 |
| 3258.7566428 | 1 |
| 3258.7571932 | 2 |
| 3258.7572032 | 0 |
| 3258.7573658 | 3 |
| 3258.7573795 | 1 |
| 3258.7578678 | 6 |
| 3258.7578768 | 4 |
| 3258.7583827 | 1 |
| 3258.75852 | 3 |
| 3258.7585423 | 2 |
| 3258.7585469 | 1 |
| 3258.7589437 | 4 |
| 3258.7593182 | 0 |
| 3258.7596277 | 4 |
| 3258.7597058 | 4 |
| 3258.7599465 | 0 |
| 3258.7606408 | 2 |
| 3258.7613224 | 3 |
| 3258.7622228 | 1 |
| 3258.7623206 | 3 |
| 3258.7628516 | 3 |
| 3258.7641624 | 1 |
| 3258.764223 | 1 |
| 3258.7648929 | 4 |
| 3258.7657557 | 3 |
| 3258.7665188 | 4 |
| 3258.7666862 | 1 |
| 3258.7679213 | 5 |
| 3258.7685189 | 1 |
| 3258.7685439 | 1 |
| 3258.7692577 | 1 |

| | |
|---|---|
| 3258.7701334 | 1 |
| 3258.77065 | 2 |
| 3258.7714499 | 6 |
| 3258.7719549 | 0 |
| 3258.7730057 | 2 |
| 3258.7738164 | 5 |
| 3258.7742095 | 1 |
| 3258.7751787 | 3 |
| 3258.7753755 | 4 |
| 3258.7762316 | 1 |
| 3258.776342 | 4 |
| 3258.7769479 | 1 |
| 3258.7778955 | 2 |
| 3258.7782684 | 3 |
| 3258.7786861 | 4 |
| 3258.7795436 | 3 |
| 3258.7808603 | 2 |
| 3258.7812377 | 3 |
| 3258.7815629 | 1 |
| 3258.78196 | 6 |
| 3258.782507 | 2 |
| 3258.7826033 | 3 |
| 3258.7836917 | 1 |
| 3258.7848882 | 2 |
| 3258.785579 | 3 |
| 3258.7861165 | 3 |
| 3258.7871361 | 2 |
| 3258.7875365 | 4 |
| 3258.7876296 | 4 |
| 3258.7882898 | 0 |
| 3258.7886915 | 2 |
| 3258.789368 | 3 |
| 3258.7907577 | 2 |
| 3258.7907842 | 0 |
| 3258.7915669 | 4 |
| 3258.7920252 | 4 |
| 3258.7927655 | 3 |
| 3258.7935043 | 2 |
| 3258.7936802 | 2 |
| 3258.7940606 | 4 |
| 3258.7948797 | 5 |
| 3258.7963563 | 0 |
| 3258.7966638 | 2 |
| 3258.7968229 | 3 |
| 3258.7976525 | 2 |
| 3258.7982322 | 0 |
| 3258.7988903 | 4 |
| 3258.7995987 | 2 |
| 3258.7998284 | 2 |
| 3258.8000825 | 1 |
| 3258.8014978 | 2 |
| 3258.8015481 | 3 |
| 3258.8025471 | 3 |
| 3258.8027945 | 2 |
| 3258.8040172 | 4 |
| 3258.80451 | 1 |
| 3258.8049341 | 6 |
| 3258.8054562 | 3 |
| 3258.8065771 | 2 |
| 3258.8068528 | 1 |
| 3258.8073093 | 0 |
| 3258.8088126 | 1 |
| 3258.8088772 | 4 |
| 3258.8091207 | 1 |
| 3258.8095175 | 1 |
| 3258.8106165 | 2 |
| 3258.8110261 | 4 |
| 3258.8113334 | 1 |
| 3258.8122845 | 2 |
| 3258.8130532 | 6 |
| 3258.814296 | 3 |
| 3258.8147513 | 3 |
| 3258.8153399 | 5 |
| 3258.8154667 | 3 |
| 3258.8162489 | 2 |
| 3258.8173082 | 2 |
| 3258.8173278 | 4 |
| 3258.8180357 | 1 |
| 3258.8185739 | 3 |
| 3258.8201833 | 3 |
| 3258.8202013 | 0 |
| 3258.8210294 | 4 |
| 3258.8217835 | 4 |
| 3258.8223529 | 0 |
| 3258.8223688 | 5 |
| 3258.8230653 | 4 |
| 3258.823259 | 2 |
| 3258.8247057 | 2 |
| 3258.8249095 | 3 |
| 3258.826686 | 2 |
| 3258.8270707 | 2 |
| 3258.8277279 | 2 |
| 3258.8283532 | 0 |
| 3258.8284384 | 0 |
| 3258.8293931 | 1 |
| 3258.8295226 | 4 |
| 3258.8303106 | 2 |
| 3258.8306804 | 2 |
| 3258.8318185 | 4 |
| 3258.8326278 | 1 |
| 3258.8329383 | 2 |
| 3258.8334273 | 2 |
| 3258.8343416 | 1 |
| 3258.8349818 | 3 |
| 3258.8352401 | 4 |
| 3258.8358148 | 1 |
| 3258.836233 | 2 |
| 3258.8374712 | 1 |
| 3258.8376236 | 5 |
| 3258.8380411 | 2 |
| 3258.8383532 | 4 |
| 3258.8393789 | 3 |
| 3258.8399881 | 3 |
| 3258.8410415 | 3 |
| 3258.8415256 | 1 |
| 3258.8417618 | 4 |
| 3258.8426844 | 3 |
| 3258.8429416 | 3 |
| 3258.8430309 | 3 |
| 3258.8443824 | 2 |
| 3258.8451563 | 4 |
| 3258.8457851 | 1 |
| 3258.8458373 | 3 |
| 3258.847129 | 6 |
| 3258.8478608 | 1 |
| 3258.8480282 | 0 |
| 3258.8488465 | 3 |
| 3258.8496533 | 2 |
| 3258.8499493 | 2 |
| 3258.8499729 | 0 |
| 3258.8502178 | 3 |
| 3258.8506761 | 1 |
| 3258.8509013 | 2 |
| 3258.851299 | 2 |
| 3258.8520403 | 3 |
| 3258.8523772 | 2 |
| 3258.8531143 | 1 |
| 3258.853326 | 2 |
| 3258.8537194 | 0 |
| 3258.854152 | 2 |
| 3258.8541632 | 6 |
| 3258.8545313 | 0 |
| 3258.855165 | 2 |
| 3258.855545 | 5 |
| 3258.8556205 | 5 |
| 3258.8561301 | 4 |
| 3258.8561381 | 1 |
| 3258.8567448 | 0 |
| 3258.8568153 | 4 |
| 3258.8576951 | 3 |
| 3258.8578278 | 2 |
| 3258.8585643 | 2 |
| 3258.8587667 | 2 |
| 3258.8590327 | 0 |
| 3258.8596048 | 3 |
| 3258.859666 | 2 |
| 3258.8602827 | 1 |
| 3258.8603335 | 1 |
| 3258.860776 | 2 |
| 3258.8613923 | 1 |
| 3258.8614438 | 2 |
| 3258.8623254 | 2 |
| 3258.862411 | 5 |
| 3258.862781 | 1 |
| 3258.863342 | 3 |
| 3258.8638772 | 2 |
| 3258.8639343 | 0 |
| 3258.8642119 | 3 |
| 3258.8645022 | 2 |
| 3258.8650381 | 3 |
| 3258.8655155 | 2 |
| 3258.8657118 | 4 |
| 3258.8658458 | 3 |
| 3258.8670314 | 2 |
| 3258.8671222 | 1 |
| 3258.8675733 | 2 |
| 3258.8679125 | 4 |
| 3258.8681935 | 3 |
| 3258.8686052 | 4 |
| 3258.8688941 | 3 |
| 3258.8688986 | 2 |
| 3258.8699959 | 2 |
| 3258.8702758 | 3 |
| 3258.8707195 | 4 |
| 3258.8709081 | 2 |
| 3258.8709636 | 6 |
| 3258.8717598 | 2 |
| 3258.8721879 | 2 |
| 3258.8724955 | 4 |
| 3258.8725786 | 1 |
| 3258.873008 | 1 |
| 3258.8736209 | 1 |
| 3258.8737135 | 3 |
| 3258.874449 | 2 |
| 3258.8746265 | 2 |
| 3258.8748429 | 1 |
| 3258.87552 | 1 |
| 3258.875655 | 2 |
| 3258.8759881 | 0 |
| 3258.8768207 | 4 |
| 3258.8768429 | 2 |
| 3258.8772962 | 3 |
| 3258.8776548 | 2 |
| 3258.8781523 | 1 |
| 3258.8783834 | 3 |
| 3258.8786023 | 2 |
| 3258.8794384 | 2 |
| 3258.8795186 | 1 |
| 3258.8797495 | 4 |
| 3258.8803294 | 2 |
| 3258.8805987 | 1 |
| 3258.8806925 | 3 |
| 3258.88096 | 2 |
| 3258.8819707 | 2 |
| 3258.8820997 | 1 |
| 3258.8821537 | 3 |
| 3258.8826068 | 3 |
| 3258.8834051 | 2 |
| 3258.8837779 | 2 |
| 3258.8841 | 1 |
| 3258.8842037 | 2 |
| 3258.8849599 | 0 |
| 3258.8853373 | 2 |
| 3258.8856048 | 2 |
| 3258.8859855 | 2 |
| 3258.8862067 | 4 |
| 3258.8866763 | 0 |
| 3258.8868473 | 0 |
| 3258.887017 | 0 |
| 3258.8877973 | 1 |
| 3258.8879905 | 3 |
| 3258.8885702 | 1 |
| 3258.8889558 | 1 |
| 3258.8889603 | 0 |
| 3258.8890754 | 3 |
| 3258.8895344 | 2 |
| 3258.8895549 | 4 |
| 3258.8899029 | 1 |
| 3258.8908526 | 4 |
| 3258.8917203 | 3 |
| 3258.8917652 | 2 |
| 3258.8919276 | 0 |
| 3258.8924513 | 4 |
| 3258.8926973 | 1 |
| 3258.8932863 | 2 |
| 3258.8935888 | 1 |
| 3258.8940368 | 0 |
| 3258.8946465 | 1 |
| 3258.8950057 | 1 |
| 3258.8951133 | 1 |
| 3258.8953419 | 2 |
| 3258.8957704 | 2 |
| 3258.8961169 | 2 |
| 3258.8961301 | 0 |
| 3258.8961483 | 2 |
| 3258.8969202 | 1 |
| 3258.8976062 | 1 |
| 3258.8979334 | 1 |
| 3258.8982812 | 0 |
| 3258.8985242 | 3 |
| 3258.8988007 | 1 |
| 3258.8992959 | 1 |
| 3258.8997861 | 2 |
| 3258.9004415 | 1 |
| 3258.9005308 | 0 |
| 3258.9006312 | 3 |
| 3258.9014083 | 1 |
| 3258.9016981 | 3 |
| 3258.9020002 | 4 |
| 3258.9022281 | 2 |
| 3258.9027383 | 4 |
| 3258.9031174 | 2 |
| 3258.9033399 | 1 |
| 3258.9039179 | 2 |
| 3258.9045349 | 1 |
| 3258.9046412 | 5 |
| 3258.9050624 | 2 |
| 3258.9055396 | 1 |
| 3258.9061471 | 0 |
| 3258.9064357 | 3 |
| 3258.9066856 | 2 |
| 3258.9068396 | 2 |
| 3258.9069672 | 1 |
| 3258.9074649 | 2 |
| 3258.9076794 | 8 |
| 3258.9088694 | 0 |
| 3258.9089859 | 0 |
| 3258.9092306 | 1 |
| 3258.9095194 | 2 |
| 3258.9096553 | 1 |
| 3258.9100068 | 3 |
| 3258.9111715 | 1 |
| 3258.9115494 | 3 |
| 3258.9116611 | 1 |
| 3258.9117116 | 4 |
| 3258.9127052 | 3 |
| 3258.9131799 | 2 |
| 3258.9132051 | 5 |
| 3258.9135058 | 4 |
| 3258.9142993 | 3 |
| 3258.9143064 | 2 |
| 3258.9145499 | 2 |
| 3258.9148097 | 0 |
| 3258.9150074 | 5 |
| 3258.9159394 | 3 |
| 3258.9159645 | 2 |
| 3258.9161444 | 3 |
| 3258.9168546 | 2 |
| 3258.9172356 | 1 |
| 3258.9177256 | 3 |
| 3258.9177443 | 2 |
| 3258.9182483 | 4 |
| 3258.918949 | 1 |
| 3258.9194664 | 0 |
| 3258.9194823 | 3 |
| 3258.9196333 | 2 |
| 3258.9202315 | 2 |
| 3258.9205281 | 1 |
| 3258.9207444 | 5 |
| 3258.9216487 | 6 |
| 3258.9217551 | 2 |
| 3258.9218647 | 2 |
| 3258.9225619 | 0 |
| 3258.9227143 | 6 |
| 3258.9230392 | 2 |
| 3258.9232636 | 1 |
| 3258.923422 | 4 |
| 3258.9246258 | 3 |
| 3258.9247222 | 1 |
| 3258.9249467 | 3 |
| 3258.9257923 | 1 |
| 3258.9260601 | 2 |
| 3258.9263404 | 4 |
| 3258.9266772 | 1 |
| 3258.9270122 | 1 |
| 3258.9273341 | 5 |
| 3258.9275728 | 1 |
| 3258.9281262 | 2 |
| 3258.9282832 | 0 |
| 3258.9288418 | 4 |
| 3258.929479 | 2 |
| 3258.9295674 | 3 |
| 3258.9302176 | 3 |
| 3258.930435 | 1 |
| 3258.930645 | 3 |
| 3258.9313599 | 2 |
| 3258.9315629 | 1 |
| 3258.9318405 | 4 |
| 3258.931998 | 0 |
| 3258.9323235 | 2 |
| 3258.9328403 | 3 |
| 3258.9332166 | 5 |
| 3258.9338472 | 3 |
| 3258.9340648 | 3 |
| 3258.9347017 | 2 |
| 3258.9350668 | 2 |
| 3258.9351302 | 2 |
| 3258.9353086 | 2 |
| 3258.9357752 | 5 |
| 3258.9362171 | 1 |
| 3258.9362937 | 1 |
| 3258.9372429 | 2 |
| 3258.9382255 | 0 |

| | |
|---|---|
| 3258.9385365 | 0 |
| 3258.9386251 | 4 |
| 3258.9387948 | 1 |
| 3258.9392629 | 2 |
| 3258.9396209 | 4 |
| 3258.9398006 | 2 |
| 3258.9403665 | 2 |
| 3258.9405902 | 2 |
| 3258.9412738 | 3 |
| 3258.9414643 | 0 |
| 3258.9421213 | 2 |
| 3258.9425027 | 3 |
| 3258.9432982 | 4 |
| 3258.9441592 | 3 |
| 3258.945597 | 3 |
| 3258.9464414 | 2 |
| 3258.9472435 | 2 |
| 3258.9482739 | 1 |
| 3258.9487825 | 1 |
| 3258.9494329 | 4 |
| 3258.9511246 | 1 |
| 3258.9518518 | 2 |
| 3258.953537 | 2 |
| 3258.9535379 | 2 |
| 3258.9545956 | 2 |
| 3258.9555395 | 2 |
| 3258.9562211 | 1 |
| 3258.9573817 | 2 |
| 3258.9581026 | 0 |
| 3258.9599817 | 5 |
| 3258.9600721 | 3 |
| 3258.9611787 | 3 |
| 3258.9624413 | 1 |
| 3258.9631939 | 2 |
| 3258.9643623 | 0 |
| 3258.964788 | 3 |
| 3258.9660662 | 1 |
| 3258.9671686 | 1 |
| 3258.9680765 | 3 |
| 3258.9686778 | 0 |
| 3258.970212 | 1 |
| 3258.9709493 | 1 |
| 3258.9721546 | 1 |
| 3258.9725527 | 0 |
| 3258.9733829 | 1 |
| 3258.9746608 | 2 |
| 3258.9751423 | 3 |
| 3258.9761966 | 3 |
| 3258.9772618 | 3 |
| 3258.9781924 | 3 |
| 3258.9787536 | 3 |
| 3258.9797802 | 1 |
| 3258.9810862 | 4 |
| 3258.981388 | 4 |
| 3258.9824193 | 5 |
| 3258.9831893 | 2 |
| 3258.9853063 | 3 |
| 3258.9857212 | 0 |
| 3258.9862519 | 1 |
| 3258.9878511 | 3 |
| 3258.9882436 | 2 |
| 3258.9892323 | 1 |
| 3258.9895505 | 1 |
| 3258.990713 | 3 |
| 3258.9920108 | 5 |
| 3258.9920185 | 3 |
| 3258.993465 | 2 |
| 3258.9946965 | 4 |
| 3258.9955624 | 5 |
| 3258.996555 | 3 |
| 3258.9968808 | 2 |
| 3258.9984749 | 4 |
| 3258.9992242 | 2 |
| 3258.9998437 | 4 |
| 3259.0007173 | 3 |
| 3259.0007732 | 1 |
| 3259.0014865 | 0 |
| 3259.00264 | 2 |
| 3259.0026892 | 2 |
| 3259.0036465 | 0 |
| 3259.004616 | 0 |
| 3259.005287 | 2 |
| 3259.0053346 | 0 |
| 3259.0065132 | 1 |
| 3259.0070773 | 0 |
| 3259.0073747 | 4 |
| 3259.0082236 | 1 |
| 3259.0090351 | 1 |
| 3259.0098278 | 6 |
| 3259.0102002 | 14 |
| 3259.0103625 | 9 |
| 3259.0114974 | 28 |
| 3259.0120368 | 14 |
| 3259.0126806 | 23 |
| 3259.0135003 | 27 |
| 3259.0138282 | 16 |
| 3259.0146532 | 15 |
| 3259.0147212 | 24 |
| 3259.0157427 | 22 |
| 3259.016086 | 18 |
| 3259.0167726 | 12 |
| 3259.0175853 | 24 |
| 3259.0178302 | 19 |
| 3259.0181609 | 24 |
| 3259.0182866 | 21 |
| 3259.0189885 | 14 |
| 3259.0190575 | 25 |
| 3259.0193094 | 21 |
| 3259.0195582 | 14 |
| 3259.0200058 | 13 |
| 3259.0204561 | 12 |
| 3259.0206959 | 9 |
| 3259.0207367 | 6 |
| 3259.0213169 | 1 |
| 3259.0213323 | 7 |
| 3259.0218635 | 4 |
| 3259.0218753 | 2 |
| 3259.0219523 | 5 |
| 3259.022315 | 2 |
| 3259.0224737 | 2 |
| 3259.0228323 | 2 |
| 3259.0231784 | 3 |
| 3259.023354 | 0 |
| 3259.0234785 | 3 |
| 3259.0237045 | 4 |
| 3259.0242714 | 4 |
| 3259.024314 | 2 |
| 3259.0243655 | 5 |
| 3259.024717 | 1 |
| 3259.0249195 | 1 |
| 3259.025401 | 3 |
| 3259.0260314 | 5 |
| 3259.0260377 | 0 |
| 3259.0262839 | 1 |
| 3259.0264539 | 0 |
| 3259.0266263 | 9 |
| 3259.0268636 | 3 |
| 3259.0271393 | 1 |
| 3259.0272476 | 4 |
| 3259.0273386 | 1 |
| 3259.0278392 | 8 |
| 3259.0278758 | 5 |
| 3259.0280622 | 8 |
| 3259.0283774 | 16 |
| 3259.028888 | 10 |
| 3259.0293156 | 25 |
| 3259.0293397 | 22 |
| 3259.0294052 | 33 |
| 3259.0295673 | 38 |
| 3259.0298954 | 43 |
| 3259.0301039 | 39 |
| 3259.0305792 | 51 |
| 3259.0308227 | 38 |
| 3259.0311934 | 37 |
| 3259.03129 | 53 |
| 3259.0313821 | 65 |
| 3259.0316314 | 51 |
| 3259.0317515 | 51 |
| 3259.03181 | 56 |
| 3259.0321977 | 57 |
| 3259.0322361 | 62 |
| 3259.032651 | 49 |
| 3259.032909 | 48 |
| 3259.0333749 | 47 |
| 3259.0335 | 73 |
| 3259.0335101 | 42 |
| 3259.0337211 | 58 |
| 3259.0337302 | 66 |
| 3259.0340302 | 39 |
| 3259.0345726 | 50 |
| 3259.0350153 | 56 |
| 3259.0351158 | 61 |
| 3259.0352225 | 56 |
| 3259.0353921 | 58 |
| 3259.0356551 | 42 |
| 3259.0357716 | 60 |
| 3259.0359973 | 65 |
| 3259.0364374 | 47 |
| 3259.0365625 | 45 |
| 3259.0369113 | 51 |
| 3259.0370926 | 26 |
| 3259.0374118 | 37 |
| 3259.037449 | 20 |
| 3259.0374492 | 48 |
| 3259.037651 | 31 |
| 3259.0380818 | 21 |
| 3259.0382601 | 12 |
| 3259.0388227 | 7 |
| 3259.0391513 | 8 |
| 3259.0393052 | 10 |
| 3259.0393284 | 3 |
| 3259.0400891 | 2 |
| 3259.0401022 | 3 |
| 3259.0401605 | 1 |
| 3259.0401965 | 4 |
| 3259.0405378 | 1 |
| 3259.0406974 | 1 |
| 3259.0407101 | 4 |
| 3259.0413761 | 3 |
| 3259.0413874 | 5 |
| 3259.0417645 | 2 |
| 3259.0419033 | 0 |
| 3259.0421478 | 5 |
| 3259.0422308 | 2 |
| 3259.0424285 | 8 |
| 3259.0428532 | 4 |
| 3259.0430378 | 2 |
| 3259.0430639 | 2 |
| 3259.043877 | 0 |
| 3259.0440664 | 4 |
| 3259.0441792 | 4 |
| 3259.0442854 | 2 |
| 3259.0447311 | 2 |
| 3259.0448453 | 2 |
| 3259.0449783 | 1 |
| 3259.0449822 | 1 |
| 3259.0454095 | 3 |
| 3259.0456254 | 2 |
| 3259.0457155 | 4 |
| 3259.0463104 | 1 |
| 3259.0463836 | 2 |
| 3259.0466145 | 1 |
| 3259.0469975 | 2 |
| 3259.0471671 | 0 |
| 3259.0488383 | 1 |
| 3259.0501546 | 2 |
| 3259.0503461 | 4 |
| 3259.0515289 | 4 |
| 3259.0522067 | 4 |
| 3259.0533404 | 1 |
| 3259.05453 | 4 |
| 3259.0553762 | 3 |
| 3259.0566243 | 2 |
| 3259.0569397 | 1 |
| 3259.0583997 | 1 |
| 3259.0589697 | 1 |
| 3259.0598271 | 3 |
| 3259.0610656 | 3 |
| 3259.0616111 | 1 |
| 3259.0632368 | 3 |
| 3259.0637663 | 2 |
| 3259.0645249 | 5 |
| 3259.0658109 | 1 |
| 3259.0661191 | 3 |
| 3259.0674898 | 1 |
| 3259.0684916 | 2 |
| 3259.0697151 | 3 |
| 3259.0704229 | 4 |
| 3259.0715815 | 3 |
| 3259.0726962 | 1 |
| 3259.0728 | 0 |
| 3259.074118 | 1 |
| 3259.0750815 | 5 |
| 3259.0754521 | 3 |
| 3259.076842 | 3 |
| 3259.0775399 | 1 |
| 3259.0788452 | 1 |
| 3259.0801634 | 0 |
| 3259.0806199 | 4 |
| 3259.0816026 | 1 |
| 3259.0818185 | 0 |
| 3259.0830862 | 1 |
| 3259.0845694 | 4 |
| 3259.0846258 | 4 |
| 3259.0866825 | 2 |
| 3259.0873433 | 1 |
| 3259.0877401 | 1 |
| 3259.0889351 | 0 |
| 3259.0898766 | 2 |
| 3259.090711 | 3 |
| 3259.0916444 | 1 |
| 3259.0925038 | 3 |
| 3259.0941715 | 0 |
| 3259.0947263 | 0 |
| 3259.0956773 | 1 |
| 3259.0963646 | 2 |
| 3259.0971706 | 0 |
| 3259.0985672 | 1 |
| 3259.0997568 | 4 |
| 3259.1005421 | 2 |
| 3259.1010446 | 4 |
| 3259.1020764 | 1 |
| 3259.1027749 | 3 |
| 3259.1041419 | 0 |
| 3259.1050171 | 5 |
| 3259.1060631 | 1 |
| 3259.1072223 | 3 |
| 3259.1078697 | 2 |
| 3259.1089742 | 0 |
| 3259.1102619 | 1 |
| 3259.1107151 | 1 |
| 3259.1118669 | 5 |
| 3259.1123027 | 4 |
| 3259.1137317 | 4 |
| 3259.1146082 | 2 |
| 3259.115162 | 2 |
| 3259.1160429 | 4 |
| 3259.1172349 | 4 |
| 3259.1182436 | 4 |
| 3259.1194699 | 1 |
| 3259.1198526 | 3 |
| 3259.1210766 | 2 |
| 3259.1222283 | 3 |
| 3259.1226304 | 6 |
| 3259.1235263 | 2 |
| 3259.1245557 | 3 |
| 3259.1260481 | 3 |
| 3259.1261871 | 5 |
| 3259.1270269 | 3 |
| 3259.1287824 | 1 |
| 3259.1291575 | 1 |
| 3259.1303508 | 1 |
| 3259.1312286 | 4 |
| 3259.1324151 | 2 |
| 3259.133423 | 2 |
| 3259.1339106 | 0 |
| 3259.1348846 | 3 |
| 3259.135255 | 4 |
| 3259.1367949 | 2 |
| 3259.1378569 | 2 |
| 3259.1379955 | 4 |
| 3259.1399676 | 2 |
| 3259.1401616 | 2 |
| 3259.1413436 | 1 |
| 3259.1421115 | 3 |
| 3259.1433572 | 1 |
| 3259.1444897 | 4 |
| 3259.1449285 | 2 |
| 3259.1460497 | 3 |
| 3259.1476054 | 2 |
| 3259.1480828 | 4 |
| 3259.1488043 | 4 |
| 3259.1498723 | 3 |
| 3259.1515192 | 1 |
| 3259.1520205 | 0 |
| 3259.1528744 | 0 |
| 3259.1541431 | 3 |
| 3259.154592 | 3 |
| 3259.1558293 | 3 |
| 3259.1559059 | 2 |
| 3259.1571838 | 1 |
| 3259.1584012 | 2 |
| 3259.159386 | 2 |
| 3259.1602356 | 2 |
| 3259.1609431 | 2 |
| 3259.1623829 | 0 |
| 3259.163989 | 0 |
| 3259.1640767 | 4 |
| 3259.1652465 | 2 |
| 3259.1657275 | 1 |
| 3259.1667251 | 4 |
| 3259.1670728 | 5 |
| 3259.1687799 | 2 |
| 3259.1700351 | 4 |
| 3259.1701232 | 5 |
| 3259.1710675 | 3 |
| 3259.1721539 | 2 |
| 3259.1729549 | 1 |
| 3259.1745577 | 1 |
| 3259.1748372 | 2 |
| 3259.1757693 | 4 |
| 3259.1770027 | 3 |
| 3259.1776383 | 4 |

| | |
|---|---|
| 3259.1788077 | 1 |
| 3259.1801758 | 2 |
| 3259.1806475 | 0 |
| 3259.1821838 | 2 |
| 3259.1827273 | 3 |
| 3259.1838188 | 3 |
| 3259.1842909 | 1 |
| 3259.1851619 | 0 |
| 3259.186504 | 0 |
| 3259.1872782 | 0 |
| 3259.1880467 | 2 |
| 3259.1893916 | 3 |
| 3259.1900087 | 1 |
| 3259.190384 | 3 |
| 3259.1920795 | 1 |
| 3259.1934163 | 3 |
| 3259.1938432 | 3 |
| 3259.1952804 | 0 |
| 3259.1958802 | 2 |
| 3259.1966741 | 1 |
| 3259.1978547 | 0 |
| 3259.1983239 | 2 |
| 3259.1990674 | 1 |
| 3259.2004613 | 2 |
| 3259.2010373 | 1 |
| 3259.2023338 | 3 |
| 3259.2034271 | 1 |
| 3259.2048227 | 4 |
| 3259.2049475 | 1 |
| 3259.2056672 | 0 |
| 3259.2074564 | 1 |
| 3259.2076199 | 2 |
| 3259.2090038 | 1 |
| 3259.2096596 | 2 |
| 3259.2104246 | 3 |
| 3259.2116474 | 1 |
| 3259.2123288 | 0 |
| 3259.2136341 | 3 |
| 3259.2137769 | 2 |
| 3259.2161837 | 2 |
| 3259.2167648 | 1 |
| 3259.217232 | 1 |
| 3259.2182566 | 1 |
| 3259.2188814 | 3 |
| 3259.2204313 | 2 |
| 3259.2204848 | 1 |
| 3259.221608 | 0 |
| 3259.2234574 | 1 |
| 3259.2235886 | 2 |
| 3259.2247136 | 2 |
| 3259.2259402 | 3 |
| 3259.2266448 | 0 |
| 3259.2274517 | 3 |
| 3259.228366 | 3 |
| 3259.2294671 | 5 |
| 3259.2295073 | 3 |
| 3259.2310771 | 1 |
| 3259.2322479 | 4 |
| 3259.2331122 | 1 |
| 3259.2342557 | 0 |
| 3259.2343003 | 1 |
| 3259.2356019 | 3 |
| 3259.237127 | 1 |
| 3259.2376459 | 2 |
| 3259.2384735 | 3 |
| 3259.2397266 | 2 |
| 3259.2407933 | 1 |
| 3259.2419479 | 2 |
| 3259.2419798 | 0 |
| 3259.2430091 | 1 |
| 3259.2444469 | 3 |
| 3259.2451638 | 0 |
| 3259.2462585 | 2 |
| 3259.2473065 | 2 |
| 3259.2484518 | 1 |
| 3259.2485342 | 1 |
| 3259.2500046 | 2 |
| 3259.2513793 | 0 |
| 3259.2516308 | 2 |
| 3259.2528118 | 2 |
| 3259.2536021 | 4 |
| 3259.2543785 | 3 |
| 3259.2554503 | 1 |
| 3259.2570846 | 2 |
| 3259.2576068 | 4 |
| 3259.2584527 | 0 |
| 3259.2589925 | 2 |
| 3259.2602066 | 1 |
| 3259.2605899 | 3 |
| 3259.2622402 | 8 |
| 3259.262859 | 0 |
| 3259.2641411 | 1 |
| 3259.265374 | 4 |
| 3259.2659455 | 1 |
| 3259.2669513 | 3 |
| 3259.2672838 | 2 |
| 3259.2686529 | 2 |
| 3259.2688334 | 1 |
| 3259.2703798 | 0 |
| 3259.2717098 | 1 |
| 3259.272119 | 4 |
| 3259.2730388 | 4 |
| 3259.2737962 | 1 |
| 3259.2751922 | 4 |
| 3259.2761417 | 2 |
| 3259.2768614 | 0 |
| 3259.2779851 | 1 |
| 3259.2785829 | 1 |
| 3259.2799861 | 1 |
| 3259.2801868 | 2 |
| 3259.2813637 | 1 |
| 3259.2822383 | 1 |
| 3259.2831083 | 0 |
| 3259.2844921 | 3 |
| 3259.2851815 | 1 |
| 3259.2871015 | 1 |
| 3259.2876664 | 0 |
| 3259.2877905 | 1 |
| 3259.2895607 | 2 |
| 3259.2905169 | 1 |
| 3259.290753 | 0 |
| 3259.2923535 | 2 |
| 3259.2930281 | 2 |
| 3259.2941612 | 2 |
| 3259.2946311 | 1 |
| 3259.2956884 | 0 |
| 3259.2963077 | 2 |
| 3259.2971645 | 0 |
| 3259.2983232 | 2 |
| 3259.2996265 | 1 |
| 3259.3008436 | 2 |
| 3259.301589 | 3 |
| 3259.3016303 | 2 |
| 3259.3030085 | 3 |
| 3259.3036594 | 3 |
| 3259.3047999 | 3 |
| 3259.3059332 | 0 |
| 3259.3068763 | 1 |
| 3259.3082743 | 0 |
| 3259.3087157 | 2 |
| 3259.3097016 | 4 |
| 3259.3109265 | 3 |
| 3259.310929 | 1 |
| 3259.3125905 | 1 |
| 3259.3137074 | 0 |
| 3259.3142838 | 0 |
| 3259.3152186 | 0 |
| 3259.3162267 | 4 |
| 3259.3167466 | 1 |
| 3259.3177662 | 1 |
| 3259.3189538 | 1 |
| 3259.3200616 | 3 |
| 3259.3205737 | 4 |
| 3259.3218116 | 2 |
| 3259.322198 | 3 |
| 3259.3231233 | 3 |
| 3259.3247298 | 3 |
| 3259.3254276 | 0 |
| 3259.3264994 | 5 |
| 3259.3273564 | 2 |
| 3259.3288481 | 4 |
| 3259.3289904 | 3 |
| 3259.3295352 | 3 |
| 3259.3311842 | 1 |
| 3259.3318156 | 2 |
| 3259.3335174 | 2 |
| 3259.3341905 | 1 |
| 3259.3344099 | 2 |
| 3259.3356527 | 1 |
| 3259.3367213 | 2 |
| 3259.3377697 | 1 |
| 3259.3389507 | 1 |
| 3259.3395515 | 3 |
| 3259.3409056 | 2 |
| 3259.3409933 | 0 |
| 3259.3420567 | 1 |
| 3259.3428685 | 1 |
| 3259.3444851 | 2 |
| 3259.3450218 | 1 |
| 3259.345386 | 4 |
| 3259.3466386 | 2 |
| 3259.3484796 | 1 |
| 3259.3485291 | 2 |
| 3259.3498256 | 0 |
| 3259.3503803 | 4 |
| 3259.3511587 | 1 |
| 3259.3527614 | 1 |
| 3259.3527855 | 0 |
| 3259.3544433 | 1 |
| 3259.354981 | 1 |
| 3259.3560388 | 5 |
| 3259.3564464 | 1 |
| 3259.3577361 | 1 |
| 3259.3587775 | 3 |
| 3259.3594013 | 1 |
| 3259.3601729 | 2 |
| 3259.3613033 | 4 |
| 3259.3627352 | 1 |
| 3259.3634352 | 0 |
| 3259.364407 | 1 |
| 3259.3653482 | 2 |
| 3259.3656359 | 3 |
| 3259.3672788 | 0 |
| 3259.3684336 | 2 |
| 3259.3685809 | 2 |
| 3259.3700285 | 3 |
| 3259.3709077 | 1 |
| 3259.3719671 | 1 |
| 3259.3734192 | 2 |
| 3259.3736598 | 1 |
| 3259.3744604 | 2 |
| 3259.3757944 | 4 |
| 3259.376498 | 2 |
| 3259.3774221 | 5 |
| 3259.3784174 | 1 |
| 3259.3798571 | 1 |
| 3259.3799065 | 6 |
| 3259.3814758 | 4 |
| 3259.3819717 | 1 |
| 3259.3834164 | 1 |
| 3259.3840495 | 5 |
| 3259.3853624 | 7 |
| 3259.3862095 | 4 |
| 3259.3870582 | 4 |
| 3259.3874398 | 7 |
| 3259.3887964 | 4 |
| 3259.3893557 | 2 |
| 3259.3894261 | 0 |
| 3259.3896766 | 3 |
| 3259.3899457 | 3 |
| 3259.3899827 | 2 |
| 3259.3902829 | 2 |
| 3259.3913501 | 3 |
| 3259.3914505 | 3 |
| 3259.3915683 | 0 |
| 3259.3921144 | 2 |
| 3259.3923046 | 2 |
| 3259.3927468 | 1 |
| 3259.3927569 | 0 |
| 3259.3933928 | 1 |
| 3259.3935657 | 0 |
| 3259.3937402 | 1 |
| 3259.394248 | 1 |
| 3259.3943459 | 2 |
| 3259.3945817 | 5 |
| 3259.3951649 | 2 |
| 3259.3955117 | 0 |
| 3259.3955549 | 1 |
| 3259.3959217 | 2 |
| 3259.3962133 | 3 |
| 3259.3963995 | 2 |
| 3259.3967895 | 2 |
| 3259.3973863 | 3 |
| 3259.3976395 | 1 |
| 3259.3977146 | 0 |
| 3259.397936 | 2 |
| 3259.3984632 | 1 |
| 3259.3986892 | 0 |
| 3259.3989736 | 1 |
| 3259.3994999 | 1 |
| 3259.399693 | 5 |
| 3259.3999226 | 3 |
| 3259.4001275 | 1 |
| 3259.400586 | 0 |
| 3259.4007039 | 0 |
| 3259.4009309 | 3 |
| 3259.4014691 | 6 |
| 3259.4018594 | 5 |
| 3259.4019753 | 14 |
| 3259.4024585 | 11 |
| 3259.4024963 | 10 |
| 3259.4033883 | 36 |
| 3259.4034323 | 30 |
| 3259.4036512 | 24 |
| 3259.4036932 | 42 |
| 3259.4042673 | 49 |
| 3259.4049823 | 37 |
| 3259.4049918 | 32 |
| 3259.4050691 | 45 |
| 3259.4052289 | 37 |
| 3259.4057488 | 32 |
| 3259.406027 | 25 |
| 3259.4063056 | 28 |
| 3259.4071034 | 31 |
| 3259.4072508 | 31 |
| 3259.4076733 | 36 |
| 3259.4077719 | 34 |
| 3259.4081633 | 25 |
| 3259.4081897 | 40 |
| 3259.4088146 | 36 |
| 3259.4089397 | 39 |
| 3259.4090255 | 37 |
| 3259.4094048 | 33 |
| 3259.4097085 | 35 |
| 3259.4098818 | 38 |
| 3259.4101053 | 35 |
| 3259.4105091 | 22 |
| 3259.4108137 | 41 |
| 3259.4111237 | 32 |
| 3259.4113489 | 29 |
| 3259.4122823 | 7 |
| 3259.4122977 | 19 |
| 3259.4124897 | 21 |
| 3259.4126104 | 15 |
| 3259.412665 | 23 |
| 3259.413546 | 2 |
| 3259.4140346 | 6 |
| 3259.4141409 | 3 |
| 3259.4142592 | 2 |
| 3259.4147114 | 3 |
| 3259.414838 | 0 |
| 3259.4149551 | 4 |
| 3259.4154556 | 3 |
| 3259.4157565 | 0 |
| 3259.4164997 | 1 |
| 3259.4167019 | 0 |
| 3259.4174484 | 1 |
| 3259.4176387 | 2 |
| 3259.4176802 | 0 |
| 3259.418204 | 5 |
| 3259.4184879 | 2 |
| 3259.4187122 | 2 |
| 3259.4190575 | 3 |
| 3259.4191263 | 3 |
| 3259.4191977 | 0 |
| 3259.4194875 | 1 |
| 3259.4199006 | 3 |
| 3259.4201949 | 5 |
| 3259.4204936 | 2 |
| 3259.4209887 | 2 |
| 3259.4213137 | 2 |
| 3259.4220001 | 2 |
| 3259.4230421 | 2 |
| 3259.4244292 | 6 |
| 3259.4249848 | 5 |
| 3259.4264798 | 0 |
| 3259.4267401 | 4 |
| 3259.4272392 | 1 |
| 3259.4286642 | 2 |
| 3259.4293948 | 2 |
| 3259.4301677 | 0 |
| 3259.4313156 | 0 |
| 3259.4322347 | 0 |
| 3259.4335261 | 1 |
| 3259.4339588 | 3 |
| 3259.4350527 | 1 |
| 3259.4362459 | 2 |
| 3259.4365394 | 1 |
| 3259.4382869 | 2 |
| 3259.4384427 | 3 |
| 3259.4400216 | 4 |
| 3259.4402675 | 2 |
| 3259.4413207 | 0 |
| 3259.4428735 | 0 |
| 3259.4435353 | 1 |
| 3259.4445054 | 3 |
| 3259.444978 | 3 |
| 3259.4464542 | 2 |
| 3259.447373 | 2 |
| 3259.4477015 | 3 |
| 3259.4489057 | 7 |
| 3259.4497893 | 2 |
| 3259.4513 | 2 |

| | |
|---|---|
| 3259.4521765 | 1 |
| 3259.4525681 | 4 |
| 3259.4540008 | 4 |
| 3259.4543607 | 0 |
| 3259.4552532 | 2 |
| 3259.4567301 | 2 |
| 3259.4572893 | 5 |
| 3259.45868 | 2 |
| 3259.4590969 | 2 |
| 3259.4603151 | 2 |
| 3259.4609995 | 0 |
| 3259.4616308 | 1 |
| 3259.4625145 | 1 |
| 3259.4625474 | 1 |
| 3259.4635548 | 2 |
| 3259.4644334 | 1 |
| 3259.4648184 | 4 |
| 3259.4650079 | 1 |
| 3259.4657828 | 1 |
| 3259.4666702 | 2 |
| 3259.4676331 | 5 |
| 3259.4677806 | 4 |
| 3259.4686271 | 3 |
| 3259.4690981 | 5 |
| 3259.4699191 | 0 |
| 3259.4703816 | 1 |
| 3259.4711501 | 2 |
| 3259.4718277 | 2 |
| 3259.4720009 | 0 |
| 3259.4727895 | 3 |
| 3259.4734813 | 5 |
| 3259.4743184 | 2 |
| 3259.474683 | 1 |
| 3259.4753468 | 2 |
| 3259.4762849 | 2 |
| 3259.4762944 | 1 |
| 3259.4768963 | 1 |
| 3259.4780443 | 2 |
| 3259.4780664 | 0 |
| 3259.4791162 | 2 |
| 3259.4801318 | 2 |
| 3259.4803395 | 2 |
| 3259.4804261 | 4 |
| 3259.4811007 | 1 |
| 3259.4819944 | 0 |
| 3259.4828064 | 1 |
| 3259.4834902 | 2 |
| 3259.4837189 | 0 |
| 3259.4843248 | 4 |
| 3259.4850083 | 2 |
| 3259.4859572 | 2 |
| 3259.4862931 | 1 |
| 3259.4869769 | 1 |
| 3259.4874092 | 6 |
| 3259.488724 | 0 |
| 3259.4889163 | 0 |
| 3259.4895493 | 2 |
| 3259.4903125 | 1 |
| 3259.4906806 | 2 |
| 3259.4915078 | 0 |
| 3259.4922796 | 3 |
| 3259.4928799 | 3 |
| 3259.4932854 | 1 |
| 3259.4940356 | 0 |
| 3259.4940655 | 0 |
| 3259.4948743 | 1 |
| 3259.4957585 | 2 |
| 3259.496231 | 3 |
| 3259.4974305 | 1 |
| 3259.4974635 | 1 |
| 3259.4979422 | 2 |
| 3259.4986479 | 1 |
| 3259.4993362 | 4 |
| 3259.499758 | 3 |
| 3259.5004089 | 0 |
| 3259.5011563 | 2 |
| 3259.5022046 | 2 |
| 3259.5023861 | 2 |
| 3259.5034195 | 2 |
| 3259.5039912 | 1 |
| 3259.5041075 | 2 |
| 3259.5049127 | 4 |
| 3259.505612 | 2 |
| 3259.5065187 | 0 |
| 3259.5068005 | 1 |
| 3259.507435 | 2 |
| 3259.5082579 | 1 |
| 3259.5087729 | 0 |
| 3259.5090063 | 1 |
| 3259.5090404 | 2 |
| 3259.5091067 | 0 |
| 3259.5097207 | 5 |
| 3259.5097761 | 2 |
| 3259.5098516 | 1 |
| 3259.5101839 | 2 |
| 3259.5103574 | 1 |
| 3259.5106362 | 1 |
| 3259.5108321 | 3 |
| 3259.5108328 | 3 |
| 3259.5113179 | 0 |
| 3259.5113943 | 1 |
| 3259.5116789 | 2 |
| 3259.5117922 | 2 |
| 3259.5119548 | 1 |
| 3259.5125643 | 0 |
| 3259.5126222 | 0 |
| 3259.5129545 | 2 |
| 3259.5130347 | 1 |
| 3259.5130411 | 6 |
| 3259.513636 | 1 |
| 3259.5140166 | 4 |
| 3259.514024 | 2 |
| 3259.5141156 | 4 |
| 3259.5141989 | 2 |
| 3259.5148009 | 1 |
| 3259.5153299 | 3 |
| 3259.515612 | 1 |
| 3259.5156264 | 0 |
| 3259.5158086 | 4 |
| 3259.5160184 | 3 |
| 3259.5163044 | 1 |
| 3259.5166018 | 2 |
| 3259.5168025 | 5 |
| 3259.5169992 | 5 |
| 3259.5171715 | 1 |
| 3259.5172443 | 0 |
| 3259.5177823 | 2 |
| 3259.5178414 | 2 |
| 3259.5179017 | 0 |
| 3259.518039 | 1 |
| 3259.5186781 | 1 |
| 3259.5189036 | 1 |
| 3259.5189302 | 2 |
| 3259.5192918 | 3 |
| 3259.5194324 | 11 |
| 3259.5195059 | 4 |
| 3259.5198039 | 4 |
| 3259.5201223 | 8 |
| 3259.52032 | 8 |
| 3259.5203533 | 14 |
| 3259.520767 | 19 |
| 3259.5208921 | 13 |
| 3259.5212849 | 43 |
| 3259.5214428 | 16 |
| 3259.5219724 | 31 |
| 3259.5220354 | 56 |
| 3259.5224831 | 50 |
| 3259.5226773 | 59 |
| 3259.5231009 | 72 |
| 3259.5231614 | 58 |
| 3259.5233 | 63 |
| 3259.5233952 | 65 |
| 3259.5234654 | 63 |
| 3259.5238896 | 76 |
| 3259.523937 | 78 |
| 3259.5242845 | 73 |
| 3259.5244335 | 75 |
| 3259.5250272 | 49 |
| 3259.5250483 | 54 |
| 3259.525252 | 51 |
| 3259.525604 | 47 |
| 3259.525878 | 13 |
| 3259.5260286 | 40 |
| 3259.5262888 | 22 |
| 3259.5265777 | 26 |
| 3259.5266489 | 15 |
| 3259.5267828 | 22 |
| 3259.5270369 | 15 |
| 3259.5274856 | 17 |
| 3259.5276176 | 7 |
| 3259.52779 | 13 |
| 3259.5280168 | 33 |
| 3259.5283228 | 21 |
| 3259.5283988 | 24 |
| 3259.5285843 | 41 |
| 3259.5291378 | 65 |
| 3259.5291412 | 58 |
| 3259.5296676 | 89 |
| 3259.5298029 | 64 |
| 3259.5299282 | 88 |
| 3259.5304263 | 103 |
| 3259.530446 | 79 |
| 3259.5307829 | 103 |
| 3259.5308614 | 86 |
| 3259.5312229 | 80 |
| 3259.5312935 | 85 |
| 3259.5313465 | 85 |
| 3259.5314544 | 98 |
| 3259.5318986 | 86 |
| 3259.5325591 | 90 |
| 3259.5326 | 92 |
| 3259.532958 | 87 |
| 3259.5329807 | 67 |
| 3259.5330023 | 71 |
| 3259.5331149 | 49 |
| 3259.5336713 | 51 |
| 3259.533795 | 35 |
| 3259.5339075 | 54 |
| 3259.5344546 | 16 |
| 3259.5344923 | 50 |
| 3259.5348388 | 24 |
| 3259.5349812 | 27 |
| 3259.5352136 | 23 |
| 3259.5355543 | 6 |
| 3259.5357315 | 10 |
| 3259.5359359 | 7 |
| 3259.5361533 | 3 |
| 3259.5362498 | 6 |
| 3259.5365851 | 4 |
| 3259.5366572 | 2 |
| 3259.5368629 | 2 |
| 3259.5372378 | 1 |
| 3259.5375271 | 1 |
| 3259.5376219 | 3 |
| 3259.5380005 | 0 |
| 3259.5380983 | 1 |
| 3259.5382816 | 1 |
| 3259.5387066 | 0 |
| 3259.5388839 | 5 |
| 3259.538901 | 3 |
| 3259.5393199 | 1 |
| 3259.5393946 | 1 |
| 3259.5395566 | 2 |
| 3259.5402907 | 1 |
| 3259.5404207 | 2 |
| 3259.5405625 | 0 |
| 3259.5406344 | 3 |
| 3259.5407028 | 2 |
| 3259.5412 | 1 |
| 3259.5412289 | 3 |
| 3259.5415929 | 2 |
| 3259.5417326 | 1 |
| 3259.5423318 | 3 |
| 3259.5424168 | 1 |
| 3259.5428164 | 8 |
| 3259.5429964 | 0 |
| 3259.5432437 | 1 |
| 3259.5435298 | 2 |
| 3259.5437258 | 1 |
| 3259.544103 | 2 |
| 3259.5441106 | 1 |
| 3259.5441705 | 1 |
| 3259.5443082 | 0 |
| 3259.5448015 | 2 |
| 3259.5448927 | 2 |
| 3259.5454657 | 1 |
| 3259.5457815 | 5 |
| 3259.5458191 | 2 |
| 3259.5459267 | 2 |
| 3259.5460945 | 3 |
| 3259.5466246 | 2 |
| 3259.5470111 | 2 |
| 3259.5470476 | 3 |
| 3259.5473591 | 2 |
| 3259.5473744 | 3 |
| 3259.5474173 | 1 |
| 3259.5480079 | 2 |
| 3259.5483893 | 4 |
| 3259.5488147 | 3 |
| 3259.5496675 | 3 |
| 3259.5505605 | 3 |
| 3259.551576 | 1 |
| 3259.5525895 | 2 |
| 3259.5533791 | 1 |
| 3259.5541867 | 0 |
| 3259.5555567 | 4 |
| 3259.5568224 | 1 |
| 3259.5577747 | 6 |
| 3259.5584592 | 1 |
| 3259.5596108 | 0 |
| 3259.5599596 | 0 |
| 3259.5612303 | 5 |
| 3259.5624356 | 1 |
| 3259.5631188 | 0 |
| 3259.564117 | 3 |
| 3259.5644168 | 1 |
| 3259.5660467 | 1 |
| 3259.566337 | 2 |
| 3259.5681325 | 1 |
| 3259.5689811 | 2 |
| 3259.5693487 | 1 |
| 3259.5706757 | 2 |
| 3259.5707895 | 2 |
| 3259.571916 | 2 |
| 3259.572964 | 3 |
| 3259.5741498 | 1 |
| 3259.5750417 | 2 |
| 3259.5754862 | 3 |
| 3259.5771405 | 1 |
| 3259.5775658 | 1 |
| 3259.5787172 | 2 |
| 3259.5799339 | 0 |
| 3259.5807221 | 2 |
| 3259.5813608 | 2 |
| 3259.5826377 | 3 |
| 3259.583716 | 2 |
| 3259.5843735 | 3 |
| 3259.5858119 | 3 |
| 3259.5859658 | 1 |
| 3259.5875884 | 1 |
| 3259.5880251 | 1 |
| 3259.5894369 | 3 |
| 3259.5904495 | 2 |
| 3259.5907184 | 2 |
| 3259.5922906 | 3 |
| 3259.5925543 | 0 |
| 3259.5930693 | 1 |
| 3259.5948479 | 1 |
| 3259.5960198 | 0 |
| 3259.5970162 | 1 |
| 3259.5971507 | 2 |
| 3259.5986826 | 3 |
| 3259.5987879 | 4 |
| 3259.6003572 | 3 |
| 3259.6004169 | 1 |
| 3259.6007278 | 1 |
| 3259.6015248 | 4 |
| 3259.6015985 | 2 |
| 3259.6018303 | 3 |
| 3259.6019564 | 1 |
| 3259.6020232 | 0 |
| 3259.6024541 | 5 |
| 3259.6025543 | 0 |
| 3259.6029393 | 3 |
| 3259.6032426 | 1 |
| 3259.6033214 | 1 |
| 3259.6038632 | 5 |
| 3259.6039401 | 2 |
| 3259.6042038 | 3 |
| 3259.6046393 | 3 |
| 3259.6047033 | 2 |
| 3259.6050842 | 4 |
| 3259.6053263 | 4 |
| 3259.6060479 | 7 |
| 3259.6062172 | 1 |
| 3259.6063073 | 1 |
| 3259.6065341 | 3 |
| 3259.6072248 | 0 |
| 3259.6073058 | 1 |
| 3259.6073578 | 0 |
| 3259.6073976 | 2 |
| 3259.6082753 | 0 |
| 3259.6083316 | 1 |
| 3259.6084689 | 1 |
| 3259.6084762 | 3 |
| 3259.6086902 | 2 |
| 3259.6087009 | 2 |
| 3259.6089862 | 0 |
| 3259.6090318 | 2 |
| 3259.6095252 | 1 |
| 3259.6095426 | 2 |
| 3259.609721 | 5 |
| 3259.6099183 | 1 |
| 3259.6101887 | 2 |
| 3259.6104401 | 3 |
| 3259.6105925 | 1 |
| 3259.61072 | 1 |
| 3259.6111908 | 0 |
| 3259.6111963 | 0 |
| 3259.6112956 | 2 |
| 3259.611297 | 0 |
| 3259.6116283 | 2 |
| 3259.6118198 | 5 |

| | |
|---|---|
| 3259.6121759 | 4 |
| 3259.6123695 | 0 |
| 3259.6127526 | 1 |
| 3259.6127902 | 3 |
| 3259.6128283 | 0 |
| 3259.613214 | 2 |
| 3259.613259 | 1 |
| 3259.6133326 | 1 |
| 3259.6134174 | 4 |
| 3259.613799 | 4 |
| 3259.6138307 | 4 |
| 3259.6138465 | 3 |
| 3259.614106 | 5 |
| 3259.6141228 | 7 |
| 3259.6144316 | 6 |
| 3259.6146938 | 4 |
| 3259.6149146 | 17 |
| 3259.6149784 | 28 |
| 3259.6149807 | 9 |
| 3259.6153585 | 9 |
| 3259.6153637 | 25 |
| 3259.6154407 | 35 |
| 3259.6156737 | 44 |
| 3259.6158828 | 28 |
| 3259.6159989 | 33 |
| 3259.6163359 | 42 |
| 3259.6163975 | 36 |
| 3259.6164847 | 53 |
| 3259.6166468 | 66 |
| 3259.6166952 | 52 |
| 3259.6172049 | 47 |
| 3259.6173477 | 64 |
| 3259.6175177 | 68 |
| 3259.61757 | 62 |
| 3259.6175955 | 59 |
| 3259.6176593 | 48 |
| 3259.6181716 | 60 |
| 3259.6183686 | 71 |
| 3259.6184896 | 57 |
| 3259.6185172 | 63 |
| 3259.6186719 | 73 |
| 3259.6190524 | 77 |
| 3259.619239 | 78 |
| 3259.619248 | 66 |
| 3259.619379 | 78 |
| 3259.6195018 | 65 |
| 3259.619755 | 68 |
| 3259.6199399 | 65 |
| 3259.620048 | 79 |
| 3259.6204294 | 59 |
| 3259.6205499 | 63 |
| 3259.6207377 | 52 |
| 3259.6207571 | 72 |
| 3259.6208964 | 55 |
| 3259.6211951 | 72 |
| 3259.6216136 | 61 |
| 3259.6217335 | 57 |
| 3259.6218886 | 77 |
| 3259.6219556 | 72 |
| 3259.6221221 | 64 |
| 3259.6222861 | 79 |
| 3259.6223467 | 74 |
| 3259.6227785 | 72 |
| 3259.6227989 | 71 |
| 3259.6228364 | 68 |
| 3259.6231009 | 68 |
| 3259.6231566 | 72 |
| 3259.6232386 | 67 |
| 3259.6236003 | 64 |
| 3259.623748 | 68 |
| 3259.6239528 | 38 |
| 3259.6240832 | 28 |
| 3259.6242423 | 59 |
| 3259.6244978 | 55 |
| 3259.6247659 | 25 |
| 3259.6248691 | 17 |
| 3259.6249078 | 18 |
| 3259.6249417 | 49 |
| 3259.6251523 | 20 |
| 3259.6252438 | 9 |
| 3259.6254716 | 12 |
| 3259.6257794 | 17 |
| 3259.6258232 | 6 |
| 3259.6259297 | 6 |
| 3259.6261979 | 3 |
| 3259.6264445 | 0 |
| 3259.6265011 | 6 |
| 3259.6268078 | 2 |
| 3259.626955 | 0 |
| 3259.6271737 | 2 |
| 3259.6272301 | 4 |
| 3259.6273908 | 2 |
| 3259.6274906 | 1 |
| 3259.6275711 | 0 |
| 3259.6276514 | 3 |
| 3259.62785 | 2 |
| 3259.6281639 | 1 |
| 3259.628514 | 0 |
| 3259.6290355 | 2 |
| 3259.6301573 | 1 |
| 3259.6310434 | 2 |
| 3259.6319345 | 1 |
| 3259.6333119 | 2 |
| 3259.6338893 | 0 |
| 3259.6346811 | 2 |
| 3259.6355659 | 0 |
| 3259.6363977 | 0 |
| 3259.6373221 | 2 |
| 3259.6385278 | 3 |
| 3259.6398223 | 1 |
| 3259.6403419 | 0 |
| 3259.6418551 | 1 |
| 3259.6418807 | 2 |
| 3259.6434143 | 4 |
| 3259.6442519 | 2 |
| 3259.6450047 | 2 |
| 3259.6463868 | 3 |
| 3259.6472779 | 2 |
| 3259.648512 | 1 |
| 3259.648821 | 2 |
| 3259.6500828 | 0 |
| 3259.6514753 | 3 |
| 3259.6516724 | 2 |
| 3259.6529178 | 0 |
| 3259.654743 | 3 |
| 3259.6547726 | 3 |
| 3259.655948 | 1 |
| 3259.6562807 | 0 |
| 3259.6577779 | 0 |
| 3259.6579618 | 4 |
| 3259.6592 | 1 |
| 3259.6602337 | 1 |
| 3259.6610439 | 1 |
| 3259.6623583 | 3 |
| 3259.66353 | 2 |
| 3259.663702 | 4 |
| 3259.665038 | 2 |
| 3259.6652273 | 2 |
| 3259.6668281 | 2 |
| 3259.6672688 | 2 |
| 3259.6692026 | 2 |
| 3259.6699239 | 1 |
| 3259.6699958 | 2 |
| 3259.6715292 | 3 |
| 3259.6719325 | 1 |
| 3259.6729473 | 2 |
| 3259.6742772 | 1 |
| 3259.6748773 | 0 |
| 3259.6761604 | 0 |
| 3259.6765912 | 4 |
| 3259.6778162 | 2 |
| 3259.6784585 | 2 |
| 3259.6796231 | 1 |
| 3259.6807005 | 2 |
| 3259.6816129 | 1 |
| 3259.682485 | 0 |
| 3259.6834255 | 2 |
| 3259.6838689 | 1 |
| 3259.6858631 | 4 |
| 3259.6860504 | 3 |
| 3259.6875008 | 3 |
| 3259.6881119 | 2 |
| 3259.6892249 | 1 |
| 3259.6900815 | 3 |
| 3259.6911278 | 0 |
| 3259.6924204 | 1 |
| 3259.6930283 | 1 |
| 3259.6938629 | 2 |
| 3259.6951588 | 2 |
| 3259.6956465 | 1 |
| 3259.696967 | 5 |
| 3259.6981488 | 0 |
| 3259.6986613 | 4 |
| 3259.7000168 | 2 |
| 3259.7000672 | 0 |
| 3259.70151 | 1 |
| 3259.7024426 | 1 |
| 3259.7033349 | 2 |
| 3259.7045614 | 1 |
| 3259.705296 | 1 |
| 3259.70632 | 3 |
| 3259.7066398 | 1 |
| 3259.7081465 | 4 |
| 3259.7095425 | 2 |
| 3259.7095966 | 2 |
| 3259.710934 | 2 |
| 3259.7118283 | 1 |
| 3259.712586 | 1 |
| 3259.7130937 | 0 |
| 3259.7142342 | 2 |
| 3259.7158118 | 2 |
| 3259.7170655 | 0 |
| 3259.7171916 | 1 |
| 3259.7184528 | 4 |
| 3259.7186349 | 1 |
| 3259.7207533 | 1 |
| 3259.720891 | 3 |
| 3259.7220492 | 1 |
| 3259.7233649 | 1 |
| 3259.7240037 | 0 |
| 3259.7250506 | 3 |
| 3259.7254727 | 1 |
| 3259.7272317 | 3 |
| 3259.7281518 | 1 |
| 3259.7288962 | 1 |
| 3259.7296562 | 1 |
| 3259.7306294 | 1 |
| 3259.7312393 | 3 |
| 3259.7325818 | 0 |
| 3259.7331423 | 1 |
| 3259.7342901 | 3 |
| 3259.7353414 | 2 |
| 3259.7368398 | 5 |
| 3259.7370861 | 1 |
| 3259.7380604 | 4 |
| 3259.7385536 | 3 |
| 3259.7398138 | 3 |
| 3259.7408029 | 1 |
| 3259.7418953 | 1 |
| 3259.7428764 | 2 |
| 3259.7443469 | 0 |
| 3259.7449052 | 1 |
| 3259.7460911 | 0 |
| 3259.746115 | 2 |
| 3259.7479136 | 1 |
| 3259.7481044 | 3 |
| 3259.7496018 | 2 |
| 3259.7509566 | 3 |
| 3259.7512341 | 3 |
| 3259.751954 | 1 |
| 3259.7529763 | 1 |
| 3259.754121 | 3 |
| 3259.7551296 | 1 |
| 3259.7559726 | 3 |
| 3259.7571254 | 2 |
| 3259.7572365 | 0 |
| 3259.7581552 | 0 |
| 3259.7596727 | 0 |
| 3259.7601795 | 3 |
| 3259.7614271 | 1 |
| 3259.7624304 | 3 |
| 3259.7636572 | 0 |
| 3259.7642187 | 4 |
| 3259.7648826 | 2 |
| 3259.7665428 | 3 |
| 3259.7666865 | 5 |
| 3259.7685282 | 1 |
| 3259.7687869 | 1 |
| 3259.7702401 | 2 |
| 3259.7708336 | 2 |
| 3259.7712783 | 1 |
| 3259.7729627 | 4 |
| 3259.7736117 | 1 |
| 3259.7739448 | 3 |
| 3259.7752453 | 1 |
| 3259.7759076 | 0 |
| 3259.7774675 | 2 |
| 3259.7786016 | 0 |
| 3259.778744 | 2 |
| 3259.7802707 | 2 |
| 3259.7815894 | 3 |
| 3259.7820124 | 2 |
| 3259.7823788 | 2 |
| 3259.7840403 | 3 |
| 3259.7853053 | 1 |
| 3259.7858 | 0 |
| 3259.7868656 | 0 |
| 3259.7878357 | 3 |
| 3259.7894845 | 2 |
| 3259.7903669 | 0 |
| 3259.7906419 | 2 |
| 3259.7925838 | 2 |
| 3259.7926936 | 4 |
| 3259.7928066 | 0 |
| 3259.794743 | 0 |
| 3259.7955266 | 3 |
| 3259.7964976 | 0 |
| 3259.7975176 | 0 |
| 3259.7981426 | 2 |
| 3259.7993611 | 2 |
| 3259.7997949 | 2 |
| 3259.8016258 | 3 |
| 3259.8016654 | 1 |
| 3259.8027284 | 0 |
| 3259.8038285 | 2 |
| 3259.8047066 | 1 |
| 3259.8056634 | 2 |
| 3259.8061684 | 2 |
| 3259.8075944 | 0 |
| 3259.8087177 | 2 |
| 3259.8091199 | 1 |
| 3259.8109241 | 1 |
| 3259.8113801 | 2 |
| 3259.8126035 | 0 |
| 3259.8134526 | 3 |
| 3259.8139513 | 2 |
| 3259.8150593 | 3 |
| 3259.8154971 | 4 |
| 3259.816644 | 3 |
| 3259.8177435 | 1 |
| 3259.8185656 | 0 |
| 3259.8198101 | 3 |
| 3259.8201375 | 6 |
| 3259.8218031 | 6 |
| 3259.8228767 | 1 |
| 3259.8231505 | 2 |
| 3259.8248208 | 2 |
| 3259.8256701 | 0 |
| 3259.8262643 | 1 |
| 3259.8272508 | 2 |
| 3259.8287055 | 2 |
| 3259.8293943 | 1 |
| 3259.8301787 | 3 |
| 3259.8315245 | 3 |
| 3259.8317556 | 0 |
| 3259.8330895 | 2 |
| 3259.8341075 | 1 |
| 3259.8350189 | 3 |
| 3259.835774 | 2 |
| 3259.8366067 | 1 |
| 3259.8378335 | 4 |
| 3259.8388555 | 4 |
| 3259.839387 | 1 |
| 3259.8407501 | 3 |
| 3259.8418124 | 4 |
| 3259.8423031 | 4 |
| 3259.8434988 | 0 |
| 3259.8444984 | 2 |
| 3259.8448167 | 2 |
| 3259.8460792 | 3 |
| 3259.8466536 | 3 |
| 3259.8480049 | 3 |
| 3259.8484621 | 0 |
| 3259.8497231 | 5 |
| 3259.8504591 | 1 |
| 3259.8511904 | 3 |
| 3259.8530137 | 1 |
| 3259.85321 | 2 |
| 3259.8546183 | 3 |
| 3259.8550444 | 0 |
| 3259.8562447 | 3 |
| 3259.8565896 | 2 |
| 3259.8581098 | 0 |
| 3259.8592936 | 1 |
| 3259.8601097 | 1 |
| 3259.8610704 | 3 |
| 3259.8621837 | 2 |
| 3259.8622502 | 0 |
| 3259.8641194 | 0 |
| 3259.8646295 | 1 |
| 3259.8651984 | 1 |
| 3259.8662966 | 0 |
| 3259.867384 | 2 |
| 3259.8686278 | 5 |
| 3259.8689406 | 2 |
| 3259.8705673 | 0 |
| 3259.8706651 | 1 |
| 3259.8722343 | 1 |
| 3259.8738229 | 1 |
| 3259.8739505 | 0 |
| 3259.8752098 | 1 |
| 3259.8761857 | 0 |
| 3259.8772828 | 4 |

| | |
|---|---|
| 3259.8780932 | 4 |
| 3259.8783966 | 3 |
| 3259.879743 | 2 |
| 3259.8802447 | 2 |
| 3259.8821149 | 2 |
| 3259.8825275 | 3 |
| 3259.8834912 | 2 |
| 3259.8846692 | 2 |
| 3259.885608 | 3 |
| 3259.8861275 | 1 |
| 3259.8873878 | 2 |
| 3259.8882354 | 1 |
| 3259.8894528 | 0 |
| 3259.8895214 | 3 |
| 3259.8912022 | 0 |
| 3259.8919199 | 1 |
| 3259.8925289 | 3 |
| 3259.893936 | 1 |
| 3259.8947065 | 1 |
| 3259.895292 | 3 |
| 3259.8964098 | 2 |
| 3259.8970531 | 0 |
| 3259.8985623 | 2 |
| 3259.8993858 | 2 |
| 3259.9002487 | 1 |
| 3259.9013363 | 3 |
| 3259.9020856 | 1 |
| 3259.9028446 | 1 |
| 3259.9033381 | 5 |
| 3259.9050971 | 2 |
| 3259.906087 | 1 |
| 3259.906542 | 2 |
| 3259.9076141 | 1 |
| 3259.9084267 | 1 |
| 3259.9098509 | 1 |
| 3259.9110494 | 1 |
| 3259.9111465 | 0 |
| 3259.9126103 | 2 |
| 3259.913032 | 2 |
| 3259.9139312 | 0 |
| 3259.9145129 | 2 |
| 3259.9148208 | 2 |
| 3259.9149757 | 2 |
| 3259.9150747 | 2 |
| 3259.9153852 | 3 |
| 3259.9161886 | 1 |
| 3259.9164003 | 5 |
| 3259.9165267 | 3 |
| 3259.9167247 | 1 |
| 3259.916816 | 1 |
| 3259.9169752 | 3 |
| 3259.917018 | 0 |
| 3259.9170209 | 1 |
| 3259.9176296 | 1 |
| 3259.9178162 | 2 |
| 3259.9180349 | 2 |
| 3259.9181476 | 5 |
| 3259.9182084 | 0 |
| 3259.9187949 | 2 |
| 3259.9191714 | 2 |
| 3259.9196059 | 1 |
| 3259.9199402 | 1 |
| 3259.9201304 | 4 |
| 3259.9201555 | 0 |
| 3259.9202415 | 3 |
| 3259.9204545 | 0 |
| 3259.9207013 | 3 |
| 3259.9209063 | 1 |
| 3259.9212165 | 4 |
| 3259.9215536 | 2 |
| 3259.9216261 | 2 |
| 3259.9218759 | 1 |
| 3259.9220182 | 0 |
| 3259.9221986 | 2 |
| 3259.9224571 | 3 |
| 3259.9226405 | 2 |
| 3259.9228375 | 1 |
| 3259.9229237 | 0 |
| 3259.9230439 | 2 |
| 3259.9233847 | 1 |
| 3259.924242 | 1 |
| 3259.924269 | 1 |
| 3259.9242792 | 3 |
| 3259.9250033 | 1 |
| 3259.9250883 | 2 |
| 3259.9252602 | 5 |
| 3259.9253311 | 2 |
| 3259.9254705 | 3 |
| 3259.9260673 | 1 |
| 3259.9260938 | 3 |
| 3259.9261536 | 0 |
| 3259.9265419 | 0 |
| 3259.9265432 | 4 |
| 3259.9269849 | 7 |
| 3259.9270641 | 2 |
| 3259.9275385 | 5 |
| 3259.927746 | 2 |
| 3259.9279461 | 5 |
| 3259.9281653 | 2 |
| 3259.9285345 | 9 |
| 3259.9285833 | 6 |
| 3259.9287125 | 11 |
| 3259.9290666 | 16 |
| 3259.9291194 | 16 |
| 3259.9293112 | 46 |
| 3259.9295342 | 22 |
| 3259.9303414 | 56 |
| 3259.9304027 | 41 |
| 3259.9305246 | 32 |
| 3259.9308357 | 46 |
| 3259.9312112 | 48 |
| 3259.9314835 | 58 |
| 3259.9314979 | 55 |
| 3259.9315944 | 53 |
| 3259.9316148 | 73 |
| 3259.9319301 | 49 |
| 3259.9325554 | 39 |
| 3259.9326532 | 38 |
| 3259.9328615 | 54 |
| 3259.9330799 | 24 |
| 3259.9337501 | 33 |
| 3259.9338128 | 43 |
| 3259.9338514 | 44 |
| 3259.9340489 | 48 |
| 3259.9343988 | 56 |
| 3259.9345086 | 33 |
| 3259.934699 | 43 |
| 3259.935039 | 58 |
| 3259.9352506 | 52 |
| 3259.9352922 | 70 |
| 3259.9359717 | 45 |
| 3259.9360268 | 42 |
| 3259.9360915 | 50 |
| 3259.9361291 | 50 |
| 3259.9363843 | 37 |
| 3259.9370413 | 52 |
| 3259.9370617 | 40 |
| 3259.937171 | 55 |
| 3259.937612 | 32 |
| 3259.9378279 | 37 |
| 3259.9379618 | 36 |
| 3259.938098 | 39 |
| 3259.9382346 | 42 |
| 3259.9384883 | 29 |
| 3259.9386585 | 41 |
| 3259.9387218 | 32 |
| 3259.9389434 | 23 |
| 3259.9392631 | 26 |
| 3259.9397391 | 15 |
| 3259.940028 | 8 |
| 3259.9402787 | 3 |
| 3259.9403526 | 4 |
| 3259.9405531 | 2 |
| 3259.9407461 | 7 |
| 3259.9414513 | 1 |
| 3259.941575 | 0 |
| 3259.941595 | 4 |
| 3259.9419864 | 1 |
| 3259.9420817 | 3 |
| 3259.9423264 | 2 |
| 3259.942688 | 2 |
| 3259.9427764 | 0 |
| 3259.9428399 | 4 |
| 3259.9431346 | 0 |
| 3259.9435305 | 3 |
| 3259.9436716 | 6 |
| 3259.9437487 | 1 |
| 3259.9437856 | 1 |
| 3259.9443299 | 2 |
| 3259.9450514 | 0 |
| 3259.9450798 | 3 |
| 3259.9456134 | 1 |
| 3259.9456443 | 5 |
| 3259.9457049 | 4 |
| 3259.9457452 | 2 |
| 3259.9457476 | 0 |
| 3259.9461698 | 1 |
| 3259.9464834 | 1 |
| 3259.9467351 | 3 |
| 3259.9470108 | 1 |
| 3259.9472247 | 0 |
| 3259.9472953 | 1 |
| 3259.9473906 | 3 |
| 3259.9474381 | 3 |
| 3259.9478094 | 4 |
| 3259.9478835 | 4 |
| 3259.9485814 | 3 |
| 3259.948703 | 1 |
| 3259.9490229 | 2 |
| 3259.9492386 | 0 |
| 3259.9492925 | 1 |
| 3259.9496641 | 3 |
| 3259.9499636 | 3 |
| 3259.9507711 | 2 |
| 3259.9513637 | 1 |
| 3259.9527323 | 1 |
| 3259.9534207 | 0 |
| 3259.9537511 | 1 |
| 3259.9553303 | 0 |
| 3259.9559702 | 1 |
| 3259.9570911 | 2 |
| 3259.9582359 | 4 |
| 3259.9582467 | 0 |
| 3259.9597983 | 0 |
| 3259.9614601 | 1 |
| 3259.9614887 | 2 |
| 3259.9627744 | 0 |
| 3259.9637004 | 2 |
| 3259.9646091 | 0 |
| 3259.9657319 | 2 |
| 3259.9661308 | 2 |
| 3259.9670259 | 1 |
| 3259.9682132 | 2 |
| 3259.9695739 | 2 |
| 3259.9701651 | 1 |
| 3259.971372 | 2 |
| 3259.9719401 | 0 |
| 3259.9723592 | 2 |
| 3259.9738802 | 1 |
| 3259.9750376 | 2 |
| 3259.9759503 | 0 |
| 3259.9765808 | 0 |
| 3259.9771769 | 4 |
| 3259.9787966 | 2 |
| 3259.9798721 | 3 |
| 3259.9799616 | 3 |
| 3259.9813093 | 2 |
| 3259.9823856 | 0 |
| 3259.9828189 | 3 |
| 3259.9836049 | 1 |
| 3259.9850307 | 0 |
| 3259.9863699 | 2 |
| 3259.986546 | 3 |
| 3259.9875065 | 1 |
| 3259.9887959 | 3 |
| 3259.9894164 | 1 |
| 3259.9906726 | 0 |
| 3259.9913727 | 2 |
| 3259.9923521 | 2 |
| 3259.9927286 | 0 |
| 3259.9951407 | 1 |
| 3259.9953594 | 1 |
| 3259.9954829 | 4 |
| 3259.9964278 | 2 |
| 3259.9978551 | 2 |
| 3259.9983781 | 4 |
| 3259.9990494 | 3 |
| 3260.0003298 | 2 |
| 3260.001547 | 3 |
| 3260.0024804 | 3 |
| 3260.0033188 | 0 |
| 3260.0043347 | 2 |
| 3260.0046935 | 2 |
| 3260.006265 | 4 |
| 3260.0068556 | 1 |
| 3260.0079905 | 2 |
| 3260.009646 | 1 |
| 3260.0100623 | 2 |
| 3260.0106833 | 4 |
| 3260.0107225 | 2 |
| 3260.0124753 | 2 |
| 3260.0142971 | 3 |
| 3260.0148987 | 0 |
| 3260.0159807 | 3 |
| 3260.0167209 | 2 |
| 3260.0174013 | 1 |
| 3260.0179631 | 4 |
| 3260.0190016 | 2 |
| 3260.0198154 | 0 |
| 3260.0205995 | 0 |
| 3260.0217741 | 1 |
| 3260.022867 | 2 |
| 3260.0237267 | 1 |
| 3260.0249468 | 3 |
| 3260.0252391 | 3 |
| 3260.026389 | 3 |
| 3260.0281112 | 2 |
| 3260.0284714 | 1 |
| 3260.0294599 | 4 |
| 3260.0296205 | 0 |
| 3260.0313283 | 1 |
| 3260.031764 | 1 |
| 3260.0331168 | 0 |
| 3260.0339579 | 3 |
| 3260.0344741 | 1 |
| 3260.0354632 | 5 |
| 3260.0361218 | 0 |
| 3260.0365639 | 3 |
| 3260.0384499 | 1 |
| 3260.0388192 | 1 |
| 3260.0397895 | 1 |
| 3260.0408003 | 1 |
| 3260.04226 | 0 |
| 3260.0422652 | 0 |
| 3260.044187 | 1 |
| 3260.0446106 | 2 |
| 3260.044696 | 3 |
| 3260.0458556 | 2 |
| 3260.0470032 | 5 |
| 3260.0474569 | 1 |
| 3260.0490962 | 0 |
| 3260.0499735 | 0 |
| 3260.0508372 | 0 |
| 3260.0513634 | 5 |
| 3260.0529678 | 1 |
| 3260.0537554 | 1 |
| 3260.0539377 | 1 |
| 3260.055421 | 0 |
| 3260.056155 | 0 |
| 3260.0563948 | 0 |
| 3260.0574225 | 1 |
| 3260.0577889 | 3 |
| 3260.0581234 | 1 |
| 3260.0581501 | 1 |
| 3260.0586895 | 2 |
| 3260.0595863 | 1 |
| 3260.0597111 | 1 |
| 3260.0604125 | 1 |
| 3260.060715 | 0 |
| 3260.0608214 | 2 |
| 3260.0611075 | 1 |
| 3260.0617513 | 2 |
| 3260.0634743 | 1 |
| 3260.0640434 | 2 |
| 3260.0653901 | 0 |
| 3260.0666192 | 2 |
| 3260.0672658 | 2 |
| 3260.0675732 | 1 |
| 3260.0686491 | 0 |
| 3260.0703702 | 0 |
| 3260.0705286 | 2 |
| 3260.0719623 | 4 |
| 3260.0721433 | 3 |
| 3260.0730638 | 2 |
| 3260.0744138 | 2 |
| 3260.0750851 | 2 |
| 3260.0761688 | 0 |
| 3260.077441 | 1 |
| 3260.0780522 | 1 |
| 3260.0790456 | 1 |
| 3260.0797106 | 4 |
| 3260.0813197 | 0 |
| 3260.082609 | 3 |
| 3260.0828493 | 1 |
| 3260.0841588 | 1 |
| 3260.0842655 | 4 |
| 3260.0858902 | 2 |
| 3260.0867745 | 1 |
| 3260.0874572 | 1 |
| 3260.0883691 | 2 |
| 3260.0890521 | 2 |
| 3260.0910813 | 0 |
| 3260.0913028 | 5 |
| 3260.0924565 | 1 |
| 3260.0935435 | 2 |
| 3260.0940742 | 0 |
| 3260.0952294 | 2 |
| 3260.0957621 | 0 |
| 3260.0967724 | 1 |
| 3260.0975242 | 1 |
| 3260.0987207 | 2 |
| 3260.1000246 | 1 |
| 3260.1012312 | 5 |

| | |
|---|---|
| 3260.1017552 | 2 |
| 3260.1026523 | 5 |
| 3260.1035857 | 2 |
| 3260.1043578 | 2 |
| 3260.1050789 | 3 |
| 3260.1067006 | 0 |
| 3260.1076741 | 2 |
| 3260.1083675 | 1 |
| 3260.1093193 | 4 |
| 3260.1095884 | 0 |
| 3260.1107051 | 1 |
| 3260.1122221 | 2 |
| 3260.1129557 | 2 |
| 3260.1138873 | 1 |
| 3260.1147374 | 3 |
| 3260.1154511 | 3 |
| 3260.1168336 | 1 |
| 3260.1171016 | 2 |
| 3260.1184238 | 2 |
| 3260.1195442 | 1 |
| 3260.1209663 | 1 |
| 3260.1216306 | 2 |
| 3260.1224677 | 2 |
| 3260.1229986 | 0 |
| 3260.1231954 | 2 |
| 3260.1247948 | 2 |
| 3260.1261779 | 3 |
| 3260.1266631 | 1 |
| 3260.1278554 | 2 |
| 3260.1280668 | 1 |
| 3260.1298158 | 1 |
| 3260.1308024 | 1 |
| 3260.1314507 | 1 |
| 3260.1326247 | 1 |
| 3260.1330825 | 0 |
| 3260.134325 | 5 |
| 3260.1351511 | 0 |
| 3260.1358239 | 1 |
| 3260.1370852 | 3 |
| 3260.1373662 | 1 |
| 3260.1388191 | 1 |
| 3260.1397733 | 3 |
| 3260.1408967 | 1 |
| 3260.1423119 | 3 |
| 3260.142352 | 4 |
| 3260.1438064 | 0 |
| 3260.1444853 | 1 |
| 3260.1452849 | 3 |
| 3260.1465048 | 2 |
| 3260.1473011 | 2 |
| 3260.1483119 | 2 |
| 3260.1490397 | 3 |
| 3260.150278 | 3 |
| 3260.1513348 | 3 |
| 3260.1523144 | 0 |
| 3260.1536439 | 1 |
| 3260.1537377 | 0 |
| 3260.1554636 | 2 |
| 3260.1560435 | 1 |
| 3260.1566202 | 1 |
| 3260.157974 | 0 |
| 3260.1587165 | 1 |
| 3260.1599277 | 0 |
| 3260.1606247 | 2 |
| 3260.1613876 | 1 |
| 3260.1621461 | 0 |
| 3260.1632626 | 1 |
| 3260.1641389 | 2 |
| 3260.1651401 | 2 |
| 3260.166063 | 1 |
| 3260.1669759 | 0 |
| 3260.167933 | 0 |
| 3260.1686409 | 1 |
| 3260.1702116 | 2 |
| 3260.1702712 | 1 |
| 3260.1719407 | 1 |
| 3260.1724095 | 2 |
| 3260.1734666 | 2 |
| 3260.1737527 | 3 |
| 3260.1752665 | 2 |
| 3260.1764916 | 0 |
| 3260.1772144 | 3 |
| 3260.1785938 | 3 |
| 3260.1787504 | 0 |
| 3260.1801093 | 0 |
| 3260.181061 | 2 |
| 3260.1815514 | 3 |
| 3260.182874 | 2 |
| 3260.1837907 | 2 |
| 3260.1848649 | 1 |
| 3260.1859337 | 1 |
| 3260.1868569 | 2 |
| 3260.1877023 | 2 |
| 3260.188264 | 1 |
| 3260.1897348 | 1 |
| 3260.1907519 | 2 |
| 3260.1916525 | 1 |
| 3260.1929945 | 2 |
| 3260.1932374 | 4 |
| 3260.1943325 | 1 |
| 3260.1957375 | 2 |
| 3260.1959382 | 4 |
| 3260.1972098 | 0 |
| 3260.1981624 | 2 |
| 3260.1988851 | 3 |
| 3260.2000795 | 6 |
| 3260.2003013 | 5 |
| 3260.201988 | 3 |
| 3260.202322 | 8 |
| 3260.203757 | 2 |
| 3260.2040599 | 3 |
| 3260.2054438 | 1 |
| 3260.2064611 | 2 |
| 3260.2064621 | 5 |
| 3260.208037 | 0 |
| 3260.2092565 | 2 |
| 3260.2100803 | 1 |
| 3260.2112385 | 4 |
| 3260.2117158 | 1 |
| 3260.2127908 | 2 |
| 3260.2136048 | 2 |
| 3260.2145335 | 0 |
| 3260.2155573 | 1 |
| 3260.2168951 | 2 |
| 3260.2178067 | 2 |
| 3260.2179529 | 3 |
| 3260.2199446 | 0 |
| 3260.2199616 | 1 |
| 3260.221035 | 3 |
| 3260.2220078 | 0 |
| 3260.2230678 | 0 |
| 3260.2242314 | 3 |
| 3260.2245567 | 3 |
| 3260.2259117 | 4 |
| 3260.2265933 | 2 |
| 3260.2274059 | 1 |
| 3260.2286933 | 2 |
| 3260.2294818 | 0 |
| 3260.2308832 | 3 |
| 3260.2313257 | 5 |
| 3260.2320207 | 1 |
| 3260.2334365 | 4 |
| 3260.2339242 | 0 |
| 3260.2354514 | 1 |
| 3260.2358707 | 0 |
| 3260.2368601 | 2 |
| 3260.2382368 | 5 |
| 3260.2387857 | 3 |
| 3260.2400171 | 1 |
| 3260.2403636 | 4 |
| 3260.2416987 | 0 |
| 3260.2431534 | 1 |
| 3260.2434507 | 0 |
| 3260.2442696 | 2 |
| 3260.2453351 | 2 |
| 3260.2470954 | 2 |
| 3260.2475066 | 1 |
| 3260.2486089 | 1 |
| 3260.2498714 | 0 |
| 3260.249946 | 3 |
| 3260.2507915 | 0 |
| 3260.2518983 | 2 |
| 3260.2523522 | 3 |
| 3260.2538743 | 2 |
| 3260.2542221 | 1 |
| 3260.2555453 | 2 |
| 3260.2566065 | 6 |
| 3260.257667 | 1 |
| 3260.2588282 | 2 |
| 3260.2588636 | 2 |
| 3260.2602909 | 1 |
| 3260.2611041 | 1 |
| 3260.2615378 | 3 |
| 3260.2626997 | 4 |
| 3260.2644437 | 3 |
| 3260.2653038 | 1 |
| 3260.2656836 | 1 |
| 3260.2670339 | 2 |
| 3260.2679867 | 1 |
| 3260.2680656 | 1 |
| 3260.2699477 | 3 |
| 3260.2709956 | 1 |
| 3260.271324 | 0 |
| 3260.2723601 | 2 |
| 3260.273415 | 2 |
| 3260.2746599 | 2 |
| 3260.275093 | 0 |
| 3260.2767304 | 1 |
| 3260.2773155 | 1 |
| 3260.2776661 | 2 |
| 3260.2785161 | 3 |
| 3260.2801208 | 5 |
| 3260.2807439 | 1 |
| 3260.2818306 | 1 |
| 3260.2831744 | 3 |
| 3260.2837115 | 1 |
| 3260.2850918 | 3 |
| 3260.2852097 | 1 |
| 3260.2870692 | 1 |
| 3260.2873594 | 1 |
| 3260.2883146 | 0 |
| 3260.2890897 | 2 |
| 3260.2905648 | 2 |
| 3260.291799 | 1 |
| 3260.2921982 | 3 |
| 3260.2930725 | 2 |
| 3260.2938158 | 0 |
| 3260.2952109 | 1 |
| 3260.2956377 | 2 |
| 3260.2964073 | 0 |
| 3260.2976315 | 2 |
| 3260.2982741 | 1 |
| 3260.2990302 | 2 |
| 3260.2999925 | 4 |
| 3260.3013336 | 1 |
| 3260.3024923 | 1 |
| 3260.3026004 | 0 |
| 3260.3040052 | 2 |
| 3260.3047386 | 2 |
| 3260.3056162 | 2 |
| 3260.3068808 | 4 |
| 3260.3077264 | 2 |
| 3260.3081095 | 1 |
| 3260.3098551 | 4 |
| 3260.3099635 | 1 |
| 3260.3114553 | 0 |
| 3260.3120904 | 1 |
| 3260.3136566 | 3 |
| 3260.3137737 | 2 |
| 3260.3147748 | 2 |
| 3260.3166205 | 1 |
| 3260.3167885 | 4 |
| 3260.3177719 | 0 |
| 3260.3195996 | 0 |
| 3260.3199654 | 2 |
| 3260.3205423 | 6 |
| 3260.3219179 | 2 |
| 3260.3229644 | 1 |
| 3260.3229884 | 1 |
| 3260.3245059 | 2 |
| 3260.3258142 | 3 |
| 3260.3261972 | 1 |
| 3260.3274315 | 1 |
| 3260.3280197 | 2 |
| 3260.3291011 | 0 |
| 3260.3305826 | 2 |
| 3260.3306351 | 1 |
| 3260.3309861 | 0 |
| 3260.3313681 | 4 |
| 3260.3314226 | 1 |
| 3260.3316272 | 3 |
| 3260.3319662 | 1 |
| 3260.3325008 | 1 |
| 3260.3325101 | 1 |
| 3260.3325604 | 1 |
| 3260.3328111 | 4 |
| 3260.3330172 | 1 |
| 3260.3335409 | 5 |
| 3260.3341588 | 3 |
| 3260.334253 | 3 |
| 3260.3347674 | 5 |
| 3260.3347799 | 3 |
| 3260.3348749 | 3 |
| 3260.3353353 | 2 |
| 3260.335351 | 2 |
| 3260.3355768 | 1 |
| 3260.3358912 | 0 |
| 3260.336251 | 3 |
| 3260.3365684 | 1 |
| 3260.3368638 | 2 |
| 3260.3370378 | 0 |
| 3260.3372321 | 3 |
| 3260.3372979 | 1 |
| 3260.3376076 | 1 |
| 3260.3383044 | 2 |
| 3260.3384759 | 0 |
| 3260.3386391 | 0 |
| 3260.3390857 | 4 |
| 3260.3392049 | 3 |
| 3260.3396078 | 1 |
| 3260.3398792 | 1 |
| 3260.3399369 | 1 |
| 3260.3399912 | 3 |
| 3260.3401677 | 1 |
| 3260.3407799 | 3 |
| 3260.3409014 | 2 |
| 3260.3415934 | 3 |
| 3260.3417961 | 0 |
| 3260.3419032 | 2 |
| 3260.3419897 | 3 |
| 3260.3422423 | 3 |
| 3260.3425232 | 3 |
| 3260.3429317 | 1 |
| 3260.3430564 | 2 |
| 3260.3431927 | 2 |
| 3260.343793 | 3 |
| 3260.34381 | 1 |
| 3260.3439489 | 3 |
| 3260.3439797 | 2 |
| 3260.3440951 | 1 |
| 3260.3445672 | 1 |
| 3260.3448157 | 4 |
| 3260.3449516 | 1 |
| 3260.3452685 | 1 |
| 3260.3455136 | 1 |
| 3260.3458735 | 1 |
| 3260.3460318 | 3 |
| 3260.346214 | 4 |
| 3260.3466329 | 1 |
| 3260.3471905 | 0 |
| 3260.3474369 | 1 |
| 3260.3474712 | 0 |
| 3260.3474813 | 3 |
| 3260.3476922 | 0 |
| 3260.3482155 | 0 |
| 3260.3482932 | 2 |
| 3260.3483367 | 6 |
| 3260.3486279 | 1 |
| 3260.3487224 | 5 |
| 3260.3497491 | 2 |
| 3260.3498048 | 5 |
| 3260.349843 | 10 |
| 3260.3499086 | 14 |
| 3260.3501104 | 4 |
| 3260.3502022 | 26 |
| 3260.3503261 | 28 |
| 3260.3505698 | 26 |
| 3260.3511509 | 50 |
| 3260.3512897 | 46 |
| 3260.351498 | 71 |
| 3260.351682 | 58 |
| 3260.3519519 | 49 |
| 3260.3519568 | 67 |
| 3260.3526167 | 73 |
| 3260.3526295 | 69 |
| 3260.3528388 | 76 |
| 3260.3531604 | 66 |
| 3260.3532307 | 65 |
| 3260.3538226 | 101 |
| 3260.353881 | 70 |
| 3260.3541901 | 77 |
| 3260.3543133 | 81 |
| 3260.3547067 | 80 |
| 3260.3547243 | 77 |
| 3260.3547966 | 77 |
| 3260.3550484 | 80 |
| 3260.3553099 | 91 |
| 3260.3557932 | 76 |
| 3260.3558859 | 89 |
| 3260.3561796 | 84 |
| 3260.3563396 | 71 |
| 3260.3564949 | 92 |
| 3260.3567321 | 78 |
| 3260.3571932 | 90 |
| 3260.3573884 | 81 |
| 3260.3574989 | 86 |
| 3260.3576334 | 74 |
| 3260.357692 | 72 |
| 3260.3577266 | 81 |
| 3260.3583084 | 86 |
| 3260.3587049 | 67 |
| 3260.3587396 | 70 |
| 3260.359165 | 80 |

| | |
|---|---|
| 3260.3592249 | 46 |
| 3260.3593346 | 82 |
| 3260.3597386 | 41 |
| 3260.3598666 | 56 |
| 3260.3601929 | 52 |
| 3260.3603499 | 22 |
| 3260.3605837 | 10 |
| 3260.3609003 | 5 |
| 3260.3609377 | 14 |
| 3260.3609496 | 27 |
| 3260.3612304 | 4 |
| 3260.3612616 | 5 |
| 3260.3615263 | 3 |
| 3260.3616344 | 2 |
| 3260.3626616 | 1 |
| 3260.362688 | 4 |
| 3260.3628202 | 2 |
| 3260.3628814 | 3 |
| 3260.3634701 | 3 |
| 3260.3636808 | 0 |
| 3260.3642312 | 2 |
| 3260.3642351 | 1 |
| 3260.3643312 | 1 |
| 3260.3644709 | 2 |
| 3260.3644766 | 3 |
| 3260.3646147 | 3 |
| 3260.3653329 | 3 |
| 3260.3653613 | 3 |
| 3260.3654557 | 0 |
| 3260.3657418 | 2 |
| 3260.3657816 | 4 |
| 3260.3662591 | 2 |
| 3260.3665822 | 1 |
| 3260.3666508 | 2 |
| 3260.3673142 | 0 |
| 3260.3674085 | 2 |
| 3260.3677314 | 2 |
| 3260.3677731 | 3 |
| 3260.3682872 | 2 |
| 3260.3683291 | 0 |
| 3260.3686244 | 2 |
| 3260.368704 | 2 |
| 3260.369933 | 4 |
| 3260.3710527 | 1 |
| 3260.3720328 | 5 |
| 3260.3730021 | 2 |
| 3260.3736485 | 2 |
| 3260.3749653 | 2 |
| 3260.3754426 | 1 |
| 3260.3763901 | 1 |
| 3260.3774888 | 1 |
| 3260.3779666 | 2 |
| 3260.3791184 | 0 |
| 3260.3803617 | 1 |
| 3260.381203 | 0 |
| 3260.3822838 | 2 |
| 3260.382897 | 2 |
| 3260.3839258 | 1 |
| 3260.3852008 | 1 |
| 3260.3861101 | 0 |
| 3260.3873755 | 3 |
| 3260.3877556 | 2 |
| 3260.3888913 | 1 |
| 3260.3890181 | 0 |
| 3260.3901401 | 2 |
| 3260.3911275 | 7 |
| 3260.3922533 | 2 |
| 3260.3931542 | 4 |
| 3260.3937617 | 2 |
| 3260.3953657 | 3 |
| 3260.3964524 | 0 |
| 3260.3964689 | 2 |
| 3260.3975235 | 2 |
| 3260.3987184 | 1 |
| 3260.399336 | 2 |
| 3260.4005627 | 2 |
| 3260.401298 | 2 |
| 3260.4023958 | 1 |
| 3260.4036483 | 0 |
| 3260.4045731 | 3 |
| 3260.4056578 | 1 |
| 3260.4057288 | 2 |
| 3260.4071436 | 4 |
| 3260.4075791 | 2 |
| 3260.408981 | 3 |
| 3260.4102855 | 1 |
| 3260.410435 | 0 |
| 3260.4118627 | 3 |
| 3260.4124249 | 2 |
| 3260.4134102 | 4 |
| 3260.4142125 | 4 |
| 3260.4157021 | 1 |
| 3260.4167468 | 2 |
| 3260.4168078 | 2 |
| 3260.418358 | 1 |
| 3260.419805 | 1 |
| 3260.4198762 | 2 |
| 3260.4208969 | 1 |
| 3260.4216122 | 4 |
| 3260.4231278 | 2 |
| 3260.4241827 | 2 |
| 3260.424196 | 1 |
| 3260.4258479 | 2 |
| 3260.4264472 | 1 |
| 3260.4277799 | 0 |
| 3260.4281975 | 2 |
| 3260.4291744 | 2 |
| 3260.4305837 | 2 |
| 3260.431357 | 3 |
| 3260.4321529 | 3 |
| 3260.4329168 | 1 |
| 3260.4340576 | 1 |
| 3260.4353079 | 4 |
| 3260.4356012 | 0 |
| 3260.4369216 | 2 |
| 3260.4373048 | 0 |
| 3260.4386942 | 1 |
| 3260.4398692 | 4 |
| 3260.4404328 | 7 |
| 3260.4415667 | 1 |
| 3260.4427622 | 1 |
| 3260.4432928 | 0 |
| 3260.4445415 | 1 |
| 3260.4451755 | 3 |
| 3260.4461396 | 0 |
| 3260.4470811 | 0 |
| 3260.447963 | 1 |
| 3260.4488796 | 0 |
| 3260.4499949 | 3 |
| 3260.451189 | 1 |
| 3260.4512865 | 3 |
| 3260.4529072 | 0 |
| 3260.4534173 | 0 |
| 3260.4545583 | 2 |
| 3260.4558824 | 3 |
| 3260.4563579 | 3 |
| 3260.4578276 | 1 |
| 3260.458827 | 1 |
| 3260.4592326 | 1 |
| 3260.4602095 | 3 |
| 3260.4612681 | 1 |
| 3260.4621036 | 4 |
| 3260.4626614 | 2 |
| 3260.4641546 | 1 |
| 3260.4652841 | 4 |
| 3260.4668423 | 2 |
| 3260.4671633 | 4 |
| 3260.4678449 | 5 |
| 3260.468372 | 2 |
| 3260.4696418 | 1 |
| 3260.470288 | 2 |
| 3260.471862 | 4 |
| 3260.4726926 | 0 |
| 3260.4730867 | 5 |
| 3260.4747418 | 1 |
| 3260.4751182 | 1 |
| 3260.4765786 | 5 |
| 3260.4776377 | 1 |
| 3260.4779881 | 10 |
| 3260.4788265 | 5 |
| 3260.479936 | 2 |
| 3260.4807672 | 1 |
| 3260.481739 | 2 |
| 3260.4830265 | 3 |
| 3260.4841329 | 2 |
| 3260.4843511 | 2 |
| 3260.4856173 | 3 |
| 3260.4858989 | 1 |
| 3260.4877299 | 1 |
| 3260.4884888 | 3 |
| 3260.4890504 | 2 |
| 3260.4903963 | 3 |
| 3260.4905267 | 1 |
| 3260.4920823 | 0 |
| 3260.4933247 | 2 |
| 3260.4938774 | 1 |
| 3260.4948908 | 1 |
| 3260.4957161 | 0 |
| 3260.4966226 | 1 |
| 3260.4978044 | 4 |
| 3260.4989741 | 1 |
| 3260.4996678 | 0 |
| 3260.5004044 | 0 |
| 3260.5014712 | 0 |
| 3260.502777 | 5 |
| 3260.502912 | 2 |
| 3260.5044861 | 2 |
| 3260.5050387 | 0 |
| 3260.5050977 | 1 |
| 3260.5056422 | 1 |
| 3260.506443 | 1 |
| 3260.5075288 | 2 |
| 3260.5077923 | 1 |
| 3260.5079223 | 5 |
| 3260.5083083 | 6 |
| 3260.5092516 | 0 |
| 3260.5096574 | 2 |
| 3260.5100851 | 1 |
| 3260.5102212 | 2 |
| 3260.5103894 | 2 |
| 3260.511018 | 0 |
| 3260.5119888 | 1 |
| 3260.5122604 | 2 |
| 3260.5132211 | 0 |
| 3260.5133917 | 4 |
| 3260.5135784 | 2 |
| 3260.5140293 | 1 |
| 3260.5145861 | 2 |
| 3260.5153421 | 3 |
| 3260.5153813 | 2 |
| 3260.516268 | 1 |
| 3260.5171416 | 1 |
| 3260.5173214 | 1 |
| 3260.5174323 | 6 |
| 3260.5174753 | 0 |
| 3260.5183579 | 2 |
| 3260.5192373 | 2 |
| 3260.5194107 | 1 |
| 3260.5199784 | 1 |
| 3260.5203988 | 1 |
| 3260.5205508 | 2 |
| 3260.5212738 | 2 |
| 3260.5213341 | 2 |
| 3260.522224 | 1 |
| 3260.522514 | 1 |
| 3260.5231543 | 3 |
| 3260.5238982 | 2 |
| 3260.5240118 | 0 |
| 3260.5245535 | 3 |
| 3260.5249788 | 2 |
| 3260.5255629 | 1 |
| 3260.526133 | 1 |
| 3260.526788 | 1 |
| 3260.5275982 | 3 |
| 3260.5277338 | 1 |
| 3260.5277835 | 0 |
| 3260.5280076 | 1 |
| 3260.5284257 | 2 |
| 3260.5292414 | 1 |
| 3260.5294549 | 1 |
| 3260.5302056 | 1 |
| 3260.5309193 | 2 |
| 3260.5310122 | 3 |
| 3260.5317021 | 1 |
| 3260.5317654 | 3 |
| 3260.5324545 | 1 |
| 3260.5327534 | 1 |
| 3260.5333196 | 4 |
| 3260.5340379 | 2 |
| 3260.5345756 | 0 |
| 3260.5349344 | 2 |
| 3260.5358584 | 2 |
| 3260.5359989 | 2 |
| 3260.536244 | 1 |
| 3260.5363285 | 3 |
| 3260.5373421 | 1 |
| 3260.5373892 | 4 |
| 3260.5378026 | 1 |
| 3260.5381865 | 1 |
| 3260.5392508 | 1 |
| 3260.5392725 | 4 |
| 3260.5397133 | 0 |
| 3260.5397734 | 3 |
| 3260.54098 | 2 |
| 3260.5410251 | 6 |
| 3260.5423563 | 1 |
| 3260.5425064 | 1 |
| 3260.5425318 | 1 |
| 3260.5427896 | 0 |
| 3260.5437459 | 1 |
| 3260.5439863 | 2 |
| 3260.5443186 | 1 |
| 3260.5456757 | 1 |
| 3260.5457141 | 2 |
| 3260.5460273 | 4 |
| 3260.5464529 | 2 |
| 3260.5474255 | 3 |
| 3260.5474464 | 1 |
| 3260.5476494 | 1 |
| 3260.548294 | 1 |
| 3260.5487195 | 1 |
| 3260.5498661 | 2 |
| 3260.5500716 | 3 |
| 3260.5502772 | 3 |
| 3260.5505208 | 1 |
| 3260.5513319 | 2 |
| 3260.5519173 | 1 |
| 3260.5523003 | 2 |
| 3260.552581 | 0 |
| 3260.5532935 | 2 |
| 3260.5539676 | 1 |
| 3260.5540693 | 1 |
| 3260.5549735 | 3 |
| 3260.5553902 | 2 |
| 3260.5558254 | 1 |
| 3260.5567021 | 0 |
| 3260.5569755 | 2 |
| 3260.5570647 | 1 |
| 3260.5571757 | 2 |
| 3260.5580178 | 2 |
| 3260.5582528 | 4 |
| 3260.5591245 | 2 |
| 3260.5596677 | 0 |
| 3260.5600147 | 3 |
| 3260.5608279 | 1 |
| 3260.5608833 | 4 |
| 3260.5614437 | 2 |
| 3260.562036 | 3 |
| 3260.5620832 | 3 |
| 3260.5625617 | 0 |
| 3260.5635303 | 1 |
| 3260.563736 | 2 |
| 3260.5645779 | 1 |
| 3260.5646088 | 1 |
| 3260.5648532 | 0 |
| 3260.5660072 | 2 |
| 3260.5662067 | 2 |
| 3260.5663982 | 2 |
| 3260.5666555 | 1 |
| 3260.5678287 | 2 |
| 3260.5680943 | 0 |
| 3260.5681688 | 3 |
| 3260.5690444 | 2 |
| 3260.5694011 | 0 |
| 3260.5695007 | 1 |
| 3260.5705119 | 0 |
| 3260.5707229 | 4 |
| 3260.5712341 | 2 |
| 3260.5713217 | 2 |
| 3260.571873 | 0 |
| 3260.5724692 | 6 |
| 3260.5727485 | 2 |
| 3260.5731675 | 0 |
| 3260.5743797 | 2 |
| 3260.574398 | 5 |
| 3260.5751844 | 2 |
| 3260.5753853 | 4 |
| 3260.5756755 | 0 |
| 3260.5764996 | 2 |
| 3260.5773055 | 3 |
| 3260.5777074 | 0 |
| 3260.5778663 | 2 |
| 3260.5780655 | 1 |
| 3260.5792624 | 1 |
| 3260.5792915 | 2 |
| 3260.5793242 | 3 |
| 3260.5799038 | 3 |
| 3260.580674 | 3 |
| 3260.5809788 | 1 |
| 3260.5814854 | 2 |
| 3260.5817985 | 1 |
| 3260.5824076 | 0 |
| 3260.5826195 | 3 |
| 3260.5837718 | 1 |
| 3260.5839822 | 0 |
| 3260.5844979 | 3 |
| 3260.585358 | 1 |
| 3260.5855708 | 3 |
| 3260.5856821 | 2 |
| 3260.5864241 | 2 |
| 3260.5867252 | 2 |
| 3260.5867446 | 4 |
| 3260.5877857 | 3 |

| | |
|---|---|
| 3260.5882573 | 3 |
| 3260.5888152 | 2 |
| 3260.5888684 | 4 |
| 3260.5892166 | 0 |
| 3260.5898628 | 0 |
| 3260.5902305 | 1 |
| 3260.5910433 | 1 |
| 3260.5913895 | 1 |
| 3260.591845 | 5 |
| 3260.5921715 | 1 |
| 3260.5932658 | 5 |
| 3260.5932772 | 2 |
| 3260.593586 | 2 |
| 3260.5945017 | 6 |
| 3260.5946452 | 0 |
| 3260.5948131 | 1 |
| 3260.5959715 | 1 |
| 3260.596337 | 3 |
| 3260.596409 | 0 |
| 3260.5966068 | 3 |
| 3260.5975082 | 44 |
| 3260.5979044 | 2 |
| 3260.5981534 | 11 |
| 3260.5986979 | 29 |
| 3260.598809 | 21 |
| 3260.5995319 | 66 |
| 3260.5995483 | 42 |
| 3260.5997356 | 62 |
| 3260.6001878 | 74 |
| 3260.60086 | 80 |
| 3260.6013055 | 66 |
| 3260.6013193 | 51 |
| 3260.6016881 | 4 |
| 3260.603286 | 49 |
| 3260.6032971 | 51 |
| 3260.6033362 | 61 |
| 3260.6043688 | 49 |
| 3260.6046512 | 62 |
| 3260.6052303 | 68 |
| 3260.6053593 | 76 |
| 3260.6061454 | 65 |
| 3260.6062559 | 62 |
| 3260.6066957 | 54 |
| 3260.607465 | 51 |
| 3260.6075145 | 56 |
| 3260.6084962 | 41 |
| 3260.6092757 | 16 |
| 3260.609827 | 7 |
| 3260.6103667 | 5 |
| 3260.6110373 | 3 |
| 3260.6113924 | 1 |
| 3260.6119535 | 1 |
| 3260.6129304 | 2 |
| 3260.6136748 | 2 |
| 3260.6137093 | 5 |
| 3260.6149427 | 1 |
| 3260.6157584 | 1 |
| 3260.616565 | 1 |
| 3260.6173358 | 3 |
| 3260.6187365 | 3 |
| 3260.6201107 | 1 |
| 3260.6206991 | 2 |
| 3260.6214132 | 0 |
| 3260.6228956 | 1 |
| 3260.6237643 | 2 |
| 3260.6244319 | 1 |
| 3260.6252102 | 2 |
| 3260.6266233 | 0 |
| 3260.6269202 | 4 |
| 3260.6280019 | 3 |
| 3260.6294244 | 2 |
| 3260.6300869 | 1 |
| 3260.631307 | 3 |
| 3260.631994 | 3 |
| 3260.6330252 | 1 |
| 3260.6344582 | 2 |
| 3260.6351021 | 1 |
| 3260.6361108 | 2 |
| 3260.6368526 | 1 |
| 3260.6378579 | 4 |
| 3260.6383713 | 2 |
| 3260.6392652 | 1 |
| 3260.6410651 | 1 |
| 3260.641295 | 1 |
| 3260.6428433 | 3 |
| 3260.643413 | 1 |
| 3260.6445503 | 2 |
| 3260.6455472 | 4 |
| 3260.6464685 | 1 |
| 3260.647542 | 0 |
| 3260.647572 | 1 |
| 3260.6490404 | 3 |
| 3260.6504568 | 1 |
| 3260.6505966 | 1 |
| 3260.651821 | 1 |
| 3260.6526016 | 3 |
| 3260.6539582 | 3 |
| 3260.6552041 | 3 |
| 3260.655498 | 3 |
| 3260.6567599 | 0 |
| 3260.6574667 | 2 |
| 3260.6585485 | 4 |
| 3260.6600785 | 1 |
| 3260.660461 | 1 |
| 3260.6611486 | 0 |
| 3260.6620288 | 1 |
| 3260.6629331 | 3 |
| 3260.6637106 | 4 |
| 3260.6647751 | 2 |
| 3260.6664292 | 1 |
| 3260.666506 | 1 |
| 3260.6678537 | 0 |
| 3260.6685879 | 2 |
| 3260.6694085 | 2 |
| 3260.6708218 | 2 |
| 3260.6715517 | 2 |
| 3260.6726384 | 1 |
| 3260.6730213 | 1 |
| 3260.6746488 | 3 |
| 3260.675515 | 0 |
| 3260.6760023 | 1 |
| 3260.6773437 | 4 |
| 3260.6779029 | 3 |
| 3260.6789584 | 1 |
| 3260.6800619 | 3 |
| 3260.6812201 | 1 |
| 3260.6819953 | 1 |
| 3260.6823184 | 0 |
| 3260.6836955 | 1 |
| 3260.6842645 | 1 |
| 3260.6847599 | 0 |
| 3260.6858657 | 1 |
| 3260.6871778 | 2 |
| 3260.6883008 | 1 |
| 3260.689349 | 0 |
| 3260.6904197 | 2 |
| 3260.6912334 | 2 |
| 3260.6918392 | 0 |
| 3260.6928523 | 2 |
| 3260.6942103 | 1 |
| 3260.6950242 | 3 |
| 3260.6964832 | 1 |
| 3260.6966797 | 4 |
| 3260.698172 | 0 |
| 3260.6984366 | 1 |
| 3260.6996002 | 0 |
| 3260.7011382 | 2 |
| 3260.7012557 | 0 |
| 3260.702386 | 0 |
| 3260.703807 | 2 |
| 3260.7043581 | 0 |
| 3260.7056048 | 1 |
| 3260.7062992 | 2 |
| 3260.7074193 | 3 |
| 3260.7075427 | 0 |
| 3260.7089741 | 1 |
| 3260.7093029 | 3 |
| 3260.7102219 | 2 |
| 3260.7116517 | 1 |
| 3260.7122974 | 3 |
| 3260.71331 | 0 |
| 3260.7147285 | 2 |
| 3260.7154483 | 1 |
| 3260.7161969 | 3 |
| 3260.7168247 | 1 |
| 3260.7183959 | 0 |
| 3260.7191897 | 4 |
| 3260.7198863 | 1 |
| 3260.7210032 | 0 |
| 3260.7217436 | 1 |
| 3260.7229863 | 3 |
| 3260.723924 | 3 |
| 3260.7242702 | 3 |
| 3260.7258831 | 0 |
| 3260.726105 | 3 |
| 3260.7276162 | 2 |
| 3260.7283473 | 3 |
| 3260.7290011 | 3 |
| 3260.7302648 | 2 |
| 3260.7313441 | 4 |
| 3260.732376 | 5 |
| 3260.7330075 | 1 |
| 3260.7340823 | 1 |
| 3260.7355645 | 1 |
| 3260.7360106 | 2 |
| 3260.7371227 | 2 |
| 3260.7380682 | 0 |
| 3260.7390375 | 1 |
| 3260.7401928 | 1 |
| 3260.7405855 | 2 |
| 3260.7420078 | 4 |
| 3260.7427474 | 1 |
| 3260.7434571 | 0 |
| 3260.7449477 | 0 |
| 3260.7457369 | 1 |
| 3260.7463325 | 1 |
| 3260.7475245 | 3 |
| 3260.7482872 | 5 |
| 3260.7497235 | 0 |
| 3260.7500997 | 3 |
| 3260.750918 | 1 |
| 3260.7512806 | 0 |
| 3260.7528102 | 1 |
| 3260.7544478 | 0 |
| 3260.7547769 | 2 |
| 3260.7554538 | 2 |
| 3260.7564593 | 3 |
| 3260.7582596 | 2 |
| 3260.7585755 | 1 |
| 3260.7593388 | 1 |
| 3260.7607174 | 2 |
| 3260.7611771 | 1 |
| 3260.7617519 | 2 |
| 3260.7632117 | 1 |
| 3260.7640247 | 4 |
| 3260.7651649 | 4 |
| 3260.7657478 | 2 |
| 3260.7673359 | 2 |
| 3260.7674377 | 2 |
| 3260.7682465 | 1 |
| 3260.7700993 | 2 |
| 3260.7708231 | 2 |
| 3260.7714695 | 2 |
| 3260.7726832 | 3 |
| 3260.7738397 | 1 |
| 3260.77462 | 1 |
| 3260.775426 | 1 |
| 3260.7764953 | 1 |
| 3260.7769557 | 1 |
| 3260.7782912 | 2 |
| 3260.7797561 | 0 |
| 3260.7798127 | 1 |
| 3260.7809436 | 5 |
| 3260.7820737 | 0 |
| 3260.7831736 | 5 |
| 3260.7838876 | 5 |
| 3260.784658 | 3 |
| 3260.7859378 | 3 |
| 3260.786205 | 2 |
| 3260.7875178 | 3 |
| 3260.7882122 | 0 |
| 3260.789233 | 0 |
| 3260.7904377 | 4 |
| 3260.7911685 | 1 |
| 3260.792479 | 2 |
| 3260.7929877 | 2 |
| 3260.7946517 | 1 |
| 3260.7952279 | 0 |
| 3260.7960423 | 3 |
| 3260.7971084 | 3 |
| 3260.7973543 | 1 |
| 3260.7987472 | 0 |
| 3260.7996339 | 0 |
| 3260.8004351 | 2 |
| 3260.8013136 | 2 |
| 3260.8027397 | 3 |
| 3260.8035693 | 2 |
| 3260.8041962 | 1 |
| 3260.8045709 | 0 |
| 3260.8054771 | 1 |
| 3260.8074349 | 1 |
| 3260.8075361 | 6 |
| 3260.8093303 | 0 |
| 3260.809896 | 2 |
| 3260.8106086 | 2 |
| 3260.8114355 | 1 |
| 3260.8128468 | 1 |
| 3260.8134595 | 4 |
| 3260.8143102 | 0 |
| 3260.8152043 | 2 |
| 3260.8162035 | 1 |
| 3260.8178928 | 1 |
| 3260.8185804 | 2 |
| 3260.8188866 | 3 |
| 3260.8204025 | 1 |
| 3260.820743 | 0 |
| 3260.8221025 | 2 |
| 3260.8222106 | 1 |
| 3260.823488 | 4 |
| 3260.8247735 | 6 |
| 3260.825509 | 0 |
| 3260.8262821 | 3 |
| 3260.8272141 | 2 |
| 3260.828686 | 1 |
| 3260.8297954 | 3 |
| 3260.8298031 | 1 |
| 3260.8315061 | 2 |
| 3260.8317501 | 0 |
| 3260.8330638 | 3 |
| 3260.8342231 | 0 |
| 3260.8342966 | 3 |
| 3260.8355237 | 1 |
| 3260.8370471 | 1 |
| 3260.8380857 | 3 |
| 3260.8384399 | 2 |
| 3260.8389093 | 4 |
| 3260.8403426 | 1 |
| 3260.8406495 | 0 |
| 3260.8420034 | 3 |
| 3260.8433178 | 0 |
| 3260.8437577 | 2 |
| 3260.8449741 | 0 |
| 3260.845391 | 1 |
| 3260.8465251 | 0 |
| 3260.8476194 | 0 |
| 3260.8487892 | 2 |
| 3260.8495347 | 4 |
| 3260.8501348 | 5 |
| 3260.8514262 | 0 |
| 3260.8523989 | 1 |
| 3260.8530412 | 0 |
| 3260.8548823 | 0 |
| 3260.8549302 | 1 |
| 3260.8560007 | 2 |
| 3260.8564847 | 2 |
| 3260.8578115 | 3 |
| 3260.8586642 | 1 |
| 3260.8596512 | 3 |
| 3260.8606672 | 3 |
| 3260.861959 | 2 |
| 3260.8622222 | 1 |
| 3260.8633571 | 5 |
| 3260.8650934 | 0 |
| 3260.865503 | 4 |
| 3260.8665359 | 3 |
| 3260.8670003 | 2 |
| 3260.8683161 | 3 |
| 3260.8687382 | 1 |
| 3260.8698127 | 2 |
| 3260.8709843 | 2 |
| 3260.8718584 | 0 |
| 3260.8732965 | 0 |
| 3260.873619 | 0 |
| 3260.8744705 | 0 |
| 3260.8753541 | 3 |
| 3260.8767086 | 3 |
| 3260.877698 | 0 |
| 3260.8783786 | 1 |
| 3260.8798718 | 0 |
| 3260.8799039 | 1 |
| 3260.8808752 | 3 |
| 3260.8826558 | 1 |
| 3260.8829807 | 3 |
| 3260.8838627 | 1 |
| 3260.8845656 | 4 |
| 3260.8855185 | 2 |
| 3260.8866229 | 1 |
| 3260.8877016 | 1 |
| 3260.8888513 | 1 |
| 3260.8896207 | 2 |
| 3260.8896741 | 1 |
| 3260.8914381 | 2 |
| 3260.8916741 | 0 |
| 3260.8930173 | 0 |
| 3260.8936983 | 1 |
| 3260.894752 | 5 |
| 3260.8956889 | 0 |
| 3260.8960388 | 3 |
| 3260.8974881 | 2 |
| 3260.8986657 | 1 |
| 3260.8991849 | 0 |
| 3260.9005604 | 1 |
| 3260.9007585 | 0 |

| | |
|---|---|
| 3260.9022134 | 0 |
| 3260.9030807 | 3 |
| 3260.903453 | 4 |
| 3260.9050066 | 0 |
| 3260.9054822 | 2 |
| 3260.9068801 | 0 |
| 3260.9085467 | 0 |
| 3260.9089282 | 0 |
| 3260.9102415 | 5 |
| 3260.9105057 | 1 |
| 3260.9114662 | 1 |
| 3260.9132837 | 4 |
| 3260.9134546 | 2 |
| 3260.9144101 | 1 |
| 3260.9157166 | 0 |
| 3260.9160865 | 0 |
| 3260.9168003 | 1 |
| 3260.918308 | 3 |
| 3260.9195509 | 0 |
| 3260.9198726 | 0 |
| 3260.9209489 | 3 |
| 3260.9220656 | 1 |
| 3260.9232493 | 0 |
| 3260.9235006 | 2 |
| 3260.9247557 | 1 |
| 3260.9256014 | 1 |
| 3260.9269462 | 2 |
| 3260.9280164 | 0 |
| 3260.928783 | 1 |
| 3260.9295497 | 0 |
| 3260.9307991 | 2 |
| 3260.9314777 | 6 |
| 3260.9317083 | 2 |
| 3260.9330387 | 1 |
| 3260.9340258 | 1 |
| 3260.9354555 | 0 |
| 3260.9355028 | 3 |
| 3260.9365894 | 2 |
| 3260.9384825 | 4 |
| 3260.9386877 | 4 |
| 3260.9397856 | 4 |
| 3260.9404243 | 2 |
| 3260.9415654 | 1 |
| 3260.9419577 | 2 |
| 3260.9431474 | 3 |
| 3260.9443639 | 2 |
| 3260.945129 | 3 |
| 3260.9454647 | 0 |
| 3260.9468194 | 1 |
| 3260.9479107 | 2 |
| 3260.94833 | 1 |
| 3260.9494646 | 0 |
| 3260.9507597 | 1 |
| 3260.9515605 | 2 |
| 3260.9519827 | 1 |
| 3260.9533311 | 2 |
| 3260.9541272 | 0 |
| 3260.955171 | 2 |
| 3260.9566443 | 1 |
| 3260.9572161 | 1 |
| 3260.9586013 | 1 |
| 3260.9590307 | 3 |
| 3260.9604373 | 2 |
| 3260.9613531 | 3 |
| 3260.9616787 | 3 |
| 3260.9629933 | 4 |
| 3260.9635638 | 0 |
| 3260.9645871 | 0 |
| 3260.9656249 | 1 |
| 3260.9667141 | 3 |
| 3260.9675307 | 1 |
| 3260.9684548 | 1 |
| 3260.9695361 | 1 |
| 3260.969936 | 2 |
| 3260.9711817 | 0 |
| 3260.9720854 | 2 |
| 3260.973051 | 0 |
| 3260.9735775 | 0 |
| 3260.9753651 | 1 |
| 3260.9766685 | 0 |
| 3260.9771088 | 2 |
| 3260.977278 | 3 |
| 3260.9787807 | 2 |
| 3260.9802828 | 3 |
| 3260.9804464 | 2 |
| 3260.9815685 | 0 |
| 3260.9822581 | 3 |
| 3260.9831582 | 4 |
| 3260.9842304 | 0 |
| 3260.9849213 | 3 |
| 3260.9863198 | 1 |
| 3260.9865982 | 1 |
| 3260.9883589 | 2 |
| 3260.9887964 | 1 |
| 3260.9898228 | 3 |
| 3260.9908504 | 2 |
| 3260.991552 | 0 |
| 3260.9924961 | 1 |
| 3260.9934194 | 3 |
| 3260.994029 | 2 |
| 3260.9953395 | 2 |
| 3260.9958365 | 1 |
| 3260.9965732 | 0 |
| 3260.9977941 | 0 |
| 3260.998869 | 1 |
| 3260.9998216 | 0 |
| 3261.0005228 | 2 |
| 3261.0020624 | 3 |
| 3261.0023203 | 4 |
| 3261.0039112 | 2 |
| 3261.0049905 | 1 |
| 3261.0052399 | 3 |
| 3261.0066716 | 1 |
| 3261.0070037 | 5 |
| 3261.0086541 | 1 |
| 3261.0092007 | 2 |
| 3261.0099903 | 3 |
| 3261.01152 | 3 |
| 3261.0116565 | 1 |
| 3261.0130312 | 0 |
| 3261.0138727 | 2 |
| 3261.014649 | 4 |
| 3261.0152921 | 2 |
| 3261.0164948 | 1 |
| 3261.0176832 | 2 |
| 3261.0185703 | 3 |
| 3261.0192965 | 2 |
| 3261.0203487 | 3 |
| 3261.0205219 | 0 |
| 3261.0223184 | 1 |
| 3261.0224464 | 2 |
| 3261.0241588 | 1 |
| 3261.0251473 | 6 |
| 3261.0257023 | 0 |
| 3261.0270616 | 2 |
| 3261.0273725 | 2 |
| 3261.0292353 | 1 |
| 3261.0295166 | 2 |
| 3261.0305641 | 0 |
| 3261.031012 | 3 |
| 3261.0324787 | 2 |
| 3261.032681 | 2 |
| 3261.0342604 | 1 |
| 3261.0349615 | 4 |
| 3261.0360231 | 0 |
| 3261.0368627 | 4 |
| 3261.0378517 | 2 |
| 3261.0386976 | 5 |
| 3261.0392828 | 1 |
| 3261.0407526 | 2 |
| 3261.0418375 | 1 |
| 3261.0420634 | 1 |
| 3261.0437853 | 3 |
| 3261.0441278 | 2 |
| 3261.0451232 | 1 |
| 3261.0462937 | 2 |
| 3261.0470748 | 1 |
| 3261.0480579 | 2 |
| 3261.0488138 | 1 |
| 3261.0500617 | 0 |
| 3261.0502314 | 3 |
| 3261.0518234 | 2 |
| 3261.0529182 | 1 |
| 3261.053665 | 3 |
| 3261.0546656 | 1 |
| 3261.0553354 | 4 |
| 3261.0570373 | 2 |
| 3261.0580372 | 3 |
| 3261.0583153 | 2 |
| 3261.0590045 | 0 |
| 3261.0598163 | 3 |
| 3261.0610955 | 0 |
| 3261.0623625 | 0 |
| 3261.0629158 | 2 |
| 3261.0642989 | 0 |
| 3261.0651385 | 2 |
| 3261.0657406 | 2 |
| 3261.0666047 | 0 |
| 3261.0674336 | 3 |
| 3261.0689761 | 1 |
| 3261.0691133 | 1 |
| 3261.0707092 | 2 |
| 3261.0712834 | 2 |
| 3261.0726361 | 3 |
| 3261.0732448 | 1 |
| 3261.0736285 | 1 |
| 3261.0753627 | 1 |
| 3261.0757131 | 3 |
| 3261.0762014 | 1 |
| 3261.0776797 | 0 |
| 3261.0789505 | 1 |
| 3261.080085 | 0 |
| 3261.0803011 | 1 |
| 3261.0818111 | 3 |
| 3261.0831966 | 3 |
| 3261.0835617 | 2 |
| 3261.0848715 | 0 |
| 3261.0852954 | 1 |
| 3261.0862268 | 1 |
| 3261.0872294 | 2 |
| 3261.0883002 | 0 |
| 3261.0891883 | 1 |
| 3261.0897522 | 1 |
| 3261.0904358 | 5 |
| 3261.0921721 | 1 |
| 3261.0932222 | 1 |
| 3261.0942455 | 1 |
| 3261.0950209 | 4 |
| 3261.0961033 | 1 |
| 3261.0972031 | 2 |
| 3261.097323 | 2 |
| 3261.0990288 | 0 |
| 3261.0993493 | 4 |
| 3261.100241 | 3 |
| 3261.1014587 | 1 |
| 3261.102107 | 4 |
| 3261.1037552 | 3 |
| 3261.1053237 | 1 |
| 3261.1056107 | 3 |
| 3261.1061635 | 4 |
| 3261.1078471 | 2 |
| 3261.1084868 | 2 |
| 3261.108788 | 5 |
| 3261.1099736 | 2 |
| 3261.1105884 | 0 |
| 3261.1122035 | 6 |
| 3261.1124879 | 2 |
| 3261.1133082 | 2 |
| 3261.1145872 | 1 |
| 3261.1153117 | 4 |
| 3261.1163052 | 1 |
| 3261.1176076 | 1 |
| 3261.1185463 | 1 |
| 3261.1201141 | 0 |
| 3261.1203965 | 1 |
| 3261.1217779 | 2 |
| 3261.1219061 | 4 |
| 3261.1235211 | 1 |
| 3261.1237967 | 3 |
| 3261.1251044 | 1 |
| 3261.1255434 | 0 |
| 3261.1261375 | 2 |
| 3261.1272826 | 4 |
| 3261.1284414 | 3 |
| 3261.1299564 | 1 |
| 3261.1304549 | 1 |
| 3261.1308959 | 2 |
| 3261.1309149 | 2 |
| 3261.1312798 | 4 |
| 3261.1317908 | 2 |
| 3261.1327066 | 4 |
| 3261.1331733 | 3 |
| 3261.1335151 | 2 |
| 3261.1335284 | 1 |
| 3261.1337531 | 1 |
| 3261.1338925 | 3 |
| 3261.1340637 | 0 |
| 3261.1343272 | 2 |
| 3261.1352306 | 2 |
| 3261.1357007 | 2 |
| 3261.1361508 | 4 |
| 3261.1362479 | 1 |
| 3261.1365329 | 1 |
| 3261.1369092 | 2 |
| 3261.1374055 | 3 |
| 3261.1378635 | 3 |
| 3261.1380734 | 3 |
| 3261.1384 | 3 |
| 3261.1391233 | 1 |
| 3261.1394808 | 3 |
| 3261.1395292 | 1 |
| 3261.1397359 | 5 |
| 3261.1405099 | 3 |
| 3261.1409066 | 2 |
| 3261.1410108 | 2 |
| 3261.1412291 | 1 |
| 3261.1419889 | 3 |
| 3261.1422369 | 2 |
| 3261.1426429 | 1 |
| 3261.1429159 | 4 |
| 3261.1434389 | 2 |
| 3261.1435149 | 1 |
| 3261.1441355 | 0 |
| 3261.144744 | 1 |
| 3261.1448216 | 1 |
| 3261.1453245 | 4 |
| 3261.1454909 | 0 |
| 3261.1466071 | 2 |
| 3261.1466344 | 5 |
| 3261.1466973 | 4 |
| 3261.1469661 | 4 |
| 3261.1476248 | 1 |
| 3261.1478746 | 1 |
| 3261.1486093 | 3 |
| 3261.1487301 | 1 |
| 3261.1488236 | 4 |
| 3261.1499422 | 1 |
| 3261.1499709 | 3 |
| 3261.1503943 | 1 |
| 3261.1504138 | 1 |
| 3261.1511725 | 3 |
| 3261.1512958 | 2 |
| 3261.1513449 | 3 |
| 3261.1519833 | 4 |
| 3261.1526859 | 0 |
| 3261.1530364 | 0 |
| 3261.153419 | 5 |
| 3261.153479 | 2 |
| 3261.1535017 | 0 |
| 3261.1541023 | 2 |
| 3261.1546227 | 3 |
| 3261.1548364 | 3 |
| 3261.1555007 | 1 |
| 3261.1559867 | 1 |
| 3261.1561783 | 1 |
| 3261.1565073 | 0 |
| 3261.1566394 | 4 |
| 3261.1569184 | 0 |
| 3261.157811 | 0 |
| 3261.1579313 | 3 |
| 3261.158153 | 3 |
| 3261.1587304 | 0 |
| 3261.1590672 | 1 |
| 3261.1592928 | 3 |
| 3261.1596662 | 2 |
| 3261.1598051 | 3 |
| 3261.1608028 | 2 |
| 3261.1613046 | 2 |
| 3261.1616751 | 0 |
| 3261.1617758 | 2 |
| 3261.1622281 | 3 |
| 3261.1624697 | 2 |
| 3261.1628408 | 6 |
| 3261.1630417 | 1 |
| 3261.1632157 | 2 |
| 3261.1644445 | 4 |
| 3261.1653855 | 2 |
| 3261.1654331 | 3 |
| 3261.166053 | 1 |
| 3261.1664532 | 1 |
| 3261.16742 | 5 |
| 3261.1676809 | 3 |
| 3261.1684585 | 3 |
| 3261.1692901 | 2 |
| 3261.169816 | 0 |
| 3261.1702402 | 2 |
| 3261.1707857 | 1 |
| 3261.1714595 | 3 |
| 3261.1719333 | 1 |
| 3261.1728312 | 3 |
| 3261.1736605 | 5 |
| 3261.1737042 | 5 |
| 3261.1742212 | 4 |
| 3261.1751396 | 2 |
| 3261.1753699 | 4 |
| 3261.17617 | 1 |
| 3261.1770033 | 2 |
| 3261.1775079 | 5 |
| 3261.1781495 | 1 |
| 3261.1791139 | 1 |
| 3261.1794823 | 4 |
| 3261.1799229 | 0 |
| 3261.1807493 | 3 |

| | |
|---|---|
| 3261.1809948 | 2 |
| 3261.1812938 | 1 |
| 3261.182424 | 5 |
| 3261.1828858 | 3 |
| 3261.1831161 | 1 |
| 3261.1842414 | 1 |
| 3261.1849946 | 3 |
| 3261.1852097 | 3 |
| 3261.1861201 | 6 |
| 3261.1866579 | 5 |
| 3261.1867828 | 1 |
| 3261.1877429 | 2 |
| 3261.1888441 | 3 |
| 3261.1891404 | 5 |
| 3261.1894369 | 1 |
| 3261.1902243 | 1 |
| 3261.1904098 | 4 |
| 3261.1912616 | 3 |
| 3261.191645 | 3 |
| 3261.1925808 | 4 |
| 3261.1930856 | 1 |
| 3261.1938877 | 5 |
| 3261.1945623 | 3 |
| 3261.1954088 | 3 |
| 3261.1958689 | 1 |
| 3261.1967004 | 2 |
| 3261.1973085 | 4 |
| 3261.1976688 | 3 |
| 3261.1979634 | 1 |
| 3261.1987296 | 5 |
| 3261.1989533 | 2 |
| 3261.1998257 | 3 |
| 3261.2012495 | 2 |
| 3261.2012728 | 1 |
| 3261.2025858 | 2 |
| 3261.2027287 | 1 |
| 3261.2032726 | 1 |
| 3261.2042008 | 4 |
| 3261.2045073 | 4 |
| 3261.2050865 | 0 |
| 3261.2061989 | 1 |
| 3261.2066561 | 2 |
| 3261.2071762 | 2 |
| 3261.2073555 | 5 |
| 3261.2076278 | 0 |
| 3261.2078105 | 2 |
| 3261.2082993 | 3 |
| 3261.2085763 | 5 |
| 3261.2089629 | 2 |
| 3261.2092151 | 3 |
| 3261.2093595 | 4 |
| 3261.2094257 | 2 |
| 3261.2097033 | 3 |
| 3261.2102091 | 4 |
| 3261.210742 | 4 |
| 3261.2109035 | 3 |
| 3261.2110486 | 5 |
| 3261.2111049 | 1 |
| 3261.2112417 | 4 |
| 3261.2114477 | 2 |
| 3261.2118077 | 3 |
| 3261.212208 | 4 |
| 3261.2125208 | 3 |
| 3261.2127431 | 2 |
| 3261.2129311 | 1 |
| 3261.2131659 | 5 |
| 3261.2132996 | 3 |
| 3261.2136281 | 1 |
| 3261.2139439 | 2 |
| 3261.2140183 | 3 |
| 3261.2142925 | 4 |
| 3261.2145355 | 1 |
| 3261.2148244 | 2 |
| 3261.2153843 | 2 |
| 3261.21541 | 3 |
| 3261.2158163 | 4 |
| 3261.2164315 | 2 |
| 3261.2164336 | 5 |
| 3261.2164542 | 4 |
| 3261.2169571 | 4 |
| 3261.2170554 | 3 |
| 3261.2173521 | 2 |
| 3261.2175725 | 1 |
| 3261.2178413 | 1 |
| 3261.2180377 | 2 |
| 3261.2181252 | 1 |
| 3261.2184834 | 4 |
| 3261.2186739 | 1 |
| 3261.2190264 | 1 |
| 3261.2190401 | 6 |
| 3261.2191066 | 2 |
| 3261.2193408 | 3 |
| 3261.2199156 | 3 |
| 3261.2201844 | 0 |
| 3261.2202966 | 3 |
| 3261.2202984 | 2 |
| 3261.2206999 | 2 |
| 3261.2209274 | 2 |
| 3261.2209931 | 2 |
| 3261.221036 | 2 |
| 3261.2214892 | 0 |
| 3261.2218859 | 2 |
| 3261.2220732 | 2 |
| 3261.2224274 | 4 |
| 3261.222516 | 5 |
| 3261.222758 | 3 |
| 3261.2229526 | 2 |
| 3261.2231536 | 3 |
| 3261.2232426 | 2 |
| 3261.2234129 | 4 |
| 3261.2236431 | 4 |
| 3261.2239962 | 5 |
| 3261.224254 | 7 |
| 3261.224761 | 0 |
| 3261.224882 | 1 |
| 3261.2253135 | 1 |
| 3261.2253168 | 0 |
| 3261.2254445 | 2 |
| 3261.2255866 | 7 |
| 3261.2256944 | 2 |
| 3261.2263878 | 3 |
| 3261.2268665 | 4 |
| 3261.2269787 | 3 |
| 3261.2271587 | 5 |
| 3261.2272164 | 0 |
| 3261.2273599 | 2 |
| 3261.227437 | 4 |
| 3261.2276083 | 1 |
| 3261.2281811 | 3 |
| 3261.2284299 | 3 |
| 3261.22885 | 3 |
| 3261.2289034 | 3 |
| 3261.2290036 | 4 |
| 3261.2291317 | 3 |
| 3261.2291987 | 3 |
| 3261.2294178 | 3 |
| 3261.2297119 | 5 |
| 3261.229855 | 2 |
| 3261.229905 | 1 |
| 3261.2299227 | 2 |
| 3261.2300092 | 4 |
| 3261.2306021 | 3 |
| 3261.2312172 | 4 |
| 3261.2315864 | 5 |
| 3261.2317745 | 3 |
| 3261.2318251 | 4 |
| 3261.2318601 | 3 |
| 3261.2319734 | 2 |
| 3261.2324637 | 1 |
| 3261.2330243 | 2 |
| 3261.2333405 | 1 |
| 3261.233357 | 2 |
| 3261.2334487 | 1 |
| 3261.2336249 | 1 |
| 3261.2338235 | 2 |
| 3261.2341086 | 1 |
| 3261.2344476 | 0 |
| 3261.2345928 | 1 |
| 3261.2348422 | 1 |
| 3261.2357 | 6 |
| 3261.2357258 | 2 |
| 3261.2358567 | 4 |
| 3261.2359626 | 16 |
| 3261.2361898 | 4 |
| 3261.2363002 | 3 |
| 3261.2363089 | 8 |
| 3261.2367027 | 10 |
| 3261.2370334 | 30 |
| 3261.2370574 | 27 |
| 3261.237419 | 46 |
| 3261.2375931 | 66 |
| 3261.2378483 | 31 |
| 3261.2378935 | 55 |
| 3261.2379843 | 53 |
| 3261.2384091 | 67 |
| 3261.2385513 | 76 |
| 3261.2386027 | 74 |
| 3261.2389792 | 79 |
| 3261.2392469 | 70 |
| 3261.2393332 | 78 |
| 3261.2397046 | 81 |
| 3261.239803 | 92 |
| 3261.2406491 | 77 |
| 3261.2406749 | 98 |
| 3261.240848 | 88 |
| 3261.2410194 | 83 |
| 3261.2411825 | 85 |
| 3261.2416614 | 74 |
| 3261.2418791 | 84 |
| 3261.2424052 | 70 |
| 3261.2426144 | 86 |
| 3261.2426721 | 90 |
| 3261.2429805 | 67 |
| 3261.2430402 | 83 |
| 3261.2433927 | 82 |
| 3261.243417 | 75 |
| 3261.2438315 | 80 |
| 3261.2439049 | 91 |
| 3261.2441541 | 78 |
| 3261.244781 | 89 |
| 3261.2451483 | 75 |
| 3261.2452949 | 84 |
| 3261.2453251 | 80 |
| 3261.2454517 | 85 |
| 3261.2455704 | 106 |
| 3261.2457756 | 66 |
| 3261.2459182 | 78 |
| 3261.2465698 | 56 |
| 3261.2469884 | 32 |
| 3261.2470103 | 50 |
| 3261.2470974 | 64 |
| 3261.2473397 | 23 |
| 3261.247745 | 17 |
| 3261.2477683 | 11 |
| 3261.248071 | 16 |
| 3261.2484332 | 4 |
| 3261.2487036 | 2 |
| 3261.2487055 | 9 |
| 3261.2491109 | 4 |
| 3261.2491254 | 2 |
| 3261.2495753 | 0 |
| 3261.2496707 | 4 |
| 3261.2499247 | 5 |
| 3261.2501372 | 4 |
| 3261.2507098 | 0 |
| 3261.2509201 | 3 |
| 3261.2511923 | 1 |
| 3261.2516535 | 1 |
| 3261.2517556 | 4 |
| 3261.2518641 | 0 |
| 3261.2519548 | 2 |
| 3261.2520032 | 0 |
| 3261.2522279 | 2 |
| 3261.2525945 | 3 |
| 3261.2530284 | 1 |
| 3261.2532138 | 0 |
| 3261.253283 | 2 |
| 3261.2536387 | 1 |
| 3261.253845 | 2 |
| 3261.2540909 | 1 |
| 3261.2543323 | 4 |
| 3261.2545944 | 3 |
| 3261.2547763 | 4 |
| 3261.2548146 | 2 |
| 3261.2553304 | 6 |
| 3261.2553692 | 1 |
| 3261.2558053 | 3 |
| 3261.2558207 | 4 |
| 3261.2560371 | 1 |
| 3261.2564555 | 2 |
| 3261.2569304 | 4 |
| 3261.2569761 | 2 |
| 3261.2570621 | 5 |
| 3261.2576086 | 2 |
| 3261.2577337 | 5 |
| 3261.2577724 | 2 |
| 3261.257831 | 1 |
| 3261.2581644 | 0 |
| 3261.2583224 | 3 |
| 3261.2589103 | 1 |
| 3261.259243 | 2 |
| 3261.2592592 | 2 |
| 3261.259413 | 3 |
| 3261.2597231 | 5 |
| 3261.2598148 | 1 |
| 3261.25987 | 1 |
| 3261.260644 | 3 |
| 3261.2609576 | 3 |
| 3261.2611653 | 1 |
| 3261.2611845 | 3 |
| 3261.2613079 | 0 |
| 3261.2617066 | 0 |
| 3261.2617362 | 0 |
| 3261.2618589 | 0 |
| 3261.2622813 | 4 |
| 3261.2624882 | 2 |
| 3261.262794 | 4 |
| 3261.262804 | 0 |
| 3261.2629684 | 2 |
| 3261.2630087 | 2 |
| 3261.2636474 | 0 |
| 3261.2642497 | 2 |
| 3261.2643198 | 2 |
| 3261.2643525 | 2 |
| 3261.2644679 | 3 |
| 3261.2647008 | 3 |
| 3261.2648044 | 2 |
| 3261.2651737 | 4 |
| 3261.2652221 | 4 |
| 3261.265636 | 2 |
| 3261.2663877 | 5 |
| 3261.2676659 | 2 |
| 3261.2678107 | 1 |
| 3261.2691507 | 3 |
| 3261.2699243 | 3 |
| 3261.2700171 | 2 |
| 3261.2717816 | 1 |
| 3261.2726439 | 3 |
| 3261.2738828 | 1 |
| 3261.2744706 | 4 |
| 3261.2753031 | 1 |
| 3261.2757934 | 1 |
| 3261.2771327 | 3 |
| 3261.2779953 | 1 |
| 3261.2790465 | 3 |
| 3261.2796482 | 0 |
| 3261.2811638 | 0 |
| 3261.2815134 | 4 |
| 3261.283045 | 2 |
| 3261.2842345 | 1 |
| 3261.2848084 | 5 |
| 3261.2860617 | 3 |
| 3261.2865809 | 2 |
| 3261.2878138 | 3 |
| 3261.2882182 | 4 |
| 3261.2891487 | 1 |
| 3261.2904737 | 0 |
| 3261.291336 | 2 |
| 3261.2922096 | 3 |
| 3261.2932832 | 6 |
| 3261.2939637 | 2 |
| 3261.2954137 | 2 |
| 3261.296077 | 2 |
| 3261.297179 | 0 |
| 3261.2975838 | 1 |
| 3261.2990248 | 2 |
| 3261.3003856 | 1 |
| 3261.3009193 | 3 |
| 3261.3014467 | 1 |
| 3261.3025456 | 1 |
| 3261.3030902 | 1 |
| 3261.3035688 | 3 |
| 3261.3047478 | 2 |
| 3261.305192 | 2 |
| 3261.3055085 | 1 |
| 3261.3066261 | 3 |
| 3261.3071092 | 4 |
| 3261.3072214 | 3 |
| 3261.3075613 | 1 |
| 3261.3079926 | 2 |
| 3261.3086889 | 1 |
| 3261.3087896 | 2 |
| 3261.309587 | 4 |
| 3261.3096514 | 3 |
| 3261.3100888 | 3 |
| 3261.3103828 | 1 |
| 3261.3113887 | 1 |
| 3261.3115818 | 1 |
| 3261.3120884 | 1 |
| 3261.3125569 | 7 |
| 3261.312603 | 1 |
| 3261.3137035 | 3 |
| 3261.3144084 | 6 |
| 3261.3144406 | 5 |
| 3261.3144749 | 3 |
| 3261.3154279 | 2 |
| 3261.3168298 | 1 |
| 3261.3173472 | 0 |
| 3261.3178809 | 3 |
| 3261.3181456 | 2 |
| 3261.3189735 | 3 |
| 3261.319075 | 4 |
| 3261.3196368 | 2 |

| | |
|---|---|
| 3261.3201607 | 1 |
| 3261.3203624 | 4 |
| 3261.3210051 | 3 |
| 3261.321625 | 0 |
| 3261.3216822 | 2 |
| 3261.3222271 | 1 |
| 3261.3234543 | 3 |
| 3261.3234607 | 2 |
| 3261.324869 | 2 |
| 3261.3250056 | 0 |
| 3261.3258717 | 1 |
| 3261.3280736 | 2 |
| 3261.3286068 | 2 |
| 3261.3292925 | 3 |
| 3261.3303956 | 3 |
| 3261.3314441 | 1 |
| 3261.3322059 | 5 |
| 3261.3332091 | 4 |
| 3261.333909 | 1 |
| 3261.3351369 | 2 |
| 3261.3355201 | 2 |
| 3261.3363579 | 3 |
| 3261.3373116 | 1 |
| 3261.3386114 | 6 |
| 3261.3396749 | 4 |
| 3261.3402572 | 1 |
| 3261.3418377 | 2 |
| 3261.3431639 | 2 |
| 3261.343951 | 4 |
| 3261.3445262 | 1 |
| 3261.3458501 | 6 |
| 3261.3474471 | 2 |
| 3261.3474955 | 5 |
| 3261.3487501 | 3 |
| 3261.3499462 | 2 |
| 3261.3499903 | 2 |
| 3261.3509583 | 4 |
| 3261.3524301 | 2 |
| 3261.3532137 | 2 |
| 3261.3542866 | 0 |
| 3261.3546902 | 6 |
| 3261.3562363 | 0 |
| 3261.3564678 | 2 |
| 3261.3579068 | 1 |
| 3261.3587141 | 0 |
| 3261.3597252 | 3 |
| 3261.3598628 | 3 |
| 3261.3607503 | 0 |
| 3261.3623312 | 1 |
| 3261.3634223 | 7 |
| 3261.3642639 | 1 |
| 3261.3646066 | 1 |
| 3261.3657665 | 4 |
| 3261.3667264 | 4 |
| 3261.3677911 | 2 |
| 3261.3682047 | 3 |
| 3261.3692884 | 0 |
| 3261.3709149 | 2 |
| 3261.3709752 | 2 |
| 3261.3719577 | 6 |
| 3261.371966 | 1 |
| 3261.372301 | 1 |
| 3261.3729283 | 1 |
| 3261.3734727 | 3 |
| 3261.3736813 | 2 |
| 3261.3745236 | 0 |
| 3261.3749339 | 7 |
| 3261.3755818 | 3 |
| 3261.3760354 | 4 |
| 3261.3771265 | 2 |
| 3261.3776342 | 3 |
| 3261.3779134 | 4 |
| 3261.378467 | 3 |
| 3261.3793318 | 2 |
| 3261.3797811 | 4 |
| 3261.3798087 | 2 |
| 3261.3799908 | 1 |
| 3261.380435 | 4 |
| 3261.3816105 | 2 |
| 3261.3821011 | 2 |
| 3261.3829184 | 3 |
| 3261.3829462 | 4 |
| 3261.3832852 | 2 |
| 3261.3843027 | 3 |
| 3261.3852944 | 2 |
| 3261.3855194 | 4 |
| 3261.3856421 | 3 |
| 3261.3868384 | 2 |
| 3261.386908 | 2 |
| 3261.3869956 | 1 |
| 3261.3875464 | 2 |
| 3261.3881506 | 2 |
| 3261.3887164 | 1 |
| 3261.388721 | 2 |
| 3261.3892311 | 0 |
| 3261.3901526 | 3 |
| 3261.3904651 | 1 |
| 3261.3913941 | 2 |
| 3261.3913991 | 2 |
| 3261.3914608 | 2 |
| 3261.3925804 | 3 |
| 3261.3929738 | 5 |
| 3261.3931475 | 1 |
| 3261.3939463 | 3 |
| 3261.3946423 | 3 |
| 3261.3951473 | 3 |
| 3261.3954252 | 2 |
| 3261.3956743 | 0 |
| 3261.396153 | 2 |
| 3261.3967128 | 2 |
| 3261.3972444 | 1 |
| 3261.3975099 | 1 |
| 3261.3981723 | 3 |
| 3261.3984154 | 2 |
| 3261.3987427 | 2 |
| 3261.3995755 | 2 |
| 3261.4002488 | 2 |
| 3261.4007954 | 0 |
| 3261.4009241 | 0 |
| 3261.4015967 | 2 |
| 3261.4017271 | 3 |
| 3261.4018995 | 2 |
| 3261.4023372 | 2 |
| 3261.4027778 | 5 |
| 3261.403201 | 4 |
| 3261.4032828 | 2 |
| 3261.4036288 | 5 |
| 3261.4037906 | 3 |
| 3261.404097 | 2 |
| 3261.4041201 | 1 |
| 3261.4042377 | 4 |
| 3261.4044398 | 2 |
| 3261.4045256 | 2 |
| 3261.4046739 | 3 |
| 3261.4048576 | 1 |
| 3261.4052206 | 3 |
| 3261.4052897 | 3 |
| 3261.4055445 | 2 |
| 3261.405548 | 1 |
| 3261.4056657 | 3 |
| 3261.4061921 | 2 |
| 3261.4062527 | 5 |
| 3261.4064073 | 2 |
| 3261.4064768 | 3 |
| 3261.4067728 | 3 |
| 3261.4068069 | 2 |
| 3261.4070095 | 1 |
| 3261.4070459 | 3 |
| 3261.4073599 | 1 |
| 3261.4076212 | 2 |
| 3261.4076313 | 2 |
| 3261.4077258 | 0 |
| 3261.4080807 | 3 |
| 3261.4081581 | 6 |
| 3261.4082896 | 0 |
| 3261.4086301 | 0 |
| 3261.4088871 | 2 |
| 3261.4089285 | 4 |
| 3261.4091476 | 4 |
| 3261.4093362 | 3 |
| 3261.4095707 | 2 |
| 3261.4097039 | 3 |
| 3261.4098942 | 3 |
| 3261.4100114 | 1 |
| 3261.4100712 | 0 |
| 3261.4103628 | 4 |
| 3261.4104175 | 3 |
| 3261.4105457 | 4 |
| 3261.4107827 | 2 |
| 3261.4110484 | 2 |
| 3261.4112313 | 2 |
| 3261.411264 | 4 |
| 3261.4114247 | 3 |
| 3261.4118057 | 2 |
| 3261.4121997 | 1 |
| 3261.4123967 | 0 |
| 3261.4124339 | 6 |
| 3261.412644 | 3 |
| 3261.4127339 | 8 |
| 3261.4129535 | 3 |
| 3261.4135323 | 0 |
| 3261.4135659 | 6 |
| 3261.4136215 | 2 |
| 3261.4137894 | 1 |
| 3261.414011 | 1 |
| 3261.4141339 | 2 |
| 3261.4142614 | 4 |
| 3261.4142949 | 1 |
| 3261.4143083 | 4 |
| 3261.4143502 | 5 |
| 3261.4143927 | 3 |
| 3261.4144752 | 14 |
| 3261.4151389 | 5 |
| 3261.4152307 | 8 |
| 3261.4152839 | 10 |
| 3261.4154281 | 7 |
| 3261.4154939 | 13 |
| 3261.4155377 | 30 |
| 3261.4157991 | 60 |
| 3261.4160886 | 57 |
| 3261.4160956 | 60 |
| 3261.4161864 | 45 |
| 3261.4163215 | 51 |
| 3261.4165418 | 59 |
| 3261.4169084 | 97 |
| 3261.4169961 | 67 |
| 3261.417057 | 20 |
| 3261.4172273 | 84 |
| 3261.4174129 | 82 |
| 3261.4174666 | 76 |
| 3261.4174964 | 66 |
| 3261.4178138 | 95 |
| 3261.4178569 | 103 |
| 3261.4179419 | 105 |
| 3261.4180256 | 74 |
| 3261.4181023 | 48 |
| 3261.4181694 | 52 |
| 3261.4187733 | 41 |
| 3261.4188386 | 69 |
| 3261.419137 | 53 |
| 3261.4192351 | 65 |
| 3261.4192979 | 49 |
| 3261.4193579 | 59 |
| 3261.4195525 | 78 |
| 3261.4196552 | 57 |
| 3261.4200019 | 67 |
| 3261.4204197 | 52 |
| 3261.4204604 | 65 |
| 3261.4207141 | 65 |
| 3261.4213346 | 57 |
| 3261.42135 | 66 |
| 3261.4215849 | 63 |
| 3261.4218013 | 71 |
| 3261.4218204 | 75 |
| 3261.4219519 | 60 |
| 3261.421989 | 55 |
| 3261.4220943 | 74 |
| 3261.42246 | 72 |
| 3261.4226026 | 59 |
| 3261.4229536 | 69 |
| 3261.4235053 | 68 |
| 3261.4238511 | 58 |
| 3261.4238635 | 68 |
| 3261.4240637 | 62 |
| 3261.4240932 | 57 |
| 3261.4243934 | 57 |
| 3261.4249646 | 55 |
| 3261.425017 | 53 |
| 3261.4251882 | 23 |
| 3261.4252271 | 44 |
| 3261.4254648 | 24 |
| 3261.42583 | 14 |
| 3261.426219 | 24 |
| 3261.4262907 | 54 |
| 3261.4267079 | 17 |
| 3261.4269485 | 5 |
| 3261.4270756 | 26 |
| 3261.4272804 | 10 |
| 3261.4273526 | 10 |
| 3261.4273622 | 2 |
| 3261.4277097 | 9 |
| 3261.4279234 | 10 |
| 3261.4284507 | 1 |
| 3261.4285709 | 3 |
| 3261.4287868 | 4 |
| 3261.4293857 | 7 |
| 3261.4294921 | 2 |
| 3261.4295288 | 4 |
| 3261.4298128 | 2 |
| 3261.4299832 | 3 |
| 3261.4299881 | 4 |
| 3261.4300663 | 4 |
| 3261.4301077 | 3 |
| 3261.4305512 | 2 |
| 3261.4313679 | 4 |
| 3261.4314701 | 2 |
| 3261.4316446 | 1 |
| 3261.4316618 | 5 |
| 3261.4317701 | 2 |
| 3261.4322143 | 4 |
| 3261.4323663 | 1 |
| 3261.4325407 | 3 |
| 3261.4325417 | 0 |
| 3261.4328329 | 4 |
| 3261.4328905 | 2 |
| 3261.4333346 | 2 |
| 3261.4333432 | 3 |
| 3261.4338454 | 1 |
| 3261.4339011 | 5 |
| 3261.4343341 | 3 |
| 3261.4343994 | 1 |
| 3261.4347747 | 3 |
| 3261.4347886 | 3 |
| 3261.4350781 | 4 |
| 3261.4355445 | 3 |
| 3261.4355604 | 5 |
| 3261.4356859 | 2 |
| 3261.4357366 | 1 |
| 3261.4359083 | 3 |
| 3261.4364509 | 0 |
| 3261.4368044 | 3 |
| 3261.4368579 | 3 |
| 3261.4370877 | 7 |
| 3261.4374969 | 2 |
| 3261.4375823 | 3 |
| 3261.4377226 | 1 |
| 3261.4377338 | 1 |
| 3261.4378102 | 1 |
| 3261.4381238 | 1 |
| 3261.4389874 | 3 |
| 3261.4391223 | 3 |
| 3261.439125 | 1 |
| 3261.4393098 | 4 |
| 3261.4398451 | 7 |
| 3261.4400698 | 0 |
| 3261.4402478 | 1 |
| 3261.4402511 | 2 |
| 3261.4402544 | 4 |
| 3261.4403543 | 3 |
| 3261.440552 | 3 |
| 3261.4410884 | 1 |
| 3261.4413377 | 2 |
| 3261.4414959 | 3 |
| 3261.4415467 | 0 |
| 3261.4416301 | 2 |
| 3261.4420098 | 2 |
| 3261.4421643 | 2 |
| 3261.4426053 | 1 |
| 3261.4428433 | 3 |
| 3261.4430851 | 4 |
| 3261.4431076 | 2 |
| 3261.4433281 | 3 |
| 3261.443338 | 3 |
| 3261.4435615 | 8 |
| 3261.4437261 | 1 |
| 3261.4439601 | 1 |
| 3261.4442201 | 2 |
| 3261.4449148 | 3 |
| 3261.4450034 | 1 |
| 3261.4451432 | 2 |
| 3261.4453214 | 3 |
| 3261.4453256 | 3 |
| 3261.4457256 | 1 |
| 3261.4457873 | 3 |
| 3261.4463717 | 2 |
| 3261.4464401 | 4 |
| 3261.4465858 | 2 |
| 3261.446827 | 2 |
| 3261.4469719 | 3 |
| 3261.4474694 | 3 |
| 3261.4475027 | 4 |
| 3261.4475725 | 2 |
| 3261.4476989 | 4 |
| 3261.4478137 | 1 |
| 3261.4481977 | 2 |
| 3261.448283 | 3 |
| 3261.4488517 | 2 |
| 3261.4491045 | 3 |
| 3261.4492276 | 2 |
| 3261.4493781 | 5 |
| 3261.4495414 | 2 |
| 3261.4495797 | 0 |
| 3261.4497739 | 3 |

| | |
|---|---|
| 3261.4498972 | 1 |
| 3261.4505714 | 3 |
| 3261.4506501 | 3 |
| 3261.4506883 | 1 |
| 3261.4508198 | 2 |
| 3261.4512613 | 3 |
| 3261.4515813 | 5 |
| 3261.4515922 | 1 |
| 3261.4517855 | 4 |
| 3261.4519407 | 4 |
| 3261.4522861 | 3 |
| 3261.4524136 | 4 |
| 3261.4529967 | 3 |
| 3261.4532929 | 1 |
| 3261.4533537 | 2 |
| 3261.4533821 | 3 |
| 3261.4535508 | 6 |
| 3261.4535822 | 1 |
| 3261.4540505 | 1 |
| 3261.4546155 | 0 |
| 3261.4546621 | 2 |
| 3261.4547493 | 3 |
| 3261.4548927 | 3 |
| 3261.4551866 | 2 |
| 3261.4553132 | 0 |
| 3261.4553841 | 1 |
| 3261.4556972 | 2 |
| 3261.4559307 | 0 |
| 3261.455966 | 2 |
| 3261.4562039 | 5 |
| 3261.4563932 | 2 |
| 3261.4565535 | 5 |
| 3261.4565539 | 3 |
| 3261.4569572 | 2 |
| 3261.4571981 | 3 |
| 3261.4576422 | 2 |
| 3261.4579242 | 5 |
| 3261.4580897 | 3 |
| 3261.4581035 | 5 |
| 3261.4581457 | 5 |
| 3261.4584048 | 0 |
| 3261.458764 | 2 |
| 3261.4590551 | 2 |
| 3261.4591835 | 5 |
| 3261.4592415 | 1 |
| 3261.4593505 | 4 |
| 3261.4595222 | 1 |
| 3261.4596027 | 2 |
| 3261.4596719 | 4 |
| 3261.4602517 | 3 |
| 3261.4602949 | 2 |
| 3261.4605713 | 2 |
| 3261.4606909 | 4 |
| 3261.4609569 | 2 |
| 3261.4612444 | 1 |
| 3261.461295 | 0 |
| 3261.4614399 | 5 |
| 3261.4615457 | 2 |
| 3261.4617835 | 1 |
| 3261.4618294 | 0 |
| 3261.4620487 | 5 |
| 3261.4621296 | 2 |
| 3261.462153 | 4 |
| 3261.462514 | 3 |
| 3261.4626085 | 0 |
| 3261.4627324 | 0 |
| 3261.4629323 | 3 |
| 3261.4635142 | 3 |
| 3261.4635948 | 4 |
| 3261.463642 | 3 |
| 3261.4638405 | 2 |
| 3261.4642451 | 2 |
| 3261.4642851 | 2 |
| 3261.4644507 | 4 |
| 3261.4645038 | 2 |
| 3261.4648356 | 4 |
| 3261.4650956 | 2 |
| 3261.4651554 | 2 |
| 3261.4655255 | 2 |
| 3261.4658831 | 3 |
| 3261.4660411 | 2 |
| 3261.4662326 | 4 |
| 3261.4663978 | 3 |
| 3261.4668446 | 2 |
| 3261.4668664 | 2 |
| 3261.4668992 | 4 |
| 3261.4671981 | 9 |
| 3261.4674034 | 2 |
| 3261.4682349 | 8 |
| 3261.4682787 | 11 |
| 3261.4684246 | 2 |
| 3261.4688123 | 18 |
| 3261.4688367 | 4 |
| 3261.4689799 | 4 |
| 3261.4693892 | 19 |
| 3261.4694476 | 17 |
| 3261.4700021 | 3 |
| 3261.4701672 | 12 |
| 3261.4705872 | 0 |
| 3261.4710723 | 42 |
| 3261.4714336 | 25 |
| 3261.4714655 | 37 |
| 3261.4716911 | 34 |
| 3261.4721929 | 1 |
| 3261.4723814 | 26 |
| 3261.472622 | 19 |
| 3261.4728583 | 1 |
| 3261.473201 | 13 |
| 3261.473201 | 37 |
| 3261.4737616 | 4 |
| 3261.4737896 | 20 |
| 3261.4738453 | 10 |
| 3261.4740553 | 11 |
| 3261.4744816 | 14 |
| 3261.4747159 | 1 |
| 3261.4750781 | 8 |
| 3261.4752515 | 1 |
| 3261.475451 | 9 |
| 3261.4760167 | 6 |
| 3261.4763756 | 3 |
| 3261.4764358 | 1 |

| | |
|---|---|
| 3261.4765196 | 7 |
| 3261.476535 | 5 |
| 3261.47663 | 5 |
| 3261.4771237 | 4 |
| 3261.4777863 | 1 |
| 3261.477893 | 2 |
| 3261.4778941 | 1 |
| 3261.4780906 | 2 |
| 3261.4782266 | 3 |
| 3261.478726 | 3 |
| 3261.4787423 | 1 |
| 3261.4788649 | 5 |
| 3261.4792179 | 2 |
| 3261.4795915 | 1 |
| 3261.4798916 | 1 |
| 3261.4800938 | 2 |
| 3261.4801476 | 2 |
| 3261.4805883 | 4 |
| 3261.4806153 | 4 |
| 3261.4806456 | 3 |
| 3261.4810583 | 1 |
| 3261.4811197 | 1 |
| 3261.4814247 | 2 |
| 3261.4815679 | 2 |
| 3261.4819056 | 2 |
| 3261.4821832 | 4 |
| 3261.4823251 | 4 |
| 3261.4825372 | 1 |
| 3261.4826578 | 3 |
| 3261.4829808 | 2 |
| 3261.4831991 | 1 |
| 3261.4834807 | 2 |
| 3261.4835489 | 1 |
| 3261.4836439 | 1 |
| 3261.4843084 | 2 |
| 3261.4845571 | 0 |
| 3261.4846745 | 4 |
| 3261.4847623 | 4 |
| 3261.4847717 | 1 |
| 3261.484909 | 3 |
| 3261.4855851 | 3 |
| 3261.4856694 | 2 |
| 3261.4857388 | 0 |
| 3261.4860268 | 1 |
| 3261.4866758 | 4 |
| 3261.4869662 | 3 |
| 3261.4869752 | 1 |
| 3261.4871533 | 1 |
| 3261.4871743 | 2 |
| 3261.4872125 | 1 |
| 3261.4874768 | 4 |
| 3261.4876839 | 2 |
| 3261.487823 | 2 |
| 3261.4882699 | 2 |
| 3261.4882761 | 3 |
| 3261.4882896 | 2 |
| 3261.4884074 | 5 |
| 3261.4889639 | 3 |
| 3261.4892283 | 4 |
| 3261.4893333 | 5 |
| 3261.4895493 | 6 |
| 3261.4898219 | 0 |
| 3261.4898767 | 3 |
| 3261.4900179 | 2 |
| 3261.4907414 | 4 |
| 3261.4913313 | 1 |
| 3261.4916029 | 4 |
| 3261.4925809 | 2 |
| 3261.4931434 | 3 |
| 3261.4931841 | 2 |
| 3261.4939841 | 2 |
| 3261.4943035 | 4 |
| 3261.4948802 | 3 |
| 3261.4956732 | 3 |
| 3261.4958347 | 3 |
| 3261.4962839 | 2 |
| 3261.496873 | 3 |
| 3261.4971771 | 2 |
| 3261.4981572 | 2 |
| 3261.4988282 | 4 |
| 3261.4988513 | 1 |
| 3261.4990968 | 4 |
| 3261.5005459 | 8 |
| 3261.50074 | 0 |
| 3261.5013588 | 1 |
| 3261.5019473 | 6 |
| 3261.5021819 | 2 |
| 3261.5026817 | 2 |
| 3261.5028841 | 2 |
| 3261.5038031 | 4 |
| 3261.5038491 | 3 |
| 3261.5044927 | 1 |
| 3261.5051464 | 1 |
| 3261.5055976 | 0 |
| 3261.5057398 | 5 |
| 3261.5058312 | 1 |
| 3261.5068373 | 4 |
| 3261.5076176 | 1 |
| 3261.5090492 | 3 |
| 3261.5093485 | 5 |
| 3261.510637 | 2 |
| 3261.513012 | 1 |
| 3261.5133379 | 2 |
| 3261.5156048 | 1 |
| 3261.5165104 | 8 |
| 3261.5183012 | 3 |
| 3261.5199184 | 7 |
| 3261.5212613 | 5 |
| 3261.5228252 | 2 |
| 3261.5234994 | 2 |
| 3261.5251402 | 1 |
| 3261.5257506 | 3 |
| 3261.5281299 | 5 |
| 3261.5287156 | 5 |
| 3261.5297239 | 2 |
| 3261.5316325 | 2 |
| 3261.5329027 | 6 |
| 3261.5335319 | 3 |
| 3261.535315 | 1 |
| 3261.5361649 | 3 |
| 3261.5374874 | 3 |

| | |
|---|---|
| 3261.5392566 | 3 |
| 3261.5402743 | 3 |
| 3261.5413446 | 4 |
| 3261.5425336 | 1 |
| 3261.5438342 | 2 |
| 3261.5448282 | 4 |
| 3261.5463025 | 3 |
| 3261.5477786 | 3 |
| 3261.5485051 | 4 |
| 3261.5496775 | 3 |
| 3261.5500136 | 4 |
| 3261.5516944 | 4 |
| 3261.5524948 | 1 |
| 3261.5531182 | 2 |
| 3261.5540861 | 3 |
| 3261.5547709 | 3 |
| 3261.5562809 | 4 |
| 3261.5585505 | 1 |
| 3261.5586628 | 1 |
| 3261.5610321 | 1 |
| 3261.5633291 | 2 |
| 3261.5638106 | 1 |
| 3261.566182 | 4 |
| 3261.5672902 | 2 |
| 3261.568069 | 0 |
| 3261.5699136 | 3 |
| 3261.5705362 | 2 |
| 3261.5727882 | 5 |
| 3261.5730148 | 3 |
| 3261.5750953 | 2 |
| 3261.5759327 | 3 |
| 3261.5773322 | 3 |
| 3261.5786487 | 4 |
| 3261.5792774 | 2 |
| 3261.5810141 | 3 |
| 3261.582713 | 2 |
| 3261.5833566 | 1 |
| 3261.5848841 | 1 |
| 3261.585209 | 10 |
| 3261.5872115 | 1 |
| 3261.5876129 | 1 |
| 3261.5893734 | 2 |
| 3261.5909068 | 1 |
| 3261.5912964 | 4 |
| 3261.5928523 | 2 |
| 3261.5944045 | 5 |
| 3261.5946135 | 1 |
| 3261.5956853 | 4 |
| 3261.5967039 | 3 |
| 3261.5974142 | 3 |
| 3261.5984783 | 4 |
| 3261.6001228 | 1 |
| 3261.6013798 | 3 |
| 3261.6021201 | 1 |
| 3261.6042679 | 2 |
| 3261.604929 | 0 |
| 3261.6074203 | 4 |
| 3261.6090967 | 6 |
| 3261.6104359 | 3 |
| 3261.6117859 | 0 |
| 3261.6123588 | 4 |
| 3261.6146777 | 1 |
| 3261.6158028 | 2 |
| 3261.6169907 | 2 |
| 3261.6182456 | 2 |
| 3261.618886 | 5 |
| 3261.6202836 | 1 |
| 3261.6222257 | 3 |
| 3261.622709 | 0 |
| 3261.6243384 | 3 |
| 3261.6252488 | 4 |
| 3261.6262981 | 4 |
| 3261.6284258 | 1 |
| 3261.6285256 | 1 |
| 3261.6304937 | 4 |
| 3261.631025 | 2 |
| 3261.6330021 | 3 |
| 3261.634257 | 3 |
| 3261.6356581 | 1 |
| 3261.6359999 | 6 |
| 3261.637196 | 4 |
| 3261.6384221 | 5 |
| 3261.6388854 | 3 |
| 3261.6407943 | 1 |
| 3261.641477 | 0 |
| 3261.6424009 | 1 |
| 3261.6430499 | 2 |
| 3261.6439841 | 2 |
| 3261.6451381 | 3 |
| 3261.6469984 | 1 |
| 3261.6475638 | 2 |
| 3261.6495916 | 2 |
| 3261.6514884 | 2 |
| 3261.6525257 | 0 |
| 3261.653903 | 1 |
| 3261.6552115 | 3 |
| 3261.6572835 | 1 |
| 3261.6594324 | 1 |
| 3261.6596912 | 4 |
| 3261.6614708 | 3 |
| 3261.6619452 | 2 |
| 3261.6640112 | 1 |
| 3261.6643109 | 4 |
| 3261.666655 | 0 |
| 3261.6681204 | 4 |
| 3261.668469 | 1 |
| 3261.6704255 | 3 |
| 3261.6708175 | 5 |
| 3261.6727015 | 3 |
| 3261.6735817 | 2 |
| 3261.6749647 | 1 |
| 3261.6761048 | 1 |
| 3261.676376 | 3 |
| 3261.6779914 | 1 |
| 3261.679708 | 6 |
| 3261.6804791 | 1 |
| 3261.6820354 | 2 |
| 3261.6830039 | 4 |
| 3261.6841131 | 2 |
| 3261.6859713 | 1 |
| 3261.6860976 | 2 |

| | |
|---|---|
| 3261.6870449 | 1 |
| 3261.6876374 | 0 |
| 3261.6890956 | 1 |
| 3261.6895853 | 1 |
| 3261.690495 | 4 |
| 3261.6918684 | 0 |
| 3261.6943769 | 4 |
| 3261.6951495 | 4 |
| 3261.6973211 | 7 |
| 3261.6984372 | 0 |
| 3261.7009458 | 2 |
| 3261.7018787 | 3 |
| 3261.7034992 | 2 |
| 3261.704454 | 6 |
| 3261.7056957 | 2 |
| 3261.7070527 | 2 |
| 3261.7080393 | 4 |
| 3261.7095116 | 3 |
| 3261.7107347 | 1 |
| 3261.7115949 | 2 |
| 3261.7136485 | 2 |
| 3261.7137208 | 5 |
| 3261.7147774 | 3 |
| 3261.7173281 | 0 |
| 3261.7187833 | 5 |
| 3261.7191558 | 2 |
| 3261.7208515 | 1 |
| 3261.7221991 | 4 |
| 3261.7230797 | 3 |
| 3261.7252416 | 2 |
| 3261.7256111 | 3 |
| 3261.7272081 | 3 |
| 3261.7275317 | 3 |
| 3261.729595 | 4 |
| 3261.7305156 | 4 |
| 3261.7311566 | 1 |
| 3261.7317613 | 2 |
| 3261.7329771 | 2 |
| 3261.7342151 | 3 |
| 3261.734497 | 1 |
| 3261.7354375 | 3 |
| 3261.7368582 | 2 |
| 3261.7381386 | 3 |
| 3261.7394559 | 1 |
| 3261.7422435 | 4 |
| 3261.7432419 | 2 |
| 3261.7455548 | 3 |
| 3261.7472642 | 2 |
| 3261.7487358 | 1 |
| 3261.7494446 | 3 |
| 3261.7509399 | 6 |
| 3261.7523859 | 3 |
| 3261.7530538 | 4 |
| 3261.7548179 | 5 |
| 3261.7564101 | 0 |
| 3261.7569496 | 0 |
| 3261.7584411 | 5 |
| 3261.7594535 | 2 |
| 3261.7607037 | 5 |
| 3261.7620488 | 6 |
| 3261.7634279 | 3 |
| 3261.7646566 | 4 |
| 3261.7652906 | 1 |
| 3261.7669068 | 1 |
| 3261.7687406 | 2 |
| 3261.7693566 | 3 |
| 3261.7704751 | 3 |
| 3261.771828 | 0 |
| 3261.7732039 | 4 |
| 3261.7745324 | 2 |
| 3261.7757698 | 5 |
| 3261.7766949 | 5 |
| 3261.7769338 | 5 |
| 3261.77768 | 3 |
| 3261.7786807 | 3 |
| 3261.7792697 | 5 |
| 3261.7802911 | 0 |
| 3261.7808796 | 3 |
| 3261.7831566 | 3 |
| 3261.785345 | 0 |
| 3261.7863127 | 2 |
| 3261.7883538 | 2 |
| 3261.7891002 | 3 |
| 3261.7920914 | 3 |
| 3261.7940389 | 1 |
| 3261.7947833 | 1 |
| 3261.7960859 | 1 |
| 3261.7973463 | 5 |
| 3261.7979714 | 3 |
| 3261.7998641 | 1 |
| 3261.8004926 | 4 |
| 3261.8021625 | 3 |
| 3261.8039429 | 3 |
| 3261.8043412 | 3 |
| 3261.8060392 | 0 |
| 3261.8068042 | 0 |
| 3261.8080145 | 3 |
| 3261.8097389 | 1 |
| 3261.810644 | 4 |
| 3261.8122682 | 1 |
| 3261.8129902 | 3 |
| 3261.8142872 | 1 |
| 3261.8154399 | 2 |
| 3261.8165781 | 1 |
| 3261.8181524 | 2 |
| 3261.8194107 | 2 |
| 3261.8210378 | 1 |
| 3261.8215889 | 5 |
| 3261.8236227 | 4 |
| 3261.8241717 | 0 |
| 3261.8242271 | 3 |
| 3261.8252692 | 6 |
| 3261.8254306 | 2 |
| 3261.8265305 | 3 |
| 3261.8283312 | 6 |
| 3261.8287953 | 1 |
| 3261.830546 | 1 |
| 3261.8324571 | 3 |
| 3261.8351268 | 3 |
| 3261.8363658 | 4 |
| 3261.8376072 | 3 |
| 3261.83961 | 3 |
| 3261.8402109 | 2 |
| 3261.8422677 | 2 |
| 3261.8438449 | 0 |
| 3261.8440611 | 3 |
| 3261.8466529 | 2 |
| 3261.8468829 | 1 |
| 3261.8484408 | 1 |
| 3261.8497965 | 1 |
| 3261.8507781 | 3 |
| 3261.8525276 | 6 |
| 3261.8529852 | 2 |
| 3261.8551439 | 2 |
| 3261.8551692 | 5 |
| 3261.8568344 | 4 |
| 3261.8589758 | 2 |
| 3261.8597005 | 3 |
| 3261.8609424 | 2 |
| 3261.8618148 | 1 |
| 3261.863224 | 2 |
| 3261.8639437 | 3 |
| 3261.8654185 | 1 |
| 3261.8670489 | 4 |
| 3261.8676065 | 4 |
| 3261.868784 | 4 |
| 3261.8703586 | 3 |
| 3261.8704414 | 2 |
| 3261.8719438 | 2 |
| 3261.8722028 | 2 |
| 3261.8735727 | 3 |
| 3261.8746713 | 8 |
| 3261.8751263 | 5 |
| 3261.8782561 | 2 |
| 3261.8783034 | 3 |
| 3261.8810124 | 2 |
| 3261.8838478 | 0 |
| 3261.8844555 | 4 |
| 3261.8863984 | 1 |
| 3261.8867572 | 3 |
| 3261.8884437 | 1 |
| 3261.8899199 | 1 |
| 3261.890803 | 5 |
| 3261.8927965 | 1 |
| 3261.8928806 | 1 |
| 3261.8942802 | 1 |
| 3261.8965401 | 1 |
| 3261.897006 | 0 |
| 3261.8990298 | 1 |
| 3261.8998287 | 2 |
| 3261.9010095 | 1 |
| 3261.9028334 | 0 |
| 3261.9034001 | 2 |
| 3261.9047875 | 1 |
| 3261.9056012 | 2 |
| 3261.9072739 | 2 |
| 3261.9085236 | 6 |
| 3261.9098111 | 1 |
| 3261.9110689 | 2 |
| 3261.9121188 | 2 |
| 3261.9132761 | 0 |
| 3261.9147081 | 4 |
| 3261.9147743 | 1 |
| 3261.9162161 | 3 |
| 3261.9162228 | 2 |
| 3261.9168897 | 5 |
| 3261.9183306 | 3 |
| 3261.9186878 | 3 |
| 3261.921015 | 3 |
| 3261.9217257 | 4 |
| 3261.9239895 | 0 |
| 3261.9268345 | 2 |
| 3261.9272098 | 4 |
| 3261.9299117 | 4 |
| 3261.9299747 | 1 |
| 3261.9322822 | 1 |
| 3261.933687 | 3 |
| 3261.934917 | 1 |
| 3261.9358438 | 2 |
| 3261.9373797 | 5 |
| 3261.9388196 | 5 |
| 3261.9397796 | 3 |
| 3261.9404373 | 4 |
| 3261.9427762 | 4 |
| 3261.9431279 | 3 |
| 3261.9449545 | 1 |
| 3261.9462149 | 1 |
| 3261.9470459 | 3 |
| 3261.9483799 | 5 |
| 3261.9495516 | 3 |
| 3261.9508874 | 1 |
| 3261.9524157 | 2 |
| 3261.9533188 | 3 |
| 3261.954341 | 4 |
| 3261.9563236 | 0 |
| 3261.956696 | 3 |
| 3261.9586036 | 5 |
| 3261.9592703 | 3 |
| 3261.960488 | 1 |
| 3261.9613237 | 5 |
| 3261.9621189 | 2 |
| 3261.9628904 | 3 |
| 3261.9630187 | 3 |
| 3261.9651348 | 1 |
| 3261.9673345 | 3 |
| 3261.9694849 | 0 |
| 3261.9713522 | 3 |
| 3261.9717309 | 3 |
| 3261.9735529 | 1 |
| 3261.9741355 | 4 |
| 3261.9759065 | 3 |
| 3261.977773 | 0 |
| 3261.9782683 | 0 |
| 3261.9796973 | 2 |
| 3261.9804141 | 3 |
| 3261.9816776 | 0 |
| 3261.9837358 | 2 |
| 3261.9845459 | 1 |
| 3261.9859999 | 3 |
| 3261.9870374 | 7 |
| 3261.9880199 | 4 |
| 3261.9902923 | 2 |
| 3261.9905669 | 0 |
| 3261.9923386 | 2 |
| 3261.9935245 | 4 |
| 3261.9942212 | 2 |
| 3261.9952283 | 1 |
| 3261.9966703 | 1 |
| 3261.9980106 | 2 |
| 3261.9993708 | 4 |
| 3262.00042 | 1 |
| 3262.0017986 | 1 |
| 3262.0026247 | 6 |
| 3262.003988 | 3 |
| 3262.0061559 | 2 |
| 3262.0061952 | 3 |
| 3262.0069941 | 1 |
| 3262.0079064 | 4 |
| 3262.0092557 | 3 |
| 3262.0094823 | 2 |
| 3262.0111552 | 2 |
| 3262.0126147 | 1 |
| 3262.0133808 | 4 |
| 3262.0151878 | 2 |
| 3262.0167732 | 3 |
| 3262.0179888 | 2 |
| 3262.0195738 | 3 |
| 3262.0213041 | 3 |
| 3262.0236672 | 3 |
| 3262.0243205 | 1 |
| 3262.025919 | 1 |
| 3262.0273527 | 4 |
| 3262.0279153 | 2 |
| 3262.0295457 | 2 |
| 3262.030961 | 5 |
| 3262.0319473 | 3 |
| 3262.0337927 | 6 |
| 3262.0347303 | 1 |
| 3262.0361642 | 1 |
| 3262.0373135 | 0 |
| 3262.0377313 | 5 |
| 3262.0395861 | 2 |
| 3262.0404423 | 2 |
| 3262.0416607 | 3 |
| 3262.043041 | 1 |
| 3262.0442189 | 1 |
| 3262.0457922 | 5 |
| 3262.0464772 | 1 |
| 3262.0483079 | 2 |
| 3262.0495201 | 3 |
| 3262.0499114 | 2 |
| 3262.0519783 | 2 |
| 3262.0521462 | 6 |
| 3262.0536258 | 3 |
| 3262.0538757 | 2 |
| 3262.0554831 | 2 |
| 3262.0559764 | 2 |
| 3262.0574081 | 2 |
| 3262.0591248 | 2 |
| 3262.0594119 | 2 |
| 3262.0617414 | 2 |
| 3262.0636378 | 7 |
| 3262.0647279 | 0 |
| 3262.0664564 | 1 |
| 3262.0676368 | 2 |
| 3262.069333 | 4 |
| 3262.0704023 | 4 |
| 3262.0714573 | 4 |
| 3262.0725714 | 3 |
| 3262.0744697 | 2 |
| 3262.0755493 | 1 |
| 3262.0770367 | 0 |
| 3262.0778375 | 2 |
| 3262.078942 | 2 |
| 3262.08071 | 1 |
| 3262.0812282 | 3 |
| 3262.08292 | 3 |
| 3262.0835354 | 3 |
| 3262.0855787 | 3 |
| 3262.0871886 | 0 |
| 3262.0874161 | 3 |
| 3262.0890437 | 2 |
| 3262.0895384 | 3 |
| 3262.0905962 | 3 |
| 3262.0928137 | 5 |
| 3262.0932625 | 0 |
| 3262.0954141 | 2 |
| 3262.0959572 | 3 |
| 3262.0978308 | 2 |
| 3262.0982212 | 1 |
| 3262.0991093 | 0 |
| 3262.1001329 | 5 |
| 3262.1005827 | 1 |
| 3262.1020919 | 3 |
| 3262.1028616 | 4 |
| 3262.1047112 | 1 |
| 3262.1058226 | 2 |
| 3262.1070164 | 5 |
| 3262.1098158 | 2 |
| 3262.1119939 | 0 |
| 3262.1122155 | 5 |
| 3262.1142375 | 1 |
| 3262.1146821 | 4 |
| 3262.1161486 | 4 |
| 3262.1177315 | 1 |
| 3262.1188703 | 4 |
| 3262.120262 | 5 |
| 3262.1210196 | 14 |
| 3262.1225706 | 9 |
| 3262.1243879 | 12 |
| 3262.1250523 | 21 |
| 3262.1263646 | 8 |
| 3262.127289 | 4 |
| 3262.1285607 | 3 |
| 3262.1300686 | 4 |
| 3262.1312817 | 0 |
| 3262.1325629 | 2 |
| 3262.1334571 | 3 |
| 3262.1353851 | 7 |
| 3262.1360396 | 1 |
| 3262.1367589 | 2 |
| 3262.1392116 | 0 |
| 3262.139259 | 4 |

| | |
|---|---|
| 3262.1408696 | 3 |
| 3262.1413494 | 2 |
| 3262.1425398 | 3 |
| 3262.1425405 | 2 |
| 3262.1425794 | 1 |
| 3262.1427411 | 4 |
| 3262.1441018 | 3 |
| 3262.1442731 | 0 |
| 3262.1443 | 2 |
| 3262.1450258 | 3 |
| 3262.1452293 | 1 |
| 3262.1453846 | 4 |
| 3262.1456804 | 2 |
| 3262.1459752 | 3 |
| 3262.1468968 | 6 |
| 3262.1471198 | 1 |
| 3262.147445 | 2 |
| 3262.1486575 | 1 |
| 3262.1490235 | 1 |
| 3262.1490837 | 2 |
| 3262.1491489 | 2 |
| 3262.1501276 | 1 |
| 3262.1501638 | 2 |
| 3262.1505156 | 3 |
| 3262.1505442 | 5 |
| 3262.1515271 | 2 |
| 3262.1519899 | 2 |
| 3262.1531679 | 5 |
| 3262.1532264 | 4 |
| 3262.1537568 | 1 |
| 3262.1540329 | 1 |
| 3262.1544314 | 3 |
| 3262.1550134 | 4 |
| 3262.1558083 | 4 |
| 3262.1558454 | 3 |
| 3262.1569236 | 2 |
| 3262.1569701 | 4 |
| 3262.1574893 | 5 |
| 3262.1579289 | 1 |
| 3262.1580554 | 2 |
| 3262.1582593 | 2 |
| 3262.1585333 | 2 |
| 3262.1596006 | 1 |
| 3262.1597968 | 1 |
| 3262.1602714 | 2 |
| 3262.1605082 | 0 |
| 3262.1612875 | 1 |
| 3262.1617852 | 2 |
| 3262.1618924 | 1 |
| 3262.1622546 | 2 |
| 3262.1629044 | 1 |
| 3262.1631869 | 2 |
| 3262.1636477 | 3 |
| 3262.1646542 | 1 |
| 3262.1646723 | 4 |
| 3262.164732 | 1 |
| 3262.1657625 | 4 |
| 3262.1657826 | 1 |
| 3262.1663281 | 3 |
| 3262.1671589 | 1 |
| 3262.1675731 | 0 |
| 3262.1676024 | 4 |
| 3262.1683822 | 3 |
| 3262.1688912 | 2 |
| 3262.1690871 | 4 |
| 3262.1691991 | 2 |
| 3262.1695997 | 2 |
| 3262.1698697 | 3 |
| 3262.170807 | 2 |
| 3262.1712682 | 2 |
| 3262.1714529 | 3 |
| 3262.1722519 | 1 |
| 3262.1722976 | 4 |
| 3262.1723446 | 2 |
| 3262.1723915 | 4 |
| 3262.1741088 | 1 |
| 3262.1744191 | 1 |
| 3262.1746535 | 3 |
| 3262.1750968 | 2 |
| 3262.1757919 | 2 |
| 3262.175881 | 4 |
| 3262.1762591 | 1 |
| 3262.1763992 | 2 |
| 3262.176544 | 1 |
| 3262.1773932 | 2 |
| 3262.1784251 | 3 |
| 3262.1784575 | 2 |
| 3262.1789043 | 3 |
| 3262.179555 | 1 |
| 3262.1796028 | 2 |
| 3262.1801302 | 3 |
| 3262.1805546 | 2 |
| 3262.1808678 | 2 |
| 3262.1814616 | 3 |
| 3262.1815209 | 3 |
| 3262.1819061 | 1 |
| 3262.1824195 | 1 |
| 3262.1830488 | 4 |
| 3262.1835098 | 1 |
| 3262.1836638 | 2 |
| 3262.1839355 | 3 |
| 3262.1841824 | 3 |
| 3262.184277 | 2 |
| 3262.1849902 | 1 |
| 3262.1861365 | 2 |
| 3262.1865158 | 2 |
| 3262.1865996 | 3 |
| 3262.1868212 | 3 |
| 3262.1877146 | 3 |
| 3262.1883444 | 7 |
| 3262.1884389 | 3 |
| 3262.1885273 | 1 |
| 3262.189529 | 2 |
| 3262.1896123 | 2 |
| 3262.1898346 | 3 |
| 3262.1900638 | 1 |
| 3262.1907549 | 3 |
| 3262.1909387 | 2 |
| 3262.1911188 | 5 |
| 3262.1917388 | 2 |
| 3262.1926251 | 0 |
| 3262.1930641 | 2 |
| 3262.1930661 | 1 |
| 3262.1931816 | 6 |
| 3262.1934827 | 0 |
| 3262.1943411 | 3 |
| 3262.1946707 | 0 |
| 3262.1953597 | 2 |
| 3262.1953734 | 5 |
| 3262.1958736 | 2 |
| 3262.1958902 | 3 |
| 3262.1969612 | 2 |
| 3262.1973017 | 3 |
| 3262.1974514 | 1 |
| 3262.1977856 | 3 |
| 3262.1983014 | 2 |
| 3262.1995257 | 3 |
| 3262.1995646 | 1 |
| 3262.2000979 | 5 |
| 3262.2006302 | 4 |
| 3262.2011027 | 7 |
| 3262.2012968 | 0 |
| 3262.2017233 | 1 |
| 3262.2028028 | 3 |
| 3262.2028528 | 4 |
| 3262.2033241 | 3 |
| 3262.2034057 | 1 |
| 3262.2038295 | 3 |
| 3262.2049239 | 1 |
| 3262.2051043 | 0 |
| 3262.2051759 | 0 |
| 3262.2063898 | 2 |
| 3262.207067 | 4 |
| 3262.2071358 | 3 |
| 3262.2072234 | 1 |
| 3262.2077725 | 8 |
| 3262.2085199 | 3 |
| 3262.2086376 | 3 |
| 3262.209283 | 2 |
| 3262.209315 | 2 |
| 3262.2100762 | 1 |
| 3262.2100819 | 6 |
| 3262.210771 | 8 |
| 3262.2114449 | 4 |
| 3262.2117878 | 7 |
| 3262.2118706 | 10 |
| 3262.2128435 | 15 |
| 3262.2131066 | 26 |
| 3262.2135765 | 18 |
| 3262.2139106 | 20 |
| 3262.214539 | 18 |
| 3262.2148167 | 9 |
| 3262.2150729 | 9 |
| 3262.2156455 | 9 |
| 3262.2158257 | 7 |
| 3262.2164689 | 4 |
| 3262.216959 | 6 |
| 3262.217663 | 2 |
| 3262.2177401 | 4 |
| 3262.2184581 | 3 |
| 3262.2184703 | 1 |
| 3262.219287 | 3 |
| 3262.2193738 | 4 |
| 3262.2199677 | 3 |
| 3262.2203714 | 2 |
| 3262.2210941 | 2 |
| 3262.2216914 | 9 |
| 3262.2219809 | 2 |
| 3262.2221525 | 3 |
| 3262.2224539 | 5 |
| 3262.2225448 | 1 |
| 3262.2226817 | 1 |
| 3262.2237651 | 2 |
| 3262.2238376 | 3 |
| 3262.2246834 | 4 |
| 3262.2252298 | 2 |
| 3262.2258127 | 1 |
| 3262.2258727 | 2 |
| 3262.2258864 | 3 |
| 3262.2265257 | 5 |
| 3262.2266962 | 3 |
| 3262.2277318 | 1 |
| 3262.2281621 | 5 |
| 3262.2281696 | 1 |
| 3262.2281824 | 5 |
| 3262.2291786 | 1 |
| 3262.2296105 | 1 |
| 3262.2301215 | 3 |
| 3262.2301466 | 2 |
| 3262.2302925 | 2 |
| 3262.2310817 | 5 |
| 3262.231314 | 5 |
| 3262.2321184 | 3 |
| 3262.2323673 | 1 |
| 3262.2324498 | 2 |
| 3262.2331738 | 1 |
| 3262.233922 | 1 |
| 3262.234193 | 3 |
| 3262.2343221 | 2 |
| 3262.234869 | 7 |
| 3262.2349625 | 4 |
| 3262.2350499 | 2 |
| 3262.2362254 | 2 |
| 3262.2365278 | 1 |
| 3262.2368507 | 3 |
| 3262.2372608 | 3 |
| 3262.2376739 | 0 |
| 3262.2377413 | 2 |
| 3262.2386422 | 1 |
| 3262.2391853 | 2 |
| 3262.2393822 | 3 |
| 3262.2396753 | 1 |
| 3262.239701 | 2 |
| 3262.2402497 | 5 |
| 3262.2410351 | 1 |
| 3262.2410434 | 2 |
| 3262.2415636 | 3 |
| 3262.2424811 | 0 |
| 3262.2427848 | 4 |
| 3262.2439387 | 4 |
| 3262.2440509 | 4 |
| 3262.2440628 | 5 |
| 3262.2446044 | 2 |
| 3262.2449078 | 2 |
| 3262.2451375 | 0 |
| 3262.2460867 | 2 |
| 3262.2466153 | 3 |
| 3262.2468949 | 3 |
| 3262.247232 | 3 |
| 3262.2480871 | 2 |
| 3262.2482492 | 1 |
| 3262.2487692 | 2 |
| 3262.2492896 | 3 |
| 3262.2494982 | 0 |
| 3262.2501931 | 2 |
| 3262.2503176 | 2 |
| 3262.2506712 | 1 |
| 3262.2510935 | 2 |
| 3262.2513997 | 3 |
| 3262.2519996 | 4 |
| 3262.2524715 | 5 |
| 3262.2530842 | 2 |
| 3262.2533735 | 4 |
| 3262.2538401 | 1 |
| 3262.2547285 | 4 |
| 3262.2550173 | 1 |
| 3262.2550605 | 2 |
| 3262.2552645 | 2 |
| 3262.2563805 | 4 |
| 3262.2567853 | 1 |
| 3262.256908 | 3 |
| 3262.2570342 | 3 |
| 3262.257572 | 4 |
| 3262.2577237 | 1 |
| 3262.2580664 | 1 |
| 3262.2581747 | 4 |
| 3262.2582673 | 4 |
| 3262.2584938 | 1 |
| 3262.2590672 | 2 |
| 3262.2591932 | 2 |
| 3262.2591994 | 2 |
| 3262.2593875 | 1 |
| 3262.2595105 | 5 |
| 3262.2595771 | 7 |
| 3262.2604236 | 5 |
| 3262.2610863 | 2 |
| 3262.2612522 | 3 |
| 3262.2612915 | 2 |
| 3262.2613719 | 2 |
| 3262.2615945 | 0 |
| 3262.2616833 | 3 |
| 3262.2620617 | 7 |
| 3262.2625465 | 3 |
| 3262.2628072 | 1 |
| 3262.2630988 | 3 |
| 3262.2631048 | 0 |
| 3262.2631918 | 0 |
| 3262.2632958 | 2 |
| 3262.2633808 | 2 |
| 3262.2636826 | 2 |
| 3262.2639428 | 0 |
| 3262.2641284 | 2 |
| 3262.2641601 | 3 |
| 3262.2651892 | 3 |
| 3262.2653023 | 2 |
| 3262.2655505 | 1 |
| 3262.2657262 | 2 |
| 3262.2657264 | 2 |
| 3262.2657439 | 2 |
| 3262.2659682 | 0 |
| 3262.2660403 | 3 |
| 3262.266061 | 2 |
| 3262.26714 | 2 |
| 3262.2673063 | 2 |
| 3262.2673625 | 2 |
| 3262.2673977 | 0 |
| 3262.2675448 | 6 |
| 3262.267831 | 0 |
| 3262.2678403 | 6 |
| 3262.2678859 | 3 |
| 3262.2681776 | 2 |
| 3262.2682509 | 0 |
| 3262.2687133 | 4 |
| 3262.2690964 | 2 |
| 3262.269228 | 3 |
| 3262.2696572 | 2 |
| 3262.2696616 | 3 |
| 3262.2697084 | 2 |
| 3262.2699029 | 2 |
| 3262.2699287 | 2 |
| 3262.2707032 | 2 |
| 3262.2707217 | 5 |
| 3262.271061 | 3 |
| 3262.2710956 | 2 |
| 3262.2711487 | 3 |
| 3262.2713798 | 0 |
| 3262.2718394 | 3 |
| 3262.2719839 | 2 |
| 3262.2724448 | 1 |
| 3262.2724854 | 1 |
| 3262.2726654 | 3 |
| 3262.2727824 | 1 |
| 3262.2731242 | 3 |
| 3262.27329 | 3 |
| 3262.2734574 | 5 |
| 3262.2734798 | 2 |
| 3262.2736814 | 3 |
| 3262.273744 | 5 |
| 3262.2737876 | 1 |
| 3262.2743944 | 1 |
| 3262.274627 | 5 |
| 3262.2747879 | 4 |
| 3262.2751254 | 2 |
| 3262.2756753 | 5 |
| 3262.2756908 | 3 |
| 3262.2763342 | 7 |
| 3262.2763349 | 9 |
| 3262.2764765 | 22 |
| 3262.2767063 | 42 |
| 3262.2767637 | 4 |
| 3262.2768018 | 15 |
| 3262.2770449 | 28 |
| 3262.2775265 | 25 |

| | |
|---|---|
| 3262.2776468 | 39 |
| 3262.2777754 | 46 |
| 3262.2780728 | 51 |
| 3262.2783048 | 63 |
| 3262.2784083 | 59 |
| 3262.2790595 | 87 |
| 3262.2791159 | 77 |
| 3262.2793405 | 80 |
| 3262.279413 | 71 |
| 3262.2797322 | 73 |
| 3262.2798422 | 66 |
| 3262.2799979 | 83 |
| 3262.28 | 79 |
| 3262.2800008 | 74 |
| 3262.2800045 | 79 |
| 3262.2800843 | 75 |
| 3262.280278 | 65 |
| 3262.2807214 | 74 |
| 3262.2807632 | 76 |
| 3262.2811795 | 69 |
| 3262.2814227 | 63 |
| 3262.2816761 | 67 |
| 3262.2818165 | 70 |
| 3262.2818307 | 83 |
| 3262.2818409 | 67 |
| 3262.2820842 | 59 |
| 3262.2822494 | 87 |
| 3262.2825076 | 63 |
| 3262.2825441 | 56 |
| 3262.2826916 | 72 |
| 3262.2833245 | 102 |
| 3262.2834517 | 87 |
| 3262.2836993 | 73 |
| 3262.2837412 | 71 |
| 3262.2839661 | 80 |
| 3262.2840976 | 53 |
| 3262.2843823 | 84 |
| 3262.2844066 | 77 |
| 3262.284897 | 73 |
| 3262.2852777 | 70 |
| 3262.2852857 | 55 |
| 3262.285683 | 73 |
| 3262.2858288 | 43 |
| 3262.2859203 | 18 |
| 3262.2860792 | 48 |
| 3262.2866818 | 75 |
| 3262.2869888 | 24 |
| 3262.2870476 | 34 |
| 3262.2870644 | 6 |
| 3262.2875066 | 48 |
| 3262.2884033 | 9 |
| 3262.288423 | 31 |
| 3262.2886236 | 27 |
| 3262.2887417 | 12 |
| 3262.288856 | 10 |
| 3262.289621 | 8 |
| 3262.2896351 | 1 |
| 3262.2898524 | 1 |
| 3262.2899644 | 5 |
| 3262.2901245 | 2 |
| 3262.2904919 | 1 |
| 3262.2905189 | 3 |
| 3262.2907593 | 2 |
| 3262.2910646 | 4 |
| 3262.2915557 | 0 |
| 3262.2918651 | 1 |
| 3262.2920002 | 4 |
| 3262.2925954 | 1 |
| 3262.2927127 | 1 |
| 3262.2928183 | 4 |
| 3262.293116 | 5 |
| 3262.2935484 | 6 |
| 3262.2936144 | 2 |
| 3262.2937534 | 2 |
| 3262.2939577 | 2 |
| 3262.29414 | 6 |
| 3262.2941991 | 3 |
| 3262.2946587 | 3 |
| 3262.2948895 | 3 |
| 3262.2950773 | 3 |
| 3262.2956194 | 2 |
| 3262.2956642 | 2 |
| 3262.2960899 | 4 |
| 3262.2961778 | 3 |
| 3262.296332 | 2 |
| 3262.2965155 | 3 |
| 3262.2969526 | 4 |
| 3262.2969943 | 0 |
| 3262.2970706 | 2 |
| 3262.2979401 | 1 |
| 3262.2979471 | 0 |
| 3262.2981861 | 1 |
| 3262.2984461 | 4 |
| 3262.298595 | 3 |
| 3262.2990578 | 4 |
| 3262.2991419 | 4 |
| 3262.2991432 | 2 |
| 3262.2996019 | 0 |
| 3262.2996681 | 1 |
| 3262.2999327 | 4 |
| 3262.3002425 | 1 |
| 3262.3003069 | 3 |
| 3262.3003947 | 2 |
| 3262.3005017 | 2 |
| 3262.3010148 | 2 |
| 3262.3012313 | 3 |
| 3262.301594 | 1 |
| 3262.3016667 | 3 |
| 3262.3017292 | 1 |
| 3262.3021014 | 2 |
| 3262.3021048 | 3 |
| 3262.3021566 | 1 |
| 3262.3023653 | 3 |
| 3262.3023828 | 2 |
| 3262.3026852 | 3 |
| 3262.3031834 | 6 |
| 3262.3032949 | 0 |
| 3262.3039953 | 2 |
| 3262.304016 | 0 |
| 3262.304108 | 4 |
| 3262.304653 | 1 |
| 3262.3047387 | 2 |
| 3262.3048603 | 5 |
| 3262.3048955 | 1 |
| 3262.3053876 | 3 |
| 3262.3055142 | 1 |
| 3262.3055815 | 3 |
| 3262.305795 | 2 |
| 3262.3062328 | 0 |
| 3262.3062955 | 2 |
| 3262.3063862 | 3 |
| 3262.3066385 | 1 |
| 3262.3068245 | 2 |
| 3262.306895 | 0 |
| 3262.3072851 | 3 |
| 3262.3075173 | 2 |
| 3262.3076408 | 2 |
| 3262.3076858 | 3 |
| 3262.3085945 | 1 |
| 3262.308622 | 0 |
| 3262.3087459 | 2 |
| 3262.3088 | 2 |
| 3262.3088953 | 2 |
| 3262.3092512 | 4 |
| 3262.3093771 | 0 |
| 3262.3093833 | 1 |
| 3262.3100561 | 2 |
| 3262.3101721 | 2 |
| 3262.3102457 | 1 |
| 3262.3104811 | 3 |
| 3262.3104904 | 3 |
| 3262.3106379 | 0 |
| 3262.3112093 | 3 |
| 3262.3112127 | 2 |
| 3262.3112376 | 5 |
| 3262.3113076 | 2 |
| 3262.3115539 | 2 |
| 3262.3122103 | 2 |
| 3262.3125358 | 2 |
| 3262.3127675 | 0 |
| 3262.3129914 | 2 |
| 3262.3130221 | 0 |
| 3262.3131179 | 2 |
| 3262.3132047 | 3 |
| 3262.3135342 | 1 |
| 3262.3137123 | 4 |
| 3262.3138925 | 0 |
| 3262.314307 | 2 |
| 3262.3143208 | 3 |
| 3262.3143765 | 4 |
| 3262.3145227 | 2 |
| 3262.3148356 | 1 |
| 3262.3151812 | 3 |
| 3262.3154005 | 1 |
| 3262.3157981 | 4 |
| 3262.3158479 | 1 |
| 3262.315963 | 5 |
| 3262.3159911 | 1 |
| 3262.3164888 | 3 |
| 3262.3166091 | 2 |
| 3262.3167124 | 3 |
| 3262.3168949 | 1 |
| 3262.3171528 | 4 |
| 3262.3171743 | 2 |
| 3262.3178628 | 2 |
| 3262.3181158 | 4 |
| 3262.3183232 | 2 |
| 3262.3183488 | 4 |
| 3262.3186398 | 5 |
| 3262.3187258 | 2 |
| 3262.319206 | 1 |
| 3262.3192965 | 1 |
| 3262.3193879 | 4 |
| 3262.3194976 | 2 |
| 3262.3195281 | 2 |
| 3262.3199439 | 4 |
| 3262.3201097 | 2 |
| 3262.3204404 | 0 |
| 3262.3206634 | 0 |
| 3262.3208531 | 5 |
| 3262.3209541 | 1 |
| 3262.3211907 | 0 |
| 3262.3215314 | 2 |
| 3262.3217183 | 0 |
| 3262.3217616 | 5 |
| 3262.3219395 | 2 |
| 3262.3220528 | 1 |
| 3262.3221344 | 5 |
| 3262.3224097 | 2 |
| 3262.322472 | 1 |
| 3262.3230149 | 1 |
| 3262.3234554 | 3 |
| 3262.3234657 | 6 |
| 3262.323493 | 1 |
| 3262.3236629 | 1 |
| 3262.3239345 | 4 |
| 3262.3240674 | 3 |
| 3262.3247092 | 4 |
| 3262.3249813 | 1 |
| 3262.3250761 | 1 |
| 3262.3251076 | 1 |
| 3262.325178 | 1 |
| 3262.3252494 | 1 |
| 3262.3253703 | 4 |
| 3262.3255777 | 3 |
| 3262.3255934 | 3 |
| 3262.3260331 | 5 |
| 3262.3260596 | 2 |
| 3262.3264363 | 2 |
| 3262.3266849 | 3 |
| 3262.3267232 | 1 |
| 3262.3270129 | 1 |
| 3262.3270268 | 1 |
| 3262.3271191 | 2 |
| 3262.3271398 | 4 |
| 3262.3271945 | 1 |
| 3262.3274218 | 2 |
| 3262.3274241 | 4 |
| 3262.327565 | 3 |
| 3262.3275687 | 3 |
| 3262.3281687 | 7 |
| 3262.3282576 | 3 |
| 3262.3284009 | 5 |
| 3262.3284683 | 3 |
| 3262.3291802 | 8 |
| 3262.3293027 | 9 |
| 3262.329351 | 1 |
| 3262.3294004 | 2 |
| 3262.329924 | 11 |
| 3262.3300238 | 2 |
| 3262.3301731 | 13 |
| 3262.3303283 | 1 |
| 3262.3308605 | 20 |
| 3262.3309632 | 30 |
| 3262.3310007 | 6 |
| 3262.3311253 | 2 |
| 3262.3312167 | 19 |
| 3262.3312531 | 30 |
| 3262.3314157 | 31 |
| 3262.3320972 | 40 |
| 3262.3321049 | 55 |
| 3262.3325916 | 3 |
| 3262.3331278 | 50 |
| 3262.3335021 | 3 |
| 3262.333609 | 3 |
| 3262.3337539 | 57 |
| 3262.3342844 | 51 |
| 3262.3349582 | 50 |
| 3262.3349731 | 1 |
| 3262.3353126 | 36 |
| 3262.3354464 | 46 |
| 3262.33551 | 2 |
| 3262.3355259 | 39 |
| 3262.3356303 | 3 |
| 3262.3361749 | 37 |
| 3262.3364441 | 4 |
| 3262.3367287 | 33 |
| 3262.3368436 | 2 |
| 3262.3368782 | 23 |
| 3262.3374643 | 5 |
| 3262.3374842 | 15 |
| 3262.3375833 | 22 |
| 3262.3378316 | 1 |
| 3262.3383528 | 3 |
| 3262.3386611 | 0 |
| 3262.3388 | 5 |
| 3262.3388213 | 0 |
| 3262.3394672 | 2 |
| 3262.3396671 | 2 |
| 3262.3397388 | 0 |
| 3262.3399174 | 3 |
| 3262.3405809 | 1 |
| 3262.3406023 | 4 |
| 3262.3406404 | 4 |
| 3262.3409161 | 3 |
| 3262.3411317 | 4 |
| 3262.3411674 | 1 |
| 3262.3413828 | 1 |
| 3262.3414351 | 4 |
| 3262.3418154 | 1 |
| 3262.3422568 | 2 |
| 3262.3423837 | 4 |
| 3262.3428363 | 1 |
| 3262.3428812 | 2 |
| 3262.3428843 | 2 |
| 3262.3430119 | 1 |
| 3262.3437486 | 3 |
| 3262.3437925 | 1 |
| 3262.3438692 | 0 |
| 3262.343995 | 3 |
| 3262.3443497 | 2 |
| 3262.3444012 | 1 |
| 3262.3444451 | 4 |
| 3262.3446587 | 2 |
| 3262.3447635 | 3 |
| 3262.3449719 | 1 |
| 3262.3452969 | 4 |
| 3262.3457508 | 0 |
| 3262.3459351 | 4 |
| 3262.3461512 | 3 |
| 3262.3461987 | 2 |
| 3262.3467007 | 5 |
| 3262.3469333 | 5 |
| 3262.3470414 | 5 |
| 3262.3470543 | 2 |
| 3262.3472519 | 4 |
| 3262.347392 | 1 |
| 3262.3477574 | 2 |
| 3262.3482486 | 2 |
| 3262.3486319 | 2 |
| 3262.3487361 | 3 |
| 3262.3488504 | 3 |
| 3262.3490032 | 0 |
| 3262.3491893 | 2 |
| 3262.349425 | 3 |
| 3262.3494932 | 1 |
| 3262.3505018 | 1 |
| 3262.3505471 | 3 |
| 3262.350609 | 2 |
| 3262.3507689 | 3 |
| 3262.3508501 | 2 |
| 3262.3509088 | 6 |
| 3262.3509434 | 4 |
| 3262.3510914 | 4 |
| 3262.3516102 | 0 |
| 3262.3517966 | 4 |
| 3262.3519179 | 3 |
| 3262.3521126 | 5 |
| 3262.3521849 | 3 |
| 3262.352542 | 5 |
| 3262.3529124 | 3 |
| 3262.3531029 | 3 |
| 3262.3533587 | 2 |
| 3262.3534638 | 3 |
| 3262.3535126 | 3 |
| 3262.3535477 | 2 |
| 3262.3537582 | 0 |
| 3262.35395 | 4 |
| 3262.3544487 | 2 |
| 3262.3547197 | 3 |
| 3262.3549567 | 0 |
| 3262.3553479 | 3 |
| 3262.3554328 | 2 |

| | |
|---|---|
| 3262.3556471 | 5 |
| 3262.3556848 | 3 |
| 3262.356034 | 4 |
| 3262.3562961 | 3 |
| 3262.3563837 | 0 |
| 3262.3565875 | 0 |
| 3262.3567152 | 6 |
| 3262.35714 | 4 |
| 3262.3573475 | 4 |
| 3262.3573941 | 3 |
| 3262.3575377 | 3 |
| 3262.3580119 | 1 |
| 3262.3580442 | 1 |
| 3262.3581618 | 1 |
| 3262.3582803 | 1 |
| 3262.3583318 | 1 |
| 3262.3587865 | 3 |
| 3262.3587918 | 3 |
| 3262.3587944 | 3 |
| 3262.3591418 | 2 |
| 3262.3598144 | 1 |
| 3262.3602429 | 3 |
| 3262.360365 | 1 |
| 3262.3603805 | 3 |
| 3262.3605227 | 6 |
| 3262.3607808 | 3 |
| 3262.3608999 | 5 |
| 3262.3614485 | 1 |
| 3262.361451 | 5 |
| 3262.3614857 | 3 |
| 3262.3614968 | 3 |
| 3262.3618864 | 5 |
| 3262.3621457 | 6 |
| 3262.3626346 | 1 |
| 3262.3628312 | 0 |
| 3262.3630415 | 0 |
| 3262.3632237 | 2 |
| 3262.3632237 | 2 |
| 3262.3632553 | 2 |
| 3262.3633671 | 1 |
| 3262.3636819 | 3 |
| 3262.3637667 | 3 |
| 3262.364033 | 2 |
| 3262.3640856 | 1 |
| 3262.3642387 | 4 |
| 3262.3647194 | 2 |
| 3262.3647611 | 4 |
| 3262.36522 | 3 |
| 3262.3656998 | 4 |
| 3262.3660056 | 1 |
| 3262.366019 | 2 |
| 3262.3661023 | 5 |
| 3262.3662055 | 3 |
| 3262.3663318 | 1 |
| 3262.3664455 | 3 |
| 3262.3668272 | 3 |
| 3262.3668708 | 0 |
| 3262.3673402 | 1 |
| 3262.3674001 | 1 |
| 3262.3678034 | 5 |
| 3262.3679854 | 1 |
| 3262.3679919 | 7 |
| 3262.3682082 | 1 |
| 3262.3685762 | 1 |
| 3262.3687076 | 1 |
| 3262.3688901 | 3 |
| 3262.3689365 | 4 |
| 3262.3693712 | 2 |
| 3262.369612 | 3 |
| 3262.3699667 | 4 |
| 3262.3701366 | 3 |
| 3262.3702674 | 2 |
| 3262.3702863 | 4 |
| 3262.3706334 | 2 |
| 3262.3706459 | 2 |
| 3262.3710156 | 3 |
| 3262.3712028 | 2 |
| 3262.3713334 | 2 |
| 3262.3713992 | 4 |
| 3262.3715978 | 2 |
| 3262.3716276 | 1 |
| 3262.371811 | 3 |
| 3262.3720147 | 3 |
| 3262.3721575 | 0 |
| 3262.3721894 | 0 |
| 3262.3723888 | 1 |
| 3262.3723943 | 1 |
| 3262.3727248 | 0 |
| 3262.3727515 | 3 |
| 3262.372889 | 1 |
| 3262.3729602 | 3 |
| 3262.3734077 | 1 |
| 3262.3735417 | 2 |
| 3262.373545 | 2 |
| 3262.3737002 | 3 |
| 3262.3740441 | 4 |
| 3262.3741576 | 2 |
| 3262.3743144 | 3 |
| 3262.3746697 | 0 |
| 3262.3748424 | 2 |
| 3262.3751996 | 6 |
| 3262.3752674 | 3 |
| 3262.3752835 | 1 |
| 3262.3755463 | 3 |
| 3262.3756375 | 2 |
| 3262.3757362 | 3 |
| 3262.3760234 | 2 |
| 3262.3766724 | 7 |
| 3262.3767349 | 1 |
| 3262.3768177 | 2 |
| 3262.3770113 | 0 |
| 3262.3770968 | 1 |
| 3262.377228 | 5 |
| 3262.3775182 | 1 |
| 3262.3782792 | 5 |
| 3262.3783581 | 1 |
| 3262.3784358 | 1 |
| 3262.3784402 | 1 |
| 3262.3785575 | 1 |
| 3262.3787698 | 5 |
| 3262.3791362 | 4 |
| 3262.3795421 | 2 |
| 3262.3799341 | 0 |
| 3262.3803454 | 3 |
| 3262.3804483 | 1 |
| 3262.3805941 | 1 |
| 3262.3806529 | 3 |
| 3262.3809879 | 1 |
| 3262.3811691 | 1 |
| 3262.3823781 | 1 |
| 3262.3826124 | 2 |
| 3262.3826292 | 3 |
| 3262.3827201 | 6 |
| 3262.3828183 | 1 |
| 3262.3830133 | 2 |
| 3262.383343 | 5 |
| 3262.3834842 | 5 |
| 3262.3835525 | 0 |
| 3262.3837268 | 4 |
| 3262.3846553 | 9 |
| 3262.3849755 | 3 |
| 3262.3850635 | 4 |
| 3262.3854786 | 2 |
| 3262.3854881 | 4 |
| 3262.3859134 | 2 |
| 3262.3860446 | 7 |
| 3262.3863448 | 41 |
| 3262.3863522 | 14 |
| 3262.3864023 | 11 |
| 3262.3865093 | 31 |
| 3262.3866661 | 39 |
| 3262.3870247 | 46 |
| 3262.3875789 | 70 |
| 3262.3876047 | 109 |
| 3262.3877274 | 81 |
| 3262.387823 | 103 |
| 3262.3878885 | 63 |
| 3262.3882843 | 80 |
| 3262.3884352 | 73 |
| 3262.388522 | 53 |
| 3262.3890004 | 82 |
| 3262.3891527 | 74 |
| 3262.3891601 | 108 |
| 3262.3891809 | 80 |
| 3262.3898136 | 73 |
| 3262.3901367 | 77 |
| 3262.3902952 | 82 |
| 3262.3906504 | 79 |
| 3262.3908046 | 74 |
| 3262.3911099 | 84 |
| 3262.3911873 | 53 |
| 3262.3913746 | 62 |
| 3262.3915028 | 65 |
| 3262.3915253 | 62 |
| 3262.3921592 | 60 |
| 3262.3925934 | 83 |
| 3262.3927862 | 64 |
| 3262.3928502 | 65 |
| 3262.3931998 | 54 |
| 3262.3935517 | 75 |
| 3262.3936559 | 73 |
| 3262.3938922 | 66 |
| 3262.3940267 | 56 |
| 3262.3940277 | 53 |
| 3262.3940772 | 82 |
| 3262.3942382 | 56 |
| 3262.3943097 | 57 |
| 3262.3944653 | 78 |
| 3262.3948842 | 61 |
| 3262.3952548 | 53 |
| 3262.3953559 | 73 |
| 3262.3959025 | 27 |
| 3262.3959061 | 29 |
| 3262.3959503 | 48 |
| 3262.3962909 | 18 |
| 3262.3964891 | 28 |
| 3262.3967069 | 36 |
| 3262.3967365 | 9 |
| 3262.3973187 | 6 |
| 3262.3974664 | 8 |
| 3262.3975942 | 9 |
| 3262.3979665 | 1 |
| 3262.398111 | 3 |
| 3262.3982418 | 4 |
| 3262.3985111 | 2 |
| 3262.398515 | 3 |
| 3262.3985241 | 2 |
| 3262.3986209 | 3 |
| 3262.3989435 | 0 |
| 3262.39923 | 6 |
| 3262.3993781 | 1 |
| 3262.3999593 | 1 |
| 3262.4003846 | 3 |
| 3262.4006559 | 5 |
| 3262.4007487 | 2 |
| 3262.400902 | 3 |
| 3262.4009453 | 4 |
| 3262.4009537 | 2 |
| 3262.40103 | 1 |
| 3262.4018614 | 4 |
| 3262.4021113 | 0 |
| 3262.4021429 | 1 |
| 3262.4024017 | 1 |
| 3262.4026449 | 4 |
| 3262.4029295 | 2 |
| 3262.4030303 | 1 |
| 3262.4031746 | 2 |
| 3262.4036361 | 4 |
| 3262.4038416 | 4 |
| 3262.4038696 | 3 |
| 3262.404074 | 1 |
| 3262.4041385 | 8 |
| 3262.4043276 | 2 |
| 3262.4046029 | 2 |
| 3262.4047487 | 1 |
| 3262.4052975 | 0 |
| 3262.4055698 | 2 |
| 3262.405658 | 2 |
| 3262.4057875 | 2 |
| 3262.4060753 | 0 |
| 3262.4061215 | 3 |
| 3262.406562 | 4 |
| 3262.4066924 | 2 |
| 3262.4067631 | 0 |
| 3262.4068268 | 2 |
| 3262.4070605 | 3 |
| 3262.4070933 | 2 |
| 3262.4079083 | 1 |
| 3262.4082716 | 4 |
| 3262.4085083 | 2 |
| 3262.4097264 | 0 |
| 3262.4099461 | 1 |
| 3262.4099477 | 2 |
| 3262.4099633 | 0 |
| 3262.4100034 | 3 |
| 3262.4109045 | 5 |
| 3262.4109117 | 3 |
| 3262.4113174 | 2 |
| 3262.4115771 | 0 |
| 3262.4124999 | 4 |
| 3262.4126817 | 2 |
| 3262.4129974 | 0 |
| 3262.4135373 | 1 |
| 3262.4138235 | 4 |
| 3262.4143381 | 2 |
| 3262.4145407 | 0 |
| 3262.4145767 | 2 |
| 3262.4152354 | 2 |
| 3262.4155919 | 0 |
| 3262.4162156 | 1 |
| 3262.4166719 | 3 |
| 3262.416779 | 2 |
| 3262.4173956 | 1 |
| 3262.4174287 | 2 |
| 3262.4175076 | 2 |
| 3262.4182803 | 1 |
| 3262.4184551 | 4 |
| 3262.4189119 | 2 |
| 3262.4189131 | 2 |
| 3262.4198828 | 5 |
| 3262.4199629 | 4 |
| 3262.4199851 | 5 |
| 3262.4201735 | 2 |
| 3262.420423 | 2 |
| 3262.4205176 | 2 |
| 3262.4212154 | 1 |
| 3262.4216096 | 2 |
| 3262.4218654 | 5 |
| 3262.4223451 | 1 |
| 3262.422964 | 1 |
| 3262.4234976 | 2 |
| 3262.4235244 | 2 |
| 3262.4247137 | 1 |
| 3262.4249061 | 3 |
| 3262.4249484 | 4 |
| 3262.4251974 | 5 |
| 3262.4255819 | 3 |
| 3262.4267318 | 2 |
| 3262.4272276 | 2 |
| 3262.4276433 | 1 |
| 3262.4278779 | 2 |
| 3262.428153 | 0 |
| 3262.4286618 | 2 |
| 3262.4287026 | 2 |
| 3262.4295439 | 1 |
| 3262.4297837 | 1 |
| 3262.4298566 | 1 |
| 3262.4309675 | 4 |
| 3262.4315138 | 3 |
| 3262.4315797 | 2 |
| 3262.4316783 | 3 |
| 3262.4316877 | 2 |
| 3262.4318268 | 3 |
| 3262.4328405 | 1 |
| 3262.4332516 | 7 |
| 3262.4334229 | 2 |
| 3262.433857 | 3 |
| 3262.4343284 | 0 |
| 3262.4352335 | 0 |
| 3262.4358816 | 2 |
| 3262.4359974 | 1 |
| 3262.4364762 | 6 |
| 3262.4370629 | 3 |
| 3262.4382485 | 2 |
| 3262.438337 | 4 |
| 3262.4395954 | 1 |
| 3262.4400409 | 1 |
| 3262.4410375 | 3 |
| 3262.4414383 | 3 |
| 3262.4414468 | 4 |
| 3262.4426517 | 2 |
| 3262.4437002 | 0 |
| 3262.4441013 | 1 |
| 3262.444589 | 1 |
| 3262.4459928 | 3 |
| 3262.4462365 | 2 |
| 3262.4463554 | 4 |
| 3262.4473964 | 0 |
| 3262.4483195 | 1 |
| 3262.4485257 | 0 |
| 3262.4490205 | 2 |
| 3262.4499348 | 2 |
| 3262.4507278 | 0 |
| 3262.4509587 | 3 |
| 3262.451697 | 1 |
| 3262.4531928 | 1 |
| 3262.4532626 | 2 |
| 3262.453295 | 3 |
| 3262.4540631 | 2 |
| 3262.4549562 | 3 |
| 3262.4554453 | 1 |
| 3262.4563186 | 1 |
| 3262.457197 | 1 |
| 3262.4579651 | 2 |
| 3262.4583649 | 1 |
| 3262.4592698 | 3 |
| 3262.4596016 | 1 |
| 3262.4600148 | 5 |
| 3262.4610007 | 0 |
| 3262.4615488 | 1 |
| 3262.4616775 | 1 |
| 3262.4631252 | 0 |
| 3262.4637979 | 5 |

| | |
|---|---|
| 3262.4641976 | 2 |
| 3262.4645672 | 1 |
| 3262.465075 | 1 |
| 3262.4651376 | 2 |
| 3262.4654817 | 4 |
| 3262.4667065 | 2 |
| 3262.4672966 | 5 |
| 3262.4675033 | 0 |
| 3262.4676492 | 2 |
| 3262.4699589 | 2 |
| 3262.4709845 | 3 |
| 3262.4713036 | 3 |
| 3262.4717636 | 2 |
| 3262.4725179 | 6 |
| 3262.4733149 | 2 |
| 3262.4741428 | 0 |
| 3262.4744894 | 2 |
| 3262.4756463 | 1 |
| 3262.4759932 | 1 |
| 3262.4764025 | 4 |
| 3262.4773142 | 2 |
| 3262.4783915 | 1 |
| 3262.4784223 | 5 |
| 3262.4789676 | 7 |
| 3262.4803423 | 3 |
| 3262.4804603 | 5 |
| 3262.481624 | 3 |
| 3262.4824476 | 4 |
| 3262.4825972 | 4 |
| 3262.4828173 | 3 |
| 3262.4829862 | 2 |
| 3262.4848593 | 2 |
| 3262.4851951 | 2 |
| 3262.4862583 | 2 |
| 3262.4865582 | 1 |
| 3262.4865975 | 1 |
| 3262.4880232 | 2 |
| 3262.4891535 | 3 |
| 3262.4896135 | 0 |
| 3262.4898302 | 1 |
| 3262.4905406 | 1 |
| 3262.4918388 | 6 |
| 3262.4925056 | 0 |
| 3262.4927776 | 2 |
| 3262.4930305 | 2 |
| 3262.4933141 | 1 |
| 3262.4945967 | 3 |
| 3262.4946051 | 11 |
| 3262.495677 | 0 |
| 3262.4962793 | 1 |
| 3262.4973151 | 0 |
| 3262.4980864 | 3 |
| 3262.4981914 | 2 |
| 3262.4996871 | 2 |
| 3262.500151 | 2 |
| 3262.5005605 | 3 |
| 3262.5013349 | 2 |
| 3262.5023158 | 3 |
| 3262.5037096 | 1 |
| 3262.5040638 | 2 |
| 3262.5040899 | 4 |
| 3262.5056283 | 2 |
| 3262.506565 | 0 |
| 3262.5066786 | 3 |
| 3262.5082277 | 3 |
| 3262.5088284 | 5 |
| 3262.5099843 | 3 |
| 3262.5104701 | 2 |
| 3262.5112331 | 2 |
| 3262.5119032 | 2 |
| 3262.5125712 | 1 |
| 3262.5128229 | 1 |
| 3262.5135279 | 1 |
| 3262.5141835 | 5 |
| 3262.5148072 | 4 |
| 3262.5152245 | 5 |
| 3262.5169843 | 0 |
| 3262.5175835 | 2 |
| 3262.5181454 | 2 |
| 3262.5184788 | 3 |
| 3262.5203263 | 3 |
| 3262.5219017 | 4 |
| 3262.5223321 | 2 |
| 3262.5229927 | 1 |
| 3262.5236391 | 3 |
| 3262.5241859 | 3 |
| 3262.524663 | 3 |
| 3262.5254305 | 5 |
| 3262.5259606 | 1 |
| 3262.5277111 | 1 |
| 3262.5279146 | 1 |
| 3262.5281948 | 2 |
| 3262.5293089 | 1 |
| 3262.5298066 | 0 |
| 3262.5301438 | 2 |
| 3262.5305052 | 4 |
| 3262.532241 | 3 |
| 3262.5328134 | 5 |
| 3262.5332368 | 1 |
| 3262.5342744 | 4 |
| 3262.5347355 | 2 |
| 3262.5356176 | 0 |
| 3262.5360404 | 5 |
| 3262.536552 | 2 |
| 3262.5369289 | 2 |
| 3262.5376417 | 5 |
| 3262.5382541 | 4 |
| 3262.5395421 | 2 |
| 3262.5396612 | 1 |
| 3262.5404056 | 5 |
| 3262.5409289 | 3 |
| 3262.5418863 | 0 |
| 3262.5420418 | 4 |
| 3262.5430969 | 1 |
| 3262.5434821 | 4 |
| 3262.5443188 | 4 |
| 3262.5450965 | 4 |
| 3262.5452784 | 4 |
| 3262.5455509 | 1 |
| 3262.5476252 | 1 |
| 3262.550482 | 0 |
| 3262.55163 | 1 |
| 3262.5538089 | 2 |
| 3262.5567172 | 2 |
| 3262.5578818 | 0 |
| 3262.5611053 | 4 |
| 3262.5623272 | 1 |
| 3262.5652543 | 3 |
| 3262.5655243 | 5 |
| 3262.5691337 | 1 |
| 3262.5718813 | 1 |
| 3262.5736179 | 2 |
| 3262.5763438 | 1 |
| 3262.5779822 | 2 |
| 3262.5801403 | 2 |
| 3262.5825785 | 4 |
| 3262.5846514 | 1 |
| 3262.5868907 | 0 |
| 3262.5883612 | 1 |
| 3262.5919524 | 4 |
| 3262.5943555 | 4 |
| 3262.5953022 | 3 |
| 3262.5979867 | 1 |
| 3262.5989152 | 1 |
| 3262.6018217 | 4 |
| 3262.6034569 | 3 |
| 3262.6060586 | 1 |
| 3262.6084542 | 1 |
| 3262.6099305 | 1 |
| 3262.6126081 | 2 |
| 3262.6153192 | 2 |
| 3262.6171114 | 5 |
| 3262.61964 | 1 |
| 3262.6208287 | 5 |
| 3262.624467 | 2 |
| 3262.6267016 | 3 |
| 3262.6276979 | 2 |
| 3262.6303758 | 3 |
| 3262.6316351 | 3 |
| 3262.6346579 | 1 |
| 3262.6370166 | 3 |
| 3262.6386122 | 8 |
| 3262.6415403 | 4 |
| 3262.6424235 | 1 |
| 3262.6450812 | 2 |
| 3262.6468596 | 3 |
| 3262.6493689 | 3 |
| 3262.6524927 | 3 |
| 3262.654033 | 5 |
| 3262.6561913 | 1 |
| 3262.657687 | 3 |
| 3262.6606556 | 2 |
| 3262.6634766 | 2 |
| 3262.6647872 | 0 |
| 3262.6684975 | 5 |
| 3262.6700493 | 3 |
| 3262.6717098 | 5 |
| 3262.6744692 | 0 |
| 3262.6754099 | 7 |
| 3262.6783847 | 3 |
| 3262.6799121 | 2 |
| 3262.6822984 | 1 |
| 3262.6849958 | 4 |
| 3262.6868169 | 0 |
| 3262.6890377 | 3 |
| 3262.690351 | 1 |
| 3262.6929252 | 1 |
| 3262.6961827 | 3 |
| 3262.6966935 | 2 |
| 3262.6996774 | 4 |
| 3262.7006158 | 4 |
| 3262.7036621 | 3 |
| 3262.7063391 | 3 |
| 3262.7082117 | 2 |
| 3262.7115318 | 4 |
| 3262.7126802 | 1 |
| 3262.7152996 | 0 |
| 3262.7181134 | 6 |
| 3262.719723 | 5 |
| 3262.7217979 | 1 |
| 3262.7234245 | 2 |
| 3262.7261643 | 2 |
| 3262.7286213 | 3 |
| 3262.7302452 | 3 |
| 3262.7328081 | 2 |
| 3262.7345526 | 1 |
| 3262.7366649 | 2 |
| 3262.7398367 | 0 |
| 3262.7409487 | 2 |
| 3262.7445122 | 5 |
| 3262.7448538 | 1 |
| 3262.7480294 | 7 |
| 3262.7491767 | 5 |
| 3262.751849 | 1 |
| 3262.7550993 | 1 |
| 3262.7560902 | 4 |
| 3262.7591896 | 1 |
| 3262.7613967 | 4 |
| 3262.7632565 | 3 |
| 3262.7656368 | 1 |
| 3262.7670924 | 4 |
| 3262.7704645 | 4 |
| 3262.7729128 | 3 |
| 3262.7740673 | 1 |
| 3262.7768677 | 4 |
| 3262.7783746 | 3 |
| 3262.7809663 | 0 |
| 3262.7822326 | 0 |
| 3262.784851 | 2 |
| 3262.7877105 | 0 |
| 3262.7889559 | 2 |
| 3262.7918255 | 1 |
| 3262.7928857 | 5 |
| 3262.796209 | 3 |
| 3262.7982807 | 2 |
| 3262.8000884 | 2 |
| 3262.8031037 | 1 |
| 3262.8043172 | 2 |
| 3262.8077368 | 3 |
| 3262.8090062 | 2 |
| 3262.8114064 | 2 |
| 3262.8140307 | 3 |
| 3262.815282 | 2 |
| 3262.8181563 | 1 |
| 3262.820855 | 5 |
| 3262.8221232 | 0 |
| 3262.8248506 | 4 |
| 3262.8262352 | 1 |
| 3262.8289984 | 5 |
| 3262.8297028 | 2 |
| 3262.8327206 | 5 |
| 3262.83554 | 3 |
| 3262.8371443 | 2 |
| 3262.8397202 | 0 |
| 3262.8414241 | 2 |
| 3262.8441747 | 0 |
| 3262.8470246 | 3 |
| 3262.8478684 | 2 |
| 3262.8515301 | 0 |
| 3262.852963 | 1 |
| 3262.8554493 | 2 |
| 3262.8584865 | 4 |
| 3262.8601024 | 1 |
| 3262.8621487 | 1 |
| 3262.8639505 | 4 |
| 3262.8662315 | 3 |
| 3262.8677831 | 0 |
| 3262.8709459 | 6 |
| 3262.8732516 | 3 |
| 3262.8737815 | 2 |
| 3262.8767565 | 6 |
| 3262.8801805 | 1 |
| 3262.8812441 | 6 |
| 3262.8837742 | 2 |
| 3262.8852134 | 2 |
| 3262.8880906 | 4 |
| 3262.8898289 | 2 |
| 3262.8930909 | 2 |
| 3262.8953039 | 1 |
| 3262.8966952 | 5 |
| 3262.8992438 | 2 |
| 3262.9003116 | 5 |
| 3262.9025722 | 4 |
| 3262.9059723 | 4 |
| 3262.9074756 | 2 |
| 3262.9098255 | 2 |
| 3262.9114333 | 1 |
| 3262.9143385 | 2 |
| 3262.9164637 | 2 |
| 3262.9179032 | 4 |
| 3262.9209776 | 1 |
| 3262.9223402 | 1 |
| 3262.9247446 | 1 |
| 3262.9280861 | 2 |
| 3262.929102 | 3 |
| 3262.9318183 | 1 |
| 3262.933429 | 1 |
| 3262.9361348 | 3 |
| 3262.9392204 | 1 |
| 3262.9404646 | 3 |
| 3262.943466 | 3 |
| 3262.9447993 | 2 |
| 3262.9473144 | 4 |
| 3262.9487316 | 1 |
| 3262.9519645 | 3 |
| 3262.9537692 | 2 |
| 3262.9562682 | 1 |
| 3262.9585002 | 5 |
| 3262.9604203 | 1 |
| 3262.9619269 | 1 |
| 3262.9649425 | 2 |
| 3262.9654703 | 1 |
| 3262.9684447 | 7 |
| 3262.971555 | 2 |
| 3262.9734984 | 2 |
| 3262.9759276 | 2 |
| 3262.9774356 | 2 |
| 3262.9806807 | 1 |
| 3262.9827249 | 2 |
| 3262.9840706 | 3 |
| 3262.9870131 | 1 |
| 3262.9878663 | 1 |
| 3262.9909297 | 4 |
| 3262.9922965 | 2 |
| 3262.9949319 | 3 |
| 3262.995491 | 3 |
| 3262.9962517 | 5 |
| 3262.99818 | 2 |
| 3262.998838 | 6 |
| 3262.999501 | 2 |
| 3263.0014694 | 1 |
| 3263.00285 | 0 |
| 3263.0029676 | 0 |
| 3263.0036795 | 2 |
| 3263.0058752 | 1 |
| 3263.0067494 | 0 |
| 3263.0072369 | 3 |
| 3263.008628 | 0 |
| 3263.0099785 | 2 |
| 3263.0100854 | 5 |
| 3263.0123404 | 4 |
| 3263.0127064 | 3 |
| 3263.012816 | 1 |
| 3263.0143306 | 3 |
| 3263.0159113 | 5 |
| 3263.0163282 | 1 |
| 3263.0178709 | 5 |
| 3263.0190269 | 3 |
| 3263.0201701 | 6 |
| 3263.0209751 | 2 |
| 3263.022265 | 1 |
| 3263.0230376 | 1 |
| 3263.023796 | 1 |
| 3263.0252923 | 1 |
| 3263.0275608 | 2 |
| 3263.029047 | 2 |
| 3263.0319001 | 3 |
| 3263.0349618 | 1 |
| 3263.0363349 | 4 |
| 3263.0379638 | 2 |
| 3263.0394745 | 2 |
| 3263.0429799 | 1 |
| 3263.0457524 | 0 |

| | |
|---|---|
| 3263.0467838 | 5 |
| 3263.0495327 | 2 |
| 3263.050737 | 1 |
| 3263.0532673 | 2 |
| 3263.0568761 | 4 |
| 3263.0581555 | 3 |
| 3263.0609107 | 3 |
| 3263.0620572 | 4 |
| 3263.0641276 | 0 |
| 3263.067352 | 1 |
| 3263.0687892 | 4 |
| 3263.0710387 | 1 |
| 3263.0729844 | 0 |
| 3263.0758239 | 3 |
| 3263.0786228 | 2 |
| 3263.0800371 | 4 |
| 3263.083191 | 1 |
| 3263.0840243 | 5 |
| 3263.0873449 | 1 |
| 3263.0881252 | 1 |
| 3263.0910415 | 5 |
| 3263.0935753 | 2 |
| 3263.0949256 | 4 |
| 3263.0971355 | 6 |
| 3263.0988675 | 1 |
| 3263.102041 | 2 |
| 3263.1046367 | 1 |
| 3263.1058012 | 1 |
| 3263.1090446 | 2 |
| 3263.1097255 | 1 |
| 3263.1125659 | 3 |
| 3263.1146976 | 0 |
| 3263.1164075 | 6 |
| 3263.1191119 | 0 |
| 3263.1211439 | 2 |
| 3263.1236407 | 0 |
| 3263.1265281 | 2 |
| 3263.1275469 | 2 |
| 3263.1308045 | 2 |
| 3263.1323495 | 1 |
| 3263.1345767 | 2 |
| 3263.137294 | 1 |
| 3263.1391438 | 0 |
| 3263.1418802 | 1 |
| 3263.1431309 | 3 |
| 3263.1460366 | 1 |
| 3263.1473652 | 4 |
| 3263.1497769 | 1 |
| 3263.1524889 | 1 |
| 3263.1536176 | 1 |
| 3263.1571198 | 2 |
| 3263.1575972 | 3 |
| 3263.1601922 | 5 |
| 3263.1637435 | 0 |
| 3263.165363 | 3 |
| 3263.1674847 | 3 |
| 3263.1685205 | 2 |
| 3263.171614 | 3 |
| 3263.1733134 | 1 |
| 3263.1760651 | 6 |
| 3263.1786445 | 2 |
| 3263.1800782 | 3 |
| 3263.1837772 | 2 |
| 3263.1864166 | 2 |
| 3263.1870024 | 2 |
| 3263.1900362 | 1 |
| 3263.1909701 | 3 |
| 3263.193644 | 3 |
| 3263.1954321 | 5 |
| 3263.1982482 | 4 |
| 3263.2001742 | 3 |
| 3263.2024457 | 4 |
| 3263.2040682 | 2 |
| 3263.2069678 | 2 |
| 3263.2082461 | 2 |
| 3263.2112926 | 2 |
| 3263.2124039 | 5 |
| 3263.2159189 | 2 |
| 3263.2179595 | 2 |
| 3263.2196193 | 2 |
| 3263.2222618 | 3 |
| 3263.2236844 | 3 |
| 3263.2270513 | 2 |
| 3263.2293225 | 1 |
| 3263.2305562 | 1 |
| 3263.2342496 | 1 |
| 3263.2343562 | 0 |
| 3263.2368977 | 1 |
| 3263.2388093 | 1 |
| 3263.2412398 | 2 |
| 3263.2435713 | 1 |
| 3263.2458156 | 4 |
| 3263.2482328 | 1 |
| 3263.2496536 | 5 |
| 3263.252391 | 1 |
| 3263.2550792 | 2 |
| 3263.2566108 | 2 |
| 3263.2591061 | 2 |
| 3263.2620293 | 0 |
| 3263.2631028 | 4 |
| 3263.2657532 | 0 |
| 3263.2689055 | 1 |
| 3263.2698518 | 1 |
| 3263.2727172 | 2 |
| 3263.2743963 | 3 |
| 3263.2771413 | 6 |
| 3263.2787158 | 2 |
| 3263.2809231 | 3 |
| 3263.2831197 | 3 |
| 3263.2845801 | 2 |
| 3263.2875647 | 6 |
| 3263.290244 | 0 |
| 3263.2908676 | 4 |
| 3263.2939918 | 2 |
| 3263.2948591 | 5 |
| 3263.2982486 | 6 |
| 3263.2994588 | 1 |
| 3263.3026386 | 1 |
| 3263.3055577 | 4 |
| 3263.3066736 | 1 |
| 3263.3092534 | 2 |
| 3263.3107869 | 2 |
| 3263.3129316 | 4 |
| 3263.3164307 | 0 |
| 3263.3171673 | 1 |
| 3263.3198594 | 2 |
| 3263.3212732 | 0 |
| 3263.3243147 | 1 |
| 3263.3269916 | 2 |
| 3263.328441 | 2 |
| 3263.3309362 | 4 |
| 3263.3321492 | 4 |
| 3263.334385 | 4 |
| 3263.3380143 | 3 |
| 3263.3384068 | 3 |
| 3263.3415949 | 1 |
| 3263.3428687 | 4 |
| 3263.3452069 | 0 |
| 3263.3480075 | 3 |
| 3263.3491795 | 2 |
| 3263.352613 | 3 |
| 3263.3540714 | 0 |
| 3263.3565846 | 3 |
| 3263.3599435 | 5 |
| 3263.3607691 | 3 |
| 3263.364089 | 7 |
| 3263.3645868 | 3 |
| 3263.3675628 | 4 |
| 3263.3702023 | 1 |
| 3263.3710036 | 2 |
| 3263.3735141 | 3 |
| 3263.3754458 | 3 |
| 3263.3778883 | 2 |
| 3263.3805434 | 2 |
| 3263.3814343 | 2 |
| 3263.385308 | 1 |
| 3263.385886 | 2 |
| 3263.3889839 | 6 |
| 3263.391409 | 2 |
| 3263.3930447 | 1 |
| 3263.3965578 | 3 |
| 3263.3972509 | 2 |
| 3263.4000077 | 2 |
| 3263.4023942 | 1 |
| 3263.4038399 | 2 |
| 3263.4065226 | 4 |
| 3263.4081911 | 1 |
| 3263.4104062 | 1 |
| 3263.4131078 | 4 |
| 3263.4149742 | 3 |
| 3263.4171882 | 0 |
| 3263.4183451 | 0 |
| 3263.4216091 | 1 |
| 3263.42367 | 3 |
| 3263.4253581 | 5 |
| 3263.4282504 | 1 |
| 3263.4293948 | 3 |
| 3263.432152 | 3 |
| 3263.4345737 | 3 |
| 3263.4365772 | 3 |
| 3263.4389802 | 3 |
| 3263.4401957 | 2 |
| 3263.4428285 | 2 |
| 3263.4437717 | 3 |
| 3263.4468434 | 1 |
| 3263.4496048 | 3 |
| 3263.4509678 | 5 |
| 3263.4540801 | 2 |
| 3263.4554787 | 4 |
| 3263.4581309 | 0 |
| 3263.4604772 | 2 |
| 3263.4622461 | 5 |
| 3263.4646865 | 3 |
| 3263.4656805 | 3 |
| 3263.4688959 | 3 |
| 3263.4714769 | 4 |
| 3263.472853 | 3 |
| 3263.4756112 | 2 |
| 3263.4767572 | 2 |
| 3263.4794422 | 3 |
| 3263.4819145 | 3 |
| 3263.4833738 | 2 |
| 3263.4865164 | 2 |
| 3263.488035 | 4 |
| 3263.4903729 | 2 |
| 3263.4937371 | 6 |
| 3263.4947405 | 3 |
| 3263.4971284 | 3 |
| 3263.498785 | 4 |
| 3263.5013742 | 5 |
| 3263.502551 | 0 |
| 3263.5051112 | 2 |
| 3263.5083993 | 4 |
| 3263.5092111 | 0 |
| 3263.5122447 | 2 |
| 3263.5135948 | 4 |
| 3263.5167383 | 2 |
| 3263.5185662 | 2 |
| 3263.5200044 | 4 |
| 3263.5228739 | 2 |
| 3263.5241534 | 2 |
| 3263.5265746 | 2 |
| 3263.5293452 | 0 |
| 3263.5310718 | 1 |
| 3263.5340978 | 3 |
| 3263.5346192 | 3 |
| 3263.5379773 | 3 |
| 3263.5408227 | 1 |
| 3263.5421334 | 3 |
| 3263.544552 | 4 |
| 3263.5465251 | 0 |
| 3263.5490837 | 2 |
| 3263.5509057 | 3 |
| 3263.5520192 | 3 |
| 3263.5546008 | 5 |
| 3263.5578854 | 1 |
| 3263.5588964 | 2 |
| 3263.5616686 | 2 |
| 3263.5633749 | 1 |
| 3263.5660185 | 3 |
| 3263.5682898 | 5 |
| 3263.5700159 | 2 |
| 3263.5727012 | 3 |
| 3263.5739524 | 6 |
| 3263.57693 | 1 |
| 3263.5774626 | 1 |
| 3263.5807776 | 2 |
| 3263.5840872 | 3 |
| 3263.5845402 | 0 |
| 3263.5880124 | 2 |
| 3263.5906118 | 5 |
| 3263.5912448 | 1 |
| 3263.5946527 | 0 |
| 3263.5955333 | 1 |
| 3263.5980673 | 4 |
| 3263.5998672 | 0 |
| 3263.6022739 | 4 |
| 3263.6050945 | 3 |
| 3263.6071383 | 2 |
| 3263.609363 | 1 |
| 3263.6107417 | 1 |
| 3263.6136298 | 0 |
| 3263.6159343 | 3 |
| 3263.6170024 | 0 |
| 3263.6197635 | 2 |
| 3263.6217897 | 2 |
| 3263.6241871 | 4 |
| 3263.6264082 | 3 |
| 3263.6286483 | 0 |
| 3263.6313278 | 7 |
| 3263.6324265 | 2 |
| 3263.6352707 | 2 |
| 3263.6381137 | 0 |
| 3263.6394692 | 1 |
| 3263.6421044 | 2 |
| 3263.6434198 | 4 |
| 3263.6462389 | 4 |
| 3263.6475763 | 2 |
| 3263.6501117 | 3 |
| 3263.6526056 | 3 |
| 3263.6548357 | 1 |
| 3263.6571394 | 3 |
| 3263.6578372 | 5 |
| 3263.6609851 | 1 |
| 3263.6636268 | 4 |
| 3263.6643165 | 6 |
| 3263.6676455 | 0 |
| 3263.6691437 | 4 |
| 3263.6716896 | 2 |
| 3263.6744428 | 7 |
| 3263.6767503 | 3 |
| 3263.6785354 | 4 |
| 3263.6804371 | 2 |
| 3263.6836269 | 1 |
| 3263.6853176 | 0 |
| 3263.6874471 | 1 |
| 3263.6894419 | 0 |
| 3263.691962 | 1 |
| 3263.6943798 | 2 |
| 3263.695374 | 4 |
| 3263.6983987 | 2 |
| 3263.6994875 | 4 |
| 3263.7017643 | 0 |
| 3263.7048171 | 3 |
| 3263.7063879 | 0 |
| 3263.708929 | 0 |
| 3263.7111195 | 2 |
| 3263.712932 | 5 |
| 3263.7141013 | 5 |
| 3263.7175346 | 6 |
| 3263.7208048 | 2 |
| 3263.7224103 | 1 |
| 3263.7247857 | 0 |
| 3263.7257992 | 1 |
| 3263.7287324 | 2 |
| 3263.7311764 | 2 |
| 3263.7329825 | 0 |
| 3263.7358651 | 3 |
| 3263.7367695 | 2 |
| 3263.7394888 | 2 |
| 3263.7425552 | 4 |
| 3263.7434182 | 1 |
| 3263.7462697 | 3 |
| 3263.7478416 | 1 |
| 3263.7501477 | 1 |
| 3263.7523765 | 3 |
| 3263.7541558 | 1 |
| 3263.7574401 | 5 |
| 3263.7585007 | 0 |
| 3263.7613962 | 1 |
| 3263.7640345 | 5 |
| 3263.7652648 | 2 |
| 3263.7677287 | 3 |
| 3263.7704456 | 2 |
| 3263.7722673 | 1 |
| 3263.7754415 | 2 |
| 3263.7764843 | 4 |
| 3263.7791953 | 2 |
| 3263.7806813 | 0 |
| 3263.7833486 | 5 |
| 3263.7856044 | 2 |
| 3263.7874995 | 2 |
| 3263.7901846 | 0 |
| 3263.7916538 | 4 |
| 3263.7941563 | 2 |
| 3263.796789 | 3 |
| 3263.7980423 | 4 |
| 3263.8011642 | 1 |
| 3263.8019663 | 2 |
| 3263.8055206 | 6 |
| 3263.8083749 | 4 |
| 3263.8094275 | 3 |
| 3263.8119974 | 1 |
| 3263.8137303 | 2 |
| 3263.8160231 | 3 |
| 3263.8192194 | 3 |
| 3263.8202876 | 1 |
| 3263.822483 | 2 |
| 3263.8254144 | 3 |
| 3263.827197 | 3 |
| 3263.8293306 | 4 |
| 3263.8308129 | 3 |
| 3263.8339183 | 2 |

| | |
|---|---|
| 3263.8361359 | 5 |
| 3263.8382183 | 0 |
| 3263.8401842 | 6 |
| 3263.8420632 | 4 |
| 3263.8447973 | 1 |
| 3263.8458153 | 2 |
| 3263.8486265 | 1 |
| 3263.8515139 | 3 |
| 3263.8528776 | 2 |
| 3263.855529 | 3 |
| 3263.8568639 | 0 |
| 3263.8600264 | 4 |
| 3263.8628759 | 5 |
| 3263.8643577 | 2 |
| 3263.8669599 | 2 |
| 3263.8677824 | 1 |
| 3263.8708312 | 4 |
| 3263.8742342 | 3 |
| 3263.8745987 | 0 |
| 3263.8773656 | 1 |
| 3263.8792875 | 2 |
| 3263.8819905 | 2 |
| 3263.883426 | 4 |
| 3263.8857031 | 2 |
| 3263.8879402 | 1 |
| 3263.8893808 | 1 |
| 3263.8925767 | 1 |
| 3263.8957919 | 1 |
| 3263.8971206 | 1 |
| 3263.8995365 | 2 |
| 3263.9005536 | 2 |
| 3263.90326 | 3 |
| 3263.9067742 | 3 |
| 3263.907739 | 2 |
| 3263.9102542 | 1 |
| 3263.912361 | 1 |
| 3263.9144956 | 3 |
| 3263.9170054 | 3 |
| 3263.9188264 | 2 |
| 3263.9212578 | 5 |
| 3263.9226802 | 2 |
| 3263.9252941 | 0 |
| 3263.9265923 | 4 |
| 3263.9296631 | 2 |
| 3263.932563 | 2 |
| 3263.9337667 | 3 |
| 3263.9368601 | 3 |
| 3263.937557 | 2 |
| 3263.9404356 | 1 |
| 3263.9433788 | 3 |
| 3263.9441928 | 3 |
| 3263.9478796 | 5 |
| 3263.9496958 | 4 |
| 3263.9505216 | 4 |
| 3263.9544779 | 1 |
| 3263.9552818 | 3 |
| 3263.9579686 | 0 |
| 3263.9617397 | 2 |
| 3263.9623237 | 3 |
| 3263.964519 | 1 |
| 3263.9669675 | 1 |
| 3263.9691863 | 1 |
| 3263.9710205 | 0 |
| 3263.9732675 | 1 |
| 3263.9760965 | 1 |
| 3263.9768562 | 5 |
| 3263.9803466 | 1 |
| 3263.9818502 | 2 |
| 3263.9837477 | 6 |
| 3263.9864025 | 6 |
| 3263.9880351 | 4 |
| 3263.9910308 | 2 |
| 3263.9923626 | 4 |
| 3263.9945802 | 2 |
| 3263.9982406 | 1 |
| 3263.9995728 | 2 |
| 3264.0021723 | 1 |
| 3264.00442 | 7 |
| 3264.0061233 | 0 |
| 3264.0090786 | 4 |
| 3264.0098964 | 3 |
| 3264.0125363 | 1 |
| 3264.0134765 | 3 |
| 3264.0160366 | 1 |
| 3264.0190759 | 4 |
| 3264.0204649 | 5 |
| 3264.0241862 | 3 |
| 3264.0246741 | 6 |
| 3264.0284621 | 2 |
| 3264.0301755 | 0 |
| 3264.031559 | 1 |
| 3264.0338969 | 2 |
| 3264.0359927 | 2 |
| 3264.038462 | 1 |
| 3264.0415068 | 0 |
| 3264.0431036 | 2 |
| 3264.0452073 | 2 |
| 3264.0470451 | 1 |
| 3264.0492728 | 2 |
| 3264.0524081 | 5 |
| 3264.0534856 | 3 |
| 3264.0565376 | 0 |
| 3264.0573109 | 4 |
| 3264.0598682 | 0 |
| 3264.0630143 | 3 |
| 3264.0641706 | 2 |
| 3264.0669778 | 1 |
| 3264.0682148 | 0 |
| 3264.0703715 | 4 |
| 3264.0738984 | 1 |
| 3264.0750837 | 3 |
| 3264.0774177 | 3 |
| 3264.0795568 | 2 |
| 3264.0820275 | 3 |
| 3264.0849563 | 3 |
| 3264.0861274 | 1 |
| 3264.089274 | 4 |
| 3264.0916938 | 2 |
| 3264.0926891 | 0 |
| 3264.0954525 | 2 |
| 3264.0962675 | 4 |
| 3264.0969223 | 3 |
| 3264.0988017 | 1 |
| 3264.0993887 | 4 |
| 3264.0998632 | 1 |
| 3264.102111 | 4 |
| 3264.1030736 | 3 |
| 3264.1035446 | 3 |
| 3264.1054301 | 2 |
| 3264.1061809 | 2 |
| 3264.1067502 | 3 |
| 3264.1075387 | 2 |
| 3264.1096505 | 3 |
| 3264.1105506 | 2 |
| 3264.1109565 | 3 |
| 3264.111066 | 2 |
| 3264.1134765 | 5 |
| 3264.1141304 | 0 |
| 3264.1165044 | 1 |
| 3264.1168675 | 2 |
| 3264.1176099 | 1 |
| 3264.1176276 | 3 |
| 3264.1204464 | 1 |
| 3264.1211608 | 3 |
| 3264.1214703 | 1 |
| 3264.1218843 | 1 |
| 3264.1245181 | 3 |
| 3264.1254636 | 4 |
| 3264.12744 | 3 |
| 3264.128178 | 3 |
| 3264.1285142 | 0 |
| 3264.1293242 | 2 |
| 3264.1314494 | 1 |
| 3264.1318536 | 3 |
| 3264.1326405 | 1 |
| 3264.1337592 | 0 |
| 3264.1351568 | 2 |
| 3264.1368854 | 4 |
| 3264.1369316 | 5 |
| 3264.1379824 | 3 |
| 3264.13982 | 2 |
| 3264.1409802 | 1 |
| 3264.142453 | 1 |
| 3264.1429494 | 1 |
| 3264.1441547 | 3 |
| 3264.1442832 | 5 |
| 3264.1460504 | 4 |
| 3264.1471869 | 3 |
| 3264.1484492 | 2 |
| 3264.1490139 | 1 |
| 3264.1502162 | 1 |
| 3264.1508704 | 2 |
| 3264.1530097 | 2 |
| 3264.1537854 | 2 |
| 3264.1541863 | 4 |
| 3264.1546065 | 2 |
| 3264.1564307 | 2 |
| 3264.1579164 | 1 |
| 3264.1593709 | 1 |
| 3264.1602882 | 1 |
| 3264.1605997 | 0 |
| 3264.1612833 | 0 |
| 3264.1633828 | 2 |
| 3264.164034 | 2 |
| 3264.1651582 | 4 |
| 3264.167057 | 3 |
| 3264.1673695 | 2 |
| 3264.1680523 | 2 |
| 3264.1705546 | 4 |
| 3264.1705864 | 2 |
| 3264.1716101 | 3 |
| 3264.1725429 | 2 |
| 3264.1748768 | 3 |
| 3264.1751202 | 2 |
| 3264.1769173 | 3 |
| 3264.1770137 | 2 |
| 3264.178532 | 3 |
| 3264.1794108 | 5 |
| 3264.1816945 | 3 |
| 3264.1819105 | 3 |
| 3264.1826692 | 0 |
| 3264.1832701 | 1 |
| 3264.1857262 | 1 |
| 3264.1862673 | 1 |
| 3264.1874253 | 4 |
| 3264.1882885 | 2 |
| 3264.1895112 | 3 |
| 3264.1910697 | 4 |
| 3264.1926532 | 2 |
| 3264.1927821 | 1 |
| 3264.1933074 | 6 |
| 3264.1941203 | 1 |
| 3264.1958511 | 4 |
| 3264.1967318 | 3 |
| 3264.1970806 | 3 |
| 3264.1976042 | 2 |
| 3264.1999127 | 3 |
| 3264.2005006 | 4 |
| 3264.2025254 | 2 |
| 3264.202772 | 3 |
| 3264.203396 | 2 |
| 3264.2044332 | 1 |
| 3264.2066627 | 3 |
| 3264.2084628 | 3 |
| 3264.2110846 | 1 |
| 3264.2139302 | 3 |
| 3264.2146198 | 3 |
| 3264.2182936 | 2 |
| 3264.219478 | 2 |
| 3264.2225872 | 0 |
| 3264.2231042 | 1 |
| 3264.2254884 | 1 |
| 3264.2290735 | 2 |
| 3264.2317023 | 2 |
| 3264.232167 | 0 |
| 3264.2353129 | 5 |
| 3264.2363331 | 2 |
| 3264.2390917 | 3 |
| 3264.2405728 | 1 |
| 3264.2435319 | 2 |
| 3264.2460092 | 5 |
| 3264.2465072 | 6 |
| 3264.2497575 | 8 |
| 3264.2509996 | 2 |
| 3264.2539586 | 6 |
| 3264.2563768 | 6 |
| 3264.2580253 | 4 |
| 3264.2604975 | 3 |
| 3264.2621751 | 3 |
| 3264.2646901 | 1 |
| 3264.2676235 | 3 |
| 3264.2693244 | 0 |
| 3264.2718676 | 3 |
| 3264.2735029 | 4 |
| 3264.2755401 | 2 |
| 3264.2785909 | 5 |
| 3264.2801002 | 4 |
| 3264.2826333 | 0 |
| 3264.2837426 | 1 |
| 3264.286336 | 2 |
| 3264.2889944 | 2 |
| 3264.2897595 | 3 |
| 3264.2930198 | 4 |
| 3264.2942462 | 2 |
| 3264.2969446 | 5 |
| 3264.3001201 | 3 |
| 3264.3015725 | 4 |
| 3264.3040613 | 3 |
| 3264.3054819 | 1 |
| 3264.3072203 | 2 |
| 3264.3105193 | 1 |
| 3264.3116628 | 3 |
| 3264.3154642 | 0 |
| 3264.3173569 | 2 |
| 3264.3190704 | 1 |
| 3264.3215241 | 3 |
| 3264.3236825 | 3 |
| 3264.3255197 | 4 |
| 3264.3272258 | 1 |
| 3264.3296668 | 2 |
| 3264.3322831 | 2 |
| 3264.3337351 | 3 |
| 3264.3361042 | 3 |
| 3264.3375609 | 2 |
| 3264.3402016 | 8 |
| 3264.3425658 | 5 |
| 3264.3440608 | 3 |
| 3264.3469754 | 4 |
| 3264.3483484 | 7 |
| 3264.351128 | 3 |
| 3264.3540669 | 1 |
| 3264.355859 | 4 |
| 3264.3580703 | 3 |
| 3264.3593551 | 1 |
| 3264.362603 | 3 |
| 3264.3636428 | 1 |
| 3264.3663413 | 2 |
| 3264.3694761 | 1 |
| 3264.3705398 | 3 |
| 3264.3730619 | 2 |
| 3264.374717 | 5 |
| 3264.3765916 | 2 |
| 3264.3798457 | 1 |
| 3264.3810825 | 3 |
| 3264.3836798 | 1 |
| 3264.386453 | 6 |
| 3264.3873155 | 5 |
| 3264.3904377 | 5 |
| 3264.3910162 | 5 |
| 3264.3948104 | 3 |
| 3264.3970654 | 3 |
| 3264.3992265 | 3 |
| 3264.4016852 | 2 |
| 3264.4027971 | 3 |
| 3264.4059516 | 4 |
| 3264.4065711 | 4 |
| 3264.409282 | 4 |
| 3264.4120869 | 2 |
| 3264.4138548 | 4 |
| 3264.41701 | 4 |
| 3264.4178299 | 1 |
| 3264.4201857 | 1 |
| 3264.4235187 | 3 |
| 3264.4244219 | 2 |
| 3264.42696 | 4 |
| 3264.4280477 | 1 |
| 3264.4309221 | 1 |
| 3264.4333335 | 2 |
| 3264.4346385 | 3 |
| 3264.4381605 | 3 |
| 3264.4406786 | 3 |
| 3264.4414403 | 1 |
| 3264.4448073 | 3 |
| 3264.4456628 | 1 |
| 3264.448514 | 4 |
| 3264.4501934 | 1 |
| 3264.4530252 | 1 |
| 3264.4550716 | 0 |
| 3264.4565963 | 1 |
| 3264.4589245 | 3 |
| 3264.4623417 | 3 |
| 3264.4637615 | 3 |
| 3264.4655893 | 1 |
| 3264.4672368 | 2 |
| 3264.4700421 | 3 |
| 3264.4726574 | 3 |
| 3264.4742908 | 5 |
| 3264.4771403 | 5 |
| 3264.4783516 | 2 |
| 3264.4805522 | 3 |
| 3264.4836198 | 5 |
| 3264.4850577 | 1 |
| 3264.4879981 | 2 |
| 3264.4889412 | 1 |
| 3264.4912922 | 2 |
| 3264.49542 | 2 |
| 3264.4960623 | 4 |
| 3264.498898 | 0 |
| 3264.5006705 | 2 |
| 3264.5028671 | 3 |
| 3264.504773 | 3 |
| 3264.5072431 | 0 |
| 3264.509611 | 6 |

| | |
|---|---|
| 3264.511218 | 5 |
| 3264.5139577 | 1 |
| 3264.5166605 | 1 |
| 3264.5174571 | 2 |
| 3264.520528 | 3 |
| 3264.5229252 | 1 |
| 3264.5241334 | 0 |
| 3264.5271128 | 4 |
| 3264.5282954 | 2 |
| 3264.5307561 | 1 |
| 3264.5327061 | 2 |
| 3264.5354398 | 3 |
| 3264.5375977 | 1 |
| 3264.5399342 | 6 |
| 3264.5424708 | 2 |
| 3264.5438856 | 4 |
| 3264.5472655 | 2 |
| 3264.5496296 | 6 |
| 3264.5508758 | 1 |
| 3264.553098 | 3 |
| 3264.55464 | 2 |
| 3264.5577841 | 1 |
| 3264.558519 | 4 |
| 3264.5610324 | 3 |
| 3264.5642207 | 3 |
| 3264.5649464 | 5 |
| 3264.5678468 | 1 |
| 3264.5698662 | 0 |
| 3264.571503 | 3 |
| 3264.5744166 | 1 |
| 3264.5753475 | 3 |
| 3264.5788471 | 4 |
| 3264.5815942 | 4 |
| 3264.5839181 | 6 |
| 3264.5860601 | 1 |
| 3264.5878512 | 0 |
| 3264.5903661 | 2 |
| 3264.5929765 | 1 |
| 3264.5942442 | 3 |
| 3264.597188 | 3 |
| 3264.5984172 | 5 |
| 3264.60093 | 5 |
| 3264.6029156 | 1 |
| 3264.6052021 | 5 |
| 3264.6072902 | 7 |
| 3264.6086428 | 3 |
| 3264.6122239 | 2 |
| 3264.6135882 | 3 |
| 3264.6162657 | 3 |
| 3264.6187737 | 2 |
| 3264.62015 | 1 |
| 3264.6227496 | 2 |
| 3264.6242555 | 6 |
| 3264.6262581 | 2 |
| 3264.6301184 | 1 |
| 3264.6306497 | 3 |
| 3264.6341532 | 2 |
| 3264.6355407 | 1 |
| 3264.6389172 | 2 |
| 3264.6414597 | 2 |
| 3264.6423577 | 3 |
| 3264.6451808 | 1 |
| 3264.6466149 | 0 |
| 3264.6497956 | 4 |
| 3264.651785 | 3 |
| 3264.6537093 | 1 |
| 3264.6562595 | 2 |
| 3264.6567205 | 2 |
| 3264.6594748 | 3 |
| 3264.6624858 | 1 |
| 3264.6636388 | 3 |
| 3264.6667928 | 1 |
| 3264.6682467 | 1 |
| 3264.6713882 | 2 |
| 3264.6736411 | 3 |
| 3264.6752584 | 3 |
| 3264.6778336 | 2 |
| 3264.6794668 | 0 |
| 3264.6825019 | 3 |
| 3264.6835774 | 3 |
| 3264.6859839 | 1 |
| 3264.6890437 | 1 |
| 3264.6907187 | 2 |
| 3264.6937005 | 1 |
| 3264.694619 | 2 |
| 3264.6977586 | 2 |
| 3264.7000934 | 3 |
| 3264.7010089 | 1 |
| 3264.7041945 | 1 |
| 3264.7050517 | 3 |
| 3264.7076122 | 1 |
| 3264.7108136 | 3 |
| 3264.7118201 | 5 |
| 3264.7142007 | 3 |
| 3264.7176571 | 1 |
| 3264.7187083 | 4 |
| 3264.7216456 | 3 |
| 3264.7234695 | 1 |
| 3264.7260495 | 2 |
| 3264.7272511 | 2 |
| 3264.7305059 | 2 |
| 3264.7326856 | 3 |
| 3264.7345439 | 1 |
| 3264.7372261 | 0 |
| 3264.7381337 | 1 |
| 3264.7410447 | 3 |
| 3264.7438061 | 1 |
| 3264.7447005 | 3 |
| 3264.747234 | 8 |
| 3264.749242 | 3 |
| 3264.7512356 | 2 |
| 3264.7546646 | 5 |
| 3264.7558785 | 2 |
| 3264.7589673 | 0 |
| 3264.7594524 | 1 |
| 3264.763263 | 5 |
| 3264.7641129 | 4 |
| 3264.7671279 | 1 |
| 3264.7698334 | 1 |
| 3264.7710709 | 0 |
| 3264.7737166 | 1 |
| 3264.7762044 | 3 |
| 3264.7779293 | 1 |
| 3264.7801921 | 3 |
| 3264.7823845 | 4 |
| 3264.7846161 | 1 |
| 3264.7870907 | 3 |
| 3264.7885534 | 2 |
| 3264.7913009 | 2 |
| 3264.792657 | 6 |
| 3264.7951536 | 1 |
| 3264.7982572 | 2 |
| 3264.7991597 | 6 |
| 3264.8019274 | 3 |
| 3264.803481 | 3 |
| 3264.8056292 | 1 |
| 3264.8090313 | 2 |
| 3264.8103299 | 2 |
| 3264.8129069 | 4 |
| 3264.8145075 | 1 |
| 3264.816802 | 3 |
| 3264.8207322 | 1 |
| 3264.8213996 | 3 |
| 3264.8243143 | 1 |
| 3264.825572 | 2 |
| 3264.8282092 | 3 |
| 3264.8294272 | 2 |
| 3264.8318989 | 3 |
| 3264.8347056 | 4 |
| 3264.8357571 | 1 |
| 3264.8390585 | 2 |
| 3264.8413832 | 2 |
| 3264.8424973 | 5 |
| 3264.8460773 | 2 |
| 3264.8468407 | 3 |
| 3264.8490965 | 1 |
| 3264.8522007 | 3 |
| 3264.853812 | 1 |
| 3264.8562027 | 0 |
| 3264.8580793 | 1 |
| 3264.8605344 | 3 |
| 3264.8630912 | 2 |
| 3264.8644092 | 2 |
| 3264.8678075 | 1 |
| 3264.8688195 | 2 |
| 3264.8720067 | 3 |
| 3264.8744231 | 1 |
| 3264.8755965 | 1 |
| 3264.877897 | 0 |
| 3264.8793665 | 2 |
| 3264.8823711 | 3 |
| 3264.8850965 | 2 |
| 3264.8860849 | 5 |
| 3264.8893697 | 0 |
| 3264.8904696 | 3 |
| 3264.8933915 | 1 |
| 3264.894914 | 1 |
| 3264.898131 | 0 |
| 3264.9007897 | 5 |
| 3264.9014915 | 2 |
| 3264.9047759 | 3 |
| 3264.9071965 | 1 |
| 3264.9084133 | 3 |
| 3264.9109493 | 6 |
| 3264.9119804 | 4 |
| 3264.9153428 | 2 |
| 3264.9166545 | 2 |
| 3264.9198404 | 3 |
| 3264.9218869 | 1 |
| 3264.9234187 | 3 |
| 3264.9261487 | 3 |
| 3264.9272374 | 3 |
| 3264.9292811 | 2 |
| 3264.9324223 | 0 |
| 3264.9335177 | 2 |
| 3264.9361251 | 2 |
| 3264.9378113 | 4 |
| 3264.9403268 | 3 |
| 3264.9430902 | 5 |
| 3264.944365 | 7 |
| 3264.9475676 | 3 |
| 3264.9486193 | 3 |
| 3264.9518134 | 2 |
| 3264.9544896 | 6 |
| 3264.9558054 | 3 |
| 3264.9582803 | 1 |
| 3264.9597942 | 2 |
| 3264.9622066 | 6 |
| 3264.9639552 | 3 |
| 3264.9665576 | 2 |
| 3264.9692335 | 0 |
| 3264.9705686 | 0 |
| 3264.9734617 | 1 |
| 3264.9748759 | 2 |
| 3264.9770074 | 4 |
| 3264.9794842 | 2 |
| 3264.9821311 | 2 |
| 3264.9832439 | 1 |
| 3264.9871174 | 3 |
| 3264.9876821 | 2 |
| 3264.9906925 | 1 |
| 3264.9916672 | 3 |
| 3264.9948239 | 2 |
| 3264.9975484 | 3 |
| 3264.9985762 | 0 |
| 3265.0018596 | 1 |
| 3265.0028736 | 5 |
| 3265.0054979 | 1 |
| 3265.0083703 | 0 |
| 3265.0093479 | 3 |
| 3265.0123557 | 1 |
| 3265.0132702 | 1 |
| 3265.0162875 | 4 |
| 3265.0187353 | 3 |
| 3265.020513 | 3 |
| 3265.0229135 | 3 |
| 3265.0244455 | 2 |
| 3265.0272555 | 1 |
| 3265.0296335 | 2 |
| 3265.0308907 | 3 |
| 3265.0339684 | 2 |
| 3265.0366837 | 4 |
| 3265.0377242 | 4 |
| 3265.040423 | 2 |
| 3265.041694 | 3 |
| 3265.044536 | 0 |
| 3265.0470754 | 4 |
| 3265.0495477 | 2 |
| 3265.0516242 | 3 |
| 3265.0525525 | 4 |
| 3265.055956 | 2 |
| 3265.0565626 | 2 |
| 3265.0591562 | 1 |
| 3265.0622828 | 3 |
| 3265.0631019 | 2 |
| 3265.0662223 | 1 |
| 3265.0688267 | 2 |
| 3265.0703468 | 3 |
| 3265.0729705 | 1 |
| 3265.074115 | 3 |
| 3265.0773725 | 0 |
| 3265.0784221 | 2 |
| 3265.0809192 | 4 |
| 3265.0840526 | 4 |
| 3265.0858844 | 4 |
| 3265.0885122 | 5 |
| 3265.0898495 | 2 |
| 3265.0924635 | 0 |
| 3265.0951646 | 1 |
| 3265.0962268 | 1 |
| 3265.0995151 | 0 |
| 3265.1004174 | 5 |
| 3265.1037791 | 2 |
| 3265.1064373 | 3 |
| 3265.1072007 | 3 |
| 3265.1097554 | 5 |
| 3265.1113849 | 2 |
| 3265.1145354 | 1 |
| 3265.1160497 | 6 |
| 3265.1185898 | 1 |
| 3265.1206188 | 3 |
| 3265.1219974 | 4 |
| 3265.124844 | 1 |
| 3265.1274027 | 4 |
| 3265.1288126 | 4 |
| 3265.1317627 | 3 |
| 3265.1345529 | 3 |
| 3265.1363133 | 0 |
| 3265.1391373 | 2 |
| 3265.1401686 | 7 |
| 3265.1430163 | 1 |
| 3265.1442677 | 3 |
| 3265.1467266 | 0 |
| 3265.1492924 | 1 |
| 3265.1514259 | 6 |
| 3265.1542694 | 1 |
| 3265.1549803 | 2 |
| 3265.1579001 | 2 |
| 3265.1611136 | 2 |
| 3265.1617457 | 1 |
| 3265.1640584 | 2 |
| 3265.1670035 | 1 |
| 3265.1686559 | 3 |
| 3265.1702751 | 1 |
| 3265.1730737 | 3 |
| 3265.1756568 | 3 |
| 3265.1783451 | 3 |
| 3265.1795682 | 1 |
| 3265.1819819 | 4 |
| 3265.182289 | 3 |
| 3265.1842343 | 4 |
| 3265.1866561 | 6 |
| 3265.1874843 | 2 |
| 3265.1891641 | 1 |
| 3265.1906529 | 3 |
| 3265.1927584 | 2 |
| 3265.1942351 | 0 |
| 3265.1952228 | 1 |
| 3265.1975938 | 2 |
| 3265.1976664 | 1 |
| 3265.1996616 | 6 |
| 3265.2020093 | 2 |
| 3265.2029444 | 4 |
| 3265.2049405 | 2 |
| 3265.2059147 | 3 |
| 3265.207699 | 1 |
| 3265.2097629 | 1 |
| 3265.2107524 | 2 |
| 3265.2126599 | 4 |
| 3265.2136714 | 4 |
| 3265.21535 | 2 |
| 3265.2167705 | 0 |
| 3265.2188234 | 3 |
| 3265.2209134 | 2 |
| 3265.2218692 | 5 |
| 3265.2238158 | 4 |
| 3265.224975 | 1 |
| 3265.2268438 | 2 |
| 3265.2292256 | 4 |
| 3265.2303192 | 4 |
| 3265.2316295 | 3 |
| 3265.233006 | 2 |
| 3265.2342615 | 2 |
| 3265.2365798 | 2 |
| 3265.2378399 | 2 |
| 3265.2397311 | 1 |
| 3265.2402477 | 0 |
| 3265.2425692 | 3 |
| 3265.2448077 | 1 |
| 3265.2451749 | 2 |
| 3265.2471213 | 1 |
| 3265.2481281 | 2 |
| 3265.2499823 | 5 |
| 3265.2509603 | 0 |
| 3265.2532557 | 1 |
| 3265.254446 | 5 |
| 3265.2560307 | 2 |
| 3265.2581394 | 2 |
| 3265.2590732 | 3 |
| 3265.2614508 | 2 |
| 3265.2634266 | 0 |
| 3265.2641807 | 6 |
| 3265.2665563 | 2 |

| | |
|---|---|
| 3265.2673593 | 2 |
| 3265.2691294 | 0 |
| 3265.2708712 | 2 |
| 3265.2724701 | 1 |
| 3265.2745603 | 3 |
| 3265.2760746 | 2 |
| 3265.2772414 | 2 |
| 3265.2793688 | 2 |
| 3265.2799474 | 7 |
| 3265.281916 | 3 |
| 3265.2828948 | 3 |
| 3265.2848819 | 0 |
| 3265.286424 | 3 |
| 3265.2873985 | 2 |
| 3265.2886526 | 2 |
| 3265.2896747 | 4 |
| 3265.2897759 | 0 |
| 3265.2906236 | 6 |
| 3265.2912543 | 3 |
| 3265.2928242 | 3 |
| 3265.2928636 | 2 |
| 3265.2938535 | 5 |
| 3265.294377 | 0 |
| 3265.2948617 | 4 |
| 3265.2959182 | 2 |
| 3265.2973899 | 1 |
| 3265.2980211 | 3 |
| 3265.2980931 | 2 |
| 3265.2986924 | 6 |
| 3265.299799 | 1 |
| 3265.3003492 | 3 |
| 3265.3018574 | 3 |
| 3265.3022102 | 4 |
| 3265.3031112 | 3 |
| 3265.3036048 | 2 |
| 3265.3041508 | 6 |
| 3265.3047977 | 3 |
| 3265.3053929 | 1 |
| 3265.3069347 | 1 |
| 3265.308733 | 3 |
| 3265.3093932 | 2 |
| 3265.3111203 | 3 |
| 3265.3130191 | 3 |
| 3265.3144137 | 2 |
| 3265.3163068 | 3 |
| 3265.3179567 | 0 |
| 3265.3196627 | 2 |
| 3265.3218076 | 2 |
| 3265.3225646 | 2 |
| 3265.3246587 | 2 |
| 3265.3250375 | 3 |
| 3265.3272047 | 3 |
| 3265.328829 | 1 |
| 3265.3296454 | 1 |
| 3265.3318465 | 3 |
| 3265.3329413 | 2 |
| 3265.3345261 | 1 |
| 3265.3365823 | 2 |
| 3265.3378899 | 1 |
| 3265.3401256 | 4 |
| 3265.3407623 | 6 |
| 3265.3421614 | 3 |
| 3265.3443045 | 3 |
| 3265.3449246 | 2 |
| 3265.3473328 | 0 |
| 3265.348431 | 3 |
| 3265.3498536 | 3 |
| 3265.3520584 | 1 |
| 3265.3532262 | 1 |
| 3265.3550466 | 1 |
| 3265.3562008 | 3 |
| 3265.3579038 | 1 |
| 3265.3600181 | 2 |
| 3265.3607791 | 1 |
| 3265.3634179 | 0 |
| 3265.3643289 | 4 |
| 3265.3661877 | 4 |
| 3265.3673531 | 6 |
| 3265.3689863 | 2 |
| 3265.3706574 | 0 |
| 3265.3727138 | 0 |
| 3265.3739067 | 0 |
| 3265.3761322 | 3 |
| 3265.3764835 | 2 |
| 3265.3788372 | 1 |
| 3265.3799662 | 2 |
| 3265.381217 | 5 |
| 3265.3833842 | 2 |
| 3265.3841136 | 4 |
| 3265.385857 | 3 |
| 3265.3873727 | 2 |
| 3265.3886784 | 1 |
| 3265.3908823 | 3 |
| 3265.3920035 | 7 |
| 3265.3940975 | 3 |
| 3265.3950982 | 4 |
| 3265.3969192 | 2 |
| 3265.3992123 | 1 |
| 3265.4000002 | 3 |
| 3265.4019611 | 5 |
| 3265.4030077 | 0 |
| 3265.4057754 | 2 |
| 3265.4078188 | 1 |
| 3265.4080208 | 3 |
| 3265.4099939 | 2 |
| 3265.411768 | 4 |
| 3265.412181 | 4 |
| 3265.4145581 | 5 |
| 3265.4156692 | 2 |
| 3265.4175391 | 3 |
| 3265.4196188 | 4 |
| 3265.4201035 | 1 |
| 3265.4228992 | 3 |
| 3265.423401 | 6 |
| 3265.4246348 | 0 |
| 3265.4265484 | 0 |
| 3265.4283856 | 2 |
| 3265.4301708 | 2 |
| 3265.4313925 | 4 |
| 3265.4331332 | 3 |
| 3265.4353443 | 2 |
| 3265.4360568 | 0 |
| 3265.4376443 | 1 |
| 3265.4388666 | 6 |
| 3265.4407174 | 1 |
| 3265.4424035 | 4 |
| 3265.4436605 | 2 |
| 3265.4459806 | 4 |
| 3265.4470076 | 2 |
| 3265.449114 | 3 |
| 3265.4506863 | 1 |
| 3265.4515145 | 2 |
| 3265.4537172 | 3 |
| 3265.454675 | 2 |
| 3265.4570269 | 1 |
| 3265.4576929 | 3 |
| 3265.460109 | 1 |
| 3265.4623491 | 1 |
| 3265.4626999 | 3 |
| 3265.464921 | 5 |
| 3265.4654901 | 2 |
| 3265.4676366 | 3 |
| 3265.4688954 | 3 |
| 3265.470397 | 2 |
| 3265.4724527 | 3 |
| 3265.4741011 | 5 |
| 3265.4752722 | 3 |
| 3265.4775544 | 5 |
| 3265.4775604 | 0 |
| 3265.4793395 | 0 |
| 3265.4819553 | 2 |
| 3265.482739 | 4 |
| 3265.4850544 | 3 |
| 3265.4860065 | 1 |
| 3265.487583 | 1 |
| 3265.4883223 | 3 |
| 3265.4908403 | 3 |
| 3265.4933304 | 3 |
| 3265.4936168 | 6 |
| 3265.4961024 | 0 |
| 3265.4972156 | 3 |
| 3265.4989117 | 0 |
| 3265.5006615 | 0 |
| 3265.5018451 | 0 |
| 3265.5041338 | 3 |
| 3265.5052032 | 4 |
| 3265.5064965 | 0 |
| 3265.5089586 | 2 |
| 3265.509573 | 4 |
| 3265.5109799 | 4 |
| 3265.5134933 | 0 |
| 3265.5143014 | 1 |
| 3265.5160576 | 2 |
| 3265.5176685 | 3 |
| 3265.5195071 | 3 |
| 3265.5201408 | 1 |
| 3265.5219912 | 3 |
| 3265.5244715 | 0 |
| 3265.5246218 | 1 |
| 3265.5271013 | 2 |
| 3265.5282565 | 3 |
| 3265.5301535 | 4 |
| 3265.5316937 | 0 |
| 3265.5328102 | 4 |
| 3265.5348769 | 2 |
| 3265.5363866 | 2 |
| 3265.5376329 | 3 |
| 3265.5399913 | 2 |
| 3265.5403062 | 3 |
| 3265.5428402 | 2 |
| 3265.5433159 | 2 |
| 3265.5457738 | 1 |
| 3265.5484538 | 1 |
| 3265.5485321 | 8 |
| 3265.5503953 | 5 |
| 3265.5514923 | 3 |
| 3265.5533768 | 5 |
| 3265.5551802 | 3 |
| 3265.5568004 | 3 |
| 3265.5584957 | 2 |
| 3265.5590374 | 0 |
| 3265.5613648 | 0 |
| 3265.5636529 | 3 |
| 3265.5637924 | 0 |
| 3265.5663278 | 3 |
| 3265.5674468 | 4 |
| 3265.5697315 | 3 |
| 3265.5713767 | 3 |
| 3265.5721012 | 2 |
| 3265.5736756 | 5 |
| 3265.575942 | 3 |
| 3265.5765614 | 2 |
| 3265.5789995 | 5 |
| 3265.5794214 | 1 |
| 3265.5818234 | 2 |
| 3265.5825769 | 0 |
| 3265.5847625 | 2 |
| 3265.5868372 | 3 |
| 3265.5874945 | 1 |
| 3265.5893706 | 3 |
| 3265.5910212 | 6 |
| 3265.5925857 | 3 |
| 3265.5948072 | 6 |
| 3265.5956242 | 1 |
| 3265.5977438 | 0 |
| 3265.5981939 | 1 |
| 3265.6003246 | 1 |
| 3265.6016471 | 1 |
| 3265.6034405 | 1 |
| 3265.6054276 | 1 |
| 3265.6062368 | 3 |
| 3265.6083865 | 1 |
| 3265.6103868 | 1 |
| 3265.6109484 | 1 |
| 3265.6126563 | 2 |
| 3265.6147948 | 2 |
| 3265.615191 | 4 |
| 3265.617701 | 5 |
| 3265.6186747 | 3 |
| 3265.620307 | 1 |
| 3265.6223551 | 3 |
| 3265.6235538 | 2 |
| 3265.6254983 | 1 |
| 3265.6257592 | 3 |
| 3265.6283654 | 4 |
| 3265.6307954 | 3 |
| 3265.6308908 | 4 |
| 3265.6335584 | 3 |
| 3265.6351096 | 2 |
| 3265.6358908 | 3 |
| 3265.6377917 | 0 |
| 3265.6390614 | 3 |
| 3265.6407636 | 4 |
| 3265.6419306 | 3 |
| 3265.6443957 | 4 |
| 3265.6460917 | 3 |
| 3265.6465378 | 1 |
| 3265.6487131 | 5 |
| 3265.6497122 | 2 |
| 3265.6517417 | 0 |
| 3265.653246 | 2 |
| 3265.6545787 | 3 |
| 3265.6566526 | 3 |
| 3265.6572399 | 1 |
| 3265.6594462 | 4 |
| 3265.6614485 | 3 |
| 3265.662235 | 2 |
| 3265.6635541 | 5 |
| 3265.6650546 | 1 |
| 3265.6668358 | 3 |
| 3265.6692738 | 1 |
| 3265.6700223 | 1 |
| 3265.6717014 | 5 |
| 3265.6728992 | 1 |
| 3265.6750721 | 3 |
| 3265.6767898 | 2 |
| 3265.6785188 | 0 |
| 3265.680204 | 6 |
| 3265.682397 | 2 |
| 3265.6824043 | 4 |
| 3265.684918 | 0 |
| 3265.6853658 | 2 |
| 3265.6881423 | 1 |
| 3265.6889777 | 5 |
| 3265.6908166 | 2 |
| 3265.6924262 | 2 |
| 3265.6932202 | 1 |
| 3265.6947076 | 1 |
| 3265.6969763 | 0 |
| 3265.6981937 | 2 |
| 3265.6999062 | 2 |
| 3265.701653 | 2 |
| 3265.7033269 | 3 |
| 3265.7037971 | 4 |
| 3265.705722 | 2 |
| 3265.7076095 | 2 |
| 3265.708044 | 1 |
| 3265.7104805 | 3 |
| 3265.7117491 | 1 |
| 3265.7132639 | 1 |
| 3265.715149 | 4 |
| 3265.7165106 | 3 |
| 3265.7186877 | 2 |
| 3265.7198878 | 6 |
| 3265.7201293 | 1 |
| 3265.7212225 | 4 |
| 3265.7212901 | 4 |
| 3265.722677 | 1 |
| 3265.7230488 | 2 |
| 3265.7233352 | 2 |
| 3265.7242353 | 1 |
| 3265.724359 | 4 |
| 3265.7262588 | 7 |
| 3265.7263681 | 1 |
| 3265.7276252 | 4 |
| 3265.7276734 | 2 |
| 3265.7283878 | 3 |
| 3265.7290201 | 1 |
| 3265.729211 | 2 |
| 3265.730902 | 3 |
| 3265.7313251 | 3 |
| 3265.7315359 | 3 |
| 3265.7316335 | 2 |
| 3265.7337119 | 7 |
| 3265.7339845 | 3 |
| 3265.7350183 | 1 |
| 3265.7350388 | 2 |
| 3265.7351542 | 3 |
| 3265.7369664 | 0 |
| 3265.7369736 | 2 |
| 3265.7387285 | 3 |
| 3265.7391593 | 3 |
| 3265.7397881 | 3 |
| 3265.740099 | 0 |
| 3265.7402925 | 6 |
| 3265.7415532 | 2 |
| 3265.7426059 | 6 |
| 3265.7426176 | 0 |
| 3265.7433928 | 5 |
| 3265.7435823 | 0 |
| 3265.7442068 | 1 |
| 3265.7464102 | 3 |
| 3265.7470336 | 4 |
| 3265.7470955 | 3 |
| 3265.7475185 | 2 |
| 3265.7478631 | 3 |
| 3265.7489428 | 1 |
| 3265.7496868 | 5 |
| 3265.7507042 | 0 |
| 3265.7513111 | 4 |
| 3265.7520733 | 3 |
| 3265.7521055 | 2 |
| 3265.7528009 | 2 |
| 3265.7534861 | 2 |
| 3265.7542644 | 3 |
| 3265.7551138 | 6 |
| 3265.7560629 | 2 |
| 3265.7565621 | 4 |
| 3265.7567063 | 4 |
| 3265.7574331 | 1 |
| 3265.7587661 | 1 |
| 3265.7597006 | 2 |
| 3265.7601165 | 0 |

| | |
|---|---|
| 3265.7605477 | 3 |
| 3265.7611676 | 2 |
| 3265.761591 | 4 |
| 3265.7629625 | 3 |
| 3265.7630471 | 1 |
| 3265.7643412 | 0 |
| 3265.7644188 | 2 |
| 3265.7649745 | 3 |
| 3265.7658914 | 0 |
| 3265.7670899 | 2 |
| 3265.7679207 | 0 |
| 3265.7679638 | 1 |
| 3265.7685729 | 4 |
| 3265.7698045 | 2 |
| 3265.7706843 | 0 |
| 3265.7707295 | 1 |
| 3265.7721886 | 5 |
| 3265.7724184 | 4 |
| 3265.7732081 | 1 |
| 3265.7738648 | 6 |
| 3265.7747661 | 4 |
| 3265.775457 | 14 |
| 3265.7763836 | 9 |
| 3265.7768583 | 7 |
| 3265.7772471 | 11 |
| 3265.7780087 | 7 |
| 3265.7780214 | 11 |
| 3265.7784795 | 6 |
| 3265.7800624 | 8 |
| 3265.7803488 | 9 |
| 3265.7815788 | 2 |
| 3265.7824719 | 5 |
| 3265.7832395 | 4 |
| 3265.7832473 | 0 |
| 3265.7845786 | 1 |
| 3265.7846608 | 1 |
| 3265.7851143 | 3 |
| 3265.7858192 | 5 |
| 3265.7862911 | 3 |
| 3265.7879662 | 3 |
| 3265.7882482 | 1 |
| 3265.7892099 | 4 |
| 3265.789323 | 1 |
| 3265.7893523 | 2 |
| 3265.7907068 | 3 |
| 3265.7912888 | 3 |
| 3265.7924511 | 4 |
| 3265.7927449 | 2 |
| 3265.7940111 | 1 |
| 3265.7942071 | 5 |
| 3265.7955181 | 1 |
| 3265.7962139 | 3 |
| 3265.7964721 | 4 |
| 3265.7967822 | 2 |
| 3265.7968988 | 0 |
| 3265.7984736 | 2 |
| 3265.7986102 | 2 |
| 3265.8003256 | 1 |
| 3265.8003941 | 2 |
| 3265.8014098 | 1 |
| 3265.8016601 | 5 |
| 3265.8021923 | 1 |
| 3265.803515 | 4 |
| 3265.8035933 | 2 |
| 3265.8048837 | 2 |
| 3265.8049591 | 6 |
| 3265.8062816 | 3 |
| 3265.8064423 | 2 |
| 3265.8064918 | 3 |
| 3265.8083675 | 1 |
| 3265.8088898 | 3 |
| 3265.8094314 | 5 |
| 3265.8100707 | 2 |
| 3265.8104405 | 4 |
| 3265.8119908 | 3 |
| 3265.8127956 | 3 |
| 3265.8134233 | 2 |
| 3265.8137721 | 3 |
| 3265.8139174 | 0 |
| 3265.8146132 | 0 |
| 3265.8157959 | 1 |
| 3265.8163829 | 4 |
| 3265.816997 | 2 |
| 3265.8179168 | 3 |
| 3265.8187134 | 4 |
| 3265.8192058 | 1 |
| 3265.8192226 | 1 |
| 3265.8209665 | 3 |
| 3265.8217331 | 2 |
| 3265.8218055 | 0 |
| 3265.8219313 | 3 |
| 3265.8235088 | 5 |
| 3265.8240493 | 2 |
| 3265.825216 | 2 |
| 3265.825663 | 1 |
| 3265.8268529 | 2 |
| 3265.8270743 | 2 |
| 3265.8273751 | 3 |
| 3265.8278434 | 7 |
| 3265.8283325 | 2 |
| 3265.8292797 | 0 |
| 3265.8301268 | 4 |
| 3265.8309162 | 3 |
| 3265.8311111 | 2 |
| 3265.8315611 | 3 |
| 3265.8330455 | 1 |
| 3265.8340635 | 4 |
| 3265.8345347 | 1 |
| 3265.8346272 | 4 |
| 3265.8353047 | 3 |
| 3265.8368438 | 2 |
| 3265.8376811 | 5 |
| 3265.8377262 | 4 |
| 3265.8377281 | 0 |
| 3265.8393411 | 3 |
| 3265.8399748 | 4 |
| 3265.840475 | 14 |
| 3265.8415608 | 16 |
| 3265.8416413 | 17 |
| 3265.8424283 | 27 |
| 3265.8429277 | 5 |
| 3265.8435016 | 11 |
| 3265.8442968 | 2 |
| 3265.8449882 | 2 |
| 3265.8462924 | 4 |
| 3265.8472235 | 1 |
| 3265.84741 | 2 |
| 3265.8478862 | 2 |
| 3265.8481578 | 1 |
| 3265.849484 | 3 |
| 3265.8500091 | 2 |
| 3265.8507142 | 6 |
| 3265.8516561 | 0 |
| 3265.8517504 | 3 |
| 3265.852789 | 1 |
| 3265.8540554 | 0 |
| 3265.8548378 | 2 |
| 3265.8554226 | 3 |
| 3265.855694 | 2 |
| 3265.8561257 | 4 |
| 3265.856216 | 2 |
| 3265.8587179 | 2 |
| 3265.8587355 | 3 |
| 3265.8589244 | 0 |
| 3265.8597937 | 3 |
| 3265.8606975 | 2 |
| 3265.8612908 | 5 |
| 3265.8625758 | 4 |
| 3265.8630729 | 2 |
| 3265.8637907 | 2 |
| 3265.8639764 | 0 |
| 3265.8642508 | 1 |
| 3265.8662593 | 3 |
| 3265.8668416 | 8 |
| 3265.8670418 | 3 |
| 3265.8676488 | 5 |
| 3265.8685435 | 2 |
| 3265.8687758 | 1 |
| 3265.8688763 | 3 |
| 3265.8708553 | 3 |
| 3265.8717036 | 1 |
| 3265.8720072 | 2 |
| 3265.872403 | 3 |
| 3265.8734521 | 3 |
| 3265.8736384 | 2 |
| 3265.8744178 | 2 |
| 3265.8752795 | 3 |
| 3265.8761164 | 1 |
| 3265.8764573 | 3 |
| 3265.8764703 | 3 |
| 3265.8771454 | 2 |
| 3265.8784264 | 2 |
| 3265.8789958 | 0 |
| 3265.8793619 | 2 |
| 3265.8803518 | 3 |
| 3265.8813609 | 2 |
| 3265.8818311 | 1 |
| 3265.8820698 | 2 |
| 3265.8835545 | 2 |
| 3265.8840821 | 2 |
| 3265.8841277 | 2 |
| 3265.8856815 | 1 |
| 3265.8861916 | 2 |
| 3265.8870458 | 0 |
| 3265.8874491 | 2 |
| 3265.8883197 | 2 |
| 3265.8889433 | 4 |
| 3265.8895663 | 3 |
| 3265.8904217 | 3 |
| 3265.8905671 | 0 |
| 3265.8923179 | 2 |
| 3265.8927729 | 3 |
| 3265.8937092 | 1 |
| 3265.8938098 | 1 |
| 3265.8944807 | 7 |
| 3265.8954976 | 6 |
| 3265.8962983 | 2 |
| 3265.89701 | 2 |
| 3265.8970414 | 2 |
| 3265.8977326 | 1 |
| 3265.8977376 | 1 |
| 3265.8984785 | 4 |
| 3265.8997438 | 2 |
| 3265.900213 | 5 |
| 3265.9016833 | 4 |
| 3265.9019643 | 1 |
| 3265.9026959 | 3 |
| 3265.9038351 | 2 |
| 3265.9046957 | 2 |
| 3265.9049232 | 0 |
| 3265.9066065 | 4 |
| 3265.9095956 | 2 |
| 3265.911718 | 3 |
| 3265.9145256 | 1 |
| 3265.9171348 | 4 |
| 3265.9187613 | 3 |
| 3265.9221016 | 2 |
| 3265.9248362 | 0 |
| 3265.9265409 | 1 |
| 3265.9301907 | 2 |
| 3265.9314513 | 0 |
| 3265.9345854 | 0 |
| 3265.9372484 | 4 |
| 3265.9388473 | 0 |
| 3265.9423864 | 4 |
| 3265.9432738 | 0 |
| 3265.9466917 | 1 |
| 3265.9501351 | 2 |
| 3265.9513637 | 1 |
| 3265.9546481 | 0 |
| 3265.9563482 | 2 |
| 3265.9597795 | 3 |
| 3265.9621451 | 2 |
| 3265.9638963 | 2 |
| 3265.9671665 | 1 |
| 3265.968913 | 2 |
| 3265.97203 | 1 |
| 3265.9726611 | 4 |
| 3265.9763491 | 0 |
| 3265.9800338 | 6 |
| 3265.9804343 | 3 |
| 3265.9842305 | 2 |
| 3265.9868351 | 5 |
| 3265.9884676 | 3 |
| 3265.9911445 | 2 |
| 3265.9943607 | 3 |
| 3265.9962673 | 2 |
| 3265.9993113 | 6 |
| 3266.0015562 | 5 |
| 3266.0040928 | 1 |
| 3266.0072468 | 3 |
| 3266.0093244 | 1 |
| 3266.0121791 | 2 |
| 3266.0132466 | 4 |
| 3266.0173378 | 2 |
| 3266.0200232 | 0 |
| 3266.0214721 | 2 |
| 3266.0248438 | 4 |
| 3266.0258927 | 0 |
| 3266.02872 | 1 |
| 3266.032469 | 1 |
| 3266.0334933 | 2 |
| 3266.0368858 | 2 |
| 3266.0386793 | 3 |
| 3266.0416106 | 1 |
| 3266.0450848 | 1 |
| 3266.0467596 | 2 |
| 3266.0500505 | 2 |
| 3266.051512 | 0 |
| 3266.0545748 | 3 |
| 3266.0568935 | 3 |
| 3266.0593059 | 3 |
| 3266.0620237 | 3 |
| 3266.0641377 | 2 |
| 3266.0670153 | 1 |
| 3266.0701679 | 0 |
| 3266.0720047 | 1 |
| 3266.0744652 | 4 |
| 3266.0765236 | 0 |
| 3266.079373 | 3 |
| 3266.0816943 | 1 |
| 3266.0841282 | 3 |
| 3266.0871641 | 4 |
| 3266.09091 | 0 |
| 3266.0922562 | 4 |
| 3266.0952304 | 4 |
| 3266.0970923 | 2 |
| 3266.0999765 | 9 |
| 3266.101823 | 2 |
| 3266.1054047 | 4 |
| 3266.1082132 | 2 |
| 3266.1096341 | 2 |
| 3266.1123252 | 3 |
| 3266.1137519 | 2 |
| 3266.1169872 | 3 |
| 3266.1207465 | 3 |
| 3266.1220755 | 2 |
| 3266.1257634 | 4 |
| 3266.126612 | 3 |
| 3266.1298466 | 1 |
| 3266.1325965 | 1 |
| 3266.1343155 | 1 |
| 3266.1376031 | 2 |
| 3266.139701 | 3 |
| 3266.1426954 | 0 |
| 3266.1455753 | 3 |
| 3266.1472836 | 2 |
| 3266.1501737 | 2 |
| 3266.152493 | 3 |
| 3266.1557734 | 2 |
| 3266.158211 | 4 |
| 3266.1606577 | 2 |
| 3266.1624823 | 3 |
| 3266.1637743 | 4 |
| 3266.166394 | 3 |
| 3266.1701069 | 1 |
| 3266.1711833 | 0 |
| 3266.1750989 | 1 |
| 3266.1763649 | 1 |
| 3266.179762 | 2 |
| 3266.1827644 | 3 |
| 3266.1844401 | 2 |
| 3266.1877413 | 1 |
| 3266.1893614 | 0 |
| 3266.1925163 | 3 |
| 3266.1948885 | 4 |
| 3266.1972039 | 1 |
| 3266.2002277 | 1 |
| 3266.2017748 | 3 |
| 3266.2042581 | 2 |
| 3266.2080781 | 0 |
| 3266.2094504 | 1 |
| 3266.2121586 | 1 |
| 3266.2140623 | 1 |
| 3266.217654 | 0 |
| 3266.2191329 | 2 |
| 3266.2220822 | 5 |
| 3266.2255017 | 3 |
| 3266.2267619 | 1 |
| 3266.2303383 | 4 |
| 3266.2329823 | 2 |
| 3266.2348131 | 5 |
| 3266.2378505 | 3 |
| 3266.2392826 | 1 |
| 3266.2426758 | 1 |
| 3266.2437533 | 6 |
| 3266.2472148 | 2 |
| 3266.2505811 | 1 |
| 3266.2517749 | 4 |
| 3266.2551608 | 2 |
| 3266.2566845 | 5 |
| 3266.2596056 | 3 |
| 3266.2620133 | 0 |
| 3266.2639271 | 5 |
| 3266.2677354 | 3 |
| 3266.2689874 | 2 |
| 3266.2721368 | 1 |
| 3266.275853 | 2 |
| 3266.2767972 | 4 |
| 3266.2801142 | 4 |
| 3266.2813742 | 1 |
| 3266.2847082 | 0 |
| 3266.2881781 | 2 |

| | |
|---|---|
| 3266.2897182 | 2 |
| 3266.2923488 | 3 |
| 3266.2940342 | 1 |
| 3266.2973637 | 1 |
| 3266.2991012 | 3 |
| 3266.3019057 | 5 |
| 3266.3048637 | 3 |
| 3266.307022 | 0 |
| 3266.3097805 | 2 |
| 3266.3109874 | 2 |
| 3266.3144679 | 4 |
| 3266.3185206 | 1 |
| 3266.3193036 | 4 |
| 3266.3227658 | 3 |
| 3266.3234767 | 2 |
| 3266.3260797 | 1 |
| 3266.32979 | 4 |
| 3266.3320648 | 5 |
| 3266.3337904 | 3 |
| 3266.3368919 | 1 |
| 3266.3386504 | 6 |
| 3266.3423207 | 2 |
| 3266.343395 | 2 |
| 3266.3464408 | 1 |
| 3266.3494514 | 2 |
| 3266.3507254 | 1 |
| 3266.3537076 | 2 |
| 3266.3557019 | 5 |
| 3266.3588407 | 0 |
| 3266.3619761 | 2 |
| 3266.3637747 | 4 |
| 3266.3670849 | 1 |
| 3266.3683298 | 1 |
| 3266.3712421 | 1 |
| 3266.3727437 | 0 |
| 3266.3755987 | 4 |
| 3266.379395 | 3 |
| 3266.3806367 | 2 |
| 3266.3835514 | 0 |
| 3266.3855054 | 6 |
| 3266.3884876 | 0 |
| 3266.3917327 | 4 |
| 3266.3933536 | 2 |
| 3266.395912 | 3 |
| 3266.3970702 | 3 |
| 3266.4000455 | 0 |
| 3266.4028297 | 3 |
| 3266.4045925 | 2 |
| 3266.407935 | 1 |
| 3266.4114088 | 2 |
| 3266.4133421 | 4 |
| 3266.4160245 | 0 |
| 3266.4173947 | 2 |
| 3266.4212799 | 3 |
| 3266.4220787 | 3 |
| 3266.4252849 | 2 |
| 3266.4284949 | 2 |
| 3266.4293323 | 1 |
| 3266.4327607 | 0 |
| 3266.4343498 | 1 |
| 3266.4374071 | 4 |
| 3266.4405436 | 1 |
| 3266.4426744 | 2 |
| 3266.4456928 | 3 |
| 3266.4463854 | 1 |
| 3266.4502194 | 2 |
| 3266.4513227 | 0 |
| 3266.4536922 | 2 |
| 3266.4545273 | 2 |
| 3266.4548767 | 0 |
| 3266.4554847 | 4 |
| 3266.4583133 | 3 |
| 3266.4592867 | 3 |
| 3266.459768 | 4 |
| 3266.460923 | 4 |
| 3266.4624876 | 3 |
| 3266.4643788 | 4 |
| 3266.4652992 | 1 |
| 3266.4666598 | 1 |
| 3266.4671827 | 0 |
| 3266.4693319 | 4 |
| 3266.469952 | 5 |
| 3266.4710356 | 2 |
| 3266.4718797 | 3 |
| 3266.4730742 | 1 |
| 3266.4744737 | 2 |
| 3266.4767291 | 0 |
| 3266.4777057 | 2 |
| 3266.4794721 | 2 |
| 3266.4801585 | 3 |
| 3266.4819485 | 3 |
| 3266.4822008 | 3 |
| 3266.4834152 | 3 |
| 3266.4844557 | 2 |
| 3266.4855667 | 3 |
| 3266.4870191 | 3 |
| 3266.4892163 | 4 |
| 3266.4898378 | 4 |
| 3266.490956 | 1 |
| 3266.4920465 | 0 |
| 3266.4927402 | 2 |
| 3266.4939555 | 2 |
| 3266.4953725 | 2 |
| 3266.4963407 | 2 |
| 3266.4986677 | 4 |
| 3266.4996857 | 3 |
| 3266.5001037 | 4 |
| 3266.5004082 | 0 |
| 3266.5045842 | 2 |
| 3266.5055338 | 3 |
| 3266.5089723 | 4 |
| 3266.5103163 | 1 |
| 3266.5136181 | 0 |
| 3266.5173542 | 3 |
| 3266.5182806 | 2 |
| 3266.5212476 | 3 |
| 3266.5227 | 3 |
| 3266.5262518 | 0 |
| 3266.5292382 | 5 |
| 3266.5305817 | 3 |
| 3266.533714 | 3 |
| 3266.5355884 | 2 |
| 3266.5391161 | 1 |
| 3266.5395237 | 2 |
| 3266.5443513 | 1 |
| 3266.5463413 | 2 |
| 3266.5473996 | 2 |
| 3266.5510522 | 4 |
| 3266.5525374 | 5 |
| 3266.5556964 | 1 |
| 3266.5588165 | 3 |
| 3266.5605136 | 1 |
| 3266.5631856 | 3 |
| 3266.5648502 | 0 |
| 3266.5681863 | 0 |
| 3266.5711633 | 4 |
| 3266.5728347 | 0 |
| 3266.5755653 | 2 |
| 3266.5767551 | 1 |
| 3266.5804622 | 0 |
| 3266.5831014 | 3 |
| 3266.58463 | 3 |
| 3266.5875055 | 2 |
| 3266.5903592 | 3 |
| 3266.5919515 | 4 |
| 3266.5955661 | 2 |
| 3266.5975286 | 2 |
| 3266.5999916 | 1 |
| 3266.6034347 | 2 |
| 3266.6047169 | 2 |
| 3266.6081181 | 2 |
| 3266.6098745 | 5 |
| 3266.6126064 | 4 |
| 3266.6142194 | 2 |
| 3266.6173757 | 5 |
| 3266.6209261 | 3 |
| 3266.6214532 | 2 |
| 3266.6252531 | 5 |
| 3266.6263747 | 2 |
| 3266.6292491 | 3 |
| 3266.6326154 | 2 |
| 3266.6345317 | 2 |
| 3266.637841 | 4 |
| 3266.63878 | 1 |
| 3266.6420881 | 5 |
| 3266.6450752 | 2 |
| 3266.6470929 | 6 |
| 3266.6497572 | 0 |
| 3266.6526945 | 1 |
| 3266.6543632 | 3 |
| 3266.6572611 | 3 |
| 3266.6591226 | 1 |
| 3266.6616684 | 3 |
| 3266.6632126 | 0 |
| 3266.6668651 | 2 |
| 3266.6698533 | 1 |
| 3266.6707893 | 2 |
| 3266.6739622 | 4 |
| 3266.6754711 | 1 |
| 3266.6781275 | 2 |
| 3266.6811184 | 2 |
| 3266.6828996 | 3 |
| 3266.6863066 | 3 |
| 3266.6878538 | 3 |
| 3266.6923355 | 2 |
| 3266.6946881 | 3 |
| 3266.6960886 | 2 |
| 3266.6987694 | 4 |
| 3266.7002834 | 3 |
| 3266.7033376 | 6 |
| 3266.7065059 | 1 |
| 3266.7084296 | 2 |
| 3266.7111167 | 2 |
| 3266.712503 | 1 |
| 3266.7161279 | 2 |
| 3266.7181602 | 3 |
| 3266.7202657 | 3 |
| 3266.7223101 | 2 |
| 3266.7246414 | 2 |
| 3266.7275567 | 4 |
| 3266.7310713 | 3 |
| 3266.732619 | 1 |
| 3266.7363703 | 1 |
| 3266.7378871 | 1 |
| 3266.7404712 | 2 |
| 3266.7442894 | 4 |
| 3266.745344 | 4 |
| 3266.7487125 | 4 |
| 3266.7501045 | 2 |
| 3266.7538107 | 3 |
| 3266.7545317 | 3 |
| 3266.7579273 | 2 |
| 3266.7600289 | 2 |
| 3266.7620687 | 4 |
| 3266.7648206 | 3 |
| 3266.7679179 | 5 |
| 3266.7703048 | 1 |
| 3266.7729225 | 1 |
| 3266.7750353 | 5 |
| 3266.778457 | 2 |
| 3266.7791335 | 1 |
| 3266.7798486 | 1 |
| 3266.7814133 | 1 |
| 3266.783253 | 0 |
| 3266.7838202 | 5 |
| 3266.7860508 | 4 |
| 3266.7864562 | 2 |
| 3266.7875728 | 1 |
| 3266.7880255 | 3 |
| 3266.7911269 | 3 |
| 3266.7911414 | 1 |
| 3266.792548 | 7 |
| 3266.7943746 | 2 |
| 3266.7956348 | 3 |
| 3266.7958881 | 2 |
| 3266.7973419 | 2 |
| 3266.7987734 | 4 |
| 3266.800256 | 2 |
| 3266.8007284 | 3 |
| 3266.8013466 | 2 |
| 3266.8038566 | 6 |
| 3266.8054176 | 1 |
| 3266.8091133 | 2 |
| 3266.8108564 | 1 |
| 3266.8132525 | 3 |
| 3266.8158321 | 1 |
| 3266.8189034 | 1 |
| 3266.8207385 | 1 |
| 3266.8238475 | 7 |
| 3266.8251021 | 3 |
| 3266.8292285 | 1 |
| 3266.8316407 | 1 |
| 3266.8329438 | 5 |
| 3266.8363559 | 1 |
| 3266.8372743 | 1 |
| 3266.8405072 | 1 |
| 3266.8436604 | 5 |
| 3266.8460212 | 8 |
| 3266.847806 | 0 |
| 3266.8506137 | 1 |
| 3266.8535353 | 3 |
| 3266.8546898 | 4 |
| 3266.8577494 | 3 |
| 3266.861203 | 3 |
| 3266.8620942 | 1 |
| 3266.8661152 | 1 |
| 3266.867434 | 0 |
| 3266.8714485 | 7 |
| 3266.8738682 | 1 |
| 3266.8752142 | 0 |
| 3266.8786991 | 1 |
| 3266.8809145 | 2 |
| 3266.8824988 | 0 |
| 3266.8825388 | 5 |
| 3266.8851143 | 4 |
| 3266.8869913 | 2 |
| 3266.8899252 | 5 |
| 3266.8930283 | 2 |
| 3266.8949857 | 2 |
| 3266.8978263 | 3 |
| 3266.8993297 | 3 |
| 3266.9027187 | 2 |
| 3266.9043092 | 5 |
| 3266.906846 | 2 |
| 3266.9105364 | 1 |
| 3266.9120407 | 5 |
| 3266.9144596 | 2 |
| 3266.9186649 | 2 |
| 3266.9200629 | 1 |
| 3266.923181 | 1 |
| 3266.9243656 | 1 |
| 3266.9273217 | 1 |
| 3266.9293772 | 0 |
| 3266.9331712 | 1 |
| 3266.9351233 | 3 |
| 3266.936986 | 3 |
| 3266.9395096 | 2 |
| 3266.9425262 | 4 |
| 3266.9442016 | 4 |
| 3266.9470182 | 1 |
| 3266.9486251 | 2 |
| 3266.9522549 | 1 |
| 3266.9544255 | 4 |
| 3266.9548074 | 1 |
| 3266.9561154 | 3 |
| 3266.9598026 | 3 |
| 3266.9622136 | 2 |
| 3266.9643981 | 3 |
| 3266.9659525 | 3 |
| 3266.9692575 | 4 |
| 3266.9716972 | 1 |
| 3266.9736522 | 2 |
| 3266.9766049 | 2 |
| 3266.9781412 | 0 |
| 3266.9816115 | 1 |
| 3266.9846255 | 4 |
| 3266.9857577 | 0 |
| 3266.988815 | 0 |
| 3266.9904388 | 2 |
| 3266.9930566 | 2 |
| 3266.9968479 | 1 |
| 3266.9986408 | 2 |
| 3267.0019072 | 6 |
| 3267.0028648 | 1 |
| 3267.0067469 | 1 |
| 3267.0090537 | 0 |
| 3267.0108531 | 2 |
| 3267.0145625 | 5 |
| 3267.0155707 | 1 |
| 3267.0189496 | 1 |
| 3267.0196183 | 1 |
| 3267.0233847 | 4 |
| 3267.0267584 | 1 |
| 3267.0278357 | 3 |
| 3267.0307493 | 1 |
| 3267.0333251 | 2 |
| 3267.0347589 | 3 |
| 3267.038403 | 1 |
| 3267.0401949 | 0 |
| 3267.042342 | 1 |
| 3267.0463146 | 2 |
| 3267.0478082 | 4 |
| 3267.0516042 | 3 |
| 3267.0527383 | 5 |
| 3267.0553736 | 2 |
| 3267.0585485 | 3 |
| 3267.0600698 | 5 |
| 3267.063085 | 0 |
| 3267.064403 | 3 |
| 3267.0655476 | 4 |
| 3267.0679561 | 2 |
| 3267.0692845 | 5 |
| 3267.0721338 | 4 |
| 3267.0758119 | 3 |
| 3267.0770527 | 2 |
| 3267.0799158 | 4 |
| 3267.0818197 | 2 |
| 3267.0844474 | 3 |
| 3267.0866437 | 1 |
| 3267.0881012 | 4 |
| 3267.0897524 | 1 |
| 3267.0925174 | 1 |
| 3267.0946965 | 2 |

| | |
|---|---|
| 3267.0969311 | 2 |
| 3267.0990266 | 1 |
| 3267.1021712 | 3 |
| 3267.104496 | 9 |
| 3267.1072836 | 3 |
| 3267.10984 | 4 |
| 3267.1122268 | 3 |
| 3267.1141807 | 1 |
| 3267.1169512 | 3 |
| 3267.11735 | 2 |
| 3267.1177574 | 3 |
| 3267.1186078 | 3 |
| 3267.1197363 | 4 |
| 3267.1214607 | 3 |
| 3267.1216849 | 3 |
| 3267.1218747 | 4 |
| 3267.1251263 | 0 |
| 3267.1255406 | 3 |
| 3267.1264867 | 2 |
| 3267.1265438 | 2 |
| 3267.12957 | 0 |
| 3267.1302438 | 1 |
| 3267.1310902 | 1 |
| 3267.1314947 | 6 |
| 3267.1340542 | 2 |
| 3267.1348415 | 2 |
| 3267.136688 | 2 |
| 3267.1381023 | 2 |
| 3267.1387997 | 4 |
| 3267.1391635 | 0 |
| 3267.1416993 | 1 |
| 3267.1421757 | 1 |
| 3267.1438052 | 5 |
| 3267.1458165 | 2 |
| 3267.1467935 | 3 |
| 3267.1476189 | 2 |
| 3267.1500519 | 4 |
| 3267.1509015 | 0 |
| 3267.1516761 | 4 |
| 3267.1521283 | 1 |
| 3267.1551983 | 1 |
| 3267.1552017 | 2 |
| 3267.1557079 | 3 |
| 3267.1564461 | 3 |
| 3267.1570234 | 3 |
| 3267.1590363 | 1 |
| 3267.1598308 | 0 |
| 3267.1616064 | 3 |
| 3267.1625731 | 0 |
| 3267.1634814 | 6 |
| 3267.1645117 | 2 |
| 3267.16604 | 4 |
| 3267.167982 | 2 |
| 3267.1692732 | 1 |
| 3267.169328 | 0 |
| 3267.1694307 | 4 |
| 3267.1696996 | 1 |
| 3267.1701156 | 2 |
| 3267.170648 | 1 |
| 3267.1721809 | 1 |
| 3267.1739578 | 3 |
| 3267.1748348 | 4 |
| 3267.1785097 | 1 |
| 3267.181756 | 2 |
| 3267.1831714 | 1 |
| 3267.1864519 | 1 |
| 3267.1901378 | 4 |
| 3267.1902014 | 2 |
| 3267.1911693 | 4 |
| 3267.1925738 | 2 |
| 3267.1940732 | 1 |
| 3267.195454 | 5 |
| 3267.1958679 | 4 |
| 3267.1987452 | 3 |
| 3267.2003779 | 5 |
| 3267.2033142 | 5 |
| 3267.2064574 | 3 |
| 3267.207984 | 3 |
| 3267.2108295 | 2 |
| 3267.2143269 | 3 |
| 3267.2158297 | 3 |
| 3267.2183906 | 1 |
| 3267.2191664 | 2 |
| 3267.2203192 | 2 |
| 3267.2236148 | 5 |
| 3267.224778 | 2 |
| 3267.2279116 | 1 |
| 3267.2312617 | 2 |
| 3267.233035 | 3 |
| 3267.2364132 | 3 |
| 3267.2381571 | 1 |
| 3267.2412326 | 2 |
| 3267.2440534 | 1 |
| 3267.2455005 | 2 |
| 3267.2492633 | 0 |
| 3267.2501666 | 3 |
| 3267.2537843 | 2 |
| 3267.2561189 | 5 |
| 3267.2572054 | 1 |
| 3267.2611877 | 2 |
| 3267.2624613 | 3 |
| 3267.2653858 | 5 |
| 3267.2685788 | 1 |
| 3267.2707064 | 2 |
| 3267.2733393 | 2 |
| 3267.2757864 | 3 |
| 3267.278378 | 5 |
| 3267.2812915 | 1 |
| 3267.2829653 | 6 |
| 3267.2863588 | 3 |
| 3267.2878426 | 0 |
| 3267.2909149 | 1 |
| 3267.2938989 | 3 |
| 3267.2955382 | 3 |
| 3267.2984851 | 1 |
| 3267.3006648 | 3 |
| 3267.3032522 | 0 |
| 3267.305802 | 2 |
| 3267.3079808 | 5 |
| 3267.3106821 | 2 |
| 3267.3126903 | 5 |
| 3267.3158636 | 3 |
| 3267.3167769 | 6 |
| 3267.3204961 | 2 |
| 3267.3244844 | 1 |
| 3267.3254383 | 4 |
| 3267.3285665 | 4 |
| 3267.330628 | 2 |
| 3267.3334347 | 2 |
| 3267.3368334 | 2 |
| 3267.3385457 | 1 |
| 3267.341385 | 2 |
| 3267.343857 | 2 |
| 3267.3448945 | 0 |
| 3267.3491711 | 4 |
| 3267.3506365 | 5 |
| 3267.3527852 | 1 |
| 3267.3564236 | 4 |
| 3267.3579651 | 2 |
| 3267.3608875 | 4 |
| 3267.3631984 | 3 |
| 3267.3662296 | 2 |
| 3267.3674504 | 2 |
| 3267.3714705 | 3 |
| 3267.3745344 | 1 |
| 3267.3754952 | 4 |
| 3267.3789144 | 3 |
| 3267.3820509 | 3 |
| 3267.3832992 | 1 |
| 3267.3860256 | 3 |
| 3267.3881157 | 2 |
| 3267.3916187 | 1 |
| 3267.3926982 | 2 |
| 3267.3961539 | 5 |
| 3267.3988671 | 1 |
| 3267.3999646 | 2 |
| 3267.4033035 | 2 |
| 3267.4050079 | 1 |
| 3267.4083441 | 4 |
| 3267.4109964 | 2 |
| 3267.4126359 | 4 |
| 3267.4152342 | 2 |
| 3267.4188863 | 2 |
| 3267.4208914 | 4 |
| 3267.423733 | 1 |
| 3267.4252174 | 2 |
| 3267.4285009 | 1 |
| 3267.4317004 | 4 |
| 3267.4332705 | 1 |
| 3267.4365004 | 2 |
| 3267.4388971 | 1 |
| 3267.4406866 | 3 |
| 3267.4433575 | 5 |
| 3267.4448483 | 1 |
| 3267.4483621 | 2 |
| 3267.4498748 | 1 |
| 3267.4535811 | 3 |
| 3267.4559011 | 2 |
| 3267.4584241 | 1 |
| 3267.4614533 | 3 |
| 3267.4624671 | 2 |
| 3267.466223 | 3 |
| 3267.4693717 | 5 |
| 3267.4708201 | 5 |
| 3267.4735019 | 0 |
| 3267.4752689 | 4 |
| 3267.4782783 | 2 |
| 3267.4812381 | 2 |
| 3267.4833741 | 5 |
| 3267.4860539 | 3 |
| 3267.4876945 | 4 |
| 3267.4907071 | 3 |
| 3267.4941341 | 5 |
| 3267.4947788 | 3 |
| 3267.4983371 | 3 |
| 3267.4992576 | 2 |
| 3267.502719 | 1 |
| 3267.506542 | 2 |
| 3267.5079137 | 4 |
| 3267.5111823 | 0 |
| 3267.5128752 | 5 |
| 3267.5156915 | 3 |
| 3267.5174077 | 4 |
| 3267.5204326 | 2 |
| 3267.5240448 | 1 |
| 3267.5252743 | 4 |
| 3267.5281381 | 5 |
| 3267.5308497 | 0 |
| 3267.5323748 | 3 |
| 3267.5363246 | 4 |
| 3267.5378643 | 2 |
| 3267.5409575 | 3 |
| 3267.5421953 | 1 |
| 3267.545668 | 6 |
| 3267.5492178 | 0 |
| 3267.5508825 | 0 |
| 3267.552772 | 4 |
| 3267.5546177 | 2 |
| 3267.5583379 | 1 |
| 3267.5608566 | 2 |
| 3267.5626432 | 4 |
| 3267.5658409 | 1 |
| 3267.5670662 | 0 |
| 3267.5703295 | 1 |
| 3267.5715575 | 2 |
| 3267.5750666 | 3 |
| 3267.5777479 | 2 |
| 3267.5797837 | 3 |
| 3267.5829179 | 0 |
| 3267.5853714 | 1 |
| 3267.5874078 | 3 |
| 3267.5903887 | 4 |
| 3267.5931717 | 1 |
| 3267.5945811 | 2 |
| 3267.5978529 | 2 |
| 3267.5996758 | 1 |
| 3267.6030912 | 4 |
| 3267.6045676 | 1 |
| 3267.6079501 | 5 |
| 3267.610484 | 0 |
| 3267.6122402 | 1 |
| 3267.6154712 | 2 |
| 3267.6160882 | 0 |
| 3267.6199782 | 4 |
| 3267.622916 | 8 |
| 3267.6243246 | 7 |
| 3267.6271143 | 2 |
| 3267.6290861 | 2 |
| 3267.6320873 | 1 |
| 3267.634942 | 1 |
| 3267.6375774 | 2 |
| 3267.6410686 | 3 |
| 3267.6417568 | 0 |
| 3267.64474 | 0 |
| 3267.6460997 | 2 |
| 3267.6488117 | 3 |
| 3267.6519564 | 3 |
| 3267.6542605 | 3 |
| 3267.6569823 | 3 |
| 3267.660227 | 1 |
| 3267.6614198 | 2 |
| 3267.6642661 | 4 |
| 3267.6663679 | 3 |
| 3267.6697656 | 5 |
| 3267.670966 | 2 |
| 3267.6739429 | 4 |
| 3267.6773174 | 1 |
| 3267.6782203 | 2 |
| 3267.6810851 | 4 |
| 3267.684827 | 1 |
| 3267.6856404 | 9 |
| 3267.6890446 | 2 |
| 3267.6906486 | 2 |
| 3267.6941712 | 1 |
| 3267.6971024 | 1 |
| 3267.6989277 | 6 |
| 3267.702679 | 5 |
| 3267.7032262 | 2 |
| 3267.7070763 | 3 |
| 3267.7102316 | 3 |
| 3267.7107243 | 4 |
| 3267.7138735 | 3 |
| 3267.7161432 | 6 |
| 3267.7182889 | 2 |
| 3267.7208132 | 4 |
| 3267.7238444 | 3 |
| 3267.7263018 | 1 |
| 3267.7273498 | 1 |
| 3267.7321239 | 5 |
| 3267.7347089 | 3 |
| 3267.7363128 | 2 |
| 3267.7387967 | 2 |
| 3267.7409123 | 4 |
| 3267.7435206 | 2 |
| 3267.7467231 | 2 |
| 3267.7482558 | 3 |
| 3267.7510315 | 2 |
| 3267.754066 | 3 |
| 3267.756075 | 2 |
| 3267.7592265 | 2 |
| 3267.7594547 | 2 |
| 3267.7630015 | 5 |
| 3267.7664193 | 3 |
| 3267.7680575 | 0 |
| 3267.7716133 | 3 |
| 3267.7731776 | 0 |
| 3267.7756132 | 0 |
| 3267.7774275 | 0 |
| 3267.7807049 | 4 |
| 3267.7845391 | 2 |
| 3267.7854419 | 0 |
| 3267.7889874 | 2 |
| 3267.7900006 | 6 |
| 3267.7935689 | 4 |
| 3267.7961272 | 5 |
| 3267.798468 | 4 |
| 3267.8014338 | 4 |
| 3267.8027695 | 1 |
| 3267.8059423 | 2 |
| 3267.8091019 | 5 |
| 3267.8093797 | 1 |
| 3267.8095055 | 3 |
| 3267.8096616 | 1 |
| 3267.8098083 | 2 |
| 3267.8103074 | 2 |
| 3267.8103984 | 3 |
| 3267.8105389 | 0 |
| 3267.8108922 | 2 |
| 3267.8110289 | 2 |
| 3267.8113733 | 1 |
| 3267.8113937 | 4 |
| 3267.8116506 | 2 |
| 3267.8116609 | 3 |
| 3267.8118596 | 1 |
| 3267.8120052 | 2 |
| 3267.8122963 | 1 |
| 3267.8127686 | 1 |
| 3267.8130911 | 2 |
| 3267.8131631 | 4 |
| 3267.8135064 | 1 |
| 3267.8138434 | 1 |
| 3267.8140006 | 3 |
| 3267.8140505 | 1 |
| 3267.8145259 | 1 |
| 3267.8145319 | 1 |
| 3267.8147724 | 4 |
| 3267.8148538 | 3 |
| 3267.8150275 | 0 |
| 3267.8153697 | 3 |
| 3267.8161998 | 3 |
| 3267.8162863 | 1 |
| 3267.8165723 | 1 |
| 3267.8166347 | 5 |
| 3267.816979 | 0 |
| 3267.8170703 | 0 |
| 3267.8173841 | 3 |
| 3267.8175966 | 3 |
| 3267.8181085 | 6 |
| 3267.8183131 | 2 |
| 3267.8183616 | 2 |
| 3267.8185238 | 6 |
| 3267.8189085 | 3 |
| 3267.8191511 | 3 |

| | |
|---|---|
| 3267.8193352 | 2 |
| 3267.8194488 | 1 |
| 3267.8197451 | 1 |
| 3267.8201452 | 3 |
| 3267.8208041 | 1 |
| 3267.8209193 | 3 |
| 3267.8209277 | 0 |
| 3267.821003 | 3 |
| 3267.8210439 | 2 |
| 3267.82158 | 5 |
| 3267.8220915 | 4 |
| 3267.822403 | 3 |
| 3267.8224345 | 3 |
| 3267.8224689 | 3 |
| 3267.8225049 | 3 |
| 3267.8225232 | 4 |
| 3267.8228574 | 7 |
| 3267.822985 | 3 |
| 3267.8237808 | 9 |
| 3267.8240496 | 2 |
| 3267.8240617 | 7 |
| 3267.8240648 | 6 |
| 3267.8244053 | 2 |
| 3267.8247805 | 9 |
| 3267.8248587 | 11 |
| 3267.8249276 | 11 |
| 3267.825333 | 13 |
| 3267.825627 | 20 |
| 3267.8262166 | 13 |
| 3267.8262734 | 30 |
| 3267.8262766 | 30 |
| 3267.8263048 | 35 |
| 3267.8267837 | 42 |
| 3267.8270029 | 32 |
| 3267.8275723 | 34 |
| 3267.8277244 | 50 |
| 3267.8277562 | 47 |
| 3267.8280014 | 50 |
| 3267.828356 | 53 |
| 3267.8284101 | 34 |
| 3267.8287714 | 45 |
| 3267.8288954 | 45 |
| 3267.8290027 | 40 |
| 3267.8292792 | 49 |
| 3267.8294082 | 49 |
| 3267.8297793 | 58 |
| 3267.8302565 | 39 |
| 3267.8305208 | 47 |
| 3267.8305482 | 48 |
| 3267.8308964 | 45 |
| 3267.8313232 | 42 |
| 3267.8313453 | 38 |
| 3267.8315766 | 49 |
| 3267.8320016 | 26 |
| 3267.8321175 | 39 |
| 3267.8322323 | 39 |
| 3267.8323107 | 45 |
| 3267.8326431 | 15 |
| 3267.832718 | 56 |
| 3267.8332463 | 28 |
| 3267.83364 | 7 |
| 3267.8338057 | 27 |
| 3267.8341027 | 8 |
| 3267.8341465 | 11 |
| 3267.8345926 | 3 |
| 3267.8346588 | 4 |
| 3267.8350829 | 2 |
| 3267.8353824 | 3 |
| 3267.8354854 | 3 |
| 3267.8356863 | 2 |
| 3267.8358192 | 2 |
| 3267.8360332 | 2 |
| 3267.8365357 | 4 |
| 3267.8366287 | 3 |
| 3267.8366313 | 1 |
| 3267.8368649 | 6 |
| 3267.8369355 | 3 |
| 3267.8374663 | 1 |
| 3267.8376624 | 1 |
| 3267.8381134 | 0 |
| 3267.8383125 | 1 |
| 3267.838356 | 3 |
| 3267.83849 | 4 |
| 3267.8386702 | 2 |
| 3267.8387591 | 1 |
| 3267.8397179 | 0 |
| 3267.8397422 | 2 |
| 3267.8398714 | 3 |
| 3267.8403094 | 7 |
| 3267.8403208 | 3 |
| 3267.8414462 | 4 |
| 3267.8427911 | 0 |
| 3267.8434582 | 2 |
| 3267.8447874 | 1 |
| 3267.8450369 | 0 |
| 3267.8463324 | 6 |
| 3267.8470763 | 4 |
| 3267.8481446 | 3 |
| 3267.8488572 | 4 |
| 3267.8492067 | 0 |
| 3267.8508485 | 2 |
| 3267.8520201 | 1 |
| 3267.8522791 | 5 |
| 3267.8535064 | 4 |
| 3267.8550822 | 2 |
| 3267.8554543 | 1 |
| 3267.8563697 | 2 |
| 3267.8574216 | 2 |
| 3267.8582335 | 0 |
| 3267.8585025 | 3 |
| 3267.8600574 | 6 |
| 3267.8606857 | 3 |
| 3267.861857 | 1 |
| 3267.8636526 | 3 |
| 3267.863738 | 1 |
| 3267.8645937 | 1 |
| 3267.8654312 | 3 |
| 3267.8665314 | 4 |
| 3267.8676838 | 2 |
| 3267.869094 | 1 |
| 3267.8694234 | 1 |
| 3267.871051 | 3 |
| 3267.8715714 | 2 |
| 3267.872821 | 1 |
| 3267.8731126 | 2 |
| 3267.8742493 | 5 |
| 3267.8759028 | 4 |
| 3267.8764306 | 2 |
| 3267.8772263 | 4 |
| 3267.8782828 | 3 |
| 3267.8788794 | 1 |
| 3267.8799576 | 4 |
| 3267.880851 | 1 |
| 3267.8819573 | 4 |
| 3267.8824742 | 2 |
| 3267.8836497 | 3 |
| 3267.8848762 | 3 |
| 3267.8849551 | 3 |
| 3267.8870563 | 4 |
| 3267.8874777 | 1 |
| 3267.888659 | 1 |
| 3267.8895624 | 1 |
| 3267.8904142 | 2 |
| 3267.8909766 | 0 |
| 3267.8920108 | 2 |
| 3267.8935843 | 3 |
| 3267.8937916 | 1 |
| 3267.8947545 | 3 |
| 3267.8962229 | 0 |
| 3267.8963497 | 3 |
| 3267.8980581 | 5 |
| 3267.898272 | 2 |
| 3267.8998397 | 4 |
| 3267.9004143 | 8 |
| 3267.9017231 | 1 |
| 3267.9026867 | 0 |
| 3267.9029075 | 1 |
| 3267.9042467 | 3 |
| 3267.9048677 | 2 |
| 3267.9065195 | 6 |
| 3267.9070974 | 4 |
| 3267.9076783 | 2 |
| 3267.9093038 | 4 |
| 3267.9096873 | 2 |
| 3267.9113332 | 1 |
| 3267.9118605 | 2 |
| 3267.912479 | 0 |
| 3267.9141712 | 3 |
| 3267.9145433 | 2 |
| 3267.9157131 | 7 |
| 3267.9170173 | 1 |
| 3267.9171542 | 0 |
| 3267.9183692 | 4 |
| 3267.9188388 | 3 |
| 3267.9199607 | 1 |
| 3267.9212237 | 3 |
| 3267.9223253 | 1 |
| 3267.9235642 | 3 |
| 3267.9242117 | 1 |
| 3267.925584 | 1 |
| 3267.9264183 | 2 |
| 3267.927529 | 1 |
| 3267.927986 | 0 |
| 3267.9283781 | 1 |
| 3267.9301816 | 2 |
| 3267.9304995 | 3 |
| 3267.9307052 | 1 |
| 3267.9311517 | 3 |
| 3267.9316396 | 1 |
| 3267.9325727 | 5 |
| 3267.9333421 | 3 |
| 3267.9338904 | 1 |
| 3267.9343071 | 2 |
| 3267.9343904 | 2 |
| 3267.935469 | 3 |
| 3267.9357621 | 2 |
| 3267.9358819 | 4 |
| 3267.9371595 | 2 |
| 3267.9373599 | 3 |
| 3267.9376056 | 4 |
| 3267.9381829 | 4 |
| 3267.9394913 | 1 |
| 3267.9395759 | 2 |
| 3267.9403953 | 0 |
| 3267.9404556 | 2 |
| 3267.94066 | 1 |
| 3267.9419933 | 0 |
| 3267.9426385 | 1 |
| 3267.9426873 | 3 |
| 3267.9433301 | 2 |
| 3267.9434151 | 2 |
| 3267.9443507 | 1 |
| 3267.9450144 | 2 |
| 3267.9454349 | 0 |
| 3267.9457035 | 1 |
| 3267.9459641 | 3 |
| 3267.9472826 | 5 |
| 3267.94731 | 3 |
| 3267.9479494 | 1 |
| 3267.9488415 | 4 |
| 3267.9495926 | 5 |
| 3267.9498347 | 2 |
| 3267.9499786 | 1 |
| 3267.9506025 | 1 |
| 3267.9513204 | 3 |
| 3267.9516908 | 3 |
| 3267.9521358 | 2 |
| 3267.9532032 | 4 |
| 3267.9537984 | 2 |
| 3267.9538434 | 3 |
| 3267.9546117 | 0 |
| 3267.954616 | 3 |
| 3267.9560946 | 1 |
| 3267.956443 | 3 |
| 3267.956765 | 3 |
| 3267.9569654 | 2 |
| 3267.957828 | 3 |
| 3267.958851 | 0 |
| 3267.9592608 | 2 |
| 3267.9597897 | 1 |
| 3267.9598094 | 1 |
| 3267.9609767 | 0 |
| 3267.960986 | 1 |
| 3267.9613146 | 0 |
| 3267.9622306 | 4 |
| 3267.9623495 | 1 |
| 3267.9636693 | 1 |
| 3267.9640058 | 1 |
| 3267.964438 | 2 |
| 3267.9645892 | 1 |
| 3267.9658082 | 1 |
| 3267.9663447 | 1 |
| 3267.9668603 | 1 |
| 3267.9670592 | 1 |
| 3267.9676704 | 3 |
| 3267.9686487 | 3 |
| 3267.9692361 | 1 |
| 3267.9692927 | 5 |
| 3267.9693598 | 2 |
| 3267.9706599 | 2 |
| 3267.9709189 | 1 |
| 3267.9713239 | 2 |
| 3267.9716057 | 1 |
| 3267.9722338 | 4 |
| 3267.9725639 | 2 |
| 3267.9729291 | 5 |
| 3267.9743919 | 3 |
| 3267.975027 | 2 |
| 3267.9753382 | 3 |
| 3267.9754315 | 1 |
| 3267.975946 | 6 |
| 3267.9767755 | 2 |
| 3267.9768455 | 1 |
| 3267.9776366 | 3 |
| 3267.9781004 | 1 |
| 3267.9786834 | 4 |
| 3267.9797679 | 4 |
| 3267.9802037 | 3 |
| 3267.9804515 | 4 |
| 3267.9809407 | 3 |
| 3267.9812038 | 0 |
| 3267.9813002 | 3 |
| 3267.9828952 | 1 |
| 3267.9829061 | 1 |
| 3267.9837803 | 1 |
| 3267.9839389 | 2 |
| 3267.9843769 | 0 |
| 3267.9844652 | 2 |
| 3267.9857586 | 1 |
| 3267.986429 | 2 |
| 3267.9867599 | 4 |
| 3267.9871378 | 1 |
| 3267.9873662 | 4 |
| 3267.9875175 | 5 |
| 3267.9888862 | 1 |
| 3267.9904621 | 2 |
| 3267.9911696 | 1 |
| 3267.9931733 | 0 |
| 3267.9939373 | 5 |
| 3267.9955019 | 2 |
| 3267.9961414 | 3 |
| 3267.9974145 | 1 |
| 3267.9992952 | 4 |
| 3267.999401 | 3 |
| 3268.0016119 | 2 |
| 3268.00318 | 9 |
| 3268.0034643 | 31 |
| 3268.005169 | 60 |
| 3268.0064899 | 76 |
| 3268.0073059 | 53 |
| 3268.0085718 | 45 |
| 3268.0102654 | 48 |
| 3268.0115473 | 44 |
| 3268.0126306 | 46 |
| 3268.0137397 | 45 |
| 3268.0152423 | 14 |
| 3268.0163688 | 0 |
| 3268.0180052 | 2 |
| 3268.018722 | 2 |
| 3268.0198888 | 2 |
| 3268.0213683 | 3 |
| 3268.0221125 | 1 |
| 3268.0241946 | 2 |
| 3268.0245751 | 1 |
| 3268.0259744 | 1 |
| 3268.0274929 | 3 |
| 3268.0284872 | 1 |
| 3268.0301168 | 2 |
| 3268.0306005 | 3 |
| 3268.0322448 | 1 |
| 3268.0332917 | 1 |
| 3268.0345774 | 1 |
| 3268.0360309 | 3 |
| 3268.0369245 | 2 |
| 3268.0385502 | 5 |
| 3268.0391471 | 6 |
| 3268.0410066 | 1 |
| 3268.0432277 | 1 |
| 3268.0435588 | 2 |
| 3268.045151 | 0 |
| 3268.0459033 | 3 |
| 3268.0478587 | 0 |
| 3268.048735 | 2 |
| 3268.0502346 | 2 |
| 3268.0514975 | 3 |
| 3268.05274 | 3 |
| 3268.0538077 | 3 |
| 3268.0551912 | 0 |
| 3268.0560565 | 0 |
| 3268.0572836 | 1 |
| 3268.0584615 | 4 |
| 3268.060158 | 2 |
| 3268.0615496 | 9 |
| 3268.062335 | 8 |
| 3268.0638463 | 3 |
| 3268.0638466 | 10 |
| 3268.066219 | 1 |
| 3268.067537 | 2 |
| 3268.0677718 | 2 |
| 3268.069751 | 1 |
| 3268.0701916 | 1 |
| 3268.0721682 | 2 |
| 3268.0735427 | 4 |

| | |
|---|---|
| 3268.0740826 | 3 |
| 3268.0759419 | 1 |
| 3268.0767039 | 2 |
| 3268.077641 | 2 |
| 3268.0798224 | 2 |
| 3268.0805771 | 2 |
| 3268.0824461 | 1 |
| 3268.0828193 | 1 |
| 3268.0842876 | 5 |
| 3268.0859873 | 1 |
| 3268.086671 | 4 |
| 3268.0882556 | 3 |
| 3268.0895204 | 3 |
| 3268.0914636 | 0 |
| 3268.0915728 | 1 |
| 3268.093266 | 2 |
| 3268.0950734 | 2 |
| 3268.0964928 | 1 |
| 3268.0969292 | 1 |
| 3268.0985951 | 5 |
| 3268.0994445 | 1 |
| 3268.1007309 | 2 |
| 3268.1015828 | 4 |
| 3268.1039715 | 1 |
| 3268.1051464 | 2 |
| 3268.105518 | 2 |
| 3268.1073726 | 2 |
| 3268.1079027 | 1 |
| 3268.1097503 | 3 |
| 3268.1109525 | 1 |
| 3268.111612 | 1 |
| 3268.1133932 | 4 |
| 3268.1147173 | 1 |
| 3268.1152363 | 1 |
| 3268.1161379 | 2 |
| 3268.1181021 | 3 |
| 3268.1199124 | 3 |
| 3268.1204418 | 2 |
| 3268.1224344 | 2 |
| 3268.1224799 | 2 |
| 3268.1242317 | 2 |
| 3268.1262626 | 1 |
| 3268.1264145 | 4 |
| 3268.1277908 | 1 |
| 3268.1300763 | 1 |
| 3268.130165 | 0 |
| 3268.1326661 | 3 |
| 3268.1340783 | 4 |
| 3268.1347503 | 4 |
| 3268.1357423 | 3 |
| 3268.1365596 | 2 |
| 3268.1384258 | 6 |
| 3268.1394071 | 5 |
| 3268.1405147 | 2 |
| 3268.1421489 | 2 |
| 3268.1434126 | 1 |
| 3268.1449492 | 0 |
| 3268.1457233 | 2 |
| 3268.1473313 | 3 |
| 3268.1492537 | 2 |
| 3268.1493475 | 2 |
| 3268.1510254 | 2 |
| 3268.1517209 | 1 |
| 3268.1535328 | 1 |
| 3268.1538959 | 2 |
| 3268.1556144 | 0 |
| 3268.1578186 | 1 |
| 3268.1579017 | 2 |
| 3268.1595708 | 2 |
| 3268.1608586 | 1 |
| 3268.1615867 | 3 |
| 3268.163221 | 3 |
| 3268.164129 | 2 |
| 3268.1653285 | 3 |
| 3268.1665853 | 1 |
| 3268.168273 | 3 |
| 3268.1696604 | 2 |
| 3268.1706206 | 5 |
| 3268.1719795 | 1 |
| 3268.1727204 | 1 |
| 3268.17456 | 5 |
| 3268.1757573 | 3 |
| 3268.1770961 | 4 |
| 3268.1783774 | 3 |
| 3268.1797368 | 0 |
| 3268.1810551 | 1 |
| 3268.1820941 | 4 |
| 3268.1828847 | 0 |
| 3268.1849407 | 2 |
| 3268.1860555 | 1 |
| 3268.1875674 | 1 |
| 3268.1888075 | 2 |
| 3268.1899228 | 3 |
| 3268.1914748 | 1 |
| 3268.1926394 | 1 |
| 3268.193437 | 0 |
| 3268.1950768 | 1 |
| 3268.1958122 | 2 |
| 3268.1973048 | 4 |
| 3268.1981233 | 1 |
| 3268.2000403 | 2 |
| 3268.2015453 | 4 |
| 3268.2016336 | 2 |
| 3268.2037177 | 0 |
| 3268.2044443 | 3 |
| 3268.2059935 | 2 |
| 3268.2075135 | 2 |
| 3268.2081832 | 3 |
| 3268.2094156 | 3 |
| 3268.210994 | 2 |
| 3268.2116373 | 4 |
| 3268.213429 | 0 |
| 3268.2140604 | 2 |
| 3268.2161651 | 2 |
| 3268.2177995 | 3 |
| 3268.2186501 | 3 |
| 3268.2195537 | 1 |
| 3268.2205679 | 0 |
| 3268.2222272 | 6 |
| 3268.2232388 | 0 |
| 3268.2245912 | 2 |
| 3268.2267468 | 4 |
| 3268.2277161 | 0 |
| 3268.2292795 | 2 |
| 3268.2295368 | 3 |
| 3268.2312173 | 2 |
| 3268.2331757 | 6 |
| 3268.2331944 | 1 |
| 3268.2356763 | 3 |
| 3268.2361531 | 5 |
| 3268.2377109 | 2 |
| 3268.2393711 | 2 |
| 3268.2397542 | 1 |
| 3268.2415122 | 3 |
| 3268.2426272 | 2 |
| 3268.2441221 | 5 |
| 3268.2453297 | 3 |
| 3268.2463934 | 1 |
| 3268.2474382 | 2 |
| 3268.2484148 | 1 |
| 3268.25014 | 1 |
| 3268.2520657 | 4 |
| 3268.2527854 | 1 |
| 3268.2542756 | 3 |
| 3268.2549112 | 4 |
| 3268.2562816 | 1 |
| 3268.2577717 | 1 |
| 3268.2587548 | 1 |
| 3268.2600867 | 1 |
| 3268.2610934 | 2 |
| 3268.2624177 | 2 |
| 3268.2645729 | 2 |
| 3268.265311 | 2 |
| 3268.2666668 | 5 |
| 3268.2673974 | 3 |
| 3268.2688781 | 4 |
| 3268.270147 | 1 |
| 3268.2714476 | 2 |
| 3268.2727416 | 2 |
| 3268.2741564 | 5 |
| 3268.2755929 | 6 |
| 3268.2770947 | 3 |
| 3268.2784681 | 3 |
| 3268.279948 | 2 |
| 3268.2800944 | 1 |
| 3268.2815764 | 2 |
| 3268.2825625 | 1 |
| 3268.2846055 | 2 |
| 3268.2856433 | 0 |
| 3268.2868278 | 2 |
| 3268.2879696 | 2 |
| 3268.2895714 | 2 |
| 3268.2906201 | 5 |
| 3268.2920114 | 1 |
| 3268.2933717 | 3 |
| 3268.2940765 | 1 |
| 3268.2954506 | 0 |
| 3268.2965495 | 2 |
| 3268.2976107 | 3 |
| 3268.2994259 | 2 |
| 3268.2997878 | 1 |
| 3268.3020477 | 4 |
| 3268.3029575 | 4 |
| 3268.3048252 | 2 |
| 3268.3055225 | 5 |
| 3268.306231 | 1 |
| 3268.3083074 | 3 |
| 3268.3091353 | 0 |
| 3268.3099157 | 3 |
| 3268.311655 | 2 |
| 3268.3130942 | 4 |
| 3268.3147146 | 1 |
| 3268.3152686 | 3 |
| 3268.3175247 | 2 |
| 3268.3175377 | 0 |
| 3268.3196355 | 3 |
| 3268.3215188 | 0 |
| 3268.3215934 | 3 |
| 3268.323806 | 3 |
| 3268.3249086 | 2 |
| 3268.3254847 | 2 |
| 3268.3274696 | 4 |
| 3268.3277968 | 1 |
| 3268.3297815 | 1 |
| 3268.3315106 | 3 |
| 3268.3316601 | 0 |
| 3268.3339225 | 1 |
| 3268.3345335 | 1 |
| 3268.3363223 | 2 |
| 3268.3368086 | 0 |
| 3268.338515 | 1 |
| 3268.339839 | 1 |
| 3268.3412346 | 3 |
| 3268.3422184 | 1 |
| 3268.3433702 | 6 |
| 3268.3446294 | 2 |
| 3268.3455428 | 0 |
| 3268.3468737 | 2 |
| 3268.3484235 | 4 |
| 3268.3500323 | 2 |
| 3268.3505185 | 4 |
| 3268.3521443 | 0 |
| 3268.3529268 | 2 |
| 3268.3544933 | 3 |
| 3268.3563639 | 4 |
| 3268.3567941 | 1 |
| 3268.3584639 | 4 |
| 3268.3590203 | 1 |
| 3268.3609347 | 1 |
| 3268.3625078 | 0 |
| 3268.3635635 | 0 |
| 3268.3648828 | 1 |
| 3268.3657566 | 1 |
| 3268.3671575 | 3 |
| 3268.369168 | 5 |
| 3268.369838 | 2 |
| 3268.3706821 | 2 |
| 3268.3726547 | 1 |
| 3268.3735242 | 2 |
| 3268.3746337 | 2 |
| 3268.3759366 | 3 |
| 3268.3772267 | 3 |
| 3268.3787311 | 1 |
| 3268.3800674 | 2 |
| 3268.3816326 | 5 |
| 3268.3819101 | 1 |
| 3268.3846821 | 1 |
| 3268.3851531 | 1 |
| 3268.385994 | 4 |
| 3268.3873052 | 2 |
| 3268.3885021 | 1 |
| 3268.3897706 | 5 |
| 3268.391127 | 1 |
| 3268.392122 | 1 |
| 3268.3936583 | 2 |
| 3268.3941983 | 2 |
| 3268.3960413 | 4 |
| 3268.3969872 | 0 |
| 3268.3988077 | 2 |
| 3268.3997375 | 2 |
| 3268.4008914 | 1 |
| 3268.402257 | 1 |
| 3268.403638 | 2 |
| 3268.4042221 | 1 |
| 3268.4063956 | 2 |
| 3268.4065882 | 0 |
| 3268.408867 | 2 |
| 3268.4107785 | 1 |
| 3268.4108393 | 2 |
| 3268.4124854 | 4 |
| 3268.4131376 | 3 |
| 3268.4149824 | 1 |
| 3268.4167634 | 2 |
| 3268.4174707 | 1 |
| 3268.4188389 | 0 |
| 3268.4202307 | 1 |
| 3268.4211656 | 4 |
| 3268.4221834 | 2 |
| 3268.4235819 | 0 |
| 3268.424873 | 1 |
| 3268.4260705 | 1 |
| 3268.4277999 | 1 |
| 3268.4284155 | 4 |
| 3268.4294382 | 4 |
| 3268.4313641 | 3 |
| 3268.4318192 | 3 |
| 3268.4336174 | 2 |
| 3268.4338702 | 1 |
| 3268.4356876 | 4 |
| 3268.4377657 | 1 |
| 3268.4380813 | 1 |
| 3268.4397337 | 1 |
| 3268.4402261 | 1 |
| 3268.4421193 | 1 |
| 3268.4436658 | 4 |
| 3268.4446332 | 1 |
| 3268.4461971 | 4 |
| 3268.4468175 | 6 |
| 3268.4479686 | 2 |
| 3268.4489496 | 4 |
| 3268.4504028 | 4 |
| 3268.4527065 | 1 |
| 3268.4539682 | 0 |
| 3268.4549216 | 0 |
| 3268.4564556 | 4 |
| 3268.4572123 | 2 |
| 3268.4584826 | 2 |
| 3268.4597481 | 0 |
| 3268.4609876 | 1 |
| 3268.4625284 | 0 |
| 3268.4635353 | 2 |
| 3268.4652226 | 0 |
| 3268.4656349 | 2 |
| 3268.4677564 | 3 |
| 3268.4691821 | 0 |
| 3268.4695063 | 2 |
| 3268.4712867 | 3 |
| 3268.4718365 | 0 |
| 3268.473566 | 2 |
| 3268.4748664 | 3 |
| 3268.4762022 | 0 |
| 3268.4778033 | 0 |
| 3268.4785795 | 3 |
| 3268.4796088 | 1 |
| 3268.480445 | 0 |
| 3268.481734 | 5 |
| 3268.4834348 | 6 |
| 3268.4841114 | 7 |
| 3268.4856735 | 7 |
| 3268.4870012 | 3 |
| 3268.4876532 | 3 |
| 3268.489442 | 0 |
| 3268.4898819 | 0 |
| 3268.4916232 | 3 |
| 3268.4935107 | 0 |
| 3268.4941186 | 9 |
| 3268.4959653 | 0 |
| 3268.4966605 | 5 |
| 3268.4982618 | 5 |
| 3268.49959 | 2 |
| 3268.5003989 | 3 |
| 3268.5024381 | 4 |
| 3268.5034837 | 4 |
| 3268.5051739 | 1 |
| 3268.5056775 | 3 |
| 3268.5069859 | 4 |
| 3268.5084329 | 2 |
| 3268.5095731 | 4 |
| 3268.5112999 | 0 |
| 3268.5118895 | 0 |
| 3268.5138556 | 3 |
| 3268.515201 | 3 |
| 3268.5156622 | 1 |
| 3268.5175701 | 1 |
| 3268.5178722 | 1 |
| 3268.5193693 | 3 |
| 3268.5208493 | 2 |
| 3268.5214008 | 2 |
| 3268.5226651 | 3 |
| 3268.5237843 | 0 |
| 3268.525509 | 1 |
| 3268.5268559 | 1 |
| 3268.5277696 | 2 |

| | |
|---|---|
| 3268.5296547 | 0 |
| 3268.529961 | 3 |
| 3268.5321989 | 5 |
| 3268.5330965 | 1 |
| 3268.5339422 | 3 |
| 3268.5355511 | 0 |
| 3268.5359561 | 1 |
| 3268.5377346 | 3 |
| 3268.5392574 | 1 |
| 3268.5395979 | 1 |
| 3268.5421106 | 2 |
| 3268.5424092 | 2 |
| 3268.5425825 | 4 |
| 3268.5439871 | 2 |
| 3268.5455199 | 3 |
| 3268.5461581 | 2 |
| 3268.5465405 | 3 |
| 3268.5473927 | 2 |
| 3268.5480834 | 1 |
| 3268.5490773 | 3 |
| 3268.5506155 | 5 |
| 3268.5506423 | 4 |
| 3268.551435 | 2 |
| 3268.5520602 | 3 |
| 3268.5529659 | 3 |
| 3268.5545757 | 3 |
| 3268.5548149 | 5 |
| 3268.5548693 | 1 |
| 3268.5561152 | 1 |
| 3268.5568183 | 2 |
| 3268.5577916 | 3 |
| 3268.5587592 | 3 |
| 3268.5593828 | 2 |
| 3268.5605203 | 1 |
| 3268.5613097 | 2 |
| 3268.5617416 | 3 |
| 3268.5626526 | 1 |
| 3268.5634012 | 0 |
| 3268.5642532 | 4 |
| 3268.5650242 | 2 |
| 3268.5651112 | 1 |
| 3268.5665065 | 0 |
| 3268.5675174 | 1 |
| 3268.568161 | 1 |
| 3268.5687564 | 1 |
| 3268.5693926 | 1 |
| 3268.5707104 | 2 |
| 3268.5712552 | 3 |
| 3268.5719648 | 3 |
| 3268.5729709 | 3 |
| 3268.5730138 | 2 |
| 3268.5732582 | 1 |
| 3268.5748476 | 0 |
| 3268.5761278 | 1 |
| 3268.5766593 | 3 |
| 3268.5770879 | 4 |
| 3268.577887 | 3 |
| 3268.5786739 | 3 |
| 3268.5796115 | 4 |
| 3268.5803219 | 1 |
| 3268.5810958 | 2 |
| 3268.5820741 | 1 |
| 3268.5828667 | 2 |
| 3268.5832877 | 1 |
| 3268.5842698 | 5 |
| 3268.5852711 | 2 |
| 3268.5859152 | 2 |
| 3268.5872956 | 3 |
| 3268.5874462 | 1 |
| 3268.5879151 | 2 |
| 3268.5881839 | 2 |
| 3268.5902547 | 4 |
| 3268.5907133 | 2 |
| 3268.5917593 | 5 |
| 3268.5921037 | 1 |
| 3268.5936972 | 5 |
| 3268.5942277 | 4 |
| 3268.594838 | 2 |
| 3268.5955184 | 5 |
| 3268.5961466 | 2 |
| 3268.5973115 | 3 |
| 3268.5977028 | 5 |
| 3268.5986155 | 2 |
| 3268.5998918 | 1 |
| 3268.6008544 | 1 |
| 3268.6009996 | 2 |
| 3268.6019979 | 2 |
| 3268.6028243 | 2 |
| 3268.604124 | 0 |
| 3268.6050783 | 4 |
| 3268.6051816 | 5 |
| 3268.6061468 | 1 |
| 3268.6061761 | 3 |
| 3268.6069802 | 1 |
| 3268.6088827 | 2 |
| 3268.6091193 | 4 |
| 3268.6100683 | 2 |
| 3268.6103815 | 3 |
| 3268.6109148 | 3 |
| 3268.6126061 | 2 |
| 3268.6128906 | 1 |
| 3268.6130341 | 5 |
| 3268.6142806 | 5 |
| 3268.6149333 | 1 |
| 3268.615972 | 3 |
| 3268.6169778 | 4 |
| 3268.6171076 | 0 |
| 3268.6184648 | 2 |
| 3268.6191096 | 1 |
| 3268.6191821 | 1 |
| 3268.6207951 | 1 |
| 3268.6219943 | 0 |
| 3268.6226772 | 2 |
| 3268.6227421 | 2 |
| 3268.6232613 | 3 |
| 3268.6250637 | 2 |
| 3268.6252369 | 2 |
| 3268.6263863 | 2 |
| 3268.6265274 | 6 |
| 3268.6272342 | 3 |
| 3268.6278199 | 2 |
| 3268.6292395 | 4 |
| 3268.6300986 | 3 |
| 3268.6303867 | 4 |
| 3268.631557 | 0 |
| 3268.632881 | 3 |
| 3268.633177 | 0 |
| 3268.6339014 | 2 |
| 3268.6349354 | 1 |
| 3268.6355171 | 4 |
| 3268.6370975 | 4 |
| 3268.637286 | 1 |
| 3268.6376307 | 2 |
| 3268.6387336 | 0 |
| 3268.6394439 | 2 |
| 3268.6410659 | 4 |
| 3268.6411398 | 1 |
| 3268.6421055 | 5 |
| 3268.643469 | 1 |
| 3268.6445623 | 5 |
| 3268.6445794 | 2 |
| 3268.645935 | 3 |
| 3268.6460215 | 6 |
| 3268.6473296 | 2 |
| 3268.6482367 | 2 |
| 3268.6484675 | 5 |
| 3268.6496603 | 5 |
| 3268.6501581 | 2 |
| 3268.6504053 | 2 |
| 3268.6519165 | 3 |
| 3268.6521526 | 2 |
| 3268.6534832 | 6 |
| 3268.653976 | 5 |
| 3268.6542957 | 1 |
| 3268.6560655 | 1 |
| 3268.6570133 | 1 |
| 3268.6571925 | 1 |
| 3268.6590288 | 2 |
| 3268.6590326 | 3 |
| 3268.6594071 | 2 |
| 3268.6609692 | 3 |
| 3268.6610021 | 4 |
| 3268.6618488 | 3 |
| 3268.6621833 | 0 |
| 3268.663099 | 1 |
| 3268.6637392 | 7 |
| 3268.6643966 | 4 |
| 3268.66518 | 1 |
| 3268.6667583 | 3 |
| 3268.6673205 | 2 |
| 3268.6681576 | 2 |
| 3268.6696896 | 4 |
| 3268.6702622 | 4 |
| 3268.6710499 | 2 |
| 3268.6716119 | 3 |
| 3268.6716697 | 7 |
| 3268.6732124 | 2 |
| 3268.6736655 | 0 |
| 3268.6737104 | 1 |
| 3268.675398 | 1 |
| 3268.6754864 | 6 |
| 3268.6771405 | 7 |
| 3268.6776973 | 3 |
| 3268.6781047 | 1 |
| 3268.6792249 | 3 |
| 3268.6797284 | 2 |
| 3268.6805599 | 3 |
| 3268.6815894 | 1 |
| 3268.6816883 | 4 |
| 3268.6827955 | 2 |
| 3268.6838671 | 0 |
| 3268.6850412 | 3 |
| 3268.6858707 | 2 |
| 3268.6859043 | 3 |
| 3268.6874652 | 0 |
| 3268.6879316 | 3 |
| 3268.6883926 | 4 |
| 3268.6893783 | 5 |
| 3268.6906242 | 4 |
| 3268.6917973 | 4 |
| 3268.6918827 | 2 |
| 3268.6931869 | 2 |
| 3268.6937157 | 1 |
| 3268.6947132 | 5 |
| 3268.695747 | 2 |
| 3268.6957687 | 2 |
| 3268.6968725 | 3 |
| 3268.6979729 | 1 |
| 3268.6979839 | 2 |
| 3268.6988805 | 1 |
| 3268.7003864 | 3 |
| 3268.7008726 | 3 |
| 3268.7016697 | 3 |
| 3268.7026793 | 3 |
| 3268.7030208 | 3 |
| 3268.7039478 | 3 |
| 3268.7041063 | 2 |
| 3268.7052558 | 2 |
| 3268.7062293 | 2 |
| 3268.7072012 | 3 |
| 3268.7079482 | 1 |
| 3268.7084698 | 0 |
| 3268.7091304 | 1 |
| 3268.7109621 | 1 |
| 3268.7112813 | 3 |
| 3268.7120435 | 3 |
| 3268.7132337 | 4 |
| 3268.7134775 | 3 |
| 3268.7148252 | 3 |
| 3268.7150704 | 4 |
| 3268.7151713 | 5 |
| 3268.7164565 | 2 |
| 3268.7175436 | 2 |
| 3268.7183305 | 1 |
| 3268.7185368 | 0 |
| 3268.7186327 | 2 |
| 3268.7200964 | 1 |
| 3268.7217717 | 5 |
| 3268.7221252 | 2 |
| 3268.7234741 | 2 |
| 3268.7237477 | 3 |
| 3268.724873 | 3 |
| 3268.7257097 | 2 |
| 3268.7260809 | 1 |
| 3268.7268221 | 0 |
| 3268.7274974 | 2 |
| 3268.7285 | 4 |
| 3268.7291682 | 1 |
| 3268.7299578 | 2 |
| 3268.7304773 | 0 |
| 3268.731842 | 0 |
| 3268.7326885 | 2 |
| 3268.73343 | 2 |
| 3268.7342031 | 1 |
| 3268.7343147 | 1 |
| 3268.7362547 | 2 |
| 3268.7369737 | 5 |
| 3268.7376737 | 7 |
| 3268.7386374 | 1 |
| 3268.73997 | 4 |
| 3268.7405651 | 4 |
| 3268.7408264 | 4 |
| 3268.7413643 | 5 |
| 3268.7421745 | 1 |
| 3268.743381 | 1 |
| 3268.7438605 | 2 |
| 3268.7439328 | 3 |
| 3268.7456144 | 2 |
| 3268.7485855 | 2 |
| 3268.7512027 | 1 |
| 3268.7527277 | 4 |
| 3268.7553296 | 0 |
| 3268.7556921 | 6 |
| 3268.7587429 | 3 |
| 3268.7610091 | 3 |
| 3268.7634026 | 5 |
| 3268.7654984 | 4 |
| 3268.7682068 | 1 |
| 3268.7700927 | 0 |
| 3268.7724576 | 2 |
| 3268.7740093 | 1 |
| 3268.7765784 | 3 |
| 3268.7777672 | 2 |
| 3268.7809078 | 3 |
| 3268.7842205 | 2 |
| 3268.7851903 | 3 |
| 3268.7877293 | 2 |
| 3268.789094 | 2 |
| 3268.7920378 | 3 |
| 3268.7943514 | 1 |
| 3268.796663 | 3 |
| 3268.7988889 | 3 |
| 3268.7997919 | 6 |
| 3268.8034627 | 3 |
| 3268.8059275 | 1 |
| 3268.8065324 | 2 |
| 3268.8095785 | 1 |
| 3268.8116098 | 2 |
| 3268.8139041 | 0 |
| 3268.8147483 | 1 |
| 3268.8181514 | 2 |
| 3268.8208707 | 3 |
| 3268.8215425 | 3 |
| 3268.8245479 | 2 |
| 3268.8272735 | 0 |
| 3268.828484 | 3 |
| 3268.8314102 | 3 |
| 3268.8332791 | 5 |
| 3268.8352029 | 4 |
| 3268.836954 | 1 |
| 3268.8399114 | 4 |
| 3268.8426407 | 2 |
| 3268.8435808 | 2 |
| 3268.8466103 | 4 |
| 3268.8493333 | 1 |
| 3268.8502267 | 2 |
| 3268.8525406 | 2 |
| 3268.8545017 | 2 |
| 3268.8572467 | 1 |
| 3268.8590054 | 6 |
| 3268.8620203 | 3 |
| 3268.8644002 | 1 |
| 3268.865315 | 3 |
| 3268.8686094 | 4 |
| 3268.8696388 | 1 |
| 3268.8723917 | 2 |
| 3268.8752876 | 1 |
| 3268.8765136 | 3 |
| 3268.879522 | 0 |
| 3268.8802539 | 2 |
| 3268.8832923 | 4 |
| 3268.8864915 | 4 |
| 3268.8875028 | 3 |
| 3268.8905444 | 2 |
| 3268.892024 | 1 |
| 3268.8942178 | 3 |
| 3268.8954065 | 3 |
| 3268.89842 | 2 |
| 3268.9005399 | 3 |
| 3268.9018781 | 2 |
| 3268.9041421 | 3 |
| 3268.9070781 | 3 |
| 3268.9093793 | 4 |
| 3268.9115976 | 1 |
| 3268.9132546 | 2 |
| 3268.9164178 | 1 |
| 3268.9171522 | 3 |
| 3268.9198956 | 1 |
| 3268.9228007 | 3 |
| 3268.9249517 | 2 |
| 3268.9266764 | 5 |
| 3268.9285342 | 0 |
| 3268.9311055 | 3 |
| 3268.9336603 | 5 |
| 3268.9347879 | 2 |
| 3268.9378754 | 0 |
| 3268.9392204 | 3 |
| 3268.9419991 | 4 |
| 3268.9445492 | 2 |
| 3268.9459429 | 0 |
| 3268.9486248 | 2 |
| 3268.9496783 | 1 |
| 3268.9523738 | 1 |

| | |
|---|---|
| 3268.9539 | 3 |
| 3268.9566723 | 3 |
| 3268.9595516 | 3 |
| 3268.9605226 | 1 |
| 3268.9631652 | 0 |
| 3268.966424 | 1 |
| 3268.9671545 | 4 |
| 3268.9702987 | 0 |
| 3268.9714655 | 0 |
| 3268.9744048 | 3 |
| 3268.9769948 | 2 |
| 3268.9778602 | 1 |
| 3268.9805839 | 3 |
| 3268.9825737 | 0 |
| 3268.9848323 | 2 |
| 3268.9876233 | 3 |
| 3268.9888677 | 5 |
| 3268.9921395 | 6 |
| 3268.9922116 | 1 |
| 3268.9952548 | 2 |
| 3268.9984755 | 1 |
| 3268.9998698 | 7 |
| 3269.0026073 | 3 |
| 3269.0047521 | 4 |
| 3269.00663 | 5 |
| 3269.0097214 | 2 |
| 3269.0107405 | 1 |
| 3269.0134159 | 2 |
| 3269.0148774 | 2 |
| 3269.0175264 | 1 |
| 3269.0202541 | 0 |
| 3269.0218426 | 2 |
| 3269.0242098 | 0 |
| 3269.0259175 | 2 |
| 3269.0276133 | 3 |
| 3269.0314717 | 3 |
| 3269.0320356 | 0 |
| 3269.0350638 | 2 |
| 3269.0364325 | 3 |
| 3269.0391092 | 1 |
| 3269.0402557 | 2 |
| 3269.0429063 | 1 |
| 3269.0449659 | 0 |
| 3269.0465404 | 1 |
| 3269.0502141 | 1 |
| 3269.0524273 | 2 |
| 3269.0534548 | 2 |
| 3269.056399 | 1 |
| 3269.057669 | 1 |
| 3269.0602043 | 1 |
| 3269.0629506 | 1 |
| 3269.0642408 | 2 |
| 3269.067293 | 2 |
| 3269.0684028 | 1 |
| 3269.071229 | 2 |
| 3269.074222 | 2 |
| 3269.0754708 | 3 |
| 3269.0776329 | 2 |
| 3269.0796274 | 3 |
| 3269.08218 | 3 |
| 3269.083363 | 2 |
| 3269.0862987 | 1 |
| 3269.0887642 | 2 |
| 3269.0898477 | 2 |
| 3269.0928087 | 2 |
| 3269.0939865 | 2 |
| 3269.0969761 | 3 |
| 3269.0998403 | 1 |
| 3269.1004825 | 1 |
| 3269.1035473 | 1 |
| 3269.1065395 | 0 |
| 3269.107782 | 3 |
| 3269.1097593 | 3 |
| 3269.1116179 | 3 |
| 3269.1145592 | 3 |
| 3269.1171695 | 1 |
| 3269.118297 | 2 |
| 3269.1215102 | 1 |
| 3269.1224502 | 2 |
| 3269.1249118 | 1 |
| 3269.1268692 | 2 |
| 3269.1283534 | 2 |
| 3269.1317923 | 4 |
| 3269.1332672 | 1 |
| 3269.1360425 | 4 |
| 3269.137909 | 2 |
| 3269.1398069 | 2 |
| 3269.1426258 | 2 |
| 3269.1439007 | 6 |
| 3269.1466576 | 0 |
| 3269.1494982 | 3 |
| 3269.1502009 | 0 |
| 3269.1531542 | 3 |
| 3269.1552182 | 1 |
| 3269.1576581 | 1 |
| 3269.1600612 | 3 |
| 3269.1619846 | 4 |
| 3269.1642117 | 3 |
| 3269.165191 | 2 |
| 3269.1684597 | 3 |
| 3269.171685 | 1 |
| 3269.1722926 | 1 |
| 3269.1747648 | 3 |
| 3269.1763998 | 1 |
| 3269.1787332 | 3 |
| 3269.1814241 | 1 |
| 3269.1827349 | 1 |
| 3269.1857461 | 1 |
| 3269.1871318 | 1 |
| 3269.1894459 | 3 |
| 3269.1924831 | 2 |
| 3269.1938668 | 2 |
| 3269.1966626 | 2 |
| 3269.1984682 | 1 |
| 3269.201247 | 2 |
| 3269.2037785 | 3 |
| 3269.2052371 | 3 |
| 3269.2075413 | 1 |
| 3269.2084152 | 1 |
| 3269.2110562 | 1 |
| 3269.2140647 | 2 |
| 3269.2151061 | 3 |
| 3269.2182597 | 2 |
| 3269.2193662 | 0 |
| 3269.2224205 | 3 |
| 3269.2247485 | 0 |
| 3269.2265722 | 1 |
| 3269.2292091 | 3 |
| 3269.230116 | 2 |
| 3269.2329688 | 3 |
| 3269.2354499 | 2 |
| 3269.2366633 | 2 |
| 3269.2395544 | 1 |
| 3269.2423523 | 4 |
| 3269.2440263 | 8 |
| 3269.2471921 | 3 |
| 3269.2478615 | 4 |
| 3269.2508996 | 3 |
| 3269.2537263 | 2 |
| 3269.2545974 | 3 |
| 3269.2576141 | 2 |
| 3269.2587616 | 2 |
| 3269.2614268 | 0 |
| 3269.2627179 | 0 |
| 3269.2654791 | 2 |
| 3269.268118 | 2 |
| 3269.2688566 | 3 |
| 3269.2717927 | 5 |
| 3269.2751434 | 2 |
| 3269.2759456 | 4 |
| 3269.2794888 | 2 |
| 3269.2805372 | 2 |
| 3269.2836915 | 2 |
| 3269.2854695 | 0 |
| 3269.2879256 | 3 |
| 3269.2901757 | 0 |
| 3269.2921901 | 1 |
| 3269.2943079 | 0 |
| 3269.2969955 | 1 |
| 3269.2979533 | 3 |
| 3269.3012529 | 3 |
| 3269.3016144 | 1 |
| 3269.3046857 | 3 |
| 3269.3075383 | 0 |
| 3269.308837 | 0 |
| 3269.3115535 | 4 |
| 3269.3133765 | 1 |
| 3269.3164076 | 4 |
| 3269.3192845 | 3 |
| 3269.3197612 | 5 |
| 3269.3226749 | 3 |
| 3269.3235805 | 2 |
| 3269.3262018 | 1 |
| 3269.3293099 | 6 |
| 3269.3311268 | 3 |
| 3269.3336131 | 2 |
| 3269.3351717 | 3 |
| 3269.338296 | 3 |
| 3269.340905 | 5 |
| 3269.3414607 | 4 |
| 3269.3451064 | 4 |
| 3269.3461749 | 3 |
| 3269.3493846 | 2 |
| 3269.3501438 | 2 |
| 3269.3527794 | 1 |
| 3269.3552006 | 1 |
| 3269.3561875 | 4 |
| 3269.3591018 | 2 |
| 3269.3610845 | 1 |
| 3269.3638513 | 4 |
| 3269.3668263 | 1 |
| 3269.3679209 | 1 |
| 3269.3712178 | 3 |
| 3269.3720541 | 3 |
| 3269.3746012 | 2 |
| 3269.3770631 | 2 |
| 3269.3781415 | 2 |
| 3269.3792075 | 3 |
| 3269.3815922 | 3 |
| 3269.3816936 | 2 |
| 3269.3837166 | 3 |
| 3269.384386 | 5 |
| 3269.3865851 | 3 |
| 3269.387177 | 3 |
| 3269.3883011 | 1 |
| 3269.3901147 | 3 |
| 3269.3907044 | 4 |
| 3269.3919322 | 4 |
| 3269.3931776 | 2 |
| 3269.3943922 | 2 |
| 3269.3953639 | 3 |
| 3269.3973504 | 3 |
| 3269.3985007 | 3 |
| 3269.3994166 | 0 |
| 3269.4005766 | 3 |
| 3269.4024503 | 6 |
| 3269.402516 | 1 |
| 3269.4047497 | 3 |
| 3269.4053752 | 2 |
| 3269.4075005 | 4 |
| 3269.4086285 | 1 |
| 3269.4092377 | 1 |
| 3269.4111844 | 0 |
| 3269.4125141 | 0 |
| 3269.4126771 | 1 |
| 3269.4147696 | 1 |
| 3269.4155834 | 5 |
| 3269.4169636 | 1 |
| 3269.4181297 | 6 |
| 3269.4200515 | 4 |
| 3269.4213099 | 4 |
| 3269.4218363 | 7 |
| 3269.4235442 | 7 |
| 3269.4255339 | 14 |
| 3269.4255434 | 6 |
| 3269.4273921 | 4 |
| 3269.4292427 | 3 |
| 3269.4292548 | 1 |
| 3269.4313704 | 1 |
| 3269.4320152 | 2 |
| 3269.4341283 | 1 |
| 3269.4347232 | 0 |
| 3269.4363199 | 3 |
| 3269.4379584 | 8 |
| 3269.4387735 | 3 |
| 3269.4403107 | 0 |
| 3269.440822 | 1 |
| 3269.4420239 | 9 |
| 3269.4434673 | 9 |
| 3269.4442648 | 9 |
| 3269.4464737 | 3 |
| 3269.4468947 | 1 |
| 3269.4475261 | 1 |
| 3269.4500551 | 3 |
| 3269.450729 | 4 |
| 3269.4517622 | 4 |
| 3269.453487 | 3 |
| 3269.4547531 | 3 |
| 3269.4549473 | 7 |
| 3269.4570119 | 1 |
| 3269.4588794 | 1 |
| 3269.459017 | 4 |
| 3269.4604772 | 1 |
| 3269.4618426 | 3 |
| 3269.4631029 | 1 |
| 3269.4651356 | 0 |
| 3269.4651828 | 0 |
| 3269.467374 | 3 |
| 3269.4691277 | 5 |
| 3269.4691833 | 2 |
| 3269.4709869 | 2 |
| 3269.4720088 | 5 |
| 3269.473891 | 0 |
| 3269.4750434 | 2 |
| 3269.4759765 | 3 |
| 3269.4775548 | 1 |
| 3269.4785891 | 2 |
| 3269.4799181 | 3 |
| 3269.4814127 | 3 |
| 3269.4827221 | 1 |
| 3269.4836004 | 5 |
| 3269.4849425 | 3 |
| 3269.4867462 | 0 |
| 3269.487729 | 0 |
| 3269.4886594 | 2 |
| 3269.4898693 | 3 |
| 3269.4899091 | 2 |
| 3269.4922704 | 2 |
| 3269.4932456 | 1 |
| 3269.4939413 | 2 |
| 3269.4966906 | 1 |
| 3269.4971901 | 0 |
| 3269.4980313 | 3 |
| 3269.4996363 | 4 |
| 3269.5005476 | 2 |
| 3269.5025919 | 0 |
| 3269.5028463 | 2 |
| 3269.5052575 | 2 |
| 3269.5054646 | 4 |
| 3269.5070652 | 1 |
| 3269.5082585 | 2 |
| 3269.5091694 | 1 |
| 3269.5106713 | 3 |
| 3269.5126365 | 2 |
| 3269.5133805 | 2 |
| 3269.515254 | 4 |
| 3269.515683 | 3 |
| 3269.5172057 | 2 |
| 3269.5195448 | 4 |
| 3269.5198363 | 1 |
| 3269.5208529 | 5 |
| 3269.5217192 | 4 |
| 3269.5237602 | 3 |
| 3269.5249174 | 2 |
| 3269.5259089 | 2 |
| 3269.5274481 | 1 |
| 3269.5282173 | 1 |
| 3269.5297474 | 2 |
| 3269.5307578 | 0 |
| 3269.5321142 | 2 |
| 3269.5334848 | 4 |
| 3269.5342845 | 3 |
| 3269.536199 | 6 |
| 3269.5370157 | 1 |
| 3269.5387928 | 1 |
| 3269.5399807 | 3 |
| 3269.5403845 | 1 |
| 3269.5420277 | 2 |
| 3269.5435618 | 5 |
| 3269.543896 | 0 |
| 3269.5460403 | 2 |
| 3269.5476309 | 3 |
| 3269.5489676 | 4 |
| 3269.5496423 | 2 |
| 3269.5507065 | 1 |
| 3269.5522427 | 2 |
| 3269.5528086 | 7 |
| 3269.5546799 | 2 |
| 3269.5558504 | 1 |
| 3269.5567054 | 0 |
| 3269.5586704 | 3 |
| 3269.5593905 | 0 |
| 3269.5596527 | 1 |
| 3269.5602125 | 3 |
| 3269.5605196 | 1 |
| 3269.5607577 | 6 |
| 3269.5610596 | 3 |
| 3269.5613321 | 3 |
| 3269.5615646 | 3 |
| 3269.5621078 | 1 |
| 3269.5624159 | 1 |
| 3269.5627313 | 1 |
| 3269.5629971 | 5 |
| 3269.5632868 | 3 |
| 3269.5634274 | 2 |
| 3269.5638648 | 2 |
| 3269.5639005 | 2 |
| 3269.5639788 | 3 |
| 3269.5643115 | 3 |
| 3269.5646848 | 0 |
| 3269.5648633 | 1 |
| 3269.5653862 | 4 |
| 3269.5658537 | 1 |

| | |
|---|---|
| 3269.5660366 | 5 |
| 3269.5662636 | 3 |
| 3269.5664124 | 2 |
| 3269.566655 | 1 |
| 3269.5670657 | 4 |
| 3269.5672683 | 5 |
| 3269.5673814 | 2 |
| 3269.567696 | 2 |
| 3269.5680389 | 0 |
| 3269.5680445 | 2 |
| 3269.5684313 | 6 |
| 3269.5684927 | 4 |
| 3269.5686191 | 1 |
| 3269.5691209 | 2 |
| 3269.569225 | 1 |
| 3269.5702307 | 3 |
| 3269.5702453 | 2 |
| 3269.5703076 | 3 |
| 3269.5703229 | 2 |
| 3269.5708756 | 3 |
| 3269.5710464 | 3 |
| 3269.571223 | 2 |
| 3269.5715534 | 2 |
| 3269.5721866 | 0 |
| 3269.5723397 | 3 |
| 3269.5723779 | 1 |
| 3269.5727437 | 3 |
| 3269.5727651 | 3 |
| 3269.5732556 | 0 |
| 3269.573405 | 5 |
| 3269.5738915 | 2 |
| 3269.5740673 | 1 |
| 3269.5741138 | 2 |
| 3269.5746673 | 2 |
| 3269.57481 | 1 |
| 3269.5752109 | 2 |
| 3269.5753064 | 0 |
| 3269.5754189 | 3 |
| 3269.5754307 | 7 |
| 3269.5764244 | 2 |
| 3269.5766362 | 9 |
| 3269.5768877 | 1 |
| 3269.5769141 | 3 |
| 3269.5769687 | 4 |
| 3269.5769779 | 12 |
| 3269.5772934 | 9 |
| 3269.5777993 | 24 |
| 3269.5778713 | 21 |
| 3269.5781554 | 26 |
| 3269.5787447 | 42 |
| 3269.5792482 | 38 |
| 3269.5792545 | 50 |
| 3269.579325 | 34 |
| 3269.5795633 | 44 |
| 3269.5802832 | 49 |
| 3269.580331 | 53 |
| 3269.5803643 | 48 |
| 3269.5803726 | 38 |
| 3269.58096 | 61 |
| 3269.5812117 | 38 |
| 3269.5812672 | 54 |
| 3269.581644 | 53 |
| 3269.5818951 | 42 |
| 3269.5821109 | 44 |
| 3269.582776 | 52 |
| 3269.5827978 | 46 |
| 3269.5827989 | 38 |
| 3269.5828344 | 52 |
| 3269.5829337 | 43 |
| 3269.5835197 | 39 |
| 3269.5837456 | 54 |
| 3269.5839621 | 59 |
| 3269.5840444 | 53 |
| 3269.584402 | 55 |
| 3269.5848247 | 61 |
| 3269.584887 | 50 |
| 3269.5849764 | 46 |
| 3269.5854463 | 44 |
| 3269.5856369 | 40 |
| 3269.5857345 | 18 |
| 3269.5857555 | 31 |
| 3269.5859383 | 9 |
| 3269.5860111 | 53 |
| 3269.5863123 | 14 |
| 3269.5870631 | 12 |
| 3269.5871103 | 3 |
| 3269.5872874 | 4 |
| 3269.5873689 | 19 |
| 3269.5877436 | 3 |
| 3269.5885896 | 5 |
| 3269.5886277 | 5 |
| 3269.5886383 | 1 |
| 3269.5888492 | 1 |
| 3269.588993 | 1 |
| 3269.5891065 | 3 |
| 3269.5892183 | 2 |
| 3269.5897928 | 5 |
| 3269.5900258 | 3 |
| 3269.5901118 | 2 |
| 3269.590304 | 2 |
| 3269.5909175 | 2 |
| 3269.5909661 | 3 |
| 3269.590984 | 1 |
| 3269.5913262 | 4 |
| 3269.5914813 | 3 |
| 3269.5915309 | 0 |
| 3269.5919344 | 1 |
| 3269.5920048 | 4 |
| 3269.5925096 | 6 |
| 3269.592685 | 4 |
| 3269.5936755 | 5 |
| 3269.5940879 | 1 |
| 3269.5953776 | 1 |
| 3269.5964921 | 1 |
| 3269.5969112 | 3 |
| 3269.5975821 | 4 |
| 3269.5996342 | 1 |
| 3269.6002704 | 2 |
| 3269.6008293 | 1 |
| 3269.6025554 | 1 |
| 3269.603298 | 3 |
| 3269.6039136 | 0 |
| 3269.604824 | 0 |
| 3269.6062274 | 2 |
| 3269.6064433 | 2 |
| 3269.6082037 | 3 |
| 3269.6083389 | 2 |
| 3269.6091811 | 5 |
| 3269.6107708 | 2 |
| 3269.6109148 | 1 |
| 3269.6129712 | 1 |
| 3269.6132492 | 2 |
| 3269.614398 | 1 |
| 3269.615548 | 2 |
| 3269.6162771 | 1 |
| 3269.6175086 | 4 |
| 3269.6180714 | 2 |
| 3269.6189195 | 3 |
| 3269.6199781 | 1 |
| 3269.6204578 | 0 |
| 3269.6218082 | 2 |
| 3269.6220065 | 3 |
| 3269.6233898 | 1 |
| 3269.6243446 | 1 |
| 3269.6246149 | 1 |
| 3269.6262969 | 3 |
| 3269.6270778 | 1 |
| 3269.6273703 | 1 |
| 3269.6293156 | 3 |
| 3269.6302372 | 3 |
| 3269.6308875 | 3 |
| 3269.6316203 | 1 |
| 3269.6324842 | 0 |
| 3269.6336739 | 2 |
| 3269.6337745 | 0 |
| 3269.6358417 | 2 |
| 3269.6359006 | 3 |
| 3269.6376841 | 0 |
| 3269.6381878 | 1 |
| 3269.6383494 | 0 |
| 3269.6399653 | 2 |
| 3269.6406137 | 3 |
| 3269.6418503 | 2 |
| 3269.6434697 | 3 |
| 3269.6435189 | 2 |
| 3269.644959 | 3 |
| 3269.6450681 | 1 |
| 3269.6470456 | 4 |
| 3269.6475483 | 2 |
| 3269.6486213 | 3 |
| 3269.6495908 | 1 |
| 3269.6501516 | 1 |
| 3269.6512997 | 3 |
| 3269.651648 | 2 |
| 3269.6530065 | 2 |
| 3269.6545881 | 1 |
| 3269.6548481 | 3 |
| 3269.6556323 | 3 |
| 3269.656845 | 4 |
| 3269.6581295 | 0 |
| 3269.6588718 | 1 |
| 3269.6597192 | 0 |
| 3269.6612804 | 0 |
| 3269.6621393 | 1 |
| 3269.6623709 | 3 |
| 3269.6635056 | 1 |
| 3269.6651321 | 2 |
| 3269.6652047 | 2 |
| 3269.6667251 | 2 |
| 3269.6676734 | 2 |
| 3269.6687586 | 2 |
| 3269.6691996 | 2 |
| 3269.6699367 | 0 |
| 3269.6710051 | 4 |
| 3269.671253 | 3 |
| 3269.6726933 | 2 |
| 3269.6737483 | 1 |
| 3269.6750976 | 2 |
| 3269.6760011 | 2 |
| 3269.6765089 | 4 |
| 3269.677773 | 0 |
| 3269.678103 | 3 |
| 3269.6793223 | 1 |
| 3269.6799152 | 2 |
| 3269.6807977 | 1 |
| 3269.6823627 | 1 |
| 3269.6834146 | 3 |
| 3269.683866 | 3 |
| 3269.6848612 | 2 |
| 3269.6853019 | 2 |
| 3269.6866069 | 1 |
| 3269.6872253 | 3 |
| 3269.6884269 | 4 |
| 3269.6896111 | 3 |
| 3269.6908494 | 1 |
| 3269.691616 | 1 |
| 3269.6923127 | 0 |
| 3269.6934785 | 3 |
| 3269.6950535 | 2 |
| 3269.6951313 | 4 |
| 3269.6964452 | 2 |
| 3269.6968567 | 4 |
| 3269.6981097 | 1 |
| 3269.699451 | 2 |
| 3269.6999955 | 1 |
| 3269.7013394 | 4 |
| 3269.702193 | 2 |
| 3269.7029219 | 1 |
| 3269.7040552 | 1 |
| 3269.704587 | 5 |
| 3269.7061357 | 2 |
| 3269.7061809 | 4 |
| 3269.7078986 | 2 |
| 3269.7086844 | 4 |
| 3269.7093842 | 3 |
| 3269.7104342 | 1 |
| 3269.7110336 | 3 |
| 3269.7122623 | 4 |
| 3269.7136955 | 5 |
| 3269.7140501 | 3 |
| 3269.7146993 | 2 |
| 3269.7152527 | 1 |
| 3269.7166583 | 2 |
| 3269.717148 | 1 |
| 3269.7177513 | 1 |
| 3269.7195039 | 1 |
| 3269.7195506 | 4 |
| 3269.7212838 | 3 |
| 3269.7226386 | 3 |
| 3269.7228716 | 2 |
| 3269.7244876 | 4 |
| 3269.7247489 | 3 |
| 3269.7256141 | 0 |
| 3269.7273784 | 2 |
| 3269.7275668 | 1 |
| 3269.7291193 | 7 |
| 3269.7297737 | 3 |
| 3269.7308259 | 7 |
| 3269.7310461 | 3 |
| 3269.7328433 | 7 |
| 3269.7341455 | 4 |
| 3269.7342159 | 8 |
| 3269.7360437 | 4 |
| 3269.7364254 | 1 |
| 3269.7373156 | 4 |
| 3269.7387339 | 2 |
| 3269.7390708 | 1 |
| 3269.7407055 | 0 |
| 3269.7410332 | 2 |
| 3269.7416131 | 0 |
| 3269.7429376 | 0 |
| 3269.7437611 | 1 |
| 3269.7451341 | 2 |
| 3269.7462481 | 2 |
| 3269.7478003 | 3 |
| 3269.7480689 | 2 |
| 3269.7487167 | 3 |
| 3269.7496123 | 4 |
| 3269.7499057 | 5 |
| 3269.7506496 | 2 |
| 3269.75228 | 1 |
| 3269.7525046 | 5 |
| 3269.7528013 | 3 |
| 3269.752895 | 2 |
| 3269.7542213 | 2 |
| 3269.7553628 | 1 |
| 3269.7555802 | 3 |
| 3269.7573007 | 4 |
| 3269.7583344 | 1 |
| 3269.7584737 | 2 |
| 3269.7602605 | 3 |
| 3269.7608769 | 3 |
| 3269.7609645 | 0 |
| 3269.7610922 | 1 |
| 3269.7635617 | 2 |
| 3269.7641215 | 2 |
| 3269.7642717 | 5 |
| 3269.7651713 | 2 |
| 3269.7662848 | 1 |
| 3269.7670805 | 0 |
| 3269.7673823 | 1 |
| 3269.7673904 | 4 |
| 3269.7676509 | 2 |
| 3269.7693213 | 4 |
| 3269.7694173 | 2 |
| 3269.7696933 | 4 |
| 3269.7700805 | 1 |
| 3269.7701688 | 1 |
| 3269.7705876 | 2 |
| 3269.7707365 | 2 |
| 3269.7725517 | 2 |
| 3269.7727959 | 4 |
| 3269.773022 | 4 |
| 3269.773075 | 0 |
| 3269.7732181 | 2 |
| 3269.7745797 | 3 |
| 3269.7747717 | 1 |
| 3269.7751267 | 1 |
| 3269.7765602 | 2 |
| 3269.777046 | 2 |
| 3269.7771112 | 2 |
| 3269.7773281 | 1 |
| 3269.7784836 | 4 |
| 3269.778782 | 1 |
| 3269.7791711 | 3 |
| 3269.7794207 | 2 |
| 3269.7801425 | 3 |
| 3269.7804834 | 4 |
| 3269.7805022 | 2 |
| 3269.7819733 | 2 |
| 3269.7820924 | 1 |
| 3269.7824203 | 3 |
| 3269.7828737 | 0 |
| 3269.7839577 | 3 |
| 3269.784266 | 4 |
| 3269.7847461 | 2 |
| 3269.7849677 | 4 |
| 3269.7853841 | 4 |
| 3269.7857161 | 4 |
| 3269.7857712 | 2 |
| 3269.7861135 | 1 |
| 3269.7869293 | 3 |
| 3269.787326 | 9 |
| 3269.7881536 | 0 |
| 3269.7887854 | 2 |
| 3269.7894801 | 0 |
| 3269.7896067 | 2 |
| 3269.7899561 | 3 |
| 3269.7902602 | 1 |
| 3269.7905077 | 3 |
| 3269.7918042 | 0 |
| 3269.7921281 | 3 |
| 3269.7921607 | 2 |
| 3269.7928421 | 0 |
| 3269.7932999 | 1 |
| 3269.7934178 | 1 |
| 3269.793755 | 3 |
| 3269.7947043 | 1 |
| 3269.7952024 | 1 |
| 3269.795386 | 2 |
| 3269.7963867 | 0 |
| 3269.7964534 | 4 |
| 3269.797404 | 2 |

| | |
|---|---|
| 3269.7975636 | 1 |
| 3269.7980944 | 1 |
| 3269.7984145 | 5 |
| 3269.7988068 | 2 |
| 3269.7992032 | 1 |
| 3269.7993806 | 2 |
| 3269.7999137 | 8 |
| 3269.8007899 | 3 |
| 3269.8009428 | 0 |
| 3269.8019647 | 1 |
| 3269.8021897 | 4 |
| 3269.8024139 | 3 |
| 3269.803155 | 1 |
| 3269.8035654 | 1 |
| 3269.803861 | 3 |
| 3269.8040941 | 3 |
| 3269.8043567 | 1 |
| 3269.8049204 | 1 |
| 3269.8062997 | 4 |
| 3269.8066787 | 7 |
| 3269.8067006 | 2 |
| 3269.8069218 | 4 |
| 3269.8072874 | 1 |
| 3269.8077471 | 1 |
| 3269.8081832 | 3 |
| 3269.8085193 | 0 |
| 3269.8089069 | 8 |
| 3269.8097418 | 3 |
| 3269.8098898 | 4 |
| 3269.8108919 | 3 |
| 3269.8113392 | 3 |
| 3269.8115432 | 4 |
| 3269.8121766 | 1 |
| 3269.8123532 | 1 |
| 3269.8127437 | 4 |
| 3269.8131373 | 1 |
| 3269.8136006 | 1 |
| 3269.8136426 | 5 |
| 3269.8147877 | 5 |
| 3269.8153674 | 3 |
| 3269.8154075 | 1 |
| 3269.8156368 | 1 |
| 3269.8161573 | 1 |
| 3269.8170823 | 2 |
| 3269.8172766 | 0 |
| 3269.8174356 | 2 |
| 3269.8183813 | 6 |
| 3269.8194931 | 4 |
| 3269.8195775 | 3 |
| 3269.8198247 | 2 |
| 3269.820792 | 4 |
| 3269.820837 | 0 |
| 3269.8208626 | 3 |
| 3269.8210847 | 4 |
| 3269.8224634 | 2 |
| 3269.8228778 | 3 |
| 3269.8230738 | 0 |
| 3269.8233659 | 1 |
| 3269.8243854 | 15 |
| 3269.8251872 | 50 |
| 3269.8252594 | 10 |
| 3269.825346 | 42 |
| 3269.8253479 | 10 |
| 3269.825667 | 61 |
| 3269.8265198 | 65 |
| 3269.8272018 | 72 |
| 3269.8275312 | 63 |
| 3269.8288973 | 65 |
| 3269.8292266 | 58 |
| 3269.8294494 | 44 |
| 3269.8295248 | 53 |
| 3269.8297548 | 48 |
| 3269.830456 | 51 |
| 3269.8315095 | 56 |
| 3269.8319014 | 50 |
| 3269.8321141 | 48 |
| 3269.8327881 | 65 |
| 3269.8329661 | 54 |
| 3269.8337753 | 52 |
| 3269.8349812 | 51 |
| 3269.8350713 | 56 |
| 3269.8353341 | 42 |
| 3269.8357012 | 14 |
| 3269.8358579 | 13 |
| 3269.8361337 | 20 |
| 3269.8367719 | 2 |
| 3269.8372923 | 4 |
| 3269.8380162 | 2 |
| 3269.8383641 | 5 |
| 3269.8384472 | 6 |
| 3269.8388385 | 2 |
| 3269.8400555 | 1 |
| 3269.8401081 | 1 |
| 3269.8409375 | 3 |
| 3269.8418223 | 1 |
| 3269.8418346 | 2 |
| 3269.8423062 | 3 |
| 3269.842513 | 3 |
| 3269.8426521 | 1 |
| 3269.8436588 | 1 |
| 3269.8445423 | 1 |
| 3269.8446538 | 1 |
| 3269.8446668 | 1 |
| 3269.8454423 | 0 |
| 3269.8458599 | 2 |
| 3269.8463977 | 1 |
| 3269.8468334 | 4 |
| 3269.8476932 | 3 |
| 3269.8479159 | 4 |
| 3269.848581 | 2 |
| 3269.8488925 | 2 |
| 3269.8489943 | 1 |
| 3269.8491089 | 1 |
| 3269.8499239 | 0 |
| 3269.8503593 | 3 |
| 3269.8509154 | 1 |
| 3269.8513394 | 3 |
| 3269.8513596 | 2 |
| 3269.8524653 | 1 |
| 3269.8525695 | 2 |
| 3269.8530804 | 5 |
| 3269.8536429 | 2 |
| 3269.8540877 | 4 |
| 3269.8546284 | 1 |
| 3269.8546537 | 2 |
| 3269.8550953 | 1 |
| 3269.8558914 | 7 |
| 3269.8564392 | 1 |
| 3269.8565743 | 2 |
| 3269.8570478 | 4 |
| 3269.8571653 | 2 |
| 3269.8572171 | 0 |
| 3269.8589512 | 2 |
| 3269.8590088 | 2 |
| 3269.8593339 | 3 |
| 3269.8600168 | 4 |
| 3269.8621464 | 2 |
| 3269.8637654 | 4 |
| 3269.8661676 | 3 |
| 3269.8682548 | 1 |
| 3269.8701109 | 0 |
| 3269.8719006 | 1 |
| 3269.8734591 | 4 |
| 3269.8757152 | 2 |
| 3269.8767824 | 1 |
| 3269.8791752 | 1 |
| 3269.8811681 | 2 |
| 3269.8827344 | 5 |
| 3269.8856614 | 1 |
| 3269.886582 | 2 |
| 3269.8893895 | 3 |
| 3269.8916305 | 1 |
| 3269.8924471 | 0 |
| 3269.8943688 | 3 |
| 3269.895595 | 1 |
| 3269.898093 | 3 |
| 3269.8991509 | 1 |
| 3269.9014119 | 1 |
| 3269.9038086 | 1 |
| 3269.9055462 | 0 |
| 3269.9066812 | 0 |
| 3269.9094883 | 2 |
| 3269.9103804 | 3 |
| 3269.9124512 | 1 |
| 3269.9144491 | 4 |
| 3269.9165626 | 3 |
| 3269.9189214 | 3 |
| 3269.9195186 | 4 |
| 3269.9229229 | 8 |
| 3269.9233896 | 3 |
| 3269.9257826 | 2 |
| 3269.9286781 | 5 |
| 3269.929787 | 3 |
| 3269.9324469 | 2 |
| 3269.9332104 | 2 |
| 3269.9356514 | 3 |
| 3269.9367177 | 0 |
| 3269.9390183 | 4 |
| 3269.9412594 | 5 |
| 3269.9439415 | 1 |
| 3269.9448649 | 1 |
| 3269.9470888 | 1 |
| 3269.9485442 | 3 |
| 3269.9509337 | 3 |
| 3269.9512085 | 3 |
| 3269.9535403 | 2 |
| 3269.9566951 | 5 |
| 3269.957078 | 2 |
| 3269.960142 | 2 |
| 3269.9609849 | 3 |
| 3269.9636582 | 3 |
| 3269.9658039 | 5 |
| 3269.9665364 | 3 |
| 3269.9696433 | 2 |
| 3269.9705786 | 1 |
| 3269.9733285 | 1 |
| 3269.975033 | 1 |
| 3269.9768236 | 0 |
| 3269.9789735 | 4 |
| 3269.9805637 | 2 |
| 3269.982516 | 1 |
| 3269.9851002 | 0 |
| 3269.9855514 | 2 |
| 3269.988206 | 1 |
| 3269.9896477 | 6 |
| 3269.9916079 | 2 |
| 3269.9927678 | 5 |
| 3269.995406 | 4 |
| 3269.9971702 | 1 |
| 3269.9981897 | 4 |
| 3270.0000986 | 4 |
| 3270.0031195 | 4 |
| 3270.0042076 | 0 |
| 3270.0067135 | 1 |
| 3270.0085404 | 3 |
| 3270.0103131 | 4 |
| 3270.0114372 | 3 |
| 3270.014493 | 1 |
| 3270.0169996 | 0 |
| 3270.0176732 | 4 |
| 3270.020307 | 1 |
| 3270.0211785 | 2 |
| 3270.0236719 | 0 |
| 3270.0259276 | 0 |
| 3270.0270031 | 0 |
| 3270.0291098 | 3 |
| 3270.0314514 | 0 |
| 3270.032832 | 3 |
| 3270.0343999 | 4 |
| 3270.0357903 | 1 |
| 3270.0384355 | 1 |
| 3270.0411288 | 3 |
| 3270.0414705 | 1 |
| 3270.0443949 | 2 |
| 3270.04554 | 1 |
| 3270.0468962 | 1 |
| 3270.0496447 | 2 |
| 3270.0506662 | 3 |
| 3270.053584 | 1 |
| 3270.0543777 | 1 |
| 3270.0568621 | 1 |
| 3270.0599114 | 4 |
| 3270.0612807 | 1 |
| 3270.0630606 | 4 |
| 3270.065082 | 3 |
| 3270.0672811 | 1 |
| 3270.0677507 | 5 |
| 3270.0705003 | 1 |
| 3270.0730686 | 2 |
| 3270.0751809 | 0 |
| 3270.0756665 | 3 |
| 3270.0781085 | 5 |
| 3270.0789566 | 1 |
| 3270.0816052 | 4 |
| 3270.082796 | 2 |
| 3270.0853762 | 2 |
| 3270.0877032 | 2 |
| 3270.0886537 | 1 |
| 3270.0901115 | 0 |
| 3270.0922569 | 3 |
| 3270.0944255 | 1 |
| 3270.0964147 | 4 |
| 3270.097853 | 6 |
| 3270.1006389 | 3 |
| 3270.1013426 | 5 |
| 3270.1037041 | 1 |
| 3270.1062224 | 2 |
| 3270.107506 | 2 |
| 3270.109935 | 4 |
| 3270.1112226 | 1 |
| 3270.1136028 | 5 |
| 3270.1162797 | 2 |
| 3270.1170128 | 5 |
| 3270.1191715 | 3 |
| 3270.120535 | 2 |
| 3270.1225195 | 2 |
| 3270.1248673 | 1 |
| 3270.1265736 | 1 |
| 3270.1282825 | 2 |
| 3270.1292434 | 3 |
| 3270.1316899 | 1 |
| 3270.1342691 | 2 |
| 3270.1354699 | 5 |
| 3270.1377414 | 2 |
| 3270.1393227 | 2 |
| 3270.1411995 | 2 |
| 3270.142302 | 1 |
| 3270.1445001 | 3 |
| 3270.1466733 | 2 |
| 3270.1488283 | 2 |
| 3270.1506924 | 2 |
| 3270.15307 | 2 |
| 3270.1545193 | 0 |
| 3270.1563532 | 2 |
| 3270.1580029 | 3 |
| 3270.1599713 | 3 |
| 3270.1621747 | 0 |
| 3270.163641 | 2 |
| 3270.1655933 | 1 |
| 3270.1667088 | 5 |
| 3270.1693927 | 3 |
| 3270.1717437 | 0 |
| 3270.1723066 | 3 |
| 3270.1753458 | 0 |
| 3270.1759119 | 2 |
| 3270.1791159 | 2 |
| 3270.1810341 | 5 |
| 3270.1823275 | 1 |
| 3270.1845102 | 2 |
| 3270.1855185 | 0 |
| 3270.1880143 | 1 |
| 3270.189844 | 2 |
| 3270.1918082 | 0 |
| 3270.1932103 | 2 |
| 3270.1952208 | 1 |
| 3270.1967209 | 2 |
| 3270.1994765 | 1 |
| 3270.1998568 | 5 |
| 3270.2032255 | 3 |
| 3270.2054677 | 4 |
| 3270.2061957 | 2 |
| 3270.2087548 | 1 |
| 3270.20974 | 3 |
| 3270.212126 | 4 |
| 3270.2143763 | 0 |
| 3270.2153167 | 0 |
| 3270.218082 | 2 |
| 3270.2191114 | 1 |
| 3270.2211624 | 1 |
| 3270.2235321 | 1 |
| 3270.2244167 | 3 |
| 3270.2271572 | 2 |
| 3270.227804 | 3 |
| 3270.2310204 | 3 |
| 3270.231434 | 0 |
| 3270.2341615 | 3 |
| 3270.2364802 | 1 |
| 3270.2379148 | 2 |
| 3270.2400161 | 1 |
| 3270.2417222 | 1 |
| 3270.2433708 | 0 |
| 3270.2461834 | 1 |
| 3270.2467095 | 1 |
| 3270.2492434 | 1 |
| 3270.2515727 | 1 |
| 3270.2523758 | 1 |
| 3270.254423 | 0 |
| 3270.2561632 | 3 |
| 3270.2582821 | 2 |
| 3270.2610631 | 1 |
| 3270.2619277 | 1 |
| 3270.263274 | 2 |
| 3270.2641591 | 4 |
| 3270.2666824 | 1 |
| 3270.2694018 | 1 |
| 3270.2702177 | 1 |
| 3270.2728078 | 1 |
| 3270.2740431 | 0 |
| 3270.2757919 | 2 |
| 3270.2788876 | 5 |
| 3270.2799616 | 6 |
| 3270.282237 | 3 |
| 3270.2837693 | 5 |

| | |
|---|---|
| 3270.2862134 | 5 |
| 3270.2873649 | 0 |
| 3270.2892376 | 0 |
| 3270.2918189 | 1 |
| 3270.2931945 | 1 |
| 3270.2950255 | 2 |
| 3270.2977785 | 3 |
| 3270.2989565 | 4 |
| 3270.3008714 | 3 |
| 3270.3024179 | 2 |
| 3270.3047132 | 7 |
| 3270.3055677 | 1 |
| 3270.3077652 | 3 |
| 3270.3105061 | 0 |
| 3270.3111772 | 0 |
| 3270.3141967 | 1 |
| 3270.3156389 | 0 |
| 3270.3170961 | 3 |
| 3270.31918 | 3 |
| 3270.3196015 | 2 |
| 3270.3218525 | 1 |
| 3270.3240814 | 2 |
| 3270.3263787 | 1 |
| 3270.3284694 | 2 |
| 3270.3298659 | 2 |
| 3270.3325916 | 6 |
| 3270.3333225 | 1 |
| 3270.3358755 | 4 |
| 3270.3384623 | 1 |
| 3270.3390467 | 3 |
| 3270.3424577 | 2 |
| 3270.3429479 | 0 |
| 3270.3451319 | 2 |
| 3270.3463968 | 3 |
| 3270.3492511 | 3 |
| 3270.3514696 | 3 |
| 3270.3515346 | 0 |
| 3270.354748 | 1 |
| 3270.3554703 | 2 |
| 3270.3577989 | 0 |
| 3270.3601044 | 2 |
| 3270.3611784 | 3 |
| 3270.3634292 | 1 |
| 3270.364898 | 4 |
| 3270.3663757 | 1 |
| 3270.3692821 | 1 |
| 3270.3707435 | 1 |
| 3270.3735812 | 3 |
| 3270.3750447 | 3 |
| 3270.3762532 | 3 |
| 3270.3782278 | 4 |
| 3270.379963 | 0 |
| 3270.3821726 | 3 |
| 3270.3835669 | 5 |
| 3270.3860652 | 3 |
| 3270.3883071 | 1 |
| 3270.3890258 | 2 |
| 3270.3920344 | 3 |
| 3270.3924128 | 4 |
| 3270.395146 | 3 |
| 3270.3972119 | 4 |
| 3270.399361 | 2 |
| 3270.4005933 | 1 |
| 3270.4025665 | 1 |
| 3270.4046795 | 3 |
| 3270.4071019 | 0 |
| 3270.4078508 | 2 |
| 3270.4103444 | 1 |
| 3270.4110079 | 0 |
| 3270.4135288 | 5 |
| 3270.4155313 | 6 |
| 3270.4166176 | 1 |
| 3270.4200364 | 1 |
| 3270.4204219 | 2 |
| 3270.4228582 | 6 |
| 3270.4252142 | 7 |
| 3270.426681 | 3 |
| 3270.4290836 | 5 |
| 3270.4303224 | 3 |
| 3270.4324942 | 3 |
| 3270.4345233 | 2 |
| 3270.4352246 | 3 |
| 3270.4379135 | 3 |
| 3270.4392903 | 2 |
| 3270.4412685 | 0 |
| 3270.4435518 | 0 |
| 3270.4450467 | 0 |
| 3270.4466212 | 1 |
| 3270.448265 | 6 |
| 3270.4508526 | 1 |
| 3270.452746 | 3 |
| 3270.4539819 | 0 |
| 3270.4574016 | 5 |
| 3270.4576391 | 4 |
| 3270.4598424 | 1 |
| 3270.4622767 | 1 |
| 3270.4632872 | 2 |
| 3270.4651302 | 4 |
| 3270.4677084 | 2 |
| 3270.4691227 | 1 |
| 3270.4719903 | 7 |
| 3270.4736887 | 3 |
| 3270.4753942 | 3 |
| 3270.4765592 | 1 |
| 3270.4785247 | 4 |
| 3270.4813877 | 3 |
| 3270.4821809 | 1 |
| 3270.4847263 | 1 |
| 3270.4865115 | 3 |
| 3270.4878281 | 3 |
| 3270.4904482 | 2 |
| 3270.4914867 | 1 |
| 3270.4941657 | 0 |
| 3270.496109 | 6 |
| 3270.4968661 | 0 |
| 3270.5000355 | 1 |
| 3270.5007143 | 3 |
| 3270.5028625 | 4 |
| 3270.505911 | 2 |
| 3270.506253 | 1 |
| 3270.5079152 | 2 |
| 3270.5090206 | 5 |
| 3270.5097647 | 0 |
| 3270.5115512 | 3 |
| 3270.5122994 | 2 |
| 3270.5147582 | 2 |
| 3270.5147849 | 2 |
| 3270.5155258 | 3 |
| 3270.5160662 | 3 |
| 3270.5184202 | 3 |
| 3270.5185855 | 2 |
| 3270.5195087 | 6 |
| 3270.5202474 | 5 |
| 3270.5221109 | 2 |
| 3270.5229332 | 5 |
| 3270.5244836 | 1 |
| 3270.5252331 | 4 |
| 3270.5258587 | 1 |
| 3270.527078 | 1 |
| 3270.5283904 | 4 |
| 3270.5296257 | 1 |
| 3270.529692 | 1 |
| 3270.5314186 | 3 |
| 3270.5319533 | 4 |
| 3270.5336047 | 4 |
| 3270.5337938 | 1 |
| 3270.5346297 | 3 |
| 3270.5364945 | 2 |
| 3270.5365186 | 2 |
| 3270.5377261 | 2 |
| 3270.5392816 | 4 |
| 3270.5400328 | 4 |
| 3270.5403742 | 2 |
| 3270.542566 | 4 |
| 3270.5429269 | 2 |
| 3270.5433162 | 3 |
| 3270.5436105 | 2 |
| 3270.5465089 | 3 |
| 3270.5472943 | 1 |
| 3270.5478627 | 3 |
| 3270.5485284 | 2 |
| 3270.5497263 | 0 |
| 3270.5507153 | 1 |
| 3270.5524521 | 1 |
| 3270.5534552 | 2 |
| 3270.5541292 | 1 |
| 3270.5551394 | 4 |
| 3270.5559026 | 4 |
| 3270.5581533 | 3 |
| 3270.5583723 | 2 |
| 3270.5593898 | 1 |
| 3270.5594012 | 3 |
| 3270.5617971 | 1 |
| 3270.561995 | 1 |
| 3270.5632689 | 1 |
| 3270.564203 | 2 |
| 3270.5658264 | 4 |
| 3270.5659139 | 1 |
| 3270.5664969 | 2 |
| 3270.5686678 | 1 |
| 3270.5695053 | 4 |
| 3270.5703883 | 3 |
| 3270.5719209 | 1 |
| 3270.5730924 | 2 |
| 3270.5732011 | 3 |
| 3270.5755178 | 4 |
| 3270.5762206 | 2 |
| 3270.5764814 | 1 |
| 3270.5766369 | 1 |
| 3270.5787878 | 3 |
| 3270.5792674 | 3 |
| 3270.5809257 | 3 |
| 3270.5810684 | 1 |
| 3270.5822553 | 2 |
| 3270.5838779 | 1 |
| 3270.5847398 | 3 |
| 3270.58646 | 1 |
| 3270.5866907 | 3 |
| 3270.5875873 | 0 |
| 3270.5882149 | 2 |
| 3270.590147 | 3 |
| 3270.5902929 | 1 |
| 3270.5913649 | 1 |
| 3270.5916848 | 2 |
| 3270.5930057 | 2 |
| 3270.5943971 | 2 |
| 3270.5957934 | 2 |
| 3270.5968229 | 3 |
| 3270.597333 | 6 |
| 3270.5979893 | 1 |
| 3270.6002399 | 1 |
| 3270.601321 | 2 |
| 3270.6017485 | 4 |
| 3270.6024048 | 2 |
| 3270.6026447 | 5 |
| 3270.6054535 | 3 |
| 3270.6082445 | 3 |
| 3270.6093615 | 2 |
| 3270.6118007 | 2 |
| 3270.6132314 | 2 |
| 3270.6163785 | 2 |
| 3270.6187927 | 0 |
| 3270.6206364 | 3 |
| 3270.6229551 | 2 |
| 3270.6245252 | 2 |
| 3270.6270238 | 4 |
| 3270.6296086 | 2 |
| 3270.630502 | 3 |
| 3270.6336951 | 4 |
| 3270.6346731 | 2 |
| 3270.6373643 | 3 |
| 3270.6405214 | 7 |
| 3270.6420906 | 5 |
| 3270.6444596 | 2 |
| 3270.6458831 | 3 |
| 3270.6492084 | 2 |
| 3270.650645 | 3 |
| 3270.6531526 | 6 |
| 3270.6555963 | 4 |
| 3270.6568662 | 4 |
| 3270.659657 | 3 |
| 3270.6622968 | 1 |
| 3270.6642558 | 1 |
| 3270.6662986 | 0 |
| 3270.6675878 | 2 |
| 3270.6702171 | 3 |
| 3270.6736785 | 2 |
| 3270.6741416 | 3 |
| 3270.6773071 | 2 |
| 3270.6776581 | 2 |
| 3270.6805554 | 1 |
| 3270.6834198 | 5 |
| 3270.6847888 | 4 |
| 3270.6878506 | 1 |
| 3270.6890231 | 3 |
| 3270.6924485 | 2 |
| 3270.6944716 | 4 |
| 3270.6960996 | 1 |
| 3270.698487 | 4 |
| 3270.7005393 | 2 |
| 3270.702903 | 2 |
| 3270.7042098 | 1 |
| 3270.7072339 | 4 |
| 3270.7102467 | 4 |
| 3270.7107602 | 2 |
| 3270.7140384 | 0 |
| 3270.7143195 | 3 |
| 3270.7176153 | 3 |
| 3270.7202915 | 3 |
| 3270.721784 | 2 |
| 3270.724016 | 2 |
| 3270.7262622 | 2 |
| 3270.7285259 | 0 |
| 3270.7311443 | 3 |
| 3270.7326366 | 2 |
| 3270.7349252 | 2 |
| 3270.7375982 | 2 |
| 3270.7395301 | 0 |
| 3270.7421041 | 0 |
| 3270.7435557 | 4 |
| 3270.7463024 | 1 |
| 3270.7474659 | 3 |
| 3270.7503829 | 5 |
| 3270.7526353 | 1 |
| 3270.7543329 | 2 |
| 3270.7575867 | 0 |
| 3270.7577247 | 5 |
| 3270.7610464 | 4 |
| 3270.7635444 | 3 |
| 3270.7642252 | 1 |
| 3270.7670269 | 3 |
| 3270.7686127 | 1 |
| 3270.7715653 | 1 |
| 3270.7743641 | 4 |
| 3270.7760746 | 1 |
| 3270.7785271 | 4 |
| 3270.7803879 | 2 |
| 3270.7826163 | 4 |
| 3270.7838732 | 3 |
| 3270.7867522 | 3 |
| 3270.7897364 | 1 |
| 3270.7900614 | 4 |
| 3270.7935358 | 1 |
| 3270.7963522 | 3 |
| 3270.7973914 | 2 |
| 3270.799864 | 1 |
| 3270.8014008 | 1 |
| 3270.8037272 | 2 |
| 3270.8053882 | 1 |
| 3270.8078896 | 5 |
| 3270.8106223 | 6 |
| 3270.8120882 | 3 |
| 3270.8147773 | 2 |
| 3270.8161363 | 2 |
| 3270.8184654 | 2 |
| 3270.8217623 | 0 |
| 3270.8231436 | 2 |
| 3270.8257684 | 1 |
| 3270.8266695 | 5 |
| 3270.8299148 | 5 |
| 3270.8326291 | 2 |
| 3270.8342103 | 1 |
| 3270.837212 | 4 |
| 3270.8381646 | 2 |
| 3270.8406656 | 1 |
| 3270.8432598 | 3 |
| 3270.8443434 | 2 |
| 3270.8467443 | 1 |
| 3270.8486907 | 0 |
| 3270.8509988 | 4 |
| 3270.8542832 | 2 |
| 3270.8555253 | 6 |
| 3270.8582162 | 3 |
| 3270.8597733 | 4 |
| 3270.8625978 | 4 |
| 3270.864693 | 3 |
| 3270.866112 | 1 |
| 3270.8692271 | 2 |
| 3270.8702372 | 7 |
| 3270.8733469 | 2 |
| 3270.8759497 | 2 |
| 3270.8770127 | 2 |
| 3270.8800085 | 2 |
| 3270.8824299 | 4 |
| 3270.88361 | 4 |
| 3270.8863471 | 1 |
| 3270.887427 | 3 |
| 3270.8903251 | 0 |
| 3270.8923362 | 2 |
| 3270.8952032 | 2 |
| 3270.8977564 | 3 |
| 3270.8988412 | 1 |
| 3270.9013869 | 0 |
| 3270.9021614 | 0 |
| 3270.9052361 | 4 |
| 3270.9084988 | 2 |
| 3270.909365 | 3 |
| 3270.9128378 | 1 |
| 3270.9138073 | 1 |
| 3270.9172265 | 3 |
| 3270.9190518 | 3 |
| 3270.9209067 | 0 |
| 3270.9236277 | 4 |

| | |
|---|---|
| 3270.9248196 | 2 |
| 3270.9273741 | 3 |
| 3270.9285368 | 0 |
| 3270.931233 | 3 |
| 3270.9329944 | 3 |
| 3270.9353581 | 2 |
| 3270.9378358 | 3 |
| 3270.9404735 | 1 |
| 3270.9419059 | 2 |
| 3270.9447324 | 7 |
| 3270.9457979 | 14 |
| 3270.9487455 | 1 |
| 3270.9520184 | 1 |
| 3270.9528045 | 2 |
| 3270.9558101 | 1 |
| 3270.9572838 | 6 |
| 3270.9594891 | 2 |
| 3270.9620112 | 3 |
| 3270.964627 | 2 |
| 3270.966753 | 3 |
| 3270.967923 | 1 |
| 3270.9714413 | 2 |
| 3270.9740621 | 5 |
| 3270.974837 | 1 |
| 3270.9777957 | 3 |
| 3270.9789444 | 1 |
| 3270.9814934 | 1 |
| 3270.9846258 | 3 |
| 3270.9860062 | 1 |
| 3270.9882492 | 2 |
| 3270.9894282 | 4 |
| 3270.9924215 | 2 |
| 3270.9953919 | 6 |
| 3270.9964916 | 4 |
| 3270.9993599 | 3 |
| 3271.0007352 | 3 |
| 3271.0039405 | 6 |
| 3271.0063542 | 5 |
| 3271.0084498 | 3 |
| 3271.010329 | 0 |
| 3271.0121459 | 4 |
| 3271.0146064 | 2 |
| 3271.01672 | 6 |
| 3271.0185766 | 0 |
| 3271.0216911 | 3 |
| 3271.0228917 | 1 |
| 3271.0262765 | 3 |
| 3271.0277483 | 1 |
| 3271.0288553 | 4 |
| 3271.0322488 | 3 |
| 3271.0330862 | 2 |
| 3271.0360111 | 0 |
| 3271.0380713 | 1 |
| 3271.040448 | 2 |
| 3271.0423791 | 2 |
| 3271.044638 | 0 |
| 3271.0471915 | 5 |
| 3271.0497772 | 3 |
| 3271.0514963 | 4 |
| 3271.053709 | 3 |
| 3271.0550363 | 2 |
| 3271.0580544 | 2 |
| 3271.0598382 | 3 |
| 3271.0626054 | 3 |
| 3271.0653462 | 6 |
| 3271.0667044 | 3 |
| 3271.0693746 | 1 |
| 3271.0705651 | 4 |
| 3271.072786 | 0 |
| 3271.0754932 | 1 |
| 3271.0768821 | 2 |
| 3271.0793749 | 2 |
| 3271.0823184 | 1 |
| 3271.0843452 | 2 |
| 3271.0866337 | 5 |
| 3271.08818 | 3 |
| 3271.091366 | 2 |
| 3271.0935771 | 2 |
| 3271.094892 | 1 |
| 3271.0975112 | 1 |
| 3271.0991948 | 2 |
| 3271.1013737 | 3 |
| 3271.1032485 | 3 |
| 3271.1063689 | 4 |
| 3271.1095044 | 3 |
| 3271.1101687 | 4 |
| 3271.1130145 | 2 |
| 3271.1148068 | 0 |
| 3271.1173382 | 2 |
| 3271.1196006 | 1 |
| 3271.1214377 | 1 |
| 3271.1244433 | 3 |
| 3271.1251476 | 1 |
| 3271.1278581 | 1 |
| 3271.1305431 | 2 |
| 3271.131574 | 1 |
| 3271.13444 | 1 |
| 3271.1370237 | 2 |
| 3271.1390157 | 2 |
| 3271.1420861 | 2 |
| 3271.1425872 | 3 |
| 3271.1458306 | 3 |
| 3271.1474081 | 3 |
| 3271.1502896 | 3 |
| 3271.1529856 | 2 |
| 3271.1545051 | 3 |
| 3271.1572019 | 2 |
| 3271.1594623 | 0 |
| 3271.1608861 | 5 |
| 3271.1632123 | 2 |
| 3271.1648606 | 3 |
| 3271.1677504 | 3 |
| 3271.1691901 | 0 |
| 3271.1716195 | 2 |
| 3271.1750008 | 3 |
| 3271.1757709 | 3 |
| 3271.1786662 | 3 |
| 3271.1795962 | 2 |
| 3271.1825876 | 0 |
| 3271.185023 | 2 |
| 3271.1867865 | 4 |
| 3271.1895664 | 2 |
| 3271.1905652 | 2 |
| 3271.1940493 | 0 |
| 3271.1967849 | 3 |
| 3271.1973583 | 3 |
| 3271.2006876 | 3 |
| 3271.2025488 | 2 |
| 3271.2044566 | 1 |
| 3271.2073846 | 1 |
| 3271.2092026 | 4 |
| 3271.2116382 | 1 |
| 3271.2124018 | 4 |
| 3271.2150623 | 4 |
| 3271.2180195 | 5 |
| 3271.2196851 | 3 |
| 3271.2220474 | 1 |
| 3271.2251547 | 2 |
| 3271.2263547 | 2 |
| 3271.2291345 | 1 |
| 3271.2305495 | 3 |
| 3271.2333879 | 2 |
| 3271.2345931 | 5 |
| 3271.2379466 | 2 |
| 3271.2403785 | 3 |
| 3271.2414282 | 3 |
| 3271.2448241 | 0 |
| 3271.2454026 | 3 |
| 3271.248315 | 1 |
| 3271.2513742 | 4 |
| 3271.2523153 | 2 |
| 3271.2555233 | 6 |
| 3271.2570742 | 5 |
| 3271.2595899 | 2 |
| 3271.2604238 | 4 |
| 3271.2631929 | 0 |
| 3271.265732 | 2 |
| 3271.267257 | 2 |
| 3271.2705032 | 4 |
| 3271.2722678 | 2 |
| 3271.2740034 | 1 |
| 3271.2768295 | 3 |
| 3271.2790083 | 3 |
| 3271.2804716 | 4 |
| 3271.2832707 | 2 |
| 3271.2847053 | 1 |
| 3271.2883148 | 3 |
| 3271.2907744 | 1 |
| 3271.2916934 | 2 |
| 3271.294721 | 1 |
| 3271.2961785 | 2 |
| 3271.2984824 | 4 |
| 3271.3009393 | 2 |
| 3271.3025572 | 4 |
| 3271.3051465 | 1 |
| 3271.3064057 | 0 |
| 3271.3094308 | 2 |
| 3271.3101881 | 3 |
| 3271.3129386 | 4 |
| 3271.3159724 | 3 |
| 3271.3169571 | 5 |
| 3271.3202162 | 2 |
| 3271.3214236 | 0 |
| 3271.3244231 | 6 |
| 3271.3273781 | 13 |
| 3271.3286418 | 10 |
| 3271.3306791 | 1 |
| 3271.3318077 | 1 |
| 3271.3352981 | 1 |
| 3271.3380701 | 4 |
| 3271.3391483 | 3 |
| 3271.3421172 | 1 |
| 3271.3435206 | 1 |
| 3271.3464042 | 4 |
| 3271.3487528 | 1 |
| 3271.3504834 | 0 |
| 3271.3532143 | 3 |
| 3271.3544853 | 0 |
| 3271.3570963 | 3 |
| 3271.3600664 | 2 |
| 3271.3612238 | 6 |
| 3271.363514 | 3 |
| 3271.3652171 | 7 |
| 3271.3677828 | 5 |
| 3271.3701492 | 3 |
| 3271.3717541 | 2 |
| 3271.3750715 | 0 |
| 3271.3759875 | 2 |
| 3271.3785337 | 1 |
| 3271.3798277 | 4 |
| 3271.3828803 | 1 |
| 3271.3859947 | 2 |
| 3271.3870974 | 1 |
| 3271.3904588 | 1 |
| 3271.3912694 | 2 |
| 3271.3942211 | 3 |
| 3271.396311 | 0 |
| 3271.3977982 | 3 |
| 3271.4001915 | 1 |
| 3271.4018223 | 2 |
| 3271.4043303 | 5 |
| 3271.4070682 | 4 |
| 3271.4088331 | 2 |
| 3271.4113984 | 3 |
| 3271.4142566 | 1 |
| 3271.4151077 | 3 |
| 3271.418275 | 1 |
| 3271.4183264 | 1 |
| 3271.418838 | 1 |
| 3271.4195612 | 2 |
| 3271.4224139 | 1 |
| 3271.4235123 | 3 |
| 3271.426465 | 1 |
| 3271.428421 | 1 |
| 3271.4287982 | 1 |
| 3271.4290427 | 2 |
| 3271.4297425 | 2 |
| 3271.4328196 | 1 |
| 3271.433044 | 2 |
| 3271.4343737 | 0 |
| 3271.4347459 | 2 |
| 3271.4348938 | 3 |
| 3271.4364303 | 0 |
| 3271.4375029 | 2 |
| 3271.4383638 | 0 |
| 3271.4403115 | 2 |
| 3271.4403734 | 4 |
| 3271.4411915 | 2 |
| 3271.4412612 | 7 |
| 3271.4420923 | 3 |
| 3271.4438632 | 1 |
| 3271.4440408 | 2 |
| 3271.4452078 | 3 |
| 3271.4458724 | 1 |
| 3271.446526 | 1 |
| 3271.4476676 | 2 |
| 3271.4477154 | 2 |
| 3271.44952 | 4 |
| 3271.4497794 | 3 |
| 3271.4499519 | 1 |
| 3271.4517446 | 2 |
| 3271.4525475 | 1 |
| 3271.4536887 | 1 |
| 3271.4547132 | 0 |
| 3271.4554721 | 2 |
| 3271.4560734 | 3 |
| 3271.4594097 | 1 |
| 3271.4597074 | 3 |
| 3271.4604752 | 3 |
| 3271.4622284 | 1 |
| 3271.4625228 | 1 |
| 3271.4627912 | 3 |
| 3271.4647165 | 1 |
| 3271.4652838 | 3 |
| 3271.4664817 | 2 |
| 3271.466716 | 3 |
| 3271.4700853 | 3 |
| 3271.4723133 | 2 |
| 3271.4732312 | 1 |
| 3271.4744309 | 3 |
| 3271.4748248 | 1 |
| 3271.4751609 | 3 |
| 3271.4752842 | 3 |
| 3271.4756756 | 5 |
| 3271.4757465 | 4 |
| 3271.4762661 | 5 |
| 3271.4762924 | 2 |
| 3271.476571 | 0 |
| 3271.4770497 | 3 |
| 3271.4773555 | 4 |
| 3271.4774295 | 3 |
| 3271.4776314 | 1 |
| 3271.477865 | 4 |
| 3271.4780061 | 1 |
| 3271.4781538 | 0 |
| 3271.4782591 | 2 |
| 3271.4786448 | 4 |
| 3271.4789239 | 0 |
| 3271.4793488 | 2 |
| 3271.479494 | 3 |
| 3271.4797313 | 4 |
| 3271.4798632 | 2 |
| 3271.4799961 | 4 |
| 3271.4803406 | 3 |
| 3271.4806215 | 0 |
| 3271.4806469 | 0 |
| 3271.480652 | 1 |
| 3271.4809259 | 3 |
| 3271.4814177 | 2 |
| 3271.4814418 | 2 |
| 3271.4815767 | 1 |
| 3271.4819856 | 1 |
| 3271.4821504 | 1 |
| 3271.4826172 | 4 |
| 3271.4827789 | 1 |
| 3271.4829708 | 1 |
| 3271.4831656 | 4 |
| 3271.4831818 | 3 |
| 3271.483244 | 6 |
| 3271.4835857 | 5 |
| 3271.4836144 | 2 |
| 3271.4837289 | 2 |
| 3271.4842243 | 3 |
| 3271.4842724 | 25 |
| 3271.4843393 | 5 |
| 3271.4849732 | 11 |
| 3271.4853637 | 5 |
| 3271.4853936 | 21 |
| 3271.485505 | 16 |
| 3271.4858108 | 26 |
| 3271.4859248 | 9 |
| 3271.4861243 | 43 |
| 3271.4862132 | 33 |
| 3271.4865395 | 52 |
| 3271.4867396 | 45 |
| 3271.486841 | 47 |
| 3271.4869404 | 4 |
| 3271.4869575 | 31 |
| 3271.4871315 | 37 |
| 3271.4873333 | 43 |
| 3271.4874537 | 34 |
| 3271.4876169 | 38 |
| 3271.4881659 | 50 |
| 3271.4882086 | 50 |
| 3271.4884691 | 43 |
| 3271.4886719 | 69 |
| 3271.4887536 | 53 |
| 3271.4892796 | 52 |
| 3271.4893053 | 53 |
| 3271.4893193 | 54 |
| 3271.4895223 | 43 |
| 3271.4896911 | 46 |
| 3271.4900699 | 51 |
| 3271.4902238 | 48 |
| 3271.4904107 | 65 |
| 3271.4908925 | 72 |
| 3271.4909834 | 50 |
| 3271.4911261 | 45 |
| 3271.4911979 | 56 |
| 3271.4912753 | 45 |
| 3271.4913846 | 51 |
| 3271.4916125 | 45 |
| 3271.4916726 | 65 |

| | |
|---|---|
| 3271.4921154 | 48 |
| 3271.492123 | 56 |
| 3271.4926061 | 53 |
| 3271.4930121 | 61 |
| 3271.4930277 | 44 |
| 3271.4930311 | 35 |
| 3271.4933924 | 36 |
| 3271.4935816 | 38 |
| 3271.4939763 | 35 |
| 3271.4939878 | 18 |
| 3271.4940829 | 21 |
| 3271.4942556 | 16 |
| 3271.4943925 | 16 |
| 3271.4946538 | 6 |
| 3271.4951741 | 8 |
| 3271.4953583 | 6 |
| 3271.4953641 | 5 |
| 3271.4955558 | 1 |
| 3271.4959196 | 2 |
| 3271.4960375 | 4 |
| 3271.4960799 | 6 |
| 3271.4962199 | 2 |
| 3271.4964217 | 1 |
| 3271.4966325 | 8 |
| 3271.4967404 | 1 |
| 3271.497137 | 5 |
| 3271.4974823 | 3 |
| 3271.4975481 | 1 |
| 3271.497627 | 6 |
| 3271.497708 | 2 |
| 3271.4982001 | 3 |
| 3271.4984832 | 2 |
| 3271.49899 | 3 |
| 3271.4993154 | 0 |
| 3271.4993353 | 3 |
| 3271.4993356 | 2 |
| 3271.4996758 | 3 |
| 3271.4998425 | 1 |
| 3271.4999508 | 3 |
| 3271.4999898 | 2 |
| 3271.500341 | 0 |
| 3271.5004939 | 4 |
| 3271.5005334 | 2 |
| 3271.5006898 | 2 |
| 3271.5009404 | 2 |
| 3271.5010437 | 1 |
| 3271.5014823 | 2 |
| 3271.5016746 | 1 |
| 3271.5017302 | 1 |
| 3271.5019933 | 3 |
| 3271.5021379 | 1 |
| 3271.5024119 | 0 |
| 3271.5025537 | 3 |
| 3271.5027058 | 3 |
| 3271.5027697 | 2 |
| 3271.5029563 | 2 |
| 3271.5034492 | 3 |
| 3271.5036023 | 3 |
| 3271.5036745 | 3 |
| 3271.5038585 | 1 |
| 3271.5039167 | 2 |
| 3271.5040774 | 1 |
| 3271.5042192 | 1 |
| 3271.5046351 | 0 |
| 3271.5046868 | 5 |
| 3271.504801 | 1 |
| 3271.5048193 | 2 |
| 3271.5050217 | 2 |
| 3271.5053929 | 1 |
| 3271.5053988 | 4 |
| 3271.5057031 | 3 |
| 3271.5058484 | 2 |
| 3271.5068207 | 2 |
| 3271.5068498 | 1 |
| 3271.5069527 | 1 |
| 3271.507231 | 5 |
| 3271.5073679 | 2 |
| 3271.5074143 | 2 |
| 3271.5074371 | 3 |
| 3271.5076823 | 3 |
| 3271.5077344 | 2 |
| 3271.50813 | 3 |
| 3271.5086195 | 4 |
| 3271.5090212 | 3 |
| 3271.509079 | 0 |
| 3271.5091476 | 6 |
| 3271.5093175 | 1 |
| 3271.5093726 | 3 |
| 3271.5093961 | 2 |
| 3271.5102213 | 2 |
| 3271.5103165 | 2 |
| 3271.5104677 | 1 |
| 3271.5107288 | 1 |
| 3271.5107932 | 7 |
| 3271.5108718 | 4 |
| 3271.5111297 | 2 |
| 3271.5115545 | 3 |
| 3271.5117322 | 1 |
| 3271.5118552 | 3 |
| 3271.5118928 | 2 |
| 3271.512164 | 1 |
| 3271.5121968 | 4 |
| 3271.5124635 | 1 |
| 3271.5128316 | 0 |
| 3271.5128726 | 1 |
| 3271.5135989 | 1 |
| 3271.5146717 | 0 |
| 3271.5151759 | 1 |
| 3271.5158872 | 7 |
| 3271.5159069 | 2 |
| 3271.5162148 | 3 |
| 3271.5165322 | 2 |
| 3271.5168964 | 1 |
| 3271.5173914 | 3 |
| 3271.5183063 | 4 |
| 3271.5183251 | 3 |
| 3271.5191858 | 1 |
| 3271.5196215 | 2 |
| 3271.520605 | 1 |
| 3271.5208877 | 2 |
| 3271.5213747 | 2 |
| 3271.5213891 | 2 |
| 3271.5218615 | 4 |
| 3271.5221763 | 3 |
| 3271.5229699 | 1 |
| 3271.5232293 | 4 |
| 3271.5245166 | 3 |
| 3271.5247967 | 5 |
| 3271.5249062 | 3 |
| 3271.5253273 | 1 |
| 3271.5262137 | 3 |
| 3271.5266101 | 2 |
| 3271.5269468 | 3 |
| 3271.5274251 | 2 |
| 3271.5275055 | 4 |
| 3271.5275205 | 4 |
| 3271.5286503 | 1 |
| 3271.5288373 | 4 |
| 3271.5292476 | 2 |
| 3271.5297176 | 2 |
| 3271.5302676 | 5 |
| 3271.5305009 | 3 |
| 3271.5306802 | 4 |
| 3271.5312631 | 1 |
| 3271.532074 | 1 |
| 3271.5323704 | 2 |
| 3271.5330774 | 1 |
| 3271.533501 | 4 |
| 3271.5336863 | 0 |
| 3271.5341988 | 5 |
| 3271.5351012 | 2 |
| 3271.5356445 | 5 |
| 3271.5359847 | 1 |
| 3271.5366395 | 1 |
| 3271.536875 | 2 |
| 3271.5375141 | 0 |
| 3271.5377384 | 2 |
| 3271.538491 | 2 |
| 3271.5385214 | 3 |
| 3271.5393533 | 4 |
| 3271.5395083 | 2 |
| 3271.5402702 | 1 |
| 3271.5405354 | 3 |
| 3271.5405702 | 3 |
| 3271.5412627 | 4 |
| 3271.5415948 | 3 |
| 3271.5422272 | 3 |
| 3271.5426453 | 0 |
| 3271.543247 | 2 |
| 3271.5433855 | 2 |
| 3271.5444369 | 1 |
| 3271.5444929 | 0 |
| 3271.5449372 | 5 |
| 3271.545419 | 3 |
| 3271.545663 | 4 |
| 3271.546573 | 2 |
| 3271.5469827 | 4 |
| 3271.5471435 | 3 |
| 3271.5479041 | 0 |
| 3271.5487096 | 3 |
| 3271.5490527 | 1 |
| 3271.5490533 | 2 |
| 3271.5490895 | 4 |
| 3271.5502994 | 3 |
| 3271.5503565 | 3 |
| 3271.5511904 | 0 |
| 3271.5517613 | 2 |
| 3271.5519644 | 4 |
| 3271.5530855 | 1 |
| 3271.5531708 | 3 |
| 3271.5537575 | 1 |
| 3271.5537904 | 3 |
| 3271.554105 | 0 |
| 3271.5551005 | 2 |
| 3271.5551715 | 2 |
| 3271.5556484 | 1 |
| 3271.5558583 | 0 |
| 3271.556802 | 4 |
| 3271.5574088 | 3 |
| 3271.5576849 | 1 |
| 3271.5581204 | 1 |
| 3271.5588358 | 5 |
| 3271.5590522 | 3 |
| 3271.5595973 | 2 |
| 3271.5601799 | 7 |
| 3271.5603354 | 5 |
| 3271.5607537 | 0 |
| 3271.561391 | 2 |
| 3271.5620029 | 4 |
| 3271.5628147 | 0 |
| 3271.5632062 | 3 |
| 3271.5633148 | 4 |
| 3271.5636076 | 5 |
| 3271.5647719 | 3 |
| 3271.564919 | 1 |
| 3271.5655202 | 1 |
| 3271.5661312 | 3 |
| 3271.5661316 | 3 |
| 3271.5670147 | 3 |
| 3271.5676447 | 1 |
| 3271.5681367 | 2 |
| 3271.5684437 | 1 |
| 3271.5687272 | 1 |
| 3271.5697317 | 0 |
| 3271.5697558 | 3 |
| 3271.5698112 | 1 |
| 3271.5706839 | 0 |
| 3271.5713148 | 2 |
| 3271.5717488 | 3 |
| 3271.572093 | 1 |
| 3271.5724421 | 2 |
| 3271.5725424 | 3 |
| 3271.5727779 | 3 |
| 3271.5733006 | 4 |
| 3271.5738575 | 1 |
| 3271.5749199 | 3 |
| 3271.5751266 | 4 |
| 3271.5759188 | 2 |
| 3271.5774986 | 1 |
| 3271.5776591 | 3 |
| 3271.5779112 | 1 |
| 3271.5792491 | 3 |
| 3271.5794832 | 4 |
| 3271.580748 | 0 |
| 3271.581858 | 3 |
| 3271.583287 | 6 |
| 3271.5835817 | 1 |
| 3271.5840531 | 3 |
| 3271.5841233 | 7 |
| 3271.5858252 | 1 |
| 3271.5860732 | 1 |
| 3271.5861931 | 1 |
| 3271.5876523 | 2 |
| 3271.5877078 | 1 |
| 3271.5895311 | 1 |
| 3271.5903075 | 1 |
| 3271.5908695 | 4 |
| 3271.5911229 | 2 |
| 3271.5924759 | 1 |
| 3271.5928119 | 1 |
| 3271.5937493 | 3 |
| 3271.5941551 | 2 |
| 3271.5944172 | 2 |
| 3271.5953804 | 0 |
| 3271.5956545 | 3 |
| 3271.5969498 | 2 |
| 3271.5971049 | 0 |
| 3271.5971384 | 1 |
| 3271.5979234 | 1 |
| 3271.5984174 | 3 |
| 3271.5996276 | 1 |
| 3271.6007247 | 2 |
| 3271.6007908 | 5 |
| 3271.6024731 | 2 |
| 3271.6035271 | 3 |
| 3271.6037686 | 2 |
| 3271.6055729 | 0 |
| 3271.6069285 | 0 |
| 3271.6082203 | 3 |
| 3271.6099502 | 2 |
| 3271.6121696 | 4 |
| 3271.6125977 | 1 |
| 3271.6141155 | 2 |
| 3271.6144292 | 0 |
| 3271.6147039 | 5 |
| 3271.6162012 | 3 |
| 3271.618137 | 2 |
| 3271.6183586 | 4 |
| 3271.6184359 | 3 |
| 3271.6193379 | 2 |
| 3271.6194189 | 2 |
| 3271.6205072 | 3 |
| 3271.6211082 | 2 |
| 3271.6218262 | 3 |
| 3271.6226647 | 0 |
| 3271.6240115 | 4 |
| 3271.6242436 | 2 |
| 3271.6247011 | 2 |
| 3271.625451 | 3 |
| 3271.6263926 | 1 |
| 3271.6275768 | 3 |
| 3271.6279554 | 2 |
| 3271.6284515 | 3 |
| 3271.6286185 | 0 |
| 3271.6302503 | 0 |
| 3271.6310951 | 3 |
| 3271.6324685 | 3 |
| 3271.6330038 | 7 |
| 3271.6334075 | 4 |
| 3271.6348748 | 1 |
| 3271.6350871 | 2 |
| 3271.6355333 | 3 |
| 3271.6362184 | 1 |
| 3271.6366129 | 1 |
| 3271.638052 | 1 |
| 3271.6397315 | 6 |
| 3271.640932 | 1 |
| 3271.6409723 | 3 |
| 3271.6416811 | 1 |
| 3271.6421768 | 0 |
| 3271.6425116 | 2 |
| 3271.6440067 | 2 |
| 3271.6448102 | 2 |
| 3271.6466686 | 1 |
| 3271.6466747 | 2 |
| 3271.6467194 | 0 |
| 3271.647888 | 1 |
| 3271.6488337 | 3 |
| 3271.6490884 | 2 |
| 3271.6504688 | 2 |
| 3271.6522685 | 2 |
| 3271.6523403 | 2 |
| 3271.65303 | 2 |
| 3271.653611 | 2 |
| 3271.6551307 | 3 |
| 3271.655305 | 1 |
| 3271.6555451 | 4 |
| 3271.6569129 | 4 |
| 3271.6577315 | 1 |
| 3271.6587433 | 5 |
| 3271.6593081 | 2 |
| 3271.6597681 | 1 |
| 3271.6600594 | 2 |
| 3271.6603338 | 2 |
| 3271.662389 | 2 |
| 3271.663419 | 3 |
| 3271.6640335 | 3 |
| 3271.6648498 | 1 |
| 3271.6653994 | 1 |
| 3271.6661664 | 0 |
| 3271.667847 | 1 |
| 3271.6680656 | 1 |
| 3271.6695489 | 1 |
| 3271.6703076 | 2 |
| 3271.6707309 | 3 |
| 3271.6717754 | 0 |
| 3271.6719567 | 1 |
| 3271.6724832 | 1 |
| 3271.6741873 | 0 |
| 3271.674539 | 2 |
| 3271.6749477 | 2 |
| 3271.6760723 | 1 |
| 3271.6768175 | 2 |

| | |
|---|---|
| 3271.6782464 | 4 |
| 3271.6789165 | 2 |
| 3271.6798716 | 2 |
| 3271.6801004 | 0 |
| 3271.680297 | 1 |
| 3271.6814242 | 2 |
| 3271.6825379 | 0 |
| 3271.6830397 | 2 |
| 3271.6845659 | 4 |
| 3271.6846551 | 1 |
| 3271.6863519 | 1 |
| 3271.6865057 | 2 |
| 3271.6865956 | 3 |
| 3271.6877803 | 0 |
| 3271.6886906 | 3 |
| 3271.6896513 | 3 |
| 3271.6906907 | 3 |
| 3271.6907696 | 1 |
| 3271.6917269 | 4 |
| 3271.6927373 | 4 |
| 3271.6928775 | 2 |
| 3271.6949916 | 1 |
| 3271.695069 | 1 |
| 3271.6958177 | 4 |
| 3271.697025 | 2 |
| 3271.6983567 | 3 |
| 3271.6989583 | 1 |
| 3271.6998333 | 1 |
| 3271.6999155 | 0 |
| 3271.701328 | 1 |
| 3271.701744 | 4 |
| 3271.7026789 | 2 |
| 3271.7029491 | 0 |
| 3271.70344 | 2 |
| 3271.7042065 | 3 |
| 3271.7051584 | 3 |
| 3271.7053538 | 2 |
| 3271.7055342 | 7 |
| 3271.7075709 | 3 |
| 3271.707628 | 2 |
| 3271.7085924 | 2 |
| 3271.7087445 | 1 |
| 3271.7088971 | 3 |
| 3271.7102754 | 2 |
| 3271.7111902 | 5 |
| 3271.7112092 | 2 |
| 3271.7115762 | 1 |
| 3271.7124104 | 4 |
| 3271.7126766 | 7 |
| 3271.7135898 | 3 |
| 3271.7144 | 20 |
| 3271.7145979 | 15 |
| 3271.7148483 | 1 |
| 3271.7159528 | 1 |
| 3271.7166591 | 2 |
| 3271.7167399 | 13 |
| 3271.7184424 | 4 |
| 3271.7187038 | 1 |
| 3271.7196012 | 1 |
| 3271.7205099 | 7 |
| 3271.7226161 | 1 |
| 3271.7234293 | 2 |
| 3271.7259466 | 1 |
| 3271.7272436 | 2 |
| 3271.7295231 | 2 |
| 3271.7313054 | 0 |
| 3271.7333934 | 2 |
| 3271.7344769 | 1 |
| 3271.7353108 | 4 |
| 3271.7355845 | 5 |
| 3271.7388958 | 3 |
| 3271.7392921 | 0 |
| 3271.7403142 | 2 |
| 3271.7434492 | 1 |
| 3271.7452595 | 4 |
| 3271.7484969 | 4 |
| 3271.7495982 | 1 |
| 3271.7523178 | 3 |
| 3271.7540754 | 2 |
| 3271.7552653 | 1 |
| 3271.7575381 | 1 |
| 3271.7591887 | 1 |
| 3271.7611967 | 2 |
| 3271.7638968 | 2 |
| 3271.7647756 | 2 |
| 3271.7666783 | 0 |
| 3271.7685723 | 1 |
| 3271.7694428 | 2 |
| 3271.7719618 | 3 |
| 3271.7732666 | 1 |
| 3271.7756681 | 2 |
| 3271.7781076 | 6 |
| 3271.7791915 | 3 |
| 3271.7818541 | 0 |
| 3271.7824136 | 2 |
| 3271.7843028 | 1 |
| 3271.7872786 | 3 |
| 3271.7899331 | 3 |
| 3271.7910487 | 1 |
| 3271.7913845 | 3 |
| 3271.7923711 | 2 |
| 3271.7969723 | 5 |
| 3271.7970048 | 3 |
| 3271.8004845 | 40 |
| 3271.8012081 | 42 |
| 3271.8039292 | 44 |
| 3271.8053147 | 62 |
| 3271.8075439 | 46 |
| 3271.8102323 | 24 |
| 3271.8109652 | 3 |
| 3271.8129035 | 2 |
| 3271.8141567 | 0 |
| 3271.8148703 | 1 |
| 3271.8167837 | 2 |
| 3271.8178762 | 4 |
| 3271.8180217 | 1 |
| 3271.8190338 | 2 |
| 3271.8220805 | 8 |
| 3271.8248838 | 3 |
| 3271.8252591 | 4 |
| 3271.8285596 | 1 |
| 3271.8288881 | 2 |
| 3271.8316339 | 5 |
| 3271.8328523 | 3 |
| 3271.8361367 | 2 |
| 3271.8378221 | 1 |
| 3271.8501838 | 1 |
| 3271.8513571 | 2 |
| 3271.8545389 | 3 |
| 3271.8566215 | 2 |
| 3271.8577157 | 2 |
| 3271.8588852 | 0 |
| 3271.8594958 | 2 |
| 3271.8612356 | 1 |
| 3271.8634413 | 3 |
| 3271.8654096 | 0 |
| 3271.8669341 | 2 |
| 3271.8690988 | 4 |
| 3271.8706796 | 2 |
| 3271.8714291 | 3 |
| 3271.871496 | 2 |
| 3271.871554 | 2 |
| 3271.8720673 | 1 |
| 3271.8736984 | 0 |
| 3271.8746085 | 0 |
| 3271.8757118 | 1 |
| 3271.8763774 | 1 |
| 3271.876728 | 4 |
| 3271.8807167 | 1 |
| 3271.8823376 | 0 |
| 3271.8845129 | 2 |
| 3271.8856512 | 2 |
| 3271.8870504 | 4 |
| 3271.8887232 | 3 |
| 3271.8933927 | 1 |
| 3271.904146 | 3 |
| 3271.9064123 | 2 |
| 3271.9080323 | 2 |
| 3271.9085365 | 3 |
| 3271.9098808 | 2 |
| 3271.9112926 | 2 |
| 3271.9123508 | 7 |
| 3271.9125637 | 3 |
| 3271.9129244 | 9 |
| 3271.9129297 | 1 |
| 3271.9153 | 0 |
| 3271.9169561 | 16 |
| 3271.9195704 | 11 |
| 3271.9301895 | 2 |
| 3271.9318005 | 2 |
| 3271.9321886 | 3 |
| 3271.9327023 | 4 |
| 3271.9350083 | 2 |
| 3271.9371746 | 2 |
| 3271.9387549 | 2 |
| 3271.9411425 | 3 |
| 3271.9413262 | 1 |
| 3271.9418913 | 0 |
| 3271.9437567 | 1 |
| 3271.9462536 | 3 |
| 3271.9473189 | 3 |
| 3271.9496669 | 1 |
| 3271.9498782 | 2 |
| 3271.953709 | 5 |
| 3271.9565458 | 1 |
| 3271.9572662 | 2 |
| 3271.9593482 | 1 |
| 3271.9596105 | 1 |
| 3271.9628183 | 3 |
| 3271.964726 | 6 |
| 3271.966028 | 2 |
| 3271.9686536 | 2 |
| 3271.9686989 | 1 |
| 3271.9710923 | 4 |
| 3271.9714097 | 7 |
| 3271.9730584 | 1 |
| 3271.9761745 | 3 |
| 3271.9774524 | 5 |
| 3271.9834817 | 3 |
| 3271.9858726 | 3 |
| 3271.9869305 | 5 |
| 3271.9883613 | 2 |
| 3271.9900122 | 0 |
| 3271.9905443 | 3 |
| 3271.9925957 | 2 |
| 3271.9949671 | 3 |
| 3271.9970237 | 2 |
| 3271.9983386 | 0 |
| 3272.0011828 | 3 |
| 3272.0023212 | 1 |
| 3272.0030804 | 7 |
| 3272.0058164 | 3 |
| 3272.006791 | 1 |
| 3272.0096948 | 5 |
| 3272.0118759 | 1 |
| 3272.01334 | 3 |
| 3272.0160596 | 0 |
| 3272.0180153 | 3 |
| 3272.0193452 | 5 |
| 3272.0218461 | 2 |
| 3272.0229898 | 6 |
| 3272.0257251 | 3 |
| 3272.0283141 | 2 |
| 3272.0290397 | 1 |
| 3272.0305014 | 1 |
| 3272.0315538 | 4 |
| 3272.0344126 | 3 |
| 3272.0364977 | 5 |
| 3272.0384256 | 2 |
| 3272.0411262 | 0 |
| 3272.0413526 | 1 |
| 3272.0430261 | 3 |
| 3272.0441157 | 5 |
| 3272.0466587 | 4 |
| 3272.0468952 | 1 |
| 3272.0491598 | 0 |
| 3272.0515548 | 4 |
| 3272.0527283 | 2 |
| 3272.0544492 | 1 |
| 3272.0564009 | 3 |
| 3272.0575407 | 1 |
| 3272.0599335 | 1 |
| 3272.06304 | 0 |
| 3272.0653375 | 2 |
| 3272.0665963 | 1 |
| 3272.0691585 | 5 |
| 3272.0696914 | 2 |
| 3272.0727103 | 2 |
| 3272.0730368 | 1 |
| 3272.0750646 | 3 |
| 3272.0756382 | 1 |
| 3272.0768814 | 3 |
| 3272.0791218 | 3 |
| 3272.0802456 | 0 |
| 3272.0819155 | 1 |
| 3272.0836224 | 0 |
| 3272.0874329 | 6 |
| 3272.0876898 | 1 |
| 3272.0877899 | 3 |
| 3272.089462 | 2 |
| 3272.0916796 | 0 |
| 3272.0921873 | 2 |
| 3272.0949485 | 0 |
| 3272.0970034 | 1 |
| 3272.0980654 | 0 |
| 3272.1015657 | 3 |
| 3272.1099973 | 1 |
| 3272.1111106 | 1 |
| 3272.1159883 | 4 |
| 3272.1171116 | 1 |
| 3272.1199435 | 1 |
| 3272.1219175 | 3 |
| 3272.1229082 | 1 |
| 3272.1251403 | 2 |
| 3272.1260601 | 2 |
| 3272.1266959 | 4 |
| 3272.1287456 | 1 |
| 3272.1300901 | 2 |
| 3272.1330165 | 3 |
| 3272.1332649 | 1 |
| 3272.1346295 | 3 |
| 3272.1363132 | 2 |
| 3272.1369687 | 1 |
| 3272.1378693 | 4 |
| 3272.1413517 | 5 |
| 3272.1436175 | 2 |
| 3272.1451132 | 2 |
| 3272.1454728 | 2 |
| 3272.1472015 | 2 |
| 3272.1481761 | 2 |
| 3272.1500682 | 5 |
| 3272.1507799 | 4 |
| 3272.1546464 | 1 |
| 3272.164695 | 1 |
| 3272.1648682 | 2 |
| 3272.1657217 | 1 |
| 3272.1663609 | 1 |
| 3272.1675392 | 0 |
| 3272.1701105 | 5 |
| 3272.1721779 | 1 |
| 3272.173756 | 3 |
| 3272.1757473 | 5 |
| 3272.1764405 | 3 |
| 3272.1787452 | 4 |
| 3272.180277 | 5 |
| 3272.1826423 | 2 |
| 3272.1840408 | 2 |
| 3272.1842555 | 2 |
| 3272.1862177 | 3 |
| 3272.1878121 | 1 |
| 3272.1889565 | 4 |
| 3272.1903517 | 2 |
| 3272.1939693 | 1 |
| 3272.195482 | 4 |
| 3272.1975304 | 0 |
| 3272.1989452 | 1 |
| 3272.2014754 | 1 |
| 3272.2043101 | 1 |
| 3272.2043552 | 2 |
| 3272.2076862 | 3 |
| 3272.2083485 | 4 |
| 3272.2095339 | 1 |
| 3272.2116855 | 1 |
| 3272.2123254 | 4 |
| 3272.2161888 | 5 |
| 3272.2170722 | 0 |
| 3272.2206666 | 3 |
| 3272.2209266 | 1 |
| 3272.2222939 | 2 |
| 3272.2233325 | 1 |
| 3272.2233597 | 0 |
| 3272.2256103 | 3 |
| 3272.2260162 | 1 |
| 3272.227124 | 0 |
| 3272.2294188 | 1 |
| 3272.2312495 | 4 |
| 3272.2448965 | 2 |
| 3272.2476506 | 1 |
| 3272.2485147 | 5 |
| 3272.2486905 | 2 |
| 3272.2501918 | 1 |
| 3272.250488 | 3 |
| 3272.2513021 | 1 |
| 3272.2518397 | 6 |
| 3272.2536307 | 4 |
| 3272.2549524 | 1 |
| 3272.2576619 | 4 |
| 3272.2597066 | 4 |
| 3272.2605072 | 1 |
| 3272.2630905 | 5 |
| 3272.2631312 | 6 |
| 3272.2653213 | 1 |
| 3272.2667051 | 2 |
| 3272.267563 | 3 |
| 3272.2696385 | 2 |
| 3272.2723932 | 0 |
| 3272.2745255 | 3 |
| 3272.2753456 | 3 |
| 3272.2763964 | 2 |
| 3272.2788787 | 2 |
| 3272.281336 | 1 |
| 3272.2823428 | 1 |
| 3272.2850606 | 4 |

| | |
|---|---|
| 3272.286684 | 5 |
| 3272.2881886 | 2 |
| 3272.2898507 | 3 |
| 3272.2904549 | 2 |
| 3272.2933707 | 2 |
| 3272.2958398 | 0 |
| 3272.2967778 | 3 |
| 3272.300489 | 0 |
| 3272.3015828 | 3 |
| 3272.3035485 | 4 |
| 3272.3057721 | 7 |
| 3272.3071005 | 1 |
| 3272.3091643 | 2 |
| 3272.3100007 | 3 |
| 3272.3127089 | 3 |
| 3272.3134943 | 4 |
| 3272.3163075 | 3 |
| 3272.3175611 | 6 |
| 3272.3194828 | 0 |
| 3272.3208783 | 1 |
| 3272.3219132 | 2 |
| 3272.32604 | 3 |
| 3272.3273643 | 4 |
| 3272.3287701 | 6 |
| 3272.331548 | 4 |
| 3272.3323348 | 1 |
| 3272.3345164 | 1 |
| 3272.3364558 | 2 |
| 3272.3383958 | 1 |
| 3272.3408627 | 2 |
| 3272.3417163 | 5 |
| 3272.3421603 | 2 |
| 3272.3434297 | 2 |
| 3272.3465946 | 1 |
| 3272.3498121 | 4 |
| 3272.3505317 | 2 |
| 3272.3531355 | 2 |
| 3272.3556786 | 6 |
| 3272.3562665 | 2 |
| 3272.3586866 | 1 |
| 3272.3600712 | 1 |
| 3272.3618701 | 2 |
| 3272.3644305 | 0 |
| 3272.3654941 | 2 |
| 3272.3682059 | 2 |
| 3272.3688112 | 4 |
| 3272.3702306 | 4 |
| 3272.3708602 | 0 |
| 3272.3727182 | 4 |
| 3272.3738575 | 4 |
| 3272.3739325 | 3 |
| 3272.3754949 | 2 |
| 3272.3768737 | 3 |
| 3272.378559 | 1 |
| 3272.3810546 | 2 |
| 3272.3832716 | 1 |
| 3272.3843228 | 5 |
| 3272.3868762 | 1 |
| 3272.3989609 | 2 |
| 3272.3997381 | 4 |
| 3272.4029128 | 1 |
| 3272.4052535 | 4 |
| 3272.4062011 | 2 |
| 3272.4081533 | 0 |
| 3272.4084199 | 1 |
| 3272.4101602 | 1 |
| 3272.411316 | 1 |
| 3272.4117117 | 1 |
| 3272.4139814 | 1 |
| 3272.4140435 | 4 |
| 3272.4147254 | 4 |
| 3272.4167635 | 1 |
| 3272.4198588 | 1 |
| 3272.4201065 | 5 |
| 3272.4210949 | 2 |
| 3272.4225615 | 3 |
| 3272.4263532 | 3 |
| 3272.4265246 | 1 |
| 3272.4309508 | 1 |
| 3272.432666 | 4 |
| 3272.4339085 | 1 |
| 3272.4361712 | 4 |
| 3272.4379499 | 3 |
| 3272.4401389 | 3 |
| 3272.4423308 | 0 |
| 3272.443608 | 4 |
| 3272.4459575 | 3 |
| 3272.446232 | 2 |
| 3272.4483668 | 2 |
| 3272.4500358 | 3 |
| 3272.4519368 | 0 |
| 3272.4543466 | 1 |
| 3272.4570901 | 1 |
| 3272.4579821 | 1 |
| 3272.4607283 | 3 |
| 3272.4611114 | 1 |
| 3272.4637035 | 3 |
| 3272.4662018 | 1 |
| 3272.4681376 | 3 |
| 3272.4698946 | 4 |
| 3272.4712292 | 5 |
| 3272.4728658 | 1 |
| 3272.4732409 | 0 |
| 3272.4755329 | 5 |
| 3272.4783157 | 2 |
| 3272.4801372 | 3 |
| 3272.4827337 | 2 |
| 3272.4847792 | 1 |
| 3272.4865889 | 4 |
| 3272.4887708 | 2 |
| 3272.4896634 | 1 |
| 3272.492398 | 4 |
| 3272.4930293 | 1 |
| 3272.4961367 | 4 |
| 3272.4984152 | 3 |
| 3272.4989451 | 1 |
| 3272.5006705 | 0 |
| 3272.5014682 | 3 |
| 3272.5037127 | 2 |
| 3272.5069746 | 0 |
| 3272.5081964 | 3 |
| 3272.5107713 | 1 |
| 3272.5116088 | 2 |
| 3272.5137098 | 0 |
| 3272.5163801 | 5 |
| 3272.5172162 | 5 |
| 3272.5195684 | 1 |
| 3272.5212466 | 1 |
| 3272.5238821 | 1 |
| 3272.5255899 | 2 |
| 3272.5269375 | 0 |
| 3272.5284874 | 5 |
| 3272.5313595 | 4 |
| 3272.5317574 | 2 |
| 3272.535727 | 0 |
| 3272.5361914 | 2 |
| 3272.5382334 | 6 |
| 3272.5408284 | 0 |
| 3272.5419742 | 2 |
| 3272.5442566 | 0 |
| 3272.5450146 | 0 |
| 3272.5475061 | 2 |
| 3272.5489933 | 3 |
| 3272.5503164 | 6 |
| 3272.5528814 | 4 |
| 3272.5546497 | 2 |
| 3272.5571883 | 1 |
| 3272.5593505 | 0 |
| 3272.5604738 | 3 |
| 3272.5629149 | 1 |
| 3272.563827 | 2 |
| 3272.5663587 | 5 |
| 3272.5680363 | 2 |
| 3272.5695267 | 1 |
| 3272.5725846 | 3 |
| 3272.5731244 | 1 |
| 3272.5748416 | 3 |
| 3272.5772281 | 1 |
| 3272.5787448 | 2 |
| 3272.5806183 | 1 |
| 3272.5824128 | 2 |
| 3272.5857014 | 1 |
| 3272.5876081 | 3 |
| 3272.5878944 | 5 |
| 3272.5904294 | 6 |
| 3272.591743 | 3 |
| 3272.5940128 | 5 |
| 3272.5953357 | 1 |
| 3272.598214 | 3 |
| 3272.599953 | 0 |
| 3272.6003063 | 4 |
| 3272.6030518 | 6 |
| 3272.604656 | 2 |
| 3272.6048206 | 2 |
| 3272.6058465 | 6 |
| 3272.6066424 | 2 |
| 3272.6068135 | 0 |
| 3272.6093321 | 0 |
| 3272.6100011 | 5 |
| 3272.6123189 | 1 |
| 3272.6124974 | 1 |
| 3272.6141465 | 1 |
| 3272.615309 | 1 |
| 3272.6170621 | 2 |
| 3272.618424 | 2 |
| 3272.6191277 | 2 |
| 3272.6200858 | 3 |
| 3272.620925 | 1 |
| 3272.6225252 | 1 |
| 3272.6231181 | 2 |
| 3272.6237198 | 4 |
| 3272.6249197 | 1 |
| 3272.6265866 | 2 |
| 3272.6272468 | 5 |
| 3272.6284447 | 1 |
| 3272.6289665 | 1 |
| 3272.629574 | 0 |
| 3272.6306213 | 0 |
| 3272.6315629 | 1 |
| 3272.6322046 | 1 |
| 3272.63508 | 0 |
| 3272.6363593 | 2 |
| 3272.6399955 | 4 |
| 3272.6414895 | 2 |
| 3272.6438777 | 1 |
| 3272.6478105 | 0 |
| 3272.6485919 | 3 |
| 3272.6516616 | 1 |
| 3272.6535734 | 3 |
| 3272.6560431 | 0 |
| 3272.6598292 | 2 |
| 3272.6616354 | 1 |
| 3272.6644913 | 5 |
| 3272.6655119 | 2 |
| 3272.6703182 | 1 |
| 3272.6727498 | 5 |
| 3272.6737772 | 2 |
| 3272.6766597 | 1 |
| 3272.6787199 | 2 |
| 3272.6811168 | 2 |
| 3272.6833242 | 5 |
| 3272.6861094 | 1 |
| 3272.689226 | 3 |
| 3272.6928444 | 5 |
| 3272.6939459 | 0 |
| 3272.6967597 | 0 |
| 3272.6983327 | 1 |
| 3272.7017832 | 0 |
| 3272.7034362 | 2 |
| 3272.7063178 | 0 |
| 3272.7098433 | 2 |
| 3272.711413 | 1 |
| 3272.7151136 | 1 |
| 3272.7160102 | 6 |
| 3272.7194719 | 2 |
| 3272.7223064 | 1 |
| 3272.7236203 | 3 |
| 3272.7265925 | 4 |
| 3272.7280537 | 1 |
| 3272.7307567 | 2 |
| 3272.7334709 | 2 |
| 3272.7357055 | 3 |
| 3272.7388584 | 3 |
| 3272.7405991 | 2 |
| 3272.7445729 | 3 |
| 3272.7457457 | 0 |
| 3272.7496125 | 4 |
| 3272.7519069 | 1 |
| 3272.7534476 | 2 |
| 3272.7565935 | 1 |
| 3272.7593221 | 0 |
| 3272.7610822 | 3 |
| 3272.7644217 | 4 |
| 3272.7656318 | 4 |
| 3272.7692373 | 1 |
| 3272.7725759 | 3 |
| 3272.7740917 | 5 |
| 3272.7769838 | 4 |
| 3272.7781403 | 4 |
| 3272.781105 | 1 |
| 3272.7826685 | 1 |
| 3272.7855007 | 2 |
| 3272.7893508 | 5 |
| 3272.7910191 | 2 |
| 3272.7941693 | 1 |
| 3272.7974934 | 2 |
| 3272.798722 | 4 |
| 3272.8022823 | 3 |
| 3272.8028339 | 2 |
| 3272.8062367 | 2 |
| 3272.8083808 | 1 |
| 3272.8114078 | 2 |
| 3272.8139978 | 3 |
| 3272.8157611 | 2 |
| 3272.8191534 | 1 |
| 3272.8218726 | 0 |
| 3272.8235881 | 1 |
| 3272.8267552 | 3 |
| 3272.8283401 | 3 |
| 3272.8317043 | 5 |
| 3272.8345984 | 2 |
| 3272.8360589 | 3 |
| 3272.8395655 | 1 |
| 3272.8404232 | 1 |
| 3272.8439879 | 4 |
| 3272.8475108 | 2 |
| 3272.8485691 | 3 |
| 3272.8519335 | 3 |
| 3272.8543611 | 3 |
| 3272.8570601 | 1 |
| 3272.8579019 | 3 |
| 3272.8615494 | 3 |
| 3272.8640573 | 0 |
| 3272.8655853 | 3 |
| 3272.8691752 | 2 |
| 3272.8720431 | 0 |
| 3272.8741117 | 2 |
| 3272.8771917 | 0 |
| 3272.8783389 | 1 |
| 3272.8820574 | 2 |
| 3272.8832611 | 3 |
| 3272.8865179 | 4 |
| 3272.8902525 | 1 |
| 3272.8911371 | 3 |
| 3272.8943089 | 3 |
| 3272.8971221 | 5 |
| 3272.8987413 | 1 |
| 3272.901982 | 3 |
| 3272.903682 | 2 |
| 3272.9066651 | 3 |
| 3272.9078795 | 1 |
| 3272.9110568 | 1 |
| 3272.9141148 | 2 |
| 3272.9152716 | 2 |
| 3272.9190744 | 1 |
| 3272.921246 | 2 |
| 3272.9243471 | 5 |
| 3272.9269541 | 2 |
| 3272.9286072 | 2 |
| 3272.9316965 | 3 |
| 3272.9333416 | 1 |
| 3272.936261 | 1 |
| 3272.9397558 | 1 |
| 3272.9410153 | 2 |
| 3272.9445831 | 1 |
| 3272.9460696 | 3 |
| 3272.949333 | 1 |
| 3272.9518326 | 0 |
| 3272.9535818 | 4 |
| 3272.9568035 | 1 |
| 3272.9578518 | 2 |
| 3272.9616962 | 5 |
| 3272.9654939 | 2 |
| 3272.9662316 | 5 |
| 3272.9692593 | 2 |
| 3272.970634 | 2 |
| 3272.9744616 | 4 |
| 3272.9761603 | 3 |
| 3272.9789109 | 3 |
| 3272.9819742 | 1 |
| 3272.9838127 | 3 |
| 3272.9874551 | 2 |
| 3272.9874612 | 2 |
| 3272.991074 | 2 |
| 3272.9944233 | 2 |
| 3272.9954628 | 3 |
| 3272.9984433 | 3 |
| 3273.001621 | 5 |
| 3273.0033522 | 14 |
| 3273.0058789 | 4 |
| 3273.0082006 | 1 |
| 3273.0115698 | 5 |
| 3273.0148644 | 3 |
| 3273.0161415 | 2 |
| 3273.0190592 | 3 |
| 3273.0202841 | 0 |
| 3273.0232701 | 5 |
| 3273.0258671 | 4 |
| 3273.0280359 | 0 |
| 3273.0317937 | 4 |
| 3273.0337006 | 1 |
| 3273.0361634 | 2 |
| 3273.0392485 | 2 |

| | |
|---|---|
| 3273.0405422 | 2 |
| 3273.0441685 | 3 |
| 3273.0459992 | 1 |
| 3273.0487451 | 4 |
| 3273.0504659 | 5 |
| 3273.0539499 | 7 |
| 3273.0559903 | 3 |
| 3273.0582348 | 3 |
| 3273.0617254 | 3 |
| 3273.0639683 | 4 |
| 3273.0649837 | 0 |
| 3273.0692209 | 4 |
| 3273.0714515 | 2 |
| 3273.0730536 | 1 |
| 3273.0763298 | 0 |
| 3273.0778756 | 4 |
| 3273.0808308 | 3 |
| 3273.0835478 | 1 |
| 3273.0852825 | 1 |
| 3273.0889296 | 0 |
| 3273.0896314 | 2 |
| 3273.093488 | 1 |
| 3273.0960454 | 2 |
| 3273.0975165 | 1 |
| 3273.1012632 | 2 |
| 3273.1021583 | 3 |
| 3273.1054786 | 4 |
| 3273.1088159 | 2 |
| 3273.1102176 | 3 |
| 3273.1135606 | 3 |
| 3273.1151068 | 3 |
| 3273.118711 | 2 |
| 3273.1216355 | 2 |
| 3273.1228557 | 3 |
| 3273.1258694 | 2 |
| 3273.1274847 | 1 |
| 3273.1301432 | 3 |
| 3273.132816 | 1 |
| 3273.1350125 | 4 |
| 3273.1375421 | 0 |
| 3273.1410345 | 2 |
| 3273.1430998 | 1 |
| 3273.1456112 | 1 |
| 3273.147146 | 3 |
| 3273.1503519 | 3 |
| 3273.1514756 | 0 |
| 3273.1552113 | 2 |
| 3273.1583636 | 1 |
| 3273.1599152 | 4 |
| 3273.1631827 | 2 |
| 3273.1655874 | 2 |
| 3273.1678069 | 2 |
| 3273.1706465 | 0 |
| 3273.1725914 | 2 |
| 3273.1748759 | 3 |
| 3273.1788152 | 3 |
| 3273.1800197 | 1 |
| 3273.1825837 | 5 |
| 3273.1844048 | 3 |
| 3273.1879861 | 3 |
| 3273.1891323 | 3 |
| 3273.1917172 | 2 |
| 3273.1949435 | 0 |
| 3273.1958529 | 0 |
| 3273.1999757 | 4 |
| 3273.20303 | 2 |
| 3273.2041874 | 1 |
| 3273.2076299 | 4 |
| 3273.2093969 | 3 |
| 3273.2123974 | 1 |
| 3273.2139259 | 0 |
| 3273.2175997 | 2 |
| 3273.2209423 | 2 |
| 3273.2221988 | 1 |
| 3273.2248389 | 4 |
| 3273.227903 | 0 |
| 3273.2289195 | 2 |
| 3273.2313903 | 0 |
| 3273.2332314 | 0 |
| 3273.2371657 | 2 |
| 3273.2399042 | 3 |
| 3273.2417917 | 1 |
| 3273.2455234 | 6 |
| 3273.2461234 | 3 |
| 3273.2495593 | 2 |
| 3273.2525591 | 1 |
| 3273.2542845 | 1 |
| 3273.2575382 | 1 |
| 3273.2590476 | 6 |
| 3273.2620843 | 3 |
| 3273.2636799 | 3 |
| 3273.2667921 | 5 |
| 3273.270034 | 3 |
| 3273.2711574 | 3 |
| 3273.274915 | 1 |
| 3273.2760758 | 4 |
| 3273.279333 | 4 |
| 3273.2821831 | 2 |
| 3273.2842205 | 7 |
| 3273.2874298 | 4 |
| 3273.2884068 | 4 |
| 3273.2910734 | 0 |
| 3273.2950611 | 4 |
| 3273.2959455 | 0 |
| 3273.2996301 | 6 |
| 3273.3024107 | 1 |
| 3273.3043598 | 2 |
| 3273.3075508 | 1 |
| 3273.3088573 | 4 |
| 3273.3114843 | 3 |
| 3273.3131402 | 5 |
| 3273.31698 | 2 |
| 3273.3202063 | 2 |
| 3273.3218015 | 1 |
| 3273.3241195 | 2 |
| 3273.3257539 | 3 |
| 3273.3288573 | 1 |
| 3273.332523 | 2 |
| 3273.3335324 | 2 |
| 3273.3367695 | 1 |
| 3273.3384236 | 3 |
| 3273.3414552 | 2 |
| 3273.3452216 | 1 |
| 3273.3464329 | 1 |
| 3273.3497233 | 4 |
| 3273.3508509 | 0 |
| 3273.3541671 | 5 |
| 3273.3571481 | 3 |
| 3273.3581939 | 2 |
| 3273.3616508 | 0 |
| 3273.3633559 | 3 |
| 3273.3657876 | 4 |
| 3273.3695859 | 4 |
| 3273.3704661 | 1 |
| 3273.3735411 | 3 |
| 3273.3752411 | 4 |
| 3273.3787633 | 2 |
| 3273.3815251 | 1 |
| 3273.3838828 | 3 |
| 3273.3867091 | 3 |
| 3273.3898702 | 4 |
| 3273.3910404 | 4 |
| 3273.394176 | 2 |
| 3273.395623 | 2 |
| 3273.3987462 | 2 |
| 3273.4014375 | 0 |
| 3273.4033294 | 3 |
| 3273.4072287 | 4 |
| 3273.4081636 | 1 |
| 3273.4118927 | 1 |
| 3273.4150356 | 2 |
| 3273.4157843 | 3 |
| 3273.4190374 | 1 |
| 3273.4214409 | 4 |
| 3273.4240058 | 0 |
| 3273.4256497 | 3 |
| 3273.4290822 | 4 |
| 3273.4322157 | 0 |
| 3273.4342919 | 3 |
| 3273.4362349 | 3 |
| 3273.4378875 | 0 |
| 3273.4414215 | 1 |
| 3273.4450771 | 5 |
| 3273.4461688 | 3 |
| 3273.4494712 | 1 |
| 3273.4508695 | 1 |
| 3273.4541457 | 5 |
| 3273.4575343 | 0 |
| 3273.4584938 | 3 |
| 3273.4617521 | 2 |
| 3273.4635895 | 3 |
| 3273.4663515 | 4 |
| 3273.4698795 | 2 |
| 3273.4713022 | 4 |
| 3273.4749193 | 2 |
| 3273.4764613 | 6 |
| 3273.4791189 | 2 |
| 3273.4815352 | 2 |
| 3273.4839795 | 2 |
| 3273.4873262 | 2 |
| 3273.4888549 | 4 |
| 3273.4925054 | 3 |
| 3273.4953197 | 4 |
| 3273.4959596 | 3 |
| 3273.5001598 | 3 |
| 3273.5014292 | 0 |
| 3273.5040335 | 1 |
| 3273.5060291 | 3 |
| 3273.5079309 | 3 |
| 3273.5088758 | 4 |
| 3273.5092449 | 1 |
| 3273.5101518 | 2 |
| 3273.5101989 | 3 |
| 3273.5107426 | 1 |
| 3273.5119957 | 2 |
| 3273.512191 | 3 |
| 3273.5129441 | 1 |
| 3273.5132316 | 2 |
| 3273.5140801 | 4 |
| 3273.5146479 | 3 |
| 3273.5156478 | 2 |
| 3273.5157835 | 4 |
| 3273.5166749 | 4 |
| 3273.518112 | 0 |
| 3273.5186115 | 1 |
| 3273.5186188 | 3 |
| 3273.5188371 | 3 |
| 3273.5199198 | 0 |
| 3273.5203141 | 3 |
| 3273.521082 | 0 |
| 3273.5221583 | 2 |
| 3273.5224015 | 4 |
| 3273.5229937 | 2 |
| 3273.5236639 | 2 |
| 3273.5248203 | 0 |
| 3273.5251071 | 0 |
| 3273.5255003 | 2 |
| 3273.5265729 | 4 |
| 3273.527001 | 3 |
| 3273.5274664 | 0 |
| 3273.5277091 | 0 |
| 3273.5277342 | 2 |
| 3273.528221 | 2 |
| 3273.5283484 | 0 |
| 3273.5284904 | 2 |
| 3273.5293489 | 5 |
| 3273.529439 | 2 |
| 3273.5297594 | 1 |
| 3273.5297965 | 3 |
| 3273.5299923 | 2 |
| 3273.530116 | 0 |
| 3273.5303088 | 1 |
| 3273.5304175 | 0 |
| 3273.5308185 | 2 |
| 3273.5308675 | 2 |
| 3273.5312014 | 3 |
| 3273.5322049 | 0 |
| 3273.5322632 | 0 |
| 3273.5324296 | 3 |
| 3273.5324432 | 2 |
| 3273.5325823 | 2 |
| 3273.5331457 | 2 |
| 3273.5334898 | 1 |
| 3273.533755 | 2 |
| 3273.5338178 | 2 |
| 3273.5342138 | 3 |
| 3273.5346924 | 2 |
| 3273.5347222 | 2 |
| 3273.5347264 | 4 |
| 3273.5347301 | 1 |
| 3273.5347632 | 0 |
| 3273.5353875 | 1 |
| 3273.5357182 | 2 |
| 3273.536095 | 2 |
| 3273.5363381 | 4 |
| 3273.5365889 | 1 |
| 3273.5367389 | 3 |
| 3273.5370268 | 1 |
| 3273.5370942 | 1 |
| 3273.5376124 | 0 |
| 3273.5376563 | 1 |
| 3273.5379025 | 2 |
| 3273.5385908 | 4 |
| 3273.5387335 | 3 |
| 3273.5388906 | 4 |
| 3273.5388985 | 4 |
| 3273.5393319 | 3 |
| 3273.5398071 | 1 |
| 3273.5398168 | 4 |
| 3273.5399978 | 1 |
| 3273.5403382 | 3 |
| 3273.5404797 | 0 |
| 3273.5404811 | 4 |
| 3273.5406519 | 4 |
| 3273.5410658 | 2 |
| 3273.5411295 | 3 |
| 3273.541394 | 2 |
| 3273.5419041 | 1 |
| 3273.5420578 | 2 |
| 3273.5422965 | 4 |
| 3273.5424545 | 3 |
| 3273.5431094 | 2 |
| 3273.5432965 | 4 |
| 3273.5433843 | 1 |
| 3273.5433949 | 2 |
| 3273.5443257 | 2 |
| 3273.544384 | 1 |
| 3273.5446714 | 7 |
| 3273.5450618 | 3 |
| 3273.5453031 | 7 |
| 3273.5455789 | 3 |
| 3273.5457749 | 9 |
| 3273.5458014 | 11 |
| 3273.5458088 | 11 |
| 3273.546085 | 10 |
| 3273.5464514 | 19 |
| 3273.546636 | 21 |
| 3273.5466957 | 17 |
| 3273.5470664 | 27 |
| 3273.5473902 | 37 |
| 3273.5476759 | 32 |
| 3273.5477939 | 28 |
| 3273.5478646 | 35 |
| 3273.548113 | 38 |
| 3273.5481219 | 40 |
| 3273.5485285 | 46 |
| 3273.548678 | 44 |
| 3273.5492363 | 30 |
| 3273.5493265 | 38 |
| 3273.5499321 | 46 |
| 3273.5500867 | 57 |
| 3273.5507193 | 41 |
| 3273.5507361 | 34 |
| 3273.5512231 | 40 |
| 3273.5514863 | 33 |
| 3273.55181 | 36 |
| 3273.5518388 | 35 |
| 3273.552002 | 44 |
| 3273.5521861 | 40 |
| 3273.5525089 | 40 |
| 3273.5527485 | 46 |
| 3273.5530208 | 54 |
| 3273.5533361 | 48 |
| 3273.5535024 | 48 |
| 3273.553797 | 56 |
| 3273.5539686 | 39 |
| 3273.5542614 | 40 |
| 3273.5544153 | 23 |
| 3273.5547912 | 21 |
| 3273.5548913 | 34 |
| 3273.555147 | 7 |
| 3273.5551732 | 17 |
| 3273.5554456 | 11 |
| 3273.5556425 | 12 |
| 3273.5561531 | 3 |
| 3273.5565641 | 5 |
| 3273.5568531 | 6 |
| 3273.5568967 | 1 |
| 3273.5572323 | 6 |
| 3273.5575704 | 3 |
| 3273.5578209 | 1 |
| 3273.5579625 | 2 |
| 3273.55831 | 1 |
| 3273.5590819 | 2 |
| 3273.5590852 | 0 |
| 3273.5591986 | 2 |
| 3273.5594673 | 1 |
| 3273.5596809 | 4 |
| 3273.5598238 | 4 |
| 3273.5600529 | 2 |
| 3273.5600632 | 1 |
| 3273.5601671 | 2 |
| 3273.5609784 | 2 |
| 3273.5610976 | 4 |
| 3273.5611921 | 2 |
| 3273.5614293 | 2 |
| 3273.5616093 | 6 |
| 3273.5620599 | 1 |
| 3273.5620956 | 3 |
| 3273.5622357 | 0 |
| 3273.5624652 | 2 |
| 3273.5625392 | 0 |
| 3273.5632219 | 1 |

| | |
|---|---|
| 3273.5634416 | 2 |
| 3273.5638115 | 3 |
| 3273.5640349 | 0 |
| 3273.5643675 | 0 |
| 3273.5647567 | 5 |
| 3273.5657183 | 0 |
| 3273.5674611 | 2 |
| 3273.568661 | 4 |
| 3273.5706781 | 4 |
| 3273.5718326 | 3 |
| 3273.5720385 | 2 |
| 3273.573974 | 0 |
| 3273.5740603 | 2 |
| 3273.5760611 | 5 |
| 3273.5772645 | 2 |
| 3273.5783136 | 1 |
| 3273.580023 | 4 |
| 3273.5805138 | 3 |
| 3273.5821001 | 1 |
| 3273.5836792 | 0 |
| 3273.5847068 | 0 |
| 3273.5862759 | 4 |
| 3273.5870271 | 4 |
| 3273.5886994 | 0 |
| 3273.5898809 | 4 |
| 3273.590908 | 2 |
| 3273.5922353 | 5 |
| 3273.5928471 | 1 |
| 3273.5946512 | 1 |
| 3273.5959748 | 3 |
| 3273.5962185 | 4 |
| 3273.5978793 | 1 |
| 3273.599722 | 3 |
| 3273.6006854 | 1 |
| 3273.6023151 | 3 |
| 3273.6031316 | 3 |
| 3273.6044601 | 3 |
| 3273.605523 | 2 |
| 3273.6071072 | 2 |
| 3273.6086692 | 3 |
| 3273.6096719 | 2 |
| 3273.6114945 | 2 |
| 3273.6117363 | 1 |
| 3273.6135393 | 0 |
| 3273.6151166 | 4 |
| 3273.6154597 | 3 |
| 3273.6174523 | 4 |
| 3273.6182511 | 2 |
| 3273.6197043 | 2 |
| 3273.6207188 | 6 |
| 3273.6225695 | 4 |
| 3273.6232785 | 1 |
| 3273.6246336 | 4 |
| 3273.6265217 | 5 |
| 3273.6276983 | 0 |
| 3273.6277434 | 1 |
| 3273.6307235 | 1 |
| 3273.6313918 | 3 |
| 3273.631562 | 1 |
| 3273.6338021 | 0 |
| 3273.6340548 | 2 |
| 3273.6360304 | 1 |
| 3273.6365308 | 1 |
| 3273.6385067 | 5 |
| 3273.6395777 | 4 |
| 3273.6399546 | 4 |
| 3273.6415544 | 5 |
| 3273.6433541 | 2 |
| 3273.64389 | 2 |
| 3273.6453938 | 4 |
| 3273.645656 | 0 |
| 3273.6482248 | 1 |
| 3273.6489209 | 1 |
| 3273.6500504 | 4 |
| 3273.6519275 | 1 |
| 3273.6522393 | 4 |
| 3273.654732 | 2 |
| 3273.6551133 | 1 |
| 3273.6567022 | 0 |
| 3273.6585192 | 2 |
| 3273.658794 | 2 |
| 3273.6603106 | 3 |
| 3273.6619142 | 6 |
| 3273.6622178 | 1 |
| 3273.6642583 | 1 |
| 3273.6652905 | 1 |
| 3273.6668259 | 7 |
| 3273.6685922 | 2 |
| 3273.669395 | 2 |
| 3273.6704181 | 1 |
| 3273.6712819 | 5 |
| 3273.6728374 | 1 |
| 3273.6735783 | 1 |
| 3273.6749039 | 7 |
| 3273.6769188 | 4 |
| 3273.6769212 | 3 |
| 3273.6784912 | 5 |
| 3273.6804351 | 1 |
| 3273.6807302 | 1 |
| 3273.68234 | 2 |
| 3273.6829579 | 1 |
| 3273.6847353 | 2 |
| 3273.6866321 | 1 |
| 3273.6870196 | 3 |
| 3273.6889085 | 0 |
| 3273.6892494 | 1 |
| 3273.6910789 | 0 |
| 3273.6927074 | 7 |
| 3273.69323 | 3 |
| 3273.6949366 | 0 |
| 3273.6961555 | 1 |
| 3273.6969801 | 2 |
| 3273.6989189 | 2 |
| 3273.6998076 | 1 |
| 3273.7007579 | 2 |
| 3273.7010839 | 2 |
| 3273.7041696 | 1 |
| 3273.7057209 | 2 |
| 3273.7058689 | 2 |
| 3273.7070295 | 0 |
| 3273.7091264 | 0 |
| 3273.7097488 | 2 |
| 3273.7116231 | 4 |
| 3273.7122586 | 0 |
| 3273.7135104 | 1 |
| 3273.7151421 | 3 |
| 3273.7158846 | 2 |
| 3273.7171186 | 2 |
| 3273.7178897 | 1 |
| 3273.7197342 | 3 |
| 3273.7207912 | 2 |
| 3273.7210716 | 1 |
| 3273.7221617 | 4 |
| 3273.7225629 | 3 |
| 3273.7229312 | 1 |
| 3273.723524 | 5 |
| 3273.7239976 | 2 |
| 3273.7244142 | 5 |
| 3273.7259163 | 1 |
| 3273.7263444 | 2 |
| 3273.7270912 | 1 |
| 3273.7277752 | 2 |
| 3273.7279805 | 5 |
| 3273.728566 | 1 |
| 3273.7294281 | 3 |
| 3273.7299445 | 3 |
| 3273.7304114 | 2 |
| 3273.7305959 | 0 |
| 3273.7309386 | 0 |
| 3273.7320874 | 1 |
| 3273.7330295 | 1 |
| 3273.7336991 | 2 |
| 3273.7343428 | 0 |
| 3273.7345276 | 3 |
| 3273.7356892 | 1 |
| 3273.7360096 | 2 |
| 3273.7363668 | 2 |
| 3273.736837 | 2 |
| 3273.7380342 | 3 |
| 3273.7382973 | 4 |
| 3273.7384571 | 3 |
| 3273.7392081 | 1 |
| 3273.7400293 | 5 |
| 3273.7404578 | 4 |
| 3273.7417504 | 1 |
| 3273.7423296 | 2 |
| 3273.7426466 | 1 |
| 3273.7431207 | 3 |
| 3273.7434775 | 1 |
| 3273.7444131 | 3 |
| 3273.744651 | 1 |
| 3273.7460205 | 3 |
| 3273.746285 | 1 |
| 3273.7467323 | 3 |
| 3273.747591 | 0 |
| 3273.748793 | 2 |
| 3273.7490127 | 2 |
| 3273.7495652 | 0 |
| 3273.7505844 | 3 |
| 3273.750702 | 4 |
| 3273.7510696 | 3 |
| 3273.7517053 | 1 |
| 3273.7528835 | 0 |
| 3273.7531795 | 3 |
| 3273.7542093 | 2 |
| 3273.7542689 | 1 |
| 3273.7548844 | 3 |
| 3273.7551332 | 2 |
| 3273.7561195 | 3 |
| 3273.7568263 | 2 |
| 3273.7569012 | 3 |
| 3273.7575241 | 2 |
| 3273.7587292 | 2 |
| 3273.759569 | 2 |
| 3273.75967 | 2 |
| 3273.7605358 | 0 |
| 3273.7610171 | 0 |
| 3273.7616211 | 2 |
| 3273.7620675 | 2 |
| 3273.7631325 | 4 |
| 3273.7638367 | 1 |
| 3273.7638749 | 4 |
| 3273.764948 | 6 |
| 3273.7654459 | 1 |
| 3273.7667521 | 3 |
| 3273.766758 | 3 |
| 3273.7672516 | 3 |
| 3273.7676604 | 3 |
| 3273.7689911 | 4 |
| 3273.7690618 | 2 |
| 3273.7690859 | 3 |
| 3273.7703622 | 4 |
| 3273.7704722 | 3 |
| 3273.7717754 | 1 |
| 3273.7725601 | 1 |
| 3273.7729883 | 1 |
| 3273.7732125 | 3 |
| 3273.7738037 | 1 |
| 3273.7741189 | 1 |
| 3273.7750499 | 5 |
| 3273.7754304 | 3 |
| 3273.7756576 | 1 |
| 3273.7767921 | 4 |
| 3273.7778257 | 1 |
| 3273.7782314 | 3 |
| 3273.779455 | 3 |
| 3273.779491 | 1 |
| 3273.7796451 | 2 |
| 3273.7808703 | 3 |
| 3273.7811282 | 1 |
| 3273.7813675 | 4 |
| 3273.7820737 | 3 |
| 3273.7833296 | 0 |
| 3273.7833334 | 1 |
| 3273.7833714 | 1 |
| 3273.7848971 | 3 |
| 3273.7856144 | 2 |
| 3273.7862892 | 0 |
| 3273.7871123 | 1 |
| 3273.7879422 | 5 |
| 3273.7883725 | 2 |
| 3273.7885068 | 1 |
| 3273.7885838 | 2 |
| 3273.7903128 | 2 |
| 3273.7910637 | 1 |
| 3273.7914954 | 3 |
| 3273.7916396 | 1 |
| 3273.7927305 | 0 |
| 3273.7929127 | 1 |
| 3273.7938908 | 5 |
| 3273.7938963 | 2 |
| 3273.7939777 | 0 |
| 3273.7954109 | 3 |
| 3273.7968293 | 3 |
| 3273.7973999 | 2 |
| 3273.7974898 | 4 |
| 3273.7976614 | 2 |
| 3273.7990452 | 3 |
| 3273.7991873 | 1 |
| 3273.800399 | 3 |
| 3273.8005884 | 3 |
| 3273.8015189 | 2 |
| 3273.8015972 | 2 |
| 3273.8030518 | 2 |
| 3273.8030591 | 2 |
| 3273.8035115 | 4 |
| 3273.8042174 | 3 |
| 3273.8054024 | 1 |
| 3273.8058482 | 1 |
| 3273.8065536 | 4 |
| 3273.8068291 | 1 |
| 3273.8078884 | 0 |
| 3273.8080848 | 2 |
| 3273.8089247 | 2 |
| 3273.8091923 | 1 |
| 3273.8101765 | 2 |
| 3273.8103533 | 2 |
| 3273.8108999 | 5 |
| 3273.8122583 | 2 |
| 3273.8126582 | 1 |
| 3273.8128591 | 1 |
| 3273.813349 | 2 |
| 3273.8136716 | 4 |
| 3273.8151547 | 4 |
| 3273.8158201 | 2 |
| 3273.8174175 | 1 |
| 3273.8190707 | 1 |
| 3273.8198353 | 4 |
| 3273.8211914 | 1 |
| 3273.8222965 | 4 |
| 3273.8240436 | 2 |
| 3273.8253379 | 2 |
| 3273.8258716 | 1 |
| 3273.8275048 | 1 |
| 3273.8286284 | 3 |
| 3273.8300238 | 2 |
| 3273.8318579 | 3 |
| 3273.8323397 | 4 |
| 3273.8344515 | 1 |
| 3273.8351786 | 3 |
| 3273.8363559 | 2 |
| 3273.8376288 | 1 |
| 3273.8391292 | 2 |
| 3273.8403693 | 1 |
| 3273.8404537 | 0 |
| 3273.8428608 | 1 |
| 3273.8434504 | 4 |
| 3273.8450589 | 4 |
| 3273.847289 | 2 |
| 3273.847554 | 1 |
| 3273.8495644 | 2 |
| 3273.8503245 | 2 |
| 3273.8516254 | 2 |
| 3273.8531009 | 4 |
| 3273.8539803 | 4 |
| 3273.855054 | 1 |
| 3273.855968 | 2 |
| 3273.8576422 | 1 |
| 3273.858841 | 4 |
| 3273.8605096 | 0 |
| 3273.8614006 | 3 |
| 3273.8623454 | 3 |
| 3273.8643303 | 0 |
| 3273.8654283 | 3 |
| 3273.8658537 | 2 |
| 3273.8677209 | 1 |
| 3273.8682697 | 4 |
| 3273.8700279 | 2 |
| 3273.8711421 | 2 |
| 3273.8725298 | 1 |
| 3273.8743493 | 3 |
| 3273.8745455 | 3 |
| 3273.8763047 | 0 |
| 3273.8769482 | 4 |
| 3273.8785267 | 4 |
| 3273.8797312 | 2 |
| 3273.881547 | 2 |
| 3273.882981 | 3 |
| 3273.8842588 | 5 |
| 3273.8852866 | 4 |
| 3273.8868926 | 1 |
| 3273.8872041 | 3 |
| 3273.8894812 | 1 |
| 3273.8907347 | 0 |
| 3273.8911943 | 2 |
| 3273.8931341 | 2 |
| 3273.8942908 | 1 |
| 3273.8953389 | 3 |
| 3273.8967062 | 3 |
| 3273.8980412 | 1 |
| 3273.8995868 | 1 |
| 3273.9001463 | 4 |
| 3273.901353 | 3 |
| 3273.90253 | 0 |
| 3273.903962 | 2 |
| 3273.9057392 | 0 |
| 3273.9067631 | 1 |
| 3273.9082877 | 3 |
| 3273.9091903 | 0 |
| 3273.9104987 | 1 |
| 3273.912201 | 1 |
| 3273.9125072 | 1 |
| 3273.9141783 | 3 |

| | |
|---|---|
| 3273.9153921 | 5 |
| 3273.9161503 | 3 |
| 3273.9176916 | 4 |
| 3273.9191722 | 2 |
| 3273.920662 | 26 |
| 3273.9212653 | 41 |
| 3273.9226293 | 36 |
| 3273.9247576 | 33 |
| 3273.9250855 | 39 |
| 3273.9264466 | 41 |
| 3273.9279283 | 36 |
| 3273.929115 | 27 |
| 3273.9303423 | 16 |
| 3273.9322235 | 4 |
| 3273.9333897 | 2 |
| 3273.9340101 | 3 |
| 3273.9355118 | 2 |
| 3273.9376761 | 2 |
| 3273.9378936 | 3 |
| 3273.9398352 | 3 |
| 3273.9407111 | 3 |
| 3273.9424913 | 4 |
| 3273.9426896 | 2 |
| 3273.9445832 | 1 |
| 3273.9463316 | 0 |
| 3273.9468411 | 2 |
| 3273.9487255 | 2 |
| 3273.9491111 | 2 |
| 3273.9505275 | 2 |
| 3273.952224 | 3 |
| 3273.9530534 | 0 |
| 3273.9540093 | 0 |
| 3273.9556211 | 0 |
| 3273.9572098 | 2 |
| 3273.9580105 | 1 |
| 3273.9599307 | 2 |
| 3273.9614282 | 1 |
| 3273.9618343 | 2 |
| 3273.9630506 | 1 |
| 3273.9645944 | 3 |
| 3273.9658561 | 4 |
| 3273.9663987 | 2 |
| 3273.9680317 | 1 |
| 3273.9696523 | 0 |
| 3273.971073 | 3 |
| 3273.9718838 | 2 |
| 3273.9737567 | 1 |
| 3273.9744894 | 1 |
| 3273.9759018 | 2 |
| 3273.9778067 | 2 |
| 3273.9787715 | 5 |
| 3273.9802365 | 3 |
| 3273.9809651 | 2 |
| 3273.9828993 | 3 |
| 3273.9840409 | 2 |
| 3273.984894 | 2 |
| 3273.9858876 | 1 |
| 3273.9873427 | 4 |
| 3273.9891368 | 1 |
| 3273.9896536 | 4 |
| 3273.9911834 | 4 |
| 3273.9929815 | 3 |
| 3273.9935533 | 1 |
| 3273.9954335 | 0 |
| 3273.9956824 | 1 |
| 3273.9977107 | 1 |
| 3273.9988249 | 1 |
| 3273.9999915 | 3 |
| 3274.0011822 | 1 |
| 3274.0023221 | 4 |
| 3274.0035925 | 3 |
| 3274.0049919 | 5 |
| 3274.0062531 | 1 |
| 3274.0078323 | 7 |
| 3274.0084352 | 6 |
| 3274.0100927 | 1 |
| 3274.0115585 | 4 |
| 3274.0121769 | 2 |
| 3274.014075 | 2 |
| 3274.0148612 | 2 |
| 3274.0158083 | 3 |
| 3274.0176851 | 4 |
| 3274.0182993 | 3 |
| 3274.0202029 | 1 |
| 3274.0215076 | 0 |
| 3274.0226905 | 0 |
| 3274.0233196 | 6 |
| 3274.0250803 | 2 |
| 3274.026913 | 0 |
| 3274.027427 | 2 |
| 3274.0287975 | 4 |
| 3274.0298568 | 2 |
| 3274.0312597 | 0 |
| 3274.0326142 | 1 |
| 3274.0333464 | 2 |
| 3274.0354465 | 2 |
| 3274.0367887 | 5 |
| 3274.0378072 | 2 |
| 3274.0394799 | 0 |
| 3274.0398136 | 4 |
| 3274.0418378 | 2 |
| 3274.0418414 | 2 |
| 3274.0436945 | 2 |
| 3274.0456347 | 3 |
| 3274.0463167 | 1 |
| 3274.0478539 | 3 |
| 3274.0486907 | 2 |
| 3274.050212 | 4 |
| 3274.0515642 | 1 |
| 3274.0518522 | 1 |
| 3274.0543481 | 1 |
| 3274.0552903 | 0 |
| 3274.0558986 | 2 |
| 3274.0575647 | 5 |
| 3274.0587206 | 1 |
| 3274.060103 | 3 |
| 3274.0615858 | 2 |
| 3274.0627114 | 0 |
| 3274.0648833 | 3 |
| 3274.065029 | 3 |
| 3274.0664137 | 7 |
| 3274.0677798 | 1 |
| 3274.0692486 | 4 |
| 3274.0706561 | 0 |
| 3274.0715156 | 3 |
| 3274.0729914 | 1 |
| 3274.074325 | 0 |
| 3274.0756596 | 2 |
| 3274.0770123 | 1 |
| 3274.0780012 | 0 |
| 3274.0784782 | 0 |
| 3274.0797117 | 3 |
| 3274.0808552 | 3 |
| 3274.0812968 | 1 |
| 3274.0819754 | 2 |
| 3274.0824912 | 2 |
| 3274.0837308 | 2 |
| 3274.0837364 | 3 |
| 3274.0838749 | 0 |
| 3274.0858035 | 2 |
| 3274.0863687 | 1 |
| 3274.0874665 | 2 |
| 3274.0876568 | 4 |
| 3274.0882883 | 1 |
| 3274.0888387 | 8 |
| 3274.0892258 | 3 |
| 3274.0903324 | 1 |
| 3274.0913215 | 2 |
| 3274.0922973 | 0 |
| 3274.0927424 | 3 |
| 3274.0937589 | 0 |
| 3274.0946029 | 2 |
| 3274.0946933 | 1 |
| 3274.0959419 | 2 |
| 3274.09666 | 2 |
| 3274.0967478 | 4 |
| 3274.097808 | 3 |
| 3274.0979767 | 2 |
| 3274.0988576 | 0 |
| 3274.1000541 | 1 |
| 3274.1005462 | 2 |
| 3274.1017695 | 2 |
| 3274.1024237 | 2 |
| 3274.1029761 | 0 |
| 3274.1039552 | 1 |
| 3274.104778 | 2 |
| 3274.1055748 | 4 |
| 3274.1065212 | 3 |
| 3274.106849 | 3 |
| 3274.107444 | 0 |
| 3274.1085319 | 2 |
| 3274.1094186 | 1 |
| 3274.1099894 | 1 |
| 3274.111229 | 2 |
| 3274.1116107 | 2 |
| 3274.1131097 | 1 |
| 3274.1137139 | 1 |
| 3274.1139759 | 1 |
| 3274.1144213 | 3 |
| 3274.1159974 | 0 |
| 3274.11604 | 2 |
| 3274.1169948 | 1 |
| 3274.1172608 | 2 |
| 3274.1184173 | 2 |
| 3274.1193986 | 0 |
| 3274.1203411 | 0 |
| 3274.1213138 | 3 |
| 3274.1221039 | 4 |
| 3274.1222472 | 2 |
| 3274.1233297 | 4 |
| 3274.1239581 | 1 |
| 3274.1241015 | 0 |
| 3274.125212 | 1 |
| 3274.1260822 | 1 |
| 3274.1264868 | 1 |
| 3274.1269073 | 2 |
| 3274.1289043 | 5 |
| 3274.1290419 | 2 |
| 3274.1297033 | 2 |
| 3274.1306782 | 3 |
| 3274.1311338 | 1 |
| 3274.1316767 | 1 |
| 3274.1322404 | 4 |
| 3274.1331039 | 2 |
| 3274.1338455 | 2 |
| 3274.1349981 | 3 |
| 3274.1365861 | 2 |
| 3274.1369081 | 3 |
| 3274.1370661 | 3 |
| 3274.1370678 | 2 |
| 3274.1384712 | 4 |
| 3274.1391542 | 1 |
| 3274.139949 | 3 |
| 3274.1403619 | 0 |
| 3274.1413036 | 3 |
| 3274.1424449 | 2 |
| 3274.1433576 | 4 |
| 3274.1434025 | 2 |
| 3274.14439 | 2 |
| 3274.1447841 | 0 |
| 3274.1459328 | 1 |
| 3274.146867 | 0 |
| 3274.1472115 | 2 |
| 3274.1486655 | 4 |
| 3274.1490434 | 2 |
| 3274.149574 | 2 |
| 3274.1502374 | 2 |
| 3274.1511299 | 2 |
| 3274.1516533 | 0 |
| 3274.1531681 | 4 |
| 3274.1534317 | 0 |
| 3274.154466 | 2 |
| 3274.1545425 | 2 |
| 3274.1556933 | 1 |
| 3274.156378 | 2 |
| 3274.157642 | 1 |
| 3274.1584475 | 3 |
| 3274.1588373 | 1 |
| 3274.1599604 | 6 |
| 3274.1601113 | 1 |
| 3274.1602644 | 2 |
| 3274.1620019 | 3 |
| 3274.162386 | 1 |
| 3274.1637786 | 3 |
| 3274.1639096 | 3 |
| 3274.1662374 | 3 |
| 3274.1695017 | 2 |
| 3274.1697327 | 3 |
| 3274.1720027 | 3 |
| 3274.1733965 | 2 |
| 3274.1750068 | 1 |
| 3274.1776827 | 1 |
| 3274.1787738 | 2 |
| 3274.1807771 | 1 |
| 3274.1819654 | 4 |
| 3274.1842447 | 3 |
| 3274.1859484 | 2 |
| 3274.1878884 | 4 |
| 3274.1898939 | 1 |
| 3274.1913052 | 3 |
| 3274.1938274 | 1 |
| 3274.1965673 | 0 |
| 3274.1973805 | 4 |
| 3274.1998337 | 1 |
| 3274.2008367 | 4 |
| 3274.202922 | 1 |
| 3274.2059384 | 1 |
| 3274.2071992 | 2 |
| 3274.2097631 | 2 |
| 3274.2102348 | 4 |
| 3274.2128976 | 1 |
| 3274.2152796 | 3 |
| 3274.2166746 | 3 |
| 3274.21846 | 3 |
| 3274.2198553 | 3 |
| 3274.2220968 | 3 |
| 3274.2235921 | 5 |
| 3274.2250763 | 2 |
| 3274.2275266 | 0 |
| 3274.2281947 | 0 |
| 3274.2309117 | 2 |
| 3274.2328702 | 6 |
| 3274.2345141 | 4 |
| 3274.2366118 | 3 |
| 3274.2383111 | 1 |
| 3274.2408149 | 4 |
| 3274.2433124 | 2 |
| 3274.2437503 | 4 |
| 3274.2468871 | 1 |
| 3274.2476087 | 0 |
| 3274.2495456 | 1 |
| 3274.2521213 | 4 |
| 3274.2538433 | 3 |
| 3274.2560586 | 3 |
| 3274.2568272 | 4 |
| 3274.2596551 | 4 |
| 3274.2614661 | 3 |
| 3274.262219 | 3 |
| 3274.265008 | 3 |
| 3274.2665524 | 1 |
| 3274.2688156 | 2 |
| 3274.2710878 | 3 |
| 3274.27185 | 1 |
| 3274.2742171 | 4 |
| 3274.2754189 | 2 |
| 3274.2772157 | 2 |
| 3274.2796638 | 2 |
| 3274.281113 | 3 |
| 3274.2838741 | 1 |
| 3274.284781 | 2 |
| 3274.287587 | 2 |
| 3274.2885795 | 2 |
| 3274.2906677 | 1 |
| 3274.2938822 | 2 |
| 3274.2944981 | 2 |
| 3274.2972922 | 1 |
| 3274.2988751 | 1 |
| 3274.3003328 | 1 |
| 3274.3022808 | 2 |
| 3274.3038509 | 2 |
| 3274.3058726 | 3 |
| 3274.3087247 | 1 |
| 3274.3093546 | 3 |
| 3274.3118689 | 4 |
| 3274.312945 | 1 |
| 3274.3153039 | 4 |
| 3274.3167683 | 1 |
| 3274.3168207 | 2 |
| 3274.3176142 | 1 |
| 3274.3183043 | 2 |
| 3274.3184706 | 3 |
| 3274.3196622 | 2 |
| 3274.3212491 | 1 |
| 3274.3214948 | 3 |
| 3274.3221269 | 2 |
| 3274.322435 | 1 |
| 3274.3247854 | 3 |
| 3274.3249827 | 5 |
| 3274.3260888 | 5 |
| 3274.3262147 | 1 |
| 3274.3272138 | 2 |
| 3274.3276781 | 1 |
| 3274.3280413 | 2 |
| 3274.328736 | 0 |
| 3274.3299605 | 4 |
| 3274.3304194 | 2 |
| 3274.3308789 | 1 |
| 3274.3311014 | 1 |
| 3274.3318874 | 3 |
| 3274.3331493 | 1 |
| 3274.3339557 | 0 |
| 3274.335296 | 3 |
| 3274.335811 | 2 |
| 3274.3361147 | 4 |
| 3274.3369119 | 2 |
| 3274.3382776 | 4 |
| 3274.3385868 | 1 |
| 3274.3400126 | 1 |
| 3274.3402805 | 0 |
| 3274.340922 | 4 |
| 3274.3419449 | 2 |
| 3274.3426645 | 0 |
| 3274.3430483 | 5 |

| | |
|---|---|
| 3274.3436535 | 4 |
| 3274.3443256 | 2 |
| 3274.344368 | 3 |
| 3274.3459211 | 5 |
| 3274.346436 | 2 |
| 3274.3464891 | 5 |
| 3274.3471596 | 1 |
| 3274.3480556 | 3 |
| 3274.3481833 | 4 |
| 3274.3487605 | 5 |
| 3274.3491511 | 5 |
| 3274.3499866 | 3 |
| 3274.3511962 | 6 |
| 3274.3514606 | 6 |
| 3274.3515673 | 11 |
| 3274.3519216 | 28 |
| 3274.3522568 | 35 |
| 3274.352558 | 28 |
| 3274.3532319 | 27 |
| 3274.354772 | 12 |
| 3274.3548963 | 20 |
| 3274.3550752 | 6 |
| 3274.3556173 | 6 |
| 3274.3568885 | 2 |
| 3274.3570618 | 4 |
| 3274.3571671 | 3 |
| 3274.3582423 | 4 |
| 3274.358352 | 2 |
| 3274.3591026 | 1 |
| 3274.3600973 | 2 |
| 3274.3606749 | 2 |
| 3274.3609245 | 2 |
| 3274.3617791 | 1 |
| 3274.3623773 | 2 |
| 3274.3630678 | 2 |
| 3274.363462 | 3 |
| 3274.3640582 | 3 |
| 3274.364595 | 3 |
| 3274.3649381 | 0 |
| 3274.3654351 | 2 |
| 3274.3661961 | 2 |
| 3274.3662087 | 2 |
| 3274.3668673 | 1 |
| 3274.3676098 | 4 |
| 3274.3683306 | 0 |
| 3274.3684723 | 4 |
| 3274.3691159 | 1 |
| 3274.3694911 | 2 |
| 3274.3701084 | 4 |
| 3274.3709075 | 4 |
| 3274.3714965 | 1 |
| 3274.3724841 | 1 |
| 3274.3727086 | 2 |
| 3274.373261 | 2 |
| 3274.3741025 | 1 |
| 3274.3754147 | 3 |
| 3274.3757633 | 2 |
| 3274.375932 | 2 |
| 3274.3771275 | 2 |
| 3274.3772176 | 0 |
| 3274.3776791 | 5 |
| 3274.3785167 | 0 |
| 3274.3786741 | 1 |
| 3274.3798656 | 4 |
| 3274.3803053 | 1 |
| 3274.3806184 | 3 |
| 3274.381797 | 3 |
| 3274.3819628 | 5 |
| 3274.3826104 | 1 |
| 3274.3830016 | 2 |
| 3274.3830941 | 4 |
| 3274.3849156 | 4 |
| 3274.3851057 | 2 |
| 3274.385646 | 1 |
| 3274.3864224 | 3 |
| 3274.3865474 | 2 |
| 3274.3866569 | 1 |
| 3274.3867977 | 8 |
| 3274.3887826 | 1 |
| 3274.3890599 | 2 |
| 3274.3890999 | 3 |
| 3274.3900205 | 1 |
| 3274.3900395 | 2 |
| 3274.3910841 | 5 |
| 3274.3921982 | 2 |
| 3274.3925275 | 4 |
| 3274.3931158 | 1 |
| 3274.3938504 | 1 |
| 3274.3948001 | 4 |
| 3274.3950336 | 1 |
| 3274.3954232 | 2 |
| 3274.3963376 | 3 |
| 3274.396575 | 4 |
| 3274.3978404 | 2 |
| 3274.3985927 | 1 |
| 3274.3987092 | 1 |
| 3274.3993233 | 1 |
| 3274.399437 | 0 |
| 3274.399656 | 4 |
| 3274.4001037 | 1 |
| 3274.4012744 | 0 |
| 3274.4024834 | 1 |
| 3274.4025708 | 5 |
| 3274.4031289 | 4 |
| 3274.4038062 | 0 |
| 3274.4041928 | 5 |
| 3274.4042064 | 6 |
| 3274.4057191 | 5 |
| 3274.4075417 | 3 |
| 3274.4100296 | 1 |
| 3274.413488 | 2 |
| 3274.4152343 | 1 |
| 3274.4177513 | 1 |
| 3274.4195304 | 2 |
| 3274.4228525 | 3 |
| 3274.4255175 | 3 |
| 3274.4282021 | 3 |
| 3274.4316166 | 2 |
| 3274.4326971 | 2 |
| 3274.4364135 | 2 |
| 3274.4373178 | 2 |
| 3274.4401589 | 2 |
| 3274.4436634 | 2 |
| 3274.445215 | 6 |
| 3274.4484694 | 5 |
| 3274.4514515 | 2 |
| 3274.4532846 | 4 |
| 3274.4563909 | 3 |
| 3274.4574701 | 2 |
| 3274.4600429 | 7 |
| 3274.4621049 | 5 |
| 3274.4654209 | 2 |
| 3274.4690707 | 0 |
| 3274.4699752 | 3 |
| 3274.4731245 | 1 |
| 3274.476536 | 5 |
| 3274.4780259 | 3 |
| 3274.4808663 | 1 |
| 3274.4831025 | 2 |
| 3274.4866103 | 3 |
| 3274.4873827 | 2 |
| 3274.4910904 | 3 |
| 3274.4943823 | 1 |
| 3274.495213 | 2 |
| 3274.498498 | 4 |
| 3274.5002611 | 5 |
| 3274.5029585 | 3 |
| 3274.5060612 | 3 |
| 3274.5075787 | 4 |
| 3274.5106175 | 2 |
| 3274.5140515 | 3 |
| 3274.5154553 | 5 |
| 3274.5195702 | 3 |
| 3274.5203312 | 6 |
| 3274.5237508 | 0 |
| 3274.5272762 | 5 |
| 3274.5290161 | 2 |
| 3274.5320683 | 2 |
| 3274.5331153 | 3 |
| 3274.5365939 | 2 |
| 3274.5376965 | 3 |
| 3274.5411467 | 2 |
| 3274.5437811 | 4 |
| 3274.5453893 | 2 |
| 3274.5487959 | 0 |
| 3274.5504563 | 1 |
| 3274.5531283 | 4 |
| 3274.5568783 | 1 |
| 3274.5582775 | 4 |
| 3274.561228 | 4 |
| 3274.5616322 | 1 |
| 3274.5620327 | 2 |
| 3274.562466 | 4 |
| 3274.5628418 | 5 |
| 3274.5645106 | 3 |
| 3274.5655669 | 2 |
| 3274.5662299 | 2 |
| 3274.5664385 | 4 |
| 3274.5676481 | 2 |
| 3274.5679705 | 3 |
| 3274.5695508 | 3 |
| 3274.5705664 | 5 |
| 3274.5708597 | 4 |
| 3274.5713872 | 4 |
| 3274.5727529 | 3 |
| 3274.5744314 | 5 |
| 3274.5746052 | 2 |
| 3274.5750213 | 2 |
| 3274.5756664 | 5 |
| 3274.5768157 | 2 |
| 3274.5783479 | 3 |
| 3274.5785136 | 2 |
| 3274.5791151 | 0 |
| 3274.5793438 | 1 |
| 3274.5819352 | 0 |
| 3274.5821035 | 2 |
| 3274.5828213 | 0 |
| 3274.5830723 | 1 |
| 3274.5845284 | 2 |
| 3274.5860274 | 0 |
| 3274.5865266 | 2 |
| 3274.586681 | 2 |
| 3274.5880233 | 1 |
| 3274.5884417 | 1 |
| 3274.5891195 | 6 |
| 3274.5906512 | 3 |
| 3274.5907553 | 2 |
| 3274.5913278 | 1 |
| 3274.5925401 | 2 |
| 3274.5928381 | 3 |
| 3274.594088 | 1 |
| 3274.5950031 | 3 |
| 3274.5960198 | 2 |
| 3274.5967428 | 3 |
| 3274.5970994 | 4 |
| 3274.5979071 | 1 |
| 3274.5989147 | 2 |
| 3274.5995914 | 2 |
| 3274.6006133 | 0 |
| 3274.601344 | 5 |
| 3274.602668 | 0 |
| 3274.6028699 | 2 |
| 3274.6035332 | 1 |
| 3274.6044771 | 4 |
| 3274.6054379 | 6 |
| 3274.6058311 | 5 |
| 3274.6079047 | 4 |
| 3274.6082882 | 3 |
| 3274.6087938 | 1 |
| 3274.6090945 | 3 |
| 3274.610957 | 6 |
| 3274.6121443 | 3 |
| 3274.612641 | 6 |
| 3274.6137943 | 1 |
| 3274.6143256 | 1 |
| 3274.6158405 | 1 |
| 3274.6164952 | 3 |
| 3274.6166166 | 3 |
| 3274.6171334 | 2 |
| 3274.6180603 | 2 |
| 3274.6194536 | 3 |
| 3274.6207129 | 2 |
| 3274.6209904 | 3 |
| 3274.6214144 | 1 |
| 3274.6225035 | 1 |
| 3274.623522 | 1 |
| 3274.6247402 | 3 |
| 3274.6252567 | 3 |
| 3274.6261679 | 2 |
| 3274.6272611 | 3 |
| 3274.6282268 | 1 |
| 3274.6286923 | 0 |
| 3274.6298158 | 4 |
| 3274.6298293 | 5 |
| 3274.6309585 | 5 |
| 3274.6319326 | 2 |
| 3274.6330992 | 3 |
| 3274.6337045 | 2 |
| 3274.6339935 | 2 |
| 3274.6349225 | 2 |
| 3274.6366441 | 1 |
| 3274.6366685 | 2 |
| 3274.6372572 | 3 |
| 3274.6377811 | 2 |
| 3274.6390607 | 2 |
| 3274.6403724 | 1 |
| 3274.6409969 | 6 |
| 3274.6413218 | 0 |
| 3274.64187 | 1 |
| 3274.6426955 | 2 |
| 3274.6430038 | 4 |
| 3274.6449918 | 10 |
| 3274.6462659 | 15 |
| 3274.6469142 | 15 |
| 3274.6479934 | 26 |
| 3274.6485138 | 32 |
| 3274.6497663 | 8 |
| 3274.6498956 | 23 |
| 3274.6510038 | 3 |
| 3274.6517149 | 4 |
| 3274.6525053 | 1 |
| 3274.652739 | 2 |
| 3274.6540519 | 3 |
| 3274.6543067 | 3 |
| 3274.6556445 | 1 |
| 3274.656109 | 4 |
| 3274.657626 | 2 |
| 3274.6580267 | 2 |
| 3274.6591925 | 3 |
| 3274.6606696 | 1 |
| 3274.6611714 | 0 |
| 3274.6614178 | 2 |
| 3274.6628217 | 4 |
| 3274.6641658 | 2 |
| 3274.6641684 | 3 |
| 3274.6657001 | 6 |
| 3274.6657492 | 1 |
| 3274.6666596 | 2 |
| 3274.6668221 | 1 |
| 3274.6681556 | 5 |
| 3274.669512 | 6 |
| 3274.6698794 | 0 |
| 3274.670062 | 2 |
| 3274.671582 | 2 |
| 3274.6732498 | 1 |
| 3274.6733948 | 2 |
| 3274.6742222 | 3 |
| 3274.674608 | 5 |
| 3274.6756675 | 6 |
| 3274.6763299 | 2 |
| 3274.6774558 | 1 |
| 3274.6777005 | 1 |
| 3274.6786134 | 3 |
| 3274.6808555 | 3 |
| 3274.6824664 | 2 |
| 3274.6854839 | 4 |
| 3274.6885232 | 1 |
| 3274.690016 | 3 |
| 3274.6928351 | 2 |
| 3274.6946023 | 1 |
| 3274.6982851 | 1 |
| 3274.7009662 | 4 |
| 3274.7035562 | 3 |
| 3274.705647 | 1 |
| 3274.7076385 | 0 |
| 3274.7111826 | 2 |
| 3274.7137325 | 3 |
| 3274.7158488 | 0 |
| 3274.7185733 | 3 |
| 3274.7204073 | 2 |
| 3274.7236282 | 1 |
| 3274.726376 | 2 |
| 3274.7274298 | 2 |
| 3274.7308526 | 1 |
| 3274.7320961 | 3 |
| 3274.7350066 | 1 |
| 3274.7382297 | 2 |
| 3274.7394197 | 4 |
| 3274.7425661 | 6 |
| 3274.7457265 | 0 |
| 3274.7474928 | 0 |
| 3274.7511847 | 2 |
| 3274.7522179 | 0 |
| 3274.7564222 | 2 |
| 3274.7569381 | 0 |
| 3274.7603058 | 2 |
| 3274.7632426 | 2 |
| 3274.7646817 | 2 |
| 3274.7678998 | 2 |
| 3274.7695602 | 2 |
| 3274.7721203 | 1 |
| 3274.775273 | 1 |
| 3274.7763453 | 0 |
| 3274.7793572 | 3 |
| 3274.7808183 | 3 |
| 3274.7841451 | 2 |
| 3274.787794 | 0 |
| 3274.7893689 | 3 |
| 3274.7923777 | 6 |
| 3274.79375 | 3 |
| 3274.7972784 | 3 |
| 3274.7992006 | 5 |
| 3274.8021711 | 4 |

| | |
|---|---|
| 3274.804474 | 4 |
| 3274.8065675 | 3 |
| 3274.8096351 | 4 |
| 3274.8122525 | 1 |
| 3274.8141097 | 1 |
| 3274.8169029 | 2 |
| 3274.8180933 | 4 |
| 3274.8212111 | 2 |
| 3274.8244407 | 3 |
| 3274.8259449 | 4 |
| 3274.8289381 | 0 |
| 3274.8308622 | 4 |
| 3274.8345038 | 2 |
| 3274.8364094 | 4 |
| 3274.8385708 | 2 |
| 3274.8414088 | 2 |
| 3274.8427273 | 2 |
| 3274.8462428 | 1 |
| 3274.8496183 | 1 |
| 3274.8508453 | 4 |
| 3274.8541147 | 2 |
| 3274.8563003 | 2 |
| 3274.8593422 | 3 |
| 3274.8601747 | 3 |
| 3274.8636574 | 5 |
| 3274.8657758 | 2 |
| 3274.8667271 | 1 |
| 3274.870521 | 1 |
| 3274.8735235 | 1 |
| 3274.8749998 | 2 |
| 3274.8780526 | 0 |
| 3274.8796303 | 4 |
| 3274.8830341 | 2 |
| 3274.8839223 | 3 |
| 3274.8881187 | 2 |
| 3274.8913872 | 2 |
| 3274.8929413 | 3 |
| 3274.8960406 | 4 |
| 3274.8973878 | 2 |
| 3274.8999314 | 3 |
| 3274.9033991 | 1 |
| 3274.9046808 | 7 |
| 3274.90734 | 4 |
| 3274.9090484 | 2 |
| 3274.9122942 | 2 |
| 3274.9148595 | 1 |
| 3274.916726 | 3 |
| 3274.9202224 | 3 |
| 3274.9214801 | 3 |
| 3274.9249566 | 1 |
| 3274.9283402 | 3 |
| 3274.9293672 | 5 |
| 3274.9332069 | 1 |
| 3274.9344759 | 3 |
| 3274.9383801 | 4 |
| 3274.9404576 | 2 |
| 3274.9422182 | 2 |
| 3274.9453284 | 0 |
| 3274.9463278 | 3 |
| 3274.949891 | 3 |
| 3274.9516766 | 5 |
| 3274.9544778 | 2 |
| 3274.957245 | 3 |
| 3274.9588182 | 2 |
| 3274.9616182 | 3 |
| 3274.9646711 | 0 |
| 3274.9660131 | 2 |
| 3274.9696866 | 1 |
| 3274.971266 | 0 |
| 3274.9742002 | 4 |
| 3274.977221 | 0 |
| 3274.9789943 | 1 |
| 3274.9832094 | 3 |
| 3274.9841383 | 3 |
| 3274.987092 | 4 |
| 3274.9906805 | 2 |
| 3274.9918328 | 6 |
| 3274.9948502 | 3 |
| 3274.9961802 | 2 |
| 3274.999202 | 1 |
| 3275.0023137 | 1 |
| 3275.0037279 | 3 |
| 3275.0075627 | 0 |
| 3275.0085123 | 4 |
| 3275.011581 | 2 |
| 3275.0144209 | 3 |
| 3275.016327 | 2 |
| 3275.0189478 | 4 |
| 3275.0215593 | 0 |
| 3275.0245846 | 5 |
| 3275.0258938 | 0 |
| 3275.0295 | 3 |
| 3275.0327205 | 4 |
| 3275.0328171 | 4 |
| 3275.0370152 | 1 |
| 3275.040233 | 2 |
| 3275.041375 | 1 |
| 3275.0439659 | 2 |
| 3275.0460666 | 1 |
| 3275.0486995 | 1 |
| 3275.0503271 | 0 |
| 3275.0541663 | 3 |
| 3275.0565714 | 3 |
| 3275.0581063 | 1 |
| 3275.0610706 | 2 |
| 3275.0644403 | 2 |
| 3275.0665023 | 2 |
| 3275.0694415 | 3 |
| 3275.0709864 | 1 |
| 3275.0745066 | 1 |
| 3275.0778224 | 2 |
| 3275.0787081 | 3 |
| 3275.0821616 | 1 |
| 3275.0835135 | 1 |
| 3275.0864173 | 5 |
| 3275.0897494 | 1 |
| 3275.0912111 | 3 |
| 3275.0941794 | 5 |
| 3275.0960229 | 3 |
| 3275.0985282 | 0 |
| 3275.1004255 | 3 |
| 3275.103836 | 3 |
| 3275.1068551 | 2 |
| 3275.1079041 | 2 |
| 3275.111623 | 1 |
| 3275.1133162 | 2 |
| 3275.1168339 | 3 |
| 3275.1199957 | 1 |
| 3275.121465 | 0 |
| 3275.1244494 | 1 |
| 3275.1270791 | 1 |
| 3275.1289146 | 3 |
| 3275.1322535 | 0 |
| 3275.13344 | 1 |
| 3275.136388 | 2 |
| 3275.1399228 | 0 |
| 3275.1410628 | 5 |
| 3275.1438311 | 7 |
| 3275.1456938 | 5 |
| 3275.1492367 | 1 |
| 3275.1505077 | 3 |
| 3275.1535588 | 1 |
| 3275.1563488 | 2 |
| 3275.1586671 | 3 |
| 3275.1617725 | 4 |
| 3275.1635153 | 1 |
| 3275.1669183 | 2 |
| 3275.1696768 | 3 |
| 3275.1711098 | 2 |
| 3275.1745052 | 3 |
| 3275.1765694 | 2 |
| 3275.1795476 | 4 |
| 3275.1821406 | 4 |
| 3275.183843 | 5 |
| 3275.1862913 | 4 |
| 3275.1872285 | 1 |
| 3275.1909515 | 3 |
| 3275.194142 | 0 |
| 3275.1958428 | 3 |
| 3275.1987984 | 0 |
| 3275.201476 | 1 |
| 3275.2043876 | 2 |
| 3275.2074562 | 1 |
| 3275.2090503 | 2 |
| 3275.2123663 | 2 |
| 3275.2141705 | 2 |
| 3275.2168348 | 3 |
| 3275.2199862 | 5 |
| 3275.2215136 | 2 |
| 3275.2247206 | 0 |
| 3275.2271458 | 2 |
| 3275.2286106 | 4 |
| 3275.2319759 | 2 |
| 3275.2331812 | 1 |
| 3275.2364412 | 0 |
| 3275.2388008 | 5 |
| 3275.2416054 | 3 |
| 3275.2435257 | 6 |
| 3275.2445452 | 1 |
| 3275.2463381 | 5 |
| 3275.2465885 | 2 |
| 3275.2480072 | 2 |
| 3275.2493184 | 4 |
| 3275.2507686 | 3 |
| 3275.2510822 | 1 |
| 3275.2545307 | 5 |
| 3275.2545983 | 0 |
| 3275.2557991 | 2 |
| 3275.2578661 | 0 |
| 3275.2588192 | 2 |
| 3275.2594759 | 1 |
| 3275.2621699 | 3 |
| 3275.2625028 | 0 |
| 3275.2636403 | 3 |
| 3275.2640136 | 6 |
| 3275.2667926 | 1 |
| 3275.2668442 | 0 |
| 3275.268085 | 1 |
| 3275.2702768 | 0 |
| 3275.2711903 | 2 |
| 3275.2716046 | 5 |
| 3275.273384 | 2 |
| 3275.2750511 | 2 |
| 3275.2759922 | 3 |
| 3275.2763684 | 3 |
| 3275.278919 | 6 |
| 3275.2792054 | 1 |
| 3275.2800642 | 3 |
| 3275.2809303 | 10 |
| 3275.2832975 | 3 |
| 3275.2842876 | 3 |
| 3275.2849165 | 1 |
| 3275.2867591 | 3 |
| 3275.2877371 | 4 |
| 3275.288387 | 2 |
| 3275.2891884 | 2 |
| 3275.2909612 | 4 |
| 3275.2919362 | 3 |
| 3275.295801 | 4 |
| 3275.2990618 | 0 |
| 3275.299842 | 6 |
| 3275.3032713 | 2 |
| 3275.3050969 | 2 |
| 3275.3077025 | 2 |
| 3275.3113553 | 1 |
| 3275.3138276 | 1 |
| 3275.3159875 | 1 |
| 3275.3176441 | 5 |
| 3275.3209652 | 6 |
| 3275.3218381 | 1 |
| 3275.3256583 | 5 |
| 3275.3280664 | 3 |
| 3275.3294837 | 2 |
| 3275.3338787 | 1 |
| 3275.3342763 | 3 |
| 3275.3382659 | 1 |
| 3275.3409561 | 2 |
| 3275.3419684 | 3 |
| 3275.3450782 | 1 |
| 3275.3474692 | 5 |
| 3275.3508344 | 3 |
| 3275.3518287 | 2 |
| 3275.3543595 | 0 |
| 3275.3573456 | 2 |
| 3275.3613365 | 3 |
| 3275.3616628 | 5 |
| 3275.3653059 | 2 |
| 3275.366876 | 0 |
| 3275.3700162 | 3 |
| 3275.3729202 | 5 |
| 3275.3753321 | 4 |
| 3275.3778165 | 3 |
| 3275.3795018 | 4 |
| 3275.3824226 | 1 |
| 3275.3848969 | 2 |
| 3275.3859018 | 1 |
| 3275.3900811 | 3 |
| 3275.3912159 | 4 |
| 3275.3943503 | 3 |
| 3275.3975327 | 4 |
| 3275.3989745 | 4 |
| 3275.4021277 | 0 |
| 3275.4041825 | 0 |
| 3275.4069428 | 4 |
| 3275.410213 | 2 |
| 3275.4117857 | 3 |
| 3275.4154559 | 2 |
| 3275.4160446 | 4 |
| 3275.4196578 | 6 |
| 3275.4217732 | 2 |
| 3275.4231463 | 5 |
| 3275.4262998 | 3 |
| 3275.4281718 | 3 |
| 3275.430591 | 6 |
| 3275.4340844 | 5 |
| 3275.4364987 | 0 |
| 3275.4387676 | 5 |
| 3275.4404507 | 4 |
| 3275.4441918 | 3 |
| 3275.4462626 | 0 |
| 3275.4481908 | 1 |
| 3275.4514352 | 5 |
| 3275.453972 | 5 |
| 3275.4557756 | 3 |
| 3275.4588397 | 3 |
| 3275.4599529 | 1 |
| 3275.4632773 | 4 |
| 3275.4660628 | 1 |
| 3275.4686248 | 3 |
| 3275.4710173 | 4 |
| 3275.4730992 | 1 |
| 3275.4763804 | 3 |
| 3275.479306 | 3 |
| 3275.4803223 | 3 |
| 3275.4838762 | 3 |
| 3275.4845211 | 0 |
| 3275.4888933 | 4 |
| 3275.4899115 | 0 |
| 3275.4925924 | 0 |
| 3275.496454 | 2 |
| 3275.4977293 | 2 |
| 3275.5005855 | 1 |
| 3275.5030459 | 4 |
| 3275.5057351 | 2 |
| 3275.5087177 | 4 |
| 3275.509532 | 1 |
| 3275.5133884 | 1 |
| 3275.5140412 | 4 |
| 3275.5174906 | 5 |
| 3275.5211255 | 3 |
| 3275.5223208 | 2 |
| 3275.5249593 | 3 |
| 3275.5288172 | 2 |
| 3275.5299824 | 1 |
| 3275.5330054 | 0 |
| 3275.5352718 | 3 |
| 3275.5383079 | 3 |
| 3275.5394815 | 2 |
| 3275.5428056 | 0 |
| 3275.5459706 | 2 |
| 3275.5469805 | 0 |
| 3275.5506895 | 1 |
| 3275.5519545 | 4 |
| 3275.5546401 | 2 |
| 3275.5554242 | 3 |
| 3275.5558823 | 0 |
| 3275.5573674 | 2 |
| 3275.5581538 | 1 |
| 3275.5581634 | 1 |
| 3275.5596874 | 4 |
| 3275.5603574 | 2 |
| 3275.5615383 | 2 |
| 3275.562451 | 1 |
| 3275.5634217 | 2 |
| 3275.5642823 | 5 |
| 3275.5643008 | 3 |
| 3275.5660517 | 5 |
| 3275.5664643 | 2 |
| 3275.5672884 | 2 |
| 3275.5691432 | 4 |
| 3275.5699275 | 4 |
| 3275.5699925 | 1 |
| 3275.571731 | 1 |
| 3275.5727262 | 1 |
| 3275.5729791 | 3 |
| 3275.5744892 | 3 |
| 3275.5752051 | 2 |
| 3275.5760009 | 5 |
| 3275.5761673 | 1 |
| 3275.578112 | 1 |
| 3275.5789088 | 1 |
| 3275.5806432 | 2 |
| 3275.5806595 | 1 |
| 3275.5817868 | 1 |
| 3275.5824943 | 2 |
| 3275.5831894 | 1 |
| 3275.5851714 | 2 |
| 3275.5858959 | 1 |
| 3275.5859667 | 3 |
| 3275.5878485 | 1 |
| 3275.5885735 | 1 |
| 3275.5896727 | 1 |
| 3275.5897286 | 3 |

| | |
|---|---|
| 3275.5917348 | 1 |
| 3275.592089 | 4 |
| 3275.592815 | 1 |
| 3275.5938489 | 4 |
| 3275.5942501 | 2 |
| 3275.5957933 | 1 |
| 3275.5963926 | 3 |
| 3275.5978174 | 2 |
| 3275.5988139 | 4 |
| 3275.5992947 | 1 |
| 3275.6005665 | 4 |
| 3275.6012515 | 2 |
| 3275.6017958 | 0 |
| 3275.6028086 | 0 |
| 3275.6035254 | 0 |
| 3275.6045219 | 0 |
| 3275.6060999 | 3 |
| 3275.6065553 | 2 |
| 3275.6078623 | 2 |
| 3275.609097 | 1 |
| 3275.6096915 | 4 |
| 3275.6103523 | 1 |
| 3275.6122233 | 1 |
| 3275.612741 | 2 |
| 3275.6129457 | 3 |
| 3275.6141997 | 6 |
| 3275.6148682 | 1 |
| 3275.6154248 | 2 |
| 3275.6172527 | 2 |
| 3275.6181753 | 1 |
| 3275.6185812 | 2 |
| 3275.6198274 | 2 |
| 3275.6210758 | 5 |
| 3275.6216816 | 2 |
| 3275.6229266 | 2 |
| 3275.6239961 | 5 |
| 3275.6246995 | 2 |
| 3275.6252756 | 4 |
| 3275.6267456 | 0 |
| 3275.6276786 | 6 |
| 3275.6287889 | 3 |
| 3275.6295398 | 0 |
| 3275.6297644 | 5 |
| 3275.6315986 | 3 |
| 3275.631992 | 2 |
| 3275.6330804 | 4 |
| 3275.63453 | 1 |
| 3275.6352431 | 3 |
| 3275.6362117 | 3 |
| 3275.6368314 | 3 |
| 3275.6379594 | 1 |
| 3275.6397661 | 1 |
| 3275.6397742 | 2 |
| 3275.6407126 | 1 |
| 3275.641475 | 3 |
| 3275.6424146 | 1 |
| 3275.6434498 | 3 |
| 3275.6442826 | 0 |
| 3275.6450794 | 2 |
| 3275.6462249 | 1 |
| 3275.6470214 | 3 |
| 3275.6473177 | 0 |
| 3275.6488188 | 0 |
| 3275.6503046 | 1 |
| 3275.6506546 | 4 |
| 3275.6517592 | 2 |
| 3275.6519392 | 0 |
| 3275.6525958 | 2 |
| 3275.6546029 | 2 |
| 3275.655043 | 3 |
| 3275.6567791 | 2 |
| 3275.6571184 | 3 |
| 3275.6580343 | 5 |
| 3275.6595643 | 2 |
| 3275.660263 | 0 |
| 3275.6603506 | 3 |
| 3275.661953 | 2 |
| 3275.6623685 | 3 |
| 3275.6641379 | 3 |
| 3275.6643344 | 2 |
| 3275.6651357 | 2 |
| 3275.6662532 | 1 |
| 3275.6674317 | 2 |
| 3275.6685677 | 6 |
| 3275.6694999 | 5 |
| 3275.6702792 | 2 |
| 3275.671725 | 2 |
| 3275.6725047 | 3 |
| 3275.6738921 | 2 |
| 3275.6747333 | 3 |
| 3275.6756303 | 3 |
| 3275.6760293 | 4 |
| 3275.6773952 | 1 |
| 3275.6783887 | 1 |
| 3275.6791637 | 2 |
| 3275.680699 | 2 |
| 3275.6810777 | 1 |
| 3275.68175 | 1 |
| 3275.6828706 | 1 |
| 3275.6833545 | 3 |
| 3275.685105 | 1 |
| 3275.6860389 | 4 |
| 3275.686539 | 2 |
| 3275.687952 | 5 |
| 3275.6886721 | 2 |
| 3275.6893219 | 2 |
| 3275.6902105 | 3 |
| 3275.6912563 | 2 |
| 3275.6921908 | 2 |
| 3275.6929008 | 5 |
| 3275.6944599 | 2 |
| 3275.6945841 | 3 |
| 3275.6958793 | 3 |
| 3275.6969637 | 1 |
| 3275.6972349 | 2 |
| 3275.6977349 | 2 |
| 3275.6993283 | 2 |
| 3275.7002919 | 3 |
| 3275.7012024 | 4 |
| 3275.7018026 | 2 |
| 3275.7033952 | 2 |
| 3275.7035565 | 3 |
| 3275.704712 | 3 |
| 3275.7061386 | 0 |
| 3275.7062108 | 4 |
| 3275.7080055 | 4 |
| 3275.7080927 | 3 |
| 3275.7103402 | 1 |
| 3275.7107874 | 2 |
| 3275.7114518 | 1 |
| 3275.7131887 | 5 |
| 3275.7141197 | 3 |
| 3275.7144836 | 2 |
| 3275.7155125 | 2 |
| 3275.7166828 | 4 |
| 3275.7179264 | 1 |
| 3275.7181148 | 4 |
| 3275.7197842 | 3 |
| 3275.7207163 | 3 |
| 3275.7209078 | 6 |
| 3275.7216703 | 4 |
| 3275.7235735 | 2 |
| 3275.7241336 | 2 |
| 3275.7254932 | 1 |
| 3275.7260081 | 1 |
| 3275.7267936 | 1 |
| 3275.7281953 | 2 |
| 3275.729268 | 0 |
| 3275.7298873 | 1 |
| 3275.7313448 | 4 |
| 3275.7322927 | 1 |
| 3275.7323119 | 3 |
| 3275.7331269 | 5 |
| 3275.7346946 | 4 |
| 3275.735026 | 1 |
| 3275.7358093 | 1 |
| 3275.7370491 | 4 |
| 3275.738588 | 3 |
| 3275.7390921 | 3 |
| 3275.7402562 | 4 |
| 3275.7411176 | 2 |
| 3275.7413276 | 2 |
| 3275.7429101 | 0 |
| 3275.7436969 | 3 |
| 3275.7445747 | 3 |
| 3275.7456148 | 2 |
| 3275.7460208 | 3 |
| 3275.7474517 | 1 |
| 3275.7486796 | 3 |
| 3275.7490452 | 2 |
| 3275.7503498 | 0 |
| 3275.7509765 | 3 |
| 3275.7526737 | 5 |
| 3275.7533889 | 2 |
| 3275.7540091 | 2 |
| 3275.7551229 | 2 |
| 3275.7555628 | 2 |
| 3275.7567249 | 0 |
| 3275.7573474 | 5 |
| 3275.7579385 | 10 |
| 3275.7596102 | 28 |
| 3275.7610453 | 27 |
| 3275.7618423 | 34 |
| 3275.7625486 | 33 |
| 3275.7638485 | 22 |
| 3275.7648852 | 25 |
| 3275.7655369 | 32 |
| 3275.7663486 | 22 |
| 3275.7672893 | 11 |
| 3275.7682305 | 6 |
| 3275.7695867 | 4 |
| 3275.77002 | 1 |
| 3275.77106 | 2 |
| 3275.7724604 | 2 |
| 3275.7725407 | 1 |
| 3275.7738434 | 1 |
| 3275.7743245 | 1 |
| 3275.7758359 | 2 |
| 3275.7759894 | 2 |
| 3275.7778739 | 2 |
| 3275.7784912 | 2 |
| 3275.7793481 | 0 |
| 3275.7805363 | 2 |
| 3275.781135 | 4 |
| 3275.7819802 | 4 |
| 3275.7833585 | 4 |
| 3275.784629 | 2 |
| 3275.7852302 | 1 |
| 3275.7859779 | 2 |
| 3275.7875394 | 2 |
| 3275.788099 | 1 |
| 3275.7887026 | 2 |
| 3275.7889367 | 3 |
| 3275.7901376 | 2 |
| 3275.7907779 | 3 |
| 3275.7921552 | 2 |
| 3275.793605 | 4 |
| 3275.7945869 | 2 |
| 3275.7948685 | 1 |
| 3275.7968631 | 2 |
| 3275.7972684 | 2 |
| 3275.7973496 | 4 |
| 3275.7994416 | 3 |
| 3275.7998252 | 1 |
| 3275.8007869 | 5 |
| 3275.801618 | 2 |
| 3275.8030768 | 2 |
| 3275.8038646 | 1 |
| 3275.8044921 | 1 |
| 3275.8051032 | 2 |
| 3275.8067374 | 1 |
| 3275.8077574 | 4 |
| 3275.808568 | 3 |
| 3275.8096479 | 5 |
| 3275.8108503 | 3 |
| 3275.810911 | 1 |
| 3275.8129776 | 4 |
| 3275.813983 | 2 |
| 3275.8139986 | 3 |
| 3275.8157183 | 3 |
| 3275.8160781 | 1 |
| 3275.8175005 | 6 |
| 3275.8181648 | 5 |
| 3275.8190354 | 2 |
| 3275.8193747 | 0 |
| 3275.8208887 | 2 |
| 3275.8227034 | 3 |
| 3275.8232082 | 1 |
| 3275.8240679 | 2 |
| 3275.824991 | 3 |
| 3275.8251411 | 1 |
| 3275.8264447 | 0 |
| 3275.8269372 | 3 |
| 3275.8288434 | 1 |
| 3275.8294516 | 4 |
| 3275.8302331 | 2 |
| 3275.8313862 | 3 |
| 3275.8317898 | 4 |
| 3275.8321836 | 1 |
| 3275.8334538 | 2 |
| 3275.8335254 | 0 |
| 3275.8350029 | 4 |
| 3275.8368081 | 2 |
| 3275.8371513 | 2 |
| 3275.8383049 | 1 |
| 3275.8395269 | 0 |
| 3275.8406702 | 0 |
| 3275.8414592 | 2 |
| 3275.8426316 | 2 |
| 3275.8431221 | 0 |
| 3275.8441706 | 1 |
| 3275.8447434 | 1 |
| 3275.8458683 | 1 |
| 3275.8471929 | 0 |
| 3275.8484135 | 3 |
| 3275.8486381 | 0 |
| 3275.8496903 | 1 |
| 3275.850597 | 1 |
| 3275.8514676 | 4 |
| 3275.8523174 | 1 |
| 3275.8529965 | 9 |
| 3275.854745 | 2 |
| 3275.855305 | 2 |
| 3275.8562551 | 2 |
| 3275.857459 | 3 |
| 3275.8581147 | 5 |
| 3275.8592761 | 0 |
| 3275.8598338 | 3 |
| 3275.861072 | 2 |
| 3275.8617902 | 2 |
| 3275.8634248 | 4 |
| 3275.8635198 | 1 |
| 3275.8647416 | 1 |
| 3275.8659772 | 3 |
| 3275.8664627 | 4 |
| 3275.8676897 | 8 |
| 3275.8680954 | 6 |
| 3275.8696305 | 6 |
| 3275.8705958 | 0 |
| 3275.871488 | 1 |
| 3275.8724231 | 0 |
| 3275.8733801 | 1 |
| 3275.8738276 | 3 |
| 3275.8748927 | 5 |
| 3275.8762216 | 5 |
| 3275.8772617 | 1 |
| 3275.8780304 | 1 |
| 3275.878717 | 0 |
| 3275.8801228 | 1 |
| 3275.8810683 | 7 |
| 3275.8811995 | 0 |
| 3275.8818944 | 1 |
| 3275.8832409 | 3 |
| 3275.8844428 | 2 |
| 3275.8849871 | 3 |
| 3275.8864188 | 0 |
| 3275.8870522 | 6 |
| 3275.8876381 | 1 |
| 3275.8885332 | 1 |
| 3275.8905632 | 1 |
| 3275.8912032 | 4 |
| 3275.8918201 | 4 |
| 3275.8920884 | 3 |
| 3275.8933155 | 2 |
| 3275.8953561 | 2 |
| 3275.895534 | 4 |
| 3275.8971709 | 1 |
| 3275.8979001 | 1 |
| 3275.8981388 | 3 |
| 3275.8997221 | 0 |
| 3275.9010096 | 3 |
| 3275.9018533 | 1 |
| 3275.9021709 | 3 |
| 3275.902923 | 3 |
| 3275.904564 | 2 |
| 3275.9052336 | 1 |
| 3275.9063081 | 5 |
| 3275.9064513 | 2 |
| 3275.9086947 | 1 |
| 3275.9095698 | 2 |
| 3275.9098664 | 2 |
| 3275.910131 | 3 |
| 3275.911901 | 2 |
| 3275.9129789 | 3 |
| 3275.9132745 | 4 |
| 3275.914936 | 2 |
| 3275.915591 | 2 |
| 3275.9159719 | 4 |
| 3275.9166229 | 5 |
| 3275.9184949 | 3 |
| 3275.9190322 | 2 |
| 3275.919577 | 1 |
| 3275.9205618 | 3 |
| 3275.9213913 | 3 |
| 3275.9228716 | 2 |
| 3275.9238779 | 1 |
| 3275.9251174 | 3 |
| 3275.925835 | 2 |
| 3275.9265816 | 7 |
| 3275.9268597 | 1 |
| 3275.9272964 | 6 |
| 3275.9292607 | 1 |
| 3275.9299303 | 3 |

| | |
|---|---|
| 3275.9316835 | 5 |
| 3275.9323614 | 1 |
| 3275.9328759 | 2 |
| 3275.9340763 | 4 |
| 3275.934343 | 4 |
| 3275.9357465 | 7 |
| 3275.9365777 | 1 |
| 3275.9379849 | 2 |
| 3275.9382398 | 3 |
| 3275.9393238 | 4 |
| 3275.94103 | 5 |
| 3275.9417709 | 1 |
| 3275.9419301 | 1 |
| 3275.9438373 | 4 |
| 3275.9441264 | 3 |
| 3275.9449168 | 3 |
| 3275.9461667 | 1 |
| 3275.9471299 | 1 |
| 3275.947423 | 0 |
| 3275.9488708 | 2 |
| 3275.9503461 | 1 |
| 3275.9505517 | 0 |
| 3275.951922 | 4 |
| 3275.9530398 | 3 |
| 3275.9534065 | 0 |
| 3275.9550688 | 1 |
| 3275.9554354 | 2 |
| 3275.9565996 | 3 |
| 3275.9573501 | 1 |
| 3275.9577138 | 2 |
| 3275.9594787 | 1 |
| 3275.959725 | 3 |
| 3275.9606145 | 2 |
| 3275.9620514 | 2 |
| 3275.9629897 | 3 |
| 3275.9635311 | 2 |
| 3275.9644266 | 5 |
| 3275.9648316 | 1 |
| 3275.9662029 | 2 |
| 3275.966942 | 3 |
| 3275.9685129 | 2 |
| 3275.9688587 | 1 |
| 3275.9707232 | 2 |
| 3275.9708803 | 1 |
| 3275.9714045 | 1 |
| 3275.9722404 | 4 |
| 3275.9735272 | 2 |
| 3275.9742829 | 1 |
| 3275.9758068 | 3 |
| 3275.9760449 | 5 |
| 3275.9770661 | 1 |
| 3275.9785579 | 2 |
| 3275.9794138 | 4 |
| 3275.9802538 | 3 |
| 3275.9815496 | 4 |
| 3275.9819534 | 4 |
| 3275.9829898 | 3 |
| 3275.9845514 | 3 |
| 3275.9858139 | 0 |
| 3275.9860523 | 1 |
| 3275.9863939 | 1 |
| 3275.9876246 | 2 |
| 3275.9882337 | 1 |
| 3275.9894792 | 3 |
| 3275.9904489 | 4 |
| 3275.9917368 | 3 |
| 3275.9923561 | 2 |
| 3275.9933126 | 2 |
| 3275.9944772 | 5 |
| 3275.9949748 | 1 |
| 3275.9961743 | 1 |
| 3275.9979274 | 2 |
| 3275.9982608 | 3 |
| 3275.9989995 | 2 |
| 3275.9997935 | 3 |
| 3276.0013357 | 1 |
| 3276.0021611 | 0 |
| 3276.0029194 | 3 |
| 3276.0041116 | 1 |
| 3276.0042569 | 6 |
| 3276.0055729 | 4 |
| 3276.0067858 | 3 |
| 3276.0068371 | 4 |
| 3276.0081662 | 3 |
| 3276.0089039 | 1 |
| 3276.010457 | 2 |
| 3276.0112525 | 2 |
| 3276.0115336 | 3 |
| 3276.012329 | 0 |
| 3276.0133117 | 1 |
| 3276.0148891 | 2 |
| 3276.0151204 | 4 |
| 3276.0165826 | 4 |
| 3276.0173448 | 2 |
| 3276.0186377 | 3 |
| 3276.0186588 | 2 |
| 3276.0191004 | 1 |
| 3276.019171 | 3 |
| 3276.0200706 | 2 |
| 3276.0204142 | 0 |
| 3276.0206963 | 2 |
| 3276.020821 | 6 |
| 3276.0218811 | 3 |
| 3276.0220575 | 0 |
| 3276.0229862 | 3 |
| 3276.0232563 | 1 |
| 3276.0234381 | 1 |
| 3276.0240051 | 2 |
| 3276.0246771 | 7 |
| 3276.0249446 | 1 |
| 3276.02538 | 1 |
| 3276.0259706 | 7 |
| 3276.0266349 | 3 |
| 3276.0266665 | 3 |
| 3276.0267944 | 3 |
| 3276.0269714 | 1 |
| 3276.0287586 | 2 |
| 3276.0293326 | 3 |
| 3276.0300616 | 0 |
| 3276.0302657 | 1 |
| 3276.0305163 | 0 |
| 3276.0306473 | 0 |
| 3276.0318132 | 1 |
| 3276.0321253 | 3 |
| 3276.032315 | 1 |
| 3276.0327867 | 4 |
| 3276.0332152 | 3 |
| 3276.0336562 | 1 |
| 3276.0342685 | 5 |
| 3276.0345155 | 1 |
| 3276.0346678 | 0 |
| 3276.0355803 | 3 |
| 3276.0364287 | 2 |
| 3276.0368687 | 2 |
| 3276.0369952 | 1 |
| 3276.0372472 | 2 |
| 3276.0376882 | 1 |
| 3276.0377779 | 2 |
| 3276.0384916 | 3 |
| 3276.0388709 | 3 |
| 3276.0392449 | 3 |
| 3276.0393538 | 3 |
| 3276.0402824 | 3 |
| 3276.0411158 | 2 |
| 3276.0420536 | 3 |
| 3276.0422645 | 1 |
| 3276.0425027 | 3 |
| 3276.0431911 | 2 |
| 3276.0435396 | 1 |
| 3276.0442486 | 4 |
| 3276.0442569 | 4 |
| 3276.0446869 | 3 |
| 3276.045323 | 0 |
| 3276.0456504 | 1 |
| 3276.0457724 | 0 |
| 3276.0471492 | 0 |
| 3276.0471659 | 0 |
| 3276.0474772 | 2 |
| 3276.0480417 | 1 |
| 3276.0485031 | 1 |
| 3276.0486138 | 4 |
| 3276.0492958 | 3 |
| 3276.0499844 | 0 |
| 3276.05033 | 5 |
| 3276.0510461 | 2 |
| 3276.0511917 | 2 |
| 3276.0518028 | 2 |
| 3276.0521189 | 1 |
| 3276.0522232 | 1 |
| 3276.0529072 | 9 |
| 3276.0538646 | 0 |
| 3276.0539497 | 1 |
| 3276.0545674 | 1 |
| 3276.0553212 | 1 |
| 3276.0554544 | 2 |
| 3276.0558171 | 2 |
| 3276.0565182 | 2 |
| 3276.0567954 | 0 |
| 3276.057545 | 1 |
| 3276.057819 | 0 |
| 3276.0585302 | 2 |
| 3276.0587775 | 4 |
| 3276.0590543 | 1 |
| 3276.0596235 | 2 |
| 3276.0601923 | 2 |
| 3276.0604985 | 3 |
| 3276.061268 | 3 |
| 3276.0613963 | 1 |
| 3276.0614036 | 2 |
| 3276.0621322 | 3 |
| 3276.0625778 | 0 |
| 3276.0630405 | 2 |
| 3276.0639512 | 2 |
| 3276.0640661 | 4 |
| 3276.065118 | 1 |
| 3276.0653885 | 2 |
| 3276.0655949 | 0 |
| 3276.0660227 | 2 |
| 3276.0660655 | 0 |
| 3276.0672381 | 4 |
| 3276.068096 | 1 |
| 3276.0682057 | 2 |
| 3276.0683684 | 1 |
| 3276.068822 | 1 |
| 3276.069428 | 3 |
| 3276.0694791 | 1 |
| 3276.069866 | 0 |
| 3276.0701328 | 3 |
| 3276.0712132 | 1 |
| 3276.0712625 | 5 |
| 3276.0715367 | 2 |
| 3276.072725 | 2 |
| 3276.0729339 | 1 |
| 3276.0731567 | 3 |
| 3276.0746636 | 1 |
| 3276.0749617 | 1 |
| 3276.0752874 | 2 |
| 3276.075472 | 0 |
| 3276.0761248 | 0 |
| 3276.0763123 | 0 |
| 3276.0767327 | 2 |
| 3276.0775752 | 8 |
| 3276.0777116 | 2 |
| 3276.077822 | 0 |
| 3276.0784498 | 1 |
| 3276.0794652 | 2 |
| 3276.0797781 | 0 |
| 3276.0805418 | 0 |
| 3276.0807976 | 1 |
| 3276.0810085 | 3 |
| 3276.0817208 | 3 |
| 3276.0822901 | 2 |
| 3276.0825479 | 1 |
| 3276.0830085 | 1 |
| 3276.0838344 | 1 |
| 3276.0844246 | 0 |
| 3276.0844305 | 1 |
| 3276.0846524 | 2 |
| 3276.0856397 | 3 |
| 3276.0858821 | 1 |
| 3276.0862208 | 5 |
| 3276.086968 | 3 |
| 3276.0872207 | 3 |
| 3276.0879331 | 2 |
| 3276.0884006 | 1 |
| 3276.088833 | 1 |
| 3276.0890827 | 3 |
| 3276.089544 | 2 |
| 3276.0899367 | 7 |
| 3276.0902454 | 0 |
| 3276.0911603 | 2 |
| 3276.0913675 | 0 |
| 3276.0922256 | 1 |
| 3276.0924084 | 3 |
| 3276.0932295 | 1 |
| 3276.093496 | 3 |
| 3276.0940623 | 3 |
| 3276.0942618 | 4 |
| 3276.0951764 | 2 |
| 3276.0951919 | 1 |
| 3276.0954627 | 1 |
| 3276.0960916 | 3 |
| 3276.0970076 | 1 |
| 3276.0970721 | 4 |
| 3276.097751 | 0 |
| 3276.09801 | 0 |
| 3276.0980973 | 1 |
| 3276.0981684 | 2 |
| 3276.0994572 | 4 |
| 3276.099618 | 0 |
| 3276.1001698 | 3 |
| 3276.1005939 | 1 |
| 3276.101019 | 1 |
| 3276.1016653 | 2 |
| 3276.1017132 | 0 |
| 3276.1019525 | 0 |
| 3276.1031603 | 3 |
| 3276.1032035 | 2 |
| 3276.1041678 | 0 |
| 3276.1043098 | 3 |
| 3276.104929 | 0 |
| 3276.105216 | 0 |
| 3276.105728 | 0 |
| 3276.1063217 | 0 |
| 3276.1071126 | 2 |
| 3276.1071329 | 5 |
| 3276.107347 | 6 |
| 3276.1073886 | 7 |
| 3276.1085877 | 3 |
| 3276.1092122 | 0 |
| 3276.1094197 | 0 |
| 3276.109634 | 2 |
| 3276.110538 | 4 |
| 3276.1108357 | 0 |
| 3276.1110735 | 1 |
| 3276.11133 | 0 |
| 3276.1120211 | 4 |
| 3276.1127446 | 2 |
| 3276.1133717 | 5 |
| 3276.1134648 | 1 |
| 3276.113797 | 1 |
| 3276.1142278 | 2 |
| 3276.1154187 | 1 |
| 3276.115642 | 2 |
| 3276.1156892 | 2 |
| 3276.1157925 | 4 |
| 3276.1168593 | 2 |
| 3276.1170761 | 2 |
| 3276.1179667 | 3 |
| 3276.1182241 | 1 |
| 3276.1188404 | 3 |
| 3276.1191808 | 1 |
| 3276.1197009 | 1 |
| 3276.119963 | 0 |
| 3276.1206412 | 4 |
| 3276.1213838 | 3 |
| 3276.1214735 | 1 |
| 3276.1220789 | 4 |
| 3276.12314 | 8 |
| 3276.1231627 | 8 |
| 3276.1234431 | 2 |
| 3276.1239017 | 4 |
| 3276.1244747 | 3 |
| 3276.1245779 | 3 |
| 3276.1251335 | 5 |
| 3276.1257611 | 5 |
| 3276.1262177 | 3 |
| 3276.1264847 | 3 |
| 3276.1268217 | 1 |
| 3276.1276871 | 2 |
| 3276.128181 | 3 |
| 3276.1284951 | 1 |
| 3276.1289039 | 3 |
| 3276.1304013 | 3 |
| 3276.1304623 | 0 |
| 3276.1304739 | 1 |
| 3276.1305701 | 1 |
| 3276.1315761 | 1 |
| 3276.1316434 | 2 |
| 3276.1325747 | 3 |
| 3276.1330069 | 1 |
| 3276.1335231 | 2 |
| 3276.1341073 | 3 |
| 3276.1345353 | 1 |
| 3276.1347471 | 2 |
| 3276.13492 | 2 |
| 3276.1352389 | 1 |
| 3276.1357197 | 2 |
| 3276.1366173 | 0 |
| 3276.1366945 | 3 |
| 3276.137594 | 0 |
| 3276.1376895 | 5 |
| 3276.1378782 | 2 |
| 3276.1390179 | 0 |
| 3276.1397065 | 2 |
| 3276.1397255 | 0 |
| 3276.1397967 | 6 |
| 3276.1403116 | 0 |
| 3276.1412452 | 3 |
| 3276.1418069 | 3 |
| 3276.1421987 | 0 |
| 3276.1422782 | 0 |
| 3276.1430431 | 1 |

| | |
|---|---|
| 3276.1436342 | 0 |
| 3276.1439687 | 2 |
| 3276.1442705 | 3 |
| 3276.1447787 | 6 |
| 3276.145231 | 0 |
| 3276.146231 | 3 |
| 3276.1462374 | 1 |
| 3276.1463462 | 7 |
| 3276.1470025 | 1 |
| 3276.1475026 | 2 |
| 3276.1482082 | 2 |
| 3276.1482571 | 2 |
| 3276.149157 | 2 |
| 3276.1493197 | 1 |
| 3276.1499149 | 3 |
| 3276.1502706 | 4 |
| 3276.1506202 | 2 |
| 3276.150831 | 2 |
| 3276.1516066 | 1 |
| 3276.1527818 | 1 |
| 3276.153024 | 0 |
| 3276.1532268 | 2 |
| 3276.1537197 | 3 |
| 3276.1543039 | 1 |
| 3276.1549314 | 1 |
| 3276.1553886 | 1 |
| 3276.155407 | 1 |
| 3276.1555444 | 4 |
| 3276.1563602 | 2 |
| 3276.1568324 | 3 |
| 3276.1571257 | 2 |
| 3276.1580511 | 3 |
| 3276.1583911 | 0 |
| 3276.1585487 | 5 |
| 3276.1591739 | 2 |
| 3276.1592886 | 2 |
| 3276.1603123 | 4 |
| 3276.1605321 | 0 |
| 3276.1609872 | 3 |
| 3276.1612527 | 1 |
| 3276.1621489 | 2 |
| 3276.1623085 | 2 |
| 3276.1632029 | 1 |
| 3276.1634015 | 1 |
| 3276.1639861 | 3 |
| 3276.164345 | 2 |
| 3276.1643856 | 0 |
| 3276.1647595 | 3 |
| 3276.1657894 | 1 |
| 3276.1663778 | 1 |
| 3276.1664638 | 2 |
| 3276.1665446 | 2 |
| 3276.1673717 | 1 |
| 3276.1678294 | 4 |
| 3276.1686648 | 1 |
| 3276.1689018 | 2 |
| 3276.1693941 | 3 |
| 3276.1694942 | 3 |
| 3276.1708475 | 3 |
| 3276.1711566 | 1 |
| 3276.1713558 | 3 |
| 3276.1714823 | 1 |
| 3276.1726057 | 1 |
| 3276.1726474 | 1 |
| 3276.1737344 | 1 |
| 3276.1737507 | 2 |
| 3276.1745038 | 2 |
| 3276.1747596 | 0 |
| 3276.1756758 | 1 |
| 3276.175883 | 3 |
| 3276.1759088 | 1 |
| 3276.1761483 | 2 |
| 3276.1774871 | 1 |
| 3276.1775276 | 2 |
| 3276.1778306 | 2 |
| 3276.1784558 | 3 |
| 3276.1790895 | 2 |
| 3276.1794675 | 4 |
| 3276.1799418 | 5 |
| 3276.18039 | 5 |
| 3276.1805157 | 4 |
| 3276.1817194 | 17 |
| 3276.181916 | 13 |
| 3276.1821637 | 23 |
| 3276.1823662 | 28 |
| 3276.1831815 | 36 |
| 3276.1837103 | 17 |
| 3276.1843218 | 24 |
| 3276.1846648 | 28 |
| 3276.1849279 | 39 |
| 3276.1850581 | 30 |
| 3276.185936 | 18 |
| 3276.1865272 | 17 |
| 3276.187406 | 42 |
| 3276.1876242 | 19 |
| 3276.1879579 | 30 |
| 3276.1880747 | 32 |
| 3276.1886789 | 16 |
| 3276.1891068 | 15 |
| 3276.1891952 | 26 |
| 3276.1895093 | 23 |
| 3276.1901685 | 20 |
| 3276.1903399 | 15 |
| 3276.1907608 | 12 |
| 3276.1913638 | 4 |
| 3276.1923956 | 4 |
| 3276.1926111 | 10 |
| 3276.1931007 | 0 |
| 3276.1934235 | 2 |
| 3276.1937541 | 0 |
| 3276.1943518 | 2 |
| 3276.1949509 | 3 |
| 3276.1951745 | 1 |
| 3276.1957578 | 1 |
| 3276.1962424 | 0 |
| 3276.1968476 | 0 |
| 3276.1975837 | 2 |
| 3276.1980385 | 1 |
| 3276.1980539 | 5 |
| 3276.1987706 | 0 |
| 3276.1991139 | 0 |
| 3276.1996187 | 4 |
| 3276.2002487 | 3 |
| 3276.2008432 | 2 |
| 3276.2009065 | 2 |
| 3276.2012105 | 1 |
| 3276.2012522 | 0 |
| 3276.2018435 | 2 |
| 3276.2027135 | 0 |
| 3276.2033889 | 6 |
| 3276.2035052 | 1 |
| 3276.2042683 | 1 |
| 3276.2047507 | 1 |
| 3276.2049785 | 1 |
| 3276.2052564 | 3 |
| 3276.2058415 | 5 |
| 3276.2069956 | 4 |
| 3276.2071329 | 3 |
| 3276.2075287 | 2 |
| 3276.2077442 | 3 |
| 3276.2083962 | 1 |
| 3276.2088548 | 2 |
| 3276.2089099 | 2 |
| 3276.210253 | 4 |
| 3276.2106918 | 3 |
| 3276.2113265 | 0 |
| 3276.2116194 | 1 |
| 3276.2120869 | 1 |
| 3276.2122663 | 0 |
| 3276.2126459 | 4 |
| 3276.2133548 | 0 |
| 3276.213544 | 1 |
| 3276.2139213 | 0 |
| 3276.2143086 | 3 |
| 3276.2158158 | 0 |
| 3276.2163453 | 2 |
| 3276.2164315 | 1 |
| 3276.2166139 | 4 |
| 3276.2169763 | 0 |
| 3276.2178488 | 4 |
| 3276.2180329 | 2 |
| 3276.2184154 | 1 |
| 3276.2190163 | 4 |
| 3276.2194089 | 5 |
| 3276.2195207 | 0 |
| 3276.2201173 | 2 |
| 3276.220296 | 3 |
| 3276.2208649 | 3 |
| 3276.2220429 | 2 |
| 3276.2222428 | 1 |
| 3276.2230975 | 3 |
| 3276.2232341 | 2 |
| 3276.2236394 | 1 |
| 3276.2245982 | 4 |
| 3276.2246004 | 4 |
| 3276.2253892 | 3 |
| 3276.2254628 | 1 |
| 3276.2258505 | 1 |
| 3276.2264097 | 0 |
| 3276.2266308 | 5 |
| 3276.2271525 | 2 |
| 3276.2273122 | 1 |
| 3276.2283492 | 2 |
| 3276.2291335 | 2 |
| 3276.2294099 | 1 |
| 3276.2294899 | 1 |
| 3276.2295891 | 1 |
| 3276.2305058 | 3 |
| 3276.231037 | 1 |
| 3276.2315079 | 2 |
| 3276.2320801 | 2 |
| 3276.2321116 | 3 |
| 3276.2327378 | 2 |
| 3276.2329606 | 3 |
| 3276.2343986 | 2 |
| 3276.2347993 | 3 |
| 3276.2350547 | 1 |
| 3276.235064 | 1 |
| 3276.236091 | 0 |
| 3276.236267 | 0 |
| 3276.2369575 | 0 |
| 3276.2370683 | 3 |
| 3276.2379031 | 3 |
| 3276.2383489 | 0 |
| 3276.238593 | 1 |
| 3276.239238 | 2 |
| 3276.2394589 | 0 |
| 3276.2400742 | 6 |
| 3276.2401529 | 1 |
| 3276.2408015 | 3 |
| 3276.2409814 | 1 |
| 3276.241447 | 3 |
| 3276.2424828 | 4 |
| 3276.2425025 | 1 |
| 3276.2428579 | 0 |
| 3276.2435889 | 0 |
| 3276.2442934 | 1 |
| 3276.2443848 | 7 |
| 3276.2447592 | 0 |
| 3276.245044 | 2 |
| 3276.2455868 | 1 |
| 3276.2462634 | 1 |
| 3276.246624 | 1 |
| 3276.2471479 | 1 |
| 3276.2474484 | 0 |
| 3276.2477425 | 4 |
| 3276.2483784 | 5 |
| 3276.2488675 | 1 |
| 3276.2489621 | 3 |
| 3276.2501235 | 1 |
| 3276.2505828 | 5 |
| 3276.2511611 | 1 |
| 3276.2519381 | 1 |
| 3276.2521221 | 3 |
| 3276.2526082 | 1 |
| 3276.2531355 | 0 |
| 3276.2532711 | 1 |
| 3276.25366 | 0 |
| 3276.2545288 | 1 |
| 3276.2549926 | 4 |
| 3276.2551453 | 1 |
| 3276.2553188 | 0 |
| 3276.2558334 | 1 |
| 3276.2567014 | 2 |
| 3276.2567926 | 1 |
| 3276.2577917 | 2 |
| 3276.2582073 | 3 |
| 3276.258401 | 1 |
| 3276.2587575 | 2 |
| 3276.25911 | 1 |
| 3276.259601 | 1 |
| 3276.2608111 | 2 |
| 3276.2609104 | 0 |
| 3276.2611748 | 0 |
| 3276.2614611 | 2 |
| 3276.2628127 | 1 |
| 3276.2629294 | 1 |
| 3276.2630074 | 1 |
| 3276.2638085 | 0 |
| 3276.2644931 | 0 |
| 3276.2645215 | 2 |
| 3276.2647456 | 3 |
| 3276.2653888 | 0 |
| 3276.2661538 | 2 |
| 3276.266629 | 3 |
| 3276.2670652 | 3 |
| 3276.2673208 | 1 |
| 3276.2676947 | 2 |
| 3276.2688271 | 0 |
| 3276.2692112 | 1 |
| 3276.2695308 | 1 |
| 3276.2704497 | 3 |
| 3276.270509 | 2 |
| 3276.2712293 | 2 |
| 3276.2716163 | 1 |
| 3276.2717789 | 1 |
| 3276.2722679 | 1 |
| 3276.2725654 | 2 |
| 3276.2731708 | 0 |
| 3276.2739264 | 2 |
| 3276.2741522 | 1 |
| 3276.2748324 | 1 |
| 3276.2751041 | 6 |
| 3276.2752773 | 1 |
| 3276.2754532 | 0 |
| 3276.2764313 | 0 |
| 3276.2766071 | 2 |
| 3276.2766894 | 3 |
| 3276.2776398 | 1 |
| 3276.2779741 | 2 |
| 3276.2785028 | 1 |
| 3276.2790142 | 0 |
| 3276.2796002 | 0 |
| 3276.280536 | 2 |
| 3276.2806338 | 1 |
| 3276.281455 | 3 |
| 3276.2815154 | 6 |
| 3276.2819795 | 2 |
| 3276.2825071 | 3 |
| 3276.2832146 | 0 |
| 3276.2833368 | 2 |
| 3276.2838505 | 0 |
| 3276.2847864 | 1 |
| 3276.2850245 | 1 |
| 3276.2852433 | 0 |
| 3276.2857296 | 0 |
| 3276.2864386 | 2 |
| 3276.2866211 | 5 |
| 3276.2876381 | 3 |
| 3276.2883857 | 1 |
| 3276.2889865 | 1 |
| 3276.2890287 | 4 |
| 3276.2894066 | 4 |
| 3276.2898525 | 2 |
| 3276.2905071 | 2 |
| 3276.2911962 | 0 |
| 3276.2915932 | 1 |
| 3276.2923238 | 0 |
| 3276.2926104 | 0 |
| 3276.2932747 | 4 |
| 3276.2937307 | 2 |
| 3276.2944617 | 3 |
| 3276.294785 | 4 |
| 3276.2950779 | 4 |
| 3276.2953164 | 0 |
| 3276.2963614 | 3 |
| 3276.2967043 | 1 |
| 3276.2974974 | 0 |
| 3276.2980508 | 0 |
| 3276.2980994 | 5 |
| 3276.2993811 | 1 |
| 3276.2995173 | 1 |
| 3276.299565 | 0 |
| 3276.3004426 | 1 |
| 3276.3021582 | 1 |
| 3276.3023413 | 2 |
| 3276.3024009 | 2 |
| 3276.3028046 | 0 |
| 3276.3033027 | 0 |
| 3276.3034787 | 1 |
| 3276.3049179 | 1 |
| 3276.3049683 | 3 |
| 3276.3059155 | 2 |
| 3276.3061649 | 1 |
| 3276.3063443 | 2 |
| 3276.307283 | 2 |
| 3276.3075122 | 1 |
| 3276.308171 | 1 |
| 3276.3090396 | 0 |
| 3276.3098715 | 2 |
| 3276.3099211 | 2 |
| 3276.3102831 | 4 |
| 3276.311401 | 2 |
| 3276.3118364 | 3 |
| 3276.3122017 | 4 |
| 3276.3122767 | 0 |
| 3276.3130173 | 4 |
| 3276.3130624 | 3 |
| 3276.3144868 | 0 |
| 3276.3149414 | 0 |
| 3276.3150518 | 3 |
| 3276.3159947 | 1 |
| 3276.3165253 | 1 |

| | |
|---|---|
| 3276.3172707 | 1 |
| 3276.3180144 | 1 |
| 3276.3183108 | 2 |
| 3276.3187863 | 4 |
| 3276.3189651 | 1 |
| 3276.3198599 | 1 |
| 3276.3206207 | 3 |
| 3276.3206247 | 0 |
| 3276.3207244 | 1 |
| 3276.3221722 | 1 |
| 3276.3223209 | 3 |
| 3276.3233964 | 3 |
| 3276.3235641 | 1 |
| 3276.3240844 | 0 |
| 3276.3246853 | 1 |
| 3276.3246857 | 6 |
| 3276.3261076 | 1 |
| 3276.3261374 | 2 |
| 3276.326251 | 2 |
| 3276.3274481 | 5 |
| 3276.3275592 | 0 |
| 3276.3283589 | 1 |
| 3276.3285779 | 2 |
| 3276.3289494 | 4 |
| 3276.3300539 | 2 |
| 3276.3300989 | 4 |
| 3276.3311686 | 4 |
| 3276.3313083 | 1 |
| 3276.3324618 | 3 |
| 3276.3325507 | 3 |
| 3276.332809 | 1 |
| 3276.3331825 | 1 |
| 3276.3341801 | 5 |
| 3276.334681 | 2 |
| 3276.3348122 | 5 |
| 3276.3351084 | 2 |
| 3276.3359462 | 4 |
| 3276.3366118 | 1 |
| 3276.3370623 | 0 |
| 3276.3371713 | 2 |
| 3276.3377379 | 3 |
| 3276.338815 | 3 |
| 3276.3394951 | 1 |
| 3276.3403574 | 1 |
| 3276.3404071 | 0 |
| 3276.3405524 | 0 |
| 3276.3407565 | 1 |
| 3276.3422743 | 2 |
| 3276.3424836 | 1 |
| 3276.3433076 | 0 |
| 3276.3439112 | 5 |
| 3276.3444018 | 0 |
| 3276.3447982 | 2 |
| 3276.3448623 | 1 |
| 3276.34661 | 3 |
| 3276.346729 | 2 |
| 3276.3468213 | 2 |
| 3276.3469165 | 2 |
| 3276.3480056 | 4 |
| 3276.3481296 | 1 |
| 3276.3488288 | 0 |
| 3276.3495339 | 2 |
| 3276.3498011 | 0 |
| 3276.3514304 | 2 |
| 3276.3515635 | 4 |
| 3276.3516873 | 1 |
| 3276.351709 | 1 |
| 3276.353064 | 1 |
| 3276.3534133 | 2 |
| 3276.3536073 | 2 |
| 3276.3543886 | 2 |
| 3276.3555559 | 3 |
| 3276.3557163 | 1 |
| 3276.3560403 | 1 |
| 3276.3562196 | 3 |
| 3276.3573518 | 4 |
| 3276.3575836 | 1 |
| 3276.3583897 | 1 |
| 3276.3590589 | 1 |
| 3276.3591778 | 3 |
| 3276.3596777 | 0 |
| 3276.3607462 | 4 |
| 3276.3612474 | 3 |
| 3276.3616671 | 1 |
| 3276.3620671 | 1 |
| 3276.3622853 | 5 |
| 3276.3634804 | 3 |
| 3276.3641804 | 1 |
| 3276.3641989 | 0 |
| 3276.3643616 | 8 |
| 3276.3653006 | 1 |
| 3276.3660065 | 1 |
| 3276.3660969 | 1 |
| 3276.3676716 | 3 |
| 3276.3679993 | 6 |
| 3276.368126 | 1 |
| 3276.368155 | 3 |
| 3276.3688513 | 3 |
| 3276.3700892 | 5 |
| 3276.370235 | 1 |
| 3276.3703828 | 1 |
| 3276.3709146 | 4 |
| 3276.3715809 | 2 |
| 3276.3721436 | 1 |
| 3276.3722436 | 0 |
| 3276.3724263 | 1 |
| 3276.3731818 | 1 |
| 3276.3743381 | 1 |
| 3276.3743783 | 0 |
| 3276.3753345 | 1 |
| 3276.3760113 | 2 |
| 3276.3761561 | 2 |
| 3276.376419 | 1 |
| 3276.3768104 | 2 |
| 3276.3780372 | 1 |
| 3276.37865 | 2 |
| 3276.3788658 | 0 |
| 3276.3793427 | 1 |
| 3276.3803557 | 2 |
| 3276.3807759 | 3 |
| 3276.3808281 | 3 |
| 3276.3813659 | 2 |
| 3276.3814591 | 1 |
| 3276.3823142 | 3 |
| 3276.3835248 | 0 |
| 3276.3839814 | 1 |
| 3276.3842386 | 2 |
| 3276.3850167 | 0 |
| 3276.3850182 | 0 |
| 3276.3854414 | 1 |
| 3276.3859302 | 2 |
| 3276.386649 | 2 |
| 3276.3867028 | 2 |
| 3276.3870569 | 2 |
| 3276.3871188 | 5 |
| 3276.3875852 | 3 |
| 3276.3888519 | 2 |
| 3276.3891484 | 2 |
| 3276.3893143 | 4 |
| 3276.3894361 | 3 |
| 3276.3895416 | 1 |
| 3276.3899966 | 1 |
| 3276.3905605 | 2 |
| 3276.3908117 | 3 |
| 3276.3911216 | 3 |
| 3276.3911358 | 1 |
| 3276.3913224 | 2 |
| 3276.3916234 | 1 |
| 3276.3918546 | 0 |
| 3276.3922846 | 3 |
| 3276.3931804 | 1 |
| 3276.3932754 | 2 |
| 3276.3934181 | 3 |
| 3276.3935519 | 0 |
| 3276.3936565 | 4 |
| 3276.3938768 | 4 |
| 3276.3940596 | 1 |
| 3276.3941478 | 2 |
| 3276.3944249 | 3 |
| 3276.3950666 | 2 |
| 3276.3951666 | 3 |
| 3276.3954253 | 4 |
| 3276.3956306 | 3 |
| 3276.396143 | 1 |
| 3276.3964091 | 0 |
| 3276.3964313 | 3 |
| 3276.3968265 | 5 |
| 3276.3968456 | 1 |
| 3276.3971835 | 3 |
| 3276.3974475 | 5 |
| 3276.3978994 | 3 |
| 3276.3979915 | 4 |
| 3276.3984922 | 1 |
| 3276.3985352 | 0 |
| 3276.3990204 | 5 |
| 3276.3997353 | 1 |
| 3276.3997846 | 2 |
| 3276.3999394 | 2 |
| 3276.4000494 | 1 |
| 3276.400214 | 3 |
| 3276.4004846 | 4 |
| 3276.4008815 | 3 |
| 3276.4015068 | 4 |
| 3276.401614 | 3 |
| 3276.4017987 | 0 |
| 3276.4023808 | 0 |
| 3276.4023908 | 1 |
| 3276.4027884 | 2 |
| 3276.4029195 | 1 |
| 3276.4032317 | 1 |
| 3276.4034717 | 1 |
| 3276.4039685 | 0 |
| 3276.4039699 | 1 |
| 3276.4039976 | 3 |
| 3276.4042515 | 3 |
| 3276.4045995 | 3 |
| 3276.4050151 | 2 |
| 3276.4056661 | 2 |
| 3276.4058154 | 5 |
| 3276.4060974 | 1 |
| 3276.4062405 | 4 |
| 3276.4064316 | 2 |
| 3276.4064685 | 0 |
| 3276.4065664 | 5 |
| 3276.4077444 | 4 |
| 3276.4077518 | 10 |
| 3276.4078287 | 4 |
| 3276.4078702 | 5 |
| 3276.408104 | 3 |
| 3276.4085998 | 6 |
| 3276.408687 | 10 |
| 3276.4091376 | 25 |
| 3276.4091847 | 5 |
| 3276.4094765 | 27 |
| 3276.409812 | 33 |
| 3276.4099289 | 39 |
| 3276.4105008 | 26 |
| 3276.410846 | 24 |
| 3276.4112151 | 30 |
| 3276.4114347 | 35 |
| 3276.4120408 | 15 |
| 3276.4121411 | 15 |
| 3276.4122241 | 18 |
| 3276.4125158 | 20 |
| 3276.4129363 | 13 |
| 3276.413003 | 7 |
| 3276.4131475 | 11 |
| 3276.413774 | 11 |
| 3276.4142677 | 24 |
| 3276.4143729 | 15 |
| 3276.4144805 | 36 |
| 3276.414532 | 10 |
| 3276.4146656 | 11 |
| 3276.4148185 | 12 |
| 3276.4150356 | 23 |
| 3276.4151319 | 19 |
| 3276.4156977 | 18 |
| 3276.4165254 | 25 |
| 3276.4165591 | 18 |
| 3276.4166229 | 16 |
| 3276.416913 | 20 |
| 3276.4170992 | 11 |
| 3276.4172567 | 14 |
| 3276.4173878 | 28 |
| 3276.4178619 | 15 |
| 3276.4180606 | 15 |
| 3276.4183866 | 13 |
| 3276.4185144 | 9 |
| 3276.4188017 | 7 |
| 3276.4195216 | 16 |
| 3276.4199275 | 16 |
| 3276.4202341 | 16 |
| 3276.4202484 | 15 |
| 3276.4202489 | 9 |
| 3276.4204346 | 8 |
| 3276.4209819 | 6 |
| 3276.4211219 | 3 |
| 3276.4211523 | 2 |
| 3276.4212506 | 2 |
| 3276.4213919 | 9 |
| 3276.4223429 | 2 |
| 3276.4223505 | 1 |
| 3276.4226629 | 1 |
| 3276.4226885 | 3 |
| 3276.4230582 | 1 |
| 3276.4232459 | 4 |
| 3276.4234727 | 3 |
| 3276.4240034 | 2 |
| 3276.4240093 | 1 |
| 3276.4244475 | 2 |
| 3276.4252889 | 1 |
| 3276.4254617 | 5 |
| 3276.4256168 | 0 |
| 3276.4258663 | 2 |
| 3276.4259569 | 2 |
| 3276.4263736 | 5 |
| 3276.4264352 | 0 |
| 3276.4266719 | 2 |
| 3276.4268812 | 1 |
| 3276.4270181 | 3 |
| 3276.4271794 | 2 |
| 3276.4274851 | 0 |
| 3276.4277003 | 2 |
| 3276.4279212 | 2 |
| 3276.4281379 | 4 |
| 3276.4285869 | 2 |
| 3276.4287816 | 3 |
| 3276.4289075 | 0 |
| 3276.4289406 | 2 |
| 3276.4294361 | 0 |
| 3276.4299361 | 1 |
| 3276.4300726 | 3 |
| 3276.4307583 | 0 |
| 3276.4311132 | 3 |
| 3276.4312662 | 2 |
| 3276.4315435 | 4 |
| 3276.4315794 | 4 |
| 3276.432103 | 2 |
| 3276.432463 | 3 |
| 3276.4326211 | 6 |
| 3276.432808 | 2 |
| 3276.4328637 | 2 |
| 3276.4332125 | 3 |
| 3276.4336293 | 4 |
| 3276.4337322 | 0 |
| 3276.4337491 | 3 |
| 3276.4340794 | 1 |
| 3276.4347494 | 1 |
| 3276.4349943 | 2 |
| 3276.4350073 | 3 |
| 3276.4350646 | 2 |
| 3276.4355996 | 2 |
| 3276.4356289 | 2 |
| 3276.4357606 | 5 |
| 3276.4366944 | 1 |
| 3276.4367037 | 3 |
| 3276.4367719 | 1 |
| 3276.4368962 | 3 |
| 3276.437001 | 0 |
| 3276.4372715 | 2 |
| 3276.4375064 | 1 |
| 3276.4376181 | 3 |
| 3276.4386447 | 1 |
| 3276.4387377 | 0 |
| 3276.4388093 | 6 |
| 3276.4394515 | 3 |
| 3276.4395039 | 2 |
| 3276.439939 | 3 |
| 3276.4400886 | 1 |
| 3276.4401586 | 2 |
| 3276.4402777 | 3 |
| 3276.4408235 | 1 |
| 3276.4410355 | 3 |
| 3276.4413507 | 1 |
| 3276.4416492 | 1 |
| 3276.4420494 | 4 |
| 3276.4424971 | 1 |
| 3276.4426316 | 5 |
| 3276.4427825 | 3 |
| 3276.4433364 | 0 |
| 3276.4434659 | 2 |
| 3276.4438526 | 4 |
| 3276.4439646 | 0 |
| 3276.44436 | 3 |
| 3276.4444317 | 1 |
| 3276.4444388 | 1 |
| 3276.4450151 | 1 |
| 3276.4456211 | 0 |
| 3276.4463622 | 1 |
| 3276.4474688 | 1 |
| 3276.447617 | 0 |
| 3276.448629 | 4 |
| 3276.4489172 | 0 |
| 3276.4500189 | 3 |
| 3276.4502409 | 1 |
| 3276.4510361 | 3 |
| 3276.4513918 | 1 |
| 3276.45228 | 1 |
| 3276.4526897 | 0 |
| 3276.4529364 | 1 |
| 3276.4535042 | 0 |
| 3276.4549015 | 1 |
| 3276.4561924 | 1 |

| | |
|---|---|
| 3276.4570745 | 1 |
| 3276.4588535 | 1 |
| 3276.4593482 | 0 |
| 3276.4606017 | 0 |
| 3276.4624773 | 5 |
| 3276.4638435 | 2 |
| 3276.4647356 | 0 |
| 3276.4661227 | 0 |
| 3276.4671281 | 0 |
| 3276.4690361 | 0 |
| 3276.4698862 | 0 |
| 3276.4709996 | 1 |
| 3276.4723292 | 2 |
| 3276.4727367 | 1 |
| 3276.4744406 | 1 |
| 3276.4753075 | 0 |
| 3276.4769386 | 1 |
| 3276.47859 | 2 |
| 3276.4787994 | 2 |
| 3276.4805564 | 5 |
| 3276.4811574 | 3 |
| 3276.4832507 | 1 |
| 3276.4839965 | 4 |
| 3276.4860698 | 0 |
| 3276.4871277 | 1 |
| 3276.4881963 | 2 |
| 3276.4896099 | 1 |
| 3276.4903004 | 4 |
| 3276.4916678 | 2 |
| 3276.4937092 | 4 |
| 3276.4944598 | 0 |
| 3276.4954141 | 3 |
| 3276.4966484 | 2 |
| 3276.4986525 | 1 |
| 3276.4999633 | 0 |
| 3276.5007025 | 2 |
| 3276.5026683 | 1 |
| 3276.503053 | 1 |
| 3276.5043053 | 2 |
| 3276.5052788 | 0 |
| 3276.5072016 | 0 |
| 3276.5083526 | 1 |
| 3276.5090641 | 1 |
| 3276.5112034 | 0 |
| 3276.5112367 | 0 |
| 3276.5137101 | 3 |
| 3276.5138126 | 2 |
| 3276.5155076 | 1 |
| 3276.5169164 | 3 |
| 3276.5172784 | 1 |
| 3276.5194953 | 0 |
| 3276.5201233 | 0 |
| 3276.5218321 | 3 |
| 3276.5237052 | 2 |
| 3276.5237885 | 1 |
| 3276.5259803 | 1 |
| 3276.5264617 | 2 |
| 3276.5284708 | 2 |
| 3276.5291851 | 3 |
| 3276.5303099 | 1 |
| 3276.5325393 | 0 |
| 3276.5329002 | 0 |
| 3276.534267 | 0 |
| 3276.535323 | 2 |
| 3276.5368939 | 1 |
| 3276.5381183 | 1 |
| 3276.5392558 | 2 |
| 3276.5411722 | 0 |
| 3276.5420444 | 2 |
| 3276.5436598 | 2 |
| 3276.5450485 | 0 |
| 3276.5455199 | 3 |
| 3276.54709 | 4 |
| 3276.548146 | 0 |
| 3276.5500045 | 1 |
| 3276.550879 | 1 |
| 3276.5519901 | 5 |
| 3276.5537695 | 1 |
| 3276.5540316 | 1 |
| 3276.5558181 | 1 |
| 3276.5568254 | 1 |
| 3276.5587693 | 2 |
| 3276.5600497 | 0 |
| 3276.5608372 | 1 |
| 3276.5624274 | 3 |
| 3276.5625121 | 0 |
| 3276.5642442 | 0 |
| 3276.5662426 | 1 |
| 3276.5664015 | 1 |
| 3276.5689754 | 1 |
| 3276.569342 | 1 |
| 3276.5709656 | 2 |
| 3276.5723506 | 4 |
| 3276.5732147 | 3 |
| 3276.5749539 | 1 |
| 3276.5756297 | 2 |
| 3276.5774293 | 3 |
| 3276.5782615 | 0 |
| 3276.5795953 | 0 |
| 3276.5817459 | 1 |
| 3276.5821087 | 0 |
| 3276.5837563 | 2 |
| 3276.5845046 | 1 |
| 3276.5863377 | 1 |
| 3276.5876986 | 0 |
| 3276.5883295 | 2 |
| 3276.5905672 | 1 |
| 3276.5907714 | 1 |
| 3276.5923269 | 1 |
| 3276.5942079 | 3 |
| 3276.594782 | 0 |
| 3276.5962677 | 1 |
| 3276.5973275 | 0 |
| 3276.5986751 | 3 |
| 3276.5996705 | 2 |
| 3276.6008554 | 1 |
| 3276.6029358 | 2 |
| 3276.6030108 | 0 |
| 3276.6050689 | 3 |
| 3276.6060712 | 2 |
| 3276.6075183 | 1 |
| 3276.6085313 | 2 |
| 3276.610085 | 1 |
| 3276.6118268 | 1 |
| 3276.6118609 | 2 |
| 3276.613418 | 3 |
| 3276.6150543 | 0 |
| 3276.6159637 | 2 |
| 3276.6175906 | 0 |
| 3276.6184047 | 0 |
| 3276.6205807 | 1 |
| 3276.6208539 | 1 |
| 3276.6229253 | 2 |
| 3276.6246942 | 1 |
| 3276.6249238 | 1 |
| 3276.627049 | 0 |
| 3276.6278619 | 3 |
| 3276.6298672 | 0 |
| 3276.6299964 | 3 |
| 3276.6315465 | 1 |
| 3276.6327235 | 2 |
| 3276.6337139 | 3 |
| 3276.6357654 | 0 |
| 3276.6367175 | 0 |
| 3276.6382136 | 1 |
| 3276.639664 | 2 |
| 3276.6398328 | 1 |
| 3276.6407871 | 2 |
| 3276.6428894 | 0 |
| 3276.6435706 | 1 |
| 3276.6453325 | 2 |
| 3276.6462474 | 3 |
| 3276.6479198 | 2 |
| 3276.6487811 | 0 |
| 3276.6502591 | 1 |
| 3276.6514887 | 5 |
| 3276.6532222 | 4 |
| 3276.6541899 | 1 |
| 3276.6555002 | 1 |
| 3276.6566723 | 1 |
| 3276.6573644 | 1 |
| 3276.6583628 | 3 |
| 3276.6603354 | 3 |
| 3276.6613279 | 0 |
| 3276.6628636 | 1 |
| 3276.6638249 | 1 |
| 3276.6654741 | 1 |
| 3276.6671641 | 1 |
| 3276.6673712 | 2 |
| 3276.6693045 | 1 |
| 3276.6702323 | 2 |
| 3276.6717151 | 0 |
| 3276.6732479 | 0 |
| 3276.674399 | 1 |
| 3276.6754918 | 1 |
| 3276.6764185 | 0 |
| 3276.6780711 | 2 |
| 3276.6801429 | 0 |
| 3276.6803206 | 1 |
| 3276.6821756 | 3 |
| 3276.6826105 | 0 |
| 3276.6842416 | 0 |
| 3276.6854649 | 1 |
| 3276.6868603 | 1 |
| 3276.6880867 | 1 |
| 3276.6886929 | 2 |
| 3276.6910106 | 1 |
| 3276.6913798 | 0 |
| 3276.6928188 | 1 |
| 3276.6933106 | 0 |
| 3276.6949699 | 1 |
| 3276.6964042 | 2 |
| 3276.6971896 | 2 |
| 3276.6990266 | 0 |
| 3276.7005545 | 0 |
| 3276.7014325 | 1 |
| 3276.7022028 | 1 |
| 3276.7035161 | 0 |
| 3276.7046044 | 1 |
| 3276.7066349 | 3 |
| 3276.7072457 | 3 |
| 3276.7094511 | 2 |
| 3276.7096462 | 2 |
| 3276.7111611 | 2 |
| 3276.7131526 | 0 |
| 3276.7133811 | 2 |
| 3276.7155902 | 1 |
| 3276.7161918 | 2 |
| 3276.7167963 | 1 |
| 3276.718565 | 0 |
| 3276.7197597 | 2 |
| 3276.7217242 | 1 |
| 3276.722628 | 1 |
| 3276.7242051 | 1 |
| 3276.7248019 | 1 |
| 3276.7270028 | 3 |
| 3276.7274067 | 1 |
| 3276.7291355 | 1 |
| 3276.7299637 | 1 |
| 3276.7313647 | 2 |
| 3276.7319592 | 1 |
| 3276.733763 | 1 |
| 3276.7349531 | 0 |
| 3276.7366606 | 0 |
| 3276.7371735 | 0 |
| 3276.739216 | 0 |
| 3276.739862 | 1 |
| 3276.7410703 | 3 |
| 3276.742146 | 0 |
| 3276.7435023 | 1 |
| 3276.744929 | 0 |
| 3276.7455502 | 2 |
| 3276.7472405 | 2 |
| 3276.7486778 | 0 |
| 3276.7496154 | 2 |
| 3276.7509008 | 2 |
| 3276.7522836 | 0 |
| 3276.7529512 | 0 |
| 3276.7541143 | 1 |
| 3276.7561315 | 4 |
| 3276.7575461 | 0 |
| 3276.7584515 | 0 |
| 3276.760374 | 0 |
| 3276.760454 | 2 |
| 3276.7626399 | 0 |
| 3276.764145 | 0 |
| 3276.7646066 | 1 |
| 3276.7664296 | 2 |
| 3276.7670017 | 0 |
| 3276.7687447 | 0 |
| 3276.7698145 | 2 |
| 3276.7710061 | 1 |
| 3276.772269 | 2 |
| 3276.7730441 | 0 |
| 3276.7750354 | 2 |
| 3276.7755157 | 0 |
| 3276.7769174 | 2 |
| 3276.7787601 | 0 |
| 3276.7800986 | 3 |
| 3276.7809433 | 2 |
| 3276.7819751 | 2 |
| 3276.7834551 | 4 |
| 3276.7844054 | 0 |
| 3276.7852282 | 1 |
| 3276.7873201 | 1 |
| 3276.7875251 | 1 |
| 3276.7888899 | 4 |
| 3276.7910862 | 2 |
| 3276.7919594 | 0 |
| 3276.7932489 | 1 |
| 3276.7940684 | 1 |
| 3276.7957729 | 0 |
| 3276.7963094 | 1 |
| 3276.7975869 | 2 |
| 3276.7998855 | 2 |
| 3276.8000368 | 1 |
| 3276.8012442 | 0 |
| 3276.8028195 | 0 |
| 3276.8043333 | 2 |
| 3276.8054698 | 0 |
| 3276.8062812 | 1 |
| 3276.808184 | 0 |
| 3276.8087848 | 1 |
| 3276.8106564 | 0 |
| 3276.8121414 | 6 |
| 3276.8133766 | 0 |
| 3276.8146932 | 1 |
| 3276.8152354 | 1 |
| 3276.816545 | 0 |
| 3276.8174617 | 1 |
| 3276.819406 | 2 |
| 3276.8207254 | 0 |
| 3276.8216934 | 1 |
| 3276.8230316 | 0 |
| 3276.8238885 | 1 |
| 3276.825251 | 1 |
| 3276.826968 | 3 |
| 3276.827096 | 3 |
| 3276.8286341 | 1 |
| 3276.8295012 | 2 |
| 3276.8308449 | 1 |
| 3276.8325314 | 1 |
| 3276.8339038 | 1 |
| 3276.8351287 | 0 |
| 3276.8363878 | 0 |
| 3276.8373278 | 2 |
| 3276.8378945 | 2 |
| 3276.8397798 | 0 |
| 3276.8411913 | 2 |
| 3276.8420004 | 1 |
| 3276.8437514 | 1 |
| 3276.8444583 | 4 |
| 3276.846519 | 0 |
| 3276.847151 | 1 |
| 3276.8486781 | 0 |
| 3276.8502814 | 2 |
| 3276.850698 | 0 |
| 3276.8525527 | 4 |
| 3276.8534666 | 2 |
| 3276.8551434 | 2 |
| 3276.8561794 | 0 |
| 3276.8567421 | 0 |
| 3276.858772 | 1 |
| 3276.8597347 | 0 |
| 3276.8609387 | 1 |
| 3276.8623446 | 0 |
| 3276.8636225 | 1 |
| 3276.864759 | 0 |
| 3276.8653967 | 2 |
| 3276.8669586 | 1 |
| 3276.8684907 | 1 |
| 3276.8693256 | 0 |
| 3276.870939 | 1 |
| 3276.8719656 | 2 |
| 3276.8726956 | 4 |
| 3276.8740213 | 1 |
| 3276.8749431 | 0 |
| 3276.8770308 | 3 |
| 3276.8778119 | 2 |
| 3276.879069 | 0 |
| 3276.8806068 | 3 |
| 3276.8822258 | 1 |
| 3276.8823159 | 0 |
| 3276.8836344 | 0 |
| 3276.885176 | 1 |
| 3276.8857679 | 0 |
| 3276.8878539 | 1 |
| 3276.8891111 | 0 |
| 3276.8898199 | 2 |
| 3276.8913734 | 0 |
| 3276.891775 | 0 |
| 3276.8935626 | 3 |
| 3276.8957028 | 0 |
| 3276.8963398 | 1 |
| 3276.8983808 | 0 |
| 3276.8988251 | 1 |
| 3276.900284 | 0 |
| 3276.9009153 | 0 |
| 3276.9025172 | 1 |
| 3276.904297 | 2 |
| 3276.9053991 | 0 |
| 3276.9066822 | 1 |
| 3276.9070888 | 0 |

| | |
|---|---|
| 3276.9088109 | 2 |
| 3276.9090674 | 2 |
| 3276.9108276 | 0 |
| 3276.9127751 | 1 |
| 3276.9136261 | 2 |
| 3276.9148538 | 0 |
| 3276.9158024 | 1 |
| 3276.9175464 | 1 |
| 3276.9190096 | 2 |
| 3276.9192401 | 2 |
| 3276.9206119 | 0 |
| 3276.9224903 | 2 |
| 3276.9229516 | 0 |
| 3276.9249705 | 1 |
| 3276.9252819 | 0 |
| 3276.9272727 | 2 |
| 3276.9276887 | 0 |
| 3276.9295729 | 0 |
| 3276.9310724 | 1 |
| 3276.932509 | 2 |
| 3276.9327338 | 1 |
| 3276.9338109 | 1 |
| 3276.9354818 | 1 |
| 3276.9365514 | 1 |
| 3276.9377434 | 0 |
| 3276.939933 | 1 |
| 3276.9402511 | 1 |
| 3276.9419065 | 0 |
| 3276.9433942 | 3 |
| 3276.9443216 | 0 |
| 3276.945657 | 0 |
| 3276.9464362 | 3 |
| 3276.9477879 | 0 |
| 3276.9487901 | 1 |
| 3276.9500991 | 1 |
| 3276.9518546 | 3 |
| 3276.9524983 | 3 |
| 3276.9541918 | 0 |
| 3276.9545907 | 3 |
| 3276.9567952 | 0 |
| 3276.9577369 | 2 |
| 3276.958224 | 0 |
| 3276.9604739 | 3 |
| 3276.9617249 | 1 |
| 3276.9632743 | 2 |
| 3276.9633361 | 0 |
| 3276.9644984 | 1 |
| 3276.9661616 | 2 |
| 3276.966449 | 2 |
| 3276.9682884 | 0 |
| 3276.9698976 | 2 |
| 3276.9707355 | 1 |
| 3276.9722673 | 0 |
| 3276.9722936 | 1 |
| 3276.9753286 | 0 |
| 3276.9760832 | 1 |
| 3276.9771372 | 0 |
| 3276.9784182 | 1 |
| 3276.9793224 | 2 |
| 3276.9805594 | 1 |
| 3276.9814507 | 1 |
| 3276.9833225 | 2 |
| 3276.9838943 | 2 |
| 3276.985769 | 0 |
| 3276.9875357 | 0 |
| 3276.9878102 | 3 |
| 3276.9902058 | 1 |
| 3276.9919072 | 1 |
| 3276.9924838 | 0 |
| 3276.9937913 | 2 |
| 3276.9939963 | 2 |
| 3276.9959742 | 2 |
| 3276.9964008 | 1 |
| 3276.998128 | 1 |
| 3276.9995949 | 0 |
| 3277.0006129 | 2 |
| 3277.0022052 | 2 |
| 3277.0033011 | 2 |
| 3277.0041299 | 0 |
| 3277.0056615 | 0 |
| 3277.0066254 | 3 |
| 3277.0078106 | 1 |
| 3277.0089772 | 4 |
| 3277.0104314 | 1 |
| 3277.011769 | 0 |
| 3277.0123218 | 2 |
| 3277.0141067 | 1 |
| 3277.0149691 | 1 |
| 3277.0162692 | 1 |
| 3277.0173244 | 0 |
| 3277.0185611 | 2 |
| 3277.0200258 | 1 |
| 3277.0215916 | 1 |
| 3277.0226923 | 1 |
| 3277.023977 | 1 |
| 3277.0251938 | 1 |
| 3277.026358 | 2 |
| 3277.0275056 | 2 |
| 3277.029639 | 1 |
| 3277.0298936 | 2 |
| 3277.031718 | 0 |
| 3277.0321384 | 2 |
| 3277.0338226 | 0 |
| 3277.0354366 | 3 |
| 3277.0363538 | 4 |
| 3277.0379997 | 1 |
| 3277.0382358 | 0 |
| 3277.0400883 | 3 |
| 3277.0412234 | 1 |
| 3277.0424459 | 1 |
| 3277.0445199 | 1 |
| 3277.0451263 | 0 |
| 3277.0459989 | 2 |
| 3277.0468926 | 1 |
| 3277.0489508 | 0 |
| 3277.049888 | 2 |
| 3277.0507988 | 0 |
| 3277.0521884 | 1 |
| 3277.0541843 | 1 |
| 3277.0550292 | 3 |
| 3277.0566647 | 0 |
| 3277.0572035 | 0 |
| 3277.059317 | 1 |
| 3277.0597553 | 2 |
| 3277.0610841 | 3 |
| 3277.0622358 | 0 |
| 3277.0636562 | 0 |
| 3277.0647707 | 1 |
| 3277.0662228 | 2 |
| 3277.0675446 | 3 |
| 3277.0677017 | 3 |
| 3277.0698189 | 3 |
| 3277.0714189 | 2 |
| 3277.0716868 | 3 |
| 3277.0734803 | 0 |
| 3277.0750292 | 2 |
| 3277.0761185 | 0 |
| 3277.0765917 | 2 |
| 3277.0785326 | 1 |
| 3277.0803364 | 1 |
| 3277.0812499 | 2 |
| 3277.0823238 | 0 |
| 3277.0838168 | 0 |
| 3277.0847042 | 1 |
| 3277.0856164 | 1 |
| 3277.0869416 | 1 |
| 3277.0881419 | 2 |
| 3277.0899222 | 2 |
| 3277.0909029 | 2 |
| 3277.0919807 | 1 |
| 3277.0929713 | 1 |
| 3277.0938412 | 2 |
| 3277.0941188 | 2 |
| 3277.0945158 | 2 |
| 3277.096259 | 2 |
| 3277.0964904 | 1 |
| 3277.0965777 | 6 |
| 3277.0973063 | 1 |
| 3277.0989203 | 0 |
| 3277.0990701 | 1 |
| 3277.1003153 | 0 |
| 3277.1003738 | 0 |
| 3277.102466 | 0 |
| 3277.1024764 | 0 |
| 3277.1026499 | 1 |
| 3277.1038959 | 2 |
| 3277.1047757 | 3 |
| 3277.1054938 | 0 |
| 3277.1060561 | 1 |
| 3277.1065198 | 2 |
| 3277.1066189 | 1 |
| 3277.1078455 | 0 |
| 3277.1086669 | 0 |
| 3277.1092111 | 2 |
| 3277.1102115 | 0 |
| 3277.1116413 | 4 |
| 3277.1121934 | 1 |
| 3277.1123087 | 3 |
| 3277.113015 | 1 |
| 3277.1143682 | 0 |
| 3277.1148968 | 0 |
| 3277.1150275 | 0 |
| 3277.1158479 | 1 |
| 3277.1172272 | 3 |
| 3277.1181079 | 3 |
| 3277.1182256 | 2 |
| 3277.1197639 | 1 |
| 3277.1206215 | 1 |
| 3277.1208395 | 6 |
| 3277.1214948 | 0 |
| 3277.1223497 | 3 |
| 3277.1224589 | 2 |
| 3277.124825 | 0 |
| 3277.1249139 | 5 |
| 3277.1254043 | 1 |
| 3277.1261433 | 3 |
| 3277.1266639 | 0 |
| 3277.127901 | 2 |
| 3277.1287567 | 2 |
| 3277.1288149 | 0 |
| 3277.1306166 | 2 |
| 3277.1308515 | 1 |
| 3277.1315638 | 2 |
| 3277.1326264 | 0 |
| 3277.132893 | 0 |
| 3277.1346516 | 1 |
| 3277.1346827 | 3 |
| 3277.1347348 | 1 |
| 3277.135006 | 1 |
| 3277.1369131 | 2 |
| 3277.1372529 | 1 |
| 3277.138348 | 1 |
| 3277.1394223 | 3 |
| 3277.1395577 | 2 |
| 3277.1407356 | 1 |
| 3277.1407663 | 0 |
| 3277.1413839 | 0 |
| 3277.1423757 | 4 |
| 3277.143087 | 2 |
| 3277.1446438 | 2 |
| 3277.1450138 | 2 |
| 3277.1454817 | 1 |
| 3277.1456163 | 3 |
| 3277.1472946 | 1 |
| 3277.1473384 | 2 |
| 3277.1481347 | 6 |
| 3277.1493492 | 2 |
| 3277.1496215 | 2 |
| 3277.1506877 | 1 |
| 3277.1512728 | 1 |
| 3277.1528438 | 2 |
| 3277.1530605 | 1 |
| 3277.1532517 | 3 |
| 3277.1536567 | 0 |
| 3277.1551278 | 1 |
| 3277.1560822 | 2 |
| 3277.15612 | 1 |
| 3277.1574804 | 2 |
| 3277.1580111 | 2 |
| 3277.1586184 | 1 |
| 3277.1593456 | 2 |
| 3277.1598349 | 0 |
| 3277.1611695 | 4 |
| 3277.1613051 | 1 |
| 3277.1627048 | 0 |
| 3277.1630292 | 3 |
| 3277.1637273 | 0 |
| 3277.1643641 | 2 |
| 3277.1660858 | 1 |
| 3277.1663731 | 3 |
| 3277.1668665 | 4 |
| 3277.1678805 | 2 |
| 3277.1680375 | 0 |
| 3277.1687687 | 0 |
| 3277.1705009 | 2 |
| 3277.1705222 | 2 |
| 3277.1713727 | 4 |
| 3277.1720688 | 0 |
| 3277.1726975 | 1 |
| 3277.1738877 | 0 |
| 3277.1747148 | 1 |
| 3277.175992 | 2 |
| 3277.1760114 | 0 |
| 3277.1762899 | 1 |
| 3277.1773064 | 1 |
| 3277.1780533 | 1 |
| 3277.1787057 | 3 |
| 3277.1797239 | 4 |
| 3277.1805065 | 2 |
| 3277.1812176 | 1 |
| 3277.1822295 | 1 |
| 3277.1824894 | 1 |
| 3277.1833986 | 3 |
| 3277.1843913 | 1 |
| 3277.1850157 | 5 |
| 3277.1857607 | 1 |
| 3277.1869414 | 0 |
| 3277.187361 | 3 |
| 3277.1882697 | 1 |
| 3277.1889039 | 3 |
| 3277.188905 | 1 |
| 3277.1893202 | 3 |
| 3277.191275 | 0 |
| 3277.1914329 | 7 |
| 3277.192277 | 2 |
| 3277.1930366 | 0 |
| 3277.1934649 | 3 |
| 3277.1946305 | 1 |
| 3277.1947424 | 9 |
| 3277.1957727 | 3 |
| 3277.1961726 | 0 |
| 3277.1980319 | 1 |
| 3277.1981591 | 2 |
| 3277.1985237 | 0 |
| 3277.2002992 | 1 |
| 3277.2010184 | 4 |
| 3277.2011198 | 1 |
| 3277.2012761 | 4 |
| 3277.2025554 | 1 |
| 3277.203898 | 2 |
| 3277.2043441 | 1 |
| 3277.2045326 | 0 |
| 3277.2048639 | 2 |
| 3277.2062743 | 1 |
| 3277.2067259 | 1 |
| 3277.2076468 | 1 |
| 3277.2090162 | 0 |
| 3277.2094915 | 2 |
| 3277.2098602 | 0 |
| 3277.2115038 | 3 |
| 3277.2120272 | 0 |
| 3277.2130472 | 2 |
| 3277.2131673 | 1 |
| 3277.2140177 | 2 |
| 3277.2145211 | 1 |
| 3277.2152282 | 2 |
| 3277.2162321 | 2 |
| 3277.2168124 | 2 |
| 3277.2173076 | 0 |
| 3277.2192342 | 3 |
| 3277.2194822 | 1 |
| 3277.2195353 | 2 |
| 3277.2207262 | 1 |
| 3277.2213295 | 4 |
| 3277.2229076 | 0 |
| 3277.2230988 | 0 |
| 3277.2234985 | 1 |
| 3277.2236339 | 0 |
| 3277.2247007 | 4 |
| 3277.2254067 | 2 |
| 3277.2268523 | 1 |
| 3277.2268912 | 3 |
| 3277.2284386 | 2 |
| 3277.2294487 | 4 |
| 3277.2317738 | 1 |
| 3277.2329166 | 3 |
| 3277.2359653 | 2 |
| 3277.2377676 | 4 |
| 3277.238918 | 3 |
| 3277.2403745 | 2 |
| 3277.2423058 | 1 |
| 3277.244357 | 2 |
| 3277.2466286 | 0 |
| 3277.2480334 | 3 |
| 3277.2501602 | 1 |
| 3277.2512149 | 5 |
| 3277.2534273 | 0 |
| 3277.2556958 | 3 |
| 3277.2566124 | 3 |
| 3277.2599945 | 2 |
| 3277.2602927 | 4 |
| 3277.2625958 | 3 |
| 3277.265859 | 6 |
| 3277.2661871 | 2 |
| 3277.268569 | 1 |
| 3277.2703647 | 2 |
| 3277.2718167 | 4 |
| 3277.275074 | 3 |
| 3277.276103 | 3 |
| 3277.2777248 | 1 |
| 3277.2791317 | 0 |
| 3277.2821789 | 3 |
| 3277.282537 | 1 |

| | |
|---|---|
| 3277.2846392 | 2 |
| 3277.2872561 | 1 |
| 3277.2885362 | 2 |
| 3277.2905146 | 4 |
| 3277.291855 | 2 |
| 3277.2940981 | 1 |
| 3277.296631 | 3 |
| 3277.297634 | 1 |
| 3277.3001295 | 1 |
| 3277.3013064 | 0 |
| 3277.3030322 | 3 |
| 3277.3054675 | 3 |
| 3277.3064414 | 1 |
| 3277.3093994 | 6 |
| 3277.3111036 | 2 |
| 3277.3129867 | 3 |
| 3277.3151035 | 4 |
| 3277.3156458 | 0 |
| 3277.3192271 | 3 |
| 3277.3215389 | 4 |
| 3277.3219792 | 2 |
| 3277.3247279 | 1 |
| 3277.3252734 | 1 |
| 3277.3278252 | 3 |
| 3277.3290604 | 2 |
| 3277.331086 | 4 |
| 3277.3331328 | 1 |
| 3277.3349768 | 3 |
| 3277.3375298 | 4 |
| 3277.3390018 | 1 |
| 3277.3405471 | 1 |
| 3277.3433371 | 4 |
| 3277.3444331 | 2 |
| 3277.3461782 | 3 |
| 3277.3465288 | 0 |
| 3277.3493175 | 0 |
| 3277.3521412 | 3 |
| 3277.3534544 | 2 |
| 3277.3557562 | 0 |
| 3277.3579404 | 2 |
| 3277.3588559 | 0 |
| 3277.3617095 | 2 |
| 3277.3629556 | 6 |
| 3277.3646567 | 3 |
| 3277.3658156 | 3 |
| 3277.3691309 | 1 |
| 3277.3711711 | 2 |
| 3277.3718119 | 5 |
| 3277.374109 | 3 |
| 3277.3756761 | 1 |
| 3277.3774299 | 4 |
| 3277.379282 | 0 |
| 3277.3813186 | 1 |
| 3277.3838776 | 5 |
| 3277.3856372 | 4 |
| 3277.3870595 | 5 |
| 3277.3896247 | 6 |
| 3277.3902136 | 2 |
| 3277.3924111 | 2 |
| 3277.3956539 | 5 |
| 3277.3958663 | 2 |
| 3277.3979793 | 0 |
| 3277.40017 | 4 |
| 3277.4022879 | 2 |
| 3277.4048402 | 3 |
| 3277.4062038 | 3 |
| 3277.4079882 | 3 |
| 3277.4089838 | 1 |
| 3277.4111082 | 2 |
| 3277.4138431 | 0 |
| 3277.4151645 | 0 |
| 3277.417959 | 4 |
| 3277.4181919 | 2 |
| 3277.4209564 | 2 |
| 3277.4230323 | 4 |
| 3277.4234053 | 4 |
| 3277.4260354 | 1 |
| 3277.4283603 | 3 |
| 3277.4290886 | 1 |
| 3277.4315075 | 0 |
| 3277.4340427 | 5 |
| 3277.4350524 | 3 |
| 3277.4383847 | 1 |
| 3277.4396216 | 1 |
| 3277.4420328 | 4 |
| 3277.4425035 | 1 |
| 3277.4448516 | 3 |
| 3277.448115 | 1 |
| 3277.4482423 | 2 |
| 3277.4509173 | 0 |
| 3277.4524002 | 2 |
| 3277.4545553 | 2 |
| 3277.4567774 | 1 |
| 3277.4587761 | 2 |
| 3277.4600627 | 2 |
| 3277.4613308 | 5 |
| 3277.4642106 | 3 |
| 3277.4657098 | 2 |
| 3277.4675385 | 3 |
| 3277.4694925 | 1 |
| 3277.470973 | 7 |
| 3277.4732322 | 3 |
| 3277.4747376 | 5 |
| 3277.475735 | 3 |
| 3277.4783996 | 1 |
| 3277.4792659 | 3 |
| 3277.4819106 | 3 |
| 3277.4846805 | 3 |
| 3277.4860344 | 2 |
| 3277.4880759 | 1 |
| 3277.4899349 | 1 |
| 3277.4915605 | 2 |
| 3277.4941994 | 4 |
| 3277.4950777 | 0 |
| 3277.4975394 | 3 |
| 3277.4986254 | 1 |
| 3277.5013589 | 1 |
| 3277.5034253 | 2 |
| 3277.5039437 | 2 |
| 3277.5072805 | 3 |
| 3277.507771 | 3 |
| 3277.5108539 | 0 |
| 3277.5133136 | 2 |
| 3277.5134338 | 4 |
| 3277.516717 | 5 |
| 3277.5178583 | 3 |
| 3277.5194966 | 3 |
| 3277.5214572 | 4 |
| 3277.5228645 | 3 |
| 3277.5254563 | 4 |
| 3277.5264228 | 3 |
| 3277.5294136 | 3 |
| 3277.5318472 | 4 |
| 3277.5319226 | 5 |
| 3277.5344731 | 2 |
| 3277.536058 | 3 |
| 3277.5380081 | 2 |
| 3277.5412074 | 3 |
| 3277.5422322 | 1 |
| 3277.5441147 | 3 |
| 3277.5461943 | 2 |
| 3277.5485266 | 3 |
| 3277.5499395 | 6 |
| 3277.5516041 | 3 |
| 3277.554689 | 2 |
| 3277.5560443 | 1 |
| 3277.557522 | 1 |
| 3277.560045 | 0 |
| 3277.5608322 | 2 |
| 3277.5629446 | 2 |
| 3277.5638803 | 1 |
| 3277.5667834 | 4 |
| 3277.568423 | 2 |
| 3277.5698458 | 3 |
| 3277.5723656 | 2 |
| 3277.5740736 | 2 |
| 3277.5760175 | 2 |
| 3277.5780675 | 2 |
| 3277.5799472 | 3 |
| 3277.5817553 | 4 |
| 3277.5836901 | 0 |
| 3277.5852476 | 2 |
| 3277.5867744 | 1 |
| 3277.5890964 | 1 |
| 3277.5912638 | 3 |
| 3277.5928538 | 4 |
| 3277.59487 | 1 |
| 3277.5972291 | 1 |
| 3277.5985263 | 2 |
| 3277.6007969 | 0 |
| 3277.6019862 | 3 |
| 3277.6039656 | 4 |
| 3277.606772 | 4 |
| 3277.6076194 | 2 |
| 3277.6097848 | 3 |
| 3277.6117467 | 3 |
| 3277.6137994 | 4 |
| 3277.6148652 | 5 |
| 3277.6167608 | 3 |
| 3277.6194108 | 1 |
| 3277.6203652 | 1 |
| 3277.6223997 | 0 |
| 3277.6256732 | 4 |
| 3277.6268071 | 3 |
| 3277.6288445 | 1 |
| 3277.6301931 | 1 |
| 3277.6324595 | 6 |
| 3277.6347415 | 3 |
| 3277.6361426 | 2 |
| 3277.6381611 | 0 |
| 3277.6392016 | 4 |
| 3277.6418616 | 2 |
| 3277.6445237 | 1 |
| 3277.6458505 | 1 |
| 3277.6477271 | 6 |
| 3277.6488516 | 1 |
| 3277.6517429 | 3 |
| 3277.6523489 | 0 |
| 3277.6542947 | 3 |
| 3277.6567517 | 5 |
| 3277.6584987 | 1 |
| 3277.6610375 | 2 |
| 3277.6618667 | 2 |
| 3277.6635171 | 0 |
| 3277.6663501 | 2 |
| 3277.6669064 | 3 |
| 3277.6697727 | 6 |
| 3277.6720235 | 2 |
| 3277.67332 | 1 |
| 3277.6761473 | 4 |
| 3277.6770385 | 2 |
| 3277.6797538 | 1 |
| 3277.681006 | 3 |
| 3277.681862 | 2 |
| 3277.6834404 | 1 |
| 3277.6846611 | 2 |
| 3277.6858777 | 3 |
| 3277.6870382 | 1 |
| 3277.6874921 | 3 |
| 3277.6901327 | 4 |
| 3277.6909841 | 1 |
| 3277.6923854 | 3 |
| 3277.6928581 | 1 |
| 3277.694971 | 1 |
| 3277.6960604 | 3 |
| 3277.6962663 | 3 |
| 3277.698225 | 1 |
| 3277.6985189 | 1 |
| 3277.7011202 | 3 |
| 3277.7024059 | 1 |
| 3277.7029806 | 1 |
| 3277.7038606 | 2 |
| 3277.7060826 | 3 |
| 3277.7065266 | 5 |
| 3277.7080454 | 0 |
| 3277.7086806 | 2 |
| 3277.7108104 | 1 |
| 3277.7119217 | 3 |
| 3277.7137382 | 3 |
| 3277.714378 | 4 |
| 3277.7153635 | 3 |
| 3277.7164814 | 3 |
| 3277.7186331 | 3 |
| 3277.7187612 | 2 |
| 3277.7209593 | 4 |
| 3277.7215503 | 5 |
| 3277.7230279 | 1 |
| 3277.724667 | 2 |
| 3277.726194 | 6 |
| 3277.7269078 | 2 |
| 3277.7278381 | 3 |
| 3277.7300161 | 5 |
| 3277.7314005 | 2 |
| 3277.7319694 | 5 |
| 3277.7338041 | 2 |
| 3277.7343615 | 7 |
| 3277.7358853 | 2 |
| 3277.7373841 | 6 |
| 3277.7381544 | 2 |
| 3277.7388842 | 2 |
| 3277.7406907 | 3 |
| 3277.7418462 | 3 |
| 3277.7427438 | 6 |
| 3277.7445952 | 1 |
| 3277.7461382 | 3 |
| 3277.7466105 | 1 |
| 3277.7478002 | 4 |
| 3277.7496396 | 3 |
| 3277.7503878 | 2 |
| 3277.7514438 | 1 |
| 3277.7529508 | 5 |
| 3277.7536911 | 3 |
| 3277.7558543 | 2 |
| 3277.7569685 | 0 |
| 3277.7586086 | 0 |
| 3277.7597091 | 1 |
| 3277.7602539 | 1 |
| 3277.7620134 | 3 |
| 3277.7626073 | 4 |
| 3277.7643396 | 1 |
| 3277.7661842 | 4 |
| 3277.7665752 | 4 |
| 3277.767887 | 0 |
| 3277.7694827 | 2 |
| 3277.7705675 | 3 |
| 3277.7723834 | 4 |
| 3277.7729791 | 2 |
| 3277.7746426 | 2 |
| 3277.7757066 | 1 |
| 3277.7765607 | 4 |
| 3277.7780687 | 0 |
| 3277.7793889 | 3 |
| 3277.7802508 | 2 |
| 3277.7810208 | 0 |
| 3277.7813009 | 5 |
| 3277.7813895 | 3 |
| 3277.7814933 | 1 |
| 3277.7817756 | 3 |
| 3277.7820306 | 1 |
| 3277.7833649 | 1 |
| 3277.7835513 | 5 |
| 3277.7839504 | 1 |
| 3277.7840007 | 2 |
| 3277.7840309 | 0 |
| 3277.784986 | 3 |
| 3277.7851172 | 3 |
| 3277.7854877 | 2 |
| 3277.7856994 | 1 |
| 3277.7860773 | 3 |
| 3277.7872676 | 7 |
| 3277.7878599 | 1 |
| 3277.7881501 | 4 |
| 3277.7886743 | 3 |
| 3277.7887103 | 0 |
| 3277.7890987 | 5 |
| 3277.7896091 | 4 |
| 3277.7907469 | 3 |
| 3277.790861 | 2 |
| 3277.7908616 | 1 |
| 3277.7919025 | 3 |
| 3277.7922944 | 4 |
| 3277.7927506 | 2 |
| 3277.7929489 | 1 |
| 3277.793911 | 5 |
| 3277.7941323 | 2 |
| 3277.7942966 | 1 |
| 3277.7947188 | 3 |
| 3277.795971 | 1 |
| 3277.7963197 | 5 |
| 3277.7965843 | 4 |
| 3277.7966327 | 4 |
| 3277.7970025 | 0 |
| 3277.7978395 | 2 |
| 3277.798554 | 0 |
| 3277.7985544 | 6 |
| 3277.7990378 | 1 |
| 3277.7996242 | 3 |
| 3277.8005679 | 0 |
| 3277.800652 | 1 |
| 3277.8010747 | 3 |
| 3277.8012429 | 2 |
| 3277.8016693 | 2 |
| 3277.8029972 | 1 |
| 3277.8031218 | 1 |
| 3277.8036941 | 4 |
| 3277.8038437 | 4 |
| 3277.8044938 | 5 |
| 3277.8046863 | 1 |
| 3277.8046928 | 0 |
| 3277.8050586 | 2 |
| 3277.8061974 | 6 |
| 3277.8065036 | 6 |
| 3277.8066312 | 37 |
| 3277.8076162 | 19 |
| 3277.8076563 | 37 |
| 3277.8086265 | 54 |
| 3277.8096794 | 48 |
| 3277.8096933 | 39 |
| 3277.8099331 | 48 |
| 3277.8103809 | 38 |
| 3277.8108086 | 23 |
| 3277.8115037 | 12 |
| 3277.8120952 | 13 |

| | |
|---|---|
| 3277.812687 | 2 |
| 3277.8130829 | 5 |
| 3277.8132931 | 3 |
| 3277.8137219 | 7 |
| 3277.8147384 | 0 |
| 3277.815367 | 3 |
| 3277.8153702 | 1 |
| 3277.815624 | 7 |
| 3277.8160337 | 6 |
| 3277.8162688 | 4 |
| 3277.8165932 | 1 |
| 3277.8178505 | 2 |
| 3277.8179861 | 0 |
| 3277.8180354 | 4 |
| 3277.8193327 | 3 |
| 3277.8194345 | 1 |
| 3277.8197421 | 5 |
| 3277.8204096 | 1 |
| 3277.8207639 | 3 |
| 3277.8212838 | 2 |
| 3277.8220831 | 1 |
| 3277.8223508 | 1 |
| 3277.8226634 | 6 |
| 3277.823133 | 3 |
| 3277.8236924 | 0 |
| 3277.8243032 | 1 |
| 3277.824765 | 3 |
| 3277.8252229 | 2 |
| 3277.8253054 | 6 |
| 3277.8257131 | 3 |
| 3277.8262395 | 2 |
| 3277.8268786 | 6 |
| 3277.827786 | 2 |
| 3277.8282922 | 1 |
| 3277.8286313 | 2 |
| 3277.8297959 | 1 |
| 3277.8321185 | 4 |
| 3277.8334585 | 3 |
| 3277.8356334 | 5 |
| 3277.8382297 | 2 |
| 3277.8396154 | 2 |
| 3277.8414259 | 2 |
| 3277.8422148 | 1 |
| 3277.8447119 | 0 |
| 3277.8475453 | 3 |
| 3277.8486416 | 2 |
| 3277.8504551 | 3 |
| 3277.8523794 | 4 |
| 3277.8543529 | 2 |
| 3277.8565414 | 2 |
| 3277.8580276 | 4 |
| 3277.8598153 | 5 |
| 3277.8615137 | 1 |
| 3277.8639675 | 1 |
| 3277.8661252 | 2 |
| 3277.8670697 | 2 |
| 3277.8691884 | 1 |
| 3277.8705306 | 1 |
| 3277.8728596 | 3 |
| 3277.8756516 | 2 |
| 3277.8765436 | 3 |
| 3277.8780442 | 5 |
| 3277.879871 | 7 |
| 3277.8820127 | 3 |
| 3277.8843217 | 2 |
| 3277.8855591 | 2 |
| 3277.8878427 | 1 |
| 3277.8893379 | 4 |
| 3277.89148 | 2 |
| 3277.8941277 | 3 |
| 3277.8945183 | 1 |
| 3277.8972822 | 2 |
| 3277.8981108 | 2 |
| 3277.9004717 | 3 |
| 3277.9034969 | 2 |
| 3277.9043403 | 2 |
| 3277.9073226 | 1 |
| 3277.9092087 | 2 |
| 3277.9099957 | 2 |
| 3277.9123359 | 0 |
| 3277.9134577 | 2 |
| 3277.9157633 | 0 |
| 3277.9177686 | 5 |
| 3277.9191045 | 5 |
| 3277.9219243 | 3 |
| 3277.9231997 | 2 |
| 3277.9254564 | 3 |
| 3277.9255919 | 4 |
| 3277.9288349 | 2 |
| 3277.9305476 | 2 |
| 3277.9329345 | 1 |
| 3277.9330124 | 2 |
| 3277.9366097 | 2 |
| 3277.9376419 | 2 |
| 3277.9380212 | 1 |
| 3277.9384046 | 3 |
| 3277.938712 | 1 |
| 3277.9388695 | 0 |
| 3277.9389218 | 1 |
| 3277.9390413 | 1 |
| 3277.9391466 | 3 |
| 3277.9395852 | 2 |
| 3277.939619 | 3 |
| 3277.9398541 | 2 |
| 3277.9401151 | 2 |
| 3277.9401669 | 1 |
| 3277.9404808 | 1 |
| 3277.9405224 | 3 |
| 3277.940599 | 0 |
| 3277.9406417 | 0 |
| 3277.9406893 | 2 |
| 3277.9408257 | 2 |
| 3277.9409633 | 2 |
| 3277.9409748 | 1 |
| 3277.9412745 | 1 |
| 3277.9415755 | 1 |
| 3277.941752 | 0 |
| 3277.9417543 | 0 |
| 3277.941799 | 1 |
| 3277.9418951 | 1 |
| 3277.9421009 | 3 |
| 3277.9421131 | 2 |
| 3277.9423578 | 2 |
| 3277.9424732 | 2 |
| 3277.9427537 | 0 |
| 3277.9427918 | 2 |
| 3277.9432543 | 3 |
| 3277.9433175 | 1 |
| 3277.9435267 | 3 |
| 3277.9436691 | 2 |
| 3277.9437337 | 2 |
| 3277.9437597 | 2 |
| 3277.9438217 | 2 |
| 3277.9440556 | 1 |
| 3277.9440896 | 2 |
| 3277.9441142 | 1 |
| 3277.9444219 | 1 |
| 3277.944471 | 1 |
| 3277.9444959 | 2 |
| 3277.9448022 | 3 |
| 3277.9450613 | 3 |
| 3277.9450712 | 0 |
| 3277.9453667 | 1 |
| 3277.945643 | 2 |
| 3277.9458845 | 1 |
| 3277.9462461 | 2 |
| 3277.9462589 | 1 |
| 3277.946283 | 3 |
| 3277.946496 | 5 |
| 3277.946693 | 3 |
| 3277.9467139 | 2 |
| 3277.9467266 | 5 |
| 3277.9470956 | 2 |
| 3277.9471541 | 1 |
| 3277.9472338 | 3 |
| 3277.947398 | 2 |
| 3277.9475541 | 3 |
| 3277.9475623 | 8 |
| 3277.9476497 | 0 |
| 3277.9476802 | 1 |
| 3277.9478763 | 4 |
| 3277.9478785 | 0 |
| 3277.9482206 | 3 |
| 3277.9483823 | 6 |
| 3277.9486937 | 1 |
| 3277.9487623 | 4 |
| 3277.9489984 | 2 |
| 3277.9489987 | 1 |
| 3277.9491896 | 3 |
| 3277.9493046 | 2 |
| 3277.9496113 | 3 |
| 3277.949672 | 2 |
| 3277.9497417 | 0 |
| 3277.9498769 | 1 |
| 3277.9499702 | 2 |
| 3277.950015 | 1 |
| 3277.9501964 | 1 |
| 3277.9502237 | 1 |
| 3277.9505486 | 3 |
| 3277.9505514 | 0 |
| 3277.9505875 | 5 |
| 3277.9507675 | 2 |
| 3277.950902 | 3 |
| 3277.9510138 | 1 |
| 3277.9512819 | 2 |
| 3277.9515532 | 1 |
| 3277.9515706 | 5 |
| 3277.9517561 | 3 |
| 3277.9522552 | 4 |
| 3277.9522756 | 3 |
| 3277.9524037 | 3 |
| 3277.9524368 | 0 |
| 3277.9524819 | 1 |
| 3277.9526681 | 1 |
| 3277.9528119 | 2 |
| 3277.9528533 | 2 |
| 3277.9529569 | 3 |
| 3277.9531657 | 3 |
| 3277.9531968 | 1 |
| 3277.9538556 | 2 |
| 3277.9539434 | 1 |
| 3277.9540495 | 2 |
| 3277.9541027 | 1 |
| 3277.9541527 | 3 |
| 3277.9544875 | 0 |
| 3277.9546511 | 1 |
| 3277.9546578 | 2 |
| 3277.9547704 | 3 |
| 3277.9548554 | 1 |
| 3277.9551898 | 1 |
| 3277.9552908 | 2 |
| 3277.9553218 | 3 |
| 3277.9555026 | 1 |
| 3277.955572 | 3 |
| 3277.955727 | 3 |
| 3277.955763 | 1 |
| 3277.9561912 | 3 |
| 3277.9563847 | 1 |
| 3277.9565375 | 2 |
| 3277.9566782 | 0 |
| 3277.9566989 | 2 |
| 3277.9567035 | 0 |
| 3277.9567693 | 0 |
| 3277.9568873 | 2 |
| 3277.9569846 | 4 |
| 3277.9572013 | 7 |
| 3277.9574245 | 2 |
| 3277.9575691 | 1 |
| 3277.9577281 | 2 |
| 3277.9580309 | 2 |
| 3277.9584177 | 3 |
| 3277.9584618 | 3 |
| 3277.9586061 | 2 |
| 3277.958609 | 2 |
| 3277.9586209 | 0 |
| 3277.958776 | 2 |
| 3277.9588009 | 1 |
| 3277.9590783 | 3 |
| 3277.9591744 | 2 |
| 3277.9592997 | 1 |
| 3277.9593907 | 0 |
| 3277.959563 | 4 |
| 3277.9596737 | 3 |
| 3277.9597281 | 2 |
| 3277.959868 | 1 |
| 3277.960055 | 6 |
| 3277.9602552 | 1 |
| 3277.9603326 | 2 |
| 3277.9605832 | 2 |
| 3277.9606528 | 3 |
| 3277.9609011 | 0 |
| 3277.9610588 | 2 |
| 3277.9612142 | 4 |
| 3277.9613253 | 3 |
| 3277.9613431 | 2 |
| 3277.9614998 | 0 |
| 3277.9615388 | 2 |
| 3277.9616546 | 1 |
| 3277.9619497 | 5 |
| 3277.9623158 | 2 |
| 3277.9623349 | 3 |
| 3277.9624892 | 1 |
| 3277.9627538 | 1 |
| 3277.9628768 | 1 |
| 3277.9629583 | 2 |
| 3277.9629962 | 3 |
| 3277.9630157 | 0 |
| 3277.9632865 | 2 |
| 3277.9635728 | 1 |
| 3277.9636201 | 2 |
| 3277.9637988 | 3 |
| 3277.9638747 | 1 |
| 3277.964071 | 2 |
| 3277.9641665 | 1 |
| 3277.9641983 | 2 |
| 3277.964237 | 2 |
| 3277.9643616 | 1 |
| 3277.9644807 | 2 |
| 3277.9646583 | 3 |
| 3277.9649143 | 0 |
| 3277.965189 | 1 |
| 3277.9652123 | 3 |
| 3277.9653547 | 2 |
| 3277.9655497 | 3 |
| 3277.9656363 | 1 |
| 3277.9657344 | 0 |
| 3277.9658 | 0 |
| 3277.9658084 | 2 |
| 3277.9661575 | 1 |
| 3277.9663834 | 1 |
| 3277.966435 | 0 |
| 3277.9667303 | 1 |
| 3277.9667535 | 1 |
| 3277.9667679 | 1 |
| 3277.9669662 | 0 |
| 3277.9671052 | 1 |
| 3277.9672088 | 0 |
| 3277.9675626 | 0 |
| 3277.9675695 | 2 |
| 3277.9677533 | 1 |
| 3277.9678957 | 3 |
| 3277.968088 | 0 |
| 3277.9683845 | 2 |
| 3277.968568 | 3 |
| 3277.9686416 | 1 |
| 3277.9687034 | 3 |
| 3277.9687239 | 3 |
| 3277.9689821 | 3 |
| 3277.969106 | 2 |
| 3277.9691222 | 0 |
| 3277.9692733 | 2 |
| 3277.9692985 | 6 |
| 3277.9694372 | 2 |
| 3277.9695723 | 1 |
| 3277.9697774 | 2 |
| 3277.9699449 | 3 |
| 3277.9700543 | 1 |
| 3277.9702048 | 4 |
| 3277.970362 | 3 |
| 3277.9705553 | 2 |
| 3277.970824 | 2 |
| 3277.9709244 | 0 |
| 3277.971216 | 2 |
| 3277.9712803 | 1 |
| 3277.9712886 | 1 |
| 3277.9713202 | 0 |
| 3277.9716919 | 2 |
| 3277.9721582 | 0 |
| 3277.9721681 | 1 |
| 3277.9721908 | 3 |
| 3277.9722524 | 2 |
| 3277.9723907 | 3 |
| 3277.9723916 | 0 |
| 3277.9726054 | 2 |
| 3277.972697 | 3 |
| 3277.9727253 | 0 |
| 3277.972761 | 2 |
| 3277.972886 | 3 |
| 3277.9731213 | 1 |
| 3277.9734167 | 2 |
| 3277.9734233 | 1 |
| 3277.9734633 | 1 |
| 3277.9737755 | 0 |
| 3277.9738203 | 4 |
| 3277.9738811 | 4 |
| 3277.9739283 | 3 |
| 3277.9740721 | 1 |
| 3277.9742223 | 2 |
| 3277.9743749 | 0 |
| 3277.9747374 | 2 |
| 3277.9747676 | 1 |
| 3277.9747873 | 1 |
| 3277.974789 | 0 |
| 3277.9749151 | 4 |
| 3277.975013 | 4 |
| 3277.9752028 | 1 |
| 3277.9754166 | 0 |
| 3277.9755125 | 2 |
| 3277.9755979 | 0 |
| 3277.9757479 | 0 |
| 3277.975875 | 1 |
| 3277.9759688 | 3 |
| 3277.9760215 | 2 |

| | |
|---|---|
| 3277.9761908 | 0 |
| 3277.9763535 | 2 |
| 3277.9766672 | 0 |
| 3277.976774 | 2 |
| 3277.9768099 | 2 |
| 3277.9768303 | 1 |
| 3277.9768572 | 6 |
| 3277.9769065 | 1 |
| 3277.9769601 | 2 |
| 3277.9770751 | 4 |
| 3277.9770769 | 1 |
| 3277.9771946 | 3 |
| 3277.9774308 | 3 |
| 3277.9779005 | 2 |
| 3277.9779079 | 5 |
| 3277.9781154 | 2 |
| 3277.9781261 | 5 |
| 3277.978204 | 4 |
| 3277.9783107 | 2 |
| 3277.9783353 | 2 |
| 3277.9784106 | 4 |
| 3277.9787689 | 2 |
| 3277.9788398 | 2 |
| 3277.9790839 | 1 |
| 3277.9790924 | 2 |
| 3277.9793039 | 1 |
| 3277.979446 | 2 |
| 3277.9794694 | 3 |
| 3277.9795754 | 3 |
| 3277.9796792 | 2 |
| 3277.9797876 | 1 |
| 3277.9798542 | 4 |
| 3277.9801435 | 2 |
| 3277.9801544 | 2 |
| 3277.9802953 | 2 |
| 3277.9803908 | 1 |
| 3277.980431 | 4 |
| 3277.9805689 | 3 |
| 3277.9806699 | 2 |
| 3277.9807649 | 3 |
| 3277.9810746 | 1 |
| 3277.9813063 | 2 |
| 3277.9813263 | 2 |
| 3277.9813401 | 3 |
| 3277.9813439 | 1 |
| 3277.981346 | 1 |
| 3277.9814513 | 3 |
| 3277.9815339 | 1 |
| 3277.9815827 | 1 |
| 3277.981778 | 3 |
| 3277.981871 | 1 |
| 3277.9819902 | 0 |
| 3277.9821734 | 0 |
| 3277.9822527 | 3 |
| 3277.9824182 | 2 |
| 3277.9824302 | 1 |
| 3277.9828255 | 2 |
| 3277.9828263 | 2 |
| 3277.9828345 | 1 |
| 3277.9829083 | 1 |
| 3277.9831732 | 1 |
| 3277.9832153 | 4 |
| 3277.983228 | 4 |
| 3277.9835909 | 1 |
| 3277.9836437 | 3 |
| 3277.9837533 | 0 |
| 3277.9837894 | 0 |
| 3277.9838879 | 1 |
| 3277.9839379 | 2 |
| 3277.984068 | 1 |
| 3277.9840996 | 2 |
| 3277.9842755 | 3 |
| 3277.9844189 | 6 |
| 3277.9845755 | 1 |
| 3277.9847972 | 2 |
| 3277.9850125 | 0 |
| 3277.9851292 | 0 |
| 3277.985272 | 0 |
| 3277.9852865 | 5 |
| 3277.9853047 | 2 |
| 3277.9854997 | 4 |
| 3277.9855263 | 1 |
| 3277.9856468 | 3 |
| 3277.9858713 | 0 |
| 3277.985911 | 5 |
| 3277.985944 | 2 |
| 3277.9864602 | 1 |
| 3277.9864948 | 0 |
| 3277.9866481 | 1 |
| 3277.9866711 | 1 |
| 3277.9866889 | 4 |
| 3277.9866973 | 0 |
| 3277.9867138 | 2 |
| 3277.9867863 | 4 |
| 3277.9869195 | 0 |
| 3277.9869961 | 2 |
| 3277.9873832 | 1 |
| 3277.9874454 | 2 |
| 3277.9875434 | 0 |
| 3277.987594 | 0 |
| 3277.9876469 | 0 |
| 3277.9878215 | 0 |
| 3277.9878381 | 0 |
| 3277.9880257 | 4 |
| 3277.9881207 | 1 |
| 3277.9884786 | 4 |
| 3277.9885131 | 1 |
| 3277.9885202 | 3 |
| 3277.9885523 | 0 |
| 3277.9887027 | 3 |
| 3277.988755 | 1 |
| 3277.9888991 | 3 |
| 3277.988936 | 1 |
| 3277.9890032 | 3 |
| 3277.9892579 | 1 |
| 3277.9895215 | 1 |
| 3277.989524 | 2 |
| 3277.9896969 | 4 |
| 3277.9898581 | 1 |
| 3277.9902974 | 1 |
| 3277.9902978 | 3 |
| 3277.990344 | 2 |
| 3277.9906486 | 2 |
| 3277.9907046 | 1 |
| 3277.9907952 | 4 |
| 3277.9908015 | 4 |
| 3277.9909157 | 3 |
| 3277.9909724 | 1 |
| 3277.9910749 | 2 |
| 3277.9911652 | 1 |
| 3277.9913763 | 1 |
| 3277.9914187 | 2 |
| 3277.9914836 | 3 |
| 3277.9914943 | 1 |
| 3277.9916763 | 3 |
| 3277.9917725 | 3 |
| 3277.9917792 | 4 |
| 3277.9918526 | 3 |
| 3277.9921596 | 1 |
| 3277.992267 | 3 |
| 3277.9922743 | 4 |
| 3277.992412 | 4 |
| 3277.9924941 | 0 |
| 3277.9926647 | 4 |
| 3277.9930407 | 0 |
| 3277.9931506 | 4 |
| 3277.9934155 | 1 |
| 3277.993521 | 3 |
| 3277.993571 | 1 |
| 3277.9938204 | 3 |
| 3277.9938462 | 2 |
| 3277.9940365 | 1 |
| 3277.9941029 | 4 |
| 3277.9941478 | 2 |
| 3277.9946785 | 1 |
| 3277.9946832 | 5 |
| 3277.9947021 | 2 |
| 3277.9949151 | 2 |
| 3277.9949583 | 3 |
| 3277.9951286 | 0 |
| 3277.9951399 | 1 |
| 3277.9951542 | 0 |
| 3277.995247 | 3 |
| 3277.9952857 | 0 |
| 3277.9953163 | 4 |
| 3277.9955292 | 1 |
| 3277.9955337 | 0 |
| 3277.9957101 | 2 |
| 3277.9958194 | 2 |
| 3277.9958641 | 3 |
| 3277.9960803 | 4 |
| 3277.9961287 | 2 |
| 3277.996181 | 0 |
| 3277.9964959 | 0 |
| 3277.9966682 | 3 |
| 3277.9967015 | 5 |
| 3277.9969261 | 1 |
| 3277.9970043 | 5 |
| 3277.9970183 | 1 |
| 3277.9971986 | 1 |
| 3277.9976662 | 3 |
| 3277.9979679 | 1 |
| 3277.9979957 | 1 |
| 3277.9980639 | 1 |
| 3277.9980689 | 2 |
| 3277.9982499 | 1 |
| 3277.9982694 | 0 |
| 3277.998302 | 1 |
| 3277.9984112 | 0 |
| 3277.9984167 | 2 |
| 3277.9985614 | 4 |
| 3277.998812 | 0 |
| 3277.998923 | 4 |
| 3277.9990808 | 3 |
| 3277.9991771 | 0 |
| 3277.9992854 | 2 |
| 3277.9993859 | 4 |
| 3277.999531 | 1 |
| 3277.9995471 | 0 |
| 3277.999639 | 1 |
| 3277.9996977 | 2 |
| 3277.9998252 | 4 |
| 3277.9999878 | 2 |
| 3278.0000233 | 3 |
| 3278.0003467 | 5 |
| 3278.0004365 | 4 |
| 3278.000458 | 2 |
| 3278.0004585 | 3 |
| 3278.0005737 | 3 |
| 3278.0007083 | 0 |
| 3278.000736 | 0 |
| 3278.0008466 | 1 |
| 3278.0012068 | 1 |
| 3278.0012232 | 2 |
| 3278.0013548 | 2 |
| 3278.0015462 | 3 |
| 3278.0015937 | 3 |
| 3278.0015948 | 2 |
| 3278.0016975 | 0 |
| 3278.0018741 | 3 |
| 3278.001902 | 1 |
| 3278.0021683 | 0 |
| 3278.0023198 | 1 |
| 3278.0023223 | 2 |
| 3278.0025112 | 3 |
| 3278.0025799 | 0 |
| 3278.0026593 | 2 |
| 3278.0026689 | 2 |
| 3278.0027941 | 2 |
| 3278.0033158 | 1 |
| 3278.0035117 | 0 |
| 3278.0035341 | 1 |
| 3278.003541 | 1 |
| 3278.0035478 | 3 |
| 3278.0035907 | 0 |
| 3278.003821 | 2 |
| 3278.0039698 | 1 |
| 3278.0040934 | 2 |
| 3278.0041988 | 1 |
| 3278.0042838 | 2 |
| 3278.0044009 | 3 |
| 3278.0044353 | 0 |
| 3278.0044511 | 2 |
| 3278.0044656 | 2 |
| 3278.0045007 | 1 |
| 3278.0046916 | 3 |
| 3278.0048269 | 2 |
| 3278.0052914 | 3 |
| 3278.005343 | 5 |
| 3278.005546 | 1 |
| 3278.0057374 | 1 |
| 3278.0058005 | 3 |
| 3278.0058681 | 0 |
| 3278.0058793 | 1 |
| 3278.0058849 | 4 |
| 3278.0059483 | 3 |
| 3278.005963 | 2 |
| 3278.0060843 | 2 |
| 3278.0061227 | 1 |
| 3278.0062106 | 2 |
| 3278.0062198 | 2 |
| 3278.0065656 | 1 |
| 3278.0066145 | 0 |
| 3278.0069231 | 5 |
| 3278.007055 | 1 |
| 3278.0071462 | 3 |
| 3278.0072599 | 1 |
| 3278.0072846 | 0 |
| 3278.0073616 | 1 |
| 3278.0077862 | 1 |
| 3278.0078947 | 0 |
| 3278.0080696 | 2 |
| 3278.0081367 | 3 |
| 3278.008332 | 2 |
| 3278.0083841 | 3 |
| 3278.0083982 | 1 |
| 3278.0086501 | 4 |
| 3278.0087025 | 2 |
| 3278.0088029 | 2 |
| 3278.0088802 | 1 |
| 3278.0089941 | 4 |
| 3278.0090422 | 1 |
| 3278.0092294 | 2 |
| 3278.0092471 | 4 |
| 3278.0095586 | 2 |
| 3278.009598 | 4 |
| 3278.0096062 | 0 |
| 3278.0100911 | 2 |
| 3278.0101208 | 3 |
| 3278.0101414 | 2 |
| 3278.0103266 | 1 |
| 3278.0103623 | 3 |
| 3278.0103928 | 3 |
| 3278.010537 | 2 |
| 3278.0105736 | 5 |
| 3278.0106268 | 1 |
| 3278.0106429 | 0 |
| 3278.01065 | 2 |
| 3278.0106921 | 3 |
| 3278.0107207 | 2 |
| 3278.0112745 | 3 |
| 3278.0113157 | 2 |
| 3278.0113914 | 1 |
| 3278.0115765 | 2 |
| 3278.0118832 | 3 |
| 3278.0119587 | 2 |
| 3278.0119789 | 4 |
| 3278.0120462 | 3 |
| 3278.0121454 | 2 |
| 3278.0121459 | 6 |
| 3278.012313 | 11 |
| 3278.0123733 | 2 |
| 3278.0124767 | 3 |
| 3278.012696 | 2 |
| 3278.0128677 | 3 |
| 3278.0129119 | 3 |
| 3278.0131476 | 9 |
| 3278.0132513 | 2 |
| 3278.0134208 | 9 |
| 3278.0134839 | 3 |
| 3278.0134958 | 8 |
| 3278.0135997 | 0 |
| 3278.0138712 | 5 |
| 3278.0139059 | 14 |
| 3278.014069 | 11 |
| 3278.0143445 | 29 |
| 3278.0143744 | 18 |
| 3278.0144236 | 19 |
| 3278.0145574 | 19 |
| 3278.0146766 | 7 |
| 3278.014889 | 33 |
| 3278.015007 | 25 |
| 3278.015248 | 22 |
| 3278.0154134 | 49 |
| 3278.0154245 | 31 |
| 3278.0157302 | 26 |
| 3278.0157856 | 25 |
| 3278.0158085 | 45 |
| 3278.0158924 | 40 |
| 3278.0159059 | 48 |
| 3278.0160767 | 58 |
| 3278.016107 | 46 |
| 3278.0162835 | 38 |
| 3278.0163253 | 49 |
| 3278.0163444 | 51 |
| 3278.016346 | 49 |
| 3278.0164218 | 40 |
| 3278.0165332 | 42 |
| 3278.0165748 | 51 |
| 3278.0168709 | 33 |
| 3278.0169925 | 39 |
| 3278.0172705 | 32 |
| 3278.0172919 | 39 |
| 3278.0174246 | 24 |
| 3278.0176867 | 38 |
| 3278.0177206 | 31 |
| 3278.0179325 | 14 |
| 3278.0180411 | 16 |
| 3278.0181019 | 20 |
| 3278.0181418 | 21 |
| 3278.0181519 | 10 |
| 3278.0183262 | 15 |
| 3278.0183844 | 8 |

| | |
|---|---|
| 3278.0186054 | 4 |
| 3278.0186228 | 30 |
| 3278.0186286 | 2 |
| 3278.018782 | 1 |
| 3278.0188494 | 5 |
| 3278.0189001 | 16 |
| 3278.0190182 | 12 |
| 3278.0192187 | 3 |
| 3278.0192544 | 4 |
| 3278.0194638 | 5 |
| 3278.0195155 | 3 |
| 3278.0195788 | 5 |
| 3278.0196886 | 0 |
| 3278.0198646 | 2 |
| 3278.0198828 | 3 |
| 3278.020269 | 0 |
| 3278.0203625 | 2 |
| 3278.0204698 | 3 |
| 3278.0205932 | 2 |
| 3278.0207138 | 0 |
| 3278.020836 | 1 |
| 3278.0208486 | 2 |
| 3278.0208541 | 1 |
| 3278.0208739 | 1 |
| 3278.0209687 | 2 |
| 3278.0210372 | 1 |
| 3278.0212864 | 3 |
| 3278.0213728 | 0 |
| 3278.0214081 | 1 |
| 3278.0218542 | 0 |
| 3278.0218776 | 1 |
| 3278.0219619 | 1 |
| 3278.0220589 | 2 |
| 3278.022181 | 1 |
| 3278.022184 | 2 |
| 3278.0222081 | 5 |
| 3278.0225338 | 4 |
| 3278.0226315 | 1 |
| 3278.0226884 | 1 |
| 3278.0227547 | 0 |
| 3278.0230098 | 3 |
| 3278.023028 | 2 |
| 3278.0231957 | 1 |
| 3278.0232738 | 2 |
| 3278.0232751 | 0 |
| 3278.0237788 | 3 |
| 3278.0238853 | 1 |
| 3278.0239484 | 2 |
| 3278.0239926 | 0 |
| 3278.0240254 | 2 |
| 3278.024098 | 1 |
| 3278.0241213 | 2 |
| 3278.0241598 | 0 |
| 3278.0241862 | 3 |
| 3278.0244457 | 1 |
| 3278.0245555 | 2 |
| 3278.0247547 | 1 |
| 3278.0248385 | 4 |
| 3278.0248828 | 1 |
| 3278.0248969 | 3 |
| 3278.025242 | 0 |
| 3278.0252607 | 2 |
| 3278.0253743 | 1 |
| 3278.0253892 | 1 |
| 3278.0257483 | 4 |
| 3278.0257807 | 2 |
| 3278.0260373 | 1 |
| 3278.0262941 | 3 |
| 3278.026795 | 0 |
| 3278.0268365 | 1 |
| 3278.0269295 | 2 |
| 3278.0269891 | 2 |
| 3278.0270192 | 1 |
| 3278.0270759 | 2 |
| 3278.0271493 | 1 |
| 3278.0271682 | 2 |
| 3278.027463 | 0 |
| 3278.0275132 | 0 |
| 3278.027608 | 0 |
| 3278.0281024 | 1 |
| 3278.0281386 | 1 |
| 3278.0281964 | 5 |
| 3278.0283545 | 2 |
| 3278.0284025 | 5 |
| 3278.0284232 | 1 |
| 3278.0285168 | 2 |
| 3278.0286276 | 3 |
| 3278.0287289 | 3 |
| 3278.0288106 | 2 |
| 3278.0289334 | 3 |
| 3278.0290699 | 2 |
| 3278.0293775 | 1 |
| 3278.0294044 | 2 |
| 3278.0295378 | 0 |
| 3278.0296081 | 4 |
| 3278.0297129 | 1 |
| 3278.0298249 | 2 |
| 3278.0298811 | 2 |
| 3278.0299724 | 3 |
| 3278.0302379 | 0 |
| 3278.0303274 | 3 |
| 3278.0303593 | 2 |
| 3278.03042 | 1 |
| 3278.030517 | 0 |
| 3278.0305813 | 1 |
| 3278.0305891 | 3 |
| 3278.0310457 | 1 |
| 3278.0313885 | 2 |
| 3278.031408 | 4 |
| 3278.031489 | 2 |
| 3278.0315115 | 1 |
| 3278.0316326 | 2 |
| 3278.0316768 | 0 |
| 3278.0318874 | 2 |
| 3278.0319683 | 5 |
| 3278.0321983 | 1 |
| 3278.0322666 | 0 |
| 3278.0323071 | 1 |
| 3278.0323083 | 2 |
| 3278.0324269 | 2 |
| 3278.032465 | 0 |
| 3278.0327209 | 3 |
| 3278.0327656 | 1 |
| 3278.032792 | 1 |
| 3278.0328036 | 2 |
| 3278.0329293 | 4 |
| 3278.0332175 | 4 |
| 3278.033584 | 2 |
| 3278.0336325 | 4 |
| 3278.0336823 | 2 |
| 3278.0339102 | 2 |
| 3278.0340028 | 3 |
| 3278.0340789 | 1 |
| 3278.0341503 | 2 |
| 3278.0341786 | 1 |
| 3278.0341855 | 1 |
| 3278.0342204 | 3 |
| 3278.0342423 | 1 |
| 3278.0343243 | 1 |
| 3278.0345864 | 0 |
| 3278.03462 | 2 |
| 3278.0348288 | 2 |
| 3278.0349779 | 2 |
| 3278.0354253 | 1 |
| 3278.035474 | 1 |
| 3278.035475 | 2 |
| 3278.0357563 | 3 |
| 3278.0359 | 0 |
| 3278.0359348 | 1 |
| 3278.0360586 | 3 |
| 3278.0361205 | 2 |
| 3278.0361761 | 1 |
| 3278.0363516 | 1 |
| 3278.0365274 | 3 |
| 3278.0368911 | 1 |
| 3278.0369703 | 2 |
| 3278.0370092 | 2 |
| 3278.0373096 | 4 |
| 3278.0375023 | 2 |
| 3278.0375994 | 2 |
| 3278.0376497 | 2 |
| 3278.0378743 | 2 |
| 3278.0379055 | 1 |
| 3278.0379763 | 2 |
| 3278.0380037 | 2 |
| 3278.0383386 | 2 |
| 3278.0384778 | 2 |
| 3278.0384863 | 1 |
| 3278.0386791 | 0 |
| 3278.0388412 | 6 |
| 3278.0391121 | 2 |
| 3278.0391563 | 2 |
| 3278.0392357 | 3 |
| 3278.0393629 | 1 |
| 3278.039404 | 3 |
| 3278.0396283 | 2 |
| 3278.0396611 | 3 |
| 3278.0397118 | 2 |
| 3278.0398883 | 2 |
| 3278.040051 | 2 |
| 3278.0401281 | 1 |
| 3278.0402265 | 0 |
| 3278.0402886 | 2 |
| 3278.0408798 | 1 |
| 3278.0411461 | 4 |
| 3278.0412905 | 1 |
| 3278.0415156 | 3 |
| 3278.0416078 | 0 |
| 3278.0416101 | 0 |
| 3278.0416518 | 0 |
| 3278.0416835 | 1 |
| 3278.0417883 | 0 |
| 3278.0420256 | 3 |
| 3278.0423347 | 1 |
| 3278.042424 | 2 |
| 3278.0425595 | 1 |
| 3278.042684 | 0 |
| 3278.0426889 | 3 |
| 3278.042822 | 2 |
| 3278.0429822 | 2 |
| 3278.0431022 | 0 |
| 3278.0431668 | 3 |
| 3278.0433284 | 6 |
| 3278.0437514 | 1 |
| 3278.0440134 | 2 |
| 3278.0441873 | 3 |
| 3278.0444235 | 4 |
| 3278.0444417 | 1 |
| 3278.0445078 | 3 |
| 3278.0445307 | 4 |
| 3278.0446181 | 1 |
| 3278.0446615 | 1 |
| 3278.0447923 | 2 |
| 3278.0451886 | 0 |
| 3278.0454523 | 3 |
| 3278.0455664 | 2 |
| 3278.0459047 | 2 |
| 3278.0460303 | 3 |
| 3278.0463822 | 0 |
| 3278.0464834 | 0 |
| 3278.0465179 | 0 |
| 3278.0465473 | 1 |
| 3278.0465511 | 2 |
| 3278.0465685 | 0 |
| 3278.0466975 | 2 |
| 3278.0468949 | 4 |
| 3278.0469049 | 5 |
| 3278.0470751 | 2 |
| 3278.0473303 | 3 |
| 3278.0473679 | 0 |
| 3278.0474439 | 0 |
| 3278.0476362 | 3 |
| 3278.0479612 | 1 |
| 3278.0479866 | 0 |
| 3278.0480737 | 3 |
| 3278.0484488 | 1 |
| 3278.0486473 | 2 |
| 3278.0487992 | 2 |
| 3278.0488142 | 2 |
| 3278.0490415 | 4 |
| 3278.0492754 | 3 |
| 3278.0495457 | 7 |
| 3278.0496953 | 3 |
| 3278.0497152 | 5 |
| 3278.0497415 | 6 |
| 3278.0497803 | 1 |
| 3278.0499968 | 0 |
| 3278.050034 | 2 |
| 3278.0501987 | 2 |
| 3278.050444 | 2 |
| 3278.0506021 | 0 |
| 3278.050791 | 0 |
| 3278.0510413 | 2 |
| 3278.0511452 | 0 |
| 3278.051146 | 3 |
| 3278.0511901 | 2 |
| 3278.0512051 | 2 |
| 3278.0513079 | 0 |
| 3278.0513509 | 1 |
| 3278.0514044 | 0 |
| 3278.0516344 | 2 |
| 3278.0522221 | 0 |
| 3278.0523121 | 0 |
| 3278.0524111 | 1 |
| 3278.0527813 | 0 |
| 3278.0528989 | 2 |
| 3278.0530436 | 3 |
| 3278.0530606 | 3 |
| 3278.0530669 | 1 |
| 3278.0531817 | 1 |
| 3278.0534398 | 2 |
| 3278.0536188 | 3 |
| 3278.053769 | 2 |
| 3278.0539744 | 0 |
| 3278.0542924 | 2 |
| 3278.0545059 | 4 |
| 3278.0546919 | 1 |
| 3278.054768 | 2 |
| 3278.0548636 | 1 |
| 3278.0549026 | 1 |
| 3278.054959 | 0 |
| 3278.0550012 | 6 |
| 3278.0550926 | 1 |
| 3278.0553232 | 0 |
| 3278.0554855 | 2 |
| 3278.0556971 | 1 |
| 3278.0559347 | 0 |
| 3278.0562002 | 2 |
| 3278.0562805 | 3 |
| 3278.0563299 | 1 |
| 3278.056466 | 0 |
| 3278.0565479 | 0 |
| 3278.0565877 | 1 |
| 3278.0566022 | 5 |
| 3278.0568126 | 4 |
| 3278.0569349 | 3 |
| 3278.0569928 | 1 |
| 3278.0570551 | 2 |
| 3278.0570777 | 3 |
| 3278.0570779 | 1 |
| 3278.0573204 | 2 |
| 3278.0575373 | 2 |
| 3278.0577537 | 3 |
| 3278.0580242 | 1 |
| 3278.0581619 | 0 |
| 3278.0582337 | 1 |
| 3278.0582619 | 3 |
| 3278.058347 | 3 |
| 3278.0588663 | 3 |
| 3278.0590633 | 0 |
| 3278.0591467 | 4 |
| 3278.0592483 | 0 |
| 3278.0592485 | 1 |
| 3278.0595643 | 1 |
| 3278.059572 | 3 |
| 3278.0599908 | 2 |
| 3278.0600583 | 1 |
| 3278.0601275 | 1 |
| 3278.0603596 | 4 |
| 3278.0605588 | 1 |
| 3278.0606498 | 1 |
| 3278.0607176 | 0 |
| 3278.0608528 | 4 |
| 3278.0609183 | 4 |
| 3278.0613894 | 2 |
| 3278.0613992 | 1 |
| 3278.0614514 | 2 |
| 3278.0614598 | 3 |
| 3278.0614906 | 1 |
| 3278.0616198 | 1 |
| 3278.0617309 | 0 |
| 3278.0618031 | 0 |
| 3278.06201 | 4 |
| 3278.0622275 | 3 |
| 3278.0624184 | 0 |
| 3278.0625716 | 4 |
| 3278.0627866 | 3 |
| 3278.0629018 | 2 |
| 3278.0631168 | 2 |
| 3278.0631587 | 3 |
| 3278.0633234 | 5 |
| 3278.0635133 | 1 |
| 3278.0636079 | 0 |
| 3278.0637431 | 1 |
| 3278.063951 | 4 |
| 3278.0641141 | 3 |
| 3278.0641632 | 1 |
| 3278.064325 | 1 |
| 3278.064442 | 1 |
| 3278.0645181 | 2 |
| 3278.0646265 | 5 |
| 3278.0651373 | 3 |
| 3278.0651734 | 0 |
| 3278.0652965 | 2 |
| 3278.0653059 | 3 |
| 3278.065322 | 0 |
| 3278.0655206 | 5 |
| 3278.0655312 | 2 |
| 3278.0659193 | 0 |
| 3278.0659472 | 2 |
| 3278.0659896 | 2 |
| 3278.0661218 | 1 |
| 3278.0662312 | 3 |

| | |
|---|---|
| 3278.0666253 | 0 |
| 3278.0666541 | 4 |
| 3278.0668305 | 0 |
| 3278.0668542 | 1 |
| 3278.0669475 | 0 |
| 3278.0670084 | 3 |
| 3278.0671896 | 8 |
| 3278.0675173 | 2 |
| 3278.0676456 | 1 |
| 3278.067896 | 0 |
| 3278.0680664 | 1 |
| 3278.0681358 | 2 |
| 3278.0682453 | 3 |
| 3278.0684381 | 0 |
| 3278.0684662 | 2 |
| 3278.0687052 | 3 |
| 3278.0690433 | 2 |
| 3278.0691209 | 3 |
| 3278.0692375 | 5 |
| 3278.0693476 | 1 |
| 3278.0694914 | 3 |
| 3278.0695793 | 4 |
| 3278.0696844 | 3 |
| 3278.0699367 | 0 |
| 3278.0699639 | 3 |
| 3278.0700582 | 1 |
| 3278.0701881 | 3 |
| 3278.0702316 | 2 |
| 3278.0702415 | 1 |
| 3278.0702494 | 4 |
| 3278.0707225 | 3 |
| 3278.0709061 | 3 |
| 3278.0709658 | 2 |
| 3278.071253 | 1 |
| 3278.0716265 | 1 |
| 3278.0717266 | 1 |
| 3278.0717984 | 0 |
| 3278.0718053 | 2 |
| 3278.0718452 | 1 |
| 3278.0718852 | 4 |
| 3278.0721898 | 3 |
| 3278.0722029 | 1 |
| 3278.0722619 | 0 |
| 3278.0723893 | 2 |
| 3278.0726976 | 2 |
| 3278.0729175 | 2 |
| 3278.0729617 | 8 |
| 3278.073197 | 2 |
| 3278.0732413 | 2 |
| 3278.0736938 | 1 |
| 3278.0737639 | 5 |
| 3278.0738308 | 0 |
| 3278.073965 | 1 |
| 3278.0742622 | 2 |
| 3278.0742707 | 2 |
| 3278.0744691 | 4 |
| 3278.0744755 | 1 |
| 3278.0745178 | 4 |
| 3278.0747526 | 2 |
| 3278.0748001 | 4 |
| 3278.0749803 | 3 |
| 3278.0751064 | 1 |
| 3278.0752652 | 2 |
| 3278.0753731 | 3 |
| 3278.0756393 | 1 |
| 3278.0757367 | 0 |
| 3278.0759011 | 2 |
| 3278.0760275 | 1 |
| 3278.0760676 | 2 |
| 3278.0763937 | 1 |
| 3278.0768006 | 0 |
| 3278.0768166 | 1 |
| 3278.0768304 | 1 |
| 3278.0769715 | 3 |
| 3278.0772287 | 1 |
| 3278.0774662 | 0 |
| 3278.0775295 | 1 |
| 3278.0776167 | 5 |
| 3278.0778064 | 3 |
| 3278.0778509 | 3 |
| 3278.078028 | 4 |
| 3278.0780899 | 0 |
| 3278.078172 | 4 |
| 3278.0782291 | 2 |
| 3278.0782732 | 3 |
| 3278.0786511 | 2 |
| 3278.0786555 | 2 |
| 3278.0790743 | 1 |
| 3278.0792255 | 2 |
| 3278.0794228 | 1 |
| 3278.0796178 | 3 |
| 3278.0796946 | 3 |
| 3278.0797439 | 0 |
| 3278.0798441 | 1 |
| 3278.0800809 | 2 |
| 3278.0802583 | 2 |
| 3278.0804575 | 2 |
| 3278.0806735 | 2 |
| 3278.0810487 | 0 |
| 3278.0810554 | 1 |
| 3278.0812298 | 1 |
| 3278.0812586 | 2 |
| 3278.0814905 | 2 |
| 3278.08177 | 1 |
| 3278.0818233 | 0 |
| 3278.0818686 | 4 |
| 3278.0819073 | 3 |
| 3278.0819562 | 1 |
| 3278.0822745 | 1 |
| 3278.0824746 | 1 |
| 3278.0825266 | 2 |
| 3278.0825658 | 1 |
| 3278.082612 | 0 |
| 3278.0827911 | 1 |
| 3278.082854 | 0 |
| 3278.0831133 | 4 |
| 3278.08331 | 3 |
| 3278.0834232 | 1 |
| 3278.0837399 | 1 |
| 3278.0838789 | 2 |
| 3278.084096 | 2 |
| 3278.0841032 | 4 |
| 3278.0841715 | 0 |
| 3278.0845294 | 1 |
| 3278.0845484 | 4 |
| 3278.0846046 | 3 |
| 3278.0846079 | 1 |
| 3278.0847217 | 4 |
| 3278.0853146 | 2 |
| 3278.0854187 | 2 |
| 3278.0858254 | 1 |
| 3278.0859374 | 1 |
| 3278.0859747 | 2 |
| 3278.086081 | 4 |
| 3278.086122 | 3 |
| 3278.0861527 | 3 |
| 3278.0862303 | 1 |
| 3278.0862718 | 2 |
| 3278.0864504 | 2 |
| 3278.0866524 | 1 |
| 3278.0869751 | 0 |
| 3278.0870906 | 5 |
| 3278.0873141 | 2 |
| 3278.0874775 | 2 |
| 3278.0876169 | 3 |
| 3278.0876268 | 2 |
| 3278.0878215 | 2 |
| 3278.0878697 | 2 |
| 3278.0878963 | 5 |
| 3278.0880392 | 1 |
| 3278.0883227 | 1 |
| 3278.0883238 | 1 |
| 3278.0885757 | 4 |
| 3278.0887105 | 3 |
| 3278.0888659 | 3 |
| 3278.0889101 | 4 |
| 3278.0892391 | 3 |
| 3278.0893469 | 5 |
| 3278.0896883 | 2 |
| 3278.0898073 | 1 |
| 3278.0898922 | 2 |
| 3278.0900981 | 1 |
| 3278.090254 | 2 |
| 3278.0903459 | 1 |
| 3278.0905664 | 1 |
| 3278.0905999 | 1 |
| 3278.090967 | 2 |
| 3278.0910242 | 1 |
| 3278.0911825 | 1 |
| 3278.0911904 | 2 |
| 3278.091287 | 1 |
| 3278.0913171 | 1 |
| 3278.0916208 | 1 |
| 3278.0917569 | 2 |
| 3278.091905 | 2 |
| 3278.0920696 | 1 |
| 3278.09229 | 1 |
| 3278.0923496 | 1 |
| 3278.0923512 | 2 |
| 3278.0925736 | 0 |
| 3278.0926142 | 2 |
| 3278.0927307 | 0 |
| 3278.0929691 | 3 |
| 3278.0929793 | 1 |
| 3278.0930964 | 4 |
| 3278.0934129 | 3 |
| 3278.0936154 | 3 |
| 3278.0936367 | 2 |
| 3278.0936477 | 1 |
| 3278.0937588 | 1 |
| 3278.0938979 | 0 |
| 3278.0939278 | 2 |
| 3278.0940251 | 1 |
| 3278.0940629 | 2 |
| 3278.0942435 | 1 |
| 3278.094254 | 3 |
| 3278.0944608 | 2 |
| 3278.0944964 | 3 |
| 3278.0947328 | 2 |
| 3278.0948175 | 3 |
| 3278.0949915 | 1 |
| 3278.0950016 | 2 |
| 3278.0950364 | 1 |
| 3278.095091 | 0 |
| 3278.0952515 | 2 |
| 3278.0954245 | 3 |
| 3278.0954904 | 3 |
| 3278.0955316 | 0 |
| 3278.0955944 | 0 |
| 3278.0956182 | 5 |
| 3278.0958518 | 1 |
| 3278.0960386 | 2 |
| 3278.0960474 | 0 |
| 3278.0963253 | 0 |
| 3278.0963325 | 0 |
| 3278.0967728 | 1 |
| 3278.0968071 | 0 |
| 3278.0969105 | 1 |
| 3278.0969154 | 2 |
| 3278.0969232 | 2 |
| 3278.0970001 | 2 |
| 3278.0970071 | 3 |
| 3278.0970367 | 1 |
| 3278.0971684 | 3 |
| 3278.0972254 | 1 |
| 3278.0975773 | 3 |
| 3278.0976251 | 1 |
| 3278.0979639 | 3 |
| 3278.0980373 | 1 |
| 3278.0981356 | 0 |
| 3278.0982353 | 1 |
| 3278.098264 | 5 |
| 3278.0984268 | 3 |
| 3278.0984277 | 3 |
| 3278.0987574 | 1 |
| 3278.0988823 | 1 |
| 3278.0989105 | 4 |
| 3278.0989394 | 3 |
| 3278.0990951 | 0 |
| 3278.0991564 | 1 |
| 3278.0993534 | 2 |
| 3278.0994141 | 1 |
| 3278.0994573 | 2 |
| 3278.0995068 | 2 |
| 3278.0996161 | 1 |
| 3278.0996316 | 1 |
| 3278.0998984 | 2 |
| 3278.0999062 | 4 |
| 3278.100095 | 2 |
| 3278.1001543 | 4 |
| 3278.1001637 | 1 |
| 3278.1003339 | 4 |
| 3278.1003401 | 0 |
| 3278.1008088 | 0 |
| 3278.1008664 | 3 |
| 3278.1008831 | 0 |
| 3278.1009128 | 0 |
| 3278.1010284 | 1 |
| 3278.1010344 | 3 |
| 3278.1013744 | 2 |
| 3278.1014063 | 2 |
| 3278.101517 | 2 |
| 3278.1017361 | 4 |
| 3278.1017369 | 5 |
| 3278.1017565 | 1 |
| 3278.1020472 | 4 |
| 3278.1021272 | 1 |
| 3278.1022046 | 1 |
| 3278.102306 | 0 |
| 3278.102337 | 0 |
| 3278.1023972 | 5 |
| 3278.1027338 | 3 |
| 3278.1028473 | 2 |
| 3278.1028831 | 2 |
| 3278.1029411 | 2 |
| 3278.103003 | 3 |
| 3278.1030131 | 1 |
| 3278.1030404 | 2 |
| 3278.1030478 | 2 |
| 3278.1032586 | 5 |
| 3278.1032875 | 2 |
| 3278.1035199 | 3 |
| 3278.1036069 | 5 |
| 3278.1036869 | 3 |
| 3278.1036917 | 2 |
| 3278.1041296 | 2 |
| 3278.1042133 | 4 |
| 3278.1044725 | 6 |
| 3278.1044769 | 2 |
| 3278.1045373 | 2 |
| 3278.1045612 | 2 |
| 3278.1046411 | 5 |
| 3278.1046499 | 1 |
| 3278.1046894 | 1 |
| 3278.10477 | 2 |
| 3278.1048646 | 2 |
| 3278.1049284 | 1 |
| 3278.1049423 | 2 |
| 3278.105096 | 1 |
| 3278.1051835 | 1 |
| 3278.1052434 | 1 |
| 3278.105671 | 3 |
| 3278.105759 | 0 |
| 3278.105861 | 2 |
| 3278.1058746 | 1 |
| 3278.1058848 | 5 |
| 3278.1062446 | 3 |
| 3278.1062677 | 2 |
| 3278.1063356 | 2 |
| 3278.1063969 | 3 |
| 3278.1064087 | 1 |
| 3278.1064496 | 2 |
| 3278.1066498 | 3 |
| 3278.1068632 | 1 |
| 3278.1069384 | 3 |
| 3278.1071033 | 2 |
| 3278.1072032 | 3 |
| 3278.1072131 | 1 |
| 3278.1074086 | 1 |
| 3278.107478 | 3 |
| 3278.1075576 | 3 |
| 3278.1075708 | 0 |
| 3278.1076681 | 2 |
| 3278.1077216 | 0 |
| 3278.107913 | 1 |
| 3278.1079733 | 3 |
| 3278.1080577 | 5 |
| 3278.1080717 | 0 |
| 3278.1083806 | 0 |
| 3278.1083854 | 5 |
| 3278.1084882 | 3 |
| 3278.1085936 | 2 |
| 3278.1088211 | 3 |
| 3278.1089683 | 2 |
| 3278.1089748 | 3 |
| 3278.1090685 | 1 |
| 3278.1091176 | 0 |
| 3278.1092857 | 4 |
| 3278.1093556 | 3 |
| 3278.1093761 | 0 |
| 3278.1094627 | 2 |
| 3278.1095303 | 3 |
| 3278.1096731 | 3 |
| 3278.1098989 | 4 |
| 3278.1100131 | 2 |
| 3278.1100722 | 2 |
| 3278.1102635 | 2 |
| 3278.1103807 | 3 |
| 3278.1104246 | 3 |
| 3278.1107387 | 0 |
| 3278.1107703 | 1 |
| 3278.1108276 | 0 |
| 3278.1109063 | 5 |
| 3278.1109418 | 1 |
| 3278.1112047 | 3 |
| 3278.1112509 | 0 |
| 3278.111273 | 1 |
| 3278.1112742 | 3 |
| 3278.1113432 | 5 |
| 3278.1114095 | 3 |
| 3278.1114783 | 1 |
| 3278.1115441 | 0 |
| 3278.1116429 | 2 |

| | |
|---|---|
| 3278.1116495 | 2 |
| 3278.1117011 | 1 |
| 3278.1118075 | 2 |
| 3278.1118309 | 2 |
| 3278.1123049 | 1 |
| 3278.1124223 | 0 |
| 3278.1125526 | 3 |
| 3278.1125687 | 1 |
| 3278.1126616 | 4 |
| 3278.1126739 | 0 |
| 3278.1129938 | 1 |
| 3278.1130722 | 4 |
| 3278.1130979 | 4 |
| 3278.1133644 | 1 |
| 3278.1135233 | 1 |
| 3278.1135843 | 4 |
| 3278.1138117 | 2 |
| 3278.1138349 | 1 |
| 3278.1138647 | 1 |
| 3278.1139205 | 1 |
| 3278.1139862 | 1 |
| 3278.1141859 | 2 |
| 3278.1142556 | 1 |
| 3278.1143457 | 3 |
| 3278.1143732 | 0 |
| 3278.1146621 | 0 |
| 3278.1147182 | 3 |
| 3278.1148031 | 1 |
| 3278.1149541 | 1 |
| 3278.115049 | 1 |
| 3278.1150689 | 2 |
| 3278.1151729 | 2 |
| 3278.1151878 | 4 |
| 3278.1152251 | 1 |
| 3278.1153377 | 3 |
| 3278.1156413 | 1 |
| 3278.1157254 | 0 |
| 3278.1157782 | 1 |
| 3278.1159078 | 0 |
| 3278.1159775 | 1 |
| 3278.1160213 | 0 |
| 3278.1160473 | 3 |
| 3278.1161686 | 2 |
| 3278.1163314 | 3 |
| 3278.1164486 | 3 |
| 3278.1165616 | 4 |
| 3278.1168517 | 9 |
| 3278.1168618 | 2 |
| 3278.1169471 | 3 |
| 3278.1171843 | 4 |
| 3278.1172136 | 8 |
| 3278.1173451 | 6 |
| 3278.117565 | 8 |
| 3278.1175964 | 8 |
| 3278.1177323 | 0 |
| 3278.117758 | 16 |
| 3278.1177968 | 12 |
| 3278.1178978 | 19 |
| 3278.118065 | 6 |
| 3278.1181908 | 9 |
| 3278.1181914 | 8 |
| 3278.1182366 | 12 |
| 3278.1182759 | 9 |
| 3278.1183165 | 15 |
| 3278.1183542 | 19 |
| 3278.1189173 | 5 |
| 3278.1189193 | 11 |
| 3278.1191429 | 16 |
| 3278.1192624 | 16 |
| 3278.119435 | 27 |
| 3278.1194587 | 23 |
| 3278.1195777 | 23 |
| 3278.1196572 | 23 |
| 3278.1196736 | 29 |
| 3278.1197002 | 13 |
| 3278.1197075 | 15 |
| 3278.1197304 | 15 |
| 3278.119732 | 15 |
| 3278.119943 | 16 |
| 3278.1199642 | 12 |
| 3278.1201148 | 15 |
| 3278.1205188 | 14 |
| 3278.1205275 | 11 |
| 3278.1205364 | 18 |
| 3278.120719 | 20 |
| 3278.1207234 | 18 |
| 3278.1210388 | 20 |
| 3278.1211357 | 22 |
| 3278.1213139 | 13 |
| 3278.1213494 | 14 |
| 3278.1213597 | 19 |
| 3278.1215377 | 20 |
| 3278.1216035 | 18 |
| 3278.121858 | 10 |
| 3278.1219477 | 20 |
| 3278.121999 | 20 |
| 3278.1221009 | 15 |
| 3278.1221529 | 26 |
| 3278.1222943 | 26 |
| 3278.1224116 | 11 |
| 3278.1224992 | 16 |
| 3278.1226254 | 17 |
| 3278.122628 | 25 |
| 3278.1226636 | 16 |
| 3278.1228234 | 19 |
| 3278.1230952 | 19 |
| 3278.1231504 | 15 |
| 3278.1232359 | 14 |
| 3278.1234562 | 17 |
| 3278.1236913 | 14 |
| 3278.1237826 | 35 |
| 3278.1237891 | 14 |
| 3278.1239935 | 24 |
| 3278.1241091 | 12 |
| 3278.124258 | 16 |
| 3278.1242736 | 21 |
| 3278.1242882 | 18 |
| 3278.1243288 | 22 |
| 3278.1244462 | 25 |
| 3278.1245521 | 20 |
| 3278.1245694 | 17 |
| 3278.12462 | 16 |
| 3278.1248619 | 35 |
| 3278.1249123 | 14 |
| 3278.1249171 | 20 |
| 3278.1250144 | 31 |
| 3278.1250708 | 14 |
| 3278.1252854 | 9 |
| 3278.125463 | 16 |
| 3278.1256131 | 17 |
| 3278.1256354 | 10 |
| 3278.1257267 | 13 |
| 3278.1257532 | 14 |
| 3278.125917 | 6 |
| 3278.1259316 | 7 |
| 3278.1259412 | 17 |
| 3278.1263042 | 12 |
| 3278.1263304 | 6 |
| 3278.1263462 | 8 |
| 3278.1264089 | 5 |
| 3278.1265294 | 2 |
| 3278.1265353 | 3 |
| 3278.1266352 | 6 |
| 3278.1266855 | 7 |
| 3278.1268258 | 18 |
| 3278.1270243 | 1 |
| 3278.12717 | 4 |
| 3278.1272276 | 2 |
| 3278.1272287 | 5 |
| 3278.1274482 | 2 |
| 3278.1278778 | 1 |
| 3278.1278967 | 1 |
| 3278.127977 | 0 |
| 3278.1279981 | 3 |
| 3278.1280293 | 4 |
| 3278.1280311 | 5 |
| 3278.1280355 | 2 |
| 3278.1282848 | 1 |
| 3278.1284538 | 1 |
| 3278.1284618 | 3 |
| 3278.1285118 | 1 |
| 3278.128692 | 3 |
| 3278.1290082 | 1 |
| 3278.1290251 | 5 |
| 3278.1291416 | 1 |
| 3278.1292085 | 2 |
| 3278.1292806 | 1 |
| 3278.1293399 | 5 |
| 3278.1294463 | 3 |
| 3278.1295427 | 1 |
| 3278.1296652 | 1 |
| 3278.129885 | 1 |
| 3278.1300705 | 2 |
| 3278.1302485 | 3 |
| 3278.1303707 | 1 |
| 3278.1304123 | 1 |
| 3278.1304857 | 8 |
| 3278.1305007 | 1 |
| 3278.1305714 | 3 |
| 3278.1307406 | 2 |
| 3278.1308867 | 0 |
| 3278.1309608 | 2 |
| 3278.131014 | 1 |
| 3278.1310173 | 3 |
| 3278.1310431 | 3 |
| 3278.1314079 | 1 |
| 3278.1314129 | 2 |
| 3278.131434 | 1 |
| 3278.1315496 | 5 |
| 3278.1316384 | 2 |
| 3278.1316757 | 5 |
| 3278.1316951 | 2 |
| 3278.1318588 | 3 |
| 3278.1322712 | 2 |
| 3278.1322959 | 3 |
| 3278.132299 | 4 |
| 3278.132408 | 3 |
| 3278.1324866 | 0 |
| 3278.1325476 | 3 |
| 3278.1326604 | 2 |
| 3278.1327136 | 2 |
| 3278.1328541 | 0 |
| 3278.1328967 | 2 |
| 3278.133024 | 2 |
| 3278.1331633 | 3 |
| 3278.1333492 | 3 |
| 3278.1334069 | 2 |
| 3278.1335574 | 4 |
| 3278.1339234 | 3 |
| 3278.1339701 | 0 |
| 3278.1342736 | 2 |
| 3278.1343854 | 1 |
| 3278.1344045 | 2 |
| 3278.1345354 | 0 |
| 3278.1345973 | 2 |
| 3278.1347606 | 2 |
| 3278.1347852 | 2 |
| 3278.1348434 | 3 |
| 3278.1350905 | 3 |
| 3278.1353168 | 0 |
| 3278.1354127 | 1 |
| 3278.1354694 | 1 |
| 3278.1355568 | 3 |
| 3278.1355925 | 2 |
| 3278.1356274 | 4 |
| 3278.1356463 | 4 |
| 3278.1359441 | 3 |
| 3278.1361184 | 2 |
| 3278.1361201 | 3 |
| 3278.1361277 | 1 |
| 3278.1362279 | 4 |
| 3278.1363508 | 4 |
| 3278.1365147 | 2 |
| 3278.1366048 | 0 |
| 3278.1369012 | 1 |
| 3278.1369829 | 0 |
| 3278.1370235 | 1 |
| 3278.1375132 | 5 |
| 3278.1375146 | 1 |
| 3278.1375446 | 1 |
| 3278.1375503 | 1 |
| 3278.1376386 | 2 |
| 3278.1378001 | 1 |
| 3278.1380166 | 2 |
| 3278.1380478 | 4 |
| 3278.1382043 | 2 |
| 3278.1382886 | 4 |
| 3278.1384214 | 1 |
| 3278.1384305 | 3 |
| 3278.1384537 | 8 |
| 3278.1386191 | 4 |
| 3278.1386238 | 0 |
| 3278.1387687 | 5 |
| 3278.1389468 | 3 |
| 3278.1389723 | 5 |
| 3278.1390943 | 2 |
| 3278.1393443 | 4 |
| 3278.1394926 | 0 |
| 3278.1395766 | 1 |
| 3278.1398941 | 7 |
| 3278.1398985 | 1 |
| 3278.1399788 | 0 |
| 3278.1401483 | 3 |
| 3278.1401996 | 1 |
| 3278.1402583 | 0 |
| 3278.1402756 | 2 |
| 3278.1404505 | 2 |
| 3278.1406888 | 2 |
| 3278.1407345 | 3 |
| 3278.1408205 | 1 |
| 3278.1412032 | 2 |
| 3278.1412532 | 1 |
| 3278.1413162 | 0 |
| 3278.1413805 | 1 |
| 3278.1416225 | 2 |
| 3278.1416907 | 3 |
| 3278.1418424 | 4 |
| 3278.1418838 | 0 |
| 3278.1420164 | 3 |
| 3278.1421767 | 1 |
| 3278.1422538 | 1 |
| 3278.142336 | 4 |
| 3278.1423632 | 2 |
| 3278.1428735 | 1 |
| 3278.1429965 | 2 |
| 3278.1430166 | 2 |
| 3278.1430288 | 1 |
| 3278.1433013 | 0 |
| 3278.1434653 | 2 |
| 3278.1434819 | 1 |
| 3278.1435324 | 0 |
| 3278.1436861 | 4 |
| 3278.1439397 | 1 |
| 3278.144372 | 2 |
| 3278.1444377 | 1 |
| 3278.1444482 | 1 |
| 3278.1447285 | 0 |
| 3278.1449187 | 1 |
| 3278.1449235 | 0 |
| 3278.144992 | 3 |
| 3278.1450182 | 1 |
| 3278.1452392 | 3 |
| 3278.1452448 | 0 |
| 3278.1452685 | 0 |
| 3278.1453864 | 2 |
| 3278.1459156 | 2 |
| 3278.1460354 | 1 |
| 3278.146097 | 2 |
| 3278.1461886 | 5 |
| 3278.1461919 | 1 |
| 3278.1462565 | 0 |
| 3278.1463463 | 4 |
| 3278.146543 | 1 |
| 3278.1466281 | 1 |
| 3278.14668 | 1 |
| 3278.1469261 | 1 |
| 3278.1470565 | 0 |
| 3278.147303 | 1 |
| 3278.1477041 | 1 |
| 3278.1478882 | 0 |
| 3278.1479199 | 0 |
| 3278.1480222 | 4 |
| 3278.148026 | 2 |
| 3278.1482924 | 1 |
| 3278.1484186 | 1 |
| 3278.1484405 | 3 |
| 3278.1485336 | 0 |
| 3278.1487942 | 0 |
| 3278.1488781 | 1 |
| 3278.1490333 | 2 |
| 3278.1493347 | 1 |
| 3278.1493976 | 4 |
| 3278.1494398 | 1 |
| 3278.1497437 | 1 |
| 3278.1500388 | 3 |
| 3278.1500502 | 2 |
| 3278.150078 | 2 |
| 3278.1501981 | 2 |
| 3278.150212 | 2 |
| 3278.1505217 | 3 |
| 3278.1505311 | 1 |
| 3278.1507596 | 0 |
| 3278.1508033 | 0 |
| 3278.1508905 | 1 |
| 3278.1508946 | 0 |
| 3278.1510765 | 0 |
| 3278.1513307 | 1 |
| 3278.1514021 | 3 |
| 3278.1515992 | 2 |
| 3278.1517213 | 2 |
| 3278.1518611 | 1 |
| 3278.1520201 | 2 |
| 3278.1521875 | 3 |
| 3278.1522885 | 1 |
| 3278.1523512 | 2 |
| 3278.1526412 | 1 |
| 3278.152982 | 3 |
| 3278.1531188 | 1 |
| 3278.1533041 | 6 |
| 3278.1533286 | 4 |
| 3278.1534784 | 3 |
| 3278.1535143 | 2 |
| 3278.1535611 | 1 |

| | |
|---|---|
| 3278.1536138 | 1 |
| 3278.1536146 | 0 |
| 3278.1537124 | 1 |
| 3278.1540518 | 2 |
| 3278.1541186 | 0 |
| 3278.1541801 | 4 |
| 3278.1543885 | 1 |
| 3278.1547809 | 1 |
| 3278.1548447 | 2 |
| 3278.1551952 | 1 |
| 3278.1552134 | 3 |
| 3278.1552265 | 1 |
| 3278.1554965 | 1 |
| 3278.1555865 | 5 |
| 3278.1557836 | 1 |
| 3278.1558339 | 0 |
| 3278.1558396 | 2 |
| 3278.1561429 | 1 |
| 3278.1563526 | 1 |
| 3278.1564136 | 2 |
| 3278.156441 | 3 |
| 3278.1566703 | 0 |
| 3278.1568144 | 4 |
| 3278.1568168 | 1 |
| 3278.1568588 | 1 |
| 3278.1570443 | 2 |
| 3278.1573431 | 1 |
| 3278.1575489 | 0 |
| 3278.157563 | 1 |
| 3278.1576503 | 1 |
| 3278.1579472 | 1 |
| 3278.1579787 | 1 |
| 3278.158136 | 2 |
| 3278.1583201 | 2 |
| 3278.1583982 | 1 |
| 3278.1584039 | 1 |
| 3278.1587249 | 1 |
| 3278.158767 | 0 |
| 3278.1589951 | 1 |
| 3278.159131 | 4 |
| 3278.1592637 | 1 |
| 3278.1592998 | 4 |
| 3278.1593213 | 1 |
| 3278.1600213 | 0 |
| 3278.1600586 | 1 |
| 3278.160123 | 2 |
| 3278.1601594 | 1 |
| 3278.1602387 | 0 |
| 3278.160298 | 4 |
| 3278.1606279 | 2 |
| 3278.1606322 | 0 |
| 3278.1608619 | 1 |
| 3278.1609541 | 1 |
| 3278.1610026 | 3 |
| 3278.1610894 | 4 |
| 3278.1611236 | 1 |
| 3278.1614477 | 2 |
| 3278.1615667 | 1 |
| 3278.1616508 | 4 |
| 3278.1619312 | 3 |
| 3278.1619856 | 3 |
| 3278.16199 | 2 |
| 3278.1621032 | 1 |
| 3278.1621352 | 3 |
| 3278.1621534 | 0 |
| 3278.1623498 | 1 |
| 3278.1623622 | 0 |
| 3278.1624382 | 5 |
| 3278.1624872 | 4 |
| 3278.1624951 | 2 |
| 3278.1626241 | 0 |
| 3278.1626367 | 0 |
| 3278.1626521 | 5 |
| 3278.1627934 | 1 |
| 3278.1628113 | 2 |
| 3278.1628856 | 2 |
| 3278.1629456 | 3 |
| 3278.1630796 | 0 |
| 3278.1630799 | 2 |
| 3278.1631401 | 2 |
| 3278.1631901 | 2 |
| 3278.1632485 | 0 |
| 3278.1632968 | 0 |
| 3278.1633618 | 3 |
| 3278.1633845 | 0 |
| 3278.1635555 | 2 |
| 3278.1636998 | 1 |
| 3278.1637142 | 3 |
| 3278.16374 | 3 |
| 3278.1637444 | 1 |
| 3278.1638231 | 4 |
| 3278.1640098 | 0 |
| 3278.1640423 | 0 |
| 3278.164212 | 5 |
| 3278.1642545 | 2 |
| 3278.1643316 | 3 |
| 3278.1643663 | 1 |
| 3278.1644605 | 1 |
| 3278.1645688 | 1 |
| 3278.1645869 | 2 |
| 3278.1645943 | 2 |
| 3278.1646613 | 5 |
| 3278.1647086 | 1 |
| 3278.1647502 | 3 |
| 3278.164841 | 4 |
| 3278.1650195 | 2 |
| 3278.1650644 | 1 |
| 3278.1650681 | 6 |
| 3278.1652011 | 4 |
| 3278.1652044 | 5 |
| 3278.1652501 | 2 |
| 3278.165255 | 5 |
| 3278.1653357 | 1 |
| 3278.165397 | 1 |
| 3278.1655908 | 1 |
| 3278.1656714 | 0 |
| 3278.165709 | 1 |
| 3278.1657311 | 1 |
| 3278.1659816 | 0 |
| 3278.1660639 | 1 |
| 3278.1661491 | 2 |
| 3278.1661631 | 4 |
| 3278.1661964 | 1 |
| 3278.166319 | 0 |
| 3278.1663393 | 1 |
| 3278.1663951 | 0 |
| 3278.166426 | 3 |
| 3278.166521 | 2 |
| 3278.1667035 | 6 |
| 3278.1667831 | 0 |
| 3278.1668159 | 0 |
| 3278.1668206 | 0 |
| 3278.1668918 | 0 |
| 3278.1670393 | 0 |
| 3278.1671314 | 1 |
| 3278.1671933 | 1 |
| 3278.1672785 | 4 |
| 3278.1672998 | 0 |
| 3278.1673535 | 0 |
| 3278.1673806 | 2 |
| 3278.167572 | 5 |
| 3278.1676616 | 1 |
| 3278.1676718 | 4 |
| 3278.1677165 | 1 |
| 3278.1677306 | 1 |
| 3278.1677544 | 3 |
| 3278.1678301 | 3 |
| 3278.167864 | 2 |
| 3278.1680107 | 1 |
| 3278.1680118 | 2 |
| 3278.1680272 | 2 |
| 3278.1682279 | 1 |
| 3278.1682996 | 1 |
| 3278.1685755 | 1 |
| 3278.1686174 | 2 |
| 3278.1687224 | 1 |
| 3278.1687283 | 4 |
| 3278.168787 | 1 |
| 3278.1688637 | 3 |
| 3278.1688948 | 0 |
| 3278.1691054 | 1 |
| 3278.1692412 | 2 |
| 3278.1693918 | 2 |
| 3278.1694391 | 6 |
| 3278.1694492 | 4 |
| 3278.1694696 | 1 |
| 3278.1695225 | 3 |
| 3278.1695314 | 2 |
| 3278.1695717 | 5 |
| 3278.1696108 | 0 |
| 3278.1696513 | 2 |
| 3278.1699148 | 2 |
| 3278.170049 | 1 |
| 3278.170106 | 2 |
| 3278.1701771 | 0 |
| 3278.1702275 | 0 |
| 3278.170287 | 2 |
| 3278.1704822 | 7 |
| 3278.1705207 | 1 |
| 3278.1705469 | 2 |
| 3278.1706111 | 3 |
| 3278.1706275 | 4 |
| 3278.1706778 | 4 |
| 3278.170772 | 0 |
| 3278.1709205 | 1 |
| 3278.1710241 | 3 |
| 3278.1710973 | 3 |
| 3278.1711934 | 3 |
| 3278.1712473 | 3 |
| 3278.1712907 | 1 |
| 3278.1713511 | 2 |
| 3278.1713813 | 2 |
| 3278.1713843 | 1 |
| 3278.1714422 | 2 |
| 3278.1715289 | 2 |
| 3278.171629 | 1 |
| 3278.1716416 | 3 |
| 3278.1717889 | 1 |
| 3278.1718556 | 1 |
| 3278.1719932 | 3 |
| 3278.1721452 | 1 |
| 3278.1722495 | 3 |
| 3278.1722525 | 2 |
| 3278.1722915 | 1 |
| 3278.1723308 | 2 |
| 3278.1724333 | 1 |
| 3278.172479 | 1 |
| 3278.1726159 | 4 |
| 3278.1726386 | 1 |
| 3278.1726851 | 2 |
| 3278.1727621 | 0 |
| 3278.1729706 | 3 |
| 3278.17312 | 2 |
| 3278.1731789 | 1 |
| 3278.1732825 | 2 |
| 3278.173312 | 1 |
| 3278.1733405 | 0 |
| 3278.1734247 | 1 |
| 3278.1734963 | 0 |
| 3278.1735621 | 3 |
| 3278.1735848 | 2 |
| 3278.1736473 | 2 |
| 3278.173716 | 1 |
| 3278.1738109 | 2 |
| 3278.1738381 | 0 |
| 3278.1739504 | 0 |
| 3278.1740647 | 1 |
| 3278.1742538 | 2 |
| 3278.1742761 | 1 |
| 3278.1743335 | 3 |
| 3278.1743412 | 1 |
| 3278.1744221 | 2 |
| 3278.1744737 | 1 |
| 3278.1746359 | 5 |
| 3278.1746539 | 2 |
| 3278.1747045 | 3 |
| 3278.1748364 | 2 |
| 3278.1748476 | 2 |
| 3278.1749127 | 5 |
| 3278.1749682 | 7 |
| 3278.1750743 | 1 |
| 3278.1751496 | 0 |
| 3278.1752902 | 1 |
| 3278.1753314 | 5 |
| 3278.175427 | 1 |
| 3278.1754656 | 3 |
| 3278.1754933 | 0 |
| 3278.175568 | 1 |
| 3278.1755882 | 1 |
| 3278.1757533 | 0 |
| 3278.1758508 | 3 |
| 3278.1759589 | 3 |
| 3278.1760211 | 3 |
| 3278.1760297 | 1 |
| 3278.1762089 | 1 |
| 3278.1762094 | 3 |
| 3278.1763879 | 4 |
| 3278.1764392 | 1 |
| 3278.1764588 | 3 |
| 3278.1765414 | 1 |
| 3278.1765661 | 1 |
| 3278.1766143 | 2 |
| 3278.1766166 | 4 |
| 3278.1766174 | 2 |
| 3278.1766506 | 1 |
| 3278.1766889 | 5 |
| 3278.1767989 | 2 |
| 3278.1770212 | 2 |
| 3278.1770486 | 0 |
| 3278.1770733 | 2 |
| 3278.1772547 | 1 |
| 3278.1773392 | 3 |
| 3278.177406 | 1 |
| 3278.1775221 | 4 |
| 3278.1777331 | 1 |
| 3278.1777741 | 2 |
| 3278.1777789 | 1 |
| 3278.177785 | 0 |
| 3278.1778184 | 4 |
| 3278.1778504 | 4 |
| 3278.1778645 | 2 |
| 3278.1778682 | 1 |
| 3278.1778799 | 1 |
| 3278.1779163 | 1 |
| 3278.1779369 | 0 |
| 3278.1782163 | 1 |
| 3278.1782288 | 1 |
| 3278.1783937 | 2 |
| 3278.17841 | 1 |
| 3278.1784418 | 4 |
| 3278.1785222 | 1 |
| 3278.1785438 | 1 |
| 3278.1785871 | 0 |
| 3278.1786318 | 3 |
| 3278.1787696 | 3 |
| 3278.1787987 | 0 |
| 3278.1788442 | 4 |
| 3278.1789999 | 3 |
| 3278.1790036 | 2 |
| 3278.1790314 | 2 |
| 3278.1790502 | 4 |
| 3278.1790893 | 2 |
| 3278.179154 | 1 |
| 3278.1791621 | 2 |
| 3278.1791874 | 1 |
| 3278.1791936 | 4 |
| 3278.1792642 | 2 |
| 3278.1793794 | 0 |
| 3278.1793944 | 2 |
| 3278.1794318 | 2 |
| 3278.1794641 | 5 |
| 3278.179498 | 4 |
| 3278.1796095 | 7 |
| 3278.1796288 | 1 |
| 3278.1796776 | 0 |
| 3278.1798171 | 4 |
| 3278.1798893 | 2 |
| 3278.1799329 | 0 |
| 3278.1799467 | 2 |
| 3278.1799475 | 0 |
| 3278.1799513 | 1 |
| 3278.179995 | 0 |
| 3278.1800493 | 4 |
| 3278.1800495 | 1 |
| 3278.1800902 | 3 |
| 3278.1801165 | 1 |
| 3278.1802082 | 3 |
| 3278.1802098 | 2 |
| 3278.1803105 | 1 |
| 3278.1803534 | 1 |
| 3278.1803625 | 3 |
| 3278.1804549 | 4 |
| 3278.1805018 | 0 |
| 3278.1805503 | 3 |
| 3278.1807048 | 2 |
| 3278.1808616 | 1 |
| 3278.1809017 | 1 |
| 3278.1809077 | 0 |
| 3278.1809482 | 3 |
| 3278.1809596 | 1 |
| 3278.1810635 | 1 |
| 3278.1810724 | 2 |
| 3278.1810821 | 1 |
| 3278.1810896 | 2 |
| 3278.1811501 | 5 |
| 3278.1811538 | 0 |
| 3278.1811827 | 3 |
| 3278.1812526 | 2 |
| 3278.1812837 | 1 |
| 3278.1813233 | 2 |
| 3278.1815127 | 2 |
| 3278.1815278 | 2 |
| 3278.1815459 | 3 |
| 3278.1815645 | 5 |
| 3278.1816029 | 2 |
| 3278.1816622 | 2 |
| 3278.1816969 | 5 |
| 3278.1817402 | 2 |
| 3278.1818012 | 3 |
| 3278.1818585 | 3 |
| 3278.181918 | 2 |
| 3278.1819643 | 2 |
| 3278.1820797 | 6 |

| | |
|---|---|
| 3278.1821631 | 6 |
| 3278.1822269 | 2 |
| 3278.1822486 | 4 |
| 3278.1822929 | 1 |
| 3278.1823763 | 2 |
| 3278.1824359 | 4 |
| 3278.1824519 | 0 |
| 3278.1825047 | 3 |
| 3278.182513 | 5 |
| 3278.182548 | 1 |
| 3278.1825702 | 0 |
| 3278.1826668 | 3 |
| 3278.1828056 | 2 |
| 3278.1828848 | 1 |
| 3278.182894 | 0 |
| 3278.1829368 | 3 |
| 3278.1829475 | 1 |
| 3278.1829651 | 2 |
| 3278.1829788 | 1 |
| 3278.1829913 | 4 |
| 3278.1830401 | 3 |
| 3278.1830552 | 2 |
| 3278.1830955 | 2 |
| 3278.1831167 | 1 |
| 3278.1833028 | 3 |
| 3278.1834663 | 0 |
| 3278.1834738 | 2 |
| 3278.1835308 | 1 |
| 3278.1835554 | 2 |
| 3278.1836065 | 3 |
| 3278.1836238 | 2 |
| 3278.1836303 | 3 |
| 3278.1836431 | 1 |
| 3278.1837046 | 2 |
| 3278.1837274 | 0 |
| 3278.1838068 | 1 |
| 3278.183831 | 3 |
| 3278.1838364 | 2 |
| 3278.1838413 | 1 |
| 3278.1838761 | 4 |
| 3278.1839584 | 3 |
| 3278.1839626 | 1 |
| 3278.1839943 | 2 |
| 3278.1840458 | 3 |
| 3278.1840824 | 4 |
| 3278.1842497 | 2 |
| 3278.1843096 | 2 |
| 3278.1843991 | 1 |
| 3278.1844002 | 1 |
| 3278.1844599 | 2 |
| 3278.1845069 | 2 |
| 3278.184558 | 3 |
| 3278.1845832 | 3 |
| 3278.1846113 | 3 |
| 3278.1846156 | 0 |
| 3278.1847151 | 3 |
| 3278.1848113 | 4 |
| 3278.1848191 | 6 |
| 3278.1848209 | 0 |
| 3278.1848304 | 2 |
| 3278.1849446 | 3 |
| 3278.1850533 | 1 |
| 3278.1850687 | 1 |
| 3278.1852119 | 4 |
| 3278.1852871 | 0 |
| 3278.1852873 | 1 |
| 3278.1853114 | 5 |
| 3278.1853305 | 4 |
| 3278.1853863 | 1 |
| 3278.1854786 | 0 |
| 3278.1856567 | 3 |
| 3278.1856636 | 4 |
| 3278.1857465 | 0 |
| 3278.1857559 | 2 |
| 3278.1857977 | 2 |
| 3278.1858319 | 1 |
| 3278.1859081 | 3 |
| 3278.1859752 | 1 |
| 3278.1859825 | 3 |
| 3278.1860583 | 6 |
| 3278.1860841 | 1 |
| 3278.1862583 | 1 |
| 3278.1863171 | 5 |
| 3278.1863364 | 1 |
| 3278.1863496 | 4 |
| 3278.18653 | 0 |
| 3278.1865627 | 1 |
| 3278.1865864 | 1 |
| 3278.1865898 | 0 |
| 3278.1865939 | 4 |
| 3278.1866674 | 1 |
| 3278.1866823 | 3 |
| 3278.1867047 | 1 |
| 3278.1867558 | 1 |
| 3278.1867872 | 2 |
| 3278.186847 | 5 |
| 3278.1868859 | 1 |
| 3278.1870479 | 1 |
| 3278.1870767 | 2 |
| 3278.1871028 | 4 |
| 3278.1871102 | 2 |
| 3278.1871246 | 2 |
| 3278.1871653 | 2 |
| 3278.1871855 | 3 |
| 3278.1872526 | 1 |
| 3278.1872883 | 0 |
| 3278.187352 | 4 |
| 3278.1874658 | 2 |
| 3278.1875008 | 0 |
| 3278.1875848 | 1 |
| 3278.1876302 | 1 |
| 3278.1876598 | 2 |
| 3278.1877181 | 3 |
| 3278.1878224 | 0 |
| 3278.1878254 | 4 |
| 3278.188005 | 1 |
| 3278.1880101 | 3 |
| 3278.1880132 | 2 |
| 3278.1880252 | 1 |
| 3278.1880874 | 0 |
| 3278.1881674 | 3 |
| 3278.1881783 | 2 |
| 3278.1881918 | 1 |
| 3278.1882347 | 2 |
| 3278.1883853 | 2 |
| 3278.1885049 | 3 |
| 3278.1885123 | 0 |
| 3278.1886479 | 2 |
| 3278.1886721 | 2 |
| 3278.1886932 | 1 |
| 3278.1887105 | 0 |
| 3278.1887787 | 1 |
| 3278.1888068 | 2 |
| 3278.1888129 | 2 |
| 3278.1888222 | 2 |
| 3278.1888421 | 2 |
| 3278.1888789 | 3 |
| 3278.1889169 | 2 |
| 3278.1891257 | 2 |
| 3278.1891574 | 3 |
| 3278.1892883 | 0 |
| 3278.1893185 | 1 |
| 3278.1893468 | 2 |
| 3278.1893549 | 1 |
| 3278.1893892 | 3 |
| 3278.1894397 | 2 |
| 3278.1894814 | 1 |
| 3278.1894908 | 0 |
| 3278.1894912 | 0 |
| 3278.1895044 | 4 |
| 3278.189545 | 4 |
| 3278.189586 | 0 |
| 3278.1895904 | 5 |
| 3278.1896303 | 3 |
| 3278.1896412 | 4 |
| 3278.1896831 | 2 |
| 3278.1896875 | 4 |
| 3278.1897417 | 2 |
| 3278.1897841 | 2 |
| 3278.1898499 | 1 |
| 3278.1900856 | 3 |
| 3278.1901585 | 3 |
| 3278.1902101 | 2 |
| 3278.1902295 | 4 |
| 3278.190265 | 3 |
| 3278.1902925 | 0 |
| 3278.1903596 | 2 |
| 3278.1904516 | 2 |
| 3278.1905015 | 1 |
| 3278.190588 | 2 |
| 3278.1906666 | 1 |
| 3278.1906827 | 5 |
| 3278.1907138 | 3 |
| 3278.1907436 | 1 |
| 3278.1907542 | 4 |
| 3278.1908426 | 1 |
| 3278.1909248 | 0 |
| 3278.1911596 | 2 |
| 3278.1912017 | 0 |
| 3278.1912078 | 1 |
| 3278.191238 | 1 |
| 3278.1913085 | 0 |
| 3278.1914084 | 3 |
| 3278.1914416 | 2 |
| 3278.1914677 | 3 |
| 3278.1914871 | 3 |
| 3278.1915033 | 3 |
| 3278.1915081 | 1 |
| 3278.1915788 | 0 |
| 3278.1916067 | 4 |
| 3278.1916608 | 2 |
| 3278.1916748 | 2 |
| 3278.1916797 | 1 |
| 3278.1916965 | 5 |
| 3278.1917723 | 2 |
| 3278.1919189 | 2 |
| 3278.191982 | 3 |
| 3278.1920816 | 4 |
| 3278.1920859 | 1 |
| 3278.1920902 | 4 |
| 3278.1921811 | 1 |
| 3278.1922197 | 1 |
| 3278.1922208 | 2 |
| 3278.19224 | 1 |
| 3278.1922469 | 0 |
| 3278.1922512 | 3 |
| 3278.1922745 | 1 |
| 3278.1923923 | 6 |
| 3278.1924212 | 0 |
| 3278.1924556 | 2 |
| 3278.1925205 | 1 |
| 3278.1925609 | 2 |
| 3278.1926376 | 4 |
| 3278.1927208 | 4 |
| 3278.192766 | 0 |
| 3278.1929048 | 1 |
| 3278.1929232 | 2 |
| 3278.1929965 | 2 |
| 3278.1931238 | 4 |
| 3278.1931339 | 1 |
| 3278.1931551 | 3 |
| 3278.1932669 | 2 |
| 3278.193295 | 2 |
| 3278.1933216 | 2 |
| 3278.1933524 | 1 |
| 3278.1933534 | 0 |
| 3278.1933727 | 3 |
| 3278.1933754 | 2 |
| 3278.1933957 | 2 |
| 3278.193439 | 4 |
| 3278.1934656 | 1 |
| 3278.1936628 | 3 |
| 3278.1937003 | 2 |
| 3278.193843 | 3 |
| 3278.1938842 | 1 |
| 3278.1939206 | 3 |
| 3278.1939333 | 4 |
| 3278.1939793 | 0 |
| 3278.1939861 | 6 |
| 3278.1940245 | 1 |
| 3278.1940262 | 2 |
| 3278.1941217 | 1 |
| 3278.1941233 | 2 |
| 3278.1941607 | 1 |
| 3278.1942154 | 2 |
| 3278.1942438 | 1 |
| 3278.1942493 | 2 |
| 3278.194315 | 3 |
| 3278.1943726 | 1 |
| 3278.1944328 | 1 |
| 3278.1945527 | 1 |
| 3278.194561 | 6 |
| 3278.19467 | 3 |
| 3278.1947448 | 3 |
| 3278.1947918 | 1 |
| 3278.1948229 | 2 |
| 3278.1948353 | 3 |
| 3278.1948839 | 1 |
| 3278.1950489 | 2 |
| 3278.1951316 | 2 |
| 3278.1951697 | 3 |
| 3278.1952164 | 3 |
| 3278.1952414 | 2 |
| 3278.1953018 | 2 |
| 3278.1953717 | 1 |
| 3278.1953948 | 1 |
| 3278.1954274 | 3 |
| 3278.1954467 | 0 |
| 3278.1954649 | 2 |
| 3278.1955286 | 2 |
| 3278.1955976 | 2 |
| 3278.195666 | 4 |
| 3278.1956996 | 4 |
| 3278.1959091 | 1 |
| 3278.1959369 | 2 |
| 3278.1959832 | 1 |
| 3278.1960514 | 0 |
| 3278.1961028 | 3 |
| 3278.1961132 | 0 |
| 3278.1961625 | 4 |
| 3278.1962332 | 4 |
| 3278.1962376 | 0 |
| 3278.1962404 | 1 |
| 3278.1962736 | 2 |
| 3278.19628 | 3 |
| 3278.1963269 | 1 |
| 3278.196352 | 0 |
| 3278.1963853 | 0 |
| 3278.1965348 | 3 |
| 3278.196545 | 0 |
| 3278.1965519 | 5 |
| 3278.1965757 | 2 |
| 3278.1966055 | 1 |
| 3278.1966676 | 0 |
| 3278.1967478 | 2 |
| 3278.1967603 | 2 |
| 3278.196795 | 2 |
| 3278.1968178 | 5 |
| 3278.1968615 | 2 |
| 3278.1969552 | 4 |
| 3278.1969906 | 2 |
| 3278.197007 | 2 |
| 3278.1970791 | 1 |
| 3278.1971821 | 1 |
| 3278.1972587 | 0 |
| 3278.1974117 | 4 |
| 3278.1974731 | 3 |
| 3278.197475 | 2 |
| 3278.1974834 | 2 |
| 3278.1974902 | 0 |
| 3278.1975363 | 4 |
| 3278.1975484 | 0 |
| 3278.1975912 | 3 |
| 3278.197636 | 5 |
| 3278.1977269 | 2 |
| 3278.1977335 | 4 |
| 3278.1977739 | 1 |
| 3278.1979744 | 1 |
| 3278.1979746 | 2 |
| 3278.1979764 | 1 |
| 3278.1979912 | 3 |
| 3278.1980517 | 4 |
| 3278.1980666 | 3 |
| 3278.1980783 | 1 |
| 3278.1981813 | 2 |
| 3278.198184 | 0 |
| 3278.198254 | 1 |
| 3278.1982865 | 7 |
| 3278.1983305 | 1 |
| 3278.1983769 | 1 |
| 3278.1984194 | 3 |
| 3278.1984948 | 5 |
| 3278.1985732 | 2 |
| 3278.1986703 | 0 |
| 3278.1986955 | 1 |
| 3278.1987414 | 2 |
| 3278.1987545 | 0 |
| 3278.1988028 | 3 |
| 3278.198854 | 4 |
| 3278.1989454 | 1 |
| 3278.1990032 | 3 |
| 3278.1990051 | 1 |
| 3278.19904 | 5 |
| 3278.1990561 | 4 |
| 3278.1990713 | 1 |
| 3278.1991066 | 3 |
| 3278.1991535 | 4 |
| 3278.1993124 | 2 |
| 3278.1993701 | 3 |
| 3278.1994881 | 3 |
| 3278.1995293 | 0 |
| 3278.1995457 | 2 |
| 3278.1995703 | 0 |
| 3278.1995841 | 5 |
| 3278.1996433 | 0 |
| 3278.1996575 | 2 |
| 3278.1997061 | 2 |
| 3278.1997075 | 1 |
| 3278.1997279 | 0 |
| 3278.1998271 | 3 |
| 3278.1998394 | 2 |
| 3278.1999068 | 2 |
| 3278.19994 | 1 |

| | |
|---|---|
| 3278.1999974 | 3 |
| 3278.2000491 | 2 |
| 3278.2001093 | 1 |
| 3278.200162 | 3 |
| 3278.2001645 | 3 |
| 3278.2003065 | 1 |
| 3278.2003269 | 3 |
| 3278.2003351 | 3 |
| 3278.2004364 | 3 |
| 3278.2004679 | 2 |
| 3278.2006064 | 1 |
| 3278.2006695 | 1 |
| 3278.200674 | 4 |
| 3278.2006882 | 3 |
| 3278.2007138 | 2 |
| 3278.2008607 | 3 |
| 3278.2009188 | 1 |
| 3278.200993 | 3 |
| 3278.2010092 | 1 |
| 3278.2011697 | 3 |
| 3278.2011871 | 0 |
| 3278.2012239 | 4 |
| 3278.2012772 | 5 |
| 3278.2013095 | 0 |
| 3278.2013124 | 1 |
| 3278.2013477 | 1 |
| 3278.2013697 | 3 |
| 3278.2013856 | 1 |
| 3278.2015035 | 4 |
| 3278.2015291 | 1 |
| 3278.2016844 | 1 |
| 3278.2016947 | 3 |
| 3278.2017392 | 1 |
| 3278.201832 | 6 |
| 3278.2019097 | 2 |
| 3278.2019157 | 3 |
| 3278.2019209 | 1 |
| 3278.2019907 | 2 |
| 3278.2020582 | 2 |
| 3278.2020977 | 2 |
| 3278.2021168 | 2 |
| 3278.2021344 | 2 |
| 3278.2021398 | 2 |
| 3278.2021693 | 2 |
| 3278.2022702 | 0 |
| 3278.2022802 | 3 |
| 3278.2023144 | 6 |
| 3278.2023393 | 1 |
| 3278.2023436 | 2 |
| 3278.2023492 | 0 |
| 3278.2024173 | 1 |
| 3278.202468 | 5 |
| 3278.2025281 | 2 |
| 3278.2026255 | 1 |
| 3278.2026294 | 2 |
| 3278.2026489 | 2 |
| 3278.2026898 | 2 |
| 3278.2027185 | 1 |
| 3278.2027847 | 0 |
| 3278.2028426 | 2 |
| 3278.2028464 | 2 |
| 3278.2029663 | 5 |
| 3278.2030598 | 6 |
| 3278.2032164 | 2 |
| 3278.2033037 | 2 |
| 3278.2033062 | 0 |
| 3278.2033156 | 2 |
| 3278.2033537 | 4 |
| 3278.203408 | 4 |
| 3278.2034878 | 3 |
| 3278.2035051 | 1 |
| 3278.2035244 | 4 |
| 3278.203566 | 2 |
| 3278.203589 | 7 |
| 3278.20374 | 3 |
| 3278.2037694 | 2 |
| 3278.2037738 | 1 |
| 3278.203886 | 2 |
| 3278.2039968 | 1 |
| 3278.2040476 | 1 |
| 3278.2040691 | 0 |
| 3278.2040878 | 1 |
| 3278.2041306 | 0 |
| 3278.2041327 | 1 |
| 3278.2041675 | 3 |
| 3278.2041789 | 1 |
| 3278.2044039 | 1 |
| 3278.2044232 | 2 |
| 3278.2044326 | 3 |
| 3278.2044659 | 1 |
| 3278.2045249 | 4 |
| 3278.2045659 | 4 |
| 3278.2045783 | 5 |
| 3278.2046606 | 2 |
| 3278.2047006 | 1 |
| 3278.2047702 | 4 |
| 3278.2048085 | 2 |
| 3278.2048186 | 2 |
| 3278.204931 | 0 |
| 3278.2049346 | 0 |
| 3278.2049351 | 1 |
| 3278.204946 | 4 |
| 3278.2049668 | 0 |
| 3278.2050419 | 4 |
| 3278.2050773 | 2 |
| 3278.2050808 | 0 |
| 3278.205157 | 1 |
| 3278.2052403 | 1 |
| 3278.2052567 | 2 |
| 3278.205266 | 2 |
| 3278.2052949 | 2 |
| 3278.2053355 | 2 |
| 3278.2053545 | 2 |
| 3278.2053632 | 3 |
| 3278.2055077 | 3 |
| 3278.205524 | 1 |
| 3278.2058106 | 3 |
| 3278.2058182 | 3 |
| 3278.2058224 | 2 |
| 3278.206048 | 2 |
| 3278.2061101 | 1 |
| 3278.2061148 | 2 |
| 3278.2061392 | 1 |
| 3278.2061611 | 1 |
| 3278.2062286 | 4 |
| 3278.2063011 | 1 |
| 3278.2063322 | 1 |
| 3278.2063787 | 1 |
| 3278.2063801 | 2 |
| 3278.2063893 | 3 |
| 3278.2063985 | 0 |
| 3278.2064507 | 2 |
| 3278.2064683 | 1 |
| 3278.2064711 | 1 |
| 3278.206478 | 2 |
| 3278.2064787 | 0 |
| 3278.206588 | 2 |
| 3278.2066656 | 1 |
| 3278.2067491 | 1 |
| 3278.2068705 | 1 |
| 3278.2069093 | 4 |
| 3278.2069505 | 3 |
| 3278.2069775 | 2 |
| 3278.2069893 | 2 |
| 3278.2070559 | 3 |
| 3278.2071214 | 2 |
| 3278.2073419 | 2 |
| 3278.2075309 | 4 |
| 3278.2075321 | 6 |
| 3278.2075485 | 2 |
| 3278.2076197 | 3 |
| 3278.2076635 | 1 |
| 3278.2076946 | 2 |
| 3278.207711 | 0 |
| 3278.2077538 | 1 |
| 3278.2078387 | 2 |
| 3278.2079672 | 1 |
| 3278.2080828 | 1 |
| 3278.2080883 | 4 |
| 3278.2081169 | 3 |
| 3278.2081654 | 0 |
| 3278.2082692 | 1 |
| 3278.2083007 | 5 |
| 3278.2084165 | 2 |
| 3278.2084705 | 2 |
| 3278.2084924 | 0 |
| 3278.2085077 | 2 |
| 3278.2085178 | 3 |
| 3278.2085555 | 1 |
| 3278.2085968 | 3 |
| 3278.2086247 | 1 |
| 3278.2086392 | 1 |
| 3278.2086576 | 3 |
| 3278.2086609 | 1 |
| 3278.2086803 | 2 |
| 3278.2087551 | 2 |
| 3278.2087698 | 3 |
| 3278.2088728 | 5 |
| 3278.2091556 | 1 |
| 3278.2091715 | 2 |
| 3278.209174 | 4 |
| 3278.2091858 | 3 |
| 3278.2092582 | 3 |
| 3278.2092633 | 1 |
| 3278.2092641 | 4 |
| 3278.2092954 | 1 |
| 3278.2093187 | 3 |
| 3278.2095483 | 2 |
| 3278.2095714 | 1 |
| 3278.2095727 | 3 |
| 3278.2095758 | 1 |
| 3278.2095762 | 1 |
| 3278.2095935 | 0 |
| 3278.2096369 | 2 |
| 3278.2096599 | 2 |
| 3278.2096674 | 4 |
| 3278.2097538 | 2 |
| 3278.209801 | 2 |
| 3278.2099005 | 1 |
| 3278.2099269 | 3 |
| 3278.2100902 | 1 |
| 3278.2102772 | 4 |
| 3278.2102958 | 3 |
| 3278.2103561 | 3 |
| 3278.2103832 | 1 |
| 3278.2104923 | 1 |
| 3278.2104988 | 1 |
| 3278.2105387 | 3 |
| 3278.210611 | 4 |
| 3278.2106119 | 3 |
| 3278.2106928 | 4 |
| 3278.2107217 | 0 |
| 3278.2107246 | 1 |
| 3278.2107686 | 1 |
| 3278.2108013 | 1 |
| 3278.2108185 | 0 |
| 3278.2108237 | 4 |
| 3278.2108632 | 0 |
| 3278.2110113 | 3 |
| 3278.211076 | 2 |
| 3278.2112533 | 2 |
| 3278.2112903 | 1 |
| 3278.211333 | 4 |
| 3278.2113346 | 3 |
| 3278.2113365 | 1 |
| 3278.211432 | 2 |
| 3278.2114763 | 2 |
| 3278.2114865 | 2 |
| 3278.2115137 | 2 |
| 3278.2115957 | 1 |
| 3278.2117506 | 3 |
| 3278.2117985 | 1 |
| 3278.21186 | 1 |
| 3278.2118857 | 1 |
| 3278.2119113 | 4 |
| 3278.2119165 | 4 |
| 3278.2119176 | 1 |
| 3278.2119246 | 4 |
| 3278.2119629 | 1 |
| 3278.212061 | 2 |
| 3278.2121006 | 3 |
| 3278.2121074 | 0 |
| 3278.2121736 | 2 |
| 3278.2121823 | 1 |
| 3278.2121935 | 2 |
| 3278.2122672 | 7 |
| 3278.2122869 | 3 |
| 3278.2122906 | 4 |
| 3278.2123158 | 2 |
| 3278.2123378 | 1 |
| 3278.2123452 | 1 |
| 3278.2123918 | 3 |
| 3278.2124729 | 2 |
| 3278.212483 | 1 |
| 3278.212493 | 1 |
| 3278.2125632 | 1 |
| 3278.2126053 | 3 |
| 3278.2126488 | 2 |
| 3278.2126709 | 1 |
| 3278.2126752 | 2 |
| 3278.2127007 | 2 |
| 3278.2127427 | 1 |
| 3278.2128221 | 0 |
| 3278.212823 | 4 |
| 3278.2129827 | 2 |
| 3278.2130053 | 5 |
| 3278.2130402 | 1 |
| 3278.2131676 | 3 |
| 3278.2132566 | 2 |
| 3278.2134267 | 3 |
| 3278.2134418 | 2 |
| 3278.2134465 | 0 |
| 3278.2134585 | 4 |
| 3278.2134649 | 2 |
| 3278.213479 | 1 |
| 3278.2136493 | 2 |
| 3278.2137166 | 0 |
| 3278.213731 | 7 |
| 3278.2138955 | 3 |
| 3278.2139417 | 4 |
| 3278.2139498 | 3 |
| 3278.2139789 | 1 |
| 3278.2140291 | 0 |
| 3278.2140968 | 3 |
| 3278.2141471 | 1 |
| 3278.2141974 | 3 |
| 3278.2142262 | 2 |
| 3278.2143101 | 0 |
| 3278.214361 | 2 |
| 3278.2144714 | 2 |
| 3278.2145809 | 6 |
| 3278.2145857 | 4 |
| 3278.2145991 | 4 |
| 3278.2146636 | 1 |
| 3278.2147153 | 2 |
| 3278.2147577 | 2 |
| 3278.2147678 | 1 |
| 3278.2148667 | 1 |
| 3278.2149112 | 2 |
| 3278.2150181 | 1 |
| 3278.2150943 | 0 |
| 3278.215213 | 1 |
| 3278.2152944 | 2 |
| 3278.2153099 | 1 |
| 3278.2153221 | 5 |
| 3278.2153455 | 4 |
| 3278.2155818 | 1 |
| 3278.2157219 | 1 |
| 3278.2157621 | 1 |
| 3278.2158119 | 1 |
| 3278.2159178 | 2 |
| 3278.215922 | 2 |
| 3278.2159878 | 4 |
| 3278.2160261 | 3 |
| 3278.2160664 | 1 |
| 3278.2161065 | 3 |
| 3278.2161287 | 0 |
| 3278.2161444 | 2 |
| 3278.2162489 | 2 |
| 3278.2164313 | 2 |
| 3278.2165481 | 1 |
| 3278.2165529 | 1 |
| 3278.2166873 | 2 |
| 3278.2167924 | 3 |
| 3278.2168337 | 1 |
| 3278.2171396 | 2 |
| 3278.2171599 | 1 |
| 3278.2171805 | 2 |
| 3278.2173348 | 0 |
| 3278.2173709 | 1 |
| 3278.2174393 | 2 |
| 3278.2174677 | 1 |
| 3278.2175301 | 1 |
| 3278.2175405 | 1 |
| 3278.2175798 | 1 |
| 3278.2175898 | 2 |
| 3278.2176718 | 0 |
| 3278.2177254 | 0 |
| 3278.2178484 | 4 |
| 3278.2178621 | 1 |
| 3278.2179767 | 4 |
| 3278.2179868 | 6 |
| 3278.2180471 | 1 |
| 3278.2180756 | 2 |
| 3278.2182454 | 0 |
| 3278.2184256 | 2 |
| 3278.2184733 | 0 |
| 3278.2184816 | 0 |
| 3278.2184965 | 5 |
| 3278.2185643 | 5 |
| 3278.2188502 | 2 |
| 3278.2189376 | 4 |
| 3278.2189879 | 4 |
| 3278.2190469 | 1 |
| 3278.2191026 | 4 |
| 3278.2191836 | 0 |
| 3278.2192325 | 2 |
| 3278.2192874 | 0 |
| 3278.2193344 | 1 |
| 3278.2194483 | 4 |
| 3278.219534 | 5 |
| 3278.2196826 | 3 |
| 3278.2196864 | 2 |

| | |
|---|---|
| 3278.2197501 | 0 |
| 3278.2198683 | 4 |
| 3278.2199073 | 3 |
| 3278.2199939 | 2 |
| 3278.2201743 | 2 |
| 3278.2201963 | 0 |
| 3278.2202151 | 4 |
| 3278.2205412 | 3 |
| 3278.2205828 | 2 |
| 3278.2206448 | 2 |
| 3278.2206782 | 1 |
| 3278.2207472 | 3 |
| 3278.2207548 | 1 |
| 3278.2208957 | 2 |
| 3278.2209416 | 0 |
| 3278.2209466 | 2 |
| 3278.2209543 | 5 |
| 3278.2210015 | 3 |
| 3278.2210642 | 2 |
| 3278.2211135 | 1 |
| 3278.2211192 | 1 |
| 3278.2211459 | 0 |
| 3278.2213516 | 2 |
| 3278.2214929 | 1 |
| 3278.2215418 | 1 |
| 3278.2216595 | 2 |
| 3278.2216737 | 3 |
| 3278.2218028 | 0 |
| 3278.2218047 | 1 |
| 3278.2219384 | 1 |
| 3278.22195 | 1 |
| 3278.2219723 | 2 |
| 3278.2220831 | 0 |
| 3278.2221065 | 1 |
| 3278.2221133 | 4 |
| 3278.2221272 | 0 |
| 3278.2222832 | 2 |
| 3278.2225056 | 4 |
| 3278.2225684 | 3 |
| 3278.2226937 | 2 |
| 3278.2228022 | 4 |
| 3278.2228576 | 2 |
| 3278.2228819 | 2 |
| 3278.222976 | 2 |
| 3278.2230896 | 1 |
| 3278.2232157 | 0 |
| 3278.2234082 | 4 |
| 3278.2234341 | 1 |
| 3278.2234373 | 1 |
| 3278.22346 | 2 |
| 3278.2236813 | 1 |
| 3278.223704 | 1 |
| 3278.2237293 | 5 |
| 3278.2237583 | 1 |
| 3278.2238484 | 4 |
| 3278.2239569 | 1 |
| 3278.2239838 | 1 |
| 3278.224015 | 1 |
| 3278.2240461 | 1 |
| 3278.2241081 | 1 |
| 3278.2241983 | 4 |
| 3278.2242449 | 2 |
| 3278.224327 | 0 |
| 3278.224557 | 1 |
| 3278.2246829 | 2 |
| 3278.2247657 | 3 |
| 3278.2248417 | 0 |
| 3278.2248905 | 1 |
| 3278.224973 | 0 |
| 3278.2250119 | 3 |
| 3278.2250746 | 0 |
| 3278.2250775 | 1 |
| 3278.2251546 | 2 |
| 3278.225216 | 2 |
| 3278.2252604 | 1 |
| 3278.2253691 | 0 |
| 3278.2254144 | 0 |
| 3278.2254898 | 4 |
| 3278.2254957 | 1 |
| 3278.2255698 | 2 |
| 3278.2256366 | 1 |
| 3278.2258007 | 1 |
| 3278.2258056 | 0 |
| 3278.2258643 | 0 |
| 3278.2258709 | 1 |
| 3278.2260721 | 2 |
| 3278.2261956 | 3 |
| 3278.2262644 | 0 |
| 3278.2263879 | 1 |
| 3278.2264622 | 2 |
| 3278.2265783 | 2 |
| 3278.2266233 | 2 |
| 3278.2266508 | 2 |
| 3278.2268251 | 3 |
| 3278.2269803 | 1 |
| 3278.2271483 | 2 |
| 3278.2272069 | 2 |
| 3278.2272851 | 2 |
| 3278.2272912 | 1 |
| 3278.2273541 | 0 |
| 3278.2274732 | 1 |
| 3278.2275744 | 1 |
| 3278.2275917 | 3 |
| 3278.2276134 | 1 |
| 3278.2276499 | 2 |
| 3278.2277111 | 2 |
| 3278.2277595 | 1 |
| 3278.2277648 | 3 |
| 3278.2277938 | 3 |
| 3278.2278642 | 1 |
| 3278.2279469 | 1 |
| 3278.2281481 | 1 |
| 3278.2281537 | 0 |
| 3278.2282754 | 1 |
| 3278.2282807 | 5 |
| 3278.2282822 | 1 |
| 3278.2283134 | 1 |
| 3278.2283147 | 2 |
| 3278.2283611 | 2 |
| 3278.2284834 | 2 |
| 3278.2286291 | 1 |
| 3278.2286495 | 1 |
| 3278.2286959 | 0 |
| 3278.2289354 | 3 |
| 3278.2290026 | 4 |
| 3278.2291285 | 1 |
| 3278.2291743 | 3 |
| 3278.2291841 | 0 |
| 3278.2292023 | 0 |
| 3278.2292144 | 0 |
| 3278.2293524 | 4 |
| 3278.229434 | 4 |
| 3278.2294442 | 4 |
| 3278.2294586 | 4 |
| 3278.229617 | 2 |
| 3278.2297981 | 4 |
| 3278.2298328 | 3 |
| 3278.2301861 | 1 |
| 3278.2302464 | 4 |
| 3278.2303122 | 0 |
| 3278.2303535 | 2 |
| 3278.2304029 | 1 |
| 3278.2304845 | 3 |
| 3278.230506 | 3 |
| 3278.2305918 | 3 |
| 3278.2307002 | 3 |
| 3278.2307437 | 1 |
| 3278.230858 | 3 |
| 3278.2308736 | 5 |
| 3278.2309613 | 1 |
| 3278.2310918 | 0 |
| 3278.231098 | 0 |
| 3278.2311575 | 0 |
| 3278.2313673 | 2 |
| 3278.2314894 | 1 |
| 3278.231504 | 3 |
| 3278.231542 | 2 |
| 3278.2316272 | 0 |
| 3278.2316537 | 2 |
| 3278.2317139 | 2 |
| 3278.2317173 | 1 |
| 3278.2317765 | 2 |
| 3278.2318583 | 2 |
| 3278.231989 | 4 |
| 3278.2320405 | 4 |
| 3278.232056 | 4 |
| 3278.2320794 | 0 |
| 3278.2320936 | 1 |
| 3278.2321526 | 1 |
| 3278.2321662 | 3 |
| 3278.2321849 | 1 |
| 3278.2323628 | 1 |
| 3278.2324415 | 3 |
| 3278.2326891 | 4 |
| 3278.2327273 | 0 |
| 3278.2327692 | 4 |
| 3278.2328407 | 1 |
| 3278.2329596 | 1 |
| 3278.2329938 | 2 |
| 3278.2330169 | 1 |
| 3278.233207 | 1 |
| 3278.233299 | 0 |
| 3278.2334689 | 2 |
| 3278.233543 | 3 |
| 3278.2335616 | 1 |
| 3278.2336093 | 2 |
| 3278.2336311 | 1 |
| 3278.2336567 | 4 |
| 3278.2336798 | 3 |
| 3278.2336849 | 1 |
| 3278.233731 | 1 |
| 3278.2339526 | 4 |
| 3278.2340394 | 1 |
| 3278.234148 | 2 |
| 3278.2341904 | 5 |
| 3278.2342903 | 1 |
| 3278.234385 | 4 |
| 3278.234408 | 1 |
| 3278.2344131 | 0 |
| 3278.2344655 | 4 |
| 3278.2346078 | 1 |
| 3278.2346397 | 2 |
| 3278.234662 | 4 |
| 3278.234711 | 2 |
| 3278.2348754 | 0 |
| 3278.2348793 | 0 |
| 3278.2348807 | 2 |
| 3278.2348861 | 2 |
| 3278.2348961 | 4 |
| 3278.2350768 | 2 |
| 3278.2351353 | 2 |
| 3278.2352709 | 1 |
| 3278.2353339 | 2 |
| 3278.2353646 | 3 |
| 3278.2353972 | 1 |
| 3278.2354945 | 2 |
| 3278.2355548 | 1 |
| 3278.2358083 | 0 |
| 3278.2359089 | 3 |
| 3278.2359291 | 5 |
| 3278.2360708 | 2 |
| 3278.2360743 | 1 |
| 3278.2361375 | 2 |
| 3278.2361874 | 2 |
| 3278.2362277 | 1 |
| 3278.2363332 | 0 |
| 3278.2364553 | 4 |
| 3278.2365018 | 0 |
| 3278.2365673 | 2 |
| 3278.2365702 | 2 |
| 3278.2365704 | 4 |
| 3278.2367029 | 2 |
| 3278.2367647 | 5 |
| 3278.2369452 | 1 |
| 3278.2369925 | 0 |
| 3278.2372114 | 6 |
| 3278.2373427 | 0 |
| 3278.2373995 | 2 |
| 3278.2374106 | 3 |
| 3278.2375343 | 2 |
| 3278.2375985 | 1 |
| 3278.2376818 | 0 |
| 3278.2376983 | 2 |
| 3278.2377595 | 0 |
| 3278.2377865 | 1 |
| 3278.2378015 | 5 |
| 3278.2379701 | 1 |
| 3278.2380418 | 2 |
| 3278.2380667 | 6 |
| 3278.2382016 | 2 |
| 3278.2382394 | 2 |
| 3278.2382449 | 1 |
| 3278.2384484 | 2 |
| 3278.2384769 | 3 |
| 3278.2384836 | 0 |
| 3278.2386656 | 1 |
| 3278.238679 | 2 |
| 3278.2387183 | 1 |
| 3278.2387727 | 2 |
| 3278.2387748 | 0 |
| 3278.2388152 | 2 |
| 3278.2388169 | 2 |
| 3278.2391296 | 3 |
| 3278.2392667 | 1 |
| 3278.2393094 | 1 |
| 3278.2393169 | 0 |
| 3278.2393521 | 1 |
| 3278.2394147 | 2 |
| 3278.2394745 | 0 |
| 3278.239644 | 3 |
| 3278.2396453 | 1 |
| 3278.2396739 | 0 |
| 3278.2398445 | 3 |
| 3278.2398775 | 0 |
| 3278.2399218 | 4 |
| 3278.2401168 | 3 |
| 3278.2403685 | 1 |
| 3278.2403964 | 2 |
| 3278.2404021 | 2 |
| 3278.2404417 | 2 |
| 3278.2405902 | 3 |
| 3278.240621 | 2 |
| 3278.2406685 | 1 |
| 3278.2408613 | 0 |
| 3278.2408925 | 2 |
| 3278.2410029 | 2 |
| 3278.2410078 | 0 |
| 3278.2410104 | 2 |
| 3278.2411656 | 2 |
| 3278.2412038 | 5 |
| 3278.2412531 | 2 |
| 3278.2412541 | 3 |
| 3278.2412907 | 2 |
| 3278.2413506 | 0 |
| 3278.2414204 | 0 |
| 3278.2414555 | 1 |
| 3278.241519 | 3 |
| 3278.2416082 | 2 |
| 3278.2416659 | 0 |
| 3278.241729 | 1 |
| 3278.2418917 | 2 |
| 3278.2419164 | 0 |
| 3278.2419503 | 1 |
| 3278.2419527 | 2 |
| 3278.241959 | 1 |
| 3278.2420106 | 5 |
| 3278.2421936 | 0 |
| 3278.2422057 | 0 |
| 3278.2423005 | 1 |
| 3278.242347 | 3 |
| 3278.2423824 | 4 |
| 3278.2425117 | 2 |
| 3278.2425154 | 1 |
| 3278.2426428 | 1 |
| 3278.2429002 | 4 |
| 3278.2430417 | 2 |
| 3278.2430924 | 3 |
| 3278.2431282 | 1 |
| 3278.2431872 | 1 |
| 3278.2431988 | 2 |
| 3278.24328 | 4 |
| 3278.2432856 | 1 |
| 3278.2434757 | 1 |
| 3278.2435677 | 5 |
| 3278.243646 | 1 |
| 3278.243691 | 1 |
| 3278.2437131 | 2 |
| 3278.2438098 | 0 |
| 3278.2439545 | 2 |
| 3278.2439933 | 2 |
| 3278.2440418 | 2 |
| 3278.2441209 | 2 |
| 3278.2441759 | 0 |
| 3278.2443156 | 2 |
| 3278.2443403 | 0 |
| 3278.2443922 | 2 |
| 3278.244418 | 1 |
| 3278.2445595 | 2 |
| 3278.2447165 | 1 |
| 3278.2448202 | 3 |
| 3278.2448333 | 1 |
| 3278.2448387 | 3 |
| 3278.2448745 | 3 |
| 3278.244904 | 1 |
| 3278.2450542 | 5 |
| 3278.2450817 | 0 |
| 3278.2451199 | 2 |
| 3278.2453602 | 3 |
| 3278.2454642 | 3 |
| 3278.2454669 | 2 |
| 3278.2456463 | 2 |
| 3278.245671 | 2 |
| 3278.2457033 | 4 |
| 3278.2458283 | 5 |
| 3278.2459355 | 3 |
| 3278.2461176 | 5 |
| 3278.2461734 | 2 |
| 3278.2462873 | 2 |
| 3278.2464426 | 1 |
| 3278.2464448 | 1 |
| 3278.2464694 | 1 |
| 3278.2464822 | 3 |
| 3278.2465253 | 0 |

| | |
|---|---|
| 3278.2465419 | 2 |
| 3278.2466031 | 0 |
| 3278.2466809 | 0 |
| 3278.2468321 | 1 |
| 3278.2468477 | 0 |
| 3278.2468635 | 1 |
| 3278.2469143 | 3 |
| 3278.247021 | 3 |
| 3278.2470289 | 2 |
| 3278.2470518 | 3 |
| 3278.2470569 | 3 |
| 3278.2471029 | 1 |
| 3278.247358 | 3 |
| 3278.2473896 | 3 |
| 3278.2476866 | 5 |
| 3278.2477299 | 4 |
| 3278.2477397 | 3 |
| 3278.2478221 | 4 |
| 3278.2479078 | 0 |
| 3278.2479557 | 1 |
| 3278.2479745 | 4 |
| 3278.2480222 | 2 |
| 3278.2480379 | 5 |
| 3278.2481123 | 0 |
| 3278.2485217 | 2 |
| 3278.2485615 | 2 |
| 3278.2486252 | 4 |
| 3278.2486887 | 3 |
| 3278.2488565 | 0 |
| 3278.2488752 | 2 |
| 3278.248879 | 2 |
| 3278.2489048 | 4 |
| 3278.2490159 | 1 |
| 3278.2492317 | 1 |
| 3278.2492386 | 0 |
| 3278.2493129 | 1 |
| 3278.2493383 | 4 |
| 3278.2494834 | 3 |
| 3278.2495418 | 2 |
| 3278.2495693 | 3 |
| 3278.2496666 | 2 |
| 3278.2496822 | 2 |
| 3278.2497646 | 1 |
| 3278.2497689 | 0 |
| 3278.2497955 | 2 |
| 3278.2499255 | 1 |
| 3278.2499465 | 1 |
| 3278.2500853 | 2 |
| 3278.2501423 | 2 |
| 3278.2503312 | 1 |
| 3278.250379 | 6 |
| 3278.2504037 | 3 |
| 3278.2504157 | 2 |
| 3278.2505989 | 2 |
| 3278.2507572 | 0 |
| 3278.2507613 | 3 |
| 3278.250773 | 1 |
| 3278.2507834 | 0 |
| 3278.2508115 | 2 |
| 3278.2508632 | 0 |
| 3278.2508807 | 1 |
| 3278.2511029 | 1 |
| 3278.2511276 | 1 |
| 3278.2512048 | 3 |
| 3278.2512361 | 0 |
| 3278.2512397 | 0 |
| 3278.2514439 | 5 |
| 3278.251468 | 1 |
| 3278.2515338 | 2 |
| 3278.2516167 | 2 |
| 3278.2516871 | 4 |
| 3278.2517278 | 3 |
| 3278.251762 | 2 |
| 3278.2518091 | 1 |
| 3278.2518385 | 1 |
| 3278.2519325 | 2 |
| 3278.2522791 | 2 |
| 3278.2523511 | 2 |
| 3278.2523712 | 3 |
| 3278.2524767 | 4 |
| 3278.2524983 | 0 |
| 3278.2525519 | 2 |
| 3278.2526771 | 1 |
| 3278.252751 | 0 |
| 3278.2527836 | 2 |
| 3278.252915 | 4 |
| 3278.2529754 | 3 |
| 3278.2530115 | 2 |
| 3278.2531449 | 2 |
| 3278.2531674 | 1 |
| 3278.2532238 | 1 |
| 3278.253269 | 1 |
| 3278.2534198 | 2 |
| 3278.2535732 | 1 |
| 3278.2536196 | 1 |
| 3278.2536211 | 1 |
| 3278.2537472 | 0 |
| 3278.2537638 | 0 |
| 3278.253791 | 2 |
| 3278.2538115 | 0 |
| 3278.2538922 | 1 |
| 3278.2539709 | 2 |
| 3278.2540712 | 2 |
| 3278.2540899 | 0 |
| 3278.2541152 | 0 |
| 3278.2542282 | 3 |
| 3278.2543195 | 2 |
| 3278.2543486 | 4 |
| 3278.2544582 | 4 |
| 3278.2546098 | 3 |
| 3278.254639 | 1 |
| 3278.2546643 | 1 |
| 3278.2548245 | 0 |
| 3278.2548525 | 1 |
| 3278.2549482 | 2 |
| 3278.2549523 | 2 |
| 3278.2549602 | 0 |
| 3278.2549906 | 2 |
| 3278.255096 | 0 |
| 3278.2551463 | 0 |
| 3278.2553128 | 1 |
| 3278.2553766 | 4 |
| 3278.2553862 | 1 |
| 3278.2553898 | 1 |
| 3278.2553923 | 1 |
| 3278.2556399 | 0 |
| 3278.2556848 | 2 |
| 3278.2559122 | 0 |
| 3278.2559349 | 1 |
| 3278.256107 | 2 |
| 3278.2561288 | 3 |
| 3278.2561329 | 4 |
| 3278.2561839 | 1 |
| 3278.256193 | 1 |
| 3278.2562144 | 1 |
| 3278.2562626 | 3 |
| 3278.2565446 | 0 |
| 3278.2566297 | 4 |
| 3278.2568159 | 3 |
| 3278.256829 | 2 |
| 3278.2568571 | 0 |
| 3278.2569226 | 6 |
| 3278.2571541 | 5 |
| 3278.2571798 | 0 |
| 3278.2572307 | 2 |
| 3278.2572944 | 0 |
| 3278.2573308 | 1 |
| 3278.2573309 | 1 |
| 3278.2574147 | 2 |
| 3278.2574248 | 4 |
| 3278.257472 | 1 |
| 3278.2574813 | 3 |
| 3278.2577684 | 3 |
| 3278.2577933 | 1 |
| 3278.2578763 | 4 |
| 3278.2579475 | 1 |
| 3278.25797 | 1 |
| 3278.2580857 | 3 |
| 3278.2580895 | 1 |
| 3278.2581609 | 2 |
| 3278.2585614 | 5 |
| 3278.2586005 | 0 |
| 3278.258611 | 2 |
| 3278.2586908 | 7 |
| 3278.2587955 | 1 |
| 3278.2588144 | 3 |
| 3278.2588884 | 6 |
| 3278.2589054 | 2 |
| 3278.2590117 | 4 |
| 3278.2590586 | 4 |
| 3278.2590716 | 1 |
| 3278.2591584 | 0 |
| 3278.2592113 | 2 |
| 3278.2593213 | 2 |
| 3278.2594587 | 2 |
| 3278.2595202 | 0 |
| 3278.2595555 | 1 |
| 3278.2595978 | 0 |
| 3278.2595992 | 3 |
| 3278.2597195 | 4 |
| 3278.2600306 | 3 |
| 3278.2601953 | 5 |
| 3278.2602042 | 1 |
| 3278.2602669 | 1 |
| 3278.2603121 | 2 |
| 3278.260363 | 0 |
| 3278.2604203 | 2 |
| 3278.2605123 | 7 |
| 3278.2605602 | 3 |
| 3278.2606014 | 1 |
| 3278.2606901 | 1 |
| 3278.2607553 | 2 |
| 3278.2607793 | 2 |
| 3278.2609061 | 2 |
| 3278.2612581 | 2 |
| 3278.2612919 | 1 |
| 3278.2613515 | 0 |
| 3278.261384 | 2 |
| 3278.2614283 | 0 |
| 3278.261605 | 1 |
| 3278.2616299 | 0 |
| 3278.2617357 | 0 |
| 3278.2617381 | 4 |
| 3278.2617525 | 2 |
| 3278.2617907 | 2 |
| 3278.2618536 | 3 |
| 3278.2618662 | 1 |
| 3278.2620227 | 2 |
| 3278.2621395 | 3 |
| 3278.262213 | 1 |
| 3278.2622152 | 2 |
| 3278.2622753 | 2 |
| 3278.2623129 | 3 |
| 3278.2624453 | 2 |
| 3278.2627142 | 3 |
| 3278.2627226 | 3 |
| 3278.2628628 | 3 |
| 3278.2632305 | 2 |
| 3278.2634472 | 1 |
| 3278.2634474 | 1 |
| 3278.2637601 | 3 |
| 3278.2637793 | 3 |
| 3278.2638271 | 3 |
| 3278.2639751 | 3 |
| 3278.2640728 | 4 |
| 3278.2640916 | 2 |
| 3278.2641943 | 2 |
| 3278.2646467 | 2 |
| 3278.2647368 | 3 |
| 3278.2648675 | 2 |
| 3278.2648978 | 1 |
| 3278.2649786 | 1 |
| 3278.2651934 | 2 |
| 3278.2653855 | 3 |
| 3278.2655529 | 2 |
| 3278.2657834 | 3 |
| 3278.2658634 | 0 |
| 3278.2658709 | 3 |
| 3278.2659518 | 2 |
| 3278.2660663 | 0 |
| 3278.2661136 | 4 |
| 3278.2663753 | 2 |
| 3278.2665335 | 3 |
| 3278.2665673 | 2 |
| 3278.2667881 | 6 |
| 3278.2670797 | 2 |
| 3278.2671025 | 2 |
| 3278.2673406 | 1 |
| 3278.2675698 | 0 |
| 3278.2677085 | 4 |
| 3278.2678711 | 4 |
| 3278.2680085 | 3 |
| 3278.2680514 | 2 |
| 3278.2681444 | 1 |
| 3278.2682147 | 2 |
| 3278.2683754 | 2 |
| 3278.2686722 | 2 |
| 3278.2689852 | 1 |
| 3278.2691669 | 0 |
| 3278.2692113 | 4 |
| 3278.269332 | 0 |
| 3278.2694176 | 2 |
| 3278.2694243 | 5 |
| 3278.2696354 | 2 |
| 3278.2697082 | 2 |
| 3278.2699351 | 3 |
| 3278.2699497 | 2 |
| 3278.2702694 | 3 |
| 3278.2703017 | 1 |
| 3278.2705908 | 3 |
| 3278.2706083 | 2 |
| 3278.2707457 | 0 |
| 3278.2708699 | 2 |
| 3278.2709966 | 2 |
| 3278.2710015 | 0 |
| 3278.2710479 | 0 |
| 3278.2712946 | 2 |
| 3278.2715871 | 1 |
| 3278.2716416 | 2 |
| 3278.2716751 | 3 |
| 3278.2719307 | 3 |
| 3278.2720555 | 2 |
| 3278.272318 | 1 |
| 3278.2726875 | 4 |
| 3278.2726923 | 1 |
| 3278.272757 | 1 |
| 3278.2728941 | 1 |
| 3278.2732388 | 3 |
| 3278.2732606 | 4 |
| 3278.2733125 | 0 |
| 3278.2733498 | 2 |
| 3278.2734279 | 1 |
| 3278.2734545 | 1 |
| 3278.2735057 | 1 |
| 3278.2738533 | 1 |
| 3278.2739892 | 2 |
| 3278.2740937 | 4 |
| 3278.2743155 | 2 |
| 3278.2743442 | 2 |
| 3278.2746832 | 4 |
| 3278.2747989 | 3 |
| 3278.2751063 | 0 |
| 3278.2751266 | 1 |
| 3278.2752849 | 2 |
| 3278.2754908 | 1 |
| 3278.2755515 | 1 |
| 3278.275775 | 1 |
| 3278.2759259 | 0 |
| 3278.2760095 | 1 |
| 3278.2760558 | 3 |
| 3278.2761149 | 1 |
| 3278.2762436 | 1 |
| 3278.2764805 | 3 |
| 3278.2766132 | 2 |
| 3278.2767327 | 1 |
| 3278.2768943 | 4 |
| 3278.2769314 | 3 |
| 3278.2769379 | 2 |
| 3278.2769527 | 1 |
| 3278.2773294 | 4 |
| 3278.2773488 | 2 |
| 3278.2773803 | 3 |
| 3278.2780773 | 1 |
| 3278.2781132 | 2 |
| 3278.2781312 | 2 |
| 3278.2781455 | 1 |
| 3278.2782366 | 0 |
| 3278.2782487 | 3 |
| 3278.2789085 | 3 |
| 3278.278996 | 2 |
| 3278.2792815 | 0 |
| 3278.2793216 | 1 |
| 3278.2793793 | 2 |
| 3278.279387 | 0 |
| 3278.2795292 | 2 |
| 3278.2796258 | 6 |
| 3278.2796271 | 2 |
| 3278.2796943 | 3 |
| 3278.2798869 | 2 |
| 3278.2799589 | 1 |
| 3278.2803605 | 3 |
| 3278.280538 | 1 |
| 3278.2808795 | 3 |
| 3278.2810714 | 1 |
| 3278.2811482 | 3 |
| 3278.2812579 | 0 |
| 3278.2813722 | 1 |
| 3278.2813793 | 2 |
| 3278.2814221 | 4 |
| 3278.2817963 | 2 |
| 3278.2818875 | 2 |
| 3278.2819547 | 1 |
| 3278.2820001 | 3 |
| 3278.2820792 | 0 |
| 3278.2822778 | 0 |
| 3278.2822893 | 1 |
| 3278.2824247 | 1 |
| 3278.2824834 | 1 |
| 3278.2824839 | 2 |
| 3278.2830119 | 1 |
| 3278.2830407 | 1 |
| 3278.2830643 | 4 |
| 3278.2832688 | 2 |

| | |
|---|---|
| 3278.283483 | 2 |
| 3278.2837073 | 0 |
| 3278.2837254 | 1 |
| 3278.2840513 | 1 |
| 3278.2841225 | 0 |
| 3278.2842505 | 2 |
| 3278.284407 | 2 |
| 3278.2844666 | 1 |
| 3278.2845109 | 2 |
| 3278.2845154 | 4 |
| 3278.2846345 | 3 |
| 3278.2848694 | 1 |
| 3278.2849115 | 3 |
| 3278.2853659 | 3 |
| 3278.2856824 | 4 |
| 3278.2858841 | 3 |
| 3278.2861356 | 2 |
| 3278.2863237 | 4 |
| 3278.2863513 | 5 |
| 3278.2864015 | 6 |
| 3278.2864313 | 3 |
| 3278.2864409 | 2 |
| 3278.2864411 | 1 |
| 3278.2865961 | 3 |
| 3278.286647 | 2 |
| 3278.2867962 | 3 |
| 3278.2868402 | 6 |
| 3278.2872737 | 3 |
| 3278.2873577 | 5 |
| 3278.2875159 | 3 |
| 3278.2876434 | 5 |
| 3278.2878098 | 2 |
| 3278.2878354 | 4 |
| 3278.2879324 | 0 |
| 3278.2881788 | 3 |
| 3278.2881987 | 4 |
| 3278.2882362 | 6 |
| 3278.2883075 | 4 |
| 3278.2884837 | 0 |
| 3278.2885896 | 3 |
| 3278.2886582 | 3 |
| 3278.2888075 | 2 |
| 3278.2890938 | 1 |
| 3278.289101 | 2 |
| 3278.2891196 | 2 |
| 3278.2892413 | 3 |
| 3278.2896151 | 1 |
| 3278.289702 | 2 |
| 3278.2900348 | 2 |
| 3278.2901162 | 2 |
| 3278.2902138 | 1 |
| 3278.2902173 | 1 |
| 3278.2902291 | 2 |
| 3278.2902311 | 2 |
| 3278.290451 | 1 |
| 3278.2906808 | 3 |
| 3278.2907131 | 1 |
| 3278.2907693 | 0 |
| 3278.2908623 | 2 |
| 3278.2909041 | 1 |
| 3278.2912081 | 0 |
| 3278.2912451 | 3 |
| 3278.2915718 | 1 |
| 3278.2916448 | 4 |
| 3278.2917197 | 1 |
| 3278.2920919 | 1 |
| 3278.2923885 | 0 |
| 3278.2923912 | 1 |
| 3278.2925614 | 3 |
| 3278.292596 | 2 |
| 3278.292724 | 1 |
| 3278.2929233 | 2 |
| 3278.2930401 | 1 |
| 3278.2931542 | 1 |
| 3278.2932929 | 2 |
| 3278.2933879 | 2 |
| 3278.293401 | 3 |
| 3278.2934661 | 3 |
| 3278.2937731 | 2 |
| 3278.2938115 | 1 |
| 3278.2939658 | 3 |
| 3278.2939784 | 3 |
| 3278.2940739 | 0 |
| 3278.2943497 | 0 |
| 3278.2947435 | 2 |
| 3278.2947438 | 2 |
| 3278.2947539 | 3 |
| 3278.2947675 | 2 |
| 3278.2947976 | 1 |
| 3278.295144 | 1 |
| 3278.2951544 | 2 |
| 3278.2952678 | 1 |
| 3278.2954864 | 0 |
| 3278.2958196 | 3 |
| 3278.2958277 | 3 |
| 3278.2961822 | 1 |
| 3278.2962016 | 1 |
| 3278.2962572 | 1 |
| 3278.296591 | 2 |
| 3278.2965955 | 3 |
| 3278.2969539 | 1 |
| 3278.2969897 | 4 |
| 3278.2969921 | 3 |
| 3278.2974137 | 3 |
| 3278.29743 | 0 |
| 3278.2974395 | 2 |
| 3278.2974978 | 1 |
| 3278.2976252 | 2 |
| 3278.2979904 | 1 |
| 3278.2981993 | 2 |
| 3278.2984825 | 2 |
| 3278.2985017 | 2 |
| 3278.2986941 | 1 |
| 3278.298717 | 4 |
| 3278.2988821 | 1 |
| 3278.299011 | 1 |
| 3278.2990567 | 0 |
| 3278.2990882 | 5 |
| 3278.2991137 | 3 |
| 3278.2993037 | 2 |
| 3278.2996321 | 2 |
| 3278.2996647 | 2 |
| 3278.2996711 | 1 |
| 3278.299824 | 2 |
| 3278.3001821 | 0 |
| 3278.3003294 | 2 |
| 3278.3004606 | 0 |
| 3278.3005315 | 3 |
| 3278.3005539 | 1 |
| 3278.300772 | 3 |
| 3278.3008138 | 1 |
| 3278.3008455 | 1 |
| 3278.3011534 | 1 |
| 3278.3011616 | 0 |
| 3278.3015049 | 6 |
| 3278.301543 | 1 |
| 3278.3015486 | 2 |
| 3278.3017548 | 0 |
| 3278.3019318 | 3 |
| 3278.3019704 | 4 |
| 3278.3023689 | 2 |
| 3278.3024025 | 1 |
| 3278.3025266 | 3 |
| 3278.3025486 | 3 |
| 3278.302747 | 2 |
| 3278.3028258 | 1 |
| 3278.3030726 | 3 |
| 3278.3031996 | 2 |
| 3278.3032508 | 3 |
| 3278.3035808 | 0 |
| 3278.3036607 | 1 |
| 3278.3037627 | 1 |
| 3278.3039058 | 3 |
| 3278.3039101 | 2 |
| 3278.3041803 | 3 |
| 3278.3045684 | 2 |
| 3278.3045981 | 4 |
| 3278.3046435 | 1 |
| 3278.3047568 | 2 |
| 3278.3049673 | 2 |
| 3278.3051753 | 1 |
| 3278.3052188 | 1 |
| 3278.3053075 | 4 |
| 3278.3053393 | 1 |
| 3278.3057503 | 4 |
| 3278.3057998 | 2 |
| 3278.3059484 | 2 |
| 3278.3060898 | 1 |
| 3278.3061434 | 0 |
| 3278.3063218 | 7 |
| 3278.3068505 | 1 |
| 3278.3070058 | 3 |
| 3278.3070187 | 2 |
| 3278.3071005 | 4 |
| 3278.3072369 | 3 |
| 3278.3074333 | 1 |
| 3278.3074381 | 0 |
| 3278.3075284 | 3 |
| 3278.3075827 | 1 |
| 3278.3075971 | 0 |
| 3278.3079589 | 2 |
| 3278.3081206 | 3 |
| 3278.3083078 | 4 |
| 3278.3083827 | 1 |
| 3278.3086395 | 1 |
| 3278.3092687 | 3 |
| 3278.3092843 | 1 |
| 3278.3093793 | 1 |
| 3278.3095396 | 1 |
| 3278.3096872 | 0 |
| 3278.3097682 | 1 |
| 3278.3098214 | 2 |
| 3278.3098502 | 1 |
| 3278.3098987 | 2 |
| 3278.3100682 | 1 |
| 3278.3103247 | 1 |
| 3278.3103406 | 0 |
| 3278.3108364 | 2 |
| 3278.3108827 | 1 |
| 3278.3110094 | 1 |
| 3278.3110182 | 2 |
| 3278.3111778 | 1 |
| 3278.311197 | 3 |
| 3278.3113775 | 3 |
| 3278.3120698 | 4 |
| 3278.3121044 | 2 |
| 3278.3122683 | 1 |
| 3278.3124681 | 1 |
| 3278.3124929 | 1 |
| 3278.3126476 | 3 |
| 3278.3129108 | 1 |
| 3278.3130112 | 2 |
| 3278.3131415 | 0 |
| 3278.3132957 | 0 |
| 3278.3133073 | 3 |
| 3278.3134118 | 0 |
| 3278.3138254 | 3 |
| 3278.3138266 | 2 |
| 3278.3139005 | 3 |
| 3278.3139529 | 1 |
| 3278.3141427 | 1 |
| 3278.3143603 | 1 |
| 3278.3145929 | 3 |
| 3278.3146282 | 4 |
| 3278.3148244 | 2 |
| 3278.3148464 | 2 |
| 3278.3153794 | 0 |
| 3278.3154374 | 3 |
| 3278.3157845 | 0 |
| 3278.3160907 | 1 |
| 3278.3161384 | 0 |
| 3278.3162693 | 3 |
| 3278.3164802 | 2 |
| 3278.3165372 | 2 |
| 3278.3166632 | 2 |
| 3278.3167561 | 2 |
| 3278.3168069 | 1 |
| 3278.3169085 | 3 |
| 3278.317219 | 2 |
| 3278.3173139 | 1 |
| 3278.3175573 | 2 |
| 3278.3176356 | 1 |
| 3278.317842 | 3 |
| 3278.3180223 | 1 |
| 3278.3180999 | 1 |
| 3278.3184794 | 1 |
| 3278.3185584 | 1 |
| 3278.3186067 | 4 |
| 3278.318716 | 1 |
| 3278.3190665 | 1 |
| 3278.3191175 | 2 |
| 3278.3191578 | 1 |
| 3278.3193004 | 2 |
| 3278.3194582 | 0 |
| 3278.3195472 | 2 |
| 3278.3195883 | 3 |
| 3278.3198515 | 2 |
| 3278.3198827 | 0 |
| 3278.3202351 | 1 |
| 3278.3202799 | 0 |
| 3278.3206409 | 2 |
| 3278.3210407 | 2 |
| 3278.3210881 | 1 |
| 3278.32125 | 0 |
| 3278.321422 | 0 |
| 3278.3215277 | 1 |
| 3278.3216287 | 1 |
| 3278.3217797 | 2 |
| 3278.3219058 | 2 |
| 3278.3220855 | 2 |
| 3278.3221299 | 2 |
| 3278.3222994 | 2 |
| 3278.3224071 | 2 |
| 3278.3225452 | 0 |
| 3278.3226323 | 1 |
| 3278.3227875 | 1 |
| 3278.3228107 | 0 |
| 3278.3232573 | 2 |
| 3278.3232803 | 1 |
| 3278.3233892 | 2 |
| 3278.3235317 | 2 |
| 3278.3235557 | 1 |
| 3278.3238988 | 3 |
| 3278.3240555 | 0 |
| 3278.324237 | 2 |
| 3278.3243825 | 2 |
| 3278.3245652 | 2 |
| 3278.3247385 | 1 |
| 3278.3247995 | 3 |
| 3278.3251832 | 1 |
| 3278.3252279 | 2 |
| 3278.3254214 | 4 |
| 3278.3255583 | 0 |
| 3278.3255685 | 4 |
| 3278.3258731 | 2 |
| 3278.3259964 | 0 |
| 3278.3260222 | 1 |
| 3278.3262302 | 3 |
| 3278.3262443 | 3 |
| 3278.3265717 | 1 |
| 3278.3266692 | 2 |
| 3278.3268571 | 3 |
| 3278.3269425 | 1 |
| 3278.3270909 | 3 |
| 3278.3271337 | 3 |
| 3278.3275945 | 2 |
| 3278.3276999 | 2 |
| 3278.3277837 | 2 |
| 3278.3277995 | 3 |
| 3278.3282417 | 1 |
| 3278.3285265 | 1 |
| 3278.328608 | 0 |
| 3278.3286921 | 2 |
| 3278.3288608 | 1 |
| 3278.328915 | 1 |
| 3278.3289301 | 1 |
| 3278.3291622 | 2 |
| 3278.3293509 | 2 |
| 3278.3295423 | 0 |
| 3278.32973 | 0 |
| 3278.3300979 | 1 |
| 3278.3302685 | 0 |
| 3278.3302952 | 0 |
| 3278.330334 | 3 |
| 3278.3303812 | 3 |
| 3278.3304365 | 1 |
| 3278.3304892 | 2 |
| 3278.3308081 | 2 |
| 3278.3308187 | 1 |
| 3278.3308796 | 0 |
| 3278.3314179 | 5 |
| 3278.3315553 | 2 |
| 3278.3316406 | 1 |
| 3278.3316985 | 2 |
| 3278.3321637 | 1 |
| 3278.3321736 | 3 |
| 3278.332223 | 2 |
| 3278.3323243 | 0 |
| 3278.3325093 | 1 |
| 3278.3325698 | 1 |
| 3278.3326381 | 0 |
| 3278.3328892 | 1 |
| 3278.3330973 | 2 |
| 3278.3334198 | 1 |
| 3278.3335014 | 0 |
| 3278.3338415 | 2 |
| 3278.3339368 | 2 |
| 3278.3342679 | 3 |
| 3278.3343281 | 1 |
| 3278.3343288 | 0 |
| 3278.3344482 | 1 |
| 3278.3346096 | 2 |
| 3278.3346693 | 1 |
| 3278.3346731 | 1 |
| 3278.3349114 | 1 |
| 3278.3351484 | 1 |
| 3278.3351568 | 2 |
| 3278.3356218 | 3 |
| 3278.3357021 | 1 |
| 3278.3360025 | 1 |
| 3278.3362031 | 3 |
| 3278.3362295 | 4 |
| 3278.3362417 | 2 |

| | |
|---|---|
| 3278.3365868 | 1 |
| 3278.3369077 | 3 |
| 3278.3369506 | 3 |
| 3278.3369789 | 6 |
| 3278.337033 | 1 |
| 3278.3371781 | 1 |
| 3278.3372023 | 4 |
| 3278.3372741 | 3 |
| 3278.337769 | 1 |
| 3278.3378054 | 1 |
| 3278.3379545 | 1 |
| 3278.3381199 | 2 |
| 3278.338426 | 2 |
| 3278.3385502 | 1 |
| 3278.338696 | 0 |
| 3278.3388546 | 1 |
| 3278.3391185 | 1 |
| 3278.3391578 | 0 |
| 3278.339514 | 2 |
| 3278.3396278 | 1 |
| 3278.3396473 | 6 |
| 3278.3398076 | 0 |
| 3278.3399609 | 3 |
| 3278.3400594 | 1 |
| 3278.3402784 | 0 |
| 3278.3403537 | 0 |
| 3278.3403671 | 0 |
| 3278.3404749 | 1 |
| 3278.3408561 | 2 |
| 3278.341051 | 2 |
| 3278.3410612 | 2 |
| 3278.3410761 | 0 |
| 3278.3413204 | 1 |
| 3278.341681 | 1 |
| 3278.3418406 | 1 |
| 3278.3419631 | 0 |
| 3278.3420694 | 3 |
| 3278.3421585 | 1 |
| 3278.3422087 | 1 |
| 3278.3422734 | 2 |
| 3278.342345 | 1 |
| 3278.34253 | 1 |
| 3278.343051 | 1 |
| 3278.343136 | 1 |
| 3278.3431918 | 3 |
| 3278.3432495 | 2 |
| 3278.3432924 | 1 |
| 3278.3436033 | 0 |
| 3278.3437454 | 2 |
| 3278.3439033 | 1 |
| 3278.3440067 | 2 |
| 3278.344066 | 2 |
| 3278.3442051 | 0 |
| 3278.3442849 | 1 |
| 3278.3445167 | 1 |
| 3278.3449072 | 2 |
| 3278.3449339 | 3 |
| 3278.3453832 | 3 |
| 3278.3453904 | 1 |
| 3278.3456938 | 3 |
| 3278.3457227 | 0 |
| 3278.3458817 | 3 |
| 3278.3459033 | 1 |
| 3278.3459935 | 2 |
| 3278.3461815 | 1 |
| 3278.3463596 | 4 |
| 3278.3464689 | 2 |
| 3278.3464911 | 0 |
| 3278.3469258 | 1 |
| 3278.3469509 | 1 |
| 3278.3472466 | 2 |
| 3278.3474132 | 2 |
| 3278.3477238 | 2 |
| 3278.3477559 | 3 |
| 3278.3478659 | 1 |
| 3278.3479269 | 2 |
| 3278.3479609 | 1 |
| 3278.3481728 | 1 |
| 3278.3483798 | 1 |
| 3278.3486138 | 1 |
| 3278.3489453 | 1 |
| 3278.3489622 | 1 |
| 3278.3492433 | 2 |
| 3278.3492574 | 0 |
| 3278.3494402 | 2 |
| 3278.349505 | 0 |
| 3278.3495908 | 1 |
| 3278.3497313 | 1 |
| 3278.3499074 | 1 |
| 3278.3504123 | 3 |
| 3278.3504402 | 1 |
| 3278.3504571 | 2 |
| 3278.3509433 | 2 |
| 3278.3511093 | 1 |
| 3278.3512067 | 0 |
| 3278.3512093 | 0 |
| 3278.351281 | 4 |
| 3278.351323 | 1 |
| 3278.3514098 | 3 |
| 3278.3517297 | 3 |
| 3278.3518329 | 0 |
| 3278.3523215 | 0 |
| 3278.3524119 | 0 |
| 3278.3525329 | 2 |
| 3278.3528646 | 5 |
| 3278.3528649 | 0 |
| 3278.3531328 | 0 |
| 3278.3531424 | 2 |
| 3278.3532488 | 1 |
| 3278.3533733 | 0 |
| 3278.3534875 | 3 |
| 3278.3535217 | 3 |
| 3278.353615 | 1 |
| 3278.3537227 | 4 |
| 3278.3537568 | 3 |
| 3278.3538066 | 3 |
| 3278.354293 | 3 |
| 3278.3547046 | 3 |
| 3278.354724 | 0 |
| 3278.3551942 | 2 |
| 3278.3552814 | 0 |
| 3278.3553396 | 3 |
| 3278.3556855 | 2 |
| 3278.3556908 | 4 |
| 3278.3557304 | 0 |
| 3278.355994 | 0 |
| 3278.3560267 | 3 |
| 3278.3561555 | 1 |
| 3278.3562802 | 1 |
| 3278.3563625 | 2 |
| 3278.356477 | 1 |
| 3278.3564994 | 2 |
| 3278.3568077 | 5 |
| 3278.3570772 | 4 |
| 3278.3574659 | 2 |
| 3278.3575999 | 1 |
| 3278.3576408 | 1 |
| 3278.357878 | 2 |
| 3278.3579358 | 3 |
| 3278.3579403 | 0 |
| 3278.3579771 | 2 |
| 3278.3580032 | 1 |
| 3278.3583166 | 0 |
| 3278.3585581 | 2 |
| 3278.358705 | 6 |
| 3278.3587811 | 2 |
| 3278.3590475 | 2 |
| 3278.3591002 | 0 |
| 3278.3593374 | 1 |
| 3278.3594481 | 3 |
| 3278.3597928 | 1 |
| 3278.359826 | 4 |
| 3278.3598603 | 0 |
| 3278.3600241 | 1 |
| 3278.3604361 | 2 |
| 3278.3606675 | 1 |
| 3278.3608192 | 5 |
| 3278.3608241 | 2 |
| 3278.361215 | 4 |
| 3278.3612398 | 4 |
| 3278.3612407 | 2 |
| 3278.3615401 | 1 |
| 3278.3615892 | 1 |
| 3278.3618423 | 0 |
| 3278.3620008 | 4 |
| 3278.3621519 | 2 |
| 3278.3624444 | 3 |
| 3278.3624812 | 3 |
| 3278.3625704 | 1 |
| 3278.3626297 | 1 |
| 3278.3630308 | 1 |
| 3278.3630356 | 0 |
| 3278.3631414 | 1 |
| 3278.3633208 | 3 |
| 3278.3636963 | 0 |
| 3278.3638656 | 1 |
| 3278.363871 | 1 |
| 3278.3640173 | 1 |
| 3278.3643011 | 2 |
| 3278.3644037 | 1 |
| 3278.3645855 | 0 |
| 3278.3647373 | 3 |
| 3278.3647596 | 4 |
| 3278.3648255 | 0 |
| 3278.3650642 | 2 |
| 3278.3651682 | 1 |
| 3278.365198 | 1 |
| 3278.3651983 | 0 |
| 3278.3658511 | 3 |
| 3278.3659748 | 1 |
| 3278.3661382 | 1 |
| 3278.3661976 | 0 |
| 3278.3662782 | 0 |
| 3278.3664219 | 3 |
| 3278.3665476 | 2 |
| 3278.366647 | 2 |
| 3278.366769 | 3 |
| 3278.3668825 | 1 |
| 3278.3672633 | 4 |
| 3278.3674302 | 1 |
| 3278.367558 | 1 |
| 3278.3678214 | 1 |
| 3278.368094 | 1 |
| 3278.3680966 | 3 |
| 3278.3681912 | 0 |
| 3278.3683143 | 2 |
| 3278.3685351 | 4 |
| 3278.3685852 | 0 |
| 3278.3685854 | 2 |
| 3278.3687543 | 4 |
| 3278.3689437 | 4 |
| 3278.3690264 | 0 |
| 3278.3691281 | 2 |
| 3278.3695176 | 1 |
| 3278.3697462 | 1 |
| 3278.3699565 | 1 |
| 3278.3701479 | 0 |
| 3278.3701624 | 3 |
| 3278.3702993 | 3 |
| 3278.3706128 | 7 |
| 3278.370753 | 2 |
| 3278.3708695 | 1 |
| 3278.3709574 | 2 |
| 3278.371027 | 2 |
| 3278.3714088 | 2 |
| 3278.3715438 | 3 |
| 3278.3716221 | 1 |
| 3278.3716822 | 1 |
| 3278.3717245 | 4 |
| 3278.3717711 | 3 |
| 3278.3721626 | 2 |
| 3278.3722913 | 2 |
| 3278.3723916 | 0 |
| 3278.3724499 | 1 |
| 3278.3724526 | 1 |
| 3278.372592 | 2 |
| 3278.3729758 | 1 |
| 3278.3732996 | 1 |
| 3278.3733135 | 3 |
| 3278.3733808 | 1 |
| 3278.373428 | 1 |
| 3278.3735513 | 1 |
| 3278.3736017 | 2 |
| 3278.3741209 | 1 |
| 3278.3743716 | 1 |
| 3278.3743812 | 2 |
| 3278.3744364 | 0 |
| 3278.3745056 | 1 |
| 3278.3747936 | 3 |
| 3278.3748893 | 0 |
| 3278.3750506 | 1 |
| 3278.3752682 | 3 |
| 3278.3754692 | 2 |
| 3278.3756656 | 1 |
| 3278.3757506 | 1 |
| 3278.3759112 | 2 |
| 3278.3759736 | 0 |
| 3278.3763981 | 1 |
| 3278.3764657 | 2 |
| 3278.3769175 | 2 |
| 3278.3770067 | 4 |
| 3278.3770256 | 3 |
| 3278.3771873 | 0 |
| 3278.3772637 | 1 |
| 3278.3773291 | 0 |
| 3278.3773417 | 1 |
| 3278.377553 | 0 |
| 3278.3776316 | 1 |
| 3278.3780236 | 0 |
| 3278.3782793 | 3 |
| 3278.3783298 | 3 |
| 3278.3784363 | 1 |
| 3278.3784721 | 1 |
| 3278.3785295 | 1 |
| 3278.378852 | 2 |
| 3278.3790054 | 2 |
| 3278.3792398 | 4 |
| 3278.3792581 | 3 |
| 3278.3795181 | 3 |
| 3278.379522 | 0 |
| 3278.3796382 | 2 |
| 3278.3796557 | 1 |
| 3278.3797574 | 1 |
| 3278.3799882 | 0 |
| 3278.3800414 | 1 |
| 3278.3804037 | 1 |
| 3278.3807047 | 2 |
| 3278.38071 | 1 |
| 3278.380962 | 1 |
| 3278.3811497 | 3 |
| 3278.3815679 | 3 |
| 3278.3817439 | 3 |
| 3278.3819135 | 3 |
| 3278.382056 | 4 |
| 3278.3821073 | 0 |
| 3278.382181 | 1 |
| 3278.3824337 | 4 |
| 3278.3824748 | 1 |
| 3278.3825211 | 4 |
| 3278.3825293 | 0 |
| 3278.3829495 | 4 |
| 3278.3830129 | 3 |
| 3278.3832073 | 1 |
| 3278.383357 | 1 |
| 3278.3836011 | 1 |
| 3278.3838211 | 3 |
| 3278.3840736 | 4 |
| 3278.3842176 | 1 |
| 3278.384226 | 2 |
| 3278.384304 | 2 |
| 3278.3846237 | 0 |
| 3278.3847475 | 3 |
| 3278.3848342 | 1 |
| 3278.3850753 | 0 |
| 3278.3852234 | 4 |
| 3278.3852535 | 2 |
| 3278.3853197 | 2 |
| 3278.3854713 | 3 |
| 3278.3855131 | 0 |
| 3278.3856703 | 4 |
| 3278.3859024 | 1 |
| 3278.3862965 | 0 |
| 3278.3863299 | 0 |
| 3278.3864646 | 2 |
| 3278.3864801 | 2 |
| 3278.3866848 | 0 |
| 3278.3869164 | 0 |
| 3278.3869277 | 2 |
| 3278.3873642 | 3 |
| 3278.3874484 | 1 |
| 3278.3876063 | 1 |
| 3278.3876255 | 3 |
| 3278.3877833 | 2 |
| 3278.3879999 | 1 |
| 3278.3881125 | 1 |
| 3278.3881541 | 0 |
| 3278.3885858 | 1 |
| 3278.3887557 | 1 |
| 3278.3888366 | 1 |
| 3278.3889438 | 1 |
| 3278.3889944 | 2 |
| 3278.3891233 | 2 |
| 3278.389136 | 3 |
| 3278.3893182 | 3 |
| 3278.3894288 | 1 |
| 3278.3897501 | 3 |
| 3278.3897596 | 1 |
| 3278.3900369 | 1 |
| 3278.3902832 | 0 |
| 3278.39037 | 3 |
| 3278.3905662 | 3 |
| 3278.3908 | 4 |
| 3278.3909141 | 5 |
| 3278.3909381 | 4 |
| 3278.3910323 | 3 |
| 3278.3910334 | 1 |
| 3278.3910512 | 0 |
| 3278.3917922 | 4 |
| 3278.3918275 | 2 |
| 3278.3918526 | 4 |
| 3278.3918999 | 2 |
| 3278.3919424 | 1 |
| 3278.3925275 | 1 |

| | |
|---|---|
| 3278.3925474 | 1 |
| 3278.3927202 | 1 |
| 3278.3930681 | 3 |
| 3278.3931135 | 2 |
| 3278.3934547 | 0 |
| 3278.3935013 | 1 |
| 3278.3937483 | 3 |
| 3278.3937601 | 3 |
| 3278.3937805 | 1 |
| 3278.3939597 | 2 |
| 3278.3942463 | 3 |
| 3278.3942668 | 1 |
| 3278.3942806 | 1 |
| 3278.3943618 | 2 |
| 3278.3945324 | 1 |
| 3278.3946135 | 1 |
| 3278.3947593 | 2 |
| 3278.3949184 | 1 |
| 3278.3950668 | 2 |
| 3278.3953469 | 3 |
| 3278.3956193 | 0 |
| 3278.3956935 | 2 |
| 3278.3959082 | 0 |
| 3278.3960878 | 2 |
| 3278.3963659 | 0 |
| 3278.3965145 | 2 |
| 3278.3965171 | 0 |
| 3278.3966879 | 1 |
| 3278.396812 | 2 |
| 3278.3968169 | 0 |
| 3278.3970968 | 0 |
| 3278.3973217 | 2 |
| 3278.3976425 | 0 |
| 3278.3976493 | 5 |
| 3278.3978957 | 1 |
| 3278.3983328 | 0 |
| 3278.3983437 | 0 |
| 3278.3983489 | 2 |
| 3278.3983596 | 4 |
| 3278.3984521 | 2 |
| 3278.3986316 | 2 |
| 3278.3988425 | 2 |
| 3278.3989323 | 2 |
| 3278.3991046 | 0 |
| 3278.3991882 | 3 |
| 3278.399283 | 1 |
| 3278.3995225 | 0 |
| 3278.3998271 | 1 |
| 3278.3999099 | 0 |
| 3278.4002073 | 2 |
| 3278.4005179 | 1 |
| 3278.4009635 | 0 |
| 3278.4010229 | 2 |
| 3278.4010365 | 5 |
| 3278.4010566 | 3 |
| 3278.4013234 | 1 |
| 3278.4013924 | 1 |
| 3278.4015171 | 0 |
| 3278.4015476 | 5 |
| 3278.4016329 | 1 |
| 3278.401642 | 0 |
| 3278.4020505 | 2 |
| 3278.4020836 | 2 |
| 3278.4023379 | 2 |
| 3278.4024923 | 2 |
| 3278.4025637 | 2 |
| 3278.4028713 | 1 |
| 3278.4029578 | 1 |
| 3278.4032162 | 4 |
| 3278.4035153 | 0 |
| 3278.4035575 | 2 |
| 3278.4036558 | 0 |
| 3278.4038155 | 0 |
| 3278.4038837 | 2 |
| 3278.404038 | 2 |
| 3278.4041358 | 1 |
| 3278.4044955 | 4 |
| 3278.4045273 | 1 |
| 3278.4051704 | 4 |
| 3278.4052248 | 5 |
| 3278.4053673 | 1 |
| 3278.4053854 | 1 |
| 3278.4054251 | 3 |
| 3278.4056527 | 4 |
| 3278.4056803 | 1 |
| 3278.4057108 | 1 |
| 3278.4061912 | 1 |
| 3278.4062742 | 2 |
| 3278.4063685 | 2 |
| 3278.4064473 | 1 |
| 3278.4064701 | 2 |
| 3278.4065002 | 0 |
| 3278.4066622 | 1 |
| 3278.4070429 | 0 |
| 3278.4071192 | 1 |
| 3278.4071993 | 2 |
| 3278.4075794 | 0 |
| 3278.4077161 | 1 |
| 3278.4077459 | 0 |
| 3278.4079812 | 2 |
| 3278.4083395 | 1 |
| 3278.4084705 | 1 |
| 3278.4085359 | 3 |
| 3278.4086484 | 2 |
| 3278.408763 | 3 |
| 3278.4087837 | 3 |
| 3278.4091683 | 2 |
| 3278.409284 | 2 |
| 3278.4093585 | 3 |
| 3278.4094058 | 2 |
| 3278.4094903 | 2 |
| 3278.410052 | 5 |
| 3278.4100949 | 1 |
| 3278.4102835 | 3 |
| 3278.4103107 | 1 |
| 3278.4103166 | 3 |
| 3278.4107787 | 1 |
| 3278.4108831 | 3 |
| 3278.4109273 | 0 |
| 3278.4111224 | 3 |
| 3278.4113026 | 0 |
| 3278.4113649 | 2 |
| 3278.4114858 | 2 |
| 3278.4117008 | 2 |
| 3278.4118219 | 3 |
| 3278.4119948 | 4 |
| 3278.4122313 | 2 |
| 3278.4123209 | 2 |
| 3278.4124673 | 2 |
| 3278.4124808 | 1 |
| 3278.4125703 | 3 |
| 3278.4130996 | 1 |
| 3278.4133044 | 1 |
| 3278.4133142 | 1 |
| 3278.4133314 | 3 |
| 3278.4135478 | 1 |
| 3278.413954 | 1 |
| 3278.4140834 | 1 |
| 3278.4141134 | 0 |
| 3278.4141911 | 1 |
| 3278.4142075 | 0 |
| 3278.4144793 | 1 |
| 3278.4146553 | 2 |
| 3278.4147812 | 3 |
| 3278.4149147 | 1 |
| 3278.4149815 | 2 |
| 3278.4150471 | 2 |
| 3278.4151242 | 2 |
| 3278.4153464 | 1 |
| 3278.4157754 | 1 |
| 3278.4158126 | 2 |
| 3278.4160779 | 3 |
| 3278.4161892 | 2 |
| 3278.4163513 | 1 |
| 3278.4164068 | 0 |
| 3278.4164829 | 1 |
| 3278.416544 | 1 |
| 3278.4170014 | 2 |
| 3278.4170609 | 1 |
| 3278.4171084 | 2 |
| 3278.4171844 | 1 |
| 3278.4172142 | 1 |
| 3278.4173666 | 1 |
| 3278.4175778 | 0 |
| 3278.4177191 | 0 |
| 3278.4178352 | 2 |
| 3278.4181498 | 2 |
| 3278.418352 | 1 |
| 3278.4185168 | 1 |
| 3278.4186846 | 1 |
| 3278.4187244 | 2 |
| 3278.4188012 | 2 |
| 3278.4192088 | 3 |
| 3278.4194663 | 1 |
| 3278.4197596 | 6 |
| 3278.4198207 | 3 |
| 3278.4199393 | 5 |
| 3278.4199782 | 1 |
| 3278.4200247 | 1 |
| 3278.4201224 | 0 |
| 3278.420293 | 2 |
| 3278.4204899 | 2 |
| 3278.4207423 | 3 |
| 3278.4208486 | 0 |
| 3278.4210632 | 1 |
| 3278.4212277 | 1 |
| 3278.4212761 | 1 |
| 3278.4213061 | 0 |
| 3278.4213782 | 1 |
| 3278.42196 | 2 |
| 3278.4220031 | 2 |
| 3278.4220138 | 5 |
| 3278.4220642 | 0 |
| 3278.4223134 | 2 |
| 3278.4223989 | 1 |
| 3278.422676 | 2 |
| 3278.4228173 | 4 |
| 3278.4229408 | 3 |
| 3278.4230328 | 1 |
| 3278.4231628 | 2 |
| 3278.4233253 | 2 |
| 3278.4237408 | 2 |
| 3278.4238203 | 2 |
| 3278.423829 | 1 |
| 3278.4241577 | 2 |
| 3278.4242992 | 2 |
| 3278.424487 | 0 |
| 3278.4245656 | 2 |
| 3278.4247534 | 0 |
| 3278.4248009 | 3 |
| 3278.4250847 | 0 |
| 3278.4251129 | 1 |
| 3278.4251952 | 4 |
| 3278.4254344 | 1 |
| 3278.4255279 | 4 |
| 3278.4256249 | 2 |
| 3278.4256885 | 2 |
| 3278.4257886 | 1 |
| 3278.4262835 | 3 |
| 3278.426412 | 3 |
| 3278.4267934 | 2 |
| 3278.4268288 | 1 |
| 3278.4269186 | 1 |
| 3278.426988 | 2 |
| 3278.4272594 | 2 |
| 3278.427612 | 0 |
| 3278.4278973 | 0 |
| 3278.4279364 | 0 |
| 3278.4279847 | 2 |
| 3278.4280824 | 0 |
| 3278.4280989 | 2 |
| 3278.4280994 | 3 |
| 3278.42821 | 2 |
| 3278.4284702 | 2 |
| 3278.4286005 | 2 |
| 3278.4287758 | 0 |
| 3278.4292731 | 2 |
| 3278.4293277 | 0 |
| 3278.4293509 | 2 |
| 3278.4296067 | 2 |
| 3278.4296767 | 2 |
| 3278.4298578 | 0 |
| 3278.4300455 | 2 |
| 3278.4300984 | 2 |
| 3278.4304158 | 0 |
| 3278.4304178 | 0 |
| 3278.4305238 | 2 |
| 3278.4305341 | 1 |
| 3278.4307907 | 1 |
| 3278.4309817 | 1 |
| 3278.4310466 | 0 |
| 3278.4314494 | 4 |
| 3278.4315918 | 3 |
| 3278.4317542 | 2 |
| 3278.431892 | 4 |
| 3278.4320021 | 1 |
| 3278.4320035 | 1 |
| 3278.4321637 | 1 |
| 3278.4324876 | 2 |
| 3278.4326272 | 1 |
| 3278.4326733 | 0 |
| 3278.4327832 | 1 |
| 3278.4328338 | 2 |
| 3278.4331487 | 1 |
| 3278.433151 | 0 |
| 3278.433252 | 2 |
| 3278.4336532 | 1 |
| 3278.4336658 | 1 |
| 3278.4337898 | 0 |
| 3278.4340458 | 0 |
| 3278.4340526 | 0 |
| 3278.4342303 | 3 |
| 3278.4344422 | 4 |
| 3278.4346731 | 2 |
| 3278.435012 | 2 |
| 3278.4351229 | 2 |
| 3278.4351246 | 1 |
| 3278.435214 | 0 |
| 3278.4354065 | 0 |
| 3278.4354965 | 1 |
| 3278.4356705 | 2 |
| 3278.4357442 | 2 |
| 3278.4357635 | 2 |
| 3278.4357866 | 3 |
| 3278.4361345 | 1 |
| 3278.4364361 | 1 |
| 3278.4365192 | 1 |
| 3278.4368207 | 1 |
| 3278.4372149 | 4 |
| 3278.4372226 | 2 |
| 3278.4373384 | 0 |
| 3278.4375025 | 1 |
| 3278.4376221 | 2 |
| 3278.437678 | 3 |
| 3278.437793 | 1 |
| 3278.4378064 | 1 |
| 3278.4385485 | 0 |
| 3278.4385724 | 0 |
| 3278.4386754 | 1 |
| 3278.4387172 | 2 |
| 3278.4389933 | 1 |
| 3278.4390509 | 3 |
| 3278.4390619 | 4 |
| 3278.4391341 | 1 |
| 3278.4394198 | 1 |
| 3278.4398054 | 2 |
| 3278.4398181 | 2 |
| 3278.4399367 | 1 |
| 3278.43999 | 2 |
| 3278.4401375 | 1 |
| 3278.4401686 | 0 |
| 3278.4405492 | 1 |
| 3278.4408271 | 0 |
| 3278.4408543 | 1 |
| 3278.4409886 | 4 |
| 3278.4413683 | 0 |
| 3278.4415015 | 1 |
| 3278.4415452 | 1 |
| 3278.441767 | 2 |
| 3278.4417696 | 5 |
| 3278.4418807 | 0 |
| 3278.4420222 | 2 |
| 3278.442077 | 2 |
| 3278.4421092 | 1 |
| 3278.4424094 | 3 |
| 3278.4424966 | 2 |
| 3278.4424973 | 1 |
| 3278.4430216 | 1 |
| 3278.4431824 | 1 |
| 3278.4432758 | 1 |
| 3278.4436041 | 1 |
| 3278.4437362 | 1 |
| 3278.4437997 | 2 |
| 3278.4438821 | 2 |
| 3278.4440676 | 1 |
| 3278.4442038 | 2 |
| 3278.4444041 | 2 |
| 3278.4446589 | 2 |
| 3278.4449206 | 0 |
| 3278.4449376 | 2 |
| 3278.445157 | 2 |
| 3278.4453419 | 0 |
| 3278.4453716 | 2 |
| 3278.4454226 | 0 |
| 3278.4455006 | 1 |
| 3278.4459016 | 2 |
| 3278.4460232 | 0 |
| 3278.4462062 | 1 |
| 3278.4462378 | 2 |
| 3278.4462881 | 1 |
| 3278.4463253 | 1 |
| 3278.4465365 | 2 |
| 3278.4468471 | 4 |
| 3278.4468476 | 3 |
| 3278.4471112 | 0 |
| 3278.4474774 | 2 |
| 3278.4477533 | 3 |
| 3278.447973 | 1 |
| 3278.4479785 | 2 |
| 3278.4480268 | 2 |
| 3278.4481116 | 4 |
| 3278.4483974 | 3 |
| 3278.4486078 | 2 |

| | |
|---|---|
| 3278.4486888 | 3 |
| 3278.4487219 | 1 |
| 3278.4487494 | 1 |
| 3278.4492997 | 0 |
| 3278.4493749 | 3 |
| 3278.4494194 | 3 |
| 3278.4496271 | 0 |
| 3278.4497218 | 3 |
| 3278.4499257 | 1 |
| 3278.4500398 | 2 |
| 3278.4501313 | 2 |
| 3278.4503278 | 4 |
| 3278.4503377 | 1 |
| 3278.4503518 | 1 |
| 3278.4509764 | 4 |
| 3278.4510999 | 2 |
| 3278.4515107 | 1 |
| 3278.4516419 | 0 |
| 3278.4516531 | 2 |
| 3278.451764 | 1 |
| 3278.4518118 | 1 |
| 3278.4521545 | 2 |
| 3278.4521646 | 1 |
| 3278.4522797 | 2 |
| 3278.4526357 | 2 |
| 3278.4526655 | 3 |
| 3278.4527157 | 2 |
| 3278.4528075 | 1 |
| 3278.4528598 | 1 |
| 3278.4531375 | 1 |
| 3278.4532409 | 1 |
| 3278.453504 | 1 |
| 3278.4536716 | 2 |
| 3278.4537609 | 1 |
| 3278.4537793 | 0 |
| 3278.454038 | 2 |
| 3278.4542276 | 5 |
| 3278.4545609 | 1 |
| 3278.4547216 | 1 |
| 3278.4547952 | 2 |
| 3278.454883 | 2 |
| 3278.454925 | 1 |
| 3278.4552373 | 0 |
| 3278.455277 | 4 |
| 3278.4552806 | 2 |
| 3278.4556975 | 1 |
| 3278.4557782 | 2 |
| 3278.4560818 | 2 |
| 3278.4562873 | 1 |
| 3278.4563838 | 3 |
| 3278.4566222 | 4 |
| 3278.4567559 | 3 |
| 3278.4568501 | 0 |
| 3278.456925 | 0 |
| 3278.4570139 | 0 |
| 3278.4572253 | 3 |
| 3278.4573708 | 1 |
| 3278.4575514 | 0 |
| 3278.4575875 | 1 |
| 3278.4576048 | 1 |
| 3278.4579862 | 3 |
| 3278.458075 | 1 |
| 3278.4581726 | 2 |
| 3278.4581752 | 2 |
| 3278.4587688 | 2 |
| 3278.458882 | 1 |
| 3278.4589611 | 1 |
| 3278.4593861 | 0 |
| 3278.4594243 | 0 |
| 3278.4595096 | 1 |
| 3278.4595289 | 3 |
| 3278.4595356 | 5 |
| 3278.4596604 | 4 |
| 3278.4597621 | 1 |
| 3278.4600612 | 1 |
| 3278.460107 | 1 |
| 3278.4602407 | 2 |
| 3278.4603459 | 0 |
| 3278.4606662 | 2 |
| 3278.4608711 | 1 |
| 3278.4613575 | 0 |
| 3278.4613614 | 3 |
| 3278.4614316 | 7 |
| 3278.4615116 | 0 |
| 3278.461521 | 1 |
| 3278.4617017 | 1 |
| 3278.4619049 | 1 |
| 3278.462091 | 2 |
| 3278.4622264 | 3 |
| 3278.4627133 | 0 |
| 3278.4627442 | 2 |
| 3278.4628085 | 1 |
| 3278.4628319 | 2 |
| 3278.4631758 | 0 |
| 3278.4632096 | 2 |
| 3278.4634112 | 0 |
| 3278.4635989 | 0 |
| 3278.4636802 | 2 |
| 3278.4638594 | 1 |
| 3278.463932 | 0 |
| 3278.4639893 | 3 |
| 3278.4643617 | 4 |
| 3278.4643708 | 2 |
| 3278.4645341 | 2 |
| 3278.4649479 | 0 |
| 3278.4650843 | 3 |
| 3278.4651012 | 1 |
| 3278.4651529 | 1 |
| 3278.4653409 | 2 |
| 3278.4653794 | 2 |
| 3278.4654176 | 2 |
| 3278.4654649 | 2 |
| 3278.4658074 | 1 |
| 3278.4659132 | 3 |
| 3278.466025 | 2 |
| 3278.4664792 | 2 |
| 3278.4664878 | 2 |
| 3278.4665349 | 2 |
| 3278.4667585 | 2 |
| 3278.4669007 | 4 |
| 3278.4673405 | 4 |
| 3278.4674662 | 3 |
| 3278.4674688 | 0 |
| 3278.4675311 | 3 |
| 3278.4676525 | 2 |
| 3278.4678423 | 2 |
| 3278.4678576 | 3 |
| 3278.4681452 | 1 |
| 3278.4681953 | 4 |
| 3278.4683702 | 0 |
| 3278.4685949 | 3 |
| 3278.4687139 | 2 |
| 3278.4687306 | 2 |
| 3278.4687627 | 2 |
| 3278.4687681 | 1 |
| 3278.4693049 | 2 |
| 3278.4693312 | 0 |
| 3278.4695989 | 1 |
| 3278.4696888 | 1 |
| 3278.4700563 | 2 |
| 3278.4702031 | 1 |
| 3278.4702275 | 4 |
| 3278.4702781 | 2 |
| 3278.4702857 | 1 |
| 3278.4705311 | 4 |
| 3278.4706329 | 1 |
| 3278.4708022 | 0 |
| 3278.4708146 | 3 |
| 3278.4709291 | 3 |
| 3278.4717117 | 1 |
| 3278.4719935 | 2 |
| 3278.4720191 | 3 |
| 3278.4721267 | 0 |
| 3278.4721763 | 2 |
| 3278.4721893 | 1 |
| 3278.4724285 | 1 |
| 3278.4725502 | 0 |
| 3278.4726928 | 1 |
| 3278.4728145 | 2 |
| 3278.472982 | 1 |
| 3278.4729868 | 0 |
| 3278.4730073 | 2 |
| 3278.4735869 | 3 |
| 3278.473594 | 3 |
| 3278.4737733 | 2 |
| 3278.4741381 | 3 |
| 3278.4741717 | 3 |
| 3278.4741733 | 2 |
| 3278.4742815 | 1 |
| 3278.4746326 | 2 |
| 3278.4747307 | 1 |
| 3278.474827 | 1 |
| 3278.4751298 | 2 |
| 3278.475272 | 2 |
| 3278.4753045 | 0 |
| 3278.4753364 | 0 |
| 3278.4754016 | 0 |
| 3278.4755394 | 1 |
| 3278.4757551 | 2 |
| 3278.4763323 | 2 |
| 3278.4764229 | 0 |
| 3278.4764397 | 1 |
| 3278.4764719 | 1 |
| 3278.476562 | 1 |
| 3278.476798 | 1 |
| 3278.4771389 | 3 |
| 3278.4771716 | 0 |
| 3278.4773399 | 1 |
| 3278.4775432 | 0 |
| 3278.4775913 | 1 |
| 3278.4776276 | 0 |
| 3278.477786 | 0 |
| 3278.4779651 | 2 |
| 3278.4780055 | 2 |
| 3278.4783724 | 2 |
| 3278.4784151 | 2 |
| 3278.478773 | 2 |
| 3278.478831 | 1 |
| 3278.4788991 | 1 |
| 3278.4792264 | 2 |
| 3278.4793357 | 0 |
| 3278.4793794 | 1 |
| 3278.4794341 | 1 |
| 3278.4795792 | 1 |
| 3278.4796417 | 5 |
| 3278.4798749 | 2 |
| 3278.4801021 | 2 |
| 3278.4801521 | 1 |
| 3278.4803864 | 1 |
| 3278.4804033 | 1 |
| 3278.4806794 | 0 |
| 3278.4811163 | 3 |
| 3278.4811179 | 2 |
| 3278.4811362 | 3 |
| 3278.4812698 | 3 |
| 3278.4814739 | 4 |
| 3278.4814879 | 1 |
| 3278.4816418 | 2 |
| 3278.4820164 | 0 |
| 3278.4821771 | 3 |
| 3278.4822586 | 1 |
| 3278.4823247 | 1 |
| 3278.4824152 | 1 |
| 3278.4826529 | 0 |
| 3278.4828995 | 0 |
| 3278.4830698 | 1 |
| 3278.483279 | 1 |
| 3278.4835991 | 0 |
| 3278.4836175 | 0 |
| 3278.4836226 | 2 |
| 3278.483819 | 1 |
| 3278.4840763 | 0 |
| 3278.4843714 | 1 |
| 3278.4843957 | 1 |
| 3278.4844059 | 2 |
| 3278.4845262 | 0 |
| 3278.4846389 | 0 |
| 3278.4847607 | 2 |
| 3278.4849831 | 2 |
| 3278.4850414 | 5 |
| 3278.4854426 | 2 |
| 3278.4854472 | 1 |
| 3278.4855149 | 2 |
| 3278.4856211 | 1 |
| 3278.4858973 | 0 |
| 3278.4859433 | 2 |
| 3278.4863681 | 0 |
| 3278.4863766 | 1 |
| 3278.4865617 | 0 |
| 3278.4867099 | 2 |
| 3278.4868654 | 0 |
| 3278.4871151 | 2 |
| 3278.4873135 | 2 |
| 3278.4874221 | 2 |
| 3278.4876482 | 0 |
| 3278.4876524 | 2 |
| 3278.4878089 | 2 |
| 3278.4879282 | 3 |
| 3278.4882774 | 2 |
| 3278.4883709 | 2 |
| 3278.4883859 | 0 |
| 3278.4886521 | 0 |
| 3278.4886929 | 0 |
| 3278.4888664 | 2 |
| 3278.4891988 | 0 |
| 3278.4893999 | 5 |
| 3278.4894846 | 0 |
| 3278.4896102 | 1 |
| 3278.4896223 | 2 |
| 3278.4897301 | 6 |
| 3278.4898253 | 1 |
| 3278.4900343 | 0 |
| 3278.4900723 | 1 |
| 3278.4901371 | 2 |
| 3278.4904275 | 2 |
| 3278.4905598 | 0 |
| 3278.4906659 | 4 |
| 3278.4911154 | 0 |
| 3278.4912692 | 1 |
| 3278.4915141 | 2 |
| 3278.4916849 | 1 |
| 3278.4916871 | 5 |
| 3278.4917378 | 3 |
| 3278.4917403 | 2 |
| 3278.4918446 | 1 |
| 3278.4923356 | 1 |
| 3278.4925425 | 1 |
| 3278.4925862 | 1 |
| 3278.4927029 | 3 |
| 3278.4928053 | 2 |
| 3278.493154 | 0 |
| 3278.4933277 | 0 |
| 3278.4933738 | 0 |
| 3278.4937503 | 1 |
| 3278.4937586 | 2 |
| 3278.4938708 | 0 |
| 3278.493886 | 5 |
| 3278.4939262 | 4 |
| 3278.4940402 | 2 |
| 3278.4942448 | 1 |
| 3278.4943929 | 0 |
| 3278.4952404 | 3 |
| 3278.4952416 | 6 |
| 3278.495542 | 3 |
| 3278.4955848 | 4 |
| 3278.4957309 | 0 |
| 3278.4957999 | 0 |
| 3278.4960439 | 2 |
| 3278.4962805 | 2 |
| 3278.4964645 | 0 |
| 3278.496516 | 0 |
| 3278.4966328 | 2 |
| 3278.4966443 | 0 |
| 3278.4966707 | 1 |
| 3278.4968031 | 0 |
| 3278.4968313 | 2 |
| 3278.4970665 | 0 |
| 3278.4970679 | 3 |
| 3278.4971856 | 3 |
| 3278.497365 | 2 |
| 3278.4975199 | 3 |
| 3278.4976092 | 3 |
| 3278.4980051 | 1 |
| 3278.4980748 | 0 |
| 3278.4981668 | 3 |
| 3278.4984879 | 3 |
| 3278.4986603 | 1 |
| 3278.4987035 | 4 |
| 3278.4988239 | 4 |
| 3278.4990048 | 2 |
| 3278.4990537 | 4 |
| 3278.4990932 | 1 |
| 3278.4995745 | 2 |
| 3278.4997497 | 0 |
| 3278.4998824 | 3 |
| 3278.5003588 | 2 |
| 3278.5005183 | 1 |
| 3278.500666 | 1 |
| 3278.5007451 | 1 |
| 3278.5007451 | 0 |
| 3278.5008387 | 0 |
| 3278.5009388 | 1 |
| 3278.5012969 | 3 |
| 3278.5013099 | 4 |
| 3278.5016883 | 1 |
| 3278.5017625 | 3 |
| 3278.5018736 | 0 |
| 3278.5019271 | 0 |
| 3278.5022167 | 4 |
| 3278.5023551 | 2 |
| 3278.5025493 | 1 |
| 3278.5026618 | 2 |
| 3278.502905 | 1 |
| 3278.5030642 | 2 |
| 3278.5030678 | 1 |
| 3278.5031191 | 0 |
| 3278.5034431 | 2 |
| 3278.50347 | 1 |
| 3278.5036251 | 0 |
| 3278.5038765 | 1 |
| 3278.5041143 | 2 |
| 3278.5041731 | 2 |
| 3278.5042233 | 3 |

| | |
|---|---|
| 3278.5044967 | 1 |
| 3278.5046816 | 2 |
| 3278.5048066 | 1 |
| 3278.5049546 | 2 |
| 3278.5049835 | 1 |
| 3278.5053455 | 1 |
| 3278.5053937 | 2 |
| 3278.5055503 | 1 |
| 3278.5055893 | 3 |
| 3278.5059162 | 2 |
| 3278.5062486 | 1 |
| 3278.506402 | 3 |
| 3278.5064433 | 4 |
| 3278.5066134 | 4 |
| 3278.5067794 | 0 |
| 3278.5067942 | 0 |
| 3278.5068312 | 2 |
| 3278.5069839 | 0 |
| 3278.5070374 | 2 |
| 3278.5074083 | 0 |
| 3278.5074305 | 2 |
| 3278.5079154 | 2 |
| 3278.5079166 | 2 |
| 3278.508035 | 2 |
| 3278.5080772 | 2 |
| 3278.5081392 | 0 |
| 3278.5083128 | 3 |
| 3278.5088036 | 2 |
| 3278.5088126 | 3 |
| 3278.5088731 | 1 |
| 3278.5090262 | 3 |
| 3278.509059 | 3 |
| 3278.5090757 | 4 |
| 3278.5092186 | 0 |
| 3278.509476 | 0 |
| 3278.5099379 | 2 |
| 3278.5100026 | 2 |
| 3278.5100446 | 3 |
| 3278.5102785 | 2 |
| 3278.5105211 | 1 |
| 3278.5106198 | 2 |
| 3278.5106988 | 1 |
| 3278.510764 | 1 |
| 3278.5108093 | 0 |
| 3278.5112006 | 1 |
| 3278.511342 | 2 |
| 3278.5114125 | 2 |
| 3278.5116078 | 3 |
| 3278.5116751 | 1 |
| 3278.5119344 | 0 |
| 3278.5119685 | 2 |
| 3278.5120526 | 1 |
| 3278.5121728 | 1 |
| 3278.5123921 | 1 |
| 3278.5126103 | 1 |
| 3278.5129333 | 4 |
| 3278.513119 | 0 |
| 3278.5131767 | 2 |
| 3278.5133038 | 2 |
| 3278.5134329 | 1 |
| 3278.5134519 | 1 |
| 3278.5135204 | 5 |
| 3278.5139912 | 3 |
| 3278.514287 | 1 |
| 3278.5143083 | 1 |
| 3278.5143466 | 2 |
| 3278.5143634 | 2 |
| 3278.5145203 | 0 |
| 3278.5146919 | 0 |
| 3278.5148102 | 0 |
| 3278.5149482 | 3 |
| 3278.515058 | 0 |
| 3278.5150933 | 2 |
| 3278.5152855 | 2 |
| 3278.5156085 | 3 |
| 3278.5159989 | 0 |
| 3278.5161388 | 4 |
| 3278.5161477 | 1 |
| 3278.5161645 | 2 |
| 3278.5164141 | 1 |
| 3278.5166844 | 0 |
| 3278.5169185 | 3 |
| 3278.517061 | 0 |
| 3278.5174025 | 6 |
| 3278.5174296 | 1 |
| 3278.517433 | 3 |
| 3278.5177778 | 0 |
| 3278.5177872 | 0 |
| 3278.5179351 | 0 |
| 3278.5181422 | 0 |
| 3278.5181578 | 0 |
| 3278.5182885 | 2 |
| 3278.5183643 | 0 |
| 3278.5183846 | 2 |
| 3278.5189162 | 2 |
| 3278.5189856 | 1 |
| 3278.5191074 | 0 |
| 3278.5193772 | 2 |
| 3278.5193821 | 0 |
| 3278.5195724 | 2 |
| 3278.519758 | 2 |
| 3278.5197654 | 2 |
| 3278.5198006 | 1 |
| 3278.5199497 | 0 |
| 3278.5200466 | 1 |
| 3278.5200661 | 2 |
| 3278.5202935 | 0 |
| 3278.5204402 | 0 |
| 3278.5207541 | 2 |
| 3278.5209708 | 2 |
| 3278.5210461 | 1 |
| 3278.5211277 | 2 |
| 3278.5214498 | 2 |
| 3278.5215248 | 2 |
| 3278.5216149 | 1 |
| 3278.521667 | 1 |
| 3278.5217775 | 0 |
| 3278.5219721 | 2 |
| 3278.5220788 | 2 |
| 3278.522699 | 0 |
| 3278.5227327 | 0 |
| 3278.5227749 | 1 |
| 3278.5228172 | 1 |
| 3278.5229043 | 0 |
| 3278.5229948 | 4 |
| 3278.5234381 | 2 |
| 3278.5235059 | 2 |
| 3278.5235976 | 1 |
| 3278.5237209 | 2 |
| 3278.5239392 | 0 |
| 3278.5239623 | 2 |
| 3278.5240084 | 3 |
| 3278.5243455 | 3 |
| 3278.5243848 | 2 |
| 3278.5249617 | 0 |
| 3278.5249773 | 2 |
| 3278.5250181 | 1 |
| 3278.525074 | 2 |
| 3278.5252738 | 2 |
| 3278.5257808 | 1 |
| 3278.5259276 | 0 |
| 3278.5259633 | 0 |
| 3278.5261781 | 1 |
| 3278.5261945 | 1 |
| 3278.5262585 | 0 |
| 3278.5264472 | 1 |
| 3278.5266794 | 1 |
| 3278.5267254 | 1 |
| 3278.5267916 | 0 |
| 3278.5268388 | 2 |
| 3278.5272198 | 2 |
| 3278.5275324 | 1 |
| 3278.5275693 | 2 |
| 3278.5278167 | 2 |
| 3278.5278275 | 3 |
| 3278.5278781 | 3 |
| 3278.5279691 | 1 |
| 3278.52809 | 3 |
| 3278.528113 | 2 |
| 3278.5281807 | 2 |
| 3278.5283839 | 2 |
| 3278.528457 | 3 |
| 3278.5288556 | 2 |
| 3278.5290333 | 1 |
| 3278.52941 | 0 |
| 3278.5296345 | 1 |
| 3278.5298673 | 0 |
| 3278.5300093 | 3 |
| 3278.530076 | 4 |
| 3278.5301402 | 2 |
| 3278.5301781 | 2 |
| 3278.5304432 | 2 |
| 3278.5304689 | 1 |
| 3278.5305206 | 0 |
| 3278.5308996 | 1 |
| 3278.5309794 | 2 |
| 3278.5310513 | 3 |
| 3278.5314502 | 1 |
| 3278.531459 | 2 |
| 3278.5315861 | 1 |
| 3278.531597 | 1 |
| 3278.5316743 | 1 |
| 3278.5317168 | 4 |
| 3278.5319634 | 0 |
| 3278.532236 | 1 |
| 3278.5325191 | 0 |
| 3278.5328841 | 2 |
| 3278.5328876 | 0 |
| 3278.5329758 | 1 |
| 3278.5332234 | 1 |
| 3278.5334603 | 2 |
| 3278.5334645 | 1 |
| 3278.533693 | 1 |
| 3278.5336976 | 2 |
| 3278.5338009 | 0 |
| 3278.5339159 | 4 |
| 3278.5341263 | 1 |
| 3278.5343875 | 2 |
| 3278.5344478 | 1 |
| 3278.5348459 | 2 |
| 3278.5350276 | 0 |
| 3278.5350324 | 1 |
| 3278.5352824 | 0 |
| 3278.53568 | 3 |
| 3278.5356905 | 0 |
| 3278.5357008 | 3 |
| 3278.5358087 | 2 |
| 3278.5359031 | 3 |
| 3278.5359892 | 1 |
| 3278.5360601 | 4 |
| 3278.5363002 | 1 |
| 3278.5367398 | 3 |
| 3278.5369062 | 2 |
| 3278.536973 | 3 |
| 3278.5370071 | 1 |
| 3278.5371122 | 0 |
| 3278.5372461 | 0 |
| 3278.5375631 | 2 |
| 3278.537629 | 4 |
| 3278.5380871 | 2 |
| 3278.5381724 | 3 |
| 3278.538175 | 2 |
| 3278.5384544 | 3 |
| 3278.5385827 | 1 |
| 3278.538592 | 1 |
| 3278.5386377 | 1 |
| 3278.5390534 | 0 |
| 3278.5391805 | 0 |
| 3278.5392327 | 1 |
| 3278.5393979 | 1 |
| 3278.5394674 | 1 |
| 3278.5395139 | 3 |
| 3278.5397256 | 0 |
| 3278.5399153 | 2 |
| 3278.5400131 | 0 |
| 3278.5402378 | 0 |
| 3278.540307 | 0 |
| 3278.5405236 | 3 |
| 3278.5407602 | 1 |
| 3278.5408587 | 1 |
| 3278.5408888 | 0 |
| 3278.5413004 | 0 |
| 3278.5415053 | 2 |
| 3278.5417121 | 3 |
| 3278.5417732 | 2 |
| 3278.5419856 | 1 |
| 3278.5422034 | 3 |
| 3278.5422691 | 1 |
| 3278.5422874 | 2 |
| 3278.542322 | 1 |
| 3278.5427112 | 1 |
| 3278.5427999 | 0 |
| 3278.5428989 | 4 |
| 3278.5429764 | 1 |
| 3278.5431005 | 2 |
| 3278.5433978 | 3 |
| 3278.5435393 | 4 |
| 3278.5435924 | 2 |
| 3278.5443014 | 1 |
| 3278.5443824 | 2 |
| 3278.5444445 | 5 |
| 3278.544704 | 0 |
| 3278.5447941 | 1 |
| 3278.5448206 | 3 |
| 3278.5448228 | 2 |
| 3278.5449914 | 0 |
| 3278.545278 | 1 |
| 3278.545482 | 1 |
| 3278.5454974 | 2 |
| 3278.5455938 | 1 |
| 3278.5457301 | 1 |
| 3278.5458237 | 3 |
| 3278.5458429 | 7 |
| 3278.5459371 | 2 |
| 3278.5459946 | 3 |
| 3278.5460419 | 1 |
| 3278.5460847 | 1 |
| 3278.5470627 | 1 |
| 3278.5470842 | 2 |
| 3278.5471525 | 1 |
| 3278.5472917 | 0 |
| 3278.5473488 | 3 |
| 3278.5476678 | 2 |
| 3278.5478344 | 0 |
| 3278.547864 | 2 |
| 3278.548242 | 1 |
| 3278.5483462 | 2 |
| 3278.5484218 | 2 |
| 3278.5484482 | 3 |
| 3278.5484962 | 1 |
| 3278.5488196 | 0 |
| 3278.5488246 | 0 |
| 3278.5491673 | 4 |
| 3278.5492626 | 0 |
| 3278.5494516 | 0 |
| 3278.5495814 | 2 |
| 3278.5497059 | 1 |
| 3278.5498568 | 0 |
| 3278.5500636 | 3 |
| 3278.5502722 | 0 |
| 3278.5503876 | 5 |
| 3278.550658 | 1 |
| 3278.5507968 | 4 |
| 3278.5508289 | 0 |
| 3278.5509329 | 1 |
| 3278.5509667 | 3 |
| 3278.5511418 | 0 |
| 3278.5512801 | 1 |
| 3278.5516256 | 2 |
| 3278.5516822 | 1 |
| 3278.5517014 | 0 |
| 3278.5517975 | 1 |
| 3278.5518049 | 6 |
| 3278.5519009 | 3 |
| 3278.5519116 | 1 |
| 3278.5524159 | 1 |
| 3278.5525689 | 2 |
| 3278.552928 | 2 |
| 3278.5529285 | 1 |
| 3278.5530026 | 1 |
| 3278.5532191 | 1 |
| 3278.5532628 | 1 |
| 3278.5536757 | 1 |
| 3278.5538381 | 2 |
| 3278.5539048 | 2 |
| 3278.5541031 | 1 |
| 3278.5541355 | 1 |
| 3278.5542448 | 1 |
| 3278.554255 | 2 |
| 3278.5546704 | 0 |
| 3278.5548852 | 3 |
| 3278.5549129 | 2 |
| 3278.5549708 | 0 |
| 3278.5551753 | 2 |
| 3278.5552793 | 0 |
| 3278.5554451 | 3 |
| 3278.5556727 | 3 |
| 3278.5558453 | 1 |
| 3278.5559585 | 4 |
| 3278.5560288 | 1 |
| 3278.5561288 | 1 |
| 3278.5565839 | 3 |
| 3278.5566843 | 3 |
| 3278.5568826 | 2 |
| 3278.5570102 | 1 |
| 3278.5571031 | 5 |
| 3278.5575942 | 2 |
| 3278.5577447 | 4 |
| 3278.5577545 | 2 |
| 3278.557879 | 3 |
| 3278.5580618 | 0 |
| 3278.5580931 | 4 |
| 3278.5580973 | 2 |
| 3278.5581261 | 0 |
| 3278.5582549 | 4 |
| 3278.5586071 | 0 |
| 3278.5589244 | 3 |
| 3278.5591803 | 0 |
| 3278.5593226 | 2 |
| 3278.5593668 | 2 |
| 3278.5594107 | 2 |
| 3278.5594946 | 2 |
| 3278.5599854 | 2 |

| | |
|---|---|
| 3278.5600696 | 4 |
| 3278.5601904 | 0 |
| 3278.560224 | 1 |
| 3278.5602705 | 1 |
| 3278.560365 | 2 |
| 3278.5604351 | 0 |
| 3278.5604783 | 2 |
| 3278.560977 | 0 |
| 3278.5612625 | 3 |
| 3278.5612947 | 2 |
| 3278.5613239 | 1 |
| 3278.5615667 | 0 |
| 3278.5616672 | 2 |
| 3278.5619608 | 1 |
| 3278.5619898 | 1 |
| 3278.5620083 | 3 |
| 3278.5625354 | 1 |
| 3278.5626396 | 6 |
| 3278.5627517 | 0 |
| 3278.5628918 | 1 |
| 3278.562921 | 1 |
| 3278.5631104 | 1 |
| 3278.5631721 | 3 |
| 3278.5631981 | 2 |
| 3278.5632005 | 0 |
| 3278.5633998 | 0 |
| 3278.5638855 | 1 |
| 3278.5640109 | 2 |
| 3278.5640535 | 1 |
| 3278.5642404 | 0 |
| 3278.5645465 | 2 |
| 3278.5647083 | 0 |
| 3278.5648767 | 2 |
| 3278.5651614 | 2 |
| 3278.5652271 | 3 |
| 3278.5652585 | 2 |
| 3278.5653535 | 1 |
| 3278.5656801 | 3 |
| 3278.5657104 | 0 |
| 3278.5658114 | 0 |
| 3278.5659274 | 0 |
| 3278.5660316 | 2 |
| 3278.5661294 | 3 |
| 3278.5661755 | 1 |
| 3278.5663975 | 2 |
| 3278.5668376 | 4 |
| 3278.5669233 | 1 |
| 3278.5670632 | 2 |
| 3278.5672887 | 4 |
| 3278.5673879 | 1 |
| 3278.5674644 | 6 |
| 3278.5678648 | 5 |
| 3278.5680567 | 5 |
| 3278.5681688 | 6 |
| 3278.5682388 | 5 |
| 3278.5684814 | 3 |
| 3278.5684942 | 5 |
| 3278.5685668 | 9 |
| 3278.5688126 | 10 |
| 3278.5690369 | 16 |
| 3278.5691993 | 9 |
| 3278.5692191 | 12 |
| 3278.5692791 | 9 |
| 3278.5693346 | 17 |
| 3278.5696872 | 14 |
| 3278.570044 | 27 |
| 3278.5700771 | 12 |
| 3278.5703102 | 8 |
| 3278.5704672 | 22 |
| 3278.5705235 | 18 |
| 3278.5705333 | 18 |
| 3278.5708132 | 19 |
| 3278.5709185 | 16 |
| 3278.5710304 | 15 |
| 3278.5710875 | 15 |
| 3278.5715749 | 16 |
| 3278.5716236 | 14 |
| 3278.5716253 | 13 |
| 3278.5716408 | 12 |
| 3278.572096 | 13 |
| 3278.5720997 | 14 |
| 3278.572165 | 8 |
| 3278.5721818 | 13 |
| 3278.572468 | 13 |
| 3278.5726118 | 12 |
| 3278.5730571 | 17 |
| 3278.5731503 | 17 |
| 3278.5732433 | 14 |
| 3278.5732494 | 16 |
| 3278.5734088 | 20 |
| 3278.5735568 | 15 |
| 3278.5739228 | 12 |
| 3278.5739691 | 13 |
| 3278.574021 | 17 |
| 3278.5740997 | 22 |
| 3278.574106 | 19 |
| 3278.5741186 | 12 |
| 3278.5749048 | 10 |
| 3278.5749768 | 21 |
| 3278.575016 | 15 |
| 3278.5750381 | 23 |
| 3278.5751387 | 19 |
| 3278.5754047 | 13 |
| 3278.5756761 | 8 |
| 3278.5757226 | 15 |
| 3278.5758208 | 11 |
| 3278.5759981 | 14 |
| 3278.576107 | 18 |
| 3278.5761675 | 9 |
| 3278.5764278 | 11 |
| 3278.5764885 | 12 |
| 3278.5766866 | 12 |
| 3278.576848 | 11 |
| 3278.5768642 | 10 |
| 3278.5772991 | 12 |
| 3278.5773842 | 11 |
| 3278.5774711 | 10 |
| 3278.5777305 | 7 |
| 3278.5778369 | 11 |
| 3278.5778549 | 7 |
| 3278.5779376 | 6 |
| 3278.5780903 | 6 |
| 3278.5783065 | 5 |
| 3278.5783519 | 7 |
| 3278.5787347 | 2 |
| 3278.578786 | 7 |
| 3278.5788798 | 3 |
| 3278.5789096 | 1 |
| 3278.5790551 | 1 |
| 3278.5793247 | 1 |
| 3278.5794575 | 2 |
| 3278.5798035 | 1 |
| 3278.5799931 | 2 |
| 3278.5802922 | 2 |
| 3278.5803812 | 0 |
| 3278.5804732 | 3 |
| 3278.5804973 | 1 |
| 3278.5808462 | 1 |
| 3278.5810026 | 4 |
| 3278.5813695 | 3 |
| 3278.5814098 | 1 |
| 3278.5817091 | 2 |
| 3278.5818873 | 3 |
| 3278.581925 | 2 |
| 3278.5820936 | 2 |
| 3278.582228 | 2 |
| 3278.5824924 | 0 |
| 3278.5825861 | 2 |
| 3278.5826893 | 3 |
| 3278.5829242 | 2 |
| 3278.583053 | 2 |
| 3278.5830571 | 0 |
| 3278.5831319 | 2 |
| 3278.5831882 | 3 |
| 3278.5835281 | 3 |
| 3278.5835663 | 1 |
| 3278.5837988 | 3 |
| 3278.5840521 | 3 |
| 3278.5841647 | 2 |
| 3278.5842416 | 1 |
| 3278.5842561 | 1 |
| 3278.5846045 | 1 |
| 3278.5847434 | 1 |
| 3278.5848886 | 3 |
| 3278.5849149 | 1 |
| 3278.5851548 | 0 |
| 3278.5854012 | 0 |
| 3278.5854572 | 0 |
| 3278.5856852 | 1 |
| 3278.5858353 | 1 |
| 3278.5860351 | 7 |
| 3278.5861659 | 2 |
| 3278.5865772 | 1 |
| 3278.5865841 | 3 |
| 3278.5866399 | 1 |
| 3278.5866475 | 0 |
| 3278.5866825 | 2 |
| 3278.5868413 | 1 |
| 3278.5870918 | 4 |
| 3278.5871737 | 2 |
| 3278.5872978 | 1 |
| 3278.587521 | 1 |
| 3278.587523 | 3 |
| 3278.5880708 | 0 |
| 3278.5881419 | 1 |
| 3278.5882275 | 1 |
| 3278.588536 | 1 |
| 3278.5888039 | 1 |
| 3278.5889439 | 1 |
| 3278.5890184 | 4 |
| 3278.5890235 | 2 |
| 3278.5894294 | 0 |
| 3278.5895344 | 2 |
| 3278.5895349 | 2 |
| 3278.5898054 | 0 |
| 3278.5898686 | 2 |
| 3278.5899148 | 2 |
| 3278.5902966 | 0 |
| 3278.5903075 | 2 |
| 3278.5905142 | 2 |
| 3278.5906726 | 3 |
| 3278.590754 | 1 |
| 3278.5910526 | 0 |
| 3278.591083 | 1 |
| 3278.5914561 | 0 |
| 3278.5914828 | 0 |
| 3278.5918107 | 3 |
| 3278.5918977 | 1 |
| 3278.5919413 | 1 |
| 3278.5919895 | 0 |
| 3278.5921042 | 5 |
| 3278.5922452 | 1 |
| 3278.5922647 | 3 |
| 3278.5926506 | 4 |
| 3278.592895 | 1 |
| 3278.5930011 | 2 |
| 3278.5931496 | 1 |
| 3278.5932024 | 3 |
| 3278.5933124 | 4 |
| 3278.5935338 | 2 |
| 3278.5936691 | 2 |
| 3278.5939388 | 1 |
| 3278.5942763 | 3 |
| 3278.5942789 | 2 |
| 3278.5943347 | 1 |
| 3278.5943601 | 2 |
| 3278.5944981 | 2 |
| 3278.5945831 | 1 |
| 3278.5948321 | 2 |
| 3278.5949664 | 0 |
| 3278.5954171 | 2 |
| 3278.5955907 | 1 |
| 3278.5957256 | 2 |
| 3278.5958892 | 2 |
| 3278.5959529 | 2 |
| 3278.596207 | 1 |
| 3278.5962403 | 2 |
| 3278.5964379 | 1 |
| 3278.5964694 | 0 |
| 3278.5965528 | 3 |
| 3278.5968734 | 3 |
| 3278.597019 | 1 |
| 3278.5970458 | 1 |
| 3278.5975123 | 2 |
| 3278.5975307 | 1 |
| 3278.5975587 | 0 |
| 3278.5975689 | 5 |
| 3278.5977609 | 0 |
| 3278.5977662 | 2 |
| 3278.5983523 | 0 |
| 3278.5984057 | 3 |
| 3278.5985223 | 2 |
| 3278.5986281 | 2 |
| 3278.5986661 | 2 |
| 3278.5988414 | 2 |
| 3278.5988696 | 0 |
| 3278.5989353 | 2 |
| 3278.5995399 | 2 |
| 3278.5996169 | 1 |
| 3278.5996197 | 1 |
| 3278.5996358 | 3 |
| 3278.599764 | 2 |
| 3278.5998713 | 1 |
| 3278.5999217 | 4 |
| 3278.6002724 | 0 |
| 3278.6007521 | 0 |
| 3278.6009667 | 0 |
| 3278.6011773 | 0 |
| 3278.6012017 | 2 |
| 3278.6012121 | 1 |
| 3278.6013415 | 2 |
| 3278.6014332 | 1 |
| 3278.6014575 | 1 |
| 3278.6015354 | 0 |
| 3278.6019339 | 1 |
| 3278.6021185 | 3 |
| 3278.6021822 | 0 |
| 3278.6022719 | 2 |
| 3278.6023268 | 2 |
| 3278.6027326 | 5 |
| 3278.6029234 | 2 |
| 3278.6033248 | 0 |
| 3278.6033307 | 1 |
| 3278.6034286 | 1 |
| 3278.6034297 | 1 |
| 3278.6035685 | 2 |
| 3278.6036301 | 0 |
| 3278.6038032 | 2 |
| 3278.6038901 | 0 |
| 3278.6040288 | 0 |
| 3278.6044749 | 1 |
| 3278.6046724 | 1 |
| 3278.6047693 | 3 |
| 3278.6047883 | 4 |
| 3278.6048581 | 2 |
| 3278.6052578 | 0 |
| 3278.6054484 | 1 |
| 3278.6055437 | 1 |
| 3278.6055617 | 1 |
| 3278.6055975 | 3 |
| 3278.6059147 | 0 |
| 3278.606118 | 4 |
| 3278.6065112 | 2 |
| 3278.6066091 | 2 |
| 3278.6066727 | 3 |
| 3278.6067934 | 1 |
| 3278.6068115 | 2 |
| 3278.60695 | 3 |
| 3278.6070791 | 0 |
| 3278.6071803 | 1 |
| 3278.607656 | 1 |
| 3278.6077767 | 3 |
| 3278.6077879 | 2 |
| 3278.607859 | 2 |
| 3278.6080271 | 2 |
| 3278.6080423 | 3 |
| 3278.6082471 | 0 |
| 3278.6082478 | 2 |
| 3278.6083275 | 2 |
| 3278.6091443 | 1 |
| 3278.6091654 | 0 |
| 3278.6093041 | 0 |
| 3278.6093058 | 1 |
| 3278.6094227 | 1 |
| 3278.6094585 | 1 |
| 3278.6094737 | 1 |
| 3278.6095835 | 2 |
| 3278.6099188 | 0 |
| 3278.6100451 | 0 |
| 3278.6103478 | 1 |
| 3278.610571 | 4 |
| 3278.6106193 | 0 |
| 3278.6107783 | 4 |
| 3278.6108927 | 1 |
| 3278.6109102 | 3 |
| 3278.6110982 | 3 |
| 3278.611612 | 1 |
| 3278.6117141 | 2 |
| 3278.6117467 | 2 |
| 3278.6119207 | 2 |
| 3278.6120418 | 2 |
| 3278.6121964 | 0 |
| 3278.6122111 | 1 |
| 3278.6123022 | 0 |
| 3278.6124507 | 1 |
| 3278.6125967 | 2 |
| 3278.6128059 | 1 |
| 3278.613093 | 1 |
| 3278.6130979 | 3 |
| 3278.6130979 | 2 |
| 3278.6135326 | 2 |
| 3278.6135596 | 1 |
| 3278.6138082 | 0 |
| 3278.6138634 | 5 |
| 3278.613894 | 2 |
| 3278.6139596 | 1 |
| 3278.6143968 | 1 |
| 3278.6145566 | 2 |
| 3278.6151103 | 0 |
| 3278.6152003 | 1 |
| 3278.615211 | 0 |
| 3278.615216 | 0 |
| 3278.6152831 | 0 |

| | |
|---|---|
| 3278.6153365 | 3 |
| 3278.6157107 | 3 |
| 3278.6157358 | 2 |
| 3278.6159909 | 3 |
| 3278.6160863 | 0 |
| 3278.6164363 | 0 |
| 3278.6164445 | 1 |
| 3278.6166593 | 5 |
| 3278.6168674 | 3 |
| 3278.6168848 | 2 |
| 3278.6169247 | 1 |
| 3278.616963 | 0 |
| 3278.6170547 | 1 |
| 3278.6170707 | 2 |
| 3278.6174844 | 3 |
| 3278.6178468 | 3 |
| 3278.6180424 | 2 |
| 3278.6181684 | 0 |
| 3278.6182664 | 1 |
| 3278.6182845 | 3 |
| 3278.6183441 | 4 |
| 3278.6185032 | 0 |
| 3278.6185385 | 0 |
| 3278.6192192 | 0 |
| 3278.6192334 | 1 |
| 3278.6194969 | 2 |
| 3278.6195294 | 1 |
| 3278.6196584 | 1 |
| 3278.6199707 | 1 |
| 3278.6203537 | 1 |
| 3278.6205018 | 2 |
| 3278.6205977 | 0 |
| 3278.6206263 | 4 |
| 3278.6206471 | 1 |
| 3278.6208633 | 0 |
| 3278.6209997 | 4 |
| 3278.6212111 | 1 |
| 3278.6212582 | 1 |
| 3278.6216426 | 3 |
| 3278.6218978 | 1 |
| 3278.6219664 | 4 |
| 3278.6221708 | 0 |
| 3278.622198 | 0 |
| 3278.6222654 | 4 |
| 3278.6223183 | 0 |
| 3278.6223357 | 0 |
| 3278.6224474 | 0 |
| 3278.6226283 | 4 |
| 3278.6226499 | 0 |
| 3278.6230816 | 1 |
| 3278.6233304 | 2 |
| 3278.6234015 | 1 |
| 3278.6234582 | 1 |
| 3278.6237006 | 2 |
| 3278.6237015 | 0 |
| 3278.6240326 | 0 |
| 3278.6242133 | 1 |
| 3278.6242437 | 1 |
| 3278.6244334 | 1 |
| 3278.6247901 | 3 |
| 3278.6251336 | 1 |
| 3278.6252213 | 2 |
| 3278.6253068 | 1 |
| 3278.6253502 | 2 |
| 3278.6258098 | 1 |
| 3278.625812 | 4 |
| 3278.6258965 | 0 |
| 3278.6259007 | 2 |
| 3278.6259975 | 0 |
| 3278.6261758 | 1 |
| 3278.6262682 | 1 |
| 3278.6262926 | 0 |
| 3278.6267753 | 0 |
| 3278.6270894 | 3 |
| 3278.6271155 | 1 |
| 3278.6272066 | 1 |
| 3278.6273262 | 3 |
| 3278.6273666 | 0 |
| 3278.627393 | 2 |
| 3278.6277787 | 1 |
| 3278.627876 | 2 |
| 3278.6280143 | 1 |
| 3278.6280945 | 5 |
| 3278.6284461 | 1 |
| 3278.6284503 | 2 |
| 3278.6288083 | 1 |
| 3278.6289546 | 0 |
| 3278.629153 | 0 |
| 3278.6292894 | 2 |
| 3278.6292999 | 3 |
| 3278.6293127 | 1 |
| 3278.6293193 | 2 |
| 3278.6296365 | 0 |
| 3278.6298207 | 3 |
| 3278.6301196 | 0 |
| 3278.6302414 | 1 |
| 3278.6303276 | 4 |
| 3278.6304792 | 0 |
| 3278.630669 | 0 |
| 3278.630753 | 0 |
| 3278.6308536 | 1 |
| 3278.6308696 | 1 |
| 3278.6312371 | 1 |
| 3278.6314788 | 2 |
| 3278.6317478 | 2 |
| 3278.6317496 | 3 |
| 3278.6319485 | 1 |
| 3278.6320377 | 1 |
| 3278.6320451 | 4 |
| 3278.6322883 | 1 |
| 3278.6322933 | 2 |
| 3278.6326575 | 2 |
| 3278.6327046 | 2 |
| 3278.6329157 | 1 |
| 3278.6329559 | 2 |
| 3278.6333103 | 4 |
| 3278.6336525 | 3 |
| 3278.6336674 | 3 |
| 3278.6337779 | 3 |
| 3278.633779 | 2 |
| 3278.6340053 | 1 |
| 3278.6342202 | 3 |
| 3278.6342347 | 2 |
| 3278.6344575 | 1 |
| 3278.6348489 | 0 |
| 3278.6352081 | 1 |
| 3278.6352673 | 1 |
| 3278.6353823 | 1 |
| 3278.6354783 | 2 |
| 3278.6355051 | 0 |
| 3278.6358826 | 3 |
| 3278.6359245 | 0 |
| 3278.6360397 | 1 |
| 3278.6364563 | 4 |
| 3278.636505 | 1 |
| 3278.6365222 | 0 |
| 3278.6366129 | 2 |
| 3278.6369182 | 4 |
| 3278.6369567 | 0 |
| 3278.6372512 | 1 |
| 3278.637291 | 1 |
| 3278.6373277 | 1 |
| 3278.6374463 | 0 |
| 3278.6375365 | 1 |
| 3278.6380858 | 3 |
| 3278.6382199 | 1 |
| 3278.6382292 | 2 |
| 3278.6384162 | 2 |
| 3278.6384815 | 3 |
| 3278.6387561 | 1 |
| 3278.6388098 | 2 |
| 3278.6388622 | 1 |
| 3278.63906 | 2 |
| 3278.6391062 | 1 |
| 3278.6392289 | 1 |
| 3278.6395138 | 0 |
| 3278.6395453 | 1 |
| 3278.6399182 | 2 |
| 3278.6399817 | 1 |
| 3278.6400366 | 1 |
| 3278.6404794 | 2 |
| 3278.6406766 | 0 |
| 3278.6406993 | 3 |
| 3278.6408207 | 4 |
| 3278.6410303 | 1 |
| 3278.6410555 | 4 |
| 3278.6410946 | 1 |
| 3278.6417546 | 2 |
| 3278.6418234 | 2 |
| 3278.641926 | 2 |
| 3278.6419292 | 2 |
| 3278.6420308 | 1 |
| 3278.6423804 | 0 |
| 3278.6424677 | 0 |
| 3278.6424795 | 2 |
| 3278.6425529 | 1 |
| 3278.6425566 | 1 |
| 3278.6431623 | 1 |
| 3278.6432194 | 2 |
| 3278.6433877 | 0 |
| 3278.6436671 | 0 |
| 3278.6437196 | 1 |
| 3278.6439403 | 1 |
| 3278.6440708 | 0 |
| 3278.6443283 | 3 |
| 3278.6446544 | 1 |
| 3278.6446831 | 1 |
| 3278.6449063 | 1 |
| 3278.6449673 | 2 |
| 3278.645031 | 1 |
| 3278.6450903 | 4 |
| 3278.6451613 | 0 |
| 3278.6453078 | 1 |
| 3278.6454163 | 1 |
| 3278.6454824 | 0 |
| 3278.645838 | 1 |
| 3278.6458775 | 0 |
| 3278.6459834 | 0 |
| 3278.646204 | 1 |
| 3278.6463321 | 1 |
| 3278.6465294 | 1 |
| 3278.6466288 | 0 |
| 3278.6467151 | 1 |
| 3278.6467714 | 0 |
| 3278.6470589 | 0 |
| 3278.6470955 | 1 |
| 3278.647283 | 2 |
| 3278.6477054 | 0 |
| 3278.6477802 | 1 |
| 3278.6478654 | 1 |
| 3278.648006 | 2 |
| 3278.6480073 | 1 |
| 3278.648283 | 1 |
| 3278.6483595 | 1 |
| 3278.648453 | 1 |
| 3278.6485642 | 2 |
| 3278.6489075 | 2 |
| 3278.6489505 | 1 |
| 3278.649152 | 2 |
| 3278.6493768 | 4 |
| 3278.6497207 | 3 |
| 3278.6497681 | 2 |
| 3278.6498595 | 4 |
| 3278.6498767 | 0 |
| 3278.6499245 | 1 |
| 3278.6501327 | 3 |
| 3278.6501732 | 0 |
| 3278.6504795 | 2 |
| 3278.6507065 | 2 |
| 3278.6509882 | 3 |
| 3278.6509975 | 3 |
| 3278.6510021 | 0 |
| 3278.6514539 | 0 |
| 3278.6514911 | 0 |
| 3278.6515757 | 2 |
| 3278.6518327 | 2 |
| 3278.6519775 | 1 |
| 3278.6522698 | 1 |
| 3278.6522896 | 2 |
| 3278.6523225 | 0 |
| 3278.6524731 | 2 |
| 3278.653082 | 0 |
| 3278.6531122 | 2 |
| 3278.6531993 | 1 |
| 3278.6534457 | 1 |
| 3278.6535364 | 0 |
| 3278.6535594 | 1 |
| 3278.653622 | 0 |
| 3278.6537741 | 1 |
| 3278.6538539 | 1 |
| 3278.6540595 | 1 |
| 3278.6544602 | 2 |
| 3278.6544925 | 1 |
| 3278.6547708 | 2 |
| 3278.6549581 | 1 |
| 3278.65503 | 0 |
| 3278.6550822 | 1 |
| 3278.6551314 | 0 |
| 3278.655252 | 2 |
| 3278.655382 | 2 |
| 3278.6560864 | 4 |
| 3278.6560982 | 3 |
| 3278.6563202 | 3 |
| 3278.6564142 | 2 |
| 3278.6564509 | 1 |
| 3278.6564567 | 3 |
| 3278.6566043 | 3 |
| 3278.656611 | 2 |
| 3278.6569321 | 2 |
| 3278.6569772 | 0 |
| 3278.6570166 | 4 |
| 3278.6570478 | 3 |
| 3278.6575018 | 2 |
| 3278.6575389 | 1 |
| 3278.6575681 | 4 |
| 3278.6581204 | 2 |
| 3278.6584318 | 2 |
| 3278.6584606 | 1 |
| 3278.658488 | 4 |
| 3278.6587462 | 1 |
| 3278.6587492 | 2 |
| 3278.6587638 | 0 |
| 3278.6588329 | 1 |
| 3278.6589547 | 4 |
| 3278.6589881 | 1 |
| 3278.6591612 | 3 |
| 3278.659206 | 0 |
| 3278.6594358 | 1 |
| 3278.6598455 | 1 |
| 3278.6600107 | 0 |
| 3278.6600325 | 2 |
| 3278.6600968 | 1 |
| 3278.6604259 | 1 |
| 3278.6607258 | 1 |
| 3278.6607309 | 4 |
| 3278.661167 | 1 |
| 3278.6612114 | 4 |
| 3278.6612266 | 0 |
| 3278.661514 | 1 |
| 3278.6616085 | 3 |
| 3278.6617347 | 0 |
| 3278.6618396 | 0 |
| 3278.6620985 | 2 |
| 3278.6624754 | 1 |
| 3278.662614 | 3 |
| 3278.6626908 | 1 |
| 3278.6628108 | 3 |
| 3278.662823 | 1 |
| 3278.6628547 | 2 |
| 3278.6628646 | 1 |
| 3278.6629379 | 3 |
| 3278.6629604 | 4 |
| 3278.6631674 | 1 |
| 3278.6633787 | 1 |
| 3278.6636226 | 0 |
| 3278.6637723 | 0 |
| 3278.6638105 | 2 |
| 3278.6639703 | 3 |
| 3278.6639789 | 3 |
| 3278.6643803 | 0 |
| 3278.6644494 | 0 |
| 3278.6644871 | 1 |
| 3278.6646651 | 3 |
| 3278.6646662 | 2 |
| 3278.6649367 | 2 |
| 3278.6652265 | 3 |
| 3278.6653946 | 1 |
| 3278.6654967 | 1 |
| 3278.66555 | 3 |
| 3278.6656465 | 0 |
| 3278.6656547 | 1 |
| 3278.6659621 | 2 |
| 3278.6662358 | 1 |
| 3278.6664759 | 2 |
| 3278.6664875 | 2 |
| 3278.6665592 | 4 |
| 3278.6666078 | 0 |
| 3278.6666555 | 1 |
| 3278.6669561 | 0 |
| 3278.6671449 | 0 |
| 3278.6672076 | 3 |
| 3278.6673243 | 1 |
| 3278.6673895 | 0 |
| 3278.6676005 | 0 |
| 3278.667829 | 1 |
| 3278.6680712 | 2 |
| 3278.668079 | 3 |
| 3278.6681118 | 4 |
| 3278.6683961 | 1 |
| 3278.6685238 | 1 |
| 3278.6685272 | 2 |
| 3278.6688494 | 3 |
| 3278.6688924 | 1 |
| 3278.6690894 | 2 |
| 3278.6693331 | 3 |
| 3278.669385 | 0 |
| 3278.6695179 | 3 |
| 3278.6696683 | 0 |
| 3278.6696799 | 1 |
| 3278.669933 | 1 |
| 3278.6702205 | 1 |
| 3278.6702944 | 1 |
| 3278.6703669 | 0 |

| | |
|---|---|
| 3278.6705792 | 3 |
| 3278.6706772 | 5 |
| 3278.6708017 | 2 |
| 3278.6708531 | 0 |
| 3278.6710612 | 1 |
| 3278.6710791 | 3 |
| 3278.671566 | 2 |
| 3278.671742 | 2 |
| 3278.6717584 | 3 |
| 3278.6719333 | 0 |
| 3278.6720313 | 1 |
| 3278.6720718 | 1 |
| 3278.6721925 | 2 |
| 3278.672391 | 2 |
| 3278.6727268 | 1 |
| 3278.6728163 | 3 |
| 3278.6728303 | 2 |
| 3278.6732142 | 1 |
| 3278.6735138 | 1 |
| 3278.6735422 | 0 |
| 3278.6735953 | 1 |
| 3278.67368 | 0 |
| 3278.6737216 | 1 |
| 3278.6737272 | 0 |
| 3278.673966 | 1 |
| 3278.6741942 | 1 |
| 3278.6745127 | 0 |
| 3278.6747045 | 1 |
| 3278.674713 | 0 |
| 3278.6749442 | 0 |
| 3278.6750391 | 3 |
| 3278.6750704 | 2 |
| 3278.6752408 | 3 |
| 3278.6753359 | 0 |
| 3278.6753562 | 2 |
| 3278.6753778 | 1 |
| 3278.6757319 | 1 |
| 3278.675893 | 1 |
| 3278.6759399 | 1 |
| 3278.6761802 | 1 |
| 3278.676318 | 1 |
| 3278.6763469 | 0 |
| 3278.6764091 | 1 |
| 3278.6764845 | 3 |
| 3278.6766287 | 0 |
| 3278.6769361 | 1 |
| 3278.6770255 | 1 |
| 3278.677305 | 1 |
| 3278.6773167 | 2 |
| 3278.6773652 | 1 |
| 3278.6779509 | 2 |
| 3278.6780052 | 3 |
| 3278.6781366 | 2 |
| 3278.6784069 | 3 |
| 3278.6785536 | 1 |
| 3278.6788097 | 1 |
| 3278.6788528 | 1 |
| 3278.6789477 | 2 |
| 3278.6791596 | 1 |
| 3278.6792181 | 2 |
| 3278.6796061 | 1 |
| 3278.6796922 | 1 |
| 3278.6797849 | 1 |
| 3278.679822 | 4 |
| 3278.6798581 | 4 |
| 3278.6798636 | 4 |
| 3278.679918 | 0 |
| 3278.6799434 | 1 |
| 3278.6799752 | 2 |
| 3278.6801448 | 1 |
| 3278.6807086 | 2 |
| 3278.6807931 | 4 |
| 3278.6809278 | 3 |
| 3278.6809902 | 1 |
| 3278.6810693 | 2 |
| 3278.681163 | 1 |
| 3278.6814152 | 2 |
| 3278.6815154 | 2 |
| 3278.6817186 | 2 |
| 3278.6817824 | 1 |
| 3278.6821348 | 0 |
| 3278.682165 | 1 |
| 3278.6824524 | 2 |
| 3278.6826707 | 0 |
| 3278.6826871 | 0 |
| 3278.6827551 | 3 |
| 3278.6827707 | 4 |
| 3278.6828315 | 0 |
| 3278.6830634 | 2 |
| 3278.6831252 | 0 |
| 3278.6834853 | 1 |
| 3278.6838442 | 0 |
| 3278.683846 | 4 |
| 3278.6844398 | 2 |
| 3278.6845031 | 0 |
| 3278.6846639 | 1 |
| 3278.6847553 | 2 |
| 3278.6851689 | 0 |
| 3278.6853139 | 1 |
| 3278.6858287 | 3 |
| 3278.6859311 | 0 |
| 3278.6860616 | 1 |
| 3278.686622 | 0 |
| 3278.6867862 | 2 |
| 3278.6868973 | 0 |
| 3278.6874114 | 2 |
| 3278.6881115 | 1 |
| 3278.6881574 | 1 |
| 3278.6881941 | 0 |
| 3278.6887504 | 0 |
| 3278.6887683 | 1 |
| 3278.6893534 | 1 |
| 3278.6894041 | 1 |
| 3278.6894787 | 2 |
| 3278.6895104 | 6 |
| 3278.6898206 | 3 |
| 3278.6899872 | 1 |
| 3278.6904116 | 1 |
| 3278.6906159 | 0 |
| 3278.6907711 | 1 |
| 3278.6912355 | 1 |
| 3278.6913266 | 2 |
| 3278.6913875 | 2 |
| 3278.6919511 | 1 |
| 3278.6924728 | 3 |
| 3278.6925843 | 1 |
| 3278.6926821 | 0 |
| 3278.693098 | 1 |
| 3278.693184 | 1 |
| 3278.6937066 | 0 |
| 3278.6937285 | 0 |
| 3278.6938011 | 2 |
| 3278.6940417 | 4 |
| 3278.6943457 | 0 |
| 3278.6947934 | 1 |
| 3278.694872 | 0 |
| 3278.6952776 | 3 |
| 3278.6954757 | 2 |
| 3278.695807 | 0 |
| 3278.6963405 | 2 |
| 3278.6964452 | 4 |
| 3278.6969583 | 1 |
| 3278.6970305 | 2 |
| 3278.6974507 | 1 |
| 3278.6975029 | 1 |
| 3278.6978639 | 3 |
| 3278.6979693 | 2 |
| 3278.6980514 | 2 |
| 3278.6980838 | 0 |
| 3278.6983152 | 0 |
| 3278.6985535 | 0 |
| 3278.6988574 | 2 |
| 3278.6993439 | 0 |
| 3278.6994725 | 2 |
| 3278.7000104 | 0 |
| 3278.7001496 | 2 |
| 3278.7004797 | 2 |
| 3278.7005372 | 1 |
| 3278.7009786 | 2 |
| 3278.7011342 | 0 |
| 3278.7012941 | 0 |
| 3278.7018078 | 3 |
| 3278.7022673 | 3 |
| 3278.7024358 | 2 |
| 3278.7027271 | 1 |
| 3278.7027893 | 1 |
| 3278.702797 | 0 |
| 3278.702946 | 0 |
| 3278.7034476 | 1 |
| 3278.7037124 | 1 |
| 3278.7038771 | 1 |
| 3278.7043279 | 1 |
| 3278.7045311 | 0 |
| 3278.7046136 | 1 |
| 3278.7048052 | 1 |
| 3278.7052174 | 0 |
| 3278.7053418 | 1 |
| 3278.7054701 | 2 |
| 3278.7056936 | 1 |
| 3278.705933 | 1 |
| 3278.7060045 | 4 |
| 3278.7067546 | 2 |
| 3278.7071717 | 2 |
| 3278.7074291 | 1 |
| 3278.707854 | 1 |
| 3278.7079169 | 1 |
| 3278.7082324 | 3 |
| 3278.7082467 | 1 |
| 3278.708612 | 2 |
| 3278.7088287 | 0 |
| 3278.7088404 | 2 |
| 3278.709201 | 4 |
| 3278.7097788 | 1 |
| 3278.7103219 | 1 |
| 3278.7108251 | 0 |
| 3278.710916 | 5 |
| 3278.7111649 | 0 |
| 3278.7112237 | 1 |
| 3278.7112364 | 1 |
| 3278.7117123 | 2 |
| 3278.712247 | 0 |
| 3278.712294 | 1 |
| 3278.7126051 | 0 |
| 3278.7126741 | 0 |
| 3278.7131787 | 1 |
| 3278.7131942 | 0 |
| 3278.7133626 | 2 |
| 3278.7134942 | 3 |
| 3278.7138092 | 1 |
| 3278.7140221 | 1 |
| 3278.7142827 | 2 |
| 3278.7144597 | 0 |
| 3278.7147905 | 1 |
| 3278.7152068 | 1 |
| 3278.7156891 | 1 |
| 3278.7158256 | 1 |
| 3278.7158886 | 1 |
| 3278.7159707 | 2 |
| 3278.7165509 | 1 |
| 3278.7168972 | 0 |
| 3278.7173312 | 1 |
| 3278.7173854 | 0 |
| 3278.7173923 | 2 |
| 3278.7179412 | 1 |
| 3278.7182459 | 2 |
| 3278.7182609 | 3 |
| 3278.7185769 | 1 |
| 3278.7188438 | 1 |
| 3278.7188541 | 2 |
| 3278.7190475 | 1 |
| 3278.7191126 | 2 |
| 3278.7199066 | 1 |
| 3278.7202646 | 1 |
| 3278.7206005 | 4 |
| 3278.7207026 | 1 |
| 3278.7209968 | 0 |
| 3278.7213622 | 2 |
| 3278.7214023 | 2 |
| 3278.7218414 | 3 |
| 3278.7220346 | 1 |
| 3278.72237 | 1 |
| 3278.7223727 | 3 |
| 3278.7230157 | 1 |
| 3278.7231274 | 2 |
| 3278.7234246 | 1 |
| 3278.7235667 | 1 |
| 3278.7237047 | 1 |
| 3278.7245009 | 0 |
| 3278.7246255 | 0 |
| 3278.7248515 | 1 |
| 3278.7252237 | 0 |
| 3278.7252311 | 2 |
| 3278.7252768 | 2 |
| 3278.7253702 | 4 |
| 3278.7260956 | 3 |
| 3278.7261495 | 2 |
| 3278.7262174 | 0 |
| 3278.7264624 | 3 |
| 3278.7270201 | 2 |
| 3278.727382 | 3 |
| 3278.7274449 | 0 |
| 3278.7277835 | 3 |
| 3278.728261 | 1 |
| 3278.72836 | 0 |
| 3278.7286141 | 3 |
| 3278.7291496 | 3 |
| 3278.7293641 | 1 |
| 3278.7295429 | 0 |
| 3278.7296915 | 1 |
| 3278.7297387 | 0 |
| 3278.7298125 | 1 |
| 3278.7302555 | 2 |
| 3278.7305354 | 4 |
| 3278.7308461 | 3 |
| 3278.731035 | 2 |
| 3278.7311823 | 4 |
| 3278.7314333 | 2 |
| 3278.7321676 | 1 |
| 3278.7322416 | 1 |
| 3278.7330349 | 2 |
| 3278.733093 | 0 |
| 3278.7332989 | 2 |
| 3278.7333042 | 1 |
| 3278.733487 | 0 |
| 3278.7340134 | 2 |
| 3278.7341249 | 0 |
| 3278.7342795 | 1 |
| 3278.735067 | 1 |
| 3278.7350715 | 5 |
| 3278.7352674 | 1 |
| 3278.7357547 | 0 |
| 3278.7358184 | 3 |
| 3278.7360452 | 1 |
| 3278.7361589 | 1 |
| 3278.7361865 | 1 |
| 3278.7367626 | 2 |
| 3278.7367973 | 2 |
| 3278.7369331 | 1 |
| 3278.7373994 | 3 |
| 3278.7374762 | 1 |
| 3278.7381133 | 2 |
| 3278.7382726 | 0 |
| 3278.7388053 | 0 |
| 3278.7391014 | 3 |
| 3278.7391813 | 1 |
| 3278.7391954 | 0 |
| 3278.7395606 | 3 |
| 3278.7398527 | 0 |
| 3278.7399384 | 2 |
| 3278.7401434 | 1 |
| 3278.740248 | 4 |
| 3278.7405232 | 1 |
| 3278.7410977 | 3 |
| 3278.7411657 | 0 |
| 3278.7417844 | 1 |
| 3278.7418519 | 3 |
| 3278.7418895 | 2 |
| 3278.7420683 | 2 |
| 3278.7425738 | 2 |
| 3278.7426844 | 4 |
| 3278.742787 | 0 |
| 3278.7431447 | 1 |
| 3278.7431571 | 0 |
| 3278.7435797 | 0 |
| 3278.7440567 | 0 |
| 3278.7441086 | 2 |
| 3278.7446374 | 1 |
| 3278.7447104 | 2 |
| 3278.7449577 | 2 |
| 3278.7450036 | 1 |
| 3278.745366 | 2 |
| 3278.7456792 | 2 |
| 3278.7459877 | 1 |
| 3278.7468859 | 3 |
| 3278.7468916 | 1 |
| 3278.7469017 | 2 |
| 3278.7471701 | 2 |
| 3278.7473949 | 2 |
| 3278.7474606 | 1 |
| 3278.7478616 | 2 |
| 3278.7481461 | 2 |
| 3278.7485154 | 2 |
| 3278.748727 | 2 |
| 3278.7488117 | 2 |
| 3278.7492066 | 1 |
| 3278.7493467 | 1 |
| 3278.7498214 | 1 |
| 3278.7499842 | 0 |
| 3278.7503552 | 0 |
| 3278.7503893 | 3 |
| 3278.7508134 | 1 |
| 3278.7511489 | 0 |
| 3278.7512306 | 0 |
| 3278.7512748 | 0 |
| 3278.751749 | 2 |
| 3278.7522247 | 2 |
| 3278.7525635 | 2 |
| 3278.7527627 | 0 |
| 3278.7529122 | 2 |
| 3278.7530872 | 4 |
| 3278.7533829 | 1 |
| 3278.7534707 | 1 |
| 3278.753769 | 1 |

| | |
|---|---|
| 3278.7538735 | 2 |
| 3278.7540845 | 0 |
| 3278.7547724 | 2 |
| 3278.7547767 | 1 |
| 3278.7552988 | 1 |
| 3278.7554304 | 1 |
| 3278.7555127 | 1 |
| 3278.755669 | 5 |
| 3278.755963 | 2 |
| 3278.7562684 | 0 |
| 3278.7564108 | 2 |
| 3278.7565248 | 1 |
| 3278.7568202 | 0 |
| 3278.7573821 | 0 |
| 3278.7573875 | 0 |
| 3278.757535 | 2 |
| 3278.7582437 | 1 |
| 3278.7584144 | 7 |
| 3278.7584225 | 4 |
| 3278.7587599 | 1 |
| 3278.7587941 | 2 |
| 3278.7591657 | 2 |
| 3278.7596938 | 3 |
| 3278.7602168 | 2 |
| 3278.760774 | 0 |
| 3278.760818 | 0 |
| 3278.7608284 | 1 |
| 3278.7608378 | 1 |
| 3278.7609046 | 1 |
| 3278.7609302 | 0 |
| 3278.7615775 | 0 |
| 3278.7621913 | 3 |
| 3278.7622965 | 2 |
| 3278.762699 | 0 |
| 3278.7628507 | 2 |
| 3278.762852 | 2 |
| 3278.7634503 | 2 |
| 3278.7635793 | 0 |
| 3278.7639533 | 5 |
| 3278.7641945 | 1 |
| 3278.7643461 | 1 |
| 3278.7644792 | 1 |
| 3278.7650584 | 2 |
| 3278.7655512 | 2 |
| 3278.7656354 | 0 |
| 3278.7658325 | 2 |
| 3278.7658468 | 2 |
| 3278.7660604 | 1 |
| 3278.7663531 | 2 |
| 3278.7667394 | 1 |
| 3278.7673548 | 0 |
| 3278.7674781 | 1 |
| 3278.7675666 | 0 |
| 3278.7676667 | 3 |
| 3278.7678832 | 2 |
| 3278.7684257 | 3 |
| 3278.7685823 | 0 |
| 3278.7690772 | 0 |
| 3278.7691609 | 1 |
| 3278.7692459 | 2 |
| 3278.7697685 | 2 |
| 3278.7702536 | 3 |
| 3278.7706359 | 1 |
| 3278.7708479 | 0 |
| 3278.7709495 | 1 |
| 3278.7711045 | 1 |
| 3278.7712924 | 2 |
| 3278.7716895 | 2 |
| 3278.771849 | 0 |
| 3278.7724088 | 0 |
| 3278.772577 | 0 |
| 3278.772973 | 0 |
| 3278.772994 | 0 |
| 3278.773318 | 0 |
| 3278.7733645 | 1 |
| 3278.7735582 | 0 |
| 3278.7739375 | 1 |
| 3278.7741727 | 3 |
| 3278.7743228 | 3 |
| 3278.7744016 | 0 |
| 3278.7744835 | 1 |
| 3278.7750051 | 1 |
| 3278.7750857 | 2 |
| 3278.7753134 | 0 |
| 3278.7760194 | 0 |
| 3278.7761673 | 1 |
| 3278.77636 | 1 |
| 3278.7765361 | 0 |
| 3278.7767413 | 0 |
| 3278.7768846 | 2 |
| 3278.7776246 | 0 |
| 3278.777946 | 0 |
| 3278.7781909 | 1 |
| 3278.7785989 | 0 |
| 3278.7788338 | 0 |
| 3278.779229 | 0 |
| 3278.7795437 | 2 |
| 3278.7795549 | 2 |
| 3278.7797228 | 0 |
| 3278.7801244 | 1 |
| 3278.7801931 | 2 |
| 3278.7803111 | 1 |
| 3278.7804572 | 2 |
| 3278.781013 | 3 |
| 3278.781104 | 0 |
| 3278.7811619 | 4 |
| 3278.7814065 | 2 |
| 3278.7814908 | 1 |
| 3278.7820361 | 2 |
| 3278.7821643 | 0 |
| 3278.7822678 | 2 |
| 3278.7830821 | 2 |
| 3278.7837192 | 3 |
| 3278.7837297 | 3 |
| 3278.7840011 | 3 |
| 3278.7840295 | 2 |
| 3278.7842487 | 0 |
| 3278.7843448 | 3 |
| 3278.7846432 | 0 |
| 3278.7848295 | 1 |
| 3278.7848417 | 2 |
| 3278.7851935 | 2 |
| 3278.7852969 | 2 |
| 3278.7858612 | 2 |
| 3278.7862152 | 1 |
| 3278.7863122 | 3 |
| 3278.786412 | 1 |
| 3278.7873032 | 1 |
| 3278.7873723 | 3 |
| 3278.7874713 | 1 |
| 3278.7880012 | 1 |
| 3278.7880038 | 0 |
| 3278.7881433 | 2 |
| 3278.7883289 | 1 |
| 3278.7886298 | 3 |
| 3278.7890094 | 3 |
| 3278.7893292 | 1 |
| 3278.7896003 | 1 |
| 3278.7899843 | 1 |
| 3278.7900579 | 2 |
| 3278.7900807 | 1 |
| 3278.790757 | 1 |
| 3278.7911994 | 2 |
| 3278.7912343 | 1 |
| 3278.7914128 | 1 |
| 3278.7916417 | 2 |
| 3278.791783 | 1 |
| 3278.7921395 | 1 |
| 3278.7925157 | 2 |
| 3278.7927432 | 1 |
| 3278.793282 | 2 |
[truncated: 1,451,060 more chars]
